# MiR156 regulates anthocyanin biosynthesis through *SPL* targets and other microRNAs in poplar

**DOI:** 10.1038/s41438-020-00341-w

**Published:** 2020-08-01

**Authors:** Yamei Wang, Wenwen Liu, Xinwei Wang, Ruijuan Yang, Zhenying Wu, Han Wang, Lei Wang, Zhubing Hu, Siyi Guo, Hailing Zhang, Jinxing Lin, Chunxiang Fu

**Affiliations:** 1 Shandong Provincial Key Laboratory of Energy Genetics, Key Laboratory of Biofuels, Chinese Academy of Sciences, Qingdao Institute of BioEnergy and Bioprocess Technology, Chinese Academy of Sciences, Qingdao, 266101 China; 2 University of Chinese Academy of Sciences, Beijing, 100049 China; 3 College of Biological Sciences & Biotechnology, Beijing Forestry University, Beijing, 10083 China; 4 Collaborative Innovation Center of Crop Stress Biology, Henan Province and Institute of Plant Stress Biology, Henan University, Kaifeng, 475001 China; 5 Grass and Science Institute of Heilongjiang Academy of Agricultural Sciences, Harbin, Heilongjiang China

**Keywords:** Secondary metabolism, Molecular engineering in plants

## Abstract

Anthocyanins biosynthesized from the flavonoid pathway are types of pigments that are involved in the protection of poplar from biotic and abiotic stresses. Previous researchers studying anthocyanin-related transcription factors and structural genes in poplar have made significant discoveries. However, little is known about the regulatory role of microRNAs in anthocyanin biosynthesis in poplar. Here, we overexpressed miR156 in poplar to study the comprehensive effects of the miR156-*SPL* module on the biosynthesis of anthocyanins. Small RNA sequencing analysis revealed 228 microRNAs differentially expressed in transgenic poplar plants with dramatically increased miR156 levels. Furthermore, integrated microRNAomic and transcriptomic analysis suggested that two microRNAs, miR160h, and miR858, have the potential to affect anthocyanin accumulation in poplar by regulating auxin response factors and MYB transcription factors, respectively. Additionally, the accumulation of miR160h and miR858 displayed a positive correlation with miR156 levels, suggesting a possible interaction between the miR156-*SPL* module and these microRNAs in poplar. Last, metabolomics analysis revealed that the levels of anthocyanins, flavones, and flavonols were substantially elevated in transgenic poplar plants overexpressing miR156 compared with the wild type, whereas the total lignin content was reduced in the transgenic plants. Taken together, our results indicate that miR156 can fine tune the anthocyanin biosynthetic pathway via multiple factors, including microRNAs, transcription factors, and the levels of structural genes, in poplar. This provides additional clues for understanding the complex regulatory network of anthocyanin biosynthesis in woody plants.

## Introduction

Anthocyanins, flavones, flavonols, and lignin are important metabolites derived from the essential phenylpropanoid pathway in higher plants^1^. These metabolites play crucial roles in plant growth and development, as well as in response to biotic and abiotic stresses^1,2^. Anthocyanins are comprise a group of abundant flavonoid compounds stored in the fruit, seed coat, leaf, bark, and flowers. They attract pollinators, protect plants from UV radiation and pathogen, and act as powerful antioxidants^3^. Numerous structural and regulatory genes participate in anthocyanin biosynthesis. Structural genes encoding enzymes including phenylalanine ammonialyase (PAL), chalcone synthase (CHS), chalcone isomerase (CHI), flavanone 3-hydroxylase (F3H), dihydroflavonol reductase (DFR), leucoanthocyanidin dioxygenase/anthocyanidin synthase (LDOX/ANS), glutathione *S*-transferase (GST), and UDP-glucose flavonoid 3-o-glycosyltransferase (UFGT) are responsible for the formation of anthocyanins^1,3^. These genes are primarily regulated by R2R3-type MYB, basic helix-loop-helix (bHLH), and WD40 repeat (WDR) transcription factors, which compose the MYB–bHLH–WDR (MBW) transcriptional complex^4^. The MBW complex usually binds the promoters of target gene to regulate their transcription levels in plants^4^. These regulators can be activated by factors such as light, temperature, cytokinins, and microbes^5^. Other transcription factors, such as jasmonate zim-domain (JAZ), constitutively photomorphogenic1 (COP1) and SQUAMOSA promoter binding protein-like 9 (SPL9) proteins, are also involved in anthocyanin biosynthesis^6–8^. Among them, miR156-targeted SPL9, a plant-specific transcription factor, negatively regulates the expression of anthocyanin biosynthetic genes by impairing the stability of the MBW complex^8^.

MicroRNAs are a class of endogenous noncoding small RNAs that negatively regulate gene expression in plants and animals^9^. MicroRNAs participate in plant growth and development, abiotic/biotic responses, metabolite biosynthesis, and other important biological processes^10^. In the past several few decades, microRNAs have been shown to play vital roles in the biosynthesis of anthocyanins in plants^8,11–13^. As an evolutionarily conserved microRNA, miR156 targets a subset of *SQUAMOSA PROMOTER BINDING PROTEIN-LIKE* (*SPL*) genes in plants. SPLs are involved in a broad range of developmental processes in *Arabidopsis*, alfalfa, rice, switchgrass, and other plant species^14–17^. They exhibit a dominant function during the transition from the plant vegetative phase to the reproductive stage. Moreover, overexpression of miR156 in *Arabidopsis* and switchgrass affects the biosynthesis of anthocyanins and lignin^8,18^. In addition to miR156, additional microRNAs involved in anthocyanin biosynthesis have been identified and characterized using high-throughput sequencing in *Arabidopsis*, grape, and sweet potato^11–13^. For example, a high level of miR858 can accelerate the accumulation of anthocyanins by regulating the expression of R2R3-type *MYB*s in grape^12^. Compared with herbaceous and tuberous plant species, there is still a lack of research on structural genes, transcription factors, and microRNAs involved in anthocyanin biosynthesis in poplar, an important woody plant species. Furthermore, the effects of miR156 overexpression on other microRNAs related to anthocyanin accumulation have not yet been studied.

Poplar is an important feedstock for biofuel production and pulping and papermaking^19^. In addition, poplar is widely used in ornamental horticulture for shade and as a protective tree due to its rapid growth, thick branches and leaves, and developed root system^19^. We integrated small RNA sequencing and transcriptomic and metabolomic analyses to investigate microRNAs and their target genes involved in anthocyanin biosynthesis in miR156-overexpressing transgenic poplar plants that had a reddish pigment on the epidermis of their stem, leaf margins, and petioles. Aside from miR156-SPLs, two microRNA-target modules, miR160h-*ARF18* and miR858-*MYB39*, were identified as potentially controlling the accumulation of anthocyanins in poplar. Moreover, the accumulation of miR160h and miR858 was positively correlated with miR156 levels in poplar, suggesting that the miR156-*SPL* module might regulate anthocyanin biosynthesis by affecting the expression of the two microRNAs in a direct or indirect manner. Our work revealed the comprehensive influences of miR156 overexpression on other microRNAs and their targets related to anthocyanin biosynthesis based on the combined analysis of microRNAomic, transcriptomic and metabolomic data. We provided additional potential targets for the bioengineering and subsequent breeding of novel poplar germplasm for horticulture, bioenergy, and paper production.

## Materials and methods

### Plant materials and growth conditions

The fast-growing poplar hybrid 84K (*Populus alba* × *P. tremula* var. *glandulosa*) was used for genetic transformation and omics analysis. Three vegetatively propagated clones of each plant were grown in 3-gallon pots containing loam soil, after which they were randomly distributed into three blocks. Plants were grown in a greenhouse under 14 h light/10 h darkness (390 µE/m^2^/s), 25 °C, and 60% relative humidity and watered three times a week.

### Gene constructs and transformation

An 829 bp stem-loop fragment of the MtmiR156b precursor was amplified by polymerase chain reaction (PCR) from the *Medicago truncatula* R108 genome using the primers pre-MtmiR156b-F and pre-MtmiR156b-R (Table S1). The DNA sequence of pre-MtmiR156b was inserted into a pCXSN vector, which carries the selectable marker hygromycin phosphotransferase gene (*hph*), after it was digested with *Xcm*I (New England Biolabs, Beverly, USA), and the insertion was confirmed by sequencing^20^. A reverse Tnos-R primer (Table S1) downstream of the MtmiR156b precursor was used for sequencing to detect the correct ligations in the vector constructs. The final binary vector pCXSN-pre-MtmiR156b was transferred into *Agrobacterium tumefaciens* strain EHA105 using the freeze-thaw method^21^. Poplar transformation was subsequently performed using the *Agrobacterium*-mediated method described by Jia et al.^22^. Hygromycin (PhytoTech Labs, Shawnee Mission, USA) was used as the selectable reagent to generate putative transgenic poplar plants overexpressing miR156. Genomic DNA was isolated from the leaves of the abovementioned hygromycin-resistant poplar lines following the modified 2× CTAB (hexadecyltrimethylammonium bromide) procedure^23^. Positive transgenic poplar plants were identified via PCR with specific *hph* and pre-MtmiR156b primers (Table S1) using the following conditions: 95 °C for 2 min (one cycle); 94 °C for 30 s, 55 °C for 30 s, 72 °C for 60 s (30 cycles); and 72 °C extension for 10 min. The expected sizes of the PCR products were 375 for hph and 834 bp for pre-MtmiR156b.

### RNA extraction and gene expression analysis

Total RNA was extracted from the plant tissues using a TRIzol kit (TransGen Biotech, Beijing, China) and was reverse into cDNA using a PrimeScript™ RT Reagent Kit with gDNA Eraser (Takara, Dalian, China) according to the manufacturer’s instructions. Quantitative real-time polymerase chain reaction (qRT-PCR) analysis was performed in a 20 μL reaction volume containing 10 μL of SYBR Premix ExTaq™ (Takara, Dalian, China), 2 μL of cDNA, and each primer at 0.5 μM. The primer pairs used for qRT-PCR are listed in Table S1. *QPto18S* was used as the reference for normalization. The cycle thresholds were determined using a Roche Light Cycler 480 II sequence detection system (Roche, Shanghai, China). The levels of mature microRNAs were detected and quantified using a highly sensitive quantitative RT-PCR method^24^. The mature microRNAs, including miR156, miR160h, miR858, and miR168, were reverse transcribed and measured using Mir-X miRNA First-Strand Synthesis and a SYBR qRT-PCR kit (Vazyme Biotech, Nanjing, China); miR168 was used as the reference for normalization. The primers used for qRT-PCR are listed in Table S1.

### Small RNA library construction, sequencing, and analysis

Young stems from the top of ten-month-old plants were collected from three wild-type plants and three miR156-overexpressing transgenic poplar lines (TGII-1, -2, and -3) for microRNAomic analysis. Sequencing libraries of microRNAs were obtained using TruSeq Small RNA Sample Prep Kits (Illumina, San Diego, USA) and sequenced on an Illumina HiSeq 2000/2500 (LC Sciences, Houston, USA). ACGT101-miR software (LC Sciences, Houston, USA) was used to analyze the microRNA sequences. Known noncoding RNAs derived from rRNAs, tRNAs, snRNAs, snoRNAs, and other repeat sequences were identified by BLASTN (http://www.ncbi.nlm.nih.gov/). The resulting small RNAs were identified by aligning them to the sequences stored in miRBase 22.1 (http://www.mirbase.org/). These sequences were then clustered to generate the listed microRNA families. Only perfectly matched sequences were designated conserved microRNAs. A unique sequence was identified as a new microRNA if it met the following criteria: (1) the fold-back structures formed hairpins as predicted by the RNAFOLD program; (2) the sequence was located in the duplex region of the hairpin structure; and (3) the corresponding miRNA^*^ could be detected in the small RNA libraries. We followed the procedures and criteria described by Allen et al.^25^ and Schwab et al.^26^ to predict microRNA-targeted genes. Differentially expressed microRNAs were selected if their |log2 (fold change)| was ≥1 and their *p*-value was ≤0.05. The Gene Ontology (GO) terms of the microRNA targets were annotated using the online GO analysis tool (http://www.geneontology.org). The statistical enrichment of the candidate target genes in Kyoto Encyclopedia of Genes and Genomes (KEGG) pathways was determined by the KOBAS software^27^.

### Transcriptomic analysis of transgenic poplar plants

Young stems from the top of 10-month-old plants were collected from three wild-type plants and three miR156-overexpressing transgenic poplar lines (TGII-1, -2, and -3) for transcriptomic analysis. Total RNA was extracted using TRIzol reagent (TransGen Biotech, Beijing, China), and the purified RNA was reverse transcribed to the cDNA and then sequenced on an Illumina HiSeq 4000 (LC Sciences, Houston, USA). Transcriptome de novo assembly of poplar 84 K was performed directly on the set of sequenced reads using Trinity 2.4.0 (Broad Institute, Boston, USA). Raw Illumina pair-end reads were subsequently trimmed using FastQC (http://www.bioinformatics.babraham.ac.uk/projects/fastqc/), encompassing the Q20, Q30, and GC content of the clean data, to obtain high-quality reads. All assembled unigenes were aligned against the nonredundant (Nr), GO, SwissProt (http://www.expasy.ch/sprot/), KEGG and eggNOG (http://eggnogdb.embl.de/) databases using DIAMOND (Crystal Impact GbR, Bonn, Germany), with a threshold of an *E*-value < 0.00001. The differentially expressed unigenes were selected if their |log2(fold change)| was ≥1 and if they were statistically significant (*p-*value ≤ 0.05) by the R package edgeR. GO and KEGG enrichment analyses were used for data mining. An integrated microRNAomic and transcriptomic analysis was used to identify microRNA-target pairs. An integrated microRNAomic and transcriptomic analysis was used to identify microRNA-target pairs. This integrative analysis was performed according to the five following steps: (1) microRNA-targeted genes were predicted by TargetFinder; (2) the microRNA data were correlated with the mRNA data; (3) the transcriptomic data and were correlated with the gene data; (4) microRNA-target pairs and regulatory relations based on significant difference (|log2 (fold change)| ≥ 1 and *p*-value ≤ 0.05) and regulatory relations; (5) GO and KEGG enrichment analysis was performed for the microRNA–target pairs.

### Metabolomic analysis of transgenic poplar plants

Three wild-type plants and three miR156-overexpressing transgenic poplar lines (TGII-1, -2, and -3) were selected for metabolomic analysis. A total of six young stem samples were collected from the top of ten-month-old plants and ground in liquid nitrogen by a MM 400 mixer mill (Retsch, Düsseldorf, German) at 30 Hz for 1.5 min. The ground lyophilized samples (100 mg) were then dissolved overnight in 0.6 ml of 70% aqueous methanol at 4 °C. The supernatant was collected using a microporous membrane (0.22-μm pore size) after it was centrifuged at 10,000*g* for 10 min. The extracts were subsequently subjected to ultra-performance liquid chromatography-tandem mass spectrometry (UPLC-MS/MS) analysis (UPLC, Shim-pack UFLC SHIMADZU CBM30A; MS/MS, Applied Biosystems 4500 QTRAP). The UPLC and mass spectrometry conditions were performed and determined as described by Chen et al.^28^. Qualitative and quantitative determination of metabolites was performed using the self-established MWDB database (MetWare Biological Science and Technology Co., Ltd.) and other public databases in accordance with standard metabolic operating procedures^29^.

### Total anthocyanin and lignin content analysis

We measured the total anthocyanin and lignin contents of the samples prepared for metabolomic analysis. Twenty milligrams of powder from each sample was dissolved in 1 ml of a hydrochloric acid:methanol [1:99 (v/v)] solution and extracted overnight, in the dark, at 4 °C. The absorbance values of the above extract were determined at 530 nm and 657 nm, respectively, by a UV–Vis spectrophotometer (METASH, Shanghai, China). The total anthocyanin content was then quantified as (A_530_-0.25 × A_657_)/fresh weight (unit/mg)^30^.

To measure the lignin content, soluble extracts were first removed from lyophilized samples by four successive extractions with chloroform:methanol [2:1 (v/v)], methanol, methanol:H_2_O [1:1 (v/v)], and water at room temperature as described by Chen and Dixon^31^. Lyophilized extract-free material was used to analyze the lignin content using the acetyl bromide (AcBr) method^32^. Approximately 20 mg of lyophilized extract-free powder was digested with 5 ml of acetyl bromide reagent [25% (v/v) acetyl bromide in glacial acetic acid] at 50 °C for 4 h. After incubation, the samples were cooled and centrifuged at 1157 g for 15 min, after which 4 ml of the upper layer was transferred to 50 ml volumetric flasks containing 10 ml of 2 M NaOH and 12 ml of acetic acid. One milliliter of 0.5 M hydroxylamine was added to each flask. The samples were diluted to 50 ml with acetic acid and then analyzed at 280 nm with a UV–Vis spectrophotometer (METASH). The total anthocyanin content was quantified as *X* = *Y*/17.20 (mg/ml) (*X*, content of AcBr lignin; *Y*, absorbance readings at 280 nm)^32^.

### Statistical analysis

Three wild-type plants and five miR156-overexpressing transgenic lines (TGI-1; TGI-2; and TGII-1, -2, and -3) were analyzed in this study. Each sample included three vegetatively propagated clones. Data from each trait were subjected to either an analysis of variance (ANOVA) or Student’s *t* test. The significance of the treatments was tested at the *p* < 0.05 level. Standard errors are provided in all tables and figures as appropriate. All statistical analyses were performed using the SPSS package (SPSS Inc., Chicago, USA).

## Results

### Overexpression of miR156 altered the morphology of poplar

**Fig. 1:**
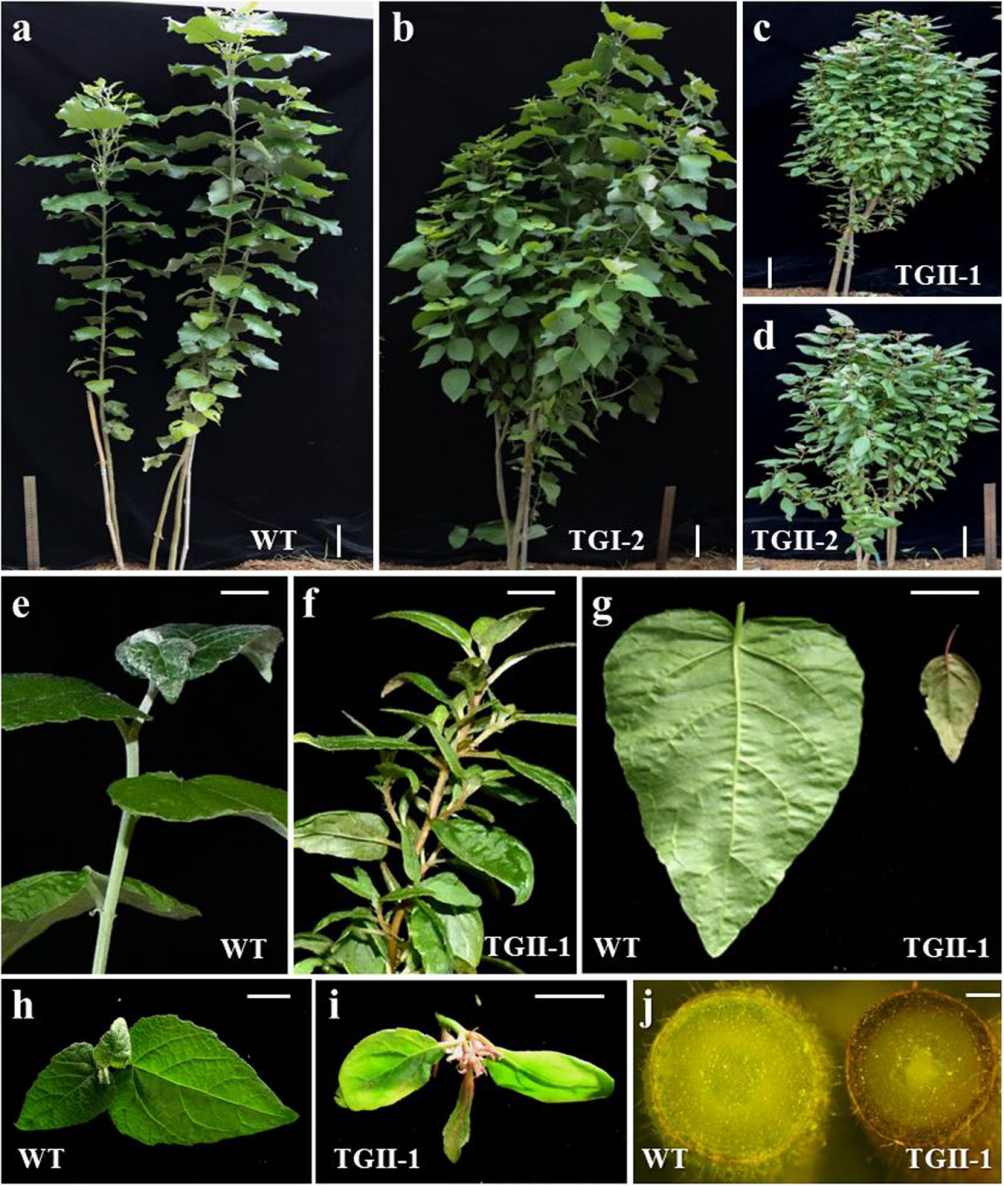
Morphological characterization of transgenic poplar plants overexpressing miR156b. **a**–**c** Representative plants from each group are shown: WT (**a**, wild type), TGI-2 (**b**, group I), TGII-1 (**c**, group II) and TGII-2 (**d**, group II). Bar = 10 cm. **e**, **f** Reddish pigment that appeared on the epidermis of the stems of TGII-1 transgenic poplar plants. Bar = 1 cm. **g** Reddish pigment that appeared on the epidermis of the leaf margins of TGII-1 transgenic poplar plants. Bar = 1 cm. **h**, **i** Reddish pigment that appeared on the epidermis of the leaf petioles of TGII-1 transgenic poplar plants. Bar = 1 cm. **j** Cross-sections of young stems of wild-type (WT) and transgenic poplar plants (TGII-1). Bar = 0.5 mm

**Fig. 2:**
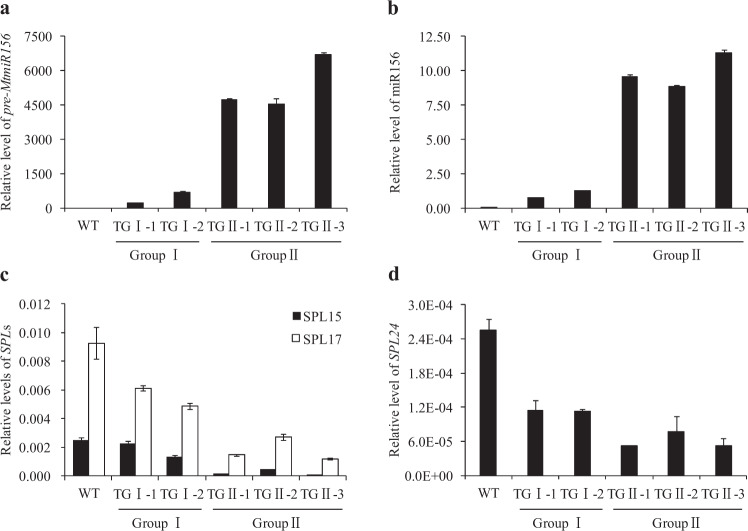
Molecular characterization of transgenic poplar plants. **a** Quantitative real-time PCR analysis of *pre-MtmiR156b* in wild-type and transgenic poplar plants. *QPto18S* was used as a reference for normalization. **b** Mature miR156 levels in wild-type and transgenic poplar plants were detected and quantified by a highly sensitive quantitative real-time PCR method. miRNA168 was used as a reference for normalization. **c**, **d** Quantitative real-time PCR analysis of miR156-targeted *SPL*s in wild-type and transgenic poplar plants. *QPto18S* was used as a reference for normalization. The values are the means ± SEs (*n* = 3). WT indicates wild type

PCR screening using *hph* and MtmiR156b precursor-specific primer pairs was used to identify positive transgenic poplar lines. The transgenic poplar plants were grown in a greenhouse (Fig. 1). Based on the expression levels of *pre-miR156b*, the transgenic poplar lines were assigned into one of two groups: group I or group II (Fig. 2a). The group I transgenic poplar lines exhibited moderately increased numbers of branches, slightly reduced leaf size, and normal plant height (Fig. 1a, b). The group II plants exhibited a dwarf phenotype that included highly increased numbers of branches; severely reduced leaf size; developed axillary buds; and a reddish pigment on the epidermis of the stems, leaf margins, and petioles (Fig. 1a, c–j). The levels of *pre-miR156b* approximately accounted for the morphology of the transgenic poplar plants (Figs. 1 and 2). The expression levels of mature miR156 and its *SPL* targets in the transgenic poplar plants were further examined. The mature miR156 levels increased 20- to 305.3-fold in the transgenic poplar lines compared to the wild type (Fig. 2b). As a consequence, the transcript abundances of miR156-targeted *SPL15*, -*17*, and -*24* exhibited corresponding decreases in the transgenic plants (Fig. 2c, d). Taken together, our results indicate that exogenous miR156b functioned efficiently in a dose-dependent manner during morphological changes of poplar.

### Small RNA sequencing uncovered various microRNAs from poplar

**Fig. 3:**
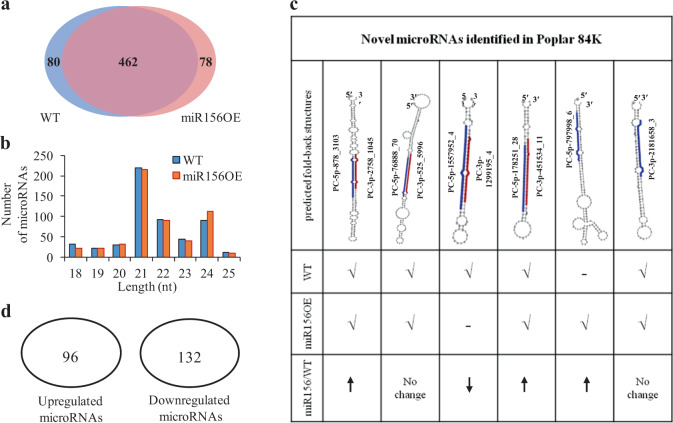
microRNAomic analysis of wild-type and group II transgenic poplar plants. **a** Venn diagram comparing the numbers of microRNAs identified in wild-type and group II transgenic poplar plants. **b** Length distribution of microRNAs in wild-type and group II transgenic poplar plants. **c** Predicted fold-back structures of six novel microRNAs identified from wild-type and group II transgenic poplar plants. The blue labels indicate mature microRNA sequences; the red labels indicate microRNA* sequences. **d** Number of differentially expressed microRNAs between the wild-type and group II transgenic poplar plants. WT indicates wild type. miR156OE indicates the group II transgenic poplar plants, including TGII-1, -2, and -3

To study the effects of miR156 overexpression on the levels of other microRNAs in poplar, we constructed small RNA libraries of wild-type and group II transgenic poplar plants (TGII-1, -2, and -3). The two small RNA libraries yielded 60,375,019 and 60,210,670 total raw reads (Table S2). After removing the reads of low quality, contaminated adapter sequences, mRNAs and Pfam RNAs, the reads with a base length between 18 and 25 nt were further analyzed as typical microRNAs. Venn diagram analysis demonstrated that 462 microRNAs were coexpressed between the two libraries (Fig. 3a). Eighty microRNAs were expressed specifically in the wild type, while 78 microRNAs were expressed specifically in the transgenic plants (Fig. 3a; Table S3). The microRNAs detected in the two libraries showed similar length and base preference, and the microRNAs with lengths of 21 and 24 nt were abundant (Fig. 3b). The analysis of nucleotide sequences revealed that uridine (U) was the most common nucleotide at the 5′ end in both libraries (Fig. S1a and b). The poplar 84K genome has not been completely sequenced to date. However, the sequences of mature microRNAs exhibit high conservation among different plant species. Thus, we performed deep sequencing of the total microRNAs and aligned these sequences to those of microRNAs deposited in miRBase to identify the conserved microRNAs. We also attempted to align the pre-microRNA sequences to the poplar 84K RNA-seq transcriptomic data. A total of 78 conserved microRNAs were retrieved from the two small RNA libraries (Table S4). The most conserved microRNAs, including miR156, miR159, miR160, miR164, miR166, miR167, miR171, miR172, miR390, miR408, and miR482, were abundantly expressed in both the wild-type and transgenic poplar plants (Table S4). Additionally, 144 nonconservative microRNAs were identified from wild-type and transgenic poplar plants (Table S4). Among them, six mature microRNAs that had more than 10 counts were identified for the first time in the libraries of the wild-type and/or transgenic poplar plants (Fig. 3c; Table S4).

### Overexpression of miR156 altered the levels of other microRNAs in poplar

The differentially expressed microRNAs (DEMs) in the wild-type and transgenic poplar plants were further studied. The conserved and novel microRNAs were subjected to the same expression analysis criteria (|log2 (fold change)| ≥ 1 and *p*-value ≤ 0.05); a total of 228 microRNAs were differentially expressed between the transgenic poplar plants and with the wild-type plants. Among them, the levels of 96 microRNAs, including miR164, miR167, miR169, miR396, miR472, miR858, PC-3p-2758_1045, and PC-5p-178251_28, were upregulated (Fig. 3c, d; Table S5). In contrast, the amounts of 132 microRNAs, including miR159, miR171, and miR172, were reduced in the transgenic poplar plants (Fig. 3c, d; Table S5). Moreover, some microRNAs involved in abiotic stress and secondary metabolic pathways were differentially expressed in the transgenic poplar plants (Table S6).

### Overexpression of miR156 had global effects on its downstream genes in poplar

**Fig. 4:**
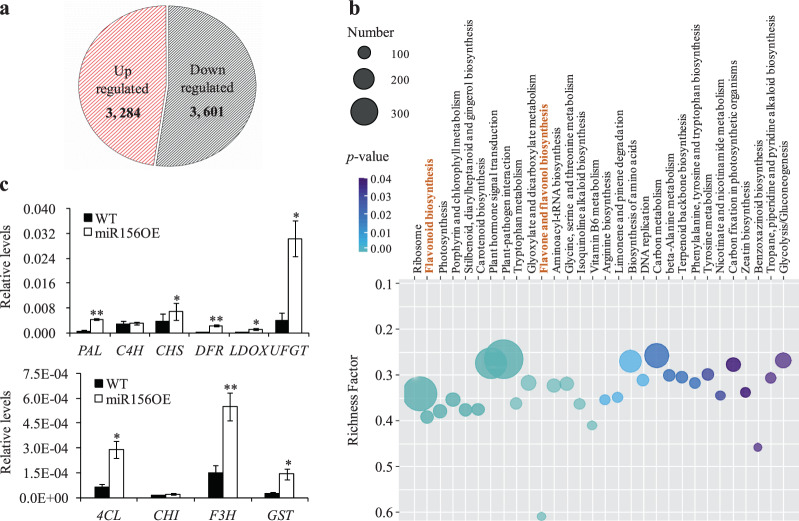
Transcriptomic analysis of wild-type and group II transgenic poplar plants. **a** Number of differentially expressed genes (DEGs) between wild-type and group II transgenic poplar plants. **b** KEGG pathway enrichment of DEGs in wild-type and group II transgenic poplar plants. The first 30 enriched pathways were identified, and the richness factor indicates the number of DEGs/total genes in this KEGG analysis. **c** Quantitative real-time PCR analysis of differentially expressed flavonoid- and anthocyanin-related genes in transgenic poplar plants. *QPto18S* was used as a reference for normalization. The values are the means ± SEs (*n* = 3). WT indicates wild type. miR156OE indicates the group II transgenic poplar plants, including TGII-1, -2, and -3. One or two asterisks indicate statistical significance at *p* < 0.05 or 0.01, respectively (Student’s *t* test)

To study the effects of miR156 overexpression on downstream genes, we examined the transcriptomes of the wild-type and group II transgenic poplar plants using the RNA-seq method. There were 6885 out of 44,189 (15.6%) genes differentially expressed in transgenic poplar plants compared to the wild type. Of these, 3284 genes were upregulated in the transgenic poplar plants, while 3601 genes were downregulated (Fig. 4a; Table S7). Additionally, 116 potential differentially expressed genes (DEGs) were identified as targets of 85 DEMs based on an integrated microRNAomic and transcriptomic analysis (Table S8). The DEGs were then subjected to KEGG pathway analysis. A total of 3105 DEGs (45.10% of 6,885 DEGs) were categorized into 137 KEGG terms (Table S9). Most enrichment pathways were related to plant-pathogen interactions; ribosomes; plant hormone signal transduction; photosynthesis; and the biosynthesis of flavones, flavonols, flavonoids, and zeatin (Fig. 4b).

Previous studies have suggested that miR156-targeted *AtSPL9/15* can regulate the biosynthesis of anthocyanins in *Arabidopsis*^8^. The miR156-targeted *SPL8*, *SPL11*, *SPL12*, *SPL17*, *SPL28*, and *SPL29* retrieved from the RNA-seq dataset of poplar 84K cultivars were orthologs of *AtSPL9/15* (Table S10). Among them, the transcript abundances of *SPL8*, *SPL11*, *SPL12*, *SPL17*, *SPL28*, and *SPL29* were significantly reduced in the transgenic poplar plants (Table S10). qRT-PCR analysis consistently revealed that all these miR156-targeted *SPL*s were also downregulated in the transgenic poplar plants (Table S10). To further investigate the molecular mechanism governing the large amounts of anthocyanins in the transgenic poplar plants, we analyzed the expression levels of genes encoding the crucial enzymes and the transcription factors involved in the flavonoid biosynthetic pathway in wild-type and group II transgenic poplar plants. qRT-PCR analysis revealed that the expression levels of most genes related to anthocyanin biosynthesis were elevated in transgenic poplar plants. In particular, the transcript abundances of *LDOX*, *UFGT*, and *GST* increased by 15.1-, 6.4-, and 5.1-fold, respectively (Fig. 4c). The expression levels of *PAL* and *DFR* increased by 6.7- and 9.2-fold, respectively, in transgenic plants compared with the wild type (Fig. 4c; Table S11). Moreover, the heat map of enriched key enzymes and transcription factors involved in the flavonoid biosynthesis pathway agreed with the results of the qRT-PCR analysis (Fig. S2).

### Accumulation of miR160h and miR858 is positively correlated with miR156 levels in poplar

**Fig. 5:**
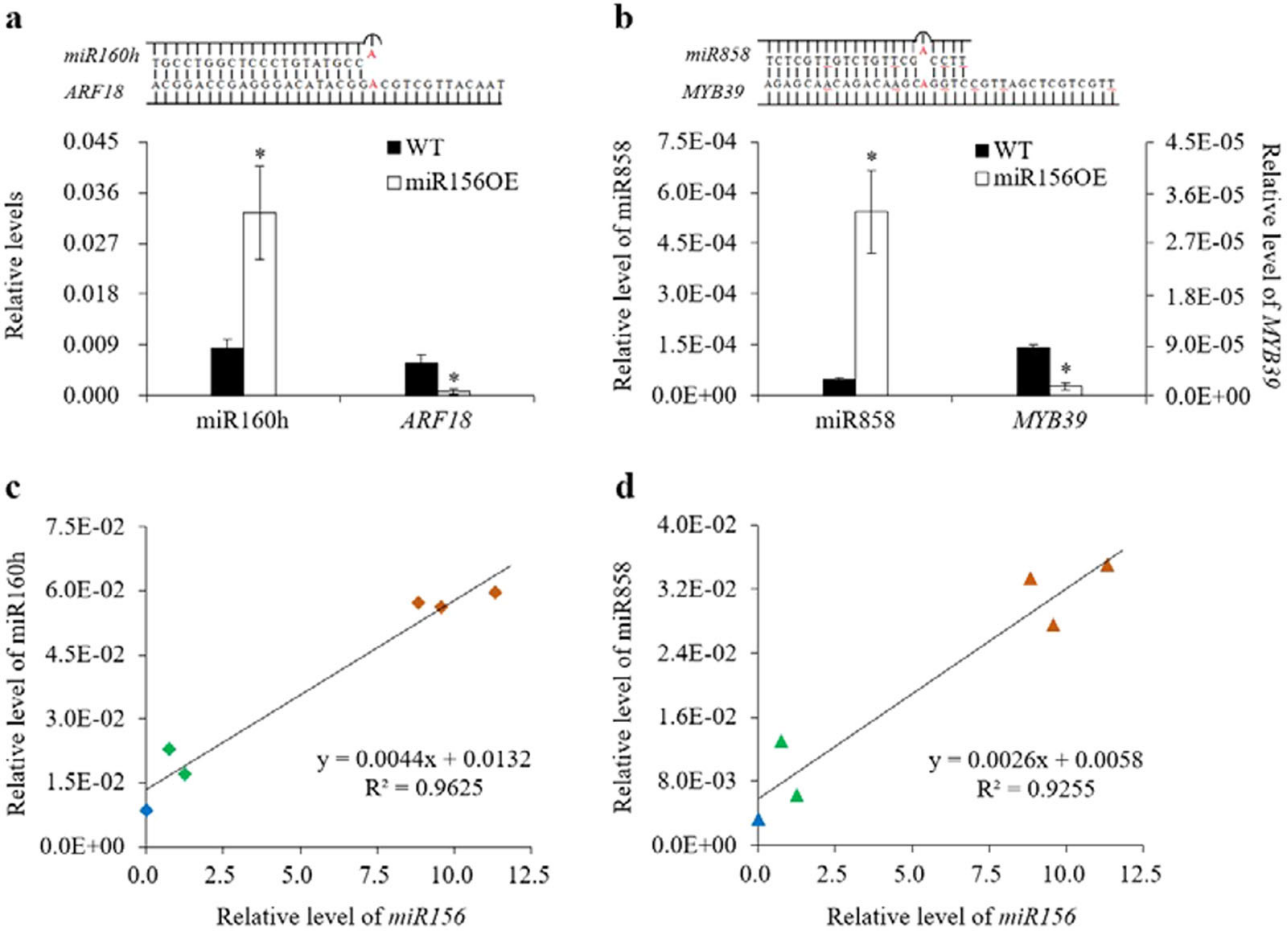
Overexpression of miR156 affected the expression levels of miR160h, miR858, and their target genes in wild-type and group II transgenic poplar plants. **a** Expression levels of mature miR160h and its target *ARF18* in wild-type and group II transgenic poplar plants. **b** Expression levels of mature miR858 and its target *MYB39* in wild-type and group II transgenic poplar plants. **c** Relationship between the expression levels of miR156 and miR160h. The blue diamonds indicate the wild type; the green diamonds indicate TGI-1 and -2; and the brown diamonds indicate TGII-1, -2, and -3. d Relationship between the expression levels of miR156 and miR858. The blue triangles indicate the wild type; the green triangles indicate TGI-1 and -2; and the brown triangles indicate TGII-1, -2, and -3. Stems were collected from wild-type, group I (TGI-1 and -2), and group II (TGII-1, -2, and -3) transgenic poplar plants. The levels of mature miR156, miR160h, and miR858 in these six samples were detected and quantified using a highly sensitive quantitative real-time PCR method. miRNA168 was used as a reference for normalization. The transcript abundances of *ARF18* and *MYB39* were quantified using a quantitative real-time PCR method. *QPto18S* was used as a reference for normalization. The values are the means ± SEs (*n* = 3). WT indicates wild type. miR156OE indicates the group II transgenic poplar plants, including TGII-1, -2, and -3. One and two asterisks indicate statistical significance at *p* < 0.05 or 0.01 (Student’s *t* test)

In addition to the miR156-*SPL* module, we identified two microRNA targets, miR160h-*ARF18* and miR858-*MYB39*, that had the potential to regulate anthocyanin biosynthesis in poplar. The levels of these two microRNAs were substantially elevated in transgenic poplar lines compared with the wild type (Fig. 5a, b), but the levels of their targets (*ARF18* and *MYB39*) were downregulated in transgenic poplar plants (Fig. 5a, b). Moreover, the expression level of *WRKY11*, a homologous gene of *AtWRKY57* and an activator of the auxin signaling pathway via interaction with IAA29, was altered in the transgenic poplar plants (Fig. S3a)^33^. The transcript abundances of the components of the MBW complex (such as *TT2*, *TT8*, and *TTG1*) were subsequently measured in wild-type and group II transgenic poplar plants. Among them, only the level of *WER*, a *TT2*-type *MYB* transcription factor, displayed significant changes in the transgenic plants (Fig. S3b). Furthermore, we examined the effects of miR156 levels on the accumulation of miR160h and miR858 in wild-type and transgenic poplar plants. Our results demonstrated that the accumulation of miR160h and miR858 displayed a strong positive correlation with miR156 levels (Fig. 5c, d).

### Overexpression of miR156 increased the accumulation of anthocyanins in transgenic poplar

**Fig. 6:**
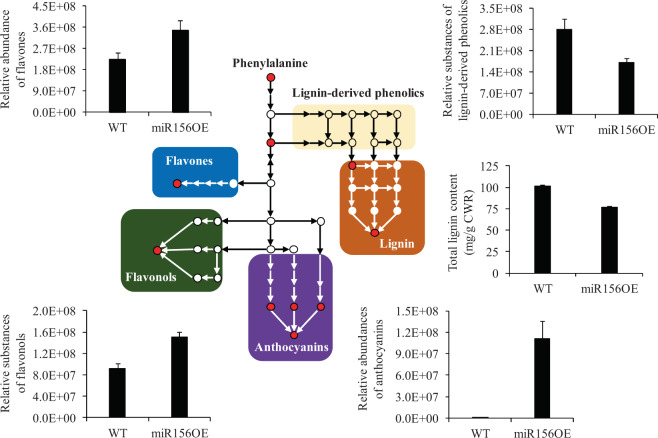
Relative abundances of metabolites derived from the phenylpropanoid pathway in wild-type and group II transgenic poplar plants. The abundance of anthocyanins, flavones, flavonols, and lignin-derived phenolics in wild-type and group II transgenic poplar plants was detected and quantified using metabolomic analysis. The total lignin contents of wild-type and group II transgenic poplar plants were determined using the AcBr method. WT indicates wild type. miR156OE indicates group II transgenic poplar plants, including TGII-1, -2, and -3

We next examined the levels of anthocyanins after a substantial amount of reddish pigment was observed on the epidermis of the stems, leaf margins, and petioles of group II transgenic poplar plants. Compared with those in the wild type, the total anthocyanin levels in the transgenic poplar plants increased by 8.3-fold (Fig. S4). Additionally, our previous study suggested that overexpression of miR156 in switchgrass can reduce lignin accumulation^17^. Furthermore, GTAC motifs required for the binding of miR156-targeted SPLs were identified within the promoter regions of lignin genes, including *CCR1*, *F5H*, and *COMT*. Given the conserved function of miR156 and the metabolic interaction between anthocyanins and lignin biosynthesis in plant species, we also measured the total lignin contents in both wild-type and transgenic poplar plants. Our results demonstrated that AcBr lignin levels were significantly reduced in transgenic poplar plants compared to the wild type (Fig. 6). Moreover, a metabolomic analysis was performed to examine the changes in the levels of various metabolites, including flavones, flavonols, and anthocyanins, in group II transgenic poplar plants. There were 228 differentially accumulated metabolites (DAMs) in the transgenic plants. Among them, 96 were upregulated, and 132 were downregulated (Fig. S5a; Table S12). KEGG analysis showed that the metabolites involved in the anthocyanin, isoflavonoid, flavonoid, flavone, flavonol, and phenylpropanoid biosynthetic pathways highly differentially accumulated (Fig. S5b). In particular, the levels of anthocyanins, including cyanidin 3-*O*-glucoside, cyanidin 3-*O*-rutinoside, and pelargonidin 3-*O*-glucoside, were substantially higher in the transgenic poplar plants than in the wild-type plants (Fig. 6; Table S12). Additionally, the levels of flavones and flavonols were significantly increased in the transgenic poplar plants compared to the wild type, whereas the levels of lignin-derived phenolics decreased in the transgenic plants (Fig. 6). In addition to anthocyanins and lignin, 130 DAMs were derived from the phenylpropanoid biosynthetic pathway in transgenic poplar plants (Table S12).

## Discussion

Mature miR156s have highly conserved sequences and functions across various plant species. The miR156b used in this study was isolated from *Medicago truncatula*, as the entire genomic sequence for the poplar 84K cultivar is currently unavailable. Previous studies have demonstrated that overexpression of miR156 can delay the juvenile-to-adult transition, increase branch/tiller numbers, reduce leaf size, and alter the accumulation of metabolites in plants^14–17^. Highly overexpressing miR156b in group II transgenic poplar lines produced plants with a spherical phenotype with a large number of branches and leaves, dramatically reduced leaf size, and reddish pigmentation, which is consistent with findings in other plant species. The morphological characterization of transgenic poplar plants can be used in ornamental horticulture. Furthermore, we used a joint microRNAomic and transcriptomic analysis to analyze the effects of overexpressing miR156 on its downstream genes. At least 85 microRNA-target modules were examined in transgenic poplar plants. Among them, the amount of miR396 was elevated in miR156-overexpressing transgenic poplar plants, while the transcript abundances of miR396-targeted *GRF*s decreased in the transgenic plants. Previous studies have indicated that overexpression of miR396 can reduce leaf width by reducing the transcript abundances of its targeted *GRF*s in *Arabidopsis*^34^. Additionally, reduced miR172 levels were observed in the transgenic poplar plants compared with the wild type. Moreover, the transcript abundances of miR172-targeted *AP2*s consistently increased in the transgenic plants. The function of the miR172-*AP2* module in both vegetative and reproductive development has been studied in various woody plant species^35^. Taken together, our results suggest that other microRNA-target modules directly or indirectly regulated by miR156-*SPL*s could also affect poplar morphology.

In addition to the typical morphological changes, overexpression of miR156 in poplar led to a high degree of anthocyanin accumulation in the epidermis of the stems, leaf margins, and petioles. Transcriptomic and metabolomic analysis revealed the structural genes that are involved in the flavonoid biosynthetic pathway and are responsible for promoting the production of anthocyanins in the miR156-overexpressing transgenic poplar plants. In particular, the transcript abundance of specific anthocyanin-related genes, including *LDOX*, *UFGT*, and *GST*, dramatically increased in the transgenic plants. Furthermore, data mining the transcriptomes revealed differentially expressed transcription factors such as *MYB39*, *ARF18*, *WRKY*11, and *WER* in the transgenic poplar plants. These transcription factors could contribute to the control of anthocyanin biosynthesis in poplar. *VvMYB114*, the *Vitis vinifera* homologous gene of *MYB39*, has been shown to regulate anthocyanin biosynthesis as a repressor^12^. The expression level of *MYB39* was reduced in the transgenic poplar plants in the present study. This is consistent with the increased expression levels of anthocyanin-specific genes observed in the transgenic poplar plants. Additionally, previous studies have demonstrated that auxin interacts with the components of the MBW transcriptional complex at the transcriptional or posttranscriptional level^36^. WRKY57 promotes auxin accumulation by interacting with the AUX/IAA protein IAA29, a repressor of the auxin signaling pathway^33^. Auxin strongly represses anthocyanin biosynthesis through ARF transcription factors in plants. In addition, both *MdARF13* and *AtWRKY57* function as repressors of anthocyanin biosynthesis^33,36^. Our transcriptomic analysis revealed that the transcript abundances of *WRKY11* and *ARF18* were significantly downregulated in the group II transgenic poplar plants, suggesting that miR156 overexpression could increase the accumulation of anthocyanins through the auxin signaling pathway. Aside from the auxin signaling pathway, miR156-targeted *AtSPL9* has the capability to impair the stability of the MBW complex via direct protein interaction in *Arabidopsis*^8^. The relationship between miR156-*SPL*s and the auxin signaling pathway is still not well understood and requires further study.

Previous studies have suggested that miR160h, miR164, miR166, miR159, miR319, miR390, miR396, and miR858 have the potential to participate in the biosynthesis of anthocyanins^13,36,37^. However, with the exceptions of those of miR160h and miR858, the predicted targets of miR164, miR166, miR159, miR319, miR390, and miR396 retrieved from the transcriptomic data of the wild-type and transgenic poplar plants were not involved in the biosynthesis of anthocyanins. Furthermore, the integrated microRNAomic and transcriptomic analysis further revealed two microRNAs, miR858 and miR160h, that have the potential to, respectively, target *MYB39* and *ARF18* in poplar. A significantly increased level of miR858 was detected in the transgenic poplar plants; seven miR858-targeted R2R3-MYBs were differentially expressed in the transgenic plants. Among them, the transcript abundance of *MYB39* exhibited a strong negative correlation with miR858 levels. Another negative correlation was found between miR160h and its target, *ARF18*. Moreover, the accumulation of miR160h and miR858 was positively correlated with miR156 levels in poplar. Together, these results suggest that the miR156-*SPL* module may directly or indirectly interact with miR160h and miR858 in poplar. Therefore, these two microRNAs could be used as novel targets for anthocyanin bioengineering in poplar.

Anthocyanins are the main bioactive compounds derived from the flavonoid biosynthetic pathway and can be used as effective antioxidants, as powerful free radical scavengers, and for natural pigmentation^2,3^. Our comprehensive analysis used microRNAomics, transcriptomics, and metabolomics and indicated that overexpression of miR156 in poplar affected anthocyanin biosynthesis through multiple factors, including microRNAs, transcription factors, and anthocyanin-specific structural genes. This basic information provides insight into the regulatory mechanism of anthocyanin biosynthesis in poplar. Given the crucial role that plant microRNAs play in a variety of processes, further investigation is needed to determine the relationship between the miR156-*SPL* module and other microRNAs and how they control anthocyanin biosynthesis in poplar.

## Supplementary information

Supporting Information

Supporting Information 2

Supporting Information 3

Supporting Information 4

Supporting Information 5

Supporting Information 6

Supporting Information 7

Supporting Information 8
