# Supplementary material for: MiR156 regulates anthocyanin biosynthesis through SPL targets and other microRNAs in poplar
Source: Hortic Res. 2020 Aug 1;7:118. doi: 10.1038/s41438-020-00341-w (PMC7395715; doi:10.1038/s41438-020-00341-w)
Supplement: Supplementary file 1 — Supporting Information [file 41438_2020_341_MOESM1_ESM.pdf]

Supplementary Information

MiR156 regulates anthocyanin biosynthesis through *SPL* targets and other microRNAs in poplar

Yamei Wang, Wenwen Liu, Xinwei Wang, Ruijuan Yang, Zhenying Wu, Han Wang, Lei Wang, Zhubing Hu, Siyi Guo, Hailing Zhang, Jinxing Lin and Chunxiang Fu

Fig. S1 Molecular characterization of microRNAs sequences identified from wild-type and group II transgenic poplar plants.

Fig. S2 Heat map of enriched key enzymes and transcription factors in the flavonoid biosynthesis pathway between wild-type and group II transgenic poplar plants.

Fig. S3 Quantitative real-time PCR analysis of *WRKY11*, *WER*, *TT8* and *TTG1* in wild-type and group II transgenic poplar plants.

Fig. S4 Analyses of anthocyanins content in stems of wild-type and group II transgenic poplar plants.

Fig. S5 Metabolome analysis of wild-type and group II transgenic poplar plants.

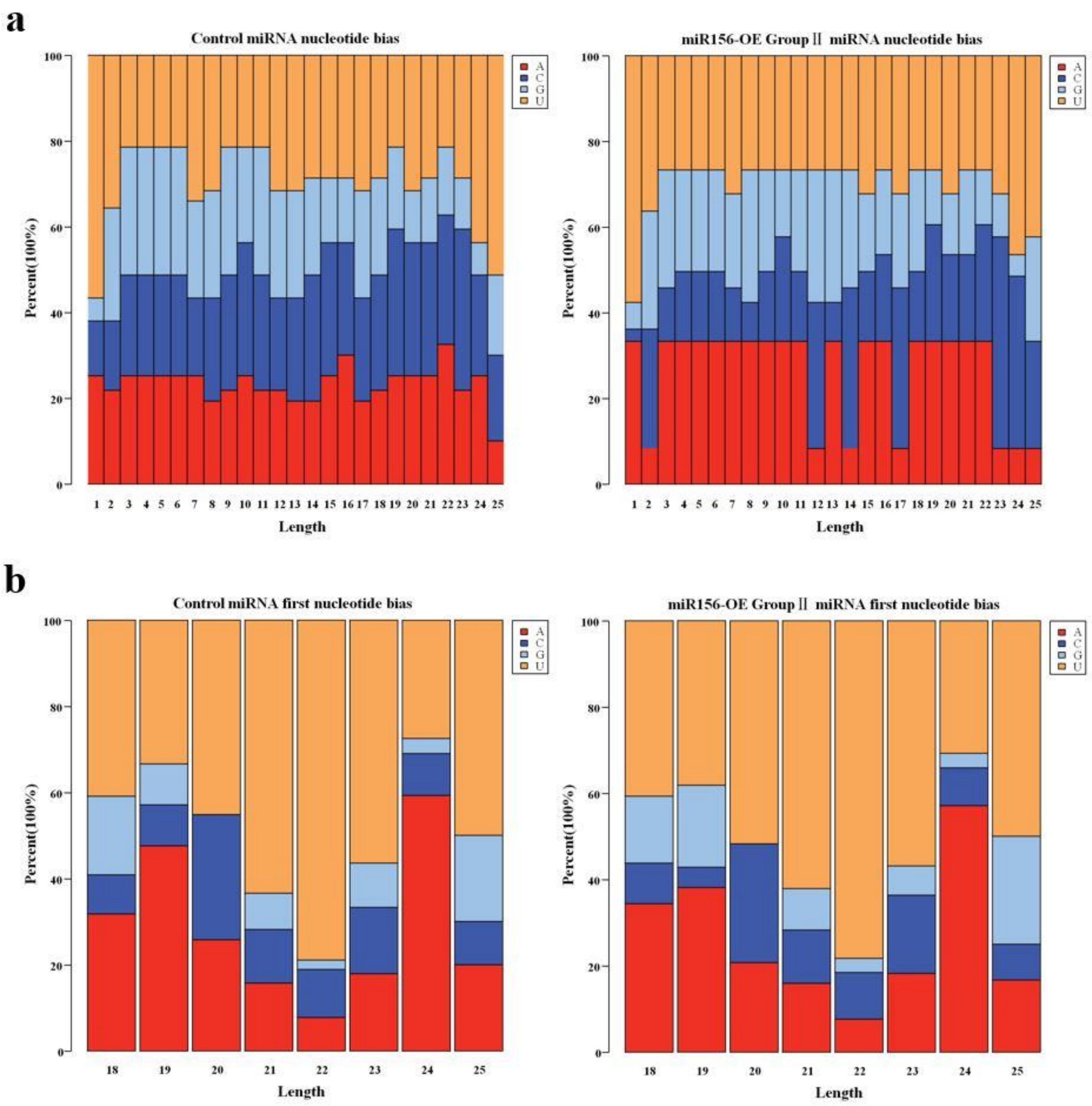

Fig. S1 Molecular characterization of microRNAs sequences identified from wild-type and group II transgenic poplar plants. **a** Nucleotide bias for each position of microRNAs in wild-type and group II transgenic poplar plants. **b** The nucleotide bias at the first position of microRNAs in wild-type and group II transgenic poplar plants.

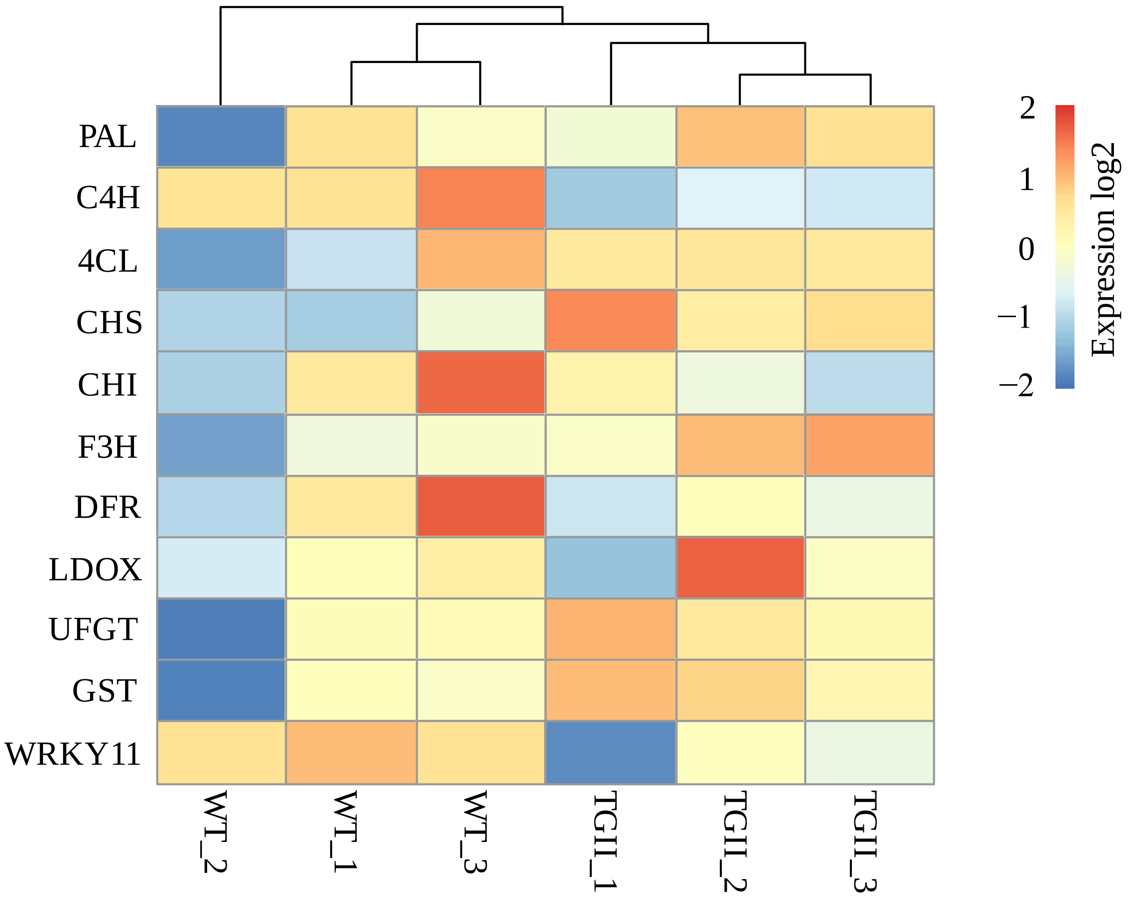

Fig. S2 Heat map of enriched key enzymes and transcription factors in the flavonoid biosynthesis pathway between wild-type and group II transgenic poplar plants. WT, wild-type. TGII-1, -2, and -3, miR156 overexpressing transgenic poplar plants (group II).

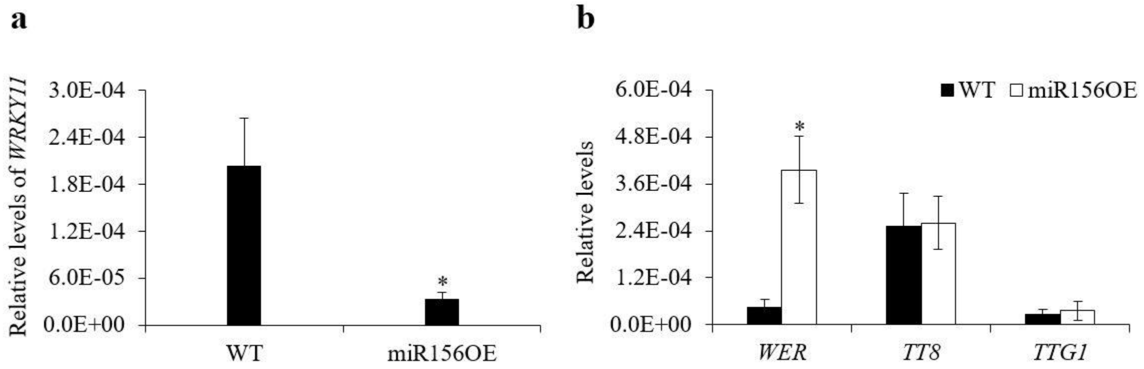

Fig. S3 Quantitative real-time PCR analysis of *WRKY11*, *WER*, *TT8* and *TTG1* in wild-type and group II transgenic poplar plants.*QPto18S* was used as the reference for normalization. Values are mean ± SE (n = 3). WT indicates wild type. miR156OE indicates the group II transgenic poplar plants including TGII-1, -2, and -3. One or two asterisks indicate statistical significance $P < 0.05$ or 0.01 (Student t-test).

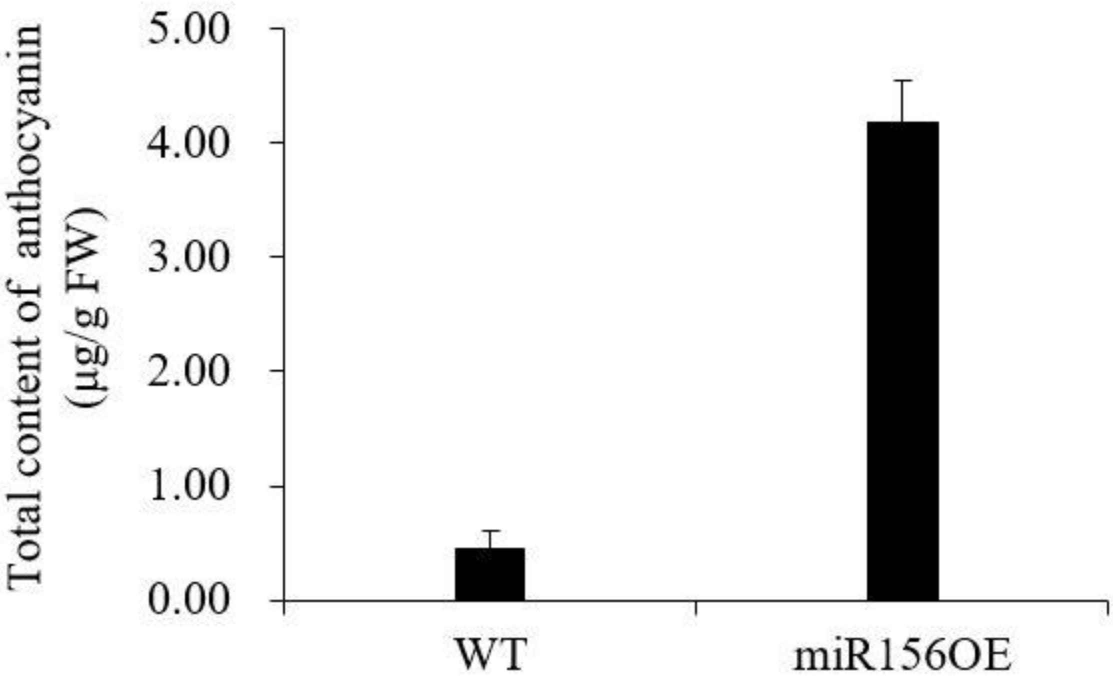

Fig. S4 Analyses of anthocyanins content in stems of wild-type and group II transgenic poplar plants. Values are mean ± SE (n = 3). WT indicates wild type. miR156OE indicates the group II transgenic poplar plants including TGII-1, -2, and -3. FW, fresh weight.

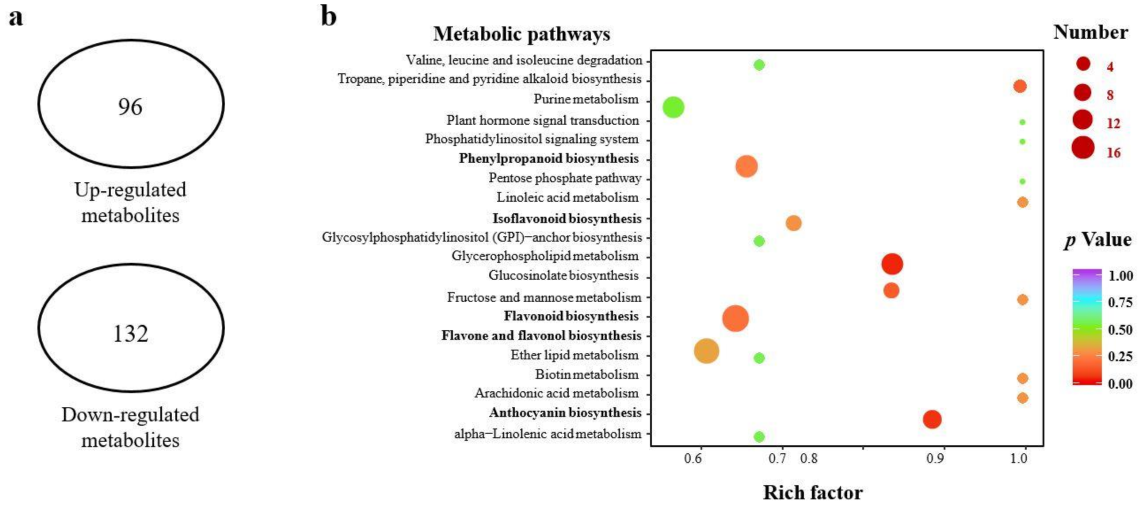

Fig. S5 Metabolome analysis of wild-type and group II transgenic poplar plants.

a The numbers of differentially accumulated metabolites (DAMs) between wild type and group II transgenic poplar plants. **b** KEGG pathway enrichment of DAMs in group II transgenic plants. The first 20 enriched pathways were selected and the rich Factor indicates that the number of DAMs/the total genes in this KEGG.
