# Supplementary material for: MiR156 regulates anthocyanin biosynthesis through SPL targets and other microRNAs in poplar
Source: Hortic Res. 2020 Aug 1;7:118. doi: 10.1038/s41438-020-00341-w (PMC7395715; doi:10.1038/s41438-020-00341-w)
Supplement: Supplementary file 2 — Supporting Information 2 [file 41438_2020_341_MOESM2_ESM.pdf]

Supplementary Information (Table S1-6)

MiR156 regulates anthocyanin biosynthesis through *SPL* targets and other microRNAs in poplar

Yamei Wang, Wenwen Liu, Xinwei Wang, Ruijuan Yang, Zhenying Wu, Han Wang, Lei Wang, Zhubing Hu, Siyi Guo, Hailing Zhang, Jinxing Lin and Chunxiang Fu

Table S1 Primers used in this study.

Table S2 Overview of microRNA reads from raw data to cleaned sequences, of wild-type and transgenic poplar plants.

Table S3 Summary of detected microRNAs between wild-type and transgenic poplar plants.

Table S4 Summary of conserved, known, and novel microRNAs in wild-type and transgenic poplar plants.

Table S5 Overview of differentially expressed microRNAs between wild-type and transgenic poplar plants.

Table S6 MicroRNAs involved in abiotic stress and secondary metabolic pathways were differentially expressed in transgenic poplar plants.

Table S1 Primers used in this study.

Gene name	Primer sequences (5'-3')
Tnos-R	TTACATGCTTAACGTAATTCAACAGAA
hph3	AAGGAATCGGTCAATACACTACATGG
hph4	AAGACCAATGCGGAGCATATACG
pre-MtmiR156b-F	CACCCTCCTCAGACAACAACAAG
pre-MtmiR156b-R	CTGGTGGTAGGATTTTTGTCA
qmiR168-RT	GTCGTATCCAGTGCAGGGTCCGAGGTATTCGCACTGGATACGACTTCCCG
qmiR156-RT	GTCGTATCCAGTGCAGGGTCCGAGGTATTCGCACTGGATACGACGTGCTC
qmiR160h-RT	GTCGTATCCAGTGCAGGGTCCGAGGTATTCGCACTGGATACGACTGGCAT
qmiR858-RT	GTCGTATCCAGTGCAGGGTCCGAGGTATTCGCACTGGATACGACAAGGTC
miR168-qRT-F	GCGTCGCTTGGTGCAGGT
miR156-qRT-F	CGCGCGTGACAGAAGAGAGT
miR160h-qRT-F	CGTGCCTGGCTCCCTGC
miR858-qRT-F	GCGCGTCTCGTTGTCTGTTC
miRNAUniversal-qRT-R	AGTGCAGGGTCCGAGGTATT
QPto18S-qRT-F	CGAAGACGATCAGATACCGTCCTA
QPto18S-qRT-R	TTTCTCATAAGGTGCTGGCGGAGT
SPL15-qRT-F	CACAAGTACAAGACTCGGATTGC
SPL15-qRT-R	GAAAAGGGGACTGAGTTATGTCG
SPL17-qRT-F	CAGTAGGACCTGTCTTAGCATC
SPL17-qRT-R	CACGAGAAGGAAAGTGTCTG
SPL24-qRT-F	GGGGTTGGTACTCAACTGAAA
SPL24-qRT-R	CCTTGGGATGGGATGCTC
MYB39-qRT-F	ACCATCTTCCCAACCCACA
MYB39-qRT-R	CCACTGCTTTGCCACTGAT
ARF18-qRT-F	TGCCTAAGTTTGCTCAGTT

ARF18-qRT-R	AATACTTTCCTCCTTCACTCC
WRKY11-qRT-F	TGTCATTCGGCAGTCAGTATTC
WRKY11-qRT-R	CTCTTATCGTCCTCTTCACCCT
PAL-qRT-F	GCTGTCTTATCGGAACTCTTGT
PAL-qRT-R	TTTGACCTGGATGGTGCTT
C4H-qRT-F	TTGCCAACAACCCTGCTAAGT
C4H-qRT-R	AATGGTGGAATGCTTCAAAATG
4CL-qRT-F	TTCCCAATTCAAAGACAACC
4CL-qRT-R	GCTCAACATCCGCATAAGT
CHS-qRT-F	CGTCCTCAGTGATTATGGC
CHS-qRT-R	GTTGTCTCCAGAACTTCGTAA
CHI-qRT-F	GACTGGATTAGTTGGCATCGC
CHI-qRT-R	AGGGAGTTGGTGGGTGAGGTA
F3H-qRT-F	ACCTGATGCTTTGAAGATAGTAGAC
F3H-qRT-R	CTGTATTCCTCGGTCACTGCTT
DFR-qRT-F	AGAAAGGTTACGCTGTCCG
DFR-qRT-R	CCATAAAGTCAAGTGGGTTG
LDOX-qRT-F	CTTGAGCAACGGGAAATAC
LDOX-qRT-R	CCAGTGGCTTGAGGATGA
GST-qRT-F	AGATTCTCTTTGTTTTATTTAGTGA
GST-qRT-R	CACCATCTTCAACAACAGGA
UFGT-qRT-F	TACAAACCAACCGATCCTG
UFGT-qRT-R	ACACCTGCCTCGCCATTA
TT8-qRT-F	GAGTGACGCATTGATAGAGTTG
TT8-qRT-R	TTCGTTCGTTGGGTTTCC
TTG1-qRT-F	ATCTCGCACAAACCAACAC
TTG1-qRT-R	GATAGTCACCAGAAGAGGCTAA
WER-qRT-F	GCTTGATAGCGAGAAGTAGCC
WER-qRT-R	AGGAACATGGTGGAAACAGTAA

Table S2 Overview of microRNA reads from raw data to cleaned sequences, of wild-type and transgenic poplar plants.

	WT_1				WT_2				WT_3				TGII-1				TGII-2				TGII-3			
**Lib**	Total	% of Total	uniq	% of uniq	Total	% of Total	uniq	% of uniq	Total	% of Total	uniq	% of uniq	Total	% of Total	uniq	% of uniq	Total	% of Total	uniq	% of uniq	Total	% of Total	uniq	% of uniq
Raw reads	16067107	100.00	3416076	100.00	24368561	100.00	4477541	100.00	19939351	100.00	3820289	100.00	20689765	100.00	2877954	100.00	19289817	100.00	2848400	100.00	20231088	100.00	2801175	100.00
3ADT& length filter	2243345	13.96	602477	17.64	3751794	15.40	968594	21.63	3477559	17.44	699043	18.30	5487464	26.52	992030	34.47	4706126	24.40	870224	30.55	5739479	28.37	976276	34.85
Junk reads	55394	0.34	31297	0.92	70181	0.29	35153	0.79	65097	0.33	33542	0.88	61225	0.30	22352	0.78	58524	0.30	23979	0.84	56883	0.28	21559	0.77
Rfam	425198	2.65	40920	1.20	804672	3.30	98507	2.20	682365	3.42	51229	1.34	1351211	6.53	83597	2.90	956653	4.96	68451	2.40	1144259	5.66	72639	2.59
Repeats	13541	0.08	1870	0.05	17869	0.07	2565	0.06	12898	0.06	1896	0.05	16900	0.08	1631	0.06	14028	0.07	1701	0.06	13228	0.07	1535	0.05
valid reads	13338728	83.02	2740188	80.21	19732977	80.98	3373539	75.34	15708342	78.78	3035201	79.45	13779109	66.60	1778847	61.81	13559403	70.29	1884564	66.16	13282049	65.65	1729638	61.75
rRNA	242880	1.51	21923	0.14	491732	2.02	54860	0.23	325845	1.63	27107	0.14	823603	3.98	50909	0.25	634479	3.29	38307	0.20	776526	3.84	44167	0.22
tRNA	120081	0.75	7034	0.04	206774	0.85	26664	0.11	264496	1.33	10015	0.05	390439	1.89	16535	0.08	200615	1.04	14171	0.07	242719	1.20	13285	0.07
snoRNA	40337	0.25	5802	0.04	65728	0.27	7433	0.03	61507	0.31	7040	0.04	67086	0.32	6934	0.03	73148	0.38	7250	0.04	61274	0.30	6658	0.03
snRNA	11882	0.07	3116	0.02	19044	0.08	3762	0.02	18199	0.09	3512	0.02	16351	0.08	3238	0.02	17076	0.09	3474	0.02	14784	0.07	3172	0.02
other Rfam RNA	10018	0.06	3045	0.02	21394	0.09	5788	0.02	12318	0.06	3555	0.02	53732	0.26	5981	0.03	31335	0.16	5249	0.03	48956	0.24	5357	0.03

Table S3 Summary of detected microRNAs between wild-type and transgenic poplar plants.

miR_name	miR_seq	Length (nt)	WT	miR156OE_group II
ptc-miR156a_R+1	TGACAGAAGAGAGTGAGCACT	21	+	+
ptc-miR156l_2ss13GA20AG	TTGACAGAAGATAGAGAGCGC	21	+	+
ath-miR157a-3p_2ss10CT13TC	GCTCTCTAGTCTCCTGTCATC	21	+	+
ptc-miR156g	TTGACAGAAGATAGAGAGCAC	21	+	+
ptc-miR156k_R+1	TGACAGAAGAGAGGGAGCACA	21	+	+
ptc-miR156k_L+1_1ss15GT	ATGACAGAAGAGAGTGAGCAC	21	+	-
ptc-miR156a	TGACAGAAGAGAGTGAGCAC	20	+	+
ptc-MIR156e-p3	TGCTCACTTCTCATTCTGTCAGC	23	+	-
ptc-miR156k	TGACAGAAGAGAGGGAGCAC	20	+	-
ptc-MIR156a-p5_1ss6AG	TGCTCGCATCTCTTCTGTCAGC	22	+	-
ptc-miR156a_L+5	GGAGGTGACAGAAGAGAGTGAGCAC	25	+	-
ptc-MIR156j-p3	TTGTGCTCTCTATGCTTCTGTC	22	+	+
ptc-miR159a_1ss21AT	TTTGGATTGAAGGGAGCTCTT	21	+	+
ptc-miR159d_L-1R-1	TTGGATTGAAGGGAGCTCC	19	+	+
ptc-MIR159b-p5_1ss21AT	AGCTCCTTGAAGTCCAATAGT	21	+	+
ptc-miR159a	TTTGGATTGAAGGGAGCTCTA	21	+	+
ptc-miR159d_R+2_2ss1CT20CA	TTTGGATTGAAGGGAGCTCATAC	23	+	+
ptc-miR160a_R+1_1ss1TG	GGCCTGGCTCCCTGTATGCCAC	22	+	+
ptc-miR160e-3p	GCATGAGGGGAGTCGAGCAGG	21	+	+
ptc-miR160e-5p	TGCCTGGCTCCCTGAATGCCA	21	+	+
ptc-MIR160e-p3	TGGCATGAGGGGAGTCGAGC	20	+	+
ptc-miR160g_L+2R-1	TCTGCCTGGCTCCCTGGATGCC	22	+	+
ptc-miR160a_R+1	TGCCTGGCTCCCTGTATGCCAC	22	+	+
ptc-miR160h_1ss15CG	TGCCTGGCTCCCTGGATGCCA	21	+	+
ptc-miR160a	TGCCTGGCTCCCTGTATGCCA	21	+	+
ptc-miR160b-3p	GCGTATGAGGAGCCATGCATA	21	+	+
ptc-miR160h	TGCCTGGCTCCCTGCATGCCA	21	+	+
ptc-miR160a_1ss21AT	TGCCTGGCTCCCTGTATGCCT	21	+	+
ptc-miR160g_1ss17TN	TGCCTGGCTCCCTGGANGCCA	21	+	+
ptc-miR160g_2ss15GT21AG	TGCCTGGCTCCCTGTATGCCG	21	+	+
eun-miR162-5p	GGAGGCAGCGGTTCATCGATC	21	+	+
ptc-miR162a	TCGATAAACCTCTGCATCCAG	21	+	+
ptc-miR162a_R+2_1ss2CT	TTGATAAACCTCTGCATCCAGTT	23	+	+
ptc-miR164f_1ss17AG	TGGAGAAGCAGGGCACGTGCT	21	+	+
ptc-miR164a_L+1	CTGGAGAAGCAGGGCACGTGCA	22	+	+
ptc-miR164a_L+1R+1_1ss22AC	CTGGAGAAGCAGGGCACGTGCCA	23	+	+
ptc-miR164a_R+1_1ss1TC	CGGAGAAGCAGGGCACGTGCAC	22	+	+
ptc-miR164a	TGGAGAAGCAGGGCACGTGCA	21	+	+
ptc-MIR164e-p3	CATGTGCCTGTCTTCCCCATC	21	+	+
ptc-miR164a_R+4	TGGAGAAGCAGGGCACGTGCATCCT	25	+	+
ptc-miR166n_R+2_2ss18CT19CT	TCGGACCAGGCTTCATTTTTTAC	23	+	+

ptc-miR166a_L+3_1ss4TC	ATCCCGGACCAGGCTTCATTCCCC	24	+	+
ptc-miR166a_L+1	CTCGGACCAGGCTTCATTCCCC	22	+	+
ptc-miR166n_L+3R-2	CTCTCGGACCAGGCTTCATTCC	22	+	+
ptc-miR166a_1ss21CA	TCGGACCAGGCTTCATTCCCA	21	+	+
ptc-MIR166f-p5	GGAATGTTGGCTGGCTCGAAG	21	+	+
ptc-miR166a	TCGGACCAGGCTTCATTCCCC	21	+	+
ptc-miR166p_1ss20TC	TCGGACCAGGCTCCATTCCCT	21	-	+
ptc-MIR166e-p5	GATTGTCGTCTGGTTCGATGT	21	+	+
ptc-MIR166j-p5	GGAATGTTGTCTGGCTCGAGG	21	+	+
ptc-MIR166q-p5	GGAATGTTGTCTGGCTCGAGG	21	+	+
ptc-miR166n_L+2R-2	TCTCGGACCAGGCTTCATTCC	21	+	+
ptc-MIR166c-p5	GGAATGTTGTCTGGCTCGAGG	21	+	+
ptc-miR167e	TGAAGCTGCCAGCATGATCTG	21	+	+
ptc-miR167f-3p_L-2_1ss13AT	ATCATGTGGCTGTTTCACC	19	+	+
ptc-miR167a_R+4_1	TGAAGCTGCCAGCATGATCTAAATT	25	+	+
ptc-miR167a_R+4_2	TGAAGCTGCCAGCATGATCTAACTT	25	+	+
ptc-miR167f-3p_L+3_1ss16AG	AACAGATCATGTGGCGGTTTCACC	24	+	+
ptc-miR167f-5p_R+1	TGAAGCTGCCAGCATGATCTTA	22	+	+
ptc-miR167f-3p	AGATCATGTGGCAGTTTCACC	21	+	+
ptc-miR167h-5p_R+2_1ss12AG	TGAAGCTGCCAGCATGATCTGAC	23	+	+
ptc-miR167f-3p_1ss13AT	AGATCATGTGGCTGTTTCACC	21	+	+
ptc-miR167e_R+2	TGAAGCTGCCAGCATGATCTGAA	23	+	+
ptc-miR168a-5p	TCGCTTGGTGCAGGTCGGGAA	21	+	+
ptc-miR168a-3p	CCCGCCTTGCATCAACTGAAT	21	+	+
ptc-miR169a	CAGCCAAGGATGACTTGCCGA	21	+	+
ptc-miR169b-3p_L+1	TGGCAGGTTGTTCTTGGCTAC	21	+	+
ptc-miR169a_R+2_1ss14CT	CAGCCAAGGATGATTTGCCGAGT	23	+	-
ptc-miR169a_1ss21AG	CAGCCAAGGATGACTTGCCGG	21	+	+
ptc-miR169n-5p_R+1_1ss2GC	TCAGCCAAGGATGACTTGCCGC	22	+	+
ptc-miR169n-3p_L+2R-1	TGGCAAGCATCCTTGGTTCTC	21	+	+
ptc-miR169d_L+1_1ss22GC	TCAGCCAAGGATGACTTGCCGC	22	+	+
ptc-miR169n-3p_L+2R-1	TGGCAAGCATCCTTGGTTCTC	21	-	+
ptc-miR169d_L+1_1ss22GC	TCAGCCAAGGATGACTTGCCGC	22	-	+
ptc-miR169o_R+1	AAGCCAAGGATGACTTGCCTGA	22	+	-
ptc-miR169v_R+3_2ss11TC21AC	TAGCCAAGGACGACTTGCCCCCTC	24	+	+
ptc-miR169ac_R+2_1ss21AC	TAGCCAAGGACGACTTGCCCCCC	23	+	+
ptc-MIR169ae-p5	TTGTTTGGTAGCCAAGGACGACT	23	+	+
ptc-miR169u-5p_R+3_1ss16TC	TAGCCAAGGACGACTCGCCTAATT	24	+	+
ptc-miR169r_R+2_2ss15TA21AT	TAGCCAAGGATGACATGCCTTTT	23	+	+
ptc-miR169aa_L+2R-2	CCGAGCCAAGAATGACTTGTC	21	+	+
ptc-MIR169s-p5_1ss5CA	CAAGATGTCCTTGGCTACAT	20	+	+
ptc-miR169ac_R+2_2ss10AG21AC	TAGCCAAGGGCGACTTGCCCCCA	23	+	+
ptc-miR169q_L+1R-1_1ss17TC	CTAGCCAAGGACGACTCGCCT	21	+	+
ptc-miR169i_1ss15TA	TAGCCAAGGATGACATGCCTG	21	+	+
ptc-MIR169m-p3	AGGCGGTCTCCTTGGCTAAC	20	+	+
ptc-miR169i_R+2	TAGCCAAGGATGACTTGCCTGTC	23	+	+

ptc-miR169x_R+2_2ss14CG19TA	TAGCCAAGGATGAGTTGCACGTT	23	+	+
ptc-MIR171i-p3_2ss17CT23GT_1	TCTTCTTTTTCTTCTTTTTCTTT	23	+	-
ptc-miR171a-3p_2ss9TC21GT	TTGAGCCGCGCCAATATCACT	21	+	+
ptc-miR171e_L+2	GCTGATTGAGCCGTGCCAATATC	23	-	+
ptc-MIR171i-p3_2ss17CT23GT_2	TCTTCTTTTTCTTCTTTTTCTTTT	24	+	-
ptc-MIR171i-p5_1ss16CT	CTTCTTTTTCTTCTTTTTCT	20	+	-
ptc-miR171g-5p_L+3	GGATGTTGGGATGGCTCAATCATG	24	-	+
ptc-miR171e_L+1	CTGATTGAGCCGTGCCAATATC	22	+	+
ptc-miR171l-5p_2ss1TC17CT	CGTGATATTGGTCCGGTTCATC	22	+	+
ptc-miR171j_R+1	CGAGCCGAATCAATATCACTC	21	+	+
ptc-miR171e	TGATTGAGCCGTGCCAATATC	21	+	+
ptc-miR171c_2ss1AT12CT	TGATTGAGCCGTGCCAATATC	21	+	+
ptc-miR171a-3p_R+1_2ss9TC21GT	TTGAGCCGCGCCAATATCACTT	22	+	+
ptc-miR171a-5p	GGATATTGGTACGGTTCAATC	21	+	+
ptc-MIR171i-p5_2ss4CG20TC	TGTGTTTCTTCTTCTTCTTCT	21	-	+
ptc-miR172a_R-1	AGAATCTTGATGATGCTGCA	20	+	+
ptc-miR172a	AGAATCTTGATGATGCTGCAT	21	-	+
ptc-miR172a_L-3	ATCTTGATGATGCTGCAT	18	+	+
ptc-miR172g-3p_1ss1GA	AGAATCTTGATGATGCTGCAG	21	-	+
ptc-miR319a_R+2_1ss20CT	TTGGACTGAAGGGAGCTCCTTT	22	+	+
ptc-MIR319a-p5	AGAGCTTTCTTCAGTCCACTC	21	+	+
ptc-miR319a	TTGGACTGAAGGGAGCTCCC	20	+	+
ptc-MIR319f-p5	AGAGCTTCCTTCAGCCCACTC	21	+	+
ptc-miR319e_R+1	TTGGACTGAAGGGAGCTCCTT	21	+	+
ptc-miR319e_R+2	TTGGACTGAAGGGAGCTCCTTT	22	+	+
ptc-miR390a	AAGCTCAGGAGGGATAGCGCC	21	+	+
ptc-MIR390a-p3	CGCTATCTATCCTGAGTTCT	20	+	+
ptc-miR390d-3p	CGCTATCCATCCTGAGTTTTA	21	+	+
ptc-miR393a-5p_R+1_1	TCCAAAGGGATCGCATTGATCC	22	+	+
ptc-miR393a-5p_R+1_2	TCCAAAGGGATCGCATTGATCT	22	+	+
ptc-MIR393c-p3_1ss12CT	ATCATGCGATCTCTTAGGAAT	21	+	+
ptc-miR393a-3p_1ss10TC	ATCATGCTACCCCTTTGGATT	21	+	+
ptc-miR394a-5p_L+1	CTTGGCATTCTGTCCACCTCC	21	+	+
ptc-miR394a-5p	TTGGCATTCTGTCCACCTCC	20	+	+
ptc-MIR394a-p3	AGCTCTGTTGGTCTCTCTTTG	21	+	+
ptc-miR394a-5p_R+1	TTGGCATTCTGTCCACCTCCT	21	+	+
ptc-miR395b	CTGAAGTGTTTGGGGGAACTC	21	+	+
ptc-miR395a	CTGAAGGGTTTGGAGGAACTC	21	+	+
ptc-MIR395a-p5_1ss21CT	TCACCCTGAGTTCCTCCTAGT	21	+	+
ptc-miR395a_L-1	TGAAGGGTTTGGAGGAACTC	20	+	+
ptc-miR396c	TTCCACAGCTTTCTTGAACTT	21	+	+
ptc-miR396e-3p_L-1	TCAAGAAAGCTGTGGGAGA	19	+	+
ptc-miR396a	TTCCACAGCTTTCTTGAACTG	21	+	+
ptc-MIR396b-p3	GTTCAATAAAGCTGTGGGAAG	21	+	+
ptc-miR396f_L+1R-3	TTTCCACGGCTTTCTTGAA	19	+	+
ptc-miR396g-3p_R-1_1ss19AG	CTCAAGAAAGCCGTGGGAGA	20	+	+

ptc-miR396c_L-3	CACAGCTTTCTTGAACTT	18	+	+
ptc-miR396g-3p_R-1_1ss13GA	CTCAAGAAAGCCATGGGAAA	20	+	+
ptc-MIR396f-p5_1ss18AT	TTTCCACGGCTTTCTTGT	18	+	-
ptc-miR397a	TCATTGAGTGCAGCGTTGATG	21	+	+
ptc-miR398c-5p_L-2R+5	AGCGACCTGAAATCACATGTGGGC	24	+	+
ptc-miR398b	TGTGTTCTCAGGTCGCCCCTG	21	+	+
ptc-miR398a_1ss8TC	TGTGTTCCCAGGTCACCCCTT	21	+	+
ptc-miR399i_R-1	TGCCAAAGGAGAGTTGCCCT	20	+	+
ptc-miR399e_1ss2GA	CACCAAAGGAGAGTTGCCCTC	21	+	+
ptc-miR399d	TGCCAAAGAAGATTTGCCCCG	21	+	-
ptc-miR399a_R-2_1ss13TG	TGCCAAAGGAGAGTTGCCC	19	+	+
ptc-miR399f	TGCCAAAGGAGAATTGCCCTG	21	+	+
ptc-miR399b	TGCCAAAGGAGATTTGCCCGG	21	+	+
ptc-miR399i_L+1R-1	TTGCCAAAGGAGAGTTGCCCT	21	+	-
ptc-miR403a_1ss20CT	TTAGATTCACGCACAAACTTG	21	+	+
ptc-MIR403b-p5	TTTGTTCGTGGATCTGACGCC	21	+	+
ptc-miR403a	TTAGATTCACGCACAAACTCG	21	+	+
ptc-MIR403a-p5	GGTTTGTGCATGAATCTAATA	21	+	+
ptc-miR403c-5p	TTTGTGCGTGGATCTGAGGCC	21	+	+
ptc-miR403a_2ss20CT21GT	TTAGATTCACGCACAAACTTT	21	+	+
hbr-miR408a_R-1_1ss6TG	AAGACGGGGAACAGGCAGAGC	21	+	+
ptc-miR408-3p	ATGCACTGCCTCTTCCCTGGC	21	+	+
ptc-MIR472b-p5_1ss1CT	TTGGATGGGTGAGTGGGGAAG	21	+	+
ptc-miR472a_L+2	TCTTTTCCCTACTCCACCCATCCC	24	+	+
ptc-MIR472b-p5	TGGGTGAGTGGGGAAGATAAC	21	+	+
ptc-miR472b	TTTTCCCAACTCCACCCATCCC	22	+	+
ptc-miR472a_L+2R+1_1ss24CA	TATTTTCCCTACTCCACCCATCCAT	25	+	+
ptc-MIR472a-p5_1ss2CT	TTGGGTGGGTGAGCGGGGAAGA	22	+	+
ptc-miR472a	TTTTCCCTACTCCACCCATCCC	22	+	+
ptc-MIR475d-p5_2	TTACAGAGTCCATTGATT	18	+	+
ptc-miR475d-3p	TTACAGAGTCCATTGATTAAG	21	+	+
ptc-MIR475d-p5_1	TCCTGATCAATGGCCATTGTA	21	+	+
ptc-MIR475d-p5_3	TTACAGAGTCCATTGATTAAG	21	+	+
ptc-miR475a-5p	AATGGCCATTGTAAGAGTAGA	21	+	+
ptc-miR475a-3p	TTACAGTGCCCATTGATTAAG	21	+	+
ptc-MIR475c-p5	TCTTGAACAATGGCCATTGTA	21	+	+
ptc-miR475c	TTACAATGTCCATTGATTAAG	21	+	+
ptc-MIR476a-p3_1ss3GA	TTACAAAGATAGATTTACTAGC	22	+	+
ptc-miR476a	TAGTAATCCTTCTTTGCAAAG	21	+	+
ptc-miR477d-5p_R-1	ATCTCCCTCAAAGGCTTCCTC	21	+	+
ptc-miR477e-5p_R+1	ACTCTCCCTCAAGGCTTCCAT	21	+	+
ptc-miR477e-3p_1ss22GA	TGAGGCCTTTGGGGGAGAGTGA	22	+	-
ptc-miR477a-5p	ATCTCCCTCAGAGGCTTCCAA	21	+	+
ptc-MIR481d-p5_2ss5AG19GA	AAAGGGCTTTCACTTGAGAGGGTG	24	+	+
ptc-MIR481d-p3_3	ATTCTTTGACATGGTATCAGAGCC	24	+	+
ptc-MIR481b-p5_1ss10TC	AAATCATATCCTGGAACCTCACCT	24	+	+

ptc-MIR481c-p3_2ss6AG19GA	ACATGGTATCAGAGCTTTAATGAC	24	+	+
ptc-MIR481b-p5_2ss13CT24AT	AACCTCACCTAATAGCTTAAGCTT	24	+	+
ptc-MIR481b-p3_2ss6AT19GA	AAGCTTTTGGGTTGAGATAGTTCT	24	+	+
ptc-MIR481d-p5_1ss6AG	ATCCTGGGACCTCACCTAACAGCT	24	+	+
ptc-MIR481d-p3_2	AAGTGGTCACGAGTTCGAATCTCA	24	+	+
ptc-MIR481b-p5_2	TGGAACCTCACCTAACAGCTT	21	+	+
ptc-MIR481d-p3_5	TTCTTTGACATGGTATCAGAGCCT	24	+	+
ptc-MIR481c-p5_2ss4CT20AG	AAGTTTTTGGGTTGAGATGGTTCT	24	+	+
ptc-MIR481b-p3_1ss21GT	TTGGGTTGAGATGGTTCTTTT	21	-	+
ptc-MIR481b-p3_2ss3TC20CA	ATCCTGGAACCTCACCTAAAAGCT	24	+	+
ptc-MIR481c-p5_1ss2CT	ATCAAGCGGTCACGAGTTCGAATC	24	+	+
ptc-MIR481d-p3_2ss14CT17AG	AAGGTAATGTGGGTCTGTGCAAGT	24	+	+
ptc-MIR481a-p3_1ss21AG	AATAATATAAATCATATCCTGGGA	24	+	+
ptc-MIR6470-p3_2ss10AG23TC	AAGTTTTTAGGTTGAGATGGTTCT	24	+	+
ptc-MIR6470-p3_2ss7GA22TC	TTTTTTAACATGATATCAGAGCTT	24	+	-
ptc-MIR481d-p3_2ss15CT17AG	AAGGTAATGTGGGCTTGTGCAAGT	24	+	+
ptc-MIR481b-p3_2ss13GA17CT	TTGGGTTGAGATAGTTTTTTGACA	24	+	+
ptc-MIR481b-p5_1	AAATCATATTCTGGAACCTCACCT	24	-	+
ptc-MIR481b-p3_2	TGGAACCTCACCTAACAGCTT	21	+	+
ptc-MIR481c-p5_2ss5CT21GA	TGAGTCTGTGCAAGTTTCAAACCC	24	+	-
ptc-MIR481a-p3_2ss13AG23TC	AAATCATATCCTGGGACCTCACCT	24	+	+
ptc-MIR481d-p5	ATTCTTTGACATGGTATCAGAGCC	24	+	+
ptc-MIR481d-p3_4	GCCCAAAGAGCTTTCACTTGAGG	23	+	+
ptc-MIR481c-p5_1ss12AG	AAGGTAATGTGGGCCTGTGCAAGT	24	+	+
ptc-MIR481d-p3_2ss11CT18GA	ACAGCTTAAGTTATTGGATTGAGA	24	-	+
ptc-MIR481c-p3_1	AGCTTAAGCTTTTGGGTTGAGATG	24	+	+
ptc-MIR481d-p5_2ss3CT23GA	AGTCCAAAGAGCTTTCACTTGAAG	24	+	+
ptc-MIR481c-p5	ACCAAGCGGTCACGAGTTCGAATC	24	+	+
ptc-MIR481c-p3_1ss4AG	AAGGGCTTTCACTTGAGGGGGTGT	24	+	+
ptc-miR481a_1ss11TC	AGGACCTCACCTAACAGCTTAAGC	24	+	+
ptc-MIR481b-p3_1ss9AT	CTTAAGCTTTTGGGTTGAGATGGT	24	+	+
ptc-MIR481d-p3_1ss1CA	ATTTGACATGGTATCAGAGCC	21	-	+
ptc-MIR481c-p5_1ss13CT	ACCAAGCGGTCATGAGTTCGAATC	24	+	+
ptc-MIR481d-p3_1ss13CT	TTTGATAAAAATTAAGCACAAGGT	24	+	+
ptc-MIR481c-p5_2ss4AG17GA	ATGGTATCAGAGCTTTAATGACCA	24	+	+
ptc-MIR481c-p3_2ss13CT19CT	ACCAAGCGGTCATGAGTTTGAATC	24	+	+
ptc-MIR481a-p5_2ss13AG23TC	AAATCATATCCTGGGACCTCACCT	24	+	+
ptc-MIR481b-p3_3	TTCTTTGACATGATATCAGAGCCT	24	+	+
ptc-MIR481a-p5_2ss16CT18AG	AATATAAATCATATCTTGGGACCT	24	+	+
ptc-MIR481b-p5_1ss3CT	AGTTTAAGCTATTGGGTTGAGAT	23	+	+
ptc-MIR481b-p3_1ss4CT	AAGTTATTGGGTTGAGATGGTTCT	24	+	+
ptc-MIR481d-p5_2ss3GA19CT	TTAACATGGTATCAGAGCTT	20	-	+
ptc-MIR481b-p3_2ss8CT20AG	ATGATATTAGAGCCTTGATGACCA	24	+	+
ptc-MIR481c-p3_2	CTTAAGCTTTTGGGTTGAGATGAT	24	+	+
ptc-miR481d_L-1R+1_1ss3GA	GAACCTCACCTAACAGCTTAAGCT	24	+	+
ptc-MIR481d-p5_2ss14CT17AG	AAGGTAATGTGGGTCTGTGCAAGT	24	+	+

ptc-MIR481b-p3_1	CTTAAGCTATTGGGTTGAGATGGT	24	+	+
ptc-MIR481d-p3_1	AAGCTATTGGGTTGAGATGATTCT	24	+	+
ptc-MIR481a-p5_2ss10CT23TC	AAATCATATTCTAGGACCTCACCT	24	-	+
ptc-MIR481b-p3_1ss3CT	TTTTTTGACATGATATCAGAGCCT	24	+	+
ptc-MIR481a-p5	TGTTAGAGAATAATATAAATCATA	24	-	+
ptc-MIR481b-p3_1ss5CT	ACAGTTTAAGCTATTGGGTTGAGA	24	+	+
ptc-MIR481b-p5_1ss11AT	TTGGGTTGAATTGATTCTTTGACA	24	+	-
ptc-MIR481b-p5_1ss3GA	AAACTATTGGGTTGAGATGGTTCT	24	+	+
ptc-MIR481c-p3_1ss12CT	ATCTCACCATCTCTATTTATTTGA	24	+	+
ptc-MIR481b-p5_1ss17CT	TTCTTTGACATGATATTAGAGCCT	24	+	+
ptc-MIR481b-p5_2ss3TC22GA	ATCCTGGAACCTCACCTAACAACT	24	+	+
ptc-MIR481b-p5_2ss3TC22GA	ATCCTGGAACCTCACCTAACAACT	24	-	+
ptc-miR482d-3p_R+1_1ss8CA	TTGCCGAACCCACCCATGCCAAA	23	+	+
ptc-MIR482d-p5_1ss9AT	GATGGAGGTCGAGGAAGTTTCT	22	+	+
ptc-MIR482d-p3_1ss14TC	ACATGGGTTGGTTCGCAAGAAAAT	24	+	+
ptc-miR482b-5p_L+2R+1	TGGGCATGAGGTGTTTGGCAAGAA	24	+	+
ptc-miR482b-3p	TTACCAATACCTCTCATGCCAA	22	+	+
ptc-miR482a.1_L+8	CCGTCTTGCCTACTCCTCCCATTCC	25	+	+
ptc-MIR482a-p5	AGATGGGAGAGTATGCAAGAAG	22	+	+
ptc-miR482a.1_L+5	TCTTGCCTACTCCTCCCATTCC	22	+	+
ptc-MIR482d-p3_1ss7AT	TGGAGGTCGAGGAAGTTTCT	20	+	-
ptc-miR482d-3p_1ss13CT	TTGCCGACCCCATCCATGCCAA	22	+	+
ptc-miR482c-5p_1ss9AC	TATGGGAGCGGCGGGAATGACT	22	+	+
ptc-miR482c-3p	TCTTTCCGAGTCCTCCCATACC	22	+	+
ptc-miR482d-3p	TTGCCGACCCCACCCATGCCAA	22	+	+
ptc-miR530a_R+1_1ss20TC_1	TGCATTTGCACCTGCACCTCA	21	+	+
ptc-miR530b_R+1	TGCATTTGCACCTGCATCTTA	21	+	+
ptc-miR530a_R+1	TGCATTTGCACCTGCACCTTA	21	+	+
ptc-MIR530a-p3	TGCAGGTGCAGGTGCAGGTGA	21	+	+
ptc-miR530b_R+3	TGCATTTGCACCTGCATCTTTG	23	+	-
ptc-miR530a_R+1_1ss20TC_2	TGCATTTGCACCTGCACCTCC	21	+	+
ptc-miR530b_L+2R-1	TCTGCATTTGCACCTGCATCT	21	+	+
ptc-miR536	CGCTCGCCAGCGTTGCACCACC	22	+	+
ptc-miR828a	TCTTGCTCAAATGAGTATTCCA	22	+	+
ptc-miR1444a_R+1	TCCACATTCGGTCAATGTTCC	21	+	+
ptc-MIR1444b-p3	CACATTCGGTCAACGTTCGAG	21	+	+
ptc-miR1446a_R-1_1ss9TC	TTCTGAACCCTCTCCCTCA	19	-	+
ptc-miR1446a_R+3_1ss9TC	TTCTGAACCCTCTCCCTCAACTT	23	+	+
ptc-miR1447_R+2	CAGAATTGCAGTGCCTTGATTTT	23	+	+
ptc-MIR1447-p5_1ss4GT	TCATGGCACTGCAATTCTAAA	21	+	+
ptc-miR1447_1ss21TC	CAGAATTGCAGTGCCTTGATC	21	+	+
ptc-MIR1448-p5_2ss19TG20AC	TATGGGAGGATTGGACAGGCC	21	+	+
ptc-miR1448_L+1R+1	TCTTTCCAACGCCTCCCATACC	22	+	+
ptc-miR1448_L-1R+5	TTTCCAACGCCTCCCATACCGGTT	24	+	+
ptc-MIR1449-p3_2ss19TC23AC	GATTAGTTGAGGTGCACGCAAGCT	24	+	+
ptc-MIR1449-p5_2ss7TC18TC	ATTAGTCGAGGTGCACGCA	19	-	+

ptc-MIR1449-p5_2ss19TC23AC	GATTAGTTGAGGTGCACGCAAGCT	24	+	+
ptc-miR1450_R-4	TTCAATGGCTCGGTCAGG	18	+	+
ptc-MIR1450-p5	CGAGCCATTGAAGACAATCATTTG	24	+	-
ptc-miR1450_2ss21AT22CT	TTCAATGGCTCGGTCAGGTTTT	22	+	+
ptc-miR1450_R-1	TTCAATGGCTCGGTCAGGTTA	21	+	+
ptc-miR1450_L-1	TCAATGGCTCGGTCAGGTTAC	21	+	+
ptc-miR2111a	TAATCTGCATCCTGAGGTTTG	21	+	+
ptc-MIR2111b-p3_2ss1GT18CT	TGTCAGGATTGGGTAATT	18	-	+
ptc-miR3627b_2ss7AG19GA	TGTCGCGGGAGAGATGGCACTA	22	+	+
ptc-MIR6421-p5	AAGTGGACTTCCCTTACAATC	21	+	+
ptc-MIR6421-p3	TTGTAAGGGAAGCCCACATGG	21	+	+
ptc-MIR6423-p5	TTCTCCGGCACCGCCGCTGTC	21	+	+
ptc-miR6425a-5p	TTGTCTTCCATGGAATAGGCAG	22	+	+
ptc-miR6425a-3p_1ss4AT	TCCTTGGAAGATAATGACTCG	21	+	+
ptc-miR6427-5p	TCGTAATGCTTCATTCTCACAA	22	+	+
ptc-miR6427-3p_1ss13AT	GTGGGAATGAACTTTATGAGA	21	+	+
ptc-MIR6430-p3	CTTTGCAGTCAAGTAATCATC	21	+	+
ptc-MIR6438b-p5_1ss11AG	TCGACTGAAAGTGAAAGCTAT	21	+	+
ptc-miR6438a	TTGTACACAGAATAGGTGAAAT	22	+	+
ptc-MIR6439b-p5_1ss20CT	AAAGAAGCAGAAGCCATCATTAGC	24	+	+
ptc-MIR6439a-p3	TCTGCTTCTTGATTTGGTGCC	21	+	+
ptc-miR6442	CTGAACGGCTTGAAGGGCACG	21	+	+
ptc-miR6443_L+3_1ss17TC	CGCGTATGATCATAGACGATGGAG	24	-	+
ptc-miR6445a_R-2	TTCATTCCTCTTCCTAAAAT	20	+	+
ptc-miR6445a	TTCATTCCTCTTCCTAAAATGG	22	+	+
ptc-miR6448_L+1R-1	ATAGGCACAGAATTAACAAGG	22	+	+
ptc-miR6456	TTGAGTCCTTCCATTAGATCC	21	+	+
ptc-miR6457b_R-1_1ss1TC	CTAGTTTGGCAGCCTCTTCT	20	+	+
ptc-MIR6457a-p3_1ss13CT	CTCAAACTTCTCTCTGACTTC	21	-	+
ptc-miR6457a_L+1R-1	ATAATCTCTCTGCAGAATGCT	21	+	+
ptc-MIR6457a-p5	TGCTGGGAGATTAGATTAGTT	21	+	+
ptc-MIR6457a-p5_2ss6TA17GT	TTTGCAGCACTCTGCTTGGAG	21	+	+
ptc-MIR6462f-p3_1ss16TC	TTATGCGTTTTTGTCCCTCGC	21	+	+
ptc-miR6457b_L+2R-2	TTTTAGTTTGGCAGCCTCTTC	21	+	+
ptc-MIR6462b-p3_1ss7AG	TTATGCGTTTTTGTCCCTCGC	21	+	+
ptc-miR6457b_R-2	TTAGTTTGGCAGCCTCTTC	19	+	+
ptc-MIR6457a-p3_1ss18AG	TTTCCTCTTCTAGTTTGGCAGCC	23	+	+
ptc-MIR6457b-p3	AGATTAGATTAGTTTCCTCTT	21	+	+
ptc-miR6459a-3p_1ss21GT	TCGAATTTGGGCTTGAGATTT	21	+	+
ptc-miR6460	TGATATGTGGCATTCAATCGA	21	+	+
ptc-MIR6460-p3	ATCGAGAATGCTGGAATAAAT	21	+	+
ptc-miR6460_R+1	TGATATGTGGCATTCAATCGAA	22	+	+
ptc-MIR6462a-p5_1ss17GA	TTTTTGTCCCTCGCTAATCTC	21	+	+
ptc-MIR6465-p3_2ss5TC24CT	TATCCTCTAACTAGTTAAACTACT	24	+	+
ptc-miR6468-5p_L-1	TTTTCCCTGAATCACTCCCA	20	+	+
ptc-miR6468-3p	GGAGTGATTCAGGGAACCCAT	21	+	+

ptc-miR6470_L+2R+1_1ss11TC	AGCTCTGATACCATATTAAAAAAT	24	+	+
ptc-miR6471	TTTGGGATCATCAGGACAGCC	21	+	+
ptc-miR6473_R-1_1ss6AT	TCCACTATCCCATCAAGACT	20	-	+
ptc-miR6476a_L+6R-1	TGCGGATCAGTGGAGATGAAACATG	25	+	+
ptc-miR6478_R+2_1ss21GA	CCGACCTTAGCTCAGTTGGTAGA	23	+	+
ptc-miR7821	AGATGGGCATCGGCATTGTGA	21	+	+
ptc-miR7823	TTGCATGCATGAACTTGAAAT	21	+	+
ptc-miR7827_L+1R-1	TGCTAGGACCAAGTTTTTTGG	21	+	+
ptc-miR7831	TACATGTAGAGACCACCAAAC	21	+	-
ptc-MIR7839-p5_1ss7CG	TGATTCGTTCCAATTCCATTTG	22	+	-
ptc-miR7839_R+1_1ss6CA	AGTGGAATTGGAGGTATCCCA	21	+	-
ptc-MIR7841-p5	TTTGACAGAAACCCCCTCATG	21	+	+
ptc-miR7841_L+4R-1_1ss11TA	ATGAGGGGGTAGCTGTCAAGCATA	24	+	+
gma-MIR156k-p5_2ss14GT17TA	AATTGACAGAAGATAGAGAGCAC	23	+	-
gma-miR167h_L+2R-4	AGATCATGCTGGCAGCTTCAAC	22	-	+
peu-MIR2916-p5_2ss5AG20TG	GGGGGCTCGAAGACGATCAGATACC	25	+	+
peu-MIR2916-p3_1ss15GC	TGTTGCTTCTAGGACTCCGCC	21	+	+
hbr-MIR6485-p5_2ss11AG20GT	TGAGTGATTCGGCAGATTTTGATT	24	+	+
hbr-MIR6485-p3	TATCCACTCATGTAGTACTT	20	+	-
stu-miR162a-3p_R+1	TCGATAAACCTCTGCATCCAGA	22	+	+
stu-miR166c-5p_L-1R+1_1ss11TC	GAATGTTGTCTGGCTCGAGGC	21	+	+
nta-miR168d_L+1	CTCGCTTGGTGCAGGTCGGGAA	22	+	+
sly-miR168b-3p_R+1	CCCGCCTTGCATCAACTGAATT	22	+	+
csi-MIR169m-p3_1ss12GT	CTTGGCTACATTTTTCTTTCT	21	+	+
stu-miR390-5p_L+1_1ss20AG	AAAGCTCAGGAGGGATAGCGCC	22	+	+
stu-miR396-3p_2ss3CT7GT	GTTCAATAAAGCTGTGGGAAA	21	+	-
lus-miR396d_L-1R-1_1ss20TA	CCCACAGCTTTATTGAACA	19	-	+
stu-miR398b-3p	TTGTGTTCTCAGGTCACCCCT	21	+	+
vvi-miR482	TCTTTCCTACTCCTCCCATTCC	22	+	+
mes-MIR482e-p3_1ss9TC	TTTCCGATCATTCCTCCCTC	20	+	+
mes-MIR482-p3_2ss10GA17TC	TTTCCGATCATTCCTCCCTCT	21	+	+
csi-miR2275a-3p_2ss5GA21TC	TTTAATTTCCTCCAATATCTCA	22	+	+
peu-MIR2913-p3_2ss10AC17CA	TCTCCTCGCCATCCCCAAGCT	21	+	+
cas-MIR11592-p5_1ss1GA	AAACCGAACCGAACCGAACCGAAA	24	+	+
stu-miR156f-5p_L-1R+1	TGACAGAAGAGAGTGAGCAT	20	+	+
ath-MIR156b-p5_1ss5CA	TTGAACTCTCTCTCTCTCTCTCTC	24	-	+
mtr-MIR156a-p3_2ss3GA21CT	TGAGAGCTCTTTCTTCTTCTTTC	23	+	-
cca-MIR156c-p5_2ss7CT17AC	AGAGCATTGAGAGGACCT	18	+	-
fve-MIR156e-p3	GCTCATTTCTCTTTCTGTCACT	22	+	-
stu-miR156f-5p_L-2R+1	GACAGAAGAGAGTGAGCAT	19	+	-
gma-miR156e	CTGACAGAAGATAGAGAGCAC	21	+	+
mtr-miR156a_L+1R-1_1ss12GT	CTGACAGAAGATAGAGAGCAC	21	+	+
fve-MIR159a-p5	AGCTGCTGAGCTATGAATT	19	+	+
mes-miR159c_R-1_1ss7GT	ATTGGATTGAAGGGAGCTCT	20	+	+
aly-MIR159c-p3_1ss5CT	TTATTTCTCTCTCTCTCTCTCTCT	24	+	-
aly-MIR159c-p3_1ss5TA	ATTAACTCTCTCTCTCTCTCTCTC	24	-	+

aly-MIR159c-p5_1ss3GA	TAAATTATCTCTCTCTCTCTCTCT	24	-	+
cme-MIR160c-p5_2ss13AG17AG	AGCCATGCATGTGTAAGT	18	+	+
cme-miR164a_2ss19GA20CA	TGGAGAAGCAGGGCACGTAAT	21	+	-
nta-MIR164a-p3	TGAGTTAGTTCTTCATGTGCC	21	-	+
mes-MIR164a-p5_2ss9GC19CA	CAACATGGCTTTCTCACCA	19	+	+
gma-miR166h-3p_L-2_1ss3TG	GCGGACCAGGCTTCATTCC	19	+	+
stu-miR166a-5p_1ss21GA	GGAATGTTGTCTGGCTCGAGA	21	+	+
cca-MIR167-p5_1ss24GA	TATTAGTGTGTGTGTGTGTGTGTA	24	+	+
ath-MIR167d-p3_2ss14TG20AT	TGTGTGTGTGTGTGTATAGTT	21	-	+
cca-MIR167-p5_2ss22GA24GA	TTAGTGTGTGTGTGTGTGTGTATA	24	-	+
cme-MIR167c-p5_2ss12AT21TC	TTTTCTCTTCTTCTTCTTCCC	21	+	+
cme-MIR167c-p5_2ss11AT18CT	TTTCTCTTCTTCTTCTTTCTC	21	+	+
ath-MIR167d-p3_2ss7TG17TG	TATATAGATAGATGTAGG	18	+	-
sly-miR167b-3p_1ss10AT	AGGTCATCTTGCAGCTTCAAT	21	+	+
cca-MIR167-p3_2ss3TA17TA	TGAGTGTGTGTGTGTGAGTGTGT	23	+	+
cca-MIR167-p3_1ss24GA	TATTAGTGTGTGTGTGTGTGTGTA	24	+	+
aau-miR168_L-3R-1_1ss23TA	CAGTTGATGCAAGGCGGGAA	20	-	+
aly-miR169j-3p_L+1R+1_2ss19TA20CG	AGGCAGTCTCCTTGGCTAAGC	21	+	+
mdm-MIR169b-p3_1ss6AG	TGACAGGCTCTTCTCTCTCATG	22	+	+
cme-MIR169b-p3_2ss6TC21TC	GCTAACTTGACAGGCTCTTTC	21	+	+
mdm-MIR169b-p5_1ss6AG	TGACAGGCTCTTCTCTCT	18	+	-
mdm-MIR169k-p3_2ss7TG22TG	TTCTCTGCTTCTTCTTCTTCTGCT	24	-	+
ppe-MIR169i-p5_2ss15GT18TA	TCTCTTCTTTCTTTTTTA	18	-	+
csi-miR169c-3p_L-2	GGCAGTCTCCTTGGCTAAG	19	-	+
mdm-MIR169k-p5_2ss1CT22TC	TTTCTCTTCTTCTTCTTCTTCCTC	24	-	+
ppe-MIR169i-p3_2ss15GT18TA	TCTCTTCTTTCTTTTTTA	18	-	+
gma-MIR169g-p3_2ss13TC20AC	TTCTTCTTCTTCCTCTTTCCC	21	+	+
aau-MIR172-p5_1ss5TC	AAAACCTCTCTCTCTCTCTCTCTC	24	+	+
aau-MIR172-p3_1ss1CA	AAAAATCTCTCTCTCTCTCTCTCT	24	-	+
nta-MIR172e-p5_2ss15CG19TA	ATTGGAACGATACAGAGAA	19	+	+
seu-miR319_R+1_1ss1CT	TGTGAATGATGCGGGAGATAGA	22	+	+
mes-MIR393b-p5_1ss21AG	CTTTGGATTCCTCCTTTGGTG	21	+	+
cpa-MIR393-p3_2ss23AG25AG	ATATATATATGTGTGTGTGTGTGTG	25	+	+
mtr-MIR396a-p5_1ss18AT	AAGAAAGCTGTGGGAGATT	19	+	-
stu-miR398a-3p_R+1_1ss2AG	TGTGTTCTCAGGTCGCCCCTGT	22	-	+
cme-MIR399c-p5_2ss16TA17GC	AAAACACACACACACACACACATA	24	-	+
cme-MIR399c-p5_2ss17TA18GC	TAAAACACACACACACACACACA	23	-	+
cme-MIR399c-p3_2ss16TA17GC	AAAACACACACACACACACACATA	24	-	+
nta-miR408_R+1	TGCACTGCCTCTTCCCTGGCTC	22	+	+
aly-MIR408-p3_1ss5TC	TTTCCCTCTCTCTCTCTCTCTCTC	24	+	-
gra-MIR482d-p5_3	TATATATACACACACACACACAC	23	+	+
gra-MIR482d-p3_1	ATATACACACACACACACACACA	23	+	+
gra-MIR482d-p5_1ss4CG	ATAGACACACACACACACACACA	23	-	+
gra-MIR482d-p3_2ss13CA21TC	CACACACACACAAACACACACACA	24	-	+
gra-MIR482d-p5_1ss4CA	ATAAACACACACACACACACACA	23	+	+
gra-MIR482d-p3_1ss1AC_1	CTACACACACACACACACACA	21	+	-

gra-MIR482d-p5_2ss23CT24AT	TATACACACACACACACACACATT	24	+	-
gra-MIR482d-p5_2ss13CA21TC	CACACACACACAAACACACACACA	24	-	+
gra-MIR482d-p3_2	TATACACACACACACACACAC	21	+	+
gra-MIR482d-p5_2ss19AT20CA	ACACACACACACACACACTAATAC	24	+	+
gra-MIR482d-p3_2ss19CT23CT	TATACACACACACACACATACATA	24	+	-
gra-MIR482d-p5_2ss13CG21TC	CACACACACACAGACACACACACA	24	-	+
gra-MIR482d-p3_1ss1AC_2	CTATACACACACACACACACACA	23	+	-
gra-MIR482d-p5_2ss20AT21CA	CACACACACACACACACACTAATA	24	+	+
gra-MIR482d-p5_2ss8CA22TC	ACACACAAACACACACACACACAC	24	+	+
gra-MIR482d-p5_2	TATACACACACACACACACACA	22	+	+
gra-MIR482d-p3_1ss5CG	TATAGACACACACACACACACACA	24	+	+
gra-MIR482d-p5_1ss5CG	TATAGACACACACACACACACACA	24	+	+
gra-MIR482d-p3_2ss21CT23AG	CACACACACACACACACATATAGA	24	-	+
gra-MIR482d-p5_2ss18AT19CA	CACACACACACACACACTAATACA	24	+	+
mdm-MIR482c-p5_2ss16AT18TC	TTTCCTAACCCTCCCTTCCC	20	+	-
gra-MIR482d-p5_1ss8CG	ATATATAGACACACACACACACAC	24	+	+
gra-MIR482d-p5_1ss6CT	ATACATACACACACACACACACAC	24	-	+
gra-MIR482d-p5_2ss19AG20CT	ACACACACACACACACATGT	20	-	+
gra-MIR482d-p5_1	ATATATACACACACACACACACA	23	+	+
gra-MIR482d-p3_3	TATATACACACACACACACACACA	24	+	+
eun-MIR530-p3_2ss3AT17CA	GATGAAGAACGTAGCGAA	18	+	+
cas-MIR5139-p3_2ss10AG18TC	GAAACTCTGGTGGAGGCC	18	+	+
aly-MIR829-p5_2ss3GA21GC	TTAAAGCTCTGATACCATGTC	21	+	+
aly-MIR829-p5_2ss18GC21GA	AAGCTCTGATACCATGTCAAAGA	23	+	+
aly-MIR829-p3_2ss18GC21GA	AAGCTCTGATACCATGTCAAAGA	23	+	+
lja-miR11138-3p_L+1_2ss17GA21AG	AAAAGCTTAAGCTGTTAGGTGAGG	24	-	+
ppe-MIR858-p5	TCTCGTTGTCTGTTCGACCTT	21	+	+
ath-miR858a_L-1R+1	TTCGTTGTCTGTTCGACCTTG	21	+	+
gma-miR1511_R-2	AACCAGGCTCTGATACCA	18	+	+
gma-MIR1536-p5_2ss7TC19CT	TGCTTACTGTGCCATATTTCC	21	-	+
cme-MIR1863-p5_2ss3TC21TA	AACCTAAAGCTCTGATACCAAGTT	24	-	+
cme-MIR1863-p5_2ss3AG21GA	AAGGCTCTGATACCATGTTAA	21	-	+
cme-MIR1863-p5_2ss3AG19TC	AAGGCTCTGATACCATGTC	19	-	+
cme-MIR1863-p5_2ss20TC22GA	TAAAGCTCTGATACCATGTCAAA	23	-	+
mtr-MIR2089-p3_1ss3AT	ATTGGTAAAACAACACTTGCA	21	+	-
mtr-MIR2592ay-p5_2ss6AG17CG	GAGTCGGGTTGTTTGGGAA	19	+	-
mtr-MIR2592bj-p5_2ss12TC19AT	ATTCCCACTGTCCCTGTCT	19	+	+
mtr-MIR2603-p3_2ss9AC17AC	GTCCCTGCCCTTTGTACA	18	+	+
mtr-MIR2603-p5_2ss9AC17AC	GTCCCTGCCCTTTGTACA	18	+	+
mtr-MIR2630x-p3_1ss8TC	TGTAGAACATTTGATTTTA	19	+	+
mtr-MIR2671c-p3_2ss11TC17AC	TTTGGTCCCTCAACTTCTTTTTT	23	+	+
peu-MIR2912b-p5_1ss9GA	TAGAACTCAAGATATGGGCTGAAC	24	+	+
peu-MIR2912b-p3_1ss6GA	AACTCAAGATATGGGCTGAACACT	24	+	+
peu-MIR2912b-p5_2ss10CT17AG	TTGTTGCTATAATTTGGACTTGAA	24	-	+
peu-miR2912a_L-2R+2	TAGAACTCGAGATATGGGCTC	21	+	+
peu-MIR2912a-p5_2ss4TA24AT	TTCAGACTTCTCCGTTGTTGCTAT	24	+	+

ghr-MIR2949a-p5_2ss1AT21CT	TTGGTTTCCAAAACCCGAGCT	21	+	+
vvi-miR3630-3p_L-1_2ss2TA22CT	ATGGGAATCTCTCTGATGCAT	21	+	+
vvi-miR3630-3p_L-1_2ss2TA22CA	ATGGGAATCTCTCTGATGCAA	21	+	+
gma-MIR4382-p5_2ss10AC18TA	TGGGGAAGTCGGAATAGA	18	+	-
gma-MIR4995-p5_1ss21GC	CAGTGGCTTGGTTAAGGGAAC	21	+	+
ath-MIR406-p3_2ss4AG18TG	AAAGCGAGTCTTCATAGG	18	+	-
gma-miR4995_L+1R-4	TAGGCAGTGGCTTGGTTA	18	+	-
gma-MIR5032-p5_2ss20AG24AC	TCCCTATGAAATGAGGCATGGAACG	25	+	-
gma-MIR5032-p3_2ss13AG17AC	GAAATGAGGCATGGAACG	18	+	-
cas-miR5139_L-1	AACCTGGCTCTGATACCA	18	+	+
stu-MIR5303j-p5_2ss10GT17CT	ATTCAAATTTCTGCCCTA	18	+	-
gma-MIR5368-p5_1ss3TC	TTCCCTGGGATTGGCTTTGGGC	22	+	+
gma-MIR5368-p3_2ss1AT18CA	TGAGATACCACTCTGGAAGAGC	22	+	+
gma-MIR5368-p5_1ss18CT	AGAATTCTAACCTTGTGTC	19	+	-
gma-MIR5371-p5_1ss1AT	TTTCTAGGAATTAGTCACT	19	+	-
mtr-MIR5241c-p3_1ss13TC	TTATGCTAATGGCCTTCCACT	21	+	+
ath-MIR5665-p5_1ss2AG	AGAGATGATGAAATCAGA	18	+	+
ath-MIR5665-p3_2ss2AG19GA	AGAGATGATGAAATCAGAAA	20	+	+
gra-MIR7486j-p3_2ss9TG17CG	ATGCGGTTGTCTGACAGG	18	+	+
ath-MIR5995b-p5_2ss4AG21TG	TGGGCATATGATCTGCATCTG	21	+	-
stu-MIR6024-p5_2ss11TC18CA	CAAGAGTTGTCTTCCCTATT	20	-	+
nta-miR6155	TAAGGTTGCCTTGCTCTTGCA	21	+	+
hbr-MIR6173-p5_2ss21AG22GC	TAGATACCCCAGTAGTCCTAGCC	23	+	+
hbr-MIR6173-p3_2ss21AG22TC	TGCGTATCGACCCGTGCAGTGC	22	+	+
hbr-MIR6173-p5_2ss19CG20AG	CGTAAACGATGGATACTGGGCG	22	+	+
hbr-MIR6173-p3_1ss4CT	CAGTGCTGTAGCTAACGCGTT	21	+	+
hbr-MIR6483-p5_1ss6TC	AGCAGCATCGTGGATAGGGAACT	23	+	-
hbr-MIR6483-p3_1ss4TC	ACCCAATTTTATTGTAGAAA	20	+	+
mtr-MIR2592ay-p3_2ss6AG17CG	GAGTCGGGTTGTTTGGGAA	19	+	-
ghr-MIR7491-p3_2ss3CT17AT	TTTCTCGGTGAATCTATA	18	+	+
ghr-MIR7505-p3_2ss3AT18CT	TCTGAAACCATCCCTTCTTTG	21	+	-
lja-MIR7528-p3_2ss15AG17CT	AAATGCTAATCTGAGGTTT	19	+	+
mtr-MIR7700-p5_2ss15TG18GA	TCCGTTGATGACTTGGTA	18	+	-
stu-MIR8005a-p3_2ss19GA20TA	AGGGTTTAGGGTTTAGGGAA	20	-	+
stu-MIR8005c-p3_2ss15AT21GA	TTTAGGGTTTAGGGTTTAGAA	21	-	+
stu-MIR8005c-p3	AGGGTTTAGGGTTTAGGGT	19	-	+
stu-MIR8005c-p5_1ss12AG	TAGGGTTTAGGGTTTAGGGTT	21	+	+
stu-MIR8005a-p3_2ss5TC19GC	AGGGCTTAGGGTTTAGGGCTTAGG	24	-	+
cas-MIR8171-p5_2ss4CG17AG	CTAGTTCCGGGTTCGAGTCC	20	+	+
gra-MIR8654b-p5_2ss10TA17AT	TTGTCACCTAGACTCATA	18	+	-
gra-MIR8658-p5_2ss15CA17AT	AAATTCAAATTTAAAATTT	19	+	-
gra-MIR8674c-p3_2ss14AT17CT	TTTTTTAACCTGATCCTT	18	+	+
gra-MIR8674b-p3_2ss11TC18AC	AAACCCTAAACCCTAAACC	19	-	+
gra-MIR8674b-p5_2ss11TC18AC	AAACCCTAAACCCTAAACC	19	-	+
gra-MIR8751b-p5_1ss1AC	CCATGGCACCGAAAATGT	18	+	-
bol-MIR9410-p5_2ss5TG18TA	CTTTGCAGACGACTTAAATA	20	+	+

sly-MIR9479-p3_2ss1GA18GC	ATGTCACGGGTTCAAATC	18	+	-
sly-MIR9479-p5_2ss1GA18GC	ATGTCACGGGTTCAAATC	18	+	-
gma-MIR10423-p3_1ss3AC	CTCTCAATGAAAGCACCA	18	+	+
gma-MIR10442-p3_2ss9GC17AC	TACATGCTCCACTTGGCT	18	+	+
mdm-MIR11004-p5_1ss1TC	CGGTGCTATCCTACCTGAGCT	21	+	+
mdm-MIR11004-p3	CTATCCTACCTGAGCTTTTTC	21	+	+
mdm-MIR11004-p3_1ss1TC	CGGTGCTATCCTACCTGAGCT	21	+	+
mdm-MIR11010-p5_1ss2AG	AGAAGAGCTGGGAGTTGTTCT	21	-	+
mdm-MIR11010-p3_1ss21TC	AGCTGGGAGTTGTTCTGCAAC	21	+	+
mdm-MIR11010-p5_2ss21TC24GC	AGCTGGGAGTTGTTCTGCAACGAC	24	+	+
cst-MIR11334-p5_2ss8TG17TC	AAGGAGTGTGTAACAACT	18	+	+
cst-MIR11334-p5_2ss9TG18TC	TAAGGAGTGTGTAACAACT	19	+	-
cas-miR11592_L+4R+1_1ss11AG	AACCGAACCGGACCGAACCGAAAC	24	+	+
cas-miR11592_L+4R+1_1ss23AC_2	AACCGAACCGAACCGAACCGAACT	24	-	+
pla-MIR11602-p5	ATCCGAGAGTTATCAGTATTTATCA	25	+	+
pla-MIR11602-p3_1ss6CG	CGGAAGGCTATTGGATCAAATGA	23	+	+
ghr-miR156a_R+2	TGACAGAAGAGAGTGAGCACTT	22	+	+
stu-miR156f-3p_L+2R-1_4ss11TC15T-22AG23TC	TGCTCACTTCCCTTCTGTCAGC	22	+	+
mtr-miR156e_R+2	TTGACAGAAGATAGAGAGCACCT	23	+	+
cas-miR159b-5p_R-1	AGCTGCTAAGCTATGGATCC	20	+	+
cas-miR159b-3p_R+1	TTTGGATTGAAGGGAGCTCTTT	22	+	+
fve-miR159c_1ss1AT	TTTGGATTGAAGGGAGCTCCC	21	+	+
ath-miR159c_R+1_1ss20CT	TTTGGATTGAAGGGAGCTCTTT	22	+	+
cpa-miR159a_R+1_1ss21AT	TTTGGATTGAAGGGAGCTCTTG	22	+	+
csi-miR160c-5p_1ss19CT	TGCCTGGCTCCCTGTATGTTT	21	+	+
csi-miR160c-3p_2ss16TA21GA	GCGTGCGAGGAGCCAAGCATA	21	+	-
cas-miR162b_L-1R+2	TCGATGAACCGCTGCCTCCAA	21	-	+
cme-miR164a_2ss19GT20CT	TGGAGAAGCAGGGCACGTTTT	21	+	+
vun-miR164_1ss6AG	TGGAGGAGGGGAGCACGTGCA	21	-	+
aly-miR167a-5p_R+1	TGAAGCTGCCAGCATGATCTAT	22	+	+
aly-miR167b-3p	GGTCATGCTCTGACAGCCTCACT	23	+	+
mes-miR167a_L-2R+3	AAGCTGCCAGCATGATCTGAGC	22	+	+
nta-miR168a_R+1_1ss21CT	TCGCTTGGTGCAGGTCGGGATT	22	+	+
sly-miR168b-3p_R+2	CCCGCCTTGCATCAACTGAATTT	23	+	+
bra-miR168b-5p_R+1_1ss21CT	TCGCTTGGTGCAGGTCGGGATT	22	+	+
aly-miR169d-5p	TGAGCCAAGGATGACTTGCCG	21	+	+
aly-miR171c-5p	AGATATTGGTGCGGTTCAATC	21	+	+
aly-miR171b-3p_R-1	TTGAGCCGTGCCAATATCAC	20	+	+
csi-miR171f-5p	TATTGGCCTGGTTCACTCAGA	21	+	+
csi-miR171c-3p_R+1	TGATTGAGCCGTGCCAATATCT	22	+	+
stu-miR171a-5p	TATTGGCCTGGTTCACTCAGA	21	+	+
sly-miR171e	TTGAGCCGCGTCAATATCTCT	21	+	+
nta-miR172d_R+1	AGAATCTTGATGATGCTGCATT	22	+	+
lus-miR319b_L+1_1ss7CT	TTTGGATTGAAGGGAGCTCCC	21	+	+
stu-miR391-5p_R+1	TACGCAGGAGAGATGATGCTGG	22	+	-
stu-miR393-5p_R+1_2ss21CT22CA	TCCAAAGGGATCGCATTGATTAT	23	+	-

csi-miR393a_L+1R+1	AATCCAAAGGGATCGCATTGATCT	24	+	+
bna-miR394a_R+2_1ss20CT	TTGGCATTCTGTCCACCTCTTT	22	+	+
aly-miR395c-3p_R+2_1ss17GA	CTGAAGTGTTTGGGGGAACTTTT	23	+	+
csi-miR396a-5p_L+4_1ss25GT	GATCTTCCACAGCTTTCTTGAACTT	25	+	+
aly-miR397a-5p_R+1	TCATTGAGTGCAGCGTTGATGT	22	-	+
gma-miR398a_L+1R-1_1ss9TC	TTGTGTTCCCAGGTCACCCCT	21	+	+
ppe-miR399b_L-2R+2	TGCCAAAGGAGAATTGCCCTG	21	+	+
aly-miR399b-3p	TGCCAAAGGAGAGTTGCCCTG	21	+	+
mes-miR399f	TGCCAAAGGAGAGTTGCCCTG	21	+	+
stu-miR408b-3p_R+3	TGCACTGCCTCTTCCCTGGCTTTC	24	-	+
mdm-miR408a_L-1R+4	TGCACTGCCTCTTCCCTGGCTTTC	24	-	+
stu-miR408a-3p	TGCACAGCCTCTTCCCTGGTT	21	+	+
sly-miR482c	TCTTGCCAATACCGCCCATTCC	22	+	+
stu-miR482e-3p	TCTTGCCAATACCGCCCATTCC	22	+	+
stu-miR482b-3p	TTACCGATTCCCCCCATTCCAA	22	+	+
gma-miR482b-3p	TCTTCCCTACACCTCCCATACC	22	+	+
stu-miR482c	TTTCCTATTCCACCCATGCCAA	22	+	+
fve-miR482c_1ss10TC	TCTTTCCTACTCCTCCCATCCC	22	+	+
csi-miR482b-3p_R+1_1ss11CT	TCTTGCCCACTCCTCCCATTCCT	23	+	+
nta-miR482d	TTCCCGACTCCCCCCATACCAC	22	+	+
aqc-miR530_L+2R-1	TCTGCATTTGCACCTGCATCT	21	+	+
csi-miR536-3p_1ss14TC	TGGTGCCACGCTGCGTGCGTC	21	+	+
nta-miR827	TTAGATGAACATCAACAAACA	21	-	+
ppe-miR858_L+2R-1	ATCTCGTTGTCTGTTCGACCTT	22	+	+
vun-miR1507a_R+1	TCTCATTCCATACATCGTCTGA	22	+	+
gma-miR1508a_L-2	TAGAAAGGGAAATAGCAGTTG	21	-	+
gma-miR1509a	TTAATCAAGGAAATCACGGTCG	22	-	+
gma-miR1510b-5p	AGGGATAGGTAAAACAACTACT	22	+	+
gma-miR1510b-3p_R+1	TGTTGTTTTACCTATTCCACCT	22	+	+
lja-miR1511-3p_R-3	AACCAGGCTCTGATACCATGA	21	+	+
csi-miR1515a	TCATTTTTGCGTGCAATGATCC	22	+	+
stu-miR1919-5p	TGTCGCAGATGACTTTCGCCC	21	+	+
stu-miR1919-3p	ACGAGAGTCATCTGTGACAGG	21	+	+
gma-miR2109-3p	GGAGGCGTAGATACTCACACC	21	+	-
gma-miR2111a_2ss10AT21GC	GTCCTTGGGTTGCAGATTACC	21	+	+
gma-miR2118a-3p_R+1	TTGCCGATTCCACCCATTCCTA	22	+	+
csi-miR2275a-3p	TTTAGTTTCCTCCAATATCTTA	22	+	+
gso-miR3522a	TGAGACCAAATGAGCAGCTGA	21	+	+
gma-miR4348d	TTTCGGTGTCGGTGAATTGCC	21	+	-
gma-miR4412-5p	TGTTGCGGGTATCTTTGCCTC	21	+	+
gma-miR4416c-3p	ACGGGTCGCTCTCACCTGGAG	21	+	-
gma-miR4996_R+1	TAGAAGCTCCCCATGTTCTCA	21	+	+
fve-miR5225_L-1R+1_1ss7TC	TGTCGCAGGAGAGATGGCGCCA	22	-	+
sly-miR5300	TCCCCAGTCCAGGCATTCCAAC	22	+	+
gma-miR5767	TGGAGGACCTTTGAAGGTGCA	21	+	-
gma-miR5761a	TTTTGTGTCGTGAAGCTTTTG	21	-	+

gma-miR5770a_L-1R+1_1ss19CT	TAGGACTATGGTTTGGATGAG	21	+	-
nta-miR6021	TTGGAAGAGGCTGCTATTGGA	21	+	-
sly-miR6022	TGGAAGGGAGAATATCCAGGA	21	+	+
nta-miR6024	TTTTAGCCAGAGTTGTTTTCCC	22	+	-
nta-miR6025a	TACCAACAATTGAGATAACATC	22	+	-
nta-miR6025b	TGCCAACTATTGAGATGACATC	22	-	+
sly-miR6026	TTCTTGGCTAGAGTTGTATTGC	22	+	-
nta-miR6149a	TTGATACGCACCTGAATCGGC	21	+	+
stu-miR6149-5p	TTGCAACACACCTGAATCGTC	21	+	+
nta-miR6155_R+3	TAAGGTTGCCTTGCTCTTGCATTC	24	-	+
nta-miR6161c_1ss13TA	AATATACTGGAGATCGGTGCACCT	24	-	+
gma-miR6300_R+5	GTCGTTGTAGTATAGTGGTGAGT	23	+	+
stu-miR7122-5p_1ss18TG	TTATACAGAGAAACCGCGGTCG	22	-	+
rgl-miR7972_1ss14TG	TTGTCAGGCTTGTGATTCTCC	21	+	+
rgl-miR7972_1ss14TG	TTGTCAGGCTTGTGATTCTCC	21	+	+
sly-miR7981a_L+2R-2	CTACCCCTTTTCGGCCTACGTGGC	24	+	-
stu-miR8036-3p	TATGTCTTTCCGATGCCTCCCA	22	+	+
ath-miR8175_L-2	TCCCCGGCAACGGCGCCA	18	+	+
gma-miR9749	TTAGCTTCTTTCACCTTTCCC	21	+	+
sly-miR10539	CTTGGAACCACAGTTACCACC	21	+	+
cas-miR11592_L+4R+1_1ss23AC_1	AACCGAACCGAACCGAACCGAACC	24	-	+
PC-5p-878_3103	CCGAGCTTGGGGATTGCGAGGAGA	24	+	+
PC-3p-2758_1045	TCGCCATCCCCAAGCTCCTGCG	22	+	+
PC-5p-76888_70	TTTGATGGGAAAGAATTATTT	21	+	+
PC-3p-525_5996	TTAGTTCTTTCCCATCAAACC	21	+	+
PC-5p-178251_28	AGGATTGGAGGGAATTAAACA	21	+	+
PC-5p-797998_6	TTATTCCCACCACTTGATTCT	21	+	-
PC-5p-1557952_4	ACTTGGTTCCTGGAAAGAATGAAA	24	-	+
PC-3p-1299195_4	TCATTCTTCCCAGGAACCGA	20	-	+
PC-3p-451534_11	TTTAATTTTCTCCAATATCTCA	22	-	+

Table S4 Summary of conserved, known, and novel microRNAs in wild-type and transgenic poplar plants.

miR_ name	miR_seq	Genome ID	Strand	pre-miRNA_seq	Type	Sequence in miRbase	Group	Hairpin Len (nt)	CG%	WT-1 (norm)	WT-2 (norm)	WT-3 (norm)	TGII-1 (norm)	TGII-2 (norm)	TGII-3 (norm)	Expression level
ptc-miR156a_R+1	TGACAGAAGAGAGTGAGCACT	TRINITY_DN26966_c0_g1	+	aaatTGACAGAAGAGAGTGAGCACAcagaggcatatttgtataaaattataccattgcttttgcgtgctcatttctctttctgtcactt	5'	Diff	gp1a	82	36.00	17.47	11.67	25.07	3329.59	2199.55	2466.82	middle
ptc-miR156l_2ss13GA20AG	TTGACAGAAGATAGAGAGCGC	TRINITY_DN30322_c0_g2	+	actgttgatgttgTTGACAGAAGATAGAGAGCACagatgatgatatgcaatggactctgcatcccactcctttgtgcTCTCTATGCTTCTGTCATCACTttcagcccc	5'	Diff	gp1a	102	45.40	2.41	4.12	2.24	13.70	13.49	18.86	middle
ath-miR157a-3p_2ss10CT13TC	GCTCTCTAGTCTCCTGTCATC	TRINITY_DN30322_c0_g1	+	aggaggcactggtgatgctgTTGACAGAAGATAGAGAGCACagatgatgaaatgcatggagcttgattgcatctcactcctttgtGCTCTCTAGTCTCCTGTCATCaccttcagcccctcact	3'	Diff	gp1a	119	48.80	1.20	1.37	10.75	999.50	734.47	762.18	middle
ptc-miR156g	TTGACAGAAGATAGAGAGCAC	TRINITY_DN30322_c0_g1	+	aggaggcactggtgatgctgTTGACAGAAGATAGAGAGCACagatgatgaaatgcatggagcttgattgcatctcactcctttgtGCTCTCTAGTCTCCTGTCATCaccttcagcccctcact	5'	Yes	gp1a	119	48.80	1247.98	1890.90	1435.97	6909.76	6602.97	5971.71	middle
ptc-miR156k_R+1	TGACAGAAGAGGGAGCACA	TRINITY_DN28110_c0_g2	-	aTGACAGAAGAGAGAGAGCACAatccgagtcttgtatttgtgttaacctgtcacagaacatactatggctacttgccccgctacttcctggc	5'	Diff	gp1a	79	46.70	0.60	0.34	0.90	5.14	7.71	2.22	low
ptc-miR156k_L+1_1ss15GT	ATGACAGAAGAGAGTGAGCAC	TRINITY_DN30506_c0_g3	-	ATGACAGAAGAGAGAGAGCACggttggagtctatgactcggtttagcccatcagagaacattttctggctgttgccactgtttccta	5'	Diff	gp1a	75	47.10	1.20	0	0	9.13	16.37	6.10	middle
ptc-miR156a	TGACAGAAGAGAGTGAGCAC	TRINITY_DN26966_c0_g2	+	cattagaaacTGACAGAAGAGAGTGAGCACacagaggcatatttgtatgaatctataccgttgcttttgcgTGCTCACTTCTCATTCTGTCAGCttccaga	5'	Yes	gp1a	92	42.60	876.96	754.99	875.82	97527.85	89692.52	83173.79	high
ptc-MIR156e-p3	TGCTCACTTCTCATTCTGTCAGC	TRINITY_DN26966_c0_g2	+	cattagaaacTGACAGAAGAGAGTGAGCACacagaggcatatttgtatgaatctataccgttgcttttgcgTGCTCACTTCTCATTCTGTCAGCttccaga	3'	New	gp1a	92	42.60	0	0	1.79	27.40	20.23	15.53	middle
ptc-miR156k	TGACAGAAGAGGGAGCAC	TRINITY_DN26455_c0_g1	-	cttgcagcacagactgagtggtgtgatttgtatgcatgggttgttcaaatgaaatagattgcggctcacgtgaaccccatgaattgttTGACAGAAGAGAGAGAGCACgatgagattctggtgctgcttc	3'	Yes	gp1a	124	46.20	0.60	0.34	0	57.65	33.23	19.97	middle
ptc-miR156k	TGACAGAAGAGGGAGCAC	TRINITY_DN22474_c1_g1	-	gcaagacttgatgctcggtttgtggagccccatggttggttTGACAGAAGAGAGAGAGCACaactcgagtctgaa	3'	Yes	gp1a	69	50.70	0.60	0.34	0	57.65	33.23	19.97	middle

ptc-MIR156a-p5_1ss6AG	TGCTCGCATCTCTTCTGTCAGC	TRINITY_DN32306_c1_g2	+	ggtgtgTGCTTGCATCTCTTCTGTCAGCttccacttccaag	5'	New	gp1a	38	51.20	0	0.69	4.48	2.28	1.93	1.11	low
ptc-miR156a_L+5	GGAGGTGACAGAAGAGAGTGAGCAC	CM000342.2	-	gtaagggaggTGACAGAAGAGAGTGAGCACacagggtactttcttgcatgacatccatgcttgaaactttgcgtgctcactctctatctgtcaccccaccagttttgaattatgtgaggaggcactggtg	5'	Diff	gp1a	92	49.50	0	0	0	2.38	1.99	0.14	low
ptc-miR156l_2ss13GA20AG	TTGACAGAAGATAGAGAGCGC	TRINITY_DN30322_c0_g1	+	atgctgTTGACAGAAGATAGAGAGCACagatgatgaaatgcatggagcttgattgcatctcactcctttgtgcTCTCTAGTCTCCTGTCATCACCttcagcccctcactccgtttaattg	5'	Diff	gp1a	143	45.60	2.41	4.12	2.24	13.70	13.49	18.86	middle
ptc-miR156k_L+1_1ss15GT	ATGACAGAAGAGAGTGAGCAC	TRINITY_DN19805_c0_g1	-	tcagctttggATGACAGAAGAGAGAGAGCACatcctgtctgcgacgctatgtatggaaatctcccagccatcaatctttcttgagaggaattccttgaaacaggatccTGAGAGCTCTTTCTTCTTCTTTCattgtgccctgccaatctcc	5'	Diff	gp1a	141	46.40	1.20	0	0	9.13	16.37	6.10	middle
ptc-miR156g	TTGACAGAAGATAGAGAGCAC	TRINITY_DN30322_c0_g2	+	tgatgttgTTGACAGAAGATAGAGAGCACagatgatgatatgcaatggactctgcatcccactcctTTGTGCTCTCTATGCTTCTGTCatcactttcagccc	5'	Yes	gp1a	98	45.10	1247.98	1890.90	1435.97	6909.76	6602.97	5971.71	middle
ptc-MIR156j-p3	TTGTGCTCTCTATGCTTCTGTC	TRINITY_DN30322_c0_g2	+	tgatgttgTTGACAGAAGATAGAGAGCACagatgatgatatgcaatggactctgcatcccactcctTTGTGCTCTCTATGCTTCTGTCatcactttcagccc	3'	New	gp1a	98	45.10	39.75	64.52	38.51	613.63	569.75	478.17	middle
ptc-miR156a_R+1	TGACAGAAGAGAGTGAGCACT	TRINITY_DN26966_c0_g2	+	ttagaaacTGACAGAAGAGAGTGAGCACAcagaggcatatttgtatgaatctataccgttgcttttgcgtgctcacttctcATTCTGTCAGCTTCCAGAGCCGGA	5'	Diff	gp1a	92	44.80	17.47	11.67	25.07	3329.59	2199.55	2466.82	middle
ptc-miR156k_L+4_1ss18GT	ATCCTGACAGAAGAGAGTGAGCAC	CM000347.2	-	ttatgcatgcTGACAGAAGAGAGGGAGCACaaccctgtaatagctaaagagagtctttgcttttgttggactgtgctttctcttcttctgtcaccaaccaa	5'	Diff	gp1a	89	43.60	0	0	0.15	0.76	1.93	0	low
ptc-miR159a_1ss21AT	TTTGGATTGAAGGGAGCTCTT	TRINITY_DN28390_c0_g1	+	AGCTGCTGAGCTATGAATCccacagccctatcaccatcagttatttgatgggcctgcggcttgcatatctcaggagctttattacctgatgttagatcttttTTTGGATTGAAGGGAGCTCTAaacctttgatctttt	3'	Diff	gp1a	79	42.40	80173.82	67314.00	58841.99	42737.22	39596.15	39277.80	high
ptc-miR159d_L-1R-1	TTGGATTGAAGGGAGCTCC	TRINITY_DN29359_c0_g2	-	ataTTGGAGTGAAGGGAGCTCCaactt	5'	Diff	gp1a	21	44.40	383.67	299.25	253.88	130.72	132.93	140.34	middle
ptc-miR159c_1ss7GT	ATTGGATTGAAGGGAGCTCGA	CM000350.2	+	gaggctcaatggagctctccccactccatgcctgaaaggagttcgatggtagaccatggctgctagttcatgaatacccttgggtgcgcagaattagcaacggtgcaggcgaagttgcgcaggctaagggtctgcatgacctaggagacgtggttaccctgaccct	3'	Diff	gp1a	200	53.20	0.60	0	0	0	0	0	low

				ttttgtATTGGAGTGAAGGGAGCTCGAtggtcttt												
ptc-MIR159b-p5_1ss21AT	AGCTCCTTGAAGTCCAATAGT	TRINITY_DN28390_c0_g1	+	gattatggagtggAGCTCCTTGAAGTCCAATAGAagctcctgctgggtagatcgagctgctgagctatgaatcccacagccctatcaccatcagttatttgatgggcctgcggcttgcatatctcaggagctttattacctgatgttagatcttttTTTGGATTGAAGGGAGCTCTAaacctttga	5'	New	gp1a	176	45.50	1387.72	1995.23	1403.28	1989.88	2153.79	1912.66	middle
ptc-miR159a	TTTGGATTGAAGGGAGCTCTA	TRINITY_DN28390_c0_g1	+	gattatggagtggAGCTCCTTGAAGTCCAATAGAagctcctgctgggtagatcgagctgctgagctatgaatcccacagccctatcaccatcagttatttgatgggcctgcggcttgcatatctcaggagctttattacctgatgttagatcttttTTTGGATTGAAGGGAGCTCTAaacctttga	3'	Yes	gp1a	176	45.50	322398.61	275157.93	237889.44	162757.06	160848.88	172233.86	high
ptc-miR159d_R+2_2ss1CT20CA	TTTGGATTGAAGGGAGCTCATAC	CM000350.2	+	ttgtaaatttgggagctttctttggttcaaaatgaggaaagaaagtgggtatactcgtctgcttgttcatggatacctctgggttgcgcaggatatattagttagggctacagcttaagggtttgcatggccaaggagacgttgcctgccttctcctttcgtttCTTGGATTGAAGGGAGCTCCTacatgtatc	3'	Diff	gp1a	191	44.80	14.15	7.89	10.40	3.56	12.28	1.39	middle
ptc-miR160a_R+1_1ss1TG	GGCCTGGCTCCCTGTATGCCAC	CM000347.2	-	aaatgggttaTGCCTGGCTCCCTGTATGCCAcagcaaagaccaatctcttctgtcttagattggctgctgccggtggcgtgcgaggagccaagcatactctct	5'	Diff	gp1a	96	53.40	0.49	5.35	0.54	1.50	4.30	0.98	low
ptc-miR160e-3p	GCATGAGGGGAGTCGAGCAGG	CM000342.2	+	aagaatggccTGCCTGGCTCCCTGAATGCCAtctaggaagcttgtcaaagagtgttggcaacctttctatttgGCATGAGGGGAGTCGAGCAGGccaatt	3'	Yes	gp1a	92	51.00	12.05	14.41	3.58	9.13	9.63	3.33	middle
ptc-miR160e-5p	TGCCTGGCTCCCTGAATGCCA	TRINITY_DN33369_c0_g1	+	aagaatggtcTGCCTGGCTCCCTGAATGCCAtctaggaagcttgtccaagagtgttggcaacctctctaacTGGCATGAGGGGAGTCACGCaggccca	5'	Yes	gp1a	90	55.10	4472.14	2089.94	3093.13	1549.20	1389.47	1497.73	middle
ptc-MIR160e-p3	TGGCATGAGGGGAGTCGAGC	TRINITY_DN33369_c0_g1	+	aagaatggtcTGCCTGGCTCCCTGAATGCCAtctaggaagcttgtccaagagtgttggcaacctctctaacTGGCATGAGGGGAGTCACGCaggccca	3'	New	gp1a	90	55.10	6.02	4.80	9.85	14.84	5.78	5.55	middle
ptc-miR160g_L+2R-1	TCTGCCTGGCTCCCTGGATGCC	TRINITY_DN33369_c0_g1	+	aatggTCTGCCTGGCTCCCTGAATGCCatctaggaagcttgtccaagagtgttggcaacctctctaactggcatgaggggagtcacgcaggcccag	5'	Diff	gp1a	90	56.20	13.25	8.24	9.85	4.57	20.23	21.63	middle
ptc-miR160a_R+1_1ss1TG	GGCCTGGCTCCCTGTATGCCA	CM000337.2	+	acatgggttaTGCCTGGCTCCCTGTATGCCAcaagcaaagaccaatcttttgttttagattggctgttgccgg	5'	Diff	gp1a	95	51.00	0.49	5.35	0.54	1.50	4.30	0.98	low

	C			tggcgtgcgaggagccaagcatactctct												
ptc-miR160a_R+1	TGCCTGGCTCCCTGTATGCCAC	TRINITY_DN21483_c0_g1	+	accatcatgcatccatatatattatatgTGCCTGGCTCCCTGTATGCCATttgcagagcccaacggatcttcgatggcctccgtggatggcgTATGAGGAGCCATGCATATTCgcatgtatattatacatggttta	5'	Diff	gp1a	130	45.60	55.41	41.87	44.78	12.56	27.93	17.20	middle
ptc-miR160h_1ss15CG	TGCCTGGCTCCCTGGATGCCA	TRINITY_DN31410_c0_g1	-	atagtgagcaTGCCTGGCTCCCTGCATGCCAgca	3'	Diff	gp1a	30	58.80	103.60	170.90	117.76	109.60	128.59	112.05	middle
ptc-miR160a	TGCCTGGCTCCCTGTATGCCA	TRINITY_DN21483_c0_g1	+	atattatatgTGCCTGGCTCCCTGTATGCCAtttgcagagcccaacggatcttcgatggcctccgtggatgGCGTATGAGGAGCCATGCATAttcgca	5'	Yes	gp1a	86	52.00	71517.45	45830.49	41687.39	10078.38	15033.68	13702.60	high
ptc-miR160b-3p	GCGTATGAGGAGCCATGCATA	TRINITY_DN21483_c0_g1	+	atattatatgTGCCTGGCTCCCTGTATGCCAtttgcagagcccaacggatcttcgatggcctccgtggatgGCGTATGAGGAGCCATGCATAttcgca	3'	Yes	gp1a	86	52.00	387.89	262.19	254.33	138.14	157.97	173.07	middle
ptc-miR160h_1ss15CG	TGCCTGGCTCCCTGGATGCCA	TRINITY_DN31659_c0_g4	-	cagtccgaagcagacctgtttgcagtttattgaggtggggatctggtagtggtagaccataatgagcaTGCCTGGCTCCCTGCATGCCAgcaggagtgttgtgaggaaga	3'	Diff	gp1a	104	52.70	103.60	170.90	117.76	109.60	128.59	112.05	middle
ptc-miR160h	TGCCTGGCTCCCTGCATGCCA	TRINITY_DN31410_c1_g1	-	cttagggaccaagttgtttccaaaggccctgtcactgaacacctgaggagcattttcacttataaagttggataaaccgaatgtggaaaaaggatctTGCCTGGCTCCCTGCATGCCA	3'	Yes	gp1a	109	46.60	18.07	40.49	13.88	69.64	74.65	56.03	middle
ptc-miR160a_1ss21AT	TGCCTGGCTCCCTGTATGCCT	TRINITY_DN32659_c1_g2	-	gaaggcgaggcagcatgatcacgtcgctgaaaatcaactgggaacagtcctgactgcagtttgttaaagtggagatctgatgaagatagttcaaattgagcaTGCCTGGCTCCCTGTATGCCTg	3'	Diff	gp1a	113	48.40	1505.17	1023.69	1079.55	404.14	388.67	396.07	middle
ptc-miR160h_L+4R-1	TGTGTGCCTGGCTCCCTGCATGCC	CM000352.2	-	gatccctgtgTGCCTGGCTCCCTGCATGCCAtttgcatagctcatcagaacgtcggtggccttggtggatggcgtgcaacgagtcatgcatgctgtga	5'	Diff	gp1a	86	57.10	0	2.75	0	0	0	0	low
ptc-miR160g_1ss17TN	TGCCTGGCTCCCTGGANGCCA	CM000350.2	-	gggatcaatcTGCCTGGCTCCCTGGATGCCAactaagaaatttgtcgagtggtcttgactcttcatagttggcatcagaggagtcatgcaggtccaac	5'	Diff	gp1a	94	50.00	3.76	4.26	4.43	0.48	5.78	1.76	low
ptc-miR160a_1ss21AT	TGCCTGGCTCCCTGTATGCCT	TRINITY_DN33886_c1_g1	-	tgagcaTGCCTGGCTCCCTGTATGCCTg	3'	Diff	gp1a	22	60.70	1505.17	1023.69	1079.55	404.14	388.67	396.07	middle
ptc-miR160g_2ss15GT21AG	TGCCTGGCTCCCTGTATGCCG	TRINITY_DN27236_c0_g1	-	tgagcaTGCCTGGCTCCCTGTATGCCTg	3'	Diff	gp1a	22	60.70	454.74	277.29	274.92	62.79	88.14	94.30	middle
eun-miR162-5p	GGAGGCAGCGGTTCATCGATC	TRINITY_DN31678_c2_g1	+	gaagacactGGAGGCAGCGGTTCATCGATCtcttcctggccaatttttgtttaacacgaaaaacatgaaccgaTCGATAAACCTCTGCATCCAGcgcttcctt	5'	Yes	gp1a	101	46.20	349.34	236.11	315.22	415.56	357.36	489.26	middle

ptc-miR162a	TCGATAAACCTCTGCATCCAG	TRINITY_DN31678_c2_g1	+	gaagacactGGAGGCAGCGGTTCATCGATCtcttcctggccaatttttgtttaacacgaaaaacatgaaccgaTCGATAAACCTCTGCATCCAGcgcttcctt	3'	Yes	gp1a	101	46.20	7159.03	9948.68	6805.06	4648.75	4570.55	4532.03	high
ptc-miR162a_R+2_1ss2CT	TTGATAAACCTCTGCATCCAGTT	CM000351.2	+	tgagaagacactggaggcagcggttcatcgatcttttcctgaagatttttttgttttacacgaacaacacgaaccgaTCGATAAACCTCTGCATCCAGtgcttccct	3'	Diff	gp1a	101	44.90	34.49	19.72	27.10	8.20	12.33	14.96	middle
ptc-miR164f_1ss17AG	TGGAGAAGCAGGGCACGTGCT	TRINITY_DN31872_c1_g4	-	aaTGGAGAAGCAGGGCACTTGCTcgtgctcatctaaattgggttgagtttggtcaaaagtgatgtatgaatccatcaatgcaggcaaagatgaagagcctgtgtcatcatagcagcttcttatg	5'	Diff	gp1a	117	43.50	108.42	176.74	136.57	536.57	325.57	371.11	middle
ptc-miR164a_L+1	CTGGAGAAGCAGGGCACGTGCA	TRINITY_DN27124_c1_g3	+	caagATGGAGAAGCAGGGCACGTGCAttactaactcatgcacacagagtgagagagacatttcttgctcgagttatgactcttacctcctatagattttgttggcctcagTGAGTTAGTTCTTCATGTGCCtgtcttccccatcat	5'	Diff	gp1a	141	45.20	238.51	326.02	223.88	798.58	796.59	584.67	middle
ptc-miR164a_L+1R+1_1ss22AC	CTGGAGAAGCAGGGCACGTGCCA	CM000338.2	+	ggttccttgcTGGAGAAGCAGGGCACGTGCAaaatcctgatgaagtgcttacactttgcacgcgctcttcttctccaacacgggc	5'	Diff	gp1a	81	54.10	0.79	0.96	0.55	2.01	1.64	0.83	low
ptc-miR164a_R+1_1ss1TC	CGGAGAAGCAGGGCACGTGCAC	CM000342.2	+	gtgagcaagaTGGAGAAGCAGGGCACGTGCActactaactcatgcacacagagagggagacgcatttcttgctggagttacgagttacgactcttacctactattgattttgttagctccagtgagttagttattcatgtgcctgtcttcctcatcatgatc	5'	Diff	gp1a	160	45.70	2.41	0.11	0.96	2.33	3.19	1.11	low
ptc-miR164a	TGGAGAAGCAGGGCACGTGCA	TRINITY_DN27124_c1_g3	+	gtgagcaagaTGGAGAAGCAGGGCACGTGCAttactaactcatgcacacagagtgagagagacatttcttgctcgagttatgactcttacctcctatagattttgttggcctcagtgagttagttcttCATGTGCCTGTCTTCCCCATCatgatc	5'	Yes	gp1a	153	45.80	27854.34	42009.56	30469.20	108185.05	107078.90	80325.14	high
ptc-MIR164e-p3	CATGTGCCTGTCTTCCCCATC	TRINITY_DN27124_c1_g3	+	gtgagcaagaTGGAGAAGCAGGGCACGTGCAttactaactcatgcacacagagtgagagagacatttcttgctcgagttatgactcttacctcctatagattttgttggcctcagtgagttagttcttCATGTGCCTGTCTTCCCCATCatgatc	3'	New	gp1a	153	45.80	193.94	264.93	217.61	592.51	478.73	524.76	middle
ptc-miR164a_R+4	TGGAGAAGCAGGGCACGTGCATCCT	CM000349.2	-	tagctcttgcTGGAGAAGCAGGGCACGTGCAagctctctcctcaggctttccttgcacgtgctccccttctccaacatgggt	5'	Diff	gp1a	80	57.30	6.81	11.21	4.06	13.15	11.24	11.93	middle
ptc-miR164a_L+1R+1_1ss22AC	CTGGAGAAGCAGGGCACGTGCCA	CM000350.2	+	tggctcacgcTGGAGAAGCAGGGCACGTGCAaaatccttctcggcttccagatgctgatgaagcactctttgcacgtgctcccctcctccaacatgagt	5'	Diff	gp1a	97	55.60	0.79	0.96	0.55	2.01	1.64	0.83	low

ptc-miR164f_1ss17AG	TGGAGAAGCAGGGCACGTGCT	TRINITY_DN31872_c1_g1	-	ttttgtgaaaaaaTGGAGAAGCAGGGCACTTGCTcgtgctcatctaaattgggttgagtttgctcataagtgatgtatgaatccaataatgcaggcaaagatgaagaacctgtgtcatcgttgcagctttccatgctaggtttggtcacaact	5'	Diff	gp1a	149	41.80	108.42	176.74	136.57	536.57	325.57	371.11	middle
ptc-miR166n_R+2_2ss18CT19CT	TCGGACCAGGCTTCATTTTTTAC	CM000355.2	-	aaggggtgtttggaatgaagtttgatccaagatccttgtctctcccgttaacttagtctctgttaccgttaggtattcattactgagtgtatttgcagccccccttgtctgattttagcatcatgagagtaggaagtgctggtgatcatagggtttggttcaagatccatttgactcttctctttatatctctcctgtgtcctagctggtaatctgtagtagtcttaattatcccttacttcttgttaatttttaggtttgatcttgcaagtaattatatttggaaaatatcataaaatgatcTCGGACCAGGCTTCATTCCTTacaccttg	3'	Diff	gp1a	326	38.40	9.17	9.10	7.35	2.38	3.61	5.29	low
ptc-miR166n_R+2_2ss18CT19CT	TCGGACCAGGCTTCATTTTTTAC	CM000349.2	+	aaggggtgtttggaatgaagtttgatccaagatccttgtctctcccgttcatttagtaatgttactgctaggtttcgctatgtattgatcagtgtatacacagcagctacgagctaggccttgtgtttcagattttagcttcaatatagagtgagagtgatagagtttgattcaagatccatctcatgtgtgctagctagcttttaaatttttaatcatatccctacttcctgttaattcttaggttttatcttgcaagttattatatttagtaaacatcatgaactgatcTCGGACCAGGCTTCATTCCTTacaccgaa	3'	Diff	gp1a	313	37.20	9.17	9.10	7.35	2.38	3.61	5.29	low
ptc-miR166a_L+3_1ss4TC	ATCCCGGACCAGGCTTCATTCCCC	CM000346.2	+	acacagttgaggggaatgctgtctgattcgagaccattcactttaagcacacattcatctttcgaatgatcTCGGACCAGGCTTCATTCCCCccaactcaa	3'	Diff	gp1a	94	46.50	0.52	0.09	0.16	0.19	0.04	0.88	low
ptc-miR166a_L+3_1ss4TC	ATCCCGGACCAGGCTTCATTCCCC	CM000344.2	-	acacagttgaggggaatgctgtctggttcgagaccattcacctgaagcgcacgcattcatcttttgagtgatcTCGGACCAGGCTTCATTCCCCccaactcaa	3'	Diff	gp1a	96	51.50	0.52	0.09	0.16	0.19	0.04	0.88	low
ptc-miR166a_L+1	CTCGGACCAGGCTTCATTCCCC	TRINITY_DN32238_c0_g6	+	aggtagcttctttttgaggGGAATGTTGTCTGGCTCGAGGactttttcttgatcaatctaatcgaactttctacctgtagatctagtatcttatttaagattgatcacgtattagggttGTCGGACCAGGCTTCATTCCCCccaatcattgcttccatc	3'	Diff	gp1a	156	41.20	2704.36	2126.32	2067.31	1048.02	926.63	1131.62	middle
ptc-miR166n_L+3R-2	CTCTCGGACCAGGCTTCATTCC	TRINITY_DN32689_c0_g1	+	aggttgagaggaatgttgtctggctcgaggtcattgaggccatgattatacagacatggcattacctgatgacagccaagaaaattcaaagtctgtgtgtatcttgcacctcgatgaTTCTCGGACCAGGCTTCATTCCttccaacaaa	3'	Diff	gp1a	144	45.00	2559.81	2131.47	1907.01	821.98	613.58	788.81	middle

ptc-miR166a_1ss21CA	TCGGACCAGGCTTCATTCCCA	TRINITY_DN24400_c1_g1	+	ctccctccccccatctctccctctcgcgtattaactgttgaggggattgtcgtctggttcgatgtcattcatgagaagctcaaacataaacgtaatattgaatgattTCGGACCAGGCTTCATTCCCCccaacaatgtttgattagcgttcacagaatggattgaggctt	3'	Diff	gp1a	164	45.90	10029.03	8074.59	7360.29	5082.00	3421.41	5013.53	high
ptc-MIR166f-p5	GGAATGTTGGCTGGCTCGAAG	TRINITY_DN32689_c0_g1	+	ggtgtgttgaggGGAATGTTGGCTGGCTCGAAGcttaagcaaagagtttcctaacatgaaacaactgttaaggctTCGGACCAGGCTTCATTCCCCtcaaacat	5'	New	gp1a	103	47.10	1273.28	744.69	897.31	804.85	704.12	867.58	middle
ptc-miR166a	TCGGACCAGGCTTCATTCCCC	TRINITY_DN32689_c0_g1	+	ggtgtgttgaggGGAATGTTGGCTGGCTCGAAGcttaagcaaagagtttcctaacatgaaacaactgttaaggctTCGGACCAGGCTTCATTCCCCtcaaacat	3'	Yes	gp1a	103	47.10	522045.65	354405.86	336998.58	178918.24	162848.48	198170.44	high
ptc-miR166a_L+3_2	TACTCGGACCAGGCTTCATTCCCC	CM000351.2	-	taacttttgtggtgaatgttgtctggttcaaggcatggccaccacatctcttggtgaatatatgtccTCGGACCAGGCTTCATTCCCCtcaattaat	3'	Diff	gp1a	86	44.30	0.72	0	0.04	0	0	0	low
ptc-miR166p_1ss20TC	TCGGACCAGGCTCCATTCCCT	CM000341.2	-	taaggttgagaggaacgctgtctgggtcgaggtcatggaggccatgattatacataaatggcattatctgatgacagcccagataatcgatgcacctgtcttgaacctaaatgattcTCGGACCAGGCTCCATTCCTTccaaccat	3'	Diff	gp1a	141	46.60	11.44	4.58	3.58	2.28	1.44	0	middle
ptc-MIR166e-p5	GATTGTCGTCTGGTTCGATGT	TRINITY_DN24400_c1_g1	+	tattaactgttgagggGATTGTCGTCTGGTTCGATGTcattcatgagaagctcaaacataaacgtaatattgaatgattTCGGACCAGGCTTCATTCCCCccaacaatgtttgatt	5'	New	gp1a	99	39.70	50.59	121.48	126.27	229.47	209.02	234.09	middle
ptc-miR166a	TCGGACCAGGCTTCATTCCCC	TRINITY_DN24400_c1_g1	+	tattaactgttgagggGATTGTCGTCTGGTTCGATGTcattcatgagaagctcaaacataaacgtaatattgaatgattTCGGACCAGGCTTCATTCCCCccaacaatgtttgatt	3'	Yes	gp1a	99	39.70	522045.65	354405.86	336998.58	178918.24	162848.48	198170.44	high
ptc-MIR166j-p5	GGAATGTTGTCTGGCTCGAGG	TRINITY_DN32238_c0_g6	+	tcttttttgaggGGAATGTTGTCTGGCTCGAGGactttttcttgatcaatctaatcgaactttctacctgtagatctagtatcttatttaagattgatcacgtattagggttgTCGGACCAGGCTTCATTCCCCccaatcatt	5'	New	gp1a	132	39.90	705.10	805.43	725.37	1339.52	945.25	1267.16	middle
ptc-miR166a	TCGGACCAGGCTTCATTCCCC	TRINITY_DN32238_c0_g6	+	tcttttttgaggGGAATGTTGTCTGGCTCGAGGactttttcttgatcaatctaatcgaactttctacctgtagatctagtatcttatttaagattgatcacgtattagggttgTCGGACCAGGCTTCATTCCCCccaatcatt	3'	Yes	gp1a	132	39.90	522045.65	354405.86	336998.58	178918.24	162848.48	198170.44	high
ptc-miR166a_1ss21CA	TCGGACCAGGCTTCATTCCCA	TRINITY_DN32689_c0_g1	+	tgggtgtgttgagggGAATGTTGGCTGGCTCGAAGCttaagcaaagagtttcctaacatgaaacaactgttaaggctTCGGACCAGGCTTCATTCCCCtcaaacatgcaag	3'	Diff	gp1a	105	47.70	10029.03	8074.59	7360.29	5082.00	3421.41	5013.53	high

ptc-MIR166q-p5	GGAATGTTGTCTGGCTCGAGG	TRINITY_DN32689_c0_g1	+	ttaggttgagaGGAATGTTGTCTGGCTCGAGGtcattgaggccatgattatacagacatggcattacctgatgacagccaagaaaattcaaagtctgtgtgtatcttgcacctcgatgatTCTCGGACCAGGCTTCATTCCttccaacaaa	5'	New	gp1a	144	44.40	705.10	805.43	725.37	1339.52	945.25	1267.16	middle
ptc-miR166n_L+2R-2	TCTCGGACCAGGCTTCATTCC	TRINITY_DN32689_c0_g1	+	ttaggttgagaGGAATGTTGTCTGGCTCGAGGtcattgaggccatgattatacagacatggcattacctgatgacagccaagaaaattcaaagtctgtgtgtatcttgcacctcgatgatTCTCGGACCAGGCTTCATTCCttccaacaaa	3'	Diff	gp1a	144	44.40	587452.68	383812.39	377880.15	122635.10	111424.05	122529.51	high
ptc-miR166a_L+3_1	AAGTCGGACCAGGCTTCATTCCCC	CM000337.2	+	tttcttttgaggggaatgttgtctggctcgaggtcactaatgggatctatgattttatctcaattgattgattttcttttaaattctagtaaattgaattgagagatatcatgatcaacttatatttaatgatgTCGGACCAGGCTTCATTCCCCccaattgtt	3'	Diff	gp1a	153	34.10	0.72	0	0.04	0	0	0	low
ptc-miR166a_L+1	CTCGGACCAGGCTTCATTCCCC	TRINITY_DN32238_c0_g5	+	tttttgaggGGAATGTTGTCTGGCTCGAGGactctttcttgatcagtccaatcaagttttctatctttagatctaacatcttagatcatgtgttagggtcGTCGGACCAGGCTTCATTCCCCccaatta	3'	Diff	gp1a	123	42.60	2704.36	2126.32	2067.31	1048.02	926.63	1131.62	middle
ptc-MIR166c-p5	GGAATGTTGTCTGGCTCGAGG	TRINITY_DN32238_c0_g5	+	ttttttgaggGGAATGTTGTCTGGCTCGAGGactctttcttgatcagtccaatcaagttttctatctttagatctaacatcttagatcatgtgttagggtcgTCGGACCAGGCTTCATTCCCCccaattatt	5'	New	gp1a	123	41.70	705.10	805.43	725.37	1339.52	945.25	1267.16	middle
ptc-miR166a	TCGGACCAGGCTTCATTCCCC	TRINITY_DN32238_c0_g5	+	ttttttgaggGGAATGTTGTCTGGCTCGAGGactctttcttgatcagtccaatcaagttttctatctttagatctaacatcttagatcatgtgttagggtcgTCGGACCAGGCTTCATTCCCCccaattatt	3'	Yes	gp1a	123	41.70	522045.65	354405.86	336998.58	178918.24	162848.48	198170.44	high
ptc-miR167e	TGAAGCTGCCAGCATGATCTG	TRINITY_DN28677_c0_g4	+	atatgttggtttgagaggtTGAAGCTGCCAGCATGATCTGGTCATCTTGCAGCTTCAATcactcattcaccaacagca	5'	Yes	gp1a	72	44.90	19263.62	23530.90	28504.88	12783.49	15428.60	15237.50	high
ptc-miR167f-3p_L-2_1ss13AT	ATCATGTGGCTGTTTCACC	TRINITY_DN34711_c2_g1	+	ATCAAGTGGCTGTTTCACCcaccacttcaagttctgctgttcgtgatccaaatataaatgatcaatcacagaatgtatctgtcaatccatcagtaattgccatgggtggaactgtttctagt	5'	Diff	gp1a	115	40.20	1.20	5.49	10.75	3.42	11.56	4.44	middle
ptc-miR167a_R+4_1	TGAAGCTGCCAGCATGATCTAAATT	CM000341.2	+	cactagcagtTGAAGCTGCCAGCATGATCTAaattaacctcttctttatcaaggatggattagatcatgtggtagtttcacctgctga	5'	Diff	gp1a	85	41.60	1.20	0.60	1.23	0.14	5.90	8.22	low
ptc-miR167a_R+4_2	TGAAGCTGCCAGCATGATCTAACTT	CM000338.2	-	cactagcagtTGAAGCTGCCAGCATGATCTAacttccttgcttctttatcaaggatggatttagatcatgtggtggtttcacctgttga	5'	Diff	gp1a	85	42.70	1.20	0.60	1.23	0.14	5.90	8.22	low

ptc-miR167e_R+3	TGAAGCTGCCAGCATGATCTGTAA	CM000341.2	+	cactagtagtTGAAGCTGCCAGCATGATCTGaactttccttaatttcctatacgggaaagactagatcatgtggtagtttcatctattga	5'	Diff	gp1a	87	38.50	0.15	0	0.61	0	0.15	0.37	low
ptc-miR167f-3p_L+3_1ss16AG	AACAGATCATGTGGCGGTTTCACC	CM000344.2	+	cactatcagtTGAAGCTGCCAGCATGATCTTaacctccctccttttgtcgaggaaagaacAGATCATGTGGCAGTTTCACCtgaagt	3'	Diff	gp1a	85	46.50	1.81	2.40	6.94	2.57	3.37	1.11	low
ptc-miR167f-5p_R+1	TGAAGCTGCCAGCATGATCTTA	TRINITY_DN23948_c2_g4	+	cactatcagtTGAAGCTGCCAGCATGATCTTAacctccctccttgtcgaggaaagaacAGATCGTGTGGCAGTTTCACCtgaagt	5'	Diff	gp1a	85	47.70	6244.73	8584.21	11583.56	11948.38	17245.75	15930.34	high
ptc-miR167f-3p	AGATCATGTGGCAGTTTCACC	TRINITY_DN23948_c2_g4	+	cactatcagtTGAAGCTGCCAGCATGATCTTAacctccctccttgtcgaggaaagaacAGATCGTGTGGCAGTTTCACCtgaagt	3'	Yes	gp1a	85	47.70	25.30	72.75	106.57	116.45	232.14	218.56	middle
ptc-miR167h-5p_R+2_1ss12AG	TGAAGCTGCCAGCATGATCTGAC	CM000349.2	+	cgctattagtTGAAGCTGCCAACATGATCTGagctttccttaatttcctatacaggaaagactAGATCATGTGGCAGTTTCACCtattga	5'	Diff	gp1a	87	39.60	15.66	31.57	29.10	11.99	23.60	28.85	middle
ptc-miR167f-3p_1ss13AT	AGATCATGTGGCTGTTTCACC	CM000344.2	+	cgctattagtTGAAGCTGCCAACATGATCTGagctttccttaatttcctatacaggaaagactAGATCATGTGGCAGTTTCACCtattga	3'	Diff	gp1a	87	39.60	115.84	374.86	455.07	422.98	496.71	514.78	middle
ptc-miR167f-3p_L+3_1ss16AG	AACAGATCATGTGGCGGTTTCACC	CM000346.2	-	ctctatcagtTGAAGCTGCCAGCATGATCTTagccttcctcctttgttgaggaaagaaacAGATCATGTGGCAGTTTCACCtgttgt	3'	Diff	gp1a	77	44.80	1.81	2.40	6.94	2.57	3.37	1.11	low
ptc-miR167f-3p_1ss13AT	AGATCATGTGGCTGTTTCACC	CM000349.2	+	tcgtgtaccactagcagaTGAAGCTGCCAGCATGATCTGagctttcgttaattatcctatacatatggatatatccagaggagagattagatcatgtggctgcttcacctgttgatggtagcatga	3'	Diff	gp1a	126	42.90	115.84	374.86	455.07	422.98	496.71	514.78	middle
ptc-miR167e	TGAAGCTGCCAGCATGATCTG	TRINITY_DN28677_c0_g3	+	tggtttgagaggtTGAAGCTGCCAGCATGATCTGgtaatgaacgtgttactctcatttatatatatatatattaacattaatcttatagcagtatctgtaggtagaaaaaatttaatgttgtcaaagatatatgtactgaatcgtggtagctag	5'	Yes	gp1a	136	31.80	19263.62	23530.90	28504.88	12783.49	15428.60	15237.50	high
ptc-miR167e_R+2	TGAAGCTGCCAGCATGATCTGAA	TRINITY_DN23948_c2_g4	+	ttatcgtgcaccactatcagtTGAAGCTGCCAGCATGATCTTAAcctccctcctttgtcgaggaaagaacAGATCGTGTGGCAGTTTCACCtgaagttggtatcacgagaa	5'	Diff	gp1a	105	46.80	20.48	69.32	86.87	77.63	86.69	137.57	middle
ptc-miR168a-5p	TCGCTTGGTGCAGGTCGGGAA	TRINITY_DN29230_c0_g2	+	ggtctctaatTCGCTTGGTGCAGGTCGGGAActgattctgcgatttcattgccaggtggctaaacacgattggctgtgaggcaaattataaaaagaaagagaattggatCCCGCCTTGCATCAACTGAATcggaga	5'	Yes	gp1a	134	46.30	35526.54	25477.40	20407.13	28261.25	25297.42	24328.75	high
ptc-miR168a-3p	CCCGCCTTGCATCAACTGAAT	TRINITY_DN29230_c0_g2	+	ggtctctaatTCGCTTGGTGCAGGTCGGGAActgattctgcgatttcattgccaggtggctaaacacgattggctgtgaggcaaattataaaaagaaag	3'	Yes	gp1a	134	46.30	1294.96	996.24	1058.51	1881.42	1699.63	1682.45	middle

				agaattggatCCCGCCTTGCATCAACTGAATcggaga												
ptc-miR168a-5p	TCGCTTGGTGCAGGTCGGGAA	TRINITY_DN29230_c0_g1	+	ggtctctgatTCGCTTGGTGCAGGTCGGGAActgattctgcgatttgattgccagatggctcgacatgactggttcttatggagaaagaaaagtaaggaaacaggaaaaaacaaagaatagcgaattggatCCCGCCTTGCATCAACTGAATcggagg	5'	Yes	gp1a	154	46.20	35526.54	25477.40	20407.13	28261.25	25297.42	24328.75	high
ptc-miR168a-3p	CCCGCCTTGCATCAACTGAAT	TRINITY_DN29230_c0_g1	+	ggtctctgatTCGCTTGGTGCAGGTCGGGAActgattctgcgatttgattgccagatggctcgacatgactggttcttatggagaaagaaaagtaaggaaacaggaaaaaacaaagaatagcgaattggatCCCGCCTTGCATCAACTGAATcggagg	3'	Yes	gp1a	154	46.20	1294.96	996.24	1058.51	1881.42	1699.63	1682.45	middle
ptc-miR169a	CAGCCAAGGATGACTTGCCGA	TRINITY_DN34666_c5_g1	+	aagattgatgCAGCCAAGGATGACTTGCCGAcgactcgcttttgcttccatcaatatacgcataattaagaagagatgaatccgtTGGCAGGTTGTTCTTGGCTACatttttc	5'	Yes	gp1a	111	42.50	59.03	98.49	55.07	174.67	199.87	249.62	middle
ptc-miR169b-3p_L+1	TGGCAGGTTGTTCTTGGCTAC	TRINITY_DN34666_c5_g1	+	aagattgatgCAGCCAAGGATGACTTGCCGAcgactcgcttttgcttccatcaatatacgcataattaagaagagatgaatccgtTGGCAGGTTGTTCTTGGCTACatttttc	3'	Diff	gp1a	111	42.50	279.47	427.60	363.58	1194.15	1006.58	1141.61	middle
ptc-miR169a_R+2_1ss14CT	CAGCCAAGGATGATTTGCCGAGT	CM000337.2	-	aagtggtatgCAGCCAAGGATGACTTGCCGActtaactgatctgtataaattaatgcatgtgtagtcaaaattactactactatatattaatctgatctgtgaccacaaattaactaactactagctagtaattagttggcaagttgtccatggctacatgctgc	5'	Diff	gp1a	163	36.40	0	0.44	0.36	0.18	0.15	0.69	low
ptc-miR169a_1ss21AG	CAGCCAAGGATGACTTGCCGG	TRINITY_DN34666_c5_g1	+	acagccttccatggaggatcataaagattgatgCAGCCAAGGATGACTTGCCGAcgactcgcttttgcttccatcaatatacgcataattaagaagagatgaatccgttggcaggttgttCTTGGCTACATTTTTCTTTCTgctcctcatgcgaggcttat	5'	Diff	gp1a	156	44.10	45.78	14.41	7.16	25.12	26.97	45.49	middle
ptc-miR169n-5p_R+1_1ss2GC	TCAGCCAAGGATGACTTGCCGC			agagtggattTGAGCCAAGGATGACTTGCCGcagatgcatggtccttgctgctgatcacataccaaggggggttttgggtgGCAAGCATCCTTGGTTCTCCttcgct	5'	Diff	gp1a	105	53.30	5.31	4.28	2.48	18.80	12.58	13.33	middle
ptc-miR169n-3p_L+2R-1	TGGCAAGCATCCTTGGTTCTC			agagtggattTGAGCCAAGGATGACTTGCCGcagatgcatggtccttgctgctgatcacataccaaggggggttttgggtgGCAAGCATCCTTGGTTCTCCttcgct	3'	Diff	gp1a	105	53.30	3.61	15.79	9.55	54.80	56.83	102.07	middle
ptc-miR169d_L+1_1ss22GC	TCAGCCAAGGATGACTTGCCGC			agagtgtgattCAGCCAAGGATGACTTGCCGGcagcaggtaagagcaaagctccgtttttggaagttcaaggatatcttaacttttccggccggcaagctgtccttggctacattgtact	5'	Diff	gp1a	118	48.30	5.31	4.28	2.48	18.80	12.58	13.33	middle

ptc-miR169ac_L-1R-1	AGCCAAGGACGACTTGCCC	TRINITY_DN24348_c0_g1	-	attgccaaagttttgtttcagtgcattatgttttcagcacctctgaccaatccatgacagcaaggctgtatggaatggcaaaggtaccattaatgcactggAGCCAAGAACGACTTGCCCtt	3'	Diff	gp1a	115	44.30	0	1.37	6.27	1.14	3.85	0	low
ptc-miR169o_R+1	AAGCCAAGGATGACTTGCCTGA	CM000337.2	-	ccttgcgtggAAGCCAAGGATGACTTGCCTGagaattttaggaaggtttctatatgaaagctttttattagttttgcaggaagtcaaccttggctttcctatag	5'	Diff	gp1a	90	40.40	0	0	0	0.25	1.44	3.33	low
ptc-miR169v_R+3_2ss11TC21AC	TAGCCAAGGACGACTTGCCCCCTC	CM000345.2	+	cgtgtttggTAGCCAAGGATGACTTGCCCActccattgaaagagtttttcaagcatatggtagtgtagaacttttctttggttctgggcagtcatcttggctatgctgac	5'	Diff	gp1a	107	44.50	5.06	6.59	8.01	21.03	19.77	23.73	middle
ptc-miR169v_R+3_2ss11TC21AC	TAGCCAAGGACGACTTGCCCCCTC	CM000337.2	+	cgtgtttggTAGCCAAGGATGACTTGCCCActctatggaaagagttctcaagcacacggcagagaggacccttacttcggctctgggcagtcaccttggctatgctgac	5'	Diff	gp1a	106	54.10	5.06	6.59	8.01	21.03	19.77	23.73	middle
ptc-miR169ac_R+2_1ss21AC	TAGCCAAGGACGACTTGCCCCCC	CM000341.2	+	cttgtttggTAGCCAAGGACGACTTGCCCAccaccatcttagttgggtccaatatattactgggcaccttccatttggttttgggcaagcaccttggctagctgaca	5'	Diff	gp1a	105	49.50	82502.34	69317.46	125256.50	167870.45	158176.07	180683.86	high
ptc-MIR169ae-p5	TTGTTTGGTAGCCAAGGACGACT	TRINITY_DN22166_c0_g2	+	gaagagaggccaagcatgaaggctaagagtcTTGTTTGGTAGCCAAGGATGACTtgcctgctcccttcgaggaggttccatgaaatgcaggcaagatctctcgaaacccctgtcgggtttcaggcggtctccttgGCTAACTTGACAGGCTCTTTCctgacatgcttagccctt	5'	New	gp1a	171	52.30	350.54	218.26	310.75	525.15	349.65	489.26	middle
ptc-miR169u-5p_R+3_1ss16TC	TAGCCAAGGACGACTCGCCTAATT	CM000351.2	+	gagggttgggTAGCCAAGGACGACTTGCCTAtttcctccatggggtcctgaaaagaatgaaatactgtcgttcagagctcattggtagggttcataGGCAGTCTCCTTTGGCTATCCtaact	5'	Diff	gp1a	119	48.40	162.62	145.16	202.57	542.28	433.07	468.79	middle
ptc-miR169r_R+2_2ss15TA21AT	TAGCCAAGGATGACATGCCTTTT	CM000351.2	+	gagggtttggTAGCCAAGGATGACTTGCCTAtttcctccataaggctttaaaaagcatgaaatgtggtttagagctcaattgaagggttcataggcagtctactttggttatcctagct	5'	Diff	gp1a	116	42.00	0.27	0.53	0.16	0.38	0.48	0.82	low
ptc-miR169d_L+1_1ss22GC	TCAGCCAAGGATGACTTGCCGC	CM000342.2	+	gagtgtaattCAGCCAAGGATGACTTGCCGGcagcacgggatctcagagcttaataactagaagatcaaggctgtcattacttttccggccggcaagttgcccttggctacattgtac	5'	Diff	gp1a	113	49.20	5.31	4.28	2.48	18.80	12.58	13.33	middle
ptc-miR169aa_L+2R-2	CCGAGCCAAGAATGACTTGTC			gtagagatcGAGCCAAGAATGACTTGTCGGcaggctagcaattgctacgtatagagcaatatgtatgctagaatgagtattccgccaggtcgttcttggctcaactttg	5'	Diff	gp1a	108	45.90	40.96	41.18	61.79	207.78	221.54	191.93	middle
ptc-MIR169s-p5_1ss5CA	CAAGATGTCCTTGGCTACAT	TRINITY_DN31963_c0_g2	-	gttCAAGATGTCTTTGGCTACATcaggattgcatgtgataataaccctagtctcgcccatgctaaaagccatg	5'	New	gp1a	80	43.00	19.27	9.61	7.16	7.99	9.63	14.42	middle

				agtcgttttgctt												
ptc-miR169ac_R+2_2ss10AG21AC	TAGCCAAGGGCGACTTGCCCCCA	CM000343.2	-	gttgttcggTAGCCAAGGACGACTTGCCCAcactatgagacaagtggttagaattagcaacttcttaattggttatgggaagtctccttggctatgctgac	5'	Diff	gp1a	95	45.50	25.41	20.97	43.43	66.79	33.76	48.26	middle
ptc-miR169q_L+1R-1_1ss17TC	CTAGCCAAGGACGACTCGCCT	CM000337.2	+	tcttgttagtTAGCCAAGGACGACTTGCCTGttcctagcaatttgggatttgccctggactaagaggcaggaccctcattccgggtttcaggcagtctccttggctaggttgaca	5'	Diff	gp1a	112	51.30	513.17	906.33	755.55	2137.15	2894.13	2672.40	middle
ptc-miR169i_L+2R+1	AATAGCCAAGGATGACTTGCCTGC	CM000337.2	-	tcttgtttgaTAGCCAAGGATGACTTGCCTGctacttgcaagagtttctgcaaagagatcagaaccaaatatgcatggaattatatgtaatgaaactcttgtttgattgccaggcagtctccttggctagcctgaca	5'	Diff	gp1a	134	41.60	0	0.13	0	0.13	0	0.50	low
ptc-miR169i_1ss15TA	TAGCCAAGGATGACATGCCTG	TRINITY_DN22166_c0_g2	+	tcttgtttggTAGCCAAGGATGACTTGCCTGctcccttcgaggaggttccatgaaatgcaggcaagatctctcgaaaccccctgtcgggtttcAGGCGGTCTCCTTGGCTAACttgacag	5'	Diff	gp1a	115	52.90	733.61	775.23	609.40	927.01	1241.13	1092.79	middle
ptc-MIR169m-p3	AGGCGGTCTCCTTGGCTAAC	TRINITY_DN22166_c0_g2	+	tcttgtttggTAGCCAAGGATGACTTGCCTGctcccttcgaggaggttccatgaaatgcaggcaagatctctcgaaaccccctgtcgggtttcAGGCGGTCTCCTTGGCTAACttgacag	3'	New	gp1a	115	52.90	110.82	46.67	47.46	87.91	92.47	78.77	middle
ptc-miR169i_R+2	TAGCCAAGGATGACTTGCCTGTC	CM000339.2	-	tcttgtttggTAGCCAAGGATGACTTGCCTGtagcctccctcggattcatgaacacgagcatttatgtggtgctcaaaagaaaaggagagaatgatcccagctggcagcgacaaggtcaatccattgaagtagcgagaagcaggtgatttatagctagaatccacaagaggttcccagcaagtgtccttggctaacgagacgg	5'	Diff	gp1a	199	48.30	7.51	10.94	11.71	10.85	11.16	12.37	middle
ptc-miR169ac_R+2_2ss10AG21AC	TAGCCAAGGGCGACTTGCCCCCA	CM000341.2	+	tcttgtttgTAGCCAAGGACGACTTGCCCAcaatatgtatcgtgagcgtttccaatttgcaacctcatacttggttgttttgggcagtctccttggctatgcagat	5'	Diff	gp1a	102	45.30	25.41	20.97	43.43	66.79	33.76	48.26	middle
ptc-miR169x_R+2_2ss14CG19TA	TAGCCAAGGATGAGTTGCACGTT	CM000343.2	-	tgctgttttcTAGCCAAGGATGACTTGCTCGttagcccttgaaagatgtttcaaatttaggctgcatatgctcagaaccttttctggcttcaggcaatcatcttggctaaatgacag	5'	Diff	gp1a	116	42.40	36.14	49.47	75.83	50.35	44.96	64.04	middle
ptc-MIR171i-p3_2ss17CT23GT_1	TCTTCTTTTTCTTCTTTTTCTTT	TRINITY_DN28915_c0_g1	-	acaaacaagaaaaacccttttaaagaaataaaaatacaaacactacagcgttgatgaataatggcagtaatccagactgaaactaatttattaaaaaactacgttcgacctttcTCTTCTTTTCCTTCTTTTTCTTTttgcaa	3'	New	gp1a	139	29.40	0	4.80	2.24	47.38	105.96	59.35	middle
ptc-miR171a-3p_2ss9TC21GT	TTGAGCCGCGCCAATATCAC	TRINITY_DN30537_c0_g1	-	ccagttgggaacagctgttgaTTGAGCCGCGCCAATATCCCTtgcgcgtgtgagaagttcccagtccctac	5'	Diff	gp1a	72	56.00	254.17	177.77	168.81	116.45	191.68	124.81	middle

	T			caac												
ptc-MIR171i-p3_2ss17CT23GT_2	TCTTCTTTTTCTTCTTTTTCTTTT	TRINITY_DN26229_c0_g6	-	ctggaggtaggaaaatcatgatcgggatagcggatccaaagctatggaacttgggtgtgggtcttttgtcgaaatagaatggccttactttttcttcttttTCTTCTTTTTCTTCTTTTTCTTTTtatttcttttttttttatttatattaaattcaaatttaaaattt	3'	New	gp1a	115	29.60	0	4.80	2.24	47.38	105.96	59.35	middle
ptc-MIR171i-p5_1ss16CT	CTTCTTTTTCTTCTTTTTCT	TRINITY_DN21562_c0_g2	-	CTTCTTTTTCTTCTTTTTCTtctgaccctcttggtcaccatcctcttgaatggtttcctctgcacctgcaggctccgcctcttgatgtttcctcttctttgaagcaccttcaggcttctttggtgtttcttcagtggatt	5'	New	gp1a	1	45.70	0	1.03	0	3.42	5.78	1.66	low
ptc-miR171e_L+2	GCTGATTGAGCCGTGCCAATATC	CM000351.2	+	gaaagtgggggaTGTTGGGATGGCTCAATCATAtcaaatctcccaaactatgatgttgggtcgtttaatcTGATTGAGCCGTGCCAATATCacactaact	3'	Diff	gp1a	93	43.00	5.17	2.27	0.18	0.23	0	0.06	low
ptc-miR171g-5p_L+3	GGATGTTGGGATGGCTCAATCATG	CM000348.2	+	gaaagtgggggaTGTTGGGATGGCTCAATCATGtcaaatctcccaaattatgatgttgggtcttttaatcTGATTGAGCCGTGCCAATATCacacttctt	5'	Diff	gp1a	95	42.00	6.02	4.80	7.16	1.14	2.89	0	low
ptc-miR171e_L+2	GCTGATTGAGCCGTGCCAATATC	CM000348.2	+	gaaagtgggggaTGTTGGGATGGCTCAATCATGtcaaatctcccaaattatgatgttgggtcttttaatcTGATTGAGCCGTGCCAATATCacacttctt	3'	Diff	gp1a	95	42.00	5.17	2.27	0.18	0.23	0	0.06	low
ptc-miR171e_L+1	CTGATTGAGCCGTGCCAATATC	CM000338.2	+	gagtgactatgatattggcctggttcactcagatcacgacttcagagcaaagtgtctttcttcttcttctttttcttcttcttcttgttttatgttcgtttgattTGATTGAGCCGTGCCAATATCtcagtactc	3'	Diff	gp1a	135	39.30	503.03	464.26	503.43	159.26	225.88	168.45	middle
ptc-miR171l-5p_2ss1TC17CT	CGTGATATTGGTCCGGTTCATC	CM000351.2	+	ggagaagtagacacggTGTGATATTGGTCCGGCTCATCttctgtgcataagcatctgaagttcttcactcatgaagaCGAGCCGAATCAATATCACTcttgtatgcttcttc	5'	Diff	gp1a	112	44.60	122.27	78.93	176.42	527.44	695.46	617.95	middle
ptc-miR171l-5p_2ss1TC17CT	CGTGATATTGGTCCGGTTCATC	CM000351.2	+	ggagaagtagacacggTGTGATATTGGTCCGGCTCATCttctgtgcataagcatctgaagttcttcactcatgaagaCGAGCCGAATCAATATCACTcttgtatgcttcttc	5'	Diff	gp1a	112	44.60	122.27	78.93	176.42	527.44	695.46	617.95	middle
ptc-miR171j_R+1	CGAGCCGAATCAATATCACTC	CM000348.2	-	ggagaagtagacacggTGTGATATTGGTCCGGCTCATCttctgtgcataagcatctgaagttcttcactcatgaagaCGAGCCGAATCAATATCACTcttgtatgcttcttc	3'	Diff	gp1a	112	44.60	10.24	4.46	12.99	25.69	41.42	19.97	middle
ptc-miR171j_R+1	CGAGCCGAATCAATATCACTC	CM000351.2	+	ggagaagtagacacggTGTGATATTGGTCCGGCTCATCttctgtgcataagcatctgaagttcttcactcatgaagaCGAGCCGAATCAATATCACTcttgtatgcttcttc	3'	Diff	gp1a	112	44.60	10.24	4.46	12.99	25.69	41.42	19.97	middle
ptc-miR171e	TGATTGAGCCGTGC	TRINITY_DN33802_	-	gTGATTGAGCCGCGCCAATATCCCT	5'	Yes	gp1a	16	56.00	12437.06	14100.08	10346.85	3540.22	5571.35	3449.78	high

	CAATATC	c0_g2														
ptc-MIR171c-p3	TGAGCCGCGCCAATATCACT	TRINITY_DN33802_c0_g2	-	gTGATTGAGCCGCGCCAATATCCCT	3'	New	gp1a	16	56.00	1.20	0.69	0	0	1.44	2.22	low
ptc-miR171c_2ss1AT12CT	TGATTGAGCCGTGCCAATATC	TRINITY_DN30909_c0_g2	-	gTGATTGAGCCGCGCCAATATCCCTT	5'	Diff	gp1a	16	53.80	12437.06	14100.08	10346.85	3540.22	5571.35	3449.78	high
ptc-miR171a-3p_R+1_2ss9TC21GT	TTGAGCCGCGCCAATATCACTT	TRINITY_DN30909_c0_g2	-	gTGATTGAGCCGCGCCAATATCCCTT	3'	Diff	gp1a	16	53.80	72.28	48.39	36.27	40.53	56.35	34.39	middle
ptc-miR171e_L+2	GCTGATTGAGCCGTGCCAATATC	CM000337.2	-	gtggaaatttgatgttggcagagctcaatcaaatcaaagcactcaatggttgggttctttcatcTGATTGAGCCGTGCCAATATCgcattaaat	3'	Diff	gp1a	75	40.40	5.17	2.27	0.18	0.23	0	0.06	low
ptc-miR171e_L+2	GCTGATTGAGCCGTGCCAATATC	CM000338.2	+	gtgtcattgcgatgttggcccggttcactcagagaaaagacaccattttttttcaagaagatcatcaaagcttgaaagaataatggtggtcggtcgtctcTGATTGAGCCGTGCCAATATCttagtgctc	3'	Diff	gp1a	126	43.80	5.17	2.27	0.18	0.23	0	0.06	low
ptc-miR171a-3p_R+1_2ss9TC21GT	TTGAGCCGCGCCAATATCACTT	TRINITY_DN32623_c3_g2	-	gttcggaacagctgttgaTTGAGCCGCGCCAATATCCCTTgcgcgtgtgagaagttcccagtccctaccaactcagctgccttgtagagctggtccaacaaggcatgctggtgctggagctggtcctggtgctggtaatgttgagttcctgaatctt	5'	Diff	gp1a	154	54.10	72.28	48.39	36.27	40.53	56.35	34.39	middle
ptc-miR171a-5p	GGATATTGGTACGGTTCAATC	CM000340.2	+	tcagagaaaacgGGATATTGGTACGGTTCAATCagaaagtaatgctcccaaaagtatagagtactattgtttgaTTGAGCCGTGCCAATATCACGtacactcat	5'	Yes	gp1a	99	39.40	191.53	224.78	229.25	77.63	140.63	98.18	middle
ptc-miR171a-3p_2ss9TC21GT	TTGAGCCGCGCCAATATCACT	TRINITY_DN33802_c0_g1	-	tgaTTGAGCCGCGCCAATATCCCTtgcgccagtaccggattcc	5'	Diff	gp1a	16	58.10	254.17	177.77	168.81	116.45	191.68	124.81	middle
ptc-MIR171i-p5_2ss4CG20TC	TGTGTTTCTTCTTCTTCTTCT	TRINITY_DN27772_c2_g4	-	TGTGTTTCTTCTTCTTCTTCTcctctgcttcaggcggccgatctggtggttgcaaaggggaccgcggctgtggtcgctgctgctgtcgtccgtcgctgctgtggagggcgatgggtcgactgttgcccggtggctgaggga	5'	New	gp1a	120	62.40	1.20	1.37	2.69	2.28	1.93	0	low
ptc-miR172a_L-3	ATCTTGATGATGCTGCAT	TRINITY_DN26957_c0_g2	+	aATCTTGATGATTCTGCATTcacct	5'	Diff	gp1a	16	37.50	3.61	8.24	0.90	0	0	0	low
ptc-miR172a_R-1	AGAATCTTGATGATGCTGCA	TRINITY_DN4804_c0_g1	-	AGAATCCTGATGATGCTGCAgcggaggacgacgacggcgacgacgacgaggaggagaagaagggcaatggcagcgtgtgaggagga	5'	Diff	gp1a	79	60.50	14.46	21.96	16.12	3.42	0.48	0.55	middle
ptc-miR172g-3p_1ss1GA	AGAATCTTGATGATGCTGCAG	TRINITY_DN33573_c0_g1	-	attgcattcgcaaggaagatgaacggctactttatagacgggttgagagggtctcgtgagacagtagaacccatttttctgcagccagttttgtggggtcctaatctgctgttAGAATCCTGATGATGCTGCAGca	3'	Diff	gp1a	133	47.10	1.81	0.34	1.34	0	1.44	0	low

ptc-miR172g-3p_R+1_1ss2GT	GTAATCTTGATGATGCTGCAGT	CM000344.2	+	ctgtttgccgatGGAGCACCATCAAGATTCACAaactttattagggctaataagtggtgatgatggtggcttttggtggtcccttcgtttcaacccaatagccatttgaattgGGAATCTTGATGATGCTGCAGcggcaataa	3'	Diff	gp1a	136	44.10	0	0.38	0.11	0	0	0	low
ptc-miR172g-3p_R+1_1ss2GT	GTAATCTTGATGATGCTGCAGT	CM000346.2	-	ctgtttgcctatGGAGCATCATCAAGATTCACAagctttattagggctagtgtgtggtgatgatggtggcttttggtggtcccttttttcaatccaatagcccttgaattgGGAATCTTGATGATGCTGCAGcggcaataa	3'	Diff	gp1a	136	43.40	0	0.38	0.11	0	0	0	low
ptc-miR172a_R-1	AGAATCTTGATGATGCTGCA	TRINITY_DN33060_c0_g1	-	gaggcagttatcgttgaagAGAATCCTGATGATGCTGCAac	3'	Diff	gp1a	35	46.30	14.46	21.96	16.12	3.42	0.48	0.55	middle
ptc-miR172a	AGAATCTTGATGATGCTGCAT	CM000345.2	-	ttgtttgcgggtGGAGCATCATCAAGATTCACAtgcaaatgcacggccggtgatgttaagagttaaatctttctttgtttctgttcatctgccaaagttctttggaagtgAGAATCTTGATGATGCTGCATcggcaataa	3'	Yes	gp1a	133	41.40	2041.82	2104.24	1808.21	124.44	188.15	132.02	middle
ptc-miR319a_R+2_1ss20CT	TTGGACTGAAGGGAGCTCCTTT	TRINITY_DN26601_c1_g1	+	AGTGAATGATGCGGGAGACaaattgaatcctaagcttcctgtacTTGGACTGAAGGGAGCTCCCTTtccttctatactatgtatctt	3'	Diff	gp1a	17	42.50	404.15	131.44	98.51	81.06	114.14	72.67	middle
ptc-MIR319a-p5	AGAGCTTTCTTCAGTCCACTC	TRINITY_DN26601_c1_g1	+	taatagctaagAGAGCTTTCTTCAGTCCACTCatgggtggtagtaggatttaattagctgccgactcattcatccaaatactgagttaaaggacaaggagattacccagtaaatgagtgaatgatgcgggagacaaattgaatcctaagcttcctgtacTTGGACTGAAGGGAGCTCCCtttcctt	5'	New	gp1a	181	42.50	3.01	2.06	1.79	10.27	30.82	2.22	middle
ptc-miR319a	TTGGACTGAAGGGAGCTCCC	TRINITY_DN26601_c1_g1	+	taatagctaagAGAGCTTTCTTCAGTCCACTCatgggtggtagtaggatttaattagctgccgactcattcatccaaatactgagttaaaggacaaggagattacccagtaaatgagtgaatgatgcgggagacaaattgaatcctaagcttcctgtacTTGGACTGAAGGGAGCTCCCtttcctt	3'	Yes	gp1a	181	42.50	7939.02	2987.69	1651.49	3209.14	3498.31	1316.90	middle
ptc-MIR319f-p5	AGAGCTTCCTTCAGCCCACTC	TRINITY_DN26601_c0_g1	+	taatggtgggagAGAGCTTCCTTCAGCCCACTCatggatagga gaaagggattgaattagctgccgactcattcattcaagcactagtagaaaaaagggggaatggatattcttttcctactgtgattgtgtgaatgatgcgggagatagtcttacatcccctctttttctgtgcTTGGACTGAAGGGAGCTCCTTccttctat	5'	New	gp1a	201	44.30	245.14	163.35	86.87	99.32	142.56	90.97	middle
ptc-miR319e_R+1	TTGGACTGAAGGGAGCTCCTT	TRINITY_DN26601_c0_g1	+	taatggtgggagAGAGCTTCCTTCAGCCCACTCatggataggagaaagggattgaattagctgccgactcattcattcaagcactagtagaaaaaagggggaatggatattcttttcctactgtgattgtgtgaatgatgcgggagatagtcttacatcccctctttttctgtgcTTGGACTG	3'	Diff	gp1a	201	44.30	6893.42	2714.52	1758.95	1251.81	1702.84	1222.59	middle

				AAGGGAGCTCCTTccttctat													
ptc-miR319e_R+2	TTGGACTGAAGGGAGCTCCTTT	TRINITY_DN26601_c0_g1	+	TGTGAATGATGCGGGAGATAGTcttacatcccctctttttctgtgcTTGGACTGAAGGGAGCTCCTTCcttctatt	3'	Diff	gp1a	23	46.10	404.15	131.44	98.51	81.06	114.14	72.67	middle
ptc-miR390a	AAGCTCAGGAGGGATAGCGCC	TRINITY_DN20472_c1_g2	+	agaatctgttAAGCTCAGGAGGGATAGCGCCatgagcatgacaaagtctatgtttgagttaatctcaacaaaatcaatccagtcatcagtggCGCTATCTATCCTGAGTTCTatgggtt	5'	Yes	gp1a	117	42.00	19719.56	10174.49	11947.59	3748.57	6710.37	5920.49	high
ptc-MIR390a-p3	CGCTATCTATCCTGAGTTCT	TRINITY_DN20472_c1_g2	+	agaatctgttAAGCTCAGGAGGGATAGCGCCatgagcatgacaaagtctatgtttgagttaatctcaacaaaatcaatccagtcatcagtggCGCTATCTATCCTGAGTTCTatgggtt	3'	New	gp1a	117	42.00	49.39	28.83	23.28	21.69	16.37	8.88	middle
ptc-miR390a	AAGCTCAGGAGGGATAGCGCC	TRINITY_DN20472_c1_g1	-	agaatctgttAAGCTCAGGAGGGATAGCGCCccaaggtaatcatgggctctttttatgtggtcttcgattctcagtggCGCTATCCATCCTGAGTTTCAttgctt	5'	Yes	gp1a	87	46.70	19719.56	10174.49	11947.59	3748.57	6710.37	5920.49	high
ptc-miR390d-3p	CGCTATCCATCCTGAGTTTA	TRINITY_DN20472_c1_g1	-	agaatctgttAAGCTCAGGAGGGATAGCGCCccaaggtaatcatgggctctttttatgtggtcttcgattctcagtggCGCTATCCATCCTGAGTTTCAttgctt	3'	Yes	gp1a	87	46.70	202.38	234.73	242.69	103.89	88.62	84.32	middle
ptc-miR390d-3p_R+2	CGCTATCCATCCTGAGTTTACC	CM000352.2	+	agaatctgttAAGCTCAGGAGGGATAGCGCCctaaggataaccatgggctctttttatttggtttttgactatcagtggCGCTATCCATCCTGAGTTTTActggtt	3'	Diff	gp1a	104	43.40	0	4.35	4.66	3.42	8.67	0	low
ptc-miR393a-5p_R+1_1	TCCAAAGGGATCGCATTGATCC	TRINITY_DN22551_c0_g1	+	atattagtgcagctggagtgtTCCAAAGGGATCGCATTGATCTaatgactctcgatgcctaaatcatattaatatgtttagttatttcgttgGATCATGCGATCTCTTAGGAATtttccagcatacagctctaaatt	5'	Diff	gp1a	131	36.50	125.28	481.82	197.91	318.52	324.61	316.19	middle
ptc-miR393a-5p_R+1_2	TCCAAAGGGATCGCATTGATCT	TRINITY_DN22551_c0_g1	+	gctggagtgtTCCAAAGGGATCGCATTGATCTaatgactctcgatgcctaaatcatattaatatgtttagttatttcgttggATCATGCGATCTCTTAGGAATtttcca	5'	Diff	gp1a	107	37.60	125.28	481.82	197.91	318.52	324.61	316.19	middle
ptc-MIR393c-p3_1ss12CT	ATCATGCGATCTCTTAGGAAT	TRINITY_DN22551_c0_g1	+	gctggagtgtTCCAAAGGGATCGCATTGATCTaatgactctcgatgcctaaatcatattaatatgtttagttatttcgttggATCATGCGATCTCTTAGGAATtttcca	3'	New	gp1a	107	37.60	74.69	175.71	60.00	94.76	87.65	110.94	middle
ptc-miR393a-3p_1ss10TC	ATCATGCTACCCCTTTGGATT	TRINITY_DN19880_c0_g1	+	ttggATCATGCTATCCCTTTGGATTtctcctttggtggcttctataacattaatgggttttagtagcggtgatgc	5'	Diff	gp1a	126	45.50	545.09	739.54	514.48	1189.59	976.72	1027.89	middle

				tccacgcactcgcccttctcatgcatcatcaacaccaacatgtttagtatggctcgccgagaaaaccaac												
ptc-miR394a-5p_L+1	CTTGGCATTCTGTCCACCTCC	TRINITY_DN24223_c0_g1	+	ctttcatgtgggtttagcaaagggtttcttacagagtttATTGGCATTCTGTCCACCTCCaatctgcagaaactacaagttgttttactttctggaggtgggcatactgccaactgagctctgttggtctcTCTTTGTAAAACCCTCGTGATta	5'	Diff	gp1a	148	42.90	1010.67	812.64	945.22	325.94	403.11	371.11	middle
ptc-miR394a-5p	TTGGCATTCTGTCCACCTCC	TRINITY_DN24223_c0_g1	+	tcatgtgggtttagcaaagggtttcttacagagtttaTTGGCATTCTGTCCACCTCCaatctgcagaaactacaagttgttttactttctggaggtgggcatactgccaactgAGCTCTGTTGGTCTCTCTTTGtaaaaccctcgtga	5'	Yes	gp1a	148	43.90	161877.51	124677.26	124118.29	40571.53	43760.70	41449.51	high
ptc-MIR394a-p3	AGCTCTGTTGGTCTCTCTTTG	TRINITY_DN24223_c0_g1	+	tcatgtgggtttagcaaagggtttcttacagagtttaTTGGCATTCTGTCCACCTCCaatctgcagaaactacaagttgttttactttctggaggtgggcatactgccaactgAGCTCTGTTGGTCTCTCTTTGtaaaaccctcgtga	3'	New	gp1a	148	43.90	114.44	137.27	202.39	108.46	158.93	146.45	middle
ptc-miR394a-5p_R+1	TTGGCATTCTGTCCACCTCCT	TRINITY_DN32467_c0_g1	-	tccatagaaatctaagcaccatatgtTTGGCATTCTGTCAACCTCCTtcc	3'	Diff	gp1a	35	42.00	214.42	100.21	175.97	70.21	66.94	63.79	middle
ptc-miR395b	CTGAAGTGTTTGGGGGAACTC	TRINITY_DN10732_c0_g1	+	cttcctcaCTGAAGTGTTTGGGGGAACTCtgggaggcatttgacactgtttagcagtcggaaactgggtgatgaagtccaatcattgttgtgttgcatcttttttacgtatttttcatctcgatttgccacacttcttctttggatct	5'	Yes	gp1a	143	42.60	310.79	288.27	247.16	2421.41	799.48	2224.41	middle
ptc-miR395a	CTGAAGGGTTTGGAGGAACTC	CM000338.2	+	ggtcaccctgagttcctcctagcttcttcagtacccgtggaaagctaatgctacaattatcttgagctggtaagactggcaggtgtcagatgtggatgtgttaaaggtaatattattgattatttacggcgccattcaagtatttgttctactgttagaggatttaatcaagtatatgtggtattactcctgatttgCTGAAGGGTTTGGAGGAACTCtaggtgct	3'	Yes	gp1a	226	41.20	4.82	0.34	2.76	3.71	0.48	1.11	low
ptc-MIR395a-p5_1ss21CT	TCACCCTGAGTTCCTCCTAGT	TRINITY_DN22579_c0_g1	+	tttgtttggggTCACCCTGAGTTCCTCCTAGCttcttcagtgcccgtggaaagctaatgctacaattatcttgagctggtaagaccggcaggtatcagatttggatgtgtTAAAGGGTTTGGAGGAACTCtaggtgctgccaaacttg	5'	New	gp1a	142	47.30	28.91	9.61	25.97	62.79	12.52	26.63	middle
ptc-miR395a_L-1	TGAAGGGTTTGGAGGAACTC	TRINITY_DN22579_c0_g1	+	tttgtttggggTCACCCTGAGTTCCTCCTAGCttcttcagtgcccgtggaaagctaatgctacaattatcttgagctggtaagaccggcaggtatcagatttggatgtgtTAAAGGGTTTGGAGGAACTCtaggtgctgccaaacttg	3'	Diff	gp1a	142	47.30	39.75	2.06	6.27	5.71	1.93	1.11	middle

ptc-miR396c	TTCCACAGCTTTCTTGAACTT	TRINITY_DN26867_c0_g2	+	atgtgaccctctttgtattcTTCCACAGCTTTCTTGAACTGcacctattagatttatgttgatgttgttgtgcgatatgccatgaccatatgacattgtattcatttttgctgcgGTTCAATAAAGCTGTGGGAAGatacaaacaggatcaaag	5'	Yes	gp1a	148	38.30	21127.16	34552.69	44189.48	45113.54	43200.09	44275.79	high
ptc-miR396f_L+3_1ss24GT	AATTTCCACGGCTTTCTTGAACTT	CM000342.2	-	cttggtgattTTCCACGGCTTTCTTGAACTGtatatattatcaatggctttttacaagactggaagatggtttccatggagaagaattgtcacaaaaacagttcaagaaagccctgaaaaattattt	5'	Diff	gp1a	121	34.60	0	0.23	0	0	1.93	0	low
ptc-miR396e-3p_L-1	TCAAGAAAGCTGTGGGAGA	TRINITY_DN26867_c0_g2	-	ctttgatcctgtttgtatcttCCCACAGCTTTATTGAACCgcagcaaaaatgaatacaatgtcatatggtcatggcatatcgcacaacaacatcaacataaatctaataggtgcagtTCAAGAAAGCTGTGGAAGAatacaaagagggtcacat	3'	Diff	gp1a	148	38.30	0.60	2.75	4.03	3.42	1.44	3.33	low
ptc-miR396a	TTCCACAGCTTTCTTGAACTG	TRINITY_DN26867_c0_g2	+	ctttgtattcTTCCACAGCTTTCTTGAACTGcacctattagatttatgttgatgttgttgtgcgatatgccatgaccatatgacattgtattcatttttgctgcgGTTCAATAAAGCTGTGGGAAGatacaa	5'	Yes	gp1a	130	37.10	4577.54	4035.75	8639.09	11281.67	9120.87	13113.49	high
ptc-MIR396b-p3	GTTCAATAAAGCTGTGGGAAG	TRINITY_DN26867_c0_g2	+	ctttgtattcTTCCACAGCTTTCTTGAACTGcacctattagatttatgttgatgttgttgtgcgatatgccatgaccatatgacattgtattcatttttgctgcgGTTCAATAAAGCTGTGGGAAGatacaa	3'	New	gp1a	130	37.10	9.64	28.83	23.28	160.97	86.69	164.20	middle
ptc-miR396e-3p_R+2	CTCAAGAAAGCTGTGGGAGAGA			ggtcatgcttTTCCACAGCTTTCTTGAACTTctttgccttgcttaatctgtgtatatatagatcactacatgtacagctcctatatatataaatatatgtatgtatagcgccatggaagCTCAAGAAAGCTGTGGGAGAacatgg	3'	Diff	gp1a	141	37.90	0.15	0	0.11	0.08	0	0.09	low
ptc-miR396f_L+1R-3	TTTCCACGGCTTTCTTGAA	TRINITY_DN27630_c0_g2	-	tatagtagtggttgatttctcaaTTTCCACTGCTTTCTTGAA	3'	Diff	gp1a	31	33.30	6.02	7.55	10.75	8.56	6.26	6.10	middle
ptc-miR396f_L+1R-3	TTTCCACGGCTTTCTTGAA	TRINITY_DN27082_c1_g3	-	tattttccaaagaccaaaaaagattcgagatttagaaaattttcaatatttctatttcctttcaaTCTCCACGGCTTTCTTGAAacca	3'	Diff	gp1a	83	29.20	6.02	7.55	10.75	8.56	6.26	6.10	middle
ptc-miR396c	TTCCACAGCTTTCTTGAACTT	TRINITY_DN26735_c0_g1	+	tgcaaatcctggtcatgcttTTCCACAGCTTTCTTGAACTTctttgccttgcttaatctgtgtatctatagatcactacatgtatagctcctatatatatatagcgccatggaagCTCAAGAAAGCTGTGGGAGAacatggcaattcaggatttttt	5'	Yes	gp1a	151	38.90	21127.16	34552.69	44189.48	45113.54	43200.09	44275.79	high
ptc-miR396g-3p_R-1_1ss19AG	CTCAAGAAAGCCGTGGGAGA	TRINITY_DN26735_c0_g1	+	tgcaaatcctggtcatgcttTTCCACAGCTTTCTTGAACTTctttgccttgcttaatctgtgtatctatagatcactacatgtatagctcctatatatatatagcgccatggaagCTCAAGAAAGCT	3'	Diff	gp1a	151	38.90	566.77	1087.87	989.55	1763.26	1357.68	1501.06	middle

				GTGGGAGAacatggcaattcaggatttttt												
ptc-miR396c_L-3	CACAGCTTTCTTGAACTT	TRINITY_DN26735_c0_g1	-	tgccatgttctccCACAGCTTTCTTGAGCTTccatggcgctatatatatataggagctatacatgtagtgatctatagatacacagattaagcaaggcaaagaaGTTCAAGAAAGCTGTGGAaaagcatgacc	5'	Diff	gp1a	127	40.60	39.75	42.90	59.55	64.50	60.68	60.46	middle
ptc-miR396c_R+2	TTCCACAGCTTTCTTGAACTTAC	CM000339.2	+	tgccatgtttTTCCACAGCTTTCTTGAACTTcctagagcctagaggtgctgctagctatacatataacttaagaagttcaagaaagccgtggaatagcatga	5'	Diff	gp1a	98	41.20	0	0	0	0.42	0.08	0	low
ptc-miR396g-3p_R-1_1ss13GA	CTCAAGAAAGCCATGGGAAA	CM000343.2	-	ttgcatgcttTTCCACGGCTTTCTTGAACTTggcactcaagagacatgagagtaaagggctaggctttcttttctattcctttcgttctttgaaattttcctgttcaaagaatccatacatatcagtctctttagtacaaagCTCAAGAAAGCCGTGGGAAAAtatga	3'	Diff	gp1a	164	38.70	156.60	289.64	252.54	318.52	332.32	319.52	middle
ptc-MIR396f-p5_1ss18AT	TTTCCACGGCTTTCTTGT	TRINITY_DN32082_c0_g10	+	TTTCCACTGCTTTCTTGTaagcatcc	5'	New	gp1a	16	42.30	0	0.34	0	1.14	3.37	0.55	low
ptc-MIR396f-p5_1ss18AT	TTTCCACGGCTTTCTTGT	TRINITY_DN32082_c0_g6	-	TTTCCACTGCTTTCTTGTaagcatcc	5'	New	gp1a	16	42.30	0	0.34	0	1.14	3.37	0.55	low
ptc-miR397a	TCATTGAGTGCAGCGTTGATG	TRINITY_DN27322_c1_g2	+	tggagaaccaTCATTGAGTGCAGCGTTGATGaaatcctacatttgtgttattaaactgttaccagccctttatggggcatggcatcatTTCACCAGCGCTGCATTCAATcatgttttc	5'	Yes	gp1a	118	41.70	16.86	21.28	21.49	5.71	4.82	1.11	middle
ptc-MIR397a-p3	TTCACCAGCGCTGCATTCAAT	TRINITY_DN27322_c1_g2	+	tggagaaccaTCATTGAGTGCAGCGTTGATGaaatcctacatttgtgttattaaactgttaccagccctttatggggcatggcatcatTTCACCAGCGCTGCATTCAATcatgttttc	3'	New	gp1a	118	41.70	0	5.49	1.79	0	0	4.44	low
ptc-miR398c-5p_L-2R+5	AGCGACCTGAAATCACATGTGGGC	TRINITY_DN31090_c0_g4	+	aggattcctacaggAGCGACCTGAAATCACATGTGGGCcgcaccccctgggttatcttgggcaacaTGTGTTCTCAGGTCGCCCCTGccgggcttt	5'	Diff	gp1a	94	58.80	6.02	19.22	10.75	5.71	2.89	2.22	middle
ptc-miR398b	TGTGTTCTCAGGTCGCCCCTG	TRINITY_DN31090_c0_g4	+	aggattcctacaggAGCGACCTGAAATCACATGTGGGCcgcaccccctgggttatcttgggcaacaTGTGTTCTCAGGTCGCCCCTGccgggcttt	3'	Yes	gp1a	94	58.80	187.32	258.75	184.03	35.39	28.90	33.28	middle
ptc-MIR398a-p5_2ss10AT21GT	AGTGGCTCCTGAGAACACAGT	TRINITY_DN14542_c0_g1	+	gtacaccccagagAGTGGCTCCTGAGAACACAGGgggttggttttctagctgcaagctacaagatggacaaagcacccTGTGTTCTCAGGTCACCCCTTtggggcacc	5'	New	gp1a	99	55.60	0	0.69	0	0	0	0	low

ptc-miR398a_1ss8TC	TGTGTTCCCAGGTCACCCCTT	TRINITY_DN14542_c0_g1	+	gtacaccccagagAGTGGCTCCTGAGAACACAGGgggttggttttctagctgcaagctacaagatggacaaagcacccTGTGTTCTCAGGTCACCCCTTggggcacc	3'	Diff	gp1a	99	55.60	88.54	99.52	63.13	92.47	81.87	80.99	middle
ptc-miR398a_L+2_1ss10TC	TCTGTGTTCCCAGGTCACCCCTT	CM000346.2	+	gtacaccccagaggagtggctccagagaacacagggggttggttttctagctgtaagctacaagatggacaaagcactcTGTGTTCTCAGGTCACCCCTTggggcacc	3'	Diff	gp1a	100	54.10	0	0.69	0	0	0	0	low
ptc-miR399i_1ss1TC	CGCCAAAGGAGAGTTGCCCTA	CM000348.2	-	aaaccagttgtagggtacctcttacttggcaagcaatggtgattgttgcaaacattgcagaatttgatctcggtgtctTGCCAAAGGAGAGTTGCCCTAtgactgttt	3'	Diff	gp1a	101	43.50	0	0	0	0.16	0.09	0	low
ptc-miR399i_R-1	TGCCAAAGGAGAGTTGCCCT	TRINITY_DN29886_c0_g3	-	aaagggataacgggaagccaactaaggacttcctaaTGCCAAAGGAGATTTGCCCTa	3'	Diff	gp1a	13	45.60	0.60	0.69	0.45	1.14	2.89	1.66	low
ptc-miR399e_1ss2GA	CACCAAAGGAGAGTTGCCCTC	CM000339.2	-	aactagttaaatggcttctcttctttggcaggtgatggtatagattacaacttatagctttaatctagtattcagttaccCGCCAAAGGAGAGTTGCCCTCtaacttcc	3'	Diff	gp1a	102	39.40	86.73	118.80	46.12	63.17	432.90	212.27	middle
ptc-miR399a_1ss21GC	TGCCAAAGGAGATTTGCCCCC	CM000340.2	-	gcaataattaccgggcaattactcttttggcagctggccactcaacggcggcaggtgatcaaattcaccaatgcaaacaagtaagctcgcTGCCAAAGGAGATTTGCCCCGcaattcatc	3'	Diff	gp1a	97	49.20	0	0.05	0	0	0	0.25	low
ptc-miR399d	TGCCAAAGAAGATTTGCCCCG	CM000340.2	-	ggatgcattactgggcaattcttctgttggcagctggtgactcatactgcactagctgagattaagcagcaagagtttgctcacTGCCAAAGAAGATTTGCCCCGcaattcctt	3'	Yes	gp1a	112	47.40	0	5.49	2.24	1.52	4.82	6.10	low
ptc-miR399a_R-2_1ss13TG	TGCCAAAGGAGAGTTGCCC	TRINITY_DN29886_c0_g3	-	gttgcagatcggagtgaagctcaacagagagaaaagaatttaacgccaaagaagatctgccctttatagggagggaacttcagtaacttTGCCAAAGGAGATTTGCCCtaaagaacttactcttaacgtctaattattactatcaactcttaaaaaggaatttatatcgccaaagaagatttgccctgc	3'	Diff	gp1a	38	38.60	2.41	0.69	0.45	13.70	21.67	17.20	middle
ptc-miR399f_L+2R+1	TTTGCCAAAGGAGAATTGCCCTGT	CM000340.2	+	taaagaataacagggctttatcctcctttggcaaacagaacatggaaataaatgcctgcatatttctgtttTGCCAAAGGAGAATTGCCCTGccattcgat	3'	Diff	gp1a	94	39.60	0	0	0	0	0.28	0	low
ptc-miR399f	TGCCAAAGGAGAATTGCCCTG	CM000340.2	+	tgatgaataactgtgcaattctcctttggcagacgtatatggcgttcggacaccaacttggacTGCCAAAGGAGAATTGCCCTGccattcaac	3'	Yes	gp1a	89	46.80	73.18	79.67	72.09	457.51	890.91	1543.87	middle
ptc-miR399b	TGCCAAAGGAGATTTGCCCGG	TRINITY_DN29886_c0_g1	-	tgccaaaggagatttgcccaaagagcaatctgaagctaaaggtagTGCCAAAGGAGATTTGCCCGGaagcaatctaagctaaatgccaaaggagatttgcccgaatagacagactatagataggtattTTGCCAAAGGAGATTTGCCCAg	5'	Yes	gp1a	52	44.00	12.65	29.17	8.96	59.37	91.51	139.23	middle

ptc-miR399i_L+1R-1	TTGCCAAAGGAGAGTTGCCCT	TRINITY_DN29886_c0_g1	-	tgccaaaggagatttgcccaaagagcaatctgaagctaaaggtagTGCCAAAGGAGATTTGCCCGGaagcaatctaagctaaatgccaaaggagatttgcccgaatagacagactatagataggtattTTGCCAAAGGAGATTTGCCCAg	3'	Diff	gp1a	52	44.00	0	0.34	0	7.99	3.37	2.77	low
ptc-MIR403c-p5	CGTGGATCTGAGGCCATC	TRINITY_DN28658_c0_g3	+	aaaagaggactcatgtctctagtttgttCGTGGATCTGACGCCATCacaaccgttcatttcacgaccatccaatggcgTTAGATTCACGCACAAACTCGcgatctgtctcctctaaa	5'	New	gp1a	111	46.20	0	0	0	0	0.96	0	low
ptc-miR403a_1ss20CT	TTAGATTCACGCACAAACTTG	TRINITY_DN28658_c0_g3	+	aaaagaggactcatgtctctagtttgttCGTGGATCTGACGCCATCacaaccgttcatttcacgaccatccaatggcgTTAGATTCACGCACAAACTCGcgatctgtctcctctaaa	3'	Diff	gp1a	111	46.20	27.71	43.24	39.85	34.82	67.91	50.48	middle
ptc-MIR403b-p5	TTTGTTCGTGGATCTGACGCC	TRINITY_DN28658_c0_g3	+	actcatgtctctagTTTGTTCGTGGATCTGACGCCatcacaaccgttcatttcacgaccatccaatggcgTTAGATTCACGCACAAACTCGcgatctgtc	5'	New	gp1a	95	48.00	391.50	628.01	667.16	799.15	710.87	697.83	middle
ptc-miR403a	TTAGATTCACGCACAAACTCG	TRINITY_DN28658_c0_g3	+	actcatgtctctagTTTGTTCGTGGATCTGACGCCatcacaaccgttcatttcacgaccatccaatggcgTTAGATTCACGCACAAACTCGcgatctgtc	3'	Yes	gp1a	95	48.00	307.78	344.89	323.73	293.97	334.24	275.69	middle
ptc-MIR403a-p5	GGTTTGTGCATGAATCTAATA	TRINITY_DN21191_c0_g1	+	aggcatatttcaGGTTTGTGCATGAATCTAATAtagctaaaaaaatcccatcaaacacaaattaaaatactagatagcactcagcactagtttagtatagtaTTAGATTCACGCACAAACTCGtaatctgtc	5'	New	gp1a	131	32.60	2.41	1.37	2.69	15.98	3.85	6.66	middle
ptc-miR403a	TTAGATTCACGCACAAACTCG	TRINITY_DN21191_c0_g1	+	aggcatatttcaGGTTTGTGCATGAATCTAATAtagctaaaaaaatcccatcaaacacaaattaaaatactagatagcactcagcactagtttagtatagtaTTAGATTCACGCACAAACTCGtaatctgtc	3'	Yes	gp1a	131	32.60	307.78	344.89	323.73	293.97	334.24	275.69	middle
ptc-miR403c-5p	TTTGTGCGTGGATCTGAGGCC	CM000344.2	+	ggTTTGTGCGTGGATCTGAGGCCatcacaaccgtccactacacgaccacccaatggctTTAGATTCACGCACAAACTCG	5'	Yes	gp1a	77	53.20	1970.75	2675.40	2596.11	4737.80	4403.91	5081.20	middle
ptc-miR403a_2ss20CT21GT	TTAGATTCACGCACAAACTTT	CM000344.2	+	ggTTTGTGCGTGGATCTGAGGCCatcacaaccgtccactacacgaccacccaatggctTTAGATTCACGCACAAACTCG	3'	Diff	gp1a	77	53.20	2.41	3.43	2.69	4.57	0.96	7.77	low
ptc-miR403a_1ss20CT	TTAGATTCACGCACAAACTTG	TRINITY_DN21191_c0_g1	+	tttaaaggaaagaggcatatttcaggtttgtgcatgaatctaatatagctaaaaaaatcccatcaaacacaaattaaaatactagatagcactcagcactagtttagtatagtaTTAGATTCACGCACAAACTCGtaatctgtcttttccaattcctttctt	3'	Diff	gp1a	156	31.50	27.71	43.24	39.85	34.82	67.91	50.48	middle
hbr-miR408a_R-1_1ss6TG	AAGACGGGGAACAGGCAG	TRINITY_DN28530_c1_g2	+	agaaagagacacatgAAGACGGGGAACAGGCAGAGCatggatggagctactaacagaagtacctgtttt	5'	Diff	gp1a	119	50.40	114.44	188.75	106.57	17.12	13.49	19.97	middle

	AGC			ggccctacccATGCACTGCCTCTTCCCTGGCttgtggctcttctttttct												
ptc-miR408-3p	ATGCACTGCCTCTTCCCTGGC	TRINITY_DN28530_c1_g2	+	agaaagagacacatgAAGACGGGGAACAGGCAGAGCatggatggagctactaacagaagtacctgttttggccctacccATGCACTGCCTCTTCCCTGGCttgtggctcttctttttct	3'	Yes	gp1a	119	50.40	4943.74	9683.06	7114.91	506.89	676.19	500.35	high
ptc-MIR472b-p5_1ss1CT	TTGGATGGGTGAGTGGGGAAG	TRINITY_DN26571_c0_g3	+	aatggagagggagtagggtgttcggaaggttaTTGGATGGGTGAGTGGGGAAGataaccaagctgttgtgcttgttattttcccaactccacccatcccataggTTTCCGATCATTCCTCCCTCTcctcat	5'	New	gp1a	124	48.90	2227.34	1435.85	1465.07	1903.11	2697.06	2251.04	middle
ptc-miR472a_L+2	TCTTTTCCCTACTCCACCCATCCC	TRINITY_DN34679_c2_g1	+	cctgaggagtggtgaccaagcagcgaatgggagatgtgcataggtttgtgagatgctacttgtTATTTTCCCTACTCCACCCATCCCataggtttccgatcattcctccct	3'	Diff	gp1a	105	49.50	133.71	111.19	125.37	146.13	129.07	142.01	middle
ptc-MIR472b-p5	TGGGTGAGTGGGGAAGATAAC	TRINITY_DN26571_c0_g3	+	tggaTGGGTGAGTGGGGAAGATAACcaagctgttgtgcttgttaTTTTCCCAACTCCACCCATCCCatag	5'	New	gp1a	64	48.60	887.20	641.74	751.79	867.64	1017.18	914.17	middle
ptc-miR472b	TTTTCCCAACTCCACCCATCCC	TRINITY_DN26571_c0_g3	+	tggaTGGGTGAGTGGGGAAGATAACcaagctgttgtgcttgttaTTTTCCCAACTCCACCCATCCCatag	3'	Yes	gp1a	64	48.60	795846.44	763827.01	715547.56	906874.45	923624.14	892089.76	high
ptc-miR472a_L+2R+1_1ss24CA	TATTTTCCCTACTCCACCCATCCAT	CM000341.2	+	ttatcgggtgggtgagcggggaagataactttggtttttgagatagtacttgttaTTTTCCCTACTCCACCCATCCCatag	3'	Diff	gp1a	79	44.40	30.12	16.82	24.63	13.70	26.97	16.64	middle
ptc-MIR472a-p5_1ss2CT	TTGGGTGGGTGAGCGGGGAAGA	TRINITY_DN26571_c0_g1	+	ttaTTGGGTGGGTGAGCGGGGAAGAtaactttaatttcacttgtgagatgctacttgtcaTTTTCCCTACTCCACCCATCCCatag	5'	New	gp1a	84	44.20	1820.78	1235.43	1486.12	2325.52	2910.89	2264.35	middle
ptc-miR472a	TTTTCCCTACTCCACCCATCCC	TRINITY_DN26571_c0_g1	+	ttaTTGGGTGGGTGAGCGGGGAAGAtaactttaatttcacttgtgagatgctacttgtcaTTTTCCCTACTCCACCCATCCCatag	3'	Yes	gp1a	84	44.20	1467594.54	1113420.53	1045855.95	1009170.93	1040794.85	958784.41	high
ptc-MIR475d-p5_2	TTACAGAGTCCATTGATT	TRINITY_DN34728_c1_g2	-	acccactTTACAGAGCCCATTGATTAAGgtgtta	5'	New	gp1a	28	41.20	32.52	36.38	35.82	51.37	67.43	57.69	middle
ptc-miR475d-3p	TTACAGAGTCCATTGATTAAG	TRINITY_DN34728_c1_g2	-	acccactTTACAGAGCCCATTGATTAAGgtgtta	3'	Yes	gp1a	28	41.20	32.52	36.38	35.82	51.37	67.43	57.69	middle
ptc-MIR475d-p5_1	TCCTGATCAATGGCCATTGTA	TRINITY_DN31868_c0_g1	+	ataacaTCTTGATCAATGGCCATTGTAagagtagaaggatccatgaagcaataactctctttgctaaaatgtgtgataaaacgttgactcctggtatttcacttcttacagtgcccattgattaagatggta	5'	New	gp1a	124	36.60	32.52	36.38	35.82	51.37	67.43	57.69	middle
ptc-MIR475d-p5_3	TTACAGAGTCCATTGATTAAG	TRINITY_DN28499_c0_g2	-	ccactTTACAGAGCCCATTGATTAAGgtggtaa	5'	New	gp1a	30	42.40	32.52	36.38	35.82	51.37	67.43	57.69	middle
ptc-miR475d-3p_L-3	CAGAGTCCATTGAT	TRINITY_DN28499_	-	ccactTTACAGAGCCCATTGATTAAGgtggtaa	3'	Diff	gp1a	30	42.40	0	1.37	3.58	0	0	0	low

	TAAG	c0_g2														
ptc-miR475a-5p	AATGGCCATTGTAAGAGTAGA	TRINITY_DN31868_c0_g1	+	gtcataacatcttgatcAATGGCCATTGTAAGAGTAGAaggatccatgaagcaataactctctttgctaaaatgtgtgataaaacgttgactcctggtatttcactcTTACAGTGCCCATTGATTAAGatggtataa	5'	Yes	gp1a	132	36.50	1208.23	1355.89	1020.00	1002.36	1257.98	1193.75	middle
ptc-miR475a-3p	TTACAGTGCCCATTGATTAAG	TRINITY_DN31868_c0_g1	+	gtcataacatcttgatcAATGGCCATTGTAAGAGTAGAaggatccatgaagcaataactctctttgctaaaatgtgtgataaaacgttgactcctggtatttcactcTTACAGTGCCCATTGATTAAGatggtataa	3'	Yes	gp1a	132	36.50	2508.01	4669.25	2587.16	3576.75	3961.30	3908.53	middle
ptc-MIR475c-p5	TCTTGAACAATGGCCATTGTA	TRINITY_DN21427_c0_g1	+	taacaTCTTGAACAATGGCCATTGTAagagtaagtggtccatgagtcaataactctctttgcagaaatgcatgacaaagcattgactcctatttcacccTTACAATGTCCATTGATTAAGa	5'	New	gp1a	116	37.20	44.57	50.45	33.13	27.40	43.35	34.39	middle
ptc-miR475c	TTACAATGTCCATTGATTAAG	TRINITY_DN21427_c0_g1	+	taacaTCTTGAACAATGGCCATTGTAagagtaagtggtccatgagtcaataactctctttgcagaaatgcatgacaaagcattgactcctatttcacccTTACAATGTCCATTGATTAAGa	3'	Yes	gp1a	116	37.20	7.23	27.45	20.60	19.41	24.56	11.09	middle
ptc-MIR476a-p3_1ss3GA	TTACAAAGATAGATTTACTAGC	TRINITY_DN24379_c1_g2	+	attaagctttatgattaagctagtaatcctttctttgcaaaatcattaattattttagctctaattaatataattaatggctTTACAAAGATAGATTTACAAGCtaaatcttaaaagcttctg	3'	New	gp1a	116	23.80	8.43	15.79	15.22	3.42	10.60	15.53	middle
ptc-miR476a	TAGTAATCCTTCTTTGCAAAG	TRINITY_DN24379_c1_g1	+	ttaagcTAGTAATCCTTCTTTGCAAAGtcatttattatttttctttaattaaTATAATCAATGACTTTGCAAAagatagatttactagctaaatctt	5'	Yes	gp1a	88	22.40	44.57	100.89	61.79	55.94	102.10	78.77	middle
ptc-MIR476a-p3_1ss19AT	TATAATCAATGACTTTGCTAA	TRINITY_DN24379_c1_g1	+	ttaagcTAGTAATCCTTCTTTGCAAAGtcatttattatttttctttaattaaTATAATCAATGACTTTGCAAAagatagatttactagctaaatctt	3'	New	gp1a	88	22.40	0	1.37	0.90	0	0	2.22	low
ptc-miR477d-5p_R-1	ATCTCCCTCAAAGGCTTCCTC	CM000351.2	+	atgctactacatggatggagaagcttccgATCTCCCTCAAAGGCTTCCTCTataattgccttacatgatggcattagTGGACTCCTTTGGGGAGATGGagagcttactccccataactcaatggcat	5'	Diff	gp1a	127	46.50	161.42	131.09	135.22	1052.59	1351.42	1761.78	middle
ptc-miR477e-5p_R+1	ACTCTCCCTCAAGGCTTCCAT	CM000350.2	+	attgttcACTCTCCCTCAAGGCTTCCAacgcaataaccagctacgtacatgtaactgtgttacggctgcaggctTGAGGCCTTTGGGGGAGAGTGGtcaa	5'	Diff	gp1a	99	51.00	38.55	15.79	19.70	67.36	130.04	113.16	middle
ptc-miR477e-3p_1ss22GA	TGAGGCCTTTGGGGGAGAGTGA	CM000350.2	+	attgttcACTCTCCCTCAAGGCTTCCAacgcaataaccagctacgtacatgtaactgtgttacggctgcaggctTGAGGCCTTTGGGGGAGAGTGGtcaa	3'	Diff	gp1a	99	51.00	0	0	0	2.28	8.67	4.44	low

ptc-miR477a-5p	ATCTCCCTCAGAGGCTTCCAA	CM000348.2	-	caATCTCCCTCAGAGGCTTCCAAtatctcagtttatagtaattgcttcaaataacgagaatgttGGATGCCTTTGGGGGAGATTG	5'	Yes	gp1a	85	41.20	33.73	33.63	49.25	323.08	92.47	125.37	middle
ptc-miR478f_L+1R-1_1ss3GA	TTAACATGTCTTCTATTTTTAGGG			aagtatcttatttttaggagccgatgtcattttttTGACATGTCTTCTATTTTTAGGGAccga	3'	Diff	gp1a	58	33.30	0	0	0	0	0	0.55	low
ptc-miR478p_L+1R-1_1ss6GA	TTAACATGTCTTCTATTTTTAGGG	CM000355.2	-	tctcctttttgggacaaacgtcaatattttagacgagtctcctaatttttagggactaatgtcattttTAACGTGTCTTCTATTTTTAGGGAcc	3'	Diff	gp1a	91	34.70	0	0	0	0	0	0.55	low
ptc-MIR481d-p5_2ss5AG19GA	AAAGGGCTTTCACTTGAGAGGGTG	TRINITY_DN29626_c0_g1	-	AAAAGGCTTTCACTTGAGAGGGTGtgttagagaataatataaatcatatcatgaaaccttacataacggcttaacctattgggttgagatgTTTCTTTGACATGGTATCAGAGCCttgatgatcaagcgattacgagttcgaatctcaccatcactatttatttg	5'	New	gp1a	14	36.40	31.92	16.13	18.36	17.12	12.04	6.10	middle
ptc-MIR481d-p3_3	ATTCTTTGACATGGTATCAGAGCC	TRINITY_DN29626_c0_g1	-	AAAAGGCTTTCACTTGAGAGGGTGtgttagagaataatataaatcatatcatgaaaccttacataacggcttaacctattgggttgagatgTTTCTTTGACATGGTATCAGAGCCttgatgatcaagcgattacgagttcgaatctcaccatcactatttatttg	3'	New	gp1a	14	36.40	186.11	116.68	144.18	60.51	57.79	62.68	middle
ptc-MIR481b-p5_1ss10TC	AAATCATATCCTGGAACCTCACCT	TRINITY_DN32021_c1_g1	-	aAAATCATATCCTAGAACCTCACCTaaaaagcttaagctattgggttgaaatggttccttgACATGGTATCAGAGCTTTGATGACtaagtgg	5'	New	gp1a	43	38.00	109.02	51.48	89.10	37.67	28.42	58.25	middle
ptc-MIR481c-p3_2ss6AG19GA	ACATGGTATCAGAGCTTTAATGAC	TRINITY_DN32021_c1_g1	-	aAAATCATATCCTAGAACCTCACCTaaaaagcttaagctattgggttgaaatggttccttgACATGGTATCAGAGCTTTGATGACtaagtgg	3'	New	gp1a	43	38.00	116.25	75.84	64.48	39.39	42.38	23.85	middle
ptc-MIR481b-p5_2ss13CT24AT	AACCTCACCTAATAGCTTAAGCTT	TRINITY_DN33283_c2_g1	-	aaactattcttagAACCTCACCTAATAGTTTAAGCTTTTGGGTTGAAATAGTTCTttaatatgggat	5'	New	gp1a	61	29.90	9.03	3.43	2.69	0.57	3.85	6.66	low
ptc-MIR481b-p3_2ss6AT19GA	AAGCTTTTGGGTTGAGATAGTTCT	TRINITY_DN33283_c2_g1	-	aaactattcttagAACCTCACCTAATAGTTTAAGCTTTTGGGTTGAAATAGTTCTttaatatgggat	3'	New	gp1a	61	29.90	22.89	17.16	42.54	6.28	12.52	21.08	middle
ptc-MIR481d-p5_1ss6AG	ATCCTGGGACCTCACCTAACAGCT	TRINITY_DN27333_c2_g1	+	aaataatATCCTAGGACCTCACCTAACAGCTtaagctattgggttgagatgactctttgacatggtatcagagttttaataactAAGTGGTCATGAGTTCGAATCTCAc	5'	New	gp1a	24	37.60	293.93	225.12	244.48	110.74	80.91	72.67	middle
ptc-MIR481d-p3_2	AAGTGGTCACGAGTTCGAATCTCA	TRINITY_DN27333_c2_g1	+	aaataatATCCTAGGACCTCACCTAACAGCTtaagctattgggttgagatgactctttgacatggtatcagagttttaataactAAGTGGTCATGAGTTCGAATCTCAc	3'	New	gp1a	24	37.60	9.64	13.38	8.96	4.57	3.85	2.77	middle

ptc-MIR481b-p5_2	TGGAACCTCACCTAACAGCTT	TRINITY_DN33936_c1_g3	+	aactgttagagaataatataaatcatacctTGGAACCTCACCTACCAGCTTaagtttttgggttgaattggTTCTTTGACATGGTATCAGAGCCTtgatgaccaa	5'	New	gp1a	78	37.10	133.11	90.94	117.31	70.21	58.28	74.33	middle
ptc-MIR481d-p3_5	TTCTTTGACATGGTATCAGAGCCT	TRINITY_DN33936_c1_g3	+	aactgttagagaataatataaatcatacctTGGAACCTCACCTACCAGCTTaagtttttgggttgaattggTTCTTTGACATGGTATCAGAGCCTtgatgaccaa	3'	New	gp1a	78	37.10	9.64	13.38	8.96	4.57	3.85	2.77	middle
ptc-MIR481c-p5_2ss4CT20AG	AAGTTTTTGGGTTGAGATGGTTCT	TRINITY_DN20566_c0_g1	+	AATTTTTTGGGTTGAGATGGTTCTTTTcagatt	5'	New	gp1a	26	30.30	6.63	4.12	1.79	1.14	4.33	4.44	low
ptc-MIR481b-p3_1ss21GT	TTGGGTTGAGATGGTTCTTTT	TRINITY_DN20566_c0_g1	+	AATTTTTTGGGTTGAGATGGTTCTTTTcagatt	3'	New	gp1a	26	30.30	1.81	2.40	3.58	2.28	1.44	0	low
ptc-MIR481b-p3_2ss3TC20CA	ATCCTGGAACCTCACCTAAAAGCT	TRINITY_DN33936_c1_g1	+	acaaggtgcagccaagaagtcatgacacttttcctatcgcgtgttttcttttgtaactcaaatagaacttctaaattgtccatgccgtcttgttagagaataatataaatcatATTCTGGAACCTCACCTAAAAGCTTaagctt	3'	New	gp1a	127	36.10	102.39	67.61	87.76	21.69	24.56	22.19	middle
ptc-MIR481b-p3_1ss21GA	AACCTCACCTAACAGCTTAAACT	TRINITY_DN27350_c0_g1	+	accactagttgttagagagttcaaataaaaaccattcttatAACCTCACCTAATAGCTTAAACTcttgggttaagatgattctttaatactagtcat	3'	New	gp1a	91	30.90	4.22	2.75	0	0	0.96	0	low
ptc-MIR481c-p5_1ss2CT	ATCAAGCGGTCACGAGTTCGAATC	TRINITY_DN31649_c1_g2	-	acctaacagcttaagctattggattgaattggttctttgacatggtatcagaaccttgataACCAAGCGGTCACGAGTTCGAATCtcaccatcctcatttatttgataaaaaattaagtacAAGGTAATGTTGGTCTGTGCAAGTttcaagcacaaataacttttacttgg	5'	New	gp1a	36	36.50	422.82	295.13	402.09	156.98	178.68	188.60	middle
ptc-MIR481d-p3_2ss14CT17AG	AAGGTAATGTGGGTCTGTGCAAGT	TRINITY_DN31649_c1_g2	-	acctaacagcttaagctattggattgaattggttctttgacatggtatcagaaccttgataACCAAGCGGTCACGAGTTCGAATCtcaccatcctcatttatttgataaaaaattaagtacAAGGTAATGTTGGTCTGTGCAAGTttcaagcacaaataacttttacttgg	3'	New	gp1a	36	36.50	435.47	275.91	361.34	204.92	125.22	196.37	middle
ptc-MIR481a-p3_1ss21AG	AATAATATAAATCATATCCTGGGA	TRINITY_DN33771_c2_g4	+	actttccaatatattaaaactgaatttaacatcttttggttattggctgttgcttttgttttactgcttttatccaccaggcaactccatactgttagagAATAATATAAATCATATCCTGGGAc	3'	New	gp1a	121	32.00	38.55	14.76	15.22	7.42	6.26	10.54	middle
ptc-MIR481a-p5_1ss4GA	AGAAAATAATATAAATCATATC	TRINITY_DN31918_c0_g1	-	AGAAAATAATATAAATCATATTttgatatctcatttaataccttAAGTTTTTAGGTTGAGATGGTTCTttaatatagtatcagaactttgctgatc	5'	New	gp1a	58	24.00	0	0	0	0	0	3.33	low
ptc-MIR6470-p3_2ss10AG23TC	AAGTTTTTAGGTTGAGATGGTTCT	TRINITY_DN31918_c0_g1	-	AGAAAATAATATAAATCATATTttgatatctcatttaataccttAAGTTTTTAGGTTGAGATGGTTCTttaatatagtatcagaac	3'	New	gp1a	58	24.00	46.38	26.08	55.97	29.11	17.34	26.07	middle

				tttgctgatc												
ptc-MIR481d-p5_1ss8GA	CCTAACAACTTAAGCTATTGG	TRINITY_DN29087_c0_g2	+	agagtgttaatataaaaccattctcgggccttaCCTAACAACTTAAGCTGTTGGgatgaatggTTTTTTAACATGACATCAGAGCTTtaatgacta	5'	New	gp1a	71	35.40	0	0	0.45	0	0	0	low
ptc-MIR6470-p3_2ss7GA22TC	TTTTTTAACATGATATCAGAGCTT	TRINITY_DN29087_c0_g2	+	agagtgttaatataaaaccattctcgggccttaCCTAACAACTTAAGCTGTTGGgatgaatggTTTTTTAACATGACATCAGAGCTTtaatgacta	3'	New	gp1a	71	35.40	0	2.06	7.16	2.28	1.93	1.11	low
ptc-MIR481b-p5_1ss1GA_2	AGGTTGAGATGGTTCTTTGACATG	TRINITY_DN32021_c0_g1	+	agcttaagctattAGGTTGAGATGGTTCTTTGACATGttatcaaagctttaatggccaagcggtcacgagttcgaatctcaccatccccatttatttgataaaattaagcacAAGGTAATGTGGACTTGTGCAAGTttcaagcccaaaggctttcacttgagggggtgtgttagagaataatataaatcatatcttggggcctcacctaaaagcttaagctattaggttgagatgattctttgaca	5'	New	gp1a	221	39.00	2.41	0.34	0	4.57	0	0.55	low
ptc-MIR481d-p3_2ss15CT17AG	AAGGTAATGTGGGCTTGTGCAAGT	TRINITY_DN32021_c0_g1	+	agcttaagctattAGGTTGAGATGGTTCTTTGACATGttatcaaagctttaatggccaagcggtcacgagttcgaatctcaccatccccatttatttgataaaattaagcacAAGGTAATGTGGACTTGTGCAAGTttcaagcccaaaggctttcacttgagggggtgtgttagagaataatataaatcatatcttggggcctcacctaaaagcttaagctattaggttgagatgattctttgaca	3'	New	gp1a	221	39.00	87.33	57.65	62.24	26.26	34.19	24.41	middle
ptc-MIR481b-p5_1ss1GA_1	AGGTGTGTTAGAGAATAATATAAA	TRINITY_DN29114_c1_g2	+	aggAGGTGTGTTAGAGAATAATGTAAAtcaagtcttagaacctcacctaacagcttacgttaTTGGGTTGAGATGGTTTTTTTGACAcc	5'	New	gp1a	27	38.60	2.41	0.34	0	4.57	0	0.55	low
ptc-MIR481b-p3_2ss13GA17CT	TTGGGTTGAGATAGTTTTTTGACA	TRINITY_DN29114_c1_g2	+	aggAGGTGTGTTAGAGAATAATGTAAAtcaagtcttagaacctcacctaacagcttacgttaTTGGGTTGAGATGGTTTTTTTGACAcc	3'	New	gp1a	27	38.60	34.33	15.79	19.70	13.70	10.60	6.66	middle
ptc-MIR481b-p5_1	AAATCATATTCTGGAACCTCACCT	TRINITY_DN33771_c2_g3	-	agtgtgttagagaataatatAAATCATATTTTGGAACCTCACCTAACAGTTTaactattggattgagatgtttcttt	5'	New	gp1a	42	28.60	15.66	5.83	4.03	0	1.93	6.10	middle
ptc-MIR481b-p3_2	TGGAACCTCACCTAACAGCTT	TRINITY_DN33771_c2_g3	-	agtgtgttagagaataatatAAATCATATTTTGGAACCTCACCTAACAGTTTaactattggattgagatgtttcttt	3'	New	gp1a	42	28.60	133.11	90.94	117.31	70.21	58.28	74.33	middle
ptc-MIR481c-p5_2ss5CT21GA	TGAGTCTGTGCAAGTTTCAAACCC	TRINITY_DN31649_c0_g1	+	agttcgaatctcatcacccttattctatttgataaaaactaagtacaagatagtgTGAGTTTGTGCAAGTTTCAAACCCaaaaggctttcacttgagagagcgtgttagagaataatatAAATC	5'	New	gp1a	65	35.90	4.82	2.06	0	4.57	0.96	0.55	low

				ATATCCTGGGACCTCACCTaa											
ptc-MIR481a-p3_2ss13AG23TC	AAATCATATCCTGGGACCTCACCT	TRINITY_DN31649_c0_g1	+	agttcgaatctcatcacccttattctatttgataaaaactaagtacaagatagtgTGAGTTTGTGCAAGTTTCAAACCCaaaaggctttcacttgagagagcgtgttagagaataatatAAATCATATCCTGGGACCTCACCTaa	3'	New	gp1a	65	35.90	1668.39	1069.34	1411.34	682.13	517.74	646.24	middle
ptc-MIR481d-p5	ATTCTTTGACATGGTATCAGAGCC	TRINITY_DN29626_c0_g1	-	ataatataaatcatatcatgaaaccttacataacggcttaacctattgggttgagatgTTTCTTTGACATGGTATCAGAGCCttgatgatcaagcgattACGAGTTCGAATCTCACCATCACTatttatttgttataaaaaattaagcacaaagtaatattggcttgtgcaaatttcaagcctaaaaggctttcacttgaga	5'	New	gp1a	64	33.20	186.11	116.68	144.18	60.51	57.79	62.68	middle
ptc-MIR481c-p3_2ss22CA23CT	ACGAGTTCGAATCTCACCATCATT	TRINITY_DN29626_c0_g1	-	ataatataaatcatatcatgaaaccttacataacggcttaacctattgggttgagatgTTTCTTTGACATGGTATCAGAGCCttgatgatcaagcgattACGAGTTCGAATCTCACCATCACTatttatttgttataaaaaattaagcacaaagtaatattggcttgtgcaaatttcaagcctaaaaggctttcacttgaga	3'	New	gp1a	64	33.20	0	0.34	0	0	0	0.55	low
ptc-MIR481c-p5_1ss2CT	ATCAAGCGGTCACGAGTTCGAATC	TRINITY_DN31649_c4_g1	+	atcttaggatctcacctaacaacttaagctattgggttgaattagttctttgacatggtatcagagccttaatgATCAAGCGGTCACGAGTTTGAATCccgctacctccattctatttgataaaaattaagcatcaggcaatgtgggtttgtgcaagtttcatGCCCAAAGAGCTTTCACTTGAGGgg	5'	New	gp1a	64	41.00	422.82	295.13	402.09	156.98	178.68	188.60	middle
ptc-MIR481d-p3_4	GCCCAAAGAGCTTTCACTTGAGG	TRINITY_DN31649_c4_g1	+	atcttaggatctcacctaacaacttaagctattgggttgaattagttctttgacatggtatcagagccttaatgATCAAGCGGTCACGAGTTTGAATCccgctacctccattctatttgataaaaattaagcatcaggcaatgtgggtttgtgcaagtttcatGCCCAAAGAGCTTTCACTTGAGGgg	3'	New	gp1a	64	41.00	9.64	13.38	8.96	4.57	3.85	2.77	middle
ptc-MIR481c-p5_1ss12AG	AAGGTAATGTGGCCTGTGCAAGT	TRINITY_DN31297_c0_g2	-	attaaacacAAGGTAATGTGGACCTGTGCAAGTttcaagcccaaagagctttcacttgaggggatgtgttacagaataatataaatcatatcttaaaatcttacctaACAGCTTAAGTTATTGGATTGAAAtgattattcagcaagaaa	5'	New	gp1a	123	32.90	663.74	440.64	595.07	279.13	214.32	267.93	middle

ptc-MIR481d-p3_2ss11CT18GA	ACAGCTTAAGTTATTGGATTGAGA	TRINITY_DN31297_c0_g2	-	attaaacacAAGGTAATGTGGACCTGTGCAAGTttcaagcccaaagagctttcacttgaggggatgtgttacagaataatataaatcatatcttaaaatcttacctaACAGCTTAAGTTATTGGATTGAAAtgattattcagcaagaaa	3'	New	gp1a	123	32.90	7.83	7.89	4.48	0.57	0	2.22	low
ptc-MIR481c-p5_1ss22GT	AACAGCTTAAGCTTTTGGGTTT	TRINITY_DN29658_c0_g1	-	attatatcttgaagcctcatctAATAGCTTAAGCTTTTGGGTTGAGATGacttttaacaaggtatcaa	5'	New	gp1a	62	32.40	1.20	2.75	0	0	0	0	low
ptc-MIR481c-p3_1	AGCTTAAGCTTTTGGGTTGAGATG	TRINITY_DN29658_c0_g1	-	attatatcttgaagcctcatctAATAGCTTAAGCTTTTGGGTTGAGATGacttttaacaaggtatcaa	3'	New	gp1a	62	32.40	9.64	9.61	19.25	4.57	10.11	7.77	middle
ptc-MIR481d-p5_2ss3CT23GA	AGTCCAAAGAGCTTTCACTTGAAG	TRINITY_DN26152_c0_g1	+	caagtttcaAGCCCAAAGAGCTTTCACTTGAAGgagcgtattagaaAATAATATAAATCATATTTTTGGAtctcatttaacagttgaaacttttgggtaag	5'	New	gp1a	88	31.70	16.86	16.47	5.82	7.99	2.41	8.88	middle
ptc-MIR481b-p3_2ss19CT23AG	AATAATATAAATCATATTTTGGGA	TRINITY_DN26152_c0_g1	+	caagtttcaAGCCCAAAGAGCTTTCACTTGAAGgagcgtattagaaAATAATATAAATCATATTTTTGGAtctcatttaacagttgaaacttttgggtaag	3'	New	gp1a	88	31.70	0	0	0	0	0.48	0	low
ptc-MIR481c-p5	ACCAAGCGGTCACGAGTTCGAATC	TRINITY_DN32021_c0_g1	+	cctcacctaaaagcttaagctattaggttgagatggttctttgacatgttatcaaagctttaatgGCCAAGCGGTCACGAGTTCGAATCtcaccatccccatttatttgataaaattaagcacaaggtaatgtggacttgtgcaagtttcaagcccAAAGGCTTTCACTTGAGGGGGTGTgttag	5'	New	gp1a	26	41.60	1621.41	1164.74	1312.83	597.08	576.98	645.69	middle
ptc-MIR481c-p3_1ss4AG	AAGGGCTTTCACTTGAGGGGGTGT	TRINITY_DN32021_c0_g1	+	cctcacctaaaagcttaagctattaggttgagatggttctttgacatgttatcaaagctttaatgGCCAAGCGGTCACGAGTTCGAATCtcaccatccccatttatttgataaaattaagcacaaggtaatgtggacttgtgcaagtttcaagcccAAAGGCTTTCACTTGAGGGGGTGTgttag	3'	New	gp1a	26	41.60	496.30	364.45	402.98	221.48	166.64	245.18	middle
ptc-miR481a_1ss11TC	AGGACCTCACCTAACAGCTTAAGC	TRINITY_DN27546_c0_g1	-	cctGGGACCTCACCTAACAGCTTAAGCTATTGGGTTGAGATGGTtctttgacattgtt	5'	Diff	gp1a	44	44.80	38.55	45.99	43.88	17.12	18.78	17.75	middle
ptc-MIR481b-p3_1ss9AT	CTTAAGCTTTTGGGTTGAGATGGT	TRINITY_DN27546_c0_g1	-	cctGGGACCTCACCTAACAGCTTAAGCTATTGGGTTGAGATGGTtctttgacattgtt	3'	New	gp1a	44	44.80	299.95	165.07	254.33	84.48	100.18	118.15	middle
ptc-MIR481d-p3_1ss1CA	ATTTGACATGGTATCAGAGCC	TRINITY_DN30884_c1_g2	+	ctctcatgtaaaggggggattcagccaATTTGACATGGTATCAGAGCTata	3'	New	gp1a	46	43.10	3.61	0.34	2.69	1.14	0.96	0	low
ptc-MIR481c-p5_1ss13CT	ACCAAGCGGTCATGAGTTCG	TRINITY_DN32021_c0_g4	-	cttgatatggtatcagagctttgatgACCAAGCGGTCATGAGTTTGAATCtcattaccctatttaTTTG	5'	New	gp1a	35	35.30	131.91	84.42	132.98	35.96	31.31	39.94	middle

	AATC			ATAAAAATTAAGCACAAGGTaatgtagacctgt												
ptc-MIR481d-p3_1ss13CT	TTTGATAAAAATTAAGCACAAGGT	TRINITY_DN32021_c0_g4	-	cttgatatggtatcagagctttgatgACCAAGCGGTCATGAGTTTGAATCtcattaccctatttaTTTGATAAAAATTAAGCACAAGGTaatgtagacctgt	3'	New	gp1a	35	35.30	9.03	6.18	8.51	1.71	10.11	3.88	middle
ptc-MIR481c-p5_2ss4AG17GA	ATGGTATCAGAGCTTTAATGACCA	TRINITY_DN30628_c1_g1	-	ggtttgttagagaataatataaatcatatcttaaaaccttacctaacagtttgggttgaattggttctttaatATGGTATTAGAGCTTTAATGACCAAGCGGTCATGAGTTTGAACCtcaccatccctatttatttgataaaaattaagcataaggtagcgtgagcctgtggaagtttcaagtc	5'	New	gp1a	30	34.20	14.46	10.98	12.99	4.57	3.37	10.54	middle
ptc-MIR481c-p3_2ss13CT19CT	ACCAAGCGGTCATGAGTTTGAATC	TRINITY_DN30628_c1_g1	-	ggtttgttagagaataatataaatcatatcttaaaaccttacctaacagtttgggttgaattggttctttaatATGGTATTAGAGCTTTAATGACCAAGCGGTCATGAGTTTGAACCtcaccatccctatttatttgataaaaattaagcataaggtagcgtgagcctgtggaagtttcaagtc	3'	New	gp1a	30	34.20	12.05	8.92	20.60	3.42	2.89	2.77	middle
ptc-MIR481a-p5_2ss13AG23TC	AAATCATATCCTGGGACCTCACCT	TRINITY_DN33771_c2_g5	-	gtaatgtgggcctgtgcaagtttcaagcccaaagggctttcacttgagggggtgtgttagagaataatatAAATCATATCCTGGGACCTCACCTaacagcttaagctattgggttgagatggTTCTTTGACATGATATCAGAGCCTtaatgaccaagcggtcacaagttcgaatctcatcatccctatttatttgataaaa	5'	New	gp1a	64	41.30	1668.39	1069.34	1411.34	682.13	517.74	646.24	middle
ptc-MIR481b-p3_3	TTCTTTGACATGATATCAGAGCCT	TRINITY_DN33771_c2_g5	-	gtaatgtgggcctgtgcaagtttcaagcccaaagggctttcacttgagggggtgtgttagagaataatatAAATCATATCCTGGGACCTCACCTaacagcttaagctattgggttgagatggTTCTTTGACATGATATCAGAGCCTtaatgaccaagcggtcacaagttcgaatctcatcatccctatttatttgataaaa	3'	New	gp1a	64	41.30	133.11	90.94	117.31	70.21	58.28	74.33	middle
ptc-MIR481a-p5_2ss16CT18AG	AATATAAATCATATCTTGGGACCT	TRINITY_DN30319_c0_g3	-	gtgttagagaatAATATAAATCATATCATGGGACCTtacctaacagCTTAAGCTATTGGGTTGAGATGGTtctttgacatca	5'	New	gp1a	11	35.40	33.13	17.16	28.66	11.99	8.67	7.21	middle
ptc-MIR481b-p3_1ss9AT	CTTAAGCTTTTGGGTTGAGATGGT	TRINITY_DN30319_c0_g3	-	gtgttagagaatAATATAAATCATATCATGGGACCTtacctaacagCTTAAGCTATTGGGTTGAGATGGTtctttgacatca	3'	New	gp1a	11	35.40	299.95	165.07	254.33	84.48	100.18	118.15	middle
ptc-MIR481b-p5_1ss3CT	AGTTTAAGCTATTGGGTTGAGAT	TRINITY_DN31297_c0_g3	+	gtgttagagaataatataaatcatattctgaagcctcacctaaaAGTTTAAGTTATTGGGTTGAGATGGTTCTtcgatatgatatcagagccttgatgacctaatgatcacg	5'	New	gp1a	57	34.80	3.61	2.06	3.58	1.71	1.44	0.55	low

ptc-MIR481b-p3_1ss4CT	AAGTTATTGGGTTGAGATGGTTCT	TRINITY_DN31297_c0_g3	+	gtgttagagaataatataaatcatattctgaagcctcacctaaaAGTTTAAGTTATTGGGTTGAGATGGTTCTtcgatatgatatcagagccttgatgacctaatgatcacg	3'	New	gp1a	57	34.80	284.29	167.81	214.03	94.19	81.87	109.83	middle
ptc-MIR481d-p5_2ss3GA19CT	TTAACATGGTATCAGAGCTT	TRINITY_DN24546_c2_g6	-	gtTTAACATGGTATCAGAGCTGgttttatggaa	5'	New	gp1a	24	36.40	2.41	3.43	4.03	0	1.93	1.11	low
ptc-MIR481b-p3_2ss8CT20AG	ATGATATTAGAGCCTTGATGACCA	TRINITY_DN28068_c0_g2	+	gtttatttgttttaacccaattagataataattttttgacATGATATTAGAGTCTTGATGACCAaat	3'	New	gp1a	11	23.90	4.22	4.12	8.06	1.14	5.78	3.33	low
ptc-MIR481a-p3_1ss1GA	ATTAGAGAATAATATAAATCATAT	TRINITY_DN33079_c0_g2	+	taactttaatataaaagctggtctgagtatATTAGAGAATAATATAAATCATATa	3'	New	gp1a	34	20.00	5.42	0.34	0	0	1.44	0	low
ptc-MIR481c-p3_2	CTTAAGCTTTTGGGTTGAGATGAT	TRINITY_DN27333_c2_g4	+	taatatatcaaagagtcatctcaacccaatagCTTAAGCTTTTAGGTTGAGATGATtttttgacataaga	3'	New	gp1a	64	30.00	9.64	9.61	19.25	4.57	10.11	7.77	middle
ptc-miR481d_L-1R+1_1ss3GA	GAACCTCACCTAACAGCTTAAGCT	TRINITY_DN27333_c1_g2	+	tattcttgGAACCTCACCTAAAAGCTTAAGCTtttgggtgaaatggttctttgacaa	5'	Diff	gp1a	51	36.80	12.05	12.35	13.88	4.57	3.37	3.88	middle
ptc-MIR481d-p5_2ss14CT17AG	AAGGTAATGTGGGTCTGTGCAAGT	TRINITY_DN27209_c1_g2	+	tcaaatctcaccatcctcatttatttgataaaaattaagcacAAGGTAATGTGGGTCTGTGCAAGTtttaagcctaaagagctttcacttgaggggggtgtgttaaagaataatataaatcatatcttgagacctcacttaacagCTTAAGCTATTGGGTTGAGATGGTtctttaacact	5'	New	gp1a	30	35.20	435.47	275.91	361.34	204.92	125.22	196.37	middle
ptc-MIR481b-p3_1	CTTAAGCTATTGGGTTGAGATGGT	TRINITY_DN27209_c1_g2	+	tcaaatctcaccatcctcatttatttgataaaaattaagcacAAGGTAATGTGGGTCTGTGCAAGTtttaagcctaaagagctttcacttgaggggggtgtgttaaagaataatataaatcatatcttgagacctcacttaacagCTTAAGCTATTGGGTTGAGATGGTtctttaacact	3'	New	gp1a	30	35.20	133.11	90.94	117.31	70.21	58.28	74.33	middle
ptc-MIR481d-p3_1	AAGCTATTGGGTTGAGATGATTCT	TRINITY_DN27333_c2_g4	-	tcttatgtcaaaaaatcatctcaacctaaaagcttAAGCTATTGGGTTGAGATGACTCTttgatatatta	3'	New	gp1a	64	30.00	9.64	13.38	8.96	4.57	3.85	2.77	middle
ptc-MIR481a-p5_2ss10CT23TC	AAATCATATTCTAGGACCTCACCT	TRINITY_DN27209_c2_g3	-	tgtctagcaatcaaccgaagctcttccttgattgcatcaacaactttggaagtgttacggaataatatAAATCATATTGTAGGACCTCACCTaatagtttaagctattgggttgagatggTTCTTTGACATGATATCAGAGCCTtaatgaccaagcggtcaca	5'	New	gp1a	31	38.70	1.20	4.80	3.58	0	0	0	low
ptc-MIR481b-p3_1ss3CT	TTTTTTGACATGATATCAGAGCCT	TRINITY_DN27209_c2_g3	-	tgtctagcaatcaaccgaagctcttccttgattgcatcaacaactttggaagtgttacggaataatatAAATCATATTGTAGGACCTCACCTaatagtttaagctattgggttgagatggTTCTT	3'	New	gp1a	31	38.70	422.22	277.97	360.89	149.55	140.63	197.48	middle

				TGACATGATATCAGAGCCTtaatgaccaagcggtcaca												
ptc-MIR481a-p5	TGTTAGAGAATAATATAAATCATA	TRINITY_DN32984_c0_g1	+	TGTTAGAGAATAATATAAATCATAtctaagaatcttacctaACAGTTTAAGCTATTGGGTTGAGTct	5'	New	gp1a	15	28.40	2.41	0.69	1.34	0	0.48	2.22	low
ptc-MIR481b-p3_1ss5CT	ACAGTTTAAGCTATTGGGTTGAGA	TRINITY_DN32984_c0_g1	+	TGTTAGAGAATAATATAAATCATAtctaagaatcttacctaACAGTTTAAGCTATTGGGTTGAGTct	3'	New	gp1a	15	28.40	7.23	6.52	10.30	4.00	6.74	2.22	middle
ptc-MIR481b-p5_1ss11AT	TTGGGTTGAATTGATTCTTTGACA	TRINITY_DN29943_c1_g3	+	TTGGGTTGAATTAATTCTTTGACAtggtataccatagtcttg	5'	New	gp1a	19	33.30	2.41	0	1.79	1.14	0.96	2.22	low
ptc-MIR481b-p5_1ss3GA	AAACTATTGGGTTGAGATGGTTCT	TRINITY_DN27209_c1_g1	-	ttgttagagaataatataaattatatcttgaaacttcacttaaaagtttAAACTATTGGGTTGAAATGGTTCTttaacatgatattagagtctcgatgacctagtgatcacgagtttaaATCTCACCATCCCTATTTATTTGAtaaaa	5'	New	gp1a	31	27.70	20.48	10.98	20.60	12.56	4.82	13.87	middle
ptc-MIR481c-p3_1ss12CT	ATCTCACCATCTCTATTTATTTGA	TRINITY_DN27209_c1_g1	-	ttgttagagaataatataaattatatcttgaaacttcacttaaaagtttAAACTATTGGGTTGAAATGGTTCTttaacatgatattagagtctcgatgacctagtgatcacgagtttaaATCTCACCATCCCTATTTATTTGAtaaaa	3'	New	gp1a	31	27.70	33.13	28.48	19.70	4.00	8.67	5.55	middle
ptc-MIR481b-p5_2ss3GA22GA	TTAACATGATATCAGAGCCTTAAT	TRINITY_DN27528_c1_g6	-	tTTAACATGATATCAAAGCCTTAATgactagacggctacaagttcgcacttgtgagggtgtattagagagtaatataattgggttgagatggtttctgacaataatgtgcttcaaactgatgggctcttatcaattatgaaaggt	5'	New	gp1a	134	36.60	1.81	1.37	1.79	0	0	0	low
ptc-MIR481b-p5_1ss17CT	TTCTTTGACATGATATTAGAGCCT	TRINITY_DN29394_c0_g1	-	tttggattgagatgaTTCTTTGACATGATATTAGAGTCTtgatgataatcaagtgattataAGTTTGAATCTCACCATCCTC	5'	New	gp1a	25	31.70	21.08	9.27	12.99	4.57	1.44	6.66	middle
ptc-MIR481b-p3_1ss14TC	AGTTTGAATCTCACCATCCTC	TRINITY_DN29394_c0_g1	-	tttggattgagatgaTTCTTTGACATGATATTAGAGTCTtgatgataatcaagtgattataAGTTTGAATCTCACCATCCTC	3'	New	gp1a	25	31.70	0	1.72	0	0	0	0	low
ptc-MIR481b-p5_2ss3TC22GA	ATCCTGGAACCTCACCTAACAACT	TRINITY_DN29114_c1_g3	+	ttttgcctattgttagagaataatataaatcatATCTTGGAACCTCACCTAACAACTtaaattattgggttgagatggTTTTTTGACATGGTATCAGAGCCTtgatgaccaagtggtcacgagttcgaatctcatca	5'	New	gp1a	31	35.80	60.23	40.15	52.84	22.83	19.26	22.19	middle
ptc-MIR481b-p3_1ss3CT	TTTTTTGACATGATATCAGAGCCT	TRINITY_DN29114_c1_g3	+	ttttgcctattgttagagaataatataaatcatATCTTGGAACCTCACCTAACAACTtaaattattgggttgagatggTTTTTTGACATGGTATCAGAGCCTtgatgaccaagt	3'	New	gp1a	31	35.80	422.22	277.97	360.89	149.55	140.63	197.48	middle

				ggtcacgagttcgaatctcatca												
ptc-miR482d-3p_R+1_1ss8CA	TTGCCGAACCCACCCATGCCAAA	CM000346.2	-	agagaagaggggaaagagatggaggacgaggaagtttctGGACATGGGTTGGTTTGCAAGAaaataagatttttgaactctctgatattgttcatggaaaacagttttcTTGCCGACCCCACCCATGCCAAtgatttcctcagcttcctctctctccctgtttccttctct	3'	Diff	gp1a	171	44.40	305.97	356.90	300.00	266.00	252.85	258.50	middle
ptc-MIR482d-p5_1ss9AT	GATGGAGGTCGAGGAAGTTTCT	TRINITY_DN30898_c0_g2	-	gaaagaGATGGAGGTCGAGGAAGTTTCTggACATGGGTTGGTTCGCAAGAAAATa	5'	New	gp1a	19	45.50	156.60	501.04	454.92	739.78	908.33	858.15	middle
ptc-MIR482d-p3_1ss14TC	ACATGGGTTGGTTCGCAAGAAAAT	TRINITY_DN30898_c0_g2	-	gaaagaGATGGAGGTCGAGGAAGTTTCTggACATGGGTTGGTTCGCAAGAAAATa	3'	New	gp1a	19	45.50	269.83	223.75	305.37	236.32	231.18	229.65	middle
ptc-miR482b-5p_L+2R+1	TGGGCATGAGGTGTTTGGCAAGAA	CM000341.2	-	gagaagagccggagggggagactgagacctactggaagttgtgGGCATGAGGTGTTTGGCAAGAaaatggatcttttccttatgatgatttcTTACCAATACCTCTCATGCCAAtgatttccagcagttcctccctttcccttctttctc	5'	Diff	gp1a	150	46.70	536.05	340.43	292.84	295.68	239.85	225.21	middle
ptc-miR482b-3p	TTACCAATACCTCTCATGCCAA	CM000341.2	-	gagaagagccggagggggagactgagacctactggaagttgtgGGCATGAGGTGTTTGGCAAGAaaatggatcttttccttatgatgatttcTTACCAATACCTCTCATGCCAAtgatttccagcagttcctccctttcccttctttctc	3'	Yes	gp1a	150	46.70	9482.74	10080.46	8148.34	4085.92	4925.98	4871.52	high
ptc-miR482a.1_L+8	CCGTCTTGCCTACTCCTCCCATTCC	CM000344.2	+	gagtcctagcaagtctttggagatgggagagtatgcaagaaggaaaaattcatgatttaatattccttcttgCCTACTCCTCCCATTCCatctgctttctgcgactc	3'	Diff	gp1a	107	43.00	31.32	29.51	44.78	59.37	48.16	28.85	middle
ptc-MIR482a-p5	AGATGGGAGAGTATGCAAGAAG	TRINITY_DN23883_c0_g1	+	gagtcttagcaagtctttggAGATGGGAGAGTATGCAAGAAGgaaaaattcatgatttaatatctctTCTTGCCTACTCCTCCCATTCCatctgctttctgcgactc	5'	New	gp1a	107	42.10	2921.19	2076.22	1681.79	1250.09	1641.35	1219.27	middle
ptc-miR482a.1_L+5	TCTTGCCTACTCCTCCCATTCC	TRINITY_DN23883_c0_g1	+	gagtcttagcaagtctttggAGATGGGAGAGTATGCAAGAAGgaaaaattcatgatttaatatctctTCTTGCCTACTCCTCCCATTCCatctgctttctgcgactc	3'	Diff	gp1a	107	42.10	572568.45	556611.77	480010.81	560046.53	524688.15	502466.46	high
ptc-MIR482d-p3_1ss7AT	TGGAGGTCGAGGAAGTTTCT	TRINITY_DN33095_c0_g3	+	ggagcttgagcagcaatttcacgaagcaaaggTGGAAGTCGAGGAAGTTTCTgctagagcata	3'	New	gp1a	57	47.60	0	0.69	0	2.28	3.85	0.55	low
ptc-miR482d-3p_1ss13CT	TTGCCGACCCCATCCATGCCAA	TRINITY_DN23340_c0_g1	-	tcatTTGCCGAACCCATCCATGCCAAtaatggaagtgatgaggcaaacac	5'	Diff	gp1a	44	46.00	1.81	1.72	2.24	1.14	0.48	2.22	low

ptc-miR482c-5p_1ss9AC	TATGGGAGCGGCGGGAATGACT	CM000353.2	-	tcctccgccgtgttggaaaggaaagggagactgctttactggaagcttccggTATGGGAGAGGCGGGAATGACTctgaaagaacttagaagagTCTTTCCGAGTCCTCCCATACCgcagatttccagtagttcctcccttacttatcttcactattccagcagctctgga	5'	Diff	gp1a	171	49.70	614.35	228.56	164.78	28.54	47.20	26.63	middle
ptc-miR482c-3p	TCTTTCCGAGTCCTCCCATACC	CM000353.2	-	tcctccgccgtgttggaaaggaaagggagactgctttactggaagcttccggTATGGGAGAGGCGGGAATGACTctgaaagaacttagaagagTCTTTCCGAGTCCTCCCATACCgcagatttccagtagttcctcccttacttatcttcactattccagcagctctgga	3'	Yes	gp1a	171	49.70	4436.60	2137.99	2048.06	397.29	366.03	298.44	middle
ptc-miR482d-3p	TTGCCGACCCCACCCATGCCAA	TRINITY_DN30898_c0_g1	+	tttcTTGCCGACCCCACCCATGCCAAtgatttcctcagcttcctctctctccctgtttccttctctcagttgtgtgctaaactctgcagatctgttcttctttttctatgctatctggtatgtagtaattaatatagggttagttc	5'	Yes	gp1a	138	43.20	20965.74	25808.56	22578.32	27519.19	20415.27	22008.92	high
ptc-miR530a_R+1_1ss20TC_1	TGCATTTGCACCTGCACCTCA	TRINITY_DN34326_c0_g1	-	agagggatttttgtaggagaagcttccatcagcgcatttaagacacaaacctttctgtggagaccaaactagttcagacagggcattggtggccgcaaatgtcttctctacacttgaaccTGCATTTGCACCTGCACCTGAatcattcttcact	3'	Diff	gp1a	150	45.50	13.25	17.16	11.64	35.39	25.04	41.05	middle
ptc-miR530b_R+1	TGCATTTGCACCTGCATCTTA	TRINITY_DN33729_c0_g1	-	ataaaaaaTGCATTTGCACCTGCATATTAgcgtatttgtc	5'	Diff	gp1a	34	32.50	10.84	45.30	38.51	123.30	262.00	266.26	middle
ptc-miR530a_R+1	TGCATTTGCACCTGCACCTTA	TRINITY_DN34326_c0_g2	-	ctagttcagacagggcattggtggccacaaatgtcatgtctacacttgaagcTGCATTTGCACCTGCACCTGAatcat	3'	Diff	gp1a	72	47.40	304.77	541.19	195.67	699.82	1021.51	830.41	middle
ptc-MIR530a-p3	TGCAGGTGCAGGTGCAGGTGA	TRINITY_DN26946_c1_g4	-	gatcaccttggcaccttcgtgaagtcctcgagcaagcccaccatgagccaatgatttcctcaacacaagctgcaaggcagtcatgatgtccataggctcaccgagagctggagcaggTGCAGGTGCAGGTGCAGGTGC	3'	New	gp1a	134	56.50	13.85	16.47	2.69	23.40	29.38	28.29	middle
ptc-miR530b_R+3	TGCATTTGCACCTGCATCTTTTG	CM000340.2	+	gccaatatgttgcctttatcTGCATTTGCACCTGCATCTTttgcgtttgttttgtttttgactccacaaacaaaatcaagttctgtcgatacagtatgtgggacaattagatatggaaactaataagcttagttgaggtgcaggtgcaagtgcaggtgaatgccattttggt	5'	Diff	gp1a	172	39.50	0	0.38	1.79	2.28	6.10	6.97	low
ptc-MIR530a-p3	TGCAGGTGCAGGTGCAGGTGA	TRINITY_DN27830_c2_g2	-	ggtgctggtgcagctggaggcggtgccggtgccggtgctggtgctggtggcggtggcggtgctggtgctggtgctggTGCAGGTGCAGGTGCAGGTGCaggtgc	3'	New	gp1a	103	72.10	13.85	16.47	2.69	23.40	29.38	28.29	middle
ptc-miR530a_R+1_1ss20TC_2	TGCATTTGCACCTGCACCTCC	TRINITY_DN18364_c0_g1	+	tagagcTGCATTTGCACCTGCACCTACagagaggaagatggagcagcaagctagcaatacgtgcttgat	5'	Diff	gp1a	143	51.00	13.25	17.16	11.64	35.39	25.04	41.05	middle

				cgctatacgctagcttcttgcacacaatgttgttcctggtcccttcctgatccctaggtgcagaggcagatgcaactg												
ptc-miR530a_R+1	TGCATTTGCACCTGCACCTTA	TRINITY_DN24739_c0_g1	-	tagttcagacagggcattggtggccacaaatgtcatgtctacacttgaagcTGCATTTGCACCTGCACCTGAatca	3'	Diff	gp1a	72	47.40	304.77	541.19	195.67	699.82	1021.51	830.41	middle
ptc-miR530b_L+2R-1	TCTGCATTTGCACCTGCATCT	CM000340.2	+	tttgccTGCATTTGCACCTGCACCTTataaatatgtacatacactcttcttcttcttcttcttcttcttatgtgtgtatatatgtaagaggtgcagaggcaagtgcaagtgaa	5'	Diff	gp1a	116	37.90	20.88	50.05	22.24	105.51	117.27	130.27	middle
ptc-miR536	CGCTCGCCAGCGTTGCACCACC	CM000337.2	-	aggaaggtttcagaCGCTCGCCAGCGTTGCACCACCaaaacttgcaaaagttctgatggtgccacgctgtgtgcgtctaaaatctctt	5'	Yes	gp1a	88	50.00	119.26	175.71	208.66	305.96	115.59	224.11	middle
ptc-miR828a	TCTTGCTCAAATGAGTATTCCA	CM000351.2	+	tctctttttaatgttTCTTGCTCAAATGAGTATTCCAacagcagtagccttatgcaacaagagcatatatttttacttgtatcactgtaatatgatgttgctggaatgctactgtttctgagatgctcatttgagcaagtaatattagaagatga	5'	Yes	gp1a	156	33.30	67.46	43.93	70.75	83.34	81.87	96.52	middle
ptc-MIR1444a-p5_1ss4GA	AATATTGACCGAATATGGATG	TRINITY_DN28736_c1_g2	+	gtttatcacttgAATATTGACCGAATATGGATGaaaagttgttctctgtttcctttccTCCACATTCGGTCAATGTTCCagtggtgagc	5'	New	gp1a	89	40.40	0	7.55	2.69	0	0	2.22	low
ptc-miR1444a_R+1	TCCACATTCGGTCAATGTTCC	TRINITY_DN28736_c1_g2	+	gtttatcacttgAATATTGACCGAATATGGATGaaaagttgttctctgtttcctttccTCCACATTCGGTCAATGTTCCagtggtgagc	3'	Diff	gp1a	89	40.40	179.49	1212.10	366.72	127.86	280.30	193.04	middle
ptc-MIR1444b-p5	TCCCTCGAATATTGATCGAAT	TRINITY_DN31089_c1_g2	+	tttaTCCCTCGAATATTGATCGAATaagtatgaaatatgatggttacatgtttcattCACATTCGGTCAACGTTCGAGtgataaa	5'	New	gp1a	85	32.90	0	3.43	6.27	0	0	0	low
ptc-MIR1444b-p3	CACATTCGGTCAACGTTCGAG	TRINITY_DN31089_c1_g2	+	tttaTCCCTCGAATATTGATCGAATaagtatgaaatatgatggttacatgtttcattCACATTCGGTCAACGTTCGAGtgataaa	3'	New	gp1a	85	32.90	231.29	544.28	363.13	25.12	14.45	17.75	middle
ptc-miR1446a_R-1_1ss9TC	TTCTGAACCCTCTCCCTCA	TRINITY_DN24504_c0_g2	-	cactgagtttgaaattcacTTCTCAACCCTCTCCCTCAc	3'	Diff	gp1a	22	46.20	1.20	10.98	3.58	1.14	0	2.22	middle
ptc-miR1446a_R+3_1ss9TC	TTCTGAACCCTCTCCCTCAACTT	CM000351.2	-	ccaacttcTTCTGAACTCTCTCCCTCAActgctactctagatcttgccaatccctgtcaagtgtttctgtgtagcagtcgaggtgagaggttcaggaaatgttgg	5'	Diff	gp1a	105	47.60	0.80	11.55	3.28	1.52	1.93	6.66	middle
ptc-miR1446a_R+3_1ss9TC	TTCTGAACCCTCTCCCTCAACTT	CM000348.2	+	ccaactttTTCTGAACTCTCTCCCTCAActgctattctagagcttcctgagctaacccttgtgtttctgtgtagcagtcaaggtgggaggctcagaaaatgttgg	5'	Diff	gp1a	105	46.70	0.80	11.55	3.28	1.52	1.93	6.66	middle

ptc-miR1446a_R+3_1ss9TC	TTCTGAACCCTCTCCCTCAACTT	CM000351.2	+	ccaactttTTCTGAACTCTCTCCCTCAActgctattctagagcttgttaatccttgtcaagtgtttccctgtagcagtcgaggcgaggggggtttcggaaaatgttgg	5'	Diff	gp1a	107	46.70	0.80	11.55	3.28	1.52	1.93	6.66	middle
ptc-miR1447_R+2	CAGAATTGCAGTGCCTTGATTTT	CM000349.2	+	gaggccctaatcagggcactgcaattctaaatgtctgttggcaagtggcgatgccagacgtttgaaatttacttatcatttattagtCAGAATTGCAGTGCCTTGATTtgggcttt	3'	Diff	gp1a	116	42.20	2.41	9.61	4.48	19.41	7.71	13.31	middle
ptc-MIR1447-p5_1ss4GT	TCATGGCACTGCAATTCTAAA	TRINITY_DN26028_c0_g1	+	gaggccctaaTCATGGCACTGCAATTCTAAAtgtctgtttgcatgtttccgatgccagacgtttgaaattgactcatttgttaatCAGAATTGCAGTGCCTTGATCagggcttt	5'	New	gp1a	114	42.10	47825.67	45437.21	41704.41	65496.92	103638.23	75759.82	high
ptc-miR1447_1ss21TC	CAGAATTGCAGTGCCTTGATC	TRINITY_DN26028_c0_g1	+	gaggccctaaTCATGGCACTGCAATTCTAAAtgtctgtttgcatgtttccgatgccagacgtttgaaattgactcatttgttaatCAGAATTGCAGTGCCTTGATCagggcttt	3'	Diff	gp1a	114	42.10	51682.85	75946.25	64522.28	122448.67	140292.18	138427.23	high
ptc-MIR1448-p5_2ss19TG20AC	TATGGGAGGATTGGACAGGCC	TRINITY_DN23883_c0_g1	+	gttacgggTATGGGAGGATTGGACAGGCCtgcttggttttaattaaccaaagtctgtTCTTTCCAACGCCTCCCATACCggt	5'	New	gp1a	79	48.80	22520.30	20532.23	20177.88	20461.58	24217.64	20270.44	high
ptc-miR1448_L+1R+1	TCTTTCCAACGCCTCCCATACC	TRINITY_DN23883_c0_g1	+	gttacgggTATGGGAGGATTGGACAGGCCtgcttggttttaattaaccaaagtctgtTCTTTCCAACGCCTCCCATACCggt	3'	Diff	gp1a	79	48.80	228308.46	239864.33	280168.17	362931.30	302964.15	330715.20	high
ptc-miR1448_L-1R+5	TTTCCAACGCCTCCCATACCGGTT	CM000344.2	+	gttacgggtatgggaggattggacagtactgcttggttttaattaaccaaagtctgttCTTTCCAACGCCTCCCATACcggtaat	3'	Diff	gp1a	85	44.70	1.20	5.49	7.16	18.27	5.78	7.77	middle
ptc-MIR1449-p3_2ss19TC23AC	GATTAGTTGAGGTGCACGCAAGCT	TRINITY_DN31091_c0_g4	+	agttttaggttcgagccttgtgattgctaatattgatagccactagagacttacctggtcgttaacttcaggacccgtggaGATTAGTTGAGGTACACGCAAGCTagcctggacac	3'	New	gp1a	110	46.60	10.24	6.18	7.16	1.14	0.48	2.22	middle
ptc-MIR1449-p5_2ss7TC18TC	ATTAGTCGAGGTGCACGCA	TRINITY_DN32569_c1_g6	-	cctgtgggATTAGTCGAGGTGCGCGCAagctagcccggacacccacg	5'	New	gp1a	43	66.00	1.81	2.40	1.79	0	0.96	0	low
ptc-MIR1449-p5_2ss7TC18TC	ATTAGTCGAGGTGCACGCA	TRINITY_DN31762_c3_g5	-	gATTAGTCGAGGTGCACGAAagttggctcgg	5'	New	gp1a	23	54.80	1.81	2.40	1.79	0	0.96	0	low
ptc-MIR1449-p5_2ss19TC23AC	GATTAGTTGAGGTGCACGCAAGCT	TRINITY_DN32673_c1_g3	-	GATTAGTTGAGGTGCGCGCAAGCTggcccgaacacc	5'	New	gp1a	28	61.10	10.24	6.18	7.16	1.14	0.48	2.22	middle
ptc-miR1450_R-4	TTCAATGGCTCGGTCAGG	TRINITY_DN27403_c0_g6	+	cgacggccactgttTTCAATGGCTCGGTGAGGa	3'	Diff	gp1a	23	57.60	37.34	39.81	36.72	23.40	21.67	24.96	middle
ptc-MIR1450-p5	CGAGCCATTGAAGACAATCATTTG	TRINITY_DN25796_c0_g1	-	CGAGCCATTGAAGAGAATCATTTGTTGGAta	5'	New	gp1a	24	38.70	1.20	2.75	0	4.57	11.56	16.64	middle
ptc-MIR1450-p3_1ss10CG	CATTGAAGAGAAT	TRINITY_DN25796_	-	CGAGCCATTGAAGAGAATCATTTGTTGGAta	3'	New	gp1a	24	38.70	0	0.69	2.69	4.57	0	0	low

	CATTTGTTGGT	c0_g1														
ptc-miR1450_2ss21AT22CT	TTCAATGGCTCGGTCAGGTTTT	CM000340.2	-	gagtcaattaggtcagttgtgtagtctgacccgagccattgaagacaatcatttgttggtgtctcattggatttgttacctgacagtgtttatccaacaaatgattctcTTCAATGGCTCGGTCAGGTTACacaagcaatctagttggctc	3'	Diff	gp1a	151	42.40	133.71	218.26	167.46	220.34	125.22	183.06	middle
ptc-miR1450_R-1	TTCAATGGCTCGGTCAGGTTA	TRINITY_DN25796_c0_g1	+	gtgtttatccaacaaatgattctcTTCAATGGCTCGGTCAGGTTAcacaagcgacctagttggctcagcatttatcgcttctgccatgccatgacctgaataactgcattgtaaagaccatatgcttggagaactacatt	5'	Diff	gp1a	137	42.90	17760.86	28511.76	17831.16	18937.49	14976.85	16918.29	high
ptc-miR1450_L-1	TCAATGGCTCGGTCAGGTTAC	TRINITY_DN27440_c3_g2	-	tacaccaaccgtacatagatatggacgttgaccagtgagaaactcacaaaagattcttgTCAATGGCTCGGTAAGGTTACactg	3'	Diff	gp1a	74	42.90	239.72	367.88	218.06	373.89	350.62	308.42	middle
ptc-miR2111a	TAATCTGCATCCTGAGGTTTG	TRINITY_DN26925_c0_g2	-	aatcagattctctagctcatcactaagttggggagcgtaggagTAATCTGCATCCTGAGGTTTTac	3'	Yes	gp1a	15	43.90	955.26	441.32	370.75	124.44	151.23	133.13	middle
ptc-MIR2111b-p3_2ss1GT18CT	TGTCAGGATTGGGTAATT	TRINITY_DN31933_c0_g1	-	gcggctgctggcaccagacttgccctccaatggatcctcgttaagggatttagattgtactcattccaattaccagactcgaagagcccggtattgttatttattgtcactacctccccgTGTCAGGATTGGGTAATTtgcgcgcctgctgccttc	3'	New	gp1a	151	50.60	7.23	4.12	4.48	6.85	0	2.22	low
ptc-miR3627b_2ss7AG19GA	TGTCGCGGGAGAGATGGCACTA	CM000353.2	+	atcctgTCGCAGGAGAGATGGCACTACCtagctttacgcatacgtaacgagatatatacgtacgtggtaaatatatatagatgagttattgatggtgtagcagctagcttgtgccatcctcctgtgac	5'	Diff	gp1a	123	43.80	500.52	485.25	250.75	37.67	25.04	28.85	middle
ptc-MIR6421-p5	AAGTGGACTTCCCTTACAATC	TRINITY_DN22586_c0_g1	+	tgaaatccAAGTGGACTTCCCTTACAATCtactcttcagtttcacagaagatccaattagattttaaggcaaacaaattgatagagcagaTTGTAAGGGAAGCCCACATGGatttca	5'	New	gp1a	115	37.60	1144.39	1645.19	1331.64	1573.18	1245.46	1316.90	middle
ptc-MIR6421-p3	TTGTAAGGGAAGCCCACATGG	TRINITY_DN22586_c0_g1	+	tgaaatccAAGTGGACTTCCCTTACAATCtactcttcagtttcacagaagatccaattagattttaaggcaaacaaattgatagagcagaTTGTAAGGGAAGCCCACATGGatttca	3'	New	gp1a	115	37.60	1792.47	2506.56	1904.77	2163.40	2202.92	1999.20	middle
ptc-miR6421-5p_L-2R+2	CCTTACAATCTACTCTTTCAG	CM000337.2	-	tgaaatccaagtggactTCCCTTACAATCTACTCTTTCagtttcacagaagatccagattagattttaaggcaaacaaattgaTAGAGCAGATTGTAAGGGAAGcccacatggatttca	5'	Diff	gp1a	117	37.80	0	2.06	0.90	0	0	2.22	low
ptc-miR6422_2ss9TA17AC	TGTGATAAAGAAGGCTCTGGT	CM000337.2	+	gtataatgccaaccatagcctccattatcacactattttgtttggtattttaccaaacaaaatggTGTGATAATGAAGGCTATGGTtggcattattgc	3'	Diff	gp1a	93	35.70	0	0	0	0	0	1.11	low
ptc-MIR6423-p5	TTCTCCGGCACCGC	TRINITY_DN32772_	+	ctcacgTTCTCCGGCACCGCCGCTGCCgccagtatctttttcc	5'	New	gp1a	59	67.60	4.82	5.49	6.27	10.27	12.52	2.22	middle

	CGCTGTC	c1_g2		gcctcacccccgtcaacgacagcggcggtgg												
ptc-miR6425a-5p	TTGTCTTCCATGGAATAGGCAG	CM000337.2	-	ggacccatgcatggcgaacaagttgagctgtagggttatgatgaacatgctaattacaagctaTTGTCTTCCATGGAATAGGCAGtgatggcatttgtttattttcctgaggtggagaaaaacaaatagaccataacactgttatTCCATGGAAGATAATGACTCGtggttgacacttgatctctccagtccagttcagattcaagctagctgctgtct	5'	Yes	gp1a	178	41.80	109.62	77.56	117.31	207.78	444.05	255.17	middle
ptc-miR6425a-3p_1ss4AT	TCCTTGGAAGATAATGACTCG	CM000337.2	-	ggacccatgcatggcgaacaagttgagctgtagggttatgatgaacatgctaattacaagctaTTGTCTTCCATGGAATAGGCAGtgatggcatttgtttattttcctgaggtggagaaaaacaaatagaccataacactgttatTCCATGGAAGATAATGACTCGtggttgacacttgatctctccagtccagttcagattcaagctagctgctgtct	3'	Diff	gp1a	178	41.80	8.43	5.49	8.06	21.69	37.57	25.52	middle
ptc-MIR6426b-p5_2ss17AC20GT	GAAGAAGAAGAAGAAGCAGTAGAA	TRINITY_DN28057_c0_g1	-	GAAGAAGAAGAAGAAGCAGAAGAAggaaatattaacatggggaacaaaaggattataaTACACACACACACACACACATACAcatgtgaagaggtcgaaatcatcggcttcgcagt	5'	New	gp1a	98	39.70	0	0	1.79	0	0.96	0	low
ptc-MIR6426b-p5_2ss13CT21GT	TTTTTTCTTCTTTTTCTTCTT	TRINITY_DN26229_c0_g6	+	TTTTTTCTTCTTTTTCTTCTTtgtgatttttttctattactattggaaagagtct	5'	New	gp1a	43	23.60	0	0	3.58	2.28	1.44	0	low
ptc-miR6427-5p	TCGTAATGCTTCATTCTCACAA	TRINITY_DN21581_c0_g1	+	tgcacgctctTCGTAATGCTTCATTCTCACAAcgtccaagtgttattgcatcatcctaatgattatcatcagccgttGTGGGAATGAACTTTATGAGActgtgta	5'	Yes	gp1a	104	41.30	248.15	595.07	367.16	729.51	1055.70	862.03	middle
ptc-miR6427-3p_1ss13AT	GTGGGAATGAACTTTATGAGA	TRINITY_DN21581_c0_g1	+	tgcacgctctTCGTAATGCTTCATTCTCACAAcgtccaagtgttattgcatcatcctaatgattatcatcagccgttGTGGGAATGAACTTTATGAGActgtgta	3'	Diff	gp1a	104	41.30	106.01	126.29	104.78	825.40	625.14	659.00	middle
ptc-MIR6430-p3	CTTTGCAGTCAAGTAATCATC	TRINITY_DN34723_c3_g1	+	caatcttagcttcttgttgtttagcttgatgattgattttctgcgaaagcaactctcttctttttttaggagaggagttgcCTTTGCAGTCAAGTAATCATCatcaagccatataccatacctgaggact	3'	New	gp1a	124	38.50	2.41	0.69	1.79	5.71	7.71	1.11	low
ptc-miR6431	TTATGTGGCATAAAAGAATCAA	CM000337.2	-	gattttgattcttTTATGTGGCATAAAAGAATCAAaatcgagttggaatctaccaatttgattttgattcttttatgcccataataaaatcaacggtc	5'	Yes	gp1a	89	29.60	2.41	0	1.79	0	0	0	low
ptc-MIR6438b-p5_1ss11AG	TCGACTGAAAGTGAAAGCTAT	TRINITY_DN29134_c0_g2	+	cttggttactgatatgattttgaaccgccatgtcaattataggttatgtaTCGACTGAAAGTGAAAGCTATttcactattctgtgcacacaattcttgaaacactaatcatgctttttgaaagtattggagcaTTGTACACAGAATAGGTGAAATaactttcattttcattcgatacgcaactaaaattgtaaatatagttcagtctca	5'	New	gp1a	157	33.60	118.05	98.83	102.09	42.24	61.65	68.78	middle

				tcttggaagccaag												
ptc-miR6438a	TTGTACACAGAATAGGTGAAAT	TRINITY_DN29134_c0_g2	+	cttggttactgatatgattttgaaccgccatgtcaattataggttatgtaTCGACTGAAAGTGAAAGCTATttcactattctgtgcacacaattcttgaaacactaatcatgctttttgaaagtattggagcaTTGTACACAGAATAGGTGAAATaactttcattttcattcgatacgcaactaaaattgtaaatatagttcagtctcatcttggaagccaag	3'	Yes	gp1a	157	33.60	225.26	175.02	140.60	85.62	97.29	79.88	middle
ptc-MIR6439b-p5_1ss20CT	AAAGAAGCAGAAGCCATCATTAGC	TRINITY_DN29354_c0_g2	+	agtgaatgctgagtaagaagccaaatAAAGAAGCAGAAGCCATCATTAGCgccgccctaattaaatgctcaacccgtcattttgatcgcaacgagttttcatctgcatatgggaagattttcatggagaagaatgctgtagcattcagtttaagtctgattgcaaacctcgtgatgtattgagcatttacttaatatgctaatggtgacgTCTGCTTCTTGATTTGGTGCCtgaatcaacattaacttttcatga	5'	New	gp1a	247	39.20	2.41	7.55	10.75	18.27	11.56	21.08	middle
ptc-MIR6439a-p3	TCTGCTTCTTGATTTGGTGCC	TRINITY_DN29354_c0_g2	+	agtgaatgctgagtaagaagccaaatAAAGAAGCAGAAGCCATCATTAGCgccgccctaattaaatgctcaacccgtcattttgatcgcaacgagttttcatctgcatatgggaagattttcatggagaagaatgctgtagcattcagtttaagtctgattgcaaacctcgtgatgtattgagcatttacttaatatgctaatggtgacgTCTGCTTCTTGATTTGGTGCCtgaatcaacattaacttttcatga	3'	New	gp1a	247	39.20	997.42	1616.36	1740.89	2117.74	1749.23	1896.02	middle
ptc-miR6442	CTGAACGGCTTGAAGGGCACG	CM000348.2	-	gtttgaacttcttgcatgtcaagccgttcaagcctagtctgaaaggtcaggagggcgtgctattcggtctaggcCTGAACGGCTTGAAGGGCACGttgttcaggc	3'	Yes	gp1a	105	53.30	26.50	34.32	34.03	38.82	65.50	32.17	middle
ptc-miR6443_L+3_1ss17TC	CGCGTATGATCATAGACGATGGAG	TRINITY_DN31860_c3_g1	+	cacaCGCGTATGATCATAGACGATGGAGgatagtccaaaccattgagaaattgggctttaacttatgtttttgggtttaatttatataaacttttagttaaggtttattaatttggttttatttgttgg	5'	Diff	gp1a	128	31.00	2.41	2.06	6.27	0.57	0	0	low
ptc-miR6443_L-1R+3_1ss11AC	TATGATCATCGATGATGGAGGGT	CM000348.2	-	tacaaactcctcatttaaatgattttgttgctgtaaagacaaatgaacattgtttcttcttgctttgagaaactagtggggacaccggtcacGTATGATCATAGATGATGGAGggta	3'	Diff	gp1a	107	37.60	1.20	0.69	0	0	0	0	low
ptc-miR6443_L+3_1ss17TC	CGCGTATGATCATAGACGATGGAG	TRINITY_DN31860_c0_g1	-	ttttctgaacatcatcaaacatgaaagtatttggtggggacaccgaacctgaaatattatttatgcaCGCGTATGATCATAGATGATGGAGgatag	3'	Diff	gp1a	90	37.50	2.41	2.06	6.27	0.57	0	0	low
ptc-miR6445a_R-2	TTCATTCCTCTTCC	TRINITY_DN25704_	-	aaTTCATTCCTCTTCCTAAAAGagacttgaaac	5'	Diff	gp1a	29	33.30	10.24	21.62	17.91	16.55	20.71	12.76	middle

	TAAAAT	c0_g1														
ptc-miR6445a_R-2	TTCATTCCTCTTCCTAAAAT	TRINITY_DN21690_c0_g1	-	aTTCATTCCTCTTCCTAAAACagggaagaaaggcacgaggcctcccttgttcttctttcgctacctgattcgagacgagttcaggaggtttgaaatcgaaagggaggagaggtgtttactgttcttactgacagaggaagg	5'	Diff	gp1a	130	46.80	10.24	21.62	17.91	16.55	20.71	12.76	middle
ptc-miR6445a	TTCATTCCTCTTCCTAAAATGG	CM000348.2	-	ggctgcttctttttctctctgggcacgttgTTCATTCCTCTTCCTAAAATGGcttcttctcctttagttgttgcaattataggagaggagaatccattttaggaagggaatgaatactgtgtcggttttacgagggagacaatc	5'	Yes	gp1a	144	42.40	1213.05	2426.94	2007.76	2723.95	3751.80	2720.33	middle
ptc-miR6448_L+1R-1	ATAGGCACAGAATTAACAAGG	CM000350.2	+	ttctagaggtaattaacatctaacatctgcagactacttgccttgttactgccgtgcctattttgaatatatatattcaaaaTAGGCACAGAATTAACAAGGCaagtattcagtagatgttagataggatgagctaaggctggag	3'	Diff	gp1a	143	36.60	13.25	9.61	5.37	5.71	11.56	21.08	middle
ptc-miR6452_1ss12CT	TGCAAAGGTAATTGAGACAAT	CM000353.2	-	tgattcttctggtattgtctcaattgcctttgcaattgatcaacaatcaaatgtagattgttgttctagatcaatTGCAAAGGTAACTGAGACAATcaaatgtagaactaagggaaca	3'	Diff	gp1a	116	33.90	1.20	1.37	0	3.42	0	0	low
ptc-miR6454_2ss17AG21AT	CTTGTAACCTGAGTAGGGGCT			tagctgggtaacgtggaCTTGTAACCTGAGTAGAGGCAataaagtgatataacattggctcgctcagcttagaaggtcgggtccagtta	5'	Diff	gp1a	89	46.10	0	0	0	4.57	0.96	0	low
ptc-miR6456	TTGAGTCCTTCCATTAGATCC	CM000355.2	-	aaaaatcagtgcaggatctagtgaaaggactcaagctcgtgatagataattattttctTTGAGTCCTTCCATTAGATCCcgcaatgatttttt	3'	Yes	gp1a	92	35.90	18.07	23.34	18.81	4.57	11.56	11.09	middle
ptc-miR6457b_R-1_1ss1TC	CTAGTTTGGCAGCCTCTTCT	CM000341.2	+	aatgagagaagagtcgagcaaaactaaaagaggaaactaatctatctccctgcaaaatgctgccaagcaacatcgtctagatcttaaatataagatcaagccgaagttggtttgctgcactttgctgggagattagattagtttcctcttTTAGTTTGGCAGCCTCTTCTCttatt	3'	Diff	gp1a	176	39.20	3991.50	6981.23	3634.47	1046.31	493.18	464.85	middle
ptc-MIR6457a-p3_1ss13CT	CTCAAACTTCTCTCTGACTTC	TRINITY_DN20100_c0_g1	-	acattgaaacccaatctcTTCAAACTTCTCTCTGACTTCct	3'	New	gp1a	20	39.00	39.75	137.96	52.84	0	10.60	3.33	middle
ptc-miR6457a_L+1R-1	ATAATCTCTCTGCAGAATGCT	CM000338.2	-	aggaagtaagagtagagttggaagaaataagagaagagtcgagccaaactaaaagaggaaactaataTAATCTCTCTGCAGAATGCTGcaaagcaacatcgtctagatctgaaacataagatcaagcagaagttggtttgctgcactctgctgggagattagattagtttcctcttctagtttgacagcctcttctcttatttcctcaaacttctccctgacttcct	5'	Diff	gp1a	227	40.10	63.84	85.45	64.03	19.41	24.08	14.42	middle
ptc-MIR6457a-p5	TGCTGGGAGATTAGATTAGTT	TRINITY_DN21772_c0_g1	-	ctcTGCTTGGAGATTAGATTAGTTtcctccttcagcttggcagcctctttgcttatttcttcaaatctttccctcacttcctcttgcccctctttaatacgtttatgccccttgctaatctccacca	5'	New	gp1a	117	44.10	17.47	26.08	17.01	6.85	2.89	6.10	middle

ptc-MIR6457a-p5_2ss6TA17GT	TTTGCAGCACTCTGCTTGGAG	TRINITY_DN23610_c0_g1	-	ctgagttgaTTTGCAGCACTCTGCTTGCAGatttgactagtttcctctttaagtttggcagcctctttacttatttcctggaatttttcccttacttccatttgaccctcccttatgcgTTTTTGACCCTCGCTAATCTC	5'	New	gp1a	35	42.10	13.85	32.26	9.40	11.42	3.85	4.44	middle
ptc-MIR6462a-p3_2ss7TA17GA	TTTTTGACCCTCGCTAATCTC	TRINITY_DN23610_c0_g1	-	ctgagttgaTTTGCAGCACTCTGCTTGCAGatttgactagtttcctctttaagtttggcagcctctttacttatttcctggaatttttcccttacttccatttgaccctcccttatgcgTTTTTGACCCTCGCTAATCTC	3'	New	gp1a	35	42.10	0	0.69	0	0	0	0	low
ptc-MIR6462f-p3_1ss16TC	TTATGCGTTTTTGTCCCTCGC	TRINITY_DN29296_c2_g3	-	gcttagagatcagatgagtctcctcTTTTAATTTGGCAGCCTCTTCacttatctcttgaaatttctccctgacttccctttgtccctctcTTATGCGTTTTTGTCCCTCGCtgatctctatca	3'	New	gp1a	35	43.10	212.01	661.99	135.67	89.62	65.98	51.03	middle
ptc-miR6457b_L+2R-2	TTTTAGTTTGGCAGCCTCTTC	TRINITY_DN29296_c2_g3	-	gcttagagatcagatgagtctcctcTTTTAATTTGGCAGCCTCTTCacttatctcttgaaatttctccctgacttccctttgtccctctcTTATGCGTTTTTGTCCCTCGCtgatctctatca	5'	Diff	gp1a	35	43.10	11.44	23.34	6.72	5.71	2.41	0.55	middle
ptc-MIR6462b-p3_1ss7AG	TTATGCGTTTTTGTCCCTCGC	TRINITY_DN25726_c1_g2	-	TCAGTTTGGCAGCCTCTTCgcttatttcttgaaatcttttcctgatttccttctgtccctctcTTATGCGTTTTTGTCCCTCGCtgatctcaaccatgtcagctcttatcgttttgacgctcc	3'	New	gp1a	54	44.70	212.01	661.99	135.67	89.62	65.98	51.03	middle
ptc-miR6457b_R-2	TTAGTTTGGCAGCCTCTTC	TRINITY_DN25726_c1_g2	-	TCAGTTTGGCAGCCTCTTCgcttatttcttgaaatcttttcctgatttccttctgtccctctcTTATGCGTTTTTGTCCCTCGCtgatctcaaccatgtcagctcttatcgttttgacgctcc	5'	Diff	gp1a	54	44.70	18.67	21.96	17.91	8.56	4.82	1.66	middle
ptc-MIR6457a-p3_1ss18AG	TTTCCTCTTCTAGTTTGGCAGCC	TRINITY_DN28158_c1_g1	-	tccgctcttgctctgaggatttgaaacataagatcaatcctgagttgatttgcagcgctctgcttggtgattagatcagTTTCCTCTTTTAGTTTGGCAGCCtctttattta	3'	New	gp1a	98	41.10	60.23	103.64	47.46	44.52	42.38	33.28	middle
ptc-MIR6457a-p5_2ss6TA17GT	TTTGCAGCACTCTGCTTGGAG	TRINITY_DN29868_c1_g2	-	TTTGCAGCGCTCTGCTTGGAGATTATATTAGTTTCCTCTTtcagtttggcagt	5'	New	gp1a	51	41.50	13.85	32.26	9.40	11.42	3.85	4.44	middle
ptc-MIR6457b-p3	AGATTAGATTAGTTTCCTCTT	TRINITY_DN29868_c1_g2	-	TTTGCAGCGCTCTGCTTGGAGATTATATTAGTTTCCTCTTtcagtttggcagt	3'	New	gp1a	51	41.50	4.82	12.70	6.27	3.42	2.41	2.77	middle
ptc-miR6459a-5p	AGCTCAAGCACAAATTCGATC	CM000339.2	+	tgcagagtagaagtcgAGCTCAAGCACAAATTCGATCttgcttaacaaggtaccggcaaagtcagaTCGAATTTGGGCTTGAGATTGtcttcttcgttgca	5'	Yes	gp1a	99	44.60	0	0	1.79	2.28	0	2.22	low
ptc-miR6459a-3p_1ss21GT	TCGAATTTGGGCTTGAGATTT	CM000339.2	+	tgcagagtagaagtcgAGCTCAAGCACAAATTCGATCttgcttaacaaggtaccggcaaagtcagaTCGAATTTGGGCTTGAGATTGtcttcttcgttgca	3'	Diff	gp1a	99	44.60	27.71	30.89	18.81	23.97	41.42	32.17	middle
ptc-miR6460	TGATATGTGGCATTCAATCGA	TRINITY_DN32193_c0_g5	-	aaTGTTATGTGGCATTCAATCGAGAATGCTGGAATAAATgat	5'	Yes	gp1a	33	33.30	6.02	12.35	12.54	31.97	23.12	26.63	middle

ptc-MIR6460-p3	ATCGAGAATGCTGGAATAAAT	TRINITY_DN32193_c0_g5	-	aaTGTTATGTGGCATTCAATCGAGAATGCTGGAATAAATgat	3'	New	gp1a	33	33.30	0	13.73	13.43	68.50	55.87	22.19	middle
ptc-miR6460_R+1	TGATATGTGGCATTCAATCGAA	CM000339.2	+	gatatctcattcatatatcatttattccagcattcttgattgaatgccacatatcattcattcctgctttttatatgaaaagtcggaatgaaTGATATGTGGCATTCAATCGAgaatgctggaataaatgatatctgaacaattgatc	3'	Diff	gp1a	148	31.80	1.20	2.75	0	1.14	3.85	4.44	low
ptc-MIR6460-p5_2ss15TC18TA	TATTCCAGCATTCTCGAATGA	TRINITY_DN32193_c0_g5	+	tcattTATTCCAGCATTCTCGATTGAatgccac	5'	New	gp1a	18	39.40	2.41	0	0.90	0	5.78	4.44	low
ptc-MIR6462a-p5_1ss17GA	TTTTTGTCCCTCGCTAATCTC	TRINITY_DN25726_c1_g1	-	TTTTTGTCCCTCGCTGATCTCtaccatatcagcttt	5'	New	gp1a	21	41.70	10.84	8.58	6.27	1.14	6.26	7.77	middle
ptc-MIR6465-p3_2ss5TC24CT	TATCCTCTAACTAGTTAAACTACT	TRINITY_DN31357_c1_g1	+	ctatttagaagatagattttaaataaggctctttaacttatgatacatttaaaaacTATCCTCTAACTAGTTAAACTACTagac	3'	New	gp1a	62	25.00	1.20	3.43	8.06	2.28	3.85	6.66	low
ptc-miR6468-5p_L-1	TTTTCCCTGAATCACTCCCA	CM000341.2	-	gtaatgctaaaagccccaaaagttggaacccaaatGTTTTCCCTGAATCACTCCCAccattgattggtgggctccatgcaccaattaaacagtgGGAGTGATTCAGGGAACCCATtcaggctcatcggactcttaggtttgattagtatgc	5'	Diff	gp1a	146	45.70	18.07	21.96	29.55	94.76	59.72	78.77	middle
ptc-miR6468-3p	GGAGTGATTCAGGGAACCCAT	CM000341.2	-	gtaatgctaaaagccccaaaagttggaacccaaatGTTTTCCCTGAATCACTCCCAccattgattggtgggctccatgcaccaattaaacagtgGGAGTGATTCAGGGAACCCATtcaggctcatcggactcttaggtttgattagtatgc	3'	Yes	gp1a	146	45.70	4.82	6.86	8.06	22.83	41.42	38.83	middle
ptc-MIR6469-p5	CCAGTTCACGAATCCTCTTTTGCC	TRINITY_DN17609_c0_g4	+	ccaaagaagcttcagataatagcacgttacatactgagcatcggaagcaagttactcttgatcCCAGTTCACGAATCCTCTTTTGCCagattcacgccgctaaagattcattgggacatgaatgtggcaaaaaaggattcgtttattgagataagcttaagagtaattttctatggcactgacgcgacgcattcttaagcaagccactttcagcatgctactcttccaatgcc	5'	New	gp1a	121	42.50	0	1.37	0	0	0	0	low
ptc-miR6470_L+2R+1_1ss11TC	AGCTCTGATACCATATTAAAAAAT	CM000342.2	-	ttcatgattgtttggttattgaagCTCTGATATCATATTAAAAAAttatcttgatctaaaatcaattaagcttttaagtttttaagttgagatggttttttgacatgatatcagagtttgttgaccaagtaattagaa	5'	Diff	gp1a	138	25.40	16.86	11.67	8.96	3.42	11.56	1.11	middle
ptc-miR6471	TTTGGGATCATCAGGACAGCC	TRINITY_DN23695_c0_g3	+	tttgttgtttaagaaccctttgatcccttaacccatcttttagcattttccaagcaaattgaatttgtatcTTTGGGATCATCAGGACAGCCcctgcatcgagaattatg	3'	Yes	gp1a	70	38.20	90.35	122.17	141.49	236.32	282.23	242.97	middle

ptc-MIR6473-p5_1ss16TA	ATTTTGACGGGATTGAGGATC	TRINITY_DN23714_c1_g1	+	tatatgttcctaaATTTTGACGGGATTGAGGATCcgaccaggtatgacacacagcggtgagaagtacctctatcttcatttttcatgtaaagcatcaagtgaaggtatggttactatatcgtaccattgtgctaatacttgtcaaaTCCACTATCCCATCAAGACTttggagcacatatgacaggttctcagaatgg	5'	New	gp1a	178	40.10	1.20	0.69	0	2.28	0	2.22	low
ptc-miR6473_R-1_1ss6AT	TCCACTATCCCATCAAGACT	TRINITY_DN23714_c1_g1	+	tatatgttcctaaATTTTGACGGGATTGAGGATCcgaccaggtatgacacacagcggtgagaagtacctctatcttcatttttcatgtaaagcatcaagtgaaggtatggttactatatcgtaccattgtgctaatacttgtcaaaTCCACTATCCCATCAAGACTttggagcacatatgacaggttctcagaatgg	3'	Diff	gp1a	178	40.10	1.20	2.06	2.69	0	1.93	3.33	low
ptc-miR6474_L-2R+2	TTCAGATCAGTAGATAGCATG	CM000344.2	-	gaaagcccctatttcaTGTTCAGATCAGTAGATAGCAtgtagtttgcttgtggtaatggtaccttgatcagactacttgctatctacggatctgcacaggttttt	5'	Diff	gp1a	105	41.00	1.20	0	1.79	0	6.74	0	low
ptc-miR6476a_L+6R-1	TGCGGATCAGTGGAGATGAAACATG	TRINITY_DN29781_c0_g2	+	tggagaTGCGGATCAGTGGAGATGAAACATGattgctacagcattgctaagtgtttaaagatgaactgtgtagttatttccttggtgaatgtattt	5'	Diff	gp1a	93	37.50	126.48	165.41	161.19	195.22	161.82	132.02	middle
ptc-miR6478_R+2_1ss21GA	CCGACCTTAGCTCAGTTGGTAGA	CM000345.2	-	aCCGACCTTAGCTCAGTTGGTGgagcggagggctgtaggtcgctggttggattccggcaaggtcggatttcaattttgtaatttcttttctcaccgaccttagttcagaagttgcattgttttgtctgttgaggtggt	5'	Diff	gp1a	138	47.80	77.10	102.95	124.48	220.34	211.91	160.87	middle
ptc-miR7817a_1ss21AT	TTTGGTTATTGTCTCGAGACT	CM000351.2	-	ctaacatccctcgggtggcgTTTGGTTATTGTCTCGAGACAgaaaagatggagacaaaaactttttggttgaagaggcagagacaaaataaatctgtctttgtgataattttagagtgaattcctgtattttcaaaacaagaagtataaggagacatgtcttatctacccccactgtctccgtctcttatgtttcgatacagtaaccaaacgctaccttgagtacaaggatga	5'	Diff	gp1a	229	38.90	2.41	1.37	0	0	0.96	2.22	low
ptc-miR7821	AGATGGGCATCGGCATTGTGA	TRINITY_DN26568_c0_g1	+	tgtcacaatgccaatacctatctagaccacaggatttgctattgcgttcacatctagtggttcAGATGGGCATCGGCATTGTGAagataggcat	3'	Yes	gp1a	82	44.70	2.41	7.55	7.16	27.40	24.08	12.20	middle
ptc-miR7823	TTGCATGCATGAACTTGAAAT	CM000340.2	+	attcaaatttatttcaagttcatgcatgcaacccaaatttataattgtccacacctgtgccaaatttaaatttcccgcattaggtaagtagatattttgtctccattcaggcaaccaaaaaaaactgaaaaaatctatattgctaaatttgacacaggtggacaaatataaattgaggTTGCATGCATGAACTTGAAATacgctctagc	3'	Yes	gp1a	191	33.00	1.20	2.75	3.58	12.56	6.74	12.20	middle

ptc-miR7825_L-1_1ss5AG	TGAGGAAAGGTAGACAGATAG	CM000339.2	-	aattaagtcctctatctatccaccttcttcaatcagatccttacgttagaataattgagagagaaaaattcacgtgttattcatttgttatgaaactatcccaatgaccaaaaagccttaaacacaaaaaacctcattcacaatgtaatgctcaggagaactagatctagttagaaaactcttcgaataataggtgtaatatttaacaccgtcttgaactgttgtcactgaagaatccgaTTGAAGAAAGGTAGACAGATAGaggacttaat	3'	Diff	gp1a	271	33.80	0	0	0	0	0.96	0	low
ptc-miR7826_L+2R-2	CTTTACCAAGTTTCAAATTCT	CM000352.2	-	ggcatatgctTTACCAAGTTTCAAATTCTCAatatatacataagcgtgtatatattgagaatttgaaacttggtaaagctctttaattaagaatgtgta	5'	Diff	gp1a	72	28.30	0	1.37	1.79	1.14	0	0	low
ptc-miR7827_L+1R-1	TGCTAGGACCAAGTTTTTTGG	CM000349.2	+	agtggaatgatttGCTAGGACCAAGTTTTTTGGAaaatgttaagattaattaatcttaacattttccagaaaacttggtcctagcaaaatagcttac	5'	Diff	gp1a	78	30.90	3.61	6.18	5.37	19.41	10.60	8.88	middle
ptc-miR7831	TACATGTAGAGACCACCAAAC	CM000346.2	+	gggaagaatacccagttagtTACATGTAGAGACCACCAAACccaaagtttcagttcactttctgacagaacgaaccctaactcaaccttttctcactcgagcagtcacagggacaaaccatcttcaaaaacccttgggcttcgattggagtggtaaaagtgagataggactctcttagaaggtgaagtttgggtttggtggccactacatgtaattggtattcttcctcgctgcc	5'	Yes	gp1a	228	45.10	2.41	0	0.90	2.28	0.96	1.11	low
ptc-miR7833_L-1R+1_1ss7GA	AATTAAAACTCATACTAGACA	TRINITY_DN26155_c1_g4	+	tcgtcttAATTAAAACTCATACTAGACAaactagtggatccttatctaatcctctagtatgagttctaattaagaggtggaaa	5'	Diff	gp1a	72	32.50	0	0	0	0	3.85	0	low
ptc-MIR7839-p5_1ss7CG	TGATTCGTTCCAATTCCATTTG	TRINITY_DN21581_c0_g1	+	aataagtaaaacaatatgtgTGATTCGTTCCAATTCCATTTGcctagtttattttcattgttcctcgaagaaatttaagaacaAGTGGAATTGGAGGTATCCCAtgtaatgctttttac	5'	New	gp1a	114	31.90	6.02	0	0	7.99	3.85	4.44	low
ptc-miR7839_R+1_1ss6CA	AGTGGAATTGGAGGTATCCCA	TRINITY_DN21581_c0_g1	+	aataagtaaaacaatatgtgTGATTCGTTCCAATTCCATTTGcctagtttattttcattgttcctcgaagaaatttaagaacaAGTGGAATTGGAGGTATCCCAtgtaatgctttttac	3'	Diff	gp1a	114	31.90	0	0.69	1.79	2.28	1.93	1.11	low
ptc-MIR7841-p5	TTTGACAGAAACCCCCTCATG	TRINITY_DN24515_c0_g2	+	aacgtattctggaattacaagctccatccataagtttttatgTTTGACAGAAACCCCCTCATGttttcaaccctaccgaggtttttgtcaaatttatatggtacagggttggaaacATGAGGGGGTAGCTGTCAAGCATAaaagcatttggatgatgcttgtaattctgcaaatacttt	5'	New	gp1a	173	38.00	48.18	85.11	47.46	138.14	140.63	145.34	middle
ptc-miR7841_L+4R-1_1ss11TA	ATGAGGGGGTAGCTGTCAAGCATA	TRINITY_DN24515_c0_g2	+	aacgtattctggaattacaagctccatccataagtttttatgTTTGACAGAAACCCCCTCATGttttcaaccctaccgaggtttttgtcaaatttatatggtacagggttggaaacATGAGGGGGTAGCTGTCAAGCATAaaagcatttggatgatgcttgtaattctgcaaata	3'	Diff	gp1a	173	38.00	32.52	17.85	30.45	62.79	70.32	54.36	middle

				cttt												
gma-MIR156k-p5_2ss14GT17TA	AATTGACAGAAGATAGAGAGCAC	TRINITY_DN26966_c0_g1	+	tgggacattagaAATTGACAGAAGAGAGTGAGCACacagaggcatatttgtataaaattataccattgcttttgcgtgctcatttctctttctgtcacttccagagccggaat	5'	New	gp1b	82	39.80	0	2.75	4.48	12.56	6.74	22.19	middle
gma-miR167h_L+2R-4	AGATCATGCTGGCAGCTTCAAC	TRINITY_DN23948_c2_g4	-	aggtgaaactgccacacgatctgttctttcctcgacaaaggagggaggttaAGATCATGCTGGCAGCTTCAACtgatagtgg	3'	Diff	gp1b	74	48.80	8.43	4.80	0.90	0	4.82	7.77	low
peu-MIR2916-p5_2ss5AG20TG	GGGGGCTCGAAGACGATCAGATACC	TRINITY_DN31933_c0_g1	+	tGGGGGCTCGAAGACGATCAGATACCgtcctagtctcaaccataaacgatgccgaccagggattggcggaTGTTGCTTCTAGGACTCCGCCa	5'	New	gp1b	90	55.40	354.16	517.51	376.12	4762.91	1854.23	5218.77	middle
peu-MIR2916-p3_1ss15GC	TGTTGCTTCTAGGACTCCGCC	TRINITY_DN31933_c0_g1	+	tGGGGGCTCGAAGACGATCAGATACCgtcctagtctcaaccataaacgatgccgaccagggattggcggaTGTTGCTTCTAGGACTCCGCCa	3'	New	gp1b	90	55.40	1376.88	1720.00	1364.77	13099.15	4877.82	13551.72	high
rgl-MIR5141-p3	CGACGCGACTGACAGATAAGCCGAC	TRINITY_DN27414_c0_g1	-	ttgagtaatgcttgagttgcaggatcaaggtctgatccaaattgagcaaaggcagcgacttctcgatattgtgccaattctagttttaaacttccgcatacttttttcatagctttaactgagcagcagaccCGACGCGACTGACAGATAAGCCGACgtta	3'	New	gp1b	27	43.20	1.20	0	3.58	0	0	0	low
hbr-MIR6485-p5_2ss11AG20GT	TGAGTGATTCGGCAGATTTTGATT	TRINITY_DN31805_c0_g2	+	acctaggatgtagaagagcataacaTGAGTGATTCGGCAGATTTTGATTcctataTATCCACTCATGTAGTACTTgacttcattatacga	5'	New	gp1b	78	36.70	12.05	8.24	12.54	55.94	42.38	32.17	middle
hbr-MIR6485-p3	TATCCACTCATGTAGTACTT	TRINITY_DN31805_c0_g2	+	acctaggatgtagaagagcataacaTGAGTGATTCGGCAGATTTTGATTcctataTATCCACTCATGTAGTACTTgacttcattatacga	3'	New	gp1b	78	36.70	1.20	0	2.69	5.71	2.89	1.11	low
tcc-MIR156g-p3_2ss1TA18TA	ATTCTGTCAGCTTCCAGAGCCGGA	TRINITY_DN26966_c0_g2	+	ttagaaacTGACAGAAGAGAGTGAGCACAcagaggcatatttgtatgaatctataccgttgcttttgcgtgctcacttctcATTCTGTCAGCTTCCAGAGCCGGA	3'	New	gp2a	92	44.80	0	0	0	1.14	0	0	low
bna-MIR156g-p3_1ss11TC	TCTCTAGTCTCCTGTCATCACT	TRINITY_DN30322_c0_g1	+	gttttgaattatgtgaggaggcactggtgatgctgTTGACAGAAGATAGAGAGCACagatgatgaaatgcatggagcttgattgcatctcactcctttgtgcTCTCTAGTCTCCTGTCATCACCttcagcccctcactccgtttaattg	3'	New	gp2a	143	45.60	0	0	0	0	0	2.22	low
ppe-MIR156h-p3	TCTCTATGCTTCTGTCATCAC	TRINITY_DN30322_c0_g2	+	actgttgatgttgTTGACAGAAGATAGAGAGCACagatgatgatatgcaatggactctgcatcccactcct	3'	New	gp2a	102	45.40	0	0	0	0	0.96	0	low

	A			ttgtgcTCTCTATGCTTCTGTCATCACTttcagcccc												
ghr-miR160_2ss18GA20AT	TATGAGGAGCCATGCATATTT	TRINITY_DN21483_c0_g1	+	accatcatgcatccatatatattatatgTGCCTGGCTCCCTGTATGCCATttgcagagcccaacggatcttcgatggcctccgtggatggcgTATGAGGAGCCATGCATATTCgcatgtatattatacatggttta	3'	Diff	gp2a	130	45.60	0	0.69	0.90	1.14	0	0	low
stu-MIR162b-p5	CAGCGGTTCATCGATCTGTTC	TRINITY_DN31678_c2_g1	+	gaagacactggaggCAGCGGTTCATCGATCTCTTCctggccaatttttgtttaacacgaaaaacatgaaccgaTCGATAAACCTCTGCATCCAGCgcttcctt	5'	New	gp2a	101	46.20	0	0.69	0.90	1.14	0	1.11	low
stu-miR162a-3p_R+1	TCGATAAACCTCTGCATCCAGA	TRINITY_DN31678_c2_g1	+	gaagacactggaggCAGCGGTTCATCGATCTCTTCctggccaatttttgtttaacacgaaaaacatgaaccgaTCGATAAACCTCTGCATCCAGCgcttcctt	3'	Diff	gp2a	101	46.20	10.84	18.53	12.54	14.84	20.23	7.77	middle
stu-miR166c-5p_L-1R+1_1ss11TC	GAATGTTGTCTGGCTCGAGGC	TRINITY_DN32689_c0_g1	+	tgggtgtgttgagggGAATGTTGGCTGGCTCGAAGCttaagcaaagagtttcctaacatgaaacaactgttaaggctTCGGACCAGGCTTCATTCCCCtcaaacatgcaag	5'	Diff	gp2a	105	47.70	4.82	2.75	6.27	3.42	3.85	3.33	low
lus-MIR167d-p3_1ss6AG	AGATCGTGTGACAGTTTCACC	TRINITY_DN23948_c2_g4	+	ttatcgtgcaccactatcagtTGAAGCTGCCAGCATGATCTTAAcctccctcctttgtcgaggaaagaacAGATCGTGTGGCAGTTTCACCtgaagttggtatcacgagaa	3'	New	gp2a	105	46.80	0	0	0	0	0.96	0	low
nta-miR168d_L+1	CTCGCTTGGTGCAGGTCGGGAA	TRINITY_DN29230_c0_g2	+	gttaccggcggtctctaaTTCGCTTGGTGCAGGTCGGGAActgattctgcgatttcattgccaggtggctaaacacgattggctgtgaggcaaattataaaaagaaagagaattggatCCCGCCTTGCATCAACTGAATCggagaccgcggtgaaga	5'	Diff	gp2a	153	49.00	143.35	130.41	103.88	115.31	98.25	134.24	middle
sly-miR168b-3p_R+1	CCCGCCTTGCATCAACTGAATT	TRINITY_DN29230_c0_g2	+	gttaccggcggtctctaaTTCGCTTGGTGCAGGTCGGGAActgattctgcgatttcattgccaggtggctaaacacgattggctgtgaggcaaattataaaaagaaagagaattggatCCCGCCTTGCATCAACTGAATCggagaccgcggtgaaga	3'	Diff	gp2a	153	49.00	102.39	110.50	116.42	364.18	279.34	310.64	middle
csi-MIR169m-p3_1ss12GT	CTTGGCTACATTTTTCTTTCT	TRINITY_DN34666_c5_g1	+	acagccttccatggaggatcataaagattgatgCAGCCAAGGATGACTTGCCGAcgactcgcttttgcttccatcaatatacgcataattaagaagagatgaatccgttggcaggttgttCTTGGCTACATTTTTCTTTCTgctcctcatgcgaggcttat	3'	New	gp2a	156	44.10	27.71	88.54	29.55	117.59	145.45	110.94	middle
stu-miR390-5p_L+1_1ss20AG	AAAGCTCAGGAGGGATAGCGCC	TRINITY_DN20472_c1_g1	-	tatggaagaatctgtTAAGCTCAGGAGGGATAGCGCCccaaggtaatcatgggctctttttatgtggtcttcgattctcagtggCGCTATCCATCCTGAGTTTCATtgcttcttcttgc	5'	Diff	gp2a	113	46.20	32.52	20.59	24.63	7.42	12.52	12.20	middle

stu-miR390-3p_R+1_1ss12TC	CGCTATCCATCCTGAGTTTTAT	TRINITY_DN20472_c1_g1	-	tatggaagaatctgtTAAGCTCAGGAGGGATAGCGCCccaaggtaatcatgggctctttttatgtggtcttcgattctcagtggCGCTATCCATCCTGAGTTTCATtgcttcttcttgc	3'	Diff	gp2a	113	46.20	0	0	0	3.42	0	0	low
stu-miR390-5p_L+1_1ss20AG	AAAGCTCAGGAGGGATAGCGCC	TRINITY_DN20472_c1_g2	+	aagtatggaagaatctgtTAAGCTCAGGAGGGATAGCGCCatgagcatgacaaagtctatgtttgagttaatctcaacaaaatcaatccagtcatcaGTGGCGCTATCTATCCTGAGTtctatgggttctccttactatt	5'	Diff	gp2a	136	40.70	32.52	20.59	24.63	7.42	12.52	12.20	middle
gma-MIR390g-p3	TTGGCGCTATCTATCCTGAGT	TRINITY_DN20472_c1_g2	+	aagtatggaagaatctgtTAAGCTCAGGAGGGATAGCGCCatgagcatgacaaagtctatgtttgagttaatctcaacaaaatcaatccagtcatcaGTGGCGCTATCTATCCTGAGTtctatgggttctccttactatt	3'	New	gp2a	136	40.70	2.41	0	0.90	0	0.96	1.11	low
stu-miR393-3p_L+1_1ss17CA	CATCATGCGATCTCTTAGGAAT	TRINITY_DN22551_c0_g1	+	atattagtgcagctggagtgtTCCAAAGGGATCGCATTGATCTaatgactctcgatgcctaaatcatattaatatgtttagttatttcgttgGATCATGCGATCTCTTAGGAATtttccagcatacagctctaaatt	3'	Diff	gp2a	131	36.50	0	1.37	0	1.14	0	0	low
csi-miR396a-3p_R-3_1ss1AG	GTTCAAGAAAGCTGTGGA	TRINITY_DN26735_c0_g1	-	tgccatgttctccCACAGCTTTCTTGAGCTTccatggcgctatatatatataggagctatacatgtagtgatctatagatacacagattaagcaaggcaaagaaGTTCAAGAAAGCTGTGGAaaagcatgacc	3'	Diff	gp2a	127	40.60	0	0	0.45	2.85	0	4.44	low
stu-miR396-3p_2ss3CT7GT	GTTCAATAAAGCTGTGGGAAA	TRINITY_DN26867_c0_g2	+	atgtgaccctctttgtattcTTCCACAGCTTTCTTGAACTGcacctattagatttatgttgatgttgttgtgcgatatgccatgaccatatgacattgtattcatttttgctgcgGTTCAATAAAGCTGTGGGAAGatacaaacaggatcaaag	3'	Diff	gp2a	148	38.30	0	4.80	0	3.42	2.89	2.22	low
lus-miR396d_L-1R-1_1ss20TA	CCCACAGCTTTATTGAACA	TRINITY_DN26867_c0_g2	-	ctttgatcctgtttgtatcttCCCACAGCTTTATTGAACCgcagcaaaaatgaatacaatgtcatatggtcatggcatatcgcacaacaacatcaacataaatctaataggtgcagtTCAAGAAAGCTGTGGAAGAatacaaagagggtcacat	5'	Diff	gp2a	148	38.30	2.41	2.06	0.90	0	0	4.44	low
stu-miR398b-3p	TTGTGTTCTCAGGTCACCCCT	TRINITY_DN14542_c0_g1	+	acgacagccggtacaccccagagagtggctcctgagaacacagggggttggttttctagctgcaagctacaagatggacaaagcaccCTGTGTTCTCAGGTCACCCCTttggggcaccctgtgga	3'	Yes	gp2a	119	56.80	24.09	24.02	17.91	21.69	19.26	38.83	middle
sly-miR403-5p_R-1	CGTTTGTGCGTGAATCTAAC	TRINITY_DN28658_c0_g3	-	tttagaggagacagatcgcgAGTTTGTGCGTGAATCTAACgccattggatggtcgtgaaatgaacggttgtgatggcgtcagatccacgaacaaactagagacatgagtcctctttt	5'	Diff	gp2a	111	46.20	1.20	3.43	0	0	3.85	5.55	low

vvi-miR482	TCTTTCCTACTCCTCCCATTCC	TRINITY_DN23883_c0_g1	+	ctttggagatgggagagtatgcaagaaggaaaaattcatgatttaatatctctTCTTGCCTACTCCTCCCATTCCatctgctttctgcgactcctctctttccttattctagacaaaagcgtgatctaaaattaatcaaggagcaagagggaaaggaaagaGGGTTGCTGGGAGTTACGGGtatgggag	5'	Yes	gp2a	73	42.90	150.58	102.27	82.39	126.72	95.36	116.49	middle
mes-MIR482c-p3_2ss14CT17TC	GGGTTGCTGGGAGTTACGGG	TRINITY_DN23883_c0_g1	+	ctttggagatgggagagtatgcaagaaggaaaaattcatgatttaatatctctTCTTGCCTACTCCTCCCATTCCatctgctttctgcgactcctctctttccttattctagacaaaagcgtgatctaaaattaatcaaggagcaagagggaaaggaaagaGGGTTGCTGGGAGTTACGGGtatgggag	3'	New	gp2a	73	42.90	0	1.37	1.79	1.14	1.93	0	low
mes-MIR482-p5_2ss17GC18AG	ATTTTCGGAAGGTTATCG	TRINITY_DN26571_c0_g1	+	aggaaatggagagggagtaggATTTTCGGAAGGTTATTGggtgggtgagcggggaagataactttaatttcacttgtgagatgctacttgtcattttccctactccacccatcccataggTTTCCGATCATTCCTCCCTCtcctcatcact	5'	New	gp2a	146	46.40	0	0	0	0	0.96	2.22	low
mes-MIR482e-p3_1ss9TC	TTTCCGATCATTCCTCCCTC	TRINITY_DN26571_c0_g1	+	aggaaatggagagggagtaggATTTTCGGAAGGTTATTGggtgggtgagcggggaagataactttaatttcacttgtgagatgctacttgtcattttccctactccacccatcccataggTTTCCGATCATTCCTCCCTCtcctcatcact	3'	New	gp2a	146	46.40	198.76	251.20	208.21	302.53	288.97	265.15	middle
mes-MIR482-p3_2ss10GA17TC	TTTCCGATCATTCCTCCCTCT	TRINITY_DN26571_c0_g3	+	aatggagagggagtagggtgttcggaaggttaTTGGATGGGTGAGTGGGGAAGataaccaagctgttgtgcttgttattttcccaactccacccatcccataggTTTCCGATCATTCCTCCCTCTcctcat	3'	New	gp2a	124	48.90	191.53	172.96	174.18	224.90	232.14	236.31	middle
csi-miR2275a-3p_2ss5GA21TC	TTTAATTTCCTCCAATATCTCA	TRINITY_DN23552_c0_g1	+	tcttggccgaatgtgaggattggagggaattaaacaagattgctttgttctcttcttactgTTTAATTTCCTCCAATATCTCAtgtctggccacct	3'	Diff	gp2a	90	40.60	24.09	24.02	25.07	31.97	19.26	17.75	middle
peu-MIR2913-p5_2ss9AG18CT	GGGATTGCGAGGAGAGCT	TRINITY_DN8199_c0_g1	+	cgcaagctggaggcctggccgacgtgtctggtgcggaccgccgagcttgGGGATTGCGAGGAGAGCTgtaccgcgtgggcgtgctgcattcagttgcggcgctcctgccaaggtgctggcTCTCCTCGCCATCCCCAAGCTcctgcgcgatgtgcccgctgccgaggcctggcctccgtcttccg	5'	New	gp2a	185	69.20	0	0	0	2.28	0.48	0	low
peu-MIR2913-p3_2ss10AC17CA	TCTCCTCGCCATCCCCAAGCT	TRINITY_DN8199_c0_g1	+	cgcaagctggaggcctggccgacgtgtctggtgcggaccgccgagcttgGGGATTGCGAGGAGAGCTgtaccgcgtgggcgtgctgcattcagttgcggcgctcctgccaaggtgctggcTCTCCTCGCCATCCCCAAGCTcctgcgcgatgtgcccgctgccgaggcctggcctccgtcttccg	3'	New	gp2a	185	69.20	9.64	5.83	12.54	6.85	10.11	12.76	middle

peu-MIR2913-p5_1ss10AG	GGGGATTGCGAGGAGAGC	TRINITY_DN8199_c0_g1	-	ctcggcagcgggcacatcgcgcaggagcttGGGGATGGCGAGGAGAGCcagcaccttggcaggagcgccgcaactgaatgcagcacgcccacgcggtacagcTCTCCTCGCAATCCCCAAGCTcggcggtccgcaccaga	5'	New	gp2a	134	67.90	0	0	0	0	0.48	0	low
peu-MIR2913-p3_2ss10AC17CA	TCTCCTCGCCATCCCCAAGCT	TRINITY_DN8199_c0_g1	-	ctcggcagcgggcacatcgcgcaggagcttGGGGATGGCGAGGAGAGCcagcaccttggcaggagcgccgcaactgaatgcagcacgcccacgcggtacagcTCTCCTCGCAATCCCCAAGCTcggcggtccgcaccaga	3'	New	gp2a	134	67.90	9.64	5.83	12.54	6.85	10.11	12.76	middle
cas-MIR11592-p5_1ss1GA	AAACCGAACCGAACCGAACCGAAA	TRINITY_DN27907_c1_g1	+	ccggtttttctaataaaaAAACCGAACCAAACCGAACCGAAAccggtcggtttgacttgatttcggttcggtttcggttttttttttgttaaaaaaataaattcggtttggttggtttttataggtaaAAACCGAACCGAACCGAAAat	5'	New	gp2a	95	36.20	42.16	33.63	46.57	22.83	20.23	25.52	middle
cas-miR11592_1ss1GA	AAACCGAACCGAACCGAAA	TRINITY_DN27907_c1_g1	+	ccggtttttctaataaaaAAACCGAACCAAACCGAACCGAAAccggtcggtttgacttgatttcggttcggtttcggttttttttttgttaaaaaaataaattcggtttggttggtttttataggtaaAAACCGAACCGAACCGAAAat	3'	Diff	gp2a	95	36.20	0	5.49	0.90	0	0	0	low
PC-5p-878_3103	CCGAGCTTGGGGATTGCGAGGAGA	TRINITY_DN8199_c0_g1	+	cgcaagctggaggcctggccgacgtgtctggtgcggaccgCCGAGCTTGGGGATTGCGAGGAGAgctgtaccgcgtgggcgtgctgcattcagttgcggcgctcctgccaaggtgctggctctccTCGCCATCCCCAAGCTCCTGCGcgatgtgcccgctgccgaggcctggcctccgtcttcc	5'	New	gp4	179	69.00	2138.19	1574.84	1890.00	1664.51	1890.35	1705.20	middle
PC-3p-2758_1045	TCGCCATCCCCAAGCTCCTGCG	TRINITY_DN8199_c0_g1	+	cgcaagctggaggcctggccgacgtgtctggtgcggaccgCCGAGCTTGGGGATTGCGAGGAGAgctgtaccgcgtgggcgtgctgcattcagttgcggcgctcctgccaaggtgctggctctccTCGCCATCCCCAAGCTCCTGCGcgatgtgcccgctgccgaggcctggcctccgtcttcc	3'	New	gp4	179	69.00	520.39	366.17	458.95	748.34	959.86	743.32	middle
PC-5p-76888_70	TTTGATGGGAAAGAATTATTT	TRINITY_DN23239_c0_g3	+	tggcatttgcatagagtgaagagagtttggtgttaaggTTTGATGGGAAAGAATTATTTgcagtgtaagcagatgaagtcgtacataaatggtattggtattcaacaatttactggaaTTAGTTCTTTCCCATCAAACCtcaacattacgctctataagtagatgaattcgtacataaata	5'	New	gp4	162	34.30	21.08	35.00	34.93	32.54	38.53	27.74	middle

PC-3p-525_5996	TTAGTTCTTTCCCATCAAACC	TRINITY_DN23239_c0_g3	+	tggcatttgcatagagtgaagagagtttggtgttaaggTTTGATGGGAAAGAATTATTTgcagtgtaagcagatgaagtcgtacataaatggtattggtattcaacaatttactggaaTTAGTTCTTTCCCATCAAACCtcaacattacgctctataagtagatgaattcgtacataaata	3'	New	gp4	162	34.30	954.66	1075.51	1171.79	1144.49	1325.89	1280.84	middle
PC-5p-1557952_4	ACTTGGTTCCTGGAAAGAATGAAA	TRINITY_DN22033_c0_g1	+	gaaaatccACTTGGTTCCTGGAAAGAATGAAAgatggcctccctctctgcagttcttTCATTCTTCCCAGGAACCGAagttagattgag	5'	New	gp4	83	44.90	1.20	1.37	0.90	0	0.96	0	low
PC-3p-1299195_4	TCATTCTTCCCAGGAACCGA	TRINITY_DN22033_c0_g1	+	gaaaatccACTTGGTTCCTGGAAAGAATGAAAgatggcctccctctctgcagttcttTCATTCTTCCCAGGAACCGAagttagattgag	3'	New	gp4	83	44.90	9.64	0.69	4.48	0	0	0	low
PC-5p-178251_28	AGGATTGGAGGGAATTAAACA	TRINITY_DN23552_c0_g1	+	cttggccgaatgtgAGGATTGGAGGGAATTAAACAagattgctttgttctcttcttactgTTTAATTTCCTCCAATATCTCAtgtctggccacc	5'	New	gp4	90	41.50	3.61	2.06	1.79	7.99	7.71	9.98	low
PC-3p-451534_11	TTTAATTTTCTCCAATATCTCA	TRINITY_DN23552_c0_g1	+	cttggccgaatgtgAGGATTGGAGGGAATTAAACAagattgctttgttctcttcttactgTTTAATTTCCTCCAATATCTCAtgtctggccacc	3'	New	gp4	90	41.50	12.05	6.18	4.48	0	1.93	4.44	middle
PC-5p-797998_6	TTATTCCCACCACTTGATTCT	TRINITY_DN21050_c0_g1	+	gttttgTTATTCCCACCACTTGATTCTcttaaaatgactaatatatcttcttcataatgtttattaaattccagtttaaaatatcctcttcatgatataaagaggttacaatagttattttaagaaagaagtggtgagaataacaat	5'	New	gp4	143	25.90	0	0.69	0.90	5.71	2.89	1.11	low
PC-3p-2181658_3	TTGCCTCTACCCACACCCTGT	TRINITY_DN32798_c0_g1	+	gttcttacaaaggagtgtgggtggggaaaaaggcagaggaagagattagtttcaatttcttctcttctcttcttcttctTTGCCTCTACCCACACCCTGTtaagttt	3'	New	gp4	101	42.10	0	2.06	0.90	2.28	0	4.44	low

Table S5 Overview of differentially expressed microRNAs between wild-type and transgenic poplar plants.

Index	miR_name	miR_seq	up/ down	fold_change (Treat(mean)/ contrast(mean))	log2 (fold_ change)	p-value (t_test)	WT (mean)	miR156OE -group II (mean)	Expression level
1	ptc-miR169aa_L+2R-2	CCGAGCCAAGAATGACTTGTC	up	4.32	2.11	1.75E-04	48	207	middle
2	ptc-miR156g	TTGACAGAAGATAGAGAGCAC	up	4.26	2.09	2.53E-04	1,525	6,495	middle
3	gma-MIR5032-p5_2ss20AG24AC	TCCCTATGAAATGAGGCATGGAACG	up	16.01	4.00	3.64E-04	8	136	middle
4	ptc-miR1450_R-4	TTCAATGGCTCGGTCAGG	down	0.62	-0.70	4.01E-04	38	23	middle
5	aly-miR399b-3p	TGCCAAAGGAGAGTTGCCCTG	up	4.22	2.08	4.92E-04	31	130	middle
6	mes-miR399f	TGCCAAAGGAGAGTTGCCCTG	up	4.22	2.08	4.92E-04	31	130	middle
7	ptc-miR481d_L-1R+1_1ss3GA	GAACCTCACCTAACAGCTTAAGCT	down	0.31	-1.70	5.53E-04	13	4	middle
8	ptc-miR169b-3p_L+1	TGGCAGGTTGTTCTTGGCTAC	up	3.12	1.64	5.93E-04	357	1,114	middle
9	ptc-miR171e_L+1	CTGATTGAGCCGTGCCAATATC	down	0.38	-1.41	6.39E-04	490	185	middle
10	ptc-miR169v_R+3_2ss11TC21AC	TAGCCAAGGACGACTTGCCCCCTC	up	3.28	1.71	7.66E-04	7	22	middle
11	aly-miR171b-3p_R-1	TTGAGCCGTGCCAATATCAC	down	0.30	-1.72	1.47E-03	217	66	middle
12	ptc-miR397a	TCATTGAGTGCAGCGTTGATG	down	0.20	-2.36	1.52E-03	20	4	middle
13	ptc-miR403c-5p	TTTGTGCGTGGATCTGAGGCC	up	1.96	0.97	1.53E-03	2,414	4,741	middle
14	ptc-miR172a	AGAATCTTGATGATGCTGCAT	down	0.07	-3.74	1.59E-03	1,985	148	middle
15	ptc-miR1447_1ss21TC	CAGAATTGCAGTGCCTTGATC	up	2.09	1.06	1.79E-03	64,050	133,723	high
16	nta-miR172d_R+1	AGAATCTTGATGATGCTGCATT	down	0.08	-3.68	1.95E-03	362	28	middle
17	aly-MIR829-p5_2ss3GA21GC	TTAAAGCTCTGATACCATGTC	down	0.23	-2.12	1.96E-03	3	1	low
18	gma-MIR5371-p5_1ss1AT	TTTCTAGGAATTAGTCACT	up	6.52	2.70	2.10E-03	1	5	low
19	ptc-miR156a	TGACAGAAGAGAGTGAGCAC	up	107.82	6.75	2.15E-03	836	90,131	high
20	ptc-miR171l-5p_2ss1TC17CT	CGTGATATTGGTCCGGTTCATC	up	4.87	2.29	2.46E-03	126	614	middle
21	ptc-miR530b_L+2R-1	TCTGCATTTGCACCTGCATCT	up	3.79	1.92	2.50E-03	31	118	middle
22	aqc-miR530_L+2R-1	TCTGCATTTGCACCTGCATCT	up	3.79	1.92	2.50E-03	31	118	middle
23	mtr-MIR156a-p3_2ss3GA21CT	TGAGAGCTCTTTCTTCTTCTTTC	up	inf	inf	2.61E-03	0	1	low
24	ptc-miR390d-3p	CGCTATCCATCCTGAGTTTTA	down	0.41	-1.30	2.66E-03	227	92	middle
25	ptc-MIR164e-p3	CATGTGCCTGTCTTCCCCATC	up	2.36	1.24	2.79E-03	225	532	middle
26	ptc-miR6471	TTTGGGATCATCAGGACAGCC	up	2.15	1.11	2.79E-03	118	254	middle
27	PC-5p-178251_28	AGGATTGGAGGGAATTAAACA	up	3.44	1.78	3.19E-03	2	9	low
28	ptc-miR169u-5p_R+3_1ss16TC	TAGCCAAGGACGACTCGCCTAATT	up	2.83	1.50	3.20E-03	170	481	middle
29	mes-MIR482-p3_2ss10GA17TC	TTTCCGATCATTCCTCCCTCT	up	1.29	0.36	4.23E-03	180	231	middle

30	cme-miR164a_2ss19GT20CT	TGGAGAAGCAGGGCACGTTTT	up	3.66	1.87	4.61E-03	54	196	middle
31	ptc-MIR156j-p3	TTGTGCTCTCTATGCTTCTGTC	up	11.64	3.54	4.61E-03	48	554	middle
32	hbr-MIR6483-p3_1ss4TC	ACCCAATTTTATTGTAGAAA	up	2.61	1.38	4.76E-03	1,070	2,794	middle
33	ptc-MIR481d-p5_1ss6AG	ATCCTGGGACCTCACCTAACAGCT	down	0.35	-1.53	4.90E-03	255	88	middle
34	ppe-MIR858-p5	TCTCGTTGTCTGTTCGACCTT	up	2.44	1.29	4.94E-03	6,051	14,765	high
35	ptc-miR394a-5p_L+1	CTTGGCATTCTGTCCACCTCC	down	0.40	-1.33	5.27E-03	923	367	middle
36	mdm-MIR169b-p3_1ss6AG	TGACAGGCTCTTCTCTCTCATG	up	4.26	2.09	5.38E-03	136	581	middle
37	ptc-miR7841_L+4R-1_1ss11TA	ATGAGGGGGTAGCTGTCAAGCATA	up	2.32	1.21	5.44E-03	27	62	middle
38	ptc-miR169q_L+1R-1_1ss17TC	CTAGCCAAGGACGACTCGCCT	up	3.54	1.82	5.45E-03	725	2,568	middle
39	ptc-miR171e	TGATTGAGCCGTGCCAATATC	down	0.34	-1.55	5.54E-03	12,295	4,187	high
40	ptc-miR171c_2ss1AT12CT	TGATTGAGCCGTGCCAATATC	down	0.34	-1.55	5.54E-03	12,295	4,187	high
41	ptc-miR481a_1ss11TC	AGGACCTCACCTAACAGCTTAAGC	down	0.42	-1.26	5.88E-03	43	18	middle
42	ptc-miR6457b_R-2	TTAGTTTGGCAGCCTCTTC	down	0.26	-1.96	6.15E-03	20	5	middle
43	ptc-miR482b-3p	TTACCAATACCTCTCATGCCAA	down	0.50	-1.00	6.25E-03	9,237	4,628	high
44	ptc-miR168a-3p	CCCGCCTTGCATCAACTGAAT	up	1.57	0.65	6.35E-03	1,117	1,755	middle
45	ptc-MIR481b-p5_2ss3GA22GA	TTAACATGATATCAGAGCCTTAAT	down	-inf	-inf	7.28E-03	2	0	low
46	ptc-miR6460	TGATATGTGGCATTCAATCGA	up	2.64	1.40	7.83E-03	10	27	middle
47	cme-miR164a_2ss19GA20CA	TGGAGAAGCAGGGCACGTAAT	up	5.62	2.49	7.94E-03	1	7	low
48	ptc-miR393a-3p_1ss10TC	ATCATGCTACCCCTTTGGATT	up	1.78	0.83	8.35E-03	600	1,065	middle
49	gra-MIR8658-p5_2ss15CA17AT	AAATTCAAATTTAAAATTT	up	13.28	3.73	8.46E-03	0	2	low
50	ptc-miR164a	TGGAGAAGCAGGGCACGTGCA	up	2.95	1.56	8.67E-03	33,444	98,530	high
51	sly-miR167b-3p_1ss10AT	AGGTCATCTTGCAGCTTCAAT	up	2.20	1.14	9.15E-03	112	247	middle
52	ptc-MIR6438b-p5_1ss11AG	TCGACTGAAAGTGAAAGCTAT	down	0.54	-0.89	9.61E-03	106	58	middle
53	ptc-miR169a	CAGCCAAGGATGACTTGCCGA	up	2.94	1.55	9.86E-03	71	208	middle
54	ptc-miR6427-3p_1ss13AT	GTGGGAATGAACTTTATGAGA	up	6.26	2.65	1.01E-02	112	703	middle
55	ath-miR157a-3p_2ss10CT13TC	GCTCTCTAGTCTCCTGTCATC	up	187.35	7.55	1.01E-02	4	832	middle
56	ptc-miR172a_R-1	AGAATCTTGATGATGCTGCA	down	0.08	-3.56	1.02E-02	18	1	middle
57	ptc-miR160a_R+1	TGCCTGGCTCCCTGTATGCCAC	down	0.41	-1.30	1.04E-02	47	19	middle
58	ptc-miR166n_L+3R-2	CTCTCGGACCAGGCTTCATTCC	down	0.34	-1.57	1.05E-02	2,199	741	middle
59	ptc-miR6457a_L+1R-1	ATAATCTCTCTGCAGAATGCT	down	0.27	-1.88	1.05E-02	71	19	middle
60	gma-MIR5032-p3_2ss13AG17AC	GAAATGAGGCATGGAACG	up	24.67	4.62	1.11E-02	1	32	middle
61	pla-MIR11602-p5	ATCCGAGAGTTATCAGTATTTATCA	up	3.52	1.81	1.13E-02	6	23	middle
62	ptc-miR171a-5p	GGATATTGGTACGGTTCAATC	down	0.49	-1.03	1.14E-02	215	105	middle

63	ptc-miR164a_L+1	CTGGAGAAGCAGGGCACGTGCA	up	2.76	1.47	1.17E-02	263	727	middle
64	sly-MIR9479-p3_2ss1GA18GC	ATGTCACGGGTTCAAATC	up	10.60	3.41	1.21E-02	0	2	low
65	sly-MIR9479-p5_2ss1GA18GC	ATGTCACGGGTTCAAATC	up	10.60	3.41	1.21E-02	0	2	low
66	sly-miR168b-3p_R+1	CCCGCCTTGCATCAACTGAATT	up	2.90	1.53	1.21E-02	110	318	middle
67	ghr-miR156a_R+2	TGACAGAAGAGAGTGAGCACTT	up	496.57	8.96	1.22E-02	4	2,056	middle
68	ptc-miR156l_2ss13GA20AG	TTGACAGAAGATAGAGAGCGC	up	5.25	2.39	1.23E-02	3	15	middle
69	ptc-miR166n_R+2_2ss18CT19CT	TCGGACCAGGCTTCATTTTTTAC	down	0.44	-1.18	1.26E-02	9	4	low
70	mtr-MIR2630x-p3_1ss8TC	TGTAGAACATTTGATTTTA	up	3.93	1.97	1.29E-02	3	10	middle
71	csi-miR171c-3p_R+1	TGATTGAGCCGTGCCAATATCT	down	0.47	-1.09	1.30E-02	101	48	middle
72	ptc-miR171g-5p_L+3	GGATGTTGGGATGGCTCAATCATG	down	0.22	-2.16	1.38E-02	6	1	low
73	ptc-miR472b	TTTTCCCAACTCCACCCATCCC	up	1.20	0.26	1.41E-02	758,407	907,529	high
74	stu-miR156f-5p_L-1R+1	TGACAGAAGAGAGTGAGCAT	up	283.92	8.15	1.42E-02	1	410	middle
75	ptc-miR399a_R-2_1ss13TG	TGCCAAAGGAGAGTTGCCC	up	14.84	3.89	1.44E-02	1	18	middle
76	mdm-MIR11010-p5_2ss21TC24GC	AGCTGGGAGTTGTTCTGCAACGAC	down	0.37	-1.42	1.48E-02	440	165	middle
77	ptc-MIR481c-p3_1ss4AG	AAGGGCTTTCACTTGAGGGGGTGT	down	0.50	-1.00	1.60E-02	421	211	middle
78	ptc-miR156a_R+1	TGACAGAAGAGAGTGAGCACT	up	147.50	7.20	1.62E-02	18	2,665	middle
79	ptc-miR394a-5p	TTGGCATTCTGTCCACCTCC	down	0.31	-1.71	1.64E-02	136,891	41,927	high
80	ptc-miR6478_R+2_1ss21GA	CCGACCTTAGCTCAGTTGGTAGA	up	1.95	0.96	1.67E-02	102	198	middle
81	ptc-MIR1449-p5_2ss7TC18TC	ATTAGTCGAGGTGCACGCA	down	0.16	-2.64	1.68E-02	2	0	low
82	ptc-miR160h	TGCCTGGCTCCCTGCATGCCA	up	2.77	1.47	1.70E-02	24	67	middle
83	cme-MIR1863-p5_2ss3AG19TC	AAGGCTCTGATACCATGTC	down	0.16	-2.62	1.71E-02	4	1	low
84	PC-3p-2758_1045	TCGCCATCCCCAAGCTCCTGCG	up	1.82	0.87	1.74E-02	449	817	middle
85	ptc-miR398b	TGTGTTCTCAGGTCGCCCCTG	down	0.15	-2.69	1.78E-02	210	33	middle
86	ptc-MIR481d-p3_2ss11CT18GA	ACAGCTTAAGTTATTGGATTGAGA	down	0.14	-2.86	1.81E-02	7	1	low
87	ptc-miR166a_L+1	CTCGGACCAGGCTTCATTCCCC	down	0.45	-1.15	1.83E-02	2,299	1,035	middle
88	sly-miR168b-3p_R+2	CCCGCCTTGCATCAACTGAATTT	up	7.85	2.97	1.84E-02	2	16	middle
89	mtr-miR156e_R+2	TTGACAGAAGATAGAGAGCACCT	up	6.38	2.67	1.86E-02	6	41	middle
90	ptc-miR166a_1ss21CA	TCGGACCAGGCTTCATTCCCA	down	0.53	-0.91	1.88E-02	8,488	4,506	high
91	ptc-miR169n-5p_R+1_1ss2GC	TCAGCCAAGGATGACTTGCCGC	up	3.71	1.89	1.88E-02	4	15	middle
92	ptc-miR169d_L+1_1ss22GC	TCAGCCAAGGATGACTTGCCGC	up	3.71	1.89	1.88E-02	4	15	middle
93	mtr-MIR396a-p5_1ss18AT	AAGAAAGCTGTGGGAGATT	up	6.14	2.62	1.94E-02	0	2	low
94	ptc-miR6456	TTGAGTCCTTCCATTAGATCC	down	0.45	-1.15	2.02E-02	20	9	middle
95	ptc-MIR7841-p5	TTTGACAGAAACCCCCTCATG	up	2.35	1.23	2.02E-02	60	141	middle

96	ptc-MIR1449-p3_2ss19TC23AC	GATTAGTTGAGGTGCACGCAAGCT	down	0.16	-2.62	2.06E-02	8	1	middle
97	ptc-MIR1449-p5_2ss19TC23AC	GATTAGTTGAGGTGCACGCAAGCT	down	0.16	-2.62	2.06E-02	8	1	middle
98	stu-miR408b-3p_R+3	TGCACTGCCTCTTCCCTGGCTTTC	down	-inf	-inf	2.17E-02	8	0	middle
99	mdm-miR408a_L-1R+4	TGCACTGCCTCTTCCCTGGCTTTC	down	-inf	-inf	2.17E-02	8	0	middle
100	ptc-miR530a_R+1	TGCATTTGCACCTGCACCTTA	up	2.45	1.29	2.23E-02	347	851	middle
101	ptc-MIR472a-p5_1ss2CT	TTGGGTGGGTGAGCGGGGAAGA	up	1.65	0.72	2.23E-02	1,514	2,500	middle
102	mdm-MIR11010-p3_1ss21TC	AGCTGGGAGTTGTTCTGCAAC	down	0.50	-1.01	2.31E-02	97	48	middle
103	ptc-MIR481c-p3_1ss12CT	ATCTCACCATCTCTATTTATTTGA	down	0.22	-2.16	2.34E-02	27	6	middle
104	ptc-miR6468-5p_L-1	TTTTCCCTGAATCACTCCCA	up	3.35	1.75	2.36E-02	23	78	middle
105	csi-miR396a-5p_L+4_1ss25GT	GATCTTCCACAGCTTTCTTGAACTT	up	1.71	0.77	2.37E-02	16	27	middle
106	ptc-MIR481b-p3_2ss3TC20CA	ATCCTGGAACCTCACCTAAAAGCT	down	0.27	-1.91	2.39E-02	86	23	middle
107	ptc-MIR6457a-p5	TGCTGGGAGATTAGATTAGTT	down	0.26	-1.93	2.41E-02	20	5	middle
108	ptc-miR1448_L+1R+1	TCTTTCCAACGCCTCCCATACC	up	1.33	0.41	2.44E-02	249,447	332,204	high
109	ptc-MIR156e-p3	TGCTCACTTCTCATTCTGTCAGC	up	35.26	5.14	2.45E-02	1	21	middle
110	ptc-MIR481d-p3_2	AAGTGGTCACGAGTTCGAATCTCA	down	0.35	-1.51	2.56E-02	11	4	middle
111	ptc-MIR481d-p3_5	TTCTTTGACATGGTATCAGAGCCT	down	0.35	-1.51	2.56E-02	11	4	middle
112	ptc-MIR481d-p3_4	GCCCAAAGAGCTTTCACTTGAGG	down	0.35	-1.51	2.56E-02	11	4	middle
113	ptc-MIR481d-p3_1	AAGCTATTGGGTTGAGATGATTCT	down	0.35	-1.51	2.56E-02	11	4	middle
114	ptc-miR477d-5p_R-1	ATCTCCCTCAAAGGCTTCCTC	up	9.74	3.28	2.59E-02	143	1,389	middle
115	ptc-miR6427-5p	TCGTAATGCTTCATTCTCACAA	up	2.19	1.13	2.64E-02	403	882	middle
116	ptc-MIR166e-p5	GATTGTCGTCTGGTTCGATGT	up	2.25	1.17	2.75E-02	99	224	middle
117	ptc-MIR481c-p5	ACCAAGCGGTCACGAGTTCGAATC	down	0.44	-1.17	2.77E-02	1,366	607	middle
118	cas-MIR11592-p5_1ss1GA	AAACCGAACCGAACCGAACCGAAA	down	0.56	-0.84	2.88E-02	41	23	middle
119	ptc-MIR475d-p5_2	TTACAGAGTCCATTGATT	up	1.69	0.75	2.96E-02	35	59	middle
120	ptc-miR475d-3p	TTACAGAGTCCATTGATTAAG	up	1.69	0.75	2.96E-02	35	59	middle
121	ptc-MIR475d-p5_1	TCCTGATCAATGGCCATTGTA	up	1.69	0.75	2.96E-02	35	59	middle
122	ptc-MIR475d-p5_3	TTACAGAGTCCATTGATTAAG	up	1.69	0.75	2.96E-02	35	59	middle
123	ptc-MIR481b-p3_1ss3CT	TTTTTTGACATGATATCAGAGCCT	down	0.46	-1.12	3.02E-02	354	163	middle
124	ptc-MIR481c-p5_1ss2CT	ATCAAGCGGTCACGAGTTCGAATC	down	0.47	-1.10	3.18E-02	373	175	middle
125	ptc-miR169ac_R+2_1ss21AC	TAGCCAAGGACGACTTGCCCCCC	up	1.83	0.87	3.23E-02	92,359	168,910	high
126	stu-miR390-5p_L+1_1ss20AG	AAAGCTCAGGAGGGATAGCGCC	down	0.41	-1.27	3.24E-02	26	11	middle
127	ptc-miR169i_1ss15TA	TAGCCAAGGATGACATGCCTG	up	1.54	0.62	3.28E-02	706	1,087	middle
128	ptc-MIR481c-p5_1ss12AG	AAGGTAATGTGGGCCTGTGCAAGT	down	0.45	-1.16	3.28E-02	566	254	middle

129	ptc-MIR481b-p5_2ss3TC22GA	ATCCTGGAACCTCACCTAACAACT	down	0.42	-1.25	3.32E-02	51	21	middle
130	ptc-MIR482d-p5_1ss9AT	GATGGAGGTCGAGGAAGTTTCT	up	2.25	1.17	3.33E-02	371	835	middle
131	ptc-miR396g-3p_R-1_1ss19AG	CTCAAGAAAGCCGTGGGAGA	up	1.75	0.81	3.34E-02	881	1,541	middle
132	cme-MIR399c-p5_2ss17TA18GC	TAAAACACACACACACACACACA	down	0.19	-2.42	3.35E-02	2	0	low
133	mes-MIR482e-p3_1ss9TC	TTTCCGATCATTCCTCCCTC	up	1.30	0.38	3.35E-02	219	286	middle
134	ptc-miR160a_1ss21AT	TGCCTGGCTCCCTGTATGCCT	down	0.33	-1.60	3.37E-02	1,203	396	middle
135	ptc-MIR481c-p5_1ss13CT	ACCAAGCGGTCATGAGTTCGAATC	down	0.31	-1.70	3.45E-02	116	36	middle
136	ptc-MIR481d-p5_2ss3GA19CT	TTAACATGGTATCAGAGCTT	down	0.31	-1.70	3.71E-02	3	1	low
137	ptc-MIR481d-p3_2ss15CT17AG	AAGGTAATGTGGGCTTGTGCAAGT	down	0.41	-1.29	3.71E-02	69	28	middle
138	hbr-MIR6485-p5_2ss11AG20GT	TGAGTGATTCGGCAGATTTTGATT	up	3.98	1.99	3.76E-02	11	43	middle
139	cas-miR159b-3p_R+1	TTTGGATTGAAGGGAGCTCTTT	down	0.71	-0.49	3.76E-02	3,775	2,684	middle
140	ath-miR159c_R+1_1ss20CT	TTTGGATTGAAGGGAGCTCTTT	down	0.71	-0.49	3.76E-02	3,775	2,684	middle
141	ptc-miR6468-3p	GGAGTGATTCAGGGAACCCAT	up	5.22	2.38	3.81E-02	7	34	middle
142	peu-MIR2912b-p3_1ss6GA	AACTCAAGATATGGGCTGAACACT	down	0.28	-1.83	3.83E-02	211	59	middle
143	cca-MIR156c-p5_2ss7CT17AC	AGAGCATTGAGAGGACCT	up	46.08	5.53	3.83E-02	1	24	middle
144	ptc-miR7823	TTGCATGCATGAACTTGAAAT	up	4.18	2.06	3.84E-02	3	11	middle
145	ptc-MIR396b-p3	GTTCAATAAAGCTGTGGGAAG	up	6.67	2.74	3.84E-02	21	137	middle
146	ptc-MIR481d-p3_2ss14CT17AG	AAGGTAATGTGGGTCTGTGCAAGT	down	0.49	-1.03	3.85E-02	358	176	middle
147	ptc-MIR481d-p5_2ss14CT17AG	AAGGTAATGTGGGTCTGTGCAAGT	down	0.49	-1.03	3.85E-02	358	176	middle
148	ptc-MIR481a-p3_2ss13AG23TC	AAATCATATCCTGGGACCTCACCT	down	0.44	-1.17	3.88E-02	1,383	615	middle
149	ptc-MIR481a-p5_2ss13AG23TC	AAATCATATCCTGGGACCTCACCT	down	0.44	-1.17	3.88E-02	1,383	615	middle
150	ptc-miR477e-5p_R+1	ACTCTCCCTCAAGGCTTCCAT	up	4.19	2.07	3.89E-02	25	104	middle
151	ptc-miR408-3p	ATGCACTGCCTCTTCCCTGGC	down	0.08	-3.69	3.93E-02	7,247	561	high
152	ptc-miR530a_R+1_1ss20TC_1	TGCATTTGCACCTGCACCTCA	up	2.41	1.27	3.97E-02	14	34	middle
153	ptc-miR530a_R+1_1ss20TC_2	TGCATTTGCACCTGCACCTCC	up	2.41	1.27	3.97E-02	14	34	middle
154	gma-MIR4382-p5_2ss10AC18TA	TGGGGAAGTCGGAATAGA	up	93.60	6.55	4.02E-02	0	28	middle
155	ptc-miR166n_L+2R-2	TCTCGGACCAGGCTTCATTCC	down	0.26	-1.92	4.04E-02	449,715	118,863	high
156	ptc-miR159a	TTTGGATTGAAGGGAGCTCTA	down	0.59	-0.75	4.12E-02	278,482	165,280	high
157	ptc-miR159a_1ss21AT	TTTGGATTGAAGGGAGCTCTT	down	0.59	-0.76	4.13E-02	68,777	40,537	high
158	gra-MIR482d-p3_1	ATATACACACACACACACACACA	down	0.56	-0.82	4.15E-02	6	4	low
159	gra-MIR482d-p3_2	TATACACACACACACACACAC	down	0.56	-0.82	4.15E-02	6	4	low
160	gra-MIR482d-p3_3	TATATACACACACACACACACACA	down	0.56	-0.82	4.15E-02	6	4	low
161	ptc-miR3627b_2ss7AG19GA	TGTCGCGGGAGAGATGGCACTA	down	0.07	-3.76	4.18E-02	412	31	middle

162	ptc-miR164f_1ss17AG	TGGAGAAGCAGGGCACGTGCT	up	2.92	1.55	4.19E-02	141	411	middle
163	gra-MIR8674b-p3_2ss11TC18AC	AAACCCTAAACCCTAAACC	down	0.08	-3.64	4.20E-02	18	1	middle
164	gra-MIR8674b-p5_2ss11TC18AC	AAACCCTAAACCCTAAACC	down	0.08	-3.64	4.20E-02	18	1	middle
165	ptc-miR159d_L-1R-1	TTGGATTGAAGGGAGCTCC	down	0.43	-1.21	4.22E-02	312	135	middle
166	csi-MIR169m-p3_1ss12GT	CTTGGCTACATTTTTCTTTCT	up	2.57	1.36	4.27E-02	49	125	middle
167	hbr-miR408a_R-1_1ss6TG	AAGACGGGGAACAGGCAGAGC	down	0.12	-3.02	4.40E-02	137	17	middle
168	ptc-miR6425a-3p_1ss4AT	TCCTTGGAAGATAATGACTCG	up	3.86	1.95	4.41E-02	7	28	middle
169	nta-miR408_R+1	TGCACTGCCTCTTCCCTGGCTC	down	0.08	-3.65	4.44E-02	131	10	middle
170	stu-miR482b-3p	TTACCGATTCCCCCCATTCCAA	up	1.73	0.79	4.49E-02	7	13	middle
171	ptc-miR396a	TTCCACAGCTTTCTTGAACTG	up	1.94	0.96	4.59E-02	5,751	11,172	high
172	cme-MIR1863-p5_2ss3AG21GA	AAGGCTCTGATACCATGTTAA	down	0.17	-2.56	4.59E-02	2	0	low
173	ptc-MIR530a-p3	TGCAGGTGCAGGTGCAGGTGA	up	2.46	1.30	4.65E-02	11	27	middle
174	ptc-miR160a	TGCCTGGCTCCCTGTATGCCA	down	0.24	-2.03	4.71E-02	53,012	12,938	high
175	ptc-MIR481d-p3_3	ATTCTTTGACATGGTATCAGAGCC	down	0.40	-1.30	4.75E-02	149	60	middle
176	ptc-MIR481d-p5	ATTCTTTGACATGGTATCAGAGCC	down	0.40	-1.30	4.75E-02	149	60	middle
177	ptc-miR160g_2ss15GT21AG	TGCCTGGCTCCCTGTATGCCG	down	0.24	-2.04	4.76E-02	336	82	middle
178	gso-miR3522a	TGAGACCAAATGAGCAGCTGA	up	1.56	0.64	4.79E-02	50	78	middle
179	ptc-MIR481b-p5_1ss3CT	AGTTTAAGCTATTGGGTTGAGAT	down	0.40	-1.32	4.79E-02	3	1	low
180	fve-miR159c_1ss1AT	TTTGGATTGAAGGGAGCTCCC	down	0.40	-1.32	4.82E-02	133	53	middle
181	lus-miR319b_L+1_1ss7CT	TTTGGATTGAAGGGAGCTCCC	down	0.40	-1.32	4.82E-02	133	53	middle
182	ptc-miR167f-5p_R+1	TGAAGCTGCCAGCATGATCTTA	up	1.71	0.77	4.83E-02	8,804	15,041	high
183	cme-MIR160c-p5_2ss13AG17AG	AGCCATGCATGTGTAAGT	up	4.64	2.21	4.85E-02	2	7	low
184	ptc-MIR481b-p5_2	TGGAACCTCACCTAACAGCTT	down	0.59	-0.75	4.95E-02	114	68	middle
185	ptc-MIR481b-p3_2	TGGAACCTCACCTAACAGCTT	down	0.59	-0.75	4.95E-02	114	68	middle
186	ptc-MIR481b-p3_3	TTCTTTGACATGATATCAGAGCCT	down	0.59	-0.75	4.95E-02	114	68	middle
187	ptc-MIR481b-p3_1	CTTAAGCTATTGGGTTGAGATGGT	down	0.59	-0.75	4.95E-02	114	68	middle

Table S6 MicroRNAs involved in abiotic stress and secondary metabolic pathways were differentially expressed in transgenic poplar plants.

miR_name	miR_seq	up/ down	Fold change	log2 (fold change)	p-value	Expression level	Targets	Functions	Anthocyanin synthesis	Hormone signal transduction	Stress response	Disease-resistant
ptc-miR156a	TGACAGAAGAGAGTGAGCAC	up	107.82	6.75	2.15E-03	high	*SPL*	Flowering under abiotic stress;<br>Shade-avoidance syndrome;<br>Heat stress memory;<br>Phosphate-deficiency response;<br>Insect resistance;<br>Drought tolerance;<br>Age-dependent vernalization;	√		√	
ptc-miR160h	TGCCTGGCTCCCTGCATGCCA	up	2.77	1.47	0.01703118	middle	ARF/LAR3	Drought tolerance;<br>Root responses to nitrate ;<br>Nodule development;<br>Heat stress tolerance;	√	√	√	
ptc-miR164a	TGGAGAAGCAGGGCACGTGCA	up	2.95	1.56	8.67E-03	high	*NAC/HD-ZIPIII*	Pathogen immune response	√	√	√	√
ptc-miR167f-5p_R+1	TGAAGCTGCCAGCATGATCTTA	up	1.71	0.77	4.83E-02	high	*ARF/MIN/TVP/LAR3*	Drought tolerance;<br>Root responses to nitrate ;<br>Nodule development;<br>Heat stress tolerance;		√	√	
ptc-miR168a-3p	CCCGCCTTGCATCAACTGAAT	up	1.57	0.65	6.35E-03	middle	*AGO1*	Pathogen immune response;			√	√
ptc-miR169q_L+1R-1_1ss17TC	CTAGCCAAGGACGACTCGCCT	up	3.54	1.82	5.45E-03	middle	*NFYA/HAP2*	Drought tolerance;<br>Nodule development;<br>Nutrient-deficiency response;<br>Pathogen immunity response;			√	√
ptc-miR393a-3p_1ss10TC	ATCATGCTACCCCTTTGGATT	up	1.78	0.83	8.35E-03	middle	*TIR1/AFB/ENOD/Bzip*	Pathogen immunity response;<br>Root responses to nitrate/aluminum stress;<br>Arbuscule/nodule development;<br>Salt/cold tolerance;Seed germination/seedling initiation under submergence;<br>Nodule development;	√	√	√	√
ptc-miR396a	TTCCACAGCTTTCTTGAACTG	up	1.94	0.96	4.59E-02	high	*GRF/WRKY/Rhodernase(NTF)*	Syncytium formation response to parasitic nematodes;<br>Leaf growth upon UV-B irradiation;<br>Pathogen immunity response;<br>Mycorrhization development;<br>WRKY6 Heat stress tolerance;	√		√	√
ptc-miR399a_R-2_1ss13TG	TGCCAAAGGAGAGTTGCCC	up	14.84	3.89	1.44E-02	middle	*LTN1/PHO2/E2-UBC(NTF)*	Phosphate-deficiency response;			√	
ptc-MIR472a-p5_1ss2CT	TTGGGTGGGTGAGCGGGGAAGA	up	1.65	0.72	2.23E-02	middle	*RPS2/RPM1/RPN7/HACD2*					√

ptc-MIR482d-p5_1ss9AT	GATGGAGGTCGAGGAAGTTTCT	up	2.25	1.17	3.33E-02	middle	*R* genes	Pathogen immunity response;	√			√
ptc-miR530a_R+1	TGCATTTGCACCTGCACCTTA	up	2.45	1.29	2.23E-02	middle	*TZP/AIB/lvr*			√		
ppe-MIR858-p5	TCTCGTTGTCTGTTCGACCTT	up	2.44	1.29	4.94E-03	high	*MYB/C1/PP1*		√			
ptc-miR159d_L-1R-1	TTGGATTGAAGGGAGCTCC	down	0.43	-1.21	4.22E-02	middle	MYB/TCP/bHLH/HIC-15	Pathogen immune response;Plant–nematode interaction;	√	√		√
ptc-miR166n_L+2R-2	TCTCGGACCAGGCTTCATTCC	down	0.26	-1.92	4.04E-02	high	HD-ZIPIII/NIT/SHR/SOR/CLP1	Pathogen immune response;<br>Drought/cold tolerance;<br>Nodule development;<br>Ion uptake and accumulation under low-nutrient conditions ;	√		√	√
ptc-miR172a	AGAATCTTGATGATGCTGCAT	down	0.07	-3.74	1.59E-03	middle	*AP2/WRKY/AP2-like*	Ambient temperature-responsive flowering;<br>Nodule development;	√			
lus-miR319b_L+1_1ss7CT	TTTGGATTGAAGGGAGCTCCC	down	0.40	-1.32	4.82E-02	middle	*TCP/MYB*	Cold tolerance;<br>Drought/salt tolerance;<br>Pathogen immunity response;<br>Plant–nematode interaction;	√		√	√
ptc-miR390d-3p	CGCTATCCATCCTGAGTTTTA	down	0.41	-1.30	2.66E-03	middle	*ARF/TAS3/RLK*	Nodule development;	√	√		
ptc-miR397a	TCATTGAGTGCAGCGTTGATG	down	0.20	-2.36	1.52E-03	middle	*LAC*	Lignin biosynthesis			√	
ptc-miR398b	TGTGTTCTCAGGTCGCCCCTG	down	0.15	-2.69	1.78E-02	middle	*CSD/CytC oxidase/SOD/SBP3*	Oxidative stress tolerance;<br>Copper-deficient response;<br>Heat stress tolerance;<br>Pathogen immunity response;			√	√
ptc-miR408-3p	ATGCACTGCCTCTTCCCTGGC	down	0.08	-3.69	3.93E-02	high	*LAC/PCL(NTF)/COX5b*	Copper-deficiency response;<br>Salt/drought/cold/oxidative/osmotic-stress responses;			√	
