# Supplementary material for: MiR156 regulates anthocyanin biosynthesis through SPL targets and other microRNAs in poplar
Source: Hortic Res. 2020 Aug 1;7:118. doi: 10.1038/s41438-020-00341-w (PMC7395715; doi:10.1038/s41438-020-00341-w)
Supplement: Supplementary file 3 — Supporting Information 3 [file 41438_2020_341_MOESM3_ESM.pdf]

Table S7 Overview of differentially expressed genes between wild-type and transgenic poplar plants.

Gene_ID	Annotation	Name	swissprot_description	TGII_mean_TPM	WT_mean_TPM	log2FC	regulation	p-value	FDR	significant
TRINITY_DN24605_c0_g7	hygromycin resistance protein, partial [Oryza sativa]	hph	Hygromycin-B 4-O-kinase OS=Escherichia coli GN=hph PE=1 SV=1	32.82	0.04	10.03	up	0.00	0.00	yes
TRINITY_DN23151_c0_g2	hypothetical protein POPTR_0009s11180g [Populus trichocarpa]	CYP82C4	Cytochrome P450 82C4 OS=Arabidopsis thaliana GN=CYP82C4 PE=2 SV=1	21.65	0.90	5.41	up	0.00	0.00	yes
TRINITY_DN15830_c0_g1	thioredoxin H [Populus tremula x Populus tremuloides]	TRX8	Thioredoxin H8 OS=Arabidopsis thaliana GN=TRX8 PE=2 SV=1	41.24	0.50	6.93	up	0.00	0.00	yes
TRINITY_DN14685_c0_g1	alcohol dehydroge family protein [Populus trichocarpa]	-	Secoisolariciresinol dehydrogenase (Fragment) OS=Podophyllum peltatum PE=1 SV=1	37.25	1.10	5.68	up	0.00	0.00	yes
TRINITY_DN27588_c0_g1	sucrose-phosphate synthase family protein [Populus trichocarpa]	SPS3	Probable sucrose-phosphate synthase 3 OS=Arabidopsis thaliana GN=SPS3 PE=2 SV=1	36.05	1.68	4.99	up	0.00	0.00	yes
TRINITY_DN15522_c0_g1	hypothetical protein POPTR_0014s11960g, partial [Populus trichocarpa]	-	-	106.11	2.79	5.86	up	0.00	0.00	yes
TRINITY_DN19978_c0_g1	galactinol synthase family protein [Populus trichocarpa]	GOLS1	Galactinol synthase 1 OS=Ajuga reptans GN=GOLS1 PE=1 SV=1	82.15	1.43	6.48	up	0.00	0.00	yes
TRINITY_DN16400_c0_g1	hypothetical protein POPTR_0010s06320g [Populus trichocarpa]	HSP21	Small heat shock protein, chloroplastic OS=Pisum sativum GN=HSP21 PE=2 SV=1	46.19	4.37	4.03	up	0.00	0.00	yes
TRINITY_DN1417_c0_g1	hypothetical protein POPTR_0017s08950g [Populus trichocarpa]	KNU	Zinc finger protein KNUCKLES OS=Arabidopsis thaliana GN=KNU PE=1 SV=1	9.69	0.02	9.22	up	0.00	0.00	yes
TRINITY_DN15087_c0_g1	hypothetical protein POPTR_0014s05870g [Populus trichocarpa]	At4g00165	Putative lipid-binding protein At4g00165 OS=Arabidopsis thaliana GN=At4g00165 PE=2 SV=1	191.71	5.32	5.77	up	0.00	0.00	yes
TRINITY_DN22731_c0_g1	PREDICTED: LOW QUALITY PROTEIN: plasma membrane ATPase 4 [Populus euphratica]	PMA4	Plasma membrane ATPase 4 OS=Nicotiana plumbaginifolia GN=PMA4 PE=2 SV=1	31.52	3.58	3.73	up	0.00	0.00	yes
TRINITY_DN12115_c0_g1	hypothetical protein POPTR_0009s06350g [Populus trichocarpa]	-	-	161.94	13.22	4.22	up	0.00	0.00	yes
TRINITY_DN20991_c0_g1	chloroplast isoprene synthase 3 [Populus alba]	ISPS	Isoprene synthase, chloroplastic OS=Populus alba GN=ISPS PE=1 SV=1	26.30	0.25	7.02	up	0.00	0.00	yes
TRINITY_DN18532_c0_g1	PREDICTED: MADS-box transcription factor 14-like isoform X1 [Populus euphratica]	CAL	Transcription factor CAULIFLOWER OS=Arabidopsis thaliana GN=CAL PE=1 SV=3	30.36	1.63	4.68	up	0.00	0.00	yes
TRINITY_DN21419_c0_g2	hypothetical protein POPTR_0005s03435g [Populus trichocarpa]	-	-	27.47	0.11	7.38	up	0.00	0.00	yes
TRINITY_DN21894_c0_g1	PREDICTED: transmembrane protein 184 homolog DDB_G0279555-like isoform X1 [Populus euphratica]	tmem184C	Transmembrane protein 184 homolog DDB_G0279555 OS=Dictyostelium discoideum GN=tmem184C PE=3 SV=1	24.46	2.87	3.70	up	0.00	0.00	yes
TRINITY_DN24443_c0_g1	hypothetical protein POPTR_0018s09660g [Populus trichocarpa]	At1g29660	GDSL esterase/lipase At1g29660 OS=Arabidopsis thaliana GN=At1g29660 PE=1 SV=1	90.09	11.63	3.60	up	0.00	0.00	yes
TRINITY_DN16734_c0_g1	zinc finger family protein [Populus trichocarpa]	MIEL1	E3 ubiquitin-protein ligase MIEL1 OS=Arabidopsis thaliana GN=MIEL1 PE=1 SV=1	11.22	0.47	4.90	up	0.00	0.00	yes
TRINITY_DN18751_c0_g1	hypothetical protein POPTR_0010s17650g [Populus trichocarpa]	ZHD11	Zinc-finger homeodomain protein 11 OS=Arabidopsis thaliana GN=ZHD11 PE=1 SV=1	25.11	2.13	4.30	up	0.00	0.00	yes
TRINITY_DN18420_c0_g1	hypothetical protein POPTR_0016s02270g [Populus trichocarpa]	UGT85A24	7-deoxyloganetin glucosyltransferase OS=Gardenia jasminoides GN=UGT85A24 PE=1 SV=1	30.15	5.07	3.37	up	0.00	0.00	yes
TRINITY_DN23190_c2_g1	xyloglucan endotransglucosylase/hydrolase protein 32 precursor [Populus trichocarpa]	XTH32	Probable xyloglucan endotransglucosylase/hydrolase protein 32 OS=Arabidopsis thaliana GN=XTH32 PE=2 SV=1	31.54	2.13	4.55	up	0.00	0.00	yes
TRINITY_DN22952_c1_g2	hypothetical protein POPTR_0003s08390g [Populus trichocarpa]	BRG1	BOI-related E3 ubiquitin-protein ligase 1 OS=Arabidopsis thaliana GN=BRG1 PE=1 SV=1	30.93	5.48	3.10	up	0.00	0.00	yes
TRINITY_DN27817_c4_g2	-	-	-	23.55	0.00	10.25	up	0.00	0.00	yes
TRINITY_DN16651_c0_g1	equilibrative nucleoside transporter family protein [Populus trichocarpa]	ENT3	Equilibrative nucleotide transporter 3 OS=Arabidopsis thaliana GN=ENT3 PE=1 SV=1	12.81	1.31	3.90	up	0.00	0.00	yes
TRINITY_DN18466_c0_g1	hypothetical protein POPTR_0018s14920g [Populus trichocarpa]	-	Alpha-galactosidase OS=Coffea arabica PE=1 SV=1	22.92	4.21	3.25	up	0.00	0.00	yes
TRINITY_DN16745_c0_g1	hypothetical protein POPTR_0014s16610g [Populus trichocarpa]	FLA2	Fasciclin-like arabinogalactan protein 2 OS=Arabidopsis thaliana GN=FLA2 PE=1 SV=1	41.77	2.96	4.38	up	0.00	0.00	yes

TRINITY_DN18480_c0_g1	Pantoate--beta-alanine ligase family protein [Populus trichocarpa]	PANC	Pantoate--beta-alanine ligase OS=Lotus japonicus GN=PANC PE=1 SV=3	17.35	2.76	3.23	up	0.00	0.00	yes		
TRINITY_DN24626_c0_g5	PREDICTED: polyamine oxidase-like [Populus euphratica]	PAO	Polyamine oxidase OS=Zea mays GN=PAO PE=1 SV=1	238.94	11.76	4.91	up	0.00	0.00	yes		
TRINITY_DN14338_c0_g1	-	-	-	6.10	0.06	7.17	up	0.00	0.00	yes		
TRINITY_DN20034_c0_g1	hypothetical protein POPTR_0006s03440g [Populus trichocarpa]	SCPL45	Serine carboxypeptidase-like 45 OS=Arabidopsis thaliana GN=SCPL45 PE=2 SV=1	16.65	2.22	3.46	up	0.00	0.00	yes		
TRINITY_DN22782_c0_g3	PREDICTED: 1-aminocyclopropane-1-carboxylate oxidase homolog 1-like [Populus euphratica]	At1g06620	1-aminocyclopropane-1-carboxylate oxidase homolog 1 OS=Arabidopsis thaliana GN=At1g06620 PE=2 SV=1	33.44	7.80	2.72	up	0.00	0.00	yes		
TRINITY_DN24850_c2_g2	hypothetical protein POPTR_0019s14280g [Populus trichocarpa]	SD18	Receptor-like serine/threonine-protein kinase SD1-8 OS=Arabidopsis thaliana GN=SD18 PE=1 SV=1	12.20	2.73	2.79	up	0.00	0.00	yes		
TRINITY_DN15487_c0_g1	methyl transferase [Populus tomentosa]	NMT2	Phosphomethylethanolamine N-methyltransferase OS=Arabidopsis thaliana GN=NMT2 PE=2 SV=2	61.40	3.01	4.95	up	0.00	0.00	yes		
TRINITY_DN17925_c1_g1	hypothetical protein POPTR_0014s04880g [Populus trichocarpa]	NDK1	Nucleoside diphosphate kinase 1 OS=Arabidopsis thaliana GN=NDK1 PE=1 SV=1	213.24	40.67	3.02	up	0.00	0.00	yes		
TRINITY_DN14585_c0_g1	hypothetical protein POPTR_0004s22120g [Populus trichocarpa]	UBL5	Ubiquitin-like protein 5 OS=Arabidopsis thaliana GN=UBL5 PE=3 SV=1	40.36	3.69	4.07	up	0.00	0.00	yes		
TRINITY_DN24990_c0_g2	alanine aminotransferase family protein [Populus simonii x Populus nigra]	ALAAT1	Alanine aminotransferase 1, mitochondrial OS=Arabidopsis thaliana GN=ALAAT1 PE=1 SV=1	51.44	7.50	3.40	up	0.00	0.00	yes		
TRINITY_DN23991_c0_g3	brassinosteroid-regulated family protein [Populus trichocarpa]	XTH16	Probable xyloglucan endotransglucosylase/hydrolase protein 16 OS=Arabidopsis thaliana GN=XTH16 PE=2 SV=2	110.57	19.64	3.09	up	0.00	0.00	yes		
TRINITY_DN22699_c1_g1	hypothetical protein POPTR_0010s05080g [Populus trichocarpa]	-	Carbonic anhydrase, chloroplastic OS=Nicotiana tabacum PE=2 SV=1	364.96	66.06	3.35	up	0.00	0.00	yes		
TRINITY_DN21312_c3_g2	hypothetical protein POPTR_0010s20780g [Populus trichocarpa]	AHL15	AT-hook motif nuclear-localized protein 15 OS=Arabidopsis thaliana GN=AHL15 PE=2 SV=1	5.44	0.29	4.80	up	0.00	0.00	yes		
TRINITY_DN22466_c0_g2	-	-	-	16.10	0.74	5.02	up	0.00	0.00	yes		
TRINITY_DN23207_c0_g2	PREDICTED: BAG family molecular chaperone regulator 2-like isoform X1 [Populus euphratica]	BAG1	BAG family molecular chaperone regulator 1 OS=Arabidopsis thaliana GN=BAG1 PE=1 SV=1	17.30	2.42	3.48	up	0.00	0.00	yes		
TRINITY_DN16749_c0_g1	hypothetical protein POPTR_0004s05080g [Populus trichocarpa]	-	-	11.74	0.02	9.09	up	0.00	0.00	yes		
TRINITY_DN20360_c1_g1	lipid-associated family protein [Populus trichocarpa]	PLAT2	PLAT domain-containing protein 2 OS=Arabidopsis thaliana GN=PLAT2 PE=2 SV=1	9.95	0.42	5.14	up	0.00	0.00	yes		
TRINITY_DN22160_c0_g1	PREDICTED: uncharacterized protein LOC105123685 [Populus euphratica]	SG1	protein SLOW GREEN 1, chloroplastic OS=Arabidopsis thaliana GN=SG1 PE=1 SV=1	145.48	37.86	2.58	up	0.00	0.00	yes		
TRINITY_DN25190_c0_g2	hypothetical protein POPTR_0007s13380g [Populus trichocarpa]	BZIP2	bZIP transcription factor 2 OS=Arabidopsis thaliana GN=BZIP2 PE=1 SV=1	15.25	2.76	3.08	up	0.00	0.00	yes		
TRINITY_DN16830_c0_g1	hypothetical protein POPTR_0001s41300g [Populus trichocarpa]	-	-	8.88	0.74	4.23	up	0.00	0.00	yes		
TRINITY_DN7763_c0_g1	cystathionine gamma-synthase [Populus tomentosa]	MGL	Methionine gamma-lyase OS=Arabidopsis thaliana GN=MGL PE=1 SV=1	18.32	0.02	10.19	up	0.00	0.00	yes		
TRINITY_DN21597_c0_g1	MLO-like protein 4 [Populus trichocarpa]	MLO4	MLO-like protein 4 OS=Arabidopsis thaliana GN=MLO4 PE=2 SV=2	28.54	3.02	3.83	up	0.00	0.00	yes		
TRINITY_DN17959_c0_g1	PREDICTED: probable S-sulfocysteine synthase, chloroplastic [Populus euphratica]	At3g03630	Probable S-sulfocysteine synthase, chloroplastic OS=Arabidopsis thaliana GN=At3g03630 PE=1 SV=1	54.54	12.96	2.77	up	0.00	0.00	yes		
TRINITY_DN18547_c0_g1	hypothetical protein POPTR_0001s19100g [Populus trichocarpa]	At3g21360	Clavaminate synthase-like protein At3g21360 OS=Arabidopsis thaliana GN=At3g21360 PE=1 SV=1	45.00	8.51	3.01	up	0.00	0.00	yes		
TRINITY_DN18765_c0_g1	hypothetical protein POPTR_0010s17420g [Populus trichocarpa]	-	-	32.83	6.11	3.33	up	0.00	0.00	yes		
TRINITY_DN17307_c0_g1	PREDICTED: thaumatin-like protein 1b [Populus euphratica]	TL1	Thaumatin-like protein 1 OS=Pyrus pyrifolia GN=TL1 PE=1 SV=1	19.77	2.44	3.62	up	0.00	0.00	yes		
TRINITY_DN23236_c1_g2	hypothetical protein POPTR_0001s40110g [Populus trichocarpa]	-	-	53.03	13.41	2.57	up	0.00	0.00	yes		
TRINITY_DN24951_c0_g1	unknown [Populus trichocarpa]	FKBP65	Peptidyl-prolyl cis-trans isomerase FKBP65 OS=Arabidopsis thaliana GN=FKBP65 PE=1 SV=1	40.05	12.23	2.63	up	0.00	0.00	yes		
TRINITY_DN20790_c0_g1	PREDICTED: cytosolic sulfotransferase 15-like [Populus euphratica]	SOT15	Cytosolic sulfotransferase 15 OS=Arabidopsis thaliana GN=SOT15 PE=1 SV=1	96.77	15.23	3.22	up	0.00	0.00	yes		

TRINITY_DN20822_c1_g1	hypothetical protein POPTR_0006s22440g [Populus trichocarpa]	PRCP	Lysosomal Pro-X carboxypeptidase OS=Pongo abelii GN=PRCP PE=2 SV=1	25.70	5.89	2.87	up	0.00	0.00	yes
TRINITY_DN24400_c0_g3	heat shock transcription factor A7a1 [Populus simonii]	HSFA6b	Heat stress transcription factor A-6b OS=Arabidopsis thaliana GN=HSFA6b PE=2 SV=1	9.71	0.94	4.57	up	0.00	0.00	yes
TRINITY_DN25476_c0_g2	PREDICTED: alanine aminotransferase 2, mitochondrial-like [Populus euphratica]	ALAAT1	Alanine aminotransferase 1, mitochondrial OS=Arabidopsis thaliana GN=ALAAT1 PE=1 SV=1	33.99	8.74	2.59	up	0.00	0.00	yes
TRINITY_DN25746_c0_g1	nodule-enhanced malate dehydrogenase family protein [Populus trichocarpa]	At3g47520	Malate dehydrogenase, chloroplastic OS=Arabidopsis thaliana GN=At3g47520 PE=1 SV=1	113.74	23.22	2.80	up	0.00	0.00	yes
TRINITY_DN21512_c1_g3	hypothetical protein POPTR_0002s19010g [Populus trichocarpa]	CAB3C	Chlorophyll a-b binding protein 3C, chloroplastic OS=Solanum lycopersicum GN=CAB3C PE=3 SV=1	25.66	4.25	3.22	up	0.00	0.00	yes
TRINITY_DN25380_c0_g1	hypothetical protein POPTR_0008s02970g [Populus trichocarpa]	DLO2	Protein DMR6-LIKE OXYGENASE 2 OS=Arabidopsis thaliana GN=DLO2 PE=2 SV=1	5.41	0.11	6.17	up	0.00	0.00	yes
TRINITY_DN20293_c0_g1	PREDICTED: protein trichome birefringence-like 25 isoform X1 [Populus euphratica]	TBL25	Protein trichome birefringence-like 25 OS=Arabidopsis thaliana GN=TBL25 PE=2 SV=1	10.87	1.30	3.70	up	0.00	0.00	yes
TRINITY_DN15227_c0_g2	PREDICTED: zeaxanthin epoxidase, chloroplastic-like [Populus euphratica]	hpxO	FAD-dependent urate hydroxylase OS=Acinetobacter baylyi (strain ATCC 33305 / BD413 / ADP1) GN=hpxO PE=1 SV=1	5.07	0.36	4.43	up	0.00	0.00	yes
TRINITY_DN16332_c1_g3	hypothetical protein POPTR_0011s15310g [Populus trichocarpa]	DLO2	Protein DMR6-LIKE OXYGENASE 2 OS=Arabidopsis thaliana GN=DLO2 PE=2 SV=1	8.29	0.36	5.06	up	0.00	0.00	yes
TRINITY_DN25145_c1_g1	-	-	-	2821.33	284.85	3.95	up	0.00	0.00	yes
TRINITY_DN14002_c0_g1	PREDICTED: probable 2-oxoglutarate-dependent dioxygenase AOP1 [Populus euphratica]	At5g51310	Gibberellin 20-oxidase-like protein OS=Arabidopsis thaliana GN=At5g51310 PE=2 SV=1	4.30	0.03	7.23	up	0.00	0.00	yes
TRINITY_DN23091_c0_g5	glutamine synthetase family protein [Populus simonii x Populus nigra]	-	Glutamine synthetase nodule isozyme OS=Vigna aconitifolia PE=2 SV=1	46.52	4.43	4.02	up	0.00	0.00	yes
TRINITY_DN21101_c1_g2	hypothetical protein POPTR_0012s13320g [Populus trichocarpa]	JAL19	Jacalin-related lectin 19 OS=Arabidopsis thaliana GN=JAL19 PE=2 SV=1	12.42	1.88	3.47	up	0.00	0.00	yes
TRINITY_DN16533_c0_g2	IQ domain-containing family protein [Populus trichocarpa]	BAG6	BAG family molecular chaperone regulator 6 OS=Arabidopsis thaliana GN=BAG6 PE=1 SV=1	5.20	0.26	4.96	up	0.00	0.00	yes
TRINITY_DN21747_c0_g1	hypothetical protein POPTR_0015s12250g [Populus trichocarpa]	GDH2	Glutamate dehydrogenase 2 OS=Arabidopsis thaliana GN=GDH2 PE=1 SV=1	29.79	5.40	3.00	up	0.00	0.00	yes
TRINITY_DN15373_c0_g1	pathogenesis-related family protein [Populus trichocarpa]	PR-1	Pathogenesis-related protein PR-1 OS=Medicago truncatula GN=PR-1 PE=2 SV=1	42.69	3.64	4.19	up	0.00	0.00	yes
TRINITY_DN19165_c0_g3	hypothetical protein POPTR_0008s14480g [Populus trichocarpa]	-	-	137.41	30.03	2.81	up	0.00	0.00	yes
TRINITY_DN22169_c0_g3	hypothetical protein POPTR_0016s13300g [Populus trichocarpa]	mcfF	Mitoferrin OS=Dictyostelium discoideum GN=mcfF PE=3 SV=1	27.25	2.26	4.20	up	0.00	0.00	yes
TRINITY_DN16713_c0_g2	PREDICTED: L-type lectin-domain containing receptor kinase VIII.1-like [Populus euphratica]	LECRKS7	Probable L-type lectin-domain containing receptor kinase S.7 OS=Arabidopsis thaliana GN=LECRKS7 PE=2 SV=1	11.35	2.08	3.08	up	0.00	0.00	yes
TRINITY_DN18660_c0_g1	hypothetical protein POPTR_0008s00200g [Populus trichocarpa]	-	-	4.79	0.15	5.50	up	0.00	0.00	yes
TRINITY_DN28790_c0_g1	PREDICTED: patatin-like protein 2 [Populus euphratica]	PLP2	Patatin-like protein 2 OS=Arabidopsis thaliana GN=PLP2 PE=1 SV=1	4.91	0.17	5.46	up	0.00	0.00	yes
TRINITY_DN22307_c0_g3	PREDICTED: 60S ribosomal protein L7a-like [Populus euphratica]	RPL7A-1	60S ribosomal protein L7a-1 OS=Oryza sativa subsp. japonica GN=RPL7A-1 PE=2 SV=1	89.49	22.39	2.62	up	0.00	0.00	yes
TRINITY_DN17431_c0_g1	adenylosuccinate synthetase family protein [Populus trichocarpa]	PURA	Adenylosuccinate synthetase, chloroplastic OS=Populus trichocarpa GN=PURA PE=3 SV=1	82.22	24.66	2.34	up	0.00	0.00	yes
TRINITY_DN21582_c0_g1	PREDICTED: fructokinase-like 2, chloroplastic [Populus euphratica]	FLN2	Fructokinase-like 2, chloroplastic OS=Arabidopsis thaliana GN=FLN2 PE=1 SV=2	83.87	26.93	2.42	up	0.00	0.00	yes
TRINITY_DN22195_c0_g3	hypothetical protein POPTR_0010s16150g [Populus trichocarpa]	DTX37	Protein DETOXIFICATION 37 OS=Arabidopsis thaliana GN=DTX37 PE=2 SV=1	55.28	4.09	4.36	up	0.00	0.00	yes
TRINITY_DN24090_c0_g1	20 kDa chaperonin family protein [Populus trichocarpa]	CPN20	20 kDa chaperonin, chloroplastic OS=Arabidopsis thaliana GN=CPN20 PE=1 SV=2	1590.59	395.72	2.62	up	0.00	0.00	yes
TRINITY_DN27157_c0_g1	inorganic pyrophosphatase family protein-2 [Populus tomentosa]	AVP1	Pyrophosphate-energized vacuolar membrane proton pump 1 OS=Arabidopsis thaliana GN=AVP1 PE=1 SV=1	170.71	25.99	3.33	up	0.00	0.00	yes
TRINITY_DN21056_c0_g1	Sulfate transporter 3.2 family protein [Populus trichocarpa]	SULTR3;1	Sulfate transporter 3.1 OS=Arabidopsis thaliana GN=SULTR3;1 PE=2 SV=1	15.18	3.04	2.72	up	0.00	0.00	yes
TRINITY_DN23619_c0_g4	PREDICTED: uncharacterized protein LOC105113419 isoform X1 [Populus euphratica]	-	-	45.04	6.09	3.49	up	0.00	0.00	yes

TRINITY_DN20315_c0_g3	hypothetical protein POPTR_0005s15610g [Populus trichocarpa]	-	-	42.07	11.07	2.57	up	0.00	0.00	yes
TRINITY_DN20660_c2_g2	harpin-induced family protein [Populus trichocarpa]	NHL12	NDR1/HIN1-like protein 12 OS=Arabidopsis thaliana GN=NHL12 PE=2 SV=1	101.02	32.66	2.24	up	0.00	0.00	yes
TRINITY_DN22037_c0_g2	PREDICTED: tubulin beta chain-like [Populus euphratica]	-	Tubulin beta-7 chain OS=Gossypium hirsutum PE=2 SV=1	30.97	6.16	2.95	up	0.00	0.00	yes
TRINITY_DN27842_c1_g2	PREDICTED: elongation factor 1-alpha-like [Populus euphratica]	EF1	Elongation factor 1-alpha OS=Manihot esculenta GN=EF1 PE=3 SV=1	935.57	293.79	2.29	up	0.00	0.00	yes
TRINITY_DN14478_c0_g1	hypothetical protein POPTR_0011s10880g [Populus trichocarpa]	-	-	21.18	1.35	4.57	up	0.00	0.00	yes
TRINITY_DN15389_c0_g1	heat shock family protein [Populus trichocarpa]	HSP90-5	Heat shock protein 90-5, chloroplastic OS=Arabidopsis thaliana GN=HSP90-5 PE=1 SV=1	934.53	246.19	2.54	up	0.00	0.00	yes
TRINITY_DN20284_c0_g1	pyridoxin biosynthesis PDX1-like protein 3 [Populus trichocarpa]	PDX1	Probable pyridoxal 5'-phosphate synthase subunit PDX1 OS=Hevea brasiliensis GN=PDX1 PE=2 SV=1	308.68	89.41	2.57	up	0.00	0.00	yes
TRINITY_DN13304_c0_g1	Ferredoxin 2 family protein [Populus trichocarpa]	AP1	Ferredoxin, chloroplastic OS=Capsicum annuum GN=AP1 PE=1 SV=1	11.33	0.36	5.61	up	0.00	0.00	yes
TRINITY_DN17272_c0_g2	hypothetical protein POPTR_0002s08270g [Populus trichocarpa]	RR4	Two-component response regulator ORR4 OS=Oryza sativa subsp. indica GN=RR4 PE=2 SV=1	57.50	9.49	3.19	up	0.00	0.00	yes
TRINITY_DN24150_c3_g3	hypothetical protein POPTR_0002s01390g [Populus trichocarpa]	LOG5	Cytokinin riboside 5'-monophosphate phosphoribohydrolase LOG5 OS=Arabidopsis thaliana GN=LOG5 PE=1 SV=1	10.44	1.05	3.82	up	0.00	0.00	yes
TRINITY_DN18751_c0_g2	hypothetical protein POPTR_0008s08560g [Populus trichocarpa]	ZHD11	Zinc-finger homeodomain protein 11 OS=Arabidopsis thaliana GN=ZHD11 PE=1 SV=1	3.04	0.23	4.25	up	0.00	0.00	yes
TRINITY_DN20720_c0_g1	PREDICTED: probable N-acetyl-gamma-glutamyl-phosphate reductase, chloroplastic isoform X2 [Populus euphratica]	At2g19940	Probable N-acetyl-gamma-glutamyl-phosphate reductase, chloroplastic OS=Arabidopsis thaliana GN=At2g19940 PE=1 SV=2	42.82	15.35	2.14	up	0.00	0.00	yes
TRINITY_DN24532_c0_g1	hypothetical protein POPTR_0001s24920g [Populus trichocarpa]	-	-	48.17	15.01	2.28	up	0.00	0.00	yes
TRINITY_DN25304_c3_g2	-	-	-	5.17	0.20	5.17	up	0.00	0.00	yes
TRINITY_DN17558_c0_g1	unknown [Populus trichocarpa x Populus deltoides]	-	-	75.20	8.45	3.72	up	0.00	0.00	yes
TRINITY_DN24326_c0_g2	hypothetical protein POPTR_0006s13340g [Populus trichocarpa]	TIM14-1	Mitochondrial import inner membrane translocase subunit TIM14-1 OS=Arabidopsis thaliana GN=TIM14-1 PE=1 SV=1	77.46	25.75	2.20	up	0.00	0.00	yes
TRINITY_DN26854_c0_g1	sulfite reductase family protein [Populus trichocarpa]	SIR1	Sulfite reductase 1 [ferredoxin], chloroplastic OS=Nicotiana tabacum GN=SIR1 PE=1 SV=1	94.07	29.35	2.31	up	0.00	0.00	yes
TRINITY_DN21914_c0_g1	ABC transporter family protein [Populus trichocarpa]	ABCI6	ABC transporter I family member 6, chloroplastic OS=Arabidopsis thaliana GN=ABCI6 PE=1 SV=1	71.47	24.13	2.29	up	0.00	0.00	yes
TRINITY_DN19961_c0_g1	PREDICTED: maltose excess protein 1, chloroplastic-like [Populus euphratica]	MEX1	Maltose excess protein 1, chloroplastic OS=Arabidopsis thaliana GN=MEX1 PE=2 SV=2	60.86	19.63	2.27	up	0.00	0.00	yes
TRINITY_DN18792_c1_g3	hypothetical protein POPTR_0013s11340g [Populus trichocarpa]	ndhT	NAD(P)H-quinone oxidoreductase subunit T, chloroplastic OS=Arabidopsis thaliana GN=ndhT PE=1 SV=1	94.30	24.72	2.55	up	0.00	0.00	yes
TRINITY_DN22269_c0_g2	glutathione S-transferase F8 [Populus yatungensis]	GSTF12	Glutathione S-transferase F12 OS=Arabidopsis thaliana GN=GSTF12 PE=1 SV=1	67.95	7.46	3.81	up	0.00	0.00	yes
TRINITY_DN14248_c0_g1	hypothetical protein POPTR_0006s09290g [Populus trichocarpa]	-	Dynein light chain LC6, flagellar outer arm OS=Heliocidaris crassispina PE=3 SV=1	11.97	0.23	6.20	up	0.00	0.00	yes
TRINITY_DN27662_c5_g1	cytokinin receptor 1A [Populus trichocarpa]	AHK4	Histidine kinase 4 OS=Arabidopsis thaliana GN=AHK4 PE=1 SV=1	22.72	6.44	2.10	up	0.00	0.00	yes
TRINITY_DN18689_c0_g1	PREDICTED: strictosidine synthase 3-like isoform X1 [Populus euphratica]	SSL2	Protein STRICTOSIDINE SYNTHASE-LIKE 2 OS=Arabidopsis thaliana GN=SSL2 PE=2 SV=1	19.71	2.54	3.51	up	0.00	0.00	yes
TRINITY_DN19971_c0_g1	hypothetical protein POPTR_0009s11190g [Populus trichocarpa]	petM	Cytochrome b6-f complex subunit 7 (Fragment) OS=Spinacia oleracea GN=petM PE=1 SV=1	39.22	9.69	2.59	up	0.00	0.00	yes
TRINITY_DN18806_c0_g2	PREDICTED: S-adenosylmethionine decarboxylase proenzyme-like isoform X3 [Solanum pennellii]	SAMDC	S-adenosylmethionine decarboxylase proenzyme OS=Datura stramonium GN=SAMDC PE=2 SV=1	161.94	56.16	2.12	up	0.00	0.00	yes
TRINITY_DN26425_c0_g1	PREDICTED: chaperonin 60 subunit beta 2, chloroplastic [Populus euphratica]	CPN60B1	Chaperonin 60 subunit beta 1, chloroplastic OS=Arabidopsis thaliana GN=CPN60B1 PE=1 SV=3	886.93	288.11	2.34	up	0.00	0.00	yes
TRINITY_DN21357_c0_g1	PREDICTED: uncharacterized protein LOC105107529 isoform X1 [Populus euphratica]	-	-	68.77	21.72	2.30	up	0.00	0.00	yes

TRINITY_DN22895_c0_g2	hypothetical protein POPTR_0098s00240g [Populus trichocarpa]	UGT85A24	7-deoxyloganetin glucosyltransferase OS=Gardenia jasminoides GN=UGT85A24 PE=1 SV=1	32.62	4.49	3.42	up	0.00	0.00	yes
TRINITY_DN20322_c0_g3	Ferredoxin--nitrite reductase family protein [Populus trichocarpa]	NIR1	Ferredoxin--nitrite reductase, chloroplastic OS=Betula pendula GN=NIR1 PE=2 SV=1	75.08	13.81	3.04	up	0.00	0.00	yes
TRINITY_DN18526_c0_g3	RNA-binding family protein [Populus trichocarpa]	CP31A	31 kDa ribonucleoprotein, chloroplastic OS=Arabidopsis thaliana GN=CP31A PE=1 SV=1	468.44	105.67	2.65	up	0.00	0.00	yes
TRINITY_DN17691_c0_g1	fasciclin-like arabinogalactan-protein 9 [Populus trichocarpa]	FLA6	Fasciclin-like arabinogalactan protein 6 OS=Arabidopsis thaliana GN=FLA6 PE=2 SV=2	97.90	12.34	3.54	up	0.00	0.00	yes
TRINITY_DN18501_c1_g1	PREDICTED: phosphoserine aminotransferase 2, chloroplastic-like [Populus euphratica]	PSAT1	Phosphoserine aminotransferase 1, chloroplastic OS=Arabidopsis thaliana GN=PSAT1 PE=1 SV=1	32.17	9.92	2.31	up	0.00	0.00	yes
TRINITY_DN13505_c0_g1	PREDICTED: protein FAM32A-like [Populus euphratica]	fam32a	Protein FAM32A OS=Xenopus tropicalis GN=fam32a PE=3 SV=1	5.25	0.22	5.45	up	0.00	0.00	yes
TRINITY_DN18270_c0_g1	hypothetical protein POPTR_0017s07840g [Populus trichocarpa]	PUR7	Phosphoribosylaminoimidazole-succinocarboxamide synthase, chloroplastic OS=Arabidopsis thaliana GN=PUR7 PE=2 SV=2	28.13	10.64	2.00	up	0.00	0.00	yes
TRINITY_DN26837_c0_g1	hypothetical protein POPTR_0010s15200g [Populus trichocarpa]	LPD2	Dihydrolipoyl dehydrogenase 2, chloroplastic OS=Arabidopsis thaliana GN=LPD2 PE=2 SV=2	81.17	29.93	2.09	up	0.00	0.00	yes
TRINITY_DN20203_c0_g3	PREDICTED: uncharacterized protein LOC105127640 [Populus euphratica]	BPG2	GTP-binding protein BRASSINAZOLE INSENSITIVE PALE GREEN 2, chloroplastic OS=Arabidopsis thaliana GN=BPG2 PE=1 SV=1	34.27	11.75	2.14	up	0.00	0.00	yes
TRINITY_DN17303_c0_g1	hypothetical protein POPTR_0005s26400g [Populus trichocarpa]	ptges2	Prostaglandin E synthase 2 OS=Danio rerio GN=ptges2 PE=2 SV=1	35.95	12.21	2.23	up	0.00	0.00	yes
TRINITY_DN25192_c0_g1	hypothetical protein POPTR_0010s02860g [Populus trichocarpa]	PPD	Pyruvate, phosphate dikinase, chloroplastic OS=Mesembryanthemum crystallinum GN=PPD PE=2 SV=1	24.30	5.86	2.93	up	0.00	0.00	yes
TRINITY_DN17100_c0_g1	PREDICTED: serine carboxypeptidase-like 51 [Populus euphratica]	SCPL51	Serine carboxypeptidase-like 51 OS=Arabidopsis thaliana GN=SCPL51 PE=2 SV=2	19.47	7.44	2.00	up	0.00	0.00	yes
TRINITY_DN17440_c0_g2	hypothetical protein POPTR_0002s00360g [Populus trichocarpa]	yjbQ	UPF0047 protein YjbQ OS=Escherichia coli O157:H7 GN=yjbQ PE=3 SV=1	169.98	48.56	2.42	up	0.00	0.00	yes
TRINITY_DN14984_c0_g1	PREDICTED: uncharacterized protein LOC105117923 [Populus euphratica]	-	-	49.34	15.09	2.32	up	0.00	0.00	yes
TRINITY_DN25131_c0_g1	hypothetical protein POPTR_0001s24160g [Populus trichocarpa]	RIBA3	Monofunctional riboflavin biosynthesis protein RIBA 3, chloroplastic OS=Arabidopsis thaliana GN=RIBA3 PE=1 SV=1	20.85	7.51	2.28	up	0.00	0.00	yes
TRINITY_DN26247_c0_g1	PREDICTED: dicarboxylate transporter 1, chloroplastic-like [Populus euphratica]	DIT1	Dicarboxylate transporter 1, chloroplastic OS=Arabidopsis thaliana GN=DIT1 PE=1 SV=1	88.63	34.97	2.09	up	0.00	0.00	yes
TRINITY_DN22142_c0_g2	60s acidic ribosomal family protein [Populus trichocarpa]	-	60S acidic ribosomal protein P2 OS=Parthenium argentatum PE=3 SV=1	231.62	72.40	2.31	up	0.00	0.00	yes
TRINITY_DN20435_c0_g2	hypothetical protein POPTR_0001s24740g [Populus trichocarpa]	-	-	30.54	2.51	4.20	up	0.00	0.00	yes
TRINITY_DN27124_c0_g2	hypothetical protein POPTR_0019s00800g [Populus trichocarpa]	Os05g0361200	Ferrochelatase-2, chloroplastic OS=Oryza sativa subsp. japonica GN=Os05g0361200 PE=2 SV=1	115.18	31.86	2.22	up	0.00	0.00	yes
TRINITY_DN25099_c0_g3	-	-	-	9.88	0.35	5.48	up	0.00	0.00	yes
TRINITY_DN21477_c1_g5	PREDICTED: glutathione S-transferase F11-like [Populus euphratica]	GSTF11	Glutathione S-transferase F11 OS=Arabidopsis thaliana GN=GSTF11 PE=2 SV=1	3486.29	837.49	2.64	up	0.00	0.00	yes
TRINITY_DN5190_c0_g2	CBL-interacting protein kinase 20 [Populus trichocarpa]	CIPK20	CBL-interacting serine/threonine-protein kinase 20 OS=Arabidopsis thaliana GN=CIPK20 PE=1 SV=1	2.51	0.07	5.55	up	0.00	0.00	yes
TRINITY_DN23091_c0_g3	glutamate-ammonia ligase family protein [Populus trichocarpa]	-	Glutamine synthetase OS=Nicotiana plumbaginifolia PE=2 SV=1	47.80	3.92	4.22	up	0.00	0.00	yes
TRINITY_DN23760_c1_g1	unknown [Populus trichocarpa x Populus deltoides]	-	-	15.41	2.03	3.72	up	0.00	0.00	yes
TRINITY_DN25540_c0_g1	hypothetical protein POPTR_0004s24220g [Populus trichocarpa]	PAE8	Pectin acetylesterase 8 OS=Arabidopsis thaliana GN=PAE8 PE=2 SV=1	39.42	13.08	2.51	up	0.00	0.00	yes
TRINITY_DN16581_c0_g1	PREDICTED: putative lipid-binding protein At4g00165 [Vitis vinifera]	-	14 kDa proline-rich protein DC2.15 OS=Daucus carota PE=2 SV=1	521.12	114.10	2.79	up	0.00	0.00	yes
TRINITY_DN16387_c0_g1	glycosyl hydrolase family 1 family protein [Populus trichocarpa]	BGLU47	Beta-glucosidase 47 OS=Arabidopsis thaliana GN=BGLU47 PE=3 SV=2	7.38	0.93	3.61	up	0.00	0.00	yes
TRINITY_DN14914_c0_g1	hypothetical protein POPTR_0001s06590g [Populus trichocarpa]	CITRX	Thioredoxin-like protein CITRX, chloroplastic OS=Arabidopsis thaliana GN=CITRX PE=1 SV=1	221.18	62.83	2.42	up	0.00	0.00	yes

TRINITY_DN27424_c0_g2	hypothetical protein POPTR_0003s04990g [Populus trichocarpa]	-	-	105.32	33.44	2.27	up	0.00	0.00	yes
TRINITY_DN20724_c2_g2	Gamma-tonoplast intrinsic protein 3 [Populus trichocarpa]	TIP1-3	Aquaporin TIP1-3 OS=Arabidopsis thaliana GN=TIP1-3 PE=1 SV=1	39.51	12.12	2.30	up	0.00	0.00	yes
TRINITY_DN17805_c0_g1	hypothetical protein POPTR_0005s01590g [Populus trichocarpa]	IREG3	Solute carrier family 40 member 3, chloroplastic OS=Arabidopsis thaliana GN=IREG3 PE=1 SV=1	29.32	9.11	2.27	up	0.00	0.00	yes
TRINITY_DN17597_c0_g1	10 kDa chaperonin family protein [Populus trichocarpa]	CPN10	10 kDa chaperonin, mitochondrial OS=Arabidopsis thaliana GN=CPN10 PE=1 SV=1	155.26	60.09	1.99	up	0.00	0.00	yes
TRINITY_DN18345_c0_g4	hypothetical protein POPTR_0013s10170g [Populus trichocarpa]	OPR2	12-oxophytodienoate reductase 2 OS=Arabidopsis thaliana GN=OPR2 PE=1 SV=2	32.67	7.40	2.78	up	0.00	0.00	yes
TRINITY_DN20891_c1_g1	PREDICTED: F-box protein At2g32560-like [Populus euphratica]	At2g26850	F-box protein At2g26850 OS=Arabidopsis thaliana GN=At2g26850 PE=2 SV=1	23.97	9.10	2.08	up	0.00	0.00	yes
TRINITY_DN15330_c0_g1	hypothetical protein POPTR_0010s10500g [Populus trichocarpa]	PRIN2	Protein PLASTID REDOX INSENSITIVE 2 OS=Arabidopsis thaliana GN=PRIN2 PE=1 SV=1	151.61	40.11	2.56	up	0.00	0.00	yes
TRINITY_DN17865_c0_g1	plastid serine hydroxymethyltransferase [Populus tremuloides]	SHM3	Serine hydroxymethyltransferase 3, chloroplastic OS=Arabidopsis thaliana GN=SHM3 PE=1 SV=2	53.46	17.12	2.27	up	0.00	0.00	yes
TRINITY_DN17613_c0_g1	hypothetical protein POPTR_0010s05230g [Populus trichocarpa]	At1g23400	CRS2-associated factor 2, chloroplastic OS=Arabidopsis thaliana GN=At1g23400 PE=2 SV=1	90.21	27.36	2.33	up	0.00	0.00	yes
TRINITY_DN16572_c0_g1	hypothetical protein POPTR_0016s06300g [Populus trichocarpa]	RPS28C	40S ribosomal protein S28-2 OS=Arabidopsis thaliana GN=RPS28C PE=3 SV=1	169.16	63.49	2.12	up	0.00	0.00	yes
TRINITY_DN18459_c0_g1	PREDICTED: uncharacterized protein LOC105139820 [Populus euphratica]	MTERF8	Transcription termination factor MTERF8, chloroplastic OS=Arabidopsis thaliana GN=MTERF8 PE=1 SV=1	11.18	3.13	2.46	up	0.00	0.00	yes
TRINITY_DN27393_c1_g1	PREDICTED: heat shock protein 83-like [Populus euphratica]	HSP90-5	Heat shock protein 90-5, chloroplastic OS=Arabidopsis thaliana GN=HSP90-5 PE=1 SV=1	375.24	129.58	2.11	up	0.00	0.00	yes
TRINITY_DN27276_c0_g1	hypothetical protein POPTR_0056s00220g [Populus trichocarpa]	-	-	20.25	15.85	2.70	up	0.00	0.00	yes
TRINITY_DN19787_c0_g1	hypothetical protein POPTR_0001s08320g [Populus trichocarpa]	CYP76B6	Geraniol 8-hydroxylase OS=Catharanthus roseus GN=CYP76B6 PE=1 SV=1	7.17	1.45	2.95	up	0.00	0.00	yes
TRINITY_DN18344_c0_g2	hypothetical protein POPTR_0007s13000g [Populus trichocarpa]	RL3	Protein RADIALIS-like 3 OS=Arabidopsis thaliana GN=RL3 PE=2 SV=1	86.69	15.79	3.11	up	0.00	0.00	yes
TRINITY_DN19419_c0_g1	hypothetical protein POPTR_0006s02220g [Populus trichocarpa]	SCL23	Scarecrow-like protein 23 OS=Arabidopsis thaliana GN=SCL23 PE=1 SV=1	4.58	0.82	3.78	up	0.00	0.00	yes
TRINITY_DN18106_c0_g1	ADR12-2 family protein [Populus trichocarpa]	-	Elongation factor 1-alpha OS=Solanum lycopersicum PE=2 SV=1	281.61	105.51	2.05	up	0.00	0.00	yes
TRINITY_DN23678_c1_g1	putative oxoglutarate-malate-translocase family protein [Populus trichocarpa]	DIT2	Dicarboxylate transporter 2, chloroplastic OS=Spinacia oleracea GN=DIT2 PE=1 SV=1	56.95	19.63	2.09	up	0.00	0.00	yes
TRINITY_DN22607_c0_g1	hypothetical protein POPTR_0004s01990g [Populus trichocarpa]	THI1-2	Thiamine thiazole synthase 2, chloroplastic OS=Vitis vinifera GN=THI1-2 PE=3 SV=1	2811.82	419.38	3.36	up	0.00	0.00	yes
TRINITY_DN17528_c0_g1	ribose-phosphate pyrophosphokinase family protein [Populus trichocarpa]	PRS5	Ribose-phosphate pyrophosphokinase 5, chloroplastic OS=Arabidopsis thaliana GN=PRS5 PE=2 SV=2	63.69	21.30	2.19	up	0.00	0.00	yes
TRINITY_DN14929_c0_g1	PREDICTED: mitochondrial import receptor subunit TOM9-2 [Populus euphratica]	TOM9-2	Mitochondrial import receptor subunit TOM9-2 OS=Arabidopsis thaliana GN=TOM9-2 PE=1 SV=3	61.07	16.90	2.40	up	0.00	0.00	yes
TRINITY_DN26631_c0_g1	PREDICTED: phosphoribosylaminoimidazole carboxylase, chloroplastic-like isoform X1 [Populus euphratica]	PURKE	Phosphoribosylaminoimidazole carboxylase, chloroplastic (Fragment) OS=Vigna aconitifolia GN=PURKE PE=2 SV=1	26.81	10.47	2.04	up	0.00	0.00	yes
TRINITY_DN17173_c0_g1	Lyk 1 [Populus x canadensis]	LYK3	LysM domain receptor-like kinase 3 OS=Arabidopsis thaliana GN=LYK3 PE=2 SV=1	17.97	6.45	2.10	up	0.00	0.00	yes
TRINITY_DN20187_c0_g1	co-chaperone grpE family protein [Populus trichocarpa]	grpE	Protein GrpE OS=Synechocystis sp. (strain PCC 6803 / Kazusa) GN=grpE PE=3 SV=1	294.95	78.53	2.51	up	0.00	0.00	yes
TRINITY_DN26940_c2_g2	hypothetical protein LE_TR2973_c1_g1_i1_g.9470, partial [Noccaea caerulescens]	-	-	56.72	13.28	2.71	up	0.00	0.00	yes
TRINITY_DN17883_c0_g1	hypothetical protein POPTR_0007s13510g [Populus trichocarpa]	At4g36390	CDK5RAP1-like protein OS=Arabidopsis thaliana GN=At4g36390 PE=2 SV=1	38.57	12.73	2.19	up	0.00	0.00	yes
TRINITY_DN21552_c0_g5	60S ribosomal protein L14 [Populus trichocarpa]	RPL14A	60S ribosomal protein L14-1 OS=Arabidopsis thaliana GN=RPL14A PE=2 SV=1	114.26	33.51	2.41	up	0.00	0.00	yes
TRINITY_DN18170_c0_g1	PREDICTED: cytochrome P450 71D9-like [Populus euphratica]	CYP71D55	Premnaspirodiene oxygenase OS=Hyoscyamus muticus GN=CYP71D55 PE=1 SV=1	32.69	4.52	3.48	up	0.00	0.00	yes

TRINITY_DN18831_c0_g1	PREDICTED: uncharacterized protein LOC105141339 [Populus euphratica]	TIC56	Protein TIC 56, chloroplastic OS=Arabidopsis thaliana GN=TIC56 PE=1 SV=1	88.33	32.32	2.05	up	0.00	0.00	yes
TRINITY_DN18149_c0_g2	T10O24.12 [Arabidopsis thaliana]	NLRP3	NACHT, LRR and PYD domains-containing protein 3 OS=Bos taurus GN=NLRP3 PE=2 SV=1	81.50	32.66	2.11	up	0.00	0.00	yes
TRINITY_DN21140_c0_g2	mitochondrial lipoamide dehydrogenase [Populus tremuloides]	LPD1	Dihydrolipoyl dehydrogenase 1, mitochondrial OS=Arabidopsis thaliana GN=LPD1 PE=1 SV=2	202.39	67.53	2.24	up	0.00	0.00	yes
TRINITY_DN19790_c1_g6	PREDICTED: NHL repeat-containing protein 2 isoform X2 [Populus euphratica]	NHLRC2	NHL repeat-containing protein 2 OS=Bos taurus GN=NHLRC2 PE=2 SV=1	16.03	4.91	2.30	up	0.00	0.00	yes
TRINITY_DN23209_c0_g1	hypothetical protein POPTR_0004s21720g [Populus trichocarpa]	-	-	147.72	29.16	2.76	up	0.00	0.00	yes
TRINITY_DN15410_c0_g1	hypothetical protein POPTR_0010s06490g [Populus trichocarpa]	-	-	37.10	11.66	2.27	up	0.00	0.00	yes
TRINITY_DN14339_c0_g1	PREDICTED: uncharacterized protein LOC105124273 [Populus euphratica]	-	-	12.65	0.07	7.60	up	0.00	0.00	yes
TRINITY_DN17920_c0_g3	PREDICTED: tubulin beta chain [Vitis vinifera]	TUBB3	Tubulin beta-3 chain OS=Zea mays GN=TUBB3 PE=2 SV=1	13.65	3.00	2.87	up	0.00	0.00	yes
TRINITY_DN19580_c0_g2	hypothetical protein POPTR_0002s09060g [Populus trichocarpa]	grpE	Protein GrpE OS=Prochlorococcus marinus (strain MIT 9211) GN=grpE PE=3 SV=1	84.30	26.38	2.39	up	0.00	0.00	yes
TRINITY_DN17495_c0_g2	PREDICTED: uncharacterized methyltransferase At1g78140, chloroplastic [Populus euphratica]	At1g78140	Uncharacterized methyltransferase At1g78140, chloroplastic OS=Arabidopsis thaliana GN=At1g78140 PE=2 SV=1	30.75	13.88	2.00	up	0.00	0.00	yes
TRINITY_DN27259_c0_g2	hypothetical protein POPTR_0014s04300g [Populus trichocarpa]	SGPP	Haloacid dehalogenase-like hydrolase domain-containing protein Sgpp OS=Arabidopsis thaliana GN=SGPP PE=1 SV=2	66.51	12.23	2.88	up	0.00	0.00	yes
TRINITY_DN20831_c0_g3	PREDICTED: two-component response regulator ARR5-like [Populus euphratica]	ARR3	Two-component response regulator ARR3 OS=Arabidopsis thaliana GN=ARR3 PE=2 SV=1	47.29	13.22	2.86	up	0.00	0.00	yes
TRINITY_DN17904_c0_g2	RNA recognition motif-containing family protein [Populus trichocarpa]	rsd1	RNA-binding protein rsd1 OS=Schizosaccharomyces pombe (strain 972 / ATCC 24843) GN=rsd1 PE=1 SV=2	146.73	33.03	2.75	up	0.00	0.00	yes
TRINITY_DN21312_c3_g4	-	-	-	2.57	0.06	5.76	up	0.00	0.00	yes
TRINITY_DN17248_c0_g2	ADR12-2 family protein [Populus trichocarpa]	EF1	Elongation factor 1-alpha OS=Manihot esculenta GN=EF1 PE=3 SV=1	292.56	105.43	2.10	up	0.00	0.00	yes
TRINITY_DN21132_c0_g2	hypothetical protein POPTR_0017s01160g [Populus trichocarpa]	HSP82	Heat shock protein 82 (Fragment) OS=Nicotiana tabacum GN=HSP82 PE=2 SV=1	24.50	5.47	2.81	up	0.00	0.00	yes
TRINITY_DN13352_c0_g1	putative auxin efflux carrier protein 9 [Populus trichocarpa]	PIN5	Auxin efflux carrier component 5 OS=Arabidopsis thaliana GN=PIN5 PE=2 SV=2	2.66	0.04	6.37	up	0.00	0.00	yes
TRINITY_DN15805_c0_g1	PREDICTED: uncharacterized protein LOC105113501 [Populus euphratica]	-	-	58.15	16.50	2.44	up	0.00	0.00	yes
TRINITY_DN26802_c1_g1	hypothetical protein POPTR_0010s22320g [Populus trichocarpa]	RAP2-7	Ethylene-responsive transcription factor RAP2-7 OS=Arabidopsis thaliana GN=RAP2-7 PE=2 SV=2	28.40	9.87	2.06	up	0.00	0.00	yes
TRINITY_DN27145_c0_g1	Stromal 70 kDa heat shock-related family protein [Populus trichocarpa]	HSP70	Stromal 70 kDa heat shock-related protein, chloroplastic OS=Pisum sativum GN=HSP70 PE=2 SV=1	862.92	304.67	2.10	up	0.00	0.00	yes
TRINITY_DN24109_c1_g6	heat shock protein 70 [Populus trichocarpa]	-	Heat shock 70 kDa protein, mitochondrial OS=Phaseolus vulgaris PE=2 SV=1	73.32	25.78	2.17	up	0.00	0.00	yes
TRINITY_DN19728_c0_g2	PREDICTED: bifunctional epoxide hydrolase 2-like [Populus euphratica]	ephA	Epoxide hydrolase A OS=Mycobacterium tuberculosis (strain ATCC 25618 / H37Rv) GN=ephA PE=1 SV=1	10.52	1.94	3.11	up	0.00	0.00	yes
TRINITY_DN15463_c0_g1	-	-	-	126.92	12.75	3.92	up	0.00	0.00	yes
TRINITY_DN20279_c0_g1	PREDICTED: LOW QUALITY PROTEIN: uncharacterized protein LOC105129063 [Populus euphratica]	murE	UDP-N-acetylmuramoyl-L-alanyl-D-glutamate--2,6-diaminopimelate ligase OS=Geobacillus kaustophilus (strain HTA426) GN=murE PE=3 SV=1	55.81	18.35	2.16	up	0.00	0.00	yes
TRINITY_DN25577_c0_g1	hypothetical protein POPTR_0019s11730g [Populus trichocarpa]	IST1	IST1 homolog OS=Homo sapiens GN=IST1 PE=1 SV=1	13.61	5.46	1.95	up	0.00	0.00	yes
TRINITY_DN22245_c0_g1	PREDICTED: triosephosphate isomerase, chloroplastic [Populus euphratica]	TPIP1	Triosephosphate isomerase, chloroplastic OS=Spinacia oleracea GN=TPIP1 PE=1 SV=1	838.73	263.34	2.23	up	0.00	0.00	yes
TRINITY_DN22243_c0_g4	A-crystallin domain-containing family protein [Populus trichocarpa]	-	-	112.81	16.82	3.41	up	0.00	0.00	yes
TRINITY_DN22627_c1_g2	PREDICTED: ATP phosphoribosyltransferase 2, chloroplastic-like [Populus euphratica]	HISN1B	ATP phosphoribosyltransferase 2, chloroplastic OS=Arabidopsis thaliana GN=HISN1B PE=1 SV=1	103.92	38.62	2.05	up	0.00	0.00	yes
TRINITY_DN26575_c0_g1	hypothetical protein POPTR_0010s22750g [Populus trichocarpa]	MBR2	E3 ubiquitin-protein ligase MBR2 OS=Arabidopsis thaliana GN=MBR2 PE=1 SV=1	8.07	0.49	3.89	up	0.00	0.00	yes

TRINITY_DN24734_c0_g2	PREDICTED: putative hydrolase C777.06c [Populus euphratica]	SPCC777.06c	Putative hydrolase C777.06c OS=Schizosaccharomyces pombe (strain 972 / ATCC 24843) GN=SPCC777.06c PE=4 SV=1	24.96	10.54	2.01	up	0.00	0.00	yes
TRINITY_DN21153_c0_g2	hypothetical protein POPTR_0010s09260g [Populus trichocarpa]	-	-	11.27	3.50	2.31	up	0.00	0.00	yes
TRINITY_DN18217_c0_g1	hypothetical protein POPTR_0001s46210g [Populus trichocarpa]	ychF	Ribosome-binding ATPase YchF OS=Bacillus subtilis (strain 168) GN=ychF PE=2 SV=1	44.44	14.30	2.18	up	0.00	0.00	yes
TRINITY_DN20236_c0_g2	hypothetical protein POPTR_0017s08230g [Populus trichocarpa]	HMGB15	High mobility group B protein 15 OS=Arabidopsis thaliana GN=HMGB15 PE=2 SV=1	16.31	3.84	2.91	up	0.00	0.00	yes
TRINITY_DN18781_c0_g2	hypothetical protein POPTR_0018s06470g [Populus trichocarpa]	-	-	57.49	18.11	2.25	up	0.00	0.00	yes
TRINITY_DN20757_c1_g1	-	-	-	3.31	0.05	6.26	up	0.00	0.00	yes
TRINITY_DN19874_c0_g1	PREDICTED: uncharacterized protein LOC105122007 [Populus euphratica]	RH48	Probable DEAD-box ATP-dependent RNA helicase 48 OS=Arabidopsis thaliana GN=RH48 PE=3 SV=1	71.32	25.90	2.03	up	0.00	0.00	yes
TRINITY_DN20491_c0_g1	hypothetical protein POPTR_0014s16010g [Populus trichocarpa]	At5g35735	Cytochrome b561 and DOMON domain-containing protein At5g35735 OS=Arabidopsis thaliana GN=At5g35735 PE=2 SV=1	4.22	0.33	4.22	up	0.00	0.00	yes
TRINITY_DN19977_c2_g2	PREDICTED: carotene epsilon-monooxygenase, chloroplastic isoform X1 [Populus euphratica]	CYP97C1	Carotene epsilon-monooxygenase, chloroplastic OS=Arabidopsis thaliana GN=CYP97C1 PE=1 SV=1	40.28	14.57	2.17	up	0.00	0.00	yes
TRINITY_DN24244_c0_g2	hypothetical protein POPTR_0001s36730g [Populus trichocarpa]	PYRB1	Aspartate carbamoyltransferase 1, chloroplastic OS=Pisum sativum GN=PYRB1 PE=2 SV=1	48.33	20.26	1.86	up	0.00	0.00	yes
TRINITY_DN24652_c3_g1	PREDICTED: auxin-induced in root cultures protein 12 [Populus euphratica]	AIR12	Auxin-induced in root cultures protein 12 OS=Arabidopsis thaliana GN=AIR12 PE=1 SV=3	69.44	20.69	2.33	up	0.00	0.00	yes
TRINITY_DN22579_c0_g1	hypothetical protein POPTR_0001s07860g [Populus trichocarpa]	CYP75B2	Flavonoid 3'-monooxygenase OS=Petunia hybrida GN=CYP75B2 PE=2 SV=1	29.32	7.54	3.09	up	0.00	0.00	yes
TRINITY_DN17512_c0_g1	PREDICTED: ubiquinol oxidase 4, chloroplastic/chromoplastic isoform X1 [Populus euphratica]	AOX4	Ubiquinol oxidase 4, chloroplastic/chromoplastic OS=Arabidopsis thaliana GN=AOX4 PE=1 SV=2	34.40	13.66	1.91	up	0.00	0.00	yes
TRINITY_DN23820_c0_g1	hypothetical protein POPTR_0014s18800g [Populus trichocarpa]	yqjG	Glutathionyl-hydroquinone reductase YqjG OS=Escherichia coli (strain K12) GN=yqjG PE=1 SV=1	20.07	7.57	2.02	up	0.00	0.00	yes
TRINITY_DN25377_c0_g1	hypothetical protein POPTR_0013s11870g [Populus trichocarpa]	EMB3003	Dihydrolipoyllysine-residue acetyltransferase component 5 of pyruvate dehydrogenase complex, chloroplastic OS=Arabidopsis thaliana GN=EMB3003 PE=2 SV=1	85.23	34.17	1.91	up	0.00	0.00	yes
TRINITY_DN26787_c0_g1	PREDICTED: protein TOC75-3, chloroplastic-like isoform X2 [Populus euphratica]	TOC75-3	Protein TOC75-3, chloroplastic OS=Arabidopsis thaliana GN=TOC75-3 PE=1 SV=1	257.16	101.99	1.94	up	0.00	0.00	yes
TRINITY_DN13782_c0_g1	hypothetical protein POPTR_0002s11010g [Populus trichocarpa]	-	-	6.55	0.32	5.00	up	0.00	0.00	yes
TRINITY_DN19752_c0_g2	-	-	-	7.37	0.29	5.15	up	0.00	0.00	yes
TRINITY_DN16094_c1_g1	PREDICTED: uncharacterized protein LOC105115462 [Populus euphratica]	trmH	tRNA (guanosine(18)-2'-O)-methyltransferase OS=Aquifex aeolicus (strain VF5) GN=trmH PE=1 SV=1	42.56	14.84	2.15	up	0.00	0.00	yes
TRINITY_DN24179_c1_g1	hypothetical protein POPTR_0001s44620g [Populus trichocarpa]	TSB	Tryptophan synthase beta chain 2, chloroplastic OS=Camptotheca acuminata GN=TSB PE=2 SV=1	107.11	47.27	1.94	up	0.00	0.00	yes
TRINITY_DN26713_c0_g2	PREDICTED: probable receptor protein kinase TMK1 [Populus euphratica]	TMK1	Receptor protein kinase TMK1 OS=Arabidopsis thaliana GN=TMK1 PE=1 SV=1	5.45	0.07	6.17	up	0.00	0.00	yes
TRINITY_DN24090_c0_g2	unknown [Populus trichocarpa x Populus deltoides]	CPN20	20 kDa chaperonin, chloroplastic OS=Arabidopsis thaliana GN=CPN20 PE=1 SV=2	199.05	59.58	2.34	up	0.00	0.00	yes
TRINITY_DN27458_c1_g1	PREDICTED: transketolase, chloroplastic [Populus euphratica]	TKT3	Transketolase, chloroplastic (Fragment) OS=Craterostigma plantagineum GN=TKT3 PE=1 SV=1	1184.20	313.96	2.44	up	0.00	0.00	yes
TRINITY_DN16951_c0_g1	PREDICTED: 60S ribosomal protein L13a-4-like [Populus euphratica]	RPL13AC	60S ribosomal protein L13a-3 OS=Arabidopsis thaliana GN=RPL13AC PE=2 SV=1	169.18	59.37	2.14	up	0.00	0.00	yes
TRINITY_DN20677_c1_g1	PREDICTED: 54S ribosomal protein L10, mitochondrial-like [Populus euphratica]	rplO	50S ribosomal protein L15 OS=Xanthobacter autotrophicus (strain ATCC BAA-1158 / Py2) GN=rplO PE=3 SV=1	40.34	15.48	1.99	up	0.00	0.00	yes
TRINITY_DN26389_c0_g1	-	-	-	24.61	1.45	4.80	up	0.00	0.00	yes
TRINITY_DN13374_c0_g2	hypothetical protein POPTR_0008s19330g [Populus trichocarpa]	-	-	2.96	0.04	6.32	up	0.00	0.00	yes
TRINITY_DN27305_c0_g3	PREDICTED: uncharacterized protein LOC105141147 [Populus euphratica]	-	-	113.32	45.12	1.97	up	0.00	0.00	yes
TRINITY_DN25837_c0_g3	-	-	-	4.97	0.15	5.53	up	0.00	0.00	yes

TRINITY_DN20796_c1_g4	heat shock protein 70 [Populus trichocarpa]	MED37C	Probable mediator of RNA polymerase II transcription subunit 37c OS=Arabidopsis thaliana GN=MED37C PE=1 SV=1	58.52	8.97	3.37	up	0.00	0.00	yes
TRINITY_DN27250_c1_g1	hypothetical protein POPTR_0005s16440g [Populus trichocarpa]	-	-	23.60	3.39	3.40	up	0.00	0.00	yes
TRINITY_DN22606_c0_g2	heat shock transcription factor HSF29 family protein [Populus trichocarpa]	HSFB2B	Heat stress transcription factor B-2b OS=Oryza sativa subsp. japonica GN=HSFB2B PE=2 SV=1	21.38	6.41	2.36	up	0.00	0.00	yes
TRINITY_DN20234_c0_g3	PREDICTED: glucan endo-1,3-beta-glucosidase isoform X1 [Populus euphratica]	GLC1	Glucan endo-1,3-beta-glucosidase OS=Triticum aestivum GN=GLC1 PE=2 SV=1	16.46	2.27	3.69	up	0.00	0.00	yes
TRINITY_DN18671_c0_g1	RNA recognition motif-containing family protein [Populus trichocarpa]	cirbp	Cold-inducible RNA-binding protein OS=Xenopus tropicalis GN=cirbp PE=2 SV=1	49.37	13.94	2.42	up	0.00	0.00	yes
TRINITY_DN16493_c1_g1	unknown [Populus trichocarpa x Populus deltoides]	RCA	Ribulose bisphosphate carboxylase/oxygenase activase, chloroplastic OS=Arabidopsis thaliana GN=RCA PE=1 SV=2	2245.25	420.25	3.03	up	0.00	0.00	yes
TRINITY_DN26127_c0_g1	Chaperonin CPN60-2 family protein [Populus trichocarpa]	CPN60-2	Chaperonin CPN60-2, mitochondrial OS=Cucurbita maxima GN=CPN60-2 PE=1 SV=1	117.86	42.68	2.19	up	0.00	0.00	yes
TRINITY_DN28251_c0_g1	-	-	-	3.06	0.04	6.23	up	0.00	0.00	yes
TRINITY_DN19253_c0_g2	PREDICTED: probable sucrose-phosphate synthase 4 [Populus euphratica]	SPS4	Probable sucrose-phosphate synthase 4 OS=Arabidopsis thaliana GN=SPS4 PE=1 SV=1	3.66	0.34	4.06	up	0.00	0.00	yes
TRINITY_DN19754_c0_g1	hypothetical protein POPTR_0002s17060g [Populus trichocarpa]	RPD1	Protein ROOT PRIMORDIUM DEFECTIVE 1 OS=Arabidopsis thaliana GN=RPD1 PE=1 SV=1	91.60	36.44	1.94	up	0.00	0.00	yes
TRINITY_DN26439_c0_g1	PREDICTED: proline--tRNA ligase isoform X1 [Populus euphratica]	OVA6	Proline--tRNA ligase, chloroplastic/mitochondrial OS=Arabidopsis thaliana GN=OVA6 PE=2 SV=1	64.68	27.02	1.92	up	0.00	0.00	yes
TRINITY_DN11377_c0_g1	PREDICTED: nicotianamine synthase-like [Populus euphratica]	CHLN	Nicotianamine synthase OS=Solanum lycopersicum GN=CHLN PE=2 SV=1	1.86	0.11	4.64	up	0.00	0.00	yes
TRINITY_DN26342_c0_g1	S-adenosylmethionine decarboxylase family protein [Populus trichocarpa]	SAMDC	S-adenosylmethionine decarboxylase proenzyme OS=Ipomoea nil GN=SAMDC PE=3 SV=1	348.95	135.54	1.99	up	0.00	0.00	yes
TRINITY_DN24116_c1_g2	hypothetical protein POPTR_0018s09340g [Populus trichocarpa]	GATA	Glutamyl-tRNA(Gln) amidotransferase subunit A, chloroplastic/mitochondrial OS=Arabidopsis thaliana GN=GATA PE=2 SV=1	63.54	26.14	1.98	up	0.00	0.00	yes
TRINITY_DN21490_c0_g1	hypothetical protein POPTR_0014s14560g [Populus trichocarpa]	-	-	91.86	36.12	1.97	up	0.00	0.00	yes
TRINITY_DN26825_c0_g2	PREDICTED: beta-fructofuranosidase, soluble isoenzyme I-like [Populus euphratica]	INV*DC4	Beta-fructofuranosidase, soluble isoenzyme I OS=Daucus carota GN=INV*DC4 PE=1 SV=2	96.10	31.98	2.18	up	0.00	0.00	yes
TRINITY_DN16744_c0_g2	hypothetical protein POPTR_0016s11780g [Populus trichocarpa]	-	-	122.77	43.65	2.05	up	0.00	0.00	yes
TRINITY_DN26853_c0_g1	PREDICTED: sulfate transporter 4.1, chloroplastic-like isoform X1 [Populus euphratica]	SULTR4;1	Sulfate transporter 4.1, chloroplastic OS=Arabidopsis thaliana GN=SULTR4;1 PE=1 SV=1	14.78	7.97	1.84	up	0.00	0.00	yes
TRINITY_DN26284_c1_g1	hypothetical protein VITISV_025518 [Vitis vinifera]	-	Retrovirus-related Pol polyprotein from transposon TNT 1-94 OS=Nicotiana tabacum PE=2 SV=1	3.90	1.29	2.20	up	0.00	0.00	yes
TRINITY_DN19380_c0_g2	FLAGELLIN-SENSITIVE 2 family protein [Populus trichocarpa]	FLS2	LRR receptor-like serine/threonine-protein kinase FLS2 OS=Arabidopsis thaliana GN=FLS2 PE=1 SV=1	9.65	2.15	2.75	up	0.00	0.00	yes
TRINITY_DN20804_c0_g1	hypothetical protein POPTR_0006s09200g [Populus trichocarpa]	UPP	Uracil phosphoribosyltransferase OS=Nicotiana tabacum GN=UPP PE=2 SV=1	192.18	65.26	2.16	up	0.00	0.00	yes
TRINITY_DN26977_c1_g2	RUBISCO SUBUNIT BINDING-protein ALPHA SUBUNIT [Populus trichocarpa]	-	RuBisCO large subunit-binding protein subunit alpha, chloroplastic OS=Brassica napus PE=2 SV=1	66.21	16.76	2.56	up	0.00	0.00	yes
TRINITY_DN19384_c0_g1	rhodanese-like domain-containing family protein [Populus trichocarpa]	At5g19370	Rhodanese-like/PpiC domain-containing protein 12, chloroplastic OS=Arabidopsis thaliana GN=At5g19370 PE=1 SV=1	105.09	43.25	1.93	up	0.00	0.00	yes
TRINITY_DN21020_c0_g1	PREDICTED: uncharacterized methyltransferase At2g41040, chloroplastic-like [Populus euphratica]	At2g41040	Uncharacterized methyltransferase At2g41040, chloroplastic OS=Arabidopsis thaliana GN=At2g41040 PE=2 SV=1	22.35	8.63	2.03	up	0.00	0.00	yes
TRINITY_DN22920_c1_g6	PREDICTED: probable ribose-5-phosphate isomerase 3, chloroplastic [Populus euphratica]	RPI3	Probable ribose-5-phosphate isomerase 3, chloroplastic OS=Arabidopsis thaliana GN=RPI3 PE=1 SV=1	555.60	140.50	2.63	up	0.00	0.00	yes
TRINITY_DN26991_c0_g1	PREDICTED: DEAD-box ATP-dependent RNA helicase 31-like [Populus euphratica]	RH31	DEAD-box ATP-dependent RNA helicase 31 OS=Arabidopsis thaliana GN=RH31 PE=2 SV=2	106.93	42.87	1.93	up	0.00	0.00	yes
TRINITY_DN22498_c0_g1	benzoyl-CoA:benzylalcohol/2-phenylethanol benzoyltransferase, partial [Populus davidiana]	HSR201	Benzyl alcohol O-benzoyltransferase OS=Nicotiana tabacum GN=HSR201 PE=1 SV=1	1333.43	383.43	2.42	up	0.00	0.00	yes
TRINITY_DN26627_c0_g1	PHOSPHOGLYCERATE KINASE 1 family protein [Populus trichocarpa]	-	Phosphoglycerate kinase, chloroplastic OS=Nicotiana tabacum PE=2 SV=1	946.82	271.09	2.37	up	0.00	0.00	yes

TRINITY_DN18710_c0_g2	hypothetical protein POPTR_0010s07730g [Populus trichocarpa]	PGR5	Protein PROTON GRADIENT REGULATION 5, chloroplastic OS=Arabidopsis thaliana GN=PGR5 PE=1 SV=1	25.37	8.54	2.21	up	0.00	0.00	yes
TRINITY_DN25428_c0_g1	-	-	-	13.21	2.06	3.37	up	0.00	0.00	yes
TRINITY_DN18637_c0_g1	hypothetical protein POPTR_0007s13790g [Populus trichocarpa]	CPP1	Protein CHAPERONE-LIKE PROTEIN OF POR1, chloroplastic OS=Arabidopsis thaliana GN=CPP1 PE=1 SV=1	44.04	18.13	1.87	up	0.00	0.00	yes
TRINITY_DN19412_c0_g11	pentatricopeptide repeat-containing family protein [Populus trichocarpa]	At1g11630	Pentatricopeptide repeat-containing protein At1g11630, mitochondrial OS=Arabidopsis thaliana GN=At1g11630 PE=1 SV=1	18.56	6.23	2.21	up	0.00	0.00	yes
TRINITY_DN17790_c0_g1	hypothetical protein POPTR_0018s12000g [Populus trichocarpa]	Chia	Acidic mammalian chitinase OS=Mus musculus GN=Chia PE=1 SV=2	5.61	1.19	2.85	up	0.00	0.00	yes
TRINITY_DN25141_c0_g1	PREDICTED: dihydrolipoyllysine-residue acetyltransferase component 4 of pyruvate dehydrogenase complex, chloroplastic [Populus euphratica]	LTA2	Dihydrolipoyllysine-residue acetyltransferase component 4 of pyruvate dehydrogenase complex, chloroplastic OS=Arabidopsis thaliana GN=LTA2 PE=2 SV=1	116.62	48.47	1.90	up	0.00	0.00	yes
TRINITY_DN26427_c2_g1	PREDICTED: flavonoid 3'-monooxygenase-like [Populus euphratica]	CYP75B2	Flavonoid 3'-monooxygenase OS=Petunia hybrida GN=CYP75B2 PE=2 SV=1	3671.74	1222.88	2.19	up	0.00	0.00	yes
TRINITY_DN19763_c0_g3	PREDICTED: malate dehydrogenase, mitochondrial [Populus euphratica]	MMDH	Malate dehydrogenase, mitochondrial OS=Citrullus lanatus GN=MMDH PE=1 SV=1	247.09	106.19	1.88	up	0.00	0.00	yes
TRINITY_DN23207_c0_g3	PREDICTED: BAG family molecular chaperone regulator 2-like [Populus euphratica]	BAG1	BAG family molecular chaperone regulator 1 OS=Arabidopsis thaliana GN=BAG1 PE=1 SV=1	18.98	4.39	2.71	up	0.00	0.00	yes
TRINITY_DN17742_c0_g3	PREDICTED: quinolinate synthase, chloroplastic [Populus euphratica]	QS	Quinolinate synthase, chloroplastic OS=Arabidopsis thaliana GN=QS PE=1 SV=1	13.69	5.06	2.07	up	0.00	0.00	yes
TRINITY_DN25369_c0_g2	PREDICTED: uncharacterized protein LOC105142434 [Populus euphratica]	tsf	Elongation factor Ts OS=Prochlorococcus marinus (strain MIT 9312) GN=tsf PE=3 SV=1	198.49	66.26	2.17	up	0.00	0.00	yes
TRINITY_DN21301_c0_g1	chloroplast thylakoid lumen family protein [Populus trichocarpa]	At4g02530	Thylakoid lumenal 16.5 kDa protein, chloroplastic OS=Arabidopsis thaliana GN=At4g02530 PE=1 SV=3	208.66	46.95	2.74	up	0.00	0.00	yes
TRINITY_DN27756_c1_g1	hypothetical protein POPTR_0010s05400g [Populus trichocarpa]	R1	Alpha-glucan water dikinase, chloroplastic OS=Citrus reticulata GN=R1 PE=2 SV=1	39.54	18.18	1.88	up	0.00	0.00	yes
TRINITY_DN25737_c0_g1	semialdehyde dehydrogenase family protein [Populus trichocarpa]	asd	Aspartate-semialdehyde dehydrogenase OS=Synechocystis sp. (strain PCC 6803 / Kazusa) GN=asd PE=3 SV=2	56.96	24.76	1.82	up	0.00	0.00	yes
TRINITY_DN20895_c0_g1	hypothetical protein POPTR_0014s10620g [Populus trichocarpa]	-	-	31.91	10.77	2.17	up	0.00	0.00	yes
TRINITY_DN12667_c0_g1	hypothetical protein POPTR_0013s04220g [Populus trichocarpa]	At1g06620	1-aminocyclopropane-1-carboxylate oxidase homolog 1 OS=Arabidopsis thaliana GN=At1g06620 PE=2 SV=1	4.05	0.56	3.45	up	0.00	0.00	yes
TRINITY_DN18698_c0_g1	PREDICTED: probable protein phosphatase 2C 40 isoform X1 [Populus euphratica]	Os04g0403701	Probable protein phosphatase 2C 39 OS=Oryza sativa subsp. japonica GN=Os04g0403701 PE=2 SV=2	8.66	2.84	3.27	up	0.00	0.00	yes
TRINITY_DN22269_c0_g1	PREDICTED: adenylate kinase 5, chloroplastic [Populus euphratica]	At5g35170	Adenylate kinase 5, chloroplastic OS=Arabidopsis thaliana GN=At5g35170 PE=1 SV=1	27.73	10.48	2.02	up	0.00	0.00	yes
TRINITY_DN22684_c0_g1	glutamate decarboxylase [Populus tremula x Populus alba]	GAD4	Glutamate decarboxylase 4 OS=Arabidopsis thaliana GN=GAD4 PE=1 SV=1	5.12	0.35	4.49	up	0.00	0.00	yes
TRINITY_DN17799_c0_g2	hypothetical protein POPTR_0013s03320g [Populus trichocarpa]	-	-	19.75	4.33	2.79	up	0.00	0.00	yes
TRINITY_DN26808_c0_g1	PREDICTED: protein YLS2-like [Populus euphratica]	SSL5	Protein STRICTOSIDINE SYNTHASE-LIKE 5 OS=Arabidopsis thaliana GN=SSL5 PE=2 SV=1	63.75	30.10	1.76	up	0.00	0.00	yes
TRINITY_DN24087_c0_g1	peptidyl-tRNA hydrolase family protein [Populus trichocarpa]	At1g18440	Peptidyl-tRNA hydrolase, chloroplastic OS=Arabidopsis thaliana GN=At1g18440 PE=2 SV=2	29.29	13.93	1.87	up	0.00	0.00	yes
TRINITY_DN17171_c0_g1	unknown [Populus trichocarpa]	-	Cytochrome b-c1 complex subunit 9 OS=Solanum tuberosum PE=1 SV=1	204.69	78.64	1.96	up	0.00	0.00	yes
TRINITY_DN17248_c0_g3	-	-	-	102.48	40.68	1.96	up	0.00	0.00	yes
TRINITY_DN17643_c0_g5	60S acidic ribosomal protein P1 [Populus trichocarpa]	RPP1C	60S acidic ribosomal protein P1-3 OS=Arabidopsis thaliana GN=RPP1C PE=1 SV=2	52.58	16.59	2.31	up	0.00	0.00	yes
TRINITY_DN17413_c0_g1	hypothetical protein POPTR_0015s09600g [Populus trichocarpa]	-	-	6.95	0.65	4.04	up	0.00	0.00	yes
TRINITY_DN26210_c0_g1	S-adenosyl-L-homocysteine hydrolase [Populus tomentosa]	SAHH	Adenosylhomocysteinase OS=Catharanthus roseus GN=SAHH PE=2 SV=1	301.69	124.53	1.88	up	0.00	0.00	yes
TRINITY_DN10744_c0_g1	-	-	-	3.61	0.08	5.97	up	0.00	0.00	yes

TRINITY_DN22958_c1_g1	PREDICTED: transcription factor BIM2-like isoform X2 [Populus euphratica]	UGT75L6	Crocetin glucosyltransferase, chloroplastic OS=Gardenia jasminoides GN=UGT75L6 PE=1 SV=1	14.62	8.00	1.76	up	0.00	0.00	yes
TRINITY_DN24137_c0_g2	PREDICTED: acyl-[acyl-carrier-protein] desaturase, chloroplastic [Populus euphratica]	-	Stearoyl-[acyl-carrier-protein] 9-desaturase, chloroplastic OS=Ricinus communis PE=1 SV=1	275.31	109.07	1.93	up	0.00	0.00	yes
TRINITY_DN19943_c0_g2	hypothetical protein POPTR_0001s09830g [Populus trichocarpa]	MDAR5	Monodehydroascorbate reductase 5, mitochondrial OS=Arabidopsis thaliana GN=MDAR5 PE=1 SV=3	26.04	12.13	1.71	up	0.00	0.00	yes
TRINITY_DN20062_c1_g6	glutaredoxin family protein [Populus trichocarpa]	At5g39865	Uncharacterized protein At5g39865 OS=Arabidopsis thaliana GN=At5g39865 PE=2 SV=1	41.42	9.66	2.68	up	0.00	0.00	yes
TRINITY_DN17482_c0_g2	hypothetical protein POPTR_0003s15960g, partial [Populus trichocarpa]	RPS27B	40S ribosomal protein S27-2 OS=Arabidopsis thaliana GN=RPS27B PE=2 SV=2	494.65	200.08	1.93	up	0.00	0.00	yes
TRINITY_DN21117_c0_g1	N-acetylglutamate kinase, partial [Populus maximowiczii x Populus nigra]	NAGK	Acetylglutamate kinase, chloroplastic OS=Arabidopsis thaliana GN=NAGK PE=1 SV=1	57.23	25.99	1.76	up	0.00	0.00	yes
TRINITY_DN26046_c0_g1	PREDICTED: chaperone protein ClpB3, chloroplastic-like [Populus euphratica]	CLPB3	Chaperone protein ClpB3, chloroplastic OS=Arabidopsis thaliana GN=CLPB3 PE=1 SV=1	137.60	63.74	1.96	up	0.00	0.00	yes
TRINITY_DN13737_c0_g1	hypothetical protein POPTR_0010s05710g [Populus trichocarpa]	-	-	33.53	3.21	3.98	up	0.00	0.00	yes
TRINITY_DN19838_c1_g1	PREDICTED: S-adenosylmethionine carrier 1, chloroplastic/mitochondrial [Populus euphratica]	SAMC1	S-adenosylmethionine carrier 1, chloroplastic/mitochondrial OS=Arabidopsis thaliana GN=SAMC1 PE=1 SV=1	99.33	42.75	2.00	up	0.00	0.00	yes
TRINITY_DN20148_c0_g2	ribose 5-phosphate isomerase family protein [Populus trichocarpa]	RPI2	Probable ribose-5-phosphate isomerase 2 OS=Arabidopsis thaliana GN=RPI2 PE=1 SV=1	6.05	1.07	3.15	up	0.00	0.00	yes
TRINITY_DN18693_c0_g1	PREDICTED: rubisco accumulation factor 1, chloroplastic [Populus euphratica]	RAF1.2	Rubisco accumulation factor 1.2, chloroplastic OS=Arabidopsis thaliana GN=RAF1.2 PE=1 SV=1	236.80	77.53	2.20	up	0.00	0.00	yes
TRINITY_DN14872_c0_g1	hypothetical protein POPTR_0004s16380g [Populus trichocarpa]	-	Polyphenol oxidase, chloroplastic OS=Malus domestica PE=2 SV=1	5.62	0.83	3.38	up	0.00	0.00	yes
TRINITY_DN20603_c0_g1	hypothetical protein POPTR_0005s05760g [Populus trichocarpa]	-	-	57.37	23.17	1.97	up	0.00	0.00	yes
TRINITY_DN23716_c0_g3	ubiquinol-cytochrome C reductase iron-sulfur subunit family protein [Populus trichocarpa]	-	Cytochrome b-c1 complex subunit Rieske-2, mitochondrial OS=Nicotiana tabacum PE=2 SV=1	94.25	38.11	1.96	up	0.00	0.00	yes
TRINITY_DN23807_c0_g2	-	-	-	10.03	1.10	3.87	up	0.00	0.00	yes
TRINITY_DN18149_c0_g1	hypothetical protein POPTR_0008s19820g [Populus trichocarpa]	-	-	28.72	9.90	2.08	up	0.00	0.00	yes
TRINITY_DN22424_c0_g1	hypothetical protein POPTR_0006s05160g [Populus trichocarpa]	typA	GTP-binding protein TypA/BipA homolog OS=Synechocystis sp. (strain PCC 6803 / Kazusa) GN=typA PE=3 SV=1	79.89	24.03	2.40	up	0.00	0.00	yes
TRINITY_DN23306_c0_g2	PREDICTED: 60S ribosomal protein L17-2-like isoform X1 [Populus euphratica]	RPL17B	60S ribosomal protein L17-2 OS=Arabidopsis thaliana GN=RPL17B PE=2 SV=2	395.65	149.06	2.02	up	0.00	0.00	yes
TRINITY_DN20980_c2_g1	PREDICTED: uncharacterized protein LOC105132050 [Populus euphratica]	-	-	51.57	19.07	2.04	up	0.00	0.00	yes
TRINITY_DN18519_c0_g1	hypothetical protein POPTR_0001s34130g [Populus trichocarpa]	frr	Ribosome-recycling factor OS=Bacillus clausii (strain KSM-K16) GN=frr PE=3 SV=1	31.61	12.93	1.91	up	0.00	0.00	yes
TRINITY_DN19150_c0_g1	hypothetical protein POPTR_0001s41190g [Populus trichocarpa]	At3g15360	Thioredoxin M4, chloroplastic OS=Arabidopsis thaliana GN=At3g15360 PE=2 SV=2	289.16	70.23	2.64	up	0.00	0.00	yes
TRINITY_DN24222_c0_g1	hypothetical protein POPTR_0002s12740g [Populus trichocarpa]	trc	Serine/threonine-protein kinase tricorner OS=Drosophila melanogaster GN=trc PE=1 SV=1	21.74	8.16	2.01	up	0.00	0.00	yes
TRINITY_DN18982_c0_g1	PREDICTED: uncharacterized protein LOC105138933 isoform X2 [Populus euphratica]	tilS	tRNA(Ile)-lysidine synthase OS=Wolbachia sp. subsp. Brugia malayi (strain TRS) GN=tilS PE=3 SV=1	14.42	5.83	1.90	up	0.00	0.00	yes
TRINITY_DN27857_c5_g1	polyprotein [Solanum lycopersicum]	TY3B-I	Transposon Ty3-I Gag-Pol polyprotein OS=Saccharomyces cerevisiae (strain ATCC 204508 / S288c) GN=TY3B-I PE=3 SV=2	42.38	8.94	3.10	up	0.00	0.00	yes
TRINITY_DN14573_c0_g1	hypothetical protein POPTR_0008s11320g [Populus trichocarpa]	-	-	58.26	7.50	3.58	up	0.00	0.00	yes
TRINITY_DN15836_c0_g2	hypothetical protein POPTR_0018s05880g [Populus trichocarpa]	-	-	17.40	2.10	3.69	up	0.00	0.00	yes
TRINITY_DN24041_c0_g1	PREDICTED: D-glycerate 3-kinase, chloroplastic-like [Populus euphratica]	GLYK	D-glycerate 3-kinase, chloroplastic OS=Arabidopsis thaliana GN=GLYK PE=1 SV=2	150.03	53.16	2.06	up	0.00	0.00	yes
TRINITY_DN17413_c0_g2	hypothetical protein POPTR_0015s09600g [Populus trichocarpa]	-	-	4.69	0.23	4.76	up	0.00	0.00	yes

TRINITY_DN542_c0_g1	hypothetical protein POPTR_0011s15340g [Populus trichocarpa]	DLO2	Protein DMR6-LIKE OXYGENASE 2 OS=Arabidopsis thaliana GN=DLO2 PE=2 SV=1	5.50	0.97	3.11	up	0.00	0.00	yes
TRINITY_DN16810_c0_g1	hypothetical protein POPTR_0014s12770g [Populus trichocarpa]	-	-	16.17	5.62	2.12	up	0.00	0.00	yes
TRINITY_DN21123_c0_g3	unnamed protein product, partial [Vitis vinifera]	RPL17B	60S ribosomal protein L17-2 OS=Arabidopsis thaliana GN=RPL17B PE=2 SV=2	299.70	128.63	1.85	up	0.00	0.00	yes
TRINITY_DN20171_c1_g1	glutamate 1-semialdehyde aminotransferase family protein [Populus trichocarpa]	-	Glutamate-1-semialdehyde 2,1-aminomutase, chloroplastic OS=Solanum lycopersicum PE=2 SV=1	407.15	149.39	2.05	up	0.00	0.00	yes
TRINITY_DN24428_c0_g2	nodulin-26 family protein [Populus trichocarpa]	TIP1-1	Aquaporin TIP1-1 OS=Arabidopsis thaliana GN=TIP1-1 PE=1 SV=1	260.76	81.20	2.16	up	0.00	0.00	yes
TRINITY_DN23172_c0_g1	hypothetical protein POPTR_0017s00350g [Populus trichocarpa]	DAPB2	4-hydroxy-tetrahydrodipicolinate reductase 2, chloroplastic OS=Arabidopsis thaliana GN=DAPB2 PE=2 SV=1	54.45	26.38	1.90	up	0.00	0.00	yes
TRINITY_DN18951_c0_g3	hypothetical protein POPTR_0015s06230g [Populus trichocarpa]	CAS	Calcium sensing receptor, chloroplastic OS=Arabidopsis thaliana GN=CAS PE=1 SV=1	235.93	64.37	2.46	up	0.00	0.00	yes
TRINITY_DN20717_c0_g8	hypothetical protein EUGRSUZ_H02959 [Eucalyptus grandis]	RPL23A	60S ribosomal protein L23 OS=Arabidopsis thaliana GN=RPL23A PE=2 SV=3	724.23	311.69	1.84	up	0.00	0.00	yes
TRINITY_DN16434_c0_g1	PREDICTED: uncharacterized protein LOC105122164 [Populus euphratica]	rpsF	30S ribosomal protein S6 OS=Alcanivorax borkumensis (strain ATCC 700651 / DSM 11573 / NCIMB 13689 / SK2) GN=rpsF PE=3 SV=1	99.45	40.77	1.89	up	0.00	0.00	yes
TRINITY_DN20401_c1_g1	PREDICTED: 3-epi-6-deoxocathasterone 23-monooxygenase-like isoform X2 [Populus euphratica]	CYP90D1	3-epi-6-deoxocathasterone 23-monooxygenase OS=Arabidopsis thaliana GN=CYP90D1 PE=2 SV=1	2.81	0.14	4.90	up	0.00	0.00	yes
TRINITY_DN15000_c0_g1	hypothetical protein POPTR_0012s10470g [Populus trichocarpa]	TPD1	Protein TAPETUM DETERMINANT 1 OS=Arabidopsis thaliana GN=TPD1 PE=1 SV=1	5.42	0.70	3.15	up	0.00	0.00	yes
TRINITY_DN17002_c0_g3	hypothetical protein POPTR_0006s08240g [Populus trichocarpa]	-	-	56.58	25.91	1.75	up	0.00	0.00	yes
TRINITY_DN24990_c0_g1	-	-	-	38.31	3.78	3.92	up	0.00	0.00	yes
TRINITY_DN19518_c0_g1	hypothetical protein POPTR_0016s14360g [Populus trichocarpa]	efp	Elongation factor P OS=Synechococcus elongatus (strain PCC 7942) GN=efp PE=3 SV=1	277.40	89.48	2.25	up	0.00	0.00	yes
TRINITY_DN18005_c1_g2	-	-	-	122.36	29.49	3.14	up	0.00	0.00	yes
TRINITY_DN21678_c1_g2	PREDICTED: glutathione reductase, chloroplastic [Populus euphratica]	EMB2360	Glutathione reductase, chloroplastic OS=Arabidopsis thaliana GN=EMB2360 PE=2 SV=1	73.53	32.22	1.79	up	0.00	0.00	yes
TRINITY_DN24500_c0_g6	PREDICTED: DEAD-box ATP-dependent RNA helicase 22 isoform X2 [Populus euphratica]	RH22	DEAD-box ATP-dependent RNA helicase 22 OS=Arabidopsis thaliana GN=RH22 PE=2 SV=1	60.25	26.15	1.81	up	0.00	0.00	yes
TRINITY_DN19893_c0_g1	hypothetical protein POPTR_0006s05930g [Populus trichocarpa]	FMO1	Probable flavin-containing monooxygenase 1 OS=Arabidopsis thaliana GN=FMO1 PE=2 SV=1	8.67	2.41	2.38	up	0.00	0.00	yes
TRINITY_DN16814_c0_g1	hypothetical protein POPTR_0013s15030g [Populus trichocarpa]	At2g30320	Putative tRNA pseudouridine synthase OS=Arabidopsis thaliana GN=At2g30320 PE=3 SV=1	24.31	12.17	1.69	up	0.00	0.00	yes
TRINITY_DN25120_c1_g1	PREDICTED: ABC transporter G family member 29-like [Populus euphratica]	ABCG36	ABC transporter G family member 36 OS=Arabidopsis thaliana GN=ABCG36 PE=1 SV=1	5.88	6.33	1.78	up	0.00	0.00	yes
TRINITY_DN27079_c0_g1	receptor kinase [Populus tomentosa]	HIP	HSP-interacting protein OS=Zea mays GN=HIP PE=1 SV=1	29.33	14.86	1.66	up	0.00	0.00	yes
TRINITY_DN18595_c0_g2	hypothetical protein POPTR_0017s00380g [Populus trichocarpa]	RAP	RAP domain-containing protein, chloroplastic OS=Arabidopsis thaliana GN=RAP PE=1 SV=1	47.76	19.58	1.87	up	0.00	0.00	yes
TRINITY_DN25793_c0_g2	PREDICTED: protein REVEILLE 7-like [Populus euphratica]	RVE7	Protein REVEILLE 7 OS=Arabidopsis thaliana GN=RVE7 PE=2 SV=1	6.18	1.76	2.51	up	0.00	0.00	yes
TRINITY_DN24213_c0_g2	PREDICTED: 60S ribosomal protein L9-like [Populus euphratica]	RPL9	60S ribosomal protein L9 OS=Oryza sativa subsp. japonica GN=RPL9 PE=2 SV=3	1502.50	630.06	1.89	up	0.00	0.00	yes
TRINITY_DN18901_c0_g1	-	-	-	5.69	1.16	2.89	up	0.00	0.00	yes
TRINITY_DN21915_c1_g2	60S ribosomal protein L11 [Populus trichocarpa]	RPL11A	60S ribosomal protein L11-1 OS=Arabidopsis thaliana GN=RPL11A PE=2 SV=2	798.91	322.84	1.93	up	0.00	0.00	yes
TRINITY_DN18890_c0_g1	hypothetical protein POPTR_0010s09820g [Populus trichocarpa]	RPS24A	40S ribosomal protein S24-1 OS=Arabidopsis thaliana GN=RPS24A PE=2 SV=1	278.90	106.23	2.04	up	0.00	0.00	yes
TRINITY_DN22108_c0_g2	PREDICTED: 40S ribosomal protein S15a-1 isoform X1 [Populus euphratica]	RPS15AA	40S ribosomal protein S15a-1 OS=Arabidopsis thaliana GN=RPS15AA PE=2 SV=2	410.99	172.46	1.87	up	0.00	0.00	yes
TRINITY_DN17176_c0_g2	PREDICTED: serine/threonine-protein kinase-like protein CCR2 [Populus euphratica]	CCR2	Serine/threonine-protein kinase-like protein CCR2 OS=Arabidopsis thaliana GN=CCR2 PE=1 SV=1	5.80	1.36	2.67	up	0.00	0.00	yes

TRINITY_DN16463_c0_g1	unknown [Populus trichocarpa]	APS1	Acid phosphatase 1 OS=Solanum lycopersicum GN=APS1 PE=2 SV=1	39.70	16.01	1.92	up	0.00	0.00	yes
TRINITY_DN22425_c0_g5	Chaperonin CPN60-2 family protein [Populus trichocarpa]	CPN60-2	Chaperonin CPN60-2, mitochondrial OS=Cucurbita maxima GN=CPN60-2 PE=1 SV=1	63.97	27.86	1.83	up	0.00	0.00	yes
TRINITY_DN19012_c0_g1	porin family protein [Populus trichocarpa]	-	Mitochondrial outer membrane protein porin of 36 kDa OS=Solanum tuberosum PE=1 SV=2	356.09	156.94	1.83	up	0.00	0.00	yes
TRINITY_DN21298_c0_g1	hypothetical protein POPTR_0009s05130g [Populus trichocarpa]	PLC4	Phosphoinositide phospholipase C 4 OS=Arabidopsis thaliana GN=PLC4 PE=2 SV=2	4.63	0.37	3.71	up	0.00	0.00	yes
TRINITY_DN24822_c0_g1	cation efflux family protein [Populus trichocarpa]	MTP11	Metal tolerance protein 11 OS=Arabidopsis thaliana GN=MTP11 PE=2 SV=1	17.50	7.44	1.82	up	0.00	0.00	yes
TRINITY_DN16505_c0_g1	60S ribosomal protein L27 [Populus trichocarpa]	RPL27C	60S ribosomal protein L27-3 OS=Arabidopsis thaliana GN=RPL27C PE=2 SV=2	593.16	254.52	1.85	up	0.00	0.00	yes
TRINITY_DN23450_c0_g1	hypothetical protein POPTR_0006s13480g [Populus trichocarpa]	RPL4	60S ribosomal protein L4 OS=Prunus armeniaca GN=RPL4 PE=2 SV=1	338.50	141.38	1.89	up	0.00	0.00	yes
TRINITY_DN14573_c1_g1	hypothetical protein POPTR_0008s11320g [Populus trichocarpa]	-	-	101.65	25.02	2.66	up	0.00	0.00	yes
TRINITY_DN18188_c1_g4	hypothetical protein POPTR_0005s08250g [Populus trichocarpa]	RPL30	60S ribosomal protein L30 OS=Lupinus luteus GN=RPL30 PE=3 SV=1	80.17	29.95	2.04	up	0.00	0.00	yes
TRINITY_DN17574_c0_g1	aldose 1-epimerase family protein [Populus trichocarpa]	-	Putative glucose-6-phosphate 1-epimerase OS=Cenchrus ciliaris PE=2 SV=1	13.53	5.15	1.98	up	0.00	0.00	yes
TRINITY_DN22415_c0_g7	-	-	-	21.20	0.00	10.64	up	0.00	0.00	yes
TRINITY_DN25318_c0_g2	hypothetical protein POPTR_0007s07680g [Populus trichocarpa]	HEMC	Porphobilinogen deaminase, chloroplastic OS=Pisum sativum GN=HEMC PE=1 SV=1	138.48	53.82	1.95	up	0.00	0.00	yes
TRINITY_DN17427_c0_g3	PREDICTED: coiled-coil domain-containing protein 9 [Populus euphratica]	-	-	216.34	79.59	2.04	up	0.00	0.00	yes
TRINITY_DN26192_c2_g2	cysteine proteinase superfamily protein [Populus tomentosa]	At3g57810	OTU domain-containing protein At3g57810 OS=Arabidopsis thaliana GN=At3g57810 PE=2 SV=1	34.40	15.35	1.88	up	0.00	0.00	yes
TRINITY_DN23249_c0_g3	hypothetical protein POPTR_0009s16340g [Populus trichocarpa]	-	-	369.82	123.40	2.21	up	0.00	0.00	yes
TRINITY_DN27124_c0_g1	hypothetical protein POPTR_0019s00800g [Populus trichocarpa]	Os05g0361200	Ferrochelatase-2, chloroplastic OS=Oryza sativa subsp. japonica GN=Os05g0361200 PE=2 SV=1	243.09	81.07	2.22	up	0.00	0.00	yes
TRINITY_DN16542_c0_g1	hypothetical protein POPTR_0008s12950g [Populus trichocarpa]	CPN10	10 kDa chaperonin, mitochondrial OS=Arabidopsis thaliana GN=CPN10 PE=1 SV=1	48.73	18.59	2.02	up	0.00	0.00	yes
TRINITY_DN22575_c0_g2	hypothetical protein POPTR_0017s13490g [Populus trichocarpa]	-	-	52.63	10.65	2.77	up	0.00	0.00	yes
TRINITY_DN13137_c0_g1	hypothetical protein POPTR_0014s11950g [Populus trichocarpa]	-	-	4.01	0.27	4.47	up	0.00	0.00	yes
TRINITY_DN19408_c0_g1	PREDICTED: uncharacterized protein LOC105636463 [Jatropha curcas]	-	-	153.92	48.79	2.25	up	0.00	0.00	yes
TRINITY_DN18931_c0_g2	FLAVODOXIN-LIKE QUINONE REDUCTASE 1 family protein [Populus trichocarpa]	FQR1	NAD(P)H dehydrogenase (quinone) FQR1 OS=Arabidopsis thaliana GN=FQR1 PE=1 SV=1	27.75	10.92	1.97	up	0.00	0.00	yes
TRINITY_DN22098_c0_g2	hypothetical protein POPTR_0019s10900g, partial [Populus trichocarpa]	BHLH80	Transcription factor bHLH80 OS=Arabidopsis thaliana GN=BHLH80 PE=2 SV=1	19.91	8.63	1.80	up	0.00	0.00	yes
TRINITY_DN15842_c0_g2	unknown [Populus trichocarpa]	-	-	68.83	33.62	1.64	up	0.00	0.00	yes
TRINITY_DN16259_c0_g1	PREDICTED: uncharacterized protein LOC105121730 [Populus euphratica]	-	-	53.03	15.43	2.39	up	0.00	0.00	yes
TRINITY_DN19201_c0_g1	PREDICTED: uncharacterized protein LOC105126414 isoform X1 [Populus euphratica]	SG1	protein SLOW GREEN 1, chloroplastic OS=Arabidopsis thaliana GN=SG1 PE=1 SV=1	28.85	13.42	1.74	up	0.00	0.00	yes
TRINITY_DN23793_c0_g2	PREDICTED: caffeic acid 3-O-methyltransferase-like [Populus euphratica]	-	Anthranilate N-methyltransferase OS=Ruta graveolens PE=1 SV=1	23.55	0.92	5.23	up	0.00	0.00	yes
TRINITY_DN21482_c0_g1	PREDICTED: peptidyl-prolyl cis-trans isomerase FKBP18, chloroplastic isoform X2 [Populus euphratica]	FKBP18	Peptidyl-prolyl cis-trans isomerase FKBP18, chloroplastic OS=Arabidopsis thaliana GN=FKBP18 PE=1 SV=2	89.84	21.97	2.53	up	0.00	0.00	yes
TRINITY_DN23462_c0_g2	hypothetical protein POPTR_0015s11410g [Populus trichocarpa]	TIM8	Mitochondrial import inner membrane translocase subunit TIM8 OS=Arabidopsis thaliana GN=TIM8 PE=1 SV=1	69.77	32.03	1.74	up	0.00	0.00	yes

TRINITY_DN16324_c0_g1	xyloglucan:xyloglucosyl transferase family protein [Populus trichocarpa]	XTH8	Probable xyloglucan endotransglucosylase/hydrolase protein 8 OS=Arabidopsis thaliana GN=XTH8 PE=2 SV=2	13.44	4.07	2.36	up	0.00	0.00	yes
TRINITY_DN21838_c1_g2	triacylglycerol lipase [Populus tomentosa]	-	Lipase OS=Rhizopus niveus PE=1 SV=1	17.48	4.72	2.45	up	0.00	0.00	yes
TRINITY_DN26008_c1_g1	SQUINT family protein [Populus trichocarpa]	CYP40	Peptidyl-prolyl cis-trans isomerase CYP40 OS=Arabidopsis thaliana GN=CYP40 PE=2 SV=1	44.13	20.58	1.90	up	0.00	0.00	yes
TRINITY_DN20772_c0_g1	hypothetical protein POPTR_0006s10670g [Populus trichocarpa]	-	-	3.58	0.06	6.80	up	0.00	0.00	yes
TRINITY_DN25526_c0_g1	lectin family protein [Populus trichocarpa]	PP2A9	Protein PHLOEM PROTEIN 2-LIKE A9 OS=Arabidopsis thaliana GN=PP2A9 PE=2 SV=1	36.11	13.57	2.10	up	0.00	0.00	yes
TRINITY_DN21222_c3_g2	PREDICTED: uncharacterized protein LOC108994565 [Juglans regia]	-	-	3.37	0.28	4.09	up	0.00	0.00	yes
TRINITY_DN11372_c0_g1	hypothetical protein POPTR_0011s15150g [Populus trichocarpa]	At5g64700	WAT1-related protein At5g64700 OS=Arabidopsis thaliana GN=At5g64700 PE=2 SV=1	4.92	0.36	3.83	up	0.00	0.00	yes
TRINITY_DN6367_c0_g1	hypothetical protein VITISV_026680 [Vitis vinifera]	-	-	7.95	0.91	3.75	up	0.00	0.00	yes
TRINITY_DN19931_c0_g1	PREDICTED: rhodanese-like domain-containing protein 14, chloroplastic [Populus euphratica]	At4g27700	Rhodanese-like domain-containing protein 14, chloroplastic OS=Arabidopsis thaliana GN=At4g27700 PE=2 SV=1	65.56	20.36	2.05	up	0.00	0.00	yes
TRINITY_DN19138_c0_g1	PREDICTED: cytochrome b561 and DOMON domain-containing protein At3g25290 [Populus euphratica]	At3g25290	Cytochrome b561 and DOMON domain-containing protein At3g25290 OS=Arabidopsis thaliana GN=At3g25290 PE=2 SV=1	55.66	25.83	1.72	up	0.00	0.00	yes
TRINITY_DN24279_c0_g1	unknown [Populus trichocarpa x Populus deltoides]	-	Phosphoenolpyruvate carboxylase 2 OS=Sorghum bicolor PE=3 SV=1	85.38	34.72	1.93	up	0.00	0.00	yes
TRINITY_DN13732_c0_g2	hypothetical protein POPTR_0002s09980g [Populus trichocarpa]	PUP1	Purine permease 1 OS=Arabidopsis thaliana GN=PUP1 PE=1 SV=1	4.18	0.36	4.15	up	0.00	0.00	yes
TRINITY_DN25709_c0_g2	hypothetical protein POPTR_0005s27800g [Populus trichocarpa]	PPD3	PsbP domain-containing protein 3, chloroplastic OS=Arabidopsis thaliana GN=PPD3 PE=1 SV=2	171.64	33.52	2.93	up	0.00	0.00	yes
TRINITY_DN23207_c0_g1	-	-	-	7.15	0.84	3.65	up	0.00	0.00	yes
TRINITY_DN26388_c0_g1	hypothetical protein POPTR_0019s12320g [Populus trichocarpa]	CCD4	Probable carotenoid cleavage dioxygenase 4, chloroplastic OS=Arabidopsis thaliana GN=CCD4 PE=1 SV=1	28.94	10.07	2.13	up	0.00	0.00	yes
TRINITY_DN19412_c0_g1	hypothetical protein POPTR_0004s01730g [Populus trichocarpa]	CYP725A1	Taxane 10-beta-hydroxylase OS=Taxus cuspidata GN=CYP725A1 PE=1 SV=1	30.47	11.30	2.36	up	0.00	0.00	yes
TRINITY_DN24175_c1_g1	unknown [Populus trichocarpa]	EFM	Myb family transcription factor EFM OS=Arabidopsis thaliana GN=EFM PE=1 SV=2	34.75	6.93	2.96	up	0.00	0.00	yes
TRINITY_DN21589_c1_g1	PREDICTED: pentatricopeptide repeat-containing protein At3g59040 [Populus euphratica]	At3g59040	Pentatricopeptide repeat-containing protein At3g59040 OS=Arabidopsis thaliana GN=At3g59040 PE=2 SV=2	65.29	27.09	1.86	up	0.00	0.00	yes
TRINITY_DN17343_c0_g2	hypothetical protein POPTR_0016s01510g [Populus trichocarpa]	-	-	123.49	51.49	1.85	up	0.00	0.00	yes
TRINITY_DN26920_c0_g3	hypothetical protein POPTR_0017s03040g [Populus trichocarpa]	-	-	21.61	4.14	2.95	up	0.00	0.00	yes
TRINITY_DN26128_c0_g3	PREDICTED: probable amino-acid acetyltransferase NAGS2, chloroplastic isoform X1 [Populus euphratica]	NAGS1	Probable amino-acid acetyltransferase NAGS1, chloroplastic OS=Arabidopsis thaliana GN=NAGS1 PE=2 SV=1	26.59	11.97	1.77	up	0.00	0.00	yes
TRINITY_DN18573_c0_g1	hypothetical protein POPTR_0015s06810g [Populus trichocarpa]	RBG3	Glycine-rich RNA-binding protein 3, mitochondrial OS=Arabidopsis thaliana GN=RBG3 PE=2 SV=1	310.87	143.26	1.72	up	0.00	0.00	yes
TRINITY_DN27695_c1_g1	hypothetical protein POPTR_0017s01160g [Populus trichocarpa]	HSP83A	Heat shock protein 83 OS=Ipomoea nil GN=HSP83A PE=2 SV=1	42.99	6.84	3.35	up	0.00	0.00	yes
TRINITY_DN19918_c0_g4	PREDICTED: glycine-rich RNA-binding protein 4, mitochondrial [Populus euphratica]	RZ1A	Glycine-rich RNA-binding protein RZ1A OS=Arabidopsis thaliana GN=RZ1A PE=1 SV=1	119.64	53.63	1.77	up	0.00	0.00	yes
TRINITY_DN23710_c0_g1	PREDICTED: uncharacterized protein LOC105117269 [Populus euphratica]	HIPP05	Heavy metal-associated isoprenylated plant protein 5 OS=Arabidopsis thaliana GN=HIPP05 PE=1 SV=2	3.00	0.55	3.03	up	0.00	0.00	yes
TRINITY_DN19069_c0_g3	wrp15a family protein [Populus trichocarpa]	RPS15AA	40S ribosomal protein S15a-1 OS=Arabidopsis thaliana GN=RPS15AA PE=2 SV=2	854.22	394.65	1.75	up	0.00	0.00	yes
TRINITY_DN19638_c0_g2	PREDICTED: 60S ribosomal protein L23a-like [Populus euphratica]	RPL23A	60S ribosomal protein L23a OS=Daucus carota GN=RPL23A PE=2 SV=1	487.27	241.23	1.64	up	0.00	0.00	yes
TRINITY_DN22320_c0_g1	PREDICTED: thylakoid lumenal 29 kDa protein, chloroplastic isoform X1 [Populus euphratica]	CLEB3J9	Thylakoid lumenal 29 kDa protein, chloroplastic OS=Solanum lycopersicum GN=CLEB3J9 PE=3 SV=1	312.91	116.47	2.05	up	0.00	0.00	yes

TRINITY_DN25056_c0_g5	HSP90 [Populus tomentosa]	HSC80	Heat shock cognate protein 80 OS=Solanum lycopersicum GN=HSC80 PE=2 SV=1	199.35	84.67	1.87	up	0.00	0.00	yes
TRINITY_DN14956_c0_g1	-	-	-	4.41	0.32	4.42	up	0.00	0.00	yes
TRINITY_DN25243_c0_g1	hypothetical protein POPTR_0014s03200g [Populus trichocarpa]	SDH5	Succinate dehydrogenase subunit 5, mitochondrial OS=Arabidopsis thaliana GN=SDH5 PE=1 SV=1	59.37	29.24	1.63	up	0.00	0.00	yes
TRINITY_DN21664_c0_g3	PREDICTED: uncharacterized protein LOC105107486 [Populus euphratica]	BURP3	BURP domain-containing protein 3 OS=Oryza sativa subsp. japonica GN=BURP3 PE=2 SV=1	295.37	86.58	2.39	up	0.00	0.00	yes
TRINITY_DN18472_c0_g1	PREDICTED: uncharacterized protein LOC105134874 [Populus euphratica]	-	-	114.26	42.49	2.06	up	0.00	0.00	yes
TRINITY_DN16250_c0_g1	PREDICTED: RING-H2 finger protein ATL70-like [Populus euphratica]	ATL71	Putative RING-H2 finger protein ATL71 OS=Arabidopsis thaliana GN=ATL71 PE=3 SV=1	6.51	0.52	3.86	up	0.00	0.00	yes
TRINITY_DN25936_c0_g1	hypothetical protein POPTR_0602s00200g [Populus trichocarpa]	VEP1	3-oxo-Delta(4,5)-steroid 5-beta-reductase OS=Arabidopsis thaliana GN=VEP1 PE=1 SV=1	385.96	159.52	1.75	up	0.00	0.00	yes
TRINITY_DN16376_c0_g1	hypothetical protein POPTR_0014s16990g [Populus trichocarpa]	AGPS1	Glucose-1-phosphate adenylyltransferase small subunit, chloroplastic OS=Brassica napus GN=AGPS1 PE=2 SV=1	212.37	90.30	1.84	up	0.00	0.00	yes
TRINITY_DN19557_c1_g2	PREDICTED: polyadenylate-binding protein RBP47-like [Populus euphratica]	RBP47C	Polyadenylate-binding protein RBP47C OS=Arabidopsis thaliana GN=RBP47C PE=2 SV=1	71.15	32.39	1.71	up	0.00	0.00	yes
TRINITY_DN22361_c1_g3	PREDICTED: uncharacterized protein LOC105125696 [Populus euphratica]	YLMG1-1	YlmG homolog protein 1-1, chloroplastic OS=Arabidopsis thaliana GN=YLMG1-1 PE=2 SV=1	361.49	134.26	2.02	up	0.00	0.00	yes
TRINITY_DN19829_c0_g1	hypothetical protein POPTR_0001s03980g [Populus trichocarpa]	-	RuBisCO large subunit-binding protein subunit beta, chloroplastic OS=Pisum sativum PE=1 SV=2	1118.96	411.30	2.04	up	0.00	0.00	yes
TRINITY_DN21889_c0_g2	PREDICTED: ribulose bisphosphate carboxylase small chain, chloroplastic-like [Populus euphratica]	RBCS	Ribulose bisphosphate carboxylase small chain, chloroplastic OS=Glycine tabacina GN=RBCS PE=3 SV=1	9592.31	1398.04	3.34	up	0.00	0.00	yes
TRINITY_DN14874_c0_g1	hypothetical protein POPTR_0007s07330g [Populus trichocarpa]	PER65	Peroxidase 65 OS=Arabidopsis thaliana GN=PER65 PE=2 SV=2	3.81	0.40	3.80	up	0.00	0.00	yes
TRINITY_DN24185_c0_g4	hypothetical protein POPTR_0017s14180g [Populus trichocarpa]	RPS8	40S ribosomal protein S8 OS=Oryza sativa subsp. japonica GN=RPS8 PE=2 SV=2	182.32	84.89	1.73	up	0.00	0.00	yes
TRINITY_DN15901_c0_g1	hypothetical protein POPTR_0004s00300g [Populus trichocarpa]	-	-	4.66	0.51	3.76	up	0.00	0.00	yes
TRINITY_DN22013_c0_g2	PREDICTED: riboflavin synthase-like [Populus euphratica]	rib5	Riboflavin synthase OS=Schizosaccharomyces pombe (strain 972 / ATCC 24843) GN=rib5 PE=1 SV=1	81.46	37.42	1.69	up	0.00	0.00	yes
TRINITY_DN15656_c0_g1	CRE1-1 [Populus tomentosa]	AHK3	Histidine kinase 3 OS=Arabidopsis thaliana GN=AHK3 PE=1 SV=1	9.41	1.76	3.02	up	0.00	0.00	yes
TRINITY_DN18395_c0_g1	PREDICTED: uncharacterized protein LOC105128587 [Populus euphratica]	SDH7B	Succinate dehydrogenase subunit 7B, mitochondrial OS=Arabidopsis thaliana GN=SDH7B PE=1 SV=1	124.26	55.35	1.81	up	0.00	0.00	yes
TRINITY_DN23422_c1_g4	PREDICTED: ATP synthase delta chain, chloroplastic-like [Populus euphratica]	ATPD	ATP synthase delta chain, chloroplastic OS=Nicotiana tabacum GN=ATPD PE=2 SV=1	1050.78	305.83	2.37	up	0.00	0.00	yes
TRINITY_DN20242_c0_g1	hypothetical protein POPTR_0001s41530g [Populus trichocarpa]	CYP37	Peptidyl-prolyl cis-trans isomerase CYP37, chloroplastic OS=Arabidopsis thaliana GN=CYP37 PE=1 SV=3	92.41	35.60	1.98	up	0.00	0.00	yes
TRINITY_DN16091_c0_g1	PREDICTED: xylulose 5-phosphate/phosphate translocator, chloroplastic [Populus euphratica]	XPT	Xylulose 5-phosphate/phosphate translocator, chloroplastic OS=Arabidopsis thaliana GN=XPT PE=1 SV=1	27.10	13.24	1.63	up	0.00	0.00	yes
TRINITY_DN23271_c0_g1	hypothetical protein POPTR_0001s35080g [Populus trichocarpa]	At5g14590	Isocitrate dehydrogenase [NADP], chloroplastic/mitochondrial OS=Arabidopsis thaliana GN=At5g14590 PE=1 SV=1	37.34	19.20	1.57	up	0.00	0.00	yes
TRINITY_DN19638_c0_g4	PREDICTED: probable zinc metalloprotease EGY1, chloroplastic isoform X1 [Populus euphratica]	EGY1	Probable zinc metalloprotease EGY1, chloroplastic OS=Arabidopsis thaliana GN=EGY1 PE=2 SV=1	54.88	21.86	1.92	up	0.00	0.00	yes
TRINITY_DN26802_c1_g3	SOUL heme-binding family protein [Populus trichocarpa]	At3g10130	Heme-binding-like protein At3g10130, chloroplastic OS=Arabidopsis thaliana GN=At3g10130 PE=2 SV=1	25.80	8.68	2.19	up	0.00	0.00	yes
TRINITY_DN19513_c0_g1	hypothetical protein POPTR_0001s23400g [Populus trichocarpa]	cysG	Siroheme synthase OS=Azotobacter vinelandii (strain DJ / ATCC BAA-1303) GN=cysG PE=3 SV=1	15.09	7.09	1.70	up	0.00	0.00	yes
TRINITY_DN15277_c0_g1	HSP90 [Populus tomentosa]	HSP90-2	Heat shock protein 90-2 OS=Arabidopsis thaliana GN=HSP90-2 PE=1 SV=1	515.73	209.22	1.94	up	0.00	0.00	yes
TRINITY_DN17318_c0_g1	hypothetical protein POPTR_0009s06860g [Populus trichocarpa]	RPL31	60S ribosomal protein L31 OS=Perilla frutescens GN=RPL31 PE=2 SV=1	353.57	150.56	1.89	up	0.00	0.00	yes
TRINITY_DN18447_c0_g1	ferredoxin-related family protein [Populus trichocarpa]	PNSB3	Photosynthetic NDH subunit of subcomplex B 3, chloroplastic OS=Arabidopsis thaliana GN=PNSB3 PE=2 SV=1	58.31	25.98	1.84	up	0.00	0.00	yes
TRINITY_DN20755_c0_g3	hypothetical protein POPTR_0007s00290g [Populus trichocarpa]	-	-	34.99	6.80	2.94	up	0.00	0.00	yes

TRINITY_DN21204_c0_g1	hypothetical protein POPTR_0002s05220g [Populus trichocarpa]	RPS18A	40S ribosomal protein S18 OS=Arabidopsis thaliana GN=RPS18A PE=1 SV=1	587.19	270.27	1.78	up	0.00	0.00	yes
TRINITY_DN20965_c0_g1	hypothetical protein POPTR_0013s06350g [Populus trichocarpa]	VAR3	Zinc finger protein VAR3, chloroplastic OS=Arabidopsis thaliana GN=VAR3 PE=1 SV=2	53.23	25.66	1.67	up	0.00	0.00	yes
TRINITY_DN21069_c0_g2	PREDICTED: rhodanese-like domain-containing protein 4A, chloroplastic isoform X1 [Populus euphratica]	STR4A	Rhodanese-like domain-containing protein 4A, chloroplastic OS=Arabidopsis thaliana GN=STR4A PE=2 SV=1	87.04	37.76	1.96	up	0.00	0.00	yes
TRINITY_DN16795_c0_g1	hydroxyisourate hydrolase family protein [Populus trichocarpa]	BGLU40	Beta-glucosidase 40 OS=Arabidopsis thaliana GN=BGLU40 PE=2 SV=1	3.65	0.76	2.85	up	0.00	0.00	yes
TRINITY_DN19271_c0_g1	PREDICTED: uncharacterized protein LOC105119782 isoform X1 [Populus euphratica]	MRM1	rRNA methyltransferase 1, mitochondrial OS=Homo sapiens GN=MRM1 PE=1 SV=1	29.55	13.53	1.73	up	0.00	0.00	yes
TRINITY_DN21919_c0_g3	glycosyl transferase family 8 family protein [Populus trichocarpa]	GATL7	Probable galacturonosyltransferase-like 7 OS=Arabidopsis thaliana GN=GATL7 PE=2 SV=1	34.19	15.23	1.75	up	0.00	0.00	yes
TRINITY_DN23093_c0_g1	3-oxoacyl-[acyl-carrier-protein] synthase I [Populus trichocarpa]	KAS1	3-oxoacyl-[acyl-carrier-protein] synthase I, chloroplastic OS=Arabidopsis thaliana GN=KAS1 PE=1 SV=2	128.96	55.68	1.81	up	0.00	0.00	yes
TRINITY_DN24077_c0_g1	hypothetical protein POPTR_0005s24670g [Populus trichocarpa]	RHON1	Rho-N domain-containing protein 1, chloroplastic OS=Arabidopsis thaliana GN=RHON1 PE=1 SV=1	167.31	73.78	1.76	up	0.00	0.00	yes
TRINITY_DN18247_c0_g7	hypothetical protein CARUB_v10018208mg [Capsella rubella]	-	Ubiquitin-60S ribosomal protein L40 OS=Brassica rapa subsp. pekinensis PE=2 SV=2	262.28	111.61	1.87	up	0.00	0.00	yes
TRINITY_DN20278_c0_g3	hypothetical protein POPTR_0002s09160g [Populus trichocarpa]	At4g37920	Uncharacterized protein At4g37920, chloroplastic OS=Arabidopsis thaliana GN=At4g37920 PE=1 SV=2	55.91	25.36	1.75	up	0.00	0.00	yes
TRINITY_DN21240_c0_g1	PREDICTED: 28 kDa ribonucleoprotein, chloroplastic-like [Populus euphratica]	-	33 kDa ribonucleoprotein, chloroplastic OS=Nicotiana sylvestris PE=1 SV=1	151.80	51.26	2.16	up	0.00	0.00	yes
TRINITY_DN16333_c0_g1	ribosomal protein L27 [Populus trichocarpa]	RPL27	60S ribosomal protein L27 OS=Pisum sativum GN=RPL27 PE=2 SV=1	285.84	118.12	1.93	up	0.00	0.00	yes
TRINITY_DN19911_c0_g1	hypothetical protein POPTR_0012s14150g [Populus trichocarpa]	FUC1	Alpha-L-fucosidase 1 OS=Arabidopsis thaliana GN=FUC1 PE=1 SV=2	73.32	23.17	2.18	up	0.00	0.00	yes
TRINITY_DN26966_c0_g1	zeaxanthin epoxidase family protein [Populus trichocarpa]	-	Zeaxanthin epoxidase, chloroplastic OS=Prunus armeniaca PE=2 SV=1	47.61	21.92	1.72	up	0.00	0.00	yes
TRINITY_DN21364_c1_g1	-	-	-	8.18	0.33	5.23	up	0.00	0.00	yes
TRINITY_DN21335_c0_g1	hypothetical protein POPTR_0014s06470g [Populus trichocarpa]	-	-	23.94	7.48	2.14	up	0.00	0.00	yes
TRINITY_DN21474_c0_g4	PREDICTED: 40S ribosomal protein S12-like [Populus euphratica]	RPS12	40S ribosomal protein S12 OS=Hordeum vulgare GN=RPS12 PE=2 SV=1	392.34	166.54	1.88	up	0.00	0.00	yes
TRINITY_DN23009_c0_g1	hypothetical protein POPTR_0008s19340g [Populus trichocarpa]	-	-	101.83	31.75	2.27	up	0.00	0.00	yes
TRINITY_DN15808_c0_g2	-	-	-	5.90	0.44	4.31	up	0.00	0.00	yes
TRINITY_DN15749_c0_g2	hypothetical protein POPTR_0013s12750g [Populus trichocarpa]	typA	GTP-binding protein TypA/BipA homolog OS=Helicobacter pylori (strain J99 / ATCC 700824) GN=typA PE=3 SV=1	51.25	22.20	1.85	up	0.00	0.00	yes
TRINITY_DN19378_c0_g1	isoflavone reductase-related family protein [Populus trichocarpa]	DVR	Divinyl chlorophyllide a 8-vinyl-reductase, chloroplastic OS=Cucumis sativus GN=DVR PE=1 SV=1	55.10	24.15	1.78	up	0.00	0.00	yes
TRINITY_DN16683_c0_g1	hypothetical protein POPTR_0010s20070g [Populus trichocarpa]	purB	Adenylosuccinate lyase OS=Haemophilus influenzae (strain ATCC 51907 / DSM 11121 / KW20 / Rd) GN=purB PE=3 SV=1	30.27	14.40	1.71	up	0.00	0.00	yes
TRINITY_DN15241_c0_g1	PREDICTED: monothiol glutaredoxin-S16, chloroplastic [Populus euphratica]	GRXS16	Bifunctional monothiol glutaredoxin-S16, chloroplastic OS=Arabidopsis thaliana GN=GRXS16 PE=1 SV=2	157.10	61.63	1.95	up	0.00	0.00	yes
TRINITY_DN23214_c0_g1	hypothetical protein POPTR_0008s02250g [Populus trichocarpa]	RCOM_1506700	Probable aspartyl aminopeptidase OS=Ricinus communis GN=RCOM_1506700 PE=2 SV=2	24.27	10.93	1.72	up	0.00	0.00	yes
TRINITY_DN21445_c0_g2	hypothetical protein POPTR_0018s04990g [Populus trichocarpa]	-	-	245.63	111.02	1.74	up	0.00	0.00	yes
TRINITY_DN25599_c0_g1	unknown [Populus trichocarpa]	PBL28	Probable serine/threonine-protein kinase PBL28 OS=Arabidopsis thaliana GN=PBL28 PE=2 SV=1	35.13	16.21	1.71	up	0.00	0.00	yes
TRINITY_DN22303_c0_g6	hypothetical protein POPTR_0001s34980g [Populus trichocarpa]	Os02g0134400	L-aspartate oxidase, chloroplastic OS=Oryza sativa subsp. japonica GN=Os02g0134400 PE=3 SV=1	14.33	5.07	2.12	up	0.00	0.00	yes
TRINITY_DN24213_c0_g1	PREDICTED: 60S ribosomal protein L9 [Populus euphratica]	RPL9	60S ribosomal protein L9 OS=Pisum sativum GN=RPL9 PE=2 SV=1	910.77	393.74	1.76	up	0.00	0.00	yes

TRINITY_DN21216_c0_g1	hypothetical protein POPTR_0005s06460g [Populus trichocarpa]	-	-	132.71	46.26	2.14	up	0.00	0.00	yes
TRINITY_DN19430_c0_g1	hypothetical protein POPTR_0007s13020g [Populus trichocarpa]	BEE2	Transcription factor BEE 2 OS=Arabidopsis thaliana GN=BEE2 PE=2 SV=1	13.11	2.53	2.61	up	0.00	0.00	yes
TRINITY_DN17555_c0_g1	ADR12-2 family protein [Populus trichocarpa]	EF1	Elongation factor 1-alpha OS=Manihot esculenta GN=EF1 PE=3 SV=1	966.86	513.85	1.71	up	0.00	0.00	yes
TRINITY_DN16542_c0_g2	hypothetical protein POPTR_0010s12180g [Populus trichocarpa]	CPN10	10 kDa chaperonin, mitochondrial OS=Arabidopsis thaliana GN=CPN10 PE=1 SV=1	54.51	14.26	2.63	up	0.00	0.00	yes
TRINITY_DN19324_c0_g1	hypothetical protein POPTR_0005s09190g [Populus trichocarpa]	FSD3	Superoxide dismutase [Fe] 3, chloroplastic OS=Arabidopsis thaliana GN=FSD3 PE=1 SV=1	68.66	31.28	1.73	up	0.00	0.00	yes
TRINITY_DN19661_c0_g1	unknown [Populus trichocarpa]	-	Isoflavone reductase homolog OS=Solanum tuberosum PE=2 SV=1	168.92	61.85	2.12	up	0.00	0.00	yes
TRINITY_DN16743_c0_g2	hypothetical protein POPTR_0002s04170g [Populus trichocarpa]	At1g06620	1-aminocyclopropane-1-carboxylate oxidase homolog 1 OS=Arabidopsis thaliana GN=At1g06620 PE=2 SV=1	9.10	1.89	2.52	up	0.00	0.00	yes
TRINITY_DN14357_c0_g1	hypothetical protein POPTR_0008s02680g [Populus trichocarpa]	GAT1	GABA transporter 1 OS=Arabidopsis thaliana GN=GAT1 PE=1 SV=1	8.41	2.44	2.78	up	0.00	0.00	yes
TRINITY_DN18188_c1_g3	hypothetical protein POPTR_0007s06050g [Populus trichocarpa]	RPL30	60S ribosomal protein L30 OS=Euphorbia esula GN=RPL30 PE=3 SV=1	88.34	43.50	1.63	up	0.00	0.00	yes
TRINITY_DN18084_c0_g1	hypothetical protein POPTR_0014s04510g [Populus trichocarpa]	FKBP20-2	Peptidyl-prolyl cis-trans isomerase FKBP20-2, chloroplastic OS=Arabidopsis thaliana GN=FKBP20-2 PE=1 SV=1	128.09	37.89	2.34	up	0.00	0.00	yes
TRINITY_DN26890_c1_g1	hypothetical protein POPTR_0007s11410g [Populus trichocarpa]	-	-	29.44	5.94	2.98	up	0.00	0.00	yes
TRINITY_DN21481_c0_g2	hypothetical protein POPTR_0003s08800g [Populus trichocarpa]	At5g46580	Pentatricopeptide repeat-containing protein At5g46580, chloroplastic OS=Arabidopsis thaliana GN=At5g46580 PE=2 SV=1	189.34	75.77	1.91	up	0.00	0.00	yes
TRINITY_DN23665_c0_g1	PREDICTED: low molecular weight phosphotyrosine protein phosphatase [Populus euphratica]	slr0328	Putative low molecular weight protein-tyrosine-phosphatase slr0328 OS=Synechocystis sp. (strain PCC 6803 / Kazusa) GN=slr0328 PE=3 SV=1	165.63	68.99	1.79	up	0.00	0.00	yes
TRINITY_DN21222_c3_g1	hypothetical protein POPTR_0018s06310g [Populus trichocarpa]	-	-	7.31	0.77	3.56	up	0.00	0.00	yes
TRINITY_DN25208_c0_g5	hypothetical protein POPTR_0007s13200g [Populus trichocarpa]	era	GTPase Era OS=Enterococcus faecalis (strain ATCC 700802 / V583) GN=era PE=3 SV=1	56.97	24.26	1.83	up	0.00	0.00	yes
TRINITY_DN23514_c0_g3	PREDICTED: ferredoxin-like [Populus euphratica]	AP1	Ferredoxin, chloroplastic OS=Capsicum annuum GN=AP1 PE=1 SV=1	4060.18	921.95	2.72	up	0.00	0.00	yes
TRINITY_DN18045_c0_g1	proton-dependent oligopeptide transport family protein [Populus trichocarpa]	NPF5.7	Protein NRT1/ PTR FAMILY 5.7 OS=Arabidopsis thaliana GN=NPF5.7 PE=2 SV=2	2.68	0.42	3.28	up	0.00	0.00	yes
TRINITY_DN16407_c0_g1	PREDICTED: heme-binding protein 2 [Populus euphratica]	At3g10130	Heme-binding-like protein At3g10130, chloroplastic OS=Arabidopsis thaliana GN=At3g10130 PE=2 SV=1	295.90	72.31	2.64	up	0.00	0.00	yes
TRINITY_DN3649_c0_g1	UDP-glucoronosyl/UDP-glucosyl transferase family protein [Populus trichocarpa]	UGT71K1	UDP-glycosyltransferase 71K1 OS=Malus domestica GN=UGT71K1 PE=1 SV=1	16.41	5.74	2.13	up	0.00	0.00	yes
TRINITY_DN15249_c0_g1	signal recognition particle 14 kDa family protein [Populus trichocarpa]	SRP14	Signal recognition particle 14 kDa protein OS=Arabidopsis thaliana GN=SRP14 PE=2 SV=2	47.78	22.86	1.67	up	0.00	0.00	yes
TRINITY_DN20592_c0_g1	hypothetical protein POPTR_0002s07910g [Populus trichocarpa]	-	-	85.52	37.68	1.77	up	0.00	0.00	yes
TRINITY_DN16166_c0_g1	PREDICTED: 50S ribosomal protein L24, chloroplastic [Populus euphratica]	rplX	50S ribosomal protein L24 OS=Thermobifida fusca (strain YX) GN=rplX PE=3 SV=1	76.76	37.83	1.65	up	0.00	0.00	yes
TRINITY_DN18058_c0_g1	voltage dependent anion channel 1 [Populus tomentosa]	-	Mitochondrial outer membrane protein porin of 34 kDa OS=Solanum tuberosum PE=1 SV=2	253.27	122.66	1.64	up	0.00	0.00	yes
TRINITY_DN23219_c0_g1	hypothetical protein POPTR_0013s15690g [Populus trichocarpa]	slr1780	Ycf54-like protein OS=Synechocystis sp. (strain PCC 6803 / Kazusa) GN=slr1780 PE=3 SV=1	420.28	138.33	2.17	up	0.00	0.00	yes
TRINITY_DN23611_c0_g3	PREDICTED: uncharacterized protein LOC105136985 [Populus euphratica]	-	-	28.32	10.10	2.08	up	0.00	0.00	yes
TRINITY_DN22367_c0_g1	PREDICTED: uncharacterized protein LOC105116556 isoform X3 [Populus euphratica]	PTAC12	Protein PLASTID TRANSCRIPTIONALLY ACTIVE 12 OS=Arabidopsis thaliana GN=PTAC12 PE=1 SV=1	86.46	37.25	1.84	up	0.00	0.00	yes
TRINITY_DN17275_c0_g1	hypothetical protein POPTR_0004s15280g [Populus trichocarpa]	-	-	71.58	28.75	1.94	up	0.00	0.00	yes

TRINITY_DN17903_c0_g1	PREDICTED: phosphopantetheine adenylyltransferase [Populus euphratica]	COAD	Phosphopantetheine adenylyltransferase OS=Arabidopsis thaliana GN=COAD PE=1 SV=1	27.44	12.41	1.83	up	0.00	0.00	yes
TRINITY_DN18874_c0_g2	hypothetical protein POPTR_0006s20430g [Populus trichocarpa]	CYP28	Peptidyl-prolyl cis-trans isomerase CYP28, chloroplastic OS=Arabidopsis thaliana GN=CYP28 PE=1 SV=1	91.46	37.16	1.88	up	0.00	0.00	yes
TRINITY_DN15273_c0_g1	PREDICTED: ubiquitin-conjugating enzyme E2 10-like [Populus euphratica]	UBC10	Ubiquitin-conjugating enzyme E2 10 OS=Arabidopsis thaliana GN=UBC10 PE=1 SV=1	8.51	1.68	2.95	up	0.00	0.00	yes
TRINITY_DN21005_c0_g2	hypothetical protein POPTR_0006s25980g [Populus trichocarpa]	EMB2001	GTP-binding protein At2g22870 OS=Arabidopsis thaliana GN=EMB2001 PE=2 SV=2	70.33	27.48	1.99	up	0.00	0.00	yes
TRINITY_DN15594_c0_g1	PREDICTED: fatty acid desaturase 4, chloroplastic-like [Populus euphratica]	FAD4	Fatty acid desaturase 4, chloroplastic OS=Arabidopsis thaliana GN=FAD4 PE=1 SV=1	3.99	0.68	3.17	up	0.00	0.00	yes
TRINITY_DN18739_c0_g1	annexin 4 family protein [Populus trichocarpa]	ANN4	Annexin D4 OS=Arabidopsis thaliana GN=ANN4 PE=2 SV=1	72.55	21.08	2.59	up	0.00	0.00	yes
TRINITY_DN22686_c0_g1	PREDICTED: 40S ribosomal protein S9-2-like isoform X1 [Populus euphratica]	RPS9C	40S ribosomal protein S9-2 OS=Arabidopsis thaliana GN=RPS9C PE=1 SV=1	1100.23	510.72	1.73	up	0.00	0.00	yes
TRINITY_DN23854_c0_g2	mitochondrial phosphate transporter family protein [Populus trichocarpa]	MPT3	Mitochondrial phosphate carrier protein 3, mitochondrial OS=Arabidopsis thaliana GN=MPT3 PE=1 SV=1	199.88	97.18	1.63	up	0.00	0.00	yes
TRINITY_DN11676_c0_g1	GDSL-motif lipase/hydrolase family protein [Populus trichocarpa]	At2g04570	GDSL esterase/lipase At2g04570 OS=Arabidopsis thaliana GN=At2g04570 PE=2 SV=1	3.76	0.45	3.67	up	0.00	0.00	yes
TRINITY_DN22874_c0_g2	hypothetical protein POPTR_0011s07310g [Populus trichocarpa]	RD21B	Probable cysteine protease RD21B OS=Arabidopsis thaliana GN=RD21B PE=1 SV=1	360.34	149.46	1.88	up	0.00	0.00	yes
TRINITY_DN26907_c0_g1	PREDICTED: ferritin-3, chloroplastic [Populus euphratica]	FER2	Ferritin-2, chloroplastic OS=Nicotiana tabacum GN=FER2 PE=2 SV=1	262.82	112.50	1.72	up	0.00	0.00	yes
TRINITY_DN19330_c1_g5	hypothetical protein POPTR_0004s23720g [Populus trichocarpa]	-	-	98.82	45.60	1.73	up	0.00	0.00	yes
TRINITY_DN18896_c0_g1	adenosine 5' phosphosulfate reductase [Populus tremula x Populus alba]	APR1	5'-adenylylsulfate reductase 1, chloroplastic OS=Arabidopsis thaliana GN=APR1 PE=1 SV=2	276.48	56.13	2.92	up	0.00	0.00	yes
TRINITY_DN19843_c0_g3	PREDICTED: serine carboxypeptidase-like 45 [Populus euphratica]	SCPL45	Serine carboxypeptidase-like 45 OS=Arabidopsis thaliana GN=SCPL45 PE=2 SV=1	3.08	0.40	3.54	up	0.00	0.00	yes
TRINITY_DN19742_c0_g1	hypothetical protein POPTR_0002s10640g [Populus trichocarpa]	At5g10920	Argininosuccinate lyase, chloroplastic OS=Arabidopsis thaliana GN=At5g10920 PE=2 SV=1	43.81	21.04	1.68	up	0.00	0.00	yes
TRINITY_DN20251_c0_g3	AP2 domain transcription factor family protein [Populus trichocarpa]	RAP2-3	Ethylene-responsive transcription factor RAP2-3 OS=Arabidopsis thaliana GN=RAP2-3 PE=1 SV=2	108.48	38.78	2.08	up	0.00	0.00	yes
TRINITY_DN24804_c0_g1	hypothetical protein POPTR_1776s00200g [Populus trichocarpa]	RD22	BURP domain protein RD22 OS=Arabidopsis thaliana GN=RD22 PE=2 SV=1	466.94	171.71	2.02	up	0.00	0.00	yes
TRINITY_DN23590_c0_g2	PREDICTED: transcription factor bHLH130-like [Populus euphratica]	BHLH130	Transcription factor bHLH130 OS=Arabidopsis thaliana GN=BHLH130 PE=1 SV=1	6.54	1.68	2.37	up	0.00	0.00	yes
TRINITY_DN25829_c0_g2	PHOSPHORIBOSYLANTHRANILATE ISOMERASE 3 family protein [Populus trichocarpa]	PAI1	N-(5'-phosphoribosyl)anthranilate isomerase 1, chloroplastic OS=Arabidopsis thaliana GN=PAI1 PE=2 SV=1	43.17	21.30	1.62	up	0.00	0.00	yes
TRINITY_DN17643_c0_g3	PREDICTED: 60S acidic ribosomal protein P1-like [Populus euphratica]	RPP1C	60S acidic ribosomal protein P1-3 OS=Arabidopsis thaliana GN=RPP1C PE=1 SV=2	92.14	41.60	1.79	up	0.00	0.00	yes
TRINITY_DN26977_c1_g1	PREDICTED: ruBisCO large subunit-binding protein subunit alpha [Populus euphratica]	-	RuBisCO large subunit-binding protein subunit alpha, chloroplastic OS=Pisum sativum PE=1 SV=2	888.17	398.63	1.72	up	0.00	0.00	yes
TRINITY_DN21651_c0_g1	cyclophilin family protein [Populus trichocarpa]	CYP20-3	Peptidyl-prolyl cis-trans isomerase CYP20-3, chloroplastic OS=Arabidopsis thaliana GN=CYP20-3 PE=1 SV=1	359.12	135.82	2.09	up	0.00	0.00	yes
TRINITY_DN17026_c0_g1	rhodanese-like domain-containing family protein [Populus trichocarpa]	STR9	Rhodanese-like domain-containing protein 9, chloroplastic OS=Arabidopsis thaliana GN=STR9 PE=2 SV=1	142.23	56.64	1.98	up	0.00	0.00	yes
TRINITY_DN16419_c0_g3	hypothetical protein POPTR_0002s08920g [Populus trichocarpa]	CML29	Probable calcium-binding protein CML29 OS=Oryza sativa subsp. japonica GN=CML29 PE=2 SV=1	20.31	5.00	2.60	up	0.00	0.00	yes
TRINITY_DN20332_c0_g1	PREDICTED: 60S ribosomal protein L15-like [Populus euphratica]	RPL15A	60S ribosomal protein L15-1 OS=Arabidopsis thaliana GN=RPL15A PE=2 SV=1	437.96	171.77	1.77	up	0.00	0.00	yes
TRINITY_DN18526_c0_g2	hypothetical protein POPTR_0012s09200g [Populus trichocarpa]	-	28 kDa ribonucleoprotein, chloroplastic OS=Spinacia oleracea PE=1 SV=1	3062.24	1238.07	1.89	up	0.00	0.00	yes
TRINITY_DN22661_c1_g1	short-chain dehydrogenase Tic32 family protein [Populus trichocarpa]	TIC32	Short-chain dehydrogenase TIC 32, chloroplastic OS=Pisum sativum GN=TIC32 PE=1 SV=1	44.77	19.53	1.82	up	0.00	0.00	yes
TRINITY_DN12624_c0_g1	hypothetical protein POPTR_0013s01580g [Populus trichocarpa]	-	-	6.30	0.70	3.83	up	0.00	0.00	yes
TRINITY_DN22348_c0_g6	hypothetical protein POPTR_0013s13150g [Populus trichocarpa]	OASB	Cysteine synthase, chloroplastic/chromoplastic OS=Arabidopsis thaliana GN=OASB PE=1 SV=2	146.50	66.04	1.74	up	0.00	0.00	yes

TRINITY_DN16715_c0_g1	hypothetical protein POPTR_0016s14970g [Populus trichocarpa]	RPL17	50S ribosomal protein L17, chloroplastic OS=Arabidopsis thaliana GN=RPL17 PE=2 SV=1	541.85	178.67	2.20	up	0.00	0.00	yes
TRINITY_DN21395_c1_g5	-	-	-	24.34	4.91	2.92	up	0.00	0.00	yes
TRINITY_DN22687_c2_g2	PREDICTED: uncharacterized protein LOC105126932 [Populus euphratica]	-	Taxadiene 5-alpha hydroxylase OS=Taxus cuspidata PE=1 SV=2	95.91	50.11	1.73	up	0.00	0.00	yes
TRINITY_DN17474_c0_g1	hypothetical protein POPTR_0002s26190g [Populus trichocarpa]	MRA_0733	Putative S-adenosyl-L-methionine-dependent methyltransferase MRA_0733 OS=Mycobacterium tuberculosis (strain ATCC 25177 / H37Ra) GN=MRA_0733 PE=3 SV=1	101.37	22.53	2.80	up	0.00	0.00	yes
TRINITY_DN16934_c0_g4	-	-	-	25.55	8.66	2.29	up	0.00	0.00	yes
TRINITY_DN22427_c2_g2	PREDICTED: uncharacterized protein LOC105142167 isoform X1 [Populus euphratica]	-	-	5.56	0.93	3.23	up	0.00	0.00	yes
TRINITY_DN15246_c0_g1	hypothetical protein POPTR_0009s17060g [Populus trichocarpa]	-	-	33.33	13.97	1.85	up	0.00	0.00	yes
TRINITY_DN23122_c0_g1	hypothetical protein POPTR_0003s08820g [Populus trichocarpa]	ABCI7	Protein ABCI7, chloroplastic OS=Arabidopsis thaliana GN=ABCI7 PE=1 SV=1	78.19	37.98	1.64	up	0.00	0.00	yes
TRINITY_DN23066_c0_g5	PREDICTED: probable 2-carboxy-D-arabinitol-1-phosphatase [Populus euphratica]	At5g22620	Probable 2-carboxy-D-arabinitol-1-phosphatase OS=Arabidopsis thaliana GN=At5g22620 PE=1 SV=1	71.98	31.71	1.83	up	0.00	0.00	yes
TRINITY_DN27695_c2_g4	HSP90 [Populus tomentosa]	HSC80	Heat shock cognate protein 80 OS=Solanum lycopersicum GN=HSC80 PE=2 SV=1	517.67	225.89	1.84	up	0.00	0.00	yes
TRINITY_DN13169_c0_g1	unknown [Populus trichocarpa]	At5g47030	ATP synthase subunit delta', mitochondrial OS=Arabidopsis thaliana GN=At5g47030 PE=1 SV=1	124.27	61.95	1.61	up	0.00	0.00	yes
TRINITY_DN22910_c0_g1	PREDICTED: uncharacterized protein LOC105109113 [Populus euphratica]	-	-	113.46	49.41	1.77	up	0.00	0.00	yes
TRINITY_DN24670_c1_g2	hypothetical protein POPTR_0005s22740g [Populus trichocarpa]	PSY	Phytoene synthase, chloroplastic OS=Daucus carota GN=PSY PE=2 SV=1	38.85	14.76	1.98	up	0.00	0.00	yes
TRINITY_DN26856_c2_g1	hypothetical protein POPTR_0005s26700g [Populus trichocarpa]	CDSP32	Thioredoxin-like protein CDSP32, chloroplastic OS=Arabidopsis thaliana GN=CDSP32 PE=1 SV=1	452.82	156.06	2.13	up	0.00	0.00	yes
TRINITY_DN23526_c2_g1	PREDICTED: B3 domain-containing protein At2g36080-like [Populus euphratica]	ARF31	B3 domain-containing protein At2g36080 OS=Arabidopsis thaliana GN=ARF31 PE=2 SV=1	6.85	1.24	2.96	up	0.00	0.00	yes
TRINITY_DN28898_c0_g1	-	-	-	3.15	0.16	4.75	up	0.00	0.00	yes
TRINITY_DN27422_c0_g2	hypothetical protein POPTR_0010s18300g [Populus trichocarpa]	YTHDF2	YTH domain-containing family protein 2 OS=Bos taurus GN=YTHDF2 PE=2 SV=1	5.50	1.34	2.54	up	0.00	0.00	yes
TRINITY_DN19402_c0_g1	hypothetical protein POPTR_0004s15020g [Populus trichocarpa]	CXE1	Probable carboxylesterase 1 OS=Arabidopsis thaliana GN=CXE1 PE=2 SV=1	12.33	1.80	3.34	up	0.00	0.00	yes
TRINITY_DN25504_c0_g1	chloroplast omega-3 desaturase family protein [Populus trichocarpa]	FAD7A-1	Omega-3 fatty acid desaturase, chloroplastic OS=Ricinus communis GN=FAD7A-1 PE=2 SV=1	178.02	89.81	1.69	up	0.00	0.00	yes
TRINITY_DN21074_c0_g2	unknown [Populus trichocarpa]	RBG2	Glycine-rich RNA-binding protein 2, mitochondrial OS=Arabidopsis thaliana GN=RBG2 PE=1 SV=1	309.14	122.31	1.84	up	0.00	0.00	yes
TRINITY_DN23281_c0_g1	PREDICTED: uncharacterized protein LOC105129843 isoform X1 [Populus euphratica]	-	-	83.47	40.21	1.69	up	0.00	0.00	yes
TRINITY_DN11229_c0_g1	hypothetical protein POPTR_0011s03550g [Populus trichocarpa]	MORF9	Multiple organellar RNA editing factor 9, chloroplastic OS=Arabidopsis thaliana GN=MORF9 PE=1 SV=1	258.45	106.27	1.90	up	0.00	0.00	yes
TRINITY_DN24738_c0_g3	hypothetical protein POPTR_0001s30040g [Populus trichocarpa]	NLP2	Protein NLP2 OS=Arabidopsis thaliana GN=NLP2 PE=2 SV=3	3.51	0.74	3.04	up	0.00	0.00	yes
TRINITY_DN23758_c0_g1	hypothetical protein POPTR_0005s27550g [Populus trichocarpa]	GAPB	Glyceraldehyde-3-phosphate dehydrogenase B, chloroplastic (Fragment) OS=Nicotiana tabacum GN=GAPB PE=2 SV=1	826.64	183.48	2.77	up	0.00	0.00	yes
TRINITY_DN15749_c0_g1	hypothetical protein POPTR_0013s12750g [Populus trichocarpa]	typA	GTP-binding protein TypA/BipA homolog OS=Bacillus subtilis (strain 168) GN=typA PE=3 SV=1	17.01	7.84	1.73	up	0.00	0.00	yes
TRINITY_DN18281_c0_g1	cupin family protein [Populus trichocarpa]	GLUA2	Glutelin type-A 2 OS=Oryza sativa subsp. japonica GN=GLUA2 PE=1 SV=1	15.55	7.08	1.69	up	0.00	0.00	yes
TRINITY_DN19971_c0_g2	PREDICTED: uncharacterized protein LOC105111889 [Populus euphratica]	petM	Cytochrome b6-f complex subunit 7 (Fragment) OS=Spinacia oleracea GN=petM PE=1 SV=1	1160.41	319.80	2.46	up	0.00	0.00	yes
TRINITY_DN26998_c2_g2	hypothetical protein POPTR_0015s08620g [Populus trichocarpa]	EX2	Protein EXECUTER 2, chloroplastic OS=Arabidopsis thaliana GN=EX2 PE=2 SV=1	96.15	41.20	1.79	up	0.00	0.00	yes
TRINITY_DN21222_c3_g6	putative polyprotein [Oryza sativa Japonica Group]	-	-	1.90	0.17	4.01	up	0.00	0.00	yes

TRINITY_DN23747_c0_g2	haloacid dehalogenase-like hydrolase family protein [Populus trichocarpa]	-	-	46.94	13.92	2.38	up	0.00	0.00	yes
TRINITY_DN14630_c0_g1	-	-	-	35.93	10.19	2.41	up	0.00	0.00	yes
TRINITY_DN25399_c0_g4	hypothetical protein POPTR_0006s07290g [Populus trichocarpa]	RPL7D	60S ribosomal protein L7-4 OS=Arabidopsis thaliana GN=RPL7D PE=2 SV=1	271.17	126.77	1.73	up	0.00	0.00	yes
TRINITY_DN17055_c0_g1	PREDICTED: serine carboxypeptidase-like 40 isoform X1 [Populus euphratica]	SCPL40	Serine carboxypeptidase-like 40 OS=Arabidopsis thaliana GN=SCPL40 PE=2 SV=2	5.66	1.87	2.20	up	0.00	0.00	yes
TRINITY_DN21330_c1_g1	unknown [Populus trichocarpa]	-	-	134.92	63.34	1.72	up	0.00	0.00	yes
TRINITY_DN20796_c0_g1	heat shock protein 70 [Populus trichocarpa]	-	Heat shock 70 kDa protein, mitochondrial OS=Phaseolus vulgaris PE=2 SV=1	111.83	45.71	1.93	up	0.00	0.00	yes
TRINITY_DN26804_c0_g1	PREDICTED: 60S ribosomal protein L28-1-like [Populus euphratica]	RPL28A	60S ribosomal protein L28-1 OS=Arabidopsis thaliana GN=RPL28A PE=1 SV=1	763.38	365.31	1.69	up	0.00	0.00	yes
TRINITY_DN24963_c0_g1	hypothetical protein POPTR_0019s13380g [Populus trichocarpa]	LIP1P-2	Lipoyl synthase 2, chloroplastic OS=Populus trichocarpa GN=LIP1P-2 PE=3 SV=1	21.89	11.85	1.51	up	0.00	0.00	yes
TRINITY_DN23240_c1_g5	PREDICTED: uncharacterized protein LOC105136996 isoform X1 [Populus euphratica]	-	-	55.20	17.84	2.27	up	0.00	0.00	yes
TRINITY_DN16722_c0_g1	PREDICTED: E3 ubiquitin-protein ligase SGR9, amyloplastic [Populus euphratica]	SGR9	E3 ubiquitin-protein ligase SGR9, amyloplastic OS=Arabidopsis thaliana GN=SGR9 PE=1 SV=1	31.83	9.80	2.42	up	0.00	0.00	yes
TRINITY_DN23450_c0_g2	unknown [Populus trichocarpa x Populus deltoides]	RPL4	60S ribosomal protein L4 OS=Prunus armeniaca GN=RPL4 PE=2 SV=1	447.19	214.51	1.69	up	0.00	0.00	yes
TRINITY_DN24089_c0_g3	hypothetical protein POPTR_0001s12920g [Populus trichocarpa]	FKBP19	Peptidyl-prolyl cis-trans isomerase FKBP19, chloroplastic OS=Arabidopsis thaliana GN=FKBP19 PE=1 SV=1	102.67	37.86	2.01	up	0.00	0.00	yes
TRINITY_DN23732_c1_g1	hypothetical protein POPTR_0013s12910g [Populus trichocarpa]	-	-	146.00	52.04	1.99	up	0.00	0.00	yes
TRINITY_DN12225_c0_g1	putative oxidoreductase-like family protein [Populus trichocarpa]	JRG21	Probable 2-oxoglutarate-dependent dioxygenase JRG21 OS=Arabidopsis thaliana GN=JRG21 PE=2 SV=1	22.80	3.06	3.22	up	0.00	0.00	yes
TRINITY_DN17272_c0_g3	-	-	-	46.03	8.36	3.04	up	0.00	0.00	yes
TRINITY_DN25386_c0_g1	anion-transporting ATPase family protein [Populus trichocarpa]	MTH_1511	Putative arsenical pump-driving ATPase OS=Methanothermobacter thermautotrophicus (strain ATCC 29096 / DSM 1053 / JCM 10044 / NBRC 100330 / Delta H) GN=MTH_1511 PE=1 SV=1	88.42	36.29	1.80	up	0.00	0.00	yes
TRINITY_DN27223_c1_g1	hypothetical protein POPTR_0003s09520g [Populus trichocarpa]	SKL2	Probable inactive shikimate kinase like 2, chloroplastic OS=Arabidopsis thaliana GN=SKL2 PE=2 SV=2	44.94	19.89	1.93	up	0.00	0.00	yes
TRINITY_DN21945_c0_g3	PREDICTED: pentatricopeptide repeat-containing protein At4g16390, chloroplastic-like [Populus euphratica]	P67	Pentatricopeptide repeat-containing protein At4g16390, chloroplastic OS=Arabidopsis thaliana GN=P67 PE=1 SV=3	97.55	43.04	1.78	up	0.00	0.00	yes
TRINITY_DN17998_c0_g2	hypothetical protein POPTR_0009s07030g [Populus trichocarpa]	PSRP2	30S ribosomal protein 2, chloroplastic OS=Spinacia oleracea GN=PSRP2 PE=1 SV=1	738.54	284.18	1.97	up	0.00	0.00	yes
TRINITY_DN25093_c0_g3	ATP synthase delta chain-related family protein [Populus trichocarpa]	ATPD	ATP synthase delta chain, chloroplastic OS=Spinacia oleracea GN=ATPD PE=1 SV=2	382.66	129.01	2.15	up	0.00	0.00	yes
TRINITY_DN26665_c0_g1	hypothetical protein POPTR_0009s15490g [Populus trichocarpa]	METE	5-methyltetrahydropteroyltriglutamate--homocysteine methyltransferase OS=Catharanthus roseus GN=METE PE=2 SV=1	216.45	108.64	1.58	up	0.00	0.00	yes
TRINITY_DN25402_c0_g2	PREDICTED: 60S acidic ribosomal protein P3-like [Populus euphratica]	RPP3A	60S acidic ribosomal protein P3-1 OS=Arabidopsis thaliana GN=RPP3A PE=3 SV=1	195.27	90.48	1.73	up	0.00	0.00	yes
TRINITY_DN17417_c0_g1	PREDICTED: ATP synthase subunit O, mitochondrial [Populus euphratica]	-	ATP synthase subunit O, mitochondrial OS=Ipomoea batatas PE=1 SV=1	222.63	112.89	1.58	up	0.00	0.00	yes
TRINITY_DN14572_c0_g1	pollen Ole e 1 allergen and extensin family protein [Populus trichocarpa]	PRP3	Proline-rich protein 3 OS=Arabidopsis thaliana GN=PRP3 PE=2 SV=1	38.49	5.26	3.46	up	0.00	0.00	yes
TRINITY_DN17745_c0_g1	hypothetical protein POPTR_0002s02460g [Populus trichocarpa]	At1g75720	WEB family protein At1g75720 OS=Arabidopsis thaliana GN=At1g75720 PE=2 SV=1	13.21	3.84	2.38	up	0.00	0.00	yes
TRINITY_DN25744_c0_g1	PREDICTED: probable L-ascorbate peroxidase 6, chloroplastic isoform X1 [Populus euphratica]	APXT	L-ascorbate peroxidase T, chloroplastic OS=Arabidopsis thaliana GN=APXT PE=2 SV=2	291.77	130.37	1.77	up	0.00	0.00	yes
TRINITY_DN16266_c0_g1	SOUL heme-binding family protein [Populus trichocarpa]	At3g10130	Heme-binding-like protein At3g10130, chloroplastic OS=Arabidopsis thaliana GN=At3g10130 PE=2 SV=1	25.15	6.11	2.65	up	0.00	0.00	yes

TRINITY_DN13131_c0_g1	PREDICTED: protein LOW PSII ACCUMULATION 2, chloroplastic [Populus euphratica]	LPA2	Protein LOW PSII ACCUMULATION 2, chloroplastic OS=Arabidopsis thaliana GN=LPA2 PE=1 SV=1	30.69	12.00	1.95	up	0.00	0.00	yes
TRINITY_DN20518_c0_g2	hypothetical protein POPTR_0001s34860g [Populus trichocarpa]	ylbH	Putative rRNA methyltransferase YlbH OS=Bacillus subtilis (strain 168) GN=ylbH PE=3 SV=2	52.27	26.66	1.62	up	0.00	0.00	yes
TRINITY_DN23758_c0_g2	hypothetical protein POPTR_0005s27560g [Populus trichocarpa]	-	-	44.50	15.76	1.96	up	0.00	0.00	yes
TRINITY_DN21476_c0_g2	PREDICTED: 60S ribosomal protein L7-2-like [Populus euphratica]	RPL7C	60S ribosomal protein L7-3 OS=Arabidopsis thaliana GN=RPL7C PE=2 SV=1	316.17	150.32	1.70	up	0.00	0.00	yes
TRINITY_DN22182_c0_g1	cytochrome c1 family protein [Populus trichocarpa]	CYC12	Cytochrome c1 2, heme protein, mitochondrial OS=Arabidopsis thaliana GN=CYC12 PE=1 SV=1	274.18	143.43	1.69	up	0.00	0.00	yes
TRINITY_DN21871_c0_g1	hypothetical protein POPTR_0001s35190g [Populus trichocarpa]	PDF1B	Peptide deformylase 1B, chloroplastic/mitochondrial OS=Arabidopsis thaliana GN=PDF1B PE=1 SV=2	68.98	29.40	1.82	up	0.00	0.00	yes
TRINITY_DN21668_c0_g1	hypothetical protein POPTR_0009s11170g [Populus trichocarpa]	VRN1	B3 domain-containing transcription factor VRN1 OS=Arabidopsis thaliana GN=VRN1 PE=1 SV=1	25.47	12.27	1.48	up	0.00	0.00	yes
TRINITY_DN17383_c0_g1	40S ribosomal protein S11-2 [Populus trichocarpa]	RPS11	40S ribosomal protein S11 OS=Zea mays GN=RPS11 PE=2 SV=1	31.53	14.94	1.70	up	0.00	0.00	yes
TRINITY_DN20564_c0_g1	Chloride channel protein CLC-a [Populus trichocarpa]	CLC-B	Chloride channel protein CLC-b OS=Arabidopsis thaliana GN=CLC-B PE=1 SV=1	12.40	1.75	3.41	up	0.00	0.00	yes
TRINITY_DN22755_c0_g2	unknown [Populus trichocarpa]	MAP1B	Methionine aminopeptidase 1B, chloroplastic OS=Arabidopsis thaliana GN=MAP1B PE=2 SV=2	261.55	115.26	1.78	up	0.00	0.00	yes
TRINITY_DN23497_c0_g1	D-3-phosphoglycerate dehydrogenase family protein [Populus trichocarpa]	PGDH1	D-3-phosphoglycerate dehydrogenase 1, chloroplastic OS=Arabidopsis thaliana GN=PGDH1 PE=1 SV=1	103.49	34.56	2.24	up	0.00	0.00	yes
TRINITY_DN26609_c0_g1	carbamoyl phosphate synthetase a family protein [Populus trichocarpa]	CARA	Carbamoyl-phosphate synthase small chain, chloroplastic OS=Arabidopsis thaliana GN=CARA PE=1 SV=1	218.53	107.54	1.61	up	0.00	0.00	yes
TRINITY_DN19848_c0_g1	phosphopyruvate hydratase family protein [Populus trichocarpa]	ENO2	Enolase 2 OS=Hevea brasiliensis GN=ENO2 PE=1 SV=1	247.16	128.86	1.56	up	0.00	0.00	yes
TRINITY_DN20754_c0_g3	hypothetical protein POPTR_0010s06230g [Populus trichocarpa]	KCR1	Very-long-chain 3-oxoacyl-CoA reductase 1 OS=Arabidopsis thaliana GN=KCR1 PE=1 SV=1	19.13	8.33	1.78	up	0.00	0.00	yes
TRINITY_DN17606_c0_g1	hypothetical protein POPTR_0013s14300g [Populus trichocarpa]	-	-	71.52	32.22	1.76	up	0.00	0.00	yes
TRINITY_DN22604_c0_g2	PREDICTED: E3 ubiquitin-protein ligase COP1-like isoform X2 [Populus euphratica]	COP1	E3 ubiquitin-protein ligase COP1 OS=Arabidopsis thaliana GN=COP1 PE=1 SV=2	33.35	12.32	2.08	up	0.00	0.00	yes
TRINITY_DN23353_c0_g1	PREDICTED: fruit protein pKIWI502-like [Populus euphratica]	pKIWI502	Fruit protein pKIWI502 OS=Actinidia deliciosa GN=pKIWI502 PE=2 SV=1	235.15	104.23	1.77	up	0.00	0.00	yes
TRINITY_DN19817_c0_g1	hypothetical protein POPTR_0017s01240g [Populus trichocarpa]	CA2	Beta-carotene hydroxylase 2, chloroplastic (Fragment) OS=Capsicum annuum GN=CA2 PE=2 SV=1	46.24	21.64	1.70	up	0.00	0.00	yes
TRINITY_DN20246_c3_g1	PREDICTED: uncharacterized protein LOC105141875 [Populus euphratica]	-	-	142.00	61.36	1.79	up	0.00	0.00	yes
TRINITY_DN15348_c0_g1	pyrophosphatase [Beta vulgaris]	AVP1	Pyrophosphate-energized vacuolar membrane proton pump 1 OS=Arabidopsis thaliana GN=AVP1 PE=1 SV=1	522.49	286.29	1.61	up	0.00	0.00	yes
TRINITY_DN22627_c1_g5	PREDICTED: ATP phosphoribosyltransferase 2, chloroplastic-like [Populus euphratica]	HISN1B	ATP phosphoribosyltransferase 2, chloroplastic OS=Arabidopsis thaliana GN=HISN1B PE=1 SV=1	58.56	24.74	1.81	up	0.00	0.00	yes
TRINITY_DN23406_c0_g1	hypothetical protein POPTR_0007s14390g [Populus trichocarpa]	DTX27	Protein DETOXIFICATION 27 OS=Arabidopsis thaliana GN=DTX27 PE=2 SV=1	10.53	2.94	2.43	up	0.00	0.00	yes
TRINITY_DN19948_c0_g2	hypothetical protein POPTR_0001s14410g [Populus trichocarpa]	sll0005	Uncharacterized protein sll0005 OS=Synechocystis sp. (strain PCC 6803 / Kazusa) GN=sll0005 PE=3 SV=1	24.77	12.71	1.58	up	0.00	0.00	yes
TRINITY_DN21698_c0_g1	PREDICTED: cytochrome c oxidase subunit 6b-1-like [Populus euphratica]	COX6B-1	Cytochrome c oxidase subunit 6b-1 OS=Arabidopsis thaliana GN=COX6B-1 PE=1 SV=1	101.21	51.17	1.58	up	0.00	0.00	yes
TRINITY_DN27842_c2_g1	PREDICTED: LOW QUALITY PROTEIN: HBS1-like protein [Populus euphratica]	EF1	Elongation factor 1-alpha OS=Manihot esculenta GN=EF1 PE=3 SV=1	1154.82	615.48	1.66	up	0.00	0.00	yes
TRINITY_DN20027_c0_g1	hypothetical protein POPTR_0012s14320g [Populus trichocarpa]	CLPT1	ATP-dependent Clp protease ATP-binding subunit CLPT1, chloroplastic OS=Arabidopsis thaliana GN=CLPT1 PE=1 SV=1	197.09	91.64	1.72	up	0.00	0.00	yes
TRINITY_DN25342_c1_g1	hypothetical protein POPTR_0012s12870g [Populus trichocarpa]	rpsI	30S ribosomal protein S9 OS=Bartonella tribocorum (strain CIP 105476 / IBS 506) GN=rpsI PE=3 SV=1	49.78	26.23	1.58	up	0.00	0.00	yes
TRINITY_DN25904_c0_g1	PREDICTED: probable plastid-lipid-associated protein 4, chloroplastic isoform X3 [Populus euphratica]	PAP4	Probable plastid-lipid-associated protein 4, chloroplastic OS=Arabidopsis thaliana GN=PAP4 PE=2 SV=1	17.98	7.06	1.97	up	0.00	0.00	yes
TRINITY_DN23705_c0_g1	hypothetical protein POPTR_0002s16500g [Populus trichocarpa]	HIPP39	Heavy metal-associated isoprenylated plant protein 39 OS=Arabidopsis thaliana GN=HIPP39 PE=2 SV=1	39.74	17.39	1.81	up	0.00	0.00	yes

TRINITY_DN6900_c0_g1	short-chain dehydrogenase/reductase family protein [Populus trichocarpa]	-	Short-chain type dehydrogenase/reductase OS=Picea abies PE=2 SV=1	21.13	8.97	1.83	up	0.00	0.00	yes
TRINITY_DN19532_c0_g1	ribosomal protein L33 [Populus trichocarpa]	RPL35AA	60S ribosomal protein L35a-1 OS=Arabidopsis thaliana GN=RPL35AA PE=3 SV=1	602.96	245.47	1.67	up	0.00	0.00	yes
TRINITY_DN22425_c0_g1	PREDICTED: F-box/kelch-repeat protein At1g67480-like [Populus euphratica]	At1g67480	F-box/kelch-repeat protein At1g67480 OS=Arabidopsis thaliana GN=At1g67480 PE=2 SV=1	15.73	5.81	2.48	up	0.00	0.00	yes
TRINITY_DN16117_c0_g1	thioredoxin family protein [Populus trichocarpa]	TRX2	Thioredoxin H2 OS=Arabidopsis thaliana GN=TRX2 PE=2 SV=2	212.80	102.72	1.57	up	0.00	0.00	yes
TRINITY_DN19924_c0_g1	hypothetical protein POPTR_0002s25280g [Populus trichocarpa]	-	-	44.45	22.85	1.57	up	0.00	0.00	yes
TRINITY_DN17180_c0_g1	PREDICTED: uncharacterized protein LOC105122694 [Populus euphratica]	CRR6	Protein CHLORORESPIRATORY REDUCTION 6, chloroplastic OS=Arabidopsis thaliana GN=CRR6 PE=2 SV=1	101.27	26.65	2.56	up	0.00	0.00	yes
TRINITY_DN14550_c0_g1	-	-	-	38.46	10.74	2.53	up	0.00	0.00	yes
TRINITY_DN21107_c0_g1	hypothetical protein POPTR_0001s07800g [Populus trichocarpa]	-	-	261.36	116.44	1.85	up	0.00	0.00	yes
TRINITY_DN23477_c0_g1	hypothetical protein POPTR_0006s06770g [Populus trichocarpa]	EBF2	EIN3-binding F-box protein 2 OS=Arabidopsis thaliana GN=EBF2 PE=1 SV=1	201.93	32.00	2.76	up	0.00	0.00	yes
TRINITY_DN25520_c1_g1	PREDICTED: phenolic glucoside malonyltransferase 1-like [Populus euphratica]	5MAT	Malonyl-CoA:anthocyanidin 5-O-glucoside-6"-O-malonyltransferase OS=Arabidopsis thaliana GN=5MAT PE=1 SV=1	138.11	49.97	2.32	up	0.00	0.00	yes
TRINITY_DN23678_c1_g5	putative oxoglutarate-malate-translocase family protein [Populus trichocarpa]	DIT2-1	Dicarboxylate transporter 2.1, chloroplastic OS=Arabidopsis thaliana GN=DIT2-1 PE=1 SV=1	51.23	24.04	1.71	up	0.00	0.00	yes
TRINITY_DN23297_c0_g6	60S ribosomal protein L15 [Populus trichocarpa]	RPL15	60S ribosomal protein L15 OS=Petunia hybrida GN=RPL15 PE=2 SV=1	179.34	77.53	1.88	up	0.00	0.00	yes
TRINITY_DN24953_c1_g1	unknown [Populus trichocarpa]	CURT1C	Protein CURVATURE THYLAKOID 1C, chloroplastic OS=Arabidopsis thaliana GN=CURT1C PE=1 SV=1	80.78	37.53	1.64	up	0.00	0.00	yes
TRINITY_DN25670_c0_g1	PREDICTED: ATPase family AAA domain-containing protein 3-like [Populus euphratica]	atad-3	ATPase family AAA domain-containing protein 3 OS=Caenorhabditis elegans GN=atad-3 PE=3 SV=2	22.58	11.89	1.63	up	0.00	0.00	yes
TRINITY_DN16239_c0_g1	hypothetical protein POPTR_0003s05320g [Populus trichocarpa]	RPL39A	60S ribosomal protein L39-1 OS=Oryza sativa subsp. japonica GN=RPL39A PE=3 SV=2	114.22	49.45	1.81	up	0.00	0.00	yes
TRINITY_DN17227_c0_g1	hypothetical protein POPTR_0001s11300g [Populus trichocarpa]	-	-	52.39	21.02	1.96	up	0.00	0.00	yes
TRINITY_DN15450_c0_g1	hypothetical protein POPTR_0012s13830g [Populus trichocarpa]	-	-	75.97	27.17	2.09	up	0.00	0.00	yes
TRINITY_DN22564_c0_g1	VDAC3.1 family protein [Populus trichocarpa]	VDAC4	Mitochondrial outer membrane protein porin 4 OS=Arabidopsis thaliana GN=VDAC4 PE=1 SV=1	56.77	29.14	1.62	up	0.00	0.00	yes
TRINITY_DN21907_c0_g2	hypothetical protein POPTR_0002s24700g [Populus trichocarpa]	FATA	Oleoyl-acyl carrier protein thioesterase 1, chloroplastic OS=Arabidopsis thaliana GN=FATA PE=1 SV=1	96.22	49.62	1.56	up	0.00	0.00	yes
TRINITY_DN23578_c0_g1	60S ribosomal protein L35 [Populus trichocarpa]	RPL35	60S ribosomal protein L35 OS=Euphorbia esula GN=RPL35 PE=2 SV=1	217.41	105.67	1.67	up	0.00	0.00	yes
TRINITY_DN16948_c0_g1	MADS-box protein GmNMH7 [Populus trichocarpa]	DEFA	Floral homeotic protein DEFICIENS OS=Antirrhinum majus GN=DEFA PE=1 SV=1	25.80	10.31	2.07	up	0.00	0.00	yes
TRINITY_DN17951_c0_g2	hypothetical protein POPTR_0001s10670g [Populus trichocarpa]	NDPK3	Nucleoside diphosphate kinase III, chloroplastic/mitochondrial OS=Arabidopsis thaliana GN=NDPK3 PE=1 SV=1	286.39	121.84	1.83	up	0.00	0.00	yes
TRINITY_DN25771_c0_g1	cell-wall invertase [Populus alba x Populus grandidentata]	INV1	Beta-fructofuranosidase, insoluble isoenzyme 1 OS=Daucus carota GN=INV1 PE=1 SV=1	101.29	37.01	2.03	up	0.00	0.00	yes
TRINITY_DN20243_c1_g4	PREDICTED: uncharacterized protein LOC105113555 [Populus euphratica]	-	-	57.90	25.21	1.79	up	0.00	0.00	yes
TRINITY_DN21144_c0_g1	hypothetical protein POPTR_0005s22020g [Populus trichocarpa]	RH39	DEAD-box ATP-dependent RNA helicase 39 OS=Arabidopsis thaliana GN=RH39 PE=2 SV=2	100.60	46.68	1.59	up	0.00	0.00	yes
TRINITY_DN22768_c0_g1	PREDICTED: pentatricopeptide repeat-containing protein At4g36680, mitochondrial-like [Populus euphratica]	At4g36680	Pentatricopeptide repeat-containing protein At4g36680, mitochondrial OS=Arabidopsis thaliana GN=At4g36680 PE=1 SV=1	5.32	1.57	2.41	up	0.00	0.00	yes
TRINITY_DN25393_c0_g1	PREDICTED: uncharacterized protein LOC105128154 [Populus euphratica]	NFD4	Protein NUCLEAR FUSION DEFECTIVE 4 OS=Arabidopsis thaliana GN=NFD4 PE=3 SV=1	11.60	4.84	1.89	up	0.00	0.00	yes
TRINITY_DN26190_c0_g4	carotenoid cleavage dioxygenase 1 family protein [Populus trichocarpa]	CCD1	Carotenoid 9,10(9',10')-cleavage dioxygenase 1 OS=Arabidopsis thaliana GN=CCD1 PE=1 SV=2	56.34	29.50	1.58	up	0.00	0.00	yes

TRINITY_DN23816_c0_g2	hypothetical protein POPTR_0015s09240g [Populus trichocarpa]	At4g26540	Probable LRR receptor-like serine/threonine-protein kinase At4g26540 OS=Arabidopsis thaliana GN=At4g26540 PE=2 SV=1	12.60	5.30	1.87	up	0.00	0.00	yes
TRINITY_DN15713_c0_g1	hypothetical protein POPTR_0271s00220g, partial [Populus trichocarpa]	RPL18	50S ribosomal protein L18, chloroplastic OS=Arabidopsis thaliana GN=RPL18 PE=2 SV=1	1387.37	448.05	2.20	up	0.00	0.00	yes
TRINITY_DN19069_c0_g4	wrp15a family protein [Populus trichocarpa]	RPS15AA	40S ribosomal protein S15a-1 OS=Arabidopsis thaliana GN=RPS15AA PE=2 SV=2	1070.52	510.47	1.73	up	0.00	0.00	yes
TRINITY_DN27340_c0_g1	PREDICTED: probable methyltransferase PMT3 [Populus euphratica]	At1g04430	Probable methyltransferase PMT8 OS=Arabidopsis thaliana GN=At1g04430 PE=2 SV=1	51.50	31.14	1.56	up	0.00	0.00	yes
TRINITY_DN24712_c0_g1	PREDICTED: ATP-dependent zinc metalloprotease FtsH [Populus euphratica]	FTSHI2	Probable inactive ATP-dependent zinc metalloprotease FTSHI 2, chloroplastic OS=Arabidopsis thaliana GN=FTSHI2 PE=1 SV=1	75.34	41.93	1.62	up	0.00	0.00	yes
TRINITY_DN21827_c0_g1	PREDICTED: transmembrane protein 120 homolog isoform X1 [Populus euphratica]	-	-	61.24	31.93	1.67	up	0.00	0.00	yes
TRINITY_DN26615_c0_g1	hypothetical protein POPTR_0019s03070g [Populus trichocarpa]	-	Squalene monooxygenase OS=Panax ginseng PE=2 SV=1	67.01	29.80	1.82	up	0.00	0.00	yes
TRINITY_DN15207_c0_g1	hypothetical protein POPTR_0006s04100g [Populus trichocarpa]	-	Pyrroline-5-carboxylate reductase OS=Glycine max PE=2 SV=1	38.39	20.18	1.59	up	0.00	0.00	yes
TRINITY_DN21301_c0_g2	PREDICTED: thylakoid lumenal 16.5 kDa protein, chloroplastic-like [Populus euphratica]	At4g02530	Thylakoid lumenal 16.5 kDa protein, chloroplastic OS=Arabidopsis thaliana GN=At4g02530 PE=1 SV=3	92.92	30.71	2.20	up	0.00	0.00	yes
TRINITY_DN23747_c0_g1	PREDICTED: NHL repeat-containing protein 2 isoform X1 [Populus euphratica]	-	-	61.86	28.80	1.68	up	0.00	0.00	yes
TRINITY_DN16272_c0_g1	hypothetical protein POPTR_0016s10710g [Populus trichocarpa]	-	-	191.68	69.61	2.07	up	0.00	0.00	yes
TRINITY_DN19206_c0_g2	methionine sulfoxide reductase A [Populus trichocarpa x Populus deltoides]	-	Peptide methionine sulfoxide reductase OS=Lactuca sativa PE=2 SV=1	72.30	32.79	1.76	up	0.00	0.00	yes
TRINITY_DN25125_c0_g3	hypothetical protein POPTR_0002s15850g [Populus trichocarpa]	FOLD4	Bifunctional protein FolD 4, chloroplastic OS=Arabidopsis thaliana GN=FOLD4 PE=1 SV=1	52.02	30.67	1.50	up	0.00	0.00	yes
TRINITY_DN22301_c0_g2	cinnamyl alcohol dehydrogenase 3 [Populus tomentosa]	CAD6	Probable cinnamyl alcohol dehydrogenase 6 OS=Oryza sativa subsp. japonica GN=CAD6 PE=2 SV=2	575.95	213.69	2.10	up	0.00	0.00	yes
TRINITY_DN19788_c0_g1	PREDICTED: mitochondrial import inner membrane translocase subunit TIM22-4-like isoform X1 [Populus euphratica]	TIM22-1	Mitochondrial import inner membrane translocase subunit TIM22-1 OS=Arabidopsis thaliana GN=TIM22-1 PE=1 SV=1	35.67	14.58	1.85	up	0.00	0.00	yes
TRINITY_DN19225_c0_g1	40S ribosomal protein S2 [Populus trichocarpa]	RPS2D	40S ribosomal protein S2-4 OS=Arabidopsis thaliana GN=RPS2D PE=2 SV=1	278.03	143.62	1.57	up	0.00	0.00	yes
TRINITY_DN24109_c1_g5	heat shock protein 70 [Populus trichocarpa]	-	Heat shock 70 kDa protein, mitochondrial OS=Phaseolus vulgaris PE=2 SV=1	69.14	24.00	2.18	up	0.00	0.00	yes
TRINITY_DN18736_c1_g5	PREDICTED: ABC transporter B family member 4-like [Populus euphratica]	ABCB11	ABC transporter B family member 11 OS=Arabidopsis thaliana GN=ABCB11 PE=2 SV=1	2.95	0.29	3.94	up	0.00	0.00	yes
TRINITY_DN14317_c0_g1	hypothetical protein POPTR_0015s01300g [Populus trichocarpa]	PTAC7	Protein PLASTID TRANSCRIPTIONALLY ACTIVE 7 OS=Arabidopsis thaliana GN=PTAC7 PE=1 SV=1	93.68	37.33	1.92	up	0.00	0.00	yes
TRINITY_DN23213_c0_g1	pyruvate dehydrogenase family protein [Populus trichocarpa]	E1	Pyruvate dehydrogenase E1 component subunit alpha-1, mitochondrial OS=Arabidopsis thaliana GN=E1 ALPHA PE=1 SV=2	125.88	67.45	1.52	up	0.00	0.00	yes
TRINITY_DN16772_c0_g1	PREDICTED: protein CURVATURE THYLAKOID 1D, chloroplastic [Populus euphratica]	CURT1D	Protein CURVATURE THYLAKOID 1D, chloroplastic OS=Arabidopsis thaliana GN=CURT1D PE=1 SV=1	189.50	92.27	1.67	up	0.00	0.00	yes
TRINITY_DN27533_c0_g1	PREDICTED: phosphoglucan, water dikinase, chloroplastic-like [Populus euphratica]	GWD3	Phosphoglucan, water dikinase, chloroplastic OS=Arabidopsis thaliana GN=GWD3 PE=1 SV=1	41.28	19.29	1.67	up	0.00	0.00	yes
TRINITY_DN17536_c0_g2	PREDICTED: pentatricopeptide repeat-containing protein At3g26782, mitochondrial-like [Populus euphratica]	DYW9	Pentatricopeptide repeat-containing protein At4g30700 OS=Arabidopsis thaliana GN=DYW9 PE=2 SV=1	2.70	0.63	2.72	up	0.00	0.00	yes
TRINITY_DN19086_c0_g2	PREDICTED: uncharacterized protein LOC105113046 [Populus euphratica]	slr0992	Putative tRNA (cytidine(34)-2'-O)-methyltransferase OS=Synechocystis sp. (strain PCC 6803 / Kazusa) GN=slr0992 PE=3 SV=1	27.77	11.34	1.89	up	0.00	0.00	yes
TRINITY_DN22846_c0_g1	PREDICTED: uncharacterized protein LOC105126210 isoform X1 [Populus euphratica]	-	-	22.57	9.25	1.88	up	0.00	0.00	yes
TRINITY_DN16449_c0_g1	PREDICTED: cytochrome P450 71A1-like isoform X1 [Populus euphratica]	CYP71A6	Cytochrome P450 71A6 (Fragment) OS=Nepeta racemosa GN=CYP71A6 PE=2 SV=1	34.70	12.40	2.09	up	0.00	0.00	yes

TRINITY_DN22837_c0_g2	sodium hydrogen antiporter family protein [Populus trichocarpa]	NHD1	Sodium/proton antiporter 1 OS=Arabidopsis thaliana GN=NHD1 PE=2 SV=1	61.07	33.75	1.64	up	0.00	0.00	yes
TRINITY_DN22246_c0_g2	hypothetical protein POPTR_0019s03500g [Populus trichocarpa]	-	-	144.86	80.32	1.68	up	0.00	0.00	yes
TRINITY_DN16622_c0_g1	hypothetical protein POPTR_0002s25810g [Populus trichocarpa]	PSB28	Photosystem II reaction center PSB28 protein, chloroplastic OS=Oryza sativa subsp. japonica GN=PSB28 PE=2 SV=2	158.06	55.46	2.12	up	0.00	0.00	yes
TRINITY_DN25855_c0_g1	PREDICTED: mechanosensitive ion channel protein 6-like [Populus euphratica]	MSL6	Mechanosensitive ion channel protein 6 OS=Arabidopsis thaliana GN=MSL6 PE=1 SV=1	4.08	0.52	3.45	up	0.00	0.00	yes
TRINITY_DN17736_c0_g2	arginine biosynthesis protein ArgJ [Populus trichocarpa]	POPTRDRAFT_	Arginine biosynthesis bifunctional protein ArgJ, chloroplastic OS=Populus trichocarpa GN=POPTRDRAFT_746969 PE=3 SV=2	59.37	31.41	1.54	up	0.00	0.00	yes
TRINITY_DN17794_c0_g2	PREDICTED: carboxymethylenebutenolidase homolog [Populus euphratica]	cmbl	Carboxymethylenebutenolidase homolog OS=Xenopus tropicalis GN=cmbl PE=2 SV=1	27.29	10.49	1.99	up	0.00	0.00	yes
TRINITY_DN20072_c0_g1	hypothetical protein POPTR_0014s08190g [Populus trichocarpa]	-	-	123.32	53.16	1.82	up	0.00	0.00	yes
TRINITY_DN22552_c0_g1	PREDICTED: cysteine--tRNA ligase isoform X1 [Populus euphratica]	SYCO	Cysteine--tRNA ligase, chloroplastic/mitochondrial OS=Arabidopsis thaliana GN=SYCO PE=2 SV=1	22.37	11.20	1.59	up	0.00	0.00	yes
TRINITY_DN27817_c4_g3	hypothetical protein POPTR_0017s01670g [Populus trichocarpa]	-	-	17.81	2.06	3.72	up	0.00	0.00	yes
TRINITY_DN27302_c0_g2	hypothetical protein POPTR_0007s01840g [Populus trichocarpa]	SYNO	Asparagine--tRNA ligase, chloroplastic/mitochondrial OS=Arabidopsis thaliana GN=SYNO PE=2 SV=3	68.86	33.67	1.67	up	0.00	0.00	yes
TRINITY_DN17543_c1_g5	hypothetical protein POPTR_0001s45760g [Populus trichocarpa]	-	-	68.30	38.49	1.44	up	0.00	0.00	yes
TRINITY_DN18608_c2_g1	PREDICTED: 5'-adenylylsulfate reductase 3, chloroplastic-like isoform X1 [Populus euphratica]	APR3	5'-adenylylsulfate reductase 3, chloroplastic OS=Arabidopsis thaliana GN=APR3 PE=2 SV=2	6.79	0.82	4.09	up	0.00	0.00	yes
TRINITY_DN21016_c1_g1	RNA recognition motif-containing family protein [Populus trichocarpa]	At2g05160	Zinc finger CCCH domain-containing protein 18 OS=Arabidopsis thaliana GN=At2g05160 PE=2 SV=1	21.09	9.21	2.05	up	0.00	0.00	yes
TRINITY_DN23349_c0_g1	Cysteine synthase C1 [Theobroma cacao]	PCAS-2	Bifunctional L-3-cyanoalanine synthase/cysteine synthase 2, mitochondrial OS=Solanum tuberosum GN=PCAS-2 PE=1 SV=1	171.68	63.08	2.04	up	0.00	0.00	yes
TRINITY_DN23864_c0_g1	29 kDa ribonucleoprotein [Populus trichocarpa]	CP29B	RNA-binding protein CP29B, chloroplastic OS=Arabidopsis thaliana GN=CP29B PE=1 SV=1	1851.11	826.20	1.73	up	0.00	0.00	yes
TRINITY_DN22989_c1_g4	PREDICTED: GTP-binding protein OBGC, chloroplastic [Populus euphratica]	OBGL	GTP-binding protein OBGC, chloroplastic OS=Arabidopsis thaliana GN=OBGL PE=2 SV=1	82.48	40.14	1.63	up	0.00	0.00	yes
TRINITY_DN20334_c0_g2	hypothetical protein POPTR_0006s21010g [Populus trichocarpa]	RPL21A	60S ribosomal protein L21-1 OS=Arabidopsis thaliana GN=RPL21A PE=2 SV=2	323.45	154.47	1.69	up	0.00	0.00	yes
TRINITY_DN22763_c0_g1	PREDICTED: COBW domain-containing protein 1-like isoform X1 [Populus euphratica]	CBWD1	COBW domain-containing protein 1 OS=Homo sapiens GN=CBWD1 PE=2 SV=1	403.89	189.02	1.66	up	0.00	0.00	yes
TRINITY_DN23572_c0_g4	hypothetical protein POPTR_0010s23890g [Populus trichocarpa]	-	Probable phytol kinase 1, chloroplastic OS=Glycine max PE=2 SV=1	26.24	12.51	1.53	up	0.00	0.00	yes
TRINITY_DN19422_c0_g2	PREDICTED: psbP domain-containing protein 5, chloroplastic [Populus euphratica]	PPD5	PsbP domain-containing protein 5, chloroplastic OS=Arabidopsis thaliana GN=PPD5 PE=1 SV=3	100.94	43.64	1.76	up	0.00	0.00	yes
TRINITY_DN25058_c0_g1	unknown [Populus trichocarpa]	LHY	Protein LHY OS=Arabidopsis thaliana GN=LHY PE=1 SV=2	5.70	1.79	2.33	up	0.00	0.00	yes
TRINITY_DN22020_c0_g2	solanesyl diphosphate synthase family protein [Populus trichocarpa]	SPS2	Solanesyl diphosphate synthase 2, chloroplastic OS=Arabidopsis thaliana GN=SPS2 PE=1 SV=1	72.51	40.62	1.82	up	0.00	0.00	yes
TRINITY_DN22903_c0_g1	PREDICTED: uridine 5'-monophosphate synthase-like [Populus euphratica]	PYRE-F	Uridine 5'-monophosphate synthase OS=Arabidopsis thaliana GN=PYRE-F PE=2 SV=2	94.37	50.65	1.52	up	0.00	0.00	yes
TRINITY_DN22233_c0_g1	hypothetical protein POPTR_0002s10420g [Populus trichocarpa]	PGI1	Glucose-6-phosphate isomerase 1, chloroplastic OS=Arabidopsis thaliana GN=PGI1 PE=1 SV=1	84.73	47.59	1.51	up	0.00	0.00	yes
TRINITY_DN21458_c0_g2	hypothetical protein POPTR_0006s04300g [Populus trichocarpa]	GAST1	Protein GAST1 OS=Solanum lycopersicum GN=GAST1 PE=2 SV=1	139.52	35.55	2.61	up	0.00	0.00	yes
TRINITY_DN18730_c0_g2	universal stress family protein [Populus trichocarpa]	-	-	23.01	5.90	2.70	up	0.00	0.00	yes
TRINITY_DN19307_c0_g1	hypothetical protein POPTR_0017s02810g [Populus trichocarpa]	HSP15.7	15.7 kDa heat shock protein, peroxisomal OS=Arabidopsis thaliana GN=HSP15.7 PE=2 SV=1	35.37	14.65	1.97	up	0.00	0.00	yes
TRINITY_DN26758_c0_g1	PREDICTED: heat shock protein 83 isoform X2 [Populus euphratica]	HSP90-6	Heat shock protein 90-6, mitochondrial OS=Arabidopsis thaliana GN=HSP90-6 PE=2 SV=1	52.31	25.64	1.70	up	0.00	0.00	yes

TRINITY_DN18595_c0_g1	PREDICTED: uncharacterized protein LOC105111021 [Populus euphratica]	RAP	RAP domain-containing protein, chloroplastic OS=Arabidopsis thaliana GN=RAP PE=1 SV=1	117.88	48.46	1.88	up	0.00	0.00	yes
TRINITY_DN23561_c0_g5	hypothetical protein POPTR_0012s10710g [Populus trichocarpa]	ACP4	Acyl carrier protein 4, chloroplastic OS=Arabidopsis thaliana GN=ACP4 PE=2 SV=1	1174.40	419.22	2.06	up	0.00	0.00	yes
TRINITY_DN24487_c0_g1	PREDICTED: UDP-glycosyltransferase 92A1-like [Populus euphratica]	UGT92A1	UDP-glycosyltransferase 92A1 OS=Arabidopsis thaliana GN=UGT92A1 PE=2 SV=1	16.37	3.93	2.10	up	0.00	0.00	yes
TRINITY_DN19445_c0_g1	PREDICTED: 36.4 kDa proline-rich protein isoform X2 [Populus euphratica]	TPRP-F1	36.4 kDa proline-rich protein OS=Solanum lycopersicum GN=TPRP-F1 PE=2 SV=1	4481.86	1973.35	1.76	up	0.00	0.00	yes
TRINITY_DN24854_c0_g1	hypothetical protein POPTR_0001s08770g [Populus trichocarpa]	TUFA	Elongation factor Tu, chloroplastic OS=Glycine max GN=TUFA PE=3 SV=1	754.02	320.58	1.82	up	0.00	0.00	yes
TRINITY_DN18929_c1_g2	PREDICTED: uncharacterized protein LOC105139703 [Populus euphratica]	-	-	72.41	33.61	1.71	up	0.00	0.00	yes
TRINITY_DN21293_c0_g2	hypothetical protein POPTR_0019s05210g [Populus trichocarpa]	MYB5	Transcription repressor MYB5 OS=Arabidopsis thaliana GN=MYB5 PE=1 SV=1	5.66	1.63	2.44	up	0.00	0.00	yes
TRINITY_DN16218_c0_g1	hypothetical protein POPTR_0016s09610g [Populus trichocarpa]	acyP	Acylphosphatase OS=Sphingomonas wittichii (strain RW1 / DSM 6014 / JCM 10273) GN=acyP PE=3 SV=1	16.99	7.54	1.77	up	0.00	0.00	yes
TRINITY_DN13626_c0_g2	maturase K (chloroplast) [Populus adenopoda]	matK	Maturase K OS=Populus alba GN=matK PE=3 SV=1	9.49	3.26	2.16	up	0.00	0.00	yes
TRINITY_DN24169_c0_g1	PREDICTED: adenosine kinase 2 isoform X1 [Populus euphratica]	ADK2	Adenosine kinase 2 OS=Arabidopsis thaliana GN=ADK2 PE=1 SV=1	170.79	90.39	1.52	up	0.00	0.00	yes
TRINITY_DN17797_c0_g1	PREDICTED: microsomal glutathione S-transferase 3 [Populus euphratica]	MGST3	Microsomal glutathione S-transferase 3 OS=Bos taurus GN=MGST3 PE=2 SV=1	33.17	16.73	1.66	up	0.00	0.00	yes
TRINITY_DN23308_c1_g1	hypothetical protein POPTR_0007s04730g [Populus trichocarpa]	-	-	77.70	42.75	1.54	up	0.00	0.00	yes
TRINITY_DN19572_c0_g2	PREDICTED: truncated transcription factor CAULIFLOWER D-like isoform X1 [Populus euphratica]	AGL19	Agamous-like MADS-box protein AGL19 OS=Arabidopsis thaliana GN=AGL19 PE=1 SV=1	76.08	17.77	2.70	up	0.00	0.00	yes
TRINITY_DN23433_c0_g2	1-deoxy-D-xylulose-5-phosphate reductoisomerase [Populus tremula x Populus alba]	DXR	1-deoxy-D-xylulose 5-phosphate reductoisomerase, chloroplastic OS=Arabidopsis thaliana GN=DXR PE=2 SV=2	84.58	38.91	1.70	up	0.00	0.00	yes
TRINITY_DN18986_c0_g3	hypothetical protein POPTR_0017s05460g [Populus trichocarpa]	RPL38A	60S ribosomal protein L38 OS=Arabidopsis thaliana GN=RPL38A PE=3 SV=1	764.68	391.32	1.57	up	0.00	0.00	yes
TRINITY_DN22348_c0_g4	O-acetylserine (thiol)lyase family protein [Populus trichocarpa]	-	Cysteine synthase OS=Citrullus lanatus PE=1 SV=1	220.16	110.90	1.61	up	0.00	0.00	yes
TRINITY_DN19738_c0_g1	hypothetical protein POPTR_0004s02950g [Populus trichocarpa]	-	-	91.14	48.15	1.63	up	0.00	0.00	yes
TRINITY_DN22522_c0_g2	PREDICTED: 30S ribosomal protein S17, chloroplastic [Populus euphratica]	RPS17	30S ribosomal protein S17, chloroplastic OS=Arabidopsis thaliana GN=RPS17 PE=1 SV=1	1151.52	402.22	2.12	up	0.00	0.00	yes
TRINITY_DN21689_c0_g1	hypothetical protein POPTR_0014s13430g [Populus trichocarpa]	-	-	117.32	44.17	1.97	up	0.00	0.00	yes
TRINITY_DN22052_c0_g3	hypothetical protein POPTR_0001s33160g [Populus trichocarpa]	At3g26840	Acyltransferase-like protein At3g26840, chloroplastic OS=Arabidopsis thaliana GN=At3g26840 PE=2 SV=1	4.81	2.48	1.98	up	0.00	0.00	yes
TRINITY_DN24038_c0_g1	PREDICTED: MATE efflux family protein 3, chloroplastic-like [Populus euphratica]	DTX45	Protein DETOXIFICATION 45, chloroplastic OS=Arabidopsis thaliana GN=DTX45 PE=2 SV=2	10.69	4.55	1.77	up	0.00	0.00	yes
TRINITY_DN20864_c0_g3	hypothetical protein POPTR_0007s07780g [Populus trichocarpa]	HCF136	Photosystem II stability/assembly factor HCF136, chloroplastic OS=Arabidopsis thaliana GN=HCF136 PE=1 SV=1	286.44	119.55	1.84	up	0.00	0.00	yes
TRINITY_DN20878_c0_g1	hypothetical protein POPTR_0004s01330g [Populus trichocarpa]	MEE40	Pentatricopeptide repeat-containing protein At3g53700, chloroplastic OS=Arabidopsis thaliana GN=MEE40 PE=2 SV=1	23.57	12.36	1.53	up	0.00	0.00	yes
TRINITY_DN19591_c1_g2	hypothetical protein POPTR_0014s17190g, partial [Populus trichocarpa]	-	-	27.53	10.58	1.99	up	0.00	0.00	yes
TRINITY_DN22075_c0_g1	PREDICTED: nifU-like protein 4, mitochondrial [Populus euphratica]	NIFU4	NifU-like protein 4, mitochondrial OS=Arabidopsis thaliana GN=NIFU4 PE=2 SV=1	32.90	17.88	1.50	up	0.00	0.00	yes
TRINITY_DN23708_c0_g2	PREDICTED: protein trichome birefringence-like 37 [Populus euphratica]	TBL37	Protein trichome birefringence-like 37 OS=Arabidopsis thaliana GN=TBL37 PE=2 SV=2	26.77	14.27	1.54	up	0.00	0.00	yes
TRINITY_DN18800_c1_g1	PREDICTED: zeaxanthin epoxidase, chloroplastic-like [Populus euphratica]	hpxO	FAD-dependent urate hydroxylase OS=Acinetobacter baylyi (strain ATCC 33305 / BD413 / ADP1) GN=hpxO PE=1 SV=1	44.90	22.57	1.72	up	0.00	0.00	yes
TRINITY_DN19044_c0_g1	hypothetical protein LR48_Vigan01g206700 [Vigna angularis]	RPL37A	60S ribosomal protein L37a OS=Gossypium hirsutum GN=RPL37A PE=3 SV=1	511.76	267.25	1.56	up	0.00	0.00	yes

TRINITY_DN19256_c3_g2	Ribosomal protein L29 [Cynara cardunculus var. scolymus]	RPL35	60S ribosomal protein L35 OS=Euphorbia esula GN=RPL35 PE=2 SV=1	374.13	195.78	1.57	up	0.00	0.00	yes
TRINITY_DN21328_c0_g1	nascent polypeptide-associated complex domain-containing family protein [Populus trichocarpa]	BTF3	Basic transcription factor 3 OS=Arabidopsis thaliana GN=BTF3 PE=1 SV=1	249.21	129.58	1.62	up	0.00	0.00	yes
TRINITY_DN27857_c5_g2	-	-	-	61.50	17.34	2.49	up	0.00	0.00	yes
TRINITY_DN20584_c0_g2	peroxisomal membrane 22 kDa family protein [Populus trichocarpa]	-	-	70.32	30.71	1.73	up	0.00	0.00	yes
TRINITY_DN26602_c0_g1	hypothetical protein POPTR_0001s28970g [Populus trichocarpa]	-	-	467.35	238.55	1.53	up	0.00	0.00	yes
TRINITY_DN18048_c0_g1	hypothetical protein POPTR_0006s13540g [Populus trichocarpa]	At2g37240	Thioredoxin-like protein AAED1, chloroplastic OS=Arabidopsis thaliana GN=At2g37240 PE=1 SV=2	35.65	12.07	2.18	up	0.00	0.00	yes
TRINITY_DN23750_c0_g2	PREDICTED: MYB-like transcription factor ETC1 [Populus euphratica]	CPC	Transcription factor CPC OS=Arabidopsis thaliana GN=CPC PE=1 SV=1	70.72	21.48	2.33	up	0.00	0.00	yes
TRINITY_DN24623_c0_g2	hypothetical protein POPTR_0008s11770g [Populus trichocarpa]	PGIC	Glucose-6-phosphate isomerase, cytosolic OS=Oenothera sinuata var. hirsuta GN=PGIC PE=3 SV=1	42.50	22.04	1.54	up	0.00	0.00	yes
TRINITY_DN14056_c0_g2	hypothetical protein POPTR_0009s15060g [Populus trichocarpa]	-	-	37.59	17.10	1.76	up	0.00	0.00	yes
TRINITY_DN23293_c0_g2	hypothetical protein POPTR_0001s45510g [Populus trichocarpa]	RPL11	50S ribosomal protein L11, chloroplastic OS=Arabidopsis thaliana GN=RPL11 PE=2 SV=1	323.44	127.58	1.93	up	0.00	0.00	yes
TRINITY_DN23090_c0_g4	PREDICTED: uncharacterized protein LOC105111744 isoform X1 [Populus euphratica]	CPP1	Protein CHAPERONE-LIKE PROTEIN OF POR1, chloroplastic OS=Arabidopsis thaliana GN=CPP1 PE=1 SV=1	356.45	165.87	1.70	up	0.00	0.00	yes
TRINITY_DN15932_c0_g1	hypothetical protein POPTR_0004s01950g [Populus trichocarpa]	-	Kirola OS=Actinidia deliciosa PE=1 SV=1	14.77	0.34	5.07	up	0.00	0.00	yes
TRINITY_DN20975_c0_g2	PREDICTED: uncharacterized protein At2g27730, mitochondrial-like isoform X1 [Populus euphratica]	At2g27730	Uncharacterized protein At2g27730, mitochondrial OS=Arabidopsis thaliana GN=At2g27730 PE=1 SV=1	77.82	42.45	1.47	up	0.00	0.00	yes
TRINITY_DN17673_c0_g1	hypothetical protein POPTR_0006s01740g [Populus trichocarpa]	-	-	196.06	102.18	1.55	up	0.00	0.00	yes
TRINITY_DN17646_c0_g3	unknown [Populus trichocarpa]	-	Polygalacturonase OS=Actinidia deliciosa PE=2 SV=1	75.56	24.78	2.20	up	0.00	0.00	yes
TRINITY_DN22072_c0_g1	hypothetical protein POPTR_0001s29700g [Populus trichocarpa]	EGY3	Probable zinc metallopeptidase EGY3, chloroplastic OS=Arabidopsis thaliana GN=EGY3 PE=1 SV=1	31.08	12.11	1.98	up	0.00	0.00	yes
TRINITY_DN21915_c1_g3	PREDICTED: elongation factor G-2, chloroplastic [Populus euphratica]	fusA2	Elongation factor G-2, chloroplastic OS=Glycine max GN=fusA2 PE=3 SV=1	224.06	85.10	2.01	up	0.00	0.00	yes
TRINITY_DN22093_c1_g1	PREDICTED: uncharacterized protein LOC105133503 [Populus euphratica]	-	-	28.08	18.04	1.58	up	0.00	0.00	yes
TRINITY_DN26158_c1_g1	sulfate transporter [Populus tremula x Populus alba]	SULTR1;3	Sulfate transporter 1.3 OS=Arabidopsis thaliana GN=SULTR1;3 PE=2 SV=1	12.32	3.69	2.35	up	0.00	0.00	yes
TRINITY_DN19791_c1_g3	hypothetical protein POPTR_0008s20300g [Populus trichocarpa]	MSRB5	Peptide methionine sulfoxide reductase B5 OS=Arabidopsis thaliana GN=MSRB5 PE=1 SV=1	178.02	79.56	1.78	up	0.00	0.00	yes
TRINITY_DN22718_c0_g1	GIANT CHLOROPLAST 1 family protein [Populus trichocarpa]	GC1	Epimerase family protein SDR39U1 homolog, chloroplastic OS=Arabidopsis thaliana GN=GC1 PE=2 SV=2	129.30	45.45	2.09	up	0.00	0.00	yes
TRINITY_DN21103_c0_g1	PREDICTED: aldehyde dehydrogenase family 2 member C4 [Populus euphratica]	ALDH2C4	Aldehyde dehydrogenase family 2 member C4 OS=Arabidopsis thaliana GN=ALDH2C4 PE=1 SV=2	66.51	28.91	1.83	up	0.00	0.00	yes
TRINITY_DN21074_c0_g1	PREDICTED: glycine-rich RNA-binding protein 2, mitochondrial-like [Populus euphratica]	RBG2	Glycine-rich RNA-binding protein 2, mitochondrial OS=Arabidopsis thaliana GN=RBG2 PE=1 SV=1	98.17	53.65	1.52	up	0.00	0.00	yes
TRINITY_DN14686_c0_g1	hypothetical protein POPTR_0018s08930g [Populus trichocarpa]	-	-	53.87	26.48	1.64	up	0.00	0.00	yes
TRINITY_DN22318_c0_g4	Chaperone DnaJ [Gossypium arboreum]	EDA3	Protein EMBRYO SAC DEVELOPMENT ARREST 3, chloroplastic OS=Arabidopsis thaliana GN=EDA3 PE=1 SV=1	66.38	17.66	2.50	up	0.00	0.00	yes
TRINITY_DN21185_c1_g9	ribosomal protein S4 [Populus alba]	rps4	30S ribosomal protein S4, chloroplastic OS=Populus alba GN=rps4 PE=3 SV=1	21.79	10.00	1.71	up	0.00	0.00	yes
TRINITY_DN23297_c0_g3	60S ribosomal protein L15 [Populus trichocarpa]	RPL15	60S ribosomal protein L15 OS=Petunia hybrida GN=RPL15 PE=2 SV=1	98.11	50.85	1.83	up	0.00	0.00	yes
TRINITY_DN25701_c2_g1	PREDICTED: uncharacterized protein LOC105140217 [Populus euphratica]	-	-	28.75	12.73	1.96	up	0.00	0.00	yes
TRINITY_DN21044_c0_g3	hypothetical protein POPTR_0014s02520g [Populus trichocarpa]	-	-	86.57	39.50	1.73	up	0.00	0.00	yes

TRINITY_DN14872_c0_g2	polyphenol oxidase-like protein [Populus trichocarpa]	AS1	Aureusidin synthase OS=Antirrhinum majus GN=AS1 PE=1 SV=1	2.86	0.52	3.04	up	0.00	0.00	yes
TRINITY_DN21847_c0_g2	PREDICTED: 6-phosphogluconate dehydrogenase, decarboxylating 2, chloroplastic [Populus euphratica]	At5g41670	6-phosphogluconate dehydrogenase, decarboxylating 2, chloroplastic OS=Arabidopsis thaliana GN=At5g41670 PE=1 SV=1	56.92	29.48	1.55	up	0.00	0.00	yes
TRINITY_DN25303_c0_g4	unknown [Populus trichocarpa]	-	-	61.95	27.21	2.06	up	0.00	0.00	yes
TRINITY_DN25367_c0_g1	hypothetical protein POPTR_0003s02770g [Populus trichocarpa]	TS2	Threonine synthase 2, chloroplastic OS=Arabidopsis thaliana GN=TS2 PE=1 SV=1	59.68	31.40	1.54	up	0.00	0.00	yes
TRINITY_DN19308_c0_g1	hypothetical protein POPTR_0013s02750g [Populus trichocarpa]	-	-	122.11	52.17	1.86	up	0.00	0.00	yes
TRINITY_DN15502_c0_g1	hypothetical protein POPTR_0004s00800g [Populus trichocarpa]	Prosc	Proline synthase co-transcribed bacterial homolog protein OS=Mus musculus GN=Prosc PE=1 SV=1	203.92	86.61	1.74	up	0.00	0.00	yes
TRINITY_DN21551_c0_g1	PREDICTED: sufE-like protein, chloroplastic [Populus euphratica]	SUFE1	SufE-like protein 1, chloroplastic/mitochondrial OS=Arabidopsis thaliana GN=SUFE1 PE=1 SV=2	49.31	25.04	1.62	up	0.00	0.00	yes
TRINITY_DN19731_c0_g4	short-chain dehydrogenase/reductase family protein [Populus trichocarpa]	IRC24	Benzil reductase ((S)-benzoin forming) IRC24 OS=Saccharomyces cerevisiae (strain ATCC 204508 / S288c) GN=IRC24 PE=1 SV=1	41.63	22.34	1.51	up	0.00	0.00	yes
TRINITY_DN13159_c0_g1	ascorbate peroxidase [Populus tomentosa]	APX3	L-ascorbate peroxidase 3, peroxisomal OS=Arabidopsis thaliana GN=APX3 PE=1 SV=1	8.23	2.11	2.54	up	0.00	0.00	yes
TRINITY_DN24894_c0_g3	hypothetical protein POPTR_0015s03960g [Populus trichocarpa]	-	-	34.92	19.04	1.50	up	0.00	0.00	yes
TRINITY_DN22651_c1_g1	hypothetical protein POPTR_0007s10760g [Populus trichocarpa]	CBSX1	CBS domain-containing protein CBSX1, chloroplastic OS=Arabidopsis thaliana GN=CBSX1 PE=1 SV=2	107.54	58.41	1.59	up	0.00	0.00	yes
TRINITY_DN22485_c0_g1	PREDICTED: uncharacterized protein LOC105127970 [Populus euphratica]	-	-	14.56	5.13	2.05	up	0.00	0.00	yes
TRINITY_DN27597_c0_g3	hypothetical protein POPTR_0005s27680g [Populus trichocarpa]	MSSP2	Monosaccharide-sensing protein 2 OS=Arabidopsis thaliana GN=MSSP2 PE=1 SV=2	39.45	22.46	1.46	up	0.00	0.00	yes
TRINITY_DN22154_c0_g2	hypothetical protein POPTR_0007s09310g [Populus trichocarpa]	AKRP	Ankyrin repeat domain-containing protein, chloroplastic OS=Arabidopsis thaliana GN=AKRP PE=1 SV=2	85.68	44.64	1.55	up	0.00	0.00	yes
TRINITY_DN23609_c0_g1	unknown [Populus trichocarpa]	At2g29150	Tropinone reductase homolog At2g29150 OS=Arabidopsis thaliana GN=At2g29150 PE=1 SV=1	110.88	58.39	1.54	up	0.00	0.00	yes
TRINITY_DN27215_c0_g1	hypothetical protein POPTR_0008s05870g [Populus trichocarpa]	RCA	Ribulose bisphosphate carboxylase/oxygenase activase, chloroplastic OS=Vigna radiata var. radiata GN=RCA PE=2 SV=2	1088.55	260.14	2.07	up	0.00	0.00	yes
TRINITY_DN19857_c0_g2	AICARFT/IMPCHase bienzyme family protein [Populus tomentosa]	purH	Bifunctional purine biosynthesis protein PurH OS=Bacillus cereus (strain ZK / E33L) GN=purH PE=3 SV=1	80.53	45.41	1.45	up	0.00	0.00	yes
TRINITY_DN20531_c0_g2	PREDICTED: homoserine kinase-like [Populus euphratica]	HSK	Homoserine kinase OS=Arabidopsis thaliana GN=HSK PE=1 SV=1	91.57	46.55	1.49	up	0.00	0.00	yes
TRINITY_DN21166_c0_g4	-	-	-	310.48	115.73	2.00	up	0.00	0.00	yes
TRINITY_DN15308_c0_g1	major pollen allergen Bet v 1k family protein [Populus trichocarpa]	-	Major allergen Pru ar 1 OS=Prunus armeniaca PE=1 SV=1	964.08	335.08	2.12	up	0.00	0.00	yes
TRINITY_DN18706_c0_g1	hypothetical protein POPTR_0014s16030g [Populus trichocarpa]	EMB2761	Threonine--tRNA ligase, chloroplastic/mitochondrial 2 OS=Arabidopsis thaliana GN=EMB2761 PE=2 SV=1	62.91	29.87	1.67	up	0.00	0.00	yes
TRINITY_DN17226_c0_g3	PREDICTED: uncharacterized protein LOC105134496 [Populus euphratica]	-	-	105.84	51.59	1.65	up	0.00	0.00	yes
TRINITY_DN24158_c0_g1	mitochondrial substrate carrier family protein [Populus trichocarpa]	MFL1	Protein MITOFERRINLIKE 1, chloroplastic OS=Arabidopsis thaliana GN=MFL1 PE=2 SV=1	42.17	24.42	1.40	up	0.00	0.00	yes
TRINITY_DN20028_c0_g1	hypothetical protein POPTR_0001s33840g [Populus trichocarpa]	htpX2	Protease HtpX homolog 2 OS=Methanosarcina acetivorans (strain ATCC 35395 / DSM 2834 / JCM 12185 / C2A) GN=htpX2 PE=3 SV=1	15.21	7.31	1.75	up	0.00	0.00	yes
TRINITY_DN19445_c0_g2	-	-	-	2893.04	1323.32	1.72	up	0.00	0.00	yes
TRINITY_DN18454_c0_g1	glutathione S-transferase F2 [Populus yatungensis]	GSTF11	Glutathione S-transferase F11 OS=Arabidopsis thaliana GN=GSTF11 PE=2 SV=1	305.90	143.62	1.69	up	0.00	0.00	yes
TRINITY_DN21312_c3_g3	PREDICTED: putative DNA-binding protein ESCAROLA [Populus euphratica]	AHL15	AT-hook motif nuclear-localized protein 15 OS=Arabidopsis thaliana GN=AHL15 PE=2 SV=1	13.91	4.22	2.31	up	0.00	0.00	yes
TRINITY_DN24602_c0_g1	hypothetical protein POPTR_0019s09190g [Populus trichocarpa]	DTX29	Protein DETOXIFICATION 29 OS=Arabidopsis thaliana GN=DTX29 PE=1 SV=1	70.73	30.81	1.70	up	0.00	0.00	yes
TRINITY_DN25428_c0_g3	-	-	-	4.16	0.22	4.62	up	0.00	0.00	yes

TRINITY_DN22273_c0_g2	PREDICTED: tRNA dimethylallyltransferase 9 [Populus euphratica]	IPT9	tRNA dimethylallyltransferase 9 OS=Arabidopsis thaliana GN=IPT9 PE=2 SV=1	80.74	46.98	1.52	up	0.00	0.00	yes
TRINITY_DN20931_c0_g3	PREDICTED: mitotic checkpoint protein BUB3.3 [Populus euphratica]	EIFSV1	Eukaryotic translation initiation factor 5A OS=Senecio vernalis GN=EIFSV1 PE=2 SV=1	255.37	136.81	1.50	up	0.00	0.00	yes
TRINITY_DN23200_c0_g2	hypothetical protein POPTR_0004s05650g [Populus trichocarpa]	-	-	5.80	4.24	1.99	up	0.00	0.00	yes
TRINITY_DN23533_c0_g1	PREDICTED: uncharacterized protein LOC105129846 [Populus euphratica]	rlmN	Probable dual-specificity RNA methyltransferase RlmN OS=Synechococcus sp. (strain JA-3-3Ab) GN=rlmN PE=3 SV=1	64.75	36.57	1.56	up	0.00	0.00	yes
TRINITY_DN27251_c0_g1	hypothetical protein POPTR_0003s02950g [Populus trichocarpa]	ABCC8	ABC transporter C family member 8 OS=Arabidopsis thaliana GN=ABCC8 PE=2 SV=3	33.01	16.26	1.65	up	0.00	0.00	yes
TRINITY_DN24605_c0_g8	-	-	-	4.25	0.00	8.38	up	0.00	0.00	yes
TRINITY_DN23701_c0_g5	plastidic aldolase family protein [Populus trichocarpa]	ALDP	Fructose-bisphosphate aldolase, chloroplastic OS=Oryza sativa subsp. japonica GN=ALDP PE=1 SV=2	7.53	2.00	2.57	up	0.00	0.00	yes
TRINITY_DN18207_c0_g1	hypothetical protein POPTR_0001s04900g [Populus trichocarpa]	PAP12	Probable plastid-lipid-associated protein 12, chloroplastic OS=Arabidopsis thaliana GN=PAP12 PE=2 SV=1	60.77	31.71	1.61	up	0.00	0.00	yes
TRINITY_DN26442_c0_g1	ATP-dependent Clp protease proteolytic subunit family protein [Populus trichocarpa]	CLPR1	ATP-dependent Clp protease proteolytic subunit-related protein 1, chloroplastic OS=Arabidopsis thaliana GN=CLPR1 PE=1 SV=1	240.33	117.24	1.69	up	0.00	0.00	yes
TRINITY_DN17848_c0_g1	PREDICTED: probable sodium/metabolite cotransporter BASS1, chloroplastic [Populus euphratica]	BASS1	Probable sodium/metabolite cotransporter BASS1, chloroplastic OS=Arabidopsis thaliana GN=BASS1 PE=2 SV=1	29.44	15.03	1.59	up	0.00	0.00	yes
TRINITY_DN18411_c0_g3	hypothetical protein POPTR_0006s12640g [Populus trichocarpa]	At3g46100	Histidine--tRNA ligase, chloroplastic/mitochondrial OS=Arabidopsis thaliana GN=At3g46100 PE=2 SV=1	101.84	53.65	1.53	up	0.00	0.00	yes
TRINITY_DN19193_c0_g1	seryl-tRNA synthetase family protein [Populus trichocarpa]	OVA7	Serine--tRNA ligase, chloroplastic/mitochondrial OS=Arabidopsis thaliana GN=OVA7 PE=2 SV=1	44.70	22.61	1.58	up	0.00	0.00	yes
TRINITY_DN22142_c0_g1	60s acidic ribosomal family protein [Populus trichocarpa]	-	60S acidic ribosomal protein P2 OS=Parthenium argentatum PE=3 SV=1	744.60	389.71	1.56	up	0.00	0.00	yes
TRINITY_DN25766_c0_g2	ABC transporter family protein [Populus trichocarpa]	ABCF5	ABC transporter F family member 5 OS=Arabidopsis thaliana GN=ABCF5 PE=2 SV=1	72.87	38.52	1.51	up	0.00	0.00	yes
TRINITY_DN18062_c0_g8	hypothetical protein POPTR_0015s12410g [Populus trichocarpa]	-	-	70.42	39.50	1.62	up	0.00	0.00	yes
TRINITY_DN24319_c0_g3	hypothetical protein POPTR_0015s00460g [Populus trichocarpa]	-	-	23.55	11.92	1.60	up	0.00	0.00	yes
TRINITY_DN22431_c0_g1	PREDICTED: uncharacterized protein LOC105129160 isoform X1 [Populus euphratica]	ynbB	Uncharacterized protein YnbB OS=Bacillus subtilis (strain 168) GN=ynbB PE=4 SV=2	33.11	17.41	1.59	up	0.00	0.00	yes
TRINITY_DN26131_c0_g2	6-phosphogluconate dehydrogenase family protein [Populus trichocarpa]	At5g41670	6-phosphogluconate dehydrogenase, decarboxylating 2, chloroplastic OS=Arabidopsis thaliana GN=At5g41670 PE=1 SV=1	63.33	33.04	1.50	up	0.00	0.00	yes
TRINITY_DN27215_c0_g3	unknown [Populus trichocarpa x Populus deltoides]	RCA	Ribulose bisphosphate carboxylase/oxygenase activase, chloroplastic OS=Malus domestica GN=RCA PE=2 SV=1	3487.30	889.50	2.44	up	0.00	0.00	yes
TRINITY_DN23540_c0_g3	hypothetical protein POPTR_0001s17980g [Populus trichocarpa]	MFP1	MAR-binding filament-like protein 1 OS=Solanum lycopersicum GN=MFP1 PE=1 SV=1	75.84	50.09	1.65	up	0.00	0.00	yes
TRINITY_DN27440_c0_g6	PREDICTED: protein DCL, chloroplastic-like [Populus euphratica]	DCL	Protein DCL, chloroplastic OS=Solanum lycopersicum GN=DCL PE=2 SV=1	67.51	32.70	1.53	up	0.00	0.00	yes
TRINITY_DN18493_c0_g1	PREDICTED: nucleoside diphosphate kinase 2, chloroplastic-like, partial [Populus euphratica]	NDPK2	Nucleoside diphosphate kinase II, chloroplastic OS=Arabidopsis thaliana GN=NDPK2 PE=1 SV=2	521.73	234.57	1.75	up	0.00	0.00	yes
TRINITY_DN14881_c0_g2	hypothetical protein POPTR_0019s10500g [Populus trichocarpa]	-	-	115.82	60.46	1.54	up	0.00	0.00	yes
TRINITY_DN22458_c0_g1	PREDICTED: pentatricopeptide repeat-containing protein At3g09650, chloroplastic-like [Populus euphratica]	HCF152	Pentatricopeptide repeat-containing protein At3g09650, chloroplastic OS=Arabidopsis thaliana GN=HCF152 PE=2 SV=1	38.84	18.91	1.66	up	0.00	0.00	yes
TRINITY_DN18631_c0_g2	GDSL-motif lipase/hydrolase family protein [Populus trichocarpa]	At1g29670	GDSL esterase/lipase At1g29670 OS=Arabidopsis thaliana GN=At1g29670 PE=2 SV=1	40.04	15.31	2.04	up	0.00	0.00	yes
TRINITY_DN18468_c0_g1	unknown [Populus trichocarpa]	Cenpv	Centromere protein V OS=Mus musculus GN=Cenpv PE=1 SV=2	75.43	40.88	1.50	up	0.00	0.00	yes
TRINITY_DN14899_c0_g1	hypothetical protein POPTR_0019s08420g [Populus trichocarpa]	-	-	6.48	1.02	3.35	up	0.00	0.00	yes

TRINITY_DN21026_c1_g1	PREDICTED: ATP-dependent Clp protease proteolytic subunit 2, mitochondrial-like [Populus euphratica]	CLPP2	ATP-dependent Clp protease proteolytic subunit 2, mitochondrial OS=Arabidopsis thaliana GN=CLPP2 PE=1 SV=1	103.26	56.38	1.46	up	0.00	0.00	yes
TRINITY_DN21854_c0_g2	PREDICTED: nucleolar GTP-binding protein 1 [Populus euphratica]	Gtpbp4	Nucleolar GTP-binding protein 1 OS=Mus musculus GN=Gtpbp4 PE=1 SV=3	47.26	29.04	1.48	up	0.00	0.00	yes
TRINITY_DN22290_c1_g1	ribosomal protein L12 [Populus trichocarpa]	RPL12	60S ribosomal protein L12 OS=Prunus armeniaca GN=RPL12 PE=2 SV=1	1118.36	600.76	1.52	up	0.00	0.00	yes
TRINITY_DN18121_c0_g2	PREDICTED: pentatricopeptide repeat-containing protein At4g18840-like [Populus euphratica]	PCMP-H12	Pentatricopeptide repeat-containing protein At1g08070, chloroplastic OS=Arabidopsis thaliana GN=PCMP-H12 PE=2 SV=1	4.64	1.81	1.98	up	0.00	0.00	yes
TRINITY_DN21469_c0_g3	unknown [Populus trichocarpa]	fabZ	3-hydroxyacyl-[acyl-carrier-protein] dehydratase FabZ OS=Thermosynechococcus elongatus (strain BP-1) GN=fabZ PE=3 SV=1	127.67	66.65	1.53	up	0.00	0.00	yes
TRINITY_DN27728_c1_g1	hypothetical protein POPTR_0007s07680g [Populus trichocarpa]	HEMC	Porphobilinogen deaminase, chloroplastic OS=Pisum sativum GN=HEMC PE=1 SV=1	57.94	21.48	2.03	up	0.00	0.00	yes
TRINITY_DN25402_c0_g4	hypothetical protein POPTR_0009s03780g [Populus trichocarpa]	RPP3B	60S acidic ribosomal protein P3-2 OS=Arabidopsis thaliana GN=RPP3B PE=2 SV=1	308.61	165.97	1.51	up	0.00	0.00	yes
TRINITY_DN22814_c0_g2	hypothetical protein POPTR_0002s19090g [Populus trichocarpa]	-	-	71.25	31.79	1.72	up	0.00	0.00	yes
TRINITY_DN20334_c0_g1	hypothetical protein POPTR_0016s06110g [Populus trichocarpa]	RPL21A	60S ribosomal protein L21-1 OS=Arabidopsis thaliana GN=RPL21A PE=2 SV=2	303.04	161.71	1.53	up	0.00	0.00	yes
TRINITY_DN21442_c0_g1	hypothetical protein POPTR_0010s10260g [Populus trichocarpa]	WHY1	Single-stranded DNA-binding protein WHY1, chloroplastic OS=Arabidopsis thaliana GN=WHY1 PE=1 SV=1	241.32	116.39	1.64	up	0.00	0.00	yes
TRINITY_DN19750_c1_g1	ribosomal protein S16 [Populus trichocarpa]	RPS16	40S ribosomal protein S16 OS=Gossypium hirsutum GN=RPS16 PE=2 SV=1	278.01	147.92	1.54	up	0.00	0.00	yes
TRINITY_DN24375_c0_g2	DEAD/DEAH box helicase family protein [Populus trichocarpa]	RH47	DEAD-box ATP-dependent RNA helicase 47, mitochondrial OS=Arabidopsis thaliana GN=RH47 PE=1 SV=2	75.03	41.22	1.46	up	0.00	0.00	yes
TRINITY_DN27506_c0_g1	chloroplast inner envelope family protein [Populus trichocarpa]	TIC110	Protein TIC110, chloroplastic OS=Arabidopsis thaliana GN=TIC110 PE=1 SV=1	115.34	59.94	1.53	up	0.00	0.00	yes
TRINITY_DN22393_c0_g2	chaperonin 10 family protein [Populus trichocarpa]	CPN10-1	10 kDa chaperonin 1, chloroplastic OS=Arabidopsis thaliana GN=CPN10-1 PE=2 SV=1	649.59	287.70	1.76	up	0.00	0.00	yes
TRINITY_DN6390_c0_g1	nodulin MtN21 family protein [Populus trichocarpa]	At5g64700	WAT1-related protein At5g64700 OS=Arabidopsis thaliana GN=At5g64700 PE=2 SV=1	1.91	0.26	3.46	up	0.00	0.00	yes
TRINITY_DN22666_c3_g1	hypothetical protein POPTR_0019s07820g [Populus trichocarpa]	At3g03100	Probable NADH dehydrogenase [ubiquinone] 1 alpha subcomplex subunit 12 OS=Arabidopsis thaliana GN=At3g03100 PE=1 SV=1	224.25	113.41	1.66	up	0.00	0.00	yes
TRINITY_DN26996_c1_g1	PREDICTED: phosphoglucomutase, chloroplastic-like [Populus euphratica]	PGMP	Phosphoglucomutase, chloroplastic OS=Pisum sativum GN=PGMP PE=2 SV=1	112.47	49.80	1.79	up	0.00	0.00	yes
TRINITY_DN19225_c0_g5	40S ribosomal protein S2 [Populus trichocarpa]	RPS2D	40S ribosomal protein S2-4 OS=Arabidopsis thaliana GN=RPS2D PE=2 SV=1	383.08	208.43	1.51	up	0.00	0.00	yes
TRINITY_DN26653_c0_g3	hypothetical protein POPTR_0016s01620g [Populus trichocarpa]	UGT71K2	UDP-glycosyltransferase 71K2 OS=Pyrus communis GN=UGT71K2 PE=1 SV=1	1363.44	562.89	1.90	up	0.00	0.00	yes
TRINITY_DN26310_c1_g1	Chloroplast ADP family protein [Populus trichocarpa]	-	Plastidic ATP/ADP-transporter OS=Solanum tuberosum PE=2 SV=2	147.13	79.20	1.51	up	0.00	0.00	yes
TRINITY_DN21686_c1_g1	PREDICTED: cytochrome P450 97B2, chloroplastic [Populus euphratica]	CYP97B2	Cytochrome P450 97B2, chloroplastic OS=Glycine max GN=CYP97B2 PE=2 SV=1	39.24	19.40	1.61	up	0.00	0.00	yes
TRINITY_DN26976_c0_g1	PREDICTED: acetolactate synthase 2, chloroplastic-like [Populus euphratica]	ALS	Acetolactate synthase 2, chloroplastic OS=Nicotiana tabacum GN=ALS SURB PE=1 SV=1	396.57	239.53	1.47	up	0.00	0.00	yes
TRINITY_DN17221_c0_g1	hypothetical protein POPTR_0013s07620g [Populus trichocarpa]	slr0537	Uncharacterized sugar kinase slr0537 OS=Synechocystis sp. (strain PCC 6803 / Kazusa) GN=slr0537 PE=3 SV=1	195.85	93.85	1.65	up	0.00	0.00	yes
TRINITY_DN16647_c0_g4	hypothetical protein POPTR_0005s16000g [Populus trichocarpa]	-	-	3.69	0.66	3.05	up	0.00	0.00	yes
TRINITY_DN21680_c0_g1	hypothetical protein POPTR_0002s22530g [Populus trichocarpa]	ISA2	Isoamylase 2, chloroplastic OS=Arabidopsis thaliana GN=ISA2 PE=1 SV=2	16.04	9.27	1.40	up	0.00	0.00	yes
TRINITY_DN19801_c0_g3	hypothetical protein POPTR_0013s11780g [Populus trichocarpa]	-	-	63.31	26.69	1.85	up	0.00	0.00	yes
TRINITY_DN21360_c0_g1	hypothetical protein POPTR_0007s12330g [Populus trichocarpa]	GGPS	Geranylgeranyl pyrophosphate synthase, chloroplastic OS=Hevea brasiliensis GN=GGPS PE=1 SV=1	81.24	38.97	1.65	up	0.00	0.00	yes

TRINITY_DN19342_c0_g1	hypothetical protein POPTR_0002s08390g [Populus trichocarpa]	-	-	9.24	3.65	2.02	up	0.00	0.00	yes
TRINITY_DN19153_c0_g1	PREDICTED: peptide methionine sulfoxide reductase B1, chloroplastic [Populus euphratica]	MSRB1	Peptide methionine sulfoxide reductase B1, chloroplastic OS=Arabidopsis thaliana GN=MSRB1 PE=1 SV=1	116.51	43.73	2.12	up	0.00	0.00	yes
TRINITY_DN19972_c0_g1	metaxin-related family protein [Populus trichocarpa]	MTX1	Mitochondrial outer membrane import complex protein METAXIN OS=Arabidopsis thaliana GN=MTX1 PE=1 SV=1	23.85	14.81	1.50	up	0.00	0.00	yes
TRINITY_DN17375_c0_g1	hypothetical protein POPTR_0006s20910g [Populus trichocarpa]	-	-	62.81	29.37	1.69	up	0.00	0.00	yes
TRINITY_DN24788_c1_g2	PREDICTED: membrane-associated 30 kDa protein, chloroplastic-like [Populus euphratica]	IM30	Membrane-associated 30 kDa protein, chloroplastic OS=Pisum sativum GN=IM30 PE=2 SV=1	180.10	86.94	1.64	up	0.00	0.00	yes
TRINITY_DN15444_c0_g1	hypothetical protein VITISV_007512 [Vitis vinifera]	SRX	Sulfiredoxin, chloroplastic/mitochondrial OS=Arabidopsis thaliana GN=SRX PE=1 SV=1	43.72	16.78	1.91	up	0.00	0.00	yes
TRINITY_DN27357_c1_g1	-	-	-	4.02	0.61	3.29	up	0.00	0.00	yes
TRINITY_DN19707_c0_g1	hypothetical protein POPTR_0002s16630g [Populus trichocarpa]	-	-	26.36	15.71	1.36	up	0.00	0.00	yes
TRINITY_DN24565_c0_g1	T-protein of the glycine decarboxylase complex [Populus trichocarpa]	GDCST	Aminomethyltransferase, mitochondrial OS=Solanum tuberosum GN=GDCST PE=2 SV=1	481.27	183.79	1.99	up	0.00	0.00	yes
TRINITY_DN17781_c0_g2	hypothetical protein POPTR_0010s17720g [Populus trichocarpa]	-	-	37.37	21.44	1.42	up	0.00	0.00	yes
TRINITY_DN18957_c1_g1	chloroplast lumen common family protein [Populus trichocarpa]	SG1	protein SLOW GREEN 1, chloroplastic OS=Arabidopsis thaliana GN=SG1 PE=1 SV=1	111.09	60.25	1.47	up	0.00	0.00	yes
TRINITY_DN14923_c0_g2	hypothetical protein POPTR_0001s25030g [Populus trichocarpa]	-	-	80.28	36.72	1.71	up	0.00	0.00	yes
TRINITY_DN19460_c0_g1	hypothetical protein POPTR_0008s11640g [Populus trichocarpa]	-	-	40.89	22.03	1.47	up	0.00	0.00	yes
TRINITY_DN15491_c0_g1	PREDICTED: 60S ribosomal protein L22-2-like [Populus euphratica]	RPL22B	60S ribosomal protein L22-2 OS=Arabidopsis thaliana GN=RPL22B PE=2 SV=1	114.75	63.48	1.45	up	0.00	0.00	yes
TRINITY_DN24025_c0_g1	PREDICTED: ATP-dependent zinc metalloprotease FTSH, chloroplastic-like [Populus euphratica]	FTSH5	ATP-dependent zinc metalloprotease FTSH 5, chloroplastic OS=Arabidopsis thaliana GN=FTSH5 PE=1 SV=1	264.69	122.59	1.71	up	0.00	0.00	yes
TRINITY_DN17643_c0_g2	hypothetical protein POPTR_0002s18010g [Populus trichocarpa]	RPP1A	60S acidic ribosomal protein P1 OS=Zea mays GN=RPP1A PE=1 SV=1	896.78	480.46	1.54	up	0.00	0.00	yes
TRINITY_DN17576_c0_g2	hypothetical protein POPTR_0003s19210g [Populus trichocarpa]	-	-	461.42	182.56	1.93	up	0.00	0.00	yes
TRINITY_DN25837_c0_g1	hypothetical protein POPTR_0010s05630g [Populus trichocarpa]	PHT2-1	Inorganic phosphate transporter 2-1, chloroplastic OS=Arabidopsis thaliana GN=PHT2-1 PE=1 SV=1	85.46	33.95	1.86	up	0.00	0.00	yes
TRINITY_DN19344_c0_g1	hypothetical protein POPTR_0002s23170g [Populus trichocarpa]	Dml	2,3-dimethylmalate lyase OS=Eubacterium barkeri GN=Dml PE=1 SV=1	12.52	6.93	1.48	up	0.00	0.00	yes
TRINITY_DN27051_c1_g3	ethylene receptor family protein [Populus trichocarpa]	-	-	2.20	0.19	4.06	up	0.00	0.00	yes
TRINITY_DN21285_c0_g2	hypothetical protein POPTR_0005s12280g [Populus trichocarpa]	GAMMACA1	Gamma carbonic anhydrase 1, mitochondrial OS=Arabidopsis thaliana GN=GAMMACA1 PE=1 SV=1	36.31	20.91	1.41	up	0.00	0.00	yes
TRINITY_DN26070_c0_g2	PREDICTED: glutamyl-tRNA(Gln) amidotransferase subunit B, chloroplastic/mitochondrial [Populus euphratica]	GATB	Glutamyl-tRNA(Gln) amidotransferase subunit B, chloroplastic/mitochondrial OS=Ricinus communis GN=GATB PE=3 SV=1	68.27	38.35	1.53	up	0.00	0.00	yes
TRINITY_DN23393_c0_g1	hypothetical protein POPTR_0001s23230g [Populus trichocarpa]	ISPE	4-diphosphocytidyl-2-C-methyl-D-erythritol kinase, chloroplastic/chromoplastic (Fragment) OS=Solanum lycopersicum GN=ISPE PE=1 SV=1	61.36	32.17	1.53	up	0.00	0.00	yes
TRINITY_DN17143_c0_g1	unknown [Populus trichocarpa x Populus deltoides]	-	-	264.27	75.39	2.38	up	0.00	0.00	yes
TRINITY_DN14098_c0_g1	glycine-rich family protein [Populus trichocarpa]	-	-	77.05	40.90	1.51	up	0.00	0.00	yes
TRINITY_DN22643_c0_g1	unknown [Populus trichocarpa]	-	-	73.71	37.50	1.59	up	0.00	0.00	yes
TRINITY_DN2195_c0_g1	-	-	-	2.46	0.00	7.37	up	0.00	0.00	yes
TRINITY_DN16821_c0_g1	PREDICTED: malate dehydrogenase, glyoxysomal [Populus euphratica]	-	Malate dehydrogenase, glyoxysomal OS=Citrullus lanatus PE=1 SV=1	248.44	104.05	1.91	up	0.00	0.00	yes
TRINITY_DN21342_c0_g1	hypothetical protein POPTR_0001s33940g [Populus trichocarpa]	pc1998	Uncharacterized RNA methyltransferase pc1998 OS=Protochlamydia amoebophila (strain UWE25) GN=pc1998 PE=3 SV=1	53.00	30.98	1.46	up	0.00	0.00	yes

TRINITY_DN19526_c0_g1	PREDICTED: uncharacterized protein LOC105126073 [Populus euphratica]	-	-	92.54	46.43	1.62	up	0.00	0.00	yes
TRINITY_DN16853_c0_g1	chromoplast-specific carotenoid-associated protein CHRC [Populus trichocarpa]	PAP	Plastid-lipid-associated protein, chloroplastic OS=Citrus unshiu GN=PAP PE=2 SV=1	49.47	24.65	1.63	up	0.00	0.00	yes
TRINITY_DN25221_c0_g1	malate dehydrogenase family protein, partial [Populus trichocarpa]	PMDH1	Malate dehydrogenase 1, peroxisomal OS=Arabidopsis thaliana GN=PMDH1 PE=2 SV=1	57.58	29.35	1.58	up	0.00	0.00	yes
TRINITY_DN19110_c1_g1	PREDICTED: uncharacterized protein LOC105126081 [Populus euphratica]	-	-	126.29	52.45	1.89	up	0.00	0.00	yes
TRINITY_DN22960_c1_g2	hypothetical protein POPTR_0013s13150g [Populus trichocarpa]	-	Cysteine synthase, chloroplastic/chromoplastic OS=Solanum tuberosum PE=2 SV=1	267.00	123.84	1.71	up	0.00	0.00	yes
TRINITY_DN22595_c0_g2	PREDICTED: phospho-2-dehydro-3-deoxyheptonate aldolase 2, chloroplastic-like [Populus euphratica]	SHKA	Phospho-2-dehydro-3-deoxyheptonate aldolase 1, chloroplastic OS=Solanum tuberosum GN=SHKA PE=1 SV=2	137.27	72.08	1.52	up	0.00	0.00	yes
TRINITY_DN26270_c0_g1	PREDICTED: RNA polymerase sigma factor sigB-like [Populus euphratica]	SIGB	RNA polymerase sigma factor sigB OS=Arabidopsis thaliana GN=SIGB PE=2 SV=2	177.29	82.45	1.70	up	0.00	0.00	yes
TRINITY_DN18586_c0_g1	ADR12-2 family protein [Populus trichocarpa]	EF1A	Elongation factor 1-alpha OS=Zea mays GN=EF1A PE=3 SV=1	572.07	278.84	1.64	up	0.00	0.00	yes
TRINITY_DN20976_c0_g1	Casbene synthase, chloroplast precursor, putative [Ricinus communis]	RCOM_1574350	Casbene synthase, chloroplastic OS=Ricinus communis GN=RCOM_1574350 PE=1 SV=1	279.70	115.31	1.86	up	0.00	0.00	yes
TRINITY_DN20828_c0_g2	Magnesium-chelatase subunit chlI family protein [Populus trichocarpa]	CHLI	Magnesium-chelatase subunit ChlI, chloroplastic OS=Glycine max GN=CHLI PE=2 SV=1	346.30	158.86	1.71	up	0.00	0.00	yes
TRINITY_DN19664_c0_g1	PREDICTED: 15-cis-phytoene desaturase, chloroplastic/chromoplastic-like [Populus euphratica]	-	-	48.42	17.78	2.04	up	0.00	0.00	yes
TRINITY_DN16077_c0_g2	40S ribosomal protein S14-3 [Populus trichocarpa]	-	40S ribosomal protein S14 OS=Zea mays PE=3 SV=1	78.32	44.68	1.43	up	0.00	0.00	yes
TRINITY_DN15716_c0_g1	hypothetical protein POPTR_0005s25470g [Populus trichocarpa]	-	-	6.35	0.00	8.08	up	0.00	0.00	yes
TRINITY_DN19303_c0_g5	hypothetical protein POPTR_0015s10490g [Populus trichocarpa]	ADK-B	Adenylate kinase 4 OS=Oryza sativa subsp. japonica GN=ADK-B PE=2 SV=1	98.70	59.24	1.33	up	0.00	0.00	yes
TRINITY_DN15107_c0_g1	hypothetical protein POPTR_0019s13590g [Populus trichocarpa]	-	-	96.55	57.40	1.35	up	0.00	0.00	yes
TRINITY_DN23603_c0_g3	hypothetical protein POPTR_0003s21140g [Populus trichocarpa]	-	-	18.14	8.27	1.72	up	0.00	0.00	yes
TRINITY_DN22886_c0_g3	-	-	-	83.63	44.51	1.52	up	0.00	0.00	yes
TRINITY_DN22402_c0_g1	PREDICTED: uncharacterized protein LOC105122445 [Populus euphratica]	SPAC24B11.05	Uncharacterized protein C24B11.05 OS=Schizosaccharomyces pombe (strain 972 / ATCC 24843) GN=SPAC24B11.05 PE=3 SV=1	68.48	38.40	1.40	up	0.00	0.00	yes
TRINITY_DN24253_c0_g4	PREDICTED: cysteine-rich receptor-like protein kinase 15 [Populus euphratica]	CRK25	Cysteine-rich receptor-like protein kinase 25 OS=Arabidopsis thaliana GN=CRK25 PE=3 SV=1	14.41	7.37	1.59	up	0.00	0.00	yes
TRINITY_DN25847_c1_g4	-	-	-	563.10	196.68	2.11	up	0.00	0.00	yes
TRINITY_DN22699_c1_g2	-	-	-	7.46	0.72	3.85	up	0.00	0.00	yes
TRINITY_DN19954_c0_g3	hypothetical protein POPTR_0002s25230g [Populus trichocarpa]	-	-	18.19	6.39	2.12	up	0.00	0.00	yes
TRINITY_DN17049_c0_g1	hypothetical protein POPTR_0002s13020g [Populus trichocarpa]	MIZ1	Protein MIZU-KUSSEI 1 OS=Arabidopsis thaliana GN=MIZ1 PE=1 SV=1	23.26	8.93	2.02	up	0.00	0.00	yes
TRINITY_DN23137_c0_g2	hypothetical protein POPTR_0010s18770g [Populus trichocarpa]	PGLP1A	Phosphoglycolate phosphatase 1A, chloroplastic OS=Arabidopsis thaliana GN=PGLP1A PE=1 SV=1	270.10	104.80	1.94	up	0.00	0.00	yes
TRINITY_DN22550_c0_g2	hypothetical protein CISIN_1g028863mg [Citrus sinensis]	-	-	421.22	131.96	2.26	up	0.00	0.00	yes
TRINITY_DN26150_c0_g1	unknown [Populus trichocarpa x Populus deltoides]	Cacybp	Calcyclin-binding protein OS=Rattus norvegicus GN=Cacybp PE=1 SV=1	32.43	15.87	1.68	up	0.00	0.00	yes
TRINITY_DN20717_c0_g2	hypothetical protein EUGRSUZ_H02959 [Eucalyptus grandis]	RPL23A	60S ribosomal protein L23 OS=Arabidopsis thaliana GN=RPL23A PE=2 SV=3	563.81	320.33	1.47	up	0.00	0.00	yes
TRINITY_DN18289_c0_g1	unknown [Populus trichocarpa]	ABP19A	Auxin-binding protein ABP19a OS=Prunus persica GN=ABP19A PE=3 SV=1	1312.41	299.48	2.73	up	0.00	0.00	yes
TRINITY_DN22534_c1_g2	PREDICTED: biotin carboxyl carrier protein of acetyl-CoA carboxylase 1, chloroplastic isoform X1 [Populus euphratica]	-	-	51.66	27.12	1.77	up	0.00	0.00	yes

TRINITY_DN24829_c1_g1	PREDICTED: chloroplast stem-loop binding protein of 41 kDa a, chloroplastic [Populus euphratica]	CSP41A	Chloroplast stem-loop binding protein of 41 kDa a, chloroplastic OS=Arabidopsis thaliana GN=CSP41A PE=1 SV=1	438.06	191.23	1.85	up	0.00	0.00	yes
TRINITY_DN22866_c0_g1	PREDICTED: mitochondrial-processing peptidase subunit alpha-like [Populus euphratica]	MPP	Mitochondrial-processing peptidase subunit alpha OS=Solanum tuberosum GN=MPP PE=1 SV=1	54.03	31.47	1.39	up	0.00	0.00	yes
TRINITY_DN19464_c0_g1	hypothetical protein POPTR_0005s08780g [Populus trichocarpa]	-	-	19.17	11.15	1.40	up	0.00	0.00	yes
TRINITY_DN21497_c0_g2	PREDICTED: sorbitol dehydrogenase [Populus euphratica]	SDH	Sorbitol dehydrogenase OS=Arabidopsis thaliana GN=SDH PE=1 SV=1	81.71	34.89	1.83	up	0.00	0.00	yes
TRINITY_DN14006_c0_g1	hypothetical protein POPTR_0001s31480g [Populus trichocarpa]	-	-	2.24	0.25	3.73	up	0.00	0.00	yes
TRINITY_DN19962_c1_g1	PREDICTED: uncharacterized protein LOC105129096 [Populus euphratica]	-	-	98.78	45.05	1.76	up	0.00	0.00	yes
TRINITY_DN22890_c1_g3	hypothetical protein POPTR_0001s46280g [Populus trichocarpa]	IJ	Protein Iojap, chloroplastic OS=Arabidopsis thaliana GN=IJ PE=2 SV=1	64.51	34.08	1.55	up	0.00	0.00	yes
TRINITY_DN19725_c1_g1	hypothetical protein POPTR_0014s18440g [Populus trichocarpa]	-	-	29.48	17.81	1.37	up	0.00	0.00	yes
TRINITY_DN23143_c1_g1	-	-	-	39.65	11.43	2.42	up	0.00	0.00	yes
TRINITY_DN22597_c0_g5	PREDICTED: purple acid phosphatase 4-like [Populus euphratica]	PAP3	Purple acid phosphatase 3 OS=Arabidopsis thaliana GN=PAP3 PE=2 SV=1	7.24	0.95	3.51	up	0.00	0.00	yes
TRINITY_DN27867_c4_g4	ATP synthase CF1 alpha chain [Populus alba]	atpA	ATP synthase subunit alpha, chloroplastic OS=Populus alba GN=atpA PE=3 SV=1	41.58	18.74	1.77	up	0.00	0.00	yes
TRINITY_DN17583_c0_g4	succinate dehydrogenase subunit 4 family protein [Populus trichocarpa]	At2g39795	Uncharacterized protein At2g39795, mitochondrial OS=Arabidopsis thaliana GN=At2g39795 PE=1 SV=1	29.20	13.25	1.79	up	0.00	0.00	yes
TRINITY_DN17679_c0_g2	PREDICTED: uncharacterized protein LOC105112706 [Populus euphratica]	RNC1	Ribonuclease III domain-containing protein RNC1, chloroplastic OS=Arabidopsis thaliana GN=RNC1 PE=2 SV=1	101.48	54.47	1.50	up	0.00	0.00	yes
TRINITY_DN23590_c0_g1	hypothetical protein POPTR_0016s05100g [Populus trichocarpa]	BHLH130	Transcription factor bHLH130 OS=Arabidopsis thaliana GN=BHLH130 PE=1 SV=1	10.37	4.31	2.13	up	0.00	0.00	yes
TRINITY_DN15062_c0_g2	hypothetical protein POPTR_0011s14460g [Populus trichocarpa]	At3g15140	Uncharacterized exonuclease domain-containing protein At3g15140 OS=Arabidopsis thaliana GN=At3g15140 PE=2 SV=1	39.60	21.17	1.51	up	0.00	0.00	yes
TRINITY_DN25260_c0_g3	glutamate-ammonia ligase family protein [Populus trichocarpa]	GS1-1	Glutamine synthetase cytosolic isozyme 1 OS=Vitis vinifera GN=GS1-1 PE=2 SV=1	11.97	1.40	3.71	up	0.00	0.00	yes
TRINITY_DN21314_c0_g1	hypothetical protein POPTR_0009s05470g [Populus trichocarpa]	-	-	52.04	25.82	1.62	up	0.00	0.00	yes
TRINITY_DN20583_c0_g3	hypothetical protein POPTR_0008s03820g [Populus trichocarpa]	SYT3	Synaptotagmin-3 OS=Arabidopsis thaliana GN=SYT3 PE=2 SV=1	4.09	1.88	2.29	up	0.00	0.00	yes
TRINITY_DN27292_c1_g2	PREDICTED: uncharacterized protein LOC105110257 [Populus euphratica]	SODB	Superoxide dismutase [Fe], chloroplastic (Fragment) OS=Nicotiana plumbaginifolia GN=SODB PE=2 SV=1	260.63	93.48	2.05	up	0.00	0.00	yes
TRINITY_DN18777_c0_g1	hypothetical protein POPTR_0005s11410g [Populus trichocarpa]	MYB308	Myb-related protein 308 OS=Antirrhinum majus GN=MYB308 PE=2 SV=1	33.53	12.89	1.82	up	0.00	0.00	yes
TRINITY_DN26342_c0_g3	-	-	-	105.43	36.68	2.24	up	0.00	0.00	yes
TRINITY_DN25178_c0_g1	60S ribosomal protein L18a-1 [Populus trichocarpa]	RPL18A	60S ribosomal protein L18a OS=Castanea sativa GN=RPL18A PE=2 SV=1	1204.98	705.71	1.45	up	0.00	0.00	yes
TRINITY_DN19761_c0_g1	PREDICTED: uncharacterized protein LOC105121461 [Populus euphratica]	-	-	46.47	23.81	1.54	up	0.00	0.00	yes
TRINITY_DN23720_c0_g1	PREDICTED: malate dehydrogenase [NADP], chloroplastic [Populus euphratica]	MDH1	Malate dehydrogenase [NADP], chloroplastic OS=Medicago sativa GN=MDH1 PE=2 SV=1	252.27	131.23	1.57	up	0.00	0.00	yes
TRINITY_DN20200_c0_g2	hypothetical protein POPTR_0008s11270g [Populus trichocarpa]	SNAT2	Serotonin N-acetyltransferase 2, chloroplastic OS=Oryza sativa subsp. japonica GN=SNAT2 PE=1 SV=1	62.39	18.96	2.33	up	0.00	0.00	yes
TRINITY_DN26344_c0_g7	hypothetical protein POPTR_0003s19425g [Populus trichocarpa]	KEG	E3 ubiquitin-protein ligase KEG OS=Arabidopsis thaliana GN=KEG PE=1 SV=2	4.77	1.04	2.84	up	0.00	0.00	yes
TRINITY_DN22318_c0_g1	PREDICTED: uncharacterized protein LOC105135002 [Populus euphratica]	EDA3	Protein EMBRYO SAC DEVELOPMENT ARREST 3, chloroplastic OS=Arabidopsis thaliana GN=EDA3 PE=1 SV=1	127.95	48.78	2.02	up	0.00	0.00	yes
TRINITY_DN22577_c0_g2	PREDICTED: PH domain-containing protein DDB_G0287875 [Populus euphratica]	OHP2	Light-harvesting complex-like protein OHP2, chloroplastic OS=Arabidopsis thaliana GN=OHP2 PE=2 SV=1	204.89	85.27	1.87	up	0.00	0.00	yes
TRINITY_DN19425_c0_g1	PREDICTED: uncharacterized protein LOC105127687 [Populus euphratica]	-	-	40.31	18.42	1.70	up	0.00	0.00	yes

TRINITY_DN15714_c0_g1	hypothetical protein POPTR_0009s10790g [Populus trichocarpa]	HIDM	2-hydroxyisoflavanone dehydratase OS=Glycyrrhiza echinata GN=HIDM PE=1 SV=1	40.95	24.47	1.33	up	0.00	0.00	yes
TRINITY_DN19084_c1_g7	hypothetical protein POPTR_0010s19380g [Populus trichocarpa]	-	-	1039.66	558.76	1.53	up	0.00	0.00	yes
TRINITY_DN22930_c0_g1	PREDICTED: uncharacterized protein LOC105111959 isoform X2 [Populus euphratica]	-	-	35.76	20.03	1.43	up	0.00	0.00	yes
TRINITY_DN24224_c0_g1	transducin family protein [Populus trichocarpa]	-	-	32.93	18.89	1.43	up	0.00	0.00	yes
TRINITY_DN22598_c1_g1	2 family protein [Populus trichocarpa]	-	2,3-bisphosphoglycerate-independent phosphoglycerate mutase OS=Ricinus communis PE=1 SV=2	136.33	77.98	1.42	up	0.00	0.00	yes
TRINITY_DN18671_c0_g7	PREDICTED: nephrocystin-3 isoform X2 [Populus euphratica]	-	-	14.57	6.97	1.69	up	0.00	0.00	yes
TRINITY_DN20946_c0_g1	ADP/ATP carrier 1 [Arabidopsis thaliana]	AAC1	ADP,ATP carrier protein 1, mitochondrial OS=Arabidopsis thaliana GN=AAC1 PE=1 SV=2	134.29	72.83	1.58	up	0.00	0.00	yes
TRINITY_DN21538_c0_g5	plastid-lipid associated protein PAP [Populus trichocarpa]	PAP6	Probable plastid-lipid-associated protein 6, chloroplastic OS=Arabidopsis thaliana GN=PAP6 PE=1 SV=1	102.15	54.74	1.50	up	0.00	0.00	yes
TRINITY_DN22430_c0_g4	hypothetical protein POPTR_0001s19990g [Populus trichocarpa]	CT0009	Uncharacterized RNA methyltransferase CT0009 OS=Chlorobium tepidum (strain ATCC 49652 / DSM 12025 / NBRC 103806 / TLS) GN=CT0009 PE=3 SV=1	52.61	30.59	1.44	up	0.00	0.00	yes
TRINITY_DN25476_c0_g4	PREDICTED: alanine aminotransferase 2-like [Populus euphratica]	ALAAT2	Alanine aminotransferase 2, mitochondrial OS=Arabidopsis thaliana GN=ALAAT2 PE=2 SV=1	21.79	7.01	2.24	up	0.00	0.00	yes
TRINITY_DN24609_c1_g3	PREDICTED: uncharacterized protein LOC105127188 [Populus euphratica]	-	-	26.52	14.26	1.50	up	0.00	0.00	yes
TRINITY_DN26030_c0_g1	unknown [Populus trichocarpa x Populus deltoides]	GAPA1	Glyceraldehyde-3-phosphate dehydrogenase GAPA1, chloroplastic OS=Arabidopsis thaliana GN=GAPA1 PE=1 SV=3	3041.90	1125.79	2.06	up	0.00	0.00	yes
TRINITY_DN22987_c1_g2	PREDICTED: nicotinate-nucleotide pyrophosphorylase [carboxylating], chloroplastic isoform X1 [Populus euphratica]	QPT	Nicotinate-nucleotide pyrophosphorylase [carboxylating], chloroplastic OS=Arabidopsis thaliana GN=QPT PE=2 SV=2	36.91	20.98	1.39	up	0.00	0.00	yes
TRINITY_DN22786_c0_g1	PREDICTED: NADPH-dependent thioredoxin reductase 3 [Populus euphratica]	NTRC	NADPH-dependent thioredoxin reductase 3 OS=Arabidopsis thaliana GN=NTRC PE=1 SV=2	86.21	44.16	1.68	up	0.00	0.00	yes
TRINITY_DN19266_c0_g1	PREDICTED: eukaryotic translation initiation factor isoform 4E-2-like [Populus euphratica]	-	Eukaryotic translation initiation factor isoform 4E-2 OS=Triticum aestivum PE=1 SV=1	65.37	36.78	1.63	up	0.00	0.00	yes
TRINITY_DN20356_c0_g1	hypothetical protein POPTR_0013s11720g [Populus trichocarpa]	RPL21	50S ribosomal protein L21, chloroplastic OS=Arabidopsis thaliana GN=RPL21 PE=2 SV=1	846.83	399.04	1.68	up	0.00	0.00	yes
TRINITY_DN26999_c0_g1	UDP-glucose pyrophosphorylase [Populus tremula x Populus tremuloides]	UGPA	UTP--glucose-1-phosphate uridylyltransferase OS=Musa acuminata GN=UGPA PE=2 SV=1	112.13	68.32	1.40	up	0.00	0.00	yes
TRINITY_DN28049_c0_g1	hypothetical protein POPTR_0001s40520g [Populus trichocarpa]	-	-	4.49	0.65	3.44	up	0.00	0.00	yes
TRINITY_DN21577_c0_g1	hypothetical protein POPTR_0012s03600g [Populus trichocarpa]	Os02g0773300	Putative D-cysteine desulfhydrase 1, mitochondrial OS=Oryza sativa subsp. japonica GN=Os02g0773300 PE=2 SV=2	43.49	26.44	1.40	up	0.00	0.00	yes
TRINITY_DN21625_c0_g2	hypothetical protein POPTR_0004s18610g [Populus trichocarpa]	GGR	Heterodimeric geranylgeranyl pyrophosphate synthase small subunit, chloroplastic OS=Arabidopsis thaliana GN=GGR PE=1 SV=2	84.37	30.74	2.04	up	0.00	0.00	yes
TRINITY_DN14033_c0_g2	-	-	-	5.61	0.51	4.03	up	0.00	0.00	yes
TRINITY_DN27918_c0_g1	hypothetical protein POPTR_0011s12350g [Populus trichocarpa]	-	-	3.65	0.85	2.70	up	0.00	0.00	yes
TRINITY_DN22590_c0_g1	hypothetical protein POPTR_0002s07330g [Populus trichocarpa]	PPD4	PsbP domain-containing protein 4, chloroplastic OS=Arabidopsis thaliana GN=PPD4 PE=1 SV=2	64.82	24.57	1.84	up	0.00	0.00	yes
TRINITY_DN12676_c0_g1	hypothetical protein POPTR_0007s09770g [Populus trichocarpa]	RPL26A	60S ribosomal protein L26-1 OS=Arabidopsis thaliana GN=RPL26A PE=2 SV=2	148.95	83.79	1.45	up	0.00	0.00	yes
TRINITY_DN25044_c0_g7	hypothetical protein POPTR_0015s00850g [Populus trichocarpa]	CYP89A9	Cytochrome P450 89A9 OS=Arabidopsis thaliana GN=CYP89A9 PE=2 SV=1	6.97	0.68	3.98	up	0.00	0.00	yes
TRINITY_DN19720_c0_g1	PREDICTED: probable inactive purple acid phosphatase 27 [Populus euphratica]	PAP27	Probable inactive purple acid phosphatase 27 OS=Arabidopsis thaliana GN=PAP27 PE=2 SV=1	25.08	14.37	1.45	up	0.00	0.00	yes
TRINITY_DN21964_c0_g2	1-deoxy-D-xylulose 5-phosphate reductoisomerase family protein [Populus trichocarpa]	DXR	1-deoxy-D-xylulose 5-phosphate reductoisomerase, chloroplastic OS=Arabidopsis thaliana GN=DXR PE=2 SV=2	40.78	19.91	1.64	up	0.00	0.00	yes

TRINITY_DN20067_c0_g1	hypothetical protein POPTR_0016s14310g [Populus trichocarpa]	At1g23740	Quinone oxidoreductase-like protein At1g23740, chloroplastic OS=Arabidopsis thaliana GN=At1g23740 PE=2 SV=2	360.20	150.22	1.89	up	0.00	0.00	yes
TRINITY_DN25625_c0_g1	PREDICTED: amidophosphoribosyltransferase, chloroplastic [Populus euphratica]	ASE2	Amidophosphoribosyltransferase 2, chloroplastic OS=Arabidopsis thaliana GN=ASE2 PE=1 SV=1	27.38	16.32	1.41	up	0.00	0.00	yes
TRINITY_DN21166_c0_g5	hypothetical protein POPTR_0008s04560g [Populus trichocarpa]	RPL10	50S ribosomal protein L10, chloroplastic OS=Nicotiana tabacum GN=RPL10 PE=2 SV=1	105.61	45.65	1.79	up	0.00	0.00	yes
TRINITY_DN23627_c1_g1	PREDICTED: cytochrome c [Pyrus x bretschneideri]	-	Cytochrome c OS=Triticum aestivum PE=1 SV=1	203.27	116.83	1.42	up	0.00	0.00	yes
TRINITY_DN16986_c0_g1	PREDICTED: uncharacterized protein LOC105107329 isoform X1 [Populus euphratica]	prfB	Peptide chain release factor 2 OS=Campylobacter concisus (strain 13826) GN=prfB PE=3 SV=1	13.50	6.79	1.60	up	0.00	0.00	yes
TRINITY_DN20864_c0_g1	hypothetical protein POPTR_0007s07780g [Populus trichocarpa]	HCF136	Photosystem II stability/assembly factor HCF136, chloroplastic OS=Arabidopsis thaliana GN=HCF136 PE=1 SV=1	251.83	109.64	1.79	up	0.00	0.00	yes
TRINITY_DN23182_c1_g2	hypothetical protein POPTR_0005s10340g [Populus trichocarpa]	CAT1	Catalase isozyme 1 OS=Gossypium hirsutum GN=CAT1 PE=2 SV=2	182.43	38.37	2.86	up	0.00	0.00	yes
TRINITY_DN23090_c0_g2	hypothetical protein POPTR_0012s05430g [Populus trichocarpa]	CPP1	Protein CHAPERONE-LIKE PROTEIN OF POR1, chloroplastic OS=Arabidopsis thaliana GN=CPP1 PE=1 SV=1	46.68	22.45	1.75	up	0.00	0.00	yes
TRINITY_DN16560_c0_g2	PREDICTED: ATP-dependent Clp protease proteolytic subunit 4, chloroplastic-like [Populus euphratica]	CLPP4	ATP-dependent Clp protease proteolytic subunit 4, chloroplastic OS=Arabidopsis thaliana GN=CLPP4 PE=1 SV=1	259.45	131.81	1.57	up	0.00	0.00	yes
TRINITY_DN16472_c0_g1	hypothetical protein POPTR_0001s11420g [Populus trichocarpa]	D27	Beta-carotene isomerase D27, chloroplastic OS=Arabidopsis thaliana GN=D27 PE=1 SV=1	105.66	45.83	1.81	up	0.00	0.00	yes
TRINITY_DN27565_c0_g1	PREDICTED: presequence protease 1, chloroplastic/mitochondrial [Populus euphratica]	PREP1	Presequence protease 1, chloroplastic/mitochondrial OS=Arabidopsis thaliana GN=PREP1 PE=1 SV=2	107.38	62.69	1.47	up	0.00	0.00	yes
TRINITY_DN27865_c1_g3	ferredoxin-dependent glutamate synthase family protein [Populus trichocarpa]	FdGOGAT	Ferredoxin-dependent glutamate synthase, chloroplastic OS=Spinacia oleracea GN=FdGOGAT PE=1 SV=3	183.61	86.98	1.68	up	0.00	0.00	yes
TRINITY_DN26499_c0_g1	PREDICTED: dnaJ homolog 1, mitochondrial-like [Populus euphratica]	DJA6	Chaperone protein dnaJ A6, chloroplastic OS=Arabidopsis thaliana GN=DJA6 PE=2 SV=2	172.38	86.88	1.57	up	0.00	0.00	yes
TRINITY_DN21980_c0_g1	60S ribosomal protein L18 [Populus trichocarpa]	RPL18B	60S ribosomal protein L18-2 OS=Arabidopsis thaliana GN=RPL18B PE=1 SV=2	854.12	474.94	1.48	up	0.00	0.00	yes
TRINITY_DN27568_c1_g1	phosphoribosylformylglycinamidine synthase family protein [Populus trichocarpa]	At1g74260	Probable phosphoribosylformylglycinamidine synthase, chloroplastic/mitochondrial OS=Arabidopsis thaliana GN=At1g74260 PE=2 SV=3	25.96	15.77	1.34	up	0.00	0.00	yes
TRINITY_DN23760_c1_g3	-	-	-	12.64	1.87	3.37	up	0.00	0.00	yes
TRINITY_DN21795_c0_g1	PREDICTED: cytochrome c biogenesis protein CCS1, chloroplastic [Populus euphratica]	CCS1	Cytochrome c biogenesis protein CCS1, chloroplastic OS=Arabidopsis thaliana GN=CCS1 PE=1 SV=1	45.14	23.11	1.57	up	0.00	0.00	yes
TRINITY_DN16465_c0_g3	40S ribosomal protein S17 [Populus trichocarpa]	RPS17D	40S ribosomal protein S17-4 OS=Arabidopsis thaliana GN=RPS17D PE=2 SV=3	766.31	422.49	1.47	up	0.00	0.00	yes
TRINITY_DN16817_c0_g1	PREDICTED: tRNA (guanine-N(7)-)-methyltransferase [Populus euphratica]	trmB	tRNA (guanine-N(7)-)-methyltransferase OS=Synechococcus sp. (strain JA-2-3B'a(2-13)) GN=trmB PE=3 SV=1	40.30	24.24	1.64	up	0.00	0.00	yes
TRINITY_DN15269_c0_g1	PREDICTED: ribosome biogenesis regulatory protein homolog [Populus euphratica]	At2g37990	Ribosome biogenesis regulatory protein homolog OS=Arabidopsis thaliana GN=At2g37990 PE=2 SV=2	60.06	31.56	1.47	up	0.00	0.00	yes
TRINITY_DN20178_c0_g1	PREDICTED: protein TRI1-like [Populus euphratica]	TRI1	Protein TRI1 OS=Saccharomyces cerevisiae (strain ATCC 204508 / S288c) GN=TRI1 PE=1 SV=1	171.22	82.66	1.65	up	0.00	0.00	yes
TRINITY_DN20507_c1_g5	60S ribosomal protein L6 [Populus trichocarpa]	RPL6	60S ribosomal protein L6 OS=Mesembryanthemum crystallinum GN=RPL6 PE=2 SV=1	941.62	558.85	1.43	up	0.00	0.00	yes
TRINITY_DN26284_c1_g3	hypothetical protein POPTR_0008s07890g [Populus trichocarpa]	HEXO3	Beta-hexosaminidase 3 OS=Arabidopsis thaliana GN=HEXO3 PE=1 SV=1	53.04	28.53	1.51	up	0.00	0.00	yes
TRINITY_DN17768_c0_g1	hypothetical protein POPTR_0009s17070g [Populus trichocarpa]	OVA3	Glutamate--tRNA ligase, chloroplastic/mitochondrial OS=Arabidopsis thaliana GN=OVA3 PE=1 SV=1	67.70	36.29	1.55	up	0.00	0.00	yes
TRINITY_DN18248_c0_g1	NADH-ubiquinone oxidoreductase 23 kDa subunit family protein [Populus trichocarpa]	At1g16700	NADH dehydrogenase [ubiquinone] iron-sulfur protein 8-B, mitochondrial OS=Arabidopsis thaliana GN=At1g16700 PE=1 SV=1	201.65	117.26	1.39	up	0.00	0.00	yes
TRINITY_DN19096_c0_g1	pop3 peptide family protein [Populus trichocarpa]	-	-	2577.88	1276.88	1.61	up	0.00	0.00	yes
TRINITY_DN21769_c2_g4	PREDICTED: oxygen-dependent coproporphyrinogen-III oxidase, chloroplastic [Populus euphratica]	CPX	Oxygen-dependent coproporphyrinogen-III oxidase, chloroplastic OS=Glycine max GN=CPX PE=2 SV=1	163.93	82.30	1.62	up	0.00	0.00	yes

TRINITY_DN22830_c0_g2	endopeptidase Clp family protein [Populus trichocarpa]	CLPB1	Chaperone protein ClpB1 OS=Arabidopsis thaliana GN=CLPB1 PE=1 SV=2	18.89	9.89	1.54	up	0.00	0.00	yes
TRINITY_DN15049_c0_g1	hypothetical protein POPTR_0008s16060g [Populus trichocarpa]	At5g43745	Putative ion channel POLLUX-like 2 OS=Arabidopsis thaliana GN=At5g43745 PE=2 SV=1	38.14	13.22	2.08	up	0.00	0.00	yes
TRINITY_DN22754_c0_g1	hypothetical protein POPTR_0005s24170g [Populus trichocarpa]	At1g66430	Probable fructokinase-6, chloroplastic OS=Arabidopsis thaliana GN=At1g66430 PE=2 SV=1	22.62	13.77	1.32	up	0.00	0.00	yes
TRINITY_DN16947_c0_g1	hypothetical protein POPTR_0010s04230g [Populus trichocarpa]	At3g06035	Uncharacterized GPI-anchored protein At3g06035 OS=Arabidopsis thaliana GN=At3g06035 PE=2 SV=1	45.36	27.43	1.33	up	0.00	0.00	yes
TRINITY_DN18276_c0_g1	PREDICTED: uncharacterized protein LOC105141657 [Populus euphratica]	-	-	55.85	27.16	1.70	up	0.00	0.00	yes
TRINITY_DN20734_c0_g1	hypothetical protein POPTR_0019s05610g [Populus trichocarpa]	CAT2	Cationic amino acid transporter 2, vacuolar OS=Arabidopsis thaliana GN=CAT2 PE=2 SV=1	25.35	14.54	1.47	up	0.00	0.00	yes
TRINITY_DN22077_c0_g2	PROHIBITIN 2 family protein [Populus trichocarpa]	PHB1	Prohibitin-1, mitochondrial OS=Arabidopsis thaliana GN=PHB1 PE=1 SV=1	156.98	88.14	1.46	up	0.00	0.00	yes
TRINITY_DN20874_c0_g1	pseudouridine synthase family protein [Populus trichocarpa]	At4g21770	RNA pseudouridine synthase 6, chloroplastic OS=Arabidopsis thaliana GN=At4g21770 PE=2 SV=1	34.59	19.20	1.48	up	0.00	0.00	yes
TRINITY_DN20494_c2_g5	PREDICTED: uncharacterized protein LOC105124247 [Populus euphratica]	-	-	92.00	36.02	1.94	up	0.00	0.00	yes
TRINITY_DN24550_c0_g2	pfkB-type carbohydrate kinase family protein [Populus trichocarpa]	At1g66430	Probable fructokinase-6, chloroplastic OS=Arabidopsis thaliana GN=At1g66430 PE=2 SV=1	100.10	51.39	1.52	up	0.00	0.00	yes
TRINITY_DN18455_c0_g1	hypothetical protein POPTR_0014s03350g [Populus trichocarpa]	HO2	Probable inactive heme oxygenase 2, chloroplastic OS=Arabidopsis thaliana GN=HO2 PE=2 SV=2	85.73	50.18	1.40	up	0.00	0.00	yes
TRINITY_DN19234_c0_g1	unknown [Populus trichocarpa]	GAMMACAL2	Gamma carbonic anhydrase-like 2, mitochondrial OS=Arabidopsis thaliana GN=GAMMACAL2 PE=1 SV=1	86.40	52.09	1.37	up	0.00	0.00	yes
TRINITY_DN26348_c0_g1	hypothetical protein POPTR_0006s01160g [Populus trichocarpa]	UGT71K1	UDP-glycosyltransferase 71K1 OS=Malus domestica GN=UGT71K1 PE=1 SV=1	31.96	24.17	1.46	up	0.00	0.00	yes
TRINITY_DN25783_c0_g2	PREDICTED: plastocyanin B, chloroplastic [Populus euphratica]	PETE	Plastocyanin B, chloroplastic OS=Populus nigra GN=PETE PE=1 SV=2	3485.86	1020.60	2.32	up	0.00	0.00	yes
TRINITY_DN21108_c0_g3	PREDICTED: 40S ribosomal protein S26-3-like [Populus euphratica]	RPS26C	40S ribosomal protein S26-3 OS=Arabidopsis thaliana GN=RPS26C PE=2 SV=1	675.14	389.54	1.41	up	0.00	0.00	yes
TRINITY_DN23901_c1_g2	PREDICTED: ferredoxin-3, chloroplastic-like isoform X1 [Populus euphratica]	FDX3	Ferredoxin-3, chloroplastic OS=Zea mays GN=FDX3 PE=2 SV=1	32.09	19.85	1.64	up	0.00	0.00	yes
TRINITY_DN15618_c0_g1	hypothetical protein POPTR_0013s03450g [Populus trichocarpa]	-	-	6.70	1.82	2.59	up	0.00	0.00	yes
TRINITY_DN18250_c0_g1	hypothetical protein POPTR_0013s02080g [Populus trichocarpa]	ZED1	Non-functional pseudokinase ZED1 OS=Arabidopsis thaliana GN=ZED1 PE=1 SV=1	18.81	8.93	1.65	up	0.00	0.00	yes
TRINITY_DN22557_c0_g6	PREDICTED: ADP-ribosylation factor 2-like isoform X1 [Populus euphratica]	ARF	ADP-ribosylation factor 2 OS=Oryza sativa subsp. japonica GN=ARF PE=2 SV=2	93.15	35.75	1.99	up	0.00	0.00	yes
TRINITY_DN27827_c1_g1	PREDICTED: LOW QUALITY PROTEIN: pyrophosphate-energized vacuolar membrane proton pump [Prunus mume]	AVP1	Pyrophosphate-energized vacuolar membrane proton pump 1 OS=Arabidopsis thaliana GN=AVP1 PE=1 SV=1	384.54	198.56	1.55	up	0.00	0.00	yes
TRINITY_DN15579_c0_g1	PREDICTED: uncharacterized protein LOC105124666 [Populus euphratica]	NFD3	Probable ribosomal protein S11, mitochondrial OS=Arabidopsis thaliana GN=NFD3 PE=2 SV=1	29.32	17.75	1.35	up	0.00	0.00	yes
TRINITY_DN25882_c0_g2	hypothetical protein POPTR_0004s22180g [Populus trichocarpa]	DBR2	Artemisinic aldehyde Delta(11(13)) reductase OS=Artemisia annua GN=DBR2 PE=1 SV=1	32.38	9.68	2.81	up	0.00	0.00	yes
TRINITY_DN18236_c0_g1	hypothetical protein POPTR_0014s11300g [Populus trichocarpa]	NIFU1	NifU-like protein 1, chloroplastic OS=Arabidopsis thaliana GN=NIFU1 PE=1 SV=1	101.58	52.71	1.54	up	0.00	0.00	yes
TRINITY_DN17591_c0_g2	hypothetical protein POPTR_0014s09030g [Populus trichocarpa]	-	-	4.84	1.52	2.26	up	0.00	0.00	yes
TRINITY_DN25448_c0_g1	PREDICTED: probable mitochondrial-processing peptidase subunit beta [Populus euphratica]	At3g02090	Probable mitochondrial-processing peptidase subunit beta, mitochondrial OS=Arabidopsis thaliana GN=At3g02090 PE=1 SV=2	128.26	74.71	1.37	up	0.00	0.00	yes
TRINITY_DN23592_c1_g1	PREDICTED: protein TRANSPARENT TESTA 12-like [Populus euphratica]	DTX40	Protein DETOXIFICATION 40 OS=Arabidopsis thaliana GN=DTX40 PE=1 SV=1	126.99	67.91	1.54	up	0.00	0.00	yes
TRINITY_DN25118_c0_g1	PREDICTED: uncharacterized protein LOC105140850 isoform X1 [Populus euphratica]	-	-	83.54	50.27	1.45	up	0.00	0.00	yes

TRINITY_DN22270_c0_g1	PREDICTED: 1,4-alpha-glucan-branching enzyme 3, chloroplastic/amyloplastic isoform X1 [Populus euphratica]	SBE3	1,4-alpha-glucan-branching enzyme 3, chloroplastic/amyloplastic OS=Arabidopsis thaliana GN=SBE3 PE=1 SV=1	14.25	8.43	1.36	up	0.00	0.00	yes
TRINITY_DN25083_c2_g1	PREDICTED: elongation factor G-2, chloroplastic [Populus euphratica]	-	-	79.98	31.58	1.95	up	0.00	0.00	yes
TRINITY_DN25205_c1_g7	photosystem I subunit VII (chloroplast) [Ficus racemosa]	psaC	Photosystem I iron-sulfur center OS=Aethionema cordifolium GN=psaC PE=3 SV=1	44.90	19.65	1.80	up	0.00	0.00	yes
TRINITY_DN26293_c1_g1	hypothetical protein POPTR_0005s14610g [Populus trichocarpa]	EMB2247	Valine--tRNA ligase, chloroplastic/mitochondrial 2 OS=Arabidopsis thaliana GN=EMB2247 PE=3 SV=1	40.82	23.33	1.40	up	0.00	0.00	yes
TRINITY_DN19880_c0_g3	methyltransferase-related family protein [Populus trichocarpa]	rsmG	Ribosomal RNA small subunit methyltransferase G OS=Alkaliphilus metalliredigens (strain QYMF) GN=rsmG PE=3 SV=1	35.97	21.94	1.35	up	0.00	0.00	yes
TRINITY_DN26126_c1_g4	hypothetical protein POPTR_0098s002202g, partial [Populus trichocarpa]	UGT85A3	UDP-glycosyltransferase 85A3 OS=Arabidopsis thaliana GN=UGT85A3 PE=2 SV=2	2.45	0.24	3.81	up	0.00	0.00	yes
TRINITY_DN21166_c0_g7	hypothetical protein POPTR_0010s22290g [Populus trichocarpa]	RPL10	50S ribosomal protein L10, chloroplastic OS=Nicotiana tabacum GN=RPL10 PE=2 SV=1	230.47	103.21	1.74	up	0.00	0.00	yes
TRINITY_DN17109_c0_g1	PREDICTED: uncharacterized protein LOC105110439 [Populus euphratica]	rd2	Rubredoxin-2 OS=Desulfovibrio desulfuricans (strain ATCC 27774 / DSM 6949) GN=rd2 PE=1 SV=3	146.70	44.29	2.32	up	0.00	0.00	yes
TRINITY_DN22393_c0_g1	hypothetical protein POPTR_0014s04370g [Populus trichocarpa]	-	-	31.60	19.54	1.38	up	0.00	0.00	yes
TRINITY_DN23320_c1_g1	hypothetical protein POPTR_0004s18390g [Populus trichocarpa]	-	-	11.54	5.67	1.59	up	0.00	0.00	yes
TRINITY_DN21016_c1_g2	PREDICTED: zinc finger CCCH domain-containing protein 18-like isoform X1 [Populus euphratica]	-	-	10.79	3.70	2.13	up	0.00	0.00	yes
TRINITY_DN26716_c1_g1	PREDICTED: phospholipid:diacylglycerol acyltransferase 1-like [Populus euphratica]	PDAT1	Phospholipid:diacylglycerol acyltransferase 1 OS=Arabidopsis thaliana GN=PDAT1 PE=2 SV=1	24.14	15.21	1.30	up	0.00	0.00	yes
TRINITY_DN17676_c1_g3	hypothetical protein POPTR_0003s06880g [Populus trichocarpa]	-	40S ribosomal protein SA OS=Glycine max PE=2 SV=1	393.62	235.38	1.47	up	0.00	0.00	yes
TRINITY_DN21215_c0_g1	PREDICTED: trigger factor-like protein TIG, Chloroplastic [Populus euphratica]	TIG	Trigger factor-like protein TIG, Chloroplastic OS=Arabidopsis thaliana GN=TIG PE=1 SV=1	184.94	89.75	1.66	up	0.00	0.00	yes
TRINITY_DN26491_c4_g2	PREDICTED: chloroplastic group IIA intron splicing facilitator CRS1, chloroplastic [Populus euphratica]	CFM2	CRM-domain containing factor CFM2, chloroplastic OS=Arabidopsis thaliana GN=CFM2 PE=1 SV=1	27.04	16.11	1.35	up	0.00	0.00	yes
TRINITY_DN18670_c0_g1	hypothetical protein POPTR_0010s25670g [Populus trichocarpa]	GLUTRBP	Glutamyl-tRNA reductase-binding protein, chloroplastic OS=Arabidopsis thaliana GN=GLUTRBP PE=1 SV=1	190.55	96.05	1.58	up	0.00	0.00	yes
TRINITY_DN18975_c0_g1	oxidoreductase family protein [Populus trichocarpa]	DLO1	Protein DMR6-LIKE OXYGENASE 1 OS=Arabidopsis thaliana GN=DLO1 PE=1 SV=1	18.29	6.07	2.10	up	0.00	0.00	yes
TRINITY_DN23351_c0_g1	hypothetical protein POPTR_0002s09320g [Populus trichocarpa]	mnmE	tRNA modification GTPase MnmE OS=Synechococcus elongatus (strain PCC 7942) GN=mnmE PE=3 SV=3	36.44	22.08	1.38	up	0.00	0.00	yes
TRINITY_DN15976_c0_g1	lecithin:cholesterol acyltransferase family protein [Populus trichocarpa]	PDAT2	Putative phospholipid:diacylglycerol acyltransferase 2 OS=Arabidopsis thaliana GN=PDAT2 PE=3 SV=1	9.17	2.97	2.23	up	0.00	0.00	yes
TRINITY_DN16584_c0_g1	PREDICTED: uncharacterized protein LOC105128231 [Populus euphratica]	At3g17800	UV-B-induced protein At3g17800, chloroplastic OS=Arabidopsis thaliana GN=At3g17800 PE=2 SV=1	29.69	16.46	1.46	up	0.00	0.00	yes
TRINITY_DN18001_c0_g1	emp24/gp25L/p24 family protein [Populus trichocarpa]	At1g09580	Transmembrane emp24 domain-containing protein p24delta3 OS=Arabidopsis thaliana GN=At1g09580 PE=1 SV=1	19.62	11.75	1.40	up	0.00	0.00	yes
TRINITY_DN23168_c0_g1	Fumarate hydratase 1 family protein [Populus trichocarpa]	FUM1	Fumarate hydratase 1, mitochondrial OS=Arabidopsis thaliana GN=FUM1 PE=1 SV=2	44.45	26.95	1.33	up	0.00	0.00	yes
TRINITY_DN22228_c0_g1	PREDICTED: zinc phosphodiesterase ELAC protein 2 [Populus euphratica]	ELAC2	Zinc phosphodiesterase ELAC protein 2 OS=Macaca fascicularis GN=ELAC2 PE=2 SV=1	17.34	10.98	1.27	up	0.00	0.00	yes
TRINITY_DN22912_c0_g6	PREDICTED: probable cytosine deaminase [Populus euphratica]	guaD	Guanine deaminase OS=Bacillus subtilis (strain 168) GN=guaD PE=1 SV=1	64.66	33.96	1.50	up	0.00	0.00	yes
TRINITY_DN19048_c0_g2	hypothetical protein POPTR_0014s01800g [Populus trichocarpa]	RPS29A	40S ribosomal protein S29 OS=Arabidopsis thaliana GN=RPS29A PE=3 SV=2	432.06	233.35	1.37	up	0.00	0.00	yes
TRINITY_DN16384_c0_g2	hypothetical protein POPTR_0001s25670g [Populus trichocarpa]	-	-	688.13	192.77	2.43	up	0.00	0.00	yes
TRINITY_DN27847_c0_g1	hypothetical protein POPTR_0008s11610g [Populus trichocarpa]	MAP1B	Methionine aminopeptidase 1B, chloroplastic OS=Arabidopsis thaliana GN=MAP1B PE=2 SV=2	34.59	16.78	1.64	up	0.00	0.00	yes

TRINITY_DN22292_c1_g2	Superoxide dismutase family protein [Populus trichocarpa]	SODA	Superoxide dismutase [Mn], mitochondrial OS=Hevea brasiliensis GN=SODA PE=2 SV=1	171.13	95.77	1.41	up	0.00	0.00	yes
TRINITY_DN23986_c0_g2	hypothetical protein POPTR_0008s12680g [Populus trichocarpa]	EAF2	ELL-associated factor 2 OS=Gallus gallus GN=EAF2 PE=2 SV=1	24.85	14.22	1.42	up	0.00	0.00	yes
TRINITY_DN18006_c0_g2	PREDICTED: uncharacterized protein LOC105110577 isoform X2 [Populus euphratica]	-	-	70.76	39.29	1.38	up	0.00	0.00	yes
TRINITY_DN18984_c0_g1	PREDICTED: tyrosine--tRNA ligase, mitochondrial [Populus euphratica]	EMB2768	Tyrosine--tRNA ligase, chloroplastic/mitochondrial OS=Arabidopsis thaliana GN=EMB2768 PE=2 SV=1	44.00	23.69	1.48	up	0.00	0.00	yes
TRINITY_DN21038_c0_g1	hypothetical protein POPTR_0019s04970g [Populus trichocarpa]	PNSB2	Photosynthetic NDH subunit of subcomplex B 2, chloroplastic OS=Arabidopsis thaliana GN=PNSB2 PE=2 SV=1	308.74	103.56	2.15	up	0.00	0.00	yes
TRINITY_DN18780_c0_g1	PREDICTED: uncharacterized protein LOC105141961 isoform X1 [Populus euphratica]	NFD6	Protein NUCLEAR FUSION DEFECTIVE 6, chloroplastic/mitochondrial OS=Arabidopsis thaliana GN=NFD6 PE=2 SV=1	102.82	59.20	1.40	up	0.00	0.00	yes
TRINITY_DN17094_c3_g2	HSP90 [Populus tomentosa]	HSP90-2	Heat shock protein 90-2 OS=Arabidopsis thaliana GN=HSP90-2 PE=1 SV=1	145.24	71.36	1.64	up	0.00	0.00	yes
TRINITY_DN23962_c0_g1	cysteine protease family protein [Populus trichocarpa]	RD21B	Probable cysteine protease RD21B OS=Arabidopsis thaliana GN=RD21B PE=1 SV=1	26.78	15.91	1.34	up	0.00	0.00	yes
TRINITY_DN17856_c0_g1	-	-	-	65.43	29.29	1.75	up	0.00	0.00	yes
TRINITY_DN27282_c0_g1	hypothetical protein POPTR_0009s01900g [Populus trichocarpa]	ISPG	4-hydroxy-3-methylbut-2-en-1-yl diphosphate synthase (ferredoxin), chloroplastic OS=Arabidopsis thaliana GN=ISPG PE=1 SV=1	120.23	67.39	1.42	up	0.00	0.00	yes
TRINITY_DN19909_c2_g1	PREDICTED: dnaJ homolog 1, mitochondrial [Populus euphratica]	dnaJ	Chaperone protein DnaJ OS=Thermosynechococcus elongatus (strain BP-1) GN=dnaJ PE=3 SV=2	78.26	45.72	1.45	up	0.00	0.00	yes
TRINITY_DN20141_c0_g4	hypothetical protein POPTR_0018s00640g [Populus trichocarpa]	infA	Translation initiation factor IF-1, chloroplastic OS=Buxus microphylla GN=infA PE=3 SV=1	49.98	18.33	2.01	up	0.00	0.00	yes
TRINITY_DN26391_c0_g4	hypothetical protein POPTR_0016s14750g [Populus trichocarpa]	RER3	Protein RETICULATA-RELATED 3, chloroplastic OS=Arabidopsis thaliana GN=RER3 PE=1 SV=1	13.85	7.55	1.46	up	0.00	0.00	yes
TRINITY_DN17718_c0_g1	chaos family protein [Populus trichocarpa]	CAO	Signal recognition particle 43 kDa protein, chloroplastic OS=Arabidopsis thaliana GN=CAO PE=1 SV=2	46.89	24.92	1.52	up	0.00	0.00	yes
TRINITY_DN21638_c0_g2	hypothetical protein POPTR_0010s21460g [Populus trichocarpa]	-	ATP synthase 6 kDa subunit, mitochondrial (Fragment) OS=Solanum tuberosum PE=1 SV=1	109.75	65.40	1.35	up	0.00	0.00	yes
TRINITY_DN18441_c0_g1	YGGT family protein [Populus trichocarpa]	YLMG2	YlmG homolog protein 2, chloroplastic OS=Arabidopsis thaliana GN=YLMG2 PE=2 SV=1	278.40	123.82	1.76	up	0.00	0.00	yes
TRINITY_DN20297_c0_g1	unknown [Populus trichocarpa x Populus deltoides]	GGCT2;2	Gamma-glutamylcyclotransferase 2-2 OS=Arabidopsis thaliana GN=GGCT2;2 PE=1 SV=1	228.91	124.07	1.52	up	0.00	0.00	yes
TRINITY_DN17543_c0_g1	hypothetical protein POPTR_0003s15410g [Populus trichocarpa]	-	-	245.95	130.89	1.57	up	0.00	0.00	yes
TRINITY_DN14193_c0_g2	-	-	-	7.15	1.16	3.10	up	0.00	0.00	yes
TRINITY_DN19915_c2_g1	hypothetical protein POPTR_0001s35310g [Populus trichocarpa]	-	-	3.84	1.64	1.86	up	0.00	0.00	yes
TRINITY_DN25171_c0_g1	hypothetical protein POPTR_0002s01220g [Populus trichocarpa]	-	-	188.14	126.72	1.46	up	0.00	0.00	yes
TRINITY_DN18443_c0_g2	hypothetical protein POPTR_0002s23610g [Populus trichocarpa]	Dctpp1	dCTP pyrophosphatase 1 OS=Mus musculus GN=Dctpp1 PE=1 SV=1	10.24	3.46	2.18	up	0.00	0.00	yes
TRINITY_DN16886_c0_g2	hypothetical protein POPTR_0016s14960g [Populus trichocarpa]	At2g38640	Protein LURP-one-related 8 OS=Arabidopsis thaliana GN=At2g38640 PE=2 SV=1	2.25	0.46	2.81	up	0.00	0.00	yes
TRINITY_DN24670_c1_g1	hypothetical protein POPTR_0002s05780g [Populus trichocarpa]	PSY	Phytoene synthase, chloroplastic OS=Daucus carota GN=PSY PE=2 SV=1	57.04	33.68	1.40	up	0.00	0.00	yes
TRINITY_DN25221_c1_g2	malate dehydrogenase family protein [Populus trichocarpa]	MDHG	Malate dehydrogenase, glyoxysomal OS=Cucumis sativus GN=MDHG PE=2 SV=1	356.58	110.81	2.25	up	0.00	0.00	yes
TRINITY_DN21580_c0_g1	PREDICTED: probable iron/ascorbate oxidoreductase DDB_G0283291 isoform X2 [Populus euphratica]	tropC	2-oxoglutarate-dependent dioxygenase tropC OS=Talaromyces stipitatus (strain ATCC 10500 / CBS 375.48 / QM 6759 / NRRL 1006) GN=tropC PE=1 SV=1	23.05	14.27	1.58	up	0.00	0.00	yes
TRINITY_DN15568_c0_g3	ribosomal protein L34 [Populus trichocarpa]	RPL34	60S ribosomal protein L34 OS=Nicotiana tabacum GN=RPL34 PE=2 SV=1	207.90	112.22	1.52	up	0.00	0.00	yes

TRINITY_DN25870_c1_g2	PREDICTED: sufE-like protein, chloroplastic [Populus euphratica]	BOLA4	Protein BOLA4, chloroplastic/mitochondrial OS=Arabidopsis thaliana GN=BOLA4 PE=1 SV=1	251.73	121.64	1.65	up	0.00	0.00	yes
TRINITY_DN21358_c0_g2	hypothetical protein POPTR_0010s19410g [Populus trichocarpa]	-	-	98.61	40.57	1.88	up	0.00	0.00	yes
TRINITY_DN27315_c1_g3	PREDICTED: polygalacturonase At1g48100-like [Populus euphratica]	At1g48100	Polygalacturonase At1g48100 OS=Arabidopsis thaliana GN=At1g48100 PE=2 SV=1	140.65	76.97	1.78	up	0.00	0.00	yes
TRINITY_DN24881_c0_g1	PREDICTED: transketolase, chloroplastic [Populus euphratica]	-	-	1790.82	677.46	1.99	up	0.00	0.00	yes
TRINITY_DN27543_c1_g1	PREDICTED: folylpolyglutamate synthase-like isoform X1 [Populus euphratica]	FPGS2	Folylpolyglutamate synthase OS=Arabidopsis thaliana GN=FPGS2 PE=1 SV=1	31.47	17.26	1.50	up	0.00	0.00	yes
TRINITY_DN25474_c0_g1	PREDICTED: diaminopimelate decarboxylase 2, chloroplastic-like [Populus euphratica]	LYSA2	Diaminopimelate decarboxylase 2, chloroplastic OS=Arabidopsis thaliana GN=LYSA2 PE=1 SV=1	144.79	88.69	1.34	up	0.00	0.00	yes
TRINITY_DN25918_c0_g1	hypothetical protein POPTR_0018s11460g [Populus trichocarpa]	At2g24020	Nucleoid-associated protein At2g24020, chloroplastic OS=Arabidopsis thaliana GN=At2g24020 PE=2 SV=2	253.54	126.94	1.60	up	0.00	0.00	yes
TRINITY_DN21170_c0_g1	thylakoid lumenal family protein [Populus trichocarpa]	TL20.3	Thylakoid lumenal protein TL20.3, chloroplastic OS=Arabidopsis thaliana GN=TL20.3 PE=1 SV=1	118.38	50.33	1.70	up	0.00	0.00	yes
TRINITY_DN22056_c1_g2	PREDICTED: cytochrome b-c1 complex subunit 6-like isoform X3 [Populus euphratica]	-	Cytochrome b-c1 complex subunit 6 OS=Solanum tuberosum PE=1 SV=2	403.72	204.98	1.40	up	0.00	0.00	yes
TRINITY_DN19334_c0_g1	peptidoglycan-binding domain-containing family protein [Populus trichocarpa]	-	-	34.78	20.85	1.36	up	0.00	0.00	yes
TRINITY_DN19353_c0_g1	PREDICTED: glucan endo-1,3-beta-glucosidase 12 [Populus euphratica]	At3g13560	Glucan endo-1,3-beta-glucosidase 4 OS=Arabidopsis thaliana GN=At3g13560 PE=1 SV=1	71.34	26.96	2.03	up	0.00	0.00	yes
TRINITY_DN24440_c0_g2	hypothetical protein POPTR_0012s09440g [Populus trichocarpa]	sll1917	Oxygen-independent coproporphyrinogen-III oxidase-like protein sll1917 OS=Synechocystis sp. (strain PCC 6803 / Kazusa) GN=sll1917 PE=2 SV=1	12.89	7.96	1.30	up	0.00	0.00	yes
TRINITY_DN23269_c1_g3	hypothetical protein POPTR_0004s21960g [Populus trichocarpa]	CLC-E	Chloride channel protein CLC-e OS=Arabidopsis thaliana GN=CLC-E PE=2 SV=2	23.88	11.93	1.64	up	0.00	0.00	yes
TRINITY_DN23435_c0_g1	hypothetical protein POPTR_0018s05190g [Populus trichocarpa]	IRT3	Fe(2+) transport protein 3, chloroplastic OS=Arabidopsis thaliana GN=IRT3 PE=2 SV=3	24.56	12.18	1.61	up	0.00	0.00	yes
TRINITY_DN19279_c0_g2	hypothetical protein POPTR_0007s11190g [Populus trichocarpa]	KRP1	Cyclin-dependent kinase inhibitor 1 OS=Arabidopsis thaliana GN=KRP1 PE=1 SV=2	14.77	4.72	2.41	up	0.00	0.00	yes
TRINITY_DN22184_c0_g2	hypothetical protein POPTR_0010s08770g [Populus trichocarpa]	CFM3	CRM-domain containing factor CFM3, chloroplastic/mitochondrial OS=Zea mays GN=CFM3 PE=1 SV=1	53.21	31.38	1.34	up	0.00	0.00	yes
TRINITY_DN25068_c0_g2	sulfate transporter [Populus tremula x Populus alba]	SULTR3;3	Probable sulfate transporter 3.3 OS=Arabidopsis thaliana GN=SULTR3;3 PE=2 SV=2	3.72	0.45	3.69	up	0.00	0.00	yes
TRINITY_DN14071_c0_g1	heat shock 22K family protein [Populus trichocarpa]	HSP21	Small heat shock protein, chloroplastic OS=Pisum sativum GN=HSP21 PE=2 SV=1	3.13	0.36	3.68	up	0.00	0.00	yes
TRINITY_DN19368_c0_g1	PREDICTED: riboflavin biosynthesis protein PYRD, chloroplastic [Populus euphratica]	PYRD	Riboflavin biosynthesis protein PYRD, chloroplastic OS=Arabidopsis thaliana GN=PYRD PE=1 SV=1	34.03	20.41	1.35	up	0.00	0.00	yes
TRINITY_DN25379_c0_g1	hypothetical protein POPTR_0001s35600g [Populus trichocarpa]	At4g13010	Putative quinone-oxidoreductase homolog, chloroplastic OS=Arabidopsis thaliana GN=At4g13010 PE=1 SV=1	112.07	58.63	1.58	up	0.00	0.00	yes
TRINITY_DN22725_c0_g1	hypothetical protein POPTR_0012s04410g [Populus trichocarpa]	ptges2	Prostaglandin E synthase 2 OS=Danio rerio GN=ptges2 PE=2 SV=1	23.89	14.90	1.31	up	0.00	0.00	yes
TRINITY_DN20065_c0_g2	PREDICTED: uncharacterized protein LOC105120731 [Populus euphratica]	-	-	46.44	24.32	1.54	up	0.00	0.00	yes
TRINITY_DN22634_c0_g1	cytochrome c oxidase family protein [Populus trichocarpa]	COX5B-2	Cytochrome c oxidase subunit 5b-2, mitochondrial OS=Arabidopsis thaliana GN=COX5B-2 PE=2 SV=1	142.26	84.38	1.37	up	0.00	0.00	yes
TRINITY_DN26417_c0_g2	Endoribonuclease Dicer family protein [Populus trichocarpa]	DCL1	Endoribonuclease Dicer homolog 1 OS=Arabidopsis thaliana GN=DCL1 PE=1 SV=2	6.69	4.27	1.25	up	0.00	0.00	yes
TRINITY_DN20803_c0_g1	40S ribosomal protein S20 [Populus trichocarpa]	RPS20A	40S ribosomal protein S20-1 OS=Arabidopsis thaliana GN=RPS20A PE=2 SV=2	489.59	282.90	1.44	up	0.00	0.00	yes
TRINITY_DN13624_c0_g1	hypothetical protein POPTR_0015s06940g [Populus trichocarpa]	TOM5	Mitochondrial import receptor subunit TOM5 homolog OS=Arabidopsis thaliana GN=TOM5 PE=1 SV=3	72.29	42.91	1.37	up	0.00	0.00	yes
TRINITY_DN15792_c0_g1	hypothetical protein POPTR_0009s14580g [Populus trichocarpa]	-	-	8.67	0.64	4.63	up	0.00	0.00	yes

TRINITY_DN16891_c0_g1	hypothetical protein POPTR_0002s10920g [Populus trichocarpa]	At5g64460	Phosphoglycerate mutase-like protein 1 OS=Arabidopsis thaliana GN=At5g64460 PE=2 SV=1	40.32	23.40	1.37	up	0.00	0.00	yes
TRINITY_DN19667_c0_g3	PREDICTED: isopentenyl-diphosphate Delta-isomerase I [Populus euphratica]	IPI2	Isopentenyl-diphosphate Delta-isomerase II OS=Camptotheca acuminata GN=IPI2 PE=2 SV=1	204.35	111.83	1.48	up	0.00	0.00	yes
TRINITY_DN20663_c0_g4	hypothetical protein POPTR_0014s05780g [Populus trichocarpa]	-	-	13.34	6.64	1.65	up	0.00	0.00	yes
TRINITY_DN27842_c1_g1	elongation factor 1-alpha [Populus davidiana x Populus alba var. pyramidalis]	EF1	Elongation factor 1-alpha OS=Manihot esculenta GN=EF1 PE=3 SV=1	788.41	466.02	1.38	up	0.00	0.00	yes
TRINITY_DN16323_c0_g3	ubiquinol-cytochrome C reductase complex ubiquinone-binding family protein [Populus trichocarpa]	-	Cytochrome b-c1 complex subunit 8 OS=Solanum tuberosum PE=1 SV=2	250.75	139.18	1.45	up	0.00	0.00	yes
TRINITY_DN19790_c1_g1	-	-	-	161.32	64.15	1.93	up	0.00	0.00	yes
TRINITY_DN12955_c0_g1	hypothetical protein POPTR_0011s05190g [Populus trichocarpa]	At5g22620	Probable 2-carboxy-D-arabinitol-1-phosphatase OS=Arabidopsis thaliana GN=At5g22620 PE=1 SV=1	15.50	6.27	1.82	up	0.00	0.00	yes
TRINITY_DN13907_c0_g1	hypothetical protein POPTR_0001s31230g [Populus trichocarpa]	At3g55350	Protein ALP1-like OS=Arabidopsis thaliana GN=At3g55350 PE=2 SV=1	2.75	0.50	3.04	up	0.00	0.00	yes
TRINITY_DN19496_c0_g1	hypothetical protein POPTR_0002s20310g [Populus trichocarpa]	-	-	14.51	6.57	1.75	up	0.00	0.00	yes
TRINITY_DN26049_c1_g1	H+-transporting two-sector ATPase family protein [Populus trichocarpa]	ATPB	ATP synthase subunit beta, mitochondrial OS=Oryza sativa subsp. japonica GN=ATPB PE=1 SV=2	510.11	302.10	1.35	up	0.00	0.00	yes
TRINITY_DN24893_c0_g2	PREDICTED: probable carboxylesterase 18 [Populus euphratica]	CXE18	Probable carboxylesterase 18 OS=Arabidopsis thaliana GN=CXE18 PE=1 SV=1	48.39	23.75	1.68	up	0.00	0.00	yes
TRINITY_DN22944_c0_g1	PREDICTED: beta-xylosidase/alpha-L-arabinofuranosidase 1-like [Populus euphratica]	Xyl2	Beta-xylosidase/alpha-L-arabinofuranosidase 2 OS=Medicago sativa subsp. varia GN=Xyl2 PE=2 SV=1	51.43	26.38	1.68	up	0.00	0.00	yes
TRINITY_DN11466_c0_g1	hypothetical protein POPTR_0010s14200g [Populus trichocarpa]	-	-	33.03	8.10	2.69	up	0.00	0.00	yes
TRINITY_DN21088_c1_g2	hypothetical protein POPTR_0005s00295g [Populus trichocarpa]	-	-	26.73	15.94	1.56	up	0.00	0.00	yes
TRINITY_DN19923_c0_g1	hypothetical protein POPTR_0017s02260g [Populus trichocarpa]	PCMP-H57	Pentatricopeptide repeat-containing protein At3g14330 OS=Arabidopsis thaliana GN=PCMP-H57 PE=2 SV=2	6.64	3.52	1.53	up	0.00	0.00	yes
TRINITY_DN17648_c0_g1	PREDICTED: uncharacterized protein At1g18480 [Populus euphratica]	SLP1	Shewanella-like protein phosphatase 1 OS=Arabidopsis thaliana GN=SLP1 PE=1 SV=1	19.45	6.55	2.26	up	0.00	0.00	yes
TRINITY_DN18387_c0_g1	hypothetical protein POPTR_0017s12240g [Populus trichocarpa]	CGS1	Cystathionine gamma-synthase 1, chloroplastic OS=Arabidopsis thaliana GN=CGS1 PE=1 SV=3	122.22	69.70	1.43	up	0.00	0.00	yes
TRINITY_DN25044_c0_g5	PREDICTED: cytochrome P450 89A2-like [Populus euphratica]	CYP89A9	Cytochrome P450 89A9 OS=Arabidopsis thaliana GN=CYP89A9 PE=2 SV=1	7.02	3.30	1.69	up	0.00	0.00	yes
TRINITY_DN20815_c0_g1	PREDICTED: ATP sulfurylase 2-like [Populus euphratica]	APS2	ATP sulfurylase 2 OS=Arabidopsis thaliana GN=APS2 PE=1 SV=1	46.46	28.77	1.41	up	0.00	0.00	yes
TRINITY_DN11701_c0_g1	PREDICTED: nudix hydrolase 8-like isoform X1 [Populus euphratica]	NUDT8	Nudix hydrolase 8 OS=Arabidopsis thaliana GN=NUDT8 PE=2 SV=2	4.03	0.00	8.16	up	0.00	0.00	yes
TRINITY_DN18049_c0_g2	hypothetical protein POPTR_0001s25590g [Populus trichocarpa]	DIVARICATA	Transcription factor DIVARICATA OS=Antirrhinum majus GN=DIVARICATA PE=2 SV=1	43.52	25.51	1.34	up	0.00	0.00	yes
TRINITY_DN18683_c2_g2	PREDICTED: probable protein Pop3 [Populus euphratica]	HS1	Stress-response A/B barrel domain-containing protein HS1 OS=Arabidopsis thaliana GN=HS1 PE=1 SV=1	680.60	338.85	1.62	up	0.00	0.00	yes
TRINITY_DN20271_c0_g1	hypothetical protein POPTR_0006s26500g [Populus trichocarpa]	NAA50	N-alpha-acetyltransferase 50 OS=Bos taurus GN=NAA50 PE=2 SV=1	70.18	40.28	1.40	up	0.00	0.00	yes
TRINITY_DN23945_c1_g1	hypothetical protein POPTR_0005s19130g [Populus trichocarpa]	slr1673	Uncharacterized tRNA/rRNA methyltransferase slr1673 OS=Synechocystis sp. (strain PCC 6803 / Kazusa) GN=slr1673 PE=3 SV=1	33.95	20.58	1.35	up	0.00	0.00	yes
TRINITY_DN16936_c0_g1	60S ribosomal protein L35 [Populus trichocarpa]	RPL35	60S ribosomal protein L35 OS=Euphorbia esula GN=RPL35 PE=2 SV=1	484.00	286.67	1.36	up	0.00	0.00	yes
TRINITY_DN21756_c0_g5	mitochondrial processing peptidase alpha subunit 2 family protein [Populus trichocarpa]	MPP	Mitochondrial-processing peptidase subunit alpha OS=Solanum tuberosum GN=MPP PE=1 SV=1	61.66	37.46	1.32	up	0.00	0.00	yes
TRINITY_DN16295_c0_g1	complex 1 family protein [Populus trichocarpa]	-	-	55.37	27.31	1.63	up	0.00	0.00	yes
TRINITY_DN17802_c0_g2	hypothetical protein POPTR_0002s10520g [Populus trichocarpa]	AHL17	AT-hook motif nuclear-localized protein 17 OS=Arabidopsis thaliana GN=AHL17 PE=2 SV=1	11.97	1.88	3.42	up	0.00	0.00	yes

TRINITY_DN19791_c1_g4	hypothetical protein POPTR_0010s02880g [Populus trichocarpa]	-	-	33.46	16.64	1.63	up	0.00	0.00	yes
TRINITY_DN19210_c0_g1	hypothetical protein POPTR_0001s44110g [Populus trichocarpa]	RPL24	50S ribosomal protein L24, chloroplastic OS=Pisum sativum GN=RPL24 PE=2 SV=1	360.84	169.37	1.69	up	0.00	0.00	yes
TRINITY_DN25616_c0_g1	PREDICTED: adoMet-dependent rRNA methyltransferase spb1 [Populus euphratica]	rlmE	Ribosomal RNA large subunit methyltransferase E OS=Halorubrum lacusprofundi (strain ATCC 49239 / DSM 5036 / JCM 8891 / ACAM 34) GN=rlmE PE=3 SV=1	25.87	11.71	1.81	up	0.00	0.00	yes
TRINITY_DN26852_c1_g1	hypothetical protein POPTR_0009s08760g [Populus trichocarpa]	At4g33760	Aspartate--tRNA ligase, chloroplastic/mitochondrial OS=Arabidopsis thaliana GN=At4g33760 PE=2 SV=1	38.26	24.17	1.39	up	0.00	0.00	yes
TRINITY_DN17905_c0_g2	hypothetical protein POPTR_0013s07570g [Populus trichocarpa]	-	-	136.44	73.29	1.50	up	0.00	0.00	yes
TRINITY_DN21417_c0_g1	SERINE ACETYLTRANSFERASE-106 family protein [Populus trichocarpa]	SAT4	Serine acetyltransferase 4 OS=Arabidopsis thaliana GN=SAT4 PE=1 SV=1	46.75	26.71	1.35	up	0.00	0.00	yes
TRINITY_DN26191_c1_g1	glycine-tRNA ligase [Populus tomentosa]	EDD1	Glycine--tRNA ligase, chloroplastic/mitochondrial 2 OS=Arabidopsis thaliana GN=EDD1 PE=1 SV=1	27.29	15.89	1.38	up	0.00	0.00	yes
TRINITY_DN17012_c0_g3	hypothetical protein POPTR_0012s02280g [Populus trichocarpa]	-	-	64.42	29.76	1.72	up	0.00	0.00	yes
TRINITY_DN20539_c0_g2	hypothetical protein POPTR_0006s15520g [Populus trichocarpa]	Os05g0200100	Thioredoxin-like 2, chloroplastic OS=Oryza sativa subsp. japonica GN=Os05g0200100 PE=2 SV=1	68.08	38.48	1.43	up	0.00	0.00	yes
TRINITY_DN24996_c0_g1	ADP-glucose pyrophosphorylase family protein [Populus trichocarpa]	ADG2	Glucose-1-phosphate adenylyltransferase large subunit 1, chloroplastic OS=Arabidopsis thaliana GN=ADG2 PE=1 SV=3	108.63	36.08	2.19	up	0.00	0.00	yes
TRINITY_DN14712_c0_g2	PREDICTED: glutamate synthase 1 [NADH], chloroplastic-like isoform X1 [Populus euphratica]	GLT1	Glutamate synthase 1 [NADH], chloroplastic OS=Arabidopsis thaliana GN=GLT1 PE=1 SV=2	12.83	3.58	2.45	up	0.00	0.00	yes
TRINITY_DN27691_c1_g1	-	-	-	2.54	0.29	3.59	up	0.00	0.00	yes
TRINITY_DN28894_c0_g1	PREDICTED: ankyrin repeat-containing protein At3g12360-like isoform X2 [Populus euphratica]	-	-	1.74	0.29	3.21	up	0.00	0.00	yes
TRINITY_DN23024_c0_g1	hypothetical protein POPTR_0009s16210g [Populus trichocarpa]	-	-	147.34	70.19	1.62	up	0.00	0.00	yes
TRINITY_DN25847_c1_g3	Chain A family protein [Populus trichocarpa]	PETH	Ferredoxin--NADP reductase, leaf-type isozyme, chloroplastic OS=Nicotiana tabacum GN=PETH PE=2 SV=1	1458.06	589.75	1.81	up	0.00	0.00	yes
TRINITY_DN20595_c0_g4	haloacid dehalogenase-like hydrolase family protein [Populus trichocarpa]	-	-	37.20	20.77	1.44	up	0.00	0.00	yes
TRINITY_DN21550_c0_g1	hypothetical protein POPTR_0013s08610g [Populus trichocarpa]	CYP97A3	Protein LUTEIN DEFICIENT 5, chloroplastic OS=Arabidopsis thaliana GN=CYP97A3 PE=1 SV=1	129.04	67.43	1.54	up	0.00	0.00	yes
TRINITY_DN25415_c0_g3	hypothetical protein POPTR_0009s08480g [Populus trichocarpa]	PPA6	Soluble inorganic pyrophosphatase 6, chloroplastic OS=Arabidopsis thaliana GN=PPA6 PE=1 SV=1	587.73	247.91	1.50	up	0.00	0.00	yes
TRINITY_DN13712_c0_g1	Mitochondrial import inner membrane translocase subunit Tim9 family protein [Populus trichocarpa]	TIM9	Mitochondrial import inner membrane translocase subunit TIM9 OS=Arabidopsis thaliana GN=TIM9 PE=1 SV=2	75.75	38.40	1.63	up	0.00	0.00	yes
TRINITY_DN18843_c0_g1	hypothetical protein POPTR_0003s18030g [Populus trichocarpa]	CIPK25	CBL-interacting serine/threonine-protein kinase 25 OS=Arabidopsis thaliana GN=CIPK25 PE=2 SV=1	14.14	4.77	2.16	up	0.00	0.00	yes
TRINITY_DN18237_c0_g2	PREDICTED: glucose-6-phosphate 1-dehydrogenase 4, chloroplastic [Populus euphratica]	At1g09420	Glucose-6-phosphate 1-dehydrogenase 4, chloroplastic OS=Arabidopsis thaliana GN=At1g09420 PE=2 SV=1	14.47	10.41	1.23	up	0.00	0.00	yes
TRINITY_DN26147_c0_g1	3-phosphoshikimate 1-carboxyvinyltransferase family protein [Populus trichocarpa]	-	3-phosphoshikimate 1-carboxyvinyltransferase, chloroplastic OS=Petunia hybrida PE=1 SV=1	288.34	147.17	1.59	up	0.00	0.00	yes
TRINITY_DN26219_c0_g1	hypothetical protein POPTR_0015s12990g [Populus trichocarpa]	PAE9	Pectin acetylesterase 9 OS=Arabidopsis thaliana GN=PAE9 PE=2 SV=1	17.91	8.68	1.57	up	0.00	0.00	yes
TRINITY_DN21108_c0_g6	PREDICTED: 40S ribosomal protein S26-3 [Populus euphratica]	RPS26C	40S ribosomal protein S26-3 OS=Arabidopsis thaliana GN=RPS26C PE=2 SV=1	224.88	130.80	1.40	up	0.00	0.00	yes
TRINITY_DN21331_c0_g1	hypothetical protein POPTR_0015s12960g [Populus trichocarpa]	RPL36A	60S ribosomal protein L36-1 OS=Arabidopsis thaliana GN=RPL36A PE=2 SV=1	1951.25	1082.52	1.46	up	0.00	0.00	yes
TRINITY_DN21563_c0_g1	hypothetical protein POPTR_0017s10940g [Populus trichocarpa]	-	-	115.85	56.00	1.63	up	0.00	0.00	yes
TRINITY_DN21443_c0_g2	PREDICTED: 40S ribosomal protein S13-like [Populus euphratica]	RPS13	40S ribosomal protein S13 OS=Glycine max GN=RPS13 PE=2 SV=1	213.24	123.96	1.42	up	0.00	0.00	yes
TRINITY_DN26121_c1_g1	PREDICTED: ATP-dependent Clp protease proteolytic subunit-related protein 2, chloroplastic [Populus euphratica]	CLPR2	ATP-dependent Clp protease proteolytic subunit-related protein 2, chloroplastic OS=Arabidopsis thaliana GN=CLPR2 PE=1 SV=1	188.91	98.38	1.53	up	0.00	0.00	yes

TRINITY_DN25080_c0_g1	Inner membrane protein ALBINO3 [Populus trichocarpa]	PPF-1	Inner membrane protein PPF-1, chloroplastic OS=Pisum sativum GN=PPF-1 PE=2 SV=2	272.25	137.16	1.68	up	0.00	0.00	yes
TRINITY_DN17248_c0_g4	PREDICTED: elongation factor 1-alpha-like [Populus euphratica]	EF1	Elongation factor 1-alpha OS=Manihot esculenta GN=EF1 PE=3 SV=1	207.42	123.76	1.37	up	0.00	0.00	yes
TRINITY_DN22217_c0_g1	hypothetical protein POPTR_0013s10050g [Populus trichocarpa]	serA	D-3-phosphoglycerate dehydrogenase OS=Archaeoglobus fulgidus (strain ATCC 49558 / VC-16 / DSM 4304 / JCM 9628 / NBRC 100126) GN=serA PE=3 SV=1	54.43	34.14	1.29	up	0.00	0.00	yes
TRINITY_DN19739_c0_g2	60S ribosomal protein L22-1 [Populus trichocarpa]	RPL22B	60S ribosomal protein L22-2 OS=Arabidopsis thaliana GN=RPL22B PE=2 SV=1	184.76	107.03	1.42	up	0.00	0.00	yes
TRINITY_DN19547_c0_g5	hypothetical protein POPTR_0002s06280g [Populus trichocarpa]	CCB3	Protein COFACTOR ASSEMBLY OF COMPLEX C SUBUNIT B CCB3, chloroplastic OS=Arabidopsis thaliana GN=CCB3 PE=1 SV=1	34.64	14.24	1.89	up	0.00	0.00	yes
TRINITY_DN20576_c1_g3	PREDICTED: 40S ribosomal protein S19-3-like isoform X1 [Populus euphratica]	RPS19C	40S ribosomal protein S19-3 OS=Arabidopsis thaliana GN=RPS19C PE=2 SV=1	69.44	43.42	1.30	up	0.00	0.00	yes
TRINITY_DN20120_c0_g1	hypothetical protein POPTR_0001s37280g [Populus trichocarpa]	MTERF9	Transcription termination factor MTERF9, chloroplastic OS=Arabidopsis thaliana GN=MTERF9 PE=2 SV=1	29.46	16.51	1.43	up	0.00	0.00	yes
TRINITY_DN15060_c0_g2	lysine decarboxylase family protein [Populus trichocarpa]	LOG8	Cytokinin riboside 5'-monophosphate phosphoribohydrolase LOG8 OS=Arabidopsis thaliana GN=LOG8 PE=1 SV=1	32.30	18.01	1.45	up	0.00	0.00	yes
TRINITY_DN20054_c0_g1	unknown [Populus trichocarpa]	RPL5	60S ribosomal protein L5 OS=Cucumis sativus GN=RPL5 PE=2 SV=1	106.19	60.79	1.50	up	0.00	0.00	yes
TRINITY_DN23272_c0_g1	hypothetical protein POPTR_0018s08760g [Populus trichocarpa]	-	-	81.17	42.74	1.54	up	0.00	0.00	yes
TRINITY_DN22356_c0_g1	hypothetical protein POPTR_0016s05550g [Populus trichocarpa]	-	-	323.37	190.12	1.39	up	0.00	0.00	yes
TRINITY_DN18322_c0_g2	ABC transporter family protein [Populus trichocarpa]	ABCI11	ABC transporter I family member 11, chloroplastic OS=Arabidopsis thaliana GN=ABCI11 PE=2 SV=1	21.91	17.48	1.61	up	0.00	0.00	yes
TRINITY_DN26427_c2_g4	-	-	-	327.07	143.55	1.82	up	0.00	0.00	yes
TRINITY_DN25235_c0_g2	unknown [Populus trichocarpa]	HDT1	Histone deacetylase HDT1 OS=Arabidopsis thaliana GN=HDT1 PE=1 SV=1	97.57	47.72	1.70	up	0.00	0.00	yes
TRINITY_DN22427_c1_g1	hypothetical protein POPTR_0015s07680g [Populus trichocarpa]	grpE	Protein GrpE OS=Bradyrhizobium sp. (strain ORS 278) GN=grpE PE=3 SV=1	24.09	13.36	1.48	up	0.00	0.00	yes
TRINITY_DN22986_c0_g3	photosystem II protein I (plastid) [Pigafetta elata]	psbI	Photosystem II reaction center protein I OS=Acorus americanus GN=psbI PE=3 SV=1	23.48	11.49	1.64	up	0.00	0.00	yes
TRINITY_DN25491_c0_g2	hypothetical protein POPTR_0003s01390g [Populus trichocarpa]	-	-	24.82	10.60	1.83	up	0.00	0.00	yes
TRINITY_DN20938_c0_g2	malate dehydrogenase family protein [Populus trichocarpa]	MDHG	Malate dehydrogenase, glyoxysomal OS=Cucumis sativus GN=MDHG PE=2 SV=1	74.92	18.32	2.63	up	0.00	0.00	yes
TRINITY_DN24693_c0_g3	PREDICTED: probable pectate lyase 13 [Populus euphratica]	PMR6	Probable pectate lyase 13 OS=Arabidopsis thaliana GN=PMR6 PE=1 SV=1	2.03	0.29	3.37	up	0.00	0.00	yes
TRINITY_DN22145_c0_g3	NADH dehydrogenase subunit 7 [Populus alba]	ndhH	NAD(P)H-quinone oxidoreductase subunit H, chloroplastic OS=Populus alba GN=ndhH PE=3 SV=1	14.79	7.17	1.65	up	0.00	0.00	yes
TRINITY_DN14132_c0_g2	PREDICTED: protein TIC 20-v, chloroplastic-like [Populus euphratica]	TIC20-V	Protein TIC 20-v, chloroplastic OS=Arabidopsis thaliana GN=TIC20-V PE=2 SV=1	160.74	64.92	1.87	up	0.00	0.00	yes
TRINITY_DN25688_c1_g2	PREDICTED: probable tyrosine-protein phosphatase At1g05000 isoform X1 [Prunus mume]	At1g05000	Probable tyrosine-protein phosphatase At1g05000 OS=Arabidopsis thaliana GN=At1g05000 PE=1 SV=1	27.27	13.52	1.79	up	0.00	0.00	yes
TRINITY_DN17169_c0_g1	hypothetical protein POPTR_0012s04250g [Populus trichocarpa]	SURF1	Surfeit locus protein 1 OS=Arabidopsis thaliana GN=SURF1 PE=2 SV=1	10.59	5.63	1.53	up	0.00	0.00	yes
TRINITY_DN15833_c0_g1	7-dimethyl-8-ribityllumazine synthase family protein [Populus trichocarpa]	At2g44050	6,7-dimethyl-8-ribityllumazine synthase, chloroplastic OS=Arabidopsis thaliana GN=At2g44050 PE=2 SV=1	128.55	74.53	1.37	up	0.00	0.00	yes
TRINITY_DN26126_c1_g2	hypothetical protein POPTR_0016s02200g [Populus trichocarpa]	UGT85A24	7-deoxyloganetin glucosyltransferase OS=Gardenia jasminoides GN=UGT85A24 PE=1 SV=1	46.02	14.35	2.06	up	0.00	0.00	yes
TRINITY_DN21760_c0_g2	PREDICTED: 3-isopropylmalate dehydrogenase, chloroplastic-like [Populus euphratica]	-	3-isopropylmalate dehydrogenase, chloroplastic OS=Brassica napus PE=2 SV=1	86.28	52.23	1.32	up	0.00	0.00	yes
TRINITY_DN19755_c0_g1	-	-	-	46.48	20.31	1.84	up	0.00	0.00	yes
TRINITY_DN21266_c2_g1	PREDICTED: uncharacterized protein LOC105120530 isoform X1 [Populus euphratica]	-	-	99.01	51.69	1.53	up	0.00	0.00	yes

TRINITY_DN24038_c0_g3	hypothetical protein POPTR_0004s19430g [Populus trichocarpa]	DTX45	Protein DETOXIFICATION 45, chloroplastic OS=Arabidopsis thaliana GN=DTX45 PE=2 SV=2	3.16	0.73	2.74	up	0.00	0.00	yes
TRINITY_DN26437_c0_g2	hypothetical protein POPTR_0014s16480g [Populus trichocarpa]	-	Glucose-6-phosphate 1-dehydrogenase, chloroplastic OS=Solanum tuberosum PE=1 SV=1	43.75	20.82	1.65	up	0.00	0.00	yes
TRINITY_DN14541_c0_g1	PREDICTED: uncharacterized protein LOC105123025 [Populus euphratica]	-	-	16.86	5.48	2.20	up	0.00	0.00	yes
TRINITY_DN24005_c0_g1	hypothetical protein POPTR_0018s13160g [Populus trichocarpa]	HO1	Heme oxygenase 1, chloroplastic OS=Arabidopsis thaliana GN=HO1 PE=1 SV=2	63.97	42.27	1.32	up	0.00	0.00	yes
TRINITY_DN20563_c0_g3	hypothetical protein POPTR_0015s04950g [Populus trichocarpa]	DJA6	Chaperone protein dnaJ A6, chloroplastic OS=Arabidopsis thaliana GN=DJA6 PE=2 SV=2	13.47	8.43	1.29	up	0.00	0.00	yes
TRINITY_DN23175_c0_g1	unknown [Populus trichocarpa x Populus deltoides]	-	-	164.73	75.76	1.63	up	0.00	0.00	yes
TRINITY_DN23543_c1_g1	hypothetical protein POPTR_0014s04300g [Populus trichocarpa]	-	-	103.05	26.13	2.60	up	0.00	0.00	yes
TRINITY_DN16022_c0_g1	hypothetical protein POPTR_0640s00200g [Populus trichocarpa]	SALAT	Salutaridinol 7-O-acetyltransferase OS=Papaver somniferum GN=SALAT PE=1 SV=1	6.27	1.56	2.65	up	0.00	0.00	yes
TRINITY_DN19931_c0_g4	photosystem II CP47 protein [Populus alba]	psbB	Photosystem II CP47 reaction center protein OS=Populus alba GN=psbB PE=3 SV=1	37.22	21.34	1.48	up	0.00	0.00	yes
TRINITY_DN16306_c0_g1	PREDICTED: 2-methoxy-6-polyprenyl-1,4-benzoquinol methylase, mitochondrial [Populus euphratica]	COQ5	2-methoxy-6-polyprenyl-1,4-benzoquinol methylase, mitochondrial OS=Arabidopsis thaliana GN=COQ5 PE=2 SV=1	19.33	10.98	1.44	up	0.00	0.00	yes
TRINITY_DN20073_c0_g1	PREDICTED: mitochondrial import inner membrane translocase subunit TIM23-3 [Populus euphratica]	TIM23-1	Mitochondrial import inner membrane translocase subunit TIM23-1 OS=Arabidopsis thaliana GN=TIM23-1 PE=2 SV=1	11.17	4.34	2.00	up	0.00	0.00	yes
TRINITY_DN20160_c2_g3	hypothetical protein POPTR_0006s06430g [Populus trichocarpa]	TPRP-F1	36.4 kDa proline-rich protein OS=Solanum lycopersicum GN=TPRP-F1 PE=2 SV=1	3035.75	1125.38	2.06	up	0.00	0.00	yes
TRINITY_DN23822_c0_g2	hypothetical protein POPTR_0004s08360g [Populus trichocarpa]	-	-	13.29	7.06	1.52	up	0.00	0.00	yes
TRINITY_DN17056_c0_g1	hypothetical protein POPTR_0015s10960g [Populus trichocarpa]	At4g24930	Thylakoid lumenal 17.9 kDa protein, chloroplastic OS=Arabidopsis thaliana GN=At4g24930 PE=1 SV=1	116.13	49.08	1.77	up	0.00	0.00	yes
TRINITY_DN26309_c0_g1	Galactokinase family protein [Populus trichocarpa]	GAL1	Galactokinase OS=Arabidopsis thaliana GN=GAL1 PE=1 SV=2	38.22	22.85	1.34	up	0.00	0.00	yes
TRINITY_DN20799_c0_g1	PREDICTED: tyrosine decarboxylase 1-like isoform X2 [Populus euphratica]	ELI5	Tyrosine decarboxylase 1 OS=Arabidopsis thaliana GN=ELI5 PE=2 SV=1	30.48	17.85	1.40	up	0.00	0.00	yes
TRINITY_DN20230_c0_g1	Csf-2 family protein [Populus trichocarpa]	MLP34	MLP-like protein 34 OS=Arabidopsis thaliana GN=MLP34 PE=2 SV=1	47.04	21.78	1.54	up	0.00	0.00	yes
TRINITY_DN19991_c0_g1	hypothetical protein POPTR_0007s11880g [Populus trichocarpa]	RPL10AB	60S ribosomal protein L10a-2 OS=Arabidopsis thaliana GN=RPL10AB PE=1 SV=1	896.89	556.97	1.36	up	0.00	0.00	yes
TRINITY_DN21964_c0_g1	PREDICTED: 1-deoxy-D-xylulose 5-phosphate reductoisomerase, chloroplastic [Populus euphratica]	DXR	1-deoxy-D-xylulose 5-phosphate reductoisomerase, chloroplastic OS=Oryza sativa subsp. japonica GN=DXR PE=2 SV=2	158.54	78.23	1.62	up	0.00	0.00	yes
TRINITY_DN21345_c1_g2	rhodanese-like domain-containing family protein [Populus trichocarpa]	At4g27700	Rhodanese-like domain-containing protein 14, chloroplastic OS=Arabidopsis thaliana GN=At4g27700 PE=2 SV=1	182.63	83.93	1.73	up	0.00	0.00	yes
TRINITY_DN26419_c0_g1	PREDICTED: protein CHUP1, chloroplastic [Populus euphratica]	CHUP1	Protein CHUP1, chloroplastic OS=Arabidopsis thaliana GN=CHUP1 PE=1 SV=1	89.57	36.59	1.87	up	0.00	0.00	yes
TRINITY_DN15104_c0_g1	hypothetical protein POPTR_0011s07530g [Populus trichocarpa]	RPL34	50S ribosomal protein L34, chloroplastic OS=Spinacia oleracea GN=RPL34 PE=1 SV=1	203.22	84.32	1.88	up	0.00	0.00	yes
TRINITY_DN19950_c0_g1	hypothetical protein POPTR_0005s03920g [Populus trichocarpa]	SUGTL2	Sugar transporter ERD6-like 3 OS=Arabidopsis thaliana GN=SUGTL2 PE=2 SV=1	59.25	14.10	2.53	up	0.00	0.00	yes
TRINITY_DN16715_c0_g3	hypothetical protein POPTR_0006s11420g [Populus trichocarpa]	RPL17	50S ribosomal protein L17, chloroplastic OS=Nicotiana tabacum GN=RPL17 PE=2 SV=1	970.08	447.60	1.70	up	0.00	0.00	yes
TRINITY_DN19596_c0_g1	hypothetical protein POPTR_0001s45520g [Populus trichocarpa]	MTPA2	Metal tolerance protein A2 OS=Arabidopsis thaliana GN=MTPA2 PE=2 SV=2	65.84	36.47	1.45	up	0.00	0.00	yes
TRINITY_DN20951_c0_g2	hypothetical protein POPTR_0005s06740g [Populus trichocarpa]	mnmG	tRNA uridine 5-carboxymethylaminomethyl modification enzyme MnmG OS=Synechococcus sp. (strain JA-2-3B'a(2-13)) GN=mnmG PE=3 SV=1	26.43	17.06	1.35	up	0.00	0.00	yes
TRINITY_DN22443_c0_g3	PREDICTED: 3-oxoacyl-[acyl-carrier-protein] synthase 3 A, chloroplastic [Populus euphratica]	KAS3A	3-oxoacyl-[acyl-carrier-protein] synthase 3 A, chloroplastic OS=Cuphea wrightii GN=KAS3A PE=2 SV=2	34.98	21.39	1.31	up	0.00	0.00	yes
TRINITY_DN19014_c0_g2	hypothetical protein POPTR_0012s03150g [Populus trichocarpa]	EMB2024	Probable 6-phosphogluconolactonase 5, chloroplastic OS=Arabidopsis thaliana GN=EMB2024 PE=2 SV=1	33.06	21.31	1.24	up	0.00	0.00	yes

TRINITY_DN25701_c2_g2	-	-	-	60.26	22.02	2.07	up	0.00	0.00	yes
TRINITY_DN19231_c0_g1	hypothetical protein POPTR_0006s11920g [Populus trichocarpa]	-	-	17.46	7.19	1.62	up	0.00	0.00	yes
TRINITY_DN14586_c0_g1	PREDICTED: uncharacterized protein LOC105137872 [Populus euphratica]	ccmE	Cytochrome c-type biogenesis protein CcmE OS=Rhodospirillum rubrum (strain ATCC 11170 / ATH 1.1.1 / DSM 467 / LMG 4362 / NCIB 8255 / S1) GN=ccmE PE=3 SV=1	10.23	4.66	1.75	up	0.00	0.00	yes
TRINITY_DN21450_c0_g1	hypothetical protein POPTR_0010s17850g [Populus trichocarpa]	-	-	18.00	10.98	1.41	up	0.00	0.00	yes
TRINITY_DN15914_c0_g1	hypothetical protein POPTR_0003s15380g [Populus trichocarpa]	-	-	40.68	25.43	1.42	up	0.00	0.00	yes
TRINITY_DN22805_c0_g1	unnamed protein product, partial [Vitis vinifera]	RPS23	40S ribosomal protein S23 OS=Fragaria ananassa GN=RPS23 PE=2 SV=1	522.11	308.49	1.37	up	0.00	0.00	yes
TRINITY_DN21931_c0_g4	hypothetical protein POPTR_0011s12050g [Populus trichocarpa]	-	-	59.33	34.36	1.37	up	0.00	0.00	yes
TRINITY_DN22350_c0_g1	unknown [Populus trichocarpa]	BAS1	2-Cys peroxiredoxin BAS1, chloroplastic OS=Arabidopsis thaliana GN=BAS1 PE=1 SV=2	1056.96	506.32	1.63	up	0.00	0.00	yes
TRINITY_DN23530_c1_g2	pyridine nucleotide-disulfide oxidoreductase family protein [Populus tomentosa]	CHLP	Geranylgeranyl diphosphate reductase, chloroplastic OS=Nicotiana tabacum GN=CHLP PE=2 SV=1	681.14	397.07	1.38	up	0.00	0.00	yes
TRINITY_DN21088_c0_g1	-	-	-	9.06	1.18	3.48	up	0.00	0.00	yes
TRINITY_DN19373_c0_g2	PREDICTED: thioredoxin-like protein CXXS1 isoform X1 [Populus euphratica]	CXXS1	Thioredoxin-like protein CXXS1 OS=Arabidopsis thaliana GN=CXXS1 PE=2 SV=2	6.41	1.26	2.96	up	0.00	0.00	yes
TRINITY_DN13846_c0_g1	hypothetical protein PRUPE_ppa000146mg [Prunus persica]	FdGOGAT	Ferredoxin-dependent glutamate synthase, chloroplastic OS=Spinacia oleracea GN=FdGOGAT PE=1 SV=3	350.17	154.52	1.79	up	0.00	0.00	yes
TRINITY_DN16559_c0_g1	30S ribosomal protein S18 [Populus trichocarpa]	-	-	32.81	20.93	1.27	up	0.00	0.00	yes
TRINITY_DN21291_c2_g2	hypothetical protein POPTR_0015s08620g [Populus trichocarpa]	EX2	Protein EXECUTER 2, chloroplastic OS=Arabidopsis thaliana GN=EX2 PE=2 SV=1	471.84	166.72	2.14	up	0.00	0.00	yes
TRINITY_DN22150_c0_g1	hypothetical protein POPTR_0005s25590g [Populus trichocarpa]	BBX21	B-box zinc finger protein 21 OS=Arabidopsis thaliana GN=BBX21 PE=1 SV=1	7.98	5.08	1.88	up	0.00	0.00	yes
TRINITY_DN18074_c0_g2	PREDICTED: nascent polypeptide-associated complex subunit alpha-like protein 1 [Jatropha curcas]	-	Nascent polypeptide-associated complex subunit alpha-like protein OS=Pinus taeda PE=2 SV=1	169.18	101.69	1.37	up	0.00	0.00	yes
TRINITY_DN26963_c0_g2	40S ribosomal protein SA [Populus trichocarpa]	LRP	40S ribosomal protein SA OS=Brassica napus GN=LRP PE=2 SV=1	427.65	256.24	1.36	up	0.00	0.00	yes
TRINITY_DN16460_c0_g1	hypothetical protein POPTR_0001s26940g [Populus trichocarpa]	-	-	30.51	14.30	1.72	up	0.00	0.00	yes
TRINITY_DN24987_c0_g2	PREDICTED: probable serine/threonine-protein kinase WNK5 isoform X1 [Populus euphratica]	WNK5	Probable serine/threonine-protein kinase WNK5 OS=Arabidopsis thaliana GN=WNK5 PE=1 SV=2	2.83	0.75	2.52	up	0.00	0.00	yes
TRINITY_DN15274_c0_g1	PREDICTED: uncharacterized protein LOC105130257 [Populus euphratica]	-	-	65.34	41.20	1.28	up	0.00	0.00	yes
TRINITY_DN16119_c0_g1	quinone reductase family protein [Populus trichocarpa]	At5g58800	Probable NAD(P)H dehydrogenase (quinone) FQR1-like 3 OS=Arabidopsis thaliana GN=At5g58800 PE=2 SV=1	14.83	6.99	1.62	up	0.00	0.00	yes
TRINITY_DN18580_c0_g3	PREDICTED: uncharacterized protein LOC105123627 [Populus euphratica]	-	-	218.79	101.88	1.71	up	0.00	0.00	yes
TRINITY_DN27385_c1_g2	PREDICTED: LOW QUALITY PROTEIN: serine/threonine-protein kinase HT1-like [Populus euphratica]	STY46	Serine/threonine-protein kinase STY46 OS=Arabidopsis thaliana GN=STY46 PE=1 SV=1	6.17	1.09	3.08	up	0.00	0.00	yes
TRINITY_DN14543_c0_g1	hypothetical protein POPTR_0012s09780g [Populus trichocarpa]	-	-	126.99	69.50	1.48	up	0.00	0.00	yes
TRINITY_DN18068_c0_g1	hypothetical protein POPTR_0006s16020g [Populus trichocarpa]	CRR21	Pentatricopeptide repeat-containing protein At5g55740, chloroplastic OS=Arabidopsis thaliana GN=CRR21 PE=2 SV=1	11.25	6.22	1.45	up	0.00	0.00	yes
TRINITY_DN27079_c0_g4	putative cytoskeletal protein mRNA [Populus trichocarpa]	HIP	HSP-interacting protein OS=Zea mays GN=HIP PE=1 SV=1	35.17	20.94	1.36	up	0.00	0.00	yes
TRINITY_DN26332_c0_g2	hypothetical protein POPTR_0011s05650g [Populus trichocarpa]	Dnajb4	DnaJ homolog subfamily B member 4 OS=Mus musculus GN=Dnajb4 PE=1 SV=1	6.69	2.62	2.01	up	0.00	0.00	yes
TRINITY_DN17272_c0_g4	PREDICTED: two-component response regulator ORR9-like isoform X1 [Tarenaya hassleriana]	RR4	Two-component response regulator ORR4 OS=Oryza sativa subsp. indica GN=RR4 PE=2 SV=1	6.30	1.20	2.59	up	0.00	0.00	yes

TRINITY_DN16679_c0_g1	hypothetical protein POPTR_0007s07630g [Populus trichocarpa]	RPS10	Ribosomal protein S10, mitochondrial OS=Pisum sativum GN=RPS10 PE=2 SV=1	33.40	20.98	1.28	up	0.00	0.00	yes
TRINITY_DN25239_c0_g1	PREDICTED: uncharacterized protein LOC105125365 [Populus euphratica]	PTAC10	Protein PLASTID TRANSCRIPTIONALLY ACTIVE 10 OS=Arabidopsis thaliana GN=PTAC10 PE=1 SV=1	116.14	71.07	1.36	up	0.00	0.00	yes
TRINITY_DN27364_c0_g5	-	-	-	8.89	1.78	2.89	up	0.00	0.00	yes
TRINITY_DN16934_c0_g1	-	-	-	446.78	255.89	1.43	up	0.00	0.00	yes
TRINITY_DN20457_c0_g3	PREDICTED: 50S ribosomal protein L31, chloroplastic [Populus euphratica]	RPL31	50S ribosomal protein L31, chloroplastic OS=Arabidopsis thaliana GN=RPL31 PE=2 SV=1	248.10	114.54	1.71	up	0.00	0.00	yes
TRINITY_DN19035_c1_g1	hypothetical protein POPTR_0005s02060g [Populus trichocarpa]	DPD1	Exonuclease DPD1, chloroplastic/mitochondrial OS=Arabidopsis thaliana GN=DPD1 PE=1 SV=1	28.57	16.43	1.43	up	0.00	0.00	yes
TRINITY_DN20543_c0_g3	hypothetical protein POPTR_0017s02980g [Populus trichocarpa]	PK	Pyridoxal kinase OS=Arabidopsis thaliana GN=PK PE=1 SV=2	33.49	20.55	1.35	up	0.00	0.00	yes
TRINITY_DN16755_c0_g1	PREDICTED: uncharacterized protein LOC105113485 [Populus euphratica]	rlmN	Dual-specificity RNA methyltransferase RlmN OS=Geobacter sulfurreducens (strain ATCC 51573 / DSM 12127 / PCA) GN=rlmN PE=3 SV=1	32.38	17.40	1.49	up	0.00	0.00	yes
TRINITY_DN17682_c0_g2	PREDICTED: glutathione S-transferase T1-like [Populus euphratica]	GSTT1	Glutathione S-transferase T1 OS=Arabidopsis thaliana GN=GSTT1 PE=2 SV=1	99.32	53.27	1.44	up	0.00	0.00	yes
TRINITY_DN15107_c0_g2	unknown [Populus trichocarpa]	-	-	111.79	68.53	1.31	up	0.00	0.00	yes
TRINITY_DN18986_c0_g7	hypothetical protein POPTR_0017s05460g [Populus trichocarpa]	RPL38A	60S ribosomal protein L38 OS=Arabidopsis thaliana GN=RPL38A PE=3 SV=1	471.11	287.73	1.33	up	0.00	0.00	yes
TRINITY_DN21038_c0_g2	PREDICTED: 40S ribosomal protein S5-like [Populus euphratica]	RPS5B	40S ribosomal protein S5-2 OS=Arabidopsis thaliana GN=RPS5B PE=1 SV=2	924.86	550.50	1.36	up	0.00	0.00	yes
TRINITY_DN16460_c0_g2	hypothetical protein POPTR_0009s06170g [Populus trichocarpa]	-	-	13.63	4.14	2.39	up	0.00	0.00	yes
TRINITY_DN19875_c0_g1	hypothetical protein POPTR_0002s01570g [Populus trichocarpa]	AMY2	Probable alpha-amylase 2 OS=Arabidopsis thaliana GN=AMY2 PE=2 SV=1	9.19	4.59	1.63	up	0.00	0.00	yes
TRINITY_DN24156_c0_g1	hypothetical protein POPTR_0013s02900g [Populus trichocarpa]	RPS10A	40S ribosomal protein S10-1 OS=Arabidopsis thaliana GN=RPS10A PE=2 SV=1	423.97	247.21	1.40	up	0.00	0.00	yes
TRINITY_DN18598_c0_g1	hypothetical protein POPTR_0008s00350g [Populus trichocarpa]	-	Serine hydroxymethyltransferase, mitochondrial OS=Pisum sativum PE=1 SV=1	468.84	197.84	1.82	up	0.00	0.00	yes
TRINITY_DN24208_c0_g1	hypothetical protein POPTR_0009s09580g [Populus trichocarpa]	-	-	76.07	46.46	1.30	up	0.00	0.00	yes
TRINITY_DN21856_c0_g1	hypothetical protein POPTR_0016s00260g [Populus trichocarpa]	At5g10770	Aspartyl protease family protein At5g10770 OS=Arabidopsis thaliana GN=At5g10770 PE=2 SV=1	70.41	42.35	1.32	up	0.00	0.00	yes
TRINITY_DN16962_c0_g1	hypothetical protein POPTR_0009s10750g [Populus trichocarpa]	HIDM	2-hydroxyisoflavanone dehydratase OS=Glycyrrhiza echinata GN=HIDM PE=1 SV=1	348.16	110.65	2.25	up	0.00	0.00	yes
TRINITY_DN17047_c0_g1	hypothetical protein POPTR_0014s15880g [Populus trichocarpa]	CPZ	Ribonuclease Z, chloroplastic OS=Arabidopsis thaliana GN=CPZ PE=2 SV=1	75.71	44.18	1.38	up	0.00	0.00	yes
TRINITY_DN18288_c2_g1	PREDICTED: epoxide hydrolase 4-like isoform X1 [Populus euphratica]	-	-	72.73	42.69	1.38	up	0.00	0.00	yes
TRINITY_DN23619_c0_g1	hypothetical protein POPTR_0001s37430g [Populus trichocarpa]	RPS10	30S ribosomal protein S10, chloroplastic OS=Arabidopsis thaliana GN=RPS10 PE=2 SV=1	481.43	235.06	1.61	up	0.00	0.00	yes
TRINITY_DN26353_c0_g1	VuP5CS family protein [Populus trichocarpa]	-	Delta-1-pyrroline-5-carboxylate synthase OS=Actinidia deliciosa PE=2 SV=1	49.12	31.04	1.30	up	0.00	0.00	yes
TRINITY_DN22026_c0_g1	PREDICTED: zinc finger CCCH domain-containing protein 18-like isoform X1 [Populus euphratica]	At2g05160	Zinc finger CCCH domain-containing protein 18 OS=Arabidopsis thaliana GN=At2g05160 PE=2 SV=1	13.64	5.07	1.81	up	0.00	0.00	yes
TRINITY_DN27267_c0_g3	ATP synthase gamma chain family protein [Populus trichocarpa]	ATPC	ATP synthase subunit gamma, mitochondrial OS=Ipomoea batatas GN=ATPC PE=1 SV=2	52.68	23.01	1.81	up	0.00	0.00	yes
TRINITY_DN20528_c1_g2	hypothetical protein POPTR_0007s07690g [Populus trichocarpa]	SCPL35	Serine carboxypeptidase-like 35 OS=Arabidopsis thaliana GN=SCPL35 PE=2 SV=1	52.93	29.17	1.58	up	0.00	0.00	yes
TRINITY_DN19686_c0_g1	hypothetical protein POPTR_0001s38600g [Populus trichocarpa]	-	-	6.82	3.15	1.77	up	0.00	0.00	yes
TRINITY_DN19743_c0_g1	PREDICTED: WAT1-related protein At4g19185-like [Populus euphratica]	At4g19185	WAT1-related protein At4g19185 OS=Arabidopsis thaliana GN=At4g19185 PE=2 SV=1	66.15	39.51	1.38	up	0.00	0.00	yes
TRINITY_DN19804_c1_g4	hypothetical protein POPTR_0006s24610g [Populus trichocarpa]	-	-	26.82	16.02	1.44	up	0.00	0.00	yes

TRINITY_DN15004_c0_g1	hypothetical protein POPTR_0011s13630g [Populus trichocarpa]	-	-	230.03	90.46	1.94	up	0.00	0.00	yes
TRINITY_DN27133_c0_g2	hypothetical protein POPTR_0014s03830g [Populus trichocarpa]	-	-	22.81	14.71	1.24	up	0.00	0.00	yes
TRINITY_DN20224_c0_g2	hypothetical protein POPTR_0006s23770g [Populus trichocarpa]	RPL4	50S ribosomal protein L4, chloroplastic OS=Arabidopsis thaliana GN=RPL4 PE=2 SV=2	639.63	329.65	1.53	up	0.00	0.00	yes
TRINITY_DN20043_c0_g1	hypothetical protein POPTR_0004s23930g [Populus trichocarpa]	ISPD	2-C-methyl-D-erythritol 4-phosphate cytidylyltransferase, chloroplastic OS=Arabidopsis thaliana GN=ISPD PE=1 SV=1	72.92	42.64	1.45	up	0.00	0.00	yes
TRINITY_DN18188_c1_g2	60S ribosomal protein L30 [Populus trichocarpa]	RPL30	60S ribosomal protein L30 OS=Euphorbia esula GN=RPL30 PE=3 SV=1	256.39	154.88	1.34	up	0.00	0.00	yes
TRINITY_DN23557_c0_g1	PREDICTED: uncharacterized protein LOC105125793 isoform X1 [Populus euphratica]	-	-	31.73	20.49	1.34	up	0.00	0.00	yes
TRINITY_DN24120_c0_g1	PREDICTED: pentatricopeptide repeat-containing protein At1g74850, chloroplastic [Populus euphratica]	PTAC2	Pentatricopeptide repeat-containing protein At1g74850, chloroplastic OS=Arabidopsis thaliana GN=PTAC2 PE=2 SV=1	35.72	20.85	1.36	up	0.00	0.00	yes
TRINITY_DN15403_c0_g1	hypothetical protein POPTR_0015s11970g [Populus trichocarpa]	SDHAF2	Succinate dehydrogenase assembly factor 2, mitochondrial OS=Arabidopsis thaliana GN=SDHAF2 PE=1 SV=1	96.73	60.22	1.33	up	0.00	0.00	yes
TRINITY_DN26958_c0_g3	PREDICTED: uncharacterized protein LOC105125138 [Populus euphratica]	-	-	35.90	21.64	1.33	up	0.00	0.00	yes
TRINITY_DN19363_c0_g1	hypothetical protein POPTR_0019s10280g [Populus trichocarpa]	-	-	168.60	76.23	1.75	up	0.00	0.00	yes
TRINITY_DN20958_c0_g1	PREDICTED: UDP-glycosyltransferase 74E1-like [Populus euphratica]	UGT74F2	UDP-glycosyltransferase 74F2 OS=Arabidopsis thaliana GN=UGT74F2 PE=1 SV=1	64.45	33.63	1.57	up	0.00	0.00	yes
TRINITY_DN12148_c0_g2	ORF45 protein (plastid) [Spinacia oleracea]	-	-	86.74	41.77	1.64	up	0.00	0.00	yes
TRINITY_DN26034_c1_g1	unknown [Populus trichocarpa]	RPS7	40S ribosomal protein S7 OS=Avicennia marina GN=RPS7 PE=2 SV=1	1128.40	639.78	1.34	up	0.00	0.00	yes
TRINITY_DN23655_c0_g1	hypothetical protein POPTR_0015s09770g [Populus trichocarpa]	STR11	Rhodanese-like domain-containing protein 11, chloroplastic OS=Arabidopsis thaliana GN=STR11 PE=2 SV=1	53.69	27.56	1.60	up	0.00	0.00	yes
TRINITY_DN18269_c0_g1	ATP-NAD kinase family protein [Populus trichocarpa]	Os09g0345700	Probable NADH kinase OS=Oryza sativa subsp. japonica GN=Os09g0345700 PE=2 SV=1	37.58	23.32	1.44	up	0.00	0.00	yes
TRINITY_DN19915_c2_g2	hypothetical protein POPTR_0008s19040g [Populus trichocarpa]	-	-	89.40	40.53	1.66	up	0.00	0.00	yes
TRINITY_DN18562_c0_g1	hypothetical protein POPTR_0014s14610g [Populus trichocarpa]	PDS	15-cis-phytoene desaturase, chloroplastic/chromoplastic OS=Capsicum annuum GN=PDS PE=1 SV=1	40.15	24.98	1.30	up	0.00	0.00	yes
TRINITY_DN16989_c0_g1	PREDICTED: uncharacterized protein LOC105138677 [Populus euphratica]	-	-	125.36	60.06	1.66	up	0.00	0.00	yes
TRINITY_DN18511_c0_g1	Protochlorophyllide reductase C family protein [Populus trichocarpa]	POR1	Protochlorophyllide reductase, chloroplastic OS=Daucus carota GN=POR1 PE=2 SV=1	294.80	155.68	1.53	up	0.00	0.00	yes
TRINITY_DN25606_c0_g1	ribosomal protein S3a [Populus trichocarpa]	GSVIVT0002003	40S ribosomal protein S3a-1 OS=Vitis vinifera GN=GSVIVT00020038001 PE=3 SV=1	637.02	375.81	1.35	up	0.00	0.00	yes
TRINITY_DN23422_c1_g1	hypothetical protein POPTR_0013s12130g [Populus trichocarpa]	ATPD	ATP synthase delta chain, chloroplastic OS=Nicotiana tabacum GN=ATPD PE=2 SV=1	344.08	129.79	1.95	up	0.00	0.00	yes
TRINITY_DN18225_c0_g3	PREDICTED: hexokinase-1-like [Populus euphratica]	HXK1	Hexokinase-1 OS=Arabidopsis thaliana GN=HXK1 PE=1 SV=2	38.97	24.40	1.29	up	0.00	0.00	yes
TRINITY_DN17609_c0_g1	hypothetical protein CICLE_v10013157mg [Citrus clementina]	RPS28	40S ribosomal protein S28 OS=Zea mays GN=RPS28 PE=3 SV=1	121.99	68.61	1.41	up	0.00	0.00	yes
TRINITY_DN13148_c0_g1	PREDICTED: uncharacterized protein At2g34460, chloroplastic [Populus euphratica]	At2g34460	Uncharacterized protein At2g34460, chloroplastic OS=Arabidopsis thaliana GN=At2g34460 PE=2 SV=1	57.16	33.19	1.39	up	0.00	0.00	yes
TRINITY_DN24320_c1_g1	PREDICTED: thioredoxin Y1, chloroplastic-like isoform X2 [Populus euphratica]	At1g76760	Thioredoxin Y1, chloroplastic OS=Arabidopsis thaliana GN=At1g76760 PE=2 SV=1	250.01	144.28	1.50	up	0.00	0.00	yes
TRINITY_DN21064_c0_g2	PREDICTED: guanine nucleotide-binding protein subunit beta-like protein [Populus euphratica]	GB1	Guanine nucleotide-binding protein subunit beta-like protein OS=Medicago sativa GN=GB1 PE=2 SV=1	501.74	303.69	1.35	up	0.00	0.00	yes
TRINITY_DN12802_c0_g1	PREDICTED: uncharacterized protein LOC105113698 [Populus euphratica]	-	-	58.36	28.13	1.68	up	0.00	0.00	yes
TRINITY_DN26455_c3_g1	-	-	-	27.39	8.85	2.24	up	0.00	0.00	yes
TRINITY_DN20741_c0_g1	hypothetical protein POPTR_0009s08150g [Populus trichocarpa]	fabD	Malonyl CoA-acyl carrier protein transacylase OS=Bacillus subtilis (strain 168) GN=fabD PE=3 SV=2	80.30	48.68	1.32	up	0.00	0.00	yes

TRINITY_DN19282_c0_g1	Thylakoid lumenal 25.6 kDa family protein [Populus trichocarpa]	PPL1	PsbP-like protein 1, chloroplastic OS=Arabidopsis thaliana GN=PPL1 PE=1 SV=1	252.70	124.52	1.64	up	0.00	0.00	yes
TRINITY_DN15407_c1_g1	PREDICTED: uncharacterized protein LOC105110455 [Populus euphratica]	-	-	17.04	9.85	1.53	up	0.00	0.00	yes
TRINITY_DN22535_c1_g2	PREDICTED: 60S ribosomal protein L35a-1 [Populus euphratica]	RPL35AA	60S ribosomal protein L35a-1 OS=Arabidopsis thaliana GN=RPL35AA PE=3 SV=1	90.01	57.36	1.34	up	0.00	0.00	yes
TRINITY_DN20576_c1_g1	40S ribosomal protein S19 [Populus trichocarpa]	RPS19C	40S ribosomal protein S19-3 OS=Arabidopsis thaliana GN=RPS19C PE=2 SV=1	811.25	497.43	1.32	up	0.00	0.00	yes
TRINITY_DN24264_c1_g1	PREDICTED: chaperone protein ClpB4, mitochondrial-like [Populus euphratica]	CLPB3	Chaperone protein ClpB3, mitochondrial OS=Oryza sativa subsp. japonica GN=CLPB3 PE=2 SV=3	10.19	6.71	1.23	up	0.00	0.00	yes
TRINITY_DN21760_c0_g1	isocitrate dehydrogenase family protein [Populus trichocarpa]	IDH5	Isocitrate dehydrogenase [NAD] catalytic subunit 5, mitochondrial OS=Arabidopsis thaliana GN=IDH5 PE=1 SV=1	53.95	39.69	1.25	up	0.00	0.00	yes
TRINITY_DN16765_c0_g1	aquaporin SIP1.2 family protein [Populus trichocarpa]	SIP1-2	Aquaporin SIP1-2 OS=Zea mays GN=SIP1-2 PE=2 SV=1	41.92	15.61	2.04	up	0.00	0.00	yes
TRINITY_DN27900_c0_g1	PREDICTED: 1-aminocyclopropane-1-carboxylate oxidase 5-like [Populus euphratica]	DLO1	Protein DMR6-LIKE OXYGENASE 1 OS=Arabidopsis thaliana GN=DLO1 PE=1 SV=1	20.23	10.42	1.54	up	0.00	0.00	yes
TRINITY_DN23877_c1_g3	PREDICTED: S-adenosylmethionine synthase 4 [Populus euphratica]	METK4	S-adenosylmethionine synthase 4 OS=Populus trichocarpa GN=METK4 PE=2 SV=1	32.62	20.75	1.26	up	0.00	0.00	yes
TRINITY_DN25370_c0_g1	hypothetical protein POPTR_0002s01500g [Populus trichocarpa]	-	-	37.28	18.66	1.63	up	0.00	0.00	yes
TRINITY_DN13872_c0_g2	peptidyl-prolyl cis-trans isomerase family protein [Populus trichocarpa]	CYP19-3	Peptidyl-prolyl cis-trans isomerase CYP19-3 OS=Arabidopsis thaliana GN=CYP19-3 PE=2 SV=2	125.54	73.01	1.39	up	0.00	0.00	yes
TRINITY_DN17020_c0_g1	hypothetical protein POPTR_0002s06890g [Populus trichocarpa]	At1g43650	WAT1-related protein At1g43650 OS=Arabidopsis thaliana GN=At1g43650 PE=2 SV=1	22.89	6.58	2.36	up	0.00	0.00	yes
TRINITY_DN26572_c0_g1	hypothetical protein POPTR_0002s20530g [Populus trichocarpa]	PHR2	Blue-light photoreceptor PHR2 OS=Arabidopsis thaliana GN=PHR2 PE=2 SV=2	430.34	248.97	1.40	up	0.00	0.00	yes
TRINITY_DN18000_c0_g7	PREDICTED: 30S ribosomal protein S20, chloroplastic [Populus euphratica]	RPS20	30S ribosomal protein S20, chloroplastic OS=Arabidopsis thaliana GN=RPS20 PE=2 SV=1	821.00	378.16	1.71	up	0.00	0.00	yes
TRINITY_DN21355_c1_g1	hypothetical protein VITISV_005279 [Vitis vinifera]	PXG4	Probable peroxygenase 4 OS=Arabidopsis thaliana GN=PXG4 PE=1 SV=1	55.49	33.47	1.40	up	0.00	0.00	yes
TRINITY_DN17109_c0_g2	rubredoxin family protein [Populus trichocarpa]	-	-	151.91	64.13	1.84	up	0.00	0.00	yes
TRINITY_DN21176_c0_g3	PREDICTED: 60S acidic ribosomal protein P0 [Populus euphratica]	-	60S acidic ribosomal protein P0 OS=Glycine max PE=2 SV=1	763.19	456.15	1.28	up	0.00	0.00	yes
TRINITY_DN18986_c0_g1	hypothetical protein PRUPE_ppa019382mg, partial [Prunus persica]	RPL38A	60S ribosomal protein L38 OS=Arabidopsis thaliana GN=RPL38A PE=3 SV=1	61.85	34.35	1.48	up	0.00	0.00	yes
TRINITY_DN27756_c0_g1	-	-	-	181.30	67.03	2.07	up	0.00	0.00	yes
TRINITY_DN18853_c2_g1	60S ribosomal protein L18 [Populus trichocarpa]	RPL18B	60S ribosomal protein L18-2 OS=Arabidopsis thaliana GN=RPL18B PE=1 SV=2	314.78	185.66	1.38	up	0.00	0.00	yes
TRINITY_DN21221_c0_g1	hypothetical protein POPTR_0016s06950g [Populus trichocarpa]	RPL27AC	60S ribosomal protein L27a-3 OS=Arabidopsis thaliana GN=RPL27AC PE=2 SV=2	416.19	246.22	1.39	up	0.00	0.00	yes
TRINITY_DN23953_c0_g1	hypothetical protein POPTR_0001s07440g [Populus trichocarpa]	RCA	Ribulose bisphosphate carboxylase/oxygenase activase, chloroplastic OS=Oryza sativa subsp. japonica GN=RCA PE=1 SV=2	14.04	8.27	1.49	up	0.00	0.00	yes
TRINITY_DN23329_c0_g1	hypothetical protein POPTR_0007s12510g [Populus trichocarpa]	At4g16580	Probable protein phosphatase 2C 55 OS=Arabidopsis thaliana GN=At4g16580 PE=2 SV=2	41.39	25.57	1.36	up	0.00	0.00	yes
TRINITY_DN17333_c0_g1	predicted protein [Arabidopsis lyrata subsp. lyrata]	-	-	19.62	10.91	1.44	up	0.00	0.00	yes
TRINITY_DN21253_c0_g1	hypothetical protein POPTR_0015s13430g [Populus trichocarpa]	CHS	Chalcone synthase OS=Vitis vinifera GN=CHS PE=2 SV=1	14.73	8.81	1.45	up	0.00	0.00	yes
TRINITY_DN20955_c1_g3	PREDICTED: phosphoglucan phosphatase LSF2, chloroplastic [Populus euphratica]	LSF2	Phosphoglucan phosphatase LSF2, chloroplastic OS=Arabidopsis thaliana GN=LSF2 PE=1 SV=1	36.15	21.91	1.32	up	0.00	0.00	yes
TRINITY_DN20822_c0_g1	hypothetical protein POPTR_0018s12030g [Populus trichocarpa]	RPL39A	60S ribosomal protein L39-1 OS=Oryza sativa subsp. japonica GN=RPL39A PE=3 SV=2	689.57	416.53	1.32	up	0.00	0.00	yes
TRINITY_DN18446_c0_g1	hypothetical protein POPTR_0006s25990g [Populus trichocarpa]	RPL37C	60S ribosomal protein L37-3 OS=Arabidopsis thaliana GN=RPL37C PE=3 SV=1	578.79	320.15	1.40	up	0.00	0.00	yes
TRINITY_DN20975_c0_g3	PREDICTED: uncharacterized protein At2g27730, mitochondrial [Populus euphratica]	At2g27730	Uncharacterized protein At2g27730, mitochondrial OS=Arabidopsis thaliana GN=At2g27730 PE=1 SV=1	68.23	40.06	1.40	up	0.00	0.00	yes

TRINITY_DN23914_c0_g2	hypothetical protein POPTR_0014s17610g [Populus trichocarpa]	BASS3	Probable sodium/metabolite cotransporter BASS3, chloroplastic OS=Arabidopsis thaliana GN=BASS3 PE=2 SV=1	47.89	26.33	1.47	up	0.00	0.00	yes
TRINITY_DN17881_c0_g1	hypothetical protein POPTR_0008s04010g [Populus trichocarpa]	VATG1	V-type proton ATPase subunit G 1 OS=Nicotiana tabacum GN=VATG1 PE=3 SV=1	237.78	141.08	1.36	up	0.00	0.00	yes
TRINITY_DN20058_c0_g1	hypothetical protein POPTR_0009s08570g [Populus trichocarpa]	DAP	LL-diaminopimelate aminotransferase, chloroplastic OS=Arabidopsis thaliana GN=DAP PE=1 SV=1	82.27	50.72	1.29	up	0.00	0.00	yes
TRINITY_DN26902_c0_g1	SPX domain-containing family protein [Populus trichocarpa]	At4g22990	SPX domain-containing membrane protein At4g22990 OS=Arabidopsis thaliana GN=At4g22990 PE=2 SV=2	18.38	11.90	1.30	up	0.00	0.00	yes
TRINITY_DN25274_c0_g1	PREDICTED: probable indole-3-acetic acid-amido synthetase GH3.5 isoform X1 [Populus euphratica]	GH3.5	Jasmonic acid-amido synthetase JAR1 OS=Oryza sativa subsp. japonica GN=GH3.5 PE=2 SV=1	8.53	1.57	2.90	up	0.00	0.00	yes
TRINITY_DN17814_c0_g3	PREDICTED: uncharacterized protein LOC105116656 isoform X1 [Populus euphratica]	-	-	152.36	67.65	1.70	up	0.00	0.00	yes
TRINITY_DN16788_c0_g1	hypothetical protein POPTR_0016s12250g [Populus trichocarpa]	immp2l	Mitochondrial inner membrane protease subunit 2 OS=Danio rerio GN=immp2l PE=2 SV=1	16.67	7.36	1.82	up	0.00	0.00	yes
TRINITY_DN21093_c0_g1	PREDICTED: uncharacterized protein LOC105123355 [Populus euphratica]	slr0575	Thylakoid membrane protein slr0575 OS=Synechocystis sp. (strain PCC 6803 / Kazusa) GN=slr0575 PE=4 SV=1	98.86	53.37	1.55	up	0.00	0.00	yes
TRINITY_DN19050_c1_g1	hypothetical protein POPTR_0019s08170g, partial [Populus trichocarpa]	BCCP1	Biotin carboxyl carrier protein of acetyl-CoA carboxylase 1, chloroplastic OS=Arabidopsis thaliana GN=BCCP1 PE=1 SV=2	123.18	74.70	1.32	up	0.00	0.00	yes
TRINITY_DN26676_c0_g1	60S ribosomal protein L13 [Populus trichocarpa]	RPL13B	60S ribosomal protein L13-1 OS=Arabidopsis thaliana GN=RPL13B PE=1 SV=1	815.99	534.53	1.40	up	0.00	0.00	yes
TRINITY_DN18318_c0_g2	unknown [Populus trichocarpa]	RPL35	50S ribosomal protein L35, chloroplastic OS=Arabidopsis thaliana GN=RPL35 PE=2 SV=1	339.70	165.15	1.63	up	0.00	0.00	yes
TRINITY_DN18165_c0_g1	GCN5-related N-acetyltransferase family protein [Populus trichocarpa]	-	-	27.16	15.08	1.45	up	0.00	0.00	yes
TRINITY_DN24235_c0_g1	PREDICTED: uncharacterized protein LOC105130729 isoform X1 [Populus euphratica]	rluB	Ribosomal large subunit pseudouridine synthase B OS=Bacillus subtilis (strain 168) GN=rluB PE=1 SV=2	126.93	69.11	1.48	up	0.00	0.00	yes
TRINITY_DN17847_c0_g1	PREDICTED: ATP synthase subunit epsilon, mitochondrial-like [Populus euphratica]	-	ATP synthase subunit epsilon, mitochondrial OS=Ipomoea batatas PE=1 SV=2	269.06	157.20	1.34	up	0.00	0.00	yes
TRINITY_DN17087_c0_g5	chaperone protein dnaJ [Populus trichocarpa]	-	-	13.30	5.62	1.86	up	0.00	0.00	yes
TRINITY_DN17127_c0_g1	hypothetical protein POPTR_0007s04100g [Populus trichocarpa]	ndhO	NAD(P)H-quinone oxidoreductase subunit O, chloroplastic OS=Arabidopsis thaliana GN=ndhO PE=2 SV=1	221.33	94.73	1.82	up	0.00	0.00	yes
TRINITY_DN19629_c0_g1	hypothetical protein POPTR_0006s12920g [Populus trichocarpa]	LOG3	Cytokinin riboside 5'-monophosphate phosphoribohydrolase LOG3 OS=Arabidopsis thaliana GN=LOG3 PE=1 SV=1	24.28	14.66	1.42	up	0.00	0.00	yes
TRINITY_DN21903_c0_g1	unknown [Populus trichocarpa]	TIF3I1	Eukaryotic translation initiation factor 3 subunit I OS=Arabidopsis thaliana GN=TIF3I1 PE=2 SV=2	81.78	51.30	1.29	up	0.00	0.00	yes
TRINITY_DN25562_c0_g1	hypothetical protein POPTR_0008s01090g [Populus trichocarpa]	ACR11	ACT domain-containing protein ACR11 OS=Arabidopsis thaliana GN=ACR11 PE=1 SV=1	627.09	246.27	1.89	up	0.00	0.00	yes
TRINITY_DN20148_c0_g1	hypothetical protein POPTR_0010s12560g [Populus trichocarpa]	RPI2	Probable ribose-5-phosphate isomerase 2 OS=Arabidopsis thaliana GN=RPI2 PE=1 SV=1	131.18	75.15	1.45	up	0.00	0.00	yes
TRINITY_DN21260_c0_g2	hypothetical protein POPTR_0018s03130g [Populus trichocarpa]	GDU3	Protein GLUTAMINE DUMPER 3 OS=Arabidopsis thaliana GN=GDU3 PE=2 SV=1	2.49	0.43	3.18	up	0.00	0.00	yes
TRINITY_DN17648_c0_g2	calcineurin-like phosphoesterase family protein [Populus trichocarpa]	SLP1	Shewanella-like protein phosphatase 1 OS=Arabidopsis thaliana GN=SLP1 PE=1 SV=1	7.85	2.39	2.31	up	0.00	0.00	yes
TRINITY_DN19154_c0_g1	PREDICTED: peroxisomal membrane protein 11B-like [Populus euphratica]	PEX11B	Peroxisomal membrane protein 11B OS=Arabidopsis thaliana GN=PEX11B PE=1 SV=1	35.95	15.31	1.84	up	0.00	0.00	yes
TRINITY_DN24886_c0_g1	PREDICTED: elongation factor 1-gamma 2-like [Populus euphratica]	Os02g0220500	Elongation factor 1-gamma 2 OS=Oryza sativa subsp. japonica GN=Os02g0220500 PE=2 SV=2	232.26	138.32	1.29	up	0.00	0.00	yes
TRINITY_DN21323_c0_g1	PREDICTED: glutamyl-tRNA(Gln) amidotransferase subunit C, chloroplastic/mitochondrial isoform X1 [Populus euphratica]	GATC	Glutamyl-tRNA(Gln) amidotransferase subunit C, chloroplastic/mitochondrial OS=Populus trichocarpa GN=GATC PE=3 SV=1	205.18	105.69	1.46	up	0.00	0.00	yes
TRINITY_DN17930_c0_g1	unknown [Populus trichocarpa x Populus deltoides]	HPR-A	Glycerate dehydrogenase OS=Cucumis sativus GN=HPR-A PE=2 SV=1	1011.61	280.35	2.42	up	0.00	0.00	yes
TRINITY_DN16168_c0_g2	hypothetical protein POPTR_0010s15190g, partial [Populus trichocarpa]	RPL29A	60S ribosomal protein L29-1 OS=Arabidopsis thaliana GN=RPL29A PE=1 SV=1	470.54	295.79	1.28	up	0.00	0.00	yes
TRINITY_DN17423_c0_g1	PREDICTED: nudix hydrolase 2-like [Populus euphratica]	NUDT2	Nudix hydrolase 2 OS=Arabidopsis thaliana GN=NUDT2 PE=1 SV=1	17.61	15.62	1.49	up	0.00	0.00	yes

TRINITY_DN15718_c0_g1	hypothetical protein POPTR_0012s08870g [Populus trichocarpa]	At4g17560	50S ribosomal protein L19-1, chloroplastic OS=Arabidopsis thaliana GN=At4g17560 PE=2 SV=1	31.89	19.20	1.41	up	0.00	0.00	yes
TRINITY_DN16333_c0_g2	ribosomal protein L27 [Populus trichocarpa]	RPL27	60S ribosomal protein L27 OS=Pisum sativum GN=RPL27 PE=2 SV=1	445.26	270.06	1.35	up	0.00	0.00	yes
TRINITY_DN20897_c0_g1	PREDICTED: putative pentatricopeptide repeat-containing protein At3g15200 [Populus euphratica]	At3g15200	Putative pentatricopeptide repeat-containing protein At3g15200 OS=Arabidopsis thaliana GN=At3g15200 PE=3 SV=1	6.37	3.46	1.49	up	0.00	0.00	yes
TRINITY_DN24156_c0_g2	-	-	-	151.55	84.93	1.44	up	0.00	0.00	yes
TRINITY_DN20380_c0_g3	PREDICTED: protein DCL, chloroplastic [Populus euphratica]	DCL	Protein DCL, chloroplastic OS=Solanum lycopersicum GN=DCL PE=2 SV=1	82.21	48.59	1.39	up	0.00	0.00	yes
TRINITY_DN16334_c0_g1	PREDICTED: uncharacterized protein LOC105110550 [Populus euphratica]	-	-	511.20	242.24	1.67	up	0.00	0.00	yes
TRINITY_DN23627_c1_g2	hypothetical protein POPTR_1028s00200g [Populus trichocarpa]	CYTC	Cytochrome c OS=Fritillaria agrestis GN=CYTC PE=3 SV=3	108.32	59.63	1.49	up	0.00	0.00	yes
TRINITY_DN19739_c0_g3	hypothetical protein POPTR_0005s02430g [Populus trichocarpa]	RPL22B	60S ribosomal protein L22-2 OS=Arabidopsis thaliana GN=RPL22B PE=2 SV=1	525.21	316.51	1.34	up	0.00	0.00	yes
TRINITY_DN23937_c0_g1	Glutamine synthetase nodule isozyme [Ananas comosus]	GLN1	Glutamine synthetase OS=Alnus glutinosa GN=GLN1 PE=2 SV=1	127.57	57.48	1.78	up	0.00	0.00	yes
TRINITY_DN24214_c1_g4	Ribosome recycling factor family protein [Populus trichocarpa]	RRF	Ribosome-recycling factor, chloroplastic OS=Spinacia oleracea GN=RRF PE=1 SV=1	220.86	121.85	1.46	up	0.00	0.00	yes
TRINITY_DN20880_c0_g1	PREDICTED: uncharacterized protein At4g37920, chloroplastic isoform X1 [Populus euphratica]	At4g37920	Uncharacterized protein At4g37920, chloroplastic OS=Arabidopsis thaliana GN=At4g37920 PE=1 SV=2	52.57	29.40	1.42	up	0.00	0.00	yes
TRINITY_DN26526_c0_g1	ACT domain-containing family protein [Populus trichocarpa]	ACR3	ACT domain-containing protein ACR3 OS=Arabidopsis thaliana GN=ACR3 PE=2 SV=1	16.75	10.39	1.27	up	0.00	0.00	yes
TRINITY_DN23789_c0_g2	PREDICTED: uncharacterized protein LOC105112215 [Populus euphratica]	At3g02060	ATP-dependent DNA helicase At3g02060, chloroplastic OS=Arabidopsis thaliana GN=At3g02060 PE=2 SV=1	14.18	8.86	1.65	up	0.00	0.00	yes
TRINITY_DN23437_c0_g3	hypothetical protein POPTR_0017s11960g [Populus trichocarpa]	NIP2-1	Aquaporin NIP2-1 OS=Oryza sativa subsp. japonica GN=NIP2-1 PE=1 SV=1	6.28	1.73	2.56	up	0.00	0.00	yes
TRINITY_DN23268_c0_g1	hypothetical protein POPTR_0006s21650g [Populus trichocarpa]	CP33	RNA-binding protein CP33, chloroplastic OS=Arabidopsis thaliana GN=CP33 PE=2 SV=1	465.66	243.37	1.52	up	0.00	0.00	yes
TRINITY_DN19445_c0_g3	-	-	-	610.14	301.43	1.59	up	0.00	0.00	yes
TRINITY_DN15649_c0_g1	PREDICTED: UPF0160 protein C27H6.8 [Populus euphratica]	Myg1	UPF0160 protein MYG1, mitochondrial OS=Rattus norvegicus GN=Myg1 PE=1 SV=1	35.57	23.46	1.28	up	0.00	0.00	yes
TRINITY_DN17607_c0_g1	hypothetical protein POPTR_0001s10030g [Populus trichocarpa]	rplY	50S ribosomal protein L25 OS=Rhodopseudomonas palustris (strain HaA2) GN=rplY PE=3 SV=1	27.23	16.71	1.34	up	0.00	0.00	yes
TRINITY_DN19010_c0_g1	hypothetical protein POPTR_0016s11690g [Populus trichocarpa]	FLN1	Fructokinase-like 1, chloroplastic OS=Arabidopsis thaliana GN=FLN1 PE=1 SV=1	93.49	53.39	1.39	up	0.00	0.00	yes
TRINITY_DN20590_c0_g2	METHIONINE AMINOPEPTIDASE 1D family protein [Populus trichocarpa]	MAP1D	Methionine aminopeptidase 1D, chloroplastic/mitochondrial OS=Arabidopsis thaliana GN=MAP1D PE=1 SV=1	79.40	44.51	1.44	up	0.00	0.00	yes
TRINITY_DN19565_c2_g2	hypothetical protein POPTR_0017s01450g, partial [Populus trichocarpa]	-	-	14.77	4.78	2.25	up	0.00	0.00	yes
TRINITY_DN26184_c0_g4	PREDICTED: 50S ribosomal protein L10, chloroplastic [Jatropha curcas]	RPL10	50S ribosomal protein L10, chloroplastic OS=Nicotiana tabacum GN=RPL10 PE=2 SV=1	56.79	23.65	1.85	up	0.00	0.00	yes
TRINITY_DN21889_c0_g3	017G114600 [Populus tomentosa]	RBCS	Ribulose bisphosphate carboxylase small chain, chloroplastic OS=Pyrus pyrifolia GN=RBCS PE=2 SV=1	20448.45	6363.37	2.27	up	0.00	0.00	yes
TRINITY_DN26633_c0_g1	hypothetical protein POPTR_0008s19040g [Populus trichocarpa]	-	-	232.96	80.74	2.13	up	0.00	0.00	yes
TRINITY_DN17408_c0_g3	hypothetical protein POPTR_0001s46750g [Populus trichocarpa]	CCB1	Protein COFACTOR ASSEMBLY OF COMPLEX C SUBUNIT B CCB1, chloroplastic OS=Arabidopsis thaliana GN=CCB1 PE=1 SV=1	127.53	52.11	1.90	up	0.00	0.00	yes
TRINITY_DN17396_c0_g2	PREDICTED: ATP-dependent Clp protease proteolytic subunit 3, chloroplastic [Populus euphratica]	CLPP3	ATP-dependent Clp protease proteolytic subunit 3, chloroplastic OS=Arabidopsis thaliana GN=CLPP3 PE=1 SV=1	158.54	87.42	1.47	up	0.00	0.00	yes
TRINITY_DN22798_c0_g3	hypothetical protein POPTR_0002s12740g [Populus trichocarpa]	STK38L	Serine/threonine-protein kinase 38-like OS=Homo sapiens GN=STK38L PE=1 SV=3	5.19	1.17	2.68	up	0.00	0.00	yes
TRINITY_DN21064_c0_g1	PREDICTED: guanine nucleotide-binding protein subunit beta-like protein [Populus euphratica]	GB1	Guanine nucleotide-binding protein subunit beta-like protein OS=Medicago sativa GN=GB1 PE=2 SV=1	224.22	138.39	1.34	up	0.00	0.00	yes

TRINITY_DN22563_c0_g1	2-oxoacid dehydrogenase family protein [Populus trichocarpa]	At4g26910	Dihydrolipoyllysine-residue succinyltransferase component of 2-oxoglutarate dehydrogenase complex 2, mitochondrial OS=Arabidopsis thaliana GN=At4g26910 PE=1 SV=2	62.68	37.12	1.23	up	0.00	0.00	yes
TRINITY_DN26163_c0_g1	PREDICTED: dihydrolipoyllysine-residue acetyltransferase component 2 of pyruvate dehydrogenase complex, mitochondrial-like [Populus euphratica]	At3g13930	Dihydrolipoyllysine-residue acetyltransferase component 2 of pyruvate dehydrogenase complex, mitochondrial OS=Arabidopsis thaliana GN=At3g13930 PE=1 SV=2	49.33	31.65	1.24	up	0.00	0.00	yes
TRINITY_DN27349_c0_g1	hypothetical protein POPTR_0001s44720g [Populus trichocarpa]	POLIA	DNA polymerase I A, chloroplastic/mitochondrial OS=Arabidopsis thaliana GN=POLIA PE=2 SV=1	31.99	22.49	1.26	up	0.00	0.00	yes
TRINITY_DN27695_c0_g1	hypothetical protein POPTR_0017s01160g [Populus trichocarpa]	HSP83A	Heat shock protein 83 OS=Ipomoea nil GN=HSP83A PE=2 SV=1	29.21	5.47	3.03	up	0.00	0.00	yes
TRINITY_DN18623_c2_g2	hypothetical protein POPTR_0004s06340g [Populus trichocarpa]	TPS1	Alpha,alpha-trehalose-phosphate synthase [UDP-forming] 1 OS=Arabidopsis thaliana GN=TPS1 PE=1 SV=1	2.59	0.56	2.80	up	0.00	0.00	yes
TRINITY_DN22954_c0_g2	TRYPTOPHAN SYNTHASE ALPHA CHAIN family protein [Populus trichocarpa]	TSA1	Tryptophan synthase alpha chain, chloroplastic OS=Arabidopsis thaliana GN=TSA1 PE=1 SV=1	98.67	70.99	1.23	up	0.00	0.00	yes
TRINITY_DN23709_c0_g2	aspartate aminotransferase 2 family protein [Populus trichocarpa]	ASP5	Aspartate aminotransferase, chloroplastic OS=Arabidopsis thaliana GN=ASP5 PE=1 SV=2	46.64	32.80	1.23	up	0.00	0.00	yes
TRINITY_DN17914_c0_g3	quinone reductase family protein [Populus trichocarpa]	At4g36750	Probable NAD(P)H dehydrogenase (quinone) FQR1-like 2 OS=Arabidopsis thaliana GN=At4g36750 PE=1 SV=1	12.02	5.14	1.82	up	0.00	0.00	yes
TRINITY_DN18964_c0_g2	PREDICTED: probable aldehyde dehydrogenase [Populus euphratica]	FIS1	Probable aldehyde dehydrogenase OS=Linum usitatissimum GN=FIS1 PE=2 SV=1	11.98	7.36	1.33	up	0.00	0.00	yes
TRINITY_DN25870_c1_g1	unknown [Populus trichocarpa]	EMB1144	Chorismate synthase, chloroplastic OS=Arabidopsis thaliana GN=EMB1144 PE=2 SV=2	296.23	175.19	1.47	up	0.00	0.00	yes
TRINITY_DN25100_c0_g1	PREDICTED: myosin-2 heavy chain-like [Populus euphratica]	-	-	47.44	30.54	1.22	up	0.00	0.00	yes
TRINITY_DN25205_c1_g5	hypothetical protein M569_00220, partial [Genlisea aurea]	rps3	30S ribosomal protein S3, chloroplastic OS=Populus alba GN=rps3 PE=3 SV=1	38.98	24.08	1.26	up	0.00	0.00	yes
TRINITY_DN22693_c0_g1	unknown [Populus trichocarpa]	YNR064C	Uncharacterized hydrolase YNR064C OS=Saccharomyces cerevisiae (strain ATCC 204508 / S288c) GN=YNR064C PE=3 SV=1	25.70	12.53	1.68	up	0.00	0.00	yes
TRINITY_DN20645_c0_g2	PREDICTED: prolyl endopeptidase-like isoform X1 [Populus euphratica]	dapb1	Dipeptidyl aminopeptidase BI OS=Pseudoxanthomonas mexicana GN=dapb1 PE=1 SV=1	3.99	1.89	1.71	up	0.00	0.00	yes
TRINITY_DN27171_c2_g1	PREDICTED: D-glycerate 3-kinase, chloroplastic-like [Populus euphratica]	GLYK	D-glycerate 3-kinase, chloroplastic OS=Arabidopsis thaliana GN=GLYK PE=1 SV=2	158.02	76.60	1.79	up	0.00	0.00	yes
TRINITY_DN23776_c0_g1	hypothetical protein POPTR_0005s14280g [Populus trichocarpa]	-	-	31.39	19.38	1.29	up	0.00	0.00	yes
TRINITY_DN22394_c0_g4	PREDICTED: embryogenesis-associated protein EMB8 [Populus euphratica]	EMB8	Embryogenesis-associated protein EMB8 OS=Picea glauca GN=EMB8 PE=2 SV=1	39.52	24.62	1.29	up	0.00	0.00	yes
TRINITY_DN17492_c0_g1	hypothetical protein POPTR_0002s25540g [Populus trichocarpa]	GRXS10	Monothiol glutaredoxin-S10 OS=Oryza sativa subsp. japonica GN=GRXS10 PE=2 SV=2	94.40	49.92	1.52	up	0.00	0.00	yes
TRINITY_DN24922_c0_g1	hypothetical protein POPTR_0789s00200g [Populus trichocarpa]	RPL8	60S ribosomal protein L8 OS=Solanum lycopersicum GN=RPL8 PE=2 SV=1	589.51	362.18	1.32	up	0.00	0.00	yes
TRINITY_DN20621_c0_g1	PREDICTED: probable isoleucine--tRNA ligase, mitochondrial isoform X2 [Populus euphratica]	OVA2	Isoleucine--tRNA ligase, chloroplastic/mitochondrial OS=Arabidopsis thaliana GN=OVA2 PE=2 SV=1	36.44	21.66	1.34	up	0.00	0.00	yes
TRINITY_DN19728_c0_g1	hypothetical protein POPTR_0010s01430g [Populus trichocarpa]	ephA	Epoxide hydrolase A OS=Mycobacterium tuberculosis (strain ATCC 25618 / H37Rv) GN=ephA PE=1 SV=1	46.16	30.18	1.25	up	0.00	0.00	yes
TRINITY_DN22886_c0_g1	RIBOSOMAL protein L23A [Populus trichocarpa]	RPL23A	60S ribosomal protein L23A OS=Fritillaria agrestis GN=RPL23A PE=2 SV=1	626.80	394.25	1.31	up	0.00	0.00	yes
TRINITY_DN17999_c0_g2	PREDICTED: NADH-cytochrome b5 reductase-like protein [Populus euphratica]	CBR2	NADH-cytochrome b5 reductase-like protein OS=Arabidopsis thaliana GN=CBR2 PE=1 SV=2	44.41	29.39	1.21	up	0.00	0.00	yes
TRINITY_DN24569_c0_g3	PREDICTED: pentatricopeptide repeat-containing protein At1g59720, mitochondrial [Populus euphratica]	PCMP-H51	Pentatricopeptide repeat-containing protein At1g59720, chloroplastic/mitochondrial OS=Arabidopsis thaliana GN=PCMP-H51 PE=2 SV=2	5.14	2.82	1.49	up	0.00	0.00	yes
TRINITY_DN19966_c0_g1	hypothetical protein POPTR_0001s35030g [Populus trichocarpa]	NRT2.7	High affinity nitrate transporter 2.7 OS=Arabidopsis thaliana GN=NRT2.7 PE=2 SV=1	5.94	1.76	2.35	up	0.00	0.00	yes

TRINITY_DN22908_c0_g1	PREDICTED: pentatricopeptide repeat-containing protein DOT4, chloroplastic-like [Populus euphratica]	PCMP-H32	Putative pentatricopeptide repeat-containing protein At3g23330 OS=Arabidopsis thaliana GN=PCMP-H32 PE=3 SV=1	5.53	3.14	1.42	up	0.00	0.00	yes
TRINITY_DN24940_c0_g2	PREDICTED: ADP,ATP carrier protein 1, mitochondrial [Populus euphratica]	ANT1	ADP,ATP carrier protein 1, mitochondrial OS=Gossypium hirsutum GN=ANT1 PE=2 SV=1	432.60	260.66	1.30	up	0.00	0.00	yes
TRINITY_DN20732_c0_g1	hypothetical protein POPTR_0014s11480g [Populus trichocarpa]	CLPP5	ATP-dependent Clp protease proteolytic subunit 5, chloroplastic OS=Arabidopsis thaliana GN=CLPP5 PE=1 SV=1	377.24	212.25	1.44	up	0.00	0.00	yes
TRINITY_DN22503_c0_g1	hypothetical protein POPTR_0018s08920g [Populus trichocarpa]	Os01g0760900	Probable protein ABIL5 OS=Oryza sativa subsp. japonica GN=Os01g0760900 PE=2 SV=1	144.60	92.65	1.32	up	0.00	0.00	yes
TRINITY_DN19997_c0_g2	PREDICTED: cytochrome P450 72A15-like isoform X1 [Populus euphratica]	CYP72A15	Cytochrome P450 72A15 OS=Arabidopsis thaliana GN=CYP72A15 PE=2 SV=1	25.07	10.83	2.21	up	0.00	0.00	yes
TRINITY_DN18339_c0_g1	PREDICTED: uncharacterized protein LOC105138036 [Populus euphratica]	HHL1	Protein HHL1, chloroplastic OS=Arabidopsis thaliana GN=HHL1 PE=1 SV=1	276.96	124.88	1.67	up	0.00	0.00	yes
TRINITY_DN19380_c0_g1	PREDICTED: LRR receptor-like serine/threonine-protein kinase FLS2 [Populus euphratica]	EFR	LRR receptor-like serine/threonine-protein kinase EFR OS=Arabidopsis thaliana GN=EFR PE=1 SV=1	2.68	0.26	3.80	up	0.00	0.00	yes
TRINITY_DN23999_c0_g1	PREDICTED: uncharacterized protein LOC105124413 isoform X1 [Populus euphratica]	-	-	56.82	36.29	1.35	up	0.00	0.00	yes
TRINITY_DN21795_c0_g5	chloroplast biogenesis family protein [Populus trichocarpa]	ISPH	4-hydroxy-3-methylbut-2-enyl diphosphate reductase, chloroplastic OS=Arabidopsis thaliana GN=ISPH PE=2 SV=1	125.22	79.24	1.27	up	0.00	0.00	yes
TRINITY_DN16657_c0_g1	PREDICTED: uncharacterized protein LOC105133394 isoform X2 [Populus euphratica]	-	-	24.31	14.76	1.36	up	0.00	0.00	yes
TRINITY_DN19482_c0_g2	30S ribosomal protein S9 [Populus trichocarpa]	RPS9	30S ribosomal protein S9, chloroplastic OS=Arabidopsis thaliana GN=RPS9 PE=2 SV=1	516.76	258.18	1.61	up	0.00	0.00	yes
TRINITY_DN21600_c0_g2	PREDICTED: acetolactate synthase small subunit 2, chloroplastic-like [Populus euphratica]	At2g31810	Acetolactate synthase small subunit 2, chloroplastic OS=Arabidopsis thaliana GN=At2g31810 PE=1 SV=1	35.74	23.13	1.23	up	0.00	0.00	yes
TRINITY_DN22914_c0_g1	hypothetical protein POPTR_0004s18310g [Populus trichocarpa]	hpxO	FAD-dependent urate hydroxylase OS=Acinetobacter baylyi (strain ATCC 33305 / BD413 / ADP1) GN=hpxO PE=1 SV=1	20.68	12.07	1.41	up	0.00	0.00	yes
TRINITY_DN17628_c0_g1	hypothetical protein POPTR_0012s14230g [Populus trichocarpa]	NEET	CDGSH iron-sulfur domain-containing protein NEET OS=Arabidopsis thaliana GN=NEET PE=1 SV=1	331.30	165.25	1.61	up	0.00	0.00	yes
TRINITY_DN20325_c0_g4	hypothetical protein POPTR_0002s04420g [Populus trichocarpa]	RPS15	40S ribosomal protein S15 OS=Oryza sativa subsp. japonica GN=RPS15 PE=2 SV=2	658.50	416.42	1.28	up	0.00	0.00	yes
TRINITY_DN16732_c0_g1	hypothetical protein POPTR_0017s07130g [Populus trichocarpa]	Mdp1	Magnesium-dependent phosphatase 1 OS=Mus musculus GN=Mdp1 PE=1 SV=1	32.81	20.70	1.28	up	0.00	0.00	yes
TRINITY_DN19700_c0_g1	PREDICTED: ATP-dependent zinc metalloprotease FTSH 12, chloroplastic [Populus euphratica]	FTSH12	ATP-dependent zinc metalloprotease FTSH 12, chloroplastic OS=Arabidopsis thaliana GN=FTSH12 PE=2 SV=2	88.02	55.24	1.29	up	0.00	0.00	yes
TRINITY_DN24238_c0_g1	unknown [Populus trichocarpa x Populus deltoides]	-	-	31.05	19.63	1.26	up	0.00	0.00	yes
TRINITY_DN25193_c0_g1	hypothetical protein POPTR_0009s14340g [Populus trichocarpa]	POPTRDRAFT_[illegible]	Alanine--tRNA ligase, chloroplastic/mitochondrial OS=Populus trichocarpa GN=POPTRDRAFT_821063 PE=3 SV=1	45.99	27.53	1.36	up	0.00	0.00	yes
TRINITY_DN17062_c0_g1	unknown [Populus trichocarpa x Populus deltoides]	At5g52970	Thylakoid lumenal 15.0 kDa protein 2, chloroplastic OS=Arabidopsis thaliana GN=At5g52970 PE=1 SV=2	40.75	21.00	1.57	up	0.00	0.00	yes
TRINITY_DN19263_c0_g4	hypothetical protein POPTR_0001s32260g [Populus trichocarpa]	-	-	135.63	87.91	1.25	up	0.00	0.00	yes
TRINITY_DN18868_c0_g1	PREDICTED: peptide deformylase 1A, chloroplastic/mitochondrial [Populus euphratica]	PDF1A	Peptide deformylase 1A, chloroplastic OS=Solanum lycopersicum GN=PDF1A PE=2 SV=1	83.75	50.49	1.34	up	0.00	0.00	yes
TRINITY_DN19079_c1_g2	hypothetical protein POPTR_0001s35910g [Populus trichocarpa]	-	-	24.52	12.07	1.64	up	0.00	0.00	yes
TRINITY_DN21885_c0_g3	PREDICTED: 7-deoxyloganetin glucosyltransferase-like [Populus euphratica]	UGT85A24	7-deoxyloganetin glucosyltransferase OS=Gardenia jasminoides GN=UGT85A24 PE=1 SV=1	23.23	13.15	1.52	up	0.00	0.00	yes
TRINITY_DN16886_c0_g3	hypothetical protein POPTR_0006s11410g [Populus trichocarpa]	-	-	2.67	0.49	3.04	up	0.00	0.00	yes
TRINITY_DN27629_c1_g1	thioredoxin m family protein [Populus trichocarpa]	-	Thioredoxin M-type, chloroplastic OS=Spinacia oleracea PE=1 SV=2	732.04	310.86	1.83	up	0.00	0.00	yes
TRINITY_DN21717_c0_g1	hypothetical protein POPTR_0002s24430g [Populus trichocarpa]	-	-	12.77	6.19	1.64	up	0.00	0.00	yes
TRINITY_DN23260_c0_g1	anthranilate synthase beta subunit 1 family protein [Populus trichocarpa]	ASB2	Anthranilate synthase beta subunit 2, chloroplastic OS=Arabidopsis thaliana GN=ASB2 PE=2 SV=1	230.33	121.36	1.32	up	0.00	0.00	yes

TRINITY_DN18544_c0_g2	hypothetical protein POPTR_0006s29050g [Populus trichocarpa]	At4g31390	Uncharacterized aarF domain-containing protein kinase At4g31390, chloroplastic OS=Arabidopsis thaliana GN=At4g31390 PE=2 SV=1	25.69	14.85	1.38	up	0.00	0.00	yes
TRINITY_DN18555_c0_g3	PREDICTED: protoheme IX farnesyltransferase, mitochondrial [Populus euphratica]	COX10	Protoheme IX farnesyltransferase, mitochondrial OS=Arabidopsis thaliana GN=COX10 PE=2 SV=4	26.04	16.92	1.21	up	0.00	0.00	yes
TRINITY_DN20102_c0_g6	PREDICTED: 50S ribosomal protein L29, chloroplastic [Populus euphratica]	RPL29	50S ribosomal protein L29, chloroplastic OS=Arabidopsis thaliana GN=RPL29 PE=1 SV=1	500.72	224.46	1.74	up	0.00	0.00	yes
TRINITY_DN21283_c2_g1	PREDICTED: uncharacterized protein LOC105114856 [Populus euphratica]	rpmH	50S ribosomal protein L34 OS=Colwellia psychrerythraea (strain 34H / ATCC BAA-681) GN=rpmH PE=3 SV=1	121.97	77.71	1.26	up	0.00	0.00	yes
TRINITY_DN20311_c0_g1	PREDICTED: programmed cell death protein 2 isoform X1 [Populus euphratica]	ERS1	Probable ethylene response sensor 1 OS=Oryza sativa subsp. indica GN=ERS1 PE=2 SV=1	25.80	14.04	1.47	up	0.00	0.00	yes
TRINITY_DN25221_c1_g5	hypothetical protein POPTR_0006s08320g [Populus trichocarpa]	-	-	8.38	2.99	2.09	up	0.00	0.00	yes
TRINITY_DN18533_c0_g2	Cf-4/9 disease resistance-like family protein [Populus trichocarpa]	-	-	3.32	1.17	2.09	up	0.00	0.00	yes
TRINITY_DN24720_c0_g1	PREDICTED: pentatricopeptide repeat-containing protein At1g02150-like [Populus euphratica]	At1g02150	Pentatricopeptide repeat-containing protein At1g02150 OS=Arabidopsis thaliana GN=At1g02150 PE=2 SV=2	105.18	60.95	1.38	up	0.00	0.00	yes
TRINITY_DN21819_c0_g1	hypothetical protein POPTR_0001s30900g [Populus trichocarpa]	-	-	47.10	25.05	1.53	up	0.00	0.00	yes
TRINITY_DN18459_c0_g2	mitochondrial transcription termination factor-related family protein [Populus trichocarpa]	-	-	8.04	1.87	2.69	up	0.00	0.00	yes
TRINITY_DN17668_c0_g2	PREDICTED: retrotransposon-like protein 1 [Populus euphratica]	-	-	8.28	2.66	2.26	up	0.00	0.00	yes
TRINITY_DN22142_c1_g1	PREDICTED: 104 kDa microneme/rhoptry antigen-like [Populus euphratica]	-	-	19.86	11.40	1.72	up	0.00	0.00	yes
TRINITY_DN17038_c0_g1	PREDICTED: transcription factor WER-like [Populus euphratica]	WER	Transcription factor WER OS=Arabidopsis thaliana GN=WER PE=1 SV=1	78.70	30.19	2.14	up	0.00	0.00	yes
TRINITY_DN18189_c0_g2	ATP synthase gamma chain 1 family protein [Populus trichocarpa]	ATPC	ATP synthase gamma chain, chloroplastic OS=Nicotiana tabacum GN=ATPC PE=1 SV=1	776.42	338.36	1.79	up	0.00	0.00	yes
TRINITY_DN24423_c1_g2	-	-	-	8.93	1.94	2.79	up	0.00	0.00	yes
TRINITY_DN20703_c0_g1	hypothetical protein POPTR_0001s06160g [Populus trichocarpa]	-	-	31.88	17.03	1.51	up	0.00	0.00	yes
TRINITY_DN19750_c1_g4	40S ribosomal protein S16 [Populus trichocarpa]	RPS16	40S ribosomal protein S16 OS=Gossypium hirsutum GN=RPS16 PE=2 SV=1	746.46	441.88	1.33	up	0.00	0.00	yes
TRINITY_DN17628_c0_g2	hypothetical protein POPTR_0015s14240g [Populus trichocarpa]	NEET	CDGSH iron-sulfur domain-containing protein NEET OS=Arabidopsis thaliana GN=NEET PE=1 SV=1	54.49	29.82	1.48	up	0.00	0.00	yes
TRINITY_DN18944_c0_g1	hypothetical protein POPTR_0001s35130g [Populus trichocarpa]	PPT2	Phosphoenolpyruvate/phosphate translocator 2, chloroplastic OS=Arabidopsis thaliana GN=PPT2 PE=2 SV=1	11.16	6.52	1.39	up	0.00	0.00	yes
TRINITY_DN26479_c0_g1	AAA-type ATPase family protein [Populus trichocarpa]	atad-3	ATPase family AAA domain-containing protein 3 OS=Caenorhabditis elegans GN=atad-3 PE=3 SV=2	23.09	19.00	1.26	up	0.00	0.00	yes
TRINITY_DN18074_c0_g3	hypothetical protein POPTR_0003s18990g [Populus trichocarpa]	At3g12390	Nascent polypeptide-associated complex subunit alpha-like protein 1 OS=Arabidopsis thaliana GN=At3g12390 PE=1 SV=1	92.72	60.22	1.24	up	0.00	0.00	yes
TRINITY_DN20346_c0_g1	PROHIBITIN 3 family protein [Populus trichocarpa]	PHB3	Prohibitin-3, mitochondrial OS=Arabidopsis thaliana GN=PHB3 PE=1 SV=1	187.25	121.07	1.26	up	0.00	0.00	yes
TRINITY_DN27871_c2_g1	hypothetical protein POPTR_0006s19380g [Populus trichocarpa]	TSS	Protein TSS OS=Arabidopsis thaliana GN=TSS PE=1 SV=1	214.80	100.94	1.69	up	0.00	0.00	yes
TRINITY_DN26495_c0_g1	PREDICTED: fructose-bisphosphate aldolase-lysine N-methyltransferase, chloroplastic [Populus euphratica]	LSMT-L	[Fructose-bisphosphate aldolase]-lysine N-methyltransferase, chloroplastic OS=Arabidopsis thaliana GN=LSMT-L PE=1 SV=1	56.21	32.00	1.41	up	0.00	0.00	yes
TRINITY_DN21588_c1_g3	ubiquinol-cytochrome C reductase iron-sulfur subunit family protein [Populus trichocarpa]	-	Cytochrome b-c1 complex subunit Rieske-4, mitochondrial OS=Nicotiana tabacum PE=2 SV=1	145.82	99.13	1.17	up	0.00	0.00	yes
TRINITY_DN19811_c0_g4	hypothetical protein POPTR_0002s19890g [Populus trichocarpa]	TMEM205	Transmembrane protein 205 OS=Homo sapiens GN=TMEM205 PE=1 SV=1	295.79	158.40	1.51	up	0.00	0.00	yes
TRINITY_DN21048_c1_g1	hypothetical protein POPTR_0002s10870g [Populus trichocarpa]	HEMA1	Glutamyl-tRNA reductase 1, chloroplastic OS=Cucumis sativus GN=HEMA1 PE=2 SV=1	151.87	89.22	1.36	up	0.00	0.00	yes
TRINITY_DN22097_c0_g1	hypothetical protein POPTR_0018s01270g [Populus trichocarpa]	-	-	28.75	15.39	1.48	up	0.00	0.00	yes

TRINITY_DN17384_c0_g1	PREDICTED: uncharacterized protein LOC105133813 isoform X2 [Populus euphratica]	-	-	86.64	42.02	1.54	up	0.00	0.00	yes
TRINITY_DN23670_c0_g1	hypothetical protein POPTR_0014s15230g [Populus trichocarpa]	ARASP	Membrane metalloprotease ARASP, chloroplastic OS=Arabidopsis thaliana GN=ARASP PE=2 SV=1	52.26	29.54	1.39	up	0.00	0.00	yes
TRINITY_DN23449_c1_g6	hypothetical protein POPTR_0012s11580g [Populus trichocarpa]	-	-	18.75	10.70	1.45	up	0.00	0.00	yes
TRINITY_DN18666_c0_g4	hypothetical protein POPTR_0005s05420g [Populus trichocarpa]	ACA13	Putative calcium-transporting ATPase 13, plasma membrane-type OS=Arabidopsis thaliana GN=ACA13 PE=3 SV=1	4.64	1.27	2.50	up	0.00	0.00	yes
TRINITY_DN19223_c0_g1	hypothetical protein POPTR_0017s07870g [Populus trichocarpa]	PA1024	Nitronate monooxygenase OS=Pseudomonas aeruginosa (strain ATCC 15692 / DSM 22644 / CIP 104116 / JCM 14847 / LMG 12228 / 1C / PRS 101 / PAO1) GN=PA1024 PE=1 SV=1	8.76	4.08	1.71	up	0.00	0.00	yes
TRINITY_DN23671_c0_g1	PREDICTED: chloroplast processing peptidase [Populus euphratica]	PLSP1	Chloroplast processing peptidase OS=Arabidopsis thaliana GN=PLSP1 PE=2 SV=2	123.40	80.67	1.34	up	0.00	0.00	yes
TRINITY_DN17841_c0_g2	PREDICTED: uncharacterized protein LOC105130722 [Populus euphratica]	-	-	105.98	51.73	1.55	up	0.00	0.00	yes
TRINITY_DN16537_c0_g2	hypothetical protein POPTR_0004s12260g [Populus trichocarpa]	-	-	204.83	128.50	1.27	up	0.00	0.00	yes
TRINITY_DN18307_c1_g3	profilin 2 family protein [Populus trichocarpa]	-	Profilin-3 OS=Hevea brasiliensis PE=1 SV=1	202.97	135.62	1.20	up	0.00	0.00	yes
TRINITY_DN26661_c0_g1	hypothetical protein CISIN_1g016748mg [Citrus sinensis]	-	Fructose-bisphosphate aldolase, cytoplasmic isozyme 1 OS=Pisum sativum PE=2 SV=1	718.86	176.98	2.62	up	0.00	0.00	yes
TRINITY_DN20672_c0_g1	hypothetical protein POPTR_0006s03190g [Populus trichocarpa]	FKBP65	Peptidyl-prolyl cis-trans isomerase FKBP65 OS=Arabidopsis thaliana GN=FKBP65 PE=1 SV=1	49.08	22.60	1.85	up	0.00	0.00	yes
TRINITY_DN17699_c0_g1	PREDICTED: fatty-acid-binding protein 3 [Populus euphratica]	FAP3	Fatty-acid-binding protein 3, chloroplastic OS=Arabidopsis thaliana GN=FAP3 PE=1 SV=1	105.66	57.49	1.45	up	0.00	0.00	yes
TRINITY_DN19823_c0_g5	hypothetical protein POPTR_0005s09370g [Populus trichocarpa]	SCOA	Succinate--CoA ligase [ADP-forming] subunit alpha-1, mitochondrial OS=Solanum lycopersicum GN=SCOA PE=1 SV=1	70.43	46.85	1.20	up	0.00	0.00	yes
TRINITY_DN14004_c0_g1	PREDICTED: uncharacterized protein LOC105130283 [Populus euphratica]	rplW	50S ribosomal protein L23 OS=Pseudomonas aeruginosa (strain PA7) GN=rplW PE=3 SV=1	65.83	41.05	1.32	up	0.00	0.00	yes
TRINITY_DN17923_c0_g1	hypothetical protein POPTR_0004s12040g [Populus trichocarpa]	MINE1	Cell division topological specificity factor homolog, chloroplastic OS=Arabidopsis thaliana GN=MINE1 PE=1 SV=1	21.17	10.10	1.68	up	0.00	0.00	yes
TRINITY_DN17779_c0_g1	hypothetical protein POPTR_0001s26450g [Populus trichocarpa]	-	-	83.38	35.66	1.81	up	0.00	0.00	yes
TRINITY_DN19792_c0_g1	ATP-binding-cassette transporter family protein [Populus trichocarpa]	ABCI8	UPF0051 protein ABCI8, chloroplastic OS=Arabidopsis thaliana GN=ABCI8 PE=2 SV=1	73.85	45.58	1.31	up	0.00	0.00	yes
TRINITY_DN24899_c0_g1	PREDICTED: 60S ribosomal protein L7-4 [Populus euphratica]	RPL7D	60S ribosomal protein L7-4 OS=Arabidopsis thaliana GN=RPL7D PE=2 SV=1	574.05	369.19	1.26	up	0.00	0.00	yes
TRINITY_DN23152_c0_g1	thylakoid lumen 18.3 kDa family protein [Populus trichocarpa]	At1g54780	UPF0603 protein At1g54780, chloroplastic OS=Arabidopsis thaliana GN=At1g54780 PE=1 SV=1	638.56	295.27	1.74	up	0.00	0.00	yes
TRINITY_DN23858_c0_g2	PREDICTED: uncharacterized protein LOC105111212 [Populus euphratica]	HBII	Hemoglobin-2 OS=Casuarina glauca GN=HBII PE=3 SV=1	23.78	10.14	1.84	up	0.00	0.00	yes
TRINITY_DN15780_c0_g1	hypothetical protein POPTR_0004s03340g [Populus trichocarpa]	-	-	66.74	26.20	1.96	up	0.00	0.00	yes
TRINITY_DN18727_c0_g1	hypothetical protein POPTR_0007s11440g [Populus trichocarpa]	rplY	50S ribosomal protein L25 OS=Ehrlichia ruminantium (strain Gardel) GN=rplY PE=3 SV=2	34.61	23.25	1.18	up	0.00	0.00	yes
TRINITY_DN22554_c0_g5	aquaporin NIP6.1 family protein [Populus trichocarpa]	NIP6-1	Aquaporin NIP6-1 OS=Arabidopsis thaliana GN=NIP6-1 PE=1 SV=1	3.16	1.23	1.99	up	0.00	0.00	yes
TRINITY_DN22978_c0_g5	PREDICTED: serine carboxypeptidase-like 20 isoform X1 [Populus euphratica]	SCPL20	Serine carboxypeptidase-like 20 OS=Arabidopsis thaliana GN=SCPL20 PE=2 SV=2	22.84	11.51	1.57	up	0.00	0.00	yes
TRINITY_DN22849_c0_g1	RNA-binding protein RNP1 precursor [Populus trichocarpa]	CP29B	RNA-binding protein CP29B, chloroplastic OS=Arabidopsis thaliana GN=CP29B PE=1 SV=1	253.11	130.90	1.56	up	0.00	0.00	yes
TRINITY_DN14981_c0_g1	single-strand-binding family protein [Populus trichocarpa]	At4g11060	Single-stranded DNA-binding protein, mitochondrial OS=Arabidopsis thaliana GN=At4g11060 PE=2 SV=1	44.95	29.84	1.20	up	0.00	0.00	yes
TRINITY_DN22952_c1_g1	hypothetical protein POPTR_0003s03410g [Populus trichocarpa]	BOI	E3 ubiquitin-protein ligase BOI OS=Arabidopsis thaliana GN=BOI PE=1 SV=1	36.90	21.29	1.39	up	0.00	0.00	yes
TRINITY_DN24696_c0_g1	PREDICTED: proteinaceous RNase P 1, chloroplastic/mitochondrial-like isoform X1 [Populus euphratica]	PRORP1	Proteinaceous RNase P 1, chloroplastic/mitochondrial OS=Arabidopsis thaliana GN=PRORP1 PE=1 SV=1	26.14	14.84	1.40	up	0.00	0.00	yes

TRINITY_DN20981_c2_g2	hypothetical protein POPTR_0012s14730g [Populus trichocarpa]	At3g12800	Peroxisomal 2,4-dienoyl-CoA reductase OS=Arabidopsis thaliana GN=At3g12800 PE=2 SV=1	31.60	20.56	1.23	up	0.00	0.00	yes
TRINITY_DN21278_c0_g2	PREDICTED: uncharacterized protein LOC105125518 isoform X3 [Populus euphratica]	-	-	41.90	27.45	1.23	up	0.00	0.00	yes
TRINITY_DN22697_c0_g3	3-PHOSPHOSERINE PHOSPHATASE family protein [Populus trichocarpa]	PSP	Phosphoserine phosphatase, chloroplastic OS=Arabidopsis thaliana GN=PSP PE=1 SV=2	51.79	35.35	1.19	up	0.00	0.00	yes
TRINITY_DN24130_c0_g1	hypothetical protein POPTR_0001s39310g [Populus trichocarpa]	RPL13	50S ribosomal protein L13, chloroplastic OS=Arabidopsis thaliana GN=RPL13 PE=2 SV=1	811.24	439.27	1.47	up	0.00	0.00	yes
TRINITY_DN23872_c0_g1	PREDICTED: 60S ribosomal protein L24-like [Populus euphratica]	RPL24B	60S ribosomal protein L24-2 OS=Arabidopsis thaliana GN=RPL24B PE=1 SV=2	570.45	355.52	1.31	up	0.00	0.00	yes
TRINITY_DN17925_c1_g2	unknown [Populus trichocarpa]	NDK1	Nucleoside diphosphate kinase 1 OS=Arabidopsis thaliana GN=NDK1 PE=1 SV=1	708.50	436.01	1.30	up	0.00	0.00	yes
TRINITY_DN22867_c0_g1	proton-dependent oligopeptide transport family protein [Populus trichocarpa]	NPF2.11	Protein NRT1/ PTR FAMILY 2.11 OS=Arabidopsis thaliana GN=NPF2.11 PE=1 SV=1	6.94	2.63	2.03	up	0.00	0.00	yes
TRINITY_DN24551_c2_g6	hypothetical protein POPTR_0012s03020g [Populus trichocarpa]	GIF3	GRF1-interacting factor 3 OS=Arabidopsis thaliana GN=GIF3 PE=1 SV=1	35.00	22.94	1.22	up	0.00	0.00	yes
TRINITY_DN21182_c0_g4	RNA polymerase alpha subunit [Populus alba]	rpoA	DNA-directed RNA polymerase subunit alpha OS=Populus alba GN=rpoA PE=3 SV=1	6.16	3.50	1.43	up	0.00	0.00	yes
TRINITY_DN24188_c1_g5	pentatricopeptide repeat superfamily protein [Populus tomentosa]	PCMP-H12	Pentatricopeptide repeat-containing protein At1g08070, chloroplastic OS=Arabidopsis thaliana GN=PCMP-H12 PE=2 SV=1	17.60	9.15	1.55	up	0.00	0.00	yes
TRINITY_DN23701_c0_g1	latex plastidic aldolase-like family protein [Populus trichocarpa]	FBA2	Fructose-bisphosphate aldolase 2, chloroplastic OS=Arabidopsis thaliana GN=FBA2 PE=1 SV=2	2289.47	879.27	1.96	up	0.00	0.00	yes
TRINITY_DN15149_c0_g1	hypothetical protein POPTR_0001s10930g [Populus trichocarpa]	RPS6	30S ribosomal protein S6 alpha, chloroplastic OS=Arabidopsis thaliana GN=RPS6 PE=2 SV=1	917.35	458.74	1.59	up	0.00	0.00	yes
TRINITY_DN19877_c1_g1	hipI-SODC1s [Populus tremula]	SODCC.2	Superoxide dismutase [Cu-Zn] 2 OS=Mesembryanthemum crystallinum GN=SODCC.2 PE=2 SV=1	76.97	44.87	1.52	up	0.00	0.00	yes
TRINITY_DN21443_c0_g3	ribosomal protein S13 [Populus trichocarpa]	RPS13	40S ribosomal protein S13 OS=Glycine max GN=RPS13 PE=2 SV=1	234.19	147.73	1.30	up	0.00	0.00	yes
TRINITY_DN25892_c1_g4	unknown [Populus trichocarpa x Populus deltoides]	BASS2	Sodium/pyruvate cotransporter BASS2, chloroplastic OS=Arabidopsis thaliana GN=BASS2 PE=1 SV=1	56.52	36.35	1.32	up	0.00	0.00	yes
TRINITY_DN22117_c0_g6	PREDICTED: uncharacterized protein LOC105114340 [Populus euphratica]	mrnC	Mini-ribonuclease 3 OS=Synechocystis sp. (strain PCC 6803 / Kazusa) GN=mrnC PE=3 SV=1	81.42	44.37	1.45	up	0.00	0.00	yes
TRINITY_DN21007_c0_g2	patched family protein [Populus trichocarpa]	NPC1	Niemann-Pick C1 protein OS=Sus scrofa GN=NPC1 PE=2 SV=1	15.84	5.74	2.08	up	0.00	0.00	yes
TRINITY_DN14911_c0_g1	hypothetical protein POPTR_0007s12190g [Populus trichocarpa]	DAR1	Protein DA1-related 1 OS=Arabidopsis thaliana GN=DAR1 PE=1 SV=3	6.07	1.23	2.88	up	0.00	0.00	yes
TRINITY_DN24131_c0_g1	PREDICTED: probable terpene synthase 9 isoform X1 [Populus euphratica]	TPS9	Probable terpene synthase 9 OS=Ricinus communis GN=TPS9 PE=3 SV=1	12.38	4.15	2.15	up	0.00	0.00	yes
TRINITY_DN25569_c0_g1	shikimate kinase family protein [Populus trichocarpa]	SK	Shikimate kinase, chloroplastic OS=Solanum lycopersicum GN=SK PE=1 SV=1	60.67	37.43	1.34	up	0.00	0.00	yes
TRINITY_DN15116_c0_g1	hypothetical protein POPTR_0014s13550g [Populus trichocarpa]	Rnf126	E3 ubiquitin-protein ligase RNF126 OS=Mus musculus GN=Rnf126 PE=1 SV=1	3.22	1.19	2.04	up	0.00	0.00	yes
TRINITY_DN23286_c0_g1	hydrolase family protein [Populus trichocarpa]	-	-	113.62	52.83	1.68	up	0.00	0.00	yes
TRINITY_DN12141_c0_g1	hypothetical protein POPTR_0001s33250g [Populus trichocarpa]	-	-	63.04	40.89	1.25	up	0.00	0.00	yes
TRINITY_DN17481_c0_g2	LIPOYLTRANSFERASE 2 family protein [Populus trichocarpa]	LIP2	Octanoyltransferase OS=Arabidopsis thaliana GN=LIP2 PE=2 SV=1	20.15	11.87	1.38	up	0.00	0.00	yes
TRINITY_DN20178_c0_g2	SWIB complex BAF60b domain-containing family protein [Populus trichocarpa]	UAF30	Upstream activation factor subunit UAF30 OS=Saccharomyces cerevisiae (strain ATCC 204508 / S288c) GN=UAF30 PE=1 SV=1	272.01	162.50	1.34	up	0.00	0.00	yes
TRINITY_DN24964_c0_g1	PREDICTED: histidinol dehydrogenase, chloroplastic-like isoform X3 [Populus euphratica]	HISN8	Histidinol dehydrogenase, chloroplastic OS=Arabidopsis thaliana GN=HISN8 PE=2 SV=1	31.19	20.54	1.27	up	0.00	0.00	yes
TRINITY_DN19196_c0_g1	PREDICTED: pentatricopeptide repeat-containing protein At1g11290 [Populus euphratica]	PCMP-H40	Pentatricopeptide repeat-containing protein At1g11290, chloroplastic OS=Arabidopsis thaliana GN=PCMP-H40 PE=2 SV=1	9.63	6.25	1.23	up	0.00	0.00	yes
TRINITY_DN27619_c7_g1	-	-	-	204.68	85.96	1.86	up	0.00	0.00	yes

TRINITY_DN21779_c0_g1	hypothetical protein POPTR_0001s40130g [Populus trichocarpa]	HEME1	Uroporphyrinogen decarboxylase 1, chloroplastic OS=Arabidopsis thaliana GN=HEME1 PE=2 SV=2	94.27	52.40	1.44	up	0.00	0.00	yes
TRINITY_DN26731_c1_g1	hypothetical protein POPTR_0010s22980g [Populus trichocarpa]	manB	Phosphomannomutase OS=Escherichia coli GN=manB PE=3 SV=1	36.09	25.10	1.19	up	0.00	0.00	yes
TRINITY_DN19614_c0_g2	hypothetical protein POPTR_0018s08840g [Populus trichocarpa]	-	-	26.40	14.74	1.51	up	0.00	0.00	yes
TRINITY_DN23702_c0_g3	-	-	-	44.04	16.90	1.98	up	0.00	0.00	yes
TRINITY_DN22393_c0_g4	hypothetical protein POPTR_0014s04370g [Populus trichocarpa]	-	-	66.94	35.74	1.50	up	0.00	0.00	yes
TRINITY_DN22580_c0_g3	hypothetical protein POPTR_0003s10720g [Populus trichocarpa]	At2g43090	3-isopropylmalate dehydratase small subunit 3 OS=Arabidopsis thaliana GN=At2g43090 PE=1 SV=1	124.71	79.50	1.24	up	0.00	0.00	yes
TRINITY_DN18389_c1_g1	PREDICTED: tropinone reductase homolog [Populus euphratica]	At2g29260	Tropinone reductase homolog At2g29260, chloroplastic OS=Arabidopsis thaliana GN=At2g29260 PE=2 SV=1	35.46	24.97	1.18	up	0.00	0.00	yes
TRINITY_DN26341_c0_g2	dihydrolipoamide S-acetyltransferase family protein [Populus trichocarpa]	LTA3	Dihydrolipoyllysine-residue acetyltransferase component 1 of pyruvate dehydrogenase complex, mitochondrial OS=Arabidopsis thaliana GN=LTA3 PE=1 SV=2	11.43	7.48	1.21	up	0.00	0.00	yes
TRINITY_DN26168_c0_g17	-	-	-	46.83	7.68	3.05	up	0.00	0.00	yes
TRINITY_DN22455_c0_g1	PREDICTED: uncharacterized protein LOC105113285 [Populus euphratica]	-	-	29.43	19.50	1.28	up	0.00	0.00	yes
TRINITY_DN19947_c0_g1	PREDICTED: 40S ribosomal protein S11 [Populus euphratica]	RPS11	40S ribosomal protein S11 OS=Euphorbia esula GN=RPS11 PE=2 SV=1	757.36	474.08	1.26	up	0.00	0.00	yes
TRINITY_DN21443_c0_g1	ribosomal protein S13 [Populus trichocarpa]	RPS13A	40S ribosomal protein S13-1 OS=Arabidopsis thaliana GN=RPS13A PE=2 SV=1	64.53	38.20	1.37	up	0.00	0.00	yes
TRINITY_DN26248_c0_g2	PREDICTED: 40S ribosomal protein S3-3 [Populus euphratica]	RPS3C	40S ribosomal protein S3-3 OS=Arabidopsis thaliana GN=RPS3C PE=1 SV=1	554.83	341.10	1.25	up	0.00	0.00	yes
TRINITY_DN19079_c1_g6	hypothetical protein POPTR_0012s09250g [Populus trichocarpa]	-	-	23.16	9.13	2.01	up	0.00	0.00	yes
TRINITY_DN22564_c0_g2	VDAC3.1 family protein [Populus trichocarpa]	VDAC4	Mitochondrial outer membrane protein porin 4 OS=Arabidopsis thaliana GN=VDAC4 PE=1 SV=1	22.64	15.12	1.20	up	0.00	0.00	yes
TRINITY_DN23227_c0_g1	hypothetical protein POPTR_0008s06640g [Populus trichocarpa]	EGY2	Probable zinc metalloprotease EGY2, chloroplastic OS=Arabidopsis thaliana GN=EGY2 PE=2 SV=1	58.86	34.97	1.40	up	0.00	0.00	yes
TRINITY_DN22141_c1_g5	CP12 domain-containing family protein [Populus trichocarpa]	CP12-2	Calvin cycle protein CP12-2, chloroplastic OS=Arabidopsis thaliana GN=CP12-2 PE=1 SV=1	1102.99	499.52	1.75	up	0.00	0.00	yes
TRINITY_DN26711_c0_g2	Sedoheptulose-1 family protein [Populus trichocarpa]	-	Sedoheptulose-1,7-bisphosphatase, chloroplastic OS=Spinacia oleracea PE=2 SV=1	1906.74	735.61	1.91	up	0.00	0.00	yes
TRINITY_DN19949_c0_g1	zinc finger family protein [Populus trichocarpa]	VAR3	Zinc finger protein VAR3, chloroplastic OS=Arabidopsis thaliana GN=VAR3 PE=1 SV=2	43.82	30.89	1.13	up	0.00	0.00	yes
TRINITY_DN16857_c0_g1	oxidoreductase family protein [Populus trichocarpa]	At4g09670	Uncharacterized oxidoreductase At4g09670 OS=Arabidopsis thaliana GN=At4g09670 PE=1 SV=1	50.64	33.84	1.18	up	0.00	0.00	yes
TRINITY_DN20407_c0_g1	plastid developmental protein DAG [Populus trichocarpa]	MORF5	Multiple organellar RNA editing factor 5, mitochondrial OS=Arabidopsis thaliana GN=MORF5 PE=2 SV=1	313.75	204.79	1.23	up	0.00	0.00	yes
TRINITY_DN17369_c0_g3	ascorbate peroxidase [Populus tomentosa]	APX2	L-ascorbate peroxidase 2, cytosolic OS=Arabidopsis thaliana GN=APX2 PE=2 SV=3	128.46	76.99	1.35	up	0.00	0.00	yes
TRINITY_DN15808_c0_g1	PREDICTED: ferredoxin-dependent glutamate synthase, chloroplastic-like [Populus euphratica]	GLU1	Ferredoxin-dependent glutamate synthase 1, chloroplastic/mitochondrial OS=Arabidopsis thaliana GN=GLU1 PE=1 SV=3	604.52	260.27	1.81	up	0.00	0.00	yes
TRINITY_DN23582_c0_g1	hypothetical protein POPTR_0006s29400g [Populus trichocarpa]	topA	DNA topoisomerase 1 OS=Rickettsia bellii (strain RML369-C) GN=topA PE=3 SV=1	44.02	30.96	1.21	up	0.00	0.00	yes
TRINITY_DN22484_c1_g1	brassinosteroid-regulated family protein [Populus trichocarpa]	-	Xyloglucan endotransglucosylase/hydrolase 2 OS=Glycine max PE=2 SV=1	76.54	24.94	1.76	up	0.00	0.00	yes
TRINITY_DN22850_c0_g3	hypothetical protein POPTR_0017s11710g [Populus trichocarpa]	NRP2	NAP1-related protein 2 OS=Arabidopsis thaliana GN=NRP2 PE=1 SV=2	38.03	24.96	1.24	up	0.00	0.00	yes
TRINITY_DN16458_c0_g1	PREDICTED: LOW QUALITY PROTEIN: uncharacterized protein LOC105136146 [Populus euphratica]	ATPG	ATP synthase subunit b', chloroplastic OS=Spinacia oleracea GN=ATPG PE=1 SV=2	1816.06	855.39	1.67	up	0.00	0.00	yes

TRINITY_DN25356_c0_g2	PREDICTED: uncharacterized protein LOC105130188 [Populus euphratica]	-	-	56.67	30.32	1.49	up	0.00	0.00	yes
TRINITY_DN26462_c0_g2	hypothetical protein POPTR_0009s05900g [Populus trichocarpa]	der	GTPase Der OS=Synechococcus elongatus (strain PCC 7942) GN=der PE=3 SV=1	36.70	23.79	1.22	up	0.00	0.00	yes
TRINITY_DN13514_c0_g2	glycine-rich family protein [Populus trichocarpa]	-	-	143.84	79.29	1.46	up	0.00	0.00	yes
TRINITY_DN26922_c0_g2	Phosphoribulokinase family protein [Populus trichocarpa]	-	Phosphoribulokinase, chloroplastic OS=Mesembryanthemum crystallinum PE=2 SV=1	951.86	414.20	1.76	up	0.00	0.00	yes
TRINITY_DN23156_c0_g1	hypothetical protein POPTR_0002s19970g [Populus trichocarpa]	PUR5	Phosphoribosylformylglycinamidine cyclo-ligase, chloroplastic OS=Arabidopsis thaliana GN=PUR5 PE=1 SV=2	53.28	34.52	1.23	up	0.00	0.00	yes
TRINITY_DN25553_c0_g3	-	-	-	20.89	6.57	2.28	up	0.00	0.00	yes
TRINITY_DN21735_c0_g1	PREDICTED: two-component response regulator ARR8-like [Populus euphratica]	ARR9	Two-component response regulator ARR9 OS=Arabidopsis thaliana GN=ARR9 PE=1 SV=1	59.85	28.73	1.66	up	0.00	0.00	yes
TRINITY_DN16103_c0_g1	pentatricopeptide repeat-containing family protein [Populus trichocarpa]	At1g55890	Pentatricopeptide repeat-containing protein At1g55890, mitochondrial OS=Arabidopsis thaliana GN=At1g55890 PE=1 SV=1	20.69	13.81	1.20	up	0.00	0.00	yes
TRINITY_DN15395_c0_g1	hypothetical protein POPTR_0010s01590g [Populus trichocarpa]	LEA5	Late embryogenesis abundant protein Lea5 OS=Citrus sinensis GN=LEA5 PE=2 SV=1	64.04	42.26	1.28	up	0.00	0.00	yes
TRINITY_DN19567_c0_g1	hypothetical protein POPTR_0006s28310g [Populus trichocarpa]	TIC55	Protein TIC 55, chloroplastic OS=Pisum sativum GN=TIC55 PE=1 SV=1	31.96	19.57	1.28	up	0.00	0.00	yes
TRINITY_DN21515_c0_g1	PREDICTED: protochlorophyllide-dependent translocon component 52, chloroplastic-like [Populus euphratica]	PTC52	Protochlorophyllide-dependent translocon component 52, chloroplastic OS=Arabidopsis thaliana GN=PTC52 PE=2 SV=1	90.87	54.59	1.39	up	0.00	0.00	yes
TRINITY_DN26318_c0_g2	PREDICTED: protein TIC 40, chloroplastic-like isoform X2 [Populus euphratica]	TIC40	Protein TIC 40, chloroplastic OS=Pisum sativum GN=TIC40 PE=1 SV=1	165.88	107.04	1.23	up	0.00	0.00	yes
TRINITY_DN23305_c1_g1	putative cytoskeletal protein mRNA [Populus trichocarpa]	HIP	HSP-interacting protein OS=Zea mays GN=HIP PE=1 SV=1	16.94	9.10	1.45	up	0.00	0.00	yes
TRINITY_DN20038_c0_g1	PREDICTED: pentatricopeptide repeat-containing protein ELI1, chloroplastic [Populus euphratica]	ELI1	Pentatricopeptide repeat-containing protein ELI1, chloroplastic OS=Arabidopsis thaliana GN=ELI1 PE=3 SV=1	14.92	9.99	1.19	up	0.00	0.00	yes
TRINITY_DN26739_c0_g3	hypothetical protein POPTR_0010s17740g [Populus trichocarpa]	-	-	19.05	16.10	1.12	up	0.00	0.00	yes
TRINITY_DN26347_c0_g1	CTR1-like protein kinase [Populus trichocarpa]	CTR1	Serine/threonine-protein kinase CTR1 OS=Arabidopsis thaliana GN=CTR1 PE=1 SV=1	35.40	16.01	1.58	up	0.00	0.00	yes
TRINITY_DN25870_c1_g3	unknown [Populus trichocarpa]	rps7	30S ribosomal protein S7, chloroplastic OS=Asarum canadense GN=rps7 PE=3 SV=1	897.20	455.35	1.57	up	0.00	0.00	yes
TRINITY_DN20143_c0_g6	PREDICTED: pentatricopeptide repeat-containing protein At4g39620, chloroplastic-like [Populus euphratica]	EMB2453	Pentatricopeptide repeat-containing protein At4g39620, chloroplastic OS=Arabidopsis thaliana GN=EMB2453 PE=2 SV=1	29.43	18.17	1.28	up	0.00	0.00	yes
TRINITY_DN20173_c0_g3	PREDICTED: protein app1-like [Populus euphratica]	-	-	12.22	5.91	1.73	up	0.00	0.00	yes
TRINITY_DN15774_c0_g1	SEC14 cytosolic factor family protein [Populus trichocarpa]	-	-	45.11	30.40	1.29	up	0.00	0.00	yes
TRINITY_DN24278_c3_g2	ATP-dependent Clp protease proteolytic subunit family protein [Populus trichocarpa]	CLPR3	ATP-dependent Clp protease proteolytic subunit-related protein 3, chloroplastic OS=Arabidopsis thaliana GN=CLPR3 PE=1 SV=1	188.59	120.10	1.30	up	0.00	0.00	yes
TRINITY_DN16988_c0_g1	putative translation elongation factor Tu family protein [Zea mays]	A1	Elongation factor 1-alpha 1 OS=Arabidopsis thaliana GN=A1 PE=1 SV=1	606.26	392.97	1.28	up	0.00	0.00	yes
TRINITY_DN25250_c0_g1	hypothetical protein POPTR_0018s11270g [Populus trichocarpa]	Cbei_0202	Uncharacterized protein Cbei_0202 OS=Clostridium beijerinckii (strain ATCC 51743 / NCIMB 8052) GN=Cbei_0202 PE=4 SV=2	16.23	10.34	1.26	up	0.00	0.00	yes
TRINITY_DN23698_c1_g1	putative galactinol synthase family protein [Populus trichocarpa]	GOLS1	Galactinol synthase 1 OS=Arabidopsis thaliana GN=GOLS1 PE=1 SV=1	17.66	4.42	2.67	up	0.00	0.00	yes
TRINITY_DN18852_c0_g1	hypothetical protein POPTR_0006s23230g [Populus trichocarpa]	-	-	53.29	36.90	1.14	up	0.00	0.00	yes
TRINITY_DN15939_c0_g1	hypothetical protein POPTR_0006s24950g, partial [Populus trichocarpa]	-	-	244.94	144.89	1.39	up	0.00	0.00	yes
TRINITY_DN24275_c0_g1	hypothetical protein POPTR_0018s12120g [Populus trichocarpa]	-	-	93.21	60.09	1.31	up	0.00	0.00	yes

TRINITY_DN24504_c0_g3	PREDICTED: type I inositol 1,4,5-trisphosphate 5-phosphatase 2-like isoform X1 [Populus euphratica]	IP5P2	Type I inositol polyphosphate 5-phosphatase 2 OS=Arabidopsis thaliana GN=IP5P2 PE=1 SV=2	2.24	0.61	2.44	up	0.00	0.00	yes
TRINITY_DN14230_c0_g1	-	-	-	11.44	3.07	2.52	up	0.00	0.00	yes
TRINITY_DN25469_c1_g2	PREDICTED: sulfate transporter 1.2-like [Populus euphratica]	SULTR1;3	Sulfate transporter 1.3 OS=Arabidopsis thaliana GN=SULTR1;3 PE=2 SV=1	10.77	3.34	2.38	up	0.00	0.00	yes
TRINITY_DN23998_c0_g1	PREDICTED: serine/threonine-protein kinase ATG1-like isoform X1 [Populus euphratica]	ATG1A	Serine/threonine-protein kinase ATG1a OS=Arabidopsis thaliana GN=ATG1A PE=1 SV=1	10.29	7.11	1.28	up	0.00	0.00	yes
TRINITY_DN21352_c0_g2	PREDICTED: tRNA-dihydrouridine(47) synthase [NAD(P)(+)]-like [Populus euphratica]	At4g38890	tRNA-dihydrouridine(47) synthase [NAD(P)(+)]-like OS=Arabidopsis thaliana GN=At4g38890 PE=1 SV=2	35.72	24.49	1.13	up	0.00	0.00	yes
TRINITY_DN13649_c0_g1	putative major latex family protein [Populus trichocarpa]	-	-	39.29	9.58	2.59	up	0.00	0.00	yes
TRINITY_DN21071_c0_g1	PREDICTED: uncharacterized protein LOC105114560 [Populus euphratica]	MAV_4873	Putative S-adenosyl-L-methionine-dependent methyltransferase MAV_4873 OS=Mycobacterium avium (strain 104) GN=MAV_4873 PE=3 SV=1	14.20	8.49	1.36	up	0.00	0.00	yes
TRINITY_DN24628_c0_g1	PREDICTED: aldehyde dehydrogenase family 3 member H1-like [Populus euphratica]	ALDH3I1	Aldehyde dehydrogenase family 3 member I1, chloroplastic OS=Arabidopsis thaliana GN=ALDH3I1 PE=1 SV=2	40.39	24.46	1.28	up	0.00	0.00	yes
TRINITY_DN17609_c0_g2	40S ribosomal protein S28 [Populus trichocarpa]	RPS28	40S ribosomal protein S28 OS=Zea mays GN=RPS28 PE=3 SV=1	271.86	165.24	1.35	up	0.00	0.00	yes
TRINITY_DN20003_c0_g1	hypothetical protein POPTR_0014s06610g [Populus trichocarpa]	At1g01970	Pentatricopeptide repeat-containing protein At1g01970 OS=Arabidopsis thaliana GN=At1g01970 PE=2 SV=1	18.65	12.73	1.16	up	0.00	0.00	yes
TRINITY_DN23579_c0_g1	PREDICTED: ATP-dependent zinc metalloprotease FtsH [Populus euphratica]	FTSHI4	Probable inactive ATP-dependent zinc metalloprotease FTSHI 4, chloroplastic OS=Arabidopsis thaliana GN=FTSHI4 PE=1 SV=1	36.90	24.29	1.20	up	0.00	0.00	yes
TRINITY_DN25351_c0_g1	PREDICTED: serine/threonine-protein kinase HT1 isoform X1 [Populus euphratica]	STY17	Serine/threonine-protein kinase STY17 OS=Arabidopsis thaliana GN=STY17 PE=1 SV=1	22.50	17.55	1.21	up	0.00	0.00	yes
TRINITY_DN16140_c0_g2	PREDICTED: uncharacterized protein At2g39795, mitochondrial [Populus euphratica]	-	-	12.78	5.72	1.82	up	0.00	0.00	yes
TRINITY_DN12148_c0_g1	NADH dehydrogenase subunit 5 [Populus alba]	ndhF	NAD(P)H-quinone oxidoreductase subunit 5, chloroplastic OS=Populus alba GN=ndhF PE=3 SV=1	12.83	4.99	1.93	up	0.00	0.00	yes
TRINITY_DN15777_c0_g1	ribosomal protein L20 [Populus trichocarpa]	rplT	50S ribosomal protein L20 OS=Rhodospirillum centenum (strain ATCC 51521 / SW) GN=rplT PE=3 SV=1	22.86	13.02	1.44	up	0.00	0.00	yes
TRINITY_DN14158_c0_g1	hypothetical protein POPTR_0011s02610g [Populus trichocarpa]	ycf23	Uncharacterized protein ycf23 OS=Porphyra purpurea GN=ycf23 PE=3 SV=1	287.87	147.90	1.55	up	0.00	0.00	yes
TRINITY_DN24953_c1_g2	PREDICTED: protein CURVATURE THYLAKOID 1C, chloroplastic-like [Populus euphratica]	-	-	167.74	90.86	1.47	up	0.00	0.00	yes
TRINITY_DN21727_c0_g1	PREDICTED: auxin-responsive protein IAA2-like [Populus euphratica]	IAA26	Auxin-responsive protein IAA26 OS=Arabidopsis thaliana GN=IAA26 PE=1 SV=2	12.92	5.91	1.61	up	0.00	0.00	yes
TRINITY_DN20663_c0_g2	hypothetical protein POPTR_0002s14390g [Populus trichocarpa]	-	-	23.04	18.43	1.16	up	0.00	0.00	yes
TRINITY_DN19599_c0_g2	PREDICTED: ATP synthase subunit d, mitochondrial-like [Populus euphratica]	At3g52300	ATP synthase subunit d, mitochondrial OS=Arabidopsis thaliana GN=At3g52300 PE=1 SV=3	166.37	106.64	1.22	up	0.00	0.00	yes
TRINITY_DN25624_c2_g1	PREDICTED: probable lactoylglutathione lyase, chloroplast [Populus euphratica]	At1g67280	Probable lactoylglutathione lyase, chloroplastic OS=Arabidopsis thaliana GN=At1g67280 PE=1 SV=1	166.29	105.77	1.26	up	0.00	0.00	yes
TRINITY_DN22728_c2_g2	hypothetical protein POPTR_0005s01380g [Populus trichocarpa]	ECH	Golgi apparatus membrane protein-like protein ECHIDNA OS=Arabidopsis thaliana GN=ECH PE=1 SV=1	36.88	24.22	1.29	up	0.00	0.00	yes
TRINITY_DN22587_c0_g1	PREDICTED: fructose-bisphosphate aldolase cytoplasmic isozyme [Populus euphratica]	FBA8	Fructose-bisphosphate aldolase 8, cytosolic OS=Arabidopsis thaliana GN=FBA8 PE=1 SV=1	371.77	247.12	1.20	up	0.00	0.00	yes
TRINITY_DN18796_c0_g1	hypothetical protein POPTR_0012s07250g [Populus trichocarpa]	NPF4.6	Protein NRT1/ PTR FAMILY 4.6 OS=Arabidopsis thaliana GN=NPF4.6 PE=1 SV=1	8.97	3.59	1.96	up	0.00	0.00	yes
TRINITY_DN26572_c0_g2	PREDICTED: blue-light photoreceptor PHR2-like [Populus euphratica]	PHR2	Blue-light photoreceptor PHR2 OS=Arabidopsis thaliana GN=PHR2 PE=2 SV=2	125.22	61.66	1.60	up	0.00	0.00	yes
TRINITY_DN23796_c0_g2	PREDICTED: pentatricopeptide repeat-containing protein At2g15820 [Populus euphratica]	OTP51	Pentatricopeptide repeat-containing protein At2g15820, chloroplastic OS=Arabidopsis thaliana GN=OTP51 PE=2 SV=3	15.79	11.39	1.13	up	0.00	0.00	yes
TRINITY_DN20380_c0_g1	hypothetical protein POPTR_0002s12730g [Populus trichocarpa]	DCL	Protein DCL, chloroplastic OS=Solanum lycopersicum GN=DCL PE=2 SV=1	82.03	52.27	1.21	up	0.00	0.00	yes
TRINITY_DN18051_c1_g3	hypothetical protein POPTR_0001s09860g [Populus trichocarpa]	ISPF	2-C-methyl-D-erythritol 2,4-cyclodiphosphate synthase, chloroplastic OS=Arabidopsis thaliana GN=ISPF PE=1 SV=1	100.06	57.21	1.42	up	0.00	0.00	yes

TRINITY_DN14719_c0_g4	hypothetical protein POPTR_0015s09880g [Populus trichocarpa]	-	-	123.62	78.61	1.25	up	0.00	0.00	yes
TRINITY_DN24710_c0_g1	hypothetical protein POPTR_0012s09890g [Populus trichocarpa]	-	-	45.43	30.25	1.19	up	0.00	0.00	yes
TRINITY_DN21457_c0_g1	hypothetical protein POPTR_0005s24430g [Populus trichocarpa]	KLCR2	Protein KINESIN LIGHT CHAIN-RELATED 2 OS=Arabidopsis thaliana GN=KLCR2 PE=1 SV=1	16.26	12.12	1.27	up	0.00	0.00	yes
TRINITY_DN25013_c0_g1	PREDICTED: uncharacterized protein LOC105765014 isoform X2 [Gossypium raimondii]	LOL1	Protein LOL1 OS=Arabidopsis thaliana GN=LOL1 PE=2 SV=1	88.84	47.84	1.53	up	0.00	0.00	yes
TRINITY_DN24185_c0_g1	hypothetical protein B456_013G156600 [Gossypium raimondii]	RPS8B	40S ribosomal protein S8-2 OS=Arabidopsis thaliana GN=RPS8B PE=2 SV=1	862.27	583.73	1.24	up	0.00	0.00	yes
TRINITY_DN24788_c1_g5	hypothetical protein POPTR_0006s12560g [Populus trichocarpa]	Os01g0895100	Probable membrane-associated 30 kDa protein, chloroplastic OS=Oryza sativa subsp. japonica GN=Os01g0895100 PE=1 SV=1	48.63	26.44	1.54	up	0.00	0.00	yes
TRINITY_DN23732_c2_g1	PREDICTED: uncharacterized protein LOC105126557 [Populus euphratica]	-	-	218.11	120.82	1.44	up	0.00	0.00	yes
TRINITY_DN19380_c0_g4	-	-	-	3.00	0.63	2.81	up	0.00	0.00	yes
TRINITY_DN20593_c0_g1	Metal transporter Nramp1 family protein [Populus trichocarpa]	NRAMP6	Metal transporter Nramp6 OS=Arabidopsis thaliana GN=NRAMP6 PE=2 SV=2	4.27	1.83	1.77	up	0.00	0.00	yes
TRINITY_DN18373_c0_g2	-	-	-	5.01	1.15	2.69	up	0.00	0.00	yes
TRINITY_DN21034_c0_g4	hypothetical protein POPTR_0013s13240g [Populus trichocarpa]	EARLI1	Lipid transfer protein EARLI 1 OS=Arabidopsis thaliana GN=EARLI1 PE=1 SV=1	52.10	27.79	1.51	up	0.00	0.00	yes
TRINITY_DN22865_c0_g3	hypothetical protein POPTR_0006s19740g [Populus trichocarpa]	-	-	11.47	4.28	2.02	up	0.00	0.00	yes
TRINITY_DN22852_c0_g2	Tic20 family protein [Populus trichocarpa]	TIC20-I	Protein TIC 20-I, chloroplastic OS=Arabidopsis thaliana GN=TIC20-I PE=1 SV=1	226.96	131.41	1.38	up	0.00	0.00	yes
TRINITY_DN22712_c0_g1	PHOTOSYNTHETIC ELECTRON TRANSFER C family protein [Populus trichocarpa]	petC	Cytochrome b6-f complex iron-sulfur subunit, chloroplastic OS=Pisum sativum GN=petC PE=2 SV=1	1514.69	688.40	1.81	up	0.00	0.00	yes
TRINITY_DN16179_c0_g1	PREDICTED: ribosome biogenesis protein 15 [Populus euphratica]	mrd1	Multiple RNA-binding domain-containing protein 1 OS=Dictyostelium discoideum GN=mrd1 PE=3 SV=1	57.18	34.88	1.33	up	0.00	0.00	yes
TRINITY_DN25355_c0_g5	PREDICTED: uncharacterized protein LOC105109055 [Populus euphratica]	-	-	197.71	118.29	1.41	up	0.00	0.00	yes
TRINITY_DN19614_c0_g1	PREDICTED: pentatricopeptide repeat-containing protein At5g21222-like isoform X1 [Populus euphratica]	ATC401	Pentatricopeptide repeat-containing protein At5g21222 OS=Arabidopsis thaliana GN=ATC401 PE=2 SV=1	14.84	8.66	1.57	up	0.00	0.00	yes
TRINITY_DN19190_c0_g1	hypothetical protein POPTR_0001s07840g [Populus trichocarpa]	-	-	20.59	11.53	1.45	up	0.00	0.00	yes
TRINITY_DN16120_c0_g1	hypothetical protein POPTR_0008s00540g [Populus trichocarpa]	-	-	16.97	9.72	1.40	up	0.00	0.00	yes
TRINITY_DN13637_c0_g1	PREDICTED: probable glutathione S-transferase [Populus euphratica]	-	Probable glutathione S-transferase OS=Nicotiana tabacum PE=2 SV=1	42.52	28.09	1.20	up	0.00	0.00	yes
TRINITY_DN20179_c0_g3	hypothetical protein POPTR_0005s14860g [Populus trichocarpa]	-	-	6.78	2.90	1.81	up	0.00	0.00	yes
TRINITY_DN18320_c0_g2	PREDICTED: phenylalanine--tRNA ligase, chloroplastic/mitochondrial [Populus euphratica]	At3g58140	Phenylalanine--tRNA ligase, chloroplastic/mitochondrial OS=Arabidopsis thaliana GN=At3g58140 PE=1 SV=1	108.32	64.98	1.34	up	0.00	0.00	yes
TRINITY_DN17087_c0_g2	PREDICTED: LOW QUALITY PROTEIN: 15-cis-zeta-carotene isomerase, chloroplastic [Populus euphratica]	Z-ISO	15-cis-zeta-carotene isomerase, chloroplastic OS=Arabidopsis thaliana GN=Z-ISO PE=1 SV=1	36.68	22.85	1.30	up	0.00	0.00	yes
TRINITY_DN26880_c0_g1	hypothetical protein POPTR_0001s34620g [Populus trichocarpa]	TLP40	Peptidyl-prolyl cis-trans isomerase, chloroplastic OS=Spinacia oleracea GN=TLP40 PE=1 SV=1	201.19	111.29	1.45	up	0.00	0.00	yes
TRINITY_DN22124_c1_g3	hypothetical protein POPTR_0006s01590g [Populus trichocarpa]	At5g47540	Putative MO25-like protein At5g47540 OS=Arabidopsis thaliana GN=At5g47540 PE=2 SV=1	126.71	94.28	1.14	up	0.00	0.00	yes
TRINITY_DN15775_c0_g1	hypothetical protein POPTR_0001s46060g [Populus trichocarpa]	PILS3	Protein PIN-LIKES 3 OS=Arabidopsis thaliana GN=PILS3 PE=2 SV=1	7.49	3.44	1.57	up	0.00	0.00	yes
TRINITY_DN20281_c1_g1	hypothetical protein POPTR_0001s14030g [Populus trichocarpa]	MEE4	NADH dehydrogenase [ubiquinone] 1 alpha subcomplex subunit 13-A OS=Arabidopsis thaliana GN=MEE4 PE=2 SV=1	166.67	102.26	1.27	up	0.00	0.00	yes
TRINITY_DN20141_c0_g3	hypothetical protein POPTR_0006s25780g [Populus trichocarpa]	infA	Translation initiation factor IF-1, chloroplastic OS=Glycine max GN=infA PE=2 SV=1	60.54	34.19	1.42	up	0.00	0.00	yes

TRINITY_DN21653_c0_g1	VDAC2.1 family protein [Populus trichocarpa]	VDAC2	Mitochondrial outer membrane protein porin 2 OS=Arabidopsis thaliana GN=VDAC2 PE=1 SV=1	109.71	73.25	1.18	up	0.00	0.00	yes
TRINITY_DN19414_c0_g1	PREDICTED: protein TIC 62, chloroplastic [Populus euphratica]	At2g34460	Uncharacterized protein At2g34460, chloroplastic OS=Arabidopsis thaliana GN=At2g34460 PE=2 SV=1	28.33	15.80	1.39	up	0.00	0.00	yes
TRINITY_DN19356_c0_g3	PREDICTED: dolichyl-diphosphooligosaccharide--protein glycosyltransferase subunit DAD1 [Populus euphratica]	DAD1	Dolichyl-diphosphooligosaccharide--protein glycosyltransferase subunit DAD1 OS=Citrus unshiu GN=DAD1 PE=3 SV=1	134.68	83.13	1.22	up	0.00	0.00	yes
TRINITY_DN19649_c0_g1	hypothetical protein POPTR_0019s09230g [Populus trichocarpa]	-	-	46.95	28.61	1.36	up	0.00	0.00	yes
TRINITY_DN18156_c0_g1	PREDICTED: glyoxylate/hydroxypyruvate reductase A HPR2-like [Populus euphratica]	HPR2	Glyoxylate/hydroxypyruvate reductase A HPR2 OS=Arabidopsis thaliana GN=HPR2 PE=1 SV=1	133.92	82.89	1.29	up	0.00	0.00	yes
TRINITY_DN12896_c0_g1	PREDICTED: ubiquitin-fold modifier-conjugating enzyme 1 [Populus euphratica]	At1g27530	Ubiquitin-fold modifier-conjugating enzyme 1 OS=Arabidopsis thaliana GN=At1g27530 PE=2 SV=1	51.11	34.04	1.19	up	0.00	0.00	yes
TRINITY_DN27545_c0_g1	PREDICTED: uncharacterized protein LOC105139443 isoform X1 [Populus euphratica]	rnj	Ribonuclease J OS=Corynebacterium glutamicum (strain ATCC 13032 / DSM 20300 / JCM 1318 / LMG 3730 / NCIMB 10025) GN=rnj PE=3 SV=2	53.33	37.03	1.23	up	0.00	0.00	yes
TRINITY_DN26748_c0_g2	hypothetical protein POPTR_0006s10460g [Populus trichocarpa]	RPS4	40S ribosomal protein S4 OS=Solanum tuberosum GN=RPS4 PE=2 SV=1	939.38	615.87	1.21	up	0.00	0.00	yes
TRINITY_DN16423_c1_g1	PREDICTED: uncharacterized protein LOC105134715 [Populus euphratica]	-	-	32.86	16.57	1.33	up	0.00	0.00	yes
TRINITY_DN23978_c0_g2	hypothetical protein POPTR_0425s00200g [Populus trichocarpa]	-	-	53.47	29.17	1.49	up	0.00	0.00	yes
TRINITY_DN18844_c0_g3	hypothetical protein POPTR_1436s00210g, partial [Populus trichocarpa]	-	-	421.08	199.32	1.67	up	0.00	0.00	yes
TRINITY_DN15309_c0_g1	hypothetical protein POPTR_0015s08340g [Populus trichocarpa]	At5g50100	Uncharacterized protein At5g50100, mitochondrial OS=Arabidopsis thaliana GN=At5g50100 PE=2 SV=1	14.29	7.29	1.62	up	0.00	0.00	yes
TRINITY_DN22358_c0_g1	PREDICTED: ATP synthase subunit gamma, mitochondrial-like [Populus euphratica]	ATPC	ATP synthase subunit gamma, mitochondrial OS=Ipomoea batatas GN=ATPC PE=1 SV=2	171.42	111.03	1.19	up	0.00	0.00	yes
TRINITY_DN27336_c1_g2	hypothetical protein POPTR_0019s08760g [Populus trichocarpa]	-	Ubiquinol-cytochrome c reductase complex 6.7 kDa protein OS=Solanum tuberosum PE=1 SV=2	269.60	168.77	1.28	up	0.00	0.00	yes
TRINITY_DN23137_c0_g4	hypothetical protein POPTR_0010s18770g [Populus trichocarpa]	PGLP1A	Phosphoglycolate phosphatase 1A, chloroplastic OS=Arabidopsis thaliana GN=PGLP1A PE=1 SV=1	257.32	102.92	1.91	up	0.00	0.00	yes
TRINITY_DN19243_c0_g1	DTW domain-containing family protein [Populus trichocarpa]	-	-	10.36	6.83	1.24	up	0.00	0.00	yes
TRINITY_DN23816_c0_g3	-	-	-	3.97	1.50	1.99	up	0.00	0.00	yes
TRINITY_DN23227_c0_g2	hypothetical protein POPTR_0010s19790g [Populus trichocarpa]	ASA1	Anthranilate synthase alpha subunit 1, chloroplastic OS=Arabidopsis thaliana GN=ASA1 PE=1 SV=1	6.63	4.18	1.27	up	0.00	0.00	yes
TRINITY_DN19303_c0_g4	hypothetical protein POPTR_0012s09720g [Populus trichocarpa]	ADK-B	Adenylate kinase 4 OS=Oryza sativa subsp. japonica GN=ADK-B PE=2 SV=1	77.17	48.93	1.26	up	0.00	0.00	yes
TRINITY_DN27686_c0_g1	SPA1-RELATED 4 family protein [Populus trichocarpa]	SPA3	Protein SPA1-RELATED 3 OS=Arabidopsis thaliana GN=SPA3 PE=1 SV=1	28.22	19.14	1.24	up	0.00	0.00	yes
TRINITY_DN24437_c0_g1	stress inducible family protein [Populus trichocarpa]	HOP2	Hsp70-Hsp90 organizing protein 2 OS=Arabidopsis thaliana GN=HOP2 PE=1 SV=1	103.66	64.11	1.35	up	0.00	0.00	yes
TRINITY_DN14077_c0_g1	PREDICTED: uncharacterized protein LOC105121464 [Populus euphratica]	-	-	32.91	14.20	1.81	up	0.00	0.00	yes
TRINITY_DN23395_c0_g1	4-coumarate--CoA ligase family protein [Populus trichocarpa]	4CLL7	4-coumarate--CoA ligase-like 7 OS=Arabidopsis thaliana GN=4CLL7 PE=1 SV=1	14.87	10.65	1.24	up	0.00	0.00	yes
TRINITY_DN20738_c0_g4	unknown [Populus trichocarpa]	ISCA	Iron-sulfur assembly protein IscA, chloroplastic OS=Arabidopsis thaliana GN=ISCA PE=2 SV=2	71.33	47.04	1.21	up	0.00	0.00	yes
TRINITY_DN26806_c1_g1	hypothetical protein POPTR_0005s00295g [Populus trichocarpa]	-	-	11.39	4.43	1.95	up	0.00	0.00	yes
TRINITY_DN26243_c0_g1	hypothetical protein POPTR_0001s34940g [Populus trichocarpa]	LSF1	Phosphoglucan phosphatase LSF1, chloroplastic OS=Arabidopsis thaliana GN=LSF1 PE=1 SV=1	23.20	15.32	1.21	up	0.00	0.00	yes
TRINITY_DN22814_c0_g1	hypothetical protein POPTR_0014s11080g [Populus trichocarpa]	-	-	17.56	10.81	1.35	up	0.00	0.00	yes
TRINITY_DN23965_c0_g1	PREDICTED: triosephosphate isomerase, cytosolic [Populus euphratica]	CTIMC	Triosephosphate isomerase, cytosolic OS=Arabidopsis thaliana GN=CTIMC PE=1 SV=2	641.46	426.80	1.23	up	0.00	0.00	yes

TRINITY_DN21050_c0_g2	unknown [Populus trichocarpa]	PXN	Peroxisomal nicotinamide adenine dinucleotide carrier OS=Arabidopsis thaliana GN=PXN PE=1 SV=1	74.04	44.50	1.36	up	0.00	0.00	yes
TRINITY_DN22376_c0_g1	unknown [Populus trichocarpa]	-	-	35.20	24.25	1.15	up	0.00	0.00	yes
TRINITY_DN18930_c0_g1	hypothetical protein POPTR_0008s08960g [Populus trichocarpa]	-	-	47.89	31.57	1.23	up	0.00	0.00	yes
TRINITY_DN24662_c0_g1	ribosomal protein L27 precursor [Populus trichocarpa]	RPL27	50S ribosomal protein L27, chloroplastic OS=Arabidopsis thaliana GN=RPL27 PE=2 SV=1	682.72	346.90	1.58	up	0.00	0.00	yes
TRINITY_DN26682_c0_g2	PREDICTED: pentatricopeptide repeat-containing protein At1g10910, chloroplastic-like isoform X1 [Populus euphratica]	At1g10910	Pentatricopeptide repeat-containing protein At1g10910, chloroplastic OS=Arabidopsis thaliana GN=At1g10910 PE=2 SV=1	31.97	27.79	1.22	up	0.00	0.00	yes
TRINITY_DN23264_c0_g1	PREDICTED: omega-6 fatty acid desaturase, chloroplastic isoform X1 [Populus euphratica]	FAD6	Omega-6 fatty acid desaturase, chloroplastic OS=Arabidopsis thaliana GN=FAD6 PE=1 SV=2	94.87	57.51	1.36	up	0.00	0.00	yes
TRINITY_DN19349_c0_g1	PREDICTED: sec-independent protein translocase protein TATB, chloroplastic-like [Populus euphratica]	TATB	Sec-independent protein translocase protein TATB, chloroplastic OS=Arabidopsis thaliana GN=TATB PE=1 SV=1	248.60	144.95	1.38	up	0.00	0.00	yes
TRINITY_DN18204_c0_g1	hypothetical protein POPTR_0006s29130g [Populus trichocarpa]	PAP8	Probable plastid-lipid-associated protein 8, chloroplastic OS=Arabidopsis thaliana GN=PAP8 PE=1 SV=1	195.51	98.22	1.60	up	0.00	0.00	yes
TRINITY_DN23764_c0_g1	In2-1 family protein [Populus trichocarpa]	GSTL1	Glutathione S-transferase L1 OS=Arabidopsis thaliana GN=GSTL1 PE=2 SV=1	688.86	380.28	1.49	up	0.00	0.00	yes
TRINITY_DN14732_c0_g2	hypothetical protein POPTR_0016s09330g [Populus trichocarpa]	-	-	200.63	129.73	1.23	up	0.00	0.00	yes
TRINITY_DN20068_c0_g1	PREDICTED: uncharacterized protein LOC105121984 [Populus euphratica]	nusG	Transcription termination/antitermination protein NusG OS=Thermus thermophilus (strain HB8 / ATCC 27634 / DSM 579) GN=nusG PE=1 SV=1	97.32	56.60	1.35	up	0.00	0.00	yes
TRINITY_DN17305_c0_g1	hypothetical protein POPTR_0013s02770g [Populus trichocarpa]	-	-	17.59	10.43	1.47	up	0.00	0.00	yes
TRINITY_DN21165_c2_g2	PREDICTED: ribulose-1,5 bisphosphate carboxylase/oxygenase large subunit N-methyltransferase, chloroplastic isoform X2 [Populus euphratica]	-	-	94.86	77.99	1.26	up	0.00	0.00	yes
TRINITY_DN27867_c2_g2	-	-	-	2.14	0.28	3.47	up	0.00	0.00	yes
TRINITY_DN15934_c0_g2	PREDICTED: uncharacterized protein LOC105114368 [Populus euphratica]	-	-	26.83	13.90	1.57	up	0.00	0.00	yes
TRINITY_DN25754_c0_g1	PREDICTED: flavonoid 3'-monooxygenase-like [Populus euphratica]	CYP98A2	Cytochrome P450 98A2 OS=Glycine max GN=CYP98A2 PE=2 SV=1	1925.81	889.78	1.73	up	0.00	0.00	yes
TRINITY_DN21221_c0_g2	hypothetical protein POPTR_0008s19110g [Populus trichocarpa]	RPL27AC	60S ribosomal protein L27a-3 OS=Arabidopsis thaliana GN=RPL27AC PE=2 SV=2	542.34	339.17	1.25	up	0.00	0.00	yes
TRINITY_DN22554_c0_g1	hypothetical protein POPTR_0003s21070g [Populus trichocarpa]	DTC	Mitochondrial dicarboxylate/tricarboxylate transporter DTC OS=Arabidopsis thaliana GN=DTC PE=1 SV=1	330.82	220.36	1.22	up	0.00	0.00	yes
TRINITY_DN19269_c1_g1	PREDICTED: uncharacterized protein LOC105111558 isoform X1 [Populus euphratica]	RER6	Protein RETICULATA-RELATED 6, chloroplastic OS=Arabidopsis thaliana GN=RER6 PE=2 SV=1	7.47	4.48	1.32	up	0.00	0.00	yes
TRINITY_DN21062_c0_g1	PREDICTED: hexokinase-1-like [Populus euphratica]	HXK1	Hexokinase-1 OS=Nicotiana tabacum GN=HXK1 PE=2 SV=1	9.26	6.04	1.22	up	0.00	0.00	yes
TRINITY_DN17094_c3_g1	hypothetical protein POPTR_0004s07190g [Populus trichocarpa]	HSP83A	Heat shock protein 83 OS=Ipomoea nil GN=HSP83A PE=2 SV=1	10.86	4.99	2.21	up	0.00	0.00	yes
TRINITY_DN21018_c1_g1	hypothetical protein POPTR_0004s14170g [Populus trichocarpa]	-	-	54.61	31.86	1.39	up	0.00	0.00	yes
TRINITY_DN17644_c0_g1	PREDICTED: NADP-dependent D-sorbitol-6-phosphate dehydrogenase-like [Populus euphratica]	S6PDH	NADP-dependent D-sorbitol-6-phosphate dehydrogenase OS=Malus domestica GN=S6PDH PE=2 SV=1	152.43	100.73	1.22	up	0.00	0.00	yes
TRINITY_DN18244_c0_g1	unknown [Populus trichocarpa]	At2g39020	Probable acetyltransferase NATA1-like OS=Arabidopsis thaliana GN=At2g39020 PE=2 SV=1	49.63	33.97	1.14	up	0.00	0.00	yes
TRINITY_DN27871_c3_g2	hypothetical protein POPTR_0006s19380g [Populus trichocarpa]	TSS	Protein TSS OS=Arabidopsis thaliana GN=TSS PE=1 SV=1	59.72	24.52	1.70	up	0.00	0.00	yes
TRINITY_DN18032_c0_g1	hypothetical protein POPTR_0006s14820g [Populus trichocarpa]	UBA1C	UBP1-associated proteins 1C OS=Arabidopsis thaliana GN=UBA1C PE=3 SV=2	23.46	13.61	1.38	up	0.00	0.00	yes
TRINITY_DN26363_c0_g1	hypothetical protein POPTR_0003s15750g [Populus trichocarpa]	HGSNAT	Heparan-alpha-glucosaminide N-acetyltransferase OS=Homo sapiens GN=HGSNAT PE=1 SV=2	50.15	39.16	1.20	up	0.00	0.00	yes

TRINITY_DN18070_c0_g2	hypothetical protein POPTR_0010s18370g [Populus trichocarpa]	-	-	43.48	24.25	1.46	up	0.00	0.00	yes
TRINITY_DN18951_c0_g4	-	-	-	6.04	2.33	1.95	up	0.00	0.00	yes
TRINITY_DN26567_c0_g1	PREDICTED: mitochondrial carnitine/acylcarnitine carrier-like protein isoform X1 [Populus euphratica]	BOU	Mitochondrial carnitine/acylcarnitine carrier-like protein OS=Arabidopsis thaliana GN=BOU PE=1 SV=1	356.33	145.88	1.87	up	0.00	0.00	yes
TRINITY_DN23005_c0_g2	hypothetical protein POPTR_0011s01420g [Populus trichocarpa]	EMB2369	Leucine--tRNA ligase, chloroplastic/mitochondrial OS=Arabidopsis thaliana GN=EMB2369 PE=2 SV=1	35.29	23.06	1.25	up	0.00	0.00	yes
TRINITY_DN28632_c0_g1	-	-	-	20.57	3.30	3.19	up	0.00	0.00	yes
TRINITY_DN23365_c0_g1	PREDICTED: uncharacterized protein At5g03900, chloroplastic [Populus euphratica]	At5g03900	Uncharacterized protein At5g03900, chloroplastic OS=Arabidopsis thaliana GN=At5g03900 PE=2 SV=2	56.43	36.05	1.24	up	0.00	0.00	yes
TRINITY_DN22886_c0_g6	RIBOSOMAL protein L23A [Populus trichocarpa]	RPL23AA	60S ribosomal protein L23a-1 OS=Arabidopsis thaliana GN=RPL23AA PE=2 SV=2	44.78	24.69	1.49	up	0.00	0.00	yes
TRINITY_DN17454_c0_g1	PREDICTED: putative E3 ubiquitin-protein ligase LIN-1 isoform X1 [Populus euphratica]	LIN	Putative E3 ubiquitin-protein ligase LIN OS=Medicago truncatula GN=LIN PE=2 SV=1	2.65	0.45	3.16	up	0.00	0.00	yes
TRINITY_DN20500_c0_g2	hypothetical protein POPTR_0006s22590g, partial [Populus trichocarpa]	-	-	23.70	13.05	1.52	up	0.00	0.00	yes
TRINITY_DN22011_c1_g5	ribosomal protein L33 [Populus trichocarpa]	rpmG	50S ribosomal protein L33 OS=Orientia tsutsugamushi (strain Ikeda) GN=rpmG PE=3 SV=1	110.25	71.30	1.27	up	0.00	0.00	yes
TRINITY_DN24015_c0_g2	PREDICTED: adenylate kinase, chloroplastic-like [Populus euphratica]	At5g47840	Adenylate kinase 2, chloroplastic OS=Arabidopsis thaliana GN=At5g47840 PE=1 SV=1	70.20	43.65	1.27	up	0.00	0.00	yes
TRINITY_DN27280_c1_g1	patched family protein [Populus trichocarpa]	NPC1	Niemann-Pick C1 protein OS=Sus scrofa GN=NPC1 PE=2 SV=1	39.38	41.33	1.15	up	0.00	0.00	yes
TRINITY_DN16327_c0_g1	hypothetical protein POPTR_0017s13830g [Populus trichocarpa]	-	-	33.60	22.80	1.18	up	0.00	0.00	yes
TRINITY_DN156_c0_g1	-	-	-	21.74	4.96	2.76	up	0.00	0.00	yes
TRINITY_DN24924_c0_g1	PREDICTED: chloroplastic group IIA intron splicing facilitator CRS1, chloroplastic, partial [Populus euphratica]	CFM3	CRM-domain containing factor CFM3, chloroplastic/mitochondrial OS=Zea mays GN=CFM3 PE=1 SV=1	19.06	12.73	1.18	up	0.00	0.00	yes
TRINITY_DN16523_c0_g1	unknown [Populus trichocarpa]	RPS29A	40S ribosomal protein S29 OS=Arabidopsis thaliana GN=RPS29A PE=3 SV=2	235.77	155.66	1.25	up	0.00	0.00	yes
TRINITY_DN20532_c1_g1	rubredoxin family protein [Populus trichocarpa]	-	-	228.32	108.21	1.67	up	0.00	0.00	yes
TRINITY_DN19265_c2_g1	PREDICTED: 60S ribosomal protein L44 [Theobroma cacao]	RPL44	60S ribosomal protein L44 OS=Gossypium hirsutum GN=RPL44 PE=3 SV=3	814.87	538.68	1.29	up	0.00	0.00	yes
TRINITY_DN15716_c0_g2	hypothetical protein POPTR_0005s25470g [Populus trichocarpa]	DREB2D	Dehydration-responsive element-binding protein 2D OS=Arabidopsis thaliana GN=DREB2D PE=2 SV=1	8.26	1.38	4.85	up	0.00	0.00	yes
TRINITY_DN23373_c0_g2	hypothetical protein POPTR_0014s16070g [Populus trichocarpa]	-	-	11.83	5.52	1.75	up	0.00	0.00	yes
TRINITY_DN24057_c1_g1	PREDICTED: uncharacterized protein LOC105123050 [Populus euphratica]	-	-	64.99	40.29	1.29	up	0.00	0.00	yes
TRINITY_DN21720_c0_g1	PREDICTED: S-type anion channel SLAH2-like [Populus euphratica]	SLAH3	S-type anion channel SLAH3 OS=Arabidopsis thaliana GN=SLAH3 PE=1 SV=1	8.14	2.18	2.04	up	0.00	0.00	yes
TRINITY_DN16039_c0_g1	PREDICTED: protein Asterix [Populus euphratica]	At5g07960	Protein Asterix OS=Arabidopsis thaliana GN=At5g07960 PE=3 SV=1	25.57	15.82	1.30	up	0.00	0.00	yes
TRINITY_DN16155_c0_g1	hypothetical protein POPTR_0004s12440g [Populus trichocarpa]	Mfla_2099	UPF0301 protein Mfla_2099 OS=Methylobacillus flagellatus (strain KT / ATCC 51484 / DSM 6875) GN=Mfla_2099 PE=3 SV=1	53.21	18.16	2.15	up	0.00	0.00	yes
TRINITY_DN21228_c0_g1	hypothetical protein POPTR_0001s35070g [Populus trichocarpa]	PNP2	Polyribonucleotide nucleotidyltransferase 2, mitochondrial OS=Arabidopsis thaliana GN=PNP2 PE=2 SV=1	4.70	2.42	1.59	up	0.00	0.00	yes
TRINITY_DN18652_c0_g2	hypothetical protein POPTR_0003s17150g [Populus trichocarpa]	-	-	141.32	91.00	1.24	up	0.00	0.00	yes
TRINITY_DN25996_c0_g1	heat shock protein 70 [Populus trichocarpa]	HSP70-8	Heat shock 70 kDa protein 8 OS=Arabidopsis thaliana GN=HSP70-8 PE=1 SV=1	6.48	3.97	1.35	up	0.00	0.00	yes
TRINITY_DN17105_c0_g2	PREDICTED: cytochrome b-c1 complex subunit 7-2-like [Populus euphratica]	QCR7-2	Cytochrome b-c1 complex subunit 7-2 OS=Arabidopsis thaliana GN=QCR7-2 PE=1 SV=1	67.61	44.77	1.19	up	0.00	0.00	yes
TRINITY_DN18568_c0_g1	PREDICTED: 60S ribosomal protein L13a-2 [Populus euphratica]	RPL13AD	60S ribosomal protein L13a-4 OS=Arabidopsis thaliana GN=RPL13AD PE=2 SV=1	641.02	423.98	1.20	up	0.00	0.00	yes

TRINITY_DN8327_c0_g1	unknown [Populus trichocarpa]	FOLD2	Bifunctional protein FolD 2 OS=Arabidopsis thaliana GN=FOLD2 PE=2 SV=1	17.80	11.35	1.29	up	0.00	0.00	yes
TRINITY_DN18731_c0_g3	unknown [Populus trichocarpa x Populus deltoides]	RPL28	50S ribosomal protein L28, chloroplastic OS=Arabidopsis thaliana GN=RPL28 PE=2 SV=2	496.17	250.77	1.58	up	0.00	0.00	yes
TRINITY_DN19330_c1_g4	hypothetical protein POPTR_0004s23710g [Populus trichocarpa]	At1g10310	NADPH-dependent pterin aldehyde reductase OS=Arabidopsis thaliana GN=At1g10310 PE=1 SV=1	22.29	13.72	1.30	up	0.00	0.00	yes
TRINITY_DN19736_c0_g1	DISPROPORTIONATING ENZYME family protein [Populus trichocarpa]	DPEP	4-alpha-glucanotransferase, chloroplastic/amyloplastic OS=Solanum tuberosum GN=DPEP PE=1 SV=1	13.53	9.85	1.19	up	0.00	0.00	yes
TRINITY_DN14846_c0_g2	unknown [Populus trichocarpa]	COX6A	Cytochrome c oxidase subunit 6a, mitochondrial OS=Arabidopsis thaliana GN=COX6A PE=2 SV=1	185.89	118.98	1.24	up	0.00	0.00	yes
TRINITY_DN21210_c0_g6	homoserine dehydrogenase family protein [Populus trichocarpa]	thrA	Bifunctional aspartokinase/homoserine dehydrogenase 1 OS=Escherichia coli (strain K12) GN=thrA PE=1 SV=2	16.38	11.90	1.13	up	0.00	0.00	yes
TRINITY_DN20832_c0_g1	PREDICTED: ATP synthase subunit beta, mitochondrial [Populus euphratica]	ATPB	ATP synthase subunit beta, mitochondrial OS=Oryza sativa subsp. japonica GN=ATPB PE=1 SV=2	314.72	184.88	1.37	up	0.00	0.00	yes
TRINITY_DN19526_c1_g1	hypothetical protein POPTR_0003s20830g [Populus trichocarpa]	BCDH	2-oxoisovalerate dehydrogenase subunit beta 1, mitochondrial OS=Arabidopsis thaliana GN=BCDH BETA1 PE=2 SV=1	33.01	24.69	1.13	up	0.00	0.00	yes
TRINITY_DN23261_c0_g4	ABC1 family protein [Populus trichocarpa]	At1g79600	Uncharacterized aarF domain-containing protein kinase At1g79600, chloroplastic OS=Arabidopsis thaliana GN=At1g79600 PE=2 SV=1	39.15	25.95	1.19	up	0.00	0.00	yes
TRINITY_DN17879_c0_g2	hypothetical protein POPTR_0002s11020g [Populus trichocarpa]	At1g09900	Pentatricopeptide repeat-containing protein At1g09900 OS=Arabidopsis thaliana GN=At1g09900 PE=2 SV=1	31.50	20.04	1.26	up	0.00	0.00	yes
TRINITY_DN22257_c1_g1	PREDICTED: uncharacterized protein LOC105112774 isoform X2 [Populus euphratica]	-	-	37.51	25.29	1.16	up	0.00	0.00	yes
TRINITY_DN25671_c0_g1	ENOYL-ACP REDUCTASE 1 family protein [Populus trichocarpa]	MOD1	Enoyl-[acyl-carrier-protein] reductase [NADH], chloroplastic OS=Arabidopsis thaliana GN=MOD1 PE=1 SV=1	122.50	78.93	1.18	up	0.00	0.00	yes
TRINITY_DN15882_c0_g1	hypothetical protein POPTR_0018s05150g [Populus trichocarpa]	GDCSP	Glycine dehydrogenase (decarboxylating), mitochondrial OS=Pisum sativum GN=GDCSP PE=1 SV=1	16.28	3.88	2.67	up	0.00	0.00	yes
TRINITY_DN17171_c0_g3	unknown [Populus trichocarpa]	-	Cytochrome b-c1 complex subunit 9 OS=Solanum tuberosum PE=1 SV=1	116.10	78.28	1.14	up	0.00	0.00	yes
TRINITY_DN26175_c0_g1	PREDICTED: hexokinase-1-like [Populus euphratica]	HXK1	Hexokinase-1 OS=Spinacia oleracea GN=HXK1 PE=2 SV=1	24.35	14.67	1.33	up	0.00	0.00	yes
TRINITY_DN22810_c0_g1	hypothetical protein POPTR_0018s13680g [Populus trichocarpa]	OXA1	Mitochondrial inner membrane protein OXA1 OS=Arabidopsis thaliana GN=OXA1 PE=2 SV=2	16.05	10.85	1.25	up	0.00	0.00	yes
TRINITY_DN18356_c0_g1	hypothetical protein POPTR_0006s01320g [Populus trichocarpa]	-	-	39.77	18.09	1.77	up	0.00	0.00	yes
TRINITY_DN19483_c0_g2	PREDICTED: uncharacterized protein LOC105117153 [Populus euphratica]	-	-	243.27	130.47	1.48	up	0.00	0.00	yes
TRINITY_DN18222_c1_g1	PREDICTED: UDP-sugar pyrophosphorylase [Populus euphratica]	USP	UDP-sugar pyrophosphorylase OS=Cucumis melo GN=USP PE=1 SV=1	28.36	19.26	1.16	up	0.00	0.00	yes
TRINITY_DN26311_c0_g1	mRNA for protoporphyrinogen oxidase family protein [Populus trichocarpa]	PPOX1	Protoporphyrinogen oxidase 1, chloroplastic OS=Arabidopsis thaliana GN=PPOX1 PE=1 SV=1	101.12	61.44	1.31	up	0.00	0.00	yes
TRINITY_DN17098_c0_g1	hypothetical protein POPTR_0007s13060g [Populus trichocarpa]	-	Putative glucose-6-phosphate 1-epimerase OS=Cenchrus ciliaris PE=2 SV=1	103.84	56.53	1.47	up	0.00	0.00	yes
TRINITY_DN23873_c0_g1	PREDICTED: cell division protein FtsY homolog, chloroplastic [Populus euphratica]	CPFTSY	Cell division protein FtsY homolog, chloroplastic OS=Arabidopsis thaliana GN=CPFTSY PE=1 SV=2	77.71	49.65	1.27	up	0.00	0.00	yes
TRINITY_DN18413_c0_g1	Heat shock 22 kDa family protein [Populus trichocarpa]	HSP23.6	23.6 kDa heat shock protein, mitochondrial OS=Arabidopsis thaliana GN=HSP23.6 PE=2 SV=1	12.50	5.08	2.31	up	0.00	0.00	yes
TRINITY_DN23840_c0_g1	peptidyl-prolyl cis-trans isomerase cyclophilin-type family protein [Populus trichocarpa]	PNSL5	Photosynthetic NDH subunit of lumenal location 5, chloroplastic OS=Arabidopsis thaliana GN=PNSL5 PE=1 SV=1	722.73	421.37	1.34	up	0.00	0.00	yes
TRINITY_DN20771_c6_g1	PREDICTED: uncharacterized protein LOC105113655 [Populus euphratica]	-	Ycf3-interacting protein 1, chloroplastic OS=Nicotiana tabacum PE=1 SV=2	82.13	47.73	1.39	up	0.00	0.00	yes
TRINITY_DN25124_c0_g3	PREDICTED: ferredoxin-thioredoxin reductase, variable chain-like [Populus euphratica]	-	Ferredoxin-thioredoxin reductase, variable chain OS=Zea mays PE=1 SV=1	25.21	13.59	1.51	up	0.00	0.00	yes
TRINITY_DN15880_c0_g3	unknown [Populus trichocarpa]	At1g28150	UPF0426 protein At1g28150, chloroplastic OS=Arabidopsis thaliana GN=At1g28150 PE=2 SV=1	51.44	27.78	1.49	up	0.00	0.00	yes
TRINITY_DN25640_c0_g1	hypothetical protein POPTR_0002s22560g [Populus trichocarpa]	FTSH2	ATP-dependent zinc metalloprotease FTSH 2, chloroplastic OS=Arabidopsis thaliana GN=FTSH2 PE=1 SV=1	279.75	162.70	1.39	up	0.00	0.00	yes

TRINITY_DN17951_c0_g1	PREDICTED: nucleoside diphosphate kinase 3 isoform X1 [Populus euphratica]	NDPK3	Nucleoside diphosphate kinase III, chloroplastic/mitochondrial OS=Arabidopsis thaliana GN=NDPK3 PE=1 SV=1	91.53	60.90	1.19	up	0.00	0.00	yes
TRINITY_DN18641_c0_g1	CDK-activating kinase 1at family protein [Populus trichocarpa]	CDKF-1	Cyclin-dependent kinase F-1 OS=Arabidopsis thaliana GN=CDKF-1 PE=1 SV=1	12.62	8.40	1.20	up	0.00	0.00	yes
TRINITY_DN24341_c0_g1	PREDICTED: alpha-glucosidase-like [Populus euphratica]	-	Alpha-glucosidase OS=Spinacia oleracea PE=1 SV=1	5.68	2.81	1.63	up	0.00	0.00	yes
TRINITY_DN26743_c0_g1	RecName: Full=Phosphoglucomutase, cytoplasmic; Short=PGM; AltName: Full=Glucose phosphomutase	PGM1	Phosphoglucomutase, cytoplasmic OS=Populus tremula GN=PGM1 PE=2 SV=1	74.05	53.71	1.15	up	0.00	0.00	yes
TRINITY_DN18528_c0_g1	hypothetical protein POPTR_0005s21670g [Populus trichocarpa]	COG0212	5-formyltetrahydrofolate cyclo-ligase-like protein COG0212 OS=Arabidopsis thaliana GN=COG0212 PE=2 SV=1	18.78	13.99	1.16	up	0.00	0.00	yes
TRINITY_DN26991_c0_g2	DEAD box RNA helicase family protein [Populus trichocarpa]	RH31	DEAD-box ATP-dependent RNA helicase 31 OS=Arabidopsis thaliana GN=RH31 PE=2 SV=2	23.57	16.72	1.30	up	0.00	0.00	yes
TRINITY_DN23862_c0_g1	ribosomal protein 1 [Populus trichocarpa]	RPL3	60S ribosomal protein L3 OS=Oryza sativa subsp. japonica GN=RPL3 PE=2 SV=2	623.84	415.86	1.20	up	0.00	0.00	yes
TRINITY_DN26815_c0_g1	beta-amylase family protein [Populus trichocarpa]	BAM3	Beta-amylase 3, chloroplastic OS=Arabidopsis thaliana GN=BAM3 PE=1 SV=3	150.16	101.01	1.59	up	0.00	0.00	yes
TRINITY_DN17015_c0_g1	hypothetical protein POPTR_0009s01810g [Populus trichocarpa]	PDX2	Probable pyridoxal 5'-phosphate synthase subunit PDX2 OS=Arabidopsis thaliana GN=PDX2 PE=1 SV=1	88.79	57.90	1.23	up	0.00	0.00	yes
TRINITY_DN27585_c1_g2	serine carboxypeptidase S10 family protein [Populus trichocarpa]	SCPL2	Serine carboxypeptidase-like 2 OS=Arabidopsis thaliana GN=SCPL2 PE=2 SV=1	8.97	3.59	1.90	up	0.00	0.00	yes
TRINITY_DN16825_c0_g1	actin-depolymerizing factor 6 family protein [Populus trichocarpa]	-	Actin-depolymerizing factor OS=Vitis vinifera PE=2 SV=1	60.96	43.23	1.08	up	0.00	0.00	yes
TRINITY_DN23909_c2_g1	hypothetical protein POPTR_0001s05980g [Populus trichocarpa]	-	-	86.96	47.74	1.39	up	0.00	0.00	yes
TRINITY_DN15922_c1_g1	PREDICTED: peroxiredoxin-2F, mitochondrial [Populus euphratica]	PRXIIF	Peroxiredoxin-2F, mitochondrial OS=Arabidopsis thaliana GN=PRXIIF PE=1 SV=2	104.19	69.35	1.19	up	0.00	0.00	yes
TRINITY_DN21638_c0_g4	hypothetical protein POPTR_0008s05310g [Populus trichocarpa]	-	-	75.66	51.61	1.14	up	0.00	0.00	yes
TRINITY_DN17627_c0_g1	PREDICTED: carbon catabolite repressor protein 4 homolog 4 isoform X2 [Populus euphratica]	CCR4-4	Carbon catabolite repressor protein 4 homolog 4 OS=Arabidopsis thaliana GN=CCR4-4 PE=1 SV=1	20.79	14.48	1.18	up	0.00	0.00	yes
TRINITY_DN13061_c0_g1	hypothetical protein POPTR_0001s02960g [Populus trichocarpa]	-	-	63.60	35.92	1.44	up	0.00	0.00	yes
TRINITY_DN21625_c0_g1	PREDICTED: heterodimeric geranylgeranyl pyrophosphate synthase small subunit, chloroplastic [Populus euphratica]	GGR	Heterodimeric geranylgeranyl pyrophosphate synthase small subunit, chloroplastic OS=Arabidopsis thaliana GN=GGR PE=1 SV=2	47.23	29.10	1.31	up	0.00	0.00	yes
TRINITY_DN26795_c0_g3	PREDICTED: CBL-interacting serine/threonine-protein kinase 21-like [Populus euphratica]	CIPK21	CBL-interacting serine/threonine-protein kinase 21 OS=Arabidopsis thaliana GN=CIPK21 PE=1 SV=1	22.32	13.52	1.40	up	0.00	0.00	yes
TRINITY_DN17709_c0_g1	SULFITE OXIDASE family protein [Populus trichocarpa]	SOX	Sulfite oxidase OS=Arabidopsis thaliana GN=SOX PE=1 SV=1	69.74	47.16	1.17	up	0.00	0.00	yes
TRINITY_DN24740_c1_g1	Chlorophyll a-b binding protein CP29.3 [Populus trichocarpa]	LHCB4.3	Chlorophyll a-b binding protein CP29.3, chloroplastic OS=Arabidopsis thaliana GN=LHCB4.3 PE=2 SV=1	1015.65	449.23	1.79	up	0.00	0.00	yes
TRINITY_DN22011_c1_g2	hypothetical protein POPTR_0017s02030g [Populus trichocarpa]	-	-	7.45	3.78	1.56	up	0.00	0.00	yes
TRINITY_DN20055_c0_g9	hypothetical protein POPTR_0012s09340g [Populus trichocarpa]	EMB1006	Pentatricopeptide repeat-containing protein At5g50280, chloroplastic OS=Arabidopsis thaliana GN=EMB1006 PE=2 SV=1	35.26	22.30	1.27	up	0.00	0.00	yes
TRINITY_DN13158_c0_g1	PREDICTED: nucleolar protein 16 [Populus euphratica]	-	-	44.15	28.12	1.29	up	0.00	0.00	yes
TRINITY_DN26554_c0_g1	hypothetical protein POPTR_0010s17680g [Populus trichocarpa]	-	-	56.74	31.18	1.45	up	0.00	0.00	yes
TRINITY_DN27319_c1_g2	hypothetical protein POPTR_0019s10910g [Populus trichocarpa]	FTIP1	FT-interacting protein 1 OS=Arabidopsis thaliana GN=FTIP1 PE=1 SV=1	7.71	3.24	1.85	up	0.00	0.00	yes
TRINITY_DN20321_c0_g2	hypothetical protein POPTR_0001s29740g [Populus trichocarpa]	rsmE	Ribosomal RNA small subunit methyltransferase E OS=Rickettsia conorii (strain ATCC VR-613 / Malish 7) GN=rsmE PE=3 SV=2	12.52	8.46	1.35	up	0.00	0.00	yes
TRINITY_DN20718_c0_g1	hypothetical protein POPTR_0008s12000g [Populus trichocarpa]	RPL15	50S ribosomal protein L15, chloroplastic (Fragment) OS=Pisum sativum GN=RPL15 PE=2 SV=1	337.73	182.53	1.48	up	0.00	0.00	yes

TRINITY_DN20354_c0_g4	hypothetical protein POPTR_0006s07490g [Populus trichocarpa]	CIA2	Protein CHLOROPLAST IMPORT APPARATUS 2 OS=Arabidopsis thaliana GN=CIA2 PE=2 SV=1	24.54	15.53	1.25	up	0.00	0.00	yes
TRINITY_DN26421_c0_g1	the aldehyde dehydrogenase cp-ADH from C.plantagineum family protein [Populus trichocarpa]	ALDH3H1	Aldehyde dehydrogenase family 3 member H1 OS=Arabidopsis thaliana GN=ALDH3H1 PE=1 SV=2	58.13	34.64	1.32	up	0.00	0.00	yes
TRINITY_DN27298_c0_g1	PREDICTED: phosphomethylpyrimidine synthase, chloroplastic [Populus euphratica]	THIC	Phosphomethylpyrimidine synthase, chloroplastic OS=Arabidopsis thaliana GN=THIC PE=1 SV=1	350.59	177.84	1.57	up	0.00	0.00	yes
TRINITY_DN25593_c1_g1	PREDICTED: chloroplastic group IIA intron splicing facilitator CRS1, chloroplastic isoform X1 [Populus euphratica]	CFM3	CRM-domain containing factor CFM3, chloroplastic/mitochondrial OS=Zea mays GN=CFM3 PE=1 SV=1	47.20	31.54	1.16	up	0.00	0.00	yes
TRINITY_DN15986_c0_g1	PREDICTED: glutaredoxin-C1 [Populus euphratica]	GRXC1	Glutaredoxin-C1 OS=Arabidopsis thaliana GN=GRXC1 PE=2 SV=2	57.17	33.95	1.37	up	0.00	0.00	yes
TRINITY_DN26265_c0_g1	hypothetical protein POPTR_0013s10300g, partial [Populus trichocarpa]	-	Cytochrome c OS=Sambucus nigra PE=1 SV=1	33.29	19.38	1.39	up	0.00	0.00	yes
TRINITY_DN23371_c2_g2	hypothetical protein GLYMA_11G032800 [Glycine max]	RPS30A	40S ribosomal protein S30 OS=Arabidopsis thaliana GN=RPS30A PE=3 SV=3	401.15	257.07	1.18	up	0.00	0.00	yes
TRINITY_DN20285_c0_g2	hypothetical protein POPTR_0014s18940g [Populus trichocarpa]	RPL32A	60S ribosomal protein L32-1 OS=Arabidopsis thaliana GN=RPL32A PE=2 SV=2	849.10	568.63	1.21	up	0.00	0.00	yes
TRINITY_DN15767_c0_g1	-	-	-	115.22	76.76	1.18	up	0.00	0.00	yes
TRINITY_DN24122_c0_g1	PREDICTED: uncharacterized protein LOC105119383 [Populus euphratica]	BF2056	UPF0301 protein BF2056 OS=Bacteroides fragilis (strain YCH46) GN=BF2056 PE=3 SV=1	79.62	51.81	1.21	up	0.00	0.00	yes
TRINITY_DN15170_c0_g1	-	-	-	8.77	1.67	2.90	up	0.00	0.00	yes
TRINITY_DN19814_c0_g1	hypothetical protein POPTR_0001s21690g [Populus trichocarpa]	PAP4	Probable plastid-lipid-associated protein 4, chloroplastic OS=Arabidopsis thaliana GN=PAP4 PE=2 SV=1	134.77	67.93	1.48	up	0.00	0.00	yes
TRINITY_DN25512_c0_g2	hypothetical protein POPTR_0010s06180g [Populus trichocarpa]	ALB3L1	ALBINO3-like protein 1, chloroplastic OS=Arabidopsis thaliana GN=ALB3L1 PE=2 SV=3	47.66	30.80	1.21	up	0.00	0.00	yes
TRINITY_DN14754_c0_g1	hypothetical protein POPTR_0008s15330g [Populus trichocarpa]	SECE1	Preprotein translocase subunit SECE1 OS=Arabidopsis thaliana GN=SECE1 PE=1 SV=1	72.88	36.56	1.59	up	0.00	0.00	yes
TRINITY_DN21313_c0_g1	dehydration-responsive family protein [Populus trichocarpa]	At4g00750	Probable methyltransferase PMT15 OS=Arabidopsis thaliana GN=At4g00750 PE=2 SV=1	17.51	10.28	1.35	up	0.00	0.00	yes
TRINITY_DN16751_c0_g1	PREDICTED: photosystem II core complex proteins psbY, chloroplastic-like [Populus euphratica]	PSBY	Photosystem II core complex proteins psbY, chloroplastic OS=Spinacia oleracea GN=PSBY PE=1 SV=2	1155.38	570.16	1.61	up	0.00	0.00	yes
TRINITY_DN25289_c0_g2	PREDICTED: methionine--tRNA ligase, mitochondrial [Populus euphratica]	OVA1	Methionine--tRNA ligase, chloroplastic/mitochondrial OS=Arabidopsis thaliana GN=OVA1 PE=2 SV=1	47.66	32.26	1.21	up	0.00	0.00	yes
TRINITY_DN16998_c0_g1	PREDICTED: uncharacterized protein LOC105125891 [Populus euphratica]	-	-	36.10	17.83	1.64	up	0.00	0.00	yes
TRINITY_DN22348_c0_g1	O-acetylserine (thiol)lyase family protein [Populus trichocarpa]	-	Cysteine synthase OS=Citrullus lanatus PE=1 SV=1	18.41	6.25	2.11	up	0.00	0.00	yes
TRINITY_DN22776_c0_g1	50S ribosomal protein L12-2 [Populus trichocarpa]	RPL12	50S ribosomal protein L12, chloroplastic OS=Spinacia oleracea GN=RPL12 PE=1 SV=2	1729.35	825.41	1.66	up	0.00	0.00	yes
TRINITY_DN26989_c0_g1	PREDICTED: serine/threonine-protein kinase STN7, chloroplastic [Populus euphratica]	STN7	Serine/threonine-protein kinase STN7, chloroplastic OS=Arabidopsis thaliana GN=STN7 PE=1 SV=1	49.69	31.34	1.30	up	0.00	0.00	yes
TRINITY_DN20052_c1_g3	hypothetical protein POPTR_0001s35620g [Populus trichocarpa]	At5g14450	GDSL esterase/lipase At5g14450 OS=Arabidopsis thaliana GN=At5g14450 PE=2 SV=1	30.84	19.96	1.22	up	0.00	0.00	yes
TRINITY_DN17176_c0_g3	PREDICTED: serine/threonine-protein kinase-like protein CCR2 [Populus euphratica]	CCR2	Serine/threonine-protein kinase-like protein CCR2 OS=Arabidopsis thaliana GN=CCR2 PE=1 SV=1	3.52	0.83	2.64	up	0.00	0.00	yes
TRINITY_DN21910_c0_g1	hypothetical protein POPTR_0004s16520g [Populus trichocarpa]	CYB5	Cytochrome b5 OS=Brassica oleracea var. botrytis GN=CYB5 PE=1 SV=1	923.39	456.94	1.60	up	0.00	0.00	yes
TRINITY_DN25791_c0_g1	hypothetical protein POPTR_0010s18400g [Populus trichocarpa]	ABCC2	ABC transporter D family member 2, chloroplastic OS=Arabidopsis thaliana GN=ABCC2 PE=1 SV=1	15.98	11.25	1.16	up	0.00	0.00	yes
TRINITY_DN16561_c0_g1	PREDICTED: LOW QUALITY PROTEIN: aarF domain-containing protein kinase 4 [Populus euphratica]	ABC1	Protein ABC transporter 1, mitochondrial OS=Arabidopsis thaliana GN=ABC1 PE=2 SV=1	8.38	5.48	1.24	up	0.00	0.00	yes
TRINITY_DN27236_c0_g1	PREDICTED: uncharacterized protein LOC105139901 isoform X1 [Populus euphratica]	rps1b	30S ribosomal protein S1 homolog B OS=Synechocystis sp. (strain PCC 6803 / Kazusa) GN=rps1b PE=3 SV=1	56.01	35.39	1.25	up	0.00	0.00	yes
TRINITY_DN11899_c0_g1	hypothetical protein POPTR_0012s01400g [Populus trichocarpa]	SWEET3	Bidirectional sugar transporter SWEET3 OS=Arabidopsis thaliana GN=SWEET3 PE=1 SV=1	6.87	0.87	3.55	up	0.00	0.00	yes

TRINITY_DN23418_c0_g1	PREDICTED: protein sym-1-like [Populus euphratica]	sym-1	Protein sym-1 OS=Neurospora crassa (strain ATCC 24698 / 74-OR23-1A / CBS 708.71 / DSM 1257 / FGSC 987) GN=sym-1 PE=3 SV=2	46.34	33.91	1.11	up	0.00	0.00	yes
TRINITY_DN21916_c4_g1	PREDICTED: uncharacterized protein LOC105113662 [Populus euphratica]	-	-	132.75	88.40	1.19	up	0.00	0.00	yes
TRINITY_DN21390_c0_g3	PREDICTED: 60S ribosomal protein L19-3-like [Populus euphratica]	RPL19C	60S ribosomal protein L19-3 OS=Arabidopsis thaliana GN=RPL19C PE=2 SV=3	527.62	353.97	1.21	up	0.00	0.00	yes
TRINITY_DN27893_c10_g1	PREDICTED: anthranilate phosphoribosyltransferase, chloroplastic-like isoform X1 [Populus euphratica]	PAT1	Anthranilate phosphoribosyltransferase, chloroplastic OS=Arabidopsis thaliana GN=PAT1 PE=2 SV=1	53.94	35.34	1.22	up	0.00	0.00	yes
TRINITY_DN26004_c0_g2	PREDICTED: E3 ubiquitin-protein ligase RMA1H1 [Populus euphratica]	RMA1H1	E3 ubiquitin-protein ligase RMA1H1 OS=Capsicum annuum GN=RMA1H1 PE=1 SV=1	20.19	5.45	2.59	up	0.00	0.00	yes
TRINITY_DN15625_c0_g1	hypothetical protein POPTR_0009s06720g [Populus trichocarpa]	At3g63540	Thylakoid lumenal 19 kDa protein, chloroplastic OS=Arabidopsis thaliana GN=At3g63540 PE=1 SV=2	298.42	161.42	1.48	up	0.00	0.00	yes
TRINITY_DN16326_c0_g1	PREDICTED: mitochondrial import receptor subunit TOM20 [Populus euphratica]	TOM20	Mitochondrial import receptor subunit TOM20 OS=Solanum tuberosum GN=TOM20 PE=1 SV=1	53.04	36.64	1.13	up	0.00	0.00	yes
TRINITY_DN19918_c0_g3	PREDICTED: glycine-rich RNA-binding protein 4, mitochondrial-like [Populus euphratica]	RBG3	Glycine-rich RNA-binding protein 3, mitochondrial OS=Arabidopsis thaliana GN=RBG3 PE=2 SV=1	71.78	49.39	1.15	up	0.00	0.00	yes
TRINITY_DN16409_c0_g4	hypothetical protein POPTR_0001s46010g [Populus trichocarpa]	-	-	116.27	61.42	1.50	up	0.00	0.00	yes
TRINITY_DN12822_c0_g1	PREDICTED: LOW QUALITY PROTEIN: uncharacterized protein LOC105138331 [Populus euphratica]	-	-	36.65	25.74	1.13	up	0.00	0.00	yes
TRINITY_DN23019_c0_g2	PREDICTED: psbQ-like protein 2, chloroplastic [Populus euphratica]	PNSL3	Photosynthetic NDH subunit of lumenal location 3, chloroplastic OS=Arabidopsis thaliana GN=PNSL3 PE=1 SV=1	168.82	67.76	1.89	up	0.00	0.00	yes
TRINITY_DN17663_c0_g3	unknown [Populus trichocarpa x Populus deltoides]	GAPC	Glyceraldehyde-3-phosphate dehydrogenase, cytosolic OS=Antirrhinum majus GN=GAPC PE=2 SV=1	373.94	255.99	1.15	up	0.00	0.00	yes
TRINITY_DN26378_c0_g1	Toc64 family protein [Populus trichocarpa]	OEP64	Outer envelope protein 64, chloroplastic OS=Arabidopsis thaliana GN=OEP64 PE=1 SV=1	55.18	35.77	1.25	up	0.00	0.00	yes
TRINITY_DN18710_c0_g1	hypothetical protein POPTR_0008s17090g [Populus trichocarpa]	PGR5	Protein PROTON GRADIENT REGULATION 5, chloroplastic OS=Arabidopsis thaliana GN=PGR5 PE=1 SV=1	13.05	5.99	1.73	up	0.00	0.00	yes
TRINITY_DN26711_c0_g1	Sedoheptulose-1 family protein [Populus trichocarpa]	At3g55800	Sedoheptulose-1,7-bisphosphatase, chloroplastic OS=Arabidopsis thaliana GN=At3g55800 PE=2 SV=1	85.33	41.36	1.68	up	0.00	0.00	yes
TRINITY_DN13315_c0_g1	-	-	-	12.26	3.62	2.36	up	0.00	0.00	yes
TRINITY_DN19030_c0_g2	hypothetical protein POPTR_0018s02280g [Populus trichocarpa]	ERF003	Ethylene-responsive transcription factor ERF003 OS=Arabidopsis thaliana GN=ERF003 PE=2 SV=1	21.55	12.38	1.34	up	0.00	0.00	yes
TRINITY_DN26640_c0_g3	PREDICTED: frataxin, mitochondrial [Populus euphratica]	FH	Frataxin, mitochondrial OS=Arabidopsis thaliana GN=FH PE=1 SV=2	23.30	14.64	1.29	up	0.00	0.00	yes
TRINITY_DN18864_c0_g4	hypothetical protein POPTR_0009s16560g [Populus trichocarpa]	-	-	22.33	6.91	2.29	up	0.00	0.00	yes
TRINITY_DN23651_c0_g1	sterol 4-alpha-methyl-oxidase 1 family protein [Populus trichocarpa]	SMO2-2	Methylsterol monooxygenase 2-2 OS=Arabidopsis thaliana GN=SMO2-2 PE=2 SV=1	41.44	30.62	1.05	up	0.00	0.00	yes
TRINITY_DN21839_c0_g1	hypothetical protein POPTR_0012s06360g, partial [Populus trichocarpa]	RPL6	50S ribosomal protein L6, chloroplastic OS=Arabidopsis thaliana GN=RPL6 PE=2 SV=1	473.60	256.93	1.47	up	0.00	0.00	yes
TRINITY_DN19448_c1_g1	PREDICTED: cytochrome P450 89A2-like [Populus euphratica]	CYP89A9	Cytochrome P450 89A9 OS=Arabidopsis thaliana GN=CYP89A9 PE=2 SV=1	39.33	17.69	1.73	up	0.00	0.00	yes
TRINITY_DN18397_c0_g1	hypothetical protein POPTR_0010s26180g [Populus trichocarpa]	-	-	112.47	69.93	1.31	up	0.00	0.00	yes
TRINITY_DN22895_c0_g5	hypothetical protein POPTR_0016s021501g, partial [Populus trichocarpa]	UGT85A23	7-deoxyloganetin glucosyltransferase OS=Catharanthus roseus GN=UGT85A23 PE=1 SV=1	9.84	3.87	1.93	up	0.00	0.00	yes
TRINITY_DN16503_c0_g1	hypothetical protein POPTR_0017s11510g [Populus trichocarpa]	-	-	7.83	3.56	1.76	up	0.00	0.00	yes
TRINITY_DN18164_c0_g1	PREDICTED: uncharacterized protein At5g02240 [Populus euphratica]	At2g37660	Uncharacterized protein At2g37660, chloroplastic OS=Arabidopsis thaliana GN=At2g37660 PE=1 SV=2	304.88	180.79	1.34	up	0.00	0.00	yes
TRINITY_DN24025_c0_g5	PREDICTED: peptidyl-prolyl cis-trans isomerase FKBP17-2, chloroplastic-like isoform X1 [Populus euphratica]	FKBP17-2	Peptidyl-prolyl cis-trans isomerase FKBP17-2, chloroplastic OS=Arabidopsis thaliana GN=FKBP17-2 PE=1 SV=1	466.40	197.15	1.82	up	0.00	0.00	yes

TRINITY_DN26451_c2_g5	PREDICTED: probable receptor-like protein kinase At5g39030 [Populus euphratica]	LRK10L-2.2	LEAF RUST 10 DISEASE-RESISTANCE LOCUS RECEPTOR-LIKE PROTEIN KINASE-like 2.2 OS=Arabidopsis thaliana GN=LRK10L-2.2 PE=3 SV=2	8.72	1.89	2.92	up	0.00	0.00	yes
TRINITY_DN15018_c0_g1	hypothetical protein POPTR_0007s01450g [Populus trichocarpa]	At1g62350	Pentatricopeptide repeat-containing protein At1g62350 OS=Arabidopsis thaliana GN=At1g62350 PE=2 SV=1	29.37	18.34	1.31	up	0.00	0.00	yes
TRINITY_DN21499_c0_g3	putative plasma membrane H+ ATPase family protein [Populus trichocarpa]	AHA10	ATPase 10, plasma membrane-type OS=Arabidopsis thaliana GN=AHA10 PE=2 SV=2	91.83	53.55	1.35	up	0.00	0.00	yes
TRINITY_DN27114_c0_g1	signal recognition particle 54 kDa subunit precursor family protein [Populus trichocarpa]	FFC	Signal recognition particle 54 kDa protein, chloroplastic OS=Arabidopsis thaliana GN=FFC PE=1 SV=1	129.89	73.75	1.34	up	0.00	0.00	yes
TRINITY_DN17179_c0_g2	PREDICTED: uncharacterized protein LOC105109440 [Populus euphratica]	UP3	Stress-response A/B barrel domain-containing protein UP3 OS=Arabidopsis thaliana GN=UP3 PE=2 SV=1	30.00	20.81	1.13	up	0.00	0.00	yes
TRINITY_DN20101_c0_g1	hypothetical protein POPTR_0016s03160g [Populus trichocarpa]	CYP94A1	Cytochrome P450 94A1 OS=Vicia sativa GN=CYP94A1 PE=2 SV=2	10.12	5.77	1.43	up	0.00	0.00	yes
TRINITY_DN26336_c0_g3	PREDICTED: probable WRKY transcription factor 31 isoform X2 [Populus euphratica]	WRKY6	WRKY transcription factor 6 OS=Arabidopsis thaliana GN=WRKY6 PE=1 SV=1	34.63	22.78	1.19	up	0.00	0.00	yes
TRINITY_DN27865_c1_g4	-	-	-	61.36	32.80	1.51	up	0.00	0.00	yes
TRINITY_DN24520_c0_g2	hypothetical protein POPTR_0016s06010g [Populus trichocarpa]	Ahsa2	Activator of 90 kDa heat shock protein ATPase homolog 2 OS=Mus musculus GN=Ahsa2 PE=1 SV=2	56.23	28.20	1.23	up	0.00	0.00	yes
TRINITY_DN21816_c0_g1	PREDICTED: peter Pan-like protein [Populus euphratica]	PPAN	Peter Pan-like protein OS=Arabidopsis thaliana GN=PPAN PE=1 SV=1	24.99	17.40	1.15	up	0.00	0.00	yes
TRINITY_DN20620_c1_g3	unknown [Populus trichocarpa]	-	3-oxo-Delta(4,5)-steroid 5-beta-reductase OS=Digitalis lanata PE=1 SV=1	72.81	51.40	1.10	up	0.00	0.00	yes
TRINITY_DN24599_c5_g4	PREDICTED: cytochrome P450 714A1-like [Populus euphratica]	CYP714A1	Cytochrome P450 714A1 OS=Arabidopsis thaliana GN=CYP714A1 PE=2 SV=1	4.63	0.20	4.92	up	0.00	0.00	yes
TRINITY_DN25325_c0_g1	PREDICTED: uncharacterized protein LOC105133067 [Populus euphratica]	-	-	46.58	35.85	1.17	up	0.00	0.00	yes
TRINITY_DN14831_c0_g1	hypothetical protein POPTR_0007s12890g [Populus trichocarpa]	RPL6	60S ribosomal protein L6, mitochondrial OS=Marchantia polymorpha GN=RPL6 PE=3 SV=2	37.57	22.49	1.35	up	0.00	0.00	yes
TRINITY_DN19917_c0_g1	PREDICTED: uncharacterized protein LOC105113649 [Populus euphratica]	-	-	44.98	28.98	1.23	up	0.00	0.00	yes
TRINITY_DN16002_c0_g1	hypothetical protein POPTR_0014s14430g, partial [Populus trichocarpa]	RPS30A	40S ribosomal protein S30 OS=Arabidopsis thaliana GN=RPS30A PE=3 SV=3	442.90	307.62	1.26	up	0.00	0.00	yes
TRINITY_DN19917_c0_g4	hypothetical protein POPTR_0010s13740g [Populus trichocarpa]	At1g68590	30S ribosomal protein 3-1, chloroplastic OS=Arabidopsis thaliana GN=At1g68590 PE=2 SV=1	371.72	209.47	1.41	up	0.00	0.00	yes
TRINITY_DN17231_c0_g1	hypothetical protein POPTR_0009s11500g [Populus trichocarpa]	-	-	23.67	3.94	3.17	up	0.00	0.00	yes
TRINITY_DN19771_c0_g1	hypothetical protein POPTR_0010s22250g [Populus trichocarpa]	ADK	Adenylate kinase 1, chloroplastic OS=Arabidopsis thaliana GN=ADK PE=1 SV=1	53.45	36.30	1.21	up	0.00	0.00	yes
TRINITY_DN25890_c0_g1	PREDICTED: 50S ribosomal protein L1, chloroplastic [Populus euphratica]	rplA	50S ribosomal protein L1 OS=Rhizobium meliloti (strain 1021) GN=rplA PE=3 SV=1	23.24	17.09	1.09	up	0.00	0.00	yes
TRINITY_DN21607_c0_g1	PREDICTED: NAC domain-containing protein 86-like [Populus euphratica]	NAC086	NAC domain-containing protein 86 OS=Arabidopsis thaliana GN=NAC086 PE=2 SV=1	3.39	1.09	2.26	up	0.00	0.00	yes
TRINITY_DN15671_c0_g1	hypothetical protein POPTR_0007s13530g [Populus trichocarpa]	-	-	64.61	45.48	1.14	up	0.00	0.00	yes
TRINITY_DN21181_c0_g1	hypothetical protein POPTR_0002s08900g [Populus trichocarpa]	RPS13	30S ribosomal protein S13, chloroplastic OS=Arabidopsis thaliana GN=RPS13 PE=2 SV=1	660.13	352.10	1.51	up	0.00	0.00	yes
TRINITY_DN20774_c0_g1	hypothetical protein EUGRSUZ_J00099 [Eucalyptus grandis]	RPL10	60S ribosomal protein L10 OS=Vitis riparia GN=RPL10 PE=2 SV=1	985.72	659.04	1.18	up	0.00	0.00	yes
TRINITY_DN17533_c0_g1	hypothetical protein POPTR_0013s08220g [Populus trichocarpa]	MTACP2	Acyl carrier protein 2, mitochondrial OS=Arabidopsis thaliana GN=MTACP2 PE=1 SV=1	132.64	89.90	1.19	up	0.00	0.00	yes
TRINITY_DN16792_c0_g1	hypothetical protein POPTR_0003s09430g [Populus trichocarpa]	-	-	16.67	10.39	1.30	up	0.00	0.00	yes
TRINITY_DN17065_c0_g1	hypothetical protein POPTR_0010s07380g [Populus trichocarpa]	-	-	27.35	15.53	1.39	up	0.00	0.00	yes
TRINITY_DN23901_c1_g4	PREDICTED: ferredoxin-3, chloroplastic-like isoform X1 [Populus euphratica]	FDX3	Ferredoxin-3, chloroplastic OS=Zea mays GN=FDX3 PE=2 SV=1	126.81	55.15	1.43	up	0.00	0.00	yes

TRINITY_DN15694_c0_g2	hypothetical protein POPTR_0014s11560g [Populus trichocarpa]	COX5C	Cytochrome c oxidase subunit 5C OS=Oryza sativa subsp. japonica GN=COX5C PE=3 SV=3	251.46	161.21	1.24	up	0.00	0.00	yes
TRINITY_DN23839_c0_g2	PREDICTED: uncharacterized protein LOC105134600 isoform X1 [Populus euphratica]	-	-	31.37	17.79	1.40	up	0.00	0.00	yes
TRINITY_DN7900_c0_g3	retrovirus-related Pol polyprotein from transposon TNT 1-94 [Dorcoceras hygrometricum]	GIP	Copia protein OS=Drosophila melanogaster GN=GIP PE=1 SV=3	2.91	1.22	1.88	up	0.00	0.00	yes
TRINITY_DN18219_c0_g1	ubiquitin-40S ribosomal protein S27a-like [Solanum tuberosum]	UBI3	Ubiquitin-40S ribosomal protein S27a OS=Solanum lycopersicum GN=UBI3 PE=3 SV=2	766.87	524.72	1.17	up	0.00	0.00	yes
TRINITY_DN28132_c0_g1	hypothetical protein POPTR_0011s00470g [Populus trichocarpa]	NTF2	Nuclear transport factor 2 OS=Arabidopsis thaliana GN=NTF2 PE=1 SV=1	4.79	1.26	2.50	up	0.00	0.00	yes
TRINITY_DN22578_c1_g2	hypothetical protein POPTR_0015s12790g [Populus trichocarpa]	MED36B	Probable mediator of RNA polymerase II transcription subunit 36b OS=Arabidopsis thaliana GN=MED36B PE=1 SV=1	127.24	69.53	1.44	up	0.00	0.00	yes
TRINITY_DN19512_c0_g1	mitochondrial substrate carrier family protein [Populus trichocarpa]	SFC1	Mitochondrial succinate-fumarate transporter 1 OS=Arabidopsis thaliana GN=SFC1 PE=2 SV=1	12.18	7.45	1.30	up	0.00	0.00	yes
TRINITY_DN18160_c0_g1	inositol monophosphatase family protein [Populus trichocarpa]	IMPL1	Phosphatase IMPL1, chloroplastic OS=Arabidopsis thaliana GN=IMPL1 PE=1 SV=2	15.50	10.06	1.23	up	0.00	0.00	yes
TRINITY_DN23297_c0_g7	PREDICTED: 60S ribosomal protein L15-like [Populus euphratica]	RPL15	60S ribosomal protein L15 OS=Petunia hybrida GN=RPL15 PE=2 SV=1	334.41	234.14	1.14	up	0.00	0.00	yes
TRINITY_DN22178_c2_g4	hypothetical protein POPTR_0001s37650g [Populus trichocarpa]	FLA1	Fasciclin-like arabinogalactan protein 1 OS=Arabidopsis thaliana GN=FLA1 PE=1 SV=1	81.83	56.14	1.14	up	0.00	0.00	yes
TRINITY_DN27724_c1_g1	PREDICTED: protein DA1-related 1-like [Populus euphratica]	DAR1	Protein DA1-related 1 OS=Arabidopsis thaliana GN=DAR1 PE=1 SV=3	29.63	22.85	1.13	up	0.00	0.00	yes
TRINITY_DN19521_c1_g1	hypothetical protein POPTR_0016s10850g, partial [Populus trichocarpa]	-	-	7.29	4.85	2.04	up	0.00	0.00	yes
TRINITY_DN22940_c0_g1	PREDICTED: uncharacterized protein LOC105108056 [Populus euphratica]	-	-	21.59	15.28	1.08	up	0.00	0.00	yes
TRINITY_DN25221_c1_g3	PREDICTED: malate dehydrogenase, glyoxysomal [Populus euphratica]	MDHG	Malate dehydrogenase, glyoxysomal OS=Cucumis sativus GN=MDHG PE=2 SV=1	643.81	285.37	1.80	up	0.00	0.00	yes
TRINITY_DN14709_c0_g1	hypothetical protein POPTR_0006s12500g [Populus trichocarpa]	Os04g0560200	Thioredoxin-like 3-3 OS=Oryza sativa subsp. japonica GN=Os04g0560200 PE=2 SV=2	75.50	41.79	1.37	up	0.00	0.00	yes
TRINITY_DN21034_c0_g1	hypothetical protein POPTR_0019s13040g [Populus trichocarpa]	RPL5	60S ribosomal protein L5 OS=Cucumis sativus GN=RPL5 PE=2 SV=1	1026.65	691.90	1.14	up	0.00	0.00	yes
TRINITY_DN15755_c0_g3	hypothetical protein POPTR_0001s26960g [Populus trichocarpa]	RER4	Protein RETICULATA-RELATED 4, chloroplastic OS=Arabidopsis thaliana GN=RER4 PE=2 SV=1	73.87	49.39	1.19	up	0.00	0.00	yes
TRINITY_DN25877_c1_g1	50S ribosomal protein L3-2 [Populus trichocarpa]	RPL3B	50S ribosomal protein L3-2, chloroplastic OS=Arabidopsis thaliana GN=RPL3B PE=2 SV=1	35.13	24.19	1.16	up	0.00	0.00	yes
TRINITY_DN22890_c1_g1	unknown [Populus trichocarpa]	IJ	Protein Iojap, chloroplastic OS=Arabidopsis thaliana GN=IJ PE=2 SV=1	53.04	31.86	1.45	up	0.00	0.00	yes
TRINITY_DN20938_c0_g1	PREDICTED: malate dehydrogenase, glyoxysomal [Populus euphratica]	PMDH1	Malate dehydrogenase 1, peroxisomal OS=Arabidopsis thaliana GN=PMDH1 PE=2 SV=1	118.58	72.32	1.33	up	0.00	0.00	yes
TRINITY_DN16977_c0_g1	hypothetical protein POPTR_0013s09550g [Populus trichocarpa]	-	-	231.70	120.70	1.54	up	0.00	0.00	yes
TRINITY_DN26158_c0_g1	PREDICTED: sulfate transporter 1.2-like [Populus euphratica]	SULTR1;2	Sulfate transporter 1.2 OS=Arabidopsis thaliana GN=SULTR1;2 PE=1 SV=1	3.20	0.92	2.40	up	0.00	0.00	yes
TRINITY_DN16340_c0_g1	hypothetical protein POPTR_0018s13940g [Populus trichocarpa]	-	-	113.98	78.71	1.13	up	0.00	0.00	yes
TRINITY_DN18555_c0_g2	Ycf2 [Populus alba]	ycf2-A	Protein Ycf2 OS=Populus alba GN=ycf2-A PE=3 SV=1	7.83	2.72	2.13	up	0.00	0.00	yes
TRINITY_DN24029_c0_g2	hypothetical protein POPTR_0006s20610g [Populus trichocarpa]	-	-	24.36	16.64	1.14	up	0.00	0.00	yes
TRINITY_DN21664_c0_g2	hypothetical protein POPTR_0019s00670g, partial [Populus trichocarpa]	-	-	7.51	3.64	1.73	up	0.00	0.00	yes
TRINITY_DN22268_c0_g3	PREDICTED: geranylgeranyl pyrophosphate synthase 7, chloroplastic-like [Populus euphratica]	GGPPS1	Heterodimeric geranylgeranyl pyrophosphate synthase large subunit 1, chloroplastic OS=Arabidopsis thaliana GN=GGPPS1 PE=1 SV=2	4.43	2.07	1.70	up	0.00	0.00	yes
TRINITY_DN18861_c0_g1	hypothetical protein POPTR_0001s30240g [Populus trichocarpa]	EMB506	Ankyrin repeat domain-containing protein EMB506, chloroplastic OS=Arabidopsis thaliana GN=EMB506 PE=1 SV=1	54.29	39.08	1.16	up	0.00	0.00	yes

TRINITY_DN23380_c0_g1	hypothetical protein POPTR_0017s04010g [Populus trichocarpa]	OEP37	Outer envelope pore protein 37, chloroplastic OS=Pisum sativum GN=OEP37 PE=1 SV=1	86.43	60.06	1.17	up	0.00	0.00	yes
TRINITY_DN18591_c0_g2	PREDICTED: homogentisate solanesyltransferase, chloroplastic [Populus euphratica]	HPT2	Probable homogentisate phytyltransferase 2, chloroplastic OS=Oryza sativa subsp. japonica GN=HPT2 PE=3 SV=2	50.03	31.91	1.26	up	0.00	0.00	yes
TRINITY_DN26243_c0_g2	hypothetical protein POPTR_0001s19210g [Populus trichocarpa]	CHUP1	Protein CHUP1, chloroplastic OS=Arabidopsis thaliana GN=CHUP1 PE=1 SV=1	99.68	63.13	1.24	up	0.00	0.00	yes
TRINITY_DN23470_c0_g1	hypothetical protein POPTR_0001s41350g [Populus trichocarpa]	BRIX1-2	Ribosome biogenesis protein BRX1 homolog 2 OS=Arabidopsis thaliana GN=BRIX1-2 PE=2 SV=1	50.38	30.01	1.17	up	0.00	0.00	yes
TRINITY_DN17958_c0_g4	hypothetical protein POPTR_0013s11500g [Populus trichocarpa]	-	-	86.94	56.70	1.19	up	0.00	0.00	yes
TRINITY_DN27448_c0_g1	glycolate oxidase family protein [Populus trichocarpa]	-	Peroxisomal (S)-2-hydroxy-acid oxidase OS=Spinacia oleracea PE=1 SV=1	1633.96	640.54	1.92	up	0.00	0.00	yes
TRINITY_DN26715_c0_g1	glutathione S-transferase U33 [Populus yatungensis]	-	Probable glutathione S-transferase OS=Nicotiana tabacum PE=2 SV=1	640.88	395.99	1.32	up	0.00	0.00	yes
TRINITY_DN21106_c1_g1	-	-	-	80.05	53.31	1.18	up	0.00	0.00	yes
TRINITY_DN22033_c0_g1	Chain D family protein [Populus trichocarpa]	FBP	Fructose-1,6-bisphosphatase, chloroplastic OS=Pisum sativum GN=FBP PE=1 SV=2	364.35	179.65	1.60	up	0.00	0.00	yes
TRINITY_DN26190_c0_g5	carotenoid cleavage dioxygenase 1 family protein [Populus trichocarpa]	CCD1	Carotenoid 9,10(9',10')-cleavage dioxygenase 1 OS=Pisum sativum GN=CCD1 PE=2 SV=1	85.35	48.80	1.41	up	0.00	0.00	yes
TRINITY_DN24733_c1_g1	hypothetical protein POPTR_0007s15080g [Populus trichocarpa]	-	-	29.34	18.86	1.27	up	0.00	0.00	yes
TRINITY_DN27572_c1_g1	PREDICTED: lon protease homolog 1, mitochondrial-like [Populus euphratica]	LON1	Lon protease homolog 1, mitochondrial OS=Arabidopsis thaliana GN=LON1 PE=1 SV=2	27.46	19.45	1.18	up	0.00	0.00	yes
TRINITY_DN19663_c2_g5	hypothetical protein POPTR_0014s00450g [Populus trichocarpa]	-	-	4.49	1.36	2.38	up	0.00	0.00	yes
TRINITY_DN27060_c0_g1	PREDICTED: GDSL esterase/lipase At5g62930 [Populus euphratica]	At5g62930	GDSL esterase/lipase At5g62930 OS=Arabidopsis thaliana GN=At5g62930 PE=2 SV=3	43.86	29.47	1.17	up	0.00	0.00	yes
TRINITY_DN21046_c0_g3	ATP-dependent Clp protease proteolytic subunit family protein [Populus trichocarpa]	CLPR4	ATP-dependent Clp protease proteolytic subunit-related protein 4, chloroplastic OS=Arabidopsis thaliana GN=CLPR4 PE=1 SV=1	102.01	67.98	1.18	up	0.00	0.00	yes
TRINITY_DN22549_c0_g1	hypothetical protein POPTR_0010s13310g [Populus trichocarpa]	-	-	42.74	27.53	1.23	up	0.00	0.00	yes
TRINITY_DN19644_c0_g1	hypothetical protein POPTR_0013s14200g [Populus trichocarpa]	-	-	24.37	12.22	1.64	up	0.00	0.00	yes
TRINITY_DN27827_c1_g2	Pyrophosphate-energized vacuolar membrane proton pump family protein [Populus trichocarpa]	-	Pyrophosphate-energized vacuolar membrane proton pump OS=Vigna radiata var. radiata PE=1 SV=4	116.65	64.76	1.44	up	0.00	0.00	yes
TRINITY_DN22066_c0_g1	hypothetical protein POPTR_0003s12000g [Populus trichocarpa]	NRS/ER	Bifunctional dTDP-4-dehydrorhamnose 3,5-epimerase/dTDP-4-dehydrorhamnose reductase OS=Arabidopsis thaliana GN=NRS/ER PE=1 SV=1	72.83	51.13	1.09	up	0.00	0.00	yes
TRINITY_DN19907_c0_g1	hypothetical protein POPTR_0001s24210g [Populus trichocarpa]	HACD2	Very-long-chain (3R)-3-hydroxyacyl-CoA dehydratase 2 OS=Bos taurus GN=HACD2 PE=2 SV=2	29.87	18.49	1.28	up	0.00	0.00	yes
TRINITY_DN24133_c0_g4	hypothetical protein POPTR_0013s04620g [Populus trichocarpa]	FTSHI5	Probable inactive ATP-dependent zinc metalloprotease FTSHI 5, chloroplastic OS=Arabidopsis thaliana GN=FTSHI5 PE=2 SV=1	28.53	19.92	1.11	up	0.00	0.00	yes
TRINITY_DN22665_c0_g3	hypothetical protein POPTR_0013s03080g [Populus trichocarpa]	EMB3004	Bifunctional 3-dehydroquinate dehydratase/shikimate dehydrogenase, chloroplastic OS=Arabidopsis thaliana GN=EMB3004 PE=1 SV=1	7.83	2.17	2.46	up	0.00	0.00	yes
TRINITY_DN23011_c0_g1	nitrate transporter family protein [Populus trichocarpa]	NPF6.3	Protein NRT1/ PTR FAMILY 6.3 OS=Arabidopsis thaliana GN=NPF6.3 PE=1 SV=1	12.72	7.99	1.29	up	0.00	0.00	yes
TRINITY_DN26359_c0_g1	PREDICTED: DEAD-box ATP-dependent RNA helicase 51-like [Populus euphratica]	Os03g0802700	DEAD-box ATP-dependent RNA helicase 27 OS=Oryza sativa subsp. japonica GN=Os03g0802700 PE=3 SV=1	42.84	25.49	1.32	up	0.00	0.00	yes
TRINITY_DN16981_c0_g1	PREDICTED: quinone oxidoreductase-like protein 2 homolog [Populus euphratica]	v1g238856	Quinone oxidoreductase-like protein 2 homolog OS=Nematostella vectensis GN=v1g238856 PE=3 SV=1	35.67	25.53	1.11	up	0.00	0.00	yes
TRINITY_DN21280_c0_g1	-	-	-	12.89	3.47	2.54	up	0.00	0.00	yes
TRINITY_DN15650_c0_g1	hypothetical protein POPTR_0016s11470g [Populus trichocarpa]	ALY2	THO complex subunit 4B OS=Arabidopsis thaliana GN=ALY2 PE=1 SV=1	42.47	30.58	1.09	up	0.00	0.00	yes

TRINITY_DN25759_c0_g1	hypothetical protein POPTR_0015s00930g [Populus trichocarpa]	PLR1	Pyridoxal reductase, chloroplastic OS=Arabidopsis thaliana GN=PLR1 PE=1 SV=1	25.95	15.09	1.28	up	0.00	0.00	yes
TRINITY_DN19453_c0_g2	PREDICTED: pentatricopeptide repeat-containing protein At2g18940, chloroplastic [Populus euphratica]	At2g18940	Pentatricopeptide repeat-containing protein At2g18940, chloroplastic OS=Arabidopsis thaliana GN=At2g18940 PE=2 SV=1	27.51	17.60	1.27	up	0.00	0.00	yes
TRINITY_DN18268_c0_g1	hypothetical protein POPTR_0006s01850g [Populus trichocarpa]	TDL1	TPD1 protein homolog 1 OS=Arabidopsis thaliana GN=TDL1 PE=2 SV=1	64.39	38.88	1.33	up	0.00	0.00	yes
TRINITY_DN23297_c0_g5	PREDICTED: 60S ribosomal protein L15-like [Populus euphratica]	RPL15	60S ribosomal protein L15 OS=Petunia hybrida GN=RPL15 PE=2 SV=1	300.57	208.45	1.14	up	0.00	0.00	yes
TRINITY_DN14033_c0_g1	hypothetical protein POPTR_0005s03910g [Populus trichocarpa]	-	-	28.00	7.11	2.67	up	0.00	0.00	yes
TRINITY_DN18471_c0_g1	ATP-dependent Clp protease proteolytic subunit family protein [Populus trichocarpa]	CLPP6	ATP-dependent Clp protease proteolytic subunit 6, chloroplastic OS=Arabidopsis thaliana GN=CLPP6 PE=1 SV=1	145.96	97.75	1.21	up	0.00	0.00	yes
TRINITY_DN19266_c0_g2	eukaryotic translation initiation factor 4E isoform family protein [Populus trichocarpa]	-	Eukaryotic translation initiation factor isoform 4E-2 OS=Triticum aestivum PE=1 SV=1	20.47	13.56	1.21	up	0.00	0.00	yes
TRINITY_DN16633_c0_g2	hypothetical protein POPTR_0009s00700g [Populus trichocarpa]	-	-	11.91	6.92	1.40	up	0.00	0.00	yes
TRINITY_DN25348_c1_g4	hypothetical protein POPTR_0025s00770g [Populus trichocarpa]	At5g20260	Probable glycosyltransferase At5g20260 OS=Arabidopsis thaliana GN=At5g20260 PE=3 SV=3	8.27	4.28	1.55	up	0.00	0.00	yes
TRINITY_DN17853_c0_g1	PREDICTED: 1-aminocyclopropane-1-carboxylate oxidase homolog 1-like [Populus euphratica]	At1g06620	1-aminocyclopropane-1-carboxylate oxidase homolog 1 OS=Arabidopsis thaliana GN=At1g06620 PE=2 SV=1	9.07	5.51	1.34	up	0.00	0.00	yes
TRINITY_DN22695_c0_g4	aconitase family protein [Populus trichocarpa]	IIL1	3-isopropylmalate dehydratase large subunit, chloroplastic OS=Arabidopsis thaliana GN=IIL1 PE=1 SV=1	151.04	102.95	1.16	up	0.00	0.00	yes
TRINITY_DN21751_c0_g1	plastid-lipid associated protein PAP [Populus trichocarpa]	PAP10	Probable plastid-lipid-associated protein 10, chloroplastic OS=Arabidopsis thaliana GN=PAP10 PE=2 SV=1	35.99	22.41	1.28	up	0.00	0.00	yes
TRINITY_DN11369_c0_g1	PREDICTED: ethylene-responsive transcription factor ERF034 [Populus euphratica]	ERF034	Ethylene-responsive transcription factor ERF034 OS=Arabidopsis thaliana GN=ERF034 PE=2 SV=2	3.26	1.22	2.01	up	0.00	0.00	yes
TRINITY_DN13075_c0_g1	vacuolar H+-pyrophosphatase [Prunus persica]	-	Pyrophosphate-energized vacuolar membrane proton pump OS=Vigna radiata var. radiata PE=1 SV=4	377.71	245.83	1.23	up	0.00	0.00	yes
TRINITY_DN24590_c0_g3	hypothetical protein POPTR_0005s23230g [Populus trichocarpa]	-	-	59.23	36.42	1.31	up	0.00	0.00	yes
TRINITY_DN23645_c0_g1	hypothetical protein POPTR_0008s17880g [Populus trichocarpa]	CLKR27	3-oxoacyl-[acyl-carrier-protein] reductase, chloroplastic OS=Cuphea lanceolata GN=CLKR27 PE=2 SV=1	115.75	81.25	1.12	up	0.00	0.00	yes
TRINITY_DN24014_c0_g1	SALT OVERLY SENSITIVE 1 family protein [Populus trichocarpa]	-	-	27.49	9.79	2.10	up	0.00	0.00	yes
TRINITY_DN21945_c0_g1	hypothetical protein POPTR_0006s01990g [Populus trichocarpa]	P67	Pentatricopeptide repeat-containing protein At4g16390, chloroplastic OS=Arabidopsis thaliana GN=P67 PE=1 SV=3	16.01	10.84	1.16	up	0.00	0.00	yes
TRINITY_DN26103_c0_g4	hypothetical protein POPTR_0322s00200g [Populus trichocarpa]	UGT74E2	UDP-glycosyltransferase 74E2 OS=Arabidopsis thaliana GN=UGT74E2 PE=1 SV=1	65.51	31.51	1.68	up	0.00	0.00	yes
TRINITY_DN24765_c0_g1	peroxisomal biogenesis factor 11 family protein [Populus trichocarpa]	PEX11D	Peroxisomal membrane protein 11D OS=Arabidopsis thaliana GN=PEX11D PE=1 SV=2	475.01	301.42	1.27	up	0.00	0.00	yes
TRINITY_DN24537_c0_g4	PREDICTED: sucrose-phosphatase 1-like isoform X1 [Populus euphratica]	SPP1	Sucrose-phosphatase 1 OS=Nicotiana tabacum GN=SPP1 PE=2 SV=1	49.51	32.86	1.19	up	0.00	0.00	yes
TRINITY_DN15586_c0_g1	hypothetical protein POPTR_0005s06430g [Populus trichocarpa]	GSVIVT0002692	Probable polygalacturonase OS=Vitis vinifera GN=GSVIVT00026920001 PE=1 SV=1	2.82	1.17	2.45	up	0.00	0.00	yes
TRINITY_DN17168_c0_g3	PREDICTED: ATP-dependent zinc metalloprotease FtsH isoform X1 [Populus euphratica]	FTSHI3	Probable inactive ATP-dependent zinc metalloprotease FTSHI 3, chloroplastic OS=Arabidopsis thaliana GN=FTSHI3 PE=1 SV=1	20.53	14.38	1.11	up	0.00	0.00	yes
TRINITY_DN21390_c0_g1	60S ribosomal protein L19-2 [Populus trichocarpa]	RPL19C	60S ribosomal protein L19-3 OS=Arabidopsis thaliana GN=RPL19C PE=2 SV=3	291.74	205.26	1.18	up	0.00	0.00	yes
TRINITY_DN22535_c1_g1	ribosomal protein L33 [Populus trichocarpa]	RPL35AA	60S ribosomal protein L35a-1 OS=Arabidopsis thaliana GN=RPL35AA PE=3 SV=1	1111.68	712.11	1.17	up	0.00	0.00	yes
TRINITY_DN15812_c0_g1	PREDICTED: mitochondrial outer membrane protein porin 2-like [Populus euphratica]	VDAC2	Mitochondrial outer membrane protein porin 2 OS=Arabidopsis thaliana GN=VDAC2 PE=1 SV=1	13.90	9.05	1.31	up	0.00	0.00	yes
TRINITY_DN24482_c0_g4	seed maturation protein PM37 [Populus trichocarpa]	-	DnaJ protein homolog ANJ1 OS=Atriplex nummularia PE=2 SV=1	28.60	18.11	1.33	up	0.00	0.00	yes

TRINITY_DN17542_c0_g1	hypothetical protein POPTR_0004s07100g [Populus trichocarpa]	lhpI	Delta(1)-pyrroline-2-carboxylate reductase OS=Colwellia psychrerythraea (strain 34H / ATCC BAA-681) GN=lhpI PE=1 SV=1	7.14	3.70	1.56	up	0.00	0.00	yes
TRINITY_DN15725_c0_g2	PREDICTED: uncharacterized protein LOC105111613 [Populus euphratica]	-	-	157.33	109.14	1.14	up	0.00	0.00	yes
TRINITY_DN16992_c0_g1	ribosomal protein L12 [Populus trichocarpa]	rplL	50S ribosomal protein L7/L12 OS=Liberibacter africanus subsp. capensis GN=rplL PE=3 SV=1	19.80	13.97	1.12	up	0.00	0.00	yes
TRINITY_DN26593_c1_g4	2-dehydro-3-deoxyphosphoheptonate aldolase family protein [Populus trichocarpa]	DHAPS-1	Phospho-2-dehydro-3-deoxyheptonate aldolase 1, chloroplastic OS=Nicotiana tabacum GN=DHAPS-1 PE=2 SV=1	647.76	427.17	1.29	up	0.00	0.00	yes
TRINITY_DN26528_c0_g1	PREDICTED: D-3-phosphoglycerate dehydrogenase 3, chloroplastic-like [Populus euphratica]	PGDH1	D-3-phosphoglycerate dehydrogenase 1, chloroplastic OS=Arabidopsis thaliana GN=PGDH1 PE=1 SV=1	74.65	55.30	1.13	up	0.00	0.00	yes
TRINITY_DN22141_c1_g1	PREDICTED: calvin cycle protein CP12-1, chloroplastic [Populus euphratica]	CP12-2	Calvin cycle protein CP12-2, chloroplastic OS=Arabidopsis thaliana GN=CP12-2 PE=1 SV=1	2160.91	1096.61	1.58	up	0.00	0.00	yes
TRINITY_DN15149_c0_g4	hypothetical protein POPTR_0001s10930g [Populus trichocarpa]	RPS6	30S ribosomal protein S6 alpha, chloroplastic (Fragment) OS=Spinacia oleracea GN=RPS6 PE=1 SV=2	610.58	326.04	1.49	up	0.00	0.00	yes
TRINITY_DN21981_c0_g4	PREDICTED: peptide methionine sulfoxide reductase B5 [Populus euphratica]	MSRB2	Peptide methionine sulfoxide reductase B2, chloroplastic OS=Arabidopsis thaliana GN=MSRB2 PE=1 SV=1	146.64	95.32	1.25	up	0.00	0.00	yes
TRINITY_DN20450_c0_g2	PREDICTED: methionyl-tRNA formyltransferase, mitochondrial isoform X2 [Populus euphratica]	fmt	Methionyl-tRNA formyltransferase OS=Kosmotoga olearia (strain TBF 19.5.1) GN=fmt PE=3 SV=1	53.40	39.72	1.18	up	0.00	0.00	yes
TRINITY_DN19346_c0_g1	hypothetical protein POPTR_0001s10850g [Populus trichocarpa]	BXL6	Probable beta-D-xylosidase 6 OS=Arabidopsis thaliana GN=BXL6 PE=2 SV=1	31.58	21.33	1.13	up	0.00	0.00	yes
TRINITY_DN22067_c0_g2	PREDICTED: uncharacterized protein LOC100263114 [Vitis vinifera]	-	-	436.53	221.02	1.57	up	0.00	0.00	yes
TRINITY_DN14787_c0_g1	hypothetical protein POPTR_0012s13810g [Populus trichocarpa]	ycf20	Uncharacterized protein ycf20 OS=Pyropia yezoensis GN=ycf20 PE=3 SV=1	29.01	15.27	1.52	up	0.00	0.00	yes
TRINITY_DN16057_c0_g1	hypothetical protein POPTR_0001s31680g [Populus trichocarpa]	PAP7	Probable plastid-lipid-associated protein 7, chloroplastic OS=Arabidopsis thaliana GN=PAP7 PE=2 SV=1	45.05	24.98	1.39	up	0.00	0.00	yes
TRINITY_DN25703_c0_g1	PREDICTED: cytochrome P450 71A1-like [Populus euphratica]	CYP98A2	Cytochrome P450 98A2 OS=Glycine max GN=CYP98A2 PE=2 SV=1	3184.19	1450.94	1.74	up	0.00	0.00	yes
TRINITY_DN18209_c1_g1	PREDICTED: pyruvate kinase, cytosolic isozyme-like [Populus euphratica]	OsI_37456	Pyruvate kinase 2, cytosolic OS=Oryza sativa subsp. indica GN=OsI_37456 PE=3 SV=1	19.18	14.26	1.05	up	0.00	0.00	yes
TRINITY_DN23514_c0_g1	PREDICTED: ferredoxin-A-like [Populus euphratica]	AP1	Ferredoxin, chloroplastic OS=Capsicum annuum GN=AP1 PE=1 SV=1	903.88	450.99	1.59	up	0.00	0.00	yes
TRINITY_DN25763_c0_g1	Lysyl-tRNA synthetase family protein [Populus trichocarpa]	At3g11710	Lysine--tRNA ligase, cytoplasmic OS=Arabidopsis thaliana GN=At3g11710 PE=1 SV=1	76.40	64.24	1.13	up	0.00	0.00	yes
TRINITY_DN25941_c0_g1	PREDICTED: glutamine synthetase leaf isozyme, chloroplastic [Populus euphratica]	GS2	Glutamine synthetase leaf isozyme, chloroplastic OS=Pisum sativum GN=GS2 PE=2 SV=2	986.31	589.65	1.37	up	0.00	0.00	yes
TRINITY_DN22492_c1_g1	PREDICTED: uncharacterized protein LOC105131096 [Populus euphratica]	-	-	40.31	28.55	1.18	up	0.00	0.00	yes
TRINITY_DN18224_c0_g1	iron-sulfur cluster assembly complex family protein [Populus trichocarpa]	ISU1	Iron-sulfur cluster assembly protein 1 OS=Arabidopsis thaliana GN=ISU1 PE=2 SV=1	301.08	200.01	1.20	up	0.00	0.00	yes
TRINITY_DN19521_c0_g3	-	-	-	149.08	108.48	1.13	up	0.00	0.00	yes
TRINITY_DN24687_c0_g2	PREDICTED: uncharacterized protein ycf39-like [Populus euphratica]	ycf39	Uncharacterized protein ycf39 OS=Cyanophora paradoxa GN=ycf39 PE=3 SV=1	127.95	76.05	1.37	up	0.00	0.00	yes
TRINITY_DN18298_c0_g2	Mitochondrial import receptor subunit TOM6 family protein [Populus trichocarpa]	TOM6	Mitochondrial import receptor subunit TOM6 homolog OS=Arabidopsis thaliana GN=TOM6 PE=1 SV=1	32.88	19.44	1.38	up	0.00	0.00	yes
TRINITY_DN26029_c0_g1	MGDG synthase type A family protein [Populus trichocarpa]	MGD	Probable monogalactosyldiacylglycerol synthase, chloroplastic OS=Glycine max GN=MGD A PE=2 SV=1	31.79	20.27	1.24	up	0.00	0.00	yes
TRINITY_DN23754_c0_g1	hypothetical protein POPTR_0002s04790g [Populus trichocarpa]	-	2-methyl-6-phytyl-1,4-hydroquinone methyltransferase, chloroplastic OS=Spinacia oleracea PE=1 SV=1	281.29	177.06	1.22	up	0.00	0.00	yes
TRINITY_DN18071_c0_g1	PREDICTED: monothiol glutaredoxin-S7, chloroplastic [Populus euphratica]	GRXS14	Monothiol glutaredoxin-S14, chloroplastic OS=Arabidopsis thaliana GN=GRXS14 PE=1 SV=2	78.22	48.42	1.30	up	0.00	0.00	yes
TRINITY_DN18636_c0_g2	unknown [Populus trichocarpa]	FAX4	Protein FATTY ACID EXPORT 4, chloroplastic OS=Arabidopsis thaliana GN=FAX4 PE=2 SV=1	57.58	35.23	1.32	up	0.00	0.00	yes
TRINITY_DN23462_c0_g5	hypothetical protein POPTR_0012s10520g [Populus trichocarpa]	TIM8	Mitochondrial import inner membrane translocase subunit TIM8 OS=Arabidopsis thaliana GN=TIM8 PE=1 SV=1	36.28	18.68	1.51	up	0.00	0.00	yes

TRINITY_DN27009_c0_g1	hypothetical protein POPTR_0002s22720g [Populus trichocarpa]	-	Phosphoenolpyruvate carboxylase 2 OS=Sorghum bicolor PE=3 SV=1	10.34	5.23	1.60	up	0.00	0.00	yes
TRINITY_DN24627_c0_g1	PREDICTED: peroxisomal fatty acid beta-oxidation multifunctional protein AIM1 isoform X1 [Populus euphratica]	AIM1	Peroxisomal fatty acid beta-oxidation multifunctional protein AIM1 OS=Arabidopsis thaliana GN=AIM1 PE=1 SV=1	227.07	150.71	1.20	up	0.00	0.00	yes
TRINITY_DN16032_c0_g1	PREDICTED: uncharacterized protein LOC105126496 [Populus euphratica]	-	-	274.99	134.30	1.63	up	0.00	0.00	yes
TRINITY_DN23043_c0_g1	PREDICTED: lysosomal beta glucosidase-like [Populus euphratica]	BACOVA_02659	Beta-glucosidase BoGH3B OS=Bacteroides ovatus (strain ATCC 8483 / DSM 1896 / JCM 5824 / NCTC 11153) GN=BACOVA_02659 PE=1 SV=1	13.89	10.05	1.04	up	0.00	0.00	yes
TRINITY_DN25044_c0_g1	hypothetical protein POPTR_0015s00850g [Populus trichocarpa]	CYP89A9	Cytochrome P450 89A9 OS=Arabidopsis thaliana GN=CYP89A9 PE=2 SV=1	9.30	3.87	1.87	up	0.00	0.00	yes
TRINITY_DN27297_c0_g4	PREDICTED: protein Mpv17-like [Populus euphratica]	sym-1	Protein sym-1 OS=Neurospora crassa (strain ATCC 24698 / 74-OR23-1A / CBS 708.71 / DSM 1257 / FGSC 987) GN=sym-1 PE=3 SV=2	6.75	3.71	1.47	up	0.00	0.00	yes
TRINITY_DN21091_c0_g1	PREDICTED: ER membrane protein complex subunit 8/9 homolog [Populus euphratica]	EMB2731	ER membrane protein complex subunit 8/9 homolog OS=Arabidopsis thaliana GN=EMB2731 PE=2 SV=1	45.25	32.70	1.05	up	0.00	0.00	yes
TRINITY_DN24723_c0_g4	hypothetical protein POPTR_0015s04810g [Populus trichocarpa]	-	-	79.63	43.44	1.47	up	0.00	0.00	yes
TRINITY_DN22158_c0_g2	photosystem 2 reaction center PsbP family protein [Populus trichocarpa]	PNSL1	Photosynthetic NDH subunit of lumenal location 1, chloroplastic OS=Arabidopsis thaliana GN=PNSL1 PE=1 SV=2	238.39	102.06	1.75	up	0.00	0.00	yes
TRINITY_DN22125_c0_g2	PREDICTED: cytochrome P450 71A1-like [Populus euphratica]	CYP71A1	Cytochrome P450 71A1 OS=Persea americana GN=CYP71A1 PE=1 SV=2	59.53	32.43	1.35	up	0.00	0.00	yes
TRINITY_DN22891_c0_g1	PREDICTED: LOW QUALITY PROTEIN: phosphoglucan, water dikinase, chloroplastic-like [Populus euphratica]	GWD3	Phosphoglucan, water dikinase, chloroplastic OS=Arabidopsis thaliana GN=GWD3 PE=1 SV=1	8.15	3.01	2.02	up	0.00	0.00	yes
TRINITY_DN16520_c0_g1	hypothetical protein POPTR_0018s02310g [Populus trichocarpa]	-	-	13.09	7.13	1.52	up	0.00	0.00	yes
TRINITY_DN22105_c0_g1	PREDICTED: uncharacterized protein LOC105132640 [Populus euphratica]	PNSB1	Photosynthetic NDH subunit of subcomplex B 1, chloroplastic OS=Arabidopsis thaliana GN=PNSB1 PE=2 SV=1	158.99	73.19	1.70	up	0.00	0.00	yes
TRINITY_DN20755_c0_g1	ARGOS-like protein, partial [Populus nigra]	-	-	31.40	14.75	1.71	up	0.00	0.00	yes
TRINITY_DN24759_c0_g1	biotin/lipoyl attachment domain-containing family protein [Populus trichocarpa]	-	-	140.38	101.66	1.11	up	0.00	0.00	yes
TRINITY_DN20688_c0_g3	PREDICTED: alpha-glucan phosphorylase, H isozyme [Populus euphratica]	-	Alpha-glucan phosphorylase, H isozyme OS=Solanum tuberosum PE=1 SV=1	31.16	22.07	1.12	up	0.00	0.00	yes
TRINITY_DN26313_c1_g1	unknown [Populus trichocarpa]	At2g29340	Tropinone reductase homolog At2g29340 OS=Arabidopsis thaliana GN=At2g29340 PE=2 SV=1	76.73	48.46	1.31	up	0.00	0.00	yes
TRINITY_DN19877_c1_g2	putative CuZn-superoxide dismutase [Populus tremula x Populus tremuloides]	CSD3	Superoxide dismutase [Cu-Zn] 3 OS=Arabidopsis thaliana GN=CSD3 PE=1 SV=1	23.67	12.08	1.59	up	0.00	0.00	yes
TRINITY_DN19089_c0_g1	60S ribosomal protein L26B [Populus trichocarpa]	RPL26A	60S ribosomal protein L26-1 OS=Arabidopsis thaliana GN=RPL26A PE=2 SV=2	727.41	501.12	1.17	up	0.00	0.00	yes
TRINITY_DN23716_c0_g4	ubiquinol-cytochrome C reductase iron-sulfur subunit family protein [Populus trichocarpa]	-	Cytochrome b-c1 complex subunit Rieske-1, mitochondrial (Fragment) OS=Nicotiana tabacum PE=2 SV=1	88.26	58.55	1.21	up	0.00	0.00	yes
TRINITY_DN25773_c3_g2	PREDICTED: uncharacterized protein LOC105133193 [Populus euphratica]	-	-	41.87	29.40	1.12	up	0.00	0.00	yes
TRINITY_DN16308_c0_g2	universal stress family protein [Populus trichocarpa]	-	-	41.45	28.10	1.19	up	0.00	0.00	yes
TRINITY_DN22010_c0_g3	hypothetical protein POPTR_0015s07840g [Populus trichocarpa]	HIPL2	HIPL2 protein OS=Arabidopsis thaliana GN=HIPL2 PE=1 SV=2	6.99	2.77	1.93	up	0.00	0.00	yes
TRINITY_DN19688_c0_g1	hypothetical protein POPTR_0001s22620g [Populus trichocarpa]	-	40S ribosomal protein S14 OS=Zea mays PE=3 SV=1	941.89	640.14	1.19	up	0.00	0.00	yes
TRINITY_DN24178_c0_g3	lipid phosphate phosphatase 2 [Populus tomentosa]	LPP2	Lipid phosphate phosphatase 2 OS=Arabidopsis thaliana GN=LPP2 PE=2 SV=1	19.00	13.25	1.11	up	0.00	0.00	yes
TRINITY_DN22283_c0_g1	hypothetical protein POPTR_0002s09390g [Populus trichocarpa]	gpmA	2,3-bisphosphoglycerate-dependent phosphoglycerate mutase OS=Protochlamydia amoebophila (strain UWE25) GN=gpmA PE=3 SV=1	139.18	61.68	1.79	up	0.00	0.00	yes

TRINITY_DN13355_c0_g1	hypothetical protein POPTR_0018s11150g [Populus trichocarpa]	TSS	Protein TSS OS=Arabidopsis thaliana GN=TSS PE=1 SV=1	10.25	2.85	2.44	up	0.00	0.00	yes
TRINITY_DN25521_c1_g2	PREDICTED: APO protein 1, chloroplastic isoform X1 [Populus euphratica]	APO1	APO protein 1, chloroplastic OS=Arabidopsis thaliana GN=APO1 PE=2 SV=1	21.99	15.44	1.13	up	0.00	0.00	yes
TRINITY_DN25402_c0_g1	hypothetical protein POPTR_0016s12620g [Populus trichocarpa]	HCC1	Protein SCO1 homolog 1, mitochondrial OS=Arabidopsis thaliana GN=HCC1 PE=2 SV=1	14.02	9.55	1.25	up	0.00	0.00	yes
TRINITY_DN20925_c0_g1	PREDICTED: eukaryotic translation initiation factor 3 subunit H [Populus euphratica]	TIF3H1	Eukaryotic translation initiation factor 3 subunit H OS=Arabidopsis thaliana GN=TIF3H1 PE=1 SV=2	88.80	63.07	1.08	up	0.00	0.00	yes
TRINITY_DN23265_c0_g1	hypothetical protein POPTR_0014s18550g [Populus trichocarpa]	-	-	31.43	19.38	1.32	up	0.00	0.00	yes
TRINITY_DN17571_c0_g4	fasciclin-like AGP 12 [Populus tremula x Populus alba]	FLA7	Fasciclin-like arabinogalactan protein 7 OS=Arabidopsis thaliana GN=FLA7 PE=1 SV=1	25.74	17.27	1.19	up	0.00	0.00	yes
TRINITY_DN24395_c0_g2	hypothetical protein POPTR_0005s26230g [Populus trichocarpa]	-	-	66.57	46.27	1.21	up	0.00	0.00	yes
TRINITY_DN22427_c1_g2	hypothetical protein POPTR_0011s09130g [Populus trichocarpa]	grpE	Protein GrpE OS=Nitrobacter winogradskyi (strain ATCC 25391 / DSM 10237 / CIP 104748 / NCIMB 11846 / Nb-255) GN=grpE PE=3 SV=1	8.04	4.67	1.40	up	0.00	0.00	yes
TRINITY_DN23677_c0_g1	PREDICTED: probable RNA 3'-terminal phosphate cyclase-like protein isoform X1 [Populus euphratica]	At5g22100	Probable RNA 3'-terminal phosphate cyclase-like protein OS=Arabidopsis thaliana GN=At5g22100 PE=2 SV=1	15.94	10.08	1.30	up	0.00	0.00	yes
TRINITY_DN17675_c0_g2	tetratricopeptide repeat-like superfamily protein [Populus tomentosa]	-	-	23.63	16.47	1.11	up	0.00	0.00	yes
TRINITY_DN18147_c0_g2	hypothetical protein POPTR_0005s11300g [Populus trichocarpa]	DHQS	3-dehydroquinate synthase, chloroplastic OS=Actinidia chinensis GN=DHQS PE=1 SV=2	170.60	105.98	1.29	up	0.00	0.00	yes
TRINITY_DN18666_c0_g3	PREDICTED: calcium-transporting ATPase 12, plasma membrane-type-like [Populus euphratica]	ACA13	Putative calcium-transporting ATPase 13, plasma membrane-type OS=Arabidopsis thaliana GN=ACA13 PE=3 SV=1	2.45	1.22	1.61	up	0.00	0.00	yes
TRINITY_DN19547_c0_g4	hypothetical protein POPTR_0005s22090g [Populus trichocarpa]	CCB3	Protein COFACTOR ASSEMBLY OF COMPLEX C SUBUNIT B CCB3, chloroplastic OS=Arabidopsis thaliana GN=CCB3 PE=1 SV=1	41.28	19.95	1.66	up	0.00	0.00	yes
TRINITY_DN20175_c0_g1	peroxiredoxin Q family protein [Populus trichocarpa]	PRXQ	Peroxiredoxin Q, chloroplastic OS=Populus jackii GN=PRXQ PE=1 SV=1	1020.77	504.37	1.59	up	0.00	0.00	yes
TRINITY_DN22841_c0_g1	PREDICTED: LOW QUALITY PROTEIN: aminoacylase-1 [Populus euphratica]	Acy1	Aminoacylase-1 OS=Mus musculus GN=Acy1 PE=1 SV=1	87.12	56.00	1.22	up	0.00	0.00	yes
TRINITY_DN19372_c0_g1	PREDICTED: pentatricopeptide repeat-containing protein PNM1, mitochondrial [Populus euphratica]	PNM1	Pentatricopeptide repeat-containing protein PNM1, mitochondrial OS=Arabidopsis thaliana GN=PNM1 PE=1 SV=1	31.55	21.89	1.12	up	0.00	0.00	yes
TRINITY_DN22282_c0_g1	hypothetical protein POPTR_0010s18960g [Populus trichocarpa]	-	-	18.50	8.98	1.65	up	0.00	0.00	yes
TRINITY_DN18073_c1_g1	PREDICTED: reticulon-like protein B1 [Populus euphratica]	RTNLB1	Reticulon-like protein B1 OS=Arabidopsis thaliana GN=RTNLB1 PE=1 SV=1	90.00	61.98	1.18	up	0.00	0.00	yes
TRINITY_DN25439_c0_g1	biotin carboxylase precursor family protein [Populus trichocarpa]	POPTR_0018s14	Biotin carboxylase 2, chloroplastic OS=Populus trichocarpa GN=POPTR_0018s14250g PE=2 SV=2	85.63	60.20	1.11	up	0.00	0.00	yes
TRINITY_DN12446_c0_g1	hypothetical protein POPTR_0004s17270g [Populus trichocarpa]	-	-	6.44	1.45	2.77	up	0.00	0.00	yes
TRINITY_DN20517_c0_g1	chorismate synthase family protein [Populus trichocarpa]	EMB1144	Chorismate synthase, chloroplastic OS=Arabidopsis thaliana GN=EMB1144 PE=2 SV=2	289.88	166.51	1.45	up	0.00	0.00	yes
TRINITY_DN15731_c0_g1	-	-	-	7.00	2.12	2.31	up	0.00	0.00	yes
TRINITY_DN17329_c0_g1	hypothetical protein POPTR_0002s25400g [Populus trichocarpa]	ZNHIT3	Zinc finger HIT domain-containing protein 3 OS=Homo sapiens GN=ZNHIT3 PE=1 SV=2	16.84	10.08	1.35	up	0.00	0.00	yes
TRINITY_DN24539_c0_g2	hypothetical protein POPTR_0010s18100g [Populus trichocarpa]	rlmI	Ribosomal RNA large subunit methyltransferase I OS=Salmonella arizonae (strain ATCC BAA-731 / CDC346-86 / RSK2980) GN=rlmI PE=3 SV=1	24.88	15.40	1.25	up	0.00	0.00	yes
TRINITY_DN20246_c2_g3	hypothetical protein POPTR_0013s08200g [Populus trichocarpa]	-	-	69.63	44.62	1.21	up	0.00	0.00	yes
TRINITY_DN23070_c0_g1	monodehydroascorbate reductase [Populus alba x Populus glandulosa]	AFRR	Monodehydroascorbate reductase OS=Solanum lycopersicum GN=AFRR PE=1 SV=1	169.27	118.14	1.14	up	0.00	0.00	yes
TRINITY_DN18146_c0_g1	PREDICTED: magnesium transporter MRS2-11, chloroplastic isoform X2 [Populus euphratica]	MRS2-11	Magnesium transporter MRS2-11, chloroplastic OS=Arabidopsis thaliana GN=MRS2-11 PE=1 SV=1	13.82	10.33	1.11	up	0.00	0.00	yes

TRINITY_DN22629_c0_g1	hypothetical protein POPTR_0010s16360g [Populus trichocarpa]	EMB2750	Pentatricopeptide repeat-containing protein At3g06430, chloroplastic OS=Arabidopsis thaliana GN=EMB2750 PE=2 SV=1	80.13	56.88	1.13	up	0.00	0.00	yes
TRINITY_DN23403_c0_g1	hypothetical protein POPTR_0005s23960g [Populus trichocarpa]	OSB1	Protein OSB1, mitochondrial OS=Arabidopsis thaliana GN=OSB1 PE=1 SV=1	80.55	57.95	1.10	up	0.00	0.00	yes
TRINITY_DN20742_c0_g1	FKBP-type peptidyl-prolyl cis-trans isomerase 3 family protein [Populus trichocarpa]	FKBP13	Peptidyl-prolyl cis-trans isomerase FKBP13, chloroplastic OS=Arabidopsis thaliana GN=FKBP13 PE=1 SV=2	166.22	81.22	1.62	up	0.00	0.00	yes
TRINITY_DN23096_c0_g1	unknown [Populus trichocarpa x Populus deltoides]	PNSL4	Photosynthetic NDH subunit of lumenal location 4, chloroplastic OS=Arabidopsis thaliana GN=PNSL4 PE=1 SV=1	91.97	35.51	1.75	up	0.00	0.00	yes
TRINITY_DN23333_c0_g2	PREDICTED: uncharacterized protein LOC105131439 isoform X1 [Populus euphratica]	-	-	4.34	1.91	1.81	up	0.00	0.00	yes
TRINITY_DN25650_c0_g4	PREDICTED: uncharacterized protein At3g17611 [Populus euphratica]	RBL14	Rhomboid-like protein 14, mitochondrial OS=Arabidopsis thaliana GN=RBL14 PE=2 SV=1	7.50	4.40	1.39	up	0.00	0.00	yes
TRINITY_DN20401_c1_g2	cytochrome P450 family protein [Populus trichocarpa]	CYP90D1	3-epi-6-deoxocathasterone 23-monooxygenase OS=Arabidopsis thaliana GN=CYP90D1 PE=2 SV=1	7.41	2.50	1.59	up	0.00	0.00	yes
TRINITY_DN23000_c0_g1	hypothetical protein POPTR_0001s37830g [Populus trichocarpa]	NOP2	Probable 28S rRNA (cytosine(4447)-C(5))-methyltransferase OS=Homo sapiens GN=NOP2 PE=1 SV=2	26.94	15.37	1.45	up	0.00	0.00	yes
TRINITY_DN20407_c0_g2	hypothetical protein POPTR_0010s07890g [Populus trichocarpa]	MORF5	Multiple organellar RNA editing factor 5, mitochondrial OS=Arabidopsis thaliana GN=MORF5 PE=2 SV=1	232.02	140.49	1.21	up	0.00	0.00	yes
TRINITY_DN16280_c0_g1	nodulin 35 family protein [Populus trichocarpa]	-	Uricase-2 isozyme 1 OS=Canavalia lineata PE=2 SV=1	36.34	29.20	1.20	up	0.00	0.00	yes
TRINITY_DN12746_c0_g1	PREDICTED: protein translation factor SUI1 homolog 2 [Populus euphratica]	At1g54290	Protein translation factor SUI1 homolog 2 OS=Arabidopsis thaliana GN=At1g54290 PE=3 SV=1	156.11	85.71	1.50	up	0.00	0.00	yes
TRINITY_DN22658_c0_g1	PREDICTED: probable anion transporter 2, chloroplastic [Populus euphratica]	PHT4;4	Ascorbate transporter, chloroplastic OS=Arabidopsis thaliana GN=PHT4;4 PE=1 SV=1	57.16	41.87	1.20	up	0.00	0.00	yes
TRINITY_DN22701_c0_g1	shikimate kinase family protein [Populus trichocarpa]	SKL1	Probable inactive shikimate kinase like 1, chloroplastic OS=Arabidopsis thaliana GN=SKL1 PE=2 SV=1	108.38	67.24	1.35	up	0.00	0.00	yes
TRINITY_DN23812_c0_g1	hypothetical protein POPTR_0019s05800g [Populus trichocarpa]	-	-	33.77	20.60	1.40	up	0.00	0.00	yes
TRINITY_DN26840_c0_g1	hypothetical protein POPTR_0019s06020g [Populus trichocarpa]	SIGA	RNA polymerase sigma factor sigA OS=Arabidopsis thaliana GN=SIGA PE=1 SV=1	87.19	56.38	1.22	up	0.00	0.00	yes
TRINITY_DN13893_c0_g1	SPFH/Band 7/PHB domain-containing membrane-associated protein [Populus tomentosa]	HIR1	Hypersensitive-induced response protein 1 OS=Arabidopsis thaliana GN=HIR1 PE=1 SV=1	11.96	4.97	1.87	up	0.00	0.00	yes
TRINITY_DN23136_c0_g1	PREDICTED: haloacid dehalogenase-like hydrolase domain-containing protein At3g48420 isoform X1 [Populus euphratica]	At3g48420	Haloacid dehalogenase-like hydrolase domain-containing protein At3g48420 OS=Arabidopsis thaliana GN=At3g48420 PE=1 SV=1	493.00	231.60	1.64	up	0.00	0.00	yes
TRINITY_DN20638_c0_g1	PREDICTED: FGGY carbohydrate kinase domain-containing protein isoform X1 [Populus euphratica]	Fggy	FGGY carbohydrate kinase domain-containing protein OS=Mus musculus GN=Fggy PE=1 SV=1	29.59	21.24	1.13	up	0.00	0.00	yes
TRINITY_DN23901_c1_g3	hypothetical protein POPTR_0004s16450g [Populus trichocarpa]	RPS25	40S ribosomal protein S25 OS=Solanum lycopersicum GN=RPS25 PE=3 SV=1	472.63	332.91	1.13	up	0.00	0.00	yes
TRINITY_DN22388_c1_g1	PREDICTED: ferredoxin isoform X1 [Populus euphratica]	petF2	Ferredoxin-2 OS=Synechococcus sp. (strain ATCC 27144 / PCC 6301 / SAUG 1402/1) GN=petF2 PE=3 SV=2	256.46	159.05	1.28	up	0.00	0.00	yes
TRINITY_DN20694_c0_g1	hypothetical protein POPTR_0002s16440g [Populus trichocarpa]	LCKB2	Sphingoid long-chain bases kinase 2, mitochondrial OS=Arabidopsis thaliana GN=LCKB2 PE=2 SV=1	19.99	18.02	1.09	up	0.00	0.00	yes
TRINITY_DN16685_c0_g1	PREDICTED: uncharacterized protein LOC105130744 [Populus euphratica]	dnlz	DNL-type zinc finger protein OS=Xenopus laevis GN=dnlz PE=2 SV=1	20.69	12.58	1.34	up	0.00	0.00	yes
TRINITY_DN23614_c0_g1	PREDICTED: transcription factor ILR3-like [Populus euphratica]	ILR3	Transcription factor ILR3 OS=Arabidopsis thaliana GN=ILR3 PE=1 SV=1	80.20	56.38	1.16	up	0.00	0.00	yes
TRINITY_DN24926_c0_g1	Elongation factor Tu family protein [Populus trichocarpa]	TUFA	Elongation factor Tu, mitochondrial OS=Arabidopsis thaliana GN=TUFA PE=1 SV=1	70.61	52.16	1.11	up	0.00	0.00	yes
TRINITY_DN15968_c0_g2	hypothetical protein POPTR_0009s10520g [Populus trichocarpa]	SCO2	Protein disulfide-isomerase SCO2 OS=Arabidopsis thaliana GN=SCO2 PE=1 SV=1	17.06	12.70	1.21	up	0.00	0.00	yes
TRINITY_DN12934_c0_g1	-	-	-	4.86	1.30	2.51	up	0.00	0.00	yes
TRINITY_DN20092_c0_g1	PREDICTED: uncharacterized protein LOC105134626 [Populus euphratica]	-	-	88.98	57.17	1.35	up	0.00	0.00	yes
TRINITY_DN17540_c0_g1	hypothetical protein POPTR_0003s15130g [Populus trichocarpa]	uqcc1	Ubiquinol-cytochrome-c reductase complex assembly factor 1 OS=Xenopus laevis GN=uqcc1 PE=2 SV=1	15.11	10.27	1.17	up	0.00	0.00	yes

TRINITY_DN21025_c0_g1	cytochrome b5 domain-containing family protein [Populus trichocarpa]	MSBP1	Membrane steroid-binding protein 1 OS=Arabidopsis thaliana GN=MSBP1 PE=1 SV=2	328.18	207.36	1.31	up	0.00	0.00	yes
TRINITY_DN22865_c0_g2	PREDICTED: APO protein 2, chloroplastic-like [Populus euphratica]	APO2	APO protein 2, chloroplastic OS=Arabidopsis thaliana GN=APO2 PE=2 SV=1	86.24	50.32	1.37	up	0.00	0.00	yes
TRINITY_DN16480_c0_g1	hypothetical protein POPTR_0004s07130g [Populus trichocarpa]	At5g52780	Uncharacterized protein PAM68-like OS=Arabidopsis thaliana GN=At5g52780 PE=2 SV=1	361.22	147.15	1.89	up	0.00	0.00	yes
TRINITY_DN17969_c0_g2	ribosomal protein L37 [Populus trichocarpa]	RPL37C	60S ribosomal protein L37-3 OS=Arabidopsis thaliana GN=RPL37C PE=3 SV=1	113.41	74.68	1.24	up	0.00	0.00	yes
TRINITY_DN21552_c0_g1	PREDICTED: 60S ribosomal protein L14-1-like [Populus euphratica]	RPL14A	60S ribosomal protein L14-1 OS=Arabidopsis thaliana GN=RPL14A PE=2 SV=1	180.10	121.87	1.19	up	0.00	0.00	yes
TRINITY_DN27563_c0_g1	PREDICTED: uncharacterized protein LOC105142581 isoform X1 [Populus euphratica]	-	-	37.71	13.84	2.04	up	0.00	0.00	yes
TRINITY_DN23082_c0_g2	hypothetical protein POPTR_0003s15390g [Populus trichocarpa]	PTAC14	Protein PLASTID TRANSCRIPTIONALLY ACTIVE 14 OS=Arabidopsis thaliana GN=PTAC14 PE=1 SV=1	61.38	40.90	1.22	up	0.00	0.00	yes
TRINITY_DN19085_c0_g2	PREDICTED: protein CURVATURE THYLAKOID 1A, chloroplastic-like [Populus euphratica]	CURT1A	Protein CURVATURE THYLAKOID 1A, chloroplastic OS=Arabidopsis thaliana GN=CURT1A PE=1 SV=1	1357.21	729.16	1.43	up	0.00	0.00	yes
TRINITY_DN26214_c0_g3	-	-	-	7.22	1.61	2.48	up	0.00	0.00	yes
TRINITY_DN22183_c0_g1	hypothetical protein POPTR_0008s02290g [Populus trichocarpa]	MORF5	Multiple organellar RNA editing factor 5, mitochondrial OS=Arabidopsis thaliana GN=MORF5 PE=2 SV=1	46.84	32.01	1.17	up	0.00	0.00	yes
TRINITY_DN15688_c0_g3	PREDICTED: organic cation/carnitine transporter 4 [Populus euphratica]	OCT4	Organic cation/carnitine transporter 4 OS=Arabidopsis thaliana GN=OCT4 PE=2 SV=1	3.42	1.55	1.76	up	0.00	0.00	yes
TRINITY_DN27842_c2_g4	ADR12-2 family protein [Populus trichocarpa]	-	Elongation factor 1-alpha OS=Daucus carota PE=2 SV=1	203.14	117.25	1.41	up	0.00	0.00	yes
TRINITY_DN23394_c0_g6	PREDICTED: uncharacterized protein LOC105139468 [Populus euphratica]	-	-	24.79	14.42	1.39	up	0.00	0.00	yes
TRINITY_DN17559_c0_g1	NADPH:QUINONE OXIDOREDUCTASE family protein [Populus trichocarpa]	NQR	NADPH:quinone oxidoreductase OS=Arabidopsis thaliana GN=NQR PE=1 SV=1	40.63	29.82	1.04	up	0.00	0.00	yes
TRINITY_DN16797_c0_g1	hypothetical protein POPTR_0017s12880g [Populus trichocarpa]	WDR12	Ribosome biogenesis protein WDR12 homolog OS=Arabidopsis thaliana GN=WDR12 PE=1 SV=1	17.47	11.81	1.20	up	0.00	0.00	yes
TRINITY_DN15297_c0_g1	hypothetical protein POPTR_0012s08820g [Populus trichocarpa]	-	-	4.21	0.80	3.02	up	0.00	0.00	yes
TRINITY_DN21361_c0_g3	hypothetical protein POPTR_0014s16720g [Populus trichocarpa]	-	-	111.11	72.58	1.25	up	0.00	0.00	yes
TRINITY_DN22370_c0_g1	hypothetical protein POPTR_0006s28060g [Populus trichocarpa]	PGR3	Pentatricopeptide repeat-containing protein At4g31850, chloroplastic OS=Arabidopsis thaliana GN=PGR3 PE=1 SV=1	28.82	19.95	1.17	up	0.00	0.00	yes
TRINITY_DN27150_c0_g1	ABC1 family protein [Populus trichocarpa]	At1g71810	Uncharacterized aarF domain-containing protein kinase At1g71810, chloroplastic OS=Arabidopsis thaliana GN=At1g71810 PE=2 SV=1	13.39	8.40	1.23	up	0.00	0.00	yes
TRINITY_DN2409_c0_g1	-	-	-	3.70	0.18	4.70	up	0.00	0.00	yes
TRINITY_DN25318_c0_g1	hypothetical protein POPTR_0003s17710g [Populus trichocarpa]	PRT1	E3 ubiquitin-protein ligase PRT1 OS=Arabidopsis thaliana GN=PRT1 PE=2 SV=2	358.91	170.10	1.30	up	0.00	0.00	yes
TRINITY_DN17618_c0_g1	PREDICTED: mediator-associated protein 2 [Populus euphratica]	At5g64680	Mediator-associated protein 2 OS=Arabidopsis thaliana GN=At5g64680 PE=1 SV=1	21.90	14.38	1.25	up	0.00	0.00	yes
TRINITY_DN16445_c0_g1	dehydroascorbate reductase [Populus tomentosa]	DHAR3	Glutathione S-transferase DHAR3, chloroplastic OS=Arabidopsis thaliana GN=DHAR3 PE=1 SV=1	179.37	113.67	1.25	up	0.00	0.00	yes
TRINITY_DN17746_c0_g2	zinc-binding family protein [Populus trichocarpa]	ZBP14	14 kDa zinc-binding protein OS=Zea mays GN=ZBP14 PE=1 SV=1	88.29	59.40	1.18	up	0.00	0.00	yes
TRINITY_DN18714_c0_g2	PREDICTED: uncharacterized protein LOC104611813 [Nelumbo nucifera]	-	-	3.96	1.39	2.22	up	0.00	0.00	yes
TRINITY_DN7728_c0_g2	hypothetical protein POPTR_0006s14130g [Populus trichocarpa]	BGAL7	Beta-galactosidase 7 OS=Arabidopsis thaliana GN=BGAL7 PE=2 SV=2	1.77	0.56	2.29	up	0.00	0.00	yes
TRINITY_DN21407_c0_g1	PREDICTED: NO-associated protein 1, chloroplastic/mitochondrial [Populus euphratica]	NOA1	NO-associated protein 1, chloroplastic/mitochondrial OS=Arabidopsis thaliana GN=NOA1 PE=1 SV=1	55.57	40.90	1.12	up	0.00	0.00	yes
TRINITY_DN13012_c0_g2	hypothetical protein POPTR_0012s09880g [Populus trichocarpa]	CYP94B1	Cytochrome P450 94B1 OS=Arabidopsis thaliana GN=CYP94B1 PE=2 SV=1	2.12	0.24	3.69	up	0.00	0.00	yes
TRINITY_DN16423_c1_g3	hypothetical protein POPTR_0004s16220g [Populus trichocarpa]	-	-	19.69	10.53	1.54	up	0.00	0.00	yes

TRINITY_DN19164_c0_g1	unknown [Populus trichocarpa]	TIM10	Mitochondrial import inner membrane translocase subunit TIM10 OS=Arabidopsis thaliana GN=TIM10 PE=1 SV=1	37.87	24.14	1.26	up	0.00	0.00	yes
TRINITY_DN26040_c1_g3	Ycf1 (chloroplast) [Populus adenopoda]	TIC214	Protein TIC 214 OS=Populus alba GN=TIC214 PE=3 SV=1	2.52	0.61	2.61	up	0.00	0.00	yes
TRINITY_DN26907_c0_g2	-	-	-	62.26	37.90	1.35	up	0.00	0.00	yes
TRINITY_DN26834_c1_g3	hypothetical protein POPTR_0015s12780g [Populus trichocarpa]	DTX35	Protein DETOXIFICATION 35 OS=Arabidopsis thaliana GN=DTX35 PE=2 SV=1	23.14	16.48	1.10	up	0.00	0.00	yes
TRINITY_DN27561_c0_g1	subtilase family protein [Populus trichocarpa]	SBT1.4	Subtilisin-like protease SBT1.4 OS=Arabidopsis thaliana GN=SBT1.4 PE=2 SV=1	28.53	21.68	1.16	up	0.00	0.00	yes
TRINITY_DN19274_c0_g1	hypothetical protein POPTR_0001s29510g [Populus trichocarpa]	At3g49720	Uncharacterized protein At3g49720 OS=Arabidopsis thaliana GN=At3g49720 PE=2 SV=1	74.48	55.73	1.06	up	0.00	0.00	yes
TRINITY_DN21474_c0_g1	unknown [Populus trichocarpa x Populus deltoides]	RPS12	40S ribosomal protein S12 OS=Hordeum vulgare GN=RPS12 PE=2 SV=1	246.97	177.04	1.14	up	0.00	0.00	yes
TRINITY_DN27543_c1_g3	PREDICTED: folylpolyglutamate synthase-like isoform X1 [Populus euphratica]	FPGS2	Folylpolyglutamate synthase OS=Arabidopsis thaliana GN=FPGS2 PE=1 SV=1	38.72	29.47	1.09	up	0.00	0.00	yes
TRINITY_DN23325_c0_g1	PREDICTED: 40S ribosomal protein SA-like isoform X1 [Populus euphratica]	RAP40	40S ribosomal protein SA OS=Cicer arietinum GN=RAP40 PE=2 SV=1	58.46	40.60	1.14	up	0.00	0.00	yes
TRINITY_DN17258_c0_g1	hypothetical protein POPTR_0001s34410g [Populus trichocarpa]	COR413IM1	Cold-regulated 413 inner membrane protein 1, chloroplastic OS=Arabidopsis thaliana GN=COR413IM1 PE=1 SV=1	69.25	44.15	1.24	up	0.00	0.00	yes
TRINITY_DN20990_c0_g6	ATP synthase CF1 beta subunit (chloroplast) [Citrus platymamma]	atpB	ATP synthase subunit beta, chloroplastic OS=Populus alba GN=atpB PE=3 SV=1	11.47	8.92	1.51	up	0.00	0.00	yes
TRINITY_DN18881_c0_g2	plastid-specific ribosomal family protein [Populus trichocarpa]	PSRP6	50S ribosomal protein 6, chloroplastic OS=Arabidopsis thaliana GN=PSRP6 PE=3 SV=1	245.27	116.05	1.67	up	0.00	0.00	yes
TRINITY_DN24307_c0_g1	PREDICTED: biotin carboxyl carrier protein of acetyl-CoA carboxylase, chloroplastic-like [Populus euphratica]	BCCP1	Biotin carboxyl carrier protein of acetyl-CoA carboxylase 1, chloroplastic OS=Arabidopsis thaliana GN=BCCP1 PE=1 SV=2	73.06	52.43	1.05	up	0.00	0.00	yes
TRINITY_DN16614_c0_g1	hypothetical protein POPTR_0017s03460g [Populus trichocarpa]	Mettl21A	Protein N-lysine methyltransferase METTL21A OS=Mus musculus GN=Mettl21A PE=2 SV=1	12.18	8.10	1.21	up	0.00	0.00	yes
TRINITY_DN16075_c0_g1	hypothetical protein POPTR_0010s17280g [Populus trichocarpa]	-	-	30.79	21.60	1.13	up	0.00	0.00	yes
TRINITY_DN23047_c1_g1	PREDICTED: haloacid dehalogenase-like hydrolase domain-containing protein At4g39970 isoform X1 [Populus euphratica]	At4g39970	Haloacid dehalogenase-like hydrolase domain-containing protein At4g39970 OS=Arabidopsis thaliana GN=At4g39970 PE=2 SV=1	501.61	302.05	1.31	up	0.00	0.00	yes
TRINITY_DN22997_c0_g3	hypothetical protein POPTR_0003s12330g [Populus trichocarpa]	RPL24	60S ribosomal protein L24 OS=Prunus avium GN=RPL24 PE=2 SV=1	250.51	176.74	1.12	up	0.00	0.00	yes
TRINITY_DN24685_c1_g3	photosystem II 44 kDa protein (chloroplast) [Populus tremula]	psbC	Photosystem II CP43 reaction center protein OS=Populus alba GN=psbC PE=3 SV=1	19.93	11.90	1.39	up	0.00	0.00	yes
TRINITY_DN19385_c0_g1	hypothetical protein POPTR_0008s20010g [Populus trichocarpa]	ADT2	Arogenate dehydratase/prephenate dehydratase 2, chloroplastic OS=Arabidopsis thaliana GN=ADT2 PE=1 SV=1	12.27	8.86	1.11	up	0.00	0.00	yes
TRINITY_DN27203_c1_g4	aldo/keto reductase family protein [Populus trichocarpa]	At1g06690	Uncharacterized oxidoreductase At1g06690, chloroplastic OS=Arabidopsis thaliana GN=At1g06690 PE=1 SV=1	18.63	10.82	1.48	up	0.00	0.00	yes
TRINITY_DN23653_c0_g1	hypothetical protein POPTR_0018s09500g [Populus trichocarpa]	At4g29120	Probable 3-hydroxyisobutyrate dehydrogenase-like 1, mitochondrial OS=Arabidopsis thaliana GN=At4g29120 PE=1 SV=1	54.02	38.03	1.10	up	0.00	0.00	yes
TRINITY_DN23121_c1_g1	hypothetical protein POPTR_0014s14580g [Populus trichocarpa]	rpsA	30S ribosomal protein S1 OS=Mycobacterium smegmatis (strain ATCC 700084 / mc(2)155) GN=rpsA PE=1 SV=1	67.13	39.26	1.41	up	0.00	0.00	yes
TRINITY_DN22512_c0_g1	hypothetical protein POPTR_0018s02570g [Populus trichocarpa]	At4g31810	3-hydroxyisobutyryl-CoA hydrolase-like protein 2, mitochondrial OS=Arabidopsis thaliana GN=At4g31810 PE=2 SV=1	23.51	17.76	1.04	up	0.00	0.00	yes
TRINITY_DN22536_c0_g7	PREDICTED: uncharacterized protein LOC105124797 isoform X1 [Populus euphratica]	-	-	2.91	1.32	1.74	up	0.00	0.00	yes
TRINITY_DN22628_c1_g4	PREDICTED: uncharacterized protein At4g22160-like [Populus euphratica]	-	-	44.88	30.19	1.17	up	0.00	0.00	yes
TRINITY_DN18029_c0_g1	hypothetical protein POPTR_0013s09410g [Populus trichocarpa]	GLY1	Glycerol-3-phosphate dehydrogenase [NAD(+)] 2, chloroplastic OS=Arabidopsis thaliana GN=GLY1 PE=1 SV=1	19.79	14.57	1.19	up	0.00	0.00	yes
TRINITY_DN21052_c0_g1	hypothetical protein POPTR_0002s15770g [Populus trichocarpa]	SDR1	(+)-neomenthol dehydrogenase OS=Arabidopsis thaliana GN=SDR1 PE=1 SV=1	7.17	4.84	2.07	up	0.00	0.00	yes

TRINITY_DN27215_c0_g7	-	-	-	9.88	2.82	2.39	up	0.00	0.00	yes
TRINITY_DN25430_c0_g1	PREDICTED: non-specific phospholipase C2 [Populus euphratica]	NPC2	Non-specific phospholipase C2 OS=Arabidopsis thaliana GN=NPC2 PE=2 SV=1	91.42	57.95	1.19	up	0.00	0.00	yes
TRINITY_DN16516_c0_g2	hypothetical protein POPTR_0014s14730g [Populus trichocarpa]	IBH1	Transcription factor IBH1 OS=Arabidopsis thaliana GN=IBH1 PE=1 SV=1	1.94	0.47	2.65	up	0.00	0.00	yes
TRINITY_DN17077_c0_g1	PREDICTED: phenolic glucoside malonyltransferase 1-like [Populus euphratica]	PMAT1	Phenolic glucoside malonyltransferase 1 OS=Arabidopsis thaliana GN=PMAT1 PE=1 SV=1	121.68	73.71	1.36	up	0.00	0.00	yes
TRINITY_DN22325_c0_g1	hypothetical protein POPTR_0015s01470g [Populus trichocarpa]	SS1	Starch synthase 1, chloroplastic/amyloplastic OS=Arabidopsis thaliana GN=SS1 PE=2 SV=1	21.86	16.50	1.02	up	0.00	0.00	yes
TRINITY_DN19468_c0_g2	PREDICTED: uncharacterized GPI-anchored protein At3g06035-like [Populus euphratica]	At3g06035	Uncharacterized GPI-anchored protein At3g06035 OS=Arabidopsis thaliana GN=At3g06035 PE=2 SV=1	3.43	0.98	2.39	up	0.00	0.00	yes
TRINITY_DN27198_c1_g1	aconitate hydratase family protein [Populus trichocarpa]	ACO2	Aconitate hydratase 2, mitochondrial OS=Arabidopsis thaliana GN=ACO2 PE=1 SV=2	47.60	32.18	1.09	up	0.00	0.00	yes
TRINITY_DN26300_c0_g1	hypothetical protein POPTR_0001s27210g [Populus trichocarpa]	dusA	tRNA-dihydrouridine(20/20a) synthase OS=Escherichia coli O157:H7 GN=dusA PE=3 SV=3	65.74	45.81	1.18	up	0.00	0.00	yes
TRINITY_DN14687_c0_g1	hypothetical protein POPTR_0001s23270g [Populus trichocarpa]	At5g42850	Thioredoxin-like protein Clot OS=Arabidopsis thaliana GN=At5g42850 PE=2 SV=1	84.07	59.57	1.12	up	0.00	0.00	yes
TRINITY_DN13628_c0_g1	PREDICTED: uncharacterized protein LOC105138897 isoform X3 [Populus euphratica]	NFD6	Protein NUCLEAR FUSION DEFECTIVE 6, chloroplastic/mitochondrial OS=Arabidopsis thaliana GN=NFD6 PE=2 SV=1	39.69	26.79	1.24	up	0.00	0.00	yes
TRINITY_DN14978_c0_g1	hypothetical protein POPTR_0011s15710g [Populus trichocarpa]	-	-	6.42	1.59	2.39	up	0.00	0.00	yes
TRINITY_DN27393_c0_g1	heat shock family protein [Populus trichocarpa]	HSP90-5	Heat shock protein 90-5, chloroplastic OS=Arabidopsis thaliana GN=HSP90-5 PE=1 SV=1	39.01	15.44	1.91	up	0.00	0.00	yes
TRINITY_DN22793_c0_g1	hypothetical protein POPTR_0008s12930g [Populus trichocarpa]	STL2P	SEC12-like protein 2 OS=Arabidopsis thaliana GN=STL2P PE=1 SV=4	23.18	18.78	1.02	up	0.00	0.00	yes
TRINITY_DN17245_c0_g1	PREDICTED: sec-independent protein translocase protein TATC, chloroplastic [Populus euphratica]	TATC	Sec-independent protein translocase protein TATC, chloroplastic OS=Pisum sativum GN=TATC PE=1 SV=1	72.17	45.59	1.27	up	0.00	0.00	yes
TRINITY_DN22015_c0_g12	mitochondrial import inner membrane translocase subunit Tim17/Tim22/Tim23 family protein [Populus trichocarpa]	OEP163	Outer envelope pore protein 16-3, chloroplastic/mitochondrial OS=Arabidopsis thaliana GN=OEP163 PE=1 SV=1	111.72	78.02	1.12	up	0.00	0.00	yes
TRINITY_DN20889_c0_g1	cysteine desulfurase family protein [Populus trichocarpa]	NFS2	Cysteine desulfurase 1, chloroplastic OS=Arabidopsis thaliana GN=NFS2 PE=1 SV=1	68.73	46.60	1.15	up	0.00	0.00	yes
TRINITY_DN16887_c0_g3	hypothetical protein POPTR_0010s22580g [Populus trichocarpa]	-	-	24.03	16.84	1.14	up	0.00	0.00	yes
TRINITY_DN25938_c0_g2	hypothetical protein POPTR_0017s01370g [Populus trichocarpa]	PRORP1	Proteinaceous RNase P 1, chloroplastic/mitochondrial OS=Arabidopsis thaliana GN=PRORP1 PE=1 SV=1	9.62	6.67	1.15	up	0.00	0.00	yes
TRINITY_DN15894_c0_g1	adenine phosphoribosyltransferase 1 family protein [Populus trichocarpa]	APT1	Adenine phosphoribosyltransferase 1, chloroplastic OS=Arabidopsis thaliana GN=APT1 PE=1 SV=2	84.24	61.79	1.05	up	0.00	0.00	yes
TRINITY_DN20792_c0_g1	PREDICTED: pentatricopeptide repeat-containing protein At2g01860 [Populus euphratica]	EMB975	Pentatricopeptide repeat-containing protein At2g01860 OS=Arabidopsis thaliana GN=EMB975 PE=2 SV=1	14.63	10.43	1.10	up	0.00	0.00	yes
TRINITY_DN23541_c1_g1	hypothetical protein POPTR_0013s05000g [Populus trichocarpa]	VDE1	Violaxanthin de-epoxidase, chloroplastic OS=Spinacia oleracea GN=VDE1 PE=1 SV=2	164.90	97.86	1.37	up	0.00	0.00	yes
TRINITY_DN17159_c0_g1	hypothetical protein POPTR_0008s20440g [Populus trichocarpa]	ABCI12	Protein ABCI12, chloroplastic OS=Arabidopsis thaliana GN=ABCI12 PE=1 SV=1	12.73	8.60	1.16	up	0.00	0.00	yes
TRINITY_DN25225_c0_g2	hypothetical protein POPTR_0019s05340g [Populus trichocarpa]	BACOVA_02659	Beta-glucosidase BoGH3B OS=Bacteroides ovatus (strain ATCC 8483 / DSM 1896 / JCM 5824 / NCTC 11153) GN=BACOVA_02659 PE=1 SV=1	20.38	13.56	1.17	up	0.00	0.00	yes
TRINITY_DN21541_c1_g4	PREDICTED: proline-, glutamic acid- and leucine-rich protein 1 [Populus euphratica]	OCP3	Protein OVEREXPRESSOR OF CATIONIC PEROXIDASE 3 OS=Arabidopsis thaliana GN=OCP3 PE=1 SV=1	37.94	27.01	1.14	up	0.00	0.00	yes
TRINITY_DN22632_c0_g4	26S proteasome non-ATPase regulatory subunit family protein [Populus trichocarpa]	RPN12A	26S proteasome non-ATPase regulatory subunit 8 homolog A OS=Arabidopsis thaliana GN=RPN12A PE=1 SV=1	60.85	46.51	1.05	up	0.00	0.00	yes
TRINITY_DN17710_c0_g1	hypothetical protein POPTR_0012s01280g [Populus trichocarpa]	-	Flavonol sulfotransferase-like OS=Flaveria bidentis PE=2 SV=1	51.45	22.69	1.71	up	0.00	0.00	yes
TRINITY_DN17625_c0_g1	hypothetical protein POPTR_0008s16570g [Populus trichocarpa]	ETFB	Electron transfer flavoprotein subunit beta, mitochondrial OS=Arabidopsis thaliana GN=ETFB PE=1 SV=1	48.37	33.31	1.25	up	0.00	0.00	yes

TRINITY_DN21385_c0_g1	glutathione S-transferase F4 [Populus yatungensis]	-	Glutathione S-transferase OS=Hyoscyamus muticus PE=1 SV=1	313.16	159.31	1.67	up	0.00	0.00	yes
TRINITY_DN21116_c0_g9	hypothetical protein POPTR_0014s15570g [Populus trichocarpa]	-	-	121.25	86.35	1.11	up	0.00	0.00	yes
TRINITY_DN19109_c0_g1	translational elongation factor 1 subunit Bbeta family protein [Populus trichocarpa]	At1g30230	Elongation factor 1-delta 1 OS=Arabidopsis thaliana GN=At1g30230 PE=1 SV=2	420.52	297.01	1.12	up	0.00	0.00	yes
TRINITY_DN23916_c0_g1	chloroplast import receptor p36 family protein [Populus trichocarpa]	TPT	Triose phosphate/phosphate translocator TPT, chloroplastic OS=Arabidopsis thaliana GN=TPT PE=1 SV=1	583.12	369.56	1.26	up	0.00	0.00	yes
TRINITY_DN27054_c0_g2	PREDICTED: protein TOC75-3, chloroplastic-like [Populus euphratica]	TOC75-3	Protein TOC75-3, chloroplastic OS=Arabidopsis thaliana GN=TOC75-3 PE=1 SV=1	161.75	73.00	1.72	up	0.00	0.00	yes
TRINITY_DN21049_c0_g1	PREDICTED: probable transcriptional regulatory protein At2g25830 isoform X1 [Populus euphratica]	At2g25830	Probable transcriptional regulatory protein At2g25830 OS=Arabidopsis thaliana GN=At2g25830 PE=2 SV=2	15.78	11.57	1.09	up	0.00	0.00	yes
TRINITY_DN19294_c0_g3	hypothetical protein POPTR_0006s16050g [Populus trichocarpa]	At4g13360	3-hydroxyisobutyryl-CoA hydrolase-like protein 3, mitochondrial OS=Arabidopsis thaliana GN=At4g13360 PE=1 SV=2	91.23	67.60	1.09	up	0.00	0.00	yes
TRINITY_DN17507_c0_g1	hypothetical protein POPTR_0006s23850g [Populus trichocarpa]	ndhU	NAD(P)H-quinone oxidoreductase subunit U, chloroplastic OS=Arabidopsis thaliana GN=ndhU PE=1 SV=1	414.68	200.17	1.67	up	0.00	0.00	yes
TRINITY_DN17658_c0_g1	oxidoreductase family protein [Populus trichocarpa]	rtn4ip1	Reticulon-4-interacting protein 1 homolog, mitochondrial OS=Danio rerio GN=rtn4ip1 PE=2 SV=2	26.19	19.35	1.05	up	0.00	0.00	yes
TRINITY_DN19734_c0_g2	PREDICTED: ribulose-phosphate 3-epimerase, chloroplastic [Populus euphratica]	RPE	Ribulose-phosphate 3-epimerase, chloroplastic OS=Spinacia oleracea GN=RPE PE=1 SV=1	648.43	374.61	1.39	up	0.00	0.00	yes
TRINITY_DN20629_c0_g2	tatD-related deoxyribonuclease family protein [Populus trichocarpa]	tatdn1	Putative deoxyribonuclease TATDN1 OS=Xenopus laevis GN=tatdn1 PE=2 SV=1	28.11	22.07	1.01	up	0.00	0.00	yes
TRINITY_DN26433_c0_g1	hypothetical protein POPTR_0017s04880g [Populus trichocarpa]	FKBP16-3	Peptidyl-prolyl cis-trans isomerase FKBP16-3, chloroplastic OS=Arabidopsis thaliana GN=FKBP16-3 PE=1 SV=2	281.70	153.62	1.37	up	0.00	0.00	yes
TRINITY_DN17297_c0_g1	PREDICTED: selenoprotein O [Populus euphratica]	azo1574	UPF0061 protein azo1574 OS=Azoarcus sp. (strain BH72) GN=azo1574 PE=3 SV=1	25.11	18.70	1.03	up	0.00	0.00	yes
TRINITY_DN13181_c0_g1	hypothetical protein POPTR_0006s24080g [Populus trichocarpa]	At4g33100	Uncharacterized protein At4g33100 OS=Arabidopsis thaliana GN=At4g33100 PE=3 SV=1	13.33	6.69	1.59	up	0.00	0.00	yes
TRINITY_DN23702_c0_g4	-	-	-	3.83	0.52	3.34	up	0.00	0.00	yes
TRINITY_DN20914_c0_g3	hypothetical protein POPTR_0006s07470g [Populus trichocarpa]	-	Macrophage migration inhibitory factor homolog OS=Trichuris trichiura PE=1 SV=2	89.52	50.66	1.36	up	0.00	0.00	yes
TRINITY_DN14992_c0_g2	(2R)-phospho-3-sulfolactate synthase-related family protein [Populus trichocarpa]	HSA32	Protein HEAT-STRESS-ASSOCIATED 32 OS=Arabidopsis thaliana GN=HSA32 PE=2 SV=1	8.95	5.34	1.36	up	0.00	0.00	yes
TRINITY_DN21427_c0_g1	hypothetical protein POPTR_0016s11621g [Populus trichocarpa]	FBP	Fructose-1,6-bisphosphatase, chloroplastic OS=Arabidopsis thaliana GN=FBP PE=1 SV=2	98.65	56.05	1.40	up	0.00	0.00	yes
TRINITY_DN16945_c0_g2	hypothetical protein POPTR_0019s00230g [Populus trichocarpa]	CLC-C	Chloride channel protein CLC-c OS=Arabidopsis thaliana GN=CLC-C PE=1 SV=1	4.06	1.81	1.79	up	0.00	0.00	yes
TRINITY_DN24195_c1_g1	PREDICTED: protein ESKIMO 1-like isoform X1 [Populus euphratica]	ESK1	Protein ESKIMO 1 OS=Arabidopsis thaliana GN=ESK1 PE=1 SV=1	12.27	6.76	1.34	up	0.00	0.00	yes
TRINITY_DN23430_c0_g2	hypothetical protein POPTR_0017s04920g, partial [Populus trichocarpa]	-	-	52.79	36.31	1.13	up	0.00	0.00	yes
TRINITY_DN20525_c0_g1	thioredoxin m family protein [Populus trichocarpa]	-	Thioredoxin M-type, chloroplastic OS=Pisum sativum PE=2 SV=1	637.53	308.78	1.64	up	0.00	0.00	yes
TRINITY_DN15437_c0_g2	PREDICTED: uncharacterized protein LOC105123357 [Populus euphratica]	-	-	7.46	1.94	2.75	up	0.00	0.00	yes
TRINITY_DN16204_c0_g1	PREDICTED: zinc finger protein ZAT9-like [Populus euphratica]	ZAT9	Zinc finger protein ZAT9 OS=Arabidopsis thaliana GN=ZAT9 PE=2 SV=1	3.51	0.80	2.69	up	0.00	0.00	yes
TRINITY_DN26126_c1_g5	hypothetical protein POPTR_0016s021501g, partial [Populus trichocarpa]	UGT85A7	UDP-glycosyltransferase 85A7 OS=Arabidopsis thaliana GN=UGT85A7 PE=2 SV=1	21.67	8.78	1.87	up	0.00	0.00	yes
TRINITY_DN24626_c0_g6	hypothetical protein POPTR_0001s12870g [Populus trichocarpa]	At4g14100	Uncharacterized protein At4g14100 OS=Arabidopsis thaliana GN=At4g14100 PE=2 SV=1	62.86	38.28	1.30	up	0.00	0.00	yes
TRINITY_DN24701_c0_g2	PREDICTED: pentatricopeptide repeat-containing protein At5g28460-like [Populus euphratica]	At3g61520	Pentatricopeptide repeat-containing protein At3g61520, mitochondrial OS=Arabidopsis thaliana GN=At3g61520 PE=2 SV=1	5.84	4.10	1.14	up	0.00	0.00	yes
TRINITY_DN21085_c0_g1	unknown [Populus trichocarpa]	LIL3.1	Light-harvesting complex-like protein 3 isotype 1, chloroplastic OS=Arabidopsis thaliana GN=LIL3.1 PE=1 SV=1	438.91	256.02	1.37	up	0.00	0.00	yes

TRINITY_DN14362_c0_g1	PREDICTED: uncharacterized protein LOC105109024 isoform X1 [Populus euphratica]	-	-	134.77	89.23	1.18	up	0.00	0.00	yes
TRINITY_DN17836_c0_g1	PREDICTED: clustered mitochondria protein homolog [Populus euphratica]	-	-	188.13	81.65	1.80	up	0.00	0.00	yes
TRINITY_DN26366_c0_g2	hypothetical protein POPTR_0002s22410g [Populus trichocarpa]	PED1	3-ketoacyl-CoA thiolase 2, peroxisomal OS=Arabidopsis thaliana GN=PED1 PE=1 SV=2	361.54	171.53	1.69	up	0.00	0.00	yes
TRINITY_DN19198_c0_g1	hypothetical protein POPTR_0002s24970g [Populus trichocarpa]	FKBP65	Peptidyl-prolyl cis-trans isomerase FKBP65 OS=Arabidopsis thaliana GN=FKBP65 PE=1 SV=1	135.65	95.80	1.14	up	0.00	0.00	yes
TRINITY_DN27044_c0_g1	pectate lyase 5 precursor family protein [Populus trichocarpa]	-	Pectate lyase OS=Zinnia violacea PE=1 SV=1	13.33	6.45	1.63	up	0.00	0.00	yes
TRINITY_DN21092_c0_g1	PREDICTED: NAD-dependent protein deacetylase SRT2 isoform X3 [Populus euphratica]	SRT2	NAD-dependent protein deacylase SRT2 OS=Arabidopsis thaliana GN=SRT2 PE=2 SV=1	12.55	9.15	1.18	up	0.00	0.00	yes
TRINITY_DN20325_c0_g6	ribosomal S15 family protein [Populus trichocarpa]	RPS15D	40S ribosomal protein S15-4 OS=Arabidopsis thaliana GN=RPS15D PE=2 SV=1	87.33	60.14	1.23	up	0.00	0.00	yes
TRINITY_DN15747_c0_g1	hypothetical protein POPTR_0006s24400g [Populus trichocarpa]	6-FEH	Fructan 6-exohydrolase OS=Beta vulgaris GN=6-FEH PE=1 SV=1	4.17	1.74	2.03	up	0.00	0.00	yes
TRINITY_DN22132_c0_g1	pentatricopeptide repeat-containing family protein [Populus trichocarpa]	EMB2654	Pentatricopeptide repeat-containing protein At2g41720 OS=Arabidopsis thaliana GN=EMB2654 PE=2 SV=1	2.60	0.87	2.30	up	0.00	0.00	yes
TRINITY_DN25808_c0_g1	PREDICTED: solanesyl diphosphate synthase 3, chloroplastic/mitochondrial-like isoform X1 [Populus euphratica]	SPS3	Solanesyl diphosphate synthase 3, chloroplastic/mitochondrial OS=Arabidopsis thaliana GN=SPS3 PE=1 SV=1	19.66	16.76	1.01	up	0.00	0.00	yes
TRINITY_DN21382_c0_g2	hypothetical protein POPTR_0008s16710g [Populus trichocarpa]	tas	Protein tas OS=Escherichia coli (strain K12) GN=tas PE=1 SV=1	118.77	80.72	1.23	up	0.00	0.00	yes
TRINITY_DN17684_c0_g1	hypothetical protein POPTR_0014s09800g [Populus trichocarpa]	-	-	350.16	195.05	1.44	up	0.00	0.00	yes
TRINITY_DN20278_c0_g2	hypothetical protein POPTR_0005s16410g [Populus trichocarpa]	At4g37920	Uncharacterized protein At4g37920, chloroplastic OS=Arabidopsis thaliana GN=At4g37920 PE=1 SV=2	36.58	26.96	1.02	up	0.00	0.00	yes
TRINITY_DN25398_c1_g1	PREDICTED: DEAD-box ATP-dependent RNA helicase 3, chloroplastic-like isoform X2 [Populus euphratica]	RH3	DEAD-box ATP-dependent RNA helicase 3, chloroplastic OS=Arabidopsis thaliana GN=RH3 PE=1 SV=2	571.63	379.40	1.21	up	0.00	0.00	yes
TRINITY_DN21538_c0_g4	unknown [Populus trichocarpa x Populus deltoides]	PSBR	Photosystem II 10 kDa polypeptide, chloroplastic OS=Solanum tuberosum GN=PSBR PE=2 SV=1	4735.55	2437.81	1.55	up	0.00	0.00	yes
TRINITY_DN26034_c1_g4	-	-	-	21.42	10.10	1.64	up	0.00	0.00	yes
TRINITY_DN23793_c0_g1	hypothetical protein POPTR_0002s18110g [Populus trichocarpa]	CURT1B	Protein CURVATURE THYLAKOID 1B, chloroplastic OS=Arabidopsis thaliana GN=CURT1B PE=1 SV=2	1585.22	868.08	1.53	up	0.00	0.00	yes
TRINITY_DN24230_c0_g1	PREDICTED: uncharacterized protein LOC105107868 [Populus euphratica]	-	-	89.25	56.95	1.17	up	0.00	0.00	yes
TRINITY_DN26016_c0_g1	clp protease proteolytic subunit (chloroplast) [Populus ilicifolia]	clpP	ATP-dependent Clp protease proteolytic subunit OS=Populus alba GN=clpP PE=3 SV=1	7.07	3.93	1.50	up	0.00	0.00	yes
TRINITY_DN21496_c0_g3	hypothetical protein POPTR_0012s00690g [Populus trichocarpa]	At3g49470	Nascent polypeptide-associated complex subunit alpha-like protein 2 OS=Arabidopsis thaliana GN=At3g49470 PE=2 SV=2	336.16	222.58	1.24	up	0.00	0.00	yes
TRINITY_DN23827_c0_g1	unknown [Populus trichocarpa x Populus deltoides]	CSP41B	Chloroplast stem-loop binding protein of 41 kDa b, chloroplastic OS=Arabidopsis thaliana GN=CSP41B PE=1 SV=1	759.62	376.99	1.67	up	0.00	0.00	yes
TRINITY_DN23842_c0_g2	PREDICTED: UDP-glucuronate 4-epimerase 6-like [Populus euphratica]	GAE6	UDP-glucuronate 4-epimerase 6 OS=Arabidopsis thaliana GN=GAE6 PE=1 SV=1	59.05	37.92	1.20	up	0.00	0.00	yes
TRINITY_DN20313_c0_g1	hypothetical protein CISIN_1g031166mg [Citrus sinensis]	SPA	Protein SPA, chloroplastic OS=Solanum lycopersicum GN=SPA PE=2 SV=1	383.99	191.57	1.50	up	0.00	0.00	yes
TRINITY_DN16993_c0_g2	PREDICTED: uncharacterized protein LOC105140125 [Populus euphratica]	-	-	24.80	12.25	1.63	up	0.00	0.00	yes
TRINITY_DN24969_c2_g1	Succinyl-CoA ligase beta-chain family protein [Populus trichocarpa]	At2g20420	Succinate--CoA ligase [ADP-forming] subunit beta, mitochondrial OS=Arabidopsis thaliana GN=At2g20420 PE=1 SV=1	98.12	74.47	1.05	up	0.00	0.00	yes
TRINITY_DN26195_c0_g6	PREDICTED: protein NRT1/ PTR FAMILY 6.4-like isoform X2 [Populus euphratica]	NPF6.4	Protein NRT1/ PTR FAMILY 6.4 OS=Arabidopsis thaliana GN=NPF6.4 PE=1 SV=1	8.14	5.84	1.26	up	0.00	0.00	yes
TRINITY_DN4202_c0_g1	-	-	-	12.84	2.71	2.86	up	0.00	0.00	yes

TRINITY_DN17105_c0_g1	hypothetical protein POPTR_0018s01370g [Populus trichocarpa]	QCR7-2	Cytochrome b-c1 complex subunit 7-2 OS=Arabidopsis thaliana GN=QCR7-2 PE=1 SV=1	94.98	68.89	1.07	up	0.00	0.00	yes
TRINITY_DN25741_c0_g1	aspartyl aminopeptidase family protein [Populus trichocarpa]	RCOM_1506700	Probable aspartyl aminopeptidase OS=Ricinus communis GN=RCOM_1506700 PE=2 SV=2	56.57	43.09	1.02	up	0.00	0.00	yes
TRINITY_DN24393_c0_g1	hypothetical protein POPTR_0001s24710g [Populus trichocarpa]	-	Triosephosphate isomerase, cytosolic OS=Coptis japonica PE=2 SV=1	198.18	106.97	1.31	up	0.00	0.00	yes
TRINITY_DN26018_c1_g3	PREDICTED: NADH dehydrogenase [ubiquinone] flavoprotein 2, mitochondrial-like [Populus euphratica]	At4g02580	NADH dehydrogenase [ubiquinone] flavoprotein 2, mitochondrial OS=Arabidopsis thaliana GN=At4g02580 PE=1 SV=3	125.95	93.13	1.03	up	0.00	0.00	yes
TRINITY_DN16476_c0_g1	emp24/gp25L/p24 family protein [Populus trichocarpa]	At3g07680	Transmembrane emp24 domain-containing protein p24beta2 OS=Arabidopsis thaliana GN=At3g07680 PE=1 SV=1	95.66	68.49	1.08	up	0.00	0.00	yes
TRINITY_DN24846_c0_g1	PREDICTED: bifunctional nitrilase/nitrile hydratase NIT4A [Populus euphratica]	NIT4A	Bifunctional nitrilase/nitrile hydratase NIT4A OS=Nicotiana tabacum GN=NIT4A PE=2 SV=1	57.16	28.44	1.60	up	0.00	0.00	yes
TRINITY_DN18193_c1_g1	ribosomal protein L32 (chloroplast) [Populus alba]	rpl32	50S ribosomal protein L32, chloroplastic OS=Daucus carota GN=rpl32 PE=3 SV=1	515.22	296.15	1.37	up	0.00	0.00	yes
TRINITY_DN19793_c0_g1	hypothetical protein POPTR_0019s14180g [Populus trichocarpa]	-	-	16.45	11.50	1.12	up	0.00	0.00	yes
TRINITY_DN18491_c0_g1	hypothetical protein POPTR_0010s12030g [Populus trichocarpa]	CRR3	Probable NAD(P)H dehydrogenase subunit CRR3, chloroplastic OS=Arabidopsis thaliana GN=CRR3 PE=2 SV=1	222.90	105.40	1.64	up	0.00	0.00	yes
TRINITY_DN19102_c0_g1	hypothetical protein POPTR_0015s01030g [Populus trichocarpa]	Myg1	UPF0160 protein MYG1, mitochondrial OS=Rattus norvegicus GN=Myg1 PE=1 SV=1	6.40	3.72	1.40	up	0.00	0.00	yes
TRINITY_DN19435_c0_g1	PREDICTED: anthocyanidin 3-O-glucosyltransferase 7-like [Populus euphratica]	UFGT	Anthocyanidin 3-O-glucosyltransferase 2 OS=Vitis vinifera GN=UFGT PE=1 SV=2	20.65	14.21	1.17	up	0.00	0.00	yes
TRINITY_DN22711_c0_g1	hypothetical protein POPTR_0011s13820g [Populus trichocarpa]	THI1	Thiamine thiazole synthase, chloroplastic OS=Citrus sinensis GN=THI1 PE=2 SV=1	450.01	318.88	1.10	up	0.00	0.00	yes
TRINITY_DN23562_c0_g6	photosystem 2 family protein [Populus trichocarpa]	PSB27-1	Photosystem II repair protein PSB27-H1, chloroplastic OS=Arabidopsis thaliana GN=PSB27-1 PE=1 SV=1	467.73	251.32	1.50	up	0.00	0.00	yes
TRINITY_DN16443_c1_g3	ribosomal protein S14 mitochondrial, partial [Populus trichocarpa]	RPS14	Ribosomal protein S14, mitochondrial OS=Oenothera berteroana GN=RPS14 PE=3 SV=2	94.71	68.99	1.09	up	0.00	0.00	yes
TRINITY_DN20260_c0_g1	PREDICTED: uncharacterized protein LOC105137001 isoform X1 [Populus euphratica]	MTERF6	Transcription termination factor MTERF6, chloroplastic/mitochondrial OS=Arabidopsis thaliana GN=MTERF6 PE=2 SV=1	94.97	59.85	1.27	up	0.00	0.00	yes
TRINITY_DN19585_c0_g1	hypothetical protein POPTR_0004s23400g [Populus trichocarpa]	-	-	33.70	24.19	1.19	up	0.00	0.00	yes
TRINITY_DN17619_c0_g1	PREDICTED: developmentally-regulated G-protein 3 [Populus euphratica]	DRG3	Developmentally-regulated G-protein 3 OS=Arabidopsis thaliana GN=DRG3 PE=1 SV=1	39.06	29.19	1.02	up	0.00	0.00	yes
TRINITY_DN22055_c0_g1	PREDICTED: uncharacterized protein LOC105121156 isoform X2 [Populus euphratica]	-	-	28.12	21.83	1.14	up	0.00	0.00	yes
TRINITY_DN18885_c0_g3	hypothetical protein POPTR_0006s22820g [Populus trichocarpa]	FFC	Signal recognition particle 54 kDa protein, chloroplastic OS=Arabidopsis thaliana GN=FFC PE=1 SV=1	21.93	12.66	1.38	up	0.00	0.00	yes
TRINITY_DN23336_c0_g1	hypothetical protein POPTR_0002s25720g [Populus trichocarpa]	At2g20360	NADH dehydrogenase [ubiquinone] 1 alpha subcomplex subunit 9, mitochondrial OS=Arabidopsis thaliana GN=At2g20360 PE=1 SV=2	118.25	83.78	1.05	up	0.00	0.00	yes
TRINITY_DN22459_c0_g1	PREDICTED: protein THYLAKOID FORMATION1, chloroplastic-like isoform X1 [Populus euphratica]	THF1	Protein THYLAKOID FORMATION1, chloroplastic OS=Solanum tuberosum GN=THF1 PE=2 SV=1	370.75	246.63	1.21	up	0.00	0.00	yes
TRINITY_DN27710_c0_g1	hypothetical protein POPTR_0006s22480g [Populus trichocarpa]	-	-	20.79	12.04	1.40	up	0.00	0.00	yes
TRINITY_DN17377_c1_g3	unknown [Populus trichocarpa x Populus deltoides]	-	-	103.18	76.95	1.03	up	0.00	0.00	yes
TRINITY_DN26587_c0_g2	PREDICTED: uncharacterized protein LOC105127025 [Populus euphratica]	-	-	17.04	8.49	1.55	up	0.00	0.00	yes
TRINITY_DN18859_c0_g3	aconitate hydratase family protein [Populus trichocarpa]	ACO	Aconitate hydratase (Fragment) OS=Cucumis melo var. conomon GN=ACO PE=2 SV=1	80.30	54.25	1.18	up	0.00	0.00	yes
TRINITY_DN25063_c0_g1	hypothetical protein POPTR_0001s31980g [Populus trichocarpa]	SCPL18	Serine carboxypeptidase-like 18 OS=Arabidopsis thaliana GN=SCPL18 PE=2 SV=2	82.58	54.84	1.29	up	0.00	0.00	yes
TRINITY_DN24769_c0_g1	PREDICTED: pentatricopeptide repeat-containing protein At5g02830, chloroplastic [Populus euphratica]	At5g02830	Pentatricopeptide repeat-containing protein At5g02830, chloroplastic OS=Arabidopsis thaliana GN=At5g02830 PE=2 SV=3	35.12	22.47	1.29	up	0.00	0.00	yes

TRINITY_DN18790_c0_g1	pore protein 24K chain [Populus trichocarpa]	OEP24A	Outer envelope pore protein 24A, chloroplastic OS=Arabidopsis thaliana GN=OEP24A PE=1 SV=1	101.15	70.90	1.13	up	0.00	0.00	yes
TRINITY_DN23825_c0_g1	eukaryotic translation initiation factor 3E family protein [Populus trichocarpa]	TIF3E1	Eukaryotic translation initiation factor 3 subunit E OS=Arabidopsis thaliana GN=TIF3E1 PE=1 SV=1	98.86	75.68	1.08	up	0.00	0.00	yes
TRINITY_DN20301_c0_g3	hypothetical protein POPTR_0019s02680g [Populus trichocarpa]	-	-	2.75	1.28	1.72	up	0.00	0.00	yes
TRINITY_DN21748_c0_g2	-	-	-	214.25	111.11	1.59	up	0.00	0.00	yes
TRINITY_DN16212_c0_g2	PREDICTED: uncharacterized protein LOC105142021 [Populus euphratica]	-	-	2.16	0.97	1.76	up	0.00	0.00	yes
TRINITY_DN19431_c0_g1	hypothetical protein POPTR_0005s09200g [Populus trichocarpa]	PYRD	Dihydroorotate dehydrogenase (quinone), mitochondrial OS=Arabidopsis thaliana GN=PYRD PE=1 SV=2	15.59	11.37	1.11	up	0.00	0.00	yes
TRINITY_DN15437_c0_g1	PREDICTED: uncharacterized protein LOC105123357 [Populus euphratica]	-	-	4.76	1.40	2.34	up	0.00	0.00	yes
TRINITY_DN20485_c1_g2	PREDICTED: uncharacterized protein LOC105130254 isoform X1 [Populus euphratica]	-	-	25.71	11.78	1.72	up	0.00	0.00	yes
TRINITY_DN23091_c0_g4	glutamate-ammonia ligase family protein [Populus trichocarpa]	-	Glutamine synthetase nodule isozyme OS=Vigna aconitifolia PE=2 SV=1	63.46	29.26	1.81	up	0.00	0.00	yes
TRINITY_DN18682_c1_g2	hypothetical protein POPTR_0018s10440g [Populus trichocarpa]	ROG1	Putative lipase ROG1 OS=Saccharomyces cerevisiae (strain ATCC 204508 / S288c) GN=ROG1 PE=1 SV=1	12.07	8.76	1.08	up	0.00	0.00	yes
TRINITY_DN20207_c0_g2	DNAJ chaperone C-terminal domain-containing family protein [Populus trichocarpa]	DNAJB4	DnaJ homolog subfamily B member 4 OS=Homo sapiens GN=DNAJB4 PE=1 SV=1	5.65	2.62	1.73	up	0.00	0.00	yes
TRINITY_DN18297_c0_g1	PREDICTED: tRNA pseudouridine synthase-like 1 isoform X1 [Populus euphratica]	truA1	tRNA pseudouridine synthase A 1 OS=Protochlamydia amoebophila (strain UWE25) GN=truA1 PE=3 SV=1	47.54	32.05	1.17	up	0.00	0.00	yes
TRINITY_DN22022_c1_g1	ceramidase family protein [Populus trichocarpa]	At2g38010	Neutral ceramidase OS=Arabidopsis thaliana GN=At2g38010 PE=3 SV=1	12.10	6.91	1.42	up	0.00	0.00	yes
TRINITY_DN27075_c0_g1	hypothetical protein POPTR_0010s16770g [Populus trichocarpa]	STP-1	Alpha-1,4 glucan phosphorylase L-2 isozyme, chloroplastic/amyloplastic OS=Solanum tuberosum GN=STP-1 PE=1 SV=1	41.94	26.45	1.26	up	0.00	0.00	yes
TRINITY_DN23562_c0_g2	photosystem 2 family protein [Populus trichocarpa]	PSB27-1	Photosystem II repair protein PSB27-H1, chloroplastic OS=Arabidopsis thaliana GN=PSB27-1 PE=1 SV=1	171.51	97.36	1.43	up	0.00	0.00	yes
TRINITY_DN19186_c0_g1	hypothetical protein POPTR_0007s09900g [Populus trichocarpa]	pfh1	ATP-dependent DNA helicase pfh1 OS=Schizosaccharomyces pombe (strain 972 / ATCC 24843) GN=pfh1 PE=1 SV=1	7.92	5.14	1.23	up	0.00	0.00	yes
TRINITY_DN18160_c0_g3	PREDICTED: protein PHLOEM PROTEIN 2-LIKE A1-like [Populus euphratica]	PP2A1	Protein PHLOEM PROTEIN 2-LIKE A1 OS=Arabidopsis thaliana GN=PP2A1 PE=2 SV=1	3.27	1.17	2.07	up	0.00	0.00	yes
TRINITY_DN22616_c0_g2	unknown [Populus trichocarpa]	ETR2	Ethylene receptor 2 OS=Arabidopsis thaliana GN=ETR2 PE=1 SV=2	13.61	7.11	1.51	up	0.00	0.00	yes
TRINITY_DN19279_c0_g1	hypothetical protein POPTR_0005s18080g [Populus trichocarpa]	-	-	25.03	19.06	1.15	up	0.00	0.00	yes
TRINITY_DN21876_c0_g1	tRNA synthetase class II family protein [Populus trichocarpa]	OVA5	Lysine--tRNA ligase, chloroplastic/mitochondrial OS=Arabidopsis thaliana GN=OVA5 PE=2 SV=1	52.95	36.23	1.17	up	0.00	0.00	yes
TRINITY_DN17479_c2_g1	hypothetical protein POPTR_0006s09800g [Populus trichocarpa]	-	-	39.84	21.95	1.45	up	0.00	0.00	yes
TRINITY_DN22250_c0_g1	hypothetical protein POPTR_0001s18200g [Populus trichocarpa]	-	-	48.00	24.56	1.62	up	0.00	0.00	yes
TRINITY_DN26844_c0_g1	hypothetical protein POPTR_0002s01200g [Populus trichocarpa]	At5g42310	Pentatricopeptide repeat-containing protein At5g42310, mitochondrial OS=Arabidopsis thaliana GN=At5g42310 PE=2 SV=1	25.43	18.01	1.09	up	0.00	0.00	yes
TRINITY_DN23754_c0_g2	-	-	-	173.12	86.25	1.64	up	0.00	0.00	yes
TRINITY_DN26175_c0_g4	PREDICTED: probable LRR receptor-like serine/threonine-protein kinase MRH1 isoform X1 [Populus euphratica]	MDIS2	Protein MALE DISCOVERER 2 OS=Arabidopsis thaliana GN=MDIS2 PE=2 SV=1	1.95	0.91	1.70	up	0.00	0.00	yes
TRINITY_DN22813_c0_g4	aspartate transaminase family protein [Populus trichocarpa]	ASP3	Aspartate aminotransferase 3, chloroplastic OS=Arabidopsis thaliana GN=ASP3 PE=1 SV=1	25.11	18.98	1.08	up	0.00	0.00	yes
TRINITY_DN23662_c0_g1	hypothetical protein POPTR_0015s05360g [Populus trichocarpa]	At3g17800	UV-B-induced protein At3g17800, chloroplastic OS=Arabidopsis thaliana GN=At3g17800 PE=2 SV=1	94.20	71.37	1.03	up	0.00	0.00	yes

TRINITY_DN24381_c0_g1	PREDICTED: patatin-like phospholipase domain-containing protein 2 isoform X2 [Populus euphratica]	PNPLA2	Patatin-like phospholipase domain-containing protein 2 OS=Bos taurus GN=PNPLA2 PE=2 SV=1	15.94	12.22	1.07	up	0.00	0.00	yes
TRINITY_DN21552_c0_g6	60S ribosomal protein L14 [Populus trichocarpa]	RPL14B	60S ribosomal protein L14-2 OS=Arabidopsis thaliana GN=RPL14B PE=1 SV=1	1111.47	757.20	1.09	up	0.00	0.00	yes
TRINITY_DN18166_c0_g1	unknown [Populus trichocarpa]	-	-	293.77	212.52	1.14	up	0.00	0.00	yes
TRINITY_DN17116_c0_g1	hypothetical protein POPTR_0003s16740g [Populus trichocarpa]	ABCG28	ABC transporter G family member 28 OS=Arabidopsis thaliana GN=ABCG28 PE=3 SV=1	3.22	1.40	1.82	up	0.00	0.00	yes
TRINITY_DN14967_c0_g1	PREDICTED: receptor-like cytosolic serine/threonine-protein kinase RBK2 isoform X1 [Populus euphratica]	RBK2	Receptor-like cytosolic serine/threonine-protein kinase RBK2 OS=Arabidopsis thaliana GN=RBK2 PE=1 SV=1	4.16	2.26	1.72	up	0.00	0.00	yes
TRINITY_DN22812_c0_g2	Serine hydroxymethyltransferase family protein [Populus trichocarpa]	-	Serine hydroxymethyltransferase 2, mitochondrial OS=Flaveria pringlei PE=2 SV=1	785.78	366.57	1.66	up	0.00	0.00	yes
TRINITY_DN17368_c0_g1	hypothetical protein POPTR_0004s22660g [Populus trichocarpa]	RPL9	50S ribosomal protein L9, chloroplastic OS=Arabidopsis thaliana GN=RPL9 PE=2 SV=1	352.63	222.03	1.27	up	0.00	0.00	yes
TRINITY_DN21963_c0_g1	PREDICTED: uncharacterized protein LOC105139138 isoform X1 [Populus euphratica]	-	-	22.05	16.38	1.09	up	0.00	0.00	yes
TRINITY_DN25230_c0_g1	PREDICTED: oxygen-evolving enhancer protein 1, chloroplastic [Populus euphratica]	PSBO	Oxygen-evolving enhancer protein 1, chloroplastic OS=Nicotiana tabacum GN=PSBO PE=2 SV=1	2105.50	1291.76	1.31	up	0.00	0.00	yes
TRINITY_DN15233_c0_g1	hypothetical protein POPTR_0008s10960g [Populus trichocarpa]	At5g08180	H/ACA ribonucleoprotein complex subunit 2-like protein OS=Arabidopsis thaliana GN=At5g08180 PE=1 SV=1	154.94	109.81	1.11	up	0.00	0.00	yes
TRINITY_DN21977_c0_g2	PREDICTED: putative aryl-alcohol dehydrogenase C750.01 isoform X2 [Populus euphratica]	-	-	163.39	108.29	1.24	up	0.00	0.00	yes
TRINITY_DN23974_c0_g1	putative progesterone 5beta-reductase [Populus trichocarpa]	-	3-oxo-Delta(4,5)-steroid 5-beta-reductase OS=Digitalis lanata PE=1 SV=1	1329.19	820.82	1.32	up	0.00	0.00	yes
TRINITY_DN16211_c2_g1	hypothetical protein POPTR_0016s13210g [Populus trichocarpa]	RPS4	40S ribosomal protein S4 OS=Prunus armeniaca GN=RPS4 PE=2 SV=1	535.58	373.37	1.15	up	0.00	0.00	yes
TRINITY_DN20923_c0_g1	pseudouridine synthase family protein [Populus trichocarpa]	At1g76050	RNA pseudouridine synthase 2, chloroplastic OS=Arabidopsis thaliana GN=At1g76050 PE=2 SV=1	19.14	16.20	1.08	up	0.00	0.00	yes
TRINITY_DN22812_c0_g3	mitochondrial serine hydroxymethyltransferase [Populus tremuloides]	-	Serine hydroxymethyltransferase, mitochondrial OS=Solanum tuberosum PE=2 SV=1	59.98	26.65	1.77	up	0.00	0.00	yes
TRINITY_DN18648_c0_g1	Methylcrotonyl-CoA carboxylase beta chain family protein [Populus trichocarpa]	MCCB	Methylcrotonoyl-CoA carboxylase beta chain, mitochondrial OS=Arabidopsis thaliana GN=MCCB PE=2 SV=1	22.90	15.67	1.13	up	0.00	0.00	yes
TRINITY_DN27763_c0_g2	translation initiation factor IF-2 family protein [Populus trichocarpa]	At1g17220	Translation initiation factor IF-2, chloroplastic OS=Arabidopsis thaliana GN=At1g17220 PE=2 SV=2	78.52	54.70	1.10	up	0.00	0.00	yes
TRINITY_DN18555_c0_g4	Ycf2 [Populus alba]	ycf2-A	Protein Ycf2 OS=Populus alba GN=ycf2-A PE=3 SV=1	3.15	0.93	2.33	up	0.00	0.00	yes
TRINITY_DN24658_c0_g1	hypothetical protein POPTR_0004s18030g [Populus trichocarpa]	APX3	L-ascorbate peroxidase 3, peroxisomal OS=Arabidopsis thaliana GN=APX3 PE=1 SV=1	976.13	588.02	1.35	up	0.00	0.00	yes
TRINITY_DN19819_c0_g1	hypothetical protein POPTR_0010s23850g [Populus trichocarpa]	DSP4	Phosphoglucan phosphatase DSP4, amyloplastic OS=Castanea sativa GN=DSP4 PE=1 SV=1	100.28	80.32	1.05	up	0.00	0.00	yes
TRINITY_DN21642_c0_g1	hypothetical protein POPTR_0009s02170g [Populus trichocarpa]	At5g22090	Protein FAF-like, chloroplastic OS=Arabidopsis thaliana GN=At5g22090 PE=2 SV=1	6.28	4.91	1.38	up	0.00	0.00	yes
TRINITY_DN23066_c0_g1	PREDICTED: probable ribosome biogenesis protein RLP24 [Populus euphratica]	At2g44860	Probable ribosome biogenesis protein RLP24 OS=Arabidopsis thaliana GN=At2g44860 PE=1 SV=1	52.34	39.06	1.07	up	0.00	0.00	yes
TRINITY_DN20750_c0_g4	PREDICTED: probable ribosome-binding factor A, chloroplastic [Populus euphratica]	At4g34730	Probable ribosome-binding factor A, chloroplastic OS=Arabidopsis thaliana GN=At4g34730 PE=2 SV=2	127.20	80.02	1.26	up	0.00	0.00	yes
TRINITY_DN14990_c0_g1	hypothetical protein POPTR_0013s13870g [Populus trichocarpa]	EPHX2	Bifunctional epoxide hydrolase 2 OS=Homo sapiens GN=EPHX2 PE=1 SV=2	20.13	9.04	1.78	up	0.00	0.00	yes
TRINITY_DN22470_c0_g1	oxidoreductase family protein [Populus trichocarpa]	GA2OX8	Gibberellin 2-beta-dioxygenase 8 OS=Arabidopsis thaliana GN=GA2OX8 PE=1 SV=2	61.30	40.18	1.20	up	0.00	0.00	yes
TRINITY_DN14132_c0_g1	hypothetical protein POPTR_0004s20320g [Populus trichocarpa]	TIC20-V	Protein TIC 20-v, chloroplastic OS=Arabidopsis thaliana GN=TIC20-V PE=2 SV=1	46.96	21.23	1.76	up	0.00	0.00	yes
TRINITY_DN17093_c0_g2	cytokinin response 1 family protein [Populus trichocarpa]	AHK4	Histidine kinase 4 OS=Arabidopsis thaliana GN=AHK4 PE=1 SV=1	2.05	0.40	2.92	up	0.00	0.00	yes
TRINITY_DN22292_c1_g3	PREDICTED: superoxide dismutase [Fe], chloroplastic isoform X1 [Populus euphratica]	FSD2	Superoxide dismutase [Fe] 2, chloroplastic OS=Arabidopsis thaliana GN=FSD2 PE=1 SV=1	351.06	231.45	1.19	up	0.00	0.00	yes

TRINITY_DN12743_c0_g1	hypothetical protein POPTR_0006s24180g [Populus trichocarpa]	PUB34	U-box domain-containing protein 34 OS=Arabidopsis thaliana GN=PUB34 PE=3 SV=1	2.99	1.40	1.79	up	0.00	0.00	yes
TRINITY_DN21802_c0_g1	hypothetical protein POPTR_0017s12470g [Populus trichocarpa]	CM1	Chorismate mutase 1, chloroplastic OS=Arabidopsis thaliana GN=CM1 PE=1 SV=3	144.20	95.20	1.24	up	0.00	0.00	yes
TRINITY_DN20660_c2_g1	hypothetical protein POPTR_0004s14560g [Populus trichocarpa]	Os05g0361200	Ferrochelatase-2, chloroplastic OS=Oryza sativa subsp. japonica GN=Os05g0361200 PE=2 SV=1	10.84	3.67	2.48	up	0.00	0.00	yes
TRINITY_DN14165_c0_g1	unknown [Populus trichocarpa]	hpt	Hypoxanthine-guanine phosphoribosyltransferase OS=Enterococcus faecalis (strain ATCC 700802 / V583) GN=hpt PE=3 SV=1	21.82	14.59	1.21	up	0.00	0.00	yes
TRINITY_DN26590_c0_g1	PREDICTED: acyl-CoA dehydrogenase family member 10-like isoform X2 [Populus euphratica]	IBR3	Probable acyl-CoA dehydrogenase IBR3 OS=Arabidopsis thaliana GN=IBR3 PE=1 SV=1	17.06	13.06	1.01	up	0.00	0.00	yes
TRINITY_DN15352_c1_g1	hypothetical protein POPTR_0006s15820g [Populus trichocarpa]	AMP1	Probable glutamate carboxypeptidase 2 OS=Arabidopsis thaliana GN=AMP1 PE=1 SV=3	2.43	0.89	2.04	up	0.00	0.00	yes
TRINITY_DN25667_c0_g1	PREDICTED: plasminogen activator inhibitor 1 RNA-binding protein-like isoform X2 [Populus euphratica]	RGGA	RGG repeats nuclear RNA binding protein A OS=Arabidopsis thaliana GN=RGGA PE=1 SV=1	202.35	140.58	1.14	up	0.00	0.00	yes
TRINITY_DN21121_c0_g1	PREDICTED: pentatricopeptide repeat-containing protein At5g66520-like [Populus euphratica]	PCMP-H38	Pentatricopeptide repeat-containing protein At5g48910 OS=Arabidopsis thaliana GN=PCMP-H38 PE=2 SV=1	18.30	12.37	1.14	up	0.00	0.00	yes
TRINITY_DN20820_c1_g4	hypothetical protein POPTR_0009s05010g [Populus trichocarpa]	At5g58770	Dehydrodolichyl diphosphate synthase 2 OS=Arabidopsis thaliana GN=At5g58770 PE=2 SV=2	35.10	25.40	1.07	up	0.00	0.00	yes
TRINITY_DN18265_c0_g1	PREDICTED: V-type proton ATPase subunit H isoform X2 [Populus euphratica]	VHA-H	V-type proton ATPase subunit H OS=Arabidopsis thaliana GN=VHA-H PE=1 SV=1	55.97	42.26	1.01	up	0.00	0.00	yes
TRINITY_DN21931_c0_g1	hypothetical protein POPTR_0011s12050g [Populus trichocarpa]	-	-	48.22	33.94	1.20	up	0.00	0.00	yes
TRINITY_DN26180_c0_g1	unknown [Populus trichocarpa]	RGGA	RGG repeats nuclear RNA binding protein A OS=Arabidopsis thaliana GN=RGGA PE=1 SV=1	295.36	220.27	1.03	up	0.00	0.00	yes
TRINITY_DN23656_c1_g7	pentatricopeptide repeat-containing family protein [Populus trichocarpa]	PCMP-H56	Pentatricopeptide repeat-containing protein At3g22690 OS=Arabidopsis thaliana GN=PCMP-H56 PE=2 SV=1	2.92	1.79	1.33	up	0.00	0.00	yes
TRINITY_DN23713_c1_g1	PREDICTED: ATP-dependent RNA helicase DHX36-like isoform X1 [Populus euphratica]	At1g48650	DExH-box ATP-dependent RNA helicase DExH3 OS=Arabidopsis thaliana GN=At1g48650 PE=2 SV=1	7.45	5.71	1.00	up	0.00	0.00	yes
TRINITY_DN15141_c0_g1	peptidase S41 family protein [Populus trichocarpa]	CTPA1	Carboxyl-terminal-processing peptidase 1, chloroplastic OS=Arabidopsis thaliana GN=CTPA1 PE=1 SV=1	15.82	12.20	1.05	up	0.00	0.00	yes
TRINITY_DN24958_c0_g1	Sugar carrier protein A [Populus trichocarpa]	STA	Sugar carrier protein A OS=Ricinus communis GN=STA PE=2 SV=1	17.18	10.82	1.19	up	0.00	0.00	yes
TRINITY_DN25062_c1_g8	phytanoyl-CoA dioxygenase family protein [Populus trichocarpa]	PAHX	Phytanoyl-CoA dioxygenase OS=Arabidopsis thaliana GN=PAHX PE=2 SV=2	43.85	31.82	1.06	up	0.00	0.00	yes
TRINITY_DN19583_c0_g3	PREDICTED: 60S ribosomal protein L37a [Ricinus communis]	RPL37A	60S ribosomal protein L37a OS=Gossypium hirsutum GN=RPL37A PE=3 SV=1	241.15	163.82	1.12	up	0.00	0.00	yes
TRINITY_DN19393_c0_g1	PREDICTED: uncharacterized protein LOC105117451 [Populus euphratica]	-	-	69.41	43.55	1.33	up	0.00	0.00	yes
TRINITY_DN23445_c1_g1	PREDICTED: F-box/kelch-repeat protein SKIP25-like [Populus euphratica]	SKIP25	F-box/kelch-repeat protein SKIP25 OS=Arabidopsis thaliana GN=SKIP25 PE=1 SV=1	2.94	1.34	1.75	up	0.00	0.00	yes
TRINITY_DN7787_c0_g1	PREDICTED: uncharacterized protein LOC105141219 [Populus euphratica]	-	-	3.35	1.62	1.86	up	0.00	0.00	yes
TRINITY_DN24296_c0_g5	putative CuZn-superoxide dismutase (chloroplast) [Populus tremula x Populus tremuloides]	SODCP	Superoxide dismutase [Cu-Zn], chloroplastic OS=Vitis vinifera GN=SODCP PE=2 SV=1	1020.62	621.71	1.30	up	0.00	0.00	yes
TRINITY_DN21650_c0_g3	-	-	-	2.40	0.73	2.31	up	0.00	0.00	yes
TRINITY_DN19823_c0_g2	hypothetical protein POPTR_0004s11580g [Populus trichocarpa]	C9orf85	Uncharacterized protein C9orf85 OS=Homo sapiens GN=C9orf85 PE=1 SV=1	31.67	23.60	1.06	up	0.00	0.00	yes
TRINITY_DN23823_c0_g1	PREDICTED: cellulose synthase-like protein E6 [Populus euphratica]	CSLE6	Cellulose synthase-like protein E6 OS=Oryza sativa subsp. japonica GN=CSLE6 PE=2 SV=1	29.77	17.92	1.36	up	0.00	0.00	yes
TRINITY_DN24151_c0_g1	hypothetical protein POPTR_0014s18440g [Populus trichocarpa]	-	-	41.01	26.39	1.23	up	0.00	0.00	yes
TRINITY_DN14423_c0_g1	subtilase family protein [Populus trichocarpa]	SBT1.9	Subtilisin-like protease SBT1.9 OS=Arabidopsis thaliana GN=SBT1.9 PE=2 SV=1	7.88	3.00	1.98	up	0.00	0.00	yes
TRINITY_DN24692_c0_g3	core region of GTP cyclohydrolase I family protein [Populus trichocarpa]	GCH1	GTP cyclohydrolase 1 OS=Solanum lycopersicum GN=GCH1 PE=1 SV=1	21.23	13.55	1.25	up	0.00	0.00	yes

TRINITY_DN15503_c0_g1	hypothetical protein POPTR_0016s10280g [Populus trichocarpa]	RE	Protein RETICULATA, chloroplastic OS=Arabidopsis thaliana GN=RE PE=1 SV=1	44.96	33.69	1.02	up	0.00	0.00	yes
TRINITY_DN23049_c0_g4	hypothetical protein POPTR_0030s00320g [Populus trichocarpa]	TSR2	Pre-rRNA-processing protein TSR2 homolog OS=Bos taurus GN=TSR2 PE=2 SV=1	36.30	24.29	1.24	up	0.00	0.00	yes
TRINITY_DN23239_c0_g3	hypothetical protein POPTR_0015s03440g [Populus trichocarpa]	At5g07830	Heparanase-like protein 1 OS=Arabidopsis thaliana GN=At5g07830 PE=2 SV=1	61.43	43.63	1.09	up	0.00	0.00	yes
TRINITY_DN26977_c1_g3	hypothetical protein POPTR_0015s15270g [Populus trichocarpa]	CPN60A2	Chaperonin 60 subunit alpha 2, chloroplastic OS=Arabidopsis thaliana GN=CPN60A2 PE=2 SV=1	17.42	12.03	1.29	up	0.00	0.00	yes
TRINITY_DN15492_c0_g1	apolipoprotein D [Populus trichocarpa]	CHL	Chloroplastic lipocalin OS=Arabidopsis thaliana GN=CHL PE=1 SV=1	71.80	46.67	1.22	up	0.00	0.00	yes
TRINITY_DN17111_c0_g1	hypothetical protein POPTR_0007s07260g [Populus trichocarpa]	RIBF2	FAD synthetase 2, chloroplastic OS=Arabidopsis thaliana GN=RIBF2 PE=1 SV=1	14.82	9.63	1.24	up	0.00	0.00	yes
TRINITY_DN15768_c0_g2	hypothetical protein POPTR_0001s23810g [Populus trichocarpa]	At3g19950	E3 ubiquitin-protein ligase RING1-like OS=Arabidopsis thaliana GN=At3g19950 PE=1 SV=1	19.62	13.18	1.18	up	0.00	0.00	yes
TRINITY_DN18849_c0_g1	PREDICTED: uncharacterized protein OsI_027940-like [Populus euphratica]	OsI_027940	Uncharacterized protein OsI_027940 OS=Oryza sativa subsp. indica GN=OsI_027940 PE=1 SV=2	131.23	99.42	1.02	up	0.00	0.00	yes
TRINITY_DN26022_c0_g1	PREDICTED: uncharacterized protein LOC105129218 [Populus euphratica]	-	-	90.57	62.11	1.13	up	0.00	0.00	yes
TRINITY_DN27465_c0_g1	PREDICTED: ATP synthase subunit alpha, mitochondrial [Populus euphratica]	ATPA	ATP synthase subunit alpha, mitochondrial OS=Phaseolus vulgaris GN=ATPA PE=3 SV=1	10.52	6.87	1.27	up	0.00	0.00	yes
TRINITY_DN16775_c0_g1	hypothetical protein POPTR_0012s07940g [Populus trichocarpa]	ATL48	RING-H2 finger protein ATL48 OS=Arabidopsis thaliana GN=ATL48 PE=1 SV=2	42.34	27.23	1.27	up	0.00	0.00	yes
TRINITY_DN25767_c0_g3	PREDICTED: isoamylase 3, chloroplastic-like isoform X2 [Populus euphratica]	ISA3	Isoamylase 3, chloroplastic OS=Arabidopsis thaliana GN=ISA3 PE=1 SV=2	46.51	33.16	1.00	up	0.00	0.00	yes
TRINITY_DN22894_c2_g1	hypothetical protein POPTR_0017s01860g [Populus trichocarpa]	At1g73230	Nascent polypeptide-associated complex subunit beta OS=Arabidopsis thaliana GN=At1g73230 PE=2 SV=1	342.46	251.09	1.06	up	0.00	0.00	yes
TRINITY_DN19084_c1_g1	-	-	-	541.38	383.67	1.11	up	0.00	0.00	yes
TRINITY_DN20530_c0_g8	plasma membrane H+ ATPase family protein [Populus trichocarpa]	PMA4	Plasma membrane ATPase 4 OS=Nicotiana plumbaginifolia GN=PMA4 PE=2 SV=1	3.65	3.21	2.89	up	0.00	0.00	yes
TRINITY_DN21044_c0_g2	hypothetical protein POPTR_0002s12540g, partial [Populus trichocarpa]	-	-	2.68	0.41	3.20	up	0.00	0.00	yes
TRINITY_DN24278_c2_g1	PREDICTED: uncharacterized protein LOC105112230 [Populus euphratica]	-	-	38.55	19.27	1.58	up	0.00	0.00	yes
TRINITY_DN20717_c0_g4	hypothetical protein EUGRSUZ_H02959 [Eucalyptus grandis]	RPL23A	60S ribosomal protein L23 OS=Arabidopsis thaliana GN=RPL23A PE=2 SV=3	123.63	87.58	1.13	up	0.00	0.00	yes
TRINITY_DN14369_c0_g2	PREDICTED: clustered mitochondria protein homolog [Populus euphratica]	-	-	90.77	61.03	1.19	up	0.00	0.00	yes
TRINITY_DN17705_c0_g2	hypothetical protein POPTR_0001s37860g [Populus trichocarpa]	PCMP-H12	Pentatricopeptide repeat-containing protein At1g08070, chloroplastic OS=Arabidopsis thaliana GN=PCMP-H12 PE=2 SV=1	8.10	5.27	1.24	up	0.00	0.00	yes
TRINITY_DN20681_c0_g1	PREDICTED: pentatricopeptide repeat-containing protein At3g49240 isoform X2 [Populus euphratica]	EMB1796	Pentatricopeptide repeat-containing protein At3g49240 OS=Arabidopsis thaliana GN=EMB1796 PE=2 SV=1	24.34	16.25	1.21	up	0.00	0.00	yes
TRINITY_DN14283_c0_g1	hypothetical protein POPTR_0018s11150g [Populus trichocarpa]	TSS	Protein TSS OS=Arabidopsis thaliana GN=TSS PE=1 SV=1	36.68	15.37	1.82	up	0.00	0.00	yes
TRINITY_DN20113_c0_g3	hypothetical protein POPTR_0007s14280g [Populus trichocarpa]	At1g04910	Uncharacterized protein At1g04910 OS=Arabidopsis thaliana GN=At1g04910 PE=2 SV=1	91.83	69.21	1.02	up	0.00	0.00	yes
TRINITY_DN20587_c0_g4	hypothetical protein POPTR_0001s30780g [Populus trichocarpa]	TIC21	Protein TIC 21, chloroplastic OS=Arabidopsis thaliana GN=TIC21 PE=1 SV=1	45.41	33.82	1.03	up	0.00	0.00	yes
TRINITY_DN24150_c3_g4	F-box family protein [Populus trichocarpa]	At2g39490	F-box protein At2g39490 OS=Arabidopsis thaliana GN=At2g39490 PE=2 SV=1	4.25	1.71	1.97	up	0.00	0.00	yes
TRINITY_DN23463_c0_g3	polyphenol oxidase [Populus tremuloides]	-	Polyphenol oxidase, chloroplastic OS=Malus domestica PE=2 SV=1	153.33	73.81	1.61	up	0.00	0.00	yes
TRINITY_DN22629_c0_g2	PREDICTED: 2-isopropylmalate synthase 2, chloroplastic-like [Populus euphratica]	IPMS2	2-isopropylmalate synthase 2, chloroplastic OS=Arabidopsis thaliana GN=IPMS2 PE=1 SV=1	65.58	50.13	1.01	up	0.00	0.00	yes
TRINITY_DN22135_c0_g4	RNA-binding family protein [Populus trichocarpa]	RBG2	Glycine-rich RNA-binding protein 2, mitochondrial OS=Arabidopsis thaliana GN=RBG2 PE=1 SV=1	39.18	32.64	1.22	up	0.00	0.00	yes

TRINITY_DN26209_c0_g1	hypothetical protein SETIT_022310mg [Setaria italica]	FBPban1	Fructose-1,6-bisphosphatase, cytosolic OS=Musa acuminata GN=FBPban1 PE=2 SV=1	186.50	89.03	1.84	up	0.00	0.00	yes
TRINITY_DN19085_c0_g1	calmodulin 2 family protein [Populus trichocarpa]	CURT1A	Protein CURVATURE THYLAKOID 1A, chloroplastic OS=Arabidopsis thaliana GN=CURT1A PE=1 SV=1	1002.22	568.00	1.40	up	0.00	0.00	yes
TRINITY_DN24677_c0_g1	hypothetical protein POPTR_0015s10770g [Populus trichocarpa]	RPL21M	50S ribosomal protein L21, mitochondrial OS=Arabidopsis thaliana GN=RPL21M PE=2 SV=1	29.27	23.21	1.03	up	0.00	0.00	yes
TRINITY_DN18425_c0_g1	PREDICTED: coatomer subunit zeta-2-like [Populus euphratica]	At3g09800	Coatomer subunit zeta-2 OS=Arabidopsis thaliana GN=At3g09800 PE=2 SV=1	76.03	54.48	1.09	up	0.00	0.00	yes
TRINITY_DN18761_c1_g3	PREDICTED: uncharacterized protein LOC105112681 isoform X2 [Populus euphratica]	-	-	26.77	18.00	1.24	up	0.00	0.00	yes
TRINITY_DN22607_c0_g2	ROP-INTERACTIVE CRIB MOTIF-CONTAINING protein 10 [Populus trichocarpa]	RIC10	CRIB domain-containing protein RIC10 OS=Arabidopsis thaliana GN=RIC10 PE=2 SV=1	8.28	4.28	1.54	up	0.00	0.00	yes
TRINITY_DN17716_c0_g1	PREDICTED: pentatricopeptide repeat-containing protein At3g46610 [Populus euphratica]	At3g46610	Pentatricopeptide repeat-containing protein At3g46610 OS=Arabidopsis thaliana GN=At3g46610 PE=2 SV=1	20.00	14.53	1.05	up	0.00	0.00	yes
TRINITY_DN24648_c1_g1	hypothetical protein POPTR_0005s12850g [Populus trichocarpa]	OST48	Dolichyl-diphosphooligosaccharide--protein glycosyltransferase 48 kDa subunit OS=Arabidopsis thaliana GN=OST48 PE=2 SV=1	99.94	79.78	1.01	up	0.00	0.00	yes
TRINITY_DN20350_c0_g1	hypothetical protein POPTR_0009s12570g [Populus trichocarpa]	DTX46	Protein DETOXIFICATION 46, chloroplastic OS=Arabidopsis thaliana GN=DTX46 PE=2 SV=1	14.62	8.50	1.09	up	0.00	0.00	yes
TRINITY_DN17042_c0_g2	hypothetical protein POPTR_0014s16130g [Populus trichocarpa]	At2g04740	BTB/POZ domain-containing protein At2g04740 OS=Arabidopsis thaliana GN=At2g04740 PE=2 SV=2	27.20	17.87	1.22	up	0.00	0.00	yes
TRINITY_DN24046_c1_g1	PREDICTED: transcription factor bHLH77-like [Populus euphratica]	BHLH62	Transcription factor bHLH62 OS=Arabidopsis thaliana GN=BHLH62 PE=2 SV=1	12.83	8.62	1.19	up	0.00	0.00	yes
TRINITY_DN22836_c0_g5	PREDICTED: probable lactoylglutathione lyase, chloroplast [Populus euphratica]	At1g67280	Probable lactoylglutathione lyase, chloroplastic OS=Arabidopsis thaliana GN=At1g67280 PE=1 SV=1	35.98	22.20	1.29	up	0.00	0.00	yes
TRINITY_DN19088_c0_g1	peroxiredoxin family protein [Populus trichocarpa]	PRXIIB	Peroxiredoxin-2B OS=Arabidopsis thaliana GN=PRXIIB PE=1 SV=1	393.76	285.85	1.09	up	0.00	0.00	yes
TRINITY_DN16139_c0_g2	hypothetical protein POPTR_0005s27650g [Populus trichocarpa]	OEP21B	Outer envelope pore protein 21B, chloroplastic OS=Arabidopsis thaliana GN=OEP21B PE=2 SV=1	122.53	85.34	1.12	up	0.00	0.00	yes
TRINITY_DN26324_c0_g1	hypothetical protein POPTR_0012s04160g [Populus trichocarpa]	XA21	Receptor kinase-like protein Xa21 OS=Oryza sativa subsp. japonica GN=XA21 PE=1 SV=1	6.71	2.72	1.62	up	0.00	0.00	yes
TRINITY_DN15523_c0_g1	PREDICTED: reticulon-like protein B12 [Populus euphratica]	RTNLB12	Reticulon-like protein B12 OS=Arabidopsis thaliana GN=RTNLB12 PE=2 SV=1	14.14	9.19	1.29	up	0.00	0.00	yes
TRINITY_DN16310_c0_g1	PREDICTED: 39S ribosomal protein L28, mitochondrial isoform X1 [Populus euphratica]	rpmB	50S ribosomal protein L28 OS=Magnetococcus marinus (strain ATCC BAA-1437 / JCM 17883 / MC-1) GN=rpmB PE=3 SV=1	26.57	19.47	1.08	up	0.00	0.00	yes
TRINITY_DN25473_c2_g3	hypothetical protein POPTR_0006s13180g [Populus trichocarpa]	-	-	45.02	17.60	1.95	up	0.00	0.00	yes
TRINITY_DN15512_c0_g1	hypothetical protein POPTR_0016s07800g [Populus trichocarpa]	-	-	18.06	13.11	1.06	up	0.00	0.00	yes
TRINITY_DN22205_c0_g1	nucleolar essential family protein [Populus trichocarpa]	Y39A1A.14	Ribosomal RNA small subunit methyltransferase nep-1 OS=Caenorhabditis elegans GN=Y39A1A.14 PE=3 SV=1	61.19	46.60	1.02	up	0.00	0.00	yes
TRINITY_DN19027_c1_g1	hypothetical protein POPTR_0002s17590g [Populus trichocarpa]	-	-	8.55	5.39	1.86	up	0.00	0.00	yes
TRINITY_DN17464_c0_g2	hydrolase family protein [Populus trichocarpa]	D14	Strigolactone esterase D14 OS=Oryza sativa subsp. japonica GN=D14 PE=1 SV=1	68.37	46.14	1.19	up	0.00	0.00	yes
TRINITY_DN21393_c0_g2	unknown [Populus trichocarpa]	RPS7A	40S ribosomal protein S7-1 OS=Arabidopsis thaliana GN=RPS7A PE=2 SV=1	27.58	21.40	1.01	up	0.00	0.00	yes
TRINITY_DN27585_c1_g1	serine carboxypeptidase S10 family protein [Populus trichocarpa]	SCPL18	Serine carboxypeptidase-like 18 OS=Arabidopsis thaliana GN=SCPL18 PE=2 SV=2	76.41	37.45	1.25	up	0.00	0.00	yes
TRINITY_DN17583_c0_g2	succinate dehydrogenase subunit 4 family protein [Populus trichocarpa]	At2g39795	Uncharacterized protein At2g39795, mitochondrial OS=Arabidopsis thaliana GN=At2g39795 PE=1 SV=1	21.63	14.23	1.24	up	0.00	0.00	yes
TRINITY_DN18343_c0_g2	IAA-amino acid hydrolase 1 family protein [Populus trichocarpa]	ILR1	IAA-amino acid hydrolase ILR1 OS=Arabidopsis thaliana GN=ILR1 PE=1 SV=2	5.55	3.28	1.37	up	0.00	0.00	yes
TRINITY_DN26881_c1_g1	PREDICTED: uncharacterized protein LOC105112215 [Populus euphratica]	At3g02060	ATP-dependent DNA helicase At3g02060, chloroplastic OS=Arabidopsis thaliana GN=At3g02060 PE=2 SV=1	88.96	53.75	1.20	up	0.00	0.00	yes
TRINITY_DN17867_c1_g2	rRNA processing protein EBP2 [Populus trichocarpa]	EBP2	Probable rRNA-processing protein EBP2 homolog OS=Arabidopsis thaliana GN=EBP2 PE=1 SV=1	36.54	26.21	1.12	up	0.00	0.00	yes

TRINITY_DN19164_c0_g3	Mitochondrial import inner membrane translocase subunit Tim10 family protein [Populus trichocarpa]	TIM10	Mitochondrial import inner membrane translocase subunit TIM10 OS=Arabidopsis thaliana GN=TIM10 PE=1 SV=1	106.69	77.60	1.10	up	0.00	0.00	yes
TRINITY_DN24629_c0_g5	PREDICTED: arginine--tRNA ligase, cytoplasmic-like isoform X1 [Populus euphratica]	EMB1027	Arginine--tRNA ligase, chloroplastic/mitochondrial OS=Arabidopsis thaliana GN=EMB1027 PE=1 SV=1	55.80	43.32	1.02	up	0.00	0.00	yes
TRINITY_DN13430_c0_g1	hypothetical protein POPTR_0005s11710g [Populus trichocarpa]	-	-	169.97	96.38	1.42	up	0.00	0.00	yes
TRINITY_DN22007_c0_g1	hypothetical protein POPTR_0018s06580g [Populus trichocarpa]	-	-	112.04	78.04	1.13	up	0.00	0.00	yes
TRINITY_DN15895_c0_g2	-	-	-	13.61	4.30	2.27	up	0.00	0.00	yes
TRINITY_DN25133_c1_g10	PREDICTED: copper-transporting ATPase PAA1, chloroplastic-like [Populus euphratica]	PAA1	Copper-transporting ATPase PAA1, chloroplastic OS=Arabidopsis thaliana GN=PAA1 PE=2 SV=1	24.54	17.17	1.15	up	0.00	0.00	yes
TRINITY_DN23973_c0_g1	hypothetical protein POPTR_0013s06790g [Populus trichocarpa]	-	-	81.63	51.61	1.24	up	0.00	0.00	yes
TRINITY_DN26455_c3_g3	-	-	-	4.42	1.19	2.53	up	0.00	0.00	yes
TRINITY_DN23993_c0_g1	PREDICTED: formyltetrahydrofolate deformylase 1, mitochondrial-like isoform X3 [Populus euphratica]	PURU1	Formyltetrahydrofolate deformylase 1, mitochondrial OS=Arabidopsis thaliana GN=PURU1 PE=1 SV=1	44.23	28.78	1.14	up	0.00	0.00	yes
TRINITY_DN27089_c0_g1	PREDICTED: imidazole glycerol phosphate synthase hisHF, chloroplastic-like isoform X1 [Populus euphratica]	HISN4	Imidazole glycerol phosphate synthase hisHF, chloroplastic OS=Arabidopsis thaliana GN=HISN4 PE=2 SV=1	33.93	31.04	1.00	up	0.00	0.00	yes
TRINITY_DN18554_c0_g1	hypothetical protein POPTR_0007s11880g [Populus trichocarpa]	RPL10AA	60S ribosomal protein L10a-1 OS=Arabidopsis thaliana GN=RPL10AA PE=1 SV=1	182.04	134.60	1.06	up	0.00	0.00	yes
TRINITY_DN19437_c0_g3	hypothetical protein POPTR_0016s09990g [Populus trichocarpa]	-	-	51.84	36.40	1.15	up	0.00	0.00	yes
TRINITY_DN25133_c1_g7	hypothetical protein POPTR_0003s01860g [Populus trichocarpa]	PAA1	Copper-transporting ATPase PAA1, chloroplastic OS=Arabidopsis thaliana GN=PAA1 PE=2 SV=1	15.41	11.21	1.10	up	0.00	0.00	yes
TRINITY_DN24109_c1_g4	heat shock protein 70 [Saussurea medusa]	HSC-2	Heat shock cognate 70 kDa protein 2 OS=Solanum lycopersicum GN=HSC-2 PE=2 SV=1	466.89	227.77	1.75	up	0.00	0.00	yes
TRINITY_DN27292_c1_g1	hypothetical protein POPTR_0015s12190g [Populus trichocarpa]	FSD2	Superoxide dismutase [Fe] 2, chloroplastic OS=Arabidopsis thaliana GN=FSD2 PE=1 SV=1	53.97	35.99	1.16	up	0.00	0.00	yes
TRINITY_DN16889_c0_g1	PREDICTED: ABC transporter I family member 10, chloroplastic-like [Populus euphratica]	ABCI10	ABC transporter I family member 10, chloroplastic OS=Arabidopsis thaliana GN=ABCI10 PE=2 SV=1	35.50	25.35	1.08	up	0.00	0.00	yes
TRINITY_DN19714_c0_g1	PREDICTED: uncharacterized protein LOC105109943 [Populus euphratica]	truB	tRNA pseudouridine synthase B OS=Flavobacterium johnsoniae (strain ATCC 17061 / DSM 2064 / UW101) GN=truB PE=3 SV=1	44.68	33.33	1.04	up	0.00	0.00	yes
TRINITY_DN14329_c0_g3	Potassium channel SKOR family protein [Populus trichocarpa]	SKOR	Potassium channel SKOR OS=Arabidopsis thaliana GN=SKOR PE=1 SV=1	2.53	0.73	2.43	up	0.00	0.00	yes
TRINITY_DN20779_c0_g1	PREDICTED: pentatricopeptide repeat-containing protein At3g26630, chloroplastic [Populus euphratica]	PCMP-A6	Pentatricopeptide repeat-containing protein At3g26630, chloroplastic OS=Arabidopsis thaliana GN=PCMP-A6 PE=2 SV=1	8.48	6.51	1.18	up	0.00	0.00	yes
TRINITY_DN22956_c0_g3	galactosyltransferase family protein [Populus trichocarpa]	B3GALT14	Probable beta-1,3-galactosyltransferase 14 OS=Arabidopsis thaliana GN=B3GALT14 PE=2 SV=1	2.97	0.88	2.30	up	0.00	0.00	yes
TRINITY_DN26565_c0_g1	hypothetical protein POPTR_0005s19990g [Populus trichocarpa]	LACS9	Long chain acyl-CoA synthetase 9, chloroplastic OS=Arabidopsis thaliana GN=LACS9 PE=1 SV=1	60.48	36.43	1.03	up	0.00	0.00	yes
TRINITY_DN23151_c0_g1	PREDICTED: cytochrome P450 CYP82D47-like [Populus euphratica]	CYP82C4	Cytochrome P450 82C4 OS=Arabidopsis thaliana GN=CYP82C4 PE=2 SV=1	10.65	5.56	1.61	up	0.00	0.00	yes
TRINITY_DN11993_c0_g1	hypothetical protein POPTR_0014s01280g [Populus trichocarpa]	CBSDUF3	DUF21 domain-containing protein At2g14520 OS=Arabidopsis thaliana GN=CBSDUF3 PE=2 SV=2	2.15	0.80	2.02	up	0.00	0.00	yes
TRINITY_DN26658_c0_g2	hypothetical protein POPTR_0001s32510g [Populus trichocarpa]	METTL13	Methyltransferase-like protein 13 OS=Bos taurus GN=METTL13 PE=2 SV=1	7.93	5.87	1.05	up	0.00	0.00	yes
TRINITY_DN21884_c0_g1	hypothetical protein POPTR_0005s08280g [Populus trichocarpa]	MT1988	Epoxide hydrolase B OS=Mycobacterium tuberculosis (strain CDC 1551 / Oshkosh) GN=MT1988 PE=1 SV=2	132.32	84.75	1.24	up	0.00	0.00	yes
TRINITY_DN20012_c0_g1	PREDICTED: alternative NAD(P)H-ubiquinone oxidoreductase C1, chloroplastic/mitochondrial isoform X2 [Populus euphratica]	NDC1	Alternative NAD(P)H-ubiquinone oxidoreductase C1, chloroplastic/mitochondrial OS=Arabidopsis thaliana GN=NDC1 PE=1 SV=2	35.98	24.49	1.16	up	0.00	0.00	yes

TRINITY_DN24709_c0_g1	PREDICTED: pyruvate dehydrogenase E1 component subunit beta-3, chloroplastic-like [Populus euphratica]	E1-BETA-2	Pyruvate dehydrogenase E1 component subunit beta-3, chloroplastic OS=Arabidopsis thaliana GN=E1-BETA-2 PE=2 SV=1	176.40	133.88	1.03	up	0.00	0.00	yes
TRINITY_DN21010_c0_g1	hypothetical protein POPTR_0013s00790g [Populus trichocarpa]	PPD1	PsbP domain-containing protein 1, chloroplastic OS=Arabidopsis thaliana GN=PPD1 PE=1 SV=1	87.68	59.57	1.17	up	0.00	0.00	yes
TRINITY_DN18196_c0_g1	pyridine nucleotide-disulfide oxidoreductase family protein [Populus trichocarpa]	aifB	Apoptosis-inducing factor homolog B OS=Dictyostelium discoideum GN=aifB PE=3 SV=1	9.81	6.47	1.21	up	0.00	0.00	yes
TRINITY_DN27804_c3_g1	PREDICTED: uncharacterized protein LOC105107686 [Populus euphratica]	SPP	Stromal processing peptidase, chloroplastic OS=Arabidopsis thaliana GN=SPP PE=2 SV=1	33.87	24.84	1.05	up	0.00	0.00	yes
TRINITY_DN17742_c0_g2	PREDICTED: quinolinate synthase, chloroplastic [Populus euphratica]	QS	Quinolinate synthase, chloroplastic OS=Arabidopsis thaliana GN=QS PE=1 SV=1	11.15	6.24	1.46	up	0.00	0.00	yes
TRINITY_DN26554_c0_g2	hypothetical protein POPTR_0010s17680g [Populus trichocarpa]	-	-	38.73	23.36	1.33	up	0.00	0.00	yes
TRINITY_DN20767_c0_g2	hypothetical protein POPTR_0008s01880g [Populus trichocarpa]	NUDT26	Nudix hydrolase 26, chloroplastic OS=Arabidopsis thaliana GN=NUDT26 PE=1 SV=1	39.99	29.80	1.05	up	0.00	0.00	yes
TRINITY_DN26302_c0_g1	Glycyl-tRNA synthetase family protein [Populus trichocarpa]	At1g29880	Glycine--tRNA ligase, mitochondrial 1 OS=Arabidopsis thaliana GN=At1g29880 PE=1 SV=1	49.36	36.52	1.03	up	0.00	0.00	yes
TRINITY_DN19791_c1_g5	hypothetical protein POPTR_0018s06830g [Populus trichocarpa]	-	-	360.41	216.05	1.34	up	0.00	0.00	yes
TRINITY_DN27587_c1_g1	hypothetical protein POPTR_0012s10770g [Populus trichocarpa]	CD4B	ATP-dependent Clp protease ATP-binding subunit ClpA homolog CD4B, chloroplastic OS=Solanum lycopersicum GN=CD4B PE=3 SV=1	781.57	534.86	1.14	up	0.00	0.00	yes
TRINITY_DN22238_c0_g3	hypothetical protein POPTR_0014s16700g [Populus trichocarpa]	IGPS	Indole-3-glycerol phosphate synthase, chloroplastic OS=Arabidopsis thaliana GN=IGPS PE=1 SV=2	59.72	45.45	1.04	up	0.00	0.00	yes
TRINITY_DN24845_c0_g1	hypothetical protein POPTR_0006s11810g [Populus trichocarpa]	CSLA9	Glucomannan 4-beta-mannosyltransferase 9 OS=Arabidopsis thaliana GN=CSLA9 PE=2 SV=1	38.44	24.66	1.17	up	0.00	0.00	yes
TRINITY_DN27311_c0_g1	hypothetical protein POPTR_0002s02440g [Populus trichocarpa]	-	-	18.87	9.61	1.55	up	0.00	0.00	yes
TRINITY_DN23702_c0_g1	hypothetical protein POPTR_0001s07480g [Populus trichocarpa]	-	Putative glucose-6-phosphate 1-epimerase OS=Cenchrus ciliaris PE=2 SV=1	45.77	26.38	1.17	up	0.00	0.00	yes
TRINITY_DN20776_c0_g1	PREDICTED: heterogeneous nuclear ribonucleoprotein 1-like [Populus euphratica]	RNP1	Heterogeneous nuclear ribonucleoprotein 1 OS=Arabidopsis thaliana GN=RNP1 PE=1 SV=1	33.67	26.31	1.04	up	0.00	0.00	yes
TRINITY_DN15693_c0_g1	hypothetical protein POPTR_0015s07560g [Populus trichocarpa]	-	-	5.82	3.50	1.36	up	0.00	0.00	yes
TRINITY_DN14369_c0_g1	hypothetical protein POPTR_0014s09410g [Populus trichocarpa]	-	-	155.18	100.91	1.21	up	0.00	0.00	yes
TRINITY_DN18970_c0_g6	PREDICTED: uncharacterized protein LOC105122137 [Populus euphratica]	-	-	613.02	329.96	1.49	up	0.00	0.00	yes
TRINITY_DN24328_c0_g1	PREDICTED: uncharacterized protein LOC105117914 [Populus euphratica]	PNSB4	Photosynthetic NDH subunit of subcomplex B 4, chloroplastic OS=Arabidopsis thaliana GN=PNSB4 PE=2 SV=1	575.57	296.65	1.52	up	0.00	0.00	yes
TRINITY_DN15895_c0_g1	-	-	-	13.86	5.06	2.06	up	0.00	0.00	yes
TRINITY_DN26427_c2_g3	hypothetical protein POPTR_0008s10210g [Populus trichocarpa]	PAPS3	Nuclear poly(A) polymerase 3 OS=Arabidopsis thaliana GN=PAPS3 PE=1 SV=1	4.96	2.95	1.30	up	0.00	0.00	yes
TRINITY_DN15159_c0_g1	hypothetical protein POPTR_0004s00950g, partial [Populus trichocarpa]	PMP22	Peroxisomal membrane protein PMP22 OS=Arabidopsis thaliana GN=PMP22 PE=1 SV=1	62.31	40.95	1.10	up	0.00	0.00	yes
TRINITY_DN17326_c0_g1	ribosomal protein L12 [Populus trichocarpa]	rplL	50S ribosomal protein L7/L12 OS=Liberibacter africanus GN=rplL PE=3 SV=1	63.54	45.90	1.10	up	0.00	0.00	yes
TRINITY_DN18792_c1_g4	photosystem II protein D (chloroplast) [Ficus racemosa]	psbA	Photosystem II protein D1 OS=Eucalyptus globulus subsp. globulus GN=psbA PE=3 SV=1	141.61	58.77	1.93	up	0.00	0.00	yes
TRINITY_DN25083_c2_g3	hypothetical protein POPTR_0001s31270g [Populus trichocarpa]	POPTRDRAFT_[illegible]	Translation factor GUF1 homolog, chloroplastic OS=Populus trichocarpa GN=POPTRDRAFT_815670 PE=3 SV=2	51.17	33.03	1.16	up	0.00	0.00	yes
TRINITY_DN20349_c0_g2	PREDICTED: myb-related protein Myb4-like [Populus euphratica]	MYB4	Myb-related protein Myb4 OS=Oryza sativa subsp. japonica GN=MYB4 PE=2 SV=2	24.94	12.17	1.47	up	0.00	0.00	yes
TRINITY_DN25129_c1_g2	3-phosphoglycerate kinase [Populus tremuloides]	-	Phosphoglycerate kinase, cytosolic OS=Nicotiana tabacum PE=2 SV=1	153.72	116.35	1.01	up	0.00	0.00	yes
TRINITY_DN16605_c0_g1	oxidoreductase family protein [Populus trichocarpa]	DLO2	Protein DMR6-LIKE OXYGENASE 2 OS=Arabidopsis thaliana GN=DLO2 PE=2 SV=1	3.89	1.45	2.02	up	0.00	0.00	yes

TRINITY_DN17059_c0_g2	UDP-glucose pyrophosphorylase [Populus deltoides]	UGP2	UTP--glucose-1-phosphate uridylyltransferase 2 OS=Arabidopsis thaliana GN=UGP2 PE=1 SV=1	101.22	72.02	1.13	up	0.00	0.00	yes
TRINITY_DN16624_c0_g1	unknown [Populus trichocarpa x Populus deltoides]	FKBP15-1	Peptidyl-prolyl cis-trans isomerase FKBP15-1 OS=Arabidopsis thaliana GN=FKBP15-1 PE=2 SV=2	184.06	137.19	1.03	up	0.00	0.00	yes
TRINITY_DN18475_c0_g1	hypothetical protein POPTR_0014s05560g [Populus trichocarpa]	-	-	31.41	19.04	1.41	up	0.00	0.00	yes
TRINITY_DN26275_c1_g1	hypothetical protein POPTR_0019s09060g [Populus trichocarpa]	Gspt1	Eukaryotic peptide chain release factor GTP-binding subunit ERF3A OS=Mus musculus GN=Gspt1 PE=1 SV=2	77.76	62.55	1.01	up	0.00	0.00	yes
TRINITY_DN14131_c0_g2	hypothetical protein POPTR_0006s19380g [Populus trichocarpa]	TSS	Protein TSS OS=Arabidopsis thaliana GN=TSS PE=1 SV=1	51.91	28.88	1.42	up	0.00	0.00	yes
TRINITY_DN15121_c0_g2	PREDICTED: L-type lectin-domain containing receptor kinase S.4-like isoform X2 [Populus euphratica]	LECRKS4	L-type lectin-domain containing receptor kinase S.4 OS=Arabidopsis thaliana GN=LECRKS4 PE=2 SV=1	3.09	1.59	1.57	up	0.00	0.00	yes
TRINITY_DN21602_c0_g2	hypothetical protein POPTR_0016s02190g [Populus trichocarpa]	UGT85A24	7-deoxyloganetin glucosyltransferase OS=Gardenia jasminoides GN=UGT85A24 PE=1 SV=1	3.61	0.96	2.46	up	0.00	0.00	yes
TRINITY_DN26675_c0_g1	PREDICTED: protein TIC 62, chloroplastic isoform X1 [Populus euphratica]	TIC62	Protein TIC 62, chloroplastic OS=Pisum sativum GN=TIC62 PE=1 SV=2	125.21	64.98	1.53	up	0.00	0.00	yes
TRINITY_DN26216_c0_g4	photosystem I P700 apoprotein A2 [Populus alba]	psaB	Photosystem I P700 chlorophyll a apoprotein A2 OS=Populus alba GN=psaB PE=3 SV=1	13.34	5.38	1.98	up	0.00	0.00	yes
TRINITY_DN19963_c0_g1	hypothetical protein POPTR_0008s00510g [Populus trichocarpa]	-	-	9.90	4.87	1.39	up	0.00	0.00	yes
TRINITY_DN25335_c0_g4	ascorbate oxidase precursor family protein [Populus trichocarpa]	AAO	L-ascorbate oxidase OS=Nicotiana tabacum GN=AAO PE=2 SV=1	2.96	1.21	1.91	up	0.00	0.00	yes
TRINITY_DN21065_c0_g1	hypothetical protein POPTR_0006s15500g [Populus trichocarpa]	-	-	78.59	51.58	1.24	up	0.00	0.00	yes
TRINITY_DN26497_c0_g2	hypothetical protein POPTR_0011s07381g [Populus trichocarpa]	Os01g0208600	SCAR-like protein 2 OS=Oryza sativa subsp. japonica GN=Os01g0208600 PE=2 SV=1	1.66	0.60	2.05	up	0.00	0.00	yes
TRINITY_DN19756_c0_g2	hypothetical protein POPTR_0017s00450g [Populus trichocarpa]	MLO1	MLO-like protein 1 OS=Arabidopsis thaliana GN=MLO1 PE=1 SV=1	24.42	18.32	1.05	up	0.00	0.00	yes
TRINITY_DN25363_c0_g1	hypothetical protein POPTR_0009s07790g [Populus trichocarpa]	FKFBP	6-phosphofructo-2-kinase/fructose-2,6-bisphosphatase OS=Arabidopsis thaliana GN=FKFBP PE=1 SV=1	26.30	18.23	1.07	up	0.00	0.00	yes
TRINITY_DN20348_c0_g2	4-coumarate: coenzyme A ligase 4 [Populus tomentosa]	4CL	4-coumarate--CoA ligase OS=Vanilla planifolia GN=4CL PE=3 SV=1	41.80	31.54	1.31	up	0.00	0.00	yes
TRINITY_DN19590_c0_g1	hypothetical protein POPTR_0002s15600g [Populus trichocarpa]	RPL5	50S ribosomal protein L5, chloroplastic OS=Arabidopsis thaliana GN=RPL5 PE=2 SV=1	388.73	254.29	1.19	up	0.00	0.00	yes
TRINITY_DN16185_c1_g1	hypothetical protein POPTR_0001s16970g [Populus trichocarpa]	WER	Transcription factor WER OS=Arabidopsis thaliana GN=WER PE=1 SV=1	14.46	6.11	1.66	up	0.00	0.00	yes
TRINITY_DN17108_c0_g1	phospholipase/carboxylesterase family protein [Populus trichocarpa]	AN8748	Acyl-protein thioesterase 1 OS=Emericella nidulans (strain FGSC A4 / ATCC 38163 / CBS 112.46 / NRRL 194 / M139) GN=AN8748 PE=3 SV=1	3.37	1.26	2.12	up	0.00	0.00	yes
TRINITY_DN26069_c0_g1	putative pectin methylesterase [Populus tremula x Populus tremuloides]	PMEU1	Pectinesterase/pectinesterase inhibitor U1 OS=Solanum lycopersicum GN=PMEU1 PE=2 SV=1	104.43	71.85	1.07	up	0.00	0.00	yes
TRINITY_DN24794_c0_g1	PREDICTED: probable tocopherol O-methyltransferase, chloroplastic isoform X1 [Populus euphratica]	VTE4	Probable tocopherol O-methyltransferase, chloroplastic OS=Oryza sativa subsp. japonica GN=VTE4 PE=2 SV=1	86.31	69.39	1.03	up	0.00	0.00	yes
TRINITY_DN15191_c0_g1	hypothetical protein POPTR_0007s13210g [Populus trichocarpa]	RH16	DEAD-box ATP-dependent RNA helicase 16 OS=Arabidopsis thaliana GN=RH16 PE=2 SV=1	8.75	5.83	1.23	up	0.00	0.00	yes
TRINITY_DN17265_c0_g1	hypothetical protein POPTR_0001s23730g [Populus trichocarpa]	-	-	36.23	22.79	1.25	up	0.00	0.00	yes
TRINITY_DN26522_c0_g1	hypothetical protein POPTR_0010s19320g [Populus trichocarpa]	UGP3	UTP--glucose-1-phosphate uridylyltransferase 3, chloroplastic OS=Arabidopsis thaliana GN=UGP3 PE=1 SV=1	28.97	20.34	1.05	up	0.00	0.00	yes
TRINITY_DN22716_c0_g1	CPRD49 family protein [Populus trichocarpa]	CPRD49	GDSL esterase/lipase CPRD49 OS=Arabidopsis thaliana GN=CPRD49 PE=2 SV=1	24.18	12.58	1.59	up	0.00	0.00	yes
TRINITY_DN23732_c1_g2	cytochrome P450 family protein [Populus trichocarpa]	CYP82G1	Cytochrome P450 82G1 OS=Arabidopsis thaliana GN=CYP82G1 PE=1 SV=1	2.23	0.83	2.06	up	0.00	0.00	yes
TRINITY_DN17569_c0_g1	hypothetical protein POPTR_0008s10880g [Populus trichocarpa]	-	-	31.74	14.40	1.52	up	0.00	0.00	yes

TRINITY_DN20268_c0_g1	PREDICTED: transmembrane emp24 domain-containing protein p24delta9-like [Populus euphratica]	At1g26690	Transmembrane emp24 domain-containing protein p24delta9 OS=Arabidopsis thaliana GN=At1g26690 PE=2 SV=1	36.50	29.96	1.03	up	0.00	0.00	yes
TRINITY_DN14945_c0_g1	PREDICTED: magnesium-chelatase subunit ChlH, chloroplastic-like [Populus euphratica]	CHLH	Magnesium-chelatase subunit ChlH, chloroplastic OS=Arabidopsis thaliana GN=CHLH PE=1 SV=1	294.88	199.79	1.18	up	0.00	0.00	yes
TRINITY_DN3436_c0_g1	hypothetical protein POPTR_0011s07560g [Populus trichocarpa]	-	-	9.50	5.95	1.28	up	0.00	0.00	yes
TRINITY_DN20595_c0_g2	hypothetical protein POPTR_0010s00730g [Populus trichocarpa]	RH50	DEAD-box ATP-dependent RNA helicase 50 OS=Arabidopsis thaliana GN=RH50 PE=2 SV=2	66.67	41.07	1.02	up	0.00	0.00	yes
TRINITY_DN15453_c0_g1	hypothetical protein POPTR_0006s06590g [Populus trichocarpa]	At3g15810	Protein LURP-one-related 12 OS=Arabidopsis thaliana GN=At3g15810 PE=2 SV=1	7.26	2.52	2.14	up	0.00	0.00	yes
TRINITY_DN15980_c0_g1	unknown [Populus trichocarpa]	-	-	68.51	49.90	1.05	up	0.00	0.00	yes
TRINITY_DN19931_c0_g11	cytochrome b6/f complex subunit IV (chloroplast) [Populus euphratica]	petD	Cytochrome b6-f complex subunit 4 OS=Populus alba GN=petD PE=3 SV=1	6.85	3.19	1.62	up	0.00	0.00	yes
TRINITY_DN18873_c0_g2	translation initiation factor eIF-1A family protein [Populus trichocarpa]	-	Eukaryotic translation initiation factor 1A OS=Onobrychis viciifolia PE=2 SV=2	211.89	166.21	1.04	up	0.00	0.00	yes
TRINITY_DN25796_c0_g2	PREDICTED: uncharacterized protein LOC105111698 [Populus euphratica]	PRXIIE	Peroxiredoxin-2E, chloroplastic OS=Arabidopsis thaliana GN=PRXIIE PE=1 SV=2	199.34	130.10	1.20	up	0.00	0.00	yes
TRINITY_DN18425_c0_g2	PREDICTED: coatomer subunit zeta-1-like [Populus euphratica]	At1g60970	Coatomer subunit zeta-1 OS=Arabidopsis thaliana GN=At1g60970 PE=2 SV=1	25.43	18.44	1.06	up	0.00	0.00	yes
TRINITY_DN19371_c0_g1	PREDICTED: branched-chain-amino-acid aminotransferase-like protein 1 [Populus euphratica]	EEF2KMT	Protein-lysine N-methyltransferase EEF2KMT OS=Homo sapiens GN=EEF2KMT PE=1 SV=2	15.43	10.00	1.12	up	0.00	0.00	yes
TRINITY_DN23287_c0_g1	hypothetical protein POPTR_0001s34380g [Populus trichocarpa]	rimM	Ribosome maturation factor RimM OS=Synechococcus sp. (strain WH7803) GN=rimM PE=3 SV=1	55.40	39.31	1.08	up	0.00	0.00	yes
TRINITY_DN14417_c0_g1	unknown [Populus trichocarpa]	KRP7	Cyclin-dependent kinase inhibitor 7 OS=Arabidopsis thaliana GN=KRP7 PE=1 SV=2	11.44	7.11	1.29	up	0.00	0.00	yes
TRINITY_DN19468_c0_g1	hypothetical protein POPTR_0081s00220g [Populus trichocarpa]	-	-	55.47	38.35	1.15	up	0.00	0.00	yes
TRINITY_DN17668_c0_g4	hypothetical protein POPTR_0019s11250g, partial [Populus trichocarpa]	-	-	4.52	1.70	2.01	up	0.00	0.00	yes
TRINITY_DN22984_c0_g2	hypothetical protein POPTR_0017s09760g [Populus trichocarpa]	lvr	Levodione reductase OS=Leifsonia aquatica GN=lvr PE=1 SV=1	24.83	15.86	1.24	up	0.00	0.00	yes
TRINITY_DN23922_c0_g1	PREDICTED: protein translocase subunit SecA, chloroplastic isoform X2 [Populus euphratica]	SECA1	Protein translocase subunit SECA1, chloroplastic OS=Arabidopsis thaliana GN=SECA1 PE=1 SV=2	96.97	70.38	1.12	up	0.00	0.00	yes
TRINITY_DN16769_c0_g1	GmMYB12 family protein [Populus trichocarpa]	MYB108	Transcription factor MYB108 OS=Arabidopsis thaliana GN=MYB108 PE=1 SV=1	4.17	6.44	1.87	up	0.00	0.00	yes
TRINITY_DN19680_c0_g2	PREDICTED: isoprenylcysteine alpha-carbonyl methylesterase ICME-like [Populus euphratica]	ICME	Isoprenylcysteine alpha-carbonyl methylesterase ICME OS=Arabidopsis thaliana GN=ICME PE=2 SV=1	19.58	14.25	1.07	up	0.00	0.00	yes
TRINITY_DN27742_c1_g1	peptidase M1 family protein [Populus trichocarpa]	MPA1	Puromycin-sensitive aminopeptidase OS=Arabidopsis thaliana GN=MPA1 PE=2 SV=1	169.39	130.95	1.02	up	0.00	0.00	yes
TRINITY_DN9484_c0_g2	PREDICTED: transcription factor CYCLOIDEA [Populus euphratica]	CYC	Transcription factor CYCLOIDEA (Fragment) OS=Antirrhinum majus subsp. cirrhigerum GN=CYC PE=3 SV=1	1.60	0.47	2.36	up	0.00	0.00	yes
TRINITY_DN27763_c0_g1	translation initiation factor IF-2 family protein [Populus trichocarpa]	At1g17220	Translation initiation factor IF-2, chloroplastic OS=Arabidopsis thaliana GN=At1g17220 PE=2 SV=2	106.96	76.07	1.09	up	0.00	0.00	yes
TRINITY_DN21398_c0_g1	hypothetical protein POPTR_0004s04550g [Populus trichocarpa]	rps5	30S ribosomal protein S5, chloroplastic OS=Arabidopsis thaliana GN=rps5 PE=2 SV=1	565.72	361.32	1.23	up	0.00	0.00	yes
TRINITY_DN20084_c1_g1	Photosystem II 22 kDa family protein [Populus trichocarpa]	PSBS	Photosystem II 22 kDa protein, chloroplastic OS=Spinacia oleracea GN=PSBS PE=1 SV=1	1435.69	793.79	1.45	up	0.00	0.00	yes
TRINITY_DN26983_c0_g7	PREDICTED: uncharacterized protein LOC105115405 [Populus euphratica]	-	-	31.96	26.29	1.10	up	0.00	0.00	yes
TRINITY_DN12911_c0_g1	-	-	-	8.90	2.32	2.57	up	0.00	0.00	yes
TRINITY_DN26567_c0_g2	PREDICTED: mitochondrial carnitine/acylcarnitine carrier-like protein isoform X1 [Populus euphratica]	BOU	Mitochondrial carnitine/acylcarnitine carrier-like protein OS=Arabidopsis thaliana GN=BOU PE=1 SV=1	3.30	1.03	2.26	up	0.00	0.00	yes
TRINITY_DN18203_c0_g5	hypothetical protein POPTR_0007s14910g [Populus trichocarpa]	FRO1	NADH dehydrogenase [ubiquinone] iron-sulfur protein 4, mitochondrial OS=Arabidopsis thaliana GN=FRO1 PE=2 SV=1	166.17	124.84	1.03	up	0.00	0.00	yes

TRINITY_DN25298_c0_g1	PREDICTED: putative E3 ubiquitin-protein ligase LIN isoform X1 [Populus euphratica]	LIN	Putative E3 ubiquitin-protein ligase LIN-2 OS=Lotus japonicus GN=LIN PE=1 SV=1	15.63	9.25	1.17	up	0.00	0.00	yes
TRINITY_DN21330_c1_g6	unknown [Populus trichocarpa]	-	-	69.16	52.78	1.02	up	0.00	0.00	yes
TRINITY_DN20352_c0_g1	hypothetical protein POPTR_0004s19490g [Populus trichocarpa]	GALT29A	Beta-1,6-galactosyltransferase GALT29A OS=Arabidopsis thaliana GN=GALT29A PE=1 SV=1	17.68	13.24	1.15	up	0.00	0.00	yes
TRINITY_DN16053_c0_g1	ferric reductase-like transmembrane component family protein [Populus trichocarpa]	FRO6	Ferric reduction oxidase 6 OS=Arabidopsis thaliana GN=FRO6 PE=2 SV=1	11.32	8.32	1.06	up	0.00	0.00	yes
TRINITY_DN20600_c0_g1	exostosin family protein [Populus trichocarpa]	XLT2	Xyloglucan galactosyltransferase XLT2 OS=Arabidopsis thaliana GN=XLT2 PE=1 SV=1	7.60	5.39	1.11	up	0.00	0.00	yes
TRINITY_DN18735_c0_g1	Oxygen-evolving enhancer protein 3-1 [Populus trichocarpa]	PSBQ1	Oxygen-evolving enhancer protein 3-1, chloroplastic OS=Arabidopsis thaliana GN=PSBQ1 PE=1 SV=3	1461.45	876.16	1.30	up	0.00	0.00	yes
TRINITY_DN14368_c0_g1	hypothetical protein POPTR_0010s08470g [Populus trichocarpa]	-	-	36.12	19.49	1.54	up	0.00	0.00	yes
TRINITY_DN18117_c0_g1	hypothetical protein POPTR_0012s13890g [Populus trichocarpa]	-	-	108.23	69.74	1.32	up	0.00	0.00	yes
TRINITY_DN25265_c0_g1	PREDICTED: pentatricopeptide repeat-containing protein At3g04760, chloroplastic-like [Populus euphratica]	At3g04760	Pentatricopeptide repeat-containing protein At3g04760, chloroplastic OS=Arabidopsis thaliana GN=At3g04760 PE=2 SV=1	26.23	16.84	1.20	up	0.00	0.00	yes
TRINITY_DN23305_c1_g2	GTL1 [Populus tremula x Populus alba]	GTL1	Trihelix transcription factor GTL1 OS=Arabidopsis thaliana GN=GTL1 PE=1 SV=2	50.69	55.61	1.03	up	0.00	0.00	yes
TRINITY_DN21469_c0_g2	hypothetical protein POPTR_0003s01450g [Populus trichocarpa]	CAP10A	Chlorophyll a-b binding protein CP24 10A, chloroplastic OS=Solanum lycopersicum GN=CAP10A PE=3 SV=1	441.89	284.86	1.24	up	0.00	0.00	yes
TRINITY_DN12991_c0_g1	hypothetical protein POPTR_0006s19380g [Populus trichocarpa]	TSS	Protein TSS OS=Arabidopsis thaliana GN=TSS PE=1 SV=1	199.61	106.60	1.52	up	0.00	0.00	yes
TRINITY_DN15680_c0_g1	PREDICTED: diphthamide biosynthesis protein 7 homolog isoform X9 [Populus euphratica]	wdr85	Diphthine methyltransferase homolog OS=Dictyostelium discoideum GN=wdr85 PE=3 SV=1	6.87	4.10	1.38	up	0.00	0.00	yes
TRINITY_DN19483_c0_g1	ribosomal protein S5 [Populus trichocarpa]	rpsE	30S ribosomal protein S5 OS=Blochmannia pennsylvanicus (strain BPEN) GN=rpsE PE=3 SV=1	18.63	14.40	1.00	up	0.00	0.00	yes
TRINITY_DN22478_c0_g1	hypothetical protein POPTR_0016s03360g [Populus trichocarpa]	IQM3	IQ domain-containing protein IQM3 OS=Arabidopsis thaliana GN=IQM3 PE=2 SV=1	164.22	121.65	1.06	up	0.00	0.00	yes
TRINITY_DN26342_c0_g4	-	-	-	13.43	5.18	1.95	up	0.00	0.00	yes
TRINITY_DN27051_c1_g1	PREDICTED: ethylene receptor 2-like isoform X1 [Populus euphratica]	ETR2	Ethylene receptor 2 OS=Arabidopsis thaliana GN=ETR2 PE=1 SV=2	24.09	17.42	1.53	up	0.00	0.00	yes
TRINITY_DN21291_c1_g1	PREDICTED: uncharacterized protein LOC105135456 isoform X1 [Populus euphratica]	-	-	9.18	6.04	1.15	up	0.00	0.00	yes
TRINITY_DN19435_c0_g2	PREDICTED: anthocyanidin 3-O-glucosyltransferase 7-like [Populus euphratica]	UFGT	Anthocyanidin 3-O-glucosyltransferase 2 OS=Vitis vinifera GN=UFGT PE=1 SV=2	28.71	12.23	1.89	up	0.00	0.00	yes
TRINITY_DN27783_c0_g1	PREDICTED: clustered mitochondria protein [Populus euphratica]	FMT	Clustered mitochondria protein OS=Arabidopsis thaliana GN=FMT PE=2 SV=1	26.06	21.16	1.00	up	0.00	0.00	yes
TRINITY_DN25274_c1_g1	hypothetical protein POPTR_0014s09120g [Populus trichocarpa]	GH3.5	Jasmonic acid-amido synthetase JAR1 OS=Oryza sativa subsp. japonica GN=GH3.5 PE=2 SV=1	14.82	8.16	1.18	up	0.00	0.00	yes
TRINITY_DN25264_c0_g1	PREDICTED: pentatricopeptide repeat-containing protein At1g71060, mitochondrial [Populus euphratica]	At1g71060	Pentatricopeptide repeat-containing protein At1g71060, mitochondrial OS=Arabidopsis thaliana GN=At1g71060 PE=2 SV=1	10.22	6.81	1.12	up	0.00	0.00	yes
TRINITY_DN21808_c0_g1	hypothetical protein POPTR_0022s00470g [Populus trichocarpa]	CRTISO	Prolycopene isomerase, chloroplastic OS=Daucus carota GN=CRTISO PE=2 SV=1	36.66	21.99	1.34	up	0.00	0.00	yes
TRINITY_DN18363_c0_g1	hypothetical protein POPTR_0012s05590g [Populus trichocarpa]	EIF2B4	Translation initiation factor eIF-2B subunit delta OS=Homo sapiens GN=EIF2B4 PE=1 SV=2	7.01	5.04	1.09	up	0.00	0.00	yes
TRINITY_DN19531_c1_g1	PREDICTED: protein SSUH2 homolog [Populus euphratica]	-	-	19.32	18.15	1.06	up	0.00	0.00	yes
TRINITY_DN16385_c0_g1	hypothetical protein POPTR_0013s06710g [Populus trichocarpa]	RPL3	50S ribosomal protein L3, chloroplastic (Fragment) OS=Nicotiana tabacum GN=RPL3 PE=2 SV=1	463.92	291.23	1.26	up	0.00	0.00	yes
TRINITY_DN18096_c0_g1	PREDICTED: NHP2-like protein 1 [Populus euphratica]	snu13	NHP2-like protein 1 OS=Xenopus laevis GN=snu13 PE=2 SV=1	202.04	145.54	1.16	up	0.00	0.00	yes
TRINITY_DN21445_c0_g6	hypothetical protein POPTR_0006s24790g [Populus trichocarpa]	-	-	58.27	43.31	1.03	up	0.00	0.00	yes

TRINITY_DN27440_c0_g2	PREDICTED: glutamine synthetase leaf isozyme, chloroplastic [Populus euphratica]	GLN2	Glutamine synthetase, chloroplastic OS=Daucus carota GN=GLN2 PE=2 SV=1	559.41	306.88	1.46	up	0.00	0.00	yes
TRINITY_DN23596_c1_g1	hypothetical protein POPTR_0013s10100g [Populus trichocarpa]	AAP1	Amino acid permease 1 OS=Arabidopsis thaliana GN=AAP1 PE=1 SV=1	12.79	8.71	1.13	up	0.00	0.00	yes
TRINITY_DN20152_c0_g1	hypothetical protein POPTR_0003s13830g [Populus trichocarpa]	-	-	32.55	24.59	1.06	up	0.00	0.00	yes
TRINITY_DN22576_c0_g1	hypothetical protein POPTR_0019s05080g [Populus trichocarpa]	-	-	44.25	36.34	1.01	up	0.00	0.00	yes
TRINITY_DN15082_c0_g1	cytokinin receptor 1A [Populus trichocarpa]	AHK4	Histidine kinase 4 OS=Arabidopsis thaliana GN=AHK4 PE=1 SV=1	6.96	2.99	1.80	up	0.00	0.00	yes
TRINITY_DN26042_c0_g1	PREDICTED: uncharacterized protein LOC105137440 [Populus euphratica]	LIP	Lipase OS=Thermomyces lanuginosus GN=LIP PE=1 SV=1	35.18	26.83	1.08	up	0.00	0.00	yes
TRINITY_DN16176_c0_g3	hypothetical protein POPTR_0019s10050g [Populus trichocarpa]	-	-	60.63	42.12	1.12	up	0.00	0.00	yes
TRINITY_DN18785_c0_g1	proton extrusion family protein [Populus trichocarpa]	cemA	Chloroplast envelope membrane protein OS=Guillardia theta GN=cemA PE=3 SV=1	53.45	31.20	1.32	up	0.00	0.00	yes
TRINITY_DN18656_c0_g1	hypothetical protein POPTR_0008s08550g [Populus trichocarpa]	ung	Uracil-DNA glycosylase OS=Dichelobacter nodosus (strain VCS1703A) GN=ung PE=3 SV=1	2.75	1.16	1.85	up	0.00	0.00	yes
TRINITY_DN22729_c0_g1	mitochondrial transcription termination factor family protein [Populus trichocarpa]	MTERF4	Transcription termination factor MTERF4, chloroplastic OS=Arabidopsis thaliana GN=MTERF4 PE=1 SV=1	23.23	18.98	1.02	up	0.00	0.00	yes
TRINITY_DN24813_c0_g2	hypothetical protein POPTR_0010s09620g [Populus trichocarpa]	PCMP-H3	Pentatricopeptide repeat-containing protein At4g14820 OS=Arabidopsis thaliana GN=PCMP-H3 PE=2 SV=1	9.00	6.87	1.21	up	0.00	0.00	yes
TRINITY_DN25845_c0_g3	hypothetical protein POPTR_0002s04980g [Populus trichocarpa]	At4g15470	BI1-like protein OS=Arabidopsis thaliana GN=At4g15470 PE=2 SV=1	59.12	44.54	1.02	up	0.00	0.00	yes
TRINITY_DN18806_c0_g1	-	-	-	70.32	28.39	1.94	up	0.00	0.00	yes
TRINITY_DN16436_c0_g2	hypothetical protein POPTR_0010s19770g [Populus trichocarpa]	CLC2	Clathrin light chain 2 OS=Arabidopsis thaliana GN=CLC2 PE=1 SV=1	63.56	46.85	1.03	up	0.00	0.00	yes
TRINITY_DN19304_c0_g2	peptidyl-tRNA hydrolase family protein [Populus trichocarpa]	CRS2A	Chloroplastic group IIB intron splicing facilitator CRS2-A, chloroplastic OS=Arabidopsis thaliana GN=CRS2A PE=2 SV=1	92.47	63.58	1.17	up	0.00	0.00	yes
TRINITY_DN23854_c0_g4	hypothetical protein POPTR_0017s08780g [Populus trichocarpa]	MPT3	Mitochondrial phosphate carrier protein 3, mitochondrial OS=Arabidopsis thaliana GN=MPT3 PE=1 SV=1	28.65	20.80	1.08	up	0.00	0.00	yes
TRINITY_DN22663_c0_g2	hypothetical protein POPTR_0010s05530g [Populus trichocarpa]	GGAT1	Glutamate--glyoxylate aminotransferase 1 OS=Arabidopsis thaliana GN=GGAT1 PE=1 SV=1	360.33	205.54	1.38	up	0.00	0.00	yes
TRINITY_DN20650_c0_g1	hypothetical protein GLYMA_20G228100 [Glycine max]	RPS24A	40S ribosomal protein S24-1 OS=Arabidopsis thaliana GN=RPS24A PE=2 SV=1	727.58	551.01	1.00	up	0.00	0.00	yes
TRINITY_DN15506_c0_g1	PREDICTED: uncharacterized protein LOC105121200 [Populus euphratica]	-	-	3.68	1.27	2.18	up	0.00	0.00	yes
TRINITY_DN21085_c0_g2	lil3 family protein [Populus trichocarpa]	LIL3.1	Light-harvesting complex-like protein 3 isotype 1, chloroplastic OS=Arabidopsis thaliana GN=LIL3.1 PE=1 SV=1	98.96	70.50	1.08	up	0.00	0.00	yes
TRINITY_DN19217_c1_g3	hypothetical protein POPTR_0018s09490g [Populus trichocarpa]	TIM17-2	Mitochondrial import inner membrane translocase subunit TIM17-2 OS=Arabidopsis thaliana GN=TIM17-2 PE=1 SV=2	22.34	16.43	1.06	up	0.00	0.00	yes
TRINITY_DN26040_c1_g5	ycf1 [Populus alba]	TIC214	Protein TIC 214 OS=Populus alba GN=TIC214 PE=3 SV=1	33.94	20.99	1.29	up	0.00	0.00	yes
TRINITY_DN18375_c0_g1	hypothetical protein POPTR_0030s00520g [Populus trichocarpa]	MIK2	MDIS1-interacting receptor like kinase 2 OS=Arabidopsis thaliana GN=MIK2 PE=1 SV=3	19.25	7.08	2.13	up	0.00	0.00	yes
TRINITY_DN26755_c0_g3	hypothetical protein POPTR_0015s15270g [Populus trichocarpa]	CPN60A2	Chaperonin 60 subunit alpha 2, chloroplastic OS=Arabidopsis thaliana GN=CPN60A2 PE=2 SV=1	40.82	29.41	1.04	up	0.00	0.00	yes
TRINITY_DN19645_c0_g1	hypothetical protein POPTR_0010s16900g [Populus trichocarpa]	CLPF	Clp protease adapter protein ClpF, chloroplastic OS=Arabidopsis thaliana GN=CLPF PE=1 SV=1	40.27	30.31	1.04	up	0.00	0.00	yes
TRINITY_DN24262_c0_g2	translation initiation factor IF-2 family protein [Populus trichocarpa]	At1g17220	Translation initiation factor IF-2, chloroplastic OS=Arabidopsis thaliana GN=At1g17220 PE=2 SV=2	41.50	27.87	1.18	up	0.00	0.00	yes
TRINITY_DN19518_c0_g2	PREDICTED: putative pentatricopeptide repeat-containing protein At3g16710, mitochondrial [Populus euphratica]	-	-	5.77	3.63	1.27	up	0.00	0.00	yes
TRINITY_DN24809_c0_g1	CAAX amino terminal protease family protein [Populus trichocarpa]	-	-	7.75	5.34	1.14	up	0.00	0.00	yes

TRINITY_DN23265_c0_g3	PREDICTED: photosystem I reaction center subunit IV A, chloroplastic-like [Populus euphratica]	PSAEA	Photosystem I reaction center subunit IV A, chloroplastic OS=Nicotiana sylvestris GN=PSAEA PE=1 SV=1	596.74	377.27	1.26	up	0.00	0.00	yes
TRINITY_DN24112_c0_g1	pentatricopeptide repeat-containing protein [Populus tomentosa]	PCMP-E95	Pentatricopeptide repeat-containing protein At3g22150, chloroplastic OS=Arabidopsis thaliana GN=PCMP-E95 PE=2 SV=1	10.95	11.67	1.05	up	0.00	0.00	yes
TRINITY_DN24309_c0_g1	hypothetical protein POPTR_0012s02950g [Populus trichocarpa]	GRF6	Growth-regulating factor 6 OS=Oryza sativa subsp. japonica GN=GRF6 PE=2 SV=2	30.15	21.58	1.06	up	0.00	0.00	yes
TRINITY_DN17556_c3_g1	-	-	-	5.92	2.60	1.91	up	0.00	0.00	yes
TRINITY_DN27479_c2_g1	PREDICTED: heat shock cognate 70 kDa protein 2-like [Populus euphratica]	MED37C	Probable mediator of RNA polymerase II transcription subunit 37c OS=Arabidopsis thaliana GN=MED37C PE=1 SV=1	249.29	121.53	1.63	up	0.00	0.00	yes
TRINITY_DN20397_c0_g1	PREDICTED: pentatricopeptide repeat-containing protein At1g28690, mitochondrial [Populus euphratica]	PCMP-E34	Pentatricopeptide repeat-containing protein At1g28690, mitochondrial OS=Arabidopsis thaliana GN=PCMP-E34 PE=2 SV=2	2.91	1.60	1.46	up	0.00	0.00	yes
TRINITY_DN18173_c0_g1	PREDICTED: OTU domain-containing protein At3g57810-like isoform X1 [Populus euphratica]	At3g57810	OTU domain-containing protein At3g57810 OS=Arabidopsis thaliana GN=At3g57810 PE=2 SV=1	9.67	6.40	1.44	up	0.00	0.00	yes
TRINITY_DN16092_c0_g1	hypothetical protein POPTR_0014s13450g [Populus trichocarpa]	PAC	Protein PALE CRESS, chloroplastic OS=Arabidopsis thaliana GN=PAC PE=2 SV=1	188.85	130.75	1.12	up	0.00	0.00	yes
TRINITY_DN21168_c0_g1	PREDICTED: phytoene dehydrogenase, chloroplastic/chromoplastic [Phoenix dactylifera]	PDS	Phytoene dehydrogenase, chloroplastic/chromoplastic OS=Solanum lycopersicum GN=PDS PE=2 SV=1	81.89	55.65	1.14	up	0.00	0.00	yes
TRINITY_DN19113_c2_g3	hypothetical protein POPTR_0002s25880g [Populus trichocarpa]	nxt3	Putative G3BP-like protein OS=Schizosaccharomyces pombe (strain 972 / ATCC 24843) GN=nxt3 PE=1 SV=1	32.11	23.96	1.01	up	0.00	0.00	yes
TRINITY_DN17931_c0_g2	unknown [Populus trichocarpa]	MORF8	Multiple organellar RNA editing factor 8, chloroplastic/mitochondrial OS=Arabidopsis thaliana GN=MORF8 PE=1 SV=1	189.83	143.29	1.02	up	0.00	0.00	yes
TRINITY_DN19619_c0_g1	LOB domain protein 39 [Populus trichocarpa]	LBD37	LOB domain-containing protein 37 OS=Arabidopsis thaliana GN=LBD37 PE=2 SV=1	39.32	26.06	1.26	up	0.00	0.00	yes
TRINITY_DN18321_c0_g3	PREDICTED: GTP-binding protein SAR1A [Populus euphratica]	SAR1A	GTP-binding protein SAR1A OS=Arabidopsis thaliana GN=SAR1A PE=2 SV=1	90.19	67.08	1.05	up	0.00	0.00	yes
TRINITY_DN20951_c1_g1	PREDICTED: ethylene-responsive transcription factor ERF113-like [Populus euphratica]	ERF113	Ethylene-responsive transcription factor ERF113 OS=Arabidopsis thaliana GN=ERF113 PE=2 SV=1	9.27	4.79	1.86	up	0.00	0.00	yes
TRINITY_DN23171_c1_g1	phytochrome kinase substrate-related family protein [Populus trichocarpa]	PKS4	Protein PHYTOCHROME KINASE SUBSTRATE 4 OS=Arabidopsis thaliana GN=PKS4 PE=1 SV=1	5.54	3.56	1.26	up	0.00	0.00	yes
TRINITY_DN18081_c0_g1	hypothetical protein POPTR_0014s18410g [Populus trichocarpa]	PCS3	Glutathione gamma-glutamylcysteinyltransferase 3 OS=Lotus japonicus GN=PCS3 PE=2 SV=1	13.68	10.10	1.00	up	0.00	0.00	yes
TRINITY_DN23896_c0_g3	PREDICTED: probable carboxylesterase 7 [Populus euphratica]	CXE12	Probable carboxylesterase 12 OS=Arabidopsis thaliana GN=CXE12 PE=1 SV=1	825.28	598.17	1.03	up	0.00	0.00	yes
TRINITY_DN21479_c0_g2	PREDICTED: phosphoglycerate mutase-like protein 1 isoform X1 [Populus euphratica]	At5g64460	Phosphoglycerate mutase-like protein 1 OS=Arabidopsis thaliana GN=At5g64460 PE=2 SV=1	126.16	93.07	1.07	up	0.00	0.00	yes
TRINITY_DN22088_c0_g1	hypothetical protein POPTR_0009s12310g [Populus trichocarpa]	-	-	61.10	21.83	2.08	up	0.00	0.00	yes
TRINITY_DN27723_c2_g2	hypothetical protein POPTR_0010s08910g [Populus trichocarpa]	At5g43745	Putative ion channel POLLUX-like 2 OS=Arabidopsis thaliana GN=At5g43745 PE=2 SV=1	96.38	64.35	1.26	up	0.00	0.00	yes
TRINITY_DN21063_c0_g3	hypothetical protein POPTR_0019s13430g [Populus trichocarpa]	-	-	11.90	8.62	1.07	up	0.00	0.00	yes
TRINITY_DN15216_c0_g1	myb family transcription factor family protein [Populus trichocarpa]	RL2	Protein RADIALIS-like 2 OS=Arabidopsis thaliana GN=RL2 PE=2 SV=1	14.59	5.27	2.22	up	0.00	0.00	yes
TRINITY_DN20581_c0_g1	PREDICTED: uncharacterized protein LOC105120812 [Populus euphratica]	-	-	63.75	44.85	1.11	up	0.00	0.00	yes
TRINITY_DN25009_c0_g1	pyrophosphate-dependent phosphofructokinase alpha subunit family protein [Populus trichocarpa]	PFP-ALPHA	Pyrophosphate--fructose 6-phosphate 1-phosphotransferase subunit alpha OS=Ricinus communis GN=PFP-ALPHA PE=3 SV=1	61.09	45.64	1.05	up	0.00	0.00	yes
TRINITY_DN24566_c0_g1	AMP-dependent synthetase and ligase family protein [Populus trichocarpa]	AAE12	Probable acyl-activating enzyme 12, peroxisomal OS=Arabidopsis thaliana GN=AAE12 PE=2 SV=1	395.20	247.14	1.29	up	0.00	0.00	yes
TRINITY_DN20840_c0_g1	hypothetical protein POPTR_0003s13800g [Populus trichocarpa]	AS	Hydroquinone glucosyltransferase OS=Rauvolfia serpentina GN=AS PE=1 SV=1	48.71	33.37	1.12	up	0.00	0.00	yes
TRINITY_DN24224_c1_g1	chloroplast inner membrane import protein Tic22 [Populus trichocarpa]	TIC22	Protein TIC 22, chloroplastic OS=Arabidopsis thaliana GN=TIC22 PE=1 SV=1	133.82	95.56	1.10	up	0.00	0.00	yes

TRINITY_DN22454_c0_g1	PREDICTED: protein YLS2-like [Populus euphratica]	SSL4	Protein STRICTOSIDINE SYNTHASE-LIKE 4 OS=Arabidopsis thaliana GN=SSL4 PE=1 SV=1	58.39	35.19	1.32	up	0.00	0.00	yes
TRINITY_DN25478_c0_g1	carbonic anhydrase [Populus tremula x Populus tremuloides]	BCA1	Beta carbonic anhydrase 1, chloroplastic OS=Arabidopsis thaliana GN=BCA1 PE=1 SV=2	6672.48	2098.66	2.24	up	0.00	0.00	yes
TRINITY_DN16504_c0_g1	PREDICTED: uroporphyrinogen decarboxylase [Populus euphratica]	DCUP	Uroporphyrinogen decarboxylase, chloroplastic OS=Nicotiana tabacum GN=DCUP PE=1 SV=1	215.24	147.34	1.13	up	0.00	0.00	yes
TRINITY_DN21866_c0_g6	GTP-binding family protein [Populus trichocarpa]	RABA1F	Ras-related protein RABA1f OS=Arabidopsis thaliana GN=RABA1F PE=2 SV=1	54.96	41.84	1.01	up	0.00	0.00	yes
TRINITY_DN16115_c0_g2	hypothetical protein POPTR_0001s29680g [Populus trichocarpa]	-	-	11.24	7.15	1.26	up	0.00	0.00	yes
TRINITY_DN17456_c1_g2	hypothetical protein POPTR_0006s00920g [Populus trichocarpa]	GT3	Putative UDP-glucose flavonoid 3-O-glucosyltransferase 3 OS=Fragaria ananassa GN=GT3 PE=2 SV=1	30.98	15.42	1.62	up	0.00	0.00	yes
TRINITY_DN25221_c1_g4	PREDICTED: LOW QUALITY PROTEIN: dihydrolipoyllysine-residue acetyltransferase component 5 of pyruvate dehydrogenase complex, chloroplastic-like, partial [Populus euphratica]	-	-	9.32	3.34	2.08	up	0.00	0.00	yes
TRINITY_DN21754_c0_g3	PREDICTED: uncharacterized protein LOC105123443 [Populus euphratica]	-	-	25.54	16.23	1.24	up	0.00	0.00	yes
TRINITY_DN27865_c0_g1	hypothetical protein POPTR_0016s03630g [Populus trichocarpa]	GLU1	Ferredoxin-dependent glutamate synthase 1, chloroplastic/mitochondrial OS=Arabidopsis thaliana GN=GLU1 PE=1 SV=3	23.70	13.44	1.40	up	0.00	0.00	yes
TRINITY_DN19205_c1_g4	PREDICTED: lycopene epsilon cyclase, chloroplastic isoform X1 [Populus euphratica]	LUT2	Lycopene epsilon cyclase, chloroplastic OS=Arabidopsis thaliana GN=LUT2 PE=1 SV=2	74.68	51.95	1.11	up	0.00	0.00	yes
TRINITY_DN24442_c0_g1	Lycopene beta cyclase family protein [Populus trichocarpa]	LCY1	Lycopene beta cyclase, chloroplastic/chromoplastic OS=Capsicum annuum GN=LCY1 PE=2 SV=1	35.80	23.66	1.19	up	0.00	0.00	yes
TRINITY_DN26183_c0_g1	leucine-rich repeat transmembrane protein kinase [Populus trichocarpa]	PRK3	Pollen receptor-like kinase 3 OS=Arabidopsis thaliana GN=PRK3 PE=1 SV=1	267.84	151.64	1.39	up	0.00	0.00	yes
TRINITY_DN14539_c0_g1	MATE efflux family protein [Populus trichocarpa]	DTX48	Protein DETOXIFICATION 48 OS=Arabidopsis thaliana GN=DTX48 PE=2 SV=1	2.40	0.88	2.06	up	0.00	0.00	yes
TRINITY_DN17168_c0_g1	PREDICTED: ATP-dependent zinc metalloprotease FtsH isoform X1 [Populus euphratica]	FTSHI3	Probable inactive ATP-dependent zinc metalloprotease FTSHI 3, chloroplastic OS=Arabidopsis thaliana GN=FTSHI3 PE=1 SV=1	54.81	37.45	1.15	up	0.00	0.00	yes
TRINITY_DN19392_c2_g4	hypothetical protein POPTR_0012s07240g [Populus trichocarpa]	-	-	189.16	119.98	1.26	up	0.00	0.00	yes
TRINITY_DN25630_c0_g1	aldehyde dehydrogenase 1 precursor family protein [Populus trichocarpa]	ALDH2B4	Aldehyde dehydrogenase family 2 member B4, mitochondrial OS=Arabidopsis thaliana GN=ALDH2B4 PE=2 SV=1	120.35	73.18	1.30	up	0.00	0.00	yes
TRINITY_DN25551_c1_g1	hypothetical protein POPTR_0009s04840g [Populus trichocarpa]	ANTR1	Sodium-dependent phosphate transport protein 1, chloroplastic OS=Arabidopsis thaliana GN=ANTR1 PE=1 SV=1	35.01	28.76	1.04	up	0.00	0.00	yes
TRINITY_DN25612_c0_g2	reticulon family protein [Populus trichocarpa]	RTNLB8	Reticulon-like protein B8 OS=Arabidopsis thaliana GN=RTNLB8 PE=2 SV=1	143.62	87.46	1.29	up	0.00	0.00	yes
TRINITY_DN24207_c0_g3	PREDICTED: uncharacterized protein LOC105132451 [Populus euphratica]	-	-	12.50	7.97	1.26	up	0.00	0.00	yes
TRINITY_DN19065_c0_g1	hypothetical protein POPTR_0015s06600g [Populus trichocarpa]	PCO3	Plant cysteine oxidase 3 OS=Arabidopsis thaliana GN=PCO3 PE=1 SV=1	38.06	27.94	1.11	up	0.00	0.00	yes
TRINITY_DN19540_c0_g3	hypothetical protein POPTR_0001s45280g [Populus trichocarpa]	At1g32780	Alcohol dehydrogenase-like 3 OS=Arabidopsis thaliana GN=At1g32780 PE=2 SV=1	3.69	1.58	1.78	up	0.00	0.00	yes
TRINITY_DN16995_c0_g1	Cyanate hydratase family protein [Populus trichocarpa]	CYN	Cyanate hydratase OS=Populus trichocarpa GN=CYN PE=3 SV=1	122.02	91.70	1.00	up	0.00	0.00	yes
TRINITY_DN16905_c0_g1	hypothetical protein POPTR_0018s04600g [Populus trichocarpa]	-	(S)-coclaurine N-methyltransferase OS=Thalictrum flavum subsp. glaucum PE=1 SV=1	49.91	37.20	1.02	up	0.00	0.00	yes
TRINITY_DN19801_c0_g1	-	-	-	5.05	2.09	1.85	up	0.00	0.00	yes
TRINITY_DN18198_c0_g3	6a-hydroxymaackiain methyltransferase family protein [Populus trichocarpa]	ROMT	Trans-resveratrol di-O-methyltransferase OS=Vitis vinifera GN=ROMT PE=1 SV=2	26.78	17.08	1.26	up	0.00	0.00	yes
TRINITY_DN18348_c1_g1	hypothetical protein POPTR_0001s18740g [Populus trichocarpa]	PNSB3	Photosynthetic NDH subunit of subcomplex B 3, chloroplastic OS=Arabidopsis thaliana GN=PNSB3 PE=2 SV=1	179.65	103.73	1.36	up	0.00	0.00	yes
TRINITY_DN21831_c1_g1	hypothetical protein POPTR_0022s00770g [Populus trichocarpa]	NIFU2	NifU-like protein 2, chloroplastic OS=Arabidopsis thaliana GN=NIFU2 PE=1 SV=1	100.78	76.67	1.03	up	0.00	0.00	yes

TRINITY_DN17915_c0_g1	hypothetical protein POPTR_0007s11740g [Populus trichocarpa]	PNAE	Polyneuridine-aldehyde esterase OS=Rauvolfia serpentina GN=PNAE PE=1 SV=1	42.52	24.99	1.39	up	0.00	0.00	yes
TRINITY_DN24238_c0_g2	phosphoglycerate/bisphosphoglycerate mutase family protein [Populus trichocarpa]	-	-	15.62	7.45	1.66	up	0.00	0.00	yes
TRINITY_DN21562_c1_g1	hypothetical protein POPTR_0006s22950g [Populus trichocarpa]	-	-	195.66	148.86	1.04	up	0.00	0.00	yes
TRINITY_DN21622_c0_g1	NAC domain transcription factor [Populus tomentosa]	NAC083	NAC domain-containing protein 83 OS=Arabidopsis thaliana GN=NAC083 PE=1 SV=1	18.53	13.96	1.02	up	0.00	0.00	yes
TRINITY_DN22933_c0_g2	PREDICTED: cell wall / vacuolar inhibitor of fructosidase 2-like [Populus euphratica]	-	-	21.75	14.17	1.21	up	0.00	0.00	yes
TRINITY_DN17369_c0_g1	ascorbate peroxidase [Populus tomentosa]	APX1	L-ascorbate peroxidase, cytosolic OS=Pisum sativum GN=APX1 PE=1 SV=2	210.14	152.72	1.12	up	0.00	0.00	yes
TRINITY_DN17153_c0_g2	hypothetical protein POPTR_0011s00390g [Populus trichocarpa]	ETC1	MYB-like transcription factor ETC1 OS=Arabidopsis thaliana GN=ETC1 PE=2 SV=1	7.47	2.94	1.97	up	0.00	0.00	yes
TRINITY_DN26353_c0_g3	PREDICTED: LOW QUALITY PROTEIN: uncharacterized protein At2g39795, mitochondrial [Populus euphratica]	At2g39795	Uncharacterized protein At2g39795, mitochondrial OS=Arabidopsis thaliana GN=At2g39795 PE=1 SV=1	55.06	37.88	1.18	up	0.00	0.00	yes
TRINITY_DN20069_c0_g6	hypothetical protein POPTR_0016s09060g [Populus trichocarpa]	CCR1	Serine/threonine-protein kinase-like protein CCR1 OS=Arabidopsis thaliana GN=CCR1 PE=1 SV=1	1.76	0.73	1.89	up	0.00	0.00	yes
TRINITY_DN25160_c0_g1	hypothetical protein POPTR_0008s04660g [Populus trichocarpa]	GSTL2	Glutathione S-transferase L2, chloroplastic OS=Arabidopsis thaliana GN=GSTL2 PE=2 SV=1	57.49	44.52	1.03	up	0.00	0.00	yes
TRINITY_DN16718_c0_g1	hypothetical protein POPTR_0010s08380g [Populus trichocarpa]	At1g06650	1-aminocyclopropane-1-carboxylate oxidase homolog 3 OS=Arabidopsis thaliana GN=At1g06650 PE=2 SV=1	32.67	20.40	1.26	up	0.00	0.00	yes
TRINITY_DN26462_c0_g5	PLASTID-SPECIFIC RIBOSOMAL protein 4 [Populus trichocarpa]	RPS31	30S ribosomal protein S31, chloroplastic OS=Arabidopsis thaliana GN=RPS31 PE=1 SV=1	453.99	274.55	1.32	up	0.00	0.00	yes
TRINITY_DN19690_c0_g1	PREDICTED: uncharacterized protein LOC105136408 [Populus euphratica]	-	-	17.05	12.89	1.13	up	0.00	0.00	yes
TRINITY_DN17084_c0_g1	cell division family protein [Populus trichocarpa]	FTSZ1	Cell division protein FtsZ homolog 1, chloroplastic OS=Arabidopsis thaliana GN=FTSZ1 PE=1 SV=2	97.29	72.67	1.02	up	0.00	0.00	yes
TRINITY_DN23971_c1_g1	PREDICTED: eukaryotic translation initiation factor 3 subunit D-like [Populus euphratica]	TIF3D1	Eukaryotic translation initiation factor 3 subunit D OS=Arabidopsis thaliana GN=TIF3D1 PE=1 SV=1	58.86	45.10	1.00	up	0.00	0.00	yes
TRINITY_DN13028_c0_g1	-	-	-	2.59	0.82	2.22	up	0.00	0.00	yes
TRINITY_DN22729_c0_g2	hypothetical protein POPTR_0014s13350g [Populus trichocarpa]	-	-	117.38	77.62	1.18	up	0.00	0.00	yes
TRINITY_DN25628_c0_g1	hypothetical protein POPTR_0001s24900g [Populus trichocarpa]	ufd1	Ubiquitin fusion degradation protein 1 homolog OS=Dictyostelium discoideum GN=ufd1 PE=3 SV=1	9.44	6.48	1.14	up	0.00	0.00	yes
TRINITY_DN13623_c0_g1	PREDICTED: putative E3 ubiquitin-protein ligase LIN-1 [Populus euphratica]	LIN	Putative E3 ubiquitin-protein ligase LIN OS=Medicago truncatula GN=LIN PE=2 SV=1	1.61	0.59	2.04	up	0.00	0.00	yes
TRINITY_DN18988_c0_g1	hypothetical protein POPTR_0012s01130g [Populus trichocarpa]	UGT91A1	UDP-glycosyltransferase 91A1 OS=Arabidopsis thaliana GN=UGT91A1 PE=2 SV=1	6.88	4.94	1.09	up	0.00	0.00	yes
TRINITY_DN16471_c0_g1	PREDICTED: peroxisomal (S)-2-hydroxy-acid oxidase-like [Phoenix dactylifera]	GLO5	Peroxisomal (S)-2-hydroxy-acid oxidase GLO5 OS=Oryza sativa subsp. indica GN=GLO5 PE=3 SV=1	1059.17	482.14	1.72	up	0.00	0.00	yes
TRINITY_DN22895_c0_g3	hypothetical protein POPTR_0098s00310g [Populus trichocarpa]	UGT85A23	7-deoxyloganetin glucosyltransferase OS=Catharanthus roseus GN=UGT85A23 PE=1 SV=1	23.05	12.58	1.47	up	0.00	0.00	yes
TRINITY_DN16211_c2_g2	hypothetical protein POPTR_0015s09110g [Populus trichocarpa]	RPS4	40S ribosomal protein S4 OS=Prunus armeniaca GN=RPS4 PE=2 SV=1	267.35	195.87	1.07	up	0.00	0.00	yes
TRINITY_DN23159_c0_g1	PREDICTED: mediator of RNA polymerase II transcription subunit 33A-like [Populus euphratica]	MED33A	Mediator of RNA polymerase II transcription subunit 33A OS=Arabidopsis thaliana GN=MED33A PE=1 SV=1	22.84	14.43	1.08	up	0.00	0.00	yes
TRINITY_DN27395_c0_g1	aminotransferase 2 family protein [Populus trichocarpa]	AGT1	Serine--glyoxylate aminotransferase OS=Arabidopsis thaliana GN=AGT1 PE=1 SV=2	980.92	451.86	1.70	up	0.00	0.00	yes
TRINITY_DN4499_c0_g1	hypothetical protein POPTR_0019s00990g, partial [Populus trichocarpa]	-	TMV resistance protein N OS=Nicotiana glutinosa GN=N PE=1 SV=1	3.25	1.49	1.73	up	0.00	0.01	yes
TRINITY_DN18350_c0_g3	PREDICTED: glucan endo-1,3-beta-glucosidase 12-like [Populus euphratica]	At1g11820	Glucan endo-1,3-beta-glucosidase 1 OS=Arabidopsis thaliana GN=At1g11820 PE=1 SV=3	7.30	3.93	1.51	up	0.00	0.01	yes
TRINITY_DN18078_c0_g1	PAP-specific phosphatase family protein [Populus trichocarpa]	At4g05090	Putative PAP-specific phosphatase, mitochondrial OS=Arabidopsis thaliana GN=At4g05090 PE=2 SV=1	29.85	20.66	1.16	up	0.00	0.01	yes

TRINITY_DN19375_c0_g1	hypothetical protein POPTR_0010s18960g [Populus trichocarpa]	-	-	31.64	21.55	1.03	up	0.00	0.01	yes
TRINITY_DN22208_c1_g3	PREDICTED: uncharacterized protein LOC105124084 [Populus euphratica]	-	-	137.57	102.01	1.03	up	0.00	0.01	yes
TRINITY_DN22892_c1_g1	PREDICTED: NAD(P)H-quinone oxidoreductase subunit M, chloroplastic [Populus euphratica]	ndhM	NAD(P)H-quinone oxidoreductase subunit M, chloroplastic OS=Populus jackii GN=ndhM PE=2 SV=1	227.22	123.62	1.48	up	0.00	0.01	yes
TRINITY_DN18545_c0_g1	PREDICTED: RNA polymerase sigma factor sigF, chloroplastic isoform X2 [Populus euphratica]	SIGF	RNA polymerase sigma factor sigF, chloroplastic OS=Arabidopsis thaliana GN=SIGF PE=1 SV=1	60.41	43.89	1.06	up	0.00	0.01	yes
TRINITY_DN24939_c0_g2	hypothetical protein POPTR_0009s15720g [Populus trichocarpa]	-	-	68.28	33.62	1.69	up	0.00	0.01	yes
TRINITY_DN22048_c2_g2	hypothetical protein POPTR_0015s12380g [Populus trichocarpa]	CYCU4-1	Cyclin-U4-1 OS=Arabidopsis thaliana GN=CYCU4-1 PE=1 SV=1	3.05	1.20	1.93	up	0.00	0.01	yes
TRINITY_DN18022_c4_g4	-	-	-	3.55	1.32	2.01	up	0.00	0.01	yes
TRINITY_DN25773_c2_g1	hypothetical protein POPTR_0004s18430g [Populus trichocarpa]	-	-	32.10	21.16	1.15	up	0.00	0.01	yes
TRINITY_DN17631_c0_g1	PREDICTED: thioredoxin-like protein HCF164, chloroplastic isoform X2 [Populus euphratica]	HCF164	Thioredoxin-like protein HCF164, chloroplastic OS=Arabidopsis thaliana GN=HCF164 PE=1 SV=2	53.11	40.17	1.10	up	0.00	0.01	yes
TRINITY_DN19208_c0_g1	PREDICTED: protein BCCIP homolog isoform X1 [Populus euphratica]	At2g44510	Protein BCCIP homolog OS=Arabidopsis thaliana GN=At2g44510 PE=1 SV=2	9.22	5.84	1.28	up	0.00	0.01	yes
TRINITY_DN25876_c0_g1	hypothetical protein POPTR_0018s01360g [Populus trichocarpa]	AKT1	Potassium channel AKT1 OS=Arabidopsis thaliana GN=AKT1 PE=1 SV=2	5.73	2.94	1.56	up	0.00	0.01	yes
TRINITY_DN22065_c0_g1	PREDICTED: inactive rhomboid protein 1-like [Populus euphratica]	RBL1	RHOMBOID-like protein 1 OS=Arabidopsis thaliana GN=RBL1 PE=2 SV=1	5.41	3.36	1.32	up	0.00	0.01	yes
TRINITY_DN25902_c0_g1	hypothetical protein POPTR_0007s11280g [Populus trichocarpa]	-	-	19.29	13.48	1.10	up	0.00	0.01	yes
TRINITY_DN24428_c0_g3	PREDICTED: aquaporin TIP1-1-like [Populus euphratica]	TIP1-1	Aquaporin TIP1-1 OS=Zea mays GN=TIP1-1 PE=2 SV=1	165.14	122.17	1.05	up	0.00	0.01	yes
TRINITY_DN18121_c1_g2	hypothetical protein POPTR_0098s00280g [Populus trichocarpa]	UGT85A23	7-deoxyloganetin glucosyltransferase OS=Catharanthus roseus GN=UGT85A23 PE=1 SV=1	19.49	7.07	1.85	up	0.00	0.01	yes
TRINITY_DN26559_c0_g3	PREDICTED: uncharacterized protein LOC105110830 isoform X1 [Populus euphratica]	-	-	52.98	35.41	1.18	up	0.00	0.01	yes
TRINITY_DN4520_c0_g1	unknown [Populus trichocarpa]	-	-	159.83	120.06	1.02	up	0.00	0.01	yes
TRINITY_DN26190_c0_g6	PREDICTED: carotenoid 9,10(9',10')-cleavage dioxygenase 1-like [Populus euphratica]	CCD1	Carotenoid 9,10(9',10')-cleavage dioxygenase 1 OS=Arabidopsis thaliana GN=CCD1 PE=1 SV=2	7.02	4.20	1.35	up	0.00	0.01	yes
TRINITY_DN22803_c0_g3	hypothetical protein POPTR_0001s07250g, partial [Populus trichocarpa]	-	-	39.49	25.87	1.17	up	0.00	0.01	yes
TRINITY_DN20383_c0_g1	CAAX amino terminal protease family protein [Populus trichocarpa]	-	-	75.95	52.99	1.17	up	0.00	0.01	yes
TRINITY_DN21136_c0_g1	PREDICTED: GDT1-like protein 1, chloroplastic isoform X1 [Populus euphratica]	At1g64150	GDT1-like protein 1, chloroplastic OS=Arabidopsis thaliana GN=At1g64150 PE=2 SV=2	69.55	50.48	1.14	up	0.00	0.01	yes
TRINITY_DN12652_c0_g1	hypothetical protein POPTR_0008s13880g, partial [Populus trichocarpa]	-	-	10.21	3.80	1.88	up	0.00	0.01	yes
TRINITY_DN17739_c0_g2	PREDICTED: uncharacterized protein LOC105109948 [Populus euphratica]	-	-	5.05	3.25	1.26	up	0.00	0.01	yes
TRINITY_DN27834_c1_g1	PREDICTED: CTP synthase-like [Populus euphratica]	ctps	CTP synthase OS=Dictyostelium discoideum GN=ctps PE=3 SV=1	31.65	23.97	1.03	up	0.00	0.01	yes
TRINITY_DN27021_c0_g1	Pyrophosphate-energized vacuolar membrane proton pump family protein [Populus trichocarpa]	-	Pyrophosphate-energized vacuolar membrane proton pump OS=Vigna radiata var. radiata PE=1 SV=4	58.91	27.17	1.72	up	0.00	0.01	yes
TRINITY_DN18504_c0_g1	hypothetical protein POPTR_0013s02340g [Populus trichocarpa]	-	-	23.18	16.76	1.10	up	0.00	0.01	yes
TRINITY_DN16526_c0_g1	hypothetical protein POPTR_0004s02210g [Populus trichocarpa]	PDRP1	Probable pyruvate, phosphate dikinase regulatory protein, chloroplastic OS=Oryza sativa subsp. indica GN=PDRP1 PE=3 SV=1	6.99	4.72	1.18	up	0.00	0.01	yes
TRINITY_DN26128_c0_g4	PREDICTED: probable amino-acid acetyltransferase NAGS2, chloroplastic isoform X1 [Populus euphratica]	NAGS1	Probable amino-acid acetyltransferase NAGS1, chloroplastic OS=Arabidopsis thaliana GN=NAGS1 PE=2 SV=1	2.29	0.74	2.19	up	0.00	0.01	yes

TRINITY_DN15499_c0_g1	-	-	-	4.02	1.63	2.02	up	0.00	0.01	yes
TRINITY_DN18383_c0_g1	unknown [Populus trichocarpa x Populus deltoides]	psaD	Photosystem I reaction center subunit II, chloroplastic OS=Cucumis sativus GN=psaD PE=1 SV=1	2480.53	1514.85	1.33	up	0.00	0.01	yes
TRINITY_DN26640_c0_g2	hypothetical protein POPTR_0019s14180g [Populus trichocarpa]	-	-	42.95	26.51	1.31	up	0.00	0.01	yes
TRINITY_DN15820_c0_g2	hypothetical protein POPTR_0014s02920g [Populus trichocarpa]	At1g07700	Thioredoxin-like 4, chloroplastic OS=Arabidopsis thaliana GN=At1g07700 PE=2 SV=1	57.71	39.25	1.16	up	0.00	0.01	yes
TRINITY_DN20539_c0_g1	PREDICTED: thioredoxin-like 2, chloroplastic [Populus euphratica]	At4g26160	Thioredoxin-like 2-1, chloroplastic OS=Arabidopsis thaliana GN=At4g26160 PE=2 SV=2	16.48	11.94	1.05	up	0.00	0.01	yes
TRINITY_DN17543_c0_g2	PREDICTED: uncharacterized protein LOC102589628 [Solanum tuberosum]	-	-	33.98	21.12	1.32	up	0.00	0.01	yes
TRINITY_DN15305_c0_g2	PREDICTED: ankyrin repeat domain-containing protein 50-like [Populus euphratica]	-	-	3.12	1.25	1.92	up	0.00	0.01	yes
TRINITY_DN16462_c0_g1	hypothetical protein POPTR_0014s18360g [Populus trichocarpa]	-	-	104.10	79.36	1.02	up	0.00	0.01	yes
TRINITY_DN19983_c0_g1	pentatricopeptide repeat-containing family protein [Populus trichocarpa]	PCMP-H38	Pentatricopeptide repeat-containing protein At5g48910 OS=Arabidopsis thaliana GN=PCMP-H38 PE=2 SV=1	8.95	6.44	1.13	up	0.00	0.01	yes
TRINITY_DN25281_c0_g1	PREDICTED: rhodanese-like domain-containing protein 4, chloroplastic [Populus euphratica]	STR4	Rhodanese-like domain-containing protein 4, chloroplastic OS=Arabidopsis thaliana GN=STR4 PE=1 SV=2	353.68	246.13	1.16	up	0.00	0.01	yes
TRINITY_DN18296_c0_g1	senescence-associated family protein [Populus trichocarpa]	-	-	193.38	102.71	1.38	up	0.00	0.01	yes
TRINITY_DN25333_c1_g1	PREDICTED: zeta-carotene desaturase, chloroplastic/chromoplastic-like [Populus euphratica]	ZDS	Zeta-carotene desaturase, chloroplastic/chromoplastic OS=Solanum lycopersicum GN=ZDS PE=2 SV=1	73.76	55.47	1.06	up	0.00	0.01	yes
TRINITY_DN24425_c1_g7	RecName: Full=DNA-directed RNA polymerase subunit beta'; AltName: Full=PEP; AltName: Full=Plastid-encoded RNA polymerase subunit beta'; Short=RNA polymerase subunit beta'	rpoC1	DNA-directed RNA polymerase subunit beta' OS=Sinapis alba GN=rpoC1 PE=1 SV=2	5.92	2.43	1.89	up	0.00	0.01	yes
TRINITY_DN26850_c0_g2	PREDICTED: uncharacterized protein LOC105133582 isoform X1 [Populus euphratica]	SMXL7	Protein SMAX1-LIKE 7 OS=Arabidopsis thaliana GN=SMXL7 PE=1 SV=1	8.63	6.51	1.04	up	0.00	0.01	yes
TRINITY_DN22220_c0_g1	beta-ketoacyl-CoA synthase family protein [Populus trichocarpa]	KCS7	3-ketoacyl-CoA synthase 7 OS=Arabidopsis thaliana GN=KCS7 PE=2 SV=1	15.47	13.48	1.13	up	0.00	0.01	yes
TRINITY_DN23839_c0_g1	hypothetical protein POPTR_0013s13200g [Populus trichocarpa]	-	-	103.25	65.71	1.22	up	0.00	0.01	yes
TRINITY_DN18234_c0_g3	-	-	-	3.80	1.24	2.31	up	0.00	0.01	yes
TRINITY_DN24973_c0_g4	hypothetical protein POPTR_0013s10400g [Populus trichocarpa]	-	-	3.08	1.28	1.89	up	0.00	0.01	yes
TRINITY_DN18287_c1_g3	-	-	-	17.14	8.95	1.53	up	0.00	0.01	yes
TRINITY_DN24423_c1_g1	ribosomal protein L19 [Populus trichocarpa]	At4g17560	50S ribosomal protein L19-1, chloroplastic OS=Arabidopsis thaliana GN=At4g17560 PE=2 SV=1	497.84	333.85	1.17	up	0.00	0.01	yes
TRINITY_DN27809_c1_g1	PREDICTED: magnesium-chelatase subunit ChlH, chloroplastic-like [Populus euphratica]	CHLH	Magnesium-chelatase subunit ChlH, chloroplastic OS=Arabidopsis thaliana GN=CHLH PE=1 SV=1	80.77	63.74	1.01	up	0.00	0.01	yes
TRINITY_DN16566_c0_g1	hypothetical protein POPTR_0014s13600g [Populus trichocarpa]	-	-	11.99	7.86	1.19	up	0.00	0.01	yes
TRINITY_DN16083_c0_g1	hypothetical protein POPTR_0009s01400g [Populus trichocarpa]	-	-	37.62	23.35	1.24	up	0.00	0.01	yes
TRINITY_DN18416_c0_g2	BiP isoform A family protein [Populus trichocarpa]	BIP4	Luminal-binding protein 4 OS=Nicotiana tabacum GN=BIP4 PE=2 SV=1	3.54	2.09	1.40	up	0.00	0.01	yes
TRINITY_DN22112_c0_g2	PREDICTED: Werner Syndrome-like exonuclease [Populus euphratica]	WEX	Werner Syndrome-like exonuclease OS=Arabidopsis thaliana GN=WEX PE=1 SV=1	9.53	6.74	1.32	up	0.00	0.01	yes
TRINITY_DN21981_c0_g1	PREDICTED: peptide methionine sulfoxide reductase B5-like [Populus euphratica]	MSRB2	Peptide methionine sulfoxide reductase B2, chloroplastic OS=Arabidopsis thaliana GN=MSRB2 PE=1 SV=1	193.71	129.41	1.18	up	0.00	0.01	yes
TRINITY_DN19171_c0_g1	PREDICTED: early nodulin-like protein 2 [Populus euphratica]	ENODL1	Early nodulin-like protein 1 OS=Oryza sativa subsp. japonica GN=ENODL1 PE=1 SV=1	4.12	1.17	2.41	up	0.00	0.01	yes
TRINITY_DN23869_c0_g2	PREDICTED: ethylene-responsive transcription factor RAP2-12-like isoform X2 [Populus euphratica]	RAP2-3	Ethylene-responsive transcription factor RAP2-3 OS=Arabidopsis thaliana GN=RAP2-3 PE=1 SV=2	18.61	11.90	1.26	up	0.00	0.01	yes

TRINITY_DN27525_c0_g1	hypothetical protein POPTR_0006s01590g [Populus trichocarpa]	At5g47540	Putative MO25-like protein At5g47540 OS=Arabidopsis thaliana GN=At5g47540 PE=2 SV=1	176.88	122.84	1.08	up	0.00	0.01	yes
TRINITY_DN27861_c3_g1	hypothetical protein POPTR_0014s09410g [Populus trichocarpa]	TSS	Protein TSS OS=Arabidopsis thaliana GN=TSS PE=1 SV=1	116.73	81.03	1.08	up	0.00	0.01	yes
TRINITY_DN18927_c0_g2	hypothetical protein POPTR_0001s08140g [Populus trichocarpa]	CLPT2	ATP-dependent Clp protease ATP-binding subunit CLPT2, chloroplastic OS=Arabidopsis thaliana GN=CLPT2 PE=1 SV=1	129.13	92.58	1.07	up	0.00	0.01	yes
TRINITY_DN16960_c0_g1	glyoxal oxidase-related family protein [Populus trichocarpa]	GLOX	Aldehyde oxidase GLOX OS=Vitis pseudoreticulata GN=GLOX PE=2 SV=1	18.67	10.99	1.33	up	0.00	0.01	yes
TRINITY_DN23278_c1_g1	PREDICTED: glutamate--glyoxylate aminotransferase 2 isoform X1 [Populus euphratica]	GGAT2	Glutamate--glyoxylate aminotransferase 2 OS=Arabidopsis thaliana GN=GGAT2 PE=1 SV=1	580.61	318.60	1.44	up	0.00	0.01	yes
TRINITY_DN18119_c1_g1	hypothetical protein POPTR_0013s06360g [Populus trichocarpa]	CBR1	NADH--cytochrome b5 reductase 1 OS=Arabidopsis thaliana GN=CBR1 PE=1 SV=1	131.23	89.11	1.17	up	0.00	0.01	yes
TRINITY_DN21156_c0_g5	nitroreductase family protein [Populus trichocarpa]	-	-	5.91	4.44	1.02	up	0.00	0.01	yes
TRINITY_DN20234_c0_g1	-	-	-	5.06	1.28	2.52	up	0.00	0.01	yes
TRINITY_DN18933_c0_g1	PREDICTED: psbP domain-containing protein 2, chloroplastic [Populus euphratica]	PPD2	PsbP domain-containing protein 2, chloroplastic OS=Arabidopsis thaliana GN=PPD2 PE=1 SV=1	3.35	1.56	1.79	up	0.00	0.01	yes
TRINITY_DN27203_c1_g3	aldo/keto reductase family protein [Populus trichocarpa]	At1g06690	Uncharacterized oxidoreductase At1g06690, chloroplastic OS=Arabidopsis thaliana GN=At1g06690 PE=1 SV=1	97.45	56.97	1.38	up	0.00	0.01	yes
TRINITY_DN23299_c1_g2	PREDICTED: calcineurin B-like protein 10 isoform X1 [Populus euphratica]	CBL10	Calcineurin B-like protein 10 OS=Arabidopsis thaliana GN=CBL10 PE=1 SV=1	36.75	27.64	1.05	up	0.00	0.01	yes
TRINITY_DN20951_c0_g3	PREDICTED: uncharacterized protein LOC105110678 isoform X1 [Populus euphratica]	murG	UDP-N-acetylglucosamine--N-acetylmuramyl-(pentapeptide) pyrophosphoryl-undecaprenol N-acetylglucosamine transferase OS=Salinibacter ruber (strain DSM 13855 / M31) GN=murG PE=3 SV=1	9.62	6.75	1.14	up	0.00	0.01	yes
TRINITY_DN20870_c0_g2	hypothetical protein POPTR_0012s02830g [Populus trichocarpa]	-	-	12.88	9.68	1.01	up	0.00	0.01	yes
TRINITY_DN25107_c0_g1	HXXXD-type acyl-transferase family protein [Populus tomentosa]	At3g50280	Uncharacterized acetyltransferase At3g50280 OS=Arabidopsis thaliana GN=At3g50280 PE=3 SV=1	31.54	21.30	1.16	up	0.00	0.01	yes
TRINITY_DN18587_c1_g1	PREDICTED: probable carboxylesterase 15 isoform X1 [Ricinus communis]	-	-	38.07	25.58	1.18	up	0.00	0.01	yes
TRINITY_DN18030_c1_g4	oxygen evolving enhancer 3 family protein [Populus trichocarpa]	PNSL2	Photosynthetic NDH subunit of lumenal location 2, chloroplastic OS=Arabidopsis thaliana GN=PNSL2 PE=1 SV=1	306.45	163.48	1.49	up	0.00	0.01	yes
TRINITY_DN20752_c1_g4	hypothetical protein POPTR_0016s05290g [Populus trichocarpa]	UGT75L6	Crocetin glucosyltransferase, chloroplastic OS=Gardenia jasminoides GN=UGT75L6 PE=1 SV=1	2.74	1.01	2.25	up	0.00	0.01	yes
TRINITY_DN16384_c0_g1	hypothetical protein POPTR_0009s04820g [Populus trichocarpa]	-	-	87.61	52.51	1.35	up	0.00	0.01	yes
TRINITY_DN25889_c0_g2	unknown [Populus trichocarpa x Populus deltoides]	BGAL1	Beta-galactosidase 1 OS=Arabidopsis thaliana GN=BGAL1 PE=2 SV=1	384.22	194.87	1.59	up	0.00	0.01	yes
TRINITY_DN16397_c0_g2	hypothetical protein POPTR_0001s11360g [Populus trichocarpa]	CYP78A9	Cytochrome P450 78A9 OS=Arabidopsis thaliana GN=CYP78A9 PE=2 SV=1	3.14	1.59	1.60	up	0.00	0.01	yes
TRINITY_DN16197_c0_g1	PREDICTED: CTP synthase [Populus euphratica]	-	-	62.94	46.27	1.09	up	0.00	0.01	yes
TRINITY_DN24032_c2_g2	PREDICTED: probable N-acetyltransferase HLS1 [Populus euphratica]	HLS1	Probable N-acetyltransferase HLS1 OS=Arabidopsis thaliana GN=HLS1 PE=1 SV=1	1.95	0.57	2.36	up	0.00	0.01	yes
TRINITY_DN19959_c0_g1	hypothetical protein POPTR_0005s25810g [Populus trichocarpa]	-	-	31.81	22.34	1.17	up	0.00	0.01	yes
TRINITY_DN12359_c0_g1	hypothetical protein POPTR_0001s41660g [Populus trichocarpa]	-	-	32.48	18.12	1.45	up	0.00	0.01	yes
TRINITY_DN13558_c0_g2	hypothetical protein POPTR_0004s23900g [Populus trichocarpa]	At1g14450	NADH dehydrogenase [ubiquinone] 1 beta subcomplex subunit 3-B OS=Arabidopsis thaliana GN=At1g14450 PE=3 SV=1	81.07	59.14	1.05	up	0.00	0.01	yes
TRINITY_DN26697_c0_g2	unknown [Populus trichocarpa]	Bp10	L-ascorbate oxidase homolog OS=Brassica napus GN=Bp10 PE=2 SV=1	28.25	21.14	1.03	up	0.00	0.01	yes
TRINITY_DN17780_c0_g1	hypothetical protein POPTR_0010s23280g [Populus trichocarpa]	Slc32a1	Vesicular inhibitory amino acid transporter OS=Mus musculus GN=Slc32a1 PE=1 SV=3	4.94	1.56	2.34	up	0.00	0.01	yes
TRINITY_DN19469_c0_g3	Ku70-binding family protein [Populus trichocarpa]	ATP23	Mitochondrial inner membrane protease ATP23 OS=Cryptococcus neoformans var. neoformans serotype D (strain JEC21 / ATCC MYA-565) GN=ATP23 PE=3 SV=1	19.57	14.30	1.10	up	0.00	0.01	yes

TRINITY_DN17822_c1_g11	-	-	-	10.86	5.20	1.67	up	0.00	0.01	yes
TRINITY_DN23229_c0_g2	PREDICTED: plastidial lipoyltransferase 2-like [Populus euphratica]	At1g47578	Putative lipoyltransferase-like protein, chloroplastic OS=Arabidopsis thaliana GN=At1g47578 PE=3 SV=1	3.15	1.81	1.41	up	0.00	0.01	yes
TRINITY_DN24421_c0_g6	PREDICTED: keratin, type I cytoskeletal 16-like isoform X2 [Populus euphratica]	-	-	17.71	10.40	1.39	up	0.00	0.01	yes
TRINITY_DN16751_c0_g2	Photosystem II core complex proteins psbY [Populus trichocarpa]	PSBY	Photosystem II core complex proteins psbY, chloroplastic OS=Spinacia oleracea GN=PSBY PE=1 SV=2	1306.26	779.10	1.35	up	0.00	0.01	yes
TRINITY_DN24692_c0_g1	core region of GTP cyclohydrolase I family protein [Populus trichocarpa]	GCH1	GTP cyclohydrolase 1 OS=Solanum lycopersicum GN=GCH1 PE=1 SV=1	21.04	12.47	1.34	up	0.00	0.01	yes
TRINITY_DN23777_c1_g1	hypothetical protein POPTR_0014s08880g [Populus trichocarpa]	COQ3	Ubiquinone biosynthesis O-methyltransferase, mitochondrial OS=Arabidopsis thaliana GN=COQ3 PE=2 SV=2	21.90	17.49	1.05	up	0.00	0.01	yes
TRINITY_DN25623_c0_g2	PREDICTED: uncharacterized protein LOC105122890 [Populus euphratica]	-	-	262.57	174.86	1.24	up	0.00	0.01	yes
TRINITY_DN17969_c0_g1	ribosomal protein L37 [Populus trichocarpa]	RPL37B	60S ribosomal protein L37-2 OS=Arabidopsis thaliana GN=RPL37B PE=3 SV=2	301.06	223.50	1.04	up	0.00	0.01	yes
TRINITY_DN23308_c1_g4	hypothetical protein POPTR_0007s04730g [Populus trichocarpa]	-	-	15.71	10.50	1.19	up	0.00	0.01	yes
TRINITY_DN17576_c0_g1	hypothetical protein POPTR_0001s06950g [Populus trichocarpa]	-	-	6.99	2.77	1.71	up	0.00	0.01	yes
TRINITY_DN25902_c0_g3	hypothetical protein POPTR_0005s18000g [Populus trichocarpa]	-	-	12.53	7.95	1.27	up	0.00	0.01	yes
TRINITY_DN23622_c0_g2	-	-	-	85.94	61.57	1.10	up	0.00	0.01	yes
TRINITY_DN20221_c0_g4	thylakoid assembly family protein [Populus trichocarpa]	TATA	Sec-independent protein translocase protein TATA, chloroplastic OS=Arabidopsis thaliana GN=TATA PE=1 SV=1	134.86	96.73	1.08	up	0.00	0.01	yes
TRINITY_DN24321_c0_g1	ammonium transporter family protein [Populus trichocarpa]	AMT1-1	Ammonium transporter 1 member 1 OS=Arabidopsis thaliana GN=AMT1-1 PE=1 SV=1	95.05	70.18	1.12	up	0.00	0.01	yes
TRINITY_DN17878_c0_g1	PREDICTED: NADH dehydrogenase [ubiquinone] complex I, assembly factor 7 homolog [Populus euphratica]	-	-	8.25	5.44	1.29	up	0.00	0.01	yes
TRINITY_DN12277_c1_g1	ycf1 [Populus alba]	TIC214	Protein TIC 214 OS=Populus alba GN=TIC214 PE=3 SV=1	7.07	2.43	2.10	up	0.00	0.01	yes
TRINITY_DN18603_c0_g1	hypothetical protein POPTR_0003s14190g [Populus trichocarpa]	KAS1	3-oxoacyl-[acyl-carrier-protein] synthase I, chloroplastic OS=Arabidopsis thaliana GN=KAS1 PE=1 SV=2	6.90	4.98	1.08	up	0.00	0.01	yes
TRINITY_DN25162_c0_g1	hypothetical protein POPTR_0006s28550g [Populus trichocarpa]	Slc35d3	Solute carrier family 35 member D3 OS=Mus musculus GN=Slc35d3 PE=2 SV=1	17.21	11.32	1.18	up	0.00	0.01	yes
TRINITY_DN24243_c0_g2	PREDICTED: probable receptor-like protein kinase At5g59700 [Populus euphratica]	HERK1	Receptor-like protein kinase HERK 1 OS=Arabidopsis thaliana GN=HERK1 PE=1 SV=1	3.96	2.10	1.50	up	0.00	0.01	yes
TRINITY_DN18807_c0_g6	leucine-rich repeat family protein [Populus trichocarpa]	PSY1R	Tyrosine-sulfated glycopeptide receptor 1 OS=Arabidopsis thaliana GN=PSY1R PE=1 SV=1	4.80	2.97	1.30	up	0.00	0.01	yes
TRINITY_DN20371_c0_g2	PREDICTED: uncharacterized protein C6G9.01c [Populus euphratica]	-	-	42.10	33.03	1.02	up	0.00	0.01	yes
TRINITY_DN26335_c0_g1	PREDICTED: 50S ribosomal protein L1, chloroplastic-like [Populus euphratica]	RPL1	50S ribosomal protein L1, chloroplastic OS=Arabidopsis thaliana GN=RPL1 PE=1 SV=1	574.73	389.40	1.12	up	0.00	0.01	yes
TRINITY_DN4864_c0_g2	hypothetical protein POPTR_0011s00570g, partial [Populus trichocarpa]	-	-	8.71	5.13	1.38	up	0.00	0.01	yes
TRINITY_DN20520_c1_g6	-	-	-	7.95	2.88	2.05	up	0.00	0.01	yes
TRINITY_DN13852_c0_g1	hypothetical protein POPTR_0002s05910g [Populus trichocarpa]	-	-	7.17	3.74	1.54	up	0.00	0.01	yes
TRINITY_DN20799_c0_g4	-	-	-	59.98	26.22	1.82	up	0.00	0.01	yes
TRINITY_DN14382_c0_g1	hypothetical protein POPTR_0010s21170g [Populus trichocarpa]	-	-	12.49	7.45	1.35	up	0.00	0.01	yes
TRINITY_DN22611_c0_g2	hypothetical protein POPTR_0019s09820g [Populus trichocarpa]	-	-	49.65	35.58	1.09	up	0.00	0.01	yes
TRINITY_DN19472_c0_g2	hypothetical protein POPTR_0007s01130g [Populus trichocarpa]	At5g53970	Probable aminotransferase TAT2 OS=Arabidopsis thaliana GN=At5g53970 PE=2 SV=1	8.54	5.46	1.32	up	0.00	0.01	yes
TRINITY_DN24112_c0_g4	hypothetical protein POPTR_0545s00210g [Populus trichocarpa]	GT6	UDP-glucose flavonoid 3-O-glucosyltransferase 6 OS=Fragaria ananassa GN=GT6 PE=1 SV=1	22.08	15.72	1.11	up	0.00	0.01	yes

TRINITY_DN25430_c0_g2	-	-	-	2.06	0.70	2.15	up	0.00	0.01	yes
TRINITY_DN26828_c0_g4	hypothetical protein POPTR_0012s12240g [Populus trichocarpa]	-	-	3.80	1.52	1.92	up	0.00	0.01	yes
TRINITY_DN27079_c0_g6	-	-	-	26.65	15.30	1.40	up	0.00	0.01	yes
TRINITY_DN22811_c0_g6	hypothetical protein POPTR_0010s03090g [Populus trichocarpa]	PCMP-E42	Pentatricopeptide repeat-containing protein At5g19020, mitochondrial OS=Arabidopsis thaliana GN=PCMP-E42 PE=2 SV=1	2.86	1.78	1.30	up	0.00	0.01	yes
TRINITY_DN16025_c1_g2	-	-	-	90.62	56.74	1.25	up	0.00	0.01	yes
TRINITY_DN16529_c0_g1	DnaJ-like family protein [Populus trichocarpa]	ATJ11	Chaperone protein dnaJ 11, chloroplastic OS=Arabidopsis thaliana GN=ATJ11 PE=1 SV=2	21.38	8.21	1.97	up	0.00	0.01	yes
TRINITY_DN21650_c0_g1	hypothetical protein POPTR_0014s05970g [Populus trichocarpa]	At1g05030	Probable plastidic glucose transporter 1 OS=Arabidopsis thaliana GN=At1g05030 PE=2 SV=2	12.34	8.13	1.20	up	0.00	0.01	yes
TRINITY_DN14667_c0_g1	hypothetical protein POPTR_0012s03380g [Populus trichocarpa]	BOLA1	Protein BOLA1, chloroplastic OS=Arabidopsis thaliana GN=BOLA1 PE=1 SV=1	20.86	13.33	1.26	up	0.00	0.01	yes
TRINITY_DN19319_c0_g1	hypothetical protein POPTR_0014s11630g [Populus trichocarpa]	DIM1A	Ribosomal RNA small subunit methyltransferase OS=Arabidopsis thaliana GN=DIM1A PE=1 SV=1	13.26	9.69	1.05	up	0.00	0.01	yes
TRINITY_DN12877_c0_g1	hypothetical protein POPTR_0011s10030g [Populus trichocarpa]	-	-	12.67	9.04	1.04	up	0.00	0.01	yes
TRINITY_DN6433_c0_g1	expressed protein [Arabidopsis lyrata subsp. lyrata]	-	-	10.60	4.84	1.77	up	0.00	0.01	yes
TRINITY_DN17832_c3_g3	PREDICTED: uncharacterized protein LOC105108965 isoform X3 [Populus euphratica]	-	-	37.02	9.20	3.01	up	0.00	0.01	yes
TRINITY_DN16330_c0_g1	brix domain-containing family protein [Populus trichocarpa]	Os01g0513800	Ribosome production factor 2 homolog OS=Oryza sativa subsp. japonica GN=Os01g0513800 PE=2 SV=1	23.19	17.32	1.06	up	0.00	0.01	yes
TRINITY_DN17264_c0_g1	hypothetical protein POPTR_0012s09990g [Populus trichocarpa]	-	-	22.20	11.39	1.57	up	0.00	0.01	yes
TRINITY_DN19477_c0_g1	hypothetical protein POPTR_0001s07070g [Populus trichocarpa]	kdgA	KHG/KDPG aldolase OS=Bacillus subtilis (strain 168) GN=kdgA PE=2 SV=1	35.39	22.84	1.18	up	0.00	0.01	yes
TRINITY_DN12803_c0_g1	hypothetical protein POPTR_0013s09480g [Populus trichocarpa]	-	-	5.84	1.69	2.37	up	0.00	0.01	yes
TRINITY_DN21234_c1_g4	leucine-rich repeat transmembrane protein kinase [Populus trichocarpa]	RCH2	Receptor-like protein kinase 2 OS=Arabidopsis thaliana GN=RCH2 PE=1 SV=1	1.86	0.82	1.77	up	0.00	0.01	yes
TRINITY_DN23686_c0_g2	hypothetical protein POPTR_0005s04710g [Populus trichocarpa]	NOP5-1	Probable nucleolar protein 5-1 OS=Arabidopsis thaliana GN=NOP5-1 PE=1 SV=2	84.77	60.50	1.14	up	0.00	0.01	yes
TRINITY_DN26922_c0_g4	-	-	-	5.82	2.33	1.92	up	0.00	0.01	yes
TRINITY_DN16318_c0_g1	PREDICTED: uncharacterized protein LOC105121500 [Populus euphratica]	-	-	33.00	24.04	1.04	up	0.00	0.01	yes
TRINITY_DN26586_c0_g1	hypothetical protein POPTR_0006s19380g [Populus trichocarpa]	-	-	206.75	122.16	1.37	up	0.00	0.01	yes
TRINITY_DN22194_c0_g3	pyridoxin biosynthesis PDX1-like protein 2 [Populus trichocarpa]	PDX12	Pyridoxal 5'-phosphate synthase-like subunit PDX1.2 OS=Arabidopsis thaliana GN=PDX12 PE=1 SV=1	8.07	5.60	1.15	up	0.00	0.01	yes
TRINITY_DN17620_c0_g1	hypothetical protein POPTR_0006s14620g, partial [Populus trichocarpa]	RPA1A	Replication protein A 70 kDa DNA-binding subunit A OS=Arabidopsis thaliana GN=RPA1A PE=1 SV=1	1053.56	627.95	1.37	up	0.00	0.01	yes
TRINITY_DN16165_c0_g2	PREDICTED: uncharacterized protein LOC105141681 isoform X1 [Populus euphratica]	-	-	236.04	151.59	1.22	up	0.00	0.01	yes
TRINITY_DN16993_c0_g1	hypothetical protein POPTR_0002s02650g [Populus trichocarpa]	-	-	30.38	22.23	1.06	up	0.00	0.01	yes
TRINITY_DN26012_c0_g6	hypothetical protein POPTR_0014s17730g [Populus trichocarpa]	-	-	38.20	21.23	1.44	up	0.00	0.01	yes
TRINITY_DN16279_c0_g1	UDP-glucoronosyl/UDP-glucosyl transferase family protein [Populus trichocarpa]	UGT92A1	UDP-glycosyltransferase 92A1 OS=Arabidopsis thaliana GN=UGT92A1 PE=2 SV=1	24.39	16.78	1.13	up	0.00	0.01	yes
TRINITY_DN24102_c0_g2	hypothetical protein POPTR_0013s04510g [Populus trichocarpa]	AATP1	AAA-ATPase ASD, mitochondrial OS=Arabidopsis thaliana GN=AATP1 PE=1 SV=1	5.18	3.54	1.17	up	0.00	0.01	yes
TRINITY_DN16620_c0_g1	remorin family protein [Populus trichocarpa]	-	Remorin OS=Solanum tuberosum PE=1 SV=1	15.62	8.91	1.50	up	0.00	0.01	yes
TRINITY_DN21518_c1_g2	hypothetical protein POPTR_0014s10430g, partial [Populus trichocarpa]	-	-	8.09	3.86	1.63	up	0.00	0.01	yes

TRINITY_DN22171_c2_g1	PREDICTED: S-adenosylmethionine synthase 3 [Populus euphratica]	METK3	S-adenosylmethionine synthase 3 OS=Populus trichocarpa GN=METK3 PE=2 SV=1	228.61	137.13	1.24	up	0.00	0.01	yes
TRINITY_DN16778_c0_g1	hypothetical protein POPTR_0006s24590g [Populus trichocarpa]	ycf36	Uncharacterized protein ycf36 OS=Cyanophora paradoxa GN=ycf36 PE=3 SV=1	36.74	27.51	1.03	up	0.00	0.01	yes
TRINITY_DN20530_c0_g7	protease HhoA family protein [Populus trichocarpa]	DEGP5	Protease Do-like 5, chloroplastic OS=Arabidopsis thaliana GN=DEGP5 PE=1 SV=3	46.86	32.87	1.10	up	0.00	0.01	yes
TRINITY_DN21015_c0_g1	hypothetical protein POPTR_0008s04760g [Populus trichocarpa]	-	-	104.25	68.37	1.22	up	0.00	0.01	yes
TRINITY_DN20235_c0_g1	PREDICTED: protein trichome berefringence-like 7 isoform X1 [Populus euphratica]	TBL7	Protein trichome berefringence-like 7 OS=Arabidopsis thaliana GN=TBL7 PE=3 SV=1	3.71	1.87	1.56	up	0.00	0.01	yes
TRINITY_DN20494_c2_g2	PREDICTED: uncharacterized protein LOC105127308 [Populus euphratica]	-	-	47.45	34.89	1.03	up	0.00	0.01	yes
TRINITY_DN23588_c0_g2	hypothetical protein POPTR_0012s02130g [Populus trichocarpa]	-	-	5.55	2.72	1.60	up	0.00	0.01	yes
TRINITY_DN18813_c1_g7	hypothetical protein POPTR_0002s213902g, partial [Populus trichocarpa]	-	-	80.82	53.81	1.18	up	0.00	0.01	yes
TRINITY_DN20140_c2_g1	PREDICTED: uncharacterized protein LOC105135125 isoform X2 [Populus euphratica]	-	-	53.06	39.06	1.05	up	0.00	0.01	yes
TRINITY_DN27367_c0_g1	PREDICTED: uncharacterized aarF domain-containing protein kinase At1g79600, chloroplastic-like isoform X1 [Populus euphratica]	sll1770	Uncharacterized protein sll1770 OS=Synechocystis sp. (strain PCC 6803 / Kazusa) GN=sll1770 PE=3 SV=1	257.25	204.05	1.01	up	0.00	0.01	yes
TRINITY_DN20394_c0_g1	PREDICTED: histidine biosynthesis bifunctional protein hisIE, chloroplastic [Populus euphratica]	HISN2	Histidine biosynthesis bifunctional protein hisIE, chloroplastic OS=Arabidopsis thaliana GN=HISN2 PE=1 SV=1	84.52	63.75	1.01	up	0.00	0.01	yes
TRINITY_DN21356_c0_g1	hypothetical protein POPTR_0015s13920g [Populus trichocarpa]	DAPB3	Dihydrodipicolinate reductase-like protein CRR1, chloroplastic OS=Arabidopsis thaliana GN=DAPB3 PE=2 SV=1	186.83	114.64	1.21	up	0.00	0.01	yes
TRINITY_DN19224_c0_g1	hypothetical protein POPTR_0004s09550g [Populus trichocarpa]	PMAT1	Phenolic glucoside malonyltransferase 1 OS=Arabidopsis thaliana GN=PMAT1 PE=1 SV=1	55.14	35.39	1.22	up	0.00	0.01	yes
TRINITY_DN18790_c0_g2	PREDICTED: outer envelope pore protein 24A, chloroplastic [Populus euphratica]	OEP24A	Outer envelope pore protein 24A, chloroplastic OS=Arabidopsis thaliana GN=OEP24A PE=1 SV=1	57.06	41.27	1.09	up	0.00	0.01	yes
TRINITY_DN23596_c1_g2	hypothetical protein POPTR_0005s07470g [Populus trichocarpa]	-	-	7.67	5.51	1.09	up	0.00	0.01	yes
TRINITY_DN22830_c0_g1	PREDICTED: chaperone protein ClpC, chloroplastic [Populus euphratica]	CLPC1	Chaperone protein ClpC1, chloroplastic OS=Arabidopsis thaliana GN=CLPC1 PE=1 SV=1	338.74	232.98	1.11	up	0.00	0.01	yes
TRINITY_DN21189_c1_g1	PREDICTED: glycine-rich protein 2-like [Populus euphratica]	GRP-2	Glycine-rich protein 2 OS=Nicotiana sylvestris GN=GRP-2 PE=2 SV=1	28.80	17.96	1.28	up	0.00	0.01	yes
TRINITY_DN25974_c0_g7	-	-	-	28.52	12.42	1.81	up	0.00	0.01	yes
TRINITY_DN16020_c0_g1	hypothetical protein POPTR_0006s02610g [Populus trichocarpa]	-	-	55.59	31.56	1.40	up	0.00	0.01	yes
TRINITY_DN21013_c0_g3	hypothetical protein POPTR_0008s15720g [Populus trichocarpa]	VIT_05s0020g04	1,2-dihydroxy-3-keto-5-methylthiopentene dioxygenase 1 OS=Vitis vinifera GN=VIT_05s0020g04070 PE=3 SV=1	79.15	58.37	1.06	up	0.00	0.01	yes
TRINITY_DN22717_c0_g1	short-chain dehydrogenase Tic32 family protein [Populus trichocarpa]	TIC32	Short-chain dehydrogenase TIC 32, chloroplastic OS=Pisum sativum GN=TIC32 PE=1 SV=1	31.66	18.43	1.24	up	0.00	0.01	yes
TRINITY_DN26463_c0_g1	hypothetical protein POPTR_0010s19710g [Populus trichocarpa]	CYP90A1	Cytochrome P450 90A1 OS=Arabidopsis thaliana GN=CYP90A1 PE=2 SV=1	37.16	25.77	1.00	up	0.00	0.01	yes
TRINITY_DN17325_c0_g1	hypothetical protein POPTR_0010s00630g [Populus trichocarpa]	-	-	38.54	24.37	1.26	up	0.00	0.01	yes
TRINITY_DN22920_c1_g7	hypothetical protein POPTR_0004s05320g [Populus trichocarpa]	PCMP-E34	Pentatricopeptide repeat-containing protein At1g28690, mitochondrial OS=Arabidopsis thaliana GN=PCMP-E34 PE=2 SV=2	5.46	2.68	1.63	up	0.00	0.01	yes
TRINITY_DN1476_c0_g1	hypothetical protein POPTR_0002s09850g [Populus trichocarpa]	UGT85A24	7-deoxyloganetin glucosyltransferase OS=Gardenia jasminoides GN=UGT85A24 PE=1 SV=1	10.24	5.93	1.40	up	0.00	0.01	yes
TRINITY_DN22383_c0_g1	hypothetical protein POPTR_1173s00200g [Populus trichocarpa]	-	-	49.90	35.05	1.08	up	0.00	0.01	yes
TRINITY_DN21807_c1_g1	PREDICTED: pentatricopeptide repeat-containing protein At3g46790, chloroplastic [Populus euphratica]	CRR2	Pentatricopeptide repeat-containing protein At3g46790, chloroplastic OS=Arabidopsis thaliana GN=CRR2 PE=2 SV=1	6.02	4.47	1.05	up	0.00	0.01	yes

TRINITY_DN20469_c0_g1	3' exoribonuclease domain 1-containing family protein [Populus trichocarpa]	RRP41L	Exosome complex component RRP41-like OS=Arabidopsis thaliana GN=RRP41L PE=2 SV=1	25.29	19.75	1.03	up	0.00	0.01	yes
TRINITY_DN15387_c0_g1	translation initiation factor IF-2 family protein [Populus trichocarpa]	At1g17220	Translation initiation factor IF-2, chloroplastic OS=Arabidopsis thaliana GN=At1g17220 PE=2 SV=2	202.78	134.85	1.20	up	0.00	0.01	yes
TRINITY_DN22768_c0_g2	PREDICTED: pentatricopeptide repeat-containing protein At4g36680, mitochondrial [Populus euphratica]	At4g36680	Pentatricopeptide repeat-containing protein At4g36680, mitochondrial OS=Arabidopsis thaliana GN=At4g36680 PE=1 SV=1	5.96	3.84	1.26	up	0.00	0.01	yes
TRINITY_DN18469_c0_g1	PREDICTED: uncharacterized protein LOC105125046 [Populus euphratica]	-	-	73.74	52.79	1.08	up	0.00	0.01	yes
TRINITY_DN26004_c0_g1	PREDICTED: E3 ubiquitin-protein ligase RMA1H1 [Populus euphratica]	RMA1H1	E3 ubiquitin-protein ligase RMA1H1 OS=Capsicum annuum GN=RMA1H1 PE=1 SV=1	42.42	15.41	2.02	up	0.00	0.01	yes
TRINITY_DN26451_c2_g1	PREDICTED: probable receptor-like protein kinase At5g39030 [Populus euphratica]	LRK10	Rust resistance kinase Lr10 OS=Triticum aestivum GN=LRK10 PE=2 SV=1	5.27	2.06	2.24	up	0.00	0.01	yes
TRINITY_DN21361_c0_g1	hypothetical protein VIGAN_07134100 [Vigna angularis var. angularis]	-	-	24.75	11.47	1.70	up	0.00	0.01	yes
TRINITY_DN24910_c0_g1	hypothetical protein POPTR_0018s06210g [Populus trichocarpa]	HCF101	Fe-S cluster assembly factor HCF101, chloroplastic OS=Oryza sativa subsp. japonica GN=HCF101 PE=3 SV=3	69.34	42.65	1.22	up	0.00	0.01	yes
TRINITY_DN27264_c1_g1	PREDICTED: kynurenine--oxoglutarate transaminase 1-like isoform X1 [Populus euphratica]	GLU1	Endoglucanase 9 OS=Oryza sativa subsp. japonica GN=GLU1 PE=2 SV=1	32.80	22.92	1.03	up	0.00	0.01	yes
TRINITY_DN20870_c0_g1	PREDICTED: uncharacterized protein LOC105110056 isoform X2 [Populus euphratica]	-	-	9.67	7.09	1.03	up	0.00	0.01	yes
TRINITY_DN27055_c1_g4	hypothetical protein POPTR_0005s00200g [Populus trichocarpa]	RH3	DEAD-box ATP-dependent RNA helicase 3, chloroplastic OS=Arabidopsis thaliana GN=RH3 PE=1 SV=2	99.43	64.90	1.19	up	0.00	0.01	yes
TRINITY_DN27448_c0_g2	PREDICTED: peroxisomal (S)-2-hydroxy-acid oxidase [Populus euphratica]	GLO2	Peroxisomal (S)-2-hydroxy-acid oxidase GLO2 OS=Arabidopsis thaliana GN=GLO2 PE=1 SV=1	519.75	233.20	1.73	up	0.00	0.01	yes
TRINITY_DN26746_c2_g1	PREDICTED: translation initiation factor IF-3, chloroplastic [Populus euphratica]	infC	Translation initiation factor IF-3 OS=Microcystis aeruginosa (strain NIES-843) GN=infC PE=3 SV=1	141.21	101.36	1.07	up	0.00	0.01	yes
TRINITY_DN25290_c0_g4	hypothetical protein POPTR_0002s20100g [Populus trichocarpa]	-	-	26.37	13.63	1.72	up	0.00	0.01	yes
TRINITY_DN15912_c0_g1	hypothetical protein POPTR_0001s26020g [Populus trichocarpa]	-	-	6.03	2.09	2.16	up	0.00	0.01	yes
TRINITY_DN22925_c1_g1	HSP80 family protein [Populus trichocarpa]	HSP81-1	Heat shock protein 81-1 OS=Oryza sativa subsp. indica GN=HSP81-1 PE=2 SV=1	349.08	238.31	1.15	up	0.00	0.01	yes
TRINITY_DN18553_c0_g2	Photosystem I reaction center subunit V family protein [Populus trichocarpa]	PSAG	Photosystem I reaction center subunit V, chloroplastic OS=Arabidopsis thaliana GN=PSAG PE=2 SV=1	1021.10	673.82	1.20	up	0.00	0.01	yes
TRINITY_DN15602_c0_g1	hypothetical protein POPTR_0010s19120g [Populus trichocarpa]	PYL4	Abscisic acid receptor PYL4 OS=Arabidopsis thaliana GN=PYL4 PE=1 SV=1	2.12	0.96	1.77	up	0.00	0.01	yes
TRINITY_DN15632_c0_g2	histidine kinase 1 family protein [Populus trichocarpa]	AHK1	Histidine kinase 1 OS=Arabidopsis thaliana GN=AHK1 PE=1 SV=2	3.32	1.52	1.91	up	0.00	0.01	yes
TRINITY_DN15934_c0_g1	hypothetical protein POPTR_0001s33570g [Populus trichocarpa]	-	-	46.21	34.80	1.02	up	0.00	0.01	yes
TRINITY_DN20981_c2_g6	-	-	-	7.60	2.87	2.01	up	0.00	0.01	yes
TRINITY_DN22349_c0_g1	PREDICTED: 2-oxoisovalerate dehydrogenase subunit alpha 2, mitochondrial [Populus euphratica]	At5g09300	2-oxoisovalerate dehydrogenase subunit alpha 2, mitochondrial OS=Arabidopsis thaliana GN=At5g09300 PE=1 SV=1	16.36	12.76	1.04	up	0.00	0.01	yes
TRINITY_DN24692_c0_g2	core region of GTP cyclohydrolase I family protein [Populus trichocarpa]	GCH1	GTP cyclohydrolase 1 OS=Solanum lycopersicum GN=GCH1 PE=1 SV=1	12.52	6.66	1.49	up	0.00	0.01	yes
TRINITY_DN25032_c0_g1	PREDICTED: fasciclin-like arabinogalactan protein 17 [Populus euphratica]	FLA17	Fasciclin-like arabinogalactan protein 17 OS=Arabidopsis thaliana GN=FLA17 PE=2 SV=1	15.28	11.01	1.10	up	0.00	0.01	yes
TRINITY_DN27813_c2_g1	PREDICTED: uncharacterized protein LOC105116545 [Populus euphratica]	RRS1	Disease resistance protein RRS1 OS=Arabidopsis thaliana GN=RRS1 PE=1 SV=1	9.05	13.90	1.01	up	0.00	0.01	yes
TRINITY_DN24457_c2_g5	-	-	-	3.32	1.20	2.15	up	0.00	0.01	yes
TRINITY_DN17591_c0_g1	PREDICTED: uncharacterized protein LOC105124793 [Populus euphratica]	-	-	3.65	1.75	1.93	up	0.00	0.01	yes
TRINITY_DN21293_c0_g5	hypothetical protein POPTR_0013s05300g [Populus trichocarpa]	MYB5	Transcription repressor MYB5 OS=Arabidopsis thaliana GN=MYB5 PE=1 SV=1	6.38	3.94	1.29	up	0.00	0.01	yes

TRINITY_DN18730_c0_g1	PREDICTED: universal stress protein A-like protein [Populus euphratica]	-	-	77.68	40.18	1.46	up	0.00	0.01	yes
TRINITY_DN13193_c0_g1	hypothetical protein POPTR_0003s19740g [Populus trichocarpa]	-	-	12.58	6.86	1.49	up	0.00	0.01	yes
TRINITY_DN20112_c0_g1	unknown [Populus trichocarpa x Populus deltoides]	-	-	4.68	3.16	1.20	up	0.00	0.01	yes
TRINITY_DN15011_c0_g1	-	-	-	2.83	1.36	1.77	up	0.00	0.01	yes
TRINITY_DN23936_c0_g1	PREDICTED: pentatricopeptide repeat-containing protein At1g18485 [Populus euphratica]	PCMP-H8	Pentatricopeptide repeat-containing protein At1g18485 OS=Arabidopsis thaliana GN=PCMP-H8 PE=2 SV=2	3.58	2.66	1.03	up	0.00	0.01	yes
TRINITY_DN16232_c0_g2	PREDICTED: uncharacterized protein LOC105124761 [Populus euphratica]	yuiD	Uncharacterized membrane protein YuiD OS=Bacillus subtilis (strain 168) GN=yuiD PE=4 SV=1	8.85	6.15	1.20	up	0.00	0.01	yes
TRINITY_DN23962_c0_g4	ATP-dependent protease La domain-containing family protein [Populus trichocarpa]	-	-	142.07	101.78	1.08	up	0.00	0.01	yes
TRINITY_DN17721_c0_g1	hypothetical protein POPTR_0010s14910g [Populus trichocarpa]	-	Cytochrome P450 CYP749A22 OS=Panax ginseng PE=2 SV=1	6.20	3.46	1.49	up	0.00	0.01	yes
TRINITY_DN3854_c0_g2	hypothetical protein POPTR_0006s13080g [Populus trichocarpa]	At1g30760	Berberine bridge enzyme-like 13 OS=Arabidopsis thaliana GN=At1g30760 PE=1 SV=1	1.75	0.47	2.46	up	0.00	0.01	yes
TRINITY_DN23744_c0_g2	hypothetical protein POPTR_0019s11970g, partial [Populus trichocarpa]	ESK1	Protein ESKIMO 1 OS=Arabidopsis thaliana GN=ESK1 PE=1 SV=1	2.08	2.69	1.69	up	0.00	0.01	yes
TRINITY_DN25623_c0_g5	hypothetical protein POPTR_0013s11250g [Populus trichocarpa]	-	-	103.05	57.58	1.42	up	0.00	0.01	yes
TRINITY_DN26184_c0_g1	PREDICTED: RNA-binding protein 24-like isoform X1 [Populus euphratica]	ARP1	Probable RNA-binding protein ARP1 OS=Arabidopsis thaliana GN=ARP1 PE=2 SV=1	22.91	16.26	1.15	up	0.00	0.01	yes
TRINITY_DN25124_c0_g5	hypothetical protein POPTR_0005s02360g [Populus trichocarpa]	-	-	2.51	0.95	1.97	up	0.00	0.01	yes
TRINITY_DN26687_c0_g3	hypothetical protein POPTR_0006s00750g [Populus trichocarpa]	MTACP2	Acyl carrier protein 3, mitochondrial OS=Arabidopsis thaliana GN=MTACP2 PE=2 SV=1	22.82	16.54	1.07	up	0.00	0.01	yes
TRINITY_DN27210_c0_g2	hypothetical protein POPTR_0008s14270g [Populus trichocarpa]	-	-	10.44	6.60	1.42	up	0.00	0.01	yes
TRINITY_DN17518_c0_g1	PREDICTED: probable mitochondrial adenine nucleotide transporter BTL3 [Populus euphratica]	At5g64970	Probable mitochondrial adenine nucleotide transporter BTL3 OS=Arabidopsis thaliana GN=At5g64970 PE=2 SV=1	7.89	5.94	1.02	up	0.00	0.01	yes
TRINITY_DN25509_c0_g2	hypothetical protein POPTR_0017s08780g [Populus trichocarpa]	MPT3	Mitochondrial phosphate carrier protein 3, mitochondrial OS=Arabidopsis thaliana GN=MPT3 PE=1 SV=1	27.00	20.65	1.04	up	0.00	0.01	yes
TRINITY_DN1679_c0_g1	PREDICTED: CASP-like protein 1F1 [Populus euphratica]	POPTRDRAFT_8	CASP-like protein 1F1 OS=Populus trichocarpa GN=POPTRDRAFT_824792 PE=2 SV=1	2.88	1.12	1.98	up	0.00	0.01	yes
TRINITY_DN24740_c0_g1	hypothetical protein POPTR_0004s22690g [Populus trichocarpa]	-	-	30.45	21.98	1.09	up	0.00	0.01	yes
TRINITY_DN16347_c0_g2	GNS1/SUR4 membrane family protein [Populus trichocarpa]	HOS3	Elongation of fatty acids protein 3-like OS=Arabidopsis thaliana GN=HOS3 PE=2 SV=1	17.42	12.48	1.08	up	0.00	0.01	yes
TRINITY_DN25023_c0_g1	PREDICTED: U3 small nucleolar RNA-associated protein 15 homolog [Populus euphratica]	SWA1	Protein SLOW WALKER 1 OS=Arabidopsis thaliana GN=SWA1 PE=2 SV=1	11.67	8.96	1.01	up	0.00	0.01	yes
TRINITY_DN26323_c1_g2	hypothetical protein POPTR_0014s19720g [Populus trichocarpa]	-	-	107.13	61.39	1.43	up	0.00	0.01	yes
TRINITY_DN16501_c0_g1	hypothetical protein POPTR_0006s14500g [Populus trichocarpa]	MOB1-A	MOB kinase activator-like 1A OS=Medicago sativa subsp. falcata GN=MOB1-A PE=2 SV=2	11.22	6.00	1.53	up	0.00	0.01	yes
TRINITY_DN19148_c0_g6	hypothetical protein POPTR_0005s02760g [Populus trichocarpa]	-	-	151.13	109.18	1.10	up	0.00	0.01	yes
TRINITY_DN26940_c2_g7	hypothetical protein ZEAMMB73_588552 [Zea mays]	-	-	1161.42	883.39	1.07	up	0.00	0.01	yes
TRINITY_DN27053_c0_g2	-	-	-	24.26	13.51	1.45	up	0.00	0.01	yes
TRINITY_DN21866_c0_g1	hypothetical protein POPTR_0016s12440g [Populus trichocarpa]	At5g05600	Probable 2-oxoglutarate-dependent dioxygenase At5g05600 OS=Arabidopsis thaliana GN=At5g05600 PE=2 SV=1	40.27	34.46	1.05	up	0.00	0.01	yes
TRINITY_DN22850_c0_g2	PREDICTED: protein SUPPRESSOR OF GENE SILENCING 3-like isoform X3 [Populus euphratica]	SGS3	Protein SUPPRESSOR OF GENE SILENCING 3 OS=Solanum lycopersicum GN=SGS3 PE=1 SV=1	26.52	19.00	1.16	up	0.00	0.01	yes
TRINITY_DN18777_c0_g3	hypothetical protein POPTR_0005s11400g [Populus trichocarpa]	-	-	36.57	25.22	1.21	up	0.00	0.01	yes

TRINITY_DN23197_c0_g2	hypothetical protein POPTR_0011s15050g [Populus trichocarpa]	GTL1	Trihelix transcription factor GTL1 OS=Arabidopsis thaliana GN=GTL1 PE=1 SV=2	7.96	3.57	1.74	up	0.00	0.01	yes
TRINITY_DN21566_c2_g1	hypothetical protein POPTR_0001s08230g [Populus trichocarpa]	sll0608	Ycf49-like protein OS=Synechocystis sp. (strain PCC 6803 / Kazusa) GN=sll0608 PE=3 SV=1	72.37	46.65	1.20	up	0.00	0.01	yes
TRINITY_DN25165_c0_g2	PREDICTED: glycerol-3-phosphate acyltransferase, chloroplastic [Populus euphratica]	-	Glycerol-3-phosphate acyltransferase, chloroplastic OS=Cucumis sativus PE=2 SV=1	66.47	43.88	1.20	up	0.00	0.01	yes
TRINITY_DN20048_c1_g1	PREDICTED: phospho-2-dehydro-3-deoxyheptonate aldolase 1, chloroplastic-like [Populus euphratica]	DHS1	Phospho-2-dehydro-3-deoxyheptonate aldolase 1, chloroplastic OS=Arabidopsis thaliana GN=DHS1 PE=2 SV=2	289.54	201.11	1.11	up	0.00	0.01	yes
TRINITY_DN24914_c2_g1	PREDICTED: zinc finger protein 2-like [Populus euphratica]	ZFP2	Zinc finger protein 2 OS=Arabidopsis thaliana GN=ZFP2 PE=2 SV=1	3.58	1.44	1.97	up	0.00	0.01	yes
TRINITY_DN20441_c1_g6	hypothetical protein POPTR_0007s10140g [Populus trichocarpa]	ycf36	Uncharacterized protein ycf36 OS=Porphyra purpurea GN=ycf36 PE=3 SV=1	21.44	13.32	1.29	up	0.00	0.01	yes
TRINITY_DN20180_c1_g4	-	-	-	3.64	2.05	1.86	up	0.00	0.01	yes
TRINITY_DN22789_c2_g6	hypothetical protein POPTR_0002s08740g [Populus trichocarpa]	GRP-2	Glycine-rich protein 2 OS=Nicotiana sylvestris GN=GRP-2 PE=2 SV=1	63.04	28.85	1.72	up	0.00	0.01	yes
TRINITY_DN16897_c0_g7	PREDICTED: photosystem II reaction center W protein, chloroplastic isoform X1 [Populus euphratica]	PSBW	Photosystem II reaction center W protein, chloroplastic OS=Arabidopsis thaliana GN=PSBW PE=1 SV=2	3123.34	1949.44	1.28	up	0.00	0.01	yes
TRINITY_DN23394_c0_g1	hypothetical protein POPTR_0005s21390g [Populus trichocarpa]	-	-	7.56	4.26	1.39	up	0.00	0.01	yes
TRINITY_DN21679_c1_g1	PREDICTED: uncharacterized protein LOC105133260 [Populus euphratica]	LSMT-L	[Fructose-bisphosphate aldolase]-lysine N-methyltransferase, chloroplastic OS=Arabidopsis thaliana GN=LSMT-L PE=1 SV=1	8.37	6.96	1.00	up	0.00	0.01	yes
TRINITY_DN26784_c1_g1	PREDICTED: uncharacterized protein LOC105123089 isoform X2 [Populus euphratica]	NDX1	Protein NEOXANTHIN-DEFICIENT 1 OS=Arabidopsis thaliana GN=NDX1 PE=2 SV=1	24.14	16.39	1.45	up	0.00	0.01	yes
TRINITY_DN25819_c0_g1	PREDICTED: NADP-dependent glyceraldehyde-3-phosphate dehydrogenase-like isoform X1 [Populus euphratica]	GAPN	NADP-dependent glyceraldehyde-3-phosphate dehydrogenase OS=Nicotiana plumbaginifolia GN=GAPN PE=2 SV=1	282.53	188.30	1.14	up	0.00	0.01	yes
TRINITY_DN19275_c0_g1	PREDICTED: protein CURVATURE THYLAKOID 1C, chloroplastic-like [Populus euphratica]	CURT1C	Protein CURVATURE THYLAKOID 1C, chloroplastic OS=Arabidopsis thaliana GN=CURT1C PE=1 SV=1	143.73	92.81	1.07	up	0.00	0.01	yes
TRINITY_DN14708_c0_g1	unnamed protein product, partial [Vitis vinifera]	TSS	Protein TSS OS=Arabidopsis thaliana GN=TSS PE=1 SV=1	37.13	18.27	1.59	up	0.00	0.01	yes
TRINITY_DN27310_c0_g3	hypothetical protein POPTR_0003s12690g [Populus trichocarpa]	TUBB1	Tubulin beta-1 chain OS=Glycine max GN=TUBB1 PE=3 SV=1	6.83	4.24	2.34	up	0.00	0.01	yes
TRINITY_DN20842_c0_g1	hypothetical protein POPTR_0008s18060g [Populus trichocarpa]	-	-	8.67	6.26	1.07	up	0.00	0.01	yes
TRINITY_DN14796_c0_g1	hypothetical protein POPTR_0004s22510g [Populus trichocarpa]	-	-	5.21	2.72	1.55	up	0.00	0.01	yes
TRINITY_DN19940_c0_g1	PREDICTED: adenylyl-sulfate kinase 3-like [Populus euphratica]	APK1	Adenylyl-sulfate kinase 1, chloroplastic OS=Arabidopsis thaliana GN=APK1 PE=1 SV=1	131.63	73.66	1.32	up	0.00	0.01	yes
TRINITY_DN22186_c0_g1	alpha-hydroxynitrile lyase family protein [Populus trichocarpa]	At1g32780	Alcohol dehydrogenase-like 3 OS=Arabidopsis thaliana GN=At1g32780 PE=2 SV=1	31.37	22.10	1.05	up	0.00	0.01	yes
TRINITY_DN16284_c0_g2	hypothetical protein POPTR_0004s03870g [Populus trichocarpa]	-	Polyphenol oxidase, chloroplastic OS=Vitis vinifera PE=1 SV=1	39.71	28.71	1.06	up	0.00	0.01	yes
TRINITY_DN18344_c0_g1	hypothetical protein POPTR_0005s12390g [Populus trichocarpa]	RL3	Protein RADIALIS-like 3 OS=Arabidopsis thaliana GN=RL3 PE=2 SV=1	4.29	2.12	1.73	up	0.00	0.01	yes
TRINITY_DN14144_c0_g1	hypothetical protein POPTR_0010s26120g [Populus trichocarpa]	MIA40	Mitochondrial intermembrane space import and assembly protein 40 homolog OS=Arabidopsis thaliana GN=MIA40 PE=2 SV=1	25.68	17.69	1.08	up	0.00	0.01	yes
TRINITY_DN17246_c0_g1	hypothetical protein POPTR_0017s07040g [Populus trichocarpa]	-	Fructose-1,6-bisphosphatase, chloroplastic OS=Spinacia oleracea PE=1 SV=2	24.42	17.62	1.06	up	0.00	0.01	yes
TRINITY_DN16361_c0_g1	hypothetical protein POPTR_0002s17200g [Populus trichocarpa]	TSS	Protein TSS OS=Arabidopsis thaliana GN=TSS PE=1 SV=1	34.06	24.64	1.06	up	0.00	0.01	yes
TRINITY_DN18313_c0_g2	hypothetical protein POPTR_0002s15450g [Populus trichocarpa]	At3g61080	Protein-ribulosamine 3-kinase, chloroplastic OS=Arabidopsis thaliana GN=At3g61080 PE=1 SV=2	39.01	29.05	1.00	up	0.00	0.01	yes
TRINITY_DN25309_c0_g1	hydroxyethylthiazole kinase family protein [Populus trichocarpa]	THIM	Hydroxyethylthiazole kinase OS=Arabidopsis thaliana GN=THIM PE=1 SV=1	8.19	7.36	1.17	up	0.00	0.01	yes

TRINITY_DN18520_c0_g1	hypothetical protein POPTR_0008s19520g [Populus trichocarpa]	-	-	33.10	22.01	1.13	up	0.00	0.01	yes
TRINITY_DN16247_c0_g1	hypothetical protein POPTR_0002s16990g [Populus trichocarpa]	-	-	6.95	4.38	1.23	up	0.00	0.01	yes
TRINITY_DN20390_c0_g1	PREDICTED: ribosomal L1 domain-containing protein 1-like [Populus euphratica]	RSL1D1	Ribosomal L1 domain-containing protein 1 OS=Homo sapiens GN=RSL1D1 PE=1 SV=3	13.97	10.24	1.09	up	0.00	0.01	yes
TRINITY_DN13008_c0_g1	PREDICTED: uncharacterized protein LOC105111497 isoform X1 [Populus euphratica]	-	-	2.74	1.12	1.89	up	0.00	0.01	yes
TRINITY_DN13195_c0_g1	hypothetical protein POPTR_0019s10390g [Populus trichocarpa]	-	Alcohol-forming fatty acyl-CoA reductase OS=Simmondsia chinensis PE=1 SV=1	4.32	3.51	1.88	up	0.00	0.01	yes
TRINITY_DN22424_c0_g2	PREDICTED: translation factor GUF1 homolog, chloroplastic [Populus euphratica]	typA	GTP-binding protein TypA/BipA homolog OS=Synechocystis sp. (strain PCC 6803 / Kazusa) GN=typA PE=3 SV=1	2.07	0.86	1.84	up	0.00	0.01	yes
TRINITY_DN26913_c0_g1	1L-myo-inositol 1-phosphate synthase family protein [Populus trichocarpa]	INPS1	Inositol-3-phosphate synthase OS=Nicotiana paniculata GN=INPS1 PE=2 SV=1	252.36	147.60	1.27	up	0.00	0.01	yes
TRINITY_DN21326_c0_g2	peptidyl-prolyl cis-trans isomerase cyclophilin-type family protein [Populus trichocarpa]	CYP26-2	Peptidyl-prolyl cis-trans isomerase CYP26-2, chloroplastic OS=Arabidopsis thaliana GN=CYP26-2 PE=2 SV=1	97.73	66.86	1.11	up	0.00	0.01	yes
TRINITY_DN24174_c0_g2	PREDICTED: pentatricopeptide repeat-containing protein At5g13770, chloroplastic-like [Populus euphratica]	At5g13770	Pentatricopeptide repeat-containing protein At5g13770, chloroplastic OS=Arabidopsis thaliana GN=At5g13770 PE=2 SV=1	3.41	1.90	1.44	up	0.00	0.01	yes
TRINITY_DN18748_c1_g3	hypothetical protein POPTR_0009s07130g [Populus trichocarpa]	-	-	22.81	9.87	1.82	up	0.00	0.01	yes
TRINITY_DN26320_c0_g5	hypothetical protein POPTR_0010s13170g [Populus trichocarpa]	LACTB2	Endoribonuclease LACTB2 OS=Bos taurus GN=LACTB2 PE=2 SV=1	6.62	5.09	1.01	up	0.00	0.01	yes
TRINITY_DN23066_c0_g6	60S ribosomal protein L24 [Populus trichocarpa]	At2g44860	Probable ribosome biogenesis protein RLP24 OS=Arabidopsis thaliana GN=At2g44860 PE=1 SV=1	33.03	23.21	1.13	up	0.00	0.01	yes
TRINITY_DN23224_c0_g1	hypothetical protein POPTR_0004s07270g [Populus trichocarpa]	RVE1	Protein REVEILLE 1 OS=Arabidopsis thaliana GN=RVE1 PE=2 SV=1	38.11	26.44	1.10	up	0.00	0.01	yes
TRINITY_DN27218_c0_g1	PREDICTED: protein TIC 62, chloroplastic-like [Populus euphratica]	PTAC16	Protein plastid transcriptionally active 16, chloroplastic OS=Arabidopsis thaliana GN=PTAC16 PE=1 SV=1	482.75	317.89	1.16	up	0.00	0.01	yes
TRINITY_DN27364_c0_g4	pectinesterase family protein [Populus trichocarpa]	PME51	Probable pectinesterase/pectinesterase inhibitor 51 OS=Arabidopsis thaliana GN=PME51 PE=2 SV=1	2.46	1.01	1.88	up	0.00	0.01	yes
TRINITY_DN17279_c0_g1	PREDICTED: dnaJ homolog subfamily B member 8 isoform X1 [Populus euphratica]	dnaJ	Chaperone protein DnaJ OS=Bacillus velezensis (strain DSM 23117 / BGSC 10A6 / FZB42) GN=dnaJ PE=3 SV=1	16.32	12.67	1.03	up	0.00	0.01	yes
TRINITY_DN21448_c0_g3	PREDICTED: uncharacterized protein LOC105129237 isoform X1 [Populus euphratica]	prmA	Ribosomal protein L11 methyltransferase OS=Paraburkholderia phymatum (strain DSM 17167 / CIP 108236 / LMG 21445 / STM815) GN=prmA PE=3 SV=1	10.08	7.34	1.06	up	0.00	0.01	yes
TRINITY_DN22208_c0_g1	hypothetical protein POPTR_0008s07960g [Populus trichocarpa]	-	-	55.08	37.35	1.20	up	0.00	0.01	yes
TRINITY_DN14305_c0_g1	hypothetical protein POPTR_0018s11150g [Populus trichocarpa]	TSS	Protein TSS OS=Arabidopsis thaliana GN=TSS PE=1 SV=1	14.66	5.62	1.94	up	0.00	0.01	yes
TRINITY_DN16211_c2_g3	-	-	-	5.56	2.37	1.89	up	0.00	0.01	yes
TRINITY_DN22683_c0_g1	hypothetical protein POPTR_0007s13190g [Populus trichocarpa]	MAN7	Mannan endo-1,4-beta-mannosidase 7 OS=Arabidopsis thaliana GN=MAN7 PE=2 SV=1	8.04	5.40	1.44	up	0.00	0.01	yes
TRINITY_DN15811_c0_g1	hypothetical protein POPTR_0005s11490g [Populus trichocarpa]	TL1	Thaumatin-like protein 1 OS=Pyrus pyrifolia GN=TL1 PE=1 SV=1	3.50	1.42	1.88	up	0.00	0.01	yes
TRINITY_DN19548_c0_g1	kinase family protein [Populus trichocarpa]	ATG1A	Serine/threonine-protein kinase ATG1a OS=Arabidopsis thaliana GN=ATG1A PE=1 SV=1	4.82	1.85	2.00	up	0.00	0.01	yes
TRINITY_DN22483_c2_g1	hypothetical protein POPTR_0019s04930g [Populus trichocarpa]	-	Early nodulin-93 OS=Glycine max PE=2 SV=1	16.28	11.82	1.05	up	0.00	0.01	yes
TRINITY_DN19913_c0_g1	PREDICTED: hydroxymethylglutaryl-CoA lyase, mitochondrial-like isoform X1 [Populus euphratica]	HMGCL	Hydroxymethylglutaryl-CoA lyase, mitochondrial OS=Arabidopsis thaliana GN=HMGCL PE=1 SV=2	13.16	7.43	1.05	up	0.00	0.01	yes
TRINITY_DN16137_c0_g1	hypothetical protein POPTR_0006s14510g [Populus trichocarpa]	ABP20	Auxin-binding protein ABP20 OS=Prunus persica GN=ABP20 PE=2 SV=1	1187.86	752.12	1.26	up	0.00	0.01	yes
TRINITY_DN18954_c0_g2	PREDICTED: pentatricopeptide repeat-containing protein At4g30825, chloroplastic-like [Populus euphratica]	At4g30825	Pentatricopeptide repeat-containing protein At4g30825, chloroplastic OS=Arabidopsis thaliana GN=At4g30825 PE=2 SV=2	9.13	6.58	1.07	up	0.00	0.01	yes

TRINITY_DN14883_c0_g1	PREDICTED: probable carboxylesterase 12 [Populus euphratica]	CXE12	Probable carboxylesterase 12 OS=Arabidopsis thaliana GN=CXE12 PE=1 SV=1	4.04	2.25	1.47	up	0.00	0.01	yes
TRINITY_DN20865_c0_g3	PREDICTED: hemK methyltransferase family member 1 [Populus euphratica]	prmC	Release factor glutamine methyltransferase OS=Thermosynechococcus elongatus (strain BP-1) GN=prmC PE=3 SV=1	30.29	24.84	1.04	up	0.00	0.01	yes
TRINITY_DN25565_c0_g10	BnaUnng00820D [Brassica napus]	-	-	525.72	242.67	1.78	up	0.00	0.01	yes
TRINITY_DN27574_c0_g1	glycine decarboxylase P-protein 1 [Arabidopsis thaliana]	GLDP1	Glycine dehydrogenase (decarboxylating) 1, mitochondrial OS=Arabidopsis thaliana GN=GLDP1 PE=1 SV=2	428.07	261.89	1.31	up	0.00	0.01	yes
TRINITY_DN26164_c0_g1	DHFS-FPGS B family protein [Populus trichocarpa]	FPGS1	Folylpolyglutamate synthase OS=Arabidopsis thaliana GN=FPGS1 PE=1 SV=1	45.14	26.03	1.34	up	0.00	0.01	yes
TRINITY_DN14634_c0_g1	hypothetical protein POPTR_0009s11030g [Populus trichocarpa]	-	-	5.05	2.71	1.49	up	0.00	0.01	yes
TRINITY_DN18836_c0_g2	acidic ribosomal protein P0 [Populus trichocarpa]	mrt4	Ribosome assembly factor mrt4 OS=Dictyostelium discoideum GN=mrt4 PE=3 SV=1	37.82	30.11	1.02	up	0.00	0.01	yes
TRINITY_DN27334_c0_g1	putative NADPH-cytochrome P450 reductase family protein [Populus trichocarpa]	CPR	NADPH--cytochrome P450 reductase OS=Catharanthus roseus GN=CPR PE=2 SV=1	195.19	138.75	1.13	up	0.00	0.01	yes
TRINITY_DN14120_c0_g1	hypothetical protein MANES_12G030900 [Manihot esculenta]	-	-	4.14	1.75	2.09	up	0.00	0.01	yes
TRINITY_DN18538_c0_g1	unknown [Populus trichocarpa]	At5g03700	PAN domain-containing protein At5g03700 OS=Arabidopsis thaliana GN=At5g03700 PE=1 SV=1	3.06	1.01	2.21	up	0.00	0.01	yes
TRINITY_DN25594_c0_g1	PREDICTED: 30S ribosomal protein S1, chloroplastic-like [Populus euphratica]	RPS1	30S ribosomal protein S1, chloroplastic OS=Spinacia oleracea GN=RPS1 PE=1 SV=1	490.21	352.74	1.08	up	0.00	0.01	yes
TRINITY_DN21379_c0_g1	inorganic carbon transport family protein [Populus trichocarpa]	ndhL	NAD(P)H-quinone oxidoreductase subunit L, chloroplastic OS=Arabidopsis thaliana GN=ndhL PE=2 SV=1	300.63	159.85	1.49	up	0.00	0.01	yes
TRINITY_DN25723_c0_g2	hypothetical protein POPTR_0878s002002g, partial [Populus trichocarpa]	-	-	3.86	1.92	1.62	up	0.00	0.01	yes
TRINITY_DN23265_c0_g4	PREDICTED: protein Mpv17 isoform X1 [Populus euphratica]	mpv17	Protein Mpv17 OS=Xenopus laevis GN=mpv17 PE=2 SV=2	9.80	6.43	1.22	up	0.00	0.01	yes
TRINITY_DN11879_c0_g1	hypothetical protein POPTR_0001s31380g [Populus trichocarpa]	-	-	7.05	3.51	1.63	up	0.00	0.01	yes
TRINITY_DN21112_c0_g1	PREDICTED: probable monodehydroascorbate reductase, cytoplasmic isoform 2 [Populus euphratica]	MDAR4	Monodehydroascorbate reductase 4, peroxisomal OS=Arabidopsis thaliana GN=MDAR4 PE=1 SV=1	146.08	103.50	1.12	up	0.00	0.01	yes
TRINITY_DN19490_c0_g1	putative histidine phosphotransfer protein 5 [Populus x canadensis]	AHP5	Histidine-containing phosphotransfer protein 5 OS=Arabidopsis thaliana GN=AHP5 PE=1 SV=2	9.00	6.64	1.26	up	0.00	0.01	yes
TRINITY_DN15919_c0_g1	adenylate dimethylallyltransferase 5b [Populus x canadensis]	IPT5	Adenylate isopentenyltransferase 5, chloroplastic OS=Arabidopsis thaliana GN=IPT5 PE=1 SV=2	5.06	3.28	1.73	up	0.00	0.01	yes
TRINITY_DN21902_c0_g3	PREDICTED: uncharacterized protein LOC105121040 isoform X1 [Populus euphratica]	-	-	45.76	34.67	1.00	up	0.00	0.01	yes
TRINITY_DN22888_c0_g3	CCAAT-binding transcription factor family protein [Populus trichocarpa]	NFYA3	Nuclear transcription factor Y subunit A-3 OS=Arabidopsis thaliana GN=NFYA3 PE=2 SV=2	7.64	5.10	1.19	up	0.00	0.01	yes
TRINITY_DN18651_c1_g2	hypothetical protein POPTR_0011s12770g [Populus trichocarpa]	At4g27340	tRNA (guanine(37)-N1)-methyltransferase 2 OS=Arabidopsis thaliana GN=At4g27340 PE=2 SV=1	10.77	6.78	1.25	up	0.00	0.01	yes
TRINITY_DN19931_c0_g8	RNA polymerase beta'' subunit [Populus alba]	rpoC2	DNA-directed RNA polymerase subunit beta'' OS=Populus alba GN=rpoC2 PE=3 SV=1	18.11	8.96	1.61	up	0.00	0.01	yes
TRINITY_DN15907_c0_g1	hypothetical protein POPTR_0002s06190g [Populus trichocarpa]	SK1	Shikimate kinase 1, chloroplastic OS=Oryza sativa subsp. japonica GN=SK1 PE=1 SV=1	5.64	2.06	1.59	up	0.00	0.01	yes
TRINITY_DN18181_c1_g2	hypothetical protein POPTR_0006s19110g [Populus trichocarpa]	-	-	10.20	5.29	1.52	up	0.00	0.01	yes
TRINITY_DN17217_c0_g3	PREDICTED: NEDD8-activating enzyme E1 regulatory subunit [Populus euphratica]	AXR1	NEDD8-activating enzyme E1 regulatory subunit AXR1 OS=Arabidopsis thaliana GN=AXR1 PE=1 SV=1	7.92	3.61	1.73	up	0.00	0.01	yes
TRINITY_DN17196_c0_g2	hypothetical protein POPTR_0015s10720g [Populus trichocarpa]	PCMP-H58	Pentatricopeptide repeat-containing protein At5g50390, chloroplastic OS=Arabidopsis thaliana GN=PCMP-H58 PE=2 SV=1	3.12	1.90	1.33	up	0.00	0.01	yes
TRINITY_DN14302_c0_g1	hypothetical protein POPTR_0013s08990g [Populus trichocarpa]	PK1	Putative receptor protein kinase ZmPK1 OS=Zea mays GN=PK1 PE=2 SV=2	2.35	1.00	1.87	up	0.00	0.01	yes
TRINITY_DN21652_c0_g1	tRNA-binding region domain-containing family protein [Populus trichocarpa]	AIMP1	Aminoacyl tRNA synthase complex-interacting multifunctional protein 1 OS=Cricetulus griseus GN=AIMP1 PE=2 SV=1	105.82	79.42	1.01	up	0.00	0.01	yes

TRINITY_DN16647_c0_g1	-	-	-	133.70	59.60	1.53	up	0.00	0.01	yes
TRINITY_DN23617_c0_g1	hypothetical protein POPTR_0004s20960g [Populus trichocarpa]	PSAO	Photosystem I subunit O OS=Arabidopsis thaliana GN=PSAO PE=1 SV=1	1038.82	690.16	1.17	up	0.00	0.01	yes
TRINITY_DN22526_c0_g1	hypothetical protein POPTR_0008s17400g [Populus trichocarpa]	CTPA2	Carboxyl-terminal-processing peptidase 2, chloroplastic OS=Arabidopsis thaliana GN=CTPA2 PE=1 SV=1	75.34	54.69	1.09	up	0.00	0.01	yes
TRINITY_DN17822_c1_g3	ribosomal protein S18 [Populus alba]	rps18	30S ribosomal protein S18, chloroplastic OS=Populus alba GN=rps18 PE=3 SV=1	8.70	5.11	1.40	up	0.00	0.01	yes
TRINITY_DN18355_c0_g1	hypothetical protein POPTR_0001s33380g [Populus trichocarpa]	-	-	51.47	35.99	1.14	up	0.00	0.01	yes
TRINITY_DN16876_c0_g2	-	-	-	6.55	4.04	1.48	up	0.00	0.01	yes
TRINITY_DN22869_c0_g3	photosystem I P700 apoprotein A1 (chloroplast) [Ficus racemosa]	psaA	Photosystem I P700 chlorophyll a apoprotein A1 OS=Populus alba GN=psaA PE=3 SV=1	8.78	3.54	2.00	up	0.00	0.01	yes
TRINITY_DN11334_c0_g1	hypothetical protein POPTR_0009s11230g [Populus trichocarpa]	DPH3	Diphthamide biosynthesis protein 3 OS=Kluyveromyces lactis (strain ATCC 8585 / CBS 2359 / DSM 70799 / NBRC 1267 / NRRL Y-1140 / WM37) GN=DPH3 PE=3 SV=1	9.96	6.00	1.38	up	0.00	0.01	yes
TRINITY_DN24175_c1_g2	hypothetical protein POPTR_0004s15220g [Populus trichocarpa]	EFM	Myb family transcription factor EFM OS=Arabidopsis thaliana GN=EFM PE=1 SV=2	4.10	1.72	1.90	up	0.00	0.01	yes
TRINITY_DN26852_c1_g2	PREDICTED: aspartate--tRNA ligase, mitochondrial isoform X1 [Populus euphratica]	At4g33760	Aspartate--tRNA ligase, chloroplastic/mitochondrial OS=Arabidopsis thaliana GN=At4g33760 PE=2 SV=1	59.04	40.14	1.16	up	0.00	0.01	yes
TRINITY_DN27710_c1_g1	PREDICTED: LOW QUALITY PROTEIN: protein trichome birefringence-like 8 [Populus euphratica]	TBL8	Protein trichome birefringence-like 8 OS=Arabidopsis thaliana GN=TBL8 PE=2 SV=1	78.96	57.83	1.06	up	0.00	0.01	yes
TRINITY_DN16930_c0_g1	invertase/pectin methylesterase inhibitor family protein [Populus trichocarpa]	-	21 kDa protein OS=Daucus carota PE=2 SV=1	3.03	1.02	2.20	up	0.00	0.01	yes
TRINITY_DN15897_c0_g3	PREDICTED: tRNA pseudouridine(38/39) synthase isoform X1 [Populus euphratica]	PUS3	tRNA pseudouridine(38/39) synthase OS=Homo sapiens GN=PUS3 PE=1 SV=3	2.85	1.14	1.90	up	0.00	0.01	yes
TRINITY_DN23795_c1_g1	PREDICTED: glyoxylate/succinic semialdehyde reductase 2, chloroplastic-like [Populus euphratica]	GLYR2	Glyoxylate/succinic semialdehyde reductase 2, chloroplastic OS=Arabidopsis thaliana GN=GLYR2 PE=1 SV=1	219.09	158.29	1.13	up	0.00	0.01	yes
TRINITY_DN22010_c0_g1	-	-	-	29.16	17.78	1.37	up	0.00	0.01	yes
TRINITY_DN23165_c0_g1	hypothetical protein POPTR_0003s19560g [Populus trichocarpa]	-	-	41.92	26.73	1.19	up	0.00	0.01	yes
TRINITY_DN15943_c0_g3	PREDICTED: uncharacterized protein LOC105132913 [Populus euphratica]	-	-	17.60	12.51	1.10	up	0.00	0.01	yes
TRINITY_DN20369_c0_g1	hypothetical protein POPTR_0007s12880g [Populus trichocarpa]	DDB_G0281937	Maf-like protein DDB_G0281937 OS=Dictyostelium discoideum GN=DDB_G0281937 PE=3 SV=1	12.02	8.73	1.32	up	0.00	0.01	yes
TRINITY_DN14791_c0_g1	hypothetical protein POPTR_0003s16900g [Populus trichocarpa]	-	-	18.32	11.71	1.25	up	0.00	0.01	yes
TRINITY_DN19519_c0_g3	hypothetical protein POPTR_0009s11140g [Populus trichocarpa]	MEE14	CCG-binding protein 1 OS=Arabidopsis thaliana GN=MEE14 PE=1 SV=1	98.09	65.06	1.16	up	0.00	0.01	yes
TRINITY_DN27301_c0_g1	unknown [Populus trichocarpa]	ARP2	60S ribosomal protein L3-2 OS=Arabidopsis thaliana GN=ARP2 PE=2 SV=4	48.44	35.28	1.11	up	0.00	0.01	yes
TRINITY_DN14131_c0_g1	unnamed protein product, partial [Vitis vinifera]	TSS	Protein TSS OS=Arabidopsis thaliana GN=TSS PE=1 SV=1	12.75	5.99	1.67	up	0.00	0.01	yes
TRINITY_DN17339_c0_g1	unknown [Populus trichocarpa]	PSAH	Photosystem I reaction center subunit VI, chloroplastic OS=Brassica campestris GN=PSAH PE=2 SV=1	1856.73	1211.18	1.23	up	0.00	0.01	yes
TRINITY_DN11154_c0_g3	hypothetical protein POPTR_0002s18210g [Populus trichocarpa]	At2g46850	Probably inactive receptor-like protein kinase At2g46850 OS=Arabidopsis thaliana GN=At2g46850 PE=3 SV=1	1.55	0.64	1.91	up	0.00	0.01	yes
TRINITY_DN17270_c0_g1	PREDICTED: momilactone A synthase-like [Populus euphratica]	-	Secoisolariciresinol dehydrogenase (Fragment) OS=Forsythia intermedia PE=1 SV=1	4.74	2.60	1.47	up	0.00	0.01	yes
TRINITY_DN19709_c2_g7	hypothetical protein POPTR_0011s03570g [Populus trichocarpa]	At4g27270	Probable NAD(P)H dehydrogenase (quinone) FQR1-like 1 OS=Arabidopsis thaliana GN=At4g27270 PE=2 SV=1	22.58	14.26	1.25	up	0.00	0.01	yes
TRINITY_DN18753_c0_g2	PREDICTED: cytochrome P450 716B1-like [Populus euphratica]	CYP716B1	Cytochrome P450 716B1 OS=Picea sitchensis GN=CYP716B1 PE=2 SV=1	7.50	5.03	1.20	up	0.00	0.01	yes
TRINITY_DN16949_c0_g1	PREDICTED: uncharacterized protein LOC105115061 isoform X2 [Populus euphratica]	NDF5	Protein NDH-DEPENDENT CYCLIC ELECTRON FLOW 5 OS=Arabidopsis thaliana GN=NDF5 PE=2 SV=1	112.93	77.36	1.15	up	0.00	0.01	yes
TRINITY_DN16431_c0_g1	Uncharacterized protein TCM_036777 [Theobroma cacao]	-	-	16.42	10.23	1.25	up	0.00	0.01	yes

TRINITY_DN27602_c1_g5	hypothetical protein F511_11970 [Dorcoceras hygrometricum]	Nlrc3	Protein NLRC3 OS=Mus musculus GN=Nlrc3 PE=2 SV=2	1669.44	1014.67	1.32	up	0.00	0.01	yes
TRINITY_DN26808_c0_g2	hypothetical protein POPTR_0365s002101g, partial [Populus trichocarpa]	-	-	68.43	40.66	1.37	up	0.00	0.01	yes
TRINITY_DN16604_c0_g1	FtsH protease family protein [Populus trichocarpa]	FTSH6	ATP-dependent zinc metalloprotease FTSH 6, chloroplastic OS=Arabidopsis thaliana GN=FTSH6 PE=2 SV=1	3.02	1.53	1.61	up	0.00	0.01	yes
TRINITY_DN23322_c1_g1	PREDICTED: catalase isozyme 1-like [Populus euphratica]	CAT1	Catalase isozyme 1 OS=Gossypium hirsutum GN=CAT1 PE=2 SV=2	422.64	304.47	1.07	up	0.00	0.01	yes
TRINITY_DN21880_c0_g1	hypothetical protein POPTR_0007s04160g [Populus trichocarpa]	PSAN	Photosystem I reaction center subunit N, chloroplastic OS=Arabidopsis thaliana GN=PSAN PE=1 SV=2	1115.78	684.71	1.28	up	0.00	0.01	yes
TRINITY_DN26609_c0_g3	-	-	-	7.53	3.80	1.55	up	0.00	0.01	yes
TRINITY_DN6216_c0_g1	hypothetical protein POPTR_0017s03030g [Populus trichocarpa]	-	-	10.07	3.13	2.24	up	0.00	0.01	yes
TRINITY_DN26451_c2_g6	hypothetical protein POPTR_0007s02230g [Populus trichocarpa]	-	-	6.30	1.46	2.86	up	0.00	0.01	yes
TRINITY_DN20669_c0_g1	hypothetical protein POPTR_0009s12520g [Populus trichocarpa]	-	-	32.88	25.31	1.06	up	0.00	0.01	yes
TRINITY_DN7900_c0_g1	Retrovirus-related Pol polyprotein from transposon TNT 1-94 [Cajanus cajan]	-	Retrovirus-related Pol polyprotein from transposon TNT 1-94 OS=Nicotiana tabacum PE=2 SV=1	2.25	1.02	1.75	up	0.00	0.01	yes
TRINITY_DN26820_c0_g1	hypothetical protein POPTR_0017s09915g [Populus trichocarpa]	-	-	56.11	35.30	1.22	up	0.00	0.01	yes
TRINITY_DN17042_c0_g1	PREDICTED: BTB/POZ domain-containing protein At2g04740 [Populus euphratica]	At2g04740	BTB/POZ domain-containing protein At2g04740 OS=Arabidopsis thaliana GN=At2g04740 PE=2 SV=2	6.55	4.64	1.10	up	0.00	0.01	yes
TRINITY_DN22843_c0_g2	photosystem II 11 kDa family protein [Populus trichocarpa]	PSB27-2	Photosystem II D1 precursor processing protein PSB27-H2, chloroplastic OS=Arabidopsis thaliana GN=PSB27-2 PE=1 SV=1	47.84	33.32	1.11	up	0.00	0.01	yes
TRINITY_DN22598_c0_g1	hypothetical protein POPTR_0009s08760g [Populus trichocarpa]	-	-	24.44	10.24	1.88	up	0.00	0.01	yes
TRINITY_DN24360_c1_g3	PREDICTED: G-type lectin S-receptor-like serine/threonine-protein kinase CES101 isoform X2 [Populus euphratica]	B120	G-type lectin S-receptor-like serine/threonine-protein kinase B120 OS=Arabidopsis thaliana GN=B120 PE=2 SV=1	5.43	2.63	1.30	up	0.00	0.01	yes
TRINITY_DN21945_c0_g2	hypothetical protein POPTR_0006s01990g [Populus trichocarpa]	P67	Pentatricopeptide repeat-containing protein At4g16390, chloroplastic OS=Arabidopsis thaliana GN=P67 PE=1 SV=3	8.26	4.78	1.38	up	0.00	0.01	yes
TRINITY_DN24894_c0_g4	hypothetical protein POPTR_0015s03960g [Populus trichocarpa]	-	-	54.04	27.32	1.58	up	0.00	0.01	yes
TRINITY_DN17659_c0_g3	PREDICTED: myb family transcription factor APL-like [Populus euphratica]	PHL11	Myb family transcription factor PHL11 OS=Arabidopsis thaliana GN=PHL11 PE=2 SV=1	7.32	5.27	1.10	up	0.00	0.01	yes
TRINITY_DN15628_c0_g1	molybdenum cofactor synthesis family protein [Populus trichocarpa]	At4g10100	Molybdopterin synthase sulfur carrier subunit OS=Arabidopsis thaliana GN=At4g10100 PE=2 SV=1	18.69	12.84	1.14	up	0.00	0.01	yes
TRINITY_DN25128_c1_g2	hypothetical protein POPTR_0015s09980g [Populus trichocarpa]	-	-	54.09	22.75	1.83	up	0.00	0.01	yes
TRINITY_DN15028_c0_g1	hypothetical protein POPTR_0007s07520g [Populus trichocarpa]	ATHX	Thioredoxin X, chloroplastic OS=Arabidopsis thaliana GN=ATHX PE=2 SV=2	93.18	52.23	1.42	up	0.00	0.01	yes
TRINITY_DN24329_c2_g3	PREDICTED: protein CHLOROPLAST IMPORT APPARATUS 2-like isoform X1 [Populus euphratica]	CIA2	Protein CHLOROPLAST IMPORT APPARATUS 2 OS=Arabidopsis thaliana GN=CIA2 PE=2 SV=1	39.04	24.04	1.30	up	0.00	0.01	yes
TRINITY_DN24032_c2_g3	constitutive photomorphogenic 3 family protein [Populus trichocarpa]	At2g23060	Probable N-acetyltransferase HLS1-like OS=Arabidopsis thaliana GN=At2g23060 PE=2 SV=1	3.33	1.41	1.85	up	0.00	0.01	yes
TRINITY_DN20578_c0_g1	starch synthase family protein [Populus trichocarpa]	WAXY	Granule-bound starch synthase 1, chloroplastic/amyloplastic OS=Manihot esculenta GN=WAXY PE=2 SV=1	5.03	2.06	1.96	up	0.00	0.01	yes
TRINITY_DN22598_c1_g4	NAD(P)H-quinone oxidoreductase subunit 2 [Medicago truncatula]	ndhB1	NAD(P)H-quinone oxidoreductase subunit 2 A, chloroplastic OS=Coffea arabica GN=ndhB1 PE=3 SV=1	8.88	5.74	1.24	up	0.00	0.01	yes
TRINITY_DN15406_c0_g1	early-responsive to dehydration family protein [Populus trichocarpa]	RXW8	CSC1-like protein RXW8 OS=Arabidopsis thaliana GN=RXW8 PE=2 SV=1	3.43	1.95	1.43	up	0.00	0.01	yes
TRINITY_DN20493_c0_g1	PREDICTED: transcription factor WER-like [Populus euphratica]	C1	Anthocyanin regulatory C1 protein OS=Zea mays GN=C1 PE=2 SV=1	46.96	33.83	1.03	up	0.00	0.01	yes
TRINITY_DN18485_c0_g2	hypothetical protein POPTR_0016s10010g [Populus trichocarpa]	UGT709C2	7-deoxyloganetic acid glucosyltransferase OS=Catharanthus roseus GN=UGT709C2 PE=1 SV=1	58.58	34.89	1.35	up	0.00	0.01	yes

TRINITY_DN27597_c0_g1	hypothetical protein POPTR_0002s00760g [Populus trichocarpa]	MSSP1	Monosaccharide-sensing protein 1 OS=Arabidopsis thaliana GN=MSSP1 PE=1 SV=2	14.59	10.01	1.12	up	0.00	0.01	yes
TRINITY_DN21615_c0_g3	unknown [Populus trichocarpa x Populus deltoides]	RPS16-1	30S ribosomal protein S16-1, chloroplastic OS=Arabidopsis thaliana GN=RPS16-1 PE=2 SV=1	516.99	349.08	1.16	up	0.00	0.01	yes
TRINITY_DN24256_c0_g3	hypothetical protein POPTR_0019s07790g [Populus trichocarpa]	-	-	11.80	8.03	1.12	up	0.00	0.01	yes
TRINITY_DN25703_c0_g3	hypothetical protein POPTR_0001s16790g [Populus trichocarpa]	CYP750A1	Cytochrome P450 750A1 OS=Pinus taeda GN=CYP750A1 PE=2 SV=1	343.75	216.36	1.29	up	0.00	0.01	yes
TRINITY_DN18918_c0_g1	PREDICTED: ricin-like [Populus euphratica]	-	Ricin OS=Ricinus communis PE=1 SV=1	13.39	3.88	1.64	up	0.00	0.01	yes
TRINITY_DN19070_c0_g1	unknown [Populus trichocarpa x Populus deltoides]	ndhN	NAD(P)H-quinone oxidoreductase subunit N, chloroplastic OS=Arabidopsis thaliana GN=ndhN PE=2 SV=1	88.57	53.61	1.33	up	0.00	0.01	yes
TRINITY_DN23756_c0_g1	hypothetical protein POPTR_0001s00480g [Populus trichocarpa]	-	-	4.40	1.37	1.62	up	0.00	0.01	yes
TRINITY_DN23606_c0_g2	mitogen-activated protein kinase kinase [Populus trichocarpa]	MKK9	Mitogen-activated protein kinase kinase 9 OS=Arabidopsis thaliana GN=MKK9 PE=1 SV=1	51.70	35.83	1.10	up	0.00	0.01	yes
TRINITY_DN27670_c0_g1	PQ-loop repeat family protein [Populus trichocarpa]	YPQ1	Probable vacuolar amino acid transporter YPQ1 OS=Saccharomyces cerevisiae (strain ATCC 204508 / S288c) GN=YPQ1 PE=1 SV=1	15.17	9.02	1.30	up	0.00	0.01	yes
TRINITY_DN26661_c0_g2	hypothetical protein VITISV_019194 [Vitis vinifera]	-	-	2.90	1.14	1.95	up	0.00	0.01	yes
TRINITY_DN19931_c0_g15	RNA polymerase beta" subunit [Populus alba]	rpoC2	DNA-directed RNA polymerase subunit beta" OS=Populus alba GN=rpoC2 PE=3 SV=1	3.76	2.38	1.32	up	0.00	0.01	yes
TRINITY_DN22665_c0_g1	hypothetical protein POPTR_0013s03080g [Populus trichocarpa]	EMB3004	Bifunctional 3-dehydroquinate dehydratase/shikimate dehydrogenase, chloroplastic OS=Arabidopsis thaliana GN=EMB3004 PE=1 SV=1	2.11	0.85	1.91	up	0.00	0.01	yes
TRINITY_DN18815_c0_g1	hypothetical protein POPTR_0002s25030g [Populus trichocarpa]	HINT3	Adenylylsulfatase HINT3 OS=Arabidopsis thaliana GN=HINT3 PE=1 SV=1	63.36	44.47	1.05	up	0.00	0.01	yes
TRINITY_DN25290_c0_g2	glycine cleavage system protein H precursor [Populus trichocarpa]	GDCSH	Glycine cleavage system H protein, mitochondrial (Fragment) OS=Flaveria pubescens GN=GDCSH PE=2 SV=1	1509.85	901.22	1.33	up	0.00	0.01	yes
TRINITY_DN24931_c0_g1	hypothetical protein POPTR_0004s23300g [Populus trichocarpa]	rplL	50S ribosomal protein L7/L12 OS=Prochlorococcus marinus (strain NATL1A) GN=rplL PE=3 SV=1	46.22	27.38	1.04	up	0.00	0.01	yes
TRINITY_DN22015_c0_g2	hydroxyproline-rich glycoprotein [Populus trichocarpa]	-	-	18.21	11.15	1.27	up	0.00	0.01	yes
TRINITY_DN23760_c1_g2	PREDICTED: nifU-like protein 3, chloroplastic [Populus euphratica]	NIFU3	NifU-like protein 3, chloroplastic OS=Arabidopsis thaliana GN=NIFU3 PE=2 SV=1	94.81	66.55	1.10	up	0.00	0.01	yes
TRINITY_DN19992_c0_g1	hypothetical protein POPTR_0005s23080g [Populus trichocarpa]	FZL	Probable transmembrane GTPase FZO-like, chloroplastic OS=Arabidopsis thaliana GN=FZL PE=1 SV=1	86.94	61.51	1.09	up	0.00	0.01	yes
TRINITY_DN20500_c0_g1	hypothetical protein POPTR_0016s07700g [Populus trichocarpa]	-	-	16.76	12.35	1.45	up	0.00	0.01	yes
TRINITY_DN21339_c1_g2	33 kDa ribonucleoprotein [Populus trichocarpa]	-	28 kDa ribonucleoprotein, chloroplastic OS=Nicotiana sylvestris PE=1 SV=1	122.13	88.36	1.07	up	0.00	0.01	yes
TRINITY_DN17552_c1_g1	S2-RNase family protein [Populus trichocarpa]	RNS1	Ribonuclease 1 OS=Arabidopsis thaliana GN=RNS1 PE=1 SV=1	6.85	1.98	2.30	up	0.00	0.01	yes
TRINITY_DN22214_c0_g2	hypothetical protein POPTR_0004s06010g [Populus trichocarpa]	-	-	8.08	6.40	1.14	up	0.00	0.01	yes
TRINITY_DN16612_c0_g1	hypothetical protein POPTR_0005s27840g [Populus trichocarpa]	-	-	19.12	14.82	1.01	up	0.00	0.01	yes
TRINITY_DN19580_c0_g1	PREDICTED: uncharacterized protein LOC105112341 isoform X1 [Populus euphratica]	-	-	15.43	11.72	1.01	up	0.00	0.01	yes
TRINITY_DN19206_c0_g1	hypothetical protein POPTR_0012s11630g [Populus trichocarpa]	MSRA4	Peptide methionine sulfoxide reductase A4, chloroplastic OS=Oryza sativa subsp. japonica GN=MSRA4 PE=2 SV=2	536.60	393.46	1.04	up	0.00	0.01	yes
TRINITY_DN25723_c0_g1	tau class glutathione transferase GSTU33 [Populus trichocarpa]	-	Probable glutathione S-transferase OS=Nicotiana tabacum PE=2 SV=1	334.79	243.56	1.05	up	0.00	0.01	yes
TRINITY_DN25099_c0_g6	plastocyanin-like domain-containing family protein [Populus trichocarpa]	At5g25090	Early nodulin-like protein 3 OS=Arabidopsis thaliana GN=At5g25090 PE=1 SV=2	5.19	2.74	1.56	up	0.00	0.01	yes
TRINITY_DN23261_c0_g2	hypothetical protein POPTR_0005s08630g [Populus trichocarpa]	-	-	87.47	61.87	1.09	up	0.00	0.01	yes

TRINITY_DN12335_c0_g2	PREDICTED: heparanase-like protein 2 [Populus euphratica]	At5g07830	Heparanase-like protein 1 OS=Arabidopsis thaliana GN=At5g07830 PE=2 SV=1	4.06	2.34	1.44	up	0.00	0.01	yes
TRINITY_DN19710_c0_g1	PREDICTED: electron transfer flavoprotein-ubiquinone oxidoreductase, mitochondrial [Populus euphratica]	ETFQO	Electron transfer flavoprotein-ubiquinone oxidoreductase, mitochondrial OS=Arabidopsis thaliana GN=ETFQO PE=2 SV=1	24.11	22.06	1.02	up	0.00	0.01	yes
TRINITY_DN16456_c0_g1	PREDICTED: PRA1 family protein H-like isoform X1 [Populus euphratica]	PRA1H	PRA1 family protein H OS=Arabidopsis thaliana GN=PRA1H PE=2 SV=1	7.41	4.82	1.33	up	0.00	0.01	yes
TRINITY_DN24306_c3_g1	hypothetical protein POPTR_0014s05630g [Populus trichocarpa]	-	-	17.60	9.88	1.43	up	0.00	0.01	yes
TRINITY_DN15562_c0_g1	histidine kinase receptor family protein [Populus trichocarpa]	AHK3	Histidine kinase 3 OS=Arabidopsis thaliana GN=AHK3 PE=1 SV=1	13.30	8.10	1.28	up	0.00	0.01	yes
TRINITY_DN25289_c0_g3	PREDICTED: uncharacterized protein LOC105130203 [Populus euphratica]	-	-	5.97	3.23	1.52	up	0.00	0.01	yes
TRINITY_DN14290_c0_g1	hypothetical protein POPTR_0002s17200g [Populus trichocarpa]	-	-	125.76	94.50	1.02	up	0.00	0.01	yes
TRINITY_DN20478_c0_g4	PREDICTED: growth-regulating factor 1 isoform X1 [Populus euphratica]	GRF1	Growth-regulating factor 1 OS=Oryza sativa subsp. indica GN=GRF1 PE=2 SV=1	7.41	4.30	1.54	up	0.00	0.02	yes
TRINITY_DN18593_c0_g1	hypothetical protein POPTR_0067s00270g [Populus trichocarpa]	-	-	15.75	11.22	1.10	up	0.00	0.02	yes
TRINITY_DN17651_c0_g1	hypothetical protein POPTR_0012s02310g [Populus trichocarpa]	DMS3	Protein DEFECTIVE IN MERISTEM SILENCING 3 OS=Arabidopsis thaliana GN=DMS3 PE=1 SV=1	3.08	2.20	1.10	up	0.00	0.02	yes
TRINITY_DN18161_c0_g1	rhodanese-like domain-containing family protein [Populus trichocarpa]	STR10	Rhodanese-like domain-containing protein 10 OS=Arabidopsis thaliana GN=STR10 PE=2 SV=1	71.81	54.04	1.09	up	0.00	0.02	yes
TRINITY_DN14743_c0_g1	hypothetical protein POPTR_0006s25420g [Populus trichocarpa]	-	-	18.28	12.74	1.11	up	0.00	0.02	yes
TRINITY_DN20696_c1_g2	hypothetical protein POPTR_0009s07130g [Populus trichocarpa]	-	-	7.57	2.56	2.07	up	0.00	0.02	yes
TRINITY_DN18552_c0_g1	Chain A family protein [Populus trichocarpa]	MES17	Methylesterase 17 OS=Arabidopsis thaliana GN=MES17 PE=1 SV=1	13.27	9.06	1.21	up	0.00	0.02	yes
TRINITY_DN26475_c0_g1	PREDICTED: magnesium-chelatase subunit ChlD, chloroplastic [Populus euphratica]	CHLD	Magnesium-chelatase subunit ChlD, chloroplastic OS=Nicotiana tabacum GN=CHLD PE=2 SV=1	105.95	77.46	1.03	up	0.00	0.02	yes
TRINITY_DN19532_c1_g1	hypothetical protein POPTR_0006s19010g [Populus trichocarpa]	-	-	152.42	90.14	1.11	up	0.00	0.02	yes
TRINITY_DN15794_c0_g1	PREDICTED: zinc finger CCCH domain-containing protein 39-like [Populus euphratica]	At3g19360	Zinc finger CCCH domain-containing protein 39 OS=Arabidopsis thaliana GN=At3g19360 PE=2 SV=1	3.32	1.47	1.81	up	0.00	0.02	yes
TRINITY_DN15021_c0_g5	protease inhibitor/seed storage/lipid transfer family protein [Populus trichocarpa]	-	-	4.50	2.24	1.62	up	0.00	0.02	yes
TRINITY_DN25005_c0_g2	hypothetical protein DCAR_001521 [Daucus carota subsp. sativus]	ycf68-1	Uncharacterized protein ycf68 OS=Eucalyptus globulus subsp. globulus GN=ycf68-1 PE=3 SV=1	1973.44	854.16	1.89	up	0.00	0.02	yes
TRINITY_DN26856_c2_g6	hypothetical protein POPTR_0007s14770g [Populus trichocarpa]	PP2A10	Protein PHLOEM PROTEIN 2-LIKE A10 OS=Arabidopsis thaliana GN=PP2A10 PE=2 SV=1	7.29	5.16	1.08	up	0.00	0.02	yes
TRINITY_DN18591_c0_g1	-	-	-	4.36	1.69	1.95	up	0.00	0.02	yes
TRINITY_DN15297_c0_g2	hypothetical protein POPTR_0015s09580g [Populus trichocarpa]	-	-	26.05	11.94	1.74	up	0.00	0.02	yes
TRINITY_DN11055_c0_g1	hypothetical protein VITISV_037104 [Vitis vinifera]	GIP	Copia protein OS=Drosophila melanogaster GN=GIP PE=1 SV=3	2.79	1.03	1.98	up	0.00	0.02	yes
TRINITY_DN19638_c0_g1	hypothetical protein POPTR_0009s03210g [Populus trichocarpa]	RPL23A	60S ribosomal protein L23A OS=Fritillaria agrestis GN=RPL23A PE=2 SV=1	10.61	5.94	1.45	up	0.00	0.02	yes
TRINITY_DN23225_c0_g1	hypothetical protein POPTR_0006s03280g [Populus trichocarpa]	PPD6	PsbP domain-containing protein 6, chloroplastic OS=Arabidopsis thaliana GN=PPD6 PE=1 SV=1	66.81	44.10	1.11	up	0.00	0.02	yes
TRINITY_DN22102_c0_g1	hypothetical protein POPTR_0014s15500g [Populus trichocarpa]	-	-	25.95	18.84	1.04	up	0.00	0.02	yes
TRINITY_DN16469_c0_g1	hypothetical protein POPTR_0006s00970g [Populus trichocarpa]	-	-	11.26	7.43	1.37	up	0.00	0.02	yes
TRINITY_DN16164_c0_g1	hypothetical protein POPTR_0001s44070g [Populus trichocarpa]	-	-	6.40	3.08	1.65	up	0.00	0.02	yes
TRINITY_DN14065_c0_g1	-	-	-	11.74	7.40	1.27	up	0.00	0.02	yes

TRINITY_DN15442_c0_g1	hypothetical protein POPTR_0005s00720g [Populus trichocarpa]	At1g48100	Polygalacturonase At1g48100 OS=Arabidopsis thaliana GN=At1g48100 PE=2 SV=1	2.88	1.51	1.51	up	0.00	0.02	yes
TRINITY_DN16538_c0_g3	ORF126 [Jatropha curcas]	ycf15-A	Putative uncharacterized protein ycf15 OS=Zea mays GN=ycf15-A PE=5 SV=1	3.25	1.50	1.78	up	0.00	0.02	yes
TRINITY_DN16748_c0_g1	PREDICTED: 28 kDa heat- and acid-stable phosphoprotein-like isoform X2 [Populus euphratica]	PDAP1	28 kDa heat- and acid-stable phosphoprotein OS=Homo sapiens GN=PDAP1 PE=1 SV=1	7.20	4.02	1.32	up	0.00	0.02	yes
TRINITY_DN22115_c0_g2	hypothetical protein POPTR_0001s47400g [Populus trichocarpa]	At1g30700	Berberine bridge enzyme-like 8 OS=Arabidopsis thaliana GN=At1g30700 PE=2 SV=1	5.14	3.20	1.27	up	0.00	0.02	yes
TRINITY_DN14741_c0_g1	hypothetical protein POPTR_0009s10020g [Populus trichocarpa]	kynB	Kynurenine formamidase OS=Geobacillus thermodenitrificans (strain NG80-2) GN=kynB PE=3 SV=1	8.28	5.78	1.12	up	0.00	0.02	yes
TRINITY_DN18409_c0_g4	NADH dehydrogenase subunit 5 [Populus alba]	ndhF	NAD(P)H-quinone oxidoreductase subunit 5, chloroplastic OS=Populus alba GN=ndhF PE=3 SV=1	5.12	1.54	2.30	up	0.00	0.02	yes
TRINITY_DN24142_c0_g4	hypothetical protein POPTR_0004s16310g [Populus trichocarpa]	-	-	9.19	2.13	1.51	up	0.00	0.02	yes
TRINITY_DN11038_c0_g2	ycf1 [Populus alba]	TIC214	Protein TIC 214 OS=Populus alba GN=TIC214 PE=3 SV=1	5.53	2.78	1.58	up	0.00	0.02	yes
TRINITY_DN26687_c0_g4	unknown [Populus trichocarpa]	MTACP2	Acyl carrier protein 3, mitochondrial OS=Arabidopsis thaliana GN=MTACP2 PE=2 SV=1	10.92	7.40	1.17	up	0.00	0.02	yes
TRINITY_DN26793_c0_g3	dehydration-responsive family protein [Populus trichocarpa]	At5g64030	Probable methyltransferase PMT26 OS=Arabidopsis thaliana GN=At5g64030 PE=2 SV=1	7.67	4.69	1.31	up	0.00	0.02	yes
TRINITY_DN17044_c0_g1	-	-	-	11.59	8.38	1.29	up	0.00	0.02	yes
TRINITY_DN20055_c0_g7	-	-	-	26.95	19.51	1.11	up	0.00	0.02	yes
TRINITY_DN15745_c1_g1	Thioredoxin M-type 1 family protein [Populus trichocarpa]	At4g03520	Thioredoxin M2, chloroplastic OS=Arabidopsis thaliana GN=At4g03520 PE=1 SV=2	22.04	14.86	1.03	up	0.00	0.02	yes
TRINITY_DN27619_c1_g2	-	-	-	14.26	9.61	1.20	up	0.00	0.02	yes
TRINITY_DN20663_c0_g1	PREDICTED: photosystem II 5 kDa protein, chloroplastic-like [Populus euphratica]	-	Photosystem II 5 kDa protein, chloroplastic OS=Petunia hybrida PE=1 SV=1	1174.60	737.98	1.26	up	0.00	0.02	yes
TRINITY_DN17750_c0_g1	PREDICTED: probable (S)-N-methylcoclaurine 3'-hydroxylase isozyme 2 [Populus euphratica]	CYP80B2	Probable (S)-N-methylcoclaurine 3'-hydroxylase isozyme 2 OS=Coptis japonica GN=CYP80B2 PE=2 SV=1	3.51	2.03	1.39	up	0.00	0.02	yes
TRINITY_DN14655_c0_g1	PREDICTED: folylpolyglutamate synthase-like isoform X1 [Populus euphratica]	FPGS2	Folylpolyglutamate synthase OS=Arabidopsis thaliana GN=FPGS2 PE=1 SV=1	26.10	16.23	1.28	up	0.00	0.02	yes
TRINITY_DN25013_c0_g4	PREDICTED: uncharacterized protein LOC105765014 isoform X2 [Gossypium raimondii]	LOL1	Protein LOL1 OS=Arabidopsis thaliana GN=LOL1 PE=2 SV=1	22.44	14.74	1.05	up	0.00	0.02	yes
TRINITY_DN19619_c0_g2	LOB domain protein 39 [Populus trichocarpa]	LBD37	LOB domain-containing protein 37 OS=Arabidopsis thaliana GN=LBD37 PE=2 SV=1	34.58	17.58	1.64	up	0.00	0.02	yes
TRINITY_DN14781_c0_g1	peroxidase [Populus davidiana x Populus alba var. pyramidalis]	PER21	Peroxidase 21 OS=Arabidopsis thaliana GN=PER21 PE=1 SV=1	1.86	0.63	2.09	up	0.00	0.02	yes
TRINITY_DN17534_c1_g1	PREDICTED: uncharacterized protein LOC105129740 [Populus euphratica]	-	-	10.33	7.30	1.11	up	0.00	0.02	yes
TRINITY_DN22488_c0_g1	PREDICTED: uncharacterized protein LOC105110362 isoform X1 [Populus euphratica]	bsn	Extracellular ribonuclease OS=Bacillus amyloliquefaciens GN=bsn PE=1 SV=1	20.43	13.78	1.03	up	0.00	0.02	yes
TRINITY_DN25815_c0_g3	hypothetical protein POPTR_0003s14070g [Populus trichocarpa]	-	-	12.70	8.05	1.20	up	0.00	0.02	yes
TRINITY_DN13666_c0_g1	hypothetical protein POPTR_0016s03630g [Populus trichocarpa]	FdGOGAT	Ferredoxin-dependent glutamate synthase, chloroplastic OS=Spinacia oleracea GN=FdGOGAT PE=1 SV=3	53.03	25.42	1.63	up	0.01	0.02	yes
TRINITY_DN20439_c0_g1	hypothetical protein POPTR_0002s24830g [Populus trichocarpa]	-	-	7.21	4.34	1.23	up	0.01	0.02	yes
TRINITY_DN14333_c0_g1	hypothetical protein POPTR_0008s09560g [Populus trichocarpa]	-	-	5.88	3.35	1.41	up	0.01	0.02	yes
TRINITY_DN7356_c0_g1	PREDICTED: transmembrane protein 45A [Populus euphratica]	-	-	1.98	0.62	2.29	up	0.01	0.02	yes
TRINITY_DN21291_c2_g3	-	-	-	24.58	19.92	1.04	up	0.01	0.02	yes
TRINITY_DN19489_c0_g1	hypothetical protein POPTR_0013s11030g [Populus trichocarpa]	-	-	36.46	27.16	1.02	up	0.01	0.02	yes
TRINITY_DN19392_c2_g2	PREDICTED: probable carboxylesterase 5 isoform X1 [Populus euphratica]	CXE5	Probable carboxylesterase 5 OS=Arabidopsis thaliana GN=CXE5 PE=2 SV=1	5.63	3.24	1.41	up	0.01	0.02	yes

TRINITY_DN25808_c0_g2	hypothetical protein POPTR_0006s13760g [Populus trichocarpa]	SPS3	Solanesyl diphosphate synthase 3, chloroplastic/mitochondrial OS=Arabidopsis thaliana GN=SPS3 PE=1 SV=1	8.70	6.45	1.02	up	0.01	0.02	yes
TRINITY_DN21131_c0_g1	hypothetical protein POPTR_0010s02330g [Populus trichocarpa]	-	-	14.25	15.29	1.01	up	0.01	0.02	yes
TRINITY_DN22557_c0_g4	STRESS ENHANCED protein 1 [Populus trichocarpa]	SEP1	Stress enhanced protein 1, chloroplastic OS=Arabidopsis thaliana GN=SEP1 PE=2 SV=1	41.80	29.86	1.07	up	0.01	0.02	yes
TRINITY_DN26282_c1_g2	PREDICTED: WD repeat-containing protein 44-like [Populus euphratica]	WDR44	WD repeat-containing protein 44 OS=Bos taurus GN=WDR44 PE=1 SV=1	2.37	1.47	1.32	up	0.01	0.02	yes
TRINITY_DN21185_c1_g5	-	-	-	10.29	4.42	1.74	up	0.01	0.02	yes
TRINITY_DN24243_c0_g1	hypothetical protein POPTR_0010s22010g [Populus trichocarpa]	At2g39360	Probable receptor-like protein kinase At2g39360 OS=Arabidopsis thaliana GN=At2g39360 PE=2 SV=1	9.82	6.77	1.15	up	0.01	0.02	yes
TRINITY_DN16964_c0_g1	hypothetical protein POPTR_0007s02050g [Populus trichocarpa]	NAC076	NAC domain-containing protein 76 OS=Arabidopsis thaliana GN=NAC076 PE=1 SV=1	4.55	1.43	2.34	up	0.01	0.02	yes
TRINITY_DN14428_c0_g1	hypothetical protein POPTR_0010s04960g [Populus trichocarpa]	-	-	1.57	0.61	1.93	up	0.01	0.02	yes
TRINITY_DN16827_c0_g1	PREDICTED: uncharacterized mitochondrial protein ymf11 [Populus euphratica]	YMF11	Uncharacterized mitochondrial protein ymf11 OS=Marchantia polymorpha GN=YMF11 PE=3 SV=1	2.86	2.10	1.06	up	0.01	0.02	yes
TRINITY_DN17178_c0_g1	PREDICTED: uncharacterized protein LOC105123259 [Populus euphratica]	-	-	60.57	40.94	1.08	up	0.01	0.02	yes
TRINITY_DN26752_c0_g2	PREDICTED: uncharacterized protein At3g49140-like isoform X1 [Populus euphratica]	At3g49140	Uncharacterized protein At3g49140 OS=Arabidopsis thaliana GN=At3g49140 PE=1 SV=2	51.38	38.20	1.00	up	0.01	0.02	yes
TRINITY_DN24207_c0_g2	hypothetical protein POPTR_0019s05690g [Populus trichocarpa]	-	-	8.74	5.33	1.25	up	0.01	0.02	yes
TRINITY_DN14019_c0_g1	hypothetical protein POPTR_0007s15070g [Populus trichocarpa]	-	-	39.99	25.81	1.19	up	0.01	0.02	yes
TRINITY_DN19644_c0_g2	-	-	-	2.02	0.79	1.93	up	0.01	0.02	yes
TRINITY_DN19965_c0_g4	hypothetical protein POPTR_0021s00800g [Populus trichocarpa]	DTX40	Protein DETOXIFICATION 40 OS=Arabidopsis thaliana GN=DTX40 PE=1 SV=1	3.91	2.24	1.38	up	0.01	0.02	yes
TRINITY_DN18085_c0_g1	PREDICTED: alpha-L-fucosidase 3-like [Populus euphratica]	At3g26430	GDSL esterase/lipase At3g26430 OS=Arabidopsis thaliana GN=At3g26430 PE=2 SV=1	14.27	9.57	1.20	up	0.01	0.02	yes
TRINITY_DN24157_c0_g2	hypothetical protein POPTR_0003s05250g [Populus trichocarpa]	-	-	9.90	7.22	1.06	up	0.01	0.02	yes
TRINITY_DN22330_c0_g3	PREDICTED: caffeoylshikimate esterase-like [Populus euphratica]	CSE	Caffeoylshikimate esterase OS=Arabidopsis thaliana GN=CSE PE=1 SV=1	6.38	4.44	1.14	up	0.01	0.02	yes
TRINITY_DN13033_c0_g1	hypothetical protein POPTR_0016s03630g [Populus trichocarpa]	FdGOGAT	Ferredoxin-dependent glutamate synthase, chloroplastic OS=Spinacia oleracea GN=FdGOGAT PE=1 SV=3	21.05	11.13	1.49	up	0.01	0.02	yes
TRINITY_DN25201_c0_g2	-	-	-	2.76	1.10	1.93	up	0.01	0.02	yes
TRINITY_DN12720_c0_g2	PREDICTED: vinorine synthase-like [Populus euphratica]	ACT	Vinorine synthase OS=Rauvolfia serpentina GN=ACT PE=1 SV=2	7.62	4.66	1.29	up	0.01	0.02	yes
TRINITY_DN16908_c0_g1	hypothetical protein POPTR_0012s02510g [Populus trichocarpa]	TL17	Thylakoid lumenal 17.4 kDa protein, chloroplastic OS=Arabidopsis thaliana GN=TL17 PE=1 SV=2	201.77	132.90	1.19	up	0.01	0.02	yes
TRINITY_DN26354_c0_g2	hypothetical protein POPTR_0004s14050g, partial [Populus trichocarpa]	-	-	52.39	35.67	1.26	up	0.01	0.02	yes
TRINITY_DN19553_c0_g2	PREDICTED: DNA polymerase zeta processivity subunit [Populus euphratica]	REV7	DNA polymerase zeta processivity subunit OS=Arabidopsis thaliana GN=REV7 PE=2 SV=1	24.91	18.35	1.02	up	0.01	0.02	yes
TRINITY_DN20397_c1_g1	hypothetical protein POPTR_0018s06910g [Populus trichocarpa]	PCMP-H77	Putative pentatricopeptide repeat-containing protein At3g49142 OS=Arabidopsis thaliana GN=PCMP-H77 PE=3 SV=1	7.16	5.57	1.00	up	0.01	0.02	yes
TRINITY_DN17776_c1_g3	-	-	-	10.74	5.71	1.62	up	0.01	0.02	yes
TRINITY_DN20008_c0_g1	PREDICTED: MLO-like protein 13 isoform X1 [Populus euphratica]	MLO13	MLO-like protein 13 OS=Arabidopsis thaliana GN=MLO13 PE=2 SV=1	5.17	3.22	1.12	up	0.01	0.02	yes
TRINITY_DN16897_c0_g1	glycosyl transferase family 8 family protein [Populus trichocarpa]	GAUT5	Probable galacturonosyltransferase 5 OS=Arabidopsis thaliana GN=GAUT5 PE=2 SV=1	8.61	10.24	1.03	up	0.01	0.02	yes
TRINITY_DN22831_c0_g1	PREDICTED: uncharacterized protein LOC105122122 [Populus euphratica]	-	-	66.90	53.70	1.07	up	0.01	0.02	yes

TRINITY_DN17236_c0_g1	hypothetical protein POPTR_0006s22660g [Populus trichocarpa]	-	-	3.20	1.65	1.42	up	0.01	0.02	yes
TRINITY_DN22596_c0_g1	IAA-amino acid hydrolase, partial [Populus tomentosa]	ILL6	IAA-amino acid hydrolase ILR1-like 6 OS=Arabidopsis thaliana GN=ILL6 PE=2 SV=2	21.68	11.51	1.45	up	0.01	0.02	yes
TRINITY_DN15860_c0_g1	PREDICTED: putative endo-1,3(4)-beta-glucanase 2 [Populus euphratica]	ARB_01444	Probable endo-1,3(4)-beta-glucanase ARB_01444 OS=Arthroderma benhamiae (strain ATCC MYA-4681 / CBS 112371) GN=ARB_01444 PE=1 SV=1	5.18	3.16	1.30	up	0.01	0.02	yes
TRINITY_DN20107_c0_g2	PREDICTED: protein LHCP TRANSLOCATION DEFECT [Populus euphratica]	LTD	Protein LHCP TRANSLOCATION DEFECT OS=Oryza sativa subsp. indica GN=LTD PE=3 SV=1	231.30	162.28	1.11	up	0.01	0.02	yes
TRINITY_DN26091_c0_g1	PREDICTED: peptidyl-prolyl cis-trans isomerase FKBP16-1, chloroplastic isoform X2 [Populus euphratica]	FKBP16-1	Peptidyl-prolyl cis-trans isomerase FKBP16-1, chloroplastic OS=Arabidopsis thaliana GN=FKBP16-1 PE=2 SV=1	46.03	34.08	1.06	up	0.01	0.02	yes
TRINITY_DN19412_c0_g5	-	-	-	20.33	11.44	1.41	up	0.01	0.02	yes
TRINITY_DN18567_c0_g1	PREDICTED: pentatricopeptide repeat-containing protein At3g48250, chloroplastic [Populus euphratica]	At3g48250	Pentatricopeptide repeat-containing protein At3g48250, chloroplastic OS=Arabidopsis thaliana GN=At3g48250 PE=2 SV=1	3.13	2.10	1.20	up	0.01	0.02	yes
TRINITY_DN23453_c0_g4	hypothetical protein POPTR_0011s08570g [Populus trichocarpa]	-	-	11.99	7.28	1.34	up	0.01	0.02	yes
TRINITY_DN18372_c0_g2	PREDICTED: cytochrome c oxidase assembly factor 6-like isoform X3 [Populus euphratica]	-	-	21.11	15.54	1.07	up	0.01	0.02	yes
TRINITY_DN26806_c2_g1	hypothetical protein POPTR_0019s05610g [Populus trichocarpa]	CAT4	Cationic amino acid transporter 4, vacuolar OS=Arabidopsis thaliana GN=CAT4 PE=2 SV=1	4.89	2.91	1.35	up	0.01	0.02	yes
TRINITY_DN21122_c0_g3	PREDICTED: oleosin 1-like [Populus euphratica]	At4g25140	Oleosin 18.5 kDa OS=Arabidopsis thaliana GN=At4g25140 PE=2 SV=1	4.16	2.58	1.31	up	0.01	0.02	yes
TRINITY_DN17353_c0_g1	PREDICTED: LOW QUALITY PROTEIN: methylecgonone reductase-like [Populus euphratica]	-	Methylecgonone reductase OS=Erythroxylum coca PE=1 SV=1	9.67	7.02	1.22	up	0.01	0.02	yes
TRINITY_DN25290_c0_g3	mitochondrial glycine decarboxylase complex H-protein [Populus tremuloides]	GDCSH	Glycine cleavage system H protein, mitochondrial OS=Flaveria anomala GN=GDCSH PE=2 SV=1	254.42	155.84	1.35	up	0.01	0.02	yes
TRINITY_DN22413_c1_g10	hypothetical protein VITISV_005279 [Vitis vinifera]	-	Retrovirus-related Pol polyprotein from transposon TNT 1-94 OS=Nicotiana tabacum PE=2 SV=1	2.39	0.85	2.03	up	0.01	0.02	yes
TRINITY_DN19725_c0_g1	PREDICTED: uncharacterized protein LOC105137020 [Populus euphratica]	-	-	5.45	3.29	1.34	up	0.01	0.02	yes
TRINITY_DN23752_c0_g1	hypothetical protein POPTR_0001s40410g [Populus trichocarpa]	-	-	63.85	47.00	1.07	up	0.01	0.02	yes
TRINITY_DN19989_c0_g4	PREDICTED: methyltransferase-like protein 13 isoform X2 [Populus euphratica]	-	-	1.93	0.91	1.66	up	0.01	0.02	yes
TRINITY_DN21354_c0_g1	hypothetical protein [Populus tomentosa]	-	-	9.23	5.28	1.41	up	0.01	0.02	yes
TRINITY_DN10094_c0_g1	PREDICTED: uncharacterized protein LOC105116494 isoform X1 [Populus euphratica]	-	-	14.78	10.71	1.08	up	0.01	0.02	yes
TRINITY_DN19484_c0_g1	PREDICTED: histone-lysine N-methyltransferase ATXR4 isoform X1 [Populus euphratica]	ATXR4	Histone-lysine N-methyltransferase ATXR4 OS=Arabidopsis thaliana GN=ATXR4 PE=2 SV=2	4.97	3.55	1.08	up	0.01	0.02	yes
TRINITY_DN14413_c0_g1	hypothetical protein POPTR_0011s15620g [Populus trichocarpa]	-	-	3.34	1.71	1.61	up	0.01	0.02	yes
TRINITY_DN17408_c0_g2	hypothetical protein POPTR_0001s46750g [Populus trichocarpa]	-	-	2.29	1.06	1.71	up	0.01	0.02	yes
TRINITY_DN26418_c0_g2	ferritin precursor family protein [Populus trichocarpa]	PFE	Ferritin, chloroplastic OS=Phaseolus vulgaris GN=PFE PE=2 SV=1	73.66	43.58	1.51	up	0.01	0.02	yes
TRINITY_DN14593_c0_g1	hypothetical protein POPTR_0019s14690g [Populus trichocarpa]	At2g30270	Protein LURP-one-related 7 OS=Arabidopsis thaliana GN=At2g30270 PE=2 SV=1	7.96	5.62	1.24	up	0.01	0.02	yes
TRINITY_DN13894_c0_g2	heavy-metal-associated domain-containing family protein [Populus trichocarpa]	-	-	4.85	2.36	2.12	up	0.01	0.02	yes
TRINITY_DN23690_c0_g1	PREDICTED: phosphopantothenoylcysteine decarboxylase subunit VHS3 [Populus euphratica]	-	-	69.67	32.01	1.87	up	0.01	0.02	yes
TRINITY_DN23411_c2_g4	hypothetical protein POPTR_0001s16790g [Populus trichocarpa]	CYP71A1	Cytochrome P450 71A1 OS=Persea americana GN=CYP71A1 PE=1 SV=2	215.54	128.14	1.38	up	0.01	0.02	yes

TRINITY_DN25082_c0_g1	phenylalanine ammonia-lyase [Populus tomentosa]	PAL	Phenylalanine ammonia-lyase OS=Populus trichocarpa GN=PAL PE=2 SV=1	144.67	96.35	1.19	up	0.01	0.02	yes
TRINITY_DN18131_c0_g1	zinc finger family protein [Populus trichocarpa]	ZAT5	Zinc finger protein ZAT5 OS=Arabidopsis thaliana GN=ZAT5 PE=2 SV=1	4.40	2.12	1.67	up	0.01	0.02	yes
TRINITY_DN27833_c2_g1	PREDICTED: uncharacterized protein LOC105128518 isoform X1 [Populus euphratica]	-	-	73.55	51.50	1.10	up	0.01	0.02	yes
TRINITY_DN17534_c1_g2	PREDICTED: uncharacterized protein LOC105111417 [Populus euphratica]	-	-	23.14	17.00	1.06	up	0.01	0.02	yes
TRINITY_DN17904_c0_g1	-	-	-	5.05	2.56	1.58	up	0.01	0.02	yes
TRINITY_DN18678_c1_g3	PREDICTED: zinc finger protein 4-like [Populus euphratica]	-	-	8.98	4.63	1.52	up	0.01	0.02	yes
TRINITY_DN27621_c0_g4	6-phosphogluconate dehydrogenase family protein [Populus trichocarpa]	At1g64190	6-phosphogluconate dehydrogenase, decarboxylating 1, chloroplastic OS=Arabidopsis thaliana GN=At1g64190 PE=1 SV=1	11.51	5.70	1.61	up	0.01	0.02	yes
TRINITY_DN27587_c1_g3	hypothetical protein POPTR_0012s10770g [Populus trichocarpa]	CLPC1	Chaperone protein ClpC1, chloroplastic OS=Arabidopsis thaliana GN=CLPC1 PE=1 SV=1	148.97	101.24	1.09	up	0.01	0.02	yes
TRINITY_DN18640_c0_g1	unknown [Populus trichocarpa x Populus deltoides]	FTRC	Ferredoxin-thioredoxin reductase catalytic chain, chloroplastic OS=Arabidopsis thaliana GN=FTRC PE=2 SV=1	127.73	93.63	1.06	up	0.01	0.02	yes
TRINITY_DN19200_c0_g1	-	-	-	6.48	4.04	1.27	up	0.01	0.02	yes
TRINITY_DN20247_c0_g5	hypothetical protein POPTR_0017s03150g [Populus trichocarpa]	-	-	3.41	2.21	1.53	up	0.01	0.02	yes
TRINITY_DN22761_c0_g2	hypothetical protein POPTR_0019s00610g [Populus trichocarpa]	-	-	2.01	1.06	1.52	up	0.01	0.02	yes
TRINITY_DN24803_c0_g2	PREDICTED: LOW QUALITY PROTEIN: transcription factor PIF1 [Populus euphratica]	PIF1	Transcription factor PIF1 OS=Arabidopsis thaliana GN=PIF1 PE=1 SV=1	10.70	7.89	1.01	up	0.01	0.02	yes
TRINITY_DN17902_c0_g1	hypothetical protein POPTR_0010s08410g [Populus trichocarpa]	At1g06620	1-aminocyclopropane-1-carboxylate oxidase homolog 1 OS=Arabidopsis thaliana GN=At1g06620 PE=2 SV=1	5.63	4.23	1.02	up	0.01	0.02	yes
TRINITY_DN7104_c0_g1	PREDICTED: uncharacterized protein LOC109019659 [Juglans regia]	-	-	6.19	1.93	2.27	up	0.01	0.02	yes
TRINITY_DN27695_c2_g1	HSP80 family protein [Populus trichocarpa]	HSC80	Heat shock cognate protein 80 OS=Solanum lycopersicum GN=HSC80 PE=2 SV=1	176.25	130.70	1.11	up	0.01	0.02	yes
TRINITY_DN20753_c0_g7	integral membrane family protein [Populus trichocarpa]	POPTRDRAFT_8	CASP-like protein 1E1 OS=Populus trichocarpa GN=POPTRDRAFT_820934 PE=3 SV=2	10.33	6.62	1.21	up	0.01	0.02	yes
TRINITY_DN27204_c0_g1	pentatricopeptide repeat-containing family protein [Populus trichocarpa]	EMB1270	Pentatricopeptide repeat-containing protein At3g18110, chloroplastic OS=Arabidopsis thaliana GN=EMB1270 PE=2 SV=2	4.73	3.05	1.07	up	0.01	0.02	yes
TRINITY_DN18219_c0_g2	ubiquitin-40S ribosomal protein S27a [Solanum tuberosum]	UBI3	Ubiquitin-40S ribosomal protein S27a OS=Solanum lycopersicum GN=UBI3 PE=3 SV=2	49.38	38.48	1.01	up	0.01	0.02	yes
TRINITY_DN25047_c1_g2	hypothetical protein POPTR_0003s08840g [Populus trichocarpa]	-	-	21.66	14.19	1.21	up	0.01	0.02	yes
TRINITY_DN25550_c0_g1	PREDICTED: filament-like plant protein isoform X2 [Populus euphratica]	FPP3	Filament-like plant protein 3 OS=Arabidopsis thaliana GN=FPP3 PE=2 SV=2	7.05	5.96	1.02	up	0.01	0.02	yes
TRINITY_DN21391_c0_g1	PREDICTED: uncharacterized protein LOC105124970 [Populus euphratica]	-	-	49.29	33.42	1.12	up	0.01	0.02	yes
TRINITY_DN23877_c1_g1	hypothetical protein POPTR_0008s09870g [Populus trichocarpa]	METK3	S-adenosylmethionine synthase 3 OS=Populus trichocarpa GN=METK3 PE=2 SV=1	162.62	115.69	1.12	up	0.01	0.02	yes
TRINITY_DN26905_c0_g2	IQ domain-containing family protein [Populus trichocarpa]	BAG5	BAG family molecular chaperone regulator 5, mitochondrial OS=Arabidopsis thaliana GN=BAG5 PE=1 SV=1	17.91	13.24	1.05	up	0.01	0.02	yes
TRINITY_DN25082_c0_g3	-	-	-	2.94	1.02	2.05	up	0.01	0.02	yes
TRINITY_DN19373_c0_g5	hypothetical protein POPTR_0011s03370g [Populus trichocarpa]	CXXS1	Thioredoxin-like protein CXXS1 OS=Arabidopsis thaliana GN=CXXS1 PE=2 SV=2	7.26	4.24	1.41	up	0.01	0.02	yes
TRINITY_DN1671_c0_g1	hypothetical protein POPTR_0019s13630g [Populus trichocarpa]	TIC20-IV	Protein TIC 20-IV, chloroplastic OS=Arabidopsis thaliana GN=TIC20-IV PE=2 SV=1	3.05	1.34	1.79	up	0.01	0.02	yes
TRINITY_DN14354_c0_g1	PREDICTED: pentatricopeptide repeat-containing protein At3g62890-like [Fragaria vesca subsp. vesca]	PCMP-H87	Pentatricopeptide repeat-containing protein At3g24000, mitochondrial OS=Arabidopsis thaliana GN=PCMP-H87 PE=3 SV=1	3.97	2.03	1.59	up	0.01	0.02	yes

TRINITY_DN21100_c0_g1	hypothetical protein POPTR_0003s14610g [Populus trichocarpa]	-	-	7.46	4.83	1.36	up	0.01	0.02	yes
TRINITY_DN25116_c0_g5	PREDICTED: uncharacterized protein LOC100782481 isoform X1 [Glycine max]	-	-	3.89	2.56	1.57	up	0.01	0.02	yes
TRINITY_DN23006_c1_g2	hypothetical protein POPTR_0002s25140g, partial [Populus trichocarpa]	EPFL9	EPIDERMAL PATTERNING FACTOR-like protein 9 OS=Arabidopsis thaliana GN=EPFL9 PE=1 SV=1	136.71	92.42	1.19	up	0.01	0.02	yes
TRINITY_DN19450_c0_g2	hypothetical protein POPTR_0002s01620g [Populus trichocarpa]	PCMP-H15	Pentatricopeptide repeat-containing protein At5g40410, mitochondrial OS=Arabidopsis thaliana GN=PCMP-H15 PE=2 SV=1	3.25	2.32	1.10	up	0.01	0.02	yes
TRINITY_DN22413_c1_g6	hypothetical protein VITISV_013624 [Vitis vinifera]	-	Retrovirus-related Pol polyprotein from transposon TNT 1-94 OS=Nicotiana tabacum PE=2 SV=1	2.05	0.86	1.81	up	0.01	0.02	yes
TRINITY_DN16646_c0_g1	PREDICTED: glycine-rich RNA-binding protein 4, mitochondrial [Populus euphratica]	RBG4	Glycine-rich RNA-binding protein 4, mitochondrial OS=Arabidopsis thaliana GN=RBG4 PE=2 SV=1	13.39	10.78	1.25	up	0.01	0.02	yes
TRINITY_DN17934_c2_g5	hypothetical protein POPTR_0011s00700g [Populus trichocarpa]	W02F12.2	Alkaline ceramidase OS=Caenorhabditis elegans GN=W02F12.2 PE=3 SV=2	3.53	2.32	1.23	up	0.01	0.02	yes
TRINITY_DN16269_c0_g1	GDSL-motif lipase/hydrolase family protein [Populus trichocarpa]	At5g45950	GDSL esterase/lipase At5g45950 OS=Arabidopsis thaliana GN=At5g45950 PE=2 SV=1	4.71	2.76	1.35	up	0.01	0.02	yes
TRINITY_DN24249_c0_g1	PREDICTED: ribulose bisphosphate carboxylase large chain [Populus euphratica]	rbcL	Ribulose bisphosphate carboxylase large chain OS=Populus alba GN=rbcL PE=3 SV=1	86.19	43.64	1.66	up	0.01	0.03	yes
TRINITY_DN3813_c0_g1	hypothetical protein POPTR_0011s05880g [Populus trichocarpa]	SPBC1703.11	OPA3-like protein OS=Schizosaccharomyces pombe (strain 972 / ATCC 24843) GN=SPBC1703.11 PE=3 SV=1	5.06	3.39	1.18	up	0.01	0.03	yes
TRINITY_DN19064_c0_g1	Photosystem I reaction center subunit XI family protein [Populus trichocarpa]	PSAL	Photosystem I reaction center subunit XI, chloroplastic OS=Cucumis sativus GN=PSAL PE=2 SV=1	3429.37	2570.38	1.05	up	0.01	0.03	yes
TRINITY_DN22845_c0_g2	-	-	-	4.51	2.33	1.55	up	0.01	0.03	yes
TRINITY_DN17822_c1_g6	cytochrome f [Populus alba]	petA	Cytochrome f OS=Populus alba GN=petA PE=3 SV=1	3.71	2.11	1.46	up	0.01	0.03	yes
TRINITY_DN17185_c0_g1	PREDICTED: serine/threonine-protein kinase CDG1-like isoform X1 [Populus euphratica]	ALE2	Receptor-like serine/threonine-protein kinase ALE2 OS=Arabidopsis thaliana GN=ALE2 PE=1 SV=1	3.04	1.69	1.44	up	0.01	0.03	yes
TRINITY_DN18279_c0_g1	hypothetical protein POPTR_0001s10650g [Populus trichocarpa]	ndhS	NAD(P)H-quinone oxidoreductase subunit S, chloroplastic OS=Arabidopsis thaliana GN=ndhS PE=1 SV=1	121.34	81.46	1.16	up	0.01	0.03	yes
TRINITY_DN18781_c0_g1	-	-	-	3.13	2.43	1.41	up	0.01	0.03	yes
TRINITY_DN19656_c0_g2	PREDICTED: G-type lectin S-receptor-like serine/threonine-protein kinase RLK1 [Populus euphratica]	RLK1	G-type lectin S-receptor-like serine/threonine-protein kinase RLK1 OS=Arabidopsis thaliana GN=RLK1 PE=2 SV=2	3.74	2.35	1.26	up	0.01	0.03	yes
TRINITY_DN22392_c0_g5	-	-	-	2.69	1.02	1.97	up	0.01	0.03	yes
TRINITY_DN17624_c0_g1	hypothetical protein POPTR_0012s03000g [Populus trichocarpa]	-	-	18.08	12.61	1.14	up	0.01	0.03	yes
TRINITY_DN20724_c2_g1	gamma-tonoplast intrinsic protein 2 [Populus trichocarpa]	TIP1-1	Aquaporin TIP1-1 OS=Arabidopsis thaliana GN=TIP1-1 PE=1 SV=1	491.95	333.70	1.10	up	0.01	0.03	yes
TRINITY_DN24995_c1_g3	glutathione peroxidase [Populus euphratica]	GPX2	Probable glutathione peroxidase 2 OS=Arabidopsis thaliana GN=GPX2 PE=1 SV=1	10.51	6.15	1.35	up	0.01	0.03	yes
TRINITY_DN22466_c0_g3	hypothetical protein POPTR_0001s10460g [Populus trichocarpa]	BAN	Anthocyanidin reductase OS=Arabidopsis thaliana GN=BAN PE=1 SV=2	6.63	3.91	1.36	up	0.01	0.03	yes
TRINITY_DN17787_c0_g2	PREDICTED: 30S ribosomal protein S17, chloroplastic-like [Populus euphratica]	rpsQ	30S ribosomal protein S17 OS=Peptoclostridium difficile (strain 630) GN=rpsQ PE=3 SV=1	17.39	12.04	1.04	up	0.01	0.03	yes
TRINITY_DN22510_c0_g1	hypothetical protein POPTR_0007s03960g [Populus trichocarpa]	EMB2271	U3 snoRNP-associated protein-like EMB2271 OS=Arabidopsis thaliana GN=EMB2271 PE=2 SV=1	10.61	7.87	1.09	up	0.01	0.03	yes
TRINITY_DN18592_c1_g8	PREDICTED: pentatricopeptide repeat-containing protein At1g15510, chloroplastic [Populus euphratica]	PCMP-H73	Pentatricopeptide repeat-containing protein At1g15510, chloroplastic OS=Arabidopsis thaliana GN=PCMP-H73 PE=3 SV=1	2.88	2.16	1.05	up	0.01	0.03	yes
TRINITY_DN26984_c2_g4	PREDICTED: LOW QUALITY PROTEIN: probable cyclic nucleotide-gated ion channel 14 [Populus euphratica]	CNGC17	Cyclic nucleotide-gated ion channel 17 OS=Arabidopsis thaliana GN=CNGC17 PE=1 SV=1	6.71	4.48	1.22	up	0.01	0.03	yes
TRINITY_DN26913_c0_g2	hypothetical protein POPTR_0005s08050g [Populus trichocarpa]	-	Inositol-3-phosphate synthase OS=Nicotiana tabacum PE=2 SV=1	14.60	7.42	1.63	up	0.01	0.03	yes
TRINITY_DN20454_c0_g1	-	-	-	4.33	2.55	1.35	up	0.01	0.03	yes

TRINITY_DN15236_c0_g1	Kunitz-type trypsin inhibitor [Populus nigra]	KTI2	Kunitz trypsin inhibitor 2 OS=Arabidopsis thaliana GN=KTI2 PE=2 SV=1	5.65	2.67	1.64	up	0.01	0.03	yes
TRINITY_DN16048_c0_g1	hypothetical protein POPTR_0006s08850g [Populus trichocarpa]	OHP1	Light-harvesting complex-like protein OHP1, chloroplastic OS=Arabidopsis thaliana GN=OHP1 PE=1 SV=1	124.80	88.50	1.08	up	0.01	0.03	yes
TRINITY_DN18938_c0_g1	PREDICTED: ABC transporter A family member 7-like [Populus euphratica]	ABCA7	ABC transporter A family member 7 OS=Arabidopsis thaliana GN=ABCA7 PE=3 SV=2	3.26	1.53	1.66	up	0.01	0.03	yes
TRINITY_DN27309_c0_g2	hypothetical protein BVRB_041110, partial [Beta vulgaris subsp. vulgaris]	-	Uncharacterized protein ORF91 OS=Phalaenopsis aphrodite subsp. formosana PE=4 SV=1	2593.66	1111.34	1.90	up	0.01	0.03	yes
TRINITY_DN17194_c0_g1	PREDICTED: very-long-chain enoyl-CoA reductase-like [Populus euphratica]	DET2	Steroid 5-alpha-reductase DET2 OS=Gossypium hirsutum GN=DET2 PE=1 SV=1	8.23	5.68	1.17	up	0.01	0.03	yes
TRINITY_DN23319_c0_g4	PREDICTED: probable aquaporin PIP2-2 [Amborella trichopoda]	PIP2-4	Probable aquaporin PIP2-4 OS=Arabidopsis thaliana GN=PIP2-4 PE=1 SV=1	134.55	85.24	1.15	up	0.01	0.03	yes
TRINITY_DN21976_c0_g4	PREDICTED: uncharacterized protein LOC105130182 [Populus euphratica]	petM	Cytochrome b6-f complex subunit 7 (Fragment) OS=Spinacia oleracea GN=petM PE=1 SV=1	70.73	52.68	1.03	up	0.01	0.03	yes
TRINITY_DN18467_c0_g6	hypothetical protein M569_00483, partial [Genlisea aurea]	psbJ	Photosystem II reaction center protein J OS=Eucalyptus globulus subsp. globulus GN=psbJ PE=3 SV=1	8.55	5.09	1.36	up	0.01	0.03	yes
TRINITY_DN26016_c0_g4	hypothetical protein L484_022812 [Morus notabilis]	-	-	8.87	5.44	1.31	up	0.01	0.03	yes
TRINITY_DN18060_c0_g1	PREDICTED: uncharacterized protein LOC105122593 [Populus euphratica]	-	-	80.18	55.43	1.06	up	0.01	0.03	yes
TRINITY_DN24590_c0_g5	hypothetical protein POPTR_0016s03510g [Populus trichocarpa]	At2g26850	F-box protein At2g26850 OS=Arabidopsis thaliana GN=At2g26850 PE=2 SV=1	16.01	9.79	1.30	up	0.01	0.03	yes
TRINITY_DN21957_c0_g1	cyclin d2 family protein [Populus trichocarpa]	CYCD2-1	Cyclin-D2-1 OS=Arabidopsis thaliana GN=CYCD2-1 PE=1 SV=3	4.92	3.29	1.05	up	0.01	0.03	yes
TRINITY_DN18756_c0_g3	hypothetical protein POPTR_0005s04150g [Populus trichocarpa]	TR	Tropinone reductase OS=Cochlearia officinalis GN=TR PE=1 SV=1	2.80	1.20	1.80	up	0.01	0.03	yes
TRINITY_DN391_c0_g1	hypothetical protein POPTR_0340s00200g [Populus trichocarpa]	-	Pelargonidin 3-O-(6-caffeoylglucoside) 5-O-(6-O-malonylglucoside) 4'''-malonyltransferase OS=Salvia splendens PE=1 SV=1	62.66	39.30	1.28	up	0.01	0.03	yes
TRINITY_DN21393_c0_g1	unknown [Populus trichocarpa]	RPS7A	40S ribosomal protein S7-1 OS=Arabidopsis thaliana GN=RPS7A PE=2 SV=1	24.00	17.89	1.03	up	0.01	0.03	yes
TRINITY_DN26681_c1_g2	PREDICTED: probable LRR receptor-like serine/threonine-protein kinase At4g26540 [Populus euphratica]	At4g26540	Probable LRR receptor-like serine/threonine-protein kinase At4g26540 OS=Arabidopsis thaliana GN=At4g26540 PE=2 SV=1	5.17	2.83	1.47	up	0.01	0.03	yes
TRINITY_DN4154_c0_g1	hypothetical protein POPTR_0006s10380g [Populus trichocarpa]	-	-	6.06	2.11	2.12	up	0.01	0.03	yes
TRINITY_DN23945_c2_g5	hypothetical protein POPTR_0008s02590g [Populus trichocarpa]	-	-	10.09	7.21	1.07	up	0.01	0.03	yes
TRINITY_DN15853_c0_g2	hypothetical protein POPTR_0008s13480g [Populus trichocarpa]	-	-	60.32	40.19	1.28	up	0.01	0.03	yes
TRINITY_DN10019_c0_g1	glutaredoxin family protein [Populus trichocarpa]	GRXS11	Monothiol glutaredoxin-S11 OS=Arabidopsis thaliana GN=GRXS11 PE=3 SV=1	3.70	1.46	1.94	up	0.01	0.03	yes
TRINITY_DN16025_c1_g1	-	-	-	65.82	48.04	1.08	up	0.01	0.03	yes
TRINITY_DN26755_c0_g1	hypothetical protein POPTR_0015s15120g [Populus trichocarpa]	CPN60A2	Chaperonin 60 subunit alpha 2, chloroplastic OS=Arabidopsis thaliana GN=CPN60A2 PE=2 SV=1	32.72	23.64	1.07	up	0.01	0.03	yes
TRINITY_DN26587_c0_g1	PREDICTED: uncharacterized protein LOC105134389 [Populus euphratica]	-	-	2.82	1.43	1.56	up	0.01	0.03	yes
TRINITY_DN13841_c0_g1	Pyrophosphate-energized vacuolar membrane proton pump family protein-2 [Populus tomentosa]	AVP1	Pyrophosphate-energized vacuolar membrane proton pump 1 OS=Arabidopsis thaliana GN=AVP1 PE=1 SV=1	80.63	49.50	1.30	up	0.01	0.03	yes
TRINITY_DN16162_c0_g1	hypothetical protein POPTR_0012s05150g [Populus trichocarpa]	-	-	1.99	1.18	1.35	up	0.01	0.03	yes
TRINITY_DN22760_c0_g1	PREDICTED: uncharacterized protein LOC105109291 [Populus euphratica]	MG002	DnaJ-like protein MG002 OS=Mycoplasma genitalium (strain ATCC 33530 / G-37 / NCTC 10195) GN=MG002 PE=3 SV=1	9.97	6.31	1.25	up	0.01	0.03	yes
TRINITY_DN19674_c1_g2	beta-galactosidase family protein [Populus trichocarpa]	Os03g0255100	Beta-galactosidase 6 OS=Oryza sativa subsp. japonica GN=Os03g0255100 PE=1 SV=2	9.86	4.84	1.61	up	0.01	0.03	yes
TRINITY_DN24921_c0_g1	-	-	-	3.97	2.00	1.78	up	0.01	0.03	yes
TRINITY_DN27104_c0_g2	hypothetical protein POPTR_0002s07170g [Populus trichocarpa]	-	-	1.91	0.75	1.89	up	0.01	0.03	yes

TRINITY_DN23314_c1_g1	DWARF IN LIGHT 2 family protein [Populus trichocarpa]	GH3.5	Jasmonic acid-amido synthetase JAR1 OS=Oryza sativa subsp. japonica GN=GH3.5 PE=2 SV=1	13.83	9.87	1.11	up	0.01	0.03	yes
TRINITY_DN12716_c0_g1	PREDICTED: formimidoyltransferase-cyclodeaminase-like [Populus euphratica]	-	-	7.80	5.25	1.18	up	0.01	0.03	yes
TRINITY_DN27055_c1_g3	hypothetical protein POPTR_0005s00240g [Populus trichocarpa]	RH3	DEAD-box ATP-dependent RNA helicase 3, chloroplastic OS=Arabidopsis thaliana GN=RH3 PE=1 SV=2	81.72	55.42	1.13	up	0.01	0.03	yes
TRINITY_DN19084_c1_g4	-	-	-	13.67	7.09	1.62	up	0.01	0.03	yes
TRINITY_DN27401_c0_g2	hypothetical protein POPTR_0009s13380g [Populus trichocarpa]	TOC132	Translocase of chloroplast 132, chloroplastic OS=Arabidopsis thaliana GN=TOC132 PE=1 SV=1	31.21	23.15	1.02	up	0.01	0.03	yes
TRINITY_DN14263_c0_g1	harpin-induced family protein [Populus trichocarpa]	NHL3	NDR1/HIN1-Like protein 3 OS=Arabidopsis thaliana GN=NHL3 PE=1 SV=1	4.01	2.08	1.57	up	0.01	0.03	yes
TRINITY_DN23671_c0_g2	hypothetical protein POPTR_0006s16300g [Populus trichocarpa]	-	-	26.91	16.73	1.28	up	0.01	0.03	yes
TRINITY_DN22906_c0_g1	MGDG synthase type A family protein [Populus trichocarpa]	MGD	Monogalactosyldiacylglycerol synthase, chloroplastic OS=Spinacia oleracea GN=MGD A PE=1 SV=1	7.93	5.80	1.04	up	0.01	0.03	yes
TRINITY_DN26983_c0_g6	tetrachloro-p-hydroquinone reductive dehalogenase-related family protein [Populus trichocarpa]	TCHQD	Glutathione S-transferase TCHQD OS=Arabidopsis thaliana GN=TCHQD PE=2 SV=1	6.69	5.09	1.02	up	0.01	0.03	yes
TRINITY_DN19323_c0_g3	hypothetical protein POPTR_0013s04750g [Populus trichocarpa]	-	-	7.91	5.79	1.08	up	0.01	0.03	yes
TRINITY_DN14656_c0_g1	hypothetical protein POPTR_0002s17200g [Populus trichocarpa]	-	-	174.80	118.30	1.16	up	0.01	0.03	yes
TRINITY_DN21377_c1_g3	hypothetical protein POPTR_0005s09750g [Populus trichocarpa]	-	-	44.30	32.20	1.05	up	0.01	0.03	yes
TRINITY_DN17954_c0_g1	PREDICTED: putative RNA methyltransferase At5g10620 isoform X1 [Populus euphratica]	At5g10620	Putative RNA methyltransferase At5g10620 OS=Arabidopsis thaliana GN=At5g10620 PE=3 SV=1	16.98	14.25	1.10	up	0.01	0.03	yes
TRINITY_DN22956_c0_g1	galactosyltransferase family protein [Populus trichocarpa]	B3GALT14	Probable beta-1,3-galactosyltransferase 14 OS=Arabidopsis thaliana GN=B3GALT14 PE=2 SV=1	4.29	3.02	1.09	up	0.01	0.03	yes
TRINITY_DN16683_c0_g2	hypothetical protein POPTR_0010s20070g [Populus trichocarpa]	-	-	49.51	30.46	1.35	up	0.01	0.03	yes
TRINITY_DN15903_c1_g1	PREDICTED: dof zinc finger protein DOF1.2 [Populus euphratica]	DOF1.2	Dof zinc finger protein DOF1.2 OS=Arabidopsis thaliana GN=DOF1.2 PE=2 SV=1	3.96	2.29	1.47	up	0.01	0.03	yes
TRINITY_DN20924_c0_g1	transferase family protein [Populus trichocarpa]	DCR	BAHD acyltransferase DCR OS=Arabidopsis thaliana GN=DCR PE=2 SV=1	32.99	19.55	1.28	up	0.01	0.03	yes
TRINITY_DN16760_c0_g1	unknown [Populus trichocarpa]	-	-	45.53	32.14	1.10	up	0.01	0.03	yes
TRINITY_DN18676_c0_g2	hypothetical protein POPTR_0012s13290g [Populus trichocarpa]	RUP2	WD repeat-containing protein RUP2 OS=Arabidopsis thaliana GN=RUP2 PE=1 SV=1	10.14	4.79	1.69	up	0.01	0.03	yes
TRINITY_DN22833_c0_g1	hypothetical protein POPTR_0003s09960g [Populus trichocarpa]	-	-	39.62	27.72	1.01	up	0.01	0.03	yes
TRINITY_DN24109_c1_g1	hypothetical protein POPTR_0010s21280g [Populus trichocarpa]	HSC-I	Heat shock cognate 70 kDa protein 1 OS=Solanum lycopersicum GN=HSC-I PE=2 SV=1	548.40	356.38	1.30	up	0.01	0.03	yes
TRINITY_DN21919_c0_g2	-	-	-	3.39	2.24	1.23	up	0.01	0.03	yes
TRINITY_DN18039_c0_g1	PREDICTED: uncharacterized protein LOC107023401 [Solanum pennellii]	-	-	2.51	1.61	1.23	up	0.01	0.03	yes
TRINITY_DN22791_c0_g1	NADH-plastoquinone oxidoreductase subunit K (chloroplast) [Populus euphratica]	ndhK	NAD(P)H-quinone oxidoreductase subunit K, chloroplastic OS=Populus alba GN=ndhK PE=3 SV=1	2.53	1.58	1.30	up	0.01	0.03	yes
TRINITY_DN23394_c0_g7	hypothetical protein POPTR_0002s06930g [Populus trichocarpa]	-	-	2.26	1.12	1.59	up	0.01	0.03	yes
TRINITY_DN15382_c0_g2	PREDICTED: probable cellulose synthase A catalytic subunit 3 [UDP-forming] [Populus euphratica]	CESA6	Probable cellulose synthase A catalytic subunit 6 [UDP-forming] OS=Oryza sativa subsp. japonica GN=CESA6 PE=2 SV=1	22.15	15.51	1.10	up	0.01	0.03	yes
TRINITY_DN20247_c0_g2	-	-	-	2.69	1.34	1.65	up	0.01	0.03	yes
TRINITY_DN19183_c0_g1	hypothetical protein POPTR_0018s12090g [Populus trichocarpa]	-	-	5.24	3.53	1.19	up	0.01	0.03	yes
TRINITY_DN25037_c0_g2	PREDICTED: uncharacterized protein LOC105108783 [Populus euphratica]	-	-	14.43	9.60	1.21	up	0.01	0.03	yes

TRINITY_DN17712_c0_g1	PREDICTED: grpE protein homolog, mitochondrial isoform X1 [Populus euphratica]	grpE	Protein GrpE OS=Nitrobacter winogradskyi (strain ATCC 25391 / DSM 10237 / CIP 104748 / NCIMB 11846 / Nb-255) GN=grpE PE=3 SV=1	8.98	6.94	1.03	up	0.01	0.03	yes
TRINITY_DN26778_c0_g2	PREDICTED: uncharacterized protein LOC105134744 [Populus euphratica]	MTERF1	Transcription termination factor MTEF1, chloroplastic OS=Arabidopsis thaliana GN=MTERF1 PE=2 SV=2	24.03	17.30	1.06	up	0.01	0.03	yes
TRINITY_DN23112_c0_g2	hypothetical protein POPTR_0016s00410g [Populus trichocarpa]	RFS2	Probable galactinol--sucrose galactosyltransferase 2 OS=Arabidopsis thaliana GN=RFS2 PE=2 SV=2	32.21	20.47	1.23	up	0.01	0.03	yes
TRINITY_DN20976_c0_g6	glycosyl transferase family 8 family protein [Populus trichocarpa]	GAUT6	Probable galacturonosyltransferase 6 OS=Arabidopsis thaliana GN=GAUT6 PE=2 SV=1	2.69	1.76	1.22	up	0.01	0.03	yes
TRINITY_DN23074_c0_g3	PREDICTED: two-component response regulator ARR5-like isoform X2 [Populus euphratica]	ARR5	Two-component response regulator ARR5 OS=Arabidopsis thaliana GN=ARR5 PE=1 SV=2	25.18	15.42	1.38	up	0.01	0.03	yes
TRINITY_DN19404_c0_g1	PREDICTED: uncharacterized protein LOC105119160 [Populus euphratica]	-	-	2.72	2.19	1.22	up	0.01	0.03	yes
TRINITY_DN26201_c0_g2	014G029700 [Populus tomentosa]	LHCA5	Photosystem I chlorophyll a/b-binding protein 5, chloroplastic OS=Arabidopsis thaliana GN=LHCA5 PE=1 SV=1	48.28	37.09	1.12	up	0.01	0.03	yes
TRINITY_DN21848_c0_g3	hypothetical protein POPTR_0002s02160g [Populus trichocarpa]	RH28	DEAD-box ATP-dependent RNA helicase 28 OS=Arabidopsis thaliana GN=RH28 PE=2 SV=1	1.44	0.49	2.11	up	0.01	0.03	yes
TRINITY_DN19534_c0_g1	hypothetical protein POPTR_0010s09740g [Populus trichocarpa]	-	Ferredoxin-1 OS=Equisetum telmateia PE=1 SV=1	203.71	135.05	1.17	up	0.01	0.03	yes
TRINITY_DN19448_c1_g3	hypothetical protein POPTR_0015s00850g [Populus trichocarpa]	CYP89A9	Cytochrome P450 89A9 OS=Arabidopsis thaliana GN=CYP89A9 PE=2 SV=1	5.89	3.57	1.29	up	0.01	0.03	yes
TRINITY_DN18957_c1_g2	-	-	-	6.27	3.54	1.30	up	0.01	0.03	yes
TRINITY_DN23194_c0_g4	hypothetical protein POPTR_0012s02370g [Populus trichocarpa]	-	-	1.93	1.24	1.26	up	0.01	0.03	yes
TRINITY_DN15746_c0_g1	hypothetical protein POPTR_0013s13110g [Populus trichocarpa]	FAM136A	Protein FAM136A OS=Homo sapiens GN=FAM136A PE=1 SV=1	14.79	11.47	1.02	up	0.01	0.03	yes
TRINITY_DN21695_c1_g3	RNA recognition motif-containing family protein [Populus trichocarpa]	-	-	10.69	6.95	1.26	up	0.01	0.03	yes
TRINITY_DN19039_c1_g4	hypothetical protein POPTR_0012s04420g [Populus trichocarpa]	MTERF15	Transcription termination factor MTERF15, mitochondrial OS=Arabidopsis thaliana GN=MTERF15 PE=2 SV=1	10.68	7.52	1.08	up	0.01	0.03	yes
TRINITY_DN19032_c0_g2	aux/IAA protein [Populus tremula x Populus tremuloides]	AUX22B	Auxin-induced protein 22B OS=Vigna radiata var. radiata GN=AUX22B PE=2 SV=1	8.60	5.63	1.09	up	0.01	0.03	yes
TRINITY_DN14804_c0_g1	hypothetical protein POPTR_0002s17870g [Populus trichocarpa]	-	-	20.45	14.30	1.12	up	0.01	0.03	yes
TRINITY_DN7646_c0_g1	hypothetical protein POPTR_0009s09180g [Populus trichocarpa]	-	-	3.40	2.54	1.04	up	0.01	0.03	yes
TRINITY_DN18234_c0_g5	PREDICTED: S-norcoclaurine synthase 1-like isoform X1 [Populus euphratica]	NCS1	S-norcoclaurine synthase 1 OS=Coptis japonica GN=NCS1 PE=1 SV=1	2.25	1.00	2.18	up	0.01	0.03	yes
TRINITY_DN27108_c0_g1	hypothetical protein POPTR_0007s07920g, partial [Populus trichocarpa]	MSP1	Protein MSP1 OS=Saccharomyces cerevisiae (strain ATCC 204508 / S288c) GN=MSP1 PE=1 SV=2	6.71	5.08	1.00	up	0.01	0.04	yes
TRINITY_DN17945_c0_g2	hypothetical protein POPTR_0006s06140g [Populus trichocarpa]	TYRAAT1	Arogenate dehydrogenase 1, chloroplastic OS=Arabidopsis thaliana GN=TYRAAT1 PE=1 SV=1	3.08	1.24	1.24	up	0.01	0.04	yes
TRINITY_DN18736_c1_g1	hypothetical protein POPTR_0002s18860g [Populus trichocarpa]	ABCB11	ABC transporter B family member 11 OS=Arabidopsis thaliana GN=ABCB11 PE=2 SV=1	3.93	2.53	1.23	up	0.01	0.04	yes
TRINITY_DN16313_c0_g2	hypothetical protein POPTR_0010s00740g [Populus trichocarpa]	CHX19	Cation/H(+) antiporter 19 OS=Arabidopsis thaliana GN=CHX19 PE=2 SV=1	3.76	2.60	1.18	up	0.01	0.04	yes
TRINITY_DN18189_c0_g1	ATP synthase gamma chain 1 family protein [Populus trichocarpa]	ATPC	ATP synthase gamma chain, chloroplastic OS=Nicotiana tabacum GN=ATPC PE=1 SV=1	5.06	3.77	1.01	up	0.01	0.04	yes
TRINITY_DN21002_c0_g2	PREDICTED: transcription factor bHLH30-like [Populus euphratica]	BHLH30	Transcription factor bHLH30 OS=Arabidopsis thaliana GN=BHLH30 PE=1 SV=1	3.87	2.34	1.30	up	0.01	0.04	yes
TRINITY_DN25602_c0_g2	PREDICTED: uncharacterized protein LOC105140529 [Populus euphratica]	-	-	4.01	3.15	1.02	up	0.01	0.04	yes
TRINITY_DN24285_c0_g5	PREDICTED: WAT1-related protein At2g37460-like isoform X1 [Populus euphratica]	At2g37460	WAT1-related protein At2g37460 OS=Arabidopsis thaliana GN=At2g37460 PE=2 SV=1	2.63	1.46	1.46	up	0.01	0.04	yes
TRINITY_DN20924_c0_g2	transferase family protein [Populus trichocarpa]	DCR	BAHD acyltransferase DCR OS=Arabidopsis thaliana GN=DCR PE=2 SV=1	14.61	6.33	1.79	up	0.01	0.04	yes

TRINITY_DN17174_c0_g1	PREDICTED: GDSL esterase/lipase At1g33811 [Populus euphratica]	At1g33811	GDSL esterase/lipase At1g33811 OS=Arabidopsis thaliana GN=At1g33811 PE=2 SV=1	4.85	2.09	1.76	up	0.01	0.04	yes
TRINITY_DN25120_c0_g1	hypothetical protein POPTR_0018s08220g [Populus trichocarpa]	-	-	6.32	4.33	1.18	up	0.01	0.04	yes
TRINITY_DN19557_c1_g3	-	-	-	9.00	4.96	1.48	up	0.01	0.04	yes
TRINITY_DN22577_c0_g5	-	-	-	6.65	4.49	1.42	up	0.01	0.04	yes
TRINITY_DN21396_c1_g1	hypothetical protein POPTR_0009s14820g [Populus trichocarpa]	-	-	21.54	16.30	1.00	up	0.01	0.04	yes
TRINITY_DN17447_c0_g1	hypothetical protein POPTR_0004s06720g [Populus trichocarpa]	-	-	52.76	35.58	1.22	up	0.01	0.04	yes
TRINITY_DN3610_c0_g1	hypothetical protein POPTR_0004s05380g [Populus trichocarpa]	DSEL	Phospholipase A1-IIgamma OS=Arabidopsis thaliana GN=DSEL PE=1 SV=1	53.62	22.92	1.74	up	0.01	0.04	yes
TRINITY_DN12510_c0_g1	-	-	-	5.27	1.85	2.06	up	0.01	0.04	yes
TRINITY_DN22015_c0_g8	-	-	-	3.67	1.95	1.52	up	0.01	0.04	yes
TRINITY_DN16237_c0_g1	PREDICTED: uncharacterized protein LOC105137839 [Populus euphratica]	-	-	18.01	12.99	1.06	up	0.01	0.04	yes
TRINITY_DN14576_c0_g1	PREDICTED: CSC1-like protein At1g32090 [Populus euphratica]	At1g32090	CSC1-like protein At1g32090 OS=Arabidopsis thaliana GN=At1g32090 PE=1 SV=1	19.05	12.90	1.16	up	0.01	0.04	yes
TRINITY_DN16311_c0_g1	-	-	-	3.12	1.84	1.34	up	0.01	0.04	yes
TRINITY_DN24878_c0_g2	camphor resistance CrcB family protein [Populus trichocarpa]	-	-	13.75	7.91	1.53	up	0.01	0.04	yes
TRINITY_DN21849_c0_g1	mitochondrial glycine decarboxylase complex P-protein [Populus tremuloides]	GDCSP	Glycine dehydrogenase (decarboxylating), mitochondrial OS=Solanum tuberosum GN=GDCSP PE=2 SV=1	172.09	109.30	1.18	up	0.01	0.04	yes
TRINITY_DN17244_c0_g2	hypothetical protein POPTR_0001s30660g [Populus trichocarpa]	At3g19508	LYR motif-containing protein At3g19508 OS=Arabidopsis thaliana GN=At3g19508 PE=3 SV=1	6.17	3.28	1.53	up	0.01	0.04	yes
TRINITY_DN14410_c1_g1	hypothetical protein POPTR_0007s12980g [Populus trichocarpa]	-	-	2.72	1.92	1.25	up	0.01	0.04	yes
TRINITY_DN19221_c0_g1	PREDICTED: uracil permease [Populus euphratica]	NCS1	Purine-uracil permease NCS1 OS=Arabidopsis thaliana GN=NCS1 PE=1 SV=1	6.14	4.27	1.10	up	0.01	0.04	yes
TRINITY_DN16592_c0_g1	hypothetical protein POPTR_0001s28870g [Populus trichocarpa]	UGT87A2	UDP-glycosyltransferase 87A2 OS=Arabidopsis thaliana GN=UGT87A2 PE=1 SV=1	6.31	4.78	1.00	up	0.01	0.04	yes
TRINITY_DN17100_c0_g2	unknown [Populus trichocarpa]	-	-	3.58	1.80	1.59	up	0.01	0.04	yes
TRINITY_DN18742_c0_g1	hypothetical protein POPTR_0014s12250g, partial [Populus trichocarpa]	SAG21	Protein SENESCENCE-ASSOCIATED GENE 21, mitochondrial OS=Arabidopsis thaliana GN=SAG21 PE=2 SV=1	125.46	90.77	1.11	up	0.01	0.04	yes
TRINITY_DN17685_c0_g1	-	-	-	6.00	2.96	1.65	up	0.01	0.04	yes
TRINITY_DN27435_c0_g1	hypothetical protein M569_00222, partial [Genlisea aurea]	-	-	2330.06	1061.18	1.83	up	0.01	0.04	yes
TRINITY_DN13872_c0_g3	-	-	-	3.89	2.45	1.28	up	0.01	0.04	yes
TRINITY_DN27206_c0_g5	hypothetical protein POPTR_0019s12520g [Populus trichocarpa]	-	-	6.29	3.55	1.50	up	0.01	0.04	yes
TRINITY_DN26284_c1_g2	-	-	-	3.58	1.87	1.55	up	0.01	0.04	yes
TRINITY_DN22027_c0_g2	hypothetical protein POPTR_0019s00800g [Populus trichocarpa]	Os05g0361200	Ferrochelatase-2, chloroplastic OS=Oryza sativa subsp. japonica GN=Os05g0361200 PE=2 SV=1	5.26	2.81	1.44	up	0.01	0.04	yes
TRINITY_DN22831_c0_g2	hypothetical protein POPTR_0006s23740g [Populus trichocarpa]	-	-	31.20	22.41	1.09	up	0.01	0.04	yes
TRINITY_DN25128_c1_g3	-	-	-	20494.45	12279.62	1.37	up	0.01	0.04	yes
TRINITY_DN13219_c0_g1	PREDICTED: serine/threonine-protein kinase HT1-like isoform X1 [Populus euphratica]	HT1	Serine/threonine-protein kinase HT1 OS=Arabidopsis thaliana GN=HT1 PE=1 SV=1	1.77	0.93	1.54	up	0.01	0.04	yes
TRINITY_DN27198_c1_g2	aconitate hydratase family protein [Populus trichocarpa]	-	Aconitate hydratase, cytoplasmic OS=Cucurbita maxima PE=2 SV=1	16.99	12.62	1.02	up	0.01	0.04	yes
TRINITY_DN15139_c0_g1	-	-	-	65.17	50.00	1.00	up	0.01	0.04	yes
TRINITY_DN17833_c0_g4	hypothetical protein POPTR_0008s02960g [Populus trichocarpa]	-	-	3.39	2.07	1.35	up	0.01	0.04	yes

TRINITY_DN18250_c0_g2	hypothetical protein POPTR_0013s02080g [Populus trichocarpa]	-	-	13.51	6.96	1.34	up	0.01	0.04	yes
TRINITY_DN20160_c2_g1	-	-	-	25.34	13.88	1.44	up	0.01	0.04	yes
TRINITY_DN20180_c1_g1	-	-	-	6.80	4.69	1.21	up	0.01	0.04	yes
TRINITY_DN27825_c1_g1	hypothetical protein POPTR_0003s14070g [Populus trichocarpa]	-	-	2.30	1.40	1.32	up	0.01	0.04	yes
TRINITY_DN19205_c1_g3	PREDICTED: uncharacterized protein LOC109230342 [Nicotiana attenuata]	-	-	1.56	0.83	1.49	up	0.01	0.04	yes
TRINITY_DN14875_c0_g1	-	-	-	3.57	1.80	1.58	up	0.01	0.04	yes
TRINITY_DN19591_c1_g7	-	-	-	1.95	0.97	1.58	up	0.01	0.04	yes
TRINITY_DN17000_c0_g1	hypothetical protein POPTR_0009s06800g [Populus trichocarpa]	-	Anthocyanin 5-aromatic acyltransferase OS=Gentiana triflora PE=1 SV=1	3.15	2.14	1.16	up	0.01	0.04	yes
TRINITY_DN21797_c1_g1	PREDICTED: protein TRANSPARENT TESTA 12-like [Populus euphratica]	DTX24	Protein DETOXIFICATION 24 OS=Arabidopsis thaliana GN=DTX24 PE=2 SV=1	32.05	15.81	1.52	up	0.01	0.04	yes
TRINITY_DN26202_c0_g2	pyrophosphate--fructose-6-phosphate 1-phosphotransferase beta subunit family protein [Populus trichocarpa]	PFP-BETA	Pyrophosphate--fructose 6-phosphate 1-phosphotransferase subunit beta OS=Ricinus communis GN=PFP-BETA PE=3 SV=1	30.47	22.09	1.07	up	0.01	0.04	yes
TRINITY_DN18933_c0_g2	hypothetical protein POPTR_0007s04650g [Populus trichocarpa]	PPD2	PsbP domain-containing protein 2, chloroplastic OS=Arabidopsis thaliana GN=PPD2 PE=1 SV=1	24.33	18.15	1.03	up	0.01	0.04	yes
TRINITY_DN18066_c0_g1	pentatricopeptide repeat-containing family protein [Populus trichocarpa]	At2g20710	Pentatricopeptide repeat-containing protein At2g20710, mitochondrial OS=Arabidopsis thaliana GN=At2g20710 PE=2 SV=1	1.90	1.27	1.21	up	0.01	0.04	yes
TRINITY_DN18496_c0_g1	hypothetical protein POPTR_0006s29370g [Populus trichocarpa]	At4g31240	Probable nucleoredoxin 3 OS=Arabidopsis thaliana GN=At4g31240 PE=2 SV=1	7.82	6.63	1.14	up	0.01	0.04	yes
TRINITY_DN25205_c1_g8	NADH dehydrogenase subunit 4 [Populus alba]	ndhD	NAD(P)H-quinone oxidoreductase chain 4, chloroplastic OS=Populus alba GN=ndhD PE=3 SV=1	4.94	3.65	1.06	up	0.01	0.04	yes
TRINITY_DN20938_c0_g5	PREDICTED: uncharacterized protein LOC105109708 [Populus euphratica]	-	-	1.72	0.87	1.61	up	0.01	0.04	yes
TRINITY_DN26121_c1_g2	hypothetical protein POPTR_0005s01610g [Populus trichocarpa]	-	-	24.20	17.21	1.15	up	0.01	0.04	yes
TRINITY_DN26750_c0_g3	-	-	-	1.71	0.86	1.55	up	0.01	0.04	yes
TRINITY_DN14359_c0_g1	-	-	-	3.36	1.26	1.93	up	0.02	0.04	yes
TRINITY_DN23573_c0_g4	PREDICTED: uncharacterized protein LOC105110829 [Populus euphratica]	-	-	7.95	4.55	1.44	up	0.02	0.04	yes
TRINITY_DN18651_c2_g3	hypothetical protein POPTR_0015s10250g [Populus trichocarpa]	-	-	3.65	2.44	1.14	up	0.02	0.05	yes
TRINITY_DN21859_c0_g2	hypothetical protein POPTR_0014s16530g [Populus trichocarpa]	-	-	291.11	211.14	1.05	up	0.02	0.05	yes
TRINITY_DN14451_c0_g1	hypothetical protein POPTR_0010s08390g [Populus trichocarpa]	At1g06620	1-aminocyclopropane-1-carboxylate oxidase homolog 1 OS=Arabidopsis thaliana GN=At1g06620 PE=2 SV=1	3.88	2.15	1.45	up	0.02	0.05	yes
TRINITY_DN19940_c0_g2	PREDICTED: adenylyl-sulfate kinase 3-like [Populus euphratica]	-	-	26.43	13.84	1.65	up	0.02	0.05	yes
TRINITY_DN15033_c0_g1	hypothetical protein POPTR_0015s00280g [Populus trichocarpa]	LSU2	Protein RESPONSE TO LOW SULFUR 2 OS=Arabidopsis thaliana GN=LSU2 PE=2 SV=1	97.42	56.62	1.36	up	0.02	0.05	yes
TRINITY_DN24890_c1_g2	-	-	-	2.39	1.20	1.56	up	0.02	0.05	yes
TRINITY_DN18416_c0_g3	BiP isoform A family protein [Populus trichocarpa]	BIP5	Luminal-binding protein 5 OS=Nicotiana tabacum GN=BIP5 PE=2 SV=1	31.78	24.33	1.00	up	0.02	0.05	yes
TRINITY_DN23578_c1_g1	unknown [Populus trichocarpa]	AIG2LD	AIG2-like protein D OS=Arabidopsis thaliana GN=AIG2LD PE=2 SV=1	6.57	5.88	1.11	up	0.02	0.05	yes
TRINITY_DN22063_c1_g2	hypothetical protein POPTR_0001s04020g [Populus trichocarpa]	-	-	1.74	0.91	1.52	up	0.02	0.05	yes
TRINITY_DN21702_c0_g3	PREDICTED: jacalin-related lectin 3-like isoform X1 [Populus euphratica]	JAL3	Jacalin-related lectin 3 OS=Arabidopsis thaliana GN=JAL3 PE=2 SV=1	2.42	1.49	1.35	up	0.02	0.05	yes
TRINITY_DN18206_c0_g1	PREDICTED: transcription factor bHLH51-like [Populus euphratica]	BHLH51	Transcription factor bHLH51 OS=Arabidopsis thaliana GN=BHLH51 PE=2 SV=1	3.32	1.31	1.89	up	0.02	0.05	yes

TRINITY_DN15883_c0_g1	PREDICTED: DTW domain-containing protein 2 [Populus euphratica]	-	-	8.74	6.41	1.04	up	0.02	0.05	yes
TRINITY_DN26716_c0_g1	PREDICTED: pentatricopeptide repeat-containing protein At5g50280, chloroplastic [Populus euphratica]	-	-	25.09	15.99	1.25	up	0.02	0.05	yes
TRINITY_DN20918_c0_g1	hypothetical protein POPTR_0003s04350g [Populus trichocarpa]	NAP1;2	Nucleosome assembly protein 1;2 OS=Oryza sativa subsp. indica GN=NAP1;2 PE=1 SV=1	4.98	3.41	1.25	up	0.02	0.05	yes
TRINITY_DN13485_c0_g1	hypothetical protein POPTR_0017s03340g [Populus trichocarpa]	-	-	42.49	28.13	1.23	up	0.02	0.05	yes
TRINITY_DN14465_c0_g1	PREDICTED: ABC transporter D family member 1-like [Populus euphratica]	ABCC1	ABC transporter D family member 1 OS=Arabidopsis thaliana GN=ABCC1 PE=1 SV=1	43.30	30.56	1.15	up	0.02	0.05	yes
TRINITY_DN27825_c0_g1	hypothetical protein POPTR_0005s22120g [Populus trichocarpa]	-	-	2.58	1.62	1.29	up	0.02	0.05	yes
TRINITY_DN22219_c0_g1	mov34 family protein [Populus trichocarpa]	AMSH2	AMSH-like ubiquitin thioesterase 2 OS=Arabidopsis thaliana GN=AMSH2 PE=2 SV=1	6.60	4.92	1.18	up	0.02	0.05	yes
TRINITY_DN18350_c0_g1	hypothetical protein POPTR_0005s00550g [Populus trichocarpa]	-	-	3.06	1.72	1.47	up	0.02	0.05	yes
TRINITY_DN23001_c0_g1	naphthoate synthase family protein [Populus trichocarpa]	MENB	1,4-dihydroxy-2-naphthoyl-CoA synthase, peroxisomal OS=Arabidopsis thaliana GN=MENB PE=1 SV=2	103.94	76.36	1.07	up	0.02	0.05	yes
TRINITY_DN17659_c0_g2	PREDICTED: myb family transcription factor APL-like isoform X1 [Populus euphratica]	PHL11	Myb family transcription factor PHL11 OS=Arabidopsis thaliana GN=PHL11 PE=2 SV=1	4.94	3.74	1.02	up	0.02	0.05	yes
TRINITY_DN25711_c1_g3	PREDICTED: ethylene-responsive transcription factor 2-like [Populus euphratica]	ERF2	Ethylene-responsive transcription factor 2 OS=Nicotiana sylvestris GN=ERF2 PE=2 SV=1	8.83	4.91	1.40	up	0.02	0.05	yes
TRINITY_DN20570_c0_g1	PREDICTED: 18.1 kDa class I heat shock protein-like [Populus euphratica]	HSP18.5-C	18.5 kDa class I heat shock protein OS=Glycine max GN=HSP18.5-C PE=3 SV=1	18.87	8.08	1.68	up	0.02	0.05	yes

Gene_ID	Annotation	Name	swissprot_description	Treat_mean_TPM	contrast_mean_TPM	log2FC	regulation	pvalue	FDR	significant
TRINITY_DN21586_c0_g3	patatin-related family protein [Populus trichocarpa]	PLP9	Probable inactive patatin-like protein 9 OS=Arabidopsis thaliana GN=PLP9 PE=2 SV=1	0.15	51.07	-7.77	down	0.00	0.00	yes
TRINITY_DN20647_c2_g1	hypothetical protein POPTR_0014s07010g, partial [Populus trichocarpa]	SOC1	MADS-box protein SOC1 OS=Arabidopsis thaliana GN=SOC1 PE=1 SV=1	0.46	88.39	-7.66	down	0.00	0.00	yes
TRINITY_DN19354_c0_g1	PREDICTED: glucan endo-1,3-beta-glucosidase, basic isoform-like [Populus euphratica]	GNS1	Glucan endo-1,3-beta-glucosidase, basic isoform OS=Prunus persica GN=GNS1 PE=3 SV=1	0.57	84.08	-6.54	down	0.00	0.00	yes
TRINITY_DN18012_c0_g1	hypothetical protein POPTR_0012s13980g [Populus trichocarpa]	-	-	0.08	36.93	-8.21	down	0.00	0.00	yes
TRINITY_DN24070_c1_g1	MADS-box protein PTM5 [Populus tremuloides]	-	-	0.32	170.44	-8.33	down	0.00	0.00	yes
TRINITY_DN23279_c0_g1	hypothetical protein POPTR_0014s05680g [Populus trichocarpa]	SPL8	Squamosa promoter-binding-like protein 8 OS=Arabidopsis thaliana GN=SPL8 PE=1 SV=2	0.38	41.84	-6.32	down	0.00	0.00	yes
TRINITY_DN24070_c0_g1	hypothetical protein POPTR_0014s07010g, partial [Populus trichocarpa]	SOC1	MADS-box protein SOC1 OS=Arabidopsis thaliana GN=SOC1 PE=1 SV=1	0.08	51.58	-8.92	down	0.00	0.00	yes
TRINITY_DN23130_c0_g2	PREDICTED: MADS-box protein JOINTLESS-like [Populus euphratica]	J	MADS-box protein JOINTLESS OS=Solanum lycopersicum GN=J PE=1 SV=1	0.00	24.68	-11.64	down	0.00	0.00	yes
TRINITY_DN13277_c0_g1	hypothetical protein POPTR_0006s06090g [Populus trichocarpa]	-	-	4.03	608.38	-6.66	down	0.00	0.00	yes
TRINITY_DN26461_c0_g5	cytochrome P450 family protein [Populus trichocarpa]	CYP81E8	Cytochrome P450 81E8 OS=Medicago truncatula GN=CYP81E8 PE=2 SV=1	0.57	30.30	-5.12	down	0.00	0.00	yes
TRINITY_DN19876_c0_g1	Pathogenesis-related family protein [Populus trichocarpa]	PRB1	Pathogenesis-related protein 1 OS=Arabidopsis thaliana GN=PRB1 PE=2 SV=1	5.51	478.26	-5.82	down	0.00	0.00	yes
TRINITY_DN19360_c0_g1	pathogenesis related protein-5 [Populus tomentosa]	tlp	Thaumatin-like protein OS=Actinidia deliciosa GN=tlp PE=1 SV=2	3.56	184.27	-5.19	down	0.00	0.00	yes
TRINITY_DN18292_c0_g2	unknown [Populus trichocarpa]	EP3	Endochitinase EP3 OS=Arabidopsis thaliana GN=EP3 PE=1 SV=1	5.75	143.32	-4.23	down	0.00	0.00	yes
TRINITY_DN20693_c0_g1	RNA recognition motif-containing family protein [Populus trichocarpa]	ARP1	Probable RNA-binding protein ARP1 OS=Arabidopsis thaliana GN=ARP1 PE=2 SV=1	1.12	38.18	-4.68	down	0.00	0.00	yes
TRINITY_DN936_c0_g1	hypothetical protein POPTR_0018s10730g [Populus trichocarpa]	CjBAp12	EG45-like domain containing protein OS=Citrus jambhiri GN=CjBAp12 PE=1 SV=1	1.72	84.24	-5.00	down	0.00	0.00	yes

TRINITY_DN20061_c0_g2	hypothetical protein POPTR_0003s11960g [Populus trichocarpa]	AGL19	Agamous-like MADS-box protein AGL19 OS=Arabidopsis thaliana GN=AGL19 PE=1 SV=1	0.75	45.02	-5.23	down	0.00	0.00	yes
TRINITY_DN24351_c0_g4	hypothetical protein POPTR_0003s21440g [Populus trichocarpa]	-	-	0.07	13.34	-6.88	down	0.00	0.00	yes
TRINITY_DN16978_c0_g1	chitinase 2 [Populus x canadensis]	Chit1	Chitotriosidase-1 OS=Mus musculus GN=Chit1 PE=1 SV=2	1.45	62.24	-4.84	down	0.00	0.00	yes
TRINITY_DN24801_c0_g1	ORF family protein [Populus trichocarpa]	-	Nitrate reductase [NADH] OS=Cucurbita maxima PE=2 SV=1	1.59	22.62	-3.23	down	0.00	0.00	yes
TRINITY_DN25966_c0_g3	hypothetical protein POPTR_0001s12120g [Populus trichocarpa]	-	-	0.13	19.53	-6.33	down	0.00	0.00	yes
TRINITY_DN21321_c0_g4	PREDICTED: uncharacterized protein LOC105134749 isoform X2 [Populus euphratica]	-	-	0.04	5.12	-6.38	down	0.00	0.00	yes
TRINITY_DN19602_c0_g1	hypothetical protein POPTR_0006s00440g [Populus trichocarpa]	AMP2-1	Vicilin-like antimicrobial peptides 2-1 OS=Macadamia integrifolia GN=AMP2-1 PE=2 SV=1	2.08	37.22	-3.51	down	0.00	0.00	yes
TRINITY_DN25590_c0_g1	hypothetical protein POPTR_0010s17300g [Populus trichocarpa]	MYB122	Transcription factor MYB122 OS=Arabidopsis thaliana GN=MYB122 PE=1 SV=1	0.04	18.89	-8.33	down	0.00	0.00	yes
TRINITY_DN18787_c0_g1	hypothetical protein POPTR_0009s14720g [Populus trichocarpa]	FAR3	Fatty acyl-CoA reductase 3 OS=Arabidopsis thaliana GN=FAR3 PE=2 SV=1	0.09	17.28	-6.64	down	0.00	0.00	yes
TRINITY_DN15723_c0_g1	hypothetical protein POPTR_0010s06740g [Populus trichocarpa]	Trpa1	Transient receptor potential cation channel subfamily A member 1 OS=Rattus norvegicus GN=Trpa1 PE=2 SV=1	0.12	7.89	-5.48	down	0.00	0.00	yes
TRINITY_DN24851_c0_g1	exostosin family protein [Populus trichocarpa]	ARAD1	Probable arabinosyltransferase ARAD1 OS=Arabidopsis thaliana GN=ARAD1 PE=1 SV=1	4.32	59.50	-3.19	down	0.00	0.00	yes
TRINITY_DN23054_c0_g1	hypothetical protein POPTR_0005s07540g [Populus trichocarpa]	lip3	Lipase 3 OS=Moraxella sp. (strain TA144) GN=lip3 PE=1 SV=1	0.39	15.30	-4.75	down	0.00	0.00	yes
TRINITY_DN23425_c0_g1	PREDICTED: GDSL esterase/lipase At1g29670-like [Populus euphratica]	At1g29670	GDSL esterase/lipase At1g29670 OS=Arabidopsis thaliana GN=At1g29670 PE=2 SV=1	1.04	73.66	-5.51	down	0.00	0.00	yes
TRINITY_DN23948_c0_g3	PREDICTED: CLAVATA3/ESR (CLE)-related protein TDIF-like [Populus euphratica]	-	-	0.32	15.49	-5.03	down	0.00	0.00	yes
TRINITY_DN18072_c0_g2	class 4 pathogenesis-related family protein [Populus trichocarpa]	HEV1	Pro-hevein OS=Hevea brasiliensis GN=HEV1 PE=1 SV=2	7.11	158.62	-3.95	down	0.00	0.00	yes
TRINITY_DN22250_c0_g7	hypothetical protein POPTR_0001s10510g [Populus trichocarpa]	-	-	0.00	10.67	-9.97	down	0.00	0.00	yes
TRINITY_DN27112_c1_g2	unknown [Populus trichocarpa]	MARD1	Protein MARD1 OS=Arabidopsis thaliana GN=MARD1 PE=2 SV=2	0.29	30.16	-5.18	down	0.00	0.00	yes
TRINITY_DN24365_c0_g1	hypothetical protein POPTR_0005s25950g [Populus trichocarpa]	-	-	4.47	52.34	-2.94	down	0.00	0.00	yes
TRINITY_DN23821_c0_g1	PREDICTED: AP2-like ethylene-responsive transcription factor AIL5 [Populus euphratica]	AIL5	AP2-like ethylene-responsive transcription factor AIL5 OS=Arabidopsis thaliana GN=AIL5 PE=2 SV=2	4.10	87.43	-3.69	down	0.00	0.00	yes
TRINITY_DN27214_c2_g1	FLAVIN-BINDING KELCH DOMAIN F BOX family protein [Populus trichocarpa]	ADO3	Adagio protein 3 OS=Arabidopsis thaliana GN=ADO3 PE=1 SV=1	3.86	72.07	-3.58	down	0.00	0.00	yes
TRINITY_DN15578_c0_g1	SABATH methyltransferase 3 [Populus trichocarpa]	JMT	Jasmonate O-methyltransferase OS=Brassica rapa subsp. pekinensis GN=JMT PE=1 SV=1	0.00	7.21	-10.44	down	0.00	0.00	yes
TRINITY_DN16427_c0_g1	PREDICTED: uncharacterized protein At5g22580-like [Populus euphratica]	At5g22580	Stress-response A/B barrel domain-containing protein At5g22580 OS=Arabidopsis thaliana GN=At5g22580 PE=1 SV=1	24.29	314.78	-3.15	down	0.00	0.00	yes
TRINITY_DN14075_c0_g1	PREDICTED: UPF0481 protein At3g47200-like isoform X1 [Populus euphratica]	At3g47200	UPF0481 protein At3g47200 OS=Arabidopsis thaliana GN=At3g47200 PE=2 SV=1	0.02	7.54	-7.73	down	0.00	0.00	yes
TRINITY_DN23412_c1_g9	PREDICTED: squamosa promoter-binding-like protein 3 [Populus euphratica]	SBP1	Squamosa promoter-binding protein 1 OS=Antirrhinum majus GN=SBP1 PE=2 SV=1	0.67	27.23	-5.03	down	0.00	0.00	yes
TRINITY_DN22085_c0_g1	chitinase family protein [Populus trichocarpa]	SE2	Acidic endochitinase SE2 OS=Beta vulgaris GN=SE2 PE=1 SV=1	0.89	158.15	-6.92	down	0.00	0.00	yes
TRINITY_DN26908_c0_g1	PREDICTED: uncharacterized protein LOC105139757 isoform X2 [Populus euphratica]	JMJ25	Lysine-specific demethylase JMJ25 OS=Arabidopsis thaliana GN=JMJ25 PE=1 SV=1	0.59	12.30	-3.48	down	0.00	0.00	yes
TRINITY_DN15178_c0_g1	unknown [Populus trichocarpa]	-	-	0.82	33.95	-4.79	down	0.00	0.00	yes
TRINITY_DN24369_c0_g3	PREDICTED: transcription factor MYB44-like [Populus euphratica]	MYB44	Transcription factor MYB44 OS=Arabidopsis thaliana GN=MYB44 PE=2 SV=1	8.76	97.04	-2.87	down	0.00	0.00	yes
TRINITY_DN20094_c0_g1	PREDICTED: protein NRT1/ PTR FAMILY 2.13 [Populus euphratica]	NPF2.13	Protein NRT1/ PTR FAMILY 2.13 OS=Arabidopsis thaliana GN=NPF2.13 PE=1 SV=1	0.26	17.06	-4.85	down	0.00	0.00	yes
TRINITY_DN17557_c0_g1	AAA-type ATPase family protein [Populus trichocarpa]	HSR4	Protein HYPER-SENSITIVITY-RELATED 4 OS=Arabidopsis thaliana GN=HSR4 PE=2 SV=1	0.06	6.84	-6.12	down	0.00	0.00	yes

TRINITY_DN24059_c0_g1	PREDICTED: probable membrane-associated kinase regulator 2 [Populus euphratica]	MAKR2	Probable membrane-associated kinase regulator 2 OS=Arabidopsis thaliana GN=MAKR2 PE=2 SV=1	0.94	29.63	-4.27	down	0.00	0.00	yes
TRINITY_DN16872_c0_g1	hypothetical protein POPTR_0002s04430g [Populus trichocarpa]	ERF9	Ethylene-responsive transcription factor 9 OS=Arabidopsis thaliana GN=ERF9 PE=2 SV=1	0.45	31.91	-5.53	down	0.00	0.00	yes
TRINITY_DN17824_c0_g4	hypothetical protein POPTR_0001s10310g [Populus trichocarpa]	-	-	9.97	145.02	-3.29	down	0.00	0.00	yes
TRINITY_DN15545_c0_g1	hypothetical protein POPTR_0013s00570g [Populus trichocarpa]	-	-	0.07	24.63	-7.82	down	0.00	0.00	yes
TRINITY_DN18734_c0_g1	PREDICTED: uncharacterized protein LOC105124423 [Populus euphratica]	-	-	1.69	44.30	-4.17	down	0.00	0.00	yes
TRINITY_DN15545_c0_g2	hypothetical protein POPTR_0505s00220g [Populus trichocarpa]	-	-	0.00	18.26	-10.28	down	0.00	0.00	yes
TRINITY_DN21267_c0_g1	PREDICTED: D-amino-acid transaminase, chloroplastic-like [Populus euphratica]	DAAT	D-amino-acid transaminase, chloroplastic OS=Arabidopsis thaliana GN=DAAT PE=1 SV=1	0.85	19.24	-3.88	down	0.00	0.00	yes
TRINITY_DN20076_c0_g1	PREDICTED: thaumatin-like protein 1 isoform X1 [Populus euphratica]	TL1	Thaumatin-like protein 1 OS=Pyrus pyrifolia GN=TL1 PE=1 SV=1	1.09	67.15	-5.18	down	0.00	0.00	yes
TRINITY_DN12280_c0_g3	PREDICTED: probable ADP-ribosylation factor GTPase-activating protein AGD13 isoform X3 [Populus euphratica]	CAR10	Protein C2-DOMAIN ABA-RELATED 10 OS=Arabidopsis thaliana GN=CAR10 PE=2 SV=1	0.87	25.75	-4.27	down	0.00	0.00	yes
TRINITY_DN16677_c0_g1	hypothetical protein POPTR_0007s15050g [Populus trichocarpa]	-	-	0.16	11.75	-6.00	down	0.00	0.00	yes
TRINITY_DN24032_c3_g3	hypothetical protein POPTR_0001s09640g [Populus trichocarpa]	-	-	5.18	47.53	-2.56	down	0.00	0.00	yes
TRINITY_DN22570_c0_g3	PREDICTED: probable LRR receptor-like serine/threonine-protein kinase At1g56140 isoform X1 [Populus euphratica]	At1g56140	Probable LRR receptor-like serine/threonine-protein kinase At1g56140 OS=Arabidopsis thaliana GN=At1g56140 PE=2 SV=2	0.77	10.97	-3.17	down	0.00	0.00	yes
TRINITY_DN18313_c0_g1	PREDICTED: uncharacterized protein LOC105112272 [Populus euphratica]	SMR9	Cyclin-dependent protein kinase inhibitor SMR9 OS=Arabidopsis thaliana GN=SMR9 PE=3 SV=1	2.56	51.44	-3.76	down	0.00	0.00	yes
TRINITY_DN20689_c0_g1	exostosin family protein [Populus trichocarpa]	IRX7	Probable glucuronoxylan glucuronosyltransferase IRX7 OS=Arabidopsis thaliana GN=IRX7 PE=2 SV=1	1.72	18.74	-2.85	down	0.00	0.00	yes
TRINITY_DN24453_c0_g2	hypothetical protein POPTR_0004s18760g [Populus trichocarpa]	-	-	0.58	20.85	-5.01	down	0.00	0.00	yes
TRINITY_DN23669_c0_g1	PREDICTED: uncharacterized protein LOC105131118 isoform X1 [Populus euphratica]	-	-	1.70	25.14	-3.31	down	0.00	0.00	yes
TRINITY_DN19850_c1_g3	hypothetical protein POPTR_0001s40870g [Populus trichocarpa]	SPL4	Squamosa promoter-binding-like protein 4 OS=Arabidopsis thaliana GN=SPL4 PE=1 SV=1	0.55	19.30	-4.95	down	0.00	0.00	yes
TRINITY_DN17581_c0_g1	ribonuclease 3 family protein [Populus trichocarpa]	NFD2	Protein NUCLEAR FUSION DEFECTIVE 2 OS=Arabidopsis thaliana GN=NFD2 PE=2 SV=1	4.34	48.90	-2.86	down	0.00	0.00	yes
TRINITY_DN16236_c0_g1	hypothetical protein POPTR_0004s21060g [Populus trichocarpa]	-	-	0.00	11.25	-10.01	down	0.00	0.00	yes
TRINITY_DN27112_c1_g3	unknown [Populus trichocarpa x Populus deltoides]	MARD1	Protein MARD1 OS=Arabidopsis thaliana GN=MARD1 PE=2 SV=2	0.14	12.63	-6.04	down	0.00	0.00	yes
TRINITY_DN26155_c0_g3	expansin [Populus tomentosa]	EXPA4	Expansin-A4 OS=Arabidopsis thaliana GN=EXPA4 PE=1 SV=1	0.73	26.44	-4.52	down	0.00	0.00	yes
TRINITY_DN26051_c1_g1	hypothetical protein POPTR_0442s00200g [Populus trichocarpa]	LRK10L-1.3	LEAF RUST 10 DISEASE-RESISTANCE LOCUS RECEPTOR-LIKE PROTEIN KINASE-like 1.3 OS=Arabidopsis thaliana GN=LRK10L-1.3 PE=2 SV=1	0.87	13.27	-4.91	down	0.00	0.00	yes
TRINITY_DN17494_c0_g4	leucine-rich repeat transmembrane protein kinase [Populus trichocarpa]	At2g24230	Probable LRR receptor-like serine/threonine-protein kinase At2g24230 OS=Arabidopsis thaliana GN=At2g24230 PE=2 SV=1	0.85	12.27	-3.88	down	0.00	0.00	yes
TRINITY_DN19472_c0_g13	-	-	-	0.00	6.86	-9.58	down	0.00	0.00	yes
TRINITY_DN27063_c0_g1	integrase-type DNA-binding superfamily protein [Populus tomentosa]	ANT	AP2-like ethylene-responsive transcription factor ANT OS=Arabidopsis thaliana GN=ANT PE=1 SV=2	7.52	69.47	-2.83	down	0.00	0.00	yes
TRINITY_DN14278_c0_g1	hypothetical protein POPTR_0009s16300g [Populus trichocarpa]	-	-	0.03	11.40	-7.30	down	0.00	0.00	yes
TRINITY_DN24838_c0_g1	PREDICTED: putative cyclin-A3-1 [Populus euphratica]	CYCA3-4	Cyclin-A3-4 OS=Arabidopsis thaliana GN=CYCA3-4 PE=1 SV=1	6.27	51.85	-2.45	down	0.00	0.00	yes

TRINITY_DN22684_c0_g7	glutamate decarboxylase 1 family protein [Populus trichocarpa]	GAD	Glutamate decarboxylase OS=Petunia hybrida GN=GAD PE=1 SV=1	0.08	6.22	-5.56	down	0.00	0.00	yes
TRINITY_DN23704_c1_g1	hypothetical protein POPTR_0001s07880g [Populus trichocarpa]	At1g62810	Primary amine oxidase OS=Arabidopsis thaliana GN=At1g62810 PE=2 SV=1	1.87	24.42	-3.03	down	0.00	0.00	yes
TRINITY_DN24562_c0_g1	hypothetical protein POPTR_0002s05470g [Populus trichocarpa]	-	-	0.06	7.30	-6.15	down	0.00	0.00	yes
TRINITY_DN16418_c0_g1	1,3-beta-D-glucanase GH17_39 [Populus tremula x Populus tremuloides]	VIT_06s0061g00	Glucan endo-1,3-beta-glucosidase OS=Vitis vinifera GN=VIT_06s0061g00120 PE=1 SV=2	0.68	49.75	-5.63	down	0.00	0.00	yes
TRINITY_DN27359_c0_g1	hypothetical protein POPTR_0014s05290g [Populus trichocarpa]	At3g47200	UPF0481 protein At3g47200 OS=Arabidopsis thaliana GN=At3g47200 PE=2 SV=1	16.56	129.41	-2.71	down	0.00	0.00	yes
TRINITY_DN25488_c0_g1	hypothetical protein POPTR_0018s04720g [Populus trichocarpa]	At2g19810	Zinc finger CCCH domain-containing protein 20 OS=Arabidopsis thaliana GN=At2g19810 PE=2 SV=1	14.32	200.73	-3.08	down	0.00	0.00	yes
TRINITY_DN23310_c0_g1	PREDICTED: uncharacterized protein LOC105130453 isoform X2 [Populus euphratica]	-	-	2.25	19.82	-2.55	down	0.00	0.00	yes
TRINITY_DN18292_c0_g1	PREDICTED: endochitinase PR4-like [Populus euphratica]	EP3	Endochitinase EP3 OS=Arabidopsis thaliana GN=EP3 PE=1 SV=1	0.17	8.89	-5.00	down	0.00	0.00	yes
TRINITY_DN21068_c0_g3	PREDICTED: aldose 1-epimerase-like [Populus euphratica]	mro	Aldose 1-epimerase OS=Acinetobacter calcoaceticus GN=mro PE=1 SV=1	0.45	14.22	-4.42	down	0.00	0.00	yes
TRINITY_DN21305_c1_g1	hypothetical protein POPTR_0001s29950g [Populus trichocarpa]	-	-	0.81	22.59	-4.19	down	0.00	0.00	yes
TRINITY_DN22648_c0_g5	hypothetical protein POPTR_0007s12700g [Populus trichocarpa]	-	-	0.46	17.84	-4.92	down	0.00	0.00	yes
TRINITY_DN20118_c0_g2	nucleoid DNA-binding family protein [Populus trichocarpa]	At5g10770	Aspartyl protease family protein At5g10770 OS=Arabidopsis thaliana GN=At5g10770 PE=2 SV=1	0.01	4.49	-7.89	down	0.00	0.00	yes
TRINITY_DN25906_c0_g1	hypothetical protein POPTR_0010s06940g [Populus trichocarpa]	At1g60420	Probable nucleoredoxin 1 OS=Arabidopsis thaliana GN=At1g60420 PE=1 SV=1	9.27	169.20	-3.60	down	0.00	0.00	yes
TRINITY_DN24638_c0_g1	PREDICTED: uncharacterized protein LOC105130979 [Populus euphratica]	-	-	0.46	15.60	-4.39	down	0.00	0.00	yes
TRINITY_DN25417_c0_g1	PREDICTED: patellin-6 [Populus euphratica]	PATL6	Patellin-6 OS=Arabidopsis thaliana GN=PATL6 PE=2 SV=1	4.95	87.34	-3.53	down	0.00	0.00	yes
TRINITY_DN24288_c0_g2	hypothetical protein POPTR_0018s05840g [Populus trichocarpa]	-	-	0.10	14.95	-6.02	down	0.00	0.00	yes
TRINITY_DN21703_c0_g3	hypothetical protein POPTR_0004s03750g [Populus trichocarpa]	-	-	6.88	82.50	-2.85	down	0.00	0.00	yes
TRINITY_DN20933_c0_g1	hypothetical protein POPTR_0002s19330g [Populus trichocarpa]	LACS1	Long chain acyl-CoA synthetase 1 OS=Arabidopsis thaliana GN=LACS1 PE=2 SV=1	0.65	13.67	-3.74	down	0.00	0.00	yes
TRINITY_DN26401_c1_g1	PREDICTED: protein CHUP1, chloroplastic-like [Populus euphratica]	CHUP1	Protein CHUP1, chloroplastic OS=Arabidopsis thaliana GN=CHUP1 PE=1 SV=1	1.52	20.79	-3.17	down	0.00	0.00	yes
TRINITY_DN16446_c1_g2	hypothetical protein POPTR_0006s11890g [Populus trichocarpa]	ATHB-51	Putative homeobox-leucine zipper protein ATHB-51 OS=Arabidopsis thaliana GN=ATHB-51 PE=2 SV=2	0.13	6.66	-5.02	down	0.00	0.00	yes
TRINITY_DN19852_c0_g1	PREDICTED: thaumatin-like protein [Populus euphratica]	tlp	Thaumatin-like protein OS=Actinidia deliciosa GN=tlp PE=1 SV=2	0.42	31.36	-5.65	down	0.00	0.00	yes
TRINITY_DN18190_c0_g1	PREDICTED: protein TORNADO 2-like [Populus euphratica]	TRN2	Protein TORNADO 2 OS=Arabidopsis thaliana GN=TRN2 PE=1 SV=1	0.64	12.10	-3.63	down	0.00	0.00	yes
TRINITY_DN17112_c0_g1	Leucoanthocyanidin reductase family protein [Populus trichocarpa]	ANR	Anthocyanidin reductase ((2S)-flavan-3-ol-forming) OS=Vitis vinifera GN=ANR PE=3 SV=1	3.70	139.01	-4.56	down	0.00	0.00	yes
TRINITY_DN15730_c0_g1	hypothetical protein POPTR_0016s14290g [Populus trichocarpa]	-	Non-specific lipid-transfer protein 3 OS=Prunus dulcis PE=2 SV=1	2.97	118.78	-4.72	down	0.00	0.00	yes
TRINITY_DN21593_c0_g3	hypothetical protein POPTR_0008s11540g [Populus trichocarpa]	LAR	Leucoanthocyanidin reductase OS=Desmodium uncinatum GN=LAR PE=1 SV=1	0.44	28.13	-5.38	down	0.00	0.00	yes
TRINITY_DN26408_c0_g1	PREDICTED: zinc finger protein CONSTANS-LIKE 15-like isoform X1 [Populus euphratica]	COL14	Zinc finger protein CONSTANS-LIKE 14 OS=Arabidopsis thaliana GN=COL14 PE=2 SV=2	1.53	37.46	-3.75	down	0.00	0.00	yes
TRINITY_DN22928_c0_g1	PREDICTED: tubby-like protein 8 [Populus euphratica]	TULP8	Tubby-like protein 8 OS=Arabidopsis thaliana GN=TULP8 PE=2 SV=1	7.66	59.47	-2.27	down	0.00	0.00	yes
TRINITY_DN24663_c0_g2	PREDICTED: abscisic acid 8'-hydroxylase 2 [Populus euphratica]	CYP707A2	Abscisic acid 8'-hydroxylase 2 OS=Arabidopsis thaliana GN=CYP707A2 PE=2 SV=1	3.48	34.77	-2.82	down	0.00	0.00	yes
TRINITY_DN26173_c0_g2	hypothetical protein POPTR_0001s23420g [Populus trichocarpa]	At1g04910	Uncharacterized protein At1g04910 OS=Arabidopsis thaliana GN=At1g04910 PE=2 SV=1	1.75	18.91	-2.84	down	0.00	0.00	yes

TRINITY_DN21186_c0_g1	MYB transcription factor R2R3-like protein [Populus tremuloides]	C1	Anthocyanin regulatory C1 protein OS=Zea mays GN=C1 PE=2 SV=1	18.09	301.52	-3.53	down	0.00	0.00	yes
TRINITY_DN19620_c1_g4	histone H2B family protein [Populus trichocarpa]	At2g28720	Histone H2B.3 OS=Arabidopsis thaliana GN=At2g28720 PE=1 SV=3	69.98	620.83	-2.56	down	0.00	0.00	yes
TRINITY_DN25399_c0_g2	hypothetical protein POPTR_0013s07500g [Populus trichocarpa]	GRF3	Growth-regulating factor 3 OS=Oryza sativa subsp. japonica GN=GRF3 PE=3 SV=2	0.86	18.11	-3.78	down	0.00	0.00	yes
TRINITY_DN15222_c0_g1	hypothetical protein POPTR_0016s12410g [Populus trichocarpa]	-	-	1.81	119.57	-5.11	down	0.00	0.00	yes
TRINITY_DN16171_c0_g1	hypothetical protein POPTR_0008s20200g [Populus trichocarpa]	-	-	0.03	3.55	-5.96	down	0.00	0.00	yes
TRINITY_DN22303_c0_g1	hypothetical protein POPTR_0001s34980g [Populus trichocarpa]	AO	L-aspartate oxidase, chloroplastic OS=Arabidopsis thaliana GN=AO PE=1 SV=1	8.51	87.80	-2.21	down	0.00	0.00	yes
TRINITY_DN16642_c0_g1	AP2 domain-containing transcription factor family protein [Populus trichocarpa]	WIN1	Ethylene-responsive transcription factor WIN1 OS=Arabidopsis thaliana GN=WIN1 PE=2 SV=1	0.02	5.54	-7.12	down	0.00	0.00	yes
TRINITY_DN23194_c0_g1	hypothetical protein POPTR_0015s01430g [Populus trichocarpa]	-	-	1.23	11.38	-2.61	down	0.00	0.00	yes
TRINITY_DN15461_c0_g1	terminal ear1-like 2 protein [Populus tremula x Populus alba]	TE1	Protein terminal ear1 OS=Zea mays GN=TE1 PE=2 SV=1	0.03	5.86	-7.18	down	0.00	0.00	yes
TRINITY_DN20963_c0_g5	senescence-associated family protein [Populus trichocarpa]	-	-	2.03	29.25	-3.29	down	0.00	0.00	yes
TRINITY_DN18653_c0_g2	hypothetical protein B456_004G005600 [Gossypium raimondii]	-	Histone H4 variant TH011 OS=Triticum aestivum PE=3 SV=2	24.63	188.72	-2.34	down	0.00	0.00	yes
TRINITY_DN25125_c0_g4	WOX1b [Populus tomentosa]	WOX1	WUSCHEL-related homeobox 1 OS=Arabidopsis thaliana GN=WOX1 PE=2 SV=2	0.77	11.57	-3.33	down	0.00	0.00	yes
TRINITY_DN25242_c0_g1	PREDICTED: probable protein S-acyltransferase 6 isoform X1 [Populus euphratica]	PAT07	Probable protein S-acyltransferase 7 OS=Arabidopsis thaliana GN=PAT07 PE=1 SV=1	3.78	33.04	-2.63	down	0.00	0.00	yes
TRINITY_DN20520_c0_g1	hypothetical protein POPTR_0007s04940g [Populus trichocarpa]	-	-	0.37	7.48	-3.85	down	0.00	0.00	yes
TRINITY_DN19684_c0_g4	hypothetical protein POPTR_0006s10950g [Populus trichocarpa]	WRKY70	Probable WRKY transcription factor 70 OS=Arabidopsis thaliana GN=WRKY70 PE=2 SV=1	0.74	37.00	-5.01	down	0.00	0.00	yes
TRINITY_DN18811_c0_g4	hypothetical protein POPTR_0009s05160g [Populus trichocarpa]	-	-	1.52	17.06	-2.87	down	0.00	0.00	yes
TRINITY_DN17633_c0_g2	hypothetical protein POPTR_0001s31880g [Populus trichocarpa]	ABCG11	ABC transporter G family member 11 OS=Arabidopsis thaliana GN=ABCG11 PE=1 SV=1	0.02	3.83	-6.96	down	0.00	0.00	yes
TRINITY_DN22088_c1_g2	hypothetical protein POPTR_0004s02550g [Populus trichocarpa]	CRK29	Cysteine-rich receptor-like protein kinase 29 OS=Arabidopsis thaliana GN=CRK29 PE=2 SV=1	0.24	5.78	-4.03	down	0.00	0.00	yes
TRINITY_DN26186_c0_g2	cytokinin oxidase 6 family protein [Populus trichocarpa]	CKX5	Cytokinin dehydrogenase 5 OS=Arabidopsis thaliana GN=CKX5 PE=2 SV=1	0.41	24.89	-5.46	down	0.00	0.00	yes
TRINITY_DN14748_c0_g1	hypothetical protein POPTR_0010s08290g [Populus trichocarpa]	-	-	26.63	795.18	-4.29	down	0.00	0.00	yes
TRINITY_DN27166_c1_g1	PREDICTED: protein tesmin/TSO1-like CXC 2 isoform X2 [Populus euphratica]	TCX2	Protein tesmin/TSO1-like CXC 2 OS=Arabidopsis thaliana GN=TCX2 PE=1 SV=1	6.78	39.48	-2.49	down	0.00	0.00	yes
TRINITY_DN23688_c0_g3	hypothetical protein POPTR_0005s19000g, partial [Populus trichocarpa]	-	-	0.00	16.68	-9.12	down	0.00	0.00	yes
TRINITY_DN18302_c0_g2	hypothetical protein POPTR_0008s03410g [Populus trichocarpa]	-	-	0.06	5.58	-5.73	down	0.00	0.00	yes
TRINITY_DN20439_c0_g8	PREDICTED: myb-related protein 308-like [Populus euphratica]	-	-	8.37	152.40	-3.59	down	0.00	0.00	yes
TRINITY_DN25885_c1_g5	-	-	-	0.13	10.02	-5.41	down	0.00	0.00	yes
TRINITY_DN18443_c0_g3	hypothetical protein POPTR_0014s14550g [Populus trichocarpa]	-	-	12.11	87.01	-2.26	down	0.00	0.00	yes
TRINITY_DN20906_c0_g2	hypothetical protein POPTR_0014s09560g [Populus trichocarpa]	ARF11	Auxin response factor 11 OS=Arabidopsis thaliana GN=ARF11 PE=2 SV=3	0.56	22.40	-4.70	down	0.00	0.00	yes
TRINITY_DN21206_c0_g2	hypothetical protein POPTR_0001s41900g [Populus trichocarpa]	DGK5	Diacylglycerol kinase 5 OS=Arabidopsis thaliana GN=DGK5 PE=2 SV=1	0.36	6.24	-3.49	down	0.00	0.00	yes
TRINITY_DN23773_c0_g1	hypothetical protein POPTR_0009s16390g [Populus trichocarpa]	At4g16563	Probable aspartyl protease At4g16563 OS=Arabidopsis thaliana GN=At4g16563 PE=2 SV=1	5.24	40.15	-2.35	down	0.00	0.00	yes

TRINITY_DN26861_c0_g2	hypothetical protein POPTR_0002s15500g [Populus trichocarpa]	-	-	1.40	11.79	-2.48	down	0.00	0.00	yes
TRINITY_DN26534_c0_g1	myb family transcription factor family protein [Populus trichocarpa]	KAN2	Probable transcription factor KAN2 OS=Arabidopsis thaliana GN=KAN2 PE=2 SV=1	1.96	20.92	-2.88	down	0.00	0.00	yes
TRINITY_DN21102_c0_g4	hypothetical protein PHAVU_011G163600g, partial [Phaseolus vulgaris]	-	Histone H4 variant TH011 OS=Triticum aestivum PE=3 SV=2	75.89	610.79	-2.42	down	0.00	0.00	yes
TRINITY_DN20867_c0_g1	hypothetical protein POPTR_0019s15190g [Populus trichocarpa]	PTI6	Pathogenesis-related genes transcriptional activator PTI6 OS=Solanum lycopersicum GN=PTI6 PE=2 SV=1	0.52	17.28	-4.46	down	0.00	0.00	yes
TRINITY_DN22380_c0_g1	bark storage protein 1+2, partial [Populus tremula x Populus alba]	BSPA	Bark storage protein A OS=Populus deltoides GN=BSPA PE=2 SV=1	0.22	22.27	-6.04	down	0.00	0.00	yes
TRINITY_DN18715_c0_g1	PREDICTED: gibberellin 20 oxidase 2-like [Populus euphratica]	20ox2	Gibberellin 20 oxidase 2 OS=Oryza sativa subsp. indica GN=20ox2 PE=1 SV=1	0.93	16.65	-3.55	down	0.00	0.00	yes
TRINITY_DN20948_c0_g1	armadillo/beta-catenin repeat family protein [Populus trichocarpa]	PUB19	U-box domain-containing protein 19 OS=Arabidopsis thaliana GN=PUB19 PE=2 SV=1	0.52	76.70	-6.53	down	0.00	0.00	yes
TRINITY_DN15604_c0_g1	PREDICTED: desiccation-related protein PCC13-62-like [Juglans regia]	-	Desiccation-related protein PCC13-62 OS=Craterostigma plantagineum PE=2 SV=1	1.79	74.38	-4.79	down	0.00	0.00	yes
TRINITY_DN14386_c0_g1	hypothetical protein POPTR_0015s07660g [Populus trichocarpa]	-	(-)-isopiperitenol/(-)-carveol dehydrogenase, mitochondrial OS=Mentha piperita PE=1 SV=1	6.27	67.75	-2.86	down	0.00	0.00	yes
TRINITY_DN23600_c0_g2	hypothetical protein POPTR_0011s15750g [Populus trichocarpa]	-	Alpha-glucosidase OS=Beta vulgaris PE=1 SV=1	1.22	10.68	-2.70	down	0.00	0.00	yes
TRINITY_DN20153_c0_g1	hypothetical protein POPTR_0003s02700g [Populus trichocarpa]	At3g15810	Protein LURP-one-related 12 OS=Arabidopsis thaliana GN=At3g15810 PE=2 SV=1	12.23	92.95	-2.31	down	0.00	0.00	yes
TRINITY_DN18631_c0_g1	GDSL-motif lipase/hydrolase family protein [Populus trichocarpa]	At1g29670	GDSL esterase/lipase At1g29670 OS=Arabidopsis thaliana GN=At1g29670 PE=2 SV=1	0.77	35.68	-4.86	down	0.00	0.00	yes
TRINITY_DN24272_c0_g1	hypothetical protein POPTR_0019s08640g [Populus trichocarpa]	-	-	4.54	32.41	-2.23	down	0.00	0.00	yes
TRINITY_DN20670_c0_g1	SEC14 cytosolic factor family protein [Populus trichocarpa]	PDR17	Phosphatidylinositol transfer protein PDR17 OS=Saccharomyces cerevisiae (strain ATCC 204508 / S288c) GN=PDR17 PE=1 SV=1	5.42	43.28	-2.34	down	0.00	0.00	yes
TRINITY_DN21232_c0_g2	hypothetical protein POPTR_0006s225601g [Populus trichocarpa]	LWD1	WD repeat-containing protein LWD1 OS=Arabidopsis thaliana GN=LWD1 PE=2 SV=1	0.07	8.17	-6.12	down	0.00	0.00	yes
TRINITY_DN16903_c0_g2	cytochrome P450 family protein [Populus trichocarpa]	CYP77A3	Cytochrome P450 77A3 OS=Glycine max GN=CYP77A3 PE=2 SV=1	0.00	5.21	-10.01	down	0.00	0.00	yes
TRINITY_DN15849_c0_g1	hypothetical protein POPTR_0008s08850g [Populus trichocarpa]	NAC029	NAC transcription factor 29 OS=Arabidopsis thaliana GN=NAC029 PE=2 SV=1	0.02	4.56	-7.35	down	0.00	0.00	yes
TRINITY_DN27331_c0_g5	phosphate transporter family protein [Populus trichocarpa]	PHT1-9	Probable inorganic phosphate transporter 1-9 OS=Arabidopsis thaliana GN=PHT1-9 PE=2 SV=1	0.09	4.39	-4.95	down	0.00	0.00	yes
TRINITY_DN23948_c0_g1	PREDICTED: CLAVATA3/ESR (CLE)-related protein TDIF-like [Populus euphratica]	-	-	8.11	59.57	-2.88	down	0.00	0.00	yes
TRINITY_DN18012_c0_g3	-	-	-	0.00	3.66	-8.66	down	0.00	0.00	yes
TRINITY_DN26537_c0_g1	dehydrin 3 [Populus alba x Populus glandulosa]	-	-	6.10	187.17	-4.87	down	0.00	0.00	yes
TRINITY_DN19963_c0_g4	hypothetical protein POPTR_0008s00530g [Populus trichocarpa]	TPK1	Two-pore potassium channel 1 OS=Arabidopsis thaliana GN=TPK1 PE=1 SV=2	3.52	29.37	-2.43	down	0.00	0.00	yes
TRINITY_DN24453_c0_g5	hypothetical protein POPTR_0009s14270g [Populus trichocarpa]	-	-	2.06	52.27	-3.67	down	0.00	0.00	yes
TRINITY_DN14058_c0_g1	hypothetical protein POPTR_0001s16920g, partial [Populus trichocarpa]	GRF5	Growth-regulating factor 5 OS=Arabidopsis thaliana GN=GRF5 PE=1 SV=1	0.00	2.85	-8.74	down	0.00	0.00	yes
TRINITY_DN26581_c0_g1	PREDICTED: auxin response factor 5-like [Populus euphratica]	ARF5	Auxin response factor 5 OS=Arabidopsis thaliana GN=ARF5 PE=1 SV=3	2.67	35.76	-3.15	down	0.00	0.00	yes
TRINITY_DN17961_c0_g4	IAA-amido synthetase GH3-1 [Populus davidiana x Populus alba var. pyramidalis]	GH3.1	Probable indole-3-acetic acid-amido synthetase GH3.1 OS=Arabidopsis thaliana GN=GH3.1 PE=2 SV=1	0.07	8.40	-6.34	down	0.00	0.00	yes
TRINITY_DN8172_c0_g1	PREDICTED: oligopeptide transporter 9-like [Populus euphratica]	OPT9	Oligopeptide transporter 9 OS=Arabidopsis thaliana GN=OPT9 PE=2 SV=1	0.08	4.40	-5.12	down	0.00	0.00	yes
TRINITY_DN25990_c0_g2	hypothetical protein POPTR_0007s01260g [Populus trichocarpa]	DNAJB13	DnaJ homolog subfamily B member 13 OS=Homo sapiens GN=DNAJB13 PE=1 SV=1	1.46	25.84	-4.32	down	0.00	0.00	yes

TRINITY_DN25690_c0_g1	PREDICTED: uncharacterized protein LOC105136793 isoform X2 [Populus euphratica]	ACR10	ACT domain-containing protein ACR10 OS=Arabidopsis thaliana GN=ACR10 PE=2 SV=1	8.18	63.59	-2.42	down	0.00	0.00	yes
TRINITY_DN14415_c0_g1	hypothetical protein POPTR_0006s03510g [Populus trichocarpa]	-	-	0.28	7.65	-4.17	down	0.00	0.00	yes
TRINITY_DN26182_c0_g6	hypothetical protein POPTR_0014s01260g [Populus trichocarpa]	ANT	AP2-like ethylene-responsive transcription factor ANT OS=Arabidopsis thaliana GN=ANT PE=1 SV=2	2.04	28.29	-3.20	down	0.00	0.00	yes
TRINITY_DN19216_c0_g2	PREDICTED: scopoletin glucosyltransferase-like [Populus euphratica]	TOGT1	Scopoletin glucosyltransferase OS=Nicotiana tabacum GN=TOGT1 PE=1 SV=1	1.65	19.96	-2.98	down	0.00	0.00	yes
TRINITY_DN16350_c0_g1	hypothetical protein MANES_10G045100 [Manihot esculenta]	-	-	0.09	16.71	-4.88	down	0.00	0.00	yes
TRINITY_DN25681_c0_g1	leucine-rich repeat transmembrane protein kinase [Populus trichocarpa]	At1g53420	Probable LRR receptor-like serine/threonine-protein kinase At1g53420 OS=Arabidopsis thaliana GN=At1g53420 PE=2 SV=2	6.49	48.42	-2.33	down	0.00	0.00	yes
TRINITY_DN18048_c0_g2	hypothetical protein POPTR_0005s01470g [Populus trichocarpa]	-	-	13.44	120.13	-2.64	down	0.00	0.00	yes
TRINITY_DN17362_c0_g1	PREDICTED: 18 kDa seed maturation protein [Populus euphratica]	GMPM1	18 kDa seed maturation protein OS=Glycine max GN=GMPM1 PE=2 SV=1	1.99	122.31	-5.40	down	0.00	0.00	yes
TRINITY_DN24758_c0_g2	DNA-binding family protein [Populus trichocarpa]	AHL5	AT-hook motif nuclear-localized protein 5 OS=Arabidopsis thaliana GN=AHL5 PE=1 SV=1	18.42	132.52	-2.25	down	0.00	0.00	yes
TRINITY_DN17083_c0_g1	hypothetical protein POPTR_0001s26230g [Populus trichocarpa]	-	-	0.22	7.60	-4.61	down	0.00	0.00	yes
TRINITY_DN14579_c0_g1	PREDICTED: protein FD [Populus euphratica]	FD	Protein FD OS=Arabidopsis thaliana GN=FD PE=1 SV=1	0.14	5.73	-5.06	down	0.00	0.00	yes
TRINITY_DN15571_c0_g1	PREDICTED: protein RALF-like 34 [Populus euphratica]	RALFL34	Protein RALF-like 34 OS=Arabidopsis thaliana GN=RALFL34 PE=2 SV=1	12.23	78.65	-2.08	down	0.00	0.00	yes
TRINITY_DN19850_c1_g2	squamosa promoter-binding protein 25 [Populus tomentosa]	SPL5	Squamosa promoter-binding-like protein 5 OS=Arabidopsis thaliana GN=SPL5 PE=2 SV=1	2.35	20.09	-2.98	down	0.00	0.00	yes
TRINITY_DN16803_c0_g1	-	-	-	0.95	39.06	-4.75	down	0.00	0.00	yes
TRINITY_DN18378_c0_g1	PREDICTED: uncharacterized protein LOC105120737 isoform X6 [Populus euphratica]	-	-	0.56	17.66	-3.63	down	0.00	0.00	yes
TRINITY_DN19263_c0_g2	PREDICTED: MADS-box protein JOINTLESS [Populus euphratica]	AGL24	MADS-box protein AGL24 OS=Arabidopsis thaliana GN=AGL24 PE=1 SV=1	13.75	116.97	-2.45	down	0.00	0.00	yes
TRINITY_DN21957_c1_g2	PREDICTED: HMG-Y-related protein B-like [Populus euphratica]	-	HMG-Y-related protein A OS=Glycine max PE=2 SV=1	92.99	693.03	-2.27	down	0.00	0.00	yes
TRINITY_DN21374_c0_g2	hypothetical protein POPTR_0008s14170g [Populus trichocarpa]	-	-	0.81	11.98	-3.30	down	0.00	0.00	yes
TRINITY_DN23891_c0_g1	PREDICTED: histone H1-like isoform X2 [Populus euphratica]	At2g30620	Histone H1.2 OS=Arabidopsis thaliana GN=At2g30620 PE=1 SV=1	53.38	433.87	-2.46	down	0.00	0.00	yes
TRINITY_DN21081_c0_g2	PREDICTED: uncharacterized protein LOC105112918 [Populus euphratica]	-	-	0.05	2.86	-5.12	down	0.00	0.00	yes
TRINITY_DN19230_c0_g2	hypothetical protein POPTR_0016s05690g [Populus trichocarpa]	ABCG15	ABC transporter G family member 15 OS=Arabidopsis thaliana GN=ABCG15 PE=2 SV=2	1.41	16.40	-3.05	down	0.00	0.00	yes
TRINITY_DN21521_c0_g1	hypothetical protein POPTR_0007s07830g [Populus trichocarpa]	NFYC2	Nuclear transcription factor Y subunit C-2 OS=Arabidopsis thaliana GN=NFYC2 PE=2 SV=2	4.44	45.77	-2.76	down	0.00	0.00	yes
TRINITY_DN23582_c0_g2	PREDICTED: serine/threonine-protein kinase ATM-like [Populus euphratica]	ATM	Serine/threonine-protein kinase ATM OS=Arabidopsis thaliana GN=ATM PE=2 SV=1	5.43	44.51	-2.44	down	0.00	0.00	yes
TRINITY_DN13380_c0_g1	PREDICTED: glutaredoxin-C1 [Populus euphratica]	GRXC3	Glutaredoxin-C3 OS=Oryza sativa subsp. japonica GN=GRXC3 PE=2 SV=1	0.32	14.01	-4.85	down	0.00	0.00	yes
TRINITY_DN22705_c0_g1	PREDICTED: meiotic recombination protein DMC1 homolog [Populus euphratica]	-	Meiotic recombination protein DMC1 homolog OS=Glycine max PE=2 SV=1	2.15	20.22	-2.58	down	0.00	0.00	yes
TRINITY_DN24091_c0_g2	hypothetical protein POPTR_0005s19950g [Populus trichocarpa]	-	-	2.23	23.29	-2.79	down	0.00	0.00	yes
TRINITY_DN20278_c0_g1	PREDICTED: uncharacterized protein LOC105109680 [Populus euphratica]	-	-	0.56	33.29	-4.98	down	0.00	0.00	yes
TRINITY_DN22880_c0_g1	flavonoid 3-O-galactosyl transferase family protein [Populus trichocarpa]	FGT	Anthocyanidin 3-O-glucosyltransferase 2 OS=Fragaria ananassa GN=FGT PE=1 SV=1	3.21	70.71	-3.85	down	0.00	0.00	yes
TRINITY_DN16871_c0_g1	PREDICTED: probable histone H2A.5 [Populus euphratica]	OsI_002060	Probable histone H2A.5 OS=Oryza sativa subsp. indica GN=OsI_002060 PE=3 SV=1	115.07	795.39	-2.23	down	0.00	0.00	yes

TRINITY_DN15975_c0_g2	hypothetical protein POPTR_0017s11760g [Populus trichocarpa]	PRE1	Transcription factor PRE1 OS=Arabidopsis thaliana GN=PRE1 PE=1 SV=1	2.07	36.53	-3.51	down	0.00	0.00	yes
TRINITY_DN21232_c0_g1	WD40-repeat protein [Populus tremula x Populus tremuloides]	LWD1	WD repeat-containing protein LWD1 OS=Arabidopsis thaliana GN=LWD1 PE=2 SV=1	0.07	6.36	-5.70	down	0.00	0.00	yes
TRINITY_DN5364_c0_g1	-	-	-	0.00	4.48	-8.40	down	0.00	0.00	yes
TRINITY_DN25087_c0_g5	hypothetical protein POPTR_0003s16620g [Populus trichocarpa]	ACR4	ACT domain-containing protein ACR4 OS=Arabidopsis thaliana GN=ACR4 PE=2 SV=1	1.48	19.34	-3.12	down	0.00	0.00	yes
TRINITY_DN6724_c0_g1	-	-	-	0.00	35.26	-10.81	down	0.00	0.00	yes
TRINITY_DN15531_c0_g2	hypothetical protein POPTR_0016s14020g [Populus trichocarpa]	-	-	2.65	50.97	-3.67	down	0.00	0.00	yes
TRINITY_DN21497_c0_g3	Ethylene responsive element binding factor 4 family protein [Populus trichocarpa]	ERF4	Ethylene-responsive transcription factor 4 OS=Arabidopsis thaliana GN=ERF4 PE=1 SV=1	10.72	198.84	-3.63	down	0.00	0.00	yes
TRINITY_DN21009_c0_g2	PREDICTED: interactor of constitutive active ROPs 4-like [Populus euphratica]	ICR4	Interactor of constitutive active ROPs 4 OS=Arabidopsis thaliana GN=ICR4 PE=1 SV=2	9.42	58.30	-2.03	down	0.00	0.00	yes
TRINITY_DN24316_c0_g3	PREDICTED: lupeol synthase-like isoform X1 [Populus euphratica]	-	Lupeol synthase OS=Ricinus communis PE=1 SV=1	0.18	8.11	-4.54	down	0.00	0.00	yes
TRINITY_DN23459_c0_g1	PREDICTED: flavonoid 3',5'-hydroxylase 2-like [Populus euphratica]	CYP75A1	Flavonoid 3',5'-hydroxylase 1 OS=Petunia hybrida GN=CYP75A1 PE=2 SV=1	0.03	14.96	-8.35	down	0.00	0.00	yes
TRINITY_DN21687_c0_g2	hypothetical protein POPTR_0002s08180g [Populus trichocarpa]	KIN7E	Kinesin-like protein KIN-7E, chloroplastic OS=Oryza sativa subsp. japonica GN=KIN7E PE=3 SV=2	3.19	21.15	-2.15	down	0.00	0.00	yes
TRINITY_DN21951_c0_g2	PREDICTED: probable inactive receptor kinase At5g67200 [Populus euphratica]	At5g67200	Probable inactive receptor kinase At5g67200 OS=Arabidopsis thaliana GN=At5g67200 PE=1 SV=1	3.00	40.89	-3.23	down	0.00	0.00	yes
TRINITY_DN16363_c0_g1	hypothetical protein POPTR_0006s23580g, partial [Populus trichocarpa]	COPT5.1	Copper transporter 5.1 OS=Oryza sativa subsp. japonica GN=COPT5.1 PE=2 SV=1	5.40	53.88	-2.74	down	0.00	0.00	yes
TRINITY_DN22004_c0_g1	hypothetical protein POPTR_0010s23520g [Populus trichocarpa]	ACR10	ACT domain-containing protein ACR10 OS=Arabidopsis thaliana GN=ACR10 PE=2 SV=1	0.25	10.30	-4.77	down	0.00	0.00	yes
TRINITY_DN23047_c1_g2	PREDICTED: EPIDERMAL PATTERNING FACTOR-like protein 1 [Populus euphratica]	EPFL1	EPIDERMAL PATTERNING FACTOR-like protein 1 OS=Arabidopsis thaliana GN=EPFL1 PE=1 SV=1	0.04	14.07	-6.59	down	0.00	0.00	yes
TRINITY_DN16929_c0_g1	PREDICTED: snakin-2-like isoform X2 [Populus euphratica]	GASA3	Gibberellin-regulated protein 3 OS=Arabidopsis thaliana GN=GASA3 PE=2 SV=1	11.69	285.83	-4.00	down	0.00	0.00	yes
TRINITY_DN26172_c0_g1	PREDICTED: uncharacterized protein LOC105134970 isoform X1 [Populus euphratica]	-	-	32.00	215.87	-2.10	down	0.00	0.00	yes
TRINITY_DN20674_c0_g1	xyloglucan endotransglycosylase/hydrolase precursor XTH-30 [Populus tremula]	XTH9	Xyloglucan endotransglucosylase/hydrolase protein 9 OS=Arabidopsis thaliana GN=XTH9 PE=2 SV=2	76.15	558.99	-2.35	down	0.00	0.00	yes
TRINITY_DN24379_c0_g2	PREDICTED: uncharacterized protein LOC105120687 [Populus euphratica]	-	-	34.51	222.74	-2.07	down	0.00	0.00	yes
TRINITY_DN23575_c0_g1	hypothetical protein POPTR_0014s10700g [Populus trichocarpa]	PIL1	Transcription factor PIL1 OS=Arabidopsis thaliana GN=PIL1 PE=1 SV=1	0.89	10.81	-3.00	down	0.00	0.00	yes
TRINITY_DN24781_c0_g1	hypothetical protein POPTR_0001s34120g [Populus trichocarpa]	-	-	3.24	21.41	-2.11	down	0.00	0.00	yes
TRINITY_DN20325_c0_g1	unknown [Populus trichocarpa]	At2g30620	Histone H1.2 OS=Arabidopsis thaliana GN=At2g30620 PE=1 SV=1	69.31	495.35	-2.25	down	0.00	0.00	yes
TRINITY_DN3089_c0_g1	PREDICTED: pathogenesis-related protein 1-like [Populus euphratica]	At2g14610	Pathogenesis-related protein 1 OS=Arabidopsis thaliana GN=At2g14610 PE=1 SV=1	0.75	17.80	-3.94	down	0.00	0.00	yes
TRINITY_DN27125_c0_g7	PREDICTED: beta-amyrin synthase-like [Populus euphratica]	OSCPNY2	Beta-Amyrin Synthase 2 OS=Panax ginseng GN=OSCPNY2 PE=2 SV=1	0.02	4.13	-6.63	down	0.00	0.00	yes
TRINITY_DN20021_c0_g1	hypothetical protein POPTR_0014s07740g [Populus trichocarpa]	-	-	0.22	6.88	-4.42	down	0.00	0.00	yes
TRINITY_DN24328_c0_g2	hypothetical protein POPTR_0017s07940g [Populus trichocarpa]	UGT85A24	7-deoxyloganetin glucosyltransferase OS=Gardenia jasminoides GN=UGT85A24 PE=1 SV=1	4.15	25.32	-3.10	down	0.00	0.00	yes
TRINITY_DN16806_c0_g1	PREDICTED: uncharacterized protein LOC105127206 isoform X2 [Populus euphratica]	-	-	1.44	36.48	-4.13	down	0.00	0.00	yes
TRINITY_DN21705_c0_g1	hypothetical protein POPTR_0014s07200g [Populus trichocarpa]	-	-	1.33	18.81	-3.35	down	0.00	0.00	yes
TRINITY_DN20135_c0_g1	PREDICTED: uncharacterized protein LOC105136427 isoform X1 [Populus euphratica]	-	-	0.15	6.69	-4.16	down	0.00	0.00	yes

TRINITY_DN23102_c0_g1	hypothetical protein POPTR_0018s10820g, partial [Populus trichocarpa]	-	-	0.50	18.61	-4.56	down	0.00	0.00	yes
TRINITY_DN19456_c0_g1	PREDICTED: putative Myb family transcription factor At1g14600 [Populus euphratica]	At1g14600	Putative Myb family transcription factor At1g14600 OS=Arabidopsis thaliana GN=At1g14600 PE=2 SV=2	0.80	26.04	-4.50	down	0.00	0.00	yes
TRINITY_DN18424_c0_g3	hypothetical protein POPTR_0006s18630g [Populus trichocarpa]	DTX16	Protein DETOXIFICATION 16 OS=Arabidopsis thaliana GN=DTX16 PE=2 SV=1	2.15	18.04	-2.46	down	0.00	0.00	yes
TRINITY_DN20426_c0_g2	hypothetical protein POPTR_0005s24970g [Populus trichocarpa]	PUB5	U-box domain-containing protein 5 OS=Arabidopsis thaliana GN=PUB5 PE=3 SV=3	0.17	5.63	-4.04	down	0.00	0.00	yes
TRINITY_DN25153_c0_g1	hypothetical protein POPTR_0002s05000g [Populus trichocarpa]	VAB	VAN3-binding protein OS=Arabidopsis thaliana GN=VAB PE=1 SV=1	2.60	35.36	-3.18	down	0.00	0.00	yes
TRINITY_DN26215_c0_g1	hypothetical protein POPTR_0005s19950g [Populus trichocarpa]	-	-	9.04	95.89	-2.83	down	0.00	0.00	yes
TRINITY_DN16067_c0_g1	PREDICTED: uncharacterized protein LOC105141753 isoform X2 [Populus euphratica]	-	-	0.10	12.53	-5.99	down	0.00	0.00	yes
TRINITY_DN16677_c0_g2	hypothetical protein POPTR_0007s15050g [Populus trichocarpa]	-	-	0.00	5.66	-8.29	down	0.00	0.00	yes
TRINITY_DN18405_c0_g1	PREDICTED: gibberellin-regulated protein 14-like [Populus euphratica]	-	-	3.94	178.83	-4.88	down	0.00	0.00	yes
TRINITY_DN21404_c2_g1	PREDICTED: uncharacterized protein LOC105116401 [Populus euphratica]	-	-	0.36	7.01	-3.83	down	0.00	0.00	yes
TRINITY_DN23266_c0_g2	hypothetical protein POPTR_0014s06550g [Populus trichocarpa]	AHL1	AT-hook motif nuclear-localized protein 1 OS=Arabidopsis thaliana GN=AHL1 PE=1 SV=1	15.37	99.46	-2.08	down	0.00	0.00	yes
TRINITY_DN16288_c0_g2	family II extracellular lipase 3 family protein [Populus trichocarpa]	EXL3	GDSL esterase/lipase EXL3 OS=Arabidopsis thaliana GN=EXL3 PE=2 SV=1	0.40	10.00	-5.63	down	0.00	0.00	yes
TRINITY_DN22397_c0_g1	CBL-interacting protein kinase 1 [Populus trichocarpa]	CIPK1	CBL-interacting serine/threonine-protein kinase 1 OS=Arabidopsis thaliana GN=CIPK1 PE=1 SV=2	2.73	28.37	-2.80	down	0.00	0.00	yes
TRINITY_DN17500_c0_g1	unknown [Populus trichocarpa]	DRM1	Dormancy-associated protein 1 OS=Arabidopsis thaliana GN=DRM1 PE=1 SV=1	5.53	246.20	-4.87	down	0.00	0.00	yes
TRINITY_DN21016_c2_g1	hypothetical protein POPTR_0010s22870g [Populus trichocarpa]	-	-	1.07	12.73	-3.31	down	0.00	0.00	yes
TRINITY_DN19120_c0_g2	hypothetical protein POPTR_0001s266801g, partial [Populus trichocarpa]	-	-	0.77	49.44	-5.33	down	0.00	0.00	yes
TRINITY_DN18510_c0_g1	hypothetical protein POPTR_0007s09870g [Populus trichocarpa]	FH6	Formin-like protein 6 OS=Arabidopsis thaliana GN=FH6 PE=2 SV=1	0.00	6.56	-9.02	down	0.00	0.00	yes
TRINITY_DN27644_c0_g1	hypothetical protein POPTR_0006s10970g [Populus trichocarpa]	ALA1	Phospholipid-transporting ATPase 1 OS=Arabidopsis thaliana GN=ALA1 PE=2 SV=1	2.95	18.87	-2.08	down	0.00	0.00	yes
TRINITY_DN20089_c0_g6	hypothetical protein POPTR_0017s04300g [Populus trichocarpa]	-	-	0.02	3.31	-6.46	down	0.00	0.00	yes
TRINITY_DN22121_c0_g2	hypothetical protein POPTR_0001s04700g [Populus trichocarpa]	CMT3	DNA (cytosine-5)-methyltransferase CMT3 OS=Arabidopsis thaliana GN=CMT3 PE=1 SV=2	12.32	86.92	-2.23	down	0.00	0.00	yes
TRINITY_DN23324_c0_g2	hypothetical protein POPTR_0005s24980g [Populus trichocarpa]	NFD5	Protein NUCLEAR FUSION DEFECTIVE 5, mitochondrial OS=Arabidopsis thaliana GN=NFD5 PE=2 SV=1	0.15	3.93	-4.07	down	0.00	0.00	yes
TRINITY_DN28349_c0_g1	PREDICTED: tropinone reductase-like 1 [Populus euphratica]	-	Tropinone reductase-like 1 OS=Erythroxylum coca PE=2 SV=1	0.00	3.71	-8.78	down	0.00	0.00	yes
TRINITY_DN25876_c0_g2	PREDICTED: potassium channel AKT1 isoform X1 [Populus euphratica]	AKT1	Potassium channel AKT1 OS=Arabidopsis thaliana GN=AKT1 PE=1 SV=2	4.40	7.05	-2.59	down	0.00	0.00	yes
TRINITY_DN24789_c0_g4	PREDICTED: uncharacterized protein LOC105123234 isoform X1 [Populus euphratica]	-	-	0.43	8.34	-3.22	down	0.00	0.00	yes
TRINITY_DN20567_c0_g1	hypothetical protein POPTR_0015s04680g [Populus trichocarpa]	At3g47570	Probable LRR receptor-like serine/threonine-protein kinase At3g47570 OS=Arabidopsis thaliana GN=At3g47570 PE=2 SV=1	0.57	12.42	-3.80	down	0.00	0.00	yes
TRINITY_DN24947_c1_g1	-	-	-	0.70	18.21	-3.87	down	0.00	0.00	yes
TRINITY_DN24816_c2_g1	PREDICTED: nucleobase-ascorbate transporter 2 [Populus euphratica]	NAT2	Nucleobase-ascorbate transporter 2 OS=Arabidopsis thaliana GN=NAT2 PE=2 SV=2	7.15	53.47	-2.30	down	0.00	0.00	yes
TRINITY_DN20258_c0_g1	hypothetical protein POPTR_0007s09840g [Populus trichocarpa]	BT4	BTB/POZ and TAZ domain-containing protein 4 OS=Arabidopsis thaliana GN=BT4 PE=1 SV=1	3.79	29.05	-2.32	down	0.00	0.00	yes
TRINITY_DN20452_c1_g3	PREDICTED: myb-related protein 308-like [Populus euphratica]	MYB308	Myb-related protein 308 OS=Antirrhinum majus GN=MYB308 PE=2 SV=1	0.00	5.40	-9.06	down	0.00	0.00	yes

TRINITY_DN18653_c0_g1	hypothetical protein CISIN_1g047769mg, partial [Citrus sinensis]	-	Histone H4 variant TH011 OS=Triticum aestivum PE=3 SV=2	200.40	1291.85	-2.11	down	0.00	0.00	yes
TRINITY_DN25914_c0_g2	PREDICTED: cytochrome P450 90B1 [Populus euphratica]	CYP90B1	Cytochrome P450 90B1 OS=Arabidopsis thaliana GN=CYP90B1 PE=1 SV=2	4.82	34.07	-2.26	down	0.00	0.00	yes
TRINITY_DN18332_c0_g1	C2 domain-containing family protein [Populus trichocarpa]	FTIP1	FT-interacting protein 1 OS=Arabidopsis thaliana GN=FTIP1 PE=1 SV=1	0.07	4.25	-5.33	down	0.00	0.00	yes
TRINITY_DN21023_c0_g1	hypothetical protein POPTR_0006s24290g [Populus trichocarpa]	IQM4	IQ domain-containing protein IQM4 OS=Arabidopsis thaliana GN=IQM4 PE=2 SV=1	4.36	55.76	-3.05	down	0.00	0.00	yes
TRINITY_DN20841_c0_g1	hypothetical protein POPTR_0002s00860g [Populus trichocarpa]	At4g29360	Glucan endo-1,3-beta-glucosidase 12 OS=Arabidopsis thaliana GN=At4g29360 PE=1 SV=1	7.43	72.76	-2.69	down	0.00	0.00	yes
TRINITY_DN26798_c0_g2	hypothetical protein POPTR_0004s09510g [Populus trichocarpa]	-	Non-specific lipid-transfer protein 2 OS=Prunus armeniaca PE=1 SV=1	10.06	106.48	-2.82	down	0.00	0.00	yes
TRINITY_DN21590_c1_g3	PREDICTED: auxin-responsive protein IAA13-like [Populus euphratica]	IAA12	Auxin-responsive protein IAA12 OS=Arabidopsis thaliana GN=IAA12 PE=1 SV=1	1.24	13.54	-2.93	down	0.00	0.00	yes
TRINITY_DN24271_c0_g2	PREDICTED: uncharacterized protein LOC105139490 isoform X1 [Populus euphratica]	-	-	4.74	32.89	-2.19	down	0.00	0.00	yes
TRINITY_DN24851_c0_g2	-	-	-	0.80	14.19	-4.18	down	0.00	0.00	yes
TRINITY_DN18627_c0_g7	hydroxyproline-rich glycoprotein [Populus trichocarpa]	-	-	0.26	16.23	-5.31	down	0.00	0.00	yes
TRINITY_DN26873_c0_g1	PREDICTED: glutamate receptor 3.7 [Populus euphratica]	GLR3.7	Glutamate receptor 3.7 OS=Arabidopsis thaliana GN=GLR3.7 PE=2 SV=2	1.32	12.18	-2.61	down	0.00	0.00	yes
TRINITY_DN22125_c0_g6	hypothetical protein POPTR_0016s14440g [Populus trichocarpa]	CYP71A1	Cytochrome P450 71A1 OS=Persea americana GN=CYP71A1 PE=1 SV=2	0.24	15.20	-5.41	down	0.00	0.00	yes
TRINITY_DN26798_c0_g1	PREDICTED: uncharacterized protein LOC105128937 isoform X1 [Populus euphratica]	-	-	1.63	13.19	-2.39	down	0.00	0.00	yes
TRINITY_DN23317_c0_g4	hypothetical protein POPTR_0002s00650g [Populus trichocarpa]	AHL10	AT-hook motif nuclear-localized protein 10 OS=Arabidopsis thaliana GN=AHL10 PE=1 SV=2	6.24	39.73	-2.06	down	0.00	0.00	yes
TRINITY_DN15652_c0_g1	PREDICTED: uncharacterized protein LOC105127652 [Populus euphratica]	-	-	0.18	12.07	-4.53	down	0.00	0.00	yes
TRINITY_DN19042_c0_g1	PREDICTED: protein FEZ-like [Populus euphratica]	NAC035	NAC domain-containing protein 35 OS=Arabidopsis thaliana GN=NAC035 PE=1 SV=2	0.38	9.93	-4.05	down	0.00	0.00	yes
TRINITY_DN19850_c1_g4	hypothetical protein POPTR_0015s07140g [Populus trichocarpa]	SPL6	Squamosa promoter-binding-like protein 6 OS=Arabidopsis thaliana GN=SPL6 PE=2 SV=2	1.16	10.15	-2.51	down	0.00	0.00	yes
TRINITY_DN25966_c0_g2	hypothetical protein POPTR_0672s00210g [Populus trichocarpa]	-	-	6.97	66.30	-2.62	down	0.00	0.00	yes
TRINITY_DN16293_c0_g1	hypothetical protein POPTR_0012s04950g, partial [Populus trichocarpa]	-	-	0.55	7.20	-3.24	down	0.00	0.00	yes
TRINITY_DN26222_c1_g3	hypothetical protein POPTR_0019s01470g [Populus trichocarpa]	AMT3-1	Ammonium transporter 3 member 1 OS=Oryza sativa subsp. japonica GN=AMT3-1 PE=2 SV=1	0.08	5.31	-5.36	down	0.00	0.00	yes
TRINITY_DN25060_c0_g2	PREDICTED: glucan endo-1,3-beta-glucosidase 12-like [Populus euphratica]	At4g29360	Glucan endo-1,3-beta-glucosidase 12 OS=Arabidopsis thaliana GN=At4g29360 PE=1 SV=1	10.35	91.23	-2.57	down	0.00	0.00	yes
TRINITY_DN17151_c0_g2	hypothetical protein POPTR_0001s08310g [Populus trichocarpa]	GRF12	Growth-regulating factor 12 OS=Oryza sativa subsp. japonica GN=GRF12 PE=2 SV=1	2.54	32.87	-3.14	down	0.00	0.00	yes
TRINITY_DN24359_c0_g2	hypothetical protein POPTR_0006s05660g [Populus trichocarpa]	PYM	Protein POLYCHOME OS=Arabidopsis thaliana GN=PYM PE=1 SV=1	13.93	86.17	-2.00	down	0.00	0.00	yes
TRINITY_DN18437_c0_g1	remorin family protein [Populus trichocarpa]	-	-	0.79	8.32	-2.79	down	0.00	0.00	yes
TRINITY_DN21497_c0_g4	ERF4 [Populus x canadensis]	ERF4	Ethylene-responsive transcription factor 4 OS=Arabidopsis thaliana GN=ERF4 PE=1 SV=1	11.08	166.25	-3.32	down	0.00	0.00	yes
TRINITY_DN21890_c0_g6	hypothetical protein POPTR_0007s10490g [Populus trichocarpa]	-	-	1.51	30.82	-3.56	down	0.00	0.00	yes
TRINITY_DN17088_c0_g1	hypothetical protein POPTR_0005s11220g [Populus trichocarpa]	At4g35930	F-box protein At4g35930 OS=Arabidopsis thaliana GN=At4g35930 PE=2 SV=1	3.32	26.36	-2.39	down	0.00	0.00	yes
TRINITY_DN23615_c0_g15	-	-	-	5.95	37.55	-2.06	down	0.00	0.00	yes
TRINITY_DN15428_c0_g1	PREDICTED: F-box only protein 13 [Populus euphratica]	FBX13	F-box only protein 13 OS=Arabidopsis thaliana GN=FBX13 PE=2 SV=2	2.42	20.68	-2.47	down	0.00	0.00	yes

TRINITY_DN25548_c1_g1	hypothetical protein POPTR_0001s29120g [Populus trichocarpa]	-	-	0.00	4.65	-8.20	down	0.00	0.00	yes
TRINITY_DN15653_c0_g3	hypothetical protein POPTR_0007s02820g [Populus trichocarpa]	-	-	1.06	14.38	-3.33	down	0.00	0.00	yes
TRINITY_DN19882_c0_g2	hypothetical protein POPTR_0010s16600g [Populus trichocarpa]	-	-	1.66	33.10	-3.72	down	0.00	0.00	yes
TRINITY_DN27292_c0_g1	-	-	-	0.39	14.25	-5.38	down	0.00	0.00	yes
TRINITY_DN15930_c1_g5	hypothetical protein POPTR_0004s05840g [Populus trichocarpa]	-	-	0.47	14.01	-4.29	down	0.00	0.00	yes
TRINITY_DN17769_c0_g1	hypothetical protein POPTR_0005s07220g [Populus trichocarpa]	ATL55	E3 ubiquitin-protein ligase RING1 OS=Arabidopsis thaliana GN=ATL55 PE=1 SV=1	0.41	9.75	-4.46	down	0.00	0.00	yes
TRINITY_DN928_c0_g1	kunitz trypsin inhibitor [Populus tremula]	GWIN3	Wound-responsive protein GWIN3 OS=Populus sp. GN=GWIN3 PE=2 SV=1	4.39	110.54	-4.14	down	0.00	0.00	yes
TRINITY_DN18016_c0_g1	hypothetical protein POPTR_0013s10110g, partial [Populus trichocarpa]	AAP2	Amino acid permease 2 OS=Arabidopsis thaliana GN=AAP2 PE=1 SV=1	0.20	3.93	-3.69	down	0.00	0.00	yes
TRINITY_DN22342_c0_g1	PREDICTED: protein CUP-SHAPED COTYLEDON 3-like isoform X3 [Populus euphratica]	NAC062	NAC domain-containing protein 62 OS=Arabidopsis thaliana GN=NAC062 PE=1 SV=1	0.65	15.24	-3.72	down	0.00	0.00	yes
TRINITY_DN27487_c1_g1	PREDICTED: probable L-type lectin-domain containing receptor kinase V.3 isoform X2 [Populus euphratica]	LRK10	Rust resistance kinase Lr10 OS=Triticum aestivum GN=LRK10 PE=2 SV=1	1.30	13.85	-2.85	down	0.00	0.00	yes
TRINITY_DN20403_c0_g3	PREDICTED: cinnamoyl-CoA reductase 1-like [Populus euphratica]	A1	Dihydroflavonol 4-reductase OS=Zea mays GN=A1 PE=3 SV=1	0.12	13.67	-6.23	down	0.00	0.00	yes
TRINITY_DN20164_c0_g1	hypothetical protein POPTR_0019s14110g [Populus trichocarpa]	CYCD6-1	Putative cyclin-D6-1 OS=Arabidopsis thaliana GN=CYCD6-1 PE=3 SV=1	0.99	12.70	-3.06	down	0.00	0.00	yes
TRINITY_DN18419_c0_g1	peroxidase [Populus alba x Populus glandulosa]	PER52	Peroxidase 52 OS=Arabidopsis thaliana GN=PER52 PE=2 SV=1	0.43	31.58	-5.65	down	0.00	0.00	yes
TRINITY_DN23906_c0_g1	PREDICTED: uncharacterized protein LOC105128946 [Populus euphratica]	ACR8	ACT domain-containing protein ACR8 OS=Arabidopsis thaliana GN=ACR8 PE=2 SV=1	2.12	24.53	-2.88	down	0.00	0.00	yes
TRINITY_DN19312_c0_g1	-	-	-	0.07	44.80	-7.62	down	0.00	0.00	yes
TRINITY_DN18421_c0_g1	PREDICTED: cation/calcium exchanger 2-like [Populus euphratica]	CCX2	Cation/calcium exchanger 2 OS=Arabidopsis thaliana GN=CCX2 PE=3 SV=1	1.70	26.52	-3.14	down	0.00	0.00	yes
TRINITY_DN19456_c0_g5	PREDICTED: putative Myb family transcription factor At1g14600 [Populus euphratica]	At1g14600	Putative Myb family transcription factor At1g14600 OS=Arabidopsis thaliana GN=At1g14600 PE=2 SV=2	1.21	42.06	-4.79	down	0.00	0.00	yes
TRINITY_DN18139_c0_g1	hypothetical protein POPTR_0002s17460g [Populus trichocarpa]	MYB3	Transcription factor MYB3 OS=Arabidopsis thaliana GN=MYB3 PE=1 SV=1	1.20	88.95	-4.91	down	0.00	0.00	yes
TRINITY_DN15696_c0_g2	-	-	-	0.08	7.71	-5.88	down	0.00	0.00	yes
TRINITY_DN17642_c0_g1	kunitz trypsin inhibitor [Populus tremula]	ASP	21 kDa seed protein OS=Theobroma cacao GN=ASP PE=2 SV=1	0.97	38.99	-4.64	down	0.00	0.00	yes
TRINITY_DN17821_c0_g1	PREDICTED: uncharacterized protein LOC105121376 [Populus euphratica]	-	-	15.27	86.62	-1.91	down	0.00	0.00	yes
TRINITY_DN17795_c0_g2	hypothetical protein POPTR_0005s26720g [Populus trichocarpa]	-	-	0.00	4.22	-8.43	down	0.00	0.00	yes
TRINITY_DN26007_c0_g1	PREDICTED: growth-regulating factor 1-like [Populus euphratica]	GRF1	Growth-regulating factor 1 OS=Arabidopsis thaliana GN=GRF1 PE=1 SV=1	4.82	33.44	-2.15	down	0.00	0.00	yes
TRINITY_DN22355_c0_g1	alcohol oxidase-related family protein [Populus trichocarpa]	FAO1	Long-chain-alcohol oxidase FAO1 OS=Lotus japonicus GN=FAO1 PE=1 SV=1	3.50	20.53	-2.11	down	0.00	0.00	yes
TRINITY_DN22929_c0_g2	hypothetical protein POPTR_0001s19140g [Populus trichocarpa]	-	-	7.41	47.62	-2.00	down	0.00	0.00	yes
TRINITY_DN19768_c0_g3	hypothetical protein POPTR_0005s12930g [Populus trichocarpa]	CML41	Probable calcium-binding protein CML41 OS=Arabidopsis thaliana GN=CML41 PE=2 SV=2	5.99	64.08	-2.83	down	0.00	0.00	yes
TRINITY_DN18864_c0_g2	hypothetical protein POPTR_0005s22480g [Populus trichocarpa]	WRKY28	Probable WRKY transcription factor 28 OS=Arabidopsis thaliana GN=WRKY28 PE=2 SV=1	0.15	7.47	-5.06	down	0.00	0.00	yes
TRINITY_DN27893_c3_g2	hypothetical protein POPTR_0001s28330g [Populus trichocarpa]	At1g56140	Probable LRR receptor-like serine/threonine-protein kinase At1g56140 OS=Arabidopsis thaliana GN=At1g56140 PE=2 SV=2	0.02	3.05	-6.27	down	0.00	0.00	yes
TRINITY_DN26249_c0_g4	PREDICTED: uncharacterized protein LOC105125011 [Populus euphratica]	IDM2	Increased DNA methylation 2 OS=Arabidopsis thaliana GN=IDM2 PE=1 SV=1	0.46	9.39	-3.49	down	0.00	0.00	yes
TRINITY_DN21332_c0_g1	hypothetical protein POPTR_0001s05650g [Populus trichocarpa]	HMA5	Probable copper-transporting ATPase HMA5 OS=Arabidopsis thaliana GN=HMA5 PE=1 SV=2	1.89	14.82	-2.36	down	0.00	0.00	yes

TRINITY_DN23524_c0_g2	hypothetical protein POPTR_0007s10490g [Populus trichocarpa]	MYB44	Transcription factor MYB44 OS=Arabidopsis thaliana GN=MYB44 PE=2 SV=1	5.56	48.91	-2.54	down	0.00	0.00	yes
TRINITY_DN21311_c0_g1	proton-dependent oligopeptide transport family protein [Populus trichocarpa]	NPF5.2	Protein NRT1/ PTR FAMILY 5.2 OS=Arabidopsis thaliana GN=NPF5.2 PE=2 SV=1	0.98	18.31	-2.66	down	0.00	0.00	yes
TRINITY_DN20428_c0_g3	hypothetical protein POPTR_0019s04150g [Populus trichocarpa]	At5g33370	GDSL esterase/lipase At5g33370 OS=Arabidopsis thaliana GN=At5g33370 PE=2 SV=1	4.68	47.93	-2.75	down	0.00	0.00	yes
TRINITY_DN15789_c1_g1	PREDICTED: uncharacterized protein LOC105135007 [Populus euphratica]	-	-	1.24	36.79	-4.34	down	0.00	0.00	yes
TRINITY_DN11265_c0_g1	hypothetical protein POPTR_0013s03350g [Populus trichocarpa]	-	Metalloendoproteinase 1 OS=Glycine max PE=1 SV=2	0.03	4.10	-6.24	down	0.00	0.00	yes
TRINITY_DN26707_c0_g1	hypothetical protein POPTR_0005s02810g [Populus trichocarpa]	HST	Shikimate O-hydroxycinnamoyltransferase OS=Nicotiana tabacum GN=HST PE=1 SV=1	11.02	80.41	-2.45	down	0.00	0.00	yes
TRINITY_DN16888_c0_g1	PREDICTED: ABC transporter B family member 27-like [Populus euphratica]	ABCB27	ABC transporter B family member 27 OS=Arabidopsis thaliana GN=ABCB27 PE=1 SV=1	6.50	36.64	-1.94	down	0.00	0.00	yes
TRINITY_DN21920_c1_g4	PREDICTED: NAC domain-containing protein 90-like [Populus euphratica]	NAC090	NAC domain-containing protein 90 OS=Arabidopsis thaliana GN=NAC090 PE=2 SV=1	3.38	40.20	-3.03	down	0.00	0.00	yes
TRINITY_DN19576_c0_g1	PREDICTED: cell division control protein 45 homolog [Populus euphratica]	sna41	Cell division control protein 45 homolog OS=Schizosaccharomyces pombe (strain 972 / ATCC 24843) GN=sna41 PE=1 SV=1	1.82	13.78	-2.31	down	0.00	0.00	yes
TRINITY_DN25826_c0_g2	PREDICTED: uncharacterized protein LOC105142305 [Populus euphratica]	At2g16365	F-box protein At2g16365 OS=Arabidopsis thaliana GN=At2g16365 PE=2 SV=2	0.01	2.72	-6.70	down	0.00	0.00	yes
TRINITY_DN19059_c0_g3	hypothetical protein POPTR_0015s05780g [Populus trichocarpa]	At5g24080	G-type lectin S-receptor-like serine/threonine-protein kinase At5g24080 OS=Arabidopsis thaliana GN=At5g24080 PE=2 SV=1	0.09	4.40	-6.09	down	0.00	0.00	yes
TRINITY_DN17288_c0_g1	PREDICTED: F-box/WD repeat-containing protein 11-like [Populus euphratica]	JGB	Protein JINGUBANG OS=Arabidopsis thaliana GN=JGB PE=1 SV=1	0.15	4.02	-4.11	down	0.00	0.00	yes
TRINITY_DN15542_c0_g1	hypothetical protein POPTR_0013s08130g [Populus trichocarpa]	GSVIVT0002396	Peroxidase 4 OS=Vitis vinifera GN=GSVIVT00023967001 PE=1 SV=1	0.32	10.60	-4.43	down	0.00	0.00	yes
TRINITY_DN26007_c0_g2	hypothetical protein POPTR_0007s14720g [Populus trichocarpa]	GRF1	Growth-regulating factor 1 OS=Arabidopsis thaliana GN=GRF1 PE=1 SV=1	7.53	42.84	-1.90	down	0.00	0.00	yes
TRINITY_DN22795_c0_g2	hypothetical protein POPTR_0011s14530g [Populus trichocarpa]	At1g56130	Probable LRR receptor-like serine/threonine-protein kinase At1g56130 OS=Arabidopsis thaliana GN=At1g56130 PE=2 SV=2	1.83	16.99	-2.55	down	0.00	0.00	yes
TRINITY_DN15631_c0_g1	hypothetical protein POPTR_0001s14200g, partial [Populus trichocarpa]	-	-	0.35	10.78	-4.30	down	0.00	0.00	yes
TRINITY_DN23279_c0_g2	hypothetical protein POPTR_0002s14320g [Populus trichocarpa]	SPL8	Squamosa promoter-binding-like protein 8 OS=Arabidopsis thaliana GN=SPL8 PE=1 SV=2	0.25	11.72	-4.67	down	0.00	0.00	yes
TRINITY_DN20418_c0_g2	PREDICTED: vacuolar cation/proton exchanger 3-like [Populus euphratica]	CAX3	Vacuolar cation/proton exchanger 3 OS=Arabidopsis thaliana GN=CAX3 PE=1 SV=1	0.49	12.35	-4.11	down	0.00	0.00	yes
TRINITY_DN19437_c0_g6	hypothetical protein POPTR_0006s09470g [Populus trichocarpa]	-	-	0.40	6.52	-3.41	down	0.00	0.00	yes
TRINITY_DN19195_c0_g1	PREDICTED: uncharacterized protein LOC105128097 [Populus euphratica]	-	-	2.19	19.24	-2.49	down	0.00	0.00	yes
TRINITY_DN22482_c0_g1	hypothetical protein POPTR_0003s19550g [Populus trichocarpa]	SRS6	Protein SHI RELATED SEQUENCE 6 OS=Arabidopsis thaliana GN=SRS6 PE=2 SV=1	1.09	8.43	-3.38	down	0.00	0.00	yes
TRINITY_DN22660_c0_g1	transcription factor family protein [Populus trichocarpa]	E2FE	E2F transcription factor-like E2FE OS=Arabidopsis thaliana GN=E2FE PE=2 SV=1	3.78	27.43	-2.00	down	0.00	0.00	yes
TRINITY_DN23014_c0_g2	hypothetical protein POPTR_0002s16940g [Populus trichocarpa]	-	-	4.28	55.09	-3.06	down	0.00	0.00	yes
TRINITY_DN21164_c0_g1	basic leucine zipper transcription factor family protein [Populus trichocarpa]	DPBF2	ABSCISIC ACID-INSENSITIVE 5-like protein 1 OS=Arabidopsis thaliana GN=DPBF2 PE=1 SV=1	0.77	13.70	-3.58	down	0.00	0.00	yes
TRINITY_DN21713_c1_g1	hypothetical protein POPTR_0010s20930g [Populus trichocarpa]	-	-	2.06	29.19	-3.20	down	0.00	0.00	yes
TRINITY_DN25978_c0_g2	hypothetical protein POPTR_0006s05210g [Populus trichocarpa]	NFYA7	Nuclear transcription factor Y subunit A-7 OS=Arabidopsis thaliana GN=NFYA7 PE=2 SV=1	0.03	2.37	-5.78	down	0.00	0.00	yes
TRINITY_DN21482_c1_g1	PREDICTED: uncharacterized protein LOC105121986 isoform X1 [Populus euphratica]	-	-	1.69	17.67	-2.76	down	0.00	0.00	yes
TRINITY_DN22076_c0_g2	PREDICTED: FHA domain-containing protein At4g14490 [Populus euphratica]	At4g14490	FHA domain-containing protein At4g14490 OS=Arabidopsis thaliana GN=At4g14490 PE=1 SV=1	1.96	13.52	-2.26	down	0.00	0.00	yes

TRINITY_DN19125_c0_g1	leucine-rich repeat family protein [Populus trichocarpa]	At5g49770	Probable leucine-rich repeat receptor-like protein kinase At5g49770 OS=Arabidopsis thaliana GN=At5g49770 PE=2 SV=1	4.07	23.95	-2.19	down	0.00	0.00	yes
TRINITY_DN15304_c0_g1	hypothetical protein POPTR_0015s09150g [Populus trichocarpa]	-	-	5.19	33.68	-2.09	down	0.00	0.00	yes
TRINITY_DN27639_c1_g2	leucine-rich repeat family protein [Populus trichocarpa]	At1g67720	Probable LRR receptor-like serine/threonine-protein kinase At1g67720 OS=Arabidopsis thaliana GN=At1g67720 PE=2 SV=1	4.41	22.79	-2.45	down	0.00	0.00	yes
TRINITY_DN23481_c0_g2	PREDICTED: histone H2A variant 1 [Populus euphratica]	H2AV	Histone H2A variant 1 OS=Arabidopsis thaliana GN=H2AV PE=1 SV=1	21.87	126.80	-1.93	down	0.00	0.00	yes
TRINITY_DN27002_c0_g1	PREDICTED: kinesin-4 isoform X2 [Populus euphratica]	KIN14J	Kinesin-like protein KIN-14J OS=Arabidopsis thaliana GN=KIN14J PE=1 SV=1	5.50	33.18	-1.85	down	0.00	0.00	yes
TRINITY_DN23927_c0_g1	hypothetical protein POPTR_0006s11500g [Populus trichocarpa]	SCR	Protein SCARECROW OS=Pisum sativum GN=SCR PE=2 SV=1	6.28	42.85	-2.16	down	0.00	0.00	yes
TRINITY_DN12186_c0_g1	PREDICTED: probable metal-nicotianamine transporter YSL7 [Populus euphratica]	YSL7	Probable metal-nicotianamine transporter YSL7 OS=Arabidopsis thaliana GN=YSL7 PE=2 SV=1	0.00	2.24	-8.37	down	0.00	0.00	yes
TRINITY_DN26182_c0_g1	PREDICTED: AP2-like ethylene-responsive transcription factor ANT isoform X1 [Populus euphratica]	ANT	AP2-like ethylene-responsive transcription factor ANT OS=Arabidopsis thaliana GN=ANT PE=1 SV=2	0.62	11.36	-3.59	down	0.00	0.00	yes
TRINITY_DN26003_c2_g1	hypothetical protein POPTR_0006s23800g [Populus trichocarpa]	GT-2	Trihelix transcription factor GT-2 OS=Arabidopsis thaliana GN=GT-2 PE=2 SV=1	4.42	23.50	-1.83	down	0.00	0.00	yes
TRINITY_DN27577_c1_g2	hypothetical protein POPTR_0019s00330g [Populus trichocarpa]	At1g33420	PHD finger protein At1g33420 OS=Arabidopsis thaliana GN=At1g33420 PE=1 SV=1	9.45	53.83	-1.89	down	0.00	0.00	yes
TRINITY_DN19892_c0_g1	PREDICTED: calcium-binding and coiled-coil domain-containing protein 2-like isoform X2 [Populus euphratica]	-	-	1.09	9.62	-2.54	down	0.00	0.00	yes
TRINITY_DN15374_c1_g1	PREDICTED: truncated transcription factor CAULIFLOWER A-like isoform X2 [Populus euphratica]	AP1	Floral homeotic protein APETALA 1 OS=Arabidopsis thaliana GN=AP1 PE=1 SV=2	0.78	14.42	-3.60	down	0.00	0.00	yes
TRINITY_DN12181_c0_g1	hypothetical protein POPTR_0002s07700g [Populus trichocarpa]	-	-	0.32	10.42	-4.36	down	0.00	0.00	yes
TRINITY_DN14944_c0_g1	chitinase family protein [Populus trichocarpa]	-	Basic endochitinase OS=Nicotiana tabacum PE=2 SV=1	0.85	23.24	-4.22	down	0.00	0.00	yes
TRINITY_DN20409_c1_g4	VQ motif-containing family protein [Populus trichocarpa]	VQ9	VQ motif-containing protein 9 OS=Arabidopsis thaliana GN=VQ9 PE=1 SV=1	2.96	25.76	-2.53	down	0.00	0.00	yes
TRINITY_DN18662_c0_g1	unknown [Populus trichocarpa]	PUB26	U-box domain-containing protein 26 OS=Arabidopsis thaliana GN=PUB26 PE=2 SV=1	4.29	33.47	-2.44	down	0.00	0.00	yes
TRINITY_DN25652_c0_g2	hypothetical protein POPTR_0013s05520g [Populus trichocarpa]	-	-	4.69	30.88	-2.14	down	0.00	0.00	yes
TRINITY_DN20261_c0_g5	PREDICTED: protein PRD1 [Populus euphratica]	PRD1	Protein PRD1 OS=Arabidopsis thaliana GN=PRD1 PE=1 SV=3	0.84	8.43	-2.92	down	0.00	0.00	yes
TRINITY_DN23220_c1_g1	PREDICTED: AP2-like ethylene-responsive transcription factor ANT [Populus euphratica]	ANT	AP2-like ethylene-responsive transcription factor ANT OS=Arabidopsis thaliana GN=ANT PE=1 SV=2	0.59	13.50	-3.93	down	0.00	0.00	yes
TRINITY_DN24667_c0_g1	hypothetical protein POPTR_0019s08010g [Populus trichocarpa]	-	-	1.34	12.91	-2.64	down	0.00	0.00	yes
TRINITY_DN20173_c0_g1	PREDICTED: vegetative cell wall protein gp1-like [Populus euphratica]	-	-	0.00	15.81	-9.23	down	0.00	0.00	yes
TRINITY_DN21102_c0_g2	PREDICTED: uncharacterized protein LOC108468504 [Gossypium arboreum]	-	Histone H4 variant TH011 OS=Triticum aestivum PE=3 SV=2	18.47	131.87	-2.13	down	0.00	0.00	yes
TRINITY_DN26483_c0_g1	DECREASED DNA METHYLATION 1 family protein [Populus trichocarpa]	DDM1	ATP-dependent DNA helicase DDM1 OS=Arabidopsis thaliana GN=DDM1 PE=1 SV=1	6.28	37.32	-1.98	down	0.00	0.00	yes
TRINITY_DN15707_c0_g5	hypothetical protein POPTR_0125s00210g [Populus trichocarpa]	FER	Receptor-like protein kinase FERONIA OS=Arabidopsis thaliana GN=FER PE=1 SV=1	0.09	9.82	-4.70	down	0.00	0.00	yes
TRINITY_DN22582_c0_g1	calmodulin-binding family protein [Populus trichocarpa]	IQD1	Protein IQ-DOMAIN 1 OS=Arabidopsis thaliana GN=IQD1 PE=1 SV=1	3.22	20.97	-2.09	down	0.00	0.00	yes
TRINITY_DN14287_c0_g3	histone H2A family protein [Populus trichocarpa]	HIS2A	Histone H2AX OS=Cicer arietinum GN=HIS2A PE=2 SV=1	25.79	143.46	-1.84	down	0.00	0.00	yes
TRINITY_DN27620_c1_g2	PREDICTED: probable disease resistance protein At5g66900 [Populus euphratica]	At5g66900	Probable disease resistance protein At5g66900 OS=Arabidopsis thaliana GN=At5g66900 PE=3 SV=1	1.87	17.98	-2.53	down	0.00	0.00	yes
TRINITY_DN25792_c0_g1	PREDICTED: double-stranded RNA-binding protein 3 [Populus euphratica]	DRB3	Double-stranded RNA-binding protein 3 OS=Arabidopsis thaliana GN=DRB3 PE=1 SV=1	7.15	40.08	-1.88	down	0.00	0.00	yes

TRINITY_DN26705_c0_g1	PREDICTED: patellin-3-like [Populus euphratica]	PATL3	Patellin-3 OS=Arabidopsis thaliana GN=PATL3 PE=1 SV=2	9.13	60.60	-2.16	down	0.00	0.00	yes
TRINITY_DN20935_c0_g1	hypothetical protein POPTR_0016s00760g [Populus trichocarpa]	GT-2	Trihelix transcription factor GT-2 OS=Arabidopsis thaliana GN=GT-2 PE=2 SV=1	2.76	19.67	-2.23	down	0.00	0.00	yes
TRINITY_DN20753_c0_g3	hypothetical protein POPTR_0005s11890g [Populus trichocarpa]	-	-	0.84	22.14	-4.13	down	0.00	0.00	yes
TRINITY_DN21150_c0_g1	PREDICTED: histone H2AX-like [Populus euphratica]	At1g54690	Probable histone H2AXb OS=Arabidopsis thaliana GN=At1g54690 PE=1 SV=1	87.59	512.50	-1.92	down	0.00	0.00	yes
TRINITY_DN24577_c0_g2	pathogenesis-related thaumatin family protein [Populus trichocarpa]	TL1	Thaumatin-like protein 1 OS=Pyrus pyrifolia GN=TL1 PE=1 SV=1	1.47	15.01	-2.76	down	0.00	0.00	yes
TRINITY_DN20684_c0_g1	PREDICTED: chlorophyllase-1, chloroplastic-like [Populus euphratica]	CACLH	Chlorophyllase type 0 OS=Chenopodium album GN=CACLH PE=1 SV=1	0.12	5.70	-4.91	down	0.00	0.00	yes
TRINITY_DN25469_c0_g2	hypothetical protein POPTR_0012s05440g [Populus trichocarpa]	-	-	0.28	7.43	-4.14	down	0.00	0.00	yes
TRINITY_DN27893_c5_g1	hypothetical protein POPTR_0016s07860g [Populus trichocarpa]	-	-	0.02	2.92	-6.40	down	0.00	0.00	yes
TRINITY_DN25946_c0_g2	PREDICTED: chalcone synthase 1-like isoform X1 [Populus euphratica]	PKS5	Polyketide synthase 5 OS=Rubus idaeus GN=PKS5 PE=1 SV=1	3.16	68.59	-3.86	down	0.00	0.00	yes
TRINITY_DN23220_c0_g2	PREDICTED: AP2-like ethylene-responsive transcription factor AIL1 isoform X1 [Populus euphratica]	AIL1	AP2-like ethylene-responsive transcription factor AIL1 OS=Arabidopsis thaliana GN=AIL1 PE=2 SV=1	0.14	9.03	-5.79	down	0.00	0.00	yes
TRINITY_DN24812_c0_g2	hypothetical protein POPTR_0013s00520g, partial [Populus trichocarpa]	HASPIN	Serine/threonine-protein kinase haspin homolog OS=Arabidopsis thaliana GN=HASPIN PE=1 SV=1	0.35	4.23	-3.19	down	0.00	0.00	yes
TRINITY_DN23680_c0_g2	hypothetical protein POPTR_0011s12270g [Populus trichocarpa]	TSJT1	Stem-specific protein TSJT1 OS=Nicotiana tabacum GN=TSJT1 PE=2 SV=1	5.34	68.69	-3.10	down	0.00	0.00	yes
TRINITY_DN19717_c1_g1	hypothetical protein POPTR_0005s26610g [Populus trichocarpa]	Os01g0794400	Probable nucleoredoxin 2 OS=Oryza sativa subsp. japonica GN=Os01g0794400 PE=2 SV=1	8.44	75.38	-2.56	down	0.00	0.00	yes
TRINITY_DN26110_c0_g9	PREDICTED: probable RNA-dependent RNA polymerase 1 [Populus euphratica]	RDR1	RNA-dependent RNA polymerase 1 OS=Arabidopsis thaliana GN=RDR1 PE=2 SV=1	0.54	5.99	-2.86	down	0.00	0.00	yes
TRINITY_DN24288_c0_g1	hypothetical protein POPTR_0003s11810g [Populus trichocarpa]	CYP76B10	Geraniol 8-hydroxylase OS=Swertia mussotii GN=CYP76B10 PE=1 SV=1	2.78	11.84	-2.74	down	0.00	0.00	yes
TRINITY_DN21432_c1_g1	PREDICTED: fatty acid amide hydrolase-like [Populus euphratica]	FAAH	Fatty acid amide hydrolase OS=Arabidopsis thaliana GN=FAAH PE=1 SV=1	1.04	8.62	-3.30	down	0.00	0.00	yes
TRINITY_DN21102_c0_g3	PREDICTED: uncharacterized protein LOC105050582 [Elaeis guineensis]	-	Histone H4 variant TH011 OS=Triticum aestivum PE=3 SV=2	105.80	719.69	-2.19	down	0.00	0.00	yes
TRINITY_DN15329_c1_g1	-	-	-	0.00	4.44	-8.24	down	0.00	0.00	yes
TRINITY_DN16013_c0_g4	hypothetical protein POPTR_0005s25840g [Populus trichocarpa]	SAUR50	Auxin-responsive protein SAUR50 OS=Arabidopsis thaliana GN=SAUR50 PE=1 SV=1	0.57	9.43	-3.42	down	0.00	0.00	yes
TRINITY_DN24383_c0_g3	P-glycoprotein [Populus trichocarpa]	ABCB10	ABC transporter B family member 10 OS=Arabidopsis thaliana GN=ABCB10 PE=1 SV=2	0.26	9.12	-4.47	down	0.00	0.00	yes
TRINITY_DN19620_c1_g1	PREDICTED: histone H2B [Ricinus communis]	H2B-3	Histone H2B.3 (Fragment) OS=Solanum lycopersicum GN=H2B-3 PE=2 SV=1	2.12	17.97	-2.48	down	0.00	0.00	yes
TRINITY_DN20901_c0_g2	PREDICTED: uncharacterized protein LOC105136108 [Populus euphratica]	-	-	11.98	137.83	-2.91	down	0.00	0.00	yes
TRINITY_DN11544_c0_g1	PREDICTED: uncharacterized protein LOC107880749 isoform X1 [Prunus mume]	-	-	0.13	4.63	-4.56	down	0.00	0.00	yes
TRINITY_DN20300_c1_g1	unknown [Populus trichocarpa]	AAP7	Probable amino acid permease 7 OS=Arabidopsis thaliana GN=AAP7 PE=2 SV=1	1.14	14.61	-2.85	down	0.00	0.00	yes
TRINITY_DN26876_c0_g1	hypothetical protein POPTR_0008s15680g [Populus trichocarpa]	VAB	VAN3-binding protein OS=Arabidopsis thaliana GN=VAB PE=1 SV=1	7.34	51.48	-2.21	down	0.00	0.00	yes
TRINITY_DN24595_c2_g3	-	-	-	0.00	3.90	-7.82	down	0.00	0.00	yes
TRINITY_DN23925_c0_g3	UDP-glucuronic acid decarboxylase 2 [Populus tomentosa]	UXS4	UDP-glucuronic acid decarboxylase 4 OS=Arabidopsis thaliana GN=UXS4 PE=2 SV=1	5.90	48.05	-2.42	down	0.00	0.00	yes
TRINITY_DN25181_c0_g2	PREDICTED: uncharacterized protein LOC105130933 [Populus euphratica]	CDC7	Cell division cycle 7-related protein kinase OS=Homo sapiens GN=CDC7 PE=1 SV=1	2.13	14.77	-2.19	down	0.00	0.00	yes
TRINITY_DN22594_c0_g2	hypothetical protein POPTR_0004s14730g [Populus trichocarpa]	At5g67130	PI-PLC X domain-containing protein At5g67130 OS=Arabidopsis thaliana GN=At5g67130 PE=1 SV=1	0.30	6.46	-3.80	down	0.00	0.00	yes

TRINITY_DN24981_c0_g2	peroxidase precursor family protein [Populus trichocarpa]	PER12	Peroxidase 12 OS=Arabidopsis thaliana GN=PER12 PE=1 SV=1	28.66	164.68	-1.95	down	0.00	0.00	yes
TRINITY_DN27438_c0_g1	PREDICTED: mechanosensitive ion channel protein 10-like [Populus euphratica]	MSL4	Mechanosensitive ion channel protein 4 OS=Arabidopsis thaliana GN=MSL4 PE=3 SV=1	12.02	74.03	-2.57	down	0.00	0.00	yes
TRINITY_DN21186_c0_g2	hypothetical protein POPTR_0018s05530g [Populus trichocarpa]	C1	Anthocyanin regulatory C1 protein OS=Zea mays GN=C1 PE=2 SV=1	0.32	15.24	-5.40	down	0.00	0.00	yes
TRINITY_DN15585_c0_g2	hypothetical protein POPTR_0015s09940g [Populus trichocarpa]	At5g03795	Probable glycosyltransferase At5g03795 OS=Arabidopsis thaliana GN=At5g03795 PE=3 SV=2	0.79	7.28	-2.81	down	0.00	0.00	yes
TRINITY_DN21789_c0_g1	hypothetical protein POPTR_0015s00830g [Populus trichocarpa]	IQD14	Protein IQ-DOMAIN 14 OS=Arabidopsis thaliana GN=IQD14 PE=1 SV=1	0.46	8.65	-3.15	down	0.00	0.00	yes
TRINITY_DN27852_c3_g4	hypothetical protein POPTR_0016s03460g [Populus trichocarpa]	BHLH106	Transcription factor bHLH106 OS=Arabidopsis thaliana GN=BHLH106 PE=2 SV=1	1.62	13.96	-2.51	down	0.00	0.00	yes
TRINITY_DN22347_c1_g1	hypothetical protein POPTR_0013s00500g [Populus trichocarpa]	-	-	4.13	34.41	-2.47	down	0.00	0.00	yes
TRINITY_DN16116_c0_g1	PREDICTED: apoptosis 1 inhibitor-like [Populus euphratica]	-	-	0.21	7.19	-4.45	down	0.00	0.00	yes
TRINITY_DN24079_c0_g2	PREDICTED: uncharacterized protein LOC105121611 [Populus euphratica]	-	-	1.20	10.97	-2.49	down	0.00	0.00	yes
TRINITY_DN13584_c0_g1	PREDICTED: calcium-binding protein PBP1-like [Populus euphratica]	PBP1	Calcium-binding protein PBP1 OS=Arabidopsis thaliana GN=PBP1 PE=1 SV=1	0.06	6.65	-5.95	down	0.00	0.00	yes
TRINITY_DN23650_c0_g1	PREDICTED: phosphoglycerate mutase-like protein 1 isoform X1 [Populus euphratica]	At5g64460	Phosphoglycerate mutase-like protein 1 OS=Arabidopsis thaliana GN=At5g64460 PE=2 SV=1	4.20	40.69	-2.72	down	0.00	0.00	yes
TRINITY_DN18686_c0_g1	PREDICTED: inorganic phosphate transporter 1-4-like [Populus euphratica]	PHT1-4	Inorganic phosphate transporter 1-4 OS=Arabidopsis thaliana GN=PHT1-4 PE=1 SV=1	0.54	5.74	-2.80	down	0.00	0.00	yes
TRINITY_DN26403_c0_g1	hypothetical protein POPTR_0006s24310g [Populus trichocarpa]	IQD1	Protein IQ-DOMAIN 1 OS=Arabidopsis thaliana GN=IQD1 PE=1 SV=1	20.07	109.29	-2.11	down	0.00	0.00	yes
TRINITY_DN23192_c0_g1	hypothetical protein POPTR_0004s19720g [Populus trichocarpa]	AGC1-5	Serine/threonine-protein kinase AGC1-5 OS=Arabidopsis thaliana GN=AGC1-5 PE=1 SV=1	3.73	27.49	-2.27	down	0.00	0.00	yes
TRINITY_DN18360_c0_g1	PREDICTED: uncharacterized protein LOC105115115 [Populus euphratica]	-	-	0.37	7.90	-3.77	down	0.00	0.00	yes
TRINITY_DN25761_c1_g2	PREDICTED: probable receptor-like protein kinase At5g47070 [Populus euphratica]	PIX7	Probable serine/threonine-protein kinase PIX7 OS=Arabidopsis thaliana GN=PIX7 PE=1 SV=1	4.84	25.74	-1.95	down	0.00	0.00	yes
TRINITY_DN27848_c2_g1	GIGANTEA-like protein c [Populus alba x Populus glandulosa]	GI	Protein GIGANTEA OS=Arabidopsis thaliana GN=GI PE=1 SV=2	11.27	82.69	-2.24	down	0.00	0.00	yes
TRINITY_DN21792_c1_g5	hypothetical protein POPTR_0013s01200g [Populus trichocarpa]	CBP60B	Calmodulin-binding protein 60 B OS=Arabidopsis thaliana GN=CBP60B PE=2 SV=1	0.50	8.29	-3.40	down	0.00	0.00	yes
TRINITY_DN25927_c2_g1	RNA-directed RNA Polymerase family protein [Populus trichocarpa]	RDR1	RNA-dependent RNA polymerase 1 OS=Arabidopsis thaliana GN=RDR1 PE=2 SV=1	1.22	10.07	-2.45	down	0.00	0.00	yes
TRINITY_DN21494_c1_g3	hypothetical protein B456_008G236500 [Gossypium raimondii]	YAB3	Protein YABBY 3 OS=Oryza sativa subsp. japonica GN=YAB3 PE=2 SV=1	2.66	33.45	-3.04	down	0.00	0.00	yes
TRINITY_DN24683_c0_g1	hypothetical protein POPTR_0018s10830g [Populus trichocarpa]	CER3	Protein ECERIFERUM 3 OS=Arabidopsis thaliana GN=CER3 PE=1 SV=1	2.04	34.86	-3.53	down	0.00	0.00	yes
TRINITY_DN19362_c1_g4	PREDICTED: histone H3.2-like [Zea mays]	H3-1.1	Histone H3.2 OS=Medicago sativa GN=H3-1.1 PE=1 SV=2	92.37	503.02	-1.86	down	0.00	0.00	yes
TRINITY_DN22772_c0_g5	PREDICTED: probable auxin efflux carrier component 6 [Populus euphratica]	PIN6	Auxin efflux carrier component 6 OS=Arabidopsis thaliana GN=PIN6 PE=2 SV=2	0.49	9.37	-3.64	down	0.00	0.00	yes
TRINITY_DN24298_c0_g1	PREDICTED: uncharacterized protein LOC105121999 isoform X1 [Populus euphratica]	-	-	3.23	23.62	-2.12	down	0.00	0.00	yes
TRINITY_DN16939_c0_g3	hypothetical protein POPTR_0007s06570g [Populus trichocarpa]	CYP78A7	Cytochrome P450 78A7 OS=Arabidopsis thaliana GN=CYP78A7 PE=2 SV=1	0.04	2.84	-5.27	down	0.00	0.00	yes
TRINITY_DN21126_c0_g2	PREDICTED: probable polygalacturonase isoform X1 [Populus euphratica]	GSVIVT0002692	Probable polygalacturonase OS=Vitis vinifera GN=GSVIVT00026920001 PE=1 SV=1	14.88	112.78	-2.31	down	0.00	0.00	yes
TRINITY_DN17231_c0_g2	hypothetical protein POPTR_0391s00200g [Populus trichocarpa]	-	-	3.48	55.67	-3.49	down	0.00	0.00	yes
TRINITY_DN19830_c0_g1	hypothetical protein POPTR_0005s24920g [Populus trichocarpa]	-	-	1.06	11.79	-2.85	down	0.00	0.00	yes
TRINITY_DN21666_c0_g5	PREDICTED: axial regulator YABBY 1 [Populus euphratica]	YAB1	Axial regulator YABBY 1 OS=Arabidopsis thaliana GN=YAB1 PE=1 SV=1	9.90	76.21	-2.35	down	0.00	0.00	yes

TRINITY_DN17900_c1_g3	hypothetical protein POPTR_0009s15690g [Populus trichocarpa]	WAKL11	Putative wall-associated receptor kinase-like 11 OS=Arabidopsis thaliana GN=WAKL11 PE=3 SV=2	0.43	7.28	-3.63	down	0.00	0.00	yes
TRINITY_DN27118_c0_g1	PREDICTED: condensin-2 complex subunit H2-like [Populus euphratica]	CAPH2	Condensin-2 complex subunit H2 OS=Arabidopsis thaliana GN=CAPH2 PE=2 SV=1	5.97	27.77	-1.80	down	0.00	0.00	yes
TRINITY_DN22772_c0_g6	PIN1-like auxin transport protein [Populus tremula x Populus tremuloides]	PIN1C	Probable auxin efflux carrier component 1c OS=Oryza sativa subsp. japonica GN=PIN1C PE=2 SV=1	0.54	26.50	-4.05	down	0.00	0.00	yes
TRINITY_DN24348_c0_g3	PREDICTED: serine carboxypeptidase-like 34 [Populus euphratica]	SCPL34	Serine carboxypeptidase-like 34 OS=Arabidopsis thaliana GN=SCPL34 PE=2 SV=2	2.05	16.12	-2.36	down	0.00	0.00	yes
TRINITY_DN22196_c0_g1	class III peroxidase [Populus trichocarpa]	PER3	Peroxidase 3 OS=Arabidopsis thaliana GN=PER3 PE=2 SV=1	3.21	22.70	-2.23	down	0.00	0.00	yes
TRINITY_DN13843_c0_g1	PREDICTED: protein NRT1/ PTR FAMILY 7.2-like isoform X1 [Populus euphratica]	NPF7.3	Protein NRT1/ PTR FAMILY 7.3 OS=Arabidopsis thaliana GN=NPF7.3 PE=1 SV=2	0.02	2.82	-6.24	down	0.00	0.00	yes
TRINITY_DN15298_c0_g1	hypothetical protein POPTR_0012s05200g [Populus trichocarpa]	MYB108	Transcription factor MYB108 OS=Arabidopsis thaliana GN=MYB108 PE=1 SV=1	0.19	5.29	-4.15	down	0.00	0.00	yes
TRINITY_DN15908_c0_g2	hypothetical protein POPTR_0004s09630g [Populus trichocarpa]	LRK10L-2.8	LEAF RUST 10 DISEASE-RESISTANCE LOCUS RECEPTOR-LIKE PROTEIN KINASE-like 2.8 OS=Arabidopsis thaliana GN=LRK10L-2.8 PE=2 SV=2	0.00	2.55	-8.12	down	0.00	0.00	yes
TRINITY_DN26417_c0_g1	xyloglucan endotransglycosylase/hydrolase precursor XTH-36 [Populus tremula x Populus tremuloides]	XTH6	Probable xyloglucan endotransglucosylase/hydrolase protein 6 OS=Arabidopsis thaliana GN=XTH6 PE=2 SV=2	8.56	51.01	-1.97	down	0.00	0.00	yes
TRINITY_DN14567_c0_g1	GAST-like gene product family protein [Populus trichocarpa]	GASA1	Gibberellin-regulated protein 1 OS=Arabidopsis thaliana GN=GASA1 PE=2 SV=2	0.81	13.48	-3.42	down	0.00	0.00	yes
TRINITY_DN27358_c0_g2	PREDICTED: uncharacterized protein LOC105112197 isoform X1 [Populus euphratica]	-	-	5.69	42.49	-2.18	down	0.00	0.00	yes
TRINITY_DN23808_c0_g2	hypothetical protein POPTR_0012s00240g [Populus trichocarpa]	-	-	0.20	4.35	-4.84	down	0.00	0.00	yes
TRINITY_DN16682_c0_g1	ripening-related family protein [Populus trichocarpa]	-	21 kDa protein OS=Daucus carota PE=2 SV=1	4.32	32.23	-2.32	down	0.00	0.00	yes
TRINITY_DN19214_c0_g1	PREDICTED: transcription factor RAX2-like [Populus euphratica]	RAX3	Transcription factor RAX3 OS=Arabidopsis thaliana GN=RAX3 PE=2 SV=1	0.78	9.92	-2.89	down	0.00	0.00	yes
TRINITY_DN23721_c0_g1	hypothetical protein POPTR_0007s09810g [Populus trichocarpa]	Ncapg2	Condensin-2 complex subunit G2 OS=Mus musculus GN=Ncapg2 PE=1 SV=2	2.58	14.77	-1.93	down	0.00	0.00	yes
TRINITY_DN20040_c0_g2	PREDICTED: uncharacterized protein LOC105122113 isoform X2 [Populus euphratica]	-	-	19.01	134.72	-2.25	down	0.00	0.00	yes
TRINITY_DN19362_c1_g6	hypothetical protein CARUB_v10021660mg, partial [Capsella rubella]	HTR2	Histone H3.2 OS=Arabidopsis thaliana GN=HTR2 PE=1 SV=2	105.61	580.15	-1.87	down	0.00	0.00	yes
TRINITY_DN20173_c0_g5	PREDICTED: protein app1-like [Populus euphratica]	-	-	0.04	9.64	-7.07	down	0.00	0.00	yes
TRINITY_DN14561_c0_g1	benzodiazepine receptor-related family protein [Populus trichocarpa]	TSPO	Translocator protein homolog OS=Arabidopsis thaliana GN=TSPO PE=1 SV=1	0.24	7.48	-4.93	down	0.00	0.00	yes
TRINITY_DN17670_c1_g2	PREDICTED: secretory carrier-associated membrane protein 3 [Populus euphratica]	SCAMP1	Putative secretory carrier-associated membrane protein 1 OS=Oryza sativa subsp. indica GN=SCAMP1 PE=3 SV=1	6.24	32.97	-1.81	down	0.00	0.00	yes
TRINITY_DN15101_c0_g1	argonaute family protein [Populus trichocarpa]	AGO10	Protein argonaute 10 OS=Arabidopsis thaliana GN=AGO10 PE=2 SV=1	1.23	14.77	-2.97	down	0.00	0.00	yes
TRINITY_DN27578_c0_g1	PREDICTED: probable receptor-like protein kinase At5g24010 [Populus euphratica]	At5g24010	Probable receptor-like protein kinase At5g24010 OS=Arabidopsis thaliana GN=At5g24010 PE=1 SV=1	3.81	20.53	-1.72	down	0.00	0.00	yes
TRINITY_DN26044_c0_g1	PREDICTED: probable choline kinase 3 [Populus euphratica]	At4g09760	Probable choline kinase 3 OS=Arabidopsis thaliana GN=At4g09760 PE=2 SV=1	2.97	34.90	-2.99	down	0.00	0.00	yes
TRINITY_DN16785_c0_g1	hypothetical protein POPTR_0010s08910g [Populus trichocarpa]	At5g02940	Putative ion channel POLLUX-like 1 OS=Arabidopsis thaliana GN=At5g02940 PE=2 SV=1	6.95	59.09	-2.60	down	0.00	0.00	yes
TRINITY_DN24271_c0_g1	hypothetical protein POPTR_0002s06700g [Populus trichocarpa]	-	-	3.20	17.67	-1.89	down	0.00	0.00	yes
TRINITY_DN20467_c0_g2	PREDICTED: uncharacterized protein LOC105136876 [Populus euphratica]	-	-	1.36	12.67	-2.59	down	0.00	0.00	yes
TRINITY_DN14850_c0_g1	PREDICTED: uncharacterized protein LOC105125693 [Populus euphratica]	-	-	0.88	16.60	-3.59	down	0.00	0.00	yes
TRINITY_DN15872_c0_g1	hypothetical protein POPTR_0015s13410g [Populus trichocarpa]	-	-	0.46	11.67	-4.03	down	0.00	0.00	yes
TRINITY_DN21942_c0_g3	hypothetical protein POPTR_0001s40030g [Populus trichocarpa]	-	Pathogen-related protein OS=Hordeum vulgare PE=2 SV=2	3.70	26.78	-2.30	down	0.00	0.00	yes

TRINITY_DN23072_c0_g1	-	-	-	0.02	2.78	-5.84	down	0.00	0.00	yes
TRINITY_DN25895_c0_g1	leucine-rich repeat transmembrane protein kinase [Populus trichocarpa]	ZAR1	Receptor protein kinase-like protein ZAR1 OS=Arabidopsis thaliana GN=ZAR1 PE=1 SV=1	1.65	15.78	-2.61	down	0.00	0.00	yes
TRINITY_DN23017_c2_g2	hypothetical protein POPTR_0012s04520g [Populus trichocarpa]	At1g18250	Thaumatin-like protein OS=Arabidopsis thaliana GN=At1g18250 PE=2 SV=2	66.78	341.77	-1.78	down	0.00	0.00	yes
TRINITY_DN14755_c0_g1	PREDICTED: uncharacterized protein ECU03_1610-like [Populus euphratica]	-	-	0.17	5.94	-4.44	down	0.00	0.00	yes
TRINITY_DN23253_c0_g1	hypothetical protein POPTR_0005s02640g [Populus trichocarpa]	FH11	Formin-like protein 11 OS=Arabidopsis thaliana GN=FH11 PE=2 SV=1	1.29	11.05	-2.41	down	0.00	0.00	yes
TRINITY_DN26444_c0_g1	hypothetical protein POPTR_0017s00570g [Populus trichocarpa]	RPPL1	Putative disease resistance RPP13-like protein 1 OS=Arabidopsis thaliana GN=RPPL1 PE=3 SV=1	1.38	11.27	-2.84	down	0.00	0.00	yes
TRINITY_DN24607_c0_g1	hypothetical protein POPTR_0001s05450g [Populus trichocarpa]	HTR2	Histone H3.2 OS=Arabidopsis thaliana GN=HTR2 PE=1 SV=2	95.86	582.55	-1.93	down	0.00	0.00	yes
TRINITY_DN21665_c0_g1	hypothetical protein POPTR_0007s12700g [Populus trichocarpa]	-	-	0.31	8.90	-4.13	down	0.00	0.00	yes
TRINITY_DN25946_c0_g1	PREDICTED: chalcone synthase 1-like [Populus euphratica]	CHS1	Chalcone synthase 1 OS=Camellia sinensis GN=CHS1 PE=2 SV=1	17.29	205.36	-3.03	down	0.00	0.00	yes
TRINITY_DN19733_c0_g1	leucine-rich repeat transmembrane protein kinase [Populus trichocarpa]	At1g68400	Probable leucine-rich repeat receptor-like protein kinase At1g68400 OS=Arabidopsis thaliana GN=At1g68400 PE=2 SV=1	1.46	11.31	-2.33	down	0.00	0.00	yes
TRINITY_DN24282_c0_g2	PREDICTED: suppressor protein SRP40 isoform X1 [Populus euphratica]	-	-	4.18	23.46	-1.91	down	0.00	0.00	yes
TRINITY_DN23585_c0_g1	hypothetical protein POPTR_0010s20760g [Populus trichocarpa]	-	-	0.77	9.63	-3.03	down	0.00	0.00	yes
TRINITY_DN7105_c0_g1	PREDICTED: putative DNA-binding protein ESCAROLA [Populus euphratica]	AHL17	AT-hook motif nuclear-localized protein 17 OS=Arabidopsis thaliana GN=AHL17 PE=2 SV=1	0.00	3.67	-8.97	down	0.00	0.00	yes
TRINITY_DN21635_c1_g2	SEC14 cytosolic factor family protein [Populus trichocarpa]	-	-	5.54	32.41	-2.07	down	0.00	0.00	yes
TRINITY_DN19367_c0_g4	PREDICTED: probable calcium-binding protein CML35 [Populus euphratica]	CML35	Probable calcium-binding protein CML35 OS=Arabidopsis thaliana GN=CML35 PE=2 SV=2	13.38	87.54	-2.08	down	0.00	0.00	yes
TRINITY_DN20173_c0_g6	hypothetical protein POPTR_0017s07430g [Populus trichocarpa]	-	-	0.00	29.18	-8.36	down	0.00	0.00	yes
TRINITY_DN20375_c1_g1	PREDICTED: putative cyclin-D6-1 [Populus euphratica]	CYCD6-1	Putative cyclin-D6-1 OS=Arabidopsis thaliana GN=CYCD6-1 PE=3 SV=1	7.94	62.48	-2.46	down	0.00	0.00	yes
TRINITY_DN22899_c1_g1	PREDICTED: GATA transcription factor 5-like [Populus euphratica]	GATA5	GATA transcription factor 5 OS=Arabidopsis thaliana GN=GATA5 PE=2 SV=1	9.57	49.50	-1.79	down	0.00	0.00	yes
TRINITY_DN22126_c0_g1	PREDICTED: nematode resistance protein-like HSPRO2 [Populus euphratica]	HSPRO2	Nematode resistance protein-like HSPRO2 OS=Arabidopsis thaliana GN=HSPRO2 PE=1 SV=1	4.39	88.58	-3.72	down	0.00	0.00	yes
TRINITY_DN20943_c1_g1	hypothetical protein POPTR_0017s04350g [Populus trichocarpa]	ADR2	Disease resistance protein ADR2 OS=Arabidopsis thaliana GN=ADR2 PE=2 SV=1	0.90	16.53	-2.89	down	0.00	0.00	yes
TRINITY_DN26168_c0_g1	hypothetical protein POPTR_0017s03310g [Populus trichocarpa]	-	-	5.81	33.38	-1.91	down	0.00	0.00	yes
TRINITY_DN8020_c0_g1	hypothetical protein POPTR_0015s03360g [Populus trichocarpa]	LAR	Leucoanthocyanidin reductase OS=Desmodium uncinatum GN=LAR PE=1 SV=1	0.03	6.44	-7.14	down	0.00	0.00	yes
TRINITY_DN15796_c0_g1	PREDICTED: LRR receptor-like serine/threonine-protein kinase ERL1 [Populus euphratica]	ERL1	LRR receptor-like serine/threonine-protein kinase ERL1 OS=Arabidopsis thaliana GN=ERL1 PE=1 SV=1	2.77	36.86	-3.09	down	0.00	0.00	yes
TRINITY_DN26436_c0_g7	-	-	-	14.76	72.91	-1.74	down	0.00	0.00	yes
TRINITY_DN13515_c0_g2	hypothetical protein POPTR_0001s11960g [Populus trichocarpa]	-	-	0.06	4.64	-5.45	down	0.00	0.00	yes
TRINITY_DN21508_c0_g1	PREDICTED: mucin-2-like [Populus euphratica]	-	-	5.52	28.92	-1.83	down	0.00	0.00	yes
TRINITY_DN25496_c0_g2	PREDICTED: uncharacterized protein LOC105127785 isoform X2 [Populus euphratica]	-	-	0.84	8.53	-2.79	down	0.00	0.00	yes
TRINITY_DN27088_c0_g1	hypothetical protein POPTR_0004s09750g [Populus trichocarpa]	-	-	5.15	26.87	-1.80	down	0.00	0.00	yes
TRINITY_DN19626_c0_g2	hypothetical protein POPTR_0016s13500g [Populus trichocarpa]	-	-	5.67	39.97	-2.23	down	0.00	0.00	yes

TRINITY_DN14501_c0_g1	hypothetical protein POPTR_0003s01410g [Populus trichocarpa]	DDB_G0272012	Putative elongation of fatty acids protein DDB_G0272012 OS=Dictyostelium discoideum GN=DDB_G0272012 PE=3 SV=1	0.03	4.29	-6.23	down	0.00	0.00	yes
TRINITY_DN27893_c12_g1	PREDICTED: protein ECERIFERUM 26-like [Populus euphratica]	CER26	Protein ECERIFERUM 26 OS=Arabidopsis thaliana GN=CER26 PE=2 SV=1	0.08	4.60	-5.22	down	0.00	0.00	yes
TRINITY_DN26628_c0_g1	PREDICTED: uncharacterized protein LOC105122046 [Populus euphratica]	-	-	4.24	20.19	-1.74	down	0.00	0.00	yes
TRINITY_DN24599_c0_g1	hypothetical protein POPTR_0003s01420g [Populus trichocarpa]	AMAT	Methanol O-anthraniloyltransferase OS=Vitis labrusca GN=AMAT PE=1 SV=1	0.18	9.27	-5.32	down	0.00	0.00	yes
TRINITY_DN23056_c0_g1	hypothetical protein POPTR_0014s17370g [Populus trichocarpa]	ZIP4	TPR repeat-containing protein ZIP4 OS=Arabidopsis thaliana GN=ZIP4 PE=2 SV=1	0.71	6.03	-2.46	down	0.00	0.00	yes
TRINITY_DN24273_c0_g3	PREDICTED: uncharacterized protein LOC105137969 [Populus euphratica]	-	-	5.28	25.93	-1.66	down	0.00	0.00	yes
TRINITY_DN15215_c0_g1	hypothetical protein POPTR_0001s30500g [Populus trichocarpa]	SN1	Snakin-1 OS=Solanum tuberosum GN=SN1 PE=1 SV=1	0.04	25.16	-8.04	down	0.00	0.00	yes
TRINITY_DN18888_c0_g1	proline-rich family protein [Populus trichocarpa]	-	-	2.25	18.64	-2.50	down	0.00	0.00	yes
TRINITY_DN14849_c0_g1	xyloglucan endotransglucosylase/hydrolase protein 31 precursor [Populus trichocarpa]	XTH32	Probable xyloglucan endotransglucosylase/hydrolase protein 32 OS=Arabidopsis thaliana GN=XTH32 PE=2 SV=1	0.06	3.64	-5.24	down	0.00	0.00	yes
TRINITY_DN20283_c0_g3	PREDICTED: CASP-like protein 3A2, partial [Populus euphratica]	POPTRDRAFT_	CASP-like protein 3A2 OS=Populus trichocarpa GN=POPTRDRAFT_751837 PE=2 SV=2	2.19	26.55	-2.93	down	0.00	0.00	yes
TRINITY_DN17151_c0_g1	hypothetical protein POPTR_0003s11800g [Populus trichocarpa]	GRF12	Growth-regulating factor 12 OS=Oryza sativa subsp. japonica GN=GRF12 PE=2 SV=1	0.71	19.45	-4.15	down	0.00	0.00	yes
TRINITY_DN25049_c1_g1	heavy-metal-associated domain-containing family protein [Populus trichocarpa]	HIPP37	Heavy metal-associated isoprenylated plant protein 37 OS=Arabidopsis thaliana GN=HIPP37 PE=2 SV=1	33.77	174.23	-1.73	down	0.00	0.00	yes
TRINITY_DN25548_c1_g2	PREDICTED: MADS-box transcription factor 23-like isoform X1 [Populus euphratica]	AGL16	Agamous-like MADS-box protein AGL16 OS=Arabidopsis thaliana GN=AGL16 PE=1 SV=1	13.36	52.86	-1.84	down	0.00	0.00	yes
TRINITY_DN19437_c0_g4	hypothetical protein POPTR_0006s09470g [Populus trichocarpa]	-	-	0.12	3.18	-4.05	down	0.00	0.00	yes
TRINITY_DN18541_c0_g1	hypothetical protein POPTR_0016s14080g [Populus trichocarpa]	At2g40480	WEB family protein At2g40480 OS=Arabidopsis thaliana GN=At2g40480 PE=2 SV=1	7.89	44.35	-1.91	down	0.00	0.00	yes
TRINITY_DN21068_c0_g2	hypothetical protein POPTR_0004s13470g [Populus trichocarpa]	GALM	Aldose 1-epimerase OS=Bos taurus GN=GALM PE=2 SV=1	6.77	46.35	-2.13	down	0.00	0.00	yes
TRINITY_DN21951_c0_g3	Leucine-rich repeat protein kinase family protein isoform 1 [Theobroma cacao]	At5g67200	Probable inactive receptor kinase At5g67200 OS=Arabidopsis thaliana GN=At5g67200 PE=1 SV=1	0.57	10.80	-3.76	down	0.00	0.00	yes
TRINITY_DN23047_c0_g1	PREDICTED: EPIDERMAL PATTERNING FACTOR-like protein 1 [Populus euphratica]	EPFL1	EPIDERMAL PATTERNING FACTOR-like protein 1 OS=Arabidopsis thaliana GN=EPFL1 PE=1 SV=1	1.20	11.36	-2.67	down	0.00	0.00	yes
TRINITY_DN19620_c1_g3	PREDICTED: uncharacterized protein LOC106778661 [Vigna radiata var. radiata]	At5g02570	Histone H2B.9 OS=Arabidopsis thaliana GN=At5g02570 PE=1 SV=3	348.57	1649.64	-1.74	down	0.00	0.00	yes
TRINITY_DN17315_c0_g1	hypothetical protein POPTR_0001s33760g [Populus trichocarpa]	-	-	0.53	5.49	-2.74	down	0.00	0.00	yes
TRINITY_DN24626_c0_g1	unknown [Populus trichocarpa]	UGT85A23	7-deoxyloganetin glucosyltransferase OS=Catharanthus roseus GN=UGT85A23 PE=1 SV=1	3.88	27.85	-2.15	down	0.00	0.00	yes
TRINITY_DN17083_c0_g2	hypothetical protein POPTR_0009s05500g [Populus trichocarpa]	-	-	0.04	5.60	-6.32	down	0.00	0.00	yes
TRINITY_DN17604_c0_g3	hypothetical protein POPTR_0002s07480g [Populus trichocarpa]	-	-	0.11	4.12	-4.52	down	0.00	0.00	yes
TRINITY_DN25286_c1_g1	hypothetical protein POPTR_0005s25010g [Populus trichocarpa]	TULP5	Tubby-like F-box protein 5 OS=Oryza sativa subsp. japonica GN=TULP5 PE=2 SV=1	2.72	17.25	-2.06	down	0.00	0.00	yes
TRINITY_DN20358_c0_g2	hypothetical protein POPTR_0018s02140g [Populus trichocarpa]	-	-	7.75	60.69	-2.32	down	0.00	0.00	yes
TRINITY_DN20664_c0_g1	hypothetical protein POPTR_0015s09250g [Populus trichocarpa]	-	-	13.60	58.67	-1.64	down	0.00	0.00	yes
TRINITY_DN21247_c0_g1	leucine-rich repeat transmembrane protein kinase [Populus trichocarpa]	PXC1	Leucine-rich repeat receptor-like protein kinase PXC1 OS=Arabidopsis thaliana GN=PXC1 PE=1 SV=1	2.07	16.36	-2.37	down	0.00	0.00	yes
TRINITY_DN18129_c0_g4	PREDICTED: histone H3.2-like [Gossypium hirsutum]	HTR2	Histone H3.2 OS=Arabidopsis thaliana GN=HTR2 PE=1 SV=2	50.39	262.39	-1.79	down	0.00	0.00	yes
TRINITY_DN27093_c0_g1	hypothetical protein POPTR_0009s09590g [Populus trichocarpa]	GH3.1	Probable indole-3-acetic acid-amido synthetase GH3.1 OS=Arabidopsis thaliana GN=GH3.1 PE=2 SV=1	0.75	34.57	-4.88	down	0.00	0.00	yes

TRINITY_DN24462_c1_g1	PREDICTED: two-component response regulator-like APRR9 isoform X2 [Populus euphratica]	APRR5	Two-component response regulator-like APRR5 OS=Arabidopsis thaliana GN=APRR5 PE=1 SV=2	1.99	14.54	-2.20	down	0.00	0.00	yes
TRINITY_DN15439_c0_g1	hsr203J family protein [Populus trichocarpa]	CXE15	Probable carboxylesterase 15 OS=Arabidopsis thaliana GN=CXE15 PE=2 SV=1	0.18	4.70	-4.01	down	0.00	0.00	yes
TRINITY_DN23023_c0_g1	unknown [Populus trichocarpa]	-	Probable histone H2B.1 OS=Medicago truncatula PE=3 SV=3	72.20	358.36	-1.70	down	0.00	0.00	yes
TRINITY_DN27160_c0_g1	hypothetical protein POPTR_0017s13600g [Populus trichocarpa]	At1g04910	Uncharacterized protein At1g04910 OS=Arabidopsis thaliana GN=At1g04910 PE=2 SV=1	6.33	36.23	-1.91	down	0.00	0.00	yes
TRINITY_DN19604_c2_g3	hypothetical protein POPTR_0001s11920g [Populus trichocarpa]	-	-	0.09	7.66	-5.70	down	0.00	0.00	yes
TRINITY_DN17039_c0_g1	PREDICTED: uncharacterized protein At4g26485-like [Populus euphratica]	At4g26485	Uncharacterized protein At4g26485 OS=Arabidopsis thaliana GN=At4g26485 PE=4 SV=1	0.51	6.69	-3.00	down	0.00	0.00	yes
TRINITY_DN15930_c1_g6	hypothetical protein POPTR_0011s07020g [Populus trichocarpa]	-	-	1.25	14.83	-2.93	down	0.00	0.00	yes
TRINITY_DN19680_c0_g1	unknown [Populus trichocarpa x Populus deltoides]	-	-	1.13	16.16	-3.22	down	0.00	0.00	yes
TRINITY_DN22965_c0_g1	Ca(2+)-dependent DNase [Populus tomentosa]	CAN2	Staphylococcal-like nuclease CAN2 OS=Arabidopsis thaliana GN=CAN2 PE=1 SV=1	2.69	19.38	-2.27	down	0.00	0.00	yes
TRINITY_DN26254_c0_g1	hypothetical protein POPTR_0012s12820g [Populus trichocarpa]	At1g65710	Uncharacterized protein At1g65710 OS=Arabidopsis thaliana GN=At1g65710 PE=2 SV=1	10.70	57.52	-1.79	down	0.00	0.00	yes
TRINITY_DN21383_c0_g2	zinc finger family protein [Populus trichocarpa]	-	-	1.40	14.88	-2.81	down	0.00	0.00	yes
TRINITY_DN15580_c0_g2	hypothetical protein POPTR_0009s15930g [Populus trichocarpa]	LSH2	Protein LIGHT-DEPENDENT SHORT HYPOCOTYLS 2 OS=Arabidopsis thaliana GN=LSH2 PE=1 SV=1	0.06	3.33	-5.00	down	0.00	0.00	yes
TRINITY_DN15683_c0_g1	CBL-interacting protein kinase 4 [Populus trichocarpa]	CIPK4	CBL-interacting serine/threonine-protein kinase 4 OS=Arabidopsis thaliana GN=CIPK4 PE=1 SV=1	2.18	23.46	-2.85	down	0.00	0.00	yes
TRINITY_DN22730_c0_g1	hypothetical protein POPTR_0006s12390g [Populus trichocarpa]	At3g53190	Probable pectate lyase 12 OS=Arabidopsis thaliana GN=At3g53190 PE=2 SV=2	7.19	56.40	-2.58	down	0.00	0.00	yes
TRINITY_DN17699_c0_g6	PREDICTED: probable protein phosphatase 2C 6 isoform X2 [Populus euphratica]	Os01g0583100	Probable protein phosphatase 2C 6 OS=Oryza sativa subsp. japonica GN=Os01g0583100 PE=1 SV=1	0.13	6.72	-5.00	down	0.00	0.00	yes
TRINITY_DN19859_c0_g1	hydroxyproline-rich glycoprotein [Populus trichocarpa]	FRL4A	FRIGIDA-like protein 4a OS=Arabidopsis thaliana GN=FRL4A PE=2 SV=1	62.93	330.51	-1.80	down	0.00	0.00	yes
TRINITY_DN18107_c0_g2	hypothetical protein POPTR_0004s16850g [Populus trichocarpa]	SBT3.7	Subtilisin-like protease SBT3.7 OS=Arabidopsis thaliana GN=SBT3.7 PE=3 SV=2	0.19	3.98	-3.75	down	0.00	0.00	yes
TRINITY_DN19602_c0_g2	-	-	-	0.96	25.23	-4.05	down	0.00	0.00	yes
TRINITY_DN25815_c0_g5	-	-	-	1.38	21.25	-2.75	down	0.00	0.00	yes
TRINITY_DN20341_c0_g1	Zinc finger protein, putative [Theobroma cacao]	GLIP5	GDSL esterase/lipase 5 OS=Arabidopsis thaliana GN=GLIP5 PE=2 SV=2	4.28	22.90	-1.82	down	0.00	0.00	yes
TRINITY_DN22481_c1_g1	hypothetical protein POPTR_0403s00200g [Populus trichocarpa]	At3g47200	UPF0481 protein At3g47200 OS=Arabidopsis thaliana GN=At3g47200 PE=2 SV=1	6.57	62.04	-2.66	down	0.00	0.00	yes
TRINITY_DN13037_c0_g1	PREDICTED: MLO-like protein 3 [Populus euphratica]	MLO3	MLO-like protein 3 OS=Arabidopsis thaliana GN=MLO3 PE=2 SV=1	0.22	4.22	-3.67	down	0.00	0.00	yes
TRINITY_DN25415_c0_g5	hypothetical protein POPTR_0002s24050g [Populus trichocarpa]	-	-	0.07	2.84	-4.54	down	0.00	0.00	yes
TRINITY_DN21313_c0_g2	hypothetical protein POPTR_0014s07170g [Populus trichocarpa]	PUX9	Plant UBX domain-containing protein 9 OS=Arabidopsis thaliana GN=PUX9 PE=1 SV=1	4.63	24.85	-1.72	down	0.00	0.00	yes
TRINITY_DN26813_c0_g1	hypothetical protein POPTR_0012s09580g [Populus trichocarpa]	-	-	12.28	56.78	-1.79	down	0.00	0.00	yes
TRINITY_DN19597_c1_g5	hypothetical protein POPTR_0008s02440g [Populus trichocarpa]	IQM3	IQ domain-containing protein IQM3 OS=Arabidopsis thaliana GN=IQM3 PE=2 SV=1	0.14	6.88	-4.05	down	0.00	0.00	yes
TRINITY_DN22963_c0_g1	PREDICTED: UPF0481 protein At3g47200-like [Populus euphratica]	At3g47200	UPF0481 protein At3g47200 OS=Arabidopsis thaliana GN=At3g47200 PE=2 SV=1	8.80	45.37	-1.95	down	0.00	0.00	yes
TRINITY_DN17243_c0_g1	PREDICTED: uncharacterized protein LOC105127915 [Populus euphratica]	-	-	0.09	3.58	-4.64	down	0.00	0.00	yes
TRINITY_DN15307_c0_g1	PREDICTED: probable glycosyltransferase At5g11130 [Populus euphratica]	At5g20260	Probable glycosyltransferase At5g20260 OS=Arabidopsis thaliana GN=At5g20260 PE=3 SV=3	0.02	2.20	-5.58	down	0.00	0.00	yes
TRINITY_DN27190_c0_g2	hypothetical protein POPTR_0002s22130g [Populus trichocarpa]	TK	Thymidine kinase OS=Oryza sativa subsp. japonica GN=TK PE=2 SV=2	3.55	26.41	-2.22	down	0.00	0.00	yes

TRINITY_DN22699_c0_g1	hypothetical protein POPTR_0004s05910g [Populus trichocarpa]	-	-	0.37	10.91	-4.16	down	0.00	0.00	yes
TRINITY_DN20225_c0_g1	hypothetical protein POPTR_0001s28860g [Populus trichocarpa]	UGT87A1	UDP-glycosyltransferase 87A1 OS=Arabidopsis thaliana GN=UGT87A1 PE=2 SV=1	1.93	14.45	-2.28	down	0.00	0.00	yes
TRINITY_DN12553_c0_g1	hypothetical protein POPTR_0006s28740g [Populus trichocarpa]	UGT73B4	UDP-glycosyltransferase 73B4 OS=Arabidopsis thaliana GN=UGT73B4 PE=2 SV=1	0.00	2.79	-8.82	down	0.00	0.00	yes
TRINITY_DN24877_c0_g1	auxin influx carrier family protein [Populus trichocarpa]	LAX5	Auxin transporter-like protein 5 OS=Medicago truncatula GN=LAX5 PE=2 SV=1	8.03	48.65	-2.26	down	0.00	0.00	yes
TRINITY_DN21647_c0_g2	AP2 domain-containing transcription factor family protein [Populus trichocarpa]	RAP2-4	Ethylene-responsive transcription factor RAP2-4 OS=Arabidopsis thaliana GN=RAP2-4 PE=1 SV=1	10.47	110.53	-2.74	down	0.00	0.00	yes
TRINITY_DN18264_c0_g1	putative MYB transcription factor family protein [Populus trichocarpa]	MYB39	Transcription factor MYB39 OS=Arabidopsis thaliana GN=MYB39 PE=2 SV=1	0.37	5.85	-3.37	down	0.00	0.00	yes
TRINITY_DN21169_c0_g1	hypothetical protein POPTR_0001s25810g [Populus trichocarpa]	DDB2	DNA damage-binding protein 2 OS=Oryza sativa subsp. japonica GN=DDB2 PE=1 SV=1	1.00	8.71	-2.50	down	0.00	0.00	yes
TRINITY_DN15085_c0_g1	NBS-LRR resistance gene-like protein ARGH34 [Populus trichocarpa]	-	-	0.30	12.71	-4.79	down	0.00	0.00	yes
TRINITY_DN20240_c0_g1	hypothetical protein POPTR_0004s00900g [Populus trichocarpa]	-	Aspartic proteinase OS=Cucurbita pepo PE=2 SV=1	3.55	22.32	-2.06	down	0.00	0.00	yes
TRINITY_DN13906_c0_g1	hypothetical protein POPTR_0003s08480g [Populus trichocarpa]	-	Putative invertase inhibitor OS=Platanus acerifolia PE=1 SV=1	0.31	6.95	-3.85	down	0.00	0.00	yes
TRINITY_DN25823_c0_g1	hypothetical protein POPTR_0015s09830g [Populus trichocarpa]	SOBIR1	Leucine-rich repeat receptor-like serine/threonine/tyrosine-protein kinase SOBIR1 OS=Arabidopsis thaliana GN=SOBIR1 PE=1 SV=1	1.74	15.66	-2.56	down	0.00	0.00	yes
TRINITY_DN21426_c0_g1	PREDICTED: pto-interacting protein 1 isoform X2 [Populus euphratica]	PTI1	Pto-interacting protein 1 OS=Solanum lycopersicum GN=PTI1 PE=1 SV=2	1.06	10.54	-2.47	down	0.00	0.00	yes
TRINITY_DN24359_c0_g3	PREDICTED: protein POLYCHOME isoform X1 [Populus euphratica]	PYM	Protein POLYCHOME OS=Arabidopsis thaliana GN=PYM PE=1 SV=1	9.91	46.20	-1.63	down	0.00	0.00	yes
TRINITY_DN21823_c0_g3	PREDICTED: phosphate transporter PHO1 homolog 9 [Populus euphratica]	PHO1-H9	Phosphate transporter PHO1 homolog 9 OS=Arabidopsis thaliana GN=PHO1-H9 PE=2 SV=1	1.41	7.91	-1.88	down	0.00	0.00	yes
TRINITY_DN25440_c0_g1	PREDICTED: kinesin KP1-like isoform X1 [Populus euphratica]	KIN14F	Kinesin-like protein KIN-14F OS=Arabidopsis thaliana GN=KIN14F PE=1 SV=2	1.23	7.55	-2.01	down	0.00	0.00	yes
TRINITY_DN23225_c0_g2	hypothetical protein POPTR_0013s02680g [Populus trichocarpa]	BHLH94	Transcription factor bHLH94 OS=Arabidopsis thaliana GN=BHLH94 PE=2 SV=2	2.91	17.81	-1.96	down	0.00	0.00	yes
TRINITY_DN23937_c0_g3	PREDICTED: LOW QUALITY PROTEIN: glutamine synthetase cytosolic isozyme 2 [Populus euphratica]	GS1-2	Glutamine synthetase cytosolic isozyme 2 OS=Vitis vinifera GN=GS1-2 PE=2 SV=1	0.50	8.51	-3.47	down	0.00	0.00	yes
TRINITY_DN22490_c0_g1	hypothetical protein POPTR_0017s00570g [Populus trichocarpa]	RGA4	Putative disease resistance protein RGA4 OS=Solanum bulbocastanum GN=RGA4 PE=2 SV=1	3.99	29.64	-2.28	down	0.00	0.00	yes
TRINITY_DN23884_c1_g3	hypothetical protein POPTR_0010s13990g [Populus trichocarpa]	LAR	Leucoanthocyanidin reductase OS=Desmodium uncinatum GN=LAR PE=1 SV=1	1.23	28.18	-3.93	down	0.00	0.00	yes
TRINITY_DN17238_c0_g1	hypothetical protein POPTR_0005s13990g [Populus trichocarpa]	DOF3.4	Dof zinc finger protein DOF3.4 OS=Arabidopsis thaliana GN=DOF3.4 PE=1 SV=2	0.41	6.28	-3.34	down	0.00	0.00	yes
TRINITY_DN24495_c1_g1	hypothetical protein POPTR_0008s13190g [Populus trichocarpa]	-	21 kDa protein OS=Daucus carota PE=2 SV=1	8.58	56.96	-2.08	down	0.00	0.00	yes
TRINITY_DN17312_c0_g1	PREDICTED: transcription factor MYB44-like [Populus euphratica]	MYB44	Transcription factor MYB44 OS=Arabidopsis thaliana GN=MYB44 PE=2 SV=1	1.29	11.69	-2.59	down	0.00	0.00	yes
TRINITY_DN16439_c0_g1	PREDICTED: GDSL esterase/lipase At1g74460 [Populus euphratica]	At1g74460	GDSL esterase/lipase At1g74460 OS=Arabidopsis thaliana GN=At1g74460 PE=2 SV=1	0.06	7.74	-6.31	down	0.00	0.00	yes
TRINITY_DN14260_c0_g2	hypothetical protein POPTR_0006s00390g [Populus trichocarpa]	HIPP47	Heavy metal-associated isoprenylated plant protein 47 OS=Arabidopsis thaliana GN=HIPP47 PE=3 SV=1	0.04	5.38	-5.81	down	0.00	0.00	yes
TRINITY_DN15130_c0_g1	hypothetical protein POPTR_0002s23410g [Populus trichocarpa]	At1g05835	Uncharacterized protein At1g05835 OS=Arabidopsis thaliana GN=At1g05835 PE=2 SV=1	0.36	7.82	-3.82	down	0.00	0.00	yes
TRINITY_DN15459_c0_g1	fatty acid biosynthetic process transferase [Populus tomentosa]	KCS19	3-ketoacyl-CoA synthase 19 OS=Arabidopsis thaliana GN=KCS19 PE=2 SV=1	0.05	4.51	-5.82	down	0.00	0.00	yes
TRINITY_DN24595_c2_g4	-	-	-	17.88	82.56	-1.60	down	0.00	0.00	yes
TRINITY_DN18632_c0_g2	hypothetical protein POPTR_0014s13780g [Populus trichocarpa]	HSFA4B	Heat stress transcription factor A-4b OS=Oryza sativa subsp. japonica GN=HSFA4B PE=2 SV=1	0.94	7.21	-2.32	down	0.00	0.00	yes

TRINITY_DN22648_c0_g7	hypothetical protein POPTR_0012s05340g [Populus trichocarpa]	LBD41	LOB domain-containing protein 41 OS=Arabidopsis thaliana GN=LBD41 PE=2 SV=1	0.89	24.49	-4.20	down	0.00	0.00	yes
TRINITY_DN17060_c0_g1	nitrate transporter family protein [Populus trichocarpa]	NPF4.6	Protein NRT1/ PTR FAMILY 4.6 OS=Arabidopsis thaliana GN=NPF4.6 PE=1 SV=1	0.41	4.82	-2.98	down	0.00	0.00	yes
TRINITY_DN13000_c0_g1	hypothetical protein POPTR_0010s09530g [Populus trichocarpa]	-	-	0.03	5.27	-6.68	down	0.00	0.00	yes
TRINITY_DN25559_c0_g1	hypothetical protein POPTR_0007s04520g, partial [Populus trichocarpa]	AIR3	Subtilisin-like protease SBT5.3 OS=Arabidopsis thaliana GN=AIR3 PE=2 SV=1	2.90	22.44	-2.64	down	0.00	0.00	yes
TRINITY_DN25675_c0_g3	hypothetical protein POPTR_0008s02900g [Populus trichocarpa]	At5g04500	Glycosyltransferase family protein 64 protein C5 OS=Arabidopsis thaliana GN=At5g04500 PE=2 SV=1	1.18	12.77	-2.90	down	0.00	0.00	yes
TRINITY_DN14836_c0_g1	hypothetical protein POPTR_0013s03070g [Populus trichocarpa]	EMB3004	Bifunctional 3-dehydroquinate dehydratase/shikimate dehydrogenase, chloroplastic OS=Arabidopsis thaliana GN=EMB3004 PE=1 SV=1	1.13	12.60	-2.77	down	0.00	0.00	yes
TRINITY_DN25361_c2_g2	hypothetical protein POPTR_0019s13890g [Populus trichocarpa]	PVA42	Vesicle-associated protein 4-2 OS=Arabidopsis thaliana GN=PVA42 PE=1 SV=1	9.02	48.58	-1.83	down	0.00	0.00	yes
TRINITY_DN21991_c0_g3	hypothetical protein POPTR_0002s11320g [Populus trichocarpa]	-	-	7.10	30.96	-1.57	down	0.00	0.00	yes
TRINITY_DN19230_c0_g1	PREDICTED: ABC transporter G family member 15-like [Populus euphratica]	ABCG15	ABC transporter G family member 15 OS=Arabidopsis thaliana GN=ABCG15 PE=2 SV=2	0.57	12.52	-3.88	down	0.00	0.00	yes
TRINITY_DN6170_c0_g1	myb family transcription factor family protein [Populus trichocarpa]	RL5	Protein RADIALIS-like 5 OS=Arabidopsis thaliana GN=RL5 PE=3 SV=1	0.00	4.20	-7.86	down	0.00	0.00	yes
TRINITY_DN23440_c0_g3	hypothetical protein POPTR_0018s01210g [Populus trichocarpa]	YUC4	Probable indole-3-pyruvate monooxygenase YUCCA4 OS=Arabidopsis thaliana GN=YUC4 PE=1 SV=1	0.25	3.88	-3.46	down	0.00	0.00	yes
TRINITY_DN22309_c0_g1	PREDICTED: uncharacterized protein LOC105122564 isoform X1 [Populus euphratica]	SMXL5	Protein SMAX1-LIKE 5 OS=Arabidopsis thaliana GN=SMXL5 PE=2 SV=1	2.83	24.98	-2.39	down	0.00	0.00	yes
TRINITY_DN21694_c0_g2	putative wall-associated kinase family protein [Populus trichocarpa]	WAK4	Wall-associated receptor kinase 4 OS=Arabidopsis thaliana GN=WAK4 PE=2 SV=1	0.18	3.31	-3.53	down	0.00	0.00	yes
TRINITY_DN15472_c0_g1	PREDICTED: homeobox-leucine zipper protein HOX3-like isoform X1 [Populus euphratica]	HOX3	Homeobox-leucine zipper protein HOX3 OS=Oryza sativa subsp. indica GN=HOX3 PE=1 SV=1	0.25	5.55	-3.80	down	0.00	0.00	yes
TRINITY_DN23416_c0_g2	hypothetical protein POPTR_0004s17890g [Populus trichocarpa]	At1g75040	Pathogenesis-related protein 5 OS=Arabidopsis thaliana GN=At1g75040 PE=1 SV=1	16.13	111.83	-2.11	down	0.00	0.00	yes
TRINITY_DN24369_c0_g2	PREDICTED: transcription factor MYB44-like [Populus euphratica]	MYB44	Transcription factor MYB44 OS=Arabidopsis thaliana GN=MYB44 PE=2 SV=1	12.62	87.60	-2.19	down	0.00	0.00	yes
TRINITY_DN11284_c0_g1	hypothetical protein POPTR_0019s13880g [Populus trichocarpa]	-	-	130.42	709.40	-1.84	down	0.00	0.00	yes
TRINITY_DN27033_c2_g2	hypothetical protein POPTR_0016s06760g [Populus trichocarpa]	YAB2	Putative axial regulator YABBY 2 OS=Arabidopsis thaliana GN=YAB2 PE=1 SV=1	5.90	42.22	-2.23	down	0.00	0.00	yes
TRINITY_DN17238_c0_g2	PREDICTED: LOW QUALITY PROTEIN: dof zinc finger protein DOF3.4 [Populus euphratica]	DOF3.4	Dof zinc finger protein DOF3.4 OS=Arabidopsis thaliana GN=DOF3.4 PE=1 SV=2	1.11	26.07	-3.97	down	0.00	0.00	yes
TRINITY_DN16678_c0_g1	hypothetical protein POPTR_0001s13440g [Populus trichocarpa]	GRXC3	Glutaredoxin-C3 OS=Oryza sativa subsp. japonica GN=GRXC3 PE=2 SV=1	0.16	10.55	-5.24	down	0.00	0.00	yes
TRINITY_DN27159_c0_g4	hypothetical protein POPTR_0017s12990g [Populus trichocarpa]	RPK2	LRR receptor-like serine/threonine-protein kinase RPK2 OS=Arabidopsis thaliana GN=RPK2 PE=2 SV=1	7.16	34.38	-1.65	down	0.00	0.00	yes
TRINITY_DN26010_c0_g3	hypothetical protein POPTR_0006s13130g [Populus trichocarpa]	JKD	Zinc finger protein JACKDAW OS=Arabidopsis thaliana GN=JKD PE=1 SV=1	6.52	33.86	-1.80	down	0.00	0.00	yes
TRINITY_DN23091_c0_g2	glutamine synthetase family protein [Populus trichocarpa]	-	Glutamine synthetase nodule isozyme OS=Vigna aconitifolia PE=2 SV=1	10.71	69.67	-2.11	down	0.00	0.00	yes
TRINITY_DN20264_c2_g2	band 7 family protein [Populus trichocarpa]	HIR4	Hypersensitive-induced response protein 4 OS=Arabidopsis thaliana GN=HIR4 PE=1 SV=1	3.18	17.21	-2.33	down	0.00	0.00	yes
TRINITY_DN22714_c0_g1	PREDICTED: growth-regulating factor 1-like isoform X3 [Populus euphratica]	GRF6	Growth-regulating factor 6 OS=Oryza sativa subsp. japonica GN=GRF6 PE=2 SV=2	9.65	79.56	-2.42	down	0.00	0.00	yes
TRINITY_DN27063_c0_g2	unknown [Populus trichocarpa]	AIL5	AP2-like ethylene-responsive transcription factor AIL5 OS=Arabidopsis thaliana GN=AIL5 PE=2 SV=2	1.65	26.20	-3.37	down	0.00	0.00	yes
TRINITY_DN24846_c0_g2	PREDICTED: uncharacterized protein At5g41620-like isoform X2 [Populus euphratica]	-	-	4.78	17.86	-1.83	down	0.00	0.00	yes
TRINITY_DN22874_c0_g1	PREDICTED: probable transcription factor KAN4 [Populus euphratica]	KAN4	Probable transcription factor KAN4 OS=Arabidopsis thaliana GN=KAN4 PE=1 SV=1	2.23	17.71	-2.09	down	0.00	0.00	yes

TRINITY_DN22403_c0_g1	hypothetical protein POPTR_0001s03040g [Populus trichocarpa]	DRP1E	Dynamin-related protein 1E OS=Arabidopsis thaliana GN=DRP1E PE=1 SV=1	5.05	25.66	-1.76	down	0.00	0.00	yes
TRINITY_DN19584_c0_g2	-	-	-	0.43	12.90	-4.30	down	0.00	0.00	yes
TRINITY_DN20524_c0_g2	bZIP with a Ring-finger motif family protein [Populus trichocarpa]	HY5	Transcription factor HY5 OS=Arabidopsis thaliana GN=HY5 PE=1 SV=1	0.90	15.43	-3.47	down	0.00	0.00	yes
TRINITY_DN23611_c1_g2	PREDICTED: putative pentatricopeptide repeat-containing protein At3g16890, mitochondrial [Citrus sinensis]	-	-	1.04	20.36	-3.54	down	0.00	0.00	yes
TRINITY_DN22321_c0_g1	PREDICTED: uncharacterized protein LOC105119307 isoform X2 [Populus euphratica]	IQM4	IQ domain-containing protein IQM4 OS=Arabidopsis thaliana GN=IQM4 PE=2 SV=1	5.08	32.17	-1.69	down	0.00	0.00	yes
TRINITY_DN15131_c0_g2	putative pectin methylesterase LuPME1 family protein [Populus trichocarpa]	PECS-2.1	Pectinesterase 2 OS=Citrus sinensis GN=PECS-2.1 PE=2 SV=1	0.02	2.96	-6.08	down	0.00	0.00	yes
TRINITY_DN19337_c0_g1	hypothetical protein POPTR_0017s14220g [Populus trichocarpa]	trpB2	Tryptophan synthase beta chain 2 OS=Aquifex aeolicus (strain VF5) GN=trpB2 PE=3 SV=1	2.64	16.29	-2.03	down	0.00	0.00	yes
TRINITY_DN26182_c0_g2	hypothetical protein POPTR_0003s06330g [Populus trichocarpa]	AIL1	AP2-like ethylene-responsive transcription factor AIL1 OS=Arabidopsis thaliana GN=AIL1 PE=2 SV=1	0.10	4.04	-4.64	down	0.00	0.00	yes
TRINITY_DN25730_c0_g1	PREDICTED: probable serine/threonine-protein kinase WNK9 isoform X1 [Populus euphratica]	WNK1	Probable serine/threonine-protein kinase WNK1 OS=Oryza sativa subsp. japonica GN=WNK1 PE=2 SV=1	18.60	91.25	-1.75	down	0.00	0.00	yes
TRINITY_DN20627_c0_g2	hypothetical protein POPTR_0001s08700g [Populus trichocarpa]	-	-	1.18	12.84	-2.95	down	0.00	0.00	yes
TRINITY_DN25661_c0_g2	1-phosphatidylinositol-4-phosphate 5-kinase, putative isoform 2 [Theobroma cacao]	-	-	0.72	7.62	-2.80	down	0.00	0.00	yes
TRINITY_DN27862_c2_g1	PREDICTED: structural maintenance of chromosomes protein 2-1-like [Populus euphratica]	SMC2-1	Structural maintenance of chromosomes protein 2-1 OS=Arabidopsis thaliana GN=SMC2-1 PE=2 SV=2	21.02	56.92	-1.69	down	0.00	0.00	yes
TRINITY_DN24282_c0_g1	hypothetical protein POPTR_0016s08360g [Populus trichocarpa]	-	-	1.63	14.16	-2.47	down	0.00	0.00	yes
TRINITY_DN19709_c2_g2	seed maturation protein PM22 [Populus trichocarpa]	LEA14-A	Late embryogenesis abundant protein Lea14-A OS=Gossypium hirsutum GN=LEA14-A PE=2 SV=1	7.99	108.45	-3.19	down	0.00	0.00	yes
TRINITY_DN19768_c0_g2	calmodulin-like gene family protein [Populus trichocarpa]	CML41	Probable calcium-binding protein CML41 OS=Arabidopsis thaliana GN=CML41 PE=2 SV=2	0.49	8.81	-3.50	down	0.00	0.00	yes
TRINITY_DN20358_c0_g3	serine-rich family protein [Populus trichocarpa]	-	-	9.42	60.73	-2.04	down	0.00	0.00	yes
TRINITY_DN23279_c0_g3	-	-	-	0.00	4.80	-7.48	down	0.00	0.00	yes
TRINITY_DN16362_c0_g1	L-asparaginase family protein [Populus trichocarpa]	At3g16150	Probable isoaspartyl peptidase/L-asparaginase 2 OS=Arabidopsis thaliana GN=At3g16150 PE=2 SV=2	37.08	202.34	-1.87	down	0.00	0.00	yes
TRINITY_DN19107_c0_g1	WRKY transcription factor 26 [(Populus tomentosa x Populus bolleana) x Populus tomentosa]	WRKY7	Probable WRKY transcription factor 7 OS=Arabidopsis thaliana GN=WRKY7 PE=2 SV=1	5.42	32.83	-1.99	down	0.00	0.00	yes
TRINITY_DN19314_c1_g4	WRKY32 [(Populus tomentosa x Populus bolleana) x Populus tomentosa]	WRKY49	Probable WRKY transcription factor 49 OS=Arabidopsis thaliana GN=WRKY49 PE=2 SV=1	0.49	5.63	-2.91	down	0.00	0.00	yes
TRINITY_DN22303_c0_g3	hypothetical protein POPTR_0006s14110g [Populus trichocarpa]	ERF016	Ethylene-responsive transcription factor ERF016 OS=Arabidopsis thaliana GN=ERF016 PE=2 SV=1	4.36	73.94	-3.44	down	0.00	0.00	yes
TRINITY_DN18651_c2_g1	PREDICTED: uncharacterized protein LOC105121794 [Populus euphratica]	-	-	2.79	20.89	-2.48	down	0.00	0.00	yes
TRINITY_DN24274_c0_g1	PREDICTED: protein WVD2-like 1 [Populus euphratica]	WVD2	Protein WAVE-DAMPENED 2 OS=Arabidopsis thaliana GN=WVD2 PE=2 SV=1	17.53	85.05	-1.67	down	0.00	0.00	yes
TRINITY_DN15120_c0_g2	hypothetical protein POPTR_0013s12390g [Populus trichocarpa]	KCS19	3-ketoacyl-CoA synthase 19 OS=Arabidopsis thaliana GN=KCS19 PE=2 SV=1	0.14	3.54	-4.03	down	0.00	0.00	yes
TRINITY_DN24365_c0_g2	zinc finger family protein [Populus trichocarpa]	-	-	3.69	29.69	-2.39	down	0.00	0.00	yes
TRINITY_DN18956_c0_g3	PREDICTED: uncharacterized protein LOC105124874 isoform X2 [Populus euphratica]	-	-	1.66	11.93	-2.23	down	0.00	0.00	yes
TRINITY_DN25707_c0_g3	hypothetical protein POPTR_0006s27540g [Populus trichocarpa]	-	-	17.71	174.14	-2.72	down	0.00	0.00	yes
TRINITY_DN18564_c0_g1	hypothetical protein POPTR_0002s11840g [Populus trichocarpa]	-	-	0.67	11.32	-3.35	down	0.00	0.00	yes
TRINITY_DN24219_c0_g1	hypothetical protein POPTR_0007s04780g [Populus trichocarpa]	NAC081	Protein ATAF2 OS=Arabidopsis thaliana GN=NAC081 PE=1 SV=1	13.17	161.47	-2.98	down	0.00	0.00	yes

TRINITY_DN23787_c0_g2	hypothetical protein POPTR_0019s13960g, partial [Populus trichocarpa]	-	-	6.00	106.47	-3.59	down	0.00	0.00	yes
TRINITY_DN24812_c0_g1	PREDICTED: serine/threonine-protein kinase haspin [Populus euphratica]	HASPIN	Serine/threonine-protein kinase haspin homolog OS=Arabidopsis thaliana GN=HASPIN PE=1 SV=1	2.93	15.40	-1.65	down	0.00	0.00	yes
TRINITY_DN26234_c1_g1	hypothetical protein POPTR_0005s19220g [Populus trichocarpa]	ANT	AP2-like ethylene-responsive transcription factor ANT OS=Arabidopsis thaliana GN=ANT PE=1 SV=2	3.39	33.40	-2.62	down	0.00	0.00	yes
TRINITY_DN22784_c0_g1	lysine and histidine specific transporter family protein [Populus trichocarpa]	LHT1	Lysine histidine transporter 1 OS=Arabidopsis thaliana GN=LHT1 PE=1 SV=1	5.03	42.63	-2.39	down	0.00	0.00	yes
TRINITY_DN26434_c0_g1	disease resistance family protein [Populus trichocarpa]	At4g33300	Probable disease resistance protein At4g33300 OS=Arabidopsis thaliana GN=At4g33300 PE=2 SV=3	9.97	73.92	-2.22	down	0.00	0.00	yes
TRINITY_DN9274_c0_g1	hypothetical protein POPTR_0007s03370g [Populus trichocarpa]	-	-	0.09	14.81	-6.57	down	0.00	0.00	yes
TRINITY_DN26860_c0_g1	PREDICTED: uncharacterized protein LOC105121825 isoform X1 [Populus euphratica]	brd9	Bromodomain-containing protein 9 OS=Xenopus tropicalis GN=brd9 PE=2 SV=1	2.95	14.28	-1.78	down	0.00	0.00	yes
TRINITY_DN17422_c0_g1	PREDICTED: protein TOO MANY MOUTHS-like [Populus euphratica]	PII-2	Piriformospora indica-insensitive protein 2 OS=Arabidopsis thaliana GN=PII-2 PE=2 SV=1	0.12	3.62	-4.16	down	0.00	0.00	yes
TRINITY_DN16972_c0_g1	hypothetical protein POPTR_0017s09250g [Populus trichocarpa]	CRK29	Cysteine-rich receptor-like protein kinase 29 OS=Arabidopsis thaliana GN=CRK29 PE=2 SV=1	3.90	24.20	-2.15	down	0.00	0.00	yes
TRINITY_DN23624_c0_g1	hypothetical protein POPTR_0011s12520g [Populus trichocarpa]	-	-	8.17	37.79	-1.64	down	0.00	0.00	yes
TRINITY_DN16405_c1_g1	hypothetical protein POPTR_0002s15520g [Populus trichocarpa]	-	-	0.26	4.02	-3.38	down	0.00	0.00	yes
TRINITY_DN20635_c0_g1	hypothetical protein POPTR_0002s12750g [Populus trichocarpa]	-	-	0.86	10.00	-2.83	down	0.00	0.00	yes
TRINITY_DN22357_c0_g1	hypothetical protein POPTR_0018s01390g [Populus trichocarpa]	-	-	21.41	104.01	-1.63	down	0.00	0.00	yes
TRINITY_DN23547_c0_g3	PREDICTED: zinc-finger homeodomain protein 5-like [Populus euphratica]	ZHD6	Zinc-finger homeodomain protein 6 OS=Arabidopsis thaliana GN=ZHD6 PE=1 SV=1	18.31	80.58	-1.54	down	0.00	0.00	yes
TRINITY_DN23688_c0_g2	hypothetical protein POPTR_0005s19000g, partial [Populus trichocarpa]	-	-	0.10	8.03	-5.17	down	0.00	0.00	yes
TRINITY_DN18498_c0_g2	hypothetical protein POPTR_0002s08440g [Populus trichocarpa]	-	-	8.09	48.39	-1.92	down	0.00	0.00	yes
TRINITY_DN22800_c0_g2	DegP protease family protein [Populus trichocarpa]	DEGP7	Protease Do-like 7 OS=Arabidopsis thaliana GN=DEGP7 PE=2 SV=1	0.36	4.20	-2.91	down	0.00	0.00	yes
TRINITY_DN15356_c0_g1	hypothetical protein POPTR_0018s08320g [Populus trichocarpa]	ERF017	Ethylene-responsive transcription factor ERF017 OS=Arabidopsis thaliana GN=ERF017 PE=2 SV=1	0.69	30.09	-4.82	down	0.00	0.00	yes
TRINITY_DN23511_c0_g2	NO POLLEN GERMINATION RELATED 1 family protein [Populus trichocarpa]	-	-	3.50	19.12	-1.85	down	0.00	0.00	yes
TRINITY_DN28981_c0_g1	PREDICTED: EPIDERMAL PATTERNING FACTOR-like protein 2 isoform X1 [Populus euphratica]	EPFL2	EPIDERMAL PATTERNING FACTOR-like protein 2 OS=Arabidopsis thaliana GN=EPFL2 PE=2 SV=1	0.63	12.72	-3.68	down	0.00	0.00	yes
TRINITY_DN17161_c0_g1	hypothetical protein POPTR_0013s15140g [Populus trichocarpa]	-	-	0.26	3.69	-3.18	down	0.00	0.00	yes
TRINITY_DN22722_c0_g1	hypothetical protein POPTR_0001s36260g [Populus trichocarpa]	WDL7	Protein WVD2-like 7 OS=Arabidopsis thaliana GN=WDL7 PE=2 SV=1	17.79	97.65	-1.88	down	0.00	0.00	yes
TRINITY_DN25451_c1_g2	PREDICTED: polygalacturonase QRT3-like [Populus euphratica]	QRT3	Polygalacturonase QRT3 OS=Arabidopsis thaliana GN=QRT3 PE=2 SV=1	0.15	3.07	-3.69	down	0.00	0.00	yes
TRINITY_DN24248_c0_g1	PREDICTED: uncharacterized protein LOC105122981 isoform X2 [Populus euphratica]	-	-	1.23	11.99	-2.67	down	0.00	0.00	yes
TRINITY_DN20557_c0_g1	hypothetical protein POPTR_0013s14190g [Populus trichocarpa]	PTO1242	Glutamate formimidoyltransferase OS=Picrophilus torridus (strain ATCC 700027 / DSM 9790 / JCM 10055 / NBRC 100828) GN=PTO1242 PE=1 SV=1	10.37	93.18	-2.43	down	0.00	0.00	yes
TRINITY_DN22118_c0_g1	hypothetical protein POPTR_0008s09920g [Populus trichocarpa]	-	-	3.71	22.58	-2.06	down	0.00	0.00	yes
TRINITY_DN27113_c0_g1	PREDICTED: cyclic nucleotide-gated ion channel 1-like [Populus euphratica]	CNGC9	Putative cyclic nucleotide-gated ion channel 9 OS=Arabidopsis thaliana GN=CNGC9 PE=2 SV=1	13.21	70.01	-1.83	down	0.00	0.00	yes

TRINITY_DN26642_c0_g1	peroxisomal membrane family protein [Populus trichocarpa]	At4g14310	KIN14B-interacting protein At4g14310 OS=Arabidopsis thaliana GN=At4g14310 PE=1 SV=1	6.45	31.84	-1.73	down	0.00	0.00	yes
TRINITY_DN20030_c0_g1	hypothetical protein POPTR_0004s09040g [Populus trichocarpa]	AATP1	AAA-ATPase ASD, mitochondrial OS=Arabidopsis thaliana GN=AATP1 PE=1 SV=1	3.12	18.15	-1.92	down	0.00	0.00	yes
TRINITY_DN26990_c0_g1	hypothetical protein POPTR_0004s17600g [Populus trichocarpa]	-	-	0.21	3.09	-3.29	down	0.00	0.00	yes
TRINITY_DN20750_c0_g2	putative leucine-rich repeat transmembrane protein kinase [Populus trichocarpa]	MIK2	MDIS1-interacting receptor like kinase 2 OS=Arabidopsis thaliana GN=MIK2 PE=1 SV=3	0.41	8.59	-3.07	down	0.00	0.00	yes
TRINITY_DN25150_c0_g3	hypothetical protein POPTR_0011s11370g [Populus trichocarpa]	NCED3	9-cis-epoxycarotenoid dioxygenase NCED3, chloroplastic OS=Arabidopsis thaliana GN=NCED3 PE=2 SV=1	4.47	76.88	-3.45	down	0.00	0.00	yes
TRINITY_DN26038_c0_g1	hypothetical protein POPTR_0013s00990g [Populus trichocarpa]	-	-	8.66	50.41	-1.94	down	0.00	0.00	yes
TRINITY_DN23984_c0_g2	F-box family protein [Populus trichocarpa]	FBL17	F-box/LRR-repeat protein 17 OS=Arabidopsis thaliana GN=FBL17 PE=1 SV=1	1.41	13.70	-2.64	down	0.00	0.00	yes
TRINITY_DN16399_c1_g1	hypothetical protein POPTR_0006s25610g [Populus trichocarpa]	JGB	Protein JINGUBANG OS=Arabidopsis thaliana GN=JGB PE=1 SV=1	0.28	5.09	-3.61	down	0.00	0.00	yes
TRINITY_DN25675_c0_g2	hypothetical protein POPTR_0010s23900g [Populus trichocarpa]	At5g04500	Glycosyltransferase family protein 64 protein C5 OS=Arabidopsis thaliana GN=At5g04500 PE=2 SV=1	0.08	3.15	-4.53	down	0.00	0.00	yes
TRINITY_DN16615_c0_g1	hypothetical protein POPTR_0002s09670g [Populus trichocarpa]	-	-	2.47	17.25	-2.16	down	0.00	0.00	yes
TRINITY_DN16293_c0_g2	hypothetical protein POPTR_0012s04950g, partial [Populus trichocarpa]	-	-	0.11	3.88	-4.50	down	0.00	0.00	yes
TRINITY_DN23679_c0_g1	hypothetical protein POPTR_0001s28980g [Populus trichocarpa]	-	-	2.64	15.23	-2.26	down	0.00	0.00	yes
TRINITY_DN15711_c0_g5	hypothetical protein POPTR_1763s00200g, partial [Populus trichocarpa]	-	-	0.07	3.58	-4.86	down	0.00	0.00	yes
TRINITY_DN23413_c0_g1	hypothetical protein POPTR_0003s17120g [Populus trichocarpa]	SPL12	Squamosa promoter-binding-like protein 12 OS=Oryza sativa subsp. indica GN=SPL12 PE=2 SV=1	6.74	26.09	-1.58	down	0.00	0.00	yes
TRINITY_DN23373_c0_g4	F-box family protein [Populus trichocarpa]	At3g07870	F-box protein At3g07870 OS=Arabidopsis thaliana GN=At3g07870 PE=2 SV=1	0.42	5.88	-3.17	down	0.00	0.00	yes
TRINITY_DN15225_c0_g1	Bet 5 1 allergen family protein [Populus trichocarpa]	MLP423	MLP-like protein 423 OS=Arabidopsis thaliana GN=MLP423 PE=2 SV=1	24.33	135.86	-1.88	down	0.00	0.00	yes
TRINITY_DN25140_c0_g3	PREDICTED: inactive leucine-rich repeat receptor-like protein kinase CORYNE [Populus euphratica]	CRN	Inactive leucine-rich repeat receptor-like protein kinase CORYNE OS=Arabidopsis thaliana GN=CRN PE=1 SV=1	1.72	16.02	-2.63	down	0.00	0.00	yes
TRINITY_DN18634_c0_g1	PREDICTED: E3 ubiquitin-protein ligase RNF8-B isoform X1 [Populus euphratica]	-	-	3.60	20.25	-1.83	down	0.00	0.00	yes
TRINITY_DN24461_c0_g2	PREDICTED: cysteine-rich receptor-like protein kinase 10 [Populus euphratica]	CRK25	Cysteine-rich receptor-like protein kinase 25 OS=Arabidopsis thaliana GN=CRK25 PE=3 SV=1	2.76	25.59	-2.63	down	0.00	0.00	yes
TRINITY_DN25699_c0_g1	PREDICTED: vacuolar-sorting receptor 6-like [Populus euphratica]	VSR6	Vacuolar-sorting receptor 6 OS=Arabidopsis thaliana GN=VSR6 PE=2 SV=3	2.73	13.35	-1.74	down	0.00	0.00	yes
TRINITY_DN14149_c0_g1	hypothetical protein POPTR_0010s23810g [Populus trichocarpa]	-	-	0.00	5.73	-7.55	down	0.00	0.00	yes
TRINITY_DN26168_c0_g10	hypothetical protein POPTR_0019s00860g [Populus trichocarpa]	-	-	25.80	114.18	-1.59	down	0.00	0.00	yes
TRINITY_DN24663_c0_g3	-	-	-	0.02	3.08	-6.08	down	0.00	0.00	yes
TRINITY_DN24841_c0_g2	hypothetical protein POPTR_0001s37690g [Populus trichocarpa]	-	-	1.78	10.00	-1.90	down	0.00	0.00	yes
TRINITY_DN22614_c0_g1	PREDICTED: protein IQ-DOMAIN 14 [Populus euphratica]	IQD14	Protein IQ-DOMAIN 14 OS=Arabidopsis thaliana GN=IQD14 PE=1 SV=1	1.13	7.86	-2.17	down	0.00	0.00	yes
TRINITY_DN27077_c0_g2	DNA/RNA polymerases superfamily protein [Theobroma cacao]	Tf2-11	Transposon Tf2-11 polyprotein OS=Schizosaccharomyces pombe (strain 972 / ATCC 24843) GN=Tf2-11 PE=3 SV=1	0.19	2.76	-3.23	down	0.00	0.00	yes
TRINITY_DN16602_c0_g1	unknown [Populus trichocarpa]	FEI1	LRR receptor-like serine/threonine-protein kinase FEI 1 OS=Arabidopsis thaliana GN=FEI1 PE=1 SV=1	4.93	31.22	-2.27	down	0.00	0.00	yes
TRINITY_DN5207_c0_g1	-	-	-	0.46	9.04	-3.69	down	0.00	0.00	yes
TRINITY_DN22180_c0_g2	PREDICTED: phylloplanin-like [Populus euphratica]	-	-	179.33	1001.71	-1.94	down	0.00	0.00	yes

TRINITY_DN16655_c0_g1	hypothetical protein POPTR_0002s05820g [Populus trichocarpa]	JUB1	Transcription factor JUNGBRUNNEN 1 OS=Arabidopsis thaliana GN=JUB1 PE=1 SV=1	0.16	3.45	-4.08	down	0.00	0.00	yes
TRINITY_DN21940_c0_g1	patatin-related family protein [Populus trichocarpa]	PLP7	Patatin-like protein 7 OS=Arabidopsis thaliana GN=PLP7 PE=2 SV=1	2.56	12.67	-1.72	down	0.00	0.00	yes
TRINITY_DN19232_c0_g1	hypothetical protein POPTR_0014s10660g [Populus trichocarpa]	-	-	9.61	44.44	-1.61	down	0.00	0.00	yes
TRINITY_DN25254_c2_g1	hypothetical protein POPTR_0017s13090g [Populus trichocarpa]	SP1L1	Protein SPIRAL1-like 1 OS=Arabidopsis thaliana GN=SP1L1 PE=2 SV=1	2.46	37.87	-3.34	down	0.00	0.00	yes
TRINITY_DN24362_c0_g5	WRKY transcription factor 3 [(Populus tomentosa x Populus bolleana) x Populus tomentosa]	WRKY14	Probable WRKY transcription factor 14 OS=Arabidopsis thaliana GN=WRKY14 PE=2 SV=2	14.22	77.46	-1.79	down	0.00	0.00	yes
TRINITY_DN24398_c0_g1	basic helix-loop-helix regulatory family protein [Populus trichocarpa]	GL3	Transcription factor GLABRA 3 OS=Arabidopsis thaliana GN=GL3 PE=1 SV=1	5.04	25.92	-1.79	down	0.00	0.00	yes
TRINITY_DN10911_c0_g1	integral membrane family protein [Populus trichocarpa]	POPTRDRAFT_[illegible]	CASP-like protein 1B1 OS=Populus trichocarpa GN=POPTRDRAFT_823125 PE=3 SV=1	0.04	4.30	-6.09	down	0.00	0.00	yes
TRINITY_DN27163_c0_g1	PREDICTED: probable LRR receptor-like serine/threonine-protein kinase At2g16250 [Populus euphratica]	At2g16250	Probable LRR receptor-like serine/threonine-protein kinase At2g16250 OS=Arabidopsis thaliana GN=At2g16250 PE=2 SV=1	4.67	22.30	-1.60	down	0.00	0.00	yes
TRINITY_DN26299_c0_g1	NBS-LRR type disease resistance protein [Populus trichocarpa]	At3g14460	Putative disease resistance protein At3g14460 OS=Arabidopsis thaliana GN=At3g14460 PE=3 SV=1	7.92	62.54	-2.52	down	0.00	0.00	yes
TRINITY_DN21502_c0_g1	hypothetical protein POPTR_0010s17100g [Populus trichocarpa]	AGO7	Protein argonaute 7 OS=Arabidopsis thaliana GN=AGO7 PE=2 SV=1	3.68	27.10	-2.28	down	0.00	0.00	yes
TRINITY_DN24119_c0_g1	hypothetical protein POPTR_0017s07210g [Populus trichocarpa]	pi041	Uncharacterized J domain-containing protein C17A3.05c OS=Schizosaccharomyces pombe (strain 972 / ATCC 24843) GN=pi041 PE=1 SV=1	4.82	25.35	-1.71	down	0.00	0.00	yes
TRINITY_DN19245_c0_g1	hypothetical protein POPTR_0003s115602g, partial [Populus trichocarpa]	-	-	2.71	20.82	-2.38	down	0.00	0.00	yes
TRINITY_DN25417_c0_g3	SEC14 cytosolic factor family protein [Populus trichocarpa]	-	-	7.71	111.18	-3.23	down	0.00	0.00	yes
TRINITY_DN20416_c2_g1	PREDICTED: uncharacterized protein LOC105139576 [Populus euphratica]	-	-	9.08	53.94	-2.01	down	0.00	0.00	yes
TRINITY_DN26921_c0_g1	PREDICTED: uncharacterized protein LOC105112790 isoform X1 [Populus euphratica]	-	-	4.08	18.53	-1.57	down	0.00	0.00	yes
TRINITY_DN27285_c1_g2	PREDICTED: uncharacterized protein LOC105120461 [Populus euphratica]	Itih5	Inter-alpha-trypsin inhibitor heavy chain H5 OS=Mus musculus GN=Itih5 PE=1 SV=1	5.19	25.19	-1.68	down	0.00	0.00	yes
TRINITY_DN21579_c0_g1	Zinc transporter 6 family protein [Populus trichocarpa]	ZIP6	Zinc transporter 6, chloroplastic OS=Arabidopsis thaliana GN=ZIP6 PE=3 SV=1	3.74	21.42	-1.95	down	0.00	0.00	yes
TRINITY_DN22316_c2_g2	leucine-rich repeat family protein [Populus trichocarpa]	At1g67720	Probable LRR receptor-like serine/threonine-protein kinase At1g67720 OS=Arabidopsis thaliana GN=At1g67720 PE=2 SV=1	6.00	39.32	-2.07	down	0.00	0.00	yes
TRINITY_DN18533_c0_g1	Cf-4/9 disease resistance-like family protein [Populus trichocarpa]	-	-	0.11	8.09	-4.41	down	0.00	0.00	yes
TRINITY_DN18391_c1_g2	hypothetical protein POPTR_0010s22650g [Populus trichocarpa]	-	-	1.75	14.23	-2.52	down	0.00	0.00	yes
TRINITY_DN26168_c0_g9	-	-	-	0.36	5.04	-3.20	down	0.00	0.00	yes
TRINITY_DN14287_c0_g1	hypothetical protein POPTR_0013s02990g [Populus trichocarpa]	HIS2A	Histone H2AX OS=Cicer arietinum GN=HIS2A PE=2 SV=1	12.12	67.05	-1.85	down	0.00	0.00	yes
TRINITY_DN27376_c0_g1	PREDICTED: two-component response regulator-like PRR73 isoform X1 [Populus euphratica]	PRR73	Two-component response regulator-like PRR73 OS=Oryza sativa subsp. indica GN=PRR73 PE=2 SV=2	9.11	41.26	-1.57	down	0.00	0.00	yes
TRINITY_DN19813_c0_g1	homeodomain transcription factor family protein [Populus trichocarpa]	HD1	Homeobox protein HD1 OS=Brassica napus GN=HD1 PE=2 SV=1	7.08	32.92	-1.61	down	0.00	0.00	yes
TRINITY_DN21789_c0_g2	hypothetical protein POPTR_0012s02940g [Populus trichocarpa]	IQD14	Protein IQ-DOMAIN 14 OS=Arabidopsis thaliana GN=IQD14 PE=1 SV=1	0.77	11.65	-2.95	down	0.00	0.00	yes
TRINITY_DN26824_c0_g3	hypothetical protein POPTR_0012s10420g [Populus trichocarpa]	-	-	26.55	135.07	-1.76	down	0.00	0.00	yes
TRINITY_DN15757_c2_g2	PREDICTED: probable receptor-like protein kinase At1g67000 [Populus euphratica]	LRK10	Rust resistance kinase Lr10 OS=Triticum aestivum GN=LRK10 PE=2 SV=1	0.41	4.26	-2.68	down	0.00	0.00	yes
TRINITY_DN23274_c0_g2	hypothetical protein POPTR_0003s07940g [Populus trichocarpa]	At4g16820	Phospholipase A1-Ibeta2, chloroplastic OS=Arabidopsis thaliana GN=At4g16820 PE=1 SV=2	0.48	24.54	-5.05	down	0.00	0.00	yes

TRINITY_DN12999_c0_g1	hypothetical protein POPTR_0012s08050g [Populus trichocarpa]	-	-	0.29	6.41	-4.03	down	0.00	0.00	yes
TRINITY_DN26295_c0_g2	ubiquitin conjugating-like enzyme family protein [Populus trichocarpa]	UBC30	Ubiquitin-conjugating enzyme E2 30 OS=Arabidopsis thaliana GN=UBC30 PE=2 SV=1	1.87	16.39	-2.61	down	0.00	0.00	yes
TRINITY_DN26997_c0_g1	PREDICTED: probable LRR receptor-like serine/threonine-protein kinase At1g34110 [Populus euphratica]	FEI1	LRR receptor-like serine/threonine-protein kinase FEI 1 OS=Arabidopsis thaliana GN=FEI1 PE=1 SV=1	13.18	55.35	-1.67	down	0.00	0.00	yes
TRINITY_DN14366_c0_g1	hypothetical protein POPTR_0018s12270g [Populus trichocarpa]	-	-	74.76	379.04	-1.59	down	0.00	0.00	yes
TRINITY_DN18869_c0_g2	PREDICTED: osmotin-like protein [Populus euphratica]	-	Osmotin-like protein OS=Solanum lycopersicum PE=1 SV=1	26.48	248.56	-2.69	down	0.00	0.00	yes
TRINITY_DN15736_c0_g1	hypothetical protein POPTR_0008s14850g [Populus trichocarpa]	CAISE5	Glucose and ribitol dehydrogenase OS=Daucus carota GN=CAISE5 PE=2 SV=1	0.00	7.88	-10.07	down	0.00	0.00	yes
TRINITY_DN25895_c0_g2	leucine-rich repeat transmembrane protein kinase [Populus trichocarpa]	ZAR1	Receptor protein kinase-like protein ZAR1 OS=Arabidopsis thaliana GN=ZAR1 PE=1 SV=1	1.06	9.71	-2.62	down	0.00	0.00	yes
TRINITY_DN24253_c0_g5	-	-	-	1.36	22.55	-3.43	down	0.00	0.00	yes
TRINITY_DN26460_c0_g1	PREDICTED: uncharacterized protein LOC105140484 isoform X2 [Populus euphratica]	-	-	1.49	8.50	-1.92	down	0.00	0.00	yes
TRINITY_DN25557_c0_g1	asparagine synthetase family protein 1 [Populus simonii x Populus nigra]	AS	Asparagine synthetase [glutamine-hydrolyzing] OS=Triphysaria versicolor GN=AS PE=2 SV=3	1.79	118.24	-5.45	down	0.00	0.00	yes
TRINITY_DN18490_c0_g2	expansin [Populus tomentosa]	EXPA4	Expansin-A4 OS=Arabidopsis thaliana GN=EXPA4 PE=1 SV=1	2.06	29.77	-3.27	down	0.00	0.00	yes
TRINITY_DN20149_c0_g2	PREDICTED: B3 domain-containing protein At3g19184-like [Populus euphratica]	At3g19184	B3 domain-containing protein At3g19184 OS=Arabidopsis thaliana GN=At3g19184 PE=2 SV=1	8.05	38.23	-1.57	down	0.00	0.00	yes
TRINITY_DN17437_c0_g2	nucleosidase-related family protein [Populus trichocarpa]	mtnN	5'-methylthioadenosine/S-adenosylhomocysteine nucleosidase OS=Vibrio tasmaniensis (strain LGP32) GN=mtnN PE=3 SV=1	1.70	11.18	-2.10	down	0.00	0.00	yes
TRINITY_DN25895_c0_g3	leucine-rich repeat transmembrane protein kinase [Populus trichocarpa]	At1g66830	Probable inactive leucine-rich repeat receptor-like protein kinase At1g66830 OS=Arabidopsis thaliana GN=At1g66830 PE=2 SV=1	0.82	9.37	-2.89	down	0.00	0.00	yes
TRINITY_DN15152_c0_g1	-	-	-	1.49	27.47	-3.53	down	0.00	0.00	yes
TRINITY_DN22601_c0_g2	PREDICTED: NAC domain-containing protein 2-like [Populus euphratica]	NAC002	NAC domain-containing protein 2 OS=Arabidopsis thaliana GN=NAC002 PE=2 SV=2	40.16	204.66	-1.73	down	0.00	0.00	yes
TRINITY_DN18326_c0_g1	hypothetical protein POPTR_0014s06750g [Populus trichocarpa]	GRF9	Growth-regulating factor 9 OS=Arabidopsis thaliana GN=GRF9 PE=1 SV=1	2.64	23.77	-2.60	down	0.00	0.00	yes
TRINITY_DN17253_c0_g1	PREDICTED: uncharacterized protein LOC105127581 [Populus euphratica]	-	-	1.51	22.15	-3.31	down	0.00	0.00	yes
TRINITY_DN25652_c0_g1	hypothetical protein POPTR_0019s04950g [Populus trichocarpa]	-	-	5.08	27.24	-1.82	down	0.00	0.00	yes
TRINITY_DN26451_c2_g4	hypothetical protein POPTR_0007s00780g [Populus trichocarpa]	LRK10	Rust resistance kinase Lr10 OS=Triticum aestivum GN=LRK10 PE=2 SV=1	0.81	11.83	-3.40	down	0.00	0.00	yes
TRINITY_DN23481_c0_g1	hypothetical protein POPTR_0002s04720g, partial [Populus trichocarpa]	At2g38810	Probable histone H2A variant 2 OS=Arabidopsis thaliana GN=At2g38810 PE=2 SV=1	117.99	559.47	-1.59	down	0.00	0.00	yes
TRINITY_DN15178_c0_g2	hypothetical protein POPTR_0004s10280g [Populus trichocarpa]	-	-	0.17	6.68	-4.35	down	0.00	0.00	yes
TRINITY_DN16926_c0_g1	senescence-associated family protein [Populus trichocarpa]	TET10	Tetraspanin-10 OS=Arabidopsis thaliana GN=TET10 PE=2 SV=1	9.24	49.61	-1.70	down	0.00	0.00	yes
TRINITY_DN15711_c0_g2	PREDICTED: probable LRR receptor-like serine/threonine-protein kinase At4g36180 isoform X1 [Populus euphratica]	-	-	0.32	24.28	-3.65	down	0.00	0.00	yes
TRINITY_DN22192_c0_g3	helicase domain-containing family protein [Populus trichocarpa]	At5g10370	ATP-dependent RNA helicase DEAH12, chloroplastic OS=Arabidopsis thaliana GN=At5g10370 PE=3 SV=1	1.41	8.87	-2.10	down	0.00	0.00	yes
TRINITY_DN20498_c0_g1	PREDICTED: monoacylglycerol lipase ABHD6 [Populus euphratica]	-	-	3.77	16.33	-2.27	down	0.00	0.00	yes
TRINITY_DN20644_c0_g1	hypothetical protein POPTR_0013s10080g [Populus trichocarpa]	EGS1	Eugenol synthase 1 OS=Ocimum basilicum GN=EGS1 PE=1 SV=1	10.85	54.73	-1.68	down	0.00	0.00	yes
TRINITY_DN22055_c0_g5	PREDICTED: tartrate-resistant acid phosphatase type 5-like [Populus euphratica]	-	-	0.30	9.68	-3.79	down	0.00	0.00	yes

TRINITY_DN20627_c0_g3	PREDICTED: uncharacterized protein LOC105132172 [Populus euphratica]	-	-	0.01	2.97	-6.75	down	0.00	0.00	yes
TRINITY_DN25657_c0_g1	hypothetical protein POPTR_0001s15660g [Populus trichocarpa]	VAB	VAN3-binding protein OS=Arabidopsis thaliana GN=VAB PE=1 SV=1	5.01	25.80	-2.70	down	0.00	0.00	yes
TRINITY_DN20173_c0_g4	-	-	-	0.06	10.84	-6.30	down	0.00	0.00	yes
TRINITY_DN17662_c0_g1	PREDICTED: L-type lectin-domain containing receptor kinase IX.1-like [Populus euphratica]	LECRK91	L-type lectin-domain containing receptor kinase IX.1 OS=Arabidopsis thaliana GN=LECRK91 PE=1 SV=1	0.28	5.70	-3.72	down	0.00	0.00	yes
TRINITY_DN26025_c0_g1	hypothetical protein POPTR_0004s18340g [Populus trichocarpa]	At4g38520	Probable protein phosphatase 2C 64 OS=Arabidopsis thaliana GN=At4g38520 PE=2 SV=1	5.60	27.70	-1.71	down	0.00	0.00	yes
TRINITY_DN13803_c0_g1	PREDICTED: probable prolyl 4-hydroxylase 9 [Populus euphratica]	P4H9	Probable prolyl 4-hydroxylase 9 OS=Arabidopsis thaliana GN=P4H9 PE=2 SV=1	0.24	3.68	-3.27	down	0.00	0.00	yes
TRINITY_DN18805_c0_g1	PREDICTED: uncharacterized protein LOC105137336 [Populus euphratica]	CAMBP25	Calmodulin-binding protein 25 OS=Arabidopsis thaliana GN=CAMBP25 PE=1 SV=1	35.77	345.15	-2.64	down	0.00	0.00	yes
TRINITY_DN23861_c0_g1	hypothetical protein POPTR_0002s20070g [Populus trichocarpa]	-	-	17.86	82.42	-1.74	down	0.00	0.00	yes
TRINITY_DN24396_c0_g1	nitrate transporter [Populus tremula x Populus tremuloides]	NRT2.5	High affinity nitrate transporter 2.5 OS=Arabidopsis thaliana GN=NRT2.5 PE=2 SV=1	0.76	11.24	-3.28	down	0.00	0.00	yes
TRINITY_DN24826_c0_g2	PREDICTED: uncharacterized protein LOC105109098 [Populus euphratica]	-	-	0.14	5.15	-4.47	down	0.00	0.00	yes
TRINITY_DN26256_c0_g1	hypothetical protein POPTR_0014s09880g [Populus trichocarpa]	-	-	1.81	15.37	-2.12	down	0.00	0.00	yes
TRINITY_DN23563_c1_g5	PREDICTED: uncharacterized protein LOC105129577 [Populus euphratica]	ATS3B	Embryo-specific protein ATS3B OS=Arabidopsis thaliana GN=ATS3B PE=1 SV=1	0.04	5.92	-5.75	down	0.00	0.00	yes
TRINITY_DN20070_c1_g1	NAC transcriptional factor [Populus tremula x Populus tremuloides]	NAC073	NAC domain-containing protein 73 OS=Arabidopsis thaliana GN=NAC073 PE=2 SV=1	0.39	3.79	-3.14	down	0.00	0.00	yes
TRINITY_DN26857_c0_g1	hypothetical protein POPTR_0001s02910g [Populus trichocarpa]	APRR2	Two-component response regulator-like APRR2 OS=Arabidopsis thaliana GN=APRR2 PE=2 SV=2	12.91	65.99	-1.72	down	0.00	0.00	yes
TRINITY_DN22993_c2_g1	hypothetical protein POPTR_0001s14680g [Populus trichocarpa]	At1g80640	Probable receptor-like protein kinase At1g80640 OS=Arabidopsis thaliana GN=At1g80640 PE=2 SV=1	1.12	13.57	-3.02	down	0.00	0.00	yes
TRINITY_DN24624_c0_g1	PREDICTED: LOW QUALITY PROTEIN: uncharacterized protein At5g41620-like [Populus euphratica]	At5g41620	Uncharacterized protein At5g41620 OS=Arabidopsis thaliana GN=At5g41620 PE=2 SV=2	1.99	10.43	-1.80	down	0.00	0.00	yes
TRINITY_DN20678_c0_g1	zinc finger family protein [Populus trichocarpa]	-	-	6.02	36.29	-1.67	down	0.00	0.00	yes
TRINITY_DN27262_c1_g2	hypothetical protein POPTR_0012s09560g [Populus trichocarpa]	-	-	4.61	77.78	-3.42	down	0.00	0.00	yes
TRINITY_DN21113_c0_g1	hypothetical protein POPTR_0014s04790g [Populus trichocarpa]	-	-	2.18	17.35	-2.20	down	0.00	0.00	yes
TRINITY_DN26436_c0_g1	hypothetical protein POPTR_0011s16560g [Populus trichocarpa]	At5g44440	Berberine bridge enzyme-like 28 OS=Arabidopsis thaliana GN=At5g44440 PE=1 SV=1	2.94	20.53	-2.07	down	0.00	0.00	yes
TRINITY_DN22388_c1_g5	PREDICTED: receptor protein kinase-like protein At4g34220 [Populus euphratica]	At4g34220	Receptor protein kinase-like protein At4g34220 OS=Arabidopsis thaliana GN=At4g34220 PE=2 SV=1	6.22	27.79	-1.57	down	0.00	0.00	yes
TRINITY_DN16811_c0_g1	hypothetical protein POPTR_0006s21140g [Populus trichocarpa]	At1g15670	F-box/kelch-repeat protein At1g15670 OS=Arabidopsis thaliana GN=At1g15670 PE=2 SV=1	1.19	19.71	-3.49	down	0.00	0.00	yes
TRINITY_DN15711_c0_g7	PREDICTED: probably inactive leucine-rich repeat receptor-like protein kinase IMK2 isoform X1 [Populus euphratica]	-	-	0.16	3.38	-3.79	down	0.00	0.00	yes
TRINITY_DN15881_c0_g1	wound-responsive family protein [Populus trichocarpa]	-	-	9.41	58.22	-2.05	down	0.00	0.00	yes
TRINITY_DN21159_c0_g1	PREDICTED: thioredoxin domain-containing protein 9 homolog [Populus euphratica]	At2g18990	Thioredoxin domain-containing protein 9 homolog OS=Arabidopsis thaliana GN=At2g18990 PE=2 SV=1	4.79	25.69	-1.80	down	0.00	0.00	yes
TRINITY_DN390_c0_g1	hypothetical protein POPTR_0011s04780g [Populus trichocarpa]	ephA	Epoxide hydrolase A OS=Mycobacterium tuberculosis (strain ATCC 25618 / H37Rv) GN=ephA PE=1 SV=1	0.86	7.69	-2.53	down	0.00	0.00	yes
TRINITY_DN19291_c0_g5	hypothetical protein POPTR_0015s06880g [Populus trichocarpa]	PDCB2	PLASMODESMATA CALLOSE-BINDING PROTEIN 2 OS=Arabidopsis thaliana GN=PDCB2 PE=1 SV=1	68.36	415.00	-2.01	down	0.00	0.00	yes
TRINITY_DN26358_c0_g1	6a-hydroxymaackiain methyltransferase family protein [Populus trichocarpa]	ROMT	Trans-resveratrol di-O-methyltransferase OS=Vitis vinifera GN=ROMT PE=1 SV=2	198.04	1408.66	-2.57	down	0.00	0.00	yes

TRINITY_DN23838_c0_g2	hypothetical protein POPTR_0007s12160g [Populus trichocarpa]	At3g50780	BTB/POZ domain-containing protein At3g50780 OS=Arabidopsis thaliana GN=At3g50780 PE=2 SV=1	7.86	42.53	-1.87	down	0.00	0.00	yes
TRINITY_DN18623_c1_g1	-	-	-	8.19	58.74	-2.34	down	0.00	0.00	yes
TRINITY_DN20066_c0_g1	hypothetical protein POPTR_0016s05640g [Populus trichocarpa]	-	-	0.90	10.66	-2.94	down	0.00	0.00	yes
TRINITY_DN16645_c0_g1	kinase family protein [Populus trichocarpa]	AUR3	Serine/threonine-protein kinase Aurora-3 OS=Arabidopsis thaliana GN=AUR3 PE=2 SV=1	5.12	26.93	-1.72	down	0.00	0.00	yes
TRINITY_DN26076_c0_g1	PREDICTED: WD repeat-containing protein WRAP73 isoform X4 [Ricinus communis]	Wrap73	WD repeat-containing protein WRAP73 OS=Mus musculus GN=Wrap73 PE=1 SV=2	7.55	29.01	-1.50	down	0.00	0.00	yes
TRINITY_DN26220_c0_g2	WD-40 repeat family protein-2 [Populus tomentosa]	TPR1	Topless-related protein 1 OS=Arabidopsis thaliana GN=TPR1 PE=1 SV=3	2.21	11.23	-1.74	down	0.00	0.00	yes
TRINITY_DN18346_c0_g1	WRKY transcription factor [Populus tremula x Populus alba]	WRKY23	Probable WRKY transcription factor 23 OS=Arabidopsis thaliana GN=WRKY23 PE=2 SV=1	1.35	14.38	-2.85	down	0.00	0.00	yes
TRINITY_DN14296_c0_g1	hypothetical protein POPTR_0008s04530g [Populus trichocarpa]	-	-	0.57	8.03	-3.20	down	0.00	0.00	yes
TRINITY_DN25089_c1_g1	hypothetical protein POPTR_0004s19910g [Populus trichocarpa]	-	-	0.92	8.16	-2.55	down	0.00	0.00	yes
TRINITY_DN27383_c0_g2	integral membrane family protein [Populus trichocarpa]	RCOM_1491260	CASP-like protein 3A1 OS=Ricinus communis GN=RCOM_1491260 PE=2 SV=1	0.15	6.83	-4.90	down	0.00	0.00	yes
TRINITY_DN21666_c0_g1	abnormal floral organs family protein [Populus trichocarpa]	YAB1	Axial regulator YABBY 1 OS=Arabidopsis thaliana GN=YAB1 PE=1 SV=1	26.15	135.10	-1.87	down	0.00	0.00	yes
TRINITY_DN20894_c0_g2	hypothetical protein POPTR_0006s13260g [Populus trichocarpa]	-	-	0.14	13.71	-5.70	down	0.00	0.00	yes
TRINITY_DN18415_c0_g1	unknown [Populus trichocarpa]	-	-	4.67	64.19	-3.17	down	0.00	0.00	yes
TRINITY_DN21456_c1_g2	WRKY transcription factor 6 [(Populus tomentosa x Populus bolleana) x Populus tomentosa]	WRKY53	Probable WRKY transcription factor 53 OS=Arabidopsis thaliana GN=WRKY53 PE=1 SV=1	3.42	37.12	-2.83	down	0.00	0.00	yes
TRINITY_DN23023_c0_g4	hypothetical protein POPTR_0008s03050g [Populus trichocarpa]	HIS2B	Histone H2B OS=Capsicum annuum GN=HIS2B PE=2 SV=3	170.04	754.95	-1.55	down	0.00	0.00	yes
TRINITY_DN18680_c0_g2	PREDICTED: uncharacterized protein LOC105120786 [Populus euphratica]	-	-	3.88	24.58	-1.79	down	0.00	0.00	yes
TRINITY_DN15307_c0_g3	hypothetical protein POPTR_0018s01950g [Populus trichocarpa]	At5g11120/At5g1	Probable glycosyltransferase At5g11130 OS=Arabidopsis thaliana GN=At5g11120/At5g11130 PE=3 SV=2	0.00	4.39	-7.21	down	0.00	0.00	yes
TRINITY_DN23391_c1_g3	PREDICTED: TMV resistance protein N-like isoform X1 [Populus euphratica]	-	-	0.32	4.24	-3.12	down	0.00	0.00	yes
TRINITY_DN19273_c0_g4	PREDICTED: uncharacterized protein LOC105139124 [Populus euphratica]	-	-	2.17	14.01	-2.08	down	0.00	0.00	yes
TRINITY_DN22061_c0_g1	hypothetical protein POPTR_0002s26070g [Populus trichocarpa]	BARD1	BRCA1-associated RING domain protein 1 OS=Arabidopsis thaliana GN=BARD1 PE=1 SV=1	4.70	26.54	-1.86	down	0.00	0.00	yes
TRINITY_DN21833_c0_g1	PREDICTED: glutamate receptor 2.8-like isoform X1 [Populus euphratica]	GLR2.8	Glutamate receptor 2.8 OS=Arabidopsis thaliana GN=GLR2.8 PE=2 SV=2	2.54	13.09	-1.68	down	0.00	0.00	yes
TRINITY_DN26754_c0_g2	UBIQUITIN-SPECIFIC PROTEASE 21 family protein [Populus trichocarpa]	UBP21	Ubiquitin carboxyl-terminal hydrolase 21 OS=Arabidopsis thaliana GN=UBP21 PE=2 SV=1	9.44	43.10	-1.65	down	0.00	0.00	yes
TRINITY_DN24513_c0_g3	hypothetical protein POPTR_0008s17960g [Populus trichocarpa]	ABCC9	ABC transporter C family member 9 OS=Arabidopsis thaliana GN=ABCC9 PE=2 SV=2	0.78	8.14	-2.76	down	0.00	0.00	yes
TRINITY_DN19623_c0_g2	hypothetical protein POPTR_0004s04280g [Populus trichocarpa]	-	-	5.28	29.70	-1.92	down	0.00	0.00	yes
TRINITY_DN23777_c2_g1	putative AUX1-like permease family protein [Populus trichocarpa]	LAX2	Auxin transporter-like protein 2 OS=Medicago truncatula GN=LAX2 PE=2 SV=1	2.50	17.87	-2.57	down	0.00	0.00	yes
TRINITY_DN21593_c0_g2	hypothetical protein POPTR_0010s13990g [Populus trichocarpa]	LAR	Leucoanthocyanidin reductase OS=Desmodium uncinatum GN=LAR PE=1 SV=1	1.46	33.85	-3.92	down	0.00	0.00	yes
TRINITY_DN17524_c0_g1	PREDICTED: high mobility group B protein 7-like [Populus euphratica]	HMGB7	High mobility group B protein 7 OS=Arabidopsis thaliana GN=HMGB7 PE=1 SV=1	17.66	87.26	-1.76	down	0.00	0.00	yes
TRINITY_DN22802_c0_g3	PREDICTED: uncharacterized protein LOC105130447 isoform X1 [Populus euphratica]	-	-	0.03	2.11	-5.22	down	0.00	0.00	yes
TRINITY_DN27013_c1_g1	hypothetical protein POPTR_0002s17350g [Populus trichocarpa]	ARF18	Auxin response factor 18 OS=Arabidopsis thaliana GN=ARF18 PE=2 SV=1	12.44	71.86	-1.78	down	0.00	0.00	yes

TRINITY_DN24827_c0_g1	unknown [Populus trichocarpa x Populus deltoides]	-	-	6.04	30.44	-1.70	down	0.00	0.00	yes
TRINITY_DN25929_c1_g3	GDSL-motif lipase/hydrolase family protein [Populus trichocarpa]	At3g26430	GDSL esterase/lipase At3g26430 OS=Arabidopsis thaliana GN=At3g26430 PE=2 SV=1	1.30	8.83	-2.18	down	0.00	0.00	yes
TRINITY_DN25140_c0_g1	PREDICTED: inactive leucine-rich repeat receptor-like protein kinase CORYNE [Populus euphratica]	CRN	Inactive leucine-rich repeat receptor-like protein kinase CORYNE OS=Arabidopsis thaliana GN=CRN PE=1 SV=1	2.42	12.66	-1.92	down	0.00	0.00	yes
TRINITY_DN13172_c0_g1	subtilase family protein [Populus trichocarpa]	SBT5.6	Subtilisin-like protease SBT5.6 OS=Arabidopsis thaliana GN=SBT5.6 PE=2 SV=1	1.87	12.20	-2.11	down	0.00	0.00	yes
TRINITY_DN20983_c0_g1	PREDICTED: LOW QUALITY PROTEIN: DNA repair protein RAD51 homolog [Populus euphratica]	RAD51	DNA repair protein RAD51 homolog 1 OS=Arabidopsis thaliana GN=RAD51 PE=1 SV=1	4.69	25.62	-1.63	down	0.00	0.00	yes
TRINITY_DN23704_c2_g1	PREDICTED: NAC transcription factor 29-like [Populus euphratica]	NAC047	NAC transcription factor 47 OS=Arabidopsis thaliana GN=NAC047 PE=2 SV=1	2.61	26.09	-2.79	down	0.00	0.00	yes
TRINITY_DN23763_c0_g2	PREDICTED: zinc-finger homeodomain protein 6 [Populus euphratica]	ZHD6	Zinc-finger homeodomain protein 6 OS=Arabidopsis thaliana GN=ZHD6 PE=1 SV=1	2.95	17.57	-1.96	down	0.00	0.00	yes
TRINITY_DN26281_c1_g1	hypothetical protein POPTR_0006s21580g [Populus trichocarpa]	-	-	5.73	25.33	-1.51	down	0.00	0.00	yes
TRINITY_DN19093_c1_g7	metallothionein 1b [Populus trichocarpa x Populus deltoides]	pKIWI504	Metallothionein-like protein type 2 OS=Actinidia deliciosa GN=pKIWI504 PE=2 SV=1	0.90	18.86	-3.80	down	0.00	0.00	yes
TRINITY_DN25130_c0_g8	hypothetical protein POPTR_0002s01560g [Populus trichocarpa]	-	-	0.81	8.22	-2.70	down	0.00	0.00	yes
TRINITY_DN24115_c0_g1	PREDICTED: kinesin heavy chain isoform X3 [Populus euphratica]	KIN6	Kinesin-like protein KIN-6 OS=Arabidopsis thaliana GN=KIN6 PE=3 SV=2	1.73	8.58	-1.71	down	0.00	0.00	yes
TRINITY_DN25513_c0_g2	PREDICTED: zinc finger protein NUTCRACKER-like isoform X5 [Populus euphratica]	IDD2	Protein indeterminate-domain 2 OS=Arabidopsis thaliana GN=IDD2 PE=2 SV=1	13.47	55.16	-1.55	down	0.00	0.00	yes
TRINITY_DN26408_c0_g3	PREDICTED: zinc finger protein CONSTANS-LIKE 15-like isoform X1 [Populus euphratica]	COL15	Zinc finger protein CONSTANS-LIKE 15 OS=Arabidopsis thaliana GN=COL15 PE=2 SV=1	3.87	26.39	-1.89	down	0.00	0.00	yes
TRINITY_DN23327_c0_g3	PREDICTED: uncharacterized protein LOC105138478 [Populus euphratica]	-	-	10.03	47.24	-1.66	down	0.00	0.00	yes
TRINITY_DN25302_c0_g1	hypothetical protein POPTR_0101s00210g [Populus trichocarpa]	-	-	0.78	10.31	-3.09	down	0.00	0.00	yes
TRINITY_DN19120_c0_g1	-	-	-	12.54	246.58	-3.90	down	0.00	0.00	yes
TRINITY_DN14792_c0_g2	zinc finger family protein [Populus trichocarpa]	ATL16	RING-H2 finger protein ATL16 OS=Arabidopsis thaliana GN=ATL16 PE=2 SV=1	0.06	3.31	-5.08	down	0.00	0.00	yes
TRINITY_DN17156_c0_g1	PREDICTED: protein ELC [Populus euphratica]	ELC	Protein ELC OS=Arabidopsis thaliana GN=ELC PE=1 SV=1	0.55	7.07	-3.06	down	0.00	0.00	yes
TRINITY_DN23756_c0_g3	PREDICTED: uncharacterized protein LOC105134286 [Populus euphratica]	-	-	1.66	15.98	-2.63	down	0.00	0.00	yes
TRINITY_DN14433_c0_g2	hypothetical protein POPTR_0315s00200g [Populus trichocarpa]	PBP1	Calcium-binding protein PBP1 OS=Arabidopsis thaliana GN=PBP1 PE=1 SV=1	0.17	5.08	-4.22	down	0.00	0.00	yes
TRINITY_DN21595_c0_g1	hypothetical protein POPTR_0013s13050g [Populus trichocarpa]	BHLH123	Transcription factor bHLH123 OS=Arabidopsis thaliana GN=BHLH123 PE=2 SV=1	4.27	30.26	-2.17	down	0.00	0.00	yes
TRINITY_DN15329_c0_g2	-	-	-	0.17	6.07	-4.60	down	0.00	0.00	yes
TRINITY_DN17267_c0_g1	heat shock transcription factor B4a [Populus simonii]	HSFB4	Heat stress transcription factor B-4 OS=Arabidopsis thaliana GN=HSFB4 PE=2 SV=1	0.29	4.67	-3.72	down	0.00	0.00	yes
TRINITY_DN26479_c0_g2	hypothetical protein POPTR_0012s04100g, partial [Populus trichocarpa]	-	-	0.28	5.82	-3.66	down	0.00	0.00	yes
TRINITY_DN25371_c3_g1	PREDICTED: 65-kDa microtubule-associated protein 4-like isoform X1 [Populus euphratica]	MAP65-4	65-kDa microtubule-associated protein 4 OS=Arabidopsis thaliana GN=MAP65-4 PE=1 SV=2	2.44	13.64	-1.87	down	0.00	0.00	yes
TRINITY_DN23415_c1_g1	hypothetical protein POPTR_0018s12990g [Populus trichocarpa]	YAB5	Axial regulator YABBY 5 OS=Arabidopsis thaliana GN=YAB5 PE=1 SV=1	25.68	133.67	-1.75	down	0.00	0.00	yes
TRINITY_DN7619_c0_g1	PREDICTED: palmitoyl-acyl carrier protein thioesterase, chloroplastic-like [Populus euphratica]	FATB	Palmitoyl-acyl carrier protein thioesterase, chloroplastic OS=Arabidopsis thaliana GN=FATB PE=1 SV=1	0.09	2.75	-4.15	down	0.00	0.00	yes
TRINITY_DN15527_c0_g1	PREDICTED: probably inactive leucine-rich repeat receptor-like protein kinase At5g48380 isoform X1 [Populus euphratica]	At1g69990	Probable LRR receptor-like serine/threonine-protein kinase At1g69990 OS=Arabidopsis thaliana GN=At1g69990 PE=2 SV=1	0.00	6.18	-7.22	down	0.00	0.00	yes
TRINITY_DN24334_c1_g5	hypothetical protein POPTR_0001s09180g [Populus trichocarpa]	TUBB4	Tubulin beta-4 chain OS=Eleusine indica GN=TUBB4 PE=2 SV=1	3.38	26.54	-2.37	down	0.00	0.00	yes

TRINITY_DN18667_c0_g1	PREDICTED: probable choline kinase 3 [Populus euphratica]	At4g09760	Probable choline kinase 3 OS=Arabidopsis thaliana GN=At4g09760 PE=2 SV=1	0.81	8.11	-2.71	down	0.00	0.00	yes
TRINITY_DN20085_c0_g1	hypothetical protein POPTR_0001s15160g [Populus trichocarpa]	-	-	1.12	9.15	-2.38	down	0.00	0.00	yes
TRINITY_DN20343_c2_g1	WRKY transcription factor 29 [(Populus tomentosa x Populus bolleana) x Populus tomentosa]	WRKY28	Probable WRKY transcription factor 28 OS=Arabidopsis thaliana GN=WRKY28 PE=2 SV=1	0.11	3.00	-3.99	down	0.00	0.00	yes
TRINITY_DN20007_c0_g1	PREDICTED: putative ubiquitin-conjugating enzyme E2 38 isoform X1 [Populus euphratica]	UBC25	Probable ubiquitin-conjugating enzyme E2 25 OS=Arabidopsis thaliana GN=UBC25 PE=2 SV=1	4.13	20.19	-1.66	down	0.00	0.00	yes
TRINITY_DN23212_c1_g3	PREDICTED: WD repeat domain-containing protein 83 [Populus euphratica]	WDR83	WD repeat domain-containing protein 83 OS=Homo sapiens GN=WDR83 PE=1 SV=1	6.67	30.00	-1.56	down	0.00	0.00	yes
TRINITY_DN27233_c0_g1	PREDICTED: probable serine/threonine-protein kinase mps1 isoform X1 [Populus euphratica]	mps1	Probable serine/threonine-protein kinase mps1 OS=Dictyostelium discoideum GN=mps1 PE=3 SV=1	5.48	22.75	-1.44	down	0.00	0.00	yes
TRINITY_DN20491_c0_g2	PREDICTED: cytochrome b561 and DOMON domain-containing protein At5g47530-like [Populus euphratica]	At5g35735	Cytochrome b561 and DOMON domain-containing protein At5g35735 OS=Arabidopsis thaliana GN=At5g35735 PE=2 SV=1	2.00	34.24	-3.59	down	0.00	0.00	yes
TRINITY_DN26474_c0_g1	hypothetical protein POPTR_0010s18540g [Populus trichocarpa]	At5g48380	Probably inactive leucine-rich repeat receptor-like protein kinase At5g48380 OS=Arabidopsis thaliana GN=At5g48380 PE=1 SV=1	7.99	31.30	-1.56	down	0.00	0.00	yes
TRINITY_DN26491_c3_g1	PREDICTED: phragmoplast orienting kinesin-1 isoform X1 [Populus euphratica]	-	-	8.65	44.05	-1.75	down	0.00	0.00	yes
TRINITY_DN23327_c0_g1	hypothetical protein POPTR_0601s00200g [Populus trichocarpa]	-	-	0.00	7.59	-8.34	down	0.00	0.00	yes
TRINITY_DN15839_c0_g1	hypothetical protein POPTR_0025s00230g, partial [Populus trichocarpa]	-	-	0.16	3.81	-4.40	down	0.00	0.00	yes
TRINITY_DN22795_c0_g1	kinase family protein [Populus trichocarpa]	At1g07650	Probable LRR receptor-like serine/threonine-protein kinase At1g07650 OS=Arabidopsis thaliana GN=At1g07650 PE=1 SV=1	0.70	7.96	-2.67	down	0.00	0.00	yes
TRINITY_DN19501_c0_g4	hypothetical protein POPTR_0010s24060g [Populus trichocarpa]	-	-	0.13	3.17	-3.90	down	0.00	0.00	yes
TRINITY_DN17225_c0_g1	hypothetical protein POPTR_0020s00240g [Populus trichocarpa]	YUC10	Probable indole-3-pyruvate monooxygenase YUCCA10 OS=Arabidopsis thaliana GN=YUC10 PE=2 SV=1	0.84	25.71	-4.02	down	0.00	0.00	yes
TRINITY_DN22824_c1_g1	vacuolar processing enzyme a [Populus tomentosa]	-	Vacuolar-processing enzyme OS=Citrus sinensis PE=2 SV=1	19.88	97.70	-1.64	down	0.00	0.00	yes
TRINITY_DN14439_c0_g1	PREDICTED: secoisolariciresinol dehydrogenase-like [Populus euphratica]	-	Secoisolariciresinol dehydrogenase (Fragment) OS=Podophyllum peltatum PE=1 SV=1	0.06	3.51	-5.08	down	0.00	0.00	yes
TRINITY_DN26908_c0_g2	-	-	-	0.24	5.86	-3.97	down	0.00	0.00	yes
TRINITY_DN18744_c0_g1	hypothetical protein POPTR_0013s12020g [Populus trichocarpa]	At5g08460	GDSL esterase/lipase At5g08460 OS=Arabidopsis thaliana GN=At5g08460 PE=2 SV=1	0.67	17.03	-4.07	down	0.00	0.00	yes
TRINITY_DN21205_c0_g1	hypothetical protein POPTR_0019s08970g [Populus trichocarpa]	NET4A	Protein NETWORKED 4A OS=Arabidopsis thaliana GN=NET4A PE=2 SV=1	10.20	52.06	-1.76	down	0.00	0.00	yes
TRINITY_DN23531_c0_g5	hypothetical protein POPTR_0436s00200g [Populus trichocarpa]	CYP81E1	Isoflavone 2'-hydroxylase OS=Glycyrrhiza echinata GN=CYP81E1 PE=1 SV=2	13.47	59.57	-1.67	down	0.00	0.00	yes
TRINITY_DN20081_c0_g1	integral membrane family protein [Populus trichocarpa]	POPTRDRAFT_[illegible]	CASP-like protein 4C2 OS=Populus trichocarpa GN=POPTRDRAFT_822486 PE=3 SV=1	2.09	16.67	-2.30	down	0.00	0.00	yes
TRINITY_DN27824_c1_g3	DNA topoisomerase family protein [Populus trichocarpa]	TOP2	DNA topoisomerase 2 OS=Arabidopsis thaliana GN=TOP2 PE=2 SV=2	28.67	121.37	-1.48	down	0.00	0.00	yes
TRINITY_DN13965_c0_g1	PREDICTED: uncharacterized protein LOC105133033 [Populus euphratica]	-	-	0.17	7.20	-4.50	down	0.00	0.00	yes
TRINITY_DN24995_c0_g1	PREDICTED: uncharacterized protein LOC105135565 [Populus euphratica]	-	-	7.27	40.24	-1.88	down	0.00	0.00	yes
TRINITY_DN21343_c0_g5	hypothetical protein TSUD_72310 [Trifolium subterraneum]	-	Retrovirus-related Pol polyprotein from transposon TNT 1-94 OS=Nicotiana tabacum PE=2 SV=1	0.74	3.53	-2.08	down	0.00	0.00	yes
TRINITY_DN15014_c0_g1	-	-	-	2.70	25.26	-2.60	down	0.00	0.00	yes
TRINITY_DN25198_c0_g5	PREDICTED: probable protein phosphatase 2C 34 [Populus euphratica]	At3g05640	Probable protein phosphatase 2C 34 OS=Arabidopsis thaliana GN=At3g05640 PE=2 SV=1	3.39	19.13	-1.87	down	0.00	0.00	yes
TRINITY_DN22428_c0_g2	PREDICTED: centromere-associated protein E-like [Populus euphratica]	KIN7O	Kinesin-like protein KIN-7O OS=Arabidopsis thaliana GN=KIN7O PE=3 SV=1	4.05	21.83	-1.47	down	0.00	0.00	yes

TRINITY_DN21275_c0_g1	unknown [Populus trichocarpa x Populus deltoides]	HIPP47	Heavy metal-associated isoprenylated plant protein 47 OS=Arabidopsis thaliana GN=HIPP47 PE=3 SV=1	0.60	13.63	-4.44	down	0.00	0.00	yes
TRINITY_DN27884_c4_g1	hypothetical protein POPTR_0003s19950g [Populus trichocarpa]	RPPL1	Putative disease resistance RPP13-like protein 1 OS=Arabidopsis thaliana GN=RPPL1 PE=3 SV=1	8.59	34.00	-1.85	down	0.00	0.00	yes
TRINITY_DN19909_c1_g2	hypothetical protein POPTR_0004s06180g [Populus trichocarpa]	RFK1	Probable LRR receptor-like serine/threonine-protein kinase At1g29720 OS=Arabidopsis thaliana GN=RFK1 PE=2 SV=3	8.84	41.26	-1.64	down	0.00	0.00	yes
TRINITY_DN21569_c0_g1	cyclin D3.1 family protein [Populus trichocarpa]	CYCD3-3	Cyclin-D3-3 OS=Arabidopsis thaliana GN=CYCD3-3 PE=2 SV=1	45.82	209.68	-1.63	down	0.00	0.00	yes
TRINITY_DN17212_c0_g1	hypothetical protein POPTR_0001s21600g [Populus trichocarpa]	ndc80	Probable kinetochore protein ndc80 OS=Neosartorya fumigata (strain ATCC MYA-4609 / Af293 / CBS 101355 / FGSC A1100) GN=ndc80 PE=3 SV=2	5.32	25.18	-1.64	down	0.00	0.00	yes
TRINITY_DN23700_c0_g2	hypothetical protein POPTR_0016s08150g [Populus trichocarpa]	At5g02620	Ankyrin repeat-containing protein At5g02620 OS=Arabidopsis thaliana GN=At5g02620 PE=1 SV=1	0.66	11.35	-3.49	down	0.00	0.00	yes
TRINITY_DN26175_c0_g5	PREDICTED: probable inactive receptor-like protein kinase At3g56050 [Populus euphratica]	At2g40270	Inactive receptor-like serine/threonine-protein kinase At2g40270 OS=Arabidopsis thaliana GN=At2g40270 PE=2 SV=2	4.06	22.52	-1.84	down	0.00	0.00	yes
TRINITY_DN18101_c0_g1	PREDICTED: gibberellin 2-beta-dioxygenase 2 [Populus euphratica]	-	-	1.45	15.83	-2.52	down	0.00	0.00	yes
TRINITY_DN24316_c0_g1	hypothetical protein POPTR_0001s14610g [Populus trichocarpa]	-	Lupeol synthase OS=Ricinus communis PE=1 SV=1	0.00	2.26	-7.47	down	0.00	0.00	yes
TRINITY_DN19267_c0_g3	PERIANTHIA family protein [Populus trichocarpa]	PAN	Transcription factor PERIANTHIA OS=Arabidopsis thaliana GN=PAN PE=1 SV=1	1.45	7.99	-2.90	down	0.00	0.00	yes
TRINITY_DN24189_c0_g1	PREDICTED: uncharacterized protein LOC105116330 [Populus euphratica]	-	-	3.61	15.82	-1.51	down	0.00	0.00	yes
TRINITY_DN22868_c1_g1	PREDICTED: uncharacterized protein LOC105127115 isoform X1 [Populus euphratica]	DDR4	DDT domain-containing protein DDR4 OS=Arabidopsis thaliana GN=DDR4 PE=1 SV=1	2.21	13.04	-1.92	down	0.00	0.00	yes
TRINITY_DN23138_c0_g4	hypothetical protein POPTR_0004s04030g [Populus trichocarpa]	At1g56140	Probable LRR receptor-like serine/threonine-protein kinase At1g56140 OS=Arabidopsis thaliana GN=At1g56140 PE=2 SV=2	3.40	17.04	-1.72	down	0.00	0.00	yes
TRINITY_DN20301_c0_g1	PREDICTED: enolase-phosphatase E1-like [Populus euphratica]	-	-	8.00	54.79	-2.10	down	0.00	0.00	yes
TRINITY_DN15187_c0_g3	hypothetical protein POPTR_0009s15210g, partial [Populus trichocarpa]	AHL1	AT-hook motif nuclear-localized protein 1 OS=Arabidopsis thaliana GN=AHL1 PE=1 SV=1	0.06	2.51	-4.48	down	0.00	0.00	yes
TRINITY_DN24797_c0_g1	calmodulin-binding protein 60-C [Populus tomentosa]	CBP60B	Calmodulin-binding protein 60 B OS=Arabidopsis thaliana GN=CBP60B PE=2 SV=1	6.63	33.00	-1.70	down	0.00	0.00	yes
TRINITY_DN21540_c0_g1	hypothetical protein POPTR_0010s23000g [Populus trichocarpa]	ARF18	Auxin response factor 18 OS=Oryza sativa subsp. japonica GN=ARF18 PE=2 SV=1	9.21	36.37	-1.79	down	0.00	0.00	yes
TRINITY_DN19795_c0_g2	hypothetical protein POPTR_0015s15640g [Populus trichocarpa]	PP2A9	Protein PHLOEM PROTEIN 2-LIKE A9 OS=Arabidopsis thaliana GN=PP2A9 PE=2 SV=1	10.64	66.24	-2.06	down	0.00	0.00	yes
TRINITY_DN25388_c0_g5	copper chaperone-related family protein [Populus trichocarpa]	HIPP35	Heavy metal-associated isoprenylated plant protein 35 OS=Arabidopsis thaliana GN=HIPP35 PE=2 SV=1	0.15	2.82	-3.57	down	0.00	0.00	yes
TRINITY_DN24353_c0_g3	lipoxygenase family protein [Populus trichocarpa]	LOX6	Lipoxygenase 6, chloroplastic OS=Arabidopsis thaliana GN=LOX6 PE=2 SV=1	1.48	7.25	-1.68	down	0.00	0.00	yes
TRINITY_DN17157_c0_g3	-	-	-	0.25	4.67	-3.56	down	0.00	0.00	yes
TRINITY_DN27335_c1_g2	-	-	-	0.09	5.28	-5.36	down	0.00	0.00	yes
TRINITY_DN19273_c0_g6	PREDICTED: uncharacterized protein LOC105110073 isoform X1 [Populus euphratica]	-	-	0.16	3.04	-3.54	down	0.00	0.00	yes
TRINITY_DN24801_c0_g5	nitrate reductase family protein [Populus trichocarpa]	INR1	Inducible nitrate reductase [NADH] 1 OS=Glycine max GN=INR1 PE=2 SV=1	0.00	4.03	-7.25	down	0.00	0.00	yes
TRINITY_DN15255_c0_g1	hypothetical protein POPTR_0012s07210g [Populus trichocarpa]	-	Mitochondrial outer membrane protein porin of 36 kDa OS=Solanum tuberosum PE=1 SV=2	0.43	4.45	-2.78	down	0.00	0.00	yes
TRINITY_DN13106_c0_g1	PREDICTED: uncharacterized protein LOC105110803 isoform X1 [Populus euphratica]	-	-	0.07	3.07	-4.72	down	0.00	0.00	yes
TRINITY_DN13644_c0_g1	aspartic proteinase 2 [Populus trichocarpa]	RAP	Aspartic proteinase OS=Oryza sativa subsp. japonica GN=RAP PE=2 SV=2	0.09	2.71	-4.22	down	0.00	0.00	yes
TRINITY_DN24204_c0_g11	hypothetical protein POPTR_0019s11610g [Populus trichocarpa]	At1g34300	G-type lectin S-receptor-like serine/threonine-protein kinase At1g34300 OS=Arabidopsis thaliana GN=At1g34300 PE=2 SV=1	0.28	4.95	-3.51	down	0.00	0.00	yes
TRINITY_DN22321_c0_g4	unknown [Populus trichocarpa]	-	-	2.07	86.51	-4.73	down	0.00	0.00	yes

TRINITY_DN21608_c0_g1	PREDICTED: cysteine proteinase 15A [Populus euphratica]	RD19D	Probable cysteine protease RD19D OS=Arabidopsis thaliana GN=RD19D PE=2 SV=1	0.00	5.08	-7.23	down	0.00	0.00	yes
TRINITY_DN21972_c0_g1	hypothetical protein POPTR_0014s09140g [Populus trichocarpa]	-	-	2.28	10.35	-1.57	down	0.00	0.00	yes
TRINITY_DN22816_c0_g1	SINA-like family protein [Populus trichocarpa]	SINAT3	E3 ubiquitin-protein ligase SINAT3 OS=Arabidopsis thaliana GN=SINAT3 PE=2 SV=1	8.81	35.64	-1.40	down	0.00	0.00	yes
TRINITY_DN24959_c0_g1	PREDICTED: QWRF motif-containing protein 2 [Populus euphratica]	QWRF2	QWRF motif-containing protein 2 OS=Arabidopsis thaliana GN=QWRF2 PE=2 SV=1	3.70	16.15	-1.53	down	0.00	0.00	yes
TRINITY_DN27600_c0_g1	hypothetical protein POPTR_0019s14870g [Populus trichocarpa]	EPFL6	EPIDERMAL PATTERNING FACTOR-like protein 6 OS=Arabidopsis thaliana GN=EPFL6 PE=1 SV=1	4.58	24.10	-1.73	down	0.00	0.00	yes
TRINITY_DN25867_c0_g2	PREDICTED: E3 ubiquitin-protein ligase RGLG2-like isoform X2 [Populus euphratica]	RGLG1	E3 ubiquitin-protein ligase RGLG1 OS=Arabidopsis thaliana GN=RGLG1 PE=1 SV=1	0.44	6.83	-3.33	down	0.00	0.00	yes
TRINITY_DN15363_c0_g2	putative serine/threonine protein kinase [Populus tomentosa]	PID2	Protein kinase PINOID 2 OS=Arabidopsis thaliana GN=PID2 PE=1 SV=1	0.62	7.03	-2.90	down	0.00	0.00	yes
TRINITY_DN17953_c1_g5	PREDICTED: scopoletin glucosyltransferase-like [Populus euphratica]	TOGT1	Scopoletin glucosyltransferase OS=Nicotiana tabacum GN=TOGT1 PE=1 SV=1	0.78	6.61	-2.47	down	0.00	0.00	yes
TRINITY_DN23221_c1_g4	PREDICTED: cyclic nucleotide-gated ion channel 1-like [Populus euphratica]	CNGC1	Cyclic nucleotide-gated ion channel 1 OS=Arabidopsis thaliana GN=CNGC1 PE=1 SV=1	31.48	139.05	-1.53	down	0.00	0.00	yes
TRINITY_DN22309_c0_g6	PREDICTED: pistil-specific extensin-like protein [Populus euphratica]	AGP31	Non-classical arabinogalactan protein 31 OS=Arabidopsis thaliana GN=AGP31 PE=1 SV=1	10.42	72.33	-2.18	down	0.00	0.00	yes
TRINITY_DN16641_c0_g1	hypothetical protein POPTR_0011s05050g [Populus trichocarpa]	TOM2AH3	Tetraspanin-19 OS=Arabidopsis thaliana GN=TOM2AH3 PE=2 SV=1	2.44	17.30	-2.18	down	0.00	0.00	yes
TRINITY_DN25419_c0_g1	PREDICTED: F-box/LRR-repeat protein 3-like [Populus euphratica]	FBL3	F-box/LRR-repeat protein 3 OS=Arabidopsis thaliana GN=FBL3 PE=2 SV=1	4.28	18.56	-2.27	down	0.00	0.00	yes
TRINITY_DN19238_c0_g2	hypothetical protein POPTR_0001s44700g [Populus trichocarpa]	-	-	0.52	4.90	-2.61	down	0.00	0.00	yes
TRINITY_DN25545_c0_g1	hypothetical protein POPTR_0002s05360g [Populus trichocarpa]	ARIA	ARM REPEAT PROTEIN INTERACTING WITH ABF2 OS=Arabidopsis thaliana GN=ARIA PE=1 SV=2	1.80	12.98	-2.24	down	0.00	0.00	yes
TRINITY_DN8146_c0_g1	unknown [Populus trichocarpa]	-	-	7.01	47.19	-2.17	down	0.00	0.00	yes
TRINITY_DN21477_c1_g9	hypothetical protein POPTR_0002s01650g [Populus trichocarpa]	-	-	0.47	5.06	-2.85	down	0.00	0.00	yes
TRINITY_DN24875_c0_g5	SNF7 family protein [Populus trichocarpa]	VPS2.1	Vacuolar protein sorting-associated protein 2 homolog 1 OS=Arabidopsis thaliana GN=VPS2.1 PE=1 SV=2	5.79	32.52	-1.88	down	0.00	0.00	yes
TRINITY_DN25425_c0_g1	hypothetical protein POPTR_0014s02420g [Populus trichocarpa]	WRKY7	Probable WRKY transcription factor 7 OS=Arabidopsis thaliana GN=WRKY7 PE=2 SV=1	14.00	57.95	-1.47	down	0.00	0.00	yes
TRINITY_DN20802_c0_g1	PREDICTED: thymidylate kinase isoform X1 [Populus euphratica]	ZEU1	Thymidylate kinase OS=Arabidopsis thaliana GN=ZEU1 PE=2 SV=1	5.85	27.95	-1.58	down	0.00	0.00	yes
TRINITY_DN17218_c0_g1	-	-	-	324.03	3247.80	-2.23	down	0.00	0.00	yes
TRINITY_DN25879_c0_g1	PREDICTED: shaggy-related protein kinase theta [Populus euphratica]	ASK8	Shaggy-related protein kinase theta OS=Arabidopsis thaliana GN=ASK8 PE=2 SV=3	11.99	48.33	-1.45	down	0.00	0.00	yes
TRINITY_DN20418_c0_g1	hypothetical protein POPTR_0001s25830g [Populus trichocarpa]	CAX1	Vacuolar cation/proton exchanger 1 OS=Arabidopsis thaliana GN=CAX1 PE=1 SV=3	0.14	9.76	-4.53	down	0.00	0.00	yes
TRINITY_DN22374_c0_g1	Cytochrome P450 86A1 family protein [Populus trichocarpa]	CYP86A1	Cytochrome P450 86A1 OS=Arabidopsis thaliana GN=CYP86A1 PE=1 SV=2	0.31	4.14	-3.14	down	0.00	0.00	yes
TRINITY_DN24287_c0_g1	-	-	-	1.03	13.39	-3.08	down	0.00	0.00	yes
TRINITY_DN26451_c2_g3	PREDICTED: probable receptor-like protein kinase At5g39020 [Populus euphratica]	LRK10	Rust resistance kinase Lr10 OS=Triticum aestivum GN=LRK10 PE=2 SV=1	0.18	4.41	-3.95	down	0.00	0.00	yes
TRINITY_DN21981_c0_g2	hypothetical protein POPTR_0001s02320g [Populus trichocarpa]	TKPR1	Tetraketide alpha-pyrone reductase 1 OS=Arabidopsis thaliana GN=TKPR1 PE=1 SV=1	0.24	5.58	-3.91	down	0.00	0.00	yes
TRINITY_DN18382_c0_g1	hypothetical protein POPTR_0011s14450g [Populus trichocarpa]	MMS21	E3 SUMO-protein ligase MMS21 OS=Arabidopsis thaliana GN=MMS21 PE=1 SV=1	7.65	35.66	-1.57	down	0.00	0.00	yes
TRINITY_DN16585_c0_g1	hypothetical protein POPTR_0002s17990g [Populus trichocarpa]	-	-	2.34	18.65	-2.32	down	0.00	0.00	yes
TRINITY_DN25438_c0_g1	endo-1,4-beta glucanase [Populus alba]	CEL1	Endoglucanase 1 OS=Persea americana GN=CEL1 PE=2 SV=1	5.85	54.28	-2.79	down	0.00	0.00	yes
TRINITY_DN15096_c0_g1	hypothetical protein POPTR_0011s09200g [Populus trichocarpa]	At5g55050	GDSL esterase/lipase At5g55050 OS=Arabidopsis thaliana GN=At5g55050 PE=2 SV=1	11.86	68.50	-2.01	down	0.00	0.00	yes

TRINITY_DN24138_c0_g2	PREDICTED: cyclic nucleotide-gated ion channel 4 [Populus euphratica]	CNGC4	Cyclic nucleotide-gated ion channel 4 OS=Arabidopsis thaliana GN=CNGC4 PE=2 SV=2	26.87	128.24	-1.62	down	0.00	0.00	yes
TRINITY_DN21506_c0_g2	PREDICTED: tryptophan aminotransferase-related protein 2-like [Populus euphratica]	TAR2	Tryptophan aminotransferase-related protein 2 OS=Arabidopsis thaliana GN=TAR2 PE=2 SV=1	1.39	8.60	-2.04	down	0.00	0.00	yes
TRINITY_DN26610_c0_g1	PREDICTED: nudix hydrolase 4-like [Populus euphratica]	NUDT4	Nudix hydrolase 4 OS=Arabidopsis thaliana GN=NUDT4 PE=1 SV=1	12.90	60.42	-1.54	down	0.00	0.00	yes
TRINITY_DN20224_c0_g7	PREDICTED: golgin subfamily A member 8A-like isoform X1 [Populus euphratica]	-	-	0.16	3.49	-3.74	down	0.00	0.00	yes
TRINITY_DN847_c0_g1	glutamate decarboxylase 1 family protein [Populus trichocarpa]	GAD	Glutamate decarboxylase OS=Petunia hybrida GN=GAD PE=1 SV=1	0.04	7.12	-7.22	down	0.00	0.00	yes
TRINITY_DN15219_c0_g1	hypothetical protein POPTR_0013s05650g [Populus trichocarpa]	LECRK4	G-type lectin S-receptor-like serine/threonine-protein kinase LECRK4 OS=Oryza sativa subsp. indica GN=LECRK4 PE=3 SV=1	0.23	2.86	-3.09	down	0.00	0.00	yes
TRINITY_DN17813_c0_g1	hypothetical protein POPTR_0007s12660g [Populus trichocarpa]	At5g49610	F-box protein At5g49610 OS=Arabidopsis thaliana GN=At5g49610 PE=1 SV=1	2.76	14.45	-1.77	down	0.00	0.00	yes
TRINITY_DN12563_c0_g1	PREDICTED: uncharacterized protein LOC105109851 [Populus euphratica]	-	-	0.70	12.14	-3.57	down	0.00	0.00	yes
TRINITY_DN25536_c0_g2	-	-	-	11.67	56.93	-1.62	down	0.00	0.00	yes
TRINITY_DN16544_c0_g2	Homocysteine S-methyltransferase 3 family protein [Populus trichocarpa]	HMT3	Homocysteine S-methyltransferase 3 OS=Arabidopsis thaliana GN=HMT3 PE=1 SV=2	0.36	6.85	-4.08	down	0.00	0.00	yes
TRINITY_DN27824_c1_g1	DNA topoisomerase family protein [Populus trichocarpa]	TOP2	DNA topoisomerase 2 OS=Pisum sativum GN=TOP2 PE=2 SV=1	23.66	101.17	-1.50	down	0.00	0.00	yes
TRINITY_DN21113_c0_g2	hypothetical protein [Populus tomentosa]	-	-	0.44	6.41	-3.07	down	0.00	0.00	yes
TRINITY_DN23725_c0_g1	hypothetical protein POPTR_0002s02630g [Populus trichocarpa]	ARF5	Auxin response factor 5 OS=Arabidopsis thaliana GN=ARF5 PE=1 SV=3	0.46	7.67	-3.44	down	0.00	0.00	yes
TRINITY_DN17522_c0_g1	PREDICTED: replication protein A 70 kDa DNA-binding subunit B [Populus euphratica]	RPA1B	Replication protein A 70 kDa DNA-binding subunit B OS=Arabidopsis thaliana GN=RPA1B PE=3 SV=1	3.63	29.40	-2.36	down	0.00	0.00	yes
TRINITY_DN16858_c0_g1	hypothetical protein POPTR_0010s17600g [Populus trichocarpa]	-	-	1.23	10.14	-2.44	down	0.00	0.00	yes
TRINITY_DN20245_c0_g1	PREDICTED: RING-H2 finger protein ATL80-like [Populus euphratica]	ATL80	RING-H2 finger protein ATL80 OS=Arabidopsis thaliana GN=ATL80 PE=2 SV=1	0.79	5.50	-2.24	down	0.00	0.00	yes
TRINITY_DN20057_c0_g1	PREDICTED: probable inactive receptor-like protein kinase At3g56050 [Populus euphratica]	At3g56050	Probable inactive receptor-like protein kinase At3g56050 OS=Arabidopsis thaliana GN=At3g56050 PE=2 SV=1	1.33	11.06	-2.35	down	0.00	0.00	yes
TRINITY_DN20798_c0_g1	PREDICTED: uncharacterized protein LOC105109812 [Populus euphratica]	-	-	6.49	42.79	-2.09	down	0.00	0.00	yes
TRINITY_DN26228_c0_g1	hypothetical protein POPTR_0003s20840g [Populus trichocarpa]	YTHDF2	YTH domain-containing family protein 2 OS=Homo sapiens GN=YTHDF2 PE=1 SV=2	161.10	680.67	-1.47	down	0.00	0.00	yes
TRINITY_DN20078_c1_g1	PREDICTED: uncharacterized protein LOC105117996 [Populus euphratica]	SMR6	Cyclin-dependent protein kinase inhibitor SMR6 OS=Arabidopsis thaliana GN=SMR6 PE=1 SV=1	1.00	13.56	-3.10	down	0.00	0.00	yes
TRINITY_DN21113_c0_g3	PREDICTED: uncharacterized protein LOC105131077 isoform X1 [Populus euphratica]	-	-	0.16	6.88	-3.89	down	0.00	0.00	yes
TRINITY_DN24539_c0_g1	PREDICTED: ALA-interacting subunit 3-like [Populus euphratica]	ALIS3	ALA-interacting subunit 3 OS=Arabidopsis thaliana GN=ALIS3 PE=1 SV=1	6.41	28.82	-1.63	down	0.00	0.00	yes
TRINITY_DN20411_c0_g3	PREDICTED: histone H2A.6 isoform X1 [Populus euphratica]	OsI_025469	Probable histone H2A.2 OS=Oryza sativa subsp. indica GN=OsI_025469 PE=3 SV=1	31.28	128.16	-1.43	down	0.00	0.00	yes
TRINITY_DN14374_c0_g1	PREDICTED: uncharacterized protein LOC105123768 isoform X1 [Populus euphratica]	-	-	0.00	2.61	-7.70	down	0.00	0.00	yes
TRINITY_DN17164_c0_g1	RAC-like GTP binding protein ARAC9 [Populus trichocarpa]	RAC2	Rac-like GTP-binding protein RAC2 OS=Lotus japonicus GN=RAC2 PE=2 SV=1	1.94	14.44	-2.35	down	0.00	0.00	yes
TRINITY_DN16177_c0_g1	PREDICTED: uncharacterized protein LOC105130613 [Populus euphratica]	-	-	0.31	3.87	-3.03	down	0.00	0.00	yes
TRINITY_DN16616_c0_g1	putative histone h2a.5 [Nicotiana attenuata]	-	Histone H2A OS=Euphorbia esula PE=2 SV=1	172.64	754.79	-1.53	down	0.00	0.00	yes
TRINITY_DN20039_c0_g3	hypothetical protein POPTR_0011s00950g [Populus trichocarpa]	At1g05000	Probable tyrosine-protein phosphatase At1g05000 OS=Arabidopsis thaliana GN=At1g05000 PE=1 SV=1	2.02	14.75	-2.28	down	0.00	0.00	yes
TRINITY_DN21821_c2_g1	PREDICTED: probable linoleate 9S-lipoxygenase 5 [Populus euphratica]	LOX1	Linoleate 9S-lipoxygenase 1 OS=Arabidopsis thaliana GN=LOX1 PE=1 SV=1	0.00	3.52	-7.59	down	0.00	0.00	yes

TRINITY_DN27831_c0_g1	PREDICTED: zinc finger protein NUTCRACKER-like [Populus euphratica]	IDD5	Protein indeterminate-domain 5, chloroplastic OS=Arabidopsis thaliana GN=IDD5 PE=1 SV=1	14.17	62.91	-1.56	down	0.00	0.00	yes
TRINITY_DN14013_c0_g1	hypothetical protein POPTR_0006s24550g [Populus trichocarpa]	-	-	0.06	2.59	-4.76	down	0.00	0.00	yes
TRINITY_DN26883_c0_g4	AP2 domain-containing transcription factor family protein [Populus trichocarpa]	RAP2-13	Ethylene-responsive transcription factor RAP2-13 OS=Arabidopsis thaliana GN=RAP2-13 PE=1 SV=1	13.54	128.65	-2.61	down	0.00	0.00	yes
TRINITY_DN17986_c1_g1	PREDICTED: probable WRKY transcription factor 48 isoform X1 [Populus euphratica]	-	-	0.09	3.69	-4.44	down	0.00	0.00	yes
TRINITY_DN17349_c1_g3	hypothetical protein POPTR_0010s12330g [Populus trichocarpa]	-	Probable aquaporin NIP-type OS=Nicotiana alata PE=2 SV=1	2.18	18.92	-2.46	down	0.00	0.00	yes
TRINITY_DN20455_c0_g4	hypothetical protein POPTR_0012s03730g [Populus trichocarpa]	IDD14	Protein indeterminate-domain 14 OS=Arabidopsis thaliana GN=IDD14 PE=1 SV=1	2.77	12.99	-1.61	down	0.00	0.00	yes
TRINITY_DN24880_c0_g1	curculin-like lectin family protein [Populus trichocarpa]	SD31	G-type lectin S-receptor-like serine/threonine-protein kinase SD3-1 OS=Arabidopsis thaliana GN=SD31 PE=3 SV=1	4.67	18.36	-1.43	down	0.00	0.00	yes
TRINITY_DN19472_c0_g3	hypothetical protein POPTR_0017s04550g [Populus trichocarpa]	naat-B	Nicotianamine aminotransferase B OS=Hordeum vulgare GN=naat-B PE=1 SV=2	0.21	3.06	-3.23	down	0.00	0.00	yes
TRINITY_DN21924_c0_g3	-	-	-	0.04	7.56	-6.50	down	0.00	0.00	yes
TRINITY_DN24293_c0_g5	hypothetical protein POPTR_0005s03580g [Populus trichocarpa]	LOX1.5	Probable linoleate 9S-lipoxygenase 5 OS=Solanum tuberosum GN=LOX1.5 PE=2 SV=1	7.72	39.30	-1.68	down	0.00	0.00	yes
TRINITY_DN20902_c0_g2	PREDICTED: putative glucose-6-phosphate 1-epimerase isoform X1 [Theobroma cacao]	-	Putative glucose-6-phosphate 1-epimerase OS=Cenchrus ciliaris PE=2 SV=1	6.26	29.13	-1.66	down	0.00	0.00	yes
TRINITY_DN16939_c0_g4	hypothetical protein POPTR_0007s06570g [Populus trichocarpa]	CYP78A7	Cytochrome P450 78A7 OS=Arabidopsis thaliana GN=CYP78A7 PE=2 SV=1	0.04	2.53	-5.19	down	0.00	0.00	yes
TRINITY_DN22356_c1_g2	PREDICTED: protein LONGIFOLIA 1-like [Populus euphratica]	LNG1	Protein LONGIFOLIA 1 OS=Arabidopsis thaliana GN=LNG1 PE=1 SV=1	0.62	6.47	-2.75	down	0.00	0.00	yes
TRINITY_DN7930_c0_g1	hypothetical protein POPTR_0019s10980g [Populus trichocarpa]	-	-	0.37	31.15	-5.56	down	0.00	0.00	yes
TRINITY_DN20770_c0_g3	putative histidine-containing phosphotransfer protein 2 [Populus trichocarpa]	AHP1	Histidine-containing phosphotransfer protein 1 OS=Arabidopsis thaliana GN=AHP1 PE=1 SV=1	3.26	20.12	-2.04	down	0.00	0.00	yes
TRINITY_DN22797_c0_g2	PREDICTED: alpha/beta hydrolase domain-containing protein 17B-like isoform X1 [Populus euphratica]	ABHD17B	Protein ABHD17B OS=Gallus gallus GN=ABHD17B PE=2 SV=1	3.08	12.57	-1.75	down	0.00	0.00	yes
TRINITY_DN19258_c0_g1	PREDICTED: agamous-like MADS-box protein AGL6 [Populus euphratica]	AGL62	Agamous-like MADS-box protein AGL62 OS=Arabidopsis thaliana GN=AGL62 PE=1 SV=1	0.31	2.55	-2.54	down	0.00	0.00	yes
TRINITY_DN24436_c0_g2	PREDICTED: cation/H(+) antiporter 15-like [Populus euphratica]	CHX15	Cation/H(+) antiporter 15 OS=Arabidopsis thaliana GN=CHX15 PE=2 SV=1	2.39	15.07	-2.03	down	0.00	0.00	yes
TRINITY_DN27358_c0_g1	hypothetical protein POPTR_0014s14320g [Populus trichocarpa]	-	-	2.06	16.64	-2.89	down	0.00	0.00	yes
TRINITY_DN20475_c0_g3	hypothetical protein POPTR_0002s15910g [Populus trichocarpa]	-	-	0.52	6.54	-3.00	down	0.00	0.00	yes
TRINITY_DN22467_c0_g3	PREDICTED: uncharacterized protein LOC105137361 [Populus euphratica]	BZIP17	bZIP transcription factor 17 OS=Arabidopsis thaliana GN=BZIP17 PE=1 SV=2	7.71	34.02	-1.54	down	0.00	0.00	yes
TRINITY_DN22262_c0_g2	PREDICTED: HMG1/2-like protein [Populus euphratica]	-	HMG1/2-like protein OS=Ipomoea nil PE=2 SV=1	2.46	15.63	-2.11	down	0.00	0.00	yes
TRINITY_DN20156_c0_g1	hypothetical protein POPTR_0017s03790g [Populus trichocarpa]	At5g48380	Probably inactive leucine-rich repeat receptor-like protein kinase At5g48380 OS=Arabidopsis thaliana GN=At5g48380 PE=1 SV=1	0.32	5.37	-3.38	down	0.00	0.00	yes
TRINITY_DN25785_c0_g2	PREDICTED: TMV resistance protein N-like isoform X1 [Populus euphratica]	-	-	3.10	22.69	-2.28	down	0.00	0.00	yes
TRINITY_DN22279_c0_g3	PREDICTED: abnormal spindle-like microcephaly-associated protein homolog isoform X1 [Populus euphratica]	ASPM	Abnormal spindle-like microcephaly-associated protein homolog (Fragment) OS=Bos taurus GN=ASPM PE=2 SV=1	12.21	49.21	-1.42	down	0.00	0.00	yes
TRINITY_DN23900_c0_g1	PREDICTED: probable protein phosphatase 2C 24 [Populus euphratica]	AIP1	Protein phosphatase 2C 3 OS=Arabidopsis thaliana GN=AIP1 PE=1 SV=1	13.75	77.95	-1.81	down	0.00	0.00	yes
TRINITY_DN22558_c2_g1	hypothetical protein POPTR_0016s02770g [Populus trichocarpa]	Os05g0239150	Zinc finger BED domain-containing protein RICESLEEPER 1 OS=Oryza sativa subsp. japonica GN=Os05g0239150 PE=3 SV=1	24.16	67.60	-1.83	down	0.00	0.00	yes
TRINITY_DN26170_c0_g1	hypothetical protein POPTR_0007s12120g [Populus trichocarpa]	-	-	6.45	26.47	-1.62	down	0.00	0.00	yes

TRINITY_DN26710_c1_g1	hypothetical protein POPTR_0003s10680g [Populus trichocarpa]	TDR	Leucine-rich repeat receptor-like protein kinase TDR OS=Arabidopsis thaliana GN=TDR PE=1 SV=1	3.71	20.93	-1.93	down	0.00	0.00	yes
TRINITY_DN22263_c0_g1	PREDICTED: uncharacterized protein LOC105124988 [Populus euphratica]	-	-	1.17	8.65	-2.25	down	0.00	0.00	yes
TRINITY_DN22515_c0_g14	-	-	-	10.72	45.54	-1.49	down	0.00	0.00	yes
TRINITY_DN17008_c0_g1	hypothetical protein POPTR_0003s17640g [Populus trichocarpa]	-	-	2.61	14.24	-1.85	down	0.00	0.00	yes
TRINITY_DN16806_c1_g3	putative calmodulin-related family protein [Populus trichocarpa]	CML27	Probable calcium-binding protein CML27 OS=Arabidopsis thaliana GN=CML27 PE=1 SV=1	21.78	225.85	-2.76	down	0.00	0.00	yes
TRINITY_DN23878_c0_g2	homeodomain-containing family protein [Populus trichocarpa]	BLH11	BEL1-like homeodomain protein 11 OS=Arabidopsis thaliana GN=BLH11 PE=2 SV=1	3.78	17.94	-1.73	down	0.00	0.00	yes
TRINITY_DN24298_c0_g2	PREDICTED: uncharacterized protein LOC105107718 isoform X1 [Populus euphratica]	-	-	2.88	17.95	-2.05	down	0.00	0.00	yes
TRINITY_DN19836_c0_g2	xanthine/uracil permease family protein [Populus trichocarpa]	AZG1	Adenine/guanine permease AZG1 OS=Arabidopsis thaliana GN=AZG1 PE=2 SV=1	0.55	5.10	-2.62	down	0.00	0.00	yes
TRINITY_DN16050_c0_g1	hypothetical protein POPTR_0001s38620g [Populus trichocarpa]	PTD	Protein PARTING DANCERS OS=Arabidopsis thaliana GN=PTD PE=1 SV=1	1.03	8.58	-2.47	down	0.00	0.00	yes
TRINITY_DN24287_c0_g4	PREDICTED: protein phosphatase 2C 29 [Populus euphratica]	PLL1	Protein phosphatase 2C 29 OS=Arabidopsis thaliana GN=PLL1 PE=1 SV=2	2.46	13.73	-1.84	down	0.00	0.00	yes
TRINITY_DN21384_c0_g1	PREDICTED: kinesin-like protein KIF22 [Populus euphratica]	KIN10B	Kinesin-like protein KIN-10B OS=Arabidopsis thaliana GN=KIN10B PE=2 SV=1	8.18	34.48	-1.53	down	0.00	0.00	yes
TRINITY_DN23946_c0_g3	bifunctional inhibitor/lipid-transfer protein/seed storage 2S albumin superfamily protein [Populus tomentosa]	-	-	0.40	6.22	-3.31	down	0.00	0.00	yes
TRINITY_DN18107_c0_g1	hypothetical protein POPTR_0004s16850g [Populus trichocarpa]	SBT3.13	Subtilisin-like protease SBT3.13 OS=Arabidopsis thaliana GN=SBT3.13 PE=2 SV=1	0.37	8.91	-4.14	down	0.00	0.00	yes
TRINITY_DN23250_c0_g4	PREDICTED: leucine-rich repeat receptor protein kinase EXS-like [Populus euphratica]	EMS1	Leucine-rich repeat receptor protein kinase EMS1 OS=Arabidopsis thaliana GN=EMS1 PE=1 SV=1	1.02	10.21	-2.32	down	0.00	0.00	yes
TRINITY_DN15692_c0_g1	hypothetical protein POPTR_0001s45240g [Populus trichocarpa]	At5g39865	Uncharacterized protein At5g39865 OS=Arabidopsis thaliana GN=At5g39865 PE=2 SV=1	4.23	22.31	-1.80	down	0.00	0.00	yes
TRINITY_DN13077_c0_g1	unknown [Populus trichocarpa]	-	-	0.30	5.95	-3.90	down	0.00	0.00	yes
TRINITY_DN22250_c0_g2	hypothetical protein POPTR_0001s17420g, partial [Populus trichocarpa]	-	-	45.32	209.29	-1.61	down	0.00	0.00	yes
TRINITY_DN18857_c0_g1	zinc finger family protein [Populus trichocarpa]	rnf144a	Probable E3 ubiquitin-protein ligase RNF144A OS=Xenopus tropicalis GN=rnf144a PE=2 SV=1	1.93	13.75	-2.20	down	0.00	0.00	yes
TRINITY_DN26624_c0_g1	PREDICTED: BTB/POZ domain-containing protein At3g22104-like [Populus euphratica]	At3g22104	BTB/POZ domain-containing protein At3g22104 OS=Arabidopsis thaliana GN=At3g22104 PE=2 SV=1	6.10	26.39	-1.49	down	0.00	0.00	yes
TRINITY_DN26883_c0_g1	hypothetical protein POPTR_0005s16690g [Populus trichocarpa]	RAP2-4	Ethylene-responsive transcription factor RAP2-4 OS=Arabidopsis thaliana GN=RAP2-4 PE=1 SV=1	15.04	123.74	-2.40	down	0.00	0.00	yes
TRINITY_DN21909_c0_g2	F-box family protein [Populus trichocarpa]	SKIP23	F-box protein SKIP23 OS=Arabidopsis thaliana GN=SKIP23 PE=1 SV=1	3.26	17.79	-2.01	down	0.00	0.00	yes
TRINITY_DN3176_c0_g1	PREDICTED: G-type lectin S-receptor-like serine/threonine-protein kinase At5g24080 [Populus euphratica]	LRK10	Rust resistance kinase Lr10 OS=Triticum aestivum GN=LRK10 PE=2 SV=1	0.13	2.33	-3.43	down	0.00	0.00	yes
TRINITY_DN15081_c0_g1	PREDICTED: uncharacterized protein At4g22758-like [Populus euphratica]	-	-	0.32	4.41	-3.16	down	0.00	0.00	yes
TRINITY_DN22952_c0_g1	PREDICTED: uncharacterized protein LOC105110150 [Populus euphratica]	-	-	2.83	13.95	-1.79	down	0.00	0.00	yes
TRINITY_DN21610_c0_g2	PREDICTED: uncharacterized protein LOC105114072 [Populus euphratica]	MIS12	Protein MIS12 homolog OS=Arabidopsis thaliana GN=MIS12 PE=3 SV=1	10.18	41.15	-1.37	down	0.00	0.00	yes
TRINITY_DN16291_c0_g1	hypothetical protein POPTR_0014s07350g [Populus trichocarpa]	UTR4	UDP-galactose/UDP-glucose transporter 4 OS=Arabidopsis thaliana GN=UTR4 PE=2 SV=1	0.33	3.99	-3.00	down	0.00	0.00	yes
TRINITY_DN25910_c0_g4	hypothetical protein POPTR_0009s03990g [Populus trichocarpa]	BOA	Transcription factor BOA OS=Arabidopsis thaliana GN=BOA PE=2 SV=1	5.07	23.12	-1.56	down	0.00	0.00	yes
TRINITY_DN22656_c1_g4	auxin-responsive family protein [Populus trichocarpa]	SAUR24	Auxin-responsive protein SAUR24 OS=Arabidopsis thaliana GN=SAUR24 PE=2 SV=1	0.05	4.11	-5.27	down	0.00	0.00	yes

TRINITY_DN21068_c1_g1	hypothetical protein POPTR_0013s07050g [Populus trichocarpa]	CYP75B2	Flavonoid 3'-monooxygenase OS=Petunia hybrida GN=CYP75B2 PE=2 SV=1	25.27	141.84	-1.96	down	0.00	0.00	yes
TRINITY_DN22121_c0_g5	PREDICTED: DNA (cytosine-5)-methyltransferase CMT3-like [Populus euphratica]	-	-	1.79	10.94	-2.01	down	0.00	0.00	yes
TRINITY_DN11615_c0_g2	hypothetical protein POPTR_0004s03330g [Populus trichocarpa]	-	-	1.38	12.96	-2.62	down	0.00	0.00	yes
TRINITY_DN27768_c0_g1	hypothetical protein POPTR_0005s03560g [Populus trichocarpa]	LOX1.5	Probable linoleate 9S-lipoxygenase 5 OS=Solanum tuberosum GN=LOX1.5 PE=2 SV=1	0.00	4.46	-7.23	down	0.00	0.00	yes
TRINITY_DN21194_c1_g1	PREDICTED: uncharacterized protein LOC105130266 [Populus euphratica]	-	-	4.36	23.20	-1.80	down	0.00	0.00	yes
TRINITY_DN19130_c0_g5	PREDICTED: kinesin KP1-like [Populus euphratica]	KIN14T	Kinesin-like protein KIN-14T OS=Arabidopsis thaliana GN=KIN14T PE=3 SV=1	0.34	3.03	-2.54	down	0.00	0.00	yes
TRINITY_DN17986_c3_g2	CLE41 [Populus tremula x Populus tremuloides]	CLE41	CLAVATA3/ESR (CLE)-related protein 41 OS=Arabidopsis thaliana GN=CLE41 PE=1 SV=1	0.52	9.44	-3.52	down	0.00	0.00	yes
TRINITY_DN18725_c0_g2	hypothetical protein POPTR_0007s12670g [Populus trichocarpa]	At4g09190	Putative F-box protein At4g09190 OS=Arabidopsis thaliana GN=At4g09190 PE=4 SV=1	0.23	4.30	-3.53	down	0.00	0.00	yes
TRINITY_DN20195_c0_g1	unknown [Populus trichocarpa]	NAC74	NAC domain-containing protein 74 OS=Oryza sativa subsp. japonica GN=NAC74 PE=2 SV=1	5.61	24.26	-1.52	down	0.00	0.00	yes
TRINITY_DN20699_c0_g2	PREDICTED: RING-H2 finger protein ATL13 isoform X1 [Populus euphratica]	ATL13	RING-H2 finger protein ATL13 OS=Arabidopsis thaliana GN=ATL13 PE=2 SV=2	4.06	17.08	-1.46	down	0.00	0.00	yes
TRINITY_DN16397_c0_g1	hypothetical protein POPTR_0003s14670g [Populus trichocarpa]	CYP78A9	Cytochrome P450 78A9 OS=Arabidopsis thaliana GN=CYP78A9 PE=2 SV=1	0.06	2.04	-4.37	down	0.00	0.00	yes
TRINITY_DN19427_c0_g1	PREDICTED: F-box protein At1g61340-like [Populus euphratica]	At1g61340	F-box protein At1g61340 OS=Arabidopsis thaliana GN=At1g61340 PE=2 SV=1	7.21	73.29	-2.77	down	0.00	0.00	yes
TRINITY_DN23014_c0_g1	bZIP transcription factor family protein [Populus trichocarpa]	BZIP60	bZIP transcription factor 60 OS=Arabidopsis thaliana GN=BZIP60 PE=1 SV=1	5.36	51.63	-2.53	down	0.00	0.00	yes
TRINITY_DN23756_c0_g2	PREDICTED: uncharacterized protein LOC105118035 [Populus euphratica]	-	-	2.72	18.01	-2.07	down	0.00	0.00	yes
TRINITY_DN21468_c0_g3	hypothetical protein POPTR_0006s06000g [Populus trichocarpa]	-	-	0.40	3.48	-2.50	down	0.00	0.00	yes
TRINITY_DN22991_c0_g1	SKP1 INTERACTING PARTNER 2 family protein [Populus trichocarpa]	SKIP2	F-box protein SKIP2 OS=Arabidopsis thaliana GN=SKIP2 PE=1 SV=1	2.64	23.78	-2.55	down	0.00	0.00	yes
TRINITY_DN27470_c0_g2	PREDICTED: myb-related protein P-like [Populus euphratica]	MYB12	Transcription factor MYB12 OS=Arabidopsis thaliana GN=MYB12 PE=2 SV=1	0.00	5.29	-8.05	down	0.00	0.00	yes
TRINITY_DN24607_c0_g2	PREDICTED: UPF0481 protein At3g47200-like [Populus euphratica]	-	-	1.92	13.49	-2.53	down	0.00	0.00	yes
TRINITY_DN19746_c0_g1	PREDICTED: probable membrane-associated kinase regulator 5 isoform X1 [Populus euphratica]	MAKR5	Probable membrane-associated kinase regulator 5 OS=Arabidopsis thaliana GN=MAKR5 PE=2 SV=1	1.24	9.69	-2.33	down	0.00	0.00	yes
TRINITY_DN17157_c0_g4	unknown [Populus trichocarpa]	-	-	30.95	139.17	-1.56	down	0.00	0.00	yes
TRINITY_DN20687_c0_g1	RING-H2 subgroup RHE protein [Populus tremula x Populus alba]	ATL2	RING-H2 finger protein ATL2 OS=Arabidopsis thaliana GN=ATL2 PE=2 SV=2	8.01	63.62	-2.39	down	0.00	0.00	yes
TRINITY_DN549_c0_g1	PREDICTED: heavy metal-associated isoprenylated plant protein 26-like [Populus euphratica]	HIPP24	Heavy metal-associated isoprenylated plant protein 24 OS=Arabidopsis thaliana GN=HIPP24 PE=1 SV=1	0.39	4.57	-3.31	down	0.00	0.00	yes
TRINITY_DN1759_c0_g1	hypothetical protein POPTR_0002s19130g [Populus trichocarpa]	-	-	0.03	2.73	-5.39	down	0.00	0.00	yes
TRINITY_DN15992_c0_g2	Kunitz-type trypsin inhibitor C3 [Populus nigra]	ASP	21 kDa seed protein OS=Theobroma cacao GN=ASP PE=2 SV=1	0.00	2.60	-7.80	down	0.00	0.00	yes
TRINITY_DN19264_c0_g1	PREDICTED: U-box domain-containing protein 12 [Populus euphratica]	PUB45	U-box domain-containing protein 45 OS=Arabidopsis thaliana GN=PUB45 PE=1 SV=1	2.64	18.79	-2.23	down	0.00	0.00	yes
TRINITY_DN13995_c0_g1	allergen-related family protein [Populus trichocarpa]	-	-	0.28	15.73	-5.04	down	0.00	0.00	yes
TRINITY_DN17380_c0_g1	PREDICTED: meiotic recombination protein SPO11-2 isoform X3 [Populus euphratica]	SPO11-2	Meiotic recombination protein SPO11-2 OS=Arabidopsis thaliana GN=SPO11-2 PE=1 SV=1	3.01	14.32	-1.67	down	0.00	0.00	yes
TRINITY_DN18956_c0_g1	hypothetical protein POPTR_0014s07880g [Populus trichocarpa]	-	-	3.29	17.70	-1.89	down	0.00	0.00	yes
TRINITY_DN27470_c0_g1	hypothetical protein POPTR_0003s14420g [Populus trichocarpa]	MYB12	Transcription factor MYB12 OS=Arabidopsis thaliana GN=MYB12 PE=2 SV=1	0.97	8.22	-2.44	down	0.00	0.00	yes

TRINITY_DN16080_c0_g1	hypothetical protein POPTR_0005s28010g [Populus trichocarpa]	SPL14	Squamosa promoter-binding-like protein 14 OS=Arabidopsis thaliana GN=SPL14 PE=1 SV=3	12.44	71.43	-1.90	down	0.00	0.00	yes
TRINITY_DN27442_c0_g1	PREDICTED: probably inactive leucine-rich repeat receptor-like protein kinase At3g28040 isoform X2 [Populus euphratica]	IRK	Probable LRR receptor-like serine/threonine-protein kinase IRK OS=Arabidopsis thaliana GN=IRK PE=1 SV=1	7.11	33.13	-1.61	down	0.00	0.00	yes
TRINITY_DN23970_c1_g3	hypothetical protein POPTR_0013s01840g [Populus trichocarpa]	HIPP36	Heavy metal-associated isoprenylated plant protein 36 OS=Arabidopsis thaliana GN=HIPP36 PE=2 SV=1	2.31	15.75	-2.12	down	0.00	0.00	yes
TRINITY_DN17272_c0_g1	hypothetical protein POPTR_0007s12240g [Populus trichocarpa]	At4g37220	Cold-regulated 413 plasma membrane protein 4 OS=Arabidopsis thaliana GN=At4g37220 PE=2 SV=2	4.19	30.22	-1.95	down	0.00	0.00	yes
TRINITY_DN20888_c0_g1	PREDICTED: putative ABC transporter C family member 15 isoform X1 [Populus euphratica]	ABCC9	ABC transporter C family member 9 OS=Arabidopsis thaliana GN=ABCC9 PE=2 SV=2	3.76	15.46	-1.45	down	0.00	0.00	yes
TRINITY_DN24968_c0_g1	hypothetical protein POPTR_0003s16000g [Populus trichocarpa]	At3g02290	E3 ubiquitin-protein ligase At3g02290 OS=Arabidopsis thaliana GN=At3g02290 PE=2 SV=1	7.38	30.98	-1.38	down	0.00	0.00	yes
TRINITY_DN25446_c0_g1	hypothetical protein POPTR_0006s20820g [Populus trichocarpa]	HAT14	Homeobox-leucine zipper protein HAT14 OS=Arabidopsis thaliana GN=HAT14 PE=2 SV=3	15.62	54.91	-1.51	down	0.00	0.00	yes
TRINITY_DN26554_c1_g4	hypothetical protein POPTR_0010s05480g [Populus trichocarpa]	PHS1	Protein POOR HOMOLOGOUS SYNAPSIS 1 OS=Arabidopsis thaliana GN=PHS1 PE=1 SV=1	1.41	9.46	-2.11	down	0.00	0.00	yes
TRINITY_DN19773_c0_g1	PREDICTED: exocyst complex component EXO70B1-like [Populus euphratica]	EXO70B1	Exocyst complex component EXO70B1 OS=Arabidopsis thaliana GN=EXO70B1 PE=1 SV=1	8.95	66.36	-2.30	down	0.00	0.00	yes
TRINITY_DN24215_c0_g1	hypothetical protein POPTR_0018s14500g [Populus trichocarpa]	WAKL1	Wall-associated receptor kinase-like 1 OS=Arabidopsis thaliana GN=WAKL1 PE=2 SV=1	3.34	20.39	-1.97	down	0.00	0.00	yes
TRINITY_DN20744_c1_g1	PREDICTED: uncharacterized protein LOC105119582 [Populus euphratica]	-	-	0.66	10.96	-3.39	down	0.00	0.00	yes
TRINITY_DN27005_c0_g1	PREDICTED: AT-rich interactive domain-containing protein 6-like [Populus euphratica]	ARID3	AT-rich interactive domain-containing protein 3 OS=Arabidopsis thaliana GN=ARID3 PE=1 SV=1	28.98	105.47	-1.47	down	0.00	0.00	yes
TRINITY_DN20169_c0_g1	PREDICTED: uncharacterized protein LOC105121450 isoform X3 [Populus euphratica]	-	-	5.94	25.22	-1.48	down	0.00	0.00	yes
TRINITY_DN18539_c0_g1	hypothetical protein POPTR_0017s04100g [Populus trichocarpa]	LRK10	Rust resistance kinase Lr10 OS=Triticum aestivum GN=LRK10 PE=2 SV=1	1.11	14.52	-3.08	down	0.00	0.00	yes
TRINITY_DN25788_c0_g2	PREDICTED: auxilin-like protein 1 isoform X2 [Populus euphratica]	AUL1	Auxilin-like protein 1 OS=Arabidopsis thaliana GN=AUL1 PE=2 SV=2	12.69	51.54	-1.39	down	0.00	0.00	yes
TRINITY_DN15278_c0_g1	BON association protein 1 [Populus trichocarpa]	BAP2	BON1-associated protein 2 OS=Arabidopsis thaliana GN=BAP2 PE=1 SV=1	1.19	23.84	-3.67	down	0.00	0.00	yes
TRINITY_DN16808_c0_g1	-	-	-	2.37	24.84	-2.73	down	0.00	0.00	yes
TRINITY_DN15556_c0_g1	hypothetical protein POPTR_0014s13270g [Populus trichocarpa]	-	-	0.20	2.37	-2.95	down	0.00	0.00	yes
TRINITY_DN18872_c0_g1	hypothetical protein POPTR_0002s06600g [Populus trichocarpa]	EMS1	Leucine-rich repeat receptor protein kinase EMS1 OS=Arabidopsis thaliana GN=EMS1 PE=1 SV=1	0.67	5.94	-2.52	down	0.00	0.00	yes
TRINITY_DN26756_c0_g2	Protein phosphatase 2C family protein [Populus trichocarpa]	PP2CA	Protein phosphatase 2C 37 OS=Arabidopsis thaliana GN=PP2CA PE=1 SV=1	23.37	111.87	-1.61	down	0.00	0.00	yes
TRINITY_DN22434_c0_g1	PREDICTED: uncharacterized protein LOC105123374 [Populus euphratica]	-	-	36.02	143.84	-1.51	down	0.00	0.00	yes
TRINITY_DN21494_c1_g4	hypothetical protein POPTR_0003s11230g [Populus trichocarpa]	YAB1	Axial regulator YABBY 1 OS=Arabidopsis thaliana GN=YAB1 PE=1 SV=1	8.17	49.29	-1.98	down	0.00	0.00	yes
TRINITY_DN16927_c0_g1	PREDICTED: probable protein phosphatase 2C 80 [Populus euphratica]	At4g16580	Probable protein phosphatase 2C 55 OS=Arabidopsis thaliana GN=At4g16580 PE=2 SV=2	0.40	6.58	-3.36	down	0.00	0.00	yes
TRINITY_DN21300_c1_g3	PREDICTED: zinc finger protein WIP2-like [Populus euphratica]	WIP6	Zinc finger protein WIP6 OS=Arabidopsis thaliana GN=WIP6 PE=2 SV=1	0.08	2.02	-4.08	down	0.00	0.00	yes
TRINITY_DN22542_c0_g2	-	-	-	0.09	5.44	-5.05	down	0.00	0.00	yes
TRINITY_DN25320_c0_g2	XH/XS domain-containing family protein [Populus trichocarpa]	FDM4	Factor of DNA methylation 4 OS=Arabidopsis thaliana GN=FDM4 PE=4 SV=1	2.79	12.45	-2.08	down	0.00	0.00	yes
TRINITY_DN21637_c0_g4	hypothetical protein POPTR_0012s03550g [Populus trichocarpa]	HDG11	Homeobox-leucine zipper protein HDG11 OS=Arabidopsis thaliana GN=HDG11 PE=1 SV=1	0.24	6.12	-4.08	down	0.00	0.00	yes
TRINITY_DN16591_c0_g1	PREDICTED: hexose carrier protein HEX6 [Populus euphratica]	HEX6	Hexose carrier protein HEX6 OS=Ricinus communis GN=HEX6 PE=2 SV=1	0.93	6.90	-2.29	down	0.00	0.00	yes

TRINITY_DN15480_c0_g1	PREDICTED: UDP-glycosyltransferase 92A1-like [Populus euphratica]	UGT92A1	UDP-glycosyltransferase 92A1 OS=Arabidopsis thaliana GN=UGT92A1 PE=2 SV=1	5.28	31.92	-1.97	down	0.00	0.00	yes
TRINITY_DN23654_c0_g1	hypothetical protein POPTR_0012s14270g [Populus trichocarpa]	-	-	0.98	6.37	-2.14	down	0.00	0.00	yes
TRINITY_DN21494_c1_g1	hypothetical protein POPTR_0001s00240g [Populus trichocarpa]	YAB1	Axial regulator YABBY 1 OS=Arabidopsis thaliana GN=YAB1 PE=1 SV=1	1.39	22.43	-3.51	down	0.00	0.00	yes
TRINITY_DN22006_c0_g1	hypothetical protein POPTR_0002s16160g [Populus trichocarpa]	PUB33	U-box domain-containing protein 33 OS=Arabidopsis thaliana GN=PUB33 PE=2 SV=2	0.93	7.28	-2.32	down	0.00	0.00	yes
TRINITY_DN23824_c0_g1	BRCT domain-containing family protein [Populus trichocarpa]	BRCA1	Protein BREAST CANCER SUSCEPTIBILITY 1 homolog OS=Arabidopsis thaliana GN=BRCA1 PE=1 SV=1	1.04	6.49	-2.00	down	0.00	0.00	yes
TRINITY_DN26613_c0_g1	hypothetical protein POPTR_0012s01760g [Populus trichocarpa]	LRK10	Rust resistance kinase Lr10 OS=Triticum aestivum GN=LRK10 PE=2 SV=1	7.84	53.29	-1.77	down	0.00	0.00	yes
TRINITY_DN26744_c0_g1	PREDICTED: homeobox-leucine zipper protein HDG5-like isoform X1 [Populus euphratica]	HDG5	Homeobox-leucine zipper protein HDG5 OS=Arabidopsis thaliana GN=HDG5 PE=2 SV=3	6.63	30.33	-1.97	down	0.00	0.00	yes
TRINITY_DN15555_c0_g1	hypothetical protein POPTR_0001s25450g [Populus trichocarpa]	-	-	0.19	5.04	-4.57	down	0.00	0.00	yes
TRINITY_DN14968_c0_g1	PREDICTED: uncharacterized protein LOC105121470 [Populus euphratica]	-	-	0.62	8.22	-3.08	down	0.00	0.00	yes
TRINITY_DN19912_c1_g2	hypothetical protein POPTR_0008s16660g [Populus trichocarpa]	MYB4	Myb-related protein Myb4 OS=Oryza sativa subsp. japonica GN=MYB4 PE=2 SV=2	0.47	14.26	-4.30	down	0.00	0.00	yes
TRINITY_DN25301_c0_g1	PREDICTED: UPF0503 protein At3g09070, chloroplastic-like [Populus euphratica]	At3g09070	UPF0503 protein At3g09070, chloroplastic OS=Arabidopsis thaliana GN=At3g09070 PE=1 SV=1	11.05	55.04	-1.58	down	0.00	0.00	yes
TRINITY_DN10821_c0_g2	-	-	-	1.97	13.40	-2.16	down	0.00	0.00	yes
TRINITY_DN23088_c0_g1	hypothetical protein POPTR_0010s00500g [Populus trichocarpa]	-	-	0.02	2.62	-5.95	down	0.00	0.00	yes
TRINITY_DN19026_c0_g1	PREDICTED: cyclin-D1-1-like isoform X2 [Populus euphratica]	CYCD1-1	Cyclin-D1-1 OS=Arabidopsis thaliana GN=CYCD1-1 PE=1 SV=3	1.27	8.13	-2.71	down	0.00	0.00	yes
TRINITY_DN24211_c0_g1	exocyst subunit EXO70 family protein [Populus trichocarpa]	EXO70B1	Exocyst complex component EXO70B1 OS=Arabidopsis thaliana GN=EXO70B1 PE=1 SV=1	6.70	42.93	-2.07	down	0.00	0.00	yes
TRINITY_DN21494_c1_g5	hypothetical protein POPTR_0014s06210g [Populus trichocarpa]	YAB1	Axial regulator YABBY 1 OS=Arabidopsis thaliana GN=YAB1 PE=1 SV=1	6.63	37.88	-1.91	down	0.00	0.00	yes
TRINITY_DN17146_c0_g2	hypothetical protein POPTR_0007s15030g [Populus trichocarpa]	-	-	0.05	2.73	-4.79	down	0.00	0.00	yes
TRINITY_DN24495_c1_g3	hypothetical protein POPTR_0010s11950g [Populus trichocarpa]	-	21 kDa protein OS=Daucus carota PE=2 SV=1	9.36	40.91	-1.66	down	0.00	0.00	yes
TRINITY_DN26672_c0_g2	hypothetical protein POPTR_0009s04420g [Populus trichocarpa]	-	-	0.64	4.42	-2.15	down	0.00	0.00	yes
TRINITY_DN2415_c0_g1	terpene synthase [Populus trichocarpa]	-	(-)-alpha-terpineol synthase OS=Vitis vinifera PE=1 SV=1	2.28	14.59	-2.03	down	0.00	0.00	yes
TRINITY_DN24970_c1_g1	PREDICTED: protein BREAST CANCER SUSCEPTIBILITY 2 homolog B-like [Populus euphratica]	BRCA2B	Protein BREAST CANCER SUSCEPTIBILITY 2 homolog B OS=Arabidopsis thaliana GN=BRCA2B PE=1 SV=1	1.43	9.39	-1.89	down	0.00	0.00	yes
TRINITY_DN19093_c1_g6	hypothetical protein POPTR_0019s13150g [Populus trichocarpa]	-	-	0.00	6.06	-7.94	down	0.00	0.00	yes
TRINITY_DN22966_c0_g1	hypothetical protein POPTR_0006s22750g [Populus trichocarpa]	-	-	12.07	52.77	-1.47	down	0.00	0.00	yes
TRINITY_DN23440_c0_g5	YUCCA family protein [Populus trichocarpa]	YUC1	Probable indole-3-pyruvate monooxygenase YUCCA1 OS=Arabidopsis thaliana GN=YUC1 PE=1 SV=1	0.00	4.17	-7.26	down	0.00	0.00	yes
TRINITY_DN21320_c0_g2	hypothetical protein POPTR_0014s13500g [Populus trichocarpa]	-	-	9.28	62.81	-1.97	down	0.00	0.00	yes
TRINITY_DN13786_c0_g1	PREDICTED: glycine-rich cell wall structural protein-like isoform X1 [Populus euphratica]	-	-	4.39	75.57	-3.39	down	0.00	0.00	yes
TRINITY_DN23437_c0_g2	hypothetical protein POPTR_0017s11950g [Populus trichocarpa]	SMC4	Structural maintenance of chromosomes protein 4 OS=Arabidopsis thaliana GN=SMC4 PE=1 SV=1	7.29	27.91	-1.59	down	0.00	0.00	yes
TRINITY_DN18934_c0_g1	hypothetical protein POPTR_0018s11100g [Populus trichocarpa]	ASHR3	Histone-lysine N-methyltransferase ASHR3 OS=Arabidopsis thaliana GN=ASHR3 PE=1 SV=1	2.87	14.58	-1.59	down	0.00	0.00	yes

TRINITY_DN26717_c1_g1	calmodulin-binding family protein [Populus trichocarpa]	IQD14	Protein IQ-DOMAIN 14 OS=Arabidopsis thaliana GN=IQD14 PE=1 SV=1	17.96	71.77	-1.38	down	0.00	0.00	yes
TRINITY_DN18728_c1_g2	PREDICTED: uncharacterized protein LOC105111195 [Populus euphratica]	HIPP03	Heavy metal-associated isoprenylated plant protein 3 OS=Arabidopsis thaliana GN=HIPP03 PE=1 SV=1	13.43	150.58	-2.63	down	0.00	0.00	yes
TRINITY_DN19162_c0_g1	hypothetical protein POPTR_0009s15230g [Populus trichocarpa]	rbrA	Probable E3 ubiquitin-protein ligase rbrA OS=Dictyostelium discoideum GN=rbrA PE=3 SV=1	4.83	14.57	-1.82	down	0.00	0.00	yes
TRINITY_DN20573_c0_g1	PREDICTED: equilibrative nucleotide transporter 3-like [Populus euphratica]	ENT3	Equilibrative nucleotide transporter 3 OS=Arabidopsis thaliana GN=ENT3 PE=1 SV=1	2.14	13.89	-2.17	down	0.00	0.00	yes
TRINITY_DN22686_c0_g3	ROTUNDIFOLIA 3 family protein [Populus trichocarpa]	ROT3	3-epi-6-deoxocathasterone 23-monooxygenase OS=Arabidopsis thaliana GN=ROT3 PE=2 SV=3	1.88	11.66	-1.82	down	0.00	0.00	yes
TRINITY_DN27423_c2_g2	hypothetical protein POPTR_0001s42040g [Populus trichocarpa]	At4g27290	G-type lectin S-receptor-like serine/threonine-protein kinase At4g27290 OS=Arabidopsis thaliana GN=At4g27290 PE=3 SV=4	1.69	6.95	-2.14	down	0.00	0.00	yes
TRINITY_DN15933_c1_g1	hypothetical protein POPTR_0002s13100g [Populus trichocarpa]	DOF1.6	Dof zinc finger protein DOF1.6 OS=Arabidopsis thaliana GN=DOF1.6 PE=2 SV=1	1.10	12.11	-2.90	down	0.00	0.00	yes
TRINITY_DN17019_c0_g1	PREDICTED: abscisic acid 8'-hydroxylase 4-like isoform X1 [Populus euphratica]	CYP707A4	Abscisic acid 8'-hydroxylase 4 OS=Arabidopsis thaliana GN=CYP707A4 PE=2 SV=2	1.16	7.13	-2.18	down	0.00	0.00	yes
TRINITY_DN26432_c0_g1	PREDICTED: uncharacterized protein LOC105134053 isoform X2 [Populus euphratica]	-	-	5.27	27.70	-1.79	down	0.00	0.00	yes
TRINITY_DN16238_c0_g1	PREDICTED: uncharacterized protein LOC105114883 isoform X1 [Populus euphratica]	-	-	2.96	20.83	-2.50	down	0.00	0.00	yes
TRINITY_DN23055_c4_g2	hypothetical protein POPTR_0007s03330g [Populus trichocarpa]	BCA5	Beta carbonic anhydrase 5, chloroplastic OS=Arabidopsis thaliana GN=BCA5 PE=2 SV=1	0.47	6.09	-3.07	down	0.00	0.00	yes
TRINITY_DN19042_c0_g2	no apical meristem family protein [Populus trichocarpa]	NAC035	NAC domain-containing protein 35 OS=Arabidopsis thaliana GN=NAC035 PE=1 SV=2	0.18	3.18	-3.42	down	0.00	0.00	yes
TRINITY_DN19362_c1_g7	-	-	-	4.32	32.52	-2.31	down	0.00	0.00	yes
TRINITY_DN13994_c0_g2	PREDICTED: uncharacterized protein LOC105139393 [Populus euphratica]	-	-	0.46	7.55	-3.35	down	0.00	0.00	yes
TRINITY_DN22469_c0_g1	DNA helicase family protein [Populus trichocarpa]	RECQL4A	ATP-dependent DNA helicase Q-like 4A OS=Arabidopsis thaliana GN=RECQL4A PE=2 SV=1	5.98	24.54	-1.37	down	0.00	0.00	yes
TRINITY_DN23027_c0_g4	putative wall-associated kinase family protein [Populus trichocarpa]	WAKL1	Wall-associated receptor kinase-like 1 OS=Arabidopsis thaliana GN=WAKL1 PE=2 SV=1	0.22	5.86	-4.40	down	0.00	0.00	yes
TRINITY_DN23867_c0_g1	epidermal differentiation family protein [Populus trichocarpa]	CR4	Serine/threonine-protein kinase-like protein CR4 OS=Oryza sativa subsp. japonica GN=CR4 PE=1 SV=1	1.34	9.01	-2.10	down	0.00	0.00	yes
TRINITY_DN24662_c0_g2	ferritin/ribonucleotide reductase-like family protein [Populus tomentosa]	-	Ribonucleoside-diphosphate reductase small chain OS=Nicotiana tabacum PE=2 SV=1	4.88	29.25	-1.93	down	0.00	0.00	yes
TRINITY_DN11597_c0_g1	universal stress family protein [Populus trichocarpa]	-	-	0.75	6.36	-2.45	down	0.00	0.00	yes
TRINITY_DN22415_c0_g10	hypothetical protein POPTR_0009s00200g, partial [Populus trichocarpa]	-	-	0.57	4.60	-2.39	down	0.00	0.00	yes
TRINITY_DN23891_c1_g1	zinc finger family protein [Populus trichocarpa]	BRXL4	Protein Brevis radix-like 4 OS=Arabidopsis thaliana GN=BRXL4 PE=2 SV=1	3.29	13.51	-1.70	down	0.00	0.00	yes
TRINITY_DN22113_c0_g3	PREDICTED: pleiotropic drug resistance protein 1-like [Populus euphratica]	PDR1	Pleiotropic drug resistance protein 1 OS=Nicotiana plumbaginifolia GN=PDR1 PE=1 SV=1	1.05	5.16	-2.10	down	0.00	0.00	yes
TRINITY_DN17411_c0_g1	PREDICTED: probable chalcone--flavonone isomerase 3 [Populus euphratica]	CHI3	Probable chalcone--flavonone isomerase 3 OS=Arabidopsis thaliana GN=CHI3 PE=1 SV=1	26.53	143.54	-1.84	down	0.00	0.00	yes
TRINITY_DN22263_c1_g2	-	-	-	2.83	33.05	-3.73	down	0.00	0.00	yes
TRINITY_DN17793_c0_g1	PREDICTED: glycerophosphodiester phosphodiesterase protein kinase domain-containing GDPDL2-like [Populus euphratica]	LRK10	Rust resistance kinase Lr10 OS=Triticum aestivum GN=LRK10 PE=2 SV=1	0.70	19.91	-4.25	down	0.00	0.00	yes
TRINITY_DN24553_c0_g3	PREDICTED: calcium-transporting ATPase 12, plasma membrane-type [Populus euphratica]	ACA12	Calcium-transporting ATPase 12, plasma membrane-type OS=Arabidopsis thaliana GN=ACA12 PE=2 SV=1	0.69	8.23	-2.52	down	0.00	0.00	yes
TRINITY_DN22472_c0_g1	CONSTANS-like protein [Populus alba x Populus glandulosa]	COL2	Zinc finger protein CONSTANS-LIKE 2 OS=Arabidopsis thaliana GN=COL2 PE=1 SV=1	29.77	122.36	-1.54	down	0.00	0.00	yes
TRINITY_DN17611_c0_g1	hypothetical protein POPTR_0017s14310g [Populus trichocarpa]	-	-	37.18	399.02	-2.74	down	0.00	0.00	yes
TRINITY_DN22648_c0_g1	-	-	-	0.45	7.81	-3.47	down	0.00	0.00	yes
TRINITY_DN26436_c0_g2	-	-	-	14.16	68.12	-1.69	down	0.00	0.00	yes

TRINITY_DN27224_c0_g2	hypothetical protein POPTR_0006s12600g [Populus trichocarpa]	-	-	0.16	4.05	-4.03	down	0.00	0.00	yes
TRINITY_DN20046_c0_g1	PREDICTED: protein MID1-COMPLEMENTING ACTIVITY 1 [Populus euphratica]	CNR13	Cell number regulator 13 OS=Zea mays GN=CNR13 PE=2 SV=1	16.65	68.97	-1.43	down	0.00	0.00	yes
TRINITY_DN14088_c0_g1	PREDICTED: indole-3-acetic acid-amido synthetase GH3.17-like [Populus euphratica]	GH3.17	Indole-3-acetic acid-amido synthetase GH3.17 OS=Arabidopsis thaliana GN=GH3.17 PE=1 SV=1	0.12	1.79	-3.21	down	0.00	0.00	yes
TRINITY_DN25633_c0_g5	PREDICTED: acidic endochitinase-like [Populus euphratica]	-	Acidic endochitinase OS=Phaseolus angularis PE=2 SV=1	0.13	4.28	-4.43	down	0.00	0.00	yes
TRINITY_DN25823_c0_g2	leucine-rich repeat transmembrane protein kinase [Populus trichocarpa]	SOBIR1	Leucine-rich repeat receptor-like serine/threonine/tyrosine-protein kinase SOBIR1 OS=Arabidopsis thaliana GN=SOBIR1 PE=1 SV=1	2.57	12.56	-1.67	down	0.00	0.00	yes
TRINITY_DN22121_c0_g4	hypothetical protein POPTR_0001s04700g [Populus trichocarpa]	CMT3	DNA (cytosine-5)-methyltransferase CMT3 OS=Oryza sativa subsp. japonica GN=CMT3 PE=2 SV=1	4.13	30.41	-2.34	down	0.00	0.00	yes
TRINITY_DN27785_c0_g1	hypothetical protein POPTR_0013s01820g [Populus trichocarpa]	-	-	4.32	20.66	-1.66	down	0.00	0.00	yes
TRINITY_DN26519_c0_g2	-	-	-	0.12	5.38	-4.52	down	0.00	0.00	yes
TRINITY_DN24663_c0_g1	-	-	-	0.00	2.22	-6.93	down	0.00	0.00	yes
TRINITY_DN25371_c3_g2	hypothetical protein POPTR_0014s06560g [Populus trichocarpa]	MAP65-3	65-kDa microtubule-associated protein 3 OS=Arabidopsis thaliana GN=MAP65-3 PE=1 SV=1	0.66	6.48	-2.69	down	0.00	0.00	yes
TRINITY_DN21865_c0_g3	WRKY transcription factor 1 [(Populus tomentosa x Populus bolleana) x Populus tomentosa]	WRKY24	WRKY transcription factor WRKY24 OS=Oryza sativa subsp. japonica GN=WRKY24 PE=2 SV=1	4.32	26.94	-2.01	down	0.00	0.00	yes
TRINITY_DN19531_c0_g2	hypothetical protein POPTR_0007s05020g [Populus trichocarpa]	-	-	2.09	31.51	-3.32	down	0.00	0.00	yes
TRINITY_DN16972_c0_g2	hypothetical protein POPTR_0017s09250g [Populus trichocarpa]	CRK26	Cysteine-rich receptor-like protein kinase 26 OS=Arabidopsis thaliana GN=CRK26 PE=2 SV=1	9.78	52.01	-1.79	down	0.00	0.00	yes
TRINITY_DN11329_c0_g1	PREDICTED: ankyrin repeat-containing protein At2g01680-like isoform X1 [Populus euphratica]	-	-	0.09	2.49	-4.05	down	0.00	0.00	yes
TRINITY_DN23886_c0_g1	PREDICTED: uncharacterized protein LOC105121779 isoform X1 [Populus euphratica]	pyrH	Uridylate kinase OS=Synechococcus sp. (strain JA-3-3Ab) GN=pyrH PE=3 SV=1	7.25	26.43	-1.38	down	0.00	0.00	yes
TRINITY_DN24730_c0_g2	PREDICTED: uncharacterized protein LOC105050582 [Elaeis guineensis]	-	Histone H4 variant TH011 OS=Triticum aestivum PE=3 SV=2	37.26	157.89	-1.50	down	0.00	0.00	yes
TRINITY_DN13943_c0_g1	homeobox leucine zipper family protein [Populus trichocarpa]	ATHB-7	Homeobox-leucine zipper protein ATHB-7 OS=Arabidopsis thaliana GN=ATHB-7 PE=1 SV=2	0.11	2.23	-3.69	down	0.00	0.00	yes
TRINITY_DN21456_c1_g1	hypothetical protein POPTR_0002s17010g [Populus trichocarpa]	WRKY53	Probable WRKY transcription factor 53 OS=Arabidopsis thaliana GN=WRKY53 PE=1 SV=1	0.59	15.05	-3.59	down	0.00	0.00	yes
TRINITY_DN17306_c0_g2	hypothetical protein POPTR_0007s13680g [Populus trichocarpa]	-	-	0.84	5.26	-2.04	down	0.00	0.00	yes
TRINITY_DN25402_c0_g5	zinc finger family protein [Populus trichocarpa]	ZHD5	Zinc-finger homeodomain protein 5 OS=Arabidopsis thaliana GN=ZHD5 PE=1 SV=1	30.27	115.78	-1.35	down	0.00	0.00	yes
TRINITY_DN27965_c0_g1	-	-	-	0.00	3.39	-7.04	down	0.00	0.00	yes
TRINITY_DN21133_c0_g1	KOW domain-containing transcription factor family protein [Populus trichocarpa]	-	-	55.69	222.90	-1.33	down	0.00	0.00	yes
TRINITY_DN24644_c0_g2	hypothetical protein POPTR_0007s14500g [Populus trichocarpa]	BAM1	Leucine-rich repeat receptor-like serine/threonine-protein kinase BAM1 OS=Arabidopsis thaliana GN=BAM1 PE=1 SV=1	14.58	64.02	-1.52	down	0.00	0.00	yes
TRINITY_DN21928_c0_g1	AP2/ERF domain-containing transcription factor [Populus tomentosa]	DREB2C	Dehydration-responsive element-binding protein 2C OS=Arabidopsis thaliana GN=DREB2C PE=2 SV=2	6.75	55.72	-2.29	down	0.00	0.00	yes
TRINITY_DN23675_c1_g5	hypothetical protein POPTR_0001s42030g [Populus trichocarpa]	SD11	G-type lectin S-receptor-like serine/threonine-protein kinase SD1-1 OS=Arabidopsis thaliana GN=SD11 PE=1 SV=1	0.11	2.22	-3.53	down	0.00	0.00	yes
TRINITY_DN14093_c0_g1	hypothetical protein POPTR_0003s05970g [Populus trichocarpa]	-	-	5.40	192.52	-4.61	down	0.00	0.00	yes
TRINITY_DN22216_c0_g1	bZIP transcription factor family protein [Populus trichocarpa]	-	-	1.71	12.47	-2.51	down	0.00	0.00	yes
TRINITY_DN23464_c0_g1	hypothetical protein POPTR_0009s10420g [Populus trichocarpa]	-	-	5.57	25.90	-1.54	down	0.00	0.00	yes
TRINITY_DN23327_c0_g2	hypothetical protein POPTR_0001s39260g [Populus trichocarpa]	BBX22	B-box zinc finger protein 22 OS=Arabidopsis thaliana GN=BBX22 PE=1 SV=2	5.16	24.12	-1.58	down	0.00	0.00	yes

TRINITY_DN26186_c0_g1	cytokinin oxidase 6 family protein [Populus trichocarpa]	CKX5	Cytokinin dehydrogenase 5 OS=Arabidopsis thaliana GN=CKX5 PE=2 SV=1	0.00	2.27	-7.20	down	0.00	0.00	yes
TRINITY_DN27735_c1_g4	PREDICTED: extra-large guanine nucleotide-binding protein 1-like isoform X1 [Populus euphratica]	XLG1	Extra-large guanine nucleotide-binding protein 1 OS=Arabidopsis thaliana GN=XLG1 PE=1 SV=2	2.06	12.37	-1.95	down	0.00	0.00	yes
TRINITY_DN19501_c0_g6	hypothetical protein POPTR_0010s24060g [Populus trichocarpa]	DME	Transcriptional activator DEMETER OS=Arabidopsis thaliana GN=DME PE=1 SV=2	0.34	4.12	-2.95	down	0.00	0.00	yes
TRINITY_DN22717_c0_g3	hypothetical protein POPTR_0003s06910g [Populus trichocarpa]	GLIP5	GDSL esterase/lipase 5 OS=Arabidopsis thaliana GN=GLIP5 PE=2 SV=2	6.82	35.25	-2.09	down	0.00	0.00	yes
TRINITY_DN21725_c0_g1	hypothetical protein POPTR_0005s26320g [Populus trichocarpa]	-	-	16.59	66.10	-1.42	down	0.00	0.00	yes
TRINITY_DN23471_c0_g5	PREDICTED: filament-like plant protein 7 [Populus euphratica]	FPP7	Filament-like plant protein 7 OS=Arabidopsis thaliana GN=FPP7 PE=3 SV=2	0.72	4.52	-2.03	down	0.00	0.00	yes
TRINITY_DN21925_c0_g1	hypothetical protein POPTR_0001s45700g [Populus trichocarpa]	-	-	0.22	2.82	-3.06	down	0.00	0.00	yes
TRINITY_DN19706_c1_g4	hypothetical protein POPTR_0012s01360g [Populus trichocarpa]	-	-	0.33	6.17	-3.56	down	0.00	0.00	yes
TRINITY_DN9098_c0_g2	PREDICTED: UPF0481 protein At3g47200-like [Populus euphratica]	At3g47200	UPF0481 protein At3g47200 OS=Arabidopsis thaliana GN=At3g47200 PE=2 SV=1	0.07	2.15	-4.36	down	0.00	0.00	yes
TRINITY_DN20653_c0_g1	DNAJ heat shock N-terminal domain-containing family protein [Populus trichocarpa]	ATJ6	Chaperone protein dnaJ 6 OS=Arabidopsis thaliana GN=ATJ6 PE=2 SV=1	6.68	30.88	-1.51	down	0.00	0.00	yes
TRINITY_DN23496_c1_g1	PHD finger family protein [Populus trichocarpa]	AL5	PHD finger protein ALFIN-LIKE 5 OS=Arabidopsis thaliana GN=AL5 PE=2 SV=1	48.57	159.86	-1.46	down	0.00	0.00	yes
TRINITY_DN28732_c0_g1	Expansin-related protein 3 precursor [Populus trichocarpa]	CjBAp12	EG45-like domain containing protein OS=Citrus jambhiri GN=CjBAp12 PE=1 SV=1	0.09	3.89	-4.63	down	0.00	0.00	yes
TRINITY_DN23448_c0_g3	MutT/nudix family protein [Populus trichocarpa]	NUDT17	Nudix hydrolase 17, mitochondrial OS=Arabidopsis thaliana GN=NUDT17 PE=2 SV=1	2.04	47.20	-4.18	down	0.00	0.00	yes
TRINITY_DN16393_c0_g1	hypothetical protein POPTR_0001s11330g [Populus trichocarpa]	-	-	0.14	3.23	-3.79	down	0.00	0.00	yes
TRINITY_DN22646_c1_g3	hypothetical protein POPTR_0011s16510g [Populus trichocarpa]	-	-	0.26	3.33	-3.08	down	0.00	0.00	yes
TRINITY_DN20004_c1_g1	-	-	-	1.39	8.96	-2.10	down	0.00	0.00	yes
TRINITY_DN17656_c1_g1	hypothetical protein POPTR_0008s10540g [Populus trichocarpa]	-	-	0.26	3.51	-3.31	down	0.00	0.00	yes
TRINITY_DN23480_c0_g1	hypothetical protein POPTR_0001s42400g [Populus trichocarpa]	-	-	34.04	132.04	-1.35	down	0.00	0.00	yes
TRINITY_DN22667_c0_g1	-	-	-	0.35	6.79	-3.44	down	0.00	0.00	yes
TRINITY_DN26865_c0_g1	minichromosome maintenance family protein [Populus trichocarpa]	MCM5	DNA replication licensing factor MCM5 OS=Arabidopsis thaliana GN=MCM5 PE=1 SV=1	5.82	40.93	-2.09	down	0.00	0.00	yes
TRINITY_DN20129_c0_g1	PREDICTED: crossover junction endonuclease MUS81 isoform X2 [Populus euphratica]	MUS81	Crossover junction endonuclease MUS81 OS=Arabidopsis thaliana GN=MUS81 PE=1 SV=1	1.13	5.96	-1.78	down	0.00	0.00	yes
TRINITY_DN20738_c0_g2	hypothetical protein POPTR_0002s00340g [Populus trichocarpa]	-	-	4.78	19.86	-1.51	down	0.00	0.00	yes
TRINITY_DN12665_c0_g1	hypothetical protein POPTR_0017s00730g [Populus trichocarpa]	At5g48380	Probably inactive leucine-rich repeat receptor-like protein kinase At5g48380 OS=Arabidopsis thaliana GN=At5g48380 PE=1 SV=1	0.10	5.38	-4.89	down	0.00	0.00	yes
TRINITY_DN19367_c0_g5	PREDICTED: probable calcium-binding protein CML36 [Populus euphratica]	CML35	Probable calcium-binding protein CML35 OS=Arabidopsis thaliana GN=CML35 PE=2 SV=2	15.93	70.30	-1.56	down	0.00	0.00	yes
TRINITY_DN17636_c0_g2	hypothetical protein POPTR_0002s00400g [Populus trichocarpa]	-	-	1.08	7.33	-2.13	down	0.00	0.00	yes
TRINITY_DN22963_c0_g2	hypothetical protein POPTR_0003s22170g [Populus trichocarpa]	At3g47200	UPF0481 protein At3g47200 OS=Arabidopsis thaliana GN=At3g47200 PE=2 SV=1	2.65	10.61	-2.19	down	0.00	0.00	yes
TRINITY_DN348_c0_g1	hypothetical protein POPTR_0018s03880g [Populus trichocarpa]	At2g30650	Probable 3-hydroxyisobutyryl-CoA hydrolase 2 OS=Arabidopsis thaliana GN=At2g30650 PE=2 SV=1	0.18	2.75	-3.27	down	0.00	0.00	yes
TRINITY_DN19608_c0_g1	hypothetical protein POPTR_0003s10380g [Populus trichocarpa]	Prim1	DNA primase small subunit OS=Mus musculus GN=Prim1 PE=1 SV=1	3.11	17.37	-1.86	down	0.00	0.00	yes

TRINITY_DN19143_c0_g1	PREDICTED: DNA polymerase alpha subunit B-like [Populus euphratica]	Pola2	DNA polymerase alpha subunit B OS=Mus musculus GN=Pola2 PE=1 SV=2	6.40	29.61	-1.71	down	0.00	0.00	yes
TRINITY_DN20438_c0_g1	PREDICTED: uncharacterized protein LOC105120948 [Populus euphratica]	SMXL4	Protein SMAX1-LIKE 4 OS=Arabidopsis thaliana GN=SMXL4 PE=1 SV=1	1.29	10.06	-2.51	down	0.00	0.00	yes
TRINITY_DN20691_c0_g2	unknown [Populus trichocarpa]	RANBP1C	Ran-binding protein 1 homolog c OS=Arabidopsis thaliana GN=RANBP1C PE=2 SV=1	13.07	52.52	-1.36	down	0.00	0.00	yes
TRINITY_DN26168_c0_g5	-	-	-	2.55	12.88	-1.80	down	0.00	0.00	yes
TRINITY_DN21920_c1_g1	NAC domain-containing protein 90 [Populus trichocarpa]	NAC090	NAC domain-containing protein 90 OS=Arabidopsis thaliana GN=NAC090 PE=2 SV=1	0.76	16.53	-3.31	down	0.00	0.00	yes
TRINITY_DN23528_c2_g4	PREDICTED: RING-H2 finger protein ATL3 [Populus euphratica]	ATL60	RING-H2 finger protein ATL60 OS=Arabidopsis thaliana GN=ATL60 PE=2 SV=1	1.34	8.74	-2.08	down	0.00	0.00	yes
TRINITY_DN21622_c0_g2	PREDICTED: NAC domain-containing protein 68 [Populus euphratica]	NAC083	NAC domain-containing protein 83 OS=Arabidopsis thaliana GN=NAC083 PE=1 SV=1	6.63	29.11	-1.52	down	0.00	0.00	yes
TRINITY_DN19988_c0_g8	laccase family protein [Populus trichocarpa]	LAC3	Laccase-3 OS=Arabidopsis thaliana GN=LAC3 PE=2 SV=2	0.07	1.97	-4.08	down	0.00	0.00	yes
TRINITY_DN27893_c11_g1	hypothetical protein POPTR_0004s22920g [Populus trichocarpa]	-	-	1.85	19.94	-2.87	down	0.00	0.00	yes
TRINITY_DN20364_c0_g2	PREDICTED: BAHD acyltransferase DCR [Populus euphratica]	DCR	BAHD acyltransferase DCR OS=Arabidopsis thaliana GN=DCR PE=2 SV=1	14.38	66.96	-1.63	down	0.00	0.00	yes
TRINITY_DN15910_c0_g1	hypothetical protein POPTR_0019s04710g [Populus trichocarpa]	-	-	0.20	3.10	-3.31	down	0.00	0.00	yes
TRINITY_DN25256_c0_g9	PREDICTED: LOW QUALITY PROTEIN: uncharacterized protein LOC105114840 [Populus euphratica]	-	-	0.67	4.86	-2.24	down	0.00	0.00	yes
TRINITY_DN16368_c0_g1	hypothetical protein POPTR_0002s26110g [Populus trichocarpa]	-	-	25.27	137.52	-1.79	down	0.00	0.00	yes
TRINITY_DN23055_c3_g1	PREDICTED: transcription repressor KAN1-like [Populus euphratica]	KAN1	Transcription repressor KAN1 OS=Arabidopsis thaliana GN=KAN1 PE=1 SV=1	0.69	4.93	-2.38	down	0.00	0.00	yes
TRINITY_DN13918_c0_g1	hypothetical protein POPTR_0008s14100g [Populus trichocarpa]	-	-	0.30	3.24	-2.76	down	0.00	0.00	yes
TRINITY_DN16711_c0_g1	hypothetical protein POPTR_0013s06450g [Populus trichocarpa]	HAG2	Histone acetyltransferase type B catalytic subunit OS=Arabidopsis thaliana GN=HAG2 PE=2 SV=1	9.60	45.43	-1.62	down	0.00	0.00	yes
TRINITY_DN23156_c0_g3	PREDICTED: uncharacterized protein LOC105137481 [Populus euphratica]	-	-	5.39	23.92	-1.53	down	0.00	0.00	yes
TRINITY_DN21511_c0_g1	hypothetical protein POPTR_0012s03500g [Populus trichocarpa]	At1g48405	Kinase-interacting family protein OS=Arabidopsis thaliana GN=At1g48405 PE=2 SV=1	18.05	65.40	-1.30	down	0.00	0.00	yes
TRINITY_DN27436_c0_g1	an N-terminal calmodulin binding autoinhibitory domain-containing family protein [Populus trichocarpa]	ACA9	Calcium-transporting ATPase 9, plasma membrane-type OS=Arabidopsis thaliana GN=ACA9 PE=2 SV=2	20.69	91.16	-1.41	down	0.00	0.00	yes
TRINITY_DN18608_c2_g8	PROLIFERA family protein [Populus trichocarpa]	MCM7	DNA replication licensing factor MCM7 OS=Oryza sativa subsp. japonica GN=MCM7 PE=2 SV=1	4.36	29.93	-2.15	down	0.00	0.00	yes
TRINITY_DN19386_c0_g1	PREDICTED: uncharacterized protein LOC105123042 [Populus euphratica]	-	-	12.22	43.65	-1.24	down	0.00	0.00	yes
TRINITY_DN6730_c0_g1	hypothetical protein POPTR_0001s23385g [Populus trichocarpa]	-	-	0.28	9.30	-4.36	down	0.00	0.00	yes
TRINITY_DN24849_c1_g6	hypothetical protein POPTR_0017s04790g [Populus trichocarpa]	HSL1	Receptor-like protein kinase HSL1 OS=Arabidopsis thaliana GN=HSL1 PE=2 SV=1	0.87	8.86	-2.71	down	0.00	0.00	yes
TRINITY_DN25610_c0_g1	PREDICTED: E3 ubiquitin-protein ligase ORTHRUS 2-like [Populus euphratica]	ORTH2	E3 ubiquitin-protein ligase ORTHRUS 2 OS=Arabidopsis thaliana GN=ORTH2 PE=1 SV=1	6.66	21.65	-1.43	down	0.00	0.00	yes
TRINITY_DN22423_c0_g2	hypothetical protein POPTR_0003s08780g, partial [Populus trichocarpa]	PLL1	Protein phosphatase 2C 29 OS=Arabidopsis thaliana GN=PLL1 PE=1 SV=2	0.43	4.61	-2.78	down	0.00	0.00	yes
TRINITY_DN22515_c0_g1	hypothetical protein POPTR_0002s00930g [Populus trichocarpa]	-	-	21.53	81.20	-1.26	down	0.00	0.00	yes
TRINITY_DN17937_c0_g1	PREDICTED: probable glutathione S-transferase [Populus euphratica]	GSTU7	Glutathione S-transferase U7 OS=Arabidopsis thaliana GN=GSTU7 PE=2 SV=1	2.39	14.46	-2.08	down	0.00	0.00	yes
TRINITY_DN15227_c0_g1	PREDICTED: zeaxanthin epoxidase, chloroplastic-like [Populus euphratica]	hpxO	FAD-dependent urate hydroxylase OS=Acinetobacter baylyi (strain ATCC 33305 / BD413 / ADP1) GN=hpxO PE=1 SV=1	0.38	3.26	-2.48	down	0.00	0.00	yes

TRINITY_DN24986_c0_g5	hypothetical protein POPTR_0018s09510g [Populus trichocarpa]	MIK1	MDIS1-interacting receptor like kinase 1 OS=Arabidopsis thaliana GN=MIK1 PE=1 SV=1	0.00	2.14	-6.68	down	0.00	0.00	yes
TRINITY_DN16255_c0_g1	PREDICTED: putative phytosulfokines 6 [Populus euphratica]	PSK6	Putative phytosulfokines 6 OS=Arabidopsis thaliana GN=PSK6 PE=2 SV=2	2.86	21.99	-2.35	down	0.00	0.00	yes
TRINITY_DN25498_c0_g1	PREDICTED: cyclin-A2-2-like isoform X1 [Populus euphratica]	CYCA2-1	Cyclin-A2-1 OS=Arabidopsis thaliana GN=CYCA2-1 PE=2 SV=3	17.75	66.33	-1.38	down	0.00	0.00	yes
TRINITY_DN15838_c0_g1	aminotransferase [Populus tomentosa]	-	-	3.16	16.91	-1.80	down	0.00	0.00	yes
TRINITY_DN16719_c0_g2	short-chain dehydrogenase/reductase family protein [Populus trichocarpa]	KCR2	Very-long-chain 3-oxoacyl-CoA reductase-like protein At1g24470 OS=Arabidopsis thaliana GN=KCR2 PE=2 SV=1	0.77	6.53	-2.32	down	0.00	0.00	yes
TRINITY_DN19922_c0_g1	TMS membrane family protein [Populus trichocarpa]	SERINC1	Serine incorporator 1 OS=Bos taurus GN=SERINC1 PE=2 SV=1	1.40	8.05	-1.87	down	0.00	0.00	yes
TRINITY_DN25347_c0_g1	hypothetical protein POPTR_0008s06230g [Populus trichocarpa]	ROPGEF5	Rop guanine nucleotide exchange factor 5 OS=Arabidopsis thaliana GN=ROPGEF5 PE=2 SV=1	16.25	64.74	-1.43	down	0.00	0.00	yes
TRINITY_DN21586_c0_g2	patatin-related family protein [Populus trichocarpa]	PLP9	Probable inactive patatin-like protein 9 OS=Arabidopsis thaliana GN=PLP9 PE=2 SV=1	0.95	10.42	-2.84	down	0.00	0.00	yes
TRINITY_DN22675_c2_g3	cold shock domain-containing protein 3 [Populus tomentosa]	-	-	7.38	34.42	-1.45	down	0.00	0.00	yes
TRINITY_DN17381_c0_g1	PREDICTED: protein ROOT HAIR DEFECTIVE 3 homolog 2-like isoform X3 [Populus euphratica]	At5g45160	Protein ROOT HAIR DEFECTIVE 3 homolog 2 OS=Arabidopsis thaliana GN=At5g45160 PE=2 SV=1	1.55	12.35	-2.33	down	0.00	0.00	yes
TRINITY_DN25311_c0_g3	hypothetical protein POPTR_0001s07410g [Populus trichocarpa]	-	-	4.60	31.01	-2.13	down	0.00	0.00	yes
TRINITY_DN27407_c1_g2	PREDICTED: signal peptide peptidase-like 4 [Populus euphratica]	SPPL4	Signal peptide peptidase-like 4 OS=Arabidopsis thaliana GN=SPPL4 PE=2 SV=1	11.96	47.44	-1.34	down	0.00	0.00	yes
TRINITY_DN18404_c0_g1	SABATH methyltransferase 28 [Populus trichocarpa]	At5g38100	Probable S-adenosylmethionine-dependent methyltransferase At5g38100 OS=Arabidopsis thaliana GN=At5g38100 PE=2 SV=1	1.88	17.92	-2.69	down	0.00	0.00	yes
TRINITY_DN16169_c0_g1	hypothetical protein POPTR_0019s03440g [Populus trichocarpa]	-	-	6.70	38.40	-1.49	down	0.00	0.00	yes
TRINITY_DN19175_c0_g2	PREDICTED: uncharacterized protein LOC105113673 isoform X2 [Populus euphratica]	-	-	2.85	17.45	-1.95	down	0.00	0.00	yes
TRINITY_DN25676_c0_g2	hypothetical protein POPTR_0005s27390g [Populus trichocarpa]	-	-	343.86	1366.92	-1.39	down	0.00	0.00	yes
TRINITY_DN15892_c0_g1	WRKY transcription factor 47 family protein [Populus trichocarpa]	-	-	0.07	2.13	-4.06	down	0.00	0.00	yes
TRINITY_DN20813_c0_g1	non-SMC condensin subunit family protein [Populus trichocarpa]	ncapd2	Condensin complex subunit 1 OS=Xenopus laevis GN=ncapd2 PE=1 SV=1	6.62	43.48	-1.53	down	0.00	0.00	yes
TRINITY_DN18728_c1_g1	hypothetical protein POPTR_0002s24360g [Populus trichocarpa]	KRP3	Cyclin-dependent kinase inhibitor 3 OS=Arabidopsis thaliana GN=KRP3 PE=1 SV=1	3.26	17.19	-1.78	down	0.00	0.00	yes
TRINITY_DN19067_c0_g1	hypothetical protein POPTR_0005s08460g [Populus trichocarpa]	-	-	1.31	13.09	-2.70	down	0.00	0.00	yes
TRINITY_DN18651_c2_g4	hypothetical protein POPTR_0003s15160g [Populus trichocarpa]	-	-	1.65	15.08	-2.56	down	0.00	0.00	yes
TRINITY_DN24453_c0_g1	PREDICTED: lysine histidine transporter-like 8 [Populus euphratica]	AATL1	Lysine histidine transporter-like 8 OS=Arabidopsis thaliana GN=AATL1 PE=1 SV=1	0.11	4.20	-5.05	down	0.00	0.00	yes
TRINITY_DN21714_c0_g1	speckle-type POZ family protein [Populus trichocarpa]	BT1	BTB/POZ and TAZ domain-containing protein 1 OS=Arabidopsis thaliana GN=BT1 PE=1 SV=1	0.98	8.88	-2.58	down	0.00	0.00	yes
TRINITY_DN25209_c0_g1	hypothetical protein POPTR_0017s02220g [Populus trichocarpa]	KAN1	Transcription repressor KAN1 OS=Arabidopsis thaliana GN=KAN1 PE=1 SV=1	6.32	28.20	-1.62	down	0.00	0.00	yes
TRINITY_DN26746_c1_g1	-	-	-	4.49	25.52	-2.10	down	0.00	0.00	yes
TRINITY_DN25295_c0_g1	PREDICTED: wall-associated receptor kinase-like 14 [Populus euphratica]	WAKL14	Wall-associated receptor kinase-like 14 OS=Arabidopsis thaliana GN=WAKL14 PE=2 SV=2	2.68	13.40	-1.67	down	0.00	0.00	yes
TRINITY_DN18665_c1_g3	PREDICTED: metallothionein-like protein type 2 [Populus euphratica]	MT1A	Metallothionein-like protein 2 OS=Trifolium repens GN=MT1A PE=3 SV=1	73.32	325.49	-1.54	down	0.00	0.00	yes
TRINITY_DN13784_c0_g1	PREDICTED: wall-associated receptor kinase-like 20 [Populus euphratica]	WAKL20	Wall-associated receptor kinase-like 20 OS=Arabidopsis thaliana GN=WAKL20 PE=2 SV=1	0.19	3.74	-3.53	down	0.00	0.00	yes
TRINITY_DN19673_c3_g1	hypothetical protein POPTR_0016s13650g [Populus trichocarpa]	-	-	0.08	2.19	-4.01	down	0.00	0.00	yes

TRINITY_DN19147_c0_g1	hypothetical protein POPTR_0013s10060g [Populus trichocarpa]	-	-	1.36	7.82	-1.93	down	0.00	0.00	yes
TRINITY_DN27725_c1_g1	PREDICTED: protein argonaute 4A-like [Populus euphratica]	AGO4A	Protein argonaute 4A OS=Oryza sativa subsp. japonica GN=AGO4A PE=2 SV=1	17.30	78.71	-1.29	down	0.00	0.00	yes
TRINITY_DN19868_c0_g2	hypothetical protein POPTR_0016s04260g [Populus trichocarpa]	-	-	1.52	8.45	-1.85	down	0.00	0.00	yes
TRINITY_DN15933_c1_g2	hypothetical protein POPTR_0014s03590g [Populus trichocarpa]	DOF1.6	Dof zinc finger protein DOF1.6 OS=Arabidopsis thaliana GN=DOF1.6 PE=2 SV=1	0.58	8.37	-3.18	down	0.00	0.00	yes
TRINITY_DN15610_c0_g2	harpin-induced family protein [Populus trichocarpa]	-	-	23.65	238.60	-2.76	down	0.00	0.00	yes
TRINITY_DN23712_c0_g2	hypothetical protein POPTR_0010s08980g [Populus trichocarpa]	-	-	1.25	6.17	-1.68	down	0.00	0.00	yes
TRINITY_DN23866_c0_g1	TIR-NBS-TIR-TIR-WRKY type disease resistance protein [Populus trichocarpa]	-	-	9.97	55.49	-1.95	down	0.00	0.00	yes
TRINITY_DN12645_c0_g1	putative alpha-dioxygenase family protein [Populus trichocarpa]	DOX1	Alpha-dioxygenase 1 OS=Arabidopsis thaliana GN=DOX1 PE=1 SV=1	0.07	2.13	-4.27	down	0.00	0.00	yes
TRINITY_DN22669_c1_g2	PREDICTED: type I inositol 1,4,5-trisphosphate 5-phosphatase CVP2-like isoform X1 [Populus euphratica]	IP5P6	Type IV inositol polyphosphate 5-phosphatase 6 OS=Arabidopsis thaliana GN=IP5P6 PE=1 SV=2	0.71	6.73	-2.51	down	0.00	0.00	yes
TRINITY_DN17063_c0_g1	hypothetical protein POPTR_0006s06640g [Populus trichocarpa]	-	-	0.09	3.70	-4.30	down	0.00	0.00	yes
TRINITY_DN24380_c0_g3	hypothetical protein POPTR_0005s00390g [Populus trichocarpa]	LTI6B	Hydrophobic protein LTI6B OS=Oryza sativa subsp. indica GN=LTI6B PE=3 SV=2	20.85	185.27	-2.63	down	0.00	0.00	yes
TRINITY_DN23876_c0_g2	hypothetical protein POPTR_0007s10880g [Populus trichocarpa]	POLA	DNA polymerase alpha catalytic subunit OS=Arabidopsis thaliana GN=POLA PE=3 SV=2	1.84	10.67	-1.91	down	0.00	0.00	yes
TRINITY_DN27772_c0_g2	PREDICTED: protein OBERON 4-like [Populus euphratica]	OBE4	Protein OBERON 4 OS=Arabidopsis thaliana GN=OBE4 PE=1 SV=2	9.74	38.90	-1.38	down	0.00	0.00	yes
TRINITY_DN26480_c0_g1	PREDICTED: uncharacterized protein LOC105137845 [Populus euphratica]	-	-	0.69	6.60	-2.57	down	0.00	0.00	yes
TRINITY_DN19631_c0_g1	PREDICTED: polyadenylation and cleavage factor homolog 5-like isoform X1 [Populus euphratica]	PCFS4	Polyadenylation and cleavage factor homolog 4 OS=Arabidopsis thaliana GN=PCFS4 PE=1 SV=1	2.04	21.61	-2.81	down	0.00	0.00	yes
TRINITY_DN21527_c0_g1	PROTODERMAL FACTOR2 family protein [Populus trichocarpa]	PDF2	Homeobox-leucine zipper protein PROTODERMAL FACTOR 2 OS=Arabidopsis thaliana GN=PDF2 PE=2 SV=1	0.41	7.20	-3.02	down	0.00	0.00	yes
TRINITY_DN21789_c0_g3	-	-	-	0.38	4.32	-2.88	down	0.00	0.00	yes
TRINITY_DN22858_c0_g1	hypothetical protein POPTR_0004s08570g [Populus trichocarpa]	-	-	1.46	7.84	-1.82	down	0.00	0.00	yes
TRINITY_DN22747_c0_g1	PREDICTED: condensin complex subunit 2 [Populus euphratica]	CAPH	Condensin complex subunit 2 OS=Arabidopsis thaliana GN=CAPH PE=1 SV=1	11.24	49.86	-1.42	down	0.00	0.00	yes
TRINITY_DN26920_c0_g1	PREDICTED: rho GTPase-activating protein REN1-like isoform X1 [Populus euphratica]	REN1	Rho GTPase-activating protein REN1 OS=Arabidopsis thaliana GN=REN1 PE=1 SV=2	3.61	8.85	-1.84	down	0.00	0.00	yes
TRINITY_DN13950_c0_g1	hypothetical protein POPTR_0003s10135g [Populus trichocarpa]	-	-	0.04	3.87	-5.02	down	0.00	0.00	yes
TRINITY_DN14544_c0_g2	hypothetical protein POPTR_0004s22380g [Populus trichocarpa]	-	-	0.20	4.80	-3.89	down	0.00	0.00	yes
TRINITY_DN20040_c0_g1	MTD1 family protein [Populus trichocarpa]	-	-	19.63	92.17	-1.59	down	0.00	0.00	yes
TRINITY_DN27166_c1_g2	PREDICTED: protein tesmin/TSO1-like CXC 2 isoform X1 [Populus euphratica]	TSO1	CRC domain-containing protein TSO1 OS=Arabidopsis thaliana GN=TSO1 PE=1 SV=1	1.24	6.87	-1.83	down	0.00	0.00	yes
TRINITY_DN16513_c0_g1	PREDICTED: wound-induced protein 1-like [Populus euphratica]	-	-	0.20	12.22	-4.89	down	0.00	0.00	yes
TRINITY_DN27302_c0_g3	-	-	-	4.87	25.52	-1.78	down	0.00	0.00	yes
TRINITY_DN25060_c0_g4	-	-	-	0.72	9.32	-3.23	down	0.00	0.00	yes
TRINITY_DN21745_c0_g1	PREDICTED: SNF1-related protein kinase regulatory subunit gamma-1-like [Populus euphratica]	KING1	SNF1-related protein kinase regulatory subunit gamma-1 OS=Arabidopsis thaliana GN=KING1 PE=1 SV=2	2.74	23.30	-2.46	down	0.00	0.00	yes
TRINITY_DN19572_c0_g1	PREDICTED: truncated transcription factor CAULIFLOWER D-like isoform X2 [Populus euphratica]	CAL	Transcription factor CAULIFLOWER OS=Arabidopsis thaliana GN=CAL PE=1 SV=3	0.23	4.59	-3.15	down	0.00	0.00	yes

TRINITY_DN19840_c0_g1	kinase family protein [Populus trichocarpa]	At1g56130	Probable LRR receptor-like serine/threonine-protein kinase At1g56130 OS=Arabidopsis thaliana GN=At1g56130 PE=2 SV=2	3.04	15.70	-1.90	down	0.00	0.00	yes
TRINITY_DN17164_c0_g3	hypothetical protein POPTR_0013s12760g [Populus trichocarpa]	RAC2	Rac-like GTP-binding protein RAC2 OS=Lotus japonicus GN=RAC2 PE=2 SV=1	3.48	17.44	-1.72	down	0.00	0.00	yes
TRINITY_DN20937_c0_g1	hypothetical protein POPTR_0018s11070g [Populus trichocarpa]	-	-	3.89	22.22	-2.09	down	0.00	0.00	yes
TRINITY_DN18145_c0_g1	hypothetical protein POPTR_0003s21830g [Populus trichocarpa]	NSE4A	Non-structural maintenance of chromosomes element 4 homolog A OS=Arabidopsis thaliana GN=NSE4A PE=2 SV=1	1.34	7.34	-1.83	down	0.00	0.00	yes
TRINITY_DN15789_c0_g1	PREDICTED: uncharacterized protein LOC105126274 [Populus euphratica]	-	-	1.52	12.20	-2.41	down	0.00	0.00	yes
TRINITY_DN20759_c1_g1	PREDICTED: uncharacterized protein LOC105110153 [Populus euphratica]	At4g27230	Probable histone H2A.3 OS=Arabidopsis thaliana GN=At4g27230 PE=1 SV=1	8.42	29.65	-1.31	down	0.00	0.00	yes
TRINITY_DN24562_c0_g2	PREDICTED: uncharacterized protein LOC105126865 [Populus euphratica]	-	-	5.78	28.39	-1.40	down	0.00	0.00	yes
TRINITY_DN22967_c0_g1	hypothetical protein POPTR_0001s13630g [Populus trichocarpa]	TGA1	Teosinte glume architecture 1 OS=Zea mays GN=TGA1 PE=3 SV=1	13.50	47.67	-1.26	down	0.00	0.00	yes
TRINITY_DN23015_c0_g1	tocopherol cyclase [Hevea brasiliensis]	VTE1	Tocopherol cyclase, chloroplastic OS=Arabidopsis thaliana GN=VTE1 PE=2 SV=1	5.86	30.45	-1.72	down	0.00	0.00	yes
TRINITY_DN25633_c0_g2	hypothetical protein POPTR_0018s11800g [Populus trichocarpa]	BHLH66	Transcription factor bHLH66 OS=Arabidopsis thaliana GN=BHLH66 PE=2 SV=1	7.63	29.20	-1.33	down	0.00	0.00	yes
TRINITY_DN19527_c0_g6	hypothetical protein POPTR_0016s04110g [Populus trichocarpa]	ERD15	Protein EARLY RESPONSIVE TO DEHYDRATION 15 OS=Arabidopsis thaliana GN=ERD15 PE=1 SV=1	42.54	163.43	-1.36	down	0.00	0.00	yes
TRINITY_DN22938_c1_g1	ATP-dependent DNA helicase family protein [Populus trichocarpa]	RECQL5	ATP-dependent DNA helicase Q-like 5 OS=Arabidopsis thaliana GN=RECQL5 PE=2 SV=2	1.17	7.57	-2.26	down	0.00	0.00	yes
TRINITY_DN16221_c1_g3	PREDICTED: lysosomal Pro-X carboxypeptidase-like isoform X1 [Populus euphratica]	-	-	0.80	8.62	-2.80	down	0.00	0.00	yes
TRINITY_DN19862_c1_g1	TIR-NBS-LRR-TIR type disease resistance protein, partial [Populus trichocarpa]	-	-	14.44	53.06	-1.76	down	0.00	0.00	yes
TRINITY_DN24727_c0_g2	hypothetical protein POPTR_0002s15960g [Populus trichocarpa]	AHL5	AT-hook motif nuclear-localized protein 5 OS=Arabidopsis thaliana GN=AHL5 PE=1 SV=1	46.37	168.33	-1.29	down	0.00	0.00	yes
TRINITY_DN21535_c0_g1	hypothetical protein POPTR_0013s14850g [Populus trichocarpa]	-	-	0.21	6.36	-4.23	down	0.00	0.00	yes
TRINITY_DN19398_c0_g1	hypothetical protein POPTR_0001s31000g [Populus trichocarpa]	IRX15-L	Protein IRX15-LIKE OS=Arabidopsis thaliana GN=IRX15-L PE=2 SV=1	4.66	19.66	-1.48	down	0.00	0.00	yes
TRINITY_DN24101_c0_g2	armadillo/beta-catenin repeat family protein [Populus trichocarpa]	PUB17	U-box domain-containing protein 17 OS=Arabidopsis thaliana GN=PUB17 PE=2 SV=1	4.98	20.33	-1.43	down	0.00	0.00	yes
TRINITY_DN22551_c0_g1	WRKY transcription factor 24 [(Populus tomentosa x Populus bolleana) x Populus tomentosa]	WRKY11	Probable WRKY transcription factor 11 OS=Arabidopsis thaliana GN=WRKY11 PE=2 SV=2	1.99	23.65	-3.19	down	0.00	0.00	yes
TRINITY_DN26325_c0_g2	PREDICTED: QWRF motif-containing protein 2-like isoform X1 [Populus euphratica]	QWRF2	QWRF motif-containing protein 2 OS=Arabidopsis thaliana GN=QWRF2 PE=2 SV=1	4.14	16.90	-1.43	down	0.00	0.00	yes
TRINITY_DN15994_c0_g1	GI1-2 [Populus tomentosa]	-	-	6.49	43.76	-2.17	down	0.00	0.00	yes
TRINITY_DN23369_c0_g2	hypothetical protein POPTR_0001s31320g [Populus trichocarpa]	-	-	1.17	4.79	-1.90	down	0.00	0.00	yes
TRINITY_DN21860_c0_g5	PREDICTED: protein CHUP1, chloroplastic [Populus euphratica]	CHUP1	Protein CHUP1, chloroplastic OS=Arabidopsis thaliana GN=CHUP1 PE=1 SV=1	9.48	34.48	-1.25	down	0.00	0.00	yes
TRINITY_DN15847_c0_g1	hypothetical protein POPTR_0003s13080g [Populus trichocarpa]	RHA1B	E3 ubiquitin-protein ligase RHA1B OS=Arabidopsis thaliana GN=RHA1B PE=2 SV=1	5.83	27.41	-1.63	down	0.00	0.00	yes
TRINITY_DN26928_c1_g1	PREDICTED: transcription factor GTE4 [Populus euphratica]	GTE4	Transcription factor GTE4 OS=Arabidopsis thaliana GN=GTE4 PE=2 SV=1	7.45	28.61	-1.34	down	0.00	0.00	yes
TRINITY_DN13979_c0_g1	DNA topoisomerase family protein [Populus trichocarpa]	TOP2	DNA topoisomerase 2 OS=Pisum sativum GN=TOP2 PE=2 SV=1	29.39	115.94	-1.38	down	0.00	0.00	yes
TRINITY_DN17926_c0_g2	PREDICTED: probable DNA helicase MCM8 isoform X1 [Populus euphratica]	MCM8	Probable DNA helicase MCM8 OS=Arabidopsis thaliana GN=MCM8 PE=2 SV=2	0.44	3.48	-2.37	down	0.00	0.00	yes
TRINITY_DN19909_c3_g1	-	-	-	4.90	91.39	-3.50	down	0.00	0.00	yes
TRINITY_DN27359_c0_g4	hypothetical protein POPTR_0003s22360g [Populus trichocarpa]	At3g47200	UPF0481 protein At3g47200 OS=Arabidopsis thaliana GN=At3g47200 PE=2 SV=1	0.12	1.83	-3.23	down	0.00	0.00	yes

TRINITY_DN24968_c0_g2	-	-	-	0.11	2.68	-3.80	down	0.00	0.00	yes
TRINITY_DN22594_c0_g1	hypothetical protein POPTR_0009s10320g [Populus trichocarpa]	At5g67130	PI-PLC X domain-containing protein At5g67130 OS=Arabidopsis thaliana GN=At5g67130 PE=1 SV=1	0.94	7.53	-2.85	down	0.00	0.00	yes
TRINITY_DN22481_c1_g2	-	-	-	10.37	44.28	-1.51	down	0.00	0.00	yes
TRINITY_DN14197_c0_g1	PREDICTED: uncharacterized protein LOC105139384 [Populus euphratica]	-	-	0.12	2.68	-3.83	down	0.00	0.00	yes
TRINITY_DN17416_c0_g1	hypothetical protein POPTR_0014s10590g [Populus trichocarpa]	-	-	0.82	8.91	-2.32	down	0.00	0.00	yes
TRINITY_DN22964_c0_g3	hypothetical protein POPTR_0014s02930g [Populus trichocarpa]	-	-	12.40	71.35	-1.90	down	0.00	0.00	yes
TRINITY_DN24098_c0_g1	NO POLLEN GERMINATION RELATED 2 family protein [Populus trichocarpa]	TTC7B	Tetratricopeptide repeat protein 7B OS=Homo sapiens GN=TTC7B PE=1 SV=3	2.36	10.58	-1.50	down	0.00	0.00	yes
TRINITY_DN24035_c0_g1	hypothetical protein POPTR_0008s01490g [Populus trichocarpa]	At3g03770	Probable inactive leucine-rich repeat receptor-like protein kinase At3g03770 OS=Arabidopsis thaliana GN=At3g03770 PE=2 SV=1	7.11	28.53	-1.40	down	0.00	0.00	yes
TRINITY_DN13199_c0_g2	hypothetical protein POPTR_0006s28160g [Populus trichocarpa]	-	-	0.50	3.87	-2.34	down	0.00	0.00	yes
TRINITY_DN7565_c0_g1	hypothetical protein POPTR_0005s12230g [Populus trichocarpa]	-	-	0.47	7.98	-3.38	down	0.00	0.00	yes
TRINITY_DN17306_c0_g1	hypothetical protein POPTR_0005s11980g [Populus trichocarpa]	-	-	1.60	9.38	-1.98	down	0.00	0.00	yes
TRINITY_DN23984_c0_g1	PREDICTED: F-box/LRR-repeat protein 17-like [Populus euphratica]	FBL17	F-box/LRR-repeat protein 17 OS=Arabidopsis thaliana GN=FBL17 PE=1 SV=1	1.50	12.76	-2.47	down	0.00	0.00	yes
TRINITY_DN24105_c1_g3	hypothetical protein TSUD_254980 [Trifolium subterraneum]	-	Retrovirus-related Pol polyprotein from transposon TNT 1-94 OS=Nicotiana tabacum PE=2 SV=1	230.28	1159.28	-2.06	down	0.00	0.00	yes
TRINITY_DN20188_c0_g5	PREDICTED: zinc finger protein NUTCRACKER-like [Populus euphratica]	-	-	18.28	66.86	-1.28	down	0.00	0.00	yes
TRINITY_DN24970_c1_g2	PREDICTED: protein BREAST CANCER SUSCEPTIBILITY 2 homolog B-like [Populus euphratica]	BRCA2B	Protein BREAST CANCER SUSCEPTIBILITY 2 homolog B OS=Arabidopsis thaliana GN=BRCA2B PE=1 SV=1	0.77	5.94	-2.32	down	0.00	0.00	yes
TRINITY_DN15136_c0_g1	hypothetical protein POPTR_0004s17810g [Populus trichocarpa]	ATJ11	Chaperone protein dnaJ 11, chloroplastic OS=Arabidopsis thaliana GN=ATJ11 PE=1 SV=2	96.74	703.41	-2.32	down	0.00	0.00	yes
TRINITY_DN19379_c0_g2	PREDICTED: origin recognition complex subunit 3 [Populus euphratica]	ORC3	Origin of replication complex subunit 3 OS=Arabidopsis thaliana GN=ORC3 PE=1 SV=1	2.37	16.42	-2.06	down	0.00	0.00	yes
TRINITY_DN20802_c0_g3	PREDICTED: zinc finger protein CONSTANS-LIKE 5-like [Populus euphratica]	COL5	Zinc finger protein CONSTANS-LIKE 5 OS=Arabidopsis thaliana GN=COL5 PE=2 SV=2	11.26	78.87	-2.23	down	0.00	0.00	yes
TRINITY_DN20746_c0_g4	-	-	-	0.61	11.55	-3.53	down	0.00	0.00	yes
TRINITY_DN18551_c0_g1	proline-rich family protein [Populus trichocarpa]	-	-	1.76	13.58	-2.25	down	0.00	0.00	yes
TRINITY_DN16199_c0_g1	hypothetical protein POPTR_0015s10150g [Populus trichocarpa]	-	-	0.59	5.20	-2.55	down	0.00	0.00	yes
TRINITY_DN23523_c0_g1	hypothetical protein POPTR_0006s09100g [Populus trichocarpa]	BHLH155	Transcription factor bHLH155 OS=Arabidopsis thaliana GN=BHLH155 PE=2 SV=1	9.34	39.39	-1.28	down	0.00	0.00	yes
TRINITY_DN21256_c1_g5	PREDICTED: tRNA (cytosine(34)-C(5))-methyltransferase [Populus euphratica]	-	-	0.44	3.61	-2.42	down	0.00	0.00	yes
TRINITY_DN22551_c1_g1	hypothetical protein POPTR_0018s13600g [Populus trichocarpa]	WRKY11	Probable WRKY transcription factor 11 OS=Arabidopsis thaliana GN=WRKY11 PE=2 SV=2	16.75	98.52	-1.79	down	0.00	0.00	yes
TRINITY_DN12280_c0_g2	hypothetical protein POPTR_0008s13070g [Populus trichocarpa]	CAR4	Protein C2-DOMAIN ABA-RELATED 4 OS=Arabidopsis thaliana GN=CAR4 PE=1 SV=1	0.12	3.83	-4.32	down	0.00	0.00	yes
TRINITY_DN17769_c0_g2	hypothetical protein POPTR_0005s07220g [Populus trichocarpa]	ATL55	E3 ubiquitin-protein ligase RING1 OS=Arabidopsis thaliana GN=ATL55 PE=1 SV=1	0.25	2.94	-2.90	down	0.00	0.00	yes
TRINITY_DN24020_c1_g1	hypothetical protein POPTR_0014s13090g [Populus trichocarpa]	ROPGAP2	Rho GTPase-activating protein 2 OS=Arabidopsis thaliana GN=ROPGAP2 PE=1 SV=1	27.48	105.76	-1.36	down	0.00	0.00	yes
TRINITY_DN27095_c1_g3	hypothetical protein POPTR_0002s09020g [Populus trichocarpa]	-	-	0.76	5.40	-2.22	down	0.00	0.00	yes
TRINITY_DN18180_c0_g1	C2 domain-containing family protein [Populus trichocarpa]	FTIP1	FT-interacting protein 1 OS=Arabidopsis thaliana GN=FTIP1 PE=1 SV=1	0.49	6.11	-3.00	down	0.00	0.00	yes

TRINITY_DN15433_c0_g1	hypothetical protein POPTR_0018s12210g [Populus trichocarpa]	-	-	3.09	28.51	-2.55	down	0.00	0.00	yes
TRINITY_DN21709_c0_g1	PREDICTED: phosphoenolpyruvate carboxylase kinase 2-like isoform X2 [Populus euphratica]	PPCK2	Phosphoenolpyruvate carboxylase kinase 2 OS=Arabidopsis thaliana GN=PPCK2 PE=1 SV=2	3.29	22.78	-2.27	down	0.00	0.00	yes
TRINITY_DN22131_c0_g1	PREDICTED: putative multidrug resistance protein isoform X1 [Populus euphratica]	Os02g0190300	Putative multidrug resistance protein OS=Oryza sativa subsp. japonica GN=Os02g0190300 PE=3 SV=1	1.18	5.42	-1.57	down	0.00	0.00	yes
TRINITY_DN21498_c0_g2	hypothetical protein POPTR_0009s07090g [Populus trichocarpa]	At5g26960	F-box/kelch-repeat protein At5g26960 OS=Arabidopsis thaliana GN=At5g26960 PE=2 SV=1	2.71	13.86	-1.77	down	0.00	0.00	yes
TRINITY_DN20475_c0_g2	PREDICTED: transcription repressor MYB6-like isoform X1 [Populus euphratica]	PP1	Myb-related protein Pp1 (Fragment) OS=Physcomitrella patens subsp. patens GN=PP1 PE=2 SV=1	0.48	15.23	-4.29	down	0.00	0.00	yes
TRINITY_DN17374_c0_g1	syntaxin 121 family protein [Populus trichocarpa]	SYP121	Syntaxin-121 OS=Arabidopsis thaliana GN=SYP121 PE=1 SV=1	1.57	10.21	-2.08	down	0.00	0.00	yes
TRINITY_DN20757_c0_g2	PREDICTED: glucan endo-1,3-beta-glucosidase 3-like [Populus euphratica]	At1g11820	Glucan endo-1,3-beta-glucosidase 1 OS=Arabidopsis thaliana GN=At1g11820 PE=1 SV=3	1.23	7.21	-1.98	down	0.00	0.00	yes
TRINITY_DN22400_c1_g2	hypothetical protein POPTR_0010s24060g [Populus trichocarpa]	DME	Transcriptional activator DEMETER OS=Arabidopsis thaliana GN=DME PE=1 SV=2	0.44	5.58	-2.51	down	0.00	0.00	yes
TRINITY_DN23564_c0_g3	PREDICTED: aspartic proteinase PCS1-like [Populus euphratica]	PCS1	Aspartic proteinase PCS1 OS=Arabidopsis thaliana GN=PCS1 PE=2 SV=1	4.28	37.74	-2.44	down	0.00	0.00	yes
TRINITY_DN22749_c0_g1	PREDICTED: probable leucine-rich repeat receptor-like serine/threonine-protein kinase At3g14840 isoform X4 [Populus euphratica]	At1g07650	Probable LRR receptor-like serine/threonine-protein kinase At1g07650 OS=Arabidopsis thaliana GN=At1g07650 PE=1 SV=1	2.99	17.72	-2.15	down	0.00	0.00	yes
TRINITY_DN23612_c0_g1	hypothetical protein POPTR_0010s24900g [Populus trichocarpa]	At4g06598	Uncharacterized protein At4g06598 OS=Arabidopsis thaliana GN=At4g06598 PE=2 SV=2	13.54	50.56	-1.25	down	0.00	0.00	yes
TRINITY_DN13312_c0_g2	hypothetical protein POPTR_2088s00200g, partial [Populus trichocarpa]	-	-	0.21	3.44	-2.76	down	0.00	0.00	yes
TRINITY_DN24129_c0_g1	hypothetical protein POPTR_0002s05450g [Populus trichocarpa]	MIRO2	Mitochondrial Rho GTPase 2 OS=Arabidopsis thaliana GN=MIRO2 PE=2 SV=1	0.53	5.39	-2.62	down	0.00	0.00	yes
TRINITY_DN26699_c0_g1	PREDICTED: auxin response factor 9-like [Populus euphratica]	ARF9	Auxin response factor 9 OS=Arabidopsis thaliana GN=ARF9 PE=1 SV=1	5.09	20.76	-1.43	down	0.00	0.00	yes
TRINITY_DN23092_c0_g3	hypothetical protein POPTR_0005s02860g [Populus trichocarpa]	-	-	0.63	5.15	-2.43	down	0.00	0.00	yes
TRINITY_DN16757_c0_g3	Leucine-rich repeat receptor protein kinase EXS precursor [Populus trichocarpa]	-	-	0.44	10.40	-3.78	down	0.00	0.00	yes
TRINITY_DN14361_c0_g1	hypothetical protein POPTR_0785s00200g [Populus trichocarpa]	-	-	0.33	5.17	-3.35	down	0.00	0.00	yes
TRINITY_DN23547_c0_g2	zinc finger family protein [Populus trichocarpa]	ZHD6	Zinc-finger homeodomain protein 6 OS=Arabidopsis thaliana GN=ZHD6 PE=1 SV=1	16.43	58.43	-1.24	down	0.00	0.00	yes
TRINITY_DN26178_c0_g1	PREDICTED: protein OBERON 3-like [Populus euphratica]	OBE3	Protein OBERON 3 OS=Arabidopsis thaliana GN=OBE3 PE=1 SV=1	8.39	31.30	-1.32	down	0.00	0.00	yes
TRINITY_DN20607_c1_g6	hypothetical protein POPTR_0005s25020g [Populus trichocarpa]	-	-	0.46	11.22	-3.94	down	0.00	0.00	yes
TRINITY_DN23873_c0_g4	Ethylene responsive element binding factor 5 family protein [Populus trichocarpa]	ERF6	Ethylene-responsive transcription factor 6 OS=Arabidopsis thaliana GN=ERF6 PE=2 SV=2	9.71	105.13	-3.01	down	0.00	0.00	yes
TRINITY_DN23863_c0_g4	hypothetical protein POPTR_0010s24910g [Populus trichocarpa]	-	-	2.69	12.96	-1.63	down	0.00	0.00	yes
TRINITY_DN23615_c0_g9	-	-	-	39.61	149.12	-1.29	down	0.00	0.00	yes
TRINITY_DN18215_c0_g1	empfindlicher im dunkelroten licht 1 family protein [Populus trichocarpa]	EID1	Phytochrome A-associated F-box protein OS=Arabidopsis thaliana GN=EID1 PE=1 SV=2	5.80	24.01	-1.46	down	0.00	0.00	yes
TRINITY_DN14861_c0_g1	hypothetical protein POPTR_0017s11350g [Populus trichocarpa]	-	-	0.39	11.01	-4.12	down	0.00	0.00	yes
TRINITY_DN21494_c1_g2	hypothetical protein POPTR_0003s11230g [Populus trichocarpa]	YAB4	Protein YABBY 4 OS=Oryza sativa subsp. indica GN=YAB4 PE=3 SV=1	15.54	116.32	-2.24	down	0.00	0.00	yes
TRINITY_DN27665_c0_g3	PREDICTED: putative 1-phosphatidylinositol-3-phosphate 5-kinase FAB1D [Populus euphratica]	FAB1D	Putative 1-phosphatidylinositol-3-phosphate 5-kinase FAB1D OS=Arabidopsis thaliana GN=FAB1D PE=3 SV=1	0.28	3.17	-2.87	down	0.00	0.00	yes
TRINITY_DN21350_c0_g3	-	-	-	10.96	50.98	-1.56	down	0.00	0.00	yes
TRINITY_DN18842_c0_g1	D5-type cyclin [Populus trichocarpa]	CYCD5-1	Cyclin-D5-1 OS=Arabidopsis thaliana GN=CYCD5-1 PE=2 SV=2	0.54	4.24	-2.42	down	0.00	0.00	yes

TRINITY_DN22360_c0_g2	hypothetical protein POPTR_0014s04460g [Populus trichocarpa]	HAT4	Homeobox-leucine zipper protein HAT4 OS=Arabidopsis thaliana GN=HAT4 PE=1 SV=1	12.37	46.35	-1.24	down	0.00	0.00	yes
TRINITY_DN26235_c0_g1	PREDICTED: LOW QUALITY PROTEIN: G-type lectin S-receptor-like serine/threonine-protein kinase RLK1 [Populus euphratica]	LECRK1	G-type lectin S-receptor-like serine/threonine-protein kinase LECRK1 OS=Oryza sativa subsp. japonica GN=LECRK1 PE=2 SV=1	6.54	32.64	-1.34	down	0.00	0.00	yes
TRINITY_DN26610_c0_g2	hypothetical protein POPTR_0015s04970g [Populus trichocarpa]	NUDT4	Nudix hydrolase 4 OS=Arabidopsis thaliana GN=NUDT4 PE=1 SV=1	15.67	66.74	-1.45	down	0.00	0.00	yes
TRINITY_DN15008_c0_g1	PREDICTED: uncharacterized protein LOC105137985 [Populus euphratica]	-	-	1.21	8.70	-2.23	down	0.00	0.00	yes
TRINITY_DN19354_c0_g2	PREDICTED: glucan endo-1,3-beta-glucosidase, basic isoform-like [Populus euphratica]	GNS1	Glucan endo-1,3-beta-glucosidase, basic isoform OS=Prunus persica GN=GNS1 PE=3 SV=1	2.00	14.93	-2.28	down	0.00	0.00	yes
TRINITY_DN19897_c0_g2	hypothetical protein POPTR_0078s00230g [Populus trichocarpa]	-	-	16.92	63.19	-1.30	down	0.00	0.00	yes
TRINITY_DN25894_c0_g2	hypothetical protein POPTR_0014s08920g [Populus trichocarpa]	At5g41620	Uncharacterized protein At5g41620 OS=Arabidopsis thaliana GN=At5g41620 PE=2 SV=2	1.86	8.36	-1.55	down	0.00	0.00	yes
TRINITY_DN23675_c1_g3	PREDICTED: probable LRR receptor-like serine/threonine-protein kinase At1g07650 isoform X2 [Populus euphratica]	At1g53440	Probable LRR receptor-like serine/threonine-protein kinase At1g53440 OS=Arabidopsis thaliana GN=At1g53440 PE=2 SV=2	1.47	13.45	-2.51	down	0.00	0.00	yes
TRINITY_DN17224_c0_g1	PREDICTED: probable LRR receptor-like serine/threonine-protein kinase At1g34110 [Populus euphratica]	-	-	3.93	18.00	-1.61	down	0.00	0.00	yes
TRINITY_DN23390_c0_g2	hypothetical protein POPTR_0004s14270g [Populus trichocarpa]	ZHD4	Zinc-finger homeodomain protein 4 OS=Arabidopsis thaliana GN=ZHD4 PE=1 SV=1	3.54	15.85	-1.57	down	0.00	0.00	yes
TRINITY_DN19656_c0_g3	hypothetical protein POPTR_0200s00220g [Populus trichocarpa]	LECRK3	G-type lectin S-receptor-like serine/threonine-protein kinase LECRK3 OS=Oryza sativa subsp. indica GN=LECRK3 PE=3 SV=2	0.09	2.49	-4.04	down	0.00	0.00	yes
TRINITY_DN16307_c0_g1	PREDICTED: uncharacterized protein LOC105112470 isoform X1 [Populus euphratica]	ATG2	Autophagy-related protein 2 OS=Arabidopsis thaliana GN=ATG2 PE=2 SV=1	15.80	56.65	-1.22	down	0.00	0.00	yes
TRINITY_DN22222_c2_g1	PREDICTED: proliferating cell nuclear antigen [Populus euphratica]	PCNA	Proliferating cell nuclear antigen OS=Nicotiana tabacum GN=PCNA PE=2 SV=1	20.04	106.79	-1.69	down	0.00	0.00	yes
TRINITY_DN24167_c0_g2	hypothetical protein POPTR_0003s01610g [Populus trichocarpa]	ESP1	Separase OS=Arabidopsis thaliana GN=ESP1 PE=2 SV=1	1.71	6.99	-1.44	down	0.00	0.00	yes
TRINITY_DN22408_c0_g1	PREDICTED: transcription factor AS1 [Populus euphratica]	AS1	Transcription factor AS1 OS=Arabidopsis thaliana GN=AS1 PE=1 SV=1	30.12	106.66	-1.24	down	0.00	0.00	yes
TRINITY_DN21860_c0_g8	hypothetical protein POPTR_0004s05410g [Populus trichocarpa]	CHUP1	Protein CHUP1, chloroplastic OS=Arabidopsis thaliana GN=CHUP1 PE=1 SV=1	1.00	5.75	-1.89	down	0.00	0.00	yes
TRINITY_DN26583_c1_g1	PREDICTED: scarecrow-like protein 34 [Populus euphratica]	SCL14	Scarecrow-like protein 14 OS=Arabidopsis thaliana GN=SCL14 PE=2 SV=2	2.88	13.19	-2.15	down	0.00	0.00	yes
TRINITY_DN16571_c0_g1	PREDICTED: uncharacterized protein LOC105116647 [Populus euphratica]	-	-	0.34	6.19	-3.55	down	0.00	0.00	yes
TRINITY_DN15235_c0_g1	hypothetical protein POPTR_0010s11930g [Populus trichocarpa]	PCR2	Protein PLANT CADMIUM RESISTANCE 2 OS=Arabidopsis thaliana GN=PCR2 PE=1 SV=1	0.21	2.56	-2.97	down	0.00	0.00	yes
TRINITY_DN21924_c0_g1	PREDICTED: cysteine proteinase inhibitor 6-like [Populus euphratica]	CYS6	Cysteine proteinase inhibitor 6 OS=Arabidopsis thaliana GN=CYS6 PE=1 SV=2	10.21	61.44	-2.20	down	0.00	0.00	yes
TRINITY_DN20010_c0_g1	caffeoyl shikimate esterase 12 [Populus tomentosa]	CSE	Caffeoylshikimate esterase OS=Arabidopsis thaliana GN=CSE PE=1 SV=1	1.15	8.85	-2.36	down	0.00	0.00	yes
TRINITY_DN19578_c0_g1	hypothetical protein POPTR_0001s35490g [Populus trichocarpa]	SOG1	SUPPRESSOR OF GAMMA RESPONSE 1 OS=Arabidopsis thaliana GN=SOG1 PE=1 SV=1	3.07	18.72	-1.93	down	0.00	0.00	yes
TRINITY_DN26797_c0_g1	C2 domain-containing family protein [Populus trichocarpa]	At1g03370	C2 and GRAM domain-containing protein At1g03370 OS=Arabidopsis thaliana GN=At1g03370 PE=2 SV=4	3.07	13.21	-1.43	down	0.00	0.00	yes
TRINITY_DN23741_c0_g2	hypothetical protein POPTR_0007s15350g, partial [Populus trichocarpa]	CHR27	Helicase-like transcription factor CHR27 OS=Arabidopsis thaliana GN=CHR27 PE=1 SV=1	1.81	6.34	-2.39	down	0.00	0.00	yes
TRINITY_DN23237_c0_g2	hypothetical protein POPTR_0015s04720g [Populus trichocarpa]	ygbJ	Uncharacterized oxidoreductase YgbJ OS=Escherichia coli (strain K12) GN=ygbJ PE=3 SV=1	2.20	9.25	-1.50	down	0.00	0.00	yes
TRINITY_DN19005_c0_g1	TIR-NBS disease resistance-like protein [Populus trichocarpa]	At4g11170	Putative disease resistance protein At4g11170 OS=Arabidopsis thaliana GN=At4g11170 PE=2 SV=1	1.52	10.97	-2.56	down	0.00	0.00	yes

TRINITY_DN13903_c0_g1	hypothetical protein POPTR_0019s14040g [Populus trichocarpa]	-	-	0.96	12.41	-3.08	down	0.00	0.00	yes
TRINITY_DN21864_c0_g1	hypothetical protein POPTR_0012s01170g [Populus trichocarpa]	-	-	0.53	4.37	-2.38	down	0.00	0.00	yes
TRINITY_DN17656_c1_g2	hypothetical protein POPTR_0010s15440g [Populus trichocarpa]	-	-	0.88	8.88	-2.71	down	0.00	0.00	yes
TRINITY_DN21640_c0_g1	hypothetical protein POPTR_0019s01500g [Populus trichocarpa]	At4g24290	MACPF domain-containing protein At4g24290 OS=Arabidopsis thaliana GN=At4g24290 PE=2 SV=1	5.06	14.44	-1.46	down	0.00	0.00	yes
TRINITY_DN16705_c0_g2	hypothetical protein POPTR_0011s11330g [Populus trichocarpa]	LRR-RLK	Probable leucine-rich repeat receptor-like serine/threonine-protein kinase At3g14840 OS=Arabidopsis thaliana GN=LRR-RLK PE=2 SV=1	2.24	10.42	-2.05	down	0.00	0.00	yes
TRINITY_DN21259_c0_g2	DNA topoisomerase family protein [Populus trichocarpa]	-	-	26.86	105.34	-1.37	down	0.00	0.00	yes
TRINITY_DN22434_c0_g4	-	-	-	13.89	55.46	-1.39	down	0.00	0.00	yes
TRINITY_DN17689_c0_g2	hypothetical protein POPTR_0009s08500g [Populus trichocarpa]	BHLH25	Transcription factor bHLH25 OS=Arabidopsis thaliana GN=BHLH25 PE=2 SV=2	0.66	5.23	-2.42	down	0.00	0.00	yes
TRINITY_DN22082_c1_g1	PREDICTED: protein CHROMATIN REMODELING 25 [Populus euphratica]	CHR25	Protein CHROMATIN REMODELING 25 OS=Arabidopsis thaliana GN=CHR25 PE=1 SV=1	3.66	15.30	-1.41	down	0.00	0.00	yes
TRINITY_DN20399_c0_g1	PREDICTED: L-ascorbate oxidase-like [Populus euphratica]	-	L-ascorbate oxidase OS=Cucumis sativus PE=1 SV=1	0.48	10.01	-3.78	down	0.00	0.00	yes
TRINITY_DN18022_c4_g7	hypothetical protein POPTR_0002s08050g [Populus trichocarpa]	-	-	0.39	3.06	-2.38	down	0.00	0.00	yes
TRINITY_DN18778_c0_g1	hypothetical protein POPTR_0014s16960g [Populus trichocarpa]	HIPP16	Heavy metal-associated isoprenylated plant protein 16 OS=Arabidopsis thaliana GN=HIPP16 PE=2 SV=1	3.28	30.37	-2.66	down	0.00	0.00	yes
TRINITY_DN22515_c0_g11	-	-	-	19.22	67.94	-1.21	down	0.00	0.00	yes
TRINITY_DN19681_c0_g2	hypothetical protein POPTR_0010s10190g [Populus trichocarpa]	FAF3	Protein FANTASTIC FOUR 3 OS=Arabidopsis thaliana GN=FAF3 PE=2 SV=1	0.31	3.32	-2.74	down	0.00	0.00	yes
TRINITY_DN15933_c1_g3	hypothetical protein POPTR_0014s03590g [Populus trichocarpa]	-	-	0.51	6.36	-3.00	down	0.00	0.00	yes
TRINITY_DN14855_c0_g1	PREDICTED: transcription repressor OFP13 [Populus euphratica]	OFP13	Transcription repressor OFP13 OS=Arabidopsis thaliana GN=OFP13 PE=2 SV=1	0.23	3.07	-3.15	down	0.00	0.00	yes
TRINITY_DN21611_c0_g1	hypothetical protein POPTR_0015s06140g [Populus trichocarpa]	-	-	3.84	18.46	-1.59	down	0.00	0.00	yes
TRINITY_DN19883_c0_g1	hypothetical protein POPTR_0014s08600g [Populus trichocarpa]	WRKY22	WRKY transcription factor 22 OS=Arabidopsis thaliana GN=WRKY22 PE=2 SV=1	5.09	26.21	-1.76	down	0.00	0.00	yes
TRINITY_DN21835_c0_g1	PREDICTED: chromosome transmission fidelity protein 18 homolog [Populus euphratica]	chtf18	Chromosome transmission fidelity protein 18 homolog OS=Xenopus laevis GN=chtf18 PE=2 SV=1	1.43	8.53	-1.94	down	0.00	0.00	yes
TRINITY_DN22248_c0_g3	alpha-amylase inhibitor alpha subunit family protein [Populus trichocarpa]	LECRK81	L-type lectin-domain containing receptor kinase VIII.1 OS=Arabidopsis thaliana GN=LECRK81 PE=2 SV=1	6.18	24.26	-1.36	down	0.00	0.00	yes
TRINITY_DN14710_c0_g1	PREDICTED: uncharacterized protein LOC105113593 [Populus euphratica]	-	-	8.41	40.68	-1.66	down	0.00	0.00	yes
TRINITY_DN16406_c1_g2	hypothetical protein POPTR_0002s21350g [Populus trichocarpa]	mkkA	Mitogen-activated protein kinase kinase kinase A OS=Dictyostelium discoideum GN=mkkA PE=1 SV=2	0.50	5.28	-2.75	down	0.00	0.00	yes
TRINITY_DN22922_c0_g1	WD-40 repeat family protein [Populus trichocarpa]	BUB3.2	Mitotic checkpoint protein BUB3.2 OS=Arabidopsis thaliana GN=BUB3.2 PE=2 SV=1	28.16	108.86	-1.28	down	0.00	0.00	yes
TRINITY_DN24974_c0_g2	microtubule-associated protein 65-5 [Populus tomentosa]	MAP65-5	65-kDa microtubule-associated protein 5 OS=Arabidopsis thaliana GN=MAP65-5 PE=1 SV=2	3.25	13.76	-1.55	down	0.00	0.00	yes
TRINITY_DN23350_c1_g2	hypothetical protein POPTR_0001s46760g [Populus trichocarpa]	At1g30760	Berberine bridge enzyme-like 13 OS=Arabidopsis thaliana GN=At1g30760 PE=1 SV=1	0.21	7.90	-4.52	down	0.00	0.00	yes
TRINITY_DN22129_c0_g1	PREDICTED: LRR receptor-like serine/threonine-protein kinase ERECTA [Populus euphratica]	ERECTA	LRR receptor-like serine/threonine-protein kinase ERECTA OS=Arabidopsis thaliana GN=ERECTA PE=1 SV=1	35.50	177.71	-1.64	down	0.00	0.00	yes
TRINITY_DN17893_c0_g1	hypothetical protein POPTR_0018s06270g [Populus trichocarpa]	-	-	14.87	54.93	-1.38	down	0.00	0.00	yes
TRINITY_DN19963_c0_g5	-	-	-	0.10	3.43	-4.29	down	0.00	0.00	yes
TRINITY_DN21527_c0_g3	PROTODERMAL FACTOR2 family protein [Populus trichocarpa]	PDF2	Homeobox-leucine zipper protein PROTODERMAL FACTOR 2 OS=Arabidopsis thaliana GN=PDF2 PE=2 SV=1	0.57	4.76	-2.45	down	0.00	0.00	yes

TRINITY_DN27709_c0_g2	hypothetical protein POPTR_0003s04650g [Populus trichocarpa]	IAA26	Auxin-responsive protein IAA26 OS=Arabidopsis thaliana GN=IAA26 PE=1 SV=2	24.18	88.92	-1.46	down	0.00	0.00	yes
TRINITY_DN27745_c0_g1	PREDICTED: phragmoplast orienting kinesin-1 isoform X1 [Populus euphratica]	KIN12E	Kinesin-like protein KIN-12E OS=Arabidopsis thaliana GN=KIN12E PE=3 SV=1	6.56	24.03	-1.35	down	0.00	0.00	yes
TRINITY_DN18079_c0_g1	PREDICTED: U-box domain-containing protein 21-like [Populus euphratica]	PUB21	U-box domain-containing protein 21 OS=Arabidopsis thaliana GN=PUB21 PE=2 SV=1	0.06	4.47	-5.35	down	0.00	0.00	yes
TRINITY_DN25060_c0_g1	glycosyl hydrolase family 17 family protein [Populus trichocarpa]	At3g13560	Glucan endo-1,3-beta-glucosidase 4 OS=Arabidopsis thaliana GN=At3g13560 PE=1 SV=1	14.49	70.13	-1.73	down	0.00	0.00	yes
TRINITY_DN16443_c0_g1	hypothetical protein POPTR_0001s34210g [Populus trichocarpa]	-	-	0.43	4.24	-2.74	down	0.00	0.00	yes
TRINITY_DN23887_c0_g1	hypothetical protein POPTR_0008s02980g [Populus trichocarpa]	-	-	0.32	3.73	-2.74	down	0.00	0.00	yes
TRINITY_DN12559_c0_g1	hypothetical protein POPTR_0001s10020g [Populus trichocarpa]	-	-	0.14	2.99	-3.65	down	0.00	0.00	yes
TRINITY_DN19083_c0_g3	hypothetical protein POPTR_0017s12290g [Populus trichocarpa]	-	-	31.64	115.19	-1.27	down	0.00	0.00	yes
TRINITY_DN22190_c0_g1	hypothetical protein POPTR_0009s09800g [Populus trichocarpa]	-	Limonoid UDP-glucosyltransferase OS=Citrus unshiu PE=2 SV=1	13.53	72.64	-1.81	down	0.00	0.00	yes
TRINITY_DN26201_c0_g1	PREDICTED: protein CYPRO4 [Populus euphratica]	CYPRO4	Protein CYPRO4 OS=Cynara cardunculus GN=CYPRO4 PE=2 SV=1	22.26	85.32	-1.47	down	0.00	0.00	yes
TRINITY_DN15508_c0_g1	hypothetical protein POPTR_0006s07310g [Populus trichocarpa]	-	-	2.12	44.33	-3.78	down	0.00	0.00	yes
TRINITY_DN18410_c0_g1	hypothetical protein POPTR_0001s01010g [Populus trichocarpa]	MEBL	Membrane protein of ER body-like protein OS=Arabidopsis thaliana GN=MEBL PE=2 SV=1	0.09	3.76	-4.49	down	0.00	0.00	yes
TRINITY_DN14255_c0_g1	hypothetical protein POPTR_0003s19260g [Populus trichocarpa]	SOT15	Cytosolic sulfotransferase 15 OS=Arabidopsis thaliana GN=SOT15 PE=1 SV=1	5.61	36.13	-2.11	down	0.00	0.00	yes
TRINITY_DN24334_c1_g2	hypothetical protein POPTR_0003s12540g [Populus trichocarpa]	TUBB5	Tubulin beta-5 chain OS=Zea mays GN=TUBB5 PE=2 SV=1	3.50	20.57	-1.95	down	0.00	0.00	yes
TRINITY_DN17771_c0_g7	PREDICTED: DELLA protein GAIP-like isoform X3 [Populus euphratica]	GAIP	DELLA protein GAIP OS=Cucurbita maxima GN=GAIP PE=2 SV=1	2.62	11.42	-1.68	down	0.00	0.00	yes
TRINITY_DN22858_c0_g2	PREDICTED: uncharacterized protein LOC105108815 isoform X3 [Populus euphratica]	-	-	0.30	3.78	-3.04	down	0.00	0.00	yes
TRINITY_DN16499_c0_g1	allergen-related family protein [Populus trichocarpa]	EPFL4	EPIDERMAL PATTERNING FACTOR-like protein 4 OS=Arabidopsis thaliana GN=EPFL4 PE=1 SV=1	0.37	10.23	-3.92	down	0.00	0.00	yes
TRINITY_DN25004_c0_g4	phosphatidylinositol-phosphatidylcholine transfer protein SEC14 Ssh1 [Populus trichocarpa]	-	-	7.95	36.52	-1.61	down	0.00	0.00	yes
TRINITY_DN27180_c0_g1	hypothetical protein POPTR_0014s18490g [Populus trichocarpa]	MIK2	MDIS1-interacting receptor like kinase 2 OS=Arabidopsis thaliana GN=MIK2 PE=1 SV=3	3.22	18.50	-1.49	down	0.00	0.00	yes
TRINITY_DN21133_c0_g2	PREDICTED: G-type lectin S-receptor-like serine/threonine-protein kinase RLK1 isoform X1 [Populus euphratica]	RLK1	G-type lectin S-receptor-like serine/threonine-protein kinase RLK1 OS=Arabidopsis thaliana GN=RLK1 PE=2 SV=2	6.50	22.69	-1.22	down	0.00	0.00	yes
TRINITY_DN22806_c0_g1	hypothetical protein POPTR_0003s10300g [Populus trichocarpa]	DUR3	Urea-proton symporter DUR3 OS=Arabidopsis thaliana GN=DUR3 PE=1 SV=1	3.00	13.94	-1.51	down	0.00	0.00	yes
TRINITY_DN26928_c0_g3	-	-	-	16.01	65.61	-1.42	down	0.00	0.00	yes
TRINITY_DN27528_c2_g3	hypothetical protein POPTR_0002s17990g [Populus trichocarpa]	ARP1	Probable RNA-binding protein ARP1 OS=Arabidopsis thaliana GN=ARP1 PE=2 SV=1	2.15	15.55	-2.00	down	0.00	0.00	yes
TRINITY_DN19161_c0_g1	hypothetical protein POPTR_0014s14900g [Populus trichocarpa]	-	-	4.50	27.25	-2.03	down	0.00	0.00	yes
TRINITY_DN23875_c0_g2	hypothetical protein POPTR_0003s01940g [Populus trichocarpa]	DRP4C	Dynamin-related protein 4C OS=Arabidopsis thaliana GN=DRP4C PE=2 SV=1	1.79	20.31	-2.34	down	0.00	0.00	yes
TRINITY_DN17725_c0_g2	hypothetical protein POPTR_0001s02890g [Populus trichocarpa]	-	-	0.99	5.15	-2.46	down	0.00	0.00	yes
TRINITY_DN17686_c0_g1	hypothetical protein POPTR_0008s16230g [Populus trichocarpa]	-	-	1.41	11.92	-2.54	down	0.00	0.00	yes
TRINITY_DN21851_c1_g4	hypothetical protein POPTR_0006s13070g [Populus trichocarpa]	-	-	12.68	53.16	-1.47	down	0.00	0.00	yes

TRINITY_DN16851_c0_g1	PREDICTED: calmodulin-like [Populus euphratica]	CML12	Calmodulin-like protein 12 OS=Arabidopsis thaliana GN=CML12 PE=1 SV=3	1.09	10.82	-2.71	down	0.00	0.00	yes
TRINITY_DN18315_c0_g1	hypothetical protein POPTR_0015s13340g [Populus trichocarpa]	ANL2	Homeobox-leucine zipper protein ANTHOCYANINLESS 2 OS=Arabidopsis thaliana GN=ANL2 PE=2 SV=1	0.22	2.46	-2.81	down	0.00	0.00	yes
TRINITY_DN13298_c0_g1	hypothetical protein POPTR_0002s00830g [Populus trichocarpa]	MLO	Protein MLO OS=Hordeum vulgare GN=MLO PE=1 SV=1	0.05	2.72	-4.76	down	0.00	0.00	yes
TRINITY_DN20819_c0_g1	hypothetical protein POPTR_0035s00341g [Populus trichocarpa]	-	-	6.23	35.69	-1.97	down	0.00	0.00	yes
TRINITY_DN27058_c0_g1	PREDICTED: probable leucine-rich repeat receptor-like serine/threonine-protein kinase At5g15730 [Populus euphratica]	PERK9	Proline-rich receptor-like protein kinase PERK9 OS=Arabidopsis thaliana GN=PERK9 PE=1 SV=1	7.17	31.57	-1.45	down	0.00	0.00	yes
TRINITY_DN22258_c1_g5	PREDICTED: cation/H(+) antiporter 18-like [Populus euphratica]	CHX18	Cation/H(+) antiporter 18 OS=Arabidopsis thaliana GN=CHX18 PE=2 SV=1	0.55	5.20	-2.59	down	0.00	0.00	yes
TRINITY_DN21392_c2_g1	PREDICTED: rho GDP-dissociation inhibitor 1 [Populus euphratica]	GDI1	Rho GDP-dissociation inhibitor 1 OS=Arabidopsis thaliana GN=GDI1 PE=1 SV=1	3.23	15.25	-1.68	down	0.00	0.00	yes
TRINITY_DN22924_c0_g2	PREDICTED: dual specificity protein kinase shkB-like isoform X1 [Populus euphratica]	STY46	Serine/threonine-protein kinase STY46 OS=Arabidopsis thaliana GN=STY46 PE=1 SV=1	3.17	15.95	-1.74	down	0.00	0.00	yes
TRINITY_DN20147_c0_g5	unknown [Populus trichocarpa]	TUBB1	Tubulin beta-1 chain OS=Lupinus albus GN=TUBB1 PE=3 SV=1	21.00	97.67	-1.32	down	0.00	0.00	yes
TRINITY_DN24280_c0_g2	kinase family protein [Populus trichocarpa]	LECRKS2	Receptor like protein kinase S.2 OS=Arabidopsis thaliana GN=LECRKS2 PE=2 SV=2	7.02	24.02	-1.20	down	0.00	0.00	yes
TRINITY_DN23366_c0_g2	hypothetical protein POPTR_0019s04020g [Populus trichocarpa]	-	-	0.16	2.26	-3.09	down	0.00	0.00	yes
TRINITY_DN14456_c0_g1	hypothetical protein POPTR_0013s00900g [Populus trichocarpa]	-	-	0.44	4.52	-2.73	down	0.00	0.00	yes
TRINITY_DN16180_c0_g1	PREDICTED: auxin response factor 2-like [Populus euphratica]	ARF2	Auxin response factor 2 OS=Arabidopsis thaliana GN=ARF2 PE=1 SV=2	13.42	43.70	-1.25	down	0.00	0.00	yes
TRINITY_DN20173_c0_g2	hydroxyproline-rich glycoprotein [Populus trichocarpa]	-	-	0.89	9.88	-3.38	down	0.00	0.00	yes
TRINITY_DN26026_c0_g1	PREDICTED: uncharacterized protein LOC105116539 [Populus euphratica]	-	-	15.28	90.36	-2.03	down	0.00	0.00	yes
TRINITY_DN23686_c0_g1	hypothetical protein POPTR_0002s26360g [Populus trichocarpa]	-	-	3.88	11.33	-2.16	down	0.00	0.00	yes
TRINITY_DN21917_c1_g1	INDOLE-3-ACETATE BETA-D-GLUCOSYLTRANSFERASE family protein [Populus trichocarpa]	UGT75L6	Crocetin glucosyltransferase, chloroplastic OS=Gardenia jasminoides GN=UGT75L6 PE=1 SV=1	4.31	21.67	-1.69	down	0.00	0.00	yes
TRINITY_DN23163_c0_g1	PREDICTED: rho GTPase-activating protein 5-like [Populus euphratica]	ROPGAP1	Rho GTPase-activating protein 1 OS=Arabidopsis thaliana GN=ROPGAP1 PE=2 SV=1	11.77	45.14	-1.34	down	0.00	0.00	yes
TRINITY_DN17003_c0_g1	-	-	-	0.45	7.46	-3.32	down	0.00	0.00	yes
TRINITY_DN27170_c0_g3	ABC transporter family protein [Populus trichocarpa]	ABCG22	ABC transporter G family member 22 OS=Arabidopsis thaliana GN=ABCG22 PE=1 SV=1	0.63	5.11	-2.43	down	0.00	0.00	yes
TRINITY_DN24028_c0_g6	multidrug resistance P-glycoprotein [Populus trichocarpa]	ABCB19	ABC transporter B family member 19 OS=Arabidopsis thaliana GN=ABCB19 PE=1 SV=1	17.04	81.03	-1.55	down	0.00	0.00	yes
TRINITY_DN19796_c0_g4	hypothetical protein POPTR_0015s05970g [Populus trichocarpa]	-	-	2.30	12.00	-1.98	down	0.00	0.00	yes
TRINITY_DN18449_c0_g1	hypothetical protein POPTR_0014s06570g [Populus trichocarpa]	-	-	5.90	22.93	-1.34	down	0.00	0.00	yes
TRINITY_DN17590_c0_g1	hypothetical protein POPTR_0007s14870g [Populus trichocarpa]	-	-	2.09	28.06	-2.48	down	0.00	0.00	yes
TRINITY_DN22496_c0_g1	PREDICTED: microtubule-associated protein TORTIFOLIA1-like [Populus euphratica]	TOR1L2	TORTIFOLIA1-like protein 2 OS=Arabidopsis thaliana GN=TOR1L2 PE=3 SV=1	5.29	21.94	-1.46	down	0.00	0.00	yes
TRINITY_DN16680_c0_g2	hypothetical protein POPTR_0008s11850g [Populus trichocarpa]	-	-	5.73	25.41	-1.54	down	0.00	0.00	yes
TRINITY_DN22082_c0_g1	-	-	-	0.63	6.00	-2.61	down	0.00	0.00	yes
TRINITY_DN14143_c0_g1	PREDICTED: uncharacterized protein LOC105130129 [Populus euphratica]	-	-	0.71	9.36	-3.03	down	0.00	0.00	yes
TRINITY_DN18592_c1_g6	-	-	-	4.24	33.35	-2.35	down	0.00	0.00	yes

TRINITY_DN18231_c0_g1	hypothetical protein POPTR_0010s00660g [Populus trichocarpa]	-	-	27.56	103.33	-1.31	down	0.00	0.00	yes
TRINITY_DN23062_c0_g1	PHD finger family protein [Populus trichocarpa]	Ubr7	Putative E3 ubiquitin-protein ligase UBR7 OS=Mus musculus GN=Ubr7 PE=1 SV=1	6.99	27.49	-1.34	down	0.00	0.00	yes
TRINITY_DN23314_c0_g1	hypothetical protein POPTR_0004s19550g [Populus trichocarpa]	-	-	0.87	8.13	-2.65	down	0.00	0.00	yes
TRINITY_DN27132_c3_g2	hypothetical protein POPTR_0001s24190g [Populus trichocarpa]	-	-	0.31	4.95	-3.34	down	0.00	0.00	yes
TRINITY_DN18162_c0_g1	PREDICTED: NAC transcription factor 29-like [Populus euphratica]	NAC072	NAC domain-containing protein 72 OS=Arabidopsis thaliana GN=NAC072 PE=2 SV=1	4.47	21.42	-1.61	down	0.00	0.00	yes
TRINITY_DN25860_c3_g2	hypothetical protein POPTR_0014s01380g [Populus trichocarpa]	KIN7N	Kinesin-like protein KIN-7N OS=Arabidopsis thaliana GN=KIN7N PE=2 SV=1	5.79	22.68	-1.37	down	0.00	0.00	yes
TRINITY_DN27365_c0_g1	PREDICTED: structural maintenance of chromosomes protein 2-1-like [Populus euphratica]	SMC2-1	Structural maintenance of chromosomes protein 2-1 OS=Arabidopsis thaliana GN=SMC2-1 PE=2 SV=2	3.38	19.49	-1.93	down	0.00	0.00	yes
TRINITY_DN21899_c0_g1	hypothetical protein POPTR_0009s08290g [Populus trichocarpa]	OPT4	Oligopeptide transporter 4 OS=Arabidopsis thaliana GN=OPT4 PE=1 SV=1	1.28	11.61	-2.56	down	0.00	0.00	yes
TRINITY_DN24675_c0_g1	hypothetical protein POPTR_0003s19800g [Populus trichocarpa]	ABCC3	ABC transporter C family member 3 OS=Arabidopsis thaliana GN=ABCC3 PE=1 SV=1	3.48	13.85	-1.48	down	0.00	0.00	yes
TRINITY_DN15962_c0_g2	hypothetical protein POPTR_0018s09970g [Populus trichocarpa]	ssx2ip-a	Afadin- and alpha-actinin-binding protein A OS=Xenopus laevis GN=ssx2ip-a PE=1 SV=1	11.97	40.65	-1.15	down	0.00	0.00	yes
TRINITY_DN21334_c0_g2	SAMT, partial [Populus x beijingensis]	SAMT	Salicylate carboxymethyltransferase OS=Clarkia breweri GN=SAMT PE=1 SV=1	0.13	3.35	-3.99	down	0.00	0.00	yes
TRINITY_DN19312_c0_g4	hypothetical protein POPTR_0007s03330g [Populus trichocarpa]	BCA5	Beta carbonic anhydrase 5, chloroplastic OS=Arabidopsis thaliana GN=BCA5 PE=2 SV=1	0.60	4.78	-2.39	down	0.00	0.00	yes
TRINITY_DN19759_c0_g1	PREDICTED: uncharacterized protein LOC105133990 isoform X1 [Populus euphratica]	alkB	Alpha-ketoglutarate-dependent dioxygenase AlkB OS=Salmonella typhimurium (strain LT2 / SGSC1412 / ATCC 700720) GN=alkB PE=3 SV=2	9.48	45.69	-1.38	down	0.00	0.00	yes
TRINITY_DN27505_c2_g1	PREDICTED: formin-like protein 1 [Populus euphratica]	-	-	0.42	4.92	-2.91	down	0.00	0.00	yes
TRINITY_DN24684_c0_g1	hypothetical protein POPTR_0005s22730g [Populus trichocarpa]	JASON	Protein JASON OS=Arabidopsis thaliana GN=JASON PE=2 SV=1	18.61	71.34	-1.39	down	0.00	0.00	yes
TRINITY_DN23656_c1_g8	hypothetical protein POPTR_0010s24300g [Populus trichocarpa]	-	-	0.24	2.59	-2.84	down	0.00	0.00	yes
TRINITY_DN19125_c0_g2	PREDICTED: wall-associated receptor kinase-like 5 isoform X1 [Populus euphratica]	CES101	G-type lectin S-receptor-like serine/threonine-protein kinase CES101 OS=Arabidopsis thaliana GN=CES101 PE=2 SV=2	1.10	8.38	-2.22	down	0.00	0.00	yes
TRINITY_DN22570_c0_g5	hypothetical protein POPTR_0001s42040g [Populus trichocarpa]	SD11	G-type lectin S-receptor-like serine/threonine-protein kinase SD1-1 OS=Arabidopsis thaliana GN=SD11 PE=1 SV=1	1.90	11.91	-2.17	down	0.00	0.00	yes
TRINITY_DN19938_c0_g1	hypothetical protein POPTR_0005s033602g, partial [Populus trichocarpa]	-	-	11.75	65.83	-1.53	down	0.00	0.00	yes
TRINITY_DN18512_c0_g3	hypothetical protein POPTR_0019s04510g [Populus trichocarpa]	-	-	4.21	16.50	-1.79	down	0.00	0.00	yes
TRINITY_DN19795_c0_g3	hypothetical protein POPTR_0006s02990g [Populus trichocarpa]	-	-	5.81	24.39	-1.46	down	0.00	0.00	yes
TRINITY_DN26227_c0_g1	receptor protein kinase-1 [Populus tomentosa]	CLV1	Receptor protein kinase CLAVATA1 OS=Arabidopsis thaliana GN=CLV1 PE=1 SV=3	5.06	20.86	-1.49	down	0.00	0.00	yes
TRINITY_DN26397_c0_g3	hypothetical protein POPTR_0017s14060g [Populus trichocarpa]	zcchc8	Zinc finger CCHC domain-containing protein 8 OS=Xenopus laevis GN=zcchc8 PE=2 SV=1	4.03	15.26	-1.27	down	0.00	0.00	yes
TRINITY_DN27097_c0_g1	PREDICTED: protein argonaute 5-like [Populus euphratica]	AGO5	Protein argonaute 5 OS=Arabidopsis thaliana GN=AGO5 PE=1 SV=2	2.16	12.72	-2.15	down	0.00	0.00	yes
TRINITY_DN27385_c1_g1	PREDICTED: tyrosine-protein kinase CSK-like isoform X1 [Populus euphratica]	STY46	Serine/threonine-protein kinase STY46 OS=Arabidopsis thaliana GN=STY46 PE=1 SV=1	6.89	25.06	-1.53	down	0.00	0.00	yes
TRINITY_DN17622_c0_g1	hypothetical protein POPTR_0010s11090g [Populus trichocarpa]	-	-	3.86	16.55	-1.87	down	0.00	0.00	yes
TRINITY_DN27398_c1_g1	PREDICTED: 125 kDa kinesin-related protein [Populus euphratica]	KIN5C	Kinesin-like protein KIN-5C OS=Nicotiana tabacum GN=KIN5C PE=1 SV=1	10.93	43.02	-1.40	down	0.00	0.00	yes
TRINITY_DN24355_c2_g4	hypothetical protein POPTR_0003s10650g [Populus trichocarpa]	-	-	4.31	16.98	-1.38	down	0.00	0.00	yes

TRINITY_DN27873_c1_g1	hypothetical protein POPTR_0019s127701g, partial [Populus trichocarpa]	-	-	0.87	11.35	-2.74	down	0.00	0.00	yes
TRINITY_DN22660_c0_g3	hypothetical protein POPTR_0015s08160g [Populus trichocarpa]	E2FE	E2F transcription factor-like E2FE OS=Arabidopsis thaliana GN=E2FE PE=2 SV=1	3.66	15.67	-1.50	down	0.00	0.00	yes
TRINITY_DN20591_c0_g2	hypothetical protein POPTR_0010s07180g [Populus trichocarpa]	-	Probable glutathione S-transferase OS=Nicotiana tabacum PE=2 SV=1	0.24	4.04	-3.45	down	0.00	0.00	yes
TRINITY_DN15982_c0_g1	PREDICTED: E3 ubiquitin-protein ligase ATL4 [Populus euphratica]	ATL4	E3 ubiquitin-protein ligase ATL4 OS=Arabidopsis thaliana GN=ATL4 PE=1 SV=1	0.47	3.97	-2.46	down	0.00	0.00	yes
TRINITY_DN22172_c0_g1	PREDICTED: cytochrome P450 705A5-like [Populus euphratica]	-	-	0.40	7.59	-3.95	down	0.00	0.00	yes
TRINITY_DN14368_c0_g2	hypothetical protein POPTR_0008s16470g [Populus trichocarpa]	At4g14450	Uncharacterized protein At4g14450, chloroplastic OS=Arabidopsis thaliana GN=At4g14450 PE=2 SV=1	13.76	68.14	-1.66	down	0.00	0.00	yes
TRINITY_DN17433_c0_g1	hypothetical protein POPTR_0010s12130g [Populus trichocarpa]	-	-	0.34	5.50	-3.37	down	0.00	0.00	yes
TRINITY_DN24219_c0_g3	no apical meristem family protein [Populus trichocarpa]	NAC072	NAC domain-containing protein 72 OS=Arabidopsis thaliana GN=NAC072 PE=2 SV=1	9.96	79.77	-2.42	down	0.00	0.00	yes
TRINITY_DN27834_c1_g2	-	-	-	0.44	3.89	-2.52	down	0.00	0.00	yes
TRINITY_DN21568_c0_g1	hypothetical protein POPTR_0007s02440g [Populus trichocarpa]	At2g43200	Probable methyltransferase PMT19 OS=Arabidopsis thaliana GN=At2g43200 PE=3 SV=1	1.96	10.21	-1.66	down	0.00	0.00	yes
TRINITY_DN19391_c0_g1	hypothetical protein POPTR_0006s01820g [Populus trichocarpa]	-	-	21.25	76.63	-1.24	down	0.00	0.00	yes
TRINITY_DN16731_c0_g1	hypothetical protein POPTR_0016s06660g [Populus trichocarpa]	-	-	0.33	2.73	-2.41	down	0.00	0.00	yes
TRINITY_DN10969_c0_g1	hypothetical protein POPTR_0012s14830g [Populus trichocarpa]	-	-	0.68	5.47	-2.37	down	0.00	0.00	yes
TRINITY_DN22248_c0_g4	PREDICTED: L-type lectin-domain containing receptor kinase VIII.1 [Populus euphratica]	LECRK81	L-type lectin-domain containing receptor kinase VIII.1 OS=Arabidopsis thaliana GN=LECRK81 PE=2 SV=1	5.32	19.99	-1.30	down	0.00	0.00	yes
TRINITY_DN27019_c0_g1	PREDICTED: uncharacterized protein LOC105115829 isoform X2 [Populus euphratica]	-	-	7.99	26.87	-1.28	down	0.00	0.00	yes
TRINITY_DN21696_c0_g2	PREDICTED: uncharacterized protein LOC105134657 [Populus euphratica]	-	-	13.19	47.74	-1.24	down	0.00	0.00	yes
TRINITY_DN19149_c0_g1	PREDICTED: myb-related protein P-like [Populus euphratica]	MYB12	Transcription factor MYB12 OS=Arabidopsis thaliana GN=MYB12 PE=2 SV=1	0.16	7.51	-5.88	down	0.00	0.00	yes
TRINITY_DN21202_c0_g5	hypothetical protein POPTR_0017s04540g [Populus trichocarpa]	-	-	16.30	159.89	-2.62	down	0.00	0.00	yes
TRINITY_DN20482_c0_g1	RNA polymerase II mediator complex family protein [Populus trichocarpa]	MED10B	Mediator of RNA polymerase II transcription subunit 10b OS=Arabidopsis thaliana GN=MED10B PE=1 SV=1	7.08	28.59	-1.42	down	0.00	0.00	yes
TRINITY_DN18752_c0_g2	PREDICTED: dynamin-related protein 5A isoform X1 [Populus euphratica]	DRP5A	Dynamin-related protein 5A OS=Arabidopsis thaliana GN=DRP5A PE=2 SV=1	5.05	18.98	-1.32	down	0.00	0.00	yes
TRINITY_DN22671_c1_g1	PREDICTED: shugoshin-1-like isoform X7 [Populus euphratica]	-	-	6.09	22.09	-1.24	down	0.00	0.00	yes
TRINITY_DN21289_c1_g6	-	-	-	0.23	3.62	-3.35	down	0.00	0.00	yes
TRINITY_DN27768_c1_g3	hypothetical protein POPTR_0005s03580g [Populus trichocarpa]	LOX1	Linoleate 9S-lipoxygenase 1 OS=Arabidopsis thaliana GN=LOX1 PE=1 SV=1	4.58	18.84	-1.73	down	0.00	0.00	yes
TRINITY_DN17241_c0_g2	DNA polymerase delta subunit 4 family protein [Populus trichocarpa]	-	-	37.87	132.98	-1.21	down	0.00	0.00	yes
TRINITY_DN9120_c0_g1	early nodulin 93 protein [Populus alba x Populus glandulosa]	-	Early nodulin-93 OS=Glycine max PE=2 SV=1	0.47	9.83	-3.65	down	0.00	0.00	yes
TRINITY_DN26452_c0_g1	PREDICTED: uncharacterized protein LOC105134285 isoform X1 [Populus euphratica]	-	-	5.79	21.12	-1.26	down	0.00	0.00	yes
TRINITY_DN20920_c0_g2	hypothetical protein POPTR_0012s02570g [Populus trichocarpa]	DOF3.7	Dof zinc finger protein DOF3.7 OS=Arabidopsis thaliana GN=DOF3.7 PE=1 SV=2	1.01	6.74	-2.17	down	0.00	0.00	yes
TRINITY_DN26725_c0_g1	zinc finger family protein [Populus trichocarpa]	SAP4	Zinc finger A20 and AN1 domain-containing stress-associated protein 4 OS=Arabidopsis thaliana GN=SAP4 PE=1 SV=1	2.89	17.70	-2.00	down	0.00	0.00	yes
TRINITY_DN25595_c0_g2	PREDICTED: uncharacterized protein LOC105128876 [Populus euphratica]	-	-	2.92	12.49	-1.72	down	0.00	0.00	yes

TRINITY_DN18736_c0_g1	-	-	-	14.24	55.68	-1.35	down	0.00	0.00	yes
TRINITY_DN18846_c0_g1	PREDICTED: serine/arginine repetitive matrix protein 1 [Populus euphratica]	-	-	14.33	56.00	-1.41	down	0.00	0.00	yes
TRINITY_DN22801_c0_g1	PREDICTED: protein PHLOEM PROTEIN 2-LIKE A10-like [Populus euphratica]	PP2A10	Protein PHLOEM PROTEIN 2-LIKE A10 OS=Arabidopsis thaliana GN=PP2A10 PE=2 SV=1	9.40	36.51	-1.31	down	0.00	0.00	yes
TRINITY_DN27471_c0_g1	PREDICTED: VIN3-like protein 1 isoform X1 [Populus euphratica]	VIL1	VIN3-like protein 1 OS=Arabidopsis thaliana GN=VIL1 PE=1 SV=1	10.56	45.13	-1.53	down	0.00	0.00	yes
TRINITY_DN15581_c0_g1	PREDICTED: dihydroflavonol-4-reductase, partial [Populus euphratica]	DFRA	Dihydroflavonol 4-reductase OS=Arabidopsis thaliana GN=DFRA PE=1 SV=2	0.50	4.03	-2.43	down	0.00	0.00	yes
TRINITY_DN22395_c0_g2	hypothetical protein POPTR_0012s01310g [Populus trichocarpa]	-	-	1.81	8.78	-1.67	down	0.00	0.00	yes
TRINITY_DN24615_c0_g4	hypothetical protein POPTR_0002s22840g [Populus trichocarpa]	GID1B	Gibberellin receptor GID1B OS=Arabidopsis thaliana GN=GID1B PE=1 SV=1	0.18	3.46	-3.54	down	0.00	0.00	yes
TRINITY_DN18152_c0_g1	PREDICTED: protein MIZU-KUSSEI 1 [Populus euphratica]	MIZ1	Protein MIZU-KUSSEI 1 OS=Arabidopsis thaliana GN=MIZ1 PE=1 SV=1	1.12	7.62	-2.06	down	0.00	0.00	yes
TRINITY_DN27101_c0_g1	PREDICTED: uncharacterized protein LOC105124962 [Populus euphratica]	dnajb14	DnaJ homolog subfamily B member 14 OS=Xenopus tropicalis GN=dnajb14 PE=2 SV=1	4.77	16.99	-1.22	down	0.00	0.00	yes
TRINITY_DN17572_c1_g1	hypothetical protein POPTR_0001s41880g [Populus trichocarpa]	-	-	1.59	16.65	-2.72	down	0.00	0.00	yes
TRINITY_DN25676_c0_g1	PREDICTED: high mobility group B protein 3-like [Populus euphratica]	HMGB2	High mobility group B protein 2 OS=Arabidopsis thaliana GN=HMGB2 PE=1 SV=1	385.41	1440.19	-1.24	down	0.00	0.00	yes
TRINITY_DN27547_c0_g1	calmodulin-binding family protein [Populus trichocarpa]	CMTA4	Calmodulin-binding transcription activator 4 OS=Arabidopsis thaliana GN=CMTA4 PE=1 SV=1	6.25	23.99	-1.35	down	0.00	0.00	yes
TRINITY_DN20712_c0_g1	C-repeat binding factor 1 [Populus tomentosa]	DREB1B	Dehydration-responsive element-binding protein 1B OS=Arabidopsis thaliana GN=DREB1B PE=2 SV=2	0.29	12.17	-4.68	down	0.00	0.00	yes
TRINITY_DN22844_c0_g1	unknown [Populus trichocarpa]	Dnajb4	DnaJ homolog subfamily B member 4 OS=Mus musculus GN=Dnajb4 PE=1 SV=1	6.75	26.35	-1.36	down	0.00	0.00	yes
TRINITY_DN20515_c0_g2	hypothetical protein POPTR_0018s093702g, partial [Populus trichocarpa]	Os01g0744400	Golgin-84 OS=Oryza sativa subsp. japonica GN=Os01g0744400 PE=2 SV=1	0.75	7.20	-2.97	down	0.00	0.00	yes
TRINITY_DN26274_c0_g4	proton-dependent oligopeptide transport family protein [Populus trichocarpa]	NPF5.1	Protein NRT1/ PTR FAMILY 5.1 OS=Arabidopsis thaliana GN=NPF5.1 PE=2 SV=2	0.31	3.44	-2.82	down	0.00	0.00	yes
TRINITY_DN19781_c0_g1	ovate family protein [Populus trichocarpa]	OFP4	Transcription repressor OFP4 OS=Arabidopsis thaliana GN=OFP4 PE=1 SV=1	3.48	15.24	-1.53	down	0.00	0.00	yes
TRINITY_DN22152_c0_g1	hypothetical protein POPTR_0007s01340g [Populus trichocarpa]	HSL1	Receptor-like protein kinase HSL1 OS=Arabidopsis thaliana GN=HSL1 PE=2 SV=1	2.36	11.98	-1.70	down	0.00	0.00	yes
TRINITY_DN24141_c0_g2	hypothetical protein POPTR_0008s01660g [Populus trichocarpa]	-	-	3.65	14.10	-1.46	down	0.00	0.00	yes
TRINITY_DN14474_c0_g3	hypothetical protein POPTR_0017s11180g [Populus trichocarpa]	D6PKL2	Serine/threonine-protein kinase D6PKL2 OS=Arabidopsis thaliana GN=D6PKL2 PE=1 SV=1	0.41	3.17	-2.29	down	0.00	0.00	yes
TRINITY_DN20249_c0_g1	hypothetical protein POPTR_0006s06840g [Populus trichocarpa]	-	-	1.41	9.56	-2.12	down	0.00	0.00	yes
TRINITY_DN18652_c0_g3	hypothetical protein POPTR_0003s17160g [Populus trichocarpa]	At1g67360	REF/SRPP-like protein At1g67360 OS=Arabidopsis thaliana GN=At1g67360 PE=2 SV=1	1.70	12.43	-2.20	down	0.00	0.00	yes
TRINITY_DN23505_c0_g6	-	-	-	7.67	40.65	-1.77	down	0.00	0.00	yes
TRINITY_DN22157_c0_g1	PREDICTED: uncharacterized protein LOC105142101 [Populus euphratica]	-	-	8.25	30.06	-1.26	down	0.00	0.00	yes
TRINITY_DN16129_c0_g4	hypothetical protein POPTR_0015s12090g [Populus trichocarpa]	-	-	0.57	4.10	-2.24	down	0.00	0.00	yes
TRINITY_DN24274_c0_g2	PREDICTED: protein WVD2-like 1 isoform X1 [Populus euphratica]	WDL2	Protein WVD2-like 2 OS=Arabidopsis thaliana GN=WDL2 PE=2 SV=1	7.02	25.48	-1.29	down	0.00	0.00	yes
TRINITY_DN24547_c0_g1	PREDICTED: serine carboxypeptidase-like 25 [Populus euphratica]	SCPL25	Serine carboxypeptidase-like 25 OS=Arabidopsis thaliana GN=SCPL25 PE=2 SV=2	4.43	25.58	-1.78	down	0.00	0.00	yes
TRINITY_DN25130_c0_g2	hypothetical protein POPTR_0002s01550g [Populus trichocarpa]	ROPGEF1	Rop guanine nucleotide exchange factor 1 OS=Arabidopsis thaliana GN=ROPGEF1 PE=1 SV=2	2.57	10.41	-1.42	down	0.00	0.00	yes
TRINITY_DN15920_c0_g2	PREDICTED: uncharacterized protein LOC105133646 [Populus euphratica]	-	-	0.08	2.74	-4.49	down	0.00	0.00	yes

TRINITY_DN18922_c0_g1	hypothetical protein POPTR_0005s24600g [Populus trichocarpa]	-	-	9.20	37.42	-1.53	down	0.00	0.00	yes
TRINITY_DN27372_c0_g3	PREDICTED: acyl-CoA-binding domain-containing protein 4-like isoform X1 [Populus euphratica]	ACBP4	Acyl-CoA-binding domain-containing protein 4 OS=Arabidopsis thaliana GN=ACBP4 PE=1 SV=1	1.04	5.69	-1.84	down	0.00	0.00	yes
TRINITY_DN20960_c0_g2	PREDICTED: protein PLANT CADMIUM RESISTANCE 2-like [Populus euphratica]	PCR2	Protein PLANT CADMIUM RESISTANCE 2 OS=Arabidopsis thaliana GN=PCR2 PE=1 SV=1	23.31	103.14	-1.54	down	0.00	0.00	yes
TRINITY_DN23915_c0_g2	hypothetical protein POPTR_0018s02750g [Populus trichocarpa]	At5g25050	Probable folate-biopterin transporter 2 OS=Arabidopsis thaliana GN=At5g25050 PE=2 SV=1	4.13	21.26	-1.46	down	0.00	0.00	yes
TRINITY_DN22974_c0_g1	unknown [Populus trichocarpa]	-	-	7.87	50.82	-2.36	down	0.00	0.00	yes
TRINITY_DN20623_c0_g2	PREDICTED: uncharacterized protein LOC105139259 isoform X1 [Populus euphratica]	-	-	0.64	4.20	-2.12	down	0.00	0.00	yes
TRINITY_DN23984_c0_g3	F-box family protein [Populus trichocarpa]	FBL17	F-box/LRR-repeat protein 17 OS=Arabidopsis thaliana GN=FBL17 PE=1 SV=1	1.58	13.73	-2.49	down	0.00	0.00	yes
TRINITY_DN26456_c0_g1	hypothetical protein POPTR_0001s39940g [Populus trichocarpa]	-	Polyphenol oxidase, chloroplastic OS=Malus domestica PE=2 SV=1	112.97	594.31	-2.06	down	0.00	0.00	yes
TRINITY_DN21855_c0_g1	nodulin family protein [Populus trichocarpa]	-	-	2.75	12.83	-1.90	down	0.00	0.00	yes
TRINITY_DN18381_c0_g1	SERINE/THREONINE protein KINASE 1 [Populus trichocarpa]	CIPK14	CBL-interacting serine/threonine-protein kinase 14 OS=Arabidopsis thaliana GN=CIPK14 PE=1 SV=1	1.14	6.90	-2.01	down	0.00	0.00	yes
TRINITY_DN19684_c0_g2	hypothetical protein POPTR_0016s14490g [Populus trichocarpa]	WRKY70	Probable WRKY transcription factor 70 OS=Arabidopsis thaliana GN=WRKY70 PE=2 SV=1	1.55	17.74	-2.89	down	0.00	0.00	yes
TRINITY_DN24453_c0_g3	-	-	-	0.11	2.08	-3.63	down	0.00	0.00	yes
TRINITY_DN18894_c0_g6	-	-	-	0.17	3.67	-3.72	down	0.00	0.00	yes
TRINITY_DN20037_c0_g1	PREDICTED: ABC transporter B family member 2-like isoform X1 [Populus euphratica]	ABCB2	ABC transporter B family member 2 OS=Arabidopsis thaliana GN=ABCB2 PE=1 SV=3	1.02	7.16	-2.03	down	0.00	0.00	yes
TRINITY_DN20294_c1_g1	hypothetical protein POPTR_0010s17470g [Populus trichocarpa]	MYB44	Transcription factor MYB44 OS=Arabidopsis thaliana GN=MYB44 PE=2 SV=1	0.43	6.94	-2.75	down	0.00	0.00	yes
TRINITY_DN26074_c0_g8	PREDICTED: axial regulator YABBY 1-like isoform X1 [Tarenaya hassleriana]	YAB3	Protein YABBY 3 OS=Oryza sativa subsp. japonica GN=YAB3 PE=2 SV=1	29.73	202.48	-1.59	down	0.00	0.00	yes
TRINITY_DN26664_c0_g2	PREDICTED: probable amino acid permease 7 isoform X1 [Populus euphratica]	AAP7	Probable amino acid permease 7 OS=Arabidopsis thaliana GN=AAP7 PE=2 SV=1	0.47	4.36	-2.59	down	0.00	0.00	yes
TRINITY_DN20550_c0_g5	-	-	-	1.68	9.12	-2.09	down	0.00	0.00	yes
TRINITY_DN24471_c0_g1	hypothetical protein POPTR_0010s14840g [Populus trichocarpa]	ACR4	ACT domain-containing protein ACR4 OS=Arabidopsis thaliana GN=ACR4 PE=2 SV=1	12.97	45.98	-1.23	down	0.00	0.00	yes
TRINITY_DN24431_c1_g2	PREDICTED: acyl-coenzyme A oxidase 4, peroxisomal-like isoform X3 [Populus euphratica]	ACX4	Acyl-coenzyme A oxidase 4, peroxisomal OS=Arabidopsis thaliana GN=ACX4 PE=1 SV=1	3.95	18.34	-1.25	down	0.00	0.00	yes
TRINITY_DN23563_c1_g8	hypothetical protein POPTR_0046s00390g [Populus trichocarpa]	-	-	0.18	3.59	-3.60	down	0.00	0.00	yes
TRINITY_DN22804_c0_g4	ERF domain protein 12 [Populus trichocarpa]	ERF12	Ethylene-responsive transcription factor 12 OS=Arabidopsis thaliana GN=ERF12 PE=2 SV=1	0.99	8.94	-2.56	down	0.00	0.00	yes
TRINITY_DN14183_c0_g1	hypothetical protein POPTR_0013s15370g, partial [Populus trichocarpa]	-	-	0.40	3.65	-3.13	down	0.00	0.00	yes
TRINITY_DN22868_c0_g1	hypothetical protein POPTR_0006s26110g [Populus trichocarpa]	At3g58900	F-box/LRR-repeat protein At3g58900 OS=Arabidopsis thaliana GN=At3g58900 PE=2 SV=1	0.22	3.09	-3.14	down	0.00	0.00	yes
TRINITY_DN20930_c0_g1	PREDICTED: E3 ubiquitin-protein ligase brl2 isoform X1 [Populus euphratica]	At4g02110	BRCT domain-containing protein At4g02110 OS=Arabidopsis thaliana GN=At4g02110 PE=3 SV=3	1.71	7.42	-1.49	down	0.00	0.00	yes
TRINITY_DN25276_c0_g2	hypothetical protein POPTR_0018s00860g [Populus trichocarpa]	TEB	Helicase and polymerase-containing protein TEBICHI OS=Arabidopsis thaliana GN=TEB PE=2 SV=1	1.70	6.52	-1.48	down	0.00	0.00	yes
TRINITY_DN25238_c0_g1	hypothetical protein POPTR_0012s00760g [Populus trichocarpa]	NAC062	NAC domain-containing protein 62 OS=Arabidopsis thaliana GN=NAC062 PE=1 SV=1	3.02	41.76	-3.15	down	0.00	0.00	yes
TRINITY_DN19912_c0_g3	PREDICTED: transcription repressor MYB6-like isoform X1 [Populus euphratica]	MYB39	Transcription factor MYB39 OS=Arabidopsis thaliana GN=MYB39 PE=2 SV=1	0.26	4.18	-3.25	down	0.00	0.00	yes
TRINITY_DN24231_c0_g3	PREDICTED: phosphatidylinositol/phosphatidylcholine transfer protein SFH1 isoform X1 [Populus euphratica]	SFH8	Phosphatidylinositol/phosphatidylcholine transfer protein SFH8 OS=Arabidopsis thaliana GN=SFH8 PE=2 SV=1	11.24	38.79	-1.20	down	0.00	0.00	yes
TRINITY_DN19073_c1_g2	PREDICTED: RING-H2 finger protein ATL56-like [Populus euphratica]	ATL56	RING-H2 finger protein ATL56 OS=Arabidopsis thaliana GN=ATL56 PE=2 SV=1	0.54	11.75	-3.78	down	0.00	0.00	yes

TRINITY_DN19130_c0_g2	hypothetical protein POPTR_0001s03800g [Populus trichocarpa]	-	-	0.32	3.65	-2.84	down	0.00	0.00	yes
TRINITY_DN26322_c0_g1	PREDICTED: methyl-CpG-binding domain-containing protein 13-like isoform X2 [Populus euphratica]	MBD13	Methyl-CpG-binding domain-containing protein 13 OS=Arabidopsis thaliana GN=MBD13 PE=2 SV=1	5.16	20.35	-1.43	down	0.00	0.00	yes
TRINITY_DN25664_c0_g1	PREDICTED: inactive leucine-rich repeat receptor-like serine/threonine-protein kinase At1g60630 [Populus euphratica]	At1g60630	Inactive leucine-rich repeat receptor-like serine/threonine-protein kinase At1g60630 OS=Arabidopsis thaliana GN=At1g60630 PE=2 SV=1	1.28	7.79	-1.86	down	0.00	0.00	yes
TRINITY_DN19059_c0_g2	PREDICTED: G-type lectin S-receptor-like serine/threonine-protein kinase At5g24080 isoform X1 [Populus euphratica]	At5g24080	G-type lectin S-receptor-like serine/threonine-protein kinase At5g24080 OS=Arabidopsis thaliana GN=At5g24080 PE=2 SV=1	1.17	7.57	-1.92	down	0.00	0.00	yes
TRINITY_DN21437_c0_g4	hypothetical protein POPTR_0007s02370g, partial [Populus trichocarpa]	-	-	0.53	4.91	-2.55	down	0.00	0.00	yes
TRINITY_DN17157_c0_g1	unknown [Populus trichocarpa]	-	-	80.90	441.01	-1.74	down	0.00	0.00	yes
TRINITY_DN23212_c1_g5	PREDICTED: uncharacterized protein LOC105136109 [Populus euphratica]	-	-	21.06	71.03	-1.18	down	0.00	0.00	yes
TRINITY_DN21705_c0_g7	PREDICTED: uncharacterized protein LOC105124916 isoform X1 [Populus euphratica]	-	-	1.15	8.14	-2.21	down	0.00	0.00	yes
TRINITY_DN18869_c0_g1	pathogenesis-related thaumatin superfamily protein [Populus tomentosa]	-	Osmotin-like protein OS=Solanum lycopersicum PE=1 SV=1	15.92	152.33	-2.67	down	0.00	0.00	yes
TRINITY_DN18022_c4_g1	-	-	-	0.73	7.73	-3.08	down	0.00	0.00	yes
TRINITY_DN19177_c0_g1	hypothetical protein POPTR_0002s09520g [Populus trichocarpa]	At1g22220	F-box protein At1g22220 OS=Arabidopsis thaliana GN=At1g22220 PE=2 SV=1	4.57	24.47	-1.84	down	0.00	0.00	yes
TRINITY_DN20149_c0_g1	hypothetical protein POPTR_0009s10600g [Populus trichocarpa]	At3g19184	B3 domain-containing protein At3g19184 OS=Arabidopsis thaliana GN=At3g19184 PE=2 SV=1	5.80	23.12	-1.44	down	0.00	0.00	yes
TRINITY_DN28655_c0_g1	hypothetical protein POPTR_0021s00410g [Populus trichocarpa]	-	-	4.77	58.79	-2.97	down	0.00	0.00	yes
TRINITY_DN20594_c0_g2	hypothetical protein POPTR_0019s05310g [Populus trichocarpa]	RPP13L3	Putative disease resistance RPP13-like protein 3 OS=Arabidopsis thaliana GN=RPP13L3 PE=3 SV=1	0.69	3.84	-1.86	down	0.00	0.00	yes
TRINITY_DN26687_c0_g2	PREDICTED: PHD finger protein ALFIN-LIKE 1-like [Populus euphratica]	AL1	PHD finger protein ALFIN-LIKE 1 OS=Arabidopsis thaliana GN=AL1 PE=1 SV=1	9.77	33.30	-1.15	down	0.00	0.00	yes
TRINITY_DN27456_c1_g1	PREDICTED: probable LRR receptor-like serine/threonine-protein kinase At4g20940 [Populus euphratica]	At4g20940	Probable LRR receptor-like serine/threonine-protein kinase At4g20940 OS=Arabidopsis thaliana GN=At4g20940 PE=1 SV=1	5.06	16.85	-1.23	down	0.00	0.00	yes
TRINITY_DN23243_c1_g1	unknown [Populus trichocarpa]	DOF5.4	Dof zinc finger protein DOF5.4 OS=Arabidopsis thaliana GN=DOF5.4 PE=2 SV=2	14.08	58.20	-1.41	down	0.00	0.00	yes
TRINITY_DN25637_c0_g1	PREDICTED: zinc finger CCCH domain-containing protein 29-like [Populus euphratica]	At2g40140	Zinc finger CCCH domain-containing protein 29 OS=Arabidopsis thaliana GN=At2g40140 PE=2 SV=1	14.77	129.31	-2.41	down	0.00	0.00	yes
TRINITY_DN18328_c0_g1	PREDICTED: fidgetin-like protein 1 isoform X1 [Populus euphratica]	FIGNL1	Fidgetin-like protein 1 OS=Homo sapiens GN=FIGNL1 PE=1 SV=2	1.38	6.65	-1.63	down	0.00	0.00	yes
TRINITY_DN21857_c0_g1	alternative oxidase [Populus tremula x Populus tremuloides]	AOX1A	Ubiquinol oxidase 1a, mitochondrial OS=Arabidopsis thaliana GN=AOX1A PE=1 SV=2	2.17	12.56	-1.95	down	0.00	0.00	yes
TRINITY_DN21863_c0_g1	Protein phosphatase 2C family protein [Populus trichocarpa]	PP2CA	Protein phosphatase 2C 37 OS=Arabidopsis thaliana GN=PP2CA PE=1 SV=1	44.56	211.95	-1.56	down	0.00	0.00	yes
TRINITY_DN22163_c0_g3	kinase family protein [Populus trichocarpa]	LYK3	LysM domain receptor-like kinase 3 OS=Arabidopsis thaliana GN=LYK3 PE=2 SV=1	2.52	12.43	-1.69	down	0.00	0.00	yes
TRINITY_DN25263_c0_g1	hypothetical protein POPTR_0007s13040g [Populus trichocarpa]	TULP5	Tubby-like F-box protein 5 OS=Oryza sativa subsp. japonica GN=TULP5 PE=2 SV=1	71.50	191.76	-1.31	down	0.00	0.00	yes
TRINITY_DN20327_c0_g2	hypothetical protein POPTR_0011s17240g, partial [Populus trichocarpa]	BRL3	Receptor-like protein kinase BRI1-like 3 OS=Arabidopsis thaliana GN=BRL3 PE=1 SV=1	0.65	3.61	-2.07	down	0.00	0.00	yes
TRINITY_DN20744_c1_g2	Calmodulin-binding family protein [Populus trichocarpa]	-	-	0.19	2.22	-2.83	down	0.00	0.00	yes
TRINITY_DN22574_c0_g1	PREDICTED: probable receptor-like protein kinase At1g80640 [Populus euphratica]	At1g80640	Probable receptor-like protein kinase At1g80640 OS=Arabidopsis thaliana GN=At1g80640 PE=2 SV=1	5.26	19.16	-1.23	down	0.00	0.00	yes
TRINITY_DN23350_c1_g3	hypothetical protein POPTR_0001s46710g [Populus trichocarpa]	MEE23	Berberine bridge enzyme-like 15 OS=Arabidopsis thaliana GN=MEE23 PE=1 SV=1	1.13	5.40	-1.65	down	0.00	0.00	yes

TRINITY_DN18813_c1_g8	zinc finger family protein [Populus trichocarpa]	SAP5	Zinc finger A20 and AN1 domain-containing stress-associated protein 5 OS=Arabidopsis thaliana GN=SAP5 PE=2 SV=1	25.14	102.37	-1.39	down	0.00	0.00	yes
TRINITY_DN24099_c0_g3	hypothetical protein POPTR_0001s32120g [Populus trichocarpa]	KRP4	Cyclin-dependent kinase inhibitor 4 OS=Arabidopsis thaliana GN=KRP4 PE=1 SV=2	28.37	96.32	-1.15	down	0.00	0.00	yes
TRINITY_DN21235_c0_g2	PREDICTED: uncharacterized protein LOC105134060 [Populus euphratica]	-	-	0.31	3.18	-2.58	down	0.00	0.00	yes
TRINITY_DN18615_c0_g1	PREDICTED: uncharacterized protein LOC105116005 isoform X1 [Populus euphratica]	-	-	0.49	3.72	-2.03	down	0.00	0.00	yes
TRINITY_DN21116_c0_g11	hypothetical protein POPTR_0019s01930g [Populus trichocarpa]	At1g53440	Probable LRR receptor-like serine/threonine-protein kinase At1g53440 OS=Arabidopsis thaliana GN=At1g53440 PE=2 SV=2	0.74	4.36	-1.93	down	0.00	0.00	yes
TRINITY_DN16727_c0_g1	GTP-binding family protein [Populus trichocarpa]	RABA3	Ras-related protein RABA3 OS=Arabidopsis thaliana GN=RABA3 PE=2 SV=1	2.53	11.58	-1.59	down	0.00	0.00	yes
TRINITY_DN16801_c0_g1	PREDICTED: probable DNA helicase MCM9 [Populus euphratica]	MCM9	Probable DNA helicase MCM9 OS=Arabidopsis thaliana GN=MCM9 PE=3 SV=1	1.33	5.67	-1.52	down	0.00	0.00	yes
TRINITY_DN14190_c0_g2	kinase family protein [Populus trichocarpa]	OXI1	Serine/threonine-protein kinase OXI1 OS=Arabidopsis thaliana GN=OXI1 PE=1 SV=1	0.45	3.82	-2.42	down	0.00	0.00	yes
TRINITY_DN19751_c0_g2	PREDICTED: probable fructokinase-1 [Populus euphratica]	At2g31390	Probable fructokinase-1 OS=Arabidopsis thaliana GN=At2g31390 PE=2 SV=1	4.28	23.17	-1.99	down	0.00	0.00	yes
TRINITY_DN20382_c0_g2	PREDICTED: U-box domain-containing protein 28-like [Populus euphratica]	PUB29	U-box domain-containing protein 29 OS=Arabidopsis thaliana GN=PUB29 PE=1 SV=1	0.97	8.24	-2.47	down	0.00	0.00	yes
TRINITY_DN25152_c0_g1	PREDICTED: CDT1-like protein a, chloroplastic [Populus euphratica]	CDT1A	CDT1-like protein a, chloroplastic OS=Arabidopsis thaliana GN=CDT1A PE=1 SV=1	1.98	18.33	-2.45	down	0.00	0.00	yes
TRINITY_DN22515_c0_g5	-	-	-	17.20	81.31	-1.64	down	0.00	0.00	yes
TRINITY_DN20017_c0_g2	hypothetical protein POPTR_0018s11350g [Populus trichocarpa]	-	-	1.64	8.46	-1.86	down	0.00	0.00	yes
TRINITY_DN24529_c1_g1	polyamine oxidase 1 [Populus tomentosa]	PAO1	Polyamine oxidase 1 OS=Arabidopsis thaliana GN=PAO1 PE=1 SV=1	4.94	37.37	-2.26	down	0.00	0.00	yes
TRINITY_DN19626_c0_g1	-	-	-	0.27	3.63	-3.08	down	0.00	0.00	yes
TRINITY_DN19083_c0_g7	hypothetical protein POPTR_0017s12290g [Populus trichocarpa]	At5g39570	Uncharacterized protein At5g39570 OS=Arabidopsis thaliana GN=At5g39570 PE=1 SV=1	33.61	139.93	-1.46	down	0.00	0.00	yes
TRINITY_DN26657_c0_g2	hypothetical protein POPTR_0015s06040g [Populus trichocarpa]	At4g23740	Probable inactive receptor kinase At4g23740 OS=Arabidopsis thaliana GN=At4g23740 PE=2 SV=1	5.31	17.39	-1.16	down	0.00	0.00	yes
TRINITY_DN16547_c0_g1	hypothetical protein POPTR_0017s12100g [Populus trichocarpa]	CML45	Probable calcium-binding protein CML45 OS=Arabidopsis thaliana GN=CML45 PE=1 SV=1	0.07	4.07	-5.02	down	0.00	0.00	yes
TRINITY_DN22669_c1_g1	hypothetical protein POPTR_0008s15550g [Populus trichocarpa]	IP5P6	Type IV inositol polyphosphate 5-phosphatase 6 OS=Arabidopsis thaliana GN=IP5P6 PE=1 SV=2	0.67	4.65	-2.16	down	0.00	0.00	yes
TRINITY_DN21609_c3_g3	hypothetical protein POPTR_0001s434902g, partial [Populus trichocarpa]	-	-	0.63	6.02	-2.62	down	0.00	0.00	yes
TRINITY_DN27423_c2_g5	hypothetical protein POPTR_0011s12880g [Populus trichocarpa]	SD11	G-type lectin S-receptor-like serine/threonine-protein kinase SD1-1 OS=Arabidopsis thaliana GN=SD11 PE=1 SV=1	0.11	2.63	-3.86	down	0.00	0.00	yes
TRINITY_DN13098_c0_g1	hypothetical protein POPTR_0016s14280g [Populus trichocarpa]	WAX9B	Non-specific lipid-transfer protein B OS=Brassica oleracea var. italica GN=WAX9B PE=3 SV=1	1.99	24.34	-2.96	down	0.00	0.00	yes
TRINITY_DN24778_c2_g4	PREDICTED: uncharacterized protein LOC105140833 [Populus euphratica]	-	-	1.97	8.84	-1.55	down	0.00	0.00	yes
TRINITY_DN15128_c0_g1	PREDICTED: protein BREAKING OF ASYMMETRY IN THE STOMATAL LINEAGE [Populus euphratica]	BASL	Protein BREAKING OF ASYMMETRY IN THE STOMATAL LINEAGE OS=Arabidopsis thaliana GN=BASL PE=2 SV=1	0.44	4.09	-2.64	down	0.00	0.00	yes
TRINITY_DN7076_c0_g1	hypothetical protein POPTR_0005s23000g [Populus trichocarpa]	ALMT2	Aluminum-activated malate transporter 2 OS=Arabidopsis thaliana GN=ALMT2 PE=2 SV=2	0.12	2.38	-3.64	down	0.00	0.00	yes
TRINITY_DN17493_c0_g5	-	-	-	1.40	9.60	-2.17	down	0.00	0.00	yes
TRINITY_DN23629_c0_g1	PREDICTED: uncharacterized protein LOC105113187 isoform X1 [Populus euphratica]	-	-	1.06	5.72	-1.75	down	0.00	0.00	yes
TRINITY_DN20370_c1_g3	hypothetical protein POPTR_0012s11070g [Populus trichocarpa]	-	-	0.65	4.53	-2.23	down	0.00	0.00	yes
TRINITY_DN17754_c0_g1	PREDICTED: pectinesterase-like [Populus euphratica]	PME40	Probable pectinesterase/pectinesterase inhibitor 40 OS=Arabidopsis thaliana GN=PME40 PE=2 SV=1	0.76	4.68	-2.02	down	0.00	0.00	yes

TRINITY_DN25444_c0_g1	PREDICTED: tyrosyl-DNA phosphodiesterase 2 isoform X8 [Populus euphratica]	-	-	5.40	20.98	-1.23	down	0.00	0.00	yes
TRINITY_DN23063_c0_g1	hypothetical protein POPTR_0001s26480g [Populus trichocarpa]	NFYA1	Nuclear transcription factor Y subunit A-1 OS=Arabidopsis thaliana GN=NFYA1 PE=2 SV=1	0.98	6.66	-2.26	down	0.00	0.00	yes
TRINITY_DN19501_c0_g5	PREDICTED: transcriptional activator DEMETER-like isoform X1 [Populus euphratica]	ROS1	Protein ROS1 OS=Arabidopsis thaliana GN=ROS1 PE=1 SV=2	1.34	6.22	-1.59	down	0.00	0.00	yes
TRINITY_DN14809_c0_g1	unknown [Populus trichocarpa x Populus deltoides]	-	-	0.86	10.85	-2.94	down	0.00	0.00	yes
TRINITY_DN24064_c0_g1	phosphatase 2C family protein [Populus trichocarpa]	At4g31860	Probable protein phosphatase 2C 60 OS=Arabidopsis thaliana GN=At4g31860 PE=2 SV=1	18.61	61.06	-1.11	down	0.00	0.00	yes
TRINITY_DN26578_c0_g1	myosin heavy chain-related family protein [Populus trichocarpa]	ICR2	Interactor of constitutive active ROPs 2, chloroplastic OS=Arabidopsis thaliana GN=ICR2 PE=1 SV=1	26.25	106.65	-1.44	down	0.00	0.00	yes
TRINITY_DN17733_c0_g1	transcriptional factor B3 family protein [Populus trichocarpa]	REM16	B3 domain-containing protein REM16 OS=Arabidopsis thaliana GN=REM16 PE=2 SV=1	1.01	7.59	-2.46	down	0.00	0.00	yes
TRINITY_DN22304_c0_g1	cucumber protein kinase CsPK3 [Populus trichocarpa]	WAG1	Serine/threonine-protein kinase WAG1 OS=Arabidopsis thaliana GN=WAG1 PE=2 SV=1	2.28	16.67	-2.25	down	0.00	0.00	yes
TRINITY_DN26239_c0_g2	Tubulin gamma-1 chain family protein [Populus trichocarpa]	TUBG2	Tubulin gamma-2 chain OS=Arabidopsis thaliana GN=TUBG2 PE=1 SV=1	9.81	35.94	-1.19	down	0.00	0.00	yes
TRINITY_DN26155_c0_g2	alpha-expansin 9 precursor family protein [Populus trichocarpa]	EXPA4	Expansin-A4 OS=Arabidopsis thaliana GN=EXPA4 PE=1 SV=1	2.23	13.61	-2.00	down	0.00	0.00	yes
TRINITY_DN18490_c0_g1	alpha-expansin 9 precursor family protein [Populus trichocarpa]	EXPA4	Expansin-A4 OS=Arabidopsis thaliana GN=EXPA4 PE=1 SV=1	5.19	17.55	-2.21	down	0.00	0.00	yes
TRINITY_DN22963_c0_g3	hypothetical protein POPTR_0001s05540g [Populus trichocarpa]	At3g47200	UPF0481 protein At3g47200 OS=Arabidopsis thaliana GN=At3g47200 PE=2 SV=1	0.34	4.93	-3.17	down	0.00	0.00	yes
TRINITY_DN17440_c0_g1	hypothetical protein POPTR_0002s00350g [Populus trichocarpa]	CML37	Calcium-binding protein CML37 OS=Arabidopsis thaliana GN=CML37 PE=2 SV=1	1.12	21.89	-3.55	down	0.00	0.00	yes
TRINITY_DN26110_c0_g3	PREDICTED: probable RNA-dependent RNA polymerase 1 [Populus euphratica]	-	-	0.11	2.00	-3.32	down	0.00	0.00	yes
TRINITY_DN19401_c0_g1	hypothetical protein POPTR_0018s00600g [Populus trichocarpa]	TCX5	Protein tesmin/TSO1-like CXC 5 OS=Arabidopsis thaliana GN=TCX5 PE=1 SV=1	1.44	7.34	-1.75	down	0.00	0.00	yes
TRINITY_DN23119_c0_g1	PREDICTED: uncharacterized protein LOC105111049 isoform X1 [Populus euphratica]	-	-	6.21	25.00	-1.50	down	0.00	0.00	yes
TRINITY_DN20475_c0_g4	hypothetical protein POPTR_0014s07700g [Populus trichocarpa]	-	-	0.49	5.32	-2.74	down	0.00	0.00	yes
TRINITY_DN19764_c0_g1	hypothetical protein POPTR_0008s06200g [Populus trichocarpa]	-	-	0.19	4.33	-4.28	down	0.00	0.00	yes
TRINITY_DN17170_c1_g1	hypothetical protein POPTR_0002s16180g [Populus trichocarpa]	SPPL4	Signal peptide peptidase-like 4 OS=Arabidopsis thaliana GN=SPPL4 PE=2 SV=1	5.45	21.95	-1.74	down	0.00	0.00	yes
TRINITY_DN3054_c0_g2	PREDICTED: nudix hydrolase 2-like isoform X1 [Populus euphratica]	NUDT2	Nudix hydrolase 2 OS=Arabidopsis thaliana GN=NUDT2 PE=1 SV=1	0.16	3.24	-3.57	down	0.00	0.00	yes
TRINITY_DN26435_c0_g1	hypothetical protein POPTR_0001s30470g [Populus trichocarpa]	-	-	6.41	25.37	-1.34	down	0.00	0.00	yes
TRINITY_DN21340_c0_g2	calmodulin-binding family protein [Populus trichocarpa]	-	-	10.99	38.42	-1.21	down	0.00	0.00	yes
TRINITY_DN16255_c0_g3	PREDICTED: putative phytosulfokines 6 [Populus euphratica]	-	-	23.71	84.82	-1.23	down	0.00	0.00	yes
TRINITY_DN24476_c0_g1	hypothetical protein POPTR_0001s05490g [Populus trichocarpa]	-	-	28.74	105.96	-1.32	down	0.00	0.00	yes
TRINITY_DN17095_c0_g1	hypothetical protein POPTR_0009s10610g [Populus trichocarpa]	Os01g0234100	B3 domain-containing protein Os01g0234100 OS=Oryza sativa subsp. japonica GN=Os01g0234100 PE=2 SV=1	0.42	2.58	-1.99	down	0.00	0.00	yes
TRINITY_DN22490_c0_g2	hypothetical protein POPTR_0017s04700g [Populus trichocarpa]	RGA4	Putative disease resistance protein RGA4 OS=Solanum bulbocastanum GN=RGA4 PE=2 SV=1	0.57	5.45	-2.81	down	0.00	0.00	yes
TRINITY_DN25978_c0_g1	CCAAT-binding transcription factor subunit B [Populus euphratica]	NFYA1	Nuclear transcription factor Y subunit A-1 OS=Arabidopsis thaliana GN=NFYA1 PE=2 SV=1	14.31	57.96	-1.38	down	0.00	0.00	yes
TRINITY_DN17757_c0_g2	PREDICTED: uncharacterized protein LOC105142247 [Populus euphratica]	EOGT	EGF domain-specific O-linked N-acetylglucosamine transferase OS=Bos taurus GN=EOGT PE=2 SV=1	1.03	5.94	-1.90	down	0.00	0.00	yes
TRINITY_DN13948_c1_g4	protease inhibitor/seed storage/lipid transfer family protein [Populus trichocarpa]	-	-	0.04	2.85	-4.93	down	0.00	0.00	yes

TRINITY_DN20117_c0_g1	dynein light chain family protein [Populus trichocarpa]	-	-	24.85	113.28	-1.57	down	0.00	0.00	yes
TRINITY_DN15134_c0_g2	hypothetical protein POPTR_0010s12630g [Populus trichocarpa]	-	-	1.54	13.17	-2.45	down	0.00	0.00	yes
TRINITY_DN27730_c1_g1	hypothetical protein POPTR_0003s02960g [Populus trichocarpa]	SELMODRAFT_	Inactive protein kinase SELMODRAFT_444075 OS=Selaginella moellendorffii GN=SELMODRAFT_444075 PE=2 SV=1	32.25	107.25	-1.13	down	0.00	0.00	yes
TRINITY_DN21060_c0_g3	MYB transcription factor [Populus tomentosa]	MYB308	Myb-related protein 308 OS=Antirrhinum majus GN=MYB308 PE=2 SV=1	10.98	56.43	-1.72	down	0.00	0.00	yes
TRINITY_DN22302_c0_g2	PREDICTED: Fanconi anemia group J protein homolog isoform X1 [Populus euphratica]	BRIP1	Fanconi anemia group J protein homolog OS=Gallus gallus GN=BRIP1 PE=2 SV=1	1.47	6.74	-1.57	down	0.00	0.00	yes
TRINITY_DN4777_c0_g1	hypothetical protein POPTR_0061s00220g [Populus trichocarpa]	At3g47200	UPF0481 protein At3g47200 OS=Arabidopsis thaliana GN=At3g47200 PE=2 SV=1	1.10	8.61	-2.41	down	0.00	0.00	yes
TRINITY_DN27382_c1_g2	PTH-1 family protein [Populus trichocarpa]	ERL1	LRR receptor-like serine/threonine-protein kinase ERL1 OS=Arabidopsis thaliana GN=ERL1 PE=1 SV=1	3.89	17.98	-1.46	down	0.00	0.00	yes
TRINITY_DN17472_c0_g1	hypothetical protein POPTR_0001s17160g [Populus trichocarpa]	-	-	0.72	12.28	-3.57	down	0.00	0.00	yes
TRINITY_DN22386_c0_g2	hypothetical protein POPTR_0001s06940g [Populus trichocarpa]	At3g28050	WAT1-related protein At3g28050 OS=Arabidopsis thaliana GN=At3g28050 PE=2 SV=1	0.18	1.90	-2.74	down	0.00	0.00	yes
TRINITY_DN18457_c0_g1	hypothetical protein POPTR_0001s12380g [Populus trichocarpa]	MCM6	DNA replication licensing factor MCM6 OS=Arabidopsis thaliana GN=MCM6 PE=1 SV=1	3.01	18.30	-1.89	down	0.00	0.00	yes
TRINITY_DN19719_c0_g1	PREDICTED: uncharacterized protein LOC105109154 isoform X1 [Populus euphratica]	LNK1	Protein LNK1 OS=Arabidopsis thaliana GN=LNK1 PE=1 SV=1	3.66	15.01	-1.41	down	0.00	0.00	yes
TRINITY_DN22202_c0_g5	PREDICTED: probable 2-oxoglutarate-dependent dioxygenase AOP1 [Populus euphratica]	AOP1.2	Probable 2-oxoglutarate-dependent dioxygenase AOP1.2 OS=Arabidopsis thaliana GN=AOP1.2 PE=2 SV=1	2.48	12.90	-1.76	down	0.00	0.00	yes
TRINITY_DN20164_c0_g3	sterile alpha motif domain-containing family protein [Populus trichocarpa]	SNM1	DNA cross-link repair protein SNM1 OS=Arabidopsis thaliana GN=SNM1 PE=2 SV=1	3.50	14.58	-1.54	down	0.00	0.00	yes
TRINITY_DN24219_c0_g2	NAC domain transcriptional regulator superfamily protein [Populus tomentosa]	NAC072	NAC domain-containing protein 72 OS=Arabidopsis thaliana GN=NAC072 PE=2 SV=1	4.72	57.22	-3.10	down	0.00	0.00	yes
TRINITY_DN20382_c0_g1	hypothetical protein POPTR_0005s21210g [Populus trichocarpa]	PUB28	U-box domain-containing protein 28 OS=Arabidopsis thaliana GN=PUB28 PE=1 SV=1	0.20	2.48	-3.03	down	0.00	0.00	yes
TRINITY_DN14266_c0_g2	-	-	-	1.50	9.39	-2.01	down	0.00	0.00	yes
TRINITY_DN1192_c0_g1	-	-	-	0.34	3.31	-2.63	down	0.00	0.00	yes
TRINITY_DN27538_c0_g1	hypothetical protein POPTR_0005s13960g [Populus trichocarpa]	FLACCA	Molybdenum cofactor sulfurase OS=Solanum lycopersicum GN=FLACCA PE=2 SV=1	11.34	36.76	-1.13	down	0.00	0.00	yes
TRINITY_DN5788_c0_g1	PREDICTED: pentatricopeptide repeat-containing protein At1g12620-like [Populus euphratica]	-	-	0.36	6.09	-3.23	down	0.00	0.00	yes
TRINITY_DN26643_c2_g1	hypothetical protein POPTR_0004s12020g [Populus trichocarpa]	-	-	3.22	14.90	-1.60	down	0.00	0.00	yes
TRINITY_DN21017_c0_g1	PREDICTED: lysM domain-containing GPI-anchored protein 2-like isoform X1 [Populus euphratica]	LYM2	LysM domain-containing GPI-anchored protein 2 OS=Arabidopsis thaliana GN=LYM2 PE=1 SV=1	14.15	51.35	-1.22	down	0.00	0.00	yes
TRINITY_DN19620_c1_g2	PREDICTED: histone H2B-like [Populus euphratica]	HIS2B	Histone H2B OS=Capsicum annuum GN=HIS2B PE=2 SV=3	30.30	109.79	-1.22	down	0.00	0.00	yes
TRINITY_DN14099_c0_g1	hypothetical protein POPTR_0006s01610g [Populus trichocarpa]	At4g17280	Cytochrome b561 and DOMON domain-containing protein At4g17280 OS=Arabidopsis thaliana GN=At4g17280 PE=2 SV=1	0.53	4.24	-2.39	down	0.00	0.00	yes
TRINITY_DN19310_c0_g1	hypothetical protein POPTR_0014s13090g [Populus trichocarpa]	ROPGAP2	Rho GTPase-activating protein 2 OS=Arabidopsis thaliana GN=ROPGAP2 PE=1 SV=1	14.11	54.62	-1.38	down	0.00	0.00	yes
TRINITY_DN25768_c0_g1	PREDICTED: uncharacterized protein At5g05190-like [Populus euphratica]	-	-	2.56	13.23	-1.66	down	0.00	0.00	yes
TRINITY_DN23700_c0_g1	hypothetical protein POPTR_0006s23110g [Populus trichocarpa]	At5g02620	Ankyrin repeat-containing protein At5g02620 OS=Arabidopsis thaliana GN=At5g02620 PE=1 SV=1	3.37	14.60	-1.57	down	0.00	0.00	yes
TRINITY_DN24746_c0_g1	hypothetical protein POPTR_0010s09610g [Populus trichocarpa]	-	-	10.12	35.15	-1.22	down	0.00	0.00	yes
TRINITY_DN20228_c0_g2	PREDICTED: WEB family protein At2g40480 isoform X1 [Populus euphratica]	At2g40480	WEB family protein At2g40480 OS=Arabidopsis thaliana GN=At2g40480 PE=2 SV=1	1.66	8.93	-1.82	down	0.00	0.00	yes
TRINITY_DN9097_c0_g1	PREDICTED: uncharacterized protein LOC108221543 [Daucus carota subsp. sativus]	-	-	0.13	7.69	-4.68	down	0.00	0.00	yes

TRINITY_DN23808_c0_g1	PREDICTED: receptor-like protein 12 isoform X1 [Populus euphratica]	-	-	2.75	18.66	-2.61	down	0.00	0.00	yes
TRINITY_DN17567_c0_g1	PREDICTED: uncharacterized protein LOC105129902 [Populus euphratica]	-	-	0.49	4.58	-2.56	down	0.00	0.00	yes
TRINITY_DN15419_c0_g1	GDSL-motif lipase/hydrolase family protein [Populus trichocarpa]	At3g48460	GDSL esterase/lipase At3g48460 OS=Arabidopsis thaliana GN=At3g48460 PE=2 SV=1	0.56	4.19	-2.33	down	0.00	0.00	yes
TRINITY_DN27495_c1_g1	kinase family protein [Populus trichocarpa]	PHOT1	Phototropin-1 OS=Arabidopsis thaliana GN=PHOT1 PE=1 SV=1	5.00	19.67	-1.39	down	0.00	0.00	yes
TRINITY_DN24338_c0_g2	hypothetical protein POPTR_0016s14350g [Populus trichocarpa]	-	-	2.24	13.12	-1.94	down	0.00	0.00	yes
TRINITY_DN23485_c0_g3	PREDICTED: cytochrome P450 71A1-like [Populus euphratica]	CYP71A1	Cytochrome P450 71A1 OS=Persea americana GN=CYP71A1 PE=1 SV=2	1.44	6.13	-1.49	down	0.00	0.00	yes
TRINITY_DN26478_c0_g2	PREDICTED: probable alpha,alpha-trehalose-phosphate synthase [UDP-forming] 11 [Populus euphratica]	TPS11	Probable alpha,alpha-trehalose-phosphate synthase [UDP-forming] 11 OS=Arabidopsis thaliana GN=TPS11 PE=2 SV=1	5.24	24.55	-1.65	down	0.00	0.00	yes
TRINITY_DN18856_c0_g6	hypothetical protein POPTR_0019s12150g [Populus trichocarpa]	-	-	9.98	32.46	-1.10	down	0.00	0.00	yes
TRINITY_DN20124_c0_g1	hypothetical protein POPTR_0005s22770g [Populus trichocarpa]	NPR2	BTB/POZ domain and ankyrin repeat-containing protein NPR2 OS=Oryza sativa subsp. japonica GN=NPR2 PE=1 SV=1	0.22	2.53	-2.90	down	0.00	0.00	yes
TRINITY_DN23493_c0_g2	BRCT domain-containing family protein [Populus trichocarpa]	At4g02110	BRCT domain-containing protein At4g02110 OS=Arabidopsis thaliana GN=At4g02110 PE=3 SV=3	1.47	7.83	-1.81	down	0.00	0.00	yes
TRINITY_DN26292_c0_g1	hypothetical protein POPTR_0017s02170g [Populus trichocarpa]	RGA3	Putative disease resistance protein RGA3 OS=Solanum bulbocastanum GN=RGA3 PE=2 SV=2	5.66	22.15	-1.38	down	0.00	0.00	yes
TRINITY_DN20963_c0_g1	hypothetical protein POPTR_0008s19510g [Populus trichocarpa]	BHLH79	Transcription factor bHLH79 OS=Arabidopsis thaliana GN=BHLH79 PE=2 SV=1	0.48	3.86	-2.68	down	0.00	0.00	yes
TRINITY_DN22426_c0_g5	hypothetical protein POPTR_0008s05120g [Populus trichocarpa]	-	-	0.26	2.85	-2.82	down	0.00	0.00	yes
TRINITY_DN22293_c1_g2	PREDICTED: receptor-like protein kinase HSL1 [Populus euphratica]	HSL1	Receptor-like protein kinase HSL1 OS=Arabidopsis thaliana GN=HSL1 PE=2 SV=1	2.27	9.17	-1.42	down	0.00	0.00	yes
TRINITY_DN20597_c1_g1	DREB70 [Populus hopeiensis]	DREB1D	Dehydration-responsive element-binding protein 1D OS=Arabidopsis thaliana GN=DREB1D PE=2 SV=1	0.56	8.11	-3.43	down	0.00	0.00	yes
TRINITY_DN26265_c0_g3	PREDICTED: zinc finger CCCH domain-containing protein 53-like isoform X2 [Populus euphratica]	Os07g0682400	Zinc finger CCCH domain-containing protein 53 OS=Oryza sativa subsp. japonica GN=Os07g0682400 PE=2 SV=1	25.37	76.55	-1.13	down	0.00	0.00	yes
TRINITY_DN20674_c0_g3	xyloglucan endo-1 family protein [Populus trichocarpa]	XTH23	Probable xyloglucan endotransglucosylase/hydrolase protein 23 OS=Arabidopsis thaliana GN=XTH23 PE=2 SV=1	6.47	32.26	-1.74	down	0.00	0.00	yes
TRINITY_DN23359_c0_g1	potassium channel tetramerisation domain-containing family protein [Populus trichocarpa]	At2g24240	BTB/POZ domain-containing protein At2g24240 OS=Arabidopsis thaliana GN=At2g24240 PE=2 SV=1	1.84	9.70	-1.74	down	0.00	0.00	yes
TRINITY_DN21321_c0_g3	PREDICTED: uncharacterized protein LOC105107246 isoform X1 [Populus euphratica]	-	-	1.71	7.59	-1.56	down	0.00	0.00	yes
TRINITY_DN27376_c0_g2	pseudo-response regulator 73 [Populus trichocarpa]	PRR73	Two-component response regulator-like PRR73 OS=Oryza sativa subsp. japonica GN=PRR73 PE=2 SV=1	5.28	18.66	-1.17	down	0.00	0.00	yes
TRINITY_DN23142_c2_g1	hypothetical protein POPTR_0002s06800g [Populus trichocarpa]	GBF4	G-box-binding factor 4 OS=Arabidopsis thaliana GN=GBF4 PE=1 SV=1	6.92	26.64	-1.34	down	0.00	0.00	yes
TRINITY_DN24570_c0_g1	hypothetical protein POPTR_0010s13980g [Populus trichocarpa]	SOG1	SUPPRESSOR OF GAMMA RESPONSE 1 OS=Arabidopsis thaliana GN=SOG1 PE=1 SV=1	14.86	52.47	-1.13	down	0.00	0.00	yes
TRINITY_DN17253_c0_g2	hypothetical protein POPTR_0016s03600g [Populus trichocarpa]	SIB1	Sigma factor binding protein 1, chloroplastic OS=Arabidopsis thaliana GN=SIB1 PE=1 SV=1	0.45	5.62	-2.94	down	0.00	0.00	yes
TRINITY_DN19764_c0_g2	PREDICTED: uncharacterized protein LOC105133657 [Populus euphratica]	-	-	1.59	23.50	-3.77	down	0.00	0.00	yes
TRINITY_DN16447_c0_g1	PREDICTED: uncharacterized protein LOC105124255 [Populus euphratica]	-	-	9.71	37.27	-1.36	down	0.00	0.00	yes
TRINITY_DN20301_c0_g4	hypothetical protein POPTR_0007s01400g [Populus trichocarpa]	GRXC9	Glutaredoxin-C9 OS=Arabidopsis thaliana GN=GRXC9 PE=1 SV=1	1.44	15.25	-2.81	down	0.00	0.00	yes
TRINITY_DN25043_c0_g1	kinase family protein [Populus trichocarpa]	-	-	0.65	5.79	-2.53	down	0.00	0.00	yes
TRINITY_DN16142_c0_g1	hypothetical protein POPTR_0009s02440g [Populus trichocarpa]	-	-	41.23	238.57	-1.98	down	0.00	0.00	yes
TRINITY_DN24359_c0_g1	-	-	-	0.34	3.98	-2.92	down	0.00	0.00	yes

TRINITY_DN22083_c0_g3	hypothetical protein POPTR_0019s13910g, partial [Populus trichocarpa]	-	-	0.49	5.10	-2.78	down	0.00	0.00	yes
TRINITY_DN17430_c0_g1	CBS domain-containing family protein [Populus trichocarpa]	CBSCBS2	SNF1-related protein kinase regulatory subunit gamma-1-like OS=Arabidopsis thaliana GN=CBSCBS2 PE=1 SV=1	3.91	19.25	-1.74	down	0.00	0.00	yes
TRINITY_DN26186_c0_g4	-	-	-	0.43	6.81	-3.45	down	0.00	0.00	yes
TRINITY_DN20449_c0_g1	PREDICTED: uncharacterized protein LOC105129425 isoform X2 [Populus euphratica]	Tgs1	Trimethylguanosine synthase OS=Rattus norvegicus GN=Tgs1 PE=1 SV=1	1.03	4.63	-1.58	down	0.00	0.00	yes
TRINITY_DN21060_c0_g1	dormancy/auxin associated family protein [Populus trichocarpa]	At1g54070	Dormancy-associated protein homolog 4 OS=Arabidopsis thaliana GN=At1g54070 PE=3 SV=1	7.84	79.93	-2.86	down	0.00	0.00	yes
TRINITY_DN14434_c0_g1	PREDICTED: probable ADP-ribosylation factor GTPase-activating protein AGD11 [Populus euphratica]	CAR4	Protein C2-DOMAIN ABA-RELATED 4 OS=Arabidopsis thaliana GN=CAR4 PE=1 SV=1	20.40	65.24	-1.08	down	0.00	0.00	yes
TRINITY_DN26441_c0_g1	PREDICTED: uncharacterized protein LOC105137111 [Populus euphratica]	Cacna2d4	Voltage-dependent calcium channel subunit alpha-2/delta-4 OS=Mus musculus GN=Cacna2d4 PE=2 SV=1	2.57	9.61	-1.30	down	0.00	0.00	yes
TRINITY_DN14308_c0_g1	hypothetical protein POPTR_0001s04040g [Populus trichocarpa]	-	-	0.14	2.24	-3.30	down	0.00	0.00	yes
TRINITY_DN11420_c0_g1	MutT/nudix family protein [Populus trichocarpa]	NUDT1	Nudix hydrolase 1 OS=Arabidopsis thaliana GN=NUDT1 PE=1 SV=1	3.24	16.62	-1.76	down	0.00	0.00	yes
TRINITY_DN19919_c0_g2	leucine-rich repeat transmembrane protein kinase [Populus trichocarpa]	TDR	Leucine-rich repeat receptor-like protein kinase TDR OS=Arabidopsis thaliana GN=TDR PE=1 SV=1	3.53	12.35	-1.25	down	0.00	0.00	yes
TRINITY_DN18724_c0_g1	PREDICTED: uncharacterized protein LOC105124611 [Populus euphratica]	-	-	1.83	7.81	-1.49	down	0.00	0.00	yes
TRINITY_DN17471_c0_g2	PREDICTED: uncharacterized protein LOC105133073 isoform X1 [Populus euphratica]	-	-	6.56	24.96	-1.30	down	0.00	0.00	yes
TRINITY_DN24045_c0_g2	heavy-metal-associated domain-containing family protein [Populus trichocarpa]	HIPP32	Heavy metal-associated isoprenylated plant protein 32 OS=Arabidopsis thaliana GN=HIPP32 PE=2 SV=1	40.04	155.28	-1.29	down	0.00	0.00	yes
TRINITY_DN22750_c0_g2	hypothetical protein POPTR_1207s00200g [Populus trichocarpa]	-	-	1.90	10.57	-2.27	down	0.00	0.00	yes
TRINITY_DN15320_c0_g1	hypothetical protein POPTR_0018s05480g [Populus trichocarpa]	-	-	2.91	45.69	-3.41	down	0.00	0.00	yes
TRINITY_DN16281_c0_g1	hypothetical protein POPTR_0003s15230g [Populus trichocarpa]	-	-	0.92	6.29	-2.40	down	0.00	0.00	yes
TRINITY_DN24441_c0_g1	hypothetical protein POPTR_0006s22190g [Populus trichocarpa]	OFP13	Transcription repressor OFP13 OS=Arabidopsis thaliana GN=OFP13 PE=2 SV=1	7.47	30.88	-1.43	down	0.00	0.00	yes
TRINITY_DN24045_c0_g4	hypothetical protein POPTR_0005s03840g [Populus trichocarpa]	HIPP32	Heavy metal-associated isoprenylated plant protein 32 OS=Arabidopsis thaliana GN=HIPP32 PE=2 SV=1	13.09	45.01	-1.17	down	0.00	0.00	yes
TRINITY_DN22699_c1_g4	GPI-anchored protein precursor [Populus trichocarpa]	LTPG1	Non-specific lipid transfer protein GPI-anchored 1 OS=Arabidopsis thaliana GN=LTPG1 PE=1 SV=1	2.11	14.90	-2.22	down	0.00	0.00	yes
TRINITY_DN25139_c1_g6	peroxidase family protein [Populus trichocarpa]	GSVIVT0002396	Peroxidase 4 OS=Vitis vinifera GN=GSVIVT00023967001 PE=1 SV=1	3.62	33.94	-2.66	down	0.00	0.00	yes
TRINITY_DN21555_c0_g6	unknown [Populus trichocarpa]	-	-	21.22	91.24	-1.49	down	0.00	0.00	yes
TRINITY_DN24756_c0_g1	hypothetical protein POPTR_0001s34390g [Populus trichocarpa]	CTDSPL2	CTD small phosphatase-like protein 2 OS=Homo sapiens GN=CTDSPL2 PE=1 SV=2	3.60	12.69	-1.29	down	0.00	0.00	yes
TRINITY_DN26587_c0_g3	nitrate transporter family protein [Populus trichocarpa]	NPF4.6	Protein NRT1/ PTR FAMILY 4.6 OS=Arabidopsis thaliana GN=NPF4.6 PE=1 SV=1	1.01	7.57	-2.64	down	0.00	0.00	yes
TRINITY_DN18120_c0_g1	1-aminocyclopropane-1-carboxylate synthase family protein [Populus trichocarpa]	ACS1	1-aminocyclopropane-1-carboxylate synthase OS=Glycine max GN=ACS1 PE=2 SV=1	2.09	14.08	-2.09	down	0.00	0.00	yes
TRINITY_DN26328_c1_g3	leucine-rich repeat family protein [Populus trichocarpa]	PXC3	Leucine-rich repeat receptor-like tyrosine-protein kinase PXC3 OS=Arabidopsis thaliana GN=PXC3 PE=2 SV=1	3.58	12.70	-1.27	down	0.00	0.00	yes
TRINITY_DN21599_c0_g1	hypothetical protein POPTR_0004s04390g [Populus trichocarpa]	-	-	7.39	38.61	-1.79	down	0.00	0.00	yes
TRINITY_DN20493_c0_g3	hypothetical protein POPTR_0017s12230g [Populus trichocarpa]	MYB39	Transcription factor MYB39 OS=Arabidopsis thaliana GN=MYB39 PE=2 SV=1	3.01	13.64	-1.60	down	0.00	0.00	yes
TRINITY_DN21925_c0_g2	hypothetical protein POPTR_0011s15230g [Populus trichocarpa]	NAC014	NAC domain-containing protein 14 OS=Arabidopsis thaliana GN=NAC014 PE=2 SV=1	4.86	17.73	-1.26	down	0.00	0.00	yes

TRINITY_DN19945_c0_g2	hypothetical protein POPTR_0006s21130g [Populus trichocarpa]	-	-	0.60	4.58	-2.34	down	0.00	0.00	yes
TRINITY_DN22288_c0_g1	PREDICTED: calcium-dependent protein kinase 32-like [Populus euphratica]	CPK32	Calcium-dependent protein kinase 32 OS=Arabidopsis thaliana GN=CPK32 PE=1 SV=1	3.94	16.40	-1.46	down	0.00	0.00	yes
TRINITY_DN16006_c0_g1	PREDICTED: mitogen-activated protein kinase kinase 6 [Populus euphratica]	MKK6	Mitogen-activated protein kinase kinase 6 OS=Arabidopsis thaliana GN=MKK6 PE=1 SV=1	10.58	36.23	-1.20	down	0.00	0.00	yes
TRINITY_DN25456_c0_g4	PREDICTED: chaperone protein ClpD, chloroplastic-like [Populus euphratica]	CLPD	Chaperone protein ClpD, chloroplastic OS=Arabidopsis thaliana GN=CLPD PE=1 SV=1	0.35	2.16	-2.01	down	0.00	0.00	yes
TRINITY_DN17541_c0_g1	hypothetical protein POPTR_0001s12020g [Populus trichocarpa]	-	-	0.46	5.15	-3.25	down	0.00	0.00	yes
TRINITY_DN23513_c0_g1	-	-	-	0.71	4.75	-2.39	down	0.00	0.00	yes
TRINITY_DN19122_c0_g1	hypothetical protein POPTR_0002s14330g [Populus trichocarpa]	SPL7	Squamosa promoter-binding-like protein 7 OS=Oryza sativa subsp. japonica GN=SPL7 PE=2 SV=2	2.37	11.06	-2.19	down	0.00	0.00	yes
TRINITY_DN19970_c0_g2	hypothetical protein POPTR_0022s00560g [Populus trichocarpa]	-	-	2.95	13.12	-1.58	down	0.00	0.00	yes
TRINITY_DN24914_c5_g1	hypothetical protein POPTR_0004s05920g [Populus trichocarpa]	WRKY65	Probable WRKY transcription factor 65 OS=Arabidopsis thaliana GN=WRKY65 PE=2 SV=1	1.28	7.25	-2.08	down	0.00	0.00	yes
TRINITY_DN24181_c0_g3	hypothetical protein POPTR_0017s12930g [Populus trichocarpa]	-	-	0.45	3.17	-2.32	down	0.00	0.00	yes
TRINITY_DN21530_c0_g2	hypothetical protein POPTR_0007s13700g [Populus trichocarpa]	GATA5	GATA transcription factor 5 OS=Arabidopsis thaliana GN=GATA5 PE=2 SV=1	28.07	93.39	-1.11	down	0.00	0.00	yes
TRINITY_DN22847_c0_g1	hypothetical protein POPTR_0014s05480g [Populus trichocarpa]	UBP12	Ubiquitin carboxyl-terminal hydrolase 12 OS=Arabidopsis thaliana GN=UBP12 PE=2 SV=2	1.55	12.00	-2.62	down	0.00	0.00	yes
TRINITY_DN2062_c0_g1	-	-	-	0.45	6.79	-3.25	down	0.00	0.00	yes
TRINITY_DN20375_c1_g2	PREDICTED: GDSL esterase/lipase At1g28580-like [Populus euphratica]	At1g28650	GDSL esterase/lipase At1g28650 OS=Arabidopsis thaliana GN=At1g28650 PE=2 SV=1	1.55	9.10	-2.00	down	0.00	0.00	yes
TRINITY_DN21601_c2_g2	-	-	-	0.48	4.14	-2.86	down	0.00	0.00	yes
TRINITY_DN15058_c0_g1	PREDICTED: basic leucine zipper 43-like [Populus euphratica]	-	-	1.11	9.16	-2.43	down	0.00	0.00	yes
TRINITY_DN23341_c1_g5	hypothetical protein POPTR_0006s01450g [Populus trichocarpa]	-	-	0.45	4.10	-2.43	down	0.00	0.00	yes
TRINITY_DN16661_c0_g1	plant disease resistance response protein, partial [Populus alba]	PI206	Disease resistance response protein 206 OS=Pisum sativum GN=PI206 PE=1 SV=2	4.22	27.44	-2.09	down	0.00	0.00	yes
TRINITY_DN24360_c1_g7	cysteine-rich receptor-like protein kinase 29 [Populus tomentosa]	CRK41	Cysteine-rich receptor-like protein kinase 41 OS=Arabidopsis thaliana GN=CRK41 PE=3 SV=2	0.25	2.69	-2.78	down	0.00	0.00	yes
TRINITY_DN23474_c0_g2	PREDICTED: ninja-family protein AFP3-like [Populus euphratica]	AFP3	Ninja-family protein AFP3 OS=Arabidopsis thaliana GN=AFP3 PE=1 SV=1	1.57	10.81	-2.18	down	0.00	0.00	yes
TRINITY_DN25682_c1_g1	hypothetical protein POPTR_0019s08290g [Populus trichocarpa]	CRT3	Calreticulin-3 OS=Arabidopsis thaliana GN=CRT3 PE=1 SV=2	12.05	54.98	-1.49	down	0.00	0.00	yes
TRINITY_DN24815_c0_g1	multifunctional protein 2 [Populus tomentosa]	-	Glyoxysomal fatty acid beta-oxidation multifunctional protein MFP-a OS=Brassica napus PE=2 SV=2	12.46	43.51	-1.19	down	0.00	0.00	yes
TRINITY_DN23197_c1_g1	PREDICTED: probable protein phosphatase 2C 75 [Populus euphratica]	AHG1	Probable protein phosphatase 2C 75 OS=Arabidopsis thaliana GN=AHG1 PE=2 SV=1	1.70	12.43	-2.23	down	0.00	0.00	yes
TRINITY_DN27517_c0_g1	F-box family protein [Populus trichocarpa]	At1g10780	F-box protein At1g10780 OS=Arabidopsis thaliana GN=At1g10780 PE=2 SV=1	9.11	30.26	-1.10	down	0.00	0.00	yes
TRINITY_DN25123_c1_g2	-	-	-	0.52	6.02	-2.88	down	0.00	0.00	yes
TRINITY_DN21102_c0_g1	hypothetical protein POPTR_0168s00200g [Populus trichocarpa]	-	Histone H4 variant TH011 OS=Triticum aestivum PE=3 SV=2	27.40	101.51	-1.16	down	0.00	0.00	yes
TRINITY_DN16940_c1_g1	hypothetical protein POPTR_0007s13120g [Populus trichocarpa]	-	-	2.49	21.65	-2.50	down	0.00	0.00	yes
TRINITY_DN22137_c2_g3	ankyrin repeat family protein [Populus trichocarpa]	XBAT31	Putative E3 ubiquitin-protein ligase XBAT31 OS=Arabidopsis thaliana GN=XBAT31 PE=2 SV=1	8.76	28.22	-1.13	down	0.00	0.00	yes
TRINITY_DN18017_c0_g1	PREDICTED: G2/mitotic-specific cyclin C13-1-like isoform X1 [Populus euphratica]	CYCA3-1	Cyclin-A3-1 OS=Oryza sativa subsp. japonica GN=CYCA3-1 PE=3 SV=1	0.59	4.35	-2.29	down	0.00	0.00	yes
TRINITY_DN26652_c0_g1	hypothetical protein POPTR_0223s00200g [Populus trichocarpa]	-	-	2.32	21.49	-2.29	down	0.00	0.00	yes

TRINITY_DN20696_c0_g1	epoxide hydrolase family protein [Populus trichocarpa]	ephA	Epoxide hydrolase A OS=Mycobacterium tuberculosis (strain ATCC 25618 / H37Rv) GN=ephA PE=1 SV=1	0.84	9.54	-2.87	down	0.00	0.00	yes
TRINITY_DN20087_c0_g1	PREDICTED: serine/threonine-protein kinase UCNL-like [Populus euphratica]	UNC	Serine/threonine-protein kinase UCN OS=Arabidopsis thaliana GN=UNC PE=1 SV=1	3.22	13.35	-1.46	down	0.00	0.00	yes
TRINITY_DN21553_c0_g4	hypothetical protein POPTR_0003s14120g [Populus trichocarpa]	At5g03810	GDSL esterase/lipase At5g03810 OS=Arabidopsis thaliana GN=At5g03810 PE=3 SV=1	0.13	2.44	-3.41	down	0.00	0.00	yes
TRINITY_DN24529_c1_g3	hypothetical protein POPTR_0001s27060g [Populus trichocarpa]	PAO1	Polyamine oxidase 1 OS=Arabidopsis thaliana GN=PAO1 PE=1 SV=1	0.40	2.77	-2.21	down	0.00	0.00	yes
TRINITY_DN17172_c0_g3	hypothetical protein POPTR_0008s16510g [Populus trichocarpa]	-	-	23.40	75.42	-1.21	down	0.00	0.00	yes
TRINITY_DN25725_c1_g1	phosphatase PP1 family protein [Populus trichocarpa]	TOPP8	Serine/threonine-protein phosphatase PP1 isozyme 8 OS=Arabidopsis thaliana GN=TOPP8 PE=2 SV=3	8.62	31.49	-1.25	down	0.00	0.00	yes
TRINITY_DN24360_c1_g1	hypothetical protein POPTR_0011s03220g [Populus trichocarpa]	CRK14	Cysteine-rich receptor-like protein kinase 14 OS=Arabidopsis thaliana GN=CRK14 PE=2 SV=2	6.00	19.41	-1.54	down	0.00	0.00	yes
TRINITY_DN20704_c1_g1	tubulin beta chain family protein [Populus trichocarpa]	TUBB3	Tubulin beta-3 chain OS=Oryza sativa subsp. japonica GN=TUBB3 PE=2 SV=2	32.81	114.19	-1.46	down	0.00	0.00	yes
TRINITY_DN18716_c0_g1	hypothetical protein POPTR_0016s07150g [Populus trichocarpa]	CTF7	Protein CHROMOSOME TRANSMISSION FIDELITY 7 OS=Arabidopsis thaliana GN=CTF7 PE=1 SV=1	1.17	7.05	-1.97	down	0.00	0.00	yes
TRINITY_DN13393_c0_g1	hypothetical protein POPTR_0006s24050g [Populus trichocarpa]	WRKY50	Probable WRKY transcription factor 50 OS=Arabidopsis thaliana GN=WRKY50 PE=2 SV=1	0.10	4.01	-3.55	down	0.00	0.00	yes
TRINITY_DN18097_c0_g3	PREDICTED: uncharacterized protein LOC105136607 [Populus euphratica]	RBOHC	Respiratory burst oxidase homolog protein C OS=Arabidopsis thaliana GN=RBOHC PE=2 SV=2	0.96	5.85	-1.82	down	0.00	0.00	yes
TRINITY_DN22285_c0_g5	kinase family protein [Populus trichocarpa]	RIPK	Serine/threonine-protein kinase RIPK OS=Arabidopsis thaliana GN=RIPK PE=1 SV=1	0.43	2.89	-2.14	down	0.00	0.00	yes
TRINITY_DN21758_c0_g2	PREDICTED: ATP-dependent DNA helicase Q-like 1 [Populus euphratica]	RECQL1	ATP-dependent DNA helicase Q-like 1 OS=Arabidopsis thaliana GN=RECQL1 PE=2 SV=1	0.97	4.91	-1.73	down	0.00	0.00	yes
TRINITY_DN25011_c0_g1	hypothetical protein POPTR_0001s30920g [Populus trichocarpa]	CYCD3-3	Cyclin-D3-3 OS=Arabidopsis thaliana GN=CYCD3-3 PE=2 SV=1	43.75	165.37	-1.15	down	0.00	0.00	yes
TRINITY_DN18803_c0_g1	hypothetical protein POPTR_0002s25260g [Populus trichocarpa]	At5g48480	Uncharacterized protein At5g48480 OS=Arabidopsis thaliana GN=At5g48480 PE=1 SV=1	248.12	859.43	-1.18	down	0.00	0.00	yes
TRINITY_DN20528_c2_g1	hypothetical protein POPTR_0006s02030g [Populus trichocarpa]	-	-	3.55	46.31	-2.85	down	0.00	0.00	yes
TRINITY_DN27815_c1_g1	hypothetical protein POPTR_0002s02230g [Populus trichocarpa]	-	-	0.48	6.68	-5.08	down	0.00	0.00	yes
TRINITY_DN24171_c0_g2	hypothetical protein POPTR_0013s03670g [Populus trichocarpa]	At5g28300	Trihelix transcription factor GTL2 OS=Arabidopsis thaliana GN=At5g28300 PE=2 SV=1	14.50	47.11	-1.11	down	0.00	0.00	yes
TRINITY_DN26919_c0_g1	PREDICTED: uncharacterized protein LOC105129062 isoform X2 [Populus euphratica]	-	-	6.50	19.61	-1.07	down	0.00	0.00	yes
TRINITY_DN22944_c0_g3	PREDICTED: probable beta-D-xylosidase 7 [Populus euphratica]	BXL7	Probable beta-D-xylosidase 7 OS=Arabidopsis thaliana GN=BXL7 PE=2 SV=2	9.58	31.73	-1.16	down	0.00	0.00	yes
TRINITY_DN24017_c0_g1	PREDICTED: uncharacterized protein At3g61260-like [Populus euphratica]	-	-	1.59	11.02	-2.12	down	0.00	0.00	yes
TRINITY_DN25066_c0_g1	hypothetical protein POPTR_0007s10500g [Populus trichocarpa]	At4g34220	Receptor protein kinase-like protein At4g34220 OS=Arabidopsis thaliana GN=At4g34220 PE=2 SV=1	0.23	3.76	-3.28	down	0.00	0.00	yes
TRINITY_DN21371_c0_g3	PREDICTED: uncharacterized protein LOC105122213 isoform X4 [Populus euphratica]	-	-	7.64	24.43	-1.05	down	0.00	0.00	yes
TRINITY_DN27775_c0_g1	PREDICTED: LOW QUALITY PROTEIN: TMV resistance protein N-like [Populus euphratica]	-	-	3.59	16.75	-1.44	down	0.00	0.00	yes
TRINITY_DN21166_c0_g1	PREDICTED: uncharacterized protein LOC105125662 [Populus euphratica]	-	-	3.12	18.15	-1.94	down	0.00	0.00	yes
TRINITY_DN6415_c0_g2	hypothetical protein POPTR_0010s19480g [Populus trichocarpa]	TBL28	Protein trichome birefringence-like 28 OS=Arabidopsis thaliana GN=TBL28 PE=2 SV=1	0.14	1.75	-2.95	down	0.00	0.00	yes
TRINITY_DN26900_c0_g1	hypothetical protein POPTR_0014s18750g [Populus trichocarpa]	-	-	1.49	7.49	-1.75	down	0.00	0.00	yes
TRINITY_DN22899_c0_g1	PREDICTED: GATA transcription factor 2-like [Populus euphratica]	GATA4	GATA transcription factor 4 OS=Arabidopsis thaliana GN=GATA4 PE=2 SV=1	1.53	8.96	-1.93	down	0.00	0.00	yes

TRINITY_DN18533_c0_g6	hypothetical protein POPTR_0011s07850g [Populus trichocarpa]	-	-	0.33	3.82	-2.86	down	0.00	0.00	yes
TRINITY_DN20174_c1_g1	PHD finger family protein [Populus trichocarpa]	ING1	PHD finger protein ING1 OS=Arabidopsis thaliana GN=ING1 PE=1 SV=1	13.32	41.74	-1.12	down	0.00	0.00	yes
TRINITY_DN14644_c0_g1	hypothetical protein POPTR_0013s12320g [Populus trichocarpa]	-	-	0.55	4.61	-2.44	down	0.00	0.00	yes
TRINITY_DN22566_c0_g2	SNF4b family protein [Populus trichocarpa]	CBSX5	CBS domain-containing protein CBSX5 OS=Arabidopsis thaliana GN=CBSX5 PE=2 SV=2	0.11	1.96	-3.46	down	0.00	0.00	yes
TRINITY_DN27849_c0_g2	PREDICTED: protein MOR1-like [Populus euphratica]	MOR1	Protein MOR1 OS=Arabidopsis thaliana GN=MOR1 PE=1 SV=1	10.48	36.43	-1.18	down	0.00	0.00	yes
TRINITY_DN22412_c0_g1	hypothetical protein POPTR_0001s37200g [Populus trichocarpa]	-	-	42.44	139.73	-1.16	down	0.00	0.00	yes
TRINITY_DN22261_c0_g2	hypothetical protein POPTR_0002s09400g [Populus trichocarpa]	SPCC23B6.04c	CRAL-TRIO domain-containing protein C23B6.04c OS=Schizosaccharomyces pombe (strain 972 / ATCC 24843) GN=SPCC23B6.04c PE=1 SV=1	24.56	81.28	-1.12	down	0.00	0.00	yes
TRINITY_DN18346_c0_g4	WRKY transcription factor 31 [(Populus tomentosa x Populus bolleana) x Populus tomentosa]	WRKY48	Probable WRKY transcription factor 48 OS=Arabidopsis thaliana GN=WRKY48 PE=2 SV=1	1.86	12.19	-2.11	down	0.00	0.00	yes
TRINITY_DN22873_c1_g1	hypothetical protein POPTR_0014s05240g [Populus trichocarpa]	RMR2	Receptor homology region, transmembrane domain- and RING domain-containing protein 2 OS=Arabidopsis thaliana GN=RMR2 PE=2 SV=1	2.02	9.22	-1.57	down	0.00	0.00	yes
TRINITY_DN24799_c0_g1	hypothetical protein POPTR_0019s12930g [Populus trichocarpa]	-	-	13.48	66.05	-1.48	down	0.00	0.00	yes
TRINITY_DN14000_c0_g1	hypothetical protein POPTR_0013s05640g [Populus trichocarpa]	LECRK4	G-type lectin S-receptor-like serine/threonine-protein kinase LECRK4 OS=Oryza sativa subsp. indica GN=LECRK4 PE=3 SV=1	0.38	5.38	-2.71	down	0.00	0.00	yes
TRINITY_DN27436_c0_g2	an N-terminal calmodulin binding autoinhibitory domain-containing family protein [Populus trichocarpa]	ACA9	Calcium-transporting ATPase 9, plasma membrane-type OS=Arabidopsis thaliana GN=ACA9 PE=2 SV=2	0.30	3.91	-2.99	down	0.00	0.00	yes
TRINITY_DN18240_c2_g2	PREDICTED: uncharacterized protein LOC105131731 [Populus euphratica]	-	-	1.75	9.51	-1.66	down	0.00	0.00	yes
TRINITY_DN16595_c0_g2	hypothetical protein POPTR_0006s13790g [Populus trichocarpa]	BHLH82	Transcription factor bHLH82 OS=Arabidopsis thaliana GN=BHLH82 PE=2 SV=1	2.32	12.81	-1.88	down	0.00	0.00	yes
TRINITY_DN17900_c1_g2	hypothetical protein POPTR_0009s15650g [Populus trichocarpa]	WAKL8	Wall-associated receptor kinase-like 8 OS=Arabidopsis thaliana GN=WAKL8 PE=2 SV=1	0.45	7.94	-2.93	down	0.00	0.00	yes
TRINITY_DN19654_c0_g1	PREDICTED: uncharacterized protein LOC105126277 [Populus euphratica]	pod	Peroxidase 15 OS=Ipomoea batatas GN=pod PE=1 SV=1	8.23	23.51	-1.34	down	0.00	0.00	yes
TRINITY_DN19370_c0_g1	PREDICTED: BAG family molecular chaperone regulator 2-like [Populus euphratica]	BAG2	BAG family molecular chaperone regulator 2 OS=Arabidopsis thaliana GN=BAG2 PE=1 SV=1	0.72	4.86	-2.12	down	0.00	0.00	yes
TRINITY_DN18153_c0_g1	PREDICTED: uncharacterized protein LOC105140504 isoform X1 [Populus euphratica]	-	-	1.79	11.04	-1.92	down	0.00	0.00	yes
TRINITY_DN17335_c0_g2	hypothetical protein POPTR_0006s04360g [Populus trichocarpa]	PAXIP1	PAX-interacting protein 1 OS=Bos taurus GN=PAXIP1 PE=2 SV=1	0.47	3.35	-2.21	down	0.00	0.00	yes
TRINITY_DN17440_c0_g4	hypothetical protein POPTR_0005s28110g [Populus trichocarpa]	CML37	Calcium-binding protein CML37 OS=Arabidopsis thaliana GN=CML37 PE=2 SV=1	3.67	72.28	-3.65	down	0.00	0.00	yes
TRINITY_DN19782_c1_g1	PREDICTED: auxin-induced protein 22D-like isoform X1 [Populus euphratica]	IAA12	Auxin-responsive protein IAA12 OS=Arabidopsis thaliana GN=IAA12 PE=1 SV=1	1.94	8.69	-1.86	down	0.00	0.00	yes
TRINITY_DN26485_c1_g2	PREDICTED: extended synaptotagmin-1 [Populus euphratica]	FTIP1	FT-interacting protein 1 OS=Arabidopsis thaliana GN=FTIP1 PE=1 SV=1	29.87	103.79	-1.14	down	0.00	0.00	yes
TRINITY_DN13817_c0_g1	hypothetical protein POPTR_0016s04180g [Populus trichocarpa]	-	-	0.13	2.51	-3.56	down	0.00	0.00	yes
TRINITY_DN24459_c0_g1	PREDICTED: uncharacterized protein LOC105133552 isoform X1 [Populus euphratica]	bub1	Probable inactive serine/threonine-protein kinase bub1 OS=Dictyostelium discoideum GN=bub1 PE=3 SV=1	3.23	13.63	-1.38	down	0.00	0.00	yes
TRINITY_DN20771_c4_g1	hypothetical protein POPTR_0010s15010g [Populus trichocarpa]	-	-	0.60	4.64	-2.25	down	0.00	0.00	yes
TRINITY_DN11211_c0_g1	hypothetical protein POPTR_0005s25340g, partial [Populus trichocarpa]	-	-	0.15	2.30	-3.36	down	0.00	0.00	yes

TRINITY_DN20166_c0_g1	PREDICTED: DNA replication licensing factor MCM4 [Populus euphratica]	MCM4	DNA replication licensing factor MCM4 OS=Oryza sativa subsp. japonica GN=MCM4 PE=3 SV=2	5.65	29.26	-1.73	down	0.00	0.00	yes
TRINITY_DN25760_c0_g2	hypothetical protein POPTR_0018s09190g [Populus trichocarpa]	At2g20050/At2g2	Protein phosphatase 2C and cyclic nucleotide-binding/kinase domain-containing protein OS=Arabidopsis thaliana GN=At2g20050/At2g20040 PE=2 SV=2	0.20	3.09	-3.29	down	0.00	0.00	yes
TRINITY_DN27492_c0_g1	SPL1-Related3 family protein [Populus trichocarpa]	SPL16	Squamosa promoter-binding-like protein 16 OS=Arabidopsis thaliana GN=SPL16 PE=2 SV=2	9.23	48.87	-1.47	down	0.00	0.00	yes
TRINITY_DN24295_c0_g1	PREDICTED: protein transport protein sec23-1-like [Populus euphratica]	SEC23	Protein transport protein SEC23 OS=Ustilago maydis (strain 521 / FGSC 9021) GN=SEC23 PE=3 SV=1	7.42	24.49	-1.12	down	0.00	0.00	yes
TRINITY_DN17726_c0_g1	hypothetical protein POPTR_0001s30260g [Populus trichocarpa]	ZAT6	Zinc finger protein ZAT6 OS=Arabidopsis thaliana GN=ZAT6 PE=2 SV=1	2.72	31.81	-2.94	down	0.00	0.00	yes
TRINITY_DN12237_c0_g1	PREDICTED: putative expansin-B2 [Populus euphratica]	EXPB2	Putative expansin-B2 OS=Arabidopsis thaliana GN=EXPB2 PE=3 SV=2	0.30	6.80	-3.78	down	0.00	0.00	yes
TRINITY_DN15032_c0_g1	zinc finger family protein [Populus trichocarpa]	dscc1	Sister chromatid cohesion protein DCC1 OS=Danio rerio GN=dscc1 PE=2 SV=1	2.61	12.29	-1.56	down	0.00	0.00	yes
TRINITY_DN14553_c0_g1	hypothetical protein POPTR_0012s12150g [Populus trichocarpa]	-	-	0.97	22.34	-3.39	down	0.00	0.00	yes
TRINITY_DN26173_c0_g3	hypothetical protein POPTR_0001s23420g [Populus trichocarpa]	-	-	0.57	8.93	-3.32	down	0.00	0.00	yes
TRINITY_DN19309_c0_g1	PREDICTED: zinc finger protein CONSTANS-LIKE 15-like isoform X1 [Populus euphratica]	COL15	Zinc finger protein CONSTANS-LIKE 15 OS=Arabidopsis thaliana GN=COL15 PE=2 SV=1	2.05	15.81	-2.32	down	0.00	0.00	yes
TRINITY_DN27701_c1_g1	hypothetical protein POPTR_0007s04450g [Populus trichocarpa]	CHR24	Protein CHROMATIN REMODELING 24 OS=Arabidopsis thaliana GN=CHR24 PE=2 SV=1	9.27	35.54	-1.14	down	0.00	0.00	yes
TRINITY_DN22309_c0_g5	kinase-like protein TMKL1 precursor [Populus trichocarpa]	TMKL1	Putative kinase-like protein TMKL1 OS=Arabidopsis thaliana GN=TMKL1 PE=1 SV=1	1.84	8.70	-1.62	down	0.00	0.00	yes
TRINITY_DN14405_c0_g4	hypothetical protein POPTR_0008s11970g [Populus trichocarpa]	-	-	0.27	3.18	-2.89	down	0.00	0.00	yes
TRINITY_DN22828_c0_g3	PREDICTED: DNA replication licensing factor MCM3 [Populus euphratica]	MCM3	DNA replication licensing factor MCM3 OS=Arabidopsis thaliana GN=MCM3 PE=1 SV=1	3.67	17.36	-1.62	down	0.00	0.00	yes
TRINITY_DN11497_c0_g1	hypothetical protein POPTR_0001s09650g, partial [Populus trichocarpa]	SR45A	Serine/arginine-rich splicing factor SR45a OS=Arabidopsis thaliana GN=SR45A PE=1 SV=1	0.25	3.27	-3.05	down	0.00	0.00	yes
TRINITY_DN22826_c0_g1	hypothetical protein POPTR_0005s11440g [Populus trichocarpa]	-	-	2.36	14.55	-1.94	down	0.00	0.00	yes
TRINITY_DN14008_c0_g1	PREDICTED: uncharacterized protein LOC105133752 isoform X2 [Populus euphratica]	DRIP2	E3 ubiquitin protein ligase DRIP2 OS=Arabidopsis thaliana GN=DRIP2 PE=1 SV=1	0.16	3.09	-3.44	down	0.00	0.00	yes
TRINITY_DN14424_c0_g2	PREDICTED: plant intracellular Ras-group-related LRR protein 3-like [Populus euphratica]	PIRL3	Plant intracellular Ras-group-related LRR protein 3 OS=Arabidopsis thaliana GN=PIRL3 PE=2 SV=1	0.38	2.48	-2.10	down	0.00	0.00	yes
TRINITY_DN15322_c0_g1	hypothetical protein POPTR_0011s16860g [Populus trichocarpa]	FER	Receptor-like protein kinase FERONIA OS=Arabidopsis thaliana GN=FER PE=1 SV=1	0.30	2.17	-2.21	down	0.00	0.00	yes
TRINITY_DN25356_c0_g1	hypothetical protein POPTR_0008s22950g [Populus trichocarpa]	GATA15	GATA transcription factor 15 OS=Arabidopsis thaliana GN=GATA15 PE=2 SV=2	8.65	32.52	-1.29	down	0.00	0.00	yes
TRINITY_DN23420_c0_g1	hypothetical protein POPTR_0006s11650g [Populus trichocarpa]	-	-	29.17	89.98	-1.07	down	0.00	0.00	yes
TRINITY_DN17604_c0_g2	PREDICTED: uncharacterized protein LOC105107569 [Populus euphratica]	-	-	0.88	8.26	-2.68	down	0.00	0.00	yes
TRINITY_DN18330_c0_g1	trans-cinnamate 4-hydroxylase [Populus trichocarpa]	-	Cytochrome P450 CYP73A100 OS=Panax ginseng PE=2 SV=1	0.62	4.40	-2.18	down	0.00	0.00	yes
TRINITY_DN20471_c0_g1	PREDICTED: inner centromere protein-like isoform X1 [Populus euphratica]	NET3A	Protein NETWORKED 3A OS=Arabidopsis thaliana GN=NET3A PE=2 SV=1	10.31	34.01	-1.13	down	0.00	0.00	yes
TRINITY_DN27310_c1_g1	beta-tubulin 5 [Salix arbutifolia]	TUBB1	Tubulin beta-1 chain OS=Lupinus albus GN=TUBB1 PE=3 SV=1	40.36	135.82	-1.15	down	0.00	0.00	yes
TRINITY_DN22256_c0_g1	hypothetical protein POPTR_0006s08160g [Populus trichocarpa]	SKIP2	F-box protein SKIP2 OS=Arabidopsis thaliana GN=SKIP2 PE=1 SV=1	11.55	38.24	-1.13	down	0.00	0.00	yes
TRINITY_DN25899_c0_g1	PREDICTED: high mobility group B protein 13-like [Populus euphratica]	HMGB6	High mobility group B protein 6 OS=Arabidopsis thaliana GN=HMGB6 PE=2 SV=1	73.89	274.64	-1.31	down	0.00	0.00	yes
TRINITY_DN19039_c1_g7	hypothetical protein POPTR_0016s00570g [Populus trichocarpa]	FPS1	Farnesyl pyrophosphate synthase 1 OS=Lupinus albus GN=FPS1 PE=2 SV=1	0.19	2.22	-3.14	down	0.00	0.00	yes

TRINITY_DN26904_c0_g1	hypothetical protein POPTR_0006s02710g [Populus trichocarpa]	ZIFL1	Protein ZINC INDUCED FACILITATOR-LIKE 1 OS=Arabidopsis thaliana GN=ZIFL1 PE=2 SV=1	5.49	20.98	-1.36	down	0.00	0.00	yes
TRINITY_DN25109_c1_g7	-	-	-	0.29	2.49	-2.50	down	0.00	0.00	yes
TRINITY_DN20147_c0_g9	hypothetical protein POPTR_0006s09610g [Populus trichocarpa]	TUBB	Tubulin beta chain (Fragment) OS=Glycine max GN=TUBB PE=2 SV=2	20.96	88.67	-1.50	down	0.00	0.00	yes
TRINITY_DN14893_c0_g1	hypothetical protein POPTR_0154s00260g [Populus trichocarpa]	GLIP3	GDSL esterase/lipase 3 OS=Arabidopsis thaliana GN=GLIP3 PE=2 SV=2	0.85	4.83	-1.88	down	0.00	0.00	yes
TRINITY_DN18979_c0_g1	F-box family protein [Populus trichocarpa]	PP2A13	F-box protein PP2-A13 OS=Arabidopsis thaliana GN=PP2A13 PE=1 SV=1	3.48	18.17	-1.75	down	0.00	0.00	yes
TRINITY_DN21362_c0_g4	hypothetical protein POPTR_0007s11620g [Populus trichocarpa]	DOF3.1	Dof zinc finger protein DOF3.1 OS=Arabidopsis thaliana GN=DOF3.1 PE=2 SV=2	1.43	9.96	-2.14	down	0.00	0.00	yes
TRINITY_DN22612_c0_g2	leucine-rich repeat family protein [Populus trichocarpa]	At4g36180	Probable LRR receptor-like serine/threonine-protein kinase At4g36180 OS=Arabidopsis thaliana GN=At4g36180 PE=2 SV=1	5.53	17.87	-1.10	down	0.00	0.00	yes
TRINITY_DN23241_c0_g1	PREDICTED: double-stranded RNA-binding protein 1-like isoform X4 [Populus euphratica]	DBR4	Double-stranded RNA-binding protein 4 OS=Arabidopsis thaliana GN=DBR4 PE=1 SV=1	8.11	30.85	-1.11	down	0.00	0.00	yes
TRINITY_DN25301_c0_g2	-	-	-	0.27	3.42	-2.98	down	0.00	0.00	yes
TRINITY_DN20834_c0_g1	PREDICTED: probable receptor-like protein kinase At5g39020 isoform X1 [Populus euphratica]	-	-	1.52	12.13	-2.53	down	0.00	0.00	yes
TRINITY_DN27647_c0_g1	PREDICTED: hornerin isoform X1 [Populus euphratica]	-	-	8.64	32.91	-1.26	down	0.00	0.00	yes
TRINITY_DN12280_c0_g1	-	-	-	0.42	9.54	-3.86	down	0.00	0.00	yes
TRINITY_DN19458_c0_g1	PREDICTED: NAC domain-containing protein 7-like isoform X2 [Populus euphratica]	NAC037	NAC domain-containing protein 37 OS=Arabidopsis thaliana GN=NAC037 PE=1 SV=1	1.58	8.63	-1.92	down	0.00	0.00	yes
TRINITY_DN20682_c1_g2	PREDICTED: uncharacterized protein LOC105138029 [Populus euphratica]	-	-	1.00	5.90	-1.91	down	0.00	0.00	yes
TRINITY_DN25704_c0_g3	hypothetical protein POPTR_0011s05360g [Populus trichocarpa]	SMG7L	Protein SMG7L OS=Arabidopsis thaliana GN=SMG7L PE=2 SV=1	0.76	3.67	-1.67	down	0.00	0.00	yes
TRINITY_DN22585_c0_g2	PREDICTED: LOW QUALITY PROTEIN: putative disease resistance protein RGA4 [Populus euphratica]	-	-	1.77	14.86	-2.34	down	0.00	0.00	yes
TRINITY_DN27591_c1_g1	homeodomain family protein [Populus trichocarpa]	ANL2	Homeobox-leucine zipper protein ANTHOCYANINLESS 2 OS=Arabidopsis thaliana GN=ANL2 PE=2 SV=1	27.16	94.11	-1.23	down	0.00	0.00	yes
TRINITY_DN18198_c0_g2	PREDICTED: trans-resveratrol di-O-methyltransferase-like [Populus euphratica]	-	Myricetin O-methyltransferase OS=Catharanthus roseus PE=1 SV=1	100.03	540.89	-1.90	down	0.00	0.00	yes
TRINITY_DN25692_c0_g2	PREDICTED: pachytene checkpoint protein 2 homolog [Populus euphratica]	At4g24710	Pachytene checkpoint protein 2 homolog OS=Arabidopsis thaliana GN=At4g24710 PE=2 SV=1	12.27	47.09	-1.31	down	0.00	0.00	yes
TRINITY_DN26152_c0_g6	PREDICTED: uncharacterized protein LOC105126156 [Populus euphratica]	-	-	0.58	3.46	-2.19	down	0.00	0.00	yes
TRINITY_DN21948_c0_g1	PREDICTED: probable serine/threonine-protein kinase At5g41260 isoform X2 [Populus euphratica]	At5g41260	Probable serine/threonine-protein kinase At5g41260 OS=Arabidopsis thaliana GN=At5g41260 PE=1 SV=1	12.83	35.61	-1.08	down	0.00	0.00	yes
TRINITY_DN20933_c0_g3	PREDICTED: long chain acyl-CoA synthetase 1 [Populus euphratica]	LACS1	Long chain acyl-CoA synthetase 1 OS=Arabidopsis thaliana GN=LACS1 PE=2 SV=1	0.62	6.55	-2.76	down	0.00	0.00	yes
TRINITY_DN14511_c0_g1	hypothetical protein POPTR_0006s13740g [Populus trichocarpa]	PME54	Probable pectinesterase/pectinesterase inhibitor 54 OS=Arabidopsis thaliana GN=PME54 PE=2 SV=1	0.95	4.88	-1.77	down	0.00	0.00	yes
TRINITY_DN21916_c3_g1	hypothetical protein POPTR_0003s13300g [Populus trichocarpa]	At5g41590	Protein LURP-one-related 17 OS=Arabidopsis thaliana GN=At5g41590 PE=2 SV=2	0.53	7.01	-2.91	down	0.00	0.00	yes
TRINITY_DN26436_c0_g3	-	-	-	16.31	51.73	-1.14	down	0.00	0.00	yes
TRINITY_DN19552_c0_g9	hypothetical protein POPTR_0003s03660g [Populus trichocarpa]	-	-	0.34	4.52	-3.10	down	0.00	0.00	yes
TRINITY_DN22800_c0_g1	PREDICTED: protease Do-like 7 [Populus euphratica]	DEGP7	Protease Do-like 7 OS=Arabidopsis thaliana GN=DEGP7 PE=2 SV=1	1.92	7.41	-1.28	down	0.00	0.00	yes
TRINITY_DN25311_c0_g2	hypothetical protein POPTR_0001s07410g [Populus trichocarpa]	-	-	15.98	116.66	-2.27	down	0.00	0.00	yes
TRINITY_DN17652_c0_g2	hypothetical protein POPTR_0003s08600g [Populus trichocarpa]	APRR2	Two-component response regulator-like APRR2 OS=Arabidopsis thaliana GN=APRR2 PE=2 SV=2	0.46	6.28	-3.08	down	0.00	0.00	yes
TRINITY_DN17842_c0_g1	hypothetical protein POPTR_0008s09510g [Populus trichocarpa]	UPS1	Ureide permease 1 OS=Arabidopsis thaliana GN=UPS1 PE=1 SV=1	1.44	6.65	-1.59	down	0.00	0.00	yes

TRINITY_DN21303_c0_g1	zinc finger family protein [Populus trichocarpa]	RDUF1	E3 ubiquitin-protein ligase RDUF1 OS=Arabidopsis thaliana GN=RDUF1 PE=1 SV=1	6.58	46.57	-2.26	down	0.00	0.00	yes
TRINITY_DN22148_c0_g1	phosphate transporter 1 family protein [Populus trichocarpa]	PHT1-4	Inorganic phosphate transporter 1-4 OS=Arabidopsis thaliana GN=PHT1-4 PE=1 SV=1	20.21	75.31	-1.46	down	0.00	0.00	yes
TRINITY_DN19946_c0_g1	PREDICTED: indole-3-acetic acid-amido synthetase GH3.6-like [Populus euphratica]	GH3.6	Indole-3-acetic acid-amido synthetase GH3.6 OS=Arabidopsis thaliana GN=GH3.6 PE=1 SV=1	0.59	3.72	-2.05	down	0.00	0.00	yes
TRINITY_DN27000_c0_g2	PREDICTED: HORMA domain-containing protein 1-like isoform X2 [Populus euphratica]	hormad1	HORMA domain-containing protein 1 OS=Xenopus laevis GN=hormad1 PE=2 SV=1	9.14	23.70	-1.28	down	0.00	0.00	yes
TRINITY_DN3843_c0_g1	hypothetical protein POPTR_0001s39190g [Populus trichocarpa]	-	-	0.20	2.49	-2.97	down	0.00	0.00	yes
TRINITY_DN19213_c0_g4	hypothetical protein POPTR_0009s02970g [Populus trichocarpa]	-	-	1.21	9.86	-2.15	down	0.00	0.00	yes
TRINITY_DN16847_c0_g1	cytochrome P450 family protein [Populus trichocarpa]	CYP94C1	Cytochrome P450 94C1 OS=Arabidopsis thaliana GN=CYP94C1 PE=2 SV=1	0.47	8.14	-3.50	down	0.00	0.00	yes
TRINITY_DN24047_c0_g2	hypothetical protein POPTR_0016s00700g [Populus trichocarpa]	CAR11	Protein C2-DOMAIN ABA-RELATED 11 OS=Arabidopsis thaliana GN=CAR11 PE=1 SV=1	7.93	37.70	-1.77	down	0.00	0.00	yes
TRINITY_DN22538_c1_g1	PREDICTED: histone H1-like [Populus euphratica]	-	Histone H1 OS=Pisum sativum PE=2 SV=1	85.64	320.68	-1.32	down	0.00	0.00	yes
TRINITY_DN17444_c0_g1	PREDICTED: uncharacterized protein LOC104888072 [Beta vulgaris subsp. vulgaris]	pol	Retrovirus-related Pol polyprotein from type-2 retrotransposable element R2DM OS=Drosophila melanogaster GN=pol PE=3 SV=1	0.40	2.38	-1.97	down	0.00	0.00	yes
TRINITY_DN24215_c0_g3	hypothetical protein POPTR_0463s00220g, partial [Populus trichocarpa]	LRK10L-1.1	LEAF RUST 10 DISEASE-RESISTANCE LOCUS RECEPTOR-LIKE PROTEIN KINASE-like 1.1 OS=Arabidopsis thaliana GN=LRK10L-1.1 PE=2 SV=1	0.63	7.15	-2.95	down	0.00	0.00	yes
TRINITY_DN20902_c0_g6	PREDICTED: uncharacterized protein LOC105134212 [Populus euphratica]	SRC2	Protein SRC2 OS=Glycine max GN=SRC2 PE=2 SV=1	0.44	4.85	-2.79	down	0.00	0.00	yes
TRINITY_DN14955_c0_g1	PREDICTED: non-specific lipid-transfer protein-like protein At5g64080 [Populus euphratica]	XYP11	Xylogen-like protein 11 OS=Arabidopsis thaliana GN=XYP11 PE=1 SV=2	0.62	5.90	-2.53	down	0.00	0.00	yes
TRINITY_DN16607_c0_g1	PREDICTED: DNA mismatch repair protein MSH7 isoform X1 [Populus euphratica]	MSH7	DNA mismatch repair protein MSH7 OS=Arabidopsis thaliana GN=MSH7 PE=1 SV=1	0.80	4.64	-1.91	down	0.00	0.00	yes
TRINITY_DN13848_c0_g1	-	-	-	0.79	6.21	-2.75	down	0.00	0.00	yes
TRINITY_DN22052_c0_g6	hypothetical protein POPTR_0012s02680g [Populus trichocarpa]	PUB44	U-box domain-containing protein 44 OS=Arabidopsis thaliana GN=PUB44 PE=1 SV=1	0.38	3.10	-2.43	down	0.00	0.00	yes
TRINITY_DN22400_c1_g1	hypothetical protein POPTR_0010s24060g [Populus trichocarpa]	DME	Transcriptional activator DEMETER OS=Arabidopsis thaliana GN=DME PE=1 SV=2	0.92	5.88	-2.22	down	0.00	0.00	yes
TRINITY_DN26106_c0_g1	PREDICTED: probable protein phosphatase 2C 52 [Populus euphratica]	At4g03415	Probable protein phosphatase 2C 52 OS=Arabidopsis thaliana GN=At4g03415 PE=2 SV=1	22.88	78.84	-1.21	down	0.00	0.00	yes
TRINITY_DN23770_c0_g1	WRKY transcription factor 12 [(Populus tomentosa x Populus bolleana) x Populus tomentosa]	WRKY41	Probable WRKY transcription factor 41 OS=Arabidopsis thaliana GN=WRKY41 PE=2 SV=2	4.49	40.11	-2.52	down	0.00	0.00	yes
TRINITY_DN27679_c1_g1	PREDICTED: protein argonaute 10 isoform X1 [Populus euphratica]	AGO10	Protein argonaute 10 OS=Arabidopsis thaliana GN=AGO10 PE=2 SV=1	14.98	53.84	-1.24	down	0.00	0.00	yes
TRINITY_DN20702_c0_g1	calcium-dependent protein kinase [Populus trichocarpa]	CRK4	CDPK-related kinase 4 OS=Arabidopsis thaliana GN=CRK4 PE=2 SV=1	2.51	13.20	-1.67	down	0.00	0.00	yes
TRINITY_DN22580_c0_g1	hypothetical protein POPTR_0003s10690g [Populus trichocarpa]	SMC6B	Structural maintenance of chromosomes protein 6B OS=Arabidopsis thaliana GN=SMC6B PE=2 SV=1	2.70	9.00	-1.13	down	0.00	0.00	yes
TRINITY_DN16267_c1_g1	APETAL2-like family protein [Populus trichocarpa]	-	-	14.36	50.69	-1.24	down	0.00	0.00	yes
TRINITY_DN23429_c0_g1	PREDICTED: flap endonuclease GEN-like 2 isoform X4 [Populus euphratica]	GEN2	Flap endonuclease GEN-like 2 OS=Arabidopsis thaliana GN=GEN2 PE=2 SV=2	5.55	23.55	-1.21	down	0.00	0.00	yes
TRINITY_DN18130_c0_g1	PREDICTED: uncharacterized protein LOC105130999 [Populus euphratica]	RER4	Protein RETICULATA-RELATED 4, chloroplastic OS=Arabidopsis thaliana GN=RER4 PE=2 SV=1	3.08	15.25	-1.70	down	0.00	0.00	yes
TRINITY_DN22677_c0_g1	alpha galactosyltransferase family protein [Populus trichocarpa]	GT7	Putative glycosyltransferase 7 OS=Arabidopsis thaliana GN=GT7 PE=2 SV=1	16.78	58.67	-1.18	down	0.00	0.00	yes
TRINITY_DN26154_c0_g2	PREDICTED: protein SCAR2-like isoform X1 [Populus euphratica]	SCAR2	Protein SCAR2 OS=Arabidopsis thaliana GN=SCAR2 PE=1 SV=1	5.40	17.64	-1.12	down	0.00	0.00	yes
TRINITY_DN26308_c0_g1	hypothetical protein POPTR_0003s19630g [Populus trichocarpa]	NRPE1	DNA-directed RNA polymerase V subunit 1 OS=Arabidopsis thaliana GN=NRPE1 PE=1 SV=1	5.58	19.18	-1.12	down	0.00	0.00	yes
TRINITY_DN27360_c1_g5	-	-	-	0.79	6.26	-2.38	down	0.00	0.00	yes

TRINITY_DN19897_c0_g10	hypothetical protein POPTR_0001s38390g [Populus trichocarpa]	-	-	9.41	32.33	-1.17	down	0.00	0.00	yes
TRINITY_DN15596_c0_g2	hypothetical protein POPTR_0009s13290g [Populus trichocarpa]	SPAC869.01	Putative amidase C869.01 OS=Schizosaccharomyces pombe (strain 972 / ATCC 24843) GN=SPAC869.01 PE=3 SV=1	0.23	2.39	-2.70	down	0.00	0.00	yes
TRINITY_DN27252_c1_g2	-	-	-	0.51	3.98	-2.35	down	0.00	0.00	yes
TRINITY_DN21503_c0_g1	PREDICTED: probable disease resistance protein At4g27220 [Populus euphratica]	-	-	1.89	9.70	-1.66	down	0.00	0.00	yes
TRINITY_DN25798_c0_g1	hypothetical protein POPTR_0008s07740g [Populus trichocarpa]	FAS1	Chromatin assembly factor 1 subunit FAS1 OS=Arabidopsis thaliana GN=FAS1 PE=1 SV=1	3.88	18.80	-1.47	down	0.00	0.00	yes
TRINITY_DN27867_c4_g1	hypothetical protein POPTR_0001s09590g [Populus trichocarpa]	At1g76660	Uncharacterized protein At1g76660 OS=Arabidopsis thaliana GN=At1g76660 PE=2 SV=1	1.89	7.44	-1.52	down	0.00	0.00	yes
TRINITY_DN19125_c0_g8	hypothetical protein POPTR_0011s13080g [Populus trichocarpa]	CRK35	Putative cysteine-rich receptor-like protein kinase 35 OS=Arabidopsis thaliana GN=CRK35 PE=3 SV=3	0.39	3.36	-2.48	down	0.00	0.00	yes
TRINITY_DN16681_c0_g2	hypothetical protein POPTR_0001s19150g [Populus trichocarpa]	-	-	0.58	3.29	-1.88	down	0.00	0.00	yes
TRINITY_DN20666_c0_g2	pyruvate decarboxylase family protein [Populus trichocarpa]	PDC1	Pyruvate decarboxylase 1 OS=Pisum sativum GN=PDC1 PE=2 SV=1	2.05	4.49	-2.84	down	0.00	0.00	yes
TRINITY_DN21712_c1_g1	hypothetical protein POPTR_0006s08460g [Populus trichocarpa]	-	-	5.83	20.15	-1.22	down	0.00	0.00	yes
TRINITY_DN22869_c0_g1	hypothetical protein POPTR_0015s13090g [Populus trichocarpa]	-	-	3.26	10.97	-1.31	down	0.00	0.00	yes
TRINITY_DN22997_c0_g4	XH/XS domain-containing family protein [Populus trichocarpa]	IDN2	Protein INVOLVED IN DE NOVO 2 OS=Arabidopsis thaliana GN=IDN2 PE=1 SV=1	4.20	13.31	-1.06	down	0.00	0.00	yes
TRINITY_DN25057_c0_g3	-	-	-	0.18	3.69	-3.70	down	0.00	0.00	yes
TRINITY_DN26953_c0_g2	PREDICTED: extended synaptotagmin-1 [Populus euphratica]	FTIP1	FT-interacting protein 1 OS=Arabidopsis thaliana GN=FTIP1 PE=1 SV=1	8.04	27.37	-1.71	down	0.00	0.00	yes
TRINITY_DN18467_c0_g2	-	-	-	1.51	14.20	-3.51	down	0.00	0.00	yes
TRINITY_DN19395_c0_g1	PREDICTED: replication protein A 70 kDa DNA-binding subunit E-like [Populus euphratica]	RPA1E	Replication protein A 70 kDa DNA-binding subunit E OS=Arabidopsis thaliana GN=RPA1E PE=2 SV=1	2.44	9.54	-1.33	down	0.00	0.00	yes
TRINITY_DN18393_c0_g1	PREDICTED: probable LRR receptor-like serine/threonine-protein kinase At1g56140 [Populus euphratica]	At1g56130	Probable LRR receptor-like serine/threonine-protein kinase At1g56130 OS=Arabidopsis thaliana GN=At1g56130 PE=2 SV=2	1.28	6.75	-1.76	down	0.00	0.00	yes
TRINITY_DN17759_c0_g1	hypothetical protein POPTR_0004s01460g, partial [Populus trichocarpa]	SD25	G-type lectin S-receptor-like serine/threonine-protein kinase SD2-5 OS=Arabidopsis thaliana GN=SD25 PE=1 SV=1	1.43	5.88	-1.68	down	0.00	0.00	yes
TRINITY_DN27331_c0_g2	PREDICTED: probable polygalacturonase [Populus euphratica]	GSVIVT0002692	Probable polygalacturonase OS=Vitis vinifera GN=GSVIVT00026920001 PE=1 SV=1	15.61	64.79	-1.09	down	0.00	0.00	yes
TRINITY_DN23957_c2_g2	PREDICTED: RNA-binding protein 1-like [Populus euphratica]	RBP1	RNA-binding protein 1 OS=Arabidopsis thaliana GN=RBP1 PE=2 SV=1	4.25	15.60	-1.16	down	0.00	0.00	yes
TRINITY_DN26322_c0_g2	PREDICTED: uncharacterized protein LOC105142633 isoform X5 [Populus euphratica]	MBD13	Methyl-CpG-binding domain-containing protein 13 OS=Arabidopsis thaliana GN=MBD13 PE=2 SV=1	2.91	10.46	-1.23	down	0.00	0.00	yes
TRINITY_DN20646_c0_g1	PREDICTED: uncharacterized protein LOC105127223 isoform X1 [Populus euphratica]	MARD1	Protein MARD1 OS=Arabidopsis thaliana GN=MARD1 PE=2 SV=2	2.02	8.37	-1.45	down	0.00	0.00	yes
TRINITY_DN21647_c0_g4	hypothetical protein POPTR_0005s07900g [Populus trichocarpa]	ERF060	Ethylene-responsive transcription factor ERF060 OS=Arabidopsis thaliana GN=ERF060 PE=2 SV=1	40.13	183.60	-1.54	down	0.00	0.00	yes
TRINITY_DN24574_c0_g1	PREDICTED: cyclin-A2-4-like isoform X1 [Populus euphratica]	CYCA2-4	Cyclin-A2-4 OS=Arabidopsis thaliana GN=CYCA2-4 PE=2 SV=1	7.64	25.30	-1.15	down	0.00	0.00	yes
TRINITY_DN21337_c0_g3	PREDICTED: multiple inositol polyphosphate phosphatase 1-like isoform X1 [Populus euphratica]	mipp1	Multiple inositol polyphosphate phosphatase 1 OS=Dictyostelium discoideum GN=mipp1 PE=1 SV=1	0.16	1.69	-2.71	down	0.00	0.00	yes
TRINITY_DN16320_c0_g1	hypothetical protein POPTR_0014s17950g [Populus trichocarpa]	-	-	0.90	5.84	-2.13	down	0.00	0.00	yes
TRINITY_DN24268_c0_g3	PREDICTED: DNA-directed RNA polymerases IV and V subunit 2-like [Populus euphratica]	NRPD2b	DNA-directed RNA polymerase D subunit 2b OS=Arabidopsis thaliana GN=NRPD2b PE=2 SV=1	0.30	2.20	-2.26	down	0.00	0.00	yes
TRINITY_DN22413_c1_g1	hypothetical protein POPTR_0005s03070g [Populus trichocarpa]	-	TMV resistance protein N OS=Nicotiana glutinosa GN=N PE=1 SV=1	0.70	6.53	-2.63	down	0.00	0.00	yes
TRINITY_DN14376_c0_g1	hypothetical protein POPTR_0001s44750g [Populus trichocarpa]	-	-	0.44	3.99	-2.58	down	0.00	0.00	yes

TRINITY_DN20462_c0_g1	cation/H+ exchanger 18 [Populus tomentosa]	CHX18	Cation/H(+) antiporter 18 OS=Arabidopsis thaliana GN=CHX18 PE=2 SV=1	2.54	13.07	-1.46	down	0.00	0.00	yes
TRINITY_DN20608_c0_g1	PREDICTED: galactose oxidase-like [Populus euphratica]	GLOX1	Aldehyde oxidase GLOX1 OS=Arabidopsis thaliana GN=GLOX1 PE=2 SV=1	5.23	19.99	-1.35	down	0.00	0.00	yes
TRINITY_DN25892_c1_g3	-	-	-	1.17	10.66	-2.70	down	0.00	0.00	yes
TRINITY_DN21946_c0_g1	hypothetical protein POPTR_0003s14540g [Populus trichocarpa]	OPT7	Oligopeptide transporter 7 OS=Arabidopsis thaliana GN=OPT7 PE=2 SV=1	1.98	8.66	-1.52	down	0.00	0.00	yes
TRINITY_DN15605_c0_g1	PREDICTED: uncharacterized protein LOC105112829 isoform X1 [Populus euphratica]	-	-	4.24	19.49	-2.05	down	0.00	0.00	yes
TRINITY_DN18011_c0_g1	hypothetical protein POPTR_0006s06240g [Populus trichocarpa]	-	-	0.78	4.25	-1.83	down	0.00	0.00	yes
TRINITY_DN15892_c0_g2	unknown [Populus trichocarpa x Populus deltoides]	WRKY42	WRKY transcription factor 42 OS=Arabidopsis thaliana GN=WRKY42 PE=2 SV=1	0.40	7.59	-2.99	down	0.00	0.00	yes
TRINITY_DN17637_c1_g3	PREDICTED: uncharacterized protein LOC105141757 [Populus euphratica]	-	-	2.35	9.85	-1.46	down	0.00	0.00	yes
TRINITY_DN19637_c0_g4	laccase [Populus trichocarpa]	LAC5	Laccase-5 OS=Arabidopsis thaliana GN=LAC5 PE=2 SV=1	0.90	5.44	-1.98	down	0.00	0.00	yes
TRINITY_DN25730_c0_g3	hypothetical protein POPTR_0010s09830g [Populus trichocarpa]	WNK1	Probable serine/threonine-protein kinase WNK1 OS=Oryza sativa subsp. indica GN=WNK1 PE=2 SV=2	8.34	40.56	-1.71	down	0.00	0.00	yes
TRINITY_DN14623_c0_g1	hypothetical protein POPTR_0001s37380g [Populus trichocarpa]	CYP71D11	Cytochrome P450 71D11 (Fragment) OS=Lotus japonicus GN=CYP71D11 PE=2 SV=1	4.89	22.95	-1.62	down	0.00	0.00	yes
TRINITY_DN22143_c0_g2	PREDICTED: uncharacterized protein LOC105108743 isoform X1 [Populus euphratica]	-	-	1.39	10.52	-2.19	down	0.00	0.00	yes
TRINITY_DN14734_c0_g1	PREDICTED: DNA topoisomerase 2-like [Populus euphratica]	-	-	22.64	84.42	-1.29	down	0.00	0.00	yes
TRINITY_DN27150_c0_g4	PREDICTED: probable LRR receptor-like serine/threonine-protein kinase At1g07650 isoform X1 [Populus euphratica]	LRR-RLK	Probable leucine-rich repeat receptor-like serine/threonine-protein kinase At3g14840 OS=Arabidopsis thaliana GN=LRR-RLK PE=2 SV=1	3.36	26.23	-2.74	down	0.00	0.00	yes
TRINITY_DN13999_c0_g1	-	-	-	0.30	3.38	-2.83	down	0.00	0.00	yes
TRINITY_DN21455_c0_g1	potassium channel tetramerisation domain-containing family protein [Populus trichocarpa]	At5g41330	BTB/POZ domain-containing protein At5g41330 OS=Arabidopsis thaliana GN=At5g41330 PE=2 SV=1	4.90	26.94	-1.84	down	0.00	0.00	yes
TRINITY_DN20061_c0_g3	MADS box transcription factor [Populus tomentosa]	AGL19	Agamous-like MADS-box protein AGL19 OS=Arabidopsis thaliana GN=AGL19 PE=1 SV=1	1.94	15.68	-2.07	down	0.00	0.00	yes
TRINITY_DN23479_c0_g1	hypothetical protein POPTR_0014s17590g [Populus trichocarpa]	SKS1	Monocopper oxidase-like protein SKS1 OS=Arabidopsis thaliana GN=SKS1 PE=1 SV=1	4.89	18.74	-1.43	down	0.00	0.00	yes
TRINITY_DN27423_c2_g3	PREDICTED: G-type lectin S-receptor-like serine/threonine-protein kinase At4g27290 [Populus euphratica]	SD11	G-type lectin S-receptor-like serine/threonine-protein kinase SD1-1 OS=Arabidopsis thaliana GN=SD11 PE=1 SV=1	0.13	3.28	-3.94	down	0.00	0.00	yes
TRINITY_DN25485_c0_g1	kinase family protein [Populus trichocarpa]	At1g80870	Putative receptor-like protein kinase At1g80870 OS=Arabidopsis thaliana GN=At1g80870 PE=3 SV=1	1.88	6.89	-1.34	down	0.00	0.00	yes
TRINITY_DN23277_c0_g1	hypothetical protein POPTR_0004s08390g [Populus trichocarpa]	ncapg	Condensin complex subunit 3 OS=Xenopus laevis GN=ncapg PE=1 SV=1	7.83	22.39	-1.08	down	0.00	0.00	yes
TRINITY_DN17142_c0_g1	aminocyclopropane carboxylate oxidase family protein [Populus trichocarpa]	ACO	1-aminocyclopropane-1-carboxylate oxidase OS=Actinidia deliciosa GN=ACO PE=2 SV=1	17.61	99.90	-1.88	down	0.00	0.00	yes
TRINITY_DN19525_c0_g2	hypothetical protein POPTR_0008s04120g [Populus trichocarpa]	TET6	Tetraspanin-6 OS=Arabidopsis thaliana GN=TET6 PE=2 SV=1	0.61	4.94	-2.44	down	0.00	0.00	yes
TRINITY_DN24457_c2_g2	hypothetical protein POPTR_0006s21000g, partial [Populus trichocarpa]	-	-	10.18	35.80	-1.27	down	0.00	0.00	yes
TRINITY_DN21638_c0_g1	PREDICTED: histone H3-like centromeric protein HTR12 [Populus euphratica]	HTR12	Histone H3-like centromeric protein HTR12 OS=Arabidopsis thaliana GN=HTR12 PE=1 SV=3	17.07	53.61	-1.04	down	0.00	0.00	yes
TRINITY_DN20245_c0_g2	PREDICTED: RING-H2 finger protein ATL80-like [Populus euphratica]	ATL80	RING-H2 finger protein ATL80 OS=Arabidopsis thaliana GN=ATL80 PE=2 SV=1	4.87	20.18	-1.43	down	0.00	0.00	yes
TRINITY_DN27090_c1_g3	hypothetical protein POPTR_0010s25850g [Populus trichocarpa]	At1g16860	Uncharacterized membrane protein At1g16860 OS=Arabidopsis thaliana GN=At1g16860 PE=1 SV=1	7.57	29.34	-1.35	down	0.00	0.00	yes
TRINITY_DN24629_c0_g4	kinase family protein [Populus trichocarpa]	At5g41260	Probable serine/threonine-protein kinase At5g41260 OS=Arabidopsis thaliana GN=At5g41260 PE=1 SV=1	4.50	20.03	-1.59	down	0.00	0.00	yes

TRINITY_DN22646_c1_g4	ethylene-responsive nuclear family protein [Populus trichocarpa]	-	-	1.42	12.65	-2.53	down	0.00	0.00	yes
TRINITY_DN19486_c0_g2	hypothetical protein POPTR_0001s19300g [Populus trichocarpa]	MBD10	Methyl-CpG-binding domain-containing protein 10 OS=Arabidopsis thaliana GN=MBD10 PE=1 SV=1	2.81	12.30	-1.49	down	0.00	0.00	yes
TRINITY_DN20875_c0_g2	-	-	-	1.24	12.74	-2.76	down	0.00	0.00	yes
TRINITY_DN23033_c0_g1	kinesin motor family protein [Populus trichocarpa]	KIN8A	Kinesin-like protein KIN-8A OS=Arabidopsis thaliana GN=KIN8A PE=3 SV=1	11.04	30.75	-1.39	down	0.00	0.00	yes
TRINITY_DN22123_c0_g1	PREDICTED: uncharacterized protein LOC105110096 [Populus euphratica]	-	-	36.59	134.62	-1.30	down	0.00	0.00	yes
TRINITY_DN15626_c0_g2	PREDICTED: metacaspase-1-like [Populus euphratica]	AMC1	Metacaspase-1 OS=Arabidopsis thaliana GN=AMC1 PE=1 SV=1	0.26	2.83	-2.72	down	0.00	0.00	yes
TRINITY_DN27639_c0_g1	leucine-rich repeat family protein [Populus trichocarpa]	At1g67720	Probable LRR receptor-like serine/threonine-protein kinase At1g67720 OS=Arabidopsis thaliana GN=At1g67720 PE=2 SV=1	4.88	38.96	-2.33	down	0.00	0.00	yes
TRINITY_DN18758_c0_g1	PREDICTED: heptahelical transmembrane protein 1-like [Populus euphratica]	HHP1	Heptahelical transmembrane protein 1 OS=Arabidopsis thaliana GN=HHP1 PE=1 SV=1	0.52	3.03	-1.93	down	0.00	0.00	yes
TRINITY_DN26053_c0_g4	hypothetical protein POPTR_0014s04210g [Populus trichocarpa]	JAC1	J domain-containing protein required for chloroplast accumulation response 1 OS=Arabidopsis thaliana GN=JAC1 PE=1 SV=1	0.40	2.82	-2.20	down	0.00	0.00	yes
TRINITY_DN25536_c1_g7	-	-	-	0.44	3.46	-2.35	down	0.00	0.00	yes
TRINITY_DN24360_c1_g4	hypothetical protein POPTR_0001s43180g [Populus trichocarpa]	SRK	G-type lectin S-receptor-like serine/threonine-protein kinase SRK OS=Arabidopsis thaliana GN=SRK PE=2 SV=1	3.62	16.53	-1.98	down	0.00	0.00	yes
TRINITY_DN24336_c0_g3	PREDICTED: derlin-1-like [Populus euphratica]	DER1	Derlin-1 OS=Arabidopsis thaliana GN=DER1 PE=2 SV=1	2.38	10.98	-1.62	down	0.00	0.00	yes
TRINITY_DN27663_c1_g1	hypothetical protein POPTR_0005s21630g [Populus trichocarpa]	-	-	1.46	6.99	-2.09	down	0.00	0.00	yes
TRINITY_DN21145_c0_g2	hypothetical protein POPTR_0001s10580g [Populus trichocarpa]	-	-	4.70	15.97	-1.16	down	0.00	0.00	yes
TRINITY_DN15904_c0_g2	PREDICTED: cytochrome b5-like [Populus euphratica]	-	Cytochrome b5 OS=Nicotiana tabacum PE=2 SV=1	5.77	66.32	-2.91	down	0.00	0.00	yes
TRINITY_DN19002_c0_g2	hypothetical protein POPTR_0008s05860g [Populus trichocarpa]	LECRKS4	L-type lectin-domain containing receptor kinase S.4 OS=Arabidopsis thaliana GN=LECRKS4 PE=2 SV=1	1.06	4.85	-1.58	down	0.00	0.00	yes
TRINITY_DN20534_c2_g1	cytochrome P450 78A3p family protein [Populus trichocarpa]	CYP78A3	Cytochrome P450 78A3 OS=Glycine max GN=CYP78A3 PE=2 SV=1	0.27	3.89	-3.15	down	0.00	0.00	yes
TRINITY_DN26112_c1_g6	hypothetical protein POPTR_0008s09730g [Populus trichocarpa]	-	-	0.55	3.04	-1.83	down	0.00	0.00	yes
TRINITY_DN20922_c0_g1	PREDICTED: endo-1,4-beta-xylanase A-like [Populus euphratica]	rsgI6	Anti-sigma-I factor RsgI6 OS=Clostridium thermocellum (strain ATCC 27405 / DSM 1237 / NBRC 103400 / NCIMB 10682 / NRRL B-4536 / VPI 7372) GN=rsgI6 PE=1 SV=1	0.82	3.92	-1.67	down	0.00	0.00	yes
TRINITY_DN26181_c1_g2	-	-	-	0.17	3.83	-3.61	down	0.00	0.00	yes
TRINITY_DN16187_c0_g2	RALF-LIKE 27 family protein [Populus trichocarpa]	-	-	109.27	437.08	-1.42	down	0.00	0.00	yes
TRINITY_DN18498_c0_g4	-	-	-	0.41	4.11	-2.63	down	0.00	0.00	yes
TRINITY_DN16332_c1_g4	hypothetical protein POPTR_0012s04540g [Populus trichocarpa]	-	-	0.23	4.26	-3.54	down	0.00	0.00	yes
TRINITY_DN23143_c1_g5	-	-	-	0.38	3.54	-2.58	down	0.00	0.00	yes
TRINITY_DN27770_c0_g2	NBS-LRR resistance gene-like protein ARGH34 [Populus trichocarpa]	-	-	0.22	2.16	-2.65	down	0.00	0.00	yes
TRINITY_DN15054_c0_g1	teosinte-branched-like protein 1 [Populus tremula x Populus alba]	TCP12	Transcription factor TCP12 OS=Arabidopsis thaliana GN=TCP12 PE=2 SV=1	0.49	3.67	-2.27	down	0.00	0.00	yes
TRINITY_DN22414_c0_g3	hypothetical protein POPTR_0007s04220g [Populus trichocarpa]	PUB26	U-box domain-containing protein 26 OS=Arabidopsis thaliana GN=PUB26 PE=2 SV=1	1.53	7.77	-1.74	down	0.00	0.00	yes
TRINITY_DN16825_c0_g2	putative actin-depolymerizing factor family protein [Populus trichocarpa]	ADF5	Actin-depolymerizing factor 5 OS=Arabidopsis thaliana GN=ADF5 PE=1 SV=1	4.48	20.08	-1.70	down	0.00	0.00	yes
TRINITY_DN21612_c1_g1	-	-	-	2.71	14.84	-1.83	down	0.00	0.00	yes
TRINITY_DN20899_c0_g1	-	-	-	0.55	4.33	-2.36	down	0.00	0.00	yes

TRINITY_DN18476_c0_g1	hypothetical protein POPTR_0006s29520g [Populus trichocarpa]	-	-	27.54	88.56	-1.09	down	0.00	0.00	yes
TRINITY_DN26021_c1_g1	hypothetical protein POPTR_0001s13110g [Populus trichocarpa]	ARF2	Auxin response factor 2 OS=Arabidopsis thaliana GN=ARF2 PE=1 SV=2	22.71	95.09	-1.46	down	0.00	0.00	yes
TRINITY_DN16121_c0_g2	PREDICTED: protein YLS9-like [Populus euphratica]	-	-	1.18	8.48	-2.28	down	0.00	0.00	yes
TRINITY_DN18044_c0_g2	homeodomain family protein [Populus trichocarpa]	ANL2	Homeobox-leucine zipper protein ANTHOCYANINLESS 2 OS=Arabidopsis thaliana GN=ANL2 PE=2 SV=1	17.21	59.25	-1.22	down	0.00	0.00	yes
TRINITY_DN20047_c0_g1	PREDICTED: mitotic checkpoint serine/threonine-protein kinase BUB1 [Populus euphratica]	BUB1	Mitotic checkpoint serine/threonine-protein kinase BUB1 OS=Arabidopsis thaliana GN=BUB1 PE=1 SV=1	6.89	21.33	-1.12	down	0.00	0.00	yes
TRINITY_DN24778_c2_g8	PREDICTED: uncharacterized protein LOC105114011 [Populus euphratica]	-	-	5.07	17.56	-1.23	down	0.00	0.00	yes
TRINITY_DN25371_c4_g1	quinate O-hydroxycinnamoyltransferase/shikimate O-hydroxycinnamoyltransferase [Populus trichocarpa]	HST	Shikimate O-hydroxycinnamoyltransferase OS=Arabidopsis thaliana GN=HST PE=2 SV=1	8.71	30.47	-1.21	down	0.00	0.00	yes
TRINITY_DN26098_c0_g2	hypothetical protein POPTR_0009s04570g [Populus trichocarpa]	IRKI	IRK-interacting protein OS=Arabidopsis thaliana GN=IRKI PE=1 SV=1	12.94	39.57	-1.00	down	0.00	0.00	yes
TRINITY_DN27059_c0_g4	-	-	-	17.70	54.69	-1.01	down	0.00	0.00	yes
TRINITY_DN19287_c0_g1	hypothetical protein POPTR_0003s21370g [Populus trichocarpa]	-	-	1.43	6.78	-1.82	down	0.00	0.00	yes
TRINITY_DN24242_c0_g1	hypothetical protein POPTR_0003s06730g [Populus trichocarpa]	Os10g0391300	Zinc finger CCCH domain-containing protein 62 OS=Oryza sativa subsp. japonica GN=Os10g0391300 PE=3 SV=2	3.03	11.55	-1.34	down	0.00	0.00	yes
TRINITY_DN22547_c1_g3	-	-	-	1.02	6.26	-1.96	down	0.00	0.00	yes
TRINITY_DN23228_c1_g1	PREDICTED: protein ROOT HAIR DEFECTIVE 3 homolog 2-like isoform X11 [Populus euphratica]	At5g45160	Protein ROOT HAIR DEFECTIVE 3 homolog 2 OS=Arabidopsis thaliana GN=At5g45160 PE=2 SV=1	2.61	15.33	-1.77	down	0.00	0.00	yes
TRINITY_DN18506_c0_g2	hypothetical protein POPTR_0004s03930g [Populus trichocarpa]	PLT4	Probable polyol transporter 4 OS=Arabidopsis thaliana GN=PLT4 PE=2 SV=1	0.88	4.50	-1.72	down	0.00	0.00	yes
TRINITY_DN17407_c0_g1	amino acid transporter family protein [Populus trichocarpa]	AATL1	Lysine histidine transporter-like 8 OS=Arabidopsis thaliana GN=AATL1 PE=1 SV=1	2.71	14.94	-1.95	down	0.00	0.00	yes
TRINITY_DN23114_c0_g2	N7 family protein [Populus trichocarpa]	FBW2	F-box protein FBW2 OS=Arabidopsis thaliana GN=FBW2 PE=1 SV=1	31.12	87.51	-1.12	down	0.00	0.00	yes
TRINITY_DN18863_c1_g4	raffinose synthase family protein [Populus tomentosa]	RFS5	Probable galactinol--sucrose galactosyltransferase 5 OS=Arabidopsis thaliana GN=RFS5 PE=1 SV=1	0.30	2.68	-2.50	down	0.00	0.00	yes
TRINITY_DN27065_c1_g1	PREDICTED: uncharacterized protein At4g38062-like isoform X1 [Populus euphratica]	At4g38062	Uncharacterized protein At4g38062 OS=Arabidopsis thaliana GN=At4g38062 PE=4 SV=1	5.39	19.68	-1.29	down	0.00	0.00	yes
TRINITY_DN26579_c0_g1	hypothetical protein POPTR_0009s06580g [Populus trichocarpa]	Os03g0698800	Zinc finger CCCH domain-containing protein 24 OS=Oryza sativa subsp. japonica GN=Os03g0698800 PE=2 SV=1	15.17	47.96	-1.11	down	0.00	0.00	yes
TRINITY_DN21486_c0_g1	hypothetical protein POPTR_0005s22280g [Populus trichocarpa]	PDF1	Protodermal factor 1 OS=Arabidopsis thaliana GN=PDF1 PE=2 SV=1	22.22	255.84	-2.90	down	0.00	0.00	yes
TRINITY_DN25814_c0_g1	hypothetical protein POPTR_0014s13440g [Populus trichocarpa]	MED33B	Mediator of RNA polymerase II transcription subunit 33B OS=Arabidopsis thaliana GN=MED33B PE=1 SV=1	5.50	17.45	-1.05	down	0.00	0.00	yes
TRINITY_DN18879_c0_g1	hypothetical protein POPTR_0003s11010g [Populus trichocarpa]	-	-	5.56	18.48	-1.04	down	0.00	0.00	yes
TRINITY_DN23316_c1_g1	hypothetical protein POPTR_0002s23910g [Populus trichocarpa]	-	-	20.76	62.27	-1.01	down	0.00	0.00	yes
TRINITY_DN21692_c0_g1	PREDICTED: probable beta-D-xylosidase 2 [Populus euphratica]	BXL2	Probable beta-D-xylosidase 2 OS=Arabidopsis thaliana GN=BXL2 PE=2 SV=1	0.65	3.58	-2.02	down	0.00	0.00	yes
TRINITY_DN27674_c0_g3	trichohyalin-related family protein [Populus trichocarpa]	AUL1	Auxilin-like protein 1 OS=Arabidopsis thaliana GN=AUL1 PE=2 SV=2	7.51	35.55	-1.38	down	0.00	0.00	yes
TRINITY_DN24100_c0_g2	hypothetical protein POPTR_0010s05300g [Populus trichocarpa]	NIK1	Protein NSP-INTERACTING KINASE 1 OS=Arabidopsis thaliana GN=NIK1 PE=1 SV=1	4.38	18.17	-1.43	down	0.00	0.00	yes
TRINITY_DN24718_c0_g1	PREDICTED: calmodulin-binding transcription activator 3-like isoform X3 [Populus euphratica]	CMTA3	Calmodulin-binding transcription activator 3 OS=Arabidopsis thaliana GN=CMTA3 PE=1 SV=1	2.73	8.94	-1.13	down	0.00	0.00	yes
TRINITY_DN24943_c0_g1	hypothetical protein POPTR_0010s14690g [Populus trichocarpa]	At1g71691	GDSL esterase/lipase At1g71691 OS=Arabidopsis thaliana GN=At1g71691 PE=2 SV=1	0.37	2.73	-2.15	down	0.00	0.00	yes
TRINITY_DN22356_c1_g3	hypothetical protein POPTR_0005s23780g [Populus trichocarpa]	-	-	1.54	6.96	-1.57	down	0.00	0.00	yes

TRINITY_DN25618_c1_g1	PREDICTED: protein tesmin/TSO1-like CXC 5 isoform X1 [Populus euphratica]	TCX5	Protein tesmin/TSO1-like CXC 5 OS=Arabidopsis thaliana GN=TCX5 PE=1 SV=1	6.41	19.44	-1.00	down	0.00	0.00	yes
TRINITY_DN25711_c1_g4	hypothetical protein POPTR_0001s11770g [Populus trichocarpa]	ERF2	Ethylene-responsive transcription factor 2 OS=Nicotiana tabacum GN=ERF2 PE=2 SV=1	0.54	4.70	-2.55	down	0.00	0.00	yes
TRINITY_DN19591_c1_g1	PREDICTED: spindle and kinetochore-associated protein 1 homolog [Populus euphratica]	At3g60660	Spindle and kinetochore-associated protein 1 homolog OS=Arabidopsis thaliana GN=At3g60660 PE=2 SV=1	3.29	12.21	-1.27	down	0.00	0.00	yes
TRINITY_DN19456_c0_g3	-	-	-	0.24	3.88	-3.20	down	0.00	0.00	yes
TRINITY_DN23466_c1_g1	PREDICTED: uncharacterized protein LOC105138874 [Populus euphratica]	-	-	0.21	2.36	-2.83	down	0.00	0.00	yes
TRINITY_DN26166_c0_g4	PREDICTED: serine/threonine-protein kinase/endoribonuclease IRE1a [Populus euphratica]	IRE1A	Serine/threonine-protein kinase/endoribonuclease IRE1a OS=Arabidopsis thaliana GN=IRE1A PE=1 SV=1	1.91	7.44	-1.36	down	0.00	0.00	yes
TRINITY_DN21253_c0_g4	hypothetical protein POPTR_0012s13440g [Populus trichocarpa]	CHS1	Chalcone synthase 1 OS=Gerbera hybrida GN=CHS1 PE=2 SV=1	0.21	2.45	-2.89	down	0.00	0.00	yes
TRINITY_DN25066_c0_g2	leucine-rich repeat transmembrane protein kinase [Populus trichocarpa]	At4g37250	Probable LRR receptor-like serine/threonine-protein kinase At4g37250 OS=Arabidopsis thaliana GN=At4g37250 PE=2 SV=1	2.46	10.51	-1.39	down	0.00	0.00	yes
TRINITY_DN18941_c1_g1	TIR-NBS type disease resistance protein [Populus trichocarpa]	-	-	2.58	14.40	-1.96	down	0.00	0.00	yes
TRINITY_DN26053_c0_g2	hypothetical protein POPTR_0014s04210g [Populus trichocarpa]	JAC1	J domain-containing protein required for chloroplast accumulation response 1 OS=Arabidopsis thaliana GN=JAC1 PE=1 SV=1	1.41	6.89	-1.66	down	0.00	0.00	yes
TRINITY_DN17850_c0_g1	zinc finger family protein [Populus trichocarpa]	ATL72	RING-H2 finger protein ATL72 OS=Arabidopsis thaliana GN=ATL72 PE=2 SV=1	0.95	5.11	-1.85	down	0.00	0.00	yes
TRINITY_DN23866_c0_g2	PREDICTED: uncharacterized protein LOC105123817 isoform X1 [Populus euphratica]	-	-	14.44	43.37	-1.01	down	0.00	0.00	yes
TRINITY_DN15626_c0_g1	hypothetical protein POPTR_0012s11420g [Populus trichocarpa]	AMC1	Metacaspase-1 OS=Arabidopsis thaliana GN=AMC1 PE=1 SV=1	0.57	9.29	-2.06	down	0.00	0.00	yes
TRINITY_DN13640_c0_g2	hypothetical protein POPTR_0010s15820g [Populus trichocarpa]	-	-	0.32	4.21	-3.01	down	0.00	0.00	yes
TRINITY_DN24495_c1_g5	PREDICTED: protein STAY-GREEN 1, chloroplastic-like [Populus euphratica]	SGR	Protein STAY-GREEN, chloroplastic OS=Pisum sativum GN=SGR PE=2 SV=1	0.40	2.85	-2.22	down	0.00	0.00	yes
TRINITY_DN19523_c0_g1	hypothetical protein POPTR_0014s05160g [Populus trichocarpa]	PBL19	Probable serine/threonine-protein kinase PBL19 OS=Arabidopsis thaliana GN=PBL19 PE=1 SV=1	0.36	2.30	-2.13	down	0.00	0.00	yes
TRINITY_DN19446_c0_g1	glycosyl hydrolase family 17 family protein [Populus trichocarpa]	GLC1	Glucan endo-1,3-beta-glucosidase OS=Triticum aestivum GN=GLC1 PE=2 SV=1	8.26	37.67	-1.56	down	0.00	0.00	yes
TRINITY_DN24008_c0_g2	delta8-sphingolipid desaturase [Populus tomentosa]	SLD2	Delta(8)-fatty-acid desaturase 2 OS=Arabidopsis thaliana GN=SLD2 PE=1 SV=1	19.94	69.52	-1.19	down	0.00	0.00	yes
TRINITY_DN12704_c0_g2	PREDICTED: BAHD acyltransferase At5g47980-like [Populus euphratica]	ACT	Vinorine synthase OS=Rauvolfia serpentina GN=ACT PE=1 SV=2	0.27	1.96	-2.23	down	0.00	0.00	yes
TRINITY_DN18342_c0_g2	hypothetical protein POPTR_0003s08160g [Populus trichocarpa]	At5g43530	Putative SWI/SNF-related matrix-associated actin-dependent regulator of chromatin subfamily A member 3-like 3 OS=Arabidopsis thaliana GN=At5g43530 PE=3 SV=1	0.61	4.82	-2.33	down	0.00	0.00	yes
TRINITY_DN15829_c1_g1	hypothetical protein POPTR_0016s05620g, partial [Populus trichocarpa]	-	-	1.16	13.50	-2.91	down	0.00	0.00	yes
TRINITY_DN24181_c0_g2	hypothetical protein POPTR_0005s13360g, partial [Populus trichocarpa]	-	-	0.14	2.24	-3.32	down	0.00	0.00	yes
TRINITY_DN18435_c0_g1	PREDICTED: uncharacterized protein LOC105108367 isoform X1 [Populus euphratica]	ACR6	ACT domain-containing protein ACR6 OS=Arabidopsis thaliana GN=ACR6 PE=2 SV=1	3.53	21.48	-1.32	down	0.00	0.00	yes
TRINITY_DN21189_c3_g1	hypothetical protein POPTR_0008s14440g [Populus trichocarpa]	-	-	7.13	30.72	-1.54	down	0.00	0.00	yes
TRINITY_DN14789_c0_g1	hypothetical protein POPTR_0015s13050g [Populus trichocarpa]	-	-	0.26	3.19	-2.99	down	0.00	0.00	yes
TRINITY_DN26014_c0_g2	PREDICTED: CRC domain-containing protein TSO1-like isoform X1 [Populus euphratica]	TCX2	Protein tesmin/TSO1-like CXC 2 OS=Arabidopsis thaliana GN=TCX2 PE=1 SV=1	3.59	13.68	-1.33	down	0.00	0.00	yes
TRINITY_DN15379_c0_g1	hypothetical protein POPTR_0006s27240g [Populus trichocarpa]	FRS6	Protein FAR1-RELATED SEQUENCE 6 OS=Arabidopsis thaliana GN=FRS6 PE=2 SV=1	0.93	4.11	-1.55	down	0.00	0.00	yes

TRINITY_DN26166_c0_g1	PREDICTED: serine/threonine-protein kinase/endoribonuclease IRE1b isoform X4 [Populus euphratica]	IRE1B	Serine/threonine-protein kinase/endoribonuclease IRE1b OS=Arabidopsis thaliana GN=IRE1B PE=2 SV=1	6.86	15.30	-1.05	down	0.00	0.00	yes
TRINITY_DN23717_c0_g2	kinase family protein [Populus trichocarpa]	At4g25390	Receptor-like serine/threonine-protein kinase At4g25390 OS=Arabidopsis thaliana GN=At4g25390 PE=2 SV=1	1.77	4.53	-1.97	down	0.00	0.00	yes
TRINITY_DN24382_c0_g2	3-hydroxy-3-methylglutaryl coenzyme A reductase family protein [Populus trichocarpa]	HMG1	3-hydroxy-3-methylglutaryl-coenzyme A reductase 1 OS=Gossypium hirsutum GN=HMG1 PE=3 SV=1	0.97	10.41	-2.82	down	0.00	0.00	yes
TRINITY_DN26224_c0_g3	PREDICTED: type I inositol 1,4,5-trisphosphate 5-phosphatase CVP2-like isoform X1 [Populus euphratica]	IP5P7	Type IV inositol polyphosphate 5-phosphatase 7 OS=Arabidopsis thaliana GN=IP5P7 PE=1 SV=1	0.71	4.72	-2.12	down	0.00	0.00	yes
TRINITY_DN18473_c0_g1	hypothetical protein POPTR_0006s11760g [Populus trichocarpa]	-	-	3.07	12.62	-1.44	down	0.00	0.00	yes
TRINITY_DN17462_c0_g1	hypothetical protein POPTR_0001s44670g [Populus trichocarpa]	spg1	Septum-promoting GTP-binding protein 1 OS=Schizosaccharomyces pombe (strain 972 / ATCC 24843) GN=spg1 PE=1 SV=1	2.25	11.62	-1.69	down	0.00	0.00	yes
TRINITY_DN24639_c1_g2	hypothetical protein POPTR_0014s09520g [Populus trichocarpa]	AIB	Transcription factor ABA-INDUCIBLE bHLH-TYPE OS=Arabidopsis thaliana GN=AIB PE=2 SV=2	19.48	74.22	-1.33	down	0.00	0.00	yes
TRINITY_DN21609_c2_g2	hypothetical protein POPTR_0019s02900g [Populus trichocarpa]	RPS4	Disease resistance protein RPS4 OS=Arabidopsis thaliana GN=RPS4 PE=1 SV=1	1.37	8.51	-2.26	down	0.00	0.00	yes
TRINITY_DN14678_c0_g1	PREDICTED: putative ion channel POLLUX-like 2 isoform X1 [Populus euphratica]	At5g43745	Putative ion channel POLLUX-like 2 OS=Arabidopsis thaliana GN=At5g43745 PE=2 SV=1	22.96	109.98	-1.68	down	0.00	0.00	yes
TRINITY_DN14829_c0_g2	hypothetical protein POPTR_0018s05900g [Populus trichocarpa]	-	-	5.07	39.68	-2.28	down	0.00	0.00	yes
TRINITY_DN23387_c0_g1	Cell division control protein 2 B [Populus trichocarpa]	CDKB1-2	Cyclin-dependent kinase B1-2 OS=Arabidopsis thaliana GN=CDKB1-2 PE=1 SV=2	37.68	134.49	-1.24	down	0.00	0.00	yes
TRINITY_DN19897_c0_g8	-	-	-	1.42	9.73	-2.18	down	0.00	0.00	yes
TRINITY_DN24789_c0_g3	hypothetical protein POPTR_0018s12690g [Populus trichocarpa]	HAT	Zinc finger BED domain-containing protein DAYSLEEPER OS=Arabidopsis thaliana GN=HAT PE=1 SV=1	10.76	37.30	-1.19	down	0.00	0.00	yes
TRINITY_DN17388_c0_g1	hypothetical protein POPTR_0003s11470g [Populus trichocarpa]	-	-	0.51	4.39	-2.52	down	0.00	0.00	yes
TRINITY_DN25764_c0_g1	hypothetical protein POPTR_0003s12760g [Populus trichocarpa]	-	-	3.81	12.25	-1.07	down	0.00	0.00	yes
TRINITY_DN15592_c0_g1	hypothetical protein POPTR_0010s16760g [Populus trichocarpa]	WRKY57	Probable WRKY transcription factor 57 OS=Arabidopsis thaliana GN=WRKY57 PE=2 SV=1	2.25	4.65	-2.13	down	0.00	0.00	yes
TRINITY_DN19938_c0_g3	PREDICTED: pentatricopeptide repeat-containing protein At3g05340-like [Populus euphratica]	-	-	1.07	9.81	-2.54	down	0.00	0.00	yes
TRINITY_DN25565_c0_g7	PREDICTED: probable 1-deoxy-D-xylulose-5-phosphate synthase 2, chloroplastic [Populus euphratica]	Os07g0190000	Probable 1-deoxy-D-xylulose-5-phosphate synthase 2, chloroplastic OS=Oryza sativa subsp. japonica GN=Os07g0190000 PE=2 SV=1	1.26	5.40	-1.49	down	0.00	0.00	yes
TRINITY_DN21243_c0_g2	bZIP transcription factor family protein [Populus trichocarpa]	BZIP44	bZIP transcription factor 44 OS=Arabidopsis thaliana GN=BZIP44 PE=1 SV=1	1.08	6.04	-1.83	down	0.00	0.00	yes
TRINITY_DN22667_c0_g4	unknown [Populus trichocarpa]	At5g64080	Non-specific lipid-transfer protein-like protein At5g64080 OS=Arabidopsis thaliana GN=At5g64080 PE=1 SV=1	25.70	78.19	-1.13	down	0.00	0.00	yes
TRINITY_DN25799_c0_g1	hypothetical protein POPTR_0014s11090g [Populus trichocarpa]	At3g62260	Probable protein phosphatase 2C 49 OS=Arabidopsis thaliana GN=At3g62260 PE=2 SV=1	11.34	56.60	-1.70	down	0.00	0.00	yes
TRINITY_DN22925_c0_g1	PREDICTED: indole-3-pyruvate monooxygenase YUCCA6-like [Populus euphratica]	YUC6	Indole-3-pyruvate monooxygenase YUCCA6 OS=Arabidopsis thaliana GN=YUC6 PE=1 SV=1	0.66	3.71	-1.90	down	0.00	0.00	yes
TRINITY_DN27432_c0_g1	hypothetical protein POPTR_0001s40950g, partial [Populus trichocarpa]	-	-	0.24	2.79	-2.71	down	0.00	0.00	yes
TRINITY_DN23972_c0_g1	hypothetical protein POPTR_0019s08400g [Populus trichocarpa]	ETG1	Mini-chromosome maintenance complex-binding protein OS=Arabidopsis thaliana GN=ETG1 PE=1 SV=1	5.18	26.32	-1.67	down	0.00	0.00	yes
TRINITY_DN17428_c0_g2	PREDICTED: probable calcium-binding protein CML43 [Populus euphratica]	BETVIII	Calcium-binding allergen Bet v 3 OS=Betula pendula GN=BETVIII PE=1 SV=1	1.69	21.28	-2.90	down	0.00	0.00	yes
TRINITY_DN19485_c0_g1	heat shock protein 70 cognate [Populus trichocarpa]	HSP70	Heat shock cognate 70 kDa protein OS=Petunia hybrida GN=HSP70 PE=2 SV=1	0.43	3.41	-2.36	down	0.00	0.00	yes
TRINITY_DN25833_c0_g1	hypothetical protein POPTR_0009s03570g [Populus trichocarpa]	PUB33	U-box domain-containing protein 33 OS=Arabidopsis thaliana GN=PUB33 PE=2 SV=2	3.89	14.00	-1.20	down	0.00	0.00	yes

TRINITY_DN24591_c0_g1	hypothetical protein POPTR_0011s04540g [Populus trichocarpa]	SRF1	Protein STRUBBELIG-RECEPTOR FAMILY 1 OS=Arabidopsis thaliana GN=SRF1 PE=2 SV=2	2.10	8.21	-1.43	down	0.00	0.00	yes
TRINITY_DN18104_c0_g2	PREDICTED: LOW QUALITY PROTEIN: uncharacterized protein LOC105111782 [Populus euphratica]	-	Retrovirus-related Pol polyprotein from transposon TNT 1-94 OS=Nicotiana tabacum PE=2 SV=1	0.51	4.24	-2.76	down	0.00	0.00	yes
TRINITY_DN15788_c0_g1	PREDICTED: CASP-like protein 1F3 [Populus euphratica]	POPTRDRAFT_	CASP-like protein 1F3 OS=Populus trichocarpa GN=POPTRDRAFT_752786 PE=3 SV=1	0.39	2.56	-2.48	down	0.00	0.00	yes
TRINITY_DN22486_c0_g2	PREDICTED: uncharacterized protein LOC105141853 isoform X1 [Populus euphratica]	At3g20280	PHD finger protein At3g20280 OS=Arabidopsis thaliana GN=At3g20280 PE=2 SV=1	4.36	13.81	-1.06	down	0.00	0.00	yes
TRINITY_DN23040_c0_g1	hypothetical protein POPTR_0004s08600g [Populus trichocarpa]	MYOB3	Myosin-binding protein 3 OS=Arabidopsis thaliana GN=MYOB3 PE=1 SV=1	2.02	8.09	-1.38	down	0.00	0.00	yes
TRINITY_DN21637_c0_g2	hypothetical protein POPTR_0015s05050g [Populus trichocarpa]	HDG11	Homeobox-leucine zipper protein HDG11 OS=Arabidopsis thaliana GN=HDG11 PE=1 SV=1	3.13	12.74	-1.12	down	0.00	0.00	yes
TRINITY_DN25383_c0_g1	PREDICTED: putative disease resistance RPP13-like protein 1 [Populus euphratica]	RGA4	Putative disease resistance protein RGA4 OS=Solanum bulbocastanum GN=RGA4 PE=2 SV=1	2.86	10.01	-1.12	down	0.00	0.00	yes
TRINITY_DN18282_c0_g1	calmodulin-like protein 6a [Populus trichocarpa]	CALM1	Calmodulin OS=Solanum lycopersicum GN=CALM1 PE=2 SV=2	10.53	36.61	-1.17	down	0.00	0.00	yes
TRINITY_DN22542_c0_g3	hypothetical protein POPTR_0005s19430g [Populus trichocarpa]	-	-	0.85	5.31	-2.07	down	0.00	0.00	yes
TRINITY_DN20381_c0_g1	hypothetical protein POPTR_0015s05670g [Populus trichocarpa]	UGT88B1	UDP-glycosyltransferase 88B1 OS=Stevia rebaudiana GN=UGT88B1 PE=2 SV=1	3.58	16.81	-1.87	down	0.00	0.00	yes
TRINITY_DN10255_c0_g1	hypothetical protein POPTR_0014s04950g [Populus trichocarpa]	-	-	0.17	3.43	-3.69	down	0.00	0.00	yes
TRINITY_DN23076_c1_g2	PREDICTED: E3 ubiquitin-protein ligase SDIR1-like isoform X4 [Populus euphratica]	SDIR1	E3 ubiquitin-protein ligase SDIR1 OS=Arabidopsis thaliana GN=SDIR1 PE=1 SV=1	6.02	23.40	-1.21	down	0.00	0.00	yes
TRINITY_DN22974_c0_g2	putative phosphatase family protein [Populus trichocarpa]	-	-	2.24	22.90	-2.37	down	0.00	0.00	yes
TRINITY_DN22292_c0_g1	hypothetical protein POPTR_0015s12180g [Populus trichocarpa]	-	-	1.38	7.75	-1.88	down	0.00	0.00	yes
TRINITY_DN20514_c0_g3	hypothetical protein POPTR_0003s22250g [Populus trichocarpa]	-	-	0.56	3.79	-2.09	down	0.00	0.00	yes
TRINITY_DN15758_c0_g1	hypothetical protein POPTR_0008s11350g [Populus trichocarpa]	NUDT1	Nudix hydrolase 1 OS=Arabidopsis thaliana GN=NUDT1 PE=1 SV=1	2.30	11.31	-1.67	down	0.00	0.00	yes
TRINITY_DN15803_c0_g1	hypothetical protein POPTR_0001s45900g [Populus trichocarpa]	At4g10390	Probable receptor-like protein kinase At4g10390 OS=Arabidopsis thaliana GN=At4g10390 PE=2 SV=1	0.30	2.75	-2.56	down	0.00	0.00	yes
TRINITY_DN15179_c0_g1	hypothetical protein POPTR_0016s14500g [Populus trichocarpa]	-	-	1.93	12.87	-2.09	down	0.00	0.00	yes
TRINITY_DN27070_c0_g1	PREDICTED: uncharacterized protein LOC105109647 isoform X2 [Populus euphratica]	-	-	8.77	29.50	-1.07	down	0.00	0.00	yes
TRINITY_DN24844_c0_g1	hypothetical protein POPTR_0008s15990g [Populus trichocarpa]	KCS20	3-ketoacyl-CoA synthase 20 OS=Arabidopsis thaliana GN=KCS20 PE=2 SV=1	6.75	33.49	-1.50	down	0.00	0.00	yes
TRINITY_DN26689_c1_g1	phototropic-responsive NPH3 family protein [Populus trichocarpa]	NPY2	BTB/POZ domain-containing protein NPY2 OS=Arabidopsis thaliana GN=NPY2 PE=2 SV=1	4.14	15.49	-1.19	down	0.00	0.00	yes
TRINITY_DN23841_c0_g3	PREDICTED: replication protein A 32 kDa subunit A-like [Populus euphratica]	RPA2A	Replication protein A 32 kDa subunit A OS=Arabidopsis thaliana GN=RPA2A PE=1 SV=2	7.83	32.50	-1.40	down	0.00	0.00	yes
TRINITY_DN23831_c1_g3	hypothetical protein GLYMA_17G255600 [Glycine max]	HTR4	Histone H3.3 OS=Arabidopsis thaliana GN=HTR4 PE=1 SV=2	949.87	2470.01	-1.10	down	0.00	0.00	yes
TRINITY_DN26519_c0_g4	hypothetical protein POPTR_0010s22070g [Populus trichocarpa]	-	-	3.09	12.97	-1.24	down	0.00	0.00	yes
TRINITY_DN25839_c1_g3	WRKY transcription factor 30 [(Populus tomentosa x Populus bolleana) x Populus tomentosa]	WRKY32	Probable WRKY transcription factor 32 OS=Arabidopsis thaliana GN=WRKY32 PE=2 SV=1	6.93	21.93	-1.09	down	0.00	0.00	yes
TRINITY_DN26344_c0_g5	hypothetical protein POPTR_0006s13270g [Populus trichocarpa]	-	-	5.97	20.11	-1.16	down	0.00	0.00	yes
TRINITY_DN16489_c0_g1	hypothetical protein POPTR_0012s02620g [Populus trichocarpa]	At4g27740	Protein yippee-like At4g27740 OS=Arabidopsis thaliana GN=At4g27740 PE=3 SV=1	3.59	16.58	-1.61	down	0.00	0.00	yes
TRINITY_DN15023_c0_g1	unknown [Populus trichocarpa x Populus deltoides]	-	Non-specific lipid-transfer protein OS=Spinacia oleracea PE=1 SV=2	350.78	1135.91	-1.07	down	0.00	0.00	yes

TRINITY_DN18436_c1_g3	PREDICTED: DNA-directed RNA polymerase III subunit RPC6-like [Populus euphratica]	POLR3F	DNA-directed RNA polymerase III subunit RPC6 OS=Homo sapiens GN=POLR3F PE=1 SV=1	0.98	5.17	-1.79	down	0.00	0.00	yes
TRINITY_DN16495_c0_g1	-	-	-	0.37	5.94	-3.19	down	0.00	0.00	yes
TRINITY_DN25985_c2_g1	hypothetical protein POPTR_0012s02740g [Populus trichocarpa]	-	-	1.80	12.18	-1.73	down	0.00	0.00	yes
TRINITY_DN27277_c0_g1	PREDICTED: 65-kDa microtubule-associated protein 3-like isoform X1 [Populus euphratica]	MAP65-3	65-kDa microtubule-associated protein 3 OS=Arabidopsis thaliana GN=MAP65-3 PE=1 SV=1	32.44	111.60	-1.16	down	0.00	0.00	yes
TRINITY_DN26555_c0_g1	hypothetical protein POPTR_0003s11770g [Populus trichocarpa]	TPX2	Protein TPX2 OS=Arabidopsis thaliana GN=TPX2 PE=1 SV=1	19.38	60.43	-1.04	down	0.00	0.00	yes
TRINITY_DN25704_c0_g2	hypothetical protein POPTR_0004s04510g [Populus trichocarpa]	SMG7L	Protein SMG7L OS=Arabidopsis thaliana GN=SMG7L PE=2 SV=1	2.77	13.56	-1.25	down	0.00	0.00	yes
TRINITY_DN27493_c1_g1	PREDICTED: homeobox-leucine zipper protein ATHB-15 [Populus euphratica]	ATHB-15	Homeobox-leucine zipper protein ATHB-15 OS=Arabidopsis thaliana GN=ATHB-15 PE=1 SV=1	10.09	30.65	-1.03	down	0.00	0.00	yes
TRINITY_DN19122_c0_g3	cinnamoyl-CoA reductase-related family protein [Populus trichocarpa]	-	-	0.82	10.61	-3.10	down	0.00	0.00	yes
TRINITY_DN18391_c1_g10	hypothetical protein POPTR_0002s06090g [Populus trichocarpa]	PAR2	Transcription factor PAR2 OS=Arabidopsis thaliana GN=PAR2 PE=3 SV=1	10.02	33.09	-1.19	down	0.00	0.00	yes
TRINITY_DN19902_c0_g2	ethylene-responsive element-binding family protein [Populus trichocarpa]	ERF105	Ethylene-responsive transcription factor ERF105 OS=Arabidopsis thaliana GN=ERF105 PE=2 SV=1	4.55	40.17	-2.54	down	0.00	0.00	yes
TRINITY_DN24231_c0_g2	phosphatidylinositol-phosphatidylcholine transfer protein SEC14 Ssh1 [Populus trichocarpa]	Sec14l4	SEC14-like protein 4 OS=Mus musculus GN=Sec14l4 PE=1 SV=1	2.32	13.28	-2.09	down	0.00	0.00	yes
TRINITY_DN24662_c0_g3	PREDICTED: ribonucleoside-diphosphate reductase small chain [Populus euphratica]	-	Ribonucleoside-diphosphate reductase small chain OS=Nicotiana tabacum PE=2 SV=1	25.18	80.64	-1.05	down	0.00	0.00	yes
TRINITY_DN27657_c0_g1	PREDICTED: calmodulin-binding transcription activator 5 isoform X1 [Populus euphratica]	CMTA5	Calmodulin-binding transcription activator 5 OS=Arabidopsis thaliana GN=CMTA5 PE=2 SV=2	12.35	44.04	-1.16	down	0.00	0.00	yes
TRINITY_DN18854_c0_g1	hypothetical protein POPTR_0012s06670g [Populus trichocarpa]	-	-	3.44	16.82	-1.66	down	0.00	0.00	yes
TRINITY_DN23288_c0_g1	hypothetical protein POPTR_0001s12700g [Populus trichocarpa]	OsI_36121	DNA replication licensing factor MCM2 OS=Oryza sativa subsp. indica GN=OsI_36121 PE=3 SV=1	3.61	17.41	-1.63	down	0.00	0.00	yes
TRINITY_DN20199_c0_g1	hypothetical protein POPTR_0013s05920g [Populus trichocarpa]	At3g03773	Uncharacterized protein At3g03773 OS=Arabidopsis thaliana GN=At3g03773 PE=1 SV=1	5.46	19.25	-1.20	down	0.00	0.00	yes
TRINITY_DN16133_c0_g1	hypothetical protein POPTR_0001s38070g [Populus trichocarpa]	-	-	1.26	6.80	-1.84	down	0.00	0.00	yes
TRINITY_DN20768_c1_g1	hypothetical protein POPTR_0013s00920g [Populus trichocarpa]	BHLH149	Transcription factor bHLH149 OS=Arabidopsis thaliana GN=BHLH149 PE=1 SV=1	14.86	46.80	-1.06	down	0.00	0.00	yes
TRINITY_DN14440_c0_g1	hypothetical protein POPTR_0010s24710g [Populus trichocarpa]	DIVARICATA	Transcription factor DIVARICATA OS=Antirrhinum majus GN=DIVARICATA PE=2 SV=1	2.67	16.78	-2.07	down	0.00	0.00	yes
TRINITY_DN25630_c0_g2	-	-	-	1.84	9.09	-1.66	down	0.00	0.00	yes
TRINITY_DN24423_c0_g1	hypothetical protein POPTR_0007s13610g [Populus trichocarpa]	SHI	Protein SHORT INTERNODES OS=Arabidopsis thaliana GN=SHI PE=1 SV=1	0.58	5.99	-2.95	down	0.00	0.00	yes
TRINITY_DN18614_c0_g1	-	-	-	1.75	9.50	-1.78	down	0.00	0.00	yes
TRINITY_DN19178_c0_g1	auxin-responsive family protein [Populus trichocarpa]	SAUR36	Auxin-responsive protein SAUR36 OS=Arabidopsis thaliana GN=SAUR36 PE=2 SV=1	1.12	20.32	-3.55	down	0.00	0.00	yes
TRINITY_DN16322_c0_g2	hypothetical protein POPTR_0008s19150g [Populus trichocarpa]	SMR6	Cyclin-dependent protein kinase inhibitor SMR6 OS=Arabidopsis thaliana GN=SMR6 PE=1 SV=1	1.19	6.37	-1.83	down	0.00	0.00	yes
TRINITY_DN16595_c0_g1	hypothetical protein POPTR_0006s13790g [Populus trichocarpa]	BHLH82	Transcription factor bHLH82 OS=Arabidopsis thaliana GN=BHLH82 PE=2 SV=1	1.12	7.33	-1.69	down	0.00	0.00	yes
TRINITY_DN23793_c0_g5	eugenol O-methyltransferase family protein [Populus trichocarpa]	COMT1	Caffeic acid 3-O-methyltransferase OS=Prunus dulcis GN=COMT1 PE=2 SV=1	2.31	10.60	-1.54	down	0.00	0.00	yes
TRINITY_DN24988_c0_g2	PREDICTED: chloride channel protein CLC-c-like [Populus euphratica]	CLC-C	Chloride channel protein CLC-c OS=Arabidopsis thaliana GN=CLC-C PE=1 SV=1	0.16	2.27	-3.10	down	0.00	0.00	yes
TRINITY_DN17149_c1_g1	hypothetical protein POPTR_0013s12190g [Populus trichocarpa]	BHLH30	Transcription factor bHLH30 OS=Arabidopsis thaliana GN=BHLH30 PE=1 SV=1	0.54	4.10	-2.27	down	0.00	0.00	yes
TRINITY_DN18394_c0_g1	unknown [Populus tremuloides]	HIPP39	Heavy metal-associated isoprenylated plant protein 39 OS=Arabidopsis thaliana GN=HIPP39 PE=2 SV=1	7.54	36.39	-1.79	down	0.00	0.00	yes

TRINITY_DN23725_c0_g3	auxin response factor 6 family protein [Populus trichocarpa]	ARF6	Auxin response factor 6 OS=Arabidopsis thaliana GN=ARF6 PE=1 SV=2	0.62	3.63	-1.94	down	0.00	0.00	yes
TRINITY_DN17583_c0_g3	MLO-like protein 11 [Populus trichocarpa]	MLO11	MLO-like protein 11 OS=Arabidopsis thaliana GN=MLO11 PE=2 SV=1	3.26	13.44	-1.42	down	0.00	0.00	yes
TRINITY_DN17928_c2_g4	TIR-NBS-LRR-TIR type disease resistance protein [Populus trichocarpa]	-	-	2.38	13.22	-1.84	down	0.00	0.00	yes
TRINITY_DN26813_c0_g4	hypothetical protein POPTR_0012s09590g [Populus trichocarpa]	GTE8	Transcription factor GTE8 OS=Arabidopsis thaliana GN=GTE8 PE=2 SV=2	22.50	70.14	-1.02	down	0.00	0.00	yes
TRINITY_DN22068_c0_g1	PREDICTED: UPF0481 protein At3g47200-like isoform X4 [Populus euphratica]	At3g47200	UPF0481 protein At3g47200 OS=Arabidopsis thaliana GN=At3g47200 PE=2 SV=1	0.69	9.16	-2.67	down	0.00	0.00	yes
TRINITY_DN16061_c0_g1	hypothetical protein POPTR_0002s15180g [Populus trichocarpa]	-	-	4.20	21.80	-1.77	down	0.00	0.00	yes
TRINITY_DN14876_c0_g1	-	-	-	0.41	3.94	-2.64	down	0.00	0.00	yes
TRINITY_DN20743_c0_g4	hypothetical protein POPTR_0008s15510g, partial [Populus trichocarpa]	-	-	0.31	3.64	-2.89	down	0.00	0.00	yes
TRINITY_DN24875_c0_g1	UDP-XYLOSE SYNTHASE 4 family protein [Populus trichocarpa]	UXS2	UDP-glucuronic acid decarboxylase 2 OS=Arabidopsis thaliana GN=UXS2 PE=1 SV=1	20.20	83.94	-1.20	down	0.00	0.00	yes
TRINITY_DN21351_c1_g3	-	-	-	0.77	7.54	-2.61	down	0.00	0.00	yes
TRINITY_DN21762_c0_g2	glutathione S-transferase U12 [Populus yatungensis]	HSP26-A	Probable glutathione S-transferase OS=Glycine max GN=HSP26-A PE=2 SV=1	5.91	24.58	-1.26	down	0.00	0.00	yes
TRINITY_DN19204_c0_g1	hypothetical protein POPTR_0006s23570g [Populus trichocarpa]	-	-	10.88	42.91	-1.53	down	0.00	0.00	yes
TRINITY_DN22973_c0_g1	-	-	-	0.49	4.66	-2.52	down	0.00	0.00	yes
TRINITY_DN22393_c1_g1	PREDICTED: ras-related protein RABC2a [Populus euphratica]	RABC2A	Ras-related protein RABC2a OS=Arabidopsis thaliana GN=RABC2A PE=1 SV=1	4.89	20.65	-1.49	down	0.00	0.00	yes
TRINITY_DN25232_c0_g1	hypothetical protein POPTR_0006s11580g [Populus trichocarpa]	KIPK2	Serine/threonine-protein kinase KIPK2 OS=Arabidopsis thaliana GN=KIPK2 PE=1 SV=1	3.34	11.12	-1.14	down	0.00	0.00	yes
TRINITY_DN16549_c0_g1	Endochitinase 2 family protein [Populus trichocarpa]	Cht10	Chitinase 10 OS=Oryza sativa subsp. japonica GN=Cht10 PE=2 SV=1	0.77	6.73	-2.65	down	0.00	0.00	yes
TRINITY_DN23683_c0_g1	hypothetical protein POPTR_0008s11700g [Populus trichocarpa]	PNS1	Protein PNS1 OS=Ustilago maydis (strain 521 / FGSC 9021) GN=PNS1 PE=3 SV=2	2.09	10.18	-1.44	down	0.00	0.00	yes
TRINITY_DN18002_c0_g2	PREDICTED: phospholipid-transporting ATPase 1-like [Populus euphratica]	ALA1	Phospholipid-transporting ATPase 1 OS=Arabidopsis thaliana GN=ALA1 PE=2 SV=1	0.20	2.37	-2.89	down	0.00	0.00	yes
TRINITY_DN18592_c1_g3	auxin-responsive family protein [Populus trichocarpa]	-	-	1.56	10.25	-2.13	down	0.00	0.00	yes
TRINITY_DN15757_c2_g1	PREDICTED: probable receptor-like protein kinase At5g39020 [Populus euphratica]	LRK10	Rust resistance kinase Lr10 OS=Triticum aestivum GN=LRK10 PE=2 SV=1	0.54	5.65	-2.60	down	0.00	0.00	yes
TRINITY_DN23957_c2_g1	PREDICTED: RNA-binding protein 1-like isoform X1 [Populus euphratica]	-	-	7.28	29.89	-1.56	down	0.00	0.00	yes
TRINITY_DN15224_c0_g1	hypothetical protein POPTR_0017s14190g [Populus trichocarpa]	-	-	0.83	5.65	-2.11	down	0.00	0.00	yes
TRINITY_DN27246_c0_g1	PREDICTED: uncharacterized protein LOC105116729 [Populus euphratica]	-	-	4.98	17.03	-1.11	down	0.00	0.00	yes
TRINITY_DN27296_c1_g4	hypothetical protein POPTR_0001s07040g [Populus trichocarpa]	RPPL1	Putative disease resistance RPP13-like protein 1 OS=Arabidopsis thaliana GN=RPPL1 PE=3 SV=1	0.25	4.55	-3.48	down	0.00	0.00	yes
TRINITY_DN27467_c1_g4	hypothetical protein CISIN_1g025930mg [Citrus sinensis]	KINUB	Kinesin-like protein KIN-UB OS=Arabidopsis thaliana GN=KINUB PE=1 SV=2	15.52	46.61	-1.01	down	0.00	0.00	yes
TRINITY_DN20301_c0_g2	hypothetical protein POPTR_0017s04860g [Populus trichocarpa]	GRXC9	Glutaredoxin-C9 OS=Arabidopsis thaliana GN=GRXC9 PE=1 SV=1	3.03	15.95	-1.66	down	0.00	0.00	yes
TRINITY_DN25550_c0_g2	hypothetical protein POPTR_0002s08400g [Populus trichocarpa]	FPP	Filament-like plant protein (Fragment) OS=Solanum lycopersicum GN=FPP PE=1 SV=1	7.26	23.63	-1.08	down	0.00	0.00	yes
TRINITY_DN19021_c0_g2	PREDICTED: transcription factor GTE12-like isoform X1 [Populus euphratica]	GTE12	Transcription factor GTE12 OS=Arabidopsis thaliana GN=GTE12 PE=2 SV=2	6.95	22.31	-1.02	down	0.00	0.00	yes
TRINITY_DN21917_c0_g1	hypothetical protein POPTR_0004s11860g [Populus trichocarpa]	-	-	1.10	8.46	-2.27	down	0.00	0.00	yes
TRINITY_DN27283_c1_g1	hypothetical protein POPTR_0001s05760g [Populus trichocarpa]	Os07g0682400	Zinc finger CCCH domain-containing protein 53 OS=Oryza sativa subsp. japonica GN=Os07g0682400 PE=2 SV=1	45.21	142.17	-1.11	down	0.00	0.00	yes

TRINITY_DN19897_c0_g13	-	-	-	47.22	150.81	-1.05	down	0.00	0.00	yes
TRINITY_DN23372_c1_g3	hydroxyproline-rich glycoprotein [Populus trichocarpa]	IKU1	Protein HAIKU1 OS=Arabidopsis thaliana GN=IKU1 PE=1 SV=1	2.52	8.92	-1.21	down	0.00	0.00	yes
TRINITY_DN27617_c0_g2	-	-	-	25.74	99.95	-1.37	down	0.00	0.00	yes
TRINITY_DN19273_c0_g2	hypothetical protein MANES_16G019400 [Manihot esculenta]	-	-	0.47	4.86	-2.74	down	0.00	0.00	yes
TRINITY_DN21198_c0_g1	PREDICTED: B3 domain-containing protein REM16-like [Populus euphratica]	REM16	B3 domain-containing protein REM16 OS=Arabidopsis thaliana GN=REM16 PE=2 SV=1	12.03	22.46	-1.57	down	0.00	0.00	yes
TRINITY_DN26813_c0_g3	hypothetical protein POPTR_0015s10340g [Populus trichocarpa]	-	-	4.37	18.38	-1.48	down	0.00	0.00	yes
TRINITY_DN22532_c0_g2	PREDICTED: protein ALTERED XYLOGLUCAN 4-like [Populus euphratica]	-	-	0.82	12.20	-3.18	down	0.00	0.00	yes
TRINITY_DN22568_c0_g1	PREDICTED: beta-galactosidase 5-like [Populus euphratica]	BGAL3	Beta-galactosidase 3 OS=Arabidopsis thaliana GN=BGAL3 PE=2 SV=1	22.12	82.08	-1.35	down	0.00	0.00	yes
TRINITY_DN22462_c0_g1	hypothetical protein POPTR_0008s00870g, partial [Populus trichocarpa]	CDC2	Cell division control protein 2 homolog OS=Oxybasis rubra GN=CDC2 PE=2 SV=1	1.78	7.87	-1.50	down	0.00	0.00	yes
TRINITY_DN17028_c0_g1	hypothetical protein POPTR_0001s24910g [Populus trichocarpa]	-	-	9.71	28.75	-1.05	down	0.00	0.00	yes
TRINITY_DN23021_c0_g1	PREDICTED: uncharacterized protein LOC105109755 isoform X1 [Populus euphratica]	-	-	2.77	12.08	-1.48	down	0.00	0.00	yes
TRINITY_DN23063_c0_g3	PREDICTED: nuclear transcription factor Y subunit A-1-like [Populus euphratica]	NFYA9	Nuclear transcription factor Y subunit A-9 OS=Arabidopsis thaliana GN=NFYA9 PE=2 SV=1	3.27	16.58	-1.37	down	0.00	0.00	yes
TRINITY_DN16965_c0_g1	hypothetical protein POPTR_0002s24380g [Populus trichocarpa]	At5g48800	BTB/POZ domain-containing protein At5g48800 OS=Arabidopsis thaliana GN=At5g48800 PE=2 SV=1	1.17	4.85	-1.45	down	0.00	0.00	yes
TRINITY_DN20685_c0_g1	PREDICTED: B3 domain-containing protein Os07g0563300-like isoform X2 [Populus euphratica]	Os07g0563300	B3 domain-containing protein Os07g0563300 OS=Oryza sativa subsp. japonica GN=Os07g0563300 PE=3 SV=2	3.06	12.28	-1.35	down	0.00	0.00	yes
TRINITY_DN23685_c0_g1	hypothetical protein POPTR_0001s39520g [Populus trichocarpa]	BEH4	BES1/BZR1 homolog protein 4 OS=Arabidopsis thaliana GN=BEH4 PE=1 SV=1	4.39	19.16	-1.44	down	0.00	0.00	yes
TRINITY_DN22048_c2_g5	hypothetical protein POPTR_0012s11610g [Populus trichocarpa]	CYCU4-1	Cyclin-U4-1 OS=Arabidopsis thaliana GN=CYCU4-1 PE=1 SV=1	1.63	8.07	-1.67	down	0.00	0.00	yes
TRINITY_DN22415_c0_g8	-	-	-	15.15	47.64	-1.08	down	0.00	0.00	yes
TRINITY_DN23691_c0_g1	hypothetical protein POPTR_0010s12340g [Populus trichocarpa]	WDL7	Protein WVD2-like 7 OS=Arabidopsis thaliana GN=WDL7 PE=2 SV=1	17.68	54.18	-1.11	down	0.00	0.00	yes
TRINITY_DN22426_c0_g1	PREDICTED: uncharacterized protein LOC105127502 [Populus euphratica]	-	-	4.35	15.55	-1.23	down	0.00	0.00	yes
TRINITY_DN24250_c0_g2	PSKR2 [Populus tomentosa]	PSKR2	Phytosulfokine receptor 2 OS=Arabidopsis thaliana GN=PSKR2 PE=2 SV=1	1.32	6.02	-1.56	down	0.00	0.00	yes
TRINITY_DN19057_c5_g1	-	-	-	0.30	2.34	-2.29	down	0.00	0.00	yes
TRINITY_DN21928_c0_g2	DREB2b [Populus hopeiensis]	DREB2C	Dehydration-responsive element-binding protein 2C OS=Arabidopsis thaliana GN=DREB2C PE=2 SV=2	1.91	8.07	-1.46	down	0.00	0.00	yes
TRINITY_DN23547_c0_g5	PREDICTED: zinc-finger homeodomain protein 4-like [Populus euphratica]	ZHD4	Zinc-finger homeodomain protein 4 OS=Arabidopsis thaliana GN=ZHD4 PE=1 SV=1	1.28	8.12	-2.08	down	0.00	0.00	yes
TRINITY_DN18072_c0_g8	PREDICTED: uncharacterized protein LOC105132865 [Populus euphratica]	-	-	1.05	5.32	-1.75	down	0.00	0.00	yes
TRINITY_DN12221_c0_g1	zinc finger family protein [Populus trichocarpa]	ZAT9	Zinc finger protein ZAT9 OS=Arabidopsis thaliana GN=ZAT9 PE=2 SV=1	0.21	2.14	-2.65	down	0.00	0.00	yes
TRINITY_DN20861_c0_g1	hypothetical protein POPTR_0001s12890g [Populus trichocarpa]	NPF6.1	Protein NRT1/ PTR FAMILY 6.1 OS=Arabidopsis thaliana GN=NPF6.1 PE=2 SV=1	1.64	6.79	-1.43	down	0.00	0.00	yes
TRINITY_DN24849_c1_g3	hypothetical protein POPTR_0007s01340g [Populus trichocarpa]	HSL1	Receptor-like protein kinase HSL1 OS=Arabidopsis thaliana GN=HSL1 PE=2 SV=1	6.30	24.29	-1.34	down	0.00	0.00	yes
TRINITY_DN14106_c0_g1	PREDICTED: uncharacterized protein LOC105119910 [Populus euphratica]	-	-	0.67	4.95	-2.25	down	0.00	0.00	yes
TRINITY_DN19259_c0_g1	PREDICTED: DNA polymerase epsilon subunit 2 [Populus euphratica]	DPB2	DNA polymerase epsilon subunit B OS=Arabidopsis thaliana GN=DPB2 PE=1 SV=1	1.72	8.35	-1.63	down	0.00	0.00	yes
TRINITY_DN27476_c0_g1	hypothetical protein POPTR_0008s19220g [Populus trichocarpa]	-	-	3.45	15.07	-1.64	down	0.00	0.00	yes

TRINITY_DN21468_c0_g4	PREDICTED: receptor-like protein 12 [Populus euphratica]	-	-	0.21	2.08	-2.79	down	0.00	0.00	yes
TRINITY_DN20786_c0_g1	hypothetical protein POPTR_0005s28050g [Populus trichocarpa]	-	-	0.96	14.03	-3.25	down	0.00	0.00	yes
TRINITY_DN26649_c0_g1	hypothetical protein POPTR_0010s21950g [Populus trichocarpa]	ITN1	Ankyrin repeat-containing protein ITN1 OS=Arabidopsis thaliana GN=ITN1 PE=1 SV=1	16.52	66.25	-1.13	down	0.00	0.00	yes
TRINITY_DN25490_c0_g1	hypothetical protein POPTR_0008s09810g [Populus trichocarpa]	SPL1	Squamosa promoter-binding-like protein 1 OS=Arabidopsis thaliana GN=SPL1 PE=1 SV=2	8.30	26.99	-1.06	down	0.00	0.00	yes
TRINITY_DN19552_c0_g3	hypothetical protein POPTR_1110s00200g [Populus trichocarpa]	-	-	7.94	30.72	-1.48	down	0.00	0.00	yes
TRINITY_DN17615_c0_g1	PREDICTED: BEL1-like homeodomain protein 9 [Populus euphratica]	BLH8	BEL1-like homeodomain protein 8 OS=Arabidopsis thaliana GN=BLH8 PE=1 SV=1	0.48	3.15	-2.23	down	0.00	0.00	yes
TRINITY_DN24395_c0_g3	PREDICTED: uncharacterized protein LOC105132954 isoform X1 [Populus euphratica]	-	-	0.69	9.76	-3.28	down	0.00	0.00	yes
TRINITY_DN25018_c0_g1	hypothetical protein POPTR_0014s16890g [Populus trichocarpa]	At3g27390	Uncharacterized membrane protein At3g27390 OS=Arabidopsis thaliana GN=At3g27390 PE=1 SV=2	1.39	9.02	-2.44	down	0.00	0.00	yes
TRINITY_DN22311_c0_g1	-	-	-	5.20	21.84	-1.57	down	0.00	0.00	yes
TRINITY_DN22206_c0_g4	PREDICTED: trihelix transcription factor ASIL1 isoform X1 [Populus euphratica]	ASIL2	Trihelix transcription factor ASIL2 OS=Arabidopsis thaliana GN=ASIL2 PE=2 SV=1	3.42	11.47	-1.15	down	0.00	0.00	yes
TRINITY_DN19617_c0_g2	hypothetical protein POPTR_0012s01500g [Populus trichocarpa]	-	-	0.85	5.92	-2.13	down	0.00	0.00	yes
TRINITY_DN19800_c2_g6	hypothetical protein POPTR_0016s01070g [Populus trichocarpa]	-	-	1.89	12.02	-1.87	down	0.00	0.00	yes
TRINITY_DN16453_c0_g1	hypothetical protein POPTR_0009s10470g [Populus trichocarpa]	ERF109	Ethylene-responsive transcription factor ERF109 OS=Arabidopsis thaliana GN=ERF109 PE=1 SV=1	0.95	23.11	-3.92	down	0.00	0.00	yes
TRINITY_DN23538_c0_g2	PREDICTED: uncharacterized protein LOC105129410 [Populus euphratica]	-	-	4.48	15.27	-1.15	down	0.00	0.00	yes
TRINITY_DN14337_c0_g1	DNA topoisomerase family protein [Populus trichocarpa]	TOP2	DNA topoisomerase 2 OS=Arabidopsis thaliana GN=TOP2 PE=2 SV=2	8.27	39.58	-1.63	down	0.00	0.00	yes
TRINITY_DN16031_c0_g1	hypothetical protein POPTR_0004s08350g, partial [Populus trichocarpa]	-	-	0.94	5.31	-1.92	down	0.00	0.00	yes
TRINITY_DN17985_c0_g1	PREDICTED: putative disease resistance protein At4g19050 isoform X1 [Populus euphratica]	At4g19050	Putative disease resistance protein At4g19050 OS=Arabidopsis thaliana GN=At4g19050 PE=3 SV=2	0.90	3.80	-1.47	down	0.00	0.00	yes
TRINITY_DN24816_c2_g2	hypothetical protein POPTR_0017s12550g [Populus trichocarpa]	CNGC2	Cyclic nucleotide-gated ion channel 2 OS=Arabidopsis thaliana GN=CNGC2 PE=1 SV=1	3.51	11.94	-1.14	down	0.00	0.00	yes
TRINITY_DN27689_c0_g3	-	-	-	0.34	3.22	-2.57	down	0.00	0.00	yes
TRINITY_DN10237_c0_g1	hypothetical protein POPTR_0004s07380g [Populus trichocarpa]	-	Universal stress protein in QAH/OAS sulfhydrylase 3'region OS=Thermus aquaticus PE=3 SV=1	0.31	2.96	-2.66	down	0.00	0.00	yes
TRINITY_DN19567_c0_g2	PREDICTED: putative F-box protein PP2-B12 [Populus euphratica]	At2g02240	F-box protein At2g02240 OS=Arabidopsis thaliana GN=At2g02240 PE=2 SV=1	1.06	6.23	-2.55	down	0.00	0.00	yes
TRINITY_DN16976_c0_g4	DNA-directed DNA polymerase epsilon catalytic subunit family protein [Populus trichocarpa]	POL2A	DNA polymerase epsilon catalytic subunit A OS=Arabidopsis thaliana GN=POL2A PE=1 SV=1	2.96	11.76	-1.35	down	0.00	0.00	yes
TRINITY_DN22179_c0_g2	PREDICTED: methyl-CpG-binding domain-containing protein 2-like isoform X2 [Populus euphratica]	MBD2	Methyl-CpG-binding domain-containing protein 2 OS=Arabidopsis thaliana GN=MBD2 PE=1 SV=1	0.88	4.90	-1.87	down	0.00	0.00	yes
TRINITY_DN24150_c3_g2	hypothetical protein POPTR_0005s27030g [Populus trichocarpa]	-	-	1.68	11.11	-2.32	down	0.00	0.00	yes
TRINITY_DN18535_c0_g1	hypothetical protein POPTR_2131s00200g [Populus trichocarpa]	-	-	14.78	55.94	-1.39	down	0.00	0.00	yes
TRINITY_DN22407_c0_g1	PREDICTED: transcription factor bHLH110-like isoform X2 [Populus euphratica]	BHLH110	Transcription factor bHLH110 OS=Arabidopsis thaliana GN=BHLH110 PE=2 SV=2	1.69	14.48	-1.76	down	0.00	0.00	yes
TRINITY_DN20084_c1_g2	phosphatase 2C family protein [Populus trichocarpa]	At4g28400	Probable protein phosphatase 2C 58 OS=Arabidopsis thaliana GN=At4g28400 PE=2 SV=1	2.74	35.19	-3.01	down	0.00	0.00	yes
TRINITY_DN24001_c0_g1	PREDICTED: wee1-like protein kinase [Populus euphratica]	WEE1	Wee1-like protein kinase OS=Arabidopsis thaliana GN=WEE1 PE=1 SV=1	1.76	10.43	-1.59	down	0.00	0.00	yes
TRINITY_DN20113_c0_g1	chalcone synthase [Populus alba]	CHS	Chalcone synthase OS=Betula pendula GN=CHS PE=2 SV=2	95.98	585.75	-1.45	down	0.00	0.00	yes

TRINITY_DN25319_c0_g1	PREDICTED: probable WRKY transcription factor 3 [Populus euphratica]	WRKY4	Probable WRKY transcription factor 4 OS=Arabidopsis thaliana GN=WRKY4 PE=1 SV=2	7.32	30.17	-1.11	down	0.00	0.00	yes
TRINITY_DN21576_c0_g1	transcription factor EREBP-like family protein [Populus trichocarpa]	RAP2-2	Ethylene-responsive transcription factor RAP2-2 OS=Arabidopsis thaliana GN=RAP2-2 PE=1 SV=2	16.49	50.53	-1.06	down	0.00	0.00	yes
TRINITY_DN28972_c0_g1	hypothetical protein POPTR_0010s07210g [Populus trichocarpa]	-	Probable glutathione S-transferase OS=Nicotiana tabacum PE=2 SV=1	0.19	1.69	-2.49	down	0.00	0.00	yes
TRINITY_DN20214_c0_g1	hypothetical protein POPTR_0002s25190g [Populus trichocarpa]	hmces	Embryonic stem cell-specific 5-hydroxymethylcytosine-binding protein OS=Xenopus tropicalis GN=hmces PE=2 SV=1	1.39	6.57	-1.62	down	0.00	0.00	yes
TRINITY_DN24182_c0_g1	PREDICTED: cysteine-rich receptor-like protein kinase 42 [Populus euphratica]	CRK42	Cysteine-rich receptor-like protein kinase 42 OS=Arabidopsis thaliana GN=CRK42 PE=2 SV=1	1.24	7.79	-2.00	down	0.00	0.00	yes
TRINITY_DN24995_c1_g4	PREDICTED: peroxidase 64-like [Populus euphratica]	PER64	Peroxidase 64 OS=Arabidopsis thaliana GN=PER64 PE=1 SV=1	7.03	18.53	-1.46	down	0.00	0.00	yes
TRINITY_DN27500_c0_g2	-	-	-	1.93	14.50	-2.51	down	0.00	0.00	yes
TRINITY_DN18290_c0_g5	octicosapeptide/Phox/Bem1p domain-containing family protein [Populus trichocarpa]	-	-	3.38	14.20	-1.44	down	0.00	0.00	yes
TRINITY_DN27053_c0_g1	PREDICTED: LRR receptor-like serine/threonine-protein kinase FLS2 isoform X2 [Populus euphratica]	-	-	0.84	5.09	-1.64	down	0.00	0.00	yes
TRINITY_DN20685_c0_g2	hypothetical protein POPTR_0004s03530g [Populus trichocarpa]	Os07g0563300	B3 domain-containing protein Os07g0563300 OS=Oryza sativa subsp. japonica GN=Os07g0563300 PE=3 SV=2	5.99	19.10	-1.06	down	0.00	0.00	yes
TRINITY_DN14463_c0_g1	unknown [Populus trichocarpa]	-	-	6.58	72.50	-2.87	down	0.00	0.00	yes
TRINITY_DN25664_c0_g4	PREDICTED: inactive leucine-rich repeat receptor-like serine/threonine-protein kinase At1g60630 [Populus euphratica]	At1g60630	Inactive leucine-rich repeat receptor-like serine/threonine-protein kinase At1g60630 OS=Arabidopsis thaliana GN=At1g60630 PE=2 SV=1	0.24	2.80	-2.90	down	0.00	0.00	yes
TRINITY_DN18752_c0_g3	-	-	-	0.56	3.52	-2.01	down	0.00	0.00	yes
TRINITY_DN14812_c0_g2	PREDICTED: calcium-binding protein PBP1-like [Populus euphratica]	PBP1	Calcium-binding protein PBP1 OS=Arabidopsis thaliana GN=PBP1 PE=1 SV=1	1.20	14.58	-2.94	down	0.00	0.00	yes
TRINITY_DN19506_c0_g1	PREDICTED: DNA repair endonuclease UVH1 [Populus euphratica]	UVH1	DNA repair endonuclease UVH1 OS=Arabidopsis thaliana GN=UVH1 PE=1 SV=2	2.88	9.43	-1.09	down	0.00	0.00	yes
TRINITY_DN24663_c0_g4	-	-	-	0.23	2.55	-2.75	down	0.00	0.00	yes
TRINITY_DN20448_c0_g1	PREDICTED: putative disease resistance protein At1g50180 [Populus euphratica]	At1g50180	Putative disease resistance protein At1g50180 OS=Arabidopsis thaliana GN=At1g50180 PE=3 SV=2	1.25	5.40	-1.50	down	0.00	0.00	yes
TRINITY_DN18769_c0_g2	hypothetical protein POPTR_0005s27980g [Populus trichocarpa]	CP12-3	Calvin cycle protein CP12-3, chloroplastic OS=Arabidopsis thaliana GN=CP12-3 PE=1 SV=1	4.02	14.87	-1.29	down	0.00	0.00	yes
TRINITY_DN20265_c0_g2	PREDICTED: cullin-1-like [Populus euphratica]	At1g43140	Putative cullin-like protein 1 OS=Arabidopsis thaliana GN=At1g43140 PE=3 SV=1	1.83	8.99	-1.84	down	0.00	0.00	yes
TRINITY_DN22282_c1_g6	kelch repeat-containing F-box family protein [Populus trichocarpa]	AFR	F-box protein AFR OS=Arabidopsis thaliana GN=AFR PE=1 SV=2	9.16	28.81	-1.04	down	0.00	0.00	yes
TRINITY_DN15783_c0_g3	hypothetical protein POPTR_0002s02190g [Populus trichocarpa]	At1g18250	Thaumatin-like protein OS=Arabidopsis thaliana GN=At1g18250 PE=2 SV=2	0.24	3.16	-3.08	down	0.00	0.00	yes
TRINITY_DN17150_c1_g4	hypothetical protein POPTR_0013s11330g [Populus trichocarpa]	ZHD1	Zinc-finger homeodomain protein 1 OS=Oryza sativa subsp. indica GN=ZHD1 PE=3 SV=1	15.14	51.27	-1.14	down	0.00	0.00	yes
TRINITY_DN25097_c0_g3	hypothetical protein POPTR_0018s08450g [Populus trichocarpa]	FAP2	Fatty-acid-binding protein 2 OS=Arabidopsis thaliana GN=FAP2 PE=2 SV=2	0.70	5.87	-1.98	down	0.00	0.00	yes
TRINITY_DN27816_c1_g1	PREDICTED: uncharacterized protein LOC105109000 isoform X2 [Populus euphratica]	-	-	24.55	103.12	-1.58	down	0.00	0.00	yes
TRINITY_DN23943_c0_g1	hypothetical protein POPTR_0006s25460g [Populus trichocarpa]	-	-	16.40	52.65	-1.08	down	0.00	0.00	yes
TRINITY_DN20044_c0_g1	forkhead-associated domain-containing family protein [Populus trichocarpa]	PS1	FHA domain-containing protein PS1 OS=Arabidopsis thaliana GN=PS1 PE=2 SV=1	5.37	18.47	-1.22	down	0.00	0.00	yes
TRINITY_DN25324_c1_g2	hypothetical protein POPTR_0001s44090g [Populus trichocarpa]	CIGR1	Chitin-inducible gibberellin-responsive protein 1 OS=Oryza sativa subsp. japonica GN=CIGR1 PE=2 SV=1	5.91	44.70	-2.31	down	0.00	0.00	yes
TRINITY_DN23089_c0_g2	hypothetical protein POPTR_0014s11100g [Populus trichocarpa]	-	-	0.74	8.07	-2.43	down	0.00	0.00	yes
TRINITY_DN23228_c1_g4	PREDICTED: protein ROOT HAIR DEFECTIVE 3 homolog 2-like isoform X4 [Populus euphratica]	Os12g0604600	Protein ROOT HAIR DEFECTIVE 3 homolog 1 OS=Oryza sativa subsp. japonica GN=Os12g0604600 PE=2 SV=1	0.21	2.05	-2.65	down	0.00	0.00	yes
TRINITY_DN25139_c1_g1	zinc finger family protein [Populus trichocarpa]	MIEL1	E3 ubiquitin-protein ligase MIEL1 OS=Arabidopsis thaliana GN=MIEL1 PE=1 SV=1	14.32	44.46	-1.03	down	0.00	0.00	yes

TRINITY_DN22496_c0_g2	GDSL-motif lipase/hydrolase family protein [Populus trichocarpa]	CPRD49	GDSL esterase/lipase CPRD49 OS=Arabidopsis thaliana GN=CPRD49 PE=2 SV=1	7.04	23.61	-1.21	down	0.00	0.00	yes
TRINITY_DN17241_c0_g1	DNA polymerase delta subunit 4 family protein [Populus trichocarpa]	-	-	4.03	15.90	-1.38	down	0.00	0.00	yes
TRINITY_DN27636_c0_g2	-	-	-	0.54	8.03	-3.31	down	0.00	0.00	yes
TRINITY_DN24484_c0_g1	PREDICTED: uncharacterized protein LOC105140737 [Populus euphratica]	-	-	1.24	5.40	-1.45	down	0.00	0.00	yes
TRINITY_DN22302_c0_g3	hypothetical protein POPTR_0002s00970g [Populus trichocarpa]	rtel1	Regulator of telomere elongation helicase 1 OS=Danio rerio GN=rtel1 PE=3 SV=1	0.20	2.55	-3.03	down	0.00	0.00	yes
TRINITY_DN20512_c0_g3	putative subtilisin precursor family protein [Populus trichocarpa]	CRSP	CO(2)-response secreted protease OS=Arabidopsis thaliana GN=CRSP PE=2 SV=1	0.26	4.33	-3.34	down	0.00	0.00	yes
TRINITY_DN27037_c0_g2	PREDICTED: probable ubiquitin-like-specific protease 2B isoform X1 [Populus euphratica]	ULP2B	Probable ubiquitin-like-specific protease 2B OS=Arabidopsis thaliana GN=ULP2B PE=1 SV=3	3.99	12.63	-1.08	down	0.00	0.00	yes
TRINITY_DN21778_c0_g1	hypothetical protein POPTR_0009s02820g [Populus trichocarpa]	-	-	4.83	16.56	-1.15	down	0.00	0.00	yes
TRINITY_DN24603_c0_g3	hypothetical protein POPTR_0463s00220g, partial [Populus trichocarpa]	LRK10L-1.1	LEAF RUST 10 DISEASE-RESISTANCE LOCUS RECEPTOR-LIKE PROTEIN KINASE-like 1.1 OS=Arabidopsis thaliana GN=LRK10L-1.1 PE=2 SV=1	0.33	2.90	-2.51	down	0.00	0.00	yes
TRINITY_DN22566_c0_g1	SNF4b family protein [Populus trichocarpa]	CBSX5	CBS domain-containing protein CBSX5 OS=Arabidopsis thaliana GN=CBSX5 PE=2 SV=2	0.69	6.38	-2.13	down	0.00	0.00	yes
TRINITY_DN14356_c0_g1	PREDICTED: uncharacterized protein LOC105126469 [Populus euphratica]	SMR13	Cyclin-dependent protein kinase inhibitor SMR13 OS=Arabidopsis thaliana GN=SMR13 PE=4 SV=1	0.59	3.59	-1.99	down	0.00	0.00	yes
TRINITY_DN22977_c0_g1	hypothetical protein POPTR_0017s10860g [Populus trichocarpa]	ATL46	RING-H2 finger protein ATL46 OS=Arabidopsis thaliana GN=ATL46 PE=2 SV=1	3.45	14.70	-1.20	down	0.00	0.00	yes
TRINITY_DN19735_c0_g1	hypothetical protein POPTR_0004s24330g [Populus trichocarpa]	DCP5-L	Decapping 5-like protein OS=Arabidopsis thaliana GN=DCP5-L PE=2 SV=1	1.08	5.22	-1.61	down	0.00	0.00	yes
TRINITY_DN9459_c0_g1	-	-	-	0.26	2.23	-2.46	down	0.00	0.00	yes
TRINITY_DN21559_c0_g1	-	-	-	2.11	11.15	-1.78	down	0.00	0.00	yes
TRINITY_DN23250_c0_g6	Leucine-rich repeat receptor protein kinase EXS precursor [Populus trichocarpa]	MSP1	Leucine-rich repeat receptor protein kinase MSP1 OS=Oryza sativa subsp. japonica GN=MSP1 PE=1 SV=1	0.36	3.06	-2.43	down	0.00	0.00	yes
TRINITY_DN24461_c0_g3	PREDICTED: cysteine-rich receptor-like protein kinase 10 [Populus euphratica]	CRK10	Cysteine-rich receptor-like protein kinase 10 OS=Arabidopsis thaliana GN=CRK10 PE=1 SV=3	2.26	9.66	-1.53	down	0.00	0.00	yes
TRINITY_DN16570_c0_g1	hypothetical protein POPTR_0014s03230g [Populus trichocarpa]	CXE2	Probable carboxylesterase 2 OS=Arabidopsis thaliana GN=CXE2 PE=2 SV=1	1.04	4.89	-1.64	down	0.00	0.00	yes
TRINITY_DN21286_c0_g1	hypothetical protein POPTR_0018s12660g [Populus trichocarpa]	-	-	0.75	5.02	-2.00	down	0.00	0.00	yes
TRINITY_DN26058_c0_g1	PREDICTED: uncharacterized protein At1g51745-like [Populus euphratica]	At1g51745	Uncharacterized protein At1g51745 OS=Arabidopsis thaliana GN=At1g51745 PE=2 SV=2	3.91	12.37	-1.06	down	0.00	0.00	yes
TRINITY_DN17729_c0_g1	PREDICTED: uncharacterized protein LOC105120396 [Populus euphratica]	-	-	8.40	26.03	-1.13	down	0.00	0.00	yes
TRINITY_DN19076_c0_g1	hypothetical protein POPTR_0013s00470g [Populus trichocarpa]	-	-	8.48	26.87	-1.19	down	0.00	0.00	yes
TRINITY_DN15596_c0_g1	hypothetical protein POPTR_0009s13300g [Populus trichocarpa]	SPAC869.01	Putative amidase C869.01 OS=Schizosaccharomyces pombe (strain 972 / ATCC 24843) GN=SPAC869.01 PE=3 SV=1	0.46	2.84	-2.20	down	0.00	0.00	yes
TRINITY_DN20289_c0_g1	hypothetical protein POPTR_0002s05760g [Populus trichocarpa]	tmem56-b	Transmembrane protein 56-B OS=Xenopus laevis GN=tmem56-b PE=2 SV=1	3.03	13.57	-1.50	down	0.00	0.00	yes
TRINITY_DN25054_c0_g3	cyclin-dependent kinase B [Populus tomentosa]	CDKB2-1	Cyclin-dependent kinase B2-1 OS=Arabidopsis thaliana GN=CDKB2-1 PE=1 SV=2	76.90	251.75	-1.18	down	0.00	0.00	yes
TRINITY_DN27186_c0_g2	PREDICTED: uncharacterized protein LOC105141197 [Populus euphratica]	-	-	5.30	18.55	-1.34	down	0.00	0.00	yes
TRINITY_DN19463_c0_g2	small nuclear ribonucleoprotein [Populus trichocarpa]	LSM8	Sm-like protein LSM8 OS=Arabidopsis thaliana GN=LSM8 PE=1 SV=1	0.51	3.86	-2.40	down	0.00	0.00	yes
TRINITY_DN16210_c0_g1	disease resistance-responsive family protein [Populus trichocarpa]	DIR20	Dirigent protein 20 OS=Arabidopsis thaliana GN=DIR20 PE=2 SV=1	2.58	11.47	-1.55	down	0.00	0.00	yes
TRINITY_DN27772_c1_g2	PREDICTED: protein OBERON 4-like [Populus euphratica]	OBE4	Protein OBERON 4 OS=Arabidopsis thaliana GN=OBE4 PE=1 SV=2	14.12	44.66	-1.27	down	0.00	0.00	yes

TRINITY_DN26009_c1_g2	hypothetical protein POPTR_0006s23660g [Populus trichocarpa]	XI-I	Myosin-15 OS=Arabidopsis thaliana GN=XI-I PE=1 SV=1	5.05	17.76	-1.05	down	0.00	0.00	yes
TRINITY_DN16406_c0_g1	kinase family protein [Populus trichocarpa]	mkkA	Mitogen-activated protein kinase kinase kinase A OS=Dictyostelium discoideum GN=mkkA PE=1 SV=2	0.36	2.95	-2.36	down	0.00	0.00	yes
TRINITY_DN19843_c0_g1	PREDICTED: serine carboxypeptidase-like 45 [Populus euphratica]	SCPL45	Serine carboxypeptidase-like 45 OS=Arabidopsis thaliana GN=SCPL45 PE=2 SV=1	4.18	14.76	-1.21	down	0.00	0.00	yes
TRINITY_DN25283_c0_g1	heat shock transcription factor A5a [Populus simonii]	HSFA5	Heat stress transcription factor A-5 OS=Arabidopsis thaliana GN=HSFA5 PE=2 SV=1	4.76	15.28	-1.07	down	0.00	0.00	yes
TRINITY_DN14064_c0_g1	-	-	-	0.32	2.96	-2.59	down	0.00	0.00	yes
TRINITY_DN17350_c0_g1	kinase family protein [Populus trichocarpa]	PHOT1	Phototropin-1 OS=Arabidopsis thaliana GN=PHOT1 PE=1 SV=1	15.80	52.30	-1.13	down	0.00	0.00	yes
TRINITY_DN23034_c3_g4	integral membrane family protein [Populus trichocarpa]	At5g47470	WAT1-related protein At5g47470 OS=Arabidopsis thaliana GN=At5g47470 PE=3 SV=1	4.03	18.36	-1.57	down	0.00	0.00	yes
TRINITY_DN16548_c0_g1	PREDICTED: probable LRR receptor-like serine/threonine-protein kinase At1g14390 isoform X1 [Populus euphratica]	At1g14390	Probable LRR receptor-like serine/threonine-protein kinase At1g14390 OS=Arabidopsis thaliana GN=At1g14390 PE=2 SV=1	0.72	4.13	-1.98	down	0.00	0.00	yes
TRINITY_DN18212_c1_g1	aldo/keto reductase family protein [Populus trichocarpa]	At1g06690	Uncharacterized oxidoreductase At1g06690, chloroplastic OS=Arabidopsis thaliana GN=At1g06690 PE=1 SV=1	63.19	221.55	-1.15	down	0.00	0.00	yes
TRINITY_DN21920_c1_g7	NAC domain-containing protein 90 [Populus trichocarpa]	NAC090	NAC domain-containing protein 90 OS=Arabidopsis thaliana GN=NAC090 PE=2 SV=1	0.76	8.06	-2.81	down	0.00	0.00	yes
TRINITY_DN25105_c0_g1	PREDICTED: glucose-6-phosphate/phosphate translocator 1, chloroplastic-like [Populus euphratica]	GPT1	Glucose-6-phosphate/phosphate translocator 1, chloroplastic OS=Arabidopsis thaliana GN=GPT1 PE=2 SV=1	4.62	16.65	-1.23	down	0.00	0.00	yes
TRINITY_DN15275_c0_g2	hypothetical protein POPTR_0006s02680g [Populus trichocarpa]	-	-	3.17	24.88	-2.37	down	0.00	0.00	yes
TRINITY_DN21609_c4_g1	PREDICTED: putative disease resistance RPP13-like protein 1 [Populus euphratica]	-	-	22.88	67.15	-1.00	down	0.00	0.00	yes
TRINITY_DN18792_c1_g1	-	-	-	7.26	31.08	-1.48	down	0.00	0.00	yes
TRINITY_DN26288_c0_g5	PREDICTED: LOW QUALITY PROTEIN: non-lysosomal glucosylceramidase-like [Populus euphratica]	CG33090	Non-lysosomal glucosylceramidase OS=Drosophila melanogaster GN=CG33090 PE=1 SV=1	0.29	2.74	-2.57	down	0.00	0.00	yes
TRINITY_DN27819_c0_g2	hypothetical protein POPTR_0008s02610g [Populus trichocarpa]	DME	Transcriptional activator DEMETER OS=Arabidopsis thaliana GN=DME PE=1 SV=2	6.83	23.35	-1.19	down	0.00	0.00	yes
TRINITY_DN23867_c0_g5	PREDICTED: serine/threonine-protein kinase-like protein ACR4 [Populus euphratica]	ACR4	Serine/threonine-protein kinase-like protein ACR4 OS=Arabidopsis thaliana GN=ACR4 PE=1 SV=1	1.09	8.19	-2.28	down	0.00	0.00	yes
TRINITY_DN19781_c0_g2	ovate family protein [Populus trichocarpa]	OFP1	Transcription repressor OFP1 OS=Arabidopsis thaliana GN=OFP1 PE=1 SV=1	2.88	11.53	-1.44	down	0.00	0.00	yes
TRINITY_DN24651_c0_g1	S-adenosyl-methionine-sterol-C- methyltransferase family protein [Populus trichocarpa]	SMT1	Cycloartenol-C-24-methyltransferase OS=Arabidopsis thaliana GN=SMT1 PE=1 SV=1	55.88	208.38	-1.09	down	0.00	0.00	yes
TRINITY_DN21703_c0_g2	hypothetical protein POPTR_0019s13480g [Populus trichocarpa]	-	-	2.93	16.78	-2.07	down	0.00	0.00	yes
TRINITY_DN21750_c0_g1	hypothetical protein POPTR_0012s01740g [Populus trichocarpa]	Os05g0572700	Probable protein phosphatase 2C 51 OS=Oryza sativa subsp. japonica GN=Os05g0572700 PE=2 SV=1	3.51	27.33	-2.52	down	0.00	0.00	yes
TRINITY_DN16013_c0_g2	hypothetical protein POPTR_0007s14060g [Populus trichocarpa]	SAUR50	Auxin-responsive protein SAUR50 OS=Arabidopsis thaliana GN=SAUR50 PE=1 SV=1	0.56	4.44	-2.33	down	0.00	0.00	yes
TRINITY_DN17792_c0_g1	PREDICTED: protein SRG1-like [Populus euphratica]	SRG1	Protein SRG1 OS=Arabidopsis thaliana GN=SRG1 PE=2 SV=1	1.35	7.32	-1.77	down	0.00	0.00	yes
TRINITY_DN16858_c0_g2	PREDICTED: uncharacterized protein LOC105117470 isoform X1 [Populus euphratica]	-	-	0.74	4.16	-1.89	down	0.00	0.00	yes
TRINITY_DN25030_c0_g1	PREDICTED: uncharacterized protein LOC105141850 [Populus euphratica]	-	-	3.29	12.91	-1.48	down	0.00	0.00	yes
TRINITY_DN20770_c0_g1	PREDICTED: histidine-containing phosphotransfer protein 1-like isoform X1 [Populus euphratica]	AHP1	Histidine-containing phosphotransfer protein 1 OS=Arabidopsis thaliana GN=AHP1 PE=1 SV=1	11.03	36.32	-1.05	down	0.00	0.00	yes
TRINITY_DN24730_c0_g1	hypothetical protein B456_013G264400, partial [Gossypium raimondii]	-	Histone H4 variant TH011 OS=Triticum aestivum PE=3 SV=2	187.99	606.76	-1.07	down	0.00	0.00	yes
TRINITY_DN19413_c0_g2	PREDICTED: receptor-like serine/threonine-protein kinase ALE2 isoform X2 [Populus euphratica]	ALE2	Receptor-like serine/threonine-protein kinase ALE2 OS=Arabidopsis thaliana GN=ALE2 PE=1 SV=1	1.76	5.92	-1.15	down	0.00	0.00	yes

TRINITY_DN24622_c0_g4	PREDICTED: type I inositol 1,4,5-trisphosphate 5-phosphatase CVP2-like isoform X1 [Populus euphratica]	IP5P8	Type I inositol polyphosphate 5-phosphatase 8 OS=Arabidopsis thaliana GN=IP5P8 PE=2 SV=1	0.22	1.92	-2.50	down	0.00	0.00	yes
TRINITY_DN28064_c0_g1	hypothetical protein POPTR_0009s02650g [Populus trichocarpa]	HSP22	Heat shock 22 kDa protein, mitochondrial OS=Pisum sativum GN=HSP22 PE=2 SV=1	0.22	1.81	-2.47	down	0.00	0.00	yes
TRINITY_DN19626_c0_g5	hypothetical protein POPTR_0016s13490g [Populus trichocarpa]	-	-	30.16	94.54	-1.05	down	0.00	0.00	yes
TRINITY_DN16890_c0_g1	hypothetical protein POPTR_0004s15530g [Populus trichocarpa]	-	-	4.79	23.01	-1.62	down	0.00	0.00	yes
TRINITY_DN15334_c0_g1	-	-	-	6.57	35.33	-1.81	down	0.00	0.00	yes
TRINITY_DN20370_c1_g4	PREDICTED: uncharacterized protein LOC105138572 isoform X1 [Populus euphratica]	-	-	0.72	3.98	-1.87	down	0.00	0.00	yes
TRINITY_DN16958_c0_g1	hypothetical protein POPTR_0004s18150g [Populus trichocarpa]	BRG3	Probable BOI-related E3 ubiquitin-protein ligase 3 OS=Arabidopsis thaliana GN=BRG3 PE=1 SV=1	0.49	4.41	-2.91	down	0.00	0.00	yes
TRINITY_DN20896_c1_g2	PREDICTED: protein SHOOT GRAVITROPISM 5 [Populus euphratica]	IDD14	Protein indeterminate-domain 14 OS=Arabidopsis thaliana GN=IDD14 PE=1 SV=1	4.99	19.09	-1.22	down	0.00	0.00	yes
TRINITY_DN19804_c1_g1	PREDICTED: zinc finger protein ZAT9-like [Populus euphratica]	ZAT9	Zinc finger protein ZAT9 OS=Arabidopsis thaliana GN=ZAT9 PE=2 SV=1	0.78	4.85	-1.97	down	0.00	0.00	yes
TRINITY_DN23469_c0_g1	PREDICTED: uncharacterized protein LOC105115352 [Populus euphratica]	-	-	4.26	13.85	-1.15	down	0.00	0.00	yes
TRINITY_DN16738_c1_g1	hypothetical protein POPTR_0009s08270g [Populus trichocarpa]	-	-	2.52	10.24	-1.41	down	0.00	0.00	yes
TRINITY_DN21206_c0_g1	hypothetical protein POPTR_0001s41900g [Populus trichocarpa]	DGK5	Diacylglycerol kinase 5 OS=Arabidopsis thaliana GN=DGK5 PE=2 SV=1	0.55	5.54	-2.64	down	0.00	0.00	yes
TRINITY_DN14637_c0_g2	-	-	-	0.37	3.65	-2.57	down	0.00	0.00	yes
TRINITY_DN23979_c0_g1	hypothetical protein POPTR_0010s05700g [Populus trichocarpa]	-	-	6.36	26.34	-1.43	down	0.00	0.00	yes
TRINITY_DN25097_c0_g2	hypothetical protein POPTR_0006s23620g [Populus trichocarpa]	FAP2	Fatty-acid-binding protein 2 OS=Arabidopsis thaliana GN=FAP2 PE=2 SV=2	1.14	5.59	-1.70	down	0.00	0.00	yes
TRINITY_DN26107_c0_g1	hydrolase family protein [Populus trichocarpa]	-	-	14.06	43.20	-1.02	down	0.00	0.00	yes
TRINITY_DN21258_c0_g1	alliinase family protein [Populus trichocarpa]	TAR4	Tryptophan aminotransferase-related protein 4 OS=Arabidopsis thaliana GN=TAR4 PE=2 SV=2	7.01	23.79	-1.04	down	0.00	0.00	yes
TRINITY_DN23974_c0_g4	hypothetical protein POPTR_0014s01950g [Populus trichocarpa]	-	3-oxo-Delta(4,5)-steroid 5-beta-reductase OS=Digitalis lanata PE=1 SV=1	1.88	9.00	-1.42	down	0.00	0.00	yes
TRINITY_DN24812_c0_g4	hypothetical protein POPTR_0013s00520g, partial [Populus trichocarpa]	HASPIN	Serine/threonine-protein kinase haspin homolog OS=Arabidopsis thaliana GN=HASPIN PE=1 SV=1	0.22	1.60	-2.19	down	0.00	0.00	yes
TRINITY_DN25000_c0_g7	-	-	-	32.32	87.48	-1.01	down	0.00	0.00	yes
TRINITY_DN24917_c0_g3	hypothetical protein POPTR_0016s14930g [Populus trichocarpa]	-	Serine/threonine-protein phosphatase PP1 isozyme 1 OS=Acetabularia peniculus PE=3 SV=1	9.73	23.49	-1.08	down	0.00	0.00	yes
TRINITY_DN26668_c1_g1	PREDICTED: lysine-specific demethylase JMJ25-like isoform X1 [Populus euphratica]	JMJ25	Lysine-specific demethylase JMJ25 OS=Arabidopsis thaliana GN=JMJ25 PE=1 SV=1	11.75	51.18	-1.04	down	0.00	0.00	yes
TRINITY_DN22396_c1_g4	hypothetical protein POPTR_0019s00705g [Populus trichocarpa]	-	-	0.53	4.59	-2.45	down	0.00	0.00	yes
TRINITY_DN22958_c1_g2	PREDICTED: transcription factor BIM2-like isoform X1 [Populus euphratica]	BIM2	Transcription factor BIM2 OS=Arabidopsis thaliana GN=BIM2 PE=1 SV=1	8.99	30.73	-1.07	down	0.00	0.00	yes
TRINITY_DN16063_c0_g4	S-locus lectin protein kinase [Populus trichocarpa]	B120	G-type lectin S-receptor-like serine/threonine-protein kinase B120 OS=Arabidopsis thaliana GN=B120 PE=2 SV=1	0.36	2.45	-2.15	down	0.00	0.00	yes
TRINITY_DN25943_c0_g3	PREDICTED: uncharacterized protein LOC105134064 [Populus euphratica]	-	-	13.63	37.80	-1.07	down	0.00	0.00	yes
TRINITY_DN18507_c0_g1	PREDICTED: uncharacterized protein LOC105137049 [Populus euphratica]	-	-	2.86	9.83	-1.19	down	0.00	0.00	yes
TRINITY_DN16442_c0_g1	HISTONE H1-3 family protein [Populus trichocarpa]	-	Histone H1 OS=Solanum pennellii PE=2 SV=1	92.28	383.16	-1.49	down	0.00	0.00	yes
TRINITY_DN20745_c0_g1	hypothetical protein POPTR_0007s03160g [Populus trichocarpa]	nuf2	Kinetochore protein nuf2 OS=Schizosaccharomyces pombe (strain 972 / ATCC 24843) GN=nuf2 PE=1 SV=1	6.22	19.55	-1.03	down	0.00	0.00	yes
TRINITY_DN15026_c0_g1	hypothetical protein POPTR_0013s14140g, partial [Populus trichocarpa]	-	-	0.12	2.73	-3.79	down	0.00	0.00	yes

TRINITY_DN18763_c0_g2	cytochrome P450 family protein [Populus trichocarpa]	CYP81E8	Cytochrome P450 81E8 OS=Medicago truncatula GN=CYP81E8 PE=2 SV=1	0.40	2.47	-2.06	down	0.00	0.00	yes
TRINITY_DN23666_c1_g1	PREDICTED: uncharacterized protein LOC105131940 [Populus euphratica]	CBP60E	Calmodulin-binding protein 60 E OS=Arabidopsis thaliana GN=CBP60E PE=2 SV=1	4.15	21.47	-1.79	down	0.00	0.00	yes
TRINITY_DN26839_c2_g2	plasma membrane intrinsic protein 2;1 [Populus tremula x Populus alba]	PIP2-7	Aquaporin PIP2-7 OS=Arabidopsis thaliana GN=PIP2-7 PE=1 SV=2	141.52	493.68	-1.20	down	0.00	0.00	yes
TRINITY_DN25952_c0_g1	PREDICTED: protein CPR-5-like [Populus euphratica]	-	-	2.57	8.17	-1.26	down	0.00	0.00	yes
TRINITY_DN27701_c1_g2	hypothetical protein POPTR_0007s04450g [Populus trichocarpa]	CHR24	Protein CHROMATIN REMODELING 24 OS=Arabidopsis thaliana GN=CHR24 PE=2 SV=1	6.83	25.81	-1.32	down	0.00	0.00	yes
TRINITY_DN25078_c0_g1	PREDICTED: probable aldo-keto reductase 1 isoform X1 [Populus euphratica]	AKR1	Probable aldo-keto reductase 1 OS=Glycine max GN=AKR1 PE=2 SV=1	17.67	45.66	-1.04	down	0.00	0.00	yes
TRINITY_DN25803_c0_g1	leucine-rich repeat transmembrane protein kinase [Populus trichocarpa]	ZAR1	Receptor protein kinase-like protein ZAR1 OS=Arabidopsis thaliana GN=ZAR1 PE=1 SV=1	19.96	65.39	-1.01	down	0.00	0.00	yes
TRINITY_DN18125_c0_g1	phospholipase C [Populus tomentosa]	PLC4	Phosphoinositide phospholipase C 4 OS=Arabidopsis thaliana GN=PLC4 PE=2 SV=2	0.58	3.28	-1.88	down	0.00	0.00	yes
TRINITY_DN22972_c0_g1	hypothetical protein POPTR_0010s23010g [Populus trichocarpa]	GATA8	GATA transcription factor 8 OS=Arabidopsis thaliana GN=GATA8 PE=2 SV=1	35.78	108.31	-1.06	down	0.00	0.00	yes
TRINITY_DN27107_c0_g2	PREDICTED: uncharacterized protein LOC105129360 [Populus euphratica]	-	-	4.80	15.63	-1.54	down	0.00	0.00	yes
TRINITY_DN21470_c1_g1	-	-	-	0.26	2.79	-2.86	down	0.00	0.00	yes
TRINITY_DN15199_c0_g2	hypothetical protein POPTR_0017s08900g [Populus trichocarpa]	-	-	0.40	2.83	-2.19	down	0.00	0.00	yes
TRINITY_DN22708_c0_g2	hypothetical protein POPTR_0017s11440g [Populus trichocarpa]	PIN8	Auxin efflux carrier component 8 OS=Arabidopsis thaliana GN=PIN8 PE=2 SV=1	0.34	2.10	-2.00	down	0.00	0.00	yes
TRINITY_DN24665_c0_g1	PREDICTED: uncharacterized protein At5g41620-like [Populus euphratica]	At5g41620	Uncharacterized protein At5g41620 OS=Arabidopsis thaliana GN=At5g41620 PE=2 SV=2	4.31	13.24	-1.01	down	0.00	0.00	yes
TRINITY_DN20071_c2_g6	hypothetical protein POPTR_0012s01020g [Populus trichocarpa]	MAKR6	Probable membrane-associated kinase regulator 6 OS=Arabidopsis thaliana GN=MAKR6 PE=2 SV=1	3.67	15.74	-1.54	down	0.00	0.00	yes
TRINITY_DN27127_c0_g1	putative phosphatidylinositol-4-phosphate 5-kinase mRNA family protein [Populus trichocarpa]	PIP5K1	Phosphatidylinositol 4-phosphate 5-kinase 1 OS=Arabidopsis thaliana GN=PIP5K1 PE=1 SV=1	6.64	21.31	-1.04	down	0.00	0.00	yes
TRINITY_DN25927_c2_g3	PREDICTED: RNA-dependent RNA polymerase 1-like isoform X1 [Populus euphratica]	RDR1	RNA-dependent RNA polymerase 1 OS=Arabidopsis thaliana GN=RDR1 PE=2 SV=1	0.21	2.09	-2.66	down	0.00	0.00	yes
TRINITY_DN27095_c0_g1	-	-	-	1.39	10.19	-2.21	down	0.00	0.00	yes
TRINITY_DN19125_c0_g7	PREDICTED: chitotriosidase-1-like [Populus euphratica]	OVGP1	Oviduct-specific glycoprotein OS=Sus scrofa GN=OVGP1 PE=2 SV=1	0.31	2.36	-2.27	down	0.00	0.00	yes
TRINITY_DN21762_c0_g1	PREDICTED: probable glutathione S-transferase [Populus euphratica]	HSP26-A	Probable glutathione S-transferase OS=Glycine max GN=HSP26-A PE=2 SV=1	1.87	12.37	-1.80	down	0.00	0.00	yes
TRINITY_DN20607_c1_g8	PREDICTED: uncharacterized protein LOC105128754 [Populus euphratica]	-	-	0.07	3.15	-4.63	down	0.00	0.00	yes
TRINITY_DN15590_c0_g1	hypothetical protein POPTR_0003s06860g [Populus trichocarpa]	-	-	6.11	26.29	-1.50	down	0.00	0.00	yes
TRINITY_DN19387_c1_g2	SET domain-containing family protein [Populus trichocarpa]	ASHH3	Histone-lysine N-methyltransferase ASHH3 OS=Arabidopsis thaliana GN=ASHH3 PE=2 SV=2	4.51	17.04	-1.34	down	0.00	0.00	yes
TRINITY_DN21851_c1_g1	senescence-associated family protein [Populus trichocarpa]	TET8	Tetraspanin-8 OS=Arabidopsis thaliana GN=TET8 PE=2 SV=1	39.29	185.18	-1.64	down	0.00	0.00	yes
TRINITY_DN25898_c1_g1	hypothetical protein POPTR_0006s07660g [Populus trichocarpa]	-	-	1.77	6.47	-1.27	down	0.00	0.00	yes
TRINITY_DN11628_c0_g1	hypothetical protein [Populus tomentosa]	At3g42800	Protein BIG GRAIN 1-like C OS=Arabidopsis thaliana GN=At3g42800 PE=2 SV=1	0.72	8.34	-2.71	down	0.00	0.00	yes
TRINITY_DN26010_c0_g4	hypothetical protein POPTR_0006s13130g [Populus trichocarpa]	IDD1	Protein indeterminate-domain 1 OS=Arabidopsis thaliana GN=IDD1 PE=1 SV=1	3.69	17.16	-1.72	down	0.00	0.00	yes
TRINITY_DN17699_c0_g3	PREDICTED: protein FANTASTIC FOUR 1-like [Populus euphratica]	-	-	20.68	50.20	-1.16	down	0.00	0.00	yes
TRINITY_DN15312_c0_g1	leafy protein [Populus tremula]	FL	Floricaula/leafy homolog OS=Populus trichocarpa GN=FL PE=2 SV=2	0.41	2.80	-2.13	down	0.00	0.00	yes

TRINITY_DN21309_c0_g2	retinoblastoma-related protein 1 [Populus tremula x Populus tremuloides]	RBL901	Retinoblastoma-related protein OS=Populus trichocarpa GN=RBL901 PE=3 SV=1	4.91	16.41	-1.12	down	0.00	0.00	yes
TRINITY_DN20862_c2_g1	hypothetical protein POPTR_0007s10270g [Populus trichocarpa]	-	-	5.81	17.99	-1.01	down	0.00	0.00	yes
TRINITY_DN20299_c0_g1	hypothetical protein POPTR_0005s19515g [Populus trichocarpa]	HVA22F	HVA22-like protein f OS=Arabidopsis thaliana GN=HVA22F PE=2 SV=1	1.80	6.58	-1.31	down	0.00	0.00	yes
TRINITY_DN19657_c0_g1	PREDICTED: KH domain-containing protein At4g18375 isoform X2 [Populus euphratica]	HEN4	KH domain-containing protein HEN4 OS=Arabidopsis thaliana GN=HEN4 PE=1 SV=1	5.36	16.98	-1.06	down	0.00	0.00	yes
TRINITY_DN25238_c0_g2	hypothetical protein POPTR_0015s00640g [Populus trichocarpa]	NAC091	NAC domain-containing protein 91 OS=Arabidopsis thaliana GN=NAC091 PE=1 SV=1	3.92	13.72	-1.20	down	0.00	0.00	yes
TRINITY_DN27680_c0_g1	hypothetical protein POPTR_0002s09230g [Populus trichocarpa]	-	-	8.97	29.50	-1.01	down	0.00	0.00	yes
TRINITY_DN24782_c0_g1	PREDICTED: probable serine/threonine-protein kinase At1g18390 [Populus euphratica]	LRK10L-1.1	LEAF RUST 10 DISEASE-RESISTANCE LOCUS RECEPTOR-LIKE PROTEIN KINASE-like 1.1 OS=Arabidopsis thaliana GN=LRK10L-1.1 PE=2 SV=1	0.52	4.51	-2.52	down	0.00	0.00	yes
TRINITY_DN17617_c0_g1	hypothetical protein POPTR_0005s02410g [Populus trichocarpa]	At1g56220	Dormancy-associated protein homolog 3 OS=Arabidopsis thaliana GN=At1g56220 PE=1 SV=1	19.66	56.87	-1.06	down	0.00	0.00	yes
TRINITY_DN26496_c0_g1	hypothetical protein POPTR_0004s06180g [Populus trichocarpa]	RKF1	Probable LRR receptor-like serine/threonine-protein kinase RFK1 OS=Arabidopsis thaliana GN=RKF1 PE=1 SV=1	3.81	11.95	-1.38	down	0.00	0.00	yes
TRINITY_DN27681_c0_g1	-	-	-	0.57	4.59	-2.36	down	0.00	0.00	yes
TRINITY_DN20223_c0_g1	zinc finger family protein [Populus trichocarpa]	-	-	5.37	24.46	-1.54	down	0.00	0.00	yes
TRINITY_DN27284_c0_g2	PREDICTED: formin-like protein 6 isoform X1 [Populus euphratica]	FH18	Formin-like protein 18 OS=Arabidopsis thaliana GN=FH18 PE=2 SV=2	6.30	19.53	-1.04	down	0.00	0.00	yes
TRINITY_DN23615_c0_g16	hypothetical protein POPTR_0017s03345g [Populus trichocarpa]	-	-	12.91	50.78	-1.35	down	0.00	0.00	yes
TRINITY_DN22592_c0_g4	hypothetical protein POPTR_0001s13050g [Populus trichocarpa]	-	-	1.68	10.08	-1.98	down	0.00	0.00	yes
TRINITY_DN26394_c2_g1	-	-	-	2.34	11.26	-1.63	down	0.00	0.00	yes
TRINITY_DN27126_c2_g3	hypothetical protein POPTR_0019s00570g [Populus trichocarpa]	-	-	3.67	17.37	-1.68	down	0.00	0.00	yes
TRINITY_DN21705_c0_g5	hypothetical protein POPTR_0014s07220g [Populus trichocarpa]	NAC014	NAC domain-containing protein 14 OS=Arabidopsis thaliana GN=NAC014 PE=2 SV=1	0.39	4.79	-2.82	down	0.00	0.00	yes
TRINITY_DN15851_c0_g2	PREDICTED: DNA replication complex GINS protein PSF2-like [Populus euphratica]	GINS2	DNA replication complex GINS protein PSF2 OS=Arabidopsis thaliana GN=GINS2 PE=2 SV=2	2.39	11.58	-1.66	down	0.00	0.00	yes
TRINITY_DN26394_c3_g7	PREDICTED: WD-40 repeat-containing protein MSI4-like [Populus euphratica]	MSI4	WD-40 repeat-containing protein MSI4 OS=Arabidopsis thaliana GN=MSI4 PE=1 SV=3	4.37	14.20	-1.09	down	0.00	0.00	yes
TRINITY_DN19600_c0_g1	PREDICTED: uncharacterized protein LOC105119493 [Populus euphratica]	WUN1	Wound-induced protein 1 OS=Solanum tuberosum GN=WUN1 PE=2 SV=1	43.83	137.63	-1.07	down	0.00	0.00	yes
TRINITY_DN27242_c0_g1	hypothetical protein POPTR_0151s00200g [Populus trichocarpa]	At2g19130	G-type lectin S-receptor-like serine/threonine-protein kinase At2g19130 OS=Arabidopsis thaliana GN=At2g19130 PE=2 SV=1	2.45	8.98	-1.23	down	0.00	0.00	yes
TRINITY_DN25266_c0_g1	endo-1 family protein [Populus trichocarpa]	CEL1	Endoglucanase 8 OS=Arabidopsis thaliana GN=CEL1 PE=2 SV=1	13.14	52.56	-1.42	down	0.00	0.00	yes
TRINITY_DN25313_c1_g5	leucine-rich repeat transmembrane protein kinase [Populus trichocarpa]	At5g67200	Probable inactive receptor kinase At5g67200 OS=Arabidopsis thaliana GN=At5g67200 PE=1 SV=1	1.99	9.18	-1.55	down	0.00	0.00	yes
TRINITY_DN23135_c0_g2	hypothetical protein POPTR_0010s16340g [Populus trichocarpa]	PDR3	Pleiotropic drug resistance protein 3 OS=Nicotiana tabacum GN=PDR3 PE=2 SV=1	2.00	7.36	-1.62	down	0.00	0.00	yes
TRINITY_DN27392_c0_g1	PREDICTED: cation/H(+) antiporter 2-like [Populus euphratica]	CHX2	Cation/H(+) antiporter 2 OS=Arabidopsis thaliana GN=CHX2 PE=2 SV=1	1.50	6.80	-1.59	down	0.00	0.00	yes
TRINITY_DN15479_c0_g1	hypothetical protein POPTR_0004s15700g [Populus trichocarpa]	-	-	2.56	11.22	-1.54	down	0.00	0.00	yes
TRINITY_DN17167_c0_g1	GHMP kinase-related family protein [Populus trichocarpa]	GLCAK1	Glucuronokinase 1 OS=Arabidopsis thaliana GN=GLCAK1 PE=1 SV=1	3.51	12.23	-1.21	down	0.00	0.00	yes
TRINITY_DN26883_c0_g2	DREB protein, partial [Populus pruinosa]	-	-	13.40	69.58	-1.75	down	0.00	0.00	yes
TRINITY_DN19811_c0_g2	PREDICTED: uncharacterized protein LOC105137961 [Populus euphratica]	-	-	4.14	19.51	-1.62	down	0.00	0.00	yes
TRINITY_DN21520_c0_g1	hypothetical protein POPTR_0014s11700g [Populus trichocarpa]	SUD1	Probable E3 ubiquitin ligase SUD1 OS=Arabidopsis thaliana GN=SUD1 PE=1 SV=1	16.16	50.24	-1.12	down	0.00	0.00	yes

TRINITY_DN23481_c0_g3	-	-	-	0.64	3.74	-1.94	down	0.00	0.00	yes
TRINITY_DN25885_c1_g3	PREDICTED: ATP-citrate synthase beta chain protein 2 [Vitis vinifera]	ACLB-1	ATP-citrate synthase beta chain protein 1 OS=Arabidopsis thaliana GN=ACLB-1 PE=2 SV=1	0.56	4.93	-2.54	down	0.00	0.00	yes
TRINITY_DN9253_c0_g1	hypothetical protein POPTR_0012s04960g [Populus trichocarpa]	-	-	0.78	5.29	-2.13	down	0.00	0.00	yes
TRINITY_DN23593_c0_g1	hypothetical protein POPTR_0009s08770g [Populus trichocarpa]	-	-	14.03	49.19	-1.20	down	0.00	0.00	yes
TRINITY_DN17588_c0_g3	hypothetical protein POPTR_0009s06710g [Populus trichocarpa]	-	-	0.41	3.07	-2.25	down	0.00	0.00	yes
TRINITY_DN19427_c0_g2	PREDICTED: F-box protein At1g61340-like [Populus euphratica]	At1g61340	F-box protein At1g61340 OS=Arabidopsis thaliana GN=At1g61340 PE=2 SV=1	0.89	5.65	-2.03	down	0.00	0.00	yes
TRINITY_DN26207_c0_g3	calcium-binding family protein [Populus trichocarpa]	KIC	Calcium-binding protein KIC OS=Arabidopsis thaliana GN=KIC PE=1 SV=2	5.82	24.48	-1.48	down	0.00	0.00	yes
TRINITY_DN20655_c0_g1	PREDICTED: putative cell division cycle ATPase [Populus euphratica]	At2g18193	AAA-ATPase At2g18193 OS=Arabidopsis thaliana GN=At2g18193 PE=2 SV=1	0.83	4.18	-1.92	down	0.00	0.00	yes
TRINITY_DN22057_c0_g1	PREDICTED: disease resistance protein RPM1-like isoform X1 [Populus euphratica]	RPP8	Disease resistance protein RPP8 OS=Arabidopsis thaliana GN=RPP8 PE=1 SV=2	3.27	10.73	-1.01	down	0.00	0.00	yes
TRINITY_DN25506_c0_g3	PREDICTED: cell division control protein 2 homolog isoform X1 [Populus euphratica]	CRK1	Cell division control protein 2 homolog 1 OS=Trypanosoma brucei brucei GN=CRK1 PE=3 SV=1	0.51	2.73	-1.83	down	0.00	0.00	yes
TRINITY_DN14119_c0_g1	hypothetical protein POPTR_0009s03100g [Populus trichocarpa]	SRG1	Protein SRG1 OS=Arabidopsis thaliana GN=SRG1 PE=2 SV=1	1.68	7.93	-1.37	down	0.00	0.00	yes
TRINITY_DN14335_c0_g1	-	-	-	0.43	5.31	-3.07	down	0.00	0.00	yes
TRINITY_DN23017_c2_g1	-	-	-	0.15	1.82	-2.88	down	0.00	0.00	yes
TRINITY_DN25440_c0_g3	hypothetical protein POPTR_0006s04720g [Populus trichocarpa]	-	-	47.61	190.42	-1.37	down	0.00	0.00	yes
TRINITY_DN25736_c2_g6	RECEPTOR-LIKE protein KINASE 1 [Populus trichocarpa]	RPK2	LRR receptor-like serine/threonine-protein kinase RPK2 OS=Arabidopsis thaliana GN=RPK2 PE=2 SV=1	2.07	8.61	-1.44	down	0.00	0.00	yes
TRINITY_DN14558_c0_g1	PREDICTED: uncharacterized protein LOC109021545 [Juglans regia]	AtMg00810	Uncharacterized mitochondrial protein AtMg00810 OS=Arabidopsis thaliana GN=AtMg00810 PE=4 SV=1	0.85	4.88	-2.01	down	0.00	0.00	yes
TRINITY_DN25591_c1_g2	hypothetical protein POPTR_0001s20570g [Populus trichocarpa]	Os01g0583100	Probable protein phosphatase 2C 6 OS=Oryza sativa subsp. japonica GN=Os01g0583100 PE=1 SV=1	0.29	2.49	-2.45	down	0.00	0.00	yes
TRINITY_DN22415_c0_g6	-	-	-	121.87	382.79	-1.05	down	0.00	0.00	yes
TRINITY_DN22303_c0_g2	hypothetical protein POPTR_0018s00700g [Populus trichocarpa]	ERF016	Ethylene-responsive transcription factor ERF016 OS=Arabidopsis thaliana GN=ERF016 PE=2 SV=1	2.23	17.05	-2.29	down	0.00	0.00	yes
TRINITY_DN26714_c0_g1	hypothetical protein POPTR_0018s04685g [Populus trichocarpa]	-	-	0.44	2.46	-1.87	down	0.00	0.00	yes
TRINITY_DN17274_c0_g3	hypothetical protein POPTR_0016s12170g [Populus trichocarpa]	At3g47110	Putative receptor-like protein kinase At3g47110 OS=Arabidopsis thaliana GN=At3g47110 PE=3 SV=1	0.67	3.41	-1.71	down	0.00	0.00	yes
TRINITY_DN23463_c0_g1	hypothetical protein POPTR_0011s09560g [Populus trichocarpa]	-	-	28.79	92.22	-1.03	down	0.00	0.00	yes
TRINITY_DN16928_c0_g2	-	-	-	0.32	2.98	-2.52	down	0.00	0.00	yes
TRINITY_DN18272_c0_g1	hypothetical protein POPTR_0008s03240g [Populus trichocarpa]	-	-	2.15	16.99	-1.92	down	0.00	0.00	yes
TRINITY_DN7156_c0_g1	hypothetical protein POPTR_0007s12090g [Populus trichocarpa]	-	-	0.33	2.37	-2.20	down	0.00	0.00	yes
TRINITY_DN26199_c1_g2	PREDICTED: cytochrome P450 714B2-like [Populus euphratica]	CYP714B1	Cytochrome P450 714B1 OS=Oryza sativa subsp. japonica GN=CYP714B1 PE=1 SV=2	5.65	19.06	-1.09	down	0.00	0.00	yes
TRINITY_DN23219_c0_g2	zinc knuckle family protein [Populus trichocarpa]	-	-	1.63	9.45	-1.90	down	0.00	0.00	yes
TRINITY_DN24312_c1_g1	PREDICTED: squamosa promoter-binding-like protein 12 isoform X3 [Populus euphratica]	SPL3	Squamosa promoter-binding-like protein 3 OS=Oryza sativa subsp. indica GN=SPL3 PE=2 SV=1	3.78	12.13	-1.20	down	0.00	0.00	yes
TRINITY_DN25057_c0_g2	PREDICTED: GATA transcription factor 1-like [Populus euphratica]	GATA1	GATA transcription factor 1 OS=Arabidopsis thaliana GN=GATA1 PE=2 SV=2	3.25	9.78	-1.00	down	0.00	0.00	yes
TRINITY_DN25241_c1_g1	hypothetical protein POPTR_0006s23780g [Populus trichocarpa]	C1	Anthocyanin regulatory C1 protein OS=Zea mays GN=C1 PE=2 SV=1	3.32	15.29	-1.60	down	0.00	0.00	yes

TRINITY_DN25912_c0_g1	PREDICTED: serine/threonine protein phosphatase 2A 57 kDa regulatory subunit B' iota isoform-like [Populus euphratica]	B'IOTA	Serine/threonine protein phosphatase 2A 57 kDa regulatory subunit B' iota isoform OS=Arabidopsis thaliana GN=B'IOTA PE=2 SV=1	7.20	22.71	-1.05	down	0.00	0.00	yes
TRINITY_DN23717_c0_g1	PREDICTED: kinesin-like protein KIF22 isoform X2 [Populus euphratica]	KIN10C	Kinesin-like protein KIN-10C OS=Arabidopsis thaliana GN=KIN10C PE=2 SV=1	7.98	22.43	-1.01	down	0.00	0.00	yes
TRINITY_DN20656_c0_g1	PREDICTED: uncharacterized protein LOC105121051 [Populus euphratica]	-	-	5.83	14.96	-1.17	down	0.00	0.00	yes
TRINITY_DN27113_c0_g2	PREDICTED: cyclic nucleotide-gated ion channel 1-like [Populus euphratica]	CNGC1	Cyclic nucleotide-gated ion channel 1 OS=Arabidopsis thaliana GN=CNGC1 PE=1 SV=1	8.34	29.60	-1.16	down	0.00	0.00	yes
TRINITY_DN22185_c0_g1	basic helix-loop-helix family protein [Populus trichocarpa]	BHLH30	Transcription factor bHLH30 OS=Arabidopsis thaliana GN=BHLH30 PE=1 SV=1	1.63	7.32	-1.57	down	0.00	0.00	yes
TRINITY_DN27442_c0_g3	leucine-rich repeat transmembrane protein kinase [Populus trichocarpa]	IRK	Probable LRR receptor-like serine/threonine-protein kinase IRK OS=Arabidopsis thaliana GN=IRK PE=1 SV=1	1.23	6.67	-1.83	down	0.00	0.00	yes
TRINITY_DN23675_c0_g1	hypothetical protein POPTR_0001s42050g [Populus trichocarpa]	At4g27290	G-type lectin S-receptor-like serine/threonine-protein kinase At4g27290 OS=Arabidopsis thaliana GN=At4g27290 PE=3 SV=4	0.38	3.34	-2.55	down	0.00	0.00	yes
TRINITY_DN27487_c1_g2	PREDICTED: probable receptor-like protein kinase At5g39020, partial [Populus euphratica]	LRK10	Rust resistance kinase Lr10 OS=Triticum aestivum GN=LRK10 PE=2 SV=1	2.68	9.82	-2.45	down	0.00	0.00	yes
TRINITY_DN23875_c0_g1	hypothetical protein POPTR_0003s01950g [Populus trichocarpa]	DRP4C	Dynamin-related protein 4C OS=Arabidopsis thaliana GN=DRP4C PE=2 SV=1	1.06	9.01	-2.53	down	0.00	0.00	yes
TRINITY_DN14700_c0_g1	hypothetical protein POPTR_0008s01650g [Populus trichocarpa]	-	-	0.80	4.63	-2.16	down	0.00	0.00	yes
TRINITY_DN25758_c0_g1	E2F TRANSCRIPTION FACTOR-3 family protein [Populus trichocarpa]	E2FA	Transcription factor E2FA OS=Arabidopsis thaliana GN=E2FA PE=1 SV=1	6.30	20.93	-1.11	down	0.00	0.00	yes
TRINITY_DN24388_c0_g1	PREDICTED: lipase-like PAD4 [Populus euphratica]	PAD4	Lipase-like PAD4 OS=Arabidopsis thaliana GN=PAD4 PE=1 SV=1	4.42	23.88	-1.87	down	0.00	0.00	yes
TRINITY_DN21952_c0_g1	hypothetical protein POPTR_0006s18580g [Populus trichocarpa]	At3g07870	F-box protein At3g07870 OS=Arabidopsis thaliana GN=At3g07870 PE=2 SV=1	0.78	8.74	-2.42	down	0.00	0.00	yes
TRINITY_DN25523_c0_g1	kinase family protein [Populus trichocarpa]	PERK1	Proline-rich receptor-like protein kinase PERK1 OS=Arabidopsis thaliana GN=PERK1 PE=2 SV=1	4.54	14.75	-1.11	down	0.00	0.00	yes
TRINITY_DN12315_c0_g1	hypothetical protein POPTR_0006s06710g [Populus trichocarpa]	-	-	0.55	4.30	-2.36	down	0.00	0.00	yes
TRINITY_DN20748_c0_g1	hypothetical protein POPTR_0004s07050g [Populus trichocarpa]	WRKY27	Probable WRKY transcription factor 27 OS=Arabidopsis thaliana GN=WRKY27 PE=2 SV=1	0.76	4.78	-1.99	down	0.00	0.00	yes
TRINITY_DN19185_c1_g6	PREDICTED: beta-amyrin 28-oxidase-like [Populus euphratica]	-	Beta-amyrin 28-oxidase OS=Panax ginseng PE=2 SV=1	0.23	2.53	-2.75	down	0.00	0.00	yes
TRINITY_DN23045_c0_g4	hypothetical protein POPTR_0014s18490g [Populus trichocarpa]	-	-	1.08	12.36	-1.64	down	0.00	0.00	yes
TRINITY_DN23068_c1_g1	PREDICTED: probable galacturonosyltransferase-like 9 [Populus euphratica]	GATL9	Probable galacturonosyltransferase-like 9 OS=Arabidopsis thaliana GN=GATL9 PE=2 SV=1	3.69	19.63	-1.79	down	0.00	0.00	yes
TRINITY_DN25866_c1_g2	hypothetical protein POPTR_0018s01030g [Populus trichocarpa]	-	-	4.79	14.14	-1.00	down	0.00	0.00	yes
TRINITY_DN21284_c0_g1	hypothetical protein POPTR_0005s02650g [Populus trichocarpa]	RECQSIM	ATP-dependent DNA helicase Q-like SIM OS=Arabidopsis thaliana GN=RECQSIM PE=2 SV=1	1.77	6.79	-1.34	down	0.00	0.00	yes
TRINITY_DN23598_c0_g3	PREDICTED: uncharacterized protein LOC105124444 isoform X1 [Populus euphratica]	-	-	7.49	26.02	-1.15	down	0.00	0.00	yes
TRINITY_DN27517_c0_g2	PREDICTED: F-box protein At1g10780-like isoform X1 [Populus euphratica]	At1g10780	F-box protein At1g10780 OS=Arabidopsis thaliana GN=At1g10780 PE=2 SV=1	6.11	21.08	-1.24	down	0.00	0.00	yes
TRINITY_DN18030_c1_g6	hypothetical protein POPTR_0001s27950g [Populus trichocarpa]	TUBB2	Tubulin beta-2 chain OS=Zea mays GN=TUBB2 PE=2 SV=1	7.30	26.00	-1.19	down	0.00	0.00	yes
TRINITY_DN18907_c0_g4	aspartyl protease family protein [Populus trichocarpa]	ASPG1	Protein ASPARTIC PROTEASE IN GUARD CELL 1 OS=Arabidopsis thaliana GN=ASPG1 PE=1 SV=1	14.64	49.75	-1.19	down	0.00	0.00	yes
TRINITY_DN25887_c0_g1	PREDICTED: uncharacterized protein LOC105138186 isoform X1 [Populus euphratica]	-	-	6.54	23.22	-1.15	down	0.00	0.00	yes
TRINITY_DN24435_c1_g1	E2F transcription factor-1 family protein [Populus trichocarpa]	E2FB	Transcription factor E2FB OS=Arabidopsis thaliana GN=E2FB PE=1 SV=1	5.02	19.25	-1.33	down	0.00	0.00	yes
TRINITY_DN23656_c1_g5	hypothetical protein POPTR_0010s24310g [Populus trichocarpa]	CYP94C1	Cytochrome P450 94C1 OS=Arabidopsis thaliana GN=CYP94C1 PE=2 SV=1	0.58	3.67	-2.04	down	0.00	0.00	yes

TRINITY_DN26973_c0_g1	hypothetical protein POPTR_0001s16780g [Populus trichocarpa]	LOX3.1	Linoleate 13S-lipoxygenase 3-1, chloroplastic OS=Solanum tuberosum GN=LOX3.1 PE=1 SV=1	4.72	22.23	-1.85	down	0.00	0.00	yes
TRINITY_DN25740_c0_g5	PREDICTED: growth-regulating factor 1-like [Populus euphratica]	GRF3	Growth-regulating factor 3 OS=Oryza sativa subsp. japonica GN=GRF3 PE=3 SV=2	0.82	5.05	-2.00	down	0.00	0.00	yes
TRINITY_DN27739_c1_g1	hypothetical protein POPTR_0019s00705g [Populus trichocarpa]	At4g27220	Probable disease resistance protein At4g27220 OS=Arabidopsis thaliana GN=At4g27220 PE=2 SV=1	2.48	12.17	-1.76	down	0.00	0.00	yes
TRINITY_DN24078_c0_g1	PREDICTED: transcription factor MYC2 isoform X1 [Populus euphratica]	MYC2	Transcription factor MYC2 OS=Arabidopsis thaliana GN=MYC2 PE=1 SV=2	12.43	60.19	-1.75	down	0.00	0.00	yes
TRINITY_DN26632_c0_g1	hypothetical protein POPTR_0012s10730g [Populus trichocarpa]	SCPL45	Serine carboxypeptidase-like 45 OS=Arabidopsis thaliana GN=SCPL45 PE=2 SV=1	14.84	44.17	-1.10	down	0.00	0.00	yes
TRINITY_DN19315_c3_g1	hypothetical protein POPTR_0013s02670g [Populus trichocarpa]	ATL6	E3 ubiquitin-protein ligase ATL6 OS=Arabidopsis thaliana GN=ATL6 PE=1 SV=2	3.46	14.68	-1.50	down	0.00	0.00	yes
TRINITY_DN27163_c0_g2	hypothetical protein POPTR_0014s18350g [Populus trichocarpa]	At2g16250	Probable LRR receptor-like serine/threonine-protein kinase At2g16250 OS=Arabidopsis thaliana GN=At2g16250 PE=2 SV=1	0.85	3.80	-1.53	down	0.00	0.00	yes
TRINITY_DN22356_c1_g1	PREDICTED: protein LONGIFOLIA 1-like isoform X1 [Populus euphratica]	LNG1	Protein LONGIFOLIA 1 OS=Arabidopsis thaliana GN=LNG1 PE=1 SV=1	4.99	17.84	-1.16	down	0.00	0.00	yes
TRINITY_DN27663_c1_g5	hypothetical protein POPTR_0002s06700g [Populus trichocarpa]	-	-	0.78	6.93	-2.48	down	0.00	0.00	yes
TRINITY_DN22657_c0_g1	WRKY transcription factor 28 [(Populus tomentosa x Populus bolleana) x Populus tomentosa]	WRKY6	WRKY transcription factor 6 OS=Arabidopsis thaliana GN=WRKY6 PE=1 SV=1	1.37	10.01	-2.28	down	0.00	0.00	yes
TRINITY_DN24973_c0_g1	hypothetical protein POPTR_0019s10350g [Populus trichocarpa]	-	-	2.08	8.44	-1.44	down	0.00	0.00	yes
TRINITY_DN16818_c0_g2	syringolide-induced protein B13-1-9 [Populus trichocarpa]	YLS9	Protein YLS9 OS=Arabidopsis thaliana GN=YLS9 PE=2 SV=1	5.72	64.56	-2.90	down	0.00	0.00	yes
TRINITY_DN19789_c0_g5	hypothetical protein POPTR_0015s13690g [Populus trichocarpa]	-	-	2.79	10.64	-1.46	down	0.00	0.00	yes
TRINITY_DN22844_c0_g2	DNAJ heat shock family protein [Populus trichocarpa]	Dnajb4	DnaJ homolog subfamily B member 4 OS=Mus musculus GN=Dnajb4 PE=1 SV=1	4.19	16.51	-1.34	down	0.00	0.00	yes
TRINITY_DN25241_c1_g2	PREDICTED: transcription initiation factor TFIID subunit 8-like [Populus euphratica]	TAF8	Transcription initiation factor TFIID subunit 8 OS=Arabidopsis thaliana GN=TAF8 PE=1 SV=1	4.28	15.18	-1.07	down	0.00	0.01	yes
TRINITY_DN24868_c1_g1	cyclin family protein [Populus trichocarpa]	CYCA1-1	Cyclin-A1-1 OS=Arabidopsis thaliana GN=CYCA1-1 PE=1 SV=1	56.44	192.52	-1.09	down	0.00	0.01	yes
TRINITY_DN26843_c0_g2	-	-	-	35.82	170.25	-1.63	down	0.00	0.01	yes
TRINITY_DN23745_c0_g2	zinc finger family protein [Populus trichocarpa]	-	-	5.57	34.25	-1.98	down	0.00	0.01	yes
TRINITY_DN24436_c0_g4	-	-	-	0.76	6.51	-2.48	down	0.00	0.01	yes
TRINITY_DN25537_c0_g1	xyloglucan endotransglycosylase/hydrolase precursor XTH-27 [Populus trichocarpa]	XTH5	Probable xyloglucan endotransglucosylase/hydrolase protein 5 OS=Arabidopsis thaliana GN=XTH5 PE=2 SV=1	33.20	109.96	-1.11	down	0.00	0.01	yes
TRINITY_DN23017_c2_g3	hypothetical protein POPTR_0009s01450g [Populus trichocarpa]	-	-	0.58	3.41	-1.94	down	0.00	0.01	yes
TRINITY_DN18180_c0_g2	C2 domain-containing family protein [Populus trichocarpa]	FTIP1	FT-interacting protein 1 OS=Arabidopsis thaliana GN=FTIP1 PE=1 SV=1	0.16	6.49	-2.97	down	0.00	0.01	yes
TRINITY_DN15573_c0_g1	alcohol dehydrogenase family protein [Populus trichocarpa]	At5g42250	Alcohol dehydrogenase-like 7 OS=Arabidopsis thaliana GN=At5g42250 PE=2 SV=1	0.77	3.49	-2.43	down	0.00	0.01	yes
TRINITY_DN15046_c0_g2	PREDICTED: homeobox-leucine zipper protein HOX11-like [Populus euphratica]	HAT14	Homeobox-leucine zipper protein HAT14 OS=Arabidopsis thaliana GN=HAT14 PE=2 SV=3	3.73	21.23	-1.87	down	0.00	0.01	yes
TRINITY_DN14897_c0_g1	PREDICTED: alpha carbonic anhydrase 4-like [Populus euphratica]	ACA4	Alpha carbonic anhydrase 4 OS=Arabidopsis thaliana GN=ACA4 PE=3 SV=1	1.18	5.78	-1.66	down	0.00	0.01	yes
TRINITY_DN16651_c0_g2	PREDICTED: equilibrative nucleotide transporter 3-like isoform X1 [Populus euphratica]	ENT3	Equilibrative nucleotide transporter 3 OS=Arabidopsis thaliana GN=ENT3 PE=1 SV=1	0.46	3.47	-2.30	down	0.00	0.01	yes
TRINITY_DN17344_c0_g1	hypothetical protein POPTR_0009s09700g [Populus trichocarpa]	-	-	1.24	6.36	-1.75	down	0.00	0.01	yes
TRINITY_DN16591_c0_g2	PREDICTED: hexose carrier protein HEX6 [Populus euphratica]	HEX6	Hexose carrier protein HEX6 OS=Ricinus communis GN=HEX6 PE=2 SV=1	0.82	6.02	-2.14	down	0.00	0.01	yes
TRINITY_DN24396_c0_g4	PREDICTED: high affinity nitrate transporter 2.5-like [Populus euphratica]	NRT2.5	High affinity nitrate transporter 2.5 OS=Arabidopsis thaliana GN=NRT2.5 PE=2 SV=1	0.65	3.93	-2.69	down	0.00	0.01	yes
TRINITY_DN14869_c0_g3	hypothetical protein POPTR_0006s02430g [Populus trichocarpa]	-	-	1.48	8.70	-1.92	down	0.00	0.01	yes

TRINITY_DN21134_c0_g3	hypothetical protein POPTR_0011s15040g [Populus trichocarpa]	-	-	0.59	2.67	-1.57	down	0.00	0.01	yes
TRINITY_DN25455_c0_g1	PREDICTED: protein NSP-INTERACTING KINASE 1-like isoform X2 [Populus euphratica]	NIK1	Protein NSP-INTERACTING KINASE 1 OS=Arabidopsis thaliana GN=NIK1 PE=1 SV=1	10.84	33.18	-1.00	down	0.00	0.01	yes
TRINITY_DN27790_c0_g1	hypothetical protein POPTR_0015s11900g [Populus trichocarpa]	-	-	2.16	14.18	-1.52	down	0.00	0.01	yes
TRINITY_DN24614_c0_g5	hypothetical protein POPTR_0018s06320g [Populus trichocarpa]	B'IOTA	Serine/threonine protein phosphatase 2A 57 kDa regulatory subunit B' iota isoform OS=Arabidopsis thaliana GN=B'IOTA PE=2 SV=1	2.11	7.39	-1.20	down	0.00	0.01	yes
TRINITY_DN21818_c0_g1	hypothetical protein POPTR_0019s05510g [Populus trichocarpa]	PCO1	Plant cysteine oxidase 1 OS=Arabidopsis thaliana GN=PCO1 PE=1 SV=1	0.26	3.14	-2.54	down	0.00	0.01	yes
TRINITY_DN19213_c0_g2	hypothetical protein POPTR_0009s02970g [Populus trichocarpa]	-	-	0.38	4.37	-2.84	down	0.00	0.01	yes
TRINITY_DN22625_c2_g3	CBL-interacting protein kinase 11 [Populus trichocarpa]	CIPK11	CBL-interacting serine/threonine-protein kinase 11 OS=Arabidopsis thaliana GN=CIPK11 PE=1 SV=1	8.45	36.81	-1.49	down	0.00	0.01	yes
TRINITY_DN18665_c1_g4	PREDICTED: uncharacterized protein LOC105115687 [Populus euphratica]	COL16	Zinc finger protein CONSTANS-LIKE 16 OS=Arabidopsis thaliana GN=COL16 PE=2 SV=2	14.53	59.68	-1.17	down	0.00	0.01	yes
TRINITY_DN23587_c0_g2	PREDICTED: TBC1 domain family member 8B-like isoform X1 [Populus euphratica]	Tbc1d8	TBC1 domain family member 8 OS=Mus musculus GN=Tbc1d8 PE=1 SV=2	2.11	7.96	-1.09	down	0.00	0.01	yes
TRINITY_DN19040_c0_g1	PREDICTED: transcription factor TGA2-like [Populus euphratica]	DOGL4	Protein DOG1-like 4 OS=Arabidopsis thaliana GN=DOGL4 PE=2 SV=1	5.09	59.54	-2.95	down	0.00	0.01	yes
TRINITY_DN19981_c0_g1	copper/topa quinone amine oxidase precursor family protein [Populus trichocarpa]	-	Primary amine oxidase OS=Pisum sativum PE=1 SV=1	1.81	6.48	-1.21	down	0.00	0.01	yes
TRINITY_DN18733_c0_g1	hypothetical protein POPTR_0002s16580g [Populus trichocarpa]	-	-	3.82	14.39	-1.27	down	0.00	0.01	yes
TRINITY_DN16599_c0_g1	PREDICTED: LRR receptor-like serine/threonine-protein kinase RPK2 [Populus euphratica]	RPK2	LRR receptor-like serine/threonine-protein kinase RPK2 OS=Arabidopsis thaliana GN=RPK2 PE=2 SV=1	1.11	7.50	-2.12	down	0.00	0.01	yes
TRINITY_DN26929_c0_g4	hypothetical protein POPTR_0013s04050g [Populus trichocarpa]	-	-	2.96	13.11	-1.54	down	0.00	0.01	yes
TRINITY_DN23947_c1_g1	hypothetical protein POPTR_0016s08030g [Populus trichocarpa]	At5g39865	Uncharacterized protein At5g39865 OS=Arabidopsis thaliana GN=At5g39865 PE=2 SV=1	6.16	19.52	-1.09	down	0.00	0.01	yes
TRINITY_DN27619_c3_g2	hypothetical protein POPTR_0010s21830g [Populus trichocarpa]	-	-	0.43	3.55	-2.39	down	0.00	0.01	yes
TRINITY_DN15715_c0_g1	hypothetical protein POPTR_0010s19830g [Populus trichocarpa]	-	-	0.55	4.16	-2.32	down	0.00	0.01	yes
TRINITY_DN22305_c0_g3	PREDICTED: uncharacterized protein LOC105117331 [Populus euphratica]	-	-	2.29	9.38	-1.43	down	0.00	0.01	yes
TRINITY_DN20937_c0_g6	-	-	-	20.65	75.33	-1.26	down	0.00	0.01	yes
TRINITY_DN22864_c0_g1	hypothetical protein POPTR_0001s29300g [Populus trichocarpa]	rmnd5a	Protein RMD5 homolog A OS=Xenopus laevis GN=rmnd5a PE=2 SV=1	3.53	12.11	-1.14	down	0.00	0.01	yes
TRINITY_DN29009_c0_g1	hypothetical protein POPTR_0009s06760g [Populus trichocarpa]	-	-	2.19	26.01	-2.90	down	0.00	0.01	yes
TRINITY_DN15875_c0_g1	-	-	-	0.14	2.98	-3.67	down	0.00	0.01	yes
TRINITY_DN18333_c0_g1	-	-	-	1.33	6.76	-1.69	down	0.00	0.01	yes
TRINITY_DN22337_c0_g1	PREDICTED: uncharacterized protein LOC105121820 [Populus euphratica]	-	-	9.33	32.21	-1.19	down	0.00	0.01	yes
TRINITY_DN18664_c1_g2	-	-	-	5.50	19.14	-1.48	down	0.00	0.01	yes
TRINITY_DN25260_c0_g4	unknown [Populus trichocarpa]	-	Glutamine synthetase nodule isozyme OS=Vigna aconitifolia PE=2 SV=1	26.98	83.37	-1.01	down	0.00	0.01	yes
TRINITY_DN27794_c1_g2	hypothetical protein POPTR_0008s04420g [Populus trichocarpa]	-	-	2.92	15.87	-1.82	down	0.00	0.01	yes
TRINITY_DN16695_c0_g1	hypothetical protein POPTR_0006s23480g [Populus trichocarpa]	ERF017	Ethylene-responsive transcription factor ERF017 OS=Arabidopsis thaliana GN=ERF017 PE=2 SV=1	0.81	11.67	-3.27	down	0.00	0.01	yes
TRINITY_DN19215_c1_g1	hypothetical protein POPTR_0007s10210g [Populus trichocarpa]	At4g33920	Probable protein phosphatase 2C 63 OS=Arabidopsis thaliana GN=At4g33920 PE=2 SV=1	3.47	12.55	-1.38	down	0.00	0.01	yes
TRINITY_DN22515_c0_g15	-	-	-	1.89	8.00	-1.48	down	0.00	0.01	yes

TRINITY_DN18642_c0_g1	hypothetical protein POPTR_0010s01550g [Populus trichocarpa]	Fanci	Fanconi anemia group I protein homolog OS=Mus musculus GN=Fanci PE=1 SV=2	1.89	8.06	-1.36	down	0.00	0.01	yes
TRINITY_DN26980_c0_g1	mitogen-activated protein kinase homologue [Populus trichocarpa]	MPK19	Mitogen-activated protein kinase 19 OS=Arabidopsis thaliana GN=MPK19 PE=2 SV=2	26.58	76.98	-1.01	down	0.00	0.01	yes
TRINITY_DN27394_c0_g1	resistance family protein [Populus trichocarpa]	At3g47570	Probable LRR receptor-like serine/threonine-protein kinase At3g47570 OS=Arabidopsis thaliana GN=At3g47570 PE=2 SV=1	0.24	2.87	-2.95	down	0.00	0.01	yes
TRINITY_DN26562_c0_g1	PREDICTED: cytochrome P450 87A3-like isoform X3 [Populus euphratica]	CYP87A3	Cytochrome P450 87A3 OS=Oryza sativa subsp. japonica GN=CYP87A3 PE=2 SV=3	2.79	10.43	-1.27	down	0.00	0.01	yes
TRINITY_DN20092_c0_g3	PREDICTED: uncharacterized protein DDB_G0271670-like isoform X1 [Populus euphratica]	VQ22	VQ motif-containing protein 22 OS=Arabidopsis thaliana GN=VQ22 PE=2 SV=1	1.46	4.37	-1.49	down	0.00	0.01	yes
TRINITY_DN25818_c2_g1	PREDICTED: putative disease resistance RPP13-like protein 1 [Populus euphratica]	At3g14460	Putative disease resistance protein At3g14460 OS=Arabidopsis thaliana GN=At3g14460 PE=3 SV=1	1.48	9.05	-2.11	down	0.00	0.01	yes
TRINITY_DN23364_c0_g1	PREDICTED: serine/threonine-protein kinase-like protein CCR4 [Populus euphratica]	CCR4	Serine/threonine-protein kinase-like protein CCR4 OS=Arabidopsis thaliana GN=CCR4 PE=1 SV=1	0.82	4.55	-1.89	down	0.00	0.01	yes
TRINITY_DN22538_c1_g2	Histone H1 family protein [Populus trichocarpa]	-	Histone H1 OS=Pisum sativum PE=2 SV=1	182.59	594.08	-1.13	down	0.00	0.01	yes
TRINITY_DN22310_c0_g2	hypothetical protein POPTR_0017s01700g [Populus trichocarpa]	FRO5	Ferric reduction oxidase 5 OS=Arabidopsis thaliana GN=FRO5 PE=2 SV=1	0.57	3.80	-2.11	down	0.00	0.01	yes
TRINITY_DN25564_c0_g1	amine oxidase family protein [Populus trichocarpa]	LDL1	Lysine-specific histone demethylase 1 homolog 1 OS=Arabidopsis thaliana GN=LDL1 PE=1 SV=1	6.24	17.85	-1.06	down	0.00	0.01	yes
TRINITY_DN17961_c0_g10	PREDICTED: formin-like protein 1 [Populus euphratica]	FH1	Formin-like protein 1 OS=Arabidopsis thaliana GN=FH1 PE=1 SV=1	0.39	4.79	-2.98	down	0.00	0.01	yes
TRINITY_DN21712_c0_g2	PREDICTED: uncharacterized protein At3g17950-like [Populus euphratica]	Y-3	Uncharacterized protein At3g17950 OS=Arabidopsis thaliana GN=Y-3 PE=1 SV=1	0.52	5.11	-2.05	down	0.00	0.01	yes
TRINITY_DN16815_c0_g1	hypothetical protein POPTR_0013s15520g [Populus trichocarpa]	At1g06840	Probable LRR receptor-like serine/threonine-protein kinase At1g06840 OS=Arabidopsis thaliana GN=At1g06840 PE=2 SV=2	0.65	2.93	-1.87	down	0.00	0.01	yes
TRINITY_DN19538_c0_g1	hypothetical protein POPTR_0013s00290g [Populus trichocarpa]	MYB86	Transcription factor MYB86 OS=Arabidopsis thaliana GN=MYB86 PE=2 SV=1	1.54	5.32	-1.44	down	0.00	0.01	yes
TRINITY_DN27183_c0_g2	unknown [Populus trichocarpa]	-	-	2.49	12.47	-1.76	down	0.00	0.01	yes
TRINITY_DN20468_c0_g1	hypothetical protein POPTR_0015s12600g [Populus trichocarpa]	-	-	9.01	37.99	-1.27	down	0.00	0.01	yes
TRINITY_DN27220_c0_g1	PREDICTED: uncharacterized protein LOC105130123 [Populus euphratica]	KIN14Q	Kinesin-like protein KIN-14Q OS=Arabidopsis thaliana GN=KIN14Q PE=2 SV=1	10.28	33.40	-1.11	down	0.00	0.01	yes
TRINITY_DN19117_c0_g1	trehalose-6-phosphate phosphatase family protein [Populus trichocarpa]	TPPJ	Probable trehalose-phosphate phosphatase J OS=Arabidopsis thaliana GN=TPPJ PE=1 SV=1	0.56	3.33	-2.20	down	0.00	0.01	yes
TRINITY_DN25761_c1_g1	PREDICTED: probable receptor-like protein kinase At1g80640 isoform X3 [Populus euphratica]	At1g80640	Probable receptor-like protein kinase At1g80640 OS=Arabidopsis thaliana GN=At1g80640 PE=2 SV=1	1.23	6.93	-2.06	down	0.00	0.01	yes
TRINITY_DN14219_c0_g1	S-adenosyl-L-methionine:carboxyl methyltransferase family protein [Populus trichocarpa]	IAMT1	Indole-3-acetate O-methyltransferase 1 OS=Arabidopsis thaliana GN=IAMT1 PE=1 SV=1	0.43	3.49	-2.35	down	0.00	0.01	yes
TRINITY_DN27705_c0_g1	hypothetical protein POPTR_0008s20040g [Populus trichocarpa]	RUK	Serine/threonine-protein kinase RUNKEL OS=Arabidopsis thaliana GN=RUK PE=1 SV=1	19.91	77.33	-1.35	down	0.00	0.01	yes
TRINITY_DN18772_c0_g1	Cel9B [Populus tremula x Populus tremuloides]	CEL3	Endoglucanase 9 OS=Arabidopsis thaliana GN=CEL3 PE=1 SV=1	1.44	7.23	-1.72	down	0.00	0.01	yes
TRINITY_DN23885_c1_g4	hypothetical protein POPTR_0005s10110g [Populus trichocarpa]	-	-	5.63	17.64	-1.42	down	0.00	0.01	yes
TRINITY_DN20133_c0_g3	GDSL-motif lipase/hydrolase family protein [Populus trichocarpa]	At1g71691	GDSL esterase/lipase At1g71691 OS=Arabidopsis thaliana GN=At1g71691 PE=2 SV=1	0.45	3.34	-2.31	down	0.00	0.01	yes
TRINITY_DN21075_c1_g3	PREDICTED: ferric reduction oxidase 4-like isoform X2 [Populus euphratica]	FRO4	Ferric reduction oxidase 4 OS=Arabidopsis thaliana GN=FRO4 PE=2 SV=1	0.85	3.76	-1.55	down	0.00	0.01	yes
TRINITY_DN26589_c0_g1	PREDICTED: G2/mitotic-specific cyclin S13-7-like [Populus euphratica]	CYCB1-4	Cyclin-B1-4 OS=Arabidopsis thaliana GN=CYCB1-4 PE=2 SV=1	23.43	75.94	-1.19	down	0.00	0.01	yes
TRINITY_DN16839_c0_g2	hypothetical protein POPTR_0018s08740g [Populus trichocarpa]	OFP8	Transcription repressor OFP8 OS=Arabidopsis thaliana GN=OFP8 PE=2 SV=1	1.40	6.67	-1.63	down	0.00	0.01	yes
TRINITY_DN23918_c0_g2	kinase family protein [Populus trichocarpa]	LYK5	Protein LYK5 OS=Arabidopsis thaliana GN=LYK5 PE=2 SV=1	2.19	11.22	-1.75	down	0.00	0.01	yes
TRINITY_DN20062_c1_g1	hypothetical protein POPTR_0007s11750g [Populus trichocarpa]	MES3	Methylesterase 3 OS=Arabidopsis thaliana GN=MES3 PE=2 SV=1	13.47	41.01	-1.15	down	0.00	0.01	yes
TRINITY_DN17457_c0_g2	ethylene-responsive family protein [Populus trichocarpa]	BHLH123	Transcription factor bHLH123 OS=Arabidopsis thaliana GN=BHLH123 PE=2 SV=1	1.13	5.78	-1.74	down	0.00	0.01	yes

TRINITY_DN21045_c0_g1	-	-	-	28.42	92.06	-1.17	down	0.00	0.01	yes
TRINITY_DN15851_c0_g1	PREDICTED: DNA replication complex GINS protein PSF2-like isoform X1 [Populus euphratica]	GINS2	DNA replication complex GINS protein PSF2 OS=Arabidopsis thaliana GN=GINS2 PE=2 SV=2	0.74	4.52	-2.13	down	0.00	0.01	yes
TRINITY_DN21048_c0_g1	-	-	-	6.10	36.53	-1.96	down	0.00	0.01	yes
TRINITY_DN14003_c0_g3	fatty acid elongase 3-ketoacyl-CoA synthase 1 family protein [Populus trichocarpa]	KCS1	3-ketoacyl-CoA synthase 1 OS=Arabidopsis thaliana GN=KCS1 PE=1 SV=1	0.44	2.93	-2.11	down	0.00	0.01	yes
TRINITY_DN22050_c0_g2	zinc finger family protein [Populus trichocarpa]	XERICO	Probable E3 ubiquitin-protein ligase XERICO OS=Arabidopsis thaliana GN=XERICO PE=1 SV=1	4.31	15.91	-1.26	down	0.00	0.01	yes
TRINITY_DN20715_c0_g2	-	-	-	0.24	2.53	-2.68	down	0.00	0.01	yes
TRINITY_DN20119_c0_g1	hypothetical protein POPTR_0009s04350g [Populus trichocarpa]	-	-	1.65	6.25	-1.31	down	0.00	0.01	yes
TRINITY_DN20852_c0_g3	NO POLLEN GERMINATION RELATED 1 family protein [Populus trichocarpa]	-	-	1.32	8.01	-2.00	down	0.00	0.01	yes
TRINITY_DN20820_c1_g1	hypothetical protein POPTR_0015s12560g [Populus trichocarpa]	-	-	1.99	8.34	-1.54	down	0.00	0.01	yes
TRINITY_DN25916_c0_g1	PREDICTED: protein SCAI homolog isoform X1 [Populus euphratica]	SCAI	Protein SCAI OS=Homo sapiens GN=SCAI PE=1 SV=2	3.40	10.77	-1.08	down	0.00	0.01	yes
TRINITY_DN20778_c3_g2	-	-	-	1.28	4.79	-1.98	down	0.00	0.01	yes
TRINITY_DN19103_c0_g1	PREDICTED: charged multivesicular body protein 7 isoform X1 [Populus euphratica]	-	-	3.93	12.95	-1.10	down	0.00	0.01	yes
TRINITY_DN7940_c0_g2	PREDICTED: uncharacterized protein LOC105111104, partial [Populus euphratica]	-	-	0.33	2.49	-2.25	down	0.00	0.01	yes
TRINITY_DN22667_c0_g5	PREDICTED: transcription factor TCP14-like [Populus euphratica]	TCP14	Transcription factor TCP14 OS=Arabidopsis thaliana GN=TCP14 PE=1 SV=1	8.12	25.93	-1.03	down	0.00	0.01	yes
TRINITY_DN16047_c0_g1	hypothetical protein POPTR_0002s07680g [Populus trichocarpa]	-	-	0.57	3.70	-2.09	down	0.00	0.01	yes
TRINITY_DN22346_c0_g2	PREDICTED: zinc finger protein CONSTANS-LIKE 7 [Populus euphratica]	CIA2	Protein CHLOROPLAST IMPORT APPARATUS 2 OS=Arabidopsis thaliana GN=CIA2 PE=2 SV=1	0.92	4.83	-1.77	down	0.00	0.01	yes
TRINITY_DN15251_c0_g2	PREDICTED: omega-hydroxypalmitate O-feruloyl transferase-like [Populus euphratica]	AMAT	Methanol O-anthraniloyltransferase OS=Vitis labrusca GN=AMAT PE=1 SV=1	0.43	2.86	-2.09	down	0.00	0.01	yes
TRINITY_DN14807_c0_g1	hypothetical protein GLYMA_11G052900 [Glycine max]	-	-	0.75	3.95	-2.46	down	0.00	0.01	yes
TRINITY_DN27663_c1_g4	hypothetical protein POPTR_0002s06700g [Populus trichocarpa]	-	-	0.29	2.28	-2.33	down	0.00	0.01	yes
TRINITY_DN16294_c0_g1	harpin-induced family protein [Populus trichocarpa]	-	-	13.72	76.93	-1.90	down	0.00	0.01	yes
TRINITY_DN25695_c0_g3	hypothetical protein POPTR_0017s05340g [Populus trichocarpa]	At3g47200	UPF0481 protein At3g47200 OS=Arabidopsis thaliana GN=At3g47200 PE=2 SV=1	0.71	8.82	-2.37	down	0.00	0.01	yes
TRINITY_DN14971_c0_g1	DREB68 [Populus hopeiensis]	DREB1A	Dehydration-responsive element-binding protein 1A OS=Arabidopsis thaliana GN=DREB1A PE=1 SV=2	0.24	11.63	-4.47	down	0.00	0.01	yes
TRINITY_DN16794_c0_g2	hypothetical protein POPTR_0010s13270g [Populus trichocarpa]	-	-	0.92	4.62	-1.65	down	0.00	0.01	yes
TRINITY_DN26393_c0_g2	PREDICTED: uncharacterized protein LOC105139336 [Populus euphratica]	Tubgcp6	Gamma-tubulin complex component 6 OS=Mus musculus GN=Tubgcp6 PE=1 SV=1	5.07	19.00	-1.00	down	0.00	0.01	yes
TRINITY_DN19582_c0_g1	PREDICTED: probable WRKY transcription factor 40 isoform X1 [Populus euphratica]	WRKY40	Probable WRKY transcription factor 40 OS=Arabidopsis thaliana GN=WRKY40 PE=1 SV=1	2.06	18.45	-2.67	down	0.00	0.01	yes
TRINITY_DN20291_c0_g1	PREDICTED: fasciclin-like arabinogalactan protein 12 [Populus euphratica]	FLA12	Fasciclin-like arabinogalactan protein 12 OS=Arabidopsis thaliana GN=FLA12 PE=2 SV=2	0.89	6.34	-1.94	down	0.00	0.01	yes
TRINITY_DN15549_c0_g1	PREDICTED: NAC domain-containing protein 90-like [Populus euphratica]	NAC090	NAC domain-containing protein 90 OS=Arabidopsis thaliana GN=NAC090 PE=2 SV=1	9.93	59.23	-2.07	down	0.00	0.01	yes
TRINITY_DN17355_c0_g1	PREDICTED: uncharacterized protein LOC105120397 [Populus euphratica]	-	-	5.43	28.90	-1.83	down	0.00	0.01	yes
TRINITY_DN27824_c1_g2	PREDICTED: DNA topoisomerase 2-like isoform X3 [Populus euphratica]	TOP2	DNA topoisomerase 2 OS=Arabidopsis thaliana GN=TOP2 PE=2 SV=2	10.70	42.44	-1.42	down	0.00	0.01	yes
TRINITY_DN27605_c0_g1	-	-	-	2.91	14.08	-1.66	down	0.00	0.01	yes

TRINITY_DN22426_c0_g3	hypothetical protein POPTR_0008s05120g [Populus trichocarpa]	-	-	2.00	8.62	-1.64	down	0.00	0.01	yes
TRINITY_DN17816_c0_g1	hypothetical protein POPTR_0006s22930g [Populus trichocarpa]	-	-	0.42	2.66	-2.07	down	0.00	0.01	yes
TRINITY_DN22980_c0_g3	-	-	-	0.30	2.31	-2.33	down	0.00	0.01	yes
TRINITY_DN20411_c0_g2	PREDICTED: nicotinamide mononucleotide adenylyltransferase-like [Populus euphratica]	NMNAT	Nicotinamide/nicotinic acid mononucleotide adenylyltransferase OS=Arabidopsis thaliana GN=NMNAT PE=2 SV=1	5.17	16.84	-1.08	down	0.00	0.01	yes
TRINITY_DN24058_c0_g1	F-box family protein [Populus trichocarpa]	FBL8	Putative F-box/LRR-repeat protein 8 OS=Arabidopsis thaliana GN=FBL8 PE=3 SV=1	8.01	24.79	-1.04	down	0.00	0.01	yes
TRINITY_DN21772_c0_g8	hypothetical protein POPTR_0008s00610g [Populus trichocarpa]	-	-	4.85	18.35	-1.31	down	0.00	0.01	yes
TRINITY_DN17260_c1_g1	hypothetical protein POPTR_0003s17180g [Populus trichocarpa]	-	-	0.72	6.74	-2.39	down	0.00	0.01	yes
TRINITY_DN23385_c0_g3	PREDICTED: serine/threonine-protein kinase CTR1 isoform X1 [Populus euphratica]	CTR1	Serine/threonine-protein kinase CTR1 OS=Arabidopsis thaliana GN=CTR1 PE=1 SV=1	0.76	4.25	-1.89	down	0.00	0.01	yes
TRINITY_DN25336_c0_g1	WRKY transcription factor 21 family protein [Populus trichocarpa]	WRKY21	Probable WRKY transcription factor 21 OS=Arabidopsis thaliana GN=WRKY21 PE=2 SV=1	7.37	24.00	-1.12	down	0.00	0.01	yes
TRINITY_DN25950_c0_g1	hypothetical protein POPTR_0003s10580g [Populus trichocarpa]	RMR1	Receptor homology region, transmembrane domain- and RING domain-containing protein 2 OS=Oryza sativa subsp. japonica GN=RMR1 PE=2 SV=2	3.89	13.18	-1.03	down	0.00	0.01	yes
TRINITY_DN26677_c1_g2	PREDICTED: phragmoplast orienting kinesin-1 isoform X1 [Populus euphratica]	KIN12C	Kinesin-like protein KIN-12C OS=Arabidopsis thaliana GN=KIN12C PE=1 SV=1	2.96	9.55	-1.11	down	0.00	0.01	yes
TRINITY_DN24856_c0_g1	hypothetical protein POPTR_0009s12460g [Populus trichocarpa]	-	-	10.73	31.13	-1.04	down	0.00	0.01	yes
TRINITY_DN19209_c0_g2	DNA replication family protein [Populus trichocarpa]	Gins4	DNA replication complex GINS protein SLD5 OS=Rattus norvegicus GN=Gins4 PE=1 SV=1	3.11	12.25	-1.35	down	0.00	0.01	yes
TRINITY_DN16214_c0_g1	hypothetical protein POPTR_0014s14320g [Populus trichocarpa]	-	-	1.46	9.29	-2.04	down	0.00	0.01	yes
TRINITY_DN16952_c0_g1	hypothetical protein POPTR_0008s02180g [Populus trichocarpa]	TBL36	Protein trichome birefringence-like 36 OS=Arabidopsis thaliana GN=TBL36 PE=2 SV=1	4.10	16.48	-1.42	down	0.00	0.01	yes
TRINITY_DN23474_c0_g3	hypothetical protein POPTR_0004s11980g, partial [Populus trichocarpa]	AFP3	Ninja-family protein AFP3 OS=Arabidopsis thaliana GN=AFP3 PE=1 SV=1	0.46	2.47	-1.81	down	0.00	0.01	yes
TRINITY_DN23773_c1_g3	brassinosteroid-6-oxidase family protein [Populus trichocarpa]	BA13	Cytochrome P450 85A OS=Phaseolus vulgaris GN=BA13 PE=3 SV=2	2.55	13.27	-1.83	down	0.00	0.01	yes
TRINITY_DN27107_c0_g1	hypothetical protein POPTR_0006s05130g [Populus trichocarpa]	RFS2	Probable galactinol--sucrose galactosyltransferase 2 OS=Arabidopsis thaliana GN=RFS2 PE=2 SV=2	10.71	55.55	-1.71	down	0.00	0.01	yes
TRINITY_DN20161_c1_g3	alpha-tubulin 7 [Salix arbutifolia]	-	Tubulin alpha-2 chain OS=Gossypium hirsutum PE=2 SV=1	172.29	554.38	-1.10	down	0.00	0.01	yes
TRINITY_DN18043_c0_g1	hypothetical protein POPTR_0020s00530g [Populus trichocarpa]	ARAD1	Probable arabinosyltransferase ARAD1 OS=Arabidopsis thaliana GN=ARAD1 PE=1 SV=1	1.34	5.32	-1.40	down	0.00	0.01	yes
TRINITY_DN26373_c0_g1	PREDICTED: zinc finger BED domain-containing protein DAYSLEEPER-like isoform X6 [Populus euphratica]	HAT	Zinc finger BED domain-containing protein DAYSLEEPER OS=Arabidopsis thaliana GN=HAT PE=1 SV=1	41.86	128.39	-1.00	down	0.00	0.01	yes
TRINITY_DN18970_c0_g1	hypothetical protein POPTR_0008s15120g [Populus trichocarpa]	ACL5	Thermospermine synthase ACAULIS5 OS=Arabidopsis thaliana GN=ACL5 PE=1 SV=1	4.62	19.39	-1.57	down	0.00	0.01	yes
TRINITY_DN17636_c0_g3	PREDICTED: uncharacterized protein LOC105132777 isoform X3 [Populus euphratica]	-	-	0.61	3.85	-2.03	down	0.00	0.01	yes
TRINITY_DN9137_c0_g1	hypothetical protein POPTR_0008s11870g [Populus trichocarpa]	-	-	1.48	10.76	-2.23	down	0.00	0.01	yes
TRINITY_DN18117_c0_g2	-	-	-	0.51	3.77	-2.20	down	0.00	0.01	yes
TRINITY_DN26143_c0_g1	PREDICTED: mitogen-activated protein kinase kinase kinase 1-like [Populus euphratica]	MEKK1	Mitogen-activated protein kinase kinase kinase 1 OS=Arabidopsis thaliana GN=MEKK1 PE=1 SV=2	13.45	42.54	-1.18	down	0.00	0.01	yes
TRINITY_DN18907_c0_g3	-	-	-	0.48	4.43	-2.54	down	0.00	0.01	yes
TRINITY_DN27876_c5_g1	hypothetical protein POPTR_0005s16540g [Populus trichocarpa]	ACC1	Acetyl-CoA carboxylase 1 OS=Arabidopsis thaliana GN=ACC1 PE=1 SV=1	2.20	11.69	-1.61	down	0.00	0.01	yes
TRINITY_DN17206_c0_g1	hypothetical protein POPTR_0016s12210g [Populus trichocarpa]	Os04g0679100	Clathrin light chain 1 OS=Oryza sativa subsp. japonica GN=Os04g0679100 PE=2 SV=1	3.70	14.65	-1.36	down	0.00	0.01	yes

TRINITY_DN15014_c0_g2	-	-	-	0.73	5.26	-2.20	down	0.00	0.01	yes
TRINITY_DN22925_c0_g4	-	-	-	0.96	7.79	-2.39	down	0.00	0.01	yes
TRINITY_DN17875_c0_g2	-	-	-	0.78	3.84	-1.74	down	0.00	0.01	yes
TRINITY_DN16757_c1_g2	-	-	-	1.33	4.43	-1.76	down	0.00	0.01	yes
TRINITY_DN27786_c1_g2	PREDICTED: transcription factor bHLH13-like [Populus euphratica]	BHLH13	Transcription factor bHLH13 OS=Arabidopsis thaliana GN=BHLH13 PE=2 SV=1	6.32	25.46	-1.44	down	0.00	0.01	yes
TRINITY_DN19247_c0_g1	PREDICTED: oxalate--CoA ligase-like [Populus euphratica]	AAE3	Oxalate--CoA ligase OS=Arabidopsis thaliana GN=AAE3 PE=1 SV=1	5.18	30.82	-1.92	down	0.00	0.01	yes
TRINITY_DN23579_c0_g2	hypothetical protein POPTR_0001s16780g [Populus trichocarpa]	LOX3	Lipoxygenase 3, chloroplastic OS=Arabidopsis thaliana GN=LOX3 PE=2 SV=1	14.61	101.79	-2.24	down	0.00	0.01	yes
TRINITY_DN21116_c0_g1	hypothetical protein POPTR_0019s01930g [Populus trichocarpa]	-	-	2.14	19.83	-2.51	down	0.00	0.01	yes
TRINITY_DN24767_c0_g2	PREDICTED: uncharacterized protein LOC105122922 [Populus euphratica]	tag	DNA-3-methyladenine glycosylase 1 OS=Escherichia coli (strain K12) GN=tag PE=1 SV=1	4.81	14.63	-1.01	down	0.00	0.01	yes
TRINITY_DN1629_c0_g1	hypothetical protein POPTR_0011s03050g [Populus trichocarpa]	-	-	0.43	4.61	-2.77	down	0.00	0.01	yes
TRINITY_DN17261_c0_g4	PREDICTED: potassium channel KAT3-like [Populus euphratica]	KAT3	Potassium channel KAT3 OS=Arabidopsis thaliana GN=KAT3 PE=1 SV=1	0.47	5.28	-2.87	down	0.00	0.01	yes
TRINITY_DN26427_c2_g6	hypothetical protein POPTR_0019s09130g [Populus trichocarpa]	-	-	0.39	2.54	-2.11	down	0.00	0.01	yes
TRINITY_DN25864_c0_g6	hypothetical protein POPTR_0004s12530g [Populus trichocarpa]	MYB306	Myb-related protein 306 OS=Antirrhinum majus GN=MYB306 PE=2 SV=1	1.97	10.50	-1.81	down	0.00	0.01	yes
TRINITY_DN14534_c0_g1	-	-	-	0.81	6.64	-2.36	down	0.00	0.01	yes
TRINITY_DN13467_c0_g1	hypothetical protein POPTR_0007s15330g [Populus trichocarpa]	-	-	0.37	3.09	-2.43	down	0.00	0.01	yes
TRINITY_DN24123_c0_g1	MYB055 [Populus tomentosa]	MYB86	Transcription factor MYB86 OS=Arabidopsis thaliana GN=MYB86 PE=2 SV=1	0.79	4.58	-1.98	down	0.00	0.01	yes
TRINITY_DN16399_c0_g2	hypothetical protein POPTR_0001s11800g [Populus trichocarpa]	ERF106	Ethylene-responsive transcription factor ERF106 OS=Arabidopsis thaliana GN=ERF106 PE=2 SV=1	3.31	12.97	-1.39	down	0.00	0.01	yes
TRINITY_DN23046_c0_g1	PIN1-like family protein [Populus trichocarpa]	PIN7	Auxin efflux carrier component 7 OS=Arabidopsis thaliana GN=PIN7 PE=1 SV=2	0.66	3.70	-1.86	down	0.00	0.01	yes
TRINITY_DN23466_c1_g6	hypothetical protein POPTR_0008s13370g [Populus trichocarpa]	-	-	3.96	6.00	-2.06	down	0.00	0.01	yes
TRINITY_DN15184_c0_g1	PREDICTED: protein MOR1-like [Populus euphratica]	MOR1	Protein MOR1 OS=Oryza sativa subsp. japonica GN=MOR1 PE=2 SV=1	5.71	21.99	-1.32	down	0.00	0.01	yes
TRINITY_DN17795_c0_g1	hypothetical protein POPTR_0002s01760g [Populus trichocarpa]	-	-	2.32	10.98	-1.36	down	0.00	0.01	yes
TRINITY_DN25161_c0_g1	-	-	-	0.34	2.69	-2.31	down	0.00	0.01	yes
TRINITY_DN24985_c0_g1	PREDICTED: probable disease resistance protein At4g27220 isoform X1 [Populus euphratica]	At5g63020	Probable disease resistance protein At5g63020 OS=Arabidopsis thaliana GN=At5g63020 PE=2 SV=2	2.94	10.83	-1.82	down	0.00	0.01	yes
TRINITY_DN21559_c0_g2	hypothetical protein POPTR_0024s00730g [Populus trichocarpa]	LRX4	Leucine-rich repeat extensin-like protein 4 OS=Arabidopsis thaliana GN=LRX4 PE=1 SV=1	1.31	5.26	-1.37	down	0.00	0.01	yes
TRINITY_DN14845_c0_g2	zinc finger family protein [Populus trichocarpa]	ZAT9	Zinc finger protein ZAT9 OS=Arabidopsis thaliana GN=ZAT9 PE=2 SV=1	0.88	4.99	-1.90	down	0.00	0.01	yes
TRINITY_DN23214_c0_g2	hypothetical protein POPTR_0010s24410g [Populus trichocarpa]	ZPR4	Protein LITTLE ZIPPER 4 OS=Arabidopsis thaliana GN=ZPR4 PE=1 SV=1	0.78	4.98	-2.04	down	0.00	0.01	yes
TRINITY_DN19795_c0_g1	hypothetical protein POPTR_0016s02850g [Populus trichocarpa]	-	-	1.15	5.52	-1.64	down	0.00	0.01	yes
TRINITY_DN13596_c0_g1	hypothetical protein POPTR_0018s03620g [Populus trichocarpa]	-	-	1.19	7.88	-2.09	down	0.00	0.01	yes
TRINITY_DN23061_c0_g1	PREDICTED: leucine-rich repeat extensin-like protein 3 isoform X1 [Populus euphratica]	LRX4	Leucine-rich repeat extensin-like protein 4 OS=Arabidopsis thaliana GN=LRX4 PE=1 SV=1	2.28	10.63	-1.72	down	0.00	0.01	yes
TRINITY_DN18461_c0_g1	hypothetical protein POPTR_0010s09130g [Populus trichocarpa]	ACA12	Calcium-transporting ATPase 12, plasma membrane-type OS=Arabidopsis thaliana GN=ACA12 PE=2 SV=1	0.30	2.22	-2.23	down	0.00	0.01	yes

TRINITY_DN24443_c0_g3	hypothetical protein POPTR_0006s18240g [Populus trichocarpa]	At5g45670	GDSL esterase/lipase At5g45670 OS=Arabidopsis thaliana GN=At5g45670 PE=2 SV=1	48.96	203.82	-1.30	down	0.00	0.01	yes
TRINITY_DN15098_c0_g1	-	-	-	3.42	13.15	-1.34	down	0.00	0.01	yes
TRINITY_DN25576_c1_g3	hypothetical protein POPTR_0018s02370g [Populus trichocarpa]	CAF1-7	Probable CCR4-associated factor 1 homolog 7 OS=Arabidopsis thaliana GN=CAF1-7 PE=2 SV=2	12.78	49.78	-1.36	down	0.00	0.01	yes
TRINITY_DN19575_c0_g1	PREDICTED: transcriptional regulator ATRX homolog [Populus euphratica]	-	-	24.06	115.90	-1.67	down	0.00	0.01	yes
TRINITY_DN26763_c0_g1	KINESIN-LIKE protein A [Populus trichocarpa]	KIN14C	Kinesin-like protein KIN-14C OS=Arabidopsis thaliana GN=KIN14C PE=2 SV=1	16.84	50.92	-1.05	down	0.00	0.01	yes
TRINITY_DN14926_c0_g1	hypothetical protein POPTR_0003s08870g [Populus trichocarpa]	IRL5	Plant intracellular Ras-group-related LRR protein 5 OS=Oryza sativa subsp. japonica GN=IRL5 PE=2 SV=1	0.47	3.31	-2.05	down	0.00	0.01	yes
TRINITY_DN20959_c0_g6	PREDICTED: zinc finger A20 and AN1 domain-containing stress-associated protein 5-like [Populus euphratica]	SAP5	Zinc finger A20 and AN1 domain-containing stress-associated protein 5 OS=Arabidopsis thaliana GN=SAP5 PE=2 SV=1	18.09	76.21	-1.41	down	0.00	0.01	yes
TRINITY_DN26052_c0_g1	hypothetical protein POPTR_0008s11780g, partial [Populus trichocarpa]	PRPF4B	Serine/threonine-protein kinase PRP4 homolog OS=Bos taurus GN=PRPF4B PE=2 SV=1	7.04	22.42	-1.02	down	0.00	0.01	yes
TRINITY_DN23002_c0_g2	PREDICTED: golgin subfamily A member 6-like protein 22 [Populus euphratica]	-	-	6.45	25.04	-1.36	down	0.00	0.01	yes
TRINITY_DN27082_c0_g1	hypothetical protein POPTR_0005s22880g [Populus trichocarpa]	PAO2	Probable polyamine oxidase 2 OS=Arabidopsis thaliana GN=PAO2 PE=2 SV=1	18.47	72.77	-1.32	down	0.00	0.01	yes
TRINITY_DN27252_c0_g1	hypothetical protein POPTR_0003s19720g [Populus trichocarpa]	HERC2	E3 ubiquitin-protein ligase HERC2 OS=Homo sapiens GN=HERC2 PE=1 SV=2	4.92	16.33	-1.15	down	0.00	0.01	yes
TRINITY_DN25440_c0_g5	PREDICTED: kinesin-4 [Populus euphratica]	KIN14L	Kinesin-like protein KIN-14L OS=Arabidopsis thaliana GN=KIN14L PE=3 SV=2	3.71	12.32	-1.14	down	0.00	0.01	yes
TRINITY_DN20973_c0_g1	PREDICTED: DNA polymerase delta catalytic subunit [Populus euphratica]	POLD1	DNA polymerase delta catalytic subunit OS=Oryza sativa subsp. japonica GN=POLD1 PE=2 SV=1	3.70	13.41	-1.20	down	0.00	0.01	yes
TRINITY_DN16562_c0_g2	hypothetical protein POPTR_0018s06250g [Populus trichocarpa]	At1g64760	Glucan endo-1,3-beta-glucosidase 8 OS=Arabidopsis thaliana GN=At1g64760 PE=1 SV=2	0.35	2.29	-2.14	down	0.00	0.01	yes
TRINITY_DN13443_c0_g1	hypothetical protein POPTR_0006s05080g [Populus trichocarpa]	-	-	9.94	48.36	-1.61	down	0.00	0.01	yes
TRINITY_DN27459_c0_g1	hypothetical protein POPTR_0005s28380g [Populus trichocarpa]	KIN4C	Kinesin-like protein KIN-4C OS=Arabidopsis thaliana GN=KIN4C PE=2 SV=2	20.97	60.76	-1.01	down	0.00	0.01	yes
TRINITY_DN25911_c1_g4	-	-	-	4.46	15.08	-1.17	down	0.00	0.01	yes
TRINITY_DN22742_c0_g1	hypothetical protein POPTR_0001s08250g [Populus trichocarpa]	-	-	7.50	23.99	-1.04	down	0.00	0.01	yes
TRINITY_DN25966_c0_g1	hypothetical protein POPTR_0010s25750g [Populus trichocarpa]	-	-	2.93	21.79	-2.26	down	0.00	0.01	yes
TRINITY_DN21482_c1_g2	PREDICTED: calmodulin-binding receptor-like cytoplasmic kinase 2 [Populus euphratica]	CRCK2	Calmodulin-binding receptor-like cytoplasmic kinase 2 OS=Arabidopsis thaliana GN=CRCK2 PE=2 SV=1	2.33	9.92	-1.35	down	0.00	0.01	yes
TRINITY_DN26427_c2_g2	-	-	-	0.20	1.91	-2.57	down	0.00	0.01	yes
TRINITY_DN16270_c0_g1	-	-	-	0.73	5.64	-2.24	down	0.00	0.01	yes
TRINITY_DN17334_c0_g1	PREDICTED: uncharacterized protein LOC105122874 [Populus euphratica]	SGS3	Protein SUPPRESSOR OF GENE SILENCING 3 OS=Solanum lycopersicum GN=SGS3 PE=1 SV=1	1.11	5.99	-2.19	down	0.00	0.01	yes
TRINITY_DN16178_c0_g2	hypothetical protein POPTR_0012s04810g [Populus trichocarpa]	DTX16	Protein DETOXIFICATION 16 OS=Arabidopsis thaliana GN=DTX16 PE=2 SV=1	0.96	4.90	-1.73	down	0.00	0.01	yes
TRINITY_DN25469_c1_g3	hypothetical protein POPTR_0010s01670g [Populus trichocarpa]	-	-	13.19	44.47	-1.15	down	0.00	0.01	yes
TRINITY_DN23547_c0_g1	PREDICTED: zinc-finger homeodomain protein 4 [Populus euphratica]	ZHD4	Zinc-finger homeodomain protein 4 OS=Arabidopsis thaliana GN=ZHD4 PE=1 SV=1	9.82	31.58	-1.13	down	0.00	0.01	yes
TRINITY_DN19537_c0_g3	PREDICTED: uncharacterized protein LOC105109677 isoform X1 [Populus euphratica]	-	-	5.42	16.71	-1.06	down	0.00	0.01	yes
TRINITY_DN20968_c0_g1	hypothetical protein POPTR_0001s30340g [Populus trichocarpa]	-	-	2.40	8.45	-1.38	down	0.00	0.01	yes
TRINITY_DN27737_c1_g1	zinc finger family protein [Populus trichocarpa]	SAP5	Zinc finger A20 and AN1 domain-containing stress-associated protein 5 OS=Arabidopsis thaliana GN=SAP5 PE=2 SV=1	21.95	99.76	-1.55	down	0.00	0.01	yes

TRINITY_DN20879_c0_g2	PREDICTED: uncharacterized protein LOC105123549 [Populus euphratica]	-	-	6.37	26.35	-1.31	down	0.00	0.01	yes
TRINITY_DN17257_c0_g1	hypothetical protein POPTR_0001s00440g [Populus trichocarpa]	-	L-lactate dehydrogenase A OS=Hordeum vulgare PE=1 SV=1	6.45	23.17	-1.22	down	0.00	0.01	yes
TRINITY_DN21666_c0_g4	PREDICTED: axial regulator YABBY 1 [Populus euphratica]	YAB4	Protein YABBY 4 OS=Oryza sativa subsp. indica GN=YAB4 PE=3 SV=1	0.24	1.94	-2.45	down	0.00	0.01	yes
TRINITY_DN14445_c0_g2	PREDICTED: glycine-rich cell wall structural protein-like, partial [Populus euphratica]	-	-	15.77	78.50	-1.71	down	0.00	0.01	yes
TRINITY_DN25014_c0_g1	GAMMA RESPONSE 1 family protein [Populus trichocarpa]	GR1	Protein gamma response 1 OS=Arabidopsis thaliana GN=GR1 PE=1 SV=2	1.74	7.06	-1.35	down	0.00	0.01	yes
TRINITY_DN24076_c0_g1	PREDICTED: uncharacterized protein LOC105122325 isoform X7 [Populus euphratica]	ASI1	Protein ANTI-SILENCING 1 OS=Arabidopsis thaliana GN=ASI1 PE=3 SV=2	2.34	8.24	-1.11	down	0.00	0.01	yes
TRINITY_DN13915_c1_g3	hypothetical protein POPTR_0013s13800g [Populus trichocarpa]	At2g20760	Clathrin light chain 1 OS=Arabidopsis thaliana GN=At2g20760 PE=2 SV=1	0.67	4.64	-2.14	down	0.00	0.01	yes
TRINITY_DN22481_c1_g3	PREDICTED: UPF0481 protein At3g47200-like isoform X1 [Populus euphratica]	At3g47200	UPF0481 protein At3g47200 OS=Arabidopsis thaliana GN=At3g47200 PE=2 SV=1	0.61	3.78	-1.97	down	0.00	0.01	yes
TRINITY_DN13216_c0_g1	-	-	-	0.28	2.31	-2.40	down	0.00	0.01	yes
TRINITY_DN24209_c0_g1	PREDICTED: cellulose synthase-like protein D3 [Populus euphratica]	CSLD2	Cellulose synthase-like protein D2 OS=Oryza sativa subsp. japonica GN=CSLD2 PE=2 SV=1	1.25	9.86	-2.35	down	0.00	0.01	yes
TRINITY_DN27889_c0_g2	TIR-NBS disease resistance-like protein [Populus trichocarpa]	-	-	3.77	20.58	-1.74	down	0.00	0.01	yes
TRINITY_DN20799_c0_g5	hypothetical protein POPTR_0013s04970g [Populus trichocarpa]	TYRDC-2	Tyrosine decarboxylase 2 OS=Petroselinum crispum GN=TYRDC-2 PE=2 SV=1	0.28	2.25	-2.32	down	0.00	0.01	yes
TRINITY_DN16864_c0_g1	-	-	-	0.80	4.83	-2.21	down	0.00	0.01	yes
TRINITY_DN22113_c0_g5	hypothetical protein POPTR_0001s14660g [Populus trichocarpa]	ABCG36	ABC transporter G family member 36 OS=Oryza sativa subsp. japonica GN=ABCG36 PE=2 SV=1	0.34	2.38	-2.55	down	0.00	0.01	yes
TRINITY_DN21601_c2_g1	-	-	-	0.27	2.65	-2.71	down	0.00	0.01	yes
TRINITY_DN23341_c1_g9	-	-	-	20.37	74.27	-1.23	down	0.00	0.01	yes
TRINITY_DN8603_c0_g3	-	-	-	0.29	2.73	-2.59	down	0.00	0.01	yes
TRINITY_DN18972_c0_g4	PREDICTED: probable LRR receptor-like serine/threonine-protein kinase RFK1 isoform X1 [Populus euphratica]	RKF1	Probable LRR receptor-like serine/threonine-protein kinase RFK1 OS=Arabidopsis thaliana GN=RKF1 PE=1 SV=1	1.48	5.13	-1.41	down	0.00	0.01	yes
TRINITY_DN21865_c0_g1	WRKY transcription factor 17 [(Populus tomentosa x Populus bolleana) x Populus tomentosa]	WRKY24	WRKY transcription factor WRKY24 OS=Oryza sativa subsp. japonica GN=WRKY24 PE=2 SV=1	2.52	17.60	-2.33	down	0.00	0.01	yes
TRINITY_DN19539_c0_g2	hypothetical protein POPTR_0010s11890g [Populus trichocarpa]	SKIP28	F-box protein SKIP28 OS=Arabidopsis thaliana GN=SKIP28 PE=1 SV=1	6.32	19.65	-1.01	down	0.00	0.01	yes
TRINITY_DN19374_c0_g2	hypothetical protein POPTR_0010s23370g [Populus trichocarpa]	KINUC	Kinesin-like protein KIN-UC OS=Arabidopsis thaliana GN=KINUC PE=1 SV=2	0.57	4.04	-2.18	down	0.00	0.01	yes
TRINITY_DN23914_c0_g1	hypothetical protein POPTR_0013s02120g [Populus trichocarpa]	ALY2	Protein ALWAYS EARLY 2 OS=Arabidopsis thaliana GN=ALY2 PE=1 SV=1	4.30	13.26	-1.00	down	0.00	0.01	yes
TRINITY_DN21203_c0_g2	hypothetical protein POPTR_0005s08900g [Populus trichocarpa]	TKI1	TSL-kinase interacting protein 1 OS=Arabidopsis thaliana GN=TKI1 PE=1 SV=2	1.50	6.39	-1.45	down	0.00	0.01	yes
TRINITY_DN22673_c1_g1	hypothetical protein POPTR_0003s07860g [Populus trichocarpa]	-	-	0.98	4.81	-1.85	down	0.00	0.01	yes
TRINITY_DN23208_c0_g1	PREDICTED: oxysterol-binding protein-related protein 1D-like [Populus euphratica]	ORP1D	Oxysterol-binding protein-related protein 1D OS=Arabidopsis thaliana GN=ORP1D PE=2 SV=1	5.04	20.81	-1.01	down	0.00	0.01	yes
TRINITY_DN23022_c0_g2	peptidoglycan-binding LysM domain-containing family protein, partial [Populus trichocarpa]	-	-	0.80	5.18	-2.07	down	0.00	0.01	yes
TRINITY_DN13794_c0_g1	-	-	-	2.66	27.58	-2.86	down	0.00	0.01	yes
TRINITY_DN17867_c1_g1	hypothetical protein POPTR_0015s14360g [Populus trichocarpa]	-	-	1.26	6.03	-1.65	down	0.00	0.01	yes
TRINITY_DN17570_c0_g1	PREDICTED: E3 ubiquitin-protein ligase MARCH7-like [Populus euphratica]	-	-	0.93	3.55	-1.38	down	0.00	0.01	yes
TRINITY_DN17072_c0_g2	PREDICTED: putative glucose-6-phosphate 1-epimerase [Populus euphratica]	-	Putative glucose-6-phosphate 1-epimerase OS=Cenchrus ciliaris PE=2 SV=1	1.75	6.41	-1.31	down	0.00	0.01	yes

TRINITY_DN20438_c0_g4	hypothetical protein POPTR_0006s07350g [Populus trichocarpa]	SMXL5	Protein SMAX1-LIKE 5 OS=Arabidopsis thaliana GN=SMXL5 PE=2 SV=1	0.90	8.33	-2.55	down	0.00	0.01	yes
TRINITY_DN22492_c1_g4	hypothetical protein POPTR_0003s19840g [Populus trichocarpa]	ASIL2	Trihelix transcription factor ASIL2 OS=Arabidopsis thaliana GN=ASIL2 PE=2 SV=1	1.57	6.57	-1.43	down	0.00	0.01	yes
TRINITY_DN25060_c0_g3	glycosyl hydrolase family 17 family protein [Populus trichocarpa]	At4g29360	Glucan endo-1,3-beta-glucosidase 12 OS=Arabidopsis thaliana GN=At4g29360 PE=1 SV=1	114.85	330.01	-1.01	down	0.00	0.01	yes
TRINITY_DN16637_c0_g1	hypothetical protein POPTR_0007s03710g [Populus trichocarpa]	TSK	Protein TONSOKU OS=Arabidopsis thaliana GN=TSK PE=1 SV=2	1.19	5.06	-1.44	down	0.00	0.01	yes
TRINITY_DN24236_c0_g3	hypothetical protein POPTR_0014s03120g [Populus trichocarpa]	At5g42700	B3 domain-containing protein At5g42700 OS=Arabidopsis thaliana GN=At5g42700 PE=2 SV=2	9.65	29.84	-1.03	down	0.00	0.01	yes
TRINITY_DN22465_c0_g3	hypothetical protein POPTR_0015s07870g [Populus trichocarpa]	AATP1	AAA-ATPase ASD, mitochondrial OS=Arabidopsis thaliana GN=AATP1 PE=1 SV=1	0.53	2.97	-1.84	down	0.00	0.01	yes
TRINITY_DN16799_c0_g1	-	-	-	0.64	5.38	-2.37	down	0.00	0.01	yes
TRINITY_DN27442_c0_g2	-	-	-	0.88	8.76	-2.62	down	0.00	0.01	yes
TRINITY_DN18367_c0_g2	PREDICTED: glutathione S-transferase F13-like [Populus euphratica]	GSTF13	Glutathione S-transferase F13 OS=Arabidopsis thaliana GN=GSTF13 PE=3 SV=1	30.78	108.77	-1.21	down	0.00	0.01	yes
TRINITY_DN24119_c0_g4	-	-	-	0.54	3.48	-2.06	down	0.00	0.01	yes
TRINITY_DN25142_c0_g2	pleckstrin homology domain-containing family protein [Populus trichocarpa]	-	-	0.32	2.56	-2.32	down	0.00	0.01	yes
TRINITY_DN23563_c1_g1	hypothetical protein MANES_15G172200 [Manihot esculenta]	-	-	0.51	4.12	-1.92	down	0.00	0.01	yes
TRINITY_DN16680_c0_g3	hypothetical protein POPTR_0010s13590g [Populus trichocarpa]	-	-	12.71	40.94	-1.06	down	0.00	0.01	yes
TRINITY_DN22141_c1_g4	-	-	-	0.51	3.16	-1.99	down	0.00	0.01	yes
TRINITY_DN14721_c0_g1	hypothetical protein POPTR_0008s14180g [Populus trichocarpa]	IDD5	Protein indeterminate-domain 5, chloroplastic OS=Arabidopsis thaliana GN=IDD5 PE=1 SV=1	12.06	37.39	-1.05	down	0.00	0.01	yes
TRINITY_DN28369_c0_g1	hypothetical protein POPTR_0011s00950g [Populus trichocarpa]	-	-	2.77	19.48	-2.16	down	0.00	0.01	yes
TRINITY_DN18280_c0_g1	PREDICTED: probable leucine-rich repeat receptor-like serine/threonine-protein kinase At3g14840 [Populus euphratica]	-	-	1.03	5.29	-2.29	down	0.00	0.01	yes
TRINITY_DN26211_c0_g3	leucine-rich repeat transmembrane protein kinase [Populus trichocarpa]	BAM3	Leucine-rich repeat receptor-like serine/threonine-protein kinase BAM3 OS=Arabidopsis thaliana GN=BAM3 PE=2 SV=3	4.01	16.95	-1.47	down	0.00	0.01	yes
TRINITY_DN22618_c1_g1	hypothetical protein POPTR_0014s07450g [Populus trichocarpa]	TCP9	Transcription factor TCP9 OS=Arabidopsis thaliana GN=TCP9 PE=1 SV=1	4.23	16.18	-1.28	down	0.00	0.01	yes
TRINITY_DN27159_c0_g1	hypothetical protein POPTR_0004s11970g [Populus trichocarpa]	RPK2	LRR receptor-like serine/threonine-protein kinase RPK2 OS=Arabidopsis thaliana GN=RPK2 PE=2 SV=1	1.63	7.70	-1.60	down	0.00	0.01	yes
TRINITY_DN24061_c0_g1	kinase family protein [Populus trichocarpa]	PUB50	Putative U-box domain-containing protein 50 OS=Arabidopsis thaliana GN=PUB50 PE=3 SV=1	1.22	5.01	-1.37	down	0.00	0.01	yes
TRINITY_DN22414_c0_g2	hypothetical protein POPTR_0005s06470g [Populus trichocarpa]	PUB25	U-box domain-containing protein 25 OS=Arabidopsis thaliana GN=PUB25 PE=2 SV=1	2.15	7.39	-1.20	down	0.00	0.01	yes
TRINITY_DN25597_c0_g4	PREDICTED: probable receptor-like serine/threonine-protein kinase At4g34500 [Populus euphratica]	At4g34500	Probable receptor-like serine/threonine-protein kinase At4g34500 OS=Arabidopsis thaliana GN=At4g34500 PE=2 SV=1	4.95	15.58	-1.04	down	0.00	0.01	yes
TRINITY_DN16968_c0_g1	125 kDa kinesin-related family protein [Populus trichocarpa]	KIN5C	Kinesin-like protein KIN-5C OS=Nicotiana tabacum GN=KIN5C PE=1 SV=1	5.11	17.25	-1.14	down	0.00	0.01	yes
TRINITY_DN23772_c0_g3	hypothetical protein POPTR_0004s07700g [Populus trichocarpa]	SCL8	Scarecrow-like protein 8 OS=Arabidopsis thaliana GN=SCL8 PE=2 SV=1	4.69	19.13	-1.41	down	0.00	0.01	yes
TRINITY_DN22040_c1_g5	PREDICTED: uncharacterized protein LOC105121103 [Populus euphratica]	-	-	39.54	129.86	-1.12	down	0.00	0.01	yes
TRINITY_DN24097_c0_g1	vacuolar ATP synthase subunit E family protein [Populus trichocarpa]	VHA-E2	V-type proton ATPase subunit E2 OS=Arabidopsis thaliana GN=VHA-E2 PE=2 SV=1	2.41	9.54	-1.40	down	0.00	0.01	yes
TRINITY_DN22515_c0_g12	-	-	-	25.21	78.02	-1.04	down	0.00	0.01	yes
TRINITY_DN22208_c1_g6	hypothetical protein POPTR_0005s10950g [Populus trichocarpa]	-	-	3.17	14.32	-1.55	down	0.00	0.01	yes
TRINITY_DN14407_c0_g1	PREDICTED: uncharacterized protein LOC105112182 [Populus euphratica]	-	-	0.29	2.57	-2.49	down	0.00	0.01	yes

TRINITY_DN27674_c0_g2	hypothetical protein POPTR_0007s13120g [Populus trichocarpa]	AUL1	Auxilin-like protein 1 OS=Arabidopsis thaliana GN=AUL1 PE=2 SV=2	2.83	21.44	-2.22	down	0.00	0.01	yes
TRINITY_DN17389_c0_g2	PREDICTED: ethylene-responsive transcription factor SHINE 2-like [Populus euphratica]	SHN2	Ethylene-responsive transcription factor SHINE 2 OS=Arabidopsis thaliana GN=SHN2 PE=2 SV=1	1.38	6.32	-1.53	down	0.00	0.01	yes
TRINITY_DN17166_c0_g1	hypothetical protein [Populus tomentosa]	IAA16	Auxin-responsive protein IAA16 OS=Arabidopsis thaliana GN=IAA16 PE=1 SV=1	4.27	19.54	-1.53	down	0.00	0.01	yes
TRINITY_DN23221_c1_g2	PREDICTED: putative cyclic nucleotide-gated ion channel 13 [Populus euphratica]	CNGC10	Probable cyclic nucleotide-gated ion channel 10 OS=Arabidopsis thaliana GN=CNGC10 PE=2 SV=2	0.99	4.50	-1.60	down	0.00	0.01	yes
TRINITY_DN26928_c1_g3	-	-	-	0.74	5.09	-2.14	down	0.00	0.01	yes
TRINITY_DN23846_c1_g1	polyubiquitin 3 [Medicago truncatula]	UBI11	Polyubiquitin OS=Nicotiana sylvestris GN=UBI11 PE=2 SV=1	117.55	341.42	-1.07	down	0.00	0.01	yes
TRINITY_DN19480_c0_g1	hypothetical protein POPTR_0001s35550g [Populus trichocarpa]	-	-	5.53	16.92	-1.00	down	0.00	0.01	yes
TRINITY_DN20821_c0_g2	PREDICTED: uncharacterized protein LOC105108615 isoform X1 [Populus euphratica]	-	-	1.05	6.17	-1.90	down	0.00	0.01	yes
TRINITY_DN17816_c0_g6	ERF domain protein 11 [Populus trichocarpa]	ERF4	Ethylene-responsive transcription factor 4 OS=Nicotiana sylvestris GN=ERF4 PE=2 SV=1	3.09	18.64	-2.04	down	0.00	0.01	yes
TRINITY_DN13859_c0_g1	PREDICTED: piriformospora indica-insensitive protein 2-like [Populus euphratica]	PII-2	Piriformospora indica-insensitive protein 2 OS=Arabidopsis thaliana GN=PII-2 PE=2 SV=1	0.50	3.47	-2.15	down	0.00	0.01	yes
TRINITY_DN24843_c0_g1	PREDICTED: probable glycosyltransferase At5g03795 [Populus euphratica]	At5g03795	Probable glycosyltransferase At5g03795 OS=Arabidopsis thaliana GN=At5g03795 PE=3 SV=2	1.75	6.75	-1.16	down	0.00	0.01	yes
TRINITY_DN27817_c3_g2	PREDICTED: calcium-transporting ATPase 9, plasma membrane-type isoform X1 [Populus euphratica]	ACA9	Calcium-transporting ATPase 9, plasma membrane-type OS=Arabidopsis thaliana GN=ACA9 PE=2 SV=2	1.08	6.59	-1.99	down	0.00	0.01	yes
TRINITY_DN17632_c0_g1	copper-binding family protein [Populus trichocarpa]	HIPP09	Heavy metal-associated isoprenylated plant protein 9 OS=Arabidopsis thaliana GN=HIPP09 PE=2 SV=1	0.46	3.14	-2.22	down	0.00	0.01	yes
TRINITY_DN21000_c0_g1	disease resistance RPP13-like protein 4 [Populus trichocarpa]	RPP13L4	Disease resistance RPP13-like protein 4 OS=Arabidopsis thaliana GN=RPP13L4 PE=1 SV=2	1.10	4.20	-1.32	down	0.00	0.01	yes
TRINITY_DN18290_c0_g1	hypothetical protein POPTR_0012s08690g [Populus trichocarpa]	-	-	2.93	16.03	-1.63	down	0.00	0.01	yes
TRINITY_DN25139_c1_g4	gibberellin 20-oxidase [Populus alba]	GA20OX1	Gibberellin 20 oxidase 1 OS=Arabidopsis thaliana GN=GA20OX1 PE=2 SV=2	3.02	13.44	-1.54	down	0.00	0.01	yes
TRINITY_DN27021_c1_g3	hypothetical protein POPTR_0004s08530g [Populus trichocarpa]	-	-	0.47	4.50	-2.61	down	0.00	0.01	yes
TRINITY_DN18814_c0_g1	PREDICTED: sister chromatid cohesion 1 protein 3 [Populus euphratica]	SYN3	Sister chromatid cohesion 1 protein 3 OS=Arabidopsis thaliana GN=SYN3 PE=2 SV=2	0.96	5.34	-1.80	down	0.00	0.01	yes
TRINITY_DN23075_c0_g1	hypothetical protein POPTR_0337s00220g [Populus trichocarpa]	At1g43910	AAA-ATPase At1g43910 OS=Arabidopsis thaliana GN=At1g43910 PE=1 SV=1	5.96	32.70	-1.39	down	0.00	0.01	yes
TRINITY_DN26710_c1_g2	hypothetical protein POPTR_0003s10680g [Populus trichocarpa]	TDR	Leucine-rich repeat receptor-like protein kinase TDR OS=Arabidopsis thaliana GN=TDR PE=1 SV=1	0.23	2.01	-2.56	down	0.00	0.01	yes
TRINITY_DN24484_c0_g2	-	-	-	0.67	5.97	-2.48	down	0.00	0.01	yes
TRINITY_DN24599_c6_g1	hypothetical protein POPTR_0007s03670g [Populus trichocarpa]	LECRK2	G-type lectin S-receptor-like serine/threonine-protein kinase LECRK2 OS=Oryza sativa subsp. indica GN=LECRK2 PE=2 SV=1	0.44	2.68	-1.97	down	0.00	0.01	yes
TRINITY_DN20752_c1_g2	INDOLE-3-ACETATE BETA-D-GLUCOSYLTRANSFERASE family protein [Populus trichocarpa]	UGT75L6	Crocetin glucosyltransferase, chloroplastic OS=Gardenia jasminoides GN=UGT75L6 PE=1 SV=1	9.89	40.30	-1.41	down	0.00	0.01	yes
TRINITY_DN18769_c0_g6	hypothetical protein POPTR_0005s27980g [Populus trichocarpa]	CP12-3	Calvin cycle protein CP12-3, chloroplastic OS=Arabidopsis thaliana GN=CP12-3 PE=1 SV=1	15.17	55.74	-1.31	down	0.00	0.01	yes
TRINITY_DN15954_c0_g1	hypothetical protein POPTR_0019s10670g [Populus trichocarpa]	-	-	6.40	20.19	-1.06	down	0.00	0.01	yes
TRINITY_DN27840_c1_g1	PREDICTED: putative disease resistance protein RGA1 [Populus euphratica]	-	-	23.92	81.78	-1.38	down	0.00	0.01	yes
TRINITY_DN19119_c1_g1	-	-	-	1.59	6.82	-1.48	down	0.00	0.01	yes
TRINITY_DN19501_c0_g1	hypothetical protein POPTR_0010s24060g [Populus trichocarpa]	-	-	0.19	1.77	-2.59	down	0.00	0.01	yes
TRINITY_DN24889_c1_g4	hypothetical protein POPTR_0018s10700g [Populus trichocarpa]	-	-	1.36	6.83	-1.70	down	0.00	0.01	yes

TRINITY_DN21220_c3_g2	hypothetical protein POPTR_0013s06100g [Populus trichocarpa]	-	-	12.55	46.34	-1.11	down	0.00	0.01	yes
TRINITY_DN27694_c0_g3	PREDICTED: protein IQ-DOMAIN 1 isoform X1 [Populus euphratica]	-	-	4.50	15.83	-1.19	down	0.00	0.01	yes
TRINITY_DN22515_c0_g2	-	-	-	6.04	22.27	-1.20	down	0.00	0.01	yes
TRINITY_DN23989_c1_g1	hypothetical protein POPTR_0007s13860g [Populus trichocarpa]	KIN7B	Putative inactive kinesin-like protein KIN-7B (Fragment) OS=Oryza sativa subsp. japonica GN=KIN7B PE=5 SV=2	1.46	7.68	-1.58	down	0.00	0.01	yes
TRINITY_DN19660_c1_g2	hypothetical protein POPTR_0005s25090g [Populus trichocarpa]	-	-	0.99	5.27	-1.82	down	0.00	0.01	yes
TRINITY_DN17771_c0_g6	PREDICTED: uncharacterized protein LOC105140768 isoform X1 [Populus euphratica]	-	-	0.42	4.18	-2.50	down	0.00	0.01	yes
TRINITY_DN22861_c0_g1	hypothetical protein POPTR_0004s04660g [Populus trichocarpa]	RING1A	Putative E3 ubiquitin-protein ligase RING1a OS=Arabidopsis thaliana GN=RING1A PE=1 SV=2	1.27	5.42	-1.37	down	0.00	0.01	yes
TRINITY_DN78_c0_g1	PREDICTED: GDSL esterase/lipase At2g42990-like [Populus euphratica]	At2g42990	GDSL esterase/lipase At2g42990 OS=Arabidopsis thaliana GN=At2g42990 PE=2 SV=1	0.33	2.30	-2.13	down	0.00	0.01	yes
TRINITY_DN24962_c1_g1	hypothetical protein POPTR_0016s14410g [Populus trichocarpa]	IRK	Probable LRR receptor-like serine/threonine-protein kinase IRK OS=Arabidopsis thaliana GN=IRK PE=1 SV=1	2.58	9.76	-1.31	down	0.00	0.01	yes
TRINITY_DN15762_c0_g2	PREDICTED: BTB/POZ domain-containing protein At3g49900 [Populus euphratica]	At3g49900	BTB/POZ domain-containing protein At3g49900 OS=Arabidopsis thaliana GN=At3g49900 PE=2 SV=1	0.33	2.48	-2.26	down	0.00	0.01	yes
TRINITY_DN17412_c0_g1	PREDICTED: probable E3 ubiquitin-protein ligase ARI8 [Populus euphratica]	ARI8	Probable E3 ubiquitin-protein ligase ARI8 OS=Arabidopsis thaliana GN=ARI8 PE=2 SV=1	1.00	3.64	-1.25	down	0.00	0.01	yes
TRINITY_DN19627_c0_g2	LUMINIDEPENDENS family protein [Populus trichocarpa]	LD	Homeobox protein LUMINIDEPENDENS OS=Arabidopsis thaliana GN=LD PE=1 SV=2	6.40	20.35	-1.04	down	0.00	0.01	yes
TRINITY_DN2232_c0_g1	hypothetical protein POPTR_0011s11520g [Populus trichocarpa]	-	-	0.97	6.92	-2.17	down	0.00	0.01	yes
TRINITY_DN19485_c0_g6	heat shock protein 70 cognate [Populus trichocarpa]	dnaK	Chaperone protein DnaK OS=Lactobacillus fermentum (strain NBRC 3956 / LMG 18251) GN=dnaK PE=3 SV=1	0.49	3.23	-2.06	down	0.00	0.01	yes
TRINITY_DN18002_c0_g6	-	-	-	1.06	6.74	-1.74	down	0.00	0.01	yes
TRINITY_DN21372_c2_g1	PREDICTED: fanconi-associated nuclease 1 homolog [Populus euphratica]	At1g48360	Fanconi-associated nuclease 1 homolog OS=Arabidopsis thaliana GN=At1g48360 PE=2 SV=2	2.08	9.61	-1.50	down	0.00	0.01	yes
TRINITY_DN16931_c0_g1	PREDICTED: uncharacterized protein C17orf53 [Populus euphratica]	-	-	0.73	3.53	-1.66	down	0.00	0.01	yes
TRINITY_DN15099_c0_g1	-	-	-	13.50	45.08	-1.42	down	0.00	0.01	yes
TRINITY_DN21231_c0_g2	armadillo/beta-catenin repeat family protein [Populus trichocarpa]	-	-	1.72	5.76	-1.15	down	0.00	0.01	yes
TRINITY_DN24894_c0_g6	putative MYB transcription factor family protein [Populus trichocarpa]	MYB108	Transcription factor MYB108 OS=Arabidopsis thaliana GN=MYB108 PE=1 SV=1	0.69	7.84	-2.92	down	0.00	0.01	yes
TRINITY_DN20209_c0_g2	hypothetical protein POPTR_0009s16310g [Populus trichocarpa]	CAF1-11	Probable CCR4-associated factor 1 homolog 11 OS=Arabidopsis thaliana GN=CAF1-11 PE=2 SV=1	2.28	10.43	-1.58	down	0.00	0.01	yes
TRINITY_DN19958_c0_g2	RecName: Full=Caffeoyl-CoA O-methyltransferase; AltName: Full=Trans-caffeoyl-CoA 3-O-methyltransferase; Short=CCoAMT; Short=CCoAOMT	-	Caffeoyl-CoA O-methyltransferase OS=Populus tremuloides PE=2 SV=1	6.52	20.89	-1.13	down	0.00	0.01	yes
TRINITY_DN21748_c0_g3	hypothetical protein POPTR_0006s02130g [Populus trichocarpa]	-	-	4.18	19.68	-1.55	down	0.00	0.01	yes
TRINITY_DN14812_c0_g1	hypothetical protein POPTR_0001s42270g [Populus trichocarpa]	PBP1	Calcium-binding protein PBP1 OS=Arabidopsis thaliana GN=PBP1 PE=1 SV=1	1.92	15.36	-2.36	down	0.00	0.01	yes
TRINITY_DN15829_c1_g2	hypothetical protein POPTR_0006s04930g [Populus trichocarpa]	-	-	4.48	33.67	-2.30	down	0.00	0.01	yes
TRINITY_DN16653_c0_g1	transducin family protein [Populus trichocarpa]	DTL	Denticleless protein homolog OS=Gallus gallus GN=DTL PE=2 SV=1	0.69	3.60	-1.72	down	0.00	0.01	yes
TRINITY_DN17452_c0_g1	SKP1 INTERACTING PARTNER 4 family protein [Populus trichocarpa]	SKIP4	F-box/kelch-repeat protein SKIP4 OS=Arabidopsis thaliana GN=SKIP4 PE=1 SV=1	5.47	17.24	-1.08	down	0.00	0.01	yes
TRINITY_DN15929_c0_g1	hypothetical protein POPTR_0008s04420g [Populus trichocarpa]	-	-	2.86	18.34	-1.79	down	0.00	0.01	yes

TRINITY_DN14196_c1_g1	hypothetical protein POPTR_0017s08910g [Populus trichocarpa]	POPTRDRAFT_	CASP-like protein 1F3 OS=Populus trichocarpa GN=POPTRDRAFT_752786 PE=3 SV=1	6.31	20.58	-1.08	down	0.00	0.01	yes
TRINITY_DN18172_c0_g1	hypothetical protein POPTR_0009s13480g [Populus trichocarpa]	TL1	Thaumatin-like protein 1 OS=Pyrus pyrifolia GN=TL1 PE=1 SV=1	1.60	6.00	-1.32	down	0.00	0.01	yes
TRINITY_DN14845_c0_g1	zinc finger family protein [Populus trichocarpa]	-	-	0.60	3.62	-2.00	down	0.00	0.01	yes
TRINITY_DN17155_c0_g2	PREDICTED: lysine-specific demethylase JMJ25-like [Populus euphratica]	JMJ25	Lysine-specific demethylase JMJ25 OS=Arabidopsis thaliana GN=JMJ25 PE=1 SV=1	1.46	7.95	-1.74	down	0.00	0.01	yes
TRINITY_DN23373_c0_g3	hypothetical protein POPTR_0002s22050g [Populus trichocarpa]	At3g07870	F-box protein At3g07870 OS=Arabidopsis thaliana GN=At3g07870 PE=2 SV=1	0.92	4.21	-1.59	down	0.00	0.01	yes
TRINITY_DN18331_c0_g2	PREDICTED: probable inactive poly [ADP-ribose] polymerase SRO5 [Populus euphratica]	SRO5	Probable inactive poly [ADP-ribose] polymerase SRO5 OS=Arabidopsis thaliana GN=SRO5 PE=1 SV=1	1.41	6.39	-1.88	down	0.00	0.01	yes
TRINITY_DN17390_c1_g1	PREDICTED: NAC transcription factor 25-like [Populus euphratica]	NAC083	NAC domain-containing protein 83 OS=Arabidopsis thaliana GN=NAC083 PE=1 SV=1	2.36	3.97	-2.09	down	0.00	0.01	yes
TRINITY_DN26116_c1_g4	PREDICTED: vacuolar amino acid transporter 1-like [Populus euphratica]	AVT4	Vacuolar amino acid transporter 4 OS=Saccharomyces cerevisiae (strain ATCC 204508 / S288c) GN=AVT4 PE=1 SV=1	3.21	11.08	-1.21	down	0.00	0.01	yes
TRINITY_DN27603_c1_g2	-	-	-	0.49	4.36	-2.49	down	0.00	0.01	yes
TRINITY_DN27719_c0_g1	-	-	-	7.92	31.45	-1.40	down	0.00	0.01	yes
TRINITY_DN12999_c0_g2	hypothetical protein POPTR_0015s08580g [Populus trichocarpa]	-	-	0.73	3.97	-1.84	down	0.00	0.01	yes
TRINITY_DN23274_c0_g1	PREDICTED: phospholipase A1-Ibeta2, chloroplastic-like [Populus euphratica]	At4g16820	Phospholipase A1-Ibeta2, chloroplastic OS=Arabidopsis thaliana GN=At4g16820 PE=1 SV=2	1.95	13.94	-2.24	down	0.00	0.01	yes
TRINITY_DN23532_c0_g2	hypothetical protein POPTR_0007s06560g [Populus trichocarpa]	RDR5	Probable RNA-dependent RNA polymerase 5 OS=Arabidopsis thaliana GN=RDR5 PE=2 SV=2	2.00	7.11	-1.21	down	0.00	0.01	yes
TRINITY_DN15331_c0_g1	hypothetical protein POPTR_0006s23120g [Populus trichocarpa]	MAKR4	Probable membrane-associated kinase regulator 4 OS=Arabidopsis thaliana GN=MAKR4 PE=3 SV=1	0.65	3.96	-1.99	down	0.00	0.01	yes
TRINITY_DN25467_c0_g2	cyclopropane-fatty-acyl-phospholipid synthase family protein [Populus trichocarpa]	-	-	0.69	4.43	-2.06	down	0.00	0.01	yes
TRINITY_DN14209_c0_g1	-	-	-	0.82	9.68	-2.93	down	0.00	0.01	yes
TRINITY_DN24247_c0_g2	PREDICTED: uncharacterized protein LOC105112517 [Populus euphratica]	-	-	15.43	51.54	-1.18	down	0.00	0.01	yes
TRINITY_DN25848_c0_g1	endo-1 family protein [Populus trichocarpa]	At4g02290	Endoglucanase 17 OS=Arabidopsis thaliana GN=At4g02290 PE=2 SV=1	29.70	110.05	-1.30	down	0.00	0.01	yes
TRINITY_DN26182_c0_g3	hypothetical protein POPTR_0001s43870g [Populus trichocarpa]	-	-	12.86	49.53	-1.37	down	0.00	0.01	yes
TRINITY_DN18105_c0_g2	PREDICTED: homologous-pairing protein 2 homolog [Populus euphratica]	HOP2	Homologous-pairing protein 2 homolog OS=Arabidopsis thaliana GN=HOP2 PE=1 SV=1	2.32	10.39	-1.58	down	0.00	0.01	yes
TRINITY_DN14818_c0_g1	pleiotropic drug resistance 6 [Populus tomentosa]	ABCG39	ABC transporter G family member 39 OS=Arabidopsis thaliana GN=ABCG39 PE=3 SV=1	0.48	2.29	-1.63	down	0.00	0.01	yes
TRINITY_DN20006_c0_g1	hypothetical protein POPTR_0005s25090g [Populus trichocarpa]	-	-	4.18	18.19	-1.46	down	0.00	0.01	yes
TRINITY_DN26026_c0_g3	hypothetical protein POPTR_0018s11180g [Populus trichocarpa]	-	-	5.77	30.53	-1.77	down	0.00	0.01	yes
TRINITY_DN19623_c0_g1	PREDICTED: putative golgin subfamily A member 6-like protein 8 [Populus euphratica]	-	-	1.60	6.33	-1.36	down	0.00	0.01	yes
TRINITY_DN22966_c0_g2	-	-	-	0.59	3.40	-2.01	down	0.00	0.01	yes
TRINITY_DN23023_c0_g2	-	-	-	3.07	13.64	-1.54	down	0.00	0.01	yes
TRINITY_DN22415_c1_g1	-	-	-	3.07	17.28	-1.88	down	0.00	0.01	yes
TRINITY_DN24400_c0_g2	methyladenine glycosylase family protein [Populus trichocarpa]	-	-	0.67	5.62	-2.42	down	0.00	0.01	yes
TRINITY_DN15581_c0_g4	PREDICTED: tetraketide alpha-pyrone reductase 2-like [Populus euphratica]	TKPR2	Tetraketide alpha-pyrone reductase 2 OS=Arabidopsis thaliana GN=TKPR2 PE=1 SV=1	17.14	70.10	-1.46	down	0.00	0.01	yes
TRINITY_DN24363_c0_g2	hypothetical protein POPTR_0002s08250g [Populus trichocarpa]	-	-	0.62	3.99	-2.25	down	0.00	0.01	yes
TRINITY_DN25285_c0_g1	PREDICTED: ribonucleoside-diphosphate reductase large subunit-like [Populus euphratica]	RNR1	Ribonucleoside-diphosphate reductase large subunit OS=Arabidopsis thaliana GN=RNR1 PE=1 SV=1	33.12	103.10	-1.11	down	0.00	0.01	yes

TRINITY_DN25381_c2_g1	hypothetical protein POPTR_0015s11230g [Populus trichocarpa]	RMI1	RecQ-mediated genome instability protein 1 OS=Arabidopsis thaliana GN=RMI1 PE=1 SV=1	0.73	3.66	-1.69	down	0.00	0.01	yes
TRINITY_DN15944_c0_g1	hypothetical protein POPTR_0003s07430g [Populus trichocarpa]	-	-	2.00	9.22	-1.74	down	0.00	0.01	yes
TRINITY_DN22372_c0_g3	hypothetical protein POPTR_0015s15280g [Populus trichocarpa]	PLP7	Patatin-like protein 7 OS=Arabidopsis thaliana GN=PLP7 PE=2 SV=1	0.42	2.44	-1.90	down	0.00	0.01	yes
TRINITY_DN21333_c0_g1	PREDICTED: transcription factor SPEECHLESS-like [Populus euphratica]	SPCH	Transcription factor SPEECHLESS OS=Arabidopsis thaliana GN=SPCH PE=1 SV=1	1.07	8.44	-2.38	down	0.00	0.01	yes
TRINITY_DN21446_c0_g1	armadillo/beta-catenin repeat family protein [Populus trichocarpa]	-	-	3.25	10.44	-1.14	down	0.00	0.01	yes
TRINITY_DN26380_c0_g3	PREDICTED: uncharacterized protein LOC105137990 [Populus euphratica]	-	-	2.64	8.49	-1.05	down	0.00	0.01	yes
TRINITY_DN22293_c1_g1	hypothetical protein POPTR_0011s05710g [Populus trichocarpa]	HSL1	Receptor-like protein kinase HSL1 OS=Arabidopsis thaliana GN=HSL1 PE=2 SV=1	1.08	4.65	-1.47	down	0.00	0.01	yes
TRINITY_DN24203_c0_g1	hypothetical protein POPTR_0014s03480g [Populus trichocarpa]	MAPKKK5	Mitogen-activated protein kinase kinase kinase 5 OS=Arabidopsis thaliana GN=MAPKKK5 PE=1 SV=1	1.89	7.86	-1.20	down	0.00	0.01	yes
TRINITY_DN26436_c0_g4	hypothetical protein POPTR_0006s01410g, partial [Populus trichocarpa]	-	-	4.50	14.30	-1.06	down	0.00	0.01	yes
TRINITY_DN27137_c0_g1	hypothetical protein POPTR_0001s40900g [Populus trichocarpa]	PSKR2	Phytosulfokine receptor 2 OS=Arabidopsis thaliana GN=PSKR2 PE=2 SV=1	3.02	15.80	-1.72	down	0.00	0.01	yes
TRINITY_DN27544_c0_g1	PREDICTED: CTP synthase-like isoform X1 [Populus euphratica]	ctps	CTP synthase OS=Dictyostelium discoideum GN=ctps PE=3 SV=1	17.08	60.80	-1.20	down	0.00	0.01	yes
TRINITY_DN25653_c0_g1	leucine-rich repeat transmembrane protein kinase [Populus trichocarpa]	HSL1	Receptor-like protein kinase HSL1 OS=Arabidopsis thaliana GN=HSL1 PE=2 SV=1	5.26	16.36	-1.05	down	0.00	0.01	yes
TRINITY_DN16806_c1_g2	putative calmodulin-related family protein [Populus trichocarpa]	CML27	Probable calcium-binding protein CML27 OS=Arabidopsis thaliana GN=CML27 PE=1 SV=1	26.99	125.77	-1.62	down	0.00	0.01	yes
TRINITY_DN26024_c0_g3	-	-	-	0.73	4.00	-1.82	down	0.00	0.01	yes
TRINITY_DN24617_c0_g3	putative calmodulin-binding family protein [Populus trichocarpa]	BAG7	BAG family molecular chaperone regulator 7 OS=Arabidopsis thaliana GN=BAG7 PE=1 SV=1	36.03	115.74	-1.06	down	0.00	0.01	yes
TRINITY_DN15135_c0_g1	PREDICTED: inactive protein kinase SELMODRAFT_444075 [Ricinus communis]	-	-	14.66	46.16	-1.03	down	0.00	0.01	yes
TRINITY_DN21987_c0_g1	sucrose synthase [Populus tomentosa]	SUS2	Sucrose synthase 2 OS=Pisum sativum GN=SUS2 PE=2 SV=1	5.57	17.49	-1.19	down	0.00	0.01	yes
TRINITY_DN19125_c0_g5	hypothetical protein POPTR_0018s002602g, partial [Populus trichocarpa]	At5g49770	Probable leucine-rich repeat receptor-like protein kinase At5g49770 OS=Arabidopsis thaliana GN=At5g49770 PE=2 SV=1	3.35	19.03	-1.59	down	0.00	0.01	yes
TRINITY_DN23774_c0_g1	hypothetical protein POPTR_0004s20080g [Populus trichocarpa]	At5g22750	Putative SWI/SNF-related matrix-associated actin-dependent regulator of chromatin subfamily A member 3-like 2 OS=Arabidopsis thaliana GN=At5g22750 PE=2 SV=1	1.48	5.19	-1.18	down	0.00	0.01	yes
TRINITY_DN26666_c0_g4	hypothetical protein POPTR_0001s06420g [Populus trichocarpa]	-	-	1.86	11.48	-1.67	down	0.00	0.01	yes
TRINITY_DN20623_c0_g1	hypothetical protein POPTR_0006s17920g [Populus trichocarpa]	-	-	3.20	10.65	-1.15	down	0.00	0.01	yes
TRINITY_DN19411_c0_g2	TRNA ISOPENTENYLTRANSFERASE family protein [Populus trichocarpa]	IPT2	tRNA dimethylallyltransferase 2 OS=Arabidopsis thaliana GN=IPT2 PE=1 SV=2	4.22	14.39	-1.11	down	0.00	0.01	yes
TRINITY_DN21626_c0_g1	kinase family protein [Populus trichocarpa]	At1g11050	Probable receptor-like protein kinase At1g11050 OS=Arabidopsis thaliana GN=At1g11050 PE=2 SV=1	10.41	35.72	-1.06	down	0.00	0.01	yes
TRINITY_DN27159_c0_g3	PREDICTED: LRR receptor-like serine/threonine-protein kinase RPK2 [Populus euphratica]	RPK2	LRR receptor-like serine/threonine-protein kinase RPK2 OS=Arabidopsis thaliana GN=RPK2 PE=2 SV=1	0.85	8.53	-2.66	down	0.00	0.01	yes
TRINITY_DN17021_c0_g1	hypothetical protein POPTR_0009s05580g [Populus trichocarpa]	ABCG15	ABC transporter G family member 15 OS=Arabidopsis thaliana GN=ABCG15 PE=2 SV=2	0.93	4.81	-1.78	down	0.00	0.01	yes
TRINITY_DN25890_c0_g2	hypothetical protein POPTR_0014s13990g [Populus trichocarpa]	PBS1	Serine/threonine-protein kinase PBS1 OS=Arabidopsis thaliana GN=PBS1 PE=1 SV=1	6.52	23.13	-1.10	down	0.00	0.01	yes
TRINITY_DN21938_c0_g1	pathogenesis-related thaumatin, partial [Populus deltoides]	OSM34	Osmotin-like protein OSM34 OS=Arabidopsis thaliana GN=OSM34 PE=2 SV=2	6.20	51.82	-2.55	down	0.00	0.01	yes
TRINITY_DN27090_c1_g1	hypothetical protein POPTR_0008s00670g [Populus trichocarpa]	At1g16860	Uncharacterized membrane protein At1g16860 OS=Arabidopsis thaliana GN=At1g16860 PE=1 SV=1	18.17	57.74	-1.04	down	0.00	0.01	yes

TRINITY_DN21841_c0_g1	PREDICTED: ATP-dependent DNA helicase 2 subunit KU80 [Populus euphratica]	KU80	ATP-dependent DNA helicase 2 subunit KU80 OS=Arabidopsis thaliana GN=KU80 PE=1 SV=1	2.85	10.71	-1.03	down	0.00	0.01	yes
TRINITY_DN20764_c0_g2	hypothetical protein POPTR_0010s06870g [Populus trichocarpa]	-	-	1.02	4.76	-1.59	down	0.00	0.01	yes
TRINITY_DN27059_c0_g2	-	-	-	8.72	44.39	-1.71	down	0.00	0.01	yes
TRINITY_DN20295_c0_g2	hypothetical protein POPTR_0007s04191g [Populus trichocarpa]	-	-	0.68	3.85	-1.86	down	0.00	0.01	yes
TRINITY_DN16111_c0_g1	hypothetical protein POPTR_0001s14450g [Populus trichocarpa]	-	-	0.43	3.74	-2.45	down	0.00	0.01	yes
TRINITY_DN25478_c0_g3	-	-	-	1.37	12.60	-2.54	down	0.00	0.01	yes
TRINITY_DN25552_c0_g1	hypothetical protein POPTR_0002s02000g [Populus trichocarpa]	FD	Protein FD OS=Arabidopsis thaliana GN=FD PE=1 SV=1	8.43	21.82	-1.29	down	0.00	0.01	yes
TRINITY_DN23822_c0_g1	hypothetical protein POPTR_0013s05610g [Populus trichocarpa]	-	-	1.16	6.64	-1.87	down	0.00	0.01	yes
TRINITY_DN14828_c0_g1	hypothetical protein POPTR_0004s18850g [Populus trichocarpa]	LOG5	Cytokinin riboside 5'-monophosphate phosphoribohydrolase LOG5 OS=Arabidopsis thaliana GN=LOG5 PE=1 SV=1	0.35	2.69	-2.30	down	0.00	0.01	yes
TRINITY_DN14060_c0_g1	hypothetical protein POPTR_0001s15250g [Populus trichocarpa]	CER26L	Protein ECERIFERUM 26-like OS=Arabidopsis thaliana GN=CER26L PE=2 SV=1	0.52	2.87	-1.83	down	0.00	0.01	yes
TRINITY_DN25183_c0_g1	hypothetical protein POPTR_0013s02220g [Populus trichocarpa]	LACS7	Long chain acyl-CoA synthetase 7, peroxisomal OS=Arabidopsis thaliana GN=LACS7 PE=1 SV=2	7.46	25.55	-1.02	down	0.00	0.01	yes
TRINITY_DN27794_c1_g5	hypothetical protein POPTR_0008s04420g [Populus trichocarpa]	-	-	2.40	9.24	-1.34	down	0.00	0.01	yes
TRINITY_DN18002_c0_g3	hypothetical protein POPTR_0010s25250g [Populus trichocarpa]	ALA1	Phospholipid-transporting ATPase 1 OS=Arabidopsis thaliana GN=ALA1 PE=2 SV=1	0.24	1.88	-2.29	down	0.00	0.01	yes
TRINITY_DN22258_c1_g2	PREDICTED: BES1/BZR1 homolog protein 4-like [Populus euphratica]	BEH4	BES1/BZR1 homolog protein 4 OS=Arabidopsis thaliana GN=BEH4 PE=1 SV=1	7.65	24.56	-1.06	down	0.00	0.01	yes
TRINITY_DN27005_c0_g3	hypothetical protein POPTR_0011s14850g [Populus trichocarpa]	ARID3	AT-rich interactive domain-containing protein 3 OS=Arabidopsis thaliana GN=ARID3 PE=1 SV=1	1.45	10.50	-1.47	down	0.00	0.01	yes
TRINITY_DN22885_c0_g1	hypothetical protein POPTR_0004s11340g [Populus trichocarpa]	At3g02290	E3 ubiquitin-protein ligase At3g02290 OS=Arabidopsis thaliana GN=At3g02290 PE=2 SV=1	4.07	12.98	-1.11	down	0.00	0.01	yes
TRINITY_DN22099_c0_g2	hypothetical protein POPTR_0003s15810g [Populus trichocarpa]	RBOHC	Respiratory burst oxidase homolog protein C OS=Solanum tuberosum GN=RBOHC PE=1 SV=2	0.33	2.99	-2.52	down	0.00	0.01	yes
TRINITY_DN21154_c0_g2	hypothetical protein POPTR_0015s11100g [Populus trichocarpa]	SPL13A	Squamosa promoter-binding-like protein 13A OS=Arabidopsis thaliana GN=SPL13A PE=2 SV=1	4.27	15.68	-1.29	down	0.00	0.01	yes
TRINITY_DN23917_c0_g1	hypothetical protein POPTR_0004s24130g [Populus trichocarpa]	PUB35	U-box domain-containing protein 35 OS=Arabidopsis thaliana GN=PUB35 PE=1 SV=2	1.68	5.32	-1.06	down	0.00	0.01	yes
TRINITY_DN24039_c0_g2	PREDICTED: dynamin-2A-like [Populus euphratica]	DRP2B	Dynamin-2B OS=Arabidopsis thaliana GN=DRP2B PE=1 SV=2	0.33	1.90	-1.95	down	0.00	0.01	yes
TRINITY_DN16649_c0_g1	PREDICTED: DNA mismatch repair protein MSH4-like isoform X1 [Populus euphratica]	MSH4	DNA mismatch repair protein MSH4 OS=Arabidopsis thaliana GN=MSH4 PE=2 SV=1	1.58	6.18	-1.48	down	0.00	0.01	yes
TRINITY_DN23710_c0_g2	PREDICTED: uncharacterized protein LOC105121704 isoform X1 [Populus euphratica]	HIPP05	Heavy metal-associated isoprenylated plant protein 5 OS=Arabidopsis thaliana GN=HIPP05 PE=1 SV=2	36.78	114.51	-1.05	down	0.00	0.01	yes
TRINITY_DN18325_c1_g2	PREDICTED: trihelix transcription factor ASIL2-like [Populus euphratica]	-	-	0.67	3.22	-1.61	down	0.00	0.01	yes
TRINITY_DN23615_c0_g10	-	-	-	9.88	33.49	-1.14	down	0.00	0.01	yes
TRINITY_DN23703_c0_g3	hypothetical protein POPTR_0005s27290g [Populus trichocarpa]	PEPKR2	Serine/threonine-protein kinase PEPKR2 OS=Arabidopsis thaliana GN=PEPKR2 PE=2 SV=1	3.20	10.35	-1.01	down	0.00	0.01	yes
TRINITY_DN25522_c1_g1	PREDICTED: zinc finger protein NUTCRACKER-like [Populus euphratica]	IDD2	Protein indeterminate-domain 2 OS=Arabidopsis thaliana GN=IDD2 PE=2 SV=1	4.97	17.73	-1.19	down	0.00	0.01	yes
TRINITY_DN20188_c0_g1	PREDICTED: zinc finger protein MAGPIE-like [Populus euphratica]	-	-	25.40	79.88	-1.06	down	0.00	0.01	yes
TRINITY_DN27663_c0_g1	hypothetical protein POPTR_0002s06700g [Populus trichocarpa]	-	-	1.38	7.70	-1.76	down	0.00	0.01	yes
TRINITY_DN28736_c0_g1	-	-	-	0.82	5.27	-2.07	down	0.00	0.01	yes
TRINITY_DN25692_c0_g1	AAA-type ATPase family protein [Populus trichocarpa]	At4g24710	Pachytene checkpoint protein 2 homolog OS=Arabidopsis thaliana GN=At4g24710 PE=2 SV=1	9.04	31.77	-1.21	down	0.00	0.01	yes

TRINITY_DN26643_c1_g2	PREDICTED: vicianin hydrolase-like [Populus euphratica]	-	Vicianin hydrolase (Fragment) OS=Vicia sativa subsp. nigra PE=1 SV=1	12.50	47.56	-1.33	down	0.00	0.01	yes
TRINITY_DN14003_c0_g2	fatty acid elongase 3-ketoacyl-CoA synthase 1 family protein [Populus trichocarpa]	KCS1	3-ketoacyl-CoA synthase 1 OS=Arabidopsis thaliana GN=KCS1 PE=1 SV=1	0.22	4.38	-2.79	down	0.00	0.01	yes
TRINITY_DN26211_c0_g2	leucine-rich repeat transmembrane protein kinase [Populus trichocarpa]	BAM3	Leucine-rich repeat receptor-like serine/threonine-protein kinase BAM3 OS=Arabidopsis thaliana GN=BAM3 PE=2 SV=3	2.19	8.98	-1.37	down	0.00	0.01	yes
TRINITY_DN17376_c0_g2	hypothetical protein POPTR_0010s02830g [Populus trichocarpa]	-	-	33.53	114.44	-1.15	down	0.00	0.01	yes
TRINITY_DN6964_c0_g2	PREDICTED: uncharacterized protein At5g08430-like isoform X2 [Populus euphratica]	-	-	0.29	1.88	-2.07	down	0.00	0.01	yes
TRINITY_DN26602_c0_g3	-	-	-	4.82	21.98	-1.40	down	0.00	0.01	yes
TRINITY_DN14100_c0_g1	hypothetical protein POPTR_0007s14780g [Populus trichocarpa]	ACR1	ACT domain-containing protein ACR1 OS=Arabidopsis thaliana GN=ACR1 PE=2 SV=1	0.78	3.85	-1.66	down	0.00	0.01	yes
TRINITY_DN23743_c1_g2	-	-	-	3.27	8.59	-1.75	down	0.00	0.01	yes
TRINITY_DN15497_c0_g2	hypothetical protein POPTR_0002s24780g [Populus trichocarpa]	-	-	0.41	1.99	-1.67	down	0.00	0.01	yes
TRINITY_DN13756_c0_g1	hypothetical protein POPTR_0014s03380g [Populus trichocarpa]	-	-	0.56	5.73	-2.70	down	0.00	0.01	yes
TRINITY_DN17421_c0_g2	hypothetical protein POPTR_0005s19360g [Populus trichocarpa]	-	-	0.86	4.25	-1.70	down	0.00	0.01	yes
TRINITY_DN27240_c1_g2	hypothetical protein POPTR_0006s09500g [Populus trichocarpa]	At5g41260	Probable serine/threonine-protein kinase At5g41260 OS=Arabidopsis thaliana GN=At5g41260 PE=1 SV=1	2.18	11.99	-1.81	down	0.00	0.01	yes
TRINITY_DN23052_c0_g1	hypothetical protein POPTR_0009s03850g [Populus trichocarpa]	SCL14	Scarecrow-like protein 14 OS=Arabidopsis thaliana GN=SCL14 PE=2 SV=2	13.01	43.55	-1.10	down	0.00	0.01	yes
TRINITY_DN19298_c0_g1	-	-	-	10.70	33.28	-1.03	down	0.00	0.01	yes
TRINITY_DN14842_c0_g1	PREDICTED: BTB/POZ domain-containing protein At3g22104-like [Populus euphratica]	At3g19850	BTB/POZ domain-containing protein At3g19850 OS=Arabidopsis thaliana GN=At3g19850 PE=2 SV=1	0.43	2.80	-2.03	down	0.00	0.01	yes
TRINITY_DN18952_c0_g1	hypothetical protein POPTR_0006s05990g [Populus trichocarpa]	-	-	2.76	9.08	-1.05	down	0.00	0.01	yes
TRINITY_DN15247_c0_g2	PREDICTED: uncharacterized protein LOC105129684 [Populus euphratica]	-	-	15.18	77.45	-1.73	down	0.00	0.01	yes
TRINITY_DN18866_c0_g2	hypothetical protein POPTR_0004s16810g, partial [Populus trichocarpa]	BBX20	B-box zinc finger protein 20 OS=Arabidopsis thaliana GN=BBX20 PE=1 SV=1	0.72	3.39	-1.61	down	0.00	0.01	yes
TRINITY_DN26877_c2_g4	hypothetical protein POPTR_0004s03570g [Populus trichocarpa]	-	-	0.34	2.66	-2.29	down	0.00	0.01	yes
TRINITY_DN24617_c0_g2	-	-	-	0.41	2.89	-2.16	down	0.00	0.01	yes
TRINITY_DN17862_c0_g1	hypothetical protein POPTR_0002s02540g [Populus trichocarpa]	-	-	2.85	9.83	-1.15	down	0.00	0.01	yes
TRINITY_DN25007_c0_g1	TIR-NBS-LRR-TIR type disease resistance protein, partial [Populus trichocarpa]	-	TMV resistance protein N OS=Nicotiana glutinosa GN=N PE=1 SV=1	12.22	41.68	-1.55	down	0.00	0.01	yes
TRINITY_DN22876_c0_g1	PREDICTED: chromatin modification-related protein MEAF6-like isoform X1 [Populus euphratica]	-	-	9.67	29.76	-1.01	down	0.00	0.01	yes
TRINITY_DN24918_c0_g3	-	-	-	0.42	2.79	-2.08	down	0.00	0.01	yes
TRINITY_DN19500_c0_g2	hypothetical protein POPTR_0011s12140g [Populus trichocarpa]	RMA3	E3 ubiquitin-protein ligase RMA3 OS=Arabidopsis thaliana GN=RMA3 PE=1 SV=1	1.71	7.64	-1.54	down	0.00	0.01	yes
TRINITY_DN21528_c1_g1	hypothetical protein POPTR_0002s09330g [Populus trichocarpa]	ACC1	Acetyl-CoA carboxylase 1 OS=Arabidopsis thaliana GN=ACC1 PE=1 SV=1	0.78	4.44	-1.91	down	0.00	0.01	yes
TRINITY_DN90_c0_g1	hypothetical protein POPTR_0014s16980g [Populus trichocarpa]	CYP724B1	Cytochrome P450 724B1 OS=Oryza sativa subsp. japonica GN=CYP724B1 PE=1 SV=1	0.28	1.81	-2.07	down	0.00	0.01	yes
TRINITY_DN19486_c0_g1	PREDICTED: glucan endo-1,3-beta-glucosidase 14-like [Populus euphratica]	At2g27500	Glucan endo-1,3-beta-glucosidase 14 OS=Arabidopsis thaliana GN=At2g27500 PE=1 SV=2	2.34	7.34	-1.20	down	0.00	0.01	yes
TRINITY_DN20695_c0_g1	-	-	-	1.31	6.52	-1.65	down	0.00	0.01	yes
TRINITY_DN22282_c1_g5	PREDICTED: defensin-like protein 1 [Populus euphratica]	-	Defensin-like protein OS=Nelumbo nucifera PE=3 SV=1	76.77	248.64	-1.09	down	0.00	0.01	yes

TRINITY_DN700_c0_g1	PREDICTED: uncharacterized protein LOC105137388 [Populus euphratica]	-	-	0.38	2.12	-1.85	down	0.00	0.01	yes
TRINITY_DN24075_c0_g1	PREDICTED: uncharacterized protein LOC105122772 [Populus euphratica]	-	-	0.43	2.90	-2.22	down	0.00	0.01	yes
TRINITY_DN20133_c0_g2	PREDICTED: GDSL esterase/lipase At1g71691-like isoform X1 [Populus euphratica]	At1g71691	GDSL esterase/lipase At1g71691 OS=Arabidopsis thaliana GN=At1g71691 PE=2 SV=1	2.12	10.83	-1.79	down	0.00	0.01	yes
TRINITY_DN15827_c0_g1	PREDICTED: zinc finger protein ZAT10-like [Populus euphratica]	ZAT6	Zinc finger protein ZAT6 OS=Arabidopsis thaliana GN=ZAT6 PE=2 SV=1	5.89	45.93	-2.38	down	0.00	0.01	yes
TRINITY_DN26041_c0_g1	PREDICTED: cysteine-rich repeat secretory protein 3-like isoform X1 [Nicotiana tomentosiformis]	-	-	3.73	14.12	-1.32	down	0.00	0.01	yes
TRINITY_DN20920_c0_g1	hypothetical protein POPTR_0011s05410g [Populus trichocarpa]	-	-	0.31	2.00	-2.05	down	0.00	0.01	yes
TRINITY_DN16547_c0_g2	calcium-binding EF hand family protein [Populus trichocarpa]	CML45	Probable calcium-binding protein CML45 OS=Arabidopsis thaliana GN=CML45 PE=1 SV=1	0.35	4.71	-3.06	down	0.00	0.01	yes
TRINITY_DN21261_c0_g1	PREDICTED: uncharacterized protein LOC105131681 isoform X1 [Populus euphratica]	-	-	3.59	14.03	-1.33	down	0.00	0.01	yes
TRINITY_DN13954_c0_g1	PREDICTED: probable mediator of RNA polymerase II transcription subunit 36b isoform X1 [Populus euphratica]	MED36B	Probable mediator of RNA polymerase II transcription subunit 36b OS=Arabidopsis thaliana GN=MED36B PE=1 SV=1	0.65	4.14	-2.15	down	0.00	0.01	yes
TRINITY_DN23006_c1_g3	PREDICTED: uncharacterized protein LOC105124556 [Populus euphratica]	-	-	1.31	5.35	-1.40	down	0.00	0.01	yes
TRINITY_DN22515_c0_g7	-	-	-	3.21	12.69	-1.37	down	0.00	0.01	yes
TRINITY_DN19945_c0_g1	hypothetical protein POPTR_0016s06230g [Populus trichocarpa]	-	-	2.41	10.95	-1.55	down	0.00	0.01	yes
TRINITY_DN26303_c0_g2	RESPONSIVE TO HIGH LIGHT 41 family protein [Populus trichocarpa]	ZAT11	Zinc finger protein ZAT11 OS=Arabidopsis thaliana GN=ZAT11 PE=2 SV=1	0.63	8.19	-3.05	down	0.00	0.01	yes
TRINITY_DN15211_c0_g2	PREDICTED: uncharacterized protein LOC105133504 [Populus euphratica]	-	-	0.21	2.58	-2.41	down	0.00	0.01	yes
TRINITY_DN22660_c0_g4	transcription factor family protein [Populus trichocarpa]	E2FE	E2F transcription factor-like E2FE OS=Arabidopsis thaliana GN=E2FE PE=2 SV=1	4.66	18.64	-1.38	down	0.00	0.01	yes
TRINITY_DN26896_c0_g1	-	-	-	9.98	50.18	-1.70	down	0.00	0.01	yes
TRINITY_DN20824_c0_g3	hypothetical protein POPTR_0001s19240g [Populus trichocarpa]	APG	GDSL esterase/lipase APG OS=Arabidopsis thaliana GN=APG PE=2 SV=1	6.89	26.31	-1.57	down	0.00	0.01	yes
TRINITY_DN27331_c0_g3	PREDICTED: high mobility group B protein 13-like [Populus euphratica]	-	-	86.37	353.11	-1.43	down	0.00	0.01	yes
TRINITY_DN17970_c0_g1	hypothetical protein POPTR_0014s02270g [Populus trichocarpa]	At4g34215	Probable carbohydrate esterase At4g34215 OS=Arabidopsis thaliana GN=At4g34215 PE=1 SV=2	8.00	22.09	-1.03	down	0.00	0.01	yes
TRINITY_DN13853_c0_g1	PREDICTED: major allergen Pru ar 1-like [Populus euphratica]	-	Major allergen Pru ar 1 OS=Prunus armeniaca PE=1 SV=1	2.68	12.94	-1.72	down	0.00	0.01	yes
TRINITY_DN18047_c0_g1	PREDICTED: ABC transporter B family member 13-like isoform X1 [Populus euphratica]	ABCB13	ABC transporter B family member 13 OS=Arabidopsis thaliana GN=ABCB13 PE=3 SV=1	1.31	4.76	-1.25	down	0.00	0.01	yes
TRINITY_DN15381_c0_g1	PREDICTED: cytochrome b561 domain-containing protein At2g30890-like isoform X2 [Populus euphratica]	At4g18260	Cytochrome b561 domain-containing protein At4g18260 OS=Arabidopsis thaliana GN=At4g18260 PE=2 SV=1	0.38	2.26	-1.92	down	0.00	0.01	yes
TRINITY_DN17782_c0_g1	PREDICTED: inositol-tetrakisphosphate 1-kinase 2-like isoform X1 [Populus euphratica]	ITPK3	Inositol-tetrakisphosphate 1-kinase 3 OS=Arabidopsis thaliana GN=ITPK3 PE=1 SV=2	2.86	14.19	-1.40	down	0.00	0.01	yes
TRINITY_DN25269_c0_g1	PREDICTED: LRR receptor-like serine/threonine-protein kinase GSO2 [Populus euphratica]	-	-	1.85	7.52	-1.65	down	0.00	0.01	yes
TRINITY_DN20959_c0_g7	hypothetical protein POPTR_0003s19470g [Populus trichocarpa]	ASIL2	Trihelix transcription factor ASIL2 OS=Arabidopsis thaliana GN=ASIL2 PE=2 SV=1	0.36	2.31	-2.11	down	0.00	0.01	yes
TRINITY_DN18703_c0_g1	basic helix-loop-helix family protein [Populus trichocarpa]	-	-	1.69	9.56	-1.86	down	0.00	0.01	yes
TRINITY_DN18278_c0_g1	vesicle-associated membrane family protein [Populus trichocarpa]	PHYL2.2	Phytolongin Phyl2.2 OS=Arabidopsis thaliana GN=PHYL2.2 PE=2 SV=1	3.07	10.07	-1.11	down	0.00	0.01	yes
TRINITY_DN15670_c0_g1	hypothetical protein POPTR_0001s19340g [Populus trichocarpa]	-	-	9.97	49.57	-1.82	down	0.00	0.01	yes

TRINITY_DN14313_c0_g1	unknown [Populus trichocarpa]	-	-	3.76	30.46	-2.34	down	0.00	0.01	yes
TRINITY_DN19106_c1_g1	-	-	-	0.52	2.76	-1.78	down	0.00	0.01	yes
TRINITY_DN22162_c0_g1	hypothetical protein POPTR_0004s01350g [Populus trichocarpa]	ankrd13c-b	Ankyrin repeat domain-containing protein 13C-B OS=Xenopus laevis GN=ankrd13c-b PE=2 SV=1	1.80	6.91	-1.34	down	0.00	0.01	yes
TRINITY_DN27691_c4_g1	-	-	-	0.77	4.38	-1.83	down	0.00	0.01	yes
TRINITY_DN22859_c0_g6	-	-	-	44.36	167.93	-1.29	down	0.00	0.01	yes
TRINITY_DN18428_c0_g4	hypothetical protein POPTR_0015s08500g [Populus trichocarpa]	RDR2	RNA-dependent RNA polymerase 2 OS=Arabidopsis thaliana GN=RDR2 PE=1 SV=1	1.78	5.87	-1.10	down	0.00	0.01	yes
TRINITY_DN27619_c4_g1	-	-	-	0.37	4.02	-2.87	down	0.00	0.01	yes
TRINITY_DN18606_c0_g1	hypothetical protein POPTR_0463s00220g, partial [Populus trichocarpa]	-	-	1.69	7.03	-2.31	down	0.00	0.01	yes
TRINITY_DN14341_c0_g2	PREDICTED: probable protein phosphatase 2C 2 [Populus euphratica]	PP2C5	Probable protein phosphatase 2C 30 OS=Arabidopsis thaliana GN=PP2C5 PE=2 SV=1	0.43	2.39	-1.83	down	0.00	0.01	yes
TRINITY_DN26890_c1_g4	PREDICTED: uncharacterized protein At1g28695-like [Populus euphratica]	At1g28695	Uncharacterized protein At1g28695 OS=Arabidopsis thaliana GN=At1g28695 PE=2 SV=1	0.38	2.42	-2.10	down	0.00	0.01	yes
TRINITY_DN17916_c0_g1	PREDICTED: transcription factor DIVARICATA-like [Populus euphratica]	DIVARICATA	Transcription factor DIVARICATA OS=Antirrhinum majus GN=DIVARICATA PE=2 SV=1	0.83	6.00	-2.23	down	0.00	0.01	yes
TRINITY_DN21094_c0_g3	hypothetical protein POPTR_0010s02240g [Populus trichocarpa]	-	-	3.77	12.81	-1.30	down	0.00	0.01	yes
TRINITY_DN17163_c0_g1	U-box domain-containing family protein [Populus trichocarpa]	PUB21	U-box domain-containing protein 21 OS=Arabidopsis thaliana GN=PUB21 PE=2 SV=1	1.45	17.90	-3.02	down	0.00	0.01	yes
TRINITY_DN22836_c0_g7	hypothetical protein POPTR_0006s25630g [Populus trichocarpa]	At1g67280	Probable lactoylglutathione lyase, chloroplastic OS=Arabidopsis thaliana GN=At1g67280 PE=1 SV=1	0.60	2.90	-1.81	down	0.00	0.01	yes
TRINITY_DN18246_c1_g1	PREDICTED: uncharacterized protein LOC105142765 isoform X1 [Populus euphratica]	-	-	0.64	5.64	-2.17	down	0.00	0.01	yes
TRINITY_DN19660_c0_g1	hypothetical protein POPTR_0006s04670g [Populus trichocarpa]	BG2	Glucan endo-1,3-beta-glucosidase, acidic isoform OS=Arabidopsis thaliana GN=BG2 PE=1 SV=2	3.59	15.42	-1.47	down	0.00	0.01	yes
TRINITY_DN10881_c0_g1	PREDICTED: protein trichome birefringence-like 43 [Populus euphratica]	TBL43	Protein trichome birefringence-like 43 OS=Arabidopsis thaliana GN=TBL43 PE=2 SV=1	1.92	9.40	-1.71	down	0.00	0.01	yes
TRINITY_DN19817_c0_g2	hypothetical protein POPTR_0004s07230g [Populus trichocarpa]	BHY	Beta-carotene 3-hydroxylase, chloroplastic OS=Gentiana lutea GN=BHY PE=2 SV=1	3.19	12.38	-1.38	down	0.00	0.01	yes
TRINITY_DN20283_c0_g2	TIR-NBS disease resistance-like protein [Populus trichocarpa]	-	-	1.82	13.67	-2.06	down	0.00	0.01	yes
TRINITY_DN20017_c0_g4	hypothetical protein POPTR_0018s11340g [Populus trichocarpa]	MPS1	Protein MULTIPOLAR SPINDLE 1 OS=Arabidopsis thaliana GN=MPS1 PE=2 SV=2	0.64	3.88	-2.01	down	0.00	0.01	yes
TRINITY_DN23575_c0_g2	hypothetical protein POPTR_0005s01980g [Populus trichocarpa]	-	-	1.52	10.86	-1.74	down	0.00	0.01	yes
TRINITY_DN23278_c1_g3	zinc finger family protein [Populus trichocarpa]	ATL8	RING-H2 finger protein ATL8 OS=Arabidopsis thaliana GN=ATL8 PE=2 SV=2	2.82	11.74	-1.30	down	0.00	0.01	yes
TRINITY_DN20150_c0_g1	hypothetical protein POPTR_0007s02320g [Populus trichocarpa]	GEX2	Protein GAMETE EXPRESSED 2 OS=Arabidopsis thaliana GN=GEX2 PE=2 SV=1	2.25	6.34	-1.10	down	0.00	0.01	yes
TRINITY_DN25097_c0_g1	PREDICTED: fatty-acid-binding protein 2-like [Populus euphratica]	-	-	0.40	2.42	-1.95	down	0.00	0.01	yes
TRINITY_DN24320_c1_g2	PREDICTED: protein SET DOMAIN GROUP 41 isoform X1 [Populus euphratica]	SDG41	Protein SET DOMAIN GROUP 41 OS=Arabidopsis thaliana GN=SDG41 PE=2 SV=1	2.54	8.27	-1.04	down	0.00	0.01	yes
TRINITY_DN21322_c0_g3	hypothetical protein POPTR_0002s05560g [Populus trichocarpa]	OEP7	Outer envelope membrane protein 7 OS=Arabidopsis thaliana GN=OEP7 PE=1 SV=1	3.35	13.00	-1.30	down	0.00	0.01	yes
TRINITY_DN23739_c0_g3	PREDICTED: E3 ubiquitin-protein ligase RNF185-like [Populus euphratica]	rnf-5	RING finger protein 5 OS=Caenorhabditis elegans GN=rnf-5 PE=1 SV=1	1.67	7.00	-1.43	down	0.00	0.01	yes
TRINITY_DN21781_c0_g1	-	-	-	8.74	34.49	-1.33	down	0.00	0.01	yes
TRINITY_DN25157_c0_g2	ABC transporter family protein [Populus trichocarpa]	ABCG5	ABC transporter G family member 5 OS=Arabidopsis thaliana GN=ABCG5 PE=2 SV=1	1.24	4.88	-1.41	down	0.00	0.01	yes
TRINITY_DN18115_c0_g5	hypothetical protein POPTR_0017s02570g [Populus trichocarpa]	-	-	5.33	37.58	-2.11	down	0.00	0.01	yes

TRINITY_DN25125_c0_g1	WOX1a [Populus tomentosa]	WOX1	WUSCHEL-related homeobox 1 OS=Arabidopsis thaliana GN=WOX1 PE=2 SV=2	9.58	34.91	-1.34	down	0.00	0.01	yes
TRINITY_DN25198_c0_g1	-	-	-	3.28	22.08	-2.09	down	0.00	0.01	yes
TRINITY_DN24362_c0_g3	hypothetical protein POPTR_0014s11490g [Populus trichocarpa]	WRKY69	Probable WRKY transcription factor 69 OS=Arabidopsis thaliana GN=WRKY69 PE=2 SV=1	3.36	15.60	-1.56	down	0.00	0.01	yes
TRINITY_DN26691_c0_g2	PREDICTED: LOW QUALITY PROTEIN: neutral ceramidase [Populus euphratica]	Os01g0624000	Neutral ceramidase OS=Oryza sativa subsp. japonica GN=Os01g0624000 PE=1 SV=1	2.18	9.58	-1.54	down	0.00	0.01	yes
TRINITY_DN20128_c0_g1	PREDICTED: dihydrofolate reductase-like [Populus euphratica]	dfr1	Dihydrofolate reductase OS=Schizosaccharomyces pombe (strain 972 / ATCC 24843) GN=dfr1 PE=2 SV=2	15.72	65.08	-1.45	down	0.00	0.01	yes
TRINITY_DN18203_c0_g1	hypothetical protein POPTR_0002s11850g [Populus trichocarpa]	-	-	0.40	2.48	-1.98	down	0.00	0.01	yes
TRINITY_DN20607_c1_g5	-	-	-	1.97	7.24	-1.54	down	0.00	0.01	yes
TRINITY_DN21575_c0_g1	PREDICTED: mitogen-activated protein kinase-binding protein 1-like isoform X2 [Populus euphratica]	mapkbp1	Mitogen-activated protein kinase-binding protein 1 OS=Xenopus laevis GN=mapkbp1 PE=2 SV=1	1.67	5.45	-1.09	down	0.00	0.01	yes
TRINITY_DN19762_c0_g2	xanthine/uracil permease family protein [Populus trichocarpa]	NAT1	Nucleobase-ascorbate transporter 1 OS=Arabidopsis thaliana GN=NAT1 PE=2 SV=1	1.03	4.10	-1.36	down	0.00	0.01	yes
TRINITY_DN20839_c0_g4	hypothetical protein POPTR_0018s06130g [Populus trichocarpa]	LIMYB	L10-interacting MYB domain-containing protein OS=Arabidopsis thaliana GN=LIMYB PE=1 SV=1	2.08	7.42	-1.26	down	0.00	0.01	yes
TRINITY_DN24579_c0_g2	hypothetical protein POPTR_0005s23270g [Populus trichocarpa]	IDM1	Increased DNA methylation 1 OS=Arabidopsis thaliana GN=IDM1 PE=1 SV=1	2.04	6.42	-1.03	down	0.00	0.01	yes
TRINITY_DN24595_c1_g1	-	-	-	0.37	2.06	-1.86	down	0.00	0.01	yes
TRINITY_DN16346_c0_g1	hypothetical protein POPTR_0006s05900g [Populus trichocarpa]	-	-	1.61	8.42	-1.73	down	0.00	0.01	yes
TRINITY_DN22279_c0_g2	PREDICTED: abnormal spindle-like microcephaly-associated protein homolog isoform X1 [Populus euphratica]	ASPM	Abnormal spindle-like microcephaly-associated protein homolog (Fragment) OS=Bos taurus GN=ASPM PE=2 SV=1	2.62	9.98	-1.35	down	0.00	0.01	yes
TRINITY_DN23199_c0_g1	hypothetical protein POPTR_0015s06910g [Populus trichocarpa]	EXO70B1	Exocyst complex component EXO70B1 OS=Arabidopsis thaliana GN=EXO70B1 PE=1 SV=1	2.95	9.19	-1.14	down	0.00	0.01	yes
TRINITY_DN24518_c0_g1	PREDICTED: LOW QUALITY PROTEIN: TMV resistance protein N-like [Populus euphratica]	-	TMV resistance protein N OS=Nicotiana glutinosa GN=N PE=1 SV=1	0.75	3.63	-1.66	down	0.00	0.01	yes
TRINITY_DN27706_c0_g2	PREDICTED: protein Jade-1 [Populus euphratica]	BRPF1	Peregrin OS=Homo sapiens GN=BRPF1 PE=1 SV=2	1.48	7.66	-1.92	down	0.00	0.01	yes
TRINITY_DN14826_c0_g1	-	-	-	11.12	51.51	-1.61	down	0.00	0.01	yes
TRINITY_DN27283_c0_g1	hypothetical protein POPTR_0003s20310g [Populus trichocarpa]	-	-	37.18	122.71	-1.11	down	0.00	0.01	yes
TRINITY_DN18483_c0_g1	PREDICTED: probable receptor-like protein kinase At5g47070 isoform X1 [Populus euphratica]	PIX7	Probable serine/threonine-protein kinase PIX7 OS=Arabidopsis thaliana GN=PIX7 PE=1 SV=1	1.48	5.38	-1.28	down	0.00	0.01	yes
TRINITY_DN26152_c0_g4	PREDICTED: MATH and LRR domain-containing protein PFE0570w-like [Populus euphratica]	-	-	0.43	2.15	-1.72	down	0.00	0.02	yes
TRINITY_DN23177_c0_g7	hypothetical protein POPTR_0009s14910g [Populus trichocarpa]	KIPK2	Serine/threonine-protein kinase KIPK2 OS=Arabidopsis thaliana GN=KIPK2 PE=1 SV=1	1.14	5.47	-1.66	down	0.00	0.02	yes
TRINITY_DN15531_c0_g1	hypothetical protein POPTR_0006s10750g [Populus trichocarpa]	-	-	21.49	64.14	-1.01	down	0.00	0.02	yes
TRINITY_DN25202_c0_g2	PREDICTED: protein TRANSPARENT TESTA 12-like [Populus euphratica]	DTX41	Protein DETOXIFICATION 41 OS=Arabidopsis thaliana GN=DTX41 PE=2 SV=1	6.65	35.09	-1.33	down	0.00	0.02	yes
TRINITY_DN28622_c0_g1	PREDICTED: kiwellin-like [Populus euphratica]	-	Kiwellin OS=Actinidia deliciosa PE=1 SV=1	0.43	2.81	-2.12	down	0.00	0.02	yes
TRINITY_DN22484_c1_g3	putative xyloglucan endotransglycosylase family protein [Populus trichocarpa]	XTH25	Probable xyloglucan endotransglucosylase/hydrolase protein 25 OS=Arabidopsis thaliana GN=XTH25 PE=2 SV=2	2.21	28.22	-3.02	down	0.00	0.02	yes
TRINITY_DN22528_c0_g2	-	-	-	3.50	13.03	-1.28	down	0.00	0.02	yes
TRINITY_DN21701_c0_g1	hypothetical protein POPTR_0005s11350g [Populus trichocarpa]	MLO4	MLO-like protein 4 OS=Arabidopsis thaliana GN=MLO4 PE=2 SV=2	0.94	4.05	-1.45	down	0.00	0.02	yes
TRINITY_DN19057_c4_g1	PREDICTED: uncharacterized protein LOC107858972 [Capsicum annuum]	-	-	2.03	6.85	-1.19	down	0.00	0.02	yes
TRINITY_DN23123_c0_g1	hypothetical protein POPTR_0014s12800g [Populus trichocarpa]	At1g03010	BTB/POZ domain-containing protein At1g03010 OS=Arabidopsis thaliana GN=At1g03010 PE=2 SV=1	2.38	7.46	-1.18	down	0.00	0.02	yes

TRINITY_DN25630_c0_g3	PREDICTED: branched-chain-amino-acid aminotransferase 2, chloroplastic-like isoform X1 [Populus euphratica]	BCAT2	Branched-chain-amino-acid aminotransferase 2, chloroplastic OS=Arabidopsis thaliana GN=BCAT2 PE=1 SV=1	0.50	3.81	-2.44	down	0.00	0.02	yes
TRINITY_DN24615_c0_g3	PREDICTED: gibberellin receptor GID1B-like [Populus euphratica]	GID1B	Gibberellin receptor GID1B OS=Arabidopsis thaliana GN=GID1B PE=1 SV=1	2.49	9.82	-1.96	down	0.00	0.02	yes
TRINITY_DN25802_c0_g2	PREDICTED: formin-like protein 5 [Populus euphratica]	FH5	Formin-like protein 5 OS=Arabidopsis thaliana GN=FH5 PE=2 SV=2	7.64	25.20	-1.12	down	0.00	0.02	yes
TRINITY_DN13358_c0_g1	hypothetical protein POPTR_0001s12800g [Populus trichocarpa]	-	-	0.44	2.17	-1.70	down	0.00	0.02	yes
TRINITY_DN13415_c0_g2	hypothetical protein POPTR_0017s07740g [Populus trichocarpa]	-	-	0.45	3.20	-2.09	down	0.00	0.02	yes
TRINITY_DN27855_c0_g2	hypothetical protein POPTR_0016s01090g [Populus trichocarpa]	-	-	1.60	6.54	-1.39	down	0.00	0.02	yes
TRINITY_DN12114_c0_g2	hypothetical protein POPTR_0012s01875g [Populus trichocarpa]	-	-	0.58	2.70	-1.60	down	0.00	0.02	yes
TRINITY_DN13760_c0_g1	unknown [Populus trichocarpa x Populus deltoides]	ATL47	RING-H2 finger protein ATL47 OS=Arabidopsis thaliana GN=ATL47 PE=2 SV=1	3.94	14.28	-1.24	down	0.00	0.02	yes
TRINITY_DN13202_c0_g1	PREDICTED: uncharacterized protein LOC105130433 isoform X1 [Populus euphratica]	-	-	1.27	6.33	-1.54	down	0.00	0.02	yes
TRINITY_DN14432_c0_g1	PREDICTED: heat stress transcription factor B-3 [Populus euphratica]	HSFB3	Heat stress transcription factor B-3 OS=Arabidopsis thaliana GN=HSFB3 PE=2 SV=1	0.19	2.03	-2.77	down	0.00	0.02	yes
TRINITY_DN16380_c0_g1	hypothetical protein POPTR_0009s11290g [Populus trichocarpa]	COR413PM2	Cold-regulated 413 plasma membrane protein 2 OS=Arabidopsis thaliana GN=COR413PM2 PE=2 SV=1	2.79	10.39	-1.36	down	0.00	0.02	yes
TRINITY_DN22342_c0_g2	hypothetical protein POPTR_0014s10320g [Populus trichocarpa]	-	-	0.79	3.37	-1.44	down	0.00	0.02	yes
TRINITY_DN25198_c0_g2	phosphatase 2C family protein [Populus trichocarpa]	PPC6-7	Probable protein phosphatase 2C 73 OS=Arabidopsis thaliana GN=PPC6-7 PE=2 SV=1	17.69	68.18	-1.22	down	0.00	0.02	yes
TRINITY_DN19215_c2_g1	PREDICTED: probable protein phosphatase 2C 63 [Populus euphratica]	At4g33920	Probable protein phosphatase 2C 63 OS=Arabidopsis thaliana GN=At4g33920 PE=2 SV=1	6.48	30.16	-1.56	down	0.00	0.02	yes
TRINITY_DN15756_c0_g1	-	-	-	1.41	6.04	-1.52	down	0.00	0.02	yes
TRINITY_DN27867_c4_g2	PREDICTED: uncharacterized protein LOC105124362 isoform X1 [Populus euphratica]	-	-	0.29	2.19	-2.13	down	0.00	0.02	yes
TRINITY_DN14458_c0_g1	hypothetical protein POPTR_0019s10680g [Populus trichocarpa]	EGS1	Eugenol synthase 1 OS=Ocimum basilicum GN=EGS1 PE=1 SV=1	1.37	5.09	-1.51	down	0.00	0.02	yes
TRINITY_DN15337_c0_g1	PREDICTED: ethylene-responsive transcription factor ERF017-like [Populus euphratica]	ERF017	Ethylene-responsive transcription factor ERF017 OS=Arabidopsis thaliana GN=ERF017 PE=2 SV=1	1.96	21.20	-2.85	down	0.00	0.02	yes
TRINITY_DN19854_c0_g4	hypothetical protein POPTR_0003s03730g [Populus trichocarpa]	-	-	2.82	10.01	-1.18	down	0.00	0.02	yes
TRINITY_DN27295_c1_g2	PREDICTED: uncharacterized protein LOC105124152 isoform X2 [Populus euphratica]	-	-	0.52	4.14	-2.40	down	0.00	0.02	yes
TRINITY_DN25498_c0_g3	hypothetical protein POPTR_0006s26340g [Populus trichocarpa]	CYCA2-2	Cyclin-A2-2 OS=Arabidopsis thaliana GN=CYCA2-2 PE=2 SV=1	7.69	23.42	-1.01	down	0.00	0.02	yes
TRINITY_DN14801_c0_g1	hypothetical protein POPTR_0018s03570g [Populus trichocarpa]	-	-	2.20	10.42	-1.60	down	0.00	0.02	yes
TRINITY_DN17483_c0_g1	hypothetical protein POPTR_0011s14800g [Populus trichocarpa]	At5g67130	PI-PLC X domain-containing protein At5g67130 OS=Arabidopsis thaliana GN=At5g67130 PE=1 SV=1	0.75	3.88	-1.51	down	0.00	0.02	yes
TRINITY_DN27674_c0_g1	PREDICTED: auxilin-like protein 1 isoform X1 [Populus euphratica]	-	-	2.79	11.46	-1.36	down	0.00	0.02	yes
TRINITY_DN19420_c0_g2	PREDICTED: rop guanine nucleotide exchange factor 3 [Populus euphratica]	ROPGEF3	Rop guanine nucleotide exchange factor 3 OS=Arabidopsis thaliana GN=ROPGEF3 PE=2 SV=1	0.79	3.86	-1.67	down	0.00	0.02	yes
TRINITY_DN27703_c1_g9	hypothetical protein POPTR_0019s00700g [Populus trichocarpa]	RPS5	Disease resistance protein RPS5 OS=Arabidopsis thaliana GN=RPS5 PE=1 SV=2	0.78	3.50	-1.63	down	0.00	0.02	yes
TRINITY_DN6640_c0_g1	-	-	-	1.08	13.26	-3.04	down	0.00	0.02	yes
TRINITY_DN15706_c0_g1	hypothetical protein POPTR_0018s06550g [Populus trichocarpa]	CYP734A1	Cytochrome P450 734A1 OS=Arabidopsis thaliana GN=CYP734A1 PE=2 SV=1	0.55	3.38	-2.00	down	0.00	0.02	yes

TRINITY_DN16483_c1_g3	SEC14 cytosolic factor family protein [Populus trichocarpa]	SFH1	Phosphatidylinositol/phosphatidylcholine transfer protein SFH1 OS=Arabidopsis thaliana GN=SFH1 PE=2 SV=1	0.54	2.86	-1.75	down	0.00	0.02	yes
TRINITY_DN27181_c1_g1	hypothetical protein POPTR_0019s01080g [Populus trichocarpa]	-	-	3.94	16.81	-1.67	down	0.00	0.02	yes
TRINITY_DN22947_c3_g1	PREDICTED: 4,5-DOPA dioxygenase extradiol-like [Populus euphratica]	DODA	4,5-DOPA dioxygenase extradiol OS=Beta vulgaris GN=DODA PE=1 SV=1	1.18	3.60	-1.72	down	0.00	0.02	yes
TRINITY_DN23876_c0_g1	hypothetical protein POPTR_0007s10880g [Populus trichocarpa]	POLA	DNA polymerase alpha catalytic subunit OS=Arabidopsis thaliana GN=POLA PE=3 SV=2	0.94	6.43	-2.14	down	0.00	0.02	yes
TRINITY_DN21321_c0_g1	hypothetical protein POPTR_0004s15720g [Populus trichocarpa]	-	-	8.63	19.23	-1.39	down	0.00	0.02	yes
TRINITY_DN21353_c0_g3	hypothetical protein POPTR_0010s08920g [Populus trichocarpa]	NFP	Serine/threonine receptor-like kinase NFP OS=Medicago truncatula GN=NFP PE=1 SV=1	0.51	4.14	-2.36	down	0.00	0.02	yes
TRINITY_DN24129_c0_g2	GTP-binding family protein [Populus trichocarpa]	MIRO2	Mitochondrial Rho GTPase 2 OS=Arabidopsis thaliana GN=MIRO2 PE=2 SV=1	2.76	11.15	-1.05	down	0.00	0.02	yes
TRINITY_DN27181_c0_g2	hypothetical protein POPTR_0019s00390g [Populus trichocarpa]	-	-	3.03	13.48	-1.49	down	0.00	0.02	yes
TRINITY_DN23887_c0_g3	-	-	-	0.36	2.00	-1.87	down	0.00	0.02	yes
TRINITY_DN22125_c0_g1	-	-	-	1.28	5.93	-1.64	down	0.00	0.02	yes
TRINITY_DN18392_c0_g1	PREDICTED: glucuronoxylan 4-O-methyltransferase 3-like [Populus euphratica]	GXM3	Glucuronoxylan 4-O-methyltransferase 3 OS=Arabidopsis thaliana GN=GXM3 PE=1 SV=1	0.53	3.12	-2.15	down	0.00	0.02	yes
TRINITY_DN22021_c0_g1	PREDICTED: calmodulin-like protein 3 isoform X3 [Populus euphratica]	CML28	Probable calcium-binding protein CML28 OS=Oryza sativa subsp. japonica GN=CML28 PE=2 SV=1	4.36	16.35	-1.34	down	0.00	0.02	yes
TRINITY_DN13612_c0_g2	PREDICTED: GEM-like protein 4 [Populus euphratica]	At5g08350	GEM-like protein 4 OS=Arabidopsis thaliana GN=At5g08350 PE=2 SV=1	1.07	6.41	-1.99	down	0.00	0.02	yes
TRINITY_DN19134_c0_g2	leucine-rich repeat transmembrane protein kinase [Populus trichocarpa]	INRPK1	Receptor-like protein kinase OS=Ipomoea nil GN=INRPK1 PE=2 SV=2	1.28	4.62	-1.23	down	0.00	0.02	yes
TRINITY_DN19143_c0_g6	PREDICTED: uncharacterized protein LOC105129502 [Populus euphratica]	-	-	0.25	1.91	-2.26	down	0.00	0.02	yes
TRINITY_DN16896_c0_g2	hypothetical protein POPTR_0011s13290g [Populus trichocarpa]	SD18	Receptor-like serine/threonine-protein kinase SD1-8 OS=Arabidopsis thaliana GN=SD18 PE=1 SV=1	0.63	4.87	-2.24	down	0.00	0.02	yes
TRINITY_DN12146_c0_g1	PREDICTED: putative disease resistance protein At4g19050 isoform X1 [Populus euphratica]	At5g45510	Probable disease resistance protein At5g45510 OS=Arabidopsis thaliana GN=At5g45510 PE=1 SV=2	0.30	2.23	-2.23	down	0.00	0.02	yes
TRINITY_DN23279_c0_g6	hypothetical protein POPTR_0019s05120g [Populus trichocarpa]	-	-	4.71	16.39	-1.23	down	0.00	0.02	yes
TRINITY_DN22396_c1_g1	PREDICTED: probable disease resistance protein At4g27220 [Populus euphratica]	-	-	0.71	3.88	-1.82	down	0.00	0.02	yes
TRINITY_DN28887_c0_g1	hypothetical protein POPTR_0004s06350g [Populus trichocarpa]	-	-	1.56	7.03	-1.52	down	0.00	0.02	yes
TRINITY_DN23219_c0_g3	PREDICTED: mediator-associated protein 1-like [Populus euphratica]	At5g28040	Probable transcription factor At5g28040 OS=Arabidopsis thaliana GN=At5g28040 PE=1 SV=1	3.55	11.26	-1.06	down	0.00	0.02	yes
TRINITY_DN13864_c0_g4	PREDICTED: uncharacterized protein LOC105111439 [Populus euphratica]	MDC1	Mediator of DNA damage checkpoint protein 1 OS=Sus scrofa GN=MDC1 PE=3 SV=1	1.63	5.43	-1.11	down	0.00	0.02	yes
TRINITY_DN21574_c0_g3	hypothetical protein POPTR_0009s12890g [Populus trichocarpa]	SAUR50	Auxin-responsive protein SAUR50 OS=Arabidopsis thaliana GN=SAUR50 PE=1 SV=1	1.15	6.50	-1.89	down	0.00	0.02	yes
TRINITY_DN27362_c1_g1	hypothetical protein POPTR_0019s14380g [Populus trichocarpa]	MIK2	MDIS1-interacting receptor like kinase 2 OS=Arabidopsis thaliana GN=MIK2 PE=1 SV=3	4.96	18.47	-1.25	down	0.00	0.02	yes
TRINITY_DN21351_c1_g11	-	-	-	44.40	169.69	-1.30	down	0.00	0.02	yes
TRINITY_DN15967_c0_g1	hypothetical protein POPTR_0015s04050g [Populus trichocarpa]	GGR	Heterodimeric geranylgeranyl pyrophosphate synthase small subunit, chloroplastic OS=Arabidopsis thaliana GN=GGR PE=1 SV=2	3.43	15.36	-1.54	down	0.00	0.02	yes
TRINITY_DN27876_c5_g5	PREDICTED: acetyl-CoA carboxylase 1-like [Populus euphratica]	ACC1	Acetyl-CoA carboxylase 1 OS=Arabidopsis thaliana GN=ACC1 PE=1 SV=1	0.87	6.39	-2.33	down	0.00	0.02	yes
TRINITY_DN25497_c1_g1	chitinase 7 [Populus x canadensis]	EP3	Endochitinase EP3 OS=Arabidopsis thaliana GN=EP3 PE=1 SV=1	5.06	22.67	-1.61	down	0.00	0.02	yes
TRINITY_DN25851_c0_g1	hypothetical protein POPTR_0006s01720g [Populus trichocarpa]	SCL13	Scarecrow-like protein 13 OS=Arabidopsis thaliana GN=SCL13 PE=2 SV=2	10.33	33.35	-1.07	down	0.00	0.02	yes

TRINITY_DN16618_c0_g1	-	-	-	5.53	19.28	-1.12	down	0.00	0.02	yes
TRINITY_DN27031_c2_g1	hypothetical protein POPTR_0006s09490g [Populus trichocarpa]	IPCS2	Phosphatidylinositol:ceramide inositolphosphotransferase 2 OS=Arabidopsis thaliana GN=IPCS2 PE=2 SV=1	44.37	151.26	-1.33	down	0.00	0.02	yes
TRINITY_DN26263_c0_g2	-	-	-	0.35	2.94	-2.46	down	0.00	0.02	yes
TRINITY_DN21027_c0_g1	phospholipid/glycerol acyltransferase family protein [Populus trichocarpa]	GPAT8	Probable glycerol-3-phosphate acyltransferase 8 OS=Arabidopsis thaliana GN=GPAT8 PE=2 SV=1	12.14	41.22	-1.16	down	0.00	0.02	yes
TRINITY_DN26866_c0_g2	PREDICTED: ankyrin repeat domain-containing protein 13C-A-like [Populus euphratica]	ANKRD13B	Ankyrin repeat domain-containing protein 13B OS=Homo sapiens GN=ANKRD13B PE=1 SV=4	5.32	18.64	-1.21	down	0.00	0.02	yes
TRINITY_DN27262_c1_g1	PREDICTED: homeobox-leucine zipper protein HDG2 isoform X2 [Populus euphratica]	HDG2	Homeobox-leucine zipper protein HDG2 OS=Arabidopsis thaliana GN=HDG2 PE=2 SV=1	4.55	16.64	-1.29	down	0.00	0.02	yes
TRINITY_DN17942_c0_g4	unknown [Populus trichocarpa]	-	-	4.85	14.93	-1.05	down	0.00	0.02	yes
TRINITY_DN17668_c0_g5	hypothetical protein POPTR_0013s11610g [Populus trichocarpa]	-	-	5.11	16.78	-1.09	down	0.00	0.02	yes
TRINITY_DN26489_c1_g1	hypothetical protein POPTR_0001s01500g [Populus trichocarpa]	tmem45b	Transmembrane protein 45B OS=Xenopus laevis GN=tmem45b PE=2 SV=1	21.82	64.86	-1.05	down	0.00	0.02	yes
TRINITY_DN18498_c0_g1	PREDICTED: uncharacterized protein LOC105107231 isoform X2 [Populus euphratica]	-	-	1.76	7.04	-1.39	down	0.00	0.02	yes
TRINITY_DN24093_c0_g3	hypothetical protein POPTR_0018s07790g [Populus trichocarpa]	At3g55350	Protein ALP1-like OS=Arabidopsis thaliana GN=At3g55350 PE=2 SV=1	11.03	101.81	-2.62	down	0.00	0.02	yes
TRINITY_DN21675_c0_g5	hypothetical protein POPTR_0010s24020g [Populus trichocarpa]	-	-	1.88	7.15	-1.34	down	0.00	0.02	yes
TRINITY_DN23005_c0_g6	-	-	-	0.63	4.06	-2.12	down	0.00	0.02	yes
TRINITY_DN22820_c0_g2	PXY [Populus tremula x Populus tremuloides]	TDR	Leucine-rich repeat receptor-like protein kinase TDR OS=Arabidopsis thaliana GN=TDR PE=1 SV=1	0.91	4.75	-1.71	down	0.00	0.02	yes
TRINITY_DN18895_c0_g1	mitochondrial aldehyde dehydrogenase family protein [Populus trichocarpa]	ALDH2B7	Aldehyde dehydrogenase family 2 member B7, mitochondrial OS=Arabidopsis thaliana GN=ALDH2B7 PE=2 SV=2	7.40	23.22	-1.06	down	0.00	0.02	yes
TRINITY_DN20088_c0_g3	-	-	-	0.55	3.03	-1.82	down	0.00	0.02	yes
TRINITY_DN20274_c0_g1	PREDICTED: protein IQ-DOMAIN 31-like [Populus euphratica]	IQD31	Protein IQ-DOMAIN 31 OS=Arabidopsis thaliana GN=IQD31 PE=1 SV=1	1.03	4.42	-1.48	down	0.00	0.02	yes
TRINITY_DN20692_c0_g1	PREDICTED: probable DNA primase large subunit [Populus euphratica]	At1g67320	Probable DNA primase large subunit OS=Arabidopsis thaliana GN=At1g67320 PE=2 SV=2	8.23	30.45	-1.11	down	0.00	0.02	yes
TRINITY_DN26299_c0_g2	PREDICTED: putative disease resistance RPP13-like protein 1 isoform X2 [Populus euphratica]	-	-	0.25	2.33	-2.51	down	0.00	0.02	yes
TRINITY_DN23135_c0_g11	hypothetical protein POPTR_0455s00200g, partial [Populus trichocarpa]	PDR3	Pleiotropic drug resistance protein 3 OS=Nicotiana tabacum GN=PDR3 PE=2 SV=1	0.38	2.69	-2.21	down	0.00	0.02	yes
TRINITY_DN24543_c0_g2	PREDICTED: gibberellin 2-beta-dioxygenase 2-like [Populus euphratica]	GA2OX2	Gibberellin 2-beta-dioxygenase 2 OS=Pisum sativum GN=GA2OX2 PE=2 SV=1	0.83	8.88	-2.47	down	0.00	0.02	yes
TRINITY_DN23036_c0_g3	PREDICTED: long chain acyl-CoA synthetase 2 isoform X1 [Populus euphratica]	LACS2	Long chain acyl-CoA synthetase 2 OS=Arabidopsis thaliana GN=LACS2 PE=2 SV=1	4.05	12.87	-1.08	down	0.00	0.02	yes
TRINITY_DN1373_c0_g1	hypothetical protein POPTR_0016s05610g [Populus trichocarpa]	-	-	0.63	4.71	-1.99	down	0.00	0.02	yes
TRINITY_DN19365_c0_g4	PREDICTED: putative GEM-like protein 8 isoform X2 [Populus euphratica]	At5g23370	Putative GEM-like protein 8 OS=Arabidopsis thaliana GN=At5g23370 PE=3 SV=1	3.73	12.35	-1.24	down	0.01	0.02	yes
TRINITY_DN15306_c0_g1	hypothetical protein CICLE_v10008271mg [Citrus clementina]	-	-	2.15	9.12	-1.46	down	0.01	0.02	yes
TRINITY_DN24556_c0_g3	-	-	-	1.64	7.20	-1.48	down	0.01	0.02	yes
TRINITY_DN17619_c0_g3	hypothetical protein POPTR_0004s15180g [Populus trichocarpa]	-	-	0.80	3.77	-1.60	down	0.01	0.02	yes
TRINITY_DN21289_c1_g1	PREDICTED: uncharacterized protein LOC108983648 [Juglans regia]	-	-	0.45	3.16	-2.22	down	0.01	0.02	yes
TRINITY_DN24138_c0_g3	-	-	-	4.17	25.90	-1.99	down	0.01	0.02	yes
TRINITY_DN20548_c0_g1	Mitogen-activated protein kinase kinase kinase 1 [Populus trichocarpa]	ANP1	Mitogen-activated protein kinase kinase kinase ANP1 OS=Arabidopsis thaliana GN=ANP1 PE=1 SV=2	3.59	12.01	-1.24	down	0.01	0.02	yes
TRINITY_DN14590_c0_g2	PREDICTED: uncharacterized protein LOC105121565 [Populus euphratica]	-	-	1.54	5.96	-1.33	down	0.01	0.02	yes

TRINITY_DN27872_c1_g2	hypothetical protein POPTR_0154s00220g [Populus trichocarpa]	RGA3	Putative disease resistance protein RGA3 OS=Solanum bulbocastanum GN=RGA3 PE=2 SV=2	4.07	18.65	-1.51	down	0.01	0.02	yes
TRINITY_DN17229_c0_g1	hypothetical protein POPTR_0014s03490g [Populus trichocarpa]	OSB3	Protein OSB3, chloroplastic/mitochondrial OS=Arabidopsis thaliana GN=OSB3 PE=2 SV=1	1.22	5.46	-1.51	down	0.01	0.02	yes
TRINITY_DN21202_c0_g2	hypothetical protein POPTR_0017s04540g [Populus trichocarpa]	ERF9	Ethylene-responsive transcription factor 9 OS=Arabidopsis thaliana GN=ERF9 PE=2 SV=1	24.81	154.42	-2.06	down	0.01	0.02	yes
TRINITY_DN20032_c1_g1	PIN1-like auxin transport protein [Populus tremula x Populus tremuloides]	PIN3	Auxin efflux carrier component 3 OS=Arabidopsis thaliana GN=PIN3 PE=1 SV=1	2.63	9.76	-1.26	down	0.01	0.02	yes
TRINITY_DN19552_c0_g11	hypothetical protein POPTR_0242s00210g [Populus trichocarpa]	-	-	1.98	7.81	-1.38	down	0.01	0.02	yes
TRINITY_DN23615_c0_g6	-	-	-	19.72	61.77	-1.03	down	0.01	0.02	yes
TRINITY_DN26517_c0_g1	hypothetical protein POPTR_0006s25460g [Populus trichocarpa]	-	-	2.85	10.78	-1.28	down	0.01	0.02	yes
TRINITY_DN21932_c0_g3	phosphatase 2C family protein [Populus trichocarpa]	At3g17090	Probable protein phosphatase 2C 42 OS=Arabidopsis thaliana GN=At3g17090 PE=2 SV=1	2.53	8.10	-1.06	down	0.01	0.02	yes
TRINITY_DN15687_c0_g1	hypothetical protein POPTR_0010s05160g [Populus trichocarpa]	MTOPVIB	Type 2 DNA topoisomerase 6 subunit B-like OS=Arabidopsis thaliana GN=MTOPVIB PE=1 SV=1	0.67	3.15	-1.61	down	0.01	0.02	yes
TRINITY_DN16816_c0_g1	pleckstrin homology domain-containing family protein [Populus trichocarpa]	ROPGAP7	Rho GTPase-activating protein 7 OS=Arabidopsis thaliana GN=ROPGAP7 PE=2 SV=1	15.05	49.14	-1.10	down	0.01	0.02	yes
TRINITY_DN27528_c2_g4	hypothetical protein POPTR_0014s10090g [Populus trichocarpa]	ARP1	Probable RNA-binding protein ARP1 OS=Arabidopsis thaliana GN=ARP1 PE=2 SV=1	0.85	3.71	-1.46	down	0.01	0.02	yes
TRINITY_DN27873_c0_g1	PREDICTED: disease resistance protein RPS6-like isoform X1 [Populus euphratica]	-	TMV resistance protein N OS=Nicotiana glutinosa GN=N PE=1 SV=1	3.91	17.23	-1.55	down	0.01	0.02	yes
TRINITY_DN26292_c0_g7	hypothetical protein POPTR_0017s02570g [Populus trichocarpa]	RGA4	Putative disease resistance protein RGA4 OS=Solanum bulbocastanum GN=RGA4 PE=2 SV=1	1.60	15.90	-2.62	down	0.01	0.02	yes
TRINITY_DN17652_c0_g1	hypothetical protein POPTR_0001s02910g [Populus trichocarpa]	-	-	2.95	16.85	-1.78	down	0.01	0.02	yes
TRINITY_DN27613_c1_g3	-	-	-	2.09	7.75	-1.50	down	0.01	0.02	yes
TRINITY_DN27426_c0_g2	-	-	-	0.47	3.23	-2.16	down	0.01	0.02	yes
TRINITY_DN11567_c0_g1	CBL-interacting serine/threonine-protein kinase 4-like [Populus euphratica]	-	-	1.69	8.47	-1.71	down	0.01	0.02	yes
TRINITY_DN13317_c0_g1	PREDICTED: WD repeat-containing protein tag-125-like [Populus euphratica]	JGB	Protein JINGUBANG OS=Arabidopsis thaliana GN=JGB PE=1 SV=1	0.32	2.12	-2.09	down	0.01	0.02	yes
TRINITY_DN26727_c0_g2	PREDICTED: uncharacterized protein LOC105134358 isoform X1 [Populus euphratica]	-	-	1.77	6.29	-1.19	down	0.01	0.02	yes
TRINITY_DN16818_c0_g1	PREDICTED: protein YLS9-like [Populus euphratica]	YLS9	Protein YLS9 OS=Arabidopsis thaliana GN=YLS9 PE=2 SV=1	2.50	21.97	-2.61	down	0.01	0.02	yes
TRINITY_DN26887_c0_g2	kinase family protein [Populus trichocarpa]	At1g49180	Serine/threonine-protein kinase ATG1t OS=Arabidopsis thaliana GN=At1g49180 PE=2 SV=1	7.21	50.16	-1.10	down	0.01	0.02	yes
TRINITY_DN16928_c0_g1	-	-	-	0.77	4.35	-1.84	down	0.01	0.02	yes
TRINITY_DN18972_c0_g6	PREDICTED: probable LRR receptor-like serine/threonine-protein kinase RFK1 isoform X1 [Populus euphratica]	RKF1	Probable LRR receptor-like serine/threonine-protein kinase RFK1 OS=Arabidopsis thaliana GN=RKF1 PE=1 SV=1	0.31	2.70	-1.91	down	0.01	0.02	yes
TRINITY_DN16287_c0_g1	PREDICTED: uncharacterized protein LOC105122418 [Populus euphratica]	-	-	5.96	19.08	-1.03	down	0.01	0.02	yes
TRINITY_DN20610_c0_g1	PREDICTED: putative phospholipid-transporting ATPase 8 isoform X1 [Populus euphratica]	ALA8	Probable phospholipid-transporting ATPase 8 OS=Arabidopsis thaliana GN=ALA8 PE=3 SV=1	0.95	3.47	-1.25	down	0.01	0.02	yes
TRINITY_DN9503_c0_g1	hypothetical protein POPTR_0014s07840g [Populus trichocarpa]	Os02g0637000	Putative ripening-related protein 2 OS=Oryza sativa subsp. japonica GN=Os02g0637000 PE=3 SV=1	1.63	8.13	-1.72	down	0.01	0.02	yes
TRINITY_DN20064_c0_g2	hypothetical protein POPTR_0009s05520g, partial [Populus trichocarpa]	Os07g0682400	Zinc finger CCCH domain-containing protein 53 OS=Oryza sativa subsp. japonica GN=Os07g0682400 PE=2 SV=1	4.89	17.34	-1.23	down	0.01	0.02	yes
TRINITY_DN23359_c0_g2	hypothetical protein POPTR_0018s11700g [Populus trichocarpa]	-	-	1.32	10.13	-2.22	down	0.01	0.02	yes
TRINITY_DN26031_c0_g4	calcium-dependent protein kinase [Populus trichocarpa]	CPK13	Calcium-dependent protein kinase 13 OS=Arabidopsis thaliana GN=CPK13 PE=1 SV=2	6.99	22.76	-1.10	down	0.01	0.02	yes
TRINITY_DN21256_c1_g8	-	-	-	0.41	2.73	-2.11	down	0.01	0.02	yes

TRINITY_DN16974_c0_g1	hypothetical protein POPTR_0007s01930g [Populus trichocarpa]	FRS5	Protein FAR1-RELATED SEQUENCE 5 OS=Arabidopsis thaliana GN=FRS5 PE=2 SV=1	1.50	5.40	-1.24	down	0.01	0.02	yes
TRINITY_DN22242_c0_g1	hypothetical protein POPTR_0004s04410g [Populus trichocarpa]	LECRK71	L-type lectin-domain containing receptor kinase VII.1 OS=Arabidopsis thaliana GN=LECRK71 PE=2 SV=1	1.74	6.75	-1.16	down	0.01	0.02	yes
TRINITY_DN18157_c0_g1	PREDICTED: receptor-like cytosolic serine/threonine-protein kinase RBK2 isoform X2 [Populus euphratica]	RBK2	Receptor-like cytosolic serine/threonine-protein kinase RBK2 OS=Arabidopsis thaliana GN=RBK2 PE=1 SV=1	1.58	5.15	-1.26	down	0.01	0.02	yes
TRINITY_DN20192_c0_g4	-	-	-	1.38	5.97	-1.52	down	0.01	0.02	yes
TRINITY_DN18394_c0_g5	hypothetical protein POPTR_0017s01100g [Populus trichocarpa]	HIPP39	Heavy metal-associated isoprenylated plant protein 39 OS=Arabidopsis thaliana GN=HIPP39 PE=2 SV=1	2.46	11.02	-1.51	down	0.01	0.02	yes
TRINITY_DN24141_c0_g1	PREDICTED: uncharacterized protein LOC105131592 [Populus euphratica]	-	-	0.78	4.69	-1.92	down	0.01	0.02	yes
TRINITY_DN28958_c0_g1	-	-	-	3.11	10.79	-1.18	down	0.01	0.02	yes
TRINITY_DN20069_c0_g9	zinc finger family protein [Populus trichocarpa]	RHC2A	Probable E3 ubiquitin-protein ligase RHC2A OS=Arabidopsis thaliana GN=RHC2A PE=2 SV=1	5.84	19.88	-1.14	down	0.01	0.02	yes
TRINITY_DN3175_c0_g1	-	-	-	0.39	1.97	-1.75	down	0.01	0.02	yes
TRINITY_DN22872_c1_g1	hypothetical protein POPTR_0012s02420g [Populus trichocarpa]	-	-	11.75	33.54	-1.00	down	0.01	0.02	yes
TRINITY_DN15837_c0_g1	hypothetical protein POPTR_0013s00340g [Populus trichocarpa]	RCI2A	Hydrophobic protein RCI2A OS=Arabidopsis thaliana GN=RCI2A PE=2 SV=1	15.29	58.26	-1.37	down	0.01	0.02	yes
TRINITY_DN16217_c0_g2	pleckstrin homology domain-containing family protein [Populus trichocarpa]	ROPGAP7	Rho GTPase-activating protein 7 OS=Arabidopsis thaliana GN=ROPGAP7 PE=2 SV=1	12.99	40.31	-1.04	down	0.01	0.02	yes
TRINITY_DN17818_c1_g6	phosphatase 2C family protein [Populus trichocarpa]	At3g23360	Putative protein phosphatase 2C-like protein 44 OS=Arabidopsis thaliana GN=At3g23360 PE=5 SV=1	0.81	4.42	-1.82	down	0.01	0.02	yes
TRINITY_DN22123_c0_g2	hypothetical protein POPTR_0008s15150g [Populus trichocarpa]	-	-	1.39	5.41	-1.37	down	0.01	0.02	yes
TRINITY_DN16009_c0_g1	-	-	-	3.33	13.91	-1.47	down	0.01	0.02	yes
TRINITY_DN25536_c1_g9	-	-	-	0.62	3.46	-1.89	down	0.01	0.02	yes
TRINITY_DN24277_c0_g2	kinase MMK4 family protein [Populus trichocarpa]	MMK1	Mitogen-activated protein kinase homolog MMK1 OS=Medicago sativa GN=MMK1 PE=1 SV=1	8.48	37.20	-1.57	down	0.01	0.02	yes
TRINITY_DN22328_c0_g1	PREDICTED: G-box-binding factor 3-like isoform X1 [Populus euphratica]	CPRF1	Common plant regulatory factor 1 OS=Petroselinum crispum GN=CPRF1 PE=2 SV=1	12.13	40.13	-1.08	down	0.01	0.02	yes
TRINITY_DN24829_c0_g3	-	-	-	0.63	2.97	-1.63	down	0.01	0.02	yes
TRINITY_DN7452_c0_g1	cystatin family protein [Populus trichocarpa]	CYS5	Cysteine proteinase inhibitor 5 OS=Arabidopsis thaliana GN=CYS5 PE=2 SV=2	78.65	270.46	-1.18	down	0.01	0.02	yes
TRINITY_DN21244_c0_g1	PREDICTED: Werner syndrome ATP-dependent helicase-like isoform X1 [Populus euphratica]	recQ	ATP-dependent DNA helicase RecQ OS=Pasteurella multocida (strain Pm70) GN=recQ PE=3 SV=1	2.33	7.40	-1.04	down	0.01	0.02	yes
TRINITY_DN23027_c0_g2	hypothetical protein POPTR_0316s00220g [Populus trichocarpa]	WAKL22	Wall-associated receptor kinase-like 22 OS=Arabidopsis thaliana GN=WAKL22 PE=2 SV=1	0.95	5.52	-1.39	down	0.01	0.02	yes
TRINITY_DN22360_c0_g1	homeobox-leucine zipper family protein [Populus trichocarpa]	HAT4	Homeobox-leucine zipper protein HAT4 OS=Arabidopsis thaliana GN=HAT4 PE=1 SV=1	4.84	17.40	-1.21	down	0.01	0.02	yes
TRINITY_DN23723_c0_g1	hypothetical protein POPTR_0001s08030g [Populus trichocarpa]	PHR1	Deoxyribodipyrimidine photo-lyase OS=Arabidopsis thaliana GN=PHR1 PE=2 SV=1	4.04	15.57	-1.12	down	0.01	0.02	yes
TRINITY_DN23399_c0_g2	adhesion of calyx edges family protein [Populus trichocarpa]	HTH	Protein HOTHEAD OS=Arabidopsis thaliana GN=HTH PE=1 SV=1	1.75	8.05	-1.59	down	0.01	0.02	yes
TRINITY_DN27694_c0_g2	hypothetical protein POPTR_0014s10040g [Populus trichocarpa]	IQD14	Protein IQ-DOMAIN 14 OS=Arabidopsis thaliana GN=IQD14 PE=1 SV=1	1.37	5.21	-1.32	down	0.01	0.02	yes
TRINITY_DN23341_c1_g2	-	-	-	1.56	7.64	-1.52	down	0.01	0.02	yes
TRINITY_DN22361_c0_g2	membrane-associated mannitol-induced family protein [Populus trichocarpa]	PVA41	Vesicle-associated protein 4-1 OS=Arabidopsis thaliana GN=PVA41 PE=2 SV=1	3.86	17.94	-1.61	down	0.01	0.02	yes
TRINITY_DN25943_c0_g1	-	-	-	0.39	2.81	-2.20	down	0.01	0.02	yes
TRINITY_DN24707_c1_g1	nodulin MtN21 family protein [Populus trichocarpa]	WAT1	Protein WALLS ARE THIN 1 OS=Arabidopsis thaliana GN=WAT1 PE=1 SV=1	14.31	50.91	-1.25	down	0.01	0.02	yes
TRINITY_DN16023_c0_g1	PREDICTED: glycine-rich cell wall structural protein 2-like isoform X1 [Populus euphratica]	-	-	1.90	10.46	-1.62	down	0.01	0.02	yes

TRINITY_DN9299_c0_g1	hypothetical protein POPTR_0256s00200g [Populus trichocarpa]	PARC	Probable glutathione S-transferase parC OS=Nicotiana tabacum GN=PARC PE=2 SV=1	1.86	6.96	-1.29	down	0.01	0.02	yes
TRINITY_DN25008_c1_g1	zinc finger family protein [Populus trichocarpa]	-	-	8.07	27.04	-1.16	down	0.01	0.02	yes
TRINITY_DN14938_c0_g2	PREDICTED: uncharacterized protein LOC105121819 [Populus euphratica]	-	-	0.48	2.82	-1.94	down	0.01	0.02	yes
TRINITY_DN21655_c0_g1	PREDICTED: dolichyl-diphosphooligosaccharide--protein glycosyltransferase subunit STT3A isoform X1 [Populus euphratica]	-	-	4.31	16.78	-1.22	down	0.01	0.02	yes
TRINITY_DN15844_c0_g3	PREDICTED: RING-H2 finger protein ATL22-like [Populus euphratica]	-	-	0.26	1.63	-1.98	down	0.01	0.02	yes
TRINITY_DN23102_c1_g1	PREDICTED: probable WRKY transcription factor 39 [Populus euphratica]	WRKY39	Probable WRKY transcription factor 39 OS=Arabidopsis thaliana GN=WRKY39 PE=2 SV=1	2.26	7.38	-1.10	down	0.01	0.02	yes
TRINITY_DN21174_c0_g1	PREDICTED: putative disease resistance protein RGA4 [Populus euphratica]	RGA4	Putative disease resistance protein RGA4 OS=Solanum bulbocastanum GN=RGA4 PE=2 SV=1	6.42	41.54	-2.24	down	0.01	0.02	yes
TRINITY_DN23085_c0_g1	PREDICTED: uncharacterized protein LOC105126242 [Populus euphratica]	-	-	2.37	8.03	-1.17	down	0.01	0.02	yes
TRINITY_DN20456_c0_g3	hypothetical protein F383_26710 [Gossypium arboreum]	-	-	0.46	2.46	-1.76	down	0.01	0.02	yes
TRINITY_DN18105_c0_g5	hypothetical protein POPTR_0010s13710g [Populus trichocarpa]	-	-	0.82	3.76	-1.61	down	0.01	0.02	yes
TRINITY_DN16700_c1_g1	hypothetical protein POPTR_0001s35040g [Populus trichocarpa]	-	-	1.13	5.30	-1.84	down	0.01	0.02	yes
TRINITY_DN24537_c0_g6	hypothetical protein MANES_16G026000 [Manihot esculenta]	At4g04930	Sphingolipid delta(4)-desaturase DES1-like OS=Arabidopsis thaliana GN=At4g04930 PE=2 SV=1	0.34	2.70	-2.31	down	0.01	0.02	yes
TRINITY_DN22414_c0_g1	hypothetical protein POPTR_0007s04220g [Populus trichocarpa]	PUB27	U-box domain-containing protein 27 OS=Arabidopsis thaliana GN=PUB27 PE=2 SV=1	0.68	3.25	-1.65	down	0.01	0.02	yes
TRINITY_DN26427_c0_g2	-	-	-	4.36	15.33	-1.22	down	0.01	0.02	yes
TRINITY_DN21634_c0_g1	-	-	-	1.39	5.41	-1.36	down	0.01	0.02	yes
TRINITY_DN17428_c0_g1	calcium-binding family protein [Populus trichocarpa]	BETVIII	Calcium-binding allergen Bet v 3 OS=Betula pendula GN=BETVIII PE=1 SV=1	1.92	16.00	-2.46	down	0.01	0.02	yes
TRINITY_DN24277_c0_g1	kinase MMK4 family protein [Populus trichocarpa]	MPK3	Mitogen-activated protein kinase 3 OS=Arabidopsis thaliana GN=MPK3 PE=1 SV=2	3.48	14.42	-1.54	down	0.01	0.02	yes
TRINITY_DN19811_c0_g1	-	-	-	1.16	8.60	-2.22	down	0.01	0.02	yes
TRINITY_DN16511_c0_g1	PREDICTED: alkaline/neutral invertase CINV2 [Populus euphratica]	INVB	Probable alkaline/neutral invertase B OS=Arabidopsis thaliana GN=INVB PE=1 SV=1	2.76	8.63	-1.03	down	0.01	0.02	yes
TRINITY_DN14610_c0_g2	PREDICTED: basic 7S globulin-like isoform X1 [Populus euphratica]	At5g10770	Aspartyl protease family protein At5g10770 OS=Arabidopsis thaliana GN=At5g10770 PE=2 SV=1	0.39	2.60	-2.05	down	0.01	0.02	yes
TRINITY_DN20682_c1_g1	PREDICTED: uncharacterized protein LOC105120705 [Populus euphratica]	-	-	4.19	13.06	-1.02	down	0.01	0.02	yes
TRINITY_DN21402_c3_g1	hypothetical protein POPTR_0014s13610g [Populus trichocarpa]	-	-	6.81	27.45	-1.38	down	0.01	0.02	yes
TRINITY_DN15134_c0_g1	PREDICTED: uncharacterized protein LOC105124599 [Populus euphratica]	-	-	1.67	14.39	-2.48	down	0.01	0.02	yes
TRINITY_DN12653_c0_g2	PREDICTED: exonuclease 1 [Populus euphratica]	EXO1	Exonuclease 1 OS=Arabidopsis thaliana GN=EXO1 PE=2 SV=2	0.76	3.12	-1.41	down	0.01	0.02	yes
TRINITY_DN23531_c0_g7	cytochrome P450 family protein [Populus trichocarpa]	CYP81E8	Cytochrome P450 81E8 OS=Medicago truncatula GN=CYP81E8 PE=2 SV=1	1.88	6.94	-1.31	down	0.01	0.02	yes
TRINITY_DN15336_c0_g1	hypothetical protein POPTR_0004s03650g [Populus trichocarpa]	-	-	1.33	9.38	-2.17	down	0.01	0.02	yes
TRINITY_DN21031_c0_g2	serine carboxypeptidase S10 family protein [Populus trichocarpa]	SCPL42	Serine carboxypeptidase-like 42 OS=Arabidopsis thaliana GN=SCPL42 PE=2 SV=1	9.81	29.23	-1.01	down	0.01	0.02	yes
TRINITY_DN15734_c0_g3	PREDICTED: aluminum-activated malate transporter 9-like [Populus euphratica]	ALMT9	Aluminum-activated malate transporter 9 OS=Arabidopsis thaliana GN=ALMT9 PE=2 SV=1	0.52	2.21	-1.48	down	0.01	0.02	yes
TRINITY_DN19113_c0_g1	auxin-induced protein aux28 [Populus trichocarpa]	AUX28	Auxin-induced protein AUX28 OS=Glycine max GN=AUX28 PE=2 SV=1	7.11	22.97	-1.11	down	0.01	0.02	yes
TRINITY_DN16875_c0_g1	-	-	-	2.52	9.22	-1.73	down	0.01	0.02	yes

TRINITY_DN22416_c0_g2	-	-	-	0.74	5.02	-2.04	down	0.01	0.02	yes
TRINITY_DN14839_c0_g1	clavata3/esr-related 25 family protein [Populus trichocarpa]	CLE25	CLAVATA3/ESR (CLE)-related protein 25 OS=Arabidopsis thaliana GN=CLE25 PE=2 SV=1	0.79	3.61	-1.54	down	0.01	0.02	yes
TRINITY_DN25169_c0_g3	PREDICTED: GATA transcription factor 5-like [Populus euphratica]	GATA5	GATA transcription factor 5 OS=Arabidopsis thaliana GN=GATA5 PE=2 SV=1	6.88	22.73	-1.10	down	0.01	0.02	yes
TRINITY_DN16894_c0_g3	hypothetical protein POPTR_0002s05210g [Populus trichocarpa]	OFP11	Transcription repressor OFP11 OS=Arabidopsis thaliana GN=OFP11 PE=2 SV=1	2.06	8.17	-1.35	down	0.01	0.02	yes
TRINITY_DN21313_c0_g3	hypothetical protein POPTR_0014s07180g [Populus trichocarpa]	At4g00750	Probable methyltransferase PMT15 OS=Arabidopsis thaliana GN=At4g00750 PE=2 SV=1	1.97	7.11	-1.27	down	0.01	0.02	yes
TRINITY_DN18623_c2_g4	-	-	-	15.67	64.01	-1.49	down	0.01	0.02	yes
TRINITY_DN22671_c0_g1	hypothetical protein POPTR_0005s10390g [Populus trichocarpa]	PIX13	Probable serine/threonine-protein kinase PIX13 OS=Arabidopsis thaliana GN=PIX13 PE=1 SV=2	1.12	6.06	-1.18	down	0.01	0.02	yes
TRINITY_DN24534_c0_g2	PREDICTED: uncharacterized rhomboid protein AN10929 [Populus euphratica]	RBL1	RHOMBOID-like protein 1 OS=Arabidopsis thaliana GN=RBL1 PE=2 SV=1	15.35	62.82	-1.50	down	0.01	0.02	yes
TRINITY_DN21539_c0_g1	PREDICTED: histone chaperone ASF1B-like [Populus euphratica]	ASF1B	Histone chaperone ASF1B OS=Arabidopsis thaliana GN=ASF1B PE=1 SV=1	4.21	14.29	-1.09	down	0.01	0.02	yes
TRINITY_DN19049_c0_g1	PREDICTED: uncharacterized protein LOC105113370 [Populus euphratica]	-	-	1.11	4.25	-1.33	down	0.01	0.02	yes
TRINITY_DN24079_c0_g1	hypothetical protein POPTR_0001s02970g [Populus trichocarpa]	-	-	0.52	2.96	-1.83	down	0.01	0.02	yes
TRINITY_DN25985_c2_g2	hypothetical protein POPTR_0012s00800g [Populus trichocarpa]	-	-	0.45	3.17	-2.24	down	0.01	0.02	yes
TRINITY_DN19748_c0_g2	PREDICTED: transcription factor bHLH35-like isoform X2 [Populus euphratica]	BHLH35	Transcription factor bHLH35 OS=Arabidopsis thaliana GN=BHLH35 PE=2 SV=1	5.09	40.00	-2.21	down	0.01	0.02	yes
TRINITY_DN19299_c0_g1	hypothetical protein POPTR_0003s10040g [Populus trichocarpa]	HIPP41	Heavy metal-associated isoprenylated plant protein 41 OS=Arabidopsis thaliana GN=HIPP41 PE=3 SV=1	1.52	5.93	-1.22	down	0.01	0.02	yes
TRINITY_DN20662_c0_g1	expansin-like family protein [Populus trichocarpa]	EXLA2	Expansin-like A2 OS=Arabidopsis thaliana GN=EXLA2 PE=2 SV=1	1.31	6.67	-1.69	down	0.01	0.02	yes
TRINITY_DN2724_c0_g1	PREDICTED: protein EXORDIUM-like 2 [Populus euphratica]	EXL2	Protein EXORDIUM-like 2 OS=Arabidopsis thaliana GN=EXL2 PE=2 SV=1	1.97	13.60	-2.16	down	0.01	0.02	yes
TRINITY_DN14427_c0_g1	hypothetical protein POPTR_0004s05290g [Populus trichocarpa]	-	-	1.48	7.38	-1.69	down	0.01	0.02	yes
TRINITY_DN9151_c0_g1	auxin-responsive family protein [Populus trichocarpa]	SAUR67	Auxin-responsive protein SAUR67 OS=Arabidopsis thaliana GN=SAUR67 PE=2 SV=1	0.46	2.77	-1.97	down	0.01	0.02	yes
TRINITY_DN19202_c3_g1	PREDICTED: RING-H2 finger protein ATL65 [Populus euphratica]	ATL65	RING-H2 finger protein ATL65 OS=Arabidopsis thaliana GN=ATL65 PE=2 SV=2	1.29	4.77	-1.24	down	0.01	0.02	yes
TRINITY_DN14975_c0_g1	hypothetical protein POPTR_0009s08540g [Populus trichocarpa]	IKU2	Receptor-like protein kinase HAIKU2 OS=Arabidopsis thaliana GN=IKU2 PE=1 SV=1	0.48	3.45	-2.28	down	0.01	0.02	yes
TRINITY_DN15247_c0_g1	hypothetical protein MANES_14G023900 [Manihot esculenta]	-	-	22.04	104.92	-1.61	down	0.01	0.02	yes
TRINITY_DN16289_c0_g1	hypothetical protein POPTR_0004s18790g [Populus trichocarpa]	AATL1	Lysine histidine transporter-like 8 OS=Arabidopsis thaliana GN=AATL1 PE=1 SV=1	0.62	3.75	-1.99	down	0.01	0.02	yes
TRINITY_DN17469_c0_g1	PREDICTED: uncharacterized protein LOC105127753 isoform X1 [Populus euphratica]	-	-	9.17	28.14	-1.08	down	0.01	0.02	yes
TRINITY_DN13236_c0_g1	leucine-rich repeat transmembrane protein kinase [Populus trichocarpa]	GSO1	LRR receptor-like serine/threonine-protein kinase GSO1 OS=Arabidopsis thaliana GN=GSO1 PE=2 SV=1	0.61	2.85	-1.65	down	0.01	0.02	yes
TRINITY_DN26928_c1_g2	PREDICTED: transcription factor GTE4-like [Populus euphratica]	GTE4	Transcription factor GTE4 OS=Arabidopsis thaliana GN=GTE4 PE=2 SV=1	3.52	12.61	-1.24	down	0.01	0.02	yes
TRINITY_DN14166_c0_g1	hypothetical protein POPTR_0009s06760g [Populus trichocarpa]	-	-	26.93	130.69	-1.66	down	0.01	0.02	yes
TRINITY_DN17587_c0_g1	hypothetical protein POPTR_0008s02060g [Populus trichocarpa]	-	-	1.65	8.11	-1.68	down	0.01	0.02	yes
TRINITY_DN19302_c0_g1	PREDICTED: probable LRR receptor-like serine/threonine-protein kinase At1g53430 [Populus euphratica]	At1g07650	Probable LRR receptor-like serine/threonine-protein kinase At1g07650 OS=Arabidopsis thaliana GN=At1g07650 PE=1 SV=1	12.74	38.87	-1.39	down	0.01	0.02	yes

TRINITY_DN27318_c1_g1	PREDICTED: putative disease resistance protein At4g10780 [Populus euphratica]	-	-	2.07	15.95	-2.27	down	0.01	0.02	yes
TRINITY_DN24099_c0_g4	hypothetical protein POPTR_0001s32120g [Populus trichocarpa]	-	-	21.57	72.44	-1.12	down	0.01	0.02	yes
TRINITY_DN21798_c0_g1	PREDICTED: uncharacterized protein LOC105134943 isoform X1 [Populus euphratica]	-	-	2.36	9.11	-1.29	down	0.01	0.02	yes
TRINITY_DN23005_c0_g1	hypothetical protein POPTR_0003s19490g [Populus trichocarpa]	AED3	Aspartyl protease AED3 OS=Arabidopsis thaliana GN=AED3 PE=1 SV=1	66.70	205.02	-1.02	down	0.01	0.02	yes
TRINITY_DN1593_c0_g1	hypothetical protein POPTR_0019s01540g [Populus trichocarpa]	BEAT	Acetyl-CoA-benzylalcohol acetyltransferase OS=Clarkia breweri GN=BEAT PE=1 SV=1	1.85	7.22	-1.37	down	0.01	0.02	yes
TRINITY_DN14958_c0_g1	PREDICTED: UPF0481 protein At3g47200-like [Populus euphratica]	At3g47200	UPF0481 protein At3g47200 OS=Arabidopsis thaliana GN=At3g47200 PE=2 SV=1	0.57	2.42	-1.49	down	0.01	0.02	yes
TRINITY_DN14347_c0_g1	PREDICTED: uncharacterized protein LOC105111043 [Populus euphratica]	-	-	1.71	7.18	-1.48	down	0.01	0.02	yes
TRINITY_DN16629_c0_g1	PREDICTED: uncharacterized protein LOC105109563 [Populus euphratica]	-	-	1.71	7.03	-1.47	down	0.01	0.02	yes
TRINITY_DN17819_c0_g1	hypothetical protein POPTR_0010s21320g [Populus trichocarpa]	ATL52	RING-H2 finger protein ATL52 OS=Arabidopsis thaliana GN=ATL52 PE=2 SV=1	0.85	7.85	-2.62	down	0.01	0.02	yes
TRINITY_DN19897_c0_g3	-	-	-	23.93	97.66	-1.50	down	0.01	0.02	yes
TRINITY_DN18361_c0_g2	PREDICTED: E3 ubiquitin-protein ligase PUB22-like [Populus euphratica]	PUB22	E3 ubiquitin-protein ligase PUB22 OS=Arabidopsis thaliana GN=PUB22 PE=1 SV=1	0.35	3.77	-2.79	down	0.01	0.02	yes
TRINITY_DN25500_c0_g2	nucleoside phosphatase family protein [Populus trichocarpa]	APY6	Probable apyrase 6 OS=Arabidopsis thaliana GN=APY6 PE=2 SV=2	6.42	19.00	-1.03	down	0.01	0.02	yes
TRINITY_DN17135_c0_g1	hypothetical protein POPTR_0006s19340g [Populus trichocarpa]	RT	Anthocyanidin 3-O-glucosyltransferase OS=Petunia hybrida GN=RT PE=2 SV=1	24.77	84.05	-1.23	down	0.01	0.02	yes
TRINITY_DN17614_c1_g1	P-loop-containing nucleoside triphosphate hydrolase superfamily protein [Populus tomentosa]	-	-	1.09	4.74	-1.55	down	0.01	0.02	yes
TRINITY_DN25850_c0_g3	hypothetical protein POPTR_0013s13820g [Populus trichocarpa]	-	-	1.51	6.49	-1.75	down	0.01	0.02	yes
TRINITY_DN26491_c2_g1	PREDICTED: mitogen-activated protein kinase 3-like [Populus euphratica]	MPK3	Mitogen-activated protein kinase 3 OS=Arabidopsis thaliana GN=MPK3 PE=1 SV=2	6.80	34.82	-2.13	down	0.01	0.02	yes
TRINITY_DN13113_c0_g1	copper-binding family protein [Populus trichocarpa]	HIPP31	Heavy metal-associated isoprenylated plant protein 31 OS=Arabidopsis thaliana GN=HIPP31 PE=2 SV=1	2.13	8.46	-1.62	down	0.01	0.02	yes
TRINITY_DN13230_c0_g2	PREDICTED: peroxisomal (S)-2-hydroxy-acid oxidase-like isoform X1 [Populus euphratica]	GLO1	Peroxisomal (S)-2-hydroxy-acid oxidase GLO1 OS=Oryza sativa subsp. indica GN=GLO1 PE=3 SV=1	0.42	2.27	-1.83	down	0.01	0.02	yes
TRINITY_DN23748_c0_g2	hypothetical protein POPTR_0010s14150g [Populus trichocarpa]	MIZ1	Protein MIZU-KUSSEI 1 OS=Arabidopsis thaliana GN=MIZ1 PE=1 SV=1	2.16	7.17	-1.12	down	0.01	0.02	yes
TRINITY_DN25716_c0_g1	MLO-like protein 11 [Populus trichocarpa]	MLO11	MLO-like protein 11 OS=Arabidopsis thaliana GN=MLO11 PE=2 SV=1	4.09	14.97	-1.12	down	0.01	0.02	yes
TRINITY_DN20534_c2_g6	permease-related family protein [Populus trichocarpa]	At3g23870	Probable magnesium transporter NIPA1 OS=Arabidopsis thaliana GN=At3g23870 PE=2 SV=1	0.44	2.64	-1.99	down	0.01	0.02	yes
TRINITY_DN27250_c2_g2	-	-	-	0.88	4.08	-1.61	down	0.01	0.02	yes
TRINITY_DN16894_c0_g1	PREDICTED: transcription repressor OFP16-like [Populus euphratica]	OFP11	Transcription repressor OFP11 OS=Arabidopsis thaliana GN=OFP11 PE=2 SV=1	0.92	4.31	-1.71	down	0.01	0.02	yes
TRINITY_DN17121_c0_g1	hypothetical protein POPTR_0005s27010g, partial [Populus trichocarpa]	-	-	1.89	7.13	-1.12	down	0.01	0.02	yes
TRINITY_DN25402_c0_g3	hypothetical protein POPTR_0018s03340g [Populus trichocarpa]	-	-	6.69	21.79	-1.17	down	0.01	0.02	yes
TRINITY_DN27433_c0_g1	hypothetical protein POPTR_0027s00200g [Populus trichocarpa]	-	-	4.52	12.49	-1.22	down	0.01	0.02	yes
TRINITY_DN16260_c0_g1	hypothetical protein POPTR_0001s33390g [Populus trichocarpa]	HHT1	Omega-hydroxypalmitate O-feruloyl transferase OS=Arabidopsis thaliana GN=HHT1 PE=1 SV=1	0.49	2.82	-1.95	down	0.01	0.02	yes
TRINITY_DN17702_c0_g1	hypothetical protein POPTR_0007s15300g [Populus trichocarpa]	-	-	2.91	9.29	-1.08	down	0.01	0.03	yes
TRINITY_DN25227_c0_g3	unknown [Populus trichocarpa x Populus deltoides]	FATB1	Palmitoyl-acyl carrier protein thioesterase, chloroplastic OS=Gossypium hirsutum GN=FATB1 PE=1 SV=1	4.77	15.05	-1.04	down	0.01	0.03	yes

TRINITY_DN22931_c0_g2	-	-	-	0.56	2.88	-1.74	down	0.01	0.03	yes
TRINITY_DN15532_c0_g1	PREDICTED: uncharacterized protein LOC105141379 [Populus euphratica]	SMXL3	Protein SMAX1-LIKE 3 OS=Arabidopsis thaliana GN=SMXL3 PE=2 SV=1	0.44	2.48	-1.83	down	0.01	0.03	yes
TRINITY_DN19314_c1_g2	hypothetical protein POPTR_0016s10610g [Populus trichocarpa]	WRKY49	Probable WRKY transcription factor 49 OS=Arabidopsis thaliana GN=WRKY49 PE=2 SV=1	5.91	17.87	-1.02	down	0.01	0.03	yes
TRINITY_DN27066_c1_g3	-	-	-	0.46	2.57	-1.89	down	0.01	0.03	yes
TRINITY_DN21281_c0_g3	-	-	-	0.22	2.07	-2.51	down	0.01	0.03	yes
TRINITY_DN16813_c0_g2	hypothetical protein POPTR_0013s14730g [Populus trichocarpa]	PBP1	Calcium-binding protein PBP1 OS=Arabidopsis thaliana GN=PBP1 PE=1 SV=1	1.77	15.69	-2.41	down	0.01	0.03	yes
TRINITY_DN22115_c1_g2	hypothetical protein POPTR_0011s16180g [Populus trichocarpa]	At4g20840	Berberine bridge enzyme-like 21 OS=Arabidopsis thaliana GN=At4g20840 PE=2 SV=1	1.82	10.37	-1.92	down	0.01	0.03	yes
TRINITY_DN14118_c0_g1	PREDICTED: basic leucine zipper 43 [Populus euphratica]	-	-	0.27	2.09	-2.32	down	0.01	0.03	yes
TRINITY_DN12679_c0_g3	PREDICTED: uncharacterized protein LOC105110252 isoform X1 [Populus euphratica]	-	-	1.20	4.04	-1.16	down	0.01	0.03	yes
TRINITY_DN21547_c0_g1	PREDICTED: LOW QUALITY PROTEIN: probable disease resistance protein At4g27220 [Populus euphratica]	-	-	4.35	8.54	-1.47	down	0.01	0.03	yes
TRINITY_DN21725_c0_g2	hypothetical protein POPTR_0002s02120g [Populus trichocarpa]	-	-	0.97	4.53	-1.66	down	0.01	0.03	yes
TRINITY_DN17243_c0_g2	hypothetical protein POPTR_0005s20360g [Populus trichocarpa]	-	-	0.35	3.81	-2.76	down	0.01	0.03	yes
TRINITY_DN24194_c0_g2	hypothetical protein POPTR_0007s12320g [Populus trichocarpa]	At2g18630	UPF0496 protein At2g18630 OS=Arabidopsis thaliana GN=At2g18630 PE=2 SV=3	2.32	7.59	-1.10	down	0.01	0.03	yes
TRINITY_DN23954_c0_g3	-	-	-	0.68	2.92	-1.49	down	0.01	0.03	yes
TRINITY_DN24195_c1_g2	PREDICTED: protein ESKIMO 1-like isoform X2 [Populus euphratica]	ESK1	Protein ESKIMO 1 OS=Arabidopsis thaliana GN=ESK1 PE=1 SV=1	2.60	8.56	-1.11	down	0.01	0.03	yes
TRINITY_DN23319_c0_g6	plasma membrane intrinsic protein 2;1 [Populus tremula x Populus alba]	PIP2-7	Aquaporin PIP2-7 OS=Arabidopsis thaliana GN=PIP2-7 PE=1 SV=2	118.71	404.12	-1.19	down	0.01	0.03	yes
TRINITY_DN25074_c0_g2	hypothetical protein POPTR_0006s12700g [Populus trichocarpa]	-	-	1.06	4.46	-1.42	down	0.01	0.03	yes
TRINITY_DN16005_c2_g1	hypothetical protein POPTR_0010s20990g [Populus trichocarpa]	TRP3	Telomere repeat-binding protein 3 OS=Arabidopsis thaliana GN=TRP3 PE=1 SV=1	6.53	20.02	-1.10	down	0.01	0.03	yes
TRINITY_DN15486_c0_g1	PREDICTED: 3beta-hydroxysteroid-dehydrogenase/decarboxylase isoform 3-like isoform X1 [Populus euphratica]	3BETAHSD/D3	3beta-hydroxysteroid-dehydrogenase/decarboxylase isoform 3 OS=Arabidopsis thaliana GN=3BETAHSD/D3 PE=2 SV=2	0.76	3.83	-1.67	down	0.01	0.03	yes
TRINITY_DN23982_c1_g1	PREDICTED: putative disease resistance protein RGA1 isoform X1 [Populus euphratica]	-	-	6.90	28.31	-1.66	down	0.01	0.03	yes
TRINITY_DN25383_c0_g2	PREDICTED: putative disease resistance RPP13-like protein 1 [Populus euphratica]	RPPL1	Putative disease resistance RPP13-like protein 1 OS=Arabidopsis thaliana GN=RPPL1 PE=3 SV=1	0.36	1.90	-1.77	down	0.01	0.03	yes
TRINITY_DN27744_c1_g2	PREDICTED: LOW QUALITY PROTEIN: DEAD-box ATP-dependent RNA helicase 14-like [Populus euphratica]	RH14	DEAD-box ATP-dependent RNA helicase 14 OS=Arabidopsis thaliana GN=RH14 PE=1 SV=2	1.52	7.36	-1.59	down	0.01	0.03	yes
TRINITY_DN20593_c1_g1	cinnamoyl-CoA reductase [Populus tomentosa]	CCR1	Cinnamoyl-CoA reductase 1 OS=Arabidopsis thaliana GN=CCR1 PE=1 SV=1	5.35	18.95	-1.21	down	0.01	0.03	yes
TRINITY_DN16063_c0_g3	S-locus lectin protein kinase [Populus trichocarpa]	B120	G-type lectin S-receptor-like serine/threonine-protein kinase B120 OS=Arabidopsis thaliana GN=B120 PE=2 SV=1	1.79	6.89	-1.33	down	0.01	0.03	yes
TRINITY_DN19965_c1_g1	-	-	-	0.44	3.67	-2.41	down	0.01	0.03	yes
TRINITY_DN14917_c0_g1	GCN5-related N-acetyltransferase family protein [Populus trichocarpa]	yjcK	Putative ribosomal-protein-alanine acetyltransferase OS=Bacillus subtilis (strain 168) GN=yjcK PE=3 SV=1	0.91	5.60	-1.99	down	0.01	0.03	yes
TRINITY_DN24865_c0_g4	PREDICTED: vesicle-associated protein 1-1-like isoform X2 [Populus euphratica]	PVA22	Vesicle-associated protein 2-2 OS=Arabidopsis thaliana GN=PVA22 PE=1 SV=1	3.17	9.94	-1.01	down	0.01	0.03	yes
TRINITY_DN13902_c0_g1	-	-	-	1.12	4.86	-1.65	down	0.01	0.03	yes
TRINITY_DN21731_c0_g2	PREDICTED: aldo-keto reductase family 4 member C9-like [Populus euphratica]	AKR4C9	Aldo-keto reductase family 4 member C9 OS=Arabidopsis thaliana GN=AKR4C9 PE=1 SV=1	3.19	10.75	-1.12	down	0.01	0.03	yes

TRINITY_DN25583_c1_g2	-	-	-	0.46	2.39	-1.78	down	0.01	0.03	yes
TRINITY_DN15123_c0_g1	hypothetical protein POPTR_0005s23070g [Populus trichocarpa]	FAF2	Protein FANTASTIC FOUR 2 OS=Arabidopsis thaliana GN=FAF2 PE=2 SV=1	1.06	5.68	-2.05	down	0.01	0.03	yes
TRINITY_DN23656_c1_g6	PREDICTED: cytochrome P450 94C1-like [Populus euphratica]	CYP94C1	Cytochrome P450 94C1 OS=Arabidopsis thaliana GN=CYP94C1 PE=2 SV=1	0.77	3.61	-1.62	down	0.01	0.03	yes
TRINITY_DN23887_c0_g5	hypothetical protein POPTR_0008s02980g [Populus trichocarpa]	-	-	0.34	2.03	-1.96	down	0.01	0.03	yes
TRINITY_DN15046_c0_g1	PREDICTED: homeobox-leucine zipper protein HOX11-like [Populus euphratica]	-	-	1.00	6.08	-2.02	down	0.01	0.03	yes
TRINITY_DN5017_c0_g1	hypothetical protein [Brassica napus]	At1g65750	Putative ribonuclease H protein At1g65750 OS=Arabidopsis thaliana GN=At1g65750 PE=3 SV=1	0.48	2.01	-1.46	down	0.01	0.03	yes
TRINITY_DN17942_c0_g2	unknown [Populus trichocarpa]	-	-	3.61	14.78	-1.43	down	0.01	0.03	yes
TRINITY_DN21110_c0_g1	hypothetical protein POPTR_0016s14030g [Populus trichocarpa]	PNC1	Cationic peroxidase 1 OS=Arachis hypogaea GN=PNC1 PE=1 SV=2	2.86	10.12	-1.22	down	0.01	0.03	yes
TRINITY_DN23211_c3_g2	leucine-rich repeat transmembrane protein kinase [Populus trichocarpa]	At2g25790	Probably inactive leucine-rich repeat receptor-like protein kinase At2g25790 OS=Arabidopsis thaliana GN=At2g25790 PE=2 SV=1	1.24	4.29	-1.20	down	0.01	0.03	yes
TRINITY_DN25428_c0_g2	ABC transporter family protein [Populus trichocarpa]	ABCC10	ABC transporter C family member 10 OS=Arabidopsis thaliana GN=ABCC10 PE=2 SV=2	4.94	22.23	-1.29	down	0.01	0.03	yes
TRINITY_DN24354_c0_g3	-	-	-	0.54	3.03	-1.83	down	0.01	0.03	yes
TRINITY_DN27819_c0_g1	PREDICTED: transcriptional activator DEMETER-like isoform X1 [Populus euphratica]	DME	Transcriptional activator DEMETER OS=Arabidopsis thaliana GN=DME PE=1 SV=2	1.50	5.12	-1.10	down	0.01	0.03	yes
TRINITY_DN15814_c0_g1	hypothetical protein POPTR_0004s01860g [Populus trichocarpa]	CYP77A3	Cytochrome P450 77A3 OS=Glycine max GN=CYP77A3 PE=2 SV=1	1.60	5.98	-1.33	down	0.01	0.03	yes
TRINITY_DN15572_c0_g1	chromosome condensation regulator family protein [Populus trichocarpa]	BRXL3	Protein Brevis radix-like 3 OS=Arabidopsis thaliana GN=BRXL3 PE=2 SV=2	3.84	14.28	-1.29	down	0.01	0.03	yes
TRINITY_DN16976_c0_g5	DNA-directed DNA polymerase epsilon catalytic subunit family protein [Populus trichocarpa]	POL2A	DNA polymerase epsilon catalytic subunit A OS=Arabidopsis thaliana GN=POL2A PE=1 SV=1	0.84	4.16	-1.67	down	0.01	0.03	yes
TRINITY_DN27007_c0_g3	-	-	-	1.58	6.51	-1.75	down	0.01	0.03	yes
TRINITY_DN22340_c0_g1	phosphatase 2C family protein [Populus trichocarpa]	At2g30020	Probable protein phosphatase 2C 25 OS=Arabidopsis thaliana GN=At2g30020 PE=1 SV=1	21.39	64.26	-1.00	down	0.01	0.03	yes
TRINITY_DN21210_c0_g3	PREDICTED: LOW QUALITY PROTEIN: alpha-mannosidase 2x [Populus euphratica]	GMII	Alpha-mannosidase 2 OS=Arabidopsis thaliana GN=GMII PE=1 SV=1	2.53	12.25	-1.73	down	0.01	0.03	yes
TRINITY_DN21906_c0_g1	hypothetical protein POPTR_0001s15560g [Populus trichocarpa]	-	-	2.04	7.02	-1.13	down	0.01	0.03	yes
TRINITY_DN19491_c0_g2	hypothetical protein POPTR_0002s11920g [Populus trichocarpa]	-	-	6.49	29.16	-1.86	down	0.01	0.03	yes
TRINITY_DN14358_c0_g1	phosphatidylinositol-4-phosphate 5-kinase family protein [Populus trichocarpa]	PIP5K6	Phosphatidylinositol 4-phosphate 5-kinase 6 OS=Arabidopsis thaliana GN=PIP5K6 PE=2 SV=1	0.42	2.23	-1.80	down	0.01	0.03	yes
TRINITY_DN15638_c1_g2	PREDICTED: RING-H2 finger protein ATL63-like [Populus euphratica]	ATL63	RING-H2 finger protein ATL63 OS=Arabidopsis thaliana GN=ATL63 PE=2 SV=1	0.60	3.33	-1.87	down	0.01	0.03	yes
TRINITY_DN26162_c0_g3	PREDICTED: protein phosphatase 2C 32-like isoform X1 [Populus euphratica]	POL	Protein phosphatase 2C 32 OS=Arabidopsis thaliana GN=POL PE=1 SV=2	1.12	5.39	-1.67	down	0.01	0.03	yes
TRINITY_DN14619_c0_g1	PREDICTED: uncharacterized protein At5g39865 [Populus euphratica]	At5g39865	Uncharacterized protein At5g39865 OS=Arabidopsis thaliana GN=At5g39865 PE=2 SV=1	0.69	3.71	-1.69	down	0.01	0.03	yes
TRINITY_DN17144_c0_g3	leucine-rich repeat family protein [Populus trichocarpa]	At1g67720	Probable LRR receptor-like serine/threonine-protein kinase At1g67720 OS=Arabidopsis thaliana GN=At1g67720 PE=2 SV=1	0.87	3.41	-1.36	down	0.01	0.03	yes
TRINITY_DN14080_c0_g1	-	-	-	272.67	855.63	-1.05	down	0.01	0.03	yes
TRINITY_DN17247_c0_g1	UDP-glucoronosyl/UDP-glucosyl transferase family protein [Populus trichocarpa]	UGT74F2	UDP-glycosyltransferase 74F2 OS=Arabidopsis thaliana GN=UGT74F2 PE=1 SV=1	7.87	26.34	-1.15	down	0.01	0.03	yes
TRINITY_DN16955_c0_g2	hypothetical protein POPTR_0014s06690g [Populus trichocarpa]	ZPR1	Protein LITTLE ZIPPER 1 OS=Arabidopsis thaliana GN=ZPR1 PE=1 SV=1	1.84	6.95	-1.29	down	0.01	0.03	yes
TRINITY_DN18267_c2_g8	PREDICTED: zinc finger MYND domain-containing protein 15 isoform X1 [Populus euphratica]	MSS51	Putative protein MSS51 homolog, mitochondrial OS=Homo sapiens GN=MSS51 PE=1 SV=2	0.85	2.86	-1.16	down	0.01	0.03	yes
TRINITY_DN22755_c0_g5	hypothetical protein POPTR_0014s06990g [Populus trichocarpa]	At2g45640	Histone deacetylase complex subunit SAP18 OS=Arabidopsis thaliana GN=At2g45640 PE=1 SV=1	0.94	5.42	-1.78	down	0.01	0.03	yes

TRINITY_DN11307_c0_g1	hypothetical protein POPTR_0016s05940g [Populus trichocarpa]	-	-	0.47	2.49	-1.78	down	0.01	0.03	yes
TRINITY_DN26715_c0_g5	hypothetical protein POPTR_0001s42770g [Populus trichocarpa]	-	Probable glutathione S-transferase OS=Nicotiana tabacum PE=2 SV=1	1.88	9.06	-1.63	down	0.01	0.03	yes
TRINITY_DN26198_c0_g4	-	-	-	1.73	7.63	-1.55	down	0.01	0.03	yes
TRINITY_DN22977_c0_g3	hypothetical protein POPTR_0001s05080g [Populus trichocarpa]	-	-	0.47	2.27	-1.65	down	0.01	0.03	yes
TRINITY_DN23026_c1_g4	PREDICTED: probable disease resistance protein At5g43730 [Populus euphratica]	At1g52660	Probable disease resistance protein At1g52660 OS=Arabidopsis thaliana GN=At1g52660 PE=3 SV=1	4.03	18.29	-1.57	down	0.01	0.03	yes
TRINITY_DN7162_c0_g2	-	-	-	0.54	2.97	-1.83	down	0.01	0.03	yes
TRINITY_DN25841_c0_g1	WRKY transcription factor 5 [(Populus tomentosa x Populus bolleana) x Populus tomentosa]	WRKY40	Probable WRKY transcription factor 40 OS=Arabidopsis thaliana GN=WRKY40 PE=1 SV=1	32.92	138.24	-1.52	down	0.01	0.03	yes
TRINITY_DN16226_c0_g1	PREDICTED: guanine nucleotide-binding protein subunit gamma 2 [Populus euphratica]	GG2	Guanine nucleotide-binding protein subunit gamma 2 OS=Arabidopsis thaliana GN=GG2 PE=1 SV=1	0.91	4.25	-1.46	down	0.01	0.03	yes
TRINITY_DN26337_c3_g2	PREDICTED: zinc finger protein NUTCRACKER-like [Populus euphratica]	IDD2	Protein indeterminate-domain 2 OS=Arabidopsis thaliana GN=IDD2 PE=2 SV=1	2.46	10.32	-1.73	down	0.01	0.03	yes
TRINITY_DN20017_c0_g1	hypothetical protein POPTR_0006s19610g [Populus trichocarpa]	MPS1	Protein MULTIPOLAR SPINDLE 1 OS=Arabidopsis thaliana GN=MPS1 PE=2 SV=2	2.34	7.88	-1.20	down	0.01	0.03	yes
TRINITY_DN25295_c0_g3	hypothetical protein POPTR_0007s11990g [Populus trichocarpa]	-	-	1.08	6.00	-1.88	down	0.01	0.03	yes
TRINITY_DN21792_c1_g1	PREDICTED: sterol 3-beta-glucosyltransferase UGT80A2 isoform X1 [Populus euphratica]	tylN	O-mycaminosyltylonolide 6-deoxyallosyltransferase OS=Streptomyces fradiae GN=tylN PE=1 SV=2	2.56	8.79	-1.16	down	0.01	0.03	yes
TRINITY_DN19634_c0_g1	PREDICTED: ethylene-responsive transcription factor CRF4-like [Populus euphratica]	CRF4	Ethylene-responsive transcription factor CRF4 OS=Arabidopsis thaliana GN=CRF4 PE=1 SV=2	3.82	14.13	-1.28	down	0.01	0.03	yes
TRINITY_DN25521_c0_g1	-	-	-	1.77	7.76	-1.53	down	0.01	0.03	yes
TRINITY_DN20883_c0_g1	exonuclease family protein [Populus trichocarpa]	SDN5	Small RNA degrading nuclease 5 OS=Arabidopsis thaliana GN=SDN5 PE=2 SV=2	1.15	5.65	-1.62	down	0.01	0.03	yes
TRINITY_DN26254_c0_g2	hypothetical protein POPTR_0015s12740g [Populus trichocarpa]	At1g65710	Uncharacterized protein At1g65710 OS=Arabidopsis thaliana GN=At1g65710 PE=2 SV=1	1.46	6.38	-1.55	down	0.01	0.03	yes
TRINITY_DN18041_c0_g1	transferase family protein [Populus trichocarpa]	SDT	Spermidine sinapoyl-CoA acyltransferase OS=Arabidopsis thaliana GN=SDT PE=1 SV=1	0.89	3.54	-1.35	down	0.01	0.03	yes
TRINITY_DN26938_c1_g1	hypothetical protein POPTR_0012s09330g [Populus trichocarpa]	At4g24790	Protein STICHEL-like 2 OS=Arabidopsis thaliana GN=At4g24790 PE=2 SV=1	1.30	4.35	-1.15	down	0.01	0.03	yes
TRINITY_DN17361_c0_g1	kinase family protein [Populus trichocarpa]	HT1	Serine/threonine-protein kinase HT1 OS=Arabidopsis thaliana GN=HT1 PE=1 SV=1	1.11	5.18	-1.23	down	0.01	0.03	yes
TRINITY_DN27469_c0_g2	PREDICTED: inter-alpha-trypsin inhibitor heavy chain H3-like isoform X1 [Populus euphratica]	ITIH3	Inter-alpha-trypsin inhibitor heavy chain H3 OS=Oryctolagus cuniculus GN=ITIH3 PE=2 SV=1	1.04	4.70	-1.45	down	0.01	0.03	yes
TRINITY_DN12500_c0_g1	hypothetical protein POPTR_0006s13170g [Populus trichocarpa]	MLO3	MLO-like protein 3 OS=Arabidopsis thaliana GN=MLO3 PE=2 SV=1	0.69	3.55	-1.75	down	0.01	0.03	yes
TRINITY_DN20339_c0_g4	-	-	-	1.75	12.39	-1.67	down	0.01	0.03	yes
TRINITY_DN23852_c0_g1	PREDICTED: RNA pseudouridine synthase 1 [Populus euphratica]	At1g56345	RNA pseudouridine synthase 1 OS=Arabidopsis thaliana GN=At1g56345 PE=2 SV=1	2.56	8.67	-1.09	down	0.01	0.03	yes
TRINITY_DN27510_c0_g1	hypothetical protein POPTR_0223s00210g [Populus trichocarpa]	-	-	4.20	17.42	-1.53	down	0.01	0.03	yes
TRINITY_DN15732_c0_g1	hypothetical protein POPTR_0002s17910g, partial [Populus trichocarpa]	GULLO6	Probable L-gulonolactone oxidase 6 OS=Arabidopsis thaliana GN=GULLO6 PE=3 SV=1	0.30	2.88	-2.60	down	0.01	0.03	yes
TRINITY_DN19134_c0_g9	-	-	-	0.98	5.13	-1.59	down	0.01	0.03	yes
TRINITY_DN22624_c0_g1	hypothetical protein POPTR_0006s13130g [Populus trichocarpa]	JKD	Zinc finger protein JACKDAW OS=Arabidopsis thaliana GN=JKD PE=1 SV=1	2.14	9.80	-1.58	down	0.01	0.03	yes
TRINITY_DN18072_c0_g9	hypothetical protein POPTR_0002s01270g [Populus trichocarpa]	-	-	2.29	12.79	-1.89	down	0.01	0.03	yes
TRINITY_DN20464_c0_g2	hypothetical protein POPTR_0017s12640g [Populus trichocarpa]	MUB2	Membrane-anchored ubiquitin-fold protein 2 OS=Arabidopsis thaliana GN=MUB2 PE=1 SV=1	2.31	7.78	-1.18	down	0.01	0.03	yes
TRINITY_DN17639_c0_g2	hypothetical protein POPTR_0005s26140g [Populus trichocarpa]	TUBB	Tubulin beta chain OS=Cicer arietinum GN=TUBB PE=2 SV=1	12.94	39.02	-1.02	down	0.01	0.03	yes

TRINITY_DN16243_c0_g1	hypothetical protein POPTR_0019s13010g [Populus trichocarpa]	-	TMV resistance protein N OS=Nicotiana glutinosa GN=N PE=1 SV=1	4.25	17.56	-1.44	down	0.01	0.03	yes
TRINITY_DN23017_c2_g4	-	-	-	0.71	3.47	-1.71	down	0.01	0.03	yes
TRINITY_DN19751_c0_g1	unknown [Populus trichocarpa]	At3g59480	Probable fructokinase-4 OS=Arabidopsis thaliana GN=At3g59480 PE=2 SV=1	5.54	20.26	-1.26	down	0.01	0.03	yes
TRINITY_DN26109_c0_g1	hypothetical protein POPTR_0004s03530g [Populus trichocarpa]	Os07g0563300	B3 domain-containing protein Os07g0563300 OS=Oryza sativa subsp. japonica GN=Os07g0563300 PE=3 SV=2	4.49	20.00	-1.02	down	0.01	0.03	yes
TRINITY_DN19656_c0_g1	PREDICTED: G-type lectin S-receptor-like serine/threonine-protein kinase RLK1 isoform X1 [Populus euphratica]	RLK1	G-type lectin S-receptor-like serine/threonine-protein kinase RLK1 OS=Arabidopsis thaliana GN=RLK1 PE=2 SV=2	0.57	2.63	-1.62	down	0.01	0.03	yes
TRINITY_DN2875_c0_g1	hypothetical protein POPTR_0001s38010g [Populus trichocarpa]	At1g63170	E3 ubiquitin-protein ligase At1g63170 OS=Arabidopsis thaliana GN=At1g63170 PE=2 SV=2	0.33	2.13	-2.07	down	0.01	0.03	yes
TRINITY_DN15966_c0_g1	calmodulin family protein [Populus trichocarpa]	CML11	Probable calcium-binding protein CML11 OS=Oryza sativa subsp. japonica GN=CML11 PE=2 SV=1	1.41	5.55	-1.63	down	0.01	0.03	yes
TRINITY_DN27003_c0_g2	Glutamate receptor 3.3 precursor family protein [Populus trichocarpa]	GLR3.3	Glutamate receptor 3.3 OS=Arabidopsis thaliana GN=GLR3.3 PE=2 SV=1	1.56	5.08	-1.07	down	0.01	0.03	yes
TRINITY_DN23957_c2_g3	PREDICTED: RNA-binding protein 1-like isoform X2 [Populus euphratica]	RNP1	Heterogeneous nuclear ribonucleoprotein 1 OS=Arabidopsis thaliana GN=RNP1 PE=1 SV=1	1.60	5.99	-1.31	down	0.01	0.03	yes
TRINITY_DN21067_c0_g2	hypothetical protein POPTR_0005s00260g [Populus trichocarpa]	-	-	0.73	3.51	-1.66	down	0.01	0.03	yes
TRINITY_DN18042_c0_g1	putative Cys2/His2 zinc finger protein, partial [Populus tremula x Populus alba]	ZAT11	Zinc finger protein ZAT11 OS=Arabidopsis thaliana GN=ZAT11 PE=2 SV=1	1.19	21.32	-2.95	down	0.01	0.03	yes
TRINITY_DN25050_c0_g1	hypothetical protein POPTR_0010s12780g [Populus trichocarpa]	At2g01130	DExH-box ATP-dependent RNA helicase DExH5, mitochondrial OS=Arabidopsis thaliana GN=At2g01130 PE=3 SV=1	2.27	7.09	-1.00	down	0.01	0.03	yes
TRINITY_DN22868_c0_g2	hypothetical protein POPTR_0006s26110g [Populus trichocarpa]	-	-	1.60	5.65	-1.19	down	0.01	0.03	yes
TRINITY_DN15103_c0_g1	hypothetical protein POPTR_0018s10700g [Populus trichocarpa]	-	-	2.15	8.64	-1.42	down	0.01	0.03	yes
TRINITY_DN21752_c0_g1	hypothetical protein POPTR_0008s05600g [Populus trichocarpa]	GLC1	Glucan endo-1,3-beta-glucosidase OS=Triticum aestivum GN=GLC1 PE=2 SV=1	16.32	82.75	-1.81	down	0.01	0.03	yes
TRINITY_DN20920_c0_g3	hypothetical protein POPTR_0004s04590g [Populus trichocarpa]	-	-	0.54	2.55	-1.59	down	0.01	0.03	yes
TRINITY_DN19108_c0_g1	PREDICTED: uncharacterized protein LOC105122797 isoform X5 [Populus euphratica]	-	-	8.27	33.34	-1.15	down	0.01	0.03	yes
TRINITY_DN21322_c0_g2	outer envelope membrane family protein [Populus trichocarpa]	OEP7	Outer envelope membrane protein 7 OS=Arabidopsis thaliana GN=OEP7 PE=1 SV=1	4.91	17.53	-1.30	down	0.01	0.03	yes
TRINITY_DN11131_c0_g2	hypothetical protein POPTR_0006s14640g [Populus trichocarpa]	MSL10	Mechanosensitive ion channel protein 10 OS=Arabidopsis thaliana GN=MSL10 PE=1 SV=1	0.31	1.75	-1.85	down	0.01	0.03	yes
TRINITY_DN27260_c0_g1	PREDICTED: receptor-like serine/threonine-protein kinase ALE2 isoform X4 [Populus euphratica]	ALE2	Receptor-like serine/threonine-protein kinase ALE2 OS=Arabidopsis thaliana GN=ALE2 PE=1 SV=1	2.68	8.76	-1.17	down	0.01	0.03	yes
TRINITY_DN13054_c0_g1	hypothetical protein POPTR_0007s12140g [Populus trichocarpa]	-	-	0.79	4.03	-1.72	down	0.01	0.03	yes
TRINITY_DN21234_c0_g1	kinase family protein [Populus trichocarpa]	WAKL9	Wall-associated receptor kinase-like 9 OS=Arabidopsis thaliana GN=WAKL9 PE=2 SV=1	0.25	1.82	-2.22	down	0.01	0.03	yes
TRINITY_DN20200_c0_g1	Glutaredoxin family protein [Populus tomentosa]	At5g39865	Uncharacterized protein At5g39865 OS=Arabidopsis thaliana GN=At5g39865 PE=2 SV=1	2.88	9.11	-1.07	down	0.01	0.03	yes
TRINITY_DN27194_c0_g3	-	-	-	2.88	9.81	-1.17	down	0.01	0.03	yes
TRINITY_DN15073_c0_g1	-	-	-	0.52	2.63	-1.72	down	0.01	0.03	yes
TRINITY_DN16804_c0_g1	hypothetical protein POPTR_0004s03890g [Populus trichocarpa]	-	-	2.14	7.18	-1.16	down	0.01	0.03	yes
TRINITY_DN24264_c1_g2	hypothetical protein POPTR_0012s00440g [Populus trichocarpa]	-	-	6.09	22.39	-1.30	down	0.01	0.03	yes
TRINITY_DN22628_c1_g5	hypothetical protein POPTR_0004s00580g [Populus trichocarpa]	At4g22160	Uncharacterized protein At4g22160 OS=Arabidopsis thaliana GN=At4g22160 PE=2 SV=2	2.20	8.66	-1.35	down	0.01	0.03	yes
TRINITY_DN25910_c0_g2	PREDICTED: transcription factor PCL1-like [Populus euphratica]	BOA	Transcription factor BOA OS=Arabidopsis thaliana GN=BOA PE=2 SV=1	4.93	16.82	-1.06	down	0.01	0.03	yes

TRINITY_DN25110_c1_g1	PREDICTED: U-box domain-containing protein 4-like [Populus euphratica]	PUB4	U-box domain-containing protein 4 OS=Arabidopsis thaliana GN=PUB4 PE=1 SV=3	4.20	15.26	-1.23	down	0.01	0.03	yes
TRINITY_DN19330_c1_g6	hypothetical protein POPTR_0010s05100g [Populus trichocarpa]	-	-	3.03	13.16	-1.46	down	0.01	0.03	yes
TRINITY_DN19056_c0_g3	hypothetical protein POPTR_0010s17810g [Populus trichocarpa]	OsI_033149	UPF0496 protein 4 OS=Oryza sativa subsp. indica GN=OsI_033149 PE=3 SV=2	7.44	24.51	-1.11	down	0.01	0.03	yes
TRINITY_DN19577_c2_g2	hypothetical protein POPTR_0003s19240g [Populus trichocarpa]	-	-	0.52	2.42	-1.57	down	0.01	0.03	yes
TRINITY_DN18421_c0_g3	-	-	-	1.10	7.66	-2.03	down	0.01	0.03	yes
TRINITY_DN17376_c0_g1	-	-	-	10.37	50.48	-1.60	down	0.01	0.03	yes
TRINITY_DN24394_c0_g2	hypothetical protein POPTR_0008s04810g [Populus trichocarpa]	At4g12130	Putative transferase At4g12130, mitochondrial OS=Arabidopsis thaliana GN=At4g12130 PE=1 SV=1	1.28	4.64	-1.13	down	0.01	0.03	yes
TRINITY_DN24105_c1_g4	Retrovirus-related Pol polyprotein from transposon TNT 1-94 [Cajanus cajan]	-	-	0.46	2.75	-1.90	down	0.01	0.03	yes
TRINITY_DN16424_c0_g1	PREDICTED: uncharacterized protein LOC105122109 [Populus euphratica]	-	-	1.93	6.14	-1.06	down	0.01	0.03	yes
TRINITY_DN25715_c1_g3	casein kinase i family protein [Populus trichocarpa]	-	-	0.43	2.53	-1.95	down	0.01	0.03	yes
TRINITY_DN26235_c0_g3	PREDICTED: G-type lectin S-receptor-like serine/threonine-protein kinase RLK1 [Populus euphratica]	LECRK1	G-type lectin S-receptor-like serine/threonine-protein kinase LECRK1 OS=Oryza sativa subsp. japonica GN=LECRK1 PE=2 SV=1	4.02	17.62	-1.48	down	0.01	0.03	yes
TRINITY_DN17748_c0_g1	hypothetical protein POPTR_0015s09670g [Populus trichocarpa]	NRT3.1	High-affinity nitrate transporter 3.1 OS=Arabidopsis thaliana GN=NRT3.1 PE=1 SV=1	2.03	7.86	-1.40	down	0.01	0.03	yes
TRINITY_DN26449_c0_g1	PREDICTED: probable protein phosphatase 2C 23 isoform X1 [Populus euphratica]	PLL4	Probable protein phosphatase 2C 23 OS=Arabidopsis thaliana GN=PLL4 PE=2 SV=1	1.27	7.95	-2.02	down	0.01	0.03	yes
TRINITY_DN21912_c0_g1	hypothetical protein POPTR_0019s09010g [Populus trichocarpa]	At5g58300	Probable inactive receptor kinase At5g58300 OS=Arabidopsis thaliana GN=At5g58300 PE=2 SV=1	3.43	10.58	-1.01	down	0.01	0.03	yes
TRINITY_DN16896_c0_g1	-	-	-	3.07	13.92	-1.35	down	0.01	0.03	yes
TRINITY_DN16741_c0_g2	class1 chitinase family protein [Populus trichocarpa]	CTL2	Chitinase-like protein 2 OS=Arabidopsis thaliana GN=CTL2 PE=2 SV=1	0.48	3.20	-2.09	down	0.01	0.03	yes
TRINITY_DN19796_c0_g2	-	-	-	2.75	13.72	-1.84	down	0.01	0.03	yes
TRINITY_DN17263_c0_g1	PREDICTED: purple acid phosphatase 15-like [Populus euphratica]	PAP15	Purple acid phosphatase 15 OS=Arabidopsis thaliana GN=PAP15 PE=1 SV=1	0.74	3.01	-1.39	down	0.01	0.03	yes
TRINITY_DN15931_c0_g1	hypothetical protein POPTR_0005s04270g [Populus trichocarpa]	-	-	1.48	5.52	-1.67	down	0.01	0.03	yes
TRINITY_DN25892_c1_g7	-	-	-	1.49	8.41	-2.26	down	0.01	0.03	yes
TRINITY_DN17398_c0_g1	hypothetical protein POPTR_0001s45800g [Populus trichocarpa]	ATL79	RING-H2 finger protein ATL79 OS=Arabidopsis thaliana GN=ATL79 PE=2 SV=1	5.47	18.55	-1.20	down	0.01	0.03	yes
TRINITY_DN23909_c3_g2	PREDICTED: uncharacterized protein LOC105124850 [Populus euphratica]	-	-	1.31	4.46	-1.13	down	0.01	0.03	yes
TRINITY_DN13110_c0_g3	-	-	-	1.01	3.43	-1.46	down	0.01	0.03	yes
TRINITY_DN19868_c0_g3	PREDICTED: uncharacterized protein LOC105131746 isoform X1 [Populus euphratica]	-	-	0.51	3.19	-2.04	down	0.01	0.03	yes
TRINITY_DN18767_c0_g1	pectinesterase family protein [Populus trichocarpa]	PME31	Pectinesterase 31 OS=Arabidopsis thaliana GN=PME31 PE=1 SV=1	3.89	13.90	-1.14	down	0.01	0.03	yes
TRINITY_DN11076_c0_g1	hypothetical protein POPTR_0007s11160g [Populus trichocarpa]	-	-	2.38	12.27	-1.78	down	0.01	0.03	yes
TRINITY_DN19552_c0_g12	hypothetical protein POPTR_0011s17290g [Populus trichocarpa]	-	-	12.61	38.06	-1.00	down	0.01	0.03	yes
TRINITY_DN27855_c0_g1	hypothetical protein POPTR_0016s01080g [Populus trichocarpa]	ACA10	Calcium-transporting ATPase 10, plasma membrane-type OS=Arabidopsis thaliana GN=ACA10 PE=1 SV=2	6.97	20.40	-1.05	down	0.01	0.03	yes
TRINITY_DN8124_c0_g1	PREDICTED: somatic embryogenesis receptor kinase 2-like isoform X1 [Populus euphratica]	SERK2	Somatic embryogenesis receptor kinase 2 OS=Arabidopsis thaliana GN=SERK2 PE=1 SV=1	0.61	2.73	-1.70	down	0.01	0.03	yes
TRINITY_DN25037_c0_g3	hypothetical protein POPTR_0015s08750g [Populus trichocarpa]	-	-	8.43	30.24	-1.24	down	0.01	0.03	yes

TRINITY_DN20193_c1_g5	PREDICTED: NAC domain-containing protein 72 [Populus euphratica]	NAC035	NAC domain-containing protein 35 OS=Arabidopsis thaliana GN=NAC035 PE=1 SV=2	6.09	18.08	-1.02	down	0.01	0.03	yes
TRINITY_DN18233_c0_g1	hypothetical protein POPTR_0005s18240g [Populus trichocarpa]	YDA	Mitogen-activated protein kinase kinase kinase YODA OS=Arabidopsis thaliana GN=YDA PE=1 SV=1	2.42	24.27	-2.71	down	0.01	0.03	yes
TRINITY_DN24552_c1_g1	COP1-interactive protein 1 [Populus trichocarpa]	-	-	1.19	3.81	-1.08	down	0.01	0.03	yes
TRINITY_DN17650_c0_g1	hypothetical protein POPTR_0019s00330g [Populus trichocarpa]	At1g33420	PHD finger protein At1g33420 OS=Arabidopsis thaliana GN=At1g33420 PE=1 SV=1	2.37	9.00	-1.35	down	0.01	0.03	yes
TRINITY_DN17502_c0_g1	hypothetical protein POPTR_0018s06230g [Populus trichocarpa]	-	-	9.39	67.58	-2.27	down	0.01	0.03	yes
TRINITY_DN20438_c0_g3	hypothetical protein POPTR_0018s13770g [Populus trichocarpa]	SMXL5	Protein SMAX1-LIKE 5 OS=Arabidopsis thaliana GN=SMXL5 PE=2 SV=1	0.81	6.29	-2.32	down	0.01	0.03	yes
TRINITY_DN23878_c0_g6	-	-	-	0.62	5.17	-2.43	down	0.01	0.03	yes
TRINITY_DN25771_c0_g2	hypothetical protein POPTR_0018s04090g [Populus trichocarpa]	HT1	Serine/threonine-protein kinase HT1 OS=Arabidopsis thaliana GN=HT1 PE=1 SV=1	5.19	19.18	-1.10	down	0.01	0.03	yes
TRINITY_DN18639_c0_g2	PREDICTED: uncharacterized protein LOC105125116 [Populus euphratica]	-	-	0.69	3.02	-1.49	down	0.01	0.03	yes
TRINITY_DN5919_c0_g1	hypothetical protein POPTR_0092s00200g [Populus trichocarpa]	-	(-)-alpha-terpineol synthase OS=Vitis vinifera PE=1 SV=1	1.33	8.19	-1.96	down	0.01	0.03	yes
TRINITY_DN24029_c1_g2	PREDICTED: uncharacterized protein LOC104603528 [Nelumbo nucifera]	-	-	2.01	5.94	-1.05	down	0.01	0.03	yes
TRINITY_DN22559_c0_g2	hypothetical protein POPTR_0008s05520g [Populus trichocarpa]	DOF3.6	Dof zinc finger protein DOF3.6 OS=Arabidopsis thaliana GN=DOF3.6 PE=1 SV=2	2.33	8.49	-1.43	down	0.01	0.03	yes
TRINITY_DN26278_c0_g2	hypothetical protein POPTR_0002s13880g [Populus trichocarpa]	VAD1	Protein VASCULAR ASSOCIATED DEATH 1, chloroplastic OS=Arabidopsis thaliana GN=VAD1 PE=1 SV=1	3.43	12.65	-1.08	down	0.01	0.03	yes
TRINITY_DN14906_c0_g1	PREDICTED: ankyrin repeat-containing protein At3g12360-like isoform X1 [Populus euphratica]	-	-	1.89	6.28	-1.10	down	0.01	0.03	yes
TRINITY_DN18465_c0_g1	PREDICTED: cell division control protein 6 homolog [Populus euphratica]	CDC6B	Cell division control protein 6 homolog B OS=Arabidopsis thaliana GN=CDC6B PE=2 SV=1	1.23	5.60	-1.53	down	0.01	0.03	yes
TRINITY_DN20179_c0_g2	hypothetical protein POPTR_0008s12630g [Populus trichocarpa]	-	-	3.31	11.62	-1.02	down	0.01	0.03	yes
TRINITY_DN17308_c0_g1	hypothetical protein POPTR_0009s08910g [Populus trichocarpa]	-	-	3.54	13.57	-1.30	down	0.01	0.03	yes
TRINITY_DN24920_c0_g4	-	-	-	1.16	4.63	-1.39	down	0.01	0.03	yes
TRINITY_DN17324_c0_g2	hypothetical protein POPTR_0001s44360g [Populus trichocarpa]	NDR1	Protein NDR1 OS=Arabidopsis thaliana GN=NDR1 PE=1 SV=1	2.10	9.34	-1.51	down	0.01	0.03	yes
TRINITY_DN19928_c1_g2	PREDICTED: calcium-transporting ATPase 2, plasma membrane-type isoform X1 [Populus euphratica]	ACA2	Calcium-transporting ATPase 2, plasma membrane-type OS=Arabidopsis thaliana GN=ACA2 PE=1 SV=1	12.94	60.17	-1.43	down	0.01	0.03	yes
TRINITY_DN18561_c0_g2	glycine-rich family protein [Populus trichocarpa]	-	-	88.42	476.47	-1.86	down	0.01	0.03	yes
TRINITY_DN13088_c0_g1	PREDICTED: uncharacterized protein LOC105123154 [Populus euphratica]	-	-	1.22	8.90	-2.17	down	0.01	0.03	yes
TRINITY_DN24184_c0_g1	hypothetical protein POPTR_0012s02230g [Populus trichocarpa]	CBL4	Calcineurin B-like protein 4 OS=Arabidopsis thaliana GN=CBL4 PE=1 SV=3	1.52	9.99	-1.18	down	0.01	0.03	yes
TRINITY_DN22049_c1_g2	PREDICTED: protein BREVIS RADIX-like [Populus euphratica]	BRXL1	Protein Brevis radix-like 1 OS=Arabidopsis thaliana GN=BRXL1 PE=2 SV=2	1.00	4.32	-1.40	down	0.01	0.03	yes
TRINITY_DN7671_c0_g1	hypothetical protein POPTR_0013s06280g [Populus trichocarpa]	-	-	2.70	9.46	-1.20	down	0.01	0.03	yes
TRINITY_DN26427_c0_g1	-	-	-	13.11	47.72	-1.28	down	0.01	0.03	yes
TRINITY_DN21756_c0_g4	PREDICTED: mitochondrial-processing peptidase subunit alpha-like [Populus euphratica]	-	-	1.35	5.17	-1.32	down	0.01	0.03	yes
TRINITY_DN23426_c0_g3	hypothetical protein POPTR_0019s05450g [Populus trichocarpa]	-	-	3.97	18.43	-1.57	down	0.01	0.03	yes
TRINITY_DN23885_c1_g3	hypothetical protein POPTR_0018s07790g [Populus trichocarpa]	-	-	13.39	107.88	-2.41	down	0.01	0.03	yes
TRINITY_DN12311_c0_g1	hypothetical protein POPTR_0010s14180g [Populus trichocarpa]	ABCG21	ABC transporter G family member 21 OS=Arabidopsis thaliana GN=ABCG21 PE=2 SV=2	0.27	1.76	-2.09	down	0.01	0.03	yes

TRINITY_DN13808_c0_g3	hypothetical protein POPTR_0001s04370g [Populus trichocarpa]	AAE18	Probable acyl-activating enzyme 18, peroxisomal OS=Arabidopsis thaliana GN=AAE18 PE=2 SV=1	0.58	3.70	-1.99	down	0.01	0.03	yes
TRINITY_DN25413_c4_g2	diacylglycerol kinase family protein [Populus trichocarpa]	DGK2	Diacylglycerol kinase 2 OS=Arabidopsis thaliana GN=DGK2 PE=1 SV=1	8.61	47.89	-1.83	down	0.01	0.03	yes
TRINITY_DN25008_c1_g4	zinc finger family protein [Populus trichocarpa]	-	-	8.15	27.86	-1.22	down	0.01	0.03	yes
TRINITY_DN23966_c0_g2	PREDICTED: ABC transporter C family member 13 isoform X1 [Populus euphratica]	ABCC13	ABC transporter C family member 13 OS=Arabidopsis thaliana GN=ABCC13 PE=2 SV=3	2.06	4.71	-1.00	down	0.01	0.03	yes
TRINITY_DN23341_c1_g1	PREDICTED: arginine/serine-rich coiled-coil protein 2 isoform X1 [Populus euphratica]	-	-	5.03	15.56	-1.15	down	0.01	0.03	yes
TRINITY_DN23918_c0_g1	hypothetical protein POPTR_0002s00370g [Populus trichocarpa]	LYK5	Protein LYK5 OS=Arabidopsis thaliana GN=LYK5 PE=2 SV=1	0.41	2.84	-2.35	down	0.01	0.03	yes
TRINITY_DN16725_c0_g1	AP2/ERF domain-containing protein [Populus nigra]	ERF026	Ethylene-responsive transcription factor ERF026 OS=Arabidopsis thaliana GN=ERF026 PE=2 SV=1	1.39	10.44	-2.27	down	0.01	0.03	yes
TRINITY_DN21607_c0_g4	-	-	-	0.40	2.94	-2.24	down	0.01	0.03	yes
TRINITY_DN17958_c0_g3	-	-	-	0.47	3.12	-2.15	down	0.01	0.03	yes
TRINITY_DN19591_c1_g5	hypothetical protein POPTR_0014s05880g, partial [Populus trichocarpa]	-	-	1.60	6.32	-1.41	down	0.01	0.03	yes
TRINITY_DN17660_c0_g1	homeobox-leucine zipper transcription factor family protein [Populus trichocarpa]	WOX4	WUSCHEL-related homeobox 4 OS=Arabidopsis thaliana GN=WOX4 PE=2 SV=1	2.24	10.14	-1.58	down	0.01	0.03	yes
TRINITY_DN27021_c1_g2	hypothetical protein POPTR_0004s08530g [Populus trichocarpa]	-	-	10.53	31.62	-1.09	down	0.01	0.03	yes
TRINITY_DN21289_c1_g5	-	-	-	0.63	3.00	-1.59	down	0.01	0.03	yes
TRINITY_DN23366_c0_g3	hypothetical protein POPTR_0019s04005g [Populus trichocarpa]	-	-	0.63	3.96	-1.96	down	0.01	0.03	yes
TRINITY_DN14028_c0_g3	putative leucine-rich repeat transmembrane protein kinase [Populus trichocarpa]	At5g63930	Probable leucine-rich repeat receptor-like protein kinase At5g63930 OS=Arabidopsis thaliana GN=At5g63930 PE=2 SV=1	0.37	2.00	-1.78	down	0.01	0.03	yes
TRINITY_DN25740_c0_g1	-	-	-	6.97	23.06	-1.01	down	0.01	0.03	yes
TRINITY_DN18678_c1_g4	hypothetical protein EUGRSUZ_J01765 [Eucalyptus grandis]	-	-	0.76	3.45	-1.60	down	0.01	0.03	yes
TRINITY_DN27528_c1_g1	-	-	-	0.67	4.35	-1.82	down	0.01	0.03	yes
TRINITY_DN15020_c0_g1	PREDICTED: uncharacterized protein LOC105110111 [Populus euphratica]	-	-	1.83	7.03	-1.35	down	0.01	0.04	yes
TRINITY_DN24278_c3_g3	-	-	-	2.16	10.50	-1.66	down	0.01	0.04	yes
TRINITY_DN19062_c0_g1	hypothetical protein POPTR_0009s05990g [Populus trichocarpa]	ASAT1	Acyl-CoA--sterol O-acyltransferase 1 OS=Arabidopsis thaliana GN=ASAT1 PE=1 SV=1	1.02	3.91	-1.33	down	0.01	0.04	yes
TRINITY_DN24974_c0_g1	hypothetical protein POPTR_0001s06990g [Populus trichocarpa]	-	-	0.88	4.33	-1.64	down	0.01	0.04	yes
TRINITY_DN12681_c0_g1	unknown [Populus trichocarpa x Populus deltoides]	VEP1	3-oxo-Delta(4,5)-steroid 5-beta-reductase OS=Arabidopsis thaliana GN=VEP1 PE=1 SV=1	0.53	3.24	-1.93	down	0.01	0.04	yes
TRINITY_DN17665_c0_g1	hydrolase family protein [Populus trichocarpa]	BDG1	Probable lysophospholipase BODYGUARD 1 OS=Arabidopsis thaliana GN=BDG1 PE=2 SV=1	1.44	6.52	-2.03	down	0.01	0.04	yes
TRINITY_DN22553_c0_g1	PREDICTED: F-box protein MAX2 [Populus euphratica]	MAX2A	F-box/LRR-repeat MAX2 homolog A OS=Petunia hybrida GN=MAX2A PE=1 SV=1	3.32	12.64	-1.33	down	0.01	0.04	yes
TRINITY_DN25984_c0_g4	hypothetical protein POPTR_0031s00260g, partial [Populus trichocarpa]	-	-	34.70	120.61	-1.19	down	0.01	0.04	yes
TRINITY_DN20312_c0_g1	hypothetical protein POPTR_0018s01760g [Populus trichocarpa]	-	-	2.15	14.97	-2.19	down	0.01	0.04	yes
TRINITY_DN17738_c0_g1	glycosyltransferase 8E [Populus tremula x Populus alba]	GATL1	Probable galacturonosyltransferase-like 1 OS=Arabidopsis thaliana GN=GATL1 PE=2 SV=1	1.69	10.09	-1.93	down	0.01	0.04	yes
TRINITY_DN24760_c0_g1	PREDICTED: diacylglycerol kinase 2-like isoform X1 [Populus euphratica]	DGK2	Diacylglycerol kinase 2 OS=Arabidopsis thaliana GN=DGK2 PE=1 SV=1	1.68	11.01	-2.11	down	0.01	0.04	yes
TRINITY_DN23392_c0_g1	PREDICTED: tankyrase-2 [Populus euphratica]	-	-	3.70	11.35	-1.01	down	0.01	0.04	yes
TRINITY_DN12345_c0_g1	hypothetical protein POPTR_0008s06330g [Populus trichocarpa]	-	-	0.43	2.20	-1.75	down	0.01	0.04	yes

TRINITY_DN24123_c0_g2	hypothetical protein POPTR_0002s18700g [Populus trichocarpa]	MYB86	Transcription factor MYB86 OS=Arabidopsis thaliana GN=MYB86 PE=2 SV=1	1.27	4.49	-1.27	down	0.01	0.04	yes
TRINITY_DN15549_c0_g2	-	-	-	12.20	82.29	-2.10	down	0.01	0.04	yes
TRINITY_DN8156_c0_g1	hypothetical protein POPTR_0002s10060g [Populus trichocarpa]	-	-	0.30	2.55	-2.42	down	0.01	0.04	yes
TRINITY_DN26053_c0_g3	heat shock protein-binding protein, putative [Medicago truncatula]	-	-	1.16	6.21	-2.19	down	0.01	0.04	yes
TRINITY_DN27892_c2_g1	-	-	-	9.50	28.27	-1.19	down	0.01	0.04	yes
TRINITY_DN22185_c0_g2	hypothetical protein POPTR_0008s11500g [Populus trichocarpa]	-	-	0.96	3.25	-1.59	down	0.01	0.04	yes
TRINITY_DN21108_c0_g4	hypothetical protein POPTR_0003s16350g [Populus trichocarpa]	TIFY9	Protein TIFY 9 OS=Arabidopsis thaliana GN=TIFY9 PE=1 SV=1	13.59	39.10	-2.03	down	0.01	0.04	yes
TRINITY_DN12346_c0_g1	PREDICTED: uncharacterized protein LOC105140669 isoform X2 [Populus euphratica]	SNI1	Negative regulator of systemic acquired resistance SNI1 OS=Arabidopsis thaliana GN=SNI1 PE=1 SV=1	0.99	3.66	-1.25	down	0.01	0.04	yes
TRINITY_DN21253_c0_g3	hypothetical protein POPTR_0012s13440g [Populus trichocarpa]	CHS2	Chalcone synthase 2 OS=Daucus carota GN=CHS2 PE=2 SV=1	0.53	2.49	-1.59	down	0.01	0.04	yes
TRINITY_DN26779_c0_g3	PREDICTED: uncharacterized protein LOC105112845 [Populus euphratica]	-	-	1.84	6.26	-1.15	down	0.01	0.04	yes
TRINITY_DN21860_c0_g4	hypothetical protein POPTR_0003s17700g [Populus trichocarpa]	LUH	Transcriptional corepressor LEUNIG_HOMOLOG OS=Arabidopsis thaliana GN=LUH PE=1 SV=1	4.41	11.88	-1.03	down	0.01	0.04	yes
TRINITY_DN21547_c0_g2	PREDICTED: probable disease resistance protein At4g27220 [Populus euphratica]	-	-	0.81	6.07	-2.24	down	0.01	0.04	yes
TRINITY_DN18813_c1_g3	hypothetical protein POPTR_0003s19470g [Populus trichocarpa]	-	-	1.75	6.53	-1.31	down	0.01	0.04	yes
TRINITY_DN2973_c0_g1	PREDICTED: uncharacterized protein LOC105126972 [Populus euphratica]	-	-	1.14	4.80	-1.43	down	0.01	0.04	yes
TRINITY_DN27318_c1_g3	PREDICTED: probable disease resistance protein At4g27220 [Populus euphratica]	-	-	0.57	2.76	-1.62	down	0.01	0.04	yes
TRINITY_DN23538_c0_g1	-	-	-	1.15	6.44	-1.29	down	0.01	0.04	yes
TRINITY_DN19013_c0_g2	PREDICTED: histidine kinase CKI1 [Populus euphratica]	CKI1	Histidine kinase CKI1 OS=Arabidopsis thaliana GN=CKI1 PE=1 SV=1	2.10	10.03	-1.35	down	0.01	0.04	yes
TRINITY_DN21262_c0_g2	hypothetical protein POPTR_0005s02740g [Populus trichocarpa]	-	-	1.01	3.39	-1.21	down	0.01	0.04	yes
TRINITY_DN24826_c0_g1	PREDICTED: uncharacterized protein LOC105138712 [Populus euphratica]	-	-	1.85	5.77	-1.05	down	0.01	0.04	yes
TRINITY_DN22050_c0_g4	zinc finger family protein [Populus trichocarpa]	ATL9	E3 ubiquitin-protein ligase ATL9 OS=Arabidopsis thaliana GN=ATL9 PE=1 SV=1	5.86	19.22	-1.10	down	0.01	0.04	yes
TRINITY_DN20035_c0_g1	hypothetical protein POPTR_0003s07460g [Populus trichocarpa]	PME68	Probable pectinesterase 68 OS=Arabidopsis thaliana GN=PME68 PE=2 SV=1	88.55	285.41	-1.14	down	0.01	0.04	yes
TRINITY_DN18874_c0_g1	hypothetical protein POPTR_0006s20390g [Populus trichocarpa]	-	-	10.85	36.39	-1.13	down	0.01	0.04	yes
TRINITY_DN15260_c0_g1	hypothetical protein POPTR_0005s04570g [Populus trichocarpa]	-	-	0.43	2.53	-1.85	down	0.01	0.04	yes
TRINITY_DN21603_c0_g1	phosphoinositide-specific phospholipase C family protein [Populus trichocarpa]	PLC6	Phosphoinositide phospholipase C 6 OS=Arabidopsis thaliana GN=PLC6 PE=2 SV=2	2.32	9.54	-1.14	down	0.01	0.04	yes
TRINITY_DN16270_c0_g3	hypothetical protein POPTR_0010s24680g [Populus trichocarpa]	Os05g0277500	Germin-like protein 5-1 OS=Oryza sativa subsp. japonica GN=Os05g0277500 PE=2 SV=1	0.62	4.01	-2.06	down	0.01	0.04	yes
TRINITY_DN17537_c0_g1	hypothetical protein POPTR_0010s18190g [Populus trichocarpa]	-	-	3.05	9.83	-1.16	down	0.01	0.04	yes
TRINITY_DN11157_c0_g1	-	-	-	0.76	2.92	-1.32	down	0.01	0.04	yes
TRINITY_DN25054_c0_g1	-	-	-	0.44	2.62	-1.98	down	0.01	0.04	yes
TRINITY_DN25043_c0_g3	PREDICTED: uncharacterized protein LOC105133583 isoform X2 [Populus euphratica]	-	-	0.60	3.20	-1.79	down	0.01	0.04	yes
TRINITY_DN18651_c2_g2	hypothetical protein POPTR_0745s00210g [Populus trichocarpa]	-	-	3.30	12.62	-1.39	down	0.01	0.04	yes

TRINITY_DN25918_c1_g3	hypothetical protein POPTR_0003s11130g [Populus trichocarpa]	-	-	0.40	1.98	-1.72	down	0.01	0.04	yes
TRINITY_DN26328_c1_g2	leucine-rich repeat family protein [Populus trichocarpa]	HSL2	LRR receptor-like serine/threonine-protein kinase HSL2 OS=Arabidopsis thaliana GN=HSL2 PE=2 SV=1	1.74	6.45	-1.24	down	0.01	0.04	yes
TRINITY_DN25324_c1_g1	hypothetical protein POPTR_0011s13460g [Populus trichocarpa]	CIGR1	Chitin-inducible gibberellin-responsive protein 1 OS=Oryza sativa subsp. japonica GN=CIGR1 PE=2 SV=1	1.40	5.45	-1.32	down	0.01	0.04	yes
TRINITY_DN16608_c0_g1	unknown [Populus trichocarpa x Populus deltoides]	-	-	73.84	262.20	-1.28	down	0.01	0.04	yes
TRINITY_DN23475_c1_g2	hypothetical protein POPTR_0008s01110g [Populus trichocarpa]	ZIFL1	Protein ZINC INDUCED FACILITATOR-LIKE 1 OS=Arabidopsis thaliana GN=ZIFL1 PE=2 SV=1	2.39	10.58	-1.39	down	0.01	0.04	yes
TRINITY_DN22415_c0_g2	-	-	-	4.48	14.21	-1.04	down	0.01	0.04	yes
TRINITY_DN15963_c0_g1	hypothetical protein POPTR_0004s04050g [Populus trichocarpa]	MED16	Mediator of RNA polymerase II transcription subunit 16 OS=Arabidopsis thaliana GN=MED16 PE=1 SV=1	7.91	29.66	-1.32	down	0.01	0.04	yes
TRINITY_DN24231_c0_g1	PREDICTED: mediator of RNA polymerase II transcription subunit 33B isoform X1 [Populus euphratica]	MED33A	Mediator of RNA polymerase II transcription subunit 33A OS=Arabidopsis thaliana GN=MED33A PE=1 SV=1	1.78	6.85	-1.31	down	0.01	0.04	yes
TRINITY_DN20905_c0_g3	p-coumarate 3-hydroxylase [Populus tomentosa]	CYP98A2	Cytochrome P450 98A2 OS=Glycine max GN=CYP98A2 PE=2 SV=1	2.28	8.17	-1.18	down	0.01	0.04	yes
TRINITY_DN16317_c0_g3	PREDICTED: uncharacterized protein LOC105132592 isoform X1 [Populus euphratica]	mprA	Response regulator MprA OS=Mycobacterium vanbaalenii (strain DSM 7251 / PYR-1) GN=mprA PE=3 SV=1	0.79	2.52	-1.32	down	0.01	0.04	yes
TRINITY_DN26143_c0_g3	-	-	-	1.05	5.14	-1.69	down	0.01	0.04	yes
TRINITY_DN23098_c0_g4	hypothetical protein POPTR_0014s14120g, partial [Populus trichocarpa]	-	-	3.89	15.12	-1.35	down	0.01	0.04	yes
TRINITY_DN23598_c0_g1	unknown [Populus trichocarpa]	-	-	2.75	10.74	-1.38	down	0.01	0.04	yes
TRINITY_DN21599_c2_g1	PREDICTED: E3 ubiquitin-protein ligase SINA-like 10 [Populus euphratica]	At5g37930	E3 ubiquitin-protein ligase SINA-like 10 OS=Arabidopsis thaliana GN=At5g37930 PE=2 SV=1	1.09	4.71	-1.46	down	0.01	0.04	yes
TRINITY_DN19582_c0_g2	WRKY transcription factor 9 [(Populus tomentosa x Populus bolleana) x Populus tomentosa]	WRKY40	Probable WRKY transcription factor 40 OS=Arabidopsis thaliana GN=WRKY40 PE=1 SV=1	2.54	14.58	-1.85	down	0.01	0.04	yes
TRINITY_DN24081_c1_g3	-	-	-	1.82	6.39	-1.18	down	0.01	0.04	yes
TRINITY_DN20942_c0_g6	PREDICTED: probable ADP-ribosylation factor GTPase-activating protein AGD14 isoform X1 [Populus euphratica]	-	-	1.23	4.19	-1.14	down	0.01	0.04	yes
TRINITY_DN14628_c0_g1	hypothetical protein POPTR_0006s26640g [Populus trichocarpa]	Copb2	Coatomer subunit beta' OS=Rattus norvegicus GN=Copb2 PE=1 SV=3	0.64	2.80	-1.49	down	0.01	0.04	yes
TRINITY_DN26166_c0_g3	hypothetical protein POPTR_0007s09440g [Populus trichocarpa]	IRE1B	Serine/threonine-protein kinase/endoribonuclease IRE1b OS=Arabidopsis thaliana GN=IRE1B PE=2 SV=1	0.71	3.10	-1.53	down	0.01	0.04	yes
TRINITY_DN23463_c0_g2	-	-	-	1.64	7.98	-1.36	down	0.01	0.04	yes
TRINITY_DN27649_c0_g1	-	-	-	7.92	24.16	-1.01	down	0.01	0.04	yes
TRINITY_DN20200_c0_g3	-	-	-	0.91	4.17	-1.55	down	0.01	0.04	yes
TRINITY_DN13625_c0_g1	Na+/H+ antiporter family protein [Populus trichocarpa]	NHX2	Sodium/hydrogen exchanger 2 OS=Arabidopsis thaliana GN=NHX2 PE=2 SV=2	0.62	2.81	-1.57	down	0.01	0.04	yes
TRINITY_DN26012_c0_g4	hypothetical protein POPTR_0007s05610g, partial [Populus trichocarpa]	DRT111	DNA-damage-repair/toleration protein DRT111, chloroplastic OS=Arabidopsis thaliana GN=DRT111 PE=1 SV=2	0.87	3.77	-1.52	down	0.01	0.04	yes
TRINITY_DN19800_c2_g5	PREDICTED: calcium-transporting ATPase 12, plasma membrane-type-like [Populus euphratica]	-	-	2.14	10.05	-1.40	down	0.01	0.04	yes
TRINITY_DN26903_c1_g3	PREDICTED: uncharacterized protein LOC105129188 [Populus euphratica]	-	-	2.16	8.05	-1.35	down	0.01	0.04	yes
TRINITY_DN23504_c0_g2	PREDICTED: calmodulin-like protein 11 [Populus euphratica]	CML11	Calmodulin-like protein 11 OS=Arabidopsis thaliana GN=CML11 PE=2 SV=1	8.28	46.91	-1.89	down	0.01	0.04	yes
TRINITY_DN16937_c0_g1	PREDICTED: protein ASPARTIC PROTEASE IN GUARD CELL 2-like [Populus euphratica]	ASPG2	Protein ASPARTIC PROTEASE IN GUARD CELL 2 OS=Arabidopsis thaliana GN=ASPG2 PE=2 SV=1	2.23	8.70	-1.15	down	0.01	0.04	yes
TRINITY_DN20452_c2_g1	hypothetical protein POPTR_0002s23750g [Populus trichocarpa]	-	-	0.77	3.39	-1.47	down	0.01	0.04	yes
TRINITY_DN18702_c0_g1	PWWP domain-containing family protein [Populus trichocarpa]	-	-	6.25	20.19	-1.00	down	0.01	0.04	yes

TRINITY_DN20548_c0_g2	Mitogen-activated protein kinase kinase kinase 1 [Populus trichocarpa]	-	-	2.61	9.38	-1.14	down	0.01	0.04	yes
TRINITY_DN20069_c0_g2	hypothetical protein POPTR_0016s09060g [Populus trichocarpa]	CCR1	Serine/threonine-protein kinase-like protein CCR1 OS=Arabidopsis thaliana GN=CCR1 PE=1 SV=1	0.55	2.14	-1.35	down	0.01	0.04	yes
TRINITY_DN26295_c0_g3	-	-	-	0.66	3.54	-1.73	down	0.01	0.04	yes
TRINITY_DN17664_c0_g2	PREDICTED: GDSL esterase/lipase CPRD49-like [Populus euphratica]	CPRD49	GDSL esterase/lipase CPRD49 OS=Arabidopsis thaliana GN=CPRD49 PE=2 SV=1	3.21	10.26	-1.06	down	0.01	0.04	yes
TRINITY_DN18394_c0_g3	PREDICTED: uncharacterized protein LOC105140760 [Populus euphratica]	HIPP39	Heavy metal-associated isoprenylated plant protein 39 OS=Arabidopsis thaliana GN=HIPP39 PE=2 SV=1	15.65	81.22	-1.65	down	0.01	0.04	yes
TRINITY_DN17063_c0_g2	PREDICTED: cucumber peeling cupredoxin-like [Populus euphratica]	-	-	13.40	57.74	-1.51	down	0.01	0.04	yes
TRINITY_DN19003_c0_g2	hypothetical protein POPTR_0006s11830g, partial [Populus trichocarpa]	CYP76A1	Cytochrome P450 76A1 (Fragment) OS=Solanum melongena GN=CYP76A1 PE=2 SV=1	0.76	3.31	-1.48	down	0.01	0.04	yes
TRINITY_DN16833_c0_g3	zinc finger family protein [Populus trichocarpa]	-	-	0.42	3.09	-2.22	down	0.01	0.04	yes
TRINITY_DN24619_c0_g3	hypothetical protein POPTR_0815s00210g [Populus trichocarpa]	EXO	Protein EXORDIUM OS=Arabidopsis thaliana GN=EXO PE=2 SV=1	22.40	123.67	-1.89	down	0.01	0.04	yes
TRINITY_DN24141_c0_g3	hypothetical protein POPTR_0162s00200g [Populus trichocarpa]	-	-	0.56	2.42	-1.45	down	0.01	0.04	yes
TRINITY_DN15394_c0_g1	PREDICTED: regulator of telomere elongation helicase 1 homolog isoform X2 [Populus euphratica]	RTEL1	Regulator of telomere elongation helicase 1 OS=Bos taurus GN=RTEL1 PE=2 SV=1	1.10	3.54	-1.12	down	0.01	0.04	yes
TRINITY_DN20100_c0_g3	unknown [Populus trichocarpa x Populus deltoides]	-	-	2.66	8.39	-1.08	down	0.01	0.04	yes
TRINITY_DN21549_c1_g1	PREDICTED: LOW QUALITY PROTEIN: endoribonuclease Dicer homolog 2-like [Populus euphratica]	At3g03300	Endoribonuclease Dicer homolog 2 OS=Arabidopsis thaliana GN=At3g03300 PE=1 SV=2	2.08	7.18	-1.04	down	0.01	0.04	yes
TRINITY_DN17426_c0_g1	transducin family protein [Populus trichocarpa]	-	-	1.31	4.35	-1.11	down	0.01	0.04	yes
TRINITY_DN16851_c0_g2	hypersensitive reaction associated Ca2+-binding family protein [Populus trichocarpa]	CML12	Calmodulin-like protein 12 OS=Arabidopsis thaliana GN=CML12 PE=1 SV=3	5.56	22.61	-1.40	down	0.01	0.04	yes
TRINITY_DN15813_c0_g1	-	-	-	0.54	2.72	-1.69	down	0.01	0.04	yes
TRINITY_DN20990_c0_g10	AtpE, partial (chloroplast) [Passiflora ciliata]	-	-	1.87	9.84	-1.61	down	0.01	0.04	yes
TRINITY_DN27892_c3_g1	hypothetical protein POPTR_0013s107402g, partial [Populus trichocarpa]	-	-	2.27	8.94	-1.37	down	0.01	0.04	yes
TRINITY_DN27873_c1_g3	hypothetical protein POPTR_0019s13010g [Populus trichocarpa]	RPS6	Disease resistance protein RPS6 OS=Arabidopsis thaliana GN=RPS6 PE=1 SV=1	5.34	17.91	-1.15	down	0.01	0.04	yes
TRINITY_DN24859_c1_g6	-	-	-	2.68	12.63	-1.58	down	0.01	0.04	yes
TRINITY_DN23002_c0_g3	-	-	-	0.98	3.95	-1.39	down	0.01	0.04	yes
TRINITY_DN26571_c0_g3	Cf-4/9 disease resistance-like family protein [Populus trichocarpa]	-	-	8.47	32.95	-1.33	down	0.01	0.04	yes
TRINITY_DN25139_c1_g2	peroxidase family protein [Populus trichocarpa]	GSVIVT0002396	Peroxidase 4 OS=Vitis vinifera GN=GSVIVT00023967001 PE=1 SV=1	3.33	16.46	-1.69	down	0.01	0.04	yes
TRINITY_DN23966_c0_g3	PREDICTED: ABC transporter C family member 13 isoform X1 [Populus euphratica]	ABCC13	ABC transporter C family member 13 OS=Arabidopsis thaliana GN=ABCC13 PE=2 SV=3	0.54	2.81	-1.76	down	0.01	0.04	yes
TRINITY_DN11452_c0_g2	hypothetical protein POPTR_0010s00830g [Populus trichocarpa]	At3g05640	Probable protein phosphatase 2C 34 OS=Arabidopsis thaliana GN=At3g05640 PE=2 SV=1	0.52	2.46	-1.59	down	0.01	0.04	yes
TRINITY_DN21952_c0_g6	PREDICTED: bifunctional dethiobiotin synthetase/7,8-diamino-pelargonic acid aminotransferase, mitochondrial-like [Populus euphratica]	BIO3-BIO1	Bifunctional dethiobiotin synthetase/7,8-diamino-pelargonic acid aminotransferase, mitochondrial OS=Arabidopsis thaliana GN=BIO3-BIO1 PE=1 SV=1	2.07	6.92	-1.14	down	0.01	0.04	yes
TRINITY_DN25181_c0_g1	PREDICTED: uncharacterized protein LOC105141889 isoform X1 [Populus euphratica]	-	-	1.59	6.89	-1.80	down	0.01	0.04	yes
TRINITY_DN19442_c0_g2	hypothetical protein POPTR_0018s08890g [Populus trichocarpa]	-	-	0.91	3.75	-1.41	down	0.01	0.04	yes
TRINITY_DN18443_c0_g1	hypothetical protein POPTR_0002s25150g [Populus trichocarpa]	Dctpp1	dCTP pyrophosphatase 1 OS=Rattus norvegicus GN=Dctpp1 PE=2 SV=1	1.30	6.00	-1.61	down	0.01	0.04	yes
TRINITY_DN26250_c1_g7	hypothetical protein POPTR_0005s19940g [Populus trichocarpa]	-	-	1.06	4.39	-1.41	down	0.01	0.04	yes

TRINITY_DN21649_c1_g2	TIR-NBS-LRR type disease resistance protein [Populus trichocarpa]	-	-	9.75	40.94	-1.42	down	0.01	0.04	yes
TRINITY_DN19594_c0_g5	hypothetical protein POPTR_0014s05730g [Populus trichocarpa]	-	-	0.71	3.59	-1.76	down	0.01	0.04	yes
TRINITY_DN21499_c0_g2	lipase class 3 family protein [Populus trichocarpa]	-	Lipase OS=Rhizopus niveus PE=1 SV=1	1.05	3.31	-1.06	down	0.01	0.04	yes
TRINITY_DN22240_c0_g4	hypothetical protein POPTR_0016s02070g [Populus trichocarpa]	UGT82A1	UDP-glycosyltransferase 82A1 OS=Arabidopsis thaliana GN=UGT82A1 PE=2 SV=1	2.17	9.60	-1.57	down	0.01	0.04	yes
TRINITY_DN26634_c0_g2	PREDICTED: uncharacterized protein LOC105113096 [Populus euphratica]	-	-	1.39	4.75	-1.16	down	0.01	0.04	yes
TRINITY_DN15172_c0_g1	hypothetical protein POPTR_0010s15460g [Populus trichocarpa]	-	-	1.57	6.44	-1.43	down	0.01	0.04	yes
TRINITY_DN25207_c0_g3	hypothetical protein POPTR_0005s15760g, partial [Populus trichocarpa]	-	-	8.36	25.45	-1.13	down	0.01	0.04	yes
TRINITY_DN15638_c1_g1	hypothetical protein POPTR_0001s28610g, partial [Populus trichocarpa]	-	-	0.49	2.50	-1.74	down	0.01	0.04	yes
TRINITY_DN24914_c5_g2	WRKY transcription factor 8 [(Populus tomentosa x Populus bolleana) x Populus tomentosa]	WRKY65	Probable WRKY transcription factor 65 OS=Arabidopsis thaliana GN=WRKY65 PE=2 SV=1	0.57	2.18	-1.35	down	0.01	0.04	yes
TRINITY_DN27662_c5_g2	-	-	-	1.44	5.84	-1.36	down	0.01	0.04	yes
TRINITY_DN26083_c1_g1	hypothetical protein POPTR_0001s15820g [Populus trichocarpa]	PGSIP8	Putative glucuronosyltransferase PGSIP8 OS=Arabidopsis thaliana GN=PGSIP8 PE=2 SV=1	1.93	8.53	-1.54	down	0.01	0.04	yes
TRINITY_DN21333_c0_g2	-	-	-	1.40	10.81	-2.29	down	0.01	0.04	yes
TRINITY_DN22931_c0_g4	hypothetical protein POPTR_0018s11780g [Populus trichocarpa]	At1g51120	AP2/ERF and B3 domain-containing transcription factor At1g51120 OS=Arabidopsis thaliana GN=At1g51120 PE=2 SV=1	0.88	3.02	-1.16	down	0.01	0.04	yes
TRINITY_DN15627_c0_g1	PREDICTED: auxin-induced protein AUX22 [Populus euphratica]	AUX22	Auxin-induced protein AUX22 OS=Glycine max GN=AUX22 PE=2 SV=1	2.52	10.20	-1.33	down	0.01	0.04	yes
TRINITY_DN19854_c0_g2	PREDICTED: protein TIFY 10A [Populus euphratica]	TIFY10A	Protein TIFY 10A OS=Arabidopsis thaliana GN=TIFY10A PE=1 SV=1	35.59	214.11	-2.03	down	0.01	0.04	yes
TRINITY_DN20496_c2_g1	hypothetical protein POPTR_0007s15230g [Populus trichocarpa]	PUB35	U-box domain-containing protein 35 OS=Arabidopsis thaliana GN=PUB35 PE=1 SV=2	1.80	6.78	-1.34	down	0.01	0.04	yes
TRINITY_DN24250_c0_g1	PREDICTED: phytosulfokine receptor 2-like [Populus euphratica]	-	-	0.61	3.19	-1.75	down	0.01	0.04	yes
TRINITY_DN18664_c1_g4	TIR-NBS type disease resistance protein [Populus trichocarpa]	-	-	2.03	8.68	-1.40	down	0.01	0.04	yes
TRINITY_DN19673_c3_g2	hypothetical protein POPTR_0006s10630g [Populus trichocarpa]	-	-	4.23	44.14	-2.77	down	0.01	0.04	yes
TRINITY_DN27743_c1_g4	-	-	-	4.32	21.96	-1.71	down	0.02	0.04	yes
TRINITY_DN17870_c0_g1	PREDICTED: probable glutathione S-transferase [Populus euphratica]	-	Probable glutathione S-transferase OS=Nicotiana tabacum PE=2 SV=1	1.73	7.15	-1.34	down	0.02	0.04	yes
TRINITY_DN24642_c0_g1	PREDICTED: 3-ketoacyl-CoA synthase 3-like [Populus euphratica]	KCS3	3-ketoacyl-CoA synthase 3 OS=Arabidopsis thaliana GN=KCS3 PE=2 SV=3	1.48	4.58	-1.04	down	0.02	0.04	yes
TRINITY_DN24619_c0_g2	hypothetical protein POPTR_0815s00210g [Populus trichocarpa]	EXO	Protein EXORDIUM OS=Arabidopsis thaliana GN=EXO PE=2 SV=1	40.77	178.92	-1.48	down	0.02	0.04	yes
TRINITY_DN20582_c0_g1	hypothetical protein POPTR_0002s17930g [Populus trichocarpa]	-	-	8.00	26.10	-1.10	down	0.02	0.05	yes
TRINITY_DN27331_c0_g1	PREDICTED: probable polygalacturonase [Populus euphratica]	-	-	6.46	20.06	-1.03	down	0.02	0.05	yes
TRINITY_DN21017_c0_g3	LysM-domain GPI-anchored protein 2 precursor [Populus trichocarpa]	LYM2	LysM domain-containing GPI-anchored protein 2 OS=Arabidopsis thaliana GN=LYM2 PE=1 SV=1	1.01	4.27	-1.50	down	0.02	0.05	yes
TRINITY_DN25785_c0_g1	PREDICTED: LOW QUALITY PROTEIN: TMV resistance protein N-like [Populus euphratica]	-	-	2.32	7.65	-1.31	down	0.02	0.05	yes
TRINITY_DN22515_c0_g6	-	-	-	2.54	8.24	-1.11	down	0.02	0.05	yes
TRINITY_DN18050_c0_g2	PREDICTED: PRA1 family protein E-like [Populus euphratica]	PRA1E	PRA1 family protein E OS=Arabidopsis thaliana GN=PRA1E PE=1 SV=1	0.92	3.02	-1.11	down	0.02	0.05	yes
TRINITY_DN26887_c0_g3	PREDICTED: serine/threonine-protein kinase ULK3 isoform X1 [Populus euphratica]	At1g49180	Serine/threonine-protein kinase ATG1t OS=Arabidopsis thaliana GN=At1g49180 PE=2 SV=1	5.57	26.50	-1.16	down	0.02	0.05	yes

TRINITY_DN21202_c0_g1	PREDICTED: ethylene-responsive transcription factor 4-like [Populus euphratica]	-	-	30.81	181.66	-1.95	down	0.02	0.05	yes
TRINITY_DN18287_c0_g1	-	-	-	2.14	8.19	-1.33	down	0.02	0.05	yes
TRINITY_DN21259_c0_g1	hypothetical protein B456_009G249000 [Gossypium raimondii]	At1g21570	Zinc finger CCCH domain-containing protein 7 OS=Arabidopsis thaliana GN=At1g21570 PE=1 SV=1	3.30	9.54	-1.31	down	0.02	0.05	yes
TRINITY_DN16405_c1_g2	hypothetical protein POPTR_0014s07230g [Populus trichocarpa]	-	-	1.05	7.27	-1.77	down	0.02	0.05	yes
TRINITY_DN22253_c0_g2	PREDICTED: putative receptor-like protein kinase At1g72540 [Populus euphratica]	At1g72540	Putative receptor-like protein kinase At1g72540 OS=Arabidopsis thaliana GN=At1g72540 PE=2 SV=1	0.97	3.55	-1.28	down	0.02	0.05	yes
TRINITY_DN25075_c2_g1	hypothetical protein POPTR_0018s07270g [Populus trichocarpa]	Rnf217	Probable E3 ubiquitin-protein ligase RNF217 OS=Mus musculus GN=Rnf217 PE=3 SV=2	3.39	9.78	-1.13	down	0.02	0.05	yes
TRINITY_DN17759_c0_g3	PREDICTED: proline-rich receptor-like protein kinase PERK3 isoform X1 [Populus euphratica]	At2g19130	G-type lectin S-receptor-like serine/threonine-protein kinase At2g19130 OS=Arabidopsis thaliana GN=At2g19130 PE=2 SV=1	0.24	1.71	-2.14	down	0.02	0.05	yes
TRINITY_DN20455_c0_g1	-	-	-	0.99	5.23	-1.75	down	0.02	0.05	yes
TRINITY_DN21935_c0_g2	-	-	-	0.84	4.25	-1.72	down	0.02	0.05	yes
TRINITY_DN20227_c0_g1	PREDICTED: malate synthase, glyoxysomal [Populus euphratica]	-	Malate synthase, glyoxysomal OS=Gossypium hirsutum PE=2 SV=1	2.85	13.95	-1.45	down	0.02	0.05	yes
TRINITY_DN23531_c0_g3	-	-	-	2.70	8.68	-1.08	down	0.02	0.05	yes
TRINITY_DN27648_c1_g3	hypothetical protein POPTR_0014s16290g [Populus trichocarpa]	-	-	2.97	10.44	-1.20	down	0.02	0.05	yes
TRINITY_DN16046_c0_g2	hypothetical protein POPTR_0001s03820g [Populus trichocarpa]	-	-	0.83	3.54	-1.54	down	0.02	0.05	yes
TRINITY_DN15876_c0_g1	PREDICTED: GATA transcription factor 18 [Populus euphratica]	GATA18	GATA transcription factor 18 OS=Arabidopsis thaliana GN=GATA18 PE=2 SV=2	1.00	7.32	-1.85	down	0.02	0.05	yes
TRINITY_DN25362_c0_g5	hypothetical protein POPTR_0004s12460g [Populus trichocarpa]	CGT	UDP-glycosyltransferase 13 OS=Mangifera indica GN=CGT PE=1 SV=1	0.47	2.61	-1.81	down	0.02	0.05	yes
TRINITY_DN27119_c0_g1	unknown [Populus trichocarpa x Populus deltoides]	VEP1	3-oxo-Delta(4,5)-steroid 5-beta-reductase OS=Arabidopsis thaliana GN=VEP1 PE=1 SV=1	0.41	1.96	-1.65	down	0.02	0.05	yes
TRINITY_DN26717_c1_g3	calmodulin-binding family protein [Populus trichocarpa]	IQD14	Protein IQ-DOMAIN 14 OS=Arabidopsis thaliana GN=IQD14 PE=1 SV=1	0.58	2.54	-1.53	down	0.02	0.05	yes
TRINITY_DN13002_c0_g1	PREDICTED: proline-rich receptor-like protein kinase PERK13 [Populus euphratica]	PERK12	Proline-rich receptor-like protein kinase PERK12 OS=Arabidopsis thaliana GN=PERK12 PE=2 SV=2	0.61	2.89	-1.57	down	0.02	0.05	yes
TRINITY_DN16783_c0_g2	PREDICTED: F-box protein At3g07870-like [Populus euphratica]	-	-	1.30	4.60	-1.18	down	0.02	0.05	yes
TRINITY_DN26360_c0_g3	hypothetical protein POPTR_0006s19290g [Populus trichocarpa]	-	-	1.76	6.40	-1.23	down	0.02	0.05	yes
TRINITY_DN13069_c0_g3	PREDICTED: uncharacterized protein LOC105111104, partial [Populus euphratica]	-	-	1.20	4.32	-1.25	down	0.02	0.05	yes
TRINITY_DN23236_c0_g3	-	-	-	2.65	9.60	-1.27	down	0.02	0.05	yes
TRINITY_DN14672_c0_g1	hypothetical protein POPTR_0006s13920g [Populus trichocarpa]	-	-	1.25	6.32	-1.65	down	0.02	0.05	yes
TRINITY_DN20370_c1_g2	hypothetical protein POPTR_0003s20570g [Populus trichocarpa]	-	-	0.85	3.93	-1.61	down	0.02	0.05	yes
TRINITY_DN27296_c0_g1	hypothetical protein POPTR_0003s20080g [Populus trichocarpa]	RPPL1	Putative disease resistance RPP13-like protein 1 OS=Arabidopsis thaliana GN=RPPL1 PE=3 SV=1	2.00	6.14	-1.17	down	0.02	0.05	yes
TRINITY_DN23743_c2_g2	hypothetical protein POPTR_2170s00200g [Populus trichocarpa]	-	-	1.87	11.38	-1.46	down	0.02	0.05	yes
TRINITY_DN24982_c1_g4	PREDICTED: ankyrin repeat domain-containing protein 13C-A-like [Populus euphratica]	-	-	3.63	12.30	-1.19	down	0.02	0.05	yes
TRINITY_DN12159_c0_g1	calcium-binding family protein [Populus trichocarpa]	CML44	Probable calcium-binding protein CML44 OS=Arabidopsis thaliana GN=CML44 PE=2 SV=2	0.41	3.24	-2.38	down	0.02	0.05	yes
TRINITY_DN25856_c0_g2	PREDICTED: acyl-coenzyme A thioesterase 9, mitochondrial-like [Populus euphratica]	-	-	2.47	8.85	-1.26	down	0.02	0.05	yes
TRINITY_DN14259_c0_g1	hypothetical protein POPTR_0001s20710g [Populus trichocarpa]	SELMODRAFT_	Inactive protein kinase SELMODRAFT_444075 OS=Selaginella moellendorffii GN=SELMODRAFT_444075 PE=2 SV=1	7.73	26.19	-1.16	down	0.02	0.05	yes

TRINITY_DN26329_c0_g3	hypothetical protein POPTR_0003s20040g [Populus trichocarpa]	-	-	0.60	2.41	-1.24	down	0.02	0.05	yes
TRINITY_DN16183_c0_g2	hypothetical protein POPTR_0006s01190g [Populus trichocarpa]	ACT	Vinorine synthase OS=Rauvolfia serpentina GN=ACT PE=1 SV=2	1.74	8.00	-1.80	down	0.02	0.05	yes
TRINITY_DN25931_c0_g1	hypothetical protein POPTR_0005s11600g [Populus trichocarpa]	F3H	Naringenin,2-oxoglutarate 3-dioxygenase OS=Vitis vinifera GN=F3H PE=2 SV=1	47.00	165.81	-1.22	down	0.02	0.05	yes
TRINITY_DN14705_c0_g2	-	-	-	0.65	2.76	-1.48	down	0.02	0.05	yes
TRINITY_DN23699_c1_g6	PREDICTED: uncharacterized protein LOC105115824 isoform X1 [Populus euphratica]	-	-	3.69	17.20	-1.21	down	0.02	0.05	yes
TRINITY_DN27766_c1_g3	hypothetical protein POPTR_0018s01080g [Populus trichocarpa]	PHF3	PHD finger protein 3 OS=Homo sapiens GN=PHF3 PE=1 SV=3	2.63	9.30	-1.20	down	0.02	0.05	yes
TRINITY_DN21604_c0_g1	hypothetical protein POPTR_0202s002001g, partial [Populus trichocarpa]	-	-	8.79	26.43	-1.01	down	0.02	0.05	yes
TRINITY_DN21667_c0_g1	cyclin family protein [Populus tomentosa]	CYCT1-3	Cyclin-T1-3 OS=Oryza sativa subsp. japonica GN=CYCT1-3 PE=3 SV=2	4.14	14.38	-1.11	down	0.02	0.05	yes
TRINITY_DN26643_c2_g8	-	-	-	0.55	2.90	-1.75	down	0.02	0.05	yes
TRINITY_DN23479_c0_g2	-	-	-	10.58	53.35	-1.75	down	0.02	0.05	yes
TRINITY_DN24741_c1_g2	PREDICTED: UPF0503 protein At3g09070, chloroplastic-like [Populus euphratica]	At3g09070	UPF0503 protein At3g09070, chloroplastic OS=Arabidopsis thaliana GN=At3g09070 PE=1 SV=1	4.63	16.28	-1.23	down	0.02	0.05	yes
TRINITY_DN23038_c0_g1	PREDICTED: THO complex subunit 5B [Populus euphratica]	THO5B	THO complex subunit 5B OS=Arabidopsis thaliana GN=THO5B PE=1 SV=1	0.55	2.38	-1.51	down	0.02	0.05	yes
TRINITY_DN17900_c1_g4	-	-	-	1.05	4.56	-1.47	down	0.02	0.05	yes
TRINITY_DN22111_c0_g1	hypothetical protein POPTR_0013s15345g [Populus trichocarpa]	ARR17	Two-component response regulator ARR17 OS=Arabidopsis thaliana GN=ARR17 PE=2 SV=1	2.02	11.10	-1.62	down	0.02	0.05	yes
TRINITY_DN17833_c0_g3	hypothetical protein POPTR_0010s04930g [Populus trichocarpa]	-	-	0.65	2.95	-1.57	down	0.02	0.05	yes
TRINITY_DN22164_c1_g3	hypothetical protein POPTR_0006s19240g [Populus trichocarpa]	GAE1	UDP-glucuronate 4-epimerase 1 OS=Arabidopsis thaliana GN=GAE1 PE=1 SV=1	45.77	151.67	-1.14	down	0.02	0.05	yes
TRINITY_DN20147_c0_g4	-	-	-	0.82	3.25	-1.38	down	0.02	0.05	yes
TRINITY_DN14377_c0_g1	-	-	-	0.43	1.99	-1.63	down	0.02	0.05	yes
TRINITY_DN27056_c0_g4	PREDICTED: uncharacterized protein LOC107062216 [Solanum tuberosum]	-	-	0.31	1.71	-1.82	down	0.02	0.05	yes
