# Supplementary material for: MiR156 regulates anthocyanin biosynthesis through SPL targets and other microRNAs in poplar
Source: Hortic Res. 2020 Aug 1;7:118. doi: 10.1038/s41438-020-00341-w (PMC7395715; doi:10.1038/s41438-020-00341-w)
Supplement: Supplementary file 4 — Supporting Information 4 [file 41438_2020_341_MOESM4_ESM.pdf]

Table S8 Integrated analysis of DEMs and DEGs between wild-type and transgenic poplar plants.

**# Correlation**	**microRNA Information**						**mRNA Information**						
**# microRNA : mRNA : Gene**	**miRNA**	**FC (TGII/WT)**	**Sig_FC**	**Regulation**	**p-value**	**Sig p-value**	**mRNA**	**FC (TGII/WT)**	**Sig_FC**	**Regulation**	**FDR**	**Sig_FDR**	**GO_term**
aly-miR399b-3p : TRINITY_DN22148_c0_g1 : TRINITY_DN22148_c0_g1	aly-miR399b-3p	4.22	yes	up	0.00	yes	TRINITY_DN22148_c0_g1	0.36	yes	down	0.00	yes	integral component of plasma membrane;substrate-specific transmembra
aly-MIR829-p5_2ss3GA21GC : TRINITY_DN21709_c0_g1 : TRINITY_DN21709_c0_g1	aly-MIR829-p5_2ss3GA21GC	0.23	yes	down	0.00	yes	TRINITY_DN21709_c0_g1	0.21	yes	down	0.00	yes	calmodulin binding;peptidyl-serine phosphorylation;cytoplasm;intracellu
aqc-miR530_L+2R-1 : TRINITY_DN22984_c0_g2 : TRINITY_DN22984_c0_g2	aqc-miR530_L+2R-1	3.79	yes	up	0.00	yes	TRINITY_DN22984_c0_g2	2.37	yes	up	0.00	yes	peroxisome;integral component of membrane;oxidoreductase activity
aqc-miR530_L+2R-1 : TRINITY_DN24639_c1_g2 : TRINITY_DN24639_c1_g2	aqc-miR530_L+2R-1	3.79	yes	up	0.00	yes	TRINITY_DN24639_c1_g2	0.40	yes	down	0.00	yes	protein dimerization activity;abscisic acid-activated signaling pathway;tr
aqc-miR530_L+2R-1 : TRINITY_DN25890_c0_g1 : TRINITY_DN25890_c0_g1	aqc-miR530_L+2R-1	3.79	yes	up	0.00	yes	TRINITY_DN25890_c0_g1	2.13	yes	up	0.00	yes	mitochondrion;RNA binding;cytosolic large ribosomal subunit;maturatio
ath-miR157a-3p_2ss10CT13TC : TRINITY_DN18121_c0_g2 : TRINITY_DN18121_c0_g2	ath-miR157a-3p_2ss10CT13TC	187.35	yes	up	0.01	yes	TRINITY_DN18121_c0_g2	3.94	yes	up	0.00	yes	chloroplast;chloroplast RNA processing;zinc ion binding;mRNA processi
ath-miR159c_R+1_1ss20CT : TRINITY_DN17636_c0_g2 : TRINITY_DN17636_c0_g2	ath-miR159c_R+1_1ss20CT	0.71	yes	down	0.04	yes	TRINITY_DN17636_c0_g2	0.23	yes	down	0.00	yes	.
cas-miR159b-3p_R+1 : TRINITY_DN17636_c0_g2 : TRINITY_DN17636_c0_g2	cas-miR159b-3p_R+1	0.71	yes	down	0.04	yes	TRINITY_DN17636_c0_g2	0.23	yes	down	0.00	yes	.
cca-MIR156c-p5_2ss7CT17AC : TRINITY_DN16376_c0_g1 : TRINITY_DN16376_c0_g1	cca-MIR156c-p5_2ss7CT17AC	46.08	yes	up	0.04	yes	TRINITY_DN16376_c0_g1	3.57	yes	up	0.00	yes	apoplast;glucose-1-phosphate adenylyltransferase activity;heterotetrame
cca-MIR156c-p5_2ss7CT17AC : TRINITY_DN20685_c0_g1 : TRINITY_DN20685_c0_g1	cca-MIR156c-p5_2ss7CT17AC	46.08	yes	up	0.04	yes	TRINITY_DN20685_c0_g1	0.39	yes	down	0.00	yes	regulation of transcription, DNA-templated;protein binding;nucleus;trans
cca-MIR156c-p5_2ss7CT17AC : TRINITY_DN23437_c0_g3 : TRINITY_DN23437_c0_g3	cca-MIR156c-p5_2ss7CT17AC	46.08	yes	up	0.04	yes	TRINITY_DN23437_c0_g3	5.90	yes	up	0.00	yes	cellular water homeostasis;glycerol channel activity;ion transmembrane t
cca-MIR156c-p5_2ss7CT17AC : TRINITY_DN24627_c0_g1 : TRINITY_DN24627_c0_g1	cca-MIR156c-p5_2ss7CT17AC	46.08	yes	up	0.04	yes	TRINITY_DN24627_c0_g1	2.29	yes	up	0.00	yes	cytoplasm;jasmonic acid biosynthetic process;flower development;seed g
cca-MIR156c-p5_2ss7CT17AC : TRINITY_DN26485_c1_g2 : TRINITY_DN26485_c1_g2	cca-MIR156c-p5_2ss7CT17AC	46.08	yes	up	0.04	yes	TRINITY_DN26485_c1_g2	0.45	yes	down	0.00	yes	plasma membrane;calcium-dependent phospholipid binding;calcium ion b
cca-MIR156c-p5_2ss7CT17AC : TRINITY_DN27533_c0_g1 : TRINITY_DN27533_c0_g1	cca-MIR156c-p5_2ss7CT17AC	46.08	yes	up	0.04	yes	TRINITY_DN27533_c0_g1	3.18	yes	up	0.00	yes	phosphoglucan, water dikinase activity;carbohydrate kinase activity;met
cca-MIR156c-p5_2ss7CT17AC : TRINITY_DN27884_c4_g1 : TRINITY_DN27884_c4_g1	cca-MIR156c-p5_2ss7CT17AC	46.08	yes	up	0.04	yes	TRINITY_DN27884_c4_g1	0.28	yes	down	0.00	yes	ADP binding;defense response;ATP binding;protein binding;extracellular
cme-MIR160c-p5_2ss13AG17AG : TRINITY_DN16725_c0_g1 : TRINITY_DN16725_c0_g1	cme-MIR160c-p5_2ss13AG17AG	4.64	yes	up	0.05	yes	TRINITY_DN16725_c0_g1	0.21	yes	down	0.03	yes	defense response to fungus;regulation of transcription, DNA-templated;t
cme-MIR160c-p5_2ss13AG17AG : TRINITY_DN16903_c0_g2 : TRINITY_DN16903_c0_g2	cme-MIR160c-p5_2ss13AG17AG	4.64	yes	up	0.05	yes	TRINITY_DN16903_c0_g2	0.00	yes	down	0.00	yes	secondary metabolite biosynthetic process;membrane;oxidoreductase acti
cme-MIR160c-p5_2ss13AG17AG : TRINITY_DN19743_c0_g1 : TRINITY_DN19743_c0_g1	cme-MIR160c-p5_2ss13AG17AG	4.64	yes	up	0.05	yes	TRINITY_DN19743_c0_g1	2.61	yes	up	0.00	yes	plasma membrane;transmembrane transporter activity;transport
cme-MIR160c-p5_2ss13AG17AG : TRINITY_DN21153_c0_g2 : TRINITY_DN21153_c0_g2	cme-MIR160c-p5_2ss13AG17AG	4.64	yes	up	0.05	yes	TRINITY_DN21153_c0_g2	4.95	yes	up	0.00	yes	anchored component of membrane

cme-MIR160c-p5_2ss13AG17AG : TRINITY_DN23227_c0_g1 : TRINITY_DN23227_c0_g1	cme-MIR160c-p5_2ss13AG17AG	4.64	yes	up	0.05	yes	TRINITY_DN23227_c0_g1	2.63	yes	up	0.00	yes	chloroplast;metalloendopeptidase activity;metallopeptidase activity;inte
cme-MIR160c-p5_2ss13AG17AG : TRINITY_DN24453_c0_g1 : TRINITY_DN24453_c0_g1	cme-MIR160c-p5_2ss13AG17AG	4.64	yes	up	0.05	yes	TRINITY_DN24453_c0_g1	0.03	yes	down	0.00	yes	amino acid transmembrane transporter activity;amino acid transmembra
cme-MIR160c-p5_2ss13AG17AG : TRINITY_DN25318_c0_g1 : TRINITY_DN25318_c0_g1	cme-MIR160c-p5_2ss13AG17AG	4.64	yes	up	0.05	yes	TRINITY_DN25318_c0_g1	2.46	yes	up	0.00	yes	proteasome-mediated ubiquitin-dependent protein catabolic process;ubiqu
cme-MIR1863-p5_2ss3AG19TC : TRINITY_DN19411_c0_g2 : TRINITY_DN19411_c0_g2	cme-MIR1863-p5_2ss3AG19TC	0.16	yes	down	0.02	yes	TRINITY_DN19411_c0_g2	0.46	yes	down	0.01	yes	mitochondrion;tRNA modification;tRNA dimethylallyltransferase activi
cme-MIR1863-p5_2ss3AG19TC : TRINITY_DN21165_c2_g2 : TRINITY_DN21165_c2_g2	cme-MIR1863-p5_2ss3AG19TC	0.16	yes	down	0.02	yes	TRINITY_DN21165_c2_g2	2.40	yes	up	0.00	yes	protein-lysine N-methyltransferase activity;peptidyl-lysine monomethyl
cme-MIR1863-p5_2ss3AG19TC : TRINITY_DN21709_c0_g1 : TRINITY_DN21709_c0_g1	cme-MIR1863-p5_2ss3AG19TC	0.16	yes	down	0.02	yes	TRINITY_DN21709_c0_g1	0.21	yes	down	0.00	yes	calmodulin binding;peptidyl-serine phosphorylation;cytoplasm;intracellu
cme-MIR1863-p5_2ss3AG19TC : TRINITY_DN25050_c0_g1 : TRINITY_DN25050_c0_g1	cme-MIR1863-p5_2ss3AG19TC	0.16	yes	down	0.02	yes	TRINITY_DN25050_c0_g1	0.50	yes	down	0.03	yes	poly(A) RNA binding;RNA processing;nucleus;cytoplasm;ATP-dependen
cme-MIR1863-p5_2ss3AG19TC : TRINITY_DN27220_c0_g1 : TRINITY_DN27220_c0_g1	cme-MIR1863-p5_2ss3AG19TC	0.16	yes	down	0.02	yes	TRINITY_DN27220_c0_g1	0.46	yes	down	0.01	yes	ATPase activity;microtubule-based movement;kinesin complex;microtub
cme-MIR1863-p5_2ss3AG21GA : TRINITY_DN21709_c0_g1 : TRINITY_DN21709_c0_g1	cme-MIR1863-p5_2ss3AG21GA	0.17	yes	down	0.05	yes	TRINITY_DN21709_c0_g1	0.21	yes	down	0.00	yes	calmodulin binding;peptidyl-serine phosphorylation;cytoplasm;intracellu
cme-MIR1863-p5_2ss3AG21GA : TRINITY_DN27220_c0_g1 : TRINITY_DN27220_c0_g1	cme-MIR1863-p5_2ss3AG21GA	0.17	yes	down	0.05	yes	TRINITY_DN27220_c0_g1	0.46	yes	down	0.01	yes	ATPase activity;microtubule-based movement;kinesin complex;microtub
cme-MIR399c-p5_2ss17TA18GC : TRINITY_DN19185_c1_g6 : TRINITY_DN19185_c1_g6	cme-MIR399c-p5_2ss17TA18GC	0.19	yes	down	0.03	yes	TRINITY_DN19185_c1_g6	0.15	yes	down	0.00	yes	sterol metabolic process;brassinosteroid homeostasis;multicellular organis
csi-MIR169m-p3_1ss12GT : TRINITY_DN18544_c0_g2 : TRINITY_DN18544_c0_g2	csi-MIR169m-p3_1ss12GT	2.57	yes	up	0.04	yes	TRINITY_DN18544_c0_g2	2.60	yes	up	0.00	yes	protein phosphorylation;chloroplast;response to photooxidative stress;p
ghr-miR156a_R+2 : TRINITY_DN19122_c0_g1 : TRINITY_DN19122_c0_g1	ghr-miR156a_R+2	496.57	yes	up	0.01	yes	TRINITY_DN19122_c0_g1	0.22	yes	down	0.00	yes	transcription factor activity, sequence-specific DNA binding;defense resp
ghr-miR156a_R+2 : TRINITY_DN19850_c1_g2 : TRINITY_DN19850_c1_g2	ghr-miR156a_R+2	496.57	yes	up	0.01	yes	TRINITY_DN19850_c1_g2	0.13	yes	down	0.00	yes	metal ion binding;transcription factor activity, sequence-specific DNA bi
ghr-miR156a_R+2 : TRINITY_DN19850_c1_g3 : TRINITY_DN19850_c1_g3	ghr-miR156a_R+2	496.57	yes	up	0.01	yes	TRINITY_DN19850_c1_g3	0.03	yes	down	0.00	yes	flower development;cytoplasm;DNA binding;transcription, DNA-templa
ghr-miR156a_R+2 : TRINITY_DN19850_c1_g4 : TRINITY_DN19850_c1_g4	ghr-miR156a_R+2	496.57	yes	up	0.01	yes	TRINITY_DN19850_c1_g4	0.18	yes	down	0.00	yes	transcription factor activity, sequence-specific DNA binding;defense resp
ghr-miR156a_R+2 : TRINITY_DN21154_c0_g2 : TRINITY_DN21154_c0_g2	ghr-miR156a_R+2	496.57	yes	up	0.01	yes	TRINITY_DN21154_c0_g2	0.41	yes	down	0.01	yes	regulation of transcription, DNA-templated;anther development;transcri
ghr-miR156a_R+2 : TRINITY_DN22967_c0_g1 : TRINITY_DN22967_c0_g1	ghr-miR156a_R+2	496.57	yes	up	0.01	yes	TRINITY_DN22967_c0_g1	0.42	yes	down	0.00	yes	transcription factor activity, sequence-specific DNA binding;anther deve
ghr-miR156a_R+2 : TRINITY_DN23412_c1_g9 : TRINITY_DN23412_c1_g9	ghr-miR156a_R+2	496.57	yes	up	0.01	yes	TRINITY_DN23412_c1_g9	0.03	yes	down	0.00	yes	regulation of transcription, DNA-templated;cell differentiation;positive
ghr-miR156a_R+2 : TRINITY_DN23413_c0_g1 : TRINITY_DN23413_c0_g1	ghr-miR156a_R+2	496.57	yes	up	0.01	yes	TRINITY_DN23413_c0_g1	0.33	yes	down	0.00	yes	transcription factor activity, sequence-specific DNA binding;anther deve
ghr-miR156a_R+2 : TRINITY_DN24312_c1_g1 : TRINITY_DN24312_c1_g1	ghr-miR156a_R+2	496.57	yes	up	0.01	yes	TRINITY_DN24312_c1_g1	0.44	yes	down	0.00	yes	regulation of transcription, DNA-templated;nucleus;transcription, DNA-
gma-MIR4382-p5_2ss10AC18TA : TRINITY_DN21427_c0_g1 : TRINITY_DN21427_c0_g1	gma-MIR4382-p5_2ss10AC18TA	93.60	yes	up	0.04	yes	TRINITY_DN21427_c0_g1	2.64	yes	up	0.00	yes	response to cold;reductive pentose-phosphate cycle;fructose 1,6-bisphos

gma-MIR4382-p5_2ss10AC18TA : TRINITY_DN22484_c1_g1 : TRINITY_DN22484_c1_g1	gma-MIR4382-p5_2ss10AC18TA	93.60	yes	up	0.04	yes	TRINITY_DN22484_c1_g1	3.40	yes	up	0.00	yes	cell wall biogenesis;hydrolase activity, acting on glycosyl bonds;extracell
gma-MIR4382-p5_2ss10AC18TA : TRINITY_DN23562_c0_g6 : TRINITY_DN23562_c0_g6	gma-MIR4382-p5_2ss10AC18TA	93.60	yes	up	0.04	yes	TRINITY_DN23562_c0_g6	2.83	yes	up	0.00	yes	photosystem II assembly;chloroplast thylakoid lumen;photosystem II re
gma-MIR4382-p5_2ss10AC18TA : TRINITY_DN25474_c0_g1 : TRINITY_DN25474_c0_g1	gma-MIR4382-p5_2ss10AC18TA	93.60	yes	up	0.04	yes	TRINITY_DN25474_c0_g1	2.54	yes	up	0.00	yes	diaminopimelate decarboxylase activity
gma-MIR4382-p5_2ss10AC18TA : TRINITY_DN26186_c0_g2 : TRINITY_DN26186_c0_g2	gma-MIR4382-p5_2ss10AC18TA	93.60	yes	up	0.04	yes	TRINITY_DN26186_c0_g2	0.02	yes	down	0.00	yes	oxidation-reduction process;cytokinin dehydrogenase activity;oxidoredu
gma-MIR5032-p3_2ss13AG17AC : TRINITY_DN16715_c0_g1 : TRINITY_DN16715_c0_g1	gma-MIR5032-p3_2ss13AG17AC	24.67	yes	up	0.01	yes	TRINITY_DN16715_c0_g1	4.58	yes	up	0.00	yes	large ribosomal subunit;structural constituent of ribosome
gma-MIR5032-p3_2ss13AG17AC : TRINITY_DN16715_c0_g3 : TRINITY_DN16715_c0_g3	gma-MIR5032-p3_2ss13AG17AC	24.67	yes	up	0.01	yes	TRINITY_DN16715_c0_g3	3.25	yes	up	0.00	yes	structural constituent of ribosome;large ribosomal subunit
gma-MIR5032-p3_2ss13AG17AC : TRINITY_DN18318_c0_g2 : TRINITY_DN18318_c0_g2	gma-MIR5032-p3_2ss13AG17AC	24.67	yes	up	0.01	yes	TRINITY_DN18318_c0_g2	3.09	yes	up	0.00	yes	ribosome;translation;chloroplast;structural constituent of ribosome
gma-MIR5032-p3_2ss13AG17AC : TRINITY_DN20870_c0_g1 : TRINITY_DN20870_c0_g1	gma-MIR5032-p3_2ss13AG17AC	24.67	yes	up	0.01	yes	TRINITY_DN20870_c0_g1	2.05	yes	up	0.01	yes	vacuole;lipid binding;mitochondrion;integral component of membrane
gma-MIR5032-p3_2ss13AG17AC : TRINITY_DN20925_c0_g1 : TRINITY_DN20925_c0_g1	gma-MIR5032-p3_2ss13AG17AC	24.67	yes	up	0.01	yes	TRINITY_DN20925_c0_g1	2.12	yes	up	0.00	yes	polysomal ribosome;response to maltose;cytoplasm;response to glucose;
gma-MIR5032-p3_2ss13AG17AC : TRINITY_DN21228_c0_g1 : TRINITY_DN21228_c0_g1	gma-MIR5032-p3_2ss13AG17AC	24.67	yes	up	0.01	yes	TRINITY_DN21228_c0_g1	3.01	yes	up	0.00	yes	mitochondrial RNA processing;3'-5'-exoribonuclease activity;nucleic acid
gma-MIR5032-p3_2ss13AG17AC : TRINITY_DN22370_c0_g1 : TRINITY_DN22370_c0_g1	gma-MIR5032-p3_2ss13AG17AC	24.67	yes	up	0.01	yes	TRINITY_DN22370_c0_g1	2.25	yes	up	0.00	yes	chloroplast
gma-MIR5032-p3_2ss13AG17AC : TRINITY_DN23972_c0_g1 : TRINITY_DN23972_c0_g1	gma-MIR5032-p3_2ss13AG17AC	24.67	yes	up	0.01	yes	TRINITY_DN23972_c0_g1	0.31	yes	down	0.00	yes	nucleus;postreplication repair;protein binding;cell division;nuclear repliso
gma-MIR5032-p3_2ss13AG17AC : TRINITY_DN26040_c1_g5 : TRINITY_DN26040_c1_g5	gma-MIR5032-p3_2ss13AG17AC	24.67	yes	up	0.01	yes	TRINITY_DN26040_c1_g5	2.45	yes	up	0.00	yes	.
gma-MIR5032-p3_2ss13AG17AC : TRINITY_DN27495_c1_g1 : TRINITY_DN27495_c1_g1	gma-MIR5032-p3_2ss13AG17AC	24.67	yes	up	0.01	yes	TRINITY_DN27495_c1_g1	0.38	yes	down	0.00	yes	cytoplasm;intracellular signal transduction;protein serine/threonine kinas
gma-MIR5032-p3_2ss13AG17AC : TRINITY_DN27857_c5_g1 : TRINITY_DN27857_c5_g1	gma-MIR5032-p3_2ss13AG17AC	24.67	yes	up	0.01	yes	TRINITY_DN27857_c5_g1	8.57	yes	up	0.00	yes	DNA integration;cytoplasm;RNA-dependent DNA biosynthetic process;
gma-MIR5032-p5_2ss20AG24AC : TRINITY_DN26040_c1_g5 : TRINITY_DN26040_c1_g5	gma-MIR5032-p5_2ss20AG24AC	16.01	yes	up	0.00	yes	TRINITY_DN26040_c1_g5	2.45	yes	up	0.00	yes	.
gma-MIR5371-p5_1ss1AT : TRINITY_DN18944_c0_g1 : TRINITY_DN18944_c0_g1	gma-MIR5371-p5_1ss1AT	6.52	yes	up	0.00	yes	TRINITY_DN18944_c0_g1	2.61	yes	up	0.00	yes	carbohydrate transmembrane transport
gma-MIR5371-p5_1ss1AT : TRINITY_DN19384_c0_g1 : TRINITY_DN19384_c0_g1	gma-MIR5371-p5_1ss1AT	6.52	yes	up	0.00	yes	TRINITY_DN19384_c0_g1	3.82	yes	up	0.00	yes	isomerase activity;response to cytokinin;chloroplast
gma-MIR5371-p5_1ss1AT : TRINITY_DN23212_c1_g5 : TRINITY_DN23212_c1_g5	gma-MIR5371-p5_1ss1AT	6.52	yes	up	0.00	yes	TRINITY_DN23212_c1_g5	0.44	yes	down	0.00	yes	nucleus;cytoplasm
gra-MIR482d-p3_1 : TRINITY_DN17038_c0_g1 : TRINITY_DN17038_c0_g1	gra-MIR482d-p3_1	0.56	yes	down	0.04	yes	TRINITY_DN17038_c0_g1	4.41	yes	up	0.00	yes	transcription, DNA-templated;nucleus;positive regulation of transcriptio
gra-MIR482d-p3_1 : TRINITY_DN18030_c1_g4 : TRINITY_DN18030_c1_g4	gra-MIR482d-p3_1	0.56	yes	down	0.04	yes	TRINITY_DN18030_c1_g4	2.81	yes	up	0.01	yes	calcium ion binding;chloroplast thylakoid;electron transporter, transferri
gra-MIR482d-p3_1 : TRINITY_DN19185_c1_g6 : TRINITY_DN19185_c1_g6	gra-MIR482d-p3_1	0.56	yes	down	0.04	yes	TRINITY_DN19185_c1_g6	0.15	yes	down	0.00	yes	sterol metabolic process;brassinosteroid homeostasis;multicellular organis

gra-MIR482d-p3_1 : TRINITY_DN21060_c0_g1 : TRINITY_DN21060_c0_g1	gra-MIR482d-p3_1	0.56	yes	down	0.04	yes	TRINITY_DN21060_c0_g1	0.14	yes	down	0.00	yes	nucleus
gra-MIR482d-p3_1 : TRINITY_DN21948_c0_g1 : TRINITY_DN21948_c0_g1	gra-MIR482d-p3_1	0.56	yes	down	0.04	yes	TRINITY_DN21948_c0_g1	0.47	yes	down	0.00	yes	protein kinase activity;protein phosphorylation;ATP binding;plasmodes
gra-MIR482d-p3_1 : TRINITY_DN23055_c3_g1 : TRINITY_DN23055_c3_g1	gra-MIR482d-p3_1	0.56	yes	down	0.04	yes	TRINITY_DN23055_c3_g1	0.19	yes	down	0.00	yes	transcription regulatory region DNA binding;nucleus;regulation of transc
gra-MIR482d-p3_1 : TRINITY_DN23299_c1_g2 : TRINITY_DN23299_c1_g2	gra-MIR482d-p3_1	0.56	yes	down	0.04	yes	TRINITY_DN23299_c1_g2	2.07	yes	up	0.01	yes	plasma membrane;chloroplast;cytoplasm;endosome;calcium ion binding;
gra-MIR482d-p3_1 : TRINITY_DN23466_c1_g1 : TRINITY_DN23466_c1_g1	gra-MIR482d-p3_1	0.56	yes	down	0.04	yes	TRINITY_DN23466_c1_g1	0.14	yes	down	0.00	yes	regulation of transcription from RNA polymerase II promoter;RNA pol
gra-MIR482d-p3_1 : TRINITY_DN24856_c0_g1 : TRINITY_DN24856_c0_g1	gra-MIR482d-p3_1	0.56	yes	down	0.04	yes	TRINITY_DN24856_c0_g1	0.49	yes	down	0.01	yes	.
gra-MIR482d-p3_2 : TRINITY_DN17038_c0_g1 : TRINITY_DN17038_c0_g1	gra-MIR482d-p3_2	0.56	yes	down	0.04	yes	TRINITY_DN17038_c0_g1	4.41	yes	up	0.00	yes	transcription, DNA-templated;nucleus;positive regulation of transcriptio
gra-MIR482d-p3_2 : TRINITY_DN18030_c1_g4 : TRINITY_DN18030_c1_g4	gra-MIR482d-p3_2	0.56	yes	down	0.04	yes	TRINITY_DN18030_c1_g4	2.81	yes	up	0.01	yes	calcium ion binding;chloroplast thylakoid;electron transporter, transferri
gra-MIR482d-p3_2 : TRINITY_DN18145_c0_g1 : TRINITY_DN18145_c0_g1	gra-MIR482d-p3_2	0.56	yes	down	0.04	yes	TRINITY_DN18145_c0_g1	0.28	yes	down	0.00	yes	DNA recombination;DNA repair;Smc5-Smc6 complex;nucleus
gra-MIR482d-p3_2 : TRINITY_DN19185_c1_g6 : TRINITY_DN19185_c1_g6	gra-MIR482d-p3_2	0.56	yes	down	0.04	yes	TRINITY_DN19185_c1_g6	0.15	yes	down	0.00	yes	sterol metabolic process;brassinosteroid homeostasis;multicellular organis
gra-MIR482d-p3_2 : TRINITY_DN20607_c1_g6 : TRINITY_DN20607_c1_g6	gra-MIR482d-p3_2	0.56	yes	down	0.04	yes	TRINITY_DN20607_c1_g6	0.07	yes	down	0.00	yes	nucleus;anaerobic respiration
gra-MIR482d-p3_2 : TRINITY_DN21060_c0_g1 : TRINITY_DN21060_c0_g1	gra-MIR482d-p3_2	0.56	yes	down	0.04	yes	TRINITY_DN21060_c0_g1	0.14	yes	down	0.00	yes	nucleus
gra-MIR482d-p3_2 : TRINITY_DN21482_c0_g1 : TRINITY_DN21482_c0_g1	gra-MIR482d-p3_2	0.56	yes	down	0.04	yes	TRINITY_DN21482_c0_g1	5.76	yes	up	0.00	yes	peptidyl-prolyl cis-trans isomerase activity;endoplasmic reticulum memb
gra-MIR482d-p3_2 : TRINITY_DN21637_c0_g2 : TRINITY_DN21637_c0_g2	gra-MIR482d-p3_2	0.56	yes	down	0.04	yes	TRINITY_DN21637_c0_g2	0.46	yes	down	0.00	yes	DNA binding;lipid binding;nucleus;transcription, DNA-templated;protein
gra-MIR482d-p3_2 : TRINITY_DN21948_c0_g1 : TRINITY_DN21948_c0_g1	gra-MIR482d-p3_2	0.56	yes	down	0.04	yes	TRINITY_DN21948_c0_g1	0.47	yes	down	0.00	yes	protein kinase activity;protein phosphorylation;ATP binding;plasmodes
gra-MIR482d-p3_2 : TRINITY_DN22814_c0_g2 : TRINITY_DN22814_c0_g2	gra-MIR482d-p3_2	0.56	yes	down	0.04	yes	TRINITY_DN22814_c0_g2	3.29	yes	up	0.00	yes	nucleus
gra-MIR482d-p3_2 : TRINITY_DN23055_c3_g1 : TRINITY_DN23055_c3_g1	gra-MIR482d-p3_2	0.56	yes	down	0.04	yes	TRINITY_DN23055_c3_g1	0.19	yes	down	0.00	yes	transcription regulatory region DNA binding;nucleus;regulation of transc
gra-MIR482d-p3_2 : TRINITY_DN23299_c1_g2 : TRINITY_DN23299_c1_g2	gra-MIR482d-p3_2	0.56	yes	down	0.04	yes	TRINITY_DN23299_c1_g2	2.07	yes	up	0.01	yes	plasma membrane;chloroplast;cytoplasm;endosome;calcium ion binding;
gra-MIR482d-p3_2 : TRINITY_DN23466_c1_g1 : TRINITY_DN23466_c1_g1	gra-MIR482d-p3_2	0.56	yes	down	0.04	yes	TRINITY_DN23466_c1_g1	0.14	yes	down	0.00	yes	regulation of transcription from RNA polymerase II promoter;RNA pol
gra-MIR482d-p3_2 : TRINITY_DN23763_c0_g2 : TRINITY_DN23763_c0_g2	gra-MIR482d-p3_2	0.56	yes	down	0.04	yes	TRINITY_DN23763_c0_g2	0.26	yes	down	0.00	yes	transcription factor activity, sequence-specific DNA binding;protein bind
gra-MIR482d-p3_2 : TRINITY_DN24856_c0_g1 : TRINITY_DN24856_c0_g1	gra-MIR482d-p3_2	0.56	yes	down	0.04	yes	TRINITY_DN24856_c0_g1	0.49	yes	down	0.01	yes	.
gra-MIR482d-p3_2 : TRINITY_DN24894_c0_g6 : TRINITY_DN24894_c0_g6	gra-MIR482d-p3_2	0.56	yes	down	0.04	yes	TRINITY_DN24894_c0_g6	0.13	yes	down	0.01	yes	response to fungus;transcription factor activity, sequence-specific DNA

gra-MIR482d-p3_2 : TRINITY_DN25478_c0_g1 : TRINITY_DN25478_c0_g1	gra-MIR482d-p3_2	0.56	yes	down	0.04	yes	TRINITY_DN25478_c0_g1	4.72	yes	up	0.00	yes	chloroplast stroma;regulation of stomatal movement;carbonate dehydrat
gra-MIR482d-p3_2 : TRINITY_DN25927_c2_g1 : TRINITY_DN25927_c2_g1	gra-MIR482d-p3_2	0.56	yes	down	0.04	yes	TRINITY_DN25927_c2_g1	0.18	yes	down	0.00	yes	wax biosynthetic process;response to virus;defense response;nucleic acid
gra-MIR482d-p3_2 : TRINITY_DN26873_c0_g1 : TRINITY_DN26873_c0_g1	gra-MIR482d-p3_2	0.56	yes	down	0.04	yes	TRINITY_DN26873_c0_g1	0.16	yes	down	0.00	yes	protein binding;plasmodesma;integral component of membrane;intracell
gra-MIR482d-p3_3 : TRINITY_DN17038_c0_g1 : TRINITY_DN17038_c0_g1	gra-MIR482d-p3_3	0.56	yes	down	0.04	yes	TRINITY_DN17038_c0_g1	4.41	yes	up	0.00	yes	transcription, DNA-templated;nucleus;positive regulation of transcriptio
gra-MIR482d-p3_3 : TRINITY_DN18030_c1_g4 : TRINITY_DN18030_c1_g4	gra-MIR482d-p3_3	0.56	yes	down	0.04	yes	TRINITY_DN18030_c1_g4	2.81	yes	up	0.01	yes	calcium ion binding;chloroplast thylakoid;electron transporter, transferri
gra-MIR482d-p3_3 : TRINITY_DN21060_c0_g1 : TRINITY_DN21060_c0_g1	gra-MIR482d-p3_3	0.56	yes	down	0.04	yes	TRINITY_DN21060_c0_g1	0.14	yes	down	0.00	yes	nucleus
gra-MIR482d-p3_3 : TRINITY_DN21948_c0_g1 : TRINITY_DN21948_c0_g1	gra-MIR482d-p3_3	0.56	yes	down	0.04	yes	TRINITY_DN21948_c0_g1	0.47	yes	down	0.00	yes	protein kinase activity;protein phosphorylation;ATP binding;plasmodes
gra-MIR482d-p3_3 : TRINITY_DN23299_c1_g2 : TRINITY_DN23299_c1_g2	gra-MIR482d-p3_3	0.56	yes	down	0.04	yes	TRINITY_DN23299_c1_g2	2.07	yes	up	0.01	yes	plasma membrane;chloroplast;cytoplasm;endosome;calcium ion binding;
gra-MIR482d-p3_3 : TRINITY_DN23453_c0_g4 : TRINITY_DN23453_c0_g4	gra-MIR482d-p3_3	0.56	yes	down	0.04	yes	TRINITY_DN23453_c0_g4	2.53	yes	up	0.02	yes	mitochondrion;integral component of membrane
gra-MIR482d-p3_3 : TRINITY_DN23466_c1_g1 : TRINITY_DN23466_c1_g1	gra-MIR482d-p3_3	0.56	yes	down	0.04	yes	TRINITY_DN23466_c1_g1	0.14	yes	down	0.00	yes	regulation of transcription from RNA polymerase II promoter;RNA pol
gra-MIR482d-p3_3 : TRINITY_DN25478_c0_g1 : TRINITY_DN25478_c0_g1	gra-MIR482d-p3_3	0.56	yes	down	0.04	yes	TRINITY_DN25478_c0_g1	4.72	yes	up	0.00	yes	chloroplast stroma;regulation of stomatal movement;carbonate dehydrat
gra-MIR8658-p5_2ss15CA17AT : TRINITY_DN15062_c0_g2 : TRINITY_DN15062_c0_g2	gra-MIR8658-p5_2ss15CA17AT	13.28	yes	up	0.01	yes	TRINITY_DN15062_c0_g2	2.84	yes	up	0.00	yes	mitochondrion;regulation of production of siRNA involved in RNA inter
gra-MIR8658-p5_2ss15CA17AT : TRINITY_DN17241_c0_g2 : TRINITY_DN17241_c0_g2	gra-MIR8658-p5_2ss15CA17AT	13.28	yes	up	0.01	yes	TRINITY_DN17241_c0_g2	0.43	yes	down	0.00	yes	delta DNA polymerase complex;DNA synthesis involved in DNA repair;
gra-MIR8658-p5_2ss15CA17AT : TRINITY_DN19010_c0_g1 : TRINITY_DN19010_c0_g1	gra-MIR8658-p5_2ss15CA17AT	13.28	yes	up	0.01	yes	TRINITY_DN19010_c0_g1	2.61	yes	up	0.00	yes	chloroplast nucleoid;chloroplast organization;chloroplast;phosphorylati
gra-MIR8658-p5_2ss15CA17AT : TRINITY_DN20283_c0_g3 : TRINITY_DN20283_c0_g3	gra-MIR8658-p5_2ss15CA17AT	13.28	yes	up	0.01	yes	TRINITY_DN20283_c0_g3	0.13	yes	down	0.00	yes	integral component of membrane;plasma membrane
gra-MIR8658-p5_2ss15CA17AT : TRINITY_DN22145_c0_g3 : TRINITY_DN22145_c0_g3	gra-MIR8658-p5_2ss15CA17AT	13.28	yes	up	0.01	yes	TRINITY_DN22145_c0_g3	3.13	yes	up	0.00	yes	plastid
gra-MIR8658-p5_2ss15CA17AT : TRINITY_DN23481_c0_g1 : TRINITY_DN23481_c0_g1	gra-MIR8658-p5_2ss15CA17AT	13.28	yes	up	0.01	yes	TRINITY_DN23481_c0_g1	0.33	yes	down	0.00	yes	DNA binding;nuclear chromatin;chromatin silencing
gra-MIR8658-p5_2ss15CA17AT : TRINITY_DN23598_c0_g3 : TRINITY_DN23598_c0_g3	gra-MIR8658-p5_2ss15CA17AT	13.28	yes	up	0.01	yes	TRINITY_DN23598_c0_g3	0.45	yes	down	0.00	yes	nucleus
gra-MIR8658-p5_2ss15CA17AT : TRINITY_DN24249_c0_g1 : TRINITY_DN24249_c0_g1	gra-MIR8658-p5_2ss15CA17AT	13.28	yes	up	0.01	yes	TRINITY_DN24249_c0_g1	3.15	yes	up	0.03	yes	calcium-dependent cell-cell adhesion via plasma membrane cell adhesion
gra-MIR8674b-p3_2ss11TC18AC : TRINITY_DN16023_c0_g1 : TRINITY_DN16023_c0_g1	gra-MIR8674b-p3_2ss11TC18AC	0.08	yes	down	0.04	yes	TRINITY_DN16023_c0_g1	0.33	yes	down	0.02	yes	.
gra-MIR8674b-p3_2ss11TC18AC : TRINITY_DN17111_c0_g1 : TRINITY_DN17111_c0_g1	gra-MIR8674b-p3_2ss11TC18AC	0.08	yes	down	0.04	yes	TRINITY_DN17111_c0_g1	2.37	yes	up	0.00	yes	FAD biosynthetic process;kinase activity;chloroplast;FMN adenylyltrans
gra-MIR8674b-p3_2ss11TC18AC : TRINITY_DN18331_c0_g2 : TRINITY_DN18331_c0_g2	gra-MIR8674b-p3_2ss11TC18AC	0.08	yes	down	0.04	yes	TRINITY_DN18331_c0_g2	0.27	yes	down	0.01	yes	nucleus;multicellular organism development;reactive oxygen species met

gra-MIR8674b-p3_2ss11TC18AC : TRINITY_DN18785_c0_g1 : TRINITY_DN18785_c0_g1	gra-MIR8674b-p3_2ss11TC18AC	0.08	yes	down	0.04	yes	TRINITY_DN18785_c0_g1	2.50	yes	up	0.00	yes	plasma membrane;integral component of membrane
gra-MIR8674b-p3_2ss11TC18AC : TRINITY_DN20148_c0_g1 : TRINITY_DN20148_c0_g1	gra-MIR8674b-p3_2ss11TC18AC	0.08	yes	down	0.04	yes	TRINITY_DN20148_c0_g1	2.73	yes	up	0.00	yes	cell death;ribose-5-phosphate isomerase activity;vegetative to reproducti
gra-MIR8674b-p3_2ss11TC18AC : TRINITY_DN22903_c0_g1 : TRINITY_DN22903_c0_g1	gra-MIR8674b-p3_2ss11TC18AC	0.08	yes	down	0.04	yes	TRINITY_DN22903_c0_g1	2.87	yes	up	0.00	yes	de novo' pyrimidine nucleobase biosynthetic process;nucleoside metaboli
gra-MIR8674b-p3_2ss11TC18AC : TRINITY_DN23341_c1_g9 : TRINITY_DN23341_c1_g9	gra-MIR8674b-p3_2ss11TC18AC	0.08	yes	down	0.04	yes	TRINITY_DN23341_c1_g9	0.43	yes	down	0.01	yes	.
gra-MIR8674b-p3_2ss11TC18AC : TRINITY_DN23970_c1_g3 : TRINITY_DN23970_c1_g3	gra-MIR8674b-p3_2ss11TC18AC	0.08	yes	down	0.04	yes	TRINITY_DN23970_c1_g3	0.23	yes	down	0.00	yes	cytoplasm;transition metal ion binding;cellular transition metal ion hom
gra-MIR8674b-p3_2ss11TC18AC : TRINITY_DN24730_c0_g2 : TRINITY_DN24730_c0_g2	gra-MIR8674b-p3_2ss11TC18AC	0.08	yes	down	0.04	yes	TRINITY_DN24730_c0_g2	0.35	yes	down	0.00	yes	nucleus;Golgi apparatus;DNA binding;plasmodesma;plasma membrane;cy
gra-MIR8674b-p3_2ss11TC18AC : TRINITY_DN27054_c0_g2 : TRINITY_DN27054_c0_g2	gra-MIR8674b-p3_2ss11TC18AC	0.08	yes	down	0.04	yes	TRINITY_DN27054_c0_g2	3.30	yes	up	0.00	yes	chloroplast;chloroplast envelope;vacuolar membrane;P-P-bond-hydrolys
gra-MIR8674b-p5_2ss11TC18AC : TRINITY_DN16023_c0_g1 : TRINITY_DN16023_c0_g1	gra-MIR8674b-p5_2ss11TC18AC	0.08	yes	down	0.04	yes	TRINITY_DN16023_c0_g1	0.33	yes	down	0.02	yes	.
gra-MIR8674b-p5_2ss11TC18AC : TRINITY_DN17111_c0_g1 : TRINITY_DN17111_c0_g1	gra-MIR8674b-p5_2ss11TC18AC	0.08	yes	down	0.04	yes	TRINITY_DN17111_c0_g1	2.37	yes	up	0.00	yes	FAD biosynthetic process;kinase activity;chloroplast;FMN adenylyltrans
gra-MIR8674b-p5_2ss11TC18AC : TRINITY_DN18331_c0_g2 : TRINITY_DN18331_c0_g2	gra-MIR8674b-p5_2ss11TC18AC	0.08	yes	down	0.04	yes	TRINITY_DN18331_c0_g2	0.27	yes	down	0.01	yes	nucleus;multicellular organism development;reactive oxygen species met
gra-MIR8674b-p5_2ss11TC18AC : TRINITY_DN18785_c0_g1 : TRINITY_DN18785_c0_g1	gra-MIR8674b-p5_2ss11TC18AC	0.08	yes	down	0.04	yes	TRINITY_DN18785_c0_g1	2.50	yes	up	0.00	yes	plasma membrane;integral component of membrane
gra-MIR8674b-p5_2ss11TC18AC : TRINITY_DN20148_c0_g1 : TRINITY_DN20148_c0_g1	gra-MIR8674b-p5_2ss11TC18AC	0.08	yes	down	0.04	yes	TRINITY_DN20148_c0_g1	2.73	yes	up	0.00	yes	cell death;ribose-5-phosphate isomerase activity;vegetative to reproducti
gra-MIR8674b-p5_2ss11TC18AC : TRINITY_DN22903_c0_g1 : TRINITY_DN22903_c0_g1	gra-MIR8674b-p5_2ss11TC18AC	0.08	yes	down	0.04	yes	TRINITY_DN22903_c0_g1	2.87	yes	up	0.00	yes	de novo' pyrimidine nucleobase biosynthetic process;nucleoside metaboli
gra-MIR8674b-p5_2ss11TC18AC : TRINITY_DN23341_c1_g9 : TRINITY_DN23341_c1_g9	gra-MIR8674b-p5_2ss11TC18AC	0.08	yes	down	0.04	yes	TRINITY_DN23341_c1_g9	0.43	yes	down	0.01	yes	.
gra-MIR8674b-p5_2ss11TC18AC : TRINITY_DN23970_c1_g3 : TRINITY_DN23970_c1_g3	gra-MIR8674b-p5_2ss11TC18AC	0.08	yes	down	0.04	yes	TRINITY_DN23970_c1_g3	0.23	yes	down	0.00	yes	cytoplasm;transition metal ion binding;cellular transition metal ion hom
gra-MIR8674b-p5_2ss11TC18AC : TRINITY_DN24730_c0_g2 : TRINITY_DN24730_c0_g2	gra-MIR8674b-p5_2ss11TC18AC	0.08	yes	down	0.04	yes	TRINITY_DN24730_c0_g2	0.35	yes	down	0.00	yes	nucleus;Golgi apparatus;DNA binding;plasmodesma;plasma membrane;cy
gra-MIR8674b-p5_2ss11TC18AC : TRINITY_DN27054_c0_g2 : TRINITY_DN27054_c0_g2	gra-MIR8674b-p5_2ss11TC18AC	0.08	yes	down	0.04	yes	TRINITY_DN27054_c0_g2	3.30	yes	up	0.00	yes	chloroplast;chloroplast envelope;vacuolar membrane;P-P-bond-hydrolys
hbr-MIR6483-p3_1ss4TC : TRINITY_DN20452_c2_g1 : TRINITY_DN20452_c2_g1	hbr-MIR6483-p3_1ss4TC	2.61	yes	up	0.00	yes	TRINITY_DN20452_c2_g1	0.36	yes	down	0.04	yes	.
hbr-MIR6483-p3_1ss4TC : TRINITY_DN21332_c0_g1 : TRINITY_DN21332_c0_g1	hbr-MIR6483-p3_1ss4TC	2.61	yes	up	0.00	yes	TRINITY_DN21332_c0_g1	0.19	yes	down	0.00	yes	copper-transporting ATPase activity;intracellular membrane-bounded or
hbr-MIR6483-p3_1ss4TC : TRINITY_DN24207_c0_g2 : TRINITY_DN24207_c0_g2	hbr-MIR6483-p3_1ss4TC	2.61	yes	up	0.00	yes	TRINITY_DN24207_c0_g2	2.38	yes	up	0.02	yes	unfolded protein binding;heat shock protein binding;chloroplast
hbr-MIR6483-p3_1ss4TC : TRINITY_DN25711_c1_g3 : TRINITY_DN25711_c1_g3	hbr-MIR6483-p3_1ss4TC	2.61	yes	up	0.00	yes	TRINITY_DN25711_c1_g3	2.64	yes	up	0.05	yes	transcription factor activity, sequence-specific DNA binding;defense resp
hbr-MIR6483-p3_1ss4TC : TRINITY_DN25711_c1_g4 : TRINITY_DN25711_c1_g4	hbr-MIR6483-p3_1ss4TC	2.61	yes	up	0.00	yes	TRINITY_DN25711_c1_g4	0.17	yes	down	0.00	yes	cell division;response to chitin;transcription factor activity, sequence-sp

mes-miR399f : TRINITY_DN22148_c0_g1 : TRINITY_DN22148_c0_g1	mes-miR399f	4.22	yes	up	0.00	yes	TRINITY_DN22148_c0_g1	0.36	yes	down	0.00	yes	integral component of plasma membrane;substrate-specific transmembra
mtr-MIR156a-p3_2ss3GA21CT : TRINITY_DN19122_c0_g1 : TRINITY_DN19122_c0_g1	mtr-MIR156a-p3_2ss3GA21CT	inf	yes	up	0.00	yes	TRINITY_DN19122_c0_g1	0.22	yes	down	0.00	yes	transcription factor activity, sequence-specific DNA binding;defense resp
mtr-MIR156a-p3_2ss3GA21CT : TRINITY_DN20438_c0_g1 : TRINITY_DN20438_c0_g1	mtr-MIR156a-p3_2ss3GA21CT	inf	yes	up	0.00	yes	TRINITY_DN20438_c0_g1	0.18	yes	down	0.00	yes	protein metabolic process;nucleus;transcription, DNA-templated;hydrola
mtr-miR156e_R+2 : TRINITY_DN19122_c0_g1 : TRINITY_DN19122_c0_g1	mtr-miR156e_R+2	6.38	yes	up	0.02	yes	TRINITY_DN19122_c0_g1	0.22	yes	down	0.00	yes	transcription factor activity, sequence-specific DNA binding;defense resp
mtr-miR156e_R+2 : TRINITY_DN23413_c0_g1 : TRINITY_DN23413_c0_g1	mtr-miR156e_R+2	6.38	yes	up	0.02	yes	TRINITY_DN23413_c0_g1	0.33	yes	down	0.00	yes	transcription factor activity, sequence-specific DNA binding;anther deve
mtr-miR156e_R+2 : TRINITY_DN24312_c1_g1 : TRINITY_DN24312_c1_g1	mtr-miR156e_R+2	6.38	yes	up	0.02	yes	TRINITY_DN24312_c1_g1	0.44	yes	down	0.00	yes	regulation of transcription, DNA-templated;nucleus;transcription, DNA-
mtr-MIR2630x-p3_1ss8TC : TRINITY_DN21752_c0_g1 : TRINITY_DN21752_c0_g1	mtr-MIR2630x-p3_1ss8TC	3.93	yes	up	0.01	yes	TRINITY_DN21752_c0_g1	0.28	yes	down	0.03	yes	polysaccharide binding;hydrolase activity, hydrolyzing O-glycosyl comp
mtr-MIR2630x-p3_1ss8TC : TRINITY_DN23822_c0_g2 : TRINITY_DN23822_c0_g2	mtr-MIR2630x-p3_1ss8TC	3.93	yes	up	0.01	yes	TRINITY_DN23822_c0_g2	2.87	yes	up	0.00	yes	.
mtr-MIR2630x-p3_1ss8TC : TRINITY_DN24253_c0_g4 : TRINITY_DN24253_c0_g4	mtr-MIR2630x-p3_1ss8TC	3.93	yes	up	0.01	yes	TRINITY_DN24253_c0_g4	3.00	yes	up	0.00	yes	plasmodesma;protein phosphorylation;defense response;protein serine/t
mtr-MIR2630x-p3_1ss8TC : TRINITY_DN24278_c3_g2 : TRINITY_DN24278_c3_g2	mtr-MIR2630x-p3_1ss8TC	3.93	yes	up	0.01	yes	TRINITY_DN24278_c3_g2	2.47	yes	up	0.00	yes	chloroplastic endopeptidase Clp complex;chloroplast;chloroplast envelo
mtr-MIR2630x-p3_1ss8TC : TRINITY_DN25890_c0_g2 : TRINITY_DN25890_c0_g2	mtr-MIR2630x-p3_1ss8TC	3.93	yes	up	0.01	yes	TRINITY_DN25890_c0_g2	0.47	yes	down	0.01	yes	integral component of membrane;protein phosphorylation;ATP binding;
mtr-MIR2630x-p3_1ss8TC : TRINITY_DN26347_c0_g1 : TRINITY_DN26347_c0_g1	mtr-MIR2630x-p3_1ss8TC	3.93	yes	up	0.01	yes	TRINITY_DN26347_c0_g1	3.00	yes	up	0.00	yes	protein serine/threonine/tyrosine kinase activity;sugar mediated signaling
mtr-MIR2630x-p3_1ss8TC : TRINITY_DN27166_c1_g2 : TRINITY_DN27166_c1_g2	mtr-MIR2630x-p3_1ss8TC	3.93	yes	up	0.01	yes	TRINITY_DN27166_c1_g2	0.28	yes	down	0.00	yes	regulation of cell division;metal ion binding;nucleus;transcription factor
mtr-MIR2630x-p3_1ss8TC : TRINITY_DN27680_c0_g1 : TRINITY_DN27680_c0_g1	mtr-MIR2630x-p3_1ss8TC	3.93	yes	up	0.01	yes	TRINITY_DN27680_c0_g1	0.50	yes	down	0.00	yes	vacuole;cytoplasm
mtr-MIR396a-p5_1ss18AT : TRINITY_DN17945_c0_g2 : TRINITY_DN17945_c0_g2	mtr-MIR396a-p5_1ss18AT	6.14	yes	up	0.02	yes	TRINITY_DN17945_c0_g2	2.36	yes	up	0.04	yes	mitochondrion;oxidation-reduction process;prephenate dehydrogenase a
mtr-MIR396a-p5_1ss18AT : TRINITY_DN21717_c0_g1 : TRINITY_DN21717_c0_g1	mtr-MIR396a-p5_1ss18AT	6.14	yes	up	0.02	yes	TRINITY_DN21717_c0_g1	3.11	yes	up	0.00	yes	integral component of membrane;chloroplast
mtr-MIR396a-p5_1ss18AT : TRINITY_DN21920_c1_g4 : TRINITY_DN21920_c1_g4	mtr-MIR396a-p5_1ss18AT	6.14	yes	up	0.02	yes	TRINITY_DN21920_c1_g4	0.12	yes	down	0.00	yes	regulation of transcription, DNA-templated;transcription factor activity
mtr-MIR396a-p5_1ss18AT : TRINITY_DN21940_c0_g1 : TRINITY_DN21940_c0_g1	mtr-MIR396a-p5_1ss18AT	6.14	yes	up	0.02	yes	TRINITY_DN21940_c0_g1	0.30	yes	down	0.00	yes	acyl-CoA hydrolase activity;response to other organism;lipid catabolic p
mtr-MIR396a-p5_1ss18AT : TRINITY_DN22143_c0_g2 : TRINITY_DN22143_c0_g2	mtr-MIR396a-p5_1ss18AT	6.14	yes	up	0.02	yes	TRINITY_DN22143_c0_g2	0.22	yes	down	0.00	yes	nucleus
mtr-MIR396a-p5_1ss18AT : TRINITY_DN23720_c0_g1 : TRINITY_DN23720_c0_g1	mtr-MIR396a-p5_1ss18AT	6.14	yes	up	0.02	yes	TRINITY_DN23720_c0_g1	2.97	yes	up	0.00	yes	malate dehydrogenase (NADP+) activity;L-malate dehydrogenase activit
mtr-MIR396a-p5_1ss18AT : TRINITY_DN25497_c1_g1 : TRINITY_DN25497_c1_g1	mtr-MIR396a-p5_1ss18AT	6.14	yes	up	0.02	yes	TRINITY_DN25497_c1_g1	0.33	yes	down	0.02	yes	somatic embryogenesis;extracellular region;defense response;polysacchar
mtr-MIR396a-p5_1ss18AT : TRINITY_DN27662_c5_g1 : TRINITY_DN27662_c5_g1	mtr-MIR396a-p5_1ss18AT	6.14	yes	up	0.02	yes	TRINITY_DN27662_c5_g1	4.30	yes	up	0.00	yes	enzyme binding;protein binding;sulfate transport;integral component of

nta-miR172d_R+1 : TRINITY_DN16267_c1_g1 : TRINITY_DN16267_c1_g1	nta-miR172d_R+1	0.08	yes	down	0.00	yes	TRINITY_DN16267_c1_g1	0.42	yes	down	0.00	yes	.
nta-miR172d_R+1 : TRINITY_DN20593_c0_g1 : TRINITY_DN20593_c0_g1	nta-miR172d_R+1	0.08	yes	down	0.00	yes	TRINITY_DN20593_c0_g1	3.40	yes	up	0.00	yes	cadmium ion transmembrane transport;cellular metal ion homeostasis;iro
nta-miR172d_R+1 : TRINITY_DN26802_c1_g1 : TRINITY_DN26802_c1_g1	nta-miR172d_R+1	0.08	yes	down	0.00	yes	TRINITY_DN26802_c1_g1	4.16	yes	up	0.00	yes	transcription factor activity, sequence-specific DNA binding;seed develo
ppe-MIR858-p5 : TRINITY_DN16185_c1_g1 : TRINITY_DN16185_c1_g1	ppe-MIR858-p5	2.44	yes	up	0.00	yes	TRINITY_DN16185_c1_g1	3.16	yes	up	0.00	yes	transcription factor activity, sequence-specific DNA binding;regulation o
ppe-MIR858-p5 : TRINITY_DN18139_c0_g1 : TRINITY_DN18139_c0_g1	ppe-MIR858-p5	2.44	yes	up	0.00	yes	TRINITY_DN18139_c0_g1	0.03	yes	down	0.00	yes	transcription factor activity, sequence-specific DNA binding;response to
ppe-MIR858-p5 : TRINITY_DN18264_c0_g1 : TRINITY_DN18264_c0_g1	ppe-MIR858-p5	2.44	yes	up	0.00	yes	TRINITY_DN18264_c0_g1	0.10	yes	down	0.00	yes	response to salt stress;transcription factor activity, sequence-specific DN
ppe-MIR858-p5 : TRINITY_DN19912_c0_g3 : TRINITY_DN19912_c0_g3	ppe-MIR858-p5	2.44	yes	up	0.00	yes	TRINITY_DN19912_c0_g3	0.11	yes	down	0.00	yes	DNA binding;response to salicylic acid;regulation of flower development;
ppe-MIR858-p5 : TRINITY_DN19912_c1_g2 : TRINITY_DN19912_c1_g2	ppe-MIR858-p5	2.44	yes	up	0.00	yes	TRINITY_DN19912_c1_g2	0.05	yes	down	0.00	yes	transcription factor activity, sequence-specific DNA binding;response to
ppe-MIR858-p5 : TRINITY_DN20475_c0_g2 : TRINITY_DN20475_c0_g2	ppe-MIR858-p5	2.44	yes	up	0.00	yes	TRINITY_DN20475_c0_g2	0.05	yes	down	0.00	yes	transcription factor activity, sequence-specific DNA binding;regulation o
ppe-MIR858-p5 : TRINITY_DN24894_c0_g6 : TRINITY_DN24894_c0_g6	ppe-MIR858-p5	2.44	yes	up	0.00	yes	TRINITY_DN24894_c0_g6	0.13	yes	down	0.01	yes	response to fungus;transcription factor activity, sequence-specific DNA
ptc-miR1447_1ss21TC : TRINITY_DN20227_c0_g1 : TRINITY_DN20227_c0_g1	ptc-miR1447_1ss21TC	2.09	yes	up	0.00	yes	TRINITY_DN20227_c0_g1	0.37	yes	down	0.05	yes	malate synthase activity;cytoplasm;glyoxylate cycle
ptc-miR1447_1ss21TC : TRINITY_DN26873_c0_g1 : TRINITY_DN26873_c0_g1	ptc-miR1447_1ss21TC	2.09	yes	up	0.00	yes	TRINITY_DN26873_c0_g1	0.16	yes	down	0.00	yes	protein binding;plasmodesma;integral component of membrane;intracell
ptc-MIR1449-p3_2ss19TC23AC : TRINITY_DN24348_c0_g3 : TRINITY_DN24348_c0_g3	ptc-MIR1449-p3_2ss19TC23AC	0.16	yes	down	0.02	yes	TRINITY_DN24348_c0_g3	0.19	yes	down	0.00	yes	serine-type carboxypeptidase activity;proteolysis involved in cellular pr
ptc-MIR1449-p5_2ss19TC23AC : TRINITY_DN24348_c0_g3 : TRINITY_DN24348_c0_g3	ptc-MIR1449-p5_2ss19TC23AC	0.16	yes	down	0.02	yes	TRINITY_DN24348_c0_g3	0.19	yes	down	0.00	yes	serine-type carboxypeptidase activity;proteolysis involved in cellular pr
ptc-MIR1449-p5_2ss7TC18TC : TRINITY_DN16897_c0_g1 : TRINITY_DN16897_c0_g1	ptc-MIR1449-p5_2ss7TC18TC	0.16	yes	down	0.02	yes	TRINITY_DN16897_c0_g1	2.04	yes	up	0.02	yes	polygalacturonate 4-alpha-galacturonosyltransferase activity;Golgi mem
ptc-MIR1449-p5_2ss7TC18TC : TRINITY_DN22171_c2_g1 : TRINITY_DN22171_c2_g1	ptc-MIR1449-p5_2ss7TC18TC	0.16	yes	down	0.02	yes	TRINITY_DN22171_c2_g1	2.36	yes	up	0.01	yes	cytosol;methionine adenosyltransferase activity
ptc-MIR1449-p5_2ss7TC18TC : TRINITY_DN24348_c0_g3 : TRINITY_DN24348_c0_g3	ptc-MIR1449-p5_2ss7TC18TC	0.16	yes	down	0.02	yes	TRINITY_DN24348_c0_g3	0.19	yes	down	0.00	yes	serine-type carboxypeptidase activity;proteolysis involved in cellular pr
ptc-MIR1449-p5_2ss7TC18TC : TRINITY_DN24626_c0_g1 : TRINITY_DN24626_c0_g1	ptc-MIR1449-p5_2ss7TC18TC	0.16	yes	down	0.02	yes	TRINITY_DN24626_c0_g1	0.22	yes	down	0.00	yes	flavonoid glucuronidation;quercetin 3-O-glucosyltransferase activity;intr
ptc-MIR1449-p5_2ss7TC18TC : TRINITY_DN25286_c1_g1 : TRINITY_DN25286_c1_g1	ptc-MIR1449-p5_2ss7TC18TC	0.16	yes	down	0.02	yes	TRINITY_DN25286_c1_g1	0.24	yes	down	0.00	yes	cilium;protein localization to cilium;phosphatidylinositol binding
ptc-miR1450_R-4 : TRINITY_DN14423_c0_g1 : TRINITY_DN14423_c0_g1	ptc-miR1450_R-4	0.62	yes	down	0.00	yes	TRINITY_DN14423_c0_g1	3.93	yes	up	0.00	yes	serine-type endopeptidase activity;cell wall
ptc-miR1450_R-4 : TRINITY_DN16778_c0_g1 : TRINITY_DN16778_c0_g1	ptc-miR1450_R-4	0.62	yes	down	0.00	yes	TRINITY_DN16778_c0_g1	2.04	yes	up	0.01	yes	integral component of membrane;chloroplast
ptc-miR1450_R-4 : TRINITY_DN22683_c0_g1 : TRINITY_DN22683_c0_g1	ptc-miR1450_R-4	0.62	yes	down	0.00	yes	TRINITY_DN22683_c0_g1	2.71	yes	up	0.01	yes	mannan catabolic process;mannan endo-1,4-beta-mannosidase activity

ptc-miR1450_R-4 : TRINITY_DN23592_c1_g1 : TRINITY_DN23592_c1_g1	ptc-miR1450_R-4	0.62	yes	down	0.00	yes	TRINITY_DN23592_c1_g1	2.92	yes	up	0.00	yes	drug transmembrane transporter activity;drug transmembrane transport
ptc-miR1450_R-4 : TRINITY_DN27506_c0_g1 : TRINITY_DN27506_c0_g1	ptc-miR1450_R-4	0.62	yes	down	0.00	yes	TRINITY_DN27506_c0_g1	2.89	yes	up	0.00	yes	plastid;protein import into chloroplast stroma;integral component of m
ptc-miR156a : TRINITY_DN17879_c0_g2 : TRINITY_DN17879_c0_g2	ptc-miR156a	107.82	yes	up	0.00	yes	TRINITY_DN17879_c0_g2	2.40	yes	up	0.00	yes	membrane;mitochondrion
ptc-miR156a : TRINITY_DN19122_c0_g1 : TRINITY_DN19122_c0_g1	ptc-miR156a	107.82	yes	up	0.00	yes	TRINITY_DN19122_c0_g1	0.22	yes	down	0.00	yes	transcription factor activity, sequence-specific DNA binding;defense resp
ptc-miR156a : TRINITY_DN19850_c1_g2 : TRINITY_DN19850_c1_g2	ptc-miR156a	107.82	yes	up	0.00	yes	TRINITY_DN19850_c1_g2	0.13	yes	down	0.00	yes	metal ion binding;transcription factor activity, sequence-specific DNA bi
ptc-miR156a : TRINITY_DN19850_c1_g3 : TRINITY_DN19850_c1_g3	ptc-miR156a	107.82	yes	up	0.00	yes	TRINITY_DN19850_c1_g3	0.03	yes	down	0.00	yes	flower development;cytoplasm;DNA binding;transcription, DNA-templa
ptc-miR156a : TRINITY_DN19850_c1_g4 : TRINITY_DN19850_c1_g4	ptc-miR156a	107.82	yes	up	0.00	yes	TRINITY_DN19850_c1_g4	0.18	yes	down	0.00	yes	transcription factor activity, sequence-specific DNA binding;defense resp
ptc-miR156a : TRINITY_DN20299_c0_g1 : TRINITY_DN20299_c0_g1	ptc-miR156a	107.82	yes	up	0.00	yes	TRINITY_DN20299_c0_g1	0.40	yes	down	0.00	yes	integral component of membrane
ptc-miR156a : TRINITY_DN21154_c0_g2 : TRINITY_DN21154_c0_g2	ptc-miR156a	107.82	yes	up	0.00	yes	TRINITY_DN21154_c0_g2	0.41	yes	down	0.01	yes	regulation of transcription, DNA-templated;anther development;transcri
ptc-miR156a : TRINITY_DN22967_c0_g1 : TRINITY_DN22967_c0_g1	ptc-miR156a	107.82	yes	up	0.00	yes	TRINITY_DN22967_c0_g1	0.42	yes	down	0.00	yes	transcription factor activity, sequence-specific DNA binding;anther deve
ptc-miR156a : TRINITY_DN23287_c0_g1 : TRINITY_DN23287_c0_g1	ptc-miR156a	107.82	yes	up	0.00	yes	TRINITY_DN23287_c0_g1	2.12	yes	up	0.00	yes	cytosol;UDP-N-acetylglucosamine diphosphorylase activity;UDP-N-ace
ptc-miR156a : TRINITY_DN23412_c1_g9 : TRINITY_DN23412_c1_g9	ptc-miR156a	107.82	yes	up	0.00	yes	TRINITY_DN23412_c1_g9	0.03	yes	down	0.00	yes	regulation of transcription, DNA-templated;cell differentiation;positive
ptc-miR156a : TRINITY_DN23413_c0_g1 : TRINITY_DN23413_c0_g1	ptc-miR156a	107.82	yes	up	0.00	yes	TRINITY_DN23413_c0_g1	0.33	yes	down	0.00	yes	transcription factor activity, sequence-specific DNA binding;anther deve
ptc-miR156a : TRINITY_DN24312_c1_g1 : TRINITY_DN24312_c1_g1	ptc-miR156a	107.82	yes	up	0.00	yes	TRINITY_DN24312_c1_g1	0.44	yes	down	0.00	yes	regulation of transcription, DNA-templated;nucleus;transcription, DNA-
ptc-miR156a_R+1 : TRINITY_DN19122_c0_g1 : TRINITY_DN19122_c0_g1	ptc-miR156a_R+1	147.50	yes	up	0.02	yes	TRINITY_DN19122_c0_g1	0.22	yes	down	0.00	yes	transcription factor activity, sequence-specific DNA binding;defense resp
ptc-miR156a_R+1 : TRINITY_DN19850_c1_g2 : TRINITY_DN19850_c1_g2	ptc-miR156a_R+1	147.50	yes	up	0.02	yes	TRINITY_DN19850_c1_g2	0.13	yes	down	0.00	yes	metal ion binding;transcription factor activity, sequence-specific DNA bi
ptc-miR156a_R+1 : TRINITY_DN19850_c1_g3 : TRINITY_DN19850_c1_g3	ptc-miR156a_R+1	147.50	yes	up	0.02	yes	TRINITY_DN19850_c1_g3	0.03	yes	down	0.00	yes	flower development;cytoplasm;DNA binding;transcription, DNA-templa
ptc-miR156a_R+1 : TRINITY_DN19850_c1_g4 : TRINITY_DN19850_c1_g4	ptc-miR156a_R+1	147.50	yes	up	0.02	yes	TRINITY_DN19850_c1_g4	0.18	yes	down	0.00	yes	transcription factor activity, sequence-specific DNA binding;defense resp
ptc-miR156a_R+1 : TRINITY_DN21154_c0_g2 : TRINITY_DN21154_c0_g2	ptc-miR156a_R+1	147.50	yes	up	0.02	yes	TRINITY_DN21154_c0_g2	0.41	yes	down	0.01	yes	regulation of transcription, DNA-templated;anther development;transcri
ptc-miR156a_R+1 : TRINITY_DN22967_c0_g1 : TRINITY_DN22967_c0_g1	ptc-miR156a_R+1	147.50	yes	up	0.02	yes	TRINITY_DN22967_c0_g1	0.42	yes	down	0.00	yes	transcription factor activity, sequence-specific DNA binding;anther deve
ptc-miR156a_R+1 : TRINITY_DN23412_c1_g9 : TRINITY_DN23412_c1_g9	ptc-miR156a_R+1	147.50	yes	up	0.02	yes	TRINITY_DN23412_c1_g9	0.03	yes	down	0.00	yes	regulation of transcription, DNA-templated;cell differentiation;positive
ptc-miR156a_R+1 : TRINITY_DN23413_c0_g1 : TRINITY_DN23413_c0_g1	ptc-miR156a_R+1	147.50	yes	up	0.02	yes	TRINITY_DN23413_c0_g1	0.33	yes	down	0.00	yes	transcription factor activity, sequence-specific DNA binding;anther deve

ptc-miR156a_R+1 : TRINITY_DN24312_c1_g1 : TRINITY_DN24312_c1_g1	ptc-miR156a_R+1	147.50	yes	up	0.02	yes	TRINITY_DN24312_c1_g1	0.44	yes	down	0.00	yes	regulation of transcription, DNA-templated;nucleus;transcription, DNA-
ptc-miR156g : TRINITY_DN15260_c0_g1 : TRINITY_DN15260_c0_g1	ptc-miR156g	4.26	yes	up	0.00	yes	TRINITY_DN15260_c0_g1	0.28	yes	down	0.04	yes	.
ptc-miR156g : TRINITY_DN19122_c0_g1 : TRINITY_DN19122_c0_g1	ptc-miR156g	4.26	yes	up	0.00	yes	TRINITY_DN19122_c0_g1	0.22	yes	down	0.00	yes	transcription factor activity, sequence-specific DNA binding;defense resp
ptc-miR156g : TRINITY_DN19850_c1_g2 : TRINITY_DN19850_c1_g2	ptc-miR156g	4.26	yes	up	0.00	yes	TRINITY_DN19850_c1_g2	0.13	yes	down	0.00	yes	metal ion binding;transcription factor activity, sequence-specific DNA bi
ptc-miR156g : TRINITY_DN19850_c1_g3 : TRINITY_DN19850_c1_g3	ptc-miR156g	4.26	yes	up	0.00	yes	TRINITY_DN19850_c1_g3	0.03	yes	down	0.00	yes	flower development;cytoplasm;DNA binding;transcription, DNA-templa
ptc-miR156g : TRINITY_DN19850_c1_g4 : TRINITY_DN19850_c1_g4	ptc-miR156g	4.26	yes	up	0.00	yes	TRINITY_DN19850_c1_g4	0.18	yes	down	0.00	yes	transcription factor activity, sequence-specific DNA binding;defense resp
ptc-miR156g : TRINITY_DN21154_c0_g2 : TRINITY_DN21154_c0_g2	ptc-miR156g	4.26	yes	up	0.00	yes	TRINITY_DN21154_c0_g2	0.41	yes	down	0.01	yes	regulation of transcription, DNA-templated;anther development;transcri
ptc-miR156g : TRINITY_DN22967_c0_g1 : TRINITY_DN22967_c0_g1	ptc-miR156g	4.26	yes	up	0.00	yes	TRINITY_DN22967_c0_g1	0.42	yes	down	0.00	yes	transcription factor activity, sequence-specific DNA binding;anther deve
ptc-miR156g : TRINITY_DN23412_c1_g9 : TRINITY_DN23412_c1_g9	ptc-miR156g	4.26	yes	up	0.00	yes	TRINITY_DN23412_c1_g9	0.03	yes	down	0.00	yes	regulation of transcription, DNA-templated;cell differentiation;positive
ptc-miR156g : TRINITY_DN23413_c0_g1 : TRINITY_DN23413_c0_g1	ptc-miR156g	4.26	yes	up	0.00	yes	TRINITY_DN23413_c0_g1	0.33	yes	down	0.00	yes	transcription factor activity, sequence-specific DNA binding;anther deve
ptc-miR156g : TRINITY_DN24312_c1_g1 : TRINITY_DN24312_c1_g1	ptc-miR156g	4.26	yes	up	0.00	yes	TRINITY_DN24312_c1_g1	0.44	yes	down	0.00	yes	regulation of transcription, DNA-templated;nucleus;transcription, DNA-
ptc-miR156l_2ss13GA20AG : TRINITY_DN10911_c0_g1 : TRINITY_DN10911_c0_g1	ptc-miR156l_2ss13GA20AG	5.25	yes	up	0.01	yes	TRINITY_DN10911_c0_g1	0.01	yes	down	0.00	yes	plasma membrane;integral component of membrane
ptc-miR156l_2ss13GA20AG : TRINITY_DN16894_c0_g3 : TRINITY_DN16894_c0_g3	ptc-miR156l_2ss13GA20AG	5.25	yes	up	0.01	yes	TRINITY_DN16894_c0_g3	0.39	yes	down	0.02	yes	transcription, DNA-templated;nucleus;negative regulation of transcriptio
ptc-miR156l_2ss13GA20AG : TRINITY_DN19122_c0_g1 : TRINITY_DN19122_c0_g1	ptc-miR156l_2ss13GA20AG	5.25	yes	up	0.01	yes	TRINITY_DN19122_c0_g1	0.22	yes	down	0.00	yes	transcription factor activity, sequence-specific DNA binding;defense resp
ptc-miR156l_2ss13GA20AG : TRINITY_DN21154_c0_g2 : TRINITY_DN21154_c0_g2	ptc-miR156l_2ss13GA20AG	5.25	yes	up	0.01	yes	TRINITY_DN21154_c0_g2	0.41	yes	down	0.01	yes	regulation of transcription, DNA-templated;anther development;transcri
ptc-miR156l_2ss13GA20AG : TRINITY_DN22967_c0_g1 : TRINITY_DN22967_c0_g1	ptc-miR156l_2ss13GA20AG	5.25	yes	up	0.01	yes	TRINITY_DN22967_c0_g1	0.42	yes	down	0.00	yes	transcription factor activity, sequence-specific DNA binding;anther deve
ptc-miR156l_2ss13GA20AG : TRINITY_DN23412_c1_g9 : TRINITY_DN23412_c1_g9	ptc-miR156l_2ss13GA20AG	5.25	yes	up	0.01	yes	TRINITY_DN23412_c1_g9	0.03	yes	down	0.00	yes	regulation of transcription, DNA-templated;cell differentiation;positive
ptc-miR156l_2ss13GA20AG : TRINITY_DN23413_c0_g1 : TRINITY_DN23413_c0_g1	ptc-miR156l_2ss13GA20AG	5.25	yes	up	0.01	yes	TRINITY_DN23413_c0_g1	0.33	yes	down	0.00	yes	transcription factor activity, sequence-specific DNA binding;anther deve
ptc-miR156l_2ss13GA20AG : TRINITY_DN24312_c1_g1 : TRINITY_DN24312_c1_g1	ptc-miR156l_2ss13GA20AG	5.25	yes	up	0.01	yes	TRINITY_DN24312_c1_g1	0.44	yes	down	0.00	yes	regulation of transcription, DNA-templated;nucleus;transcription, DNA-
ptc-miR159a_1ss21AT : TRINITY_DN17552_c1_g1 : TRINITY_DN17552_c1_g1	ptc-miR159a_1ss21AT	0.59	yes	down	0.04	yes	TRINITY_DN17552_c1_g1	4.93	yes	up	0.01	yes	RNA binding;endoribonuclease activity;ribonuclease activity;ribonuclease
ptc-miR159a_1ss21AT : TRINITY_DN17636_c0_g2 : TRINITY_DN17636_c0_g2	ptc-miR159a_1ss21AT	0.59	yes	down	0.04	yes	TRINITY_DN17636_c0_g2	0.23	yes	down	0.00	yes	.
ptc-miR159d_L-1R-1 : TRINITY_DN23142_c2_g1 : TRINITY_DN23142_c2_g1	ptc-miR159d_L-1R-1	0.43	yes	down	0.04	yes	TRINITY_DN23142_c2_g1	0.39	yes	down	0.00	yes	nucleus;transcription factor activity, sequence-specific DNA binding;sequ

ptc-miR159d_L-1R-1 : TRINITY_DN23533_c0_g1 : TRINITY_DN23533_c0_g1	ptc-miR159d_L-1R-1	0.43	yes	down	0.04	yes	TRINITY_DN23533_c0_g1	2.94	yes	up	0.00	yes	rRNA base methylation;rRNA methyltransferase activity;tRNA methylt
ptc-miR159d_L-1R-1 : TRINITY_DN25793_c0_g2 : TRINITY_DN25793_c0_g2	ptc-miR159d_L-1R-1	0.43	yes	down	0.04	yes	TRINITY_DN25793_c0_g2	5.69	yes	up	0.00	yes	nucleus;transcription, DNA-templated;DNA binding;response to cadmiu
ptc-miR160a : TRINITY_DN21540_c0_g1 : TRINITY_DN21540_c0_g1	ptc-miR160a	0.24	yes	down	0.05	yes	TRINITY_DN21540_c0_g1	0.29	yes	down	0.00	yes	miRNA binding;cell division;transcription factor activity, sequence-speci
ptc-miR160a_1ss21AT : TRINITY_DN21540_c0_g1 : TRINITY_DN21540_c0_g1	ptc-miR160a_1ss21AT	0.33	yes	down	0.03	yes	TRINITY_DN21540_c0_g1	0.29	yes	down	0.00	yes	miRNA binding;cell division;transcription factor activity, sequence-speci
ptc-miR160a_R+1 : TRINITY_DN21540_c0_g1 : TRINITY_DN21540_c0_g1	ptc-miR160a_R+1	0.41	yes	down	0.01	yes	TRINITY_DN21540_c0_g1	0.29	yes	down	0.00	yes	miRNA binding;cell division;transcription factor activity, sequence-speci
ptc-miR160g_2ss15GT21AG : TRINITY_DN21540_c0_g1 : TRINITY_DN21540_c0_g1	ptc-miR160g_2ss15GT21AG	0.24	yes	down	0.05	yes	TRINITY_DN21540_c0_g1	0.29	yes	down	0.00	yes	miRNA binding;cell division;transcription factor activity, sequence-speci
ptc-miR160h : TRINITY_DN21540_c0_g1 : TRINITY_DN21540_c0_g1	ptc-miR160h	2.77	yes	up	0.02	yes	TRINITY_DN21540_c0_g1	0.29	yes	down	0.00	yes	miRNA binding;cell division;transcription factor activity, sequence-speci
ptc-MIR164e-p3 : TRINITY_DN20771_c4_g1 : TRINITY_DN20771_c4_g1	ptc-MIR164e-p3	2.36	yes	up	0.00	yes	TRINITY_DN20771_c4_g1	0.21	yes	down	0.00	yes	root hair cell differentiation;response to stress
ptc-MIR164e-p3 : TRINITY_DN25978_c0_g1 : TRINITY_DN25978_c0_g1	ptc-MIR164e-p3	2.36	yes	up	0.00	yes	TRINITY_DN25978_c0_g1	0.38	yes	down	0.00	yes	embryo development ending in seed dormancy;DNA binding;transcriptio
ptc-MIR166e-p5 : TRINITY_DN26613_c0_g1 : TRINITY_DN26613_c0_g1	ptc-MIR166e-p5	2.25	yes	up	0.03	yes	TRINITY_DN26613_c0_g1	0.29	yes	down	0.00	yes	plasma membrane
ptc-miR169a : TRINITY_DN21854_c0_g2 : TRINITY_DN21854_c0_g2	ptc-miR169a	2.94	yes	up	0.01	yes	TRINITY_DN21854_c0_g2	2.78	yes	up	0.00	yes	integral component of membrane;nucleus;GTP binding;ferrous iron trans
ptc-miR169a : TRINITY_DN22888_c0_g3 : TRINITY_DN22888_c0_g3	ptc-miR169a	2.94	yes	up	0.01	yes	TRINITY_DN22888_c0_g3	2.28	yes	up	0.01	yes	regulation of transcription, DNA-templated;transcription factor activity
ptc-miR169a : TRINITY_DN23063_c0_g3 : TRINITY_DN23063_c0_g3	ptc-miR169a	2.94	yes	up	0.01	yes	TRINITY_DN23063_c0_g3	0.39	yes	down	0.00	yes	DNA binding;embryo development ending in seed dormancy;nucleus;tran
ptc-miR169a : TRINITY_DN25978_c0_g1 : TRINITY_DN25978_c0_g1	ptc-miR169a	2.94	yes	up	0.01	yes	TRINITY_DN25978_c0_g1	0.38	yes	down	0.00	yes	embryo development ending in seed dormancy;DNA binding;transcriptio
ptc-miR169i_1ss15TA : TRINITY_DN14474_c0_g3 : TRINITY_DN14474_c0_g3	ptc-miR169i_1ss15TA	1.54	yes	up	0.03	yes	TRINITY_DN14474_c0_g3	0.20	yes	down	0.00	yes	cytoplasm;nucleus;plasma membrane;protein serine/threonine kinase acti
ptc-miR169i_1ss15TA : TRINITY_DN22888_c0_g3 : TRINITY_DN22888_c0_g3	ptc-miR169i_1ss15TA	1.54	yes	up	0.03	yes	TRINITY_DN22888_c0_g3	2.28	yes	up	0.01	yes	regulation of transcription, DNA-templated;transcription factor activity
ptc-miR169i_1ss15TA : TRINITY_DN25978_c0_g1 : TRINITY_DN25978_c0_g1	ptc-miR169i_1ss15TA	1.54	yes	up	0.03	yes	TRINITY_DN25978_c0_g1	0.38	yes	down	0.00	yes	embryo development ending in seed dormancy;DNA binding;transcriptio
ptc-miR172a : TRINITY_DN16267_c1_g1 : TRINITY_DN16267_c1_g1	ptc-miR172a	0.07	yes	down	0.00	yes	TRINITY_DN16267_c1_g1	0.42	yes	down	0.00	yes	.
ptc-miR172a : TRINITY_DN20593_c0_g1 : TRINITY_DN20593_c0_g1	ptc-miR172a	0.07	yes	down	0.00	yes	TRINITY_DN20593_c0_g1	3.40	yes	up	0.00	yes	cadmium ion transmembrane transport;cellular metal ion homeostasis;iro
ptc-miR172a : TRINITY_DN25362_c0_g5 : TRINITY_DN25362_c0_g5	ptc-miR172a	0.07	yes	down	0.00	yes	TRINITY_DN25362_c0_g5	0.29	yes	down	0.05	yes	UDP-glycosyltransferase activity;flavonoid biosynthetic process;intracel
ptc-miR172a : TRINITY_DN26802_c1_g1 : TRINITY_DN26802_c1_g1	ptc-miR172a	0.07	yes	down	0.00	yes	TRINITY_DN26802_c1_g1	4.16	yes	up	0.00	yes	transcription factor activity, sequence-specific DNA binding;seed develo
ptc-miR172a_R-1 : TRINITY_DN16267_c1_g1 : TRINITY_DN16267_c1_g1	ptc-miR172a_R-1	0.08	yes	down	0.01	yes	TRINITY_DN16267_c1_g1	0.42	yes	down	0.00	yes	.

ptc-miR172a_R-1 : TRINITY_DN19501_c0_g6 : TRINITY_DN19501_c0_g6	ptc-miR172a_R-1	0.08	yes	down	0.01	yes	TRINITY_DN19501_c0_g6	0.13	yes	down	0.00	yes	DNA-(apurinic or apyrimidinic site) lyase activity;DNA N-glycosylase a
ptc-miR172a_R-1 : TRINITY_DN20593_c0_g1 : TRINITY_DN20593_c0_g1	ptc-miR172a_R-1	0.08	yes	down	0.01	yes	TRINITY_DN20593_c0_g1	3.40	yes	up	0.00	yes	cadmium ion transmembrane transport;cellular metal ion homeostasis;iro
ptc-miR172a_R-1 : TRINITY_DN22400_c1_g2 : TRINITY_DN22400_c1_g2	ptc-miR172a_R-1	0.08	yes	down	0.01	yes	TRINITY_DN22400_c1_g2	0.18	yes	down	0.00	yes	transcription, DNA-templated;nucleus;base-excision repair;embryo devel
ptc-miR172a_R-1 : TRINITY_DN25512_c0_g2 : TRINITY_DN25512_c0_g2	ptc-miR172a_R-1	0.08	yes	down	0.01	yes	TRINITY_DN25512_c0_g2	2.31	yes	up	0.00	yes	signal transduction;integral component of membrane;protein binding;me
ptc-miR172a_R-1 : TRINITY_DN26802_c1_g1 : TRINITY_DN26802_c1_g1	ptc-miR172a_R-1	0.08	yes	down	0.01	yes	TRINITY_DN26802_c1_g1	4.16	yes	up	0.00	yes	transcription factor activity, sequence-specific DNA binding;seed develo
ptc-miR172a_R-1 : TRINITY_DN27819_c0_g1 : TRINITY_DN27819_c0_g1	ptc-miR172a_R-1	0.08	yes	down	0.01	yes	TRINITY_DN27819_c0_g1	0.47	yes	down	0.03	yes	transcription, DNA-templated;nucleus;embryo development ending in se
ptc-miR393a-3p_1ss10TC : TRINITY_DN18975_c0_g1 : TRINITY_DN18975_c0_g1	ptc-miR393a-3p_1ss10TC	1.78	yes	up	0.01	yes	TRINITY_DN18975_c0_g1	4.28	yes	up	0.00	yes	dioxygenase activity
ptc-miR394a-5p : TRINITY_DN20638_c0_g1 : TRINITY_DN20638_c0_g1	ptc-miR394a-5p	0.31	yes	down	0.02	yes	TRINITY_DN20638_c0_g1	2.19	yes	up	0.00	yes	cytoplasm;phosphorylation;phosphotransferase activity, alcohol group a
ptc-miR394a-5p : TRINITY_DN27467_c1_g4 : TRINITY_DN27467_c1_g4	ptc-miR394a-5p	0.31	yes	down	0.02	yes	TRINITY_DN27467_c1_g4	0.50	yes	down	0.00	yes	cytoskeleton-dependent intracellular transport;microtubule-based move
ptc-miR394a-5p_L+1 : TRINITY_DN27467_c1_g4 : TRINITY_DN27467_c1_g4	ptc-miR394a-5p_L+1	0.40	yes	down	0.01	yes	TRINITY_DN27467_c1_g4	0.50	yes	down	0.00	yes	cytoskeleton-dependent intracellular transport;microtubule-based move
ptc-miR396a : TRINITY_DN14058_c0_g1 : TRINITY_DN14058_c0_g1	ptc-miR396a	1.94	yes	up	0.05	yes	TRINITY_DN14058_c0_g1	0.00	yes	down	0.00	yes	transcription, DNA-templated;leaf development;nucleus;regulation of tra
ptc-miR396a : TRINITY_DN17151_c0_g2 : TRINITY_DN17151_c0_g2	ptc-miR396a	1.94	yes	up	0.05	yes	TRINITY_DN17151_c0_g2	0.11	yes	down	0.00	yes	leaf development;nucleus
ptc-miR396a : TRINITY_DN18326_c0_g1 : TRINITY_DN18326_c0_g1	ptc-miR396a	1.94	yes	up	0.05	yes	TRINITY_DN18326_c0_g1	0.17	yes	down	0.00	yes	leaf development;nucleus
ptc-miR396a : TRINITY_DN22596_c0_g1 : TRINITY_DN22596_c0_g1	ptc-miR396a	1.94	yes	up	0.05	yes	TRINITY_DN22596_c0_g1	2.72	yes	up	0.02	yes	regulation of systemic acquired resistance;response to jasmonic acid;IAA
ptc-miR396a : TRINITY_DN22714_c0_g1 : TRINITY_DN22714_c0_g1	ptc-miR396a	1.94	yes	up	0.05	yes	TRINITY_DN22714_c0_g1	0.19	yes	down	0.00	yes	leaf development;root development;regulation of transcription, DNA-te
ptc-miR396a : TRINITY_DN24029_c0_g2 : TRINITY_DN24029_c0_g2	ptc-miR396a	1.94	yes	up	0.05	yes	TRINITY_DN24029_c0_g2	2.20	yes	up	0.00	yes	.
ptc-miR396a : TRINITY_DN24309_c0_g1 : TRINITY_DN24309_c0_g1	ptc-miR396a	1.94	yes	up	0.05	yes	TRINITY_DN24309_c0_g1	2.08	yes	up	0.00	yes	leaf development;transcription, DNA-templated;nucleus;regulation of tra
ptc-miR396a : TRINITY_DN24662_c0_g3 : TRINITY_DN24662_c0_g3	ptc-miR396a	1.94	yes	up	0.05	yes	TRINITY_DN24662_c0_g3	0.48	yes	down	0.00	yes	ribonucleoside-diphosphate reductase complex;deoxyribonucleotide biosy
ptc-miR396a : TRINITY_DN25740_c0_g5 : TRINITY_DN25740_c0_g5	ptc-miR396a	1.94	yes	up	0.05	yes	TRINITY_DN25740_c0_g5	0.25	yes	down	0.00	yes	response to gibberellin;nucleus;response to deep water
ptc-miR396a : TRINITY_DN26007_c0_g1 : TRINITY_DN26007_c0_g1	ptc-miR396a	1.94	yes	up	0.05	yes	TRINITY_DN26007_c0_g1	0.23	yes	down	0.00	yes	leaf development;root development;transcription, DNA-templated;regul
ptc-miR396a : TRINITY_DN26348_c0_g1 : TRINITY_DN26348_c0_g1	ptc-miR396a	1.94	yes	up	0.05	yes	TRINITY_DN26348_c0_g1	2.75	yes	up	0.00	yes	flavonoid biosynthetic process;quercetin 7-O-glucosyltransferase activity
ptc-miR396a : TRINITY_DN27459_c0_g1 : TRINITY_DN27459_c0_g1	ptc-miR396a	1.94	yes	up	0.05	yes	TRINITY_DN27459_c0_g1	0.50	yes	down	0.01	yes	kinesin complex;microtubule motor activity;ATPase activity;microtubul

ptc-miR396g-3p_R-1_1ss19AG : TRINITY_DN22496_c0_g1 : TRINITY_DN22496_c0_g1	ptc-miR396g-3p_R-1_1ss19AG	1.75	yes	up	0.03	yes	TRINITY_DN22496_c0_g1	0.36	yes	down	0.00	yes	cytoplasm
ptc-miR397a : TRINITY_DN24129_c0_g2 : TRINITY_DN24129_c0_g2	ptc-miR397a	0.20	yes	down	0.00	yes	TRINITY_DN24129_c0_g2	0.48	yes	down	0.02	yes	GTP binding;integral component of mitochondrial outer membrane;mito
ptc-miR399a_R-2_1ss13TG : TRINITY_DN18686_c0_g1 : TRINITY_DN18686_c0_g1	ptc-miR399a_R-2_1ss13TG	14.84	yes	up	0.01	yes	TRINITY_DN18686_c0_g1	0.14	yes	down	0.00	yes	integral component of plasma membrane;substrate-specific transmembra
ptc-miR399a_R-2_1ss13TG : TRINITY_DN19079_c1_g6 : TRINITY_DN19079_c1_g6	ptc-miR399a_R-2_1ss13TG	14.84	yes	up	0.01	yes	TRINITY_DN19079_c1_g6	4.02	yes	up	0.00	yes	.
ptc-miR399a_R-2_1ss13TG : TRINITY_DN22148_c0_g1 : TRINITY_DN22148_c0_g1	ptc-miR399a_R-2_1ss13TG	14.84	yes	up	0.01	yes	TRINITY_DN22148_c0_g1	0.36	yes	down	0.00	yes	integral component of plasma membrane;substrate-specific transmembra
ptc-miR399a_R-2_1ss13TG : TRINITY_DN24222_c0_g1 : TRINITY_DN24222_c0_g1	ptc-miR399a_R-2_1ss13TG	14.84	yes	up	0.01	yes	TRINITY_DN24222_c0_g1	4.03	yes	up	0.00	yes	peptidyl-serine phosphorylation;intracellular signal transduction;protein
ptc-miR399a_R-2_1ss13TG : TRINITY_DN24789_c0_g4 : TRINITY_DN24789_c0_g4	ptc-miR399a_R-2_1ss13TG	14.84	yes	up	0.01	yes	TRINITY_DN24789_c0_g4	0.11	yes	down	0.00	yes	.
ptc-miR403c-5p : TRINITY_DN19209_c0_g2 : TRINITY_DN19209_c0_g2	ptc-miR403c-5p	1.96	yes	up	0.00	yes	TRINITY_DN19209_c0_g2	0.39	yes	down	0.01	yes	double-strand break repair via break-induced replication;replication fork
ptc-miR408-3p : TRINITY_DN21005_c0_g2 : TRINITY_DN21005_c0_g2	ptc-miR408-3p	0.08	yes	down	0.04	yes	TRINITY_DN21005_c0_g2	3.97	yes	up	0.00	yes	chloroplast;hydrolase activity;barrier septum assembly;GTP binding
ptc-MIR472a-p5_1ss2CT : TRINITY_DN19907_c0_g1 : TRINITY_DN19907_c0_g1	ptc-MIR472a-p5_1ss2CT	1.65	yes	up	0.02	yes	TRINITY_DN19907_c0_g1	2.43	yes	up	0.00	yes	integral component of membrane
ptc-miR472b : TRINITY_DN27663_c1_g1 : TRINITY_DN27663_c1_g1	ptc-miR472b	1.20	yes	up	0.01	yes	TRINITY_DN27663_c1_g1	0.23	yes	down	0.00	yes	.
ptc-MIR475d-p5_2 : TRINITY_DN15579_c0_g1 : TRINITY_DN15579_c0_g1	ptc-MIR475d-p5_2	1.69	yes	up	0.03	yes	TRINITY_DN15579_c0_g1	2.55	yes	up	0.00	yes	maturation of SSU-rRNA from tricistronic rRNA transcript (SSU-rRNA,
ptc-MIR475d-p5_2 : TRINITY_DN19458_c0_g1 : TRINITY_DN19458_c0_g1	ptc-MIR475d-p5_2	1.69	yes	up	0.03	yes	TRINITY_DN19458_c0_g1	0.26	yes	down	0.00	yes	transcription factor activity, sequence-specific DNA binding;response to
ptc-MIR475d-p5_2 : TRINITY_DN20720_c0_g1 : TRINITY_DN20720_c0_g1	ptc-MIR475d-p5_2	1.69	yes	up	0.03	yes	TRINITY_DN20720_c0_g1	4.40	yes	up	0.00	yes	chloroplast;NAD binding;arginine biosynthetic process;cellular amino aci
ptc-MIR475d-p5_2 : TRINITY_DN21417_c0_g1 : TRINITY_DN21417_c0_g1	ptc-MIR475d-p5_2	1.69	yes	up	0.03	yes	TRINITY_DN21417_c0_g1	2.55	yes	up	0.00	yes	cysteine biosynthetic process from serine;sulfate assimilation;serine O-ac
ptc-MIR475d-p5_2 : TRINITY_DN24185_c0_g4 : TRINITY_DN24185_c0_g4	ptc-MIR475d-p5_2	1.69	yes	up	0.03	yes	TRINITY_DN24185_c0_g4	3.31	yes	up	0.00	yes	structural constituent of ribosome;maturation of SSU-rRNA from tricistr
ptc-MIR475d-p5_2 : TRINITY_DN24701_c0_g2 : TRINITY_DN24701_c0_g2	ptc-MIR475d-p5_2	1.69	yes	up	0.03	yes	TRINITY_DN24701_c0_g2	2.20	yes	up	0.00	yes	mitochondrion
ptc-MIR475d-p5_2 : TRINITY_DN24968_c0_g1 : TRINITY_DN24968_c0_g1	ptc-MIR475d-p5_2	1.69	yes	up	0.03	yes	TRINITY_DN24968_c0_g1	0.39	yes	down	0.00	yes	cytoplasm;protein polyubiquitination;nucleus;ubiquitin protein ligase acti
ptc-MIR475d-p5_2 : TRINITY_DN26109_c0_g1 : TRINITY_DN26109_c0_g1	ptc-MIR475d-p5_2	1.69	yes	up	0.03	yes	TRINITY_DN26109_c0_g1	0.49	yes	down	0.03	yes	positive regulation of seed germination;regulation of seed maturation;tra
ptc-MIR475d-p5_2 : TRINITY_DN26311_c0_g1 : TRINITY_DN26311_c0_g1	ptc-MIR475d-p5_2	1.69	yes	up	0.03	yes	TRINITY_DN26311_c0_g1	2.49	yes	up	0.00	yes	chloroplast thylakoid;protein binding;oxidation-reduction process;porph
ptc-MIR475d-p5_2 : TRINITY_DN26519_c0_g4 : TRINITY_DN26519_c0_g4	ptc-MIR475d-p5_2	1.69	yes	up	0.03	yes	TRINITY_DN26519_c0_g4	0.42	yes	down	0.00	yes	.
ptc-MIR475d-p5_2 : TRINITY_DN26642_c0_g1 : TRINITY_DN26642_c0_g1	ptc-MIR475d-p5_2	1.69	yes	up	0.03	yes	TRINITY_DN26642_c0_g1	0.30	yes	down	0.00	yes	nucleus;cell wall;protein binding

ptc-MIR475d-p5_2 : TRINITY_DN27657_c0_g1 : TRINITY_DN27657_c0_g1	ptc-MIR475d-p5_2	1.69	yes	up	0.03	yes	TRINITY_DN27657_c0_g1	0.45	yes	down	0.00	yes	nucleus;positive regulation of transcription from RNA polymerase II pro
ptc-miR477d-5p_R-1 : TRINITY_DN15461_c0_g1 : TRINITY_DN15461_c0_g1	ptc-miR477d-5p_R-1	9.74	yes	up	0.03	yes	TRINITY_DN15461_c0_g1	0.01	yes	down	0.00	yes	RNA binding;nucleic acid binding;cytoplasm;nucleotide binding
ptc-miR477d-5p_R-1 : TRINITY_DN21841_c0_g1 : TRINITY_DN21841_c0_g1	ptc-miR477d-5p_R-1	9.74	yes	up	0.03	yes	TRINITY_DN21841_c0_g1	0.49	yes	down	0.01	yes	damaged DNA binding;ATP-dependent DNA helicase activity;telomere
ptc-miR481a_1ss11TC : TRINITY_DN23422_c1_g1 : TRINITY_DN23422_c1_g1	ptc-miR481a_1ss11TC	0.42	yes	down	0.01	yes	TRINITY_DN23422_c1_g1	3.85	yes	up	0.00	yes	membrane;proton-transporting ATP synthase complex, catalytic core F
ptc-MIR481a-p3_2ss13AG23TC : TRINITY_DN23422_c1_g1 : TRINITY_DN23422_c1_g1	ptc-MIR481a-p3_2ss13AG23TC	0.44	yes	down	0.04	yes	TRINITY_DN23422_c1_g1	3.85	yes	up	0.00	yes	membrane;proton-transporting ATP synthase complex, catalytic core F
ptc-MIR481a-p5_2ss13AG23TC : TRINITY_DN23422_c1_g1 : TRINITY_DN23422_c1_g1	ptc-MIR481a-p5_2ss13AG23TC	0.44	yes	down	0.04	yes	TRINITY_DN23422_c1_g1	3.85	yes	up	0.00	yes	membrane;proton-transporting ATP synthase complex, catalytic core F
ptc-MIR481b-p3_1 : TRINITY_DN23422_c1_g1 : TRINITY_DN23422_c1_g1	ptc-MIR481b-p3_1	0.59	yes	down	0.05	yes	TRINITY_DN23422_c1_g1	3.85	yes	up	0.00	yes	membrane;proton-transporting ATP synthase complex, catalytic core F
ptc-MIR481b-p3_1ss3CT : TRINITY_DN23422_c1_g1 : TRINITY_DN23422_c1_g1	ptc-MIR481b-p3_1ss3CT	0.46	yes	down	0.03	yes	TRINITY_DN23422_c1_g1	3.85	yes	up	0.00	yes	membrane;proton-transporting ATP synthase complex, catalytic core F
ptc-MIR481b-p3_2 : TRINITY_DN23422_c1_g1 : TRINITY_DN23422_c1_g1	ptc-MIR481b-p3_2	0.59	yes	down	0.05	yes	TRINITY_DN23422_c1_g1	3.85	yes	up	0.00	yes	membrane;proton-transporting ATP synthase complex, catalytic core F
ptc-MIR481b-p3_2 : TRINITY_DN24644_c0_g2 : TRINITY_DN24644_c0_g2	ptc-MIR481b-p3_2	0.59	yes	down	0.05	yes	TRINITY_DN24644_c0_g2	0.35	yes	down	0.00	yes	anther development;plasma membrane;protein self-association;ATP bin
ptc-MIR481b-p3_2ss3TC20CA : TRINITY_DN23422_c1_g1 : TRINITY_DN23422_c1_g1	ptc-MIR481b-p3_2ss3TC20CA	0.27	yes	down	0.02	yes	TRINITY_DN23422_c1_g1	3.85	yes	up	0.00	yes	membrane;proton-transporting ATP synthase complex, catalytic core F
ptc-MIR481b-p3_3 : TRINITY_DN23422_c1_g1 : TRINITY_DN23422_c1_g1	ptc-MIR481b-p3_3	0.59	yes	down	0.05	yes	TRINITY_DN23422_c1_g1	3.85	yes	up	0.00	yes	membrane;proton-transporting ATP synthase complex, catalytic core F
ptc-MIR481b-p5_1ss3CT : TRINITY_DN23422_c1_g1 : TRINITY_DN23422_c1_g1	ptc-MIR481b-p5_1ss3CT	0.40	yes	down	0.05	yes	TRINITY_DN23422_c1_g1	3.85	yes	up	0.00	yes	membrane;proton-transporting ATP synthase complex, catalytic core F
ptc-MIR481b-p5_2 : TRINITY_DN23422_c1_g1 : TRINITY_DN23422_c1_g1	ptc-MIR481b-p5_2	0.59	yes	down	0.05	yes	TRINITY_DN23422_c1_g1	3.85	yes	up	0.00	yes	membrane;proton-transporting ATP synthase complex, catalytic core F
ptc-MIR481b-p5_2 : TRINITY_DN24644_c0_g2 : TRINITY_DN24644_c0_g2	ptc-MIR481b-p5_2	0.59	yes	down	0.05	yes	TRINITY_DN24644_c0_g2	0.35	yes	down	0.00	yes	anther development;plasma membrane;protein self-association;ATP bin
ptc-MIR481b-p5_2ss3GA22GA : TRINITY_DN23422_c1_g1 : TRINITY_DN23422_c1_g1	ptc-MIR481b-p5_2ss3GA22GA	-inf	yes	down	0.01	yes	TRINITY_DN23422_c1_g1	3.85	yes	up	0.00	yes	membrane;proton-transporting ATP synthase complex, catalytic core F
ptc-MIR481b-p5_2ss3TC22GA : TRINITY_DN23422_c1_g1 : TRINITY_DN23422_c1_g1	ptc-MIR481b-p5_2ss3TC22GA	0.42	yes	down	0.03	yes	TRINITY_DN23422_c1_g1	3.85	yes	up	0.00	yes	membrane;proton-transporting ATP synthase complex, catalytic core F
ptc-MIR481c-p3_1ss12CT : TRINITY_DN23422_c1_g1 : TRINITY_DN23422_c1_g1	ptc-MIR481c-p3_1ss12CT	0.22	yes	down	0.02	yes	TRINITY_DN23422_c1_g1	3.85	yes	up	0.00	yes	membrane;proton-transporting ATP synthase complex, catalytic core F
ptc-MIR481c-p3_1ss4AG : TRINITY_DN23422_c1_g1 : TRINITY_DN23422_c1_g1	ptc-MIR481c-p3_1ss4AG	0.50	yes	down	0.02	yes	TRINITY_DN23422_c1_g1	3.85	yes	up	0.00	yes	membrane;proton-transporting ATP synthase complex, catalytic core F
ptc-MIR481c-p5 : TRINITY_DN23422_c1_g1 : TRINITY_DN23422_c1_g1	ptc-MIR481c-p5	0.44	yes	down	0.03	yes	TRINITY_DN23422_c1_g1	3.85	yes	up	0.00	yes	membrane;proton-transporting ATP synthase complex, catalytic core F
ptc-MIR481c-p5_1ss12AG : TRINITY_DN23422_c1_g1 : TRINITY_DN23422_c1_g1	ptc-MIR481c-p5_1ss12AG	0.45	yes	down	0.03	yes	TRINITY_DN23422_c1_g1	3.85	yes	up	0.00	yes	membrane;proton-transporting ATP synthase complex, catalytic core F
ptc-MIR481c-p5_1ss13CT : TRINITY_DN23422_c1_g1 : TRINITY_DN23422_c1_g1	ptc-MIR481c-p5_1ss13CT	0.31	yes	down	0.03	yes	TRINITY_DN23422_c1_g1	3.85	yes	up	0.00	yes	membrane;proton-transporting ATP synthase complex, catalytic core F

ptc-MIR481c-p5_1ss2CT : TRINITY_DN23422_c1_g1 : TRINITY_DN23422_c1_g1	ptc-MIR481c-p5_1ss2CT	0.47	yes	down	0.03	yes	TRINITY_DN23422_c1_g1	3.85	yes	up	0.00	yes	membrane;proton-transporting ATP synthase complex, catalytic core F
ptc-miR481d_L-1R+1_1ss3GA : TRINITY_DN23422_c1_g1 : TRINITY_DN23422_c1_g1	ptc-miR481d_L-1R+1_1ss3GA	0.31	yes	down	0.00	yes	TRINITY_DN23422_c1_g1	3.85	yes	up	0.00	yes	membrane;proton-transporting ATP synthase complex, catalytic core F
ptc-MIR481d-p3_1 : TRINITY_DN23422_c1_g1 : TRINITY_DN23422_c1_g1	ptc-MIR481d-p3_1	0.35	yes	down	0.03	yes	TRINITY_DN23422_c1_g1	3.85	yes	up	0.00	yes	membrane;proton-transporting ATP synthase complex, catalytic core F
ptc-MIR481d-p3_2 : TRINITY_DN23422_c1_g1 : TRINITY_DN23422_c1_g1	ptc-MIR481d-p3_2	0.35	yes	down	0.03	yes	TRINITY_DN23422_c1_g1	3.85	yes	up	0.00	yes	membrane;proton-transporting ATP synthase complex, catalytic core F
ptc-MIR481d-p3_2ss11CT18GA : TRINITY_DN23422_c1_g1 : TRINITY_DN23422_c1_g1	ptc-MIR481d-p3_2ss11CT18GA	0.14	yes	down	0.02	yes	TRINITY_DN23422_c1_g1	3.85	yes	up	0.00	yes	membrane;proton-transporting ATP synthase complex, catalytic core F
ptc-MIR481d-p3_2ss14CT17AG : TRINITY_DN23422_c1_g1 : TRINITY_DN23422_c1_g1	ptc-MIR481d-p3_2ss14CT17AG	0.49	yes	down	0.04	yes	TRINITY_DN23422_c1_g1	3.85	yes	up	0.00	yes	membrane;proton-transporting ATP synthase complex, catalytic core F
ptc-MIR481d-p3_2ss15CT17AG : TRINITY_DN23422_c1_g1 : TRINITY_DN23422_c1_g1	ptc-MIR481d-p3_2ss15CT17AG	0.41	yes	down	0.04	yes	TRINITY_DN23422_c1_g1	3.85	yes	up	0.00	yes	membrane;proton-transporting ATP synthase complex, catalytic core F
ptc-MIR481d-p3_3 : TRINITY_DN23422_c1_g1 : TRINITY_DN23422_c1_g1	ptc-MIR481d-p3_3	0.40	yes	down	0.05	yes	TRINITY_DN23422_c1_g1	3.85	yes	up	0.00	yes	membrane;proton-transporting ATP synthase complex, catalytic core F
ptc-MIR481d-p3_4 : TRINITY_DN23422_c1_g1 : TRINITY_DN23422_c1_g1	ptc-MIR481d-p3_4	0.35	yes	down	0.03	yes	TRINITY_DN23422_c1_g1	3.85	yes	up	0.00	yes	membrane;proton-transporting ATP synthase complex, catalytic core F
ptc-MIR481d-p3_5 : TRINITY_DN23422_c1_g1 : TRINITY_DN23422_c1_g1	ptc-MIR481d-p3_5	0.35	yes	down	0.03	yes	TRINITY_DN23422_c1_g1	3.85	yes	up	0.00	yes	membrane;proton-transporting ATP synthase complex, catalytic core F
ptc-MIR481d-p5 : TRINITY_DN23422_c1_g1 : TRINITY_DN23422_c1_g1	ptc-MIR481d-p5	0.40	yes	down	0.05	yes	TRINITY_DN23422_c1_g1	3.85	yes	up	0.00	yes	membrane;proton-transporting ATP synthase complex, catalytic core F
ptc-MIR481d-p5_1ss6AG : TRINITY_DN23422_c1_g1 : TRINITY_DN23422_c1_g1	ptc-MIR481d-p5_1ss6AG	0.35	yes	down	0.00	yes	TRINITY_DN23422_c1_g1	3.85	yes	up	0.00	yes	membrane;proton-transporting ATP synthase complex, catalytic core F
ptc-MIR481d-p5_2ss14CT17AG : TRINITY_DN23422_c1_g1 : TRINITY_DN23422_c1_g1	ptc-MIR481d-p5_2ss14CT17AG	0.49	yes	down	0.04	yes	TRINITY_DN23422_c1_g1	3.85	yes	up	0.00	yes	membrane;proton-transporting ATP synthase complex, catalytic core F
ptc-MIR481d-p5_2ss3GA19CT : TRINITY_DN20065_c0_g2 : TRINITY_DN20065_c0_g2	ptc-MIR481d-p5_2ss3GA19CT	0.31	yes	down	0.04	yes	TRINITY_DN20065_c0_g2	2.90	yes	up	0.00	yes	chloroplast
ptc-MIR481d-p5_2ss3GA19CT : TRINITY_DN23422_c1_g1 : TRINITY_DN23422_c1_g1	ptc-MIR481d-p5_2ss3GA19CT	0.31	yes	down	0.04	yes	TRINITY_DN23422_c1_g1	3.85	yes	up	0.00	yes	membrane;proton-transporting ATP synthase complex, catalytic core F
ptc-miR530a_R+1 : TRINITY_DN24639_c1_g2 : TRINITY_DN24639_c1_g2	ptc-miR530a_R+1	2.45	yes	up	0.02	yes	TRINITY_DN24639_c1_g2	0.40	yes	down	0.00	yes	protein dimerization activity;abscisic acid-activated signaling pathway;tr
ptc-miR530a_R+1 : TRINITY_DN26026_c0_g1 : TRINITY_DN26026_c0_g1	ptc-miR530a_R+1	2.45	yes	up	0.02	yes	TRINITY_DN26026_c0_g1	0.24	yes	down	0.00	yes	nucleus
ptc-miR530a_R+1 : TRINITY_DN26026_c0_g3 : TRINITY_DN26026_c0_g3	ptc-miR530a_R+1	2.45	yes	up	0.02	yes	TRINITY_DN26026_c0_g3	0.29	yes	down	0.01	yes	nucleus
ptc-miR530a_R+1_1ss20TC_1 : TRINITY_DN24639_c1_g2 : TRINITY_DN24639_c1_g2	ptc-miR530a_R+1_1ss20TC_1	2.41	yes	up	0.04	yes	TRINITY_DN24639_c1_g2	0.40	yes	down	0.00	yes	protein dimerization activity;abscisic acid-activated signaling pathway;tr
ptc-miR530a_R+1_1ss20TC_1 : TRINITY_DN26026_c0_g1 : TRINITY_DN26026_c0_g1	ptc-miR530a_R+1_1ss20TC_1	2.41	yes	up	0.04	yes	TRINITY_DN26026_c0_g1	0.24	yes	down	0.00	yes	nucleus
ptc-miR530a_R+1_1ss20TC_1 : TRINITY_DN26026_c0_g3 : TRINITY_DN26026_c0_g3	ptc-miR530a_R+1_1ss20TC_1	2.41	yes	up	0.04	yes	TRINITY_DN26026_c0_g3	0.29	yes	down	0.01	yes	nucleus
ptc-miR530b_L+2R-1 : TRINITY_DN22984_c0_g2 : TRINITY_DN22984_c0_g2	ptc-miR530b_L+2R-1	3.79	yes	up	0.00	yes	TRINITY_DN22984_c0_g2	2.37	yes	up	0.00	yes	peroxisome;integral component of membrane;oxidoreductase activity

ptc-miR530b_L+2R-1 : TRINITY_DN24639_c1_g2 : TRINITY_DN24639_c1_g2	ptc-miR530b_L+2R-1	3.79	yes	up	0.00	yes	TRINITY_DN24639_c1_g2	0.40	yes	down	0.00	yes	protein dimerization activity;abscisic acid-activated signaling pathway;tr
ptc-miR530b_L+2R-1 : TRINITY_DN25890_c0_g1 : TRINITY_DN25890_c0_g1	ptc-miR530b_L+2R-1	3.79	yes	up	0.00	yes	TRINITY_DN25890_c0_g1	2.13	yes	up	0.00	yes	mitochondrion;RNA binding;cytosolic large ribosomal subunit;maturatio
ptc-miR6425a-3p_1ss4AT : TRINITY_DN23474_c0_g2 : TRINITY_DN23474_c0_g2	ptc-miR6425a-3p_1ss4AT	3.86	yes	up	0.04	yes	TRINITY_DN23474_c0_g2	0.22	yes	down	0.00	yes	nucleus;protein binding;signal transduction
ptc-miR6425a-3p_1ss4AT : TRINITY_DN27163_c0_g1 : TRINITY_DN27163_c0_g1	ptc-miR6425a-3p_1ss4AT	3.86	yes	up	0.04	yes	TRINITY_DN27163_c0_g1	0.33	yes	down	0.00	yes	transmembrane receptor protein tyrosine kinase signaling pathway;plas
ptc-miR6427-3p_1ss13AT : TRINITY_DN26040_c1_g5 : TRINITY_DN26040_c1_g5	ptc-miR6427-3p_1ss13AT	6.26	yes	up	0.01	yes	TRINITY_DN26040_c1_g5	2.45	yes	up	0.00	yes	.
ptc-miR6456 : TRINITY_DN13864_c0_g4 : TRINITY_DN13864_c0_g4	ptc-miR6456	0.45	yes	down	0.02	yes	TRINITY_DN13864_c0_g4	0.46	yes	down	0.02	yes	nucleus
ptc-miR6456 : TRINITY_DN20543_c0_g3 : TRINITY_DN20543_c0_g3	ptc-miR6456	0.45	yes	down	0.02	yes	TRINITY_DN20543_c0_g3	2.55	yes	up	0.00	yes	pyridoxal 5'-phosphate salvage;vitamin B6 metabolic process;cytosol;py
ptc-miR6457a_L+1R-1 : TRINITY_DN27861_c3_g1 : TRINITY_DN27861_c3_g1	ptc-miR6457a_L+1R-1	0.27	yes	down	0.01	yes	TRINITY_DN27861_c3_g1	2.11	yes	up	0.01	yes	cytosol;cytoplasm;nucleus
ptc-MIR6457a-p5 : TRINITY_DN18651_c2_g2 : TRINITY_DN18651_c2_g2	ptc-MIR6457a-p5	0.26	yes	down	0.02	yes	TRINITY_DN18651_c2_g2	0.38	yes	down	0.04	yes	.
ptc-miR6457b_R-2 : TRINITY_DN18651_c2_g2 : TRINITY_DN18651_c2_g2	ptc-miR6457b_R-2	0.26	yes	down	0.01	yes	TRINITY_DN18651_c2_g2	0.38	yes	down	0.04	yes	.
ptc-miR6457b_R-2 : TRINITY_DN21640_c0_g1 : TRINITY_DN21640_c0_g1	ptc-miR6457b_R-2	0.26	yes	down	0.01	yes	TRINITY_DN21640_c0_g1	0.36	yes	down	0.00	yes	extracellular region;plant-type hypersensitive response;plasma membran
ptc-miR6457b_R-2 : TRINITY_DN25550_c0_g1 : TRINITY_DN25550_c0_g1	ptc-miR6457b_R-2	0.26	yes	down	0.01	yes	TRINITY_DN25550_c0_g1	2.03	yes	up	0.02	yes	cytoplasm
ptc-miR6457b_R-2 : TRINITY_DN27295_c1_g2 : TRINITY_DN27295_c1_g2	ptc-miR6457b_R-2	0.26	yes	down	0.01	yes	TRINITY_DN27295_c1_g2	0.19	yes	down	0.02	yes	.
ptc-miR6468-5p_L-1 : TRINITY_DN19419_c0_g1 : TRINITY_DN19419_c0_g1	ptc-miR6468-5p_L-1	3.35	yes	up	0.02	yes	TRINITY_DN19419_c0_g1	13.75	yes	up	0.00	yes	nucleus;leaf development;sequence-specific DNA binding;transcription fa
ptc-miR6468-5p_L-1 : TRINITY_DN21914_c0_g1 : TRINITY_DN21914_c0_g1	ptc-miR6468-5p_L-1	3.35	yes	up	0.02	yes	TRINITY_DN21914_c0_g1	4.89	yes	up	0.00	yes	embryo development ending in seed dormancy;chloroplast stroma;transp
ptc-miR6471 : TRINITY_DN22270_c0_g1 : TRINITY_DN22270_c0_g1	ptc-miR6471	2.15	yes	up	0.00	yes	TRINITY_DN22270_c0_g1	2.56	yes	up	0.00	yes	plastid;embryo development ending in seed dormancy;chloroplast stroma
sly-MIR9479-p3_2ss1GA18GC : TRINITY_DN20240_c0_g1 : TRINITY_DN20240_c0_g1	sly-MIR9479-p3_2ss1GA18GC	10.60	yes	up	0.01	yes	TRINITY_DN20240_c0_g1	0.24	yes	down	0.00	yes	proteolysis;aspartic-type endopeptidase activity;protein catabolic proces
sly-MIR9479-p5_2ss1GA18GC : TRINITY_DN20240_c0_g1 : TRINITY_DN20240_c0_g1	sly-MIR9479-p5_2ss1GA18GC	10.60	yes	up	0.01	yes	TRINITY_DN20240_c0_g1	0.24	yes	down	0.00	yes	proteolysis;aspartic-type endopeptidase activity;protein catabolic proces
stu-miR156f-5p_L-1R+1 : TRINITY_DN18058_c0_g1 : TRINITY_DN18058_c0_g1	stu-miR156f-5p_L-1R+1	283.92	yes	up	0.01	yes	TRINITY_DN18058_c0_g1	3.12	yes	up	0.00	yes	vacuole;vacuolar membrane;response to bacterium;Golgi apparatus;plasm
stu-miR156f-5p_L-1R+1 : TRINITY_DN19122_c0_g1 : TRINITY_DN19122_c0_g1	stu-miR156f-5p_L-1R+1	283.92	yes	up	0.01	yes	TRINITY_DN19122_c0_g1	0.22	yes	down	0.00	yes	transcription factor activity, sequence-specific DNA binding;defense resp
stu-miR156f-5p_L-1R+1 : TRINITY_DN19850_c1_g2 : TRINITY_DN19850_c1_g2	stu-miR156f-5p_L-1R+1	283.92	yes	up	0.01	yes	TRINITY_DN19850_c1_g2	0.13	yes	down	0.00	yes	metal ion binding;transcription factor activity, sequence-specific DNA bi
stu-miR156f-5p_L-1R+1 : TRINITY_DN19850_c1_g3 : TRINITY_DN19850_c1_g3	stu-miR156f-5p_L-1R+1	283.92	yes	up	0.01	yes	TRINITY_DN19850_c1_g3	0.03	yes	down	0.00	yes	flower development;cytoplasm;DNA binding;transcription, DNA-templa

stu-miR156f-5p_L-1R+1 : TRINITY_DN19850_c1_g4 : TRINITY_DN19850_c1_g4	stu-miR156f-5p_L-1R+1	283.92	yes	up	0.01	yes	TRINITY_DN19850_c1_g4	0.18	yes	down	0.00	yes	transcription factor activity, sequence-specific DNA binding;defense resp
stu-miR156f-5p_L-1R+1 : TRINITY_DN20299_c0_g1 : TRINITY_DN20299_c0_g1	stu-miR156f-5p_L-1R+1	283.92	yes	up	0.01	yes	TRINITY_DN20299_c0_g1	0.40	yes	down	0.00	yes	integral component of membrane
stu-miR156f-5p_L-1R+1 : TRINITY_DN21154_c0_g2 : TRINITY_DN21154_c0_g2	stu-miR156f-5p_L-1R+1	283.92	yes	up	0.01	yes	TRINITY_DN21154_c0_g2	0.41	yes	down	0.01	yes	regulation of transcription, DNA-templated;anther development;transcri
stu-miR156f-5p_L-1R+1 : TRINITY_DN22967_c0_g1 : TRINITY_DN22967_c0_g1	stu-miR156f-5p_L-1R+1	283.92	yes	up	0.01	yes	TRINITY_DN22967_c0_g1	0.42	yes	down	0.00	yes	transcription factor activity, sequence-specific DNA binding;anther deve
stu-miR156f-5p_L-1R+1 : TRINITY_DN23287_c0_g1 : TRINITY_DN23287_c0_g1	stu-miR156f-5p_L-1R+1	283.92	yes	up	0.01	yes	TRINITY_DN23287_c0_g1	2.12	yes	up	0.00	yes	cytosol;UDP-N-acetylglucosamine diphosphorylase activity;UDP-N-ace
stu-miR156f-5p_L-1R+1 : TRINITY_DN23412_c1_g9 : TRINITY_DN23412_c1_g9	stu-miR156f-5p_L-1R+1	283.92	yes	up	0.01	yes	TRINITY_DN23412_c1_g9	0.03	yes	down	0.00	yes	regulation of transcription, DNA-templated;cell differentiation;positive
stu-miR156f-5p_L-1R+1 : TRINITY_DN23413_c0_g1 : TRINITY_DN23413_c0_g1	stu-miR156f-5p_L-1R+1	283.92	yes	up	0.01	yes	TRINITY_DN23413_c0_g1	0.33	yes	down	0.00	yes	transcription factor activity, sequence-specific DNA binding;anther deve
stu-miR156f-5p_L-1R+1 : TRINITY_DN24312_c1_g1 : TRINITY_DN24312_c1_g1	stu-miR156f-5p_L-1R+1	283.92	yes	up	0.01	yes	TRINITY_DN24312_c1_g1	0.44	yes	down	0.00	yes	regulation of transcription, DNA-templated;nucleus;transcription, DNA-
