# Supplementary material for: MiR156 regulates anthocyanin biosynthesis through SPL targets and other microRNAs in poplar
Source: Hortic Res. 2020 Aug 1;7:118. doi: 10.1038/s41438-020-00341-w (PMC7395715; doi:10.1038/s41438-020-00341-w)
Supplement: Supplementary file 5 — Supporting Information 5 [file 41438_2020_341_MOESM5_ESM.pdf]

Table S9 KEGG enrichment of differentially expressed genes between wild-type and transgenic poplar plants.

Pathway Entry	Pathway Definition	p-value	Gene_ID	Log2FC	Regulation	Annotation	TGII_mean _TPM	WT_mean _TPM
map03010	Ribosome	4.75E-12	TRINITY_DN21839_c0_g1	1.47	up	hypothetical protein POPTR_0012s06360g, partial [Populus trichocarpa]	473.60	256.93
map03010	Ribosome	4.75E-12	TRINITY_DN21915_c1_g2	1.93	up	60S ribosomal protein L11 [Populus trichocarpa]	798.91	322.84
map03010	Ribosome	4.75E-12	TRINITY_DN21980_c0_g1	1.48	up	60S ribosomal protein L18 [Populus trichocarpa]	854.12	474.94
map03010	Ribosome	4.75E-12	TRINITY_DN22011_c1_g5	1.27	up	ribosomal protein L33 [Populus trichocarpa]	110.25	71.30
map03010	Ribosome	4.75E-12	TRINITY_DN22108_c0_g2	1.87	up	PREDICTED: 40S ribosomal protein S15a-1 isoform X1 [Populus euphratica]	410.99	172.46
map03010	Ribosome	4.75E-12	TRINITY_DN22142_c0_g1	1.56	up	60s acidic ribosomal family protein [Populus trichocarpa]	744.60	389.71
map03010	Ribosome	4.75E-12	TRINITY_DN22142_c0_g2	2.31	up	60s acidic ribosomal family protein [Populus trichocarpa]	231.62	72.40
map03010	Ribosome	4.75E-12	TRINITY_DN22160_c0_g1	2.58	up	PREDICTED: uncharacterized protein LOC105123685 [Populus euphratica]	145.48	37.86
map03010	Ribosome	4.75E-12	TRINITY_DN22290_c1_g1	1.52	up	ribosomal protein L12 [Populus trichocarpa]	1118.36	600.76
map03010	Ribosome	4.75E-12	TRINITY_DN22307_c0_g3	2.62	up	PREDICTED: 60S ribosomal protein L7a-like [Populus euphratica]	89.49	22.39
map03010	Ribosome	4.75E-12	TRINITY_DN22522_c0_g2	2.12	up	PREDICTED: 30S ribosomal protein S17, chloroplastic [Populus euphratica]	1151.52	402.22
map03010	Ribosome	4.75E-12	TRINITY_DN22535_c1_g1	1.17	up	ribosomal protein L33 [Populus trichocarpa]	1111.68	712.11
map03010	Ribosome	4.75E-12	TRINITY_DN22535_c1_g2	1.34	up	PREDICTED: 60S ribosomal protein L35a-1 [Populus euphratica]	90.01	57.36
map03010	Ribosome	4.75E-12	TRINITY_DN22598_c1_g4	1.24	up	NAD(P)H-quinone oxidoreductase subunit 2 [Medicago truncatula]	8.88	5.74
map03010	Ribosome	4.75E-12	TRINITY_DN22643_c0_g1	1.59	up	unknown [Populus trichocarpa]	73.71	37.50
map03010	Ribosome	4.75E-12	TRINITY_DN22686_c0_g1	1.73	up	PREDICTED: 40S ribosomal protein S9-2-like isoform X1 [Populus euphratica]	1100.23	510.72
map03010	Ribosome	4.75E-12	TRINITY_DN22776_c0_g1	1.66	up	50S ribosomal protein L12-2 [Populus trichocarpa]	1729.35	825.41
map03010	Ribosome	4.75E-12	TRINITY_DN22805_c0_g1	1.37	up	unnamed protein product, partial [Vitis vinifera]	522.11	308.49
map03010	Ribosome	4.75E-12	TRINITY_DN22885_c0_g1	-1.11	down	hypothetical protein POPTR_0004s11340g [Populus trichocarpa]	4.07	12.98
map03010	Ribosome	4.75E-12	TRINITY_DN22886_c0_g1	1.31	up	RIBOSOMAL protein L23A [Populus trichocarpa]	626.80	394.25
map03010	Ribosome	4.75E-12	TRINITY_DN22886_c0_g6	1.49	up	RIBOSOMAL protein L23A [Populus trichocarpa]	44.78	24.69
map03010	Ribosome	4.75E-12	TRINITY_DN22997_c0_g3	1.12	up	hypothetical protein POPTR_0003s12330g [Populus trichocarpa]	250.51	176.74
map03010	Ribosome	4.75E-12	TRINITY_DN23061_c0_g1	-1.72	down	PREDICTED: leucine-rich repeat extensin-like protein 3 isoform X1 [Populus euphratica]	2.28	10.63
map03010	Ribosome	4.75E-12	TRINITY_DN23066_c0_g1	1.07	up	PREDICTED: probable ribosome biogenesis protein RLP24 [Populus euphratica]	52.34	39.06
map03010	Ribosome	4.75E-12	TRINITY_DN23066_c0_g6	1.13	up	60S ribosomal protein L24 [Populus trichocarpa]	33.03	23.21
map03010	Ribosome	4.75E-12	TRINITY_DN23293_c0_g2	1.93	up	hypothetical protein POPTR_0001s45510g [Populus trichocarpa]	323.44	127.58
map03010	Ribosome	4.75E-12	TRINITY_DN23297_c0_g3	1.83	up	60S ribosomal protein L15 [Populus trichocarpa]	98.11	50.85
map03010	Ribosome	4.75E-12	TRINITY_DN23297_c0_g5	1.14	up	PREDICTED: 60S ribosomal protein L15-like [Populus euphratica]	300.57	208.45
map03010	Ribosome	4.75E-12	TRINITY_DN23297_c0_g6	1.88	up	60S ribosomal protein L15 [Populus trichocarpa]	179.34	77.53
map03010	Ribosome	4.75E-12	TRINITY_DN23297_c0_g7	1.14	up	PREDICTED: 60S ribosomal protein L15-like [Populus euphratica]	334.41	234.14
map03010	Ribosome	4.75E-12	TRINITY_DN23306_c0_g2	2.02	up	PREDICTED: 60S ribosomal protein L17-2-like isoform X1 [Populus euphratica]	395.65	149.06
map03010	Ribosome	4.75E-12	TRINITY_DN23325_c0_g1	1.14	up	PREDICTED: 40S ribosomal protein SA-like isoform X1 [Populus euphratica]	58.46	40.60
map03010	Ribosome	4.75E-12	TRINITY_DN23371_c2_g2	1.18	up	hypothetical protein GLYMA_11G032800 [Glycine max]	401.15	257.07
map03010	Ribosome	4.75E-12	TRINITY_DN23450_c0_g1	1.89	up	hypothetical protein POPTR_0006s13480g [Populus trichocarpa]	338.50	141.38
map03010	Ribosome	4.75E-12	TRINITY_DN23450_c0_g2	1.69	up	unknown [Populus trichocarpa x Populus deltoides]	447.19	214.51
map03010	Ribosome	4.75E-12	TRINITY_DN23578_c0_g1	1.67	up	60S ribosomal protein L35 [Populus trichocarpa]	217.41	105.67
map03010	Ribosome	4.75E-12	TRINITY_DN23619_c0_g1	1.61	up	hypothetical protein POPTR_0001s37430g [Populus trichocarpa]	481.43	235.06
map03010	Ribosome	4.75E-12	TRINITY_DN23846_c1_g1	-1.07	down	polyubiquitin 3 [Medicago truncatula]	117.55	341.42
map03010	Ribosome	4.75E-12	TRINITY_DN23862_c0_g1	1.20	up	ribosomal protein 1 [Populus trichocarpa]	623.84	415.86
map03010	Ribosome	4.75E-12	TRINITY_DN23872_c0_g1	1.31	up	PREDICTED: 60S ribosomal protein L24-like [Populus euphratica]	570.45	355.52
map03010	Ribosome	4.75E-12	TRINITY_DN23901_c1_g3	1.13	up	hypothetical protein POPTR_0004s16450g [Populus trichocarpa]	472.63	332.91
map03010	Ribosome	4.75E-12	TRINITY_DN24130_c0_g1	1.47	up	hypothetical protein POPTR_0001s39310g [Populus trichocarpa]	811.24	439.27
map03010	Ribosome	4.75E-12	TRINITY_DN24156_c0_g1	1.40	up	hypothetical protein POPTR_0013s02900g [Populus trichocarpa]	423.97	247.21

map03010	Ribosome	4.75E-12	TRINITY_DN24185_c0_g1	1.24	up	hypothetical protein B456_013G156600 [Gossypium raimondii]	862.27	583.73
map03010	Ribosome	4.75E-12	TRINITY_DN24185_c0_g4	1.73	up	hypothetical protein POPTR_0017s14180g [Populus trichocarpa]	182.32	84.89
map03010	Ribosome	4.75E-12	TRINITY_DN24213_c0_g1	1.76	up	PREDICTED: 60S ribosomal protein L9 [Populus euphratica]	910.77	393.74
map03010	Ribosome	4.75E-12	TRINITY_DN24213_c0_g2	1.89	up	PREDICTED: 60S ribosomal protein L9-like [Populus euphratica]	1502.50	630.06
map03010	Ribosome	4.75E-12	TRINITY_DN24275_c0_g1	1.31	up	hypothetical protein POPTR_0018s12120g [Populus trichocarpa]	93.21	60.09
map03010	Ribosome	4.75E-12	TRINITY_DN24423_c1_g1	1.17	up	ribosomal protein L19 [Populus trichocarpa]	497.84	333.85
map03010	Ribosome	4.75E-12	TRINITY_DN24579_c0_g2	-1.03	down	hypothetical protein POPTR_0005s23270g [Populus trichocarpa]	2.04	6.42
map03010	Ribosome	4.75E-12	TRINITY_DN24662_c0_g1	1.58	up	ribosomal protein L27 precursor [Populus trichocarpa]	682.72	346.90
map03010	Ribosome	4.75E-12	TRINITY_DN24677_c0_g1	1.03	up	hypothetical protein POPTR_0015s10770g [Populus trichocarpa]	29.27	23.21
map03010	Ribosome	4.75E-12	TRINITY_DN24838_c0_g1	-2.45	down	PREDICTED: putative cyclin-A3-1 [Populus euphratica]	6.27	51.85
map03010	Ribosome	4.75E-12	TRINITY_DN24899_c0_g1	1.26	up	PREDICTED: 60S ribosomal protein L7-4 [Populus euphratica]	574.05	369.19
map03010	Ribosome	4.75E-12	TRINITY_DN24922_c0_g1	1.32	up	hypothetical protein POPTR_0789s00200g [Populus trichocarpa]	589.51	362.18
map03010	Ribosome	4.75E-12	TRINITY_DN24931_c0_g1	1.04	up	hypothetical protein POPTR_0004s23300g [Populus trichocarpa]	46.22	27.38
map03010	Ribosome	4.75E-12	TRINITY_DN24968_c0_g1	-1.38	down	hypothetical protein POPTR_0003s16000g [Populus trichocarpa]	7.38	30.98
map03010	Ribosome	4.75E-12	TRINITY_DN25152_c0_g1	-2.45	down	PREDICTED: CDT1-like protein a, chloroplastic [Populus euphratica]	1.98	18.33
map03010	Ribosome	4.75E-12	TRINITY_DN25178_c0_g1	1.45	up	60S ribosomal protein L18a-1 [Populus trichocarpa]	1204.98	705.71
map03010	Ribosome	4.75E-12	TRINITY_DN25205_c1_g5	1.26	up	hypothetical protein M569_00220, partial [Genlisea aurea]	38.98	24.08
map03010	Ribosome	4.75E-12	TRINITY_DN25342_c1_g1	1.58	up	hypothetical protein POPTR_0012s12870g [Populus trichocarpa]	49.78	26.23
map03010	Ribosome	4.75E-12	TRINITY_DN25399_c0_g4	1.73	up	hypothetical protein POPTR_0006s07290g [Populus trichocarpa]	271.17	126.77
map03010	Ribosome	4.75E-12	TRINITY_DN25402_c0_g2	1.73	up	PREDICTED: 60S acidic ribosomal protein P3-like [Populus euphratica]	195.27	90.48
map03010	Ribosome	4.75E-12	TRINITY_DN25402_c0_g4	1.51	up	hypothetical protein POPTR_0009s03780g [Populus trichocarpa]	308.61	165.97
map03010	Ribosome	4.75E-12	TRINITY_DN25498_c0_g1	-1.38	down	PREDICTED: cyclin-A2-2-like isoform X1 [Populus euphratica]	17.75	66.33
map03010	Ribosome	4.75E-12	TRINITY_DN25594_c0_g1	1.08	up	PREDICTED: 30S ribosomal protein S1, chloroplastic-like [Populus euphratica]	490.21	352.74
map03010	Ribosome	4.75E-12	TRINITY_DN25606_c0_g1	1.35	up	ribosomal protein S3a [Populus trichocarpa]	637.02	375.81
map03010	Ribosome	4.75E-12	TRINITY_DN25839_c1_g3	-1.09	down	WRKY transcription factor 30 [(Populus tomentosa x Populus bolleana) x Populus tomentosa]	6.93	21.93
map03010	Ribosome	4.75E-12	TRINITY_DN25870_c1_g3	1.57	up	unknown [Populus trichocarpa]	897.20	455.35
map03010	Ribosome	4.75E-12	TRINITY_DN25877_c1_g1	1.16	up	50S ribosomal protein L3-2 [Populus trichocarpa]	35.13	24.19
map03010	Ribosome	4.75E-12	TRINITY_DN25890_c0_g1	1.09	up	PREDICTED: 50S ribosomal protein L1, chloroplastic [Populus euphratica]	23.24	17.09
map03010	Ribosome	4.75E-12	TRINITY_DN26016_c0_g1	1.50	up	clp protease proteolytic subunit (chloroplast) [Populus ilicifolia]	7.07	3.93
map03010	Ribosome	4.75E-12	TRINITY_DN26034_c1_g1	1.34	up	unknown [Populus trichocarpa]	1128.40	639.78
map03010	Ribosome	4.75E-12	TRINITY_DN26184_c0_g4	1.85	up	PREDICTED: 50S ribosomal protein L10, chloroplastic [Jatropha curcas]	56.79	23.65
map03010	Ribosome	4.75E-12	TRINITY_DN26248_c0_g2	1.25	up	PREDICTED: 40S ribosomal protein S3-3 [Populus euphratica]	554.83	341.10
map03010	Ribosome	4.75E-12	TRINITY_DN26335_c0_g1	1.12	up	PREDICTED: 50S ribosomal protein L1, chloroplastic-like [Populus euphratica]	574.73	389.40
map03010	Ribosome	4.75E-12	TRINITY_DN26668_c1_g1	-1.04	down	PREDICTED: lysine-specific demethylase JMJ25-like isoform X1 [Populus euphratica]	11.75	51.18
map03010	Ribosome	4.75E-12	TRINITY_DN26676_c0_g1	1.40	up	60S ribosomal protein L13 [Populus trichocarpa]	815.99	534.53
map03010	Ribosome	4.75E-12	TRINITY_DN26748_c0_g2	1.21	up	hypothetical protein POPTR_0006s10460g [Populus trichocarpa]	939.38	615.87
map03010	Ribosome	4.75E-12	TRINITY_DN26804_c0_g1	1.69	up	PREDICTED: 60S ribosomal protein L28-1-like [Populus euphratica]	763.38	365.31
map03010	Ribosome	4.75E-12	TRINITY_DN26908_c0_g1	-3.48	down	PREDICTED: uncharacterized protein LOC105139757 isoform X2 [Populus euphratica]	0.59	12.30
map03010	Ribosome	4.75E-12	TRINITY_DN26963_c0_g2	1.36	up	40S ribosomal protein SA [Populus trichocarpa]	427.65	256.24
map03010	Ribosome	4.75E-12	TRINITY_DN27236_c0_g1	1.25	up	PREDICTED: uncharacterized protein LOC105139901 isoform X1 [Populus euphratica]	56.01	35.39
map03010	Ribosome	4.75E-12	TRINITY_DN27301_c0_g1	1.11	up	unknown [Populus trichocarpa]	48.44	35.28
map03010	Ribosome	4.75E-12	TRINITY_DN27525_c0_g1	1.08	up	hypothetical protein POPTR_0006s01590g [Populus trichocarpa]	176.88	122.84
map03010	Ribosome	4.75E-12	TRINITY_DN12676_c0_g1	1.45	up	hypothetical protein POPTR_0007s09770g [Populus trichocarpa]	148.95	83.79
map03010	Ribosome	4.75E-12	TRINITY_DN14004_c0_g1	1.32	up	PREDICTED: uncharacterized protein LOC105130283 [Populus euphratica]	65.83	41.05

map03010	Ribosome	4.75E-12	TRINITY_DN14831_c0_g1	1.35	up	hypothetical protein POPTR_0007s12890g [Populus trichocarpa]	37.57	22.49
map03010	Ribosome	4.75E-12	TRINITY_DN15087_c0_g1	5.77	up	hypothetical protein POPTR_0014s05870g [Populus trichocarpa]	191.71	5.32
map03010	Ribosome	4.75E-12	TRINITY_DN15104_c0_g1	1.88	up	hypothetical protein POPTR_0011s07530g [Populus trichocarpa]	203.22	84.32
map03010	Ribosome	4.75E-12	TRINITY_DN15149_c0_g1	1.59	up	hypothetical protein POPTR_0001s10930g [Populus trichocarpa]	917.35	458.74
map03010	Ribosome	4.75E-12	TRINITY_DN15149_c0_g4	1.49	up	hypothetical protein POPTR_0001s10930g [Populus trichocarpa]	610.58	326.04
map03010	Ribosome	4.75E-12	TRINITY_DN15491_c0_g1	1.45	up	PREDICTED: 60S ribosomal protein L22-2-like [Populus euphratica]	114.75	63.48
map03010	Ribosome	4.75E-12	TRINITY_DN15568_c0_g3	1.52	up	ribosomal protein L34 [Populus trichocarpa]	207.90	112.22
map03010	Ribosome	4.75E-12	TRINITY_DN15579_c0_g1	1.35	up	PREDICTED: uncharacterized protein LOC105124666 [Populus euphratica]	29.32	17.75
map03010	Ribosome	4.75E-12	TRINITY_DN15713_c0_g1	2.20	up	hypothetical protein POPTR_0271s00220g, partial [Populus trichocarpa]	1387.37	448.05
map03010	Ribosome	4.75E-12	TRINITY_DN15718_c0_g1	1.41	up	hypothetical protein POPTR_0012s08870g [Populus trichocarpa]	31.89	19.20
map03010	Ribosome	4.75E-12	TRINITY_DN15777_c0_g1	1.44	up	ribosomal protein L20 [Populus trichocarpa]	22.86	13.02
map03010	Ribosome	4.75E-12	TRINITY_DN15842_c0_g2	1.64	up	unknown [Populus trichocarpa]	68.83	33.62
map03010	Ribosome	4.75E-12	TRINITY_DN16002_c0_g1	1.26	up	hypothetical protein POPTR_0014s14430g, partial [Populus trichocarpa]	442.90	307.62
map03010	Ribosome	4.75E-12	TRINITY_DN16063_c0_g4	-2.15	down	S-locus lectin protein kinase [Populus trichocarpa]	0.36	2.45
map03010	Ribosome	4.75E-12	TRINITY_DN16077_c0_g2	1.43	up	40S ribosomal protein S14-3 [Populus trichocarpa]	78.32	44.68
map03010	Ribosome	4.75E-12	TRINITY_DN16166_c0_g1	1.65	up	PREDICTED: 50S ribosomal protein L24, chloroplastic [Populus euphratica]	76.76	37.83
map03010	Ribosome	4.75E-12	TRINITY_DN16168_c0_g2	1.28	up	hypothetical protein POPTR_0010s15190g, partial [Populus trichocarpa]	470.54	295.79
map03010	Ribosome	4.75E-12	TRINITY_DN16211_c2_g1	1.15	up	hypothetical protein POPTR_0016s13210g [Populus trichocarpa]	535.58	373.37
map03010	Ribosome	4.75E-12	TRINITY_DN16211_c2_g2	1.07	up	hypothetical protein POPTR_0015s09110g [Populus trichocarpa]	267.35	195.87
map03010	Ribosome	4.75E-12	TRINITY_DN16239_c0_g1	1.81	up	hypothetical protein POPTR_0003s05320g [Populus trichocarpa]	114.22	49.45
map03010	Ribosome	4.75E-12	TRINITY_DN16310_c0_g1	1.08	up	PREDICTED: 39S ribosomal protein L28, mitochondrial isoform X1 [Populus euphratica]	26.57	19.47
map03010	Ribosome	4.75E-12	TRINITY_DN16333_c0_g1	1.93	up	ribosomal protein L27 [Populus trichocarpa]	285.84	118.12
map03010	Ribosome	4.75E-12	TRINITY_DN16333_c0_g2	1.35	up	ribosomal protein L27 [Populus trichocarpa]	445.26	270.06
map03010	Ribosome	4.75E-12	TRINITY_DN16385_c0_g1	1.26	up	hypothetical protein POPTR_0013s06710g [Populus trichocarpa]	463.92	291.23
map03010	Ribosome	4.75E-12	TRINITY_DN16443_c1_g3	1.09	up	ribosomal protein S14 mitochondrial, partial [Populus trichocarpa]	94.71	68.99
map03010	Ribosome	4.75E-12	TRINITY_DN16463_c0_g1	1.92	up	unknown [Populus trichocarpa]	39.70	16.01
map03010	Ribosome	4.75E-12	TRINITY_DN16465_c0_g3	1.47	up	40S ribosomal protein S17 [Populus trichocarpa]	766.31	422.49
map03010	Ribosome	4.75E-12	TRINITY_DN16505_c0_g1	1.85	up	60S ribosomal protein L27 [Populus trichocarpa]	593.16	254.52
map03010	Ribosome	4.75E-12	TRINITY_DN16511_c0_g1	-1.03	down	PREDICTED: alkaline/neutral invertase CINV2 [Populus euphratica]	2.76	8.63
map03010	Ribosome	4.75E-12	TRINITY_DN16523_c0_g1	1.25	up	unknown [Populus trichocarpa]	235.77	155.66
map03010	Ribosome	4.75E-12	TRINITY_DN16559_c0_g1	1.27	up	30S ribosomal protein S18 [Populus trichocarpa]	32.81	20.93
map03010	Ribosome	4.75E-12	TRINITY_DN16572_c0_g1	2.12	up	hypothetical protein POPTR_0016s06300g [Populus trichocarpa]	169.16	63.49
map03010	Ribosome	4.75E-12	TRINITY_DN16581_c0_g1	2.79	up	PREDICTED: putative lipid-binding protein At4g00165 [Vitis vinifera]	521.12	114.10
map03010	Ribosome	4.75E-12	TRINITY_DN16679_c0_g1	1.28	up	hypothetical protein POPTR_0007s07630g [Populus trichocarpa]	33.40	20.98
map03010	Ribosome	4.75E-12	TRINITY_DN16715_c0_g1	2.20	up	hypothetical protein POPTR_0016s14970g [Populus trichocarpa]	541.85	178.67
map03010	Ribosome	4.75E-12	TRINITY_DN16715_c0_g3	1.70	up	hypothetical protein POPTR_0006s11420g [Populus trichocarpa]	970.08	447.60
map03010	Ribosome	4.75E-12	TRINITY_DN16936_c0_g1	1.36	up	60S ribosomal protein L35 [Populus trichocarpa]	484.00	286.67
map03010	Ribosome	4.75E-12	TRINITY_DN16951_c0_g1	2.14	up	PREDICTED: 60S ribosomal protein L13a-4-like [Populus euphratica]	169.18	59.37
map03010	Ribosome	4.75E-12	TRINITY_DN16992_c0_g1	1.12	up	ribosomal protein L12 [Populus trichocarpa]	19.80	13.97
map03010	Ribosome	4.75E-12	TRINITY_DN17318_c0_g1	1.89	up	hypothetical protein POPTR_0009s06860g [Populus trichocarpa]	353.57	150.56
map03010	Ribosome	4.75E-12	TRINITY_DN17326_c0_g1	1.10	up	ribosomal protein L12 [Populus trichocarpa]	63.54	45.90
map03010	Ribosome	4.75E-12	TRINITY_DN17368_c0_g1	1.27	up	hypothetical protein POPTR_0004s22660g [Populus trichocarpa]	352.63	222.03
map03010	Ribosome	4.75E-12	TRINITY_DN17383_c0_g1	1.70	up	40S ribosomal protein S11-2 [Populus trichocarpa]	31.53	14.94
map03010	Ribosome	4.75E-12	TRINITY_DN17482_c0_g2	1.93	up	hypothetical protein POPTR_0003s15960g, partial [Populus trichocarpa]	494.65	200.08
map03010	Ribosome	4.75E-12	TRINITY_DN17607_c0_g1	1.34	up	hypothetical protein POPTR_0001s10030g [Populus trichocarpa]	27.23	16.71
map03010	Ribosome	4.75E-12	TRINITY_DN17609_c0_g1	1.41	up	hypothetical protein CICLE_v10013157mg [Citrus clementina]	121.99	68.61

map03010	Ribosome	4.75E-12	TRINITY_DN17609_c0_g2	1.35	up	40S ribosomal protein S28 [Populus trichocarpa]	271.86	165.24
map03010	Ribosome	4.75E-12	TRINITY_DN17643_c0_g2	1.54	up	hypothetical protein POPTR_0002s18010g [Populus trichocarpa]	896.78	480.46
map03010	Ribosome	4.75E-12	TRINITY_DN17643_c0_g3	1.79	up	PREDICTED: 60S acidic ribosomal protein P1-like [Populus euphratica]	92.14	41.60
map03010	Ribosome	4.75E-12	TRINITY_DN17643_c0_g5	2.31	up	60S acidic ribosomal protein P1 [Populus trichocarpa]	52.58	16.59
map03010	Ribosome	4.75E-12	TRINITY_DN17676_c1_g3	1.47	up	hypothetical protein POPTR_0003s06880g [Populus trichocarpa]	393.62	235.38
map03010	Ribosome	4.75E-12	TRINITY_DN17787_c0_g2	1.04	up	PREDICTED: 30S ribosomal protein S17, chloroplastic-like [Populus euphratica]	17.39	12.04
map03010	Ribosome	4.75E-12	TRINITY_DN17822_c1_g3	1.40	up	ribosomal protein S18 [Populus alba]	8.70	5.11
map03010	Ribosome	4.75E-12	TRINITY_DN17969_c0_g1	1.04	up	ribosomal protein L37 [Populus trichocarpa]	301.06	223.50
map03010	Ribosome	4.75E-12	TRINITY_DN17969_c0_g2	1.24	up	ribosomal protein L37 [Populus trichocarpa]	113.41	74.68
map03010	Ribosome	4.75E-12	TRINITY_DN18000_c0_g7	1.71	up	PREDICTED: 30S ribosomal protein S20, chloroplastic [Populus euphratica]	821.00	378.16
map03010	Ribosome	4.75E-12	TRINITY_DN18188_c1_g2	1.34	up	60S ribosomal protein L30 [Populus trichocarpa]	256.39	154.88
map03010	Ribosome	4.75E-12	TRINITY_DN18188_c1_g3	1.63	up	hypothetical protein POPTR_0007s06050g [Populus trichocarpa]	88.34	43.50
map03010	Ribosome	4.75E-12	TRINITY_DN18188_c1_g4	2.04	up	hypothetical protein POPTR_0005s08250g [Populus trichocarpa]	80.17	29.95
map03010	Ribosome	4.75E-12	TRINITY_DN18193_c1_g1	1.37	up	ribosomal protein L32 (chloroplast) [Populus alba]	515.22	296.15
map03010	Ribosome	4.75E-12	TRINITY_DN18219_c0_g1	1.17	up	ubiquitin-40S ribosomal protein S27a-like [Solanum tuberosum]	766.87	524.72
map03010	Ribosome	4.75E-12	TRINITY_DN18219_c0_g2	1.01	up	ubiquitin-40S ribosomal protein S27a [Solanum tuberosum]	49.38	38.48
map03010	Ribosome	4.75E-12	TRINITY_DN18247_c0_g7	1.87	up	hypothetical protein CARUB_v10018208mg [Capsella rubella]	262.28	111.61
map03010	Ribosome	4.75E-12	TRINITY_DN18290_c0_g1	-1.63	down	hypothetical protein POPTR_0012s08690g [Populus trichocarpa]	2.93	16.03
map03010	Ribosome	4.75E-12	TRINITY_DN18290_c0_g5	-1.44	down	octicosapeptide/Phox/Bem1p domain-containing family protein [Populus trichocarpa]	3.38	14.20
map03010	Ribosome	4.75E-12	TRINITY_DN18318_c0_g2	1.63	up	unknown [Populus trichocarpa]	339.70	165.15
map03010	Ribosome	4.75E-12	TRINITY_DN18446_c0_g1	1.40	up	hypothetical protein POPTR_0006s25990g [Populus trichocarpa]	578.79	320.15
map03010	Ribosome	4.75E-12	TRINITY_DN18554_c0_g1	1.06	up	hypothetical protein POPTR_0007s11880g [Populus trichocarpa]	182.04	134.60
map03010	Ribosome	4.75E-12	TRINITY_DN18568_c0_g1	1.20	up	PREDICTED: 60S ribosomal protein L13a-2 [Populus euphratica]	641.02	423.98
map03010	Ribosome	4.75E-12	TRINITY_DN18727_c0_g1	1.18	up	hypothetical protein POPTR_0007s11440g [Populus trichocarpa]	34.61	23.25
map03010	Ribosome	4.75E-12	TRINITY_DN18731_c0_g3	1.58	up	unknown [Populus trichocarpa x Populus deltoides]	496.17	250.77
map03010	Ribosome	4.75E-12	TRINITY_DN18853_c2_g1	1.38	up	60S ribosomal protein L18 [Populus trichocarpa]	314.78	185.66
map03010	Ribosome	4.75E-12	TRINITY_DN18890_c0_g1	2.04	up	hypothetical protein POPTR_0010s09820g [Populus trichocarpa]	278.90	106.23
map03010	Ribosome	4.75E-12	TRINITY_DN18930_c0_g1	1.23	up	hypothetical protein POPTR_0008s08960g [Populus trichocarpa]	47.89	31.57
map03010	Ribosome	4.75E-12	TRINITY_DN18957_c1_g1	1.47	up	chloroplast lumen common family protein [Populus trichocarpa]	111.09	60.25
map03010	Ribosome	4.75E-12	TRINITY_DN18986_c0_g1	1.48	up	hypothetical protein PRUPE_ppa019382mg, partial [Prunus persica]	61.85	34.35
map03010	Ribosome	4.75E-12	TRINITY_DN18986_c0_g3	1.57	up	hypothetical protein POPTR_0017s05460g [Populus trichocarpa]	764.68	391.32
map03010	Ribosome	4.75E-12	TRINITY_DN18986_c0_g7	1.33	up	hypothetical protein POPTR_0017s05460g [Populus trichocarpa]	471.11	287.73
map03010	Ribosome	4.75E-12	TRINITY_DN19044_c0_g1	1.56	up	hypothetical protein LR48_Vigan01g206700 [Vigna angularis]	511.76	267.25
map03010	Ribosome	4.75E-12	TRINITY_DN19048_c0_g2	1.37	up	hypothetical protein POPTR_0014s01800g [Populus trichocarpa]	432.06	233.35
map03010	Ribosome	4.75E-12	TRINITY_DN19069_c0_g3	1.75	up	wrp15a family protein [Populus trichocarpa]	854.22	394.65
map03010	Ribosome	4.75E-12	TRINITY_DN19069_c0_g4	1.73	up	wrp15a family protein [Populus trichocarpa]	1070.52	510.47
map03010	Ribosome	4.75E-12	TRINITY_DN19084_c1_g7	1.53	up	hypothetical protein POPTR_0010s19380g [Populus trichocarpa]	1039.66	558.76
map03010	Ribosome	4.75E-12	TRINITY_DN19089_c0_g1	1.17	up	60S ribosomal protein L26B [Populus trichocarpa]	727.41	501.12
map03010	Ribosome	4.75E-12	TRINITY_DN19210_c0_g1	1.69	up	hypothetical protein POPTR_0001s44110g [Populus trichocarpa]	360.84	169.37
map03010	Ribosome	4.75E-12	TRINITY_DN19225_c0_g1	1.57	up	40S ribosomal protein S2 [Populus trichocarpa]	278.03	143.62
map03010	Ribosome	4.75E-12	TRINITY_DN19225_c0_g5	1.51	up	40S ribosomal protein S2 [Populus trichocarpa]	383.08	208.43
map03010	Ribosome	4.75E-12	TRINITY_DN19256_c3_g2	1.57	up	Ribosomal protein L29 [Cynara cardunculus var. scolymus]	374.13	195.78
map03010	Ribosome	4.75E-12	TRINITY_DN19265_c2_g1	1.29	up	PREDICTED: 60S ribosomal protein L44 [Theobroma cacao]	814.87	538.68
map03010	Ribosome	4.75E-12	TRINITY_DN19445_c0_g1	1.76	up	PREDICTED: 36.4 kDa proline-rich protein isoform X2 [Populus euphratica]	4481.86	1973.35
map03010	Ribosome	4.75E-12	TRINITY_DN19482_c0_g2	1.61	up	30S ribosomal protein S9 [Populus trichocarpa]	516.76	258.18
map03010	Ribosome	4.75E-12	TRINITY_DN19483_c0_g1	1.00	up	ribosomal protein S5 [Populus trichocarpa]	18.63	14.40

map03010	Ribosome	4.75E-12	TRINITY_DN19532_c0_g1	1.67	up	ribosomal protein L33 [Populus trichocarpa]	602.96	245.47
map03010	Ribosome	4.75E-12	TRINITY_DN19583_c0_g3	1.12	up	PREDICTED: 60S ribosomal protein L37a [Ricinus communis]	241.15	163.82
map03010	Ribosome	4.75E-12	TRINITY_DN19590_c0_g1	1.19	up	hypothetical protein POPTR_0002s15600g [Populus trichocarpa]	388.73	254.29
map03010	Ribosome	4.75E-12	TRINITY_DN19638_c0_g1	1.45	up	hypothetical protein POPTR_0009s03210g [Populus trichocarpa]	10.61	5.94
map03010	Ribosome	4.75E-12	TRINITY_DN19638_c0_g2	1.64	up	PREDICTED: 60S ribosomal protein L23a-like [Populus euphratica]	487.27	241.23
map03010	Ribosome	4.75E-12	TRINITY_DN19688_c0_g1	1.19	up	hypothetical protein POPTR_0001s22620g [Populus trichocarpa]	941.89	640.14
map03010	Ribosome	4.75E-12	TRINITY_DN19739_c0_g2	1.42	up	60S ribosomal protein L22-1 [Populus trichocarpa]	184.76	107.03
map03010	Ribosome	4.75E-12	TRINITY_DN19739_c0_g3	1.34	up	hypothetical protein POPTR_0005s02430g [Populus trichocarpa]	525.21	316.51
map03010	Ribosome	4.75E-12	TRINITY_DN19750_c1_g1	1.54	up	ribosomal protein S16 [Populus trichocarpa]	278.01	147.92
map03010	Ribosome	4.75E-12	TRINITY_DN19750_c1_g4	1.33	up	40S ribosomal protein S16 [Populus trichocarpa]	746.46	441.88
map03010	Ribosome	4.75E-12	TRINITY_DN19947_c0_g1	1.26	up	PREDICTED: 40S ribosomal protein S11 [Populus euphratica]	757.36	474.08
map03010	Ribosome	4.75E-12	TRINITY_DN19991_c0_g1	1.36	up	hypothetical protein POPTR_0007s11880g [Populus trichocarpa]	896.89	556.97
map03010	Ribosome	4.75E-12	TRINITY_DN20054_c0_g1	1.50	up	unknown [Populus trichocarpa]	106.19	60.79
map03010	Ribosome	4.75E-12	TRINITY_DN20102_c0_g6	1.74	up	PREDICTED: 50S ribosomal protein L29, chloroplastic [Populus euphratica]	500.72	224.46
map03010	Ribosome	4.75E-12	TRINITY_DN20141_c0_g3	1.42	up	hypothetical protein POPTR_0006s25780g [Populus trichocarpa]	60.54	34.19
map03010	Ribosome	4.75E-12	TRINITY_DN20141_c0_g4	2.01	up	hypothetical protein POPTR_0018s00640g [Populus trichocarpa]	49.98	18.33
map03010	Ribosome	4.75E-12	TRINITY_DN20224_c0_g2	1.53	up	hypothetical protein POPTR_0006s23770g [Populus trichocarpa]	639.63	329.65
map03010	Ribosome	4.75E-12	TRINITY_DN20285_c0_g2	1.21	up	hypothetical protein POPTR_0014s18940g [Populus trichocarpa]	849.10	568.63
map03010	Ribosome	4.75E-12	TRINITY_DN20325_c0_g4	1.28	up	hypothetical protein POPTR_0002s04420g [Populus trichocarpa]	658.50	416.42
map03010	Ribosome	4.75E-12	TRINITY_DN20325_c0_g6	1.23	up	ribosomal S15 family protein [Populus trichocarpa]	87.33	60.14
map03010	Ribosome	4.75E-12	TRINITY_DN20332_c0_g1	1.77	up	PREDICTED: 60S ribosomal protein L15-like [Populus euphratica]	437.96	171.77
map03010	Ribosome	4.75E-12	TRINITY_DN20334_c0_g1	1.53	up	hypothetical protein POPTR_0016s06110g [Populus trichocarpa]	303.04	161.71
map03010	Ribosome	4.75E-12	TRINITY_DN20334_c0_g2	1.69	up	hypothetical protein POPTR_0006s21010g [Populus trichocarpa]	323.45	154.47
map03010	Ribosome	4.75E-12	TRINITY_DN20356_c0_g1	1.68	up	hypothetical protein POPTR_0013s11720g [Populus trichocarpa]	846.83	399.04
map03010	Ribosome	4.75E-12	TRINITY_DN20390_c0_g1	1.09	up	PREDICTED: ribosomal L1 domain-containing protein 1-like [Populus euphratica]	13.97	10.24
map03010	Ribosome	4.75E-12	TRINITY_DN20457_c0_g3	1.71	up	PREDICTED: 50S ribosomal protein L31, chloroplastic [Populus euphratica]	248.10	114.54
map03010	Ribosome	4.75E-12	TRINITY_DN20507_c1_g5	1.43	up	60S ribosomal protein L6 [Populus trichocarpa]	941.62	558.85
map03010	Ribosome	4.75E-12	TRINITY_DN20576_c1_g1	1.32	up	40S ribosomal protein S19 [Populus trichocarpa]	811.25	497.43
map03010	Ribosome	4.75E-12	TRINITY_DN20576_c1_g3	1.30	up	PREDICTED: 40S ribosomal protein S19-3-like isoform X1 [Populus euphratica]	69.44	43.42
map03010	Ribosome	4.75E-12	TRINITY_DN20646_c0_g1	-1.45	down	PREDICTED: uncharacterized protein LOC105127223 isoform X1 [Populus euphratica]	2.02	8.37
map03010	Ribosome	4.75E-12	TRINITY_DN20650_c0_g1	1.00	up	hypothetical protein GLYMA_20G228100 [Glycine max]	727.58	551.01
map03010	Ribosome	4.75E-12	TRINITY_DN20677_c1_g1	1.99	up	PREDICTED: 54S ribosomal protein L10, mitochondrial-like [Populus euphratica]	40.34	15.48
map03010	Ribosome	4.75E-12	TRINITY_DN20717_c0_g2	1.47	up	hypothetical protein EUGRSUZ_H02959 [Eucalyptus grandis]	563.81	320.33
map03010	Ribosome	4.75E-12	TRINITY_DN20717_c0_g4	1.13	up	hypothetical protein EUGRSUZ_H02959 [Eucalyptus grandis]	123.63	87.58
map03010	Ribosome	4.75E-12	TRINITY_DN20717_c0_g8	1.84	up	hypothetical protein EUGRSUZ_H02959 [Eucalyptus grandis]	724.23	311.69
map03010	Ribosome	4.75E-12	TRINITY_DN20718_c0_g1	1.48	up	hypothetical protein POPTR_0008s12000g [Populus trichocarpa]	337.73	182.53
map03010	Ribosome	4.75E-12	TRINITY_DN20734_c0_g1	1.47	up	hypothetical protein POPTR_0019s05610g [Populus trichocarpa]	25.35	14.54
map03010	Ribosome	4.75E-12	TRINITY_DN20774_c0_g1	1.18	up	hypothetical protein EUGRSUZ_J00099 [Eucalyptus grandis]	985.72	659.04
map03010	Ribosome	4.75E-12	TRINITY_DN20803_c0_g1	1.44	up	40S ribosomal protein S20 [Populus trichocarpa]	489.59	282.90
map03010	Ribosome	4.75E-12	TRINITY_DN20822_c0_g1	1.32	up	hypothetical protein POPTR_0018s12030g [Populus trichocarpa]	689.57	416.53
map03010	Ribosome	4.75E-12	TRINITY_DN21034_c0_g1	1.14	up	hypothetical protein POPTR_0019s13040g [Populus trichocarpa]	1026.65	691.90
map03010	Ribosome	4.75E-12	TRINITY_DN21034_c0_g4	1.51	up	hypothetical protein POPTR_0013s13240g [Populus trichocarpa]	52.10	27.79
map03010	Ribosome	4.75E-12	TRINITY_DN21038_c0_g2	1.36	up	PREDICTED: 40S ribosomal protein S5-like [Populus euphratica]	924.86	550.50

map03010	Ribosome	4.75E-12	TRINITY_DN21071_c0_g1	1.36	up	PREDICTED: uncharacterized protein LOC105114560 [Populus euphratica]	14.20	8.49
map03010	Ribosome	4.75E-12	TRINITY_DN21108_c0_g3	1.41	up	PREDICTED: 40S ribosomal protein S26-3-like [Populus euphratica]	675.14	389.54
map03010	Ribosome	4.75E-12	TRINITY_DN21108_c0_g6	1.40	up	PREDICTED: 40S ribosomal protein S26-3 [Populus euphratica]	224.88	130.80
map03010	Ribosome	4.75E-12	TRINITY_DN21116_c0_g9	1.11	up	hypothetical protein POPTR_0014s15570g [Populus trichocarpa]	121.25	86.35
map03010	Ribosome	4.75E-12	TRINITY_DN21123_c0_g3	1.85	up	unnamed protein product, partial [Vitis vinifera]	299.70	128.63
map03010	Ribosome	4.75E-12	TRINITY_DN21166_c0_g4	2.00	up	-	310.48	115.73
map03010	Ribosome	4.75E-12	TRINITY_DN21166_c0_g5	1.79	up	hypothetical protein POPTR_0008s04560g [Populus trichocarpa]	105.61	45.65
map03010	Ribosome	4.75E-12	TRINITY_DN21166_c0_g7	1.74	up	hypothetical protein POPTR_0010s22290g [Populus trichocarpa]	230.47	103.21
map03010	Ribosome	4.75E-12	TRINITY_DN21176_c0_g3	1.28	up	PREDICTED: 60S acidic ribosomal protein P0 [Populus euphratica]	763.19	456.15
map03010	Ribosome	4.75E-12	TRINITY_DN21181_c0_g1	1.51	up	hypothetical protein POPTR_0002s08900g [Populus trichocarpa]	660.13	352.10
map03010	Ribosome	4.75E-12	TRINITY_DN21185_c1_g9	1.71	up	ribosomal protein S4 [Populus alba]	21.79	10.00
map03010	Ribosome	4.75E-12	TRINITY_DN21204_c0_g1	1.78	up	hypothetical protein POPTR_0002s05220g [Populus trichocarpa]	587.19	270.27
map03010	Ribosome	4.75E-12	TRINITY_DN21221_c0_g1	1.39	up	hypothetical protein POPTR_0016s06950g [Populus trichocarpa]	416.19	246.22
map03010	Ribosome	4.75E-12	TRINITY_DN21221_c0_g2	1.25	up	hypothetical protein POPTR_0008s19110g [Populus trichocarpa]	542.34	339.17
map03010	Ribosome	4.75E-12	TRINITY_DN21283_c2_g1	1.26	up	PREDICTED: uncharacterized protein LOC105114856 [Populus euphratica]	121.97	77.71
map03010	Ribosome	4.75E-12	TRINITY_DN21331_c0_g1	1.46	up	hypothetical protein POPTR_0015s12960g [Populus trichocarpa]	1951.25	1082.52
map03010	Ribosome	4.75E-12	TRINITY_DN21390_c0_g1	1.18	up	60S ribosomal protein L19-2 [Populus trichocarpa]	291.74	205.26
map03010	Ribosome	4.75E-12	TRINITY_DN21390_c0_g3	1.21	up	PREDICTED: 60S ribosomal protein L19-3-like [Populus euphratica]	527.62	353.97
map03010	Ribosome	4.75E-12	TRINITY_DN21393_c0_g1	1.03	up	unknown [Populus trichocarpa]	24.00	17.89
map03010	Ribosome	4.75E-12	TRINITY_DN21393_c0_g2	1.01	up	unknown [Populus trichocarpa]	27.58	21.40
map03010	Ribosome	4.75E-12	TRINITY_DN21398_c0_g1	1.23	up	hypothetical protein POPTR_0004s04550g [Populus trichocarpa]	565.72	361.32
map03010	Ribosome	4.75E-12	TRINITY_DN21443_c0_g1	1.37	up	ribosomal protein S13 [Populus trichocarpa]	64.53	38.20
map03010	Ribosome	4.75E-12	TRINITY_DN21443_c0_g2	1.42	up	PREDICTED: 40S ribosomal protein S13-like [Populus euphratica]	213.24	123.96
map03010	Ribosome	4.75E-12	TRINITY_DN21443_c0_g3	1.30	up	ribosomal protein S13 [Populus trichocarpa]	234.19	147.73
map03010	Ribosome	4.75E-12	TRINITY_DN21474_c0_g1	1.14	up	unknown [Populus trichocarpa x Populus deltoides]	246.97	177.04
map03010	Ribosome	4.75E-12	TRINITY_DN21474_c0_g4	1.88	up	PREDICTED: 40S ribosomal protein S12-like [Populus euphratica]	392.34	166.54
map03010	Ribosome	4.75E-12	TRINITY_DN21476_c0_g2	1.70	up	PREDICTED: 60S ribosomal protein L7-2-like [Populus euphratica]	316.17	150.32
map03010	Ribosome	4.75E-12	TRINITY_DN21552_c0_g1	1.19	up	PREDICTED: 60S ribosomal protein L14-1-like [Populus euphratica]	180.10	121.87
map03010	Ribosome	4.75E-12	TRINITY_DN21552_c0_g5	2.41	up	60S ribosomal protein L14 [Populus trichocarpa]	114.26	33.51
map03010	Ribosome	4.75E-12	TRINITY_DN21552_c0_g6	1.09	up	60S ribosomal protein L14 [Populus trichocarpa]	1111.47	757.20
map03010	Ribosome	4.75E-12	TRINITY_DN21559_c0_g2	-1.37	down	hypothetical protein POPTR_0024s00730g [Populus trichocarpa]	1.31	5.26
map03010	Ribosome	4.75E-12	TRINITY_DN21615_c0_g3	1.16	up	unknown [Populus trichocarpa x Populus deltoides]	516.99	349.08
map03010	Ribosome	4.75E-12	TRINITY_DN21638_c0_g2	1.35	up	hypothetical protein POPTR_0010s21460g [Populus trichocarpa]	109.75	65.40
map00941	Flavonoid biosynthesis	2.55E-05	TRINITY_DN21866_c0_g1	1.05	up	hypothetical protein POPTR_0016s12440g [Populus trichocarpa]	40.27	34.46
map00941	Flavonoid biosynthesis	2.55E-05	TRINITY_DN22466_c0_g3	1.36	up	hypothetical protein POPTR_0001s10460g [Populus trichocarpa]	6.63	3.91
map00941	Flavonoid biosynthesis	2.55E-05	TRINITY_DN22579_c0_g1	3.09	up	hypothetical protein POPTR_0001s07860g [Populus trichocarpa]	29.32	7.54
map00941	Flavonoid biosynthesis	2.55E-05	TRINITY_DN23459_c0_g1	-8.35	down	PREDICTED: flavonoid 3',5'-hydroxylase 2-like [Populus euphratica]	0.03	14.96
map00941	Flavonoid biosynthesis	2.55E-05	TRINITY_DN23884_c1_g3	-3.93	down	hypothetical protein POPTR_0010s13990g [Populus trichocarpa]	1.23	28.18
map00941	Flavonoid biosynthesis	2.55E-05	TRINITY_DN25107_c0_g1	1.16	up	HXXXD-type acyl-transferase family protein [Populus tomentosa]	31.54	21.30
map00941	Flavonoid biosynthesis	2.55E-05	TRINITY_DN25371_c4_g1	-1.21	down	quinate O-hydroxycinnamoyltransferase/shikimate O-hydroxycinnamoyltransferase [Populus trichocarpa]	8.71	30.47
map00941	Flavonoid biosynthesis	2.55E-05	TRINITY_DN25380_c0_g1	6.17	up	hypothetical protein POPTR_0008s02970g [Populus trichocarpa]	5.41	0.11
map00941	Flavonoid biosynthesis	2.55E-05	TRINITY_DN25931_c0_g1	-1.22	down	hypothetical protein POPTR_0005s11600g [Populus trichocarpa]	47.00	165.81
map00941	Flavonoid biosynthesis	2.55E-05	TRINITY_DN25946_c0_g1	-3.03	down	PREDICTED: chalcone synthase 1-like [Populus euphratica]	17.29	205.36
map00941	Flavonoid biosynthesis	2.55E-05	TRINITY_DN25946_c0_g2	-3.86	down	PREDICTED: chalcone synthase 1-like isoform X1 [Populus euphratica]	3.16	68.59

map00941	Flavonoid biosynthesis	2.55E-05	TRINITY_DN26707_c0_g1	-2.45	down	hypothetical protein POPTR_0005s02810g [Populus trichocarpa]	11.02	80.41
map00941	Flavonoid biosynthesis	2.55E-05	TRINITY_DN27893_c12_g1	-5.22	down	PREDICTED: protein ECERIFERUM 26-like [Populus euphratica]	0.08	4.60
map00941	Flavonoid biosynthesis	2.55E-05	TRINITY_DN27900_c0_g1	1.54	up	PREDICTED: 1-aminocyclopropane-1-carboxylate oxidase 5-like [Populus euphratica]	20.23	10.42
map00941	Flavonoid biosynthesis	2.55E-05	TRINITY_DN12704_c0_g2	-2.23	down	PREDICTED: BAHD acyltransferase At5g47980-like [Populus euphratica]	0.27	1.96
map00941	Flavonoid biosynthesis	2.55E-05	TRINITY_DN12720_c0_g2	1.29	up	PREDICTED: vinorine synthase-like [Populus euphratica]	7.62	4.66
map00941	Flavonoid biosynthesis	2.55E-05	TRINITY_DN14060_c0_g1	-1.83	down	hypothetical protein POPTR_0001s15250g [Populus trichocarpa]	0.52	2.87
map00941	Flavonoid biosynthesis	2.55E-05	TRINITY_DN14458_c0_g1	-1.51	down	hypothetical protein POPTR_0019s10680g [Populus trichocarpa]	1.37	5.09
map00941	Flavonoid biosynthesis	2.55E-05	TRINITY_DN15581_c0_g1	-2.43	down	PREDICTED: dihydroflavonol-4-reductase, partial [Populus euphratica]	0.50	4.03
map00941	Flavonoid biosynthesis	2.55E-05	TRINITY_DN1593_c0_g1	-1.37	down	hypothetical protein POPTR_0019s01540g [Populus trichocarpa]	1.85	7.22
map00941	Flavonoid biosynthesis	2.55E-05	TRINITY_DN16022_c0_g1	2.65	up	hypothetical protein POPTR_0640s00200g [Populus trichocarpa]	6.27	1.56
map00941	Flavonoid biosynthesis	2.55E-05	TRINITY_DN16183_c0_g2	-1.80	down	hypothetical protein POPTR_0006s01190g [Populus trichocarpa]	1.74	8.00
map00941	Flavonoid biosynthesis	2.55E-05	TRINITY_DN16332_c1_g3	5.06	up	hypothetical protein POPTR_0011s15310g [Populus trichocarpa]	8.29	0.36
map00941	Flavonoid biosynthesis	2.55E-05	TRINITY_DN16677_c0_g1	-6.00	down	hypothetical protein POPTR_0007s15050g [Populus trichocarpa]	0.16	11.75
map00941	Flavonoid biosynthesis	2.55E-05	TRINITY_DN17112_c0_g1	-4.56	down	Leucoanthocyanidin reductase family protein [Populus trichocarpa]	3.70	139.01
map00941	Flavonoid biosynthesis	2.55E-05	TRINITY_DN17411_c0_g1	-1.84	down	PREDICTED: probable chalcone--flavonone isomerase 3 [Populus euphratica]	26.53	143.54
map00941	Flavonoid biosynthesis	2.55E-05	TRINITY_DN17792_c0_g1	-1.77	down	PREDICTED: protein SRG1-like [Populus euphratica]	1.35	7.32
map00941	Flavonoid biosynthesis	2.55E-05	TRINITY_DN18234_c0_g5	2.18	up	PREDICTED: S-norcoclaurine synthase 1-like isoform X1 [Populus euphratica]	2.25	1.00
map00941	Flavonoid biosynthesis	2.55E-05	TRINITY_DN18302_c0_g2	-5.73	down	hypothetical protein POPTR_0008s03410g [Populus trichocarpa]	0.06	5.58
map00941	Flavonoid biosynthesis	2.55E-05	TRINITY_DN18330_c0_g1	-2.18	down	trans-cinnamate 4-hydroxylase [Populus trichocarpa]	0.62	4.40
map00941	Flavonoid biosynthesis	2.55E-05	TRINITY_DN19661_c0_g1	2.12	up	unknown [Populus trichocarpa]	168.92	61.85
map00941	Flavonoid biosynthesis	2.55E-05	TRINITY_DN19958_c0_g2	-1.13	down	RecName: Full=Caffeoyl-CoA O-methyltransferase; AltName: Full=Trans-caffeoyl-CoA 3-O-methyltransferase; Short=CCoAMT; Short=CCoAOMT	6.52	20.89
map00941	Flavonoid biosynthesis	2.55E-05	TRINITY_DN20113_c0_g1	-1.45	down	chalcone synthase [Populus alba]	95.98	585.75
map00941	Flavonoid biosynthesis	2.55E-05	TRINITY_DN20364_c0_g2	-1.63	down	PREDICTED: BAHD acyltransferase DCR [Populus euphratica]	14.38	66.96
map00941	Flavonoid biosynthesis	2.55E-05	TRINITY_DN20644_c0_g1	-1.68	down	hypothetical protein POPTR_0013s10080g [Populus trichocarpa]	10.85	54.73
map00941	Flavonoid biosynthesis	2.55E-05	TRINITY_DN20842_c0_g1	1.07	up	hypothetical protein POPTR_0008s18060g [Populus trichocarpa]	8.67	6.26
map00941	Flavonoid biosynthesis	2.55E-05	TRINITY_DN20905_c0_g3	-1.18	down	p-coumarate 3-hydroxylase [Populus tomentosa]	2.28	8.17
map00941	Flavonoid biosynthesis	2.55E-05	TRINITY_DN20924_c0_g1	1.28	up	transferase family protein [Populus trichocarpa]	32.99	19.55
map00941	Flavonoid biosynthesis	2.55E-05	TRINITY_DN20924_c0_g2	1.79	up	transferase family protein [Populus trichocarpa]	14.61	6.33
map00941	Flavonoid biosynthesis	2.55E-05	TRINITY_DN21068_c1_g1	-1.96	down	hypothetical protein POPTR_0013s07050g [Populus trichocarpa]	25.27	141.84
map00941	Flavonoid biosynthesis	2.55E-05	TRINITY_DN21253_c0_g1	1.45	up	hypothetical protein POPTR_0015s13430g [Populus trichocarpa]	14.73	8.81
map00941	Flavonoid biosynthesis	2.55E-05	TRINITY_DN21253_c0_g3	-1.59	down	hypothetical protein POPTR_0012s13440g [Populus trichocarpa]	0.53	2.49
map00941	Flavonoid biosynthesis	2.55E-05	TRINITY_DN21253_c0_g4	-2.89	down	hypothetical protein POPTR_0012s13440g [Populus trichocarpa]	0.21	2.45
map00941	Flavonoid biosynthesis	2.55E-05	TRINITY_DN21593_c0_g2	-3.92	down	hypothetical protein POPTR_0010s13990g [Populus trichocarpa]	1.46	33.85
map00941	Flavonoid biosynthesis	2.55E-05	TRINITY_DN21593_c0_g3	-5.38	down	hypothetical protein POPTR_0008s11540g [Populus trichocarpa]	0.44	28.13
map00941	Flavonoid biosynthesis	2.55E-05	TRINITY_DN391_c0_g1	1.28	up	hypothetical protein POPTR_0340s00200g [Populus trichocarpa]	62.66	39.30
map00941	Flavonoid biosynthesis	2.55E-05	TRINITY_DN542_c0_g1	3.11	up	hypothetical protein POPTR_0011s15340g [Populus trichocarpa]	5.50	0.97
map00941	Flavonoid biosynthesis	2.55E-05	TRINITY_DN8020_c0_g1	-7.14	down	hypothetical protein POPTR_0015s03360g [Populus trichocarpa]	0.03	6.44
map00195	Photosynthesis	3.00E-05	TRINITY_DN21880_c0_g1	1.28	up	hypothetical protein POPTR_0007s04160g [Populus trichocarpa]	1115.78	684.71
map00195	Photosynthesis	3.00E-05	TRINITY_DN22158_c0_g2	1.75	up	photosystem 2 reaction center PsbP family protein [Populus trichocarpa]	238.39	102.06
map00195	Photosynthesis	3.00E-05	TRINITY_DN22388_c1_g1	1.28	up	PREDICTED: ferredoxin isoform X1 [Populus euphratica]	256.46	159.05
map00195	Photosynthesis	3.00E-05	TRINITY_DN22712_c0_g1	1.81	up	PHOTOSYNTHETIC ELECTRON TRANSFER C family protein [Populus trichocarpa]	1514.69	688.40
map00195	Photosynthesis	3.00E-05	TRINITY_DN22869_c0_g3	2.00	up	photosystem I P700 apoprotein A1 (chloroplast) [Ficus racemosa]	8.78	3.54

map00195	Photosynthesis	3.00E-05	TRINITY_DN22910_c0_g1	1.77	up	PREDICTED: uncharacterized protein LOC105109113 [Populus euphratica]	113.46	49.41
map00195	Photosynthesis	3.00E-05	TRINITY_DN22986_c0_g3	1.64	up	photosystem II protein I (plastid) [Pigafetta elata]	23.48	11.49
map00195	Photosynthesis	3.00E-05	TRINITY_DN23019_c0_g2	1.89	up	PREDICTED: psbQ-like protein 2, chloroplastic [Populus euphratica]	168.82	67.76
map00195	Photosynthesis	3.00E-05	TRINITY_DN23265_c0_g3	1.26	up	PREDICTED: photosystem I reaction center subunit IV A, chloroplastic-like [Populus euphratica]	596.74	377.27
map00195	Photosynthesis	3.00E-05	TRINITY_DN23422_c1_g1	1.95	up	hypothetical protein POPTR_0013s12130g [Populus trichocarpa]	344.08	129.79
map00195	Photosynthesis	3.00E-05	TRINITY_DN23422_c1_g4	2.37	up	PREDICTED: ATP synthase delta chain, chloroplastic-like [Populus euphratica]	1050.78	305.83
map00195	Photosynthesis	3.00E-05	TRINITY_DN23514_c0_g1	1.59	up	PREDICTED: ferredoxin-A-like [Populus euphratica]	903.88	450.99
map00195	Photosynthesis	3.00E-05	TRINITY_DN23514_c0_g3	2.72	up	PREDICTED: ferredoxin-like [Populus euphratica]	4060.18	921.95
map00195	Photosynthesis	3.00E-05	TRINITY_DN23562_c0_g2	1.43	up	photosystem 2 family protein [Populus trichocarpa]	171.51	97.36
map00195	Photosynthesis	3.00E-05	TRINITY_DN23562_c0_g6	1.50	up	photosystem 2 family protein [Populus trichocarpa]	467.73	251.32
map00195	Photosynthesis	3.00E-05	TRINITY_DN23617_c0_g1	1.17	up	hypothetical protein POPTR_0004s20960g [Populus trichocarpa]	1038.82	690.16
map00195	Photosynthesis	3.00E-05	TRINITY_DN23901_c1_g2	1.64	up	PREDICTED: ferredoxin-3, chloroplastic-like isoform X1 [Populus euphratica]	32.09	19.85
map00195	Photosynthesis	3.00E-05	TRINITY_DN23901_c1_g4	1.43	up	PREDICTED: ferredoxin-3, chloroplastic-like isoform X1 [Populus euphratica]	126.81	55.15
map00195	Photosynthesis	3.00E-05	TRINITY_DN24685_c1_g3	1.39	up	photosystem II 44 kDa protein (chloroplast) [Populus tremula]	19.93	11.90
map00195	Photosynthesis	3.00E-05	TRINITY_DN25093_c0_g3	2.15	up	ATP synthase delta chain-related family protein [Populus trichocarpa]	382.66	129.01
map00195	Photosynthesis	3.00E-05	TRINITY_DN25205_c1_g7	1.80	up	photosystem I subunit VII (chloroplast) [Ficus racemosa]	44.90	19.65
map00195	Photosynthesis	3.00E-05	TRINITY_DN25230_c0_g1	1.31	up	PREDICTED: oxygen-evolving enhancer protein 1, chloroplastic [Populus euphratica]	2105.50	1291.76
map00195	Photosynthesis	3.00E-05	TRINITY_DN25369_c0_g2	2.17	up	PREDICTED: uncharacterized protein LOC105142434 [Populus euphratica]	198.49	66.26
map00195	Photosynthesis	3.00E-05	TRINITY_DN25783_c0_g2	2.32	up	PREDICTED: plastocyanin B, chloroplastic [Populus euphratica]	3485.86	1020.60
map00195	Photosynthesis	3.00E-05	TRINITY_DN25847_c1_g3	1.81	up	Chain A family protein [Populus trichocarpa]	1458.06	589.75
map00195	Photosynthesis	3.00E-05	TRINITY_DN25847_c1_g4	2.11	up	-	563.10	196.68
map00195	Photosynthesis	3.00E-05	TRINITY_DN26216_c0_g4	1.98	up	photosystem I P700 apoprotein A2 [Populus alba]	13.34	5.38
map00195	Photosynthesis	3.00E-05	TRINITY_DN27867_c4_g4	1.77	up	ATP synthase CF1 alpha chain [Populus alba]	41.58	18.74
map00195	Photosynthesis	3.00E-05	TRINITY_DN13304_c0_g1	5.61	up	Ferredoxin 2 family protein [Populus trichocarpa]	11.33	0.36
map00195	Photosynthesis	3.00E-05	TRINITY_DN16458_c0_g1	1.67	up	PREDICTED: LOW QUALITY PROTEIN: uncharacterized protein LOC105136146 [Populus euphratica]	1816.06	855.39
map00195	Photosynthesis	3.00E-05	TRINITY_DN16622_c0_g1	2.12	up	hypothetical protein POPTR_0002s25810g [Populus trichocarpa]	158.06	55.46
map00195	Photosynthesis	3.00E-05	TRINITY_DN16751_c0_g1	1.61	up	PREDICTED: photosystem II core complex proteins psbY, chloroplastic-like [Populus euphratica]	1155.38	570.16
map00195	Photosynthesis	3.00E-05	TRINITY_DN16751_c0_g2	1.35	up	Photosystem II core complex proteins psbY [Populus trichocarpa]	1306.26	779.10
map00195	Photosynthesis	3.00E-05	TRINITY_DN16897_c0_g7	1.28	up	PREDICTED: photosystem II reaction center W protein, chloroplastic isoform X1 [Populus euphratica]	3123.34	1949.44
map00195	Photosynthesis	3.00E-05	TRINITY_DN17339_c0_g1	1.23	up	unknown [Populus trichocarpa]	1856.73	1211.18
map00195	Photosynthesis	3.00E-05	TRINITY_DN17822_c1_g6	1.46	up	cytochrome f [Populus alba]	3.71	2.11
map00195	Photosynthesis	3.00E-05	TRINITY_DN18030_c1_g4	1.49	up	oxygen evolving enhancer 3 family protein [Populus trichocarpa]	306.45	163.48
map00195	Photosynthesis	3.00E-05	TRINITY_DN18189_c0_g1	1.01	up	ATP synthase gamma chain 1 family protein [Populus trichocarpa]	5.06	3.77
map00195	Photosynthesis	3.00E-05	TRINITY_DN18189_c0_g2	1.79	up	ATP synthase gamma chain 1 family protein [Populus trichocarpa]	776.42	338.36
map00195	Photosynthesis	3.00E-05	TRINITY_DN18383_c0_g1	1.33	up	unknown [Populus trichocarpa x Populus deltoides]	2480.53	1514.85
map00195	Photosynthesis	3.00E-05	TRINITY_DN18467_c0_g6	1.36	up	hypothetical protein M569_00483, partial [Genlisea aurea]	8.55	5.09
map00195	Photosynthesis	3.00E-05	TRINITY_DN18553_c0_g2	1.20	up	Photosystem I reaction center subunit V family protein [Populus trichocarpa]	1021.10	673.82
map00195	Photosynthesis	3.00E-05	TRINITY_DN18735_c0_g1	1.30	up	Oxygen-evolving enhancer protein 3-1 [Populus trichocarpa]	1461.45	876.16
map00195	Photosynthesis	3.00E-05	TRINITY_DN18792_c1_g4	1.93	up	photosystem II protein D (chloroplast) [Ficus racemosa]	141.61	58.77
map00195	Photosynthesis	3.00E-05	TRINITY_DN19064_c0_g1	1.05	up	Photosystem I reaction center subunit XI family protein [Populus trichocarpa]	3429.37	2570.38
map00195	Photosynthesis	3.00E-05	TRINITY_DN19282_c0_g1	1.64	up	Thylakoid lumenal 25.6 kDa family protein [Populus trichocarpa]	252.70	124.52
map00195	Photosynthesis	3.00E-05	TRINITY_DN19534_c0_g1	1.17	up	hypothetical protein POPTR_0010s09740g [Populus trichocarpa]	203.71	135.05
map00195	Photosynthesis	3.00E-05	TRINITY_DN19931_c0_g11	1.62	up	cytochrome b6/f complex subunit IV (chloroplast) [Populus euphratica]	6.85	3.19
map00195	Photosynthesis	3.00E-05	TRINITY_DN19931_c0_g4	1.48	up	photosystem II CP47 protein [Populus alba]	37.22	21.34
map00195	Photosynthesis	3.00E-05	TRINITY_DN20084_c1_g1	1.45	up	Photosystem II 22 kDa family protein [Populus trichocarpa]	1435.69	793.79
map00195	Photosynthesis	3.00E-05	TRINITY_DN20990_c0_g10	-1.61	down	AtpE, partial (chloroplast) [Passiflora ciliata]	1.87	9.84

map00195	Photosynthesis	3.00E-05	TRINITY_DN20990_c0_g6	1.51	up	ATP synthase CF1 beta subunit (chloroplast) [Citrus platymamma]	11.47	8.92
map00195	Photosynthesis	3.00E-05	TRINITY_DN21538_c0_g4	1.55	up	unknown [Populus trichocarpa x Populus deltoides]	4735.55	2437.81
map00860	Porphyrin and chlorophyll metabolism	5.64E-05	TRINITY_DN21769_c2_g4	1.62	up	PREDICTED: oxygen-dependent coproporphyrinogen-III oxidase, chloroplastic [Populus euphratica]	163.93	82.30
map00860	Porphyrin and chlorophyll metabolism	5.64E-05	TRINITY_DN21779_c0_g1	1.44	up	hypothetical protein POPTR_0001s40130g [Populus trichocarpa]	94.27	52.40
map00860	Porphyrin and chlorophyll metabolism	5.64E-05	TRINITY_DN22027_c0_g2	1.44	up	hypothetical protein POPTR_0019s00800g [Populus trichocarpa]	5.26	2.81
map00860	Porphyrin and chlorophyll metabolism	5.64E-05	TRINITY_DN22661_c1_g1	1.82	up	short-chain dehydrogenase Tic32 family protein [Populus trichocarpa]	44.77	19.53
map00860	Porphyrin and chlorophyll metabolism	5.64E-05	TRINITY_DN22717_c0_g3	-2.09	down	hypothetical protein POPTR_0003s06910g [Populus trichocarpa]	6.82	35.25
map00860	Porphyrin and chlorophyll metabolism	5.64E-05	TRINITY_DN23425_c0_g1	-5.51	down	PREDICTED: GDSL esterase/lipase At1g29670-like [Populus euphratica]	1.04	73.66
map00860	Porphyrin and chlorophyll metabolism	5.64E-05	TRINITY_DN23530_c1_g2	1.38	up	pyridine nucleotide-disulfide oxidoreductase family protein [Populus tomentosa]	681.14	397.07
map00860	Porphyrin and chlorophyll metabolism	5.64E-05	TRINITY_DN24005_c0_g1	1.32	up	hypothetical protein POPTR_0018s13160g [Populus trichocarpa]	63.97	42.27
map00860	Porphyrin and chlorophyll metabolism	5.64E-05	TRINITY_DN24123_c0_g1	-1.98	down	MYB055 [Populus tomentosa]	0.79	4.58
map00860	Porphyrin and chlorophyll metabolism	5.64E-05	TRINITY_DN24123_c0_g2	-1.27	down	hypothetical protein POPTR_0002s18700g [Populus trichocarpa]	1.27	4.49
map00860	Porphyrin and chlorophyll metabolism	5.64E-05	TRINITY_DN24440_c0_g2	1.30	up	hypothetical protein POPTR_0012s09440g [Populus trichocarpa]	12.89	7.96
map00860	Porphyrin and chlorophyll metabolism	5.64E-05	TRINITY_DN24443_c0_g3	-1.30	down	hypothetical protein POPTR_0006s18240g [Populus trichocarpa]	48.96	203.82
map00860	Porphyrin and chlorophyll metabolism	5.64E-05	TRINITY_DN24687_c0_g2	1.37	up	PREDICTED: uncharacterized protein ycf39-like [Populus euphratica]	127.95	76.05
map00860	Porphyrin and chlorophyll metabolism	5.64E-05	TRINITY_DN25318_c0_g2	1.95	up	hypothetical protein POPTR_0007s07680g [Populus trichocarpa]	138.48	53.82
map00860	Porphyrin and chlorophyll metabolism	5.64E-05	TRINITY_DN25590_c0_g1	-8.33	down	hypothetical protein POPTR_0010s17300g [Populus trichocarpa]	0.04	18.89
map00860	Porphyrin and chlorophyll metabolism	5.64E-05	TRINITY_DN26311_c0_g1	1.31	up	mRNA for protoporphyrinogen oxidase family protein [Populus trichocarpa]	101.12	61.44
map00860	Porphyrin and chlorophyll metabolism	5.64E-05	TRINITY_DN26475_c0_g1	1.03	up	PREDICTED: magnesium-chelatase subunit ChlD, chloroplastic [Populus euphratica]	105.95	77.46
map00860	Porphyrin and chlorophyll metabolism	5.64E-05	TRINITY_DN26640_c0_g3	1.29	up	PREDICTED: frataxin, mitochondrial [Populus euphratica]	23.30	14.64
map00860	Porphyrin and chlorophyll metabolism	5.64E-05	TRINITY_DN27124_c0_g1	2.22	up	hypothetical protein POPTR_0019s00800g [Populus trichocarpa]	243.09	81.07
map00860	Porphyrin and chlorophyll metabolism	5.64E-05	TRINITY_DN27124_c0_g2	2.22	up	hypothetical protein POPTR_0019s00800g [Populus trichocarpa]	115.18	31.86
map00860	Porphyrin and chlorophyll metabolism	5.64E-05	TRINITY_DN27728_c1_g1	2.03	up	hypothetical protein POPTR_0007s07680g [Populus trichocarpa]	57.94	21.48
map00860	Porphyrin and chlorophyll metabolism	5.64E-05	TRINITY_DN27809_c1_g1	1.01	up	PREDICTED: magnesium-chelatase subunit ChlH, chloroplastic-like [Populus euphratica]	80.77	63.74
map00860	Porphyrin and chlorophyll metabolism	5.64E-05	TRINITY_DN14945_c0_g1	1.18	up	PREDICTED: magnesium-chelatase subunit ChlH, chloroplastic-like [Populus euphratica]	294.88	199.79
map00860	Porphyrin and chlorophyll metabolism	5.64E-05	TRINITY_DN15096_c0_g1	-2.01	down	hypothetical protein POPTR_0011s09200g [Populus trichocarpa]	11.86	68.50
map00860	Porphyrin and chlorophyll metabolism	5.64E-05	TRINITY_DN15298_c0_g1	-4.15	down	hypothetical protein POPTR_0012s05200g [Populus trichocarpa]	0.19	5.29
map00860	Porphyrin and chlorophyll metabolism	5.64E-05	TRINITY_DN16439_c0_g1	-6.31	down	PREDICTED: GDSL esterase/lipase At1g74460 [Populus euphratica]	0.06	7.74
map00860	Porphyrin and chlorophyll metabolism	5.64E-05	TRINITY_DN16504_c0_g1	1.13	up	PREDICTED: uroporphyrinogen decarboxylase [Populus euphratica]	215.24	147.34
map00860	Porphyrin and chlorophyll metabolism	5.64E-05	TRINITY_DN16769_c0_g1	1.87	up	GmMYB12 family protein [Populus trichocarpa]	4.17	6.44
map00860	Porphyrin and chlorophyll metabolism	5.64E-05	TRINITY_DN17174_c0_g1	1.76	up	PREDICTED: GDSL esterase/lipase At1g33811 [Populus euphratica]	4.85	2.09
map00860	Porphyrin and chlorophyll metabolism	5.64E-05	TRINITY_DN17768_c0_g1	1.55	up	hypothetical protein POPTR_0009s17070g [Populus trichocarpa]	67.70	36.29
map00860	Porphyrin and chlorophyll metabolism	5.64E-05	TRINITY_DN18139_c0_g1	-4.91	down	hypothetical protein POPTR_0002s17460g [Populus trichocarpa]	1.20	88.95

map00860	Porphyrin and chlorophyll metabolism	5.64E-05	TRINITY_DN18264_c0_g1	-3.37	down	putative MYB transcription factor family protein [Populus trichocarpa]	0.37	5.85
map00860	Porphyrin and chlorophyll metabolism	5.64E-05	TRINITY_DN18455_c0_g1	1.40	up	hypothetical protein POPTR_0014s03350g [Populus trichocarpa]	85.73	50.18
map00860	Porphyrin and chlorophyll metabolism	5.64E-05	TRINITY_DN18511_c0_g1	1.53	up	Protochlorophyllide reductase C family protein [Populus trichocarpa]	294.80	155.68
map00860	Porphyrin and chlorophyll metabolism	5.64E-05	TRINITY_DN18552_c0_g1	1.21	up	Chain A family protein [Populus trichocarpa]	13.27	9.06
map00860	Porphyrin and chlorophyll metabolism	5.64E-05	TRINITY_DN18555_c0_g3	1.21	up	PREDICTED: protoheme IX farnesyltransferase, mitochondrial [Populus euphratica]	26.04	16.92
map00860	Porphyrin and chlorophyll metabolism	5.64E-05	TRINITY_DN18631_c0_g1	-4.86	down	GDSL-motif lipase/hydrolase family protein [Populus trichocarpa]	0.77	35.68
map00860	Porphyrin and chlorophyll metabolism	5.64E-05	TRINITY_DN18631_c0_g2	2.04	up	GDSL-motif lipase/hydrolase family protein [Populus trichocarpa]	40.04	15.31
map00860	Porphyrin and chlorophyll metabolism	5.64E-05	TRINITY_DN18744_c0_g1	-4.07	down	hypothetical protein POPTR_0013s12020g [Populus trichocarpa]	0.67	17.03
map00860	Porphyrin and chlorophyll metabolism	5.64E-05	TRINITY_DN18777_c0_g1	1.82	up	hypothetical protein POPTR_0005s11410g [Populus trichocarpa]	33.53	12.89
map00860	Porphyrin and chlorophyll metabolism	5.64E-05	TRINITY_DN18970_c0_g6	1.49	up	PREDICTED: uncharacterized protein LOC105122137 [Populus euphratica]	613.02	329.96
map00860	Porphyrin and chlorophyll metabolism	5.64E-05	TRINITY_DN19214_c0_g1	-2.89	down	PREDICTED: transcription factor RAX2-like [Populus euphratica]	0.78	9.92
map00860	Porphyrin and chlorophyll metabolism	5.64E-05	TRINITY_DN19378_c0_g1	1.78	up	isoflavone reductase-related family protein [Populus trichocarpa]	55.10	24.15
map00860	Porphyrin and chlorophyll metabolism	5.64E-05	TRINITY_DN19538_c0_g1	-1.44	down	hypothetical protein POPTR_0013s00290g [Populus trichocarpa]	1.54	5.32
map00860	Porphyrin and chlorophyll metabolism	5.64E-05	TRINITY_DN19567_c0_g1	1.28	up	hypothetical protein POPTR_0006s28310g [Populus trichocarpa]	31.96	19.57
map00860	Porphyrin and chlorophyll metabolism	5.64E-05	TRINITY_DN19912_c0_g3	-3.25	down	PREDICTED: transcription repressor MYB6-like isoform X1 [Populus euphratica]	0.26	4.18
map00860	Porphyrin and chlorophyll metabolism	5.64E-05	TRINITY_DN19912_c1_g2	-4.30	down	hypothetical protein POPTR_0008s16660g [Populus trichocarpa]	0.47	14.26
map00860	Porphyrin and chlorophyll metabolism	5.64E-05	TRINITY_DN20133_c0_g2	-1.79	down	PREDICTED: GDSL esterase/lipase At1g71691-like isoform X1 [Populus euphratica]	2.12	10.83
map00860	Porphyrin and chlorophyll metabolism	5.64E-05	TRINITY_DN20133_c0_g3	-2.31	down	GDSL-motif lipase/hydrolase family protein [Populus trichocarpa]	0.45	3.34
map00860	Porphyrin and chlorophyll metabolism	5.64E-05	TRINITY_DN20171_c1_g1	2.05	up	glutamate 1-semialdehyde aminotransferase family protein [Populus trichocarpa]	407.15	149.39
map00860	Porphyrin and chlorophyll metabolism	5.64E-05	TRINITY_DN20349_c0_g2	1.47	up	PREDICTED: myb-related protein Myb4-like [Populus euphratica]	24.94	12.17
map00860	Porphyrin and chlorophyll metabolism	5.64E-05	TRINITY_DN20428_c0_g3	-2.75	down	hypothetical protein POPTR_0019s04150g [Populus trichocarpa]	4.68	47.93
map00860	Porphyrin and chlorophyll metabolism	5.64E-05	TRINITY_DN20452_c1_g3	-9.06	down	PREDICTED: myb-related protein 308-like [Populus euphratica]	0.00	5.40
map00860	Porphyrin and chlorophyll metabolism	5.64E-05	TRINITY_DN20475_c0_g2	-4.29	down	PREDICTED: transcription repressor MYB6-like isoform X1 [Populus euphratica]	0.48	15.23
map00860	Porphyrin and chlorophyll metabolism	5.64E-05	TRINITY_DN20493_c0_g3	-1.60	down	hypothetical protein POPTR_0017s12230g [Populus trichocarpa]	3.01	13.64
map00860	Porphyrin and chlorophyll metabolism	5.64E-05	TRINITY_DN20660_c2_g1	2.48	up	hypothetical protein POPTR_0004s14560g [Populus trichocarpa]	10.84	3.67
map00860	Porphyrin and chlorophyll metabolism	5.64E-05	TRINITY_DN20684_c0_g1	-4.91	down	PREDICTED: chlorophyllase-1, chloroplastic-like [Populus euphratica]	0.12	5.70
map00860	Porphyrin and chlorophyll metabolism	5.64E-05	TRINITY_DN20828_c0_g2	1.71	up	Magnesium-chelatase subunit chlI family protein [Populus trichocarpa]	346.30	158.86
map00860	Porphyrin and chlorophyll metabolism	5.64E-05	TRINITY_DN21048_c1_g1	1.36	up	hypothetical protein POPTR_0002s10870g [Populus trichocarpa]	151.87	89.22
map00860	Porphyrin and chlorophyll metabolism	5.64E-05	TRINITY_DN21060_c0_g3	-1.72	down	MYB transcription factor [Populus tomentosa]	10.98	56.43
map00860	Porphyrin and chlorophyll metabolism	5.64E-05	TRINITY_DN21293_c0_g2	2.44	up	hypothetical protein POPTR_0019s05210g [Populus trichocarpa]	5.66	1.63
map00860	Porphyrin and chlorophyll metabolism	5.64E-05	TRINITY_DN21293_c0_g5	1.29	up	hypothetical protein POPTR_0013s05300g [Populus trichocarpa]	6.38	3.94
map00860	Porphyrin and chlorophyll metabolism	5.64E-05	TRINITY_DN21515_c0_g1	1.39	up	PREDICTED: protochlorophyllide-dependent translocon component 52, chloroplastic-like [Populus euphratica]	90.87	54.59
map00860	Porphyrin and chlorophyll metabolism	5.64E-05	TRINITY_DN21553_c0_g4	-3.41	down	hypothetical protein POPTR_0003s14120g [Populus trichocarpa]	0.13	2.44

map00945	Stilbenoid, diarylheptanoid and gingerol biosynthesis	1.36E-04	TRINITY_DN21714_c0_g1	-2.58	down	speckle-type POZ family protein [Populus trichocarpa]	0.98	8.88
map00945	Stilbenoid, diarylheptanoid and gingerol biosynthesis	1.36E-04	TRINITY_DN22125_c0_g2	1.35	up	PREDICTED: cytochrome P450 71A1-like [Populus euphratica]	59.53	32.43
map00945	Stilbenoid, diarylheptanoid and gingerol biosynthesis	1.36E-04	TRINITY_DN22125_c0_g6	-5.41	down	hypothetical protein POPTR_0016s14440g [Populus trichocarpa]	0.24	15.20
map00945	Stilbenoid, diarylheptanoid and gingerol biosynthesis	1.36E-04	TRINITY_DN23151_c0_g1	1.61	up	PREDICTED: cytochrome P450 CYP82D47-like [Populus euphratica]	10.65	5.56
map00945	Stilbenoid, diarylheptanoid and gingerol biosynthesis	1.36E-04	TRINITY_DN23151_c0_g2	5.41	up	hypothetical protein POPTR_0009s11180g [Populus trichocarpa]	21.65	0.90
map00945	Stilbenoid, diarylheptanoid and gingerol biosynthesis	1.36E-04	TRINITY_DN23531_c0_g5	-1.67	down	hypothetical protein POPTR_0436s00200g [Populus trichocarpa]	13.47	59.57
map00945	Stilbenoid, diarylheptanoid and gingerol biosynthesis	1.36E-04	TRINITY_DN23531_c0_g7	-1.31	down	cytochrome P450 family protein [Populus trichocarpa]	1.88	6.94
map00945	Stilbenoid, diarylheptanoid and gingerol biosynthesis	1.36E-04	TRINITY_DN25044_c0_g1	1.87	up	hypothetical protein POPTR_0015s00850g [Populus trichocarpa]	9.30	3.87
map00945	Stilbenoid, diarylheptanoid and gingerol biosynthesis	1.36E-04	TRINITY_DN25044_c0_g7	3.98	up	hypothetical protein POPTR_0015s00850g [Populus trichocarpa]	6.97	0.68
map00945	Stilbenoid, diarylheptanoid and gingerol biosynthesis	1.36E-04	TRINITY_DN25107_c0_g1	1.16	up	HXXXD-type acyl-transferase family protein [Populus tomentosa]	31.54	21.30
map00945	Stilbenoid, diarylheptanoid and gingerol biosynthesis	1.36E-04	TRINITY_DN25371_c4_g1	-1.21	down	quinate O-hydroxycinnamoyltransferase/shikimate O-hydroxycinnamoyltransferase [Populus trichocarpa]	8.71	30.47
map00945	Stilbenoid, diarylheptanoid and gingerol biosynthesis	1.36E-04	TRINITY_DN26358_c0_g1	-2.57	down	6a-hydroxymaackiain methyltransferase family protein [Populus trichocarpa]	198.04	1408.66
map00945	Stilbenoid, diarylheptanoid and gingerol biosynthesis	1.36E-04	TRINITY_DN26461_c0_g5	-5.12	down	cytochrome P450 family protein [Populus trichocarpa]	0.57	30.30
map00945	Stilbenoid, diarylheptanoid and gingerol biosynthesis	1.36E-04	TRINITY_DN26707_c0_g1	-2.45	down	hypothetical protein POPTR_0005s02810g [Populus trichocarpa]	11.02	80.41
map00945	Stilbenoid, diarylheptanoid and gingerol biosynthesis	1.36E-04	TRINITY_DN27893_c12_g1	-5.22	down	PREDICTED: protein ECERIFERUM 26-like [Populus euphratica]	0.08	4.60
map00945	Stilbenoid, diarylheptanoid and gingerol biosynthesis	1.36E-04	TRINITY_DN12704_c0_g2	-2.23	down	PREDICTED: BAHD acyltransferase At5g47980-like [Populus euphratica]	0.27	1.96
map00945	Stilbenoid, diarylheptanoid and gingerol biosynthesis	1.36E-04	TRINITY_DN12720_c0_g2	1.29	up	PREDICTED: vinorine synthase-like [Populus euphratica]	7.62	4.66
map00945	Stilbenoid, diarylheptanoid and gingerol biosynthesis	1.36E-04	TRINITY_DN14060_c0_g1	-1.83	down	hypothetical protein POPTR_0001s15250g [Populus trichocarpa]	0.52	2.87
map00945	Stilbenoid, diarylheptanoid and gingerol biosynthesis	1.36E-04	TRINITY_DN15814_c0_g1	-1.33	down	hypothetical protein POPTR_0004s01860g [Populus trichocarpa]	1.60	5.98
map00945	Stilbenoid, diarylheptanoid and gingerol biosynthesis	1.36E-04	TRINITY_DN1593_c0_g1	-1.37	down	hypothetical protein POPTR_0019s01540g [Populus trichocarpa]	1.85	7.22
map00945	Stilbenoid, diarylheptanoid and gingerol biosynthesis	1.36E-04	TRINITY_DN16022_c0_g1	2.65	up	hypothetical protein POPTR_0640s00200g [Populus trichocarpa]	6.27	1.56
map00945	Stilbenoid, diarylheptanoid and gingerol biosynthesis	1.36E-04	TRINITY_DN16183_c0_g2	-1.80	down	hypothetical protein POPTR_0006s01190g [Populus trichocarpa]	1.74	8.00
map00945	Stilbenoid, diarylheptanoid and gingerol biosynthesis	1.36E-04	TRINITY_DN16397_c0_g1	-4.37	down	hypothetical protein POPTR_0003s14670g [Populus trichocarpa]	0.06	2.04
map00945	Stilbenoid, diarylheptanoid and gingerol biosynthesis	1.36E-04	TRINITY_DN16397_c0_g2	1.60	up	hypothetical protein POPTR_0001s11360g [Populus trichocarpa]	3.14	1.59
map00945	Stilbenoid, diarylheptanoid and gingerol biosynthesis	1.36E-04	TRINITY_DN16677_c0_g1	-6.00	down	hypothetical protein POPTR_0007s15050g [Populus trichocarpa]	0.16	11.75
map00945	Stilbenoid, diarylheptanoid and gingerol biosynthesis	1.36E-04	TRINITY_DN16903_c0_g2	-10.01	down	cytochrome P450 family protein [Populus trichocarpa]	0.00	5.21
map00945	Stilbenoid, diarylheptanoid and gingerol biosynthesis	1.36E-04	TRINITY_DN16939_c0_g3	-5.27	down	hypothetical protein POPTR_0007s06570g [Populus trichocarpa]	0.04	2.84
map00945	Stilbenoid, diarylheptanoid and gingerol biosynthesis	1.36E-04	TRINITY_DN16939_c0_g4	-5.19	down	hypothetical protein POPTR_0007s06570g [Populus trichocarpa]	0.04	2.53
map00945	Stilbenoid, diarylheptanoid and gingerol biosynthesis	1.36E-04	TRINITY_DN18198_c0_g2	-1.90	down	PREDICTED: trans-resveratrol di-O-methyltransferase-like [Populus euphratica]	100.03	540.89
map00945	Stilbenoid, diarylheptanoid and gingerol biosynthesis	1.36E-04	TRINITY_DN18198_c0_g3	1.26	up	6a-hydroxymaackiain methyltransferase family protein [Populus trichocarpa]	26.78	17.08
map00945	Stilbenoid, diarylheptanoid and gingerol biosynthesis	1.36E-04	TRINITY_DN18302_c0_g2	-5.73	down	hypothetical protein POPTR_0008s03410g [Populus trichocarpa]	0.06	5.58
map00945	Stilbenoid, diarylheptanoid and gingerol biosynthesis	1.36E-04	TRINITY_DN18330_c0_g1	-2.18	down	trans-cinnamate 4-hydroxylase [Populus trichocarpa]	0.62	4.40

map00945	Stilbenoid, diarylheptanoid and gingerol biosynthesis	1.36E-04	TRINITY_DN18763_c0_g2	-2.06	down	cytochrome P450 family protein [Populus trichocarpa]	0.40	2.47
map00945	Stilbenoid, diarylheptanoid and gingerol biosynthesis	1.36E-04	TRINITY_DN19448_c1_g1	1.73	up	PREDICTED: cytochrome P450 89A2-like [Populus euphratica]	39.33	17.69
map00945	Stilbenoid, diarylheptanoid and gingerol biosynthesis	1.36E-04	TRINITY_DN19448_c1_g3	1.29	up	hypothetical protein POPTR_0015s00850g [Populus trichocarpa]	5.89	3.57
map00945	Stilbenoid, diarylheptanoid and gingerol biosynthesis	1.36E-04	TRINITY_DN19958_c0_g2	-1.13	down	RecName: Full=Caffeoyl-CoA O-methyltransferase; AltName: Full=Trans-caffeoyl-CoA 3-O-methyltransferase; Short=CCoAMT; Short=CCoAOMT	6.52	20.89
map00945	Stilbenoid, diarylheptanoid and gingerol biosynthesis	1.36E-04	TRINITY_DN20101_c0_g1	1.43	up	hypothetical protein POPTR_0016s03160g [Populus trichocarpa]	10.12	5.77
map00945	Stilbenoid, diarylheptanoid and gingerol biosynthesis	1.36E-04	TRINITY_DN20258_c0_g1	-2.32	down	hypothetical protein POPTR_0007s09840g [Populus trichocarpa]	3.79	29.05
map00945	Stilbenoid, diarylheptanoid and gingerol biosynthesis	1.36E-04	TRINITY_DN20364_c0_g2	-1.63	down	PREDICTED: BAHD acyltransferase DCR [Populus euphratica]	14.38	66.96
map00945	Stilbenoid, diarylheptanoid and gingerol biosynthesis	1.36E-04	TRINITY_DN20534_c2_g1	-3.15	down	cytochrome P450 78A3p family protein [Populus trichocarpa]	0.27	3.89
map00945	Stilbenoid, diarylheptanoid and gingerol biosynthesis	1.36E-04	TRINITY_DN20842_c0_g1	1.07	up	hypothetical protein POPTR_0008s18060g [Populus trichocarpa]	8.67	6.26
map00945	Stilbenoid, diarylheptanoid and gingerol biosynthesis	1.36E-04	TRINITY_DN20905_c0_g3	-1.18	down	p-coumarate 3-hydroxylase [Populus tomentosa]	2.28	8.17
map00945	Stilbenoid, diarylheptanoid and gingerol biosynthesis	1.36E-04	TRINITY_DN20924_c0_g1	1.28	up	transferase family protein [Populus trichocarpa]	32.99	19.55
map00945	Stilbenoid, diarylheptanoid and gingerol biosynthesis	1.36E-04	TRINITY_DN20924_c0_g2	1.79	up	transferase family protein [Populus trichocarpa]	14.61	6.33
map00945	Stilbenoid, diarylheptanoid and gingerol biosynthesis	1.36E-04	TRINITY_DN391_c0_g1	1.28	up	hypothetical protein POPTR_0340s00200g [Populus trichocarpa]	62.66	39.30
map00906	Carotenoid biosynthesis	2.90E-04	TRINITY_DN21686_c1_g1	1.61	up	PREDICTED: cytochrome P450 97B2, chloroplastic [Populus euphratica]	39.24	19.40
map00906	Carotenoid biosynthesis	2.90E-04	TRINITY_DN21808_c0_g1	1.34	up	hypothetical protein POPTR_0022s00470g [Populus trichocarpa]	36.66	21.99
map00906	Carotenoid biosynthesis	2.90E-04	TRINITY_DN22914_c0_g1	1.41	up	hypothetical protein POPTR_0004s18310g [Populus trichocarpa]	20.68	12.07
map00906	Carotenoid biosynthesis	2.90E-04	TRINITY_DN23541_c1_g1	1.37	up	hypothetical protein POPTR_0013s05000g [Populus trichocarpa]	164.90	97.86
map00906	Carotenoid biosynthesis	2.90E-04	TRINITY_DN24442_c0_g1	1.19	up	Lycopene beta cyclase family protein [Populus trichocarpa]	35.80	23.66
map00906	Carotenoid biosynthesis	2.90E-04	TRINITY_DN24663_c0_g2	-2.82	down	PREDICTED: abscisic acid 8'-hydroxylase 2 [Populus euphratica]	3.48	34.77
map00906	Carotenoid biosynthesis	2.90E-04	TRINITY_DN24670_c1_g1	1.40	up	hypothetical protein POPTR_0002s05780g [Populus trichocarpa]	57.04	33.68
map00906	Carotenoid biosynthesis	2.90E-04	TRINITY_DN24670_c1_g2	1.98	up	hypothetical protein POPTR_0005s22740g [Populus trichocarpa]	38.85	14.76
map00906	Carotenoid biosynthesis	2.90E-04	TRINITY_DN25150_c0_g3	-3.45	down	hypothetical protein POPTR_0011s11370g [Populus trichocarpa]	4.47	76.88
map00906	Carotenoid biosynthesis	2.90E-04	TRINITY_DN25333_c1_g1	1.06	up	PREDICTED: zeta-carotene desaturase, chloroplastic/chromoplastic-like [Populus euphratica]	73.76	55.47
map00906	Carotenoid biosynthesis	2.90E-04	TRINITY_DN26190_c0_g4	1.58	up	carotenoid cleavage dioxygenase 1 family protein [Populus trichocarpa]	56.34	29.50
map00906	Carotenoid biosynthesis	2.90E-04	TRINITY_DN26190_c0_g5	1.41	up	carotenoid cleavage dioxygenase 1 family protein [Populus trichocarpa]	85.35	48.80
map00906	Carotenoid biosynthesis	2.90E-04	TRINITY_DN26190_c0_g6	1.35	up	PREDICTED: carotenoid 9,10(9',10')-cleavage dioxygenase 1-like [Populus euphratica]	7.02	4.20
map00906	Carotenoid biosynthesis	2.90E-04	TRINITY_DN26199_c1_g2	-1.09	down	PREDICTED: cytochrome P450 714B2-like [Populus euphratica]	5.65	19.06
map00906	Carotenoid biosynthesis	2.90E-04	TRINITY_DN26388_c0_g1	2.13	up	hypothetical protein POPTR_0019s12320g [Populus trichocarpa]	28.94	10.07
map00906	Carotenoid biosynthesis	2.90E-04	TRINITY_DN26966_c0_g1	1.72	up	zeaxanthin epoxidase family protein [Populus trichocarpa]	47.61	21.92
map00906	Carotenoid biosynthesis	2.90E-04	TRINITY_DN14386_c0_g1	-2.86	down	hypothetical protein POPTR_0015s07660g [Populus trichocarpa]	6.27	67.75
map00906	Carotenoid biosynthesis	2.90E-04	TRINITY_DN14439_c0_g1	-5.08	down	PREDICTED: secoisolariciresinol dehydrogenase-like [Populus euphratica]	0.06	3.51
map00906	Carotenoid biosynthesis	2.90E-04	TRINITY_DN14685_c0_g1	5.68	up	alcohol dehydroge family protein [Populus trichocarpa]	37.25	1.10
map00906	Carotenoid biosynthesis	2.90E-04	TRINITY_DN15227_c0_g1	-2.48	down	PREDICTED: zeaxanthin epoxidase, chloroplastic-like [Populus euphratica]	0.38	3.26
map00906	Carotenoid biosynthesis	2.90E-04	TRINITY_DN15227_c0_g2	4.43	up	PREDICTED: zeaxanthin epoxidase, chloroplastic-like [Populus euphratica]	5.07	0.36
map00906	Carotenoid biosynthesis	2.90E-04	TRINITY_DN15480_c0_g1	-1.97	down	PREDICTED: UDP-glycosyltransferase 92A1-like [Populus euphratica]	5.28	31.92
map00906	Carotenoid biosynthesis	2.90E-04	TRINITY_DN16279_c0_g1	1.13	up	UDP-glucoronosyl/UDP-glucosyl transferase family protein [Populus trichocarpa]	24.39	16.78
map00906	Carotenoid biosynthesis	2.90E-04	TRINITY_DN16472_c0_g1	1.81	up	hypothetical protein POPTR_0001s11420g [Populus trichocarpa]	105.66	45.83

map00906	Carotenoid biosynthesis	2.90E-04	TRINITY_DN17019_c0_g1	-2.18	down	PREDICTED: abscisic acid 8'-hydroxylase 4-like isoform X1 [Populus euphratica]	1.16	7.13
map00906	Carotenoid biosynthesis	2.90E-04	TRINITY_DN17087_c0_g2	1.30	up	PREDICTED: LOW QUALITY PROTEIN: 15-cis-zeta-carotene isomerase, chloroplastic [Populus euphratica]	36.68	22.85
map00906	Carotenoid biosynthesis	2.90E-04	TRINITY_DN17953_c1_g5	-2.47	down	PREDICTED: scopoletin glucosyltransferase-like [Populus euphratica]	0.78	6.61
map00906	Carotenoid biosynthesis	2.90E-04	TRINITY_DN18562_c0_g1	1.30	up	hypothetical protein POPTR_0014s14610g [Populus trichocarpa]	40.15	24.98
map00906	Carotenoid biosynthesis	2.90E-04	TRINITY_DN18753_c0_g2	1.20	up	PREDICTED: cytochrome P450 716B1-like [Populus euphratica]	7.50	5.03
map00906	Carotenoid biosynthesis	2.90E-04	TRINITY_DN18800_c1_g1	1.72	up	PREDICTED: zeaxanthin epoxidase, chloroplastic-like [Populus euphratica]	44.90	22.57
map00906	Carotenoid biosynthesis	2.90E-04	TRINITY_DN19205_c1_g4	1.11	up	PREDICTED: lycopene epsilon cyclase, chloroplastic isoform X1 [Populus euphratica]	74.68	51.95
map00906	Carotenoid biosynthesis	2.90E-04	TRINITY_DN19216_c0_g2	-2.98	down	PREDICTED: scopoletin glucosyltransferase-like [Populus euphratica]	1.65	19.96
map00906	Carotenoid biosynthesis	2.90E-04	TRINITY_DN19567_c0_g2	-2.55	down	PREDICTED: putative F-box protein PP2-B12 [Populus euphratica]	1.06	6.23
map00906	Carotenoid biosynthesis	2.90E-04	TRINITY_DN19664_c0_g1	2.04	up	PREDICTED: 15-cis-phytoene desaturase, chloroplastic/chromoplastic-like [Populus euphratica]	48.42	17.78
map00906	Carotenoid biosynthesis	2.90E-04	TRINITY_DN19817_c0_g1	1.70	up	hypothetical protein POPTR_0017s01240g [Populus trichocarpa]	46.24	21.64
map00906	Carotenoid biosynthesis	2.90E-04	TRINITY_DN19817_c0_g2	-1.38	down	hypothetical protein POPTR_0004s07230g [Populus trichocarpa]	3.19	12.38
map00906	Carotenoid biosynthesis	2.90E-04	TRINITY_DN19977_c2_g2	2.17	up	PREDICTED: carotene epsilon-monooxygenase, chloroplastic isoform X1 [Populus euphratica]	40.28	14.57
map00906	Carotenoid biosynthesis	2.90E-04	TRINITY_DN20375_c1_g2	-2.00	down	PREDICTED: GDSL esterase/lipase At1g28580-like [Populus euphratica]	1.55	9.10
map00906	Carotenoid biosynthesis	2.90E-04	TRINITY_DN21168_c0_g1	1.14	up	PREDICTED: phytoene dehydrogenase, chloroplastic/chromoplastic [Phoenix dactylifera]	81.89	55.65
map00906	Carotenoid biosynthesis	2.90E-04	TRINITY_DN21550_c0_g1	1.54	up	hypothetical protein POPTR_0013s08610g [Populus trichocarpa]	129.04	67.43
map00906	Carotenoid biosynthesis	2.90E-04	TRINITY_DN28349_c0_g1	-8.78	down	PREDICTED: tropinone reductase-like 1 [Populus euphratica]	0.00	3.71
map04075	Plant hormone signal transduction	3.53E-04	TRINITY_DN21647_c0_g2	-2.74	down	AP2 domain-containing transcription factor family protein [Populus trichocarpa]	10.47	110.53
map04075	Plant hormone signal transduction	3.53E-04	TRINITY_DN21727_c0_g1	1.61	up	PREDICTED: auxin-responsive protein IAA2-like [Populus euphratica]	12.92	5.91
map04075	Plant hormone signal transduction	3.53E-04	TRINITY_DN21735_c0_g1	1.66	up	PREDICTED: two-component response regulator ARR8-like [Populus euphratica]	59.85	28.73
map04075	Plant hormone signal transduction	3.53E-04	TRINITY_DN21912_c0_g1	-1.01	down	hypothetical protein POPTR_0019s09010g [Populus trichocarpa]	3.43	10.58
map04075	Plant hormone signal transduction	3.53E-04	TRINITY_DN21928_c0_g1	-2.29	down	AP2/ERF domain-containing transcription factor [Populus tomentosa]	6.75	55.72
map04075	Plant hormone signal transduction	3.53E-04	TRINITY_DN21928_c0_g2	-1.46	down	DREB2b [Populus hopeiensis]	1.91	8.07
map04075	Plant hormone signal transduction	3.53E-04	TRINITY_DN21948_c0_g1	-1.08	down	PREDICTED: probable serine/threonine-protein kinase At5g41260 isoform X2 [Populus euphratica]	12.83	35.61
map04075	Plant hormone signal transduction	3.53E-04	TRINITY_DN21951_c0_g2	-3.23	down	PREDICTED: probable inactive receptor kinase At5g67200 [Populus euphratica]	3.00	40.89
map04075	Plant hormone signal transduction	3.53E-04	TRINITY_DN21957_c0_g1	1.05	up	cyclin d2 family protein [Populus trichocarpa]	4.92	3.29
map04075	Plant hormone signal transduction	3.53E-04	TRINITY_DN22111_c0_g1	-1.62	down	hypothetical protein POPTR_0013s15345g [Populus trichocarpa]	2.02	11.10
map04075	Plant hormone signal transduction	3.53E-04	TRINITY_DN22129_c0_g1	-1.64	down	PREDICTED: LRR receptor-like serine/threonine-protein kinase ERECTA [Populus euphratica]	35.50	177.71
map04075	Plant hormone signal transduction	3.53E-04	TRINITY_DN22152_c0_g1	-1.70	down	hypothetical protein POPTR_0007s01340g [Populus trichocarpa]	2.36	11.98
map04075	Plant hormone signal transduction	3.53E-04	TRINITY_DN22258_c1_g2	-1.06	down	PREDICTED: BES1/BZR1 homolog protein 4-like [Populus euphratica]	7.65	24.56
map04075	Plant hormone signal transduction	3.53E-04	TRINITY_DN22293_c1_g1	-1.47	down	hypothetical protein POPTR_0011s05710g [Populus trichocarpa]	1.08	4.65
map04075	Plant hormone signal transduction	3.53E-04	TRINITY_DN22293_c1_g2	-1.42	down	PREDICTED: receptor-like protein kinase HSL1 [Populus euphratica]	2.27	9.17
map04075	Plant hormone signal transduction	3.53E-04	TRINITY_DN22309_c0_g5	-1.62	down	kinase-like protein TMKL1 precursor [Populus trichocarpa]	1.84	8.70
map04075	Plant hormone signal transduction	3.53E-04	TRINITY_DN22388_c1_g5	-1.57	down	PREDICTED: receptor protein kinase-like protein At4g34220 [Populus euphratica]	6.22	27.79
map04075	Plant hormone signal transduction	3.53E-04	TRINITY_DN22397_c0_g1	-2.80	down	CBL-interacting protein kinase 1 [Populus trichocarpa]	2.73	28.37
map04075	Plant hormone signal transduction	3.53E-04	TRINITY_DN22484_c1_g1	1.76	up	brassinosteroid-regulated family protein [Populus trichocarpa]	76.54	24.94
map04075	Plant hormone signal transduction	3.53E-04	TRINITY_DN22484_c1_g3	-3.02	down	putative xyloglucan endotransglycosylase family protein [Populus trichocarpa]	2.21	28.22
map04075	Plant hormone signal transduction	3.53E-04	TRINITY_DN22574_c0_g1	-1.23	down	PREDICTED: probable receptor-like protein kinase At1g80640 [Populus euphratica]	5.26	19.16
map04075	Plant hormone signal transduction	3.53E-04	TRINITY_DN22612_c0_g2	-1.10	down	leucine-rich repeat family protein [Populus trichocarpa]	5.53	17.87
map04075	Plant hormone signal transduction	3.53E-04	TRINITY_DN22616_c0_g2	1.51	up	unknown [Populus trichocarpa]	13.61	7.11
map04075	Plant hormone signal transduction	3.53E-04	TRINITY_DN22625_c2_g3	-1.49	down	CBL-interacting protein kinase 11 [Populus trichocarpa]	8.45	36.81
map04075	Plant hormone signal transduction	3.53E-04	TRINITY_DN22656_c1_g4	-5.27	down	auxin-responsive family protein [Populus trichocarpa]	0.05	4.11

map04075	Plant hormone signal transduction	3.53E-04	TRINITY_DN22750_c0_g2	-2.27	down	hypothetical protein POPTR_1207s00200g [Populus trichocarpa]	1.90	10.57
map04075	Plant hormone signal transduction	3.53E-04	TRINITY_DN22795_c0_g1	-2.67	down	kinase family protein [Populus trichocarpa]	0.70	7.96
map04075	Plant hormone signal transduction	3.53E-04	TRINITY_DN22931_c0_g4	-1.16	down	hypothetical protein POPTR_0018s11780g [Populus trichocarpa]	0.88	3.02
map04075	Plant hormone signal transduction	3.53E-04	TRINITY_DN22991_c0_g1	-2.55	down	SKP1 INTERACTING PARTNER 2 family protein [Populus trichocarpa]	2.64	23.78
map04075	Plant hormone signal transduction	3.53E-04	TRINITY_DN23052_c0_g1	-1.10	down	hypothetical protein POPTR_0009s03850g [Populus trichocarpa]	13.01	43.55
map04075	Plant hormone signal transduction	3.53E-04	TRINITY_DN23074_c0_g3	1.38	up	PREDICTED: two-component response regulator ARR5-like isoform X2 [Populus euphratica]	25.18	15.42
map04075	Plant hormone signal transduction	3.53E-04	TRINITY_DN23142_c2_g1	-1.34	down	hypothetical protein POPTR_0002s06800g [Populus trichocarpa]	6.92	26.64
map04075	Plant hormone signal transduction	3.53E-04	TRINITY_DN23190_c2_g1	4.55	up	xyloglucan endotransglucosylase/hydrolase protein 32 precursor [Populus trichocarpa]	31.54	2.13
map04075	Plant hormone signal transduction	3.53E-04	TRINITY_DN23211_c3_g2	-1.20	down	leucine-rich repeat transmembrane protein kinase [Populus trichocarpa]	1.24	4.29
map04075	Plant hormone signal transduction	3.53E-04	TRINITY_DN23225_c0_g2	-1.96	down	hypothetical protein POPTR_0013s02680g [Populus trichocarpa]	2.91	17.81
map04075	Plant hormone signal transduction	3.53E-04	TRINITY_DN23250_c0_g4	-2.32	down	PREDICTED: leucine-rich repeat receptor protein kinase EXS-like [Populus euphratica]	1.02	10.21
map04075	Plant hormone signal transduction	3.53E-04	TRINITY_DN23314_c1_g1	1.11	up	DWARF IN LIGHT 2 family protein [Populus trichocarpa]	13.83	9.87
map04075	Plant hormone signal transduction	3.53E-04	TRINITY_DN23385_c0_g3	-1.89	down	PREDICTED: serine/threonine-protein kinase CTR1 isoform X1 [Populus euphratica]	0.76	4.25
map04075	Plant hormone signal transduction	3.53E-04	TRINITY_DN23412_c1_g9	-5.03	down	PREDICTED: squamosa promoter-binding-like protein 3 [Populus euphratica]	0.67	27.23
map04075	Plant hormone signal transduction	3.53E-04	TRINITY_DN23477_c0_g1	2.76	up	hypothetical protein POPTR_0006s06770g [Populus trichocarpa]	201.93	32.00
map04075	Plant hormone signal transduction	3.53E-04	TRINITY_DN23526_c2_g1	2.96	up	PREDICTED: B3 domain-containing protein At2g36080-like [Populus euphratica]	6.85	1.24
map04075	Plant hormone signal transduction	3.53E-04	TRINITY_DN23575_c0_g1	-3.00	down	hypothetical protein POPTR_0014s10700g [Populus trichocarpa]	0.89	10.81
map04075	Plant hormone signal transduction	3.53E-04	TRINITY_DN23590_c0_g2	2.37	up	PREDICTED: transcription factor bHLH130-like [Populus euphratica]	6.54	1.68
map04075	Plant hormone signal transduction	3.53E-04	TRINITY_DN23685_c0_g1	-1.44	down	hypothetical protein POPTR_0001s39520g [Populus trichocarpa]	4.39	19.16
map04075	Plant hormone signal transduction	3.53E-04	TRINITY_DN23725_c0_g1	-3.44	down	hypothetical protein POPTR_0002s02630g [Populus trichocarpa]	0.46	7.67
map04075	Plant hormone signal transduction	3.53E-04	TRINITY_DN23725_c0_g3	-1.94	down	auxin response factor 6 family protein [Populus trichocarpa]	0.62	3.63
map04075	Plant hormone signal transduction	3.53E-04	TRINITY_DN23772_c0_g3	-1.41	down	hypothetical protein POPTR_0004s07700g [Populus trichocarpa]	4.69	19.13
map04075	Plant hormone signal transduction	3.53E-04	TRINITY_DN23777_c2_g1	-2.57	down	putative AUX1-like permease family protein [Populus trichocarpa]	2.50	17.87
map04075	Plant hormone signal transduction	3.53E-04	TRINITY_DN23816_c0_g2	1.87	up	hypothetical protein POPTR_0015s09240g [Populus trichocarpa]	12.60	5.30
map04075	Plant hormone signal transduction	3.53E-04	TRINITY_DN23927_c0_g1	-2.16	down	hypothetical protein POPTR_0006s11500g [Populus trichocarpa]	6.28	42.85
map04075	Plant hormone signal transduction	3.53E-04	TRINITY_DN23991_c0_g3	3.09	up	brassinosteroid-regulated family protein [Populus trichocarpa]	110.57	19.64
map04075	Plant hormone signal transduction	3.53E-04	TRINITY_DN24078_c0_g1	-1.75	down	PREDICTED: transcription factor MYC2 isoform X1 [Populus euphratica]	12.43	60.19
map04075	Plant hormone signal transduction	3.53E-04	TRINITY_DN24175_c1_g2	1.90	up	hypothetical protein POPTR_0004s15220g [Populus trichocarpa]	4.10	1.72
map04075	Plant hormone signal transduction	3.53E-04	TRINITY_DN24215_c0_g1	-1.97	down	hypothetical protein POPTR_0018s14500g [Populus trichocarpa]	3.34	20.39
map04075	Plant hormone signal transduction	3.53E-04	TRINITY_DN24250_c0_g2	-1.56	down	PSKR2 [Populus tomentosa]	1.32	6.02
map04075	Plant hormone signal transduction	3.53E-04	TRINITY_DN24277_c0_g1	-1.54	down	kinase MMK4 family protein [Populus trichocarpa]	3.48	14.42
map04075	Plant hormone signal transduction	3.53E-04	TRINITY_DN24277_c0_g2	-1.57	down	kinase MMK4 family protein [Populus trichocarpa]	8.48	37.20
map04075	Plant hormone signal transduction	3.53E-04	TRINITY_DN24280_c0_g2	-1.20	down	kinase family protein [Populus trichocarpa]	7.02	24.02
map04075	Plant hormone signal transduction	3.53E-04	TRINITY_DN24312_c1_g1	-1.20	down	PREDICTED: squamosa promoter-binding-like protein 12 isoform X3 [Populus euphratica]	3.78	12.13
map04075	Plant hormone signal transduction	3.53E-04	TRINITY_DN24398_c0_g1	-1.79	down	basic helix-loop-helix regulatory family protein [Populus trichocarpa]	5.04	25.92
map04075	Plant hormone signal transduction	3.53E-04	TRINITY_DN24537_c0_g4	1.19	up	PREDICTED: sucrose-phosphatase 1-like isoform X1 [Populus euphratica]	49.51	32.86
map04075	Plant hormone signal transduction	3.53E-04	TRINITY_DN24615_c0_g3	-1.96	down	PREDICTED: gibberellin receptor GID1B-like [Populus euphratica]	2.49	9.82
map04075	Plant hormone signal transduction	3.53E-04	TRINITY_DN24615_c0_g4	-3.54	down	hypothetical protein POPTR_0002s22840g [Populus trichocarpa]	0.18	3.46
map04075	Plant hormone signal transduction	3.53E-04	TRINITY_DN24629_c0_g4	-1.59	down	kinase family protein [Populus trichocarpa]	4.50	20.03
map04075	Plant hormone signal transduction	3.53E-04	TRINITY_DN24639_c1_g2	-1.33	down	hypothetical protein POPTR_0014s09520g [Populus trichocarpa]	19.48	74.22
map04075	Plant hormone signal transduction	3.53E-04	TRINITY_DN24644_c0_g2	-1.52	down	hypothetical protein POPTR_0007s14500g [Populus trichocarpa]	14.58	64.02
map04075	Plant hormone signal transduction	3.53E-04	TRINITY_DN24803_c0_g2	1.01	up	PREDICTED: LOW QUALITY PROTEIN: transcription factor PIF1 [Populus euphratica]	10.70	7.89

map04075	Plant hormone signal transduction	3.53E-04	TRINITY_DN24877_c0_g1	-2.26	down	auxin influx carrier family protein [Populus trichocarpa]	8.03	48.65
map04075	Plant hormone signal transduction	3.53E-04	TRINITY_DN24893_c0_g2	1.68	up	PREDICTED: probable carboxylesterase 18 [Populus euphratica]	48.39	23.75
map04075	Plant hormone signal transduction	3.53E-04	TRINITY_DN24962_c1_g1	-1.31	down	hypothetical protein POPTR_0016s14410g [Populus trichocarpa]	2.58	9.76
map04075	Plant hormone signal transduction	3.53E-04	TRINITY_DN24986_c0_g5	-6.68	down	hypothetical protein POPTR_0018s09510g [Populus trichocarpa]	0.00	2.14
map04075	Plant hormone signal transduction	3.53E-04	TRINITY_DN25011_c0_g1	-1.15	down	hypothetical protein POPTR_0001s30920g [Populus trichocarpa]	43.75	165.37
map04075	Plant hormone signal transduction	3.53E-04	TRINITY_DN25066_c0_g2	-1.39	down	leucine-rich repeat transmembrane protein kinase [Populus trichocarpa]	2.46	10.51
map04075	Plant hormone signal transduction	3.53E-04	TRINITY_DN25181_c0_g2	-2.19	down	PREDICTED: uncharacterized protein LOC105130933 [Populus euphratica]	2.13	14.77
map04075	Plant hormone signal transduction	3.53E-04	TRINITY_DN25274_c0_g1	2.90	up	PREDICTED: probable indole-3-acetic acid-amido synthetase GH3.5 isoform X1 [Populus euphratica]	8.53	1.57
map04075	Plant hormone signal transduction	3.53E-04	TRINITY_DN25274_c1_g1	1.18	up	hypothetical protein POPTR_0014s09120g [Populus trichocarpa]	14.82	8.16
map04075	Plant hormone signal transduction	3.53E-04	TRINITY_DN25313_c1_g5	-1.55	down	leucine-rich repeat transmembrane protein kinase [Populus trichocarpa]	1.99	9.18
map04075	Plant hormone signal transduction	3.53E-04	TRINITY_DN25324_c1_g1	-1.32	down	hypothetical protein POPTR_0011s13460g [Populus trichocarpa]	1.40	5.45
map04075	Plant hormone signal transduction	3.53E-04	TRINITY_DN25324_c1_g2	-2.31	down	hypothetical protein POPTR_0001s44090g [Populus trichocarpa]	5.91	44.70
map04075	Plant hormone signal transduction	3.53E-04	TRINITY_DN25351_c0_g1	1.21	up	PREDICTED: serine/threonine-protein kinase HT1 isoform X1 [Populus euphratica]	22.50	17.55
map04075	Plant hormone signal transduction	3.53E-04	TRINITY_DN25455_c0_g1	-1.00	down	PREDICTED: protein NSP-INTERACTING KINASE 1-like isoform X2 [Populus euphratica]	10.84	33.18
map04075	Plant hormone signal transduction	3.53E-04	TRINITY_DN25537_c0_g1	-1.11	down	xyloglucan endotransglycosylase/hydrolase precursor XTH-27 [Populus trichocarpa]	33.20	109.96
map04075	Plant hormone signal transduction	3.53E-04	TRINITY_DN25633_c0_g2	-1.33	down	hypothetical protein POPTR_0018s11800g [Populus trichocarpa]	7.63	29.20
map04075	Plant hormone signal transduction	3.53E-04	TRINITY_DN25653_c0_g1	-1.05	down	leucine-rich repeat transmembrane protein kinase [Populus trichocarpa]	5.26	16.36
map04075	Plant hormone signal transduction	3.53E-04	TRINITY_DN25664_c0_g1	-1.86	down	PREDICTED: inactive leucine-rich repeat receptor-like serine/threonine-protein kinase At1g60630 [Populus euphratica]	1.28	7.79
map04075	Plant hormone signal transduction	3.53E-04	TRINITY_DN25664_c0_g4	-2.90	down	PREDICTED: inactive leucine-rich repeat receptor-like serine/threonine-protein kinase At1g60630 [Populus euphratica]	0.24	2.80
map04075	Plant hormone signal transduction	3.53E-04	TRINITY_DN25681_c0_g1	-2.33	down	leucine-rich repeat transmembrane protein kinase [Populus trichocarpa]	6.49	48.42
map04075	Plant hormone signal transduction	3.53E-04	TRINITY_DN25736_c2_g6	-1.44	down	RECEPTOR-LIKE protein KINASE 1 [Populus trichocarpa]	2.07	8.61
map04075	Plant hormone signal transduction	3.53E-04	TRINITY_DN25771_c0_g2	-1.10	down	hypothetical protein POPTR_0018s04090g [Populus trichocarpa]	5.19	19.18
map04075	Plant hormone signal transduction	3.53E-04	TRINITY_DN25803_c0_g1	-1.01	down	leucine-rich repeat transmembrane protein kinase [Populus trichocarpa]	19.96	65.39
map04075	Plant hormone signal transduction	3.53E-04	TRINITY_DN25823_c0_g1	-2.56	down	hypothetical protein POPTR_0015s09830g [Populus trichocarpa]	1.74	15.66
map04075	Plant hormone signal transduction	3.53E-04	TRINITY_DN25823_c0_g2	-1.67	down	leucine-rich repeat transmembrane protein kinase [Populus trichocarpa]	2.57	12.56
map04075	Plant hormone signal transduction	3.53E-04	TRINITY_DN25851_c0_g1	-1.07	down	hypothetical protein POPTR_0006s01720g [Populus trichocarpa]	10.33	33.35
map04075	Plant hormone signal transduction	3.53E-04	TRINITY_DN25879_c0_g1	-1.45	down	PREDICTED: shaggy-related protein kinase theta [Populus euphratica]	11.99	48.33
map04075	Plant hormone signal transduction	3.53E-04	TRINITY_DN25895_c0_g2	-2.62	down	leucine-rich repeat transmembrane protein kinase [Populus trichocarpa]	1.06	9.71
map04075	Plant hormone signal transduction	3.53E-04	TRINITY_DN25895_c0_g3	-2.89	down	leucine-rich repeat transmembrane protein kinase [Populus trichocarpa]	0.82	9.37
map04075	Plant hormone signal transduction	3.53E-04	TRINITY_DN25910_c0_g4	-1.56	down	hypothetical protein POPTR_0009s03990g [Populus trichocarpa]	5.07	23.12
map04075	Plant hormone signal transduction	3.53E-04	TRINITY_DN26021_c1_g1	-1.46	down	hypothetical protein POPTR_0001s13110g [Populus trichocarpa]	22.71	95.09
map04075	Plant hormone signal transduction	3.53E-04	TRINITY_DN26051_c1_g1	-4.91	down	hypothetical protein POPTR_0442s00200g [Populus trichocarpa]	0.87	13.27
map04075	Plant hormone signal transduction	3.53E-04	TRINITY_DN26052_c0_g1	-1.02	down	hypothetical protein POPTR_0008s11780g, partial [Populus trichocarpa]	7.04	22.42
map04075	Plant hormone signal transduction	3.53E-04	TRINITY_DN26175_c0_g5	-1.84	down	PREDICTED: probable inactive receptor-like protein kinase At3g56050 [Populus euphratica]	4.06	22.52
map04075	Plant hormone signal transduction	3.53E-04	TRINITY_DN26211_c0_g2	-1.37	down	leucine-rich repeat transmembrane protein kinase [Populus trichocarpa]	2.19	8.98
map04075	Plant hormone signal transduction	3.53E-04	TRINITY_DN26227_c0_g1	-1.49	down	receptor protein kinase-1 [Populus tomentosa]	5.06	20.86
map04075	Plant hormone signal transduction	3.53E-04	TRINITY_DN26328_c1_g3	-1.27	down	leucine-rich repeat family protein [Populus trichocarpa]	3.58	12.70
map04075	Plant hormone signal transduction	3.53E-04	TRINITY_DN26347_c0_g1	1.58	up	CTR1-like protein kinase [Populus trichocarpa]	35.40	16.01
map04075	Plant hormone signal transduction	3.53E-04	TRINITY_DN26417_c0_g1	-1.97	down	xyloglucan endotransglycosylase/hydrolase precursor XTH-36 [Populus tremula x Populus tremuloides]	8.56	51.01
map04075	Plant hormone signal transduction	3.53E-04	TRINITY_DN26474_c0_g1	-1.56	down	hypothetical protein POPTR_0010s18540g [Populus trichocarpa]	7.99	31.30

map04075	Plant hormone signal transduction	3.53E-04	TRINITY_DN26491_c2_g1	-2.13	down	PREDICTED: mitogen-activated protein kinase 3-like [Populus euphratica]	6.80	34.82
map04075	Plant hormone signal transduction	3.53E-04	TRINITY_DN26581_c0_g1	-3.15	down	PREDICTED: auxin response factor 5-like [Populus euphratica]	2.67	35.76
map04075	Plant hormone signal transduction	3.53E-04	TRINITY_DN26583_c1_g1	-2.15	down	PREDICTED: scarecrow-like protein 34 [Populus euphratica]	2.88	13.19
map04075	Plant hormone signal transduction	3.53E-04	TRINITY_DN26657_c0_g2	-1.16	down	hypothetical protein POPTR_0015s06040g [Populus trichocarpa]	5.31	17.39
map04075	Plant hormone signal transduction	3.53E-04	TRINITY_DN26699_c0_g1	-1.43	down	PREDICTED: auxin response factor 9-like [Populus euphratica]	5.09	20.76
map04075	Plant hormone signal transduction	3.53E-04	TRINITY_DN26710_c1_g1	-1.93	down	hypothetical protein POPTR_0003s10680g [Populus trichocarpa]	3.71	20.93
map04075	Plant hormone signal transduction	3.53E-04	TRINITY_DN26795_c0_g3	1.40	up	PREDICTED: CBL-interacting serine/threonine-protein kinase 21-like [Populus euphratica]	22.32	13.52
map04075	Plant hormone signal transduction	3.53E-04	TRINITY_DN26857_c0_g1	-1.72	down	hypothetical protein POPTR_0001s02910g [Populus trichocarpa]	12.91	65.99
map04075	Plant hormone signal transduction	3.53E-04	TRINITY_DN26883_c0_g4	-2.61	down	AP2 domain-containing transcription factor family protein [Populus trichocarpa]	13.54	128.65
map04075	Plant hormone signal transduction	3.53E-04	TRINITY_DN26980_c0_g1	-1.01	down	mitogen-activated protein kinase homologue [Populus trichocarpa]	26.58	76.98
map04075	Plant hormone signal transduction	3.53E-04	TRINITY_DN26997_c0_g1	-1.67	down	PREDICTED: probable LRR receptor-like serine/threonine-protein kinase At1g34110 [Populus euphratica]	13.18	55.35
map04075	Plant hormone signal transduction	3.53E-04	TRINITY_DN27013_c1_g1	-1.78	down	hypothetical protein POPTR_0002s17350g [Populus trichocarpa]	12.44	71.86
map04075	Plant hormone signal transduction	3.53E-04	TRINITY_DN27051_c1_g1	1.53	up	PREDICTED: ethylene receptor 2-like isoform X1 [Populus euphratica]	24.09	17.42
map04075	Plant hormone signal transduction	3.53E-04	TRINITY_DN27051_c1_g3	4.06	up	ethylene receptor family protein [Populus trichocarpa]	2.20	0.19
map04075	Plant hormone signal transduction	3.53E-04	TRINITY_DN27093_c0_g1	-4.88	down	hypothetical protein POPTR_0009s09590g [Populus trichocarpa]	0.75	34.57
map04075	Plant hormone signal transduction	3.53E-04	TRINITY_DN27137_c0_g1	-1.72	down	hypothetical protein POPTR_0001s40900g [Populus trichocarpa]	3.02	15.80
map04075	Plant hormone signal transduction	3.53E-04	TRINITY_DN27159_c0_g4	-1.65	down	hypothetical protein POPTR_0017s12990g [Populus trichocarpa]	7.16	34.38
map04075	Plant hormone signal transduction	3.53E-04	TRINITY_DN27180_c0_g1	-1.49	down	hypothetical protein POPTR_0014s18490g [Populus trichocarpa]	3.22	18.50
map04075	Plant hormone signal transduction	3.53E-04	TRINITY_DN27240_c1_g2	-1.81	down	hypothetical protein POPTR_0006s09500g [Populus trichocarpa]	2.18	11.99
map04075	Plant hormone signal transduction	3.53E-04	TRINITY_DN27362_c1_g1	-1.25	down	hypothetical protein POPTR_0019s14380g [Populus trichocarpa]	4.96	18.47
map04075	Plant hormone signal transduction	3.53E-04	TRINITY_DN27382_c1_g2	-1.46	down	PTH-1 family protein [Populus trichocarpa]	3.89	17.98
map04075	Plant hormone signal transduction	3.53E-04	TRINITY_DN27385_c1_g1	-1.53	down	PREDICTED: tyrosine-protein kinase CSK-like isoform X1 [Populus euphratica]	6.89	25.06
map04075	Plant hormone signal transduction	3.53E-04	TRINITY_DN27442_c0_g1	-1.61	down	PREDICTED: probably inactive leucine-rich repeat receptor-like protein kinase At3g28040 isoform X2 [Populus euphratica]	7.11	33.13
map04075	Plant hormone signal transduction	3.53E-04	TRINITY_DN27620_c1_g2	-2.53	down	PREDICTED: probable disease resistance protein At5g66900 [Populus euphratica]	1.87	17.98
map04075	Plant hormone signal transduction	3.53E-04	TRINITY_DN27662_c5_g1	2.10	up	cytokinin receptor 1A [Populus trichocarpa]	22.72	6.44
map04075	Plant hormone signal transduction	3.53E-04	TRINITY_DN27709_c0_g2	-1.46	down	hypothetical protein POPTR_0003s04650g [Populus trichocarpa]	24.18	88.92
map04075	Plant hormone signal transduction	3.53E-04	TRINITY_DN27893_c3_g2	-6.27	down	hypothetical protein POPTR_0001s28330g [Populus trichocarpa]	0.02	3.05
map04075	Plant hormone signal transduction	3.53E-04	TRINITY_DN11369_c0_g1	2.01	up	PREDICTED: ethylene-responsive transcription factor ERF034 [Populus euphratica]	3.26	1.22
map04075	Plant hormone signal transduction	3.53E-04	TRINITY_DN11879_c0_g1	1.63	up	hypothetical protein POPTR_0001s31380g [Populus trichocarpa]	7.05	3.51
map04075	Plant hormone signal transduction	3.53E-04	TRINITY_DN13219_c0_g1	1.54	up	PREDICTED: serine/threonine-protein kinase HT1-like isoform X1 [Populus euphratica]	1.77	0.93
map04075	Plant hormone signal transduction	3.53E-04	TRINITY_DN13236_c0_g1	-1.65	down	leucine-rich repeat transmembrane protein kinase [Populus trichocarpa]	0.61	2.85
map04075	Plant hormone signal transduction	3.53E-04	TRINITY_DN14028_c0_g3	-1.78	down	putative leucine-rich repeat transmembrane protein kinase [Populus trichocarpa]	0.37	2.00
map04075	Plant hormone signal transduction	3.53E-04	TRINITY_DN14088_c0_g1	-3.21	down	PREDICTED: indole-3-acetic acid-amido synthetase GH3.17-like [Populus euphratica]	0.12	1.79
map04075	Plant hormone signal transduction	3.53E-04	TRINITY_DN14190_c0_g2	-2.42	down	kinase family protein [Populus trichocarpa]	0.45	3.82
map04075	Plant hormone signal transduction	3.53E-04	TRINITY_DN14302_c0_g1	1.87	up	hypothetical protein POPTR_0013s08990g [Populus trichocarpa]	2.35	1.00
map04075	Plant hormone signal transduction	3.53E-04	TRINITY_DN14579_c0_g1	-5.06	down	PREDICTED: protein FD [Populus euphratica]	0.14	5.73
map04075	Plant hormone signal transduction	3.53E-04	TRINITY_DN14849_c0_g1	-5.24	down	xyloglucan endotransglucosylase/hydrolase protein 31 precursor [Populus trichocarpa]	0.06	3.64
map04075	Plant hormone signal transduction	3.53E-04	TRINITY_DN14967_c0_g1	1.72	up	PREDICTED: receptor-like cytosolic serine/threonine-protein kinase RBK2 isoform X1 [Populus euphratica]	4.16	2.26
map04075	Plant hormone signal transduction	3.53E-04	TRINITY_DN15082_c0_g1	1.80	up	cytokinin receptor 1A [Populus trichocarpa]	6.96	2.99
map04075	Plant hormone signal transduction	3.53E-04	TRINITY_DN15373_c0_g1	4.19	up	pathogenesis-related family protein [Populus trichocarpa]	42.69	3.64
map04075	Plant hormone signal transduction	3.53E-04	TRINITY_DN15439_c0_g1	-4.01	down	hsr203J family protein [Populus trichocarpa]	0.18	4.70
map04075	Plant hormone signal transduction	3.53E-04	TRINITY_DN15562_c0_g1	1.28	up	histidine kinase receptor family protein [Populus trichocarpa]	13.30	8.10

map04075	Plant hormone signal transduction	3.53E-04	TRINITY_DN15602_c0_g1	1.77	up	hypothetical protein POPTR_0010s19120g [Populus trichocarpa]	2.12	0.96
map04075	Plant hormone signal transduction	3.53E-04	TRINITY_DN15627_c0_g1	-1.33	down	PREDICTED: auxin-induced protein AUX22 [Populus euphratica]	2.52	10.20
map04075	Plant hormone signal transduction	3.53E-04	TRINITY_DN15656_c0_g1	3.02	up	CRE1-1 [Populus tomentosa]	9.41	1.76
map04075	Plant hormone signal transduction	3.53E-04	TRINITY_DN15683_c0_g1	-2.85	down	CBL-interacting protein kinase 4 [Populus trichocarpa]	2.18	23.46
map04075	Plant hormone signal transduction	3.53E-04	TRINITY_DN16013_c0_g2	-2.33	down	hypothetical protein POPTR_0007s14060g [Populus trichocarpa]	0.56	4.44
map04075	Plant hormone signal transduction	3.53E-04	TRINITY_DN16013_c0_g4	-3.42	down	hypothetical protein POPTR_0005s25840g [Populus trichocarpa]	0.57	9.43
map04075	Plant hormone signal transduction	3.53E-04	TRINITY_DN16180_c0_g1	-1.25	down	PREDICTED: auxin response factor 2-like [Populus euphratica]	13.42	43.70
map04075	Plant hormone signal transduction	3.53E-04	TRINITY_DN16324_c0_g1	2.36	up	xyloglucan:xyloglucosyl transferase family protein [Populus trichocarpa]	13.44	4.07
map04075	Plant hormone signal transduction	3.53E-04	TRINITY_DN16595_c0_g1	-1.69	down	hypothetical protein POPTR_0006s13790g [Populus trichocarpa]	1.12	7.33
map04075	Plant hormone signal transduction	3.53E-04	TRINITY_DN16602_c0_g1	-2.27	down	unknown [Populus trichocarpa]	4.93	31.22
map04075	Plant hormone signal transduction	3.53E-04	TRINITY_DN16645_c0_g1	-1.72	down	kinase family protein [Populus trichocarpa]	5.12	26.93
map04075	Plant hormone signal transduction	3.53E-04	TRINITY_DN16705_c0_g2	-2.05	down	hypothetical protein POPTR_0011s11330g [Populus trichocarpa]	2.24	10.42
map04075	Plant hormone signal transduction	3.53E-04	TRINITY_DN17093_c0_g2	2.92	up	cytokinin response 1 family protein [Populus trichocarpa]	2.05	0.40
map04075	Plant hormone signal transduction	3.53E-04	TRINITY_DN17149_c1_g1	-2.27	down	hypothetical protein POPTR_0013s12190g [Populus trichocarpa]	0.54	4.10
map04075	Plant hormone signal transduction	3.53E-04	TRINITY_DN17166_c0_g1	-1.53	down	hypothetical protein [Populus tomentosa]	4.27	19.54
map04075	Plant hormone signal transduction	3.53E-04	TRINITY_DN17272_c0_g2	3.19	up	hypothetical protein POPTR_0002s08270g [Populus trichocarpa]	57.50	9.49
map04075	Plant hormone signal transduction	3.53E-04	TRINITY_DN17272_c0_g4	2.59	up	PREDICTED: two-component response regulator ORR9-like isoform X1 [Tarenaya hassleriana]	6.30	1.20
map04075	Plant hormone signal transduction	3.53E-04	TRINITY_DN17361_c0_g1	-1.23	down	kinase family protein [Populus trichocarpa]	1.11	5.18
map04075	Plant hormone signal transduction	3.53E-04	TRINITY_DN17494_c0_g4	-3.88	down	leucine-rich repeat transmembrane protein kinase [Populus trichocarpa]	0.85	12.27
map04075	Plant hormone signal transduction	3.53E-04	TRINITY_DN17689_c0_g2	-2.42	down	hypothetical protein POPTR_0009s08500g [Populus trichocarpa]	0.66	5.23
map04075	Plant hormone signal transduction	3.53E-04	TRINITY_DN17771_c0_g6	-2.50	down	PREDICTED: uncharacterized protein LOC105140768 isoform X1 [Populus euphratica]	0.42	4.18
map04075	Plant hormone signal transduction	3.53E-04	TRINITY_DN17771_c0_g7	-1.68	down	PREDICTED: DELLA protein GAIP-like isoform X3 [Populus euphratica]	2.62	11.42
map04075	Plant hormone signal transduction	3.53E-04	TRINITY_DN17780_c0_g1	2.34	up	hypothetical protein POPTR_0010s23280g [Populus trichocarpa]	4.94	1.56
map04075	Plant hormone signal transduction	3.53E-04	TRINITY_DN17961_c0_g4	-6.34	down	IAA-amido synthetase GH3-1 [Populus davidiana x Populus alba var. pyramidalis]	0.07	8.40
map04075	Plant hormone signal transduction	3.53E-04	TRINITY_DN18375_c0_g1	2.13	up	hypothetical protein POPTR_0030s00520g [Populus trichocarpa]	19.25	7.08
map04075	Plant hormone signal transduction	3.53E-04	TRINITY_DN18381_c0_g1	-2.01	down	SERINE/THREONINE protein KINASE 1 [Populus trichocarpa]	1.14	6.90
map04075	Plant hormone signal transduction	3.53E-04	TRINITY_DN18592_c1_g3	-2.13	down	auxin-responsive family protein [Populus trichocarpa]	1.56	10.25
map04075	Plant hormone signal transduction	3.53E-04	TRINITY_DN18807_c0_g6	1.30	up	leucine-rich repeat family protein [Populus trichocarpa]	4.80	2.97
map04075	Plant hormone signal transduction	3.53E-04	TRINITY_DN18842_c0_g1	-2.42	down	D5-type cyclin [Populus trichocarpa]	0.54	4.24
map04075	Plant hormone signal transduction	3.53E-04	TRINITY_DN18843_c0_g1	2.16	up	hypothetical protein POPTR_0003s18030g [Populus trichocarpa]	14.14	4.77
map04075	Plant hormone signal transduction	3.53E-04	TRINITY_DN18872_c0_g1	-2.52	down	hypothetical protein POPTR_0002s06600g [Populus trichocarpa]	0.67	5.94
map04075	Plant hormone signal transduction	3.53E-04	TRINITY_DN19026_c0_g1	-2.71	down	PREDICTED: cyclin-D1-1-like isoform X2 [Populus euphratica]	1.27	8.13
map04075	Plant hormone signal transduction	3.53E-04	TRINITY_DN19032_c0_g2	1.09	up	aux/IAA protein [Populus tremula x Populus tremuloides]	8.60	5.63
map04075	Plant hormone signal transduction	3.53E-04	TRINITY_DN19113_c0_g1	-1.11	down	auxin-induced protein aux28 [Populus trichocarpa]	7.11	22.97
map04075	Plant hormone signal transduction	3.53E-04	TRINITY_DN19134_c0_g2	-1.23	down	leucine-rich repeat transmembrane protein kinase [Populus trichocarpa]	1.28	4.62
map04075	Plant hormone signal transduction	3.53E-04	TRINITY_DN19178_c0_g1	-3.55	down	auxin-responsive family protein [Populus trichocarpa]	1.12	20.32
map04075	Plant hormone signal transduction	3.53E-04	TRINITY_DN19267_c0_g3	-2.90	down	PERIANTHIA family protein [Populus trichocarpa]	1.45	7.99
map04075	Plant hormone signal transduction	3.53E-04	TRINITY_DN19302_c0_g1	-1.39	down	PREDICTED: probable LRR receptor-like serine/threonine-protein kinase At1g53430 [Populus euphratica]	12.74	38.87
map04075	Plant hormone signal transduction	3.53E-04	TRINITY_DN19419_c0_g1	3.78	up	hypothetical protein POPTR_0006s02220g [Populus trichocarpa]	4.58	0.82
map04075	Plant hormone signal transduction	3.53E-04	TRINITY_DN19490_c0_g1	1.26	up	putative histidine phosphotransfer protein 5 [Populus x canadensis]	9.00	6.64
map04075	Plant hormone signal transduction	3.53E-04	TRINITY_DN19733_c0_g1	-2.33	down	leucine-rich repeat transmembrane protein kinase [Populus trichocarpa]	1.46	11.31
map04075	Plant hormone signal transduction	3.53E-04	TRINITY_DN19748_c0_g2	-2.21	down	PREDICTED: transcription factor bHLH35-like isoform X2 [Populus euphratica]	5.09	40.00
map04075	Plant hormone signal transduction	3.53E-04	TRINITY_DN19782_c1_g1	-1.86	down	PREDICTED: auxin-induced protein 22D-like isoform X1 [Populus euphratica]	1.94	8.69
map04075	Plant hormone signal transduction	3.53E-04	TRINITY_DN19854_c0_g2	-2.03	down	PREDICTED: protein TIFY 10A [Populus euphratica]	35.59	214.11
map04075	Plant hormone signal transduction	3.53E-04	TRINITY_DN19876_c0_g1	-5.82	down	Pathogenesis-related family protein [Populus trichocarpa]	5.51	478.26

map04075	Plant hormone signal transduction	3.53E-04	TRINITY_DN19902_c0_g2	-2.54	down	ethylene-responsive element-binding family protein [Populus trichocarpa]	4.55	40.17
map04075	Plant hormone signal transduction	3.53E-04	TRINITY_DN19909_c1_g2	-1.64	down	hypothetical protein POPTR_0004s06180g [Populus trichocarpa]	8.84	41.26
map04075	Plant hormone signal transduction	3.53E-04	TRINITY_DN19919_c0_g2	-1.25	down	leucine-rich repeat transmembrane protein kinase [Populus trichocarpa]	3.53	12.35
map04075	Plant hormone signal transduction	3.53E-04	TRINITY_DN19946_c0_g1	-2.05	down	PREDICTED: indole-3-acetic acid-amido synthetase GH3.6-like [Populus euphratica]	0.59	3.72
map04075	Plant hormone signal transduction	3.53E-04	TRINITY_DN20124_c0_g1	-2.90	down	hypothetical protein POPTR_0005s22770g [Populus trichocarpa]	0.22	2.53
map04075	Plant hormone signal transduction	3.53E-04	TRINITY_DN20156_c0_g1	-3.38	down	hypothetical protein POPTR_0017s03790g [Populus trichocarpa]	0.32	5.37
map04075	Plant hormone signal transduction	3.53E-04	TRINITY_DN20164_c0_g1	-3.06	down	hypothetical protein POPTR_0019s14110g [Populus trichocarpa]	0.99	12.70
map04075	Plant hormone signal transduction	3.53E-04	TRINITY_DN20311_c0_g1	1.47	up	PREDICTED: programmed cell death protein 2 isoform X1 [Populus euphratica]	25.80	14.04
map04075	Plant hormone signal transduction	3.53E-04	TRINITY_DN20327_c0_g2	-2.07	down	hypothetical protein POPTR_0011s17240g, partial [Populus trichocarpa]	0.65	3.61
map04075	Plant hormone signal transduction	3.53E-04	TRINITY_DN20375_c1_g1	-2.46	down	PREDICTED: putative cyclin-D6-1 [Populus euphratica]	7.94	62.48
map04075	Plant hormone signal transduction	3.53E-04	TRINITY_DN20593_c0_g1	1.77	up	Metal transporter Nramp1 family protein [Populus trichocarpa]	4.27	1.83
map04075	Plant hormone signal transduction	3.53E-04	TRINITY_DN20674_c0_g1	-2.35	down	xyloglucan endotransglycosylase/hydrolase precursor XTH-30 [Populus tremula]	76.15	558.99
map04075	Plant hormone signal transduction	3.53E-04	TRINITY_DN20674_c0_g3	-1.74	down	xyloglucan endo-1 family protein [Populus trichocarpa]	6.47	32.26
map04075	Plant hormone signal transduction	3.53E-04	TRINITY_DN20750_c0_g2	-3.07	down	putative leucine-rich repeat transmembrane protein kinase [Populus trichocarpa]	0.41	8.59
map04075	Plant hormone signal transduction	3.53E-04	TRINITY_DN20770_c0_g1	-1.05	down	PREDICTED: histidine-containing phosphotransfer protein 1-like isoform X1 [Populus euphratica]	11.03	36.32
map04075	Plant hormone signal transduction	3.53E-04	TRINITY_DN20770_c0_g3	-2.04	down	putative histidine-containing phosphotransfer protein 2 [Populus trichocarpa]	3.26	20.12
map04075	Plant hormone signal transduction	3.53E-04	TRINITY_DN20831_c0_g3	2.86	up	PREDICTED: two-component response regulator ARR5-like [Populus euphratica]	47.29	13.22
map04075	Plant hormone signal transduction	3.53E-04	TRINITY_DN20906_c0_g2	-4.70	down	hypothetical protein POPTR_0014s09560g [Populus trichocarpa]	0.56	22.40
map04075	Plant hormone signal transduction	3.53E-04	TRINITY_DN21108_c0_g4	-2.03	down	hypothetical protein POPTR_0003s16350g [Populus trichocarpa]	13.59	39.10
map04075	Plant hormone signal transduction	3.53E-04	TRINITY_DN21164_c0_g1	-3.58	down	basic leucine zipper transcription factor family protein [Populus trichocarpa]	0.77	13.70
map04075	Plant hormone signal transduction	3.53E-04	TRINITY_DN21247_c0_g1	-2.37	down	leucine-rich repeat transmembrane protein kinase [Populus trichocarpa]	2.07	16.36
map04075	Plant hormone signal transduction	3.53E-04	TRINITY_DN21361_c0_g3	1.25	up	hypothetical protein POPTR_0014s16720g [Populus trichocarpa]	111.11	72.58
map04075	Plant hormone signal transduction	3.53E-04	TRINITY_DN21396_c1_g1	1.00	up	hypothetical protein POPTR_0009s14820g [Populus trichocarpa]	21.54	16.30
map04075	Plant hormone signal transduction	3.53E-04	TRINITY_DN21540_c0_g1	-1.79	down	hypothetical protein POPTR_0010s23000g [Populus trichocarpa]	9.21	36.37
map04075	Plant hormone signal transduction	3.53E-04	TRINITY_DN21569_c0_g1	-1.63	down	cyclin D3.1 family protein [Populus trichocarpa]	45.82	209.68
map04075	Plant hormone signal transduction	3.53E-04	TRINITY_DN21574_c0_g3	-1.89	down	hypothetical protein POPTR_0009s12890g [Populus trichocarpa]	1.15	6.50
map04075	Plant hormone signal transduction	3.53E-04	TRINITY_DN21590_c1_g3	-2.93	down	PREDICTED: auxin-responsive protein IAA13-like [Populus euphratica]	1.24	13.54
map04075	Plant hormone signal transduction	3.53E-04	TRINITY_DN21626_c0_g1	-1.06	down	kinase family protein [Populus trichocarpa]	10.41	35.72
map04075	Plant hormone signal transduction	3.53E-04	TRINITY_DN3089_c0_g1	-3.94	down	PREDICTED: pathogenesis-related protein 1-like [Populus euphratica]	0.75	17.80
map04075	Plant hormone signal transduction	3.53E-04	TRINITY_DN5190_c0_g2	5.55	up	CBL-interacting protein kinase 20 [Populus trichocarpa]	2.51	0.07
map04075	Plant hormone signal transduction	3.53E-04	TRINITY_DN8124_c0_g1	-1.70	down	PREDICTED: somatic embryogenesis receptor kinase 2-like isoform X1 [Populus euphratica]	0.61	2.73
map04075	Plant hormone signal transduction	3.53E-04	TRINITY_DN9151_c0_g1	-1.97	down	auxin-responsive family protein [Populus trichocarpa]	0.46	2.77
map04626	Plant-pathogen interaction	3.83E-04	TRINITY_DN21647_c0_g4	-1.54	down	hypothetical protein POPTR_0005s07900g [Populus trichocarpa]	40.13	183.60
map04626	Plant-pathogen interaction	3.83E-04	TRINITY_DN21694_c0_g2	-3.53	down	putative wall-associated kinase family protein [Populus trichocarpa]	0.18	3.31
map04626	Plant-pathogen interaction	3.83E-04	TRINITY_DN21702_c0_g3	1.35	up	PREDICTED: jacalin-related lectin 3-like isoform X1 [Populus euphratica]	2.42	1.49
map04626	Plant-pathogen interaction	3.83E-04	TRINITY_DN21709_c0_g1	-2.27	down	PREDICTED: phosphoenolpyruvate carboxylase kinase 2-like isoform X2 [Populus euphratica]	3.29	22.78
map04626	Plant-pathogen interaction	3.83E-04	TRINITY_DN21865_c0_g1	-2.33	down	WRKY transcription factor 17 [(Populus tomentosa x Populus bolleana) x Populus tomentosa]	2.52	17.60
map04626	Plant-pathogen interaction	3.83E-04	TRINITY_DN21865_c0_g3	-2.01	down	WRKY transcription factor 1 [(Populus tomentosa x Populus bolleana) x Populus tomentosa]	4.32	26.94
map04626	Plant-pathogen interaction	3.83E-04	TRINITY_DN22006_c0_g1	-2.32	down	hypothetical protein POPTR_0002s16160g [Populus trichocarpa]	0.93	7.28
map04626	Plant-pathogen interaction	3.83E-04	TRINITY_DN22021_c0_g1	-1.34	down	PREDICTED: calmodulin-like protein 3 isoform X3 [Populus euphratica]	4.36	16.35
map04626	Plant-pathogen interaction	3.83E-04	TRINITY_DN22057_c0_g1	-1.01	down	PREDICTED: disease resistance protein RPM1-like isoform X1 [Populus euphratica]	3.27	10.73
map04626	Plant-pathogen interaction	3.83E-04	TRINITY_DN22099_c0_g2	-2.52	down	hypothetical protein POPTR_0003s15810g [Populus trichocarpa]	0.33	2.99

map04626	Plant-pathogen interaction	3.83E-04	TRINITY_DN22163_c0_g3	-1.69	down	kinase family protein [Populus trichocarpa]	2.52	12.43
map04626	Plant-pathogen interaction	3.83E-04	TRINITY_DN22253_c0_g2	-1.28	down	PREDICTED: putative receptor-like protein kinase At1g72540 [Populus euphratica]	0.97	3.55
map04626	Plant-pathogen interaction	3.83E-04	TRINITY_DN22285_c0_g5	-2.14	down	kinase family protein [Populus trichocarpa]	0.43	2.89
map04626	Plant-pathogen interaction	3.83E-04	TRINITY_DN22288_c0_g1	-1.46	down	PREDICTED: calcium-dependent protein kinase 32-like [Populus euphratica]	3.94	16.40
map04626	Plant-pathogen interaction	3.83E-04	TRINITY_DN22303_c0_g2	-2.29	down	hypothetical protein POPTR_0018s00700g [Populus trichocarpa]	2.23	17.05
map04626	Plant-pathogen interaction	3.83E-04	TRINITY_DN22303_c0_g3	-3.44	down	hypothetical protein POPTR_0006s14110g [Populus trichocarpa]	4.36	73.94
map04626	Plant-pathogen interaction	3.83E-04	TRINITY_DN22316_c2_g2	-2.07	down	leucine-rich repeat family protein [Populus trichocarpa]	6.00	39.32
map04626	Plant-pathogen interaction	3.83E-04	TRINITY_DN22388_c1_g5	-1.57	down	PREDICTED: receptor protein kinase-like protein At4g34220 [Populus euphratica]	6.22	27.79
map04626	Plant-pathogen interaction	3.83E-04	TRINITY_DN22396_c1_g1	-1.82	down	PREDICTED: probable disease resistance protein At4g27220 [Populus euphratica]	0.71	3.88
map04626	Plant-pathogen interaction	3.83E-04	TRINITY_DN22396_c1_g4	-2.45	down	hypothetical protein POPTR_0019s00705g [Populus trichocarpa]	0.53	4.59
map04626	Plant-pathogen interaction	3.83E-04	TRINITY_DN22551_c0_g1	-3.19	down	WRKY transcription factor 24 [(Populus tomentosa x Populus bolleana) x Populus tomentosa]	1.99	23.65
map04626	Plant-pathogen interaction	3.83E-04	TRINITY_DN22551_c1_g1	-1.79	down	hypothetical protein POPTR_0018s13600g [Populus trichocarpa]	16.75	98.52
map04626	Plant-pathogen interaction	3.83E-04	TRINITY_DN22612_c0_g2	-1.10	down	leucine-rich repeat family protein [Populus trichocarpa]	5.53	17.87
map04626	Plant-pathogen interaction	3.83E-04	TRINITY_DN22657_c0_g1	-2.28	down	WRKY transcription factor 28 [(Populus tomentosa x Populus bolleana) x Populus tomentosa]	1.37	10.01
map04626	Plant-pathogen interaction	3.83E-04	TRINITY_DN22671_c0_g1	-1.18	down	hypothetical protein POPTR_0005s10390g [Populus trichocarpa]	1.12	6.06
map04626	Plant-pathogen interaction	3.83E-04	TRINITY_DN22795_c0_g1	-2.67	down	kinase family protein [Populus trichocarpa]	0.70	7.96
map04626	Plant-pathogen interaction	3.83E-04	TRINITY_DN22795_c0_g2	-2.55	down	hypothetical protein POPTR_0011s14530g [Populus trichocarpa]	1.83	16.99
map04626	Plant-pathogen interaction	3.83E-04	TRINITY_DN22925_c1_g1	1.15	up	HSP80 family protein [Populus trichocarpa]	349.08	238.31
map04626	Plant-pathogen interaction	3.83E-04	TRINITY_DN22993_c2_g1	-3.02	down	hypothetical protein POPTR_0001s14680g [Populus trichocarpa]	1.12	13.57
map04626	Plant-pathogen interaction	3.83E-04	TRINITY_DN23026_c1_g4	-1.57	down	PREDICTED: probable disease resistance protein At5g43730 [Populus euphratica]	4.03	18.29
map04626	Plant-pathogen interaction	3.83E-04	TRINITY_DN23027_c0_g2	-1.39	down	hypothetical protein POPTR_0316s00220g [Populus trichocarpa]	0.95	5.52
map04626	Plant-pathogen interaction	3.83E-04	TRINITY_DN23027_c0_g4	-4.40	down	putative wall-associated kinase family protein [Populus trichocarpa]	0.22	5.86
map04626	Plant-pathogen interaction	3.83E-04	TRINITY_DN23102_c1_g1	-1.10	down	PREDICTED: probable WRKY transcription factor 39 [Populus euphratica]	2.26	7.38
map04626	Plant-pathogen interaction	3.83E-04	TRINITY_DN23138_c0_g4	-1.72	down	hypothetical protein POPTR_0004s04030g [Populus trichocarpa]	3.40	17.04
map04626	Plant-pathogen interaction	3.83E-04	TRINITY_DN23177_c0_g7	-1.66	down	hypothetical protein POPTR_0009s14910g [Populus trichocarpa]	1.14	5.47
map04626	Plant-pathogen interaction	3.83E-04	TRINITY_DN23211_c3_g2	-1.20	down	leucine-rich repeat transmembrane protein kinase [Populus trichocarpa]	1.24	4.29
map04626	Plant-pathogen interaction	3.83E-04	TRINITY_DN23220_c0_g2	-5.79	down	PREDICTED: AP2-like ethylene-responsive transcription factor AIL1 isoform X1 [Populus euphratica]	0.14	9.03
map04626	Plant-pathogen interaction	3.83E-04	TRINITY_DN23221_c1_g2	-1.60	down	PREDICTED: putative cyclic nucleotide-gated ion channel 13 [Populus euphratica]	0.99	4.50
map04626	Plant-pathogen interaction	3.83E-04	TRINITY_DN23221_c1_g4	-1.53	down	PREDICTED: cyclic nucleotide-gated ion channel 1-like [Populus euphratica]	31.48	139.05
map04626	Plant-pathogen interaction	3.83E-04	TRINITY_DN23253_c0_g1	-2.41	down	hypothetical protein POPTR_0005s02640g [Populus trichocarpa]	1.29	11.05
map04626	Plant-pathogen interaction	3.83E-04	TRINITY_DN23261_c0_g4	1.19	up	ABC1 family protein [Populus trichocarpa]	39.15	25.95
map04626	Plant-pathogen interaction	3.83E-04	TRINITY_DN23364_c0_g1	-1.89	down	PREDICTED: serine/threonine-protein kinase-like protein CCR4 [Populus euphratica]	0.82	4.55
map04626	Plant-pathogen interaction	3.83E-04	TRINITY_DN23480_c0_g1	-1.35	down	hypothetical protein POPTR_0001s42400g [Populus trichocarpa]	34.04	132.04
map04626	Plant-pathogen interaction	3.83E-04	TRINITY_DN23481_c0_g1	-1.59	down	hypothetical protein POPTR_0002s04720g, partial [Populus trichocarpa]	117.99	559.47
map04626	Plant-pathogen interaction	3.83E-04	TRINITY_DN23481_c0_g2	-1.93	down	PREDICTED: histone H2A variant 1 [Populus euphratica]	21.87	126.80
map04626	Plant-pathogen interaction	3.83E-04	TRINITY_DN23504_c0_g2	-1.89	down	PREDICTED: calmodulin-like protein 11 [Populus euphratica]	8.28	46.91
map04626	Plant-pathogen interaction	3.83E-04	TRINITY_DN23606_c0_g2	1.10	up	mitogen-activated protein kinase kinase [Populus trichocarpa]	51.70	35.83
map04626	Plant-pathogen interaction	3.83E-04	TRINITY_DN23703_c0_g3	-1.01	down	hypothetical protein POPTR_0005s27290g [Populus trichocarpa]	3.20	10.35
map04626	Plant-pathogen interaction	3.83E-04	TRINITY_DN23717_c0_g2	-1.97	down	kinase family protein [Populus trichocarpa]	1.77	4.53
map04626	Plant-pathogen interaction	3.83E-04	TRINITY_DN23770_c0_g1	-2.52	down	WRKY transcription factor 12 [(Populus tomentosa x Populus bolleana) x Populus tomentosa]	4.49	40.11
map04626	Plant-pathogen interaction	3.83E-04	TRINITY_DN23821_c0_g1	-3.69	down	PREDICTED: AP2-like ethylene-responsive transcription factor AIL5 [Populus euphratica]	4.10	87.43
map04626	Plant-pathogen interaction	3.83E-04	TRINITY_DN23867_c0_g1	-2.10	down	epidermal differentiation family protein [Populus trichocarpa]	1.34	9.01
map04626	Plant-pathogen interaction	3.83E-04	TRINITY_DN23917_c0_g1	-1.06	down	hypothetical protein POPTR_0004s24130g [Populus trichocarpa]	1.68	5.32

map04626	Plant-pathogen interaction	3.83E-04	TRINITY_DN23918_c0_g1	-2.35	down	hypothetical protein POPTR_0002s00370g [Populus trichocarpa]	0.41	2.84
map04626	Plant-pathogen interaction	3.83E-04	TRINITY_DN23918_c0_g2	-1.75	down	kinase family protein [Populus trichocarpa]	2.19	11.22
map04626	Plant-pathogen interaction	3.83E-04	TRINITY_DN24001_c0_g1	-1.59	down	PREDICTED: wee1-like protein kinase [Populus euphratica]	1.76	10.43
map04626	Plant-pathogen interaction	3.83E-04	TRINITY_DN24035_c0_g1	-1.40	down	hypothetical protein POPTR_0008s01490g [Populus trichocarpa]	7.11	28.53
map04626	Plant-pathogen interaction	3.83E-04	TRINITY_DN24061_c0_g1	-1.37	down	kinase family protein [Populus trichocarpa]	1.22	5.01
map04626	Plant-pathogen interaction	3.83E-04	TRINITY_DN24081_c1_g3	-1.18	down	-	1.82	6.39
map04626	Plant-pathogen interaction	3.83E-04	TRINITY_DN24138_c0_g2	-1.62	down	PREDICTED: cyclic nucleotide-gated ion channel 4 [Populus euphratica]	26.87	128.24
map04626	Plant-pathogen interaction	3.83E-04	TRINITY_DN24203_c0_g1	-1.20	down	hypothetical protein POPTR_0014s03480g [Populus trichocarpa]	1.89	7.86
map04626	Plant-pathogen interaction	3.83E-04	TRINITY_DN24215_c0_g1	-1.97	down	hypothetical protein POPTR_0018s14500g [Populus trichocarpa]	3.34	20.39
map04626	Plant-pathogen interaction	3.83E-04	TRINITY_DN24215_c0_g3	-2.95	down	hypothetical protein POPTR_0463s00220g, partial [Populus trichocarpa]	0.63	7.15
map04626	Plant-pathogen interaction	3.83E-04	TRINITY_DN24222_c0_g1	2.01	up	hypothetical protein POPTR_0002s12740g [Populus trichocarpa]	21.74	8.16
map04626	Plant-pathogen interaction	3.83E-04	TRINITY_DN24243_c0_g1	1.15	up	hypothetical protein POPTR_0010s22010g [Populus trichocarpa]	9.82	6.77
map04626	Plant-pathogen interaction	3.83E-04	TRINITY_DN24243_c0_g2	1.50	up	PREDICTED: probable receptor-like protein kinase At5g59700 [Populus euphratica]	3.96	2.10
map04626	Plant-pathogen interaction	3.83E-04	TRINITY_DN24280_c0_g2	-1.20	down	kinase family protein [Populus trichocarpa]	7.02	24.02
map04626	Plant-pathogen interaction	3.83E-04	TRINITY_DN24362_c0_g3	-1.56	down	hypothetical protein POPTR_0014s11490g [Populus trichocarpa]	3.36	15.60
map04626	Plant-pathogen interaction	3.83E-04	TRINITY_DN24362_c0_g5	-1.79	down	WRKY transcription factor 3 [(Populus tomentosa x Populus bolleana) x Populus tomentosa]	14.22	77.46
map04626	Plant-pathogen interaction	3.83E-04	TRINITY_DN24388_c0_g1	-1.87	down	PREDICTED: lipase-like PAD4 [Populus euphratica]	4.42	23.88
map04626	Plant-pathogen interaction	3.83E-04	TRINITY_DN24437_c0_g1	1.35	up	stress inducible family protein [Populus trichocarpa]	103.66	64.11
map04626	Plant-pathogen interaction	3.83E-04	TRINITY_DN24591_c0_g1	-1.43	down	hypothetical protein POPTR_0011s04540g [Populus trichocarpa]	2.10	8.21
map04626	Plant-pathogen interaction	3.83E-04	TRINITY_DN24603_c0_g3	-2.51	down	hypothetical protein POPTR_0463s00220g, partial [Populus trichocarpa]	0.33	2.90
map04626	Plant-pathogen interaction	3.83E-04	TRINITY_DN24782_c0_g1	-2.52	down	PREDICTED: probable serine/threonine-protein kinase At1g18390 [Populus euphratica]	0.52	4.51
map04626	Plant-pathogen interaction	3.83E-04	TRINITY_DN24816_c2_g2	-1.14	down	hypothetical protein POPTR_0017s12550g [Populus trichocarpa]	3.51	11.94
map04626	Plant-pathogen interaction	3.83E-04	TRINITY_DN24849_c1_g3	-1.34	down	hypothetical protein POPTR_0007s01340g [Populus trichocarpa]	6.30	24.29
map04626	Plant-pathogen interaction	3.83E-04	TRINITY_DN24914_c5_g1	-2.08	down	hypothetical protein POPTR_0004s05920g [Populus trichocarpa]	1.28	7.25
map04626	Plant-pathogen interaction	3.83E-04	TRINITY_DN24914_c5_g2	-1.35	down	WRKY transcription factor 8 [(Populus tomentosa x Populus bolleana) x Populus tomentosa]	0.57	2.18
map04626	Plant-pathogen interaction	3.83E-04	TRINITY_DN24962_c1_g1	-1.31	down	hypothetical protein POPTR_0016s14410g [Populus trichocarpa]	2.58	9.76
map04626	Plant-pathogen interaction	3.83E-04	TRINITY_DN24985_c0_g1	-1.82	down	PREDICTED: probable disease resistance protein At4g27220 isoform X1 [Populus euphratica]	2.94	10.83
map04626	Plant-pathogen interaction	3.83E-04	TRINITY_DN24986_c0_g5	-6.68	down	hypothetical protein POPTR_0018s09510g [Populus trichocarpa]	0.00	2.14
map04626	Plant-pathogen interaction	3.83E-04	TRINITY_DN24987_c0_g2	2.52	up	PREDICTED: probable serine/threonine-protein kinase WNK5 isoform X1 [Populus euphratica]	2.83	0.75
map04626	Plant-pathogen interaction	3.83E-04	TRINITY_DN25056_c0_g5	1.87	up	HSP90 [Populus tomentosa]	199.35	84.67
map04626	Plant-pathogen interaction	3.83E-04	TRINITY_DN25066_c0_g2	-1.39	down	leucine-rich repeat transmembrane protein kinase [Populus trichocarpa]	2.46	10.51
map04626	Plant-pathogen interaction	3.83E-04	TRINITY_DN25181_c0_g2	-2.19	down	PREDICTED: uncharacterized protein LOC105130933 [Populus euphratica]	2.13	14.77
map04626	Plant-pathogen interaction	3.83E-04	TRINITY_DN25295_c0_g1	-1.67	down	PREDICTED: wall-associated receptor kinase-like 14 [Populus euphratica]	2.68	13.40
map04626	Plant-pathogen interaction	3.83E-04	TRINITY_DN25319_c0_g1	-1.11	down	PREDICTED: probable WRKY transcription factor 3 [Populus euphratica]	7.32	30.17
map04626	Plant-pathogen interaction	3.83E-04	TRINITY_DN25336_c0_g1	-1.12	down	WRKY transcription factor 21 family protein [Populus trichocarpa]	7.37	24.00
map04626	Plant-pathogen interaction	3.83E-04	TRINITY_DN25383_c0_g1	-1.12	down	PREDICTED: putative disease resistance RPP13-like protein 1 [Populus euphratica]	2.86	10.01
map04626	Plant-pathogen interaction	3.83E-04	TRINITY_DN25425_c0_g1	-1.47	down	hypothetical protein POPTR_0014s02420g [Populus trichocarpa]	14.00	57.95
map04626	Plant-pathogen interaction	3.83E-04	TRINITY_DN25455_c0_g1	-1.00	down	PREDICTED: protein NSP-INTERACTING KINASE 1-like isoform X2 [Populus euphratica]	10.84	33.18
map04626	Plant-pathogen interaction	3.83E-04	TRINITY_DN25523_c0_g1	-1.11	down	kinase family protein [Populus trichocarpa]	4.54	14.75
map04626	Plant-pathogen interaction	3.83E-04	TRINITY_DN25597_c0_g4	-1.04	down	PREDICTED: probable receptor-like serine/threonine-protein kinase At4g34500 [Populus euphratica]	4.95	15.58
map04626	Plant-pathogen interaction	3.83E-04	TRINITY_DN25599_c0_g1	1.71	up	unknown [Populus trichocarpa]	35.13	16.21
map04626	Plant-pathogen interaction	3.83E-04	TRINITY_DN25681_c0_g1	-2.33	down	leucine-rich repeat transmembrane protein kinase [Populus trichocarpa]	6.49	48.42
map04626	Plant-pathogen interaction	3.83E-04	TRINITY_DN25711_c1_g3	1.40	up	PREDICTED: ethylene-responsive transcription factor 2-like [Populus euphratica]	8.83	4.91

map04626	Plant-pathogen interaction	3.83E-04	TRINITY_DN25711_c1_g4	-2.55	down	hypothetical protein POPTR_0001s11770g [Populus trichocarpa]	0.54	4.70
map04626	Plant-pathogen interaction	3.83E-04	TRINITY_DN25761_c1_g1	-2.06	down	PREDICTED: probable receptor-like protein kinase At1g80640 isoform X3 [Populus euphratica]	1.23	6.93
map04626	Plant-pathogen interaction	3.83E-04	TRINITY_DN25761_c1_g2	-1.95	down	PREDICTED: probable receptor-like protein kinase At5g47070 [Populus euphratica]	4.84	25.74
map04626	Plant-pathogen interaction	3.83E-04	TRINITY_DN25833_c0_g1	-1.20	down	hypothetical protein POPTR_0009s03570g [Populus trichocarpa]	3.89	14.00
map04626	Plant-pathogen interaction	3.83E-04	TRINITY_DN25841_c0_g1	-1.52	down	WRKY transcription factor 5 [(Populus tomentosa x Populus bolleana) x Populus tomentosa]	32.92	138.24
map04626	Plant-pathogen interaction	3.83E-04	TRINITY_DN25864_c0_g6	-1.81	down	hypothetical protein POPTR_0004s12530g [Populus trichocarpa]	1.97	10.50
map04626	Plant-pathogen interaction	3.83E-04	TRINITY_DN25890_c0_g2	-1.10	down	hypothetical protein POPTR_0014s13990g [Populus trichocarpa]	6.52	23.13
map04626	Plant-pathogen interaction	3.83E-04	TRINITY_DN25895_c0_g1	-2.61	down	leucine-rich repeat transmembrane protein kinase [Populus trichocarpa]	1.65	15.78
map04626	Plant-pathogen interaction	3.83E-04	TRINITY_DN25895_c0_g3	-2.89	down	leucine-rich repeat transmembrane protein kinase [Populus trichocarpa]	0.82	9.37
map04626	Plant-pathogen interaction	3.83E-04	TRINITY_DN25984_c0_g4	-1.19	down	hypothetical protein POPTR_0031s00260g, partial [Populus trichocarpa]	34.70	120.61
map04626	Plant-pathogen interaction	3.83E-04	TRINITY_DN26031_c0_g4	-1.10	down	calcium-dependent protein kinase [Populus trichocarpa]	6.99	22.76
map04626	Plant-pathogen interaction	3.83E-04	TRINITY_DN26143_c0_g1	-1.18	down	PREDICTED: mitogen-activated protein kinase kinase kinase 1-like [Populus euphratica]	13.45	42.54
map04626	Plant-pathogen interaction	3.83E-04	TRINITY_DN26175_c0_g4	1.70	up	PREDICTED: probable LRR receptor-like serine/threonine-protein kinase MRH1 isoform X1 [Populus euphratica]	1.95	0.91
map04626	Plant-pathogen interaction	3.83E-04	TRINITY_DN26207_c0_g3	-1.48	down	calcium-binding family protein [Populus trichocarpa]	5.82	24.48
map04626	Plant-pathogen interaction	3.83E-04	TRINITY_DN26292_c0_g7	-2.62	down	hypothetical protein POPTR_0017s02570g [Populus trichocarpa]	1.60	15.90
map04626	Plant-pathogen interaction	3.83E-04	TRINITY_DN26324_c0_g1	1.62	up	hypothetical protein POPTR_0012s04160g [Populus trichocarpa]	6.71	2.72
map04626	Plant-pathogen interaction	3.83E-04	TRINITY_DN26328_c1_g2	-1.24	down	leucine-rich repeat family protein [Populus trichocarpa]	1.74	6.45
map04626	Plant-pathogen interaction	3.83E-04	TRINITY_DN26434_c0_g1	-2.22	down	disease resistance family protein [Populus trichocarpa]	9.97	73.92
map04626	Plant-pathogen interaction	3.83E-04	TRINITY_DN26496_c0_g1	-1.38	down	hypothetical protein POPTR_0004s06180g [Populus trichocarpa]	3.81	11.95
map04626	Plant-pathogen interaction	3.83E-04	TRINITY_DN26657_c0_g2	-1.16	down	hypothetical protein POPTR_0015s06040g [Populus trichocarpa]	5.31	17.39
map04626	Plant-pathogen interaction	3.83E-04	TRINITY_DN26681_c1_g2	1.47	up	PREDICTED: probable LRR receptor-like serine/threonine-protein kinase At4g26540 [Populus euphratica]	5.17	2.83
map04626	Plant-pathogen interaction	3.83E-04	TRINITY_DN26758_c0_g1	1.70	up	PREDICTED: heat shock protein 83 isoform X2 [Populus euphratica]	52.31	25.64
map04626	Plant-pathogen interaction	3.83E-04	TRINITY_DN26883_c0_g1	-2.40	down	hypothetical protein POPTR_0005s16690g [Populus trichocarpa]	15.04	123.74
map04626	Plant-pathogen interaction	3.83E-04	TRINITY_DN26984_c2_g4	1.22	up	PREDICTED: LOW QUALITY PROTEIN: probable cyclic nucleotide-gated ion channel 14 [Populus euphratica]	6.71	4.48
map04626	Plant-pathogen interaction	3.83E-04	TRINITY_DN26997_c0_g1	-1.67	down	PREDICTED: probable LRR receptor-like serine/threonine-protein kinase At1g34110 [Populus euphratica]	13.18	55.35
map04626	Plant-pathogen interaction	3.83E-04	TRINITY_DN27058_c0_g1	-1.45	down	PREDICTED: probable leucine-rich repeat receptor-like serine/threonine-protein kinase At5g15730 [Populus euphratica]	7.17	31.57
map04626	Plant-pathogen interaction	3.83E-04	TRINITY_DN27063_c0_g1	-2.83	down	integrase-type DNA-binding superfamily protein [Populus tomentosa]	7.52	69.47
map04626	Plant-pathogen interaction	3.83E-04	TRINITY_DN27113_c0_g1	-1.83	down	PREDICTED: cyclic nucleotide-gated ion channel 1-like [Populus euphratica]	13.21	70.01
map04626	Plant-pathogen interaction	3.83E-04	TRINITY_DN27113_c0_g2	-1.16	down	PREDICTED: cyclic nucleotide-gated ion channel 1-like [Populus euphratica]	8.34	29.60
map04626	Plant-pathogen interaction	3.83E-04	TRINITY_DN27126_c2_g3	-1.68	down	hypothetical protein POPTR_0019s00570g [Populus trichocarpa]	3.67	17.37
map04626	Plant-pathogen interaction	3.83E-04	TRINITY_DN27150_c0_g1	1.23	up	ABC1 family protein [Populus trichocarpa]	13.39	8.40
map04626	Plant-pathogen interaction	3.83E-04	TRINITY_DN27163_c0_g1	-1.60	down	PREDICTED: probable LRR receptor-like serine/threonine-protein kinase At2g16250 [Populus euphratica]	4.67	22.30
map04626	Plant-pathogen interaction	3.83E-04	TRINITY_DN27163_c0_g2	-1.53	down	hypothetical protein POPTR_0014s18350g [Populus trichocarpa]	0.85	3.80
map04626	Plant-pathogen interaction	3.83E-04	TRINITY_DN27181_c0_g2	-1.49	down	hypothetical protein POPTR_0019s00390g [Populus trichocarpa]	3.03	13.48
map04626	Plant-pathogen interaction	3.83E-04	TRINITY_DN27181_c1_g1	-1.67	down	hypothetical protein POPTR_0019s01080g [Populus trichocarpa]	3.94	16.81
map04626	Plant-pathogen interaction	3.83E-04	TRINITY_DN27183_c0_g2	-1.76	down	unknown [Populus trichocarpa]	2.49	12.47
map04626	Plant-pathogen interaction	3.83E-04	TRINITY_DN27233_c0_g1	-1.44	down	PREDICTED: probable serine/threonine-protein kinase mps1 isoform X1 [Populus euphratica]	5.48	22.75
map04626	Plant-pathogen interaction	3.83E-04	TRINITY_DN27260_c0_g1	-1.17	down	PREDICTED: receptor-like serine/threonine-protein kinase ALE2 isoform X4 [Populus euphratica]	2.68	8.76
map04626	Plant-pathogen interaction	3.83E-04	TRINITY_DN27318_c1_g1	-2.27	down	PREDICTED: putative disease resistance protein At4g10780 [Populus euphratica]	2.07	15.95

map04626	Plant-pathogen interaction	3.83E-04	TRINITY_DN27318_c1_g3	-1.62	down	PREDICTED: probable disease resistance protein At4g27220 [Populus euphratica]	0.57	2.76
map04626	Plant-pathogen interaction	3.83E-04	TRINITY_DN27367_c0_g1	1.01	up	PREDICTED: uncharacterized aarF domain-containing protein kinase At1g79600, chloroplastic-like isoform X1 [Populus euphratica]	257.25	204.05
map04626	Plant-pathogen interaction	3.83E-04	TRINITY_DN27393_c0_g1	1.91	up	heat shock family protein [Populus trichocarpa]	39.01	15.44
map04626	Plant-pathogen interaction	3.83E-04	TRINITY_DN27393_c1_g1	2.11	up	PREDICTED: heat shock protein 83-like [Populus euphratica]	375.24	129.58
map04626	Plant-pathogen interaction	3.83E-04	TRINITY_DN27394_c0_g1	-2.95	down	resistance family protein [Populus trichocarpa]	0.24	2.87
map04626	Plant-pathogen interaction	3.83E-04	TRINITY_DN27442_c0_g3	-1.83	down	leucine-rich repeat transmembrane protein kinase [Populus trichocarpa]	1.23	6.67
map04626	Plant-pathogen interaction	3.83E-04	TRINITY_DN27456_c1_g1	-1.23	down	PREDICTED: probable LRR receptor-like serine/threonine-protein kinase At4g20940 [Populus euphratica]	5.06	16.85
map04626	Plant-pathogen interaction	3.83E-04	TRINITY_DN27470_c0_g1	-2.44	down	hypothetical protein POPTR_0003s14420g [Populus trichocarpa]	0.97	8.22
map04626	Plant-pathogen interaction	3.83E-04	TRINITY_DN27470_c0_g2	-8.05	down	PREDICTED: myb-related protein P-like [Populus euphratica]	0.00	5.29
map04626	Plant-pathogen interaction	3.83E-04	TRINITY_DN27578_c0_g1	-1.72	down	PREDICTED: probable receptor-like protein kinase At5g24010 [Populus euphratica]	3.81	20.53
map04626	Plant-pathogen interaction	3.83E-04	TRINITY_DN27639_c0_g1	-2.33	down	leucine-rich repeat family protein [Populus trichocarpa]	4.88	38.96
map04626	Plant-pathogen interaction	3.83E-04	TRINITY_DN27695_c0_g1	3.03	up	hypothetical protein POPTR_0017s01160g [Populus trichocarpa]	29.21	5.47
map04626	Plant-pathogen interaction	3.83E-04	TRINITY_DN27695_c1_g1	3.35	up	hypothetical protein POPTR_0017s01160g [Populus trichocarpa]	42.99	6.84
map04626	Plant-pathogen interaction	3.83E-04	TRINITY_DN27695_c2_g1	1.11	up	HSP80 family protein [Populus trichocarpa]	176.25	130.70
map04626	Plant-pathogen interaction	3.83E-04	TRINITY_DN27695_c2_g4	1.84	up	HSP90 [Populus tomentosa]	517.67	225.89
map04626	Plant-pathogen interaction	3.83E-04	TRINITY_DN27703_c1_g9	-1.63	down	hypothetical protein POPTR_0019s00700g [Populus trichocarpa]	0.78	3.50
map04626	Plant-pathogen interaction	3.83E-04	TRINITY_DN27730_c1_g1	-1.13	down	hypothetical protein POPTR_0003s02960g [Populus trichocarpa]	32.25	107.25
map04626	Plant-pathogen interaction	3.83E-04	TRINITY_DN27739_c1_g1	-1.76	down	hypothetical protein POPTR_0019s00705g [Populus trichocarpa]	2.48	12.17
map04626	Plant-pathogen interaction	3.83E-04	TRINITY_DN27770_c0_g2	-2.65	down	NBS-LRR resistance gene-like protein ARGH34 [Populus trichocarpa]	0.22	2.16
map04626	Plant-pathogen interaction	3.83E-04	TRINITY_DN27813_c2_g1	1.01	up	PREDICTED: uncharacterized protein LOC105116545 [Populus euphratica]	9.05	13.90
map04626	Plant-pathogen interaction	3.83E-04	TRINITY_DN27893_c3_g2	-6.27	down	hypothetical protein POPTR_0001s28330g [Populus trichocarpa]	0.02	3.05
map04626	Plant-pathogen interaction	3.83E-04	TRINITY_DN11154_c0_g3	1.91	up	hypothetical protein POPTR_0002s18210g [Populus trichocarpa]	1.55	0.64
map04626	Plant-pathogen interaction	3.83E-04	TRINITY_DN12146_c0_g1	-2.23	down	PREDICTED: putative disease resistance protein At4g19050 isoform X1 [Populus euphratica]	0.30	2.23
map04626	Plant-pathogen interaction	3.83E-04	TRINITY_DN12159_c0_g1	-2.38	down	calcium-binding family protein [Populus trichocarpa]	0.41	3.24
map04626	Plant-pathogen interaction	3.83E-04	TRINITY_DN12743_c0_g1	1.79	up	hypothetical protein POPTR_0006s24180g [Populus trichocarpa]	2.99	1.40
map04626	Plant-pathogen interaction	3.83E-04	TRINITY_DN13002_c0_g1	-1.57	down	PREDICTED: proline-rich receptor-like protein kinase PERK13 [Populus euphratica]	0.61	2.89
map04626	Plant-pathogen interaction	3.83E-04	TRINITY_DN13393_c0_g1	-3.55	down	hypothetical protein POPTR_0006s24050g [Populus trichocarpa]	0.10	4.01
map04626	Plant-pathogen interaction	3.83E-04	TRINITY_DN13584_c0_g1	-5.95	down	PREDICTED: calcium-binding protein PBP1-like [Populus euphratica]	0.06	6.65
map04626	Plant-pathogen interaction	3.83E-04	TRINITY_DN13784_c0_g1	-3.53	down	PREDICTED: wall-associated receptor kinase-like 20 [Populus euphratica]	0.19	3.74
map04626	Plant-pathogen interaction	3.83E-04	TRINITY_DN14287_c0_g1	-1.85	down	hypothetical protein POPTR_0013s02990g [Populus trichocarpa]	12.12	67.05
map04626	Plant-pathogen interaction	3.83E-04	TRINITY_DN14287_c0_g3	-1.84	down	histone H2A family protein [Populus trichocarpa]	25.79	143.46
map04626	Plant-pathogen interaction	3.83E-04	TRINITY_DN14433_c0_g2	-4.22	down	hypothetical protein POPTR_0315s00200g [Populus trichocarpa]	0.17	5.08
map04626	Plant-pathogen interaction	3.83E-04	TRINITY_DN14474_c0_g3	-2.29	down	hypothetical protein POPTR_0017s11180g [Populus trichocarpa]	0.41	3.17
map04626	Plant-pathogen interaction	3.83E-04	TRINITY_DN14812_c0_g1	-2.36	down	hypothetical protein POPTR_0001s42270g [Populus trichocarpa]	1.92	15.36
map04626	Plant-pathogen interaction	3.83E-04	TRINITY_DN14812_c0_g2	-2.94	down	PREDICTED: calcium-binding protein PBP1-like [Populus euphratica]	1.20	14.58
map04626	Plant-pathogen interaction	3.83E-04	TRINITY_DN14967_c0_g1	1.72	up	PREDICTED: receptor-like cytosolic serine/threonine-protein kinase RBK2 isoform X1 [Populus euphratica]	4.16	2.26
map04626	Plant-pathogen interaction	3.83E-04	TRINITY_DN14971_c0_g1	-4.47	down	DREB68 [Populus hopeiensis]	0.24	11.63
map04626	Plant-pathogen interaction	3.83E-04	TRINITY_DN14975_c0_g1	-2.28	down	hypothetical protein POPTR_0009s08540g [Populus trichocarpa]	0.48	3.45
map04626	Plant-pathogen interaction	3.83E-04	TRINITY_DN15085_c0_g1	-4.79	down	NBS-LRR resistance gene-like protein ARGH34 [Populus trichocarpa]	0.30	12.71
map04626	Plant-pathogen interaction	3.83E-04	TRINITY_DN15277_c0_g1	1.94	up	HSP90 [Populus tomentosa]	515.73	209.22
map04626	Plant-pathogen interaction	3.83E-04	TRINITY_DN15337_c0_g1	-2.85	down	PREDICTED: ethylene-responsive transcription factor ERF017-like [Populus euphratica]	1.96	21.20
map04626	Plant-pathogen interaction	3.83E-04	TRINITY_DN15356_c0_g1	-4.82	down	hypothetical protein POPTR_0018s08320g [Populus trichocarpa]	0.69	30.09

map04626	Plant-pathogen interaction	3.83E-04	TRINITY_DN15373_c0_g1	4.19	up	pathogenesis-related family protein [Populus trichocarpa]	42.69	3.64
map04626	Plant-pathogen interaction	3.83E-04	TRINITY_DN15389_c0_g1	2.54	up	heat shock family protein [Populus trichocarpa]	934.53	246.19
map04626	Plant-pathogen interaction	3.83E-04	TRINITY_DN15592_c0_g1	-2.13	down	hypothetical protein POPTR_0010s16760g [Populus trichocarpa]	2.25	4.65
map04626	Plant-pathogen interaction	3.83E-04	TRINITY_DN15707_c0_g5	-4.70	down	hypothetical protein POPTR_0125s00210g [Populus trichocarpa]	0.09	9.82
map04626	Plant-pathogen interaction	3.83E-04	TRINITY_DN15716_c0_g2	4.85	up	hypothetical protein POPTR_0005s25470g [Populus trichocarpa]	8.26	1.38
map04626	Plant-pathogen interaction	3.83E-04	TRINITY_DN15796_c0_g1	-3.09	down	PREDICTED: LRR receptor-like serine/threonine-protein kinase ERL1 [Populus euphratica]	2.77	36.86
map04626	Plant-pathogen interaction	3.83E-04	TRINITY_DN15803_c0_g1	-2.56	down	hypothetical protein POPTR_0001s45900g [Populus trichocarpa]	0.30	2.75
map04626	Plant-pathogen interaction	3.83E-04	TRINITY_DN15830_c0_g1	6.93	up	thioredoxin H [Populus tremula x Populus tremuloides]	41.24	0.50
map04626	Plant-pathogen interaction	3.83E-04	TRINITY_DN15892_c0_g2	-2.99	down	unknown [Populus trichocarpa x Populus deltoides]	0.40	7.59
map04626	Plant-pathogen interaction	3.83E-04	TRINITY_DN15966_c0_g1	-1.63	down	calmodulin family protein [Populus trichocarpa]	1.41	5.55
map04626	Plant-pathogen interaction	3.83E-04	TRINITY_DN15968_c0_g2	1.21	up	hypothetical protein POPTR_0009s10520g [Populus trichocarpa]	17.06	12.70
map04626	Plant-pathogen interaction	3.83E-04	TRINITY_DN16006_c0_g1	-1.20	down	PREDICTED: mitogen-activated protein kinase kinase 6 [Populus euphratica]	10.58	36.23
map04626	Plant-pathogen interaction	3.83E-04	TRINITY_DN16053_c0_g1	1.06	up	ferric reductase-like transmembrane component family protein [Populus trichocarpa]	11.32	8.32
map04626	Plant-pathogen interaction	3.83E-04	TRINITY_DN16406_c0_g1	-2.36	down	kinase family protein [Populus trichocarpa]	0.36	2.95
map04626	Plant-pathogen interaction	3.83E-04	TRINITY_DN16406_c1_g2	-2.75	down	hypothetical protein POPTR_0002s21350g [Populus trichocarpa]	0.50	5.28
map04626	Plant-pathogen interaction	3.83E-04	TRINITY_DN16419_c0_g3	2.60	up	hypothetical protein POPTR_0002s08920g [Populus trichocarpa]	20.31	5.00
map04626	Plant-pathogen interaction	3.83E-04	TRINITY_DN16453_c0_g1	-3.92	down	hypothetical protein POPTR_0009s10470g [Populus trichocarpa]	0.95	23.11
map04626	Plant-pathogen interaction	3.83E-04	TRINITY_DN16547_c0_g1	-5.02	down	hypothetical protein POPTR_0017s12100g [Populus trichocarpa]	0.07	4.07
map04626	Plant-pathogen interaction	3.83E-04	TRINITY_DN16547_c0_g2	-3.06	down	calcium-binding EF hand family protein [Populus trichocarpa]	0.35	4.71
map04626	Plant-pathogen interaction	3.83E-04	TRINITY_DN16548_c0_g1	-1.98	down	PREDICTED: probable LRR receptor-like serine/threonine-protein kinase At1g14390 isoform X1 [Populus euphratica]	0.72	4.13
map04626	Plant-pathogen interaction	3.83E-04	TRINITY_DN16561_c0_g1	1.24	up	PREDICTED: LOW QUALITY PROTEIN: aarF domain-containing protein kinase 4 [Populus euphratica]	8.38	5.48
map04626	Plant-pathogen interaction	3.83E-04	TRINITY_DN16642_c0_g1	-7.12	down	AP2 domain-containing transcription factor family protein [Populus trichocarpa]	0.02	5.54
map04626	Plant-pathogen interaction	3.83E-04	TRINITY_DN16695_c0_g1	-3.27	down	hypothetical protein POPTR_0006s23480g [Populus trichocarpa]	0.81	11.67
map04626	Plant-pathogen interaction	3.83E-04	TRINITY_DN16705_c0_g2	-2.05	down	hypothetical protein POPTR_0011s11330g [Populus trichocarpa]	2.24	10.42
map04626	Plant-pathogen interaction	3.83E-04	TRINITY_DN16806_c1_g2	-1.62	down	putative calmodulin-related family protein [Populus trichocarpa]	26.99	125.77
map04626	Plant-pathogen interaction	3.83E-04	TRINITY_DN16806_c1_g3	-2.76	down	putative calmodulin-related family protein [Populus trichocarpa]	21.78	225.85
map04626	Plant-pathogen interaction	3.83E-04	TRINITY_DN16813_c0_g2	-2.41	down	hypothetical protein POPTR_0013s14730g [Populus trichocarpa]	1.77	15.69
map04626	Plant-pathogen interaction	3.83E-04	TRINITY_DN16815_c0_g1	-1.87	down	hypothetical protein POPTR_0013s15520g [Populus trichocarpa]	0.65	2.93
map04626	Plant-pathogen interaction	3.83E-04	TRINITY_DN16851_c0_g1	-2.71	down	PREDICTED: calmodulin-like [Populus euphratica]	1.09	10.82
map04626	Plant-pathogen interaction	3.83E-04	TRINITY_DN16851_c0_g2	-1.40	down	hypersensitive reaction associated Ca2+-binding family protein [Populus trichocarpa]	5.56	22.61
map04626	Plant-pathogen interaction	3.83E-04	TRINITY_DN17094_c3_g1	2.21	up	hypothetical protein POPTR_0004s07190g [Populus trichocarpa]	10.86	4.99
map04626	Plant-pathogen interaction	3.83E-04	TRINITY_DN17094_c3_g2	1.64	up	HSP90 [Populus tomentosa]	145.24	71.36
map04626	Plant-pathogen interaction	3.83E-04	TRINITY_DN17144_c0_g3	-1.36	down	leucine-rich repeat family protein [Populus trichocarpa]	0.87	3.41
map04626	Plant-pathogen interaction	3.83E-04	TRINITY_DN17173_c0_g1	2.10	up	Lyk 1 [Populus x canadensis]	17.97	6.45
map04626	Plant-pathogen interaction	3.83E-04	TRINITY_DN17176_c0_g2	2.67	up	PREDICTED: serine/threonine-protein kinase-like protein CCR2 [Populus euphratica]	5.80	1.36
map04626	Plant-pathogen interaction	3.83E-04	TRINITY_DN17185_c0_g1	1.44	up	PREDICTED: serine/threonine-protein kinase CDG1-like isoform X1 [Populus euphratica]	3.04	1.69
map04626	Plant-pathogen interaction	3.83E-04	TRINITY_DN17274_c0_g3	-1.71	down	hypothetical protein POPTR_0016s12170g [Populus trichocarpa]	0.67	3.41
map04626	Plant-pathogen interaction	3.83E-04	TRINITY_DN17350_c0_g1	-1.13	down	kinase family protein [Populus trichocarpa]	15.80	52.30
map04626	Plant-pathogen interaction	3.83E-04	TRINITY_DN17389_c0_g2	-1.53	down	PREDICTED: ethylene-responsive transcription factor SHINE 2-like [Populus euphratica]	1.38	6.32
map04626	Plant-pathogen interaction	3.83E-04	TRINITY_DN17428_c0_g1	-2.46	down	calcium-binding family protein [Populus trichocarpa]	1.92	16.00
map04626	Plant-pathogen interaction	3.83E-04	TRINITY_DN17428_c0_g2	-2.90	down	PREDICTED: probable calcium-binding protein CML43 [Populus euphratica]	1.69	21.28
map04626	Plant-pathogen interaction	3.83E-04	TRINITY_DN17440_c0_g1	-3.55	down	hypothetical protein POPTR_0002s00350g [Populus trichocarpa]	1.12	21.89
map04626	Plant-pathogen interaction	3.83E-04	TRINITY_DN17440_c0_g4	-3.65	down	hypothetical protein POPTR_0005s28110g [Populus trichocarpa]	3.67	72.28
map04626	Plant-pathogen interaction	3.83E-04	TRINITY_DN17559_c0_g1	1.04	up	NADPH:QUINONE OXIDOREDUCTASE family protein [Populus trichocarpa]	40.63	29.82

map04626	Plant-pathogen interaction	3.83E-04	TRINITY_DN17702_c0_g1	-1.08	down	hypothetical protein POPTR_0007s15300g [Populus trichocarpa]	2.91	9.29
map04626	Plant-pathogen interaction	3.83E-04	TRINITY_DN17900_c1_g3	-3.63	down	hypothetical protein POPTR_0009s15690g [Populus trichocarpa]	0.43	7.28
map04626	Plant-pathogen interaction	3.83E-04	TRINITY_DN17985_c0_g1	-1.47	down	PREDICTED: putative disease resistance protein At4g19050 isoform X1 [Populus euphratica]	0.90	3.80
map04626	Plant-pathogen interaction	3.83E-04	TRINITY_DN18097_c0_g3	-1.82	down	PREDICTED: uncharacterized protein LOC105136607 [Populus euphratica]	0.96	5.85
map04626	Plant-pathogen interaction	3.83E-04	TRINITY_DN18157_c0_g1	-1.26	down	PREDICTED: receptor-like cytosolic serine/threonine-protein kinase RBK2 isoform X2 [Populus euphratica]	1.58	5.15
map04626	Plant-pathogen interaction	3.83E-04	TRINITY_DN18233_c0_g1	-2.71	down	hypothetical protein POPTR_0005s18240g [Populus trichocarpa]	2.42	24.27
map04626	Plant-pathogen interaction	3.83E-04	TRINITY_DN18250_c0_g1	1.65	up	hypothetical protein POPTR_0013s02080g [Populus trichocarpa]	18.81	8.93
map04626	Plant-pathogen interaction	3.83E-04	TRINITY_DN18282_c0_g1	-1.17	down	calmodulin-like protein 6a [Populus trichocarpa]	10.53	36.61
map04626	Plant-pathogen interaction	3.83E-04	TRINITY_DN18346_c0_g1	-2.85	down	WRKY transcription factor [Populus tremula x Populus alba]	1.35	14.38
map04626	Plant-pathogen interaction	3.83E-04	TRINITY_DN18346_c0_g4	-2.11	down	WRKY transcription factor 31 [(Populus tomentosa x Populus bolleana) x Populus tomentosa]	1.86	12.19
map04626	Plant-pathogen interaction	3.83E-04	TRINITY_DN18483_c0_g1	-1.28	down	PREDICTED: probable receptor-like protein kinase At5g47070 isoform X1 [Populus euphratica]	1.48	5.38
map04626	Plant-pathogen interaction	3.83E-04	TRINITY_DN18544_c0_g2	1.38	up	hypothetical protein POPTR_0006s29050g [Populus trichocarpa]	25.69	14.85
map04626	Plant-pathogen interaction	3.83E-04	TRINITY_DN18864_c0_g2	-5.06	down	hypothetical protein POPTR_0005s22480g [Populus trichocarpa]	0.15	7.47
map04626	Plant-pathogen interaction	3.83E-04	TRINITY_DN19030_c0_g2	1.34	up	hypothetical protein POPTR_0018s02280g [Populus trichocarpa]	21.55	12.38
map04626	Plant-pathogen interaction	3.83E-04	TRINITY_DN19107_c0_g1	-1.99	down	WRKY transcription factor 26 [(Populus tomentosa x Populus bolleana) x Populus tomentosa]	5.42	32.83
map04626	Plant-pathogen interaction	3.83E-04	TRINITY_DN19125_c0_g1	-2.19	down	leucine-rich repeat family protein [Populus trichocarpa]	4.07	23.95
map04626	Plant-pathogen interaction	3.83E-04	TRINITY_DN19302_c0_g1	-1.39	down	PREDICTED: probable LRR receptor-like serine/threonine-protein kinase At1g53430 [Populus euphratica]	12.74	38.87
map04626	Plant-pathogen interaction	3.83E-04	TRINITY_DN19314_c1_g2	-1.02	down	hypothetical protein POPTR_0016s10610g [Populus trichocarpa]	5.91	17.87
map04626	Plant-pathogen interaction	3.83E-04	TRINITY_DN19314_c1_g4	-2.91	down	WRKY32 [(Populus tomentosa x Populus bolleana) x Populus tomentosa]	0.49	5.63
map04626	Plant-pathogen interaction	3.83E-04	TRINITY_DN19367_c0_g4	-2.08	down	PREDICTED: probable calcium-binding protein CML35 [Populus euphratica]	13.38	87.54
map04626	Plant-pathogen interaction	3.83E-04	TRINITY_DN19367_c0_g5	-1.56	down	PREDICTED: probable calcium-binding protein CML36 [Populus euphratica]	15.93	70.30
map04626	Plant-pathogen interaction	3.83E-04	TRINITY_DN19380_c0_g1	3.80	up	PREDICTED: LRR receptor-like serine/threonine-protein kinase FLS2 [Populus euphratica]	2.68	0.26
map04626	Plant-pathogen interaction	3.83E-04	TRINITY_DN19380_c0_g2	2.75	up	FLAGELLIN-SENSITIVE 2 family protein [Populus trichocarpa]	9.65	2.15
map04626	Plant-pathogen interaction	3.83E-04	TRINITY_DN19413_c0_g2	-1.15	down	PREDICTED: receptor-like serine/threonine-protein kinase ALE2 isoform X2 [Populus euphratica]	1.76	5.92
map04626	Plant-pathogen interaction	3.83E-04	TRINITY_DN19523_c0_g1	-2.13	down	hypothetical protein POPTR_0014s05160g [Populus trichocarpa]	0.36	2.30
map04626	Plant-pathogen interaction	3.83E-04	TRINITY_DN19582_c0_g1	-2.67	down	PREDICTED: probable WRKY transcription factor 40 isoform X1 [Populus euphratica]	2.06	18.45
map04626	Plant-pathogen interaction	3.83E-04	TRINITY_DN19582_c0_g2	-1.85	down	WRKY transcription factor 9 [(Populus tomentosa x Populus bolleana) x Populus tomentosa]	2.54	14.58
map04626	Plant-pathogen interaction	3.83E-04	TRINITY_DN19634_c0_g1	-1.28	down	PREDICTED: ethylene-responsive transcription factor CRF4-like [Populus euphratica]	3.82	14.13
map04626	Plant-pathogen interaction	3.83E-04	TRINITY_DN19663_c2_g5	2.38	up	hypothetical protein POPTR_0014s00450g [Populus trichocarpa]	4.49	1.36
map04626	Plant-pathogen interaction	3.83E-04	TRINITY_DN19684_c0_g4	-5.01	down	hypothetical protein POPTR_0006s10950g [Populus trichocarpa]	0.74	37.00
map04626	Plant-pathogen interaction	3.83E-04	TRINITY_DN19768_c0_g2	-3.50	down	calmodulin-like gene family protein [Populus trichocarpa]	0.49	8.81
map04626	Plant-pathogen interaction	3.83E-04	TRINITY_DN19768_c0_g3	-2.83	down	hypothetical protein POPTR_0005s12930g [Populus trichocarpa]	5.99	64.08
map04626	Plant-pathogen interaction	3.83E-04	TRINITY_DN19814_c0_g1	1.48	up	hypothetical protein POPTR_0001s21690g [Populus trichocarpa]	134.77	67.93
map04626	Plant-pathogen interaction	3.83E-04	TRINITY_DN19840_c0_g1	-1.90	down	kinase family protein [Populus trichocarpa]	3.04	15.70
map04626	Plant-pathogen interaction	3.83E-04	TRINITY_DN19876_c0_g1	-5.82	down	Pathogenesis-related family protein [Populus trichocarpa]	5.51	478.26
map04626	Plant-pathogen interaction	3.83E-04	TRINITY_DN19883_c0_g1	-1.76	down	hypothetical protein POPTR_0014s08600g [Populus trichocarpa]	5.09	26.21
map04626	Plant-pathogen interaction	3.83E-04	TRINITY_DN19909_c1_g2	-1.64	down	hypothetical protein POPTR_0004s06180g [Populus trichocarpa]	8.84	41.26
map04626	Plant-pathogen interaction	3.83E-04	TRINITY_DN19919_c0_g2	-1.25	down	leucine-rich repeat transmembrane protein kinase [Populus trichocarpa]	3.53	12.35
map04626	Plant-pathogen interaction	3.83E-04	TRINITY_DN19948_c0_g2	1.58	up	hypothetical protein POPTR_0001s14410g [Populus trichocarpa]	24.77	12.71
map04626	Plant-pathogen interaction	3.83E-04	TRINITY_DN20057_c0_g1	-2.35	down	PREDICTED: probable inactive receptor-like protein kinase At3g56050 [Populus euphratica]	1.33	11.06
map04626	Plant-pathogen interaction	3.83E-04	TRINITY_DN20069_c0_g6	1.89	up	hypothetical protein POPTR_0016s09060g [Populus trichocarpa]	1.76	0.73

map04626	Plant-pathogen interaction	3.83E-04	TRINITY_DN20128_c0_g1	-1.45	down	PREDICTED: dihydrofolate reductase-like [Populus euphratica]	15.72	65.08
map04626	Plant-pathogen interaction	3.83E-04	TRINITY_DN20251_c0_g3	2.08	up	AP2 domain transcription factor family protein [Populus trichocarpa]	108.48	38.78
map04626	Plant-pathogen interaction	3.83E-04	TRINITY_DN20343_c2_g1	-3.99	down	WRKY transcription factor 29 [(Populus tomentosa x Populus bolleana) x Populus tomentosa]	0.11	3.00
map04626	Plant-pathogen interaction	3.83E-04	TRINITY_DN20411_c0_g3	-1.43	down	PREDICTED: histone H2A.6 isoform X1 [Populus euphratica]	31.28	128.16
map04626	Plant-pathogen interaction	3.83E-04	TRINITY_DN20448_c0_g1	-1.50	down	PREDICTED: putative disease resistance protein At1g50180 [Populus euphratica]	1.25	5.40
map04626	Plant-pathogen interaction	3.83E-04	TRINITY_DN20496_c2_g1	-1.34	down	hypothetical protein POPTR_0007s15230g [Populus trichocarpa]	1.80	6.78
map04626	Plant-pathogen interaction	3.83E-04	TRINITY_DN20567_c0_g1	-3.80	down	hypothetical protein POPTR_0015s04680g [Populus trichocarpa]	0.57	12.42
map04626	Plant-pathogen interaction	3.83E-04	TRINITY_DN20594_c0_g2	-1.86	down	hypothetical protein POPTR_0019s05310g [Populus trichocarpa]	0.69	3.84
map04626	Plant-pathogen interaction	3.83E-04	TRINITY_DN20597_c1_g1	-3.43	down	DREB70 [Populus hopeiensis]	0.56	8.11
map04626	Plant-pathogen interaction	3.83E-04	TRINITY_DN20702_c0_g1	-1.67	down	calcium-dependent protein kinase [Populus trichocarpa]	2.51	13.20
map04626	Plant-pathogen interaction	3.83E-04	TRINITY_DN20712_c0_g1	-4.68	down	C-repeat binding factor 1 [Populus tomentosa]	0.29	12.17
map04626	Plant-pathogen interaction	3.83E-04	TRINITY_DN20748_c0_g1	-1.99	down	hypothetical protein POPTR_0004s07050g [Populus trichocarpa]	0.76	4.78
map04626	Plant-pathogen interaction	3.83E-04	TRINITY_DN20759_c1_g1	-1.31	down	PREDICTED: uncharacterized protein LOC105110153 [Populus euphratica]	8.42	29.65
map04626	Plant-pathogen interaction	3.83E-04	TRINITY_DN20867_c0_g1	-4.46	down	hypothetical protein POPTR_0019s15190g [Populus trichocarpa]	0.52	17.28
map04626	Plant-pathogen interaction	3.83E-04	TRINITY_DN20923_c0_g1	1.08	up	pseudouridine synthase family protein [Populus trichocarpa]	19.14	16.20
map04626	Plant-pathogen interaction	3.83E-04	TRINITY_DN20951_c1_g1	1.86	up	PREDICTED: ethylene-responsive transcription factor ERF113-like [Populus euphratica]	9.27	4.79
map04626	Plant-pathogen interaction	3.83E-04	TRINITY_DN21000_c0_g1	-1.32	down	disease resistance RPP13-like protein 4 [Populus trichocarpa]	1.10	4.20
map04626	Plant-pathogen interaction	3.83E-04	TRINITY_DN21017_c0_g1	-1.22	down	PREDICTED: lysM domain-containing GPI-anchored protein 2-like isoform X1 [Populus euphratica]	14.15	51.35
map04626	Plant-pathogen interaction	3.83E-04	TRINITY_DN21017_c0_g3	-1.50	down	LysM-domain GPI-anchored protein 2 precursor [Populus trichocarpa]	1.01	4.27
map04626	Plant-pathogen interaction	3.83E-04	TRINITY_DN21075_c1_g3	-1.55	down	PREDICTED: ferric reduction oxidase 4-like isoform X2 [Populus euphratica]	0.85	3.76
map04626	Plant-pathogen interaction	3.83E-04	TRINITY_DN21116_c0_g11	-1.93	down	hypothetical protein POPTR_0019s01930g [Populus trichocarpa]	0.74	4.36
map04626	Plant-pathogen interaction	3.83E-04	TRINITY_DN21132_c0_g2	2.81	up	hypothetical protein POPTR_0017s01160g [Populus trichocarpa]	24.50	5.47
map04626	Plant-pathogen interaction	3.83E-04	TRINITY_DN21150_c0_g1	-1.92	down	PREDICTED: histone H2AX-like [Populus euphratica]	87.59	512.50
map04626	Plant-pathogen interaction	3.83E-04	TRINITY_DN21174_c0_g1	-2.24	down	PREDICTED: putative disease resistance protein RGA4 [Populus euphratica]	6.42	41.54
map04626	Plant-pathogen interaction	3.83E-04	TRINITY_DN21407_c0_g1	1.12	up	PREDICTED: NO-associated protein 1, chloroplastic/mitochondrial [Populus euphratica]	55.57	40.90
map04626	Plant-pathogen interaction	3.83E-04	TRINITY_DN21426_c0_g1	-2.47	down	PREDICTED: pto-interacting protein 1 isoform X2 [Populus euphratica]	1.06	10.54
map04626	Plant-pathogen interaction	3.83E-04	TRINITY_DN21456_c1_g1	-3.59	down	hypothetical protein POPTR_0002s17010g [Populus trichocarpa]	0.59	15.05
map04626	Plant-pathogen interaction	3.83E-04	TRINITY_DN21456_c1_g2	-2.83	down	WRKY transcription factor 6 [(Populus tomentosa x Populus bolleana) x Populus tomentosa]	3.42	37.12
map04626	Plant-pathogen interaction	3.83E-04	TRINITY_DN21482_c1_g2	-1.35	down	PREDICTED: calmodulin-binding receptor-like cytoplasmic kinase 2 [Populus euphratica]	2.33	9.92
map04626	Plant-pathogen interaction	3.83E-04	TRINITY_DN21503_c0_g1	-1.66	down	PREDICTED: probable disease resistance protein At4g27220 [Populus euphratica]	1.89	9.70
map04626	Plant-pathogen interaction	3.83E-04	TRINITY_DN21547_c0_g1	-1.47	down	PREDICTED: LOW QUALITY PROTEIN: probable disease resistance protein At4g27220 [Populus euphratica]	4.35	8.54
map04626	Plant-pathogen interaction	3.83E-04	TRINITY_DN21576_c0_g1	-1.06	down	transcription factor EREBP-like family protein [Populus trichocarpa]	16.49	50.53
map04626	Plant-pathogen interaction	3.83E-04	TRINITY_DN21608_c0_g1	-7.23	down	PREDICTED: cysteine proteinase 15A [Populus euphratica]	0.00	5.08
map04626	Plant-pathogen interaction	3.83E-04	TRINITY_DN21609_c2_g2	-2.26	down	hypothetical protein POPTR_0019s02900g [Populus trichocarpa]	1.37	8.51
map04626	Plant-pathogen interaction	3.83E-04	TRINITY_DN21609_c3_g3	-2.62	down	hypothetical protein POPTR_0001s434902g, partial [Populus trichocarpa]	0.63	6.02
map04626	Plant-pathogen interaction	3.83E-04	TRINITY_DN21626_c0_g1	-1.06	down	kinase family protein [Populus trichocarpa]	10.41	35.72
map04626	Plant-pathogen interaction	3.83E-04	TRINITY_DN3089_c0_g1	-3.94	down	PREDICTED: pathogenesis-related protein 1-like [Populus euphratica]	0.75	17.80
map04626	Plant-pathogen interaction	3.83E-04	TRINITY_DN8124_c0_g1	-1.70	down	PREDICTED: somatic embryogenesis receptor kinase 2-like isoform X1 [Populus euphratica]	0.61	2.73
map00380	Tryptophan metabolism	7.59E-04	TRINITY_DN21932_c0_g3	-1.06	down	phosphatase 2C family protein [Populus trichocarpa]	2.53	8.10
map00380	Tryptophan metabolism	7.59E-04	TRINITY_DN22925_c0_g1	-1.90	down	PREDICTED: indole-3-pyruvate monooxygenase YUCCA6-like [Populus euphratica]	0.66	3.71
map00380	Tryptophan metabolism	7.59E-04	TRINITY_DN23182_c1_g2	2.86	up	hypothetical protein POPTR_0005s10340g [Populus trichocarpa]	182.43	38.37
map00380	Tryptophan metabolism	7.59E-04	TRINITY_DN23278_c1_g3	-1.30	down	zinc finger family protein [Populus trichocarpa]	2.82	11.74

map00380	Tryptophan metabolism	7.59E-04	TRINITY_DN23322_c1_g1	1.07	up	PREDICTED: catalase isozyme 1-like [Populus euphratica]	422.64	304.47
map00380	Tryptophan metabolism	7.59E-04	TRINITY_DN23411_c2_g4	1.38	up	hypothetical protein POPTR_0001s16790g [Populus trichocarpa]	215.54	128.14
map00380	Tryptophan metabolism	7.59E-04	TRINITY_DN23440_c0_g3	-3.46	down	hypothetical protein POPTR_0018s01210g [Populus trichocarpa]	0.25	3.88
map00380	Tryptophan metabolism	7.59E-04	TRINITY_DN23440_c0_g5	-7.26	down	YUCCA family protein [Populus trichocarpa]	0.00	4.17
map00380	Tryptophan metabolism	7.59E-04	TRINITY_DN23485_c0_g3	-1.49	down	PREDICTED: cytochrome P450 71A1-like [Populus euphratica]	1.44	6.13
map00380	Tryptophan metabolism	7.59E-04	TRINITY_DN24288_c0_g1	-2.74	down	hypothetical protein POPTR_0003s11810g [Populus trichocarpa]	2.78	11.84
map00380	Tryptophan metabolism	7.59E-04	TRINITY_DN24628_c0_g1	1.28	up	PREDICTED: aldehyde dehydrogenase family 3 member H1-like [Populus euphratica]	40.39	24.46
map00380	Tryptophan metabolism	7.59E-04	TRINITY_DN25630_c0_g1	1.30	up	aldehyde dehydrogenase 1 precursor family protein [Populus trichocarpa]	120.35	73.18
map00380	Tryptophan metabolism	7.59E-04	TRINITY_DN25703_c0_g1	1.74	up	PREDICTED: cytochrome P450 71A1-like [Populus euphratica]	3184.19	1450.94
map00380	Tryptophan metabolism	7.59E-04	TRINITY_DN25703_c0_g3	1.29	up	hypothetical protein POPTR_0001s16790g [Populus trichocarpa]	343.75	216.36
map00380	Tryptophan metabolism	7.59E-04	TRINITY_DN25754_c0_g1	1.73	up	PREDICTED: flavonoid 3'-monooxygenase-like [Populus euphratica]	1925.81	889.78
map00380	Tryptophan metabolism	7.59E-04	TRINITY_DN26025_c0_g1	-1.71	down	hypothetical protein POPTR_0004s18340g [Populus trichocarpa]	5.60	27.70
map00380	Tryptophan metabolism	7.59E-04	TRINITY_DN26103_c0_g4	1.68	up	hypothetical protein POPTR_0322s00200g [Populus trichocarpa]	65.51	31.51
map00380	Tryptophan metabolism	7.59E-04	TRINITY_DN26421_c0_g1	1.32	up	the aldehyde dehydrogenase cp-ADH from C.plantagineum family protein [Populus trichocarpa]	58.13	34.64
map00380	Tryptophan metabolism	7.59E-04	TRINITY_DN26427_c2_g1	2.19	up	PREDICTED: flavonoid 3'-monooxygenase-like [Populus euphratica]	3671.74	1222.88
map00380	Tryptophan metabolism	7.59E-04	TRINITY_DN15596_c0_g1	-2.20	down	hypothetical protein POPTR_0009s13300g [Populus trichocarpa]	0.46	2.84
map00380	Tryptophan metabolism	7.59E-04	TRINITY_DN15596_c0_g2	-2.70	down	hypothetical protein POPTR_0009s13290g [Populus trichocarpa]	0.23	2.39
map00380	Tryptophan metabolism	7.59E-04	TRINITY_DN16449_c0_g1	2.09	up	PREDICTED: cytochrome P450 71A1-like isoform X1 [Populus euphratica]	34.70	12.40
map00380	Tryptophan metabolism	7.59E-04	TRINITY_DN17225_c0_g1	-4.02	down	hypothetical protein POPTR_0020s00240g [Populus trichocarpa]	0.84	25.71
map00380	Tryptophan metabolism	7.59E-04	TRINITY_DN17247_c0_g1	-1.15	down	UDP-glucoronosyl/UDP-glucosyl transferase family protein [Populus trichocarpa]	7.87	26.34
map00380	Tryptophan metabolism	7.59E-04	TRINITY_DN17710_c0_g1	1.71	up	hypothetical protein POPTR_0012s01280g [Populus trichocarpa]	51.45	22.69
map00380	Tryptophan metabolism	7.59E-04	TRINITY_DN17750_c0_g1	1.39	up	PREDICTED: probable (S)-N-methylcoclaurine 3'-hydroxylase isozyme 2 [Populus euphratica]	3.51	2.03
map00380	Tryptophan metabolism	7.59E-04	TRINITY_DN17819_c0_g1	-2.62	down	hypothetical protein POPTR_0010s21320g [Populus trichocarpa]	0.85	7.85
map00380	Tryptophan metabolism	7.59E-04	TRINITY_DN18895_c0_g1	-1.06	down	mitochondrial aldehyde dehydrogenase family protein [Populus trichocarpa]	7.40	23.22
map00380	Tryptophan metabolism	7.59E-04	TRINITY_DN19003_c0_g2	-1.48	down	hypothetical protein POPTR_0006s11830g, partial [Populus trichocarpa]	0.76	3.31
map00380	Tryptophan metabolism	7.59E-04	TRINITY_DN19215_c1_g1	-1.38	down	hypothetical protein POPTR_0007s10210g [Populus trichocarpa]	3.47	12.55
map00380	Tryptophan metabolism	7.59E-04	TRINITY_DN19215_c2_g1	-1.56	down	PREDICTED: probable protein phosphatase 2C 63 [Populus euphratica]	6.48	30.16
map00380	Tryptophan metabolism	7.59E-04	TRINITY_DN19787_c0_g1	2.95	up	hypothetical protein POPTR_0001s08320g [Populus trichocarpa]	7.17	1.45
map00380	Tryptophan metabolism	7.59E-04	TRINITY_DN19893_c0_g1	2.38	up	hypothetical protein POPTR_0006s05930g [Populus trichocarpa]	8.67	2.41
map00380	Tryptophan metabolism	7.59E-04	TRINITY_DN20225_c0_g1	-2.28	down	hypothetical protein POPTR_0001s28860g [Populus trichocarpa]	1.93	14.45
map00380	Tryptophan metabolism	7.59E-04	TRINITY_DN20245_c0_g1	-2.24	down	PREDICTED: RING-H2 finger protein ATL80-like [Populus euphratica]	0.79	5.50
map00380	Tryptophan metabolism	7.59E-04	TRINITY_DN20245_c0_g2	-1.43	down	PREDICTED: RING-H2 finger protein ATL80-like [Populus euphratica]	4.87	20.18
map00380	Tryptophan metabolism	7.59E-04	TRINITY_DN20790_c0_g1	3.22	up	PREDICTED: cytosolic sulfotransferase 15-like [Populus euphratica]	96.77	15.23
map00380	Tryptophan metabolism	7.59E-04	TRINITY_DN20958_c0_g1	1.57	up	PREDICTED: UDP-glycosyltransferase 74E1-like [Populus euphratica]	64.45	33.63
map00380	Tryptophan metabolism	7.59E-04	TRINITY_DN21506_c0_g2	-2.04	down	PREDICTED: tryptophan aminotransferase-related protein 2-like [Populus euphratica]	1.39	8.60
map00380	Tryptophan metabolism	7.59E-04	TRINITY_DN2875_c0_g1	-2.07	down	hypothetical protein POPTR_0001s38010g [Populus trichocarpa]	0.33	2.13
map00630	Glyoxylate and dicarboxylate metabolism	1.01E-03	TRINITY_DN21849_c0_g1	1.18	up	mitochondrial glycine decarboxylase complex P-protein [Populus tremuloides]	172.09	109.30
map00630	Glyoxylate and dicarboxylate metabolism	1.01E-03	TRINITY_DN21889_c0_g2	3.34	up	PREDICTED: ribulose bisphosphate carboxylase small chain, chloroplastic-like [Populus euphratica]	9592.31	1398.04
map00630	Glyoxylate and dicarboxylate metabolism	1.01E-03	TRINITY_DN21889_c0_g3	2.27	up	017G114600 [Populus tomentosa]	20448.45	6363.37

map00630	Glyoxylate and dicarboxylate metabolism	1.01E-03	TRINITY_DN22048_c2_g2	1.93	up	hypothetical protein POPTR_0015s12380g [Populus trichocarpa]	3.05	1.20
map00630	Glyoxylate and dicarboxylate metabolism	1.01E-03	TRINITY_DN22048_c2_g5	-1.67	down	hypothetical protein POPTR_0012s11610g [Populus trichocarpa]	1.63	8.07
map00630	Glyoxylate and dicarboxylate metabolism	1.01E-03	TRINITY_DN22217_c0_g1	1.29	up	hypothetical protein POPTR_0013s10050g [Populus trichocarpa]	54.43	34.14
map00630	Glyoxylate and dicarboxylate metabolism	1.01E-03	TRINITY_DN22663_c0_g2	1.38	up	hypothetical protein POPTR_0010s05530g [Populus trichocarpa]	360.33	205.54
map00630	Glyoxylate and dicarboxylate metabolism	1.01E-03	TRINITY_DN22812_c0_g2	1.66	up	Serine hydroxymethyltransferase family protein [Populus trichocarpa]	785.78	366.57
map00630	Glyoxylate and dicarboxylate metabolism	1.01E-03	TRINITY_DN22812_c0_g3	1.77	up	mitochondrial serine hydroxymethyltransferase [Populus tremuloides]	59.98	26.65
map00630	Glyoxylate and dicarboxylate metabolism	1.01E-03	TRINITY_DN23091_c0_g2	-2.11	down	glutamine synthetase family protein [Populus trichocarpa]	10.71	69.67
map00630	Glyoxylate and dicarboxylate metabolism	1.01E-03	TRINITY_DN23091_c0_g3	4.22	up	glutamate-ammonia ligase family protein [Populus trichocarpa]	47.80	3.92
map00630	Glyoxylate and dicarboxylate metabolism	1.01E-03	TRINITY_DN23091_c0_g4	1.81	up	glutamate-ammonia ligase family protein [Populus trichocarpa]	63.46	29.26
map00630	Glyoxylate and dicarboxylate metabolism	1.01E-03	TRINITY_DN23091_c0_g5	4.02	up	glutamine synthetase family protein [Populus simonii x Populus nigra]	46.52	4.43
map00630	Glyoxylate and dicarboxylate metabolism	1.01E-03	TRINITY_DN23137_c0_g2	1.94	up	hypothetical protein POPTR_0010s18770g [Populus trichocarpa]	270.10	104.80
map00630	Glyoxylate and dicarboxylate metabolism	1.01E-03	TRINITY_DN23137_c0_g4	1.91	up	hypothetical protein POPTR_0010s18770g [Populus trichocarpa]	257.32	102.92
map00630	Glyoxylate and dicarboxylate metabolism	1.01E-03	TRINITY_DN23182_c1_g2	2.86	up	hypothetical protein POPTR_0005s10340g [Populus trichocarpa]	182.43	38.37
map00630	Glyoxylate and dicarboxylate metabolism	1.01E-03	TRINITY_DN23278_c1_g1	1.44	up	PREDICTED: glutamate--glyoxylate aminotransferase 2 isoform X1 [Populus euphratica]	580.61	318.60
map00630	Glyoxylate and dicarboxylate metabolism	1.01E-03	TRINITY_DN23322_c1_g1	1.07	up	PREDICTED: catalase isozyme 1-like [Populus euphratica]	422.64	304.47
map00630	Glyoxylate and dicarboxylate metabolism	1.01E-03	TRINITY_DN23653_c0_g1	1.10	up	hypothetical protein POPTR_0018s09500g [Populus trichocarpa]	54.02	38.03
map00630	Glyoxylate and dicarboxylate metabolism	1.01E-03	TRINITY_DN23795_c1_g1	1.13	up	PREDICTED: glyoxylate/succinic semialdehyde reductase 2, chloroplastic-like [Populus euphratica]	219.09	158.29
map00630	Glyoxylate and dicarboxylate metabolism	1.01E-03	TRINITY_DN23937_c0_g1	1.78	up	Glutamine synthetase nodule isozyme [Ananas comosus]	127.57	57.48
map00630	Glyoxylate and dicarboxylate metabolism	1.01E-03	TRINITY_DN23937_c0_g3	-3.47	down	PREDICTED: LOW QUALITY PROTEIN: glutamine synthetase cytosolic isozyme 2 [Populus euphratica]	0.50	8.51
map00630	Glyoxylate and dicarboxylate metabolism	1.01E-03	TRINITY_DN23993_c0_g1	1.14	up	PREDICTED: formyltetrahydrofolate deformylase 1, mitochondrial-like isoform X3 [Populus euphratica]	44.23	28.78
map00630	Glyoxylate and dicarboxylate metabolism	1.01E-03	TRINITY_DN24041_c0_g1	2.06	up	PREDICTED: D-glycerate 3-kinase, chloroplastic-like [Populus euphratica]	150.03	53.16
map00630	Glyoxylate and dicarboxylate metabolism	1.01E-03	TRINITY_DN24242_c0_g1	-1.34	down	hypothetical protein POPTR_0003s06730g [Populus trichocarpa]	3.03	11.55
map00630	Glyoxylate and dicarboxylate metabolism	1.01E-03	TRINITY_DN24249_c0_g1	1.66	up	PREDICTED: ribulose bisphosphate carboxylase large chain [Populus euphratica]	86.19	43.64
map00630	Glyoxylate and dicarboxylate metabolism	1.01E-03	TRINITY_DN24565_c0_g1	1.99	up	T-protein of the glycine decarboxylase complex [Populus trichocarpa]	481.27	183.79
map00630	Glyoxylate and dicarboxylate metabolism	1.01E-03	TRINITY_DN24894_c0_g3	1.50	up	hypothetical protein POPTR_0015s03960g [Populus trichocarpa]	34.92	19.04
map00630	Glyoxylate and dicarboxylate metabolism	1.01E-03	TRINITY_DN25221_c0_g1	1.58	up	malate dehydrogenase family protein, partial [Populus trichocarpa]	57.58	29.35
map00630	Glyoxylate and dicarboxylate metabolism	1.01E-03	TRINITY_DN25221_c1_g2	2.25	up	malate dehydrogenase family protein [Populus trichocarpa]	356.58	110.81
map00630	Glyoxylate and dicarboxylate metabolism	1.01E-03	TRINITY_DN25221_c1_g3	1.80	up	PREDICTED: malate dehydrogenase, glyoxysomal [Populus euphratica]	643.81	285.37
map00630	Glyoxylate and dicarboxylate metabolism	1.01E-03	TRINITY_DN25260_c0_g3	3.71	up	glutamate-ammonia ligase family protein [Populus trichocarpa]	11.97	1.40
map00630	Glyoxylate and dicarboxylate metabolism	1.01E-03	TRINITY_DN25260_c0_g4	-1.01	down	unknown [Populus trichocarpa]	26.98	83.37
map00630	Glyoxylate and dicarboxylate metabolism	1.01E-03	TRINITY_DN25290_c0_g2	1.33	up	glycine cleavage system protein H precursor [Populus trichocarpa]	1509.85	901.22
map00630	Glyoxylate and dicarboxylate metabolism	1.01E-03	TRINITY_DN25290_c0_g3	1.35	up	mitochondrial glycine decarboxylase complex H-protein [Populus tremuloides]	254.42	155.84
map00630	Glyoxylate and dicarboxylate metabolism	1.01E-03	TRINITY_DN25746_c0_g1	2.80	up	nodule-enhanced malate dehydrogenase family protein [Populus trichocarpa]	113.74	23.22

map00630	Glyoxylate and dicarboxylate metabolism	1.01E-03	TRINITY_DN25941_c0_g1	1.37	up	PREDICTED: glutamine synthetase leaf isozyme, chloroplastic [Populus euphratica]	986.31	589.65
map00630	Glyoxylate and dicarboxylate metabolism	1.01E-03	TRINITY_DN26837_c0_g1	2.09	up	hypothetical protein POPTR_0010s15200g [Populus trichocarpa]	81.17	29.93
map00630	Glyoxylate and dicarboxylate metabolism	1.01E-03	TRINITY_DN27171_c2_g1	1.79	up	PREDICTED: D-glycerate 3-kinase, chloroplastic-like [Populus euphratica]	158.02	76.60
map00630	Glyoxylate and dicarboxylate metabolism	1.01E-03	TRINITY_DN27198_c1_g1	1.09	up	aconitate hydratase family protein [Populus trichocarpa]	47.60	32.18
map00630	Glyoxylate and dicarboxylate metabolism	1.01E-03	TRINITY_DN27198_c1_g2	1.02	up	aconitate hydratase family protein [Populus trichocarpa]	16.99	12.62
map00630	Glyoxylate and dicarboxylate metabolism	1.01E-03	TRINITY_DN27395_c0_g1	1.70	up	aminotransferase 2 family protein [Populus trichocarpa]	980.92	451.86
map00630	Glyoxylate and dicarboxylate metabolism	1.01E-03	TRINITY_DN27440_c0_g2	1.46	up	PREDICTED: glutamine synthetase leaf isozyme, chloroplastic [Populus euphratica]	559.41	306.88
map00630	Glyoxylate and dicarboxylate metabolism	1.01E-03	TRINITY_DN27448_c0_g1	1.92	up	glycolate oxidase family protein [Populus trichocarpa]	1633.96	640.54
map00630	Glyoxylate and dicarboxylate metabolism	1.01E-03	TRINITY_DN27448_c0_g2	1.73	up	PREDICTED: peroxisomal (S)-2-hydroxy-acid oxidase [Populus euphratica]	519.75	233.20
map00630	Glyoxylate and dicarboxylate metabolism	1.01E-03	TRINITY_DN27574_c0_g1	1.31	up	glycine decarboxylase P-protein 1 [Arabidopsis thaliana]	428.07	261.89
map00630	Glyoxylate and dicarboxylate metabolism	1.01E-03	TRINITY_DN27862_c2_g1	-1.69	down	PREDICTED: structural maintenance of chromosomes protein 2-1-like [Populus euphratica]	21.02	56.92
map00630	Glyoxylate and dicarboxylate metabolism	1.01E-03	TRINITY_DN27865_c0_g1	1.40	up	hypothetical protein POPTR_0016s03630g [Populus trichocarpa]	23.70	13.44
map00630	Glyoxylate and dicarboxylate metabolism	1.01E-03	TRINITY_DN27865_c1_g3	1.68	up	ferredoxin-dependent glutamate synthase family protein [Populus trichocarpa]	183.61	86.98
map00630	Glyoxylate and dicarboxylate metabolism	1.01E-03	TRINITY_DN13033_c0_g1	1.49	up	hypothetical protein POPTR_0016s03630g [Populus trichocarpa]	21.05	11.13
map00630	Glyoxylate and dicarboxylate metabolism	1.01E-03	TRINITY_DN13230_c0_g2	-1.83	down	PREDICTED: peroxisomal (S)-2-hydroxy-acid oxidase-like isoform X1 [Populus euphratica]	0.42	2.27
map00630	Glyoxylate and dicarboxylate metabolism	1.01E-03	TRINITY_DN13666_c0_g1	1.63	up	hypothetical protein POPTR_0016s03630g [Populus trichocarpa]	53.03	25.42
map00630	Glyoxylate and dicarboxylate metabolism	1.01E-03	TRINITY_DN13782_c0_g1	5.00	up	hypothetical protein POPTR_0002s11010g [Populus trichocarpa]	6.55	0.32
map00630	Glyoxylate and dicarboxylate metabolism	1.01E-03	TRINITY_DN13846_c0_g1	1.79	up	hypothetical protein PRUPE_ppa000146mg [Prunus persica]	350.17	154.52
map00630	Glyoxylate and dicarboxylate metabolism	1.01E-03	TRINITY_DN15808_c0_g1	1.81	up	PREDICTED: ferredoxin-dependent glutamate synthase, chloroplastic-like [Populus euphratica]	604.52	260.27
map00630	Glyoxylate and dicarboxylate metabolism	1.01E-03	TRINITY_DN15882_c0_g1	2.67	up	hypothetical protein POPTR_0018s05150g [Populus trichocarpa]	16.28	3.88
map00630	Glyoxylate and dicarboxylate metabolism	1.01E-03	TRINITY_DN16471_c0_g1	1.72	up	PREDICTED: peroxisomal (S)-2-hydroxy-acid oxidase-like [Phoenix dactylifera]	1059.17	482.14
map00630	Glyoxylate and dicarboxylate metabolism	1.01E-03	TRINITY_DN16821_c0_g1	1.91	up	PREDICTED: malate dehydrogenase, glyoxysomal [Populus euphratica]	248.44	104.05
map00630	Glyoxylate and dicarboxylate metabolism	1.01E-03	TRINITY_DN17865_c0_g1	2.27	up	plastid serine hydroxymethyltransferase [Populus tremuloides]	53.46	17.12
map00630	Glyoxylate and dicarboxylate metabolism	1.01E-03	TRINITY_DN17930_c0_g1	2.42	up	unknown [Populus trichocarpa x Populus deltoides]	1011.61	280.35
map00630	Glyoxylate and dicarboxylate metabolism	1.01E-03	TRINITY_DN18156_c0_g1	1.29	up	PREDICTED: glyoxylate/hydroxypyruvate reductase A HPR2-like [Populus euphratica]	133.92	82.89
map00630	Glyoxylate and dicarboxylate metabolism	1.01E-03	TRINITY_DN18598_c0_g1	1.82	up	hypothetical protein POPTR_0008s00350g [Populus trichocarpa]	468.84	197.84
map00630	Glyoxylate and dicarboxylate metabolism	1.01E-03	TRINITY_DN18859_c0_g3	1.18	up	aconitate hydratase family protein [Populus trichocarpa]	80.30	54.25
map00630	Glyoxylate and dicarboxylate metabolism	1.01E-03	TRINITY_DN19763_c0_g3	1.88	up	PREDICTED: malate dehydrogenase, mitochondrial [Populus euphratica]	247.09	106.19
map00630	Glyoxylate and dicarboxylate metabolism	1.01E-03	TRINITY_DN20175_c0_g1	1.59	up	peroxiredoxin Q family protein [Populus trichocarpa]	1020.77	504.37
map00630	Glyoxylate and dicarboxylate metabolism	1.01E-03	TRINITY_DN20227_c0_g1	-1.45	down	PREDICTED: malate synthase, glyoxysomal [Populus euphratica]	2.85	13.95
map00630	Glyoxylate and dicarboxylate metabolism	1.01E-03	TRINITY_DN20938_c0_g1	1.33	up	PREDICTED: malate dehydrogenase, glyoxysomal [Populus euphratica]	118.58	72.32
map00630	Glyoxylate and dicarboxylate metabolism	1.01E-03	TRINITY_DN20938_c0_g2	2.63	up	malate dehydrogenase family protein [Populus trichocarpa]	74.92	18.32
map00630	Glyoxylate and dicarboxylate metabolism	1.01E-03	TRINITY_DN21140_c0_g2	2.24	up	mitochondrial lipoamide dehydrogenase [Populus tremuloides]	202.39	67.53

map00944	Flavone and flavonol biosynthesis	1.18E-03	TRINITY_DN22579_c0_g1	3.09	up	hypothetical protein POPTR_0001s07860g [Populus trichocarpa]	29.32	7.54
map00944	Flavone and flavonol biosynthesis	1.18E-03	TRINITY_DN23459_c0_g1	-8.35	down	PREDICTED: flavonoid 3',5'-hydroxylase 2-like [Populus euphratica]	0.03	14.96
map00944	Flavone and flavonol biosynthesis	1.18E-03	TRINITY_DN24487_c0_g1	2.10	up	PREDICTED: UDP-glycosyltransferase 92A1-like [Populus euphratica]	16.37	3.93
map00944	Flavone and flavonol biosynthesis	1.18E-03	TRINITY_DN25520_c1_g1	2.32	up	PREDICTED: phenolic glucoside malonyltransferase 1-like [Populus euphratica]	138.11	49.97
map00944	Flavone and flavonol biosynthesis	1.18E-03	TRINITY_DN12553_c0_g1	-8.82	down	hypothetical protein POPTR_0006s28740g [Populus trichocarpa]	0.00	2.79
map00944	Flavone and flavonol biosynthesis	1.18E-03	TRINITY_DN17000_c0_g1	1.16	up	hypothetical protein POPTR_0009s06800g [Populus trichocarpa]	3.15	2.14
map00944	Flavone and flavonol biosynthesis	1.18E-03	TRINITY_DN17077_c0_g1	1.36	up	PREDICTED: phenolic glucoside malonyltransferase 1-like [Populus euphratica]	121.68	73.71
map00944	Flavone and flavonol biosynthesis	1.18E-03	TRINITY_DN19224_c0_g1	1.22	up	hypothetical protein POPTR_0004s09550g [Populus trichocarpa]	55.14	35.39
map00944	Flavone and flavonol biosynthesis	1.18E-03	TRINITY_DN21068_c1_g1	-1.96	down	hypothetical protein POPTR_0013s07050g [Populus trichocarpa]	25.27	141.84
map00970	Aminoacyl-tRNA biosynthesis	1.39E-03	TRINITY_DN21652_c0_g1	1.01	up	tRNA-binding region domain-containing family protein [Populus trichocarpa]	105.82	79.42
map00970	Aminoacyl-tRNA biosynthesis	1.39E-03	TRINITY_DN21689_c0_g1	1.97	up	hypothetical protein POPTR_0014s13430g [Populus trichocarpa]	117.32	44.17
map00970	Aminoacyl-tRNA biosynthesis	1.39E-03	TRINITY_DN21857_c0_g1	-1.95	down	alternative oxidase [Populus tremula x Populus tremuloides]	2.17	12.56
map00970	Aminoacyl-tRNA biosynthesis	1.39E-03	TRINITY_DN21876_c0_g1	1.17	up	tRNA synthetase class II family protein [Populus trichocarpa]	52.95	36.23
map00970	Aminoacyl-tRNA biosynthesis	1.39E-03	TRINITY_DN21952_c0_g1	-2.42	down	hypothetical protein POPTR_0006s18580g [Populus trichocarpa]	0.78	8.74
map00970	Aminoacyl-tRNA biosynthesis	1.39E-03	TRINITY_DN22414_c0_g1	-1.65	down	hypothetical protein POPTR_0007s04220g [Populus trichocarpa]	0.68	3.25
map00970	Aminoacyl-tRNA biosynthesis	1.39E-03	TRINITY_DN22414_c0_g2	-1.20	down	hypothetical protein POPTR_0005s06470g [Populus trichocarpa]	2.15	7.39
map00970	Aminoacyl-tRNA biosynthesis	1.39E-03	TRINITY_DN22552_c0_g1	1.59	up	PREDICTED: cysteine--tRNA ligase isoform X1 [Populus euphratica]	22.37	11.20
map00970	Aminoacyl-tRNA biosynthesis	1.39E-03	TRINITY_DN22598_c0_g1	1.88	up	hypothetical protein POPTR_0009s08760g [Populus trichocarpa]	24.44	10.24
map00970	Aminoacyl-tRNA biosynthesis	1.39E-03	TRINITY_DN22888_c0_g3	1.19	up	CCAAT-binding transcription factor family protein [Populus trichocarpa]	7.64	5.10
map00970	Aminoacyl-tRNA biosynthesis	1.39E-03	TRINITY_DN23005_c0_g2	1.25	up	hypothetical protein POPTR_0011s01420g [Populus trichocarpa]	35.29	23.06
map00970	Aminoacyl-tRNA biosynthesis	1.39E-03	TRINITY_DN23063_c0_g1	-2.26	down	hypothetical protein POPTR_0001s26480g [Populus trichocarpa]	0.98	6.66
map00970	Aminoacyl-tRNA biosynthesis	1.39E-03	TRINITY_DN23063_c0_g3	-1.37	down	PREDICTED: nuclear transcription factor Y subunit A-1-like [Populus euphratica]	3.27	16.58
map00970	Aminoacyl-tRNA biosynthesis	1.39E-03	TRINITY_DN23373_c0_g3	-1.59	down	hypothetical protein POPTR_0002s22050g [Populus trichocarpa]	0.92	4.21
map00970	Aminoacyl-tRNA biosynthesis	1.39E-03	TRINITY_DN23373_c0_g4	-3.17	down	F-box family protein [Populus trichocarpa]	0.42	5.88
map00970	Aminoacyl-tRNA biosynthesis	1.39E-03	TRINITY_DN23430_c0_g2	1.13	up	hypothetical protein POPTR_0017s04920g, partial [Populus trichocarpa]	52.79	36.31
map00970	Aminoacyl-tRNA biosynthesis	1.39E-03	TRINITY_DN23873_c0_g4	-3.01	down	Ethylene responsive element binding factor 5 family protein [Populus trichocarpa]	9.71	105.13
map00970	Aminoacyl-tRNA biosynthesis	1.39E-03	TRINITY_DN24116_c1_g2	1.98	up	hypothetical protein POPTR_0018s09340g [Populus trichocarpa]	63.54	26.14
map00970	Aminoacyl-tRNA biosynthesis	1.39E-03	TRINITY_DN24629_c0_g5	1.02	up	PREDICTED: arginine--tRNA ligase, cytoplasmic-like isoform X1 [Populus euphratica]	55.80	43.32
map00970	Aminoacyl-tRNA biosynthesis	1.39E-03	TRINITY_DN24886_c0_g1	1.29	up	PREDICTED: elongation factor 1-gamma 2-like [Populus euphratica]	232.26	138.32
map00970	Aminoacyl-tRNA biosynthesis	1.39E-03	TRINITY_DN25193_c0_g1	1.36	up	hypothetical protein POPTR_0009s14340g [Populus trichocarpa]	45.99	27.53
map00970	Aminoacyl-tRNA biosynthesis	1.39E-03	TRINITY_DN25289_c0_g2	1.21	up	PREDICTED: methionine--tRNA ligase, mitochondrial [Populus euphratica]	47.66	32.26
map00970	Aminoacyl-tRNA biosynthesis	1.39E-03	TRINITY_DN25763_c0_g1	1.13	up	Lysyl-tRNA synthetase family protein [Populus trichocarpa]	76.40	64.24
map00970	Aminoacyl-tRNA biosynthesis	1.39E-03	TRINITY_DN25978_c0_g1	-1.38	down	CCAAT-binding transcription factor subunit B [Populus euphratica]	14.31	57.96
map00970	Aminoacyl-tRNA biosynthesis	1.39E-03	TRINITY_DN25978_c0_g2	-5.78	down	hypothetical protein POPTR_0006s05210g [Populus trichocarpa]	0.03	2.37
map00970	Aminoacyl-tRNA biosynthesis	1.39E-03	TRINITY_DN26070_c0_g2	1.53	up	PREDICTED: glutamyl-tRNA(Gln) amidotransferase subunit B, chloroplastic/mitochondrial [Populus euphratica]	68.27	38.35
map00970	Aminoacyl-tRNA biosynthesis	1.39E-03	TRINITY_DN26191_c1_g1	1.38	up	glycine-tRNA ligase [Populus tomentosa]	27.29	15.89
map00970	Aminoacyl-tRNA biosynthesis	1.39E-03	TRINITY_DN26270_c0_g1	1.70	up	PREDICTED: RNA polymerase sigma factor sigB-like [Populus euphratica]	177.29	82.45
map00970	Aminoacyl-tRNA biosynthesis	1.39E-03	TRINITY_DN26293_c1_g1	1.40	up	hypothetical protein POPTR_0005s14610g [Populus trichocarpa]	40.82	23.33
map00970	Aminoacyl-tRNA biosynthesis	1.39E-03	TRINITY_DN26302_c0_g1	1.03	up	Glycyl-tRNA synthetase family protein [Populus trichocarpa]	49.36	36.52
map00970	Aminoacyl-tRNA biosynthesis	1.39E-03	TRINITY_DN26336_c0_g3	1.19	up	PREDICTED: probable WRKY transcription factor 31 isoform X2 [Populus euphratica]	34.63	22.78

map00970	Aminoacyl-tRNA biosynthesis	1.39E-03	TRINITY_DN26378_c0_g1	1.25	up	Toc64 family protein [Populus trichocarpa]	55.18	35.77
map00970	Aminoacyl-tRNA biosynthesis	1.39E-03	TRINITY_DN26439_c0_g1	1.92	up	PREDICTED: proline--tRNA ligase isoform X1 [Populus euphratica]	64.68	27.02
map00970	Aminoacyl-tRNA biosynthesis	1.39E-03	TRINITY_DN26852_c1_g1	1.39	up	hypothetical protein POPTR_0009s08760g [Populus trichocarpa]	38.26	24.17
map00970	Aminoacyl-tRNA biosynthesis	1.39E-03	TRINITY_DN26852_c1_g2	1.16	up	PREDICTED: aspartate--tRNA ligase, mitochondrial isoform X1 [Populus euphratica]	59.04	40.14
map00970	Aminoacyl-tRNA biosynthesis	1.39E-03	TRINITY_DN27000_c0_g2	-1.28	down	PREDICTED: HORMA domain-containing protein 1-like isoform X2 [Populus euphratica]	9.14	23.70
map00970	Aminoacyl-tRNA biosynthesis	1.39E-03	TRINITY_DN27302_c0_g2	1.67	up	hypothetical protein POPTR_0007s01840g [Populus trichocarpa]	68.86	33.67
map00970	Aminoacyl-tRNA biosynthesis	1.39E-03	TRINITY_DN14926_c0_g1	-2.05	down	hypothetical protein POPTR_0003s08870g [Populus trichocarpa]	0.47	3.31
map00970	Aminoacyl-tRNA biosynthesis	1.39E-03	TRINITY_DN16210_c0_g1	-1.55	down	disease resistance-responsive family protein [Populus trichocarpa]	2.58	11.47
map00970	Aminoacyl-tRNA biosynthesis	1.39E-03	TRINITY_DN16399_c0_g2	-1.39	down	hypothetical protein POPTR_0001s11800g [Populus trichocarpa]	3.31	12.97
map00970	Aminoacyl-tRNA biosynthesis	1.39E-03	TRINITY_DN16445_c0_g1	1.25	up	dehydroascorbate reductase [Populus tomentosa]	179.37	113.67
map00970	Aminoacyl-tRNA biosynthesis	1.39E-03	TRINITY_DN16839_c0_g2	-1.63	down	hypothetical protein POPTR_0018s08740g [Populus trichocarpa]	1.40	6.67
map00970	Aminoacyl-tRNA biosynthesis	1.39E-03	TRINITY_DN17163_c0_g1	-3.02	down	U-box domain-containing family protein [Populus trichocarpa]	1.45	17.90
map00970	Aminoacyl-tRNA biosynthesis	1.39E-03	TRINITY_DN17768_c0_g1	1.55	up	hypothetical protein POPTR_0009s17070g [Populus trichocarpa]	67.70	36.29
map00970	Aminoacyl-tRNA biosynthesis	1.39E-03	TRINITY_DN18079_c0_g1	-5.35	down	PREDICTED: U-box domain-containing protein 21-like [Populus euphratica]	0.06	4.47
map00970	Aminoacyl-tRNA biosynthesis	1.39E-03	TRINITY_DN18320_c0_g2	1.34	up	PREDICTED: phenylalanine--tRNA ligase, chloroplastic/mitochondrial [Populus euphratica]	108.32	64.98
map00970	Aminoacyl-tRNA biosynthesis	1.39E-03	TRINITY_DN18361_c0_g2	-2.79	down	PREDICTED: E3 ubiquitin-protein ligase PUB22-like [Populus euphratica]	0.35	3.77
map00970	Aminoacyl-tRNA biosynthesis	1.39E-03	TRINITY_DN18411_c0_g3	1.53	up	hypothetical protein POPTR_0006s12640g [Populus trichocarpa]	101.84	53.65
map00970	Aminoacyl-tRNA biosynthesis	1.39E-03	TRINITY_DN18545_c0_g1	1.06	up	PREDICTED: RNA polymerase sigma factor sigF, chloroplastic isoform X2 [Populus euphratica]	60.41	43.89
map00970	Aminoacyl-tRNA biosynthesis	1.39E-03	TRINITY_DN18662_c0_g1	-2.44	down	unknown [Populus trichocarpa]	4.29	33.47
map00970	Aminoacyl-tRNA biosynthesis	1.39E-03	TRINITY_DN18706_c0_g1	1.67	up	hypothetical protein POPTR_0014s16030g [Populus trichocarpa]	62.91	29.87
map00970	Aminoacyl-tRNA biosynthesis	1.39E-03	TRINITY_DN18984_c0_g1	1.48	up	PREDICTED: tyrosine--tRNA ligase, mitochondrial [Populus euphratica]	44.00	23.69
map00970	Aminoacyl-tRNA biosynthesis	1.39E-03	TRINITY_DN19193_c0_g1	1.58	up	seryl-tRNA synthetase family protein [Populus trichocarpa]	44.70	22.61
map00970	Aminoacyl-tRNA biosynthesis	1.39E-03	TRINITY_DN19342_c0_g1	2.02	up	hypothetical protein POPTR_0002s08390g [Populus trichocarpa]	9.24	3.65
map00970	Aminoacyl-tRNA biosynthesis	1.39E-03	TRINITY_DN20382_c0_g2	-2.47	down	PREDICTED: U-box domain-containing protein 28-like [Populus euphratica]	0.97	8.24
map00970	Aminoacyl-tRNA biosynthesis	1.39E-03	TRINITY_DN20450_c0_g2	1.18	up	PREDICTED: methionyl-tRNA formyltransferase, mitochondrial isoform X2 [Populus euphratica]	53.40	39.72
map00970	Aminoacyl-tRNA biosynthesis	1.39E-03	TRINITY_DN20621_c0_g1	1.34	up	PREDICTED: probable isoleucine--tRNA ligase, mitochondrial isoform X2 [Populus euphratica]	36.44	21.66
map00970	Aminoacyl-tRNA biosynthesis	1.39E-03	TRINITY_DN21323_c0_g1	1.46	up	PREDICTED: glutamyl-tRNA(Gln) amidotransferase subunit C, chloroplastic/mitochondrial isoform X1 [Populus euphratica]	205.18	105.69
map00970	Aminoacyl-tRNA biosynthesis	1.39E-03	TRINITY_DN21432_c1_g1	-3.30	down	PREDICTED: fatty acid amide hydrolase-like [Populus euphratica]	1.04	8.62
map00260	Glycine, serine and threonine metabolism	2.05E-03	TRINITY_DN21849_c0_g1	1.18	up	mitochondrial glycine decarboxylase complex P-protein [Populus tremuloides]	172.09	109.30
map00260	Glycine, serine and threonine metabolism	2.05E-03	TRINITY_DN22217_c0_g1	1.29	up	hypothetical protein POPTR_0013s10050g [Populus trichocarpa]	54.43	34.14
map00260	Glycine, serine and threonine metabolism	2.05E-03	TRINITY_DN22246_c0_g2	1.68	up	hypothetical protein POPTR_0019s03500g [Populus trichocarpa]	144.86	80.32
map00260	Glycine, serine and threonine metabolism	2.05E-03	TRINITY_DN22283_c0_g1	1.79	up	hypothetical protein POPTR_0002s09390g [Populus trichocarpa]	139.18	61.68
map00260	Glycine, serine and threonine metabolism	2.05E-03	TRINITY_DN22598_c1_g1	1.42	up	2 family protein [Populus trichocarpa]	136.33	77.98
map00260	Glycine, serine and threonine metabolism	2.05E-03	TRINITY_DN22663_c0_g2	1.38	up	hypothetical protein POPTR_0010s05530g [Populus trichocarpa]	360.33	205.54
map00260	Glycine, serine and threonine metabolism	2.05E-03	TRINITY_DN22697_c0_g3	1.19	up	3-PHOSPHOSERINE PHOSPHATASE family protein [Populus trichocarpa]	51.79	35.35
map00260	Glycine, serine and threonine metabolism	2.05E-03	TRINITY_DN22812_c0_g2	1.66	up	Serine hydroxymethyltransferase family protein [Populus trichocarpa]	785.78	366.57
map00260	Glycine, serine and threonine metabolism	2.05E-03	TRINITY_DN22812_c0_g3	1.77	up	mitochondrial serine hydroxymethyltransferase [Populus tremuloides]	59.98	26.65
map00260	Glycine, serine and threonine metabolism	2.05E-03	TRINITY_DN22954_c0_g2	1.23	up	TRYPTOPHAN SYNTHASE ALPHA CHAIN family protein [Populus trichocarpa]	98.67	70.99

map00260	Glycine, serine and threonine metabolism	2.05E-03	TRINITY_DN23066_c0_g5	1.83	up	PREDICTED: probable 2-carboxy-D-arabinitol-1-phosphatase [Populus euphratica]	71.98	31.71
map00260	Glycine, serine and threonine metabolism	2.05E-03	TRINITY_DN23278_c1_g1	1.44	up	PREDICTED: glutamate--glyoxylate aminotransferase 2 isoform X1 [Populus euphratica]	580.61	318.60
map00260	Glycine, serine and threonine metabolism	2.05E-03	TRINITY_DN23497_c0_g1	2.24	up	D-3-phosphoglycerate dehydrogenase family protein [Populus trichocarpa]	103.49	34.56
map00260	Glycine, serine and threonine metabolism	2.05E-03	TRINITY_DN23704_c1_g1	-3.03	down	hypothetical protein POPTR_0001s07880g [Populus trichocarpa]	1.87	24.42
map00260	Glycine, serine and threonine metabolism	2.05E-03	TRINITY_DN24041_c0_g1	2.06	up	PREDICTED: D-glycerate 3-kinase, chloroplastic-like [Populus euphratica]	150.03	53.16
map00260	Glycine, serine and threonine metabolism	2.05E-03	TRINITY_DN24179_c1_g1	1.94	up	hypothetical protein POPTR_0001s44620g [Populus trichocarpa]	107.11	47.27
map00260	Glycine, serine and threonine metabolism	2.05E-03	TRINITY_DN24565_c0_g1	1.99	up	T-protein of the glycine decarboxylase complex [Populus trichocarpa]	481.27	183.79
map00260	Glycine, serine and threonine metabolism	2.05E-03	TRINITY_DN24599_c6_g1	-1.97	down	hypothetical protein POPTR_0007s03670g [Populus trichocarpa]	0.44	2.68
map00260	Glycine, serine and threonine metabolism	2.05E-03	TRINITY_DN24826_c0_g1	-1.05	down	PREDICTED: uncharacterized protein LOC105138712 [Populus euphratica]	1.85	5.77
map00260	Glycine, serine and threonine metabolism	2.05E-03	TRINITY_DN24826_c0_g2	-4.47	down	PREDICTED: uncharacterized protein LOC105109098 [Populus euphratica]	0.14	5.15
map00260	Glycine, serine and threonine metabolism	2.05E-03	TRINITY_DN24880_c0_g1	-1.43	down	curculin-like lectin family protein [Populus trichocarpa]	4.67	18.36
map00260	Glycine, serine and threonine metabolism	2.05E-03	TRINITY_DN24894_c0_g3	1.50	up	hypothetical protein POPTR_0015s03960g [Populus trichocarpa]	34.92	19.04
map00260	Glycine, serine and threonine metabolism	2.05E-03	TRINITY_DN25290_c0_g2	1.33	up	glycine cleavage system protein H precursor [Populus trichocarpa]	1509.85	901.22
map00260	Glycine, serine and threonine metabolism	2.05E-03	TRINITY_DN25290_c0_g3	1.35	up	mitochondrial glycine decarboxylase complex H-protein [Populus tremuloides]	254.42	155.84
map00260	Glycine, serine and threonine metabolism	2.05E-03	TRINITY_DN25367_c0_g1	1.54	up	hypothetical protein POPTR_0003s02770g [Populus trichocarpa]	59.68	31.40
map00260	Glycine, serine and threonine metabolism	2.05E-03	TRINITY_DN25737_c0_g1	1.82	up	semialdehyde dehydrogenase family protein [Populus trichocarpa]	56.96	24.76
map00260	Glycine, serine and threonine metabolism	2.05E-03	TRINITY_DN26235_c0_g1	-1.34	down	PREDICTED: LOW QUALITY PROTEIN: G-type lectin S-receptor-like serine/threonine-protein kinase RLK1 [Populus euphratica]	6.54	32.64
map00260	Glycine, serine and threonine metabolism	2.05E-03	TRINITY_DN26235_c0_g3	-1.48	down	PREDICTED: G-type lectin S-receptor-like serine/threonine-protein kinase RLK1 [Populus euphratica]	4.02	17.62
map00260	Glycine, serine and threonine metabolism	2.05E-03	TRINITY_DN26528_c0_g1	1.13	up	PREDICTED: D-3-phosphoglycerate dehydrogenase 3, chloroplastic-like [Populus euphratica]	74.65	55.30
map00260	Glycine, serine and threonine metabolism	2.05E-03	TRINITY_DN26837_c0_g1	2.09	up	hypothetical protein POPTR_0010s15200g [Populus trichocarpa]	81.17	29.93
map00260	Glycine, serine and threonine metabolism	2.05E-03	TRINITY_DN27171_c2_g1	1.79	up	PREDICTED: D-glycerate 3-kinase, chloroplastic-like [Populus euphratica]	158.02	76.60
map00260	Glycine, serine and threonine metabolism	2.05E-03	TRINITY_DN27242_c0_g1	-1.23	down	hypothetical protein POPTR_0151s00200g [Populus trichocarpa]	2.45	8.98
map00260	Glycine, serine and threonine metabolism	2.05E-03	TRINITY_DN27395_c0_g1	1.70	up	aminotransferase 2 family protein [Populus trichocarpa]	980.92	451.86
map00260	Glycine, serine and threonine metabolism	2.05E-03	TRINITY_DN27574_c0_g1	1.31	up	glycine decarboxylase P-protein 1 [Arabidopsis thaliana]	428.07	261.89
map00260	Glycine, serine and threonine metabolism	2.05E-03	TRINITY_DN12955_c0_g1	1.82	up	hypothetical protein POPTR_0011s05190g [Populus trichocarpa]	15.50	6.27
map00260	Glycine, serine and threonine metabolism	2.05E-03	TRINITY_DN13782_c0_g1	5.00	up	hypothetical protein POPTR_0002s11010g [Populus trichocarpa]	6.55	0.32
map00260	Glycine, serine and threonine metabolism	2.05E-03	TRINITY_DN14000_c0_g1	-2.71	down	hypothetical protein POPTR_0013s05640g [Populus trichocarpa]	0.38	5.38
map00260	Glycine, serine and threonine metabolism	2.05E-03	TRINITY_DN15219_c0_g1	-3.09	down	hypothetical protein POPTR_0013s05650g [Populus trichocarpa]	0.23	2.86
map00260	Glycine, serine and threonine metabolism	2.05E-03	TRINITY_DN15882_c0_g1	2.67	up	hypothetical protein POPTR_0018s05150g [Populus trichocarpa]	16.28	3.88
map00260	Glycine, serine and threonine metabolism	2.05E-03	TRINITY_DN17759_c0_g1	-1.68	down	hypothetical protein POPTR_0004s01460g, partial [Populus trichocarpa]	1.43	5.88
map00260	Glycine, serine and threonine metabolism	2.05E-03	TRINITY_DN17759_c0_g3	-2.14	down	PREDICTED: proline-rich receptor-like protein kinase PERK3 isoform X1 [Populus euphratica]	0.24	1.71
map00260	Glycine, serine and threonine metabolism	2.05E-03	TRINITY_DN17865_c0_g1	2.27	up	plastid serine hydroxymethyltransferase [Populus tremuloides]	53.46	17.12

map00260	Glycine, serine and threonine metabolism	2.05E-03	TRINITY_DN17930_c0_g1	2.42	up	unknown [Populus trichocarpa x Populus deltoides]	1011.61	280.35
map00260	Glycine, serine and threonine metabolism	2.05E-03	TRINITY_DN18156_c0_g1	1.29	up	PREDICTED: glyoxylate/hydroxypyruvate reductase A HPR2-like [Populus euphratica]	133.92	82.89
map00260	Glycine, serine and threonine metabolism	2.05E-03	TRINITY_DN18501_c1_g1	2.31	up	PREDICTED: phosphoserine aminotransferase 2, chloroplastic-like [Populus euphratica]	32.17	9.92
map00260	Glycine, serine and threonine metabolism	2.05E-03	TRINITY_DN18598_c0_g1	1.82	up	hypothetical protein POPTR_0008s00350g [Populus trichocarpa]	468.84	197.84
map00260	Glycine, serine and threonine metabolism	2.05E-03	TRINITY_DN19059_c0_g2	-1.92	down	PREDICTED: G-type lectin S-receptor-like serine/threonine-protein kinase At5g24080 isoform X1 [Populus euphratica]	1.17	7.57
map00260	Glycine, serine and threonine metabolism	2.05E-03	TRINITY_DN19059_c0_g3	-6.09	down	hypothetical protein POPTR_0015s05780g [Populus trichocarpa]	0.09	4.40
map00260	Glycine, serine and threonine metabolism	2.05E-03	TRINITY_DN19337_c0_g1	-2.03	down	hypothetical protein POPTR_0017s14220g [Populus trichocarpa]	2.64	16.29
map00260	Glycine, serine and threonine metabolism	2.05E-03	TRINITY_DN19656_c0_g1	-1.62	down	PREDICTED: G-type lectin S-receptor-like serine/threonine-protein kinase RLK1 isoform X1 [Populus euphratica]	0.57	2.63
map00260	Glycine, serine and threonine metabolism	2.05E-03	TRINITY_DN19656_c0_g2	1.26	up	PREDICTED: G-type lectin S-receptor-like serine/threonine-protein kinase RLK1 [Populus euphratica]	3.74	2.35
map00260	Glycine, serine and threonine metabolism	2.05E-03	TRINITY_DN19656_c0_g3	-4.04	down	hypothetical protein POPTR_0200s00220g [Populus trichocarpa]	0.09	2.49
map00260	Glycine, serine and threonine metabolism	2.05E-03	TRINITY_DN19981_c0_g1	-1.21	down	copper/topa quinone amine oxidase precursor family protein [Populus trichocarpa]	1.81	6.48
map00260	Glycine, serine and threonine metabolism	2.05E-03	TRINITY_DN20531_c0_g2	1.49	up	PREDICTED: homoserine kinase-like [Populus euphratica]	91.57	46.55
map00260	Glycine, serine and threonine metabolism	2.05E-03	TRINITY_DN21133_c0_g2	-1.22	down	PREDICTED: G-type lectin S-receptor-like serine/threonine-protein kinase RLK1 isoform X1 [Populus euphratica]	6.50	22.69
map00260	Glycine, serine and threonine metabolism	2.05E-03	TRINITY_DN21140_c0_g2	2.24	up	mitochondrial lipoamide dehydrogenase [Populus tremuloides]	202.39	67.53
map00260	Glycine, serine and threonine metabolism	2.05E-03	TRINITY_DN21210_c0_g6	1.13	up	homoserine dehydrogenase family protein [Populus trichocarpa]	16.38	11.90
map00260	Glycine, serine and threonine metabolism	2.05E-03	TRINITY_DN21635_c1_g2	-2.07	down	SEC14 cytosolic factor family protein [Populus trichocarpa]	5.54	32.41
map00950	Isoquinoline alkaloid biosynthesis	2.24E-03	TRINITY_DN22813_c0_g4	1.08	up	aspartate transaminase family protein [Populus trichocarpa]	25.11	18.98
map00950	Isoquinoline alkaloid biosynthesis	2.24E-03	TRINITY_DN23463_c0_g3	1.61	up	polyphenol oxidase [Populus tremuloides]	153.33	73.81
map00950	Isoquinoline alkaloid biosynthesis	2.24E-03	TRINITY_DN23704_c1_g1	-3.03	down	hypothetical protein POPTR_0001s07880g [Populus trichocarpa]	1.87	24.42
map00950	Isoquinoline alkaloid biosynthesis	2.24E-03	TRINITY_DN23709_c0_g2	1.23	up	aspartate aminotransferase 2 family protein [Populus trichocarpa]	46.64	32.80
map00950	Isoquinoline alkaloid biosynthesis	2.24E-03	TRINITY_DN24599_c6_g1	-1.97	down	hypothetical protein POPTR_0007s03670g [Populus trichocarpa]	0.44	2.68
map00950	Isoquinoline alkaloid biosynthesis	2.24E-03	TRINITY_DN24826_c0_g1	-1.05	down	PREDICTED: uncharacterized protein LOC105138712 [Populus euphratica]	1.85	5.77
map00950	Isoquinoline alkaloid biosynthesis	2.24E-03	TRINITY_DN24826_c0_g2	-4.47	down	PREDICTED: uncharacterized protein LOC105109098 [Populus euphratica]	0.14	5.15
map00950	Isoquinoline alkaloid biosynthesis	2.24E-03	TRINITY_DN24880_c0_g1	-1.43	down	curculin-like lectin family protein [Populus trichocarpa]	4.67	18.36
map00950	Isoquinoline alkaloid biosynthesis	2.24E-03	TRINITY_DN26235_c0_g1	-1.34	down	PREDICTED: LOW QUALITY PROTEIN: G-type lectin S-receptor-like serine/threonine-protein kinase RLK1 [Populus euphratica]	6.54	32.64
map00950	Isoquinoline alkaloid biosynthesis	2.24E-03	TRINITY_DN26235_c0_g3	-1.48	down	PREDICTED: G-type lectin S-receptor-like serine/threonine-protein kinase RLK1 [Populus euphratica]	4.02	17.62
map00950	Isoquinoline alkaloid biosynthesis	2.24E-03	TRINITY_DN26456_c0_g1	-2.06	down	hypothetical protein POPTR_0001s39940g [Populus trichocarpa]	112.97	594.31
map00950	Isoquinoline alkaloid biosynthesis	2.24E-03	TRINITY_DN27242_c0_g1	-1.23	down	hypothetical protein POPTR_0151s00200g [Populus trichocarpa]	2.45	8.98
map00950	Isoquinoline alkaloid biosynthesis	2.24E-03	TRINITY_DN14000_c0_g1	-2.71	down	hypothetical protein POPTR_0013s05640g [Populus trichocarpa]	0.38	5.38
map00950	Isoquinoline alkaloid biosynthesis	2.24E-03	TRINITY_DN14872_c0_g1	3.38	up	hypothetical protein POPTR_0004s16380g [Populus trichocarpa]	5.62	0.83
map00950	Isoquinoline alkaloid biosynthesis	2.24E-03	TRINITY_DN14872_c0_g2	3.04	up	polyphenol oxidase-like protein [Populus trichocarpa]	2.86	0.52
map00950	Isoquinoline alkaloid biosynthesis	2.24E-03	TRINITY_DN15219_c0_g1	-3.09	down	hypothetical protein POPTR_0013s05650g [Populus trichocarpa]	0.23	2.86
map00950	Isoquinoline alkaloid biosynthesis	2.24E-03	TRINITY_DN16284_c0_g2	1.06	up	hypothetical protein POPTR_0004s03870g [Populus trichocarpa]	39.71	28.71
map00950	Isoquinoline alkaloid biosynthesis	2.24E-03	TRINITY_DN16905_c0_g1	1.02	up	hypothetical protein POPTR_0018s04600g [Populus trichocarpa]	49.91	37.20
map00950	Isoquinoline alkaloid biosynthesis	2.24E-03	TRINITY_DN17759_c0_g1	-1.68	down	hypothetical protein POPTR_0004s01460g, partial [Populus trichocarpa]	1.43	5.88

map00950	Isoquinoline alkaloid biosynthesis	2.24E-03	TRINITY_DN17759_c0_g3	-2.14	down	PREDICTED: proline-rich receptor-like protein kinase PERK3 isoform X1 [Populus euphratica]	0.24	1.71
map00950	Isoquinoline alkaloid biosynthesis	2.24E-03	TRINITY_DN19059_c0_g2	-1.92	down	PREDICTED: G-type lectin S-receptor-like serine/threonine-protein kinase At5g24080 isoform X1 [Populus euphratica]	1.17	7.57
map00950	Isoquinoline alkaloid biosynthesis	2.24E-03	TRINITY_DN19059_c0_g3	-6.09	down	hypothetical protein POPTR_0015s05780g [Populus trichocarpa]	0.09	4.40
map00950	Isoquinoline alkaloid biosynthesis	2.24E-03	TRINITY_DN19472_c0_g2	1.32	up	hypothetical protein POPTR_0007s01130g [Populus trichocarpa]	8.54	5.46
map00950	Isoquinoline alkaloid biosynthesis	2.24E-03	TRINITY_DN19472_c0_g3	-3.23	down	hypothetical protein POPTR_0017s04550g [Populus trichocarpa]	0.21	3.06
map00950	Isoquinoline alkaloid biosynthesis	2.24E-03	TRINITY_DN19656_c0_g1	-1.62	down	PREDICTED: G-type lectin S-receptor-like serine/threonine-protein kinase RLK1 isoform X1 [Populus euphratica]	0.57	2.63
map00950	Isoquinoline alkaloid biosynthesis	2.24E-03	TRINITY_DN19656_c0_g2	1.26	up	PREDICTED: G-type lectin S-receptor-like serine/threonine-protein kinase RLK1 [Populus euphratica]	3.74	2.35
map00950	Isoquinoline alkaloid biosynthesis	2.24E-03	TRINITY_DN19656_c0_g3	-4.04	down	hypothetical protein POPTR_0200s00220g [Populus trichocarpa]	0.09	2.49
map00950	Isoquinoline alkaloid biosynthesis	2.24E-03	TRINITY_DN19981_c0_g1	-1.21	down	copper/topa quinone amine oxidase precursor family protein [Populus trichocarpa]	1.81	6.48
map00950	Isoquinoline alkaloid biosynthesis	2.24E-03	TRINITY_DN20799_c0_g1	1.40	up	PREDICTED: tyrosine decarboxylase 1-like isoform X2 [Populus euphratica]	30.48	17.85
map00950	Isoquinoline alkaloid biosynthesis	2.24E-03	TRINITY_DN20799_c0_g5	-2.32	down	hypothetical protein POPTR_0013s04970g [Populus trichocarpa]	0.28	2.25
map00950	Isoquinoline alkaloid biosynthesis	2.24E-03	TRINITY_DN21133_c0_g2	-1.22	down	PREDICTED: G-type lectin S-receptor-like serine/threonine-protein kinase RLK1 isoform X1 [Populus euphratica]	6.50	22.69
map00950	Isoquinoline alkaloid biosynthesis	2.24E-03	TRINITY_DN21391_c0_g1	1.12	up	PREDICTED: uncharacterized protein LOC105124970 [Populus euphratica]	49.29	33.42
map00750	Vitamin B6 metabolism	4.82E-03	TRINITY_DN22194_c0_g3	1.15	up	pyridoxin biosynthesis PDX1-like protein 2 [Populus trichocarpa]	8.07	5.60
map00750	Vitamin B6 metabolism	4.82E-03	TRINITY_DN22360_c0_g1	-1.21	down	homeobox-leucine zipper family protein [Populus trichocarpa]	4.84	17.40
map00750	Vitamin B6 metabolism	4.82E-03	TRINITY_DN22360_c0_g2	-1.24	down	hypothetical protein POPTR_0014s04460g [Populus trichocarpa]	12.37	46.35
map00750	Vitamin B6 metabolism	4.82E-03	TRINITY_DN25078_c0_g1	-1.04	down	PREDICTED: probable aldo-keto reductase 1 isoform X1 [Populus euphratica]	17.67	45.66
map00750	Vitamin B6 metabolism	4.82E-03	TRINITY_DN25367_c0_g1	1.54	up	hypothetical protein POPTR_0003s02770g [Populus trichocarpa]	59.68	31.40
map00750	Vitamin B6 metabolism	4.82E-03	TRINITY_DN25446_c0_g1	-1.51	down	hypothetical protein POPTR_0006s20820g [Populus trichocarpa]	15.62	54.91
map00750	Vitamin B6 metabolism	4.82E-03	TRINITY_DN25759_c0_g1	1.28	up	hypothetical protein POPTR_0015s00930g [Populus trichocarpa]	25.95	15.09
map00750	Vitamin B6 metabolism	4.82E-03	TRINITY_DN27203_c1_g3	1.38	up	aldo/keto reductase family protein [Populus trichocarpa]	97.45	56.97
map00750	Vitamin B6 metabolism	4.82E-03	TRINITY_DN13943_c0_g1	-3.69	down	homeobox leucine zipper family protein [Populus trichocarpa]	0.11	2.23
map00750	Vitamin B6 metabolism	4.82E-03	TRINITY_DN15046_c0_g2	-1.87	down	PREDICTED: homeobox-leucine zipper protein HOX11-like [Populus euphratica]	3.73	21.23
map00750	Vitamin B6 metabolism	4.82E-03	TRINITY_DN15472_c0_g1	-3.80	down	PREDICTED: homeobox-leucine zipper protein HOX3-like isoform X1 [Populus euphratica]	0.25	5.55
map00750	Vitamin B6 metabolism	4.82E-03	TRINITY_DN16446_c1_g2	-5.02	down	hypothetical protein POPTR_0006s11890g [Populus trichocarpa]	0.13	6.66
map00750	Vitamin B6 metabolism	4.82E-03	TRINITY_DN17015_c0_g1	1.23	up	hypothetical protein POPTR_0009s01810g [Populus trichocarpa]	88.79	57.90
map00750	Vitamin B6 metabolism	4.82E-03	TRINITY_DN18212_c1_g1	-1.15	down	aldo/keto reductase family protein [Populus trichocarpa]	63.19	221.55
map00750	Vitamin B6 metabolism	4.82E-03	TRINITY_DN18501_c1_g1	2.31	up	PREDICTED: phosphoserine aminotransferase 2, chloroplastic-like [Populus euphratica]	32.17	9.92
map00750	Vitamin B6 metabolism	4.82E-03	TRINITY_DN20284_c0_g1	2.57	up	pyridoxin biosynthesis PDX1-like protein 3 [Populus trichocarpa]	308.68	89.41
map00750	Vitamin B6 metabolism	4.82E-03	TRINITY_DN20543_c0_g3	1.35	up	hypothetical protein POPTR_0017s02980g [Populus trichocarpa]	33.49	20.55
map00750	Vitamin B6 metabolism	4.82E-03	TRINITY_DN21637_c0_g2	-1.12	down	hypothetical protein POPTR_0015s05050g [Populus trichocarpa]	3.13	12.74
map00220	Arginine biosynthesis	5.42E-03	TRINITY_DN21747_c0_g1	3.00	up	hypothetical protein POPTR_0015s12250g [Populus trichocarpa]	29.79	5.40
map00220	Arginine biosynthesis	5.42E-03	TRINITY_DN22663_c0_g2	1.38	up	hypothetical protein POPTR_0010s05530g [Populus trichocarpa]	360.33	205.54
map00220	Arginine biosynthesis	5.42E-03	TRINITY_DN22813_c0_g4	1.08	up	aspartate transaminase family protein [Populus trichocarpa]	25.11	18.98
map00220	Arginine biosynthesis	5.42E-03	TRINITY_DN22841_c0_g1	1.22	up	PREDICTED: LOW QUALITY PROTEIN: aminoacylase-1 [Populus euphratica]	87.12	56.00
map00220	Arginine biosynthesis	5.42E-03	TRINITY_DN23091_c0_g2	-2.11	down	glutamine synthetase family protein [Populus trichocarpa]	10.71	69.67
map00220	Arginine biosynthesis	5.42E-03	TRINITY_DN23091_c0_g3	4.22	up	glutamate-ammonia ligase family protein [Populus trichocarpa]	47.80	3.92
map00220	Arginine biosynthesis	5.42E-03	TRINITY_DN23091_c0_g4	1.81	up	glutamate-ammonia ligase family protein [Populus trichocarpa]	63.46	29.26
map00220	Arginine biosynthesis	5.42E-03	TRINITY_DN23091_c0_g5	4.02	up	glutamine synthetase family protein [Populus simonii x Populus nigra]	46.52	4.43

map00220	Arginine biosynthesis	5.42E-03	TRINITY_DN23209_c0_g1	2.76	up	hypothetical protein POPTR_0004s21720g [Populus trichocarpa]	147.72	29.16
map00220	Arginine biosynthesis	5.42E-03	TRINITY_DN23278_c1_g1	1.44	up	PREDICTED: glutamate--glyoxylate aminotransferase 2 isoform X1 [Populus euphratica]	580.61	318.60
map00220	Arginine biosynthesis	5.42E-03	TRINITY_DN23709_c0_g2	1.23	up	aspartate aminotransferase 2 family protein [Populus trichocarpa]	46.64	32.80
map00220	Arginine biosynthesis	5.42E-03	TRINITY_DN23937_c0_g1	1.78	up	Glutamine synthetase nodule isozyme [Ananas comosus]	127.57	57.48
map00220	Arginine biosynthesis	5.42E-03	TRINITY_DN23937_c0_g3	-3.47	down	PREDICTED: LOW QUALITY PROTEIN: glutamine synthetase cytosolic isozyme 2 [Populus euphratica]	0.50	8.51
map00220	Arginine biosynthesis	5.42E-03	TRINITY_DN24990_c0_g2	3.40	up	alanine aminotransferase family protein [Populus simonii x Populus nigra]	51.44	7.50
map00220	Arginine biosynthesis	5.42E-03	TRINITY_DN25260_c0_g3	3.71	up	glutamate-ammonia ligase family protein [Populus trichocarpa]	11.97	1.40
map00220	Arginine biosynthesis	5.42E-03	TRINITY_DN25260_c0_g4	-1.01	down	unknown [Populus trichocarpa]	26.98	83.37
map00220	Arginine biosynthesis	5.42E-03	TRINITY_DN25476_c0_g2	2.59	up	PREDICTED: alanine aminotransferase 2, mitochondrial-like [Populus euphratica]	33.99	8.74
map00220	Arginine biosynthesis	5.42E-03	TRINITY_DN25476_c0_g4	2.24	up	PREDICTED: alanine aminotransferase 2-like [Populus euphratica]	21.79	7.01
map00220	Arginine biosynthesis	5.42E-03	TRINITY_DN25941_c0_g1	1.37	up	PREDICTED: glutamine synthetase leaf isozyme, chloroplastic [Populus euphratica]	986.31	589.65
map00220	Arginine biosynthesis	5.42E-03	TRINITY_DN26128_c0_g3	1.77	up	PREDICTED: probable amino-acid acetyltransferase NAGS2, chloroplastic isoform X1 [Populus euphratica]	26.59	11.97
map00220	Arginine biosynthesis	5.42E-03	TRINITY_DN26128_c0_g4	2.19	up	PREDICTED: probable amino-acid acetyltransferase NAGS2, chloroplastic isoform X1 [Populus euphratica]	2.29	0.74
map00220	Arginine biosynthesis	5.42E-03	TRINITY_DN27440_c0_g2	1.46	up	PREDICTED: glutamine synthetase leaf isozyme, chloroplastic [Populus euphratica]	559.41	306.88
map00220	Arginine biosynthesis	5.42E-03	TRINITY_DN17444_c0_g1	-1.97	down	PREDICTED: uncharacterized protein LOC104888072 [Beta vulgaris subsp. vulgaris]	0.40	2.38
map00220	Arginine biosynthesis	5.42E-03	TRINITY_DN17736_c0_g2	1.54	up	arginine biosynthesis protein ArgJ [Populus trichocarpa]	59.37	31.41
map00220	Arginine biosynthesis	5.42E-03	TRINITY_DN19742_c0_g1	1.68	up	hypothetical protein POPTR_0002s10640g [Populus trichocarpa]	43.81	21.04
map00220	Arginine biosynthesis	5.42E-03	TRINITY_DN20203_c0_g3	2.14	up	PREDICTED: uncharacterized protein LOC105127640 [Populus euphratica]	34.27	11.75
map00220	Arginine biosynthesis	5.42E-03	TRINITY_DN20720_c0_g1	2.14	up	PREDICTED: probable N-acetyl-gamma-glutamyl-phosphate reductase, chloroplastic isoform X2 [Populus euphratica]	42.82	15.35
map00220	Arginine biosynthesis	5.42E-03	TRINITY_DN21117_c0_g1	1.76	up	N-acetylglutamate kinase, partial [Populus maximowiczii x Populus nigra]	57.23	25.99
map00220	Arginine biosynthesis	5.42E-03	TRINITY_DN21407_c0_g1	1.12	up	PREDICTED: NO-associated protein 1, chloroplastic/mitochondrial [Populus euphratica]	55.57	40.90
map00903	Limonene and pinene degradation	6.05E-03	TRINITY_DN21714_c0_g1	-2.58	down	speckle-type POZ family protein [Populus trichocarpa]	0.98	8.88
map00903	Limonene and pinene degradation	6.05E-03	TRINITY_DN21932_c0_g3	-1.06	down	phosphatase 2C family protein [Populus trichocarpa]	2.53	8.10
map00903	Limonene and pinene degradation	6.05E-03	TRINITY_DN22125_c0_g2	1.35	up	PREDICTED: cytochrome P450 71A1-like [Populus euphratica]	59.53	32.43
map00903	Limonene and pinene degradation	6.05E-03	TRINITY_DN22125_c0_g6	-5.41	down	hypothetical protein POPTR_0016s14440g [Populus trichocarpa]	0.24	15.20
map00903	Limonene and pinene degradation	6.05E-03	TRINITY_DN23151_c0_g1	1.61	up	PREDICTED: cytochrome P450 CYP82D47-like [Populus euphratica]	10.65	5.56
map00903	Limonene and pinene degradation	6.05E-03	TRINITY_DN23151_c0_g2	5.41	up	hypothetical protein POPTR_0009s11180g [Populus trichocarpa]	21.65	0.90
map00903	Limonene and pinene degradation	6.05E-03	TRINITY_DN23531_c0_g5	-1.67	down	hypothetical protein POPTR_0436s00200g [Populus trichocarpa]	13.47	59.57
map00903	Limonene and pinene degradation	6.05E-03	TRINITY_DN23531_c0_g7	-1.31	down	cytochrome P450 family protein [Populus trichocarpa]	1.88	6.94
map00903	Limonene and pinene degradation	6.05E-03	TRINITY_DN24628_c0_g1	1.28	up	PREDICTED: aldehyde dehydrogenase family 3 member H1-like [Populus euphratica]	40.39	24.46
map00903	Limonene and pinene degradation	6.05E-03	TRINITY_DN25044_c0_g1	1.87	up	hypothetical protein POPTR_0015s00850g [Populus trichocarpa]	9.30	3.87
map00903	Limonene and pinene degradation	6.05E-03	TRINITY_DN25044_c0_g7	3.98	up	hypothetical protein POPTR_0015s00850g [Populus trichocarpa]	6.97	0.68
map00903	Limonene and pinene degradation	6.05E-03	TRINITY_DN25630_c0_g1	1.30	up	aldehyde dehydrogenase 1 precursor family protein [Populus trichocarpa]	120.35	73.18
map00903	Limonene and pinene degradation	6.05E-03	TRINITY_DN26025_c0_g1	-1.71	down	hypothetical protein POPTR_0004s18340g [Populus trichocarpa]	5.60	27.70
map00903	Limonene and pinene degradation	6.05E-03	TRINITY_DN26421_c0_g1	1.32	up	the aldehyde dehydrogenase cp-ADH from C.plantagineum family protein [Populus trichocarpa]	58.13	34.64
map00903	Limonene and pinene degradation	6.05E-03	TRINITY_DN26461_c0_g5	-5.12	down	cytochrome P450 family protein [Populus trichocarpa]	0.57	30.30
map00903	Limonene and pinene degradation	6.05E-03	TRINITY_DN15814_c0_g1	-1.33	down	hypothetical protein POPTR_0004s01860g [Populus trichocarpa]	1.60	5.98
map00903	Limonene and pinene degradation	6.05E-03	TRINITY_DN16397_c0_g1	-4.37	down	hypothetical protein POPTR_0003s14670g [Populus trichocarpa]	0.06	2.04
map00903	Limonene and pinene degradation	6.05E-03	TRINITY_DN16397_c0_g2	1.60	up	hypothetical protein POPTR_0001s11360g [Populus trichocarpa]	3.14	1.59
map00903	Limonene and pinene degradation	6.05E-03	TRINITY_DN16903_c0_g2	-10.01	down	cytochrome P450 family protein [Populus trichocarpa]	0.00	5.21
map00903	Limonene and pinene degradation	6.05E-03	TRINITY_DN16939_c0_g3	-5.27	down	hypothetical protein POPTR_0007s06570g [Populus trichocarpa]	0.04	2.84
map00903	Limonene and pinene degradation	6.05E-03	TRINITY_DN16939_c0_g4	-5.19	down	hypothetical protein POPTR_0007s06570g [Populus trichocarpa]	0.04	2.53
map00903	Limonene and pinene degradation	6.05E-03	TRINITY_DN18763_c0_g2	-2.06	down	cytochrome P450 family protein [Populus trichocarpa]	0.40	2.47

map00903	Limonene and pinene degradation	6.05E-03	TRINITY_DN18895_c0_g1	-1.06	down	mitochondrial aldehyde dehydrogenase family protein [Populus trichocarpa]	7.40	23.22
map00903	Limonene and pinene degradation	6.05E-03	TRINITY_DN19215_c1_g1	-1.38	down	hypothetical protein POPTR_0007s10210g [Populus trichocarpa]	3.47	12.55
map00903	Limonene and pinene degradation	6.05E-03	TRINITY_DN19215_c2_g1	-1.56	down	PREDICTED: probable protein phosphatase 2C 63 [Populus euphratica]	6.48	30.16
map00903	Limonene and pinene degradation	6.05E-03	TRINITY_DN19448_c1_g1	1.73	up	PREDICTED: cytochrome P450 89A2-like [Populus euphratica]	39.33	17.69
map00903	Limonene and pinene degradation	6.05E-03	TRINITY_DN19448_c1_g3	1.29	up	hypothetical protein POPTR_0015s00850g [Populus trichocarpa]	5.89	3.57
map00903	Limonene and pinene degradation	6.05E-03	TRINITY_DN20101_c0_g1	1.43	up	hypothetical protein POPTR_0016s03160g [Populus trichocarpa]	10.12	5.77
map00903	Limonene and pinene degradation	6.05E-03	TRINITY_DN20258_c0_g1	-2.32	down	hypothetical protein POPTR_0007s09840g [Populus trichocarpa]	3.79	29.05
map00903	Limonene and pinene degradation	6.05E-03	TRINITY_DN20534_c2_g1	-3.15	down	cytochrome P450 78A3p family protein [Populus trichocarpa]	0.27	3.89
map01230	Biosynthesis of amino acids	7.14E-03	TRINITY_DN21760_c0_g1	1.25	up	isocitrate dehydrogenase family protein [Populus trichocarpa]	53.95	39.69
map01230	Biosynthesis of amino acids	7.14E-03	TRINITY_DN21760_c0_g2	1.32	up	PREDICTED: 3-isopropylmalate dehydrogenase, chloroplastic-like [Populus euphratica]	86.28	52.23
map01230	Biosynthesis of amino acids	7.14E-03	TRINITY_DN21802_c0_g1	1.24	up	hypothetical protein POPTR_0017s12470g [Populus trichocarpa]	144.20	95.20
map01230	Biosynthesis of amino acids	7.14E-03	TRINITY_DN22048_c2_g2	1.93	up	hypothetical protein POPTR_0015s12380g [Populus trichocarpa]	3.05	1.20
map01230	Biosynthesis of amino acids	7.14E-03	TRINITY_DN22048_c2_g5	-1.67	down	hypothetical protein POPTR_0012s11610g [Populus trichocarpa]	1.63	8.07
map01230	Biosynthesis of amino acids	7.14E-03	TRINITY_DN22171_c2_g1	1.24	up	PREDICTED: S-adenosylmethionine synthase 3 [Populus euphratica]	228.61	137.13
map01230	Biosynthesis of amino acids	7.14E-03	TRINITY_DN22238_c0_g3	1.04	up	hypothetical protein POPTR_0014s16700g [Populus trichocarpa]	59.72	45.45
map01230	Biosynthesis of amino acids	7.14E-03	TRINITY_DN22245_c0_g1	2.23	up	PREDICTED: triosephosphate isomerase, chloroplastic [Populus euphratica]	838.73	263.34
map01230	Biosynthesis of amino acids	7.14E-03	TRINITY_DN22246_c0_g2	1.68	up	hypothetical protein POPTR_0019s03500g [Populus trichocarpa]	144.86	80.32
map01230	Biosynthesis of amino acids	7.14E-03	TRINITY_DN22283_c0_g1	1.79	up	hypothetical protein POPTR_0002s09390g [Populus trichocarpa]	139.18	61.68
map01230	Biosynthesis of amino acids	7.14E-03	TRINITY_DN22348_c0_g1	2.11	up	O-acetylserine (thiol)lyase family protein [Populus trichocarpa]	18.41	6.25
map01230	Biosynthesis of amino acids	7.14E-03	TRINITY_DN22348_c0_g4	1.61	up	O-acetylserine (thiol)lyase family protein [Populus trichocarpa]	220.16	110.90
map01230	Biosynthesis of amino acids	7.14E-03	TRINITY_DN22348_c0_g6	1.74	up	hypothetical protein POPTR_0013s13150g [Populus trichocarpa]	146.50	66.04
map01230	Biosynthesis of amino acids	7.14E-03	TRINITY_DN22580_c0_g3	1.24	up	hypothetical protein POPTR_0003s10720g [Populus trichocarpa]	124.71	79.50
map01230	Biosynthesis of amino acids	7.14E-03	TRINITY_DN22587_c0_g1	1.20	up	PREDICTED: fructose-bisphosphate aldolase cytoplasmic isozyme [Populus euphratica]	371.77	247.12
map01230	Biosynthesis of amino acids	7.14E-03	TRINITY_DN22595_c0_g2	1.52	up	PREDICTED: phospho-2-dehydro-3-deoxyheptonate aldolase 2, chloroplastic-like [Populus euphratica]	137.27	72.08
map01230	Biosynthesis of amino acids	7.14E-03	TRINITY_DN22598_c1_g1	1.42	up	2 family protein [Populus trichocarpa]	136.33	77.98
map01230	Biosynthesis of amino acids	7.14E-03	TRINITY_DN22627_c1_g2	2.05	up	PREDICTED: ATP phosphoribosyltransferase 2, chloroplastic-like [Populus euphratica]	103.92	38.62
map01230	Biosynthesis of amino acids	7.14E-03	TRINITY_DN22627_c1_g5	1.81	up	PREDICTED: ATP phosphoribosyltransferase 2, chloroplastic-like [Populus euphratica]	58.56	24.74
map01230	Biosynthesis of amino acids	7.14E-03	TRINITY_DN22629_c0_g2	1.01	up	PREDICTED: 2-isopropylmalate synthase 2, chloroplastic-like [Populus euphratica]	65.58	50.13
map01230	Biosynthesis of amino acids	7.14E-03	TRINITY_DN22663_c0_g2	1.38	up	hypothetical protein POPTR_0010s05530g [Populus trichocarpa]	360.33	205.54
map01230	Biosynthesis of amino acids	7.14E-03	TRINITY_DN22665_c0_g1	1.91	up	hypothetical protein POPTR_0013s03080g [Populus trichocarpa]	2.11	0.85
map01230	Biosynthesis of amino acids	7.14E-03	TRINITY_DN22665_c0_g3	2.46	up	hypothetical protein POPTR_0013s03080g [Populus trichocarpa]	7.83	2.17
map01230	Biosynthesis of amino acids	7.14E-03	TRINITY_DN22695_c0_g4	1.16	up	aconitase family protein [Populus trichocarpa]	151.04	102.95
map01230	Biosynthesis of amino acids	7.14E-03	TRINITY_DN22697_c0_g3	1.19	up	3-PHOSPHOSERINE PHOSPHATASE family protein [Populus trichocarpa]	51.79	35.35
map01230	Biosynthesis of amino acids	7.14E-03	TRINITY_DN22701_c0_g1	1.35	up	shikimate kinase family protein [Populus trichocarpa]	108.38	67.24
map01230	Biosynthesis of amino acids	7.14E-03	TRINITY_DN22812_c0_g2	1.66	up	Serine hydroxymethyltransferase family protein [Populus trichocarpa]	785.78	366.57
map01230	Biosynthesis of amino acids	7.14E-03	TRINITY_DN22812_c0_g3	1.77	up	mitochondrial serine hydroxymethyltransferase [Populus tremuloides]	59.98	26.65
map01230	Biosynthesis of amino acids	7.14E-03	TRINITY_DN22813_c0_g4	1.08	up	aspartate transaminase family protein [Populus trichocarpa]	25.11	18.98
map01230	Biosynthesis of amino acids	7.14E-03	TRINITY_DN22841_c0_g1	1.22	up	PREDICTED: LOW QUALITY PROTEIN: aminoacylase-1 [Populus euphratica]	87.12	56.00
map01230	Biosynthesis of amino acids	7.14E-03	TRINITY_DN22920_c1_g6	2.63	up	PREDICTED: probable ribose-5-phosphate isomerase 3, chloroplastic [Populus euphratica]	555.60	140.50
map01230	Biosynthesis of amino acids	7.14E-03	TRINITY_DN22954_c0_g2	1.23	up	TRYPTOPHAN SYNTHASE ALPHA CHAIN family protein [Populus trichocarpa]	98.67	70.99
map01230	Biosynthesis of amino acids	7.14E-03	TRINITY_DN22960_c1_g2	1.71	up	hypothetical protein POPTR_0013s13150g [Populus trichocarpa]	267.00	123.84

map01230	Biosynthesis of amino acids	7.14E-03	TRINITY_DN23066_c0_g5	1.83	up	PREDICTED: probable 2-carboxy-D-arabinitol-1-phosphatase [Populus euphratica]	71.98	31.71
map01230	Biosynthesis of amino acids	7.14E-03	TRINITY_DN23091_c0_g2	-2.11	down	glutamine synthetase family protein [Populus trichocarpa]	10.71	69.67
map01230	Biosynthesis of amino acids	7.14E-03	TRINITY_DN23091_c0_g3	4.22	up	glutamate-ammonia ligase family protein [Populus trichocarpa]	47.80	3.92
map01230	Biosynthesis of amino acids	7.14E-03	TRINITY_DN23091_c0_g4	1.81	up	glutamate-ammonia ligase family protein [Populus trichocarpa]	63.46	29.26
map01230	Biosynthesis of amino acids	7.14E-03	TRINITY_DN23091_c0_g5	4.02	up	glutamine synthetase family protein [Populus simonii x Populus nigra]	46.52	4.43
map01230	Biosynthesis of amino acids	7.14E-03	TRINITY_DN23172_c0_g1	1.90	up	hypothetical protein POPTR_0017s00350g [Populus trichocarpa]	54.45	26.38
map01230	Biosynthesis of amino acids	7.14E-03	TRINITY_DN23209_c0_g1	2.76	up	hypothetical protein POPTR_0004s21720g [Populus trichocarpa]	147.72	29.16
map01230	Biosynthesis of amino acids	7.14E-03	TRINITY_DN23227_c0_g2	1.27	up	hypothetical protein POPTR_0010s19790g [Populus trichocarpa]	6.63	4.18
map01230	Biosynthesis of amino acids	7.14E-03	TRINITY_DN23260_c0_g1	1.32	up	anthranilate synthase beta subunit 1 family protein [Populus trichocarpa]	230.33	121.36
map01230	Biosynthesis of amino acids	7.14E-03	TRINITY_DN23271_c0_g1	1.57	up	hypothetical protein POPTR_0001s35080g [Populus trichocarpa]	37.34	19.20
map01230	Biosynthesis of amino acids	7.14E-03	TRINITY_DN23278_c1_g1	1.44	up	PREDICTED: glutamate--glyoxylate aminotransferase 2 isoform X1 [Populus euphratica]	580.61	318.60
map01230	Biosynthesis of amino acids	7.14E-03	TRINITY_DN23349_c0_g1	2.04	up	Cysteine synthase C1 [Theobroma cacao]	171.68	63.08
map01230	Biosynthesis of amino acids	7.14E-03	TRINITY_DN23497_c0_g1	2.24	up	D-3-phosphoglycerate dehydrogenase family protein [Populus trichocarpa]	103.49	34.56
map01230	Biosynthesis of amino acids	7.14E-03	TRINITY_DN23701_c0_g1	1.96	up	latex plastidic aldolase-like family protein [Populus trichocarpa]	2289.47	879.27
map01230	Biosynthesis of amino acids	7.14E-03	TRINITY_DN23701_c0_g5	2.57	up	plastidic aldolase family protein [Populus trichocarpa]	7.53	2.00
map01230	Biosynthesis of amino acids	7.14E-03	TRINITY_DN23709_c0_g2	1.23	up	aspartate aminotransferase 2 family protein [Populus trichocarpa]	46.64	32.80
map01230	Biosynthesis of amino acids	7.14E-03	TRINITY_DN23877_c1_g1	1.12	up	hypothetical protein POPTR_0008s09870g [Populus trichocarpa]	162.62	115.69
map01230	Biosynthesis of amino acids	7.14E-03	TRINITY_DN23877_c1_g3	1.26	up	PREDICTED: S-adenosylmethionine synthase 4 [Populus euphratica]	32.62	20.75
map01230	Biosynthesis of amino acids	7.14E-03	TRINITY_DN23916_c0_g1	1.26	up	chloroplast import receptor p36 family protein [Populus trichocarpa]	583.12	369.56
map01230	Biosynthesis of amino acids	7.14E-03	TRINITY_DN23937_c0_g1	1.78	up	Glutamine synthetase nodule isozyme [Ananas comosus]	127.57	57.48
map01230	Biosynthesis of amino acids	7.14E-03	TRINITY_DN23937_c0_g3	-3.47	down	PREDICTED: LOW QUALITY PROTEIN: glutamine synthetase cytosolic isozyme 2 [Populus euphratica]	0.50	8.51
map01230	Biosynthesis of amino acids	7.14E-03	TRINITY_DN23965_c0_g1	1.23	up	PREDICTED: triosephosphate isomerase, cytosolic [Populus euphratica]	641.46	426.80
map01230	Biosynthesis of amino acids	7.14E-03	TRINITY_DN24179_c1_g1	1.94	up	hypothetical protein POPTR_0001s44620g [Populus trichocarpa]	107.11	47.27
map01230	Biosynthesis of amino acids	7.14E-03	TRINITY_DN24285_c0_g5	1.46	up	PREDICTED: WAT1-related protein At2g37460-like isoform X1 [Populus euphratica]	2.63	1.46
map01230	Biosynthesis of amino acids	7.14E-03	TRINITY_DN24393_c0_g1	1.31	up	hypothetical protein POPTR_0001s24710g [Populus trichocarpa]	198.18	106.97
map01230	Biosynthesis of amino acids	7.14E-03	TRINITY_DN24707_c1_g1	-1.25	down	nodulin MtN21 family protein [Populus trichocarpa]	14.31	50.91
map01230	Biosynthesis of amino acids	7.14E-03	TRINITY_DN24881_c0_g1	1.99	up	PREDICTED: transketolase, chloroplastic [Populus euphratica]	1790.82	677.46
map01230	Biosynthesis of amino acids	7.14E-03	TRINITY_DN24894_c0_g3	1.50	up	hypothetical protein POPTR_0015s03960g [Populus trichocarpa]	34.92	19.04
map01230	Biosynthesis of amino acids	7.14E-03	TRINITY_DN24964_c0_g1	1.27	up	PREDICTED: histidinol dehydrogenase, chloroplastic-like isoform X3 [Populus euphratica]	31.19	20.54
map01230	Biosynthesis of amino acids	7.14E-03	TRINITY_DN24990_c0_g2	3.40	up	alanine aminotransferase family protein [Populus simonii x Populus nigra]	51.44	7.50
map01230	Biosynthesis of amino acids	7.14E-03	TRINITY_DN25129_c1_g2	1.01	up	3-phosphoglycerate kinase [Populus tremuloides]	153.72	116.35
map01230	Biosynthesis of amino acids	7.14E-03	TRINITY_DN25260_c0_g3	3.71	up	glutamate-ammonia ligase family protein [Populus trichocarpa]	11.97	1.40
map01230	Biosynthesis of amino acids	7.14E-03	TRINITY_DN25260_c0_g4	-1.01	down	unknown [Populus trichocarpa]	26.98	83.37
map01230	Biosynthesis of amino acids	7.14E-03	TRINITY_DN25367_c0_g1	1.54	up	hypothetical protein POPTR_0003s02770g [Populus trichocarpa]	59.68	31.40
map01230	Biosynthesis of amino acids	7.14E-03	TRINITY_DN25474_c0_g1	1.34	up	PREDICTED: diaminopimelate decarboxylase 2, chloroplastic-like [Populus euphratica]	144.79	88.69
map01230	Biosynthesis of amino acids	7.14E-03	TRINITY_DN25476_c0_g2	2.59	up	PREDICTED: alanine aminotransferase 2, mitochondrial-like [Populus euphratica]	33.99	8.74
map01230	Biosynthesis of amino acids	7.14E-03	TRINITY_DN25476_c0_g4	2.24	up	PREDICTED: alanine aminotransferase 2-like [Populus euphratica]	21.79	7.01
map01230	Biosynthesis of amino acids	7.14E-03	TRINITY_DN25569_c0_g1	1.34	up	shikimate kinase family protein [Populus trichocarpa]	60.67	37.43
map01230	Biosynthesis of amino acids	7.14E-03	TRINITY_DN25630_c0_g3	-2.44	down	PREDICTED: branched-chain-amino-acid aminotransferase 2, chloroplastic-like isoform X1 [Populus euphratica]	0.50	3.81
map01230	Biosynthesis of amino acids	7.14E-03	TRINITY_DN25737_c0_g1	1.82	up	semialdehyde dehydrogenase family protein [Populus trichocarpa]	56.96	24.76
map01230	Biosynthesis of amino acids	7.14E-03	TRINITY_DN25829_c0_g2	1.62	up	PHOSPHORIBOSYLANTHRANILATE ISOMERASE 3 family protein [Populus trichocarpa]	43.17	21.30
map01230	Biosynthesis of amino acids	7.14E-03	TRINITY_DN25870_c1_g1	1.47	up	unknown [Populus trichocarpa]	296.23	175.19
map01230	Biosynthesis of amino acids	7.14E-03	TRINITY_DN25941_c0_g1	1.37	up	PREDICTED: glutamine synthetase leaf isozyme, chloroplastic [Populus euphratica]	986.31	589.65

map01230	Biosynthesis of amino acids	7.14E-03	TRINITY_DN26128_c0_g3	1.77	up	PREDICTED: probable amino-acid acetyltransferase NAGS2, chloroplastic isoform X1 [Populus euphratica]	26.59	11.97
map01230	Biosynthesis of amino acids	7.14E-03	TRINITY_DN26128_c0_g4	2.19	up	PREDICTED: probable amino-acid acetyltransferase NAGS2, chloroplastic isoform X1 [Populus euphratica]	2.29	0.74
map01230	Biosynthesis of amino acids	7.14E-03	TRINITY_DN26147_c0_g1	1.59	up	3-phosphoshikimate 1-carboxyvinyltransferase family protein [Populus trichocarpa]	288.34	147.17
map01230	Biosynthesis of amino acids	7.14E-03	TRINITY_DN26292_c0_g1	-1.38	down	hypothetical protein POPTR_0017s02170g [Populus trichocarpa]	5.66	22.15
map01230	Biosynthesis of amino acids	7.14E-03	TRINITY_DN26353_c0_g1	1.30	up	VuP5CS family protein [Populus trichocarpa]	49.12	31.04
map01230	Biosynthesis of amino acids	7.14E-03	TRINITY_DN26528_c0_g1	1.13	up	PREDICTED: D-3-phosphoglycerate dehydrogenase 3, chloroplastic-like [Populus euphratica]	74.65	55.30
map01230	Biosynthesis of amino acids	7.14E-03	TRINITY_DN26593_c1_g4	1.29	up	2-dehydro-3-deoxyphosphoheptonate aldolase family protein [Populus trichocarpa]	647.76	427.17
map01230	Biosynthesis of amino acids	7.14E-03	TRINITY_DN26627_c0_g1	2.37	up	PHOSPHOGLYCERATE KINASE 1 family protein [Populus trichocarpa]	946.82	271.09
map01230	Biosynthesis of amino acids	7.14E-03	TRINITY_DN26661_c0_g1	2.62	up	hypothetical protein CISIN_1g016748mg [Citrus sinensis]	718.86	176.98
map01230	Biosynthesis of amino acids	7.14E-03	TRINITY_DN26665_c0_g1	1.58	up	hypothetical protein POPTR_0009s15490g [Populus trichocarpa]	216.45	108.64
map01230	Biosynthesis of amino acids	7.14E-03	TRINITY_DN26976_c0_g1	1.47	up	PREDICTED: acetolactate synthase 2, chloroplastic-like [Populus euphratica]	396.57	239.53
map01230	Biosynthesis of amino acids	7.14E-03	TRINITY_DN27089_c0_g1	1.00	up	PREDICTED: imidazole glycerol phosphate synthase hisHF, chloroplastic-like isoform X1 [Populus euphratica]	33.93	31.04
map01230	Biosynthesis of amino acids	7.14E-03	TRINITY_DN27198_c1_g1	1.09	up	aconitate hydratase family protein [Populus trichocarpa]	47.60	32.18
map01230	Biosynthesis of amino acids	7.14E-03	TRINITY_DN27198_c1_g2	1.02	up	aconitate hydratase family protein [Populus trichocarpa]	16.99	12.62
map01230	Biosynthesis of amino acids	7.14E-03	TRINITY_DN27440_c0_g2	1.46	up	PREDICTED: glutamine synthetase leaf isozyme, chloroplastic [Populus euphratica]	559.41	306.88
map01230	Biosynthesis of amino acids	7.14E-03	TRINITY_DN27458_c1_g1	2.44	up	PREDICTED: transketolase, chloroplastic [Populus euphratica]	1184.20	313.96
map01230	Biosynthesis of amino acids	7.14E-03	TRINITY_DN27893_c10_g1	1.22	up	PREDICTED: anthranilate phosphoribosyltransferase, chloroplastic-like isoform X1 [Populus euphratica]	53.94	35.34
map01230	Biosynthesis of amino acids	7.14E-03	TRINITY_DN11372_c0_g1	3.83	up	hypothetical protein POPTR_0011s15150g [Populus trichocarpa]	4.92	0.36
map01230	Biosynthesis of amino acids	7.14E-03	TRINITY_DN12955_c0_g1	1.82	up	hypothetical protein POPTR_0011s05190g [Populus trichocarpa]	15.50	6.27
map01230	Biosynthesis of amino acids	7.14E-03	TRINITY_DN13782_c0_g1	5.00	up	hypothetical protein POPTR_0002s11010g [Populus trichocarpa]	6.55	0.32
map01230	Biosynthesis of amino acids	7.14E-03	TRINITY_DN14712_c0_g2	2.45	up	PREDICTED: glutamate synthase 1 [NADH], chloroplastic-like isoform X1 [Populus euphratica]	12.83	3.58
map01230	Biosynthesis of amino acids	7.14E-03	TRINITY_DN14836_c0_g1	-2.77	down	hypothetical protein POPTR_0013s03070g [Populus trichocarpa]	1.13	12.60
map01230	Biosynthesis of amino acids	7.14E-03	TRINITY_DN15207_c0_g1	1.59	up	hypothetical protein POPTR_0006s04100g [Populus trichocarpa]	38.39	20.18
map01230	Biosynthesis of amino acids	7.14E-03	TRINITY_DN15775_c0_g1	1.57	up	hypothetical protein POPTR_0001s46060g [Populus trichocarpa]	7.49	3.44
map01230	Biosynthesis of amino acids	7.14E-03	TRINITY_DN15907_c0_g1	1.59	up	hypothetical protein POPTR_0002s06190g [Populus trichocarpa]	5.64	2.06
map01230	Biosynthesis of amino acids	7.14E-03	TRINITY_DN16732_c0_g1	1.28	up	hypothetical protein POPTR_0017s07130g [Populus trichocarpa]	32.81	20.70
map01230	Biosynthesis of amino acids	7.14E-03	TRINITY_DN16817_c0_g1	1.64	up	PREDICTED: tRNA (guanine-N(7)-)-methyltransferase [Populus euphratica]	40.30	24.24
map01230	Biosynthesis of amino acids	7.14E-03	TRINITY_DN17020_c0_g1	2.36	up	hypothetical protein POPTR_0002s06890g [Populus trichocarpa]	22.89	6.58
map01230	Biosynthesis of amino acids	7.14E-03	TRINITY_DN17444_c0_g1	-1.97	down	PREDICTED: uncharacterized protein LOC104888072 [Beta vulgaris subsp. vulgaris]	0.40	2.38
map01230	Biosynthesis of amino acids	7.14E-03	TRINITY_DN17528_c0_g1	2.19	up	ribose-phosphate pyrophosphokinase family protein [Populus trichocarpa]	63.69	21.30
map01230	Biosynthesis of amino acids	7.14E-03	TRINITY_DN17663_c0_g3	1.15	up	unknown [Populus trichocarpa x Populus deltoides]	373.94	255.99
map01230	Biosynthesis of amino acids	7.14E-03	TRINITY_DN17736_c0_g2	1.54	up	arginine biosynthesis protein ArgJ [Populus trichocarpa]	59.37	31.41
map01230	Biosynthesis of amino acids	7.14E-03	TRINITY_DN17865_c0_g1	2.27	up	plastid serine hydroxymethyltransferase [Populus tremuloides]	53.46	17.12
map01230	Biosynthesis of amino acids	7.14E-03	TRINITY_DN17945_c0_g2	1.24	up	hypothetical protein POPTR_0006s06140g [Populus trichocarpa]	3.08	1.24
map01230	Biosynthesis of amino acids	7.14E-03	TRINITY_DN17959_c0_g1	2.77	up	PREDICTED: probable S-sulfocysteine synthase, chloroplastic [Populus euphratica]	54.54	12.96
map01230	Biosynthesis of amino acids	7.14E-03	TRINITY_DN18147_c0_g2	1.29	up	hypothetical protein POPTR_0005s11300g [Populus trichocarpa]	170.60	105.98
map01230	Biosynthesis of amino acids	7.14E-03	TRINITY_DN18209_c1_g1	1.05	up	PREDICTED: pyruvate kinase, cytosolic isozyme-like [Populus euphratica]	19.18	14.26
map01230	Biosynthesis of amino acids	7.14E-03	TRINITY_DN18387_c0_g1	1.43	up	hypothetical protein POPTR_0017s12240g [Populus trichocarpa]	122.22	69.70
map01230	Biosynthesis of amino acids	7.14E-03	TRINITY_DN18501_c1_g1	2.31	up	PREDICTED: phosphoserine aminotransferase 2, chloroplastic-like [Populus euphratica]	32.17	9.92
map01230	Biosynthesis of amino acids	7.14E-03	TRINITY_DN18598_c0_g1	1.82	up	hypothetical protein POPTR_0008s00350g [Populus trichocarpa]	468.84	197.84
map01230	Biosynthesis of amino acids	7.14E-03	TRINITY_DN18859_c0_g3	1.18	up	aconitate hydratase family protein [Populus trichocarpa]	80.30	54.25

map01230	Biosynthesis of amino acids	7.14E-03	TRINITY_DN19337_c0_g1	-2.03	down	hypothetical protein POPTR_0017s14220g [Populus trichocarpa]	2.64	16.29
map01230	Biosynthesis of amino acids	7.14E-03	TRINITY_DN19385_c0_g1	1.11	up	hypothetical protein POPTR_0008s20010g [Populus trichocarpa]	12.27	8.86
map01230	Biosynthesis of amino acids	7.14E-03	TRINITY_DN19472_c0_g2	1.32	up	hypothetical protein POPTR_0007s01130g [Populus trichocarpa]	8.54	5.46
map01230	Biosynthesis of amino acids	7.14E-03	TRINITY_DN19472_c0_g3	-3.23	down	hypothetical protein POPTR_0017s04550g [Populus trichocarpa]	0.21	3.06
map01230	Biosynthesis of amino acids	7.14E-03	TRINITY_DN19518_c0_g1	2.25	up	hypothetical protein POPTR_0016s14360g [Populus trichocarpa]	277.40	89.48
map01230	Biosynthesis of amino acids	7.14E-03	TRINITY_DN19734_c0_g2	1.39	up	PREDICTED: ribulose-phosphate 3-epimerase, chloroplastic [Populus euphratica]	648.43	374.61
map01230	Biosynthesis of amino acids	7.14E-03	TRINITY_DN19742_c0_g1	1.68	up	hypothetical protein POPTR_0002s10640g [Populus trichocarpa]	43.81	21.04
map01230	Biosynthesis of amino acids	7.14E-03	TRINITY_DN19743_c0_g1	1.38	up	PREDICTED: WAT1-related protein At4g19185-like [Populus euphratica]	66.15	39.51
map01230	Biosynthesis of amino acids	7.14E-03	TRINITY_DN19848_c0_g1	1.56	up	phosphopyruvate hydratase family protein [Populus trichocarpa]	247.16	128.86
map01230	Biosynthesis of amino acids	7.14E-03	TRINITY_DN20048_c1_g1	1.11	up	PREDICTED: phospho-2-dehydro-3-deoxyheptonate aldolase 1, chloroplastic-like [Populus euphratica]	289.54	201.11
map01230	Biosynthesis of amino acids	7.14E-03	TRINITY_DN20058_c0_g1	1.29	up	hypothetical protein POPTR_0009s08570g [Populus trichocarpa]	82.27	50.72
map01230	Biosynthesis of amino acids	7.14E-03	TRINITY_DN20148_c0_g1	1.45	up	hypothetical protein POPTR_0010s12560g [Populus trichocarpa]	131.18	75.15
map01230	Biosynthesis of amino acids	7.14E-03	TRINITY_DN20148_c0_g2	3.15	up	ribose 5-phosphate isomerase family protein [Populus trichocarpa]	6.05	1.07
map01230	Biosynthesis of amino acids	7.14E-03	TRINITY_DN20394_c0_g1	1.01	up	PREDICTED: histidine biosynthesis bifunctional protein hisIE, chloroplastic [Populus euphratica]	84.52	63.75
map01230	Biosynthesis of amino acids	7.14E-03	TRINITY_DN20517_c0_g1	1.45	up	chorismate synthase family protein [Populus trichocarpa]	289.88	166.51
map01230	Biosynthesis of amino acids	7.14E-03	TRINITY_DN20531_c0_g2	1.49	up	PREDICTED: homoserine kinase-like [Populus euphratica]	91.57	46.55
map01230	Biosynthesis of amino acids	7.14E-03	TRINITY_DN20583_c0_g3	2.29	up	hypothetical protein POPTR_0008s03820g [Populus trichocarpa]	4.09	1.88
map01230	Biosynthesis of amino acids	7.14E-03	TRINITY_DN20720_c0_g1	2.14	up	PREDICTED: probable N-acetyl-gamma-glutamyl-phosphate reductase, chloroplastic isoform X2 [Populus euphratica]	42.82	15.35
map01230	Biosynthesis of amino acids	7.14E-03	TRINITY_DN21117_c0_g1	1.76	up	N-acetylglutamate kinase, partial [Populus maximowiczii x Populus nigra]	57.23	25.99
map01230	Biosynthesis of amino acids	7.14E-03	TRINITY_DN21210_c0_g6	1.13	up	homoserine dehydrogenase family protein [Populus trichocarpa]	16.38	11.90
map01230	Biosynthesis of amino acids	7.14E-03	TRINITY_DN21313_c0_g2	-1.72	down	hypothetical protein POPTR_0014s07170g [Populus trichocarpa]	4.63	24.85
map01230	Biosynthesis of amino acids	7.14E-03	TRINITY_DN21391_c0_g1	1.12	up	PREDICTED: uncharacterized protein LOC105124970 [Populus euphratica]	49.29	33.42
map01230	Biosynthesis of amino acids	7.14E-03	TRINITY_DN21417_c0_g1	1.35	up	SERINE ACETYLTRANSFERASE-106 family protein [Populus trichocarpa]	46.75	26.71
map01230	Biosynthesis of amino acids	7.14E-03	TRINITY_DN21600_c0_g2	1.23	up	PREDICTED: acetolactate synthase small subunit 2, chloroplastic-like [Populus euphratica]	35.74	23.13
map01230	Biosynthesis of amino acids	7.14E-03	TRINITY_DN21635_c1_g2	-2.07	down	SEC14 cytosolic factor family protein [Populus trichocarpa]	5.54	32.41
map01230	Biosynthesis of amino acids	7.14E-03	TRINITY_DN6390_c0_g1	3.46	up	nodulin MtN21 family protein [Populus trichocarpa]	1.91	0.26
map03030	DNA replication	1.12E-02	TRINITY_DN21835_c0_g1	-1.94	down	PREDICTED: chromosome transmission fidelity protein 18 homolog [Populus euphratica]	1.43	8.53
map03030	DNA replication	1.12E-02	TRINITY_DN22093_c1_g1	1.58	up	PREDICTED: uncharacterized protein LOC105133503 [Populus euphratica]	28.08	18.04
map03030	DNA replication	1.12E-02	TRINITY_DN22222_c2_g1	-1.69	down	PREDICTED: proliferating cell nuclear antigen [Populus euphratica]	20.04	106.79
map03030	DNA replication	1.12E-02	TRINITY_DN22828_c0_g3	-1.62	down	PREDICTED: DNA replication licensing factor MCM3 [Populus euphratica]	3.67	17.36
map03030	DNA replication	1.12E-02	TRINITY_DN23288_c0_g1	-1.63	down	hypothetical protein POPTR_0001s12700g [Populus trichocarpa]	3.61	17.41
map03030	DNA replication	1.12E-02	TRINITY_DN23841_c0_g3	-1.40	down	PREDICTED: replication protein A 32 kDa subunit A-like [Populus euphratica]	7.83	32.50
map03030	DNA replication	1.12E-02	TRINITY_DN23876_c0_g1	-2.14	down	hypothetical protein POPTR_0007s10880g [Populus trichocarpa]	0.94	6.43
map03030	DNA replication	1.12E-02	TRINITY_DN23876_c0_g2	-1.91	down	hypothetical protein POPTR_0007s10880g [Populus trichocarpa]	1.84	10.67
map03030	DNA replication	1.12E-02	TRINITY_DN24395_c0_g2	1.21	up	hypothetical protein POPTR_0005s26230g [Populus trichocarpa]	66.57	46.27
map03030	DNA replication	1.12E-02	TRINITY_DN24951_c0_g1	2.63	up	unknown [Populus trichocarpa]	40.05	12.23
map03030	DNA replication	1.12E-02	TRINITY_DN25007_c0_g1	-1.55	down	TIR-NBS-LRR-TIR type disease resistance protein, partial [Populus trichocarpa]	12.22	41.68
map03030	DNA replication	1.12E-02	TRINITY_DN25276_c0_g2	-1.48	down	hypothetical protein POPTR_0018s00860g [Populus trichocarpa]	1.70	6.52
map03030	DNA replication	1.12E-02	TRINITY_DN26865_c0_g1	-2.09	down	minichromosome maintenance family protein [Populus trichocarpa]	5.82	40.93
map03030	DNA replication	1.12E-02	TRINITY_DN27349_c0_g1	1.26	up	hypothetical protein POPTR_0001s44720g [Populus trichocarpa]	31.99	22.49

map03030	DNA replication	1.12E-02	TRINITY_DN12114_c0_g2	-1.60	down	hypothetical protein POPTR_0012s01875g [Populus trichocarpa]	0.58	2.70
map03030	DNA replication	1.12E-02	TRINITY_DN13077_c0_g1	-3.90	down	unknown [Populus trichocarpa]	0.30	5.95
map03030	DNA replication	1.12E-02	TRINITY_DN14981_c0_g1	1.20	up	single-strand-binding family protein [Populus trichocarpa]	44.95	29.84
map03030	DNA replication	1.12E-02	TRINITY_DN16624_c0_g1	1.03	up	unknown [Populus trichocarpa x Populus deltoides]	184.06	137.19
map03030	DNA replication	1.12E-02	TRINITY_DN16801_c0_g1	-1.52	down	PREDICTED: probable DNA helicase MCM9 [Populus euphratica]	1.33	5.67
map03030	DNA replication	1.12E-02	TRINITY_DN16976_c0_g4	-1.35	down	DNA-directed DNA polymerase epsilon catalytic subunit family protein [Populus trichocarpa]	2.96	11.76
map03030	DNA replication	1.12E-02	TRINITY_DN16976_c0_g5	-1.67	down	DNA-directed DNA polymerase epsilon catalytic subunit family protein [Populus trichocarpa]	0.84	4.16
map03030	DNA replication	1.12E-02	TRINITY_DN17121_c0_g1	-1.12	down	hypothetical protein POPTR_0005s27010g, partial [Populus trichocarpa]	1.89	7.13
map03030	DNA replication	1.12E-02	TRINITY_DN17241_c0_g1	-1.38	down	DNA polymerase delta subunit 4 family protein [Populus trichocarpa]	4.03	15.90
map03030	DNA replication	1.12E-02	TRINITY_DN17241_c0_g2	-1.21	down	DNA polymerase delta subunit 4 family protein [Populus trichocarpa]	37.87	132.98
map03030	DNA replication	1.12E-02	TRINITY_DN17522_c0_g1	-2.36	down	PREDICTED: replication protein A 70 kDa DNA-binding subunit B [Populus euphratica]	3.63	29.40
map03030	DNA replication	1.12E-02	TRINITY_DN17620_c0_g1	1.37	up	hypothetical protein POPTR_0006s14620g, partial [Populus trichocarpa]	1053.56	627.95
map03030	DNA replication	1.12E-02	TRINITY_DN17926_c0_g2	-2.37	down	PREDICTED: probable DNA helicase MCM8 isoform X1 [Populus euphratica]	0.44	3.48
map03030	DNA replication	1.12E-02	TRINITY_DN18457_c0_g1	-1.89	down	hypothetical protein POPTR_0001s12380g [Populus trichocarpa]	3.01	18.30
map03030	DNA replication	1.12E-02	TRINITY_DN18555_c0_g4	2.33	up	Ycf2 [Populus alba]	3.15	0.93
map03030	DNA replication	1.12E-02	TRINITY_DN18608_c2_g8	-2.15	down	PROLIFERA family protein [Populus trichocarpa]	4.36	29.93
map03030	DNA replication	1.12E-02	TRINITY_DN18970_c0_g1	-1.57	down	hypothetical protein POPTR_0008s15120g [Populus trichocarpa]	4.62	19.39
map03030	DNA replication	1.12E-02	TRINITY_DN19143_c0_g1	-1.71	down	PREDICTED: DNA polymerase alpha subunit B-like [Populus euphratica]	6.40	29.61
map03030	DNA replication	1.12E-02	TRINITY_DN19143_c0_g6	-2.26	down	PREDICTED: uncharacterized protein LOC105129502 [Populus euphratica]	0.25	1.91
map03030	DNA replication	1.12E-02	TRINITY_DN19198_c0_g1	1.14	up	hypothetical protein POPTR_0002s24970g [Populus trichocarpa]	135.65	95.80
map03030	DNA replication	1.12E-02	TRINITY_DN19259_c0_g1	-1.63	down	PREDICTED: DNA polymerase epsilon subunit 2 [Populus euphratica]	1.72	8.35
map03030	DNA replication	1.12E-02	TRINITY_DN19395_c0_g1	-1.33	down	PREDICTED: replication protein A 70 kDa DNA-binding subunit E-like [Populus euphratica]	2.44	9.54
map03030	DNA replication	1.12E-02	TRINITY_DN19537_c0_g3	-1.06	down	PREDICTED: uncharacterized protein LOC105109677 isoform X1 [Populus euphratica]	5.42	16.71
map03030	DNA replication	1.12E-02	TRINITY_DN19608_c0_g1	-1.86	down	hypothetical protein POPTR_0003s10380g [Populus trichocarpa]	3.11	17.37
map03030	DNA replication	1.12E-02	TRINITY_DN20164_c0_g3	-1.54	down	sterile alpha motif domain-containing family protein [Populus trichocarpa]	3.50	14.58
map03030	DNA replication	1.12E-02	TRINITY_DN20166_c0_g1	-1.73	down	PREDICTED: DNA replication licensing factor MCM4 [Populus euphratica]	5.65	29.26
map03030	DNA replication	1.12E-02	TRINITY_DN20672_c0_g1	1.85	up	hypothetical protein POPTR_0006s03190g [Populus trichocarpa]	49.08	22.60
map03030	DNA replication	1.12E-02	TRINITY_DN20692_c0_g1	-1.11	down	PREDICTED: probable DNA primase large subunit [Populus euphratica]	8.23	30.45
map03030	DNA replication	1.12E-02	TRINITY_DN20742_c0_g1	1.62	up	FKBP-type peptidyl-prolyl cis-trans isomerase 3 family protein [Populus trichocarpa]	166.22	81.22
map03030	DNA replication	1.12E-02	TRINITY_DN20973_c0_g1	-1.20	down	PREDICTED: DNA polymerase delta catalytic subunit [Populus euphratica]	3.70	13.41
map01200	Carbon metabolism	2.25E-02	TRINITY_DN21747_c0_g1	3.00	up	hypothetical protein POPTR_0015s12250g [Populus trichocarpa]	29.79	5.40
map01200	Carbon metabolism	2.25E-02	TRINITY_DN21760_c0_g1	1.25	up	isocitrate dehydrogenase family protein [Populus trichocarpa]	53.95	39.69
map01200	Carbon metabolism	2.25E-02	TRINITY_DN21847_c0_g2	1.55	up	PREDICTED: 6-phosphogluconate dehydrogenase, decarboxylating 2, chloroplastic [Populus euphratica]	56.92	29.48
map01200	Carbon metabolism	2.25E-02	TRINITY_DN21849_c0_g1	1.18	up	mitochondrial glycine decarboxylase complex P-protein [Populus tremuloides]	172.09	109.30
map01200	Carbon metabolism	2.25E-02	TRINITY_DN21889_c0_g2	3.34	up	PREDICTED: ribulose bisphosphate carboxylase small chain, chloroplastic-like [Populus euphratica]	9592.31	1398.04
map01200	Carbon metabolism	2.25E-02	TRINITY_DN21889_c0_g3	2.27	up	017G114600 [Populus tomentosa]	20448.45	6363.37
map01200	Carbon metabolism	2.25E-02	TRINITY_DN22033_c0_g1	1.60	up	Chain D family protein [Populus trichocarpa]	364.35	179.65
map01200	Carbon metabolism	2.25E-02	TRINITY_DN22048_c2_g2	1.93	up	hypothetical protein POPTR_0015s12380g [Populus trichocarpa]	3.05	1.20
map01200	Carbon metabolism	2.25E-02	TRINITY_DN22048_c2_g5	-1.67	down	hypothetical protein POPTR_0012s11610g [Populus trichocarpa]	1.63	8.07
map01200	Carbon metabolism	2.25E-02	TRINITY_DN22186_c0_g1	1.05	up	alpha-hydroxynitrile lyase family protein [Populus trichocarpa]	31.37	22.10
map01200	Carbon metabolism	2.25E-02	TRINITY_DN22217_c0_g1	1.29	up	hypothetical protein POPTR_0013s10050g [Populus trichocarpa]	54.43	34.14
map01200	Carbon metabolism	2.25E-02	TRINITY_DN22233_c0_g1	1.51	up	hypothetical protein POPTR_0002s10420g [Populus trichocarpa]	84.73	47.59

map01200	Carbon metabolism	2.25E-02	TRINITY_DN22245_c0_g1	2.23	up	PREDICTED: triosephosphate isomerase, chloroplastic [Populus euphratica]	838.73	263.34
map01200	Carbon metabolism	2.25E-02	TRINITY_DN22283_c0_g1	1.79	up	hypothetical protein POPTR_0002s09390g [Populus trichocarpa]	139.18	61.68
map01200	Carbon metabolism	2.25E-02	TRINITY_DN22348_c0_g1	2.11	up	O-acetylserine (thiol)lyase family protein [Populus trichocarpa]	18.41	6.25
map01200	Carbon metabolism	2.25E-02	TRINITY_DN22348_c0_g4	1.61	up	O-acetylserine (thiol)lyase family protein [Populus trichocarpa]	220.16	110.90
map01200	Carbon metabolism	2.25E-02	TRINITY_DN22348_c0_g6	1.74	up	hypothetical protein POPTR_0013s13150g [Populus trichocarpa]	146.50	66.04
map01200	Carbon metabolism	2.25E-02	TRINITY_DN22512_c0_g1	1.04	up	hypothetical protein POPTR_0018s02570g [Populus trichocarpa]	23.51	17.76
map01200	Carbon metabolism	2.25E-02	TRINITY_DN22563_c0_g1	1.23	up	2-oxoacid dehydrogenase family protein [Populus trichocarpa]	62.68	37.12
map01200	Carbon metabolism	2.25E-02	TRINITY_DN22587_c0_g1	1.20	up	PREDICTED: fructose-bisphosphate aldolase cytoplasmic isozyme [Populus euphratica]	371.77	247.12
map01200	Carbon metabolism	2.25E-02	TRINITY_DN22598_c1_g1	1.42	up	2 family protein [Populus trichocarpa]	136.33	77.98
map01200	Carbon metabolism	2.25E-02	TRINITY_DN22663_c0_g2	1.38	up	hypothetical protein POPTR_0010s05530g [Populus trichocarpa]	360.33	205.54
map01200	Carbon metabolism	2.25E-02	TRINITY_DN22697_c0_g3	1.19	up	3-PHOSPHOSERINE PHOSPHATASE family protein [Populus trichocarpa]	51.79	35.35
map01200	Carbon metabolism	2.25E-02	TRINITY_DN22755_c0_g2	1.78	up	unknown [Populus trichocarpa]	261.55	115.26
map01200	Carbon metabolism	2.25E-02	TRINITY_DN22812_c0_g2	1.66	up	Serine hydroxymethyltransferase family protein [Populus trichocarpa]	785.78	366.57
map01200	Carbon metabolism	2.25E-02	TRINITY_DN22812_c0_g3	1.77	up	mitochondrial serine hydroxymethyltransferase [Populus tremuloides]	59.98	26.65
map01200	Carbon metabolism	2.25E-02	TRINITY_DN22813_c0_g4	1.08	up	aspartate transaminase family protein [Populus trichocarpa]	25.11	18.98
map01200	Carbon metabolism	2.25E-02	TRINITY_DN22920_c1_g6	2.63	up	PREDICTED: probable ribose-5-phosphate isomerase 3, chloroplastic [Populus euphratica]	555.60	140.50
map01200	Carbon metabolism	2.25E-02	TRINITY_DN22960_c1_g2	1.71	up	hypothetical protein POPTR_0013s13150g [Populus trichocarpa]	267.00	123.84
map01200	Carbon metabolism	2.25E-02	TRINITY_DN23066_c0_g5	1.83	up	PREDICTED: probable 2-carboxy-D-arabinitol-1-phosphatase [Populus euphratica]	71.98	31.71
map01200	Carbon metabolism	2.25E-02	TRINITY_DN23137_c0_g2	1.94	up	hypothetical protein POPTR_0010s18770g [Populus trichocarpa]	270.10	104.80
map01200	Carbon metabolism	2.25E-02	TRINITY_DN23137_c0_g4	1.91	up	hypothetical protein POPTR_0010s18770g [Populus trichocarpa]	257.32	102.92
map01200	Carbon metabolism	2.25E-02	TRINITY_DN23168_c0_g1	1.33	up	Fumarate hydratase 1 family protein [Populus trichocarpa]	44.45	26.95
map01200	Carbon metabolism	2.25E-02	TRINITY_DN23182_c1_g2	2.86	up	hypothetical protein POPTR_0005s10340g [Populus trichocarpa]	182.43	38.37
map01200	Carbon metabolism	2.25E-02	TRINITY_DN23213_c0_g1	1.52	up	pyruvate dehydrogenase family protein [Populus trichocarpa]	125.88	67.45
map01200	Carbon metabolism	2.25E-02	TRINITY_DN23271_c0_g1	1.57	up	hypothetical protein POPTR_0001s35080g [Populus trichocarpa]	37.34	19.20
map01200	Carbon metabolism	2.25E-02	TRINITY_DN23278_c1_g1	1.44	up	PREDICTED: glutamate--glyoxylate aminotransferase 2 isoform X1 [Populus euphratica]	580.61	318.60
map01200	Carbon metabolism	2.25E-02	TRINITY_DN23322_c1_g1	1.07	up	PREDICTED: catalase isozyme 1-like [Populus euphratica]	422.64	304.47
map01200	Carbon metabolism	2.25E-02	TRINITY_DN23349_c0_g1	2.04	up	Cysteine synthase C1 [Theobroma cacao]	171.68	63.08
map01200	Carbon metabolism	2.25E-02	TRINITY_DN23403_c0_g1	1.10	up	hypothetical protein POPTR_0005s23960g [Populus trichocarpa]	80.55	57.95
map01200	Carbon metabolism	2.25E-02	TRINITY_DN23497_c0_g1	2.24	up	D-3-phosphoglycerate dehydrogenase family protein [Populus trichocarpa]	103.49	34.56
map01200	Carbon metabolism	2.25E-02	TRINITY_DN23653_c0_g1	1.10	up	hypothetical protein POPTR_0018s09500g [Populus trichocarpa]	54.02	38.03
map01200	Carbon metabolism	2.25E-02	TRINITY_DN23701_c0_g1	1.96	up	latex plastidic aldolase-like family protein [Populus trichocarpa]	2289.47	879.27
map01200	Carbon metabolism	2.25E-02	TRINITY_DN23701_c0_g5	2.57	up	plastidic aldolase family protein [Populus trichocarpa]	7.53	2.00
map01200	Carbon metabolism	2.25E-02	TRINITY_DN23720_c0_g1	1.57	up	PREDICTED: malate dehydrogenase [NADP], chloroplastic [Populus euphratica]	252.27	131.23
map01200	Carbon metabolism	2.25E-02	TRINITY_DN23758_c0_g1	2.77	up	hypothetical protein POPTR_0005s27550g [Populus trichocarpa]	826.64	183.48
map01200	Carbon metabolism	2.25E-02	TRINITY_DN23795_c1_g1	1.13	up	PREDICTED: glyoxylate/succinic semialdehyde reductase 2, chloroplastic-like [Populus euphratica]	219.09	158.29
map01200	Carbon metabolism	2.25E-02	TRINITY_DN23965_c0_g1	1.23	up	PREDICTED: triosephosphate isomerase, cytosolic [Populus euphratica]	641.46	426.80
map01200	Carbon metabolism	2.25E-02	TRINITY_DN24041_c0_g1	2.06	up	PREDICTED: D-glycerate 3-kinase, chloroplastic-like [Populus euphratica]	150.03	53.16
map01200	Carbon metabolism	2.25E-02	TRINITY_DN24242_c0_g1	-1.34	down	hypothetical protein POPTR_0003s06730g [Populus trichocarpa]	3.03	11.55
map01200	Carbon metabolism	2.25E-02	TRINITY_DN24249_c0_g1	1.66	up	PREDICTED: ribulose bisphosphate carboxylase large chain [Populus euphratica]	86.19	43.64
map01200	Carbon metabolism	2.25E-02	TRINITY_DN24279_c0_g1	1.93	up	unknown [Populus trichocarpa x Populus deltoides]	85.38	34.72
map01200	Carbon metabolism	2.25E-02	TRINITY_DN24285_c0_g5	1.46	up	PREDICTED: WAT1-related protein At2g37460-like isoform X1 [Populus euphratica]	2.63	1.46
map01200	Carbon metabolism	2.25E-02	TRINITY_DN24307_c0_g1	1.05	up	PREDICTED: biotin carboxyl carrier protein of acetyl-CoA carboxylase, chloroplastic-like [Populus euphratica]	73.06	52.43

map01200	Carbon metabolism	2.25E-02	TRINITY_DN24393_c0_g1	1.31	up	hypothetical protein POPTR_0001s24710g [Populus trichocarpa]	198.18	106.97
map01200	Carbon metabolism	2.25E-02	TRINITY_DN24565_c0_g1	1.99	up	T-protein of the glycine decarboxylase complex [Populus trichocarpa]	481.27	183.79
map01200	Carbon metabolism	2.25E-02	TRINITY_DN24623_c0_g2	1.54	up	hypothetical protein POPTR_0008s11770g [Populus trichocarpa]	42.50	22.04
map01200	Carbon metabolism	2.25E-02	TRINITY_DN24707_c1_g1	-1.25	down	nodulin MtN21 family protein [Populus trichocarpa]	14.31	50.91
map01200	Carbon metabolism	2.25E-02	TRINITY_DN24709_c0_g1	1.03	up	PREDICTED: pyruvate dehydrogenase E1 component subunit beta-3, chloroplastic-like [Populus euphratica]	176.40	133.88
map01200	Carbon metabolism	2.25E-02	TRINITY_DN24881_c0_g1	1.99	up	PREDICTED: transketolase, chloroplastic [Populus euphratica]	1790.82	677.46
map01200	Carbon metabolism	2.25E-02	TRINITY_DN24894_c0_g3	1.50	up	hypothetical protein POPTR_0015s03960g [Populus trichocarpa]	34.92	19.04
map01200	Carbon metabolism	2.25E-02	TRINITY_DN24969_c2_g1	1.05	up	Succinyl-CoA ligase beta-chain family protein [Populus trichocarpa]	98.12	74.47
map01200	Carbon metabolism	2.25E-02	TRINITY_DN24990_c0_g2	3.40	up	alanine aminotransferase family protein [Populus simonii x Populus nigra]	51.44	7.50
map01200	Carbon metabolism	2.25E-02	TRINITY_DN25057_c0_g2	-1.00	down	PREDICTED: GATA transcription factor 1-like [Populus euphratica]	3.25	9.78
map01200	Carbon metabolism	2.25E-02	TRINITY_DN25129_c1_g2	1.01	up	3-phosphoglycerate kinase [Populus tremuloides]	153.72	116.35
map01200	Carbon metabolism	2.25E-02	TRINITY_DN25141_c0_g1	1.90	up	PREDICTED: dihydrolipoyllysine-residue acetyltransferase component 4 of pyruvate dehydrogenase complex, chloroplastic [Populus euphratica]	116.62	48.47
map01200	Carbon metabolism	2.25E-02	TRINITY_DN25192_c0_g1	2.93	up	hypothetical protein POPTR_0010s02860g [Populus trichocarpa]	24.30	5.86
map01200	Carbon metabolism	2.25E-02	TRINITY_DN25221_c0_g1	1.58	up	malate dehydrogenase family protein, partial [Populus trichocarpa]	57.58	29.35
map01200	Carbon metabolism	2.25E-02	TRINITY_DN25221_c1_g2	2.25	up	malate dehydrogenase family protein [Populus trichocarpa]	356.58	110.81
map01200	Carbon metabolism	2.25E-02	TRINITY_DN25221_c1_g3	1.80	up	PREDICTED: malate dehydrogenase, glyoxysomal [Populus euphratica]	643.81	285.37
map01200	Carbon metabolism	2.25E-02	TRINITY_DN25221_c1_g4	2.08	up	PREDICTED: LOW QUALITY PROTEIN: dihydrolipoyllysine-residue acetyltransferase component 5 of pyruvate dehydrogenase complex, chloroplastic-like, partial [Populus euphratica]	9.32	3.34
map01200	Carbon metabolism	2.25E-02	TRINITY_DN25377_c0_g1	1.91	up	hypothetical protein POPTR_0013s11870g [Populus trichocarpa]	85.23	34.17
map01200	Carbon metabolism	2.25E-02	TRINITY_DN25439_c0_g1	1.11	up	biotin carboxylase precursor family protein [Populus trichocarpa]	85.63	60.20
map01200	Carbon metabolism	2.25E-02	TRINITY_DN25476_c0_g2	2.59	up	PREDICTED: alanine aminotransferase 2, mitochondrial-like [Populus euphratica]	33.99	8.74
map01200	Carbon metabolism	2.25E-02	TRINITY_DN25476_c0_g4	2.24	up	PREDICTED: alanine aminotransferase 2-like [Populus euphratica]	21.79	7.01
map01200	Carbon metabolism	2.25E-02	TRINITY_DN25692_c0_g1	-1.21	down	AAA-type ATPase family protein [Populus trichocarpa]	9.04	31.77
map01200	Carbon metabolism	2.25E-02	TRINITY_DN25692_c0_g2	-1.31	down	PREDICTED: pachytene checkpoint protein 2 homolog [Populus euphratica]	12.27	47.09
map01200	Carbon metabolism	2.25E-02	TRINITY_DN25746_c0_g1	2.80	up	nodule-enhanced malate dehydrogenase family protein [Populus trichocarpa]	113.74	23.22
map01200	Carbon metabolism	2.25E-02	TRINITY_DN25819_c0_g1	1.14	up	PREDICTED: NADP-dependent glyceraldehyde-3-phosphate dehydrogenase-like isoform X1 [Populus euphratica]	282.53	188.30
map01200	Carbon metabolism	2.25E-02	TRINITY_DN26030_c0_g1	2.06	up	unknown [Populus trichocarpa x Populus deltoides]	3041.90	1125.79
map01200	Carbon metabolism	2.25E-02	TRINITY_DN26131_c0_g2	1.50	up	6-phosphogluconate dehydrogenase family protein [Populus trichocarpa]	63.33	33.04
map01200	Carbon metabolism	2.25E-02	TRINITY_DN26154_c0_g2	-1.12	down	PREDICTED: protein SCAR2-like isoform X1 [Populus euphratica]	5.40	17.64
map01200	Carbon metabolism	2.25E-02	TRINITY_DN26163_c0_g1	1.24	up	PREDICTED: dihydrolipoyllysine-residue acetyltransferase component 2 of pyruvate dehydrogenase complex, mitochondrial-like [Populus euphratica]	49.33	31.65
map01200	Carbon metabolism	2.25E-02	TRINITY_DN26175_c0_g1	1.33	up	PREDICTED: hexokinase-1-like [Populus euphratica]	24.35	14.67
map01200	Carbon metabolism	2.25E-02	TRINITY_DN26209_c0_g1	1.84	up	hypothetical protein SETIT_022310mg [Setaria italica]	186.50	89.03
map01200	Carbon metabolism	2.25E-02	TRINITY_DN26341_c0_g2	1.21	up	dihydrolipoamide S-acetyltransferase family protein [Populus trichocarpa]	11.43	7.48
map01200	Carbon metabolism	2.25E-02	TRINITY_DN26437_c0_g2	1.65	up	hypothetical protein POPTR_0014s16480g [Populus trichocarpa]	43.75	20.82
map01200	Carbon metabolism	2.25E-02	TRINITY_DN26497_c0_g2	2.05	up	hypothetical protein POPTR_0011s07381g [Populus trichocarpa]	1.66	0.60
map01200	Carbon metabolism	2.25E-02	TRINITY_DN26528_c0_g1	1.13	up	PREDICTED: D-3-phosphoglycerate dehydrogenase 3, chloroplastic-like [Populus euphratica]	74.65	55.30
map01200	Carbon metabolism	2.25E-02	TRINITY_DN26572_c0_g1	1.40	up	hypothetical protein POPTR_0002s20530g [Populus trichocarpa]	430.34	248.97
map01200	Carbon metabolism	2.25E-02	TRINITY_DN26590_c0_g1	1.01	up	PREDICTED: acyl-CoA dehydrogenase family member 10-like isoform X2 [Populus euphratica]	17.06	13.06
map01200	Carbon metabolism	2.25E-02	TRINITY_DN26627_c0_g1	2.37	up	PHOSPHOGLYCERATE KINASE 1 family protein [Populus trichocarpa]	946.82	271.09
map01200	Carbon metabolism	2.25E-02	TRINITY_DN26661_c0_g1	2.62	up	hypothetical protein CISIN_1g016748mg [Citrus sinensis]	718.86	176.98
map01200	Carbon metabolism	2.25E-02	TRINITY_DN26711_c0_g1	1.68	up	Sedoheptulose-1 family protein [Populus trichocarpa]	85.33	41.36

map01200	Carbon metabolism	2.25E-02	TRINITY_DN26711_c0_g2	1.91	up	Sedoheptulose-1 family protein [Populus trichocarpa]	1906.74	735.61
map01200	Carbon metabolism	2.25E-02	TRINITY_DN26837_c0_g1	2.09	up	hypothetical protein POPTR_0010s15200g [Populus trichocarpa]	81.17	29.93
map01200	Carbon metabolism	2.25E-02	TRINITY_DN26922_c0_g2	1.76	up	Phosphoribulokinase family protein [Populus trichocarpa]	951.86	414.20
map01200	Carbon metabolism	2.25E-02	TRINITY_DN27009_c0_g1	1.60	up	hypothetical protein POPTR_0002s22720g [Populus trichocarpa]	10.34	5.23
map01200	Carbon metabolism	2.25E-02	TRINITY_DN27171_c2_g1	1.79	up	PREDICTED: D-glycerate 3-kinase, chloroplastic-like [Populus euphratica]	158.02	76.60
map01200	Carbon metabolism	2.25E-02	TRINITY_DN27198_c1_g1	1.09	up	aconitate hydratase family protein [Populus trichocarpa]	47.60	32.18
map01200	Carbon metabolism	2.25E-02	TRINITY_DN27198_c1_g2	1.02	up	aconitate hydratase family protein [Populus trichocarpa]	16.99	12.62
map01200	Carbon metabolism	2.25E-02	TRINITY_DN27395_c0_g1	1.70	up	aminotransferase 2 family protein [Populus trichocarpa]	980.92	451.86
map01200	Carbon metabolism	2.25E-02	TRINITY_DN27448_c0_g1	1.92	up	glycolate oxidase family protein [Populus trichocarpa]	1633.96	640.54
map01200	Carbon metabolism	2.25E-02	TRINITY_DN27448_c0_g2	1.73	up	PREDICTED: peroxisomal (S)-2-hydroxy-acid oxidase [Populus euphratica]	519.75	233.20
map01200	Carbon metabolism	2.25E-02	TRINITY_DN27458_c1_g1	2.44	up	PREDICTED: transketolase, chloroplastic [Populus euphratica]	1184.20	313.96
map01200	Carbon metabolism	2.25E-02	TRINITY_DN27574_c0_g1	1.31	up	glycine decarboxylase P-protein 1 [Arabidopsis thaliana]	428.07	261.89
map01200	Carbon metabolism	2.25E-02	TRINITY_DN27621_c0_g4	1.61	up	6-phosphogluconate dehydrogenase family protein [Populus trichocarpa]	11.51	5.70
map01200	Carbon metabolism	2.25E-02	TRINITY_DN27847_c0_g1	1.64	up	hypothetical protein POPTR_0008s11610g [Populus trichocarpa]	34.59	16.78
map01200	Carbon metabolism	2.25E-02	TRINITY_DN27862_c2_g1	-1.69	down	PREDICTED: structural maintenance of chromosomes protein 2-1-like [Populus euphratica]	21.02	56.92
map01200	Carbon metabolism	2.25E-02	TRINITY_DN11372_c0_g1	3.83	up	hypothetical protein POPTR_0011s15150g [Populus trichocarpa]	4.92	0.36
map01200	Carbon metabolism	2.25E-02	TRINITY_DN12955_c0_g1	1.82	up	hypothetical protein POPTR_0011s05190g [Populus trichocarpa]	15.50	6.27
map01200	Carbon metabolism	2.25E-02	TRINITY_DN13230_c0_g2	-1.83	down	PREDICTED: peroxisomal (S)-2-hydroxy-acid oxidase-like isoform X1 [Populus euphratica]	0.42	2.27
map01200	Carbon metabolism	2.25E-02	TRINITY_DN13782_c0_g1	5.00	up	hypothetical protein POPTR_0002s11010g [Populus trichocarpa]	6.55	0.32
map01200	Carbon metabolism	2.25E-02	TRINITY_DN13808_c0_g3	-1.99	down	hypothetical protein POPTR_0001s04370g [Populus trichocarpa]	0.58	3.70
map01200	Carbon metabolism	2.25E-02	TRINITY_DN14558_c0_g1	-2.01	down	PREDICTED: uncharacterized protein LOC109021545 [Juglans regia]	0.85	4.88
map01200	Carbon metabolism	2.25E-02	TRINITY_DN15573_c0_g1	-2.43	down	alcohol dehydrogenase family protein [Populus trichocarpa]	0.77	3.49
map01200	Carbon metabolism	2.25E-02	TRINITY_DN15775_c0_g1	1.57	up	hypothetical protein POPTR_0001s46060g [Populus trichocarpa]	7.49	3.44
map01200	Carbon metabolism	2.25E-02	TRINITY_DN15882_c0_g1	2.67	up	hypothetical protein POPTR_0018s05150g [Populus trichocarpa]	16.28	3.88
map01200	Carbon metabolism	2.25E-02	TRINITY_DN16471_c0_g1	1.72	up	PREDICTED: peroxisomal (S)-2-hydroxy-acid oxidase-like [Phoenix dactylifera]	1059.17	482.14
map01200	Carbon metabolism	2.25E-02	TRINITY_DN16677_c0_g2	-8.29	down	hypothetical protein POPTR_0007s15050g [Populus trichocarpa]	0.00	5.66
map01200	Carbon metabolism	2.25E-02	TRINITY_DN16732_c0_g1	1.28	up	hypothetical protein POPTR_0017s07130g [Populus trichocarpa]	32.81	20.70
map01200	Carbon metabolism	2.25E-02	TRINITY_DN16817_c0_g1	1.64	up	PREDICTED: tRNA (guanine-N(7)-)-methyltransferase [Populus euphratica]	40.30	24.24
map01200	Carbon metabolism	2.25E-02	TRINITY_DN16821_c0_g1	1.91	up	PREDICTED: malate dehydrogenase, glyoxysomal [Populus euphratica]	248.44	104.05
map01200	Carbon metabolism	2.25E-02	TRINITY_DN17020_c0_g1	2.36	up	hypothetical protein POPTR_0002s06890g [Populus trichocarpa]	22.89	6.58
map01200	Carbon metabolism	2.25E-02	TRINITY_DN17229_c0_g1	-1.51	down	hypothetical protein POPTR_0014s03490g [Populus trichocarpa]	1.22	5.46
map01200	Carbon metabolism	2.25E-02	TRINITY_DN17246_c0_g1	1.06	up	hypothetical protein POPTR_0017s07040g [Populus trichocarpa]	24.42	17.62
map01200	Carbon metabolism	2.25E-02	TRINITY_DN17464_c0_g2	1.19	up	hydrolase family protein [Populus trichocarpa]	68.37	46.14
map01200	Carbon metabolism	2.25E-02	TRINITY_DN17528_c0_g1	2.19	up	ribose-phosphate pyrophosphokinase family protein [Populus trichocarpa]	63.69	21.30
map01200	Carbon metabolism	2.25E-02	TRINITY_DN17663_c0_g3	1.15	up	unknown [Populus trichocarpa x Populus deltoides]	373.94	255.99
map01200	Carbon metabolism	2.25E-02	TRINITY_DN17865_c0_g1	2.27	up	plastid serine hydroxymethyltransferase [Populus tremuloides]	53.46	17.12
map01200	Carbon metabolism	2.25E-02	TRINITY_DN17930_c0_g1	2.42	up	unknown [Populus trichocarpa x Populus deltoides]	1011.61	280.35
map01200	Carbon metabolism	2.25E-02	TRINITY_DN17959_c0_g1	2.77	up	PREDICTED: probable S-sulfocysteine synthase, chloroplastic [Populus euphratica]	54.54	12.96
map01200	Carbon metabolism	2.25E-02	TRINITY_DN18156_c0_g1	1.29	up	PREDICTED: glyoxylate/hydroxypyruvate reductase A HPR2-like [Populus euphratica]	133.92	82.89
map01200	Carbon metabolism	2.25E-02	TRINITY_DN18209_c1_g1	1.05	up	PREDICTED: pyruvate kinase, cytosolic isozyme-like [Populus euphratica]	19.18	14.26
map01200	Carbon metabolism	2.25E-02	TRINITY_DN18225_c0_g3	1.29	up	PREDICTED: hexokinase-1-like [Populus euphratica]	38.97	24.40
map01200	Carbon metabolism	2.25E-02	TRINITY_DN18237_c0_g2	1.23	up	PREDICTED: glucose-6-phosphate 1-dehydrogenase 4, chloroplastic [Populus euphratica]	14.47	10.41
map01200	Carbon metabolism	2.25E-02	TRINITY_DN18501_c1_g1	2.31	up	PREDICTED: phosphoserine aminotransferase 2, chloroplastic-like [Populus euphratica]	32.17	9.92
map01200	Carbon metabolism	2.25E-02	TRINITY_DN18598_c0_g1	1.82	up	hypothetical protein POPTR_0008s00350g [Populus trichocarpa]	468.84	197.84

map01200	Carbon metabolism	2.25E-02	TRINITY_DN18859_c0_g3	1.18	up	aconitate hydratase family protein [Populus trichocarpa]	80.30	54.25
map01200	Carbon metabolism	2.25E-02	TRINITY_DN19014_c0_g2	1.24	up	hypothetical protein POPTR_0012s03150g [Populus trichocarpa]	33.06	21.31
map01200	Carbon metabolism	2.25E-02	TRINITY_DN19050_c1_g1	1.32	up	hypothetical protein POPTR_0019s08170g, partial [Populus trichocarpa]	123.18	74.70
map01200	Carbon metabolism	2.25E-02	TRINITY_DN19294_c0_g3	1.09	up	hypothetical protein POPTR_0006s16050g [Populus trichocarpa]	91.23	67.60
map01200	Carbon metabolism	2.25E-02	TRINITY_DN19734_c0_g2	1.39	up	PREDICTED: ribulose-phosphate 3-epimerase, chloroplastic [Populus euphratica]	648.43	374.61
map01200	Carbon metabolism	2.25E-02	TRINITY_DN19743_c0_g1	1.38	up	PREDICTED: WAT1-related protein At4g19185-like [Populus euphratica]	66.15	39.51
map01200	Carbon metabolism	2.25E-02	TRINITY_DN19763_c0_g3	1.88	up	PREDICTED: malate dehydrogenase, mitochondrial [Populus euphratica]	247.09	106.19
map01200	Carbon metabolism	2.25E-02	TRINITY_DN19823_c0_g5	1.20	up	hypothetical protein POPTR_0005s09370g [Populus trichocarpa]	70.43	46.85
map01200	Carbon metabolism	2.25E-02	TRINITY_DN19848_c0_g1	1.56	up	phosphopyruvate hydratase family protein [Populus trichocarpa]	247.16	128.86
map01200	Carbon metabolism	2.25E-02	TRINITY_DN20148_c0_g1	1.45	up	hypothetical protein POPTR_0010s12560g [Populus trichocarpa]	131.18	75.15
map01200	Carbon metabolism	2.25E-02	TRINITY_DN20148_c0_g2	3.15	up	ribose 5-phosphate isomerase family protein [Populus trichocarpa]	6.05	1.07
map01200	Carbon metabolism	2.25E-02	TRINITY_DN20175_c0_g1	1.59	up	peroxiredoxin Q family protein [Populus trichocarpa]	1020.77	504.37
map01200	Carbon metabolism	2.25E-02	TRINITY_DN20227_c0_g1	-1.45	down	PREDICTED: malate synthase, glyoxysomal [Populus euphratica]	2.85	13.95
map01200	Carbon metabolism	2.25E-02	TRINITY_DN20590_c0_g2	1.44	up	METHIONINE AMINOPEPTIDASE 1D family protein [Populus trichocarpa]	79.40	44.51
map01200	Carbon metabolism	2.25E-02	TRINITY_DN20938_c0_g1	1.33	up	PREDICTED: malate dehydrogenase, glyoxysomal [Populus euphratica]	118.58	72.32
map01200	Carbon metabolism	2.25E-02	TRINITY_DN20938_c0_g2	2.63	up	malate dehydrogenase family protein [Populus trichocarpa]	74.92	18.32
map01200	Carbon metabolism	2.25E-02	TRINITY_DN21062_c0_g1	1.22	up	PREDICTED: hexokinase-1-like [Populus euphratica]	9.26	6.04
map01200	Carbon metabolism	2.25E-02	TRINITY_DN21140_c0_g2	2.24	up	mitochondrial lipoamide dehydrogenase [Populus tremuloides]	202.39	67.53
map01200	Carbon metabolism	2.25E-02	TRINITY_DN21417_c0_g1	1.35	up	SERINE ACETYLTRANSFERASE-106 family protein [Populus trichocarpa]	46.75	26.71
map01200	Carbon metabolism	2.25E-02	TRINITY_DN21427_c0_g1	1.40	up	hypothetical protein POPTR_0016s11621g [Populus trichocarpa]	98.65	56.05
map01200	Carbon metabolism	2.25E-02	TRINITY_DN348_c0_g1	-3.27	down	hypothetical protein POPTR_0018s03880g [Populus trichocarpa]	0.18	2.75
map01200	Carbon metabolism	2.25E-02	TRINITY_DN6390_c0_g1	3.46	up	nodulin MtN21 family protein [Populus trichocarpa]	1.91	0.26
map00410	beta-Alanine metabolism	2.25E-02	TRINITY_DN21932_c0_g3	-1.06	down	phosphatase 2C family protein [Populus trichocarpa]	2.53	8.10
map00410	beta-Alanine metabolism	2.25E-02	TRINITY_DN22512_c0_g1	1.04	up	hypothetical protein POPTR_0018s02570g [Populus trichocarpa]	23.51	17.76
map00410	beta-Alanine metabolism	2.25E-02	TRINITY_DN22684_c0_g1	4.49	up	glutamate decarboxylase [Populus tremula x Populus alba]	5.12	0.35
map00410	beta-Alanine metabolism	2.25E-02	TRINITY_DN22684_c0_g7	-5.56	down	glutamate decarboxylase 1 family protein [Populus trichocarpa]	0.08	6.22
map00410	beta-Alanine metabolism	2.25E-02	TRINITY_DN23704_c1_g1	-3.03	down	hypothetical protein POPTR_0001s07880g [Populus trichocarpa]	1.87	24.42
map00410	beta-Alanine metabolism	2.25E-02	TRINITY_DN24529_c1_g1	-2.26	down	polyamine oxidase 1 [Populus tomentosa]	4.94	37.37
map00410	beta-Alanine metabolism	2.25E-02	TRINITY_DN24529_c1_g3	-2.21	down	hypothetical protein POPTR_0001s27060g [Populus trichocarpa]	0.40	2.77
map00410	beta-Alanine metabolism	2.25E-02	TRINITY_DN24599_c6_g1	-1.97	down	hypothetical protein POPTR_0007s03670g [Populus trichocarpa]	0.44	2.68
map00410	beta-Alanine metabolism	2.25E-02	TRINITY_DN24626_c0_g5	4.91	up	PREDICTED: polyamine oxidase-like [Populus euphratica]	238.94	11.76
map00410	beta-Alanine metabolism	2.25E-02	TRINITY_DN24628_c0_g1	1.28	up	PREDICTED: aldehyde dehydrogenase family 3 member H1-like [Populus euphratica]	40.39	24.46
map00410	beta-Alanine metabolism	2.25E-02	TRINITY_DN24826_c0_g1	-1.05	down	PREDICTED: uncharacterized protein LOC105138712 [Populus euphratica]	1.85	5.77
map00410	beta-Alanine metabolism	2.25E-02	TRINITY_DN24826_c0_g2	-4.47	down	PREDICTED: uncharacterized protein LOC105109098 [Populus euphratica]	0.14	5.15
map00410	beta-Alanine metabolism	2.25E-02	TRINITY_DN24880_c0_g1	-1.43	down	curculin-like lectin family protein [Populus trichocarpa]	4.67	18.36
map00410	beta-Alanine metabolism	2.25E-02	TRINITY_DN25057_c0_g2	-1.00	down	PREDICTED: GATA transcription factor 1-like [Populus euphratica]	3.25	9.78
map00410	beta-Alanine metabolism	2.25E-02	TRINITY_DN25564_c0_g1	-1.06	down	amine oxidase family protein [Populus trichocarpa]	6.24	17.85
map00410	beta-Alanine metabolism	2.25E-02	TRINITY_DN25630_c0_g1	1.30	up	aldehyde dehydrogenase 1 precursor family protein [Populus trichocarpa]	120.35	73.18
map00410	beta-Alanine metabolism	2.25E-02	TRINITY_DN26025_c0_g1	-1.71	down	hypothetical protein POPTR_0004s18340g [Populus trichocarpa]	5.60	27.70
map00410	beta-Alanine metabolism	2.25E-02	TRINITY_DN26235_c0_g1	-1.34	down	PREDICTED: LOW QUALITY PROTEIN: G-type lectin S-receptor-like serine/threonine-protein kinase RLK1 [Populus euphratica]	6.54	32.64
map00410	beta-Alanine metabolism	2.25E-02	TRINITY_DN26235_c0_g3	-1.48	down	PREDICTED: G-type lectin S-receptor-like serine/threonine-protein kinase RLK1 [Populus euphratica]	4.02	17.62
map00410	beta-Alanine metabolism	2.25E-02	TRINITY_DN26421_c0_g1	1.32	up	the aldehyde dehydrogenase cp-ADH from C.plantagineum family protein [Populus trichocarpa]	58.13	34.64

map00410	beta-Alanine metabolism	2.25E-02	TRINITY_DN26590_c0_g1	1.01	up	PREDICTED: acyl-CoA dehydrogenase family member 10-like isoform X2 [Populus euphratica]	17.06	13.06
map00410	beta-Alanine metabolism	2.25E-02	TRINITY_DN27082_c0_g1	-1.32	down	hypothetical protein POPTR_0005s22880g [Populus trichocarpa]	18.47	72.77
map00410	beta-Alanine metabolism	2.25E-02	TRINITY_DN27242_c0_g1	-1.23	down	hypothetical protein POPTR_0151s00200g [Populus trichocarpa]	2.45	8.98
map00410	beta-Alanine metabolism	2.25E-02	TRINITY_DN14000_c0_g1	-2.71	down	hypothetical protein POPTR_0013s05640g [Populus trichocarpa]	0.38	5.38
map00410	beta-Alanine metabolism	2.25E-02	TRINITY_DN15219_c0_g1	-3.09	down	hypothetical protein POPTR_0013s05650g [Populus trichocarpa]	0.23	2.86
map00410	beta-Alanine metabolism	2.25E-02	TRINITY_DN17464_c0_g2	1.19	up	hydrolase family protein [Populus trichocarpa]	68.37	46.14
map00410	beta-Alanine metabolism	2.25E-02	TRINITY_DN17759_c0_g1	-1.68	down	hypothetical protein POPTR_0004s01460g, partial [Populus trichocarpa]	1.43	5.88
map00410	beta-Alanine metabolism	2.25E-02	TRINITY_DN17759_c0_g3	-2.14	down	PREDICTED: proline-rich receptor-like protein kinase PERK3 isoform X1 [Populus euphratica]	0.24	1.71
map00410	beta-Alanine metabolism	2.25E-02	TRINITY_DN18480_c0_g1	3.23	up	Pantoate--beta-alanine ligase family protein [Populus trichocarpa]	17.35	2.76
map00410	beta-Alanine metabolism	2.25E-02	TRINITY_DN18895_c0_g1	-1.06	down	mitochondrial aldehyde dehydrogenase family protein [Populus trichocarpa]	7.40	23.22
map00410	beta-Alanine metabolism	2.25E-02	TRINITY_DN19059_c0_g2	-1.92	down	PREDICTED: G-type lectin S-receptor-like serine/threonine-protein kinase At5g24080 isoform X1 [Populus euphratica]	1.17	7.57
map00410	beta-Alanine metabolism	2.25E-02	TRINITY_DN19059_c0_g3	-6.09	down	hypothetical protein POPTR_0015s05780g [Populus trichocarpa]	0.09	4.40
map00410	beta-Alanine metabolism	2.25E-02	TRINITY_DN19215_c1_g1	-1.38	down	hypothetical protein POPTR_0007s10210g [Populus trichocarpa]	3.47	12.55
map00410	beta-Alanine metabolism	2.25E-02	TRINITY_DN19215_c2_g1	-1.56	down	PREDICTED: probable protein phosphatase 2C 63 [Populus euphratica]	6.48	30.16
map00410	beta-Alanine metabolism	2.25E-02	TRINITY_DN19294_c0_g3	1.09	up	hypothetical protein POPTR_0006s16050g [Populus trichocarpa]	91.23	67.60
map00410	beta-Alanine metabolism	2.25E-02	TRINITY_DN19656_c0_g1	-1.62	down	PREDICTED: G-type lectin S-receptor-like serine/threonine-protein kinase RLK1 isoform X1 [Populus euphratica]	0.57	2.63
map00410	beta-Alanine metabolism	2.25E-02	TRINITY_DN19656_c0_g2	1.26	up	PREDICTED: G-type lectin S-receptor-like serine/threonine-protein kinase RLK1 [Populus euphratica]	3.74	2.35
map00410	beta-Alanine metabolism	2.25E-02	TRINITY_DN19656_c0_g3	-4.04	down	hypothetical protein POPTR_0200s00220g [Populus trichocarpa]	0.09	2.49
map00410	beta-Alanine metabolism	2.25E-02	TRINITY_DN19981_c0_g1	-1.21	down	copper/topa quinone amine oxidase precursor family protein [Populus trichocarpa]	1.81	6.48
map00410	beta-Alanine metabolism	2.25E-02	TRINITY_DN21133_c0_g2	-1.22	down	PREDICTED: G-type lectin S-receptor-like serine/threonine-protein kinase RLK1 isoform X1 [Populus euphratica]	6.50	22.69
map00410	beta-Alanine metabolism	2.25E-02	TRINITY_DN348_c0_g1	-3.27	down	hypothetical protein POPTR_0018s03880g [Populus trichocarpa]	0.18	2.75
map00410	beta-Alanine metabolism	2.25E-02	TRINITY_DN847_c0_g1	-7.22	down	glutamate decarboxylase 1 family protein [Populus trichocarpa]	0.04	7.12
map00900	Terpenoid backbone biosynthesis	2.31E-02	TRINITY_DN21795_c0_g5	1.27	up	chloroplast biogenesis family protein [Populus trichocarpa]	125.22	79.24
map00900	Terpenoid backbone biosynthesis	2.31E-02	TRINITY_DN21964_c0_g1	1.62	up	PREDICTED: 1-deoxy-D-xylulose 5-phosphate reductoisomerase, chloroplastic [Populus euphratica]	158.54	78.23
map00900	Terpenoid backbone biosynthesis	2.31E-02	TRINITY_DN21964_c0_g2	1.64	up	1-deoxy-D-xylulose 5-phosphate reductoisomerase family protein [Populus trichocarpa]	40.78	19.91
map00900	Terpenoid backbone biosynthesis	2.31E-02	TRINITY_DN22020_c0_g2	1.82	up	solanesyl diphosphate synthase family protein [Populus trichocarpa]	72.51	40.62
map00900	Terpenoid backbone biosynthesis	2.31E-02	TRINITY_DN22268_c0_g3	1.70	up	PREDICTED: geranylgeranyl pyrophosphate synthase 7, chloroplastic-like [Populus euphratica]	4.43	2.07
map00900	Terpenoid backbone biosynthesis	2.31E-02	TRINITY_DN22575_c0_g2	2.77	up	hypothetical protein POPTR_0017s13490g [Populus trichocarpa]	52.63	10.65
map00900	Terpenoid backbone biosynthesis	2.31E-02	TRINITY_DN22717_c0_g3	-2.09	down	hypothetical protein POPTR_0003s06910g [Populus trichocarpa]	6.82	35.25
map00900	Terpenoid backbone biosynthesis	2.31E-02	TRINITY_DN23393_c0_g1	1.53	up	hypothetical protein POPTR_0001s23230g [Populus trichocarpa]	61.36	32.17
map00900	Terpenoid backbone biosynthesis	2.31E-02	TRINITY_DN23425_c0_g1	-5.51	down	PREDICTED: GDSL esterase/lipase At1g29670-like [Populus euphratica]	1.04	73.66
map00900	Terpenoid backbone biosynthesis	2.31E-02	TRINITY_DN23433_c0_g2	1.70	up	1-deoxy-D-xylulose-5-phosphate reductoisomerase [Populus tremula x Populus alba]	84.58	38.91
map00900	Terpenoid backbone biosynthesis	2.31E-02	TRINITY_DN23530_c1_g2	1.38	up	pyridine nucleotide-disulfide oxidoreductase family protein [Populus tomentosa]	681.14	397.07
map00900	Terpenoid backbone biosynthesis	2.31E-02	TRINITY_DN23572_c0_g4	1.53	up	hypothetical protein POPTR_0010s23890g [Populus trichocarpa]	26.24	12.51
map00900	Terpenoid backbone biosynthesis	2.31E-02	TRINITY_DN24382_c0_g2	-2.82	down	3-hydroxy-3-methylglutaryl coenzyme A reductase family protein [Populus trichocarpa]	0.97	10.41
map00900	Terpenoid backbone biosynthesis	2.31E-02	TRINITY_DN24443_c0_g3	-1.30	down	hypothetical protein POPTR_0006s18240g [Populus trichocarpa]	48.96	203.82
map00900	Terpenoid backbone biosynthesis	2.31E-02	TRINITY_DN25565_c0_g7	-1.49	down	PREDICTED: probable 1-deoxy-D-xylulose-5-phosphate synthase 2, chloroplastic [Populus euphratica]	1.26	5.40
map00900	Terpenoid backbone biosynthesis	2.31E-02	TRINITY_DN25808_c0_g1	1.01	up	PREDICTED: solanesyl diphosphate synthase 3, chloroplastic/mitochondrial-like isoform X1 [Populus euphratica]	19.66	16.76

map00900	Terpenoid backbone biosynthesis	2.31E-02	TRINITY_DN25808_c0_g2	1.02	up	hypothetical protein POPTR_0006s13760g [Populus trichocarpa]	8.70	6.45
map00900	Terpenoid backbone biosynthesis	2.31E-02	TRINITY_DN27282_c0_g1	1.42	up	hypothetical protein POPTR_0009s01900g [Populus trichocarpa]	120.23	67.39
map00900	Terpenoid backbone biosynthesis	2.31E-02	TRINITY_DN15096_c0_g1	-2.01	down	hypothetical protein POPTR_0011s09200g [Populus trichocarpa]	11.86	68.50
map00900	Terpenoid backbone biosynthesis	2.31E-02	TRINITY_DN15967_c0_g1	-1.54	down	hypothetical protein POPTR_0015s04050g [Populus trichocarpa]	3.43	15.36
map00900	Terpenoid backbone biosynthesis	2.31E-02	TRINITY_DN16439_c0_g1	-6.31	down	PREDICTED: GDSL esterase/lipase At1g74460 [Populus euphratica]	0.06	7.74
map00900	Terpenoid backbone biosynthesis	2.31E-02	TRINITY_DN17174_c0_g1	1.76	up	PREDICTED: GDSL esterase/lipase At1g33811 [Populus euphratica]	4.85	2.09
map00900	Terpenoid backbone biosynthesis	2.31E-02	TRINITY_DN18051_c1_g3	1.42	up	hypothetical protein POPTR_0001s09860g [Populus trichocarpa]	100.06	57.21
map00900	Terpenoid backbone biosynthesis	2.31E-02	TRINITY_DN18631_c0_g1	-4.86	down	GDSL-motif lipase/hydrolase family protein [Populus trichocarpa]	0.77	35.68
map00900	Terpenoid backbone biosynthesis	2.31E-02	TRINITY_DN18631_c0_g2	2.04	up	GDSL-motif lipase/hydrolase family protein [Populus trichocarpa]	40.04	15.31
map00900	Terpenoid backbone biosynthesis	2.31E-02	TRINITY_DN18744_c0_g1	-4.07	down	hypothetical protein POPTR_0013s12020g [Populus trichocarpa]	0.67	17.03
map00900	Terpenoid backbone biosynthesis	2.31E-02	TRINITY_DN19039_c1_g7	-3.14	down	hypothetical protein POPTR_0016s00570g [Populus trichocarpa]	0.19	2.22
map00900	Terpenoid backbone biosynthesis	2.31E-02	TRINITY_DN19667_c0_g3	1.48	up	PREDICTED: isopentenyl-diphosphate Delta-isomerase I [Populus euphratica]	204.35	111.83
map00900	Terpenoid backbone biosynthesis	2.31E-02	TRINITY_DN19680_c0_g2	1.07	up	PREDICTED: isoprenylcysteine alpha-carbonyl methylesterase ICME-like [Populus euphratica]	19.58	14.25
map00900	Terpenoid backbone biosynthesis	2.31E-02	TRINITY_DN20043_c0_g1	1.45	up	hypothetical protein POPTR_0004s23930g [Populus trichocarpa]	72.92	42.64
map00900	Terpenoid backbone biosynthesis	2.31E-02	TRINITY_DN20133_c0_g2	-1.79	down	PREDICTED: GDSL esterase/lipase At1g71691-like isoform X1 [Populus euphratica]	2.12	10.83
map00900	Terpenoid backbone biosynthesis	2.31E-02	TRINITY_DN20133_c0_g3	-2.31	down	GDSL-motif lipase/hydrolase family protein [Populus trichocarpa]	0.45	3.34
map00900	Terpenoid backbone biosynthesis	2.31E-02	TRINITY_DN20428_c0_g3	-2.75	down	hypothetical protein POPTR_0019s04150g [Populus trichocarpa]	4.68	47.93
map00900	Terpenoid backbone biosynthesis	2.31E-02	TRINITY_DN20820_c1_g4	1.07	up	hypothetical protein POPTR_0009s05010g [Populus trichocarpa]	35.10	25.40
map00900	Terpenoid backbone biosynthesis	2.31E-02	TRINITY_DN20991_c0_g1	7.02	up	chloroplast isoprene synthase 3 [Populus alba]	26.30	0.25
map00900	Terpenoid backbone biosynthesis	2.31E-02	TRINITY_DN21360_c0_g1	1.65	up	hypothetical protein POPTR_0007s12330g [Populus trichocarpa]	81.24	38.97
map00900	Terpenoid backbone biosynthesis	2.31E-02	TRINITY_DN21553_c0_g4	-3.41	down	hypothetical protein POPTR_0003s14120g [Populus trichocarpa]	0.13	2.44
map00900	Terpenoid backbone biosynthesis	2.31E-02	TRINITY_DN21625_c0_g1	1.31	up	PREDICTED: heterodimeric geranylgeranyl pyrophosphate synthase small subunit, chloroplastic [Populus euphratica]	47.23	29.10
map00900	Terpenoid backbone biosynthesis	2.31E-02	TRINITY_DN21625_c0_g2	2.04	up	hypothetical protein POPTR_0004s18610g [Populus trichocarpa]	84.37	30.74
map00400	Phenylalanine, tyrosine and tryptophan biosynthesis	2.37E-02	TRINITY_DN21802_c0_g1	1.24	up	hypothetical protein POPTR_0017s12470g [Populus trichocarpa]	144.20	95.20
map00400	Phenylalanine, tyrosine and tryptophan biosynthesis	2.37E-02	TRINITY_DN22238_c0_g3	1.04	up	hypothetical protein POPTR_0014s16700g [Populus trichocarpa]	59.72	45.45
map00400	Phenylalanine, tyrosine and tryptophan biosynthesis	2.37E-02	TRINITY_DN22595_c0_g2	1.52	up	PREDICTED: phospho-2-dehydro-3-deoxyheptonate aldolase 2, chloroplastic-like [Populus euphratica]	137.27	72.08
map00400	Phenylalanine, tyrosine and tryptophan biosynthesis	2.37E-02	TRINITY_DN22665_c0_g1	1.91	up	hypothetical protein POPTR_0013s03080g [Populus trichocarpa]	2.11	0.85
map00400	Phenylalanine, tyrosine and tryptophan biosynthesis	2.37E-02	TRINITY_DN22665_c0_g3	2.46	up	hypothetical protein POPTR_0013s03080g [Populus trichocarpa]	7.83	2.17
map00400	Phenylalanine, tyrosine and tryptophan biosynthesis	2.37E-02	TRINITY_DN22701_c0_g1	1.35	up	shikimate kinase family protein [Populus trichocarpa]	108.38	67.24
map00400	Phenylalanine, tyrosine and tryptophan biosynthesis	2.37E-02	TRINITY_DN22813_c0_g4	1.08	up	aspartate transaminase family protein [Populus trichocarpa]	25.11	18.98
map00400	Phenylalanine, tyrosine and tryptophan biosynthesis	2.37E-02	TRINITY_DN22954_c0_g2	1.23	up	TRYPTOPHAN SYNTHASE ALPHA CHAIN family protein [Populus trichocarpa]	98.67	70.99
map00400	Phenylalanine, tyrosine and tryptophan biosynthesis	2.37E-02	TRINITY_DN23227_c0_g2	1.27	up	hypothetical protein POPTR_0010s19790g [Populus trichocarpa]	6.63	4.18
map00400	Phenylalanine, tyrosine and tryptophan biosynthesis	2.37E-02	TRINITY_DN23260_c0_g1	1.32	up	anthranilate synthase beta subunit 1 family protein [Populus trichocarpa]	230.33	121.36
map00400	Phenylalanine, tyrosine and tryptophan biosynthesis	2.37E-02	TRINITY_DN23709_c0_g2	1.23	up	aspartate aminotransferase 2 family protein [Populus trichocarpa]	46.64	32.80
map00400	Phenylalanine, tyrosine and tryptophan biosynthesis	2.37E-02	TRINITY_DN24179_c1_g1	1.94	up	hypothetical protein POPTR_0001s44620g [Populus trichocarpa]	107.11	47.27
map00400	Phenylalanine, tyrosine and tryptophan biosynthesis	2.37E-02	TRINITY_DN25569_c0_g1	1.34	up	shikimate kinase family protein [Populus trichocarpa]	60.67	37.43
map00400	Phenylalanine, tyrosine and tryptophan biosynthesis	2.37E-02	TRINITY_DN25829_c0_g2	1.62	up	PHOSPHORIBOSYLANTHRANILATE ISOMERASE 3 family protein [Populus trichocarpa]	43.17	21.30

map00400	Phenylalanine, tyrosine and tryptophan biosynthesis	2.37E-02	TRINITY_DN25870_c1_g1	1.47	up	unknown [Populus trichocarpa]	296.23	175.19
map00400	Phenylalanine, tyrosine and tryptophan biosynthesis	2.37E-02	TRINITY_DN26147_c0_g1	1.59	up	3-phosphoshikimate 1-carboxyvinyltransferase family protein [Populus trichocarpa]	288.34	147.17
map00400	Phenylalanine, tyrosine and tryptophan biosynthesis	2.37E-02	TRINITY_DN26292_c0_g1	-1.38	down	hypothetical protein POPTR_0017s02170g [Populus trichocarpa]	5.66	22.15
map00400	Phenylalanine, tyrosine and tryptophan biosynthesis	2.37E-02	TRINITY_DN26593_c1_g4	1.29	up	2-dehydro-3-deoxyphosphoheptonate aldolase family protein [Populus trichocarpa]	647.76	427.17
map00400	Phenylalanine, tyrosine and tryptophan biosynthesis	2.37E-02	TRINITY_DN27893_c10_g1	1.22	up	PREDICTED: anthranilate phosphoribosyltransferase, chloroplastic-like isoform X1 [Populus euphratica]	53.94	35.34
map00400	Phenylalanine, tyrosine and tryptophan biosynthesis	2.37E-02	TRINITY_DN14836_c0_g1	-2.77	down	hypothetical protein POPTR_0013s03070g [Populus trichocarpa]	1.13	12.60
map00400	Phenylalanine, tyrosine and tryptophan biosynthesis	2.37E-02	TRINITY_DN15907_c0_g1	1.59	up	hypothetical protein POPTR_0002s06190g [Populus trichocarpa]	5.64	2.06
map00400	Phenylalanine, tyrosine and tryptophan biosynthesis	2.37E-02	TRINITY_DN17945_c0_g2	1.24	up	hypothetical protein POPTR_0006s06140g [Populus trichocarpa]	3.08	1.24
map00400	Phenylalanine, tyrosine and tryptophan biosynthesis	2.37E-02	TRINITY_DN18147_c0_g2	1.29	up	hypothetical protein POPTR_0005s11300g [Populus trichocarpa]	170.60	105.98
map00400	Phenylalanine, tyrosine and tryptophan biosynthesis	2.37E-02	TRINITY_DN19337_c0_g1	-2.03	down	hypothetical protein POPTR_0017s14220g [Populus trichocarpa]	2.64	16.29
map00400	Phenylalanine, tyrosine and tryptophan biosynthesis	2.37E-02	TRINITY_DN19385_c0_g1	1.11	up	hypothetical protein POPTR_0008s20010g [Populus trichocarpa]	12.27	8.86
map00400	Phenylalanine, tyrosine and tryptophan biosynthesis	2.37E-02	TRINITY_DN19472_c0_g2	1.32	up	hypothetical protein POPTR_0007s01130g [Populus trichocarpa]	8.54	5.46
map00400	Phenylalanine, tyrosine and tryptophan biosynthesis	2.37E-02	TRINITY_DN19472_c0_g3	-3.23	down	hypothetical protein POPTR_0017s04550g [Populus trichocarpa]	0.21	3.06
map00400	Phenylalanine, tyrosine and tryptophan biosynthesis	2.37E-02	TRINITY_DN20048_c1_g1	1.11	up	PREDICTED: phospho-2-dehydro-3-deoxyheptonate aldolase 1, chloroplastic-like [Populus euphratica]	289.54	201.11
map00400	Phenylalanine, tyrosine and tryptophan biosynthesis	2.37E-02	TRINITY_DN20517_c0_g1	1.45	up	chorismate synthase family protein [Populus trichocarpa]	289.88	166.51
map00400	Phenylalanine, tyrosine and tryptophan biosynthesis	2.37E-02	TRINITY_DN21391_c0_g1	1.12	up	PREDICTED: uncharacterized protein LOC105124970 [Populus euphratica]	49.29	33.42
map00350	Tyrosine metabolism	2.96E-02	TRINITY_DN22186_c0_g1	1.05	up	alpha-hydroxynitrile lyase family protein [Populus trichocarpa]	31.37	22.10
map00350	Tyrosine metabolism	2.96E-02	TRINITY_DN22813_c0_g4	1.08	up	aspartate transaminase family protein [Populus trichocarpa]	25.11	18.98
map00350	Tyrosine metabolism	2.96E-02	TRINITY_DN23463_c0_g3	1.61	up	polyphenol oxidase [Populus tremuloides]	153.33	73.81
map00350	Tyrosine metabolism	2.96E-02	TRINITY_DN23704_c1_g1	-3.03	down	hypothetical protein POPTR_0001s07880g [Populus trichocarpa]	1.87	24.42
map00350	Tyrosine metabolism	2.96E-02	TRINITY_DN23709_c0_g2	1.23	up	aspartate aminotransferase 2 family protein [Populus trichocarpa]	46.64	32.80
map00350	Tyrosine metabolism	2.96E-02	TRINITY_DN24032_c3_g3	-2.56	down	hypothetical protein POPTR_0001s09640g [Populus trichocarpa]	5.18	47.53
map00350	Tyrosine metabolism	2.96E-02	TRINITY_DN24599_c6_g1	-1.97	down	hypothetical protein POPTR_0007s03670g [Populus trichocarpa]	0.44	2.68
map00350	Tyrosine metabolism	2.96E-02	TRINITY_DN24826_c0_g1	-1.05	down	PREDICTED: uncharacterized protein LOC105138712 [Populus euphratica]	1.85	5.77
map00350	Tyrosine metabolism	2.96E-02	TRINITY_DN24826_c0_g2	-4.47	down	PREDICTED: uncharacterized protein LOC105109098 [Populus euphratica]	0.14	5.15
map00350	Tyrosine metabolism	2.96E-02	TRINITY_DN24880_c0_g1	-1.43	down	curculin-like lectin family protein [Populus trichocarpa]	4.67	18.36
map00350	Tyrosine metabolism	2.96E-02	TRINITY_DN26235_c0_g1	-1.34	down	PREDICTED: LOW QUALITY PROTEIN: G-type lectin S-receptor-like serine/threonine-protein kinase RLK1 [Populus euphratica]	6.54	32.64
map00350	Tyrosine metabolism	2.96E-02	TRINITY_DN26235_c0_g3	-1.48	down	PREDICTED: G-type lectin S-receptor-like serine/threonine-protein kinase RLK1 [Populus euphratica]	4.02	17.62
map00350	Tyrosine metabolism	2.96E-02	TRINITY_DN26456_c0_g1	-2.06	down	hypothetical protein POPTR_0001s39940g [Populus trichocarpa]	112.97	594.31
map00350	Tyrosine metabolism	2.96E-02	TRINITY_DN26905_c0_g2	1.05	up	IQ domain-containing family protein [Populus trichocarpa]	17.91	13.24
map00350	Tyrosine metabolism	2.96E-02	TRINITY_DN27242_c0_g1	-1.23	down	hypothetical protein POPTR_0151s00200g [Populus trichocarpa]	2.45	8.98
map00350	Tyrosine metabolism	2.96E-02	TRINITY_DN14000_c0_g1	-2.71	down	hypothetical protein POPTR_0013s05640g [Populus trichocarpa]	0.38	5.38
map00350	Tyrosine metabolism	2.96E-02	TRINITY_DN14872_c0_g1	3.38	up	hypothetical protein POPTR_0004s16380g [Populus trichocarpa]	5.62	0.83
map00350	Tyrosine metabolism	2.96E-02	TRINITY_DN14872_c0_g2	3.04	up	polyphenol oxidase-like protein [Populus trichocarpa]	2.86	0.52
map00350	Tyrosine metabolism	2.96E-02	TRINITY_DN15219_c0_g1	-3.09	down	hypothetical protein POPTR_0013s05650g [Populus trichocarpa]	0.23	2.86
map00350	Tyrosine metabolism	2.96E-02	TRINITY_DN15573_c0_g1	-2.43	down	alcohol dehydrogenase family protein [Populus trichocarpa]	0.77	3.49

map00350	Tyrosine metabolism	2.96E-02	TRINITY_DN16284_c0_g2	1.06	up	hypothetical protein POPTR_0004s03870g [Populus trichocarpa]	39.71	28.71
map00350	Tyrosine metabolism	2.96E-02	TRINITY_DN16533_c0_g2	4.96	up	IQ domain-containing family protein [Populus trichocarpa]	5.20	0.26
map00350	Tyrosine metabolism	2.96E-02	TRINITY_DN17759_c0_g1	-1.68	down	hypothetical protein POPTR_0004s01460g, partial [Populus trichocarpa]	1.43	5.88
map00350	Tyrosine metabolism	2.96E-02	TRINITY_DN17759_c0_g3	-2.14	down	PREDICTED: proline-rich receptor-like protein kinase PERK3 isoform X1 [Populus euphratica]	0.24	1.71
map00350	Tyrosine metabolism	2.96E-02	TRINITY_DN19059_c0_g2	-1.92	down	PREDICTED: G-type lectin S-receptor-like serine/threonine-protein kinase At5g24080 isoform X1 [Populus euphratica]	1.17	7.57
map00350	Tyrosine metabolism	2.96E-02	TRINITY_DN19059_c0_g3	-6.09	down	hypothetical protein POPTR_0015s05780g [Populus trichocarpa]	0.09	4.40
map00350	Tyrosine metabolism	2.96E-02	TRINITY_DN19472_c0_g2	1.32	up	hypothetical protein POPTR_0007s01130g [Populus trichocarpa]	8.54	5.46
map00350	Tyrosine metabolism	2.96E-02	TRINITY_DN19472_c0_g3	-3.23	down	hypothetical protein POPTR_0017s04550g [Populus trichocarpa]	0.21	3.06
map00350	Tyrosine metabolism	2.96E-02	TRINITY_DN19540_c0_g3	1.78	up	hypothetical protein POPTR_0001s45280g [Populus trichocarpa]	3.69	1.58
map00350	Tyrosine metabolism	2.96E-02	TRINITY_DN19626_c0_g2	-2.23	down	hypothetical protein POPTR_0016s13500g [Populus trichocarpa]	5.67	39.97
map00350	Tyrosine metabolism	2.96E-02	TRINITY_DN19656_c0_g1	-1.62	down	PREDICTED: G-type lectin S-receptor-like serine/threonine-protein kinase RLK1 isoform X1 [Populus euphratica]	0.57	2.63
map00350	Tyrosine metabolism	2.96E-02	TRINITY_DN19656_c0_g2	1.26	up	PREDICTED: G-type lectin S-receptor-like serine/threonine-protein kinase RLK1 [Populus euphratica]	3.74	2.35
map00350	Tyrosine metabolism	2.96E-02	TRINITY_DN19656_c0_g3	-4.04	down	hypothetical protein POPTR_0200s00220g [Populus trichocarpa]	0.09	2.49
map00350	Tyrosine metabolism	2.96E-02	TRINITY_DN19981_c0_g1	-1.21	down	copper/topa quinone amine oxidase precursor family protein [Populus trichocarpa]	1.81	6.48
map00350	Tyrosine metabolism	2.96E-02	TRINITY_DN20799_c0_g1	1.40	up	PREDICTED: tyrosine decarboxylase 1-like isoform X2 [Populus euphratica]	30.48	17.85
map00350	Tyrosine metabolism	2.96E-02	TRINITY_DN20799_c0_g5	-2.32	down	hypothetical protein POPTR_0013s04970g [Populus trichocarpa]	0.28	2.25
map00350	Tyrosine metabolism	2.96E-02	TRINITY_DN20914_c0_g3	1.36	up	hypothetical protein POPTR_0006s07470g [Populus trichocarpa]	89.52	50.66
map00350	Tyrosine metabolism	2.96E-02	TRINITY_DN21133_c0_g2	-1.22	down	PREDICTED: G-type lectin S-receptor-like serine/threonine-protein kinase RLK1 isoform X1 [Populus euphratica]	6.50	22.69
map00350	Tyrosine metabolism	2.96E-02	TRINITY_DN21391_c0_g1	1.12	up	PREDICTED: uncharacterized protein LOC105124970 [Populus euphratica]	49.29	33.42
map00760	Nicotinate and nicotinamide metabolism	3.30E-02	TRINITY_DN22065_c0_g1	1.32	up	PREDICTED: inactive rhomboid protein 1-like [Populus euphratica]	5.41	3.36
map00760	Nicotinate and nicotinamide metabolism	3.30E-02	TRINITY_DN22303_c0_g1	-2.21	down	hypothetical protein POPTR_0001s34980g [Populus trichocarpa]	8.51	87.80
map00760	Nicotinate and nicotinamide metabolism	3.30E-02	TRINITY_DN22303_c0_g6	2.12	up	hypothetical protein POPTR_0001s34980g [Populus trichocarpa]	14.33	5.07
map00760	Nicotinate and nicotinamide metabolism	3.30E-02	TRINITY_DN22402_c0_g1	1.40	up	PREDICTED: uncharacterized protein LOC105122445 [Populus euphratica]	68.48	38.40
map00760	Nicotinate and nicotinamide metabolism	3.30E-02	TRINITY_DN22974_c0_g1	-2.36	down	unknown [Populus trichocarpa]	7.87	50.82
map00760	Nicotinate and nicotinamide metabolism	3.30E-02	TRINITY_DN22974_c0_g2	-2.37	down	putative phosphatase family protein [Populus trichocarpa]	2.24	22.90
map00760	Nicotinate and nicotinamide metabolism	3.30E-02	TRINITY_DN22987_c1_g2	1.39	up	PREDICTED: nicotinate-nucleotide pyrophosphorylase [carboxylating], chloroplastic isoform X1 [Populus euphratica]	36.91	20.98
map00760	Nicotinate and nicotinamide metabolism	3.30E-02	TRINITY_DN24534_c0_g2	-1.50	down	PREDICTED: uncharacterized rhomboid protein AN10929 [Populus euphratica]	15.35	62.82
map00760	Nicotinate and nicotinamide metabolism	3.30E-02	TRINITY_DN14700_c0_g1	-2.16	down	hypothetical protein POPTR_0008s01650g [Populus trichocarpa]	0.80	4.63
map00760	Nicotinate and nicotinamide metabolism	3.30E-02	TRINITY_DN17028_c0_g1	-1.05	down	hypothetical protein POPTR_0001s24910g [Populus trichocarpa]	9.71	28.75
map00760	Nicotinate and nicotinamide metabolism	3.30E-02	TRINITY_DN17742_c0_g2	1.46	up	PREDICTED: quinolinate synthase, chloroplastic [Populus euphratica]	11.15	6.24
map00760	Nicotinate and nicotinamide metabolism	3.30E-02	TRINITY_DN17742_c0_g3	2.07	up	PREDICTED: quinolinate synthase, chloroplastic [Populus euphratica]	13.69	5.06
map00760	Nicotinate and nicotinamide metabolism	3.30E-02	TRINITY_DN18269_c0_g1	1.44	up	ATP-NAD kinase family protein [Populus trichocarpa]	37.58	23.32
map00760	Nicotinate and nicotinamide metabolism	3.30E-02	TRINITY_DN20223_c0_g1	-1.54	down	zinc finger family protein [Populus trichocarpa]	5.37	24.46
map00760	Nicotinate and nicotinamide metabolism	3.30E-02	TRINITY_DN20411_c0_g2	-1.08	down	PREDICTED: nicotinamide mononucleotide adenylyltransferase-like [Populus euphratica]	5.17	16.84

map00760	Nicotinate and nicotinamide metabolism	3.30E-02	TRINITY_DN21092_c0_g1	1.18	up	PREDICTED: NAD-dependent protein deacetylase SRT2 isoform X3 [Populus euphratica]	12.55	9.15
map00760	Nicotinate and nicotinamide metabolism	3.30E-02	TRINITY_DN21383_c0_g2	-2.81	down	zinc finger family protein [Populus trichocarpa]	1.40	14.88
map00760	Nicotinate and nicotinamide metabolism	3.30E-02	TRINITY_DN21551_c0_g1	1.62	up	PREDICTED: sufE-like protein, chloroplastic [Populus euphratica]	49.31	25.04
map00710	Carbon fixation in photosynthetic organisms	3.58E-02	TRINITY_DN21889_c0_g2	3.34	up	PREDICTED: ribulose bisphosphate carboxylase small chain, chloroplastic-like [Populus euphratica]	9592.31	1398.04
map00710	Carbon fixation in photosynthetic organisms	3.58E-02	TRINITY_DN21889_c0_g3	2.27	up	017G114600 [Populus tomentosa]	20448.45	6363.37
map00710	Carbon fixation in photosynthetic organisms	3.58E-02	TRINITY_DN22033_c0_g1	1.60	up	Chain D family protein [Populus trichocarpa]	364.35	179.65
map00710	Carbon fixation in photosynthetic organisms	3.58E-02	TRINITY_DN22245_c0_g1	2.23	up	PREDICTED: triosephosphate isomerase, chloroplastic [Populus euphratica]	838.73	263.34
map00710	Carbon fixation in photosynthetic organisms	3.58E-02	TRINITY_DN22587_c0_g1	1.20	up	PREDICTED: fructose-bisphosphate aldolase cytoplasmic isozyme [Populus euphratica]	371.77	247.12
map00710	Carbon fixation in photosynthetic organisms	3.58E-02	TRINITY_DN22663_c0_g2	1.38	up	hypothetical protein POPTR_0010s05530g [Populus trichocarpa]	360.33	205.54
map00710	Carbon fixation in photosynthetic organisms	3.58E-02	TRINITY_DN22755_c0_g2	1.78	up	unknown [Populus trichocarpa]	261.55	115.26
map00710	Carbon fixation in photosynthetic organisms	3.58E-02	TRINITY_DN22813_c0_g4	1.08	up	aspartate transaminase family protein [Populus trichocarpa]	25.11	18.98
map00710	Carbon fixation in photosynthetic organisms	3.58E-02	TRINITY_DN22920_c1_g6	2.63	up	PREDICTED: probable ribose-5-phosphate isomerase 3, chloroplastic [Populus euphratica]	555.60	140.50
map00710	Carbon fixation in photosynthetic organisms	3.58E-02	TRINITY_DN23278_c1_g1	1.44	up	PREDICTED: glutamate--glyoxylate aminotransferase 2 isoform X1 [Populus euphratica]	580.61	318.60
map00710	Carbon fixation in photosynthetic organisms	3.58E-02	TRINITY_DN23701_c0_g1	1.96	up	latex plastidic aldolase-like family protein [Populus trichocarpa]	2289.47	879.27
map00710	Carbon fixation in photosynthetic organisms	3.58E-02	TRINITY_DN23701_c0_g5	2.57	up	plastidic aldolase family protein [Populus trichocarpa]	7.53	2.00
map00710	Carbon fixation in photosynthetic organisms	3.58E-02	TRINITY_DN23720_c0_g1	1.57	up	PREDICTED: malate dehydrogenase [NADP], chloroplastic [Populus euphratica]	252.27	131.23
map00710	Carbon fixation in photosynthetic organisms	3.58E-02	TRINITY_DN23758_c0_g1	2.77	up	hypothetical protein POPTR_0005s27550g [Populus trichocarpa]	826.64	183.48
map00710	Carbon fixation in photosynthetic organisms	3.58E-02	TRINITY_DN23965_c0_g1	1.23	up	PREDICTED: triosephosphate isomerase, cytosolic [Populus euphratica]	641.46	426.80
map00710	Carbon fixation in photosynthetic organisms	3.58E-02	TRINITY_DN24249_c0_g1	1.66	up	PREDICTED: ribulose bisphosphate carboxylase large chain [Populus euphratica]	86.19	43.64
map00710	Carbon fixation in photosynthetic organisms	3.58E-02	TRINITY_DN24279_c0_g1	1.93	up	unknown [Populus trichocarpa x Populus deltoides]	85.38	34.72
map00710	Carbon fixation in photosynthetic organisms	3.58E-02	TRINITY_DN24285_c0_g5	1.46	up	PREDICTED: WAT1-related protein At2g37460-like isoform X1 [Populus euphratica]	2.63	1.46
map00710	Carbon fixation in photosynthetic organisms	3.58E-02	TRINITY_DN24393_c0_g1	1.31	up	hypothetical protein POPTR_0001s24710g [Populus trichocarpa]	198.18	106.97
map00710	Carbon fixation in photosynthetic organisms	3.58E-02	TRINITY_DN24707_c1_g1	-1.25	down	nodulin MtN21 family protein [Populus trichocarpa]	14.31	50.91
map00710	Carbon fixation in photosynthetic organisms	3.58E-02	TRINITY_DN24881_c0_g1	1.99	up	PREDICTED: transketolase, chloroplastic [Populus euphratica]	1790.82	677.46
map00710	Carbon fixation in photosynthetic organisms	3.58E-02	TRINITY_DN24990_c0_g2	3.40	up	alanine aminotransferase family protein [Populus simonii x Populus nigra]	51.44	7.50
map00710	Carbon fixation in photosynthetic organisms	3.58E-02	TRINITY_DN25129_c1_g2	1.01	up	3-phosphoglycerate kinase [Populus tremuloides]	153.72	116.35
map00710	Carbon fixation in photosynthetic organisms	3.58E-02	TRINITY_DN25192_c0_g1	2.93	up	hypothetical protein POPTR_0010s02860g [Populus trichocarpa]	24.30	5.86
map00710	Carbon fixation in photosynthetic organisms	3.58E-02	TRINITY_DN25221_c0_g1	1.58	up	malate dehydrogenase family protein, partial [Populus trichocarpa]	57.58	29.35
map00710	Carbon fixation in photosynthetic organisms	3.58E-02	TRINITY_DN25221_c1_g2	2.25	up	malate dehydrogenase family protein [Populus trichocarpa]	356.58	110.81
map00710	Carbon fixation in photosynthetic organisms	3.58E-02	TRINITY_DN25221_c1_g3	1.80	up	PREDICTED: malate dehydrogenase, glyoxysomal [Populus euphratica]	643.81	285.37
map00710	Carbon fixation in photosynthetic organisms	3.58E-02	TRINITY_DN25476_c0_g2	2.59	up	PREDICTED: alanine aminotransferase 2, mitochondrial-like [Populus euphratica]	33.99	8.74
map00710	Carbon fixation in photosynthetic organisms	3.58E-02	TRINITY_DN25476_c0_g4	2.24	up	PREDICTED: alanine aminotransferase 2-like [Populus euphratica]	21.79	7.01
map00710	Carbon fixation in photosynthetic organisms	3.58E-02	TRINITY_DN25746_c0_g1	2.80	up	nodule-enhanced malate dehydrogenase family protein [Populus trichocarpa]	113.74	23.22

map00710	Carbon fixation in photosynthetic organisms	3.58E-02	TRINITY_DN26030_c0_g1	2.06	up	unknown [Populus trichocarpa x Populus deltoides]	3041.90	1125.79
map00710	Carbon fixation in photosynthetic organisms	3.58E-02	TRINITY_DN26154_c0_g2	-1.12	down	PREDICTED: protein SCAR2-like isoform X1 [Populus euphratica]	5.40	17.64
map00710	Carbon fixation in photosynthetic organisms	3.58E-02	TRINITY_DN26209_c0_g1	1.84	up	hypothetical protein SETIT_022310mg [Setaria italica]	186.50	89.03
map00710	Carbon fixation in photosynthetic organisms	3.58E-02	TRINITY_DN26497_c0_g2	2.05	up	hypothetical protein POPTR_0011s07381g [Populus trichocarpa]	1.66	0.60
map00710	Carbon fixation in photosynthetic organisms	3.58E-02	TRINITY_DN26627_c0_g1	2.37	up	PHOSPHOGLYCERATE KINASE 1 family protein [Populus trichocarpa]	946.82	271.09
map00710	Carbon fixation in photosynthetic organisms	3.58E-02	TRINITY_DN26661_c0_g1	2.62	up	hypothetical protein CISIN_1g016748mg [Citrus sinensis]	718.86	176.98
map00710	Carbon fixation in photosynthetic organisms	3.58E-02	TRINITY_DN26711_c0_g1	1.68	up	Sedoheptulose-1 family protein [Populus trichocarpa]	85.33	41.36
map00710	Carbon fixation in photosynthetic organisms	3.58E-02	TRINITY_DN26711_c0_g2	1.91	up	Sedoheptulose-1 family protein [Populus trichocarpa]	1906.74	735.61
map00710	Carbon fixation in photosynthetic organisms	3.58E-02	TRINITY_DN26922_c0_g2	1.76	up	Phosphoribulokinase family protein [Populus trichocarpa]	951.86	414.20
map00710	Carbon fixation in photosynthetic organisms	3.58E-02	TRINITY_DN27009_c0_g1	1.60	up	hypothetical protein POPTR_0002s22720g [Populus trichocarpa]	10.34	5.23
map00710	Carbon fixation in photosynthetic organisms	3.58E-02	TRINITY_DN27458_c1_g1	2.44	up	PREDICTED: transketolase, chloroplastic [Populus euphratica]	1184.20	313.96
map00710	Carbon fixation in photosynthetic organisms	3.58E-02	TRINITY_DN27847_c0_g1	1.64	up	hypothetical protein POPTR_0008s11610g [Populus trichocarpa]	34.59	16.78
map00710	Carbon fixation in photosynthetic organisms	3.58E-02	TRINITY_DN27862_c2_g1	-1.69	down	PREDICTED: structural maintenance of chromosomes protein 2-1-like [Populus euphratica]	21.02	56.92
map00710	Carbon fixation in photosynthetic organisms	3.58E-02	TRINITY_DN11372_c0_g1	3.83	up	hypothetical protein POPTR_0011s15150g [Populus trichocarpa]	4.92	0.36
map00710	Carbon fixation in photosynthetic organisms	3.58E-02	TRINITY_DN14558_c0_g1	-2.01	down	PREDICTED: uncharacterized protein LOC109021545 [Juglans regia]	0.85	4.88
map00710	Carbon fixation in photosynthetic organisms	3.58E-02	TRINITY_DN15775_c0_g1	1.57	up	hypothetical protein POPTR_0001s46060g [Populus trichocarpa]	7.49	3.44
map00710	Carbon fixation in photosynthetic organisms	3.58E-02	TRINITY_DN16677_c0_g2	-8.29	down	hypothetical protein POPTR_0007s15050g [Populus trichocarpa]	0.00	5.66
map00710	Carbon fixation in photosynthetic organisms	3.58E-02	TRINITY_DN16817_c0_g1	1.64	up	PREDICTED: tRNA (guanine-N(7)-)-methyltransferase [Populus euphratica]	40.30	24.24
map00710	Carbon fixation in photosynthetic organisms	3.58E-02	TRINITY_DN16821_c0_g1	1.91	up	PREDICTED: malate dehydrogenase, glyoxysomal [Populus euphratica]	248.44	104.05
map00710	Carbon fixation in photosynthetic organisms	3.58E-02	TRINITY_DN17020_c0_g1	2.36	up	hypothetical protein POPTR_0002s06890g [Populus trichocarpa]	22.89	6.58
map00710	Carbon fixation in photosynthetic organisms	3.58E-02	TRINITY_DN17246_c0_g1	1.06	up	hypothetical protein POPTR_0017s07040g [Populus trichocarpa]	24.42	17.62
map00710	Carbon fixation in photosynthetic organisms	3.58E-02	TRINITY_DN17663_c0_g3	1.15	up	unknown [Populus trichocarpa x Populus deltoides]	373.94	255.99
map00710	Carbon fixation in photosynthetic organisms	3.58E-02	TRINITY_DN19734_c0_g2	1.39	up	PREDICTED: ribulose-phosphate 3-epimerase, chloroplastic [Populus euphratica]	648.43	374.61
map00710	Carbon fixation in photosynthetic organisms	3.58E-02	TRINITY_DN19743_c0_g1	1.38	up	PREDICTED: WAT1-related protein At4g19185-like [Populus euphratica]	66.15	39.51
map00710	Carbon fixation in photosynthetic organisms	3.58E-02	TRINITY_DN19763_c0_g3	1.88	up	PREDICTED: malate dehydrogenase, mitochondrial [Populus euphratica]	247.09	106.19
map00710	Carbon fixation in photosynthetic organisms	3.58E-02	TRINITY_DN20148_c0_g1	1.45	up	hypothetical protein POPTR_0010s12560g [Populus trichocarpa]	131.18	75.15
map00710	Carbon fixation in photosynthetic organisms	3.58E-02	TRINITY_DN20148_c0_g2	3.15	up	ribose 5-phosphate isomerase family protein [Populus trichocarpa]	6.05	1.07
map00710	Carbon fixation in photosynthetic organisms	3.58E-02	TRINITY_DN20175_c0_g1	1.59	up	peroxiredoxin Q family protein [Populus trichocarpa]	1020.77	504.37
map00710	Carbon fixation in photosynthetic organisms	3.58E-02	TRINITY_DN20590_c0_g2	1.44	up	METHIONINE AMINOPEPTIDASE 1D family protein [Populus trichocarpa]	79.40	44.51
map00710	Carbon fixation in photosynthetic organisms	3.58E-02	TRINITY_DN20938_c0_g1	1.33	up	PREDICTED: malate dehydrogenase, glyoxysomal [Populus euphratica]	118.58	72.32
map00710	Carbon fixation in photosynthetic organisms	3.58E-02	TRINITY_DN20938_c0_g2	2.63	up	malate dehydrogenase family protein [Populus trichocarpa]	74.92	18.32
map00710	Carbon fixation in photosynthetic organisms	3.58E-02	TRINITY_DN21427_c0_g1	1.40	up	hypothetical protein POPTR_0016s11621g [Populus trichocarpa]	98.65	56.05
map00710	Carbon fixation in photosynthetic organisms	3.58E-02	TRINITY_DN6390_c0_g1	3.46	up	nodulin MtN21 family protein [Populus trichocarpa]	1.91	0.26

map00908	Zeatin biosynthesis	3.62E-02	TRINITY_DN21885_c0_g3	1.52	up	PREDICTED: 7-deoxyloganetin glucosyltransferase-like [Populus euphratica]	23.23	13.15
map00908	Zeatin biosynthesis	3.62E-02	TRINITY_DN22273_c0_g2	1.52	up	PREDICTED: tRNA dimethylallyltransferase 9 [Populus euphratica]	80.74	46.98
map00908	Zeatin biosynthesis	3.62E-02	TRINITY_DN22895_c0_g2	3.42	up	hypothetical protein POPTR_0098s00240g [Populus trichocarpa]	32.62	4.49
map00908	Zeatin biosynthesis	3.62E-02	TRINITY_DN22895_c0_g3	1.47	up	hypothetical protein POPTR_0098s00310g [Populus trichocarpa]	23.05	12.58
map00908	Zeatin biosynthesis	3.62E-02	TRINITY_DN22895_c0_g5	1.93	up	hypothetical protein POPTR_0016s021501g, partial [Populus trichocarpa]	9.84	3.87
map00908	Zeatin biosynthesis	3.62E-02	TRINITY_DN24328_c0_g2	-3.10	down	hypothetical protein POPTR_0017s07940g [Populus trichocarpa]	4.15	25.32
map00908	Zeatin biosynthesis	3.62E-02	TRINITY_DN24599_c5_g4	4.92	up	PREDICTED: cytochrome P450 714A1-like [Populus euphratica]	4.63	0.20
map00908	Zeatin biosynthesis	3.62E-02	TRINITY_DN24626_c0_g1	-2.15	down	unknown [Populus trichocarpa]	3.88	27.85
map00908	Zeatin biosynthesis	3.62E-02	TRINITY_DN26126_c1_g2	2.06	up	hypothetical protein POPTR_0016s02200g [Populus trichocarpa]	46.02	14.35
map00908	Zeatin biosynthesis	3.62E-02	TRINITY_DN26126_c1_g5	1.87	up	hypothetical protein POPTR_0016s021501g, partial [Populus trichocarpa]	21.67	8.78
map00908	Zeatin biosynthesis	3.62E-02	TRINITY_DN26186_c0_g1	-7.20	down	cytokinin oxidase 6 family protein [Populus trichocarpa]	0.00	2.27
map00908	Zeatin biosynthesis	3.62E-02	TRINITY_DN26186_c0_g2	-5.46	down	cytokinin oxidase 6 family protein [Populus trichocarpa]	0.41	24.89
map00908	Zeatin biosynthesis	3.62E-02	TRINITY_DN1476_c0_g1	1.40	up	hypothetical protein POPTR_0002s09850g [Populus trichocarpa]	10.24	5.93
map00908	Zeatin biosynthesis	3.62E-02	TRINITY_DN15919_c0_g1	1.73	up	adenylate dimethylallyltransferase 5b [Populus x canadensis]	5.06	3.28
map00908	Zeatin biosynthesis	3.62E-02	TRINITY_DN18121_c1_g2	1.85	up	hypothetical protein POPTR_0098s00280g [Populus trichocarpa]	19.49	7.07
map00908	Zeatin biosynthesis	3.62E-02	TRINITY_DN18420_c0_g1	3.37	up	hypothetical protein POPTR_0016s02270g [Populus trichocarpa]	30.15	5.07
map00908	Zeatin biosynthesis	3.62E-02	TRINITY_DN18485_c0_g2	1.35	up	hypothetical protein POPTR_0016s10010g [Populus trichocarpa]	58.58	34.89
map00908	Zeatin biosynthesis	3.62E-02	TRINITY_DN19411_c0_g2	-1.11	down	TRNA ISOPENTENYLTRANSFERASE family protein [Populus trichocarpa]	4.22	14.39
map00908	Zeatin biosynthesis	3.62E-02	TRINITY_DN21602_c0_g2	2.46	up	hypothetical protein POPTR_0016s02190g [Populus trichocarpa]	3.61	0.96
map00402	Benzoxazinoid biosynthesis	3.84E-02	TRINITY_DN22782_c0_g3	2.72	up	PREDICTED: 1-aminocyclopropane-1-carboxylate oxidase homolog 1-like [Populus euphratica]	33.44	7.80
map00402	Benzoxazinoid biosynthesis	3.84E-02	TRINITY_DN12667_c0_g1	3.45	up	hypothetical protein POPTR_0013s04220g [Populus trichocarpa]	4.05	0.56
map00402	Benzoxazinoid biosynthesis	3.84E-02	TRINITY_DN14451_c0_g1	1.45	up	hypothetical protein POPTR_0010s08390g [Populus trichocarpa]	3.88	2.15
map00402	Benzoxazinoid biosynthesis	3.84E-02	TRINITY_DN16718_c0_g1	1.26	up	hypothetical protein POPTR_0010s08380g [Populus trichocarpa]	32.67	20.40
map00402	Benzoxazinoid biosynthesis	3.84E-02	TRINITY_DN16743_c0_g2	2.52	up	hypothetical protein POPTR_0002s04170g [Populus trichocarpa]	9.10	1.89
map00402	Benzoxazinoid biosynthesis	3.84E-02	TRINITY_DN17853_c0_g1	1.34	up	PREDICTED: 1-aminocyclopropane-1-carboxylate oxidase homolog 1-like [Populus euphratica]	9.07	5.51
map00402	Benzoxazinoid biosynthesis	3.84E-02	TRINITY_DN17902_c0_g1	1.02	up	hypothetical protein POPTR_0010s08410g [Populus trichocarpa]	5.63	4.23
map00960	Tropane, piperidine and pyridine alkaloid biosynthesis	4.00E-02	TRINITY_DN22813_c0_g4	1.08	up	aspartate transaminase family protein [Populus trichocarpa]	25.11	18.98
map00960	Tropane, piperidine and pyridine alkaloid biosynthesis	4.00E-02	TRINITY_DN23609_c0_g1	1.54	up	unknown [Populus trichocarpa]	110.88	58.39
map00960	Tropane, piperidine and pyridine alkaloid biosynthesis	4.00E-02	TRINITY_DN23704_c1_g1	-3.03	down	hypothetical protein POPTR_0001s07880g [Populus trichocarpa]	1.87	24.42
map00960	Tropane, piperidine and pyridine alkaloid biosynthesis	4.00E-02	TRINITY_DN23709_c0_g2	1.23	up	aspartate aminotransferase 2 family protein [Populus trichocarpa]	46.64	32.80
map00960	Tropane, piperidine and pyridine alkaloid biosynthesis	4.00E-02	TRINITY_DN24599_c6_g1	-1.97	down	hypothetical protein POPTR_0007s03670g [Populus trichocarpa]	0.44	2.68
map00960	Tropane, piperidine and pyridine alkaloid biosynthesis	4.00E-02	TRINITY_DN24826_c0_g1	-1.05	down	PREDICTED: uncharacterized protein LOC105138712 [Populus euphratica]	1.85	5.77
map00960	Tropane, piperidine and pyridine alkaloid biosynthesis	4.00E-02	TRINITY_DN24826_c0_g2	-4.47	down	PREDICTED: uncharacterized protein LOC105109098 [Populus euphratica]	0.14	5.15
map00960	Tropane, piperidine and pyridine alkaloid biosynthesis	4.00E-02	TRINITY_DN24880_c0_g1	-1.43	down	curculin-like lectin family protein [Populus trichocarpa]	4.67	18.36
map00960	Tropane, piperidine and pyridine alkaloid biosynthesis	4.00E-02	TRINITY_DN26235_c0_g1	-1.34	down	PREDICTED: LOW QUALITY PROTEIN: G-type lectin S-receptor-like serine/threonine-protein kinase RLK1 [Populus euphratica]	6.54	32.64
map00960	Tropane, piperidine and pyridine alkaloid biosynthesis	4.00E-02	TRINITY_DN26235_c0_g3	-1.48	down	PREDICTED: G-type lectin S-receptor-like serine/threonine-protein kinase RLK1 [Populus euphratica]	4.02	17.62
map00960	Tropane, piperidine and pyridine alkaloid biosynthesis	4.00E-02	TRINITY_DN26313_c1_g1	1.31	up	unknown [Populus trichocarpa]	76.73	48.46
map00960	Tropane, piperidine and pyridine alkaloid biosynthesis	4.00E-02	TRINITY_DN27242_c0_g1	-1.23	down	hypothetical protein POPTR_0151s00200g [Populus trichocarpa]	2.45	8.98

map00960	Tropane, piperidine and pyridine alkaloid biosynthesis	4.00E-02	TRINITY_DN14000_c0_g1	-2.71	down	hypothetical protein POPTR_0013s05640g [Populus trichocarpa]	0.38	5.38
map00960	Tropane, piperidine and pyridine alkaloid biosynthesis	4.00E-02	TRINITY_DN15219_c0_g1	-3.09	down	hypothetical protein POPTR_0013s05650g [Populus trichocarpa]	0.23	2.86
map00960	Tropane, piperidine and pyridine alkaloid biosynthesis	4.00E-02	TRINITY_DN17759_c0_g1	-1.68	down	hypothetical protein POPTR_0004s01460g, partial [Populus trichocarpa]	1.43	5.88
map00960	Tropane, piperidine and pyridine alkaloid biosynthesis	4.00E-02	TRINITY_DN17759_c0_g3	-2.14	down	PREDICTED: proline-rich receptor-like protein kinase PERK3 isoform X1 [Populus euphratica]	0.24	1.71
map00960	Tropane, piperidine and pyridine alkaloid biosynthesis	4.00E-02	TRINITY_DN18389_c1_g1	1.18	up	PREDICTED: tropinone reductase homolog [Populus euphratica]	35.46	24.97
map00960	Tropane, piperidine and pyridine alkaloid biosynthesis	4.00E-02	TRINITY_DN18756_c0_g3	1.80	up	hypothetical protein POPTR_0005s04150g [Populus trichocarpa]	2.80	1.20
map00960	Tropane, piperidine and pyridine alkaloid biosynthesis	4.00E-02	TRINITY_DN19059_c0_g2	-1.92	down	PREDICTED: G-type lectin S-receptor-like serine/threonine-protein kinase At5g24080 isoform X1 [Populus euphratica]	1.17	7.57
map00960	Tropane, piperidine and pyridine alkaloid biosynthesis	4.00E-02	TRINITY_DN19059_c0_g3	-6.09	down	hypothetical protein POPTR_0015s05780g [Populus trichocarpa]	0.09	4.40
map00960	Tropane, piperidine and pyridine alkaloid biosynthesis	4.00E-02	TRINITY_DN19472_c0_g2	1.32	up	hypothetical protein POPTR_0007s01130g [Populus trichocarpa]	8.54	5.46
map00960	Tropane, piperidine and pyridine alkaloid biosynthesis	4.00E-02	TRINITY_DN19472_c0_g3	-3.23	down	hypothetical protein POPTR_0017s04550g [Populus trichocarpa]	0.21	3.06
map00960	Tropane, piperidine and pyridine alkaloid biosynthesis	4.00E-02	TRINITY_DN19656_c0_g1	-1.62	down	PREDICTED: G-type lectin S-receptor-like serine/threonine-protein kinase RLK1 isoform X1 [Populus euphratica]	0.57	2.63
map00960	Tropane, piperidine and pyridine alkaloid biosynthesis	4.00E-02	TRINITY_DN19656_c0_g2	1.26	up	PREDICTED: G-type lectin S-receptor-like serine/threonine-protein kinase RLK1 [Populus euphratica]	3.74	2.35
map00960	Tropane, piperidine and pyridine alkaloid biosynthesis	4.00E-02	TRINITY_DN19656_c0_g3	-4.04	down	hypothetical protein POPTR_0200s00220g [Populus trichocarpa]	0.09	2.49
map00960	Tropane, piperidine and pyridine alkaloid biosynthesis	4.00E-02	TRINITY_DN19731_c0_g4	1.51	up	short-chain dehydrogenase/reductase family protein [Populus trichocarpa]	41.63	22.34
map00960	Tropane, piperidine and pyridine alkaloid biosynthesis	4.00E-02	TRINITY_DN19981_c0_g1	-1.21	down	copper/topa quinone amine oxidase precursor family protein [Populus trichocarpa]	1.81	6.48
map00960	Tropane, piperidine and pyridine alkaloid biosynthesis	4.00E-02	TRINITY_DN21133_c0_g2	-1.22	down	PREDICTED: G-type lectin S-receptor-like serine/threonine-protein kinase RLK1 isoform X1 [Populus euphratica]	6.50	22.69
map00960	Tropane, piperidine and pyridine alkaloid biosynthesis	4.00E-02	TRINITY_DN21391_c0_g1	1.12	up	PREDICTED: uncharacterized protein LOC105124970 [Populus euphratica]	49.29	33.42
map00010	Glycolysis / Gluconeogenesis	4.50E-02	TRINITY_DN21932_c0_g3	-1.06	down	phosphatase 2C family protein [Populus trichocarpa]	2.53	8.10
map00010	Glycolysis / Gluconeogenesis	4.50E-02	TRINITY_DN22033_c0_g1	1.60	up	Chain D family protein [Populus trichocarpa]	364.35	179.65
map00010	Glycolysis / Gluconeogenesis	4.50E-02	TRINITY_DN22186_c0_g1	1.05	up	alpha-hydroxynitrile lyase family protein [Populus trichocarpa]	31.37	22.10
map00010	Glycolysis / Gluconeogenesis	4.50E-02	TRINITY_DN22233_c0_g1	1.51	up	hypothetical protein POPTR_0002s10420g [Populus trichocarpa]	84.73	47.59
map00010	Glycolysis / Gluconeogenesis	4.50E-02	TRINITY_DN22245_c0_g1	2.23	up	PREDICTED: triosephosphate isomerase, chloroplastic [Populus euphratica]	838.73	263.34
map00010	Glycolysis / Gluconeogenesis	4.50E-02	TRINITY_DN22283_c0_g1	1.79	up	hypothetical protein POPTR_0002s09390g [Populus trichocarpa]	139.18	61.68
map00010	Glycolysis / Gluconeogenesis	4.50E-02	TRINITY_DN22304_c0_g1	-2.25	down	cucumber protein kinase CsPK3 [Populus trichocarpa]	2.28	16.67
map00010	Glycolysis / Gluconeogenesis	4.50E-02	TRINITY_DN22587_c0_g1	1.20	up	PREDICTED: fructose-bisphosphate aldolase cytoplasmic isozyme [Populus euphratica]	371.77	247.12
map00010	Glycolysis / Gluconeogenesis	4.50E-02	TRINITY_DN22598_c1_g1	1.42	up	2 family protein [Populus trichocarpa]	136.33	77.98
map00010	Glycolysis / Gluconeogenesis	4.50E-02	TRINITY_DN22677_c0_g1	-1.18	down	alpha galactosyltransferase family protein [Populus trichocarpa]	16.78	58.67
map00010	Glycolysis / Gluconeogenesis	4.50E-02	TRINITY_DN23066_c0_g5	1.83	up	PREDICTED: probable 2-carboxy-D-arabinitol-1-phosphatase [Populus euphratica]	71.98	31.71
map00010	Glycolysis / Gluconeogenesis	4.50E-02	TRINITY_DN23192_c0_g1	-2.27	down	hypothetical protein POPTR_0004s19720g [Populus trichocarpa]	3.73	27.49
map00010	Glycolysis / Gluconeogenesis	4.50E-02	TRINITY_DN23213_c0_g1	1.52	up	pyruvate dehydrogenase family protein [Populus trichocarpa]	125.88	67.45
map00010	Glycolysis / Gluconeogenesis	4.50E-02	TRINITY_DN23403_c0_g1	1.10	up	hypothetical protein POPTR_0005s23960g [Populus trichocarpa]	80.55	57.95
map00010	Glycolysis / Gluconeogenesis	4.50E-02	TRINITY_DN23701_c0_g1	1.96	up	latex plastidic aldolase-like family protein [Populus trichocarpa]	2289.47	879.27
map00010	Glycolysis / Gluconeogenesis	4.50E-02	TRINITY_DN23701_c0_g5	2.57	up	plastidic aldolase family protein [Populus trichocarpa]	7.53	2.00
map00010	Glycolysis / Gluconeogenesis	4.50E-02	TRINITY_DN23702_c0_g1	1.17	up	hypothetical protein POPTR_0001s07480g [Populus trichocarpa]	45.77	26.38
map00010	Glycolysis / Gluconeogenesis	4.50E-02	TRINITY_DN23965_c0_g1	1.23	up	PREDICTED: triosephosphate isomerase, cytosolic [Populus euphratica]	641.46	426.80
map00010	Glycolysis / Gluconeogenesis	4.50E-02	TRINITY_DN24285_c0_g5	1.46	up	PREDICTED: WAT1-related protein At2g37460-like isoform X1 [Populus euphratica]	2.63	1.46
map00010	Glycolysis / Gluconeogenesis	4.50E-02	TRINITY_DN24393_c0_g1	1.31	up	hypothetical protein POPTR_0001s24710g [Populus trichocarpa]	198.18	106.97

map00010	Glycolysis / Gluconeogenesis	4.50E-02	TRINITY_DN24623_c0_g2	1.54	up	hypothetical protein POPTR_0008s11770g [Populus trichocarpa]	42.50	22.04
map00010	Glycolysis / Gluconeogenesis	4.50E-02	TRINITY_DN24628_c0_g1	1.28	up	PREDICTED: aldehyde dehydrogenase family 3 member H1-like [Populus euphratica]	40.39	24.46
map00010	Glycolysis / Gluconeogenesis	4.50E-02	TRINITY_DN24707_c1_g1	-1.25	down	nodulin MtN21 family protein [Populus trichocarpa]	14.31	50.91
map00010	Glycolysis / Gluconeogenesis	4.50E-02	TRINITY_DN24709_c0_g1	1.03	up	PREDICTED: pyruvate dehydrogenase E1 component subunit beta-3, chloroplastic-like [Populus euphratica]	176.40	133.88
map00010	Glycolysis / Gluconeogenesis	4.50E-02	TRINITY_DN25129_c1_g2	1.01	up	3-phosphoglycerate kinase [Populus tremuloides]	153.72	116.35
map00010	Glycolysis / Gluconeogenesis	4.50E-02	TRINITY_DN25141_c0_g1	1.90	up	PREDICTED: dihydrolipoyllysine-residue acetyltransferase component 4 of pyruvate dehydrogenase complex, chloroplastic [Populus euphratica]	116.62	48.47
map00010	Glycolysis / Gluconeogenesis	4.50E-02	TRINITY_DN25221_c1_g4	2.08	up	PREDICTED: LOW QUALITY PROTEIN: dihydrolipoyllysine-residue acetyltransferase component 5 of pyruvate dehydrogenase complex, chloroplastic-like, partial [Populus euphratica]	9.32	3.34
map00010	Glycolysis / Gluconeogenesis	4.50E-02	TRINITY_DN25232_c0_g1	-1.14	down	hypothetical protein POPTR_0006s11580g [Populus trichocarpa]	3.34	11.12
map00010	Glycolysis / Gluconeogenesis	4.50E-02	TRINITY_DN25377_c0_g1	1.91	up	hypothetical protein POPTR_0013s11870g [Populus trichocarpa]	85.23	34.17
map00010	Glycolysis / Gluconeogenesis	4.50E-02	TRINITY_DN25630_c0_g1	1.30	up	aldehyde dehydrogenase 1 precursor family protein [Populus trichocarpa]	120.35	73.18
map00010	Glycolysis / Gluconeogenesis	4.50E-02	TRINITY_DN25692_c0_g1	-1.21	down	AAA-type ATPase family protein [Populus trichocarpa]	9.04	31.77
map00010	Glycolysis / Gluconeogenesis	4.50E-02	TRINITY_DN25692_c0_g2	-1.31	down	PREDICTED: pachytene checkpoint protein 2 homolog [Populus euphratica]	12.27	47.09
map00010	Glycolysis / Gluconeogenesis	4.50E-02	TRINITY_DN25819_c0_g1	1.14	up	PREDICTED: NADP-dependent glyceraldehyde-3-phosphate dehydrogenase-like isoform X1 [Populus euphratica]	282.53	188.30
map00010	Glycolysis / Gluconeogenesis	4.50E-02	TRINITY_DN26025_c0_g1	-1.71	down	hypothetical protein POPTR_0004s18340g [Populus trichocarpa]	5.60	27.70
map00010	Glycolysis / Gluconeogenesis	4.50E-02	TRINITY_DN26163_c0_g1	1.24	up	PREDICTED: dihydrolipoyllysine-residue acetyltransferase component 2 of pyruvate dehydrogenase complex, mitochondrial-like [Populus euphratica]	49.33	31.65
map00010	Glycolysis / Gluconeogenesis	4.50E-02	TRINITY_DN26175_c0_g1	1.33	up	PREDICTED: hexokinase-1-like [Populus euphratica]	24.35	14.67
map00010	Glycolysis / Gluconeogenesis	4.50E-02	TRINITY_DN26209_c0_g1	1.84	up	hypothetical protein SETIT_022310mg [Setaria italica]	186.50	89.03
map00010	Glycolysis / Gluconeogenesis	4.50E-02	TRINITY_DN26341_c0_g2	1.21	up	dihydrolipoamide S-acetyltransferase family protein [Populus trichocarpa]	11.43	7.48
map00010	Glycolysis / Gluconeogenesis	4.50E-02	TRINITY_DN26421_c0_g1	1.32	up	the aldehyde dehydrogenase cp-ADH from C.plantagineum family protein [Populus trichocarpa]	58.13	34.64
map00010	Glycolysis / Gluconeogenesis	4.50E-02	TRINITY_DN26627_c0_g1	2.37	up	PHOSPHOGLYCERATE KINASE 1 family protein [Populus trichocarpa]	946.82	271.09
map00010	Glycolysis / Gluconeogenesis	4.50E-02	TRINITY_DN26661_c0_g1	2.62	up	hypothetical protein CISIN_1g016748mg [Citrus sinensis]	718.86	176.98
map00010	Glycolysis / Gluconeogenesis	4.50E-02	TRINITY_DN26743_c0_g1	1.15	up	RecName: Full=Phosphoglucomutase, cytoplasmic; Short=PGM; AltName: Full=Glucose phosphomutase	74.05	53.71
map00010	Glycolysis / Gluconeogenesis	4.50E-02	TRINITY_DN26837_c0_g1	2.09	up	hypothetical protein POPTR_0010s15200g [Populus trichocarpa]	81.17	29.93
map00010	Glycolysis / Gluconeogenesis	4.50E-02	TRINITY_DN26996_c1_g1	1.79	up	PREDICTED: phosphoglucomutase, chloroplastic-like [Populus euphratica]	112.47	49.80
map00010	Glycolysis / Gluconeogenesis	4.50E-02	TRINITY_DN11372_c0_g1	3.83	up	hypothetical protein POPTR_0011s15150g [Populus trichocarpa]	4.92	0.36
map00010	Glycolysis / Gluconeogenesis	4.50E-02	TRINITY_DN12955_c0_g1	1.82	up	hypothetical protein POPTR_0011s05190g [Populus trichocarpa]	15.50	6.27
map00010	Glycolysis / Gluconeogenesis	4.50E-02	TRINITY_DN13808_c0_g3	-1.99	down	hypothetical protein POPTR_0001s04370g [Populus trichocarpa]	0.58	3.70
map00010	Glycolysis / Gluconeogenesis	4.50E-02	TRINITY_DN15363_c0_g2	-2.90	down	putative serine/threonine protein kinase [Populus tomentosa]	0.62	7.03
map00010	Glycolysis / Gluconeogenesis	4.50E-02	TRINITY_DN15573_c0_g1	-2.43	down	alcohol dehydrogenase family protein [Populus trichocarpa]	0.77	3.49
map00010	Glycolysis / Gluconeogenesis	4.50E-02	TRINITY_DN16817_c0_g1	1.64	up	PREDICTED: tRNA (guanine-N(7)-)-methyltransferase [Populus euphratica]	40.30	24.24
map00010	Glycolysis / Gluconeogenesis	4.50E-02	TRINITY_DN17020_c0_g1	2.36	up	hypothetical protein POPTR_0002s06890g [Populus trichocarpa]	22.89	6.58
map00010	Glycolysis / Gluconeogenesis	4.50E-02	TRINITY_DN17072_c0_g2	-1.31	down	PREDICTED: putative glucose-6-phosphate 1-epimerase [Populus euphratica]	1.75	6.41
map00010	Glycolysis / Gluconeogenesis	4.50E-02	TRINITY_DN17098_c0_g1	1.47	up	hypothetical protein POPTR_0007s13060g [Populus trichocarpa]	103.84	56.53
map00010	Glycolysis / Gluconeogenesis	4.50E-02	TRINITY_DN17229_c0_g1	-1.51	down	hypothetical protein POPTR_0014s03490g [Populus trichocarpa]	1.22	5.46
map00010	Glycolysis / Gluconeogenesis	4.50E-02	TRINITY_DN17246_c0_g1	1.06	up	hypothetical protein POPTR_0017s07040g [Populus trichocarpa]	24.42	17.62
map00010	Glycolysis / Gluconeogenesis	4.50E-02	TRINITY_DN17257_c0_g1	-1.22	down	hypothetical protein POPTR_0001s00440g [Populus trichocarpa]	6.45	23.17
map00010	Glycolysis / Gluconeogenesis	4.50E-02	TRINITY_DN17574_c0_g1	1.98	up	aldose 1-epimerase family protein [Populus trichocarpa]	13.53	5.15
map00010	Glycolysis / Gluconeogenesis	4.50E-02	TRINITY_DN17663_c0_g3	1.15	up	unknown [Populus trichocarpa x Populus deltoides]	373.94	255.99
map00010	Glycolysis / Gluconeogenesis	4.50E-02	TRINITY_DN18081_c0_g1	1.00	up	hypothetical protein POPTR_0014s18410g [Populus trichocarpa]	13.68	10.10
map00010	Glycolysis / Gluconeogenesis	4.50E-02	TRINITY_DN18209_c1_g1	1.05	up	PREDICTED: pyruvate kinase, cytosolic isozyme-like [Populus euphratica]	19.18	14.26

map00010	Glycolysis / Gluconeogenesis	4.50E-02	TRINITY_DN18225_c0_g3	1.29	up	PREDICTED: hexokinase-1-like [Populus euphratica]	38.97	24.40
map00010	Glycolysis / Gluconeogenesis	4.50E-02	TRINITY_DN18895_c0_g1	-1.06	down	mitochondrial aldehyde dehydrogenase family protein [Populus trichocarpa]	7.40	23.22
map00010	Glycolysis / Gluconeogenesis	4.50E-02	TRINITY_DN19215_c1_g1	-1.38	down	hypothetical protein POPTR_0007s10210g [Populus trichocarpa]	3.47	12.55
map00010	Glycolysis / Gluconeogenesis	4.50E-02	TRINITY_DN19215_c2_g1	-1.56	down	PREDICTED: probable protein phosphatase 2C 63 [Populus euphratica]	6.48	30.16
map00010	Glycolysis / Gluconeogenesis	4.50E-02	TRINITY_DN19540_c0_g3	1.78	up	hypothetical protein POPTR_0001s45280g [Populus trichocarpa]	3.69	1.58
map00010	Glycolysis / Gluconeogenesis	4.50E-02	TRINITY_DN19743_c0_g1	1.38	up	PREDICTED: WAT1-related protein At4g19185-like [Populus euphratica]	66.15	39.51
map00010	Glycolysis / Gluconeogenesis	4.50E-02	TRINITY_DN19848_c0_g1	1.56	up	phosphopyruvate hydratase family protein [Populus trichocarpa]	247.16	128.86
map00010	Glycolysis / Gluconeogenesis	4.50E-02	TRINITY_DN20087_c0_g1	-1.46	down	PREDICTED: serine/threonine-protein kinase UCNL-like [Populus euphratica]	3.22	13.35
map00010	Glycolysis / Gluconeogenesis	4.50E-02	TRINITY_DN20666_c0_g2	-2.84	down	pyruvate decarboxylase family protein [Populus trichocarpa]	2.05	4.49
map00010	Glycolysis / Gluconeogenesis	4.50E-02	TRINITY_DN20902_c0_g2	-1.66	down	PREDICTED: putative glucose-6-phosphate 1-epimerase isoform X1 [Theobroma cacao]	6.26	29.13
map00010	Glycolysis / Gluconeogenesis	4.50E-02	TRINITY_DN21062_c0_g1	1.22	up	PREDICTED: hexokinase-1-like [Populus euphratica]	9.26	6.04
map00010	Glycolysis / Gluconeogenesis	4.50E-02	TRINITY_DN21068_c0_g2	-2.13	down	hypothetical protein POPTR_0004s13470g [Populus trichocarpa]	6.77	46.35
map00010	Glycolysis / Gluconeogenesis	4.50E-02	TRINITY_DN21068_c0_g3	-4.42	down	PREDICTED: aldose 1-epimerase-like [Populus euphratica]	0.45	14.22
map00010	Glycolysis / Gluconeogenesis	4.50E-02	TRINITY_DN21140_c0_g2	2.24	up	mitochondrial lipoamide dehydrogenase [Populus tremuloides]	202.39	67.53
map00010	Glycolysis / Gluconeogenesis	4.50E-02	TRINITY_DN21337_c0_g3	-2.71	down	PREDICTED: multiple inositol polyphosphate phosphatase 1-like isoform X1 [Populus euphratica]	0.16	1.69
map00010	Glycolysis / Gluconeogenesis	4.50E-02	TRINITY_DN21427_c0_g1	1.40	up	hypothetical protein POPTR_0016s11621g [Populus trichocarpa]	98.65	56.05
map00010	Glycolysis / Gluconeogenesis	4.50E-02	TRINITY_DN6390_c0_g1	3.46	up	nodulin MtN21 family protein [Populus trichocarpa]	1.91	0.26
map04146	Peroxisome	4.88E-02	TRINITY_DN21884_c0_g1	1.24	up	hypothetical protein POPTR_0005s08280g [Populus trichocarpa]	132.32	84.75
map04146	Peroxisome	4.88E-02	TRINITY_DN22292_c1_g2	1.41	up	Superoxide dismutase family protein [Populus trichocarpa]	171.13	95.77
map04146	Peroxisome	4.88E-02	TRINITY_DN22292_c1_g3	1.19	up	PREDICTED: superoxide dismutase [Fe], chloroplastic isoform X1 [Populus euphratica]	351.06	231.45
map04146	Peroxisome	4.88E-02	TRINITY_DN22984_c0_g2	1.24	up	hypothetical protein POPTR_0017s09760g [Populus trichocarpa]	24.83	15.86
map04146	Peroxisome	4.88E-02	TRINITY_DN23036_c0_g3	-1.08	down	PREDICTED: long chain acyl-CoA synthetase 2 isoform X1 [Populus euphratica]	4.05	12.87
map04146	Peroxisome	4.88E-02	TRINITY_DN23182_c1_g2	2.86	up	hypothetical protein POPTR_0005s10340g [Populus trichocarpa]	182.43	38.37
map04146	Peroxisome	4.88E-02	TRINITY_DN23265_c0_g4	1.22	up	PREDICTED: protein Mpv17 isoform X1 [Populus euphratica]	9.80	6.43
map04146	Peroxisome	4.88E-02	TRINITY_DN23271_c0_g1	1.57	up	hypothetical protein POPTR_0001s35080g [Populus trichocarpa]	37.34	19.20
map04146	Peroxisome	4.88E-02	TRINITY_DN23322_c1_g1	1.07	up	PREDICTED: catalase isozyme 1-like [Populus euphratica]	422.64	304.47
map04146	Peroxisome	4.88E-02	TRINITY_DN23418_c0_g1	1.11	up	PREDICTED: protein sym-1-like [Populus euphratica]	46.34	33.91
map04146	Peroxisome	4.88E-02	TRINITY_DN24158_c0_g1	1.40	up	mitochondrial substrate carrier family protein [Populus trichocarpa]	42.17	24.42
map04146	Peroxisome	4.88E-02	TRINITY_DN24242_c0_g1	-1.34	down	hypothetical protein POPTR_0003s06730g [Populus trichocarpa]	3.03	11.55
map04146	Peroxisome	4.88E-02	TRINITY_DN24296_c0_g5	1.30	up	putative CuZn-superoxide dismutase (chloroplast) [Populus tremula x Populus tremuloides]	1020.62	621.71
map04146	Peroxisome	4.88E-02	TRINITY_DN24431_c1_g2	-1.25	down	PREDICTED: acyl-coenzyme A oxidase 4, peroxisomal-like isoform X3 [Populus euphratica]	3.95	18.34
map04146	Peroxisome	4.88E-02	TRINITY_DN24765_c0_g1	1.27	up	peroxisomal biogenesis factor 11 family protein [Populus trichocarpa]	475.01	301.42
map04146	Peroxisome	4.88E-02	TRINITY_DN25062_c1_g8	1.06	up	phytanoyl-CoA dioxygenase family protein [Populus trichocarpa]	43.85	31.82
map04146	Peroxisome	4.88E-02	TRINITY_DN25183_c0_g1	-1.02	down	hypothetical protein POPTR_0013s02220g [Populus trichocarpa]	7.46	25.55
map04146	Peroxisome	4.88E-02	TRINITY_DN25791_c0_g1	1.16	up	hypothetical protein POPTR_0010s18400g [Populus trichocarpa]	15.98	11.25
map04146	Peroxisome	4.88E-02	TRINITY_DN26366_c0_g2	1.69	up	hypothetical protein POPTR_0002s22410g [Populus trichocarpa]	361.54	171.53
map04146	Peroxisome	4.88E-02	TRINITY_DN26565_c0_g1	1.03	up	hypothetical protein POPTR_0005s19990g [Populus trichocarpa]	60.48	36.43
map04146	Peroxisome	4.88E-02	TRINITY_DN27292_c1_g1	1.16	up	hypothetical protein POPTR_0015s12190g [Populus trichocarpa]	53.97	35.99
map04146	Peroxisome	4.88E-02	TRINITY_DN27292_c1_g2	2.05	up	PREDICTED: uncharacterized protein LOC105110257 [Populus euphratica]	260.63	93.48
map04146	Peroxisome	4.88E-02	TRINITY_DN27297_c0_g4	1.47	up	PREDICTED: protein Mpv17-like [Populus euphratica]	6.75	3.71
map04146	Peroxisome	4.88E-02	TRINITY_DN27395_c0_g1	1.70	up	aminotransferase 2 family protein [Populus trichocarpa]	980.92	451.86
map04146	Peroxisome	4.88E-02	TRINITY_DN27448_c0_g1	1.92	up	glycolate oxidase family protein [Populus trichocarpa]	1633.96	640.54

map04146	Peroxisome	4.88E-02	TRINITY_DN27448_c0_g2	1.73	up	PREDICTED: peroxisomal (S)-2-hydroxy-acid oxidase [Populus euphratica]	519.75	233.20
map04146	Peroxisome	4.88E-02	TRINITY_DN13195_c0_g1	1.88	up	hypothetical protein POPTR_0019s10390g [Populus trichocarpa]	4.32	3.51
map04146	Peroxisome	4.88E-02	TRINITY_DN13230_c0_g2	-1.83	down	PREDICTED: peroxisomal (S)-2-hydroxy-acid oxidase-like isoform X1 [Populus euphratica]	0.42	2.27
map04146	Peroxisome	4.88E-02	TRINITY_DN14465_c0_g1	1.15	up	PREDICTED: ABC transporter D family member 1-like [Populus euphratica]	43.30	30.56
map04146	Peroxisome	4.88E-02	TRINITY_DN14990_c0_g1	1.78	up	hypothetical protein POPTR_0013s13870g [Populus trichocarpa]	20.13	9.04
map04146	Peroxisome	4.88E-02	TRINITY_DN15159_c0_g1	1.10	up	hypothetical protein POPTR_0004s00950g, partial [Populus trichocarpa]	62.31	40.95
map04146	Peroxisome	4.88E-02	TRINITY_DN15922_c1_g1	1.19	up	PREDICTED: peroxiredoxin-2F, mitochondrial [Populus euphratica]	104.19	69.35
map04146	Peroxisome	4.88E-02	TRINITY_DN16471_c0_g1	1.72	up	PREDICTED: peroxisomal (S)-2-hydroxy-acid oxidase-like [Phoenix dactylifera]	1059.17	482.14
map04146	Peroxisome	4.88E-02	TRINITY_DN18279_c0_g1	1.16	up	hypothetical protein POPTR_0001s10650g [Populus trichocarpa]	121.34	81.46
map04146	Peroxisome	4.88E-02	TRINITY_DN18787_c0_g1	-6.64	down	hypothetical protein POPTR_0009s14720g [Populus trichocarpa]	0.09	17.28
map04146	Peroxisome	4.88E-02	TRINITY_DN19088_c0_g1	1.09	up	peroxiredoxin family protein [Populus trichocarpa]	393.76	285.85
map04146	Peroxisome	4.88E-02	TRINITY_DN19154_c0_g1	1.84	up	PREDICTED: peroxisomal membrane protein 11B-like [Populus euphratica]	35.95	15.31
map04146	Peroxisome	4.88E-02	TRINITY_DN19247_c0_g1	-1.92	down	PREDICTED: oxalate--CoA ligase-like [Populus euphratica]	5.18	30.82
map04146	Peroxisome	4.88E-02	TRINITY_DN19324_c0_g1	1.73	up	hypothetical protein POPTR_0005s09190g [Populus trichocarpa]	68.66	31.28
map04146	Peroxisome	4.88E-02	TRINITY_DN19512_c0_g1	1.30	up	mitochondrial substrate carrier family protein [Populus trichocarpa]	12.18	7.45
map04146	Peroxisome	4.88E-02	TRINITY_DN19728_c0_g1	1.25	up	hypothetical protein POPTR_0010s01430g [Populus trichocarpa]	46.16	30.18
map04146	Peroxisome	4.88E-02	TRINITY_DN19728_c0_g2	3.11	up	PREDICTED: bifunctional epoxide hydrolase 2-like [Populus euphratica]	10.52	1.94
map04146	Peroxisome	4.88E-02	TRINITY_DN19877_c1_g1	1.52	up	hipI-SODC1s [Populus tremula]	76.97	44.87
map04146	Peroxisome	4.88E-02	TRINITY_DN19877_c1_g2	1.59	up	putative CuZn-superoxide dismutase [Populus tremula x Populus tremuloides]	23.67	12.08
map04146	Peroxisome	4.88E-02	TRINITY_DN19913_c0_g1	1.05	up	PREDICTED: hydroxymethylglutaryl-CoA lyase, mitochondrial-like isoform X1 [Populus euphratica]	13.16	7.43
map04146	Peroxisome	4.88E-02	TRINITY_DN20223_c0_g1	-1.54	down	zinc finger family protein [Populus trichocarpa]	5.37	24.46
map04146	Peroxisome	4.88E-02	TRINITY_DN20584_c0_g2	1.73	up	peroxisomal membrane 22 kDa family protein [Populus trichocarpa]	70.32	30.71
map04146	Peroxisome	4.88E-02	TRINITY_DN20696_c0_g1	-2.87	down	epoxide hydrolase family protein [Populus trichocarpa]	0.84	9.54
map04146	Peroxisome	4.88E-02	TRINITY_DN20891_c1_g1	2.08	up	PREDICTED: F-box protein At2g32560-like [Populus euphratica]	23.97	9.10
map04146	Peroxisome	4.88E-02	TRINITY_DN20933_c0_g1	-3.74	down	hypothetical protein POPTR_0002s19330g [Populus trichocarpa]	0.65	13.67
map04146	Peroxisome	4.88E-02	TRINITY_DN20933_c0_g3	-2.76	down	PREDICTED: long chain acyl-CoA synthetase 1 [Populus euphratica]	0.62	6.55
map04146	Peroxisome	4.88E-02	TRINITY_DN20981_c2_g2	1.23	up	hypothetical protein POPTR_0012s14730g [Populus trichocarpa]	31.60	20.56
map04146	Peroxisome	4.88E-02	TRINITY_DN21050_c0_g2	1.36	up	unknown [Populus trichocarpa]	74.04	44.50
map04146	Peroxisome	4.88E-02	TRINITY_DN21063_c0_g3	1.07	up	hypothetical protein POPTR_0019s13430g [Populus trichocarpa]	11.90	8.62
map04146	Peroxisome	4.88E-02	TRINITY_DN21232_c0_g1	-5.70	down	WD40-repeat protein [Populus tremula x Populus tremuloides]	0.07	6.36
map04146	Peroxisome	4.88E-02	TRINITY_DN21232_c0_g2	-6.12	down	hypothetical protein POPTR_0006s225601g [Populus trichocarpa]	0.07	8.17
map04146	Peroxisome	4.88E-02	TRINITY_DN21289_c1_g1	-2.22	down	PREDICTED: uncharacterized protein LOC108983648 [Juglans regia]	0.45	3.16
map04146	Peroxisome	4.88E-02	TRINITY_DN21383_c0_g2	-2.81	down	zinc finger family protein [Populus trichocarpa]	1.40	14.88
map04146	Peroxisome	4.88E-02	TRINITY_DN390_c0_g1	-2.53	down	hypothetical protein POPTR_0011s04780g [Populus trichocarpa]	0.86	7.69
map00920	Sulfur metabolism	4.92E-02	TRINITY_DN22348_c0_g1	2.11	up	O-acetylserine (thiol)lyase family protein [Populus trichocarpa]	18.41	6.25
map00920	Sulfur metabolism	4.92E-02	TRINITY_DN22348_c0_g4	1.61	up	O-acetylserine (thiol)lyase family protein [Populus trichocarpa]	220.16	110.90
map00920	Sulfur metabolism	4.92E-02	TRINITY_DN22348_c0_g6	1.74	up	hypothetical protein POPTR_0013s13150g [Populus trichocarpa]	146.50	66.04
map00920	Sulfur metabolism	4.92E-02	TRINITY_DN22960_c1_g2	1.71	up	hypothetical protein POPTR_0013s13150g [Populus trichocarpa]	267.00	123.84
map00920	Sulfur metabolism	4.92E-02	TRINITY_DN23349_c0_g1	2.04	up	Cysteine synthase C1 [Theobroma cacao]	171.68	63.08
map00920	Sulfur metabolism	4.92E-02	TRINITY_DN23627_c1_g1	1.42	up	PREDICTED: cytochrome c [Pyrus x bretschneideri]	203.27	116.83
map00920	Sulfur metabolism	4.92E-02	TRINITY_DN23627_c1_g2	1.49	up	hypothetical protein POPTR_1028s00200g [Populus trichocarpa]	108.32	59.63
map00920	Sulfur metabolism	4.92E-02	TRINITY_DN25128_c1_g2	1.83	up	hypothetical protein POPTR_0015s09980g [Populus trichocarpa]	54.09	22.75
map00920	Sulfur metabolism	4.92E-02	TRINITY_DN26265_c0_g1	1.39	up	hypothetical protein POPTR_0013s10300g, partial [Populus trichocarpa]	33.29	19.38

map00920	Sulfur metabolism	4.92E-02	TRINITY_DN26854_c0_g1	2.31	up	sulfite reductase family protein [Populus trichocarpa]	94.07	29.35
map00920	Sulfur metabolism	4.92E-02	TRINITY_DN27334_c0_g1	1.13	up	putative NADPH-cytochrome P450 reductase family protein [Populus trichocarpa]	195.19	138.75
map00920	Sulfur metabolism	4.92E-02	TRINITY_DN15986_c0_g1	1.37	up	PREDICTED: glutaredoxin-C1 [Populus euphratica]	57.17	33.95
map00920	Sulfur metabolism	4.92E-02	TRINITY_DN16732_c0_g1	1.28	up	hypothetical protein POPTR_0017s07130g [Populus trichocarpa]	32.81	20.70
map00920	Sulfur metabolism	4.92E-02	TRINITY_DN17492_c0_g1	1.52	up	hypothetical protein POPTR_0002s25540g [Populus trichocarpa]	94.40	49.92
map00920	Sulfur metabolism	4.92E-02	TRINITY_DN17709_c0_g1	1.17	up	SULFITE OXIDASE family protein [Populus trichocarpa]	69.74	47.16
map00920	Sulfur metabolism	4.92E-02	TRINITY_DN17959_c0_g1	2.77	up	PREDICTED: probable S-sulfocysteine synthase, chloroplastic [Populus euphratica]	54.54	12.96
map00920	Sulfur metabolism	4.92E-02	TRINITY_DN18078_c0_g1	1.16	up	PAP-specific phosphatase family protein [Populus trichocarpa]	29.85	20.66
map00920	Sulfur metabolism	4.92E-02	TRINITY_DN18387_c0_g1	1.43	up	hypothetical protein POPTR_0017s12240g [Populus trichocarpa]	122.22	69.70
map00920	Sulfur metabolism	4.92E-02	TRINITY_DN18608_c2_g1	4.09	up	PREDICTED: 5'-adenylylsulfate reductase 3, chloroplastic-like isoform X1 [Populus euphratica]	6.79	0.82
map00920	Sulfur metabolism	4.92E-02	TRINITY_DN18896_c0_g1	2.92	up	adenosine 5' phosphosulfate reductase [Populus tremula x Populus alba]	276.48	56.13
map00920	Sulfur metabolism	4.92E-02	TRINITY_DN19940_c0_g1	1.32	up	PREDICTED: adenylyl-sulfate kinase 3-like [Populus euphratica]	131.63	73.66
map00920	Sulfur metabolism	4.92E-02	TRINITY_DN19940_c0_g2	1.65	up	PREDICTED: adenylyl-sulfate kinase 3-like [Populus euphratica]	26.43	13.84
map00920	Sulfur metabolism	4.92E-02	TRINITY_DN20815_c0_g1	1.41	up	PREDICTED: ATP sulfurylase 2-like [Populus euphratica]	46.46	28.77
map00920	Sulfur metabolism	4.92E-02	TRINITY_DN21417_c0_g1	1.35	up	SERINE ACETYLTRANSFERASE-106 family protein [Populus trichocarpa]	46.75	26.71
map00130	Ubiquinone and other terpenoid-quinone biosynthesis	5.43E-02	TRINITY_DN23001_c0_g1	1.07	up	naphthoate synthase family protein [Populus trichocarpa]	103.94	76.36
map00130	Ubiquinone and other terpenoid-quinone biosynthesis	5.43E-02	TRINITY_DN23015_c0_g1	-1.72	down	tocopherol cyclase [Hevea brasiliensis]	5.86	30.45
map00130	Ubiquinone and other terpenoid-quinone biosynthesis	5.43E-02	TRINITY_DN23395_c0_g1	1.24	up	4-coumarate--CoA ligase family protein [Populus trichocarpa]	14.87	10.65
map00130	Ubiquinone and other terpenoid-quinone biosynthesis	5.43E-02	TRINITY_DN23754_c0_g1	1.22	up	hypothetical protein POPTR_0002s04790g [Populus trichocarpa]	281.29	177.06
map00130	Ubiquinone and other terpenoid-quinone biosynthesis	5.43E-02	TRINITY_DN23777_c1_g1	1.05	up	hypothetical protein POPTR_0014s08880g [Populus trichocarpa]	21.90	17.49
map00130	Ubiquinone and other terpenoid-quinone biosynthesis	5.43E-02	TRINITY_DN24566_c0_g1	1.29	up	AMP-dependent synthetase and ligase family protein [Populus trichocarpa]	395.20	247.14
map00130	Ubiquinone and other terpenoid-quinone biosynthesis	5.43E-02	TRINITY_DN24794_c0_g1	1.03	up	PREDICTED: probable tocopherol O-methyltransferase, chloroplastic isoform X1 [Populus euphratica]	86.31	69.39
map00130	Ubiquinone and other terpenoid-quinone biosynthesis	5.43E-02	TRINITY_DN27262_c1_g1	-1.29	down	PREDICTED: homeobox-leucine zipper protein HDG2 isoform X2 [Populus euphratica]	4.55	16.64
map00130	Ubiquinone and other terpenoid-quinone biosynthesis	5.43E-02	TRINITY_DN27591_c1_g1	-1.23	down	homeodomain family protein [Populus trichocarpa]	27.16	94.11
map00130	Ubiquinone and other terpenoid-quinone biosynthesis	5.43E-02	TRINITY_DN16119_c0_g1	1.62	up	quinone reductase family protein [Populus trichocarpa]	14.83	6.99
map00130	Ubiquinone and other terpenoid-quinone biosynthesis	5.43E-02	TRINITY_DN16306_c0_g1	1.44	up	PREDICTED: 2-methoxy-6-polyprenyl-1,4-benzoquinol methylase, mitochondrial [Populus euphratica]	19.33	10.98
map00130	Ubiquinone and other terpenoid-quinone biosynthesis	5.43E-02	TRINITY_DN17914_c0_g3	1.82	up	quinone reductase family protein [Populus trichocarpa]	12.02	5.14
map00130	Ubiquinone and other terpenoid-quinone biosynthesis	5.43E-02	TRINITY_DN18315_c0_g1	-2.81	down	hypothetical protein POPTR_0015s13340g [Populus trichocarpa]	0.22	2.46
map00130	Ubiquinone and other terpenoid-quinone biosynthesis	5.43E-02	TRINITY_DN18330_c0_g1	-2.18	down	trans-cinnamate 4-hydroxylase [Populus trichocarpa]	0.62	4.40
map00130	Ubiquinone and other terpenoid-quinone biosynthesis	5.43E-02	TRINITY_DN18591_c0_g2	1.26	up	PREDICTED: homogentisate solanesyltransferase, chloroplastic [Populus euphratica]	50.03	31.91
map00130	Ubiquinone and other terpenoid-quinone biosynthesis	5.43E-02	TRINITY_DN18931_c0_g2	1.97	up	FLAVODOXIN-LIKE QUINONE REDUCTASE 1 family protein [Populus trichocarpa]	27.75	10.92
map00130	Ubiquinone and other terpenoid-quinone biosynthesis	5.43E-02	TRINITY_DN19472_c0_g2	1.32	up	hypothetical protein POPTR_0007s01130g [Populus trichocarpa]	8.54	5.46
map00130	Ubiquinone and other terpenoid-quinone biosynthesis	5.43E-02	TRINITY_DN19472_c0_g3	-3.23	down	hypothetical protein POPTR_0017s04550g [Populus trichocarpa]	0.21	3.06
map00130	Ubiquinone and other terpenoid-quinone biosynthesis	5.43E-02	TRINITY_DN19709_c2_g7	1.25	up	hypothetical protein POPTR_0011s03570g [Populus trichocarpa]	22.58	14.26
map00130	Ubiquinone and other terpenoid-quinone biosynthesis	5.43E-02	TRINITY_DN20348_c0_g2	1.31	up	4-coumarate: coenzyme A ligase 4 [Populus tomentosa]	41.80	31.54
map00130	Ubiquinone and other terpenoid-quinone biosynthesis	5.43E-02	TRINITY_DN21391_c0_g1	1.12	up	PREDICTED: uncharacterized protein LOC105124970 [Populus euphratica]	49.29	33.42

map00130	Ubiquinone and other terpenoid-quinone biosynthesis	5.43E-02	TRINITY_DN21527_c0_g1	-3.02	down	PROTODERMAL FACTOR2 family protein [Populus trichocarpa]	0.41	7.20
map00130	Ubiquinone and other terpenoid-quinone biosynthesis	5.43E-02	TRINITY_DN21527_c0_g3	-2.45	down	PROTODERMAL FACTOR2 family protein [Populus trichocarpa]	0.57	4.76
map00360	Phenylalanine metabolism	6.66E-02	TRINITY_DN22813_c0_g4	1.08	up	aspartate transaminase family protein [Populus trichocarpa]	25.11	18.98
map00360	Phenylalanine metabolism	6.66E-02	TRINITY_DN23395_c0_g1	1.24	up	4-coumarate--CoA ligase family protein [Populus trichocarpa]	14.87	10.65
map00360	Phenylalanine metabolism	6.66E-02	TRINITY_DN23704_c1_g1	-3.03	down	hypothetical protein POPTR_0001s07880g [Populus trichocarpa]	1.87	24.42
map00360	Phenylalanine metabolism	6.66E-02	TRINITY_DN23709_c0_g2	1.23	up	aspartate aminotransferase 2 family protein [Populus trichocarpa]	46.64	32.80
map00360	Phenylalanine metabolism	6.66E-02	TRINITY_DN24032_c3_g3	-2.56	down	hypothetical protein POPTR_0001s09640g [Populus trichocarpa]	5.18	47.53
map00360	Phenylalanine metabolism	6.66E-02	TRINITY_DN24566_c0_g1	1.29	up	AMP-dependent synthetase and ligase family protein [Populus trichocarpa]	395.20	247.14
map00360	Phenylalanine metabolism	6.66E-02	TRINITY_DN24599_c6_g1	-1.97	down	hypothetical protein POPTR_0007s03670g [Populus trichocarpa]	0.44	2.68
map00360	Phenylalanine metabolism	6.66E-02	TRINITY_DN24826_c0_g1	-1.05	down	PREDICTED: uncharacterized protein LOC105138712 [Populus euphratica]	1.85	5.77
map00360	Phenylalanine metabolism	6.66E-02	TRINITY_DN24826_c0_g2	-4.47	down	PREDICTED: uncharacterized protein LOC105109098 [Populus euphratica]	0.14	5.15
map00360	Phenylalanine metabolism	6.66E-02	TRINITY_DN24880_c0_g1	-1.43	down	curculin-like lectin family protein [Populus trichocarpa]	4.67	18.36
map00360	Phenylalanine metabolism	6.66E-02	TRINITY_DN25082_c0_g1	1.19	up	phenylalanine ammonia-lyase [Populus tomentosa]	144.67	96.35
map00360	Phenylalanine metabolism	6.66E-02	TRINITY_DN26235_c0_g1	-1.34	down	PREDICTED: LOW QUALITY PROTEIN: G-type lectin S-receptor-like serine/threonine-protein kinase RLK1 [Populus euphratica]	6.54	32.64
map00360	Phenylalanine metabolism	6.66E-02	TRINITY_DN26235_c0_g3	-1.48	down	PREDICTED: G-type lectin S-receptor-like serine/threonine-protein kinase RLK1 [Populus euphratica]	4.02	17.62
map00360	Phenylalanine metabolism	6.66E-02	TRINITY_DN27242_c0_g1	-1.23	down	hypothetical protein POPTR_0151s00200g [Populus trichocarpa]	2.45	8.98
map00360	Phenylalanine metabolism	6.66E-02	TRINITY_DN14000_c0_g1	-2.71	down	hypothetical protein POPTR_0013s05640g [Populus trichocarpa]	0.38	5.38
map00360	Phenylalanine metabolism	6.66E-02	TRINITY_DN15219_c0_g1	-3.09	down	hypothetical protein POPTR_0013s05650g [Populus trichocarpa]	0.23	2.86
map00360	Phenylalanine metabolism	6.66E-02	TRINITY_DN15596_c0_g1	-2.20	down	hypothetical protein POPTR_0009s13300g [Populus trichocarpa]	0.46	2.84
map00360	Phenylalanine metabolism	6.66E-02	TRINITY_DN15596_c0_g2	-2.70	down	hypothetical protein POPTR_0009s13290g [Populus trichocarpa]	0.23	2.39
map00360	Phenylalanine metabolism	6.66E-02	TRINITY_DN17759_c0_g1	-1.68	down	hypothetical protein POPTR_0004s01460g, partial [Populus trichocarpa]	1.43	5.88
map00360	Phenylalanine metabolism	6.66E-02	TRINITY_DN17759_c0_g3	-2.14	down	PREDICTED: proline-rich receptor-like protein kinase PERK3 isoform X1 [Populus euphratica]	0.24	1.71
map00360	Phenylalanine metabolism	6.66E-02	TRINITY_DN18330_c0_g1	-2.18	down	trans-cinnamate 4-hydroxylase [Populus trichocarpa]	0.62	4.40
map00360	Phenylalanine metabolism	6.66E-02	TRINITY_DN19059_c0_g2	-1.92	down	PREDICTED: G-type lectin S-receptor-like serine/threonine-protein kinase At5g24080 isoform X1 [Populus euphratica]	1.17	7.57
map00360	Phenylalanine metabolism	6.66E-02	TRINITY_DN19059_c0_g3	-6.09	down	hypothetical protein POPTR_0015s05780g [Populus trichocarpa]	0.09	4.40
map00360	Phenylalanine metabolism	6.66E-02	TRINITY_DN19472_c0_g2	1.32	up	hypothetical protein POPTR_0007s01130g [Populus trichocarpa]	8.54	5.46
map00360	Phenylalanine metabolism	6.66E-02	TRINITY_DN19472_c0_g3	-3.23	down	hypothetical protein POPTR_0017s04550g [Populus trichocarpa]	0.21	3.06
map00360	Phenylalanine metabolism	6.66E-02	TRINITY_DN19626_c0_g2	-2.23	down	hypothetical protein POPTR_0016s13500g [Populus trichocarpa]	5.67	39.97
map00360	Phenylalanine metabolism	6.66E-02	TRINITY_DN19656_c0_g1	-1.62	down	PREDICTED: G-type lectin S-receptor-like serine/threonine-protein kinase RLK1 isoform X1 [Populus euphratica]	0.57	2.63
map00360	Phenylalanine metabolism	6.66E-02	TRINITY_DN19656_c0_g2	1.26	up	PREDICTED: G-type lectin S-receptor-like serine/threonine-protein kinase RLK1 [Populus euphratica]	3.74	2.35
map00360	Phenylalanine metabolism	6.66E-02	TRINITY_DN19656_c0_g3	-4.04	down	hypothetical protein POPTR_0200s00220g [Populus trichocarpa]	0.09	2.49
map00360	Phenylalanine metabolism	6.66E-02	TRINITY_DN19958_c0_g2	-1.13	down	RecName: Full=Caffeoyl-CoA O-methyltransferase; AltName: Full=Trans-caffeoyl-CoA 3-O-methyltransferase; Short=CCoAMT; Short=CCoAOMT	6.52	20.89
map00360	Phenylalanine metabolism	6.66E-02	TRINITY_DN19981_c0_g1	-1.21	down	copper/topa quinone amine oxidase precursor family protein [Populus trichocarpa]	1.81	6.48
map00360	Phenylalanine metabolism	6.66E-02	TRINITY_DN20348_c0_g2	1.31	up	4-coumarate: coenzyme A ligase 4 [Populus tomentosa]	41.80	31.54
map00360	Phenylalanine metabolism	6.66E-02	TRINITY_DN20914_c0_g3	1.36	up	hypothetical protein POPTR_0006s07470g [Populus trichocarpa]	89.52	50.66
map00360	Phenylalanine metabolism	6.66E-02	TRINITY_DN21133_c0_g2	-1.22	down	PREDICTED: G-type lectin S-receptor-like serine/threonine-protein kinase RLK1 isoform X1 [Populus euphratica]	6.50	22.69
map00360	Phenylalanine metabolism	6.66E-02	TRINITY_DN21391_c0_g1	1.12	up	PREDICTED: uncharacterized protein LOC105124970 [Populus euphratica]	49.29	33.42

map00561	Glycerolipid metabolism	8.38E-02	TRINITY_DN21705_c0_g5	-2.82	down	hypothetical protein POPTR_0014s07220g [Populus trichocarpa]	0.39	4.79
map00561	Glycerolipid metabolism	8.38E-02	TRINITY_DN21731_c0_g2	-1.12	down	PREDICTED: aldo-keto reductase family 4 member C9-like [Populus euphratica]	3.19	10.75
map00561	Glycerolipid metabolism	8.38E-02	TRINITY_DN21920_c1_g1	-3.31	down	NAC domain-containing protein 90 [Populus trichocarpa]	0.76	16.53
map00561	Glycerolipid metabolism	8.38E-02	TRINITY_DN21932_c0_g3	-1.06	down	phosphatase 2C family protein [Populus trichocarpa]	2.53	8.10
map00561	Glycerolipid metabolism	8.38E-02	TRINITY_DN22330_c0_g3	1.14	up	PREDICTED: caffeoylshikimate esterase-like [Populus euphratica]	6.38	4.44
map00561	Glycerolipid metabolism	8.38E-02	TRINITY_DN22906_c0_g1	1.04	up	MGDG synthase type A family protein [Populus trichocarpa]	7.93	5.80
map00561	Glycerolipid metabolism	8.38E-02	TRINITY_DN23075_c0_g1	-1.39	down	hypothetical protein POPTR_0337s00220g [Populus trichocarpa]	5.96	32.70
map00561	Glycerolipid metabolism	8.38E-02	TRINITY_DN23704_c2_g1	-2.79	down	PREDICTED: NAC transcription factor 29-like [Populus euphratica]	2.61	26.09
map00561	Glycerolipid metabolism	8.38E-02	TRINITY_DN24041_c0_g1	2.06	up	PREDICTED: D-glycerate 3-kinase, chloroplastic-like [Populus euphratica]	150.03	53.16
map00561	Glycerolipid metabolism	8.38E-02	TRINITY_DN24178_c0_g3	1.11	up	lipid phosphate phosphatase 2 [Populus tomentosa]	19.00	13.25
map00561	Glycerolipid metabolism	8.38E-02	TRINITY_DN24219_c0_g1	-2.98	down	hypothetical protein POPTR_0007s04780g [Populus trichocarpa]	13.17	161.47
map00561	Glycerolipid metabolism	8.38E-02	TRINITY_DN24443_c0_g1	3.60	up	hypothetical protein POPTR_0018s09660g [Populus trichocarpa]	90.09	11.63
map00561	Glycerolipid metabolism	8.38E-02	TRINITY_DN24628_c0_g1	1.28	up	PREDICTED: aldehyde dehydrogenase family 3 member H1-like [Populus euphratica]	40.39	24.46
map00561	Glycerolipid metabolism	8.38E-02	TRINITY_DN24718_c0_g1	-1.13	down	PREDICTED: calmodulin-binding transcription activator 3-like isoform X3 [Populus euphratica]	2.73	8.94
map00561	Glycerolipid metabolism	8.38E-02	TRINITY_DN24760_c0_g1	-2.11	down	PREDICTED: diacylglycerol kinase 2-like isoform X1 [Populus euphratica]	1.68	11.01
map00561	Glycerolipid metabolism	8.38E-02	TRINITY_DN24943_c0_g1	-2.15	down	hypothetical protein POPTR_0010s14690g [Populus trichocarpa]	0.37	2.73
map00561	Glycerolipid metabolism	8.38E-02	TRINITY_DN25165_c0_g2	1.20	up	PREDICTED: glycerol-3-phosphate acyltransferase, chloroplastic [Populus euphratica]	66.47	43.88
map00561	Glycerolipid metabolism	8.38E-02	TRINITY_DN25413_c4_g2	-1.83	down	diacylglycerol kinase family protein [Populus trichocarpa]	8.61	47.89
map00561	Glycerolipid metabolism	8.38E-02	TRINITY_DN25630_c0_g1	1.30	up	aldehyde dehydrogenase 1 precursor family protein [Populus trichocarpa]	120.35	73.18
map00561	Glycerolipid metabolism	8.38E-02	TRINITY_DN26025_c0_g1	-1.71	down	hypothetical protein POPTR_0004s18340g [Populus trichocarpa]	5.60	27.70
map00561	Glycerolipid metabolism	8.38E-02	TRINITY_DN26029_c0_g1	1.24	up	MGDG synthase type A family protein [Populus trichocarpa]	31.79	20.27
map00561	Glycerolipid metabolism	8.38E-02	TRINITY_DN26173_c0_g2	-2.84	down	hypothetical protein POPTR_0001s23420g [Populus trichocarpa]	1.75	18.91
map00561	Glycerolipid metabolism	8.38E-02	TRINITY_DN26421_c0_g1	1.32	up	the aldehyde dehydrogenase cp-ADH from C.plantagineum family protein [Populus trichocarpa]	58.13	34.64
map00561	Glycerolipid metabolism	8.38E-02	TRINITY_DN26716_c1_g1	1.30	up	PREDICTED: phospholipid:diacylglycerol acyltransferase 1-like [Populus euphratica]	24.14	15.21
map00561	Glycerolipid metabolism	8.38E-02	TRINITY_DN27171_c2_g1	1.79	up	PREDICTED: D-glycerate 3-kinase, chloroplastic-like [Populus euphratica]	158.02	76.60
map00561	Glycerolipid metabolism	8.38E-02	TRINITY_DN27547_c0_g1	-1.35	down	calmodulin-binding family protein [Populus trichocarpa]	6.25	23.99
map00561	Glycerolipid metabolism	8.38E-02	TRINITY_DN11229_c0_g1	1.90	up	hypothetical protein POPTR_0011s03550g [Populus trichocarpa]	258.45	106.27
map00561	Glycerolipid metabolism	8.38E-02	TRINITY_DN11676_c0_g1	3.67	up	GDSL-motif lipase/hydrolase family protein [Populus trichocarpa]	3.76	0.45
map00561	Glycerolipid metabolism	8.38E-02	TRINITY_DN15849_c0_g1	-7.35	down	hypothetical protein POPTR_0008s08850g [Populus trichocarpa]	0.02	4.56
map00561	Glycerolipid metabolism	8.38E-02	TRINITY_DN15976_c0_g1	2.23	up	lecithin:cholesterol acyltransferase family protein [Populus trichocarpa]	9.17	2.97
map00561	Glycerolipid metabolism	8.38E-02	TRINITY_DN16269_c0_g1	1.35	up	GDSL-motif lipase/hydrolase family protein [Populus trichocarpa]	4.71	2.76
map00561	Glycerolipid metabolism	8.38E-02	TRINITY_DN16288_c0_g2	-5.63	down	family II extracellular lipase 3 family protein [Populus trichocarpa]	0.40	10.00
map00561	Glycerolipid metabolism	8.38E-02	TRINITY_DN16655_c0_g1	-4.08	down	hypothetical protein POPTR_0002s05820g [Populus trichocarpa]	0.16	3.45
map00561	Glycerolipid metabolism	8.38E-02	TRINITY_DN17353_c0_g1	1.22	up	PREDICTED: LOW QUALITY PROTEIN: methylecgonone reductase-like [Populus euphratica]	9.67	7.02
map00561	Glycerolipid metabolism	8.38E-02	TRINITY_DN17557_c0_g1	-6.12	down	AAA-type ATPase family protein [Populus trichocarpa]	0.06	6.84
map00561	Glycerolipid metabolism	8.38E-02	TRINITY_DN17644_c0_g1	1.22	up	PREDICTED: NADP-dependent D-sorbitol-6-phosphate dehydrogenase-like [Populus euphratica]	152.43	100.73
map00561	Glycerolipid metabolism	8.38E-02	TRINITY_DN17931_c0_g2	1.02	up	unknown [Populus trichocarpa]	189.83	143.29
map00561	Glycerolipid metabolism	8.38E-02	TRINITY_DN18466_c0_g1	3.25	up	hypothetical protein POPTR_0018s14920g [Populus trichocarpa]	22.92	4.21
map00561	Glycerolipid metabolism	8.38E-02	TRINITY_DN18895_c0_g1	-1.06	down	mitochondrial aldehyde dehydrogenase family protein [Populus trichocarpa]	7.40	23.22
map00561	Glycerolipid metabolism	8.38E-02	TRINITY_DN19215_c1_g1	-1.38	down	hypothetical protein POPTR_0007s10210g [Populus trichocarpa]	3.47	12.55
map00561	Glycerolipid metabolism	8.38E-02	TRINITY_DN19215_c2_g1	-1.56	down	PREDICTED: probable protein phosphatase 2C 63 [Populus euphratica]	6.48	30.16
map00561	Glycerolipid metabolism	8.38E-02	TRINITY_DN20010_c0_g1	-2.36	down	caffeoyl shikimate esterase 12 [Populus tomentosa]	1.15	8.85

map00561	Glycerolipid metabolism	8.38E-02	TRINITY_DN20030_c0_g1	-1.92	down	hypothetical protein POPTR_0004s09040g [Populus trichocarpa]	3.12	18.15
map00561	Glycerolipid metabolism	8.38E-02	TRINITY_DN20113_c0_g3	1.02	up	hypothetical protein POPTR_0007s14280g [Populus trichocarpa]	91.83	69.21
map00561	Glycerolipid metabolism	8.38E-02	TRINITY_DN20193_c1_g5	-1.02	down	PREDICTED: NAC domain-containing protein 72 [Populus euphratica]	6.09	18.08
map00561	Glycerolipid metabolism	8.38E-02	TRINITY_DN20341_c0_g1	-1.82	down	Zinc finger protein, putative [Theobroma cacao]	4.28	22.90
map00561	Glycerolipid metabolism	8.38E-02	TRINITY_DN20407_c0_g1	1.23	up	plastid developmental protein DAG [Populus trichocarpa]	313.75	204.79
map00561	Glycerolipid metabolism	8.38E-02	TRINITY_DN20407_c0_g2	1.21	up	hypothetical protein POPTR_0010s07890g [Populus trichocarpa]	232.02	140.49
map00561	Glycerolipid metabolism	8.38E-02	TRINITY_DN20824_c0_g3	-1.57	down	hypothetical protein POPTR_0001s19240g [Populus trichocarpa]	6.89	26.31
map00561	Glycerolipid metabolism	8.38E-02	TRINITY_DN21027_c0_g1	-1.16	down	phospholipid/glycerol acyltransferase family protein [Populus trichocarpa]	12.14	41.22
map00561	Glycerolipid metabolism	8.38E-02	TRINITY_DN21206_c0_g1	-2.64	down	hypothetical protein POPTR_0001s41900g [Populus trichocarpa]	0.55	5.54
map00561	Glycerolipid metabolism	8.38E-02	TRINITY_DN21206_c0_g2	-3.49	down	hypothetical protein POPTR_0001s41900g [Populus trichocarpa]	0.36	6.24
map00561	Glycerolipid metabolism	8.38E-02	TRINITY_DN21622_c0_g1	1.02	up	NAC domain transcription factor [Populus tomentosa]	18.53	13.96
map00561	Glycerolipid metabolism	8.38E-02	TRINITY_DN21622_c0_g2	-1.52	down	PREDICTED: NAC domain-containing protein 68 [Populus euphratica]	6.63	29.11
map00561	Glycerolipid metabolism	8.38E-02	TRINITY_DN78_c0_g1	-2.13	down	PREDICTED: GDSL esterase/lipase At2g42990-like [Populus euphratica]	0.33	2.30
map01210	2-Oxocarboxylic acid metabolism	8.55E-02	TRINITY_DN21760_c0_g1	1.25	up	isocitrate dehydrogenase family protein [Populus trichocarpa]	53.95	39.69
map01210	2-Oxocarboxylic acid metabolism	8.55E-02	TRINITY_DN21760_c0_g2	1.32	up	PREDICTED: 3-isopropylmalate dehydrogenase, chloroplastic-like [Populus euphratica]	86.28	52.23
map01210	2-Oxocarboxylic acid metabolism	8.55E-02	TRINITY_DN22048_c2_g2	1.93	up	hypothetical protein POPTR_0015s12380g [Populus trichocarpa]	3.05	1.20
map01210	2-Oxocarboxylic acid metabolism	8.55E-02	TRINITY_DN22048_c2_g5	-1.67	down	hypothetical protein POPTR_0012s11610g [Populus trichocarpa]	1.63	8.07
map01210	2-Oxocarboxylic acid metabolism	8.55E-02	TRINITY_DN22580_c0_g3	1.24	up	hypothetical protein POPTR_0003s10720g [Populus trichocarpa]	124.71	79.50
map01210	2-Oxocarboxylic acid metabolism	8.55E-02	TRINITY_DN22629_c0_g2	1.01	up	PREDICTED: 2-isopropylmalate synthase 2, chloroplastic-like [Populus euphratica]	65.58	50.13
map01210	2-Oxocarboxylic acid metabolism	8.55E-02	TRINITY_DN22663_c0_g2	1.38	up	hypothetical protein POPTR_0010s05530g [Populus trichocarpa]	360.33	205.54
map01210	2-Oxocarboxylic acid metabolism	8.55E-02	TRINITY_DN22695_c0_g4	1.16	up	aconitase family protein [Populus trichocarpa]	151.04	102.95
map01210	2-Oxocarboxylic acid metabolism	8.55E-02	TRINITY_DN22813_c0_g4	1.08	up	aspartate transaminase family protein [Populus trichocarpa]	25.11	18.98
map01210	2-Oxocarboxylic acid metabolism	8.55E-02	TRINITY_DN22841_c0_g1	1.22	up	PREDICTED: LOW QUALITY PROTEIN: aminoacylase-1 [Populus euphratica]	87.12	56.00
map01210	2-Oxocarboxylic acid metabolism	8.55E-02	TRINITY_DN23209_c0_g1	2.76	up	hypothetical protein POPTR_0004s21720g [Populus trichocarpa]	147.72	29.16
map01210	2-Oxocarboxylic acid metabolism	8.55E-02	TRINITY_DN23271_c0_g1	1.57	up	hypothetical protein POPTR_0001s35080g [Populus trichocarpa]	37.34	19.20
map01210	2-Oxocarboxylic acid metabolism	8.55E-02	TRINITY_DN23278_c1_g1	1.44	up	PREDICTED: glutamate--glyoxylate aminotransferase 2 isoform X1 [Populus euphratica]	580.61	318.60
map01210	2-Oxocarboxylic acid metabolism	8.55E-02	TRINITY_DN23709_c0_g2	1.23	up	aspartate aminotransferase 2 family protein [Populus trichocarpa]	46.64	32.80
map01210	2-Oxocarboxylic acid metabolism	8.55E-02	TRINITY_DN24990_c0_g2	3.40	up	alanine aminotransferase family protein [Populus simonii x Populus nigra]	51.44	7.50
map01210	2-Oxocarboxylic acid metabolism	8.55E-02	TRINITY_DN25476_c0_g2	2.59	up	PREDICTED: alanine aminotransferase 2, mitochondrial-like [Populus euphratica]	33.99	8.74
map01210	2-Oxocarboxylic acid metabolism	8.55E-02	TRINITY_DN25476_c0_g4	2.24	up	PREDICTED: alanine aminotransferase 2-like [Populus euphratica]	21.79	7.01
map01210	2-Oxocarboxylic acid metabolism	8.55E-02	TRINITY_DN25630_c0_g3	-2.44	down	PREDICTED: branched-chain-amino-acid aminotransferase 2, chloroplastic-like isoform X1 [Populus euphratica]	0.50	3.81
map01210	2-Oxocarboxylic acid metabolism	8.55E-02	TRINITY_DN25737_c0_g1	1.82	up	semialdehyde dehydrogenase family protein [Populus trichocarpa]	56.96	24.76
map01210	2-Oxocarboxylic acid metabolism	8.55E-02	TRINITY_DN26103_c0_g4	1.68	up	hypothetical protein POPTR_0322s00200g [Populus trichocarpa]	65.51	31.51
map01210	2-Oxocarboxylic acid metabolism	8.55E-02	TRINITY_DN26128_c0_g3	1.77	up	PREDICTED: probable amino-acid acetyltransferase NAGS2, chloroplastic isoform X1 [Populus euphratica]	26.59	11.97
map01210	2-Oxocarboxylic acid metabolism	8.55E-02	TRINITY_DN26128_c0_g4	2.19	up	PREDICTED: probable amino-acid acetyltransferase NAGS2, chloroplastic isoform X1 [Populus euphratica]	2.29	0.74
map01210	2-Oxocarboxylic acid metabolism	8.55E-02	TRINITY_DN26976_c0_g1	1.47	up	PREDICTED: acetolactate synthase 2, chloroplastic-like [Populus euphratica]	396.57	239.53
map01210	2-Oxocarboxylic acid metabolism	8.55E-02	TRINITY_DN27198_c1_g1	1.09	up	aconitate hydratase family protein [Populus trichocarpa]	47.60	32.18
map01210	2-Oxocarboxylic acid metabolism	8.55E-02	TRINITY_DN27198_c1_g2	1.02	up	aconitate hydratase family protein [Populus trichocarpa]	16.99	12.62
map01210	2-Oxocarboxylic acid metabolism	8.55E-02	TRINITY_DN17247_c0_g1	-1.15	down	UDP-glucoronosyl/UDP-glucosyl transferase family protein [Populus trichocarpa]	7.87	26.34
map01210	2-Oxocarboxylic acid metabolism	8.55E-02	TRINITY_DN17444_c0_g1	-1.97	down	PREDICTED: uncharacterized protein LOC104888072 [Beta vulgaris subsp. vulgaris]	0.40	2.38
map01210	2-Oxocarboxylic acid metabolism	8.55E-02	TRINITY_DN17710_c0_g1	1.71	up	hypothetical protein POPTR_0012s01280g [Populus trichocarpa]	51.45	22.69
map01210	2-Oxocarboxylic acid metabolism	8.55E-02	TRINITY_DN17736_c0_g2	1.54	up	arginine biosynthesis protein ArgJ [Populus trichocarpa]	59.37	31.41

map01210	2-Oxocarboxylic acid metabolism	8.55E-02	TRINITY_DN18859_c0_g3	1.18	up	aconitate hydratase family protein [Populus trichocarpa]	80.30	54.25
map01210	2-Oxocarboxylic acid metabolism	8.55E-02	TRINITY_DN19518_c0_g1	2.25	up	hypothetical protein POPTR_0016s14360g [Populus trichocarpa]	277.40	89.48
map01210	2-Oxocarboxylic acid metabolism	8.55E-02	TRINITY_DN20225_c0_g1	-2.28	down	hypothetical protein POPTR_0001s28860g [Populus trichocarpa]	1.93	14.45
map01210	2-Oxocarboxylic acid metabolism	8.55E-02	TRINITY_DN20583_c0_g3	2.29	up	hypothetical protein POPTR_0008s03820g [Populus trichocarpa]	4.09	1.88
map01210	2-Oxocarboxylic acid metabolism	8.55E-02	TRINITY_DN20720_c0_g1	2.14	up	PREDICTED: probable N-acetyl-gamma-glutamyl-phosphate reductase, chloroplastic isoform X2 [Populus euphratica]	42.82	15.35
map01210	2-Oxocarboxylic acid metabolism	8.55E-02	TRINITY_DN20790_c0_g1	3.22	up	PREDICTED: cytosolic sulfotransferase 15-like [Populus euphratica]	96.77	15.23
map01210	2-Oxocarboxylic acid metabolism	8.55E-02	TRINITY_DN20958_c0_g1	1.57	up	PREDICTED: UDP-glycosyltransferase 74E1-like [Populus euphratica]	64.45	33.63
map01210	2-Oxocarboxylic acid metabolism	8.55E-02	TRINITY_DN21117_c0_g1	1.76	up	N-acetylglutamate kinase, partial [Populus maximowiczii x Populus nigra]	57.23	25.99
map01210	2-Oxocarboxylic acid metabolism	8.55E-02	TRINITY_DN21313_c0_g2	-1.72	down	hypothetical protein POPTR_0014s07170g [Populus trichocarpa]	4.63	24.85
map01210	2-Oxocarboxylic acid metabolism	8.55E-02	TRINITY_DN21600_c0_g2	1.23	up	PREDICTED: acetolactate synthase small subunit 2, chloroplastic-like [Populus euphratica]	35.74	23.13
map01210	2-Oxocarboxylic acid metabolism	8.55E-02	TRINITY_DN21635_c1_g2	-2.07	down	SEC14 cytosolic factor family protein [Populus trichocarpa]	5.54	32.41
map00232	Caffeine metabolism	8.68E-02	TRINITY_DN23485_c0_g3	-1.49	down	PREDICTED: cytochrome P450 71A1-like [Populus euphratica]	1.44	6.13
map00232	Caffeine metabolism	8.68E-02	TRINITY_DN24288_c0_g1	-2.74	down	hypothetical protein POPTR_0003s11810g [Populus trichocarpa]	2.78	11.84
map00232	Caffeine metabolism	8.68E-02	TRINITY_DN16280_c0_g1	1.20	up	nodulin 35 family protein [Populus trichocarpa]	36.34	29.20
map00232	Caffeine metabolism	8.68E-02	TRINITY_DN16449_c0_g1	2.09	up	PREDICTED: cytochrome P450 71A1-like isoform X1 [Populus euphratica]	34.70	12.40
map00232	Caffeine metabolism	8.68E-02	TRINITY_DN19787_c0_g1	2.95	up	hypothetical protein POPTR_0001s08320g [Populus trichocarpa]	7.17	1.45
map00942	Anthocyanin biosynthesis	9.61E-02	TRINITY_DN21917_c1_g1	-1.69	down	INDOLE-3-ACETATE BETA-D-GLUCOSYLTRANSFERASE family protein [Populus trichocarpa]	4.31	21.67
map00942	Anthocyanin biosynthesis	9.61E-02	TRINITY_DN22880_c0_g1	-3.85	down	flavonoid 3-O-galactosyl transferase family protein [Populus trichocarpa]	3.21	70.71
map00942	Anthocyanin biosynthesis	9.61E-02	TRINITY_DN22958_c1_g1	1.76	up	PREDICTED: transcription factor BIM2-like isoform X2 [Populus euphratica]	14.62	8.00
map00942	Anthocyanin biosynthesis	9.61E-02	TRINITY_DN26348_c0_g1	1.46	up	hypothetical protein POPTR_0006s01160g [Populus trichocarpa]	31.96	24.17
map00942	Anthocyanin biosynthesis	9.61E-02	TRINITY_DN26653_c0_g3	1.90	up	hypothetical protein POPTR_0016s01620g [Populus trichocarpa]	1363.44	562.89
map00942	Anthocyanin biosynthesis	9.61E-02	TRINITY_DN17135_c0_g1	-1.23	down	hypothetical protein POPTR_0006s19340g [Populus trichocarpa]	24.77	84.05
map00942	Anthocyanin biosynthesis	9.61E-02	TRINITY_DN18988_c0_g1	1.09	up	hypothetical protein POPTR_0012s01130g [Populus trichocarpa]	6.88	4.94
map00942	Anthocyanin biosynthesis	9.61E-02	TRINITY_DN19435_c0_g1	1.17	up	PREDICTED: anthocyanidin 3-O-glucosyltransferase 7-like [Populus euphratica]	20.65	14.21
map00942	Anthocyanin biosynthesis	9.61E-02	TRINITY_DN19435_c0_g2	1.89	up	PREDICTED: anthocyanidin 3-O-glucosyltransferase 7-like [Populus euphratica]	28.71	12.23
map00942	Anthocyanin biosynthesis	9.61E-02	TRINITY_DN20381_c0_g1	-1.87	down	hypothetical protein POPTR_0015s05670g [Populus trichocarpa]	3.58	16.81
map00942	Anthocyanin biosynthesis	9.61E-02	TRINITY_DN20752_c1_g2	-1.41	down	INDOLE-3-ACETATE BETA-D-GLUCOSYLTRANSFERASE family protein [Populus trichocarpa]	9.89	40.30
map00942	Anthocyanin biosynthesis	9.61E-02	TRINITY_DN20752_c1_g4	2.25	up	hypothetical protein POPTR_0016s05290g [Populus trichocarpa]	2.74	1.01
map00942	Anthocyanin biosynthesis	9.61E-02	TRINITY_DN3649_c0_g1	2.13	up	UDP-glucoronosyl/UDP-glucosyl transferase family protein [Populus trichocarpa]	16.41	5.74
map00061	Fatty acid biosynthesis	9.82E-02	TRINITY_DN21907_c0_g2	1.56	up	hypothetical protein POPTR_0002s24700g [Populus trichocarpa]	96.22	49.62
map00061	Fatty acid biosynthesis	9.82E-02	TRINITY_DN22443_c0_g3	1.31	up	PREDICTED: 3-oxoacyl-[acyl-carrier-protein] synthase 3 A, chloroplastic [Populus euphratica]	34.98	21.39
map00061	Fatty acid biosynthesis	9.82E-02	TRINITY_DN23036_c0_g3	-1.08	down	PREDICTED: long chain acyl-CoA synthetase 2 isoform X1 [Populus euphratica]	4.05	12.87
map00061	Fatty acid biosynthesis	9.82E-02	TRINITY_DN23093_c0_g1	1.81	up	3-oxoacyl-[acyl-carrier-protein] synthase I [Populus trichocarpa]	128.96	55.68
map00061	Fatty acid biosynthesis	9.82E-02	TRINITY_DN23645_c0_g1	1.12	up	hypothetical protein POPTR_0008s17880g [Populus trichocarpa]	115.75	81.25
map00061	Fatty acid biosynthesis	9.82E-02	TRINITY_DN24137_c0_g2	1.93	up	PREDICTED: acyl-[acyl-carrier-protein] desaturase, chloroplastic [Populus euphratica]	275.31	109.07
map00061	Fatty acid biosynthesis	9.82E-02	TRINITY_DN24307_c0_g1	1.05	up	PREDICTED: biotin carboxyl carrier protein of acetyl-CoA carboxylase, chloroplastic-like [Populus euphratica]	73.06	52.43
map00061	Fatty acid biosynthesis	9.82E-02	TRINITY_DN25183_c0_g1	-1.02	down	hypothetical protein POPTR_0013s02220g [Populus trichocarpa]	7.46	25.55
map00061	Fatty acid biosynthesis	9.82E-02	TRINITY_DN25227_c0_g3	-1.04	down	unknown [Populus trichocarpa x Populus deltoides]	4.77	15.05
map00061	Fatty acid biosynthesis	9.82E-02	TRINITY_DN25439_c0_g1	1.11	up	biotin carboxylase precursor family protein [Populus trichocarpa]	85.63	60.20

map00061	Fatty acid biosynthesis	9.82E-02	TRINITY_DN25671_c0_g1	1.18	up	ENOYL-ACP REDUCTASE 1 family protein [Populus trichocarpa]	122.50	78.93
map00061	Fatty acid biosynthesis	9.82E-02	TRINITY_DN25910_c0_g2	-1.06	down	PREDICTED: transcription factor PCL1-like [Populus euphratica]	4.93	16.82
map00061	Fatty acid biosynthesis	9.82E-02	TRINITY_DN26565_c0_g1	1.03	up	hypothetical protein POPTR_0005s19990g [Populus trichocarpa]	60.48	36.43
map00061	Fatty acid biosynthesis	9.82E-02	TRINITY_DN27876_c5_g1	-1.61	down	hypothetical protein POPTR_0005s16540g [Populus trichocarpa]	2.20	11.69
map00061	Fatty acid biosynthesis	9.82E-02	TRINITY_DN27876_c5_g5	-2.33	down	PREDICTED: acetyl-CoA carboxylase 1-like [Populus euphratica]	0.87	6.39
map00061	Fatty acid biosynthesis	9.82E-02	TRINITY_DN15736_c0_g1	-10.07	down	hypothetical protein POPTR_0008s14850g [Populus trichocarpa]	0.00	7.88
map00061	Fatty acid biosynthesis	9.82E-02	TRINITY_DN18603_c0_g1	1.08	up	hypothetical protein POPTR_0003s14190g [Populus trichocarpa]	6.90	4.98
map00061	Fatty acid biosynthesis	9.82E-02	TRINITY_DN19050_c1_g1	1.32	up	hypothetical protein POPTR_0019s08170g, partial [Populus trichocarpa]	123.18	74.70
map00061	Fatty acid biosynthesis	9.82E-02	TRINITY_DN19330_c1_g4	1.30	up	hypothetical protein POPTR_0004s23710g [Populus trichocarpa]	22.29	13.72
map00061	Fatty acid biosynthesis	9.82E-02	TRINITY_DN20741_c0_g1	1.32	up	hypothetical protein POPTR_0009s08150g [Populus trichocarpa]	80.30	48.68
map00061	Fatty acid biosynthesis	9.82E-02	TRINITY_DN20933_c0_g1	-3.74	down	hypothetical protein POPTR_0002s19330g [Populus trichocarpa]	0.65	13.67
map00061	Fatty acid biosynthesis	9.82E-02	TRINITY_DN20933_c0_g3	-2.76	down	PREDICTED: long chain acyl-CoA synthetase 1 [Populus euphratica]	0.62	6.55
map00061	Fatty acid biosynthesis	9.82E-02	TRINITY_DN21469_c0_g3	1.53	up	unknown [Populus trichocarpa]	127.67	66.65
map00061	Fatty acid biosynthesis	9.82E-02	TRINITY_DN21528_c1_g1	-1.91	down	hypothetical protein POPTR_0002s09330g [Populus trichocarpa]	0.78	4.44
map00061	Fatty acid biosynthesis	9.82E-02	TRINITY_DN6900_c0_g1	1.83	up	short-chain dehydrogenase/reductase family protein [Populus trichocarpa]	21.13	8.97
map00061	Fatty acid biosynthesis	9.82E-02	TRINITY_DN7619_c0_g1	-4.15	down	PREDICTED: palmitoyl-acyl carrier protein thioesterase, chloroplastic-like [Populus euphratica]	0.09	2.75
map00250	Alanine, aspartate and glutamate metabolism	1.08E-01	TRINITY_DN21747_c0_g1	3.00	up	hypothetical protein POPTR_0015s12250g [Populus trichocarpa]	29.79	5.40
map00250	Alanine, aspartate and glutamate metabolism	1.08E-01	TRINITY_DN22303_c0_g1	-2.21	down	hypothetical protein POPTR_0001s34980g [Populus trichocarpa]	8.51	87.80
map00250	Alanine, aspartate and glutamate metabolism	1.08E-01	TRINITY_DN22303_c0_g6	2.12	up	hypothetical protein POPTR_0001s34980g [Populus trichocarpa]	14.33	5.07
map00250	Alanine, aspartate and glutamate metabolism	1.08E-01	TRINITY_DN22663_c0_g2	1.38	up	hypothetical protein POPTR_0010s05530g [Populus trichocarpa]	360.33	205.54
map00250	Alanine, aspartate and glutamate metabolism	1.08E-01	TRINITY_DN22684_c0_g1	4.49	up	glutamate decarboxylase [Populus tremula x Populus alba]	5.12	0.35
map00250	Alanine, aspartate and glutamate metabolism	1.08E-01	TRINITY_DN22684_c0_g7	-5.56	down	glutamate decarboxylase 1 family protein [Populus trichocarpa]	0.08	6.22
map00250	Alanine, aspartate and glutamate metabolism	1.08E-01	TRINITY_DN22813_c0_g4	1.08	up	aspartate transaminase family protein [Populus trichocarpa]	25.11	18.98
map00250	Alanine, aspartate and glutamate metabolism	1.08E-01	TRINITY_DN23091_c0_g2	-2.11	down	glutamine synthetase family protein [Populus trichocarpa]	10.71	69.67
map00250	Alanine, aspartate and glutamate metabolism	1.08E-01	TRINITY_DN23091_c0_g3	4.22	up	glutamate-ammonia ligase family protein [Populus trichocarpa]	47.80	3.92
map00250	Alanine, aspartate and glutamate metabolism	1.08E-01	TRINITY_DN23091_c0_g4	1.81	up	glutamate-ammonia ligase family protein [Populus trichocarpa]	63.46	29.26
map00250	Alanine, aspartate and glutamate metabolism	1.08E-01	TRINITY_DN23091_c0_g5	4.02	up	glutamine synthetase family protein [Populus simonii x Populus nigra]	46.52	4.43
map00250	Alanine, aspartate and glutamate metabolism	1.08E-01	TRINITY_DN23278_c1_g1	1.44	up	PREDICTED: glutamate--glyoxylate aminotransferase 2 isoform X1 [Populus euphratica]	580.61	318.60
map00250	Alanine, aspartate and glutamate metabolism	1.08E-01	TRINITY_DN23709_c0_g2	1.23	up	aspartate aminotransferase 2 family protein [Populus trichocarpa]	46.64	32.80
map00250	Alanine, aspartate and glutamate metabolism	1.08E-01	TRINITY_DN23937_c0_g1	1.78	up	Glutamine synthetase nodule isozyme [Ananas comosus]	127.57	57.48
map00250	Alanine, aspartate and glutamate metabolism	1.08E-01	TRINITY_DN23937_c0_g3	-3.47	down	PREDICTED: LOW QUALITY PROTEIN: glutamine synthetase cytosolic isozyme 2 [Populus euphratica]	0.50	8.51
map00250	Alanine, aspartate and glutamate metabolism	1.08E-01	TRINITY_DN24244_c0_g2	1.86	up	hypothetical protein POPTR_0001s36730g [Populus trichocarpa]	48.33	20.26
map00250	Alanine, aspartate and glutamate metabolism	1.08E-01	TRINITY_DN24990_c0_g2	3.40	up	alanine aminotransferase family protein [Populus simonii x Populus nigra]	51.44	7.50
map00250	Alanine, aspartate and glutamate metabolism	1.08E-01	TRINITY_DN25260_c0_g3	3.71	up	glutamate-ammonia ligase family protein [Populus trichocarpa]	11.97	1.40
map00250	Alanine, aspartate and glutamate metabolism	1.08E-01	TRINITY_DN25260_c0_g4	-1.01	down	unknown [Populus trichocarpa]	26.98	83.37
map00250	Alanine, aspartate and glutamate metabolism	1.08E-01	TRINITY_DN25476_c0_g2	2.59	up	PREDICTED: alanine aminotransferase 2, mitochondrial-like [Populus euphratica]	33.99	8.74

map00250	Alanine, aspartate and glutamate metabolism	1.08E-01	TRINITY_DN25476_c0_g4	2.24	up	PREDICTED: alanine aminotransferase 2-like [Populus euphratica]	21.79	7.01
map00250	Alanine, aspartate and glutamate metabolism	1.08E-01	TRINITY_DN25557_c0_g1	-5.45	down	asparagine synthetase family protein 1 [Populus simonii x Populus nigra]	1.79	118.24
map00250	Alanine, aspartate and glutamate metabolism	1.08E-01	TRINITY_DN25625_c0_g1	1.41	up	PREDICTED: amidophosphoribosyltransferase, chloroplastic [Populus euphratica]	27.38	16.32
map00250	Alanine, aspartate and glutamate metabolism	1.08E-01	TRINITY_DN25941_c0_g1	1.37	up	PREDICTED: glutamine synthetase leaf isozyme, chloroplastic [Populus euphratica]	986.31	589.65
map00250	Alanine, aspartate and glutamate metabolism	1.08E-01	TRINITY_DN26609_c0_g1	1.61	up	carbamoyl phosphate synthetase a family protein [Populus trichocarpa]	218.53	107.54
map00250	Alanine, aspartate and glutamate metabolism	1.08E-01	TRINITY_DN27395_c0_g1	1.70	up	aminotransferase 2 family protein [Populus trichocarpa]	980.92	451.86
map00250	Alanine, aspartate and glutamate metabolism	1.08E-01	TRINITY_DN27440_c0_g2	1.46	up	PREDICTED: glutamine synthetase leaf isozyme, chloroplastic [Populus euphratica]	559.41	306.88
map00250	Alanine, aspartate and glutamate metabolism	1.08E-01	TRINITY_DN14712_c0_g2	2.45	up	PREDICTED: glutamate synthase 1 [NADH], chloroplastic-like isoform X1 [Populus euphratica]	12.83	3.58
map00250	Alanine, aspartate and glutamate metabolism	1.08E-01	TRINITY_DN16683_c0_g1	1.71	up	hypothetical protein POPTR_0010s20070g [Populus trichocarpa]	30.27	14.40
map00250	Alanine, aspartate and glutamate metabolism	1.08E-01	TRINITY_DN16683_c0_g2	1.35	up	hypothetical protein POPTR_0010s20070g [Populus trichocarpa]	49.51	30.46
map00250	Alanine, aspartate and glutamate metabolism	1.08E-01	TRINITY_DN17431_c0_g1	2.34	up	adenylosuccinate synthetase family protein [Populus trichocarpa]	82.22	24.66
map00250	Alanine, aspartate and glutamate metabolism	1.08E-01	TRINITY_DN18964_c0_g2	1.33	up	PREDICTED: probable aldehyde dehydrogenase [Populus euphratica]	11.98	7.36
map00250	Alanine, aspartate and glutamate metabolism	1.08E-01	TRINITY_DN19742_c0_g1	1.68	up	hypothetical protein POPTR_0002s10640g [Populus trichocarpa]	43.81	21.04
map00250	Alanine, aspartate and glutamate metabolism	1.08E-01	TRINITY_DN20750_c0_g4	1.26	up	PREDICTED: probable ribosome-binding factor A, chloroplastic [Populus euphratica]	127.20	80.02
map00250	Alanine, aspartate and glutamate metabolism	1.08E-01	TRINITY_DN847_c0_g1	-7.22	down	glutamate decarboxylase 1 family protein [Populus trichocarpa]	0.04	7.12
map00040	Pentose and glucuronate interconversions	1.19E-01	TRINITY_DN21731_c0_g2	-1.12	down	PREDICTED: aldo-keto reductase family 4 member C9-like [Populus euphratica]	3.19	10.75
map00040	Pentose and glucuronate interconversions	1.19E-01	TRINITY_DN21932_c0_g3	-1.06	down	phosphatase 2C family protein [Populus trichocarpa]	2.53	8.10
map00040	Pentose and glucuronate interconversions	1.19E-01	TRINITY_DN22413_c1_g10	2.03	up	hypothetical protein VITISV_005279 [Vitis vinifera]	2.39	0.85
map00040	Pentose and glucuronate interconversions	1.19E-01	TRINITY_DN22730_c0_g1	-2.58	down	hypothetical protein POPTR_0006s12390g [Populus trichocarpa]	7.19	56.40
map00040	Pentose and glucuronate interconversions	1.19E-01	TRINITY_DN23974_c0_g4	-1.42	down	hypothetical protein POPTR_0014s01950g [Populus trichocarpa]	1.88	9.00
map00040	Pentose and glucuronate interconversions	1.19E-01	TRINITY_DN24105_c1_g3	-2.06	down	hypothetical protein TSUD_254980 [Trifolium subterraneum]	230.28	1159.28
map00040	Pentose and glucuronate interconversions	1.19E-01	TRINITY_DN24122_c0_g1	1.21	up	PREDICTED: uncharacterized protein LOC105119383 [Populus euphratica]	79.62	51.81
map00040	Pentose and glucuronate interconversions	1.19E-01	TRINITY_DN24495_c1_g1	-2.08	down	hypothetical protein POPTR_0008s13190g [Populus trichocarpa]	8.58	56.96
map00040	Pentose and glucuronate interconversions	1.19E-01	TRINITY_DN24495_c1_g3	-1.66	down	hypothetical protein POPTR_0010s11950g [Populus trichocarpa]	9.36	40.91
map00040	Pentose and glucuronate interconversions	1.19E-01	TRINITY_DN24628_c0_g1	1.28	up	PREDICTED: aldehyde dehydrogenase family 3 member H1-like [Populus euphratica]	40.39	24.46
map00040	Pentose and glucuronate interconversions	1.19E-01	TRINITY_DN24693_c0_g3	3.37	up	PREDICTED: probable pectate lyase 13 [Populus euphratica]	2.03	0.29
map00040	Pentose and glucuronate interconversions	1.19E-01	TRINITY_DN25630_c0_g1	1.30	up	aldehyde dehydrogenase 1 precursor family protein [Populus trichocarpa]	120.35	73.18
map00040	Pentose and glucuronate interconversions	1.19E-01	TRINITY_DN25892_c1_g4	1.32	up	unknown [Populus trichocarpa x Populus deltoides]	56.52	36.35
map00040	Pentose and glucuronate interconversions	1.19E-01	TRINITY_DN26025_c0_g1	-1.71	down	hypothetical protein POPTR_0004s18340g [Populus trichocarpa]	5.60	27.70
map00040	Pentose and glucuronate interconversions	1.19E-01	TRINITY_DN26069_c0_g1	1.07	up	putative pectin methylesterase [Populus tremula x Populus tremuloides]	104.43	71.85
map00040	Pentose and glucuronate interconversions	1.19E-01	TRINITY_DN26284_c1_g1	2.20	up	hypothetical protein VITISV_025518 [Vitis vinifera]	3.90	1.29
map00040	Pentose and glucuronate interconversions	1.19E-01	TRINITY_DN26421_c0_g1	1.32	up	the aldehyde dehydrogenase cp-ADH from C.plantagineum family protein [Populus trichocarpa]	58.13	34.64
map00040	Pentose and glucuronate interconversions	1.19E-01	TRINITY_DN26999_c0_g1	1.40	up	UDP-glucose pyrophosphorylase [Populus tremula x Populus tremuloides]	112.13	68.32

map00040	Pentose and glucuronate interconversions	1.19E-01	TRINITY_DN27044_c0_g1	1.63	up	pectate lyase 5 precursor family protein [Populus trichocarpa]	13.33	6.45
map00040	Pentose and glucuronate interconversions	1.19E-01	TRINITY_DN27315_c1_g3	1.78	up	PREDICTED: polygalacturonase At1g48100-like [Populus euphratica]	140.65	76.97
map00040	Pentose and glucuronate interconversions	1.19E-01	TRINITY_DN27331_c0_g2	-1.09	down	PREDICTED: probable polygalacturonase [Populus euphratica]	15.61	64.79
map00040	Pentose and glucuronate interconversions	1.19E-01	TRINITY_DN11544_c0_g1	-4.56	down	PREDICTED: uncharacterized protein LOC107880749 isoform X1 [Prunus mume]	0.13	4.63
map00040	Pentose and glucuronate interconversions	1.19E-01	TRINITY_DN14333_c0_g1	1.41	up	hypothetical protein POPTR_0008s09560g [Populus trichocarpa]	5.88	3.35
map00040	Pentose and glucuronate interconversions	1.19E-01	TRINITY_DN14511_c0_g1	-1.77	down	hypothetical protein POPTR_0006s13740g [Populus trichocarpa]	0.95	4.88
map00040	Pentose and glucuronate interconversions	1.19E-01	TRINITY_DN15131_c0_g2	-6.08	down	putative pectin methylesterase LuPME1 family protein [Populus trichocarpa]	0.02	2.96
map00040	Pentose and glucuronate interconversions	1.19E-01	TRINITY_DN15216_c0_g1	2.22	up	myb family transcription factor family protein [Populus trichocarpa]	14.59	5.27
map00040	Pentose and glucuronate interconversions	1.19E-01	TRINITY_DN15442_c0_g1	1.51	up	hypothetical protein POPTR_0005s00720g [Populus trichocarpa]	2.88	1.51
map00040	Pentose and glucuronate interconversions	1.19E-01	TRINITY_DN15586_c0_g1	2.45	up	hypothetical protein POPTR_0005s06430g [Populus trichocarpa]	2.82	1.17
map00040	Pentose and glucuronate interconversions	1.19E-01	TRINITY_DN16155_c0_g1	2.15	up	hypothetical protein POPTR_0004s12440g [Populus trichocarpa]	53.21	18.16
map00040	Pentose and glucuronate interconversions	1.19E-01	TRINITY_DN16682_c0_g1	-2.32	down	ripening-related family protein [Populus trichocarpa]	4.32	32.23
map00040	Pentose and glucuronate interconversions	1.19E-01	TRINITY_DN16930_c0_g1	2.20	up	invertase/pectin methylesterase inhibitor family protein [Populus trichocarpa]	3.03	1.02
map00040	Pentose and glucuronate interconversions	1.19E-01	TRINITY_DN17059_c0_g2	1.13	up	UDP-glucose pyrophosphorylase [Populus deltoides]	101.22	72.02
map00040	Pentose and glucuronate interconversions	1.19E-01	TRINITY_DN17167_c0_g1	-1.21	down	GHMP kinase-related family protein [Populus trichocarpa]	3.51	12.23
map00040	Pentose and glucuronate interconversions	1.19E-01	TRINITY_DN17353_c0_g1	1.22	up	PREDICTED: LOW QUALITY PROTEIN: methylecgonone reductase-like [Populus euphratica]	9.67	7.02
map00040	Pentose and glucuronate interconversions	1.19E-01	TRINITY_DN17644_c0_g1	1.22	up	PREDICTED: NADP-dependent D-sorbitol-6-phosphate dehydrogenase-like [Populus euphratica]	152.43	100.73
map00040	Pentose and glucuronate interconversions	1.19E-01	TRINITY_DN17646_c0_g3	2.20	up	unknown [Populus trichocarpa]	75.56	24.78
map00040	Pentose and glucuronate interconversions	1.19E-01	TRINITY_DN17648_c0_g1	2.26	up	PREDICTED: uncharacterized protein At1g18480 [Populus euphratica]	19.45	6.55
map00040	Pentose and glucuronate interconversions	1.19E-01	TRINITY_DN17648_c0_g2	2.31	up	calcineurin-like phosphoesterase family protein [Populus trichocarpa]	7.85	2.39
map00040	Pentose and glucuronate interconversions	1.19E-01	TRINITY_DN17754_c0_g1	-2.02	down	PREDICTED: pectinesterase-like [Populus euphratica]	0.76	4.68
map00040	Pentose and glucuronate interconversions	1.19E-01	TRINITY_DN17916_c0_g1	-2.23	down	PREDICTED: transcription factor DIVARICATA-like [Populus euphratica]	0.83	6.00
map00040	Pentose and glucuronate interconversions	1.19E-01	TRINITY_DN18049_c0_g2	1.34	up	hypothetical protein POPTR_0001s25590g [Populus trichocarpa]	43.52	25.51
map00040	Pentose and glucuronate interconversions	1.19E-01	TRINITY_DN18222_c1_g1	1.16	up	PREDICTED: UDP-sugar pyrophosphorylase [Populus euphratica]	28.36	19.26
map00040	Pentose and glucuronate interconversions	1.19E-01	TRINITY_DN18344_c0_g1	1.73	up	hypothetical protein POPTR_0005s12390g [Populus trichocarpa]	4.29	2.12
map00040	Pentose and glucuronate interconversions	1.19E-01	TRINITY_DN18344_c0_g2	3.11	up	hypothetical protein POPTR_0007s13000g [Populus trichocarpa]	86.69	15.79
map00040	Pentose and glucuronate interconversions	1.19E-01	TRINITY_DN18767_c0_g1	-1.14	down	pectinesterase family protein [Populus trichocarpa]	3.89	13.90
map00040	Pentose and glucuronate interconversions	1.19E-01	TRINITY_DN18895_c0_g1	-1.06	down	mitochondrial aldehyde dehydrogenase family protein [Populus trichocarpa]	7.40	23.22
map00040	Pentose and glucuronate interconversions	1.19E-01	TRINITY_DN19215_c1_g1	-1.38	down	hypothetical protein POPTR_0007s10210g [Populus trichocarpa]	3.47	12.55
map00040	Pentose and glucuronate interconversions	1.19E-01	TRINITY_DN19215_c2_g1	-1.56	down	PREDICTED: probable protein phosphatase 2C 63 [Populus euphratica]	6.48	30.16
map00040	Pentose and glucuronate interconversions	1.19E-01	TRINITY_DN19734_c0_g2	1.39	up	PREDICTED: ribulose-phosphate 3-epimerase, chloroplastic [Populus euphratica]	648.43	374.61
map00040	Pentose and glucuronate interconversions	1.19E-01	TRINITY_DN19874_c0_g1	2.03	up	PREDICTED: uncharacterized protein LOC105122007 [Populus euphratica]	71.32	25.90
map00040	Pentose and glucuronate interconversions	1.19E-01	TRINITY_DN20035_c0_g1	-1.14	down	hypothetical protein POPTR_0003s07460g [Populus trichocarpa]	88.55	285.41

map00040	Pentose and glucuronate interconversions	1.19E-01	TRINITY_DN20067_c0_g1	1.89	up	hypothetical protein POPTR_0016s14310g [Populus trichocarpa]	360.20	150.22
map00040	Pentose and glucuronate interconversions	1.19E-01	TRINITY_DN20620_c1_g3	1.10	up	unknown [Populus trichocarpa]	72.81	51.40
map00040	Pentose and glucuronate interconversions	1.19E-01	TRINITY_DN20638_c0_g1	1.13	up	PREDICTED: FGGY carbohydrate kinase domain-containing protein isoform X1 [Populus euphratica]	29.59	21.24
map00040	Pentose and glucuronate interconversions	1.19E-01	TRINITY_DN21126_c0_g2	-2.31	down	PREDICTED: probable polygalacturonase isoform X1 [Populus euphratica]	14.88	112.78
map00040	Pentose and glucuronate interconversions	1.19E-01	TRINITY_DN21343_c0_g5	-2.08	down	hypothetical protein TSUD_72310 [Trifolium subterraneum]	0.74	3.53
map00040	Pentose and glucuronate interconversions	1.19E-01	TRINITY_DN21497_c0_g2	1.83	up	PREDICTED: sorbitol dehydrogenase [Populus euphratica]	81.71	34.89
map00040	Pentose and glucuronate interconversions	1.19E-01	TRINITY_DN6170_c0_g1	-7.86	down	myb family transcription factor family protein [Populus trichocarpa]	0.00	4.20
map00040	Pentose and glucuronate interconversions	1.19E-01	TRINITY_DN7900_c0_g1	1.75	up	Retrovirus-related Pol polyprotein from transposon TNT 1-94 [Cajanus cajan]	2.25	1.02
map00660	C5-Branched dibasic acid metabolism	1.28E-01	TRINITY_DN21760_c0_g2	1.32	up	PREDICTED: 3-isopropylmalate dehydrogenase, chloroplastic-like [Populus euphratica]	86.28	52.23
map00660	C5-Branched dibasic acid metabolism	1.28E-01	TRINITY_DN22580_c0_g3	1.24	up	hypothetical protein POPTR_0003s10720g [Populus trichocarpa]	124.71	79.50
map00660	C5-Branched dibasic acid metabolism	1.28E-01	TRINITY_DN22695_c0_g4	1.16	up	aconitase family protein [Populus trichocarpa]	151.04	102.95
map00660	C5-Branched dibasic acid metabolism	1.28E-01	TRINITY_DN26976_c0_g1	1.47	up	PREDICTED: acetolactate synthase 2, chloroplastic-like [Populus euphratica]	396.57	239.53
map00660	C5-Branched dibasic acid metabolism	1.28E-01	TRINITY_DN20583_c0_g3	2.29	up	hypothetical protein POPTR_0008s03820g [Populus trichocarpa]	4.09	1.88
map00660	C5-Branched dibasic acid metabolism	1.28E-01	TRINITY_DN21313_c0_g2	-1.72	down	hypothetical protein POPTR_0014s07170g [Populus trichocarpa]	4.63	24.85
map00660	C5-Branched dibasic acid metabolism	1.28E-01	TRINITY_DN21600_c0_g2	1.23	up	PREDICTED: acetolactate synthase small subunit 2, chloroplastic-like [Populus euphratica]	35.74	23.13
map04712	Circadian rhythm - plant	1.29E-01	TRINITY_DN22346_c0_g2	-1.77	down	PREDICTED: zinc finger protein CONSTANS-LIKE 7 [Populus euphratica]	0.92	4.83
map04712	Circadian rhythm - plant	1.29E-01	TRINITY_DN22472_c0_g1	-1.54	down	CONSTANS-like protein [Populus alba x Populus glandulosa]	29.77	122.36
map04712	Circadian rhythm - plant	1.29E-01	TRINITY_DN22559_c0_g2	-1.43	down	hypothetical protein POPTR_0008s05520g [Populus trichocarpa]	2.33	8.49
map04712	Circadian rhythm - plant	1.29E-01	TRINITY_DN22604_c0_g2	2.08	up	PREDICTED: E3 ubiquitin-protein ligase COP1-like isoform X2 [Populus euphratica]	33.35	12.32
map04712	Circadian rhythm - plant	1.29E-01	TRINITY_DN22618_c1_g1	-1.28	down	hypothetical protein POPTR_0014s07450g [Populus trichocarpa]	4.23	16.18
map04712	Circadian rhythm - plant	1.29E-01	TRINITY_DN22667_c0_g5	-1.03	down	PREDICTED: transcription factor TCP14-like [Populus euphratica]	8.12	25.93
map04712	Circadian rhythm - plant	1.29E-01	TRINITY_DN23224_c0_g1	1.10	up	hypothetical protein POPTR_0004s07270g [Populus trichocarpa]	38.11	26.44
map04712	Circadian rhythm - plant	1.29E-01	TRINITY_DN23243_c1_g1	-1.41	down	unknown [Populus trichocarpa]	14.08	58.20
map04712	Circadian rhythm - plant	1.29E-01	TRINITY_DN23575_c0_g1	-3.00	down	hypothetical protein POPTR_0014s10700g [Populus trichocarpa]	0.89	10.81
map04712	Circadian rhythm - plant	1.29E-01	TRINITY_DN23590_c0_g2	2.37	up	PREDICTED: transcription factor bHLH130-like [Populus euphratica]	6.54	1.68
map04712	Circadian rhythm - plant	1.29E-01	TRINITY_DN23750_c0_g2	2.33	up	PREDICTED: MYB-like transcription factor ETC1 [Populus euphratica]	70.72	21.48
map04712	Circadian rhythm - plant	1.29E-01	TRINITY_DN24329_c2_g3	1.30	up	PREDICTED: protein CHLOROPLAST IMPORT APPARATUS 2-like isoform X1 [Populus euphratica]	39.04	24.04
map04712	Circadian rhythm - plant	1.29E-01	TRINITY_DN24462_c1_g1	-2.20	down	PREDICTED: two-component response regulator-like APRR9 isoform X2 [Populus euphratica]	1.99	14.54
map04712	Circadian rhythm - plant	1.29E-01	TRINITY_DN24894_c0_g6	-2.92	down	putative MYB transcription factor family protein [Populus trichocarpa]	0.69	7.84
map04712	Circadian rhythm - plant	1.29E-01	TRINITY_DN25058_c0_g1	2.33	up	unknown [Populus trichocarpa]	5.70	1.79
map04712	Circadian rhythm - plant	1.29E-01	TRINITY_DN25241_c1_g1	-1.60	down	hypothetical protein POPTR_0006s23780g [Populus trichocarpa]	3.32	15.29
map04712	Circadian rhythm - plant	1.29E-01	TRINITY_DN25633_c0_g2	-1.33	down	hypothetical protein POPTR_0018s11800g [Populus trichocarpa]	7.63	29.20
map04712	Circadian rhythm - plant	1.29E-01	TRINITY_DN25793_c0_g2	2.51	up	PREDICTED: protein REVEILLE 7-like [Populus euphratica]	6.18	1.76
map04712	Circadian rhythm - plant	1.29E-01	TRINITY_DN25946_c0_g1	-3.03	down	PREDICTED: chalcone synthase 1-like [Populus euphratica]	17.29	205.36
map04712	Circadian rhythm - plant	1.29E-01	TRINITY_DN25946_c0_g2	-3.86	down	PREDICTED: chalcone synthase 1-like isoform X1 [Populus euphratica]	3.16	68.59
map04712	Circadian rhythm - plant	1.29E-01	TRINITY_DN26408_c0_g3	-1.89	down	PREDICTED: zinc finger protein CONSTANS-LIKE 15-like isoform X1 [Populus euphratica]	3.87	26.39
map04712	Circadian rhythm - plant	1.29E-01	TRINITY_DN27214_c2_g1	-3.58	down	FLAVIN-BINDING KELCH DOMAIN F BOX family protein [Populus trichocarpa]	3.86	72.07
map04712	Circadian rhythm - plant	1.29E-01	TRINITY_DN27376_c0_g1	-1.57	down	PREDICTED: two-component response regulator-like PRR73 isoform X1 [Populus euphratica]	9.11	41.26
map04712	Circadian rhythm - plant	1.29E-01	TRINITY_DN27376_c0_g2	-1.17	down	pseudo-response regulator 73 [Populus trichocarpa]	5.28	18.66

map04712	Circadian rhythm - plant	1.29E-01	TRINITY_DN27495_c1_g1	-1.39	down	kinase family protein [Populus trichocarpa]	5.00	19.67
map04712	Circadian rhythm - plant	1.29E-01	TRINITY_DN27686_c0_g1	1.24	up	SPA1-RELATED 4 family protein [Populus trichocarpa]	28.22	19.14
map04712	Circadian rhythm - plant	1.29E-01	TRINITY_DN27848_c2_g1	-2.24	down	GIGANTEA-like protein c [Populus alba x Populus glandulosa]	11.27	82.69
map04712	Circadian rhythm - plant	1.29E-01	TRINITY_DN15903_c1_g1	1.47	up	PREDICTED: dof zinc finger protein DOF1.2 [Populus euphratica]	3.96	2.29
map04712	Circadian rhythm - plant	1.29E-01	TRINITY_DN15933_c1_g1	-2.90	down	hypothetical protein POPTR_0002s13100g [Populus trichocarpa]	1.10	12.11
map04712	Circadian rhythm - plant	1.29E-01	TRINITY_DN15933_c1_g2	-3.18	down	hypothetical protein POPTR_0014s03590g [Populus trichocarpa]	0.58	8.37
map04712	Circadian rhythm - plant	1.29E-01	TRINITY_DN15994_c0_g1	-2.17	down	GI1-2 [Populus tomentosa]	6.49	43.76
map04712	Circadian rhythm - plant	1.29E-01	TRINITY_DN16185_c1_g1	1.66	up	hypothetical protein POPTR_0001s16970g [Populus trichocarpa]	14.46	6.11
map04712	Circadian rhythm - plant	1.29E-01	TRINITY_DN16595_c0_g1	-1.69	down	hypothetical protein POPTR_0006s13790g [Populus trichocarpa]	1.12	7.33
map04712	Circadian rhythm - plant	1.29E-01	TRINITY_DN16808_c0_g1	-2.73	down	-	2.37	24.84
map04712	Circadian rhythm - plant	1.29E-01	TRINITY_DN17038_c0_g1	2.14	up	PREDICTED: transcription factor WER-like [Populus euphratica]	78.70	30.19
map04712	Circadian rhythm - plant	1.29E-01	TRINITY_DN17153_c0_g2	1.97	up	hypothetical protein POPTR_0011s00390g [Populus trichocarpa]	7.47	2.94
map04712	Circadian rhythm - plant	1.29E-01	TRINITY_DN17238_c0_g1	-3.34	down	hypothetical protein POPTR_0005s13990g [Populus trichocarpa]	0.41	6.28
map04712	Circadian rhythm - plant	1.29E-01	TRINITY_DN17238_c0_g2	-3.97	down	PREDICTED: LOW QUALITY PROTEIN: dof zinc finger protein DOF3.4 [Populus euphratica]	1.11	26.07
map04712	Circadian rhythm - plant	1.29E-01	TRINITY_DN18676_c0_g2	1.69	up	hypothetical protein POPTR_0012s13290g [Populus trichocarpa]	10.14	4.79
map04712	Circadian rhythm - plant	1.29E-01	TRINITY_DN19149_c0_g1	-5.88	down	PREDICTED: myb-related protein P-like [Populus euphratica]	0.16	7.51
map04712	Circadian rhythm - plant	1.29E-01	TRINITY_DN20113_c0_g1	-1.45	down	chalcone synthase [Populus alba]	95.98	585.75
map04712	Circadian rhythm - plant	1.29E-01	TRINITY_DN20493_c0_g1	1.03	up	PREDICTED: transcription factor WER-like [Populus euphratica]	46.96	33.83
map04712	Circadian rhythm - plant	1.29E-01	TRINITY_DN20524_c0_g2	-3.47	down	bZIP with a Ring-finger motif family protein [Populus trichocarpa]	0.90	15.43
map04712	Circadian rhythm - plant	1.29E-01	TRINITY_DN20802_c0_g3	-2.23	down	PREDICTED: zinc finger protein CONSTANS-LIKE 5-like [Populus euphratica]	11.26	78.87
map04712	Circadian rhythm - plant	1.29E-01	TRINITY_DN20920_c0_g2	-2.17	down	hypothetical protein POPTR_0012s02570g [Populus trichocarpa]	1.01	6.74
map04712	Circadian rhythm - plant	1.29E-01	TRINITY_DN21186_c0_g1	-3.53	down	MYB transcription factor R2R3-like protein [Populus tremuloides]	18.09	301.52
map04712	Circadian rhythm - plant	1.29E-01	TRINITY_DN21253_c0_g1	1.45	up	hypothetical protein POPTR_0015s13430g [Populus trichocarpa]	14.73	8.81
map04712	Circadian rhythm - plant	1.29E-01	TRINITY_DN21253_c0_g3	-1.59	down	hypothetical protein POPTR_0012s13440g [Populus trichocarpa]	0.53	2.49
map04712	Circadian rhythm - plant	1.29E-01	TRINITY_DN21253_c0_g4	-2.89	down	hypothetical protein POPTR_0012s13440g [Populus trichocarpa]	0.21	2.45
map04712	Circadian rhythm - plant	1.29E-01	TRINITY_DN21362_c0_g4	-2.14	down	hypothetical protein POPTR_0007s11620g [Populus trichocarpa]	1.43	9.96
map03440	Homologous recombination	1.30E-01	TRINITY_DN21758_c0_g2	-1.73	down	PREDICTED: ATP-dependent DNA helicase Q-like 1 [Populus euphratica]	0.97	4.91
map03440	Homologous recombination	1.30E-01	TRINITY_DN22082_c1_g1	-1.41	down	PREDICTED: protein CHROMATIN REMODELING 25 [Populus euphratica]	3.66	15.30
map03440	Homologous recombination	1.30E-01	TRINITY_DN22469_c0_g1	-1.37	down	DNA helicase family protein [Populus trichocarpa]	5.98	24.54
map03440	Homologous recombination	1.30E-01	TRINITY_DN22705_c0_g1	-2.58	down	PREDICTED: meiotic recombination protein DMC1 homolog [Populus euphratica]	2.15	20.22
map03440	Homologous recombination	1.30E-01	TRINITY_DN23575_c0_g2	-1.74	down	hypothetical protein POPTR_0005s01980g [Populus trichocarpa]	1.52	10.86
map03440	Homologous recombination	1.30E-01	TRINITY_DN23582_c0_g1	1.21	up	hypothetical protein POPTR_0006s29400g [Populus trichocarpa]	44.02	30.96
map03440	Homologous recombination	1.30E-01	TRINITY_DN23741_c0_g2	-2.39	down	hypothetical protein POPTR_0007s15350g, partial [Populus trichocarpa]	1.81	6.34
map03440	Homologous recombination	1.30E-01	TRINITY_DN23774_c0_g1	-1.18	down	hypothetical protein POPTR_0004s20080g [Populus trichocarpa]	1.48	5.19
map03440	Homologous recombination	1.30E-01	TRINITY_DN23789_c0_g2	1.65	up	PREDICTED: uncharacterized protein LOC105112215 [Populus euphratica]	14.18	8.86
map03440	Homologous recombination	1.30E-01	TRINITY_DN23841_c0_g3	-1.40	down	PREDICTED: replication protein A 32 kDa subunit A-like [Populus euphratica]	7.83	32.50
map03440	Homologous recombination	1.30E-01	TRINITY_DN24395_c0_g2	1.21	up	hypothetical protein POPTR_0005s26230g [Populus trichocarpa]	66.57	46.27
map03440	Homologous recombination	1.30E-01	TRINITY_DN24914_c2_g1	1.97	up	PREDICTED: zinc finger protein 2-like [Populus euphratica]	3.58	1.44
map03440	Homologous recombination	1.30E-01	TRINITY_DN24951_c0_g1	2.63	up	unknown [Populus trichocarpa]	40.05	12.23
map03440	Homologous recombination	1.30E-01	TRINITY_DN24970_c1_g1	-1.89	down	PREDICTED: protein BREAST CANCER SUSCEPTIBILITY 2 homolog B-like [Populus euphratica]	1.43	9.39
map03440	Homologous recombination	1.30E-01	TRINITY_DN24970_c1_g2	-2.32	down	PREDICTED: protein BREAST CANCER SUSCEPTIBILITY 2 homolog B-like [Populus euphratica]	0.77	5.94

map03440	Homologous recombination	1.30E-01	TRINITY_DN25007_c0_g1	-1.55	down	TIR-NBS-LRR-TIR type disease resistance protein, partial [Populus trichocarpa]	12.22	41.68
map03440	Homologous recombination	1.30E-01	TRINITY_DN25276_c0_g2	-1.48	down	hypothetical protein POPTR_0018s00860g [Populus trichocarpa]	1.70	6.52
map03440	Homologous recombination	1.30E-01	TRINITY_DN26009_c1_g2	-1.05	down	hypothetical protein POPTR_0006s23660g [Populus trichocarpa]	5.05	17.76
map03440	Homologous recombination	1.30E-01	TRINITY_DN26881_c1_g1	1.20	up	PREDICTED: uncharacterized protein LOC105112215 [Populus euphratica]	88.96	53.75
map03440	Homologous recombination	1.30E-01	TRINITY_DN27349_c0_g1	1.26	up	hypothetical protein POPTR_0001s44720g [Populus trichocarpa]	31.99	22.49
map03440	Homologous recombination	1.30E-01	TRINITY_DN13077_c0_g1	-3.90	down	unknown [Populus trichocarpa]	0.30	5.95
map03440	Homologous recombination	1.30E-01	TRINITY_DN1417_c0_g1	9.22	up	hypothetical protein POPTR_0017s08950g [Populus trichocarpa]	9.69	0.02
map03440	Homologous recombination	1.30E-01	TRINITY_DN14981_c0_g1	1.20	up	single-strand-binding family protein [Populus trichocarpa]	44.95	29.84
map03440	Homologous recombination	1.30E-01	TRINITY_DN15020_c0_g1	-1.35	down	PREDICTED: uncharacterized protein LOC105110111 [Populus euphratica]	1.83	7.03
map03440	Homologous recombination	1.30E-01	TRINITY_DN16624_c0_g1	1.03	up	unknown [Populus trichocarpa x Populus deltoides]	184.06	137.19
map03440	Homologous recombination	1.30E-01	TRINITY_DN17121_c0_g1	-1.12	down	hypothetical protein POPTR_0005s27010g, partial [Populus trichocarpa]	1.89	7.13
map03440	Homologous recombination	1.30E-01	TRINITY_DN17241_c0_g1	-1.38	down	DNA polymerase delta subunit 4 family protein [Populus trichocarpa]	4.03	15.90
map03440	Homologous recombination	1.30E-01	TRINITY_DN17241_c0_g2	-1.21	down	DNA polymerase delta subunit 4 family protein [Populus trichocarpa]	37.87	132.98
map03440	Homologous recombination	1.30E-01	TRINITY_DN17522_c0_g1	-2.36	down	PREDICTED: replication protein A 70 kDa DNA-binding subunit B [Populus euphratica]	3.63	29.40
map03440	Homologous recombination	1.30E-01	TRINITY_DN17620_c0_g1	1.37	up	hypothetical protein POPTR_0006s14620g, partial [Populus trichocarpa]	1053.56	627.95
map03440	Homologous recombination	1.30E-01	TRINITY_DN18149_c0_g1	2.08	up	hypothetical protein POPTR_0008s19820g [Populus trichocarpa]	28.72	9.90
map03440	Homologous recombination	1.30E-01	TRINITY_DN18342_c0_g2	-2.33	down	hypothetical protein POPTR_0003s08160g [Populus trichocarpa]	0.61	4.82
map03440	Homologous recombination	1.30E-01	TRINITY_DN18970_c0_g1	-1.57	down	hypothetical protein POPTR_0008s15120g [Populus trichocarpa]	4.62	19.39
map03440	Homologous recombination	1.30E-01	TRINITY_DN19198_c0_g1	1.14	up	hypothetical protein POPTR_0002s24970g [Populus trichocarpa]	135.65	95.80
map03440	Homologous recombination	1.30E-01	TRINITY_DN19395_c0_g1	-1.33	down	PREDICTED: replication protein A 70 kDa DNA-binding subunit E-like [Populus euphratica]	2.44	9.54
map03440	Homologous recombination	1.30E-01	TRINITY_DN19537_c0_g3	-1.06	down	PREDICTED: uncharacterized protein LOC105109677 isoform X1 [Populus euphratica]	5.42	16.71
map03440	Homologous recombination	1.30E-01	TRINITY_DN20129_c0_g1	-1.78	down	PREDICTED: crossover junction endonuclease MUS81 isoform X2 [Populus euphratica]	1.13	5.96
map03440	Homologous recombination	1.30E-01	TRINITY_DN20672_c0_g1	1.85	up	hypothetical protein POPTR_0006s03190g [Populus trichocarpa]	49.08	22.60
map03440	Homologous recombination	1.30E-01	TRINITY_DN20687_c0_g1	-2.39	down	RING-H2 subgroup RHE protein [Populus tremula x Populus alba]	8.01	63.62
map03440	Homologous recombination	1.30E-01	TRINITY_DN20742_c0_g1	1.62	up	FKBP-type peptidyl-prolyl cis-trans isomerase 3 family protein [Populus trichocarpa]	166.22	81.22
map03440	Homologous recombination	1.30E-01	TRINITY_DN20973_c0_g1	-1.20	down	PREDICTED: DNA polymerase delta catalytic subunit [Populus euphratica]	3.70	13.41
map03440	Homologous recombination	1.30E-01	TRINITY_DN20983_c0_g1	-1.63	down	PREDICTED: LOW QUALITY PROTEIN: DNA repair protein RAD51 homolog [Populus euphratica]	4.69	25.62
map00785	Lipoic acid metabolism	1.33E-01	TRINITY_DN23229_c0_g2	1.41	up	PREDICTED: plastidial lipoyltransferase 2-like [Populus euphratica]	3.15	1.81
map00785	Lipoic acid metabolism	1.33E-01	TRINITY_DN24963_c0_g1	1.51	up	hypothetical protein POPTR_0019s13380g [Populus trichocarpa]	21.89	11.85
map00785	Lipoic acid metabolism	1.33E-01	TRINITY_DN25124_c0_g3	1.51	up	PREDICTED: ferredoxin-thioredoxin reductase, variable chain-like [Populus euphratica]	25.21	13.59
map00785	Lipoic acid metabolism	1.33E-01	TRINITY_DN17481_c0_g2	1.38	up	LIPOYLTRANSFERASE 2 family protein [Populus trichocarpa]	20.15	11.87
map00670	One carbon pool by folate	1.33E-01	TRINITY_DN22812_c0_g2	1.66	up	Serine hydroxymethyltransferase family protein [Populus trichocarpa]	785.78	366.57
map00670	One carbon pool by folate	1.33E-01	TRINITY_DN22812_c0_g3	1.77	up	mitochondrial serine hydroxymethyltransferase [Populus tremuloides]	59.98	26.65
map00670	One carbon pool by folate	1.33E-01	TRINITY_DN23993_c0_g1	1.14	up	PREDICTED: formyltetrahydrofolate deformylase 1, mitochondrial-like isoform X3 [Populus euphratica]	44.23	28.78
map00670	One carbon pool by folate	1.33E-01	TRINITY_DN24565_c0_g1	1.99	up	T-protein of the glycine decarboxylase complex [Populus trichocarpa]	481.27	183.79
map00670	One carbon pool by folate	1.33E-01	TRINITY_DN24894_c0_g3	1.50	up	hypothetical protein POPTR_0015s03960g [Populus trichocarpa]	34.92	19.04
map00670	One carbon pool by folate	1.33E-01	TRINITY_DN25125_c0_g3	1.50	up	hypothetical protein POPTR_0002s15850g [Populus trichocarpa]	52.02	30.67
map00670	One carbon pool by folate	1.33E-01	TRINITY_DN13782_c0_g1	5.00	up	hypothetical protein POPTR_0002s11010g [Populus trichocarpa]	6.55	0.32
map00670	One carbon pool by folate	1.33E-01	TRINITY_DN17865_c0_g1	2.27	up	plastid serine hydroxymethyltransferase [Populus tremuloides]	53.46	17.12
map00670	One carbon pool by folate	1.33E-01	TRINITY_DN18528_c0_g1	1.16	up	hypothetical protein POPTR_0005s21670g [Populus trichocarpa]	18.78	13.99

map00670	One carbon pool by folate	1.33E-01	TRINITY_DN18598_c0_g1	1.82	up	hypothetical protein POPTR_0008s00350g [Populus trichocarpa]	468.84	197.84
map00670	One carbon pool by folate	1.33E-01	TRINITY_DN19857_c0_g2	1.45	up	AICARFT/IMPCHase bienzyme family protein [Populus tomentosa]	80.53	45.41
map00670	One carbon pool by folate	1.33E-01	TRINITY_DN20450_c0_g2	1.18	up	PREDICTED: methionyl-tRNA formyltransferase, mitochondrial isoform X2 [Populus euphratica]	53.40	39.72
map00670	One carbon pool by folate	1.33E-01	TRINITY_DN8327_c0_g1	1.29	up	unknown [Populus trichocarpa]	17.80	11.35
map00020	Citrate cycle (TCA cycle)	1.39E-01	TRINITY_DN21760_c0_g1	1.25	up	isocitrate dehydrogenase family protein [Populus trichocarpa]	53.95	39.69
map00020	Citrate cycle (TCA cycle)	1.39E-01	TRINITY_DN22048_c2_g2	1.93	up	hypothetical protein POPTR_0015s12380g [Populus trichocarpa]	3.05	1.20
map00020	Citrate cycle (TCA cycle)	1.39E-01	TRINITY_DN22048_c2_g5	-1.67	down	hypothetical protein POPTR_0012s11610g [Populus trichocarpa]	1.63	8.07
map00020	Citrate cycle (TCA cycle)	1.39E-01	TRINITY_DN22563_c0_g1	1.23	up	2-oxoacid dehydrogenase family protein [Populus trichocarpa]	62.68	37.12
map00020	Citrate cycle (TCA cycle)	1.39E-01	TRINITY_DN23168_c0_g1	1.33	up	Fumarate hydratase 1 family protein [Populus trichocarpa]	44.45	26.95
map00020	Citrate cycle (TCA cycle)	1.39E-01	TRINITY_DN23213_c0_g1	1.52	up	pyruvate dehydrogenase family protein [Populus trichocarpa]	125.88	67.45
map00020	Citrate cycle (TCA cycle)	1.39E-01	TRINITY_DN23271_c0_g1	1.57	up	hypothetical protein POPTR_0001s35080g [Populus trichocarpa]	37.34	19.20
map00020	Citrate cycle (TCA cycle)	1.39E-01	TRINITY_DN24709_c0_g1	1.03	up	PREDICTED: pyruvate dehydrogenase E1 component subunit beta-3, chloroplastic-like [Populus euphratica]	176.40	133.88
map00020	Citrate cycle (TCA cycle)	1.39E-01	TRINITY_DN24969_c2_g1	1.05	up	Succinyl-CoA ligase beta-chain family protein [Populus trichocarpa]	98.12	74.47
map00020	Citrate cycle (TCA cycle)	1.39E-01	TRINITY_DN25141_c0_g1	1.90	up	PREDICTED: dihydrolipoyllysine-residue acetyltransferase component 4 of pyruvate dehydrogenase complex, chloroplastic [Populus euphratica]	116.62	48.47
map00020	Citrate cycle (TCA cycle)	1.39E-01	TRINITY_DN25221_c0_g1	1.58	up	malate dehydrogenase family protein, partial [Populus trichocarpa]	57.58	29.35
map00020	Citrate cycle (TCA cycle)	1.39E-01	TRINITY_DN25221_c1_g2	2.25	up	malate dehydrogenase family protein [Populus trichocarpa]	356.58	110.81
map00020	Citrate cycle (TCA cycle)	1.39E-01	TRINITY_DN25221_c1_g3	1.80	up	PREDICTED: malate dehydrogenase, glyoxysomal [Populus euphratica]	643.81	285.37
map00020	Citrate cycle (TCA cycle)	1.39E-01	TRINITY_DN25221_c1_g4	2.08	up	PREDICTED: LOW QUALITY PROTEIN: dihydrolipoyllysine-residue acetyltransferase component 5 of pyruvate dehydrogenase complex, chloroplastic-like, partial [Populus euphratica]	9.32	3.34
map00020	Citrate cycle (TCA cycle)	1.39E-01	TRINITY_DN25377_c0_g1	1.91	up	hypothetical protein POPTR_0013s11870g [Populus trichocarpa]	85.23	34.17
map00020	Citrate cycle (TCA cycle)	1.39E-01	TRINITY_DN25746_c0_g1	2.80	up	nodule-enhanced malate dehydrogenase family protein [Populus trichocarpa]	113.74	23.22
map00020	Citrate cycle (TCA cycle)	1.39E-01	TRINITY_DN25885_c1_g3	-2.54	down	PREDICTED: ATP-citrate synthase beta chain protein 2 [Vitis vinifera]	0.56	4.93
map00020	Citrate cycle (TCA cycle)	1.39E-01	TRINITY_DN26163_c0_g1	1.24	up	PREDICTED: dihydrolipoyllysine-residue acetyltransferase component 2 of pyruvate dehydrogenase complex, mitochondrial-like [Populus euphratica]	49.33	31.65
map00020	Citrate cycle (TCA cycle)	1.39E-01	TRINITY_DN26341_c0_g2	1.21	up	dihydrolipoamide S-acetyltransferase family protein [Populus trichocarpa]	11.43	7.48
map00020	Citrate cycle (TCA cycle)	1.39E-01	TRINITY_DN26572_c0_g1	1.40	up	hypothetical protein POPTR_0002s20530g [Populus trichocarpa]	430.34	248.97
map00020	Citrate cycle (TCA cycle)	1.39E-01	TRINITY_DN26837_c0_g1	2.09	up	hypothetical protein POPTR_0010s15200g [Populus trichocarpa]	81.17	29.93
map00020	Citrate cycle (TCA cycle)	1.39E-01	TRINITY_DN27198_c1_g1	1.09	up	aconitate hydratase family protein [Populus trichocarpa]	47.60	32.18
map00020	Citrate cycle (TCA cycle)	1.39E-01	TRINITY_DN27198_c1_g2	1.02	up	aconitate hydratase family protein [Populus trichocarpa]	16.99	12.62
map00020	Citrate cycle (TCA cycle)	1.39E-01	TRINITY_DN27862_c2_g1	-1.69	down	PREDICTED: structural maintenance of chromosomes protein 2-1-like [Populus euphratica]	21.02	56.92
map00020	Citrate cycle (TCA cycle)	1.39E-01	TRINITY_DN16821_c0_g1	1.91	up	PREDICTED: malate dehydrogenase, glyoxysomal [Populus euphratica]	248.44	104.05
map00020	Citrate cycle (TCA cycle)	1.39E-01	TRINITY_DN18859_c0_g3	1.18	up	aconitate hydratase family protein [Populus trichocarpa]	80.30	54.25
map00020	Citrate cycle (TCA cycle)	1.39E-01	TRINITY_DN19763_c0_g3	1.88	up	PREDICTED: malate dehydrogenase, mitochondrial [Populus euphratica]	247.09	106.19
map00020	Citrate cycle (TCA cycle)	1.39E-01	TRINITY_DN19823_c0_g5	1.20	up	hypothetical protein POPTR_0005s09370g [Populus trichocarpa]	70.43	46.85
map00020	Citrate cycle (TCA cycle)	1.39E-01	TRINITY_DN20175_c0_g1	1.59	up	peroxiredoxin Q family protein [Populus trichocarpa]	1020.77	504.37
map00020	Citrate cycle (TCA cycle)	1.39E-01	TRINITY_DN20938_c0_g1	1.33	up	PREDICTED: malate dehydrogenase, glyoxysomal [Populus euphratica]	118.58	72.32
map00020	Citrate cycle (TCA cycle)	1.39E-01	TRINITY_DN20938_c0_g2	2.63	up	malate dehydrogenase family protein [Populus trichocarpa]	74.92	18.32
map00020	Citrate cycle (TCA cycle)	1.39E-01	TRINITY_DN21140_c0_g2	2.24	up	mitochondrial lipoamide dehydrogenase [Populus tremuloides]	202.39	67.53
map00524	Butirosin and neomycin biosynthesis	1.41E-01	TRINITY_DN26175_c0_g1	1.33	up	PREDICTED: hexokinase-1-like [Populus euphratica]	24.35	14.67
map00524	Butirosin and neomycin biosynthesis	1.41E-01	TRINITY_DN18225_c0_g3	1.29	up	PREDICTED: hexokinase-1-like [Populus euphratica]	38.97	24.40
map00524	Butirosin and neomycin biosynthesis	1.41E-01	TRINITY_DN21062_c0_g1	1.22	up	PREDICTED: hexokinase-1-like [Populus euphratica]	9.26	6.04
map00190	Oxidative phosphorylation	1.48E-01	TRINITY_DN21698_c0_g1	1.58	up	PREDICTED: cytochrome c oxidase subunit 6b-1-like [Populus euphratica]	101.21	51.17
map00190	Oxidative phosphorylation	1.48E-01	TRINITY_DN21756_c0_g5	1.32	up	mitochondrial processing peptidase alpha subunit 2 family protein [Populus trichocarpa]	61.66	37.46

map00190	Oxidative phosphorylation	1.48E-01	TRINITY_DN22056_c1_g2	1.40	up	PREDICTED: cytochrome b-c1 complex subunit 6-like isoform X3 [Populus euphratica]	403.72	204.98
map00190	Oxidative phosphorylation	1.48E-01	TRINITY_DN22145_c0_g3	1.65	up	NADH dehydrogenase subunit 7 [Populus alba]	14.79	7.17
map00190	Oxidative phosphorylation	1.48E-01	TRINITY_DN22182_c0_g1	1.69	up	cytochrome c1 family protein [Populus trichocarpa]	274.18	143.43
map00190	Oxidative phosphorylation	1.48E-01	TRINITY_DN22358_c0_g1	1.19	up	PREDICTED: ATP synthase subunit gamma, mitochondrial-like [Populus euphratica]	171.42	111.03
map00190	Oxidative phosphorylation	1.48E-01	TRINITY_DN22634_c0_g1	1.37	up	cytochrome c oxidase family protein [Populus trichocarpa]	142.26	84.38
map00190	Oxidative phosphorylation	1.48E-01	TRINITY_DN22666_c3_g1	1.66	up	hypothetical protein POPTR_0019s07820g [Populus trichocarpa]	224.25	113.41
map00190	Oxidative phosphorylation	1.48E-01	TRINITY_DN22731_c0_g1	3.73	up	PREDICTED: LOW QUALITY PROTEIN: plasma membrane ATPase 4 [Populus euphratica]	31.52	3.58
map00190	Oxidative phosphorylation	1.48E-01	TRINITY_DN22791_c0_g1	1.30	up	NADH-plastoquinone oxidoreductase subunit K (chloroplast) [Populus euphratica]	2.53	1.58
map00190	Oxidative phosphorylation	1.48E-01	TRINITY_DN23336_c0_g1	1.05	up	hypothetical protein POPTR_0002s25720g [Populus trichocarpa]	118.25	83.78
map00190	Oxidative phosphorylation	1.48E-01	TRINITY_DN23422_c1_g1	1.95	up	hypothetical protein POPTR_0013s12130g [Populus trichocarpa]	344.08	129.79
map00190	Oxidative phosphorylation	1.48E-01	TRINITY_DN23422_c1_g4	2.37	up	PREDICTED: ATP synthase delta chain, chloroplastic-like [Populus euphratica]	1050.78	305.83
map00190	Oxidative phosphorylation	1.48E-01	TRINITY_DN23561_c0_g5	2.06	up	hypothetical protein POPTR_0012s10710g [Populus trichocarpa]	1174.40	419.22
map00190	Oxidative phosphorylation	1.48E-01	TRINITY_DN23716_c0_g3	1.96	up	ubiquinol-cytochrome C reductase iron-sulfur subunit family protein [Populus trichocarpa]	94.25	38.11
map00190	Oxidative phosphorylation	1.48E-01	TRINITY_DN23716_c0_g4	1.21	up	ubiquinol-cytochrome C reductase iron-sulfur subunit family protein [Populus trichocarpa]	88.26	58.55
map00190	Oxidative phosphorylation	1.48E-01	TRINITY_DN24097_c0_g1	-1.40	down	vacuolar ATP synthase subunit E family protein [Populus trichocarpa]	2.41	9.54
map00190	Oxidative phosphorylation	1.48E-01	TRINITY_DN24123_c0_g1	-1.98	down	MYB055 [Populus tomentosa]	0.79	4.58
map00190	Oxidative phosphorylation	1.48E-01	TRINITY_DN24123_c0_g2	-1.27	down	hypothetical protein POPTR_0002s18700g [Populus trichocarpa]	1.27	4.49
map00190	Oxidative phosphorylation	1.48E-01	TRINITY_DN24553_c0_g3	-2.52	down	PREDICTED: calcium-transporting ATPase 12, plasma membrane-type [Populus euphratica]	0.69	8.23
map00190	Oxidative phosphorylation	1.48E-01	TRINITY_DN24789_c0_g3	-1.19	down	hypothetical protein POPTR_0018s12690g [Populus trichocarpa]	10.76	37.30
map00190	Oxidative phosphorylation	1.48E-01	TRINITY_DN24974_c0_g2	-1.55	down	microtubule-associated protein 65-5 [Populus tomentosa]	3.25	13.76
map00190	Oxidative phosphorylation	1.48E-01	TRINITY_DN25093_c0_g3	2.15	up	ATP synthase delta chain-related family protein [Populus trichocarpa]	382.66	129.01
map00190	Oxidative phosphorylation	1.48E-01	TRINITY_DN25205_c1_g8	1.06	up	NADH dehydrogenase subunit 4 [Populus alba]	4.94	3.65
map00190	Oxidative phosphorylation	1.48E-01	TRINITY_DN25369_c0_g2	2.17	up	PREDICTED: uncharacterized protein LOC105142434 [Populus euphratica]	198.49	66.26
map00190	Oxidative phosphorylation	1.48E-01	TRINITY_DN25371_c3_g1	-1.87	down	PREDICTED: 65-kDa microtubule-associated protein 4-like isoform X1 [Populus euphratica]	2.44	13.64
map00190	Oxidative phosphorylation	1.48E-01	TRINITY_DN25371_c3_g2	-2.69	down	hypothetical protein POPTR_0014s06560g [Populus trichocarpa]	0.66	6.48
map00190	Oxidative phosphorylation	1.48E-01	TRINITY_DN25415_c0_g3	1.50	up	hypothetical protein POPTR_0009s08480g [Populus trichocarpa]	587.73	247.91
map00190	Oxidative phosphorylation	1.48E-01	TRINITY_DN25590_c0_g1	-8.33	down	hypothetical protein POPTR_0010s17300g [Populus trichocarpa]	0.04	18.89
map00190	Oxidative phosphorylation	1.48E-01	TRINITY_DN26018_c1_g3	1.03	up	PREDICTED: NADH dehydrogenase [ubiquinone] flavoprotein 2, mitochondrial-like [Populus euphratica]	125.95	93.13
map00190	Oxidative phosphorylation	1.48E-01	TRINITY_DN26049_c1_g1	1.35	up	H+-transporting two-sector ATPase family protein [Populus trichocarpa]	510.11	302.10
map00190	Oxidative phosphorylation	1.48E-01	TRINITY_DN26373_c0_g1	-1.00	down	PREDICTED: zinc finger BED domain-containing protein DAYSLEEPER-like isoform X6 [Populus euphratica]	41.86	128.39
map00190	Oxidative phosphorylation	1.48E-01	TRINITY_DN26572_c0_g1	1.40	up	hypothetical protein POPTR_0002s20530g [Populus trichocarpa]	430.34	248.97
map00190	Oxidative phosphorylation	1.48E-01	TRINITY_DN26658_c0_g2	1.05	up	hypothetical protein POPTR_0001s32510g [Populus trichocarpa]	7.93	5.87
map00190	Oxidative phosphorylation	1.48E-01	TRINITY_DN26687_c0_g3	1.07	up	hypothetical protein POPTR_0006s00750g [Populus trichocarpa]	22.82	16.54
map00190	Oxidative phosphorylation	1.48E-01	TRINITY_DN26687_c0_g4	1.17	up	unknown [Populus trichocarpa]	10.92	7.40
map00190	Oxidative phosphorylation	1.48E-01	TRINITY_DN27021_c0_g1	1.72	up	Pyrophosphate-energized vacuolar membrane proton pump family protein [Populus trichocarpa]	58.91	27.17
map00190	Oxidative phosphorylation	1.48E-01	TRINITY_DN27157_c0_g1	3.33	up	inorganic pyrophosphatase family protein-2 [Populus tomentosa]	170.71	25.99
map00190	Oxidative phosphorylation	1.48E-01	TRINITY_DN27267_c0_g3	1.81	up	ATP synthase gamma chain family protein [Populus trichocarpa]	52.68	23.01
map00190	Oxidative phosphorylation	1.48E-01	TRINITY_DN27277_c0_g1	-1.16	down	PREDICTED: 65-kDa microtubule-associated protein 3-like isoform X1 [Populus euphratica]	32.44	111.60
map00190	Oxidative phosphorylation	1.48E-01	TRINITY_DN27436_c0_g1	-1.41	down	an N-terminal calmodulin binding autoinhibitory domain-containing family protein [Populus trichocarpa]	20.69	91.16
map00190	Oxidative phosphorylation	1.48E-01	TRINITY_DN27465_c0_g1	1.27	up	PREDICTED: ATP synthase subunit alpha, mitochondrial [Populus euphratica]	10.52	6.87
map00190	Oxidative phosphorylation	1.48E-01	TRINITY_DN27827_c1_g1	1.55	up	PREDICTED: LOW QUALITY PROTEIN: pyrophosphate-energized vacuolar membrane proton pump [Prunus mume]	384.54	198.56

map00190	Oxidative phosphorylation	1.48E-01	TRINITY_DN27827_c1_g2	1.44	up	Pyrophosphate-energized vacuolar membrane proton pump family protein [Populus trichocarpa]	116.65	64.76
map00190	Oxidative phosphorylation	1.48E-01	TRINITY_DN27867_c4_g4	1.77	up	ATP synthase CF1 alpha chain [Populus alba]	41.58	18.74
map00190	Oxidative phosphorylation	1.48E-01	TRINITY_DN12148_c0_g1	1.93	up	NADH dehydrogenase subunit 5 [Populus alba]	12.83	4.99
map00190	Oxidative phosphorylation	1.48E-01	TRINITY_DN13075_c0_g1	1.23	up	vacuolar H+-pyrophosphatase [Prunus persica]	377.71	245.83
map00190	Oxidative phosphorylation	1.48E-01	TRINITY_DN13169_c0_g1	1.61	up	unknown [Populus trichocarpa]	124.27	61.95
map00190	Oxidative phosphorylation	1.48E-01	TRINITY_DN13841_c0_g1	1.30	up	Pyrophosphate-energized vacuolar membrane proton pump family protein-2 [Populus tomentosa]	80.63	49.50
map00190	Oxidative phosphorylation	1.48E-01	TRINITY_DN14846_c0_g2	1.24	up	unknown [Populus trichocarpa]	185.89	118.98
map00190	Oxidative phosphorylation	1.48E-01	TRINITY_DN15298_c0_g1	-4.15	down	hypothetical protein POPTR_0012s05200g [Populus trichocarpa]	0.19	5.29
map00190	Oxidative phosphorylation	1.48E-01	TRINITY_DN15348_c0_g1	1.61	up	pyrophosphatase [Beta vulgaris]	522.49	286.29
map00190	Oxidative phosphorylation	1.48E-01	TRINITY_DN16458_c0_g1	1.67	up	PREDICTED: LOW QUALITY PROTEIN: uncharacterized protein LOC105136146 [Populus euphratica]	1816.06	855.39
map00190	Oxidative phosphorylation	1.48E-01	TRINITY_DN16769_c0_g1	1.87	up	GmMYB12 family protein [Populus trichocarpa]	4.17	6.44
map00190	Oxidative phosphorylation	1.48E-01	TRINITY_DN16993_c0_g1	1.06	up	hypothetical protein POPTR_0002s02650g [Populus trichocarpa]	30.38	22.23
map00190	Oxidative phosphorylation	1.48E-01	TRINITY_DN16993_c0_g2	1.63	up	PREDICTED: uncharacterized protein LOC105140125 [Populus euphratica]	24.80	12.25
map00190	Oxidative phosphorylation	1.48E-01	TRINITY_DN17105_c0_g1	1.07	up	hypothetical protein POPTR_0018s01370g [Populus trichocarpa]	94.98	68.89
map00190	Oxidative phosphorylation	1.48E-01	TRINITY_DN17105_c0_g2	1.19	up	PREDICTED: cytochrome b-c1 complex subunit 7-2-like [Populus euphratica]	67.61	44.77
map00190	Oxidative phosphorylation	1.48E-01	TRINITY_DN17171_c0_g1	1.96	up	unknown [Populus trichocarpa]	204.69	78.64
map00190	Oxidative phosphorylation	1.48E-01	TRINITY_DN17171_c0_g3	1.14	up	unknown [Populus trichocarpa]	116.10	78.28
map00190	Oxidative phosphorylation	1.48E-01	TRINITY_DN17226_c0_g3	1.65	up	PREDICTED: uncharacterized protein LOC105134496 [Populus euphratica]	105.84	51.59
map00190	Oxidative phosphorylation	1.48E-01	TRINITY_DN17417_c0_g1	1.58	up	PREDICTED: ATP synthase subunit O, mitochondrial [Populus euphratica]	222.63	112.89
map00190	Oxidative phosphorylation	1.48E-01	TRINITY_DN17533_c0_g1	1.19	up	hypothetical protein POPTR_0013s08220g [Populus trichocarpa]	132.64	89.90
map00190	Oxidative phosphorylation	1.48E-01	TRINITY_DN17847_c0_g1	1.34	up	PREDICTED: ATP synthase subunit epsilon, mitochondrial-like [Populus euphratica]	269.06	157.20
map00190	Oxidative phosphorylation	1.48E-01	TRINITY_DN17881_c0_g1	1.36	up	hypothetical protein POPTR_0008s04010g [Populus trichocarpa]	237.78	141.08
map00190	Oxidative phosphorylation	1.48E-01	TRINITY_DN18139_c0_g1	-4.91	down	hypothetical protein POPTR_0002s17460g [Populus trichocarpa]	1.20	88.95
map00190	Oxidative phosphorylation	1.48E-01	TRINITY_DN18189_c0_g1	1.01	up	ATP synthase gamma chain 1 family protein [Populus trichocarpa]	5.06	3.77
map00190	Oxidative phosphorylation	1.48E-01	TRINITY_DN18189_c0_g2	1.79	up	ATP synthase gamma chain 1 family protein [Populus trichocarpa]	776.42	338.36
map00190	Oxidative phosphorylation	1.48E-01	TRINITY_DN18203_c0_g5	1.03	up	hypothetical protein POPTR_0007s14910g [Populus trichocarpa]	166.17	124.84
map00190	Oxidative phosphorylation	1.48E-01	TRINITY_DN18248_c0_g1	1.39	up	NADH-ubiquinone oxidoreductase 23 kDa subunit family protein [Populus trichocarpa]	201.65	117.26
map00190	Oxidative phosphorylation	1.48E-01	TRINITY_DN18264_c0_g1	-3.37	down	putative MYB transcription factor family protein [Populus trichocarpa]	0.37	5.85
map00190	Oxidative phosphorylation	1.48E-01	TRINITY_DN18265_c0_g1	1.01	up	PREDICTED: V-type proton ATPase subunit H isoform X2 [Populus euphratica]	55.97	42.26
map00190	Oxidative phosphorylation	1.48E-01	TRINITY_DN18409_c0_g4	2.30	up	NADH dehydrogenase subunit 5 [Populus alba]	5.12	1.54
map00190	Oxidative phosphorylation	1.48E-01	TRINITY_DN18461_c0_g1	-2.23	down	hypothetical protein POPTR_0010s09130g [Populus trichocarpa]	0.30	2.22
map00190	Oxidative phosphorylation	1.48E-01	TRINITY_DN18555_c0_g3	1.21	up	PREDICTED: protoheme IX farnesyltransferase, mitochondrial [Populus euphratica]	26.04	16.92
map00190	Oxidative phosphorylation	1.48E-01	TRINITY_DN18666_c0_g3	1.61	up	PREDICTED: calcium-transporting ATPase 12, plasma membrane-type-like [Populus euphratica]	2.45	1.22
map00190	Oxidative phosphorylation	1.48E-01	TRINITY_DN18777_c0_g1	1.82	up	hypothetical protein POPTR_0005s11410g [Populus trichocarpa]	33.53	12.89
map00190	Oxidative phosphorylation	1.48E-01	TRINITY_DN18970_c0_g6	1.49	up	PREDICTED: uncharacterized protein LOC105122137 [Populus euphratica]	613.02	329.96
map00190	Oxidative phosphorylation	1.48E-01	TRINITY_DN19214_c0_g1	-2.89	down	PREDICTED: transcription factor RAX2-like [Populus euphratica]	0.78	9.92
map00190	Oxidative phosphorylation	1.48E-01	TRINITY_DN19538_c0_g1	-1.44	down	hypothetical protein POPTR_0013s00290g [Populus trichocarpa]	1.54	5.32
map00190	Oxidative phosphorylation	1.48E-01	TRINITY_DN19599_c0_g2	1.22	up	PREDICTED: ATP synthase subunit d, mitochondrial-like [Populus euphratica]	166.37	106.64
map00190	Oxidative phosphorylation	1.48E-01	TRINITY_DN19912_c0_g3	-3.25	down	PREDICTED: transcription repressor MYB6-like isoform X1 [Populus euphratica]	0.26	4.18
map00190	Oxidative phosphorylation	1.48E-01	TRINITY_DN19912_c1_g2	-4.30	down	hypothetical protein POPTR_0008s16660g [Populus trichocarpa]	0.47	14.26
map00190	Oxidative phosphorylation	1.48E-01	TRINITY_DN20012_c0_g1	1.16	up	PREDICTED: alternative NAD(P)H-ubiquinone oxidoreductase C1, chloroplastic/mitochondrial isoform X2 [Populus euphratica]	35.98	24.49
map00190	Oxidative phosphorylation	1.48E-01	TRINITY_DN20281_c1_g1	1.27	up	hypothetical protein POPTR_0001s14030g [Populus trichocarpa]	166.67	102.26

map00190	Oxidative phosphorylation	1.48E-01	TRINITY_DN20349_c0_g2	1.47	up	PREDICTED: myb-related protein Myb4-like [Populus euphratica]	24.94	12.17
map00190	Oxidative phosphorylation	1.48E-01	TRINITY_DN20452_c1_g3	-9.06	down	PREDICTED: myb-related protein 308-like [Populus euphratica]	0.00	5.40
map00190	Oxidative phosphorylation	1.48E-01	TRINITY_DN20475_c0_g2	-4.29	down	PREDICTED: transcription repressor MYB6-like isoform X1 [Populus euphratica]	0.48	15.23
map00190	Oxidative phosphorylation	1.48E-01	TRINITY_DN20493_c0_g3	-1.60	down	hypothetical protein POPTR_0017s12230g [Populus trichocarpa]	3.01	13.64
map00190	Oxidative phosphorylation	1.48E-01	TRINITY_DN20530_c0_g8	2.89	up	plasma membrane H+ ATPase family protein [Populus trichocarpa]	3.65	3.21
map00190	Oxidative phosphorylation	1.48E-01	TRINITY_DN20832_c0_g1	1.37	up	PREDICTED: ATP synthase subunit beta, mitochondrial [Populus euphratica]	314.72	184.88
map00190	Oxidative phosphorylation	1.48E-01	TRINITY_DN20990_c0_g10	-1.61	down	AtpE, partial (chloroplast) [Passiflora ciliata]	1.87	9.84
map00190	Oxidative phosphorylation	1.48E-01	TRINITY_DN20990_c0_g6	1.51	up	ATP synthase CF1 beta subunit (chloroplast) [Citrus platymamma]	11.47	8.92
map00190	Oxidative phosphorylation	1.48E-01	TRINITY_DN21060_c0_g3	-1.72	down	MYB transcription factor [Populus tomentosa]	10.98	56.43
map00190	Oxidative phosphorylation	1.48E-01	TRINITY_DN21293_c0_g2	2.44	up	hypothetical protein POPTR_0019s05210g [Populus trichocarpa]	5.66	1.63
map00190	Oxidative phosphorylation	1.48E-01	TRINITY_DN21293_c0_g5	1.29	up	hypothetical protein POPTR_0013s05300g [Populus trichocarpa]	6.38	3.94
map00190	Oxidative phosphorylation	1.48E-01	TRINITY_DN21332_c0_g1	-2.36	down	hypothetical protein POPTR_0001s05650g [Populus trichocarpa]	1.89	14.82
map00190	Oxidative phosphorylation	1.48E-01	TRINITY_DN21445_c0_g2	1.74	up	hypothetical protein POPTR_0018s04990g [Populus trichocarpa]	245.63	111.02
map00190	Oxidative phosphorylation	1.48E-01	TRINITY_DN21445_c0_g6	1.03	up	hypothetical protein POPTR_0006s24790g [Populus trichocarpa]	58.27	43.31
map00190	Oxidative phosphorylation	1.48E-01	TRINITY_DN21499_c0_g3	1.35	up	putative plasma membrane H+ ATPase family protein [Populus trichocarpa]	91.83	53.55
map00190	Oxidative phosphorylation	1.48E-01	TRINITY_DN21588_c1_g3	1.17	up	ubiquinol-cytochrome C reductase iron-sulfur subunit family protein [Populus trichocarpa]	145.82	99.13
map00910	Nitrogen metabolism	1.51E-01	TRINITY_DN21747_c0_g1	3.00	up	hypothetical protein POPTR_0015s12250g [Populus trichocarpa]	29.79	5.40
map00910	Nitrogen metabolism	1.51E-01	TRINITY_DN22699_c1_g1	3.35	up	hypothetical protein POPTR_0010s05080g [Populus trichocarpa]	364.96	66.06
map00910	Nitrogen metabolism	1.51E-01	TRINITY_DN23055_c4_g2	-3.07	down	hypothetical protein POPTR_0007s03330g [Populus trichocarpa]	0.47	6.09
map00910	Nitrogen metabolism	1.51E-01	TRINITY_DN23091_c0_g2	-2.11	down	glutamine synthetase family protein [Populus trichocarpa]	10.71	69.67
map00910	Nitrogen metabolism	1.51E-01	TRINITY_DN23091_c0_g3	4.22	up	glutamate-ammonia ligase family protein [Populus trichocarpa]	47.80	3.92
map00910	Nitrogen metabolism	1.51E-01	TRINITY_DN23091_c0_g4	1.81	up	glutamate-ammonia ligase family protein [Populus trichocarpa]	63.46	29.26
map00910	Nitrogen metabolism	1.51E-01	TRINITY_DN23091_c0_g5	4.02	up	glutamine synthetase family protein [Populus simonii x Populus nigra]	46.52	4.43
map00910	Nitrogen metabolism	1.51E-01	TRINITY_DN23937_c0_g1	1.78	up	Glutamine synthetase nodule isozyme [Ananas comosus]	127.57	57.48
map00910	Nitrogen metabolism	1.51E-01	TRINITY_DN23937_c0_g3	-3.47	down	PREDICTED: LOW QUALITY PROTEIN: glutamine synthetase cytosolic isozyme 2 [Populus euphratica]	0.50	8.51
map00910	Nitrogen metabolism	1.51E-01	TRINITY_DN24008_c0_g2	-1.19	down	delta8-sphingolipid desaturase [Populus tomentosa]	19.94	69.52
map00910	Nitrogen metabolism	1.51E-01	TRINITY_DN24396_c0_g1	-3.28	down	nitrate transporter [Populus tremula x Populus tremuloides]	0.76	11.24
map00910	Nitrogen metabolism	1.51E-01	TRINITY_DN24396_c0_g4	-2.69	down	PREDICTED: high affinity nitrate transporter 2.5-like [Populus euphratica]	0.65	3.93
map00910	Nitrogen metabolism	1.51E-01	TRINITY_DN24801_c0_g1	-3.23	down	ORF family protein [Populus trichocarpa]	1.59	22.62
map00910	Nitrogen metabolism	1.51E-01	TRINITY_DN24801_c0_g5	-7.25	down	nitrate reductase family protein [Populus trichocarpa]	0.00	4.03
map00910	Nitrogen metabolism	1.51E-01	TRINITY_DN25260_c0_g3	3.71	up	glutamate-ammonia ligase family protein [Populus trichocarpa]	11.97	1.40
map00910	Nitrogen metabolism	1.51E-01	TRINITY_DN25260_c0_g4	-1.01	down	unknown [Populus trichocarpa]	26.98	83.37
map00910	Nitrogen metabolism	1.51E-01	TRINITY_DN25478_c0_g1	2.24	up	carbonic anhydrase [Populus tremula x Populus tremuloides]	6672.48	2098.66
map00910	Nitrogen metabolism	1.51E-01	TRINITY_DN25941_c0_g1	1.37	up	PREDICTED: glutamine synthetase leaf isozyme, chloroplastic [Populus euphratica]	986.31	589.65
map00910	Nitrogen metabolism	1.51E-01	TRINITY_DN27440_c0_g2	1.46	up	PREDICTED: glutamine synthetase leaf isozyme, chloroplastic [Populus euphratica]	559.41	306.88
map00910	Nitrogen metabolism	1.51E-01	TRINITY_DN27865_c0_g1	1.40	up	hypothetical protein POPTR_0016s03630g [Populus trichocarpa]	23.70	13.44
map00910	Nitrogen metabolism	1.51E-01	TRINITY_DN27865_c1_g3	1.68	up	ferredoxin-dependent glutamate synthase family protein [Populus trichocarpa]	183.61	86.98
map00910	Nitrogen metabolism	1.51E-01	TRINITY_DN13033_c0_g1	1.49	up	hypothetical protein POPTR_0016s03630g [Populus trichocarpa]	21.05	11.13
map00910	Nitrogen metabolism	1.51E-01	TRINITY_DN13666_c0_g1	1.63	up	hypothetical protein POPTR_0016s03630g [Populus trichocarpa]	53.03	25.42
map00910	Nitrogen metabolism	1.51E-01	TRINITY_DN13846_c0_g1	1.79	up	hypothetical protein PRUPE_ppa000146mg [Prunus persica]	350.17	154.52
map00910	Nitrogen metabolism	1.51E-01	TRINITY_DN14712_c0_g2	2.45	up	PREDICTED: glutamate synthase 1 [NADH], chloroplastic-like isoform X1 [Populus euphratica]	12.83	3.58
map00910	Nitrogen metabolism	1.51E-01	TRINITY_DN14897_c0_g1	-1.66	down	PREDICTED: alpha carbonic anhydrase 4-like [Populus euphratica]	1.18	5.78
map00910	Nitrogen metabolism	1.51E-01	TRINITY_DN15808_c0_g1	1.81	up	PREDICTED: ferredoxin-dependent glutamate synthase, chloroplastic-like [Populus euphratica]	604.52	260.27

map00910	Nitrogen metabolism	1.51E-01	TRINITY_DN16995_c0_g1	1.00	up	Cyanate hydratase family protein [Populus trichocarpa]	122.02	91.70
map00910	Nitrogen metabolism	1.51E-01	TRINITY_DN19312_c0_g4	-2.39	down	hypothetical protein POPTR_0007s03330g [Populus trichocarpa]	0.60	4.78
map00910	Nitrogen metabolism	1.51E-01	TRINITY_DN19966_c0_g1	2.35	up	hypothetical protein POPTR_0001s35030g [Populus trichocarpa]	5.94	1.76
map00910	Nitrogen metabolism	1.51E-01	TRINITY_DN20322_c0_g3	3.04	up	Ferredoxin--nitrite reductase family protein [Populus trichocarpa]	75.08	13.81
map00590	Arachidonic acid metabolism	1.54E-01	TRINITY_DN21884_c0_g1	1.24	up	hypothetical protein POPTR_0005s08280g [Populus trichocarpa]	132.32	84.75
map00590	Arachidonic acid metabolism	1.54E-01	TRINITY_DN22725_c0_g1	1.31	up	hypothetical protein POPTR_0012s04410g [Populus trichocarpa]	23.89	14.90
map00590	Arachidonic acid metabolism	1.54E-01	TRINITY_DN24718_c0_g1	-1.13	down	PREDICTED: calmodulin-binding transcription activator 3-like isoform X3 [Populus euphratica]	2.73	8.94
map00590	Arachidonic acid metabolism	1.54E-01	TRINITY_DN24995_c1_g3	1.35	up	glutathione peroxidase [Populus euphratica]	10.51	6.15
map00590	Arachidonic acid metabolism	1.54E-01	TRINITY_DN27547_c0_g1	-1.35	down	calmodulin-binding family protein [Populus trichocarpa]	6.25	23.99
map00590	Arachidonic acid metabolism	1.54E-01	TRINITY_DN14990_c0_g1	1.78	up	hypothetical protein POPTR_0013s13870g [Populus trichocarpa]	20.13	9.04
map00590	Arachidonic acid metabolism	1.54E-01	TRINITY_DN17303_c0_g1	2.23	up	hypothetical protein POPTR_0005s26400g [Populus trichocarpa]	35.95	12.21
map00590	Arachidonic acid metabolism	1.54E-01	TRINITY_DN18849_c0_g1	1.02	up	PREDICTED: uncharacterized protein OsI_027940-like [Populus euphratica]	131.23	99.42
map00590	Arachidonic acid metabolism	1.54E-01	TRINITY_DN19728_c0_g1	1.25	up	hypothetical protein POPTR_0010s01430g [Populus trichocarpa]	46.16	30.18
map00590	Arachidonic acid metabolism	1.54E-01	TRINITY_DN19728_c0_g2	3.11	up	PREDICTED: bifunctional epoxide hydrolase 2-like [Populus euphratica]	10.52	1.94
map00590	Arachidonic acid metabolism	1.54E-01	TRINITY_DN20199_c0_g1	-1.20	down	hypothetical protein POPTR_0013s05920g [Populus trichocarpa]	5.46	19.25
map00590	Arachidonic acid metabolism	1.54E-01	TRINITY_DN20696_c0_g1	-2.87	down	epoxide hydrolase family protein [Populus trichocarpa]	0.84	9.54
map00590	Arachidonic acid metabolism	1.54E-01	TRINITY_DN390_c0_g1	-2.53	down	hypothetical protein POPTR_0011s04780g [Populus trichocarpa]	0.86	7.69
map00620	Pyruvate metabolism	1.71E-01	TRINITY_DN21932_c0_g3	-1.06	down	phosphatase 2C family protein [Populus trichocarpa]	2.53	8.10
map00620	Pyruvate metabolism	1.71E-01	TRINITY_DN22629_c0_g2	1.01	up	PREDICTED: 2-isopropylmalate synthase 2, chloroplastic-like [Populus euphratica]	65.58	50.13
map00620	Pyruvate metabolism	1.71E-01	TRINITY_DN22677_c0_g1	-1.18	down	alpha galactosyltransferase family protein [Populus trichocarpa]	16.78	58.67
map00620	Pyruvate metabolism	1.71E-01	TRINITY_DN22755_c0_g2	1.78	up	unknown [Populus trichocarpa]	261.55	115.26
map00620	Pyruvate metabolism	1.71E-01	TRINITY_DN22836_c0_g5	1.29	up	PREDICTED: probable lactoylglutathione lyase, chloroplast [Populus euphratica]	35.98	22.20
map00620	Pyruvate metabolism	1.71E-01	TRINITY_DN22836_c0_g7	-1.81	down	hypothetical protein POPTR_0006s25630g [Populus trichocarpa]	0.60	2.90
map00620	Pyruvate metabolism	1.71E-01	TRINITY_DN23168_c0_g1	1.33	up	Fumarate hydratase 1 family protein [Populus trichocarpa]	44.45	26.95
map00620	Pyruvate metabolism	1.71E-01	TRINITY_DN23213_c0_g1	1.52	up	pyruvate dehydrogenase family protein [Populus trichocarpa]	125.88	67.45
map00620	Pyruvate metabolism	1.71E-01	TRINITY_DN23720_c0_g1	1.57	up	PREDICTED: malate dehydrogenase [NADP], chloroplastic [Populus euphratica]	252.27	131.23
map00620	Pyruvate metabolism	1.71E-01	TRINITY_DN24279_c0_g1	1.93	up	unknown [Populus trichocarpa x Populus deltoides]	85.38	34.72
map00620	Pyruvate metabolism	1.71E-01	TRINITY_DN24307_c0_g1	1.05	up	PREDICTED: biotin carboxyl carrier protein of acetyl-CoA carboxylase, chloroplastic-like [Populus euphratica]	73.06	52.43
map00620	Pyruvate metabolism	1.71E-01	TRINITY_DN24628_c0_g1	1.28	up	PREDICTED: aldehyde dehydrogenase family 3 member H1-like [Populus euphratica]	40.39	24.46
map00620	Pyruvate metabolism	1.71E-01	TRINITY_DN24709_c0_g1	1.03	up	PREDICTED: pyruvate dehydrogenase E1 component subunit beta-3, chloroplastic-like [Populus euphratica]	176.40	133.88
map00620	Pyruvate metabolism	1.71E-01	TRINITY_DN25141_c0_g1	1.90	up	PREDICTED: dihydrolipoyllysine-residue acetyltransferase component 4 of pyruvate dehydrogenase complex, chloroplastic [Populus euphratica]	116.62	48.47
map00620	Pyruvate metabolism	1.71E-01	TRINITY_DN25192_c0_g1	2.93	up	hypothetical protein POPTR_0010s02860g [Populus trichocarpa]	24.30	5.86
map00620	Pyruvate metabolism	1.71E-01	TRINITY_DN25221_c0_g1	1.58	up	malate dehydrogenase family protein, partial [Populus trichocarpa]	57.58	29.35
map00620	Pyruvate metabolism	1.71E-01	TRINITY_DN25221_c1_g2	2.25	up	malate dehydrogenase family protein [Populus trichocarpa]	356.58	110.81
map00620	Pyruvate metabolism	1.71E-01	TRINITY_DN25221_c1_g3	1.80	up	PREDICTED: malate dehydrogenase, glyoxysomal [Populus euphratica]	643.81	285.37
map00620	Pyruvate metabolism	1.71E-01	TRINITY_DN25221_c1_g4	2.08	up	PREDICTED: LOW QUALITY PROTEIN: dihydrolipoyllysine-residue acetyltransferase component 5 of pyruvate dehydrogenase complex, chloroplastic-like, partial [Populus euphratica]	9.32	3.34
map00620	Pyruvate metabolism	1.71E-01	TRINITY_DN25377_c0_g1	1.91	up	hypothetical protein POPTR_0013s11870g [Populus trichocarpa]	85.23	34.17
map00620	Pyruvate metabolism	1.71E-01	TRINITY_DN25439_c0_g1	1.11	up	biotin carboxylase precursor family protein [Populus trichocarpa]	85.63	60.20
map00620	Pyruvate metabolism	1.71E-01	TRINITY_DN25624_c2_g1	1.26	up	PREDICTED: probable lactoylglutathione lyase, chloroplast [Populus euphratica]	166.29	105.77
map00620	Pyruvate metabolism	1.71E-01	TRINITY_DN25630_c0_g1	1.30	up	aldehyde dehydrogenase 1 precursor family protein [Populus trichocarpa]	120.35	73.18
map00620	Pyruvate metabolism	1.71E-01	TRINITY_DN25746_c0_g1	2.80	up	nodule-enhanced malate dehydrogenase family protein [Populus trichocarpa]	113.74	23.22

map00620	Pyruvate metabolism	1.71E-01	TRINITY_DN26025_c0_g1	-1.71	down	hypothetical protein POPTR_0004s18340g [Populus trichocarpa]	5.60	27.70
map00620	Pyruvate metabolism	1.71E-01	TRINITY_DN26154_c0_g2	-1.12	down	PREDICTED: protein SCAR2-like isoform X1 [Populus euphratica]	5.40	17.64
map00620	Pyruvate metabolism	1.71E-01	TRINITY_DN26163_c0_g1	1.24	up	PREDICTED: dihydrolipoyllysine-residue acetyltransferase component 2 of pyruvate dehydrogenase complex, mitochondrial-like [Populus euphratica]	49.33	31.65
map00620	Pyruvate metabolism	1.71E-01	TRINITY_DN26341_c0_g2	1.21	up	dihydrolipoamide S-acetyltransferase family protein [Populus trichocarpa]	11.43	7.48
map00620	Pyruvate metabolism	1.71E-01	TRINITY_DN26421_c0_g1	1.32	up	the aldehyde dehydrogenase cp-ADH from C.plantagineum family protein [Populus trichocarpa]	58.13	34.64
map00620	Pyruvate metabolism	1.71E-01	TRINITY_DN26497_c0_g2	2.05	up	hypothetical protein POPTR_0011s07381g [Populus trichocarpa]	1.66	0.60
map00620	Pyruvate metabolism	1.71E-01	TRINITY_DN26837_c0_g1	2.09	up	hypothetical protein POPTR_0010s15200g [Populus trichocarpa]	81.17	29.93
map00620	Pyruvate metabolism	1.71E-01	TRINITY_DN27009_c0_g1	1.60	up	hypothetical protein POPTR_0002s22720g [Populus trichocarpa]	10.34	5.23
map00620	Pyruvate metabolism	1.71E-01	TRINITY_DN27847_c0_g1	1.64	up	hypothetical protein POPTR_0008s11610g [Populus trichocarpa]	34.59	16.78
map00620	Pyruvate metabolism	1.71E-01	TRINITY_DN27862_c2_g1	-1.69	down	PREDICTED: structural maintenance of chromosomes protein 2-1-like [Populus euphratica]	21.02	56.92
map00620	Pyruvate metabolism	1.71E-01	TRINITY_DN27876_c5_g1	-1.61	down	hypothetical protein POPTR_0005s16540g [Populus trichocarpa]	2.20	11.69
map00620	Pyruvate metabolism	1.71E-01	TRINITY_DN27876_c5_g5	-2.33	down	PREDICTED: acetyl-CoA carboxylase 1-like [Populus euphratica]	0.87	6.39
map00620	Pyruvate metabolism	1.71E-01	TRINITY_DN13808_c0_g3	-1.99	down	hypothetical protein POPTR_0001s04370g [Populus trichocarpa]	0.58	3.70
map00620	Pyruvate metabolism	1.71E-01	TRINITY_DN14558_c0_g1	-2.01	down	PREDICTED: uncharacterized protein LOC109021545 [Juglans regia]	0.85	4.88
map00620	Pyruvate metabolism	1.71E-01	TRINITY_DN16218_c0_g1	1.77	up	hypothetical protein POPTR_0016s09610g [Populus trichocarpa]	16.99	7.54
map00620	Pyruvate metabolism	1.71E-01	TRINITY_DN16677_c0_g2	-8.29	down	hypothetical protein POPTR_0007s15050g [Populus trichocarpa]	0.00	5.66
map00620	Pyruvate metabolism	1.71E-01	TRINITY_DN16821_c0_g1	1.91	up	PREDICTED: malate dehydrogenase, glyoxysomal [Populus euphratica]	248.44	104.05
map00620	Pyruvate metabolism	1.71E-01	TRINITY_DN17257_c0_g1	-1.22	down	hypothetical protein POPTR_0001s00440g [Populus trichocarpa]	6.45	23.17
map00620	Pyruvate metabolism	1.71E-01	TRINITY_DN17893_c0_g1	-1.38	down	hypothetical protein POPTR_0018s06270g [Populus trichocarpa]	14.87	54.93
map00620	Pyruvate metabolism	1.71E-01	TRINITY_DN18209_c1_g1	1.05	up	PREDICTED: pyruvate kinase, cytosolic isozyme-like [Populus euphratica]	19.18	14.26
map00620	Pyruvate metabolism	1.71E-01	TRINITY_DN18895_c0_g1	-1.06	down	mitochondrial aldehyde dehydrogenase family protein [Populus trichocarpa]	7.40	23.22
map00620	Pyruvate metabolism	1.71E-01	TRINITY_DN19050_c1_g1	1.32	up	hypothetical protein POPTR_0019s08170g, partial [Populus trichocarpa]	123.18	74.70
map00620	Pyruvate metabolism	1.71E-01	TRINITY_DN19213_c0_g2	-2.84	down	hypothetical protein POPTR_0009s02970g [Populus trichocarpa]	0.38	4.37
map00620	Pyruvate metabolism	1.71E-01	TRINITY_DN19213_c0_g4	-2.15	down	hypothetical protein POPTR_0009s02970g [Populus trichocarpa]	1.21	9.86
map00620	Pyruvate metabolism	1.71E-01	TRINITY_DN19215_c1_g1	-1.38	down	hypothetical protein POPTR_0007s10210g [Populus trichocarpa]	3.47	12.55
map00620	Pyruvate metabolism	1.71E-01	TRINITY_DN19215_c2_g1	-1.56	down	PREDICTED: probable protein phosphatase 2C 63 [Populus euphratica]	6.48	30.16
map00620	Pyruvate metabolism	1.71E-01	TRINITY_DN19763_c0_g3	1.88	up	PREDICTED: malate dehydrogenase, mitochondrial [Populus euphratica]	247.09	106.19
map00620	Pyruvate metabolism	1.71E-01	TRINITY_DN20175_c0_g1	1.59	up	peroxiredoxin Q family protein [Populus trichocarpa]	1020.77	504.37
map00620	Pyruvate metabolism	1.71E-01	TRINITY_DN20227_c0_g1	-1.45	down	PREDICTED: malate synthase, glyoxysomal [Populus euphratica]	2.85	13.95
map00620	Pyruvate metabolism	1.71E-01	TRINITY_DN20590_c0_g2	1.44	up	METHIONINE AMINOPEPTIDASE 1D family protein [Populus trichocarpa]	79.40	44.51
map00620	Pyruvate metabolism	1.71E-01	TRINITY_DN20938_c0_g1	1.33	up	PREDICTED: malate dehydrogenase, glyoxysomal [Populus euphratica]	118.58	72.32
map00620	Pyruvate metabolism	1.71E-01	TRINITY_DN20938_c0_g2	2.63	up	malate dehydrogenase family protein [Populus trichocarpa]	74.92	18.32
map00620	Pyruvate metabolism	1.71E-01	TRINITY_DN21140_c0_g2	2.24	up	mitochondrial lipoamide dehydrogenase [Populus tremuloides]	202.39	67.53
map00620	Pyruvate metabolism	1.71E-01	TRINITY_DN21528_c1_g1	-1.91	down	hypothetical protein POPTR_0002s09330g [Populus trichocarpa]	0.78	4.44
map00030	Pentose phosphate pathway	1.76E-01	TRINITY_DN21847_c0_g2	1.55	up	PREDICTED: 6-phosphogluconate dehydrogenase, decarboxylating 2, chloroplastic [Populus euphratica]	56.92	29.48
map00030	Pentose phosphate pathway	1.76E-01	TRINITY_DN22033_c0_g1	1.60	up	Chain D family protein [Populus trichocarpa]	364.35	179.65
map00030	Pentose phosphate pathway	1.76E-01	TRINITY_DN22233_c0_g1	1.51	up	hypothetical protein POPTR_0002s10420g [Populus trichocarpa]	84.73	47.59
map00030	Pentose phosphate pathway	1.76E-01	TRINITY_DN22587_c0_g1	1.20	up	PREDICTED: fructose-bisphosphate aldolase cytoplasmic isozyme [Populus euphratica]	371.77	247.12
map00030	Pentose phosphate pathway	1.76E-01	TRINITY_DN22920_c1_g6	2.63	up	PREDICTED: probable ribose-5-phosphate isomerase 3, chloroplastic [Populus euphratica]	555.60	140.50
map00030	Pentose phosphate pathway	1.76E-01	TRINITY_DN23403_c0_g1	1.10	up	hypothetical protein POPTR_0005s23960g [Populus trichocarpa]	80.55	57.95

map00030	Pentose phosphate pathway	1.76E-01	TRINITY_DN23701_c0_g1	1.96	up	latex plastidic aldolase-like family protein [Populus trichocarpa]	2289.47	879.27
map00030	Pentose phosphate pathway	1.76E-01	TRINITY_DN23701_c0_g5	2.57	up	plastidic aldolase family protein [Populus trichocarpa]	7.53	2.00
map00030	Pentose phosphate pathway	1.76E-01	TRINITY_DN24285_c0_g5	1.46	up	PREDICTED: WAT1-related protein At2g37460-like isoform X1 [Populus euphratica]	2.63	1.46
map00030	Pentose phosphate pathway	1.76E-01	TRINITY_DN24623_c0_g2	1.54	up	hypothetical protein POPTR_0008s11770g [Populus trichocarpa]	42.50	22.04
map00030	Pentose phosphate pathway	1.76E-01	TRINITY_DN24707_c1_g1	-1.25	down	nodulin MtN21 family protein [Populus trichocarpa]	14.31	50.91
map00030	Pentose phosphate pathway	1.76E-01	TRINITY_DN24881_c0_g1	1.99	up	PREDICTED: transketolase, chloroplastic [Populus euphratica]	1790.82	677.46
map00030	Pentose phosphate pathway	1.76E-01	TRINITY_DN25692_c0_g1	-1.21	down	AAA-type ATPase family protein [Populus trichocarpa]	9.04	31.77
map00030	Pentose phosphate pathway	1.76E-01	TRINITY_DN25692_c0_g2	-1.31	down	PREDICTED: pachytene checkpoint protein 2 homolog [Populus euphratica]	12.27	47.09
map00030	Pentose phosphate pathway	1.76E-01	TRINITY_DN25819_c0_g1	1.14	up	PREDICTED: NADP-dependent glyceraldehyde-3-phosphate dehydrogenase-like isoform X1 [Populus euphratica]	282.53	188.30
map00030	Pentose phosphate pathway	1.76E-01	TRINITY_DN26131_c0_g2	1.50	up	6-phosphogluconate dehydrogenase family protein [Populus trichocarpa]	63.33	33.04
map00030	Pentose phosphate pathway	1.76E-01	TRINITY_DN26209_c0_g1	1.84	up	hypothetical protein SETIT_022310mg [Setaria italica]	186.50	89.03
map00030	Pentose phosphate pathway	1.76E-01	TRINITY_DN26437_c0_g2	1.65	up	hypothetical protein POPTR_0014s16480g [Populus trichocarpa]	43.75	20.82
map00030	Pentose phosphate pathway	1.76E-01	TRINITY_DN26661_c0_g1	2.62	up	hypothetical protein CISIN_1g016748mg [Citrus sinensis]	718.86	176.98
map00030	Pentose phosphate pathway	1.76E-01	TRINITY_DN26743_c0_g1	1.15	up	RecName: Full=Phosphoglucomutase, cytoplasmic; Short=PGM; AltName: Full=Glucose phosphomutase	74.05	53.71
map00030	Pentose phosphate pathway	1.76E-01	TRINITY_DN26996_c1_g1	1.79	up	PREDICTED: phosphoglucomutase, chloroplastic-like [Populus euphratica]	112.47	49.80
map00030	Pentose phosphate pathway	1.76E-01	TRINITY_DN27458_c1_g1	2.44	up	PREDICTED: transketolase, chloroplastic [Populus euphratica]	1184.20	313.96
map00030	Pentose phosphate pathway	1.76E-01	TRINITY_DN27621_c0_g4	1.61	up	6-phosphogluconate dehydrogenase family protein [Populus trichocarpa]	11.51	5.70
map00030	Pentose phosphate pathway	1.76E-01	TRINITY_DN11372_c0_g1	3.83	up	hypothetical protein POPTR_0011s15150g [Populus trichocarpa]	4.92	0.36
map00030	Pentose phosphate pathway	1.76E-01	TRINITY_DN15775_c0_g1	1.57	up	hypothetical protein POPTR_0001s46060g [Populus trichocarpa]	7.49	3.44
map00030	Pentose phosphate pathway	1.76E-01	TRINITY_DN17020_c0_g1	2.36	up	hypothetical protein POPTR_0002s06890g [Populus trichocarpa]	22.89	6.58
map00030	Pentose phosphate pathway	1.76E-01	TRINITY_DN17229_c0_g1	-1.51	down	hypothetical protein POPTR_0014s03490g [Populus trichocarpa]	1.22	5.46
map00030	Pentose phosphate pathway	1.76E-01	TRINITY_DN17246_c0_g1	1.06	up	hypothetical protein POPTR_0017s07040g [Populus trichocarpa]	24.42	17.62
map00030	Pentose phosphate pathway	1.76E-01	TRINITY_DN17528_c0_g1	2.19	up	ribose-phosphate pyrophosphokinase family protein [Populus trichocarpa]	63.69	21.30
map00030	Pentose phosphate pathway	1.76E-01	TRINITY_DN18237_c0_g2	1.23	up	PREDICTED: glucose-6-phosphate 1-dehydrogenase 4, chloroplastic [Populus euphratica]	14.47	10.41
map00030	Pentose phosphate pathway	1.76E-01	TRINITY_DN19014_c0_g2	1.24	up	hypothetical protein POPTR_0012s03150g [Populus trichocarpa]	33.06	21.31
map00030	Pentose phosphate pathway	1.76E-01	TRINITY_DN19734_c0_g2	1.39	up	PREDICTED: ribulose-phosphate 3-epimerase, chloroplastic [Populus euphratica]	648.43	374.61
map00030	Pentose phosphate pathway	1.76E-01	TRINITY_DN19743_c0_g1	1.38	up	PREDICTED: WAT1-related protein At4g19185-like [Populus euphratica]	66.15	39.51
map00030	Pentose phosphate pathway	1.76E-01	TRINITY_DN20148_c0_g1	1.45	up	hypothetical protein POPTR_0010s12560g [Populus trichocarpa]	131.18	75.15
map00030	Pentose phosphate pathway	1.76E-01	TRINITY_DN20148_c0_g2	3.15	up	ribose 5-phosphate isomerase family protein [Populus trichocarpa]	6.05	1.07
map00030	Pentose phosphate pathway	1.76E-01	TRINITY_DN21427_c0_g1	1.40	up	hypothetical protein POPTR_0016s11621g [Populus trichocarpa]	98.65	56.05
map00030	Pentose phosphate pathway	1.76E-01	TRINITY_DN6390_c0_g1	3.46	up	nodulin MtN21 family protein [Populus trichocarpa]	1.91	0.26
map00480	Glutathione metabolism	1.79E-01	TRINITY_DN21678_c1_g2	1.79	up	PREDICTED: glutathione reductase, chloroplastic [Populus euphratica]	73.53	32.22
map00480	Glutathione metabolism	1.79E-01	TRINITY_DN21762_c0_g1	-1.80	down	PREDICTED: probable glutathione S-transferase [Populus euphratica]	1.87	12.37
map00480	Glutathione metabolism	1.79E-01	TRINITY_DN21762_c0_g2	-1.26	down	glutathione S-transferase U12 [Populus yatungensis]	5.91	24.58
map00480	Glutathione metabolism	1.79E-01	TRINITY_DN21847_c0_g2	1.55	up	PREDICTED: 6-phosphogluconate dehydrogenase, decarboxylating 2, chloroplastic [Populus euphratica]	56.92	29.48
map00480	Glutathione metabolism	1.79E-01	TRINITY_DN22269_c0_g2	3.81	up	glutathione S-transferase F8 [Populus yatungensis]	67.95	7.46
map00480	Glutathione metabolism	1.79E-01	TRINITY_DN22320_c0_g1	2.05	up	PREDICTED: thylakoid lumenal 29 kDa protein, chloroplastic isoform X1 [Populus euphratica]	312.91	116.47
map00480	Glutathione metabolism	1.79E-01	TRINITY_DN23271_c0_g1	1.57	up	hypothetical protein POPTR_0001s35080g [Populus trichocarpa]	37.34	19.20
map00480	Glutathione metabolism	1.79E-01	TRINITY_DN23764_c0_g1	1.49	up	In2-1 family protein [Populus trichocarpa]	688.86	380.28
map00480	Glutathione metabolism	1.79E-01	TRINITY_DN23831_c1_g3	-1.10	down	hypothetical protein GLYMA_17G255600 [Glycine max]	949.87	2470.01
map00480	Glutathione metabolism	1.79E-01	TRINITY_DN24607_c0_g1	-1.93	down	hypothetical protein POPTR_0001s05450g [Populus trichocarpa]	95.86	582.55
map00480	Glutathione metabolism	1.79E-01	TRINITY_DN24658_c0_g1	1.35	up	hypothetical protein POPTR_0004s18030g [Populus trichocarpa]	976.13	588.02
map00480	Glutathione metabolism	1.79E-01	TRINITY_DN24662_c0_g2	-1.93	down	ferritin/ribonucleotide reductase-like family protein [Populus tomentosa]	4.88	29.25

map00480	Glutathione metabolism	1.79E-01	TRINITY_DN24662_c0_g3	-1.05	down	PREDICTED: ribonucleoside-diphosphate reductase small chain [Populus euphratica]	25.18	80.64
map00480	Glutathione metabolism	1.79E-01	TRINITY_DN24995_c1_g3	1.35	up	glutathione peroxidase [Populus euphratica]	10.51	6.15
map00480	Glutathione metabolism	1.79E-01	TRINITY_DN25160_c0_g1	1.03	up	hypothetical protein POPTR_0008s04660g [Populus trichocarpa]	57.49	44.52
map00480	Glutathione metabolism	1.79E-01	TRINITY_DN25285_c0_g1	-1.11	down	PREDICTED: ribonucleoside-diphosphate reductase large subunit-like [Populus euphratica]	33.12	103.10
map00480	Glutathione metabolism	1.79E-01	TRINITY_DN25398_c1_g1	1.21	up	PREDICTED: DEAD-box ATP-dependent RNA helicase 3, chloroplastic-like isoform X2 [Populus euphratica]	571.63	379.40
map00480	Glutathione metabolism	1.79E-01	TRINITY_DN25723_c0_g1	1.05	up	tau class glutathione transferase GSTU33 [Populus trichocarpa]	334.79	243.56
map00480	Glutathione metabolism	1.79E-01	TRINITY_DN25723_c0_g2	1.62	up	hypothetical protein POPTR_0878s002002g, partial [Populus trichocarpa]	3.86	1.92
map00480	Glutathione metabolism	1.79E-01	TRINITY_DN25744_c0_g1	1.77	up	PREDICTED: probable L-ascorbate peroxidase 6, chloroplastic isoform X1 [Populus euphratica]	291.77	130.37
map00480	Glutathione metabolism	1.79E-01	TRINITY_DN26131_c0_g2	1.50	up	6-phosphogluconate dehydrogenase family protein [Populus trichocarpa]	63.33	33.04
map00480	Glutathione metabolism	1.79E-01	TRINITY_DN26437_c0_g2	1.65	up	hypothetical protein POPTR_0014s16480g [Populus trichocarpa]	43.75	20.82
map00480	Glutathione metabolism	1.79E-01	TRINITY_DN26715_c0_g1	1.32	up	glutathione S-transferase U33 [Populus yatungensis]	640.88	395.99
map00480	Glutathione metabolism	1.79E-01	TRINITY_DN26715_c0_g5	-1.63	down	hypothetical protein POPTR_0001s42770g [Populus trichocarpa]	1.88	9.06
map00480	Glutathione metabolism	1.79E-01	TRINITY_DN26983_c0_g6	1.02	up	tetrachloro-p-hydroquinone reductive dehalogenase-related family protein [Populus trichocarpa]	6.69	5.09
map00480	Glutathione metabolism	1.79E-01	TRINITY_DN27621_c0_g4	1.61	up	6-phosphogluconate dehydrogenase family protein [Populus trichocarpa]	11.51	5.70
map00480	Glutathione metabolism	1.79E-01	TRINITY_DN27742_c1_g1	1.02	up	peptidase M1 family protein [Populus trichocarpa]	169.39	130.95
map00480	Glutathione metabolism	1.79E-01	TRINITY_DN13148_c0_g1	1.39	up	PREDICTED: uncharacterized protein At2g34460, chloroplastic [Populus euphratica]	57.16	33.19
map00480	Glutathione metabolism	1.79E-01	TRINITY_DN13159_c0_g1	2.54	up	ascorbate peroxidase [Populus tomentosa]	8.23	2.11
map00480	Glutathione metabolism	1.79E-01	TRINITY_DN13637_c0_g1	1.20	up	PREDICTED: probable glutathione S-transferase [Populus euphratica]	42.52	28.09
map00480	Glutathione metabolism	1.79E-01	TRINITY_DN15028_c0_g1	1.42	up	hypothetical protein POPTR_0007s07520g [Populus trichocarpa]	93.18	52.23
map00480	Glutathione metabolism	1.79E-01	TRINITY_DN17369_c0_g1	1.12	up	ascorbate peroxidase [Populus tomentosa]	210.14	152.72
map00480	Glutathione metabolism	1.79E-01	TRINITY_DN17369_c0_g3	1.35	up	ascorbate peroxidase [Populus tomentosa]	128.46	76.99
map00480	Glutathione metabolism	1.79E-01	TRINITY_DN17682_c0_g2	1.44	up	PREDICTED: glutathione S-transferase T1-like [Populus euphratica]	99.32	53.27
map00480	Glutathione metabolism	1.79E-01	TRINITY_DN17797_c0_g1	1.66	up	PREDICTED: microsomal glutathione S-transferase 3 [Populus euphratica]	33.17	16.73
map00480	Glutathione metabolism	1.79E-01	TRINITY_DN17870_c0_g1	-1.34	down	PREDICTED: probable glutathione S-transferase [Populus euphratica]	1.73	7.15
map00480	Glutathione metabolism	1.79E-01	TRINITY_DN17937_c0_g1	-2.08	down	PREDICTED: probable glutathione S-transferase [Populus euphratica]	2.39	14.46
map00480	Glutathione metabolism	1.79E-01	TRINITY_DN18129_c0_g4	-1.79	down	PREDICTED: histone H3.2-like [Gossypium hirsutum]	50.39	262.39
map00480	Glutathione metabolism	1.79E-01	TRINITY_DN18237_c0_g2	1.23	up	PREDICTED: glucose-6-phosphate 1-dehydrogenase 4, chloroplastic [Populus euphratica]	14.47	10.41
map00480	Glutathione metabolism	1.79E-01	TRINITY_DN18367_c0_g2	-1.21	down	PREDICTED: glutathione S-transferase F13-like [Populus euphratica]	30.78	108.77
map00480	Glutathione metabolism	1.79E-01	TRINITY_DN18454_c0_g1	1.69	up	glutathione S-transferase F2 [Populus yatungensis]	305.90	143.62
map00480	Glutathione metabolism	1.79E-01	TRINITY_DN19362_c1_g4	-1.86	down	PREDICTED: histone H3.2-like [Zea mays]	92.37	503.02
map00480	Glutathione metabolism	1.79E-01	TRINITY_DN19362_c1_g6	-1.87	down	hypothetical protein CARUB_v10021660mg, partial [Capsella rubella]	105.61	580.15
map00480	Glutathione metabolism	1.79E-01	TRINITY_DN20591_c0_g2	-3.45	down	hypothetical protein POPTR_0010s07180g [Populus trichocarpa]	0.24	4.04
map00480	Glutathione metabolism	1.79E-01	TRINITY_DN21385_c0_g1	1.67	up	glutathione S-transferase F4 [Populus yatungensis]	313.16	159.31
map00480	Glutathione metabolism	1.79E-01	TRINITY_DN21477_c1_g5	2.64	up	PREDICTED: glutathione S-transferase F11-like [Populus euphratica]	3486.29	837.49
map00480	Glutathione metabolism	1.79E-01	TRINITY_DN21477_c1_g9	-2.85	down	hypothetical protein POPTR_0002s01650g [Populus trichocarpa]	0.47	5.06
map00480	Glutathione metabolism	1.79E-01	TRINITY_DN28972_c0_g1	-2.49	down	hypothetical protein POPTR_0010s07210g [Populus trichocarpa]	0.19	1.69
map00480	Glutathione metabolism	1.79E-01	TRINITY_DN9299_c0_g1	-1.29	down	hypothetical protein POPTR_0256s00200g [Populus trichocarpa]	1.86	6.96
map00740	Riboflavin metabolism	1.80E-01	TRINITY_DN22013_c0_g2	1.69	up	PREDICTED: riboflavin synthase-like [Populus euphratica]	81.46	37.42
map00740	Riboflavin metabolism	1.80E-01	TRINITY_DN23047_c1_g1	1.31	up	PREDICTED: haloacid dehalogenase-like hydrolase domain-containing protein At4g39970 isoform X1 [Populus euphratica]	501.61	302.05
map00740	Riboflavin metabolism	1.80E-01	TRINITY_DN25131_c0_g1	2.28	up	hypothetical protein POPTR_0001s24160g [Populus trichocarpa]	20.85	7.51
map00740	Riboflavin metabolism	1.80E-01	TRINITY_DN27259_c0_g2	2.88	up	hypothetical protein POPTR_0014s04300g [Populus trichocarpa]	66.51	12.23
map00740	Riboflavin metabolism	1.80E-01	TRINITY_DN15833_c0_g1	1.37	up	7-dimethyl-8-ribityllumazine synthase family protein [Populus trichocarpa]	128.55	74.53
map00740	Riboflavin metabolism	1.80E-01	TRINITY_DN19368_c0_g1	1.35	up	PREDICTED: riboflavin biosynthesis protein PYRD, chloroplastic [Populus euphratica]	34.03	20.41

map00340	Histidine metabolism	2.00E-01	TRINITY_DN21932_c0_g3	-1.06	down	phosphatase 2C family protein [Populus trichocarpa]	2.53	8.10
map00340	Histidine metabolism	2.00E-01	TRINITY_DN22627_c1_g2	2.05	up	PREDICTED: ATP phosphoribosyltransferase 2, chloroplastic-like [Populus euphratica]	103.92	38.62
map00340	Histidine metabolism	2.00E-01	TRINITY_DN22627_c1_g5	1.81	up	PREDICTED: ATP phosphoribosyltransferase 2, chloroplastic-like [Populus euphratica]	58.56	24.74
map00340	Histidine metabolism	2.00E-01	TRINITY_DN24628_c0_g1	1.28	up	PREDICTED: aldehyde dehydrogenase family 3 member H1-like [Populus euphratica]	40.39	24.46
map00340	Histidine metabolism	2.00E-01	TRINITY_DN24964_c0_g1	1.27	up	PREDICTED: histidinol dehydrogenase, chloroplastic-like isoform X3 [Populus euphratica]	31.19	20.54
map00340	Histidine metabolism	2.00E-01	TRINITY_DN25630_c0_g1	1.30	up	aldehyde dehydrogenase 1 precursor family protein [Populus trichocarpa]	120.35	73.18
map00340	Histidine metabolism	2.00E-01	TRINITY_DN26025_c0_g1	-1.71	down	hypothetical protein POPTR_0004s18340g [Populus trichocarpa]	5.60	27.70
map00340	Histidine metabolism	2.00E-01	TRINITY_DN26421_c0_g1	1.32	up	the aldehyde dehydrogenase cp-ADH from C.plantagineum family protein [Populus trichocarpa]	58.13	34.64
map00340	Histidine metabolism	2.00E-01	TRINITY_DN27089_c0_g1	1.00	up	PREDICTED: imidazole glycerol phosphate synthase hisHF, chloroplastic-like isoform X1 [Populus euphratica]	33.93	31.04
map00340	Histidine metabolism	2.00E-01	TRINITY_DN18895_c0_g1	-1.06	down	mitochondrial aldehyde dehydrogenase family protein [Populus trichocarpa]	7.40	23.22
map00340	Histidine metabolism	2.00E-01	TRINITY_DN19215_c1_g1	-1.38	down	hypothetical protein POPTR_0007s10210g [Populus trichocarpa]	3.47	12.55
map00340	Histidine metabolism	2.00E-01	TRINITY_DN19215_c2_g1	-1.56	down	PREDICTED: probable protein phosphatase 2C 63 [Populus euphratica]	6.48	30.16
map00340	Histidine metabolism	2.00E-01	TRINITY_DN20394_c0_g1	1.01	up	PREDICTED: histidine biosynthesis bifunctional protein hisIE, chloroplastic [Populus euphratica]	84.52	63.75
map00604	Glycosphingolipid biosynthesis - ganglio series	2.27E-01	TRINITY_DN22568_c0_g1	-1.35	down	PREDICTED: beta-galactosidase 5-like [Populus euphratica]	22.12	82.08
map00604	Glycosphingolipid biosynthesis - ganglio series	2.27E-01	TRINITY_DN22624_c0_g1	-1.58	down	hypothetical protein POPTR_0006s13130g [Populus trichocarpa]	2.14	9.80
map00604	Glycosphingolipid biosynthesis - ganglio series	2.27E-01	TRINITY_DN25513_c0_g2	-1.55	down	PREDICTED: zinc finger protein NUTCRACKER-like isoform X5 [Populus euphratica]	13.47	55.16
map00604	Glycosphingolipid biosynthesis - ganglio series	2.27E-01	TRINITY_DN25522_c1_g1	-1.19	down	PREDICTED: zinc finger protein NUTCRACKER-like [Populus euphratica]	4.97	17.73
map00604	Glycosphingolipid biosynthesis - ganglio series	2.27E-01	TRINITY_DN25889_c0_g2	1.59	up	unknown [Populus trichocarpa x Populus deltoides]	384.22	194.87
map00604	Glycosphingolipid biosynthesis - ganglio series	2.27E-01	TRINITY_DN26010_c0_g4	-1.72	down	hypothetical protein POPTR_0006s13130g [Populus trichocarpa]	3.69	17.16
map00604	Glycosphingolipid biosynthesis - ganglio series	2.27E-01	TRINITY_DN26284_c1_g3	1.51	up	hypothetical protein POPTR_0008s07890g [Populus trichocarpa]	53.04	28.53
map00604	Glycosphingolipid biosynthesis - ganglio series	2.27E-01	TRINITY_DN26337_c3_g2	-1.73	down	PREDICTED: zinc finger protein NUTCRACKER-like [Populus euphratica]	2.46	10.32
map00604	Glycosphingolipid biosynthesis - ganglio series	2.27E-01	TRINITY_DN14721_c0_g1	-1.05	down	hypothetical protein POPTR_0008s14180g [Populus trichocarpa]	12.06	37.39
map00604	Glycosphingolipid biosynthesis - ganglio series	2.27E-01	TRINITY_DN19674_c1_g2	1.61	up	beta-galactosidase family protein [Populus trichocarpa]	9.86	4.84
map00604	Glycosphingolipid biosynthesis - ganglio series	2.27E-01	TRINITY_DN20352_c0_g1	1.15	up	hypothetical protein POPTR_0004s19490g [Populus trichocarpa]	17.68	13.24
map00604	Glycosphingolipid biosynthesis - ganglio series	2.27E-01	TRINITY_DN20455_c0_g4	-1.61	down	hypothetical protein POPTR_0012s03730g [Populus trichocarpa]	2.77	12.99
map00604	Glycosphingolipid biosynthesis - ganglio series	2.27E-01	TRINITY_DN20896_c1_g2	-1.22	down	PREDICTED: protein SHOOT GRAVITROPISM 5 [Populus euphratica]	4.99	19.09
map00604	Glycosphingolipid biosynthesis - ganglio series	2.27E-01	TRINITY_DN7728_c0_g2	2.29	up	hypothetical protein POPTR_0006s14130g [Populus trichocarpa]	1.77	0.56
map00523	Polyketide sugar unit biosynthesis	2.31E-01	TRINITY_DN22066_c0_g1	1.09	up	hypothetical protein POPTR_0003s12000g [Populus trichocarpa]	72.83	51.13
map00430	Taurine and hypotaurine metabolism	2.53E-01	TRINITY_DN21818_c0_g1	-2.54	down	hypothetical protein POPTR_0019s05510g [Populus trichocarpa]	0.26	3.14
map00430	Taurine and hypotaurine metabolism	2.53E-01	TRINITY_DN22684_c0_g1	4.49	up	glutamate decarboxylase [Populus tremula x Populus alba]	5.12	0.35
map00430	Taurine and hypotaurine metabolism	2.53E-01	TRINITY_DN22684_c0_g7	-5.56	down	glutamate decarboxylase 1 family protein [Populus trichocarpa]	0.08	6.22
map00430	Taurine and hypotaurine metabolism	2.53E-01	TRINITY_DN19065_c0_g1	1.11	up	hypothetical protein POPTR_0015s06600g [Populus trichocarpa]	38.06	27.94
map00430	Taurine and hypotaurine metabolism	2.53E-01	TRINITY_DN847_c0_g1	-7.22	down	glutamate decarboxylase 1 family protein [Populus trichocarpa]	0.04	7.12
map00780	Biotin metabolism	2.77E-01	TRINITY_DN21952_c0_g6	-1.14	down	PREDICTED: bifunctional dethiobiotin synthetase/7,8-diamino-pelargonic acid aminotransferase, mitochondrial-like [Populus euphratica]	2.07	6.92
map00780	Biotin metabolism	2.77E-01	TRINITY_DN23093_c0_g1	1.81	up	3-oxoacyl-[acyl-carrier-protein] synthase I [Populus trichocarpa]	128.96	55.68
map00780	Biotin metabolism	2.77E-01	TRINITY_DN23645_c0_g1	1.12	up	hypothetical protein POPTR_0008s17880g [Populus trichocarpa]	115.75	81.25

map00780	Biotin metabolism	2.77E-01	TRINITY_DN25671_c0_g1	1.18	up	ENOYL-ACP REDUCTASE 1 family protein [Populus trichocarpa]	122.50	78.93
map00780	Biotin metabolism	2.77E-01	TRINITY_DN15736_c0_g1	-10.07	down	hypothetical protein POPTR_0008s14850g [Populus trichocarpa]	0.00	7.88
map00780	Biotin metabolism	2.77E-01	TRINITY_DN18603_c0_g1	1.08	up	hypothetical protein POPTR_0003s14190g [Populus trichocarpa]	6.90	4.98
map00780	Biotin metabolism	2.77E-01	TRINITY_DN19330_c1_g4	1.30	up	hypothetical protein POPTR_0004s23710g [Populus trichocarpa]	22.29	13.72
map00780	Biotin metabolism	2.77E-01	TRINITY_DN21469_c0_g3	1.53	up	unknown [Populus trichocarpa]	127.67	66.65
map00780	Biotin metabolism	2.77E-01	TRINITY_DN6900_c0_g1	1.83	up	short-chain dehydrogenase/reductase family protein [Populus trichocarpa]	21.13	8.97
map00591	Linoleic acid metabolism	2.79E-01	TRINITY_DN21821_c2_g1	-7.59	down	PREDICTED: probable linoleate 9S-lipoxygenase 5 [Populus euphratica]	0.00	3.52
map00591	Linoleic acid metabolism	2.79E-01	TRINITY_DN23485_c0_g3	-1.49	down	PREDICTED: cytochrome P450 71A1-like [Populus euphratica]	1.44	6.13
map00591	Linoleic acid metabolism	2.79E-01	TRINITY_DN23579_c0_g2	-2.24	down	hypothetical protein POPTR_0001s16780g [Populus trichocarpa]	14.61	101.79
map00591	Linoleic acid metabolism	2.79E-01	TRINITY_DN24288_c0_g1	-2.74	down	hypothetical protein POPTR_0003s11810g [Populus trichocarpa]	2.78	11.84
map00591	Linoleic acid metabolism	2.79E-01	TRINITY_DN24293_c0_g5	-1.68	down	hypothetical protein POPTR_0005s03580g [Populus trichocarpa]	7.72	39.30
map00591	Linoleic acid metabolism	2.79E-01	TRINITY_DN24353_c0_g3	-1.68	down	lipoxygenase family protein [Populus trichocarpa]	1.48	7.25
map00591	Linoleic acid metabolism	2.79E-01	TRINITY_DN24718_c0_g1	-1.13	down	PREDICTED: calmodulin-binding transcription activator 3-like isoform X3 [Populus euphratica]	2.73	8.94
map00591	Linoleic acid metabolism	2.79E-01	TRINITY_DN26973_c0_g1	-1.85	down	hypothetical protein POPTR_0001s16780g [Populus trichocarpa]	4.72	22.23
map00591	Linoleic acid metabolism	2.79E-01	TRINITY_DN27547_c0_g1	-1.35	down	calmodulin-binding family protein [Populus trichocarpa]	6.25	23.99
map00591	Linoleic acid metabolism	2.79E-01	TRINITY_DN27768_c0_g1	-7.23	down	hypothetical protein POPTR_0005s03560g [Populus trichocarpa]	0.00	4.46
map00591	Linoleic acid metabolism	2.79E-01	TRINITY_DN27768_c1_g3	-1.73	down	hypothetical protein POPTR_0005s03580g [Populus trichocarpa]	4.58	18.84
map00591	Linoleic acid metabolism	2.79E-01	TRINITY_DN16449_c0_g1	2.09	up	PREDICTED: cytochrome P450 71A1-like isoform X1 [Populus euphratica]	34.70	12.40
map00591	Linoleic acid metabolism	2.79E-01	TRINITY_DN19787_c0_g1	2.95	up	hypothetical protein POPTR_0001s08320g [Populus trichocarpa]	7.17	1.45
map03410	Base excision repair	2.91E-01	TRINITY_DN22222_c2_g1	-1.69	down	PREDICTED: proliferating cell nuclear antigen [Populus euphratica]	20.04	106.79
map03410	Base excision repair	2.91E-01	TRINITY_DN22262_c0_g2	-2.11	down	PREDICTED: HMG1/2-like protein [Populus euphratica]	2.46	15.63
map03410	Base excision repair	2.91E-01	TRINITY_DN23896_c0_g3	1.03	up	PREDICTED: probable carboxylesterase 7 [Populus euphratica]	825.28	598.17
map03410	Base excision repair	2.91E-01	TRINITY_DN24395_c0_g2	1.21	up	hypothetical protein POPTR_0005s26230g [Populus trichocarpa]	66.57	46.27
map03410	Base excision repair	2.91E-01	TRINITY_DN24400_c0_g2	-2.42	down	methyladenine glycosylase family protein [Populus trichocarpa]	0.67	5.62
map03410	Base excision repair	2.91E-01	TRINITY_DN24767_c0_g2	-1.01	down	PREDICTED: uncharacterized protein LOC105122922 [Populus euphratica]	4.81	14.63
map03410	Base excision repair	2.91E-01	TRINITY_DN24940_c0_g2	1.30	up	PREDICTED: ADP,ATP carrier protein 1, mitochondrial [Populus euphratica]	432.60	260.66
map03410	Base excision repair	2.91E-01	TRINITY_DN25276_c0_g2	-1.48	down	hypothetical protein POPTR_0018s00860g [Populus trichocarpa]	1.70	6.52
map03410	Base excision repair	2.91E-01	TRINITY_DN25676_c0_g1	-1.24	down	PREDICTED: high mobility group B protein 3-like [Populus euphratica]	385.41	1440.19
map03410	Base excision repair	2.91E-01	TRINITY_DN27349_c0_g1	1.26	up	hypothetical protein POPTR_0001s44720g [Populus trichocarpa]	31.99	22.49
map03410	Base excision repair	2.91E-01	TRINITY_DN13106_c0_g1	-4.72	down	PREDICTED: uncharacterized protein LOC105110803 isoform X1 [Populus euphratica]	0.07	3.07
map03410	Base excision repair	2.91E-01	TRINITY_DN14883_c0_g1	1.47	up	PREDICTED: probable carboxylesterase 12 [Populus euphratica]	4.04	2.25
map03410	Base excision repair	2.91E-01	TRINITY_DN15410_c0_g1	2.27	up	hypothetical protein POPTR_0010s06490g [Populus trichocarpa]	37.10	11.66
map03410	Base excision repair	2.91E-01	TRINITY_DN15714_c0_g1	1.33	up	hypothetical protein POPTR_0009s10790g [Populus trichocarpa]	40.95	24.47
map03410	Base excision repair	2.91E-01	TRINITY_DN16962_c0_g1	2.25	up	hypothetical protein POPTR_0009s10750g [Populus trichocarpa]	348.16	110.65
map03410	Base excision repair	2.91E-01	TRINITY_DN16976_c0_g4	-1.35	down	DNA-directed DNA polymerase epsilon catalytic subunit family protein [Populus trichocarpa]	2.96	11.76
map03410	Base excision repair	2.91E-01	TRINITY_DN16976_c0_g5	-1.67	down	DNA-directed DNA polymerase epsilon catalytic subunit family protein [Populus trichocarpa]	0.84	4.16
map03410	Base excision repair	2.91E-01	TRINITY_DN17241_c0_g1	-1.38	down	DNA polymerase delta subunit 4 family protein [Populus trichocarpa]	4.03	15.90
map03410	Base excision repair	2.91E-01	TRINITY_DN17241_c0_g2	-1.21	down	DNA polymerase delta subunit 4 family protein [Populus trichocarpa]	37.87	132.98
map03410	Base excision repair	2.91E-01	TRINITY_DN17524_c0_g1	-1.76	down	PREDICTED: high mobility group B protein 7-like [Populus euphratica]	17.66	87.26
map03410	Base excision repair	2.91E-01	TRINITY_DN18555_c0_g4	2.33	up	Ycf2 [Populus alba]	3.15	0.93
map03410	Base excision repair	2.91E-01	TRINITY_DN18656_c0_g1	1.85	up	hypothetical protein POPTR_0008s08550g [Populus trichocarpa]	2.75	1.16
map03410	Base excision repair	2.91E-01	TRINITY_DN19259_c0_g1	-1.63	down	PREDICTED: DNA polymerase epsilon subunit 2 [Populus euphratica]	1.72	8.35

map03410	Base excision repair	2.91E-01	TRINITY_DN19392_c2_g2	1.41	up	PREDICTED: probable carboxylesterase 5 isoform X1 [Populus euphratica]	5.63	3.24
map03410	Base excision repair	2.91E-01	TRINITY_DN19402_c0_g1	3.34	up	hypothetical protein POPTR_0004s15020g [Populus trichocarpa]	12.33	1.80
map03410	Base excision repair	2.91E-01	TRINITY_DN19537_c0_g3	-1.06	down	PREDICTED: uncharacterized protein LOC105109677 isoform X1 [Populus euphratica]	5.42	16.71
map03410	Base excision repair	2.91E-01	TRINITY_DN20164_c0_g3	-1.54	down	sterile alpha motif domain-containing family protein [Populus trichocarpa]	3.50	14.58
map03410	Base excision repair	2.91E-01	TRINITY_DN20946_c0_g1	1.58	up	ADP/ATP carrier 1 [Arabidopsis thaliana]	134.29	72.83
map03410	Base excision repair	2.91E-01	TRINITY_DN20973_c0_g1	-1.20	down	PREDICTED: DNA polymerase delta catalytic subunit [Populus euphratica]	3.70	13.41
map03410	Base excision repair	2.91E-01	TRINITY_DN21005_c0_g2	1.99	up	hypothetical protein POPTR_0006s25980g [Populus trichocarpa]	70.33	27.48
map03060	Protein export	2.98E-01	TRINITY_DN22810_c0_g1	1.25	up	hypothetical protein POPTR_0018s13680g [Populus trichocarpa]	16.05	10.85
map03060	Protein export	2.98E-01	TRINITY_DN23130_c0_g2	-11.64	down	PREDICTED: MADS-box protein JOINTLESS-like [Populus euphratica]	0.00	24.68
map03060	Protein export	2.98E-01	TRINITY_DN23240_c1_g5	2.27	up	PREDICTED: uncharacterized protein LOC105136996 isoform X1 [Populus euphratica]	55.20	17.84
map03060	Protein export	2.98E-01	TRINITY_DN23671_c0_g1	1.34	up	PREDICTED: chloroplast processing peptidase [Populus euphratica]	123.40	80.67
map03060	Protein export	2.98E-01	TRINITY_DN23671_c0_g2	1.28	up	hypothetical protein POPTR_0006s16300g [Populus trichocarpa]	26.91	16.73
map03060	Protein export	2.98E-01	TRINITY_DN23873_c0_g1	1.27	up	PREDICTED: cell division protein FtsY homolog, chloroplastic [Populus euphratica]	77.71	49.65
map03060	Protein export	2.98E-01	TRINITY_DN23922_c0_g1	1.12	up	PREDICTED: protein translocase subunit SecA, chloroplastic isoform X2 [Populus euphratica]	96.97	70.38
map03060	Protein export	2.98E-01	TRINITY_DN24070_c0_g1	-8.92	down	hypothetical protein POPTR_0014s07010g, partial [Populus trichocarpa]	0.08	51.58
map03060	Protein export	2.98E-01	TRINITY_DN24090_c0_g2	2.34	up	unknown [Populus trichocarpa x Populus deltoides]	199.05	59.58
map03060	Protein export	2.98E-01	TRINITY_DN25080_c0_g1	1.68	up	Inner membrane protein ALBINO3 [Populus trichocarpa]	272.25	137.16
map03060	Protein export	2.98E-01	TRINITY_DN25512_c0_g2	1.21	up	hypothetical protein POPTR_0010s06180g [Populus trichocarpa]	47.66	30.80
map03060	Protein export	2.98E-01	TRINITY_DN27114_c0_g1	1.34	up	signal recognition particle 54 kDa subunit precursor family protein [Populus trichocarpa]	129.89	73.75
map03060	Protein export	2.98E-01	TRINITY_DN15249_c0_g1	1.67	up	signal recognition particle 14 kDa family protein [Populus trichocarpa]	47.78	22.86
map03060	Protein export	2.98E-01	TRINITY_DN15374_c1_g1	-3.60	down	PREDICTED: truncated transcription factor CAULIFLOWER A-like isoform X2 [Populus euphratica]	0.78	14.42
map03060	Protein export	2.98E-01	TRINITY_DN16788_c0_g1	1.82	up	hypothetical protein POPTR_0016s12250g [Populus trichocarpa]	16.67	7.36
map03060	Protein export	2.98E-01	TRINITY_DN16948_c0_g1	2.07	up	MADS-box protein GmNMH7 [Populus trichocarpa]	25.80	10.31
map03060	Protein export	2.98E-01	TRINITY_DN17245_c0_g1	1.27	up	PREDICTED: sec-independent protein translocase protein TATC, chloroplastic [Populus euphratica]	72.17	45.59
map03060	Protein export	2.98E-01	TRINITY_DN18416_c0_g2	1.40	up	BiP isoform A family protein [Populus trichocarpa]	3.54	2.09
map03060	Protein export	2.98E-01	TRINITY_DN18416_c0_g3	1.00	up	BiP isoform A family protein [Populus trichocarpa]	31.78	24.33
map03060	Protein export	2.98E-01	TRINITY_DN18885_c0_g3	1.38	up	hypothetical protein POPTR_0006s22820g [Populus trichocarpa]	21.93	12.66
map03060	Protein export	2.98E-01	TRINITY_DN19263_c0_g2	-2.45	down	PREDICTED: MADS-box protein JOINTLESS [Populus euphratica]	13.75	116.97
map03060	Protein export	2.98E-01	TRINITY_DN19349_c0_g1	1.38	up	PREDICTED: sec-independent protein translocase protein TATB, chloroplastic-like [Populus euphratica]	248.60	144.95
map03060	Protein export	2.98E-01	TRINITY_DN19572_c0_g1	-3.15	down	PREDICTED: truncated transcription factor CAULIFLOWER D-like isoform X2 [Populus euphratica]	0.23	4.59
map03060	Protein export	2.98E-01	TRINITY_DN19572_c0_g2	2.70	up	PREDICTED: truncated transcription factor CAULIFLOWER D-like isoform X1 [Populus euphratica]	76.08	17.77
map03060	Protein export	2.98E-01	TRINITY_DN20061_c0_g3	-2.07	down	MADS box transcription factor [Populus tomentosa]	1.94	15.68
map03060	Protein export	2.98E-01	TRINITY_DN20221_c0_g4	1.08	up	thylakoid assembly family protein [Populus trichocarpa]	134.86	96.73
map03060	Protein export	2.98E-01	TRINITY_DN20647_c2_g1	-7.66	down	hypothetical protein POPTR_0014s07010g, partial [Populus trichocarpa]	0.46	88.39
map00230	Purine metabolism	3.00E-01	TRINITY_DN22269_c0_g1	2.02	up	PREDICTED: adenylate kinase 5, chloroplastic [Populus euphratica]	27.73	10.48
map00230	Purine metabolism	3.00E-01	TRINITY_DN22804_c0_g4	-2.56	down	ERF domain protein 12 [Populus trichocarpa]	0.99	8.94
map00230	Purine metabolism	3.00E-01	TRINITY_DN22866_c0_g1	1.39	up	PREDICTED: mitochondrial-processing peptidase subunit alpha-like [Populus euphratica]	54.03	31.47
map00230	Purine metabolism	3.00E-01	TRINITY_DN23156_c0_g1	1.23	up	hypothetical protein POPTR_0002s19970g [Populus trichocarpa]	53.28	34.52
map00230	Purine metabolism	3.00E-01	TRINITY_DN23869_c0_g2	1.26	up	PREDICTED: ethylene-responsive transcription factor RAP2-12-like isoform X2 [Populus euphratica]	18.61	11.90
map00230	Purine metabolism	3.00E-01	TRINITY_DN23876_c0_g1	-2.14	down	hypothetical protein POPTR_0007s10880g [Populus trichocarpa]	0.94	6.43
map00230	Purine metabolism	3.00E-01	TRINITY_DN23876_c0_g2	-1.91	down	hypothetical protein POPTR_0007s10880g [Populus trichocarpa]	1.84	10.67
map00230	Purine metabolism	3.00E-01	TRINITY_DN24015_c0_g2	1.27	up	PREDICTED: adenylate kinase, chloroplastic-like [Populus euphratica]	70.20	43.65

map00230	Purine metabolism	3.00E-01	TRINITY_DN24169_c0_g1	1.52	up	PREDICTED: adenosine kinase 2 isoform X1 [Populus euphratica]	170.79	90.39
map00230	Purine metabolism	3.00E-01	TRINITY_DN24268_c0_g3	-2.26	down	PREDICTED: DNA-directed RNA polymerases IV and V subunit 2-like [Populus euphratica]	0.30	2.20
map00230	Purine metabolism	3.00E-01	TRINITY_DN24355_c2_g4	-1.38	down	hypothetical protein POPTR_0003s10650g [Populus trichocarpa]	4.31	16.98
map00230	Purine metabolism	3.00E-01	TRINITY_DN24395_c0_g2	1.21	up	hypothetical protein POPTR_0005s26230g [Populus trichocarpa]	66.57	46.27
map00230	Purine metabolism	3.00E-01	TRINITY_DN24425_c1_g7	1.89	up	RecName: Full=DNA-directed RNA polymerase subunit beta'; AltName: Full=PEP; AltName: Full=Plastid-encoded RNA polymerase subunit beta'; Short=RNA polymerase subunit beta'	5.92	2.43
map00230	Purine metabolism	3.00E-01	TRINITY_DN24662_c0_g2	-1.93	down	ferritin/ribonucleotide reductase-like family protein [Populus tomentosa]	4.88	29.25
map00230	Purine metabolism	3.00E-01	TRINITY_DN24662_c0_g3	-1.05	down	PREDICTED: ribonucleoside-diphosphate reductase small chain [Populus euphratica]	25.18	80.64
map00230	Purine metabolism	3.00E-01	TRINITY_DN25128_c1_g2	1.83	up	hypothetical protein POPTR_0015s09980g [Populus trichocarpa]	54.09	22.75
map00230	Purine metabolism	3.00E-01	TRINITY_DN25276_c0_g2	-1.48	down	hypothetical protein POPTR_0018s00860g [Populus trichocarpa]	1.70	6.52
map00230	Purine metabolism	3.00E-01	TRINITY_DN25285_c0_g1	-1.11	down	PREDICTED: ribonucleoside-diphosphate reductase large subunit-like [Populus euphratica]	33.12	103.10
map00230	Purine metabolism	3.00E-01	TRINITY_DN25500_c0_g2	-1.03	down	nucleoside phosphatase family protein [Populus trichocarpa]	6.42	19.00
map00230	Purine metabolism	3.00E-01	TRINITY_DN25625_c0_g1	1.41	up	PREDICTED: amidophosphoribosyltransferase, chloroplastic [Populus euphratica]	27.38	16.32
map00230	Purine metabolism	3.00E-01	TRINITY_DN26308_c0_g1	-1.12	down	hypothetical protein POPTR_0003s19630g [Populus trichocarpa]	5.58	19.18
map00230	Purine metabolism	3.00E-01	TRINITY_DN26631_c0_g1	2.04	up	PREDICTED: phosphoribosylaminoimidazole carboxylase, chloroplastic-like isoform X1 [Populus euphratica]	26.81	10.47
map00230	Purine metabolism	3.00E-01	TRINITY_DN26743_c0_g1	1.15	up	RecName: Full=Phosphoglucomutase, cytoplasmic; Short=PGM; AltName: Full=Glucose phosphomutase	74.05	53.71
map00230	Purine metabolism	3.00E-01	TRINITY_DN26996_c1_g1	1.79	up	PREDICTED: phosphoglucomutase, chloroplastic-like [Populus euphratica]	112.47	49.80
map00230	Purine metabolism	3.00E-01	TRINITY_DN27349_c0_g1	1.26	up	hypothetical protein POPTR_0001s44720g [Populus trichocarpa]	31.99	22.49
map00230	Purine metabolism	3.00E-01	TRINITY_DN27568_c1_g1	1.34	up	phosphoribosylformylglycinamidine synthase family protein [Populus trichocarpa]	25.96	15.77
map00230	Purine metabolism	3.00E-01	TRINITY_DN14002_c0_g1	7.23	up	PREDICTED: probable 2-oxoglutarate-dependent dioxygenase AOP1 [Populus euphratica]	4.30	0.03
map00230	Purine metabolism	3.00E-01	TRINITY_DN14165_c0_g1	1.21	up	unknown [Populus trichocarpa]	21.82	14.59
map00230	Purine metabolism	3.00E-01	TRINITY_DN14700_c0_g1	-2.16	down	hypothetical protein POPTR_0008s01650g [Populus trichocarpa]	0.80	4.63
map00230	Purine metabolism	3.00E-01	TRINITY_DN14978_c0_g1	2.39	up	hypothetical protein POPTR_0011s15710g [Populus trichocarpa]	6.42	1.59
map00230	Purine metabolism	3.00E-01	TRINITY_DN15894_c0_g1	1.05	up	adenine phosphoribosyltransferase 1 family protein [Populus trichocarpa]	84.24	61.79
map00230	Purine metabolism	3.00E-01	TRINITY_DN16280_c0_g1	1.20	up	nodulin 35 family protein [Populus trichocarpa]	36.34	29.20
map00230	Purine metabolism	3.00E-01	TRINITY_DN16683_c0_g1	1.71	up	hypothetical protein POPTR_0010s20070g [Populus trichocarpa]	30.27	14.40
map00230	Purine metabolism	3.00E-01	TRINITY_DN16683_c0_g2	1.35	up	hypothetical protein POPTR_0010s20070g [Populus trichocarpa]	49.51	30.46
map00230	Purine metabolism	3.00E-01	TRINITY_DN16872_c0_g1	-5.53	down	hypothetical protein POPTR_0002s04430g [Populus trichocarpa]	0.45	31.91
map00230	Purine metabolism	3.00E-01	TRINITY_DN16976_c0_g4	-1.35	down	DNA-directed DNA polymerase epsilon catalytic subunit family protein [Populus trichocarpa]	2.96	11.76
map00230	Purine metabolism	3.00E-01	TRINITY_DN16976_c0_g5	-1.67	down	DNA-directed DNA polymerase epsilon catalytic subunit family protein [Populus trichocarpa]	0.84	4.16
map00230	Purine metabolism	3.00E-01	TRINITY_DN17241_c0_g1	-1.38	down	DNA polymerase delta subunit 4 family protein [Populus trichocarpa]	4.03	15.90
map00230	Purine metabolism	3.00E-01	TRINITY_DN17241_c0_g2	-1.21	down	DNA polymerase delta subunit 4 family protein [Populus trichocarpa]	37.87	132.98
map00230	Purine metabolism	3.00E-01	TRINITY_DN17431_c0_g1	2.34	up	adenylosuccinate synthetase family protein [Populus trichocarpa]	82.22	24.66
map00230	Purine metabolism	3.00E-01	TRINITY_DN17528_c0_g1	2.19	up	ribose-phosphate pyrophosphokinase family protein [Populus trichocarpa]	63.69	21.30
map00230	Purine metabolism	3.00E-01	TRINITY_DN17748_c0_g1	-1.40	down	hypothetical protein POPTR_0015s09670g [Populus trichocarpa]	2.03	7.86
map00230	Purine metabolism	3.00E-01	TRINITY_DN17816_c0_g6	-2.04	down	ERF domain protein 11 [Populus trichocarpa]	3.09	18.64
map00230	Purine metabolism	3.00E-01	TRINITY_DN17925_c1_g1	3.02	up	hypothetical protein POPTR_0014s04880g [Populus trichocarpa]	213.24	40.67
map00230	Purine metabolism	3.00E-01	TRINITY_DN17925_c1_g2	1.30	up	unknown [Populus trichocarpa]	708.50	436.01
map00230	Purine metabolism	3.00E-01	TRINITY_DN17951_c0_g1	1.19	up	PREDICTED: nucleoside diphosphate kinase 3 isoform X1 [Populus euphratica]	91.53	60.90
map00230	Purine metabolism	3.00E-01	TRINITY_DN17951_c0_g2	1.83	up	hypothetical protein POPTR_0001s10670g [Populus trichocarpa]	286.39	121.84
map00230	Purine metabolism	3.00E-01	TRINITY_DN18209_c1_g1	1.05	up	PREDICTED: pyruvate kinase, cytosolic isozyme-like [Populus euphratica]	19.18	14.26

map00230	Purine metabolism	3.00E-01	TRINITY_DN18270_c0_g1	2.00	up	hypothetical protein POPTR_0017s07840g [Populus trichocarpa]	28.13	10.64
map00230	Purine metabolism	3.00E-01	TRINITY_DN18436_c1_g3	-1.79	down	PREDICTED: DNA-directed RNA polymerase III subunit RPC6-like [Populus euphratica]	0.98	5.17
map00230	Purine metabolism	3.00E-01	TRINITY_DN18493_c0_g1	1.75	up	PREDICTED: nucleoside diphosphate kinase 2, chloroplastic-like, partial [Populus euphratica]	521.73	234.57
map00230	Purine metabolism	3.00E-01	TRINITY_DN19143_c0_g1	-1.71	down	PREDICTED: DNA polymerase alpha subunit B-like [Populus euphratica]	6.40	29.61
map00230	Purine metabolism	3.00E-01	TRINITY_DN19143_c0_g6	-2.26	down	PREDICTED: uncharacterized protein LOC105129502 [Populus euphratica]	0.25	1.91
map00230	Purine metabolism	3.00E-01	TRINITY_DN19259_c0_g1	-1.63	down	PREDICTED: DNA polymerase epsilon subunit 2 [Populus euphratica]	1.72	8.35
map00230	Purine metabolism	3.00E-01	TRINITY_DN19303_c0_g4	1.26	up	hypothetical protein POPTR_0012s09720g [Populus trichocarpa]	77.17	48.93
map00230	Purine metabolism	3.00E-01	TRINITY_DN19303_c0_g5	1.33	up	hypothetical protein POPTR_0015s10490g [Populus trichocarpa]	98.70	59.24
map00230	Purine metabolism	3.00E-01	TRINITY_DN19537_c0_g3	-1.06	down	PREDICTED: uncharacterized protein LOC105109677 isoform X1 [Populus euphratica]	5.42	16.71
map00230	Purine metabolism	3.00E-01	TRINITY_DN19608_c0_g1	-1.86	down	hypothetical protein POPTR_0003s10380g [Populus trichocarpa]	3.11	17.37
map00230	Purine metabolism	3.00E-01	TRINITY_DN19771_c0_g1	1.21	up	hypothetical protein POPTR_0010s22250g [Populus trichocarpa]	53.45	36.30
map00230	Purine metabolism	3.00E-01	TRINITY_DN19857_c0_g2	1.45	up	AICARFT/IMPCHase bienzyme family protein [Populus tomentosa]	80.53	45.41
map00230	Purine metabolism	3.00E-01	TRINITY_DN19931_c0_g15	1.32	up	RNA polymerase beta" subunit [Populus alba]	3.76	2.38
map00230	Purine metabolism	3.00E-01	TRINITY_DN19931_c0_g8	1.61	up	RNA polymerase beta" subunit [Populus alba]	18.11	8.96
map00230	Purine metabolism	3.00E-01	TRINITY_DN19940_c0_g1	1.32	up	PREDICTED: adenylyl-sulfate kinase 3-like [Populus euphratica]	131.63	73.66
map00230	Purine metabolism	3.00E-01	TRINITY_DN19940_c0_g2	1.65	up	PREDICTED: adenylyl-sulfate kinase 3-like [Populus euphratica]	26.43	13.84
map00230	Purine metabolism	3.00E-01	TRINITY_DN19959_c0_g1	1.17	up	hypothetical protein POPTR_0005s25810g [Populus trichocarpa]	31.81	22.34
map00230	Purine metabolism	3.00E-01	TRINITY_DN20692_c0_g1	-1.11	down	PREDICTED: probable DNA primase large subunit [Populus euphratica]	8.23	30.45
map00230	Purine metabolism	3.00E-01	TRINITY_DN20750_c0_g4	1.26	up	PREDICTED: probable ribosome-binding factor A, chloroplastic [Populus euphratica]	127.20	80.02
map00230	Purine metabolism	3.00E-01	TRINITY_DN20815_c0_g1	1.41	up	PREDICTED: ATP sulfurylase 2-like [Populus euphratica]	46.46	28.77
map00230	Purine metabolism	3.00E-01	TRINITY_DN20973_c0_g1	-1.20	down	PREDICTED: DNA polymerase delta catalytic subunit [Populus euphratica]	3.70	13.41
map00230	Purine metabolism	3.00E-01	TRINITY_DN21182_c0_g4	1.43	up	RNA polymerase alpha subunit [Populus alba]	6.16	3.50
map00230	Purine metabolism	3.00E-01	TRINITY_DN21202_c0_g2	-2.06	down	hypothetical protein POPTR_0017s04540g [Populus trichocarpa]	24.81	154.42
map00230	Purine metabolism	3.00E-01	TRINITY_DN21216_c0_g1	2.14	up	hypothetical protein POPTR_0005s06460g [Populus trichocarpa]	132.71	46.26
map00230	Purine metabolism	3.00E-01	TRINITY_DN21228_c0_g1	1.59	up	hypothetical protein POPTR_0001s35070g [Populus trichocarpa]	4.70	2.42
map00230	Purine metabolism	3.00E-01	TRINITY_DN21497_c0_g3	-3.63	down	Ethylene responsive element binding factor 4 family protein [Populus trichocarpa]	10.72	198.84
map00230	Purine metabolism	3.00E-01	TRINITY_DN21497_c0_g4	-3.32	down	ERF4 [Populus x canadensis]	11.08	166.25
map00230	Purine metabolism	3.00E-01	TRINITY_DN21521_c0_g1	-2.76	down	hypothetical protein POPTR_0007s07830g [Populus trichocarpa]	4.44	45.77
map00051	Fructose and mannose metabolism	3.13E-01	TRINITY_DN21731_c0_g2	-1.12	down	PREDICTED: aldo-keto reductase family 4 member C9-like [Populus euphratica]	3.19	10.75
map00051	Fructose and mannose metabolism	3.13E-01	TRINITY_DN22033_c0_g1	1.60	up	Chain D family protein [Populus trichocarpa]	364.35	179.65
map00051	Fructose and mannose metabolism	3.13E-01	TRINITY_DN22245_c0_g1	2.23	up	PREDICTED: triosephosphate isomerase, chloroplastic [Populus euphratica]	838.73	263.34
map00051	Fructose and mannose metabolism	3.13E-01	TRINITY_DN22587_c0_g1	1.20	up	PREDICTED: fructose-bisphosphate aldolase cytoplasmic isozyme [Populus euphratica]	371.77	247.12
map00051	Fructose and mannose metabolism	3.13E-01	TRINITY_DN22683_c0_g1	1.44	up	hypothetical protein POPTR_0007s13190g [Populus trichocarpa]	8.04	5.40
map00051	Fructose and mannose metabolism	3.13E-01	TRINITY_DN22754_c0_g1	1.32	up	hypothetical protein POPTR_0005s24170g [Populus trichocarpa]	22.62	13.77
map00051	Fructose and mannose metabolism	3.13E-01	TRINITY_DN23701_c0_g1	1.96	up	latex plastidic aldolase-like family protein [Populus trichocarpa]	2289.47	879.27
map00051	Fructose and mannose metabolism	3.13E-01	TRINITY_DN23701_c0_g5	2.57	up	plastidic aldolase family protein [Populus trichocarpa]	7.53	2.00
map00051	Fructose and mannose metabolism	3.13E-01	TRINITY_DN23965_c0_g1	1.23	up	PREDICTED: triosephosphate isomerase, cytosolic [Populus euphratica]	641.46	426.80
map00051	Fructose and mannose metabolism	3.13E-01	TRINITY_DN24285_c0_g5	1.46	up	PREDICTED: WAT1-related protein At2g37460-like isoform X1 [Populus euphratica]	2.63	1.46
map00051	Fructose and mannose metabolism	3.13E-01	TRINITY_DN24393_c0_g1	1.31	up	hypothetical protein POPTR_0001s24710g [Populus trichocarpa]	198.18	106.97
map00051	Fructose and mannose metabolism	3.13E-01	TRINITY_DN24550_c0_g2	1.52	up	pfkB-type carbohydrate kinase family protein [Populus trichocarpa]	100.10	51.39
map00051	Fructose and mannose metabolism	3.13E-01	TRINITY_DN24707_c1_g1	-1.25	down	nodulin MtN21 family protein [Populus trichocarpa]	14.31	50.91
map00051	Fructose and mannose metabolism	3.13E-01	TRINITY_DN25009_c0_g1	1.05	up	pyrophosphate-dependent phosphofructokinase alpha subunit family protein [Populus trichocarpa]	61.09	45.64

map00051	Fructose and mannose metabolism	3.13E-01	TRINITY_DN25363_c0_g1	1.07	up	hypothetical protein POPTR_0009s07790g [Populus trichocarpa]	26.30	18.23
map00051	Fructose and mannose metabolism	3.13E-01	TRINITY_DN26175_c0_g1	1.33	up	PREDICTED: hexokinase-1-like [Populus euphratica]	24.35	14.67
map00051	Fructose and mannose metabolism	3.13E-01	TRINITY_DN26202_c0_g2	1.07	up	pyrophosphate--fructose-6-phosphate 1-phosphotransferase beta subunit family protein [Populus trichocarpa]	30.47	22.09
map00051	Fructose and mannose metabolism	3.13E-01	TRINITY_DN26209_c0_g1	1.84	up	hypothetical protein SETIT_022310mg [Setaria italica]	186.50	89.03
map00051	Fructose and mannose metabolism	3.13E-01	TRINITY_DN26661_c0_g1	2.62	up	hypothetical protein CISIN_1g016748mg [Citrus sinensis]	718.86	176.98
map00051	Fructose and mannose metabolism	3.13E-01	TRINITY_DN11372_c0_g1	3.83	up	hypothetical protein POPTR_0011s15150g [Populus trichocarpa]	4.92	0.36
map00051	Fructose and mannose metabolism	3.13E-01	TRINITY_DN17020_c0_g1	2.36	up	hypothetical protein POPTR_0002s06890g [Populus trichocarpa]	22.89	6.58
map00051	Fructose and mannose metabolism	3.13E-01	TRINITY_DN17246_c0_g1	1.06	up	hypothetical protein POPTR_0017s07040g [Populus trichocarpa]	24.42	17.62
map00051	Fructose and mannose metabolism	3.13E-01	TRINITY_DN17353_c0_g1	1.22	up	PREDICTED: LOW QUALITY PROTEIN: methylecgonone reductase-like [Populus euphratica]	9.67	7.02
map00051	Fructose and mannose metabolism	3.13E-01	TRINITY_DN17644_c0_g1	1.22	up	PREDICTED: NADP-dependent D-sorbitol-6-phosphate dehydrogenase-like [Populus euphratica]	152.43	100.73
map00051	Fructose and mannose metabolism	3.13E-01	TRINITY_DN18225_c0_g3	1.29	up	PREDICTED: hexokinase-1-like [Populus euphratica]	38.97	24.40
map00051	Fructose and mannose metabolism	3.13E-01	TRINITY_DN19010_c0_g1	1.39	up	hypothetical protein POPTR_0016s11690g [Populus trichocarpa]	93.49	53.39
map00051	Fructose and mannose metabolism	3.13E-01	TRINITY_DN19743_c0_g1	1.38	up	PREDICTED: WAT1-related protein At4g19185-like [Populus euphratica]	66.15	39.51
map00051	Fructose and mannose metabolism	3.13E-01	TRINITY_DN19751_c0_g1	-1.26	down	unknown [Populus trichocarpa]	5.54	20.26
map00051	Fructose and mannose metabolism	3.13E-01	TRINITY_DN19751_c0_g2	-1.99	down	PREDICTED: probable fructokinase-1 [Populus euphratica]	4.28	23.17
map00051	Fructose and mannose metabolism	3.13E-01	TRINITY_DN20067_c0_g1	1.89	up	hypothetical protein POPTR_0016s14310g [Populus trichocarpa]	360.20	150.22
map00051	Fructose and mannose metabolism	3.13E-01	TRINITY_DN21062_c0_g1	1.22	up	PREDICTED: hexokinase-1-like [Populus euphratica]	9.26	6.04
map00051	Fructose and mannose metabolism	3.13E-01	TRINITY_DN21427_c0_g1	1.40	up	hypothetical protein POPTR_0016s11621g [Populus trichocarpa]	98.65	56.05
map00051	Fructose and mannose metabolism	3.13E-01	TRINITY_DN21497_c0_g2	1.83	up	PREDICTED: sorbitol dehydrogenase [Populus euphratica]	81.71	34.89
map00051	Fructose and mannose metabolism	3.13E-01	TRINITY_DN21582_c0_g1	2.42	up	PREDICTED: fructokinase-like 2, chloroplastic [Populus euphratica]	83.87	26.93
map00051	Fructose and mannose metabolism	3.13E-01	TRINITY_DN6390_c0_g1	3.46	up	nodulin MtN21 family protein [Populus trichocarpa]	1.91	0.26
map01051	Biosynthesis of ansamycins	3.28E-01	TRINITY_DN24881_c0_g1	1.99	up	PREDICTED: transketolase, chloroplastic [Populus euphratica]	1790.82	677.46
map01051	Biosynthesis of ansamycins	3.28E-01	TRINITY_DN27458_c1_g1	2.44	up	PREDICTED: transketolase, chloroplastic [Populus euphratica]	1184.20	313.96
map00904	Diterpenoid biosynthesis	3.34E-01	TRINITY_DN22202_c0_g5	-1.76	down	PREDICTED: probable 2-oxoglutarate-dependent dioxygenase AOP1 [Populus euphratica]	2.48	12.90
map00904	Diterpenoid biosynthesis	3.34E-01	TRINITY_DN22470_c0_g1	1.20	up	oxidoreductase family protein [Populus trichocarpa]	61.30	40.18
map00904	Diterpenoid biosynthesis	3.34E-01	TRINITY_DN23732_c1_g2	2.06	up	cytochrome P450 family protein [Populus trichocarpa]	2.23	0.83
map00904	Diterpenoid biosynthesis	3.34E-01	TRINITY_DN24543_c0_g2	-2.47	down	PREDICTED: gibberellin 2-beta-dioxygenase 2-like [Populus euphratica]	0.83	8.88
map00904	Diterpenoid biosynthesis	3.34E-01	TRINITY_DN25139_c1_g4	-1.54	down	gibberellin 20-oxidase [Populus alba]	3.02	13.44
map00904	Diterpenoid biosynthesis	3.34E-01	TRINITY_DN12225_c0_g1	3.22	up	putative oxidoreductase-like family protein [Populus trichocarpa]	22.80	3.06
map00904	Diterpenoid biosynthesis	3.34E-01	TRINITY_DN14119_c0_g1	-1.37	down	hypothetical protein POPTR_0009s03100g [Populus trichocarpa]	1.68	7.93
map00904	Diterpenoid biosynthesis	3.34E-01	TRINITY_DN16605_c0_g1	2.02	up	oxidoreductase family protein [Populus trichocarpa]	3.89	1.45
map00904	Diterpenoid biosynthesis	3.34E-01	TRINITY_DN17270_c0_g1	1.47	up	PREDICTED: momilactone A synthase-like [Populus euphratica]	4.74	2.60
map00904	Diterpenoid biosynthesis	3.34E-01	TRINITY_DN18101_c0_g1	-2.52	down	PREDICTED: gibberellin 2-beta-dioxygenase 2 [Populus euphratica]	1.45	15.83
map00904	Diterpenoid biosynthesis	3.34E-01	TRINITY_DN18715_c0_g1	-3.55	down	PREDICTED: gibberellin 20 oxidase 2-like [Populus euphratica]	0.93	16.65
map00904	Diterpenoid biosynthesis	3.34E-01	TRINITY_DN18975_c0_g1	2.10	up	oxidoreductase family protein [Populus trichocarpa]	18.29	6.07
map00904	Diterpenoid biosynthesis	3.34E-01	TRINITY_DN20976_c0_g1	1.86	up	Casbene synthase, chloroplast precursor, putative [Ricinus communis]	279.70	115.31
map00904	Diterpenoid biosynthesis	3.34E-01	TRINITY_DN21580_c0_g1	1.58	up	PREDICTED: probable iron/ascorbate oxidoreductase DDB_G0283291 isoform X2 [Populus euphratica]	23.05	14.27
map00905	Brassinosteroid biosynthesis	3.34E-01	TRINITY_DN22686_c0_g3	-1.82	down	ROTUNDIFOLIA 3 family protein [Populus trichocarpa]	1.88	11.66
map00905	Brassinosteroid biosynthesis	3.34E-01	TRINITY_DN23773_c1_g3	-1.83	down	brassinosteroid-6-oxidase family protein [Populus trichocarpa]	2.55	13.27
map00905	Brassinosteroid biosynthesis	3.34E-01	TRINITY_DN25914_c0_g2	-2.26	down	PREDICTED: cytochrome P450 90B1 [Populus euphratica]	4.82	34.07
map00905	Brassinosteroid biosynthesis	3.34E-01	TRINITY_DN26463_c0_g1	1.00	up	hypothetical protein POPTR_0010s19710g [Populus trichocarpa]	37.16	25.77
map00905	Brassinosteroid biosynthesis	3.34E-01	TRINITY_DN26562_c0_g1	-1.27	down	PREDICTED: cytochrome P450 87A3-like isoform X3 [Populus euphratica]	2.79	10.43
map00905	Brassinosteroid biosynthesis	3.34E-01	TRINITY_DN15706_c0_g1	-2.00	down	hypothetical protein POPTR_0018s06550g [Populus trichocarpa]	0.55	3.38
map00905	Brassinosteroid biosynthesis	3.34E-01	TRINITY_DN17721_c0_g1	1.49	up	hypothetical protein POPTR_0010s14910g [Populus trichocarpa]	6.20	3.46

map00905	Brassinosteroid biosynthesis	3.34E-01	TRINITY_DN18050_c0_g2	-1.11	down	PREDICTED: PRA1 family protein E-like [Populus euphratica]	0.92	3.02
map00905	Brassinosteroid biosynthesis	3.34E-01	TRINITY_DN19185_c1_g6	-2.75	down	PREDICTED: beta-amyrin 28-oxidase-like [Populus euphratica]	0.23	2.53
map00905	Brassinosteroid biosynthesis	3.34E-01	TRINITY_DN19412_c0_g1	2.36	up	hypothetical protein POPTR_0004s01730g [Populus trichocarpa]	30.47	11.30
map00905	Brassinosteroid biosynthesis	3.34E-01	TRINITY_DN19997_c0_g2	2.21	up	PREDICTED: cytochrome P450 72A15-like isoform X1 [Populus euphratica]	25.07	10.83
map00905	Brassinosteroid biosynthesis	3.34E-01	TRINITY_DN20401_c1_g1	4.90	up	PREDICTED: 3-epi-6-deoxocathasterone 23-monooxygenase-like isoform X2 [Populus euphratica]	2.81	0.14
map00905	Brassinosteroid biosynthesis	3.34E-01	TRINITY_DN20401_c1_g2	1.59	up	cytochrome P450 family protein [Populus trichocarpa]	7.41	2.50
map00905	Brassinosteroid biosynthesis	3.34E-01	TRINITY_DN90_c0_g1	-2.07	down	hypothetical protein POPTR_0014s16980g [Populus trichocarpa]	0.28	1.81
map00460	Cyanoamino acid metabolism	3.39E-01	TRINITY_DN22812_c0_g2	1.66	up	Serine hydroxymethyltransferase family protein [Populus trichocarpa]	785.78	366.57
map00460	Cyanoamino acid metabolism	3.39E-01	TRINITY_DN22812_c0_g3	1.77	up	mitochondrial serine hydroxymethyltransferase [Populus tremuloides]	59.98	26.65
map00460	Cyanoamino acid metabolism	3.39E-01	TRINITY_DN23043_c0_g1	1.04	up	PREDICTED: lysosomal beta glucosidase-like [Populus euphratica]	13.89	10.05
map00460	Cyanoamino acid metabolism	3.39E-01	TRINITY_DN23349_c0_g1	2.04	up	Cysteine synthase C1 [Theobroma cacao]	171.68	63.08
map00460	Cyanoamino acid metabolism	3.39E-01	TRINITY_DN23675_c0_g1	-2.55	down	hypothetical protein POPTR_0001s42050g [Populus trichocarpa]	0.38	3.34
map00460	Cyanoamino acid metabolism	3.39E-01	TRINITY_DN24348_c0_g3	-2.36	down	PREDICTED: serine carboxypeptidase-like 34 [Populus euphratica]	2.05	16.12
map00460	Cyanoamino acid metabolism	3.39E-01	TRINITY_DN24547_c0_g1	-1.78	down	PREDICTED: serine carboxypeptidase-like 25 [Populus euphratica]	4.43	25.58
map00460	Cyanoamino acid metabolism	3.39E-01	TRINITY_DN24846_c0_g1	1.60	up	PREDICTED: bifunctional nitrilase/nitrile hydratase NIT4A [Populus euphratica]	57.16	28.44
map00460	Cyanoamino acid metabolism	3.39E-01	TRINITY_DN24894_c0_g3	1.50	up	hypothetical protein POPTR_0015s03960g [Populus trichocarpa]	34.92	19.04
map00460	Cyanoamino acid metabolism	3.39E-01	TRINITY_DN25105_c0_g1	-1.23	down	PREDICTED: glucose-6-phosphate/phosphate translocator 1, chloroplastic-like [Populus euphratica]	4.62	16.65
map00460	Cyanoamino acid metabolism	3.39E-01	TRINITY_DN25225_c0_g2	1.17	up	hypothetical protein POPTR_0019s05340g [Populus trichocarpa]	20.38	13.56
map00460	Cyanoamino acid metabolism	3.39E-01	TRINITY_DN26632_c0_g1	-1.10	down	hypothetical protein POPTR_0012s10730g [Populus trichocarpa]	14.84	44.17
map00460	Cyanoamino acid metabolism	3.39E-01	TRINITY_DN26643_c1_g2	-1.33	down	PREDICTED: vicianin hydrolase-like [Populus euphratica]	12.50	47.56
map00460	Cyanoamino acid metabolism	3.39E-01	TRINITY_DN27220_c0_g1	-1.11	down	PREDICTED: uncharacterized protein LOC105130123 [Populus euphratica]	10.28	33.40
map00460	Cyanoamino acid metabolism	3.39E-01	TRINITY_DN27725_c1_g1	-1.29	down	PREDICTED: protein argonaute 4A-like [Populus euphratica]	17.30	78.71
map00460	Cyanoamino acid metabolism	3.39E-01	TRINITY_DN13782_c0_g1	5.00	up	hypothetical protein POPTR_0002s11010g [Populus trichocarpa]	6.55	0.32
map00460	Cyanoamino acid metabolism	3.39E-01	TRINITY_DN16091_c0_g1	1.63	up	PREDICTED: xylulose 5-phosphate/phosphate translocator, chloroplastic [Populus euphratica]	27.10	13.24
map00460	Cyanoamino acid metabolism	3.39E-01	TRINITY_DN16387_c0_g1	3.61	up	glycosyl hydrolase family 1 family protein [Populus trichocarpa]	7.38	0.93
map00460	Cyanoamino acid metabolism	3.39E-01	TRINITY_DN16795_c0_g1	2.85	up	hydroxyisourate hydrolase family protein [Populus trichocarpa]	3.65	0.76
map00460	Cyanoamino acid metabolism	3.39E-01	TRINITY_DN17055_c0_g1	2.20	up	PREDICTED: serine carboxypeptidase-like 40 isoform X1 [Populus euphratica]	5.66	1.87
map00460	Cyanoamino acid metabolism	3.39E-01	TRINITY_DN17865_c0_g1	2.27	up	plastid serine hydroxymethyltransferase [Populus tremuloides]	53.46	17.12
map00460	Cyanoamino acid metabolism	3.39E-01	TRINITY_DN18278_c0_g1	-1.11	down	vesicle-associated membrane family protein [Populus trichocarpa]	3.07	10.07
map00460	Cyanoamino acid metabolism	3.39E-01	TRINITY_DN18598_c0_g1	1.82	up	hypothetical protein POPTR_0008s00350g [Populus trichocarpa]	468.84	197.84
map00460	Cyanoamino acid metabolism	3.39E-01	TRINITY_DN18944_c0_g1	1.39	up	hypothetical protein POPTR_0001s35130g [Populus trichocarpa]	11.16	6.52
map00460	Cyanoamino acid metabolism	3.39E-01	TRINITY_DN19274_c0_g1	1.06	up	hypothetical protein POPTR_0001s29510g [Populus trichocarpa]	74.48	55.73
map00460	Cyanoamino acid metabolism	3.39E-01	TRINITY_DN19781_c0_g1	-1.53	down	ovate family protein [Populus trichocarpa]	3.48	15.24
map00460	Cyanoamino acid metabolism	3.39E-01	TRINITY_DN19781_c0_g2	-1.44	down	ovate family protein [Populus trichocarpa]	2.88	11.53
map00460	Cyanoamino acid metabolism	3.39E-01	TRINITY_DN19843_c0_g1	-1.21	down	PREDICTED: serine carboxypeptidase-like 45 [Populus euphratica]	4.18	14.76
map00460	Cyanoamino acid metabolism	3.39E-01	TRINITY_DN19843_c0_g3	3.54	up	PREDICTED: serine carboxypeptidase-like 45 [Populus euphratica]	3.08	0.40
map00460	Cyanoamino acid metabolism	3.39E-01	TRINITY_DN20034_c0_g1	3.46	up	hypothetical protein POPTR_0006s03440g [Populus trichocarpa]	16.65	2.22
map00460	Cyanoamino acid metabolism	3.39E-01	TRINITY_DN20528_c1_g2	1.58	up	hypothetical protein POPTR_0007s07690g [Populus trichocarpa]	52.93	29.17
map00460	Cyanoamino acid metabolism	3.39E-01	TRINITY_DN21031_c0_g2	-1.01	down	serine carboxypeptidase S10 family protein [Populus trichocarpa]	9.81	29.23
map00520	Amino sugar and nucleotide sugar metabolism	3.48E-01	TRINITY_DN21640_c0_g1	-1.46	down	hypothetical protein POPTR_0019s01500g [Populus trichocarpa]	5.06	14.44
map00520	Amino sugar and nucleotide sugar metabolism	3.48E-01	TRINITY_DN21692_c0_g1	-2.02	down	PREDICTED: probable beta-D-xylosidase 2 [Populus euphratica]	0.65	3.58

map00520	Amino sugar and nucleotide sugar metabolism	3.48E-01	TRINITY_DN21919_c0_g3	1.75	up	glycosyl transferase family 8 family protein [Populus trichocarpa]	34.19	15.23
map00520	Amino sugar and nucleotide sugar metabolism	3.48E-01	TRINITY_DN22066_c0_g1	1.09	up	hypothetical protein POPTR_0003s12000g [Populus trichocarpa]	72.83	51.13
map00520	Amino sugar and nucleotide sugar metabolism	3.48E-01	TRINITY_DN22085_c0_g1	-6.92	down	chitinase family protein [Populus trichocarpa]	0.89	158.15
map00520	Amino sugar and nucleotide sugar metabolism	3.48E-01	TRINITY_DN22088_c1_g2	-4.03	down	hypothetical protein POPTR_0004s02550g [Populus trichocarpa]	0.24	5.78
map00520	Amino sugar and nucleotide sugar metabolism	3.48E-01	TRINITY_DN22164_c1_g3	-1.14	down	hypothetical protein POPTR_0006s19240g [Populus trichocarpa]	45.77	151.67
map00520	Amino sugar and nucleotide sugar metabolism	3.48E-01	TRINITY_DN22233_c0_g1	1.51	up	hypothetical protein POPTR_0002s10420g [Populus trichocarpa]	84.73	47.59
map00520	Amino sugar and nucleotide sugar metabolism	3.48E-01	TRINITY_DN22242_c0_g1	-1.16	down	hypothetical protein POPTR_0004s04410g [Populus trichocarpa]	1.74	6.75
map00520	Amino sugar and nucleotide sugar metabolism	3.48E-01	TRINITY_DN22413_c1_g10	2.03	up	hypothetical protein VITISV_005279 [Vitis vinifera]	2.39	0.85
map00520	Amino sugar and nucleotide sugar metabolism	3.48E-01	TRINITY_DN22570_c0_g3	-3.17	down	PREDICTED: probable LRR receptor-like serine/threonine-protein kinase At1g56140 isoform X1 [Populus euphratica]	0.77	10.97
map00520	Amino sugar and nucleotide sugar metabolism	3.48E-01	TRINITY_DN22570_c0_g5	-2.17	down	hypothetical protein POPTR_0001s42040g [Populus trichocarpa]	1.90	11.91
map00520	Amino sugar and nucleotide sugar metabolism	3.48E-01	TRINITY_DN22754_c0_g1	1.32	up	hypothetical protein POPTR_0005s24170g [Populus trichocarpa]	22.62	13.77
map00520	Amino sugar and nucleotide sugar metabolism	3.48E-01	TRINITY_DN22944_c0_g1	1.68	up	PREDICTED: beta-xylosidase/alpha-L-arabinofuranosidase 1-like [Populus euphratica]	51.43	26.38
map00520	Amino sugar and nucleotide sugar metabolism	3.48E-01	TRINITY_DN22944_c0_g3	-1.16	down	PREDICTED: probable beta-D-xylosidase 7 [Populus euphratica]	9.58	31.73
map00520	Amino sugar and nucleotide sugar metabolism	3.48E-01	TRINITY_DN23068_c1_g1	-1.79	down	PREDICTED: probable galacturonosyltransferase-like 9 [Populus euphratica]	3.69	19.63
map00520	Amino sugar and nucleotide sugar metabolism	3.48E-01	TRINITY_DN23208_c0_g1	-1.01	down	PREDICTED: oxysterol-binding protein-related protein 1D-like [Populus euphratica]	5.04	20.81
map00520	Amino sugar and nucleotide sugar metabolism	3.48E-01	TRINITY_DN23287_c0_g1	1.08	up	hypothetical protein POPTR_0001s34380g [Populus trichocarpa]	55.40	39.31
map00520	Amino sugar and nucleotide sugar metabolism	3.48E-01	TRINITY_DN23403_c0_g1	1.10	up	hypothetical protein POPTR_0005s23960g [Populus trichocarpa]	80.55	57.95
map00520	Amino sugar and nucleotide sugar metabolism	3.48E-01	TRINITY_DN23675_c1_g3	-2.51	down	PREDICTED: probable LRR receptor-like serine/threonine-protein kinase At1g07650 isoform X2 [Populus euphratica]	1.47	13.45
map00520	Amino sugar and nucleotide sugar metabolism	3.48E-01	TRINITY_DN23675_c1_g5	-3.53	down	hypothetical protein POPTR_0001s42030g [Populus trichocarpa]	0.11	2.22
map00520	Amino sugar and nucleotide sugar metabolism	3.48E-01	TRINITY_DN23823_c0_g1	1.36	up	PREDICTED: cellulose synthase-like protein E6 [Populus euphratica]	29.77	17.92
map00520	Amino sugar and nucleotide sugar metabolism	3.48E-01	TRINITY_DN23842_c0_g2	1.20	up	PREDICTED: UDP-glucuronate 4-epimerase 6-like [Populus euphratica]	59.05	37.92
map00520	Amino sugar and nucleotide sugar metabolism	3.48E-01	TRINITY_DN23925_c0_g3	-2.42	down	UDP-glucuronic acid decarboxylase 2 [Populus tomentosa]	5.90	48.05
map00520	Amino sugar and nucleotide sugar metabolism	3.48E-01	TRINITY_DN24105_c1_g3	-2.06	down	hypothetical protein TSUD_254980 [Trifolium subterraneum]	230.28	1159.28
map00520	Amino sugar and nucleotide sugar metabolism	3.48E-01	TRINITY_DN24182_c0_g1	-2.00	down	PREDICTED: cysteine-rich receptor-like protein kinase 42 [Populus euphratica]	1.24	7.79
map00520	Amino sugar and nucleotide sugar metabolism	3.48E-01	TRINITY_DN24204_c0_g11	-3.51	down	hypothetical protein POPTR_0019s11610g [Populus trichocarpa]	0.28	4.95
map00520	Amino sugar and nucleotide sugar metabolism	3.48E-01	TRINITY_DN24209_c0_g1	-2.35	down	PREDICTED: cellulose synthase-like protein D3 [Populus euphratica]	1.25	9.86
map00520	Amino sugar and nucleotide sugar metabolism	3.48E-01	TRINITY_DN24253_c0_g4	1.59	up	PREDICTED: cysteine-rich receptor-like protein kinase 15 [Populus euphratica]	14.41	7.37
map00520	Amino sugar and nucleotide sugar metabolism	3.48E-01	TRINITY_DN24360_c1_g1	-1.54	down	hypothetical protein POPTR_0011s03220g [Populus trichocarpa]	6.00	19.41
map00520	Amino sugar and nucleotide sugar metabolism	3.48E-01	TRINITY_DN24360_c1_g3	1.30	up	PREDICTED: G-type lectin S-receptor-like serine/threonine-protein kinase CES101 isoform X2 [Populus euphratica]	5.43	2.63
map00520	Amino sugar and nucleotide sugar metabolism	3.48E-01	TRINITY_DN24360_c1_g4	-1.98	down	hypothetical protein POPTR_0001s43180g [Populus trichocarpa]	3.62	16.53
map00520	Amino sugar and nucleotide sugar metabolism	3.48E-01	TRINITY_DN24360_c1_g7	-2.78	down	cysteine-rich receptor-like protein kinase 29 [Populus tomentosa]	0.25	2.69
map00520	Amino sugar and nucleotide sugar metabolism	3.48E-01	TRINITY_DN24461_c0_g2	-2.63	down	PREDICTED: cysteine-rich receptor-like protein kinase 10 [Populus euphratica]	2.76	25.59

map00520	Amino sugar and nucleotide sugar metabolism	3.48E-01	TRINITY_DN24461_c0_g3	-1.53	down	PREDICTED: cysteine-rich receptor-like protein kinase 10 [Populus euphratica]	2.26	9.66
map00520	Amino sugar and nucleotide sugar metabolism	3.48E-01	TRINITY_DN24550_c0_g2	1.52	up	pfkB-type carbohydrate kinase family protein [Populus trichocarpa]	100.10	51.39
map00520	Amino sugar and nucleotide sugar metabolism	3.48E-01	TRINITY_DN24623_c0_g2	1.54	up	hypothetical protein POPTR_0008s11770g [Populus trichocarpa]	42.50	22.04
map00520	Amino sugar and nucleotide sugar metabolism	3.48E-01	TRINITY_DN24850_c2_g2	2.79	up	hypothetical protein POPTR_0019s14280g [Populus trichocarpa]	12.20	2.73
map00520	Amino sugar and nucleotide sugar metabolism	3.48E-01	TRINITY_DN24875_c0_g1	-1.20	down	UDP-XYLOSE SYNTHASE 4 family protein [Populus trichocarpa]	20.20	83.94
map00520	Amino sugar and nucleotide sugar metabolism	3.48E-01	TRINITY_DN24996_c0_g1	2.19	up	ADP-glucose pyrophosphorylase family protein [Populus trichocarpa]	108.63	36.08
map00520	Amino sugar and nucleotide sugar metabolism	3.48E-01	TRINITY_DN25485_c0_g1	-1.34	down	kinase family protein [Populus trichocarpa]	1.88	6.89
map00520	Amino sugar and nucleotide sugar metabolism	3.48E-01	TRINITY_DN25497_c1_g1	-1.61	down	chitinase 7 [Populus x canadensis]	5.06	22.67
map00520	Amino sugar and nucleotide sugar metabolism	3.48E-01	TRINITY_DN25633_c0_g5	-4.43	down	PREDICTED: acidic endochitinase-like [Populus euphratica]	0.13	4.28
map00520	Amino sugar and nucleotide sugar metabolism	3.48E-01	TRINITY_DN25692_c0_g1	-1.21	down	AAA-type ATPase family protein [Populus trichocarpa]	9.04	31.77
map00520	Amino sugar and nucleotide sugar metabolism	3.48E-01	TRINITY_DN25692_c0_g2	-1.31	down	PREDICTED: pachytene checkpoint protein 2 homolog [Populus euphratica]	12.27	47.09
map00520	Amino sugar and nucleotide sugar metabolism	3.48E-01	TRINITY_DN25892_c1_g4	1.32	up	unknown [Populus trichocarpa x Populus deltoides]	56.52	36.35
map00520	Amino sugar and nucleotide sugar metabolism	3.48E-01	TRINITY_DN26175_c0_g1	1.33	up	PREDICTED: hexokinase-1-like [Populus euphratica]	24.35	14.67
map00520	Amino sugar and nucleotide sugar metabolism	3.48E-01	TRINITY_DN26284_c1_g1	2.20	up	hypothetical protein VITISV_025518 [Vitis vinifera]	3.90	1.29
map00520	Amino sugar and nucleotide sugar metabolism	3.48E-01	TRINITY_DN26284_c1_g3	1.51	up	hypothetical protein POPTR_0008s07890g [Populus trichocarpa]	53.04	28.53
map00520	Amino sugar and nucleotide sugar metabolism	3.48E-01	TRINITY_DN26309_c0_g1	1.34	up	Galactokinase family protein [Populus trichocarpa]	38.22	22.85
map00520	Amino sugar and nucleotide sugar metabolism	3.48E-01	TRINITY_DN26451_c2_g1	2.24	up	PREDICTED: probable receptor-like protein kinase At5g39030 [Populus euphratica]	5.27	2.06
map00520	Amino sugar and nucleotide sugar metabolism	3.48E-01	TRINITY_DN26451_c2_g3	-3.95	down	PREDICTED: probable receptor-like protein kinase At5g39020 [Populus euphratica]	0.18	4.41
map00520	Amino sugar and nucleotide sugar metabolism	3.48E-01	TRINITY_DN26451_c2_g4	-3.40	down	hypothetical protein POPTR_0007s00780g [Populus trichocarpa]	0.81	11.83
map00520	Amino sugar and nucleotide sugar metabolism	3.48E-01	TRINITY_DN26451_c2_g5	2.92	up	PREDICTED: probable receptor-like protein kinase At5g39030 [Populus euphratica]	8.72	1.89
map00520	Amino sugar and nucleotide sugar metabolism	3.48E-01	TRINITY_DN26613_c0_g1	-1.77	down	hypothetical protein POPTR_0012s01760g [Populus trichocarpa]	7.84	53.29
map00520	Amino sugar and nucleotide sugar metabolism	3.48E-01	TRINITY_DN26743_c0_g1	1.15	up	RecName: Full=Phosphoglucomutase, cytoplasmic; Short=PGM; AltName: Full=Glucose phosphomutase	74.05	53.71
map00520	Amino sugar and nucleotide sugar metabolism	3.48E-01	TRINITY_DN26996_c1_g1	1.79	up	PREDICTED: phosphoglucomutase, chloroplastic-like [Populus euphratica]	112.47	49.80
map00520	Amino sugar and nucleotide sugar metabolism	3.48E-01	TRINITY_DN26999_c0_g1	1.40	up	UDP-glucose pyrophosphorylase [Populus tremula x Populus tremuloides]	112.13	68.32
map00520	Amino sugar and nucleotide sugar metabolism	3.48E-01	TRINITY_DN27423_c2_g2	-2.14	down	hypothetical protein POPTR_0001s42040g [Populus trichocarpa]	1.69	6.95
map00520	Amino sugar and nucleotide sugar metabolism	3.48E-01	TRINITY_DN27423_c2_g3	-3.94	down	PREDICTED: G-type lectin S-receptor-like serine/threonine-protein kinase At4g27290 [Populus euphratica]	0.13	3.28
map00520	Amino sugar and nucleotide sugar metabolism	3.48E-01	TRINITY_DN27423_c2_g5	-3.86	down	hypothetical protein POPTR_0011s12880g [Populus trichocarpa]	0.11	2.63
map00520	Amino sugar and nucleotide sugar metabolism	3.48E-01	TRINITY_DN27487_c1_g1	-2.85	down	PREDICTED: probable L-type lectin-domain containing receptor kinase V.3 isoform X2 [Populus euphratica]	1.30	13.85
map00520	Amino sugar and nucleotide sugar metabolism	3.48E-01	TRINITY_DN27487_c1_g2	-2.45	down	PREDICTED: probable receptor-like protein kinase At5g39020, partial [Populus euphratica]	2.68	9.82
map00520	Amino sugar and nucleotide sugar metabolism	3.48E-01	TRINITY_DN27639_c1_g2	-2.45	down	leucine-rich repeat family protein [Populus trichocarpa]	4.41	22.79
map00520	Amino sugar and nucleotide sugar metabolism	3.48E-01	TRINITY_DN11544_c0_g1	-4.56	down	PREDICTED: uncharacterized protein LOC107880749 isoform X1 [Prunus mume]	0.13	4.63
map00520	Amino sugar and nucleotide sugar metabolism	3.48E-01	TRINITY_DN14944_c0_g1	-4.22	down	chitinase family protein [Populus trichocarpa]	0.85	23.24

map00520	Amino sugar and nucleotide sugar metabolism	3.48E-01	TRINITY_DN15018_c0_g1	1.31	up	hypothetical protein POPTR_0007s01450g [Populus trichocarpa]	29.37	18.34
map00520	Amino sugar and nucleotide sugar metabolism	3.48E-01	TRINITY_DN15382_c0_g2	1.10	up	PREDICTED: probable cellulose synthase A catalytic subunit 3 [UDP-forming] [Populus euphratica]	22.15	15.51
map00520	Amino sugar and nucleotide sugar metabolism	3.48E-01	TRINITY_DN15757_c2_g1	-2.60	down	PREDICTED: probable receptor-like protein kinase At5g39020 [Populus euphratica]	0.54	5.65
map00520	Amino sugar and nucleotide sugar metabolism	3.48E-01	TRINITY_DN15757_c2_g2	-2.68	down	PREDICTED: probable receptor-like protein kinase At1g67000 [Populus euphratica]	0.41	4.26
map00520	Amino sugar and nucleotide sugar metabolism	3.48E-01	TRINITY_DN15908_c0_g2	-8.12	down	hypothetical protein POPTR_0004s09630g [Populus trichocarpa]	0.00	2.55
map00520	Amino sugar and nucleotide sugar metabolism	3.48E-01	TRINITY_DN16063_c0_g3	-1.33	down	S-locus lectin protein kinase [Populus trichocarpa]	1.79	6.89
map00520	Amino sugar and nucleotide sugar metabolism	3.48E-01	TRINITY_DN16376_c0_g1	1.84	up	hypothetical protein POPTR_0014s16990g [Populus trichocarpa]	212.37	90.30
map00520	Amino sugar and nucleotide sugar metabolism	3.48E-01	TRINITY_DN16549_c0_g1	-2.65	down	Endochitinase 2 family protein [Populus trichocarpa]	0.77	6.73
map00520	Amino sugar and nucleotide sugar metabolism	3.48E-01	TRINITY_DN16713_c0_g2	3.08	up	PREDICTED: L-type lectin-domain containing receptor kinase VIII.1-like [Populus euphratica]	11.35	2.08
map00520	Amino sugar and nucleotide sugar metabolism	3.48E-01	TRINITY_DN16741_c0_g2	-2.09	down	class1 chitinase family protein [Populus trichocarpa]	0.48	3.20
map00520	Amino sugar and nucleotide sugar metabolism	3.48E-01	TRINITY_DN16897_c0_g1	1.03	up	glycosyl transferase family 8 family protein [Populus trichocarpa]	8.61	10.24
map00520	Amino sugar and nucleotide sugar metabolism	3.48E-01	TRINITY_DN16972_c0_g1	-2.15	down	hypothetical protein POPTR_0017s09250g [Populus trichocarpa]	3.90	24.20
map00520	Amino sugar and nucleotide sugar metabolism	3.48E-01	TRINITY_DN16978_c0_g1	-4.84	down	chitinase 2 [Populus x canadensis]	1.45	62.24
map00520	Amino sugar and nucleotide sugar metabolism	3.48E-01	TRINITY_DN17059_c0_g2	1.13	up	UDP-glucose pyrophosphorylase [Populus deltoides]	101.22	72.02
map00520	Amino sugar and nucleotide sugar metabolism	3.48E-01	TRINITY_DN17143_c0_g1	2.38	up	unknown [Populus trichocarpa x Populus deltoides]	264.27	75.39
map00520	Amino sugar and nucleotide sugar metabolism	3.48E-01	TRINITY_DN17167_c0_g1	-1.21	down	GHMP kinase-related family protein [Populus trichocarpa]	3.51	12.23
map00520	Amino sugar and nucleotide sugar metabolism	3.48E-01	TRINITY_DN17229_c0_g1	-1.51	down	hypothetical protein POPTR_0014s03490g [Populus trichocarpa]	1.22	5.46
map00520	Amino sugar and nucleotide sugar metabolism	3.48E-01	TRINITY_DN17738_c0_g1	-1.93	down	glycosyltransferase 8E [Populus tremula x Populus alba]	1.69	10.09
map00520	Amino sugar and nucleotide sugar metabolism	3.48E-01	TRINITY_DN17790_c0_g1	2.85	up	hypothetical protein POPTR_0018s12000g [Populus trichocarpa]	5.61	1.19
map00520	Amino sugar and nucleotide sugar metabolism	3.48E-01	TRINITY_DN17793_c0_g1	-4.25	down	PREDICTED: glycerophosphodiester phosphodiesterase protein kinase domain-containing GDPDL2-like [Populus euphratica]	0.70	19.91
map00520	Amino sugar and nucleotide sugar metabolism	3.48E-01	TRINITY_DN17999_c0_g2	1.21	up	PREDICTED: NADH-cytochrome b5 reductase-like protein [Populus euphratica]	44.41	29.39
map00520	Amino sugar and nucleotide sugar metabolism	3.48E-01	TRINITY_DN18072_c0_g2	-3.95	down	class 4 pathogenesis-related family protein [Populus trichocarpa]	7.11	158.62
map00520	Amino sugar and nucleotide sugar metabolism	3.48E-01	TRINITY_DN18119_c1_g1	1.17	up	hypothetical protein POPTR_0013s06360g [Populus trichocarpa]	131.23	89.11
map00520	Amino sugar and nucleotide sugar metabolism	3.48E-01	TRINITY_DN18222_c1_g1	1.16	up	PREDICTED: UDP-sugar pyrophosphorylase [Populus euphratica]	28.36	19.26
map00520	Amino sugar and nucleotide sugar metabolism	3.48E-01	TRINITY_DN18225_c0_g3	1.29	up	PREDICTED: hexokinase-1-like [Populus euphratica]	38.97	24.40
map00520	Amino sugar and nucleotide sugar metabolism	3.48E-01	TRINITY_DN18292_c0_g1	-5.00	down	PREDICTED: endochitinase PR4-like [Populus euphratica]	0.17	8.89
map00520	Amino sugar and nucleotide sugar metabolism	3.48E-01	TRINITY_DN18292_c0_g2	-4.23	down	unknown [Populus trichocarpa]	5.75	143.32
map00520	Amino sugar and nucleotide sugar metabolism	3.48E-01	TRINITY_DN18539_c0_g1	-3.08	down	hypothetical protein POPTR_0017s04100g [Populus trichocarpa]	1.11	14.52
map00520	Amino sugar and nucleotide sugar metabolism	3.48E-01	TRINITY_DN18972_c0_g4	-1.41	down	PREDICTED: probable LRR receptor-like serine/threonine-protein kinase RFK1 isoform X1 [Populus euphratica]	1.48	5.13
map00520	Amino sugar and nucleotide sugar metabolism	3.48E-01	TRINITY_DN19010_c0_g1	1.39	up	hypothetical protein POPTR_0016s11690g [Populus trichocarpa]	93.49	53.39
map00520	Amino sugar and nucleotide sugar metabolism	3.48E-01	TRINITY_DN19125_c0_g2	-2.22	down	PREDICTED: wall-associated receptor kinase-like 5 isoform X1 [Populus euphratica]	1.10	8.38
map00520	Amino sugar and nucleotide sugar metabolism	3.48E-01	TRINITY_DN19125_c0_g7	-2.27	down	PREDICTED: chitotriosidase-1-like [Populus euphratica]	0.31	2.36

map00520	Amino sugar and nucleotide sugar metabolism	3.48E-01	TRINITY_DN19125_c0_g8	-2.48	down	hypothetical protein POPTR_0011s13080g [Populus trichocarpa]	0.39	3.36
map00520	Amino sugar and nucleotide sugar metabolism	3.48E-01	TRINITY_DN19346_c0_g1	1.13	up	hypothetical protein POPTR_0001s10850g [Populus trichocarpa]	31.58	21.33
map00520	Amino sugar and nucleotide sugar metabolism	3.48E-01	TRINITY_DN19751_c0_g1	-1.26	down	unknown [Populus trichocarpa]	5.54	20.26
map00520	Amino sugar and nucleotide sugar metabolism	3.48E-01	TRINITY_DN19751_c0_g2	-1.99	down	PREDICTED: probable fructokinase-1 [Populus euphratica]	4.28	23.17
map00520	Amino sugar and nucleotide sugar metabolism	3.48E-01	TRINITY_DN19792_c0_g1	1.31	up	ATP-binding-cassette transporter family protein [Populus trichocarpa]	73.85	45.58
map00520	Amino sugar and nucleotide sugar metabolism	3.48E-01	TRINITY_DN20515_c0_g2	-2.97	down	hypothetical protein POPTR_0018s093702g, partial [Populus trichocarpa]	0.75	7.20
map00520	Amino sugar and nucleotide sugar metabolism	3.48E-01	TRINITY_DN20976_c0_g6	1.22	up	glycosyl transferase family 8 family protein [Populus trichocarpa]	2.69	1.76
map00520	Amino sugar and nucleotide sugar metabolism	3.48E-01	TRINITY_DN21062_c0_g1	1.22	up	PREDICTED: hexokinase-1-like [Populus euphratica]	9.26	6.04
map00520	Amino sugar and nucleotide sugar metabolism	3.48E-01	TRINITY_DN21234_c0_g1	-2.22	down	kinase family protein [Populus trichocarpa]	0.25	1.82
map00520	Amino sugar and nucleotide sugar metabolism	3.48E-01	TRINITY_DN21343_c0_g5	-2.08	down	hypothetical protein TSUD_72310 [Trifolium subterraneum]	0.74	3.53
map00520	Amino sugar and nucleotide sugar metabolism	3.48E-01	TRINITY_DN21582_c0_g1	2.42	up	PREDICTED: fructokinase-like 2, chloroplastic [Populus euphratica]	83.87	26.93
map00520	Amino sugar and nucleotide sugar metabolism	3.48E-01	TRINITY_DN3176_c0_g1	-3.43	down	PREDICTED: G-type lectin S-receptor-like serine/threonine-protein kinase At5g24080 [Populus euphratica]	0.13	2.33
map00520	Amino sugar and nucleotide sugar metabolism	3.48E-01	TRINITY_DN7900_c0_g1	1.75	up	Retrovirus-related Pol polyprotein from transposon TNT 1-94 [Cajanus cajan]	2.25	1.02
map00071	Fatty acid degradation	3.59E-01	TRINITY_DN21932_c0_g3	-1.06	down	phosphatase 2C family protein [Populus trichocarpa]	2.53	8.10
map00071	Fatty acid degradation	3.59E-01	TRINITY_DN22186_c0_g1	1.05	up	alpha-hydroxynitrile lyase family protein [Populus trichocarpa]	31.37	22.10
map00071	Fatty acid degradation	3.59E-01	TRINITY_DN23036_c0_g3	-1.08	down	PREDICTED: long chain acyl-CoA synthetase 2 isoform X1 [Populus euphratica]	4.05	12.87
map00071	Fatty acid degradation	3.59E-01	TRINITY_DN24431_c1_g2	-1.25	down	PREDICTED: acyl-coenzyme A oxidase 4, peroxisomal-like isoform X3 [Populus euphratica]	3.95	18.34
map00071	Fatty acid degradation	3.59E-01	TRINITY_DN24627_c0_g1	1.20	up	PREDICTED: peroxisomal fatty acid beta-oxidation multifunctional protein AIM1 isoform X1 [Populus euphratica]	227.07	150.71
map00071	Fatty acid degradation	3.59E-01	TRINITY_DN24628_c0_g1	1.28	up	PREDICTED: aldehyde dehydrogenase family 3 member H1-like [Populus euphratica]	40.39	24.46
map00071	Fatty acid degradation	3.59E-01	TRINITY_DN24815_c0_g1	-1.19	down	multifunctional protein 2 [Populus tomentosa]	12.46	43.51
map00071	Fatty acid degradation	3.59E-01	TRINITY_DN25183_c0_g1	-1.02	down	hypothetical protein POPTR_0013s02220g [Populus trichocarpa]	7.46	25.55
map00071	Fatty acid degradation	3.59E-01	TRINITY_DN25630_c0_g1	1.30	up	aldehyde dehydrogenase 1 precursor family protein [Populus trichocarpa]	120.35	73.18
map00071	Fatty acid degradation	3.59E-01	TRINITY_DN26025_c0_g1	-1.71	down	hypothetical protein POPTR_0004s18340g [Populus trichocarpa]	5.60	27.70
map00071	Fatty acid degradation	3.59E-01	TRINITY_DN26366_c0_g2	1.69	up	hypothetical protein POPTR_0002s22410g [Populus trichocarpa]	361.54	171.53
map00071	Fatty acid degradation	3.59E-01	TRINITY_DN26421_c0_g1	1.32	up	the aldehyde dehydrogenase cp-ADH from C.plantagineum family protein [Populus trichocarpa]	58.13	34.64
map00071	Fatty acid degradation	3.59E-01	TRINITY_DN26565_c0_g1	1.03	up	hypothetical protein POPTR_0005s19990g [Populus trichocarpa]	60.48	36.43
map00071	Fatty acid degradation	3.59E-01	TRINITY_DN26590_c0_g1	1.01	up	PREDICTED: acyl-CoA dehydrogenase family member 10-like isoform X2 [Populus euphratica]	17.06	13.06
map00071	Fatty acid degradation	3.59E-01	TRINITY_DN15573_c0_g1	-2.43	down	alcohol dehydrogenase family protein [Populus trichocarpa]	0.77	3.49
map00071	Fatty acid degradation	3.59E-01	TRINITY_DN18895_c0_g1	-1.06	down	mitochondrial aldehyde dehydrogenase family protein [Populus trichocarpa]	7.40	23.22
map00071	Fatty acid degradation	3.59E-01	TRINITY_DN19215_c1_g1	-1.38	down	hypothetical protein POPTR_0007s10210g [Populus trichocarpa]	3.47	12.55
map00071	Fatty acid degradation	3.59E-01	TRINITY_DN19215_c2_g1	-1.56	down	PREDICTED: probable protein phosphatase 2C 63 [Populus euphratica]	6.48	30.16
map00071	Fatty acid degradation	3.59E-01	TRINITY_DN19540_c0_g3	1.78	up	hypothetical protein POPTR_0001s45280g [Populus trichocarpa]	3.69	1.58
map00071	Fatty acid degradation	3.59E-01	TRINITY_DN20933_c0_g1	-3.74	down	hypothetical protein POPTR_0002s19330g [Populus trichocarpa]	0.65	13.67
map00071	Fatty acid degradation	3.59E-01	TRINITY_DN20933_c0_g3	-2.76	down	PREDICTED: long chain acyl-CoA synthetase 1 [Populus euphratica]	0.62	6.55
map03430	Mismatch repair	3.78E-01	TRINITY_DN21835_c0_g1	-1.94	down	PREDICTED: chromosome transmission fidelity protein 18 homolog [Populus euphratica]	1.43	8.53
map03430	Mismatch repair	3.78E-01	TRINITY_DN22093_c1_g1	1.58	up	PREDICTED: uncharacterized protein LOC105133503 [Populus euphratica]	28.08	18.04
map03430	Mismatch repair	3.78E-01	TRINITY_DN22222_c2_g1	-1.69	down	PREDICTED: proliferating cell nuclear antigen [Populus euphratica]	20.04	106.79

map03430	Mismatch repair	3.78E-01	TRINITY_DN23841_c0_g3	-1.40	down	PREDICTED: replication protein A 32 kDa subunit A-like [Populus euphratica]	7.83	32.50
map03430	Mismatch repair	3.78E-01	TRINITY_DN24951_c0_g1	2.63	up	unknown [Populus trichocarpa]	40.05	12.23
map03430	Mismatch repair	3.78E-01	TRINITY_DN25007_c0_g1	-1.55	down	TIR-NBS-LRR-TIR type disease resistance protein, partial [Populus trichocarpa]	12.22	41.68
map03430	Mismatch repair	3.78E-01	TRINITY_DN12653_c0_g2	-1.41	down	PREDICTED: exonuclease 1 [Populus euphratica]	0.76	3.12
map03430	Mismatch repair	3.78E-01	TRINITY_DN13077_c0_g1	-3.90	down	unknown [Populus trichocarpa]	0.30	5.95
map03430	Mismatch repair	3.78E-01	TRINITY_DN14981_c0_g1	1.20	up	single-strand-binding family protein [Populus trichocarpa]	44.95	29.84
map03430	Mismatch repair	3.78E-01	TRINITY_DN16607_c0_g1	-1.91	down	PREDICTED: DNA mismatch repair protein MSH7 isoform X1 [Populus euphratica]	0.80	4.64
map03430	Mismatch repair	3.78E-01	TRINITY_DN16624_c0_g1	1.03	up	unknown [Populus trichocarpa x Populus deltoides]	184.06	137.19
map03430	Mismatch repair	3.78E-01	TRINITY_DN16649_c0_g1	-1.48	down	PREDICTED: DNA mismatch repair protein MSH4-like isoform X1 [Populus euphratica]	1.58	6.18
map03430	Mismatch repair	3.78E-01	TRINITY_DN17121_c0_g1	-1.12	down	hypothetical protein POPTR_0005s27010g, partial [Populus trichocarpa]	1.89	7.13
map03430	Mismatch repair	3.78E-01	TRINITY_DN17241_c0_g1	-1.38	down	DNA polymerase delta subunit 4 family protein [Populus trichocarpa]	4.03	15.90
map03430	Mismatch repair	3.78E-01	TRINITY_DN17241_c0_g2	-1.21	down	DNA polymerase delta subunit 4 family protein [Populus trichocarpa]	37.87	132.98
map03430	Mismatch repair	3.78E-01	TRINITY_DN17522_c0_g1	-2.36	down	PREDICTED: replication protein A 70 kDa DNA-binding subunit B [Populus euphratica]	3.63	29.40
map03430	Mismatch repair	3.78E-01	TRINITY_DN17620_c0_g1	1.37	up	hypothetical protein POPTR_0006s14620g, partial [Populus trichocarpa]	1053.56	627.95
map03430	Mismatch repair	3.78E-01	TRINITY_DN18555_c0_g4	2.33	up	Ycf2 [Populus alba]	3.15	0.93
map03430	Mismatch repair	3.78E-01	TRINITY_DN18970_c0_g1	-1.57	down	hypothetical protein POPTR_0008s15120g [Populus trichocarpa]	4.62	19.39
map03430	Mismatch repair	3.78E-01	TRINITY_DN19013_c0_g2	-1.35	down	PREDICTED: histidine kinase CKI1 [Populus euphratica]	2.10	10.03
map03430	Mismatch repair	3.78E-01	TRINITY_DN19198_c0_g1	1.14	up	hypothetical protein POPTR_0002s24970g [Populus trichocarpa]	135.65	95.80
map03430	Mismatch repair	3.78E-01	TRINITY_DN19395_c0_g1	-1.33	down	PREDICTED: replication protein A 70 kDa DNA-binding subunit E-like [Populus euphratica]	2.44	9.54
map03430	Mismatch repair	3.78E-01	TRINITY_DN19537_c0_g3	-1.06	down	PREDICTED: uncharacterized protein LOC105109677 isoform X1 [Populus euphratica]	5.42	16.71
map03430	Mismatch repair	3.78E-01	TRINITY_DN20164_c0_g3	-1.54	down	sterile alpha motif domain-containing family protein [Populus trichocarpa]	3.50	14.58
map03430	Mismatch repair	3.78E-01	TRINITY_DN20672_c0_g1	1.85	up	hypothetical protein POPTR_0006s03190g [Populus trichocarpa]	49.08	22.60
map03430	Mismatch repair	3.78E-01	TRINITY_DN20742_c0_g1	1.62	up	FKBP-type peptidyl-prolyl cis-trans isomerase 3 family protein [Populus trichocarpa]	166.22	81.22
map03430	Mismatch repair	3.78E-01	TRINITY_DN20973_c0_g1	-1.20	down	PREDICTED: DNA polymerase delta catalytic subunit [Populus euphratica]	3.70	13.41
map00290	Valine, leucine and isoleucine biosynthesis	3.80E-01	TRINITY_DN21760_c0_g2	1.32	up	PREDICTED: 3-isopropylmalate dehydrogenase, chloroplastic-like [Populus euphratica]	86.28	52.23
map00290	Valine, leucine and isoleucine biosynthesis	3.80E-01	TRINITY_DN22580_c0_g3	1.24	up	hypothetical protein POPTR_0003s10720g [Populus trichocarpa]	124.71	79.50
map00290	Valine, leucine and isoleucine biosynthesis	3.80E-01	TRINITY_DN22629_c0_g2	1.01	up	PREDICTED: 2-isopropylmalate synthase 2, chloroplastic-like [Populus euphratica]	65.58	50.13
map00290	Valine, leucine and isoleucine biosynthesis	3.80E-01	TRINITY_DN22695_c0_g4	1.16	up	aconitase family protein [Populus trichocarpa]	151.04	102.95
map00290	Valine, leucine and isoleucine biosynthesis	3.80E-01	TRINITY_DN25630_c0_g3	-2.44	down	PREDICTED: branched-chain-amino-acid aminotransferase 2, chloroplastic-like isoform X1 [Populus euphratica]	0.50	3.81
map00290	Valine, leucine and isoleucine biosynthesis	3.80E-01	TRINITY_DN26976_c0_g1	1.47	up	PREDICTED: acetolactate synthase 2, chloroplastic-like [Populus euphratica]	396.57	239.53
map00290	Valine, leucine and isoleucine biosynthesis	3.80E-01	TRINITY_DN19518_c0_g1	2.25	up	hypothetical protein POPTR_0016s14360g [Populus trichocarpa]	277.40	89.48
map00290	Valine, leucine and isoleucine biosynthesis	3.80E-01	TRINITY_DN20583_c0_g3	2.29	up	hypothetical protein POPTR_0008s03820g [Populus trichocarpa]	4.09	1.88
map00290	Valine, leucine and isoleucine biosynthesis	3.80E-01	TRINITY_DN21313_c0_g2	-1.72	down	hypothetical protein POPTR_0014s07170g [Populus trichocarpa]	4.63	24.85
map00290	Valine, leucine and isoleucine biosynthesis	3.80E-01	TRINITY_DN21600_c0_g2	1.23	up	PREDICTED: acetolactate synthase small subunit 2, chloroplastic-like [Populus euphratica]	35.74	23.13
map00902	Monoterpenoid biosynthesis	3.95E-01	TRINITY_DN23123_c0_g1	-1.18	down	hypothetical protein POPTR_0014s12800g [Populus trichocarpa]	2.38	7.46
map00902	Monoterpenoid biosynthesis	3.95E-01	TRINITY_DN24131_c0_g1	2.15	up	PREDICTED: probable terpene synthase 9 isoform X1 [Populus euphratica]	12.38	4.15
map00902	Monoterpenoid biosynthesis	3.95E-01	TRINITY_DN2415_c0_g1	-2.03	down	terpene synthase [Populus trichocarpa]	2.28	14.59
map00902	Monoterpenoid biosynthesis	3.95E-01	TRINITY_DN26689_c1_g1	-1.19	down	phototropic-responsive NPH3 family protein [Populus trichocarpa]	4.14	15.49
map00902	Monoterpenoid biosynthesis	3.95E-01	TRINITY_DN16965_c0_g1	-1.45	down	hypothetical protein POPTR_0002s24380g [Populus trichocarpa]	1.17	4.85
map00902	Monoterpenoid biosynthesis	3.95E-01	TRINITY_DN21052_c0_g1	2.07	up	hypothetical protein POPTR_0002s15770g [Populus trichocarpa]	7.17	4.84

map00902	Monoterpenoid biosynthesis	3.95E-01	TRINITY_DN5919_c0_g1	-1.96	down	hypothetical protein POPTR_0092s00200g [Populus trichocarpa]	1.33	8.19
map00300	Lysine biosynthesis	3.95E-01	TRINITY_DN23172_c0_g1	1.90	up	hypothetical protein POPTR_0017s00350g [Populus trichocarpa]	54.45	26.38
map00300	Lysine biosynthesis	3.95E-01	TRINITY_DN23916_c0_g1	1.26	up	chloroplast import receptor p36 family protein [Populus trichocarpa]	583.12	369.56
map00300	Lysine biosynthesis	3.95E-01	TRINITY_DN25474_c0_g1	1.34	up	PREDICTED: diaminopimelate decarboxylase 2, chloroplastic-like [Populus euphratica]	144.79	88.69
map00300	Lysine biosynthesis	3.95E-01	TRINITY_DN25737_c0_g1	1.82	up	semialdehyde dehydrogenase family protein [Populus trichocarpa]	56.96	24.76
map00300	Lysine biosynthesis	3.95E-01	TRINITY_DN20058_c0_g1	1.29	up	hypothetical protein POPTR_0009s08570g [Populus trichocarpa]	82.27	50.72
map00300	Lysine biosynthesis	3.95E-01	TRINITY_DN21210_c0_g6	1.13	up	homoserine dehydrogenase family protein [Populus trichocarpa]	16.38	11.90
map00300	Lysine biosynthesis	3.95E-01	TRINITY_DN21635_c1_g2	-2.07	down	SEC14 cytosolic factor family protein [Populus trichocarpa]	5.54	32.41
map00052	Galactose metabolism	4.08E-01	TRINITY_DN21731_c0_g2	-1.12	down	PREDICTED: aldo-keto reductase family 4 member C9-like [Populus euphratica]	3.19	10.75
map00052	Galactose metabolism	4.08E-01	TRINITY_DN22413_c1_g10	2.03	up	hypothetical protein VITISV_005279 [Vitis vinifera]	2.39	0.85
map00052	Galactose metabolism	4.08E-01	TRINITY_DN22568_c0_g1	-1.35	down	PREDICTED: beta-galactosidase 5-like [Populus euphratica]	22.12	82.08
map00052	Galactose metabolism	4.08E-01	TRINITY_DN22624_c0_g1	-1.58	down	hypothetical protein POPTR_0006s13130g [Populus trichocarpa]	2.14	9.80
map00052	Galactose metabolism	4.08E-01	TRINITY_DN23112_c0_g2	1.23	up	hypothetical protein POPTR_0016s00410g [Populus trichocarpa]	32.21	20.47
map00052	Galactose metabolism	4.08E-01	TRINITY_DN23600_c0_g2	-2.70	down	hypothetical protein POPTR_0011s15750g [Populus trichocarpa]	1.22	10.68
map00052	Galactose metabolism	4.08E-01	TRINITY_DN23698_c1_g1	2.67	up	putative galactinol synthase family protein [Populus trichocarpa]	17.66	4.42
map00052	Galactose metabolism	4.08E-01	TRINITY_DN24105_c1_g3	-2.06	down	hypothetical protein TSUD_254980 [Trifolium subterraneum]	230.28	1159.28
map00052	Galactose metabolism	4.08E-01	TRINITY_DN24341_c0_g1	1.63	up	PREDICTED: alpha-glucosidase-like [Populus euphratica]	5.68	2.81
map00052	Galactose metabolism	4.08E-01	TRINITY_DN25513_c0_g2	-1.55	down	PREDICTED: zinc finger protein NUTCRACKER-like isoform X5 [Populus euphratica]	13.47	55.16
map00052	Galactose metabolism	4.08E-01	TRINITY_DN25522_c1_g1	-1.19	down	PREDICTED: zinc finger protein NUTCRACKER-like [Populus euphratica]	4.97	17.73
map00052	Galactose metabolism	4.08E-01	TRINITY_DN25771_c0_g1	2.03	up	cell-wall invertase [Populus alba x Populus grandidentata]	101.29	37.01
map00052	Galactose metabolism	4.08E-01	TRINITY_DN25889_c0_g2	1.59	up	unknown [Populus trichocarpa x Populus deltoides]	384.22	194.87
map00052	Galactose metabolism	4.08E-01	TRINITY_DN25892_c1_g4	1.32	up	unknown [Populus trichocarpa x Populus deltoides]	56.52	36.35
map00052	Galactose metabolism	4.08E-01	TRINITY_DN26010_c0_g4	-1.72	down	hypothetical protein POPTR_0006s13130g [Populus trichocarpa]	3.69	17.16
map00052	Galactose metabolism	4.08E-01	TRINITY_DN26083_c1_g1	-1.54	down	hypothetical protein POPTR_0001s15820g [Populus trichocarpa]	1.93	8.53
map00052	Galactose metabolism	4.08E-01	TRINITY_DN26175_c0_g1	1.33	up	PREDICTED: hexokinase-1-like [Populus euphratica]	24.35	14.67
map00052	Galactose metabolism	4.08E-01	TRINITY_DN26284_c1_g1	2.20	up	hypothetical protein VITISV_025518 [Vitis vinifera]	3.90	1.29
map00052	Galactose metabolism	4.08E-01	TRINITY_DN26309_c0_g1	1.34	up	Galactokinase family protein [Populus trichocarpa]	38.22	22.85
map00052	Galactose metabolism	4.08E-01	TRINITY_DN26337_c3_g2	-1.73	down	PREDICTED: zinc finger protein NUTCRACKER-like [Populus euphratica]	2.46	10.32
map00052	Galactose metabolism	4.08E-01	TRINITY_DN26743_c0_g1	1.15	up	RecName: Full=Phosphoglucomutase, cytoplasmic; Short=PGM; AltName: Full=Glucose phosphomutase	74.05	53.71
map00052	Galactose metabolism	4.08E-01	TRINITY_DN26825_c0_g2	2.18	up	PREDICTED: beta-fructofuranosidase, soluble isoenzyme I-like [Populus euphratica]	96.10	31.98
map00052	Galactose metabolism	4.08E-01	TRINITY_DN26996_c1_g1	1.79	up	PREDICTED: phosphoglucomutase, chloroplastic-like [Populus euphratica]	112.47	49.80
map00052	Galactose metabolism	4.08E-01	TRINITY_DN26999_c0_g1	1.40	up	UDP-glucose pyrophosphorylase [Populus tremula x Populus tremuloides]	112.13	68.32
map00052	Galactose metabolism	4.08E-01	TRINITY_DN27107_c0_g1	-1.71	down	hypothetical protein POPTR_0006s05130g [Populus trichocarpa]	10.71	55.55
map00052	Galactose metabolism	4.08E-01	TRINITY_DN11544_c0_g1	-4.56	down	PREDICTED: uncharacterized protein LOC107880749 isoform X1 [Prunus mume]	0.13	4.63
map00052	Galactose metabolism	4.08E-01	TRINITY_DN14721_c0_g1	-1.05	down	hypothetical protein POPTR_0008s14180g [Populus trichocarpa]	12.06	37.39
map00052	Galactose metabolism	4.08E-01	TRINITY_DN15747_c0_g1	2.03	up	hypothetical protein POPTR_0006s24400g [Populus trichocarpa]	4.17	1.74
map00052	Galactose metabolism	4.08E-01	TRINITY_DN17059_c0_g2	1.13	up	UDP-glucose pyrophosphorylase [Populus deltoides]	101.22	72.02
map00052	Galactose metabolism	4.08E-01	TRINITY_DN17353_c0_g1	1.22	up	PREDICTED: LOW QUALITY PROTEIN: methylecgonone reductase-like [Populus euphratica]	9.67	7.02
map00052	Galactose metabolism	4.08E-01	TRINITY_DN17644_c0_g1	1.22	up	PREDICTED: NADP-dependent D-sorbitol-6-phosphate dehydrogenase-like [Populus euphratica]	152.43	100.73
map00052	Galactose metabolism	4.08E-01	TRINITY_DN18222_c1_g1	1.16	up	PREDICTED: UDP-sugar pyrophosphorylase [Populus euphratica]	28.36	19.26
map00052	Galactose metabolism	4.08E-01	TRINITY_DN18225_c0_g3	1.29	up	PREDICTED: hexokinase-1-like [Populus euphratica]	38.97	24.40
map00052	Galactose metabolism	4.08E-01	TRINITY_DN18466_c0_g1	3.25	up	hypothetical protein POPTR_0018s14920g [Populus trichocarpa]	22.92	4.21
map00052	Galactose metabolism	4.08E-01	TRINITY_DN18863_c1_g4	-2.50	down	raffinose synthase family protein [Populus tomentosa]	0.30	2.68

map00052	Galactose metabolism	4.08E-01	TRINITY_DN19674_c1_g2	1.61	up	beta-galactosidase family protein [Populus trichocarpa]	9.86	4.84
map00052	Galactose metabolism	4.08E-01	TRINITY_DN19978_c0_g1	6.48	up	galactinol synthase family protein [Populus trichocarpa]	82.15	1.43
map00052	Galactose metabolism	4.08E-01	TRINITY_DN20455_c0_g4	-1.61	down	hypothetical protein POPTR_0012s03730g [Populus trichocarpa]	2.77	12.99
map00052	Galactose metabolism	4.08E-01	TRINITY_DN20896_c1_g2	-1.22	down	PREDICTED: protein SHOOT GRAVITROPISM 5 [Populus euphratica]	4.99	19.09
map00052	Galactose metabolism	4.08E-01	TRINITY_DN21062_c0_g1	1.22	up	PREDICTED: hexokinase-1-like [Populus euphratica]	9.26	6.04
map00052	Galactose metabolism	4.08E-01	TRINITY_DN21068_c0_g2	-2.13	down	hypothetical protein POPTR_0004s13470g [Populus trichocarpa]	6.77	46.35
map00052	Galactose metabolism	4.08E-01	TRINITY_DN21068_c0_g3	-4.42	down	PREDICTED: aldose 1-epimerase-like [Populus euphratica]	0.45	14.22
map00052	Galactose metabolism	4.08E-01	TRINITY_DN21343_c0_g5	-2.08	down	hypothetical protein TSUD_72310 [Trifolium subterraneum]	0.74	3.53
map00052	Galactose metabolism	4.08E-01	TRINITY_DN7728_c0_g2	2.29	up	hypothetical protein POPTR_0006s14130g [Populus trichocarpa]	1.77	0.56
map00052	Galactose metabolism	4.08E-01	TRINITY_DN7900_c0_g1	1.75	up	Retrovirus-related Pol polyprotein from transposon TNT 1-94 [Cajanus cajan]	2.25	1.02
map00790	Folate biosynthesis	4.15E-01	TRINITY_DN24692_c0_g1	1.34	up	core region of GTP cyclohydrolase I family protein [Populus trichocarpa]	21.04	12.47
map00790	Folate biosynthesis	4.15E-01	TRINITY_DN24692_c0_g2	1.49	up	core region of GTP cyclohydrolase I family protein [Populus trichocarpa]	12.52	6.66
map00790	Folate biosynthesis	4.15E-01	TRINITY_DN24692_c0_g3	1.25	up	core region of GTP cyclohydrolase I family protein [Populus trichocarpa]	21.23	13.55
map00790	Folate biosynthesis	4.15E-01	TRINITY_DN26164_c0_g1	1.34	up	DHFS-FPGS B family protein [Populus trichocarpa]	45.14	26.03
map00790	Folate biosynthesis	4.15E-01	TRINITY_DN27543_c1_g1	1.50	up	PREDICTED: folylpolyglutamate synthase-like isoform X1 [Populus euphratica]	31.47	17.26
map00790	Folate biosynthesis	4.15E-01	TRINITY_DN27543_c1_g3	1.09	up	PREDICTED: folylpolyglutamate synthase-like isoform X1 [Populus euphratica]	38.72	29.47
map00790	Folate biosynthesis	4.15E-01	TRINITY_DN14655_c0_g1	1.28	up	PREDICTED: folylpolyglutamate synthase-like isoform X1 [Populus euphratica]	26.10	16.23
map00790	Folate biosynthesis	4.15E-01	TRINITY_DN15585_c0_g2	-2.81	down	hypothetical protein POPTR_0015s09940g [Populus trichocarpa]	0.79	7.28
map00790	Folate biosynthesis	4.15E-01	TRINITY_DN15628_c0_g1	1.14	up	molybdenum cofactor synthesis family protein [Populus trichocarpa]	18.69	12.84
map00790	Folate biosynthesis	4.15E-01	TRINITY_DN21267_c0_g1	-3.88	down	PREDICTED: D-amino-acid transaminase, chloroplastic-like [Populus euphratica]	0.85	19.24
map00270	Cysteine and methionine metabolism	4.19E-01	TRINITY_DN22121_c0_g2	-2.23	down	hypothetical protein POPTR_0001s04700g [Populus trichocarpa]	12.32	86.92
map00270	Cysteine and methionine metabolism	4.19E-01	TRINITY_DN22121_c0_g4	-2.34	down	hypothetical protein POPTR_0001s04700g [Populus trichocarpa]	4.13	30.41
map00270	Cysteine and methionine metabolism	4.19E-01	TRINITY_DN22121_c0_g5	-2.01	down	PREDICTED: DNA (cytosine-5)-methyltransferase CMT3-like [Populus euphratica]	1.79	10.94
map00270	Cysteine and methionine metabolism	4.19E-01	TRINITY_DN22171_c2_g1	1.24	up	PREDICTED: S-adenosylmethionine synthase 3 [Populus euphratica]	228.61	137.13
map00270	Cysteine and methionine metabolism	4.19E-01	TRINITY_DN22348_c0_g1	2.11	up	O-acetylserine (thiol)lyase family protein [Populus trichocarpa]	18.41	6.25
map00270	Cysteine and methionine metabolism	4.19E-01	TRINITY_DN22348_c0_g4	1.61	up	O-acetylserine (thiol)lyase family protein [Populus trichocarpa]	220.16	110.90
map00270	Cysteine and methionine metabolism	4.19E-01	TRINITY_DN22348_c0_g6	1.74	up	hypothetical protein POPTR_0013s13150g [Populus trichocarpa]	146.50	66.04
map00270	Cysteine and methionine metabolism	4.19E-01	TRINITY_DN22677_c0_g1	-1.18	down	alpha galactosyltransferase family protein [Populus trichocarpa]	16.78	58.67
map00270	Cysteine and methionine metabolism	4.19E-01	TRINITY_DN22813_c0_g4	1.08	up	aspartate transaminase family protein [Populus trichocarpa]	25.11	18.98
map00270	Cysteine and methionine metabolism	4.19E-01	TRINITY_DN22960_c1_g2	1.71	up	hypothetical protein POPTR_0013s13150g [Populus trichocarpa]	267.00	123.84
map00270	Cysteine and methionine metabolism	4.19E-01	TRINITY_DN23349_c0_g1	2.04	up	Cysteine synthase C1 [Theobroma cacao]	171.68	63.08
map00270	Cysteine and methionine metabolism	4.19E-01	TRINITY_DN23582_c0_g2	-2.44	down	PREDICTED: serine/threonine-protein kinase ATM-like [Populus euphratica]	5.43	44.51
map00270	Cysteine and methionine metabolism	4.19E-01	TRINITY_DN23709_c0_g2	1.23	up	aspartate aminotransferase 2 family protein [Populus trichocarpa]	46.64	32.80
map00270	Cysteine and methionine metabolism	4.19E-01	TRINITY_DN23877_c1_g1	1.12	up	hypothetical protein POPTR_0008s09870g [Populus trichocarpa]	162.62	115.69
map00270	Cysteine and methionine metabolism	4.19E-01	TRINITY_DN23877_c1_g3	1.26	up	PREDICTED: S-adenosylmethionine synthase 4 [Populus euphratica]	32.62	20.75
map00270	Cysteine and methionine metabolism	4.19E-01	TRINITY_DN25221_c0_g1	1.58	up	malate dehydrogenase family protein, partial [Populus trichocarpa]	57.58	29.35
map00270	Cysteine and methionine metabolism	4.19E-01	TRINITY_DN25221_c1_g2	2.25	up	malate dehydrogenase family protein [Populus trichocarpa]	356.58	110.81
map00270	Cysteine and methionine metabolism	4.19E-01	TRINITY_DN25221_c1_g3	1.80	up	PREDICTED: malate dehydrogenase, glyoxysomal [Populus euphratica]	643.81	285.37
map00270	Cysteine and methionine metabolism	4.19E-01	TRINITY_DN25398_c1_g1	1.21	up	PREDICTED: DEAD-box ATP-dependent RNA helicase 3, chloroplastic-like isoform X2 [Populus euphratica]	571.63	379.40
map00270	Cysteine and methionine metabolism	4.19E-01	TRINITY_DN25630_c0_g3	-2.44	down	PREDICTED: branched-chain-amino-acid aminotransferase 2, chloroplastic-like isoform X1 [Populus euphratica]	0.50	3.81
map00270	Cysteine and methionine metabolism	4.19E-01	TRINITY_DN25737_c0_g1	1.82	up	semialdehyde dehydrogenase family protein [Populus trichocarpa]	56.96	24.76

map00270	Cysteine and methionine metabolism	4.19E-01	TRINITY_DN25746_c0_g1	2.80	up	nodule-enhanced malate dehydrogenase family protein [Populus trichocarpa]	113.74	23.22
map00270	Cysteine and methionine metabolism	4.19E-01	TRINITY_DN26210_c0_g1	1.88	up	S-adenosyl-L-homocysteine hydrolase [Populus tomentosa]	301.69	124.53
map00270	Cysteine and methionine metabolism	4.19E-01	TRINITY_DN26342_c0_g1	1.99	up	S-adenosylmethionine decarboxylase family protein [Populus trichocarpa]	348.95	135.54
map00270	Cysteine and methionine metabolism	4.19E-01	TRINITY_DN26665_c0_g1	1.58	up	hypothetical protein POPTR_0009s15490g [Populus trichocarpa]	216.45	108.64
map00270	Cysteine and methionine metabolism	4.19E-01	TRINITY_DN27794_c1_g5	-1.34	down	hypothetical protein POPTR_0008s04420g [Populus trichocarpa]	2.40	9.24
map00270	Cysteine and methionine metabolism	4.19E-01	TRINITY_DN27862_c2_g1	-1.69	down	PREDICTED: structural maintenance of chromosomes protein 2-1-like [Populus euphratica]	21.02	56.92
map00270	Cysteine and methionine metabolism	4.19E-01	TRINITY_DN15789_c0_g1	-2.41	down	PREDICTED: uncharacterized protein LOC105126274 [Populus euphratica]	1.52	12.20
map00270	Cysteine and methionine metabolism	4.19E-01	TRINITY_DN15789_c1_g1	-4.34	down	PREDICTED: uncharacterized protein LOC105135007 [Populus euphratica]	1.24	36.79
map00270	Cysteine and methionine metabolism	4.19E-01	TRINITY_DN16544_c0_g2	-4.08	down	Homocysteine S-methyltransferase 3 family protein [Populus trichocarpa]	0.36	6.85
map00270	Cysteine and methionine metabolism	4.19E-01	TRINITY_DN16732_c0_g1	1.28	up	hypothetical protein POPTR_0017s07130g [Populus trichocarpa]	32.81	20.70
map00270	Cysteine and methionine metabolism	4.19E-01	TRINITY_DN16821_c0_g1	1.91	up	PREDICTED: malate dehydrogenase, glyoxysomal [Populus euphratica]	248.44	104.05
map00270	Cysteine and methionine metabolism	4.19E-01	TRINITY_DN17142_c0_g1	-1.88	down	aminocyclopropane carboxylate oxidase family protein [Populus trichocarpa]	17.61	99.90
map00270	Cysteine and methionine metabolism	4.19E-01	TRINITY_DN17257_c0_g1	-1.22	down	hypothetical protein POPTR_0001s00440g [Populus trichocarpa]	6.45	23.17
map00270	Cysteine and methionine metabolism	4.19E-01	TRINITY_DN17959_c0_g1	2.77	up	PREDICTED: probable S-sulfocysteine synthase, chloroplastic [Populus euphratica]	54.54	12.96
map00270	Cysteine and methionine metabolism	4.19E-01	TRINITY_DN18120_c0_g1	-2.09	down	1-aminocyclopropane-1-carboxylate synthase family protein [Populus trichocarpa]	2.09	14.08
map00270	Cysteine and methionine metabolism	4.19E-01	TRINITY_DN18387_c0_g1	1.43	up	hypothetical protein POPTR_0017s12240g [Populus trichocarpa]	122.22	69.70
map00270	Cysteine and methionine metabolism	4.19E-01	TRINITY_DN18806_c0_g2	2.12	up	PREDICTED: S-adenosylmethionine decarboxylase proenzyme-like isoform X3 [Solanum pennellii]	161.94	56.16
map00270	Cysteine and methionine metabolism	4.19E-01	TRINITY_DN19472_c0_g2	1.32	up	hypothetical protein POPTR_0007s01130g [Populus trichocarpa]	8.54	5.46
map00270	Cysteine and methionine metabolism	4.19E-01	TRINITY_DN19472_c0_g3	-3.23	down	hypothetical protein POPTR_0017s04550g [Populus trichocarpa]	0.21	3.06
map00270	Cysteine and methionine metabolism	4.19E-01	TRINITY_DN19763_c0_g3	1.88	up	PREDICTED: malate dehydrogenase, mitochondrial [Populus euphratica]	247.09	106.19
map00270	Cysteine and methionine metabolism	4.19E-01	TRINITY_DN20175_c0_g1	1.59	up	peroxiredoxin Q family protein [Populus trichocarpa]	1020.77	504.37
map00270	Cysteine and methionine metabolism	4.19E-01	TRINITY_DN20938_c0_g1	1.33	up	PREDICTED: malate dehydrogenase, glyoxysomal [Populus euphratica]	118.58	72.32
map00270	Cysteine and methionine metabolism	4.19E-01	TRINITY_DN20938_c0_g2	2.63	up	malate dehydrogenase family protein [Populus trichocarpa]	74.92	18.32
map00270	Cysteine and methionine metabolism	4.19E-01	TRINITY_DN21013_c0_g3	1.06	up	hypothetical protein POPTR_0008s15720g [Populus trichocarpa]	79.15	58.37
map00270	Cysteine and methionine metabolism	4.19E-01	TRINITY_DN21210_c0_g6	1.13	up	homoserine dehydrogenase family protein [Populus trichocarpa]	16.38	11.90
map00270	Cysteine and methionine metabolism	4.19E-01	TRINITY_DN21391_c0_g1	1.12	up	PREDICTED: uncharacterized protein LOC105124970 [Populus euphratica]	49.29	33.42
map00270	Cysteine and methionine metabolism	4.19E-01	TRINITY_DN21417_c0_g1	1.35	up	SERINE ACETYLTRANSFERASE-106 family protein [Populus trichocarpa]	46.75	26.71
map00270	Cysteine and methionine metabolism	4.19E-01	TRINITY_DN21635_c1_g2	-2.07	down	SEC14 cytosolic factor family protein [Populus trichocarpa]	5.54	32.41
map00270	Cysteine and methionine metabolism	4.19E-01	TRINITY_DN7763_c0_g1	10.19	up	cystathionine gamma-synthase [Populus tomentosa]	18.32	0.02
map00940	Phenylpropanoid biosynthesis	4.40E-01	TRINITY_DN21981_c0_g2	-3.91	down	hypothetical protein POPTR_0001s02320g [Populus trichocarpa]	0.24	5.58
map00940	Phenylpropanoid biosynthesis	4.40E-01	TRINITY_DN22190_c0_g1	-1.81	down	hypothetical protein POPTR_0009s09800g [Populus trichocarpa]	13.53	72.64
map00940	Phenylpropanoid biosynthesis	4.40E-01	TRINITY_DN22196_c0_g1	-2.23	down	class III peroxidase [Populus trichocarpa]	3.21	22.70
map00940	Phenylpropanoid biosynthesis	4.40E-01	TRINITY_DN22301_c0_g2	2.10	up	cinnamyl alcohol dehydrogenase 3 [Populus tomentosa]	575.95	213.69
map00940	Phenylpropanoid biosynthesis	4.40E-01	TRINITY_DN22350_c0_g1	1.63	up	unknown [Populus trichocarpa]	1056.96	506.32
map00940	Phenylpropanoid biosynthesis	4.40E-01	TRINITY_DN22978_c0_g5	1.57	up	PREDICTED: serine carboxypeptidase-like 20 isoform X1 [Populus euphratica]	22.84	11.51
map00940	Phenylpropanoid biosynthesis	4.40E-01	TRINITY_DN23043_c0_g1	1.04	up	PREDICTED: lysosomal beta glucosidase-like [Populus euphratica]	13.89	10.05
map00940	Phenylpropanoid biosynthesis	4.40E-01	TRINITY_DN23395_c0_g1	1.24	up	4-coumarate--CoA ligase family protein [Populus trichocarpa]	14.87	10.65
map00940	Phenylpropanoid biosynthesis	4.40E-01	TRINITY_DN23675_c0_g1	-2.55	down	hypothetical protein POPTR_0001s42050g [Populus trichocarpa]	0.38	3.34
map00940	Phenylpropanoid biosynthesis	4.40E-01	TRINITY_DN23793_c0_g2	5.23	up	PREDICTED: caffeic acid 3-O-methyltransferase-like [Populus euphratica]	23.55	0.92
map00940	Phenylpropanoid biosynthesis	4.40E-01	TRINITY_DN23793_c0_g5	-1.54	down	eugenol O-methyltransferase family protein [Populus trichocarpa]	2.31	10.60
map00940	Phenylpropanoid biosynthesis	4.40E-01	TRINITY_DN24014_c0_g1	2.10	up	SALT OVERLY SENSITIVE 1 family protein [Populus trichocarpa]	27.49	9.79
map00940	Phenylpropanoid biosynthesis	4.40E-01	TRINITY_DN24112_c0_g4	1.11	up	hypothetical protein POPTR_0545s00210g [Populus trichocarpa]	22.08	15.72

map00940	Phenylpropanoid biosynthesis	4.40E-01	TRINITY_DN24566_c0_g1	1.29	up	AMP-dependent synthetase and ligase family protein [Populus trichocarpa]	395.20	247.14
map00940	Phenylpropanoid biosynthesis	4.40E-01	TRINITY_DN24981_c0_g2	-1.95	down	peroxidase precursor family protein [Populus trichocarpa]	28.66	164.68
map00940	Phenylpropanoid biosynthesis	4.40E-01	TRINITY_DN24995_c1_g4	-1.46	down	PREDICTED: peroxidase 64-like [Populus euphratica]	7.03	18.53
map00940	Phenylpropanoid biosynthesis	4.40E-01	TRINITY_DN25063_c0_g1	1.29	up	hypothetical protein POPTR_0001s31980g [Populus trichocarpa]	82.58	54.84
map00940	Phenylpropanoid biosynthesis	4.40E-01	TRINITY_DN25082_c0_g1	1.19	up	phenylalanine ammonia-lyase [Populus tomentosa]	144.67	96.35
map00940	Phenylpropanoid biosynthesis	4.40E-01	TRINITY_DN25105_c0_g1	-1.23	down	PREDICTED: glucose-6-phosphate/phosphate translocator 1, chloroplastic-like [Populus euphratica]	4.62	16.65
map00940	Phenylpropanoid biosynthesis	4.40E-01	TRINITY_DN25107_c0_g1	1.16	up	HXXXD-type acyl-transferase family protein [Populus tomentosa]	31.54	21.30
map00940	Phenylpropanoid biosynthesis	4.40E-01	TRINITY_DN25139_c1_g2	-1.69	down	peroxidase family protein [Populus trichocarpa]	3.33	16.46
map00940	Phenylpropanoid biosynthesis	4.40E-01	TRINITY_DN25139_c1_g6	-2.66	down	peroxidase family protein [Populus trichocarpa]	3.62	33.94
map00940	Phenylpropanoid biosynthesis	4.40E-01	TRINITY_DN25225_c0_g2	1.17	up	hypothetical protein POPTR_0019s05340g [Populus trichocarpa]	20.38	13.56
map00940	Phenylpropanoid biosynthesis	4.40E-01	TRINITY_DN25362_c0_g5	-1.81	down	hypothetical protein POPTR_0004s12460g [Populus trichocarpa]	0.47	2.61
map00940	Phenylpropanoid biosynthesis	4.40E-01	TRINITY_DN25371_c4_g1	-1.21	down	quinate O-hydroxycinnamoyltransferase/shikimate O-hydroxycinnamoyltransferase [Populus trichocarpa]	8.71	30.47
map00940	Phenylpropanoid biosynthesis	4.40E-01	TRINITY_DN26643_c1_g2	-1.33	down	PREDICTED: vicianin hydrolase-like [Populus euphratica]	12.50	47.56
map00940	Phenylpropanoid biosynthesis	4.40E-01	TRINITY_DN26707_c0_g1	-2.45	down	hypothetical protein POPTR_0005s02810g [Populus trichocarpa]	11.02	80.41
map00940	Phenylpropanoid biosynthesis	4.40E-01	TRINITY_DN27220_c0_g1	-1.11	down	PREDICTED: uncharacterized protein LOC105130123 [Populus euphratica]	10.28	33.40
map00940	Phenylpropanoid biosynthesis	4.40E-01	TRINITY_DN27585_c1_g1	1.25	up	serine carboxypeptidase S10 family protein [Populus trichocarpa]	76.41	37.45
map00940	Phenylpropanoid biosynthesis	4.40E-01	TRINITY_DN27585_c1_g2	1.90	up	serine carboxypeptidase S10 family protein [Populus trichocarpa]	8.97	3.59
map00940	Phenylpropanoid biosynthesis	4.40E-01	TRINITY_DN27725_c1_g1	-1.29	down	PREDICTED: protein argonaute 4A-like [Populus euphratica]	17.30	78.71
map00940	Phenylpropanoid biosynthesis	4.40E-01	TRINITY_DN27893_c12_g1	-5.22	down	PREDICTED: protein ECERIFERUM 26-like [Populus euphratica]	0.08	4.60
map00940	Phenylpropanoid biosynthesis	4.40E-01	TRINITY_DN12704_c0_g2	-2.23	down	PREDICTED: BAHD acyltransferase At5g47980-like [Populus euphratica]	0.27	1.96
map00940	Phenylpropanoid biosynthesis	4.40E-01	TRINITY_DN12720_c0_g2	1.29	up	PREDICTED: vinorine synthase-like [Populus euphratica]	7.62	4.66
map00940	Phenylpropanoid biosynthesis	4.40E-01	TRINITY_DN14060_c0_g1	-1.83	down	hypothetical protein POPTR_0001s15250g [Populus trichocarpa]	0.52	2.87
map00940	Phenylpropanoid biosynthesis	4.40E-01	TRINITY_DN14781_c0_g1	2.09	up	peroxidase [Populus davidiana x Populus alba var. pyramidalis]	1.86	0.63
map00940	Phenylpropanoid biosynthesis	4.40E-01	TRINITY_DN14874_c0_g1	3.80	up	hypothetical protein POPTR_0007s07330g [Populus trichocarpa]	3.81	0.40
map00940	Phenylpropanoid biosynthesis	4.40E-01	TRINITY_DN15542_c0_g1	-4.43	down	hypothetical protein POPTR_0013s08130g [Populus trichocarpa]	0.32	10.60
map00940	Phenylpropanoid biosynthesis	4.40E-01	TRINITY_DN15581_c0_g4	-1.46	down	PREDICTED: tetraketide alpha-pyrone reductase 2-like [Populus euphratica]	17.14	70.10
map00940	Phenylpropanoid biosynthesis	4.40E-01	TRINITY_DN1593_c0_g1	-1.37	down	hypothetical protein POPTR_0019s01540g [Populus trichocarpa]	1.85	7.22
map00940	Phenylpropanoid biosynthesis	4.40E-01	TRINITY_DN16022_c0_g1	2.65	up	hypothetical protein POPTR_0640s00200g [Populus trichocarpa]	6.27	1.56
map00940	Phenylpropanoid biosynthesis	4.40E-01	TRINITY_DN16091_c0_g1	1.63	up	PREDICTED: xylulose 5-phosphate/phosphate translocator, chloroplastic [Populus euphratica]	27.10	13.24
map00940	Phenylpropanoid biosynthesis	4.40E-01	TRINITY_DN16183_c0_g2	-1.80	down	hypothetical protein POPTR_0006s01190g [Populus trichocarpa]	1.74	8.00
map00940	Phenylpropanoid biosynthesis	4.40E-01	TRINITY_DN16387_c0_g1	3.61	up	glycosyl hydrolase family 1 family protein [Populus trichocarpa]	7.38	0.93
map00940	Phenylpropanoid biosynthesis	4.40E-01	TRINITY_DN16592_c0_g1	1.00	up	hypothetical protein POPTR_0001s28870g [Populus trichocarpa]	6.31	4.78
map00940	Phenylpropanoid biosynthesis	4.40E-01	TRINITY_DN16677_c0_g1	-6.00	down	hypothetical protein POPTR_0007s15050g [Populus trichocarpa]	0.16	11.75
map00940	Phenylpropanoid biosynthesis	4.40E-01	TRINITY_DN16795_c0_g1	2.85	up	hydroxyisourate hydrolase family protein [Populus trichocarpa]	3.65	0.76
map00940	Phenylpropanoid biosynthesis	4.40E-01	TRINITY_DN17100_c0_g1	2.00	up	PREDICTED: serine carboxypeptidase-like 51 [Populus euphratica]	19.47	7.44
map00940	Phenylpropanoid biosynthesis	4.40E-01	TRINITY_DN17164_c0_g1	-2.35	down	RAC-like GTP binding protein ARAC9 [Populus trichocarpa]	1.94	14.44
map00940	Phenylpropanoid biosynthesis	4.40E-01	TRINITY_DN17164_c0_g3	-1.72	down	hypothetical protein POPTR_0013s12760g [Populus trichocarpa]	3.48	17.44
map00940	Phenylpropanoid biosynthesis	4.40E-01	TRINITY_DN17456_c1_g2	1.62	up	hypothetical protein POPTR_0006s00920g [Populus trichocarpa]	30.98	15.42
map00940	Phenylpropanoid biosynthesis	4.40E-01	TRINITY_DN18278_c0_g1	-1.11	down	vesicle-associated membrane family protein [Populus trichocarpa]	3.07	10.07
map00940	Phenylpropanoid biosynthesis	4.40E-01	TRINITY_DN18302_c0_g2	-5.73	down	hypothetical protein POPTR_0008s03410g [Populus trichocarpa]	0.06	5.58
map00940	Phenylpropanoid biosynthesis	4.40E-01	TRINITY_DN18330_c0_g1	-2.18	down	trans-cinnamate 4-hydroxylase [Populus trichocarpa]	0.62	4.40
map00940	Phenylpropanoid biosynthesis	4.40E-01	TRINITY_DN18419_c0_g1	-5.65	down	peroxidase [Populus alba x Populus glandulosa]	0.43	31.58

map00940	Phenylpropanoid biosynthesis	4.40E-01	TRINITY_DN18944_c0_g1	1.39	up	hypothetical protein POPTR_0001s35130g [Populus trichocarpa]	11.16	6.52
map00940	Phenylpropanoid biosynthesis	4.40E-01	TRINITY_DN19274_c0_g1	1.06	up	hypothetical protein POPTR_0001s29510g [Populus trichocarpa]	74.48	55.73
map00940	Phenylpropanoid biosynthesis	4.40E-01	TRINITY_DN19654_c0_g1	-1.34	down	PREDICTED: uncharacterized protein LOC105126277 [Populus euphratica]	8.23	23.51
map00940	Phenylpropanoid biosynthesis	4.40E-01	TRINITY_DN19781_c0_g1	-1.53	down	ovate family protein [Populus trichocarpa]	3.48	15.24
map00940	Phenylpropanoid biosynthesis	4.40E-01	TRINITY_DN19781_c0_g2	-1.44	down	ovate family protein [Populus trichocarpa]	2.88	11.53
map00940	Phenylpropanoid biosynthesis	4.40E-01	TRINITY_DN19958_c0_g2	-1.13	down	RecName: Full=Caffeoyl-CoA O-methyltransferase; AltName: Full=Trans-caffeoyl-CoA 3-O-methyltransferase; Short=CCoAMT; Short=CCoAOMT	6.52	20.89
map00940	Phenylpropanoid biosynthesis	4.40E-01	TRINITY_DN20348_c0_g2	1.31	up	4-coumarate: coenzyme A ligase 4 [Populus tomentosa]	41.80	31.54
map00940	Phenylpropanoid biosynthesis	4.40E-01	TRINITY_DN20364_c0_g2	-1.63	down	PREDICTED: BAHD acyltransferase DCR [Populus euphratica]	14.38	66.96
map00940	Phenylpropanoid biosynthesis	4.40E-01	TRINITY_DN20403_c0_g3	-6.23	down	PREDICTED: cinnamoyl-CoA reductase 1-like [Populus euphratica]	0.12	13.67
map00940	Phenylpropanoid biosynthesis	4.40E-01	TRINITY_DN20593_c1_g1	-1.21	down	cinnamoyl-CoA reductase [Populus tomentosa]	5.35	18.95
map00940	Phenylpropanoid biosynthesis	4.40E-01	TRINITY_DN20842_c0_g1	1.07	up	hypothetical protein POPTR_0008s18060g [Populus trichocarpa]	8.67	6.26
map00940	Phenylpropanoid biosynthesis	4.40E-01	TRINITY_DN20905_c0_g3	-1.18	down	p-coumarate 3-hydroxylase [Populus tomentosa]	2.28	8.17
map00940	Phenylpropanoid biosynthesis	4.40E-01	TRINITY_DN20924_c0_g1	1.28	up	transferase family protein [Populus trichocarpa]	32.99	19.55
map00940	Phenylpropanoid biosynthesis	4.40E-01	TRINITY_DN20924_c0_g2	1.79	up	transferase family protein [Populus trichocarpa]	14.61	6.33
map00940	Phenylpropanoid biosynthesis	4.40E-01	TRINITY_DN21103_c0_g1	1.83	up	PREDICTED: aldehyde dehydrogenase family 2 member C4 [Populus euphratica]	66.51	28.91
map00940	Phenylpropanoid biosynthesis	4.40E-01	TRINITY_DN21110_c0_g1	-1.22	down	hypothetical protein POPTR_0016s14030g [Populus trichocarpa]	2.86	10.12
map00940	Phenylpropanoid biosynthesis	4.40E-01	TRINITY_DN391_c0_g1	1.28	up	hypothetical protein POPTR_0340s00200g [Populus trichocarpa]	62.66	39.30
map00940	Phenylpropanoid biosynthesis	4.40E-01	TRINITY_DN549_c0_g1	-3.31	down	PREDICTED: heavy metal-associated isoprenylated plant protein 26-like [Populus euphratica]	0.39	4.57
map00650	Butanoate metabolism	4.42E-01	TRINITY_DN22684_c0_g1	4.49	up	glutamate decarboxylase [Populus tremula x Populus alba]	5.12	0.35
map00650	Butanoate metabolism	4.42E-01	TRINITY_DN22684_c0_g7	-5.56	down	glutamate decarboxylase 1 family protein [Populus trichocarpa]	0.08	6.22
map00650	Butanoate metabolism	4.42E-01	TRINITY_DN23653_c0_g1	1.10	up	hypothetical protein POPTR_0018s09500g [Populus trichocarpa]	54.02	38.03
map00650	Butanoate metabolism	4.42E-01	TRINITY_DN23795_c1_g1	1.13	up	PREDICTED: glyoxylate/succinic semialdehyde reductase 2, chloroplastic-like [Populus euphratica]	219.09	158.29
map00650	Butanoate metabolism	4.42E-01	TRINITY_DN26976_c0_g1	1.47	up	PREDICTED: acetolactate synthase 2, chloroplastic-like [Populus euphratica]	396.57	239.53
map00650	Butanoate metabolism	4.42E-01	TRINITY_DN19913_c0_g1	1.05	up	PREDICTED: hydroxymethylglutaryl-CoA lyase, mitochondrial-like isoform X1 [Populus euphratica]	13.16	7.43
map00650	Butanoate metabolism	4.42E-01	TRINITY_DN20583_c0_g3	2.29	up	hypothetical protein POPTR_0008s03820g [Populus trichocarpa]	4.09	1.88
map00650	Butanoate metabolism	4.42E-01	TRINITY_DN20891_c1_g1	2.08	up	PREDICTED: F-box protein At2g32560-like [Populus euphratica]	23.97	9.10
map00650	Butanoate metabolism	4.42E-01	TRINITY_DN21313_c0_g2	-1.72	down	hypothetical protein POPTR_0014s07170g [Populus trichocarpa]	4.63	24.85
map00650	Butanoate metabolism	4.42E-01	TRINITY_DN21600_c0_g2	1.23	up	PREDICTED: acetolactate synthase small subunit 2, chloroplastic-like [Populus euphratica]	35.74	23.13
map00650	Butanoate metabolism	4.42E-01	TRINITY_DN847_c0_g1	-7.22	down	glutamate decarboxylase 1 family protein [Populus trichocarpa]	0.04	7.12
map00073	Cutin, suberine and wax biosynthesis	4.42E-01	TRINITY_DN22374_c0_g1	-3.14	down	Cytochrome P450 86A1 family protein [Populus trichocarpa]	0.31	4.14
map00073	Cutin, suberine and wax biosynthesis	4.42E-01	TRINITY_DN23040_c0_g1	-1.38	down	hypothetical protein POPTR_0004s08600g [Populus trichocarpa]	2.02	8.09
map00073	Cutin, suberine and wax biosynthesis	4.42E-01	TRINITY_DN23399_c0_g2	-1.59	down	adhesion of calyx edges family protein [Populus trichocarpa]	1.75	8.05
map00073	Cutin, suberine and wax biosynthesis	4.42E-01	TRINITY_DN23463_c0_g1	-1.03	down	hypothetical protein POPTR_0011s09560g [Populus trichocarpa]	28.79	92.22
map00073	Cutin, suberine and wax biosynthesis	4.42E-01	TRINITY_DN23656_c1_g5	-2.04	down	hypothetical protein POPTR_0010s24310g [Populus trichocarpa]	0.58	3.67
map00073	Cutin, suberine and wax biosynthesis	4.42E-01	TRINITY_DN23656_c1_g6	-1.62	down	PREDICTED: cytochrome P450 94C1-like [Populus euphratica]	0.77	3.61
map00073	Cutin, suberine and wax biosynthesis	4.42E-01	TRINITY_DN24683_c0_g1	-3.53	down	hypothetical protein POPTR_0018s10830g [Populus trichocarpa]	2.04	34.86
map00073	Cutin, suberine and wax biosynthesis	4.42E-01	TRINITY_DN25044_c0_g5	1.69	up	PREDICTED: cytochrome P450 89A2-like [Populus euphratica]	7.02	3.30
map00073	Cutin, suberine and wax biosynthesis	4.42E-01	TRINITY_DN26824_c0_g3	-1.76	down	hypothetical protein POPTR_0012s10420g [Populus trichocarpa]	26.55	135.07
map00073	Cutin, suberine and wax biosynthesis	4.42E-01	TRINITY_DN13012_c0_g2	3.69	up	hypothetical protein POPTR_0012s09880g [Populus trichocarpa]	2.12	0.24
map00073	Cutin, suberine and wax biosynthesis	4.42E-01	TRINITY_DN13195_c0_g1	1.88	up	hypothetical protein POPTR_0019s10390g [Populus trichocarpa]	4.32	3.51
map00073	Cutin, suberine and wax biosynthesis	4.42E-01	TRINITY_DN15251_c0_g2	-2.09	down	PREDICTED: omega-hydroxypalmitate O-feruloyl transferase-like [Populus euphratica]	0.43	2.86
map00073	Cutin, suberine and wax biosynthesis	4.42E-01	TRINITY_DN16260_c0_g1	-1.95	down	hypothetical protein POPTR_0001s33390g [Populus trichocarpa]	0.49	2.82

map00073	Cutin, suberine and wax biosynthesis	4.42E-01	TRINITY_DN16847_c0_g1	-3.50	down	cytochrome P450 family protein [Populus trichocarpa]	0.47	8.14
map00073	Cutin, suberine and wax biosynthesis	4.42E-01	TRINITY_DN18041_c0_g1	-1.35	down	transferase family protein [Populus trichocarpa]	0.89	3.54
map00073	Cutin, suberine and wax biosynthesis	4.42E-01	TRINITY_DN18787_c0_g1	-6.64	down	hypothetical protein POPTR_0009s14720g [Populus trichocarpa]	0.09	17.28
map00073	Cutin, suberine and wax biosynthesis	4.42E-01	TRINITY_DN20089_c0_g6	-6.46	down	hypothetical protein POPTR_0017s04300g [Populus trichocarpa]	0.02	3.31
map00073	Cutin, suberine and wax biosynthesis	4.42E-01	TRINITY_DN21289_c1_g1	-2.22	down	PREDICTED: uncharacterized protein LOC108983648 [Juglans regia]	0.45	3.16
map00073	Cutin, suberine and wax biosynthesis	4.42E-01	TRINITY_DN21355_c1_g1	1.40	up	hypothetical protein VITISV_005279 [Vitis vinifera]	55.49	33.47
map00966	Glucosinolate biosynthesis	4.47E-01	TRINITY_DN26103_c0_g4	1.68	up	hypothetical protein POPTR_0322s00200g [Populus trichocarpa]	65.51	31.51
map00966	Glucosinolate biosynthesis	4.47E-01	TRINITY_DN17247_c0_g1	-1.15	down	UDP-glucoronosyl/UDP-glucosyl transferase family protein [Populus trichocarpa]	7.87	26.34
map00966	Glucosinolate biosynthesis	4.47E-01	TRINITY_DN17710_c0_g1	1.71	up	hypothetical protein POPTR_0012s01280g [Populus trichocarpa]	51.45	22.69
map00966	Glucosinolate biosynthesis	4.47E-01	TRINITY_DN20225_c0_g1	-2.28	down	hypothetical protein POPTR_0001s28860g [Populus trichocarpa]	1.93	14.45
map00966	Glucosinolate biosynthesis	4.47E-01	TRINITY_DN20790_c0_g1	3.22	up	PREDICTED: cytosolic sulfotransferase 15-like [Populus euphratica]	96.77	15.23
map00966	Glucosinolate biosynthesis	4.47E-01	TRINITY_DN20958_c0_g1	1.57	up	PREDICTED: UDP-glycosyltransferase 74E1-like [Populus euphratica]	64.45	33.63
map00640	Propanoate metabolism	4.53E-01	TRINITY_DN22349_c0_g1	1.04	up	PREDICTED: 2-oxoisovalerate dehydrogenase subunit alpha 2, mitochondrial [Populus euphratica]	16.36	12.76
map00640	Propanoate metabolism	4.53E-01	TRINITY_DN22512_c0_g1	1.04	up	hypothetical protein POPTR_0018s02570g [Populus trichocarpa]	23.51	17.76
map00640	Propanoate metabolism	4.53E-01	TRINITY_DN22677_c0_g1	-1.18	down	alpha galactosyltransferase family protein [Populus trichocarpa]	16.78	58.67
map00640	Propanoate metabolism	4.53E-01	TRINITY_DN24307_c0_g1	1.05	up	PREDICTED: biotin carboxyl carrier protein of acetyl-CoA carboxylase, chloroplastic-like [Populus euphratica]	73.06	52.43
map00640	Propanoate metabolism	4.53E-01	TRINITY_DN24969_c2_g1	1.05	up	Succinyl-CoA ligase beta-chain family protein [Populus trichocarpa]	98.12	74.47
map00640	Propanoate metabolism	4.53E-01	TRINITY_DN25057_c0_g2	-1.00	down	PREDICTED: GATA transcription factor 1-like [Populus euphratica]	3.25	9.78
map00640	Propanoate metabolism	4.53E-01	TRINITY_DN25439_c0_g1	1.11	up	biotin carboxylase precursor family protein [Populus trichocarpa]	85.63	60.20
map00640	Propanoate metabolism	4.53E-01	TRINITY_DN26590_c0_g1	1.01	up	PREDICTED: acyl-CoA dehydrogenase family member 10-like isoform X2 [Populus euphratica]	17.06	13.06
map00640	Propanoate metabolism	4.53E-01	TRINITY_DN26837_c0_g1	2.09	up	hypothetical protein POPTR_0010s15200g [Populus trichocarpa]	81.17	29.93
map00640	Propanoate metabolism	4.53E-01	TRINITY_DN27876_c5_g1	-1.61	down	hypothetical protein POPTR_0005s16540g [Populus trichocarpa]	2.20	11.69
map00640	Propanoate metabolism	4.53E-01	TRINITY_DN27876_c5_g5	-2.33	down	PREDICTED: acetyl-CoA carboxylase 1-like [Populus euphratica]	0.87	6.39
map00640	Propanoate metabolism	4.53E-01	TRINITY_DN13808_c0_g3	-1.99	down	hypothetical protein POPTR_0001s04370g [Populus trichocarpa]	0.58	3.70
map00640	Propanoate metabolism	4.53E-01	TRINITY_DN17257_c0_g1	-1.22	down	hypothetical protein POPTR_0001s00440g [Populus trichocarpa]	6.45	23.17
map00640	Propanoate metabolism	4.53E-01	TRINITY_DN17464_c0_g2	1.19	up	hydrolase family protein [Populus trichocarpa]	68.37	46.14
map00640	Propanoate metabolism	4.53E-01	TRINITY_DN19050_c1_g1	1.32	up	hypothetical protein POPTR_0019s08170g, partial [Populus trichocarpa]	123.18	74.70
map00640	Propanoate metabolism	4.53E-01	TRINITY_DN19294_c0_g3	1.09	up	hypothetical protein POPTR_0006s16050g [Populus trichocarpa]	91.23	67.60
map00640	Propanoate metabolism	4.53E-01	TRINITY_DN19526_c1_g1	1.13	up	hypothetical protein POPTR_0003s20830g [Populus trichocarpa]	33.01	24.69
map00640	Propanoate metabolism	4.53E-01	TRINITY_DN19823_c0_g5	1.20	up	hypothetical protein POPTR_0005s09370g [Populus trichocarpa]	70.43	46.85
map00640	Propanoate metabolism	4.53E-01	TRINITY_DN21140_c0_g2	2.24	up	mitochondrial lipoamide dehydrogenase [Populus tremuloides]	202.39	67.53
map00640	Propanoate metabolism	4.53E-01	TRINITY_DN21528_c1_g1	-1.91	down	hypothetical protein POPTR_0002s09330g [Populus trichocarpa]	0.78	4.44
map00640	Propanoate metabolism	4.53E-01	TRINITY_DN348_c0_g1	-3.27	down	hypothetical protein POPTR_0018s03880g [Populus trichocarpa]	0.18	2.75
map00053	Ascorbate and aldarate metabolism	4.74E-01	TRINITY_DN21932_c0_g3	-1.06	down	phosphatase 2C family protein [Populus trichocarpa]	2.53	8.10
map00053	Ascorbate and aldarate metabolism	4.74E-01	TRINITY_DN22320_c0_g1	2.05	up	PREDICTED: thylakoid lumenal 29 kDa protein, chloroplastic isoform X1 [Populus euphratica]	312.91	116.47
map00053	Ascorbate and aldarate metabolism	4.74E-01	TRINITY_DN22413_c1_g10	2.03	up	hypothetical protein VITISV_005279 [Vitis vinifera]	2.39	0.85
map00053	Ascorbate and aldarate metabolism	4.74E-01	TRINITY_DN23070_c0_g1	1.14	up	monodehydroascorbate reductase [Populus alba x Populus glandulosa]	169.27	118.14
map00053	Ascorbate and aldarate metabolism	4.74E-01	TRINITY_DN23479_c0_g1	-1.43	down	hypothetical protein POPTR_0014s17590g [Populus trichocarpa]	4.89	18.74
map00053	Ascorbate and aldarate metabolism	4.74E-01	TRINITY_DN23831_c1_g3	-1.10	down	hypothetical protein GLYMA_17G255600 [Glycine max]	949.87	2470.01
map00053	Ascorbate and aldarate metabolism	4.74E-01	TRINITY_DN24105_c1_g3	-2.06	down	hypothetical protein TSUD_254980 [Trifolium subterraneum]	230.28	1159.28
map00053	Ascorbate and aldarate metabolism	4.74E-01	TRINITY_DN24607_c0_g1	-1.93	down	hypothetical protein POPTR_0001s05450g [Populus trichocarpa]	95.86	582.55
map00053	Ascorbate and aldarate metabolism	4.74E-01	TRINITY_DN24628_c0_g1	1.28	up	PREDICTED: aldehyde dehydrogenase family 3 member H1-like [Populus euphratica]	40.39	24.46

map00053	Ascorbate and aldarate metabolism	4.74E-01	TRINITY_DN24658_c0_g1	1.35	up	hypothetical protein POPTR_0004s18030g [Populus trichocarpa]	976.13	588.02
map00053	Ascorbate and aldarate metabolism	4.74E-01	TRINITY_DN25335_c0_g4	1.91	up	ascorbate oxidase precursor family protein [Populus trichocarpa]	2.96	1.21
map00053	Ascorbate and aldarate metabolism	4.74E-01	TRINITY_DN25630_c0_g1	1.30	up	aldehyde dehydrogenase 1 precursor family protein [Populus trichocarpa]	120.35	73.18
map00053	Ascorbate and aldarate metabolism	4.74E-01	TRINITY_DN25744_c0_g1	1.77	up	PREDICTED: probable L-ascorbate peroxidase 6, chloroplastic isoform X1 [Populus euphratica]	291.77	130.37
map00053	Ascorbate and aldarate metabolism	4.74E-01	TRINITY_DN25892_c1_g4	1.32	up	unknown [Populus trichocarpa x Populus deltoides]	56.52	36.35
map00053	Ascorbate and aldarate metabolism	4.74E-01	TRINITY_DN26025_c0_g1	-1.71	down	hypothetical protein POPTR_0004s18340g [Populus trichocarpa]	5.60	27.70
map00053	Ascorbate and aldarate metabolism	4.74E-01	TRINITY_DN26284_c1_g1	2.20	up	hypothetical protein VITISV_025518 [Vitis vinifera]	3.90	1.29
map00053	Ascorbate and aldarate metabolism	4.74E-01	TRINITY_DN26421_c0_g1	1.32	up	the aldehyde dehydrogenase cp-ADH from C.plantagineum family protein [Populus trichocarpa]	58.13	34.64
map00053	Ascorbate and aldarate metabolism	4.74E-01	TRINITY_DN26697_c0_g2	1.03	up	unknown [Populus trichocarpa]	28.25	21.14
map00053	Ascorbate and aldarate metabolism	4.74E-01	TRINITY_DN26727_c0_g2	-1.19	down	PREDICTED: uncharacterized protein LOC105134358 isoform X1 [Populus euphratica]	1.77	6.29
map00053	Ascorbate and aldarate metabolism	4.74E-01	TRINITY_DN11544_c0_g1	-4.56	down	PREDICTED: uncharacterized protein LOC107880749 isoform X1 [Prunus mume]	0.13	4.63
map00053	Ascorbate and aldarate metabolism	4.74E-01	TRINITY_DN13159_c0_g1	2.54	up	ascorbate peroxidase [Populus tomentosa]	8.23	2.11
map00053	Ascorbate and aldarate metabolism	4.74E-01	TRINITY_DN15028_c0_g1	1.42	up	hypothetical protein POPTR_0007s07520g [Populus trichocarpa]	93.18	52.23
map00053	Ascorbate and aldarate metabolism	4.74E-01	TRINITY_DN17167_c0_g1	-1.21	down	GHMP kinase-related family protein [Populus trichocarpa]	3.51	12.23
map00053	Ascorbate and aldarate metabolism	4.74E-01	TRINITY_DN17369_c0_g1	1.12	up	ascorbate peroxidase [Populus tomentosa]	210.14	152.72
map00053	Ascorbate and aldarate metabolism	4.74E-01	TRINITY_DN17369_c0_g3	1.35	up	ascorbate peroxidase [Populus tomentosa]	128.46	76.99
map00053	Ascorbate and aldarate metabolism	4.74E-01	TRINITY_DN18129_c0_g4	-1.79	down	PREDICTED: histone H3.2-like [Gossypium hirsutum]	50.39	262.39
map00053	Ascorbate and aldarate metabolism	4.74E-01	TRINITY_DN18222_c1_g1	1.16	up	PREDICTED: UDP-sugar pyrophosphorylase [Populus euphratica]	28.36	19.26
map00053	Ascorbate and aldarate metabolism	4.74E-01	TRINITY_DN18895_c0_g1	-1.06	down	mitochondrial aldehyde dehydrogenase family protein [Populus trichocarpa]	7.40	23.22
map00053	Ascorbate and aldarate metabolism	4.74E-01	TRINITY_DN19215_c1_g1	-1.38	down	hypothetical protein POPTR_0007s10210g [Populus trichocarpa]	3.47	12.55
map00053	Ascorbate and aldarate metabolism	4.74E-01	TRINITY_DN19215_c2_g1	-1.56	down	PREDICTED: probable protein phosphatase 2C 63 [Populus euphratica]	6.48	30.16
map00053	Ascorbate and aldarate metabolism	4.74E-01	TRINITY_DN19362_c1_g4	-1.86	down	PREDICTED: histone H3.2-like [Zea mays]	92.37	503.02
map00053	Ascorbate and aldarate metabolism	4.74E-01	TRINITY_DN19362_c1_g6	-1.87	down	hypothetical protein CARUB_v10021660mg, partial [Capsella rubella]	105.61	580.15
map00053	Ascorbate and aldarate metabolism	4.74E-01	TRINITY_DN19943_c0_g2	1.71	up	hypothetical protein POPTR_0001s09830g [Populus trichocarpa]	26.04	12.13
map00053	Ascorbate and aldarate metabolism	4.74E-01	TRINITY_DN19988_c0_g8	-4.08	down	laccase family protein [Populus trichocarpa]	0.07	1.97
map00053	Ascorbate and aldarate metabolism	4.74E-01	TRINITY_DN20399_c0_g1	-3.78	down	PREDICTED: L-ascorbate oxidase-like [Populus euphratica]	0.48	10.01
map00053	Ascorbate and aldarate metabolism	4.74E-01	TRINITY_DN21112_c0_g1	1.12	up	PREDICTED: probable monodehydroascorbate reductase, cytoplasmic isoform 2 [Populus euphratica]	146.08	103.50
map00053	Ascorbate and aldarate metabolism	4.74E-01	TRINITY_DN21343_c0_g5	-2.08	down	hypothetical protein TSUD_72310 [Trifolium subterraneum]	0.74	3.53
map00053	Ascorbate and aldarate metabolism	4.74E-01	TRINITY_DN7900_c0_g1	1.75	up	Retrovirus-related Pol polyprotein from transposon TNT 1-94 [Cajanus cajan]	2.25	1.02
map01040	Biosynthesis of unsaturated fatty acids	5.07E-01	TRINITY_DN23645_c0_g1	1.12	up	hypothetical protein POPTR_0008s17880g [Populus trichocarpa]	115.75	81.25
map01040	Biosynthesis of unsaturated fatty acids	5.07E-01	TRINITY_DN23776_c0_g1	1.29	up	hypothetical protein POPTR_0005s14280g [Populus trichocarpa]	31.39	19.38
map01040	Biosynthesis of unsaturated fatty acids	5.07E-01	TRINITY_DN24137_c0_g2	1.93	up	PREDICTED: acyl-[acyl-carrier-protein] desaturase, chloroplastic [Populus euphratica]	275.31	109.07
map01040	Biosynthesis of unsaturated fatty acids	5.07E-01	TRINITY_DN24431_c1_g2	-1.25	down	PREDICTED: acyl-coenzyme A oxidase 4, peroxisomal-like isoform X3 [Populus euphratica]	3.95	18.34
map01040	Biosynthesis of unsaturated fatty acids	5.07E-01	TRINITY_DN25504_c0_g1	1.69	up	chloroplast omega-3 desaturase family protein [Populus trichocarpa]	178.02	89.81
map01040	Biosynthesis of unsaturated fatty acids	5.07E-01	TRINITY_DN25910_c0_g2	-1.06	down	PREDICTED: transcription factor PCL1-like [Populus euphratica]	4.93	16.82
map01040	Biosynthesis of unsaturated fatty acids	5.07E-01	TRINITY_DN26366_c0_g2	1.69	up	hypothetical protein POPTR_0002s22410g [Populus trichocarpa]	361.54	171.53
map01040	Biosynthesis of unsaturated fatty acids	5.07E-01	TRINITY_DN15736_c0_g1	-10.07	down	hypothetical protein POPTR_0008s14850g [Populus trichocarpa]	0.00	7.88
map01040	Biosynthesis of unsaturated fatty acids	5.07E-01	TRINITY_DN16719_c0_g2	-2.32	down	short-chain dehydrogenase/reductase family protein [Populus trichocarpa]	0.77	6.53
map01040	Biosynthesis of unsaturated fatty acids	5.07E-01	TRINITY_DN17194_c0_g1	1.17	up	PREDICTED: very-long-chain enoyl-CoA reductase-like [Populus euphratica]	8.23	5.68
map01040	Biosynthesis of unsaturated fatty acids	5.07E-01	TRINITY_DN19330_c1_g4	1.30	up	hypothetical protein POPTR_0004s23710g [Populus trichocarpa]	22.29	13.72
map01040	Biosynthesis of unsaturated fatty acids	5.07E-01	TRINITY_DN19907_c0_g1	1.28	up	hypothetical protein POPTR_0001s24210g [Populus trichocarpa]	29.87	18.49
map01040	Biosynthesis of unsaturated fatty acids	5.07E-01	TRINITY_DN20754_c0_g3	1.78	up	hypothetical protein POPTR_0010s06230g [Populus trichocarpa]	19.13	8.33
map01040	Biosynthesis of unsaturated fatty acids	5.07E-01	TRINITY_DN6900_c0_g1	1.83	up	short-chain dehydrogenase/reductase family protein [Populus trichocarpa]	21.13	8.97

map02010	ABC transporters	5.09E-01	TRINITY_DN21914_c0_g1	2.29	up	ABC transporter family protein [Populus trichocarpa]	71.47	24.13
map02010	ABC transporters	5.09E-01	TRINITY_DN22113_c0_g3	-2.10	down	PREDICTED: pleiotropic drug resistance protein 1-like [Populus euphratica]	1.05	5.16
map02010	ABC transporters	5.09E-01	TRINITY_DN22113_c0_g5	-2.55	down	hypothetical protein POPTR_0001s14660g [Populus trichocarpa]	0.34	2.38
map02010	ABC transporters	5.09E-01	TRINITY_DN22131_c0_g1	-1.57	down	PREDICTED: putative multidrug resistance protein isoform X1 [Populus euphratica]	1.18	5.42
map02010	ABC transporters	5.09E-01	TRINITY_DN23135_c0_g11	-2.21	down	hypothetical protein POPTR_0455s00200g, partial [Populus trichocarpa]	0.38	2.69
map02010	ABC transporters	5.09E-01	TRINITY_DN23135_c0_g2	-1.62	down	hypothetical protein POPTR_0010s16340g [Populus trichocarpa]	2.00	7.36
map02010	ABC transporters	5.09E-01	TRINITY_DN23966_c0_g2	-1.00	down	PREDICTED: ABC transporter C family member 13 isoform X1 [Populus euphratica]	2.06	4.71
map02010	ABC transporters	5.09E-01	TRINITY_DN23966_c0_g3	-1.76	down	PREDICTED: ABC transporter C family member 13 isoform X1 [Populus euphratica]	0.54	2.81
map02010	ABC transporters	5.09E-01	TRINITY_DN24028_c0_g6	-1.55	down	multidrug resistance P-glycoprotein [Populus trichocarpa]	17.04	81.03
map02010	ABC transporters	5.09E-01	TRINITY_DN24383_c0_g3	-4.47	down	P-glycoprotein [Populus trichocarpa]	0.26	9.12
map02010	ABC transporters	5.09E-01	TRINITY_DN24513_c0_g3	-2.76	down	hypothetical protein POPTR_0008s17960g [Populus trichocarpa]	0.78	8.14
map02010	ABC transporters	5.09E-01	TRINITY_DN24675_c0_g1	-1.48	down	hypothetical protein POPTR_0003s19800g [Populus trichocarpa]	3.48	13.85
map02010	ABC transporters	5.09E-01	TRINITY_DN25120_c1_g1	1.78	up	PREDICTED: ABC transporter G family member 29-like [Populus euphratica]	5.88	6.33
map02010	ABC transporters	5.09E-01	TRINITY_DN25157_c0_g2	-1.41	down	ABC transporter family protein [Populus trichocarpa]	1.24	4.88
map02010	ABC transporters	5.09E-01	TRINITY_DN25355_c0_g5	1.41	up	PREDICTED: uncharacterized protein LOC105109055 [Populus euphratica]	197.71	118.29
map02010	ABC transporters	5.09E-01	TRINITY_DN25428_c0_g2	-1.29	down	ABC transporter family protein [Populus trichocarpa]	4.94	22.23
map02010	ABC transporters	5.09E-01	TRINITY_DN25766_c0_g2	1.51	up	ABC transporter family protein [Populus trichocarpa]	72.87	38.52
map02010	ABC transporters	5.09E-01	TRINITY_DN25791_c0_g1	1.16	up	hypothetical protein POPTR_0010s18400g [Populus trichocarpa]	15.98	11.25
map02010	ABC transporters	5.09E-01	TRINITY_DN27170_c0_g3	-2.43	down	ABC transporter family protein [Populus trichocarpa]	0.63	5.11
map02010	ABC transporters	5.09E-01	TRINITY_DN27251_c0_g1	1.65	up	hypothetical protein POPTR_0003s02950g [Populus trichocarpa]	33.01	16.26
map02010	ABC transporters	5.09E-01	TRINITY_DN12311_c0_g1	-2.09	down	hypothetical protein POPTR_0010s14180g [Populus trichocarpa]	0.27	1.76
map02010	ABC transporters	5.09E-01	TRINITY_DN14465_c0_g1	1.15	up	PREDICTED: ABC transporter D family member 1-like [Populus euphratica]	43.30	30.56
map02010	ABC transporters	5.09E-01	TRINITY_DN14818_c0_g1	-1.63	down	pleiotropic drug resistance 6 [Populus tomentosa]	0.48	2.29
map02010	ABC transporters	5.09E-01	TRINITY_DN16888_c0_g1	-1.94	down	PREDICTED: ABC transporter B family member 27-like [Populus euphratica]	6.50	36.64
map02010	ABC transporters	5.09E-01	TRINITY_DN16889_c0_g1	1.08	up	PREDICTED: ABC transporter I family member 10, chloroplastic-like [Populus euphratica]	35.50	25.35
map02010	ABC transporters	5.09E-01	TRINITY_DN17021_c0_g1	-1.78	down	hypothetical protein POPTR_0009s05580g [Populus trichocarpa]	0.93	4.81
map02010	ABC transporters	5.09E-01	TRINITY_DN17633_c0_g2	-6.96	down	hypothetical protein POPTR_0001s31880g [Populus trichocarpa]	0.02	3.83
map02010	ABC transporters	5.09E-01	TRINITY_DN18047_c0_g1	-1.25	down	PREDICTED: ABC transporter B family member 13-like isoform X1 [Populus euphratica]	1.31	4.76
map02010	ABC transporters	5.09E-01	TRINITY_DN18104_c0_g2	-2.76	down	PREDICTED: LOW QUALITY PROTEIN: uncharacterized protein LOC105111782 [Populus euphratica]	0.51	4.24
map02010	ABC transporters	5.09E-01	TRINITY_DN18322_c0_g2	1.61	up	ABC transporter family protein [Populus trichocarpa]	21.91	17.48
map02010	ABC transporters	5.09E-01	TRINITY_DN18693_c0_g1	2.20	up	PREDICTED: rubisco accumulation factor 1, chloroplastic [Populus euphratica]	236.80	77.53
map02010	ABC transporters	5.09E-01	TRINITY_DN18736_c1_g1	1.23	up	hypothetical protein POPTR_0002s18860g [Populus trichocarpa]	3.93	2.53
map02010	ABC transporters	5.09E-01	TRINITY_DN18736_c1_g5	3.94	up	PREDICTED: ABC transporter B family member 4-like [Populus euphratica]	2.95	0.29
map02010	ABC transporters	5.09E-01	TRINITY_DN18938_c0_g1	1.66	up	PREDICTED: ABC transporter A family member 7-like [Populus euphratica]	3.26	1.53
map02010	ABC transporters	5.09E-01	TRINITY_DN19230_c0_g1	-3.88	down	PREDICTED: ABC transporter G family member 15-like [Populus euphratica]	0.57	12.52
map02010	ABC transporters	5.09E-01	TRINITY_DN19230_c0_g2	-3.05	down	hypothetical protein POPTR_0016s05690g [Populus trichocarpa]	1.41	16.40
map02010	ABC transporters	5.09E-01	TRINITY_DN20037_c0_g1	-2.03	down	PREDICTED: ABC transporter B family member 2-like isoform X1 [Populus euphratica]	1.02	7.16
map02010	ABC transporters	5.09E-01	TRINITY_DN20888_c0_g1	-1.45	down	PREDICTED: putative ABC transporter C family member 15 isoform X1 [Populus euphratica]	3.76	15.46
map00280	Valine, leucine and isoleucine degradation	5.15E-01	TRINITY_DN21932_c0_g3	-1.06	down	phosphatase 2C family protein [Populus trichocarpa]	2.53	8.10
map00280	Valine, leucine and isoleucine degradation	5.15E-01	TRINITY_DN22349_c0_g1	1.04	up	PREDICTED: 2-oxoisovalerate dehydrogenase subunit alpha 2, mitochondrial [Populus euphratica]	16.36	12.76
map00280	Valine, leucine and isoleucine degradation	5.15E-01	TRINITY_DN22512_c0_g1	1.04	up	hypothetical protein POPTR_0018s02570g [Populus trichocarpa]	23.51	17.76
map00280	Valine, leucine and isoleucine degradation	5.15E-01	TRINITY_DN22673_c1_g1	-1.85	down	hypothetical protein POPTR_0003s07860g [Populus trichocarpa]	0.98	4.81

map00280	Valine, leucine and isoleucine degradation	5.15E-01	TRINITY_DN23237_c0_g2	-1.50	down	hypothetical protein POPTR_0015s04720g [Populus trichocarpa]	2.20	9.25
map00280	Valine, leucine and isoleucine degradation	5.15E-01	TRINITY_DN24628_c0_g1	1.28	up	PREDICTED: aldehyde dehydrogenase family 3 member H1-like [Populus euphratica]	40.39	24.46
map00280	Valine, leucine and isoleucine degradation	5.15E-01	TRINITY_DN25057_c0_g2	-1.00	down	PREDICTED: GATA transcription factor 1-like [Populus euphratica]	3.25	9.78
map00280	Valine, leucine and isoleucine degradation	5.15E-01	TRINITY_DN25630_c0_g1	1.30	up	aldehyde dehydrogenase 1 precursor family protein [Populus trichocarpa]	120.35	73.18
map00280	Valine, leucine and isoleucine degradation	5.15E-01	TRINITY_DN25630_c0_g3	-2.44	down	PREDICTED: branched-chain-amino-acid aminotransferase 2, chloroplastic-like isoform X1 [Populus euphratica]	0.50	3.81
map00280	Valine, leucine and isoleucine degradation	5.15E-01	TRINITY_DN26025_c0_g1	-1.71	down	hypothetical protein POPTR_0004s18340g [Populus trichocarpa]	5.60	27.70
map00280	Valine, leucine and isoleucine degradation	5.15E-01	TRINITY_DN26366_c0_g2	1.69	up	hypothetical protein POPTR_0002s22410g [Populus trichocarpa]	361.54	171.53
map00280	Valine, leucine and isoleucine degradation	5.15E-01	TRINITY_DN26421_c0_g1	1.32	up	the aldehyde dehydrogenase cp-ADH from C.plantagineum family protein [Populus trichocarpa]	58.13	34.64
map00280	Valine, leucine and isoleucine degradation	5.15E-01	TRINITY_DN26590_c0_g1	1.01	up	PREDICTED: acyl-CoA dehydrogenase family member 10-like isoform X2 [Populus euphratica]	17.06	13.06
map00280	Valine, leucine and isoleucine degradation	5.15E-01	TRINITY_DN26837_c0_g1	2.09	up	hypothetical protein POPTR_0010s15200g [Populus trichocarpa]	81.17	29.93
map00280	Valine, leucine and isoleucine degradation	5.15E-01	TRINITY_DN17464_c0_g2	1.19	up	hydrolase family protein [Populus trichocarpa]	68.37	46.14
map00280	Valine, leucine and isoleucine degradation	5.15E-01	TRINITY_DN18648_c0_g1	1.13	up	Methylcrotonyl-CoA carboxylase beta chain family protein [Populus trichocarpa]	22.90	15.67
map00280	Valine, leucine and isoleucine degradation	5.15E-01	TRINITY_DN18895_c0_g1	-1.06	down	mitochondrial aldehyde dehydrogenase family protein [Populus trichocarpa]	7.40	23.22
map00280	Valine, leucine and isoleucine degradation	5.15E-01	TRINITY_DN19215_c1_g1	-1.38	down	hypothetical protein POPTR_0007s10210g [Populus trichocarpa]	3.47	12.55
map00280	Valine, leucine and isoleucine degradation	5.15E-01	TRINITY_DN19215_c2_g1	-1.56	down	PREDICTED: probable protein phosphatase 2C 63 [Populus euphratica]	6.48	30.16
map00280	Valine, leucine and isoleucine degradation	5.15E-01	TRINITY_DN19294_c0_g3	1.09	up	hypothetical protein POPTR_0006s16050g [Populus trichocarpa]	91.23	67.60
map00280	Valine, leucine and isoleucine degradation	5.15E-01	TRINITY_DN19526_c1_g1	1.13	up	hypothetical protein POPTR_0003s20830g [Populus trichocarpa]	33.01	24.69
map00280	Valine, leucine and isoleucine degradation	5.15E-01	TRINITY_DN19913_c0_g1	1.05	up	PREDICTED: hydroxymethylglutaryl-CoA lyase, mitochondrial-like isoform X1 [Populus euphratica]	13.16	7.43
map00280	Valine, leucine and isoleucine degradation	5.15E-01	TRINITY_DN20891_c1_g1	2.08	up	PREDICTED: F-box protein At2g32560-like [Populus euphratica]	23.97	9.10
map00280	Valine, leucine and isoleucine degradation	5.15E-01	TRINITY_DN21140_c0_g2	2.24	up	mitochondrial lipoamide dehydrogenase [Populus tremuloides]	202.39	67.53
map00280	Valine, leucine and isoleucine degradation	5.15E-01	TRINITY_DN348_c0_g1	-3.27	down	hypothetical protein POPTR_0018s03880g [Populus trichocarpa]	0.18	2.75
map01212	Fatty acid metabolism	5.25E-01	TRINITY_DN22443_c0_g3	1.31	up	PREDICTED: 3-oxoacyl-[acyl-carrier-protein] synthase 3 A, chloroplastic [Populus euphratica]	34.98	21.39
map01212	Fatty acid metabolism	5.25E-01	TRINITY_DN23036_c0_g3	-1.08	down	PREDICTED: long chain acyl-CoA synthetase 2 isoform X1 [Populus euphratica]	4.05	12.87
map01212	Fatty acid metabolism	5.25E-01	TRINITY_DN23093_c0_g1	1.81	up	3-oxoacyl-[acyl-carrier-protein] synthase I [Populus trichocarpa]	128.96	55.68
map01212	Fatty acid metabolism	5.25E-01	TRINITY_DN23645_c0_g1	1.12	up	hypothetical protein POPTR_0008s17880g [Populus trichocarpa]	115.75	81.25
map01212	Fatty acid metabolism	5.25E-01	TRINITY_DN23776_c0_g1	1.29	up	hypothetical protein POPTR_0005s14280g [Populus trichocarpa]	31.39	19.38
map01212	Fatty acid metabolism	5.25E-01	TRINITY_DN24137_c0_g2	1.93	up	PREDICTED: acyl-[acyl-carrier-protein] desaturase, chloroplastic [Populus euphratica]	275.31	109.07
map01212	Fatty acid metabolism	5.25E-01	TRINITY_DN24307_c0_g1	1.05	up	PREDICTED: biotin carboxyl carrier protein of acetyl-CoA carboxylase, chloroplastic-like [Populus euphratica]	73.06	52.43
map01212	Fatty acid metabolism	5.25E-01	TRINITY_DN24431_c1_g2	-1.25	down	PREDICTED: acyl-coenzyme A oxidase 4, peroxisomal-like isoform X3 [Populus euphratica]	3.95	18.34
map01212	Fatty acid metabolism	5.25E-01	TRINITY_DN24627_c0_g1	1.20	up	PREDICTED: peroxisomal fatty acid beta-oxidation multifunctional protein AIM1 isoform X1 [Populus euphratica]	227.07	150.71
map01212	Fatty acid metabolism	5.25E-01	TRINITY_DN24813_c0_g2	1.21	up	hypothetical protein POPTR_0010s09620g [Populus trichocarpa]	9.00	6.87
map01212	Fatty acid metabolism	5.25E-01	TRINITY_DN24815_c0_g1	-1.19	down	multifunctional protein 2 [Populus tomentosa]	12.46	43.51
map01212	Fatty acid metabolism	5.25E-01	TRINITY_DN25183_c0_g1	-1.02	down	hypothetical protein POPTR_0013s02220g [Populus trichocarpa]	7.46	25.55
map01212	Fatty acid metabolism	5.25E-01	TRINITY_DN25227_c0_g3	-1.04	down	unknown [Populus trichocarpa x Populus deltoides]	4.77	15.05
map01212	Fatty acid metabolism	5.25E-01	TRINITY_DN25439_c0_g1	1.11	up	biotin carboxylase precursor family protein [Populus trichocarpa]	85.63	60.20

map01212	Fatty acid metabolism	5.25E-01	TRINITY_DN25504_c0_g1	1.69	up	chloroplast omega-3 desaturase family protein [Populus trichocarpa]	178.02	89.81
map01212	Fatty acid metabolism	5.25E-01	TRINITY_DN25671_c0_g1	1.18	up	ENOYL-ACP REDUCTASE 1 family protein [Populus trichocarpa]	122.50	78.93
map01212	Fatty acid metabolism	5.25E-01	TRINITY_DN25910_c0_g2	-1.06	down	PREDICTED: transcription factor PCL1-like [Populus euphratica]	4.93	16.82
map01212	Fatty acid metabolism	5.25E-01	TRINITY_DN26366_c0_g2	1.69	up	hypothetical protein POPTR_0002s22410g [Populus trichocarpa]	361.54	171.53
map01212	Fatty acid metabolism	5.25E-01	TRINITY_DN26565_c0_g1	1.03	up	hypothetical protein POPTR_0005s19990g [Populus trichocarpa]	60.48	36.43
map01212	Fatty acid metabolism	5.25E-01	TRINITY_DN26590_c0_g1	1.01	up	PREDICTED: acyl-CoA dehydrogenase family member 10-like isoform X2 [Populus euphratica]	17.06	13.06
map01212	Fatty acid metabolism	5.25E-01	TRINITY_DN27876_c5_g1	-1.61	down	hypothetical protein POPTR_0005s16540g [Populus trichocarpa]	2.20	11.69
map01212	Fatty acid metabolism	5.25E-01	TRINITY_DN27876_c5_g5	-2.33	down	PREDICTED: acetyl-CoA carboxylase 1-like [Populus euphratica]	0.87	6.39
map01212	Fatty acid metabolism	5.25E-01	TRINITY_DN15736_c0_g1	-10.07	down	hypothetical protein POPTR_0008s14850g [Populus trichocarpa]	0.00	7.88
map01212	Fatty acid metabolism	5.25E-01	TRINITY_DN16719_c0_g2	-2.32	down	short-chain dehydrogenase/reductase family protein [Populus trichocarpa]	0.77	6.53
map01212	Fatty acid metabolism	5.25E-01	TRINITY_DN17194_c0_g1	1.17	up	PREDICTED: very-long-chain enoyl-CoA reductase-like [Populus euphratica]	8.23	5.68
map01212	Fatty acid metabolism	5.25E-01	TRINITY_DN17705_c0_g2	1.24	up	hypothetical protein POPTR_0001s37860g [Populus trichocarpa]	8.10	5.27
map01212	Fatty acid metabolism	5.25E-01	TRINITY_DN18603_c0_g1	1.08	up	hypothetical protein POPTR_0003s14190g [Populus trichocarpa]	6.90	4.98
map01212	Fatty acid metabolism	5.25E-01	TRINITY_DN19050_c1_g1	1.32	up	hypothetical protein POPTR_0019s08170g, partial [Populus trichocarpa]	123.18	74.70
map01212	Fatty acid metabolism	5.25E-01	TRINITY_DN19330_c1_g4	1.30	up	hypothetical protein POPTR_0004s23710g [Populus trichocarpa]	22.29	13.72
map01212	Fatty acid metabolism	5.25E-01	TRINITY_DN19907_c0_g1	1.28	up	hypothetical protein POPTR_0001s24210g [Populus trichocarpa]	29.87	18.49
map01212	Fatty acid metabolism	5.25E-01	TRINITY_DN19983_c0_g1	1.13	up	pentatricopeptide repeat-containing family protein [Populus trichocarpa]	8.95	6.44
map01212	Fatty acid metabolism	5.25E-01	TRINITY_DN20038_c0_g1	1.19	up	PREDICTED: pentatricopeptide repeat-containing protein ELI1, chloroplastic [Populus euphratica]	14.92	9.99
map01212	Fatty acid metabolism	5.25E-01	TRINITY_DN20741_c0_g1	1.32	up	hypothetical protein POPTR_0009s08150g [Populus trichocarpa]	80.30	48.68
map01212	Fatty acid metabolism	5.25E-01	TRINITY_DN20754_c0_g3	1.78	up	hypothetical protein POPTR_0010s06230g [Populus trichocarpa]	19.13	8.33
map01212	Fatty acid metabolism	5.25E-01	TRINITY_DN20933_c0_g1	-3.74	down	hypothetical protein POPTR_0002s19330g [Populus trichocarpa]	0.65	13.67
map01212	Fatty acid metabolism	5.25E-01	TRINITY_DN20933_c0_g3	-2.76	down	PREDICTED: long chain acyl-CoA synthetase 1 [Populus euphratica]	0.62	6.55
map01212	Fatty acid metabolism	5.25E-01	TRINITY_DN21121_c0_g1	1.14	up	PREDICTED: pentatricopeptide repeat-containing protein At5g66520-like [Populus euphratica]	18.30	12.37
map01212	Fatty acid metabolism	5.25E-01	TRINITY_DN21469_c0_g3	1.53	up	unknown [Populus trichocarpa]	127.67	66.65
map01212	Fatty acid metabolism	5.25E-01	TRINITY_DN21528_c1_g1	-1.91	down	hypothetical protein POPTR_0002s09330g [Populus trichocarpa]	0.78	4.44
map01212	Fatty acid metabolism	5.25E-01	TRINITY_DN6900_c0_g1	1.83	up	short-chain dehydrogenase/reductase family protein [Populus trichocarpa]	21.13	8.97
map01212	Fatty acid metabolism	5.25E-01	TRINITY_DN7619_c0_g1	-4.15	down	PREDICTED: palmitoyl-acyl carrier protein thioesterase, chloroplastic-like [Populus euphratica]	0.09	2.75
map00330	Arginine and proline metabolism	5.36E-01	TRINITY_DN21932_c0_g3	-1.06	down	phosphatase 2C family protein [Populus trichocarpa]	2.53	8.10
map00330	Arginine and proline metabolism	5.36E-01	TRINITY_DN22813_c0_g4	1.08	up	aspartate transaminase family protein [Populus trichocarpa]	25.11	18.98
map00330	Arginine and proline metabolism	5.36E-01	TRINITY_DN23709_c0_g2	1.23	up	aspartate aminotransferase 2 family protein [Populus trichocarpa]	46.64	32.80
map00330	Arginine and proline metabolism	5.36E-01	TRINITY_DN24529_c1_g1	-2.26	down	polyamine oxidase 1 [Populus tomentosa]	4.94	37.37
map00330	Arginine and proline metabolism	5.36E-01	TRINITY_DN24529_c1_g3	-2.21	down	hypothetical protein POPTR_0001s27060g [Populus trichocarpa]	0.40	2.77
map00330	Arginine and proline metabolism	5.36E-01	TRINITY_DN24626_c0_g5	4.91	up	PREDICTED: polyamine oxidase-like [Populus euphratica]	238.94	11.76
map00330	Arginine and proline metabolism	5.36E-01	TRINITY_DN24628_c0_g1	1.28	up	PREDICTED: aldehyde dehydrogenase family 3 member H1-like [Populus euphratica]	40.39	24.46
map00330	Arginine and proline metabolism	5.36E-01	TRINITY_DN25564_c0_g1	-1.06	down	amine oxidase family protein [Populus trichocarpa]	6.24	17.85
map00330	Arginine and proline metabolism	5.36E-01	TRINITY_DN25630_c0_g1	1.30	up	aldehyde dehydrogenase 1 precursor family protein [Populus trichocarpa]	120.35	73.18
map00330	Arginine and proline metabolism	5.36E-01	TRINITY_DN26025_c0_g1	-1.71	down	hypothetical protein POPTR_0004s18340g [Populus trichocarpa]	5.60	27.70
map00330	Arginine and proline metabolism	5.36E-01	TRINITY_DN26342_c0_g1	1.99	up	S-adenosylmethionine decarboxylase family protein [Populus trichocarpa]	348.95	135.54
map00330	Arginine and proline metabolism	5.36E-01	TRINITY_DN26353_c0_g1	1.30	up	VuP5CS family protein [Populus trichocarpa]	49.12	31.04
map00330	Arginine and proline metabolism	5.36E-01	TRINITY_DN26421_c0_g1	1.32	up	the aldehyde dehydrogenase cp-ADH from C.plantagineum family protein [Populus trichocarpa]	58.13	34.64
map00330	Arginine and proline metabolism	5.36E-01	TRINITY_DN27082_c0_g1	-1.32	down	hypothetical protein POPTR_0005s22880g [Populus trichocarpa]	18.47	72.77
map00330	Arginine and proline metabolism	5.36E-01	TRINITY_DN13803_c0_g1	-3.27	down	PREDICTED: probable prolyl 4-hydroxylase 9 [Populus euphratica]	0.24	3.68

map00330	Arginine and proline metabolism	5.36E-01	TRINITY_DN15207_c0_g1	1.59	up	hypothetical protein POPTR_0006s04100g [Populus trichocarpa]	38.39	20.18
map00330	Arginine and proline metabolism	5.36E-01	TRINITY_DN15596_c0_g1	-2.20	down	hypothetical protein POPTR_0009s13300g [Populus trichocarpa]	0.46	2.84
map00330	Arginine and proline metabolism	5.36E-01	TRINITY_DN15596_c0_g2	-2.70	down	hypothetical protein POPTR_0009s13290g [Populus trichocarpa]	0.23	2.39
map00330	Arginine and proline metabolism	5.36E-01	TRINITY_DN18806_c0_g2	2.12	up	PREDICTED: S-adenosylmethionine decarboxylase proenzyme-like isoform X3 [Solanum pennellii]	161.94	56.16
map00330	Arginine and proline metabolism	5.36E-01	TRINITY_DN18895_c0_g1	-1.06	down	mitochondrial aldehyde dehydrogenase family protein [Populus trichocarpa]	7.40	23.22
map00330	Arginine and proline metabolism	5.36E-01	TRINITY_DN18964_c0_g2	1.33	up	PREDICTED: probable aldehyde dehydrogenase [Populus euphratica]	11.98	7.36
map00330	Arginine and proline metabolism	5.36E-01	TRINITY_DN19215_c1_g1	-1.38	down	hypothetical protein POPTR_0007s10210g [Populus trichocarpa]	3.47	12.55
map00330	Arginine and proline metabolism	5.36E-01	TRINITY_DN19215_c2_g1	-1.56	down	PREDICTED: probable protein phosphatase 2C 63 [Populus euphratica]	6.48	30.16
map00330	Arginine and proline metabolism	5.36E-01	TRINITY_DN20203_c0_g3	2.14	up	PREDICTED: uncharacterized protein LOC105127640 [Populus euphratica]	34.27	11.75
map00330	Arginine and proline metabolism	5.36E-01	TRINITY_DN21407_c0_g1	1.12	up	PREDICTED: NO-associated protein 1, chloroplastic/mitochondrial [Populus euphratica]	55.57	40.90
map00965	Betalain biosynthesis	5.46E-01	TRINITY_DN22947_c3_g1	-1.72	down	PREDICTED: 4,5-DOPA dioxygenase extradiol-like [Populus euphratica]	1.18	3.60
map00471	D-Glutamine and D-glutamate metabolism	5.46E-01	TRINITY_DN21747_c0_g1	3.00	up	hypothetical protein POPTR_0015s12250g [Populus trichocarpa]	29.79	5.40
map00514	Other types of O-glycan biosynthesis	5.49E-01	TRINITY_DN22869_c0_g1	-1.31	down	hypothetical protein POPTR_0015s13090g [Populus trichocarpa]	3.26	10.97
map00514	Other types of O-glycan biosynthesis	5.49E-01	TRINITY_DN17757_c0_g2	-1.90	down	PREDICTED: uncharacterized protein LOC105142247 [Populus euphratica]	1.03	5.94
map00514	Other types of O-glycan biosynthesis	5.49E-01	TRINITY_DN20021_c0_g1	-4.42	down	hypothetical protein POPTR_0014s07740g [Populus trichocarpa]	0.22	6.88
map00531	Glycosaminoglycan degradation	5.63E-01	TRINITY_DN22568_c0_g1	-1.35	down	PREDICTED: beta-galactosidase 5-like [Populus euphratica]	22.12	82.08
map00531	Glycosaminoglycan degradation	5.63E-01	TRINITY_DN22624_c0_g1	-1.58	down	hypothetical protein POPTR_0006s13130g [Populus trichocarpa]	2.14	9.80
map00531	Glycosaminoglycan degradation	5.63E-01	TRINITY_DN23239_c0_g3	1.09	up	hypothetical protein POPTR_0015s03440g [Populus trichocarpa]	61.43	43.63
map00531	Glycosaminoglycan degradation	5.63E-01	TRINITY_DN23319_c0_g4	1.15	up	PREDICTED: probable aquaporin PIP2-2 [Amborella trichopoda]	134.55	85.24
map00531	Glycosaminoglycan degradation	5.63E-01	TRINITY_DN25513_c0_g2	-1.55	down	PREDICTED: zinc finger protein NUTCRACKER-like isoform X5 [Populus euphratica]	13.47	55.16
map00531	Glycosaminoglycan degradation	5.63E-01	TRINITY_DN25522_c1_g1	-1.19	down	PREDICTED: zinc finger protein NUTCRACKER-like [Populus euphratica]	4.97	17.73
map00531	Glycosaminoglycan degradation	5.63E-01	TRINITY_DN25889_c0_g2	1.59	up	unknown [Populus trichocarpa x Populus deltoides]	384.22	194.87
map00531	Glycosaminoglycan degradation	5.63E-01	TRINITY_DN26010_c0_g4	-1.72	down	hypothetical protein POPTR_0006s13130g [Populus trichocarpa]	3.69	17.16
map00531	Glycosaminoglycan degradation	5.63E-01	TRINITY_DN26284_c1_g3	1.51	up	hypothetical protein POPTR_0008s07890g [Populus trichocarpa]	53.04	28.53
map00531	Glycosaminoglycan degradation	5.63E-01	TRINITY_DN26337_c3_g2	-1.73	down	PREDICTED: zinc finger protein NUTCRACKER-like [Populus euphratica]	2.46	10.32
map00531	Glycosaminoglycan degradation	5.63E-01	TRINITY_DN26363_c0_g1	1.20	up	hypothetical protein POPTR_0003s15750g [Populus trichocarpa]	50.15	39.16
map00531	Glycosaminoglycan degradation	5.63E-01	TRINITY_DN26839_c2_g2	-1.20	down	plasma membrane intrinsic protein 2;1 [Populus tremula x Populus alba]	141.52	493.68
map00531	Glycosaminoglycan degradation	5.63E-01	TRINITY_DN12335_c0_g2	1.44	up	PREDICTED: heparanase-like protein 2 [Populus euphratica]	4.06	2.34
map00531	Glycosaminoglycan degradation	5.63E-01	TRINITY_DN14721_c0_g1	-1.05	down	hypothetical protein POPTR_0008s14180g [Populus trichocarpa]	12.06	37.39
map00531	Glycosaminoglycan degradation	5.63E-01	TRINITY_DN19674_c1_g2	1.61	up	beta-galactosidase family protein [Populus trichocarpa]	9.86	4.84
map00531	Glycosaminoglycan degradation	5.63E-01	TRINITY_DN20455_c0_g4	-1.61	down	hypothetical protein POPTR_0012s03730g [Populus trichocarpa]	2.77	12.99
map00531	Glycosaminoglycan degradation	5.63E-01	TRINITY_DN20724_c2_g2	2.30	up	Gamma-tonoplast intrinsic protein 3 [Populus trichocarpa]	39.51	12.12
map00531	Glycosaminoglycan degradation	5.63E-01	TRINITY_DN20896_c1_g2	-1.22	down	PREDICTED: protein SHOOT GRAVITROPISM 5 [Populus euphratica]	4.99	19.09
map00531	Glycosaminoglycan degradation	5.63E-01	TRINITY_DN7728_c0_g2	2.29	up	hypothetical protein POPTR_0006s14130g [Populus trichocarpa]	1.77	0.56
map00730	Thiamine metabolism	6.43E-01	TRINITY_DN22607_c0_g1	3.36	up	hypothetical protein POPTR_0004s01990g [Populus trichocarpa]	2811.82	419.38
map00730	Thiamine metabolism	6.43E-01	TRINITY_DN22711_c0_g1	1.10	up	hypothetical protein POPTR_0011s13820g [Populus trichocarpa]	450.01	318.88
map00730	Thiamine metabolism	6.43E-01	TRINITY_DN25309_c0_g1	1.17	up	hydroxyethylthiazole kinase family protein [Populus trichocarpa]	8.19	7.36
map00730	Thiamine metabolism	6.43E-01	TRINITY_DN25565_c0_g7	-1.49	down	PREDICTED: probable 1-deoxy-D-xylulose-5-phosphate synthase 2, chloroplastic [Populus euphratica]	1.26	5.40
map00730	Thiamine metabolism	6.43E-01	TRINITY_DN27298_c0_g1	1.57	up	PREDICTED: phosphomethylpyrimidine synthase, chloroplastic [Populus euphratica]	350.59	177.84
map00943	Isoflavonoid biosynthesis	6.43E-01	TRINITY_DN25520_c1_g1	2.32	up	PREDICTED: phenolic glucoside malonyltransferase 1-like [Populus euphratica]	138.11	49.97

map00943	Isoflavonoid biosynthesis	6.43E-01	TRINITY_DN16570_c0_g1	-1.64	down	hypothetical protein POPTR_0014s03230g [Populus trichocarpa]	1.04	4.89
map00943	Isoflavonoid biosynthesis	6.43E-01	TRINITY_DN17000_c0_g1	1.16	up	hypothetical protein POPTR_0009s06800g [Populus trichocarpa]	3.15	2.14
map00943	Isoflavonoid biosynthesis	6.43E-01	TRINITY_DN17077_c0_g1	1.36	up	PREDICTED: phenolic glucoside malonyltransferase 1-like [Populus euphratica]	121.68	73.71
map00943	Isoflavonoid biosynthesis	6.43E-01	TRINITY_DN19224_c0_g1	1.22	up	hypothetical protein POPTR_0004s09550g [Populus trichocarpa]	55.14	35.39
map00261	Monobactam biosynthesis	6.43E-01	TRINITY_DN23172_c0_g1	1.90	up	hypothetical protein POPTR_0017s00350g [Populus trichocarpa]	54.45	26.38
map00261	Monobactam biosynthesis	6.43E-01	TRINITY_DN25737_c0_g1	1.82	up	semialdehyde dehydrogenase family protein [Populus trichocarpa]	56.96	24.76
map00261	Monobactam biosynthesis	6.43E-01	TRINITY_DN20815_c0_g1	1.41	up	PREDICTED: ATP sulfurylase 2-like [Populus euphratica]	46.46	28.77
map00261	Monobactam biosynthesis	6.43E-01	TRINITY_DN21210_c0_g6	1.13	up	homoserine dehydrogenase family protein [Populus trichocarpa]	16.38	11.90
map00261	Monobactam biosynthesis	6.43E-01	TRINITY_DN21635_c1_g2	-2.07	down	SEC14 cytosolic factor family protein [Populus trichocarpa]	5.54	32.41
map00450	Selenocompound metabolism	6.45E-01	TRINITY_DN22772_c0_g5	-3.64	down	PREDICTED: probable auxin efflux carrier component 6 [Populus euphratica]	0.49	9.37
map00450	Selenocompound metabolism	6.45E-01	TRINITY_DN22772_c0_g6	-4.05	down	PIN1-like auxin transport protein [Populus tremula x Populus tremuloides]	0.54	26.50
map00450	Selenocompound metabolism	6.45E-01	TRINITY_DN22786_c0_g1	1.68	up	PREDICTED: NADPH-dependent thioredoxin reductase 3 [Populus euphratica]	86.21	44.16
map00450	Selenocompound metabolism	6.45E-01	TRINITY_DN23046_c0_g1	-1.86	down	PIN1-like family protein [Populus trichocarpa]	0.66	3.70
map00450	Selenocompound metabolism	6.45E-01	TRINITY_DN25289_c0_g2	1.21	up	PREDICTED: methionine--tRNA ligase, mitochondrial [Populus euphratica]	47.66	32.26
map00450	Selenocompound metabolism	6.45E-01	TRINITY_DN26665_c0_g1	1.58	up	hypothetical protein POPTR_0009s15490g [Populus trichocarpa]	216.45	108.64
map00450	Selenocompound metabolism	6.45E-01	TRINITY_DN18387_c0_g1	1.43	up	hypothetical protein POPTR_0017s12240g [Populus trichocarpa]	122.22	69.70
map00450	Selenocompound metabolism	6.45E-01	TRINITY_DN20815_c0_g1	1.41	up	PREDICTED: ATP sulfurylase 2-like [Populus euphratica]	46.46	28.77
map00450	Selenocompound metabolism	6.45E-01	TRINITY_DN20889_c0_g1	1.15	up	cysteine desulfurase family protein [Populus trichocarpa]	68.73	46.60
map00450	Selenocompound metabolism	6.45E-01	TRINITY_DN7763_c0_g1	10.19	up	cystathionine gamma-synthase [Populus tomentosa]	18.32	0.02
map00592	alpha-Linolenic acid metabolism	6.54E-01	TRINITY_DN22498_c0_g1	2.42	up	benzoyl-CoA:benzylalcohol/2-phenylethanol benzoyltransferase, partial [Populus davidiana]	1333.43	383.43
map00592	alpha-Linolenic acid metabolism	6.54E-01	TRINITY_DN23274_c0_g1	-2.24	down	PREDICTED: phospholipase A1-Ibeta2, chloroplastic-like [Populus euphratica]	1.95	13.94
map00592	alpha-Linolenic acid metabolism	6.54E-01	TRINITY_DN23274_c0_g2	-5.05	down	hypothetical protein POPTR_0003s07940g [Populus trichocarpa]	0.48	24.54
map00592	alpha-Linolenic acid metabolism	6.54E-01	TRINITY_DN23579_c0_g2	-2.24	down	hypothetical protein POPTR_0001s16780g [Populus trichocarpa]	14.61	101.79
map00592	alpha-Linolenic acid metabolism	6.54E-01	TRINITY_DN24353_c0_g3	-1.68	down	lipoxygenase family protein [Populus trichocarpa]	1.48	7.25
map00592	alpha-Linolenic acid metabolism	6.54E-01	TRINITY_DN24431_c1_g2	-1.25	down	PREDICTED: acyl-coenzyme A oxidase 4, peroxisomal-like isoform X3 [Populus euphratica]	3.95	18.34
map00592	alpha-Linolenic acid metabolism	6.54E-01	TRINITY_DN24599_c0_g1	-5.32	down	hypothetical protein POPTR_0003s01420g [Populus trichocarpa]	0.18	9.27
map00592	alpha-Linolenic acid metabolism	6.54E-01	TRINITY_DN24627_c0_g1	1.20	up	PREDICTED: peroxisomal fatty acid beta-oxidation multifunctional protein AIM1 isoform X1 [Populus euphratica]	227.07	150.71
map00592	alpha-Linolenic acid metabolism	6.54E-01	TRINITY_DN24718_c0_g1	-1.13	down	PREDICTED: calmodulin-binding transcription activator 3-like isoform X3 [Populus euphratica]	2.73	8.94
map00592	alpha-Linolenic acid metabolism	6.54E-01	TRINITY_DN24815_c0_g1	-1.19	down	multifunctional protein 2 [Populus tomentosa]	12.46	43.51
map00592	alpha-Linolenic acid metabolism	6.54E-01	TRINITY_DN25882_c0_g2	2.81	up	hypothetical protein POPTR_0004s22180g [Populus trichocarpa]	32.38	9.68
map00592	alpha-Linolenic acid metabolism	6.54E-01	TRINITY_DN26366_c0_g2	1.69	up	hypothetical protein POPTR_0002s22410g [Populus trichocarpa]	361.54	171.53
map00592	alpha-Linolenic acid metabolism	6.54E-01	TRINITY_DN26973_c0_g1	-1.85	down	hypothetical protein POPTR_0001s16780g [Populus trichocarpa]	4.72	22.23
map00592	alpha-Linolenic acid metabolism	6.54E-01	TRINITY_DN27547_c0_g1	-1.35	down	calmodulin-binding family protein [Populus trichocarpa]	6.25	23.99
map00592	alpha-Linolenic acid metabolism	6.54E-01	TRINITY_DN12645_c0_g1	-4.27	down	putative alpha-dioxygenase family protein [Populus trichocarpa]	0.07	2.13
map00592	alpha-Linolenic acid metabolism	6.54E-01	TRINITY_DN14219_c0_g1	-2.35	down	S-adenosyl-L-methionine:carboxyl methyltransferase family protein [Populus trichocarpa]	0.43	3.49
map00592	alpha-Linolenic acid metabolism	6.54E-01	TRINITY_DN15578_c0_g1	-10.44	down	SABATH methyltransferase 3 [Populus trichocarpa]	0.00	7.21
map00592	alpha-Linolenic acid metabolism	6.54E-01	TRINITY_DN18345_c0_g4	2.78	up	hypothetical protein POPTR_0013s10170g [Populus trichocarpa]	32.67	7.40
map00592	alpha-Linolenic acid metabolism	6.54E-01	TRINITY_DN18404_c0_g1	-2.69	down	SABATH methyltransferase 28 [Populus trichocarpa]	1.88	17.92
map00592	alpha-Linolenic acid metabolism	6.54E-01	TRINITY_DN21334_c0_g2	-3.99	down	SAMT, partial [Populus x beijingensis]	0.13	3.35
map00592	alpha-Linolenic acid metabolism	6.54E-01	TRINITY_DN3610_c0_g1	1.74	up	hypothetical protein POPTR_0004s05380g [Populus trichocarpa]	53.62	22.92
map00901	Indole alkaloid biosynthesis	6.62E-01	TRINITY_DN17915_c0_g1	1.39	up	hypothetical protein POPTR_0007s11740g [Populus trichocarpa]	42.52	24.99
map00901	Indole alkaloid biosynthesis	6.62E-01	TRINITY_DN18689_c0_g1	3.51	up	PREDICTED: strictosidine synthase 3-like isoform X1 [Populus euphratica]	19.71	2.54
map00901	Indole alkaloid biosynthesis	6.62E-01	TRINITY_DN20062_c1_g1	-1.15	down	hypothetical protein POPTR_0007s11750g [Populus trichocarpa]	13.47	41.01

map00062	Fatty acid elongation	6.64E-01	TRINITY_DN22220_c0_g1	1.13	up	beta-ketoacyl-CoA synthase family protein [Populus trichocarpa]	15.47	13.48
map00062	Fatty acid elongation	6.64E-01	TRINITY_DN23776_c0_g1	1.29	up	hypothetical protein POPTR_0005s14280g [Populus trichocarpa]	31.39	19.38
map00062	Fatty acid elongation	6.64E-01	TRINITY_DN24642_c0_g1	-1.04	down	PREDICTED: 3-ketoacyl-CoA synthase 3-like [Populus euphratica]	1.48	4.58
map00062	Fatty acid elongation	6.64E-01	TRINITY_DN24813_c0_g2	1.21	up	hypothetical protein POPTR_0010s09620g [Populus trichocarpa]	9.00	6.87
map00062	Fatty acid elongation	6.64E-01	TRINITY_DN24844_c0_g1	-1.50	down	hypothetical protein POPTR_0008s15990g [Populus trichocarpa]	6.75	33.49
map00062	Fatty acid elongation	6.64E-01	TRINITY_DN26567_c0_g1	1.87	up	PREDICTED: mitochondrial carnitine/acylcarnitine carrier-like protein isoform X1 [Populus euphratica]	356.33	145.88
map00062	Fatty acid elongation	6.64E-01	TRINITY_DN14003_c0_g2	-2.79	down	fatty acid elongase 3-ketoacyl-CoA synthase 1 family protein [Populus trichocarpa]	0.22	4.38
map00062	Fatty acid elongation	6.64E-01	TRINITY_DN14003_c0_g3	-2.11	down	fatty acid elongase 3-ketoacyl-CoA synthase 1 family protein [Populus trichocarpa]	0.44	2.93
map00062	Fatty acid elongation	6.64E-01	TRINITY_DN14501_c0_g1	-6.23	down	hypothetical protein POPTR_0003s01410g [Populus trichocarpa]	0.03	4.29
map00062	Fatty acid elongation	6.64E-01	TRINITY_DN15120_c0_g2	-4.03	down	hypothetical protein POPTR_0013s12390g [Populus trichocarpa]	0.14	3.54
map00062	Fatty acid elongation	6.64E-01	TRINITY_DN15459_c0_g1	-5.82	down	fatty acid biosynthetic process transferase [Populus tomentosa]	0.05	4.51
map00062	Fatty acid elongation	6.64E-01	TRINITY_DN16347_c0_g2	1.08	up	GNS1/SUR4 membrane family protein [Populus trichocarpa]	17.42	12.48
map00062	Fatty acid elongation	6.64E-01	TRINITY_DN16719_c0_g2	-2.32	down	short-chain dehydrogenase/reductase family protein [Populus trichocarpa]	0.77	6.53
map00062	Fatty acid elongation	6.64E-01	TRINITY_DN17194_c0_g1	1.17	up	PREDICTED: very-long-chain enoyl-CoA reductase-like [Populus euphratica]	8.23	5.68
map00062	Fatty acid elongation	6.64E-01	TRINITY_DN17705_c0_g2	1.24	up	hypothetical protein POPTR_0001s37860g [Populus trichocarpa]	8.10	5.27
map00062	Fatty acid elongation	6.64E-01	TRINITY_DN19907_c0_g1	1.28	up	hypothetical protein POPTR_0001s24210g [Populus trichocarpa]	29.87	18.49
map00062	Fatty acid elongation	6.64E-01	TRINITY_DN19983_c0_g1	1.13	up	pentatricopeptide repeat-containing family protein [Populus trichocarpa]	8.95	6.44
map00062	Fatty acid elongation	6.64E-01	TRINITY_DN20038_c0_g1	1.19	up	PREDICTED: pentatricopeptide repeat-containing protein ELI1, chloroplastic [Populus euphratica]	14.92	9.99
map00062	Fatty acid elongation	6.64E-01	TRINITY_DN20754_c0_g3	1.78	up	hypothetical protein POPTR_0010s06230g [Populus trichocarpa]	19.13	8.33
map00062	Fatty acid elongation	6.64E-01	TRINITY_DN21121_c0_g1	1.14	up	PREDICTED: pentatricopeptide repeat-containing protein At5g66520-like [Populus euphratica]	18.30	12.37
map00600	Sphingolipid metabolism	6.64E-01	TRINITY_DN21906_c0_g1	-1.13	down	hypothetical protein POPTR_0001s15560g [Populus trichocarpa]	2.04	7.02
map00600	Sphingolipid metabolism	6.64E-01	TRINITY_DN22022_c1_g1	1.42	up	ceramidase family protein [Populus trichocarpa]	12.10	6.91
map00600	Sphingolipid metabolism	6.64E-01	TRINITY_DN22240_c0_g4	-1.57	down	hypothetical protein POPTR_0016s02070g [Populus trichocarpa]	2.17	9.60
map00600	Sphingolipid metabolism	6.64E-01	TRINITY_DN22568_c0_g1	-1.35	down	PREDICTED: beta-galactosidase 5-like [Populus euphratica]	22.12	82.08
map00600	Sphingolipid metabolism	6.64E-01	TRINITY_DN22624_c0_g1	-1.58	down	hypothetical protein POPTR_0006s13130g [Populus trichocarpa]	2.14	9.80
map00600	Sphingolipid metabolism	6.64E-01	TRINITY_DN24194_c0_g2	-1.10	down	hypothetical protein POPTR_0007s12320g [Populus trichocarpa]	2.32	7.59
map00600	Sphingolipid metabolism	6.64E-01	TRINITY_DN24537_c0_g6	-2.31	down	hypothetical protein MANES_16G026000 [Manihot esculenta]	0.34	2.70
map00600	Sphingolipid metabolism	6.64E-01	TRINITY_DN25513_c0_g2	-1.55	down	PREDICTED: zinc finger protein NUTCRACKER-like isoform X5 [Populus euphratica]	13.47	55.16
map00600	Sphingolipid metabolism	6.64E-01	TRINITY_DN25522_c1_g1	-1.19	down	PREDICTED: zinc finger protein NUTCRACKER-like [Populus euphratica]	4.97	17.73
map00600	Sphingolipid metabolism	6.64E-01	TRINITY_DN25540_c0_g1	2.51	up	hypothetical protein POPTR_0004s24220g [Populus trichocarpa]	39.42	13.08
map00600	Sphingolipid metabolism	6.64E-01	TRINITY_DN25889_c0_g2	1.59	up	unknown [Populus trichocarpa x Populus deltoides]	384.22	194.87
map00600	Sphingolipid metabolism	6.64E-01	TRINITY_DN26010_c0_g4	-1.72	down	hypothetical protein POPTR_0006s13130g [Populus trichocarpa]	3.69	17.16
map00600	Sphingolipid metabolism	6.64E-01	TRINITY_DN26288_c0_g5	-2.57	down	PREDICTED: LOW QUALITY PROTEIN: non-lysosomal glucosylceramidase-like [Populus euphratica]	0.29	2.74
map00600	Sphingolipid metabolism	6.64E-01	TRINITY_DN26337_c3_g2	-1.73	down	PREDICTED: zinc finger protein NUTCRACKER-like [Populus euphratica]	2.46	10.32
map00600	Sphingolipid metabolism	6.64E-01	TRINITY_DN26691_c0_g2	-1.54	down	PREDICTED: LOW QUALITY PROTEIN: neutral ceramidase [Populus euphratica]	2.18	9.58
map00600	Sphingolipid metabolism	6.64E-01	TRINITY_DN27031_c2_g1	-1.33	down	hypothetical protein POPTR_0006s09490g [Populus trichocarpa]	44.37	151.26
map00600	Sphingolipid metabolism	6.64E-01	TRINITY_DN14721_c0_g1	-1.05	down	hypothetical protein POPTR_0008s14180g [Populus trichocarpa]	12.06	37.39
map00600	Sphingolipid metabolism	6.64E-01	TRINITY_DN17934_c2_g5	1.23	up	hypothetical protein POPTR_0011s00700g [Populus trichocarpa]	3.53	2.32
map00600	Sphingolipid metabolism	6.64E-01	TRINITY_DN18466_c0_g1	3.25	up	hypothetical protein POPTR_0018s14920g [Populus trichocarpa]	22.92	4.21
map00600	Sphingolipid metabolism	6.64E-01	TRINITY_DN19674_c1_g2	1.61	up	beta-galactosidase family protein [Populus trichocarpa]	9.86	4.84
map00600	Sphingolipid metabolism	6.64E-01	TRINITY_DN20455_c0_g4	-1.61	down	hypothetical protein POPTR_0012s03730g [Populus trichocarpa]	2.77	12.99

map00600	Sphingolipid metabolism	6.64E-01	TRINITY_DN20694_c0_g1	1.09	up	hypothetical protein POPTR_0002s16440g [Populus trichocarpa]	19.99	18.02
map00600	Sphingolipid metabolism	6.64E-01	TRINITY_DN20896_c1_g2	-1.22	down	PREDICTED: protein SHOOT GRAVITROPISM 5 [Populus euphratica]	4.99	19.09
map00600	Sphingolipid metabolism	6.64E-01	TRINITY_DN7728_c0_g2	2.29	up	hypothetical protein POPTR_0006s14130g [Populus trichocarpa]	1.77	0.56
map00254	Aflatoxin biosynthesis	7.10E-01	TRINITY_DN27876_c5_g1	-1.61	down	hypothetical protein POPTR_0005s16540g [Populus trichocarpa]	2.20	11.69
map00254	Aflatoxin biosynthesis	7.10E-01	TRINITY_DN27876_c5_g5	-2.33	down	PREDICTED: acetyl-CoA carboxylase 1-like [Populus euphratica]	0.87	6.39
map00254	Aflatoxin biosynthesis	7.10E-01	TRINITY_DN21528_c1_g1	-1.91	down	hypothetical protein POPTR_0002s09330g [Populus trichocarpa]	0.78	4.44
map03020	RNA polymerase	7.33E-01	TRINITY_DN22804_c0_g4	-2.56	down	ERF domain protein 12 [Populus trichocarpa]	0.99	8.94
map03020	RNA polymerase	7.33E-01	TRINITY_DN22866_c0_g1	1.39	up	PREDICTED: mitochondrial-processing peptidase subunit alpha-like [Populus euphratica]	54.03	31.47
map03020	RNA polymerase	7.33E-01	TRINITY_DN23869_c0_g2	1.26	up	PREDICTED: ethylene-responsive transcription factor RAP2-12-like isoform X2 [Populus euphratica]	18.61	11.90
map03020	RNA polymerase	7.33E-01	TRINITY_DN24268_c0_g3	-2.26	down	PREDICTED: DNA-directed RNA polymerases IV and V subunit 2-like [Populus euphratica]	0.30	2.20
map03020	RNA polymerase	7.33E-01	TRINITY_DN24355_c2_g4	-1.38	down	hypothetical protein POPTR_0003s10650g [Populus trichocarpa]	4.31	16.98
map03020	RNA polymerase	7.33E-01	TRINITY_DN24425_c1_g7	1.89	up	RecName: Full=DNA-directed RNA polymerase subunit beta'; AltName: Full=PEP; AltName: Full=Plastid-encoded RNA polymerase subunit beta'; Short=RNA polymerase subunit beta'	5.92	2.43
map03020	RNA polymerase	7.33E-01	TRINITY_DN26308_c0_g1	-1.12	down	hypothetical protein POPTR_0003s19630g [Populus trichocarpa]	5.58	19.18
map03020	RNA polymerase	7.33E-01	TRINITY_DN16872_c0_g1	-5.53	down	hypothetical protein POPTR_0002s04430g [Populus trichocarpa]	0.45	31.91
map03020	RNA polymerase	7.33E-01	TRINITY_DN17816_c0_g6	-2.04	down	ERF domain protein 11 [Populus trichocarpa]	3.09	18.64
map03020	RNA polymerase	7.33E-01	TRINITY_DN18436_c1_g3	-1.79	down	PREDICTED: DNA-directed RNA polymerase III subunit RPC6-like [Populus euphratica]	0.98	5.17
map03020	RNA polymerase	7.33E-01	TRINITY_DN19931_c0_g15	1.32	up	RNA polymerase beta" subunit [Populus alba]	3.76	2.38
map03020	RNA polymerase	7.33E-01	TRINITY_DN19931_c0_g8	1.61	up	RNA polymerase beta" subunit [Populus alba]	18.11	8.96
map03020	RNA polymerase	7.33E-01	TRINITY_DN19959_c0_g1	1.17	up	hypothetical protein POPTR_0005s25810g [Populus trichocarpa]	31.81	22.34
map03020	RNA polymerase	7.33E-01	TRINITY_DN21182_c0_g4	1.43	up	RNA polymerase alpha subunit [Populus alba]	6.16	3.50
map03020	RNA polymerase	7.33E-01	TRINITY_DN21202_c0_g2	-2.06	down	hypothetical protein POPTR_0017s04540g [Populus trichocarpa]	24.81	154.42
map03020	RNA polymerase	7.33E-01	TRINITY_DN21216_c0_g1	2.14	up	hypothetical protein POPTR_0005s06460g [Populus trichocarpa]	132.71	46.26
map03020	RNA polymerase	7.33E-01	TRINITY_DN21497_c0_g3	-3.63	down	Ethylene responsive element binding factor 4 family protein [Populus trichocarpa]	10.72	198.84
map03020	RNA polymerase	7.33E-01	TRINITY_DN21497_c0_g4	-3.32	down	ERF4 [Populus x canadensis]	11.08	166.25
map03020	RNA polymerase	7.33E-01	TRINITY_DN21521_c0_g1	-2.76	down	hypothetical protein POPTR_0007s07830g [Populus trichocarpa]	4.44	45.77
map00240	Pyrimidine metabolism	7.38E-01	TRINITY_DN21807_c1_g1	1.05	up	PREDICTED: pentatricopeptide repeat-containing protein At3g46790, chloroplastic [Populus euphratica]	6.02	4.47
map00240	Pyrimidine metabolism	7.38E-01	TRINITY_DN22065_c0_g1	1.32	up	PREDICTED: inactive rhomboid protein 1-like [Populus euphratica]	5.41	3.36
map00240	Pyrimidine metabolism	7.38E-01	TRINITY_DN22206_c0_g4	-1.15	down	PREDICTED: trihelix transcription factor ASIL1 isoform X1 [Populus euphratica]	3.42	11.47
map00240	Pyrimidine metabolism	7.38E-01	TRINITY_DN22492_c1_g4	-1.43	down	hypothetical protein POPTR_0003s19840g [Populus trichocarpa]	1.57	6.57
map00240	Pyrimidine metabolism	7.38E-01	TRINITY_DN22772_c0_g5	-3.64	down	PREDICTED: probable auxin efflux carrier component 6 [Populus euphratica]	0.49	9.37
map00240	Pyrimidine metabolism	7.38E-01	TRINITY_DN22772_c0_g6	-4.05	down	PIN1-like auxin transport protein [Populus tremula x Populus tremuloides]	0.54	26.50
map00240	Pyrimidine metabolism	7.38E-01	TRINITY_DN22786_c0_g1	1.68	up	PREDICTED: NADPH-dependent thioredoxin reductase 3 [Populus euphratica]	86.21	44.16
map00240	Pyrimidine metabolism	7.38E-01	TRINITY_DN22804_c0_g4	-2.56	down	ERF domain protein 12 [Populus trichocarpa]	0.99	8.94
map00240	Pyrimidine metabolism	7.38E-01	TRINITY_DN22866_c0_g1	1.39	up	PREDICTED: mitochondrial-processing peptidase subunit alpha-like [Populus euphratica]	54.03	31.47
map00240	Pyrimidine metabolism	7.38E-01	TRINITY_DN22903_c0_g1	1.52	up	PREDICTED: uridine 5'-monophosphate synthase-like [Populus euphratica]	94.37	50.65
map00240	Pyrimidine metabolism	7.38E-01	TRINITY_DN22908_c0_g1	1.42	up	PREDICTED: pentatricopeptide repeat-containing protein DOT4, chloroplastic-like [Populus euphratica]	5.53	3.14
map00240	Pyrimidine metabolism	7.38E-01	TRINITY_DN22920_c1_g7	1.63	up	hypothetical protein POPTR_0004s05320g [Populus trichocarpa]	5.46	2.68
map00240	Pyrimidine metabolism	7.38E-01	TRINITY_DN23046_c0_g1	-1.86	down	PIN1-like family protein [Populus trichocarpa]	0.66	3.70
map00240	Pyrimidine metabolism	7.38E-01	TRINITY_DN23869_c0_g2	1.26	up	PREDICTED: ethylene-responsive transcription factor RAP2-12-like isoform X2 [Populus euphratica]	18.61	11.90
map00240	Pyrimidine metabolism	7.38E-01	TRINITY_DN23876_c0_g1	-2.14	down	hypothetical protein POPTR_0007s10880g [Populus trichocarpa]	0.94	6.43
map00240	Pyrimidine metabolism	7.38E-01	TRINITY_DN23876_c0_g2	-1.91	down	hypothetical protein POPTR_0007s10880g [Populus trichocarpa]	1.84	10.67
map00240	Pyrimidine metabolism	7.38E-01	TRINITY_DN23886_c0_g1	-1.38	down	PREDICTED: uncharacterized protein LOC105121779 isoform X1 [Populus euphratica]	7.25	26.43

map00240	Pyrimidine metabolism	7.38E-01	TRINITY_DN24112_c0_g1	1.05	up	pentatricopeptide repeat-containing protein [Populus tomentosa]	10.95	11.67
map00240	Pyrimidine metabolism	7.38E-01	TRINITY_DN24244_c0_g2	1.86	up	hypothetical protein POPTR_0001s36730g [Populus trichocarpa]	48.33	20.26
map00240	Pyrimidine metabolism	7.38E-01	TRINITY_DN24268_c0_g3	-2.26	down	PREDICTED: DNA-directed RNA polymerases IV and V subunit 2-like [Populus euphratica]	0.30	2.20
map00240	Pyrimidine metabolism	7.38E-01	TRINITY_DN24355_c2_g4	-1.38	down	hypothetical protein POPTR_0003s10650g [Populus trichocarpa]	4.31	16.98
map00240	Pyrimidine metabolism	7.38E-01	TRINITY_DN24395_c0_g2	1.21	up	hypothetical protein POPTR_0005s26230g [Populus trichocarpa]	66.57	46.27
map00240	Pyrimidine metabolism	7.38E-01	TRINITY_DN24425_c1_g7	1.89	up	RecName: Full=DNA-directed RNA polymerase subunit beta'; AltName: Full=PEP; AltName: Full=Plastid-encoded RNA polymerase subunit beta'; Short=RNA polymerase subunit beta'	5.92	2.43
map00240	Pyrimidine metabolism	7.38E-01	TRINITY_DN24534_c0_g2	-1.50	down	PREDICTED: uncharacterized rhomboid protein AN10929 [Populus euphratica]	15.35	62.82
map00240	Pyrimidine metabolism	7.38E-01	TRINITY_DN24662_c0_g2	-1.93	down	ferritin/ribonucleotide reductase-like family protein [Populus tomentosa]	4.88	29.25
map00240	Pyrimidine metabolism	7.38E-01	TRINITY_DN24662_c0_g3	-1.05	down	PREDICTED: ribonucleoside-diphosphate reductase small chain [Populus euphratica]	25.18	80.64
map00240	Pyrimidine metabolism	7.38E-01	TRINITY_DN25276_c0_g2	-1.48	down	hypothetical protein POPTR_0018s00860g [Populus trichocarpa]	1.70	6.52
map00240	Pyrimidine metabolism	7.38E-01	TRINITY_DN25285_c0_g1	-1.11	down	PREDICTED: ribonucleoside-diphosphate reductase large subunit-like [Populus euphratica]	33.12	103.10
map00240	Pyrimidine metabolism	7.38E-01	TRINITY_DN25500_c0_g2	-1.03	down	nucleoside phosphatase family protein [Populus trichocarpa]	6.42	19.00
map00240	Pyrimidine metabolism	7.38E-01	TRINITY_DN26308_c0_g1	-1.12	down	hypothetical protein POPTR_0003s19630g [Populus trichocarpa]	5.58	19.18
map00240	Pyrimidine metabolism	7.38E-01	TRINITY_DN26609_c0_g1	1.61	up	carbamoyl phosphate synthetase a family protein [Populus trichocarpa]	218.53	107.54
map00240	Pyrimidine metabolism	7.38E-01	TRINITY_DN27190_c0_g2	-2.22	down	hypothetical protein POPTR_0002s22130g [Populus trichocarpa]	3.55	26.41
map00240	Pyrimidine metabolism	7.38E-01	TRINITY_DN27349_c0_g1	1.26	up	hypothetical protein POPTR_0001s44720g [Populus trichocarpa]	31.99	22.49
map00240	Pyrimidine metabolism	7.38E-01	TRINITY_DN27544_c0_g1	-1.20	down	PREDICTED: CTP synthase-like isoform X1 [Populus euphratica]	17.08	60.80
map00240	Pyrimidine metabolism	7.38E-01	TRINITY_DN27834_c1_g1	1.03	up	PREDICTED: CTP synthase-like [Populus euphratica]	31.65	23.97
map00240	Pyrimidine metabolism	7.38E-01	TRINITY_DN14700_c0_g1	-2.16	down	hypothetical protein POPTR_0008s01650g [Populus trichocarpa]	0.80	4.63
map00240	Pyrimidine metabolism	7.38E-01	TRINITY_DN16197_c0_g1	1.09	up	PREDICTED: CTP synthase [Populus euphratica]	62.94	46.27
map00240	Pyrimidine metabolism	7.38E-01	TRINITY_DN16872_c0_g1	-5.53	down	hypothetical protein POPTR_0002s04430g [Populus trichocarpa]	0.45	31.91
map00240	Pyrimidine metabolism	7.38E-01	TRINITY_DN16976_c0_g4	-1.35	down	DNA-directed DNA polymerase epsilon catalytic subunit family protein [Populus trichocarpa]	2.96	11.76
map00240	Pyrimidine metabolism	7.38E-01	TRINITY_DN16976_c0_g5	-1.67	down	DNA-directed DNA polymerase epsilon catalytic subunit family protein [Populus trichocarpa]	0.84	4.16
map00240	Pyrimidine metabolism	7.38E-01	TRINITY_DN17196_c0_g2	1.33	up	hypothetical protein POPTR_0015s10720g [Populus trichocarpa]	3.12	1.90
map00240	Pyrimidine metabolism	7.38E-01	TRINITY_DN17241_c0_g1	-1.38	down	DNA polymerase delta subunit 4 family protein [Populus trichocarpa]	4.03	15.90
map00240	Pyrimidine metabolism	7.38E-01	TRINITY_DN17241_c0_g2	-1.21	down	DNA polymerase delta subunit 4 family protein [Populus trichocarpa]	37.87	132.98
map00240	Pyrimidine metabolism	7.38E-01	TRINITY_DN17816_c0_g6	-2.04	down	ERF domain protein 11 [Populus trichocarpa]	3.09	18.64
map00240	Pyrimidine metabolism	7.38E-01	TRINITY_DN17925_c1_g1	3.02	up	hypothetical protein POPTR_0014s04880g [Populus trichocarpa]	213.24	40.67
map00240	Pyrimidine metabolism	7.38E-01	TRINITY_DN17925_c1_g2	1.30	up	unknown [Populus trichocarpa]	708.50	436.01
map00240	Pyrimidine metabolism	7.38E-01	TRINITY_DN17951_c0_g1	1.19	up	PREDICTED: nucleoside diphosphate kinase 3 isoform X1 [Populus euphratica]	91.53	60.90
map00240	Pyrimidine metabolism	7.38E-01	TRINITY_DN17951_c0_g2	1.83	up	hypothetical protein POPTR_0001s10670g [Populus trichocarpa]	286.39	121.84
map00240	Pyrimidine metabolism	7.38E-01	TRINITY_DN18436_c1_g3	-1.79	down	PREDICTED: DNA-directed RNA polymerase III subunit RPC6-like [Populus euphratica]	0.98	5.17
map00240	Pyrimidine metabolism	7.38E-01	TRINITY_DN18443_c0_g1	-1.61	down	hypothetical protein POPTR_0002s25150g [Populus trichocarpa]	1.30	6.00
map00240	Pyrimidine metabolism	7.38E-01	TRINITY_DN18443_c0_g2	2.18	up	hypothetical protein POPTR_0002s23610g [Populus trichocarpa]	10.24	3.46
map00240	Pyrimidine metabolism	7.38E-01	TRINITY_DN18443_c0_g3	-2.26	down	hypothetical protein POPTR_0014s14550g [Populus trichocarpa]	12.11	87.01
map00240	Pyrimidine metabolism	7.38E-01	TRINITY_DN18493_c0_g1	1.75	up	PREDICTED: nucleoside diphosphate kinase 2, chloroplastic-like, partial [Populus euphratica]	521.73	234.57
map00240	Pyrimidine metabolism	7.38E-01	TRINITY_DN19143_c0_g1	-1.71	down	PREDICTED: DNA polymerase alpha subunit B-like [Populus euphratica]	6.40	29.61
map00240	Pyrimidine metabolism	7.38E-01	TRINITY_DN19143_c0_g6	-2.26	down	PREDICTED: uncharacterized protein LOC105129502 [Populus euphratica]	0.25	1.91
map00240	Pyrimidine metabolism	7.38E-01	TRINITY_DN19259_c0_g1	-1.63	down	PREDICTED: DNA polymerase epsilon subunit 2 [Populus euphratica]	1.72	8.35
map00240	Pyrimidine metabolism	7.38E-01	TRINITY_DN19431_c0_g1	1.11	up	hypothetical protein POPTR_0005s09200g [Populus trichocarpa]	15.59	11.37

map00240	Pyrimidine metabolism	7.38E-01	TRINITY_DN19537_c0_g3	-1.06	down	PREDICTED: uncharacterized protein LOC105109677 isoform X1 [Populus euphratica]	5.42	16.71
map00240	Pyrimidine metabolism	7.38E-01	TRINITY_DN19608_c0_g1	-1.86	down	hypothetical protein POPTR_0003s10380g [Populus trichocarpa]	3.11	17.37
map00240	Pyrimidine metabolism	7.38E-01	TRINITY_DN19923_c0_g1	1.53	up	hypothetical protein POPTR_0017s02260g [Populus trichocarpa]	6.64	3.52
map00240	Pyrimidine metabolism	7.38E-01	TRINITY_DN19931_c0_g15	1.32	up	RNA polymerase beta" subunit [Populus alba]	3.76	2.38
map00240	Pyrimidine metabolism	7.38E-01	TRINITY_DN19931_c0_g8	1.61	up	RNA polymerase beta" subunit [Populus alba]	18.11	8.96
map00240	Pyrimidine metabolism	7.38E-01	TRINITY_DN19959_c0_g1	1.17	up	hypothetical protein POPTR_0005s25810g [Populus trichocarpa]	31.81	22.34
map00240	Pyrimidine metabolism	7.38E-01	TRINITY_DN20397_c1_g1	1.00	up	hypothetical protein POPTR_0018s06910g [Populus trichocarpa]	7.16	5.57
map00240	Pyrimidine metabolism	7.38E-01	TRINITY_DN20692_c0_g1	-1.11	down	PREDICTED: probable DNA primase large subunit [Populus euphratica]	8.23	30.45
map00240	Pyrimidine metabolism	7.38E-01	TRINITY_DN20802_c0_g1	-1.58	down	PREDICTED: thymidylate kinase isoform X1 [Populus euphratica]	5.85	27.95
map00240	Pyrimidine metabolism	7.38E-01	TRINITY_DN20804_c0_g1	2.16	up	hypothetical protein POPTR_0006s09200g [Populus trichocarpa]	192.18	65.26
map00240	Pyrimidine metabolism	7.38E-01	TRINITY_DN20959_c0_g7	-2.11	down	hypothetical protein POPTR_0003s19470g [Populus trichocarpa]	0.36	2.31
map00240	Pyrimidine metabolism	7.38E-01	TRINITY_DN20973_c0_g1	-1.20	down	PREDICTED: DNA polymerase delta catalytic subunit [Populus euphratica]	3.70	13.41
map00240	Pyrimidine metabolism	7.38E-01	TRINITY_DN21182_c0_g4	1.43	up	RNA polymerase alpha subunit [Populus alba]	6.16	3.50
map00240	Pyrimidine metabolism	7.38E-01	TRINITY_DN21202_c0_g2	-2.06	down	hypothetical protein POPTR_0017s04540g [Populus trichocarpa]	24.81	154.42
map00240	Pyrimidine metabolism	7.38E-01	TRINITY_DN21216_c0_g1	2.14	up	hypothetical protein POPTR_0005s06460g [Populus trichocarpa]	132.71	46.26
map00240	Pyrimidine metabolism	7.38E-01	TRINITY_DN21228_c0_g1	1.59	up	hypothetical protein POPTR_0001s35070g [Populus trichocarpa]	4.70	2.42
map00240	Pyrimidine metabolism	7.38E-01	TRINITY_DN21497_c0_g3	-3.63	down	Ethylene responsive element binding factor 4 family protein [Populus trichocarpa]	10.72	198.84
map00240	Pyrimidine metabolism	7.38E-01	TRINITY_DN21497_c0_g4	-3.32	down	ERF4 [Populus x canadensis]	11.08	166.25
map00240	Pyrimidine metabolism	7.38E-01	TRINITY_DN21521_c0_g1	-2.76	down	hypothetical protein POPTR_0007s07830g [Populus trichocarpa]	4.44	45.77
map00253	Tetracycline biosynthesis	7.52E-01	TRINITY_DN24307_c0_g1	1.05	up	PREDICTED: biotin carboxyl carrier protein of acetyl-CoA carboxylase, chloroplastic-like [Populus euphratica]	73.06	52.43
map00253	Tetracycline biosynthesis	7.52E-01	TRINITY_DN25439_c0_g1	1.11	up	biotin carboxylase precursor family protein [Populus trichocarpa]	85.63	60.20
map00253	Tetracycline biosynthesis	7.52E-01	TRINITY_DN19050_c1_g1	1.32	up	hypothetical protein POPTR_0019s08170g, partial [Populus trichocarpa]	123.18	74.70
map04070	Phosphatidylinositol signaling system	7.94E-01	TRINITY_DN22393_c1_g1	-1.49	down	PREDICTED: ras-related protein RABC2a [Populus euphratica]	4.89	20.65
map04070	Phosphatidylinositol signaling system	7.94E-01	TRINITY_DN22669_c1_g1	-2.16	down	hypothetical protein POPTR_0008s15550g [Populus trichocarpa]	0.67	4.65
map04070	Phosphatidylinositol signaling system	7.94E-01	TRINITY_DN22669_c1_g2	-2.51	down	PREDICTED: type I inositol 1,4,5-trisphosphate 5-phosphatase CVP2-like isoform X1 [Populus euphratica]	0.71	6.73
map04070	Phosphatidylinositol signaling system	7.94E-01	TRINITY_DN23481_c0_g1	-1.59	down	hypothetical protein POPTR_0002s04720g, partial [Populus trichocarpa]	117.99	559.47
map04070	Phosphatidylinositol signaling system	7.94E-01	TRINITY_DN23481_c0_g2	-1.93	down	PREDICTED: histone H2A variant 1 [Populus euphratica]	21.87	126.80
map04070	Phosphatidylinositol signaling system	7.94E-01	TRINITY_DN23611_c1_g2	-3.54	down	PREDICTED: putative pentatricopeptide repeat-containing protein At3g16890, mitochondrial [Citrus sinensis]	1.04	20.36
map04070	Phosphatidylinositol signaling system	7.94E-01	TRINITY_DN24045_c0_g2	-1.29	down	heavy-metal-associated domain-containing family protein [Populus trichocarpa]	40.04	155.28
map04070	Phosphatidylinositol signaling system	7.94E-01	TRINITY_DN24504_c0_g3	2.44	up	PREDICTED: type I inositol 1,4,5-trisphosphate 5-phosphatase 2-like isoform X1 [Populus euphratica]	2.24	0.61
map04070	Phosphatidylinositol signaling system	7.94E-01	TRINITY_DN24622_c0_g4	-2.50	down	PREDICTED: type I inositol 1,4,5-trisphosphate 5-phosphatase CVP2-like isoform X1 [Populus euphratica]	0.22	1.92
map04070	Phosphatidylinositol signaling system	7.94E-01	TRINITY_DN24760_c0_g1	-2.11	down	PREDICTED: diacylglycerol kinase 2-like isoform X1 [Populus euphratica]	1.68	11.01
map04070	Phosphatidylinositol signaling system	7.94E-01	TRINITY_DN25413_c4_g2	-1.83	down	diacylglycerol kinase family protein [Populus trichocarpa]	8.61	47.89
map04070	Phosphatidylinositol signaling system	7.94E-01	TRINITY_DN26207_c0_g3	-1.48	down	calcium-binding family protein [Populus trichocarpa]	5.82	24.48
map04070	Phosphatidylinositol signaling system	7.94E-01	TRINITY_DN26224_c0_g3	-2.12	down	PREDICTED: type I inositol 1,4,5-trisphosphate 5-phosphatase CVP2-like isoform X1 [Populus euphratica]	0.71	4.72
map04070	Phosphatidylinositol signaling system	7.94E-01	TRINITY_DN27077_c0_g2	-3.23	down	DNA/RNA polymerases superfamily protein [Theobroma cacao]	0.19	2.76
map04070	Phosphatidylinositol signaling system	7.94E-01	TRINITY_DN27127_c0_g1	-1.04	down	putative phosphatidylinositol-4-phosphate 5-kinase mRNA family protein [Populus trichocarpa]	6.64	21.31
map04070	Phosphatidylinositol signaling system	7.94E-01	TRINITY_DN27665_c0_g3	-2.87	down	PREDICTED: putative 1-phosphatidylinositol-3-phosphate 5-kinase FAB1D [Populus euphratica]	0.28	3.17
map04070	Phosphatidylinositol signaling system	7.94E-01	TRINITY_DN27857_c5_g1	3.10	up	polyprotein [Solanum lycopersicum]	42.38	8.94
map04070	Phosphatidylinositol signaling system	7.94E-01	TRINITY_DN13584_c0_g1	-5.95	down	PREDICTED: calcium-binding protein PBP1-like [Populus euphratica]	0.06	6.65
map04070	Phosphatidylinositol signaling system	7.94E-01	TRINITY_DN14287_c0_g1	-1.85	down	hypothetical protein POPTR_0013s02990g [Populus trichocarpa]	12.12	67.05

map04070	Phosphatidylinositol signaling system	7.94E-01	TRINITY_DN14287_c0_g3	-1.84	down	histone H2A family protein [Populus trichocarpa]	25.79	143.46
map04070	Phosphatidylinositol signaling system	7.94E-01	TRINITY_DN14358_c0_g1	-1.80	down	phosphatidylinositol-4-phosphate 5-kinase family protein [Populus trichocarpa]	0.42	2.23
map04070	Phosphatidylinositol signaling system	7.94E-01	TRINITY_DN14433_c0_g2	-4.22	down	hypothetical protein POPTR_0315s00200g [Populus trichocarpa]	0.17	5.08
map04070	Phosphatidylinositol signaling system	7.94E-01	TRINITY_DN14812_c0_g1	-2.36	down	hypothetical protein POPTR_0001s42270g [Populus trichocarpa]	1.92	15.36
map04070	Phosphatidylinositol signaling system	7.94E-01	TRINITY_DN14812_c0_g2	-2.94	down	PREDICTED: calcium-binding protein PBP1-like [Populus euphratica]	1.20	14.58
map04070	Phosphatidylinositol signaling system	7.94E-01	TRINITY_DN16419_c0_g3	2.60	up	hypothetical protein POPTR_0002s08920g [Populus trichocarpa]	20.31	5.00
map04070	Phosphatidylinositol signaling system	7.94E-01	TRINITY_DN16547_c0_g1	-5.02	down	hypothetical protein POPTR_0017s12100g [Populus trichocarpa]	0.07	4.07
map04070	Phosphatidylinositol signaling system	7.94E-01	TRINITY_DN16547_c0_g2	-3.06	down	calcium-binding EF hand family protein [Populus trichocarpa]	0.35	4.71
map04070	Phosphatidylinositol signaling system	7.94E-01	TRINITY_DN16813_c0_g2	-2.41	down	hypothetical protein POPTR_0013s14730g [Populus trichocarpa]	1.77	15.69
map04070	Phosphatidylinositol signaling system	7.94E-01	TRINITY_DN16851_c0_g1	-2.71	down	PREDICTED: calmodulin-like [Populus euphratica]	1.09	10.82
map04070	Phosphatidylinositol signaling system	7.94E-01	TRINITY_DN16851_c0_g2	-1.40	down	hypersensitive reaction associated Ca2+-binding family protein [Populus trichocarpa]	5.56	22.61
map04070	Phosphatidylinositol signaling system	7.94E-01	TRINITY_DN17782_c0_g1	-1.40	down	PREDICTED: inositol-tetrakisphosphate 1-kinase 2-like isoform X1 [Populus euphratica]	2.86	14.19
map04070	Phosphatidylinositol signaling system	7.94E-01	TRINITY_DN18160_c0_g1	1.23	up	inositol monophosphatase family protein [Populus trichocarpa]	15.50	10.06
map04070	Phosphatidylinositol signaling system	7.94E-01	TRINITY_DN18282_c0_g1	-1.17	down	calmodulin-like protein 6a [Populus trichocarpa]	10.53	36.61
map04070	Phosphatidylinositol signaling system	7.94E-01	TRINITY_DN20128_c0_g1	-1.45	down	PREDICTED: dihydrofolate reductase-like [Populus euphratica]	15.72	65.08
map04070	Phosphatidylinositol signaling system	7.94E-01	TRINITY_DN20411_c0_g3	-1.43	down	PREDICTED: histone H2A.6 isoform X1 [Populus euphratica]	31.28	128.16
map04070	Phosphatidylinositol signaling system	7.94E-01	TRINITY_DN20759_c1_g1	-1.31	down	PREDICTED: uncharacterized protein LOC105110153 [Populus euphratica]	8.42	29.65
map04070	Phosphatidylinositol signaling system	7.94E-01	TRINITY_DN21150_c0_g1	-1.92	down	PREDICTED: histone H2AX-like [Populus euphratica]	87.59	512.50
map04070	Phosphatidylinositol signaling system	7.94E-01	TRINITY_DN21206_c0_g1	-2.64	down	hypothetical protein POPTR_0001s41900g [Populus trichocarpa]	0.55	5.54
map04070	Phosphatidylinositol signaling system	7.94E-01	TRINITY_DN21206_c0_g2	-3.49	down	hypothetical protein POPTR_0001s41900g [Populus trichocarpa]	0.36	6.24
map04070	Phosphatidylinositol signaling system	7.94E-01	TRINITY_DN6216_c0_g1	2.24	up	hypothetical protein POPTR_0017s03030g [Populus trichocarpa]	10.07	3.13
map04070	Phosphatidylinositol signaling system	7.94E-01	TRINITY_DN6367_c0_g1	3.75	up	hypothetical protein VITISV_026680 [Vitis vinifera]	7.95	0.91
map00072	Synthesis and degradation of ketone bodies	8.04E-01	TRINITY_DN19913_c0_g1	1.05	up	PREDICTED: hydroxymethylglutaryl-CoA lyase, mitochondrial-like isoform X1 [Populus euphratica]	13.16	7.43
map00072	Synthesis and degradation of ketone bodies	8.04E-01	TRINITY_DN20891_c1_g1	2.08	up	PREDICTED: F-box protein At2g32560-like [Populus euphratica]	23.97	9.10
map00603	Glycosphingolipid biosynthesis - globo series	8.22E-01	TRINITY_DN26284_c1_g3	1.51	up	hypothetical protein POPTR_0008s07890g [Populus trichocarpa]	53.04	28.53
map00603	Glycosphingolipid biosynthesis - globo series	8.22E-01	TRINITY_DN18466_c0_g1	3.25	up	hypothetical protein POPTR_0018s14920g [Populus trichocarpa]	22.92	4.21
map00603	Glycosphingolipid biosynthesis - globo series	8.22E-01	TRINITY_DN20352_c0_g1	1.15	up	hypothetical protein POPTR_0004s19490g [Populus trichocarpa]	17.68	13.24
map00563	Glycosylphosphatidylinositol(GPI)-anchor biosynthesis	8.52E-01	TRINITY_DN23528_c2_g4	-2.08	down	PREDICTED: RING-H2 finger protein ATL3 [Populus euphratica]	1.34	8.74
map00563	Glycosylphosphatidylinositol(GPI)-anchor biosynthesis	8.52E-01	TRINITY_DN25950_c0_g1	-1.03	down	hypothetical protein POPTR_0003s10580g [Populus trichocarpa]	3.89	13.18
map00563	Glycosylphosphatidylinositol(GPI)-anchor biosynthesis	8.52E-01	TRINITY_DN14567_c0_g1	-3.42	down	GAST-like gene product family protein [Populus trichocarpa]	0.81	13.48
map00563	Glycosylphosphatidylinositol(GPI)-anchor biosynthesis	8.52E-01	TRINITY_DN14792_c0_g2	-5.08	down	zinc finger family protein [Populus trichocarpa]	0.06	3.31
map00563	Glycosylphosphatidylinositol(GPI)-anchor biosynthesis	8.52E-01	TRINITY_DN15638_c1_g2	-1.87	down	PREDICTED: RING-H2 finger protein ATL63-like [Populus euphratica]	0.60	3.33
map00563	Glycosylphosphatidylinositol(GPI)-anchor biosynthesis	8.52E-01	TRINITY_DN17398_c0_g1	-1.20	down	hypothetical protein POPTR_0001s45800g [Populus trichocarpa]	5.47	18.55
map00563	Glycosylphosphatidylinositol(GPI)-anchor biosynthesis	8.52E-01	TRINITY_DN17769_c0_g1	-4.46	down	hypothetical protein POPTR_0005s07220g [Populus trichocarpa]	0.41	9.75
map00563	Glycosylphosphatidylinositol(GPI)-anchor biosynthesis	8.52E-01	TRINITY_DN17769_c0_g2	-2.90	down	hypothetical protein POPTR_0005s07220g [Populus trichocarpa]	0.25	2.94
map00563	Glycosylphosphatidylinositol(GPI)-anchor biosynthesis	8.52E-01	TRINITY_DN17850_c0_g1	-1.85	down	zinc finger family protein [Populus trichocarpa]	0.95	5.11

map00563	Glycosylphosphatidylinositol(GPI)-anchor biosynthesis	8.52E-01	TRINITY_DN19202_c3_g1	-1.24	down	PREDICTED: RING-H2 finger protein ATL65 [Populus euphratica]	1.29	4.77
map00563	Glycosylphosphatidylinositol(GPI)-anchor biosynthesis	8.52E-01	TRINITY_DN19315_c3_g1	-1.50	down	hypothetical protein POPTR_0013s02670g [Populus trichocarpa]	3.46	14.68
map00563	Glycosylphosphatidylinositol(GPI)-anchor biosynthesis	8.52E-01	TRINITY_DN21458_c0_g2	2.61	up	hypothetical protein POPTR_0006s04300g [Populus trichocarpa]	139.52	35.55
map00909	Sesquiterpenoid and triterpenoid biosynthesis	8.71E-01	TRINITY_DN24316_c0_g1	-7.47	down	hypothetical protein POPTR_0001s14610g [Populus trichocarpa]	0.00	2.26
map00909	Sesquiterpenoid and triterpenoid biosynthesis	8.71E-01	TRINITY_DN24316_c0_g3	-4.54	down	PREDICTED: lupeol synthase-like isoform X1 [Populus euphratica]	0.18	8.11
map00909	Sesquiterpenoid and triterpenoid biosynthesis	8.71E-01	TRINITY_DN26615_c0_g1	1.82	up	hypothetical protein POPTR_0019s03070g [Populus trichocarpa]	67.01	29.80
map00909	Sesquiterpenoid and triterpenoid biosynthesis	8.71E-01	TRINITY_DN27125_c0_g7	-6.63	down	PREDICTED: beta-amyrin synthase-like [Populus euphratica]	0.02	4.13
map00909	Sesquiterpenoid and triterpenoid biosynthesis	8.71E-01	TRINITY_DN14623_c0_g1	-1.62	down	hypothetical protein POPTR_0001s37380g [Populus trichocarpa]	4.89	22.95
map00909	Sesquiterpenoid and triterpenoid biosynthesis	8.71E-01	TRINITY_DN18170_c0_g1	3.48	up	PREDICTED: cytochrome P450 71D9-like [Populus euphratica]	32.69	4.52
map03420	Nucleotide excision repair	9.42E-01	TRINITY_DN21835_c0_g1	-1.94	down	PREDICTED: chromosome transmission fidelity protein 18 homolog [Populus euphratica]	1.43	8.53
map03420	Nucleotide excision repair	9.42E-01	TRINITY_DN22093_c1_g1	1.58	up	PREDICTED: uncharacterized protein LOC105133503 [Populus euphratica]	28.08	18.04
map03420	Nucleotide excision repair	9.42E-01	TRINITY_DN22222_c2_g1	-1.69	down	PREDICTED: proliferating cell nuclear antigen [Populus euphratica]	20.04	106.79
map03420	Nucleotide excision repair	9.42E-01	TRINITY_DN22462_c0_g1	-1.50	down	hypothetical protein POPTR_0008s00870g, partial [Populus trichocarpa]	1.78	7.87
map03420	Nucleotide excision repair	9.42E-01	TRINITY_DN23387_c0_g1	-1.24	down	Cell division control protein 2 B [Populus trichocarpa]	37.68	134.49
map03420	Nucleotide excision repair	9.42E-01	TRINITY_DN23429_c0_g1	-1.21	down	PREDICTED: flap endonuclease GEN-like 2 isoform X4 [Populus euphratica]	5.55	23.55
map03420	Nucleotide excision repair	9.42E-01	TRINITY_DN23841_c0_g3	-1.40	down	PREDICTED: replication protein A 32 kDa subunit A-like [Populus euphratica]	7.83	32.50
map03420	Nucleotide excision repair	9.42E-01	TRINITY_DN24395_c0_g2	1.21	up	hypothetical protein POPTR_0005s26230g [Populus trichocarpa]	66.57	46.27
map03420	Nucleotide excision repair	9.42E-01	TRINITY_DN24951_c0_g1	2.63	up	unknown [Populus trichocarpa]	40.05	12.23
map03420	Nucleotide excision repair	9.42E-01	TRINITY_DN25007_c0_g1	-1.55	down	TIR-NBS-LRR-TIR type disease resistance protein, partial [Populus trichocarpa]	12.22	41.68
map03420	Nucleotide excision repair	9.42E-01	TRINITY_DN25054_c0_g3	-1.18	down	cyclin-dependent kinase B [Populus tomentosa]	76.90	251.75
map03420	Nucleotide excision repair	9.42E-01	TRINITY_DN25276_c0_g2	-1.48	down	hypothetical protein POPTR_0018s00860g [Populus trichocarpa]	1.70	6.52
map03420	Nucleotide excision repair	9.42E-01	TRINITY_DN26483_c0_g1	-1.98	down	DECREASED DNA METHYLATION 1 family protein [Populus trichocarpa]	6.28	37.32
map03420	Nucleotide excision repair	9.42E-01	TRINITY_DN27349_c0_g1	1.26	up	hypothetical protein POPTR_0001s44720g [Populus trichocarpa]	31.99	22.49
map03420	Nucleotide excision repair	9.42E-01	TRINITY_DN27701_c1_g1	-1.14	down	hypothetical protein POPTR_0007s04450g [Populus trichocarpa]	9.27	35.54
map03420	Nucleotide excision repair	9.42E-01	TRINITY_DN13077_c0_g1	-3.90	down	unknown [Populus trichocarpa]	0.30	5.95
map03420	Nucleotide excision repair	9.42E-01	TRINITY_DN13612_c0_g2	-1.99	down	PREDICTED: GEM-like protein 4 [Populus euphratica]	1.07	6.41
map03420	Nucleotide excision repair	9.42E-01	TRINITY_DN15394_c0_g1	-1.12	down	PREDICTED: regulator of telomere elongation helicase 1 homolog isoform X2 [Populus euphratica]	1.10	3.54
map03420	Nucleotide excision repair	9.42E-01	TRINITY_DN16624_c0_g1	1.03	up	unknown [Populus trichocarpa x Populus deltoides]	184.06	137.19
map03420	Nucleotide excision repair	9.42E-01	TRINITY_DN16976_c0_g4	-1.35	down	DNA-directed DNA polymerase epsilon catalytic subunit family protein [Populus trichocarpa]	2.96	11.76
map03420	Nucleotide excision repair	9.42E-01	TRINITY_DN16976_c0_g5	-1.67	down	DNA-directed DNA polymerase epsilon catalytic subunit family protein [Populus trichocarpa]	0.84	4.16
map03420	Nucleotide excision repair	9.42E-01	TRINITY_DN17121_c0_g1	-1.12	down	hypothetical protein POPTR_0005s27010g, partial [Populus trichocarpa]	1.89	7.13
map03420	Nucleotide excision repair	9.42E-01	TRINITY_DN17241_c0_g1	-1.38	down	DNA polymerase delta subunit 4 family protein [Populus trichocarpa]	4.03	15.90
map03420	Nucleotide excision repair	9.42E-01	TRINITY_DN17241_c0_g2	-1.21	down	DNA polymerase delta subunit 4 family protein [Populus trichocarpa]	37.87	132.98
map03420	Nucleotide excision repair	9.42E-01	TRINITY_DN17522_c0_g1	-2.36	down	PREDICTED: replication protein A 70 kDa DNA-binding subunit B [Populus euphratica]	3.63	29.40
map03420	Nucleotide excision repair	9.42E-01	TRINITY_DN17620_c0_g1	1.37	up	hypothetical protein POPTR_0006s14620g, partial [Populus trichocarpa]	1053.56	627.95
map03420	Nucleotide excision repair	9.42E-01	TRINITY_DN18555_c0_g4	2.33	up	Ycf2 [Populus alba]	3.15	0.93
map03420	Nucleotide excision repair	9.42E-01	TRINITY_DN18641_c0_g1	1.20	up	CDK-activating kinase 1at family protein [Populus trichocarpa]	12.62	8.40
map03420	Nucleotide excision repair	9.42E-01	TRINITY_DN18970_c0_g1	-1.57	down	hypothetical protein POPTR_0008s15120g [Populus trichocarpa]	4.62	19.39
map03420	Nucleotide excision repair	9.42E-01	TRINITY_DN19198_c0_g1	1.14	up	hypothetical protein POPTR_0002s24970g [Populus trichocarpa]	135.65	95.80
map03420	Nucleotide excision repair	9.42E-01	TRINITY_DN19259_c0_g1	-1.63	down	PREDICTED: DNA polymerase epsilon subunit 2 [Populus euphratica]	1.72	8.35

map03420	Nucleotide excision repair	9.42E-01	TRINITY_DN19365_c0_g4	-1.24	down	PREDICTED: putative GEM-like protein 8 isoform X2 [Populus euphratica]	3.73	12.35
map03420	Nucleotide excision repair	9.42E-01	TRINITY_DN19395_c0_g1	-1.33	down	PREDICTED: replication protein A 70 kDa DNA-binding subunit E-like [Populus euphratica]	2.44	9.54
map03420	Nucleotide excision repair	9.42E-01	TRINITY_DN19506_c0_g1	-1.09	down	PREDICTED: DNA repair endonuclease UVH1 [Populus euphratica]	2.88	9.43
map03420	Nucleotide excision repair	9.42E-01	TRINITY_DN19537_c0_g3	-1.06	down	PREDICTED: uncharacterized protein LOC105109677 isoform X1 [Populus euphratica]	5.42	16.71
map03420	Nucleotide excision repair	9.42E-01	TRINITY_DN20164_c0_g3	-1.54	down	sterile alpha motif domain-containing family protein [Populus trichocarpa]	3.50	14.58
map03420	Nucleotide excision repair	9.42E-01	TRINITY_DN20539_c0_g1	1.05	up	PREDICTED: thioredoxin-like 2, chloroplastic [Populus euphratica]	16.48	11.94
map03420	Nucleotide excision repair	9.42E-01	TRINITY_DN20539_c0_g2	1.43	up	hypothetical protein POPTR_0006s15520g [Populus trichocarpa]	68.08	38.48
map03420	Nucleotide excision repair	9.42E-01	TRINITY_DN20672_c0_g1	1.85	up	hypothetical protein POPTR_0006s03190g [Populus trichocarpa]	49.08	22.60
map03420	Nucleotide excision repair	9.42E-01	TRINITY_DN20742_c0_g1	1.62	up	FKBP-type peptidyl-prolyl cis-trans isomerase 3 family protein [Populus trichocarpa]	166.22	81.22
map03420	Nucleotide excision repair	9.42E-01	TRINITY_DN20973_c0_g1	-1.20	down	PREDICTED: DNA polymerase delta catalytic subunit [Populus euphratica]	3.70	13.41
map03420	Nucleotide excision repair	9.42E-01	TRINITY_DN21169_c0_g1	-2.50	down	hypothetical protein POPTR_0001s25810g [Populus trichocarpa]	1.00	8.71
map00511	Other glycan degradation	9.52E-01	TRINITY_DN22568_c0_g1	-1.35	down	PREDICTED: beta-galactosidase 5-like [Populus euphratica]	22.12	82.08
map00511	Other glycan degradation	9.52E-01	TRINITY_DN22624_c0_g1	-1.58	down	hypothetical protein POPTR_0006s13130g [Populus trichocarpa]	2.14	9.80
map00511	Other glycan degradation	9.52E-01	TRINITY_DN25513_c0_g2	-1.55	down	PREDICTED: zinc finger protein NUTCRACKER-like isoform X5 [Populus euphratica]	13.47	55.16
map00511	Other glycan degradation	9.52E-01	TRINITY_DN25522_c1_g1	-1.19	down	PREDICTED: zinc finger protein NUTCRACKER-like [Populus euphratica]	4.97	17.73
map00511	Other glycan degradation	9.52E-01	TRINITY_DN25889_c0_g2	1.59	up	unknown [Populus trichocarpa x Populus deltoides]	384.22	194.87
map00511	Other glycan degradation	9.52E-01	TRINITY_DN25929_c1_g3	-2.18	down	GDSL-motif lipase/hydrolase family protein [Populus trichocarpa]	1.30	8.83
map00511	Other glycan degradation	9.52E-01	TRINITY_DN26010_c0_g4	-1.72	down	hypothetical protein POPTR_0006s13130g [Populus trichocarpa]	3.69	17.16
map00511	Other glycan degradation	9.52E-01	TRINITY_DN26284_c1_g3	1.51	up	hypothetical protein POPTR_0008s07890g [Populus trichocarpa]	53.04	28.53
map00511	Other glycan degradation	9.52E-01	TRINITY_DN26288_c0_g5	-2.57	down	PREDICTED: LOW QUALITY PROTEIN: non-lysosomal glucosylceramidase-like [Populus euphratica]	0.29	2.74
map00511	Other glycan degradation	9.52E-01	TRINITY_DN26337_c3_g2	-1.73	down	PREDICTED: zinc finger protein NUTCRACKER-like [Populus euphratica]	2.46	10.32
map00511	Other glycan degradation	9.52E-01	TRINITY_DN14721_c0_g1	-1.05	down	hypothetical protein POPTR_0008s14180g [Populus trichocarpa]	12.06	37.39
map00511	Other glycan degradation	9.52E-01	TRINITY_DN14796_c0_g1	1.55	up	hypothetical protein POPTR_0004s22510g [Populus trichocarpa]	5.21	2.72
map00511	Other glycan degradation	9.52E-01	TRINITY_DN18085_c0_g1	1.20	up	PREDICTED: alpha-L-fucosidase 3-like [Populus euphratica]	14.27	9.57
map00511	Other glycan degradation	9.52E-01	TRINITY_DN19674_c1_g2	1.61	up	beta-galactosidase family protein [Populus trichocarpa]	9.86	4.84
map00511	Other glycan degradation	9.52E-01	TRINITY_DN19911_c0_g1	2.18	up	hypothetical protein POPTR_0012s14150g [Populus trichocarpa]	73.32	23.17
map00511	Other glycan degradation	9.52E-01	TRINITY_DN20052_c1_g3	1.22	up	hypothetical protein POPTR_0001s35620g [Populus trichocarpa]	30.84	19.96
map00511	Other glycan degradation	9.52E-01	TRINITY_DN20455_c0_g4	-1.61	down	hypothetical protein POPTR_0012s03730g [Populus trichocarpa]	2.77	12.99
map00511	Other glycan degradation	9.52E-01	TRINITY_DN20896_c1_g2	-1.22	down	PREDICTED: protein SHOOT GRAVITROPISM 5 [Populus euphratica]	4.99	19.09
map00511	Other glycan degradation	9.52E-01	TRINITY_DN7728_c0_g2	2.29	up	hypothetical protein POPTR_0006s14130g [Populus trichocarpa]	1.77	0.56
map03450	Non-homologous end-joining	9.57E-01	TRINITY_DN21841_c0_g1	-1.03	down	PREDICTED: ATP-dependent DNA helicase 2 subunit KU80 [Populus euphratica]	2.85	10.71
map00196	Photosynthesis - antenna proteins	9.66E-01	TRINITY_DN24740_c1_g1	1.79	up	Chlorophyll a-b binding protein CP29.3 [Populus trichocarpa]	1015.65	449.23
map00196	Photosynthesis - antenna proteins	9.66E-01	TRINITY_DN26183_c0_g1	1.39	up	leucine-rich repeat transmembrane protein kinase [Populus trichocarpa]	267.84	151.64
map00196	Photosynthesis - antenna proteins	9.66E-01	TRINITY_DN26201_c0_g1	-1.47	down	PREDICTED: protein CYPRO4 [Populus euphratica]	22.26	85.32
map00196	Photosynthesis - antenna proteins	9.66E-01	TRINITY_DN26201_c0_g2	1.12	up	014G029700 [Populus tomentosa]	48.28	37.09
map00196	Photosynthesis - antenna proteins	9.66E-01	TRINITY_DN21469_c0_g2	1.24	up	hypothetical protein POPTR_0003s01450g [Populus trichocarpa]	441.89	284.86
map00196	Photosynthesis - antenna proteins	9.66E-01	TRINITY_DN21512_c1_g3	3.22	up	hypothetical protein POPTR_0002s19010g [Populus trichocarpa]	25.66	4.25
map00510	N-Glycan biosynthesis	9.80E-01	TRINITY_DN21655_c0_g1	-1.22	down	PREDICTED: dolichyl-diphosphooligosaccharide--protein glycosyltransferase subunit STT3A isoform X1 [Populus euphratica]	4.31	16.78
map00510	N-Glycan biosynthesis	9.80E-01	TRINITY_DN23979_c0_g1	-1.43	down	hypothetical protein POPTR_0010s05700g [Populus trichocarpa]	6.36	26.34
map00510	N-Glycan biosynthesis	9.80E-01	TRINITY_DN24648_c1_g1	1.01	up	hypothetical protein POPTR_0005s12850g [Populus trichocarpa]	99.94	79.78
map00510	N-Glycan biosynthesis	9.80E-01	TRINITY_DN24851_c0_g1	-3.19	down	exostosin family protein [Populus trichocarpa]	4.32	59.50

map00510	N-Glycan biosynthesis	9.80E-01	TRINITY_DN28132_c0_g1	2.50	up	hypothetical protein POPTR_0011s00470g [Populus trichocarpa]	4.79	1.26
map00510	N-Glycan biosynthesis	9.80E-01	TRINITY_DN14672_c0_g1	-1.65	down	hypothetical protein POPTR_0006s13920g [Populus trichocarpa]	1.25	6.32
map00510	N-Glycan biosynthesis	9.80E-01	TRINITY_DN15590_c0_g1	-1.50	down	hypothetical protein POPTR_0003s06860g [Populus trichocarpa]	6.11	26.29
map00510	N-Glycan biosynthesis	9.80E-01	TRINITY_DN16250_c0_g1	3.86	up	PREDICTED: RING-H2 finger protein ATL70-like [Populus euphratica]	6.51	0.52
map00510	N-Glycan biosynthesis	9.80E-01	TRINITY_DN18043_c0_g1	-1.40	down	hypothetical protein POPTR_0020s00530g [Populus trichocarpa]	1.34	5.32
map00510	N-Glycan biosynthesis	9.80E-01	TRINITY_DN18652_c0_g3	-2.20	down	hypothetical protein POPTR_0003s17160g [Populus trichocarpa]	1.70	12.43
map00510	N-Glycan biosynthesis	9.80E-01	TRINITY_DN19356_c0_g3	1.22	up	PREDICTED: dolichyl-diphosphooligosaccharide--protein glycosyltransferase subunit DAD1 [Populus euphratica]	134.68	83.13
map00510	N-Glycan biosynthesis	9.80E-01	TRINITY_DN21210_c0_g3	-1.73	down	PREDICTED: LOW QUALITY PROTEIN: alpha-mannosidase 2x [Populus euphratica]	2.53	12.25
map00770	Pantothenate and CoA biosynthesis	9.80E-01	TRINITY_DN25630_c0_g3	-2.44	down	PREDICTED: branched-chain-amino-acid aminotransferase 2, chloroplastic-like isoform X1 [Populus euphratica]	0.50	3.81
map00770	Pantothenate and CoA biosynthesis	9.80E-01	TRINITY_DN26976_c0_g1	1.47	up	PREDICTED: acetolactate synthase 2, chloroplastic-like [Populus euphratica]	396.57	239.53
map00770	Pantothenate and CoA biosynthesis	9.80E-01	TRINITY_DN17903_c0_g1	1.83	up	PREDICTED: phosphopantetheine adenylyltransferase [Populus euphratica]	27.44	12.41
map00770	Pantothenate and CoA biosynthesis	9.80E-01	TRINITY_DN18480_c0_g1	3.23	up	Pantoate--beta-alanine ligase family protein [Populus trichocarpa]	17.35	2.76
map00770	Pantothenate and CoA biosynthesis	9.80E-01	TRINITY_DN19518_c0_g1	2.25	up	hypothetical protein POPTR_0016s14360g [Populus trichocarpa]	277.40	89.48
map00770	Pantothenate and CoA biosynthesis	9.80E-01	TRINITY_DN20583_c0_g3	2.29	up	hypothetical protein POPTR_0008s03820g [Populus trichocarpa]	4.09	1.88
map00770	Pantothenate and CoA biosynthesis	9.80E-01	TRINITY_DN21313_c0_g2	-1.72	down	hypothetical protein POPTR_0014s07170g [Populus trichocarpa]	4.63	24.85
map00770	Pantothenate and CoA biosynthesis	9.80E-01	TRINITY_DN21600_c0_g2	1.23	up	PREDICTED: acetolactate synthase small subunit 2, chloroplastic-like [Populus euphratica]	35.74	23.13
map04140	Regulation of autophagy	9.84E-01	TRINITY_DN22052_c0_g6	-2.43	down	hypothetical protein POPTR_0012s02680g [Populus trichocarpa]	0.38	3.10
map04140	Regulation of autophagy	9.84E-01	TRINITY_DN22798_c0_g3	2.68	up	hypothetical protein POPTR_0002s12740g [Populus trichocarpa]	5.19	1.17
map04140	Regulation of autophagy	9.84E-01	TRINITY_DN23212_c1_g3	-1.56	down	PREDICTED: WD repeat domain-containing protein 83 [Populus euphratica]	6.67	30.00
map04140	Regulation of autophagy	9.84E-01	TRINITY_DN23712_c0_g2	-1.68	down	hypothetical protein POPTR_0010s08980g [Populus trichocarpa]	1.25	6.17
map04140	Regulation of autophagy	9.84E-01	TRINITY_DN23998_c0_g1	1.28	up	PREDICTED: serine/threonine-protein kinase ATG1-like isoform X1 [Populus euphratica]	10.29	7.11
map04140	Regulation of autophagy	9.84E-01	TRINITY_DN24101_c0_g2	-1.43	down	armadillo/beta-catenin repeat family protein [Populus trichocarpa]	4.98	20.33
map04140	Regulation of autophagy	9.84E-01	TRINITY_DN25110_c1_g1	-1.23	down	PREDICTED: U-box domain-containing protein 4-like [Populus euphratica]	4.20	15.26
map04140	Regulation of autophagy	9.84E-01	TRINITY_DN26861_c0_g2	-2.48	down	hypothetical protein POPTR_0002s15500g [Populus trichocarpa]	1.40	11.79
map04140	Regulation of autophagy	9.84E-01	TRINITY_DN26887_c0_g2	-1.10	down	kinase family protein [Populus trichocarpa]	7.21	50.16
map04140	Regulation of autophagy	9.84E-01	TRINITY_DN26887_c0_g3	-1.16	down	PREDICTED: serine/threonine-protein kinase ULK3 isoform X1 [Populus euphratica]	5.57	26.50
map04140	Regulation of autophagy	9.84E-01	TRINITY_DN13358_c0_g1	-1.70	down	hypothetical protein POPTR_0001s12800g [Populus trichocarpa]	0.44	2.17
map04140	Regulation of autophagy	9.84E-01	TRINITY_DN19264_c0_g1	-2.23	down	PREDICTED: U-box domain-containing protein 12 [Populus euphratica]	2.64	18.79
map04140	Regulation of autophagy	9.84E-01	TRINITY_DN19548_c0_g1	2.00	up	kinase family protein [Populus trichocarpa]	4.82	1.85
map04140	Regulation of autophagy	9.84E-01	TRINITY_DN19928_c1_g2	-1.43	down	PREDICTED: calcium-transporting ATPase 2, plasma membrane-type isoform X1 [Populus euphratica]	12.94	60.17
map04140	Regulation of autophagy	9.84E-01	TRINITY_DN20246_c2_g3	1.21	up	hypothetical protein POPTR_0013s08200g [Populus trichocarpa]	69.63	44.62
map04140	Regulation of autophagy	9.84E-01	TRINITY_DN20948_c0_g1	-6.53	down	armadillo/beta-catenin repeat family protein [Populus trichocarpa]	0.52	76.70
map04140	Regulation of autophagy	9.84E-01	TRINITY_DN21231_c0_g2	-1.15	down	armadillo/beta-catenin repeat family protein [Populus trichocarpa]	1.72	5.76
map04140	Regulation of autophagy	9.84E-01	TRINITY_DN21446_c0_g1	-1.14	down	armadillo/beta-catenin repeat family protein [Populus trichocarpa]	3.25	10.44
map00310	Lysine degradation	9.84E-01	TRINITY_DN21932_c0_g3	-1.06	down	phosphatase 2C family protein [Populus trichocarpa]	2.53	8.10
map00310	Lysine degradation	9.84E-01	TRINITY_DN22563_c0_g1	1.23	up	2-oxoacid dehydrogenase family protein [Populus trichocarpa]	62.68	37.12
map00310	Lysine degradation	9.84E-01	TRINITY_DN24628_c0_g1	1.28	up	PREDICTED: aldehyde dehydrogenase family 3 member H1-like [Populus euphratica]	40.39	24.46
map00310	Lysine degradation	9.84E-01	TRINITY_DN25610_c0_g1	-1.43	down	PREDICTED: E3 ubiquitin-protein ligase ORTHRUS 2-like [Populus euphratica]	6.66	21.65
map00310	Lysine degradation	9.84E-01	TRINITY_DN25630_c0_g1	1.30	up	aldehyde dehydrogenase 1 precursor family protein [Populus trichocarpa]	120.35	73.18
map00310	Lysine degradation	9.84E-01	TRINITY_DN26025_c0_g1	-1.71	down	hypothetical protein POPTR_0004s18340g [Populus trichocarpa]	5.60	27.70

map00310	Lysine degradation	9.84E-01	TRINITY_DN26421_c0_g1	1.32	up	the aldehyde dehydrogenase cp-ADH from C.plantagineum family protein [Populus trichocarpa]	58.13	34.64
map00310	Lysine degradation	9.84E-01	TRINITY_DN18895_c0_g1	-1.06	down	mitochondrial aldehyde dehydrogenase family protein [Populus trichocarpa]	7.40	23.22
map00310	Lysine degradation	9.84E-01	TRINITY_DN18934_c0_g1	-1.59	down	hypothetical protein POPTR_0018s11100g [Populus trichocarpa]	2.87	14.58
map00310	Lysine degradation	9.84E-01	TRINITY_DN19215_c1_g1	-1.38	down	hypothetical protein POPTR_0007s10210g [Populus trichocarpa]	3.47	12.55
map00310	Lysine degradation	9.84E-01	TRINITY_DN19215_c2_g1	-1.56	down	PREDICTED: probable protein phosphatase 2C 63 [Populus euphratica]	6.48	30.16
map00310	Lysine degradation	9.84E-01	TRINITY_DN19387_c1_g2	-1.34	down	SET domain-containing family protein [Populus trichocarpa]	4.51	17.04
map04122	Sulfur relay system	9.85E-01	TRINITY_DN15628_c0_g1	1.14	up	molybdenum cofactor synthesis family protein [Populus trichocarpa]	18.69	12.84
map04122	Sulfur relay system	9.85E-01	TRINITY_DN18615_c0_g1	-2.03	down	PREDICTED: uncharacterized protein LOC105116005 isoform X1 [Populus euphratica]	0.49	3.72
map00100	Steroid biosynthesis	9.89E-01	TRINITY_DN23651_c0_g1	1.05	up	sterol 4-alpha-methyl-oxidase 1 family protein [Populus trichocarpa]	41.44	30.62
map00100	Steroid biosynthesis	9.89E-01	TRINITY_DN24651_c0_g1	-1.09	down	S-adenosyl-methionine-sterol-C- methyltransferase family protein [Populus trichocarpa]	55.88	208.38
map00100	Steroid biosynthesis	9.89E-01	TRINITY_DN24718_c0_g1	-1.13	down	PREDICTED: calmodulin-binding transcription activator 3-like isoform X3 [Populus euphratica]	2.73	8.94
map00100	Steroid biosynthesis	9.89E-01	TRINITY_DN26615_c0_g1	1.82	up	hypothetical protein POPTR_0019s03070g [Populus trichocarpa]	67.01	29.80
map00100	Steroid biosynthesis	9.89E-01	TRINITY_DN27547_c0_g1	-1.35	down	calmodulin-binding family protein [Populus trichocarpa]	6.25	23.99
map00100	Steroid biosynthesis	9.89E-01	TRINITY_DN13505_c0_g1	5.45	up	PREDICTED: protein FAM32A-like [Populus euphratica]	5.25	0.22
map00100	Steroid biosynthesis	9.89E-01	TRINITY_DN15486_c0_g1	-1.67	down	PREDICTED: 3beta-hydroxysteroid-dehydrogenase/decarboxylase isoform 3-like isoform X1 [Populus euphratica]	0.76	3.83
map00100	Steroid biosynthesis	9.89E-01	TRINITY_DN15523_c0_g1	1.29	up	PREDICTED: reticulon-like protein B12 [Populus euphratica]	14.14	9.19
map00100	Steroid biosynthesis	9.89E-01	TRINITY_DN18073_c1_g1	1.18	up	PREDICTED: reticulon-like protein B1 [Populus euphratica]	90.00	61.98
map00100	Steroid biosynthesis	9.89E-01	TRINITY_DN20046_c0_g1	-1.43	down	PREDICTED: protein MID1-COMPLEMENTING ACTIVITY 1 [Populus euphratica]	16.65	68.97
map00562	Inositol phosphate metabolism	9.90E-01	TRINITY_DN22245_c0_g1	2.23	up	PREDICTED: triosephosphate isomerase, chloroplastic [Populus euphratica]	838.73	263.34
map00562	Inositol phosphate metabolism	9.90E-01	TRINITY_DN22304_c0_g1	-2.25	down	cucumber protein kinase CsPK3 [Populus trichocarpa]	2.28	16.67
map00562	Inositol phosphate metabolism	9.90E-01	TRINITY_DN22393_c1_g1	-1.49	down	PREDICTED: ras-related protein RABC2a [Populus euphratica]	4.89	20.65
map00562	Inositol phosphate metabolism	9.90E-01	TRINITY_DN22669_c1_g1	-2.16	down	hypothetical protein POPTR_0008s15550g [Populus trichocarpa]	0.67	4.65
map00562	Inositol phosphate metabolism	9.90E-01	TRINITY_DN22669_c1_g2	-2.51	down	PREDICTED: type I inositol 1,4,5-trisphosphate 5-phosphatase CVP2-like isoform X1 [Populus euphratica]	0.71	6.73
map00562	Inositol phosphate metabolism	9.90E-01	TRINITY_DN23192_c0_g1	-2.27	down	hypothetical protein POPTR_0004s19720g [Populus trichocarpa]	3.73	27.49
map00562	Inositol phosphate metabolism	9.90E-01	TRINITY_DN23965_c0_g1	1.23	up	PREDICTED: triosephosphate isomerase, cytosolic [Populus euphratica]	641.46	426.80
map00562	Inositol phosphate metabolism	9.90E-01	TRINITY_DN24045_c0_g2	-1.29	down	heavy-metal-associated domain-containing family protein [Populus trichocarpa]	40.04	155.28
map00562	Inositol phosphate metabolism	9.90E-01	TRINITY_DN24393_c0_g1	1.31	up	hypothetical protein POPTR_0001s24710g [Populus trichocarpa]	198.18	106.97
map00562	Inositol phosphate metabolism	9.90E-01	TRINITY_DN24504_c0_g3	2.44	up	PREDICTED: type I inositol 1,4,5-trisphosphate 5-phosphatase 2-like isoform X1 [Populus euphratica]	2.24	0.61
map00562	Inositol phosphate metabolism	9.90E-01	TRINITY_DN24622_c0_g4	-2.50	down	PREDICTED: type I inositol 1,4,5-trisphosphate 5-phosphatase CVP2-like isoform X1 [Populus euphratica]	0.22	1.92
map00562	Inositol phosphate metabolism	9.90E-01	TRINITY_DN25232_c0_g1	-1.14	down	hypothetical protein POPTR_0006s11580g [Populus trichocarpa]	3.34	11.12
map00562	Inositol phosphate metabolism	9.90E-01	TRINITY_DN25430_c0_g1	1.19	up	PREDICTED: non-specific phospholipase C2 [Populus euphratica]	91.42	57.95
map00562	Inositol phosphate metabolism	9.90E-01	TRINITY_DN26224_c0_g3	-2.12	down	PREDICTED: type I inositol 1,4,5-trisphosphate 5-phosphatase CVP2-like isoform X1 [Populus euphratica]	0.71	4.72
map00562	Inositol phosphate metabolism	9.90E-01	TRINITY_DN26913_c0_g1	1.27	up	1L-myo-inositol 1-phosphate synthase family protein [Populus trichocarpa]	252.36	147.60
map00562	Inositol phosphate metabolism	9.90E-01	TRINITY_DN26913_c0_g2	1.63	up	hypothetical protein POPTR_0005s08050g [Populus trichocarpa]	14.60	7.42
map00562	Inositol phosphate metabolism	9.90E-01	TRINITY_DN27127_c0_g1	-1.04	down	putative phosphatidylinositol-4-phosphate 5-kinase mRNA family protein [Populus trichocarpa]	6.64	21.31
map00562	Inositol phosphate metabolism	9.90E-01	TRINITY_DN27665_c0_g3	-2.87	down	PREDICTED: putative 1-phosphatidylinositol-3-phosphate 5-kinase FAB1D [Populus euphratica]	0.28	3.17
map00562	Inositol phosphate metabolism	9.90E-01	TRINITY_DN14358_c0_g1	-1.80	down	phosphatidylinositol-4-phosphate 5-kinase family protein [Populus trichocarpa]	0.42	2.23
map00562	Inositol phosphate metabolism	9.90E-01	TRINITY_DN15363_c0_g2	-2.90	down	putative serine/threonine protein kinase [Populus tomentosa]	0.62	7.03
map00562	Inositol phosphate metabolism	9.90E-01	TRINITY_DN17782_c0_g1	-1.40	down	PREDICTED: inositol-tetrakisphosphate 1-kinase 2-like isoform X1 [Populus euphratica]	2.86	14.19
map00562	Inositol phosphate metabolism	9.90E-01	TRINITY_DN18125_c0_g1	-1.88	down	phospholipase C [Populus tomentosa]	0.58	3.28

map00562	Inositol phosphate metabolism	9.90E-01	TRINITY_DN18160_c0_g1	1.23	up	inositol monophosphatase family protein [Populus trichocarpa]	15.50	10.06
map00562	Inositol phosphate metabolism	9.90E-01	TRINITY_DN20087_c0_g1	-1.46	down	PREDICTED: serine/threonine-protein kinase UCNL-like [Populus euphratica]	3.22	13.35
map00562	Inositol phosphate metabolism	9.90E-01	TRINITY_DN21337_c0_g3	-2.71	down	PREDICTED: multiple inositol polyphosphate phosphatase 1-like isoform X1 [Populus euphratica]	0.16	1.69
map00562	Inositol phosphate metabolism	9.90E-01	TRINITY_DN21603_c0_g1	-1.14	down	phosphoinositide-specific phospholipase C family protein [Populus trichocarpa]	2.32	9.54
map00562	Inositol phosphate metabolism	9.90E-01	TRINITY_DN6216_c0_g1	2.24	up	hypothetical protein POPTR_0017s03030g [Populus trichocarpa]	10.07	3.13
map00500	Starch and sucrose metabolism	9.92E-01	TRINITY_DN21649_c1_g2	-1.42	down	TIR-NBS-LRR type disease resistance protein [Populus trichocarpa]	9.75	40.94
map00500	Starch and sucrose metabolism	9.92E-01	TRINITY_DN21692_c0_g1	-2.02	down	PREDICTED: probable beta-D-xylosidase 2 [Populus euphratica]	0.65	3.58
map00500	Starch and sucrose metabolism	9.92E-01	TRINITY_DN21752_c0_g1	-1.81	down	hypothetical protein POPTR_0008s05600g [Populus trichocarpa]	16.32	82.75
map00500	Starch and sucrose metabolism	9.92E-01	TRINITY_DN21919_c0_g3	1.75	up	glycosyl transferase family 8 family protein [Populus trichocarpa]	34.19	15.23
map00500	Starch and sucrose metabolism	9.92E-01	TRINITY_DN21987_c0_g1	-1.19	down	sucrose synthase [Populus tomentosa]	5.57	17.49
map00500	Starch and sucrose metabolism	9.92E-01	TRINITY_DN22164_c1_g3	-1.14	down	hypothetical protein POPTR_0006s19240g [Populus trichocarpa]	45.77	151.67
map00500	Starch and sucrose metabolism	9.92E-01	TRINITY_DN22233_c0_g1	1.51	up	hypothetical protein POPTR_0002s10420g [Populus trichocarpa]	84.73	47.59
map00500	Starch and sucrose metabolism	9.92E-01	TRINITY_DN22270_c0_g1	1.36	up	PREDICTED: 1,4-alpha-glucan-branching enzyme 3, chloroplastic/amyloplastic isoform X1 [Populus euphratica]	14.25	8.43
map00500	Starch and sucrose metabolism	9.92E-01	TRINITY_DN22325_c0_g1	1.02	up	hypothetical protein POPTR_0015s01470g [Populus trichocarpa]	21.86	16.50
map00500	Starch and sucrose metabolism	9.92E-01	TRINITY_DN22754_c0_g1	1.32	up	hypothetical protein POPTR_0005s24170g [Populus trichocarpa]	22.62	13.77
map00500	Starch and sucrose metabolism	9.92E-01	TRINITY_DN22811_c0_g6	1.30	up	hypothetical protein POPTR_0010s03090g [Populus trichocarpa]	2.86	1.78
map00500	Starch and sucrose metabolism	9.92E-01	TRINITY_DN22865_c0_g2	1.37	up	PREDICTED: APO protein 2, chloroplastic-like [Populus euphratica]	86.24	50.32
map00500	Starch and sucrose metabolism	9.92E-01	TRINITY_DN22944_c0_g1	1.68	up	PREDICTED: beta-xylosidase/alpha-L-arabinofuranosidase 1-like [Populus euphratica]	51.43	26.38
map00500	Starch and sucrose metabolism	9.92E-01	TRINITY_DN22944_c0_g3	-1.16	down	PREDICTED: probable beta-D-xylosidase 7 [Populus euphratica]	9.58	31.73
map00500	Starch and sucrose metabolism	9.92E-01	TRINITY_DN23043_c0_g1	1.04	up	PREDICTED: lysosomal beta glucosidase-like [Populus euphratica]	13.89	10.05
map00500	Starch and sucrose metabolism	9.92E-01	TRINITY_DN23068_c1_g1	-1.79	down	PREDICTED: probable galacturonosyltransferase-like 9 [Populus euphratica]	3.69	19.63
map00500	Starch and sucrose metabolism	9.92E-01	TRINITY_DN23208_c0_g1	-1.01	down	PREDICTED: oxysterol-binding protein-related protein 1D-like [Populus euphratica]	5.04	20.81
map00500	Starch and sucrose metabolism	9.92E-01	TRINITY_DN23403_c0_g1	1.10	up	hypothetical protein POPTR_0005s23960g [Populus trichocarpa]	80.55	57.95
map00500	Starch and sucrose metabolism	9.92E-01	TRINITY_DN23596_c1_g1	1.13	up	hypothetical protein POPTR_0013s10100g [Populus trichocarpa]	12.79	8.71
map00500	Starch and sucrose metabolism	9.92E-01	TRINITY_DN23600_c0_g2	-2.70	down	hypothetical protein POPTR_0011s15750g [Populus trichocarpa]	1.22	10.68
map00500	Starch and sucrose metabolism	9.92E-01	TRINITY_DN23656_c1_g7	1.33	up	pentatricopeptide repeat-containing family protein [Populus trichocarpa]	2.92	1.79
map00500	Starch and sucrose metabolism	9.92E-01	TRINITY_DN23675_c0_g1	-2.55	down	hypothetical protein POPTR_0001s42050g [Populus trichocarpa]	0.38	3.34
map00500	Starch and sucrose metabolism	9.92E-01	TRINITY_DN23823_c0_g1	1.36	up	PREDICTED: cellulose synthase-like protein E6 [Populus euphratica]	29.77	17.92
map00500	Starch and sucrose metabolism	9.92E-01	TRINITY_DN23842_c0_g2	1.20	up	PREDICTED: UDP-glucuronate 4-epimerase 6-like [Populus euphratica]	59.05	37.92
map00500	Starch and sucrose metabolism	9.92E-01	TRINITY_DN23925_c0_g3	-2.42	down	UDP-glucuronic acid decarboxylase 2 [Populus tomentosa]	5.90	48.05
map00500	Starch and sucrose metabolism	9.92E-01	TRINITY_DN23936_c0_g1	1.03	up	PREDICTED: pentatricopeptide repeat-containing protein At1g18485 [Populus euphratica]	3.58	2.66
map00500	Starch and sucrose metabolism	9.92E-01	TRINITY_DN23974_c0_g4	-1.42	down	hypothetical protein POPTR_0014s01950g [Populus trichocarpa]	1.88	9.00
map00500	Starch and sucrose metabolism	9.92E-01	TRINITY_DN24122_c0_g1	1.21	up	PREDICTED: uncharacterized protein LOC105119383 [Populus euphratica]	79.62	51.81
map00500	Starch and sucrose metabolism	9.92E-01	TRINITY_DN24188_c1_g5	1.55	up	pentatricopeptide repeat superfamily protein [Populus tomentosa]	17.60	9.15
map00500	Starch and sucrose metabolism	9.92E-01	TRINITY_DN24209_c0_g1	-2.35	down	PREDICTED: cellulose synthase-like protein D3 [Populus euphratica]	1.25	9.86
map00500	Starch and sucrose metabolism	9.92E-01	TRINITY_DN24341_c0_g1	1.63	up	PREDICTED: alpha-glucosidase-like [Populus euphratica]	5.68	2.81
map00500	Starch and sucrose metabolism	9.92E-01	TRINITY_DN24495_c1_g1	-2.08	down	hypothetical protein POPTR_0008s13190g [Populus trichocarpa]	8.58	56.96
map00500	Starch and sucrose metabolism	9.92E-01	TRINITY_DN24495_c1_g3	-1.66	down	hypothetical protein POPTR_0010s11950g [Populus trichocarpa]	9.36	40.91
map00500	Starch and sucrose metabolism	9.92E-01	TRINITY_DN24550_c0_g2	1.52	up	pfkB-type carbohydrate kinase family protein [Populus trichocarpa]	100.10	51.39
map00500	Starch and sucrose metabolism	9.92E-01	TRINITY_DN24569_c0_g3	1.49	up	PREDICTED: pentatricopeptide repeat-containing protein At1g59720, mitochondrial [Populus euphratica]	5.14	2.82

map00500	Starch and sucrose metabolism	9.92E-01	TRINITY_DN24623_c0_g2	1.54	up	hypothetical protein POPTR_0008s11770g [Populus trichocarpa]	42.50	22.04
map00500	Starch and sucrose metabolism	9.92E-01	TRINITY_DN24875_c0_g1	-1.20	down	UDP-XYLOSE SYNTHASE 4 family protein [Populus trichocarpa]	20.20	83.94
map00500	Starch and sucrose metabolism	9.92E-01	TRINITY_DN24996_c0_g1	2.19	up	ADP-glucose pyrophosphorylase family protein [Populus trichocarpa]	108.63	36.08
map00500	Starch and sucrose metabolism	9.92E-01	TRINITY_DN25060_c0_g1	-1.73	down	glycosyl hydrolase family 17 family protein [Populus trichocarpa]	14.49	70.13
map00500	Starch and sucrose metabolism	9.92E-01	TRINITY_DN25060_c0_g2	-2.57	down	PREDICTED: glucan endo-1,3-beta-glucosidase 12-like [Populus euphratica]	10.35	91.23
map00500	Starch and sucrose metabolism	9.92E-01	TRINITY_DN25060_c0_g3	-1.01	down	glycosyl hydrolase family 17 family protein [Populus trichocarpa]	114.85	330.01
map00500	Starch and sucrose metabolism	9.92E-01	TRINITY_DN25105_c0_g1	-1.23	down	PREDICTED: glucose-6-phosphate/phosphate translocator 1, chloroplastic-like [Populus euphratica]	4.62	16.65
map00500	Starch and sucrose metabolism	9.92E-01	TRINITY_DN25225_c0_g2	1.17	up	hypothetical protein POPTR_0019s05340g [Populus trichocarpa]	20.38	13.56
map00500	Starch and sucrose metabolism	9.92E-01	TRINITY_DN25266_c0_g1	-1.42	down	endo-1 family protein [Populus trichocarpa]	13.14	52.56
map00500	Starch and sucrose metabolism	9.92E-01	TRINITY_DN25438_c0_g1	-2.79	down	endo-1,4-beta glucanase [Populus alba]	5.85	54.28
map00500	Starch and sucrose metabolism	9.92E-01	TRINITY_DN25444_c0_g1	-1.23	down	PREDICTED: tyrosyl-DNA phosphodiesterase 2 isoform X8 [Populus euphratica]	5.40	20.98
map00500	Starch and sucrose metabolism	9.92E-01	TRINITY_DN25521_c1_g2	1.13	up	PREDICTED: APO protein 1, chloroplastic isoform X1 [Populus euphratica]	21.99	15.44
map00500	Starch and sucrose metabolism	9.92E-01	TRINITY_DN25692_c0_g1	-1.21	down	AAA-type ATPase family protein [Populus trichocarpa]	9.04	31.77
map00500	Starch and sucrose metabolism	9.92E-01	TRINITY_DN25692_c0_g2	-1.31	down	PREDICTED: pachytene checkpoint protein 2 homolog [Populus euphratica]	12.27	47.09
map00500	Starch and sucrose metabolism	9.92E-01	TRINITY_DN25771_c0_g1	2.03	up	cell-wall invertase [Populus alba x Populus grandidentata]	101.29	37.01
map00500	Starch and sucrose metabolism	9.92E-01	TRINITY_DN25848_c0_g1	-1.30	down	endo-1 family protein [Populus trichocarpa]	29.70	110.05
map00500	Starch and sucrose metabolism	9.92E-01	TRINITY_DN26069_c0_g1	1.07	up	putative pectin methylesterase [Populus tremula x Populus tremuloides]	104.43	71.85
map00500	Starch and sucrose metabolism	9.92E-01	TRINITY_DN26175_c0_g1	1.33	up	PREDICTED: hexokinase-1-like [Populus euphratica]	24.35	14.67
map00500	Starch and sucrose metabolism	9.92E-01	TRINITY_DN26478_c0_g2	-1.65	down	PREDICTED: probable alpha,alpha-trehalose-phosphate synthase [UDP-forming] 11 [Populus euphratica]	5.24	24.55
map00500	Starch and sucrose metabolism	9.92E-01	TRINITY_DN26643_c1_g2	-1.33	down	PREDICTED: vicianin hydrolase-like [Populus euphratica]	12.50	47.56
map00500	Starch and sucrose metabolism	9.92E-01	TRINITY_DN26743_c0_g1	1.15	up	RecName: Full=Phosphoglucomutase, cytoplasmic; Short=PGM; AltName: Full=Glucose phosphomutase	74.05	53.71
map00500	Starch and sucrose metabolism	9.92E-01	TRINITY_DN26815_c0_g1	1.59	up	beta-amylase family protein [Populus trichocarpa]	150.16	101.01
map00500	Starch and sucrose metabolism	9.92E-01	TRINITY_DN26825_c0_g2	2.18	up	PREDICTED: beta-fructofuranosidase, soluble isoenzyme I-like [Populus euphratica]	96.10	31.98
map00500	Starch and sucrose metabolism	9.92E-01	TRINITY_DN26996_c1_g1	1.79	up	PREDICTED: phosphoglucomutase, chloroplastic-like [Populus euphratica]	112.47	49.80
map00500	Starch and sucrose metabolism	9.92E-01	TRINITY_DN26999_c0_g1	1.40	up	UDP-glucose pyrophosphorylase [Populus tremula x Populus tremuloides]	112.13	68.32
map00500	Starch and sucrose metabolism	9.92E-01	TRINITY_DN27220_c0_g1	-1.11	down	PREDICTED: uncharacterized protein LOC105130123 [Populus euphratica]	10.28	33.40
map00500	Starch and sucrose metabolism	9.92E-01	TRINITY_DN27264_c1_g1	1.03	up	PREDICTED: kynurenine--oxoglutarate transaminase 1-like isoform X1 [Populus euphratica]	32.80	22.92
map00500	Starch and sucrose metabolism	9.92E-01	TRINITY_DN27315_c1_g3	1.78	up	PREDICTED: polygalacturonase At1g48100-like [Populus euphratica]	140.65	76.97
map00500	Starch and sucrose metabolism	9.92E-01	TRINITY_DN27331_c0_g2	-1.09	down	PREDICTED: probable polygalacturonase [Populus euphratica]	15.61	64.79
map00500	Starch and sucrose metabolism	9.92E-01	TRINITY_DN27588_c0_g1	4.99	up	sucrose-phosphate synthase family protein [Populus trichocarpa]	36.05	1.68
map00500	Starch and sucrose metabolism	9.92E-01	TRINITY_DN27725_c1_g1	-1.29	down	PREDICTED: protein argonaute 4A-like [Populus euphratica]	17.30	78.71
map00500	Starch and sucrose metabolism	9.92E-01	TRINITY_DN27775_c0_g1	-1.44	down	PREDICTED: LOW QUALITY PROTEIN: TMV resistance protein N-like [Populus euphratica]	3.59	16.75
map00500	Starch and sucrose metabolism	9.92E-01	TRINITY_DN14333_c0_g1	1.41	up	hypothetical protein POPTR_0008s09560g [Populus trichocarpa]	5.88	3.35
map00500	Starch and sucrose metabolism	9.92E-01	TRINITY_DN14511_c0_g1	-1.77	down	hypothetical protein POPTR_0006s13740g [Populus trichocarpa]	0.95	4.88
map00500	Starch and sucrose metabolism	9.92E-01	TRINITY_DN15121_c0_g2	1.57	up	PREDICTED: L-type lectin-domain containing receptor kinase S.4-like isoform X2 [Populus euphratica]	3.09	1.59
map00500	Starch and sucrose metabolism	9.92E-01	TRINITY_DN15131_c0_g2	-6.08	down	putative pectin methylesterase LuPME1 family protein [Populus trichocarpa]	0.02	2.96
map00500	Starch and sucrose metabolism	9.92E-01	TRINITY_DN15216_c0_g1	2.22	up	myb family transcription factor family protein [Populus trichocarpa]	14.59	5.27
map00500	Starch and sucrose metabolism	9.92E-01	TRINITY_DN15382_c0_g2	1.10	up	PREDICTED: probable cellulose synthase A catalytic subunit 3 [UDP-forming] [Populus euphratica]	22.15	15.51
map00500	Starch and sucrose metabolism	9.92E-01	TRINITY_DN15442_c0_g1	1.51	up	hypothetical protein POPTR_0005s00720g [Populus trichocarpa]	2.88	1.51
map00500	Starch and sucrose metabolism	9.92E-01	TRINITY_DN15586_c0_g1	2.45	up	hypothetical protein POPTR_0005s06430g [Populus trichocarpa]	2.82	1.17
map00500	Starch and sucrose metabolism	9.92E-01	TRINITY_DN15747_c0_g1	2.03	up	hypothetical protein POPTR_0006s24400g [Populus trichocarpa]	4.17	1.74
map00500	Starch and sucrose metabolism	9.92E-01	TRINITY_DN16091_c0_g1	1.63	up	PREDICTED: xylulose 5-phosphate/phosphate translocator, chloroplastic [Populus euphratica]	27.10	13.24

map00500	Starch and sucrose metabolism	9.92E-01	TRINITY_DN16155_c0_g1	2.15	up	hypothetical protein POPTR_0004s12440g [Populus trichocarpa]	53.21	18.16
map00500	Starch and sucrose metabolism	9.92E-01	TRINITY_DN16243_c0_g1	-1.44	down	hypothetical protein POPTR_0019s13010g [Populus trichocarpa]	4.25	17.56
map00500	Starch and sucrose metabolism	9.92E-01	TRINITY_DN16376_c0_g1	1.84	up	hypothetical protein POPTR_0014s16990g [Populus trichocarpa]	212.37	90.30
map00500	Starch and sucrose metabolism	9.92E-01	TRINITY_DN16387_c0_g1	3.61	up	glycosyl hydrolase family 1 family protein [Populus trichocarpa]	7.38	0.93
map00500	Starch and sucrose metabolism	9.92E-01	TRINITY_DN16418_c0_g1	-5.63	down	1,3-beta-D-glucanase GH17_39 [Populus tremula x Populus tremuloides]	0.68	49.75
map00500	Starch and sucrose metabolism	9.92E-01	TRINITY_DN16562_c0_g2	-2.14	down	hypothetical protein POPTR_0018s06250g [Populus trichocarpa]	0.35	2.29
map00500	Starch and sucrose metabolism	9.92E-01	TRINITY_DN16682_c0_g1	-2.32	down	ripening-related family protein [Populus trichocarpa]	4.32	32.23
map00500	Starch and sucrose metabolism	9.92E-01	TRINITY_DN16795_c0_g1	2.85	up	hydroxyisourate hydrolase family protein [Populus trichocarpa]	3.65	0.76
map00500	Starch and sucrose metabolism	9.92E-01	TRINITY_DN16897_c0_g1	1.03	up	glycosyl transferase family 8 family protein [Populus trichocarpa]	8.61	10.24
map00500	Starch and sucrose metabolism	9.92E-01	TRINITY_DN16930_c0_g1	2.20	up	invertase/pectin methylesterase inhibitor family protein [Populus trichocarpa]	3.03	1.02
map00500	Starch and sucrose metabolism	9.92E-01	TRINITY_DN17059_c0_g2	1.13	up	UDP-glucose pyrophosphorylase [Populus deltoides]	101.22	72.02
map00500	Starch and sucrose metabolism	9.92E-01	TRINITY_DN17229_c0_g1	-1.51	down	hypothetical protein POPTR_0014s03490g [Populus trichocarpa]	1.22	5.46
map00500	Starch and sucrose metabolism	9.92E-01	TRINITY_DN17536_c0_g2	2.72	up	PREDICTED: pentatricopeptide repeat-containing protein At3g26782, mitochondrial-like [Populus euphratica]	2.70	0.63
map00500	Starch and sucrose metabolism	9.92E-01	TRINITY_DN17646_c0_g3	2.20	up	unknown [Populus trichocarpa]	75.56	24.78
map00500	Starch and sucrose metabolism	9.92E-01	TRINITY_DN17648_c0_g1	2.26	up	PREDICTED: uncharacterized protein At1g18480 [Populus euphratica]	19.45	6.55
map00500	Starch and sucrose metabolism	9.92E-01	TRINITY_DN17648_c0_g2	2.31	up	calcineurin-like phosphoesterase family protein [Populus trichocarpa]	7.85	2.39
map00500	Starch and sucrose metabolism	9.92E-01	TRINITY_DN17662_c0_g1	-3.72	down	PREDICTED: L-type lectin-domain containing receptor kinase IX.1-like [Populus euphratica]	0.28	5.70
map00500	Starch and sucrose metabolism	9.92E-01	TRINITY_DN17738_c0_g1	-1.93	down	glycosyltransferase 8E [Populus tremula x Populus alba]	1.69	10.09
map00500	Starch and sucrose metabolism	9.92E-01	TRINITY_DN17754_c0_g1	-2.02	down	PREDICTED: pectinesterase-like [Populus euphratica]	0.76	4.68
map00500	Starch and sucrose metabolism	9.92E-01	TRINITY_DN17916_c0_g1	-2.23	down	PREDICTED: transcription factor DIVARICATA-like [Populus euphratica]	0.83	6.00
map00500	Starch and sucrose metabolism	9.92E-01	TRINITY_DN18049_c0_g2	1.34	up	hypothetical protein POPTR_0001s25590g [Populus trichocarpa]	43.52	25.51
map00500	Starch and sucrose metabolism	9.92E-01	TRINITY_DN18068_c0_g1	1.45	up	hypothetical protein POPTR_0006s16020g [Populus trichocarpa]	11.25	6.22
map00500	Starch and sucrose metabolism	9.92E-01	TRINITY_DN18121_c0_g2	1.98	up	PREDICTED: pentatricopeptide repeat-containing protein At4g18840-like [Populus euphratica]	4.64	1.81
map00500	Starch and sucrose metabolism	9.92E-01	TRINITY_DN18225_c0_g3	1.29	up	PREDICTED: hexokinase-1-like [Populus euphratica]	38.97	24.40
map00500	Starch and sucrose metabolism	9.92E-01	TRINITY_DN18278_c0_g1	-1.11	down	vesicle-associated membrane family protein [Populus trichocarpa]	3.07	10.07
map00500	Starch and sucrose metabolism	9.92E-01	TRINITY_DN18344_c0_g1	1.73	up	hypothetical protein POPTR_0005s12390g [Populus trichocarpa]	4.29	2.12
map00500	Starch and sucrose metabolism	9.92E-01	TRINITY_DN18344_c0_g2	3.11	up	hypothetical protein POPTR_0007s13000g [Populus trichocarpa]	86.69	15.79
map00500	Starch and sucrose metabolism	9.92E-01	TRINITY_DN18350_c0_g3	1.51	up	PREDICTED: glucan endo-1,3-beta-glucosidase 12-like [Populus euphratica]	7.30	3.93
map00500	Starch and sucrose metabolism	9.92E-01	TRINITY_DN18592_c1_g8	1.05	up	PREDICTED: pentatricopeptide repeat-containing protein At1g15510, chloroplastic [Populus euphratica]	2.88	2.16
map00500	Starch and sucrose metabolism	9.92E-01	TRINITY_DN18623_c2_g2	2.80	up	hypothetical protein POPTR_0004s06340g [Populus trichocarpa]	2.59	0.56
map00500	Starch and sucrose metabolism	9.92E-01	TRINITY_DN18767_c0_g1	-1.14	down	pectinesterase family protein [Populus trichocarpa]	3.89	13.90
map00500	Starch and sucrose metabolism	9.92E-01	TRINITY_DN18772_c0_g1	-1.72	down	Cel9B [Populus tremula x Populus tremuloides]	1.44	7.23
map00500	Starch and sucrose metabolism	9.92E-01	TRINITY_DN18944_c0_g1	1.39	up	hypothetical protein POPTR_0001s35130g [Populus trichocarpa]	11.16	6.52
map00500	Starch and sucrose metabolism	9.92E-01	TRINITY_DN18972_c0_g6	-1.91	down	PREDICTED: probable LRR receptor-like serine/threonine-protein kinase RFK1 isoform X1 [Populus euphratica]	0.31	2.70
map00500	Starch and sucrose metabolism	9.92E-01	TRINITY_DN19002_c0_g2	-1.58	down	hypothetical protein POPTR_0008s05860g [Populus trichocarpa]	1.06	4.85
map00500	Starch and sucrose metabolism	9.92E-01	TRINITY_DN19010_c0_g1	1.39	up	hypothetical protein POPTR_0016s11690g [Populus trichocarpa]	93.49	53.39
map00500	Starch and sucrose metabolism	9.92E-01	TRINITY_DN19117_c0_g1	-2.20	down	trehalose-6-phosphate phosphatase family protein [Populus trichocarpa]	0.56	3.33
map00500	Starch and sucrose metabolism	9.92E-01	TRINITY_DN19196_c0_g1	1.23	up	PREDICTED: pentatricopeptide repeat-containing protein At1g11290 [Populus euphratica]	9.63	6.25
map00500	Starch and sucrose metabolism	9.92E-01	TRINITY_DN19253_c0_g2	4.06	up	PREDICTED: probable sucrose-phosphate synthase 4 [Populus euphratica]	3.66	0.34
map00500	Starch and sucrose metabolism	9.92E-01	TRINITY_DN19274_c0_g1	1.06	up	hypothetical protein POPTR_0001s29510g [Populus trichocarpa]	74.48	55.73
map00500	Starch and sucrose metabolism	9.92E-01	TRINITY_DN19291_c0_g5	-2.01	down	hypothetical protein POPTR_0015s06880g [Populus trichocarpa]	68.36	415.00
map00500	Starch and sucrose metabolism	9.92E-01	TRINITY_DN19346_c0_g1	1.13	up	hypothetical protein POPTR_0001s10850g [Populus trichocarpa]	31.58	21.33

map00500	Starch and sucrose metabolism	9.92E-01	TRINITY_DN19353_c0_g1	2.03	up	PREDICTED: glucan endo-1,3-beta-glucosidase 12 [Populus euphratica]	71.34	26.96
map00500	Starch and sucrose metabolism	9.92E-01	TRINITY_DN19354_c0_g1	-6.54	down	PREDICTED: glucan endo-1,3-beta-glucosidase, basic isoform-like [Populus euphratica]	0.57	84.08
map00500	Starch and sucrose metabolism	9.92E-01	TRINITY_DN19354_c0_g2	-2.28	down	PREDICTED: glucan endo-1,3-beta-glucosidase, basic isoform-like [Populus euphratica]	2.00	14.93
map00500	Starch and sucrose metabolism	9.92E-01	TRINITY_DN19446_c0_g1	-1.56	down	glycosyl hydrolase family 17 family protein [Populus trichocarpa]	8.26	37.67
map00500	Starch and sucrose metabolism	9.92E-01	TRINITY_DN19450_c0_g2	1.10	up	hypothetical protein POPTR_0002s01620g [Populus trichocarpa]	3.25	2.32
map00500	Starch and sucrose metabolism	9.92E-01	TRINITY_DN19486_c0_g1	-1.20	down	PREDICTED: glucan endo-1,3-beta-glucosidase 14-like [Populus euphratica]	2.34	7.34
map00500	Starch and sucrose metabolism	9.92E-01	TRINITY_DN19660_c0_g1	-1.47	down	hypothetical protein POPTR_0006s04670g [Populus trichocarpa]	3.59	15.42
map00500	Starch and sucrose metabolism	9.92E-01	TRINITY_DN19736_c0_g1	1.19	up	DISPROPORTIONATING ENZYME family protein [Populus trichocarpa]	13.53	9.85
map00500	Starch and sucrose metabolism	9.92E-01	TRINITY_DN19751_c0_g1	-1.26	down	unknown [Populus trichocarpa]	5.54	20.26
map00500	Starch and sucrose metabolism	9.92E-01	TRINITY_DN19751_c0_g2	-1.99	down	PREDICTED: probable fructokinase-1 [Populus euphratica]	4.28	23.17
map00500	Starch and sucrose metabolism	9.92E-01	TRINITY_DN19781_c0_g1	-1.53	down	ovate family protein [Populus trichocarpa]	3.48	15.24
map00500	Starch and sucrose metabolism	9.92E-01	TRINITY_DN19781_c0_g2	-1.44	down	ovate family protein [Populus trichocarpa]	2.88	11.53
map00500	Starch and sucrose metabolism	9.92E-01	TRINITY_DN19792_c0_g1	1.31	up	ATP-binding-cassette transporter family protein [Populus trichocarpa]	73.85	45.58
map00500	Starch and sucrose metabolism	9.92E-01	TRINITY_DN19874_c0_g1	2.03	up	PREDICTED: uncharacterized protein LOC105122007 [Populus euphratica]	71.32	25.90
map00500	Starch and sucrose metabolism	9.92E-01	TRINITY_DN19875_c0_g1	1.63	up	hypothetical protein POPTR_0002s01570g [Populus trichocarpa]	9.19	4.59
map00500	Starch and sucrose metabolism	9.92E-01	TRINITY_DN20035_c0_g1	-1.14	down	hypothetical protein POPTR_0003s07460g [Populus trichocarpa]	88.55	285.41
map00500	Starch and sucrose metabolism	9.92E-01	TRINITY_DN20234_c0_g3	3.69	up	PREDICTED: glucan endo-1,3-beta-glucosidase isoform X1 [Populus euphratica]	16.46	2.27
map00500	Starch and sucrose metabolism	9.92E-01	TRINITY_DN20397_c0_g1	1.46	up	PREDICTED: pentatricopeptide repeat-containing protein At1g28690, mitochondrial [Populus euphratica]	2.91	1.60
map00500	Starch and sucrose metabolism	9.92E-01	TRINITY_DN20578_c0_g1	1.96	up	starch synthase family protein [Populus trichocarpa]	5.03	2.06
map00500	Starch and sucrose metabolism	9.92E-01	TRINITY_DN20620_c1_g3	1.10	up	unknown [Populus trichocarpa]	72.81	51.40
map00500	Starch and sucrose metabolism	9.92E-01	TRINITY_DN20757_c0_g2	-1.98	down	PREDICTED: glucan endo-1,3-beta-glucosidase 3-like [Populus euphratica]	1.23	7.21
map00500	Starch and sucrose metabolism	9.92E-01	TRINITY_DN20779_c0_g1	1.18	up	PREDICTED: pentatricopeptide repeat-containing protein At3g26630, chloroplastic [Populus euphratica]	8.48	6.51
map00500	Starch and sucrose metabolism	9.92E-01	TRINITY_DN20841_c0_g1	-2.69	down	hypothetical protein POPTR_0002s00860g [Populus trichocarpa]	7.43	72.76
map00500	Starch and sucrose metabolism	9.92E-01	TRINITY_DN20976_c0_g6	1.22	up	glycosyl transferase family 8 family protein [Populus trichocarpa]	2.69	1.76
map00500	Starch and sucrose metabolism	9.92E-01	TRINITY_DN21062_c0_g1	1.22	up	PREDICTED: hexokinase-1-like [Populus euphratica]	9.26	6.04
map00500	Starch and sucrose metabolism	9.92E-01	TRINITY_DN21126_c0_g2	-2.31	down	PREDICTED: probable polygalacturonase isoform X1 [Populus euphratica]	14.88	112.78
map00500	Starch and sucrose metabolism	9.92E-01	TRINITY_DN21582_c0_g1	2.42	up	PREDICTED: fructokinase-like 2, chloroplastic [Populus euphratica]	83.87	26.93
map00500	Starch and sucrose metabolism	9.92E-01	TRINITY_DN6170_c0_g1	-7.86	down	myb family transcription factor family protein [Populus trichocarpa]	0.00	4.20
map03018	RNA degradation	9.92E-01	TRINITY_DN21925_c0_g2	-1.26	down	hypothetical protein POPTR_0011s15230g [Populus trichocarpa]	4.86	17.73
map03018	RNA degradation	9.92E-01	TRINITY_DN22184_c0_g2	1.34	up	hypothetical protein POPTR_0010s08770g [Populus trichocarpa]	53.21	31.38
map03018	RNA degradation	9.92E-01	TRINITY_DN22318_c0_g1	2.02	up	PREDICTED: uncharacterized protein LOC105135002 [Populus euphratica]	127.95	48.78
map03018	RNA degradation	9.92E-01	TRINITY_DN22318_c0_g4	2.50	up	Chaperone DnaJ [Gossypium arboreum]	66.38	17.66
map03018	RNA degradation	9.92E-01	TRINITY_DN22342_c0_g1	-3.72	down	PREDICTED: protein CUP-SHAPED COTYLEDON 3-like isoform X3 [Populus euphratica]	0.65	15.24
map03018	RNA degradation	9.92E-01	TRINITY_DN22413_c1_g6	1.81	up	hypothetical protein VITISV_013624 [Vitis vinifera]	2.05	0.86
map03018	RNA degradation	9.92E-01	TRINITY_DN22425_c0_g5	1.83	up	Chaperonin CPN60-2 family protein [Populus trichocarpa]	63.97	27.86
map03018	RNA degradation	9.92E-01	TRINITY_DN22849_c0_g1	1.56	up	RNA-binding protein RNP1 precursor [Populus trichocarpa]	253.11	130.90
map03018	RNA degradation	9.92E-01	TRINITY_DN22930_c0_g1	1.43	up	PREDICTED: uncharacterized protein LOC105111959 isoform X2 [Populus euphratica]	35.76	20.03
map03018	RNA degradation	9.92E-01	TRINITY_DN22938_c1_g1	-2.26	down	ATP-dependent DNA helicase family protein [Populus trichocarpa]	1.17	7.57
map03018	RNA degradation	9.92E-01	TRINITY_DN23268_c0_g1	1.52	up	hypothetical protein POPTR_0006s21650g [Populus trichocarpa]	465.66	243.37
map03018	RNA degradation	9.92E-01	TRINITY_DN23511_c0_g2	-1.85	down	NO POLLEN GERMINATION RELATED 1 family protein [Populus trichocarpa]	3.50	19.12
map03018	RNA degradation	9.92E-01	TRINITY_DN23713_c1_g1	1.00	up	PREDICTED: ATP-dependent RNA helicase DHX36-like isoform X1 [Populus euphratica]	7.45	5.71
map03018	RNA degradation	9.92E-01	TRINITY_DN23864_c0_g1	1.73	up	29 kDa ribonucleoprotein [Populus trichocarpa]	1851.11	826.20
map03018	RNA degradation	9.92E-01	TRINITY_DN24098_c0_g1	-1.50	down	NO POLLEN GERMINATION RELATED 2 family protein [Populus trichocarpa]	2.36	10.58

map03018	RNA degradation	9.92E-01	TRINITY_DN24109_c1_g5	2.18	up	heat shock protein 70 [Populus trichocarpa]	69.14	24.00
map03018	RNA degradation	9.92E-01	TRINITY_DN24109_c1_g6	2.17	up	heat shock protein 70 [Populus trichocarpa]	73.32	25.78
map03018	RNA degradation	9.92E-01	TRINITY_DN24219_c0_g2	-3.10	down	NAC domain transcriptional regulator superfamily protein [Populus tomentosa]	4.72	57.22
map03018	RNA degradation	9.92E-01	TRINITY_DN24219_c0_g3	-2.42	down	no apical meristem family protein [Populus trichocarpa]	9.96	79.77
map03018	RNA degradation	9.92E-01	TRINITY_DN24248_c0_g1	-2.67	down	PREDICTED: uncharacterized protein LOC105122981 isoform X2 [Populus euphratica]	1.23	11.99
map03018	RNA degradation	9.92E-01	TRINITY_DN24321_c0_g1	1.12	up	ammonium transporter family protein [Populus trichocarpa]	95.05	70.18
map03018	RNA degradation	9.92E-01	TRINITY_DN24701_c0_g2	1.14	up	PREDICTED: pentatricopeptide repeat-containing protein At5g28460-like [Populus euphratica]	5.84	4.10
map03018	RNA degradation	9.92E-01	TRINITY_DN24924_c0_g1	1.18	up	PREDICTED: chloroplastic group IIA intron splicing facilitator CRS1, chloroplastic, partial [Populus euphratica]	19.06	12.73
map03018	RNA degradation	9.92E-01	TRINITY_DN25050_c0_g1	-1.00	down	hypothetical protein POPTR_0010s12780g [Populus trichocarpa]	2.27	7.09
map03018	RNA degradation	9.92E-01	TRINITY_DN25238_c0_g1	-3.15	down	hypothetical protein POPTR_0012s00760g [Populus trichocarpa]	3.02	41.76
map03018	RNA degradation	9.92E-01	TRINITY_DN25238_c0_g2	-1.20	down	hypothetical protein POPTR_0015s00640g [Populus trichocarpa]	3.92	13.72
map03018	RNA degradation	9.92E-01	TRINITY_DN25545_c0_g1	-2.24	down	hypothetical protein POPTR_0002s05360g [Populus trichocarpa]	1.80	12.98
map03018	RNA degradation	9.92E-01	TRINITY_DN25576_c1_g3	-1.36	down	hypothetical protein POPTR_0018s02370g [Populus trichocarpa]	12.78	49.78
map03018	RNA degradation	9.92E-01	TRINITY_DN25593_c1_g1	1.16	up	PREDICTED: chloroplastic group IIA intron splicing facilitator CRS1, chloroplastic isoform X1 [Populus euphratica]	47.20	31.54
map03018	RNA degradation	9.92E-01	TRINITY_DN25792_c0_g1	-1.88	down	PREDICTED: double-stranded RNA-binding protein 3 [Populus euphratica]	7.15	40.08
map03018	RNA degradation	9.92E-01	TRINITY_DN26127_c0_g1	2.19	up	Chaperonin CPN60-2 family protein [Populus trichocarpa]	117.86	42.68
map03018	RNA degradation	9.92E-01	TRINITY_DN26425_c0_g1	2.34	up	PREDICTED: chaperonin 60 subunit beta 2, chloroplastic [Populus euphratica]	886.93	288.11
map03018	RNA degradation	9.92E-01	TRINITY_DN26491_c4_g2	1.35	up	PREDICTED: chloroplastic group IIA intron splicing facilitator CRS1, chloroplastic [Populus euphratica]	27.04	16.11
map03018	RNA degradation	9.92E-01	TRINITY_DN26661_c0_g2	1.95	up	hypothetical protein VITISV_019194 [Vitis vinifera]	2.90	1.14
map03018	RNA degradation	9.92E-01	TRINITY_DN26755_c0_g3	1.04	up	hypothetical protein POPTR_0015s15270g [Populus trichocarpa]	40.82	29.41
map03018	RNA degradation	9.92E-01	TRINITY_DN26977_c1_g1	1.72	up	PREDICTED: ruBisCO large subunit-binding protein subunit alpha [Populus euphratica]	888.17	398.63
map03018	RNA degradation	9.92E-01	TRINITY_DN26977_c1_g2	2.56	up	RUBISCO SUBUNIT BINDING-protein ALPHA SUBUNIT [Populus trichocarpa]	66.21	16.76
map03018	RNA degradation	9.92E-01	TRINITY_DN26977_c1_g3	1.29	up	hypothetical protein POPTR_0015s15270g [Populus trichocarpa]	17.42	12.03
map03018	RNA degradation	9.92E-01	TRINITY_DN26998_c2_g2	1.79	up	hypothetical protein POPTR_0015s08620g [Populus trichocarpa]	96.15	41.20
map03018	RNA degradation	9.92E-01	TRINITY_DN27817_c3_g2	-1.99	down	PREDICTED: calcium-transporting ATPase 9, plasma membrane-type isoform X1 [Populus euphratica]	1.08	6.59
map03018	RNA degradation	9.92E-01	TRINITY_DN17390_c1_g1	-2.09	down	PREDICTED: NAC transcription factor 25-like [Populus euphratica]	2.36	3.97
map03018	RNA degradation	9.92E-01	TRINITY_DN17627_c0_g1	1.18	up	PREDICTED: carbon catabolite repressor protein 4 homolog 4 isoform X2 [Populus euphratica]	20.79	14.48
map03018	RNA degradation	9.92E-01	TRINITY_DN17699_c0_g1	1.45	up	PREDICTED: fatty-acid-binding protein 3 [Populus euphratica]	105.66	57.49
map03018	RNA degradation	9.92E-01	TRINITY_DN17904_c0_g2	2.75	up	RNA recognition motif-containing family protein [Populus trichocarpa]	146.73	33.03
map03018	RNA degradation	9.92E-01	TRINITY_DN17998_c0_g2	1.97	up	hypothetical protein POPTR_0009s07030g [Populus trichocarpa]	738.54	284.18
map03018	RNA degradation	9.92E-01	TRINITY_DN18162_c0_g1	-1.61	down	PREDICTED: NAC transcription factor 29-like [Populus euphratica]	4.47	21.42
map03018	RNA degradation	9.92E-01	TRINITY_DN19042_c0_g1	-4.05	down	PREDICTED: protein FEZ-like [Populus euphratica]	0.38	9.93
map03018	RNA degradation	9.92E-01	TRINITY_DN19042_c0_g2	-3.42	down	no apical meristem family protein [Populus trichocarpa]	0.18	3.18
map03018	RNA degradation	9.92E-01	TRINITY_DN19206_c0_g1	1.04	up	hypothetical protein POPTR_0012s11630g [Populus trichocarpa]	536.60	393.46
map03018	RNA degradation	9.92E-01	TRINITY_DN19206_c0_g2	1.76	up	methionine sulfoxide reductase A [Populus trichocarpa x Populus deltoides]	72.30	32.79
map03018	RNA degradation	9.92E-01	TRINITY_DN19458_c0_g1	-1.92	down	PREDICTED: NAC domain-containing protein 7-like isoform X2 [Populus euphratica]	1.58	8.63
map03018	RNA degradation	9.92E-01	TRINITY_DN19463_c0_g2	-2.40	down	small nuclear ribonucleoprotein [Populus trichocarpa]	0.51	3.86
map03018	RNA degradation	9.92E-01	TRINITY_DN19829_c0_g1	2.04	up	hypothetical protein POPTR_0001s03980g [Populus trichocarpa]	1118.96	411.30
map03018	RNA degradation	9.92E-01	TRINITY_DN19848_c0_g1	1.56	up	phosphopyruvate hydratase family protein [Populus trichocarpa]	247.16	128.86
map03018	RNA degradation	9.92E-01	TRINITY_DN19859_c0_g1	-1.80	down	hydroxyproline-rich glycoprotein [Populus trichocarpa]	62.93	330.51
map03018	RNA degradation	9.92E-01	TRINITY_DN20209_c0_g2	-1.58	down	hypothetical protein POPTR_0009s16310g [Populus trichocarpa]	2.28	10.43
map03018	RNA degradation	9.92E-01	TRINITY_DN20469_c0_g1	1.03	up	3' exoribonuclease domain 1-containing family protein [Populus trichocarpa]	25.29	19.75

map03018	RNA degradation	9.92E-01	TRINITY_DN20839_c0_g4	-1.26	down	hypothetical protein POPTR_0018s06130g [Populus trichocarpa]	2.08	7.42
map03018	RNA degradation	9.92E-01	TRINITY_DN20852_c0_g3	-2.00	down	NO POLLEN GERMINATION RELATED 1 family protein [Populus trichocarpa]	1.32	8.01
map03018	RNA degradation	9.92E-01	TRINITY_DN20918_c0_g1	1.25	up	hypothetical protein POPTR_0003s04350g [Populus trichocarpa]	4.98	3.41
map03018	RNA degradation	9.92E-01	TRINITY_DN21228_c0_g1	1.59	up	hypothetical protein POPTR_0001s35070g [Populus trichocarpa]	4.70	2.42
map03018	RNA degradation	9.92E-01	TRINITY_DN21240_c0_g1	2.16	up	PREDICTED: 28 kDa ribonucleoprotein, chloroplastic-like [Populus euphratica]	151.80	51.26
map03018	RNA degradation	9.92E-01	TRINITY_DN21244_c0_g1	-1.04	down	PREDICTED: Werner syndrome ATP-dependent helicase-like isoform X1 [Populus euphratica]	2.33	7.40
map03018	RNA degradation	9.92E-01	TRINITY_DN21284_c0_g1	-1.34	down	hypothetical protein POPTR_0005s02650g [Populus trichocarpa]	1.77	6.79
map03018	RNA degradation	9.92E-01	TRINITY_DN21291_c2_g2	2.14	up	hypothetical protein POPTR_0015s08620g [Populus trichocarpa]	471.84	166.72
map03018	RNA degradation	9.92E-01	TRINITY_DN21339_c1_g2	1.07	up	33 kDa ribonucleoprotein [Populus trichocarpa]	122.13	88.36
map03018	RNA degradation	9.92E-01	TRINITY_DN5017_c0_g1	-1.46	down	hypothetical protein [Brassica napus]	0.48	2.01
map00564	Glycerophospholipid metabolism	9.93E-01	TRINITY_DN22007_c0_g1	1.13	up	hypothetical protein POPTR_0018s06580g [Populus trichocarpa]	112.04	78.04
map00564	Glycerophospholipid metabolism	9.93E-01	TRINITY_DN22687_c2_g2	1.73	up	PREDICTED: uncharacterized protein LOC105126932 [Populus euphratica]	95.91	50.11
map00564	Glycerophospholipid metabolism	9.93E-01	TRINITY_DN23274_c0_g1	-2.24	down	PREDICTED: phospholipase A1-Ibeta2, chloroplastic-like [Populus euphratica]	1.95	13.94
map00564	Glycerophospholipid metabolism	9.93E-01	TRINITY_DN23274_c0_g2	-5.05	down	hypothetical protein POPTR_0003s07940g [Populus trichocarpa]	0.48	24.54
map00564	Glycerophospholipid metabolism	9.93E-01	TRINITY_DN24178_c0_g3	1.11	up	lipid phosphate phosphatase 2 [Populus tomentosa]	19.00	13.25
map00564	Glycerophospholipid metabolism	9.93E-01	TRINITY_DN24718_c0_g1	-1.13	down	PREDICTED: calmodulin-binding transcription activator 3-like isoform X3 [Populus euphratica]	2.73	8.94
map00564	Glycerophospholipid metabolism	9.93E-01	TRINITY_DN24760_c0_g1	-2.11	down	PREDICTED: diacylglycerol kinase 2-like isoform X1 [Populus euphratica]	1.68	11.01
map00564	Glycerophospholipid metabolism	9.93E-01	TRINITY_DN25165_c0_g2	1.20	up	PREDICTED: glycerol-3-phosphate acyltransferase, chloroplastic [Populus euphratica]	66.47	43.88
map00564	Glycerophospholipid metabolism	9.93E-01	TRINITY_DN25413_c4_g2	-1.83	down	diacylglycerol kinase family protein [Populus trichocarpa]	8.61	47.89
map00564	Glycerophospholipid metabolism	9.93E-01	TRINITY_DN25430_c0_g1	1.19	up	PREDICTED: non-specific phospholipase C2 [Populus euphratica]	91.42	57.95
map00564	Glycerophospholipid metabolism	9.93E-01	TRINITY_DN26044_c0_g1	-2.99	down	PREDICTED: probable choline kinase 3 [Populus euphratica]	2.97	34.90
map00564	Glycerophospholipid metabolism	9.93E-01	TRINITY_DN26173_c0_g2	-2.84	down	hypothetical protein POPTR_0001s23420g [Populus trichocarpa]	1.75	18.91
map00564	Glycerophospholipid metabolism	9.93E-01	TRINITY_DN26389_c0_g1	4.80	up	-	24.61	1.45
map00564	Glycerophospholipid metabolism	9.93E-01	TRINITY_DN27547_c0_g1	-1.35	down	calmodulin-binding family protein [Populus trichocarpa]	6.25	23.99
map00564	Glycerophospholipid metabolism	9.93E-01	TRINITY_DN11229_c0_g1	1.90	up	hypothetical protein POPTR_0011s03550g [Populus trichocarpa]	258.45	106.27
map00564	Glycerophospholipid metabolism	9.93E-01	TRINITY_DN15487_c0_g1	4.95	up	methyl transferase [Populus tomentosa]	61.40	3.01
map00564	Glycerophospholipid metabolism	9.93E-01	TRINITY_DN17108_c0_g1	2.12	up	phospholipase/carboxylesterase family protein [Populus trichocarpa]	3.37	1.26
map00564	Glycerophospholipid metabolism	9.93E-01	TRINITY_DN17748_c0_g1	-1.40	down	hypothetical protein POPTR_0015s09670g [Populus trichocarpa]	2.03	7.86
map00564	Glycerophospholipid metabolism	9.93E-01	TRINITY_DN17931_c0_g2	1.02	up	unknown [Populus trichocarpa]	189.83	143.29
map00564	Glycerophospholipid metabolism	9.93E-01	TRINITY_DN18029_c0_g1	1.19	up	hypothetical protein POPTR_0013s09410g [Populus trichocarpa]	19.79	14.57
map00564	Glycerophospholipid metabolism	9.93E-01	TRINITY_DN18667_c0_g1	-2.71	down	PREDICTED: probable choline kinase 3 [Populus euphratica]	0.81	8.11
map00564	Glycerophospholipid metabolism	9.93E-01	TRINITY_DN20113_c0_g3	1.02	up	hypothetical protein POPTR_0007s14280g [Populus trichocarpa]	91.83	69.21
map00564	Glycerophospholipid metabolism	9.93E-01	TRINITY_DN20407_c0_g1	1.23	up	plastid developmental protein DAG [Populus trichocarpa]	313.75	204.79
map00564	Glycerophospholipid metabolism	9.93E-01	TRINITY_DN20407_c0_g2	1.21	up	hypothetical protein POPTR_0010s07890g [Populus trichocarpa]	232.02	140.49
map00564	Glycerophospholipid metabolism	9.93E-01	TRINITY_DN21027_c0_g1	-1.16	down	phospholipid/glycerol acyltransferase family protein [Populus trichocarpa]	12.14	41.22
map00564	Glycerophospholipid metabolism	9.93E-01	TRINITY_DN21206_c0_g1	-2.64	down	hypothetical protein POPTR_0001s41900g [Populus trichocarpa]	0.55	5.54
map00564	Glycerophospholipid metabolism	9.93E-01	TRINITY_DN21206_c0_g2	-3.49	down	hypothetical protein POPTR_0001s41900g [Populus trichocarpa]	0.36	6.24
map00564	Glycerophospholipid metabolism	9.93E-01	TRINITY_DN3610_c0_g1	1.74	up	hypothetical protein POPTR_0004s05380g [Populus trichocarpa]	53.62	22.92
map00565	Ether lipid metabolism	9.99E-01	TRINITY_DN22240_c0_g4	-1.57	down	hypothetical protein POPTR_0016s02070g [Populus trichocarpa]	2.17	9.60
map00565	Ether lipid metabolism	9.99E-01	TRINITY_DN24718_c0_g1	-1.13	down	PREDICTED: calmodulin-binding transcription activator 3-like isoform X3 [Populus euphratica]	2.73	8.94
map00565	Ether lipid metabolism	9.99E-01	TRINITY_DN25430_c0_g1	1.19	up	PREDICTED: non-specific phospholipase C2 [Populus euphratica]	91.42	57.95
map00565	Ether lipid metabolism	9.99E-01	TRINITY_DN27547_c0_g1	-1.35	down	calmodulin-binding family protein [Populus trichocarpa]	6.25	23.99
map00565	Ether lipid metabolism	9.99E-01	TRINITY_DN13317_c0_g1	-2.09	down	PREDICTED: WD repeat-containing protein tag-125-like [Populus euphratica]	0.32	2.12

map00565	Ether lipid metabolism	9.99E-01	TRINITY_DN17288_c0_g1	-4.11	down	PREDICTED: F-box/WD repeat-containing protein 11-like [Populus euphratica]	0.15	4.02
map03022	Basal transcription factors	1.00E+00	TRINITY_DN22462_c0_g1	-1.50	down	hypothetical protein POPTR_0008s00870g, partial [Populus trichocarpa]	1.78	7.87
map03022	Basal transcription factors	1.00E+00	TRINITY_DN23387_c0_g1	-1.24	down	Cell division control protein 2 B [Populus trichocarpa]	37.68	134.49
map03022	Basal transcription factors	1.00E+00	TRINITY_DN25054_c0_g3	-1.18	down	cyclin-dependent kinase B [Populus tomentosa]	76.90	251.75
map03022	Basal transcription factors	1.00E+00	TRINITY_DN25074_c0_g2	-1.42	down	hypothetical protein POPTR_0006s12700g [Populus trichocarpa]	1.06	4.46
map03022	Basal transcription factors	1.00E+00	TRINITY_DN25241_c1_g2	-1.07	down	PREDICTED: transcription initiation factor TFIID subunit 8-like [Populus euphratica]	4.28	15.18
map03022	Basal transcription factors	1.00E+00	TRINITY_DN26860_c0_g1	-1.78	down	PREDICTED: uncharacterized protein LOC105121825 isoform X1 [Populus euphratica]	2.95	14.28
map03022	Basal transcription factors	1.00E+00	TRINITY_DN26928_c1_g1	-1.34	down	PREDICTED: transcription factor GTE4 [Populus euphratica]	7.45	28.61
map03022	Basal transcription factors	1.00E+00	TRINITY_DN26928_c1_g2	-1.24	down	PREDICTED: transcription factor GTE4-like [Populus euphratica]	3.52	12.61
map03022	Basal transcription factors	1.00E+00	TRINITY_DN12652_c0_g1	1.88	up	hypothetical protein POPTR_0008s13880g, partial [Populus trichocarpa]	10.21	3.80
map03022	Basal transcription factors	1.00E+00	TRINITY_DN15394_c0_g1	-1.12	down	PREDICTED: regulator of telomere elongation helicase 1 homolog isoform X2 [Populus euphratica]	1.10	3.54
map03022	Basal transcription factors	1.00E+00	TRINITY_DN18391_c1_g2	-2.52	down	hypothetical protein POPTR_0010s22650g [Populus trichocarpa]	1.75	14.23
map03022	Basal transcription factors	1.00E+00	TRINITY_DN18641_c0_g1	1.20	up	CDK-activating kinase 1at family protein [Populus trichocarpa]	12.62	8.40
map03008	Ribosome biogenesis in eukaryotes	1.00E+00	TRINITY_DN21854_c0_g2	1.48	up	PREDICTED: nucleolar GTP-binding protein 1 [Populus euphratica]	47.26	29.04
map03008	Ribosome biogenesis in eukaryotes	1.00E+00	TRINITY_DN22205_c0_g1	1.02	up	nucleolar essential family protein [Populus trichocarpa]	61.19	46.60
map03008	Ribosome biogenesis in eukaryotes	1.00E+00	TRINITY_DN22413_c1_g6	1.81	up	hypothetical protein VITISV_013624 [Vitis vinifera]	2.05	0.86
map03008	Ribosome biogenesis in eukaryotes	1.00E+00	TRINITY_DN22424_c0_g1	2.40	up	hypothetical protein POPTR_0006s05160g [Populus trichocarpa]	79.89	24.03
map03008	Ribosome biogenesis in eukaryotes	1.00E+00	TRINITY_DN22578_c1_g2	1.44	up	hypothetical protein POPTR_0015s12790g [Populus trichocarpa]	127.24	69.53
map03008	Ribosome biogenesis in eukaryotes	1.00E+00	TRINITY_DN23588_c0_g2	1.60	up	hypothetical protein POPTR_0012s02130g [Populus trichocarpa]	5.55	2.72
map03008	Ribosome biogenesis in eukaryotes	1.00E+00	TRINITY_DN23677_c0_g1	1.30	up	PREDICTED: probable RNA 3'-terminal phosphate cyclase-like protein isoform X1 [Populus euphratica]	15.94	10.08
map03008	Ribosome biogenesis in eukaryotes	1.00E+00	TRINITY_DN23686_c0_g2	1.14	up	hypothetical protein POPTR_0005s04710g [Populus trichocarpa]	84.77	60.50
map03008	Ribosome biogenesis in eukaryotes	1.00E+00	TRINITY_DN23885_c1_g3	-2.41	down	hypothetical protein POPTR_0018s07790g [Populus trichocarpa]	13.39	107.88
map03008	Ribosome biogenesis in eukaryotes	1.00E+00	TRINITY_DN24093_c0_g3	-2.62	down	hypothetical protein POPTR_0018s07790g [Populus trichocarpa]	11.03	101.81
map03008	Ribosome biogenesis in eukaryotes	1.00E+00	TRINITY_DN24102_c0_g2	1.17	up	hypothetical protein POPTR_0013s04510g [Populus trichocarpa]	5.18	3.54
map03008	Ribosome biogenesis in eukaryotes	1.00E+00	TRINITY_DN24248_c0_g1	-2.67	down	PREDICTED: uncharacterized protein LOC105122981 isoform X2 [Populus euphratica]	1.23	11.99
map03008	Ribosome biogenesis in eukaryotes	1.00E+00	TRINITY_DN24574_c0_g1	-1.15	down	PREDICTED: cyclin-A2-4-like isoform X1 [Populus euphratica]	7.64	25.30
map03008	Ribosome biogenesis in eukaryotes	1.00E+00	TRINITY_DN24868_c1_g1	-1.09	down	cyclin family protein [Populus trichocarpa]	56.44	192.52
map03008	Ribosome biogenesis in eukaryotes	1.00E+00	TRINITY_DN25023_c0_g1	1.01	up	PREDICTED: U3 small nucleolar RNA-associated protein 15 homolog [Populus euphratica]	11.67	8.96
map03008	Ribosome biogenesis in eukaryotes	1.00E+00	TRINITY_DN25083_c2_g3	1.16	up	hypothetical protein POPTR_0001s31270g [Populus trichocarpa]	51.17	33.03
map03008	Ribosome biogenesis in eukaryotes	1.00E+00	TRINITY_DN25440_c0_g1	-2.01	down	PREDICTED: kinesin KP1-like isoform X1 [Populus euphratica]	1.23	7.55
map03008	Ribosome biogenesis in eukaryotes	1.00E+00	TRINITY_DN25498_c0_g3	-1.01	down	hypothetical protein POPTR_0006s26340g [Populus trichocarpa]	7.69	23.42
map03008	Ribosome biogenesis in eukaryotes	1.00E+00	TRINITY_DN26220_c0_g2	-1.74	down	WD-40 repeat family protein-2 [Populus tomentosa]	2.21	11.23
map03008	Ribosome biogenesis in eukaryotes	1.00E+00	TRINITY_DN26282_c1_g2	1.32	up	PREDICTED: WD repeat-containing protein 44-like [Populus euphratica]	2.37	1.47
map03008	Ribosome biogenesis in eukaryotes	1.00E+00	TRINITY_DN26417_c0_g2	1.25	up	Endoribonuclease Dicer family protein [Populus trichocarpa]	6.69	4.27
map03008	Ribosome biogenesis in eukaryotes	1.00E+00	TRINITY_DN26462_c0_g2	1.22	up	hypothetical protein POPTR_0009s05900g [Populus trichocarpa]	36.70	23.79
map03008	Ribosome biogenesis in eukaryotes	1.00E+00	TRINITY_DN26763_c0_g1	-1.05	down	KINESIN-LIKE protein A [Populus trichocarpa]	16.84	50.92
map03008	Ribosome biogenesis in eukaryotes	1.00E+00	TRINITY_DN27002_c0_g1	-1.85	down	PREDICTED: kinesin-4 isoform X2 [Populus euphratica]	5.50	33.18
map03008	Ribosome biogenesis in eukaryotes	1.00E+00	TRINITY_DN13954_c0_g1	-2.15	down	PREDICTED: probable mediator of RNA polymerase II transcription subunit 36b isoform X1 [Populus euphratica]	0.65	4.14
map03008	Ribosome biogenesis in eukaryotes	1.00E+00	TRINITY_DN15233_c0_g1	1.11	up	hypothetical protein POPTR_0008s10960g [Populus trichocarpa]	154.94	109.81
map03008	Ribosome biogenesis in eukaryotes	1.00E+00	TRINITY_DN15680_c0_g1	1.38	up	PREDICTED: diphthamide biosynthesis protein 7 homolog isoform X9 [Populus euphratica]	6.87	4.10
map03008	Ribosome biogenesis in eukaryotes	1.00E+00	TRINITY_DN15749_c0_g1	1.73	up	hypothetical protein POPTR_0013s12750g [Populus trichocarpa]	17.01	7.84
map03008	Ribosome biogenesis in eukaryotes	1.00E+00	TRINITY_DN15755_c0_g3	1.19	up	hypothetical protein POPTR_0001s26960g [Populus trichocarpa]	73.87	49.39

map03008	Ribosome biogenesis in eukaryotes	1.00E+00	TRINITY_DN18017_c0_g1	-2.29	down	PREDICTED: G2/mitotic-specific cyclin C13-1-like isoform X1 [Populus euphratica]	0.59	4.35
map03008	Ribosome biogenesis in eukaryotes	1.00E+00	TRINITY_DN18096_c0_g1	1.16	up	PREDICTED: NHP2-like protein 1 [Populus euphratica]	202.04	145.54
map03008	Ribosome biogenesis in eukaryotes	1.00E+00	TRINITY_DN18130_c0_g1	-1.70	down	PREDICTED: uncharacterized protein LOC105130999 [Populus euphratica]	3.08	15.25
map03008	Ribosome biogenesis in eukaryotes	1.00E+00	TRINITY_DN19714_c0_g1	1.04	up	PREDICTED: uncharacterized protein LOC105109943 [Populus euphratica]	44.68	33.33
map03008	Ribosome biogenesis in eukaryotes	1.00E+00	TRINITY_DN19859_c0_g1	-1.80	down	hydroxyproline-rich glycoprotein [Populus trichocarpa]	62.93	330.51
map03008	Ribosome biogenesis in eukaryotes	1.00E+00	TRINITY_DN20655_c0_g1	-1.92	down	PREDICTED: putative cell division cycle ATPase [Populus euphratica]	0.83	4.18
map03008	Ribosome biogenesis in eukaryotes	1.00E+00	TRINITY_DN20883_c0_g1	-1.62	down	exonuclease family protein [Populus trichocarpa]	1.15	5.65
map03008	Ribosome biogenesis in eukaryotes	1.00E+00	TRINITY_DN21258_c0_g1	-1.04	down	alliinase family protein [Populus trichocarpa]	7.01	23.79
map03008	Ribosome biogenesis in eukaryotes	1.00E+00	TRINITY_DN21382_c0_g2	1.23	up	hypothetical protein POPTR_0008s16710g [Populus trichocarpa]	118.77	80.72
map03008	Ribosome biogenesis in eukaryotes	1.00E+00	TRINITY_DN21549_c1_g1	-1.04	down	PREDICTED: LOW QUALITY PROTEIN: endoribonuclease Dicer homolog 2-like [Populus euphratica]	2.08	7.18
map04141	Protein processing in endoplasmic reticulum	1.00E+00	TRINITY_DN21655_c0_g1	-1.22	down	PREDICTED: dolichyl-diphosphooligosaccharide--protein glycosyltransferase subunit STT3A isoform X1 [Populus euphratica]	4.31	16.78
map04141	Protein processing in endoplasmic reticulum	1.00E+00	TRINITY_DN22243_c0_g4	3.41	up	A-crystallin domain-containing family protein [Populus trichocarpa]	112.81	16.82
map04141	Protein processing in endoplasmic reticulum	1.00E+00	TRINITY_DN22413_c1_g1	-2.63	down	hypothetical protein POPTR_0005s03070g [Populus trichocarpa]	0.70	6.53
map04141	Protein processing in endoplasmic reticulum	1.00E+00	TRINITY_DN22793_c0_g1	1.02	up	hypothetical protein POPTR_0008s12930g [Populus trichocarpa]	23.18	18.78
map04141	Protein processing in endoplasmic reticulum	1.00E+00	TRINITY_DN22844_c0_g1	-1.36	down	unknown [Populus trichocarpa]	6.75	26.35
map04141	Protein processing in endoplasmic reticulum	1.00E+00	TRINITY_DN22844_c0_g2	-1.34	down	DNAJ heat shock family protein [Populus trichocarpa]	4.19	16.51
map04141	Protein processing in endoplasmic reticulum	1.00E+00	TRINITY_DN22850_c0_g2	1.16	up	PREDICTED: protein SUPPRESSOR OF GENE SILENCING 3-like isoform X3 [Populus euphratica]	26.52	19.00
map04141	Protein processing in endoplasmic reticulum	1.00E+00	TRINITY_DN22925_c1_g1	1.15	up	HSP80 family protein [Populus trichocarpa]	349.08	238.31
map04141	Protein processing in endoplasmic reticulum	1.00E+00	TRINITY_DN23240_c1_g5	2.27	up	PREDICTED: uncharacterized protein LOC105136996 isoform X1 [Populus euphratica]	55.20	17.84
map04141	Protein processing in endoplasmic reticulum	1.00E+00	TRINITY_DN23739_c0_g3	-1.43	down	PREDICTED: E3 ubiquitin-protein ligase RNF185-like [Populus euphratica]	1.67	7.00
map04141	Protein processing in endoplasmic reticulum	1.00E+00	TRINITY_DN24046_c1_g1	1.19	up	PREDICTED: transcription factor bHLH77-like [Populus euphratica]	12.83	8.62
map04141	Protein processing in endoplasmic reticulum	1.00E+00	TRINITY_DN24090_c0_g2	2.34	up	unknown [Populus trichocarpa x Populus deltoides]	199.05	59.58
map04141	Protein processing in endoplasmic reticulum	1.00E+00	TRINITY_DN24105_c1_g4	-1.90	down	Retrovirus-related Pol polyprotein from transposon TNT 1-94 [Cajanus cajan]	0.46	2.75
map04141	Protein processing in endoplasmic reticulum	1.00E+00	TRINITY_DN24109_c1_g1	1.30	up	hypothetical protein POPTR_0010s21280g [Populus trichocarpa]	548.40	356.38
map04141	Protein processing in endoplasmic reticulum	1.00E+00	TRINITY_DN24109_c1_g4	1.75	up	heat shock protein 70 [Saussurea medusa]	466.89	227.77
map04141	Protein processing in endoplasmic reticulum	1.00E+00	TRINITY_DN24119_c0_g1	-1.71	down	hypothetical protein POPTR_0017s07210g [Populus trichocarpa]	4.82	25.35
map04141	Protein processing in endoplasmic reticulum	1.00E+00	TRINITY_DN24133_c0_g4	1.11	up	hypothetical protein POPTR_0013s04620g [Populus trichocarpa]	28.53	19.92
map04141	Protein processing in endoplasmic reticulum	1.00E+00	TRINITY_DN24295_c0_g1	-1.12	down	PREDICTED: protein transport protein sec23-1-like [Populus euphratica]	7.42	24.49
map04141	Protein processing in endoplasmic reticulum	1.00E+00	TRINITY_DN24320_c1_g1	1.50	up	PREDICTED: thioredoxin Y1, chloroplastic-like isoform X2 [Populus euphratica]	250.01	144.28
map04141	Protein processing in endoplasmic reticulum	1.00E+00	TRINITY_DN24336_c0_g3	-1.62	down	PREDICTED: derlin-1-like [Populus euphratica]	2.38	10.98
map04141	Protein processing in endoplasmic reticulum	1.00E+00	TRINITY_DN24482_c0_g4	1.33	up	seed maturation protein PM37 [Populus trichocarpa]	28.60	18.11
map04141	Protein processing in endoplasmic reticulum	1.00E+00	TRINITY_DN24518_c0_g1	-1.66	down	PREDICTED: LOW QUALITY PROTEIN: TMV resistance protein N-like [Populus euphratica]	0.75	3.63
map04141	Protein processing in endoplasmic reticulum	1.00E+00	TRINITY_DN24648_c1_g1	1.01	up	hypothetical protein POPTR_0005s12850g [Populus trichocarpa]	99.94	79.78
map04141	Protein processing in endoplasmic reticulum	1.00E+00	TRINITY_DN24851_c0_g1	-3.19	down	exostosin family protein [Populus trichocarpa]	4.32	59.50
map04141	Protein processing in endoplasmic reticulum	1.00E+00	TRINITY_DN25056_c0_g5	1.87	up	HSP90 [Populus tomentosa]	199.35	84.67

map04141	Protein processing in endoplasmic reticulum	1.00E+00	TRINITY_DN25628_c0_g1	1.14	up	hypothetical protein POPTR_0001s24900g [Populus trichocarpa]	9.44	6.48
map04141	Protein processing in endoplasmic reticulum	1.00E+00	TRINITY_DN25682_c1_g1	-1.49	down	hypothetical protein POPTR_0019s08290g [Populus trichocarpa]	12.05	54.98
map04141	Protein processing in endoplasmic reticulum	1.00E+00	TRINITY_DN25990_c0_g2	-4.32	down	hypothetical protein POPTR_0007s01260g [Populus trichocarpa]	1.46	25.84
map04141	Protein processing in endoplasmic reticulum	1.00E+00	TRINITY_DN25996_c0_g1	1.35	up	heat shock protein 70 [Populus trichocarpa]	6.48	3.97
map04141	Protein processing in endoplasmic reticulum	1.00E+00	TRINITY_DN26004_c0_g1	2.02	up	PREDICTED: E3 ubiquitin-protein ligase RMA1H1 [Populus euphratica]	42.42	15.41
map04141	Protein processing in endoplasmic reticulum	1.00E+00	TRINITY_DN26004_c0_g2	2.59	up	PREDICTED: E3 ubiquitin-protein ligase RMA1H1 [Populus euphratica]	20.19	5.45
map04141	Protein processing in endoplasmic reticulum	1.00E+00	TRINITY_DN26166_c0_g1	-1.05	down	PREDICTED: serine/threonine-protein kinase/endoribonuclease IRE1b isoform X4 [Populus euphratica]	6.86	15.30
map04141	Protein processing in endoplasmic reticulum	1.00E+00	TRINITY_DN26166_c0_g3	-1.53	down	hypothetical protein POPTR_0007s09440g [Populus trichocarpa]	0.71	3.10
map04141	Protein processing in endoplasmic reticulum	1.00E+00	TRINITY_DN26166_c0_g4	-1.36	down	PREDICTED: serine/threonine-protein kinase/endoribonuclease IRE1a [Populus euphratica]	1.91	7.44
map04141	Protein processing in endoplasmic reticulum	1.00E+00	TRINITY_DN26228_c0_g1	-1.47	down	hypothetical protein POPTR_0003s20840g [Populus trichocarpa]	161.10	680.67
map04141	Protein processing in endoplasmic reticulum	1.00E+00	TRINITY_DN26295_c0_g2	-2.61	down	ubiquitin conjugating-like enzyme family protein [Populus trichocarpa]	1.87	16.39
map04141	Protein processing in endoplasmic reticulum	1.00E+00	TRINITY_DN26332_c0_g2	2.01	up	hypothetical protein POPTR_0011s05650g [Populus trichocarpa]	6.69	2.62
map04141	Protein processing in endoplasmic reticulum	1.00E+00	TRINITY_DN26479_c0_g1	1.26	up	AAA-type ATPase family protein [Populus trichocarpa]	23.09	19.00
map04141	Protein processing in endoplasmic reticulum	1.00E+00	TRINITY_DN26499_c0_g1	1.57	up	PREDICTED: dnaJ homolog 1, mitochondrial-like [Populus euphratica]	172.38	86.88
map04141	Protein processing in endoplasmic reticulum	1.00E+00	TRINITY_DN26758_c0_g1	1.70	up	PREDICTED: heat shock protein 83 isoform X2 [Populus euphratica]	52.31	25.64
map04141	Protein processing in endoplasmic reticulum	1.00E+00	TRINITY_DN27101_c0_g1	-1.22	down	PREDICTED: uncharacterized protein LOC105124962 [Populus euphratica]	4.77	16.99
map04141	Protein processing in endoplasmic reticulum	1.00E+00	TRINITY_DN27145_c0_g1	2.10	up	Stromal 70 kDa heat shock-related family protein [Populus trichocarpa]	862.92	304.67
map04141	Protein processing in endoplasmic reticulum	1.00E+00	TRINITY_DN27393_c0_g1	1.91	up	heat shock family protein [Populus trichocarpa]	39.01	15.44
map04141	Protein processing in endoplasmic reticulum	1.00E+00	TRINITY_DN27393_c1_g1	2.11	up	PREDICTED: heat shock protein 83-like [Populus euphratica]	375.24	129.58
map04141	Protein processing in endoplasmic reticulum	1.00E+00	TRINITY_DN27422_c0_g2	2.54	up	hypothetical protein POPTR_0010s18300g [Populus trichocarpa]	5.50	1.34
map04141	Protein processing in endoplasmic reticulum	1.00E+00	TRINITY_DN27479_c2_g1	1.63	up	PREDICTED: heat shock cognate 70 kDa protein 2-like [Populus euphratica]	249.29	121.53
map04141	Protein processing in endoplasmic reticulum	1.00E+00	TRINITY_DN27629_c1_g1	1.83	up	thioredoxin m family protein [Populus trichocarpa]	732.04	310.86
map04141	Protein processing in endoplasmic reticulum	1.00E+00	TRINITY_DN27695_c0_g1	3.03	up	hypothetical protein POPTR_0017s01160g [Populus trichocarpa]	29.21	5.47
map04141	Protein processing in endoplasmic reticulum	1.00E+00	TRINITY_DN27695_c1_g1	3.35	up	hypothetical protein POPTR_0017s01160g [Populus trichocarpa]	42.99	6.84
map04141	Protein processing in endoplasmic reticulum	1.00E+00	TRINITY_DN27695_c2_g1	1.11	up	HSP80 family protein [Populus trichocarpa]	176.25	130.70
map04141	Protein processing in endoplasmic reticulum	1.00E+00	TRINITY_DN27695_c2_g4	1.84	up	HSP90 [Populus tomentosa]	517.67	225.89
map04141	Protein processing in endoplasmic reticulum	1.00E+00	TRINITY_DN27873_c1_g3	-1.15	down	hypothetical protein POPTR_0019s13010g [Populus trichocarpa]	5.34	17.91
map04141	Protein processing in endoplasmic reticulum	1.00E+00	TRINITY_DN28064_c0_g1	-2.47	down	hypothetical protein POPTR_0009s02650g [Populus trichocarpa]	0.22	1.81
map04141	Protein processing in endoplasmic reticulum	1.00E+00	TRINITY_DN28132_c0_g1	2.50	up	hypothetical protein POPTR_0011s00470g [Populus trichocarpa]	4.79	1.26
map04141	Protein processing in endoplasmic reticulum	1.00E+00	TRINITY_DN13612_c0_g2	-1.99	down	PREDICTED: GEM-like protein 4 [Populus euphratica]	1.07	6.41
map04141	Protein processing in endoplasmic reticulum	1.00E+00	TRINITY_DN14071_c0_g1	3.68	up	heat shock 22K family protein [Populus trichocarpa]	3.13	0.36
map04141	Protein processing in endoplasmic reticulum	1.00E+00	TRINITY_DN14914_c0_g1	2.42	up	hypothetical protein POPTR_0001s06590g [Populus trichocarpa]	221.18	62.83

map04141	Protein processing in endoplasmic reticulum	1.00E+00	TRINITY_DN15136_c0_g1	-2.32	down	hypothetical protein POPTR_0004s17810g [Populus trichocarpa]	96.74	703.41
map04141	Protein processing in endoplasmic reticulum	1.00E+00	TRINITY_DN15273_c0_g1	2.95	up	PREDICTED: ubiquitin-conjugating enzyme E2 10-like [Populus euphratica]	8.51	1.68
map04141	Protein processing in endoplasmic reticulum	1.00E+00	TRINITY_DN15277_c0_g1	1.94	up	HSP90 [Populus tomentosa]	515.73	209.22
map04141	Protein processing in endoplasmic reticulum	1.00E+00	TRINITY_DN15389_c0_g1	2.54	up	heat shock family protein [Populus trichocarpa]	934.53	246.19
map04141	Protein processing in endoplasmic reticulum	1.00E+00	TRINITY_DN15745_c1_g1	1.03	up	Thioredoxin M-type 1 family protein [Populus trichocarpa]	22.04	14.86
map04141	Protein processing in endoplasmic reticulum	1.00E+00	TRINITY_DN16046_c0_g2	-1.54	down	hypothetical protein POPTR_0001s03820g [Populus trichocarpa]	0.83	3.54
map04141	Protein processing in endoplasmic reticulum	1.00E+00	TRINITY_DN16162_c0_g1	1.35	up	hypothetical protein POPTR_0012s05150g [Populus trichocarpa]	1.99	1.18
map04141	Protein processing in endoplasmic reticulum	1.00E+00	TRINITY_DN16250_c0_g1	3.86	up	PREDICTED: RING-H2 finger protein ATL70-like [Populus euphratica]	6.51	0.52
map04141	Protein processing in endoplasmic reticulum	1.00E+00	TRINITY_DN16400_c0_g1	4.03	up	hypothetical protein POPTR_0010s06320g [Populus trichocarpa]	46.19	4.37
map04141	Protein processing in endoplasmic reticulum	1.00E+00	TRINITY_DN16529_c0_g1	1.97	up	DnaJ-like family protein [Populus trichocarpa]	21.38	8.21
map04141	Protein processing in endoplasmic reticulum	1.00E+00	TRINITY_DN17094_c3_g1	2.21	up	hypothetical protein POPTR_0004s07190g [Populus trichocarpa]	10.86	4.99
map04141	Protein processing in endoplasmic reticulum	1.00E+00	TRINITY_DN17094_c3_g2	1.64	up	HSP90 [Populus tomentosa]	145.24	71.36
map04141	Protein processing in endoplasmic reticulum	1.00E+00	TRINITY_DN17168_c0_g1	1.15	up	PREDICTED: ATP-dependent zinc metalloprotease FtsH isoform X1 [Populus euphratica]	54.81	37.45
map04141	Protein processing in endoplasmic reticulum	1.00E+00	TRINITY_DN17279_c0_g1	1.03	up	PREDICTED: dnaJ homolog subfamily B member 8 isoform X1 [Populus euphratica]	16.32	12.67
map04141	Protein processing in endoplasmic reticulum	1.00E+00	TRINITY_DN18043_c0_g1	-1.40	down	hypothetical protein POPTR_0020s00530g [Populus trichocarpa]	1.34	5.32
map04141	Protein processing in endoplasmic reticulum	1.00E+00	TRINITY_DN18321_c0_g3	1.05	up	PREDICTED: GTP-binding protein SAR1A [Populus euphratica]	90.19	67.08
map04141	Protein processing in endoplasmic reticulum	1.00E+00	TRINITY_DN18413_c0_g1	2.31	up	Heat shock 22 kDa family protein [Populus trichocarpa]	12.50	5.08
map04141	Protein processing in endoplasmic reticulum	1.00E+00	TRINITY_DN18416_c0_g2	1.40	up	BiP isoform A family protein [Populus trichocarpa]	3.54	2.09
map04141	Protein processing in endoplasmic reticulum	1.00E+00	TRINITY_DN18416_c0_g3	1.00	up	BiP isoform A family protein [Populus trichocarpa]	31.78	24.33
map04141	Protein processing in endoplasmic reticulum	1.00E+00	TRINITY_DN18652_c0_g3	-2.20	down	hypothetical protein POPTR_0003s17160g [Populus trichocarpa]	1.70	12.43
map04141	Protein processing in endoplasmic reticulum	1.00E+00	TRINITY_DN18792_c1_g3	2.55	up	hypothetical protein POPTR_0013s11340g [Populus trichocarpa]	94.30	24.72
map04141	Protein processing in endoplasmic reticulum	1.00E+00	TRINITY_DN19005_c0_g1	-2.56	down	TIR-NBS disease resistance-like protein [Populus trichocarpa]	1.52	10.97
map04141	Protein processing in endoplasmic reticulum	1.00E+00	TRINITY_DN19150_c0_g1	2.64	up	hypothetical protein POPTR_0001s41190g [Populus trichocarpa]	289.16	70.23
map04141	Protein processing in endoplasmic reticulum	1.00E+00	TRINITY_DN19162_c0_g1	-1.82	down	hypothetical protein POPTR_0009s15230g [Populus trichocarpa]	4.83	14.57
map04141	Protein processing in endoplasmic reticulum	1.00E+00	TRINITY_DN19307_c0_g1	1.97	up	hypothetical protein POPTR_0017s02810g [Populus trichocarpa]	35.37	14.65
map04141	Protein processing in endoplasmic reticulum	1.00E+00	TRINITY_DN19356_c0_g3	1.22	up	PREDICTED: dolichyl-diphosphooligosaccharide--protein glycosyltransferase subunit DAD1 [Populus euphratica]	134.68	83.13
map04141	Protein processing in endoplasmic reticulum	1.00E+00	TRINITY_DN19365_c0_g4	-1.24	down	PREDICTED: putative GEM-like protein 8 isoform X2 [Populus euphratica]	3.73	12.35
map04141	Protein processing in endoplasmic reticulum	1.00E+00	TRINITY_DN19430_c0_g1	2.61	up	hypothetical protein POPTR_0007s13020g [Populus trichocarpa]	13.11	2.53
map04141	Protein processing in endoplasmic reticulum	1.00E+00	TRINITY_DN19485_c0_g1	-2.36	down	heat shock protein 70 cognate [Populus trichocarpa]	0.43	3.41
map04141	Protein processing in endoplasmic reticulum	1.00E+00	TRINITY_DN19485_c0_g6	-2.06	down	heat shock protein 70 cognate [Populus trichocarpa]	0.49	3.23
map04141	Protein processing in endoplasmic reticulum	1.00E+00	TRINITY_DN19500_c0_g2	-1.54	down	hypothetical protein POPTR_0011s12140g [Populus trichocarpa]	1.71	7.64
map04141	Protein processing in endoplasmic reticulum	1.00E+00	TRINITY_DN19909_c2_g1	1.45	up	PREDICTED: dnaJ homolog 1, mitochondrial [Populus euphratica]	78.26	45.72

map04141	Protein processing in endoplasmic reticulum	1.00E+00	TRINITY_DN20179_c0_g2	-1.02	down	hypothetical protein POPTR_0008s12630g [Populus trichocarpa]	3.31	11.62
map04141	Protein processing in endoplasmic reticulum	1.00E+00	TRINITY_DN20207_c0_g2	1.73	up	DNAJ chaperone C-terminal domain-containing family protein [Populus trichocarpa]	5.65	2.62
map04141	Protein processing in endoplasmic reticulum	1.00E+00	TRINITY_DN20265_c0_g2	-1.84	down	PREDICTED: cullin-1-like [Populus euphratica]	1.83	8.99
map04141	Protein processing in endoplasmic reticulum	1.00E+00	TRINITY_DN20525_c0_g1	1.64	up	thioredoxin m family protein [Populus trichocarpa]	637.53	308.78
map04141	Protein processing in endoplasmic reticulum	1.00E+00	TRINITY_DN20563_c0_g3	1.29	up	hypothetical protein POPTR_0015s04950g [Populus trichocarpa]	13.47	8.43
map04141	Protein processing in endoplasmic reticulum	1.00E+00	TRINITY_DN20570_c0_g1	1.68	up	PREDICTED: 18.1 kDa class I heat shock protein-like [Populus euphratica]	18.87	8.08
map04141	Protein processing in endoplasmic reticulum	1.00E+00	TRINITY_DN20653_c0_g1	-1.51	down	DNAJ heat shock N-terminal domain-containing family protein [Populus trichocarpa]	6.68	30.88
map04141	Protein processing in endoplasmic reticulum	1.00E+00	TRINITY_DN20796_c0_g1	1.93	up	heat shock protein 70 [Populus trichocarpa]	111.83	45.71
map04141	Protein processing in endoplasmic reticulum	1.00E+00	TRINITY_DN20796_c1_g4	3.37	up	heat shock protein 70 [Populus trichocarpa]	58.52	8.97
map04141	Protein processing in endoplasmic reticulum	1.00E+00	TRINITY_DN20819_c0_g1	-1.97	down	hypothetical protein POPTR_0035s00341g [Populus trichocarpa]	6.23	35.69
map04141	Protein processing in endoplasmic reticulum	1.00E+00	TRINITY_DN20963_c0_g1	-2.68	down	hypothetical protein POPTR_0008s19510g [Populus trichocarpa]	0.48	3.86
map04141	Protein processing in endoplasmic reticulum	1.00E+00	TRINITY_DN21132_c0_g2	2.81	up	hypothetical protein POPTR_0017s01160g [Populus trichocarpa]	24.50	5.47
map04141	Protein processing in endoplasmic reticulum	1.00E+00	TRINITY_DN21222_c3_g6	4.01	up	putative polyprotein [Oryza sativa Japonica Group]	1.90	0.17
map04141	Protein processing in endoplasmic reticulum	1.00E+00	TRINITY_DN21520_c0_g1	-1.12	down	hypothetical protein POPTR_0014s11700g [Populus trichocarpa]	16.16	50.24
map03050	Proteasome	1.00E+00	TRINITY_DN22632_c0_g4	1.05	up	26S proteasome non-ATPase regulatory subunit family protein [Populus trichocarpa]	60.85	46.51
map03050	Proteasome	1.00E+00	TRINITY_DN23579_c0_g1	1.20	up	PREDICTED: ATP-dependent zinc metalloprotease FtsH [Populus euphratica]	36.90	24.29
map03050	Proteasome	1.00E+00	TRINITY_DN24025_c0_g1	1.71	up	PREDICTED: ATP-dependent zinc metalloprotease FTSH, chloroplastic-like [Populus euphratica]	264.69	122.59
map03050	Proteasome	1.00E+00	TRINITY_DN24712_c0_g1	1.62	up	PREDICTED: ATP-dependent zinc metalloprotease FtsH [Populus euphratica]	75.34	41.93
map03050	Proteasome	1.00E+00	TRINITY_DN25640_c0_g1	1.39	up	hypothetical protein POPTR_0002s22560g [Populus trichocarpa]	279.75	162.70
map03050	Proteasome	1.00E+00	TRINITY_DN25670_c0_g1	1.63	up	PREDICTED: ATPase family AAA domain-containing protein 3-like [Populus euphratica]	22.58	11.89
map03050	Proteasome	1.00E+00	TRINITY_DN16604_c0_g1	1.61	up	FtsH protease family protein [Populus trichocarpa]	3.02	1.53
map03050	Proteasome	1.00E+00	TRINITY_DN17168_c0_g3	1.11	up	PREDICTED: ATP-dependent zinc metalloprotease FtsH isoform X1 [Populus euphratica]	20.53	14.38
map03050	Proteasome	1.00E+00	TRINITY_DN19700_c0_g1	1.29	up	PREDICTED: ATP-dependent zinc metalloprotease FTSH 12, chloroplastic [Populus euphratica]	88.02	55.24
map03050	Proteasome	1.00E+00	TRINITY_DN19764_c0_g2	-3.77	down	PREDICTED: uncharacterized protein LOC105133657 [Populus euphratica]	1.59	23.50
map03050	Proteasome	1.00E+00	TRINITY_DN21321_c0_g1	-1.39	down	hypothetical protein POPTR_0004s15720g [Populus trichocarpa]	8.63	19.23
map03050	Proteasome	1.00E+00	TRINITY_DN21322_c0_g2	-1.30	down	outer envelope membrane family protein [Populus trichocarpa]	4.91	17.53
map03050	Proteasome	1.00E+00	TRINITY_DN21322_c0_g3	-1.30	down	hypothetical protein POPTR_0002s05560g [Populus trichocarpa]	3.35	13.00
map03013	RNA transport	1.00E+00	TRINITY_DN21903_c0_g1	1.29	up	unknown [Populus trichocarpa]	81.78	51.30
map03013	RNA transport	1.00E+00	TRINITY_DN22228_c0_g1	1.27	up	PREDICTED: zinc phosphodiesterase ELAC protein 2 [Populus euphratica]	17.34	10.98
map03013	RNA transport	1.00E+00	TRINITY_DN22342_c0_g1	-3.72	down	PREDICTED: protein CUP-SHAPED COTYLEDON 3-like isoform X3 [Populus euphratica]	0.65	15.24
map03013	RNA transport	1.00E+00	TRINITY_DN22467_c0_g3	-1.54	down	PREDICTED: uncharacterized protein LOC105137361 [Populus euphratica]	7.71	34.02
map03013	RNA transport	1.00E+00	TRINITY_DN22755_c0_g5	-1.78	down	hypothetical protein POPTR_0014s06990g [Populus trichocarpa]	0.94	5.42
map03013	RNA transport	1.00E+00	TRINITY_DN22789_c2_g6	1.72	up	hypothetical protein POPTR_0002s08740g [Populus trichocarpa]	63.04	28.85
map03013	RNA transport	1.00E+00	TRINITY_DN22803_c0_g3	1.17	up	hypothetical protein POPTR_0001s07250g, partial [Populus trichocarpa]	39.49	25.87
map03013	RNA transport	1.00E+00	TRINITY_DN22849_c0_g1	1.56	up	RNA-binding protein RNP1 precursor [Populus trichocarpa]	253.11	130.90
map03013	RNA transport	1.00E+00	TRINITY_DN22922_c0_g1	-1.28	down	WD-40 repeat family protein [Populus trichocarpa]	28.16	108.86
map03013	RNA transport	1.00E+00	TRINITY_DN22930_c0_g1	1.43	up	PREDICTED: uncharacterized protein LOC105111959 isoform X2 [Populus euphratica]	35.76	20.03
map03013	RNA transport	1.00E+00	TRINITY_DN22977_c0_g3	-1.65	down	hypothetical protein POPTR_0001s05080g [Populus trichocarpa]	0.47	2.27

map03013	RNA transport	1.00E+00	TRINITY_DN23017_c2_g2	-1.78	down	hypothetical protein POPTR_0012s04520g [Populus trichocarpa]	66.78	341.77
map03013	RNA transport	1.00E+00	TRINITY_DN23038_c0_g1	-1.51	down	PREDICTED: THO complex subunit 5B [Populus euphratica]	0.55	2.38
map03013	RNA transport	1.00E+00	TRINITY_DN23268_c0_g1	1.52	up	hypothetical protein POPTR_0006s21650g [Populus trichocarpa]	465.66	243.37
map03013	RNA transport	1.00E+00	TRINITY_DN23369_c0_g2	-1.90	down	hypothetical protein POPTR_0001s31320g [Populus trichocarpa]	1.17	4.79
map03013	RNA transport	1.00E+00	TRINITY_DN23416_c0_g2	-2.11	down	hypothetical protein POPTR_0004s17890g [Populus trichocarpa]	16.13	111.83
map03013	RNA transport	1.00E+00	TRINITY_DN23588_c0_g2	1.60	up	hypothetical protein POPTR_0012s02130g [Populus trichocarpa]	5.55	2.72
map03013	RNA transport	1.00E+00	TRINITY_DN23825_c0_g1	1.08	up	eukaryotic translation initiation factor 3E family protein [Populus trichocarpa]	98.86	75.68
map03013	RNA transport	1.00E+00	TRINITY_DN23864_c0_g1	1.73	up	29 kDa ribonucleoprotein [Populus trichocarpa]	1851.11	826.20
map03013	RNA transport	1.00E+00	TRINITY_DN23885_c1_g3	-2.41	down	hypothetical protein POPTR_0018s07790g [Populus trichocarpa]	13.39	107.88
map03013	RNA transport	1.00E+00	TRINITY_DN23971_c1_g1	1.00	up	PREDICTED: eukaryotic translation initiation factor 3 subunit D-like [Populus euphratica]	58.86	45.10
map03013	RNA transport	1.00E+00	TRINITY_DN24093_c0_g3	-2.62	down	hypothetical protein POPTR_0018s07790g [Populus trichocarpa]	11.03	101.81
map03013	RNA transport	1.00E+00	TRINITY_DN24577_c0_g2	-2.76	down	pathogenesis-related thaumatin family protein [Populus trichocarpa]	1.47	15.01
map03013	RNA transport	1.00E+00	TRINITY_DN24667_c0_g1	-2.64	down	hypothetical protein POPTR_0019s08010g [Populus trichocarpa]	1.34	12.91
map03013	RNA transport	1.00E+00	TRINITY_DN24696_c0_g1	1.40	up	PREDICTED: proteinaceous RNase P 1, chloroplastic/mitochondrial-like isoform X1 [Populus euphratica]	26.14	14.84
map03013	RNA transport	1.00E+00	TRINITY_DN24926_c0_g1	1.11	up	Elongation factor Tu family protein [Populus trichocarpa]	70.61	52.16
map03013	RNA transport	1.00E+00	TRINITY_DN25388_c0_g5	-3.57	down	copper chaperone-related family protein [Populus trichocarpa]	0.15	2.82
map03013	RNA transport	1.00E+00	TRINITY_DN25595_c0_g2	-1.72	down	PREDICTED: uncharacterized protein LOC105128876 [Populus euphratica]	2.92	12.49
map03013	RNA transport	1.00E+00	TRINITY_DN25938_c0_g2	1.15	up	hypothetical protein POPTR_0017s01370g [Populus trichocarpa]	9.62	6.67
map03013	RNA transport	1.00E+00	TRINITY_DN26042_c0_g1	1.08	up	PREDICTED: uncharacterized protein LOC105137440 [Populus euphratica]	35.18	26.83
map03013	RNA transport	1.00E+00	TRINITY_DN26278_c0_g2	-1.08	down	hypothetical protein POPTR_0002s13880g [Populus trichocarpa]	3.43	12.65
map03013	RNA transport	1.00E+00	TRINITY_DN26575_c0_g1	3.89	up	hypothetical protein POPTR_0010s22750g [Populus trichocarpa]	8.07	0.49
map03013	RNA transport	1.00E+00	TRINITY_DN26990_c0_g1	-3.29	down	hypothetical protein POPTR_0004s17600g [Populus trichocarpa]	0.21	3.09
map03013	RNA transport	1.00E+00	TRINITY_DN27108_c0_g1	1.00	up	hypothetical protein POPTR_0007s07920g, partial [Populus trichocarpa]	6.71	5.08
map03013	RNA transport	1.00E+00	TRINITY_DN27392_c0_g1	-1.59	down	PREDICTED: cation/H(+) antiporter 2-like [Populus euphratica]	1.50	6.80
map03013	RNA transport	1.00E+00	TRINITY_DN27763_c0_g2	1.10	up	translation initiation factor IF-2 family protein [Populus trichocarpa]	78.52	54.70
map03013	RNA transport	1.00E+00	TRINITY_DN27842_c1_g1	1.38	up	elongation factor 1-alpha [Populus davidiana x Populus alba var. pyramidalis]	788.41	466.02
map03013	RNA transport	1.00E+00	TRINITY_DN27842_c1_g2	2.29	up	PREDICTED: elongation factor 1-alpha-like [Populus euphratica]	935.57	293.79
map03013	RNA transport	1.00E+00	TRINITY_DN27842_c2_g1	1.66	up	PREDICTED: LOW QUALITY PROTEIN: HBS1-like protein [Populus euphratica]	1154.82	615.48
map03013	RNA transport	1.00E+00	TRINITY_DN27842_c2_g4	1.41	up	ADR12-2 family protein [Populus trichocarpa]	203.14	117.25
map03013	RNA transport	1.00E+00	TRINITY_DN27873_c0_g1	-1.55	down	PREDICTED: disease resistance protein RPS6-like isoform X1 [Populus euphratica]	3.91	17.23
map03013	RNA transport	1.00E+00	TRINITY_DN12746_c0_g1	1.50	up	PREDICTED: protein translation factor SUI1 homolog 2 [Populus euphratica]	156.11	85.71
map03013	RNA transport	1.00E+00	TRINITY_DN15650_c0_g1	1.09	up	hypothetical protein POPTR_0016s11470g [Populus trichocarpa]	42.47	30.58
map03013	RNA transport	1.00E+00	TRINITY_DN15755_c0_g3	1.19	up	hypothetical protein POPTR_0001s26960g [Populus trichocarpa]	73.87	49.39
map03013	RNA transport	1.00E+00	TRINITY_DN15847_c0_g1	-1.63	down	hypothetical protein POPTR_0003s13080g [Populus trichocarpa]	5.83	27.41
map03013	RNA transport	1.00E+00	TRINITY_DN16653_c0_g1	-1.72	down	transducin family protein [Populus trichocarpa]	0.69	3.60
map03013	RNA transport	1.00E+00	TRINITY_DN16810_c0_g1	2.12	up	hypothetical protein POPTR_0014s12770g [Populus trichocarpa]	16.17	5.62
map03013	RNA transport	1.00E+00	TRINITY_DN16886_c0_g2	2.81	up	hypothetical protein POPTR_0016s14960g [Populus trichocarpa]	2.25	0.46
map03013	RNA transport	1.00E+00	TRINITY_DN16988_c0_g1	1.28	up	putative translation elongation factor Tu family protein [Zea mays]	606.26	392.97
map03013	RNA transport	1.00E+00	TRINITY_DN17047_c0_g1	1.38	up	hypothetical protein POPTR_0014s15880g [Populus trichocarpa]	75.71	44.18
map03013	RNA transport	1.00E+00	TRINITY_DN17248_c0_g2	2.10	up	ADR12-2 family protein [Populus trichocarpa]	292.56	105.43
map03013	RNA transport	1.00E+00	TRINITY_DN17248_c0_g4	1.37	up	PREDICTED: elongation factor 1-alpha-like [Populus euphratica]	207.42	123.76

map03013	RNA transport	1.00E+00	TRINITY_DN17307_c0_g1	3.62	up	PREDICTED: thaumatin-like protein 1b [Populus euphratica]	19.77	2.44
map03013	RNA transport	1.00E+00	TRINITY_DN17555_c0_g1	1.71	up	ADR12-2 family protein [Populus trichocarpa]	966.86	513.85
map03013	RNA transport	1.00E+00	TRINITY_DN17659_c0_g2	1.02	up	PREDICTED: myb family transcription factor APL-like isoform X1 [Populus euphratica]	4.94	3.74
map03013	RNA transport	1.00E+00	TRINITY_DN17659_c0_g3	1.10	up	PREDICTED: myb family transcription factor APL-like [Populus euphratica]	7.32	5.27
map03013	RNA transport	1.00E+00	TRINITY_DN17904_c0_g2	2.75	up	RNA recognition motif-containing family protein [Populus trichocarpa]	146.73	33.03
map03013	RNA transport	1.00E+00	TRINITY_DN17998_c0_g2	1.97	up	hypothetical protein POPTR_0009s07030g [Populus trichocarpa]	738.54	284.18
map03013	RNA transport	1.00E+00	TRINITY_DN18106_c0_g1	2.05	up	ADR12-2 family protein [Populus trichocarpa]	281.61	105.51
map03013	RNA transport	1.00E+00	TRINITY_DN18130_c0_g1	-1.70	down	PREDICTED: uncharacterized protein LOC105130999 [Populus euphratica]	3.08	15.25
map03013	RNA transport	1.00E+00	TRINITY_DN18162_c0_g1	-1.61	down	PREDICTED: NAC transcription factor 29-like [Populus euphratica]	4.47	21.42
map03013	RNA transport	1.00E+00	TRINITY_DN18363_c0_g1	1.09	up	hypothetical protein POPTR_0012s05590g [Populus trichocarpa]	7.01	5.04
map03013	RNA transport	1.00E+00	TRINITY_DN18459_c0_g1	2.46	up	PREDICTED: uncharacterized protein LOC105139820 [Populus euphratica]	11.18	3.13
map03013	RNA transport	1.00E+00	TRINITY_DN18586_c0_g1	1.64	up	ADR12-2 family protein [Populus trichocarpa]	572.07	278.84
map03013	RNA transport	1.00E+00	TRINITY_DN18869_c0_g2	-2.69	down	PREDICTED: osmotin-like protein [Populus euphratica]	26.48	248.56
map03013	RNA transport	1.00E+00	TRINITY_DN18873_c0_g2	1.04	up	translation initiation factor eIF-1A family protein [Populus trichocarpa]	211.89	166.21
map03013	RNA transport	1.00E+00	TRINITY_DN19039_c1_g4	1.08	up	hypothetical protein POPTR_0012s04420g [Populus trichocarpa]	10.68	7.52
map03013	RNA transport	1.00E+00	TRINITY_DN19042_c0_g1	-4.05	down	PREDICTED: protein FEZ-like [Populus euphratica]	0.38	9.93
map03013	RNA transport	1.00E+00	TRINITY_DN19266_c0_g1	1.63	up	PREDICTED: eukaryotic translation initiation factor isoform 4E-2-like [Populus euphratica]	65.37	36.78
map03013	RNA transport	1.00E+00	TRINITY_DN19266_c0_g2	1.21	up	eukaryotic translation initiation factor 4E isoform family protein [Populus trichocarpa]	20.47	13.56
map03013	RNA transport	1.00E+00	TRINITY_DN19456_c0_g1	-4.50	down	PREDICTED: putative Myb family transcription factor At1g14600 [Populus euphratica]	0.80	26.04
map03013	RNA transport	1.00E+00	TRINITY_DN19557_c1_g2	1.71	up	PREDICTED: polyadenylate-binding protein RBP47-like [Populus euphratica]	71.15	32.39
map03013	RNA transport	1.00E+00	TRINITY_DN20069_c0_g9	-1.14	down	zinc finger family protein [Populus trichocarpa]	5.84	19.88
map03013	RNA transport	1.00E+00	TRINITY_DN20076_c0_g1	-5.18	down	PREDICTED: thaumatin-like protein 1 isoform X1 [Populus euphratica]	1.09	67.15
map03013	RNA transport	1.00E+00	TRINITY_DN20153_c0_g1	-2.31	down	hypothetical protein POPTR_0003s02700g [Populus trichocarpa]	12.23	92.95
map03013	RNA transport	1.00E+00	TRINITY_DN20449_c0_g1	-1.58	down	PREDICTED: uncharacterized protein LOC105129425 isoform X2 [Populus euphratica]	1.03	4.63
map03013	RNA transport	1.00E+00	TRINITY_DN20471_c0_g1	-1.13	down	PREDICTED: inner centromere protein-like isoform X1 [Populus euphratica]	10.31	34.01
map03013	RNA transport	1.00E+00	TRINITY_DN20840_c0_g1	1.12	up	hypothetical protein POPTR_0003s13800g [Populus trichocarpa]	48.71	33.37
map03013	RNA transport	1.00E+00	TRINITY_DN20918_c0_g1	1.25	up	hypothetical protein POPTR_0003s04350g [Populus trichocarpa]	4.98	3.41
map03013	RNA transport	1.00E+00	TRINITY_DN20925_c0_g1	1.08	up	PREDICTED: eukaryotic translation initiation factor 3 subunit H [Populus euphratica]	88.80	63.07
map03013	RNA transport	1.00E+00	TRINITY_DN21009_c0_g2	-2.03	down	PREDICTED: interactor of constitutive active ROPs 4-like [Populus euphratica]	9.42	58.30
map03013	RNA transport	1.00E+00	TRINITY_DN21189_c1_g1	1.28	up	PREDICTED: glycine-rich protein 2-like [Populus euphratica]	28.80	17.96
map03013	RNA transport	1.00E+00	TRINITY_DN21205_c0_g1	-1.76	down	hypothetical protein POPTR_0019s08970g [Populus trichocarpa]	10.20	52.06
map03013	RNA transport	1.00E+00	TRINITY_DN21240_c0_g1	2.16	up	PREDICTED: 28 kDa ribonucleoprotein, chloroplastic-like [Populus euphratica]	151.80	51.26
map03013	RNA transport	1.00E+00	TRINITY_DN21303_c0_g1	-2.26	down	zinc finger family protein [Populus trichocarpa]	6.58	46.57
map03013	RNA transport	1.00E+00	TRINITY_DN21339_c1_g2	1.07	up	33 kDa ribonucleoprotein [Populus trichocarpa]	122.13	88.36
map04144	Endocytosis	1.00E+00	TRINITY_DN21687_c0_g2	-2.15	down	hypothetical protein POPTR_0002s08180g [Populus trichocarpa]	3.19	21.15
map04144	Endocytosis	1.00E+00	TRINITY_DN21860_c0_g5	-1.25	down	PREDICTED: protein CHUP1, chloroplastic [Populus euphratica]	9.48	34.48
map04144	Endocytosis	1.00E+00	TRINITY_DN21860_c0_g8	-1.89	down	hypothetical protein POPTR_0004s05410g [Populus trichocarpa]	1.00	5.75
map04144	Endocytosis	1.00E+00	TRINITY_DN21866_c0_g6	1.01	up	GTP-binding family protein [Populus trichocarpa]	54.96	41.84
map04144	Endocytosis	1.00E+00	TRINITY_DN22219_c0_g1	1.18	up	mov34 family protein [Populus trichocarpa]	6.60	4.92
map04144	Endocytosis	1.00E+00	TRINITY_DN22383_c0_g1	1.08	up	hypothetical protein POPTR_1173s00200g [Populus trichocarpa]	49.90	35.05
map04144	Endocytosis	1.00E+00	TRINITY_DN22393_c1_g1	-1.49	down	PREDICTED: ras-related protein RABC2a [Populus euphratica]	4.89	20.65
map04144	Endocytosis	1.00E+00	TRINITY_DN22403_c0_g1	-1.76	down	hypothetical protein POPTR_0001s03040g [Populus trichocarpa]	5.05	25.66
map04144	Endocytosis	1.00E+00	TRINITY_DN22413_c1_g1	-2.63	down	hypothetical protein POPTR_0005s03070g [Populus trichocarpa]	0.70	6.53

map04144	Endocytosis	1.00E+00	TRINITY_DN22428_c0_g2	-1.47	down	PREDICTED: centromere-associated protein E-like [Populus euphratica]	4.05	21.83
map04144	Endocytosis	1.00E+00	TRINITY_DN22557_c0_g6	1.99	up	PREDICTED: ADP-ribosylation factor 2-like isoform X1 [Populus euphratica]	93.15	35.75
map04144	Endocytosis	1.00E+00	TRINITY_DN22850_c0_g2	1.16	up	PREDICTED: protein SUPPRESSOR OF GENE SILENCING 3-like isoform X3 [Populus euphratica]	26.52	19.00
map04144	Endocytosis	1.00E+00	TRINITY_DN23033_c0_g1	-1.39	down	kinesin motor family protein [Populus trichocarpa]	11.04	30.75
map04144	Endocytosis	1.00E+00	TRINITY_DN23372_c1_g3	-1.21	down	hydroxyproline-rich glycoprotein [Populus trichocarpa]	2.52	8.92
map04144	Endocytosis	1.00E+00	TRINITY_DN23474_c0_g2	-2.18	down	PREDICTED: ninja-family protein AFP3-like [Populus euphratica]	1.57	10.81
map04144	Endocytosis	1.00E+00	TRINITY_DN23474_c0_g3	-1.81	down	hypothetical protein POPTR_0004s11980g, partial [Populus trichocarpa]	0.46	2.47
map04144	Endocytosis	1.00E+00	TRINITY_DN23717_c0_g1	-1.01	down	PREDICTED: kinesin-like protein KIF22 isoform X2 [Populus euphratica]	7.98	22.43
map04144	Endocytosis	1.00E+00	TRINITY_DN23875_c0_g2	-2.34	down	hypothetical protein POPTR_0003s01940g [Populus trichocarpa]	1.79	20.31
map04144	Endocytosis	1.00E+00	TRINITY_DN24039_c0_g2	-1.95	down	PREDICTED: dynamin-2A-like [Populus euphratica]	0.33	1.90
map04144	Endocytosis	1.00E+00	TRINITY_DN24047_c0_g2	-1.77	down	hypothetical protein POPTR_0016s00700g [Populus trichocarpa]	7.93	37.70
map04144	Endocytosis	1.00E+00	TRINITY_DN24079_c0_g1	-1.83	down	hypothetical protein POPTR_0001s02970g [Populus trichocarpa]	0.52	2.96
map04144	Endocytosis	1.00E+00	TRINITY_DN24079_c0_g2	-2.49	down	PREDICTED: uncharacterized protein LOC105121611 [Populus euphratica]	1.20	10.97
map04144	Endocytosis	1.00E+00	TRINITY_DN24109_c1_g1	1.30	up	hypothetical protein POPTR_0010s21280g [Populus trichocarpa]	548.40	356.38
map04144	Endocytosis	1.00E+00	TRINITY_DN24109_c1_g4	1.75	up	heat shock protein 70 [Saussurea medusa]	466.89	227.77
map04144	Endocytosis	1.00E+00	TRINITY_DN24115_c0_g1	-1.71	down	PREDICTED: kinesin heavy chain isoform X3 [Populus euphratica]	1.73	8.58
map04144	Endocytosis	1.00E+00	TRINITY_DN24453_c0_g1	-5.05	down	PREDICTED: lysine histidine transporter-like 8 [Populus euphratica]	0.11	4.20
map04144	Endocytosis	1.00E+00	TRINITY_DN24518_c0_g1	-1.66	down	PREDICTED: LOW QUALITY PROTEIN: TMV resistance protein N-like [Populus euphratica]	0.75	3.63
map04144	Endocytosis	1.00E+00	TRINITY_DN24875_c0_g5	-1.88	down	SNF7 family protein [Populus trichocarpa]	5.79	32.52
map04144	Endocytosis	1.00E+00	TRINITY_DN25013_c0_g1	1.53	up	PREDICTED: uncharacterized protein LOC105765014 isoform X2 [Gossypium raimondii]	88.84	47.84
map04144	Endocytosis	1.00E+00	TRINITY_DN25013_c0_g4	1.05	up	PREDICTED: uncharacterized protein LOC105765014 isoform X2 [Gossypium raimondii]	22.44	14.74
map04144	Endocytosis	1.00E+00	TRINITY_DN25140_c0_g1	-1.92	down	PREDICTED: inactive leucine-rich repeat receptor-like protein kinase CORYNE [Populus euphratica]	2.42	12.66
map04144	Endocytosis	1.00E+00	TRINITY_DN25140_c0_g3	-2.63	down	PREDICTED: inactive leucine-rich repeat receptor-like protein kinase CORYNE [Populus euphratica]	1.72	16.02
map04144	Endocytosis	1.00E+00	TRINITY_DN25440_c0_g5	-1.14	down	PREDICTED: kinesin-4 [Populus euphratica]	3.71	12.32
map04144	Endocytosis	1.00E+00	TRINITY_DN25559_c0_g1	-2.64	down	hypothetical protein POPTR_0007s04520g, partial [Populus trichocarpa]	2.90	22.44
map04144	Endocytosis	1.00E+00	TRINITY_DN25577_c0_g1	1.95	up	hypothetical protein POPTR_0019s11730g [Populus trichocarpa]	13.61	5.46
map04144	Endocytosis	1.00E+00	TRINITY_DN25860_c3_g2	-1.37	down	hypothetical protein POPTR_0014s01380g [Populus trichocarpa]	5.79	22.68
map04144	Endocytosis	1.00E+00	TRINITY_DN25996_c0_g1	1.35	up	heat shock protein 70 [Populus trichocarpa]	6.48	3.97
map04144	Endocytosis	1.00E+00	TRINITY_DN26401_c1_g1	-3.17	down	PREDICTED: protein CHUP1, chloroplastic-like [Populus euphratica]	1.52	20.79
map04144	Endocytosis	1.00E+00	TRINITY_DN26419_c0_g1	1.87	up	PREDICTED: protein CHUP1, chloroplastic [Populus euphratica]	89.57	36.59
map04144	Endocytosis	1.00E+00	TRINITY_DN26739_c0_g3	1.12	up	hypothetical protein POPTR_0010s17740g [Populus trichocarpa]	19.05	16.10
map04144	Endocytosis	1.00E+00	TRINITY_DN26754_c0_g2	-1.65	down	UBIQUITIN-SPECIFIC PROTEASE 21 family protein [Populus trichocarpa]	9.44	43.10
map04144	Endocytosis	1.00E+00	TRINITY_DN26797_c0_g1	-1.43	down	C2 domain-containing family protein [Populus trichocarpa]	3.07	13.21
map04144	Endocytosis	1.00E+00	TRINITY_DN27127_c0_g1	-1.04	down	putative phosphatidylinositol-4-phosphate 5-kinase mRNA family protein [Populus trichocarpa]	6.64	21.31
map04144	Endocytosis	1.00E+00	TRINITY_DN27145_c0_g1	2.10	up	Stromal 70 kDa heat shock-related family protein [Populus trichocarpa]	862.92	304.67
map04144	Endocytosis	1.00E+00	TRINITY_DN27398_c1_g1	-1.40	down	PREDICTED: 125 kDa kinesin-related protein [Populus euphratica]	10.93	43.02
map04144	Endocytosis	1.00E+00	TRINITY_DN27459_c0_g1	-1.01	down	hypothetical protein POPTR_0005s28380g [Populus trichocarpa]	20.97	60.76
map04144	Endocytosis	1.00E+00	TRINITY_DN27467_c1_g4	-1.01	down	hypothetical protein CISIN_1g025930mg [Citrus sinensis]	15.52	46.61
map04144	Endocytosis	1.00E+00	TRINITY_DN27479_c2_g1	1.63	up	PREDICTED: heat shock cognate 70 kDa protein 2-like [Populus euphratica]	249.29	121.53
map04144	Endocytosis	1.00E+00	TRINITY_DN27561_c0_g1	1.16	up	subtilase family protein [Populus trichocarpa]	28.53	21.68
map04144	Endocytosis	1.00E+00	TRINITY_DN27745_c0_g1	-1.35	down	PREDICTED: phragmoplast orienting kinesin-1 isoform X1 [Populus euphratica]	6.56	24.03
map04144	Endocytosis	1.00E+00	TRINITY_DN27790_c0_g1	-1.52	down	hypothetical protein POPTR_0015s11900g [Populus trichocarpa]	2.16	14.18
map04144	Endocytosis	1.00E+00	TRINITY_DN27873_c1_g3	-1.15	down	hypothetical protein POPTR_0019s13010g [Populus trichocarpa]	5.34	17.91

map04144	Endocytosis	1.00E+00	TRINITY_DN27918_c0_g1	2.70	up	hypothetical protein POPTR_0011s12350g [Populus trichocarpa]	3.65	0.85
map04144	Endocytosis	1.00E+00	TRINITY_DN11993_c0_g1	2.02	up	hypothetical protein POPTR_0014s01280g [Populus trichocarpa]	2.15	0.80
map04144	Endocytosis	1.00E+00	TRINITY_DN12280_c0_g2	-4.32	down	hypothetical protein POPTR_0008s13070g [Populus trichocarpa]	0.12	3.83
map04144	Endocytosis	1.00E+00	TRINITY_DN12280_c0_g3	-4.27	down	PREDICTED: probable ADP-ribosylation factor GTPase-activating protein AGD13 isoform X3 [Populus euphratica]	0.87	25.75
map04144	Endocytosis	1.00E+00	TRINITY_DN13172_c0_g1	-2.11	down	subtilase family protein [Populus trichocarpa]	1.87	12.20
map04144	Endocytosis	1.00E+00	TRINITY_DN13915_c1_g3	-2.14	down	hypothetical protein POPTR_0013s13800g [Populus trichocarpa]	0.67	4.64
map04144	Endocytosis	1.00E+00	TRINITY_DN14358_c0_g1	-1.80	down	phosphatidylinositol-4-phosphate 5-kinase family protein [Populus trichocarpa]	0.42	2.23
map04144	Endocytosis	1.00E+00	TRINITY_DN14423_c0_g1	1.98	up	subtilase family protein [Populus trichocarpa]	7.88	3.00
map04144	Endocytosis	1.00E+00	TRINITY_DN14434_c0_g1	-1.08	down	PREDICTED: probable ADP-ribosylation factor GTPase-activating protein AGD11 [Populus euphratica]	20.40	65.24
map04144	Endocytosis	1.00E+00	TRINITY_DN15247_c0_g2	-1.73	down	PREDICTED: uncharacterized protein LOC105129684 [Populus euphratica]	15.18	77.45
map04144	Endocytosis	1.00E+00	TRINITY_DN15419_c0_g1	-2.33	down	GDSL-motif lipase/hydrolase family protein [Populus trichocarpa]	0.56	4.19
map04144	Endocytosis	1.00E+00	TRINITY_DN16046_c0_g2	-1.54	down	hypothetical protein POPTR_0001s03820g [Populus trichocarpa]	0.83	3.54
map04144	Endocytosis	1.00E+00	TRINITY_DN16162_c0_g1	1.35	up	hypothetical protein POPTR_0012s05150g [Populus trichocarpa]	1.99	1.18
map04144	Endocytosis	1.00E+00	TRINITY_DN16270_c0_g3	-2.06	down	hypothetical protein POPTR_0010s24680g [Populus trichocarpa]	0.62	4.01
map04144	Endocytosis	1.00E+00	TRINITY_DN16289_c0_g1	-1.99	down	hypothetical protein POPTR_0004s18790g [Populus trichocarpa]	0.62	3.75
map04144	Endocytosis	1.00E+00	TRINITY_DN16436_c0_g2	1.03	up	hypothetical protein POPTR_0010s19770g [Populus trichocarpa]	63.56	46.85
map04144	Endocytosis	1.00E+00	TRINITY_DN16727_c0_g1	-1.59	down	GTP-binding family protein [Populus trichocarpa]	2.53	11.58
map04144	Endocytosis	1.00E+00	TRINITY_DN17156_c0_g1	-3.06	down	PREDICTED: protein ELC [Populus euphratica]	0.55	7.07
map04144	Endocytosis	1.00E+00	TRINITY_DN17206_c0_g1	-1.36	down	hypothetical protein POPTR_0016s12210g [Populus trichocarpa]	3.70	14.65
map04144	Endocytosis	1.00E+00	TRINITY_DN17407_c0_g1	-1.95	down	amino acid transporter family protein [Populus trichocarpa]	2.71	14.94
map04144	Endocytosis	1.00E+00	TRINITY_DN17462_c0_g1	-1.69	down	hypothetical protein POPTR_0001s44670g [Populus trichocarpa]	2.25	11.62
map04144	Endocytosis	1.00E+00	TRINITY_DN17471_c0_g2	-1.30	down	PREDICTED: uncharacterized protein LOC105133073 isoform X1 [Populus euphratica]	6.56	24.96
map04144	Endocytosis	1.00E+00	TRINITY_DN17518_c0_g1	1.02	up	PREDICTED: probable mitochondrial adenine nucleotide transporter BTL3 [Populus euphratica]	7.89	5.94
map04144	Endocytosis	1.00E+00	TRINITY_DN18105_c0_g5	-1.61	down	hypothetical protein POPTR_0010s13710g [Populus trichocarpa]	0.82	3.76
map04144	Endocytosis	1.00E+00	TRINITY_DN18107_c0_g1	-4.14	down	hypothetical protein POPTR_0004s16850g [Populus trichocarpa]	0.37	8.91
map04144	Endocytosis	1.00E+00	TRINITY_DN18107_c0_g2	-3.75	down	hypothetical protein POPTR_0004s16850g [Populus trichocarpa]	0.19	3.98
map04144	Endocytosis	1.00E+00	TRINITY_DN18328_c0_g1	-1.63	down	PREDICTED: fidgetin-like protein 1 isoform X1 [Populus euphratica]	1.38	6.65
map04144	Endocytosis	1.00E+00	TRINITY_DN18752_c0_g2	-1.32	down	PREDICTED: dynamin-related protein 5A isoform X1 [Populus euphratica]	5.05	18.98
map04144	Endocytosis	1.00E+00	TRINITY_DN18777_c0_g3	1.21	up	hypothetical protein POPTR_0005s11400g [Populus trichocarpa]	36.57	25.22
map04144	Endocytosis	1.00E+00	TRINITY_DN19005_c0_g1	-2.56	down	TIR-NBS disease resistance-like protein [Populus trichocarpa]	1.52	10.97
map04144	Endocytosis	1.00E+00	TRINITY_DN19103_c0_g1	-1.10	down	PREDICTED: charged multivesicular body protein 7 isoform X1 [Populus euphratica]	3.93	12.95
map04144	Endocytosis	1.00E+00	TRINITY_DN19130_c0_g5	-2.54	down	PREDICTED: kinesin KP1-like [Populus euphratica]	0.34	3.03
map04144	Endocytosis	1.00E+00	TRINITY_DN19162_c0_g1	-1.82	down	hypothetical protein POPTR_0009s15230g [Populus trichocarpa]	4.83	14.57
map04144	Endocytosis	1.00E+00	TRINITY_DN19269_c1_g1	1.32	up	PREDICTED: uncharacterized protein LOC105111558 isoform X1 [Populus euphratica]	7.47	4.48
map04144	Endocytosis	1.00E+00	TRINITY_DN19374_c0_g2	-2.18	down	hypothetical protein POPTR_0010s23370g [Populus trichocarpa]	0.57	4.04
map04144	Endocytosis	1.00E+00	TRINITY_DN19469_c0_g3	1.10	up	Ku70-binding family protein [Populus trichocarpa]	19.57	14.30
map04144	Endocytosis	1.00E+00	TRINITY_DN19485_c0_g1	-2.36	down	heat shock protein 70 cognate [Populus trichocarpa]	0.43	3.41
map04144	Endocytosis	1.00E+00	TRINITY_DN19485_c0_g6	-2.06	down	heat shock protein 70 cognate [Populus trichocarpa]	0.49	3.23
map04144	Endocytosis	1.00E+00	TRINITY_DN20409_c1_g4	-2.53	down	VQ motif-containing family protein [Populus trichocarpa]	2.96	25.76
map04144	Endocytosis	1.00E+00	TRINITY_DN20512_c0_g3	-3.34	down	putative subtilisin precursor family protein [Populus trichocarpa]	0.26	4.33
map04144	Endocytosis	1.00E+00	TRINITY_DN20796_c0_g1	1.93	up	heat shock protein 70 [Populus trichocarpa]	111.83	45.71
map04144	Endocytosis	1.00E+00	TRINITY_DN20796_c1_g4	3.37	up	heat shock protein 70 [Populus trichocarpa]	58.52	8.97
map04144	Endocytosis	1.00E+00	TRINITY_DN20820_c1_g1	-1.54	down	hypothetical protein POPTR_0015s12560g [Populus trichocarpa]	1.99	8.34

map04144	Endocytosis	1.00E+00	TRINITY_DN21384_c0_g1	-1.53	down	PREDICTED: kinesin-like protein KIF22 [Populus euphratica]	8.18	34.48
map04130	SNARE interactions in vesicular transport	1.00E+00	TRINITY_DN17374_c0_g1	-2.08	down	syntaxin 121 family protein [Populus trichocarpa]	1.57	10.21
map03015	mRNA surveillance pathway	1.00E+00	TRINITY_DN22169_c0_g3	4.20	up	hypothetical protein POPTR_0016s13300g [Populus trichocarpa]	27.25	2.26
map03015	mRNA surveillance pathway	1.00E+00	TRINITY_DN22178_c2_g4	1.14	up	hypothetical protein POPTR_0001s37650g [Populus trichocarpa]	81.83	56.14
map03015	mRNA surveillance pathway	1.00E+00	TRINITY_DN22261_c0_g2	-1.12	down	hypothetical protein POPTR_0002s09400g [Populus trichocarpa]	24.56	81.28
map03015	mRNA surveillance pathway	1.00E+00	TRINITY_DN22342_c0_g1	-3.72	down	PREDICTED: protein CUP-SHAPED COTYLEDON 3-like isoform X3 [Populus euphratica]	0.65	15.24
map03015	mRNA surveillance pathway	1.00E+00	TRINITY_DN22755_c0_g5	-1.78	down	hypothetical protein POPTR_0014s06990g [Populus trichocarpa]	0.94	5.42
map03015	mRNA surveillance pathway	1.00E+00	TRINITY_DN22849_c0_g1	1.56	up	RNA-binding protein RNP1 precursor [Populus trichocarpa]	253.11	130.90
map03015	mRNA surveillance pathway	1.00E+00	TRINITY_DN22930_c0_g1	1.43	up	PREDICTED: uncharacterized protein LOC105111959 isoform X2 [Populus euphratica]	35.76	20.03
map03015	mRNA surveillance pathway	1.00E+00	TRINITY_DN23268_c0_g1	1.52	up	hypothetical protein POPTR_0006s21650g [Populus trichocarpa]	465.66	243.37
map03015	mRNA surveillance pathway	1.00E+00	TRINITY_DN23369_c0_g2	-1.90	down	hypothetical protein POPTR_0001s31320g [Populus trichocarpa]	1.17	4.79
map03015	mRNA surveillance pathway	1.00E+00	TRINITY_DN23864_c0_g1	1.73	up	29 kDa ribonucleoprotein [Populus trichocarpa]	1851.11	826.20
map03015	mRNA surveillance pathway	1.00E+00	TRINITY_DN23957_c2_g1	-1.56	down	PREDICTED: RNA-binding protein 1-like isoform X1 [Populus euphratica]	7.28	29.89
map03015	mRNA surveillance pathway	1.00E+00	TRINITY_DN23957_c2_g2	-1.16	down	PREDICTED: RNA-binding protein 1-like [Populus euphratica]	4.25	15.60
map03015	mRNA surveillance pathway	1.00E+00	TRINITY_DN23957_c2_g3	-1.31	down	PREDICTED: RNA-binding protein 1-like isoform X2 [Populus euphratica]	1.60	5.99
map03015	mRNA surveillance pathway	1.00E+00	TRINITY_DN24614_c0_g5	-1.20	down	hypothetical protein POPTR_0018s06320g [Populus trichocarpa]	2.11	7.39
map03015	mRNA surveillance pathway	1.00E+00	TRINITY_DN24854_c0_g1	1.82	up	hypothetical protein POPTR_0001s08770g [Populus trichocarpa]	754.02	320.58
map03015	mRNA surveillance pathway	1.00E+00	TRINITY_DN24917_c0_g3	-1.08	down	hypothetical protein POPTR_0016s14930g [Populus trichocarpa]	9.73	23.49
map03015	mRNA surveillance pathway	1.00E+00	TRINITY_DN25595_c0_g2	-1.72	down	PREDICTED: uncharacterized protein LOC105128876 [Populus euphratica]	2.92	12.49
map03015	mRNA surveillance pathway	1.00E+00	TRINITY_DN25704_c0_g2	-1.25	down	hypothetical protein POPTR_0004s04510g [Populus trichocarpa]	2.77	13.56
map03015	mRNA surveillance pathway	1.00E+00	TRINITY_DN25704_c0_g3	-1.67	down	hypothetical protein POPTR_0011s05360g [Populus trichocarpa]	0.76	3.67
map03015	mRNA surveillance pathway	1.00E+00	TRINITY_DN25725_c1_g1	-1.25	down	phosphatase PP1 family protein [Populus trichocarpa]	8.62	31.49
map03015	mRNA surveillance pathway	1.00E+00	TRINITY_DN25912_c0_g1	-1.05	down	PREDICTED: serine/threonine protein phosphatase 2A 57 kDa regulatory subunit B' iota isoform-like [Populus euphratica]	7.20	22.71
map03015	mRNA surveillance pathway	1.00E+00	TRINITY_DN26275_c1_g1	1.01	up	hypothetical protein POPTR_0019s09060g [Populus trichocarpa]	77.76	62.55
map03015	mRNA surveillance pathway	1.00E+00	TRINITY_DN26403_c0_g1	-2.11	down	hypothetical protein POPTR_0006s24310g [Populus trichocarpa]	20.07	109.29
map03015	mRNA surveillance pathway	1.00E+00	TRINITY_DN26427_c2_g3	1.30	up	hypothetical protein POPTR_0008s10210g [Populus trichocarpa]	4.96	2.95
map03015	mRNA surveillance pathway	1.00E+00	TRINITY_DN13640_c0_g2	-3.01	down	hypothetical protein POPTR_0010s15820g [Populus trichocarpa]	0.32	4.21
map03015	mRNA surveillance pathway	1.00E+00	TRINITY_DN15650_c0_g1	1.09	up	hypothetical protein POPTR_0016s11470g [Populus trichocarpa]	42.47	30.58
map03015	mRNA surveillance pathway	1.00E+00	TRINITY_DN16745_c0_g1	4.38	up	hypothetical protein POPTR_0014s16610g [Populus trichocarpa]	41.77	2.96
map03015	mRNA surveillance pathway	1.00E+00	TRINITY_DN16896_c0_g2	-2.24	down	hypothetical protein POPTR_0011s13290g [Populus trichocarpa]	0.63	4.87
map03015	mRNA surveillance pathway	1.00E+00	TRINITY_DN17691_c0_g1	3.54	up	fasciclin-like arabinogalactan-protein 9 [Populus trichocarpa]	97.90	12.34
map03015	mRNA surveillance pathway	1.00E+00	TRINITY_DN17745_c0_g1	2.38	up	hypothetical protein POPTR_0002s02460g [Populus trichocarpa]	13.21	3.84
map03015	mRNA surveillance pathway	1.00E+00	TRINITY_DN17904_c0_g2	2.75	up	RNA recognition motif-containing family protein [Populus trichocarpa]	146.73	33.03
map03015	mRNA surveillance pathway	1.00E+00	TRINITY_DN17998_c0_g2	1.97	up	hypothetical protein POPTR_0009s07030g [Populus trichocarpa]	738.54	284.18
map03015	mRNA surveillance pathway	1.00E+00	TRINITY_DN18162_c0_g1	-1.61	down	PREDICTED: NAC transcription factor 29-like [Populus euphratica]	4.47	21.42
map03015	mRNA surveillance pathway	1.00E+00	TRINITY_DN19042_c0_g1	-4.05	down	PREDICTED: protein FEZ-like [Populus euphratica]	0.38	9.93
map03015	mRNA surveillance pathway	1.00E+00	TRINITY_DN19631_c0_g1	-2.81	down	PREDICTED: polyadenylation and cleavage factor homolog 5-like isoform X1 [Populus euphratica]	2.04	21.61
map03015	mRNA surveillance pathway	1.00E+00	TRINITY_DN20776_c0_g1	1.04	up	PREDICTED: heterogeneous nuclear ribonucleoprotein 1-like [Populus euphratica]	33.67	26.31
map03015	mRNA surveillance pathway	1.00E+00	TRINITY_DN20918_c0_g1	1.25	up	hypothetical protein POPTR_0003s04350g [Populus trichocarpa]	4.98	3.41
map03015	mRNA surveillance pathway	1.00E+00	TRINITY_DN21240_c0_g1	2.16	up	PREDICTED: 28 kDa ribonucleoprotein, chloroplastic-like [Populus euphratica]	151.80	51.26
map03015	mRNA surveillance pathway	1.00E+00	TRINITY_DN21339_c1_g2	1.07	up	33 kDa ribonucleoprotein [Populus trichocarpa]	122.13	88.36

map03015	mRNA surveillance pathway	1.00E+00	TRINITY_DN21340_c0_g2	-1.21	down	calmodulin-binding family protein [Populus trichocarpa]	10.99	38.42
map03040	Spliceosome	1.00E+00	TRINITY_DN21651_c0_g1	2.09	up	cyclophilin family protein [Populus trichocarpa]	359.12	135.82
map03040	Spliceosome	1.00E+00	TRINITY_DN21915_c1_g3	2.01	up	PREDICTED: elongation factor G-2, chloroplastic [Populus euphratica]	224.06	85.10
map03040	Spliceosome	1.00E+00	TRINITY_DN21981_c0_g1	1.18	up	PREDICTED: peptide methionine sulfoxide reductase B5-like [Populus euphratica]	193.71	129.41
map03040	Spliceosome	1.00E+00	TRINITY_DN21981_c0_g4	1.25	up	PREDICTED: peptide methionine sulfoxide reductase B5 [Populus euphratica]	146.64	95.32
map03040	Spliceosome	1.00E+00	TRINITY_DN21991_c0_g3	-1.57	down	hypothetical protein POPTR_0002s11320g [Populus trichocarpa]	7.10	30.96
map03040	Spliceosome	1.00E+00	TRINITY_DN22052_c0_g3	1.98	up	hypothetical protein POPTR_0001s33160g [Populus trichocarpa]	4.81	2.48
map03040	Spliceosome	1.00E+00	TRINITY_DN22135_c0_g4	1.22	up	RNA-binding family protein [Populus trichocarpa]	39.18	32.64
map03040	Spliceosome	1.00E+00	TRINITY_DN22154_c0_g2	1.55	up	hypothetical protein POPTR_0007s09310g [Populus trichocarpa]	85.68	44.64
map03040	Spliceosome	1.00E+00	TRINITY_DN22183_c0_g1	1.17	up	hypothetical protein POPTR_0008s02290g [Populus trichocarpa]	46.84	32.01
map03040	Spliceosome	1.00E+00	TRINITY_DN22192_c0_g3	-2.10	down	helicase domain-containing family protein [Populus trichocarpa]	1.41	8.87
map03040	Spliceosome	1.00E+00	TRINITY_DN22413_c1_g1	-2.63	down	hypothetical protein POPTR_0005s03070g [Populus trichocarpa]	0.70	6.53
map03040	Spliceosome	1.00E+00	TRINITY_DN22490_c0_g1	-2.28	down	hypothetical protein POPTR_0017s00570g [Populus trichocarpa]	3.99	29.64
map03040	Spliceosome	1.00E+00	TRINITY_DN22490_c0_g2	-2.81	down	hypothetical protein POPTR_0017s04700g [Populus trichocarpa]	0.57	5.45
map03040	Spliceosome	1.00E+00	TRINITY_DN22850_c0_g2	1.16	up	PREDICTED: protein SUPPRESSOR OF GENE SILENCING 3-like isoform X3 [Populus euphratica]	26.52	19.00
map03040	Spliceosome	1.00E+00	TRINITY_DN22874_c0_g1	-2.09	down	PREDICTED: probable transcription factor KAN4 [Populus euphratica]	2.23	17.71
map03040	Spliceosome	1.00E+00	TRINITY_DN23055_c3_g1	-2.38	down	PREDICTED: transcription repressor KAN1-like [Populus euphratica]	0.69	4.93
map03040	Spliceosome	1.00E+00	TRINITY_DN23369_c0_g2	-1.90	down	hypothetical protein POPTR_0001s31320g [Populus trichocarpa]	1.17	4.79
map03040	Spliceosome	1.00E+00	TRINITY_DN23475_c1_g2	-1.39	down	hypothetical protein POPTR_0008s01110g [Populus trichocarpa]	2.39	10.58
map03040	Spliceosome	1.00E+00	TRINITY_DN23840_c0_g1	1.34	up	peptidyl-prolyl cis-trans isomerase cyclophilin-type family protein [Populus trichocarpa]	722.73	421.37
map03040	Spliceosome	1.00E+00	TRINITY_DN24087_c0_g1	1.87	up	peptidyl-tRNA hydrolase family protein [Populus trichocarpa]	29.29	13.93
map03040	Spliceosome	1.00E+00	TRINITY_DN24109_c1_g1	1.30	up	hypothetical protein POPTR_0010s21280g [Populus trichocarpa]	548.40	356.38
map03040	Spliceosome	1.00E+00	TRINITY_DN24109_c1_g4	1.75	up	heat shock protein 70 [Saussurea medusa]	466.89	227.77
map03040	Spliceosome	1.00E+00	TRINITY_DN24375_c0_g2	1.46	up	DEAD/DEAH box helicase family protein [Populus trichocarpa]	75.03	41.22
map03040	Spliceosome	1.00E+00	TRINITY_DN24500_c0_g6	1.81	up	PREDICTED: DEAD-box ATP-dependent RNA helicase 22 isoform X2 [Populus euphratica]	60.25	26.15
map03040	Spliceosome	1.00E+00	TRINITY_DN24518_c0_g1	-1.66	down	PREDICTED: LOW QUALITY PROTEIN: TMV resistance protein N-like [Populus euphratica]	0.75	3.63
map03040	Spliceosome	1.00E+00	TRINITY_DN25133_c1_g10	1.15	up	PREDICTED: copper-transporting ATPase PAA1, chloroplastic-like [Populus euphratica]	24.54	17.17
map03040	Spliceosome	1.00E+00	TRINITY_DN25133_c1_g7	1.10	up	hypothetical protein POPTR_0003s01860g [Populus trichocarpa]	15.41	11.21
map03040	Spliceosome	1.00E+00	TRINITY_DN25209_c0_g1	-1.62	down	hypothetical protein POPTR_0017s02220g [Populus trichocarpa]	6.32	28.20
map03040	Spliceosome	1.00E+00	TRINITY_DN25383_c0_g2	-1.77	down	PREDICTED: putative disease resistance RPP13-like protein 1 [Populus euphratica]	0.36	1.90
map03040	Spliceosome	1.00E+00	TRINITY_DN25595_c0_g2	-1.72	down	PREDICTED: uncharacterized protein LOC105128876 [Populus euphratica]	2.92	12.49
map03040	Spliceosome	1.00E+00	TRINITY_DN25818_c2_g1	-2.11	down	PREDICTED: putative disease resistance RPP13-like protein 1 [Populus euphratica]	1.48	9.05
map03040	Spliceosome	1.00E+00	TRINITY_DN25996_c0_g1	1.35	up	heat shock protein 70 [Populus trichocarpa]	6.48	3.97
map03040	Spliceosome	1.00E+00	TRINITY_DN26008_c1_g1	1.90	up	SQUINT family protein [Populus trichocarpa]	44.13	20.58
map03040	Spliceosome	1.00E+00	TRINITY_DN26012_c0_g4	-1.52	down	hypothetical protein POPTR_0007s05610g, partial [Populus trichocarpa]	0.87	3.77
map03040	Spliceosome	1.00E+00	TRINITY_DN26184_c0_g1	1.15	up	PREDICTED: RNA-binding protein 24-like isoform X1 [Populus euphratica]	22.91	16.26
map03040	Spliceosome	1.00E+00	TRINITY_DN26299_c0_g1	-2.52	down	NBS-LRR type disease resistance protein [Populus trichocarpa]	7.92	62.54
map03040	Spliceosome	1.00E+00	TRINITY_DN26359_c0_g1	1.32	up	PREDICTED: DEAD-box ATP-dependent RNA helicase 51-like [Populus euphratica]	42.84	25.49
map03040	Spliceosome	1.00E+00	TRINITY_DN26441_c0_g1	-1.30	down	PREDICTED: uncharacterized protein LOC105137111 [Populus euphratica]	2.57	9.61
map03040	Spliceosome	1.00E+00	TRINITY_DN26444_c0_g1	-2.84	down	hypothetical protein POPTR_0017s00570g [Populus trichocarpa]	1.38	11.27
map03040	Spliceosome	1.00E+00	TRINITY_DN26534_c0_g1	-2.88	down	myb family transcription factor family protein [Populus trichocarpa]	1.96	20.92
map03040	Spliceosome	1.00E+00	TRINITY_DN26687_c0_g2	-1.15	down	PREDICTED: PHD finger protein ALFIN-LIKE 1-like [Populus euphratica]	9.77	33.30

map03040	Spliceosome	1.00E+00	TRINITY_DN26904_c0_g1	-1.36	down	hypothetical protein POPTR_0006s02710g [Populus trichocarpa]	5.49	20.98
map03040	Spliceosome	1.00E+00	TRINITY_DN26991_c0_g1	1.93	up	PREDICTED: DEAD-box ATP-dependent RNA helicase 31-like [Populus euphratica]	106.93	42.87
map03040	Spliceosome	1.00E+00	TRINITY_DN26991_c0_g2	1.30	up	DEAD box RNA helicase family protein [Populus trichocarpa]	23.57	16.72
map03040	Spliceosome	1.00E+00	TRINITY_DN27055_c1_g3	1.13	up	hypothetical protein POPTR_0005s00240g [Populus trichocarpa]	81.72	55.42
map03040	Spliceosome	1.00E+00	TRINITY_DN27055_c1_g4	1.19	up	hypothetical protein POPTR_0005s00200g [Populus trichocarpa]	99.43	64.90
map03040	Spliceosome	1.00E+00	TRINITY_DN27145_c0_g1	2.10	up	Stromal 70 kDa heat shock-related family protein [Populus trichocarpa]	862.92	304.67
map03040	Spliceosome	1.00E+00	TRINITY_DN27296_c0_g1	-1.17	down	hypothetical protein POPTR_0003s20080g [Populus trichocarpa]	2.00	6.14
map03040	Spliceosome	1.00E+00	TRINITY_DN27296_c1_g4	-3.48	down	hypothetical protein POPTR_0001s07040g [Populus trichocarpa]	0.25	4.55
map03040	Spliceosome	1.00E+00	TRINITY_DN27479_c2_g1	1.63	up	PREDICTED: heat shock cognate 70 kDa protein 2-like [Populus euphratica]	249.29	121.53
map03040	Spliceosome	1.00E+00	TRINITY_DN27528_c2_g3	-2.00	down	hypothetical protein POPTR_0002s17990g [Populus trichocarpa]	2.15	15.55
map03040	Spliceosome	1.00E+00	TRINITY_DN27528_c2_g4	-1.46	down	hypothetical protein POPTR_0014s10090g [Populus trichocarpa]	0.85	3.71
map03040	Spliceosome	1.00E+00	TRINITY_DN27744_c1_g2	-1.59	down	PREDICTED: LOW QUALITY PROTEIN: DEAD-box ATP-dependent RNA helicase 14-like [Populus euphratica]	1.52	7.36
map03040	Spliceosome	1.00E+00	TRINITY_DN27872_c1_g2	-1.51	down	hypothetical protein POPTR_0154s00220g [Populus trichocarpa]	4.07	18.65
map03040	Spliceosome	1.00E+00	TRINITY_DN27873_c1_g3	-1.15	down	hypothetical protein POPTR_0019s13010g [Populus trichocarpa]	5.34	17.91
map03040	Spliceosome	1.00E+00	TRINITY_DN27884_c4_g1	-1.85	down	hypothetical protein POPTR_0003s19950g [Populus trichocarpa]	8.59	34.00
map03040	Spliceosome	1.00E+00	TRINITY_DN11497_c0_g1	-3.05	down	hypothetical protein POPTR_0001s09650g, partial [Populus trichocarpa]	0.25	3.27
map03040	Spliceosome	1.00E+00	TRINITY_DN13872_c0_g2	1.39	up	peptidyl-prolyl cis-trans isomerase family protein [Populus trichocarpa]	125.54	73.01
map03040	Spliceosome	1.00E+00	TRINITY_DN14255_c0_g1	-2.11	down	hypothetical protein POPTR_0003s19260g [Populus trichocarpa]	5.61	36.13
map03040	Spliceosome	1.00E+00	TRINITY_DN15191_c0_g1	1.23	up	hypothetical protein POPTR_0007s13210g [Populus trichocarpa]	8.75	5.83
map03040	Spliceosome	1.00E+00	TRINITY_DN15461_c0_g1	-7.18	down	terminal ear1-like 2 protein [Populus tremula x Populus alba]	0.03	5.86
map03040	Spliceosome	1.00E+00	TRINITY_DN15650_c0_g1	1.09	up	hypothetical protein POPTR_0016s11470g [Populus trichocarpa]	42.47	30.58
map03040	Spliceosome	1.00E+00	TRINITY_DN15860_c0_g1	1.30	up	PREDICTED: putative endo-1,3(4)-beta-glucanase 2 [Populus euphratica]	5.18	3.16
map03040	Spliceosome	1.00E+00	TRINITY_DN16046_c0_g2	-1.54	down	hypothetical protein POPTR_0001s03820g [Populus trichocarpa]	0.83	3.54
map03040	Spliceosome	1.00E+00	TRINITY_DN16162_c0_g1	1.35	up	hypothetical protein POPTR_0012s05150g [Populus trichocarpa]	1.99	1.18
map03040	Spliceosome	1.00E+00	TRINITY_DN16179_c0_g1	1.33	up	PREDICTED: ribosome biogenesis protein 15 [Populus euphratica]	57.18	34.88
map03040	Spliceosome	1.00E+00	TRINITY_DN16646_c0_g1	1.25	up	PREDICTED: glycine-rich RNA-binding protein 4, mitochondrial [Populus euphratica]	13.39	10.78
map03040	Spliceosome	1.00E+00	TRINITY_DN16653_c0_g1	-1.72	down	transducin family protein [Populus trichocarpa]	0.69	3.60
map03040	Spliceosome	1.00E+00	TRINITY_DN18096_c0_g1	1.16	up	PREDICTED: NHP2-like protein 1 [Populus euphratica]	202.04	145.54
map03040	Spliceosome	1.00E+00	TRINITY_DN18526_c0_g2	1.89	up	hypothetical protein POPTR_0012s09200g [Populus trichocarpa]	3062.24	1238.07
map03040	Spliceosome	1.00E+00	TRINITY_DN18526_c0_g3	2.65	up	RNA-binding family protein [Populus trichocarpa]	468.44	105.67
map03040	Spliceosome	1.00E+00	TRINITY_DN18573_c0_g1	1.72	up	hypothetical protein POPTR_0015s06810g [Populus trichocarpa]	310.87	143.26
map03040	Spliceosome	1.00E+00	TRINITY_DN18671_c0_g1	2.42	up	RNA recognition motif-containing family protein [Populus trichocarpa]	49.37	13.94
map03040	Spliceosome	1.00E+00	TRINITY_DN18714_c0_g2	2.22	up	PREDICTED: uncharacterized protein LOC104611813 [Nelumbo nucifera]	3.96	1.39
map03040	Spliceosome	1.00E+00	TRINITY_DN19005_c0_g1	-2.56	down	TIR-NBS disease resistance-like protein [Populus trichocarpa]	1.52	10.97
map03040	Spliceosome	1.00E+00	TRINITY_DN19153_c0_g1	2.12	up	PREDICTED: peptide methionine sulfoxide reductase B1, chloroplastic [Populus euphratica]	116.51	43.73
map03040	Spliceosome	1.00E+00	TRINITY_DN19162_c0_g1	-1.82	down	hypothetical protein POPTR_0009s15230g [Populus trichocarpa]	4.83	14.57
map03040	Spliceosome	1.00E+00	TRINITY_DN19273_c0_g4	-2.08	down	PREDICTED: uncharacterized protein LOC105139124 [Populus euphratica]	2.17	14.01
map03040	Spliceosome	1.00E+00	TRINITY_DN19273_c0_g6	-3.54	down	PREDICTED: uncharacterized protein LOC105110073 isoform X1 [Populus euphratica]	0.16	3.04
map03040	Spliceosome	1.00E+00	TRINITY_DN19456_c0_g5	-4.79	down	PREDICTED: putative Myb family transcription factor At1g14600 [Populus euphratica]	1.21	42.06
map03040	Spliceosome	1.00E+00	TRINITY_DN19463_c0_g2	-2.40	down	small nuclear ribonucleoprotein [Populus trichocarpa]	0.51	3.86
map03040	Spliceosome	1.00E+00	TRINITY_DN19485_c0_g1	-2.36	down	heat shock protein 70 cognate [Populus trichocarpa]	0.43	3.41

map03040	Spliceosome	1.00E+00	TRINITY_DN19485_c0_g6	-2.06	down	heat shock protein 70 cognate [Populus trichocarpa]	0.49	3.23
map03040	Spliceosome	1.00E+00	TRINITY_DN19791_c1_g3	1.78	up	hypothetical protein POPTR_0008s20300g [Populus trichocarpa]	178.02	79.56
map03040	Spliceosome	1.00E+00	TRINITY_DN19918_c0_g3	1.15	up	PREDICTED: glycine-rich RNA-binding protein 4, mitochondrial-like [Populus euphratica]	71.78	49.39
map03040	Spliceosome	1.00E+00	TRINITY_DN19918_c0_g4	1.77	up	PREDICTED: glycine-rich RNA-binding protein 4, mitochondrial [Populus euphratica]	119.64	53.63
map03040	Spliceosome	1.00E+00	TRINITY_DN20595_c0_g2	1.02	up	hypothetical protein POPTR_0010s00730g [Populus trichocarpa]	66.67	41.07
map03040	Spliceosome	1.00E+00	TRINITY_DN20693_c0_g1	-4.68	down	RNA recognition motif-containing family protein [Populus trichocarpa]	1.12	38.18
map03040	Spliceosome	1.00E+00	TRINITY_DN20796_c0_g1	1.93	up	heat shock protein 70 [Populus trichocarpa]	111.83	45.71
map03040	Spliceosome	1.00E+00	TRINITY_DN20796_c1_g4	3.37	up	heat shock protein 70 [Populus trichocarpa]	58.52	8.97
map03040	Spliceosome	1.00E+00	TRINITY_DN21074_c0_g1	1.52	up	PREDICTED: glycine-rich RNA-binding protein 2, mitochondrial-like [Populus euphratica]	98.17	53.65
map03040	Spliceosome	1.00E+00	TRINITY_DN21074_c0_g2	1.84	up	unknown [Populus trichocarpa]	309.14	122.31
map03040	Spliceosome	1.00E+00	TRINITY_DN21144_c0_g1	1.59	up	hypothetical protein POPTR_0005s22020g [Populus trichocarpa]	100.60	46.68
map04145	Phagosome	1.00E+00	TRINITY_DN22037_c0_g2	2.95	up	PREDICTED: tubulin beta chain-like [Populus euphratica]	30.97	6.16
map04145	Phagosome	1.00E+00	TRINITY_DN22874_c0_g2	1.88	up	hypothetical protein POPTR_0011s07310g [Populus trichocarpa]	360.34	149.46
map04145	Phagosome	1.00E+00	TRINITY_DN23962_c0_g1	1.34	up	cysteine protease family protein [Populus trichocarpa]	26.78	15.91
map04145	Phagosome	1.00E+00	TRINITY_DN24097_c0_g1	-1.40	down	vacuolar ATP synthase subunit E family protein [Populus trichocarpa]	2.41	9.54
map04145	Phagosome	1.00E+00	TRINITY_DN24334_c1_g2	-1.95	down	hypothetical protein POPTR_0003s12540g [Populus trichocarpa]	3.50	20.57
map04145	Phagosome	1.00E+00	TRINITY_DN24334_c1_g5	-2.37	down	hypothetical protein POPTR_0001s09180g [Populus trichocarpa]	3.38	26.54
map04145	Phagosome	1.00E+00	TRINITY_DN25682_c1_g1	-1.49	down	hypothetical protein POPTR_0019s08290g [Populus trichocarpa]	12.05	54.98
map04145	Phagosome	1.00E+00	TRINITY_DN26239_c0_g2	-1.19	down	Tubulin gamma-1 chain family protein [Populus trichocarpa]	9.81	35.94
map04145	Phagosome	1.00E+00	TRINITY_DN27310_c0_g3	2.34	up	hypothetical protein POPTR_0003s12690g [Populus trichocarpa]	6.83	4.24
map04145	Phagosome	1.00E+00	TRINITY_DN27310_c1_g1	-1.15	down	beta-tubulin 5 [Salix arbutifolia]	40.36	135.82
map04145	Phagosome	1.00E+00	TRINITY_DN27572_c1_g1	1.18	up	PREDICTED: lon protease homolog 1, mitochondrial-like [Populus euphratica]	27.46	19.45
map04145	Phagosome	1.00E+00	TRINITY_DN27665_c0_g3	-2.87	down	PREDICTED: putative 1-phosphatidylinositol-3-phosphate 5-kinase FAB1D [Populus euphratica]	0.28	3.17
map04145	Phagosome	1.00E+00	TRINITY_DN11055_c0_g1	1.98	up	hypothetical protein VITISV_037104 [Vitis vinifera]	2.79	1.03
map04145	Phagosome	1.00E+00	TRINITY_DN17462_c0_g1	-1.69	down	hypothetical protein POPTR_0001s44670g [Populus trichocarpa]	2.25	11.62
map04145	Phagosome	1.00E+00	TRINITY_DN17639_c0_g2	-1.02	down	hypothetical protein POPTR_0005s26140g [Populus trichocarpa]	12.94	39.02
map04145	Phagosome	1.00E+00	TRINITY_DN17881_c0_g1	1.36	up	hypothetical protein POPTR_0008s04010g [Populus trichocarpa]	237.78	141.08
map04145	Phagosome	1.00E+00	TRINITY_DN17920_c0_g3	2.87	up	PREDICTED: tubulin beta chain [Vitis vinifera]	13.65	3.00
map04145	Phagosome	1.00E+00	TRINITY_DN18030_c1_g6	-1.19	down	hypothetical protein POPTR_0001s27950g [Populus trichocarpa]	7.30	26.00
map04145	Phagosome	1.00E+00	TRINITY_DN18265_c0_g1	1.01	up	PREDICTED: V-type proton ATPase subunit H isoform X2 [Populus euphratica]	55.97	42.26
map04145	Phagosome	1.00E+00	TRINITY_DN18665_c1_g4	-1.17	down	PREDICTED: uncharacterized protein LOC105115687 [Populus euphratica]	14.53	59.68
map04145	Phagosome	1.00E+00	TRINITY_DN20147_c0_g5	-1.32	down	unknown [Populus trichocarpa]	21.00	97.67
map04145	Phagosome	1.00E+00	TRINITY_DN20147_c0_g9	-1.50	down	hypothetical protein POPTR_0006s09610g [Populus trichocarpa]	20.96	88.67
map04145	Phagosome	1.00E+00	TRINITY_DN20161_c1_g3	-1.10	down	alpha-tubulin 7 [Salix arbutifolia]	172.29	554.38
map04145	Phagosome	1.00E+00	TRINITY_DN20704_c1_g1	-1.46	down	tubulin beta chain family protein [Populus trichocarpa]	32.81	114.19
map04145	Phagosome	1.00E+00	TRINITY_DN7900_c0_g3	1.88	up	retrovirus-related Pol polyprotein from transposon TNT 1-94 [Dorcoceras hygrometricum]	2.91	1.22
map04120	Ubiquitin mediated proteolysis	1.00E+00	TRINITY_DN22292_c0_g1	-1.88	down	hypothetical protein POPTR_0015s12180g [Populus trichocarpa]	1.38	7.75
map04120	Ubiquitin mediated proteolysis	1.00E+00	TRINITY_DN22604_c0_g2	2.08	up	PREDICTED: E3 ubiquitin-protein ligase COP1-like isoform X2 [Populus euphratica]	33.35	12.32
map04120	Ubiquitin mediated proteolysis	1.00E+00	TRINITY_DN22816_c0_g1	-1.40	down	SINA-like family protein [Populus trichocarpa]	8.81	35.64
map04120	Ubiquitin mediated proteolysis	1.00E+00	TRINITY_DN23022_c0_g2	-2.07	down	peptidoglycan-binding LysM domain-containing family protein, partial [Populus trichocarpa]	0.80	5.18
map04120	Ubiquitin mediated proteolysis	1.00E+00	TRINITY_DN23261_c0_g2	1.09	up	hypothetical protein POPTR_0005s08630g [Populus trichocarpa]	87.47	61.87
map04120	Ubiquitin mediated proteolysis	1.00E+00	TRINITY_DN24638_c0_g1	-4.39	down	PREDICTED: uncharacterized protein LOC105130979 [Populus euphratica]	0.46	15.60
map04120	Ubiquitin mediated proteolysis	1.00E+00	TRINITY_DN25139_c1_g1	-1.03	down	zinc finger family protein [Populus trichocarpa]	14.32	44.46
map04120	Ubiquitin mediated proteolysis	1.00E+00	TRINITY_DN25796_c0_g2	1.20	up	PREDICTED: uncharacterized protein LOC105111698 [Populus euphratica]	199.34	130.10
map04120	Ubiquitin mediated proteolysis	1.00E+00	TRINITY_DN26295_c0_g2	-2.61	down	ubiquitin conjugating-like enzyme family protein [Populus trichocarpa]	1.87	16.39

map04120	Ubiquitin mediated proteolysis	1.00E+00	TRINITY_DN27252_c0_g1	-1.15	down	hypothetical protein POPTR_0003s19720g [Populus trichocarpa]	4.92	16.33
map04120	Ubiquitin mediated proteolysis	1.00E+00	TRINITY_DN27392_c0_g1	-1.59	down	PREDICTED: cation/H(+) antiporter 2-like [Populus euphratica]	1.50	6.80
map04120	Ubiquitin mediated proteolysis	1.00E+00	TRINITY_DN27686_c0_g1	1.24	up	SPA1-RELATED 4 family protein [Populus trichocarpa]	28.22	19.14
map04120	Ubiquitin mediated proteolysis	1.00E+00	TRINITY_DN13612_c0_g2	-1.99	down	PREDICTED: GEM-like protein 4 [Populus euphratica]	1.07	6.41
map04120	Ubiquitin mediated proteolysis	1.00E+00	TRINITY_DN15273_c0_g1	2.95	up	PREDICTED: ubiquitin-conjugating enzyme E2 10-like [Populus euphratica]	8.51	1.68
map04120	Ubiquitin mediated proteolysis	1.00E+00	TRINITY_DN16323_c0_g3	1.45	up	ubiquinol-cytochrome C reductase complex ubiquinone-binding family protein [Populus trichocarpa]	250.75	139.18
map04120	Ubiquitin mediated proteolysis	1.00E+00	TRINITY_DN16399_c1_g1	-3.61	down	hypothetical protein POPTR_0006s25610g [Populus trichocarpa]	0.28	5.09
map04120	Ubiquitin mediated proteolysis	1.00E+00	TRINITY_DN16734_c0_g1	4.90	up	zinc finger family protein [Populus trichocarpa]	11.22	0.47
map04120	Ubiquitin mediated proteolysis	1.00E+00	TRINITY_DN16797_c0_g1	1.20	up	hypothetical protein POPTR_0017s12880g [Populus trichocarpa]	17.47	11.81
map04120	Ubiquitin mediated proteolysis	1.00E+00	TRINITY_DN17552_c1_g1	2.30	up	S2-RNase family protein [Populus trichocarpa]	6.85	1.98
map04120	Ubiquitin mediated proteolysis	1.00E+00	TRINITY_DN18676_c0_g2	1.69	up	hypothetical protein POPTR_0012s13290g [Populus trichocarpa]	10.14	4.79
map04120	Ubiquitin mediated proteolysis	1.00E+00	TRINITY_DN18682_c1_g2	1.08	up	hypothetical protein POPTR_0018s10440g [Populus trichocarpa]	12.07	8.76
map04120	Ubiquitin mediated proteolysis	1.00E+00	TRINITY_DN19365_c0_g4	-1.24	down	PREDICTED: putative GEM-like protein 8 isoform X2 [Populus euphratica]	3.73	12.35
map04120	Ubiquitin mediated proteolysis	1.00E+00	TRINITY_DN20007_c0_g1	-1.66	down	PREDICTED: putative ubiquitin-conjugating enzyme E2 38 isoform X1 [Populus euphratica]	4.13	20.19
map04120	Ubiquitin mediated proteolysis	1.00E+00	TRINITY_DN20265_c0_g2	-1.84	down	PREDICTED: cullin-1-like [Populus euphratica]	1.83	8.99
map04120	Ubiquitin mediated proteolysis	1.00E+00	TRINITY_DN20426_c0_g2	-4.04	down	hypothetical protein POPTR_0005s24970g [Populus trichocarpa]	0.17	5.63
map04120	Ubiquitin mediated proteolysis	1.00E+00	TRINITY_DN20539_c0_g1	1.05	up	PREDICTED: thioredoxin-like 2, chloroplastic [Populus euphratica]	16.48	11.94
map04120	Ubiquitin mediated proteolysis	1.00E+00	TRINITY_DN20539_c0_g2	1.43	up	hypothetical protein POPTR_0006s15520g [Populus trichocarpa]	68.08	38.48
map04120	Ubiquitin mediated proteolysis	1.00E+00	TRINITY_DN21169_c0_g1	-2.50	down	hypothetical protein POPTR_0001s25810g [Populus trichocarpa]	1.00	8.71
map04120	Ubiquitin mediated proteolysis	1.00E+00	TRINITY_DN21392_c2_g1	-1.68	down	PREDICTED: rho GDP-dissociation inhibitor 1 [Populus euphratica]	3.23	15.25
map04120	Ubiquitin mediated proteolysis	1.00E+00	TRINITY_DN21562_c1_g1	1.04	up	hypothetical protein POPTR_0006s22950g [Populus trichocarpa]	195.66	148.86
map04120	Ubiquitin mediated proteolysis	1.00E+00	TRINITY_DN21599_c2_g1	-1.46	down	PREDICTED: E3 ubiquitin-protein ligase SINA-like 10 [Populus euphratica]	1.09	4.71
