# Supplementary material for: MiR156 regulates anthocyanin biosynthesis through SPL targets and other microRNAs in poplar
Source: Hortic Res. 2020 Aug 1;7:118. doi: 10.1038/s41438-020-00341-w (PMC7395715; doi:10.1038/s41438-020-00341-w)
Supplement: Supplementary file 6 — Supporting Information 6 [file 41438_2020_341_MOESM6_ESM.pdf]

Table S10 Transcript abundances of miR156-targeted SPLs involved in anthocyanin biosynthesis in wild-type and group II transgenic poplar plants.

RNA-Seq data												qRT-PCR results			
**Gene_ID**	**Name**	**TGII -1**	**TGII -2**	**TGII -3**	**WT -1**	**WT -2**	**WT -3**	**TGII_mean _TPM**	**WT_mean _TPM**	**Log2FC**	**Regulation**	**WT**	**TGII-1**	**TGII-2**	**TGII-3**
TRINITY_DN19122_c0_g1	SPL8	1.25	0.81	5.05	16.61	7.47	9.10	2.37	11.06	-2.19	down	3.2152E-05±9.77701E-06	1.61716E-05±6.6271E-07	4.61349E-06±7.60325E-07	7.35884E-06±5.8613E-07
TRINITY_DN23413_c0_g1	SPL11	6.30	8.26	5.66	22.66	28.55	27.07	6.74	26.09	-1.58	down	0.000170984±3.76951E-05	5.09708E-05±1.5043E-06	6.49529E-05±2.82679E-06	2.92406E-05±1.74175E-06
TRINITY_DN22323_c1_g7	SPL12	0.58	1.09	0.90	6.27	1.53	1.01	0.86	2.94	-1.77	down	1.26088E-05±1.59614E-06	3.97505E-06±3.8759E-07	5.21238E-06±3.89517E-07	2.54469E-06±2.55992E-07
TRINITY_DN17120_c0_g1	SPL17	1.92	1.52	2.10	4.01	4.61	6.85	1.85	5.16	-0.92	down	0.00926±0.00112	0.00146±4.95E-05	0.00271±1.95E-04	0.0012±4.21E-05
TRINITY_DN19850_c1_g4	SPL28	1.28	0.88	1.32	10.23	9.20	11.03	1.16	10.15	-2.51	down	6.63402E-05±4.79153E-06	2.13988E-06±1.02998E-06	1.15209E-05±1.32763E-06	3.59E-06±5.73581E-08
TRINITY_DN24312_c1_g1	SPL29	2.03	3.62	5.69	10.86	12.09	13.43	3.78	12.13	-1.20	down	8.62059E-05±1.45782E-05	1.95564E-05±1.16748E-06	3.80989E-05±1.90546E-06	9.59607E-06±7.13535E-07
