# Supplementary material for: MiR156 regulates anthocyanin biosynthesis through SPL targets and other microRNAs in poplar
Source: Hortic Res. 2020 Aug 1;7:118. doi: 10.1038/s41438-020-00341-w (PMC7395715; doi:10.1038/s41438-020-00341-w)
Supplement: Supplementary file 8 — Supporting Information 8 [file 41438_2020_341_MOESM8_ESM.pdf]

Table S12 Overview of differentially expressed metabolites between wild-type and transgenic poplar plants.

Index	Formula	Compounds	Class I	Class II	CAS	WT-1	WT-2	WT-3	TGII-1	TGII-2	TGII-3	VIP	Fold_Change	Log2FC	Type
mws4134	C20H32N6O12S2	Oxidized Glutathione	Amino acids and derivatives	Amino acids and derivatives	121-24-4	2.88E+05	7.92E+05	4.11E+05	9.43E+04	5.08E+04	9.85E+04	1.16E+00	1.63E-01	-2.61E+00	down
mws4176	C12H16N2O3	DL-Alanyl-DL-phenylalanine	Amino acids and derivatives	Amino acids and derivatives	1999-45-7	1.50E+05	1.51E+05	1.95E+05	1.50E+04	1.54E+04	2.17E+04	1.26E+00	1.05E-01	-3.25E+00	down
mws5035	C15H22N2O3	Leucylphenylalanine	Amino acids and derivatives	Amino acids and derivatives	56217-82-4	2.53E+05	2.76E+05	3.62E+05	9.96E+04	1.33E+05	1.29E+05	1.21E+00	4.06E-01	-1.30E+00	down
pme2890	C8H16N2O4S2	L-Homocystine	Amino acids and derivatives	Amino acids and derivatives	626-72-2	3.38E+04	2.11E+04	2.84E+04	7.78E+03	8.69E+03	1.84E+04	1.07E+00	4.18E-01	-1.26E+00	down
mws0001	C4H8N2O3	L-Asparagine Anhydrous	Amino acids and derivatives	Amino acids and derivatives	70-47-3	2.08E+06	3.95E+06	2.70E+06	2.91E+07	9.76E+06	2.97E+07	1.17E+00	7.86E+00	2.97E+00	up
mws0216	C5H9NO3	Trans-4-Hydroxy-L-proline	Amino acids and derivatives	Amino acids and derivatives	51-35-4	1.02E+07	6.48E+06	3.74E+06	5.35E+07	5.14E+07	4.50E+07	1.22E+00	7.33E+00	2.87E+00	up
mws0227	C6H13NO2	L-Leucine	Amino acids and derivatives	Amino acids and derivatives	61-90-5	4.12E+05	3.21E+05	2.28E+05	2.63E+06	2.15E+06	2.06E+06	1.24E+00	7.12E+00	2.83E+00	up
mws0254	C6H9N3O2	L-Histidine	Amino acids and derivatives	Amino acids and derivatives	71-00-1	6.25E+05	2.03E+06	5.85E+05	6.68E+06	1.85E+06	5.50E+06	1.01E+00	4.33E+00	2.11E+00	up
mws0256	C5H11NO2	L-Valine	Amino acids and derivatives	Amino acids and derivatives	72-18-4	2.34E+06	1.76E+06	1.23E+06	9.73E+06	7.30E+06	9.40E+06	1.22E+00	4.95E+00	2.31E+00	up
mws0263	C5H7NO3	L-PyroglutamicAcid	Amino acids and derivatives	Amino acids and derivatives	98-79-3	5.90E+03	6.33E+03	5.68E+03	1.65E+05	1.19E+05	2.29E+05	1.25E+00	2.86E+01	4.84E+00	up
mws0582	C11H19N3O6S	S-(methyl)glutathione	Amino acids and derivatives	Amino acids and derivatives	2922-56-7	9.00E+00	9.00E+00	9.00E+00	5.64E+04	3.45E+04	1.29E+05	1.26E+00	8.14E+03	1.30E+01	up
mws0813	C5H7NO3	5-Oxoproline	Amino acids and derivatives	Amino acids and derivatives	149-87-1	3.63E+03	5.67E+03	3.62E+03	1.03E+05	4.64E+04	1.34E+05	1.23E+00	2.19E+01	4.45E+00	up
mws1550	C6H11NO2S	S-Allyl-L-cysteine	Amino acids and derivatives	Amino acids and derivatives	21593-77-1	6.95E+03	8.90E+03	5.37E+03	2.81E+04	1.25E+04	1.21E+04	1.03E+00	2.49E+00	1.31E+00	up
pme0008	C6H13N3O3	L-Citrulline	Amino acids and derivatives	Amino acids and derivatives	372-75-8	1.91E+05	3.19E+05	1.44E+05	1.15E+06	4.66E+05	1.56E+06	1.10E+00	4.85E+00	2.28E+00	up
pme0026	C6H14N2O2	L-(+)-Lysine	Amino acids and derivatives	Amino acids and derivatives	56-87-1	1.03E+05	9.67E+04	5.77E+04	2.24E+06	5.68E+05	2.72E+06	1.19E+00	2.15E+01	4.42E+00	up
pme0193	C5H10N2O3	L-Glutamine	Amino acids and derivatives	Amino acids and derivatives	56-85-9	1.26E+05	1.05E+05	7.10E+04	2.68E+06	6.80E+05	3.08E+06	1.19E+00	2.14E+01	4.42E+00	up
pme0253	C8H15NO3	N-Acetyl-L-leucine	Amino acids and derivatives	Amino acids and derivatives	1188-21-2	1.70E+04	1.14E+04	1.29E+04	2.79E+04	3.36E+04	2.51E+04	1.17E+00	2.09E+00	1.07E+00	up
pme0278	C7H14N2O4	2,6-Diaminooimelic acid	Amino acids and derivatives	Amino acids and derivatives	583-93-7	9.22E+04	9.95E+04	7.00E+04	5.13E+05	2.83E+05	4.58E+05	1.22E+00	4.79E+00	2.26E+00	up
pme1210	C5H11NO2S	L-Methionine	Amino acids and derivatives	Amino acids and derivatives	63-68-3	5.99E+05	9.80E+05	6.99E+05	2.70E+06	2.27E+06	3.06E+06	1.22E+00	3.52E+00	1.82E+00	up

pme1228	C11H12N2O3	5-Hydroxy-L-tryptophan	Amino acids and derivatives	Amino acids and derivatives	56-69-9	1.72E+04	1.46E+04	1.49E+04	4.55E+04	5.81E+04	3.59E+04	1.22E+00	2.98E+00	1.58E+00	up
pmp000967	C11H12N2O2	Tryptophan	Amino acids and derivatives	Amino acids and derivatives	73-22-3	4.12E+06	1.96E+06	1.45E+06	1.76E+07	1.55E+07	1.44E+07	1.20E+00	6.30E+00	2.66E+00	up
pmp001213	C9H11NO2	Phenylalanine	Amino acids and derivatives	Amino acids and derivatives	63-91-2	3.85E+06	3.84E+06	3.23E+06	2.06E+07	2.22E+07	1.75E+07	1.26E+00	5.52E+00	2.47E+00	up
Hmln001566	C7H6O3	Salicylic acid	Phenolic acids	Phenolic acids	69-72-7	7.57E+05	6.77E+05	9.08E+05	2.60E+05	2.63E+05	2.60E+05	1.25E+00	3.34E-01	-1.58E+00	down
Hmtn001302	C13H16O8	Glucosyloxybenzoic acid	Phenolic acids	Phenolic acids	-	3.14E+07	2.12E+07	3.95E+07	1.44E+07	1.47E+07	1.07E+07	1.13E+00	4.32E-01	-1.21E+00	down
Lmhn002573	C17H20O11	Sinapoylglucuronic acid	Phenolic acids	Phenolic acids	-	1.03E+06	1.09E+06	9.29E+05	3.79E+05	4.58E+05	3.56E+05	1.24E+00	3.92E-01	-1.35E+00	down
Lmhn002926	C13H12O7	p-Coumaroylmalic acid	Phenolic acids	Phenolic acids	-	4.05E+05	2.00E+05	2.88E+05	5.42E+04	5.62E+04	3.46E+04	1.22E+00	1.63E-01	-2.62E+00	down
Lmhn003373	C11H10O7	p-Hydroxybenzoylmalic acid	Phenolic acids	Phenolic acids	-	1.48E+05	8.55E+04	2.15E+05	1.51E+04	2.31E+04	1.16E+04	1.21E+00	1.11E-01	-3.17E+00	down
Zmhn001883	C14H18O9	Vanillic acid glycoside	Phenolic acids	Phenolic acids	-	9.97E+04	5.32E+04	7.63E+04	2.49E+04	1.99E+04	3.67E+04	1.14E+00	3.56E-01	-1.49E+00	down
Zmhn001926	C13H16O8	Salicylic acid O-glycoside	Phenolic acids	Phenolic acids	-	3.19E+07	2.12E+07	4.07E+07	1.60E+07	1.66E+07	1.11E+07	1.08E+00	4.66E-01	-1.10E+00	down
Zmxn001997	C13H16O8	Isosalicylic acid O-glycoside	Phenolic acids	Phenolic acids	-	2.86E+07	1.83E+07	3.62E+07	1.40E+07	1.42E+07	9.31E+06	1.07E+00	4.51E-01	-1.15E+00	down
mws0009	C10H10O3	Coniferaldehyde	Phenolic acids	Phenolic acids	20649-42-7	1.73E+05	2.16E+05	2.50E+05	9.00E+00	2.03E+04	9.00E+00	1.04E+00	3.19E-02	-4.97E+00	down
mws0028	C8H8O4	Vanillic acid	Phenolic acids	Phenolic acids	121-34-6	1.78E+05	1.08E+05	1.09E+05	5.64E+04	2.30E+04	6.70E+04	1.04E+00	3.71E-01	-1.43E+00	down
mws0458	C8H8O3	Vanillin	Phenolic acids	Phenolic acids	121-33-5	1.01E+06	1.20E+06	1.15E+06	3.43E+05	2.79E+05	2.40E+05	1.24E+00	2.57E-01	-1.96E+00	down
mws0459	C10H12O2	Eugenol	Phenolic acids	Phenolic acids	97-53-0	3.55E+06	1.99E+06	2.12E+06	9.00E+00	9.00E+00	9.00E+00	1.26E+00	3.52E-06	-1.81E+01	down
mws0467	C9H10O3	3-(4-Hydroxyphenyl)-propionic acid	Phenolic acids	Phenolic acids	501-97-3	6.48E+06	8.32E+06	6.49E+06	1.72E+06	1.80E+06	1.41E+06	1.25E+00	2.32E-01	-2.11E+00	down
mws0628	C7H6O2	4-Hydroxybenzaldehyde	Phenolic acids	Phenolic acids	123-08-0	1.65E+06	2.22E+06	2.54E+06	6.15E+05	7.88E+05	1.19E+06	1.14E+00	4.04E-01	-1.31E+00	down
mws0898	C10H12O2	Isoeugenol	Phenolic acids	Phenolic acids	97-54-1	2.33E+06	1.63E+06	1.30E+06	4.13E+04	4.40E+04	4.22E+04	1.26E+00	2.43E-02	-5.36E+00	down
mws0906	C16H22O8	Coniferin	Phenolic acids	Phenolic acids	531-29-3	1.52E+07	1.86E+07	2.13E+07	7.11E+06	7.27E+06	7.46E+06	1.24E+00	3.96E-01	-1.34E+00	down
mws1200	C10H10O3	Trans-4-Hydroxycinnamic Acid Methyl Ester	Phenolic acids	Phenolic acids	19367-38-5	1.84E+06	3.10E+06	1.26E+06	3.24E+05	2.79E+05	5.54E+05	1.17E+00	1.87E-01	-2.42E+00	down
mws1212	C11H12O4	Methyl ferulate	Phenolic acids	Phenolic acids	2309-07-1	6.23E+05	7.03E+05	7.22E+05	2.38E+04	2.34E+04	5.20E+04	1.25E+00	4.85E-02	-4.37E+00	down
mws1354	C10H10O4	Trans-ferulic acid	Phenolic acids	Phenolic acids	537-98-4	3.44E+05	2.21E+05	2.30E+05	8.13E+04	6.21E+04	8.84E+04	1.22E+00	2.91E-01	-1.78E+00	down
pmb0752	C17H20O9	3-O-Feruloyl quinic acid	Phenolic acids	Phenolic acids	1899-29-2	2.76E+07	2.27E+07	2.18E+07	8.84E+05	1.12E+06	7.77E+05	1.26E+00	3.86E-02	-4.70E+00	down

pmb2620	C11H12O4	3,4-Dimethoxycinnamic acid	Phenolic acids	Phenolic acids	14737-89-4	1.48E+05	1.75E+05	1.54E+05	5.18E+03	7.07E+03	1.21E+04	1.25E+00	5.09E-02	-4.30E+00	down
pmb2833	C23H30O14	3-O-Feruloyl quinic acid glucoside	Phenolic acids	Phenolic acids	-	6.73E+03	7.07E+03	5.85E+03	9.00E+00	9.00E+00	9.00E+00	1.26E+00	1.37E-03	-9.51E+00	down
pmb3068	C16H18O8	1-O-p-Coumaroyl quinic acid	Phenolic acids	Phenolic acids	-	4.82E+06	1.61E+06	2.46E+06	5.94E+04	8.95E+04	7.87E+04	1.24E+00	2.56E-02	-5.29E+00	down
pme0241	C7H6O2	Benzoic acid	Phenolic acids	Phenolic acids	65-85-0	2.34E+06	3.54E+06	3.10E+06	4.96E+05	4.60E+05	7.27E+05	1.23E+00	1.87E-01	-2.42E+00	down
pme1439	C9H8O3	p-Coumaric acid	Phenolic acids	Phenolic acids	501-98-4	1.88E+05	1.85E+05	1.63E+05	7.17E+04	9.42E+04	8.48E+04	1.23E+00	4.67E-01	-1.10E+00	down
pme3443	C11H12O4	Sinapinaldehyde	Phenolic acids	Phenolic acids	4206-58-0	2.97E+05	1.24E+05	9.15E+04	4.81E+04	6.67E+04	5.36E+04	1.02E+00	3.29E-01	-1.61E+00	down
pmf0591	C9H8O2	trans-Cinnamic acid	Phenolic acids	Phenolic acids	621-82-9	2.13E+06	1.82E+06	2.28E+06	3.68E+05	4.85E+05	5.71E+05	1.24E+00	2.29E-01	-2.13E+00	down
pmn001420	C15H18O9	1-O-[(E)-Caffeoyl]-β-D-glucopyranose	Phenolic acids	Phenolic acids	-	1.21E+07	1.92E+07	2.04E+07	5.63E+06	6.50E+06	5.24E+06	1.20E+00	3.35E-01	-1.58E+00	down
pmp000232	C16H18O8	Cis-3-p-coumaric quinic acid	Phenolic acids	Phenolic acids	-	3.48E+07	1.62E+07	2.10E+07	8.20E+05	9.76E+05	9.72E+05	1.25E+00	3.84E-02	-4.70E+00	down
Cmyp007259	C20H22O9	Poliothrysoside; Nigracin	Phenolic acids	Phenolic acids	18463-25-7	8.28E+06	1.02E+07	1.03E+07	2.04E+07	2.44E+07	2.11E+07	1.24E+00	2.29E+00	1.19E+00	up
Hmtn001120	C14H20O8	5-(2-Hydroxyethyl)-2-O-glucosylohenol	Phenolic acids	Phenolic acids	-	1.38E+06	1.89E+06	3.77E+06	5.13E+06	1.36E+07	9.13E+06	1.09E+00	3.96E+00	1.99E+00	up
Lmqp001565	C7H6O3	p-Hydroxybenzoic Acid	Phenolic acids	Phenolic acids	-	1.47E+05	2.05E+05	1.22E+05	1.06E+06	7.29E+05	1.20E+06	1.23E+00	6.30E+00	2.66E+00	up
Zmhn002227	C17H22O10	Sinapic acid-glycoside	Phenolic acids	Phenolic acids	-	1.09E+05	1.20E+05	9.00E+04	1.85E+05	4.98E+05	1.93E+05	1.02E+00	2.74E+00	1.46E+00	up
Zmhn002513	C17H22O10	Isosinapic acid-hexoside	Phenolic acids	Phenolic acids	-	8.47E+04	1.67E+05	1.24E+05	2.24E+06	2.20E+06	1.08E+06	1.24E+00	1.47E+01	3.87E+00	up
mws0444	C7H7NO3	3-Aminosalicylic acid	Phenolic acids	Phenolic acids	570-23-0	8.51E+03	1.62E+04	2.41E+04	4.59E+04	4.83E+04	4.35E+04	1.11E+00	2.82E+00	1.50E+00	up
mws1584	C25H24O12	Cynarin	Phenolic acids	Phenolic acids	30964-13-7	2.82E+05	2.35E+05	2.10E+05	5.72E+05	1.12E+06	4.71E+05	1.12E+00	2.98E+00	1.57E+00	up
pmb2940	C17H22O10	1-O-β-D-Glucopyranosyl sinapate	Phenolic acids	Phenolic acids	78185-48-5	9.00E+00	9.00E+00	9.00E+00	9.61E+04	1.11E+05	5.92E+04	1.26E+00	9.88E+03	1.33E+01	up
pme1816	C16H18O9	Neochlorogenic acid(5-O-Caffeoylquinic acid)	Phenolic acids	Phenolic acids	906-33-2	7.32E+05	1.70E+05	1.11E+05	1.57E+06	2.44E+06	2.78E+06	1.11E+00	6.71E+00	2.75E+00	up
pme2213	C9H8O4	Caffeic acid	Phenolic acids	Phenolic acids	331-39-5	5.72E+04	5.58E+04	5.98E+04	2.64E+05	2.98E+05	1.94E+05	1.25E+00	4.37E+00	2.13E+00	up
pmn001687	C13H12O8	Cis-Coutaric acid	Phenolic acids	Phenolic acids	27174-07-8	9.00E+00	9.00E+00	9.00E+00	3.36E+05	4.79E+05	3.72E+05	1.26E+00	4.40E+04	1.54E+01	up
pmn001695	C16H20O10	Trihydroxycinnamoylquinic acid	Phenolic acids	Phenolic acids	-	9.82E+05	6.93E+05	8.55E+05	3.50E+06	3.34E+06	2.15E+06	1.21E+00	3.56E+00	1.83E+00	up
pmp000544	C16H18O9	Chlorogenic Acid	Phenolic acids	Phenolic acids	327-97-9	2.18E+06	3.83E+05	2.04E+05	5.32E+06	7.27E+06	5.46E+06	1.08E+00	6.53E+00	2.71E+00	up
pmp000545	C16H18O9	4-Caffeoylquinic acid	Phenolic acids	Phenolic acids	905-99-7	2.66E+05	5.77E+04	2.56E+04	6.31E+05	9.10E+05	6.48E+05	1.08E+00	6.26E+00	2.65E+00	up

mws0248	C9H12N2O6	Uridine	Nucleotides and derivatives	Nucleotides and derivatives	58-96-8	2.20E+06	3.41E+06	2.67E+06	1.24E+06	1.16E+06	1.34E+06	1.19E+00	4.51E-01	-1.15E+00	down
mws0255	C4H5N3O	Cytosine	Nucleotides and derivatives	Nucleotides and derivatives	71-30-7	1.78E+06	2.33E+06	1.58E+06	5.00E+05	8.37E+05	9.98E+05	1.13E+00	4.11E-01	-1.28E+00	down
mws0609	C10H12N5O7P	Guanosine 3',5'-cyclic monophosphate	Nucleotides and derivatives	Nucleotides and derivatives	7665-99-8	3.84E+05	3.55E+05	3.68E+05	2.90E+04	2.33E+04	4.31E+04	1.25E+00	8.62E-02	-3.54E+00	down
mws1060	C10H12N4O5	9-(β-D-Arabinofuranosyl)hypoxanthine	Nucleotides and derivatives	Nucleotides and derivatives	7013-16-3	2.59E+05	1.79E+05	2.39E+05	7.78E+04	6.53E+04	1.74E+05	1.01E+00	4.69E-01	-1.09E+00	down
pme0033	C5H4N4O	Hypoxanthine	Nucleotides and derivatives	Nucleotides and derivatives	68-94-0	5.98E+04	1.14E+05	6.86E+04	2.40E+04	2.86E+04	1.68E+04	1.16E+00	2.87E-01	-1.80E+00	down
pme0040	C5H5N5	Adenine	Nucleotides and derivatives	Nucleotides and derivatives	73-24-5	1.20E+07	2.15E+07	1.40E+07	5.01E+06	4.98E+06	3.03E+06	1.18E+00	2.74E-01	-1.87E+00	down
pme0264	C10H14N2O5	Thymidine	Nucleotides and derivatives	Nucleotides and derivatives	50-89-5	5.20E+05	4.79E+05	5.04E+05	1.53E+05	1.70E+05	1.58E+05	1.26E+00	3.20E-01	-1.65E+00	down
pme1109	C5H5N5O	Guanine	Nucleotides and derivatives	Nucleotides and derivatives	73-40-5	3.24E+05	4.39E+05	4.28E+05	1.94E+05	1.92E+05	9.59E+04	1.11E+00	4.04E-01	-1.31E+00	down
pme1178	C10H13N5O5	Guanosine	Nucleotides and derivatives	Nucleotides and derivatives	118-00-3	4.00E+07	4.26E+07	2.90E+07	1.34E+07	1.71E+07	1.76E+07	1.19E+00	4.31E-01	-1.21E+00	down
pme1184	C10H13N5O4	Deoxyguanosine	Nucleotides and derivatives	Nucleotides and derivatives	961-07-9	1.26E+06	9.58E+05	8.47E+05	2.80E+05	3.29E+05	2.95E+05	1.24E+00	2.95E-01	-1.76E+00	down
pme1194	C9H13N3O4	Deoxycytidine	Nucleotides and derivatives	Nucleotides and derivatives	951-77-9	4.77E+05	7.04E+05	4.17E+05	3.00E+05	2.32E+05	2.38E+05	1.12E+00	4.82E-01	-1.05E+00	down
pme1474	C11H15N5O3S	5'-Deoxy-5'-(methylthio)adenosine	Nucleotides and derivatives	Nucleotides and derivatives	2457-80-9	1.22E+07	7.57E+06	8.32E+06	4.37E+06	5.40E+06	2.92E+06	1.09E+00	4.51E-01	-1.15E+00	down
pme2746	C27H33N9O15P2	Flavin adenine dinucleotide(FAD)	Nucleotides and derivatives	Nucleotides and derivatives	146-14-5	8.79E+04	6.31E+04	1.29E+05	3.96E+04	5.13E+04	3.40E+04	1.08E+00	4.46E-01	-1.17E+00	down
pme3732	C9H13N3O5	Cytidine	Nucleotides and derivatives	Nucleotides and derivatives	65-46-3	1.73E+07	1.91E+07	1.31E+07	4.49E+06	5.33E+06	6.08E+06	1.23E+00	3.21E-01	-1.64E+00	down
pme3967	C12H17N5O5	2-(Dimethylamino)guanosine	Nucleotides and derivatives	Nucleotides and derivatives	2140-67-2	4.78E+05	3.53E+05	3.58E+05	6.18E+04	2.35E+05	1.58E+05	1.02E+00	3.83E-01	-1.39E+00	down
mws0675	C11H15N2O8P	β-Nicotinamide mononucleotide	Nucleotides and derivatives	Nucleotides and derivatives	1094-61-7	7.03E+04	5.70E+04	4.64E+04	2.34E+05	1.34E+05	2.37E+05	1.19E+00	3.48E+00	1.80E+00	up
mws0847	C6H7N5	1-Methyladenine	Nucleotides and derivatives	Nucleotides and derivatives	5142-22-3	1.59E+04	2.43E+04	2.05E+04	8.03E+04	6.93E+04	7.68E+04	1.24E+00	3.73E+00	1.90E+00	up
pmb0981	C10H14N5O7P	Adenosine 5'-monophosphate	Nucleotides and derivatives	Nucleotides and derivatives	61-19-8	1.26E+04	1.49E+04	8.60E+03	6.21E+04	3.63E+04	6.00E+04	1.20E+00	4.39E+00	2.13E+00	up
pme3184	C10H14N5O6P	2'-Deoxyadenosine-5'-monophosphate	Nucleotides and derivatives	Nucleotides and derivatives	653-63-4	1.14E+05	2.39E+05	1.67E+05	3.83E+05	4.89E+05	2.73E+05	1.06E+00	2.20E+00	1.14E+00	up
**mws1179**	**C21H22O10**	**Naringenin-7-O-glucoside**	**Flavonoids**	**Dihydroflavone**	**529-55-5**	**2.27E+06**	**2.38E+06**	**1.92E+06**	**7.00E+05**	**7.38E+05**	**5.18E+05**	**1.23E+00**	**2.98E-01**	**-1.75E+00**	**down**
**pme0376**	**C15H12O5**	**Naringenin**	**Flavonoids**	**Dihydroflavone**	**480-41-1**	**2.45E+05**	**2.85E+05**	**1.60E+05**	**5.35E+04**	**3.11E+04**	**4.09E+04**	**1.22E+00**	**1.82E-01**	**-2.46E+00**	**down**
**pmp000562**	**C15H12O4**	**Pinocembrin**	**Flavonoids**	**Dihydroflavone**	**480-39-7**	**3.45E+04**	**4.30E+04**	**2.01E+04**	**9.73E+03**	**6.02E+03**	**9.98E+03**	**1.16E+00**	**2.63E-01**	**-1.92E+00**	**down**
**mws0914**	**C15H12O5**	**Pinobanksin**	**Flavonoids**	**Dihydroflavonol**	**548-82-3**	**2.53E+05**	**2.73E+05**	**1.39E+05**	**6.11E+04**	**2.09E+04**	**4.60E+04**	**1.15E+00**	**1.92E-01**	**-2.38E+00**	**down**

mws1094	C15H12O6	Dihydrokaempferol	Flavonoids	Dihydroflavonol	480-20-6	3.91E+04	3.50E+04	2.51E+04	1.60E+04	1.44E+04	1.68E+04	1.18E+00	4.75E-01	-1.07E+00	down
Lmmp002795	C22H22O11	Pratensein 7-O-glucopyranoside	Flavonoids	Flavonoid	36191-03-4	1.41E+06	9.01E+05	1.96E+06	9.00E+00	9.00E+00	9.00E+00	1.26E+00	6.33E-06	-1.73E+01	down
Zmhn003534	C21H22O10	Naringenin-O-glucoside	Flavonoids	Flavonoid	-	2.73E+06	2.48E+06	1.89E+06	8.89E+05	7.14E+05	5.10E+05	1.20E+00	2.97E-01	-1.75E+00	down
Zmhp003514	C16H12O6	6,7,8-Tetrahydroxy-5-methoxyflavone	Flavonoids	Flavonoid	-	3.12E+06	2.58E+06	2.53E+06	8.36E+05	7.36E+05	1.12E+06	1.23E+00	3.27E-01	-1.61E+00	down
Zmxp002867	C28H32O16	Chrysoeriol-di-O-glucoside	Flavonoids	Flavonoid	-	1.09E+06	8.54E+05	7.51E+05	5.20E+04	9.47E+04	5.73E+04	1.25E+00	7.57E-02	-3.72E+00	down
mws0058	C16H12O6	Diosmetin	Flavonoids	Flavonoid	520-34-3	5.12E+06	3.91E+06	3.47E+06	1.36E+06	9.40E+05	1.85E+06	1.18E+00	3.31E-01	-1.59E+00	down
mws1073	C27H30O15	Apigenin 6,8-C-diglucoside	Flavonoids	Flavonoid	23666-13-9	3.72E+06	3.39E+06	3.76E+06	7.71E+05	1.27E+06	7.28E+05	1.22E+00	2.54E-01	-1.97E+00	down
pmb0571	C26H28O14	Apigenin O-hexosyl-O-pentoside	Flavonoids	Flavonoid	-	1.15E+05	1.37E+05	1.59E+05	6.01E+04	4.52E+04	3.11E+04	1.18E+00	3.32E-01	-1.59E+00	down
pmb0587	C28H30O17	Chrysoeriol O-glucuronic acid-O-hexoside	Flavonoids	Flavonoid	-	1.04E+05	8.46E+04	1.35E+05	5.82E+04	4.62E+04	3.00E+04	1.12E+00	4.15E-01	-1.27E+00	down
pmb0588	C27H30O16	Luteolin 3',7-di-O-glucoside	Flavonoids	Flavonoid	52187-80-1	1.49E+05	1.30E+05	1.58E+05	5.36E+04	4.91E+04	4.36E+04	1.25E+00	3.35E-01	-1.58E+00	down
pmb0608	C25H24O14	Chrysoeriol O-malonylhexoside	Flavonoids	Flavonoid	-	2.24E+07	1.23E+07	1.64E+07	1.56E+05	4.17E+05	1.38E+05	1.24E+00	1.39E-02	-6.17E+00	down
pmb0613	C33H40O20	Apigenin 6-C-hexosyl-8-C-hexosyl-O-hexoside	Flavonoids	Flavonoid	-	1.66E+04	9.42E+03	1.40E+04	9.00E+00	9.00E+00	9.00E+00	1.26E+00	6.74E-04	-1.05E+01	down
pmb3006	C21H20O10	Apigenin 7-O-glucoside(Cosmosiin)	Flavonoids	Flavonoid	578-74-5	7.83E+06	7.48E+06	8.36E+06	1.13E+06	1.29E+06	1.23E+06	1.26E+00	1.54E-01	-2.70E+00	down
pmb3012	C22H22O11	Chrysoeriol 7-O-hexoside	Flavonoids	Flavonoid	-	1.35E+07	1.12E+07	1.19E+07	1.24E+06	1.27E+06	1.71E+06	1.26E+00	1.15E-01	-3.12E+00	down
pmn001713	C27H30O15	Luteolin-7-O-β-D-rutinoside	Flavonoids	Flavonoid	-	7.14E+05	6.18E+05	6.17E+05	1.06E+05	1.99E+05	1.27E+05	1.23E+00	2.22E-01	-2.17E+00	down
pmp000002	C17H14O6	Ladanein	Flavonoids	Flavonoid	10176-71-3	4.12E+04	2.11E+04	3.70E+04	5.81E+03	8.02E+03	3.80E+03	1.20E+00	1.77E-01	-2.50E+00	down
pmp000241	C27H28O17	Luteolin-6-C-2-glucuronylglucoside	Flavonoids	Flavonoid	-	2.30E+04	4.12E+04	2.15E+04	9.00E+00	9.00E+00	9.00E+00	1.26E+00	3.15E-04	-1.16E+01	down
pmp000344	C15H10O5	3’,4’,7-Trihydroxyflavone	Flavonoids	Flavonoid	2150-11-0	1.64E+04	1.39E+04	9.25E+03	2.36E+03	9.00E+00	9.00E+00	1.04E+00	6.02E-02	-4.05E+00	down
pmp000571	C15H10O5	Apigenin	Flavonoids	Flavonoid	520-36-5	2.69E+05	2.21E+05	2.74E+05	1.25E+04	1.01E+04	8.93E+03	1.26E+00	4.12E-02	-4.60E+00	down
pmp000573	C22H22O10	Acacetin-7-O-galactoside	Flavonoids	Flavonoid	-	6.93E+04	5.35E+04	7.61E+04	9.00E+00	9.00E+00	9.00E+00	1.26E+00	1.36E-04	-1.28E+01	down
pmp000579	C22H22O11	Diosmetin-7-O-galactoside	Flavonoids	Flavonoid	-	1.51E+06	1.39E+06	1.58E+06	2.47E+05	3.94E+05	2.46E+05	1.24E+00	1.98E-01	-2.34E+00	down
pmp000581	C23H22O11	Apigenin-7-O-(6'-O-acetyl)-β-D-glucoside	Flavonoids	Flavonoid	-	4.61E+06	4.03E+06	3.95E+06	7.69E+04	9.55E+04	6.43E+04	1.26E+00	1.88E-02	-5.73E+00	down
pmp000585	C24H22O13	Apigenin-7-O-(6-O-Malonyl Glucoside)	Flavonoids	Flavonoid	-	1.76E+06	1.42E+06	1.62E+06	3.58E+04	8.22E+04	4.54E+04	1.25E+00	3.40E-02	-4.88E+00	down

pmp000587	C24H22O14	Luteolin-7-O-(6'-O-malonyl)-β-D-glucoside	Flavonoids	Flavonoid	-	9.88E+05	2.01E+05	3.10E+05	2.40E+04	4.18E+04	1.40E+04	1.17E+00	5.32E-02	-4.23E+00	down
pmp000588	C25H24O14	Diosmetin-7-O-(6'-O-malonyl)-β-D-glucoside	Flavonoids	Flavonoid	-	2.25E+06	1.16E+06	1.69E+06	2.64E+04	5.01E+04	2.88E+04	1.25E+00	2.06E-02	-5.60E+00	down
pmp000592	C27H30O15	Apigenin-Glucoside-Glucoside	Flavonoids	Flavonoid	-	7.08E+06	5.05E+06	6.91E+06	1.57E+05	2.16E+05	2.23E+05	1.26E+00	3.13E-02	-5.00E+00	down
pmp000594	C28H32O15	Diosmetin-7-O-rutin	Flavonoids	Flavonoid	-	3.53E+04	8.65E+04	1.22E+05	9.00E+00	9.00E+00	9.00E+00	1.26E+00	1.11E-04	-1.31E+01	down
pmp000595	C27H30O16	Luteolin-7,3'-Di-O-β-D-Glucoside	Flavonoids	Flavonoid	257-724-7	5.43E+05	1.72E+05	2.91E+05	1.98E+04	3.94E+04	4.41E+04	1.19E+00	1.03E-01	-3.28E+00	down
pmp001127	C16H12O6	Chrysoeriol	Flavonoids	Flavonoid	491-71-4	4.00E+04	5.57E+04	8.46E+04	5.17E+03	1.01E+04	1.20E+04	1.19E+00	1.51E-01	-2.72E+00	down
Hmcp001598	C27H30O16	Isorhamnetin-O-Hexoside-O-Pentoside	Flavonoids	Flavonols	-	9.60E+04	5.87E+04	5.58E+04	1.33E+04	1.43E+04	2.48E+04	1.19E+00	2.49E-01	-2.01E+00	down
Hmln002483	C24H22O14	Kaempferol 3-O-(6′′-O-malonyl)-glucoside	Flavonoids	Flavonols	-	2.29E+07	6.37E+06	8.28E+06	1.65E+06	1.81E+06	1.99E+06	1.16E+00	1.45E-01	-2.79E+00	down
Lmbp002592	C27H30O16	Kaempferol-3,7-di-O-β-D-glucopyranoside	Flavonoids	Flavonols	-	9.67E+05	8.24E+05	1.11E+06	2.12E+05	3.32E+05	4.46E+05	1.17E+00	3.42E-01	-1.55E+00	down
Lmbp003230	C27H30O15	Kaempferol-3-O-neohesperidoside	Flavonoids	Flavonols	32602-81-6	1.27E+06	7.81E+05	1.11E+06	4.04E+05	3.45E+05	3.94E+05	1.21E+00	3.62E-01	-1.47E+00	down
Lmbp003668	C20H18O10	Kaempferol-3-arabinopyranoside	Flavonoids	Flavonols	-	1.09E+06	4.72E+05	6.87E+05	2.89E+05	3.31E+05	3.75E+05	1.06E+00	4.42E-01	-1.18E+00	down
Lnrp002296	C27H30O15	Kaempferol glc-rha	Flavonoids	Flavonols	-	1.22E+06	7.65E+05	1.23E+06	2.91E+05	3.94E+05	4.42E+05	1.19E+00	3.51E-01	-1.51E+00	down
mws0038	C17H14O6	Kumatakenin	Flavonoids	Flavonols	3301-49-3	1.00E+05	8.15E+04	1.74E+05	9.00E+00	2.91E+03	9.00E+00	1.12E+00	8.23E-03	-6.92E+00	down
mws1290	C30H26O13	Tiliroside	Flavonoids	Flavonols	20316-62-5	1.62E+06	4.85E+05	7.11E+05	9.00E+00	9.00E+00	9.00E+00	1.26E+00	9.60E-06	-1.67E+01	down
pmb3894	C17H14O7	Di-O-methylquercetin	Flavonoids	Flavonols	2068-02-2	5.89E+06	3.70E+06	5.25E+06	4.65E+05	2.06E+05	4.12E+05	1.24E+00	7.30E-02	-3.78E+00	down
pmb0663	C27H30O16	8-C-Hexosyl-luteolin O-hexoside	Flavonoids	Flavonoid carbonoside	-	1.54E+05	1.46E+05	1.97E+05	4.99E+04	8.10E+04	8.87E+04	1.14E+00	4.43E-01	-1.18E+00	down
pmp001106	C27H30O15	Vitexin-2-O-D-glucopyranoside	Flavonoids	Flavonoid carbonoside	-	1.02E+06	5.51E+05	7.62E+05	1.56E+05	2.62E+05	1.66E+05	1.20E+00	2.50E-01	-2.00E+00	down
Lmmp000897	C30H26O14	Gallocatechin-Gallocatechin	Flavonoids	Flavanols	-	7.93E+05	5.84E+05	9.83E+05	2.26E+05	2.51E+05	2.83E+05	1.22E+00	3.22E-01	-1.63E+00	down
Lmdp003577	C21H20O10	Genistin(Genistein 7-O-Glucoside)	Flavonoids	Isoflavones	-	2.30E+06	2.47E+06	2.16E+06	2.66E+05	4.15E+05	2.30E+05	1.24E+00	1.31E-01	-2.93E+00	down
pmp000191	C23H22O11	6''-O-Acetylgenistin	Flavonoids	Isoflavones	73566-30-0	8.72E+05	6.76E+05	7.69E+05	1.27E+04	1.97E+04	1.17E+04	1.26E+00	1.91E-02	-5.71E+00	down
pmp000194	C24H22O13	Malonyglygenistin	Flavonoids	Isoflavones	-	2.92E+07	2.16E+07	3.18E+07	2.14E+06	2.98E+06	2.50E+06	1.26E+00	9.22E-02	-3.44E+00	down
pme2960	C15H12O5	Naringenin chalcone	Flavonoids	Chalcones	73692-50-9	2.76E+04	3.53E+04	4.03E+04	9.37E+04	8.51E+04	8.30E+04	1.23E+00	2.54E+00	1.34E+00	up
pme1598	C22H24O11	Hesperetin 5-O-glucoside	Flavonoids	Dihydroflavonol	69651-80-5	1.14E+07	3.10E+06	3.70E+06	1.80E+07	1.60E+07	1.37E+07	1.02E+00	2.62E+00	1.39E+00	up

pmb0545	C23H25O11+	Rosinidin O-hexoside	Flavonoids	Anthocyanins	-	3.11E+04	1.80E+04	1.98E+04	9.50E+04	2.03E+05	1.10E+05	1.20E+00	5.92E+00	2.57E+00	up
pmb0550	C21H21O11+	Cyanidin 3-O-glucoside (Kuromanin)	Flavonoids	Anthocyanins	47705-70-4	6.94E+05	1.01E+05	2.69E+04	3.82E+07	2.70E+07	1.51E+07	1.19E+00	9.77E+01	6.61E+00	up
pme0444	C23H25O12+	Malvidin 3-O-glucoside (Oenin)	Flavonoids	Anthocyanins	18470-06-9	1.34E+04	1.30E+04	1.14E+04	3.38E+04	5.64E+04	3.63E+04	1.22E+00	3.35E+00	1.74E+00	up
pme1398	C21H21O12+	Delphinidin 3-O-glucoside (Mirtillin)	Flavonoids	Anthocyanins	50986-17-9	4.00E+03	5.47E+03	6.59E+03	2.14E+04	4.55E+04	3.69E+04	1.21E+00	6.46E+00	2.69E+00	up
pme1773	C27H31O15+	Cyanidin 3-O-rutinoside (Keracyanin)	Flavonoids	Anthocyanins	28338-59-2	4.01E+05	1.13E+05	2.63E+04	7.05E+07	4.95E+07	3.55E+07	1.22E+00	2.88E+02	8.17E+00	up
pme3256	C27H31O16+	Delphinidin 3-O-rutinoside	Flavonoids	Anthocyanins	15674-58-5	2.00E+03	1.41E+03	4.57E+03	1.15E+05	6.40E+04	3.18E+04	1.21E+00	2.64E+01	4.72E+00	up
pme3392	C21H21O10+	Pelargonidin 3-O-glucoside	Flavonoids	Anthocyanins	47684-27-5	2.30E+03	6.63E+03	5.04E+03	9.20E+05	4.46E+05	4.37E+05	1.25E+00	1.29E+02	7.01E+00	up
pmf0027	C21H21O11+	Cyanidin 3-O-galactoside	Flavonoids	Anthocyanins	142506-26-1	6.03E+05	1.27E+05	1.22E+04	4.85E+07	2.08E+07	2.30E+07	1.17E+00	1.24E+02	6.96E+00	up
pmf0203	C22H23O11+	Peonidin 3-O-glucoside	Flavonoids	Anthocyanins	68795-37-9	4.79E+04	2.49E+04	1.32E+04	7.20E+05	6.84E+05	3.52E+05	1.22E+00	2.04E+01	4.35E+00	up
pmf0614	C28H33O16+	Peonidin 3,5-O-diglucoside chloride	Flavonoids	Anthocyanins	47851-83-2	1.61E+03	1.27E+03	2.16E+03	4.97E+03	1.12E+04	5.77E+03	1.17E+00	4.34E+00	2.12E+00	up
Hmcp001636	C23H24O13	Limocitrin 7-glucoside	Flavonoids	Flavonoid	-	1.48E+04	2.74E+04	2.44E+04	6.74E+04	8.73E+04	1.26E+05	1.19E+00	4.21E+00	2.07E+00	up
Hmcp002268	C23H24O12	Limocitrin 3-rhamnoside	Flavonoids	Flavonoid	-	4.17E+05	3.53E+04	1.72E+05	1.13E+06	2.13E+06	9.79E+05	1.05E+00	6.80E+00	2.77E+00	up
Lmzp004885	C17H14O7	Tricin	Flavonoids	Flavonoid	520-32-1	2.58E+04	6.79E+03	8.52E+03	8.96E+04	5.57E+04	1.04E+05	1.14E+00	6.06E+00	2.60E+00	up
Zmhp002776	C21H18O10	Chrysin-7-O-Glucuronide	Flavonoids	Flavonoid	35775-49-6	1.76E+05	2.21E+05	1.52E+05	5.40E+05	8.25E+05	6.12E+05	1.23E+00	3.61E+00	1.85E+00	up
mws0040	C15H10O4	Chrysin	Flavonoids	Flavonoid	480-40-0	8.91E+03	1.09E+04	5.78E+03	8.38E+04	3.65E+04	1.61E+05	1.17E+00	1.10E+01	3.46E+00	up
pmb0736	C23H24O12	Tricin 7-O-hexoside	Flavonoids	Flavonoid	-	3.83E+05	4.26E+04	1.52E+05	9.18E+05	2.72E+06	1.07E+06	1.07E+00	8.15E+00	3.03E+00	up
pmb3026	C23H22O13	Quercetin O-acetylhexoside	Flavonoids	Flavonoid	-	5.28E+04	2.91E+03	8.71E+03	9.20E+04	4.23E+05	1.61E+05	1.04E+00	1.05E+01	3.39E+00	up
pmp000172	C23H24O11	5,2'-Dihydroxy-7,8-dimethoxyflavone glycosides	Flavonoids	Flavonoid	-	9.11E+04	8.80E+04	7.93E+04	3.17E+05	3.34E+05	3.49E+05	1.26E+00	3.87E+00	1.95E+00	up
Hmln002321	C24H22O15	Quercetin 3-O-(6′′-O-malonyl)-glucoside	Flavonoids	Flavonols	-	9.00E+00	9.00E+00	9.00E+00	1.53E+04	4.84E+04	1.48E+04	1.26E+00	2.91E+03	1.15E+01	up
Hmln002582	C23H22O12	Kaempferol 3-O-(6′′-O-acetyl)-glucoside	Flavonoids	Flavonols	-	6.33E+04	2.55E+04	5.27E+04	1.34E+05	2.31E+05	2.19E+05	1.15E+00	4.13E+00	2.05E+00	up
mws1002	C17H14O8	Syringetin	Flavonoids	Flavonols	4423-37-4	9.00E+00	9.00E+00	9.00E+00	1.10E+04	2.94E+03	1.65E+04	1.25E+00	1.13E+03	1.01E+01	up
pmb3013	C24H24O13	Isorhamnetin O-acetyl-hexoside	Flavonoids	Flavonols	-	7.03E+05	3.83E+05	4.51E+05	9.64E+05	2.03E+06	1.05E+06	1.07E+00	2.63E+00	1.40E+00	up
pmp000589	C24H22O15	Quercetin-7-O-(6'-O-malonyl)-β-D-glucoside	Flavonoids	Flavonols	-	7.53E+05	1.10E+05	1.24E+05	2.72E+06	6.13E+06	2.76E+06	1.13E+00	1.18E+01	3.56E+00	up

**pmp001310**	**C27H30O17**	**6-Hydroxykaempferol-3,6-O-Diglucoside**	**Flavonoids**	**Flavonols**	**-**	**2.28E+05**	**6.12E+04**	**7.42E+04**	**6.15E+05**	**3.06E+05**	**9.07E+05**	**1.07E+00**	**5.03E+00**	**2.33E+00**	**up**
**pme2482**	**C7H6O3**	**Protocatechuic aldehyde**	**Flavonoids**	**Flavanols**	**139-85-5**	**1.32E+05**	**2.11E+05**	**9.03E+04**	**5.98E+05**	**7.37E+05**	**8.12E+05**	**1.21E+00**	**4.96E+00**	**2.31E+00**	**up**
**mws2118**	**C21H24O10**	**Phloretin 2'-O-glucoside**	**Flavonoids**	**Isoflavones**	**60-81-1**	**8.49E+05**	**8.72E+05**	**1.03E+06**	**2.73E+06**	**2.27E+06**	**1.24E+06**	**1.07E+00**	**2.27E+00**	**1.18E+00**	**up**
**mws1077**	**C16H18O9**	**Scopolin**	**Lignans and Coumarins**	**Coumarins**	**531-44-2**	**1.72E+05**	**1.53E+05**	**1.53E+05**	**9.62E+05**	**1.11E+06**	**1.56E+06**	**1.25E+00**	**7.61E+00**	**2.93E+00**	**up**
**mws5039**	**C15H16O9**	**Esculin Hydrate**	**Lignans and Coumarins**	**Coumarins**	**66778-17-4**	**4.97E+05**	**7.23E+05**	**7.00E+05**	**6.26E+06**	**7.42E+06**	**7.20E+06**	**1.26E+00**	**1.09E+01**	**3.44E+00**	**up**
pmb0764	C6H9NOS	4-Methyl-5-thiazoleethanol	Others	Others	137-00-8	1.53E+04	2.12E+04	2.62E+04	1.16E+04	9.62E+03	9.82E+03	1.13E+00	4.95E-01	-1.01E+00	down
Lmyn000239	C6H14O6	Cordycepic acid	Others	Saccharides and Alcohols	69-65-8	3.98E+05	4.42E+05	3.94E+05	1.88E+05	1.95E+05	1.67E+05	1.25E+00	4.46E-01	-1.16E+00	down
mws0214	C6H14O6	D-Sorbitol	Others	Saccharides and Alcohols	50-70-4	2.81E+05	3.05E+05	2.48E+05	1.06E+05	1.15E+05	9.45E+04	1.24E+00	3.78E-01	-1.40E+00	down
mws0437	C5H12O5	D-Arabitol	Others	Saccharides and Alcohols	488-82-4	1.45E+05	1.34E+05	1.18E+05	4.66E+04	4.28E+04	4.23E+04	1.25E+00	3.32E-01	-1.59E+00	down
mws0438	C5H12O5	L-Arabitol	Others	Saccharides and Alcohols	7643-75-6	1.58E+05	1.51E+05	1.27E+05	5.04E+04	5.09E+04	4.08E+04	1.24E+00	3.26E-01	-1.62E+00	down
pme0513	C5H12O5	Xylitol	Others	Saccharides and Alcohols	87-99-0	1.53E+05	1.40E+05	1.38E+05	4.70E+04	4.20E+04	5.57E+04	1.25E+00	3.36E-01	-1.57E+00	down
pme0516	C6H12O6	Inositol	Others	Saccharides and Alcohols	87-89-8	3.59E+06	3.18E+06	3.50E+06	7.57E+05	5.34E+05	5.33E+05	1.25E+00	1.78E-01	-2.49E+00	down
pme0534	C6H12O7	Gluconic acid	Others	Saccharides and Alcohols	526-95-4	1.77E+06	1.73E+06	1.45E+06	9.13E+05	4.26E+05	9.25E+05	1.07E+00	4.57E-01	-1.13E+00	down
pme2237	C6H14O6	Dulcitol	Others	Saccharides and Alcohols	608-66-2	3.41E+05	3.73E+05	2.74E+05	1.30E+05	1.36E+05	1.33E+05	1.24E+00	4.04E-01	-1.31E+00	down
mws0133	C6H6N2O	Nicotinamide	Others	Vitamin	98-92-0	9.91E+05	1.26E+06	1.02E+06	3.34E+05	4.13E+05	4.37E+05	1.24E+00	3.62E-01	-1.47E+00	down
mws1337	C9H17NO5	D-Pantothenic Acid	Others	Vitamin	79-83-4	9.85E+06	1.66E+07	1.84E+07	6.22E+06	6.97E+06	4.71E+06	1.12E+00	3.99E-01	-1.32E+00	down
pme0490	C6H5NO2	Nicotinic acid	Others	Vitamin	59-67-6	1.09E+05	1.53E+05	6.63E+04	2.18E+04	4.50E+04	3.97E+04	1.09E+00	3.24E-01	-1.63E+00	down
pme2266	C10H16N2O3S	Biotin	Others	Vitamin	58-85-5	2.26E+05	2.99E+05	3.31E+05	9.53E+04	1.21E+05	1.26E+05	1.21E+00	3.99E-01	-1.32E+00	down
mws1562	C15H22O10	Catalpol	Others	Others	2415-24-9	2.61E+05	1.29E+05	1.25E+05	8.59E+05	7.71E+05	5.94E+05	1.19E+00	4.32E+00	2.11E+00	up
mws0866	C6H13O9P	D-Glucose 6-phosphate	Others	Saccharides and Alcohols	56-73-5	5.16E+05	7.09E+05	6.22E+05	1.09E+06	8.95E+05	1.78E+06	1.05E+00	2.04E+00	1.03E+00	up
pme2596	C8H9NO4	4-Pyridoxic acid	Others	Vitamin	82-82-6	4.99E+04	6.03E+04	7.09E+04	2.91E+05	3.72E+05	2.20E+05	1.23E+00	4.88E+00	2.29E+00	up
mws2218	C8H10N4O2	Caffeine	Alkaloids	Alkaloids	58-08-2	6.31E+05	6.36E+05	9.16E+05	3.85E+05	3.64E+05	3.28E+05	1.18E+00	4.94E-01	-1.02E+00	down
GQ512004	C25H33N3O5	N1-Dihydrocaffeoyl-N10-coumaroyl spermidine	Alkaloids	Phenolamine	-	1.80E+03	9.00E+00	9.00E+00	1.79E+04	1.47E+04	2.08E+04	1.07E+00	2.93E+01	4.87E+00	up

pmp001255	C18H19NO4	N-Trans-feruloyltyramine	Alkaloids	Phenolamine	66648-43-9	9.00E+00	9.00E+00	2.74E+03	1.34E+04	1.86E+04	1.73E+04	1.04E+00	1.79E+01	4.16E+00	up
Hmcp005589	C30H48O	β-Amyrenone	Terpenoids	Triterpene	638-97-1	4.39E+04	3.50E+04	3.20E+04	9.00E+00	9.00E+00	9.00E+00	1.26E+00	2.44E-04	-1.20E+01	down
mws0159	C9H8O3	Phenylpyruvic acid	Organic acids	Organic acids	156-06-9	1.04E+05	1.50E+05	2.05E+05	2.91E+04	2.05E+04	5.03E+04	1.17E+00	2.18E-01	-2.20E+00	down
mws0206	C4H8O3	(S)-2-Hydroxybutanoicacid	Organic acids	Organic acids	3347-90-8	3.45E+04	1.77E+04	1.83E+04	5.99E+03	9.08E+03	1.26E+04	1.07E+00	3.92E-01	-1.35E+00	down
mws0208	C6H10O4	Adipic Acid	Organic acids	Organic acids	124-04-9	4.24E+05	6.12E+05	4.68E+05	6.60E+04	7.88E+04	5.16E+04	1.25E+00	1.31E-01	-2.94E+00	down
mws0237	C9H16O4	Anchoic Acid	Organic acids	Organic acids	123-99-9	6.24E+05	4.87E+05	5.86E+05	2.50E+05	2.37E+05	1.39E+05	1.17E+00	3.68E-01	-1.44E+00	down
mws0473	C5H8O4	2-Methylsuccinic acid	Organic acids	Organic acids	498-21-5	2.14E+07	1.46E+07	1.68E+07	3.14E+06	1.97E+06	2.14E+06	1.24E+00	1.37E-01	-2.87E+00	down
mws0497	C9H10O3	L-(-)-3-Phenyllactic acid	Organic acids	Organic acids	20312-36-1	4.68E+06	5.97E+06	5.75E+06	1.48E+06	1.66E+06	1.27E+06	1.24E+00	2.69E-01	-1.89E+00	down
mws0574	C4H8O3	α-Hydroxyisobutyric acid	Organic acids	Organic acids	594-61-6	5.32E+04	2.74E+04	1.45E+04	7.82E+03	1.06E+04	8.38E+03	1.05E+00	2.82E-01	-1.83E+00	down
mws0576	C4H8O3	3-Hydroxybutyrate	Organic acids	Organic acids	300-85-6	1.66E+07	1.81E+07	1.09E+07	2.22E+06	1.78E+06	2.20E+06	1.24E+00	1.36E-01	-2.88E+00	down
mws0924	C6H10O4	2-Methylglutaric acid	Organic acids	Organic acids	617-62-9	3.28E+05	3.64E+05	3.10E+05	3.41E+04	6.83E+04	3.01E+04	1.23E+00	1.32E-01	-2.92E+00	down
mws0277	C7H12O6	Kinic acid	Organic acids	Organic acids	77-95-2	3.83E+06	4.96E+06	3.61E+06	9.90E+06	9.55E+06	8.13E+06	1.22E+00	2.23E+00	1.15E+00	up
mws0345	C6H11NO2	Pipecolinic acid	Organic acids	Organic acids	535-75-1	5.94E+03	1.06E+04	5.90E+03	2.31E+05	1.44E+05	3.63E+05	1.24E+00	3.29E+01	5.04E+00	up
pme0274	C6H13NO2	6-Aminocaproic acid	Organic acids	Organic acids	60-32-2	2.66E+05	1.99E+05	1.01E+05	2.12E+06	1.83E+06	1.54E+06	1.23E+00	9.69E+00	3.28E+00	up
Hmyn006303	C27H47O12P	LysoPI(18:3)	Lipids	Glycerol ester	-	1.09E+04	9.22E+03	1.27E+04	9.00E+00	9.00E+00	9.00E+00	1.26E+00	8.24E-04	-1.02E+01	down
Rfmb089	C18H34O4	9,10-Dihydroxy-12-octadecenoic acid	Lipids	Free fatty acids	263399-34-4	7.15E+04	5.50E+04	8.54E+04	9.00E+00	9.00E+00	9.00E+00	1.26E+00	1.27E-04	-1.29E+01	down
Rfmb090	C18H32O3	13-Hydroxy-9,11-octadecadienoic acid	Lipids	Free fatty acids	5204-88-6	4.94E+06	4.95E+06	5.37E+06	4.66E+05	4.06E+05	5.83E+05	1.26E+00	9.53E-02	-3.39E+00	down
Rfmb091	C18H32O3	9-Hydroxy-10,12-octadecadienoic acid	Lipids	Free fatty acids	15514-85-9	5.00E+06	5.16E+06	5.39E+06	5.33E+05	4.72E+05	6.47E+05	1.26E+00	1.06E-01	-3.24E+00	down
mws2623	C18H34O2	11-Octadecanoic acid(Vaccenic acid)	Lipids	Free fatty acids	506-17-2	5.84E+06	4.60E+06	4.32E+06	1.75E+06	2.13E+06	2.84E+06	1.17E+00	4.56E-01	-1.13E+00	down
pmb2778	C18H32O3	9,10-EODE	Lipids	Free fatty acids	65167-83-1	7.84E+06	8.19E+06	8.61E+06	1.33E+06	1.11E+06	1.51E+06	1.26E+00	1.60E-01	-2.64E+00	down
pmb2786	C18H30O3	9-HOTrE	Lipids	Free fatty acids	89886-42-0	7.81E+05	7.95E+05	8.29E+05	1.95E+05	1.71E+05	2.19E+05	1.26E+00	2.43E-01	-2.04E+00	down
pmb2787	C18H30O3	9-KODE	Lipids	Free fatty acids	54232-59-6	2.63E+05	3.14E+05	2.65E+05	6.91E+04	4.09E+04	1.17E+05	1.16E+00	2.70E-01	-1.89E+00	down
pmn001688	C18H32O3	9S-Hyroxy-10E,12E-octadecadienoic acid	Lipids	Free fatty acids	-	5.22E+06	5.14E+06	5.54E+06	5.34E+05	4.61E+05	6.46E+05	1.26E+00	1.03E-01	-3.28E+00	down

pmn001689	C18H32O4	9-Hydroxy-12-oxo-10-octadecenoic acid	Lipids	Free fatty acids	-	2.30E+04	3.21E+04	3.51E+04	1.16E+04	8.58E+03	1.48E+04	1.16E+00	3.88E-01	-1.37E+00	down
pmn001691	C18H32O5	9,12,13-Trihyroxy-10,15-octadecadienoic acid	Lipids	Free fatty acids	-	3.32E+05	2.54E+05	3.36E+05	4.67E+04	3.21E+04	4.07E+04	1.25E+00	1.30E-01	-2.95E+00	down
pmn001694	C18H34O5	9,10,13-Trihyroxy-11-octadecadienoic acid	Lipids	Free fatty acids	-	6.01E+06	3.68E+06	4.82E+06	4.48E+05	2.24E+05	3.48E+05	1.24E+00	7.03E-02	-3.83E+00	down
Hmyn007081	C22H43O9P	LysoPG(16:1)	Lipids	Glycerol ester	-	4.65E+03	6.22E+03	6.07E+03	2.59E+04	2.94E+04	2.46E+04	1.25E+00	4.72E+00	2.24E+00	up
pmp001250	C26H51NO7P+	PC(18:2)	Lipids	PC	-	6.39E+05	1.58E+06	1.53E+06	3.21E+06	3.87E+06	2.73E+06	1.09E+00	2.62E+00	1.39E+00	up
pmp001251	C26H51NO7P+	PC(18:2)isomer	Lipids	PC	-	5.83E+05	1.63E+06	1.57E+06	3.18E+06	3.87E+06	2.82E+06	1.06E+00	2.61E+00	1.39E+00	up
Hmqp006235	C21H46N3O9P	LysoPC 18:4	Lipids	LPC	-	3.28E+04	8.57E+04	9.79E+04	4.54E+05	5.95E+05	3.25E+05	1.18E+00	6.35E+00	2.67E+00	up
mws0126	C26H54NO7P	1-Stearoyl-sn-glycero-3-phosphocholine	Lipids	LPC	19420-57-6	4.18E+05	3.13E+05	3.43E+05	8.94E+05	1.09E+06	8.82E+05	1.23E+00	2.67E+00	1.42E+00	up
pmb0854	C26H48NO7P	LysoPC 18:3	Lipids	LPC	-	3.98E+05	1.04E+06	1.05E+06	2.05E+06	2.38E+06	1.58E+06	1.04E+00	2.42E+00	1.28E+00	up
pmb0855	C24H50NO7P	LysoPC 16:0	Lipids	LPC	17364-16-8	1.59E+07	1.21E+07	1.42E+07	3.66E+07	4.28E+07	3.55E+07	1.24E+00	2.73E+00	1.45E+00	up
pmb0863	C24H46NO7P	LysoPC 16:2(2n isomer)	Lipids	LPC	-	4.85E+04	1.55E+05	1.40E+05	4.01E+05	5.56E+05	2.76E+05	1.08E+00	3.59E+00	1.84E+00	up
pmb0865	C26H48NO7P	LysoPC 18:3(2n isomer)	Lipids	LPC	-	2.64E+05	6.96E+05	6.65E+05	1.35E+06	1.56E+06	1.06E+06	1.05E+00	2.45E+00	1.29E+00	up
pmb2319	C23H48NO7P	LysoPC 15:0	Lipids	LPC	-	1.50E+05	1.09E+05	1.17E+05	4.33E+05	4.81E+05	5.11E+05	1.25E+00	3.79E+00	1.92E+00	up
pmb2406	C25H52NO7P	LysoPC 17:0	Lipids	LPC	-	5.35E+04	4.30E+04	4.24E+04	2.89E+05	3.97E+05	2.80E+05	1.25E+00	6.95E+00	2.80E+00	up
pmd0132	C24H50NO7P	LysoPC 16:0(2n isomer)	Lipids	LPC	-	1.84E+06	3.90E+06	3.83E+06	8.45E+06	1.14E+07	7.10E+06	1.12E+00	2.82E+00	1.50E+00	up
pmd0136	C26H54NO7P	LysoPC 18:0	Lipids	LPC	-	3.67E+04	5.84E+04	4.94E+04	1.53E+05	2.34E+05	1.52E+05	1.21E+00	3.73E+00	1.90E+00	up
pmp001270	C24H48NO7P	LysoPC(16:1)	Lipids	LPC	-	1.10E+05	5.21E+05	3.97E+05	2.19E+06	2.98E+06	1.39E+06	1.12E+00	6.38E+00	2.67E+00	up
pmp001273	C26H50NO7P	LysoPC(18:2)	Lipids	LPC	-	6.24E+05	1.57E+06	1.56E+06	3.25E+06	3.78E+06	2.78E+06	1.08E+00	2.61E+00	1.39E+00	up
pmp001281	C26H52NO7P	LysoPC(18:1)	Lipids	LPC	-	9.12E+05	1.83E+06	2.19E+06	4.12E+06	6.35E+06	3.56E+06	1.09E+00	2.85E+00	1.51E+00	up
pmp001286	C26H54NO7P	LysoPC(18:0)	Lipids	LPC	-	4.38E+05	3.22E+05	3.14E+05	8.70E+05	1.15E+06	8.47E+05	1.21E+00	2.67E+00	1.42E+00	up
mws0289	C23H46NO7P	LysoPE 18:1	Lipids	LPE	89576-29-4	5.59E+04	8.02E+04	7.98E+04	2.38E+05	3.16E+05	2.17E+05	1.23E+00	3.57E+00	1.84E+00	up
pmb0856	C23H46NO7P	LysoPE 18:1(2n isomer)	Lipids	LPE	-	4.17E+05	9.96E+05	1.14E+06	3.54E+06	4.75E+06	2.73E+06	1.15E+00	4.31E+00	2.11E+00	up
pmb0874	C23H44NO7P	LysoPE 18:2(2n isomer)	Lipids	LPE	-	1.40E+05	3.61E+05	2.84E+05	1.10E+06	1.36E+06	1.07E+06	1.19E+00	4.49E+00	2.17E+00	up

pmb0876	C21H44NO7P	LysoPE 16:0	Lipids	LPE	53862-35-4	1.22E+06	9.96E+05	9.09E+05	5.01E+06	5.72E+06	5.56E+06	1.25E+00	5.21E+00	2.38E+00	up
pmd0160	C21H44NO7P	LysoPE 16:0(2n isomer)	Lipids	LPE	-	4.39E+04	4.65E+04	3.65E+04	1.91E+05	1.79E+05	1.38E+05	1.25E+00	4.00E+00	2.00E+00	up
